# Aberrant coordination geometries discovered in the most abundant metalloproteins

**DOI:** 10.1002/prot.25257

**Published:** 2017-03-07

**Authors:** Sen Yao, Robert M. Flight, Eric C. Rouchka, Hunter N.B. Moseley

**Affiliations:** ^1^ School of Interdisciplinary and Graduate StudiesUniversity of LouisvilleLouisvilleKentucky40292; ^2^ Department of Computer Engineering and Computer ScienceUniversity of LouisvilleLouisvilleKentucky40292; ^3^ Department of Molecular and Cellular BiochemistryUniversity of KentuckyLexingtonKentucky40356; ^4^ Markey Cancer CenterUniversity of KentuckyLexingtonKentucky40356; ^5^ Center for Environmental and Systems BiochemistryUniversity of KentuckyLexingtonKentucky40356

**Keywords:** compressed angle, 3D structure, bidentation, structural bioinformatics, structure‐function relationship

## Abstract

Metalloproteins bind and utilize metal ions for a variety of biological purposes. Due to the ubiquity of metalloprotein involvement throughout these processes across all domains of life, how proteins coordinate metal ions for different biochemical functions is of great relevance to understanding the implementation of these biological processes. Toward these ends, we have improved our methodology for structurally and functionally characterizing metal binding sites in metalloproteins. Our new ligand detection method is statistically much more robust, producing estimated false positive and false negative rates of ∼0.11% and ∼1.2%, respectively. Additional improvements expand both the range of metal ions and their coordination number that can be effectively analyzed. Also, the inclusion of additional quality control filters has significantly improved structure‐function Spearman correlations as demonstrated by rho values greater than 0.90 for several metal coordination analyses and even one rho value above 0.95. Also, improvements in bond‐length distributions have revealed bond‐length modes specific to chemical functional groups involved in multidentation. Using these improved methods, we analyzed all single metal ion binding sites with Zn, Mg, Ca, Fe, and Na ions in the wwPDB, producing statistically rigorous results supporting the existence of both a significant number of unexpected compressed angles and subsequent aberrant metal ion coordination geometries (CGs) within structurally known metalloproteins. By recognizing these aberrant CGs in our clustering analyses, high correlations are achieved between structural and functional descriptions of metal ion coordination. Moreover, distinct biochemical functions are associated with aberrant CGs versus nonaberrant CGs. Proteins 2017; 85:885–907. © 2016 Wiley Periodicals, Inc.

## INTRODUCTION

**Figure 1:**
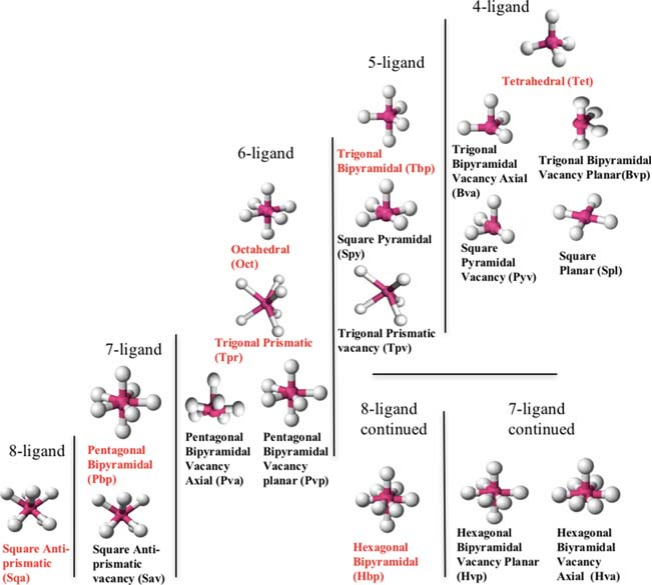
Structure of canonical CG models. For each structure, the magenta ball represents the metal ion center, and the white balls represent the binding ligands. The three‐letter code for each CG is shown in parenthesis. The major CG names are shown in red with their minor CGs follow in the same row. The minor CGs can often be viewed as missing ligands from their corresponding majors ones. CGs are also separated by lines according to their ligand numbers. [Color figure can be viewed at wileyonlinelibrary.com]

Metalloproteins are proteins that can bind at least one metal ion as a cofactor. They play various distinct functional, structural, and signal transductional roles in proteins, and are essential for all domains of life. Many proteins rely on metals to help hold their structures together,^1^, ^2^, ^3^ while others require metals to implement mechanistic steps in biochemical reactions they catalyze.^4^ However, most transition metals, such as Zn, Fe, and Cu, are highly toxic in their free ionic form, requiring tight regulation. Therefore, there are many proteins involved in the sensing, transporting, and storing of metal ions in biological systems to maintain homeostatic levels.^5^, ^6^ It is estimated that roughly 30–40% of whole proteomes across the biosphere are metalloproteins.^7^ Metal ions generally bind to proteins via coordination with electronegative atoms from the protein, such as nitrogen, oxygen, and sulfur. One of the most important aspects of metal binding is its coordination geometry (CG), which often implies functional activities. In inorganic chemistry, a metal ion can bind to its ligands almost ideally. In this context, metal ions are observed and verified to adopt a limited set of canonical CGs according to their physiochemical properties (Fig. 1). Whereas in biology, the chemical environment around metal ions is often more complicated. Since bound metal ions are often required for specific functions in metalloproteins, some metal binding sites are targeted for drug design,^8^, ^9^ providing yet another reason for their systematic study.

The applications of rapidly improving genomic sequencing technologies are generating huge amounts of gene sequence information and expression data. Various pattern recognition and expression analysis methods are identifying which biochemical and cellular functions are possible within specific tissues at specific times, addressing “What”, “Where”, and approximately “When” specific functions occur. However, “How” gene‐products implement function requires data and analyses that are focused on their three‐dimensional structure. Developing computational methods that derive and describe the linkages between structure and function represents the growing area of structural bioinformatics. This area of research utilizes the structural information accumulated in the world‐wide Protein Data Bank (wwPDB)^10^ and functional information accumulated in various knowledge bases including Uniprot^11^ and the Gene Ontology Consortium.^12^ In addition, many other structural databases and tools have been built, such as SCOP^13^ and CATH,^14^ that organize structure and relate it to function. However, for our purposes in this article, SCOP and related databases are mainly focused on the overall fold or sequence homology of a protein, while in metal binding, it is the immediate binding ligands or local environment that are more functionally relevant to “how” the metal is utilized. CheckMyMetal is a well‐maintained web‐based tool that is structurally focused on the metal binding sites of metalloproteins.^15^ It has methods for inspecting and validating metal binding sites in metalloproteins and for basic sorting of a metal's CGs. Since its main focus is on the structural aspects of metal binding sites, it does not have methods to link metalloprotein structure with function. In current efforts seeking to provide metalloproteins' structure‐function relationships, MetalPDB is one of the leading database tools.^16^ However, MetalPDB is based essentially on structural homology of a metal ion(s) and its surrounding coordination shell, and loosely summarized >17,000 structural clusters with functional details. In this study, we developed a more general structural description of metal binding sites represented mainly by their CG and having strong functional relevance.

Our previous work demonstrated that a more general CG description of a single zinc ion could be constructed based on its 3D‐structure and has high Spearman correlation (rho = 0.88, *p*‐value < 2.2 × 10^−16^) with function.^17^ Furthermore, we demonstrated that a large number of aberrant 4‐ligand CGs in zinc metalloproteins with significant deviations from canonical CGs existed due to structural constraints from the metalloprotein. These constraints, mostly in the form of bidentated ligands, and associated aberrant CGs included unique functional relationships. These controversial results generated criticism^18^ that we address in a companion perspective article.^19^ Also, these results created several new questions:

- Could similar functionally‐relevant structural descriptions of CG be constructed for other common metals, involving different numbers of ligands?
- Would similar or even new structural constraints and aberrant CGs be detected?

To address these questions in this study, we greatly expanded our methodology to allow construction of CG structural descriptions with an arbitrary number of ligands. We also had to greatly improve our detection of metal binding ligands by adding several quality control filters, compensating for crystallographic resolution, and preventing false detection of ligands. These improvements helped to detect and structurally describe single metal ion CGs and their functional relationships across the five most abundant metalloproteins.

## METHODS

### Define metal's first coordination shells (fc‐shells)

**Figure 2:**
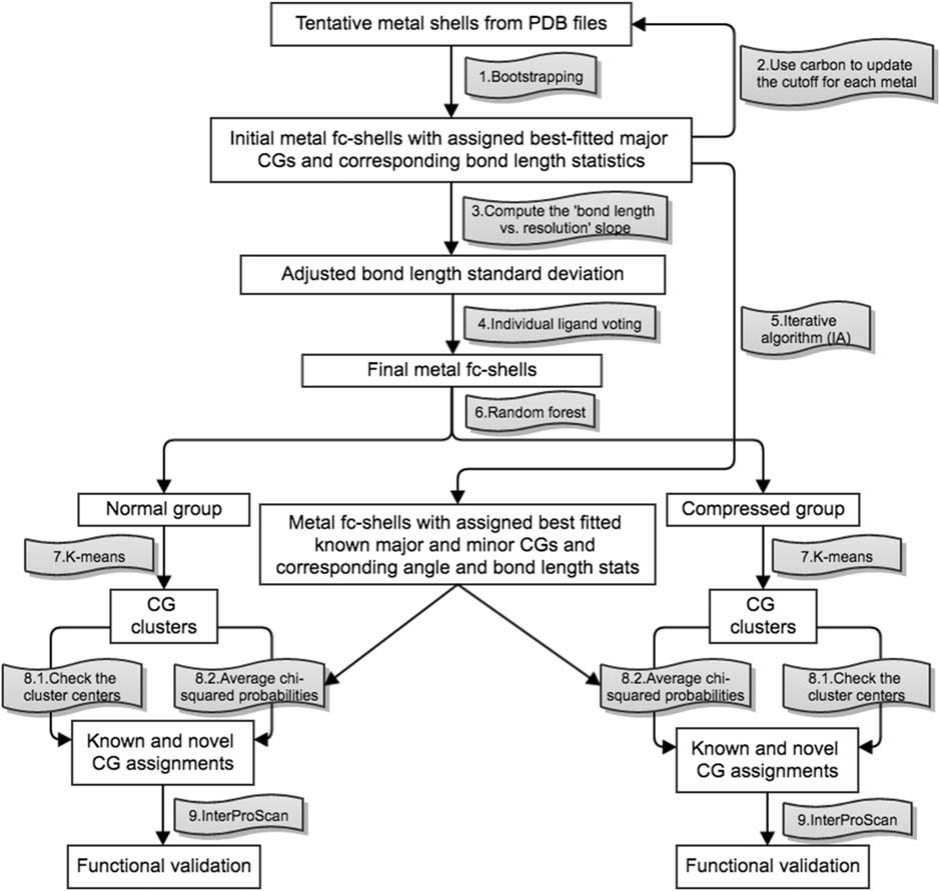
The workflow of metalloprotein CG analysis. The gray ribbons identify specific steps of the overall analysis as described in the methods.

All released structural entries were downloaded from wwPDB on Feb 25, 2015. Our metalloprotein filtering tool identified all PDB entries with at least one metal atom in the HETATM record and removed entries with fewer than 20 amino acids in the SEQRES record. Next, metal clusters were identified and removed, using two metal atoms within 3 Å as the filter. Zn, Mg, Ca, Fe, and Na were kept for the rest of the analyses in this study due to their high abundance based on Table 1. If not specified, all analyses were carried out first for each metal separately and then combined together. The overall workflow is shown in Figure 2. Since the general procedure is similar to what was performed on Zn with an older version of the wwPDB (March 13, 2013), we are mainly highlighting the extensive list of improvements here.

**Table 1:** Numbers of Metalloproteins in wwPDB as of Feb 2015

Metal	Number of PDB entries	Number of total metal sites	Metal	Number of PDB entries	Number of total metal sites
Zn	9360	26,788	Pb	48	152
Mg	9145	53,896	Gd	42	197
Ca	7762	24,335	Tl	40	261
Fe	6359	27,514	Rb	37	153
Na	4888	16,527	Sm	33	111
Mn	2266	8138	Ir	31	48
K	1673	5306	Pr	22	55
Cu	1134	4397	Rh	20	46
Ni	935	2252	Eu	19	61
Co	915	2087	Pd	19	85
Cd	758	4289	Ag	18	75
Hg	528	1923	Os	14	33
Pt	191	629	Lu	13	56
Mo	176	664	Ho	12	35
Al	158	351	Tb	11	32
V	120	364	Cr	9	21
Ba	118	311	Ga	8	10
Sr	118	3551	La	8	18
Ru	99	134	Sb	5	10
Cs	88	393	Ce	4	7
W	76	1443	Er	2	6
Yb	72	177	In	2	3
Au	64	322	Bi	1	1
Y	53	202	Dy	1	30
Li	52	88	Total	47,527	187,587

Step 1: For each metal site, we generated a list of potential non‐H shell ligands (including carbon) within a certain distance of the metal atom. The initial shell lower cutoff is 1.3 Å for all metals, and the initial upper cutoff is based on the atomic radius of the metal as shown in Table 2. To avoid the inclusion of second shell atoms due to this generous upper cutoff, the bond lengths between any atom and the metal must be smaller than 1.5 times the bond length of the metal to any other atoms in the cutoff, and also be smaller than 1.5 times the bond length between the two atoms. This ‘triangular rule’ can help exclude atoms that do not directly bind to the metal but are still part of the metal's local chemical environment. We then used the CG evaluation tools to bootstrap the best‐fit canonical CGs to identify an initial set of binding ligands. To achieve that, all subsets and combinations of the potential atoms and the corresponding ligand–metal–ligand angles (angles) were computed and compared to the ideal angles of the canonical CGs, tetrahedral (Tet), trigonal bipyramidal (Tbp), octahedral (Oct), and pentagonal bipyramidal (Pbp). Several additional filters were applied to every set of atoms before checking against the canonical CGs: (1) only the best possible alternate locations of each amino acid residue were allowed in the atom collection; (2) if any two ligand‐ligand atom pairs are smaller than 1.5 Å or >6.0 Å, they were marked as an unreasonable atom–atom bond‐length distance and eliminated; (3) if any of the atoms are symmetry‐related, unless it is from the biological multimer units indicated in the PDB file or all the symmetry‐related atoms are water, the binding site would be excluded from further analysis; (4) we also excluded the metal site if the majority of its ligands were water. These filters limit the inclusion of metal binding sites that may represent nonspecific binding or crystallographic artifacts. The set of atoms that pass all filters and have the smallest angle variance were considered the initial binding ligands.

**Table 2:** Derived Distance Cutoffs and Parameters for Defining the Coordination Shell of the Five Most Prevalent Metalloproteins in Different Steps

	Step 1		Step 2					Step 3	Step 5	Step 6
Metal	Atomic radius (pm)	Initial distance upper cutoff (Å)	The most abundant element	Bond length mean of the most abundant element (Å)	Bond length standard deviation of the most abundant element (Å)	Carbon mean peak (Å)	Element included	Updated distance upper cutoff (Å)	IA small angle removal cutoff (Å)	Random forest cutoff (degrees)
Zn	135	3.20	S	2.340	0.152	3.071	S, O, N	2.782	68	60/70
Mg	150	3.35	O	2.350	0.368	3.067	O, N	2.892	65	58/68
Ca	180	3.65	O	2.481	0.271	3.432	O	3.092	60	55/65
Fe	140	3.25	N	2.063	0.134	3.081	S, O, N	2.639	68	63/73
Na	180	3.65	O	2.697	0.369	3.568	O	3.317	60	50/65

**Figure 3:**
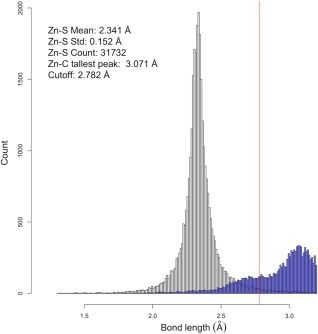
The updated upper Zn bond‐length cutoff for generating final bond‐length statistics. The gray histogram is the detected Zn‐S bond lengths based on canonical CGs. The blue histogram is the fictional Zn‐C bond lengths based on canonical CGs. The red line is the upper bond‐length cutoff used for calculating final bond‐length statistics: (Zn‐S mean + 1 Zn‐S standard deviation + the Zn‐C mode)/2. [Color figure can be viewed at wileyonlinelibrary.com]

Step 2: As the initial binding ligands were identified, bond lengths of each element type (O, S, N, …) were computed. The inclusion of carbon as binding ligands in step 1 can be used to estimate the chance of having an atom accidently aligned as well as canonical CGs in regard to other binding ligands, since carbon is a very uncommon ligand atom. This is due to increasing atom density with respect to angle space as a shell inclusion cutoff increases. A new upper cutoff was then set to be the average between bond‐length mean plus one standard deviation of the most abundant element and the main carbon distance mode (Table 2). Therefore, the updated upper cutoff is generous enough to include most of the actual binding ligands but still effective enough to exclude falsely detected ligand atoms. Taking Zn as an example, the most abundant ligand element is S, as shown in Figure 3, and the Zn‐S bond length mean and standard deviation are 2.341 Å and 0.152 Å accordingly. The main modal peak of fictional Zn‐C is 3.071 Å, so the middle point between them is (2.341 + 0.152 Å + 3.071)/2 = 2.782 (Å), which became the updated bond length cutoff for the ligand detection of zinc ions.

The computational bootstrapping step (Step 1) was then carried out again using the updated cutoffs to obtain the list of potential shell ligands. The same triangular and other filtering rules were applied. This time we only kept elements with a high occurrence (>5%), and also ignored the carbon. After the second round of bootstrapping, the tentative metal binding shells were defined, and bond length statistics were calculated for further refinement.

**Figure 4:**
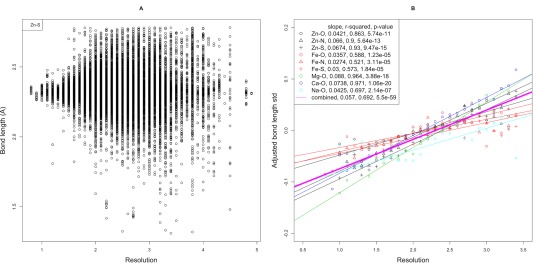
(A) Zn‐S bond length as a function of crystallographic resolution. (B) Scatter plot of bond length standard deviations versus average crystallographic resolutions by bond type. Symbols show smoothed values for individual metal‐ligand bond types. Symbols are specific to the ligand element. Symbols and regression lines are color‐coded by metal. The magenta‐color line is the combined regression with an *r* ^2^ of 0.692 and a *p*‐value of 5.5 − 10^−59^. [Color figure can be viewed at wileyonlinelibrary.com]

Step 3: It is known that the bond lengths scatter more as the crystallographic resolution worsens,^20^ as shown in Figure 4A. Our data shows that the relationship between the bond lengths standard deviation and resolution is similar regardless of the metal or the element type (Fig. 4B). Resolutions with >30 data points were kept in calculating the standard deviation specific to resolution. A resolution cutoff of 3.5 Å was used to ensure a reasonable quality of the data in this step. Considering all metal‐element pairs together, we were then able to compute the combined slope of bond length standard deviation (bl‐std) versus resolution. Then for each individual metal site, an adjusted bond length standard deviation was calculated as:

(1) $${\text{sd}}_{x}=\;m\;\left(R_{x}–\;R_{\text{avg}}\right)\;+{\text{sd}}_{\text{avg}}$$

where m is the combined slope, sd_avg_ and *R*_avg_ are the overall bond length standard deviation and the average resolution of given metal‐element type, *R_x_* is the resolution of the metal site to be calculated. The resulting adjusted bond length standard deviation, sd_*x*_, was used for the next step.

**Figure 5:**
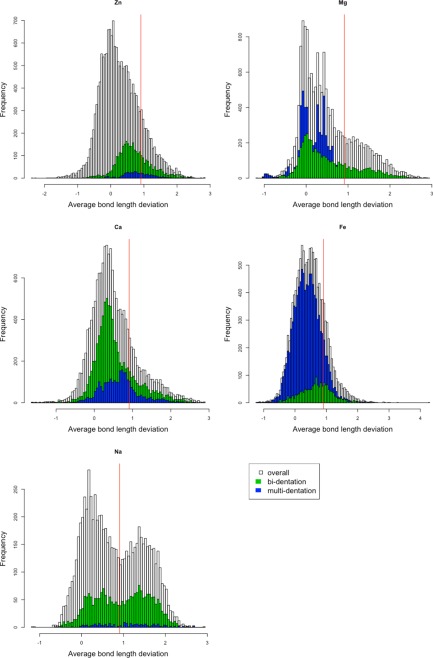
Average normalized bond length deviation histograms of five most abundant metalloproteins. The cutoff 0.91 was derived from the clear bimodal separation in Na, and was applied to all metals, which is represented as the red line in each sub‐graph. Bidentation means that two of the binding ligands come from the same molecule or residue. 3+ multidentation means that three or more of the binding ligands come from the same molecule or residue. They are the contributing factor to the shoulders in each histogram. [Color figure can be viewed at wileyonlinelibrary.com]

**Figure 6:**
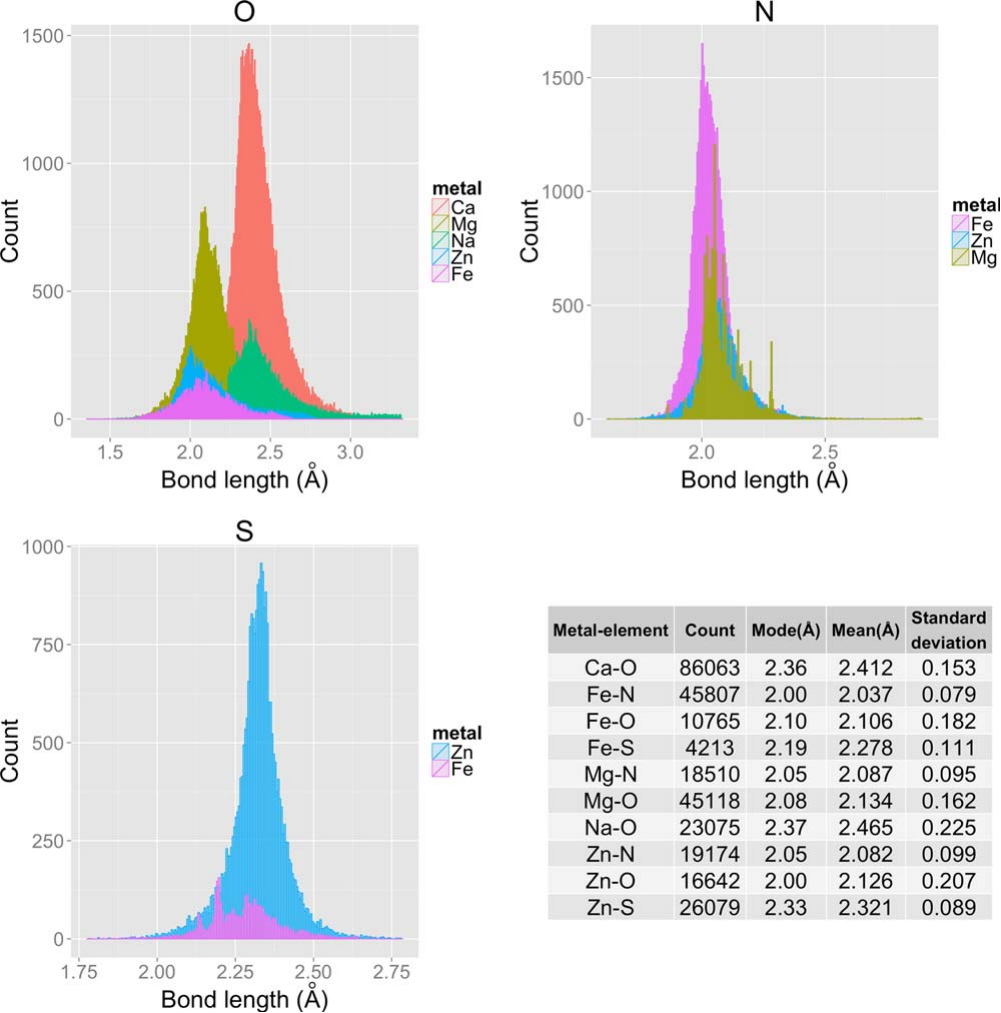
Bond length distributions and statistics of all bond types involving elements O, N, and S, which have >5% occurrence.

Step 4: With this adjusted bl‐std, all atoms within the updated cutoff were revisited and only atoms within 2.5 adjusted standard deviations of its expected value were kept. NMR structures were included and treated as structures with 2.5 Å resolution.^21^, ^22^The same set of filters as in step 1 was employed again to check the quality of the kept atoms. Thanks to the refined bl‐std, an additional filter was added at this step based on observed bimodality detected in the distribution of average bl‐std‐normalized deviations of all ligands' bond lengths to their specific bond length mode, especially for Na ion coordination (Fig. 5). This average normalized bond‐length deviation can be viewed as an average *z* scores for bond‐lengths observed in a specific metal binding site, where the expected value is the specific major bond‐length mode from a chemical perspective and the standard deviation is the refined bl‐std. If this normalized average deviation is >0.91, based on clear separation between the two modes in Na, the metal coordination is considered grossly incorrect (likely as a result of metal ion misassignment) and was thus removed from further analysis. The atoms that passed all filters composed our final metal fc‐shells for the rest of the analyses, generating the bond‐length histograms in Figure 6. Final element‐specific bond length statistics (means and variances) were calculated for each metal (Fig. 6). Finally, a nonredundant set of metal fc‐shells with a resolution better than 3 Å and an occupancy >0.9 were derived for clustering and functional analyses.

Step 5: The iterative process. All CG models shown in Figure 1 were used in this step. At each iteration, a *χ* ^2^ probability was calculated for each CG model at each metal site, using a combined angles and bond lengths vector. All combinations of the atoms within the updated cutoff defined in Step 2 were considered. We excluded the combinations if there are angles between the atoms below a cutoff specific to each metal based on its smallest angle histogram. The same set of filters in step 4 was also applied. All CG models in Figure 1 were considered. Pearson correlations between angles were estimated for each CG to calculate the *χ* ^2^ statistics. However, for trigonal prismatic (Tpr), square antiprismatic (Sqa), hexagonal bipyramidal (Hbp), and their associated minor CGs, the angle correlation matrix is large and has a very wide range of values between the elements, leading to higher error during its numerical inversion. Thus, the matrix inversion that is required for the normalized *χ* ^2^ statistics calculation is incapable of accurately capturing the angle's influence over each other. So we treated angles and bond lengths as independent variables, but with a 1.5 multiplier on the variance to counter the effect of dependency in the *χ* ^2^ statistics calculation. The CG model that possesses the highest *χ* ^2^ probability was classified as the metal site's CG. Both angle and bond length statistics of a CG were calculated at the end of an iteration, and were then used in the *χ* ^2^‐probability calculation of the next iteration. A new iteration was performed until all statistics converged.

### Cluster and assign canonical and aberrant CGs to each cluster

Step 6: Random forest^23^, ^24^ was used to separate normal versus compressed groups. Training data were composed of the main angle peaks from the smallest angle histogram. The cutoff for the normal and compressed training data was specific to each metal as shown in Table 2. The smallest angle, the two ligands composing the smallest angle, and the bidentation status of the smallest angle are the features for training the classifier.

Step 7: K‐means^25^ was employed to cluster the metal sites based on their ligand‐metal‐ligand angles. To enable the comparison of metal sites with different numbers of ligands (that is, different coordination numbers), we reduced the all‐angle space to a 6‐angle space by selecting the following angles from all angles of a given metal site: largest angle, smallest‐middle angle, 33rd‐quantile‐middle angle, 66th‐quantile‐middle angle, largest‐middle angle, and smallest‐opposite angle (Table 3). The opposite angles are those that do not share any ligands with the largest angle, and the middle angles are all angles except the largest and the smallest‐opposite angle. Except for 7‐ligand and 8‐ligand CGs (see Discussion), this reduced angle space can preserve the key information needed for separating each CG, while reducing the redundancy of the repeated angles. Four measures were used in determining the optimal number of clusters (k): (1) the Jaccard index computes how well matching clusters overlap between iterations; (2) the sum of differences indicates how close the cluster centers are to each other between iterations; (3) the Spearman's correlation coefficient rho and 4) – log(*p*‐value) indicate an average of functional correspondence across the clusters. For all four measures, a larger value denotes a better performance.

**Table 3:** 6‐angle Space for All CGs in Figure 1. [Color table can be viewed at wileyonlinelibrary.com]

CG	Largest	Ordered middle angles, with smallest‐middle, 33‐quantile‐middle, 66‐quantile‐middle, largest‐middle positions are in red	Smallest opposite
4‐ligand:			
Tet^a^	109.5	109.5, 109.5, 109.5, 109.5	109.5
Bva	120	90, 90,120, 120	90
Bvp	180	90, 90, 90, 90	120
Pyv	180	90, 90, 90, 90	90
Spl	180	90, 90, 90, 90	180
5‐ligand:			
Tbp	180	90, 90, 90, 90, 90, 90, 120, 120	120
Spy	180	90, 90, 90, 90, 90, 90, 90, 180	90
Tpv	131.8	70.6, 90, 90, 90, 90, 131.8, 131.8, 131.8	70.6
6‐ligand:			
Oct	180	90, 90, 90, 90, 90, 90, 90, 90, 90, 90, 90, 180, 180	90
Pva	144	72, 72, 72, 72, 90, 90, 90, 90, 90, 144, 144, 144, 144	72
Pvp	180	72, 72, 90, 90, 90, 90, 90, 90, 90, 90, 144, 144, 144	72
Tpr	131.8	70.6, 70.6, 90, 90, 90, 90, 90, 90, 131.8, 131.8, 131.8, 131.8, 131.8	70.6
7‐ligand:			
Pbp	180	72, 72, 72, 72, 90, 90, 90, 90, 90, 90, 90, 90, 90, 90, 144, 144, 144, 144, 144	72
Hva	180	60, 60, 60, 60, 60, 90, 90, 90, 90, 90, 90, 120, 120, 120, 120, 120, 120, 180, 180	60
Hvp	180	60, 60, 60, 90, 90, 90, 90, 90, 90, 90, 90, 90, 90, 120, 120, 120, 120, 180, 180	60
Sav	143.6	70.5, 70.5, 70.5, 70.5, 70.5, 82, 82, 82, 82, 82, 82, 109.5, 109.5, 109.5, 143.6, 143.6, 143.6, 143.6, 143.6	70.5
8‐ligand:			
Hbp	180	60, 60, 60, 60, 60, 90, 90, 90, 90, 90, 90, 90, 90, 90, 90, 90, 90, 120, 120, 120, 120, 120, 120, 180, 180, 180	60
Sqa	143.6	70.5, 70.5, 70.5, 70.5, 70.5, 70.5, 70.5, 82, 82, 82, 82, 82, 82, 82, 82, 109.5, 109.5, 109.5, 109.5, 143.6, 143.6, 143.6, 143.6, 143.6, 143.6, 143.6	70.5

a CG abbreviations are based on Figure 1.

Step 8: To characterize the clusters, we checked the cluster centers and calculated a *χ* ^2^ probability of each CG model for each metal site. The model that had the highest cluster‐average probability was then characterized as the cluster's CG.

### Functional validation of the *k*‐means clusters

Step 9: We ran InterProScan^26^, ^27^ 5.20–59.0 using the current versions of TIGRFAM,^28^ ProDom,^29^ SMART,^30^ HAMAP,^31^ Prosite‐Patterns,^32^ SuperFamily,^33^ PRINTS,^34^ Panther,^35^ Gene3d,^36^ PIRSF,^37^ PfamA,^38^ PrositeProfiles,^32^ and Coils^39^ hidden Markov models on the nonredundant sequences previously determined. We retained only those results with an InterProScan (IPR) annotation mapping and overlapping at least one ligand residue. We derived and evaluated the consistency of CG‐based structure and sequence‐based function annotation relationships between *k*‐means clusters.

For functional annotation characterization of the normal and compressed CGs, the molecular function (MF) and biological process (BP) gene ontology (GO) annotations reported with the InterProScan annotations were extracted, and the set of GO terms that are direct ancestors were added to each entry using GO.db v3.3.0. The categoryCompare2 (v0.99.158) program^40^ was used to create annotation objects based on the set of sequences annotated and to calculate hypergeometric enrichment on the normal entries and compressed entries separately. Significant GO annotations had at least two metal binding sites annotated from the normal or compressed list, and an unadjusted *p*‐value ≤ 0.05. *P*‐values were also adjusted for multiple testing via the Benjamini‐Hochberg method.^41^ The corrected *p*‐values are used to select results shown in the Supporting Information tables, while uncorrected were used for clustering groups of GO terms together. To improve interpretability, GO terms were grouped by running the cluster_walktrap algorithm from igraph (v 1.0.1 based on the Walktrap random walks algorithm^42^, ^43^) on a graph of the GO terms, where nodes are terms and edges are weights based on the number of shared annotated metal binding sites. Prior to grouping, edges with weight < 0.8 were removed. Next, enrichments were checked for consistency by examining the individual ligand groups against the “allLig” group. So, “all” metal all‐ligand was compared against all 4‐ligand, all 5‐ligand, etc, and Ca all‐ligand was compared against Ca 4‐ligand, 5‐ligand, and so forth The contributions of each metal to the GO annotations in the combined metal results were calculated from the metal specific annotations, and the maximum percentage and corresponding metal reported.

### Code and data availability

All data and code used and results generated are available from software.cesb.uky.edu or FigShare.^44^

## RESULTS AND DISCUSSION

### Defining metal binding sites

The wwPDB contains a total of 106,427 structures as of Feb 25, 2015, and 47,527 of them are metalloproteins. The number of specific metalloproteins and metal binding sites can be found in Table 1. Only the five most abundant metals, Zn, Mg, Ca, Fe, and Na are considered in this work.

Determining a metal's binding ligand is not as straightforward as one would anticipate, as first and second coordination atoms from the protein are often crowded together around the metal ion. In this situation, there is no simple rule in deciding whether an atom is metal‐binding or not. This is partly due to the limitations in structural resolution, crystallographic artifacts, and to phenomena such as the carboxylate shift^45^ that smear the metal‐ligand bond‐lengths. The determination is often achieved simultaneously with a metal binding site's CG classification. The most common approach is to use a simple distance cutoff and then select a ligand subset that best fits one of the canonical CG models.^16^ Sometimes, the bond valence model is taken into account.^15^ The dilemma of choosing the cutoff is, if it is too generous, extra second‐coordination‐shell atoms will be included, which will increase the demand for a more accurate CG fitting method. But if it is too strict, some of the loosely bound ligands will be excluded in the first step, which will hinder the fitting to the correct CG model. This methodology also precludes the existence of noncanonical, aberrant CGs.

As our previous study showed, simply matching to canonical CG models is problematic,^16^, ^17^ which makes the accurate detection of metal binding ligands even more critical for detecting and analyzing CG. In this work, we first used an initial shell cutoff based on the metal's atomic radius as shown in Table 2 to detect potential ligands that fit to canonical CGs to derive metal‐ligand bond‐length statistics for use in later steps. This first round of the bootstrap step can capture the general distribution of bond‐length for each ligand element. However, Figure 3 clearly shows that if this raw shell cutoff is the only criteria used, significant numbers of non‐ligand second‐shell atoms (represented by carbon) will be included due to the atom‐angle density issue. To get rid of these nonligand second shell atoms, we used carbon to estimate the false ligand metal distance distribution and then identified where false ligand atoms start to appear with high probability (that is, the highest carbon atom mode). In other words, we used the ubiquitous presence of carbon in protein structures to estimate the ‘accidental’ angle alignment with other ligands to fit any canonical CGs. The updated upper distance shell cutoff was also set to guarantee the inclusion of the majority of the most abundant ligand element, which is more likely to be the actual binding ligands. The red line in Figure 3 shows the cutoff used for Zn, which was the middle point between the first carbon mode (peak) and the Zn‐sulfur bond‐length mean plus one standard deviation. With these improved shell cutoffs and additional heuristics, such as the ‘triangular rule’, we generated improved bond‐length statistics for each metal (Table 2).

For accurately detecting the proper set of ligands, our next major improvement involved adjusting the bl‐stds based on crystallographic resolution. With accurate bond‐length statistics, the detection of the proper set of ligands can be performed independently, a single ligand at a time, via a statistical test. However, the bond‐lengths tend to scatter (vary) more as structure resolution worsens (that is, larger resolution value) for a specific metal‐element type.^20^ Rather than greatly restricting our analyses to structure entries with only high resolution (<1.5 Å), we are able to safely extend our analyses to structure entries with lower resolutions down to 3.0 Å by taking the crystallographic resolution into consideration in the statistical test. To do this, we shifted all the bl‐std to resolution data points along the y axis by its own overall metal‐element bl‐std to put everything on the same scale/level. Figure 4 shows that regardless of the metal and binding element, the bl‐std and resolution relationship is of the same proportion. Therefore, a combined slope can accurately describe this relationship and be used to adjust an individual metal‐atom pair's standard deviation according to the entry's resolution as shown in Equation (1). We also tested deriving similar standard deviation adjustments based on R‐factor and R‐free and combinations of R‐factor, R‐free, and resolution (data not shown). These combinations did not work well since the low density of entries prevented accurate calculation of metal‐ligand bond‐length standard deviations. However, in the future, we may have enough structural examples to reexamine combinations. But currently, the crystallographic resolution provided the highest Pearson's correlation for refining bond‐length standard deviations.

**Figure 7:**
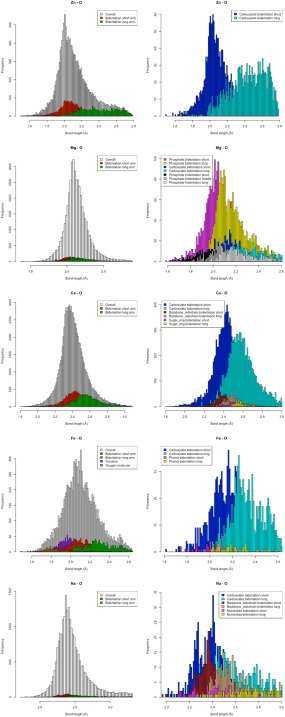
Chemical functional group and multidentation specific bond length modes. On the left is the overall bidentation short and long arms for each metal and some specific functional groups that contributing to the overall bond length histogram. On the right is a breakdown of the most abundant functional groups in the bidentation and multidentation, as they often exhibit distinct modes. [Color figure can be viewed at wileyonlinelibrary.com]

**Figure 8:**
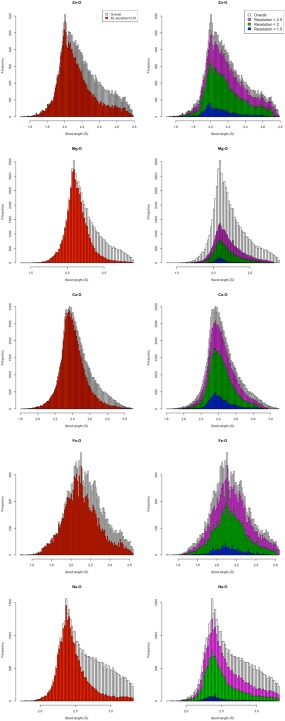
Bond length modification by two different filters: average bond length deviation (left) and X‐ray crystallography resolution (right). The average deviation filter can detect potential misassigned metal ion, and removes the skewed long tails. The resolution filter removes the whole spectrum proportionally and leaves a much smaller number of data for analysis. [Color figure can be viewed at wileyonlinelibrary.com]

The rational of the additional filter in Step 4 is that if all ligands are systematically larger than the expected value (major bond length mode), it is highly likely that the metal was incorrectly modeled or probably misassigned, from its density map. Figure 5 shows the histograms by metal ion of the average normalized deviation between the bond lengths and the major modes before we applied the filter. The cutoff was derived from the most distinctive bimodal separation seen for the Na ion, and is shown as the red line in each histogram. While bidentation and multi‐dentation metal binding sites had some shift toward higher average deviations, especially for Zn due to longer bond‐length modes present (Fig. 7), these deviations were for the most part below the cutoff used to identify incorrect modeling of the metal binding site. The reason is that gross inaccuracies manifest across all bond‐lengths in a metal binding site and not just a single bond‐length as demonstrated by the bimodal distributions in Figure 5. However, the 0.91 average normalized deviation cutoff is a tradeoff between removing large amounts of error versus including real multidentation metal ion coordination. The main effect of this filter is that it tends to remove the tailing portion in the bond length histogram more favorably than the main peak. The bond length distribution of each metal to oxygen without this filter has significant skewing with a large tail for longer bond‐lengths as shown in Figure 8. On the left, it shows how this filter eliminated potentially misassigned metal ions, reducing the skewness of the distribution. As a comparison, the right side shows the bond‐length distribution if we were to use a resolution cutoff of 2.5, 2.0, and 1.5 as the filter. These filters only reduce the tail proportionally to the overall shape (that is, no preference in filtering out the tail portion). Also, these stricter resolution filters removed too many data points, making the subsequent analyses unfeasible. From these results, it is clear that simply using higher resolution as the criterion for ‘high quality’ data is not sufficient to detect grossly inaccurate metal binding sites, probably due to metal ion misassignment. These likely detected misassignments would be due to incorrectly fitting a smaller metal ion into an electron density for a larger ion,^46^ causing the observed large average bond‐length *z* scores deviations.

Since the bond‐length histograms show an approximate normal distribution for most of the metal‐ligand bond types (Fig. 6), a simple parametric test is used to detect ligands based on bond‐length means and resolution‐adjusted standard deviations. We tested a range of ligand detection standard deviation cutoffs from 2 to 3 bl‐std. When the stricter cutoff (that is, 2 bl‐stds) is used, all downstream cluster measures tend to be higher and more stable. But on the other hand, fewer ligands will be counted as binding ligands. Due to deviations from normality, ligands in compressed angles are disproportionately lost, which leads to insufficient numbers of compressed CGs for clustering. Therefore, a 2.5 bl‐std cutoff was used for this study to compromise between the two situations. Thus, 2.5 standard deviations ensure that approximately 98.8% of the suitable ligands will be included.

Another possible way of determining the binding ligands is to use chi‐squared probability testing for the set of potential ligands together.^17^ Compared to the chi‐squared method, the single ligand testing does a much better job in identifying a higher number of ligands, as it could correctly characterize the most common number of ligands of Fe, Mg, and Na as 6 and Ca as 7, while our previously published chi‐squared probability method tended to favor 4‐ligand structures for all metals.

The filters employed in several steps throughout our analysis also helped to ensure a high quality of the structural data being analyzed. Table 4 shows the count of different number of ligand for each metal after step 4. Based on this data and the physiochemical bonding capacity of a metal ion (that is, the number of ligands a metal ion can form a bond with),^47^, ^48^, ^49^ we could estimate an error rate for our ligand detection analysis. The error rate was calculated as the number of ligands not physiochemically expected (for example, 7 and 8 for magnesium) divided by the total number of detected ligands for sites with the largest number of expected ligands. For example, the magnesium estimated ligand detection error rate is (2*2 + 69*1)/(2*8 + 69*7 + 5674*6) ≈ 0.002113. For the five metals, the estimated ligand detection error rate ranges from 0.00% to 0.21%, with an overall error rate of 0.11% across these metals. It assumed that the error rate was the same for all coordination numbers being detected, so that we could use the falsely identified and the highest true coordination number to estimate the overall error rate; but, these estimates represent only a lower limit of the real false positive rate. Overall, our analyses provide both an estimated false positive rate (∼0.11%) and an estimated false negative rate (∼1.2%) for ligand detection, indicating a very robust method. No prior protein metal binding site analysis methodology has undergone this level of statistical evaluation nor demonstrated this level of rigorous performance.

**Table 4:** Ligand Counts and Error Rates by Metal

Metal	Number of metal clusters	Number of usable metal sites (>3‐ ligand)	Number of unusable metal sites (<=3‐ligand)	4‐ligand	5‐ligand	6‐ligand	7‐ligand	8‐ligand	9‐ligand	Total	Estimated Ligand Detection Error rate	Nonredundant set
Zn	572	21,257	4959	11,380	2365	a750	b2	‐	‐	14,497	0.000443	4800
Mg	691	29,859	23,346	3595	2941	a5674	b69	b2	‐	12,281	0.002113	2813
Ca	196	21,057	3082	918	1490	4485	5399	a1258	b18	13,568	0.001760	4080
Fe	11,287	14,990	1237	1071	3929	a5804	b2	‐	‐	10,806	0.000057	2370
Na	240	11,475	4812	703	1557	1840	186	a17	‐	4303	0.000000	1184
Overall											0.001128	

a Highest coordination number considered valid for the given metal.

b Coordination numbers considered erroneous and thus used in ligand detection error estimation.

### Chemical functional group and multidentation specific bond length modes

While we used bond‐length means for the development of our ligand detection methods, the major bond‐length modes should be interpreted as expected bond lengths for monodentation ligands from a chemical perspective. These major modes of different metal‐element‐specific ligand pairs as shown in Figure 6 table agree very well with several studies based on the Cambridge Structural Database (CSD).^50^ Thus, the extensive set of quality control filters applied in this study has derived a similar level of aggregate bond‐length statistics from lower resolution wwPDB entries that was previously demonstrated from analyses of very high resolution small molecule X‐ray structures in the CSD.

Even though they do not meaningfully affect the overall statistics, the bond length distributions still exhibit skewed shoulders and long tails for certain metals and ligand elements, especially metal‐oxygen ligation. It has been known that glutamate and aspartate can bind metal ions via both of the carboxylate oxygens, causing a skew in the metal‐oxygen bond‐length distributions.^45^ As indicated in Figure 7, the carboxylate shift manifests as a bimodal distribution of the bond‐length, especially for Zn‐O. The carboxylate short bond‐length mode matches the expected monodentation bond‐length mode, while the carboxylate long bond‐length mode is distinct and broader. In addition, pyrophosphate and different nucleotides can bind metal ions with multiple atoms in a multidentation manner, which have been observed before by several independent studies.^51^, ^52^ Likewise, Figure 7 shows that these multidentating chemical functional groups also have distinctive bond length modes. The bond length modes of phosphate and carboxylate bidentation are distinct from each other as shown in Mg‐O and Na‐O. Tyrosine and molecular oxygen (O_2_) show separate bond length modes to the major mode of Fe‐O, and account for the broader peak and shoulders left to the major mode. Moreover, all of these distinctive bond‐length modes explain much of the skew and long tailness observed in the overall bond‐length distributions. Also, the existence of distinct bond‐length modes associated with multidentation is virtually unknown by the broader metalloprotein community. Thus, these derived bond‐length mode characteristics may provide additional information for future molecular simulation studies focused on understanding metal ion coordination as it relates to specific biochemical function.

During our efforts to identify different bond‐length modes that account for the skewness and long tails of the overall bond‐length distributions, we also noticed the over‐representation of certain bond‐length values. Under further investigation, we determined that these highly repetitive bond‐length values came from relatively few PDB entries with dozens and even hundreds of metal sites per PDB entry. Most of these PDB entries dealt with large and repetitive structures, like ribosomal units or chlorophyll in photoreactive centers. While causing isolated spikes when visualizing a single functional group bond‐length mode, these repetitive metal binding sites do not hinder the visual detection of the functional group bond‐length modes and do not appreciably affect the overall bond‐length distribution and derived statistics. Also, this overpopulation of certain metal binding site structures is eliminated by a sequence redundancy filter to prevent influencing the cluster analyses in subsequent steps.

### The universal existence of compressed angles among metalloproteins

**Figure 9:**
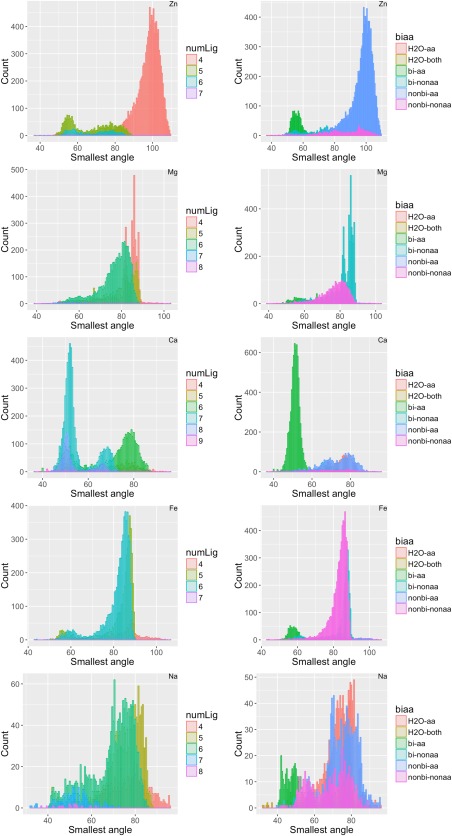
Smallest angle distributions for the five most abundant metalloproteins. The left histograms show smallest angle propensities broken down by coordination number and metal. The right histograms show smallest angle propensities broken down by ligand type and metal. bi is short for bidentation, which means that the two atoms composing the smallest angle are from the same residue or molecule. aa is short for amino acid, which means that the composing ligands are the 20 standard amino acids. Similarly, nonaa means that at least one of the composing ligands is not the 20 standard amino acids. H2O‐aa means that one of the composing ligand is the 20 standard amino acids and the other is water. And H2O‐both means that both of the composing ligands are water molecules.

Upon identifying the binding ligands, the smallest ligand‐metal‐ligand angle of individual metal sites can be computed. The smallest angle histograms (Fig. 9) show that there exists two types of angles: i) normal angles as expected from canonical CGs and ii) compressed angles, the majority of which cannot be explained by expected canonical CGs. Among the normal angles, the peaks around 72 degrees of Mg, Ca, and Fe can be justified by the Pentagonal bipyramidal (Pbp) CG, or its associate minor CGs. The peak around 90 degrees of Fe, Mg, Ca, and Na can be explained by Octahedral (Oct), Trigonal bipyramidal (Tbp), or their associated minor CGs. And the 109‐degree peak of Zn is from the Tetrahedral (Tet) as shown in Figure 9, which matches a similar graph generated from data that is two years older.^17^ Whereas the compressed angles are normally <60 degrees, and cannot be explained by any known 4‐, 5‐, and 6‐ligand canonical CGs, which are the majority ligand numbers for Zn, Mg, Fe, and Na. With the exception of Mg, these five metals contain significant numbers of compressed angles and they form a normal‐like distribution. If we associate the smallest angle based on its binding ligand's type, such as whether it is one of the 20 standard amino acids, water, or something else, or whether it is bidentated or not, most of the compressed angles consist of bidentated standard amino acid ligand residues.

Different metals have different amounts of compressed angles. Ca has the highest fraction of compressed angles partly due to its ability to bind 7or 8 ligands, which increases atom density, resulting in increased numbers of compressed angles. Hexagonal bipyramidal and its associated minor CGs have expected angles of 60°, but they only compose a small portion of calcium's CGs.^16^ Mg and Na have a much smaller proportion of compressed angles. The reason may be due to the fact that a large amount of their ligands are H_2_O, which cannot form a bidentation with the metal. Though water may not be a causal factor, the high percentage of H_2_O could limit the amount of the other possible ligands that could develop bidentation with the metal.

### Angle‐space descriptions of CG

Instead of an all‐to‐all mapping of ligands followed by comparing all corresponding angles, we first ordered the angles by finding the largest and smallest opposite angles so that the basic orientation of the metal structure was anchored at the ends of the ordered tuple. Then the middle angles were sorted from small to large to prevent any scrambling that may be introduced by ligand positioning. This ordering allows us to compare an individual metal fc‐shell not only to canonical CGs, but also to other metal fc‐shells. Thus, we were able to explore the similarity between metal structures. Moreover, different CG models possess very distinct ordered angles and are easily separable by clustering algorithms. We then further reduced the full‐angle space to a 6‐angle space so that metal sites with different numbers of ligands are comparable to each other and can be analyzed together. As shown in Table 3, this ordered angle selection method tends to capture a discriminating angle profile for each CG. The largest angle and its smallest opposite angle are kept. The middle angles are evenly sampled based on their position in the ordering to preserve the key information needed for separating each CG while reducing the redundancy.

**Figure 10:**
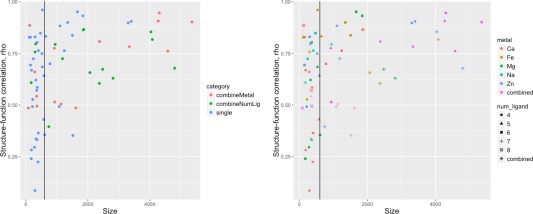
Scatter plot of the structure‐function Spearman's rank correlation coefficient (rho) as a function of data size for real datasets. On the left panel, red points represent structure‐function correlation coefficients for datasets combined by metal. Green points represent structure‐function correlation coefficients for datasets combined by coordination number. Blue points represent structure‐function correlation coefficients of datasets specific to the metal and coordination number. On the right panel is the same graph with individual metal and coordination number identified. A data size cutoff of 600 is shown as a black line on both panel. For data points with a size smaller than 600, the correlation rho and *p*‐value are not reliable. [Color figure can be viewed at wileyonlinelibrary.com]

In the test of using full‐angle space instead of 6‐angle space (results not shown), we observed very little decrease in the performance in terms of the functional tendency, especially in 5‐ and 6‐ ligand structures. This suggested that this angle space reduction was effectively picking up the functional relevant angle information, while removing the noisy redundancy coming from the structurally equivalent repeating angles. However, as the ligand number goes above 6, the collapsed 6‐angle space represents less and less of the total angle information present. This is not surprising since it is harder to capture 21 (7‐ligand) and 28 (8‐ligand) angles worth of information in just 6 representative angles. We observed a slightly unstable correlation for 7‐ and 8‐ligand Ca (see Fig. 10), which could be a synergistic contribution from both small data size and inadequate angle space representation. Since the majority of metal ions in this study have coordination numbers of 4 to 6, this effect needs further investigation as more high‐coordination‐number metal binding sites are analyzed.

### K‐means clustering and assignment

K‐means clustering was conducted with respect to each metal and each number of ligands separately, and on combined metals and combined number of ligands as well. An optimal cluster number k was manually picked for each group to maximize all four measures and to ensure a *p*‐value <0.01. Figure 10 indicates that the ability to obtain good functional relevant (high rho) clusters is largely influenced by the size of the data to be clustered. The rho increases dramatically at lower counts and plateaus at higher counts. In other words, to achieve a stable high value of rho (∼0.8), the data size should be at least 600. Therefore, in some of the groups, such as 4‐ligand compressed zinc with a size of 94, the lack of data could greatly hinder our ability to detect a sensible structure‐function relationship.

**Figure 11:**
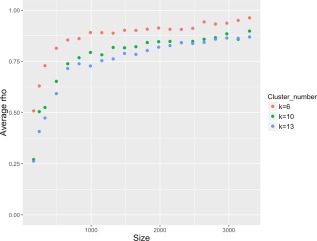
The structure‐function Spearman's rank correlation coefficient (rho) as a function of data size for subsampled zinc 4‐ligand datasets. The average rho was calculated for *k* = 6, 10, and 13 respectively based on 21 independent subsamplings of the original zinc 4‐ligand dataset at each specific dataset size. [Color figure can be viewed at wileyonlinelibrary.com]

A simulation on the 4‐ligand normal zinc sites exhibits the same trend. A series of subsets of the data were sampled without replacement. The sizes of the subset sequence were selected as 1/20, 1.5/20, 2/20, 3/20, 4/20… of the original data, and each size was repeated for 20 times. *k* = 6, 10, and 13 were used for all subsets to acquire the rho. As shown in Figure 11, the average rho increases as the size grows regardless of the selected k. Therefore, to detect a plateauing Spearman's correlation between structural and functional distance metrics, at least 600 nonredundant metal binding sites is required. In only the last few years has the structural data necessary become available to reliably detect the existence of compressed angles in CGs.^17^ Both the real and simulated data suggest that when the number of data points is <600, the derived rho value is not reliable. Therefore, for categories with <600 metal sites, the optimal k was selected based solely on the sum of absolute difference and the Jaccard index to avoid the over‐interpretation of structure‐function relationships between the clusters when the data size is insufficient for this interpretation.

In general, combining different metals with the same number of ligands (combineMetal) shows a better performance than combining different ligand numbers of the same metal (combineNumLig), even though they both enlarge the size of the group (Table 5). In particular, the 6‐ligand normal group had the second highest rho value of 0.9464 (*p*‐value < 2.2 × 10^−16^) for groups with 600+ data points. We believe this is partially due to how the 6‐angle space collapses angle information from full angle spaces of different dimensionality. Also for a given number of ligands, there are only a fixed number of possible canonical CGs and thus less heterogeneity, even with different metals together. It is interesting though that these different metals exhibit similar functional trends as long as they have similar sets of CGs. This may imply that different metals are somewhat interchangeable as long as the structure remains the same, and that the structures have higher impact on functions than the metal itself. It also provides evidence that we can combine metals with the same ligand numbers in analyzing the less abundant metals and thus have enough data to determine full structure‐function correlations (rho).

**Table 5:** Optimal k and Corresponding rho and *p*‐value for Each Metal and Ligand Number

Metal	Ligand number	Group	Size	Optimal k	Functionally mapped n_lig	Rho	*p*‐value	Category
Zn	4	normal	3300	10	4	0.8976	0.0000	single
Zn	4	compressed	94	‐	‐	‐	‐	single
Zn	4	combined	3398	11	4	0.9053	0.0000	single
aZn	5	normal	584	8	2	0.6420	0.0003	single
aZn	5	compressed	518	7	2	0.7494	0.0001	single
Zn	5	combined	1103	7	2	0.8831	0.0000	single
aZn	6	normal	150	6	2	0.6935	0.0041	single
aZn	6	compressed	128	‐	‐	‐	‐	single
aZn	6	combined	298	7	3	0.8303	0.0056	single
Zn	combined	normal	4034	7	2	0.8545	0.0000	combineNumLig
Zn	combined	compressed	741	10	3	0.3953	0.0072	combineNumLig
Zn	combined	combined	4800	9	1	0.6785	0.0000	combineNumLig
aMg	4	normal	280	7	1	0.2956	0.1933	single
aMg	4	compressed	44	‐	‐	‐	‐	single
aMg	4	combined	326	13	3	0.6215	0.0026	single
aMg	5	normal	530	5	1	0.6848	0.0351	single
Mg	5	compressed	74	‐	‐	‐	‐	single
Mg	5	combined	608	21	3	0.3553	0.0001	single
Mg	6	normal	1665	5	1	b0.9515	0.0000	single
aMg	6	compressed	173	8	1	0.2403	0.2180	single
Mg	6	combined	1843	6	1	0.9321	0.0000	single
Mg	combined	normal	2477	11	1	0.6732	0.0000	combineNumLig
aMg	combined	compressed	319	6	2	0.7964	0.0006	combineNumLig
Mg	combined	combined	2813	7	3	0.6299	0.0028	combineNumLig
aCa	4	normal	293	5	4	0.0857	0.9194	single
Ca	4	compressed	88	‐	‐	‐	‐	single
aCa	4	combined	391	5	1	0.2242	0.5367	single
aCa	5	normal	369	7	3	0.5857	0.0061	single
aCa	5	compressed	181	6	4	0.2824	0.3078	single
aCa	5	combined	575	5	1	0.4303	0.2180	single
Ca	6	normal	776	6	3	0.7000	0.0049	single
aCa	6	compressed	401	8	2	0.3645	0.0572	single
Ca	6	combined	1241	8	1	0.7630	0.0000	single
aCa	7	normal	335	8	3	0.3361	0.0809	single
Ca	7	compressed	1077	10	1	0.4929	0.0007	single
Ca	7	combined	1518	11	1	0.3527	0.0086	single
aCa	8	normal	80	‐	‐	‐	‐	single
aCa	8	compressed	123	4	3	0.8857	0.0333	single
aCa	8	combined	350	10	4	0.5710	0.0003	single
Ca	combined	normal	1853	6	2	0.8643	0.0000	combineNumLig
Ca	combined	compressed	1870	10	4	0.8664	0.0000	combineNumLig
Ca	combined	combined	4080	13	4	0.8176	0.0000	combineNumLig
aFe	4	normal	184	5	1	0.6688	0.0345	single
Fe	4	compressed	38	‐	‐	‐	‐	single
aFe	4	combined	222	5	1	0.5273	0.1228	single
aFe	5	normal	533	7	4	0.9605	0.0000	single
aFe	5	compressed	111	4	3	0.8286	0.0583	single
Fe	5	combined	644	10	4	0.8327	0.0000	single
Fe	6	normal	1349	7	1	0.9000	0.0000	single
aFe	6	compressed	149	4	2	0.8286	0.0583	single
Fe	6	combined	1503	7	1	0.8377	0.0000	single
Fe	combined	normal	2066	7	1	0.6571	0.0016	combineNumLig
aFe	combined	compressed	298	6	1	0.7571	0.0016	combineNumLig
Fe	combined	combined	2370	6	1	0.7571	0.0016	combineNumLig
aNa	4	normal	212	10	1	0.6049	0.0000	single
aNa	4	compressed	25	10	2	0.4926	0.0006	single
Na	4	combined	240	‐	‐	‐	‐	single
aNa	5	normal	360	7	3	0.7239	0.0002	single
aNa	5	compressed	37	10	1	0.3321	0.0258	single
aNa	5	combined	406	‐	‐	‐	‐	single
aNa	6	normal	362	7	2	0.7636	0.0001	single
aNa	6	compressed	82	6	3	0.8036	0.0005	single
aNa	6	combined	471	‐	‐	‐	‐	single
Na	combined	normal	946	7	3	0.8481	0.0000	combineNumLig
aNa	combined	compressed	173	5	4	0.7939	0.0098	combineNumLig
Na	combined	combined	1184	8	2	0.6085	0.0034	combineNumLig
combined	4	normal	4269	6	3	0.9071	0.0000	combineMetal
acombined	4	compressed	289	5	3	0.6606	0.0440	combineMetal
combined	4	combined	4577	7	4	0.7610	0.0001	combineMetal
combined	5	normal	2376	9	1	0.8077	0.0000	combineMetal
combined	5	compressed	921	7	4	0.7753	0.0001	combineMetal
combined	5	combined	3336	7	3	0.7831	0.0000	combineMetal
combined	6	normal	4302	6	1	0.9464	0.0000	combineMetal
combined	6	compressed	933	12	1	0.5146	0.0000	combineMetal
combined	6	combined	5356	9	1	0.9019	0.0000	combineMetal
acombined	7	normal	347	10	3	0.5428	0.0001	combineMetal
combined	7	compressed	1133	12	3	0.5049	0.0000	combineMetal
combined	7	combined	1613	12	1	0.4860	0.0000	combineMetal
acombined	8	normal	82	6	2	0.4857	0.3556	combineMetal
acombined	8	compressed	125	4	3	0.8857	0.0333	combineMetal
acombined	8	combined	360	10	2	0.4951	0.0021	combineMetal
combined	combined	normal	11376	7	4	0.7312	0.0002	combinedAll
combined	combined	compressed	3401	7	3	0.7013	0.0006	combinedAll
combined	combined	combined	15247	8	4	0.8396	0.0000	combinedAll

a The structure‐function correlation rho and associated *p*‐value is not reliable because of the low count of the data.

b Highest correlation rho value for all structure‐function analyses with data counts above 600.

We further evaluated the clustering results in comparison to our previous study on only 4‐ligand zinc sites. An additional criterion was used other than the four measures, that is, whether all known canonical CGs have at least one cluster representation. It turns out that even when *k* = 30, we did not see a square planar (Spl) CG. This is probably because fewer metal sites with Spl CG passed the extensive set of filters and the predominant CG is Tet for 4‐ligand zinc sites. Thus, if we want to detect a small Spl cluster, we need to use a *k* >30, but that will also cause the large sized CGs, such as Tet, to be broken down into smaller sub‐clusters. This unequal density of clusters is a fundamentally hard problem to solve for clustering algorithms.^53^ We also noticed that as we increase the cluster number *k*, two other small size CGs, square pyramidal vacancy (Spv) and trigonal bipyramidal vacancy planar (Bvp), started to separate when *k* = 13. What is interesting is that there is a clear peak at *k* = 13 for rho value when all four ligands in the zinc site are required to be mapped to its annotations (4‐ligand‐mapping). In comparison to the lower ligand‐mapping sites, the 4‐ligand‐mapping sites exhibit the best rho values across all *k* in the 4‐ligand zinc category. Therefore, functional mapping of all four ligands provides both the best structure‐function correlation and sensitivity to the *k* used for clustering.

**Figure 12:**
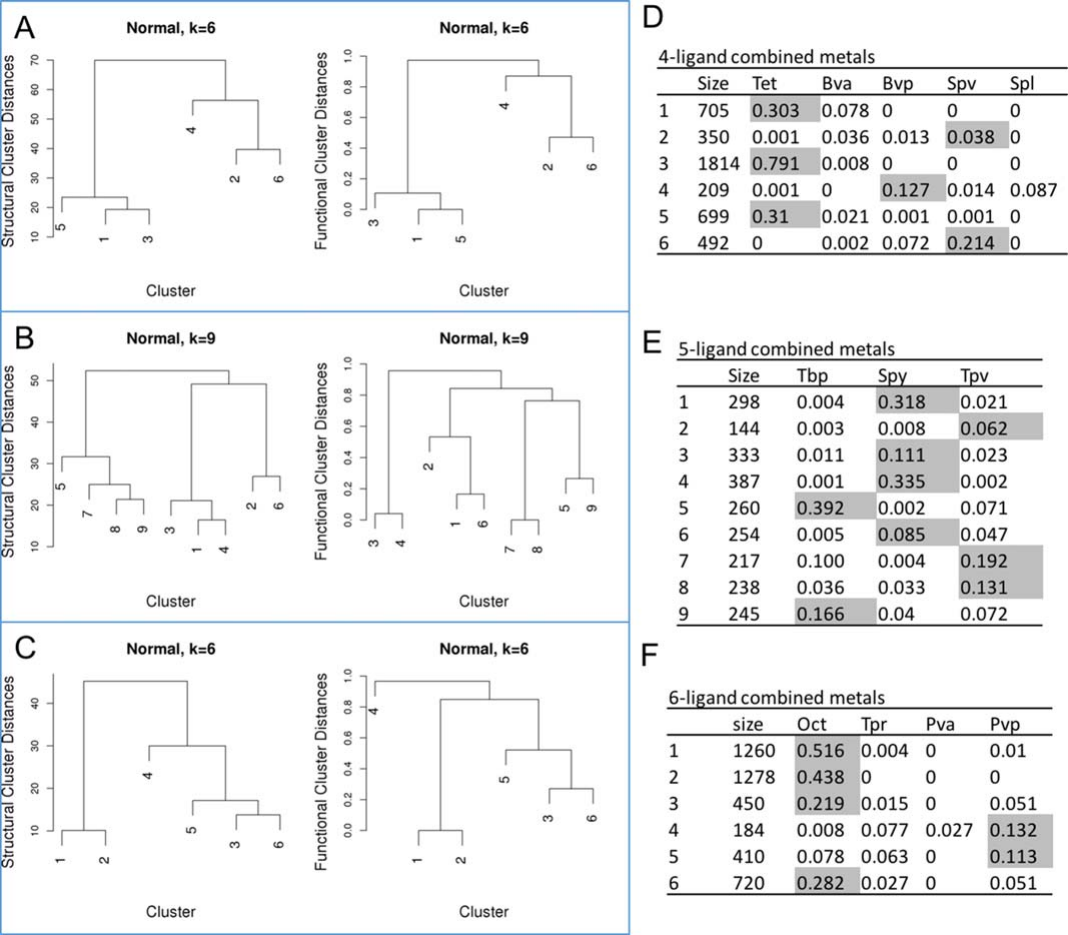
Three examples of structural versus functional dendrograms and the characteristic *χ* ^2^ probabilities of k‐means clusters. All dendrogram pairs show high similarity between each other, and also match their highest probability canonical CG model descriptions. (A, D) 4‐ligand normal combined metalloproteins. (B, E) 5‐ligand normal combined metalloproteins. (C, F) 6‐ligand normal combined metalloproteins. Similar graphs for the other metalloproteins can be found in the Supporting Information material. [Color figure can be viewed at wileyonlinelibrary.com]

For all categories, the cluster centers and a characteristic average probability of each cluster are in Supporting Information, together with the full list of metal IDs of each cluster. Figure 12A–C uses normal combined metalloproteins as examples to illustrate the structural vs. functional dendrogram comparison, since only combined metals provide enough data for evaluating both 4‐, 5‐, and 6‐ligand. The average probabilities for each cluster with respect to appropriate canonical CG models (Fig. 12D–F) provides a characterization of each cluster with respect to canonical CG models, with the highest canonical CG model probability for each cluster shaded. According to the highest *χ* ^2^ probabilities for 4‐ligand (Fig. 12A and D), clusters 1, 3, and 5 are all sub‐classes of the Tet CG, which are well identified together in the dendrograms based on both structural and functional distances. Cluster 2 and 6 are both sub‐clusters of Spv according to their *χ* ^2^ probabilities and are well grouped together both structurally and functionally. As for cluster 4, it shows the highest probability in both Bvp, and it is both structurally and functionally adjacent to the Spv group. For 5‐ligand combined metalloproteins (Fig. 12B and E), cluster 1, 3, 4, and 6 show the highest probability in Square pyramidal (Spy), and are close in both structural and functional dendrograms. Cluster 5 and 9 are both classified as Tbp, and Cluster 7 and 8 are both classified as Trigonal prismatic vacancy (Tpv). These two pairs show greater separation functionally than they do structurally. Cluster 2 is characterized as Tpv but with relatively low probability (0.62). It is closer to the Spy group both structurally and functionally, which makes it interesting to be explored further. Similarly, in 6‐ligand (Fig. 12C and F), clusters 1, 2, 3, and 6 are all sub‐clusters of Oct CG. They are also first sub‐grouped according to the order of their probabilities: Cluster 1 and 2 are grouped first with high probabilities (0.516 and 0.438), and Cluster 3 and 6 are grouped together with relatively low probabilities (0.219 and 0.282). Cluster 4 and 5 can be both characterized as Pentagonal bipyramidal vacancy planar (Pvp) based on their highest probabilities. They are also structurally and functionally related to the Oct CGs as indicated in the dendrograms. Cluster 5 is recruited first with a higher Oct probability (0.078), while Cluster 4 last with a low Oct probability (0.008). All these figures demonstrate the feasibility of analyzing all metals combined in different ligand numbers. They also revealed that our CG cluster representations have very strong functional implications, as the structural and functional distances were calculated independently from different sources of information. In particular, the normal 4‐ligand, 5‐ligand, 6‐ligand combined metal cluster analyses yielded structure‐function Spearman rho values of 0.9071, 0.8077, and 0.9464, respectively. And it is only through the CG clusters that this level of similarity is observed in the dendrograms. Likewise, similar dendrograms and patterns for the rest of the metals can be found in the Supporting Information.

### Aberrant CG clusters and their functional significance

Of the 15,150 metal binding sites analyzed, roughly 19% contain compressed angles (see Table 5); however, this is probably an underestimation due to the filtering out of some real multidentation metal binding sites by step 4. While coordination geometries that contain unexpected compressed angles would be considered aberrant, some CG clusters are clearly highly aberrant with low similarity to any canonical CGs. Table 6 shows example clusters that have the largest size for each compressed group, while Table 7 is a compilation of the most aberrant CG clusters from each metal and ligand numbers of the full CG cluster description tables in the Supporting Information. They all show abnormal deviations from the canonical CGs, and should be considered as aberrant CG descriptions, especially when they are also showing high functional associations. However, special attention needs to be paid when interpreting structure‐function correlations, when the total number of the compressed metal sites is lower than 600. These aberrant CG clusters can be found in all 4‐ to 6‐ligand metals. Some of the clusters have a small cluster size in comparison to clusters in the normal groups, simply reflecting that only 19% of the nonredundant CGs are in the compressed groups. As more nonredundant metalloprotein structures are deposited in the wwPDB, we expect the detected aberrant clusters to grow in size and potentially new aberrant clusters to emerge with distinct structure‐function propensities. Also, 7‐ and 8‐ligand metal sites tend to be less distorted from canonical CGs. This is primarily due to the presence of some small ideal angles in 7‐ and 8‐ligand CG models. Thus, the differences between expected and compressed angles are much less distinct for these metal sites.

**Table 6:** Instances of Largest Size Aberrant Clusters of the Compressed Group for Different Metals. The Complete Cluster Information can be Found in Supporting Information Material

Metal	Ligand Number	Cluster Number	Size	Angle 1^a^	Angle 2^a^	Angle 3^a^	Angle 4^a^	Angle 5^a^	Angle 6^a^	Tet^b^	Bva^b^	Bvp^b^	Spv^b^	Spl^b^
Combined	4	7	79	142.5 ± 12.1	56 ± 3.7	87.6 ± 8.1	99.1 ± 7.4	107 ± 8	101.3 ± 11	0.027	0.034	0.024	0.048	0
										Tbp^b^	Spy^b^	Tpv^b^		
Zn	5	3	128	148.5 ± 4.6	56.5 ± 2.8	93.1 ± 3.2	101.1 ± 3.4	134.6 ± 3.8	102.3 ± 3.2	0.091	0.002	0.112		
Ca	5	6	59	160.2 ± 5.8	51.8 ± 3	81.8 ± 4.7	91.1 ± 4.5	144.5 ± 5.4	79.6 ± 7.3	0.01	0.007	0.083		
Fe	5	1	41	151.1 ± 8.1	58 ± 3.5	91.1 ± 3.6	101.9 ± 3.7	134.2 ± 7.7	100.4 ± 6.8	0.008	0	0.004		
Combined	5	2	202	156.5 ± 6.8	56 ± 3.4	90.4 ± 4.2	102.8 ± 3.5	124.1 ± 4.9	105.7 ± 5.1	0.095	0	0.064		
										Oct^b^	Tpr^b^	Pvp^b^	Pva^b^	
Mg	6	8	41	175.6 ± 2.2	84.3 ± 2.7	90.2 ± 1.2	96.6 ± 2.1	158.7 ± 2.9	58.8 ± 1.9	0.342	0	0	0.166	
Ca	6	3	75	161.3 ± 4	72.9 ± 3.7	84.1 ± 3.1	104.7 ± 5.6	155.2 ± 2.8	51.3 ± 2.7	0.01	0.071	0.063	0.072	
Fe	6	2	54	172.9 ± 3.6	81.3 ± 3.5	90.2 ± 1.7	97.4 ± 2.2	164.6 ± 4.6	60.6 ± 3.5	0.025	0	0	0.004	
Combined	6	2	107	174.3 ± 3.1	82.3 ± 3.2	90.2 ± 1.8	97.2 ± 2.4	157.5 ± 2.9	58.4 ± 2.9	0.177	0.003	0	0.11	

a Angle positions are based on the 6‐angle space description.

b CG abbreviations are based on Figure 1.

**Table 7:** Instances of Highly Aberrant Clusters of the Compressed Group for Different Metals. The Complete Cluster Information can be Found in Supporting Information Material

Metal	Ligand Number	Cluster Number	Size	Angle 1^a^	Angle 2 ^a^	Angle 3 ^a^	Angle 4 ^a^	Angle 5 ^a^	Angle 6 ^a^	Tet^b^	Bva^b^	Bvp^b^	Spv^b^	Spl^b^
Combined	4	2	41	155.4 ± 10.8	54.9 ± 5.6	79.2 ± 9.1	107.3 ± 13.4	134.5 ± 10.8	90.5 ± 15.5	0	0.013	0	0.001	0
										Tbp^b^	Spy^b^	Tpv^b^		
Zn	5	5	61	164.2 ± 5.7	56.8 ± 4.7	87.3 ± 4.1	104.5 ± 3.6	123.6 ± 5.9	103.8 ± 5.7	0.038	0.001	0.022		
Ca	5	3	14	144 ± 8.4	56.1 ± 6.9	74.2 ± 5.9	86 ± 7	126.7 ± 7.6	62.6 ± 8.5	0.001	0.008	0.017		
Fe	5	3	22	147.4 ± 4.5	59.2 ± 6.1	87.2 ± 6	96 ± 4.3	141 ± 4.7	78.9 ± 8.5	0	0	0		
Combined	5	5	121	156.1 ± 8	53.1 ± 4.3	80.6 ± 5.7	95.5 ± 9	141.6 ± 7.2	75.2 ± 6	0.006	0.006	0.063		
										Oct^b^	Tpr^b^	Pvp^b^	Pva^b^	
Mg	6	4	13	166.8 ± 4.5	61.2 ± 5.8	87.1 ± 3.7	104 ± 5.8	155.5 ± 6.1	63 ± 4.4	0	0	0	0	
Ca	6	4	45	169 ± 5.2	51 ± 3.1	84.6 ± 3.7	102.2 ± 4.9	159 ± 5.9	73.5 ± 6.5	0.012	0.032	0.004	0.067	
Fe	6	4	21	162.4 ± 8	59.8 ± 3.2	87.8 ± 3	104.8 ± 4.6	155 ± 7.9	70.2 ± 6.8	0	0	0	0.001	
Combined	6	10	88	168.3 ± 4.3	52.7 ± 4.8	85.8 ± 4	104.9 ± 4.9	155.6 ± 6.5	72 ± 5.7	0.015	0.03	0.003	0.048	

a Angle positions are based on the 6‐angle space description.

b CG abbreviations are based on Figure 1.

**Figure 13:**
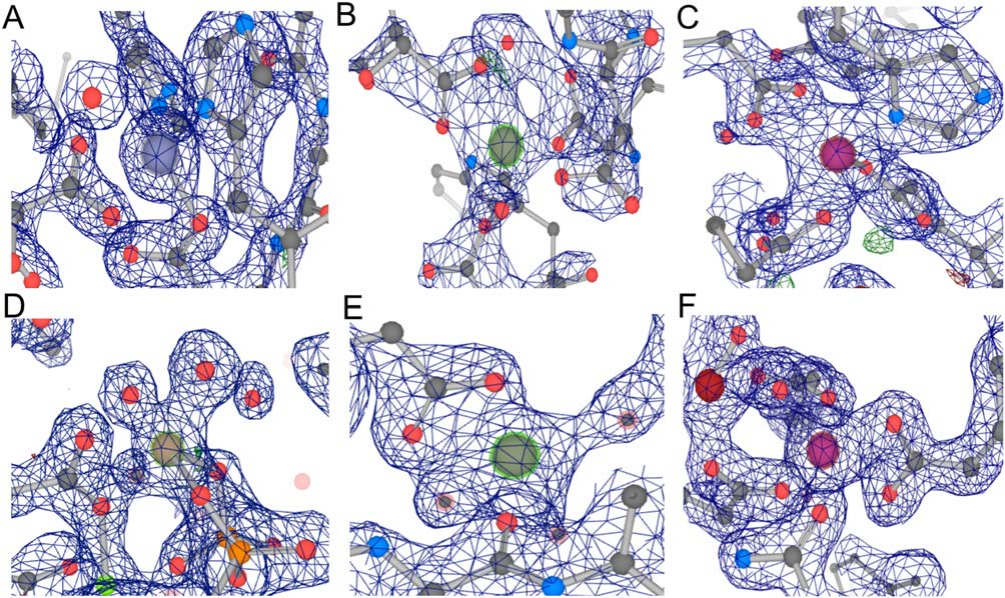
PDB structure and electron density maps of examples from clusters listed in Table VI. Aberrant CG structures are shown in balls and sticks, featured by bidentated compressed angles. These structures are also supported by their fitness to the electron density maps. All structures were generated in LiteMol Viewer 54, with 2*F*_o_ – *F*_c_ at 1.5*σ* and *F*_o_ – *F*_c_ at −3*σ* (red) and 3*σ* (green), except for panel E with 2*F*_o_ – *F*_c_ at 1.01 *σ*. Metal ions are put at the center of each subgraph with larger size, where Zn is represented as light blue, Fe as purple, and Mg and Ca as green. The cluster identifier, PDB metal site ID, and its resolutions are as follows: A, 5‐ligand Zn, cluster 3, 2B13.B.401, resolution 1.55 Å; B, 5‐ligand Ca, cluster 6, 3RYD.C.267, resolution 2.37 Å; C, 5‐ligand Fe, cluster 1, 4AM4.A.1161, resolution 1.68 Å; D, 6‐ligand Mg, cluster 8, 3ETH.A.402, resolution 1.60 Å; E, 6‐ligand Ca, cluster 3, 4P99.B.509 resolution 1.80 Å; F, 6‐ligand Fe, cluster 2, 2GYQ.B.404, resolution 1.40 Å. [Color figure can be viewed at wileyonlinelibrary.com]

**Figure 14:**
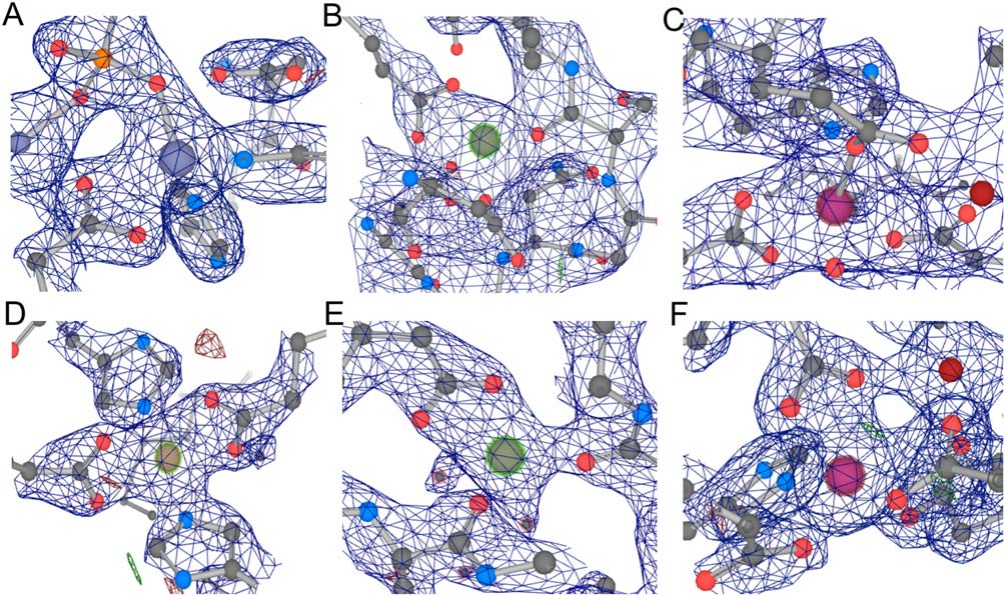
PDB structure and electron density maps of examples from clusters listed in Table VII. Aberrant CG structures are shown in balls and sticks, featured by bidentated compressed angles. These structures are also supported by their fitness to the electron density maps. All structures were generated in LiteMol Viewer 54, with 2*F*_o_ – *F*_c_ at 1.5*σ* and *F*_o_ – *F*_c_ at −3*σ* (red) and 3*σ* (green), except for panel C with 2*F*_o_ – *F*_c_ at 1.02 *σ*. Metal ions are put at the center of each subgraph with larger size, where Zn is represented as light blue, Fe as purple, and Mg and Ca as green. The cluster identifier, PDB metal site ID, and its resolution are as follows: A, 5‐ligand Zn, cluster 5, 2R2D.A.277, resolution 1.75 Å; B, 5‐ligand Ca, cluster 3, 3HR4.H.202, resolution 2.50 Å; C, 5‐ligand Fe, cluster 3, 2VZB.B.6204; resolution 2.30 Å; D, 6‐ligand Mg, cluster 4, 3CVJ.C.243, resolution 2.00 Å; E, 6‐ligand Ca, cluster 4, 1LHV.A.401, resolution 2.00 Å; F, 6‐ligand Fe, cluster 4, 3DHI.A.601, resolution 1.68 Å. [Color figure can be viewed at wileyonlinelibrary.com]

Figures 13 and 14 provide specific structural examples for each aberrant metal cluster described in Tables 6 and 7. These images were generated using LiteMol^54^ and illustrate well‐defined metal‐ion coordinating structures overlaid onto their respective electron density maps in blue mesh (2*F*o – *F*c) with very little red and green mesh present that would indicate 3+ standard deviation discrepancies between calculated and observed electron density (*F*_o_ – *F*c). Also, the structures represented in these images have crystallographic resolutions ranging from 1.40 Å to 2.50 Å, but with 9 of the 12 structures having crystallographic resolutions of 2.00 Å or below. When checking the fitness between the structure models and their electron density maps, the structural data deposited in the wwPDB are not always the best quality, as inaccuracies, misinterpretations, and even errors are often observed in different regions of a given structure. These imperfections appear in structures with normal and compressed metal binding sites. As illustrated in this study, these imperfections in PDB entries, while making detection of aberrant CG detection difficult, can be managed by using a series of quality control filters and statistical methods that are more resistant to error and outliers. Our analysis has demonstrated that these aberrant CGs are not just analytical and/or interpretive artifacts, but are true phenomena supported by rigorous statistical analyses and solid structural examples.

Furthermore, these aberrant CGs also have distinct functional propensities from normal CGs. As can be observed in the Supporting Information tables (Supporting Information Tables S149–S168), the GO terms enriched in the normal and compressed sites are completely different, implying that they are functionally distinct when considered as a group. There are no cases where the same term has a corrected or raw *p*‐value <= 0.05 in both the normal and compressed sites within a particular enrichment analysis (see Supporting Information Fig. S29). This is not to say that a particular GO term does not show up at all in both groups, however, as a function of appearing more than expected by chance, the GO terms are specific to the normal and compressed sites. This holds for each metal and number of ligands, as well as considering all of the metals together, or all of the numbers of ligands for a particular metal. However, inconsistencies between analyses are observed and marked in the analyses that combine the metals being analyzed.

Overall, a wide variety of metal‐specific annotation differences exist between the normal and compressed sites and the vast majority of enrichment results are specific to metal and ligand number (Supporting Information Tables S149–S168). In an attempt to see general annotation trends across all metals analyzed, we filtered the all‐metal, all‐ligand‐number enrichment results based on consistency with all other enrichment analyses and a max metal enrichment usage fraction of <0.5. These results are shown in Table 8. Only two general differences emerge: (a) normal metal ion coordination is enriched in biosynthetic metabolic processes and (b) compressed metal ion coordination is enriched in ion transport. While it is hard to comment on the enrichment in biosynthetic metabolic processes in normal CGs, the ion transport enrichment in compressed CGs is directly explainable. Ion transport requires transient interaction with an ion, which may be facilitated by flexible and looser binding afforded by compressed CGs.

**Table 8:** Enriched GO Terms, Having Corrected *p*‐value ≤0.05 and Consistent Enrichment Patterns in combineLig and Individual Ligand Enrichments

					Normal					Compress				
Id	Description	Type	IPR.group	Consistent	p	padjust	metal	perc	sig	p	padjust	metal	perc	sig
**GO:0044249**	Cellular biosynthetic process	BP	BO	TRUE	4.419 e −08	6.595 e −06	zn	0.4264	TRUE	1.000 e +00	1.000 e +00	mg	0.3297	FALSE
**GO:0072524**	Pyridine‐containing compound metabolic process	BP	BU	TRUE	1.518 e −03	4.483 e− 02	mg	0.4430	TRUE	9.996 e −01	1.000 e +00	mg	0.5000	FALSE
**GO:0034641**	Cellular nitrogen compound metabolic process	BP	CJ	TRUE	8.436 e −11	2.359 e −08	zn	0.4413	TRUE	1.000 e +00	1.000 e +00	mg	0.3211	FALSE
**GO:0015077**	Monovalent inorganic cation transmembrane transporter activity	MF	DE	TRUE	1.273 e −03	3.796 e− 02	fe	0.4242	TRUE	9.997 e −01	1.000 e +00	ca	0.7500	FALSE
**GO:0072593**	Reactive oxygen species metabolic process	BP	F	TRUE	2.465 e −04	9.952 e −03	fe	0.4821	TRUE	9.999 e −01	1.000 e +00	zn	1.0000	FALSE
**GO:1901566**	Organonitrogen compound biosynthetic process	BP	G	TRUE	2.530 e −05	1.320 e −03	mg	0.3933	TRUE	1.000 e +00	1.000 e +00	mg	0.4865	FALSE
**GO:0016053**	Organic acid biosynthetic process	BP	GL	TRUE	9.988 e −04	3.090 e −02	mg	0.3543	TRUE	9.996 e −01	1.000 e +00	mg	0.3077	FALSE
**GO:0044283**	Small molecule biosynthetic process	BP	GL	TRUE	1.993 e −05	1.108 e −03	mg	0.4000	TRUE	1.000 e +00	1.000 e +00	mg	0.3333	FALSE
**GO:0046394**	Carboxylic acid biosynthetic process	BP	GL	TRUE	9.988 e −04	3.090 e −02	mg	0.3543	TRUE	9.996 e −01	1.000 e +00	mg	0.3077	FALSE
**GO:0050794**	Regulation of cellular process	BP	GN	TRUE	2.047 e −06	1.459 e −04	zn	0.4454	TRUE	1.000 e +00	1.000 e +00	zn	0.4545	FALSE
**GO:0050896**	Response to stimulus	BP	GN	TRUE	2.192 e −06	1.530 e −04	mg	0.3825	TRUE	1.000 e +00	1.000 e +00	ca	0.4860	FALSE
**GO:0065008**	Regulation of biological quality	BP	CQ	TRUE	9.999 e −01	1.000 e +00	fe	0.2778	FALSE	1.661 e− 04	6.807 e −03	fe	0.3878	TRUE
**GO:0008484**	Sulfuric ester hydrolase activity	MF	FV	TRUE	9.999 e −01	1.000 e +00	ca	0.3333	FALSE	6.402 e −04	2.187 e −02	ca	0.4286	TRUE
**GO:0006811**	Ion transport	BP	I	TRUE	1.000 e +00	1.000 e +00	mg	0.3889	FALSE	7.213 e −08	6.392 e −06	ca	0.4364	TRUE

## CONCLUSIONS

We have improved our analyses and expanded their scope to cover a range of metal ions in a much wider set of coordination geometries. The inclusion of additional quality control filters has improved the quality of the results. This is especially evident by the improved Spearman correlation between functional and structural distance metrics from our previously published analysis on 4‐ligand zinc ion coordination: going from a rho of 0.88 (*p*‐value < 2.2 × 10^−16^) to 0.8976 (*p*‐value < 2.2 × 10^−16^) and the presence of multiple rho values above 0.9, including the Mg combined cluster analysis yielding a rho above 0.9515 (*p*‐value < 2.2 × 10^−16^) and the 6‐ligand combined cluster analysis yielding a rho above 0.9464 (*p*‐value < 2.2 × 10^−16^). Also, our ligand detection method is statistically rigorous, producing an estimated false positive rate of ∼0.11% and an estimated false negative rate of ∼1.2%. No prior protein metal binding site analysis methodology has undergone this level of statistical evaluation nor demonstrated this level of rigorous performance. Moreover, these results demonstrate high consistency (low unimodal variance) in metal‐ligand bond‐lengths in metalloproteins reflecting expected strong dependency on physiochemical properties of metal ion coordination. Also, distinct multidentation bond‐length modes specific to highly‐prevalent chemical functional groups were observed.

With respect to the first question posed in the introduction, can functionally‐relevant structural descriptions of CG be constructed for other common metals, involving different numbers of ligands? Results in Table 5, Figures 10 and 12 demonstrate that we can. With respect to the second question posed in the introduction, would similar or even new structural constraints and aberrant CGs be detected? Tables 5, 6, 7 and related tables in our Supporting Information material clearly indicate that roughly 19% of metal binding sites exist in aberrant CGs across the five types of metalloproteins examined in these analyses. These aberrant CG clusters are further supported by well‐defined structural examples in Figures 13 and 14. Most of these aberrant CGs are derived from the presence of unexpected compressed angles and that most of these compressed angles arise from multidentation. Moreover, these CGs with compressed angles have distinct functions from CGs without compressed angles as demonstrated in our functional annotation enrichment analyses. The reason that previous analyses have not detected these compressed angles in large numbers is due to the biased nature of prior analyses selecting ligands that fit expected canonical CGs. For instance, CheckMyMetal purposely analyzes each ligand atom and a pseudo‐atom representation of possible bidentation ligand residues and picks the “best fit” to expected canonical CGs.^15^ Clearly such a biased search will not easily find unexpected results.

In summary, the improvement in our methods and analyses provide a statistically rigorous result highly supporting the existence of large numbers of unexpected compressed angles and thus significant numbers of aberrant metal ion coordination geometries within structurally known metalloproteins. By recognizing these aberrant CGs in clustering, high correlations are achieved between structural and functional descriptions of metal ion coordination. But the broader implication is that the wide range and percentage of aberrant CGs in metalloproteins, especially with respect to bond angles, reflects metal binding site variation necessary for the implementation of a diverse set of biochemical functions.

## Supporting information

Supporting Information
