# Supplementary material for: Aberrant coordination geometries discovered in the most abundant metalloproteins
Source: Proteins. 2017 Mar 7;85(5):885–907. doi: 10.1002/prot.25257 (PMC5389913; doi:10.1002/prot.25257)
Supplement: Supplementary file 1 — Supporting Information [file PROT-85-885-s001.pdf]

Five metal supplemental graphs

Sen Yao

2016-11-09 20:00:18

Figure S1. 4-ligand Zn metalloproteins

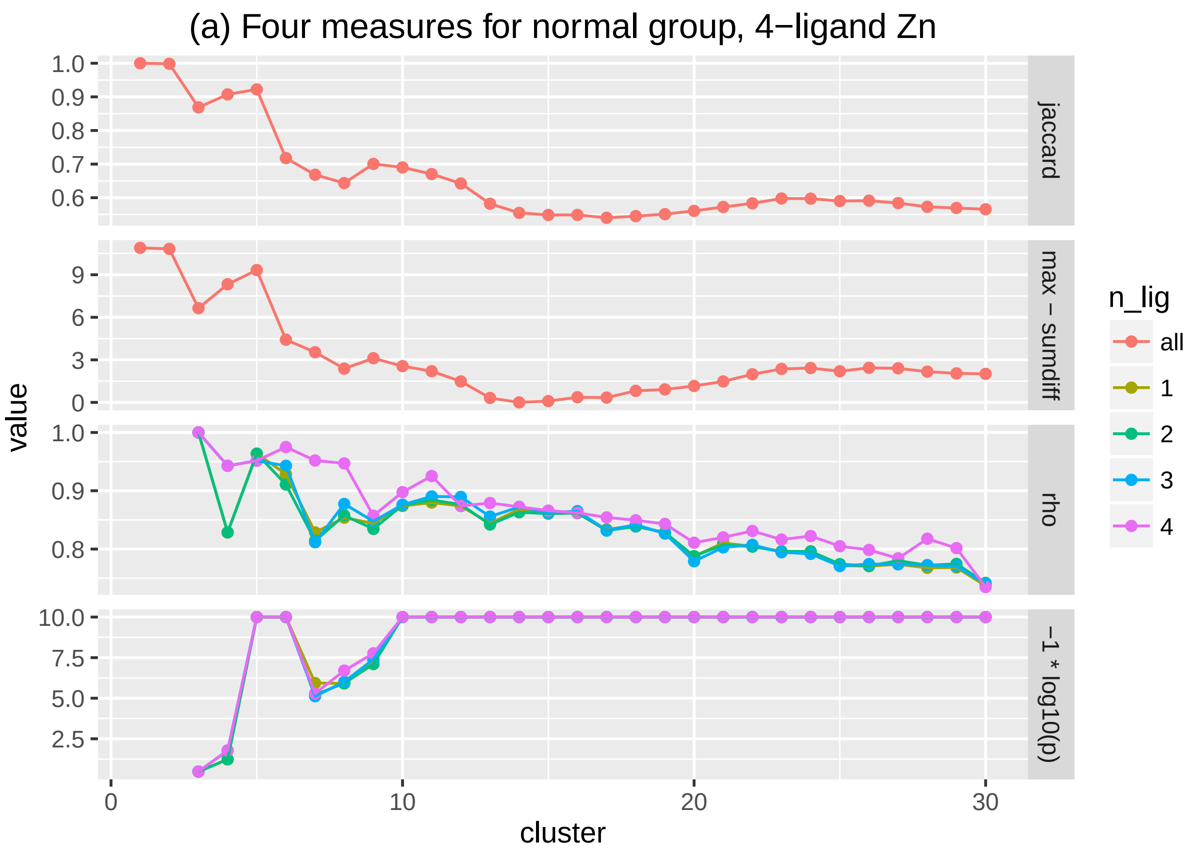

(b) Four measures for compressed group, 4−ligand Zn

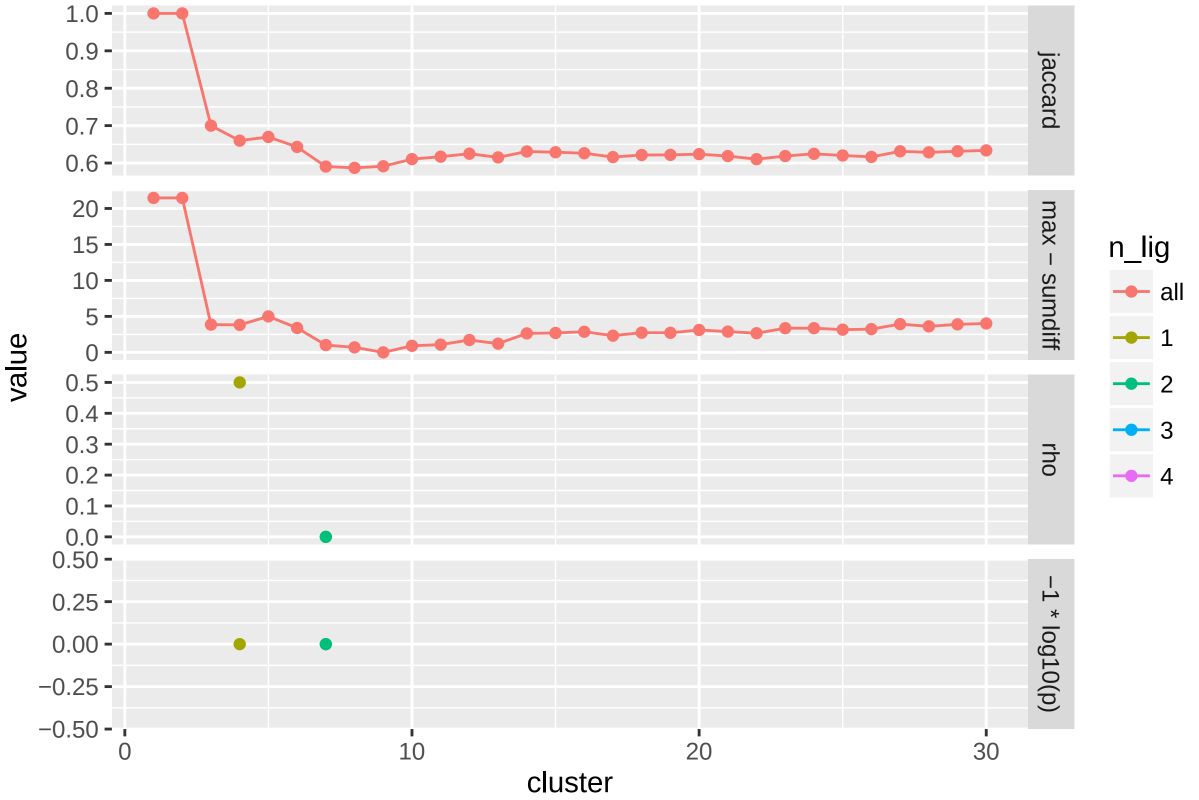

(c) Four measures for combined group, 4−ligand Zn

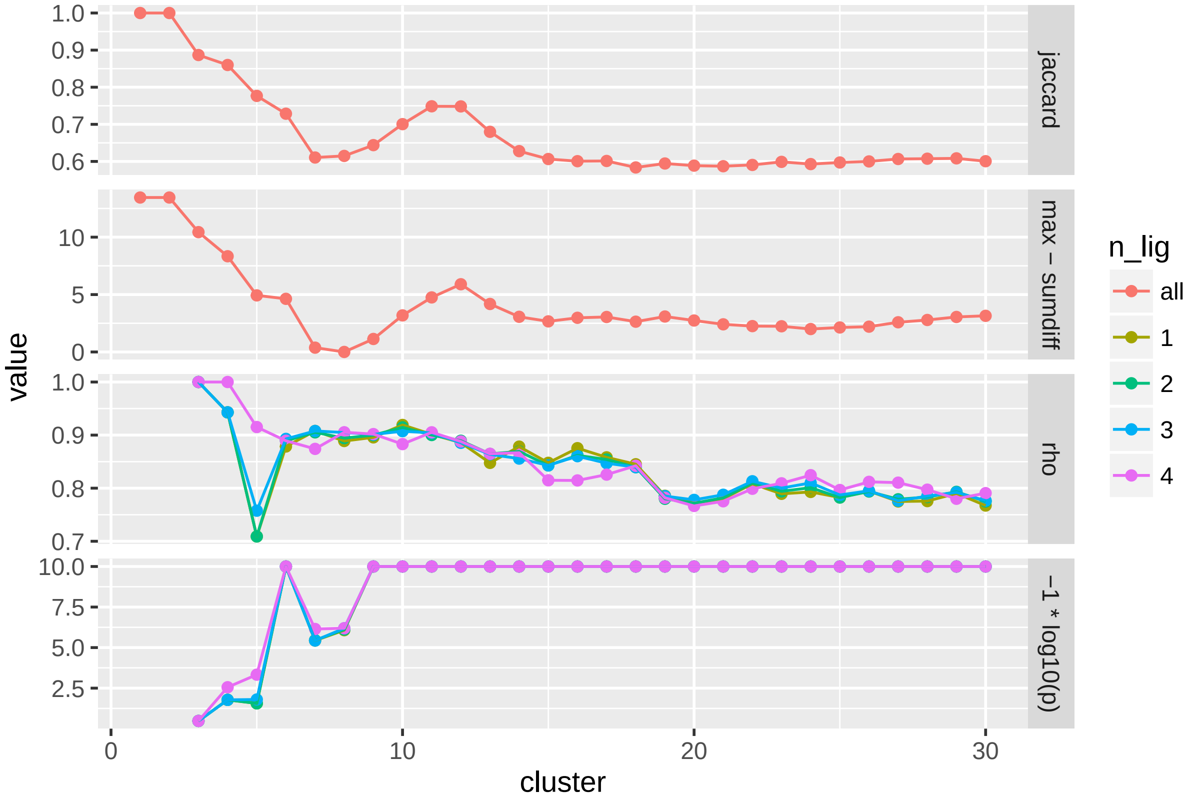

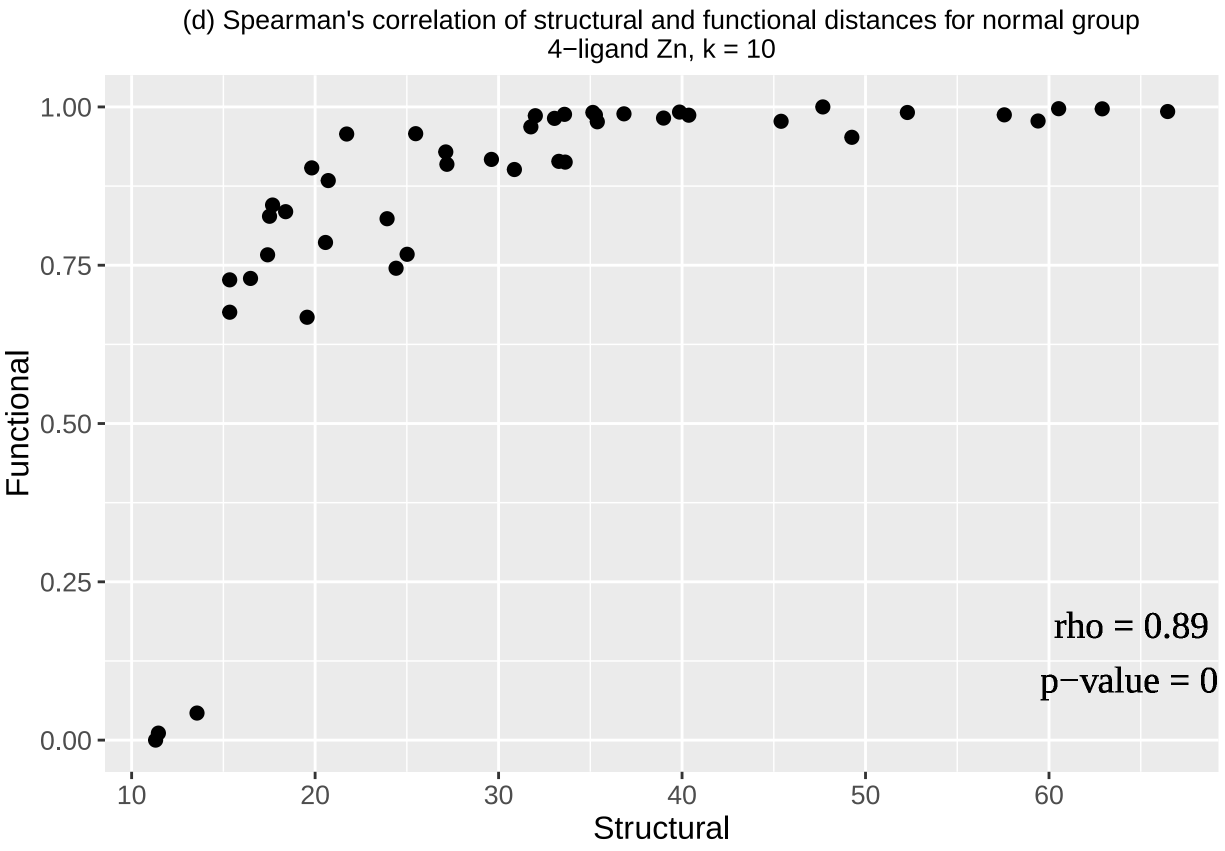

(e) Comparison between structural and functional hierarchical dendrograms for normal group 4-ligand Zn

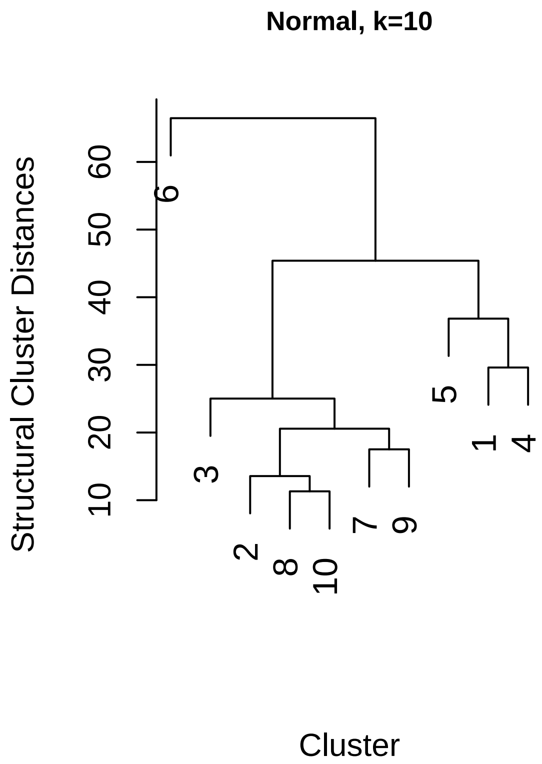

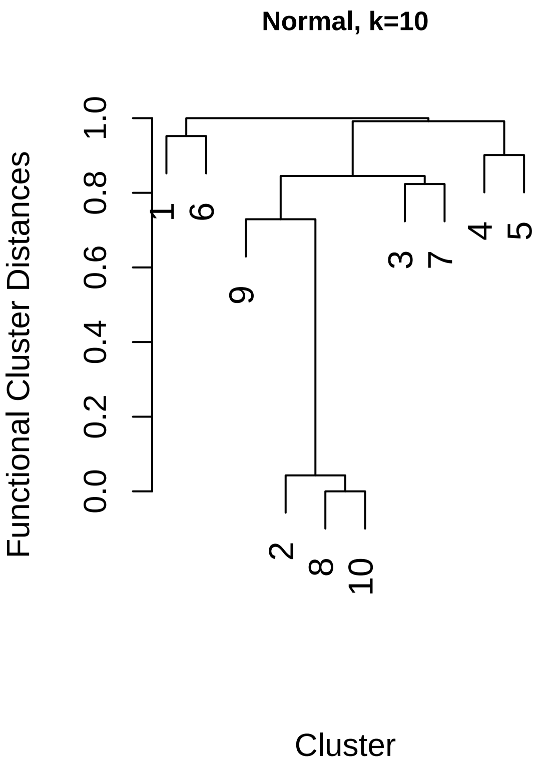

Figure S2. 5-ligand Zn metalloproteins

(a) Four measures for normal group, 5−ligand Zn

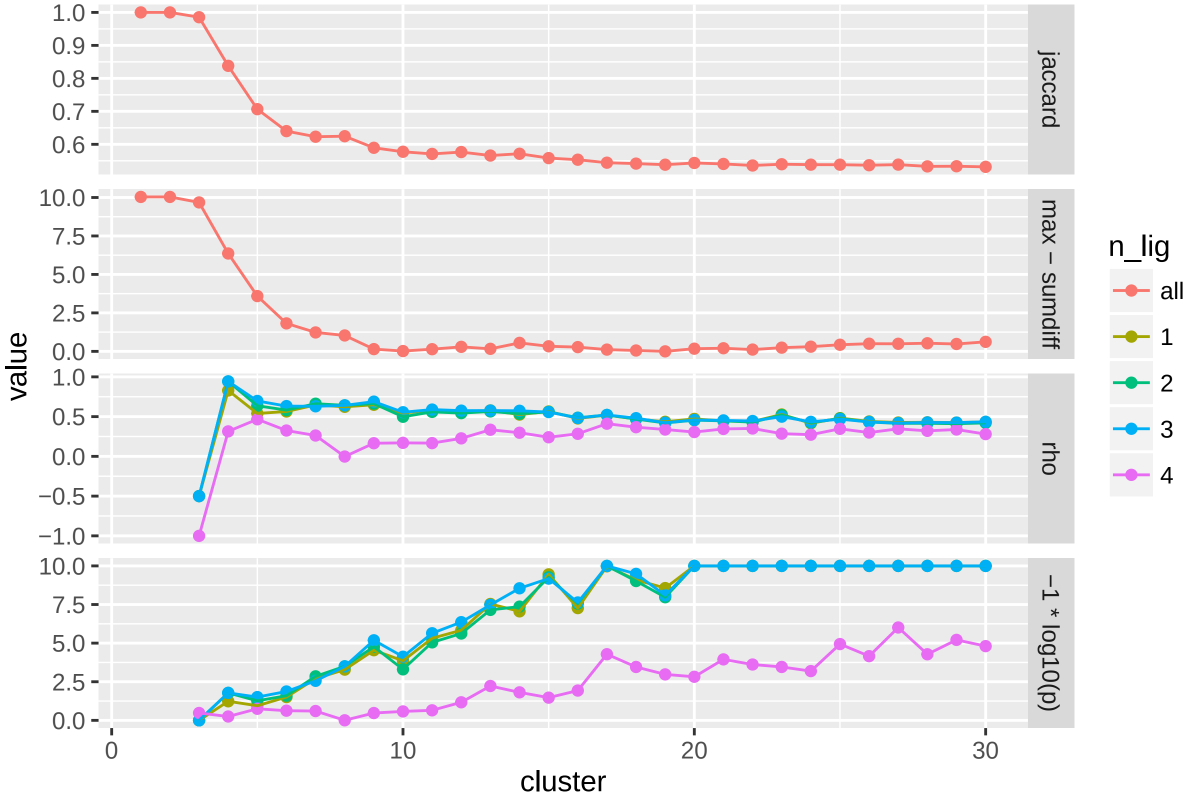

(b) Four measures for compressed group, 5−ligand Zn

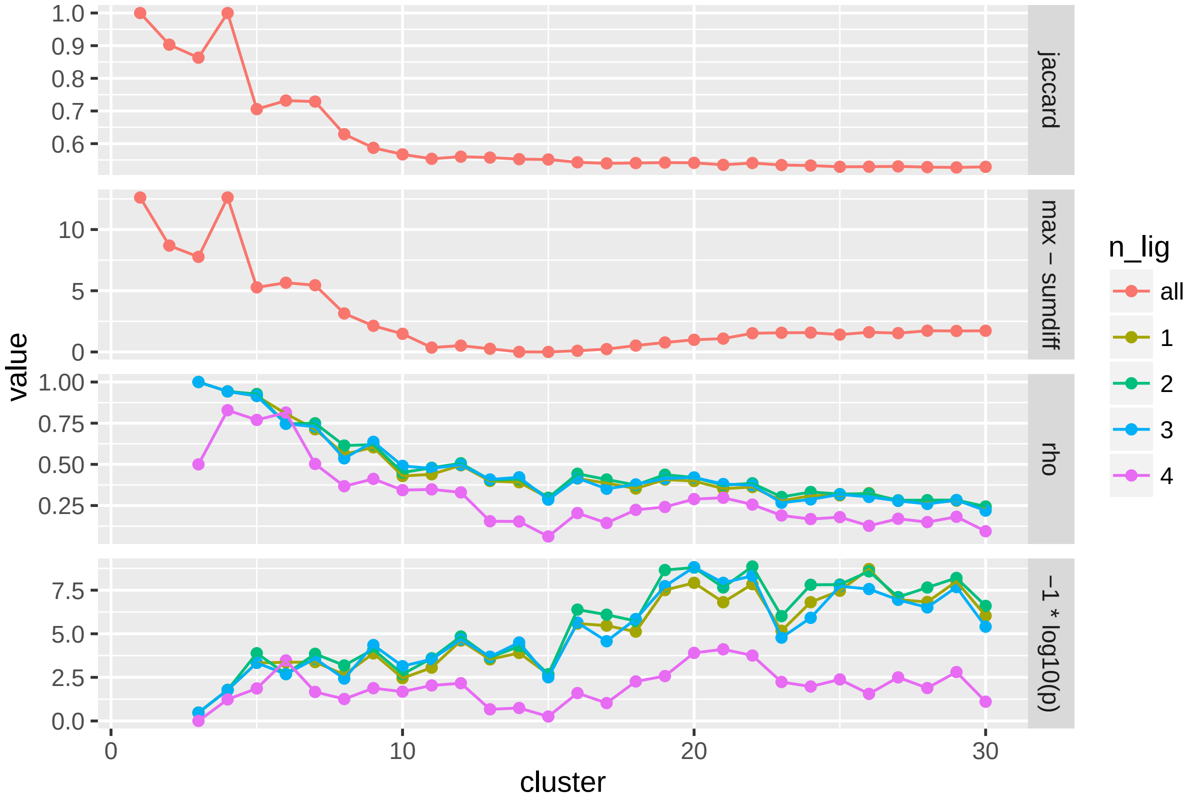

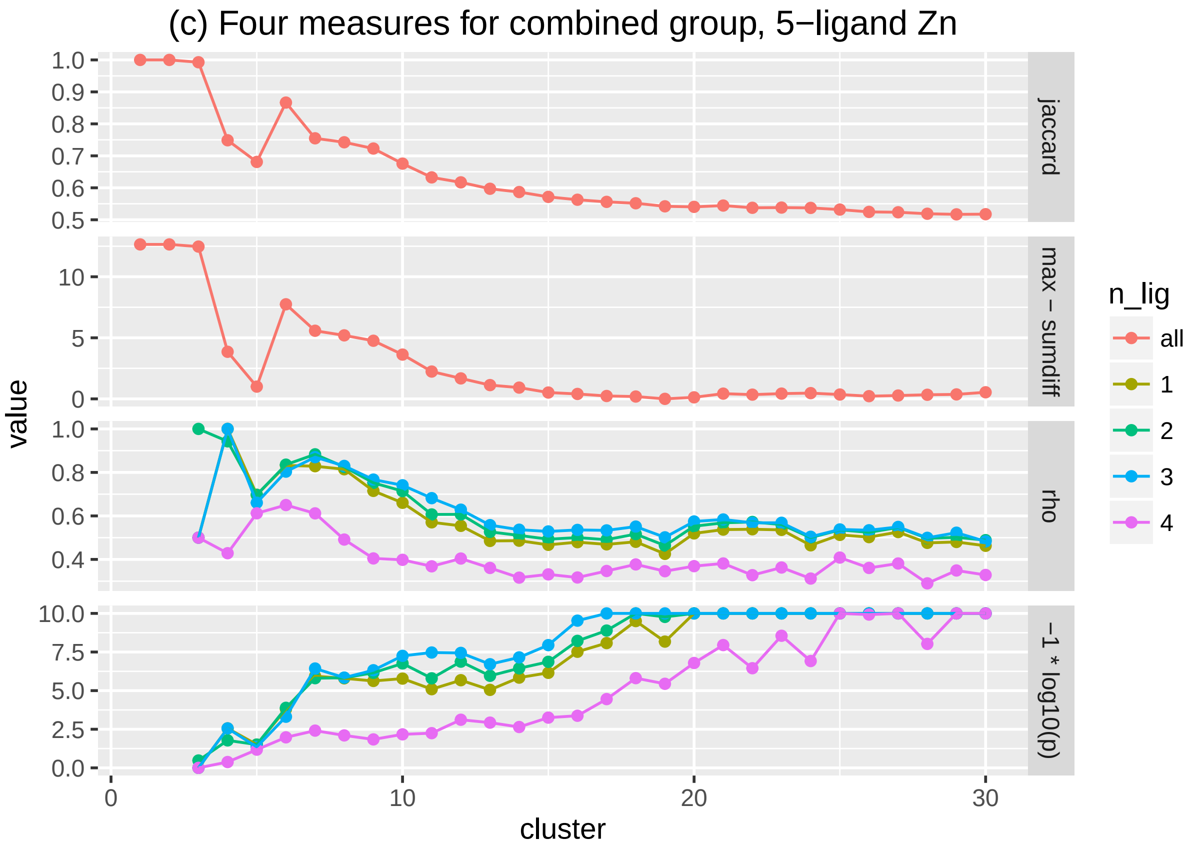
(c) Four measures for combined group, 5−ligand Zn
value
cluster
jaccard
max − sumdiff
rho
−1 * log10(p)
n_lig
all
1
2
3
4

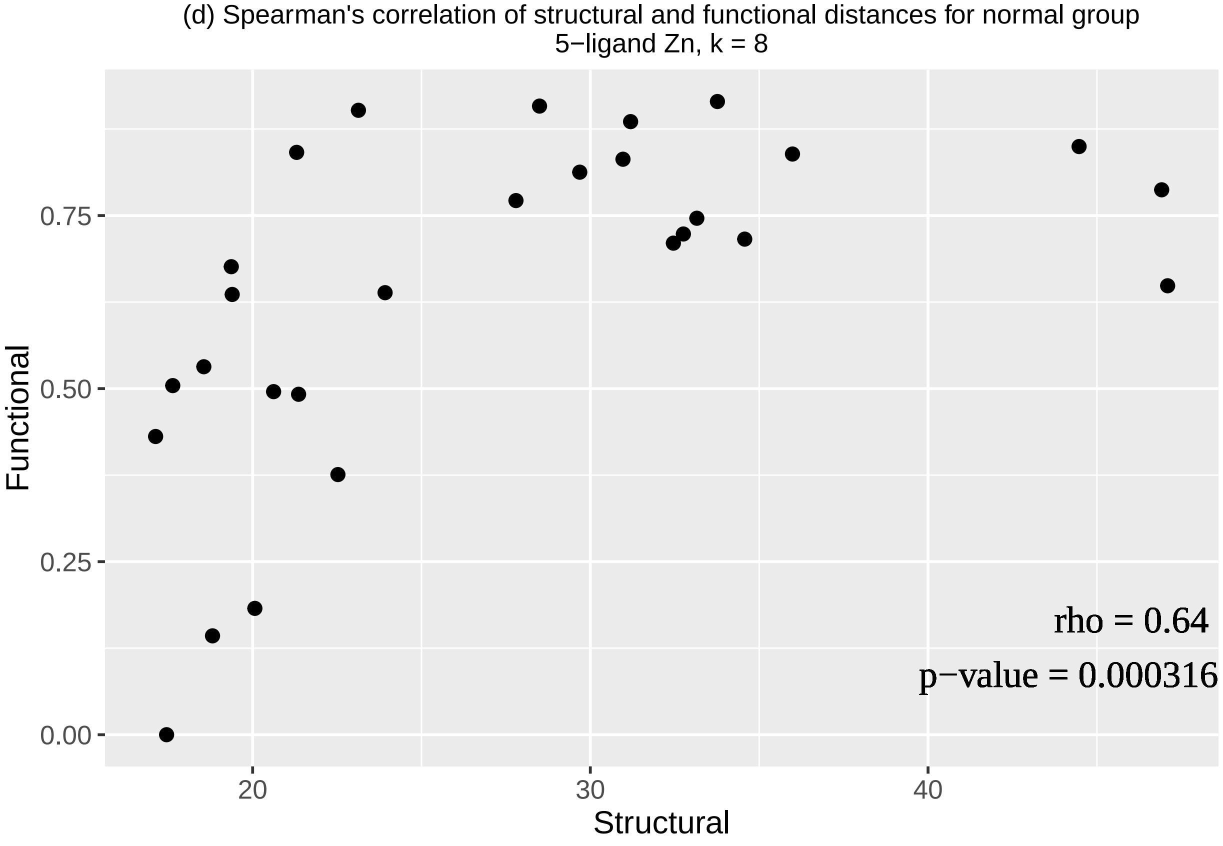
(d) Spearman's correlation of structural and functional distances for normal group
5−ligand Zn, k = 8
Functional
Structural
rho = 0.64
p−value = 0.000316

(e) Comparison between structural and functional hierarchical dendrograms for normal group 5-ligand Zn

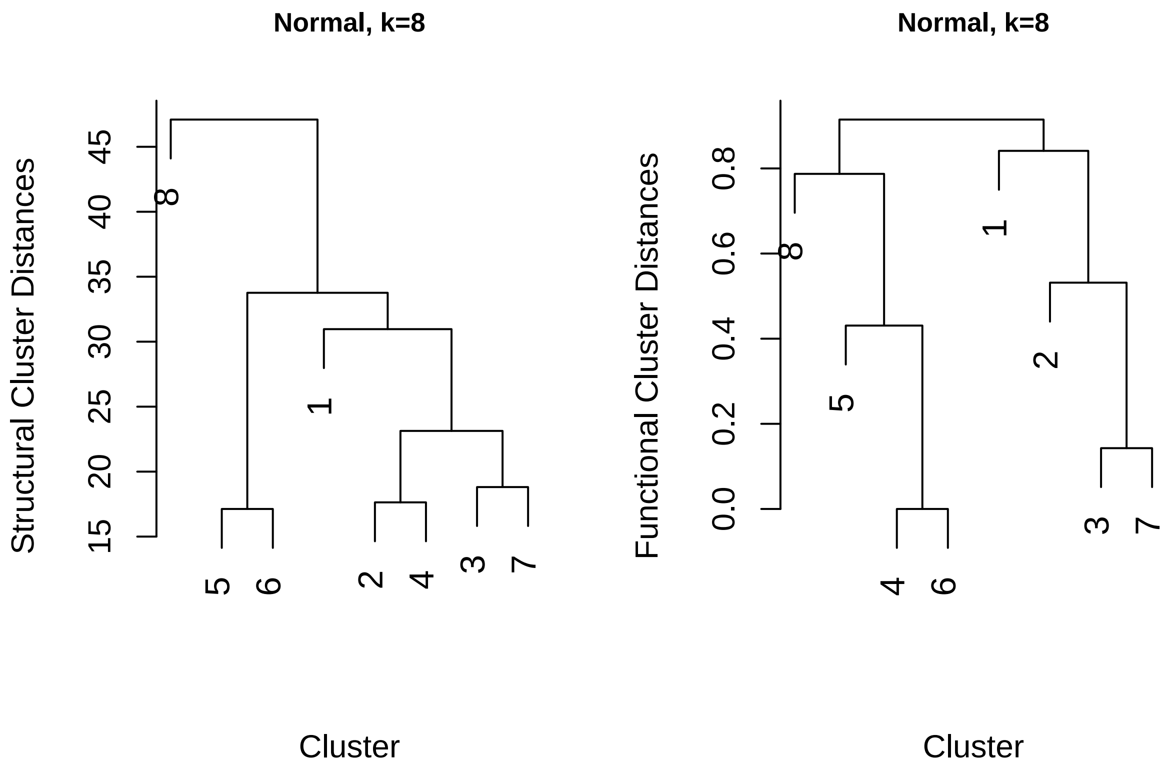

(f) Spearman's correlation of structural and functional distances for compressed group 5−ligand Zn, k = 7

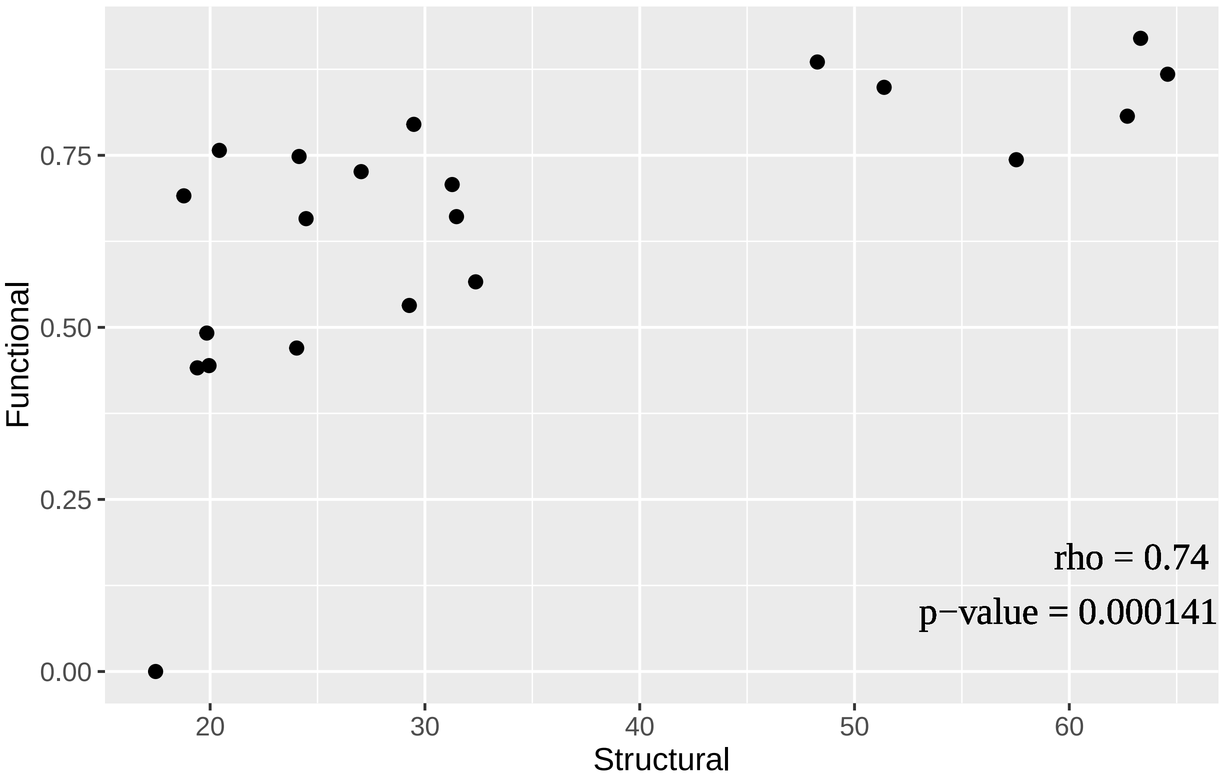

(g) Comparison between structural and functional hierarchical dendrograms for compressed group 5-ligand Zn

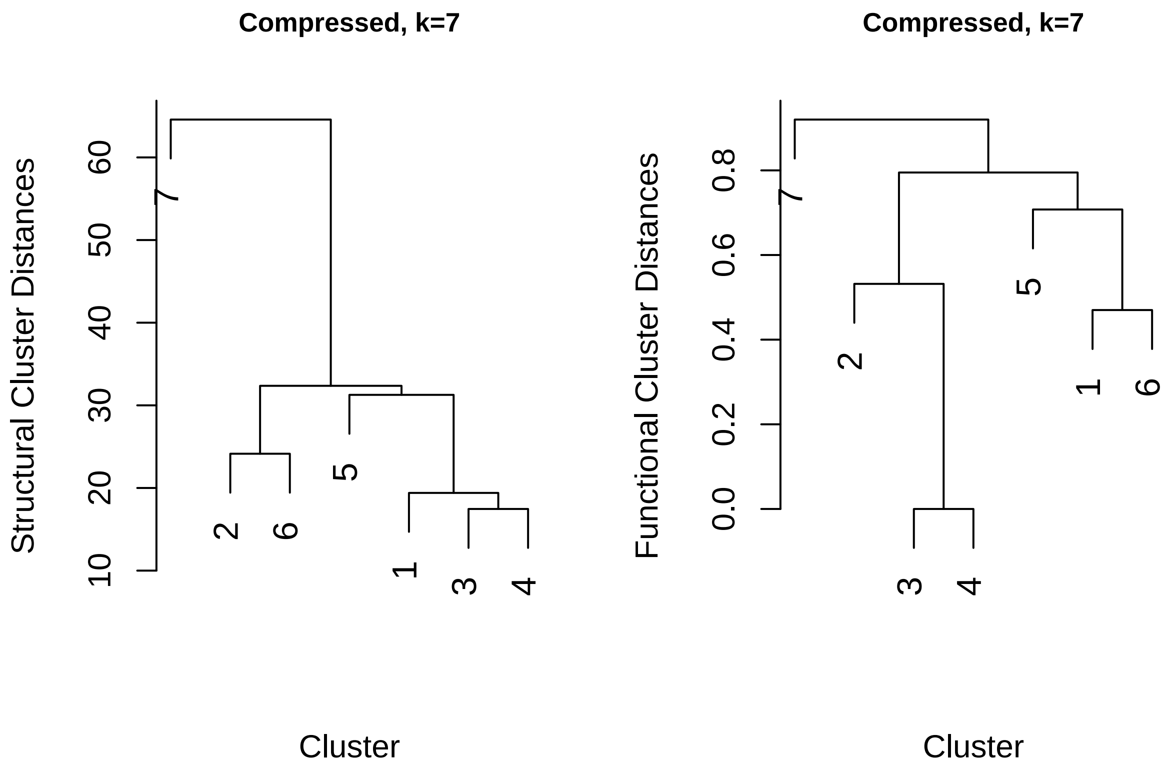

Figure S3. 6-ligand Zn metalloproteins

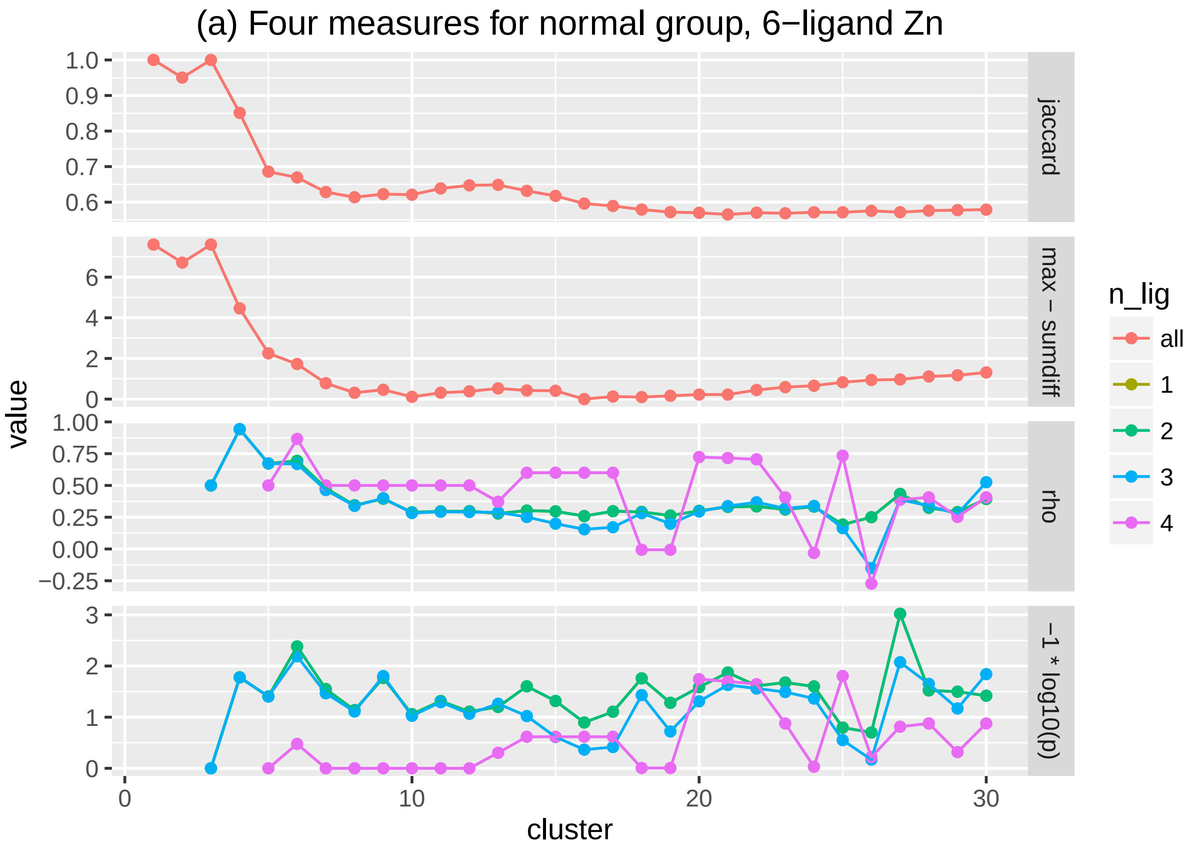
(a) Four measures for normal group, 6−ligand Zn
value
cluster
jaccard
max − sumdiff
rho
−1 * log10(p)
n_lig
all
1
2
3
4

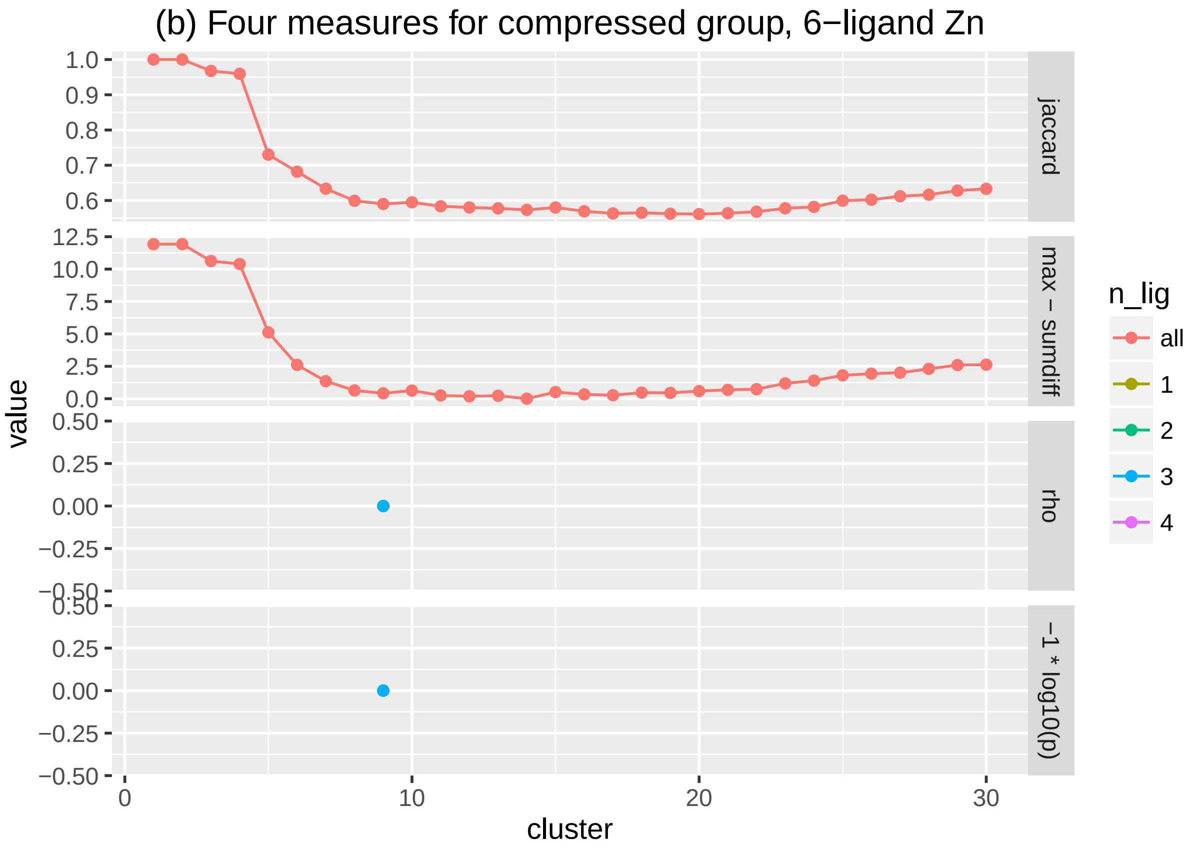
(b) Four measures for compressed group, 6−ligand Zn
value
cluster
jaccard
max − sumdiff
rho
−1 * log10(p)
n_lig
all
1
2
3
4

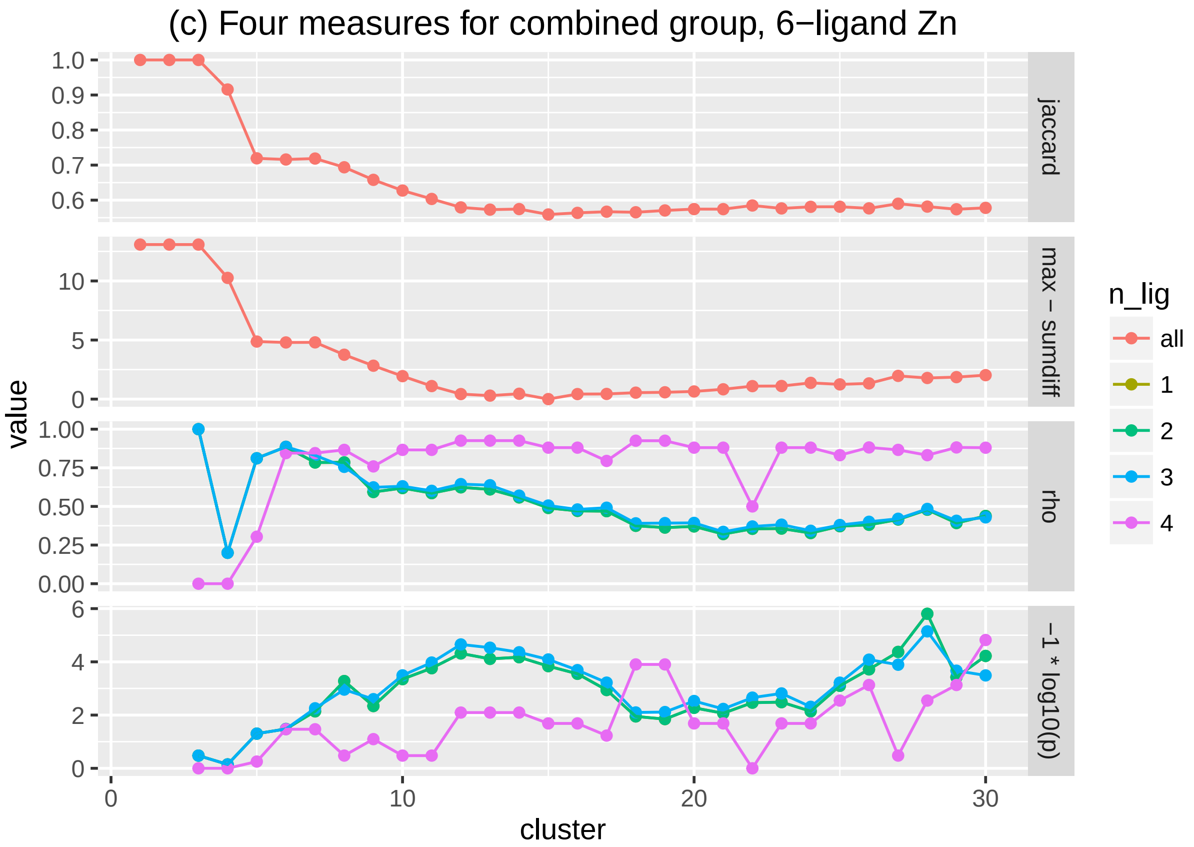
(c) Four measures for combined group, 6−ligand Zn
value
cluster
jaccard
max − sumdiff
rho
−1 * log10(p)
n_lig
all
1
2
3
4

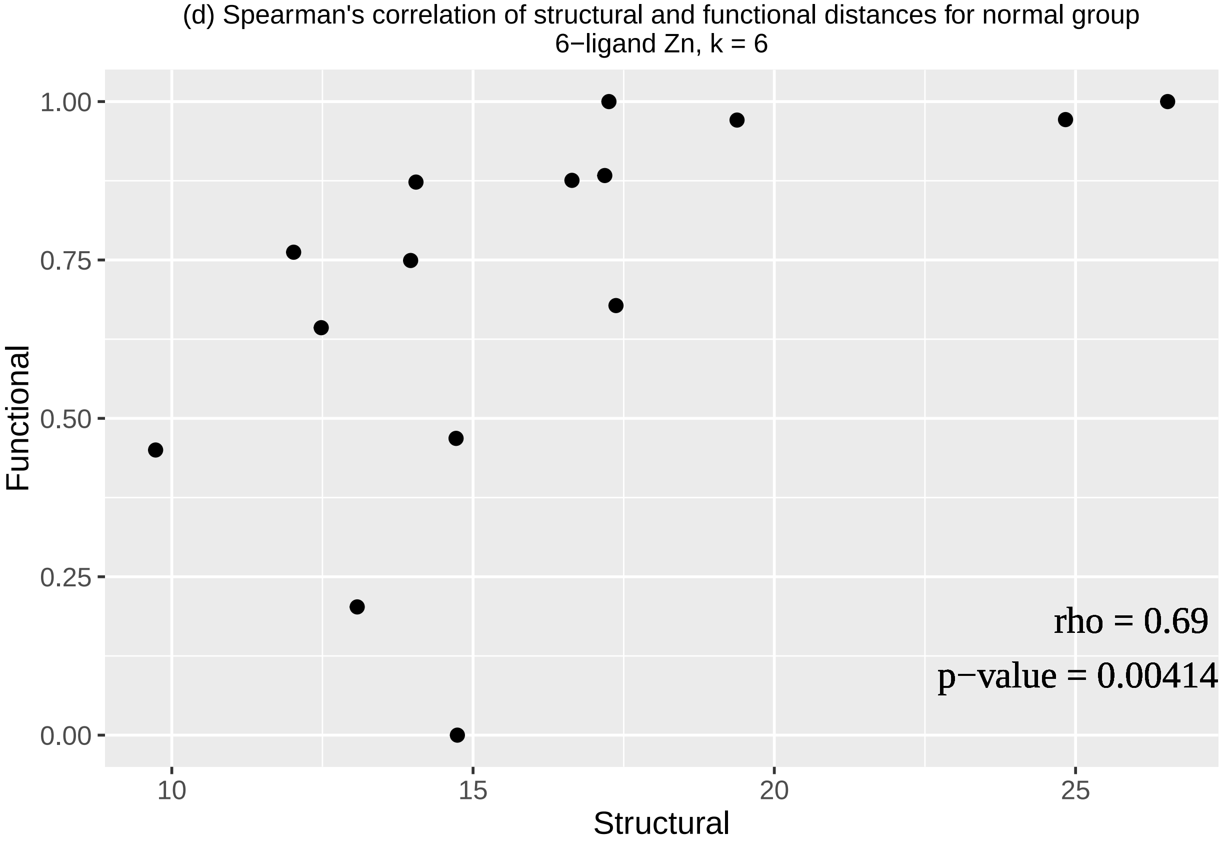
(d) Spearman's correlation of structural and functional distances for normal group
6−ligand Zn, k = 6
Functional
Structural
rho = 0.69
p−value = 0.00414

(e) Comparison between structural and functional hierarchical dendrograms for normal group 6-ligand Zn

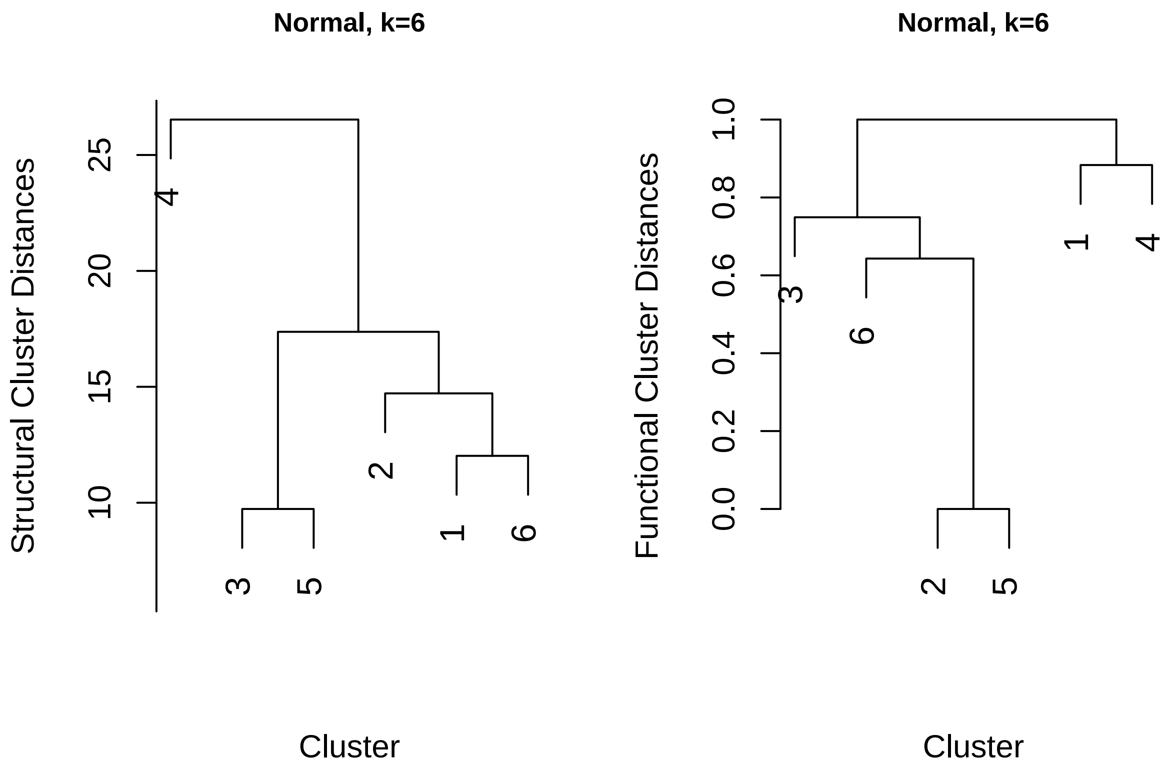

Figure S4. all-ligand Zn metalloproteins

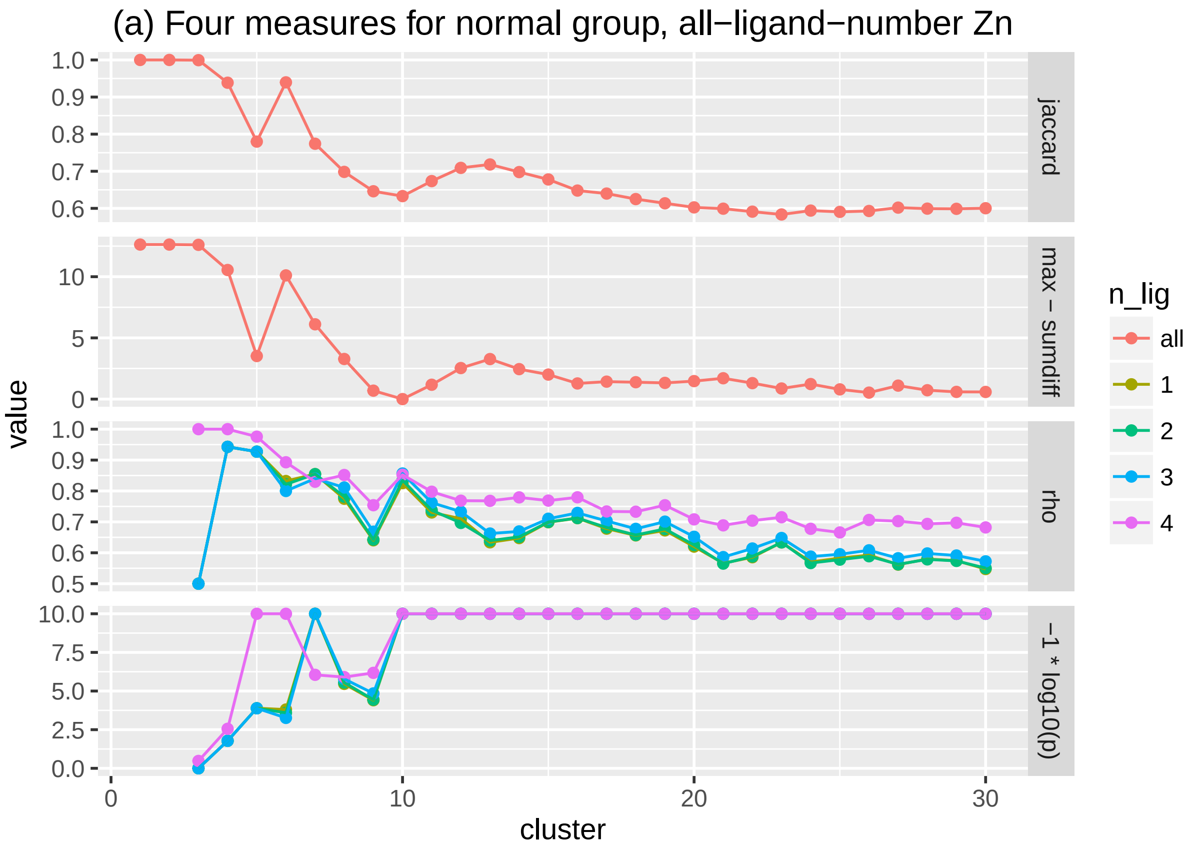
(a) Four measures for normal group, all−ligand−number Zn
jaccard
max − sumdiff
rho
−1 * log10(p)
value
cluster
n_lig
all
1
2
3
4

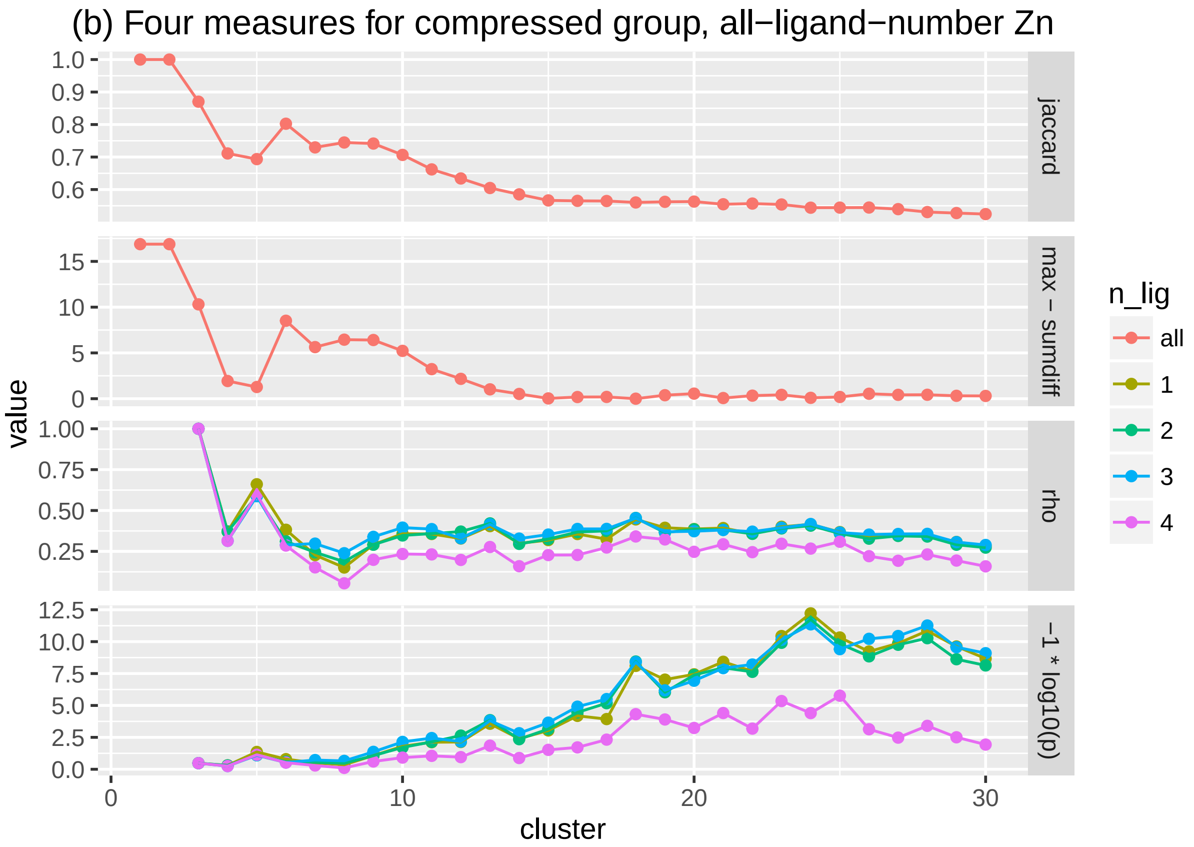
(b) Four measures for compressed group, all−ligand−number Zn
jaccard
max − sumdiff
rho
−1 * log10(p)
value
cluster
n_lig
all
1
2
3
4

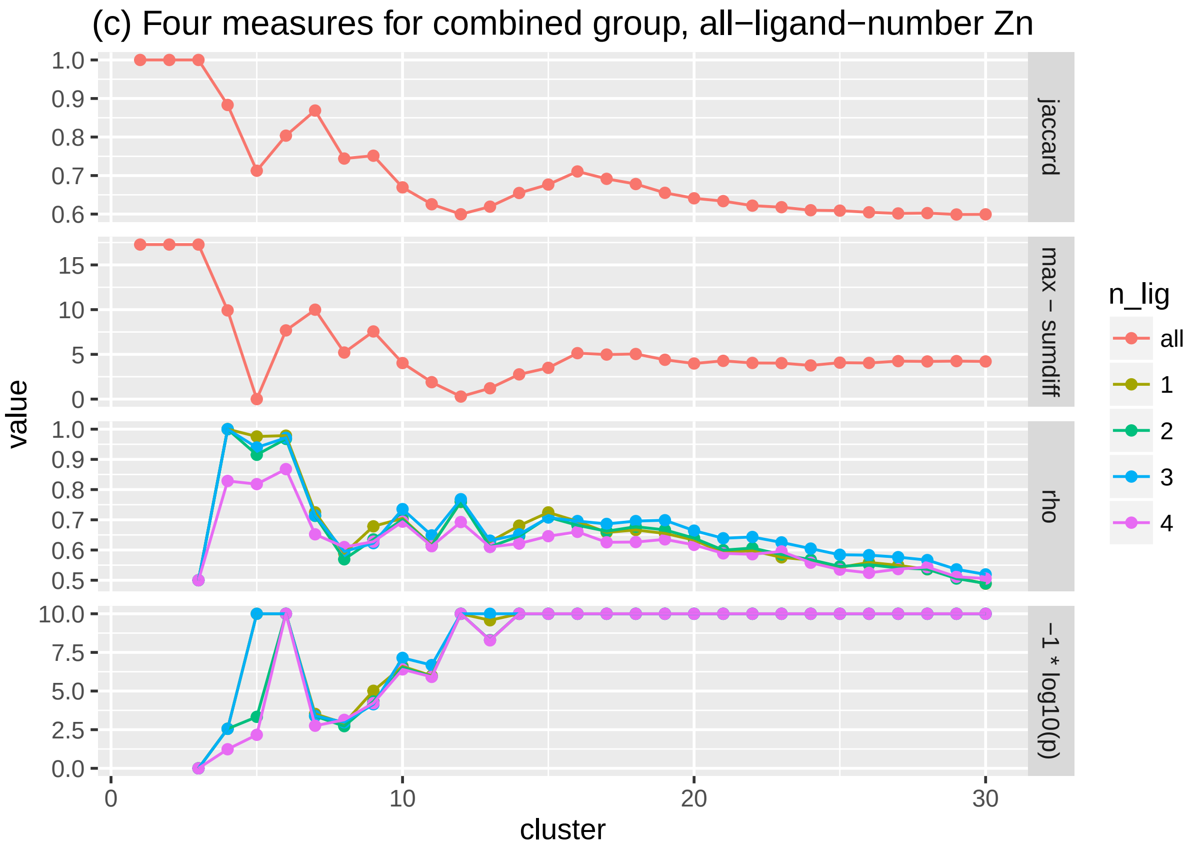
(c) Four measures for combined group, all−ligand−number Zn
value
cluster
jaccard
max − sumdiff
rho
−1 * log10(p)
n_lig
all
1
2
3
4
1.0
0.9
0.8
0.7
0.6
15
10
5
0
0.5
10.0
7.5
5.0
2.5
0.0
20
30

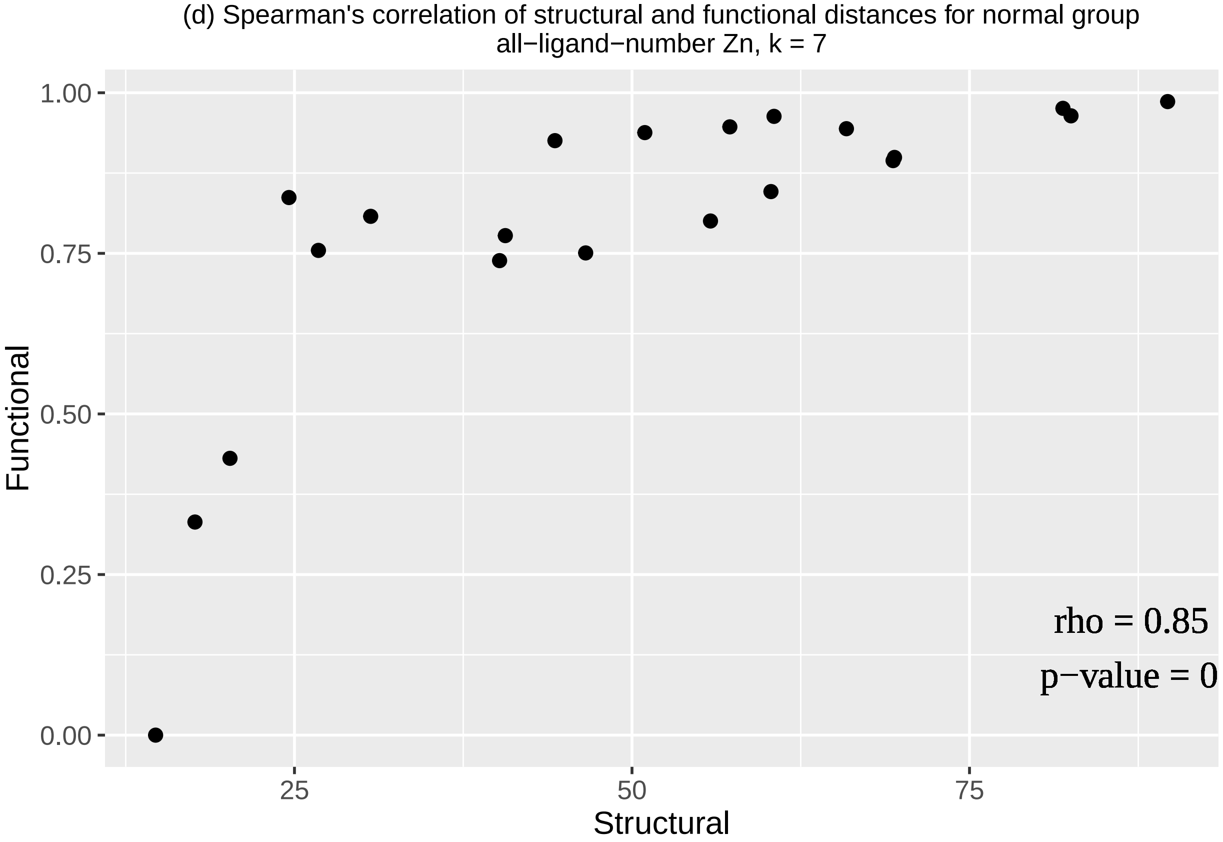
(d) Spearman's correlation of structural and functional distances for normal group
all−ligand−number Zn, k = 7
Functional
Structural
rho = 0.85
p−value = 0
1.00
0.75
0.50
0.25
0.00
25
50
75

(e) Comparison between structural and functional hierarchical dendrograms for normal group all-ligand-number Zn

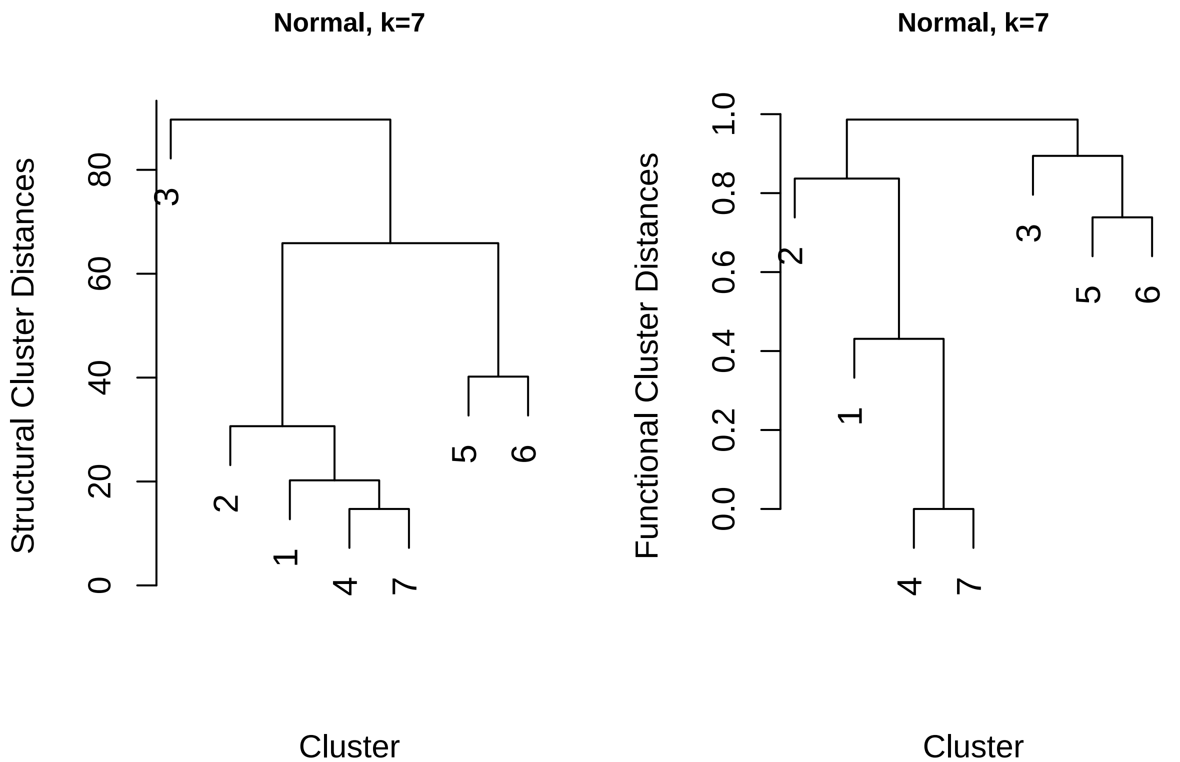

(f) Spearman's correlation of structural and functional distances for compressed group all−ligand−number Zn, k = 10

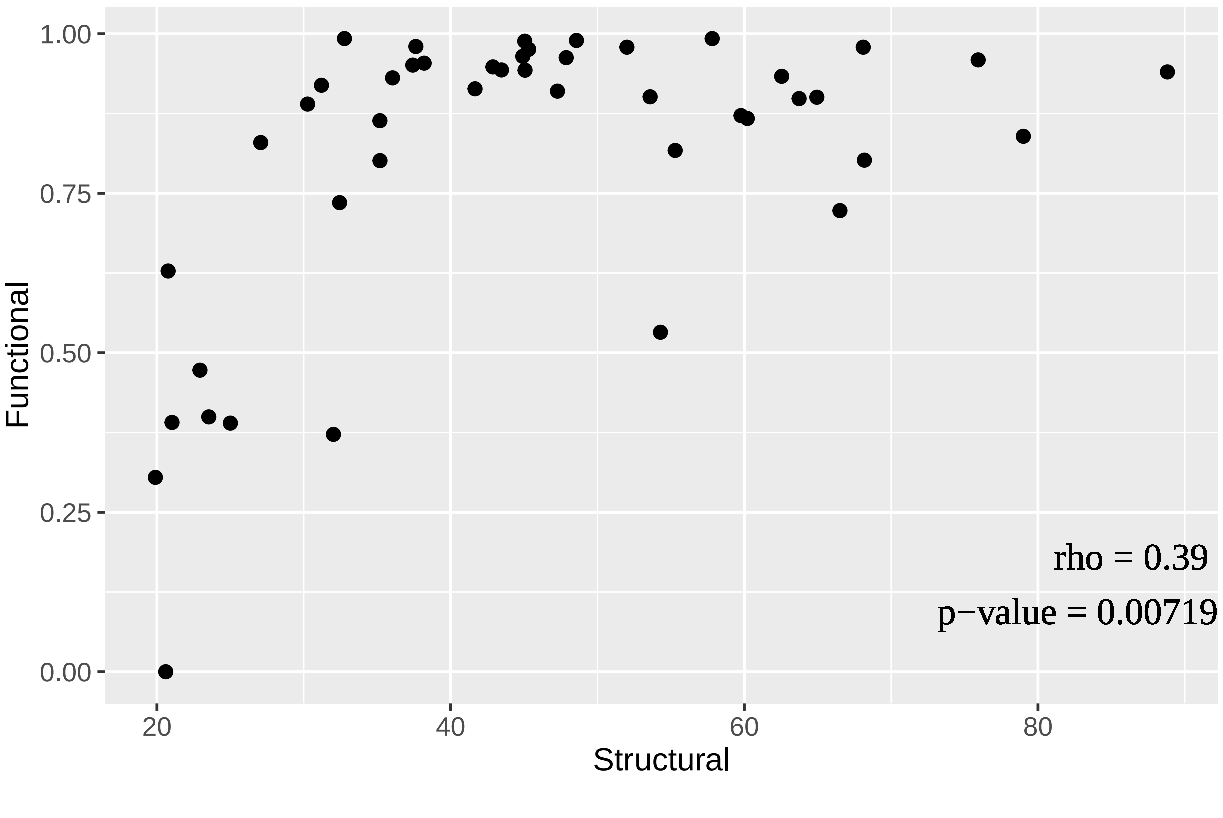

(g) Comparison between structural and functional hierarchical dendrograms for compressed group all-ligand-number Zn

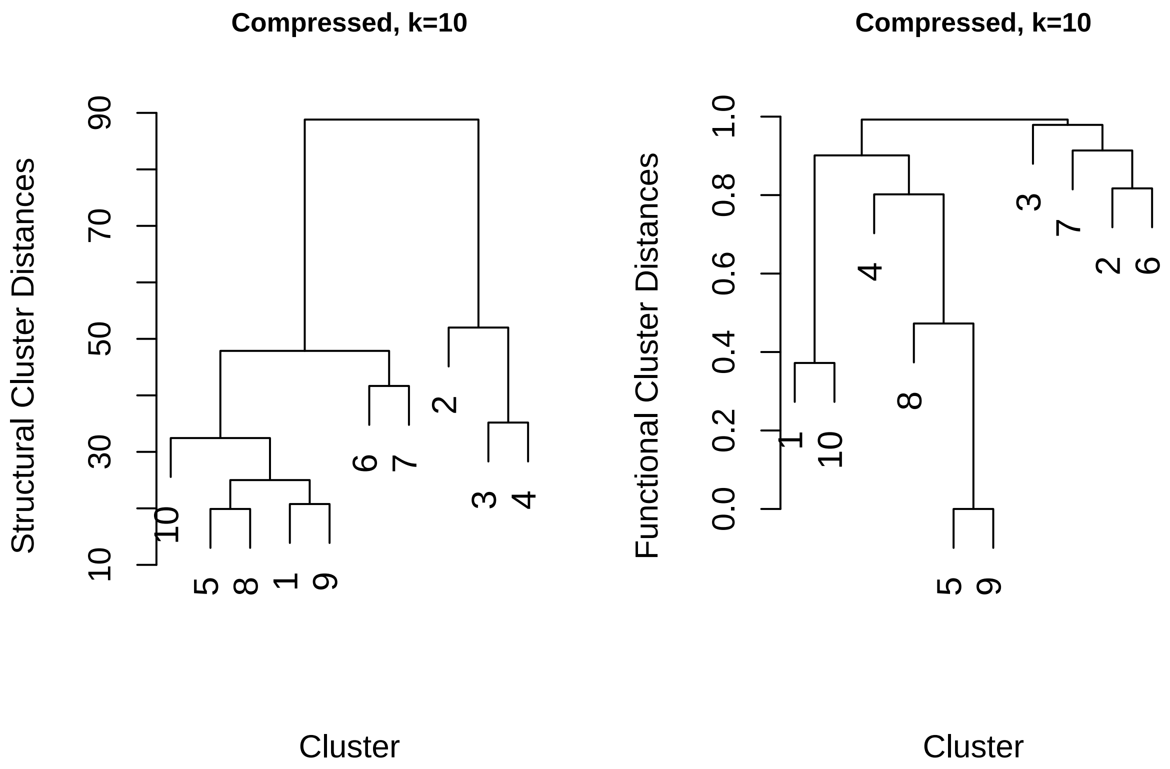

Figure S5. 4-ligand Mg metalloproteins

(a) Four measures for normal group, 4−ligand Mg

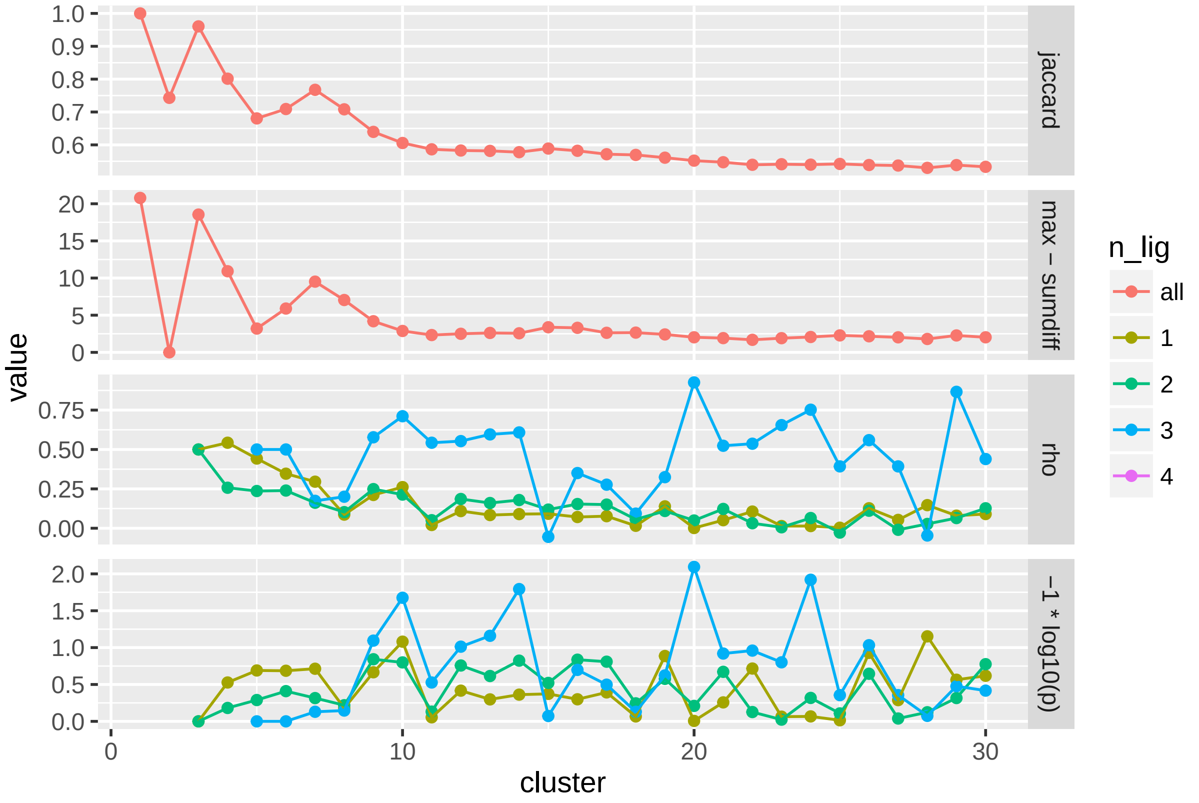

(b) Four measures for compressed group, 4−ligand Mg

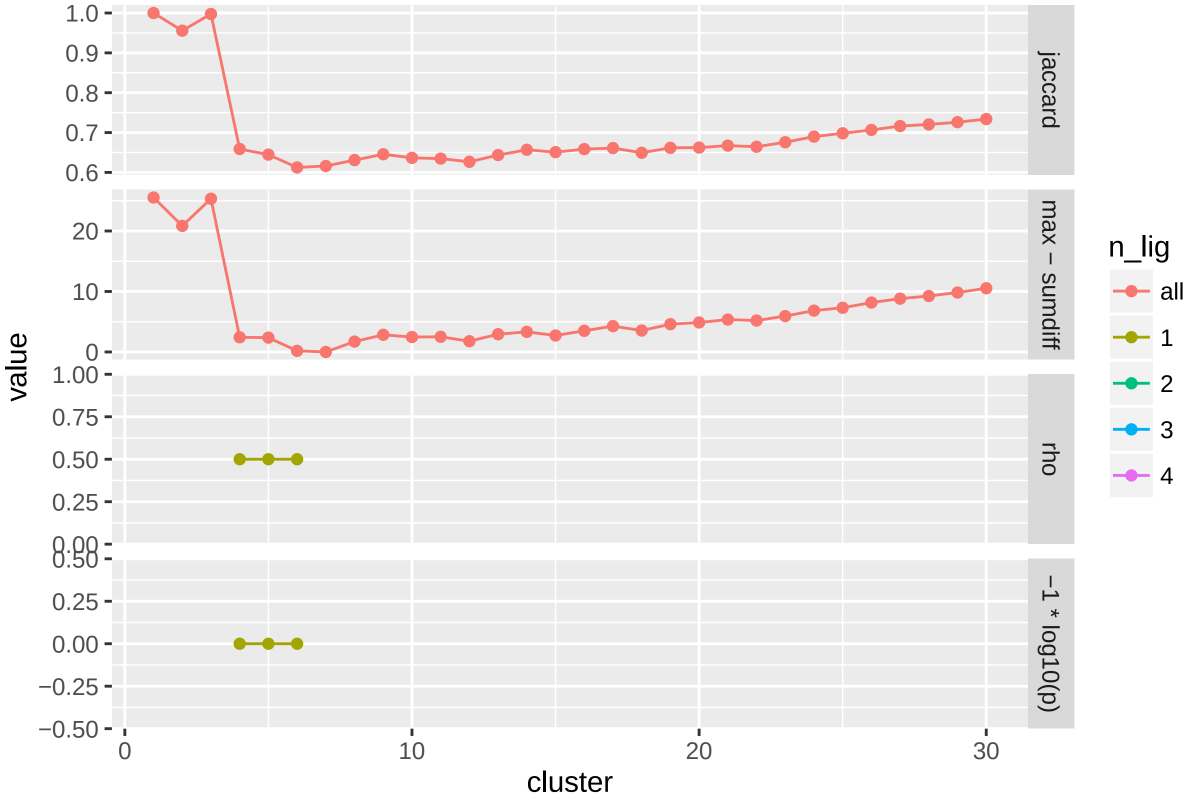

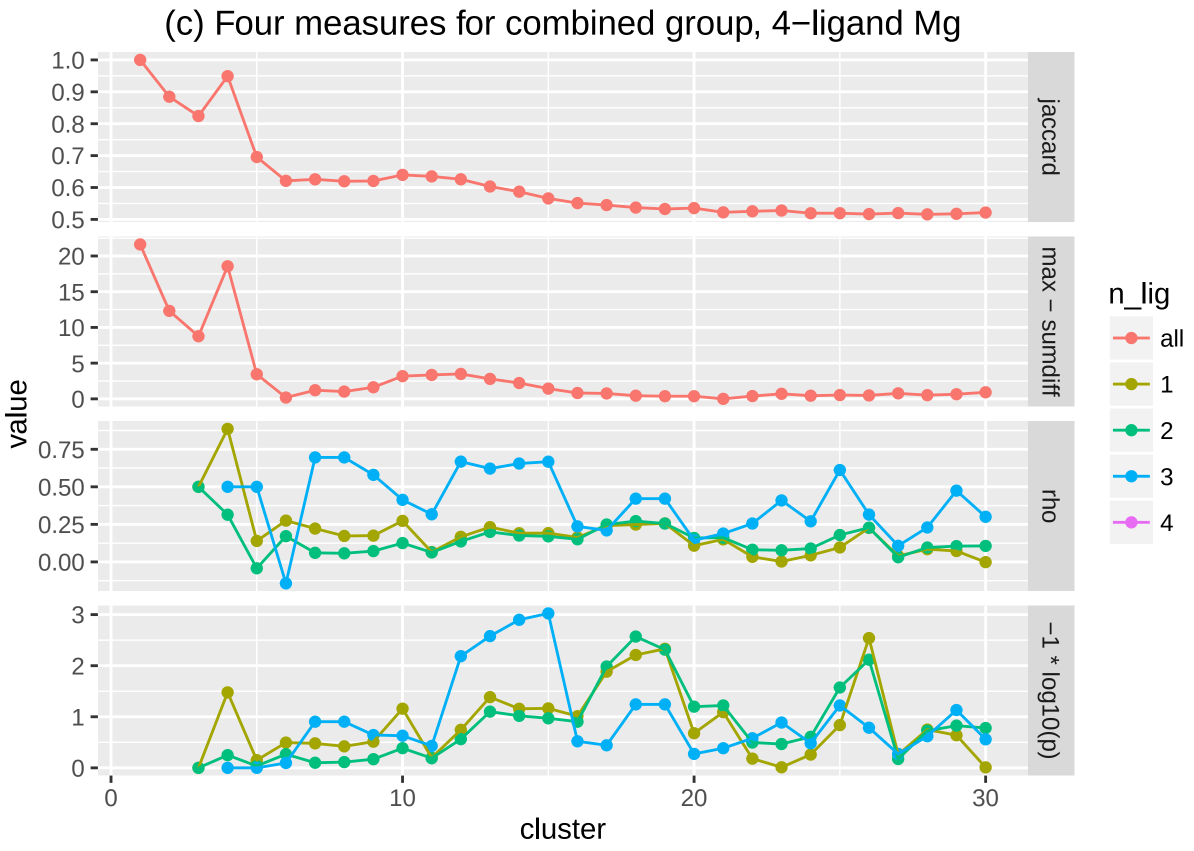
(c) Four measures for combined group, 4−ligand Mg
value
cluster
jaccard
max − sumdiff
rho
−1 * log10(p)
n_lig
all
1
2
3
4

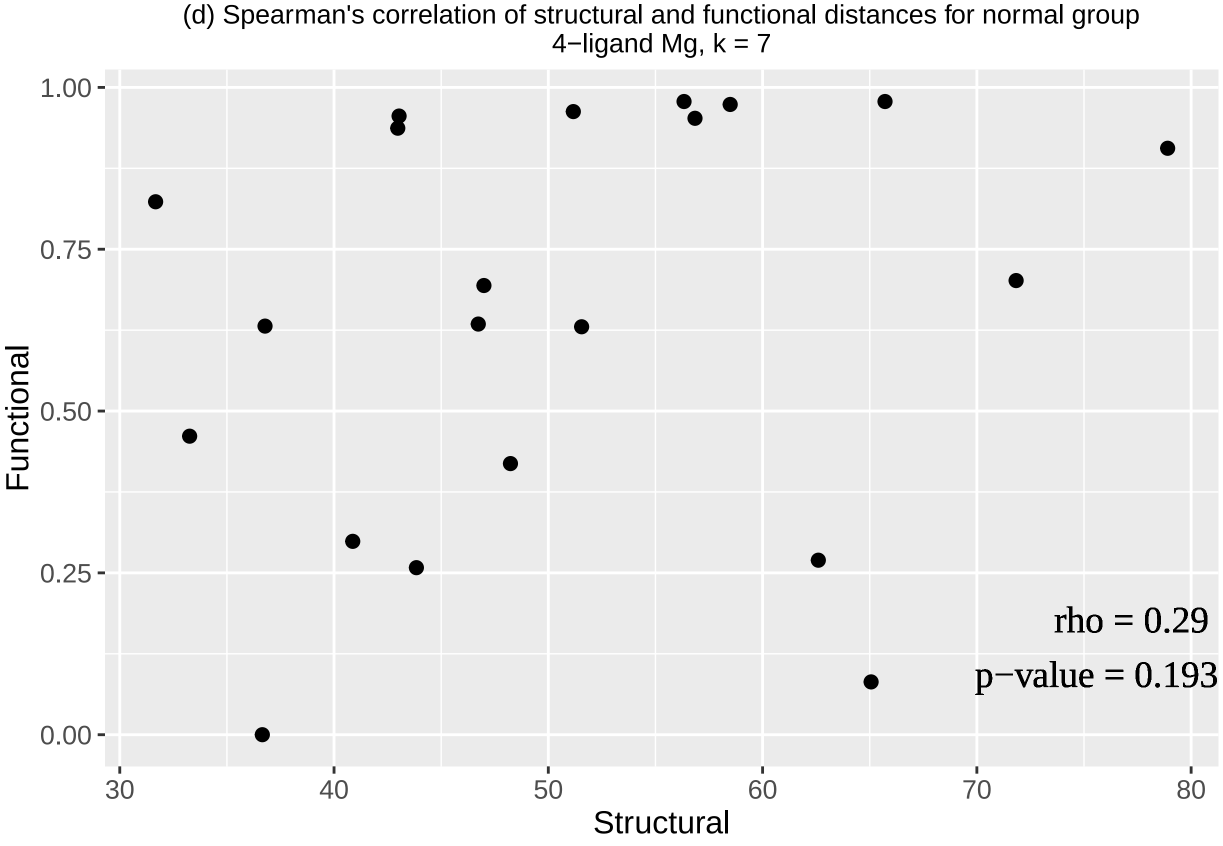
(d) Spearman's correlation of structural and functional distances for normal group
4−ligand Mg, k = 7
Functional
Structural
rho = 0.29
p−value = 0.193

(e) Comparison between structural and functional hierarchical dendrograms for normal group 4-ligand Mg

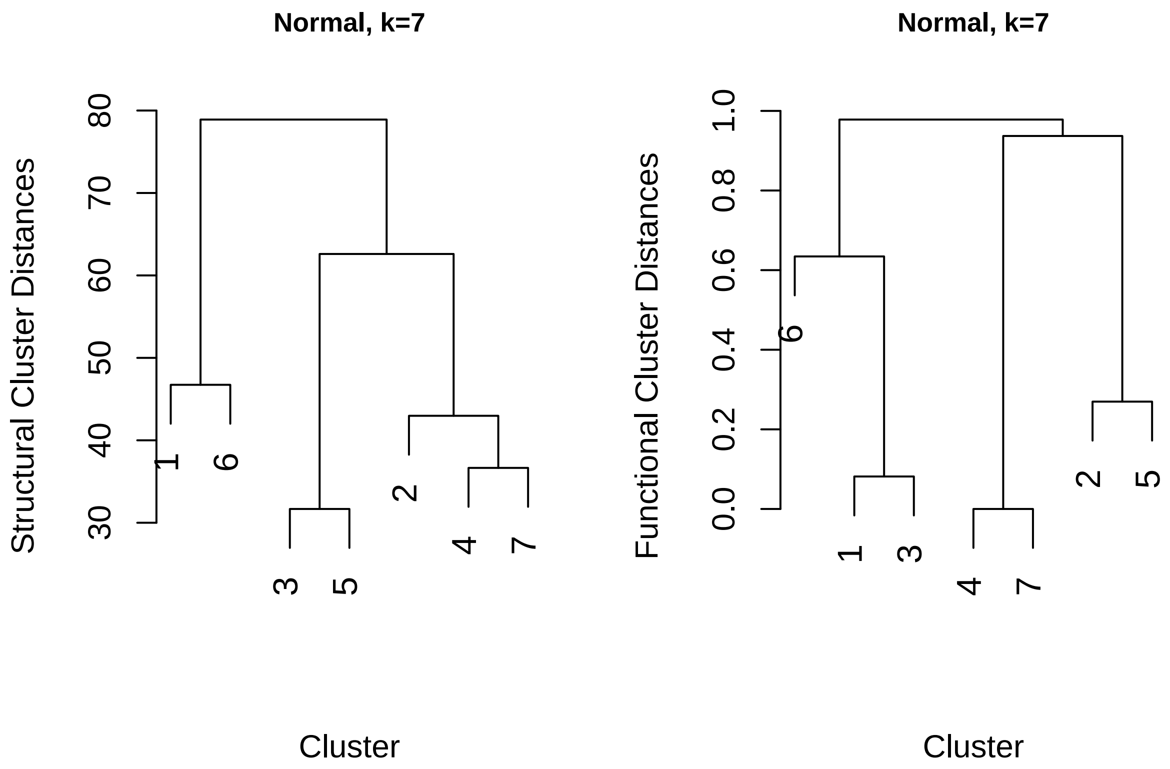

Figure S6. 5-ligand Mg metalloproteins

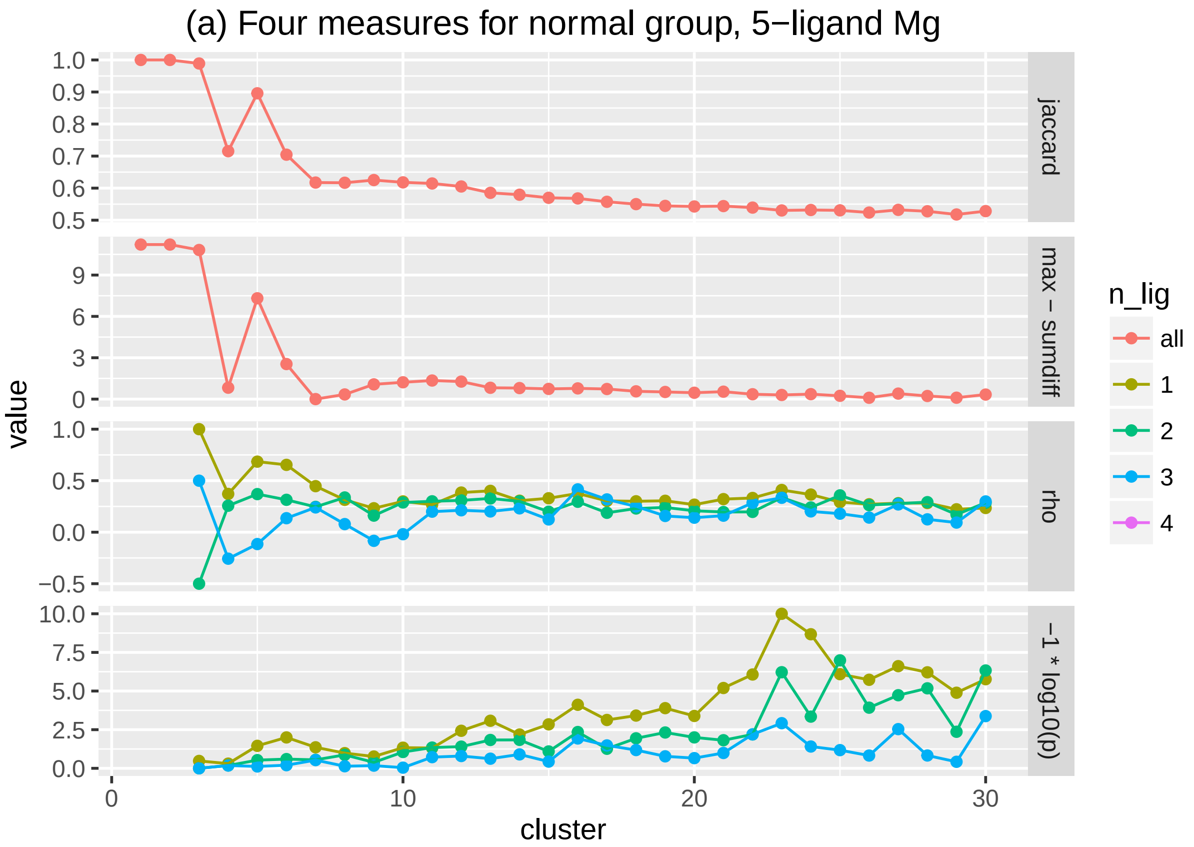
(a) Four measures for normal group, 5−ligand Mg
value
cluster
jaccard
max − sumdiff
rho
−1 * log10(p)
n_lig
all
1
2
3
4

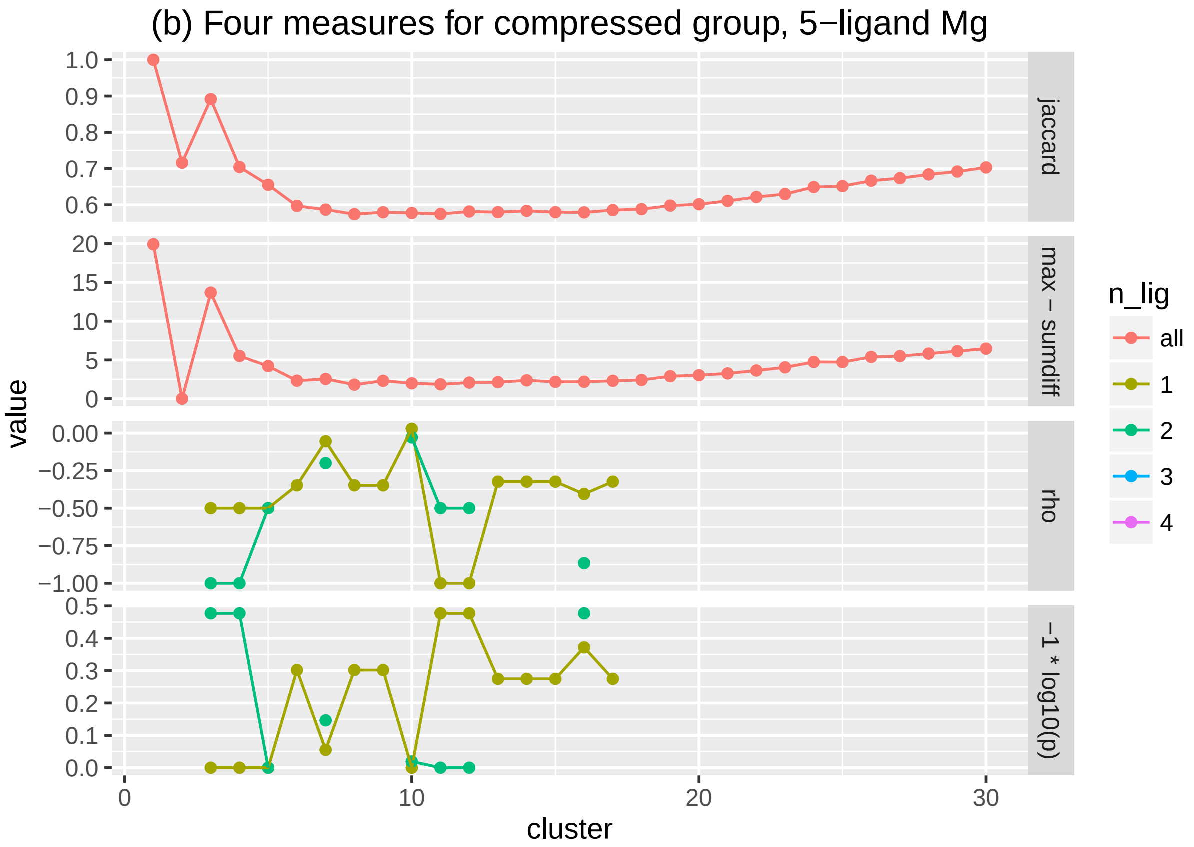
(b) Four measures for compressed group, 5−ligand Mg
value
cluster
jaccard
max − sumdiff
rho
−1 * log10(p)
n_lig
all
1
2
3
4

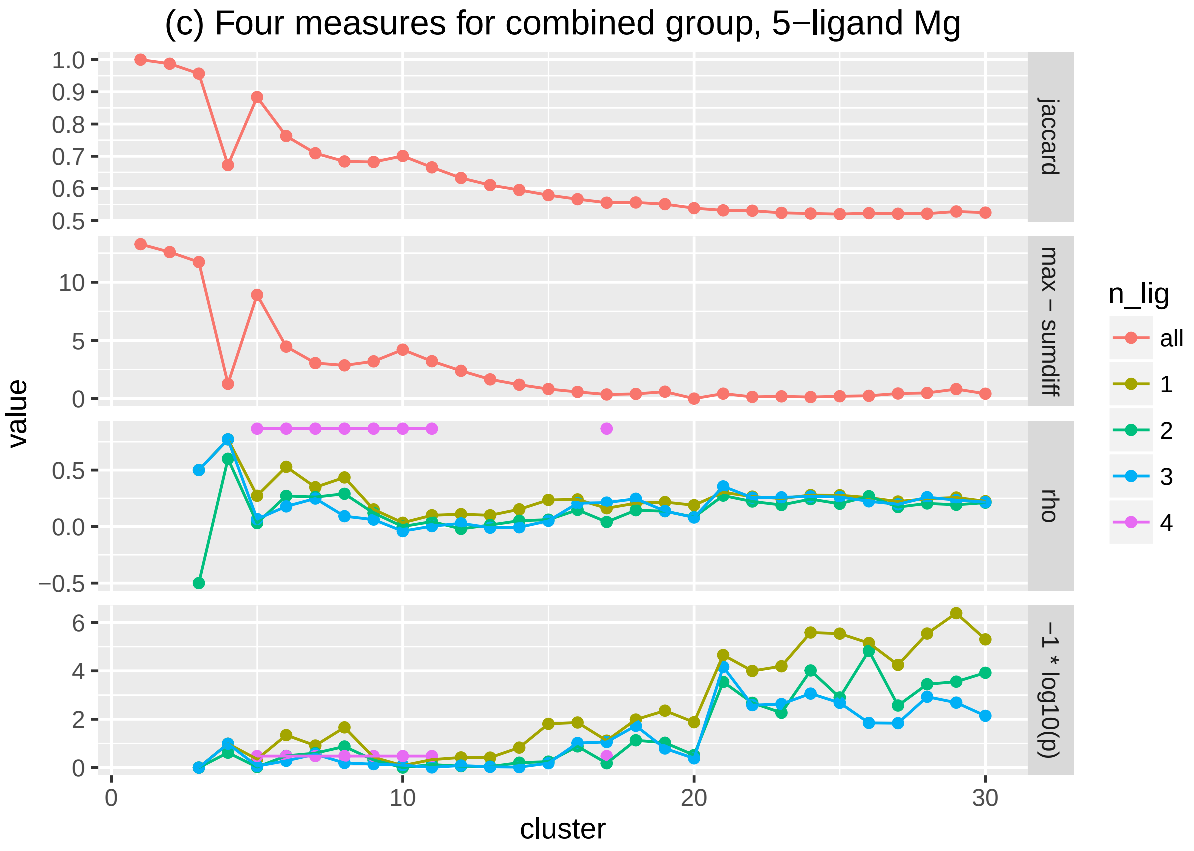
(c) Four measures for combined group, 5−ligand Mg
value
cluster
jaccard
max − sumdiff
rho
−1 * log10(p)
n_lig
all
1
2
3
4
1.0
0.9
0.8
0.7
0.6
0.5
10
5
0
0.5
0.0
−0.5
6
4
2
0
0
10
20
30

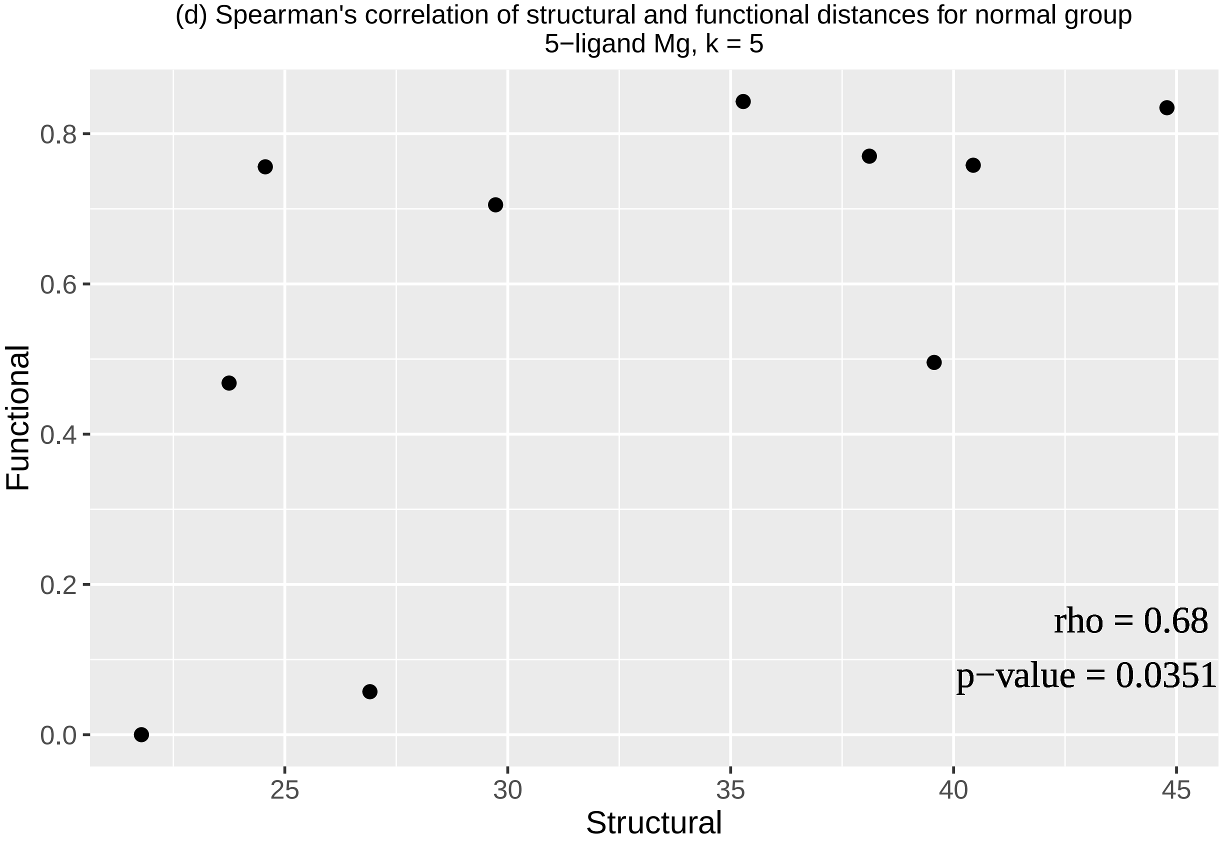
(d) Spearman's correlation of structural and functional distances for normal group
5−ligand Mg, k = 5
Functional
Structural
rho = 0.68
p−value = 0.0351
0.8
0.6
0.4
0.2
0.0
25
30
35
40
45

(e) Comparison between structural and functional hierarchical dendrograms for normal group 5-ligand Mg

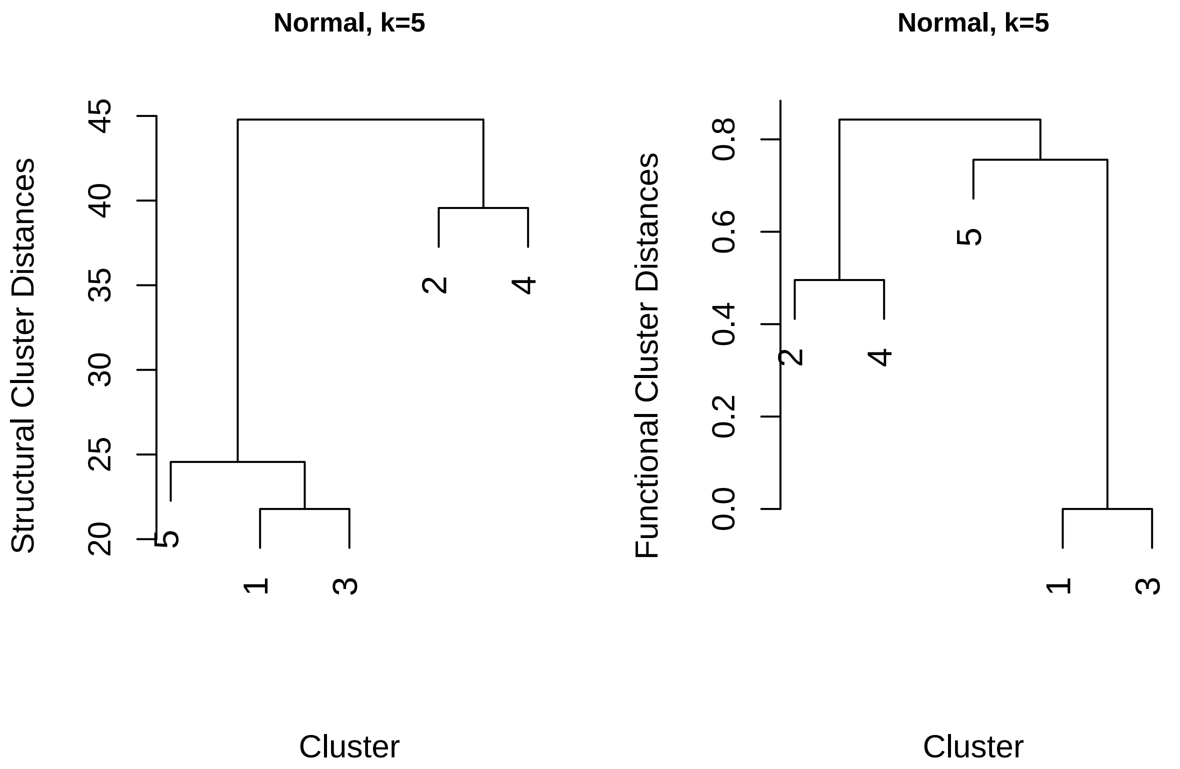

Figure S7. 6-ligand Mg metalloproteins

(a) Four measures for normal group, 6−ligand Mg

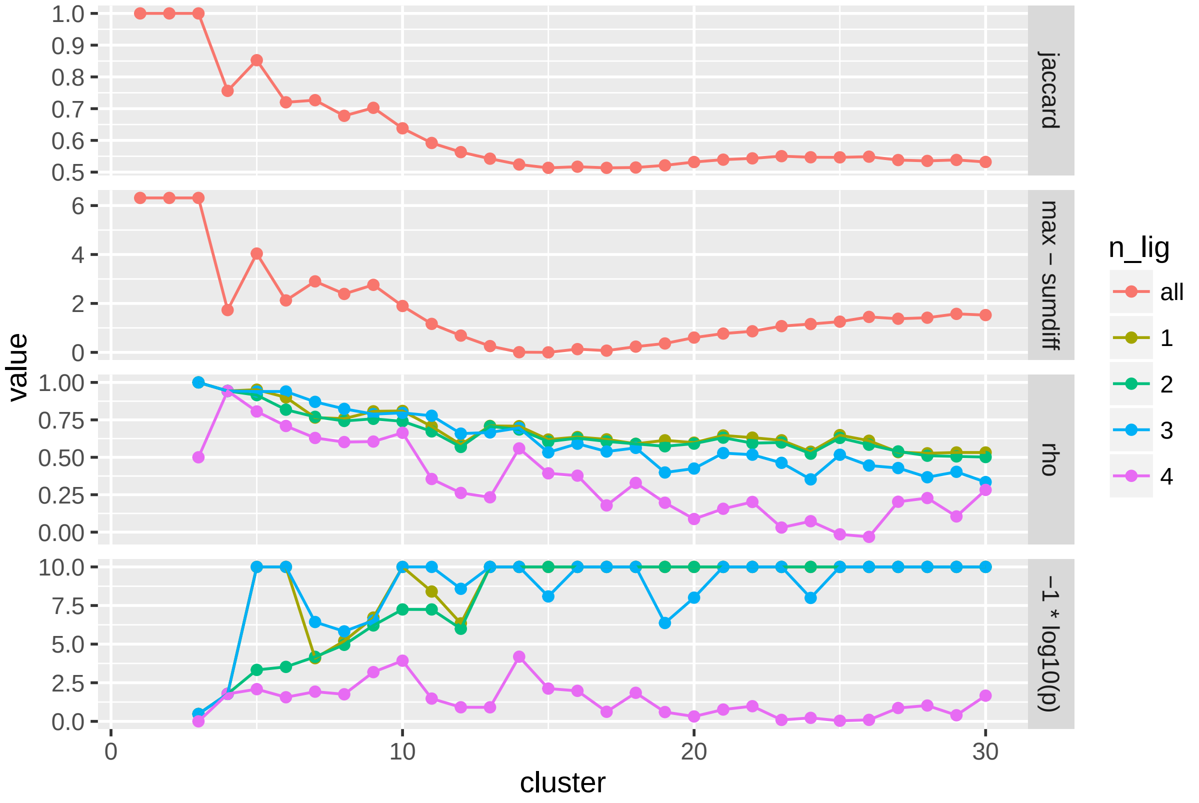

(b) Four measures for compressed group, 6−ligand Mg

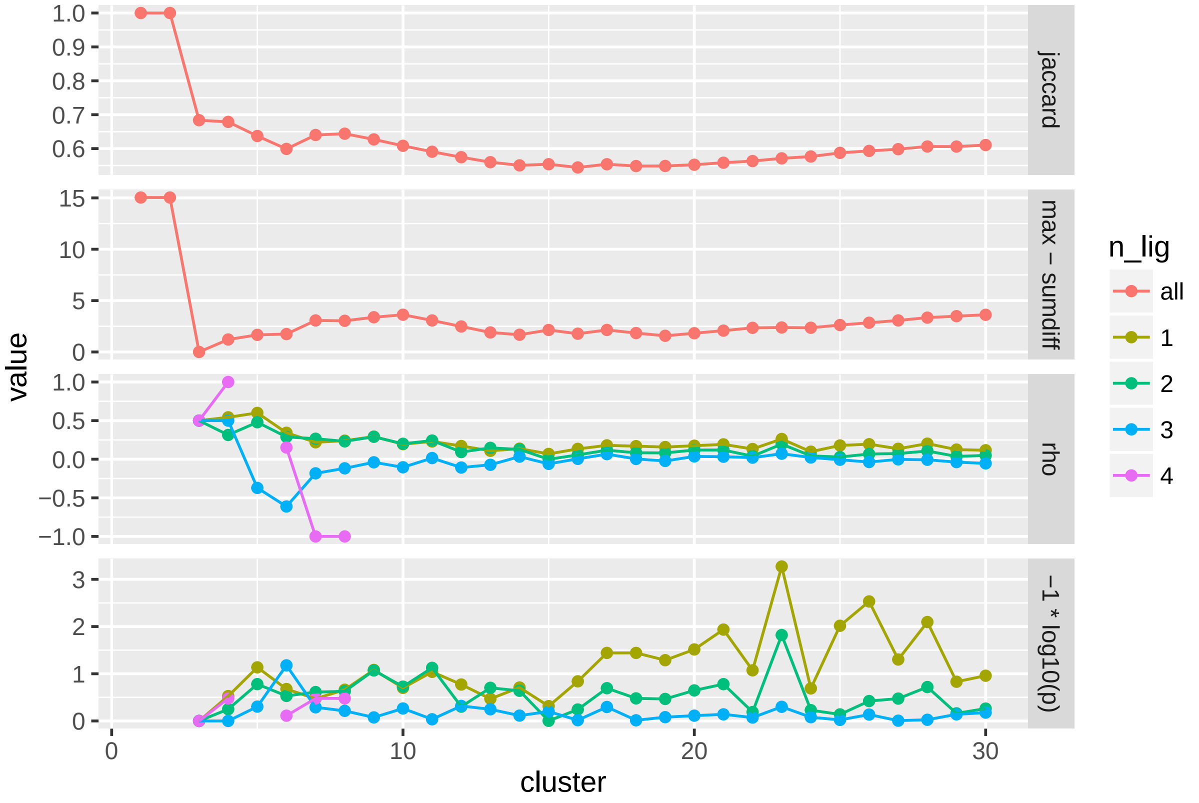

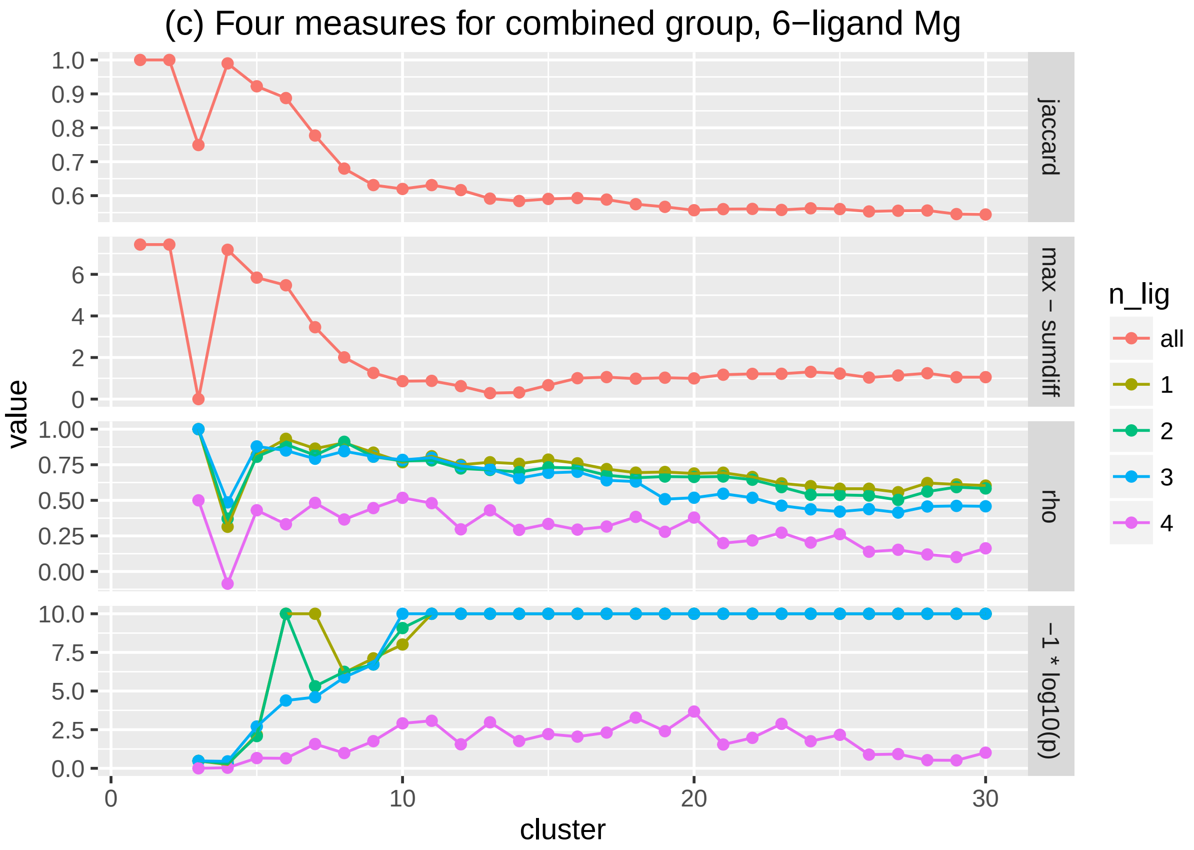
(c) Four measures for combined group, 6−ligand Mg
value
cluster
jaccard
max − sumdiff
rho
−1 * log10(p)
n_lig
all
1
2
3
4

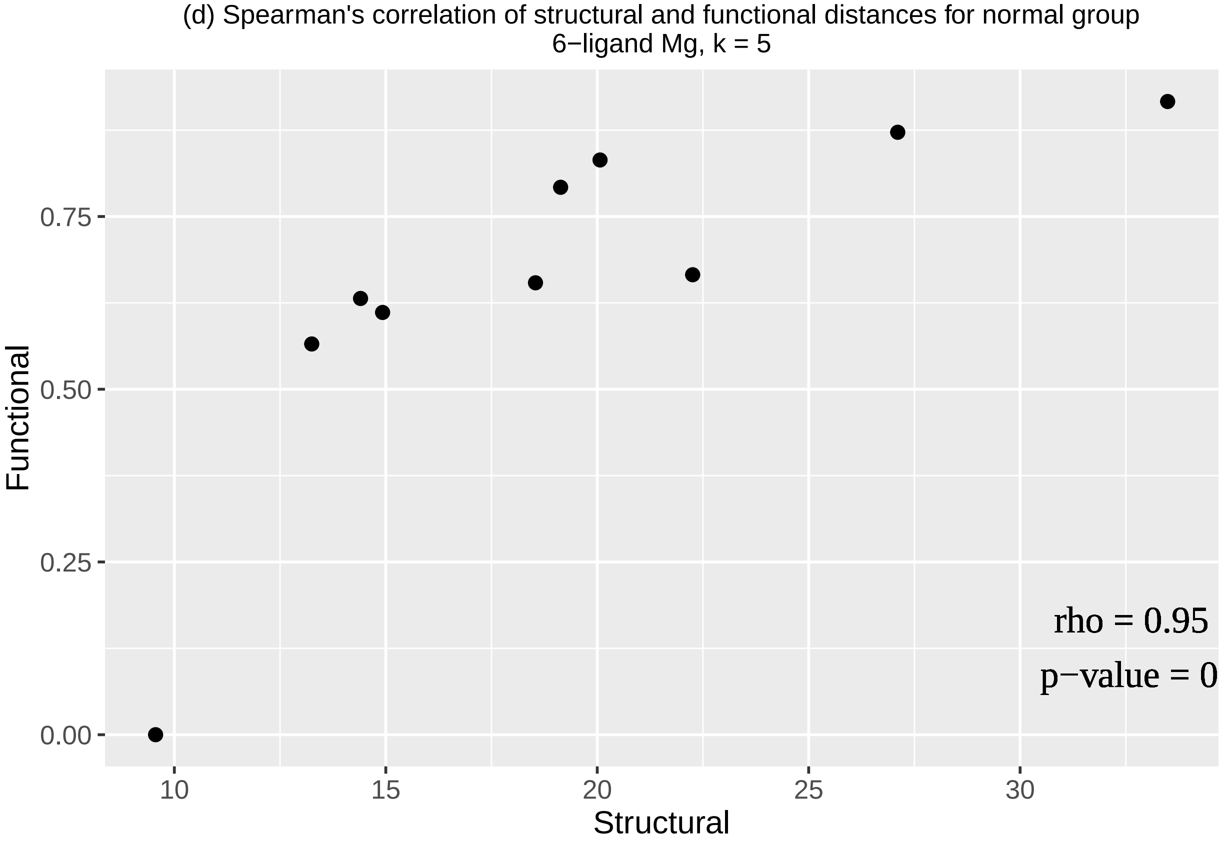
(d) Spearman's correlation of structural and functional distances for normal group
6−ligand Mg, k = 5
Functional
Structural
rho = 0.95
p−value = 0

(e) Comparison between structural and functional hierarchical dendrograms for normal group 6-ligand Mg

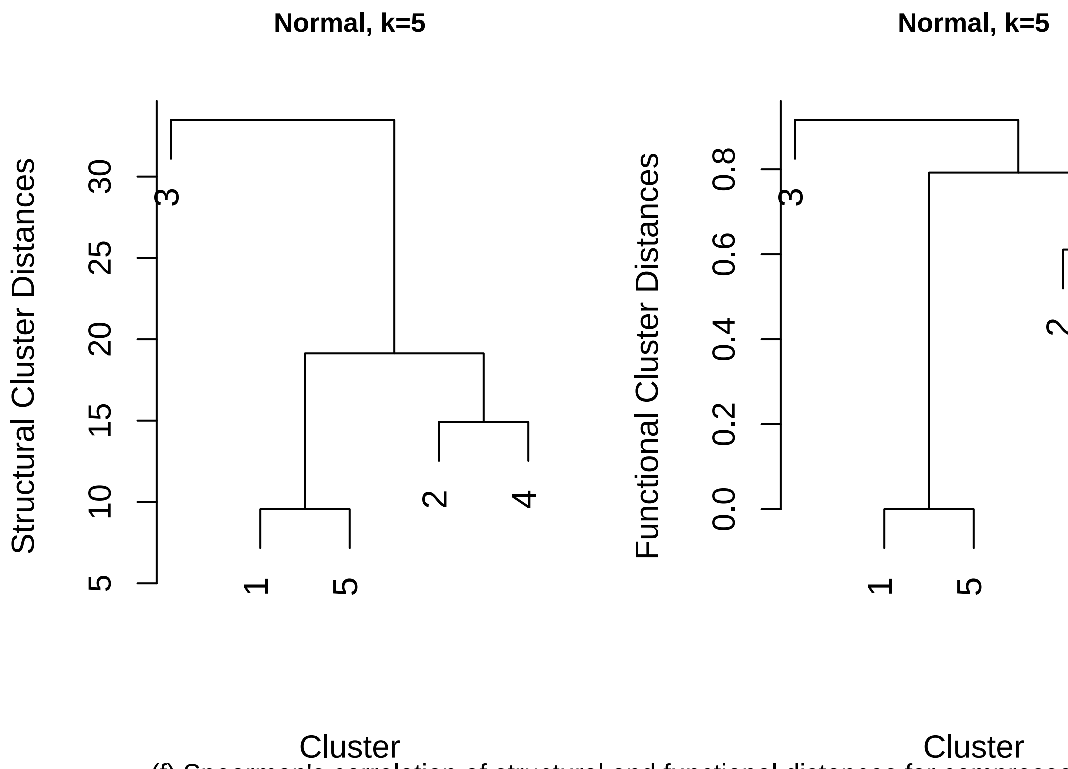

(f) Spearman's correlation of structural and functional distances for compressed group 6−ligand Mg, k = 8

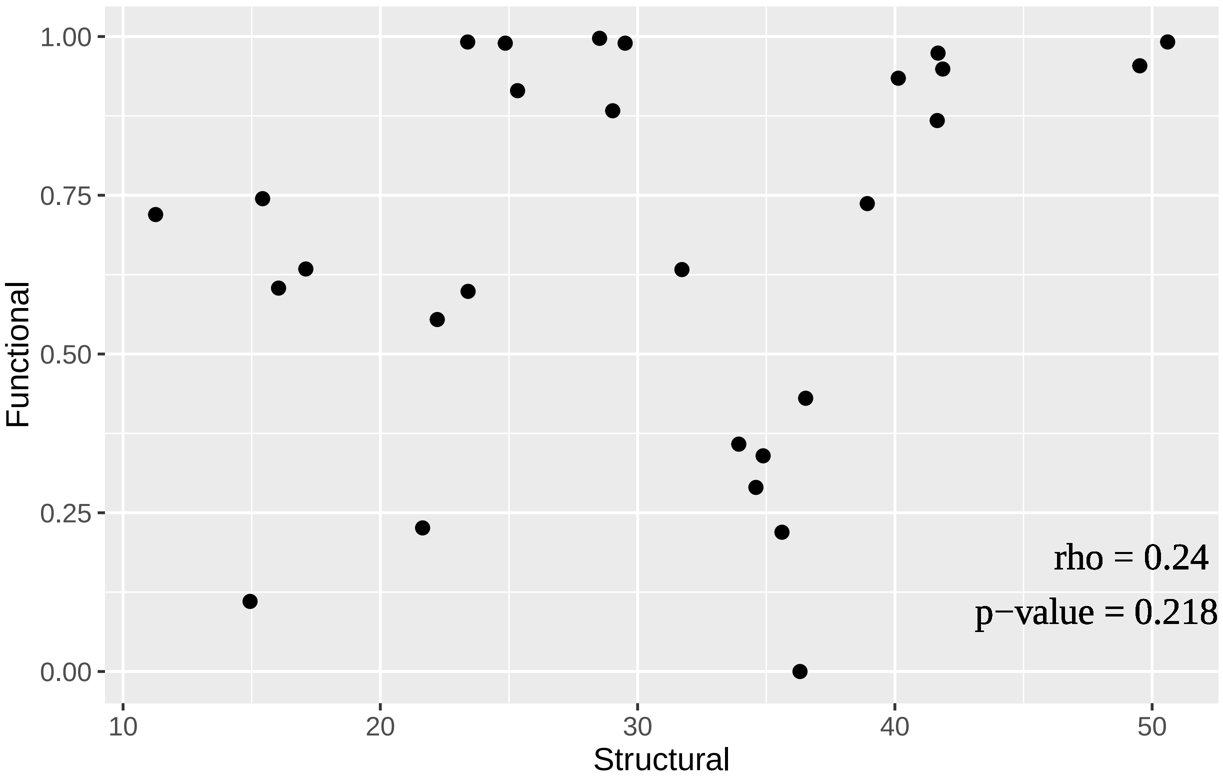

(g) Comparison between structural and functional hierarchical dendrograms for compressed group 6-ligand Mg

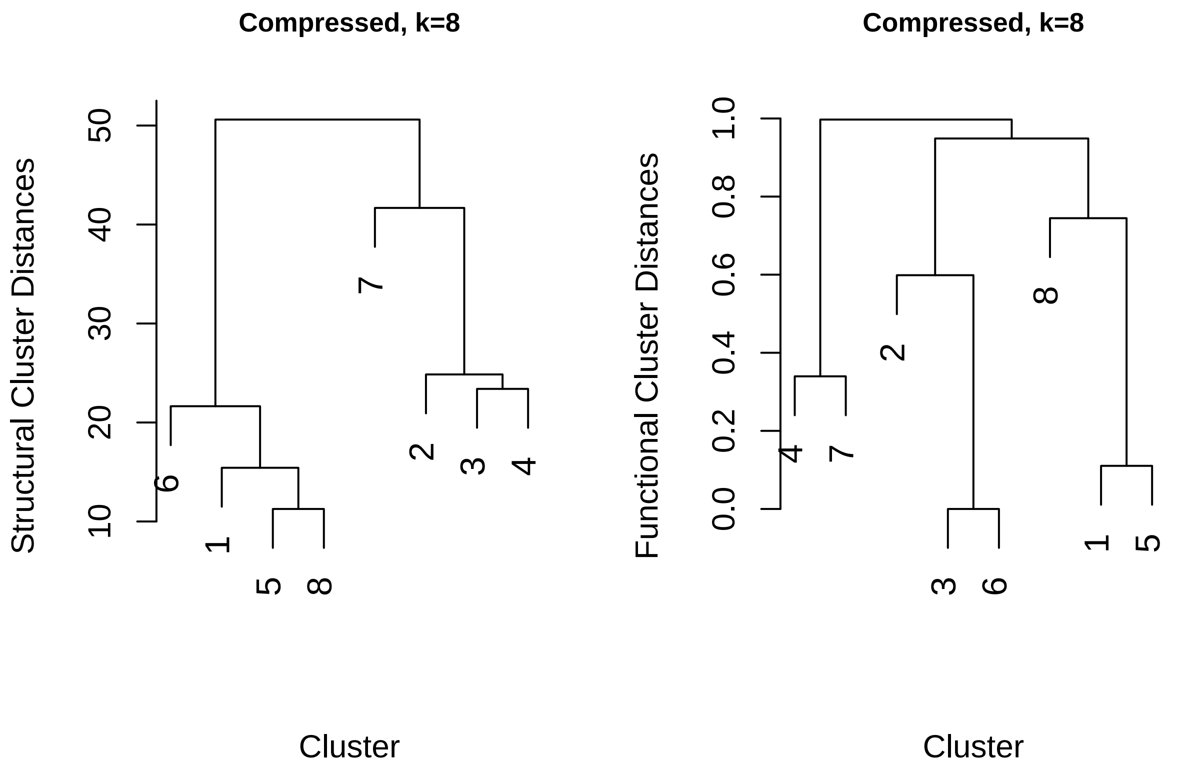

Figure S8. all-ligand Mg metalloproteins

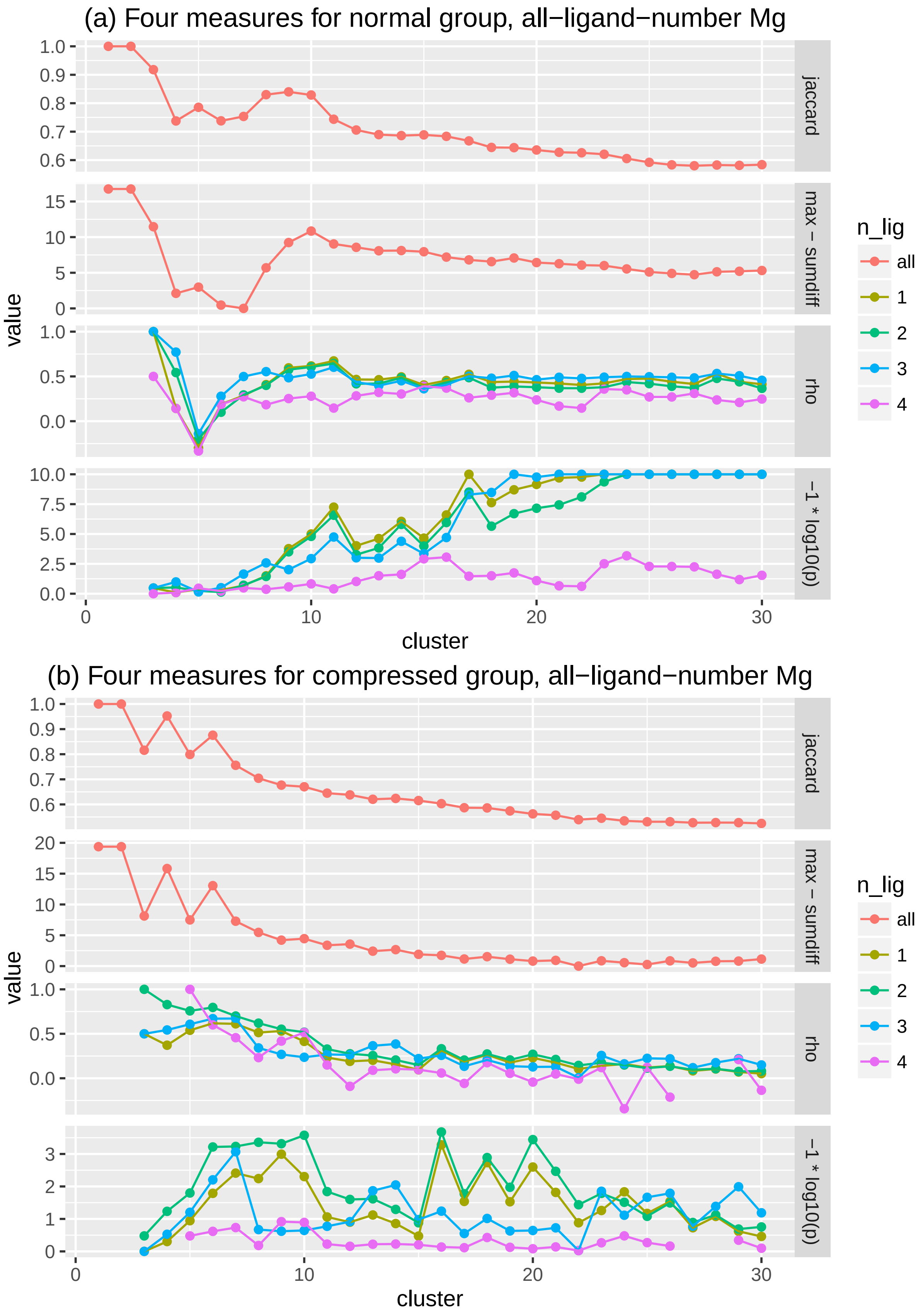
(a) Four measures for normal group, all−ligand−number Mg
jaccard
max − sumdiff
rho
−1 * log10(p)
value
cluster
n_lig
all
1
2
3
4
(b) Four measures for compressed group, all−ligand−number Mg
jaccard
max − sumdiff
rho
−1 * log10(p)
value
cluster
n_lig
all
1
2
3
4

(c) Four measures for combined group, all−ligand−number Mg

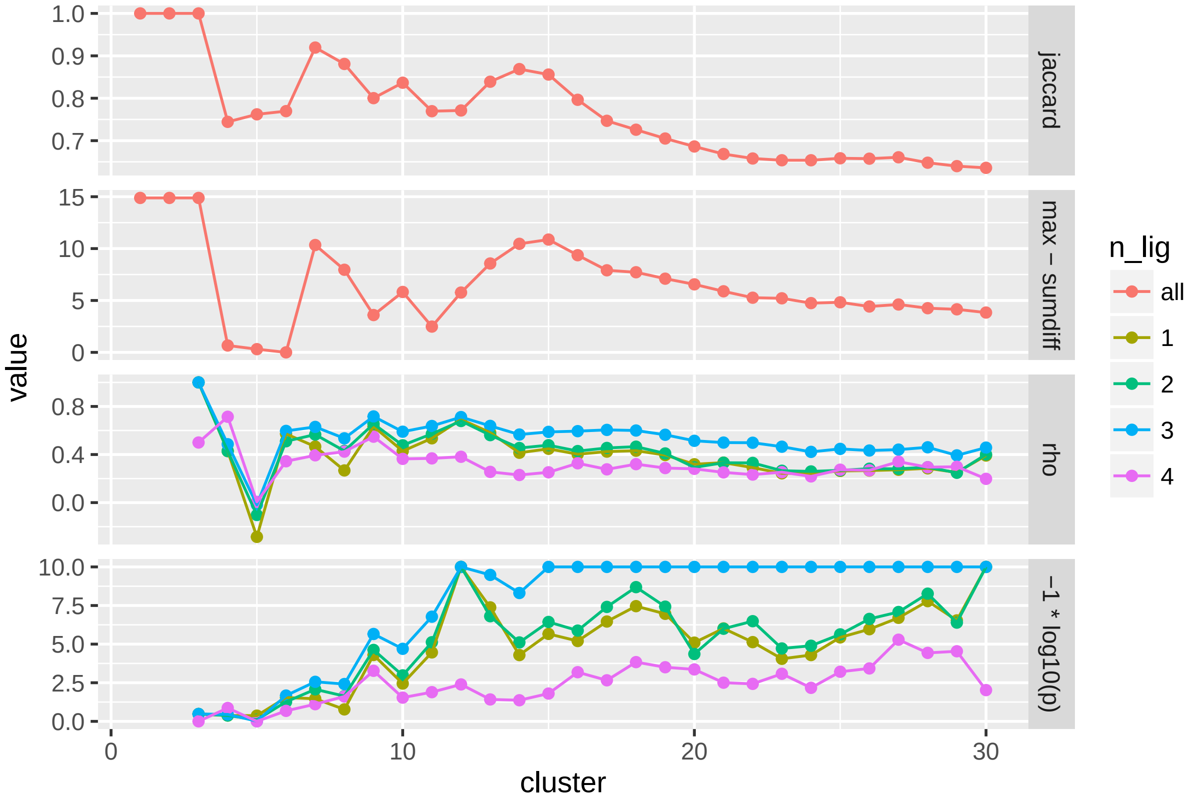

(d) Spearman's correlation of structural and functional distances for normal group
all−ligand−number Mg, k = 11

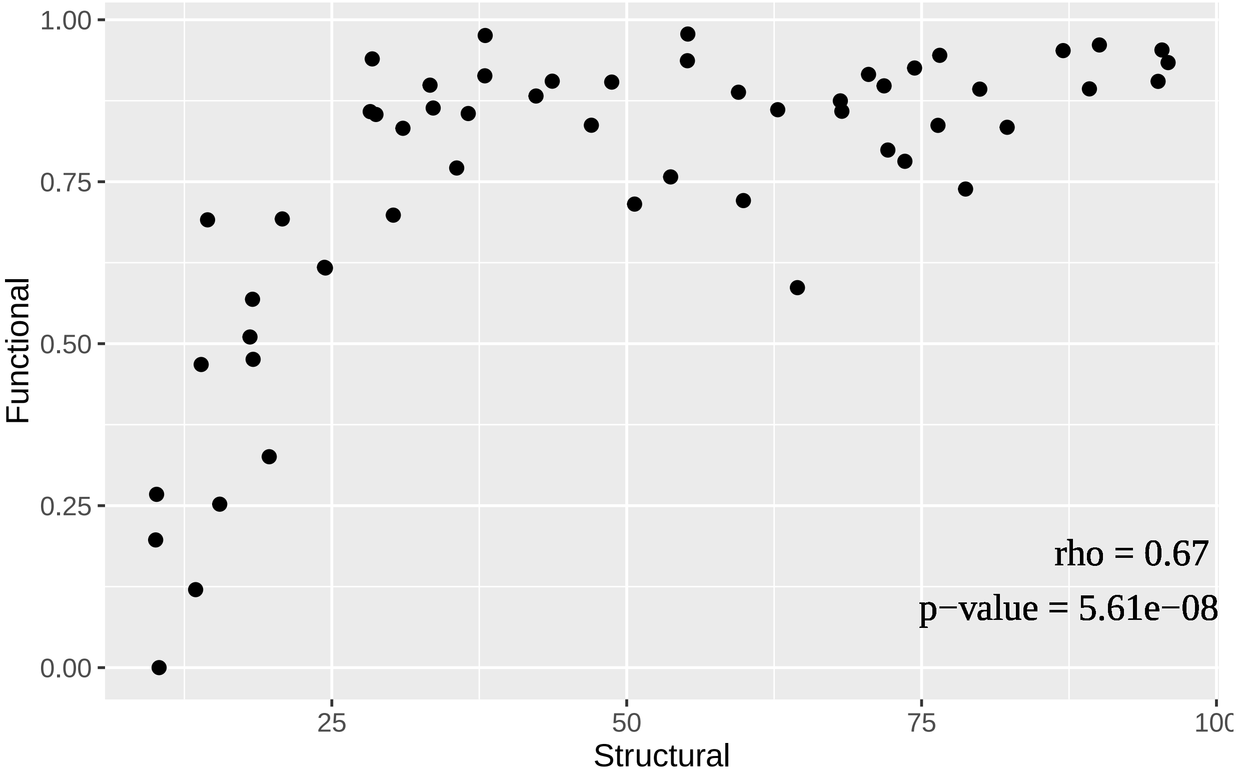

(e) Comparison between structural and functional hierarchical dendrograms for normal group all-ligand-number Mg

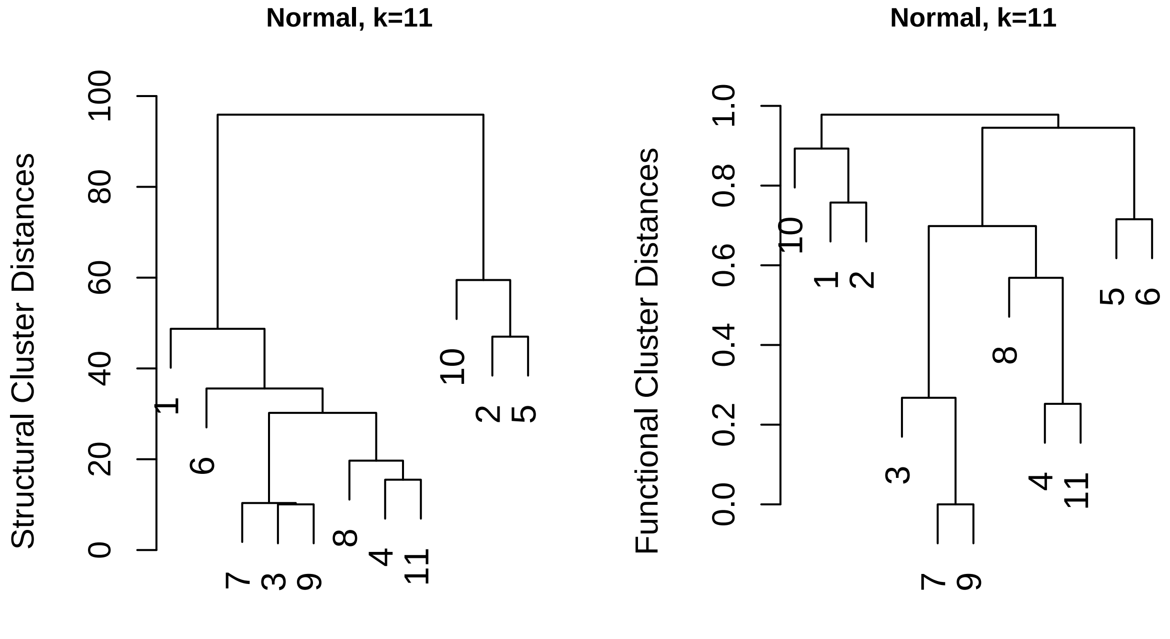

(f) Spearman's correlation of structural and functional distances for compressed group all−ligand−number Mg, k = 6

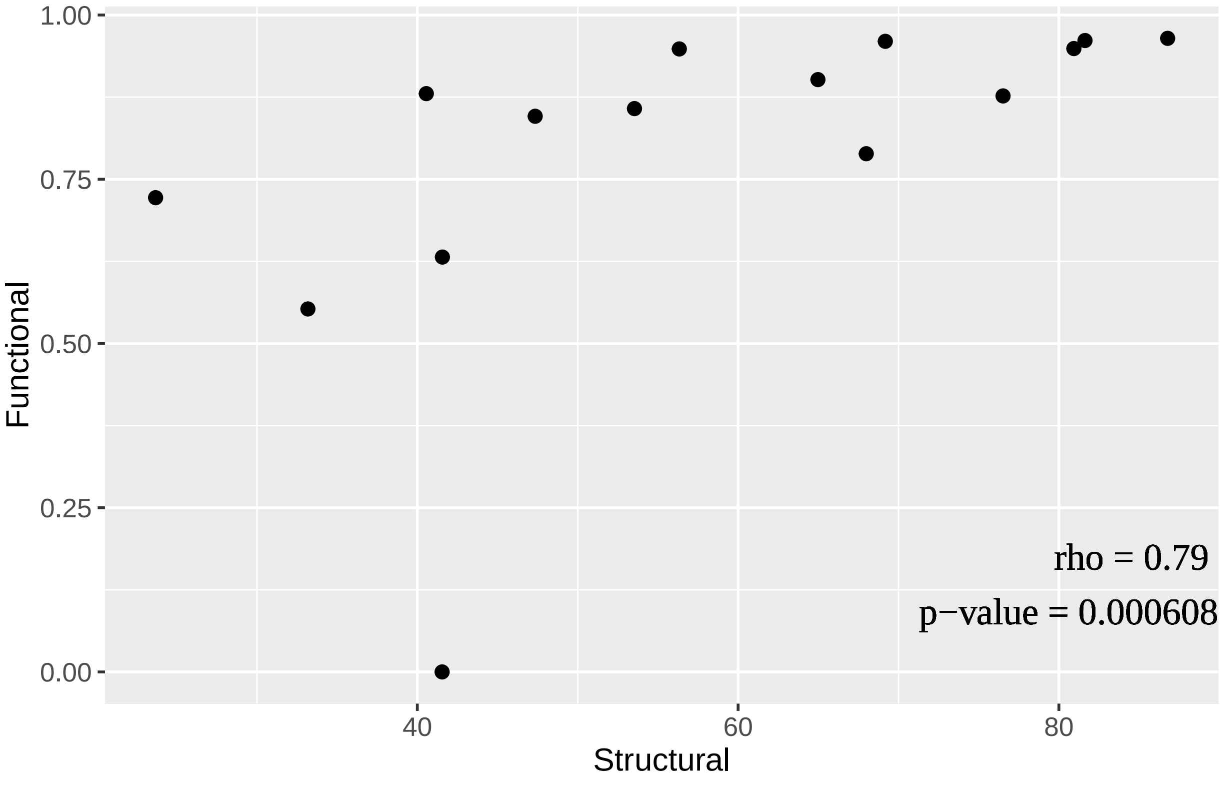

(g) Comparison between structural and functional hierarchical dendrograms for compressed group all-ligand-number Mg

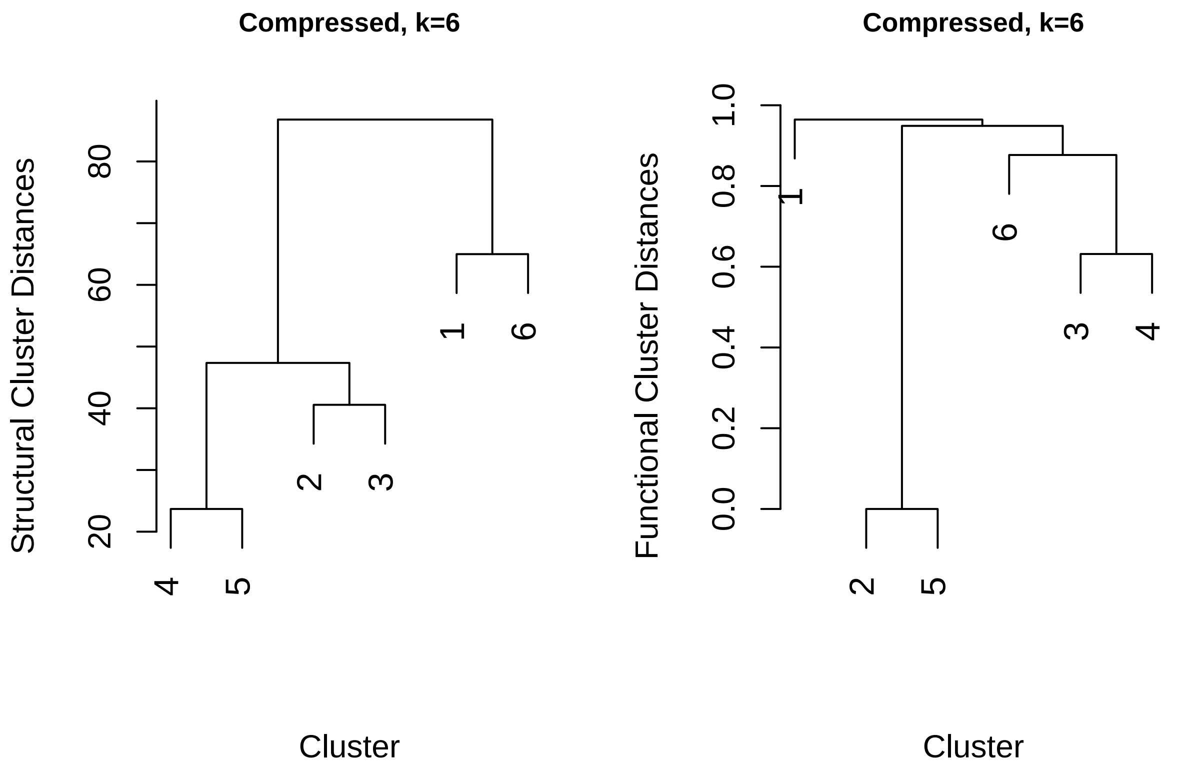

Figure S9. 4-ligand Ca metalloproteins

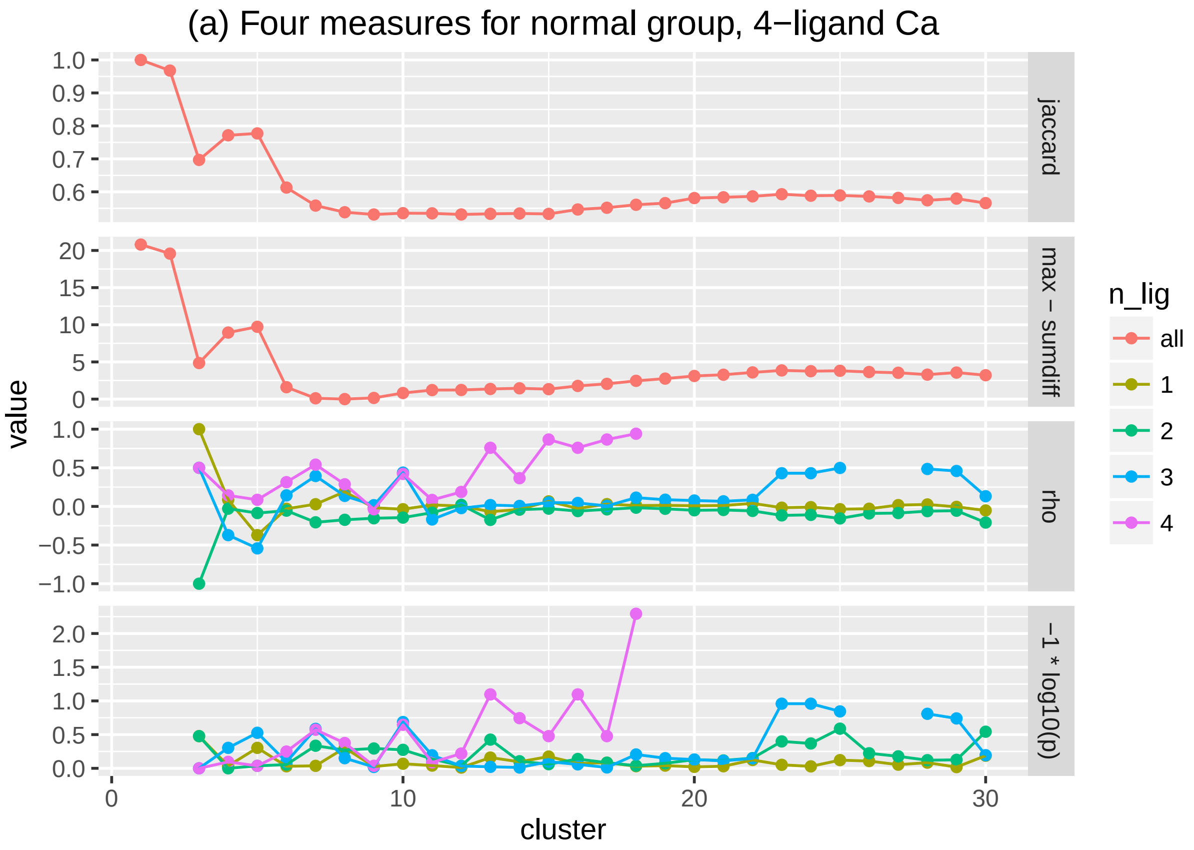
(a) Four measures for normal group, 4−ligand Ca
jaccard
max − sumdiff
rho
−1 * log10(p)
value
cluster
n_lig
all
1
2
3
4

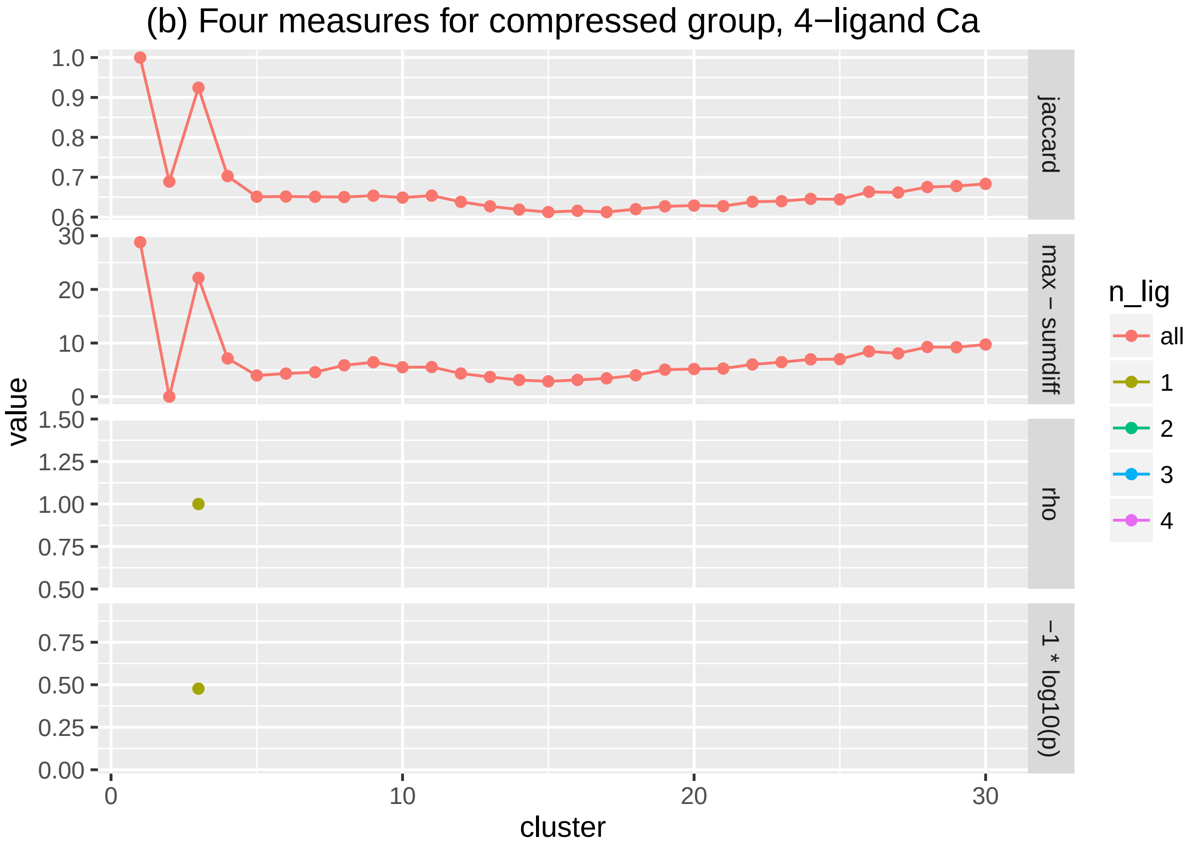
(b) Four measures for compressed group, 4−ligand Ca
jaccard
max − sumdiff
rho
−1 * log10(p)
value
cluster
n_lig
all
1
2
3
4

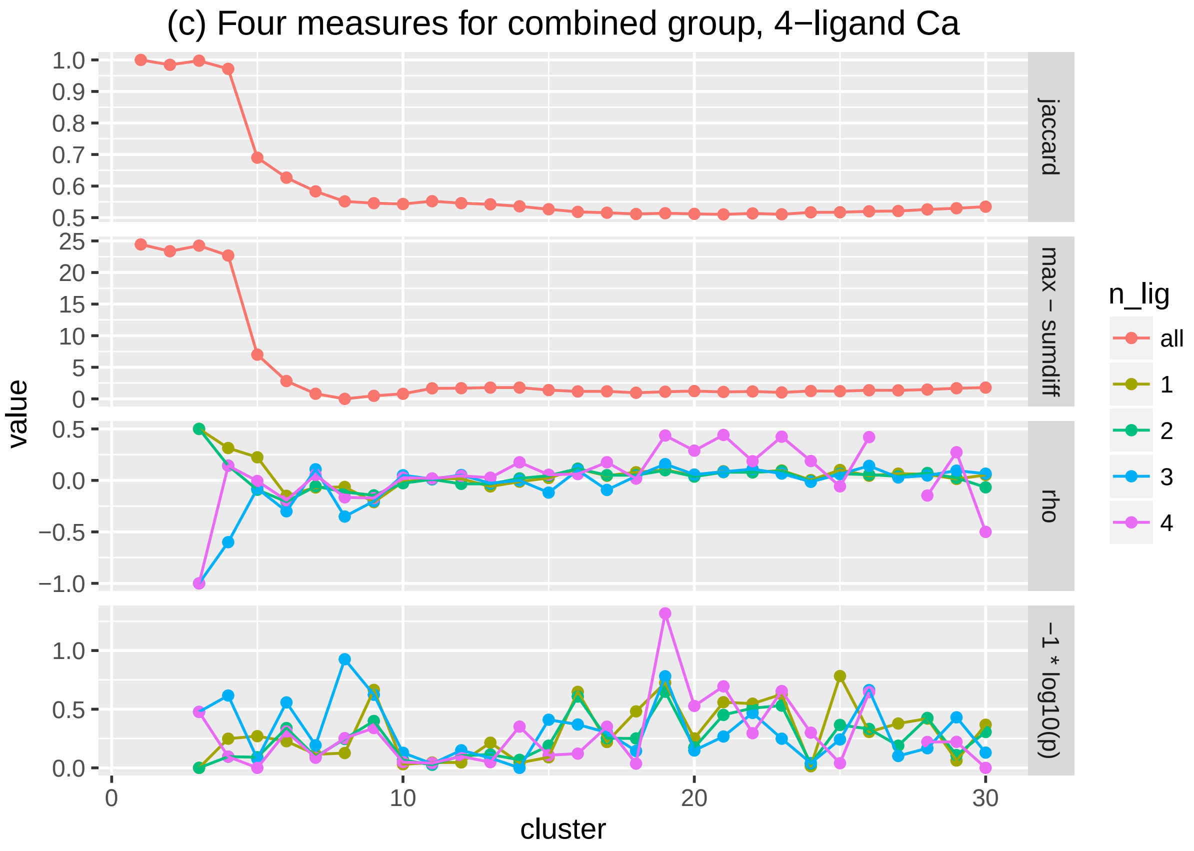
(c) Four measures for combined group, 4−ligand Ca
value
cluster
jaccard
max − sumdiff
rho
−1 * log10(p)
n_lig
all
1
2
3
4

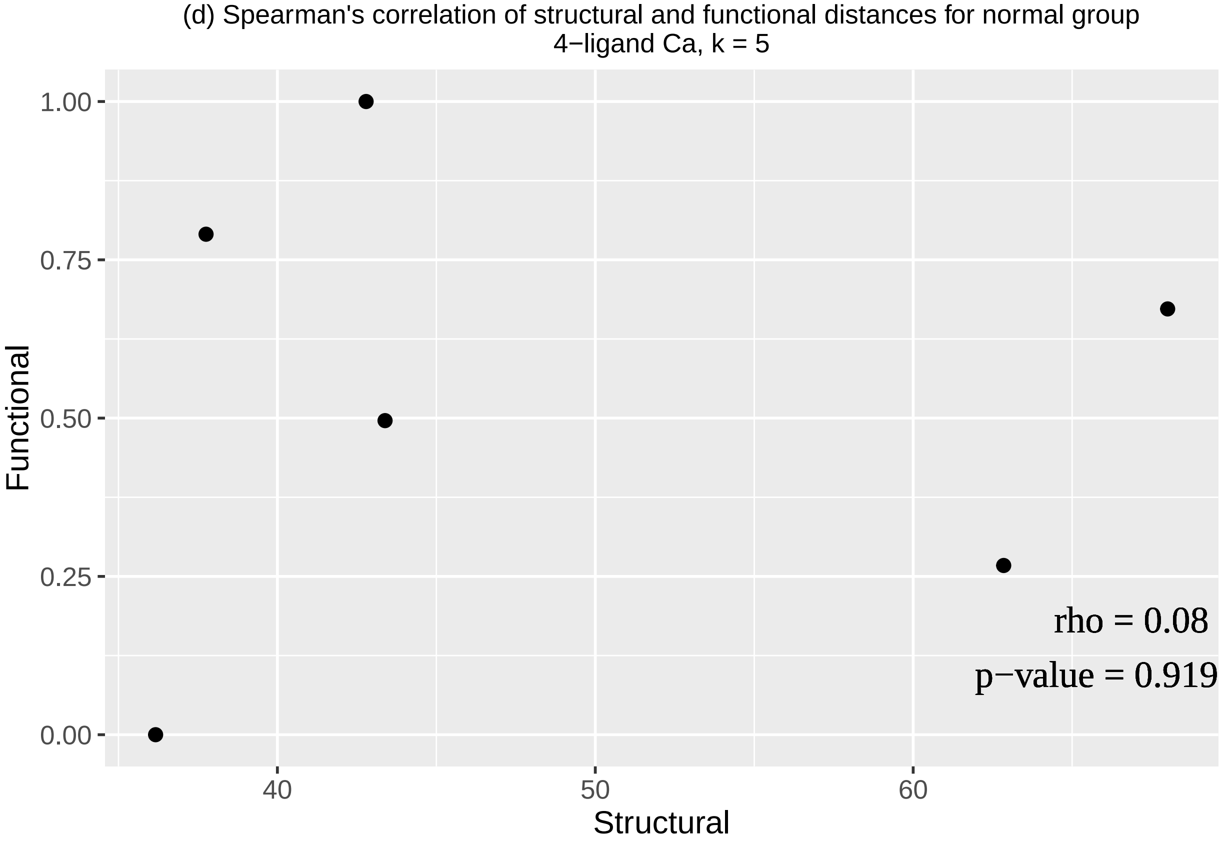
(d) Spearman's correlation of structural and functional distances for normal group
4−ligand Ca, k = 5
Functional
Structural
rho = 0.08
p−value = 0.919

(e) Comparison between structural and functional hierarchical dendrograms for normal group 4-ligand Ca

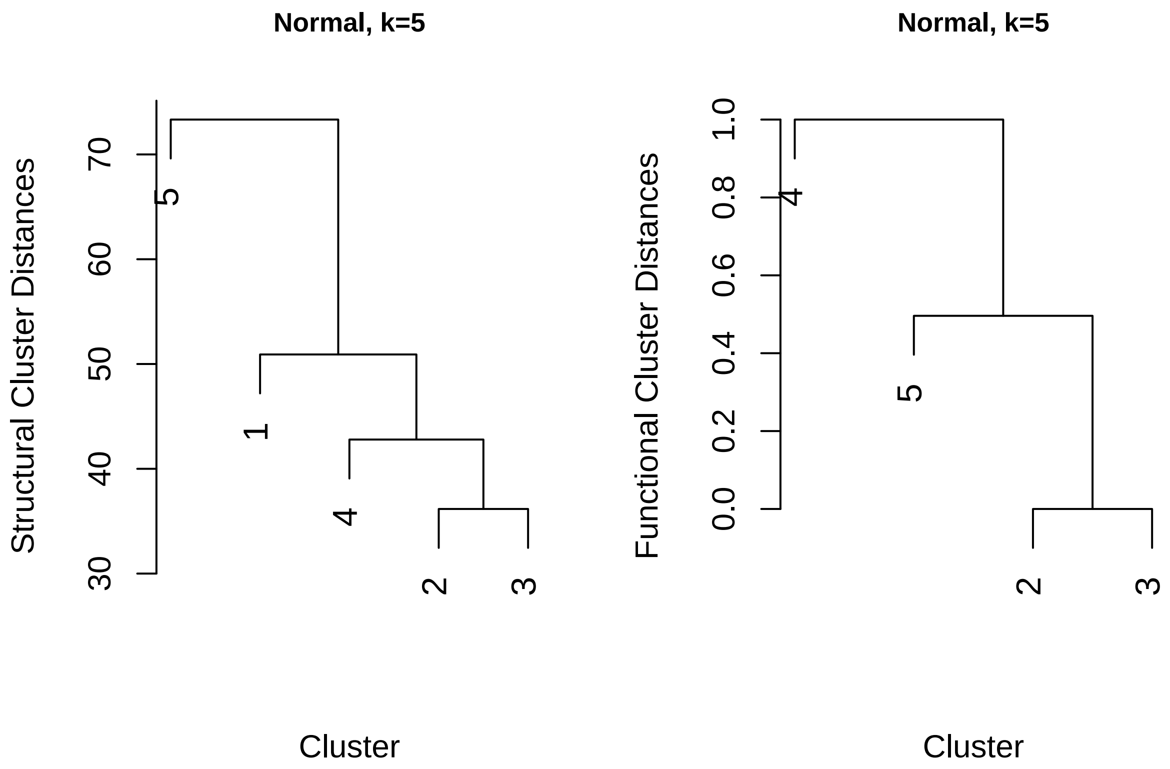

Figure S10. 5-ligand Ca metalloproteins

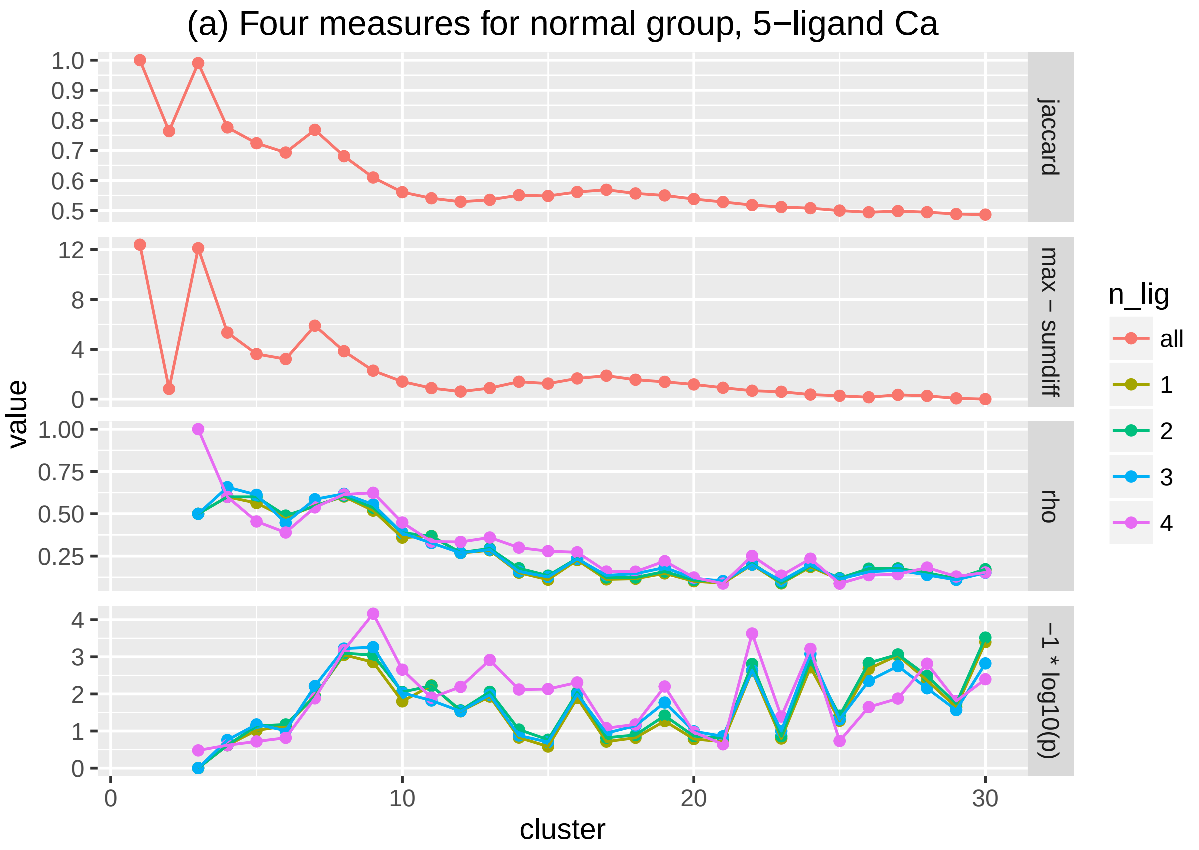
(a) Four measures for normal group, 5−ligand Ca
value
cluster
jaccard
max − sumdiff
rho
−1 * log10(p)
n_lig
all
1
2
3
4

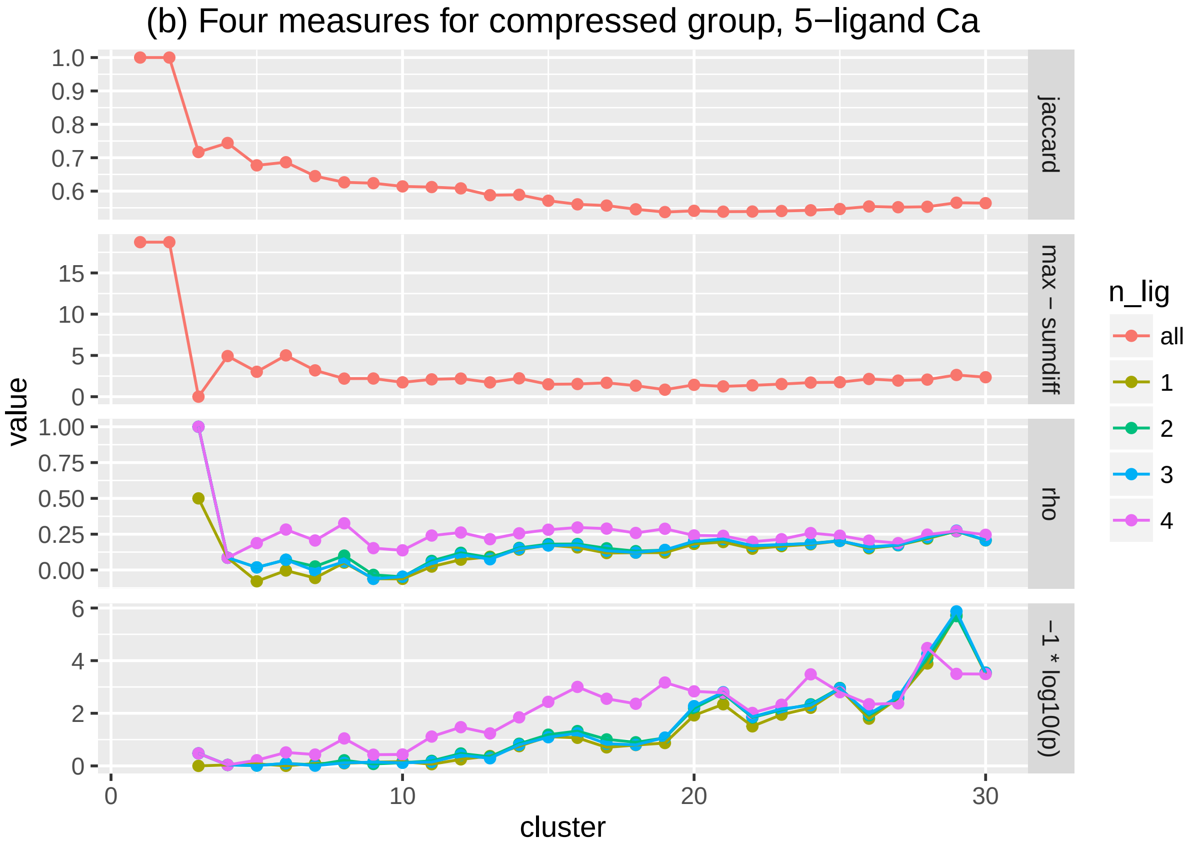
(b) Four measures for compressed group, 5−ligand Ca
value
cluster
jaccard
max − sumdiff
rho
−1 * log10(p)
n_lig
all
1
2
3
4

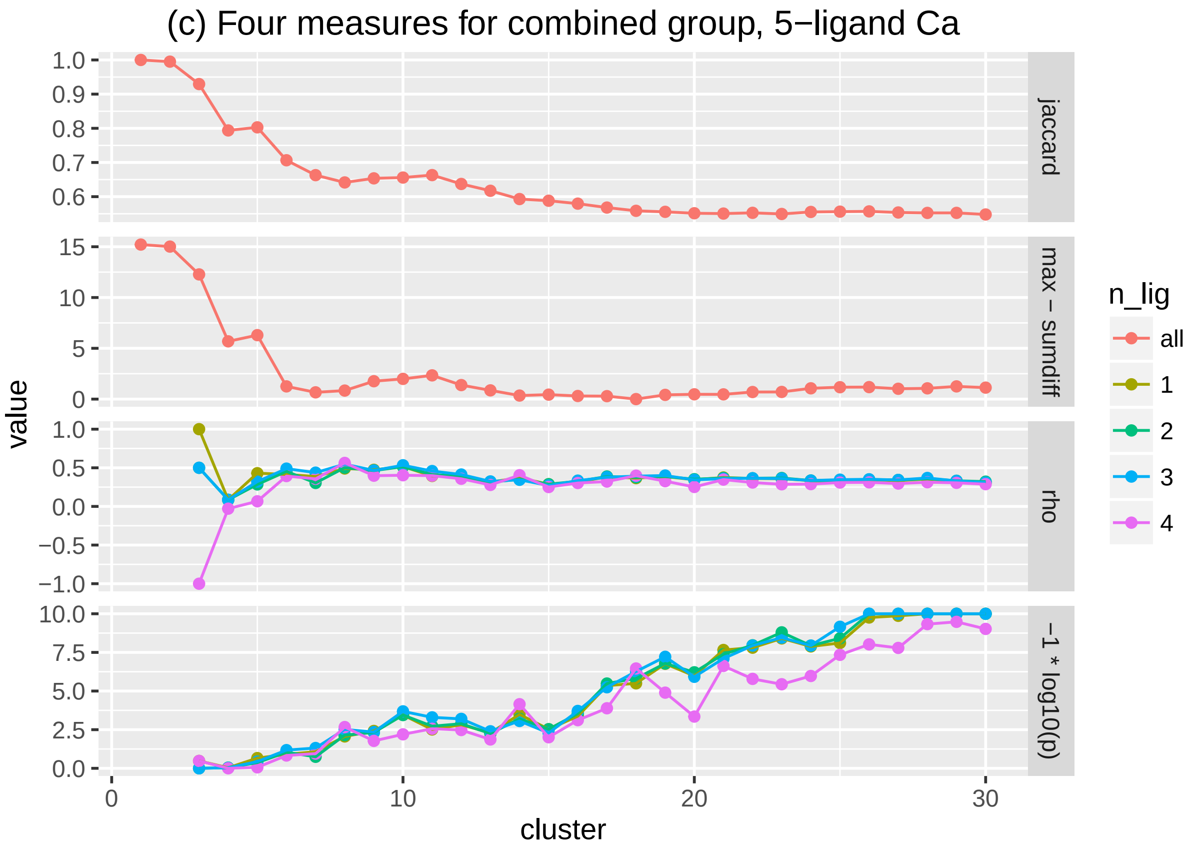
(c) Four measures for combined group, 5−ligand Ca
value
cluster
jaccard
max − sumdiff
rho
−1 * log10(p)
n_lig
all
1
2
3
4

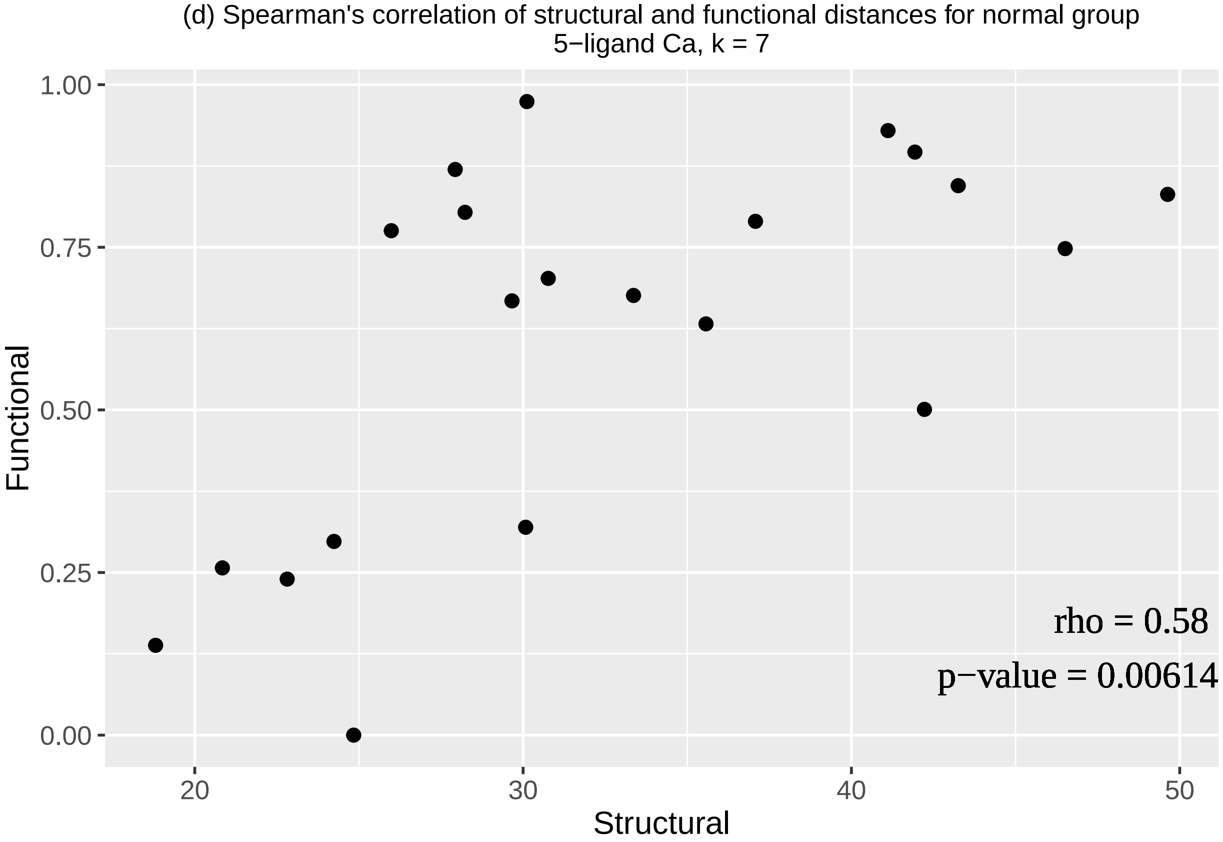
(d) Spearman's correlation of structural and functional distances for normal group
5−ligand Ca, k = 7
Functional
Structural
rho = 0.58
p−value = 0.00614

(e) Comparison between structural and functional hierarchical dendrograms for normal group 5-ligand Ca

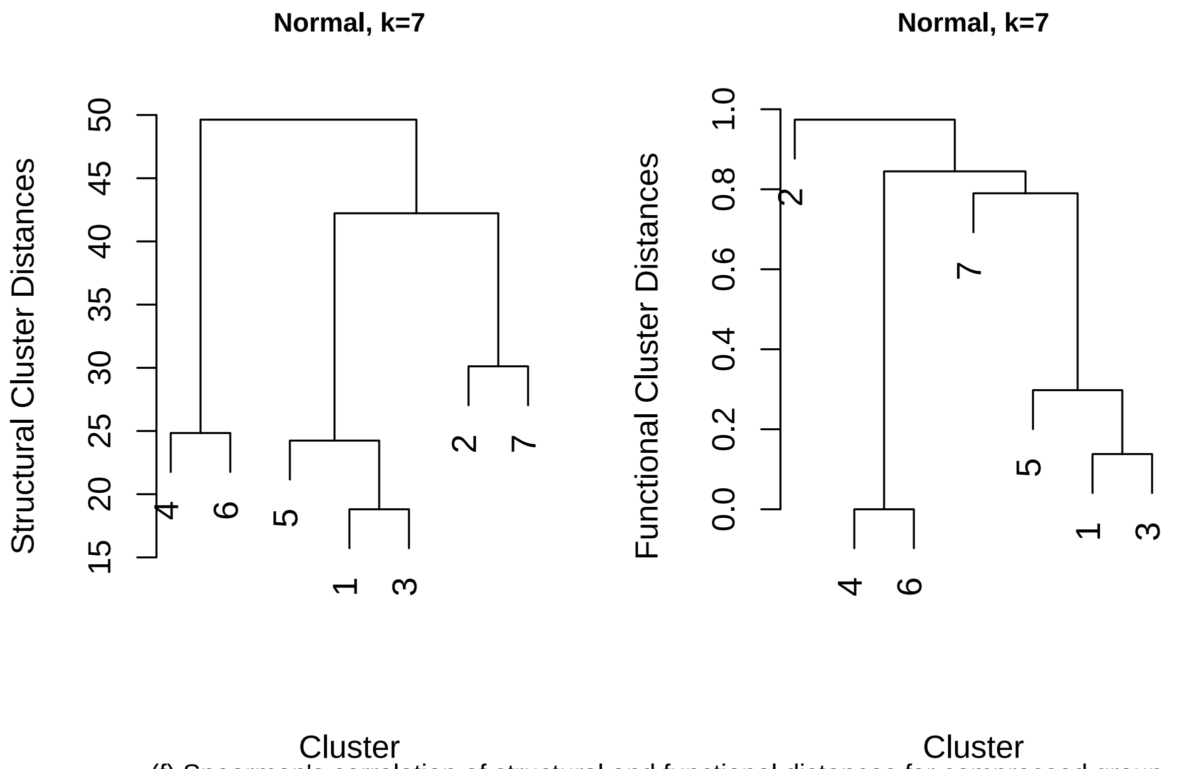

(f) Spearman's correlation of structural and functional distances for compressed group 5−ligand Ca, k = 6

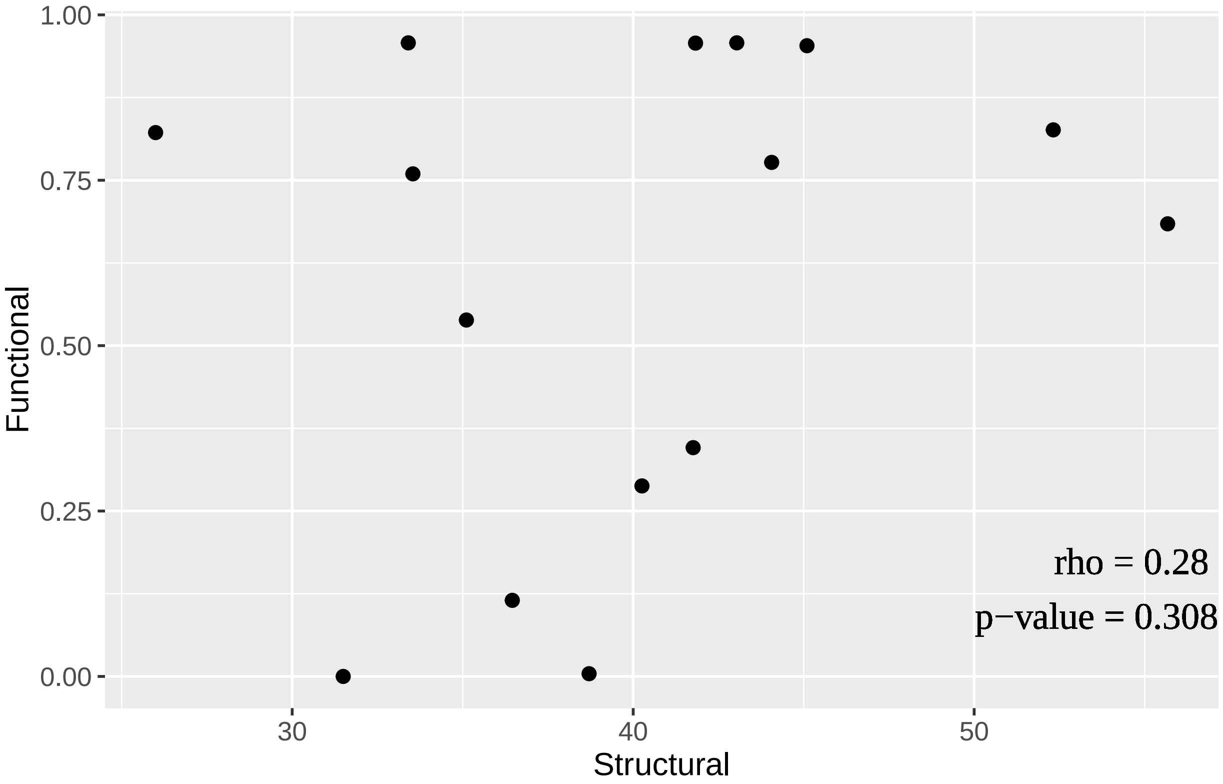

(g) Comparison between structural and functional hierarchical dendrograms for compressed group 5-ligand Ca

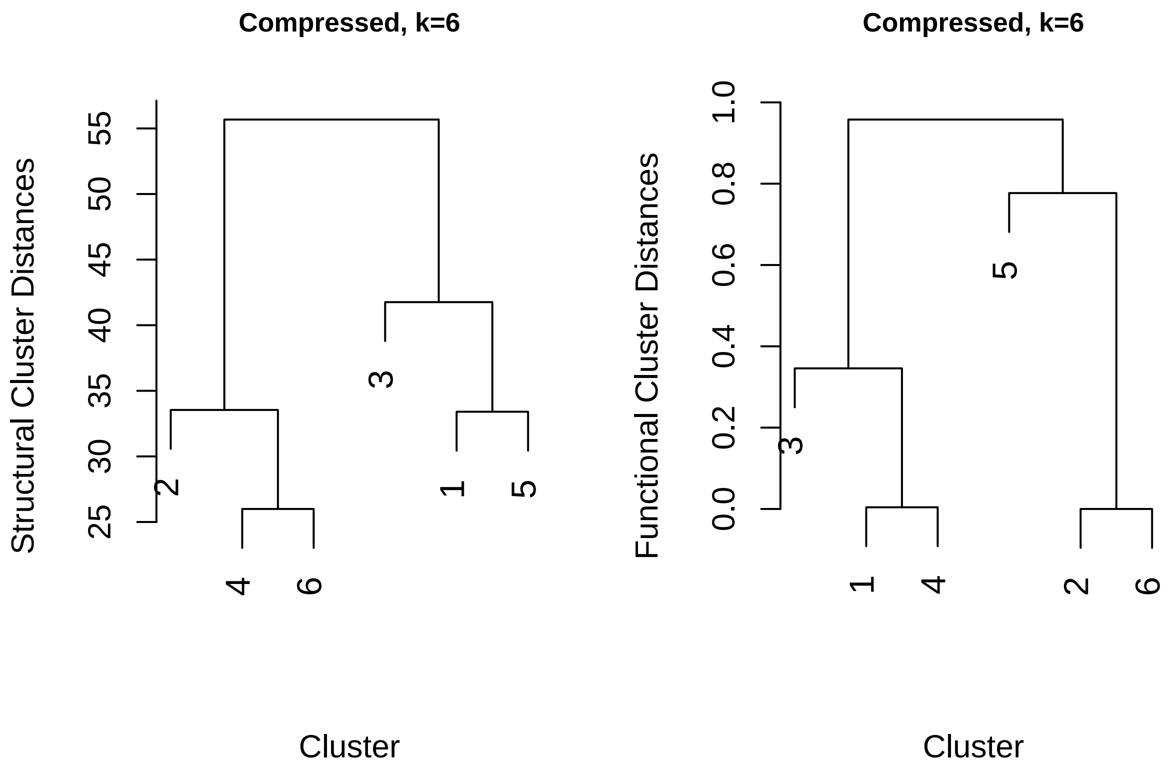

Figure S11. 6-ligand Ca metalloproteins

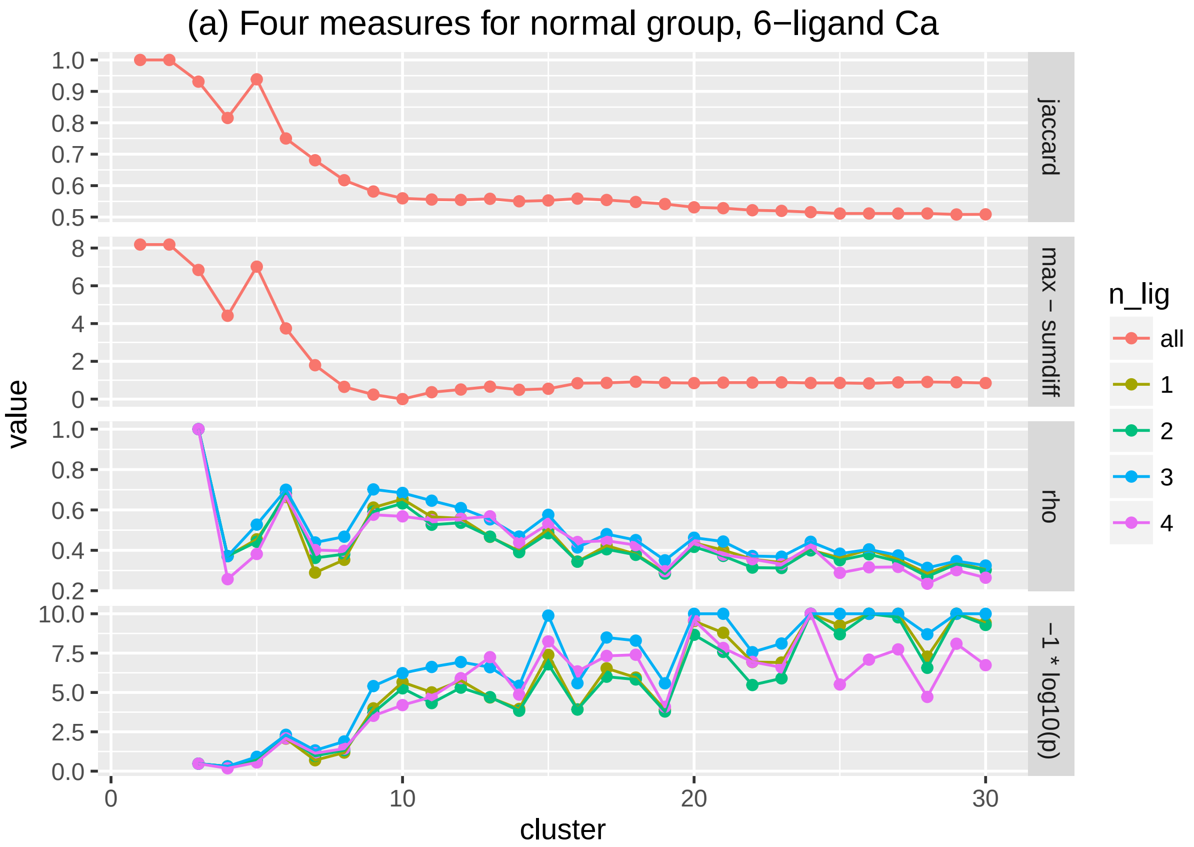
(a) Four measures for normal group, 6−ligand Ca
jaccard
max − sumdiff
rho
−1 * log10(p)
value
cluster
n_lig
all
1
2
3
4

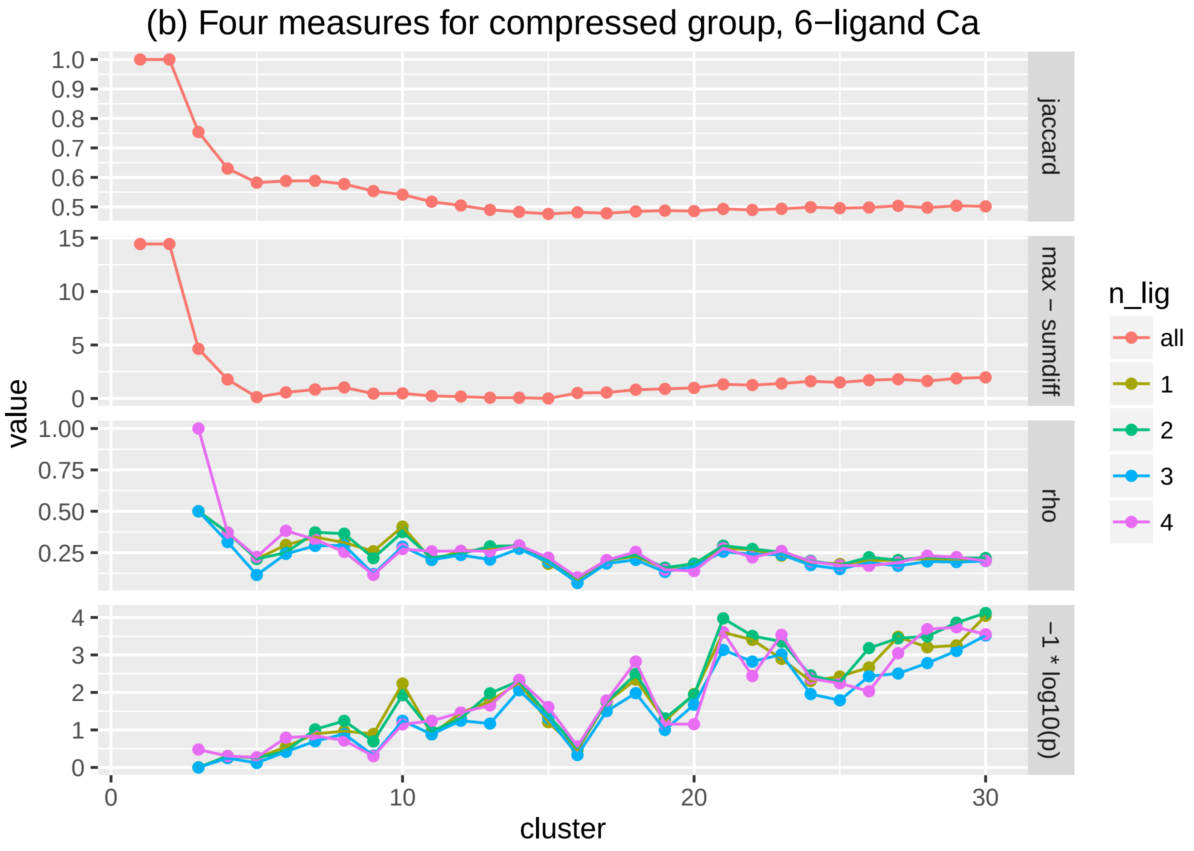
(b) Four measures for compressed group, 6−ligand Ca
jaccard
max − sumdiff
rho
−1 * log10(p)
value
cluster
n_lig
all
1
2
3
4

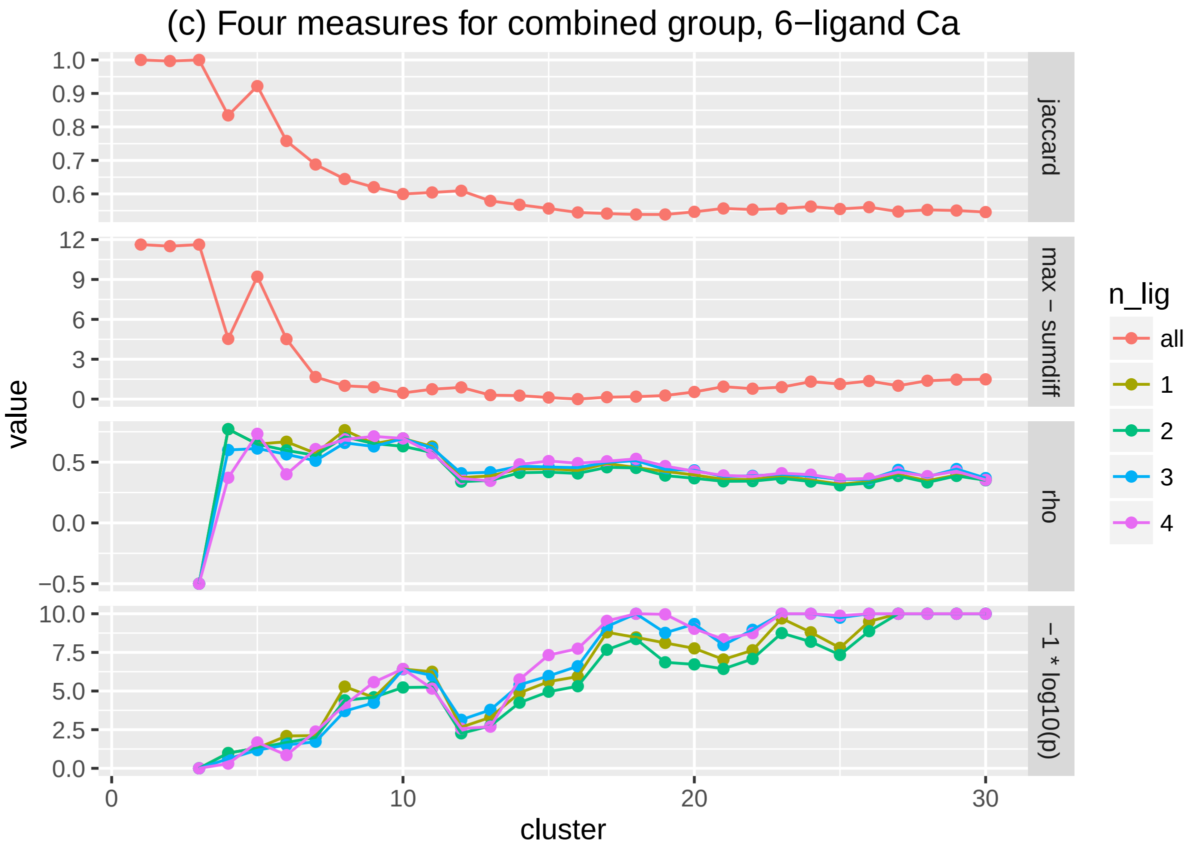
(c) Four measures for combined group, 6−ligand Ca
value
cluster
jaccard
max − sumdiff
rho
−1 * log10(p)
n_lig
all
1
2
3
4

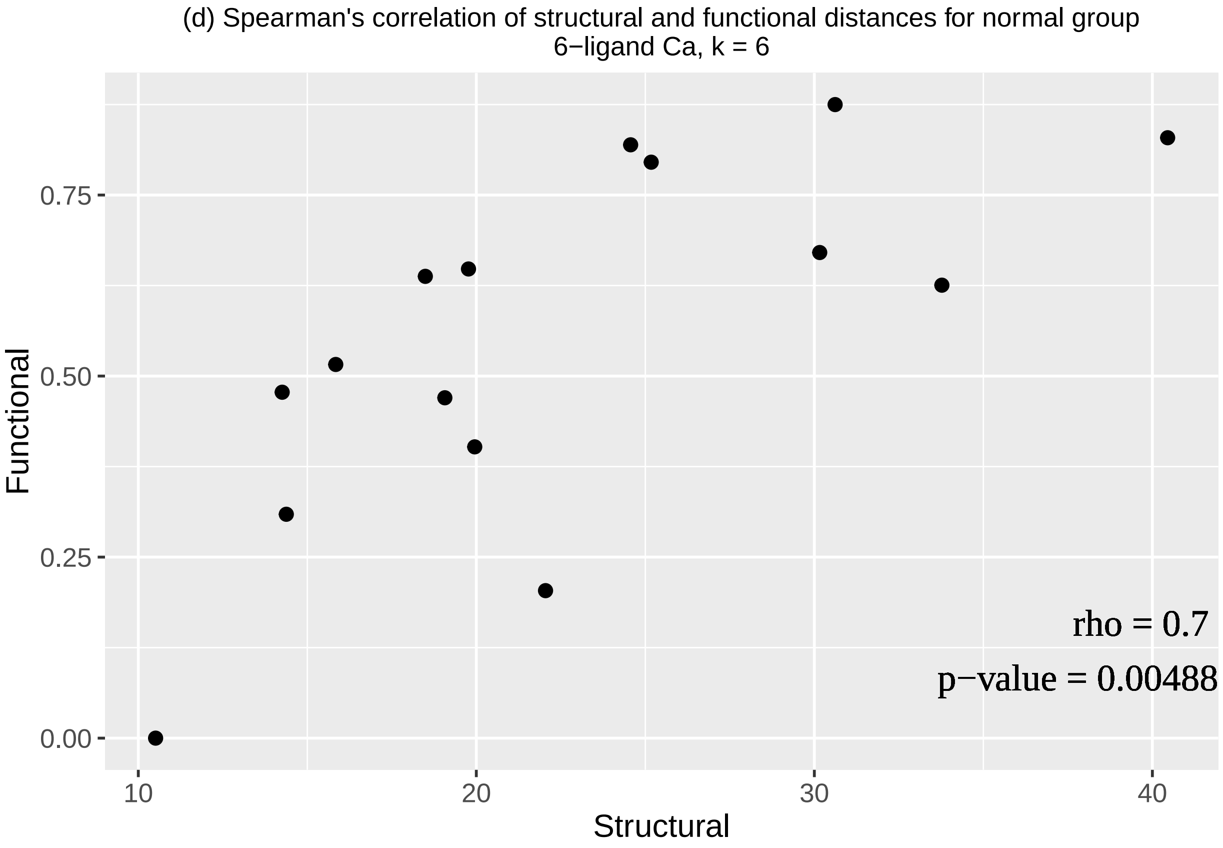
(d) Spearman's correlation of structural and functional distances for normal group
6−ligand Ca, k = 6
Functional
Structural
rho = 0.7
p−value = 0.00488

(e) Comparison between structural and functional hierarchical dendrograms for normal group 6-ligand Ca

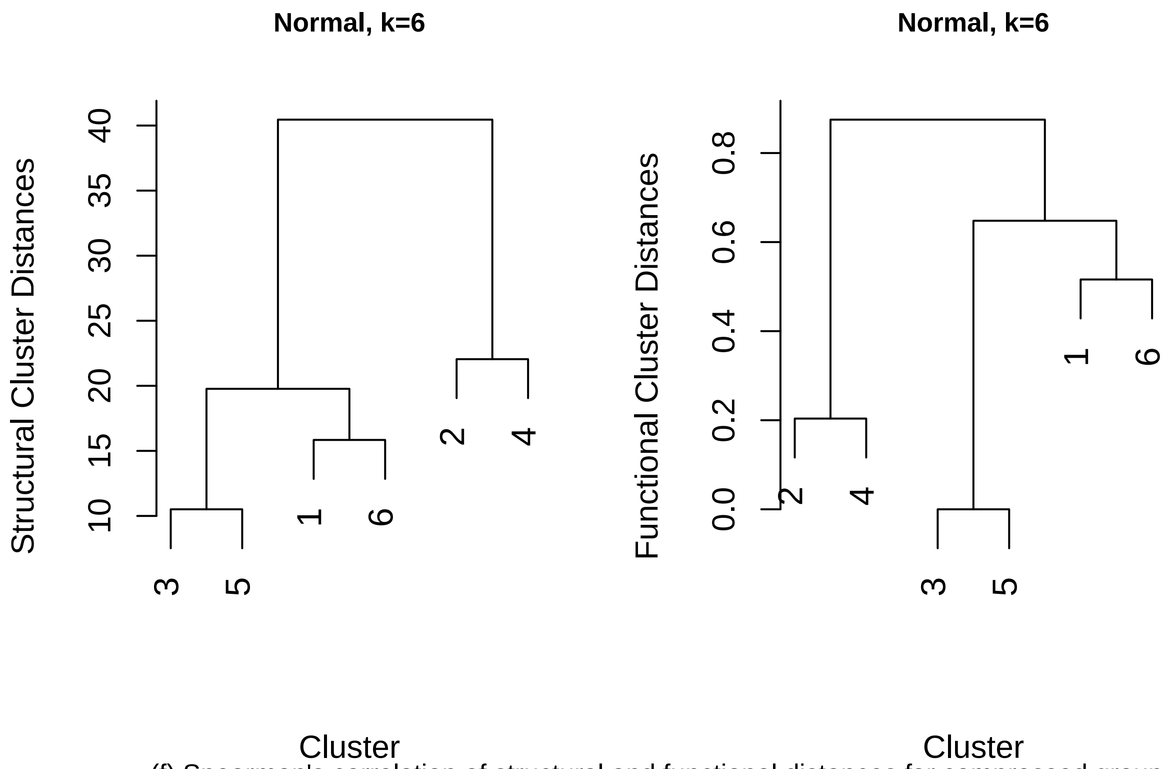

(f) Spearman's correlation of structural and functional distances for compressed group 6−ligand Ca, k = 8

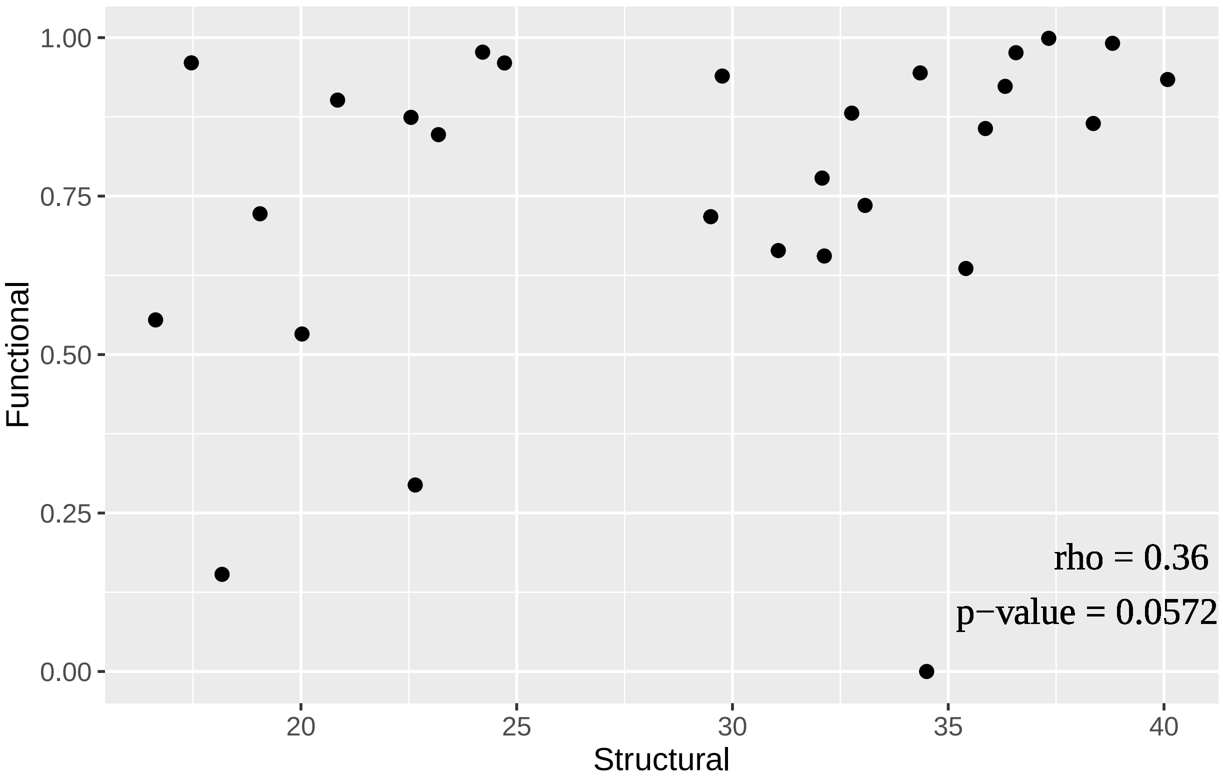

(g) Comparison between structural and functional hierarchical dendrograms for compressed group 6-ligand Ca

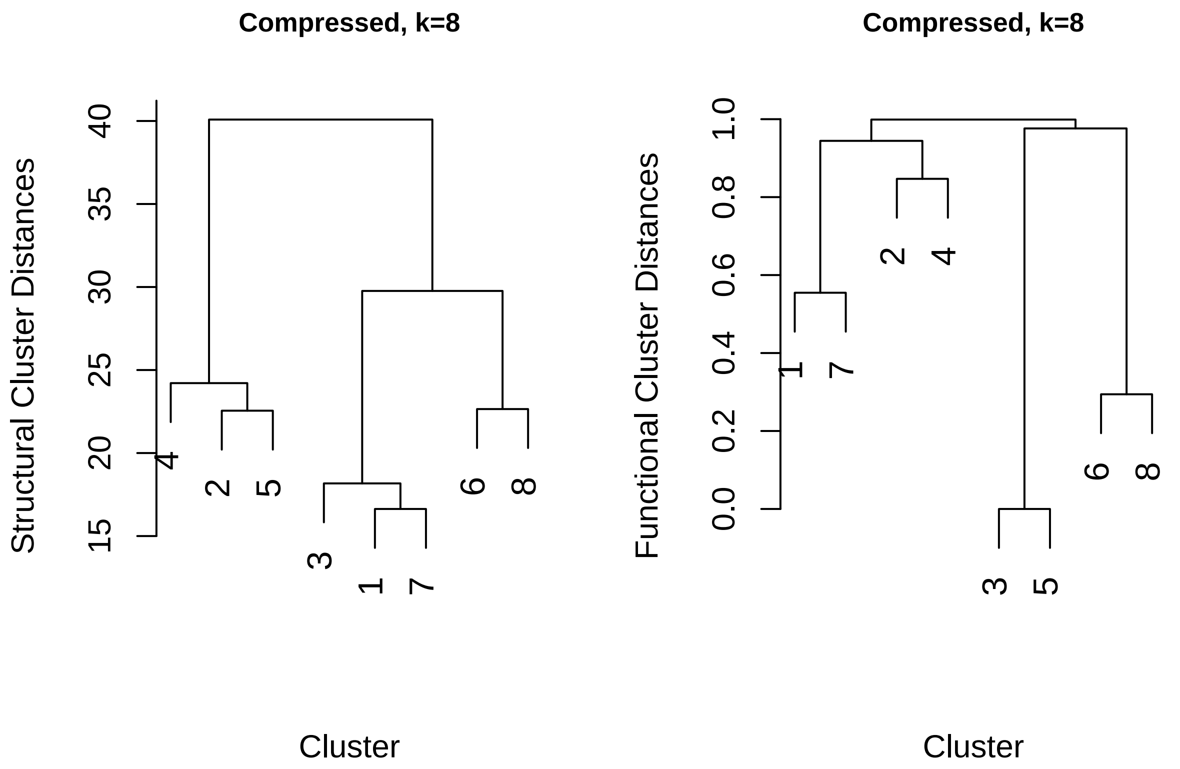

Figure S12. 7-ligand Ca metalloproteins

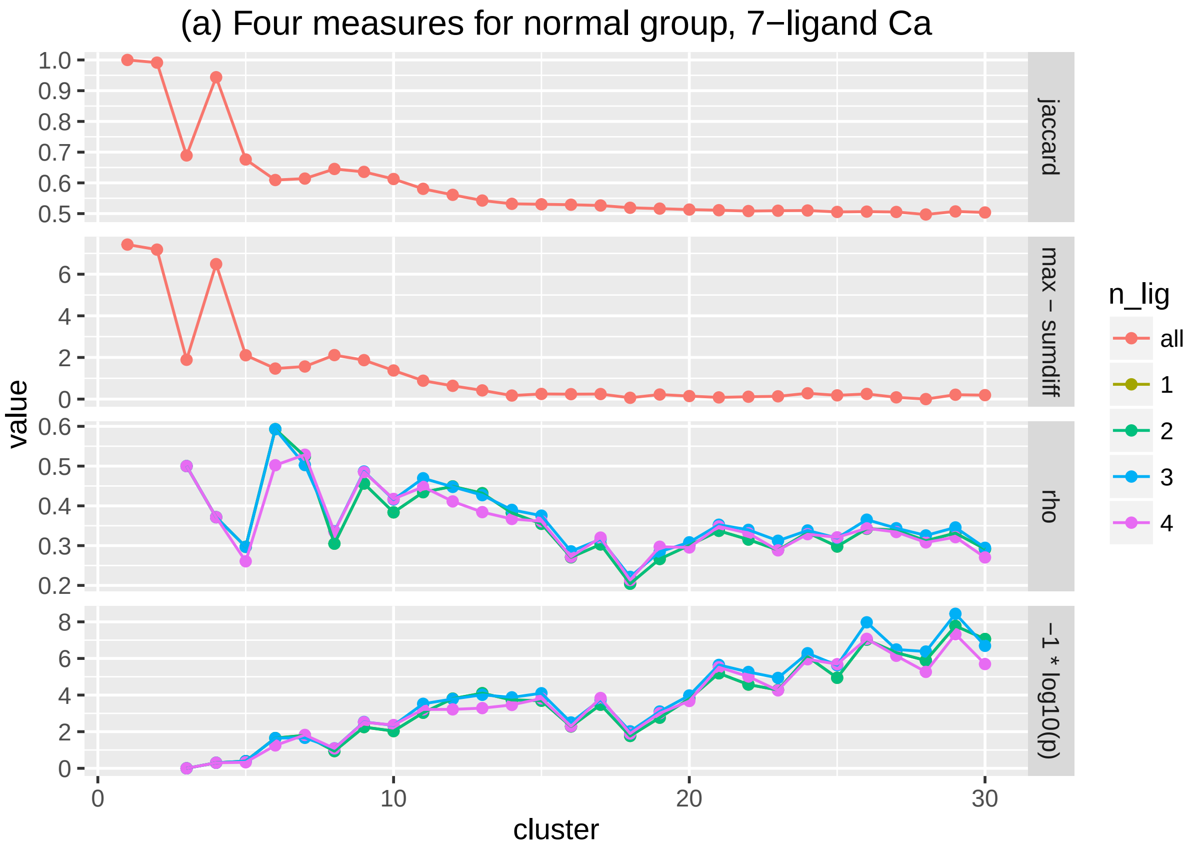
(a) Four measures for normal group, 7−ligand Ca
jaccard
max − sumdiff
rho
−1 * log10(p)
value
cluster
n_lig
all
1
2
3
4

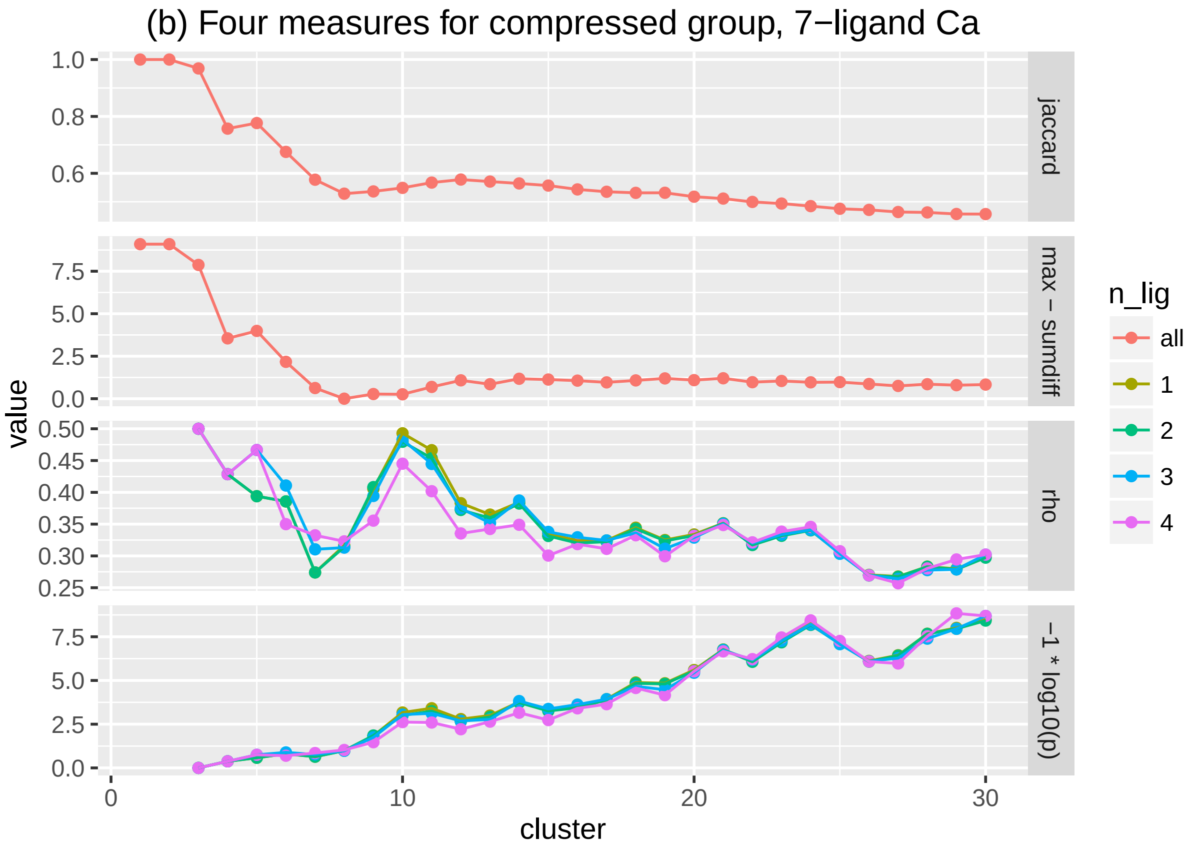
(b) Four measures for compressed group, 7−ligand Ca
jaccard
max − sumdiff
rho
−1 * log10(p)
value
cluster
n_lig
all
1
2
3
4

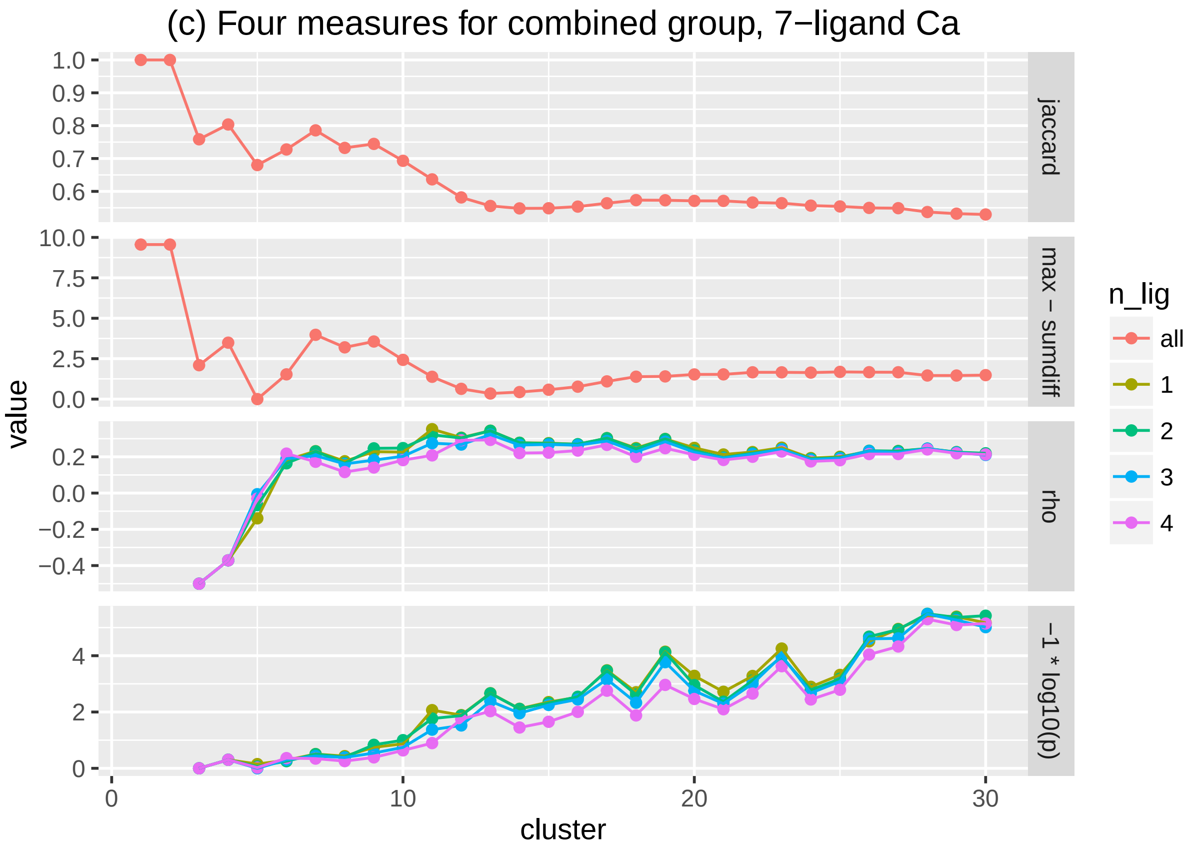
(c) Four measures for combined group, 7−ligand Ca
value
cluster
jaccard
max − sumdiff
rho
−1 * log10(p)
n_lig
all
1
2
3
4

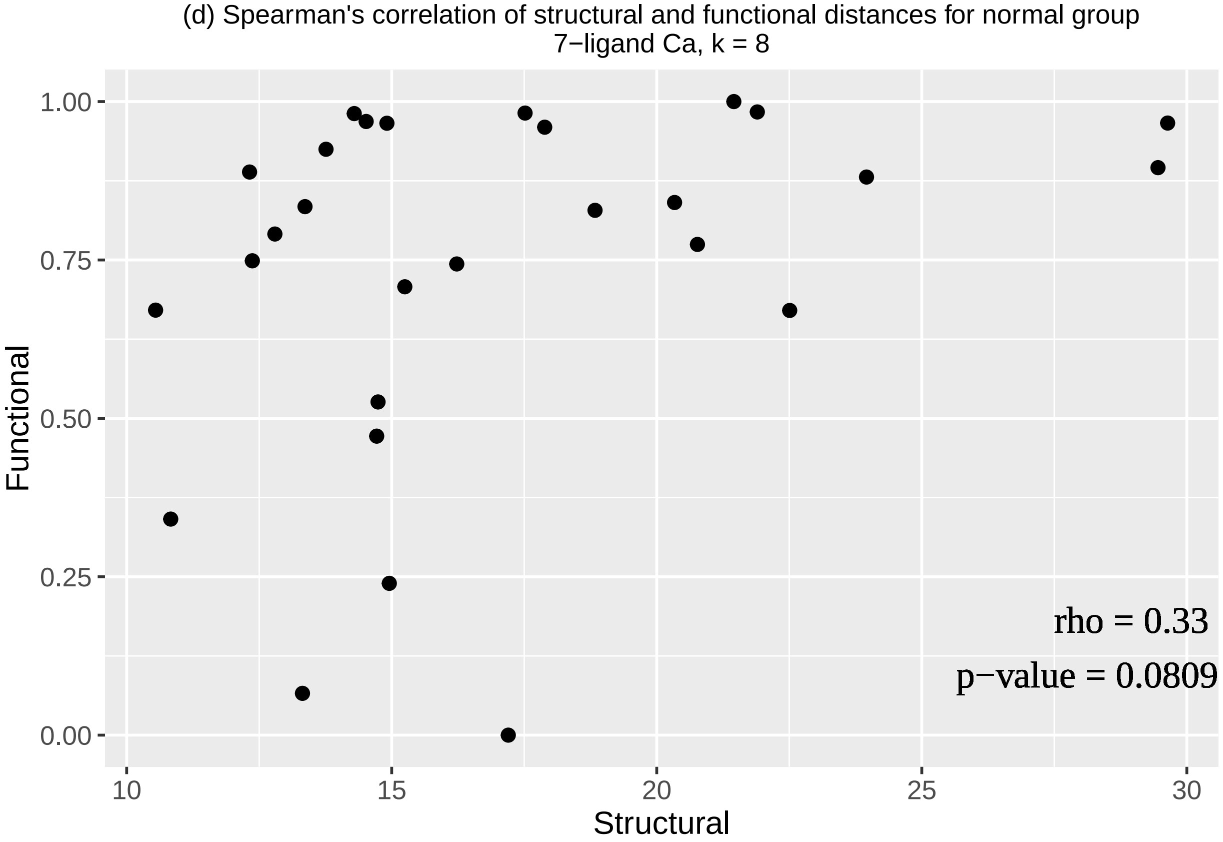
(d) Spearman's correlation of structural and functional distances for normal group
7−ligand Ca, k = 8
Functional
Structural
rho = 0.33
p−value = 0.0809

(e) Comparison between structural and functional hierarchical dendrograms for normal group 7-ligand Ca

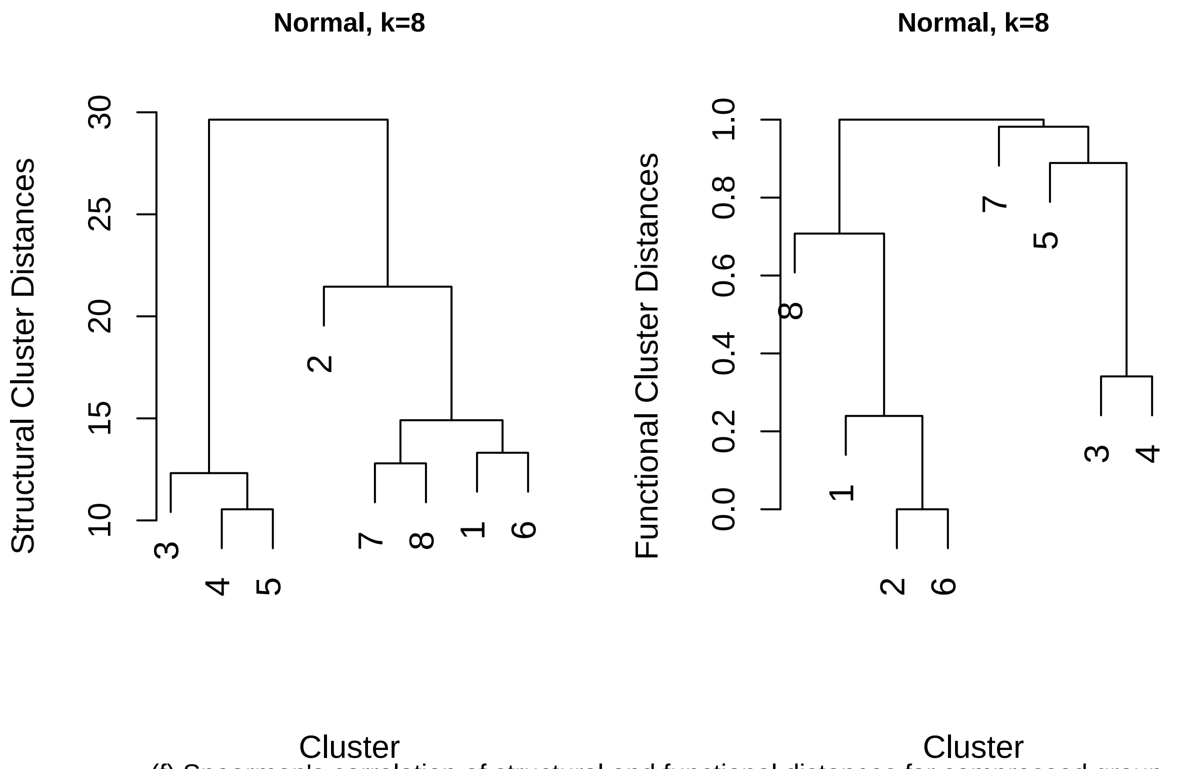

(f) Spearman's correlation of structural and functional distances for compressed group 7−ligand Ca, k = 10

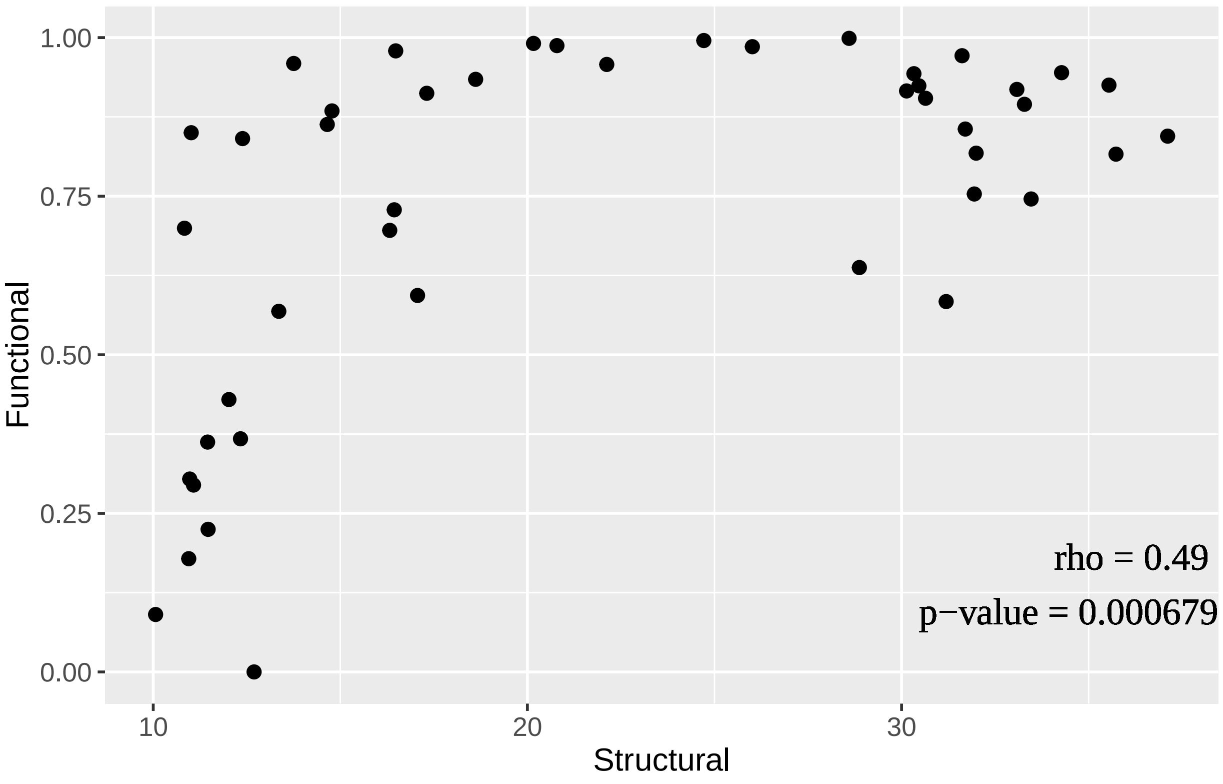

(g) Comparison between structural and functional hierarchical dendrograms for compressed group 7-ligand Ca

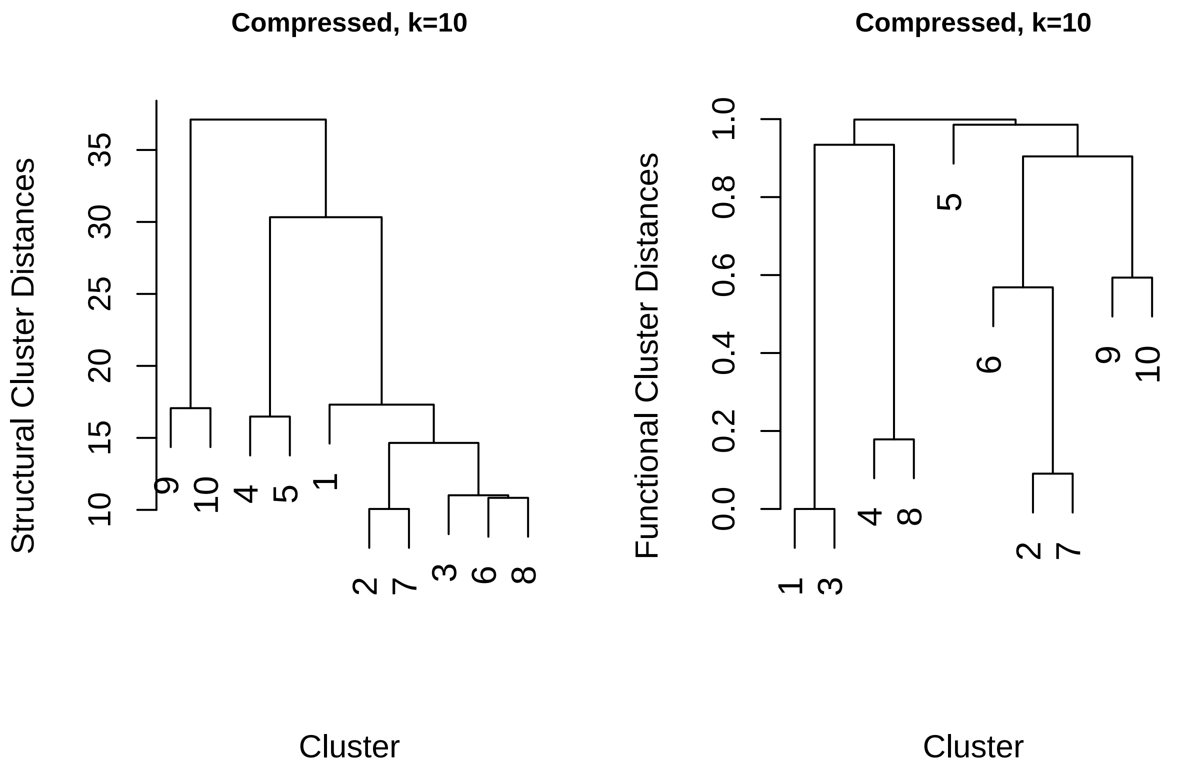

Figure S13. 8-ligand Ca metalloproteins

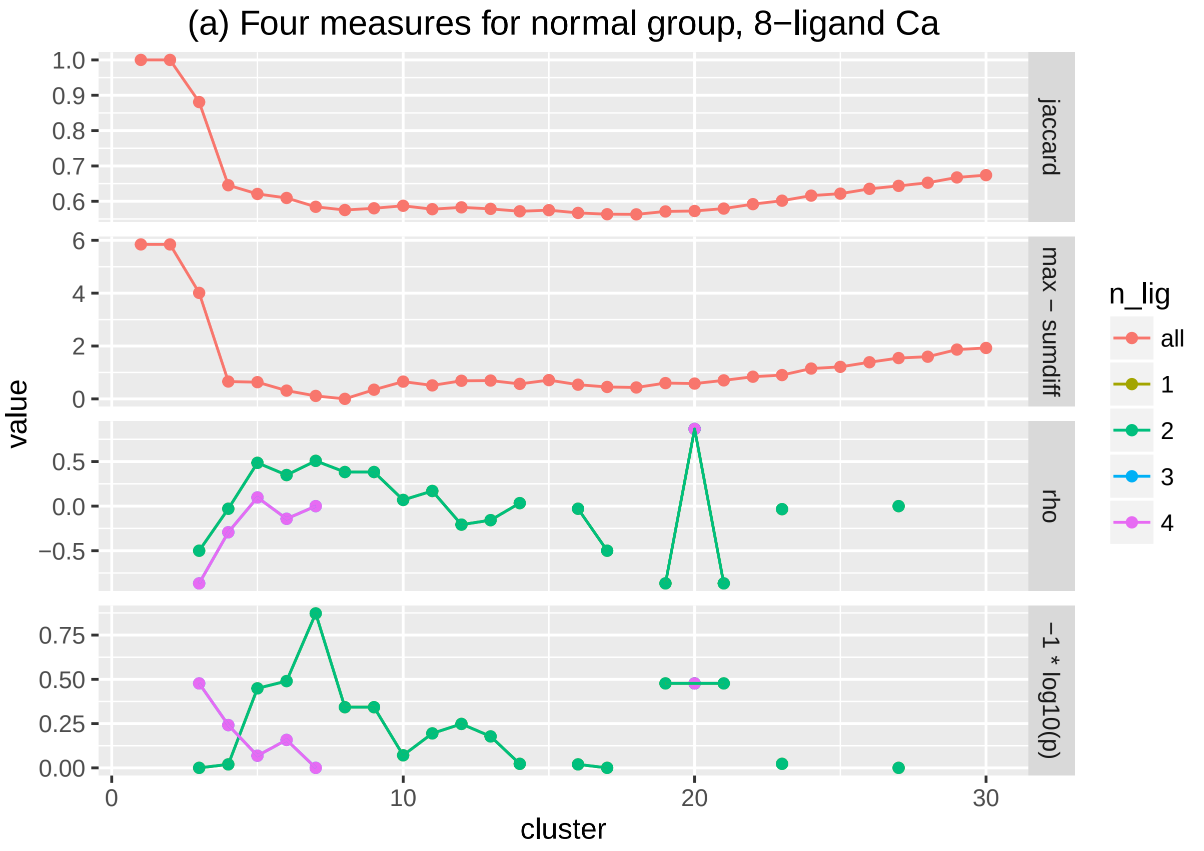
(a) Four measures for normal group, 8−ligand Ca
value
jaccard
max − sumdiff
rho
−1 * log10(p)
cluster
n_lig
all
1
2
3
4

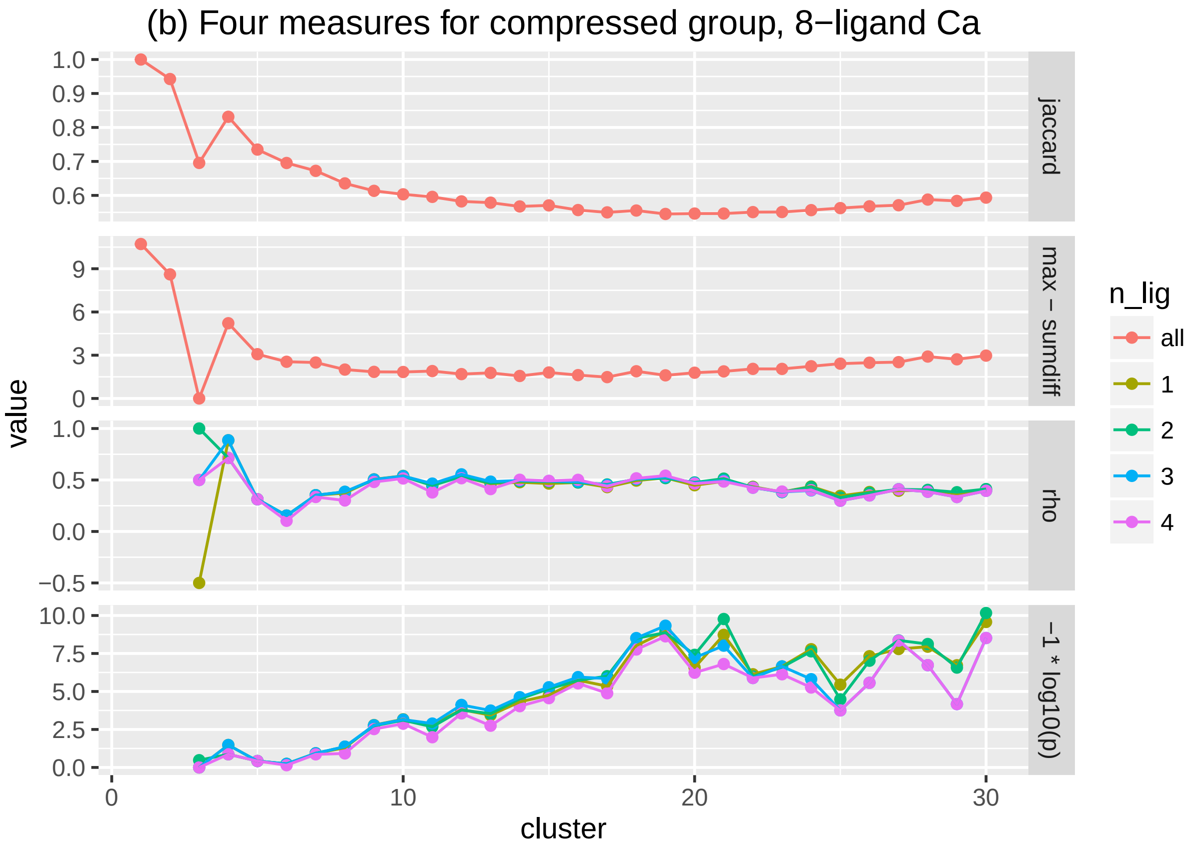
(b) Four measures for compressed group, 8−ligand Ca
value
jaccard
max − sumdiff
rho
−1 * log10(p)
cluster
n_lig
all
1
2
3
4

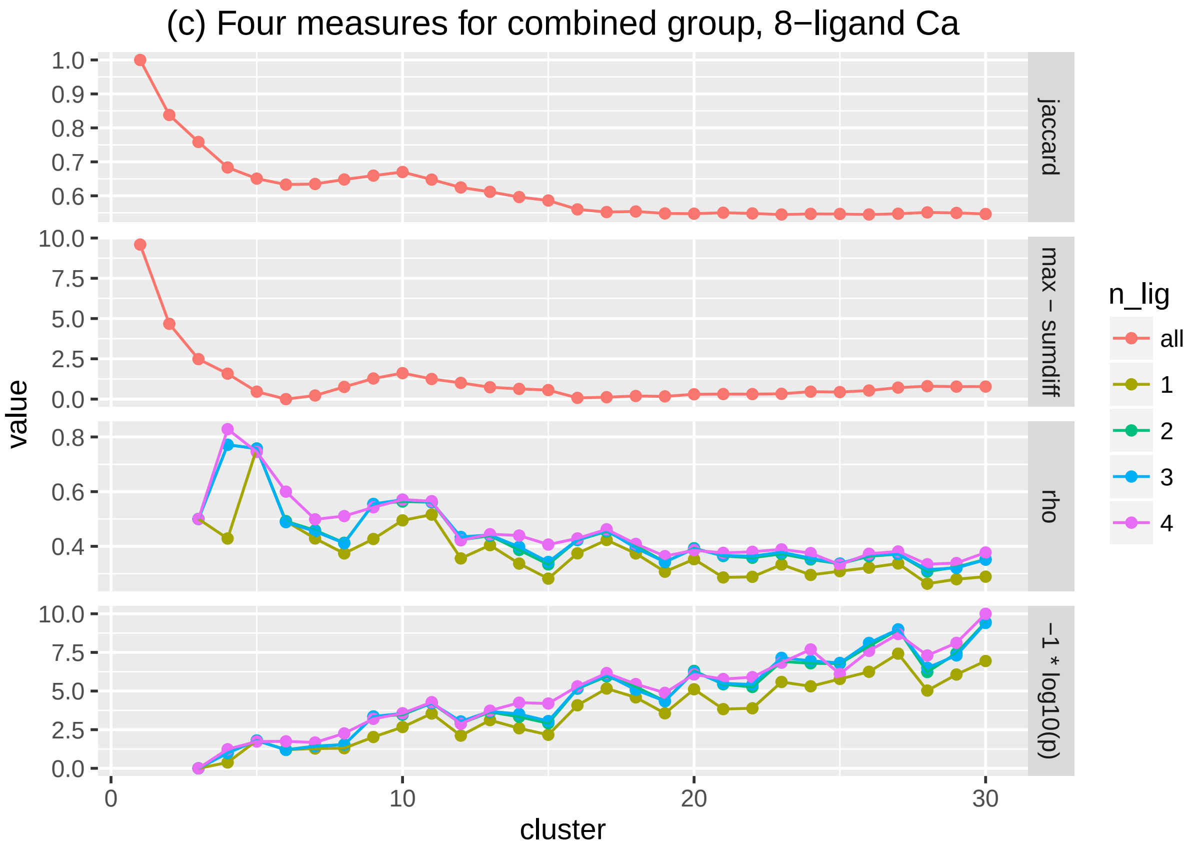
(c) Four measures for combined group, 8−ligand Ca
value
cluster
jaccard
max − sumdiff
rho
−1 * log10(p)
n_lig
all
1
2
3
4

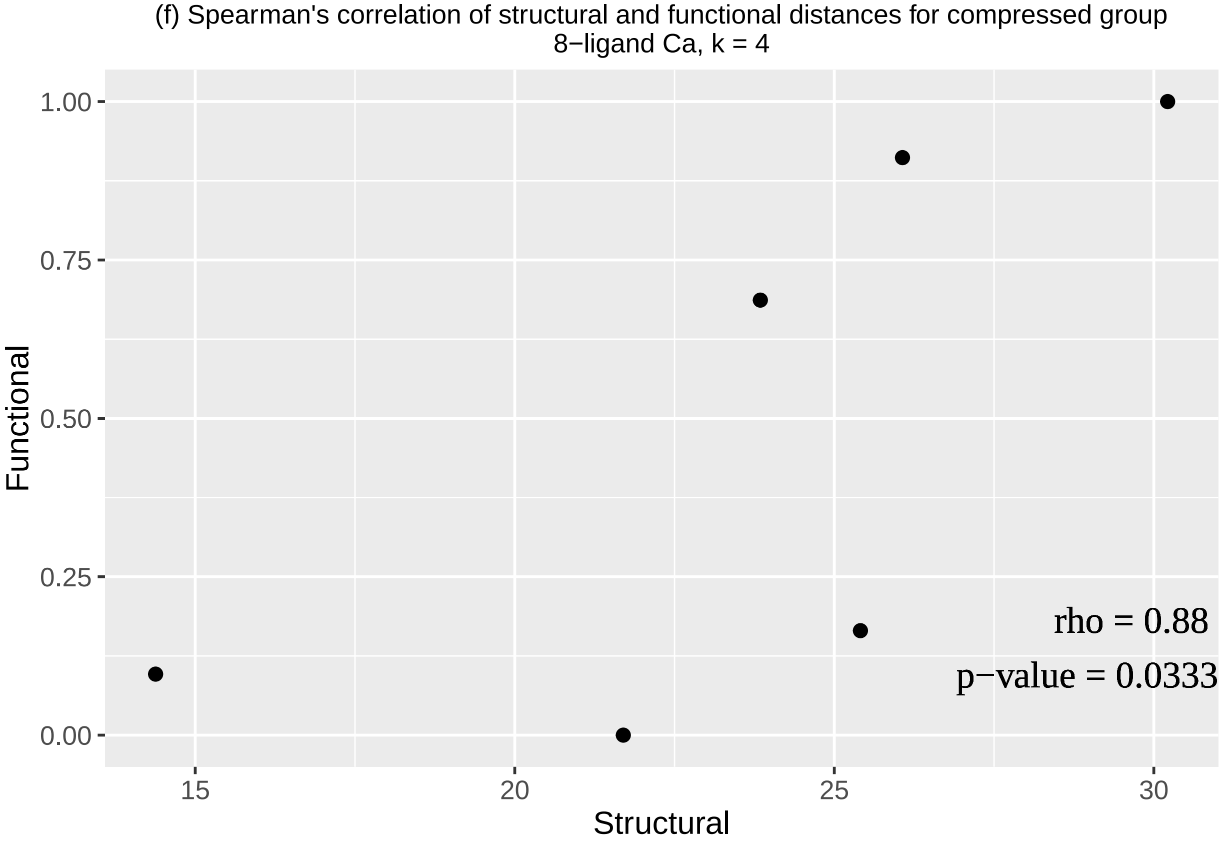
(f) Spearman's correlation of structural and functional distances for compressed group
8−ligand Ca, k = 4
Functional
Structural
rho = 0.88
p−value = 0.0333

(g) Comparison between structural and functional hierarchical dendrograms for compressed group 8-ligand Ca

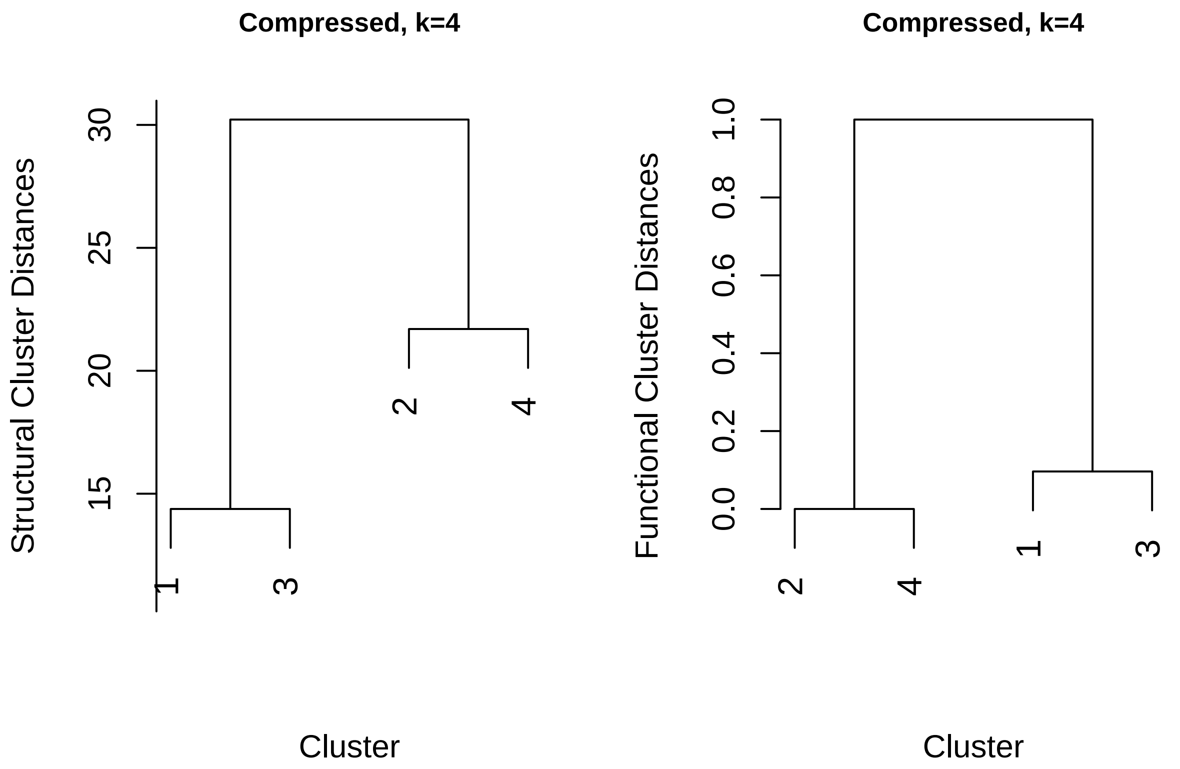

Figure S14. all-ligand Ca metalloproteins

(a) Four measures for normal group, all−ligand−number Ca

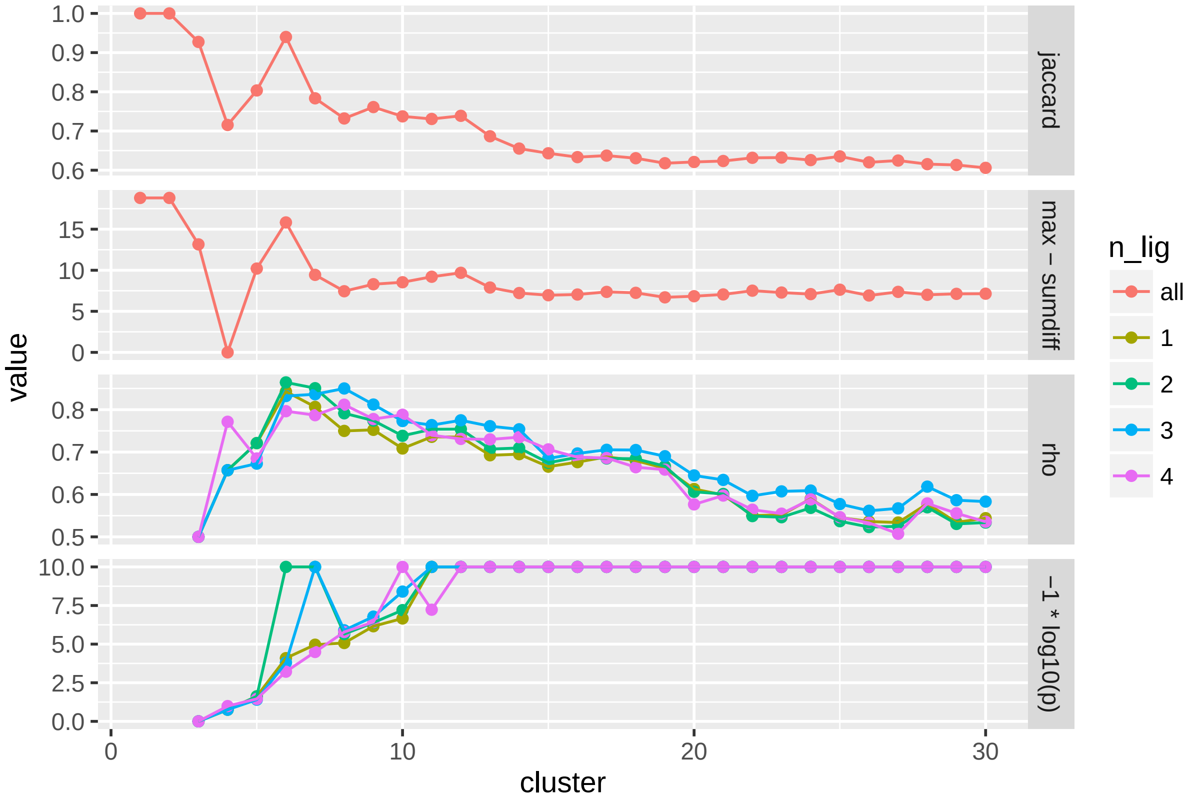

(b) Four measures for compressed group, all−ligand−number Ca

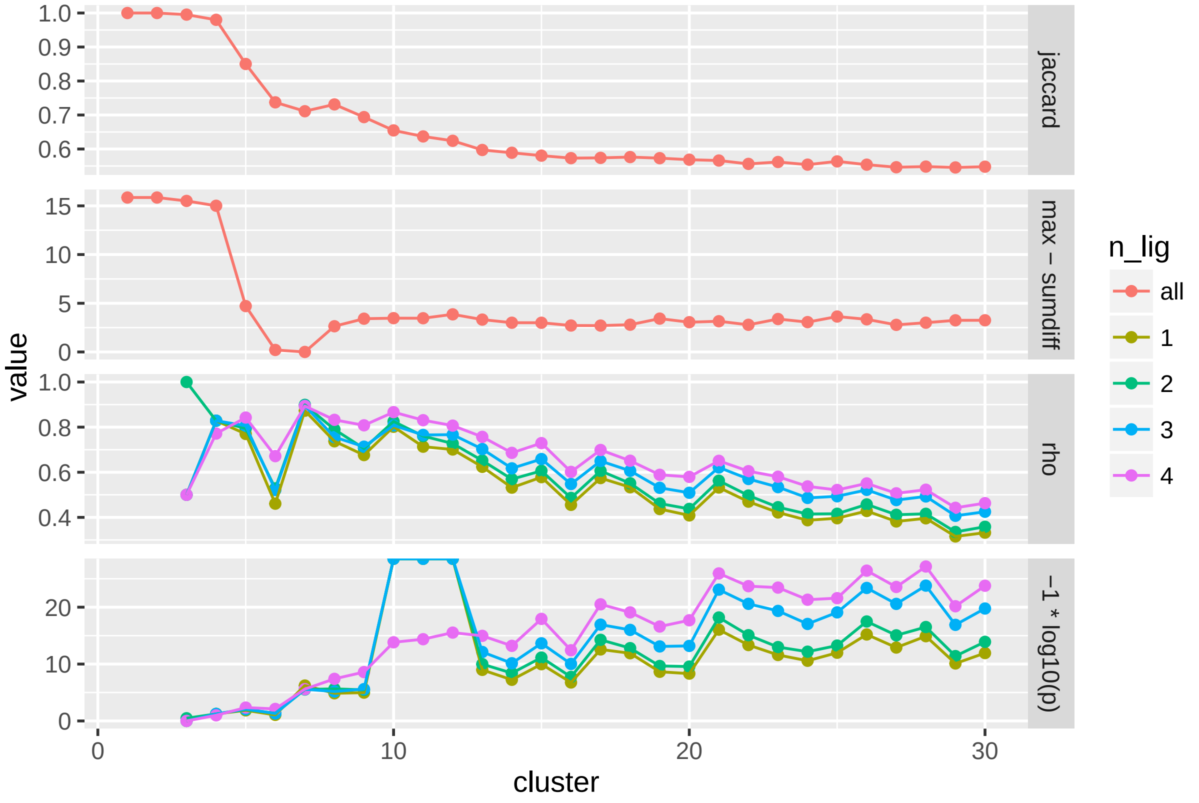

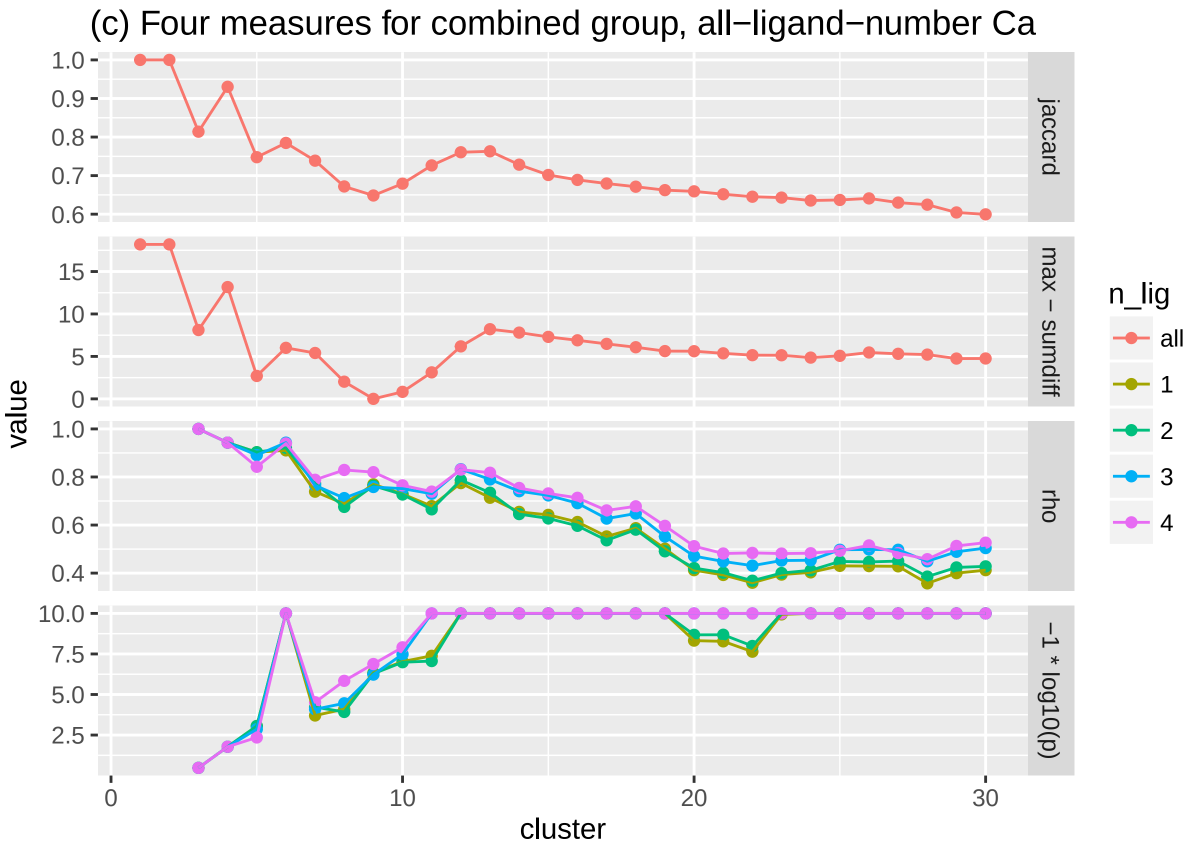

(c) Four measures for combined group, all−ligand−number Ca
value
cluster
jaccard
max − sumdiff
rho
−1 * log10(p)
n_lig
all
1
2
3
4

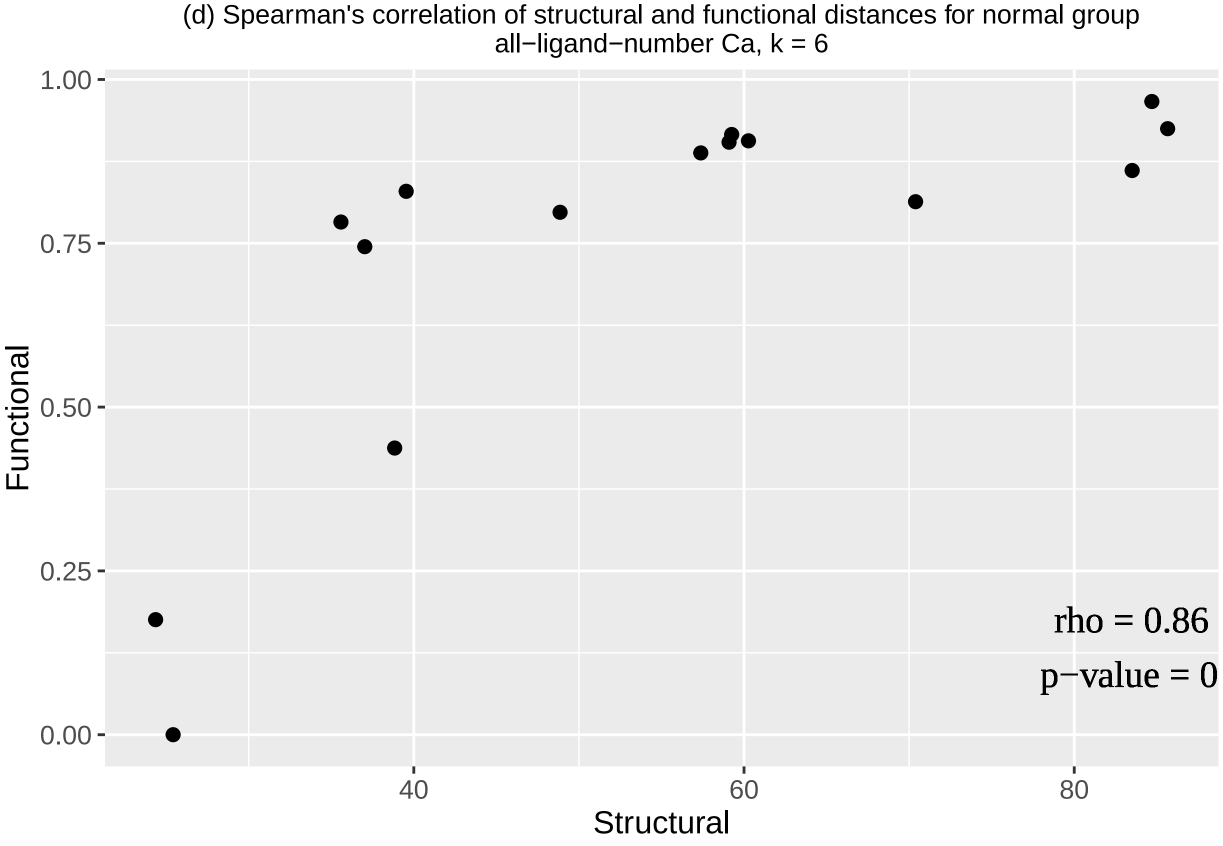

(d) Spearman's correlation of structural and functional distances for normal group
all−ligand−number Ca, k = 6
Functional
Structural
rho = 0.86
p−value = 0

(e) Comparison between structural and functional hierarchical dendrograms for normal group all-ligand-number Ca

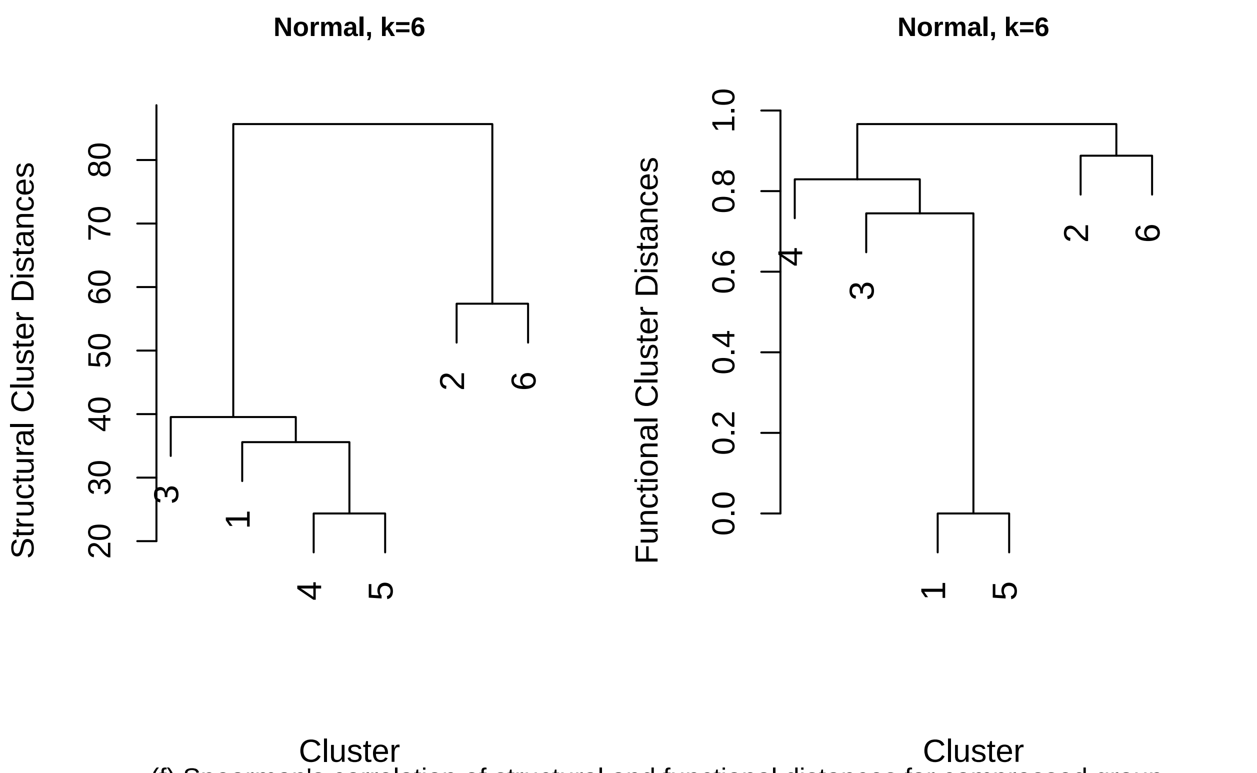

(f) Spearman's correlation of structural and functional distances for compressed group all−ligand−number Ca, k = 10

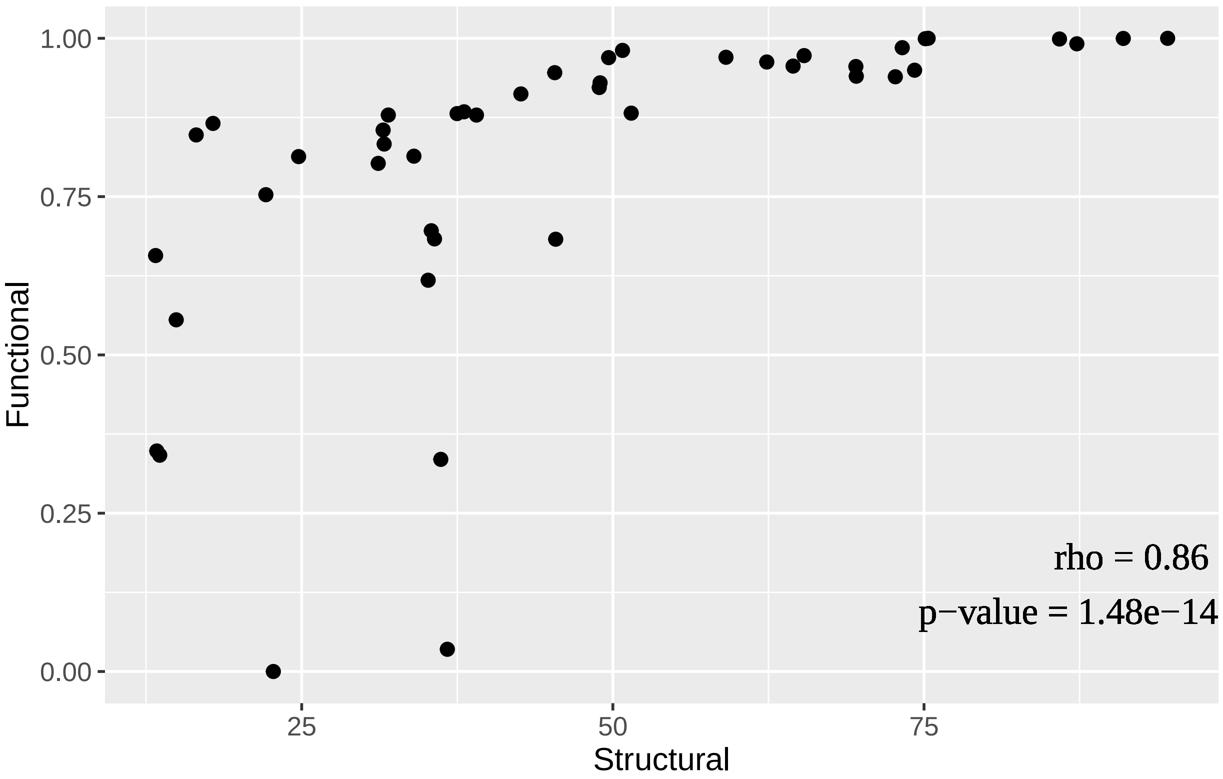

(g) Comparison between structural and functional hierarchical dendrograms for compressed group all-ligand-number Ca

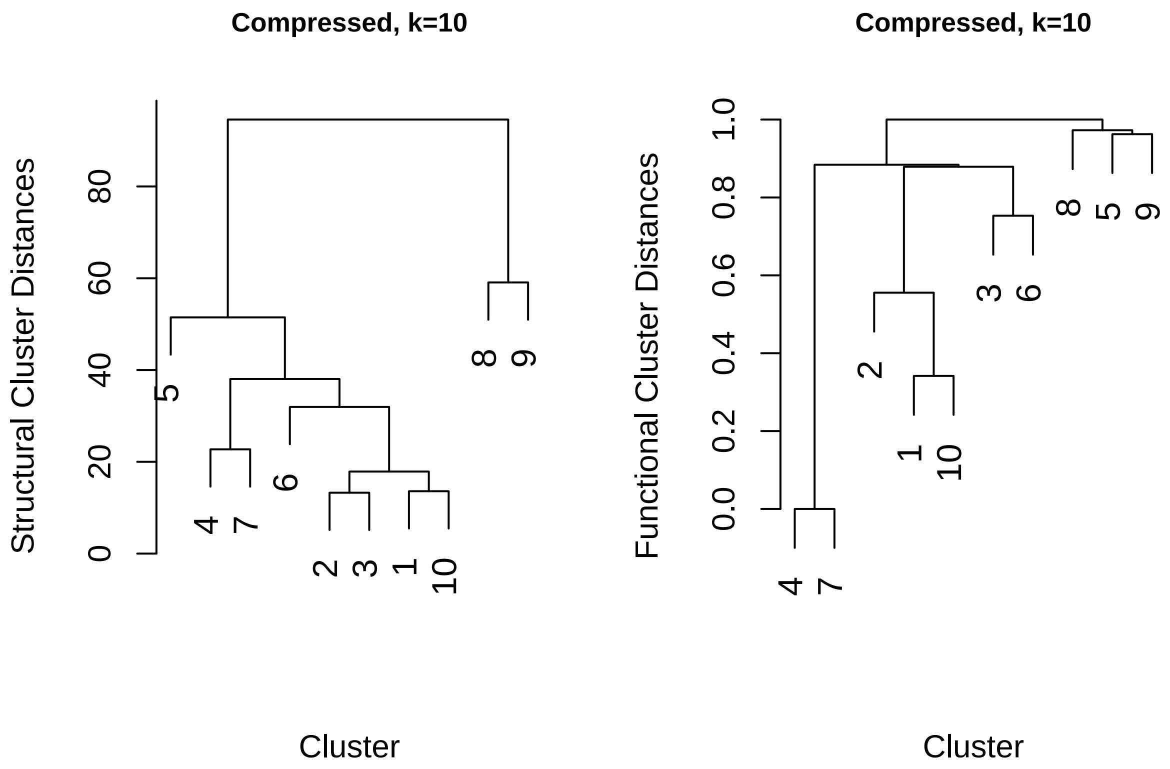

Figure S15. 4-ligand Fe metalloproteins

(a) Four measures for normal group, 4−ligand Fe

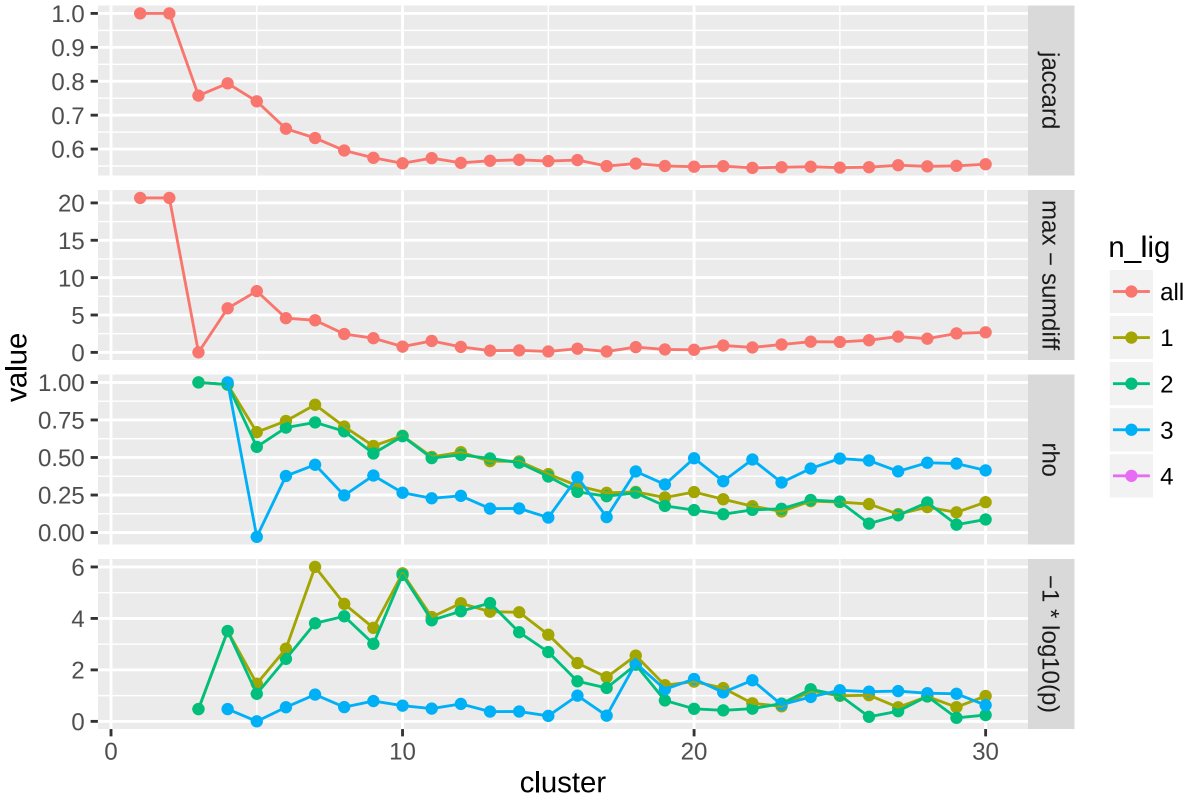

(c) Four measures for combined group, 4−ligand Fe

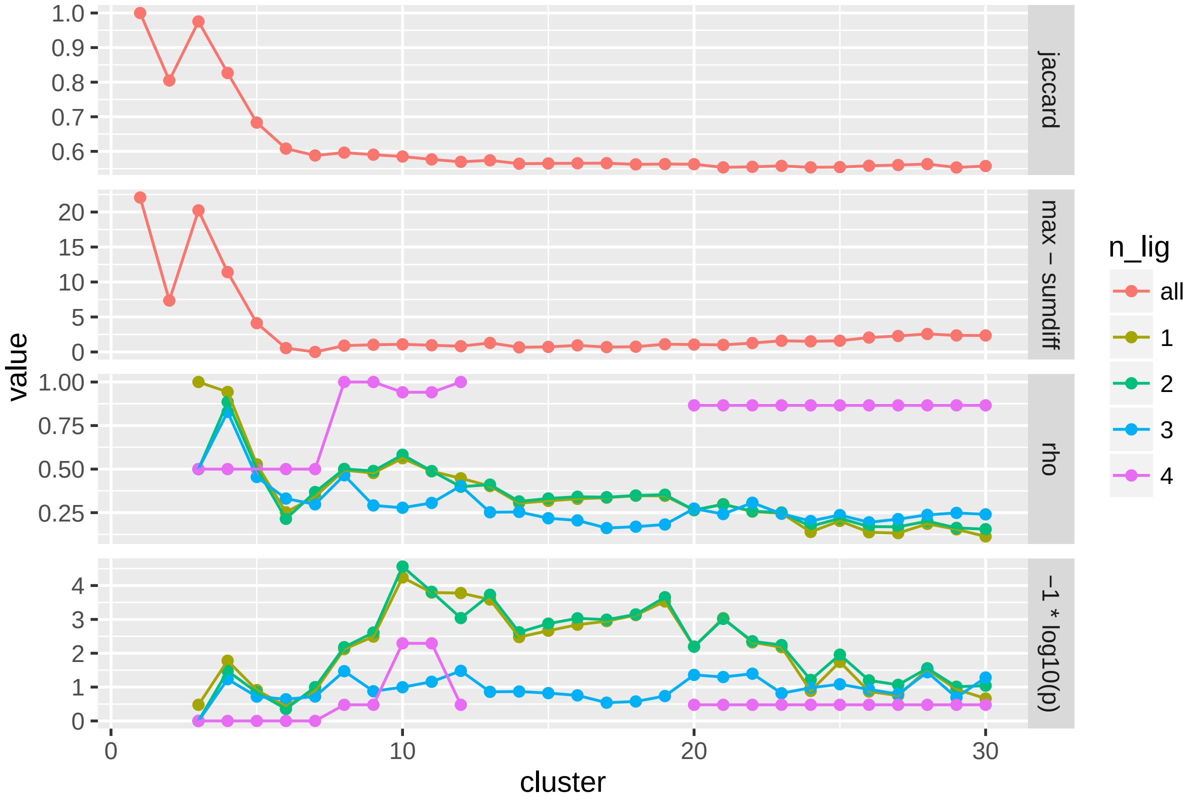

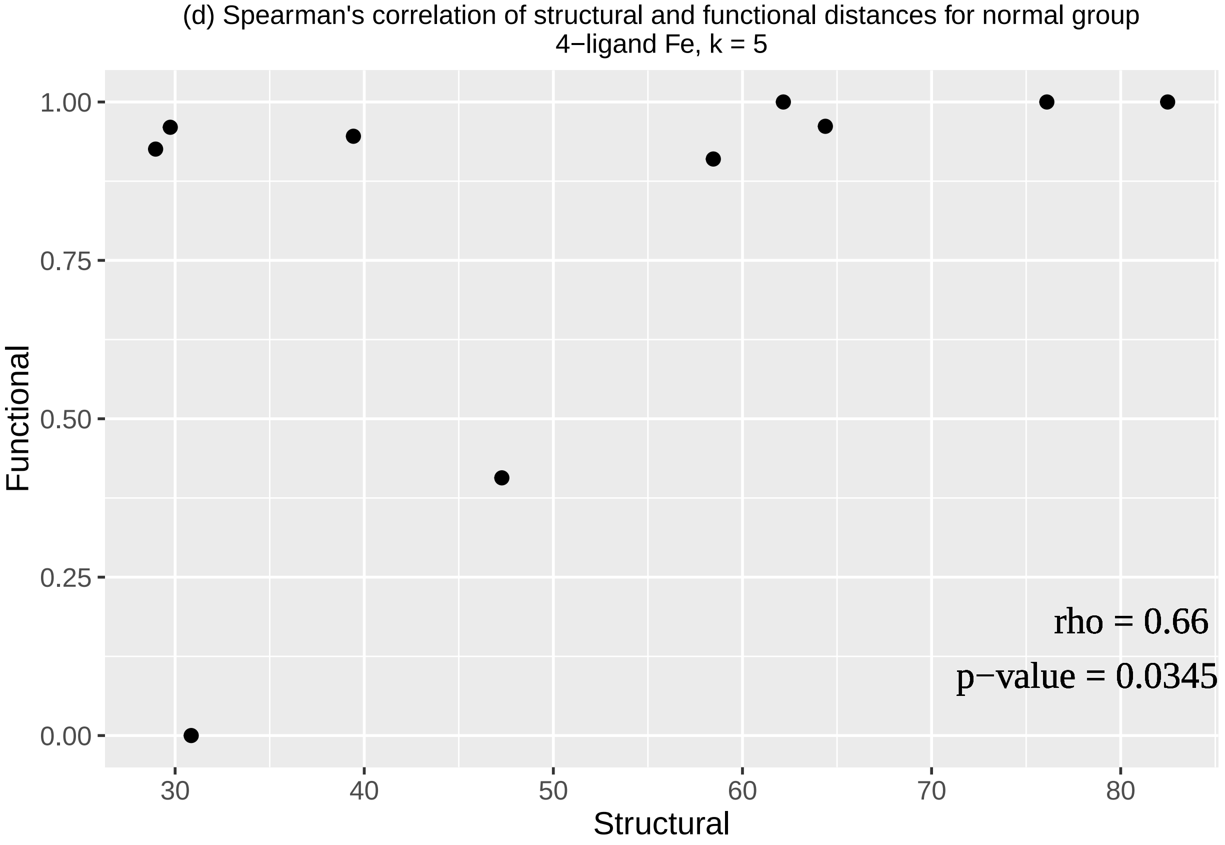

(e) Comparison between structural and functional hierarchical dendrograms for normal group 4-ligand Fe

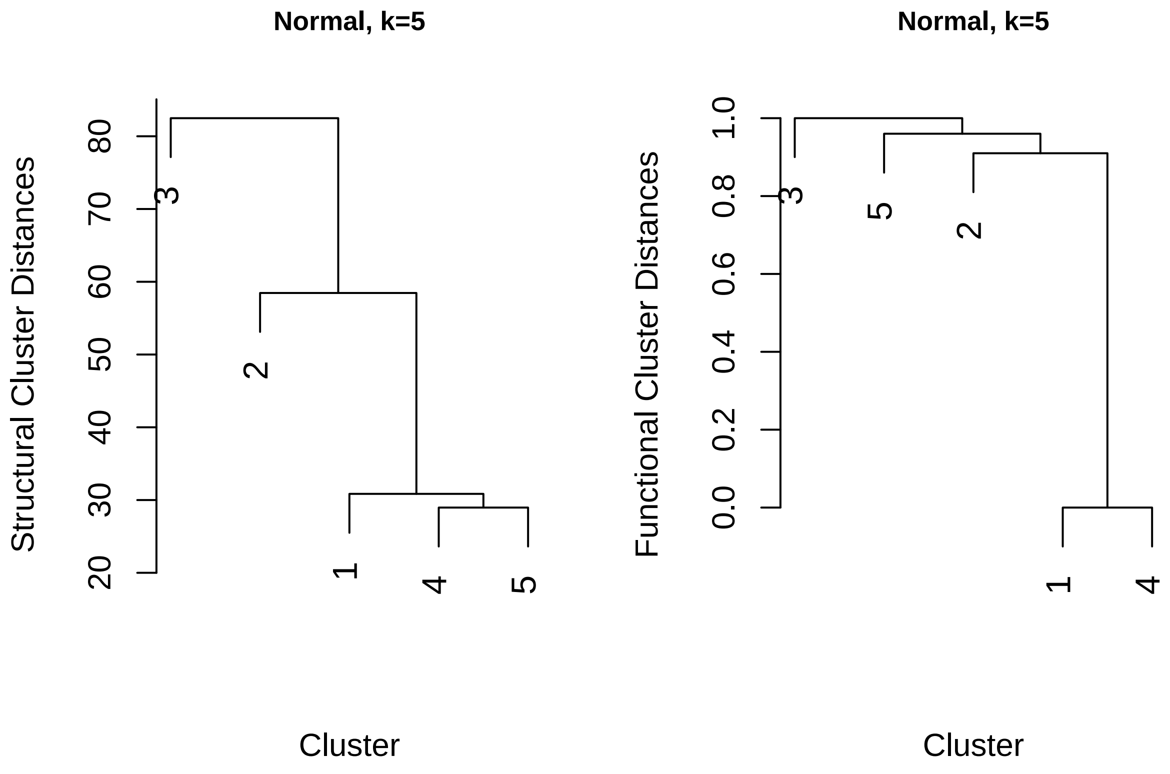

Figure S16. 5-ligand Fe metalloproteins

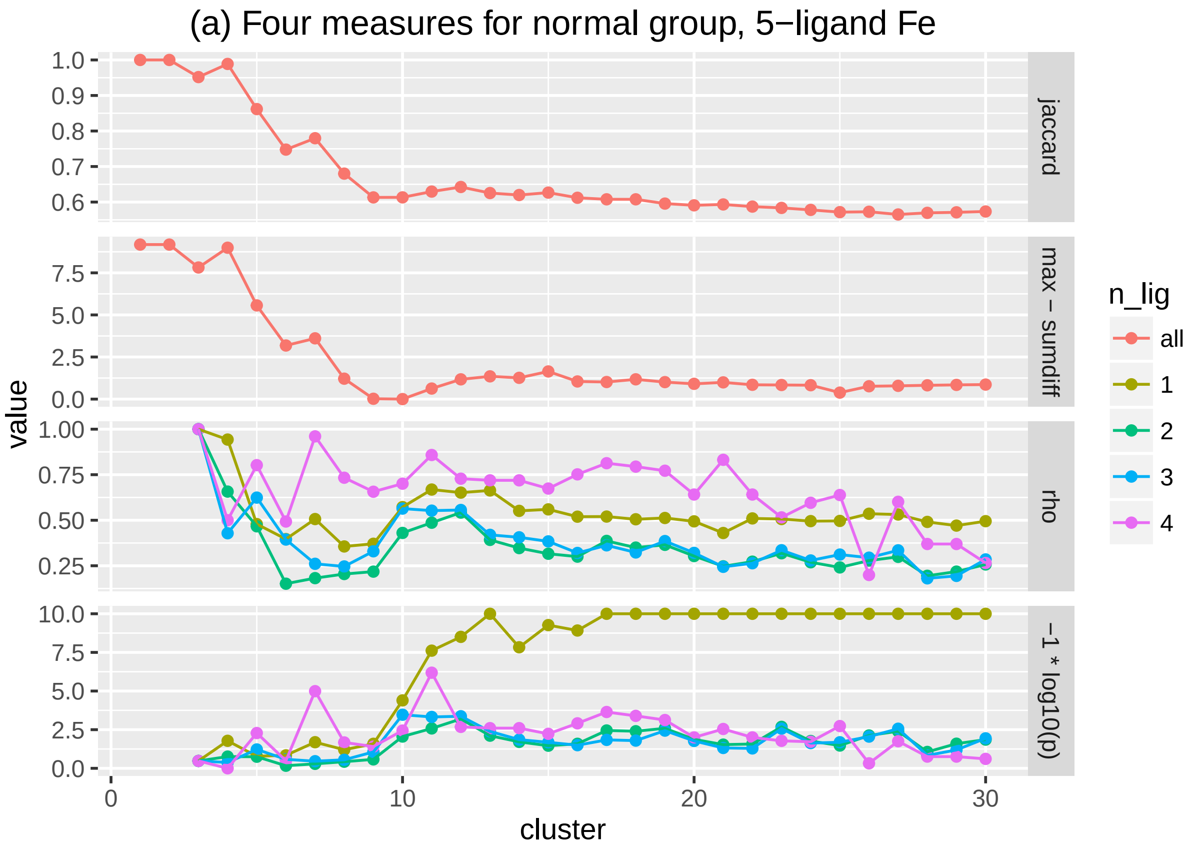
(a) Four measures for normal group, 5−ligand Fe
value
cluster
jaccard
max − sumdiff
rho
−1 * log10(p)
n_lig
all
1
2
3
4

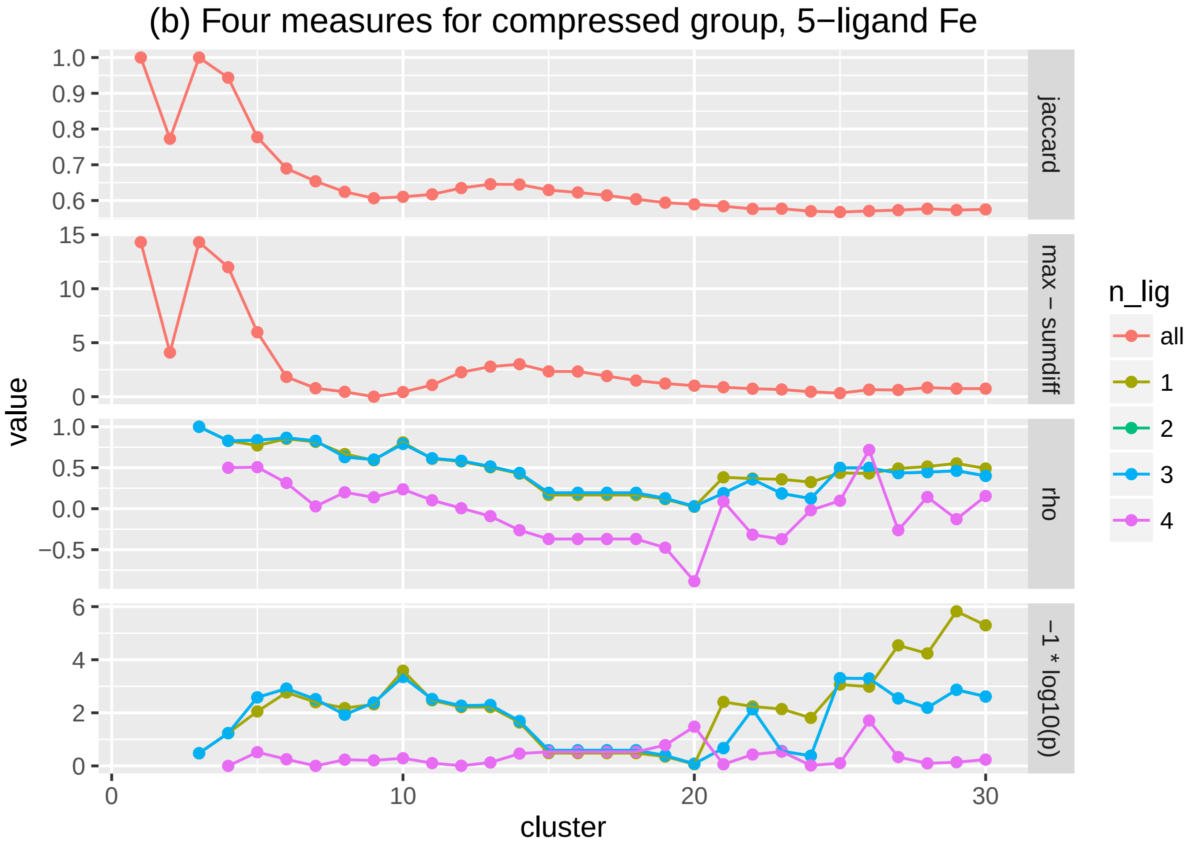
(b) Four measures for compressed group, 5−ligand Fe
value
cluster
jaccard
max − sumdiff
rho
−1 * log10(p)
n_lig
all
1
2
3
4

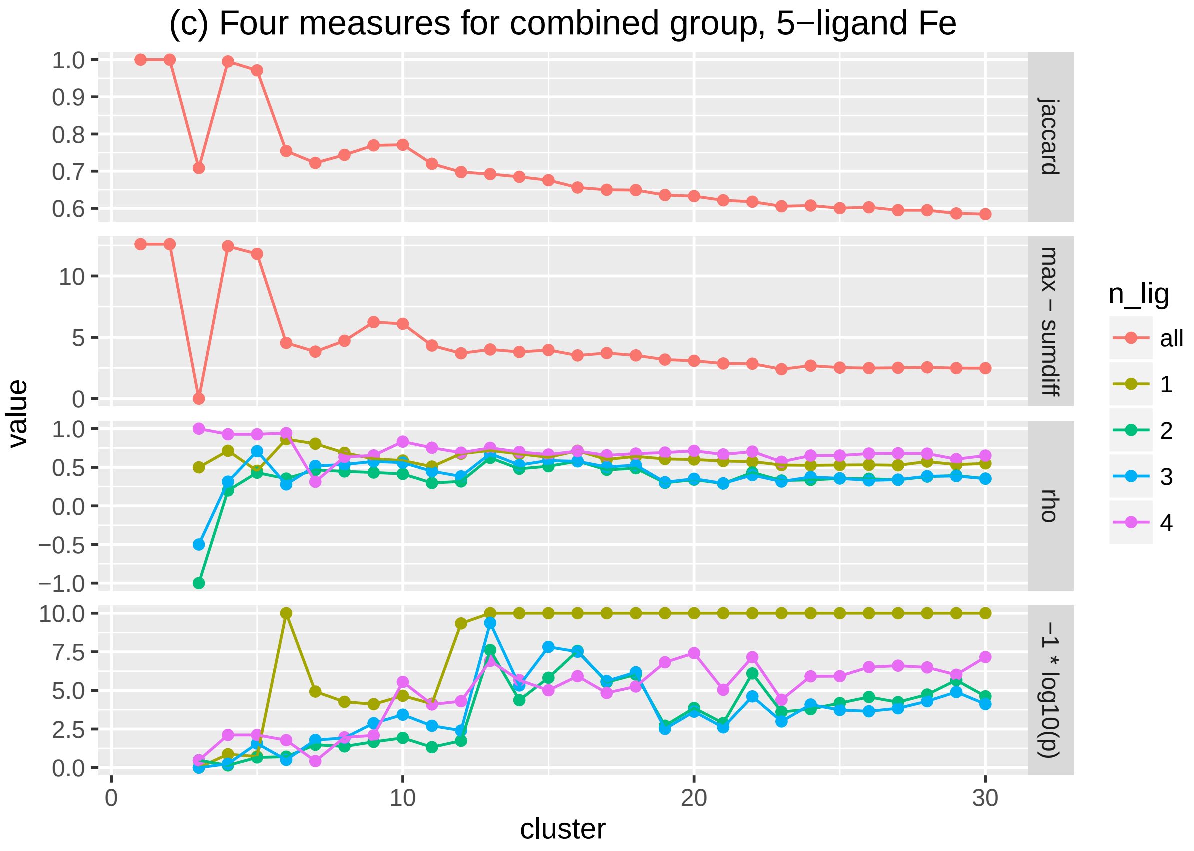
(c) Four measures for combined group, 5−ligand Fe
value
cluster
jaccard
max − sumdiff
rho
−1 * log10(p)
n_lig
all
1
2
3
4

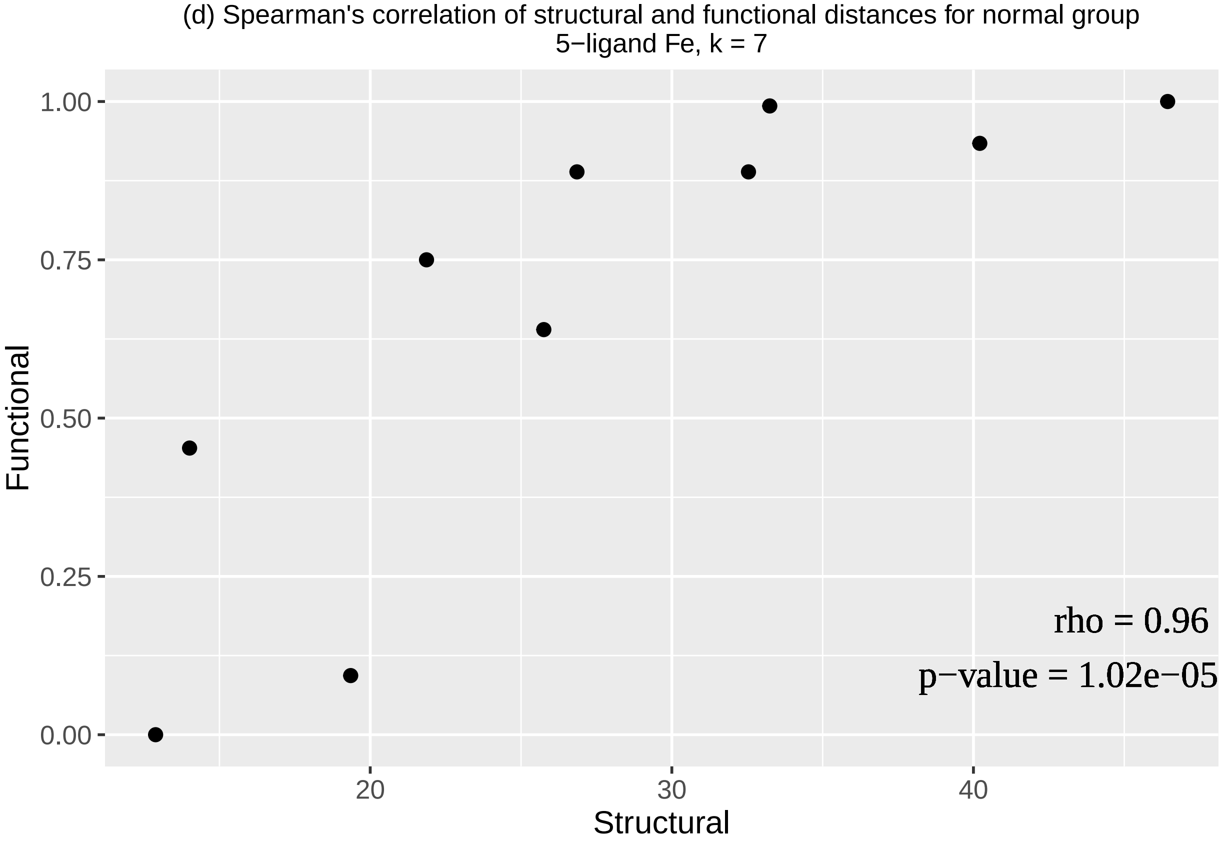
(d) Spearman's correlation of structural and functional distances for normal group
5−ligand Fe, k = 7
Functional
Structural
rho = 0.96
p−value = 1.02e−05

(e) Comparison between structural and functional hierarchical dendrograms for normal group 5-ligand Fe

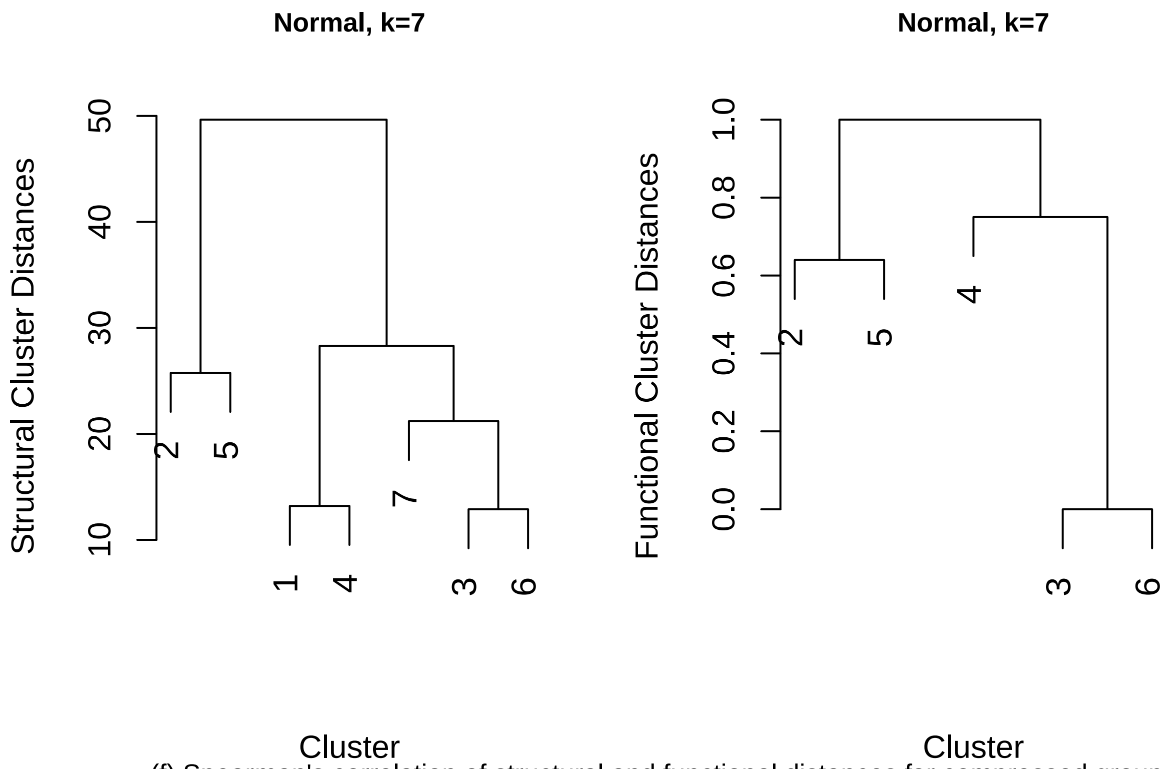

(f) Spearman's correlation of structural and functional distances for compressed group 5−ligand Fe, k = 4

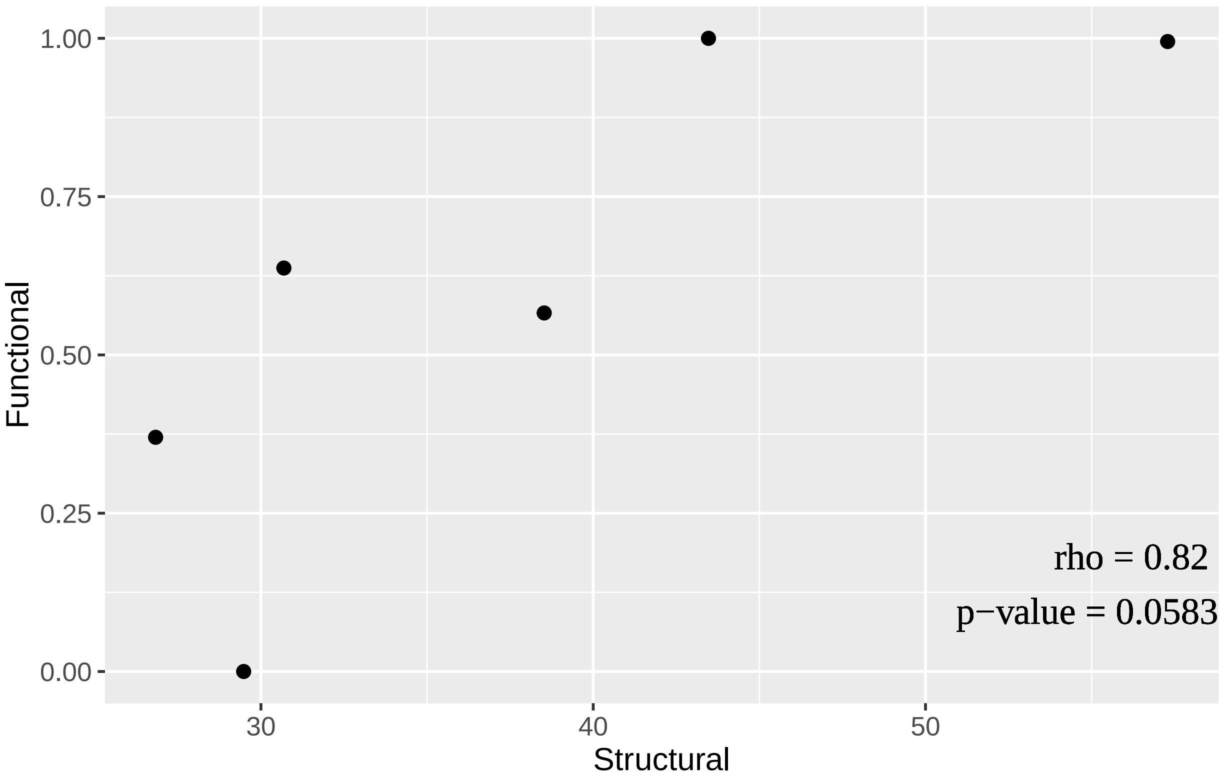

(g) Comparison between structural and functional hierarchical dendrograms for compressed group 5-ligand Fe

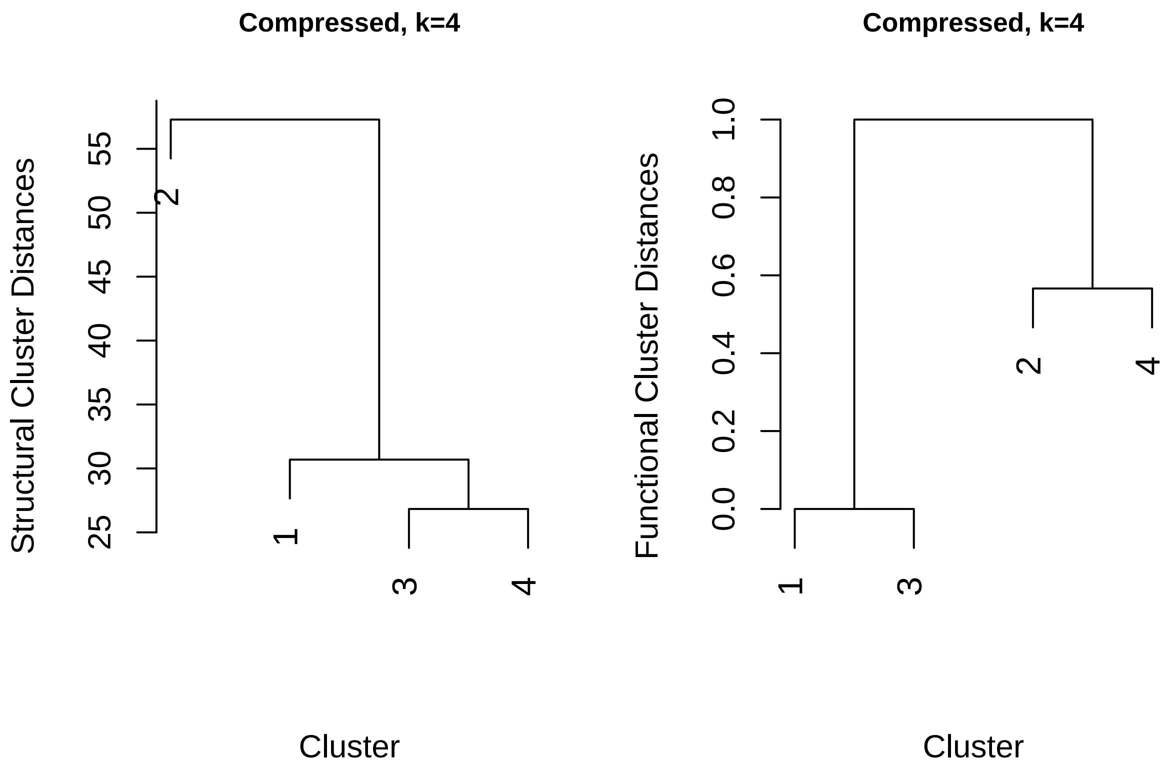

Figure S17. 6-ligand Fe metalloproteins

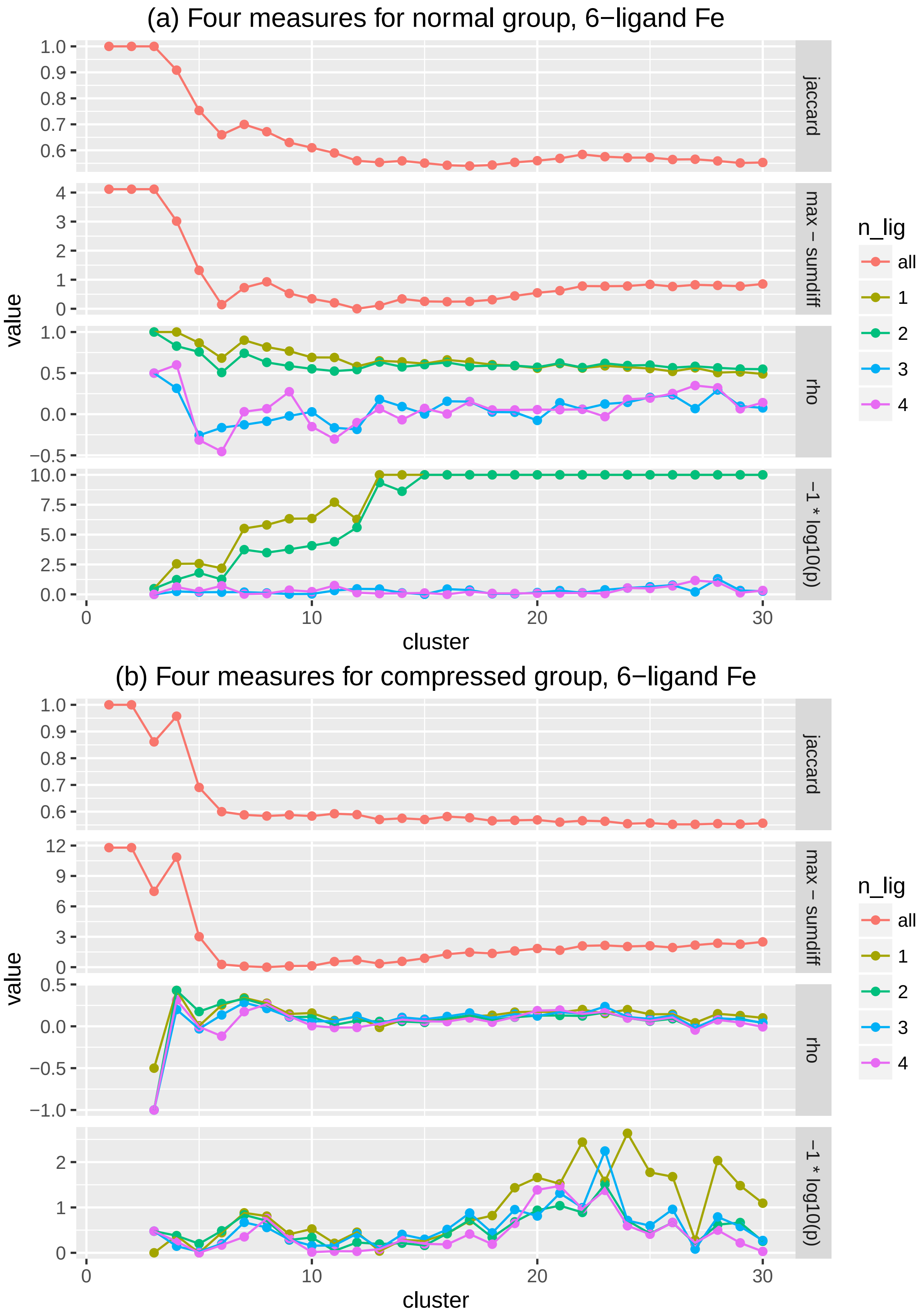
(a) Four measures for normal group, 6−ligand Fe
jaccard
max − sumdiff
rho
−1 * log10(p)
value
cluster
n_lig
all
1
2
3
4
(b) Four measures for compressed group, 6−ligand Fe
jaccard
max − sumdiff
rho
−1 * log10(p)
value
cluster
n_lig
all
1
2
3
4

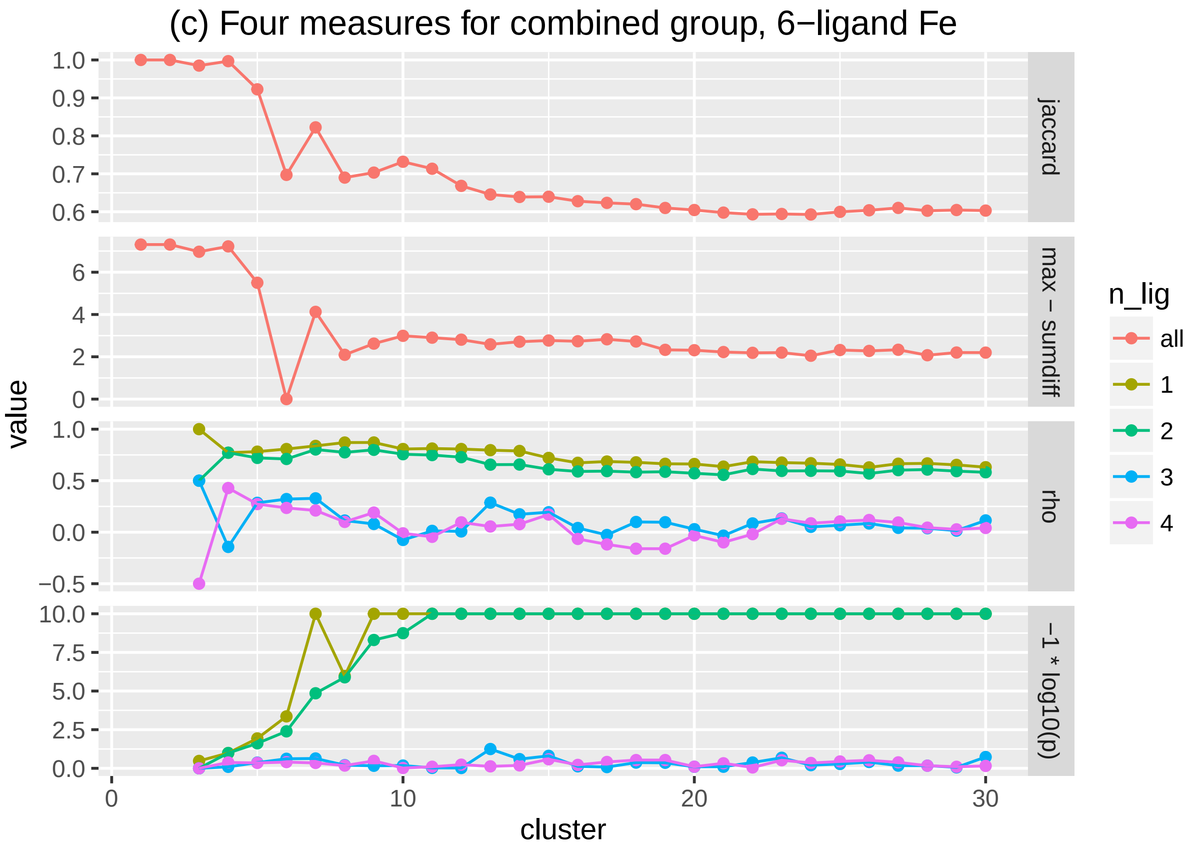

(c) Four measures for combined group, 6−ligand Fe
value
cluster
jaccard
max − sumdiff
rho
−1 * log10(p)
n_lig
all
1
2
3
4

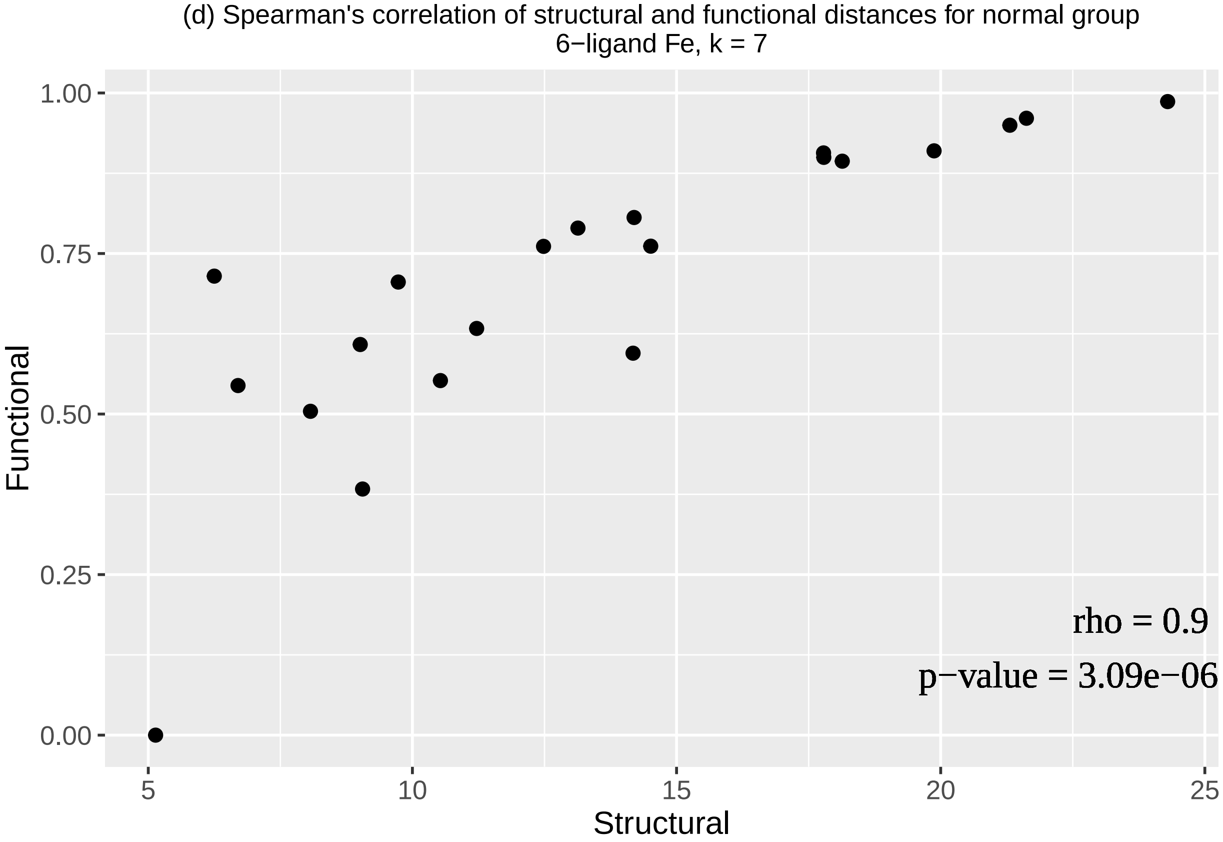

(d) Spearman's correlation of structural and functional distances for normal group
6−ligand Fe, k = 7
Functional
Structural
rho = 0.9
p−value = 3.09e−06

(e) Comparison between structural and functional hierarchical dendrograms for normal group 6-ligand Fe

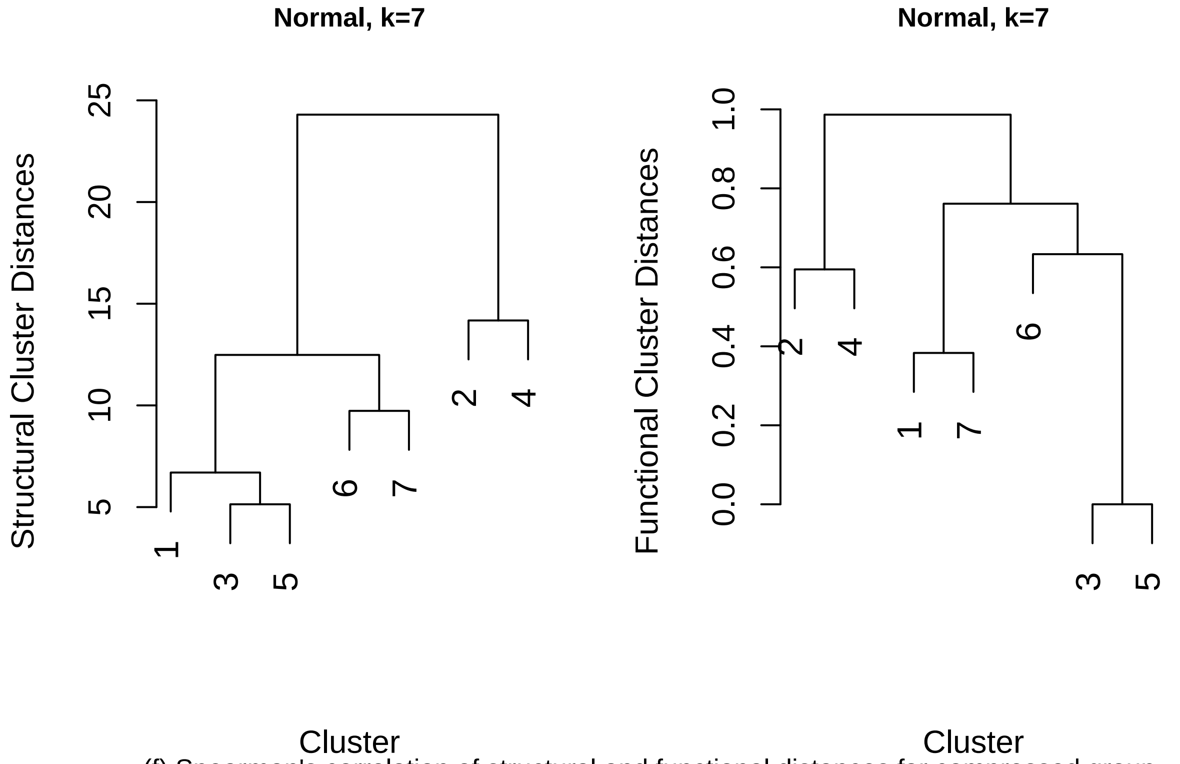

(f) Spearman's correlation of structural and functional distances for compressed group 6−ligand Fe, k = 4

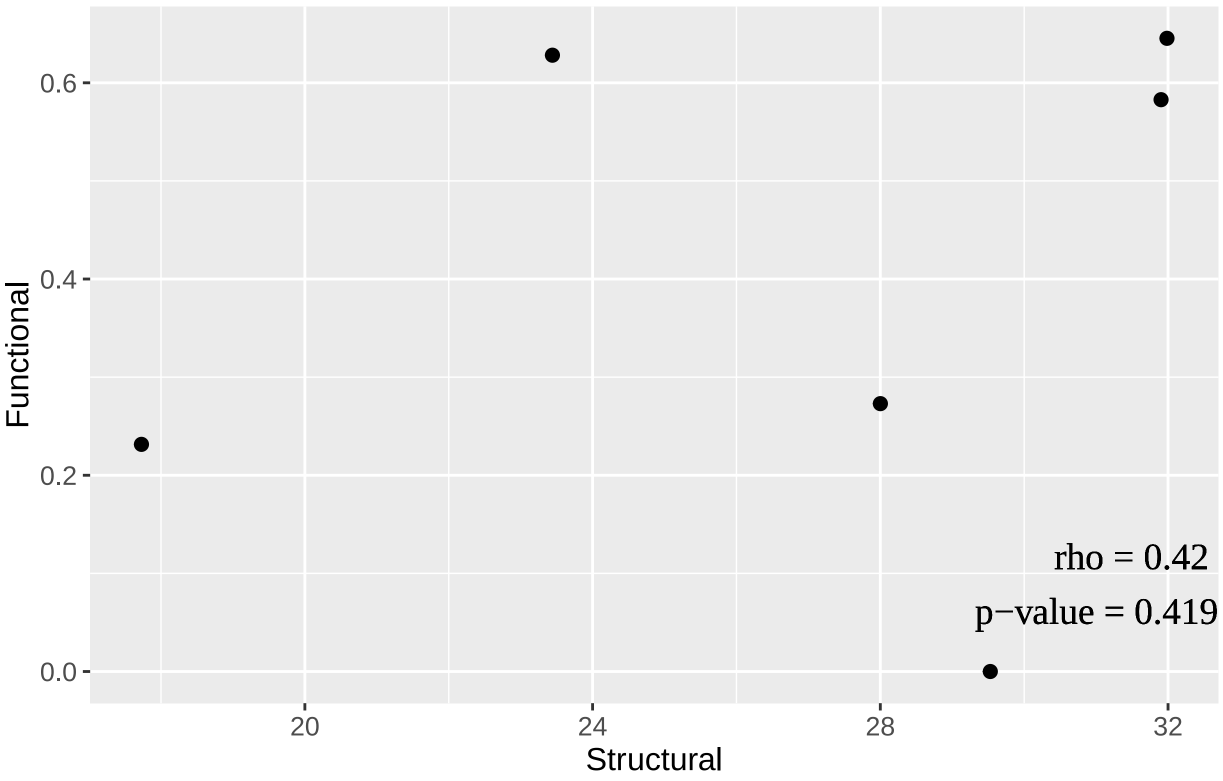

(g) Comparison between structural and functional hierarchical dendrograms for compressed group 6-ligand Fe

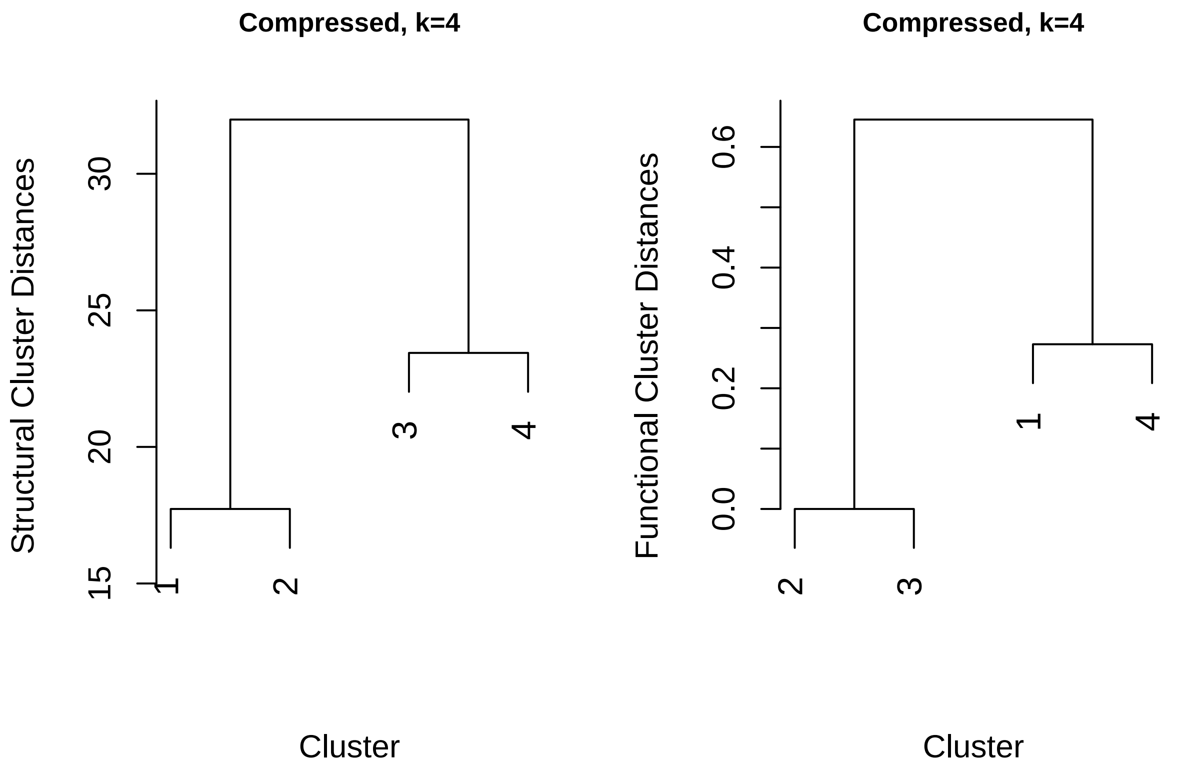

Figure S18. all-ligand Fe metalloproteins

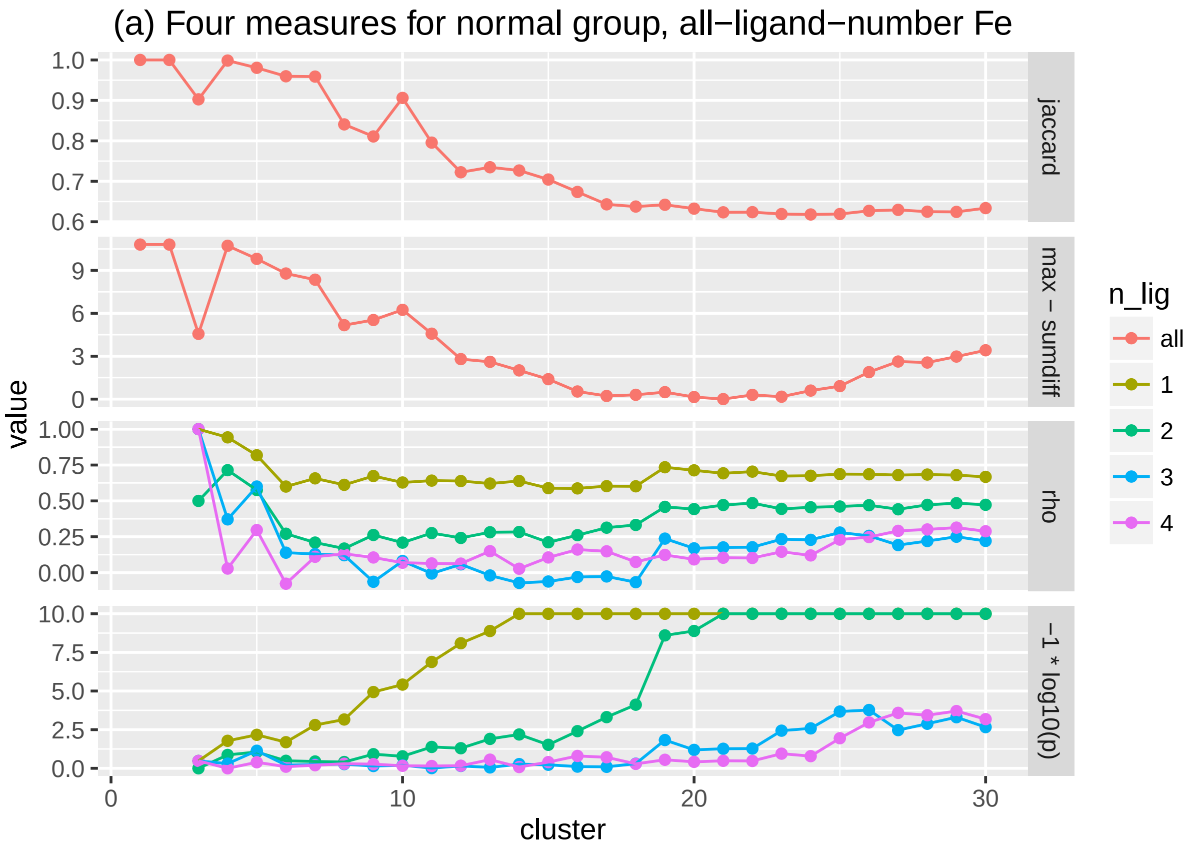
(a) Four measures for normal group, all−ligand−number Fe
jaccard
max − sumdiff
rho
−1 * log10(p)
value
cluster
n_lig
all
1
2
3
4

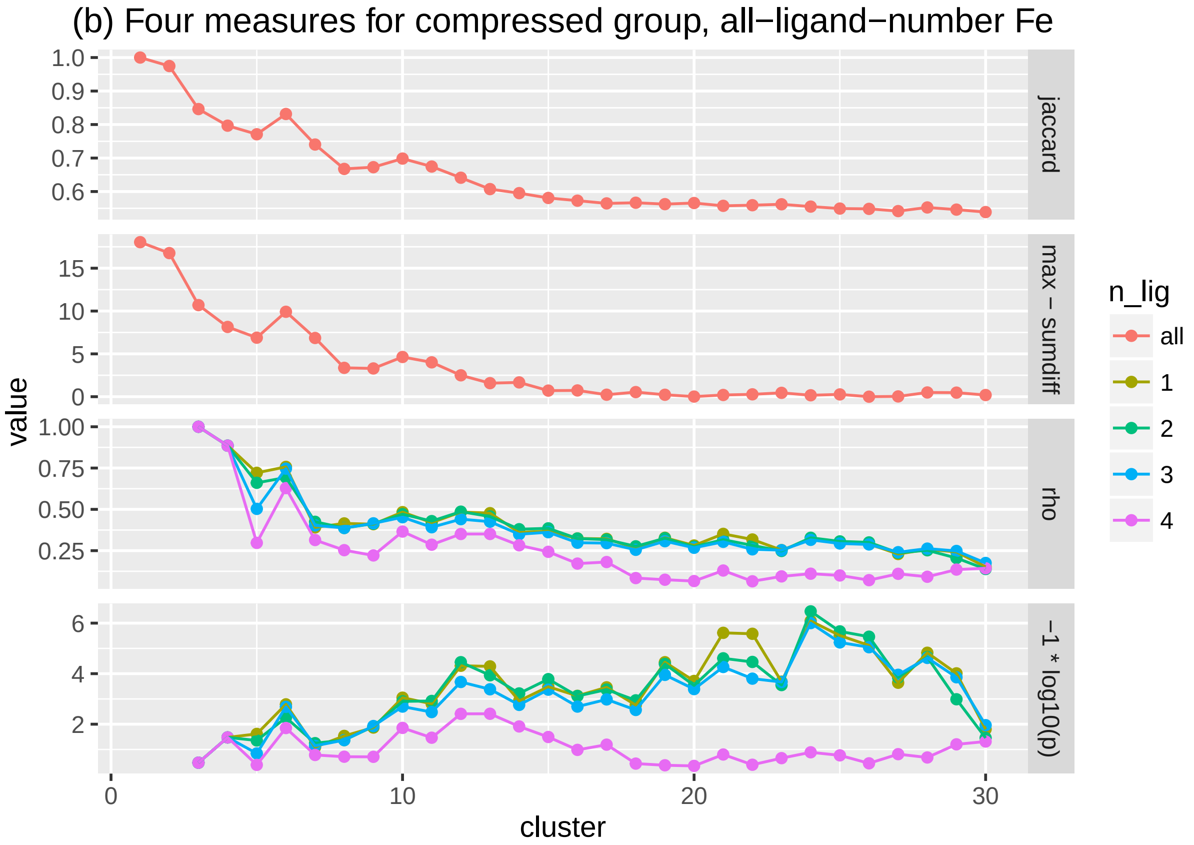
(b) Four measures for compressed group, all−ligand−number Fe
jaccard
max − sumdiff
rho
−1 * log10(p)
value
cluster
n_lig
all
1
2
3
4

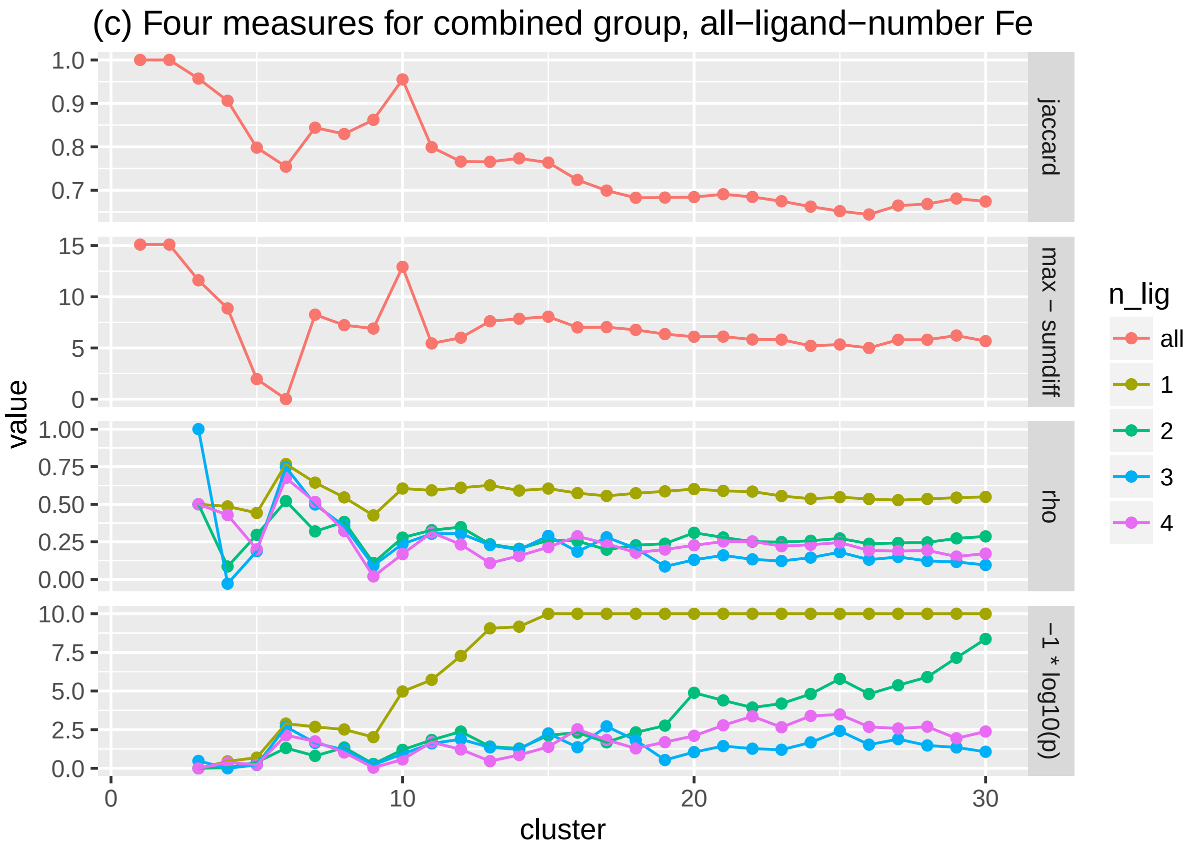
(c) Four measures for combined group, all−ligand−number Fe
value
cluster
jaccard
max − sumdiff
rho
−1 * log10(p)
n_lig
all
1
2
3
4

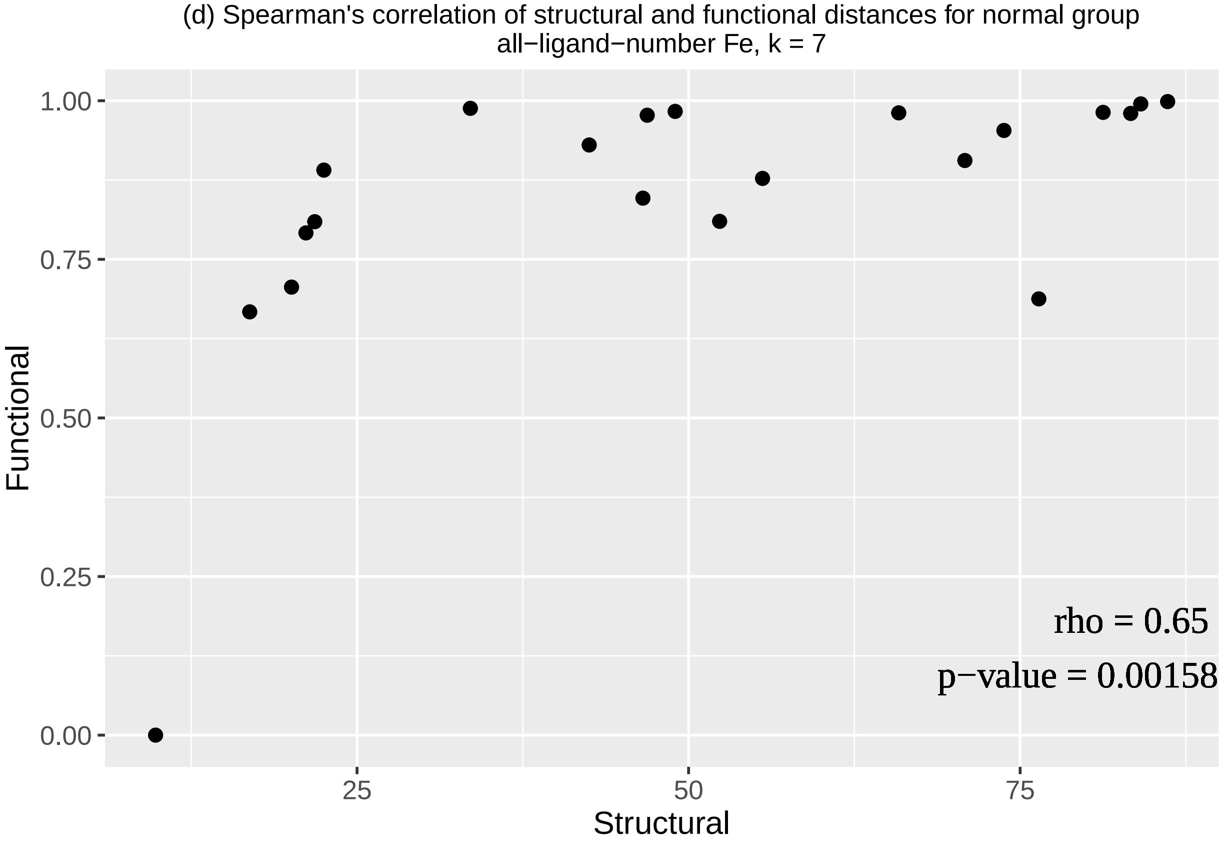
(d) Spearman's correlation of structural and functional distances for normal group
all−ligand−number Fe, k = 7
Functional
Structural
rho = 0.65
p−value = 0.00158

(e) Comparison between structural and functional hierarchical dendrograms for normal group all-ligand-number Fe

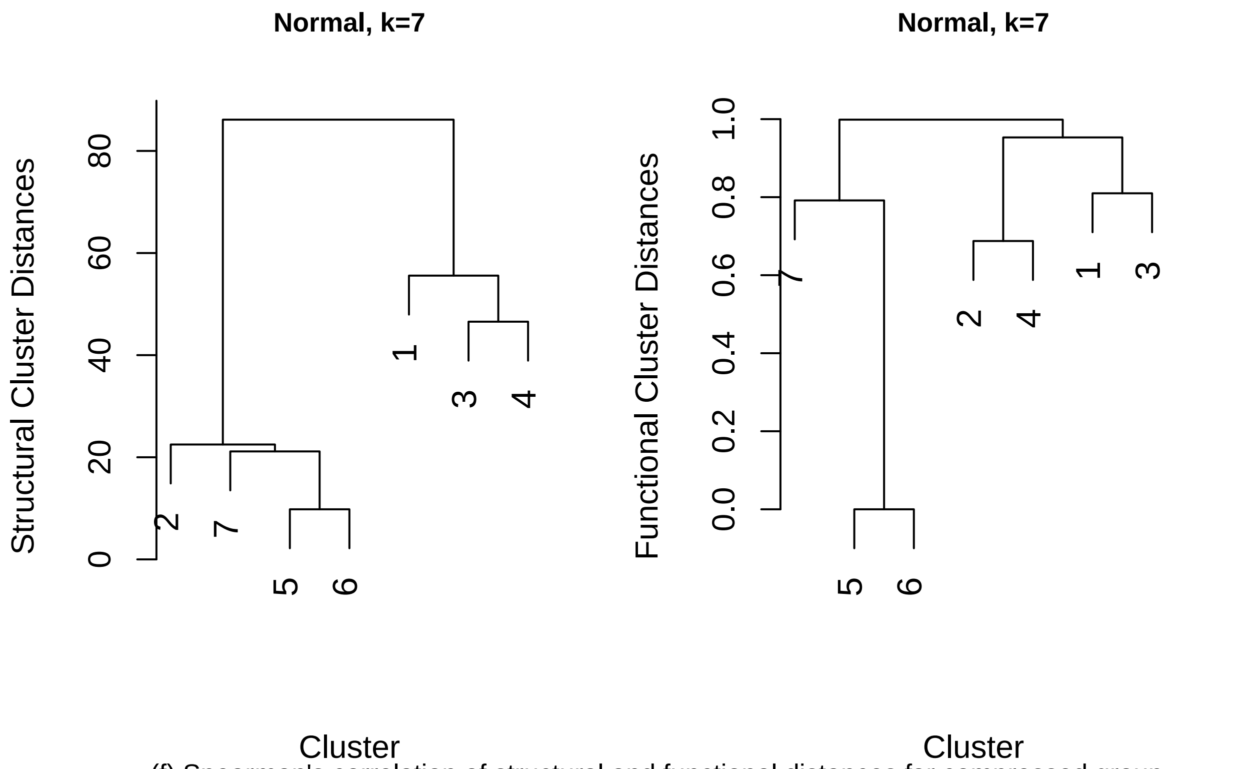

(f) Spearman's correlation of structural and functional distances for compressed group all−ligand−number Fe, k = 6

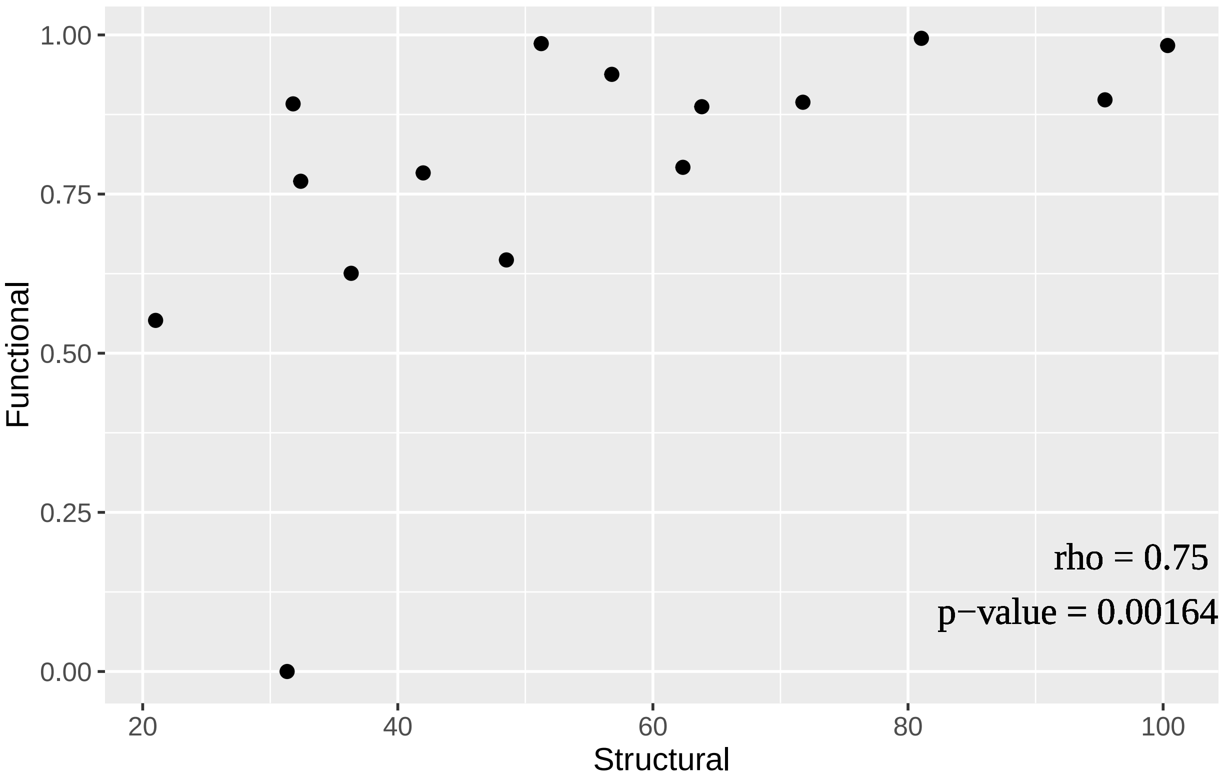

(g) Comparison between structural and functional hierarchical dendrograms for compressed group all-ligand-number Fe

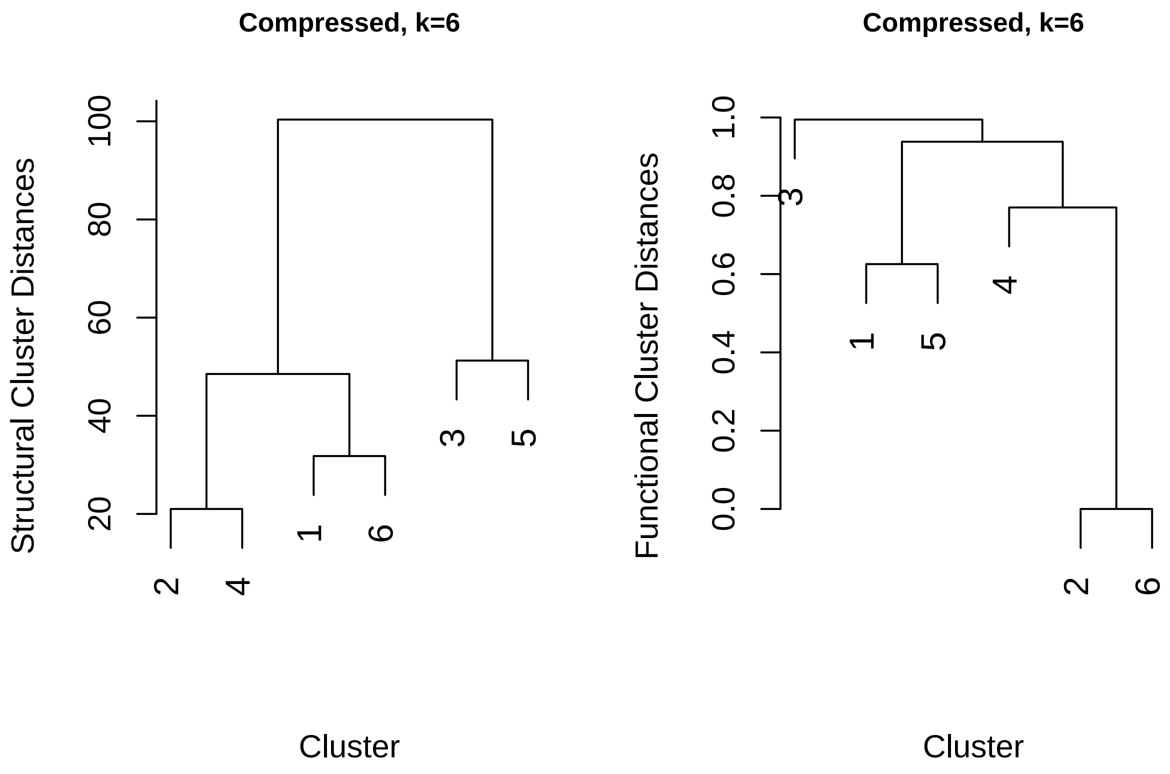

Figure S19. 4-ligand Na metalloproteins

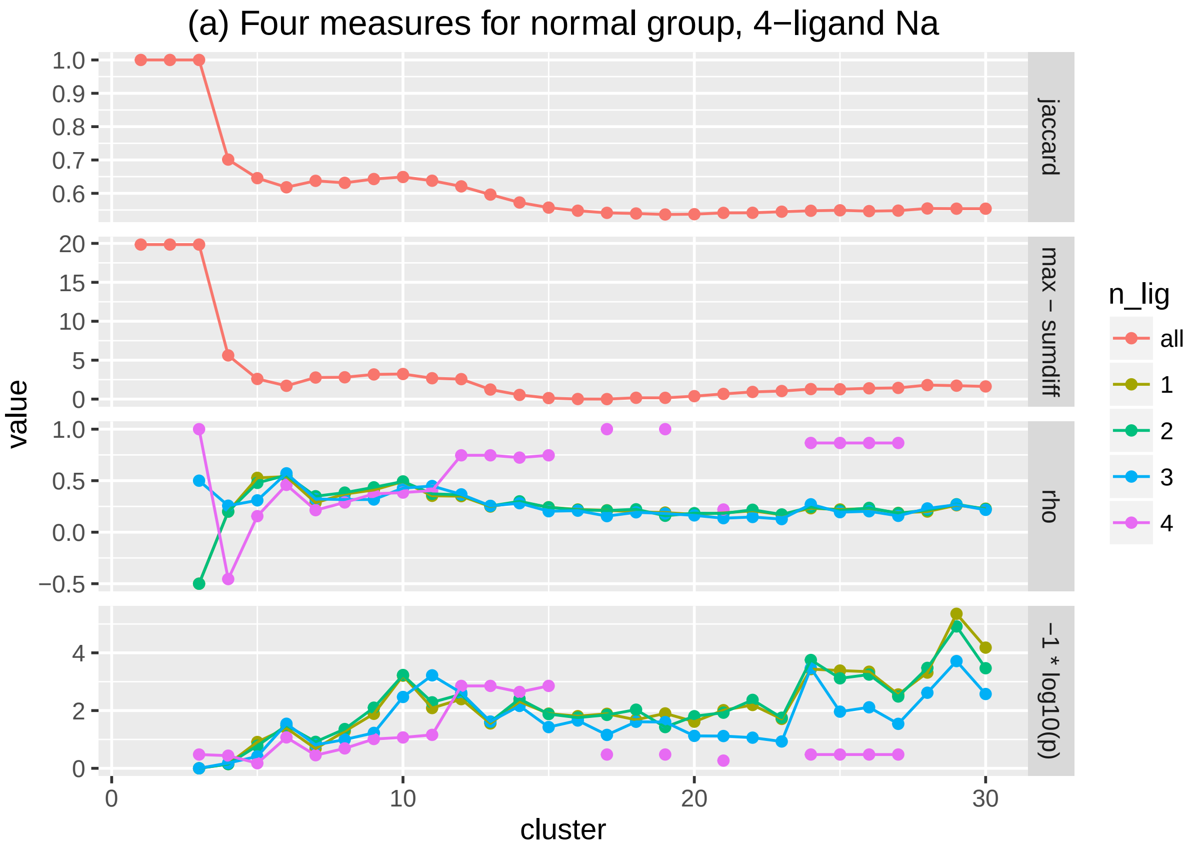
(a) Four measures for normal group, 4−ligand Na
jaccard
max − sumdiff
rho
−1 * log10(p)
value
cluster
n_lig
all
1
2
3
4

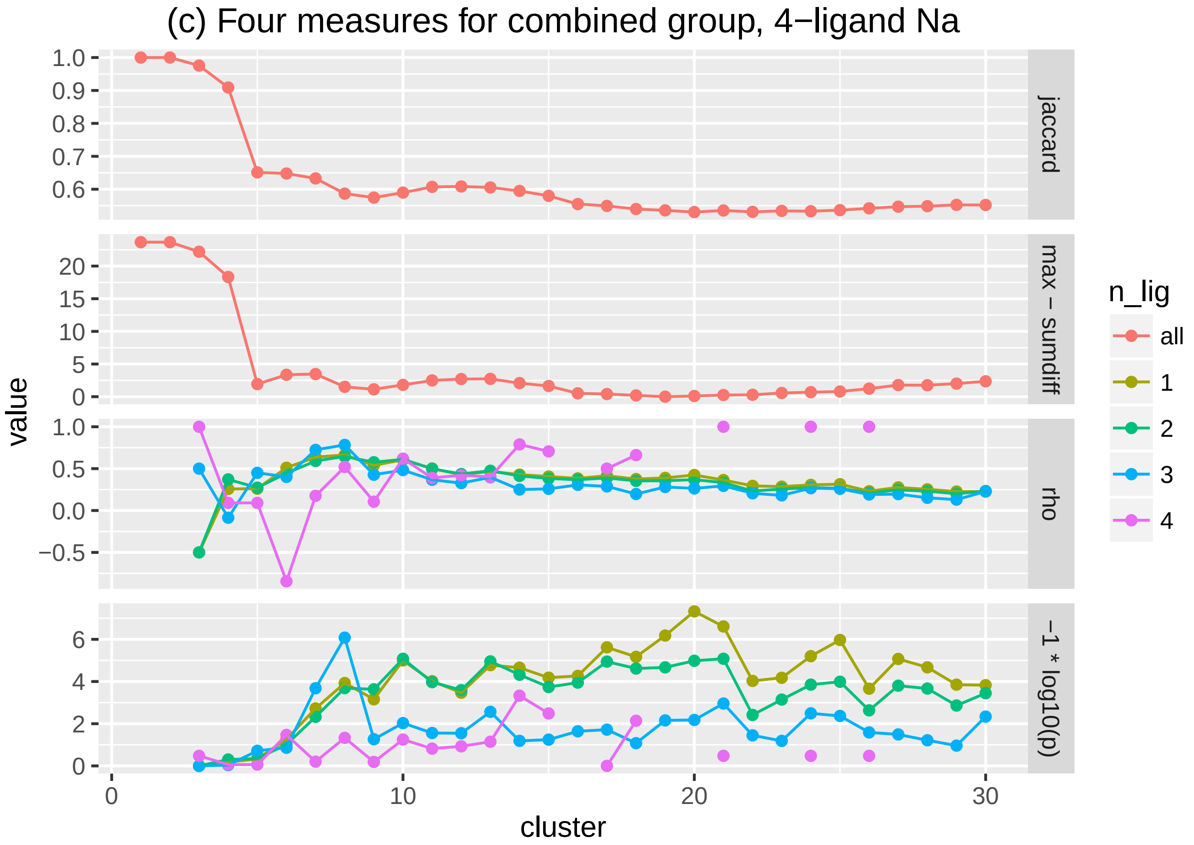
(c) Four measures for combined group, 4−ligand Na
jaccard
max − sumdiff
rho
−1 * log10(p)
value
cluster
n_lig
all
1
2
3
4

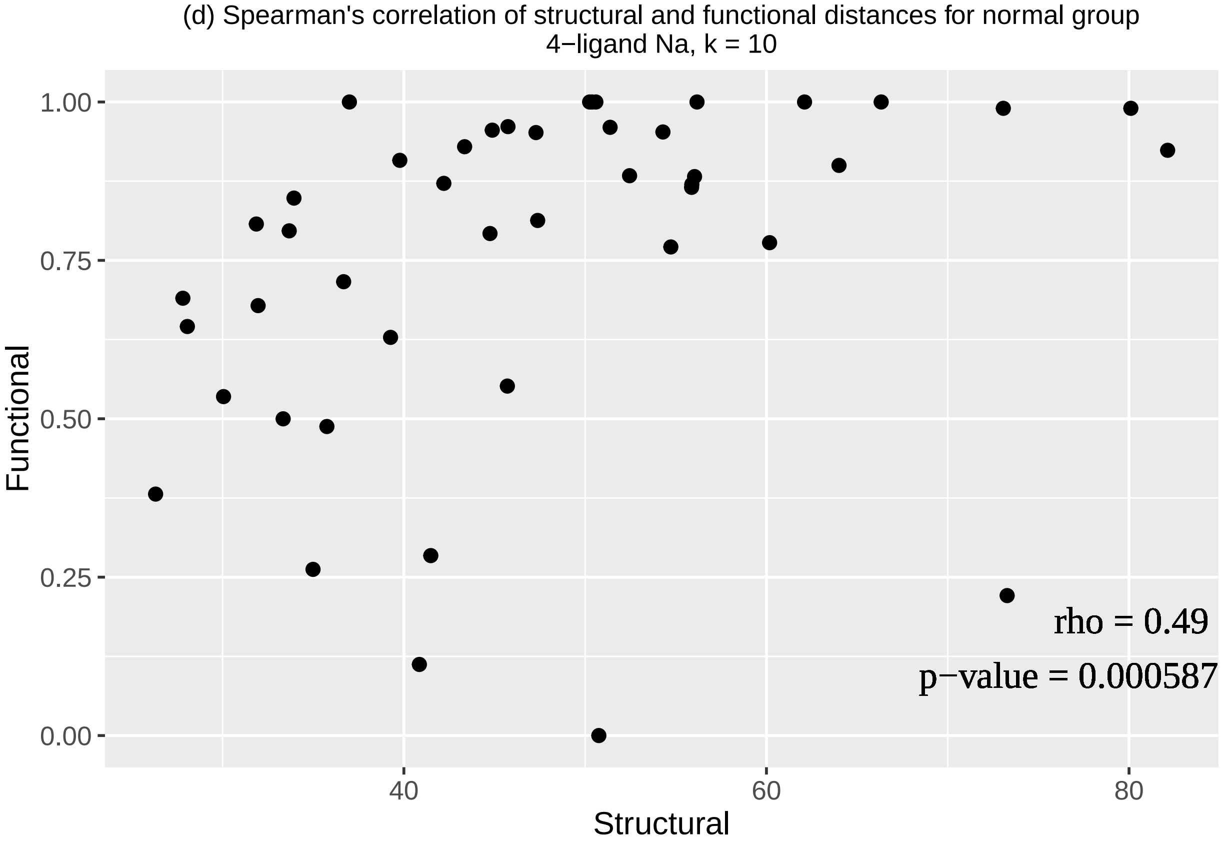

(e) Comparison between structural and functional hierarchical dendrograms for normal group 4-ligand Na

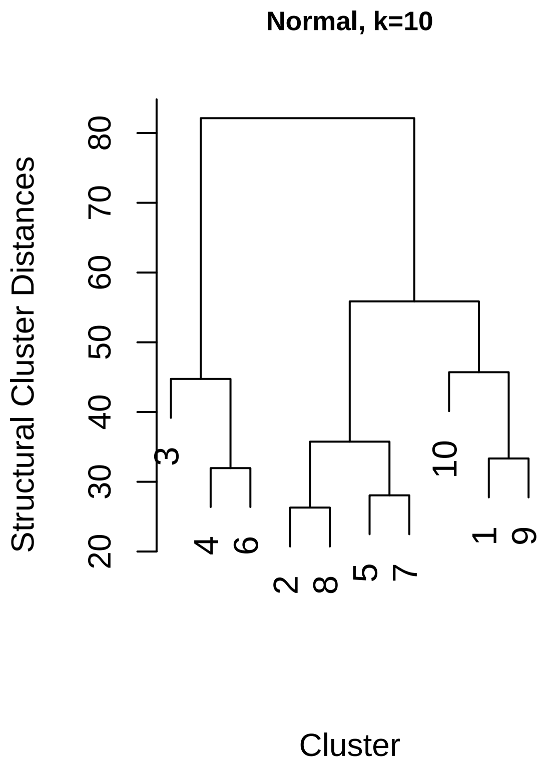

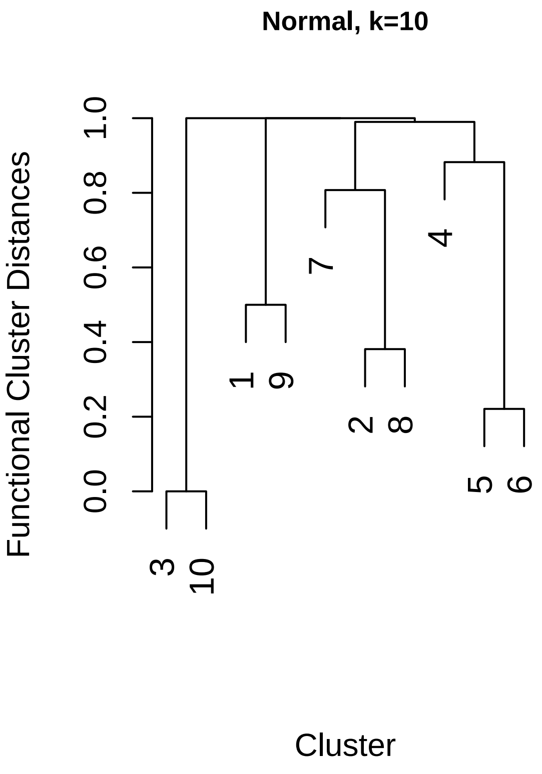

Figure S20. 5-ligand Na metalloproteins

(a) Four measures for normal group, 5−ligand Na

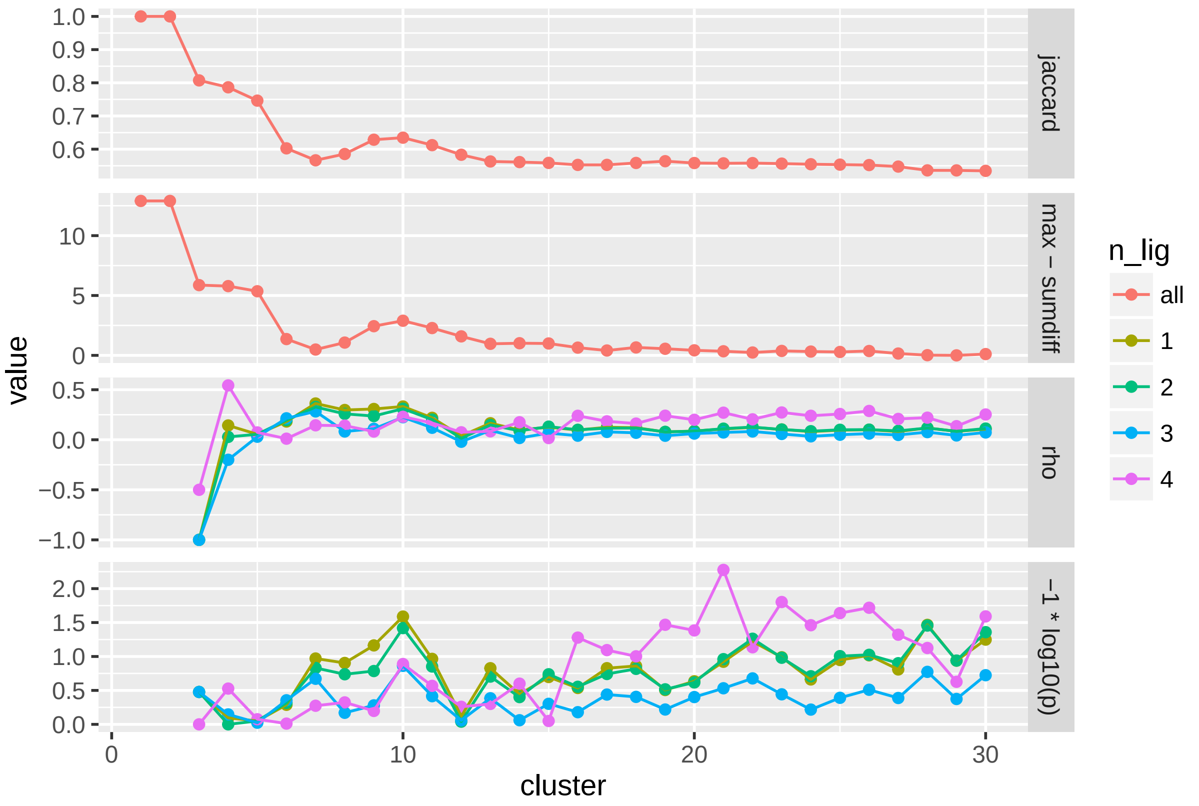

(c) Four measures for combined group, 5−ligand Na

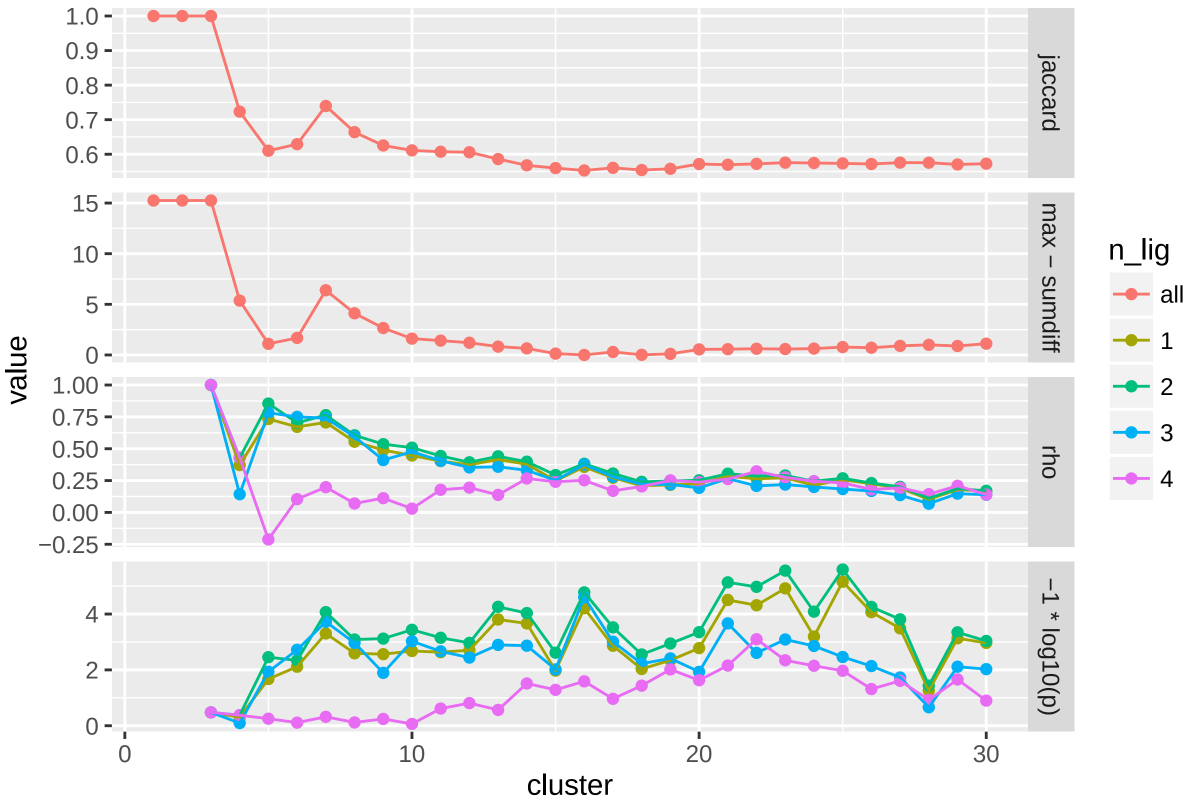

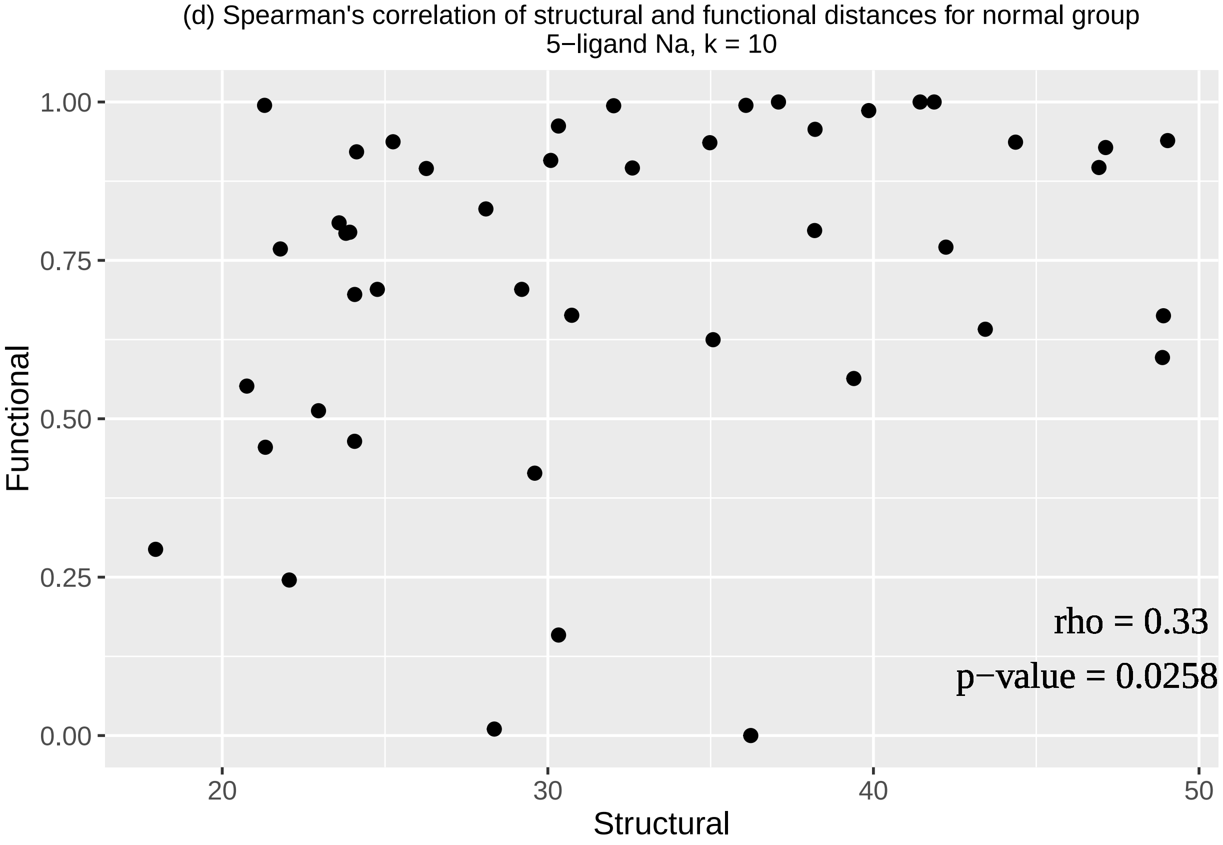

(e) Comparison between structural and functional hierarchical dendrograms for normal group 5-ligand Na

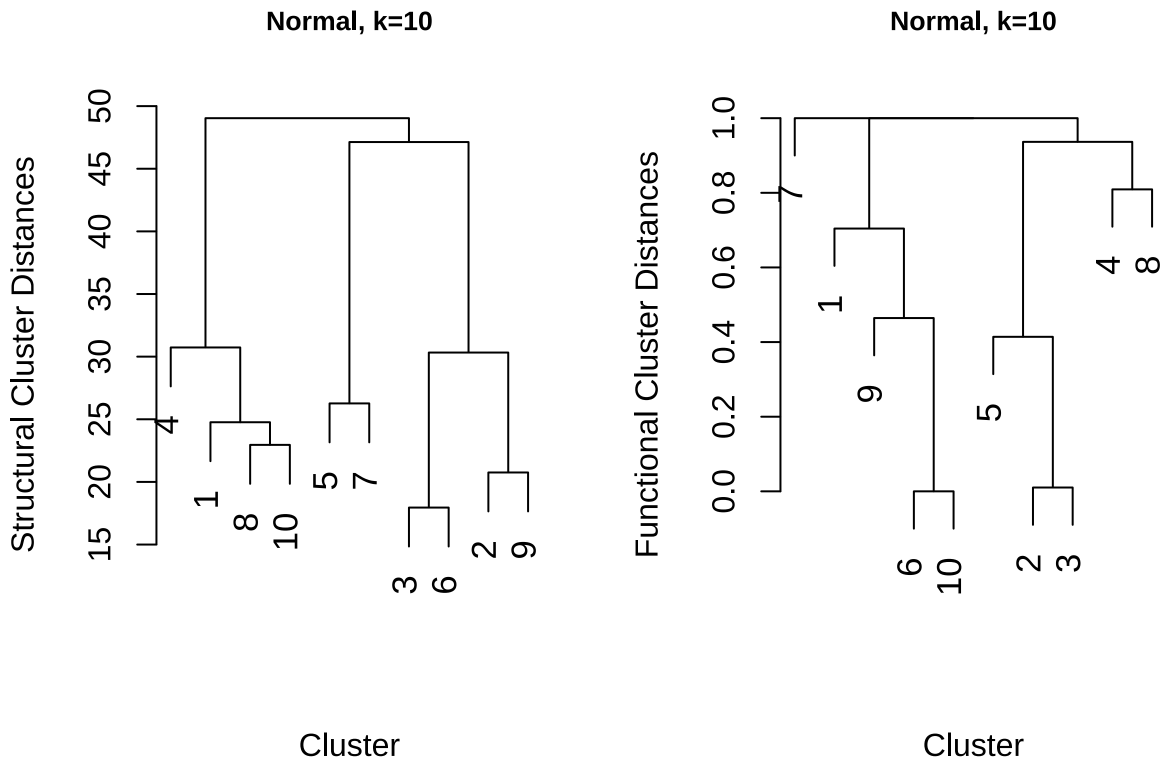

Figure S21. 6-ligand Na metalloproteins

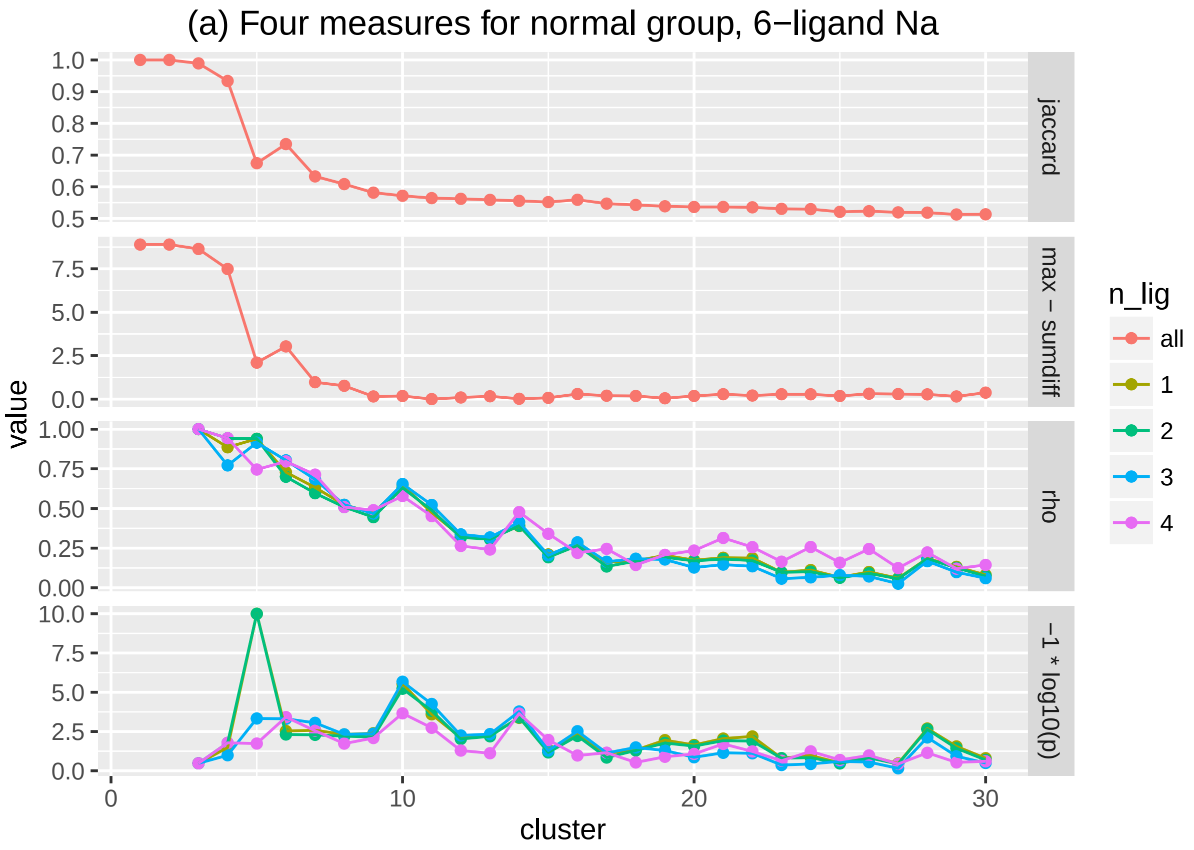
(a) Four measures for normal group, 6−ligand Na
value
cluster
jaccard
max − sumdiff
rho
−1 * log10(p)
n_lig
all
1
2
3
4

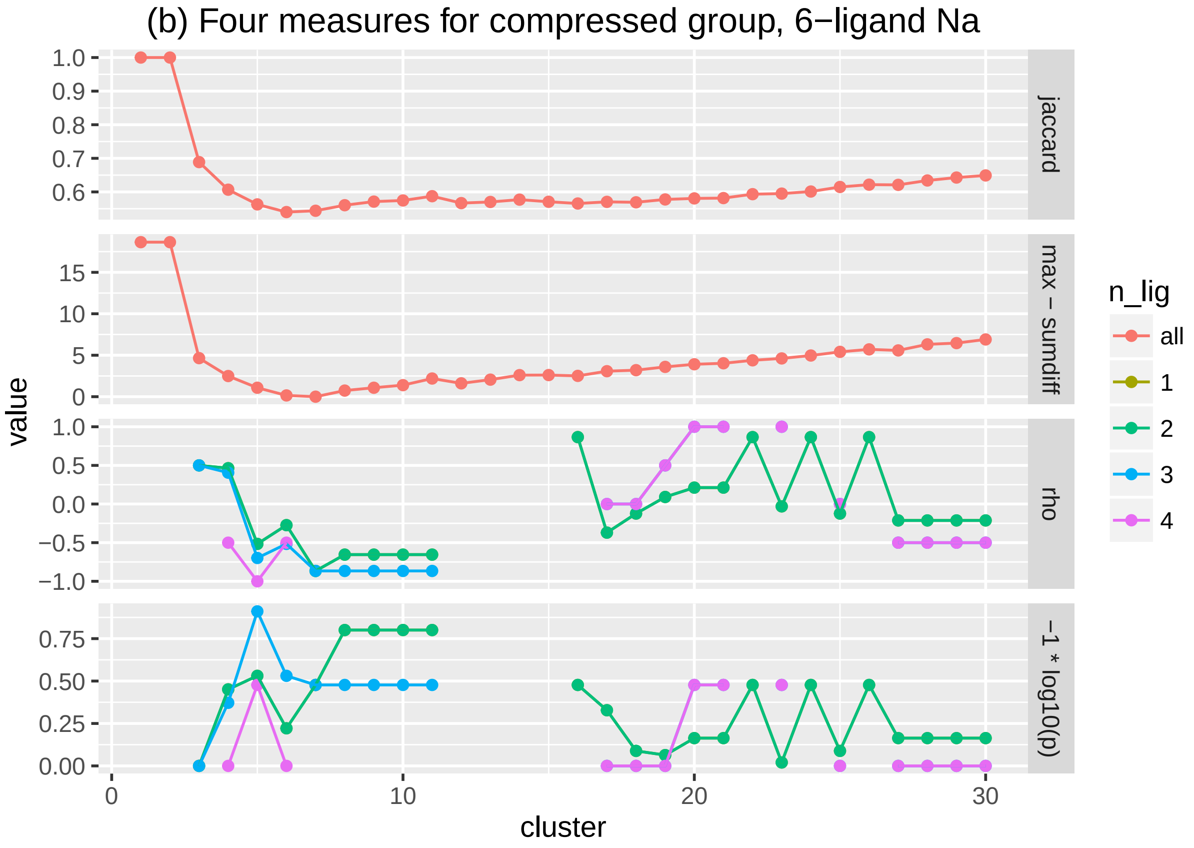
(b) Four measures for compressed group, 6−ligand Na
value
cluster
jaccard
max − sumdiff
rho
−1 * log10(p)
n_lig
all
1
2
3
4

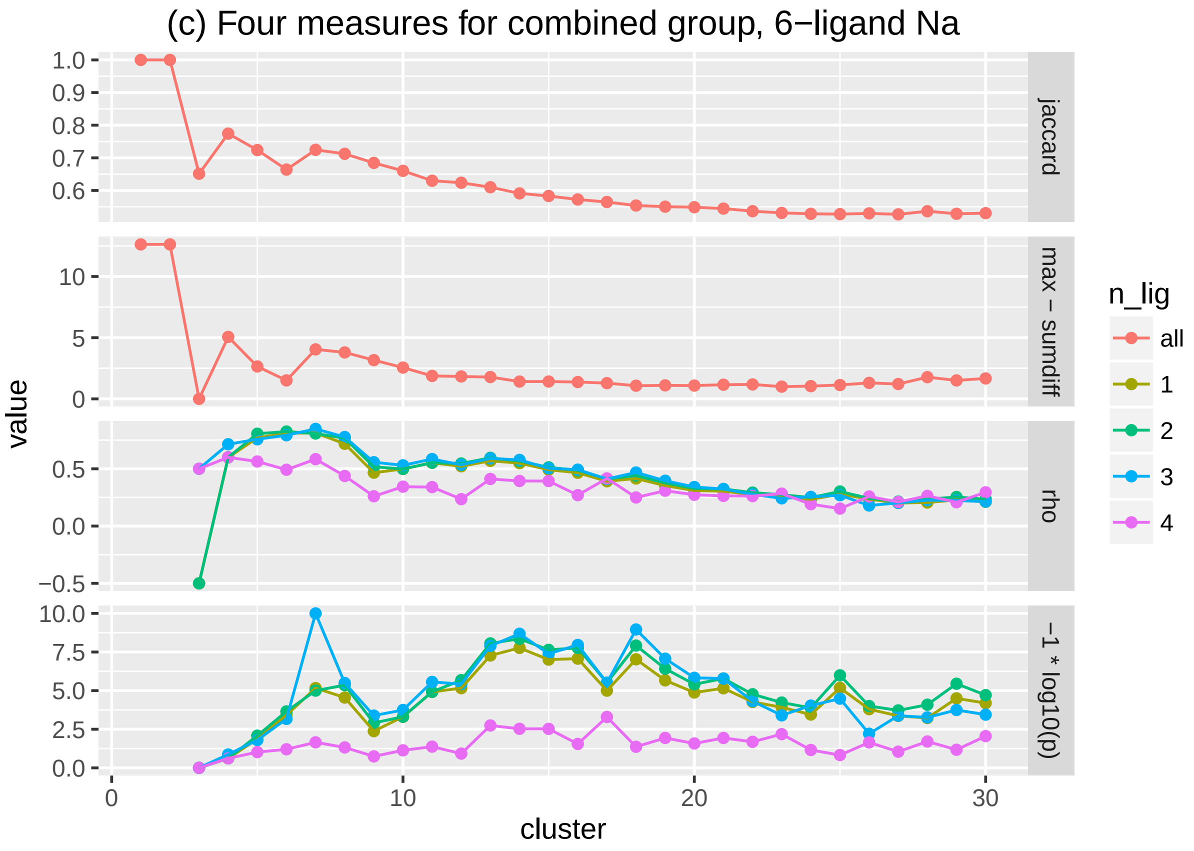
(c) Four measures for combined group, 6−ligand Na
value
cluster
jaccard
max − sumdiff
rho
−1 * log10(p)
n_lig
all
1
2
3
4

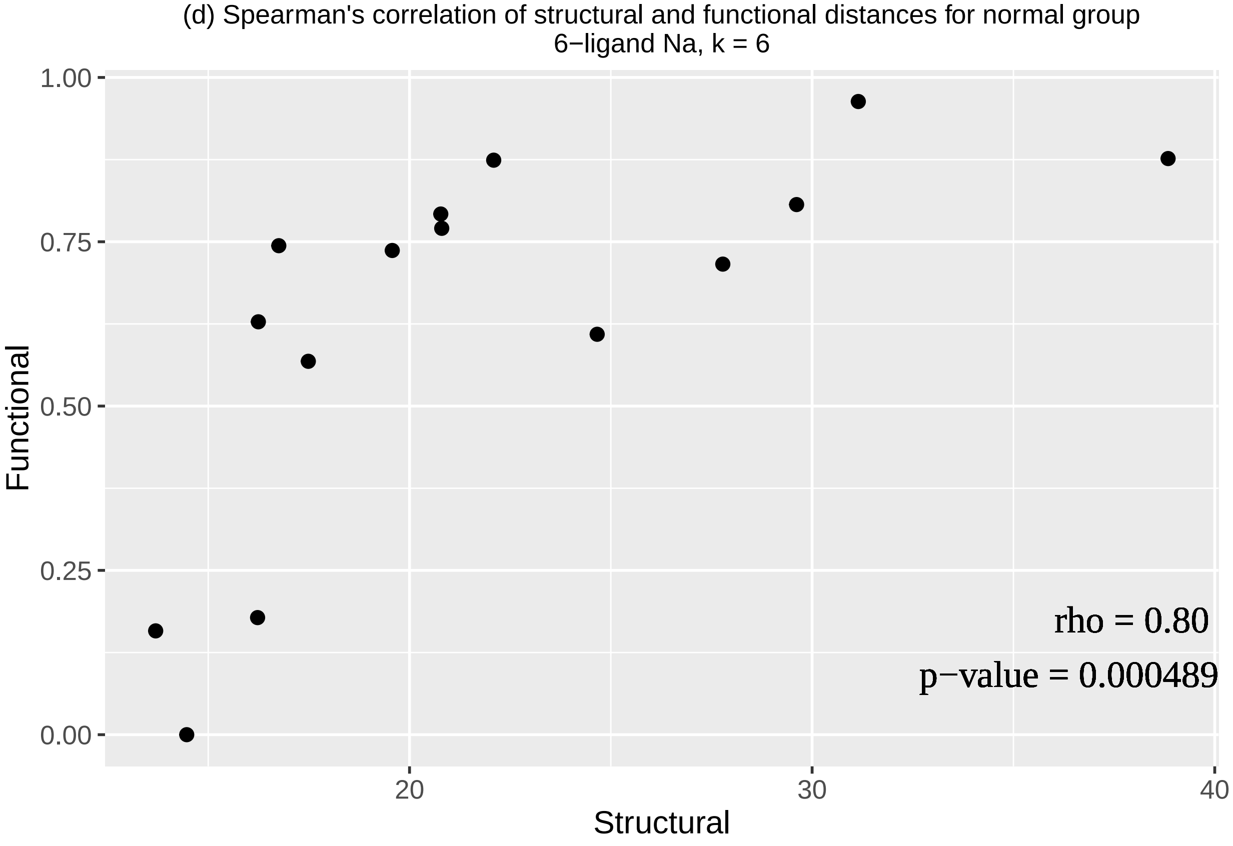
(d) Spearman's correlation of structural and functional distances for normal group
6−ligand Na, k = 6
Functional
Structural
rho = 0.80
p−value = 0.000489

(e) Comparison between structural and functional hierarchical dendrograms for normal group 6-ligand Na

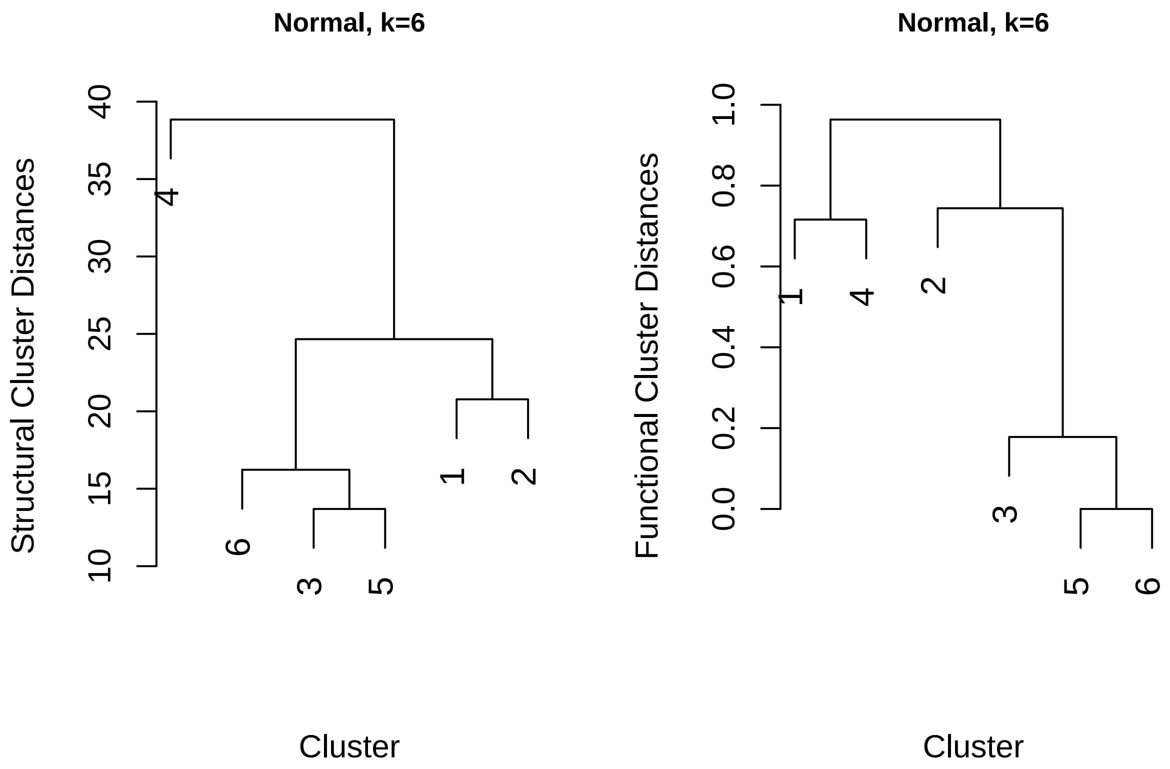

Figure S22. all-ligand Na metalloproteins

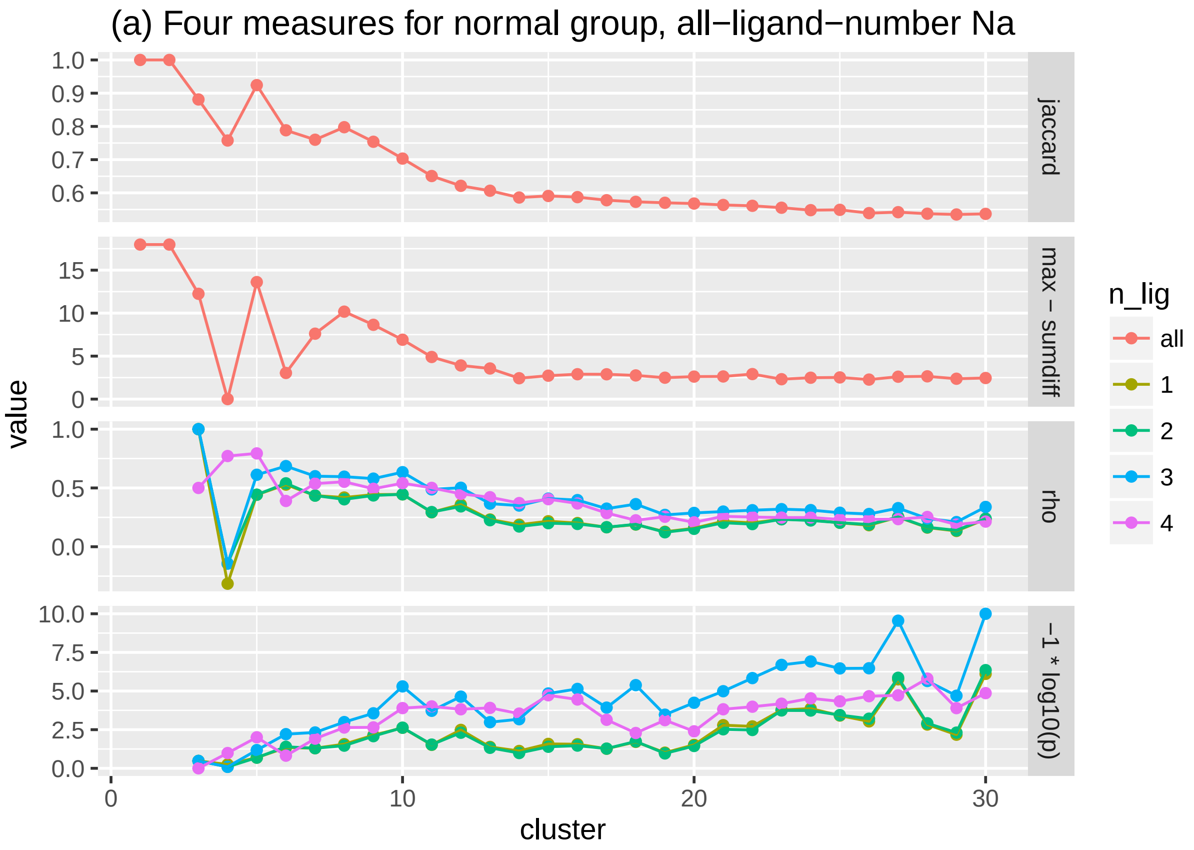

(a) Four measures for normal group, all−ligand−number Na
jaccard
max − sumdiff
rho
−1 * log10(p)
value
cluster
n_lig
all
1
2
3
4

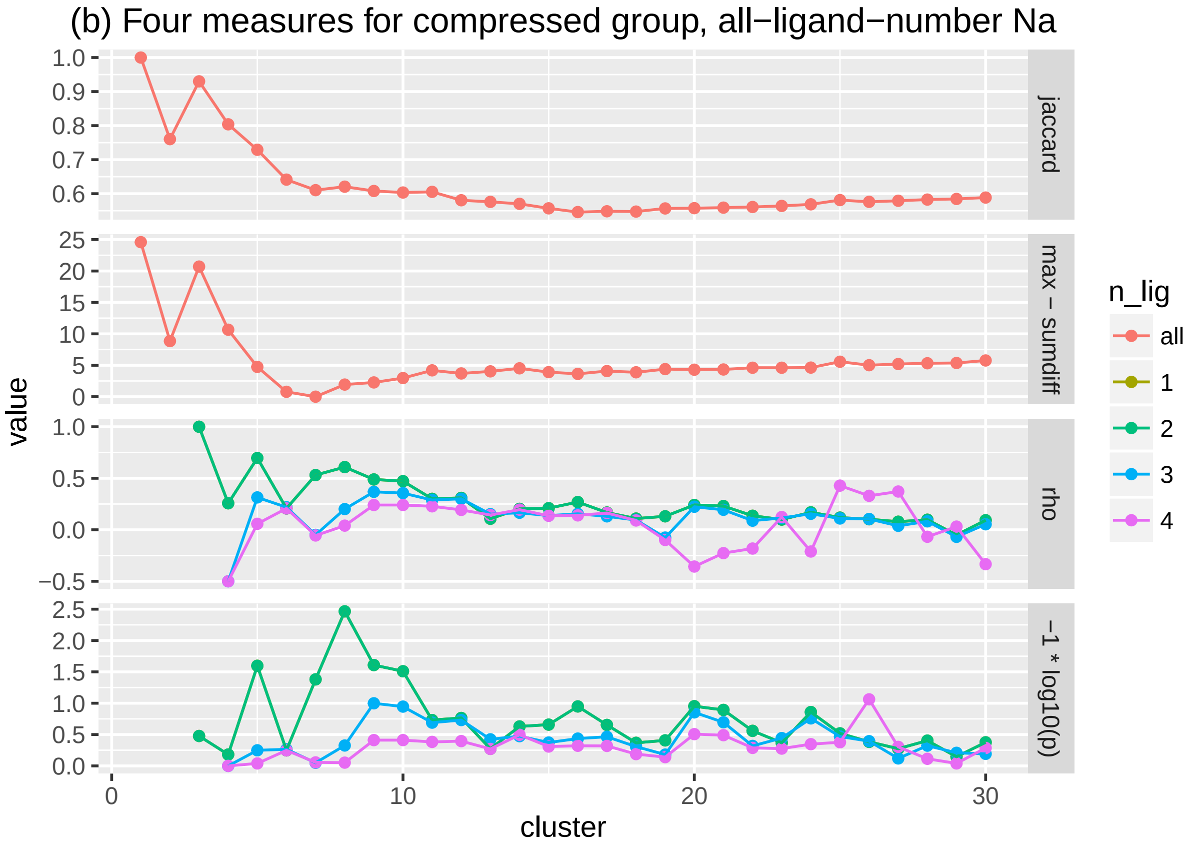

(b) Four measures for compressed group, all−ligand−number Na
jaccard
max − sumdiff
rho
−1 * log10(p)
value
cluster
n_lig
all
1
2
3
4

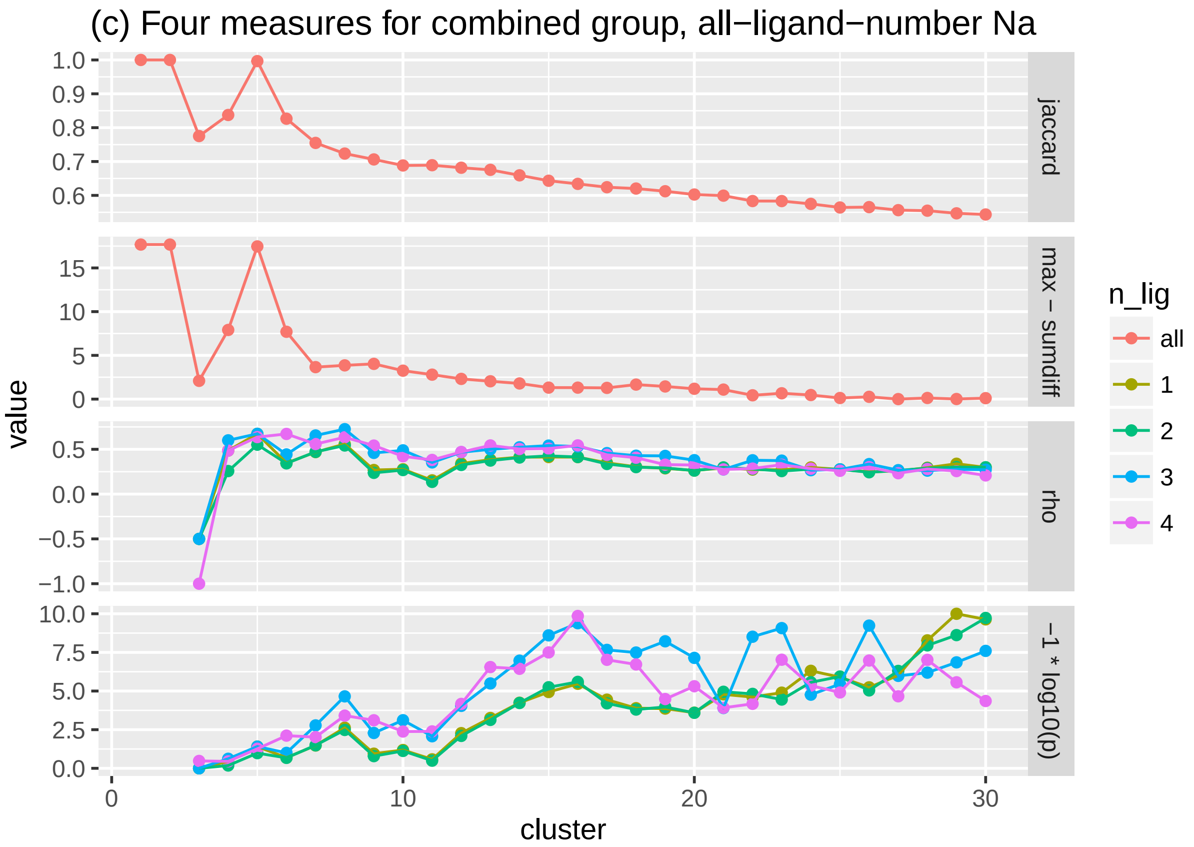
(c) Four measures for combined group, all−ligand−number Na
value
cluster
jaccard
max − sumdiff
rho
−1 * log10(p)
n_lig
all
1
2
3
4

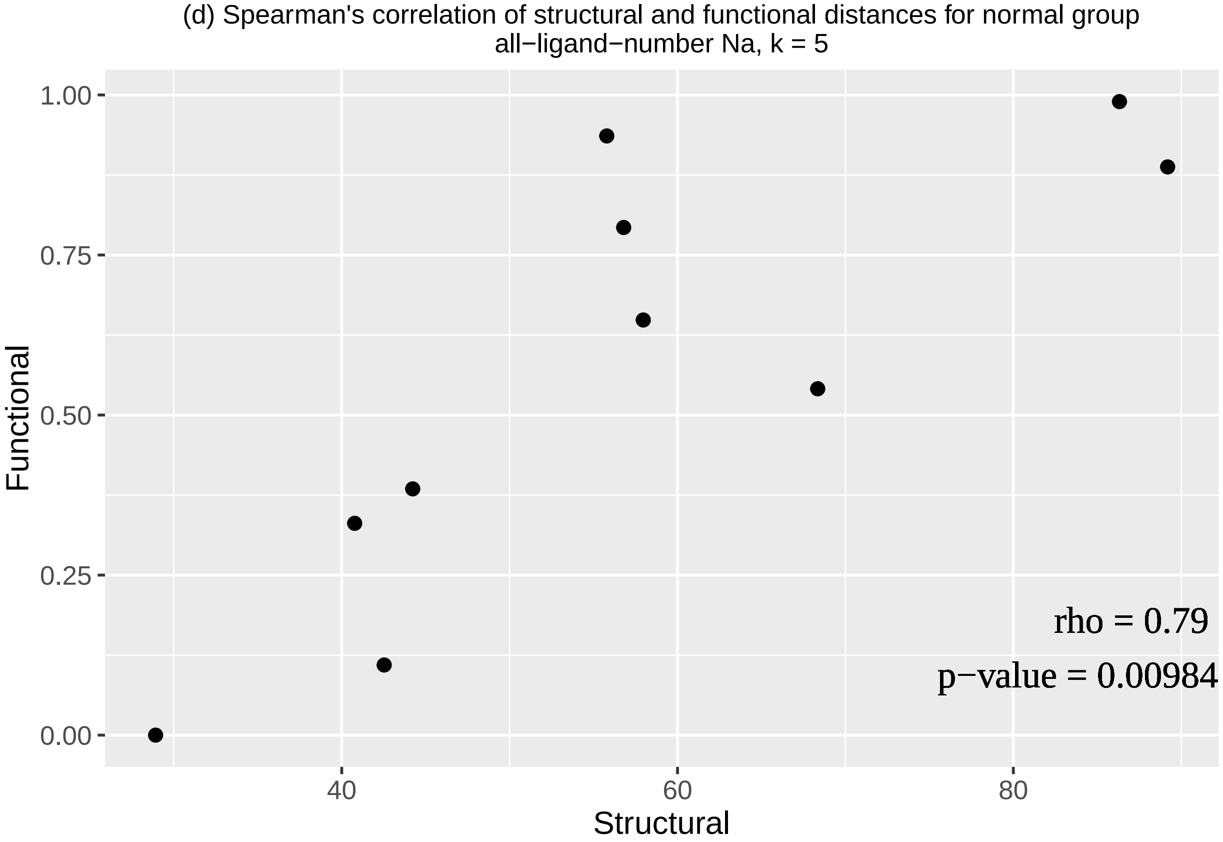
(d) Spearman's correlation of structural and functional distances for normal group
all−ligand−number Na, k = 5
Functional
Structural
rho = 0.79
p−value = 0.00984

(e) Comparison between structural and functional hierarchical dendrograms for normal group all-ligand-number Na

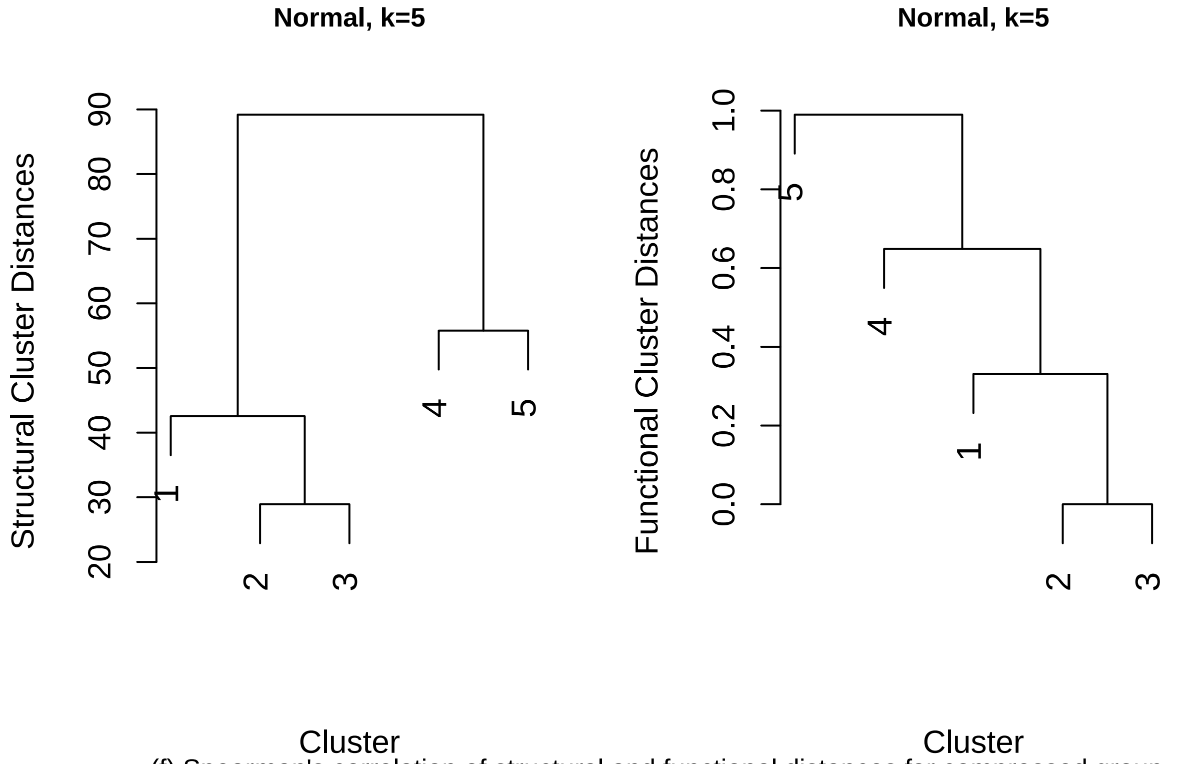

(f) Spearman's correlation of structural and functional distances for compressed group all−ligand−number Na, k = 8

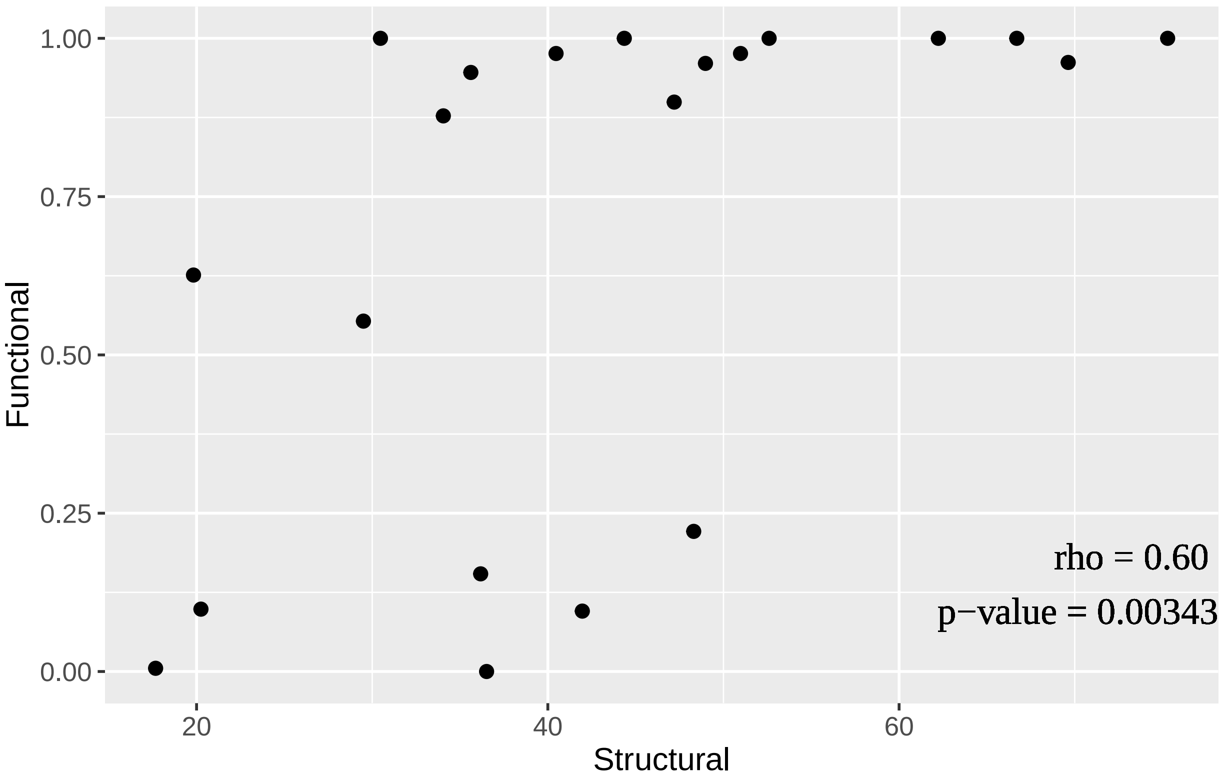

(g) Comparison between structural and functional hierarchical dendrograms for compressed group all-ligand-number Na

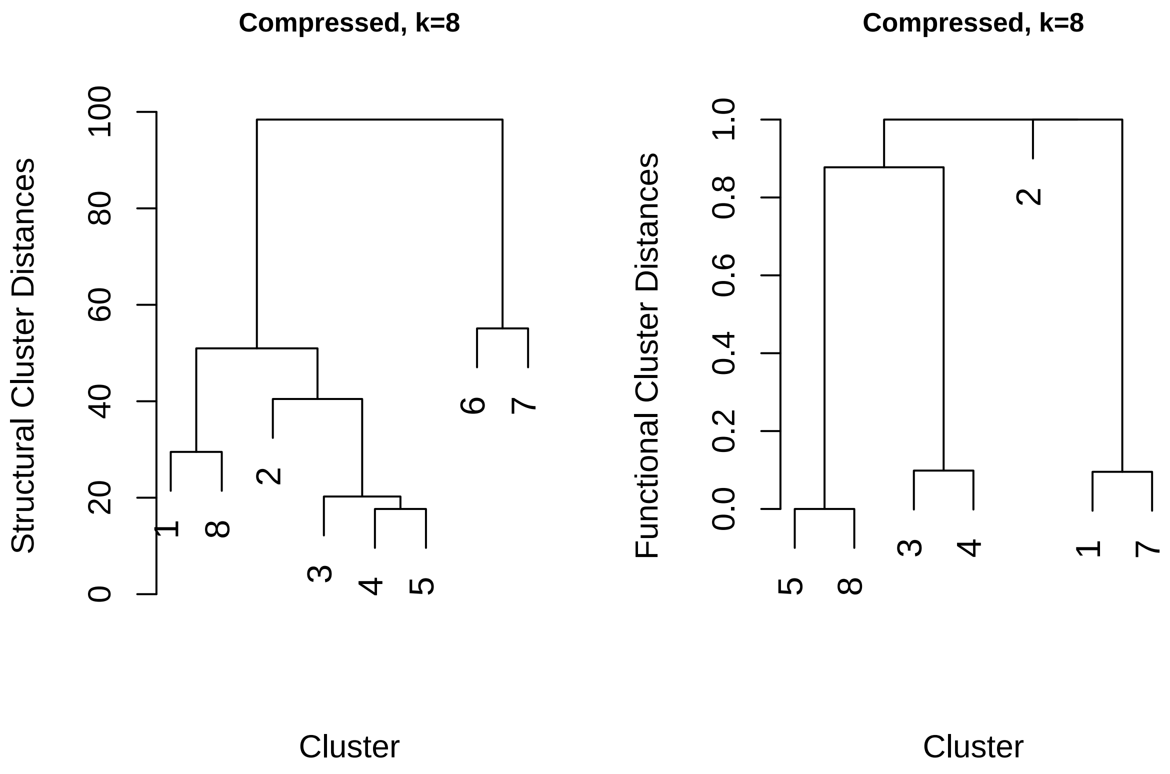

Figure S23. 4-ligand all metalloproteins

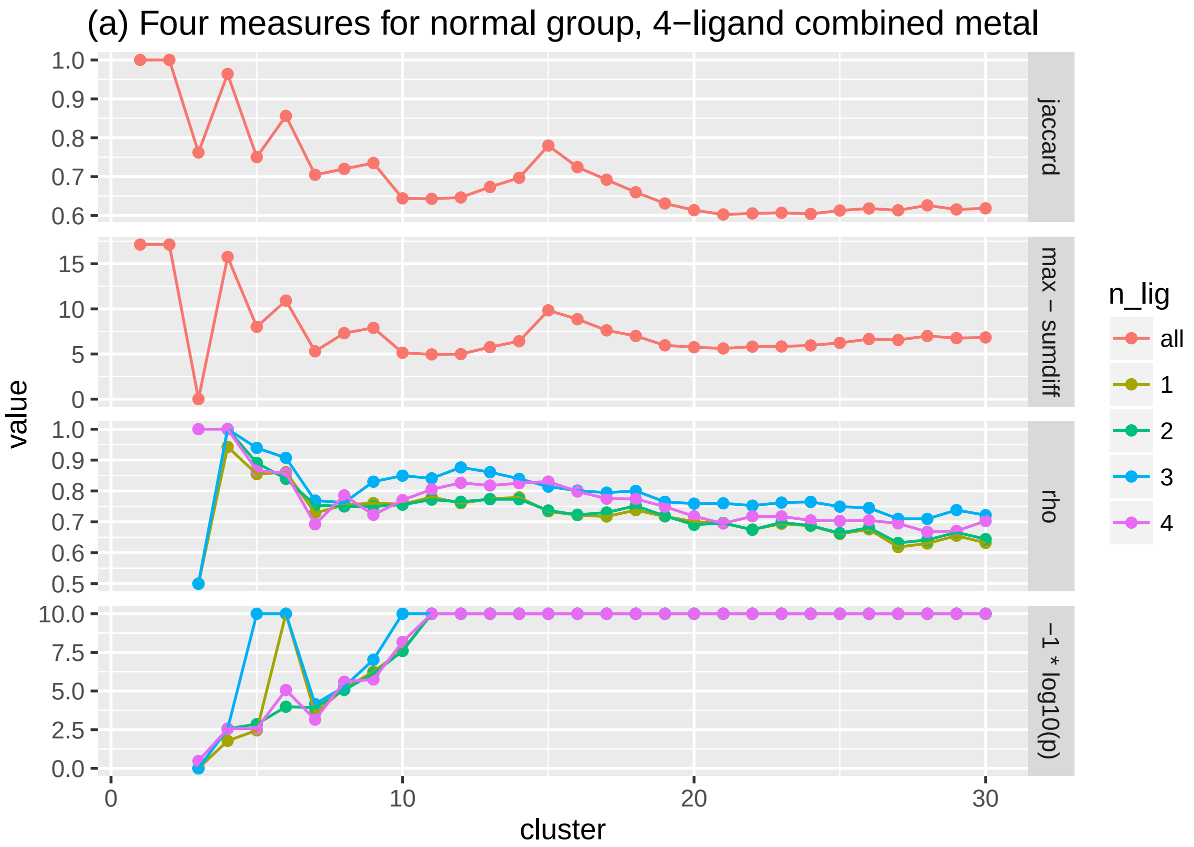
(a) Four measures for normal group, 4−ligand combined metal
jaccard
max − sumdiff
rho
−1 * log10(p)
value
cluster
n_lig
all
1
2
3
4

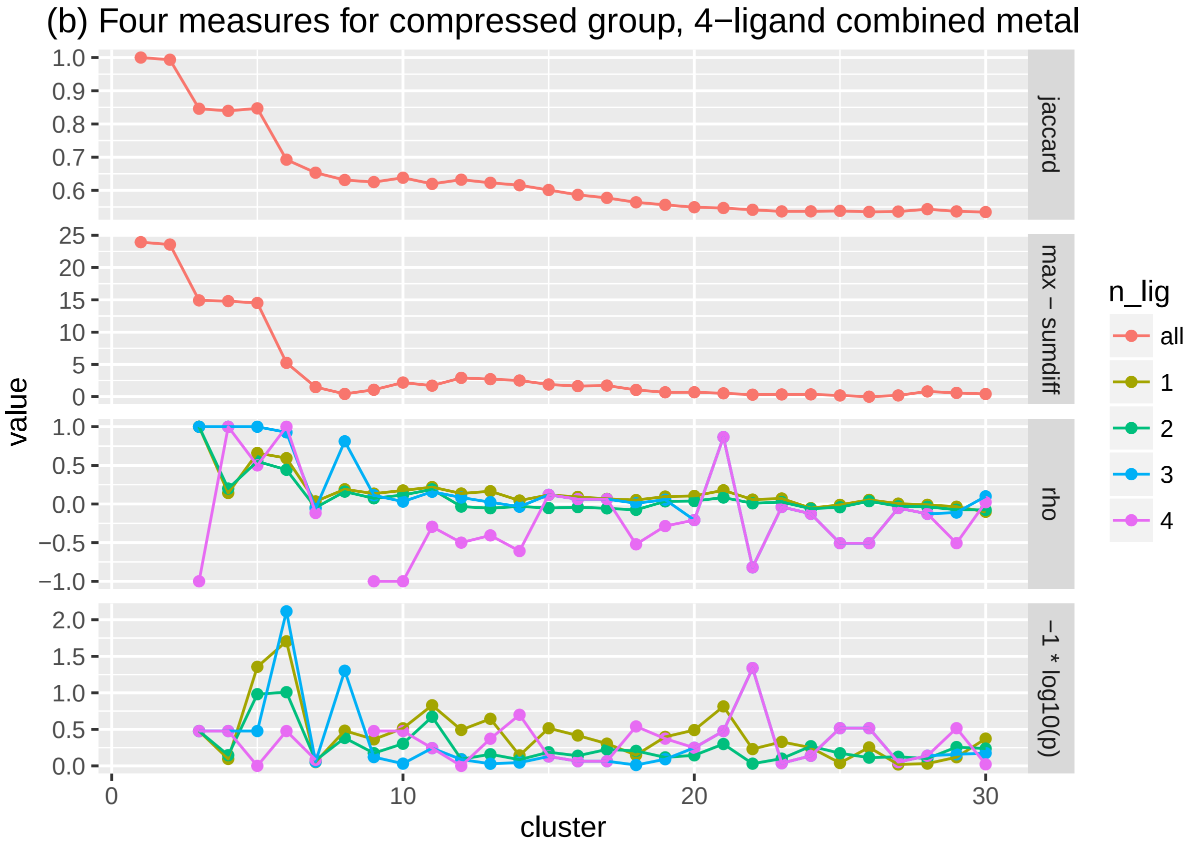
(b) Four measures for compressed group, 4−ligand combined metal
jaccard
max − sumdiff
rho
−1 * log10(p)
value
cluster
n_lig
all
1
2
3
4

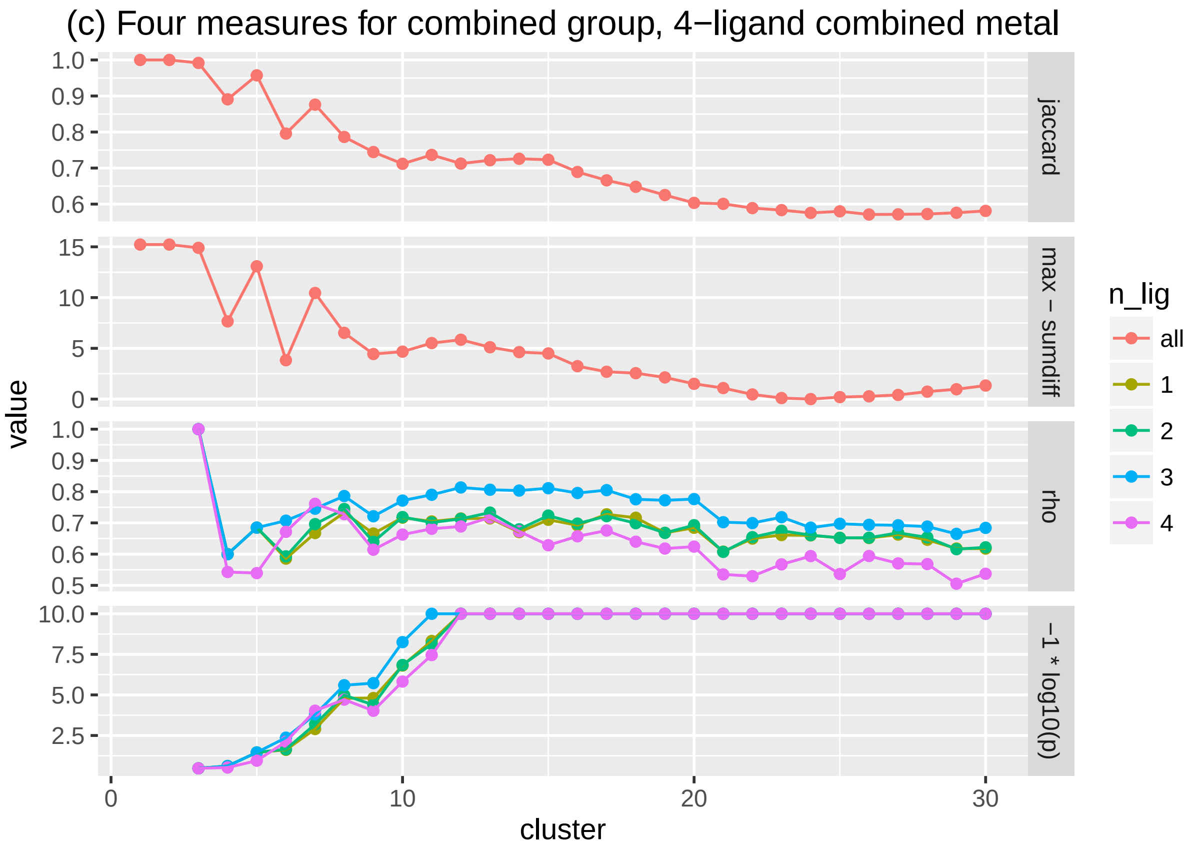
(c) Four measures for combined group, 4−ligand combined metal
value
cluster
jaccard
max − sumdiff
rho
−1 * log10(p)
n_lig
all
1
2
3
4

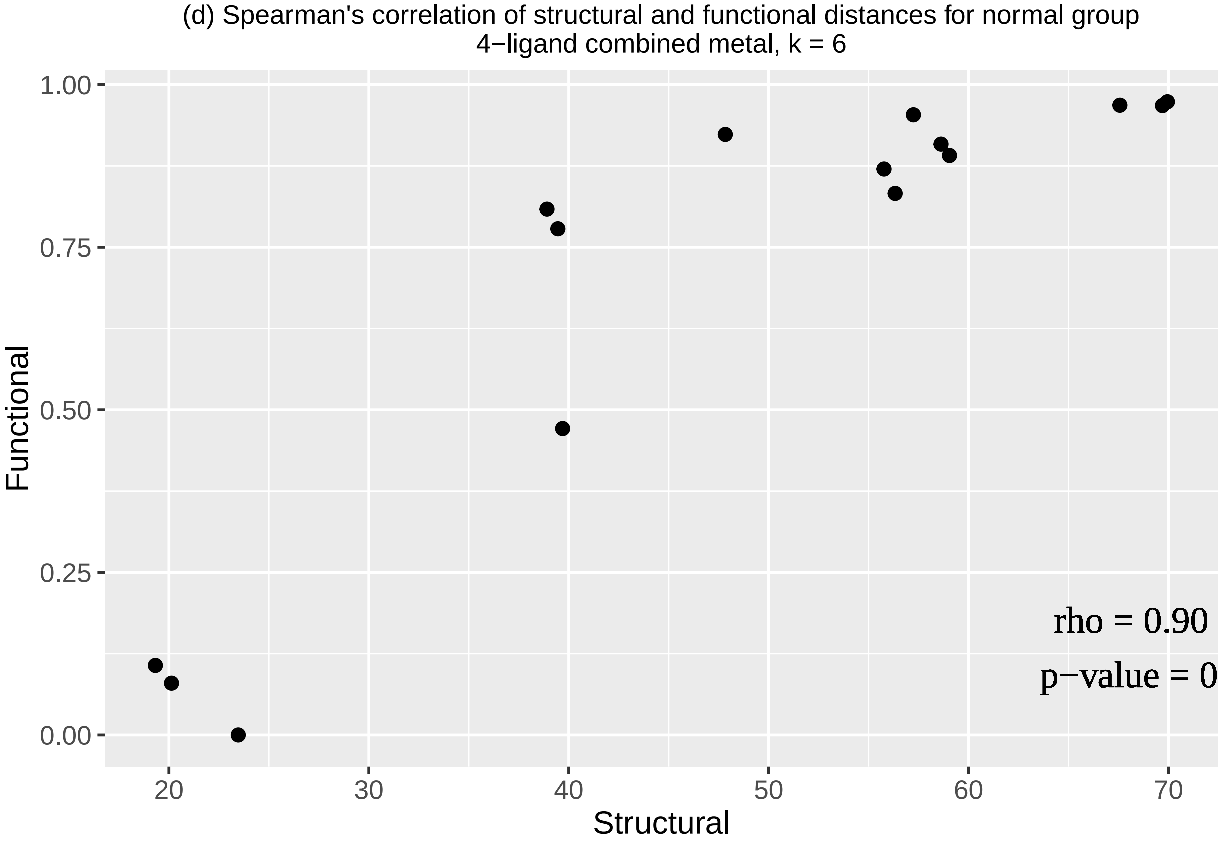
(d) Spearman's correlation of structural and functional distances for normal group
4−ligand combined metal, k = 6
Functional
Structural
rho = 0.90
p−value = 0

(e) Comparison between structural and functional hierarchical dendrograms for normal group 4-ligand combined metal

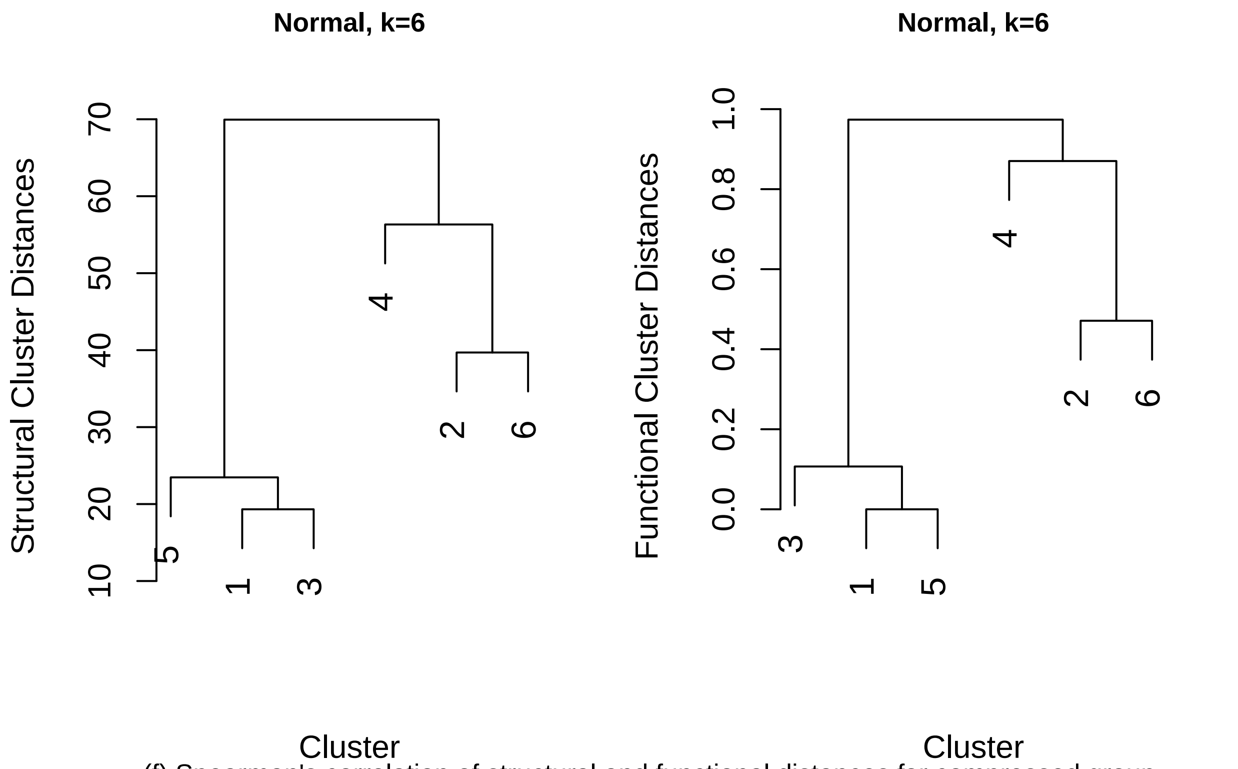

(f) Spearman's correlation of structural and functional distances for compressed group 4−ligand combined metal, k = 5

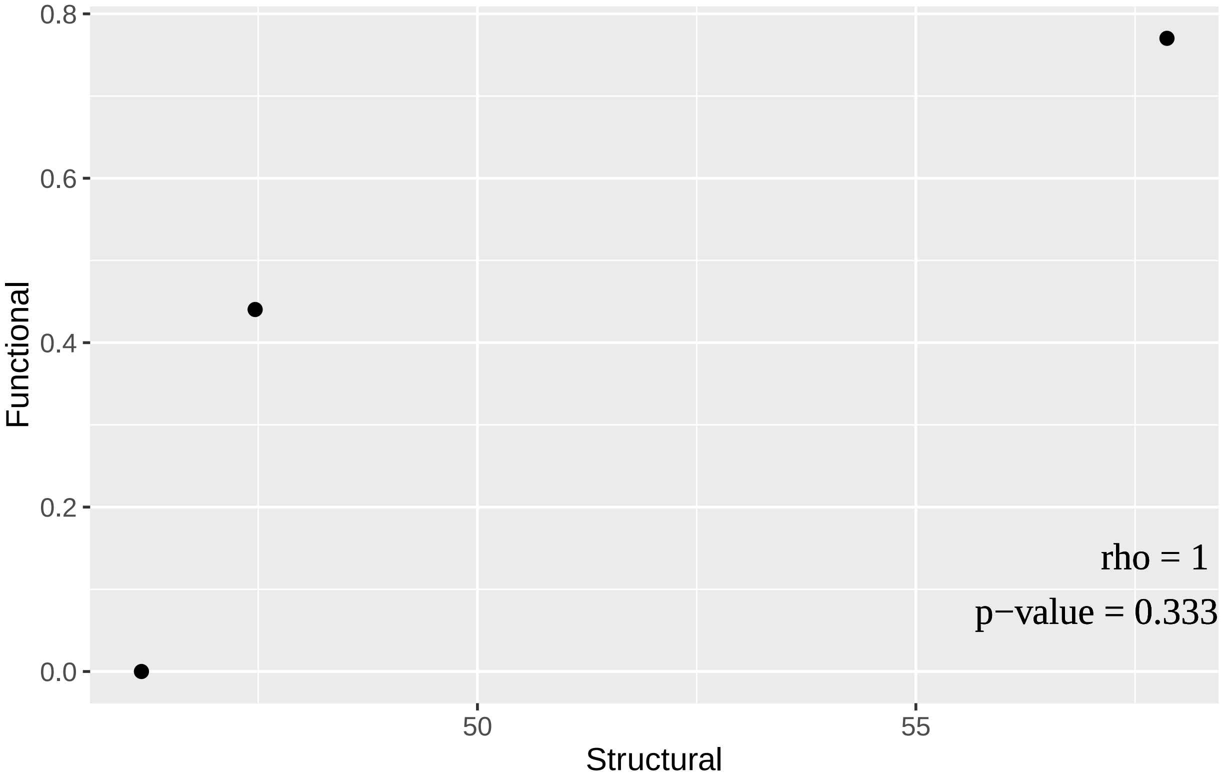

(g) Comparison between structural and functional hierarchical dendrograms for compressed group 4-ligand combined metal

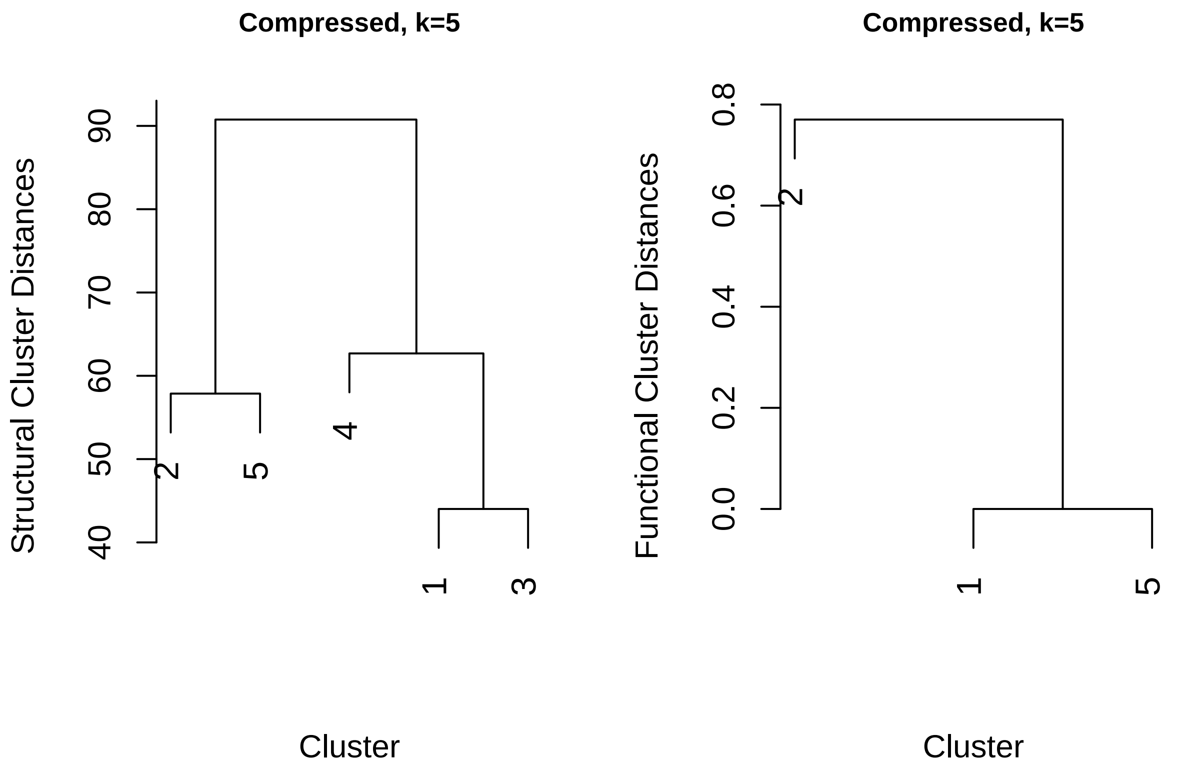

Figure S24. 5-ligand all metalloproteins

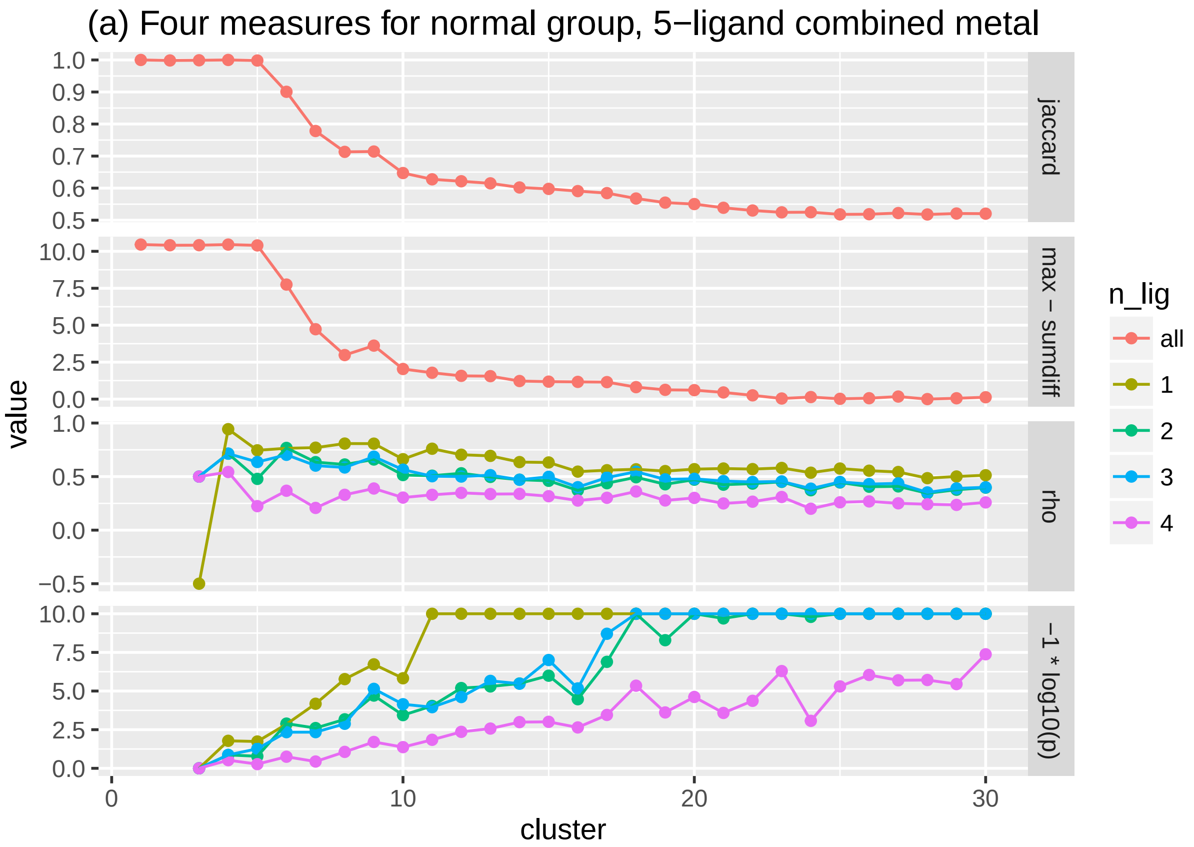
(a) Four measures for normal group, 5−ligand combined metal
value
cluster
jaccard
max − sumdiff
rho
−1 * log10(p)
n_lig
all
1
2
3
4
0
10
20
30

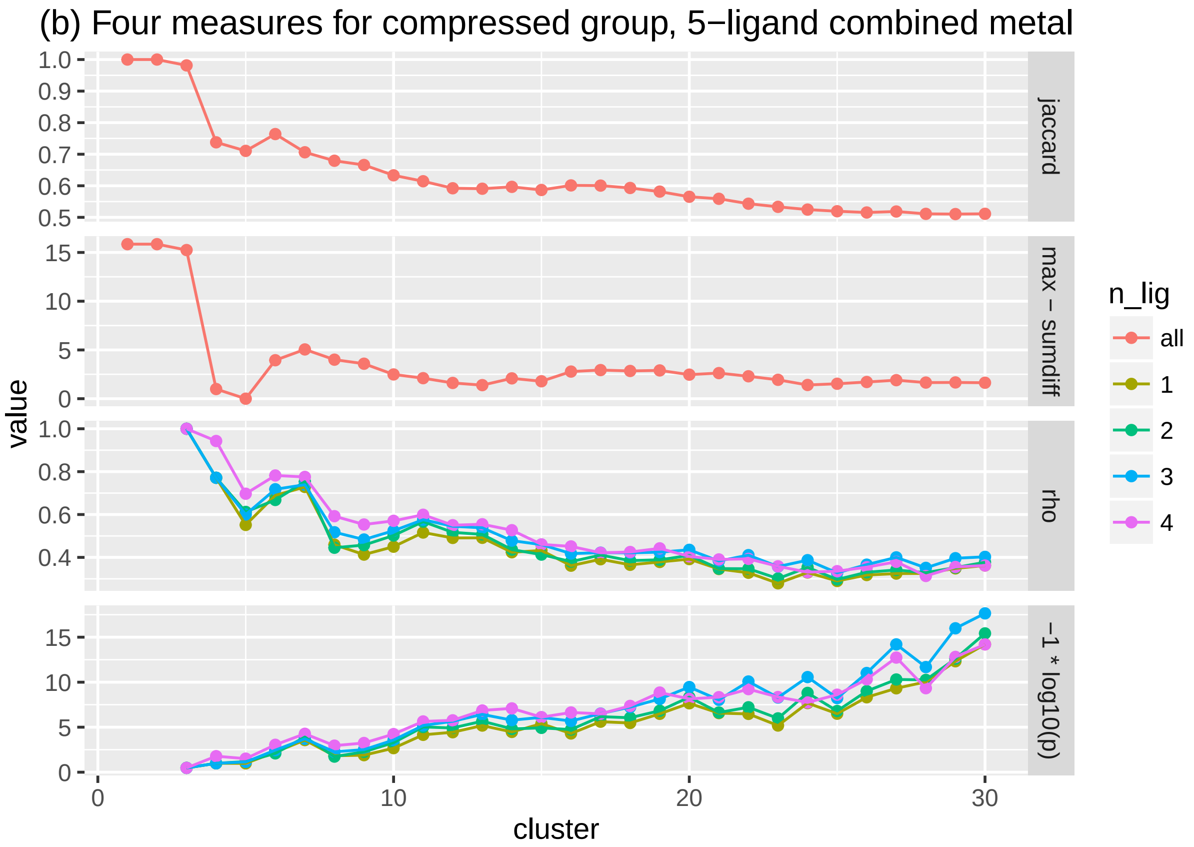
(b) Four measures for compressed group, 5−ligand combined metal
value
cluster
jaccard
max − sumdiff
rho
−1 * log10(p)
n_lig
all
1
2
3
4
0
10
20
30

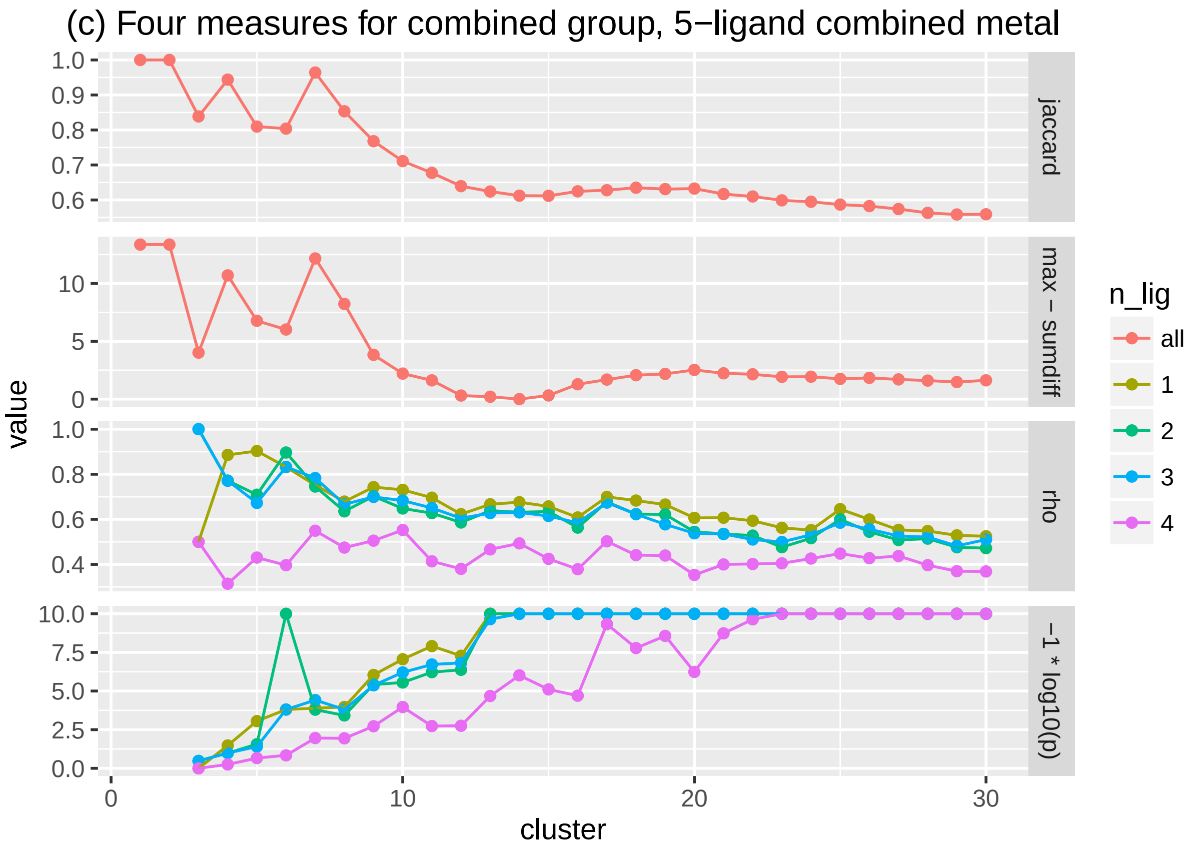
(c) Four measures for combined group, 5−ligand combined metal
value
cluster
jaccard
max − sumdiff
rho
−1 * log10(p)
n_lig
all
1
2
3
4

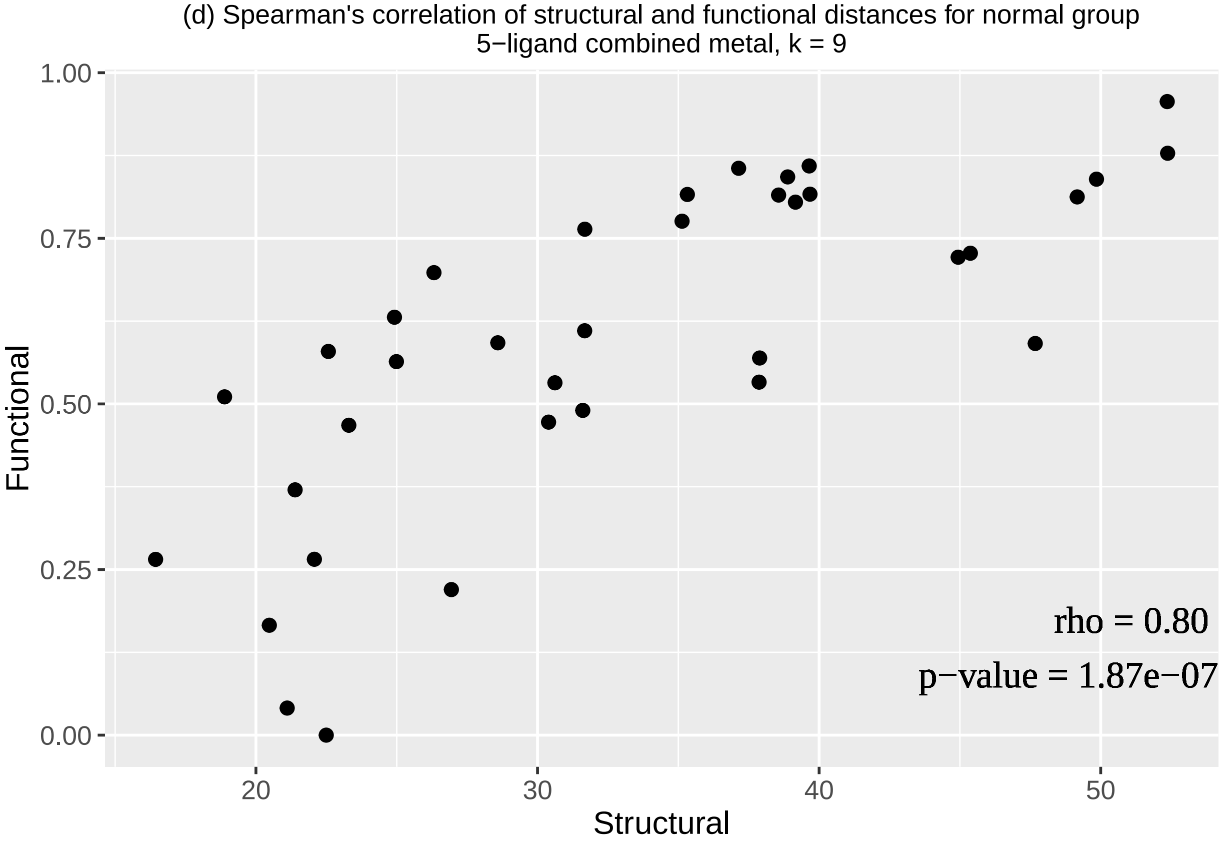
(d) Spearman's correlation of structural and functional distances for normal group
5−ligand combined metal, k = 9
Functional
Structural
rho = 0.80
p−value = 1.87e−07

(e) Comparison between structural and functional hierarchical dendrograms for normal group 5-ligand combined metal

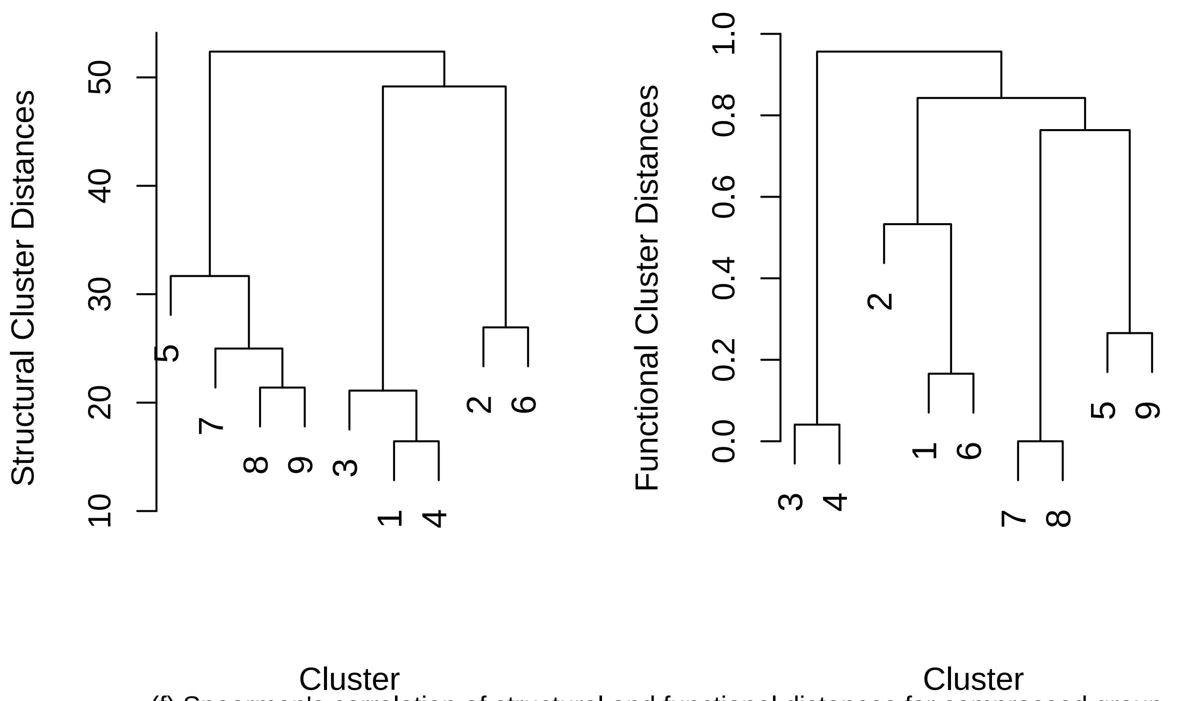

(f) Spearman's correlation of structural and functional distances for compressed group 5−ligand combined metal, k = 7

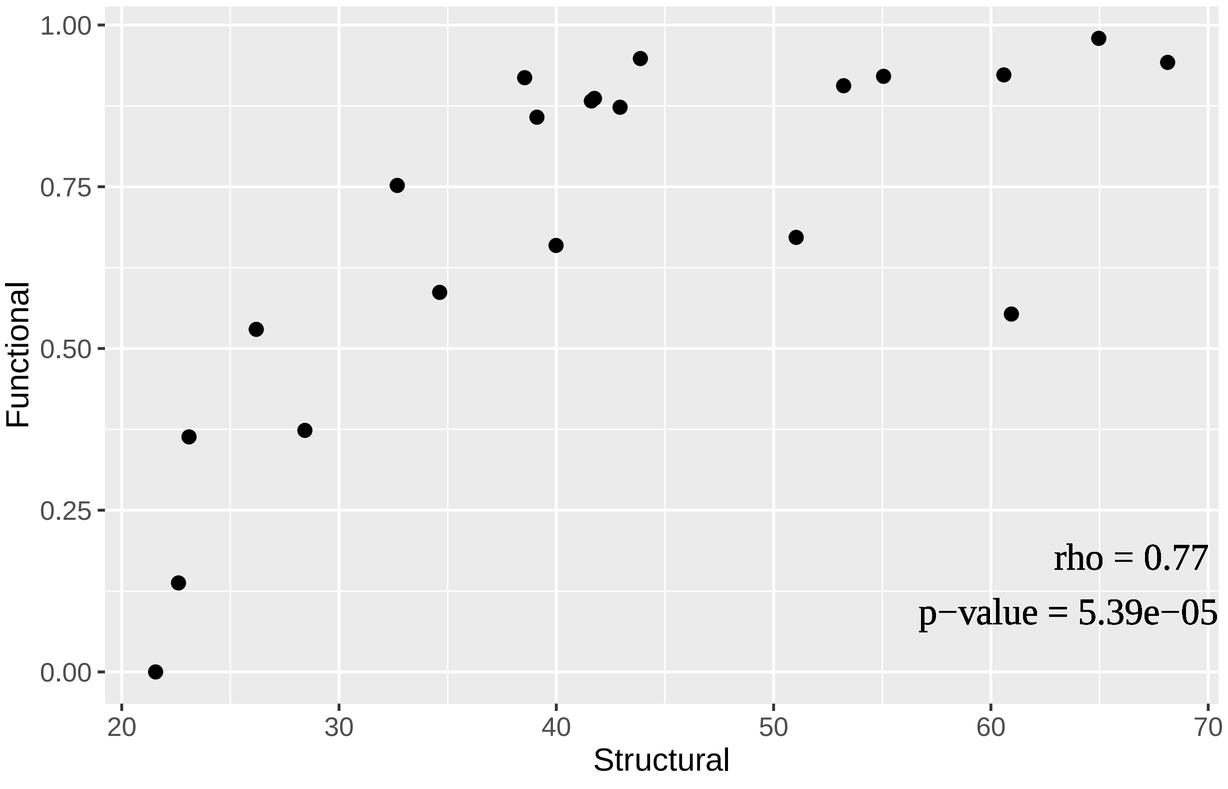

(g) Comparison between structural and functional hierarchical dendrograms for compressed group 5-ligand combined metal

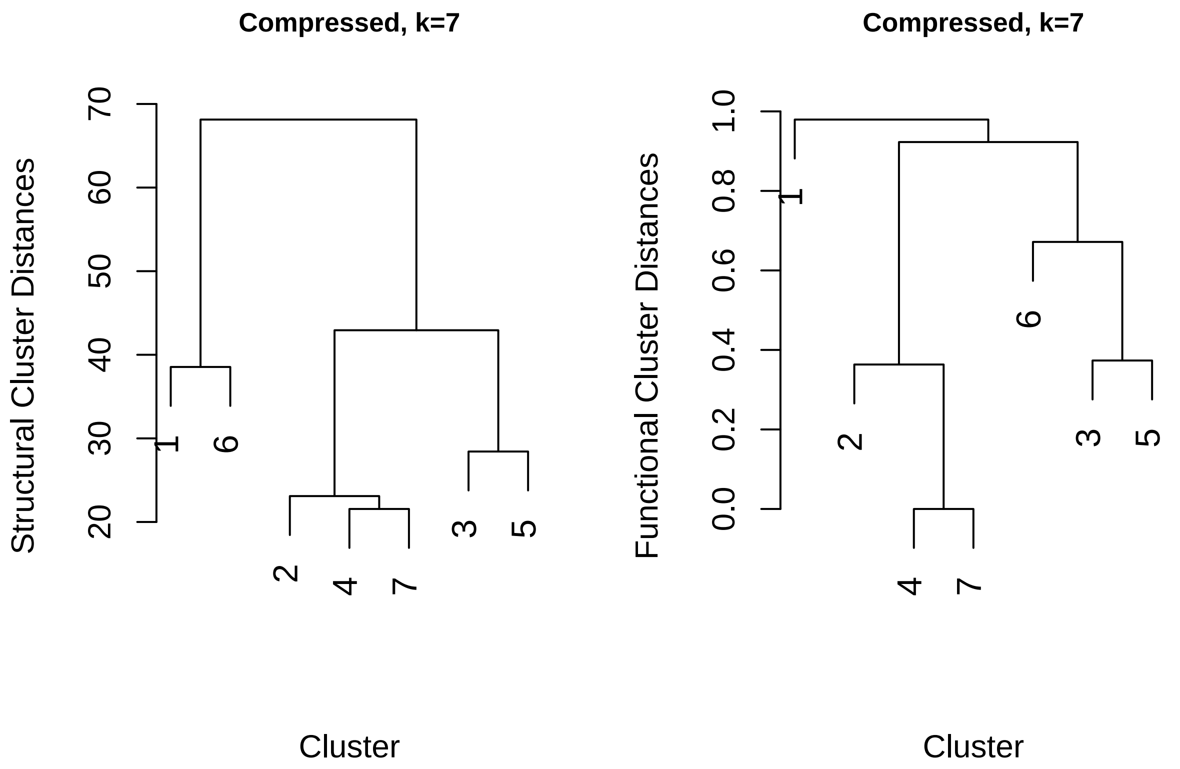

Figure S25. 6-ligand all metalloproteins

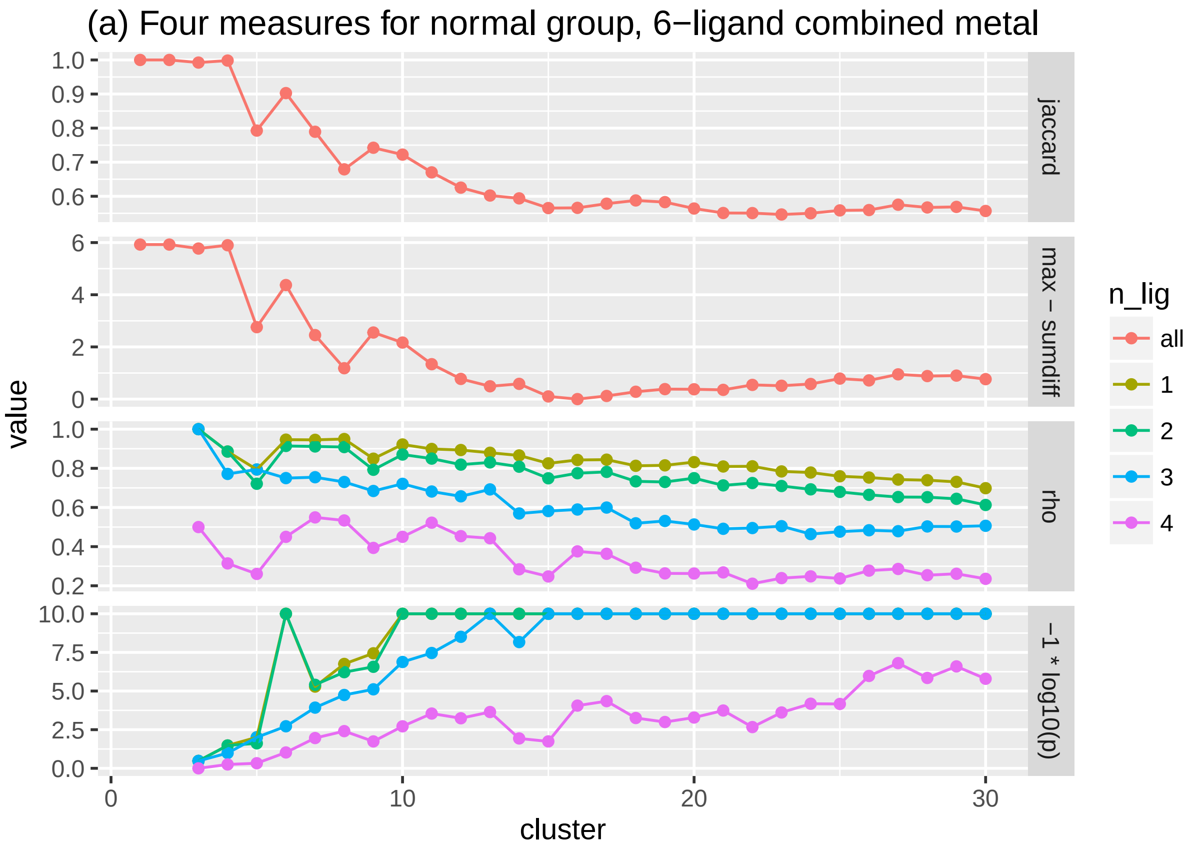
(a) Four measures for normal group, 6−ligand combined metal
jaccard
max − sumdiff
rho
−1 * log10(p)
value
cluster
n_lig
all
1
2
3
4

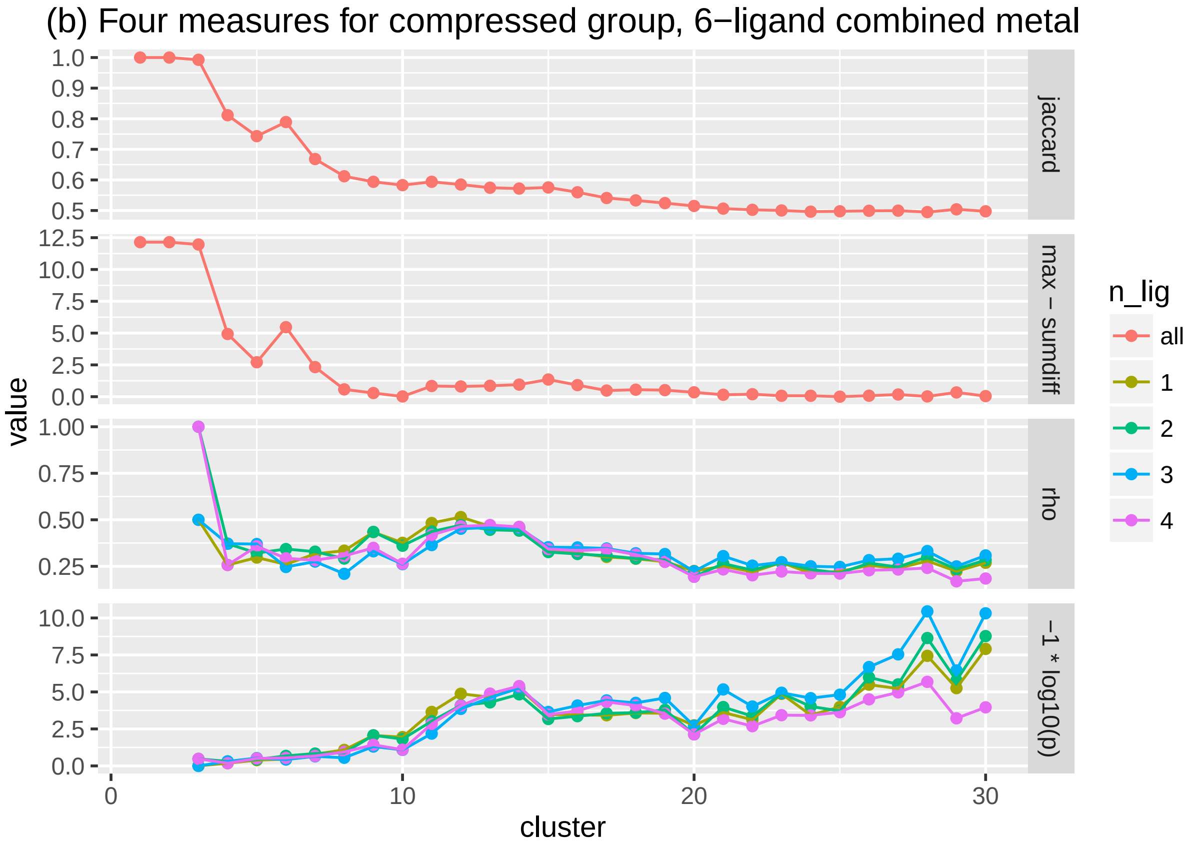
(b) Four measures for compressed group, 6−ligand combined metal
jaccard
max − sumdiff
rho
−1 * log10(p)
value
cluster
n_lig
all
1
2
3
4

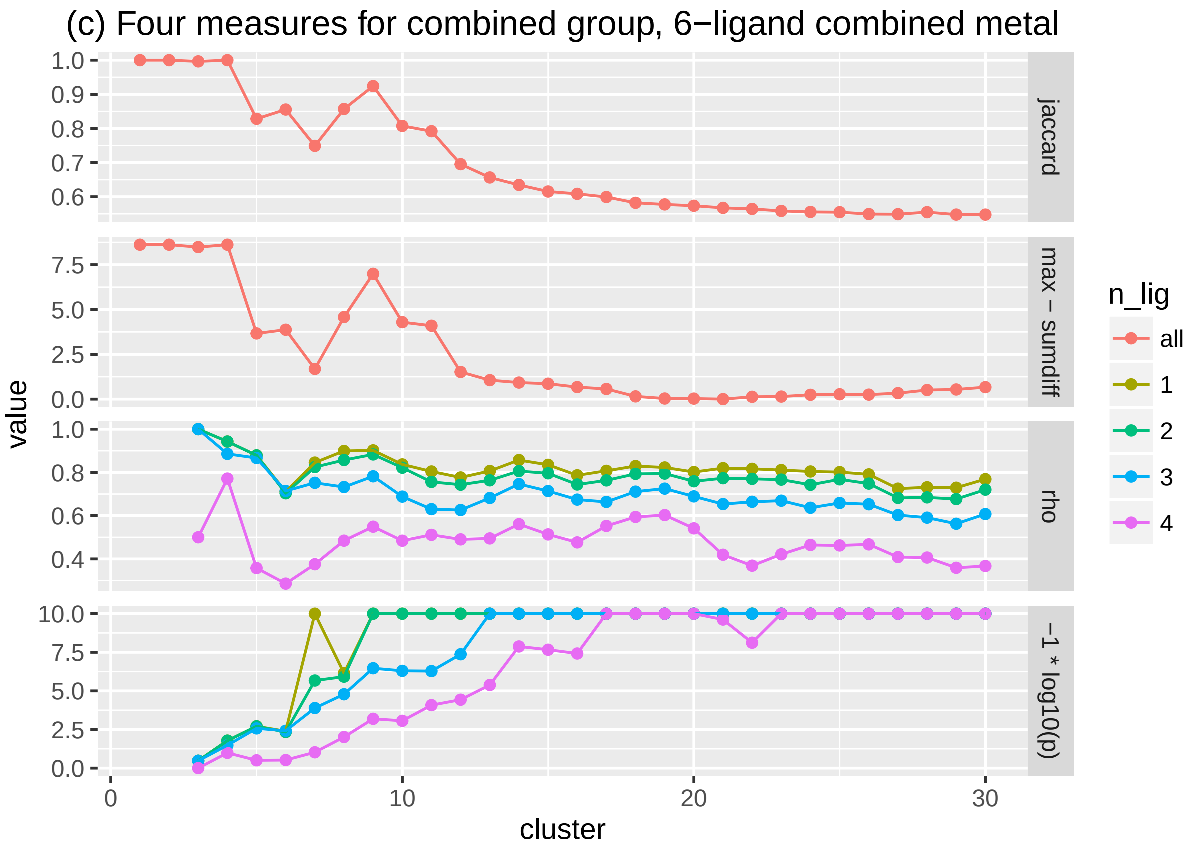

(c) Four measures for combined group, 6−ligand combined metal
jaccard
max − sumdiff
rho
−1 * log10(p)
value
cluster
n_lig
all
1
2
3
4

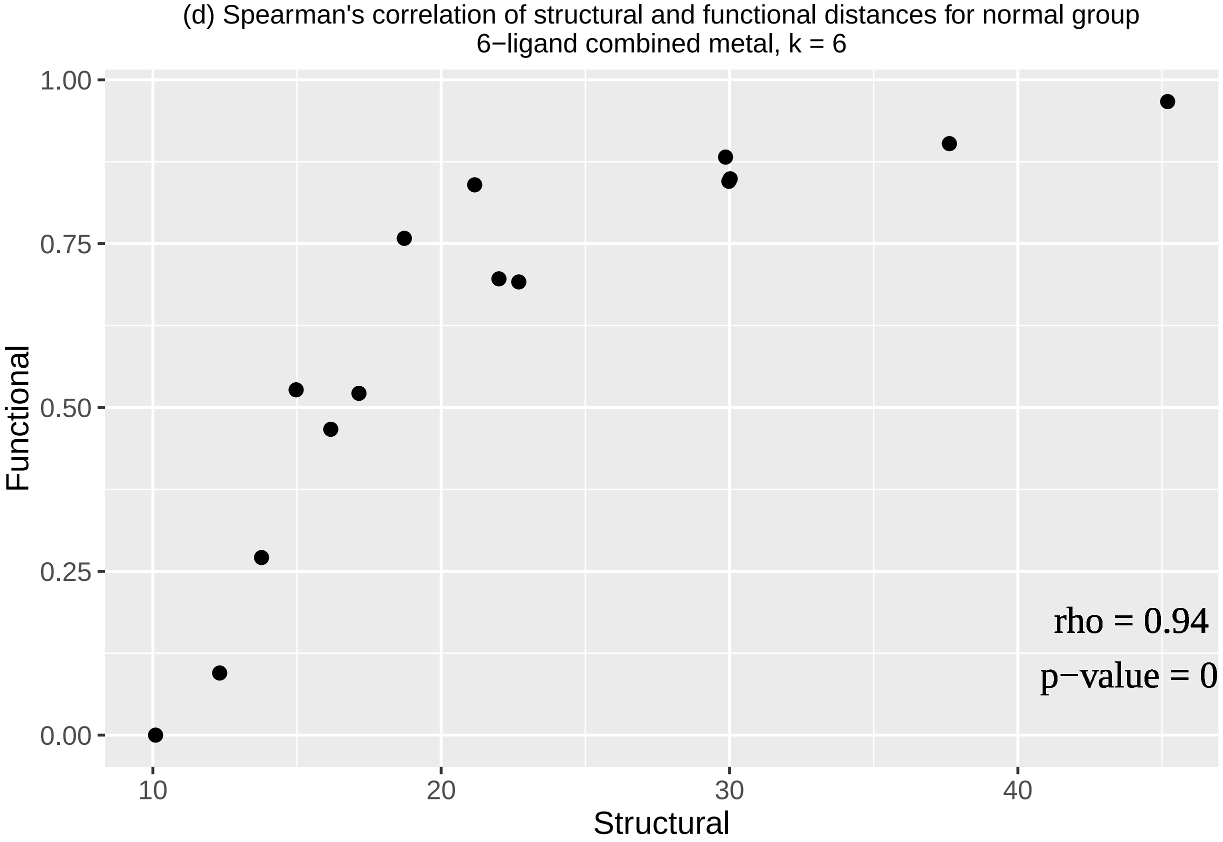

(d) Spearman's correlation of structural and functional distances for normal group
6−ligand combined metal, k = 6
Functional
Structural
rho = 0.94
p−value = 0

(e) Comparison between structural and functional hierarchical dendrograms for normal group 6-ligand combined metal

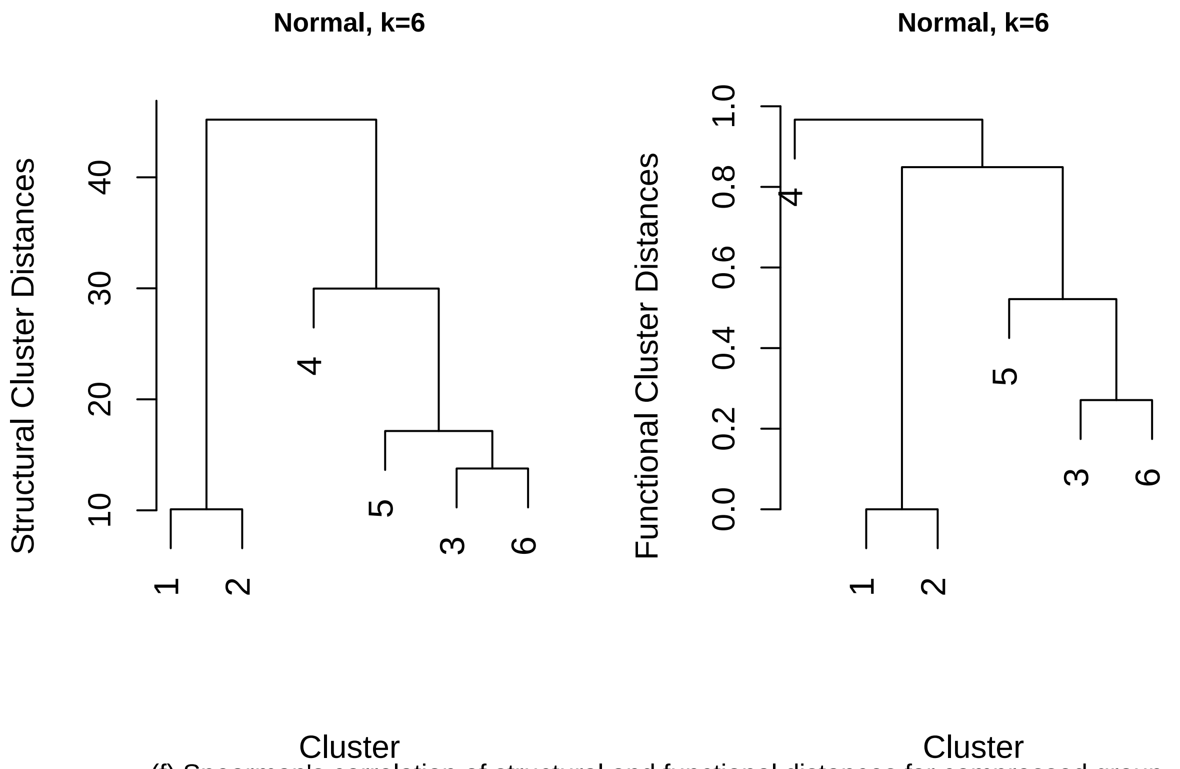

(f) Spearman's correlation of structural and functional distances for compressed group 6−ligand combined metal, k = 12

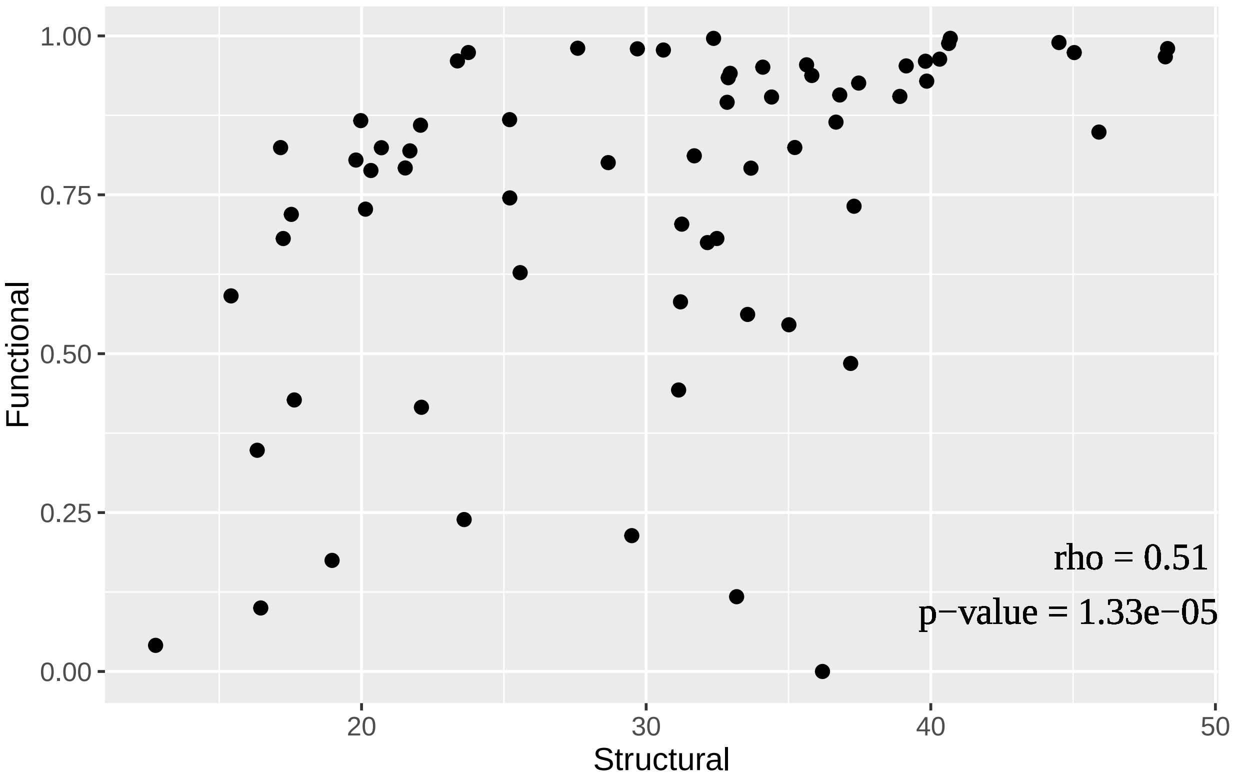

(g) Comparison between structural and functional hierarchical dendrograms for compressed group 6-ligand combined metal

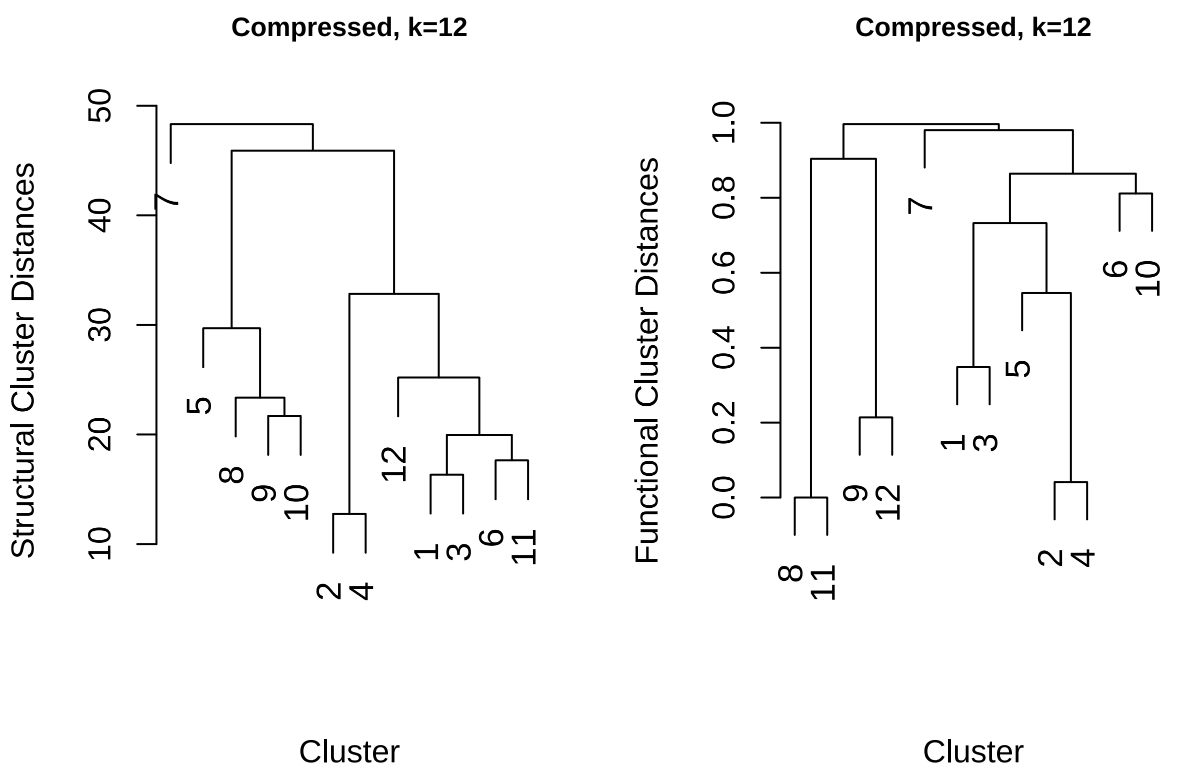

Figure S26. 7-ligand all metalloproteins

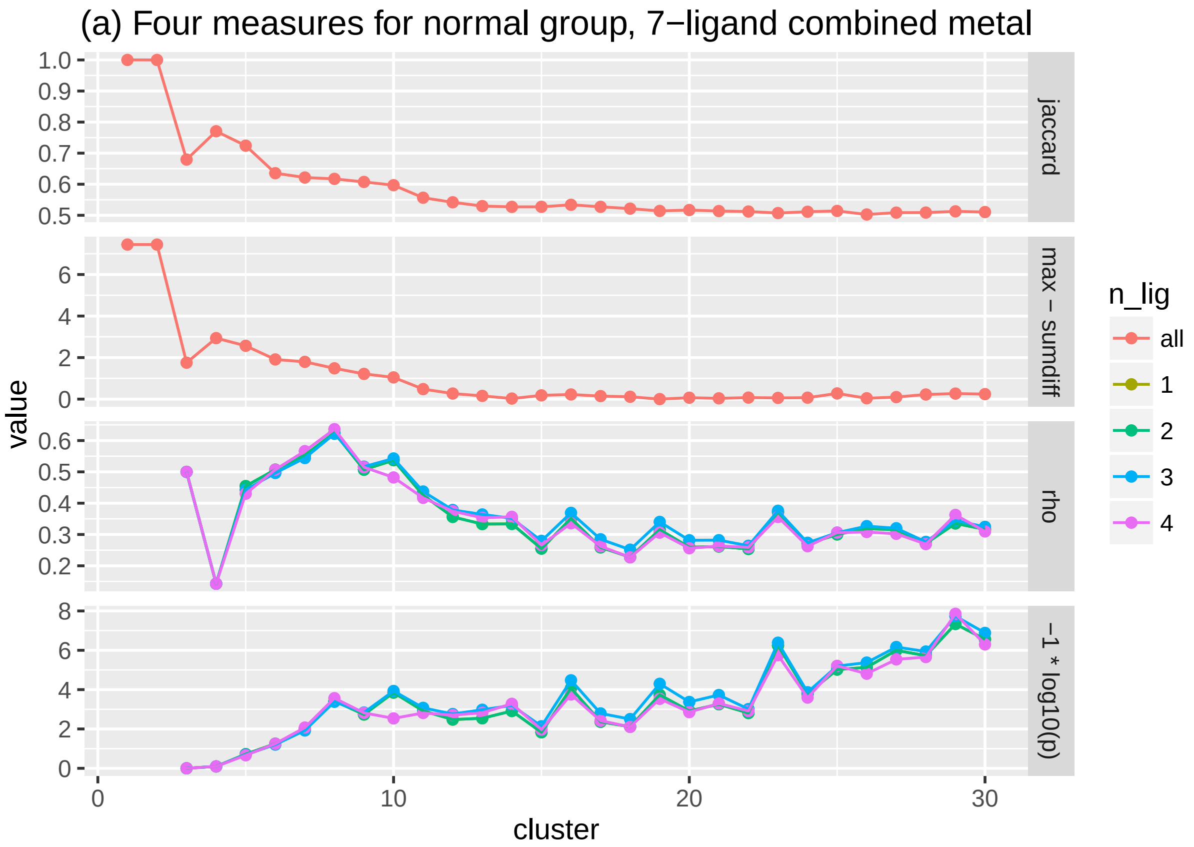
(a) Four measures for normal group, 7−ligand combined metal
value
cluster
jaccard
max − sumdiff
rho
−1 * log10(p)
n_lig
all
1
2
3
4

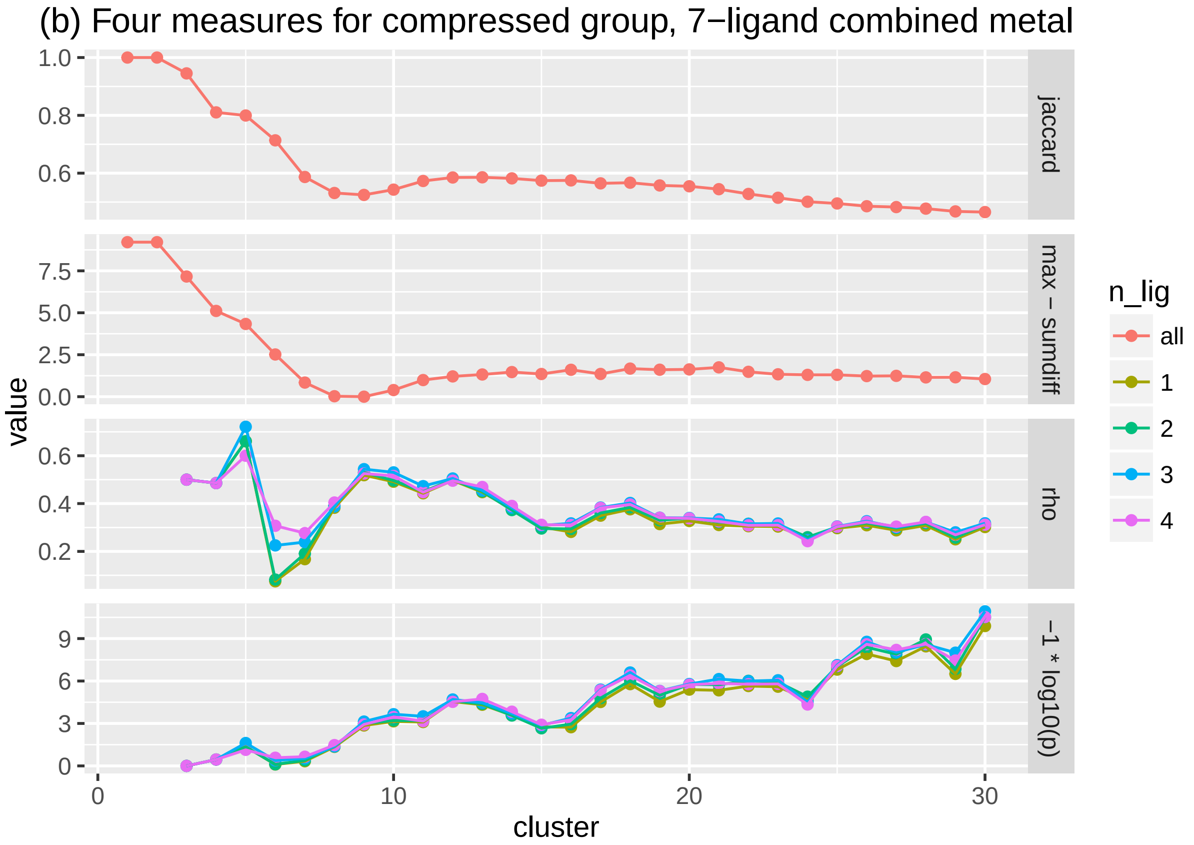
(b) Four measures for compressed group, 7−ligand combined metal
value
cluster
jaccard
max − sumdiff
rho
−1 * log10(p)
n_lig
all
1
2
3
4

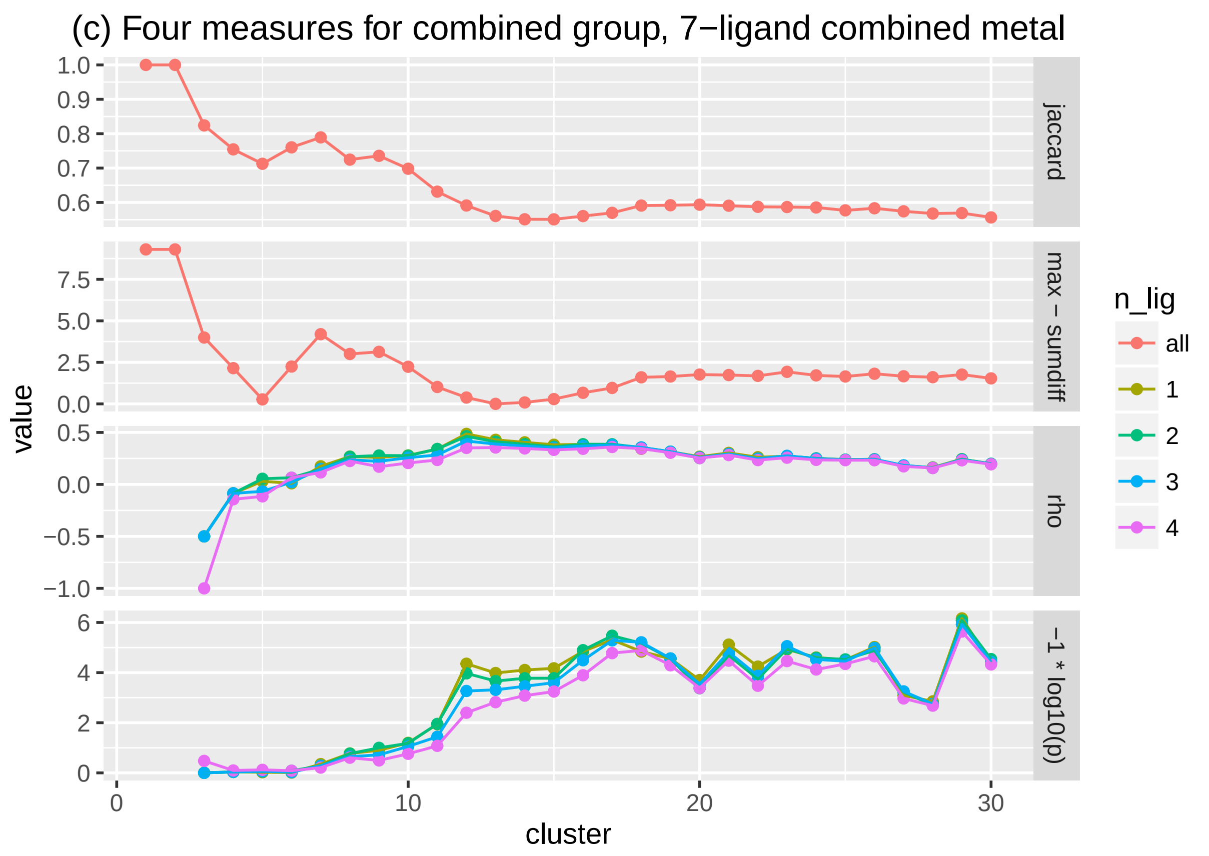
(c) Four measures for combined group, 7−ligand combined metal
value
cluster
jaccard
max − sumdiff
rho
−1 * log10(p)
n_lig
all
1
2
3
4

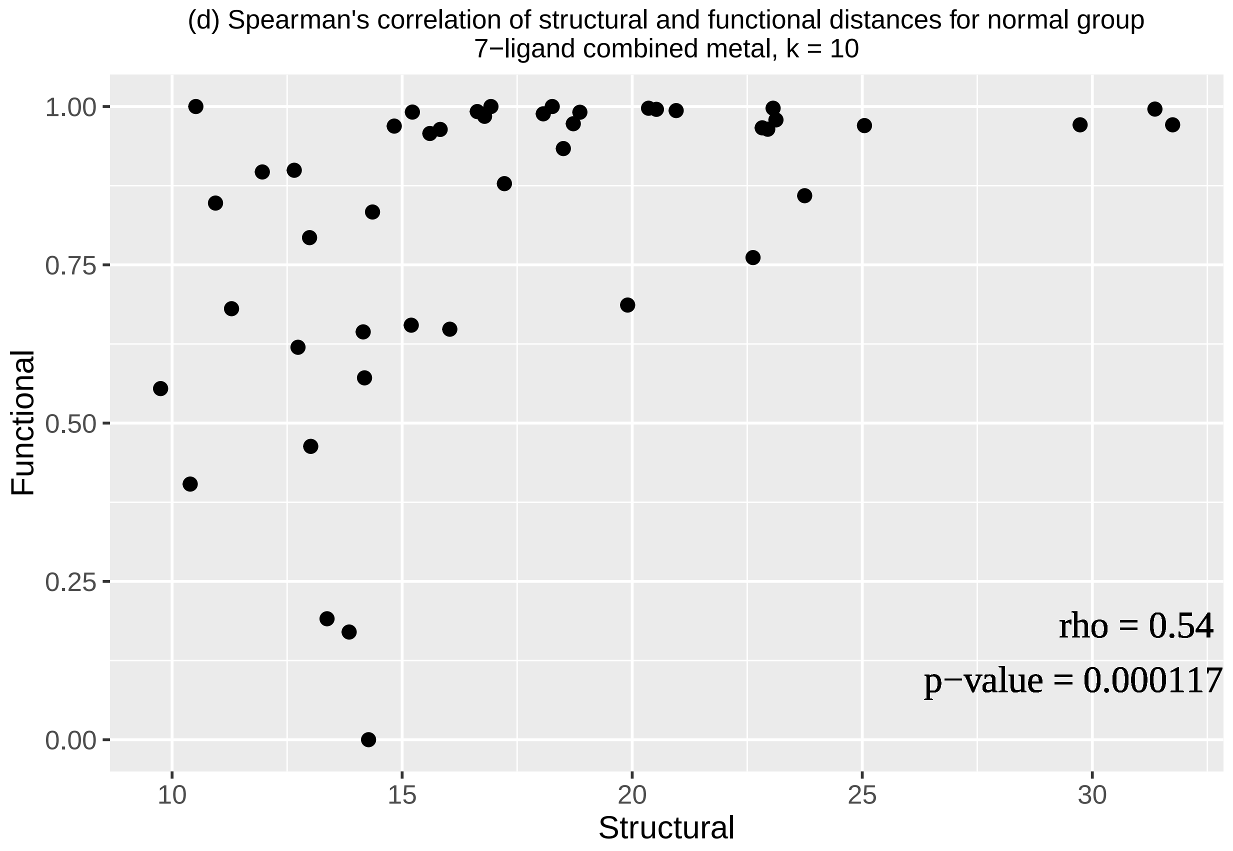
(d) Spearman's correlation of structural and functional distances for normal group
7−ligand combined metal, k = 10
Functional
Structural
rho = 0.54
p−value = 0.000117

(e) Comparison between structural and functional hierarchical dendrograms for normal group 7-ligand combined metal

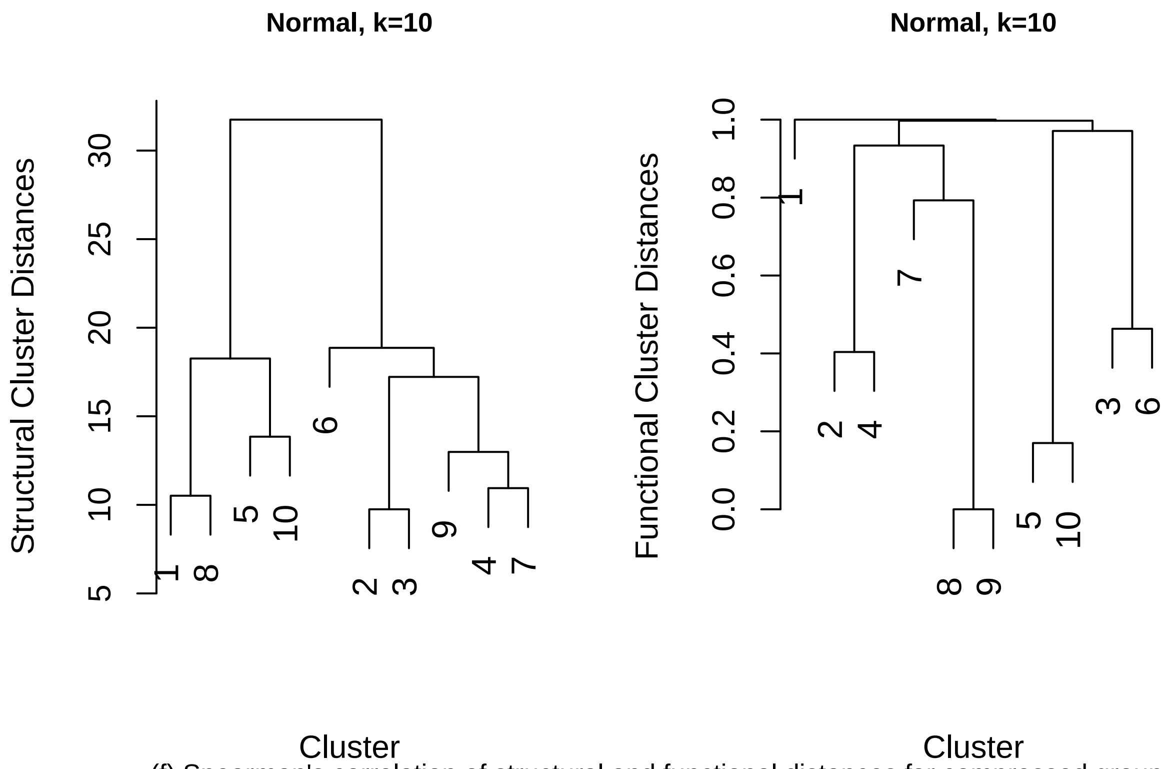

(f) Spearman's correlation of structural and functional distances for compressed group 7−ligand combined metal, k = 12

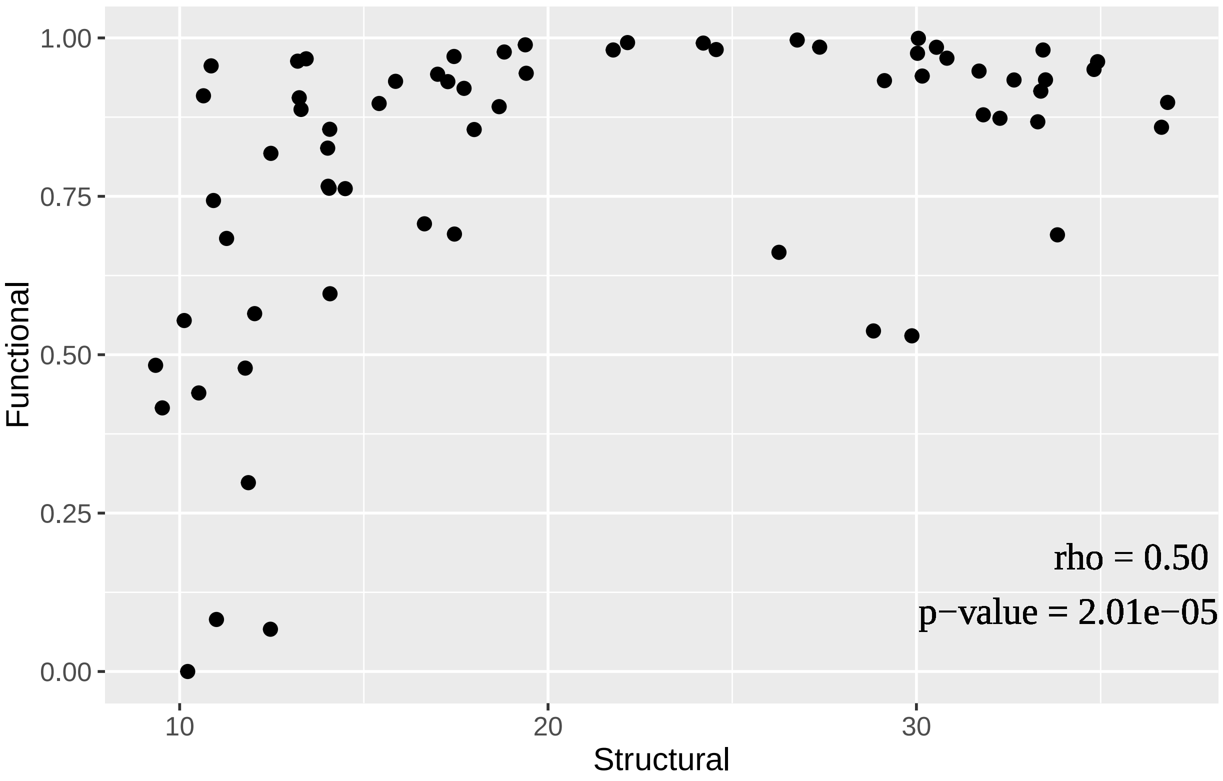

(g) Comparison between structural and functional hierarchical dendrograms for compressed group 7-ligand combined metal

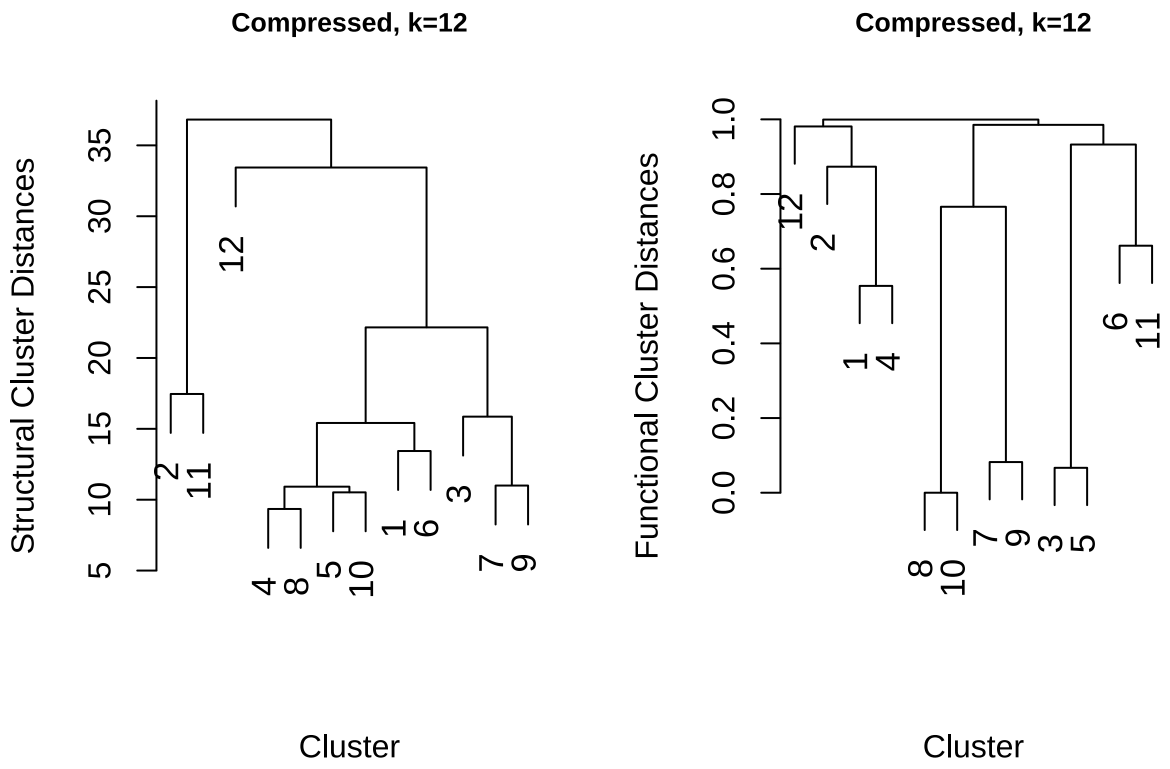

Figure S27. 8-ligand all metalloproteins

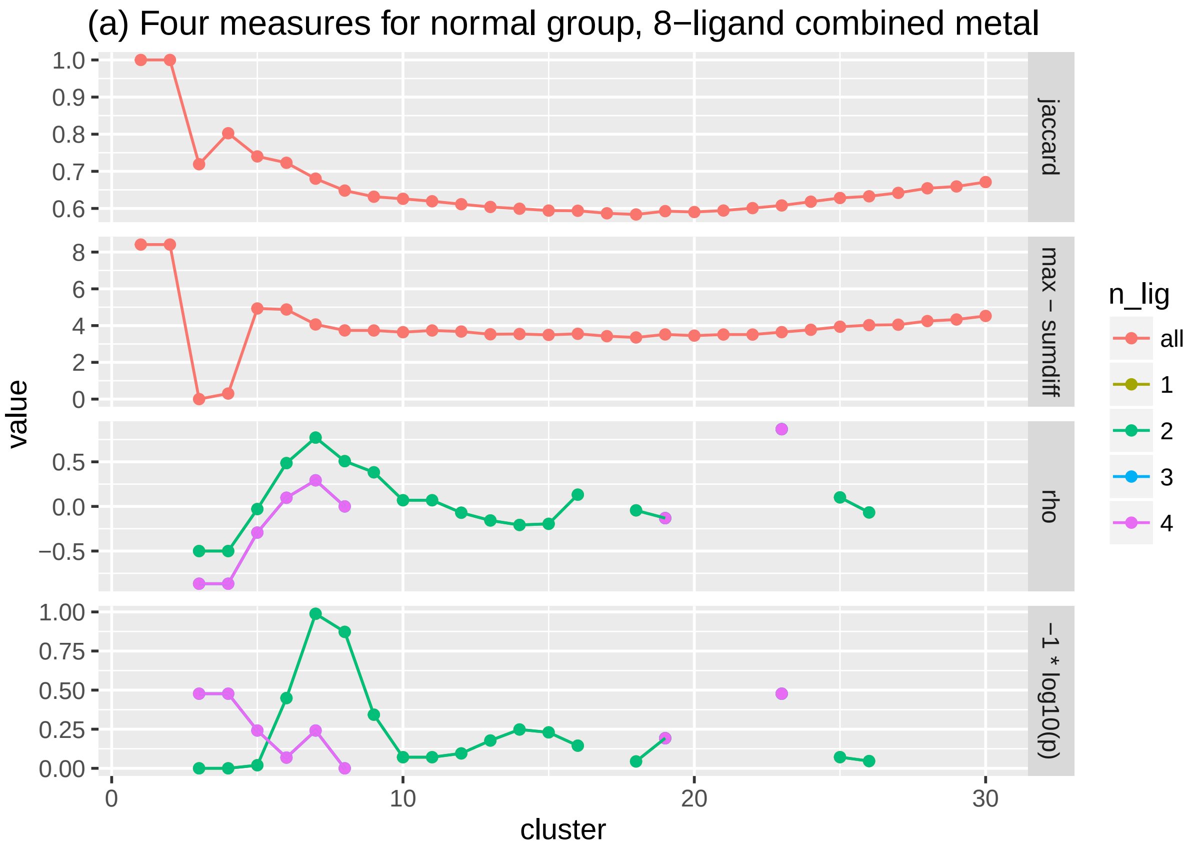
(a) Four measures for normal group, 8−ligand combined metal
value
cluster
jaccard
max − sumdiff
rho
−1 * log10(p)
n_lig
all
1
2
3
4

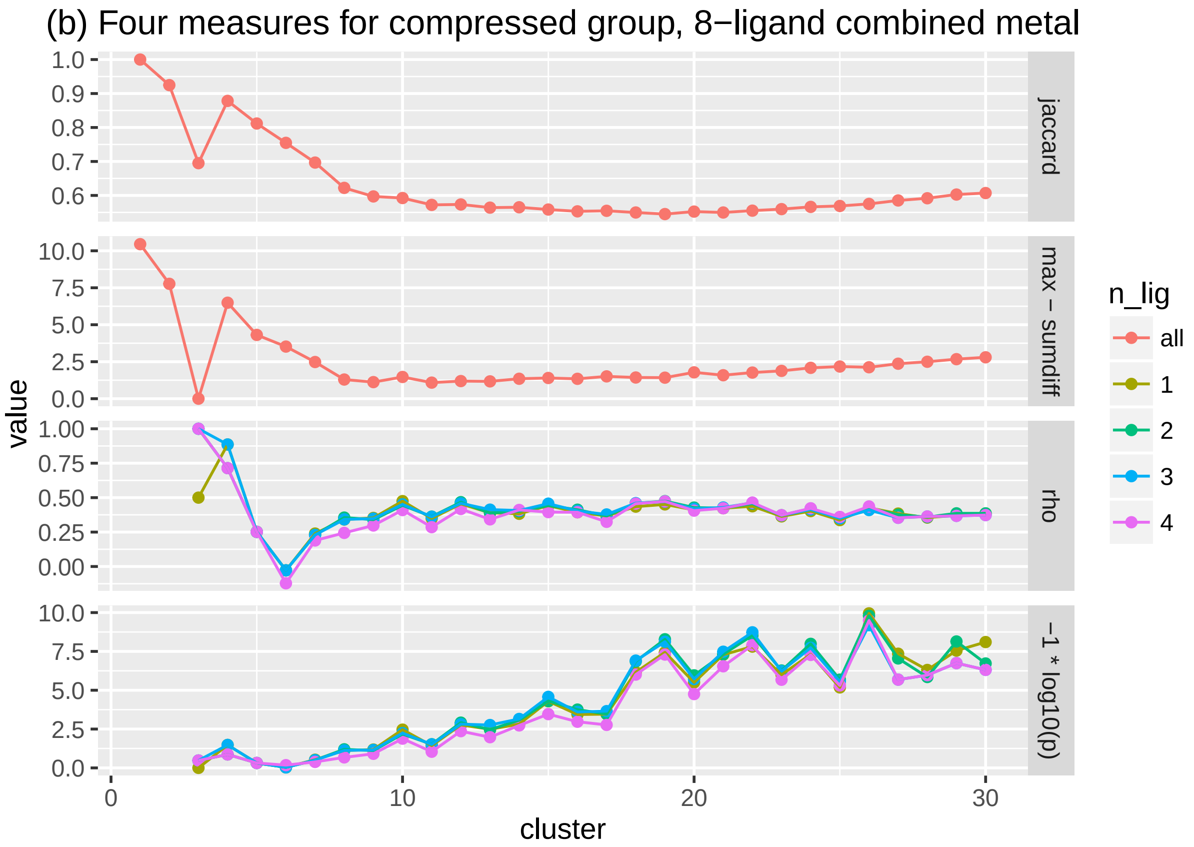
(b) Four measures for compressed group, 8−ligand combined metal
value
cluster
jaccard
max − sumdiff
rho
−1 * log10(p)
n_lig
all
1
2
3
4

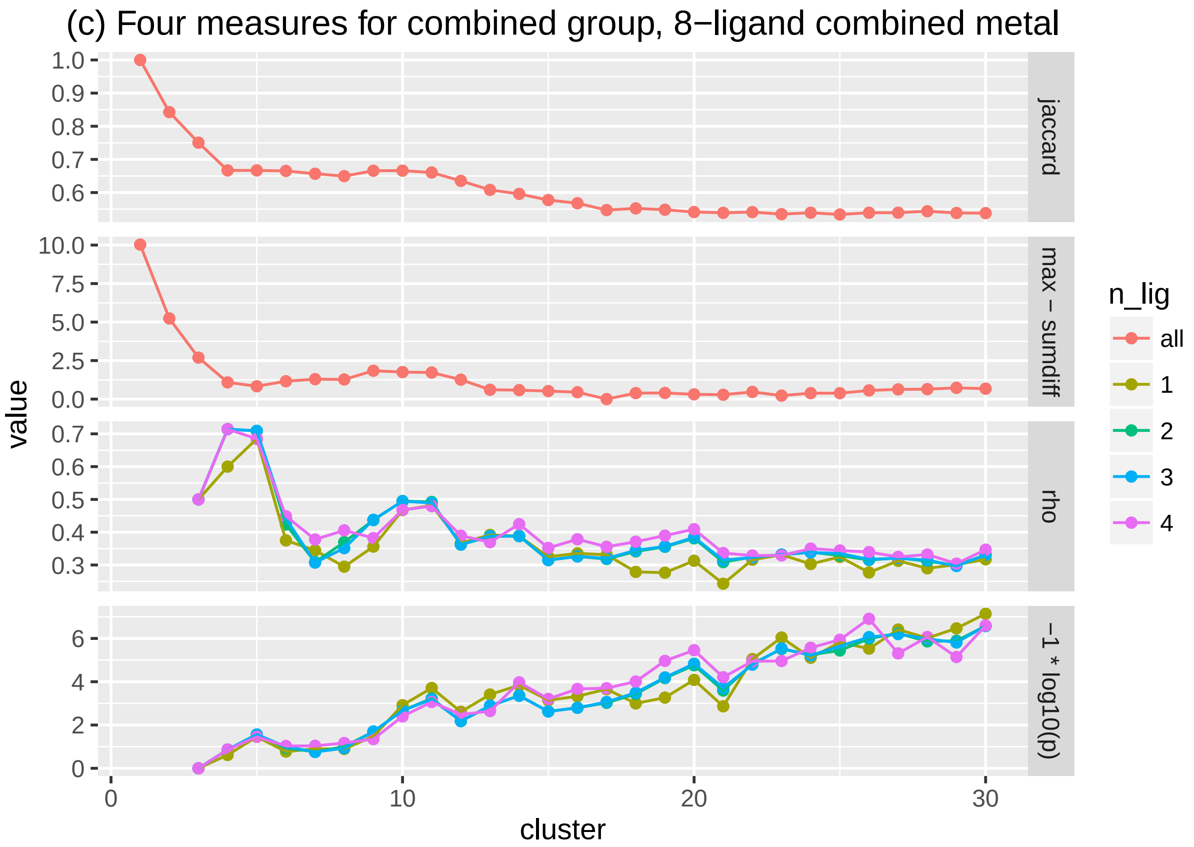

(c) Four measures for combined group, 8−ligand combined metal
value
cluster
jaccard
max − sumdiff
rho
−1 * log10(p)
n_lig
all
1
2
3
4

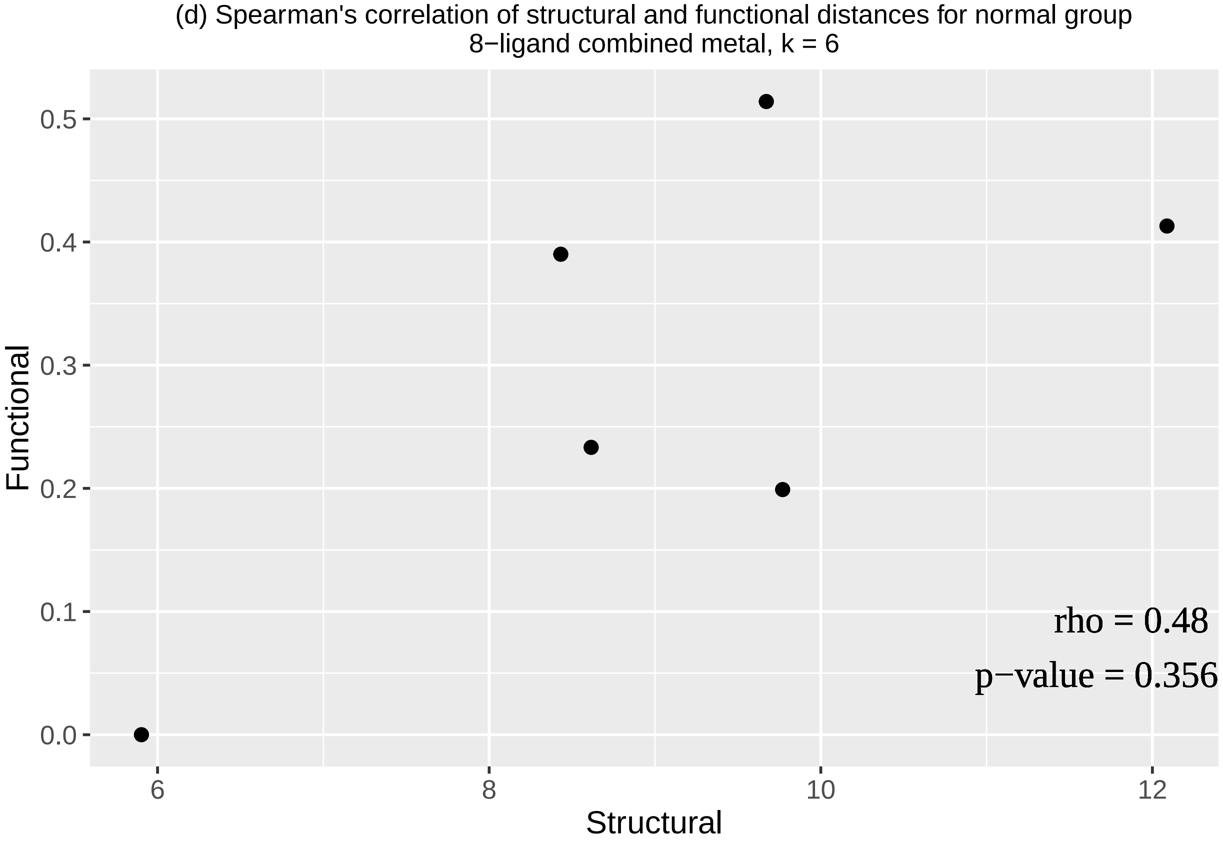

(d) Spearman's correlation of structural and functional distances for normal group
8−ligand combined metal, k = 6
Functional
Structural
rho = 0.48
p−value = 0.356

(e) Comparison between structural and functional hierarchical dendrograms for normal group 8-ligand combined metal

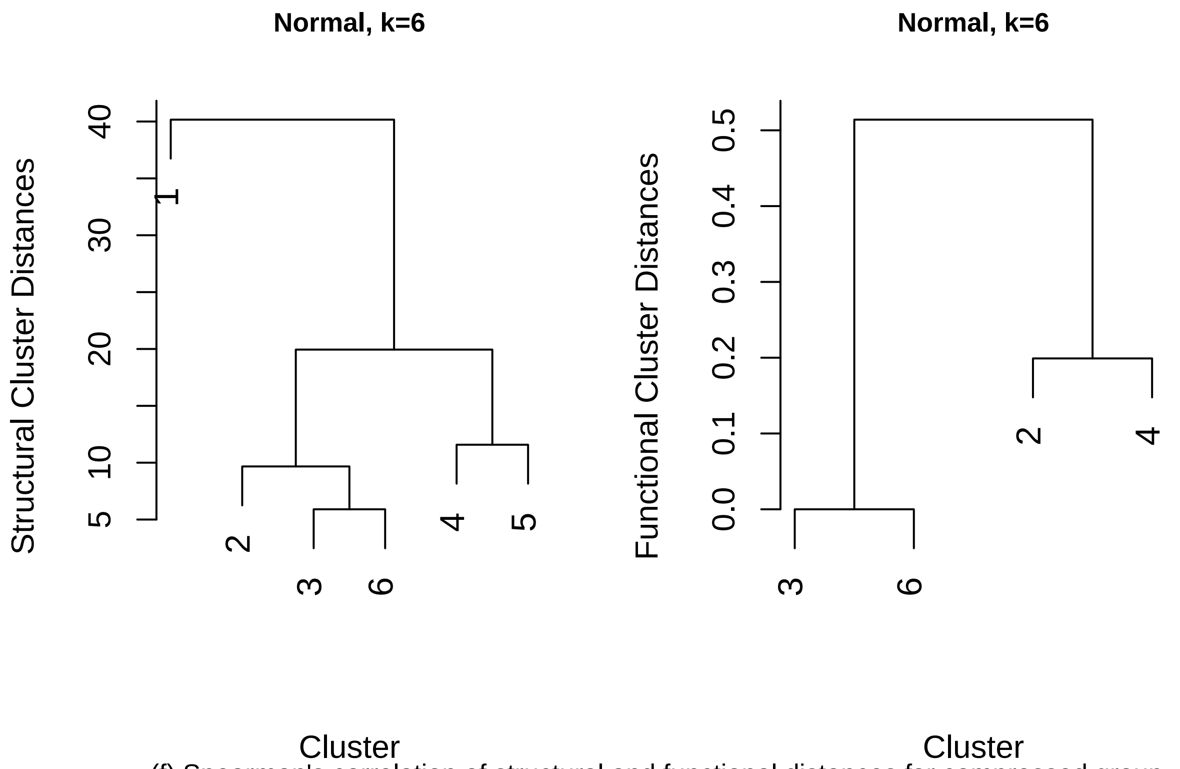

(f) Spearman's correlation of structural and functional distances for compressed group 8−ligand combined metal, k = 4

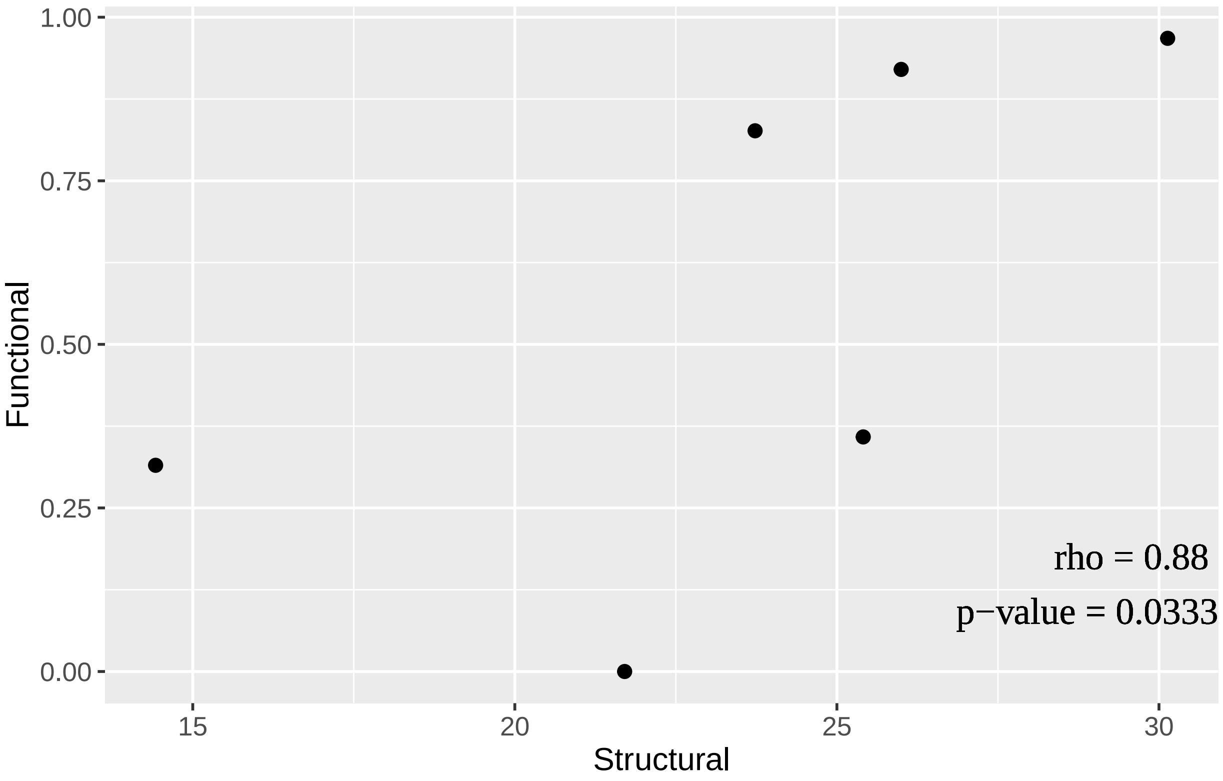

(g) Comparison between structural and functional hierarchical dendrograms for compressed group 8-ligand combined metal

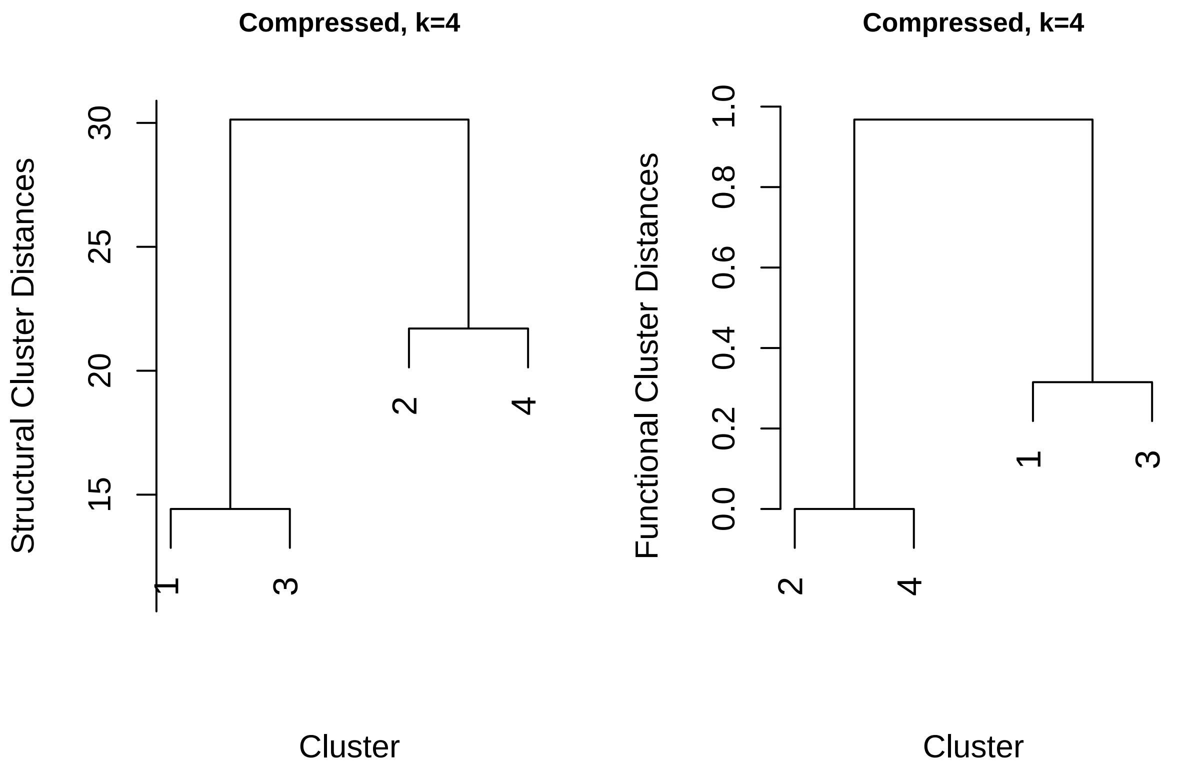

Figure S28. all-ligand all metalloproteins

(a) Four measures for normal group, all−ligand−number combined metal

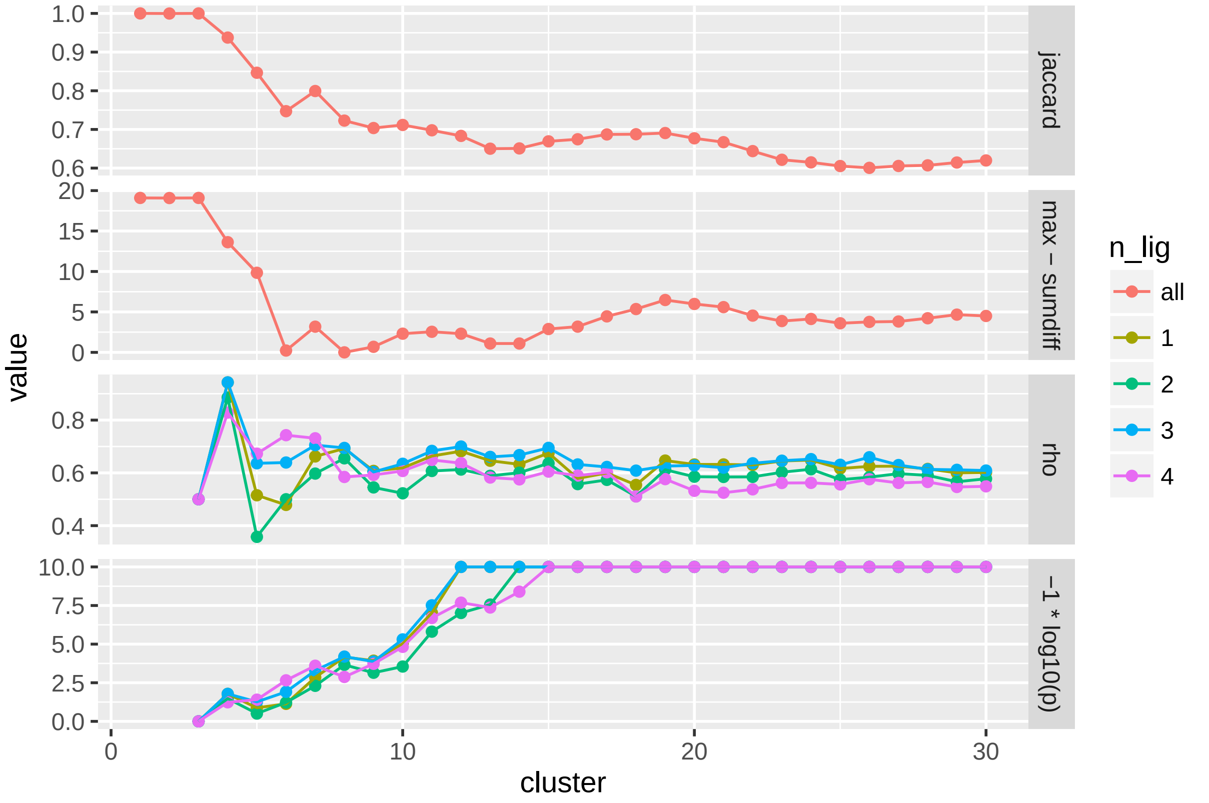

) Four measures for compressed group, all−ligand−number combined metal

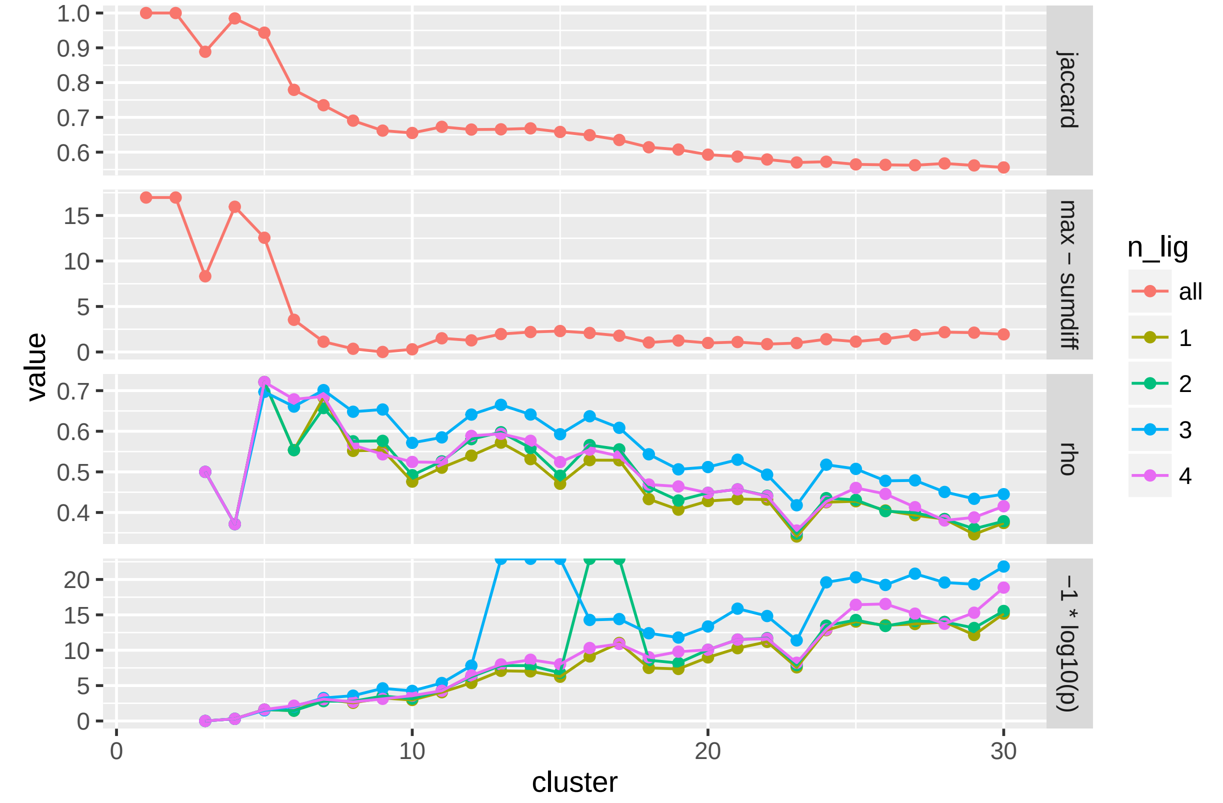

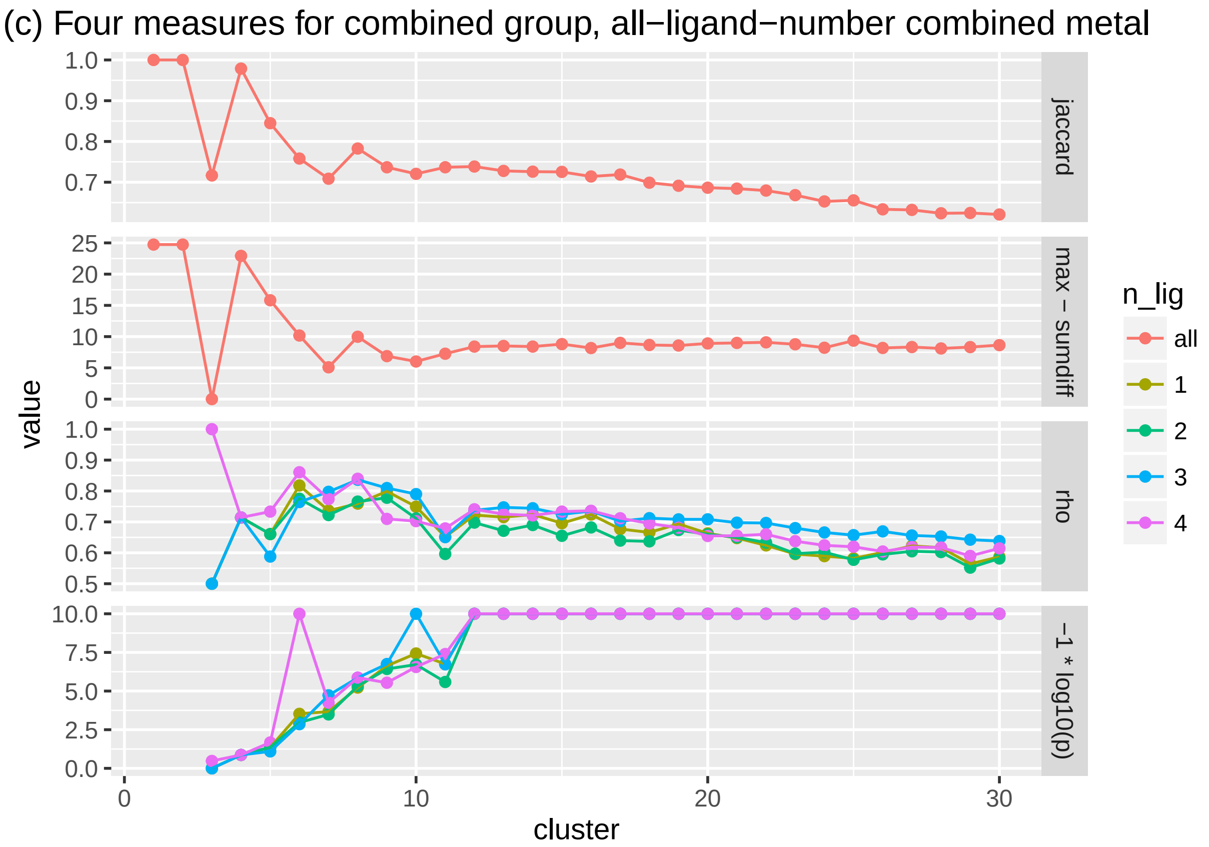
(c) Four measures for combined group, all−ligand−number combined metal
value
cluster
jaccard
max − sumdiff
rho
−1 * log10(p)
n_lig
all
1
2
3
4

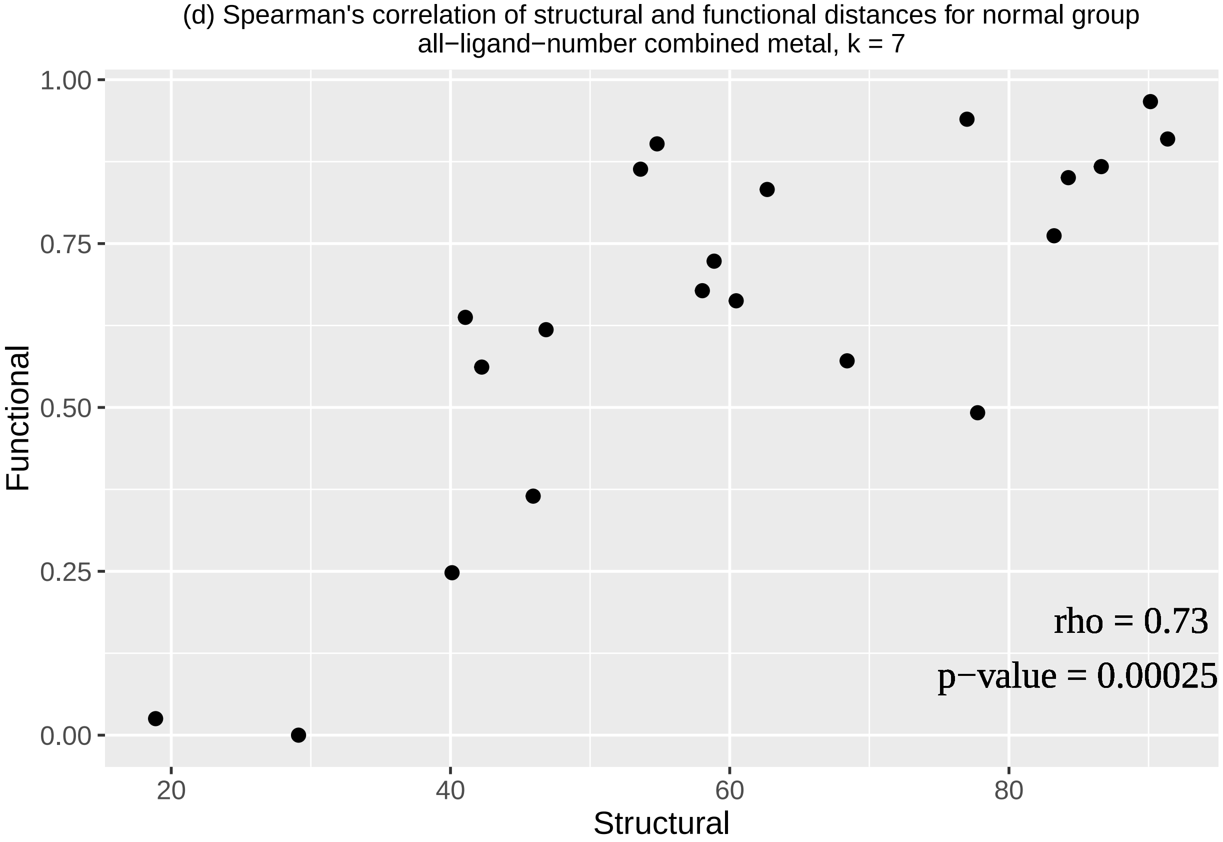
(d) Spearman's correlation of structural and functional distances for normal group
all−ligand−number combined metal, k = 7
Functional
Structural
rho = 0.73
p−value = 0.00025

(e) Comparison between structural and functional hierarchical dendrograms for normal group all-ligand-number combined metal

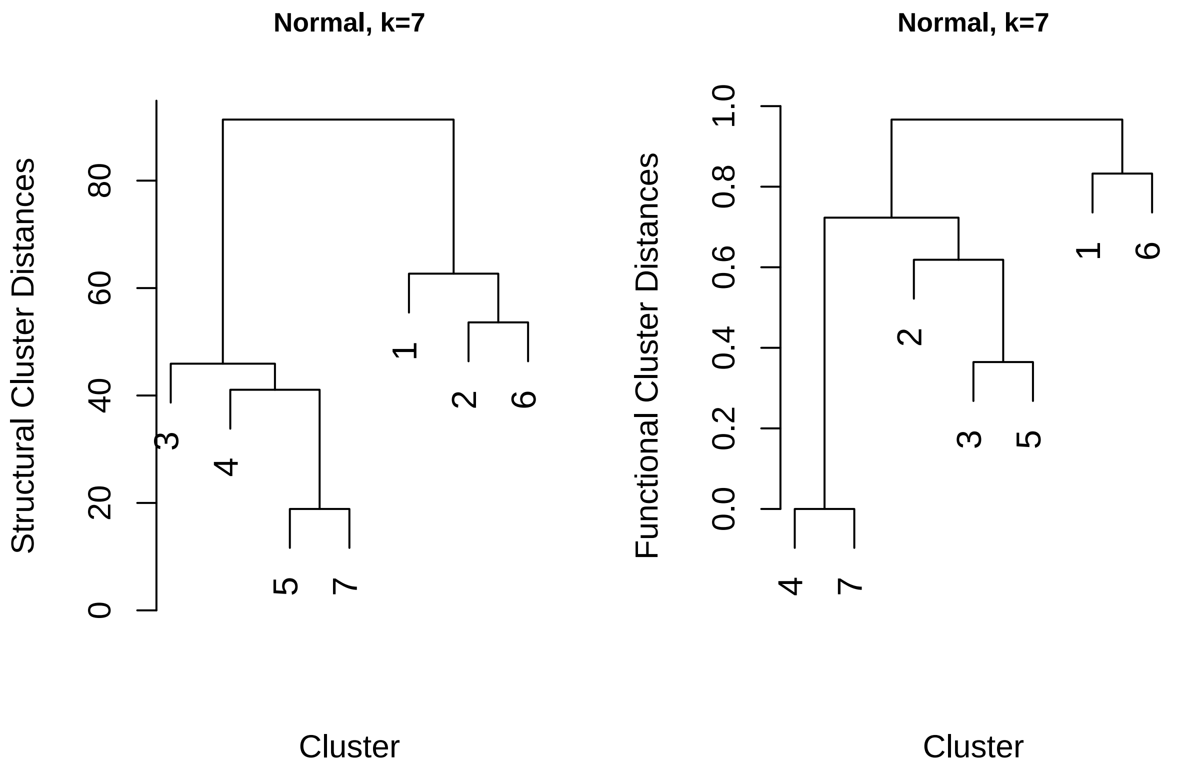

(f) Spearman's correlation of structural and functional distances for compressed group all−ligand−number combined metal, k = 7

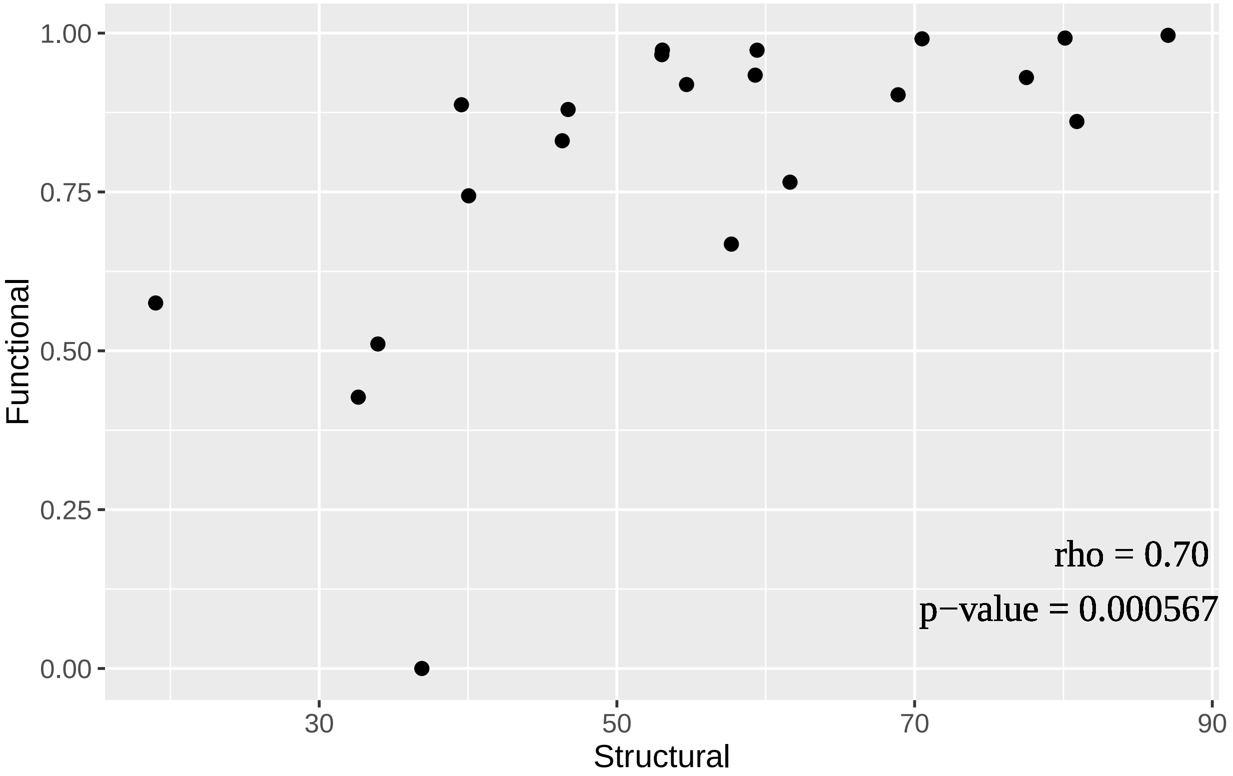

(g) Comparison between structural and functional hierarchical dendrograms for compressed group all-ligand-number combined metal

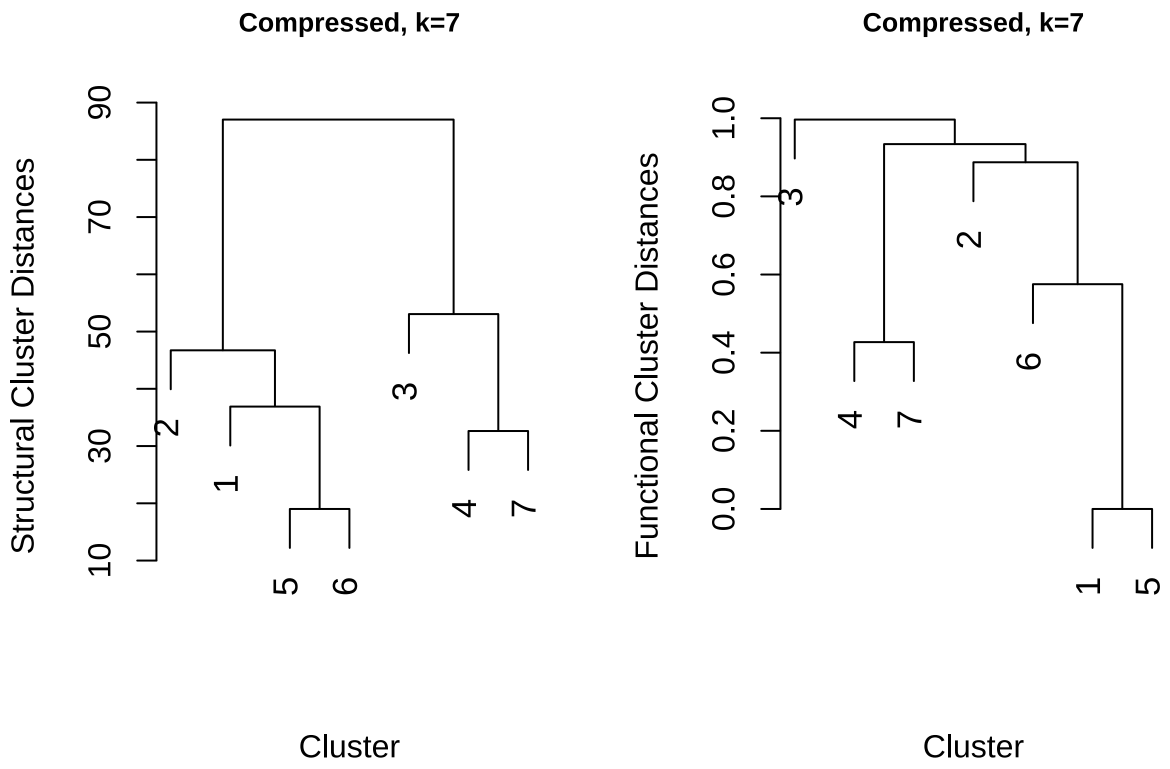

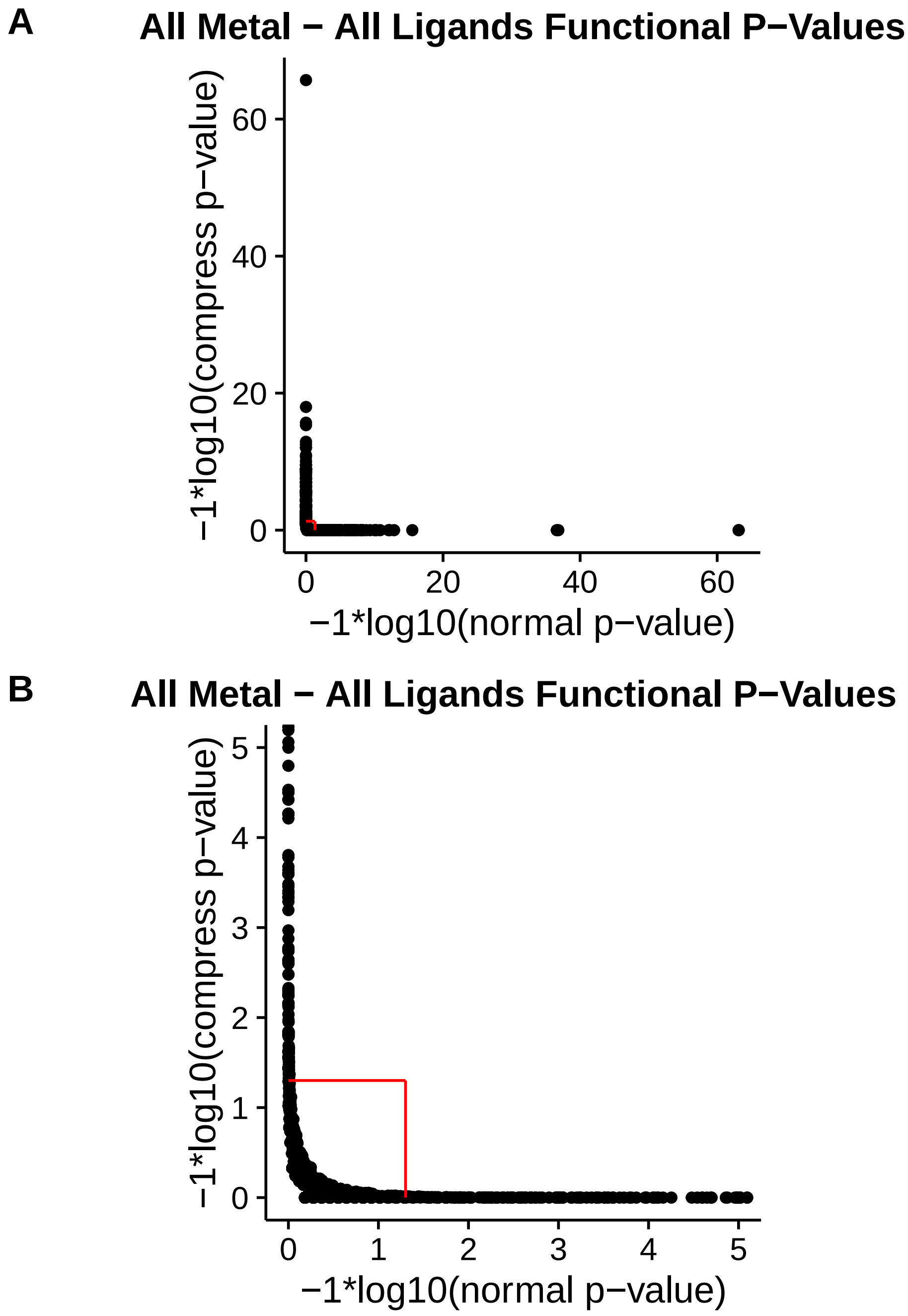

Figure S29: Log10-transformed unadjusted p-values from both compressed and normal enrichments compared. A) Full range of p-values. B) Zoomed in view of A. Red lines indicate p-value of 0.05.

Five metal supplemental tables

Sen Yao

2016-11-09 20:42:12

Table S1. 4-ligand Zn, normal group

	size	largest_angle*	middle_1*	middle_2*	middle_3*
1	"173"	"129.5+/-5.6"	"92.7+/-5.4"	"98.6+/-3.6"	"104.1+/-3.6"
2	"723"	"118.3+/-2.3"	"101.3+/-3.3"	"107.3+/-2.6"	"111.2+/-2.1"
3	"231"	"125.2+/-5.1"	"97.8+/-4.9"	"105.2+/-4.1"	"112+/-3.9"
4	"99"	"146.3+/-6.5"	"91.3+/-4.5"	"97.5+/-3.6"	"102.4+/-3.1"
5	"77"	"137.8+/-6.6"	"79.9+/-7.2"	"91.7+/-7.2"	"107.4+/-8.6"
6	"90"	"164.8+/-7.4"	"80.2+/-6.1"	"87+/-4.9"	"93.2+/-5.1"
7	"259"	"121.4+/-3.5"	"90.8+/-4.9"	"102.6+/-4.8"	"111.7+/-3.2"
8	"689"	"118.3+/-2.6"	"99.9+/-2.5"	"104.4+/-2.2"	"108.2+/-2.4"
9	"342"	"127.9+/-3.8"	"97.9+/-3.3"	"102.6+/-2.7"	"106.9+/-2.9"
10	"617"	"114+/-2.2"	"105.5+/-2"	"107.7+/-1.4"	"109.4+/-1.2"

	middle_4*	smallest_opposite_angle*	Tetrahedral
1	"112.2+/-4.7"	"120.1+/-5"	"0.235"
2	"114.2+/-1.9"	"102.8+/-3.1"	"0.68"
3	"117.2+/-3.5"	"92.4+/-5.3"	"0.208"
4	"108.5+/-4.1"	"101.4+/-9"	"0.011"
5	"123.2+/-5.7"	"96.4+/-11.1"	"0.004"
6	"100+/-7"	"107.1+/-15.1"	"0"
7	"116.8+/-2.6"	"110.7+/-4.4"	"0.338"
8	"112.7+/-2.7"	"112.9+/-2.8"	"0.739"
9	"114.2+/-4.1"	"103.9+/-3.9"	"0.324"
10	"111.3+/-1.5"	"108.7+/-2.5"	"0.93"

	TrigonalBipyramidalVA	TrigonalBipyramidalVP	SquarePyramidalV
1	"0.003"	"0.003"	"0"
2	"0.026"	"0"	"0"
3	"0.103"	"0"	"0"
4	"0.036"	"0.009"	"0.045"
5	"0.114"	"0"	"0.001"
6	"0.001"	"0.192"	"0.176"
7	"0.047"	"0"	"0"
8	"0.005"	"0"	"0"
9	"0.04"	"0"	"0"
10	"0.003"	"0"	"0"

	SquarePlanar
1	"0"
2	"0"
3	"0"
4	"0"
5	"0"
6	"0.003"
7	"0"
8	"0"
9	"0"
10	"0"

Table S2. Cluster members of 4-ligand Zn, normal group

[1] "Cluster 1"
3COQ.A.1002, 4HCC.A.504, 2KKF.A.2002, 3S14.A.1735, 3A6F.C.301, 3A6J.A.301, 3A6J.D.301, 1A7I.A.82, 1A7I.A.83, 1ADB.A.375, 1ADN.A.93, 2AF2.B.154, 2AS9.A.666, 2AS9.B.222, 1B4E.A.405, 2B5L.C.3001, 1B8T.A.196, 4BHW.A.4, 4BLB.B.910, 4BOL.A.1261, 1BP3.A.500, 4C4O.D.500, 4CBY.A.2035, 2CJL.A.1217, 1CRA.A.262, 3D2Z.A.262, 4D9W.A.408, 2DGE.A.1001, 1DMY.A.280, 1DYO.A.401, 3E24.A.230, 2E26.A.603, 1E3I.A.381, 3E3F.A.230, 3E50.A.1, 2ECI.A.401, 4EEX.B.402, 1ELX.A.451, 1ELY.A.451, 1ELZ.A.451, 1EPW.A.1291, 1ESP.A.323, 3F5L.A.1001, 1FBX.A.3316, 3FCQ.A.322, 3FIE.A.428, 3FJU.A.502, 3FJU.A.507, 3FLF.A.2004, 2FNF.X.1, 1FR7.A.262, 3FV4.A.321, 3FVP.A.321, 3FXP.A.3000, 3G42.D.500, 1G4K.A.301, 1GI4.A.409, 3GIQ.A.482, 2GSU.A.1001, 3H2P.B.154, 4H57.A.405, 1H7N.A.1342, 1HCP.A.98, 4HEW.A.301, 1HP7.A.401, 1HY7.B.801, 1HYI.A.66, 2I2X.A.524, 1I6N.A.401, 1I8J.B.400, 2I9W.A.200, 2IT4.A.561, 1JJ9.A.999, 1JM7.A.123, 2JOX.A.108, 4JOM.A.1004, 2JSD.A.275, 2JTN.A.183, 4K1R.A.502, 1K24.A.401, 1K83.I.3003, 1KHL.A.451, 4KJG.B.1001, 1KTO.A.405, 3KVE.B.489, 2L8E.A.829, 3LAT.A.215, 1LG5.A.262, 4LJP.A.1103, 3LUB.A.302, 2LUL.A.201, 2M3H.A.102, 1M65.A.300, 3MBM.A.163, 2MIU.A.303, 4MLT.A.301, 1MPO.A.376, 3MS3.A.401, 4MTW.E.401, 4MWP.E.401, 4MXJ.E.401, 4MZN.E.401, 4NOG.A.401, 4N4E.E.401, 4N5P.E.405, 4N66.E.501, 1NJ1.A.513, 1NJG.A.401, 3NQZ.B.1, 2O4H.B.401, 4OCM.E.401, 1OSO.A.600, 2OVX.A.444, 2OVZ.A.444, 1P6B.A.406, 3PBJ.D.31, 1PE8.A.317, 4Q7R.A.303, 4QEF.A.301, 1QF2.A.320, 3QH5.A.321, 3QHD.A.163, 2QL1.A.1, 4R9G.A.505, 3RCM.A.288, 2RSD.A.901, 3SSB.A.999, 3SU6.A.5, 1SW1.A.401, 3T02.A.501, 3T5Z.A.262, 3T73.A.410, 3T74.A.410, 3T8G.A.411, 3T8F.A.411, 3T87.A.326, 3T8C.A.326, 3T8D.A.325, 3T8H.A.326, 1TBN.A.1, 1THL.A.324, 3TIO.A.1, 2TMN.E.321, 6TMN.E.322, 3TTY.A.676, 1U3L.A.701, 4U4L.A.301, 3U7L.A.350, 4UA4.A.302, 1UD9.B.508, 3UKO.A.401, 2UX1.K.1173, 2VXX.A.201, 1WAA.E.1089, 4WK7.A.501, 2WWO.A.1165, 3X17.A.601, 2X4H.B.1141, 1XLL.A.399, 1XUJ.A.246, 1XWH.A.356, 2Y28.B.1181, 1Y3G.E.321, 2Y4Y.A.1172, 2Y4Y.C.1172, 1YM3.A.301, 1Z9G.E.1005, 1ZDP.E.1005, 2ZNE.B.993, 3ZPG.A.1358, 1ZSO.A.999, 1ZUD.1.501, 2ZXG.A.900

[1] "Cluster 2"
1A6Y.A.550, 2A66.A.401, 2B3J.A.2001, 3EPH.A.1, 1F2I.G.1202, 1G2D.C.302, 1G2D.C.303, 3GOX.A.302, 4HC9.A.402, 2I13.A.502, 2I13.B.507, 2JP9.A.131, 2JP9.A.134, 1K3X.A.501, 1KB2.A.150, 3KDE.C.78, 1LO1.A.196, 4M9E.A.503, 4M9E.A.504, 1MEY.C.90, 3MLN.A.501, 2OFI.A.302, 3OYM.A.393, 1OZJ.A.145, 4R2A.A.503, 4R2A.A.504, 1TF3.A.2, 3TS2.A.2, 3U6P.A.300, 3VD6.C.501, 2XQC.D.1141, 258L.A.500, 3A32.A.708, 3A6G.A.301, 4A7K.A.950, 2AC3.A.531, 4ADN.A.1223, 1AF2.A.296, 2AFZ.A.391, 2AFM.A.391, 4AIA.A.200, 1AJB.A.451, 4AJX.H.1030, 3ALR.A.602, 2AP1.A.304, 4AR9.B.1731, 2ASH.A.400, 2AW1.A.262, 2AYD.A.369, 4AYC.A.1485, 2B3Z.A.1360, 1BBO.A.61, 1BCD.A.262, 3BET.A.262, 2BE7.D.1108, 3BI1.A.1752, 3BJI.B.2, 2BL6.A.1059, 1BNN.A.262, 1BNQ.A.262, 2BNM.A.1199, 2BPO.A.1341, 1BUD.A.800, 1BV3.A.262, 1BYF.A.302, 4C09.A.351, 4C1F.A.502, 2C36.A.1311, 3C5K.A.201, 3C5K.A.203, 2C6C.A.1752, 1CAI.A.262, 1CAK.A.262, 2CBD.A.262, 4CCG.X.1375, 4CDG.A.1643, 3CE1.A.202, 2CFU.A.1002, 1CG2.C.502, 3CG7.A.299, 1CGL.A.302, 3CHQ.A.701, 3CHS.A.701, 1CIL.A.262, 1CIN.A.262, 2CKI.A.999, 3CMR.A.450, 1CNH.A.1, 2CO8.A.401, 2CON.A.201, 2COR.A.401, 4CPD.A.1200, 2CR8.A.401, 2CRC.A.401, 2CSY.A.401, 2CS3.A.400, 2CS8.A.401, 2CSH.A.300, 2CSH.A.400, 1CTT.A.296, 2CTO.A.401, 2CT7.A.401, 4CVR.A.1158, 3CZV.A.262, 2D0W.B.1207, 1D0Q.B.151, 4D0Y.A.1239, 1D1T.A.376, 3D2N.A.101, 4D6S.A.1338, 3D7F.A.1752, 2D8X.A.201, 2D8X.A.401, 2D8Y.A.201, 2D8Z.A.201, 3D8W.A.262, 2D8U.A.201, 2D9K.A.601, 2D9L.A.401, 3DCC.A.262, 1DD6.A.503, 3DDO.A.262, 3DFM.A.402, 3DI4.B.286, 2DID.A.201, 2DIP.A.20

1, 2DIP.A.401, 2DJ7.A.201, 2DJ8.A.201, 2DKT.A.191, 2DKT.A.241, 2DKT.A.291, 2DKT.A.441, 2DLO.A.201, 2DLO.A.401, 2DLQ.A.300, 2DOO.A.502, 4DZ7.A.301, 2E5R.A.401, 3E6U.B.503, 2E73.A.201, 2E73.A.401, 1E9P.B.153, 1E9Q.B.153, 2EA6.A.201, 2EBL.A.191, 2ECT.A.401, 2ECY.A.401, 2ECG.A.201, 2ECL.A.201, 2ECM.A.401, 2ECN.A.401, 3EDI.A.210, 1EE2.A.1300, 2EE8.A.301, 2EGQ.A.300, 4EGU.A.202, 3EH2.A.800, 1EI6.A.409, 2EJ4.A.401, 1EKJ.C.4003, 2ELO.A.181, 2ELR.A.181, 2ELT.A.181, 2ELM.A.181, 2EMI.A.201, 2EM0.A.200, 2EM2.A.201, 2EM6.A.201, 2EM7.A.201, 2EMB.A.201, 2EMC.A.201, 2EMK.A.201, 2EML.A.201, 2EMM.A.201, 2ENV.A.200, 2ENZ.A.400, 2EN2.A.201, 2EN6.A.181, 2EN7.A.181, 2EN8.A.181, 2EN9.A.181, 2ENA.A.181, 2ENC.A.181, 2ENE.A.181, 2ENF.A.181, 2ENN.A.300, 2ENN.A.400, 2EOR.A.201, 2EOU.A.201, 2EOV.A.201, 2EOW.A.201, 2EOX.A.201, 2EOY.A.201, 1EOU.A.300, 2EO4.A.201, 2EOK.A.201, 2EON.A.201, 2EOO.A.201, 2EPT.A.201, 2EPV.A.201, 2EPZ.A.201, 2EP1.A.201, 2EP2.A.201, 2EP3.A.201, 2EP4.A.200, 2EP4.A.300, 2EPA.A.400, 3EPZ.A.701, 2EPQ.A.201, 2EQ1.A.201, 2EQ4.A.201, 1EU3.B.402, 2EU3.A.262, 2EXU.A.501, 3EYX.A.1, 1F1G.A.4002, 1F2W.A.262, 4F3W.A.201, 3F7B.B.301, 3F7L.A.203, 3F7U.A.260, 3F9O.A.309, 1FAQ.A.1, 2FC7.A.401, 2FGY.A.620, 2FHX.A.317, 3FID.A.299, 3FLO.B.1, 4FMN.B.902, 1FN9.A.1001, 2FOQ.A.262, 2FOS.A.262, 2FOU.A.262, 2FOV.A.262, 2FOY.A.301, 4FO9.A.501, 3FPC.A.353, 3FPL.A.352, 1FQL.A.262, 1FQM.A.262, 2FR5.A.147, 2FR6.A.147, 2FSA.A.501, 3FUN.A.701, 4FU5.A.302, 4FVD.A.201, 4FVN.A.302, 4FVO.A.302, 4FVY.A.805, 3FW3.A.300, 4FWE.A.901, 4FWU.A.401, 2FYG.A.302, 2G2N.A.1001, 1G52.A.262, 1G54.A.262, 1G5C.A.1001, 1G71.A.348, 1G9K.A.600, 3GC9.B.603, 2GD8.A.262, 4GGJ.A.301, 3GJ4.D.300, 2GMN.A.805, 2GMW.A.300, 4GNE.A.1501, 4GNE.A.1502, 4GNE.A.1503, 4GNE.A.1504, 2GQJ.A.200, 4GR3.A.301, 2GSN.A.1000, 1GUP.A.350, 3GV4.A.203, 1GVY.A.1425, 1GZH.A.1293, 3GZE.B.2, 3H0L.E.902, 4H0F.A.401, 2H15.A.262, 4H2K.B.1001, 2H39.A.352, 4H3S.A.901, 2H4N.A.262, 3H7H.A.119, 3HB2.P.486, 2HBA.A.101, 1HCP.A.99, 4HCG.A.202, 3HI2.A.121, 2HJN.A.315, 3HKO.A.701, 3HKQ.A.261, 3HKT.A.261, 3HLJ.A.272, 3HNJ.B.107, 3HS4.A.301, 2HU9.A.132, 1HXP.A.350, 1HXR.A.200, 1HY7.A.302, 4I1F.A.504, 3I4C.A.400, 2I5O.A.336, 1I73.A.998, 1IA9.A.2001, 3IGP.A.262, 2IGI.A.1001, 2IGI.A.1004, 4IJD.A.501, 4IJD.A.502, 4ILO.A.301, 4ILX.A.301, 2IMZ.A.501, 2IMZ.B.502, 2IOI.A.3001, 4ITO.A.301, 4ITP.A.302, 2IUC.B.1007, 3IXE.B.302, 2IYB.E.1423, 4J1V.A.301, 1J2O.A.116, 2J7U.A.1884, 2J9U.B.1162, 1JDO.A.901, 4JEA.B.202, 4JEA.C.202, 4JEA.D.202, 4JEB.B.202, 4JIU.A.201, 1JJD.A.104, 2JLP.A.226, 1JN7.A.37, 2JOX.A.109, 1JOC.A.300, 4JSR.A.401, 4JSZ.A.301, 2JTN.A.184, 2JUN.A.222, 3JWP.A.2001, 4JXE.A.502, 1JZS.A.1101, 4JZ0.A.802, 1K2Y.X.500, 4K2H.B.201, 3K34.A.1002, 1K7I.A.486, 4K7D.A.503, 4K7D.A.506, 2KAK.A.130, 2KAK.A.150, 3KEE.A.2000, 1KH7.A.451, 1KHK.A.451, 3KHI.A.301, 2KI7.B.124, 2KKT.A.500, 3KNE.A.500, 3KNV.A.202, 4KNI.A.301, 4KNJ.A.301, 1KOL.A.1001, 4KP5.A.301, 4KP8.A.301, 3KQI.A.71, 3KQI.A.72, 4KUV.A.301, 4KUW.A.301, 3KV4.A.448, 3KV5.A.489, 4KV0.A.301, 4L05.A.203, 3L14.A.262, 4L3J.A.402, 1L6J.A.500, 4L6O.A.801, 4L6T.A.301, 2L7P.A.201, 3L8H.A.901, 3LAS.A.167, 4LBU.A.402, 2LHN.A.501, 4LHI.A.301, 2LI8.A.187, 4LJO.A.1101, 2LK5.A.37, 2LN0.A.501, 4LOF.A.401, 4LP6.A.310, 3LRQ.C.100, 4LR2.A.505, 4LW9.A.203, 4LW9.C.202, 4LW9.K.203, 3LXE.A.261, 2LXD.A.201, 2LXD.A.202, 3LYR.A.1, 3M0A.D.401, 2M1S.A.100, 3M14.A.501, 3M1D.A.1000, 3M1W.A.500, 4M2R.A.301, 4M2V.A.301, 2M3Z.A.101, 2M3Z.A.102, 2M3L.A.201, 4M3P.A.701, 3M40.A.262, 2M48.A.501, 3M5E.A.262, 3M5S.A.500, 3M67.A.263, 2M7Q.A.101, 3M79.D.107, 2M7A.A.100, 2M9Y.A.402, 3M96.A.262, 3M98.A.262, 3MBG.A.1, 3MDZ.A.263, 4MDM.A.301, 4MG3.A.201, 3MHI.A.262, 3MHL.A.262, 3MHM.A.262, 3MHS.A.472, 3MHS.A.473, 4MHN.A.400, 4MI5.A.806, 3MJH.B.70, 3ML2.A.262, 3MMF.A.262, 3MNA.A.262, 3MND.A.153, 4MO8.A.301, 3MPZ.A.300, 3MP2.A.1, 1MS7.B.3001, 3MTW.A.2, 4MT2.A.68, 4MTY.A.301, 2MUQ.A.101, 1MXG.A.437, 3MYQ.A.262, 3MZC.A.262, 4N0X.B.301, 3N2P.A.262, 3N3J.A.262, 3N4B.A.262, 3N67.A.900, 3NA7.A.300, 3NB5.A.261, 4NJ5.A.801, 2NMX.A.301, 2NNV.A.262, 2NN1.A.301, 2NN7.A.301, 4NN2.A.401, 4NN2.A.402, 4NN2.A.403, 2NNG.A.262, 2NNO.A.262, 3NQ5.A.503, 3NQY.B.520, 4NQ4.A.302, 4NQ6.A.301, 4NTM.A.201, 4NTN.A.201, 2NUP.B.1100, 3NY3.A.1, 4NZG.A.201, 3O0M.A.151, 3O36.A.1, 4O64.A.2004, 4OBI.A.201, 3OCQ.A.184, 2OFK.A.201, 3OIL.A.262, 2OIK.B.201, 3OJ7.A.114, 3OOI.A.233, 1OQ5

.A.600, 3OQ6.A.375, 2ORW.A.401, 3ORU.A.250, 2OU2.A.490, 3OY0.A.262, 3OYQ.A.262,
3OYS.A.262, 3P3H.A.261, 3P3J.A.261, 1P42.A.501, 3P58.A.262, 3PB6.X.400, 3PB8.X.4
00, 3PBE.A.391, 1PFT.A.51, 1PG5.B.500, 3PLW.A.188, 3PN3.A.1010, 3PO6.A.1, 4PQ7.A
.301, 3PSQ.B.321, 4PTB.A.901, 4PTB.A.902, 2PUY.A.355, 3PU7.A.155, 3PUQ.A.2, 3PUR
.A.3, 4PYX.A.301, 4PZH.A.302, 3Q01.A.1, 1Q0E.A.153, 3Q1D.A.202, 2Q1Q.A.262, 2Q2L
.A.1001, 1Q3A.A.465, 4Q6D.A.301, 4Q6E.A.301, 4QF2.A.1801, 4QF3.A.2001, 3QGV.A.50
0, 2QO8.A.262, 4QSJ.A.301, 1QWY.A.400, 3QYK.A.262, 3R16.A.262, 1R5T.A.150, 1R9P.
A.135, 1RAY.A.262, 3RF4.B.119, 2RHQ.A.1, 3RJ7.A.300, 4RL0.A.301, 1RM8.A.501, 1RM
D.A.119, 1RMD.A.120, 4RN4.A.301, 2RPC.A.601, 2RPP.A.201, 3RQD.A.390, 2RSI.A.103,
2RSJ.A.102, 1RUT.X.604, 3RYM.A.106, 3RYV.B.262, 3RYX.B.262, 3RYY.A.1, 3RYZ.A.1,
3RYJ.B.262, 3RZ1.B.262, 3RZ7.A.1, 3RZ8.A.1, 3S71.B.262, 3S72.B.262, 3S76.A.1, 3
S77.B.262, 3S9T.A.262, 3SAX.A.262, 3SBH.A.262, 3SBI.A.262, 1SLM.A.257, 1SML.A.27
1, 3SV6.A.4, 3T5U.A.262, 3T7L.A.2, 1T8H.A.275, 3T80.D.301, 3T84.A.261, 3T90.A.50
2, 1T9H.A.411, 3T92.A.124, 1TFI.A.51, 3TG4.A.435, 1TT5.B.1014, 4U4L.A.302, 3U5N.
A.1, 3U5N.A.2, 3U9G.A.226, 3UCK.A.228, 3UCO.A.228, 1UD9.C.509, 3UEE.A.143, 3UNT.
A.400, 4UP0.A.1383, 1USN.A.257, 3UVC.B.303, 3UVI.A.387, 1UW0.A.1118, 3V1C.A.101,
4V1T.A.776, 2V29.A.1276, 3V3G.B.301, 1V47.A.601, 3V4K.A.2, 1V51.A.602, 3V5G.A.2
62, 3V7M.A.509, 3V7X.A.2001, 2V9I.B.1275, 3VBD.A.2001, 1VDD.D.233, 1VFY.A.300, 2
VJE.B.1491, 3VPB.E.100, 2VP7.A.1399, 2VPD.A.1399, 2VPG.A.1401, 1VQ2.A.701, 2VRS.
A.1328, 3VRK.A.301, 2VR6.A.1156, 3VTH.A.802, 1VYK.A.1150, 1VYX.A.1062, 1VZY.A.12
91, 2W3N.B.1234, 2W3Q.A.1231, 2W4L.A.1170, 2W5X.A.1378, 4WAJ.A.301, 3WBH.B.505,
2WEJ.A.1262, 1WEM.A.201, 1WEO.A.401, 1WEW.A.201, 2WEO.A.1262, 3WF8.A.401, 2WGX.A
.1300, 1WIG.A.201, 1WII.A.201, 2WJV.A.3, 1WJP.A.301, 1WJV.A.401, 3WLF.A.402, 3WN
Q.A.501, 1WO3.A.26, 2WOJ.A.1353, 3WRG.A.702, 1WWR.D.204, 1X3Z.A.999, 1X4I.A.201,
1X4J.A.401, 1X4K.A.201, 1X4L.A.201, 1X4S.A.401, 2XBL.A.1197, 2XBL.C.1196, 1XC3.
A.302, 1XCR.A.1001, 4XIW.A.401, 4XIX.B.401, 1XKI.A.1003, 1XSO.A.152, 1Y02.A.161,
1Y93.A.265, 1YB0.B.160, 2YB5.A.1213, 2YHO.A.1001, 2YHW.A.1719, 2YHY.A.2000, 2YI
K.A.1533, 1YQD.A.1000, 2YQL.A.401, 2YQP.A.201, 2YRJ.A.200, 2YRT.A.201, 2YRT.A.40
1, 2YRC.A.200, 2YRE.A.501, 2YRE.A.601, 2YRG.A.401, 2YRH.A.200, 2YRM.A.201, 2YS2.
A.201, 2YSA.A.181, 2YSM.A.301, 2YSM.A.701, 2YSO.A.181, 2YTH.A.201, 2YTP.A.181, 2
YTQ.A.201, 2YTT.A.181, 2YT5.A.201, 2YT5.A.401, 2YTB.A.301, 2YTD.A.201, 2YTF.A.20
1, 2YTK.A.201, 2YTM.A.181, 2YU8.A.201, 2YYR.A.401, 2YYR.A.402, 1Z05.A.406, 2Z2Y.
A.2001, 2Z3H.A.2001, 2Z3G.A.2001, 1Z60.A.2, 2Z9J.A.902, 2ZEP.A.391, 2ZED.A.391,
2ZEE.A.391, 2ZEG.A.391, 2ZEM.A.391, 1ZFK.A.1300, 1ZFO.A.31, 1ZGE.A.1000, 1ZIN.A.
219, 1ZNB.A.1, 2ZNR.A.2, 1ZP5.A.999, 3ZP9.A.1009, 3ZQ6.A.1323, 1ZR9.A.117, 2ZTX.
A.501, 3ZVS.A.1159, 3ZXH.A.301, 1ZZU.A.900, 2ZZE.A.753
[1] "Cluster 3"
1A1G.A.201, 1A6Y.A.551, 2ER8.A.105, 4GLX.A.601, 4IFD.J.1106, 4IQR.A.403, 4NM6.A.
2002, 3VD0.A.401, 1A1R.A.901, 1A72.A.376, 1AAF.A.56, 2AB3.A.30, 2AMT.B.2900, 1AN
J.B.451, 3AY2.A.1001, 2AZH.A.150, 4B6D.A.1340, 3BJI.A.1, 3BOC.A.1001, 3BTO.C.375
, 3BYR.A.501, 2C1I.A.1465, 4C1D.A.502, 4C1G.A.301, 4C1Q.A.493, 2C2F.A.1211, 1C3I
.B.260, 1CDO.B.376, 3CJP.A.301, 2COT.A.201, 2CO8.A.201, 2COR.A.201, 1CQR.B.2301,
2CQE.A.622, 2CT0.A.201, 2CT2.A.201, 2CTT.A.401, 2CU8.A.401, 2D74.B.1002, 2D8T.A
.201, 2D8T.A.401, 2D8V.A.201, 2D9M.A.1085, 2D9N.A.456, 2DAN.A.201, 2DAR.A.201, 1
DEH.A.376, 3DHA.A.256, 2DJ8.A.401, 2DJA.A.201, 2DJA.A.401, 1DK4.A.290, 1DMT.A.75
5, 1DO5.A.28, 3E1Z.A.111, 3E6U.A.501, 3E73.A.501, 2ECT.A.201, 2ECV.A.201, 2ECW.A
.201, 2ECW.A.401, 1ED9.A.451, 4EEX.A.402, 2ELS.A.181, 2ELX.A.181, 2ELI.A.401, 2E
M9.A.201, 2EOJ.A.201, 2EOQ.A.201, 2EOH.A.201, 2EOM.A.201, 3EWF.A.400, 2EWB.A.489
, 1F35.A.306, 4FMN.B.901, 2FNF.X.2, 2FPR.A.502, 3FTN.A.354, 2GOD.A.416, 2G9Y.B.4
51, 4GRI.A.501, 4GUT.A.904, 2GZL.A.900, 4H1Q.A.302, 2HCS.A.1, 2HCS.A.2, 3HJT.A.1
, 4HMA.A.301, 1HSZ.A.1376, 1HTO.A.1376, 1HZ5.B.105, 1I73.A.999, 1IAG.A.999, 3IFU
.A.181, 2IMR.A.500, 4IOU.D.1001, 4IRO.A.201, 2IUC.A.1002, 4IUQ.A.301, 4IUW.A.701
, 2JHG.A.401, 4JH2.A.201, 4JH2.B.201, 2JIG.B.1252, 1JJD.A.103, 2JTN.A.185, 1K07.
A.1, 2KEM.A.195, 1KH5.A.451, 2KVF.A.83, 1L0Y.B.706, 2L0B.A.143, 2L0B.A.161, 3L9Y

.A.155, 1LDE.A.375, 1LDY.A.375, 1LI5.B.964, 1LLU.B.343, 1M3V.A.124, 2M48.A.504, 1M6W.A.1376, 3M8T.A.300, 3M8T.A.301, 1M9O.A.78, 3MA2.A.295, 3MDW.A.455, 3MEQ.A.401, 1MGO.A.375, 1MGO.B.375, 3MI9.C.87, 3MWM.A.141, 3N2C.E.425, 1N8K.A.375, 4NQ7.A.302, 4O64.A.2003, 2ODX.A.156, 2OHX.A.401, 2OIO.A.2, 1P1R.A.375, 1P42.A.503, 1P4Q.B.301, 2PLI.B.709, 1PV9.A.402, 1PXE.A.64, 2QDT.A.402, 3QE3.A.356, 2QIN.A.2002, 2QLO.A.53, 2QSW.A.201, 1QTW.A.301, 1QV6.A.375, 1QV7.A.375, 1R1H.A.1001, 1R37.A.500, 1R3N.B.501, 2RPC.A.801, 3RSM.A.500, 3S2E.G.500, 3S2F.E.500, 3SFH.A.403, 3SP1.A.481, 1SRD.B.156, 3SXK.B.3, 1T3K.A.201, 3T33.A.411, 1T9R.A.1, 3TEN.A.205, 1TKF.A.902, 1TWF.J.3001, 1U10.A.400, 1U3U.A.376, 1U3V.A.376, 1U40.A.160, 3UIK.A.341, 2UZG.A.131, 2V1X.A.1595, 1V5N.A.401, 1V6G.A.201, 3V96.B.302, 1VA1.A.100, 1VA2.A.100, 1VA3.A.100, 2VES.A.1297, 2VES.C.1302, 3VOW.A.201, 3VPE.A.301, 1VSH.A.281, 2W5V.B.1377, 3W5K.B.502, 3W5K.B.503, 2WBT.A.1130, 2WBT.A.1131, 1WEN.A.401, 1WEP.A.201, 1WES.A.201, 1WES.A.401, 1WFF.A.401, 1WFL.A.401, 1WFP.A.401, 1WG2.A.200, 1WIG.A.401, 1WIL.A.401, 1WIM.A.201, 1WJP.A.501, 1WJV.A.201, 3WLE.A.402, 4WOK.A.401, 1WYH.A.201, 1X3H.A.401, 1X4K.A.401, 1X61.A.201, 1X62.A.201, 1X6H.A.401, 2X8Z.A.1616, 2X95.A.1615, 2XAA.C.1346, 1XB8.A.1001, 2XML.A.1349, 2XQV.A.401, 1XRT.A.1423, 1XWY.A.401, 1Y8J.A.800, 1YC2.A.402, 1YC2.C.406, 1YLK.A.401, 2YQL.A.201, 2Z9K.A.901, 1ZLH.A.555, 3ZNC.A.1, 3ZUK.A.1664, 2ZU2.A.5517

[1] "Cluster 4"

3F2D.A.5, 3IE1.D.442, 4MTD.B.201, 1QUM.A.301, 1V15.A.1132, 3A3O.B.65, 2A97.B.2437, 1AAF.A.57, 4AA1.A.1615, 2ANH.A.451, 1BAW.A.107, 2BA1.A.201, 1BH5.A.201, 3COY.A.401, 3CE9.B.400, 2CEA.B.1606, 3D2N.A.102, 1D8M.B.801, 1DDZ.A.1, 1DE6.A.450, 2DKD.B.922, 4DLF.A.404, 1DSQ.A.144, 3E4Z.B.2, 3EBI.A.1, 2EIM.C.262, 1EYW.A.402, 1F5F.A.252, 1F8F.A.372, 3FDK.A.402, 4FVL.A.501, 2G54.A.1100, 2GA3.A.451, 3GJN.B.600, 1GYT.J.600, 3GZE.B.14, 4H01.A.602, 3H8F.B.501, 3H90.D.6, 2HSI.A.283, 1HU8.A.501, 4I28.A.602, 1IAU.A.504, 3II1.A.571, 3ISI.X.3000, 1ITU.A.401, 4JIJ.A.302, 1JM7.B.143, 1JT1.A.400, 2K2G.A.2, 4K5N.A.1101, 2K8D.A.155, 2KO8.C.54, 4KXC.A.1001, 3L8Y.A.301, 2LGV.A.110, 1LG6.A.262, 2LVR.A.101, 3LZE.A.201, 3MON.A.201, 4MLX.A.301, 1MVH.A.503, 3N2C.D.426, 1NYR.A.1002, 2O36.A.690, 2O4Q.A.2402, 2O53.B.314, 4OIW.F.501, 1OJ7.B.1389, 1OLP.D.1374, 1OS2.B.369, 2OXW.A.264, 1P5X.A.247, 3PJN.A.186, 3PJN.A.189, 3PJN.B.186, 1PS7.A.331, 4Q7R.A.306, 3QJ5.A.376, 3QVY.A.500, 1QX1.A.2004, 4R7M.D.1001, 3RAM.D.998, 2RPR.A.201, 2RPC.A.401, 1RUR.L.601, 3SZY.A.501, 3T02.A.502, 1TF9.A.901, 1TKH.A.901, 3UW2.A.474, 2V2A.A.1275, 4WD6.A.302, 2X4H.B.1142, 1XEM.A.401, 2XY9.A.1628, 1Y7W.B.285, 1YHC.A.601, 2Z26.A.400

[1] "Cluster 5"

3AUO.B.579, 4GNX.C.701, 4L8H.R.105, 2ANH.A.452, 1ARD.A.1, 1ARF.A.1, 4AR1.A.1722, 1AXG.A.401, 2AYK.A.171, 1B8T.A.193, 1B8T.A.194, 1B8T.A.195, 3BKN.A.201, 3BL5.A.300, 4BLD.D.910, 1BTG.B.902, 2C20.A.601, 3CQZ.B.3007, 1DGS.B.2701, 2E1W.A.400, 1E46.S.999, 1E67.A.129, 4FC5.E.305, 2FZ6.A.201, 1GKR.A.1452, 1GLC.F.169, 4H82.B.300, 3H90.A.291, 3H90.A.293, 2I0O.A.581, 3ID7.A.402, 3IEW.B.801, 2IWE.A.1129, 2JMD.A.66, 1KBE.A.2, 1KHN.A.452, 2KIZ.A.71, 2KV1.A.125, 2KVG.A.85, 2KVH.A.84, 1L1O.F.2, 1LD3.A.500, 4LW9.E.204, 2LXH.C.901, 2MQ1.A.102, 2MQ1.A.103, 1MVH.A.502, 1NDV.A.400, 2O04.A.6001, 1OS2.F.769, 2OW2.A.444, 1P91.B.2301, 4P9C.A.201, 1PAA.A.160, 2POJ.A.265, 2QJS.A.2002, 3SPU.D.1004, 1TBN.A.2, 1THJ.A.214, 1THJ.B.214, 1U0L.A.298, 3U7K.A.350, 4UNI.C.1697, 2V8G.C.500, 2V8V.B.1455, 1V9P.B.2701, 2W57.B.201, 3WIE.B.1001, 2X4H.A.1140, 2X5C.A.1128, 2XAM.B.700, 1XTG.A.426, 1Y0J.A.244, 2Z3I.A.2001, 2Z45.A.1003, 3ZTV.A.1599, 1ZXZ.A.198

[1] "Cluster 6"

3AF5.A.665, 2ANU.B.405, 2APO.B.501, 3B1B.A.378, 1BQQ.M.289, 3C0Z.A.101, 2CBN.A.402, 2CG3.Z.1, 2CIH.A.212, 3CQJ.B.285, 4CWM.B.433, 3D3X.A.428, 1DXW.A.301, 2EC7.A.51, 4EGE.A.411, 3EII.D.301, 1ENQ.C.238, 3EYV.L.220, 4F0R.A.501, 3FGG.A.161, 4FUK.A.401, 2GLQ.A.2002, 4H00.A.601, 2H42.A.501, 3H66.B.500, 3H90.D.5, 3HDB.A.620, 2HD1.B.101, 4HGX.B.301, 1HOV.A.165, 4IGM.A.401, 4IGM.F.401, 4IGN.A.401, 3ITM.A.1, 4J3D.A.301, 1JDI.A.301, 1JOE.A.205, 2JRP.A.150, 4JSS.A.301, 4K6T.B.403, 1KAR.A

.501, 1KAR.B.502, 2L0Z.A.487, 1L9Y.A.2, 2M7Y.A.101, 3MKV.A.425, 3MO2.D.5, 2MQ1.A
.101, 4MZ7.A.701, 4NRZ.B.301, 3O90.A.192, 4O98.A.401, 2OC7.A.901, 1OLP.A.1373, 1
OLP.A.1375, 2PJT.D.302, 2PTW.A.500, 3Q31.A.1, 1Q74.D.304, 2QFP.A.434, 3R2J.A.301
, 1RO9.A.529, 3S6L.B.185, 3SFW.A.502, 1SMP.A.472, 1T0A.B.760, 1TM6.A.23, 4TQT.A.
501, 3TVX.A.900, 3UBF.A.7, 1UR6.B.79, 1UXA.C.1367, 3V94.E.702, 3VUV.A.501, 2VXX.
D.201, 3WI2.B.801, 4X2T.D.701, 4X2T.D.702, 2X4H.A.1141, 2XR1.A.1638, 2XR1.A.1639
, 1YIX.A.601, 1YIX.B.603, 2Z2D.A.264, 1Z3J.A.264, 1ZKN.C.601, 3ZNS.A.101, 2ZNE.B
.992, 1ZSW.A.315, 3ZTV.A.1598
[1] "Cluster 7"
1F2I.G.1201, 1G2F.C.301, 2I13.A.506, 2I13.B.510, 1I3J.A.100, 4IQR.A.402, 4M9V.C.
202, 2OPF.A.501, 4R2Q.A.503, 1A42.A.262, 2ADR.A.162, 2ADR.A.163, 4AIG.A.999, 1AN
I.A.451, 3ASK.A.501, 3B6P.A.800, 1B8Y.A.301, 2B83.C.3353, 4BHW.A.1, 1BI0.A.291,
3BKN.A.202, 3BL0.A.262, 1BNL.A.179, 1BNM.A.262, 2BNN.A.1200, 4C1E.A.502, 4C1G.B.
301, 3C37.A.301, 1C8T.A.260, 3CHV.A.302, 1CO4.A.43, 2CQF.A.330, 2CRR.A.401, 2CSY
.A.201, 2CSZ.A.201, 3CSK.A.712, 2CT1.A.201, 2CT1.A.401, 2CT5.A.201, 2CT7.A.201,
2CTT.A.201, 2CTU.A.201, 2CU8.A.201, 2CUP.A.201, 2CUP.A.601, 2CUQ.A.201, 2CUQ.A.4
01, 2CUR.A.201, 2CUR.A.401, 3CX3.A.314, 3CXL.A.501, 1CXV.A.3, 1D1T.A.401, 3D7V.A
.2, 2D8S.A.201, 2D8S.A.401, 2D8U.A.401, 2D9N.A.256, 4DF9.A.503, 2DID.A.401, 2DJB
.A.201, 2DJB.A.401, 2DKT.A.391, 1DMX.A.280, 1DVB.A.194, 3E2I.A.200, 3E2U.E.102,
1E3J.A.902, 3E3I.A.230, 2E47.A.172, 2E7Y.A.1301, 2E72.A.201, 2ECI.A.201, 4EFS.A.
301, 1EKM.A.701, 2ELQ.A.181, 2EMX.A.201, 2EMY.A.201, 2EMZ.A.201, 2EN4.A.201, 2EO
I.A.201, 2EPX.A.201, 2F0Y.B.501, 3F0F.A.165, 3F4X.A.262, 4F7O.B.301, 4F9C.B.401,
1FKW.A.400, 3FTN.D.357, 4GER.A.401, 3GIQ.A.483, 4GQT.B.502, 3GRB.A.129, 4GR0.A.
301, 3GTT.A.155, 4H12.A.1802, 4HVL.A.504, 1I50.C.3002, 3I9F.B.3, 1IB5.A.201, 1IB
B.A.201, 4ICR.A.501, 2IDA.A.103, 1IF5.A.262, 3IJF.X.147, 1IML.A.77, 2INN.B.514,
1IS8.B.3109, 3IXE.B.301, 1J2T.A.302, 2J7J.A.1088, 1J9Y.A.1004, 2JBG.B.1577, 1JD5
.A.501, 1JJD.A.102, 1JQ5.A.371, 4JSA.A.301, 2JUN.A.221, 2JUN.A.223, 2JWO.A.489,
1JW9.B.250, 3K6J.A.800, 2KDP.A.1, 1KEV.B.353, 1KFI.A.700, 2KIK.B.50, 2KIZ.A.70,
4KNM.A.301, 1KWQ.A.262, 2KZM.A.1, 4L6H.A.801, 2LI8.A.188, 3LPE.B.60, 2LUY.A.301,
2LXH.C.900, 4LXL.A.403, 3M15.A.107, 3M15.B.107, 3M2N.A.263, 4M3O.A.300, 1M4M.A.
502, 2M48.A.502, 2M6M.A.202, 1MC5.A.376, 3MEN.D.400, 2MIU.A.301, 4N4F.A.1401, 1N
92.A.375, 4NJ5.A.803, 4NQ5.A.302, 1NUI.A.501, 4O1K.A.301, 3O7U.A.428, 1OHL.A.400
, 2OH3.A.300, 3OJ3.J.902, 4OWF.G.401, 3P1V.A.427, 1P42.A.502, 1P9R.A.601, 1PGU.A
.616, 3PNU.A.336, 3PZC.B.1000, 4Q0L.A.301, 1Q1A.A.701, 2Q1B.A.400, 1Q3K.A.301, 3
Q6V.A.2, 2QQ4.A.139, 3QU1.A.505, 3R0D.A.428, 1R22.A.502, 4R2Y.A.103, 2R3A.A.302,
1R4V.A.202, 3RHG.A.368, 1RJ6.A.601, 1RJW.A.402, 2RJP.B.1, 1RMD.A.117, 2RMN.A.1,
2ROW.A.602, 2RPP.A.401, 4RQU.B.402, 3RUI.A.1, 1S4I.A.802, 3SEY.C.373, 3SOU.B.8,
2SRT.A.257, 3T7E.A.252, 3TGN.A.148, 3TG4.A.437, 1U3T.A.376, 1U5S.B.139, 3UJZ.A.
1, 1V0D.A.401, 2V08.A.1090, 3V1E.A.102, 4V2W.A.502, 1V5N.A.201, 1V5R.A.201, 1V87
.A.201, 1V87.A.401, 2V9E.B.1276, 1VGN.A.301, 2VJE.B.1492, 1VK9.A.143, 2VQG.C.109
1, 2VXI.B.201, 2VXX.B.202, 3W0F.A.301, 1WAA.D.1090, 1WE9.A.201, 1WEE.A.201, 1WEM
.A.401, 1WEN.A.201, 1WEO.A.201, 1WEP.A.401, 1WEQ.A.201, 1WEQ.A.401, 1WEV.A.201,
1WEV.A.401, 1WFF.A.201, 1WFH.A.401, 1WG2.A.400, 4WH6.A.1203, 1WIL.A.201, 1WIM.A.
401, 1WJA.A.56, 1WJP.A.701, 3WL3.A.301, 1WUQ.A.1001, 1WUR.A.1001, 3WXC.A.302, 1W
YH.A.401, 1WYS.A.201, 1X3C.A.201, 1X3H.A.201, 1X4V.A.201, 1X5W.A.201, 1X6E.A.401
, 1X6F.A.201, 2XEU.A.1065, 2XOC.B.991, 2XS4.A.998, 1XUF.A.246, 2XXH.B.1303, 1XYD
.A.94, 1XYD.B.94, 2Y20.C.1331, 2Y6C.A.1267, 2Y6D.A.1267, 1YOU.A.301, 1YSB.A.501,
2YTJ.A.201, 2Z2Y.D.2004, 1Z83.B.642, 2Z94.A.901, 1ZTQ.A.550
[1] "Cluster 8"
1A1I.A.201, 2C7A.A.1641, 4CIS.A.300, 1CYQ.A.601, 2DRP.A.171, 4ESJ.A.301, 1F4S.P.
64, 1F4S.P.65, 2FF0.A.1001, 1G2F.C.303, 2HAN.A.351, 2HAN.A.352, 2HAN.B.354, 2HGH
.A.191, 4HN6.A.602, 4HP3.C.201, 4HP3.C.202, 1HWT.D.138, 2I13.A.503, 2IVH.A.1577,
1LAT.A.1515, 4LJ0.A.501, 1LLM.C.301, 1LLM.C.302, 1LO1.A.195, 3LRR.A.1, 3M9E.A.2
08, 1MEY.C.89, 2NLL.A.250, 3O9X.A.132, 3OD8.A.200, 4OLN.A.101, 4OND.A.102, 1P47.

A.203, 4PZI.A.1101, 4PZI.A.1102, 4QEN.A.802, 4QEN.A.803, 4QEN.A.804, 3QMD.A.300, 3QMD.A.301, 1TDZ.A.1001, 4TNT.A.701, 4TNT.A.702, 3TS2.A.1, 2XQC.A.1138, 2YKG.A.927, 1ZGW.A.500, 3A1B.A.3, 4A2C.A.1349, 4A46.A.65, 1A5T.A.501, 2A5H.A.421, 2A6H.D.7412, 1A71.A.402, 1A7T.A.251, 2AFW.A.996, 2AFX.A.996, 4AI5.A.200, 3ALR.A.601, 1AM6.A.262, 2APS.A.400, 2AQP.A.201, 4ARE.A.1790, 4AU7.A.1248, 3AVR.A.1502, 3AXS.A.401, 4AY8.A.600, 1AZM.A.261, 3B4F.A.262, 2B5W.A.800, 4B6D.A.1341, 2B8T.A.1218, 4BF1.A.270, 4BF6.A.1262, 3BL1.A.262, 1BN1.A.262, 1BN3.A.262, 1BN4.A.262, 1BNT.A.262, 1BNU.A.262, 1BNV.A.262, 1BNW.A.262, 3BO5.A.301, 3BOL.A.701, 1BTK.A.1, 1C2G.A.409, 4C3E.A.201, 4C3T.A.300, 4C4O.B.600, 3C5K.A.202, 3C63.A.107, 3C63.B.107, 3C63.C.107, 2C6A.A.336, 3C7P.A.262, 2C7N.A.499, 4C8E.A.1162, 3CA2.A.264, 4CA1.A.284, 1CCT.A.262, 4CCG.X.1374, 1CDO.B.377, 2CDC.A.1372, 2CEX.C.1306, 1CG2.A.502, 1CIM.A.262, 2CKL.B.1115, 1CLC.A.653, 1CNG.A.1, 1CNI.A.1, 1CNJ.A.1, 1CNW.A.262, 1CNX.A.262, 1CNY.A.262, 3COS.A.502, 4COI.A.652, 3CQZ.L.3005, 4CQ0.A.1262, 2CQE.A.822, 2CRW.A.401, 2CSZ.A.401, 2CS7.A.201, 2CT2.A.401, 2CTD.A.201, 2CTD.A.401, 2CUP.A.401, 3CXK.A.201, 3CXL.A.500, 4CYK.A.42, 1CZM.A.261, 1D1S.B.376, 4D1N.A.900, 2D5B.A.501, 2D8Y.A.401, 2D8Q.A.201, 2D8R.A.401, 2D9H.A.201, 2D9H.A.401, 3DAZ.A.262, 2DAR.A.401, 2DAS.A.101, 2DB6.A.201, 2DB6.A.401, 4DB3.A.401, 1DCQ.A.600, 3DD8.A.262, 1DDZ.A.2, 3DDT.A.46, 2DFV.A.1001, 4DLA.A.402, 3DMO.A.131, 2DMD.A.191, 2DMD.A.241, 2DPH.A.1001, 2DQ4.A.502, 1DVP.A.401, 1DVP.A.402, 3DWD.A.501, 3E1W.A.230, 4E2X.A.501, 1E3J.A.901, 2E6R.A.401, 2E6I.A.201, 2EA5.A.201, 2EBT.A.200, 2EBT.A.300, 3EB5.A.1001, 2ECJ.A.201, 2ECV.A.401, 2ECY.A.201, 2ECL.A.401, 2ECN.A.201, 2EER.A.501, 1EE2.A.1301, 1EE8.A.501, 3EED.A.194, 2EE8.A.701, 4EEZ.A.401, 1EF4.A.56, 3EFT.A.262, 2EGM.A.200, 2EGM.A.300, 3EH1.A.1269, 2ELN.A.181, 2ELW.A.181, 2ELI.A.201, 2EMJ.A.201, 2EM1.A.201, 2EMA.A.201, 2EME.A.201, 2EMH.A.201, 2EN1.A.201, 2EOS.A.201, 2EOZ.A.201, 2EOG.A.201, 2EOL.A.201, 2EOP.A.201, 2EPR.A.201, 2EPU.A.201, 2EPW.A.201, 2EPY.A.201, 2EP0.A.201, 2EPC.A.201, 2EQW.A.201, 2EQ2.A.201, 4ETS.A.302, 4EVB.A.204, 4EYL.A.303, 4EYU.A.1702, 1EZM.A.302, 3F0D.A.163, 1F18.A.155, 2F14.A.1262, 1F4T.A.369, 1F8F.A.373, 1F9X.A.999, 4F9V.A.401, 1FAQ.A.2, 4FAI.A.401, 2FEA.A.1302, 3FFP.X.262, 3FID.A.298, 3FL2.A.1002, 3FLO.B.2, 4FRC.A.302, 4FWE.A.902, 2FZW.A.375, 3G1P.A.300, 4G26.A.1001, 1G47.A.999, 1G48.A.262, 2G45.A.401, 4G7A.A.301, 2G84.A.506, 2GAG.D.101, 2GAH.D.101, 2GFJ.B.401, 2GFE.A.869, 3GJ3.B.300, 3GJ5.B.300, 3GJ7.D.300, 3GJ8.B.300, 4GR8.A.301, 2GVI.A.301, 3GZK.A.539, 1H2B.A.1362, 4H30.A.301, 3H5A.B.360, 3H5N.A.500, 2H6E.A.500, 4H84.A.301, 4H9D.A.201, 3HCI.A.1000, 3HCJ.A.1000, 1HDY.A.376, 2HD6.A.262, 4HDH.A.1002, 4HEY.A.301, 2HF1.A.102, 4HF3.A.301, 4HI8.B.101, 4HI8.B.102, 2HJH.A.800, 3HKU.A.261, 2HL4.A.262, 3HNA.A.501, 3HNA.A.504, 3HNI.A.107, 2HQH.E.1500, 1HSO.A.1376, 4HT0.A.301, 4HT2.A.301, 4HTB.A.401, 1HUG.A.261, 4HU1.A.301, 2HZ8.A.117, 2IO0.A.579, 4I1H.A.507, 2I3H.A.1001, 3I4C.A.500, 1I6P.A.301, 2I9W.A.201, 1IA6.A.1264, 3IBI.A.262, 3IBL.A.262, 3IBU.A.262, 4IBY.A.301, 3IFJ.A.201, 3IFJ.B.202, 1INN.B.167, 1IQ8.A.600, 3IR9.B.501, 4IXJ.A.301, 2IYB.E.1422, 2J1Y.A.1290, 2J6A.A.1138, 1J98.A.300, 2J9R.A.1194, 1JAZ.A.401, 2JA1.A.1192, 4JBG.A.401, 4JEA.A.202, 4JIV.D.101, 1JJE.B.251, 1JJT.B.251, 4JLW.A.401, 2JMO.A.401, 4JMY.A.201, 2JMD.A.65, 2JOX.A.110, 4JPA.A.301, 1JTK.A.137, 1JVB.A.400, 1JVB.A.500, 3JV7.A.501, 3JV7.A.502, 3JVH.A.163, 4JXE.A.501, 1JY8.A.300, 2JZ8.A.150, 3K2F.A.262, 3K5K.A.1194, 2K5C.A.96, 4K7D.A.501, 4K7D.A.505, 4K7D.A.507, 4K7W.B.101, 3KB1.A.302, 3KE1.A.163, 2KFN.A.1, 2KN9.A.82, 1KOL.A.1002, 4KUJ.A.301, 4KUY.A.301, 3KV5.A.490, 1KWG.A.806, 4KXQ.A.601, 2KZY.A.63, 3L00.A.180, 3L11.A.602, 4L56.A.401, 4L58.A.102, 2L6L.A.201, 1LBU.A.214, 2LBM.A.3, 2LCE.A.300, 2LCQ.A.162, 2LGG.A.380, 2LGG.A.382, 1LIQ.A.28, 4LIM.A.401, 3LJU.X.401, 4LJO.A.1104, 3LKM.A.904, 2LKO.A.32, 3LMI.B.1002, 1LPV.A.53, 1LPV.A.54, 4LQG.A.802, 3LSC.A.458, 3LT8.A.80, 4LU3.A.301, 1LV3.A.66, 2LWW.A.503, 3M04.A.501, 2M13.A.602, 3M2X.A.500, 3M2Y.A.500, 3M4C.B.108, 1M6H.A.1376, 3MBG.A.3, 1MBX.B.211, 3MEQ.A.501, 4ME3.A.301, 1MGO.A.376, 3MHC.A.262, 3MHH.E.97, 3MHS.A.474, 3MHS.A.475, 3MHS.A.476, 4MHQ.A.501, 4MHY.A.400, 4MI5.A.802, 4MI5.A.804, 4MI5.A.805, 2MIU.A.302, 4MJ7.A.201, 1ML9.A.1, 1ML9.A.3, 3ML5.A.263, 1MNC.A.282, 4MT2.A.67, 1M

VH.A.501, 2MWX.A.201, 1MZ8.B.600, 1MZB.A.202, 4N0L.A.401, 4N0N.A.503, 4N4F.A.140
2, 1N5N.A.401, 1N8K.A.376, 1NCS.A.61, 3NIS.A.1, 3NIS.A.3, 3NIT.A.2, 3NI5.A.262,
4NQ7.A.301, 4NS5.A.401, 1NTO.A.500, 3NY1.A.4, 3NY1.A.5, 3NY1.A.6, 3NY3.A.2, 3NY3
.A.3, 1NZJ.A.700, 2O03.A.202, 2O3K.A.401, 2O4Z.A.262, 3O4N.A.2002, 4O64.A.2001,
4O64.A.2002, 4O6I.A.601, 3O70.A.500, 3O7A.A.500, 4OAQ.A.403, 3OCA.A.300, 2OGW.A.
500, 4OGE.A.1201, 4OIF.A.701, 1OKL.A.262, 1OKM.A.262, 3OOI.A.234, 2OSM.A.262, 2O
SF.A.262, 2OUI.A.361, 1OX7.A.402, 1OXN.A.1001, 3OXF.A.440, 1OYW.A.801, 2OZU.A.80
0, 1P0F.A.2502, 2P09.A.200, 3P2A.C.151, 2P57.A.201, 1P6O.A.401, 3P8B.A.101, 3PB4
.X.400, 3PB7.X.400, 3PB9.X.400, 1PCX.A.950, 2PG3.A.300, 3PII.A.340, 3PJN.A.187,
3PJN.A.188, 1PL8.A.402, 3PN3.A.1002, 2POU.A.262, 2POW.A.262, 2POI.A.100, 2PPT.A.
300, 1PQ4.A.1002, 2PQ8.A.501, 4PQT.A.501, 3PTM.A.1001, 2PVX.A.901, 2PVE.A.301, 3
PYK.A.262, 1PZW.A.100, 4Q09.A.301, 3Q1D.A.201, 3Q7C.A.1, 4Q7R.A.301, 4Q7R.A.302,
4Q7R.B.302, 4QF2.A.1802, 2QIC.A.300, 2QIC.A.400, 3QL9.A.1, 3QL9.A.3, 4QN1.A.150
1, 2QOA.A.262, 2QP6.A.262, 4QSI.A.301, 3QU1.A.501, 3QU1.A.503, 3QWP.A.500, 3QWP.
A.501, 4R1X.B.501, 3R2N.A.135, 4R2Y.A.101, 1R79.A.201, 1R79.A.401, 3RBU.A.1752,
2RI7.A.501, 1RJQ.A.601, 1RJW.A.401, 4RM5.D.300, 1RNI.A.256, 2ROW.A.601, 2RPC.A.2
01, 4RQT.A.401, 3RSN.A.200, 2RSH.A.101, 2RSI.A.101, 2RSI.A.102, 2RSJ.A.101, 1RUT
.X.603, 3RZ0.B.262, 3RZ5.A.1, 3S2E.A.500, 3S2E.A.501, 1S3G.A.219, 3S73.B.262, 3S
75.B.262, 3SAP.A.262, 3SD9.A.2, 3SI2.A.601, 3SJG.A.1752, 3SUB.A.161, 1SX1.A.23,
1T4W.A.201, 3T82.A.261, 1TAF.A.2003, 1TEQ.X.262, 3TG4.A.436, 3TIO.B.185, 3TIO.D.
185, 3TIO.E.185, 3TMJ.A.262, 1TOT.A.53, 1TOT.A.54, 3TTC.A.1, 3TTC.A.2, 3TWO.A.34
9, 4TYT.A.302, 4TZC.A.501, 4TZU.A.503, 1U0A.A.5005, 3U1L.A.241, 1U3T.A.375, 1U3U
.A.375, 1U3W.A.375, 1U5S.B.138, 3U9G.A.227, 3U9G.A.228, 1UAQ.A.200, 3UCJ.A.228,
3UCM.A.228, 3UCN.A.228, 3UEH.A.143, 3UEI.A.143, 3UEJ.A.301, 3UKO.A.400, 4UOV.A.2
98, 4UP0.A.1384, 2USN.A.257, 4UTV.A.1299, 1UUF.A.401, 3UVC.A.301, 3UWA.A.200, 1U
X1.A.1132, 2UZG.A.133, 2V0C.A.1816, 4V0R.A.1001, 3V1F.B.703, 1V6G.A.401, 1V9E.B.
260, 2V9K.A.1533, 3VDP.A.201, 1VFY.A.301, 2VF7.A.1845, 1VGN.A.302, 3VGL.A.322, 1
VHH.A.400, 3VHS.B.51, 3VHT.B.401, 1VJ0.A.400, 1VJE.A.167, 2VKR.A.106, 2VM5.A.124
5, 3VOV.A.401, 2VO9.B.501, 2VPG.A.1400, 1VQ0.A.300, 2VQM.A.1412, 2VRW.B.1566, 1V
SR.A.201, 3VTH.A.801, 3VUW.E.801, 2VVB.X.1268, 2W0T.A.125, 1W4R.A.400, 1W5O.A.13
39, 3W5K.B.504, 4W6Z.A.402, 1WAA.A.1090, 2WB0.X.601, 2WB0.X.602, 2WCB.A.100, 2WD
2.A.1262, 2WD3.A.1263, 4WD8.B.303, 4WD8.C.303, 1WE9.A.401, 1WEE.A.401, 1WEW.A.40
1, 2WEH.A.1262, 1WFE.A.201, 1WFE.A.401, 1WFH.A.201, 1WFL.A.201, 1WFZ.A.201, 1WGE
.A.201, 1WIR.A.201, 2WJV.A.1, 1WKQ.B.202, 1WN5.A.1001, 1WO4.A.26, 3WS6.C.201, 2W
VJ.A.1193, 2WWO.A.1162, 1WYS.A.401, 1X31.D.1006, 1X4U.A.201, 1X4U.A.401, 1X4V.A.
401, 1X4W.A.201, 1X4W.A.401, 1X61.A.401, 1X64.A.201, 1X64.A.401, 1X6M.A.200, 2X7
S.A.1265, 1XAF.A.501, 1XB0.A.403, 2XB4.A.1224, 2XCM.E.1223, 1XEG.A.262, 2XOC.B.9
92, 1XTM.B.501, 1Y02.A.162, 2Y1N.A.1436, 1Y8Q.B.642, 1YO7.A.201, 1YQD.A.2000, 2Y
QM.A.201, 2YQM.A.401, 2YRK.A.201, 2YSM.A.501, 2YTG.A.201, 2YTS.A.201, 2YT9.A.201
, 2YT9.A.203, 2YTA.A.201, 2YTE.A.201, 2YTI.A.201, 2YUU.A.201, 2YVR.A.1001, 2YVR.
A.1002, 2YWW.A.504, 2YZ3.A.301, 2YZ5.B.1501, 2Z2S.B.204, 2Z3J.A.2001, 2Z45.B.100
4, 1Z5H.A.2001, 1Z6U.A.2, 1Z9N.A.201, 1Z9Y.A.300, 2Z9L.A.701, 1ZE8.A.263, 2ZEF.A
.391, 2ZEH.A.391, 2ZEL.A.391, 2ZEN.A.391, 2ZEO.A.391, 3ZFK.A.401, 1ZFQ.A.600, 1Z
GF.A.400, 3ZGO.A.400, 1ZNF.A.27, 3ZNI.A.1428, 3ZTG.A.1336, 3ZTG.A.1337, 1ZVX.A.9
99, 3ZYQ.A.1222, 3ZYQ.A.1223, 2ZZF.A.754
[1] "Cluster 9"
2ER8.A.104, 1G2F.C.302, 3HAX.C.201, 2IHX.A.235, 2JP9.A.133, 2KKF.A.2001, 4LMG.A.
201, 1MEY.C.88, 4MTD.A.201, 2NQ9.A.401, 4O6A.A.601, 1ODH.A.1171, 3PIH.A.917, 3QS
V.A.1, 3UK3.C.967, 2A5V.A.401, 1A71.A.401, 3A9J.C.1, 3A9K.C.1, 2AA4.A.1001, 2AFU
.A.391, 1ARE.A.1, 4ARF.A.1722, 3AUK.A.390, 2AU3.A.501, 4AWY.B.3228, 3AX1.A.601,
4AX0.B.3228, 4AX1.B.3228, 4AXD.A.700, 2B0P.A.400, 4B29.A.1205, 3B4N.B.712, 3B5Q.
A.500, 3B7R.L.701, 3B92.A.502, 3BHX.A.1752, 3BI0.A.1752, 2BL6.A.1060, 3BQ6.A.800
, 1BS4.A.2001, 4BT7.A.301, 3C10.A.102, 4C1D.A.501, 4C1E.A.501, 4C1F.A.501, 4C1G.
A.300, 3C2S.A.448, 4C2P.A.701, 3C52.B.401, 1C7K.A.133, 3C8Z.A.413, 4C81.A.1240,

2C9S.A.1155, 1CAQ.A.301, 1CL4.A.81, 2CLT.A.1202, 3CNG.A.508, 2COT.A.401, 3COS.A.501, 3CQZ.I.3004, 3CSQ.A.335, 1CTU.A.296, 1CVE.A.262, 2D74.A.1001, 1D9D.A.1, 3DBK.A.302, 1DCA.A.262, 1DD6.A.502, 2DH3.A.601, 3DH1.A.201, 2DJ7.A.401, 2DKT.A.341, 4DLA.A.401, 2DMD.A.291, 2DOO.A.501, 1DPM.A.801, 2DQ6.A.900, 4DR8.A.201, 1DSV.A.171, 2DSN.B.2002, 1DTH.A.901, 2DW2.A.700, 4DYG.B.307, 3E2C.A.200, 1E3I.A.380, 1E3L.A.380, 1E4B.S.999, 3E6U.C.502, 2E9H.A.301, 2EA5.A.401, 2EA6.A.401, 2ECG.A.401, 2ECL.A.601, 2ECM.A.201, 3EER.A.2004, 2EE8.A.501, 4EEZ.A.402, 3EFO.B.1034, 2EG4.A.301, 2EG4.B.302, 3EHX.A.264, 1EI6.B.408, 1EKJ.A.4001, 2ELU.A.181, 2ELV.A.181, 2EM4.A.201, 2EMF.A.201, 2EMG.A.201, 2ENH.A.181, 2EOE.A.201, 2EOF.A.201, 3EQN.B.757, 2EQ0.A.201, 2EQ3.A.201, 1EU4.A.400, 4EXS.A.302, 2FEJ.A.1, 4FGL.A.301, 2FIF.B.901, 4FKB.A.401, 4FKE.A.1024, 4FKK.A.1025, 1FLJ.A.262, 4FMP.A.400, 1FR2.B.301, 3FTW.A.701, 2FU8.A.401, 2FU9.A.401, 3FVZ.A.821, 2FZW.B.376, 4G3M.B.401, 3GAY.B.328, 2GMN.A.801, 1GO8.P.1486, 4GU1.A.905, 4GUA.A.1719, 1H19.A.701, 1H9Q.A.262, 2HB9.A.401, 3HFF.A.154, 1HK8.A.1589, 3HKN.A.261, 3HNI.G.107, 3HNI.H.107, 3HNJ.A.107, 3HNJ.C.107, 3HNJ.D.107, 4HNO.A.301, 3HPH.A.220, 3HUG.D.109, 3I1U.A.401, 3I3T.A.700, 1I5O.A.3006, 1I76.A.999, 4I7C.A.601, 2J21.A.1289, 4J3D.B.302, 4J4M.A.301, 1JAO.A.999, 4JD1.B.202, 4JE6.A.200, 2JIG.A.1253, 4JLX.A.501, 4JSW.A.301, 2JTN.A.186, 1JV0.A.261, 4K2H.A.201, 3K6I.A.202, 1K7H.A.478, 4K7D.A.504, 4K7D.A.508, 3KBF.A.159, 3KED.A.875, 1KH4.A.451, 1KK1.A.411, 1KOQ.A.301, 1KU0.A.701, 3KWO.B.161, 3KWO.C.161, 4KX8.A.1001, 4KXB.A.1001, 3KYC.B.641, 3L0V.A.1, 3L22.A.1, 4L5O.A.303, 1L7O.B.301, 4LAO.A.401, 3LE9.B.2, 3LEA.A.485, 4LEF.A.302, 4LEV.A.601, 2LFD.A.400, 2LGV.A.111, 4LGJ.A.301, 4LJQ.B.1105, 4LJQ.A.1101, 4LOE.C.401, 3LQB.A.201, 3LQH.A.1001, 3LS1.A.1, 3LS9.A.457, 2LUY.A.300, 2LVU.A.101, 2LVT.A.101, 4LW9.D.201, 4LY4.D.301, 2M0D.A.101, 2M0E.A.101, 3M3X.A.262, 3M6I.A.402, 4MB7.A.301, 3MF1.A.1000, 3MHS.E.97, 3MI9.C.88, 3MKG.A.155, 3MKV.B.426, 1MMR.A.1, 1MOO.A.262, 1MQ0.B.147, 3MWM.A.140, 3MWM.A.142, 1ND1.A.400, 3NGJ.A.250, 3NKQ.A.1001, 4NL4.H.802, 3NNQ.A.201, 1NO5.B.571, 4NQ5.A.301, 4NQ6.A.302, 2O03.A.201, 1OAL.A.152, 2OD1.A.902, 1OKN.A.262, 1ONW.A.801, 4OTE.A.304, 2OW9.B.606, 2OXZ.A.264, 2OX8.B.3, 1P1V.A.201, 1P5D.X.500, 3P5A.A.262, 3P5L.A.262, 2P53.A.401, 4P9C.B.201, 1PB0.A.1301, 1PB0.A.1303, 3PBB.A.391, 3PLW.A.187, 3PN3.A.1009, 3PN3.B.1011, 3PZC.A.1000, 1Q2L.A.963, 3Q43.A.1, 3Q44.A.1, 1Q5W.A.32, 2Q6E.A.501, 3Q94.A.301, 4QBG.B.301, 2QDT.A.401, 1QJI.A.1201, 3QNA.A.122, 2QNO.A.431, 4QP5.A.401, 1QUA.A.999, 3QVZ.D.500, 3QWO.C.500, 3R3L.A.585, 2R59.A.701, 4RF1.A.1901, 1RMD.A.118, 4RQT.A.402, 4RUW.A.501, 3RZV.A.1, 1S1G.A.152, 1S4B.P.1, 1SRP.A.920, 3SWR.A.3, 3T01.A.502, 1T3A.A.422, 3TBG.A.601, 3TGO.B.505, 1TKF.A.901, 1TTM.A.262, 1U05.A.500, 1U10.A.601, 4U10.A.401, 1U1H.A.766, 1U4G.A.9800, 4UA4.A.301, 4UA4.B.303, 3UN6.A.325, 1UUF.A.402, 2UYV.B.1276, 1UZF.A.701, 1V13.B.200, 3V1F.A.704, 1V4P.A.1001, 1V7Z.A.301, 2V9E.A.1276, 2VF7.B.1844, 2VL6.A.1266, 1VQ2.A.702, 3VQZ.A.301, 2VUT.I.1713, 1VYX.A.1061, 4W6Z.A.401, 1WAA.B.1090, 4WAI.A.101, 2WCB.B.100, 2WHG.A.1263, 1WJB.A.56, 4WKE.A.501, 1WNU.A.1001, 2WO8.C.1268, 1WY2.B.407, 2X3B.A.1341, 1X62.A.401, 2X7M.A.1174, 2X8Y.A.1616, 2X96.A.1617, 1XPZ.A.262, 1XUC.A.1261, 1Y23.A.1001, 1Y8F.A.702, 1YC5.A.1001, 1YE3.A.375, 1YEJ.L.605, 2YPU.A.1998, 2YQQ.A.201, 2YQQ.A.401, 2YSP.A.181, 2YTR.A.201, 2YTN.A.201, 2YUU.A.401, 2YXO.A.501, 2Z45.A.1001, 1Z6R.A.501, 1Z6U.A.1, 1Z84.A.603, 2ZNC.A.1, 1ZXV.B.9002

[1] "Cluster 10"

1A73.A.202, 4AA6.A.253, 2C7A.A.1642, 3CBB.A.1001, 1CYQ.A.602, 2DRP.A.172, 1DSZ.A.1121, 1DSZ.A.1122, 1DSZ.B.1222, 3EQT.A.1, 1FFY.A.1001, 2FFO.A.1002, 3G9M.A.526, 2GAT.A.67, 4GAT.A.67, 6GAT.A.67, 3GOX.A.301, 4GZN.C.203, 2HAN.B.353, 4HC9.A.401, 2HGH.A.192, 2HGH.A.193, 4HN5.A.601, 2IHX.A.236, 2JP9.A.132, 2JZW.A.56, 1K82.A.450, 2KAE.A.175, 1KB2.A.151, 2KMK.A.83, 2KMK.A.84, 2KMK.A.85, 3KMP.A.2, 1LAT.A.1514, 4LJ0.A.502, 4LJ0.A.503, 2LT7.A.701, 2LT7.A.702, 2LT7.A.703, 3M7K.A.143, 3M7K.A.144, 4M8O.A.1303, 3M9E.B.209, 4M9E.A.505, 4M9V.C.201, 3NCU.A.1, 4NDH.A.402, 2NLL.B.450, 2NLL.B.451, 2O6M.A.601, 4OLN.A.102, 4OND.A.101, 4OOR.A.102, 4QEN.A.805, 4R2A.A.505, 4R2S.A.501, 1R4O.A.526, 1TF3.A.102, 1TF3.A.3, 1UBD.C.501, 1UBD.C

.502, 1UBD.C.503, 1UBD.C.504, 3UK3.C.968, 1YUI.A.64, 1ZAA.C.203, 1ZNS.A.1500, 2A1K.A.1, 3A1B.A.1, 3A1B.A.2, 4A24.A.601, 4A24.A.602, 4A2V.A.1000, 3A43.A.701, 2A51.A.54, 2A51.A.55, 2A5H.B.421, 2A6H.D.7458, 2A8D.A.1230, 3AII.A.1001, 2AKL.A.117, 3ASL.A.3, 4AUQ.B.1299, 4AUQ.B.1300, 2AYJ.A.57, 4AYC.A.1484, 2BOO.E.698, 2B44.A.400, 2B9D.A.1002, 1BBO.A.60, 4BM9.A.1466, 4BM9.A.1469, 3BO5.A.302, 3BO5.A.303, 3BO5.A.304, 3BOF.A.701, 3BQ5.A.800, 4BS9.A.1782, 4BUE.A.2162, 2BYO.A.1209, 1BZM.A.261, 2BZ1.A.1174, 4C5W.A.403, 3C63.D.107, 3C6W.A.2, 1C9Q.A.999, 4CA1.A.283, 3CHV.A.301, 2CJS.C.201, 2CJS.C.202, 2CKL.A.1104, 2CKL.A.1105, 2CKL.B.1116, 4CPD.A.1300, 2CSV.A.200, 2CSV.A.400, 2CS2.A.200, 2CS3.A.200, 2CS8.A.601, 2CSH.A.200, 1CXX.A.1, 1DOC.A.900, 1D1T.A.375, 3D2Q.A.303, 3D2Q.A.304, 2D8Z.A.401, 2D9G.A.201, 2D9K.A.401, 3DBH.B.1, 1DFE.A.38, 3DGD.C.128, 2DLK.A.201, 2DLK.A.401, 1DL6.A.60, 2DLQ.A.200, 2DLQ.A.400, 2DLQ.A.500, 2DMI.A.200, 2DMI.A.300, 2DMJ.A.200, 2DS7.A.100, 1EOE.A.147, 2E2Z.A.101, 1E4U.A.79, 1E4U.A.80, 2E5R.A.201, 2E5S.A.201, 2E5S.A.401, 2E6R.A.201, 2E6S.A.201, 2E6S.A.401, 2E6S.A.601, 1E7L.A.1165, 2EBL.A.241, 2EBT.A.100, 2EBV.A.201, 3EBE.A.500, 2EBQ.A.201, 2EBR.A.201, 2ECJ.A.401, 3EFO.A.766, 2EGQ.A.200, 2EGP.A.200, 2EGP.A.400, 2EHE.A.200, 2EHE.A.300, 2ELY.A.200, 2ELZ.A.200, 2EL4.A.200, 2EL5.A.200, 2EL6.A.200, 2EM5.A.201, 2EM8.A.201, 2EMP.A.201, 2ENT.A.200, 2ENV.A.300, 1EN7.A.401, 2ENZ.A.300, 2EOD.A.300, 2EOD.A.400, 2EPS.A.201, 2EPA.A.300, 2EPP.A.201, 2EQE.A.201, 2EQF.A.201, 2EQG.A.201, 1ESK.A.55, 2ESL.A.4, 1EXK.A.80, 1EXK.A.81, 2F3B.A.341, 2F4M.A.501, 1F62.A.52, 1F62.A.53, 1F81.A.88, 2F9I.B.601, 4FBE.A.403, 2FC6.A.201, 2FC7.A.201, 2FE3.A.201, 2FGY.A.621, 4FKD.A.102, 3FL2.A.1001, 1FPO.A.90, 3FQM.A.901, 1FU9.A.37, 1FWQ.A.124, 4FYY.B.201, 1G25.A.66, 1G25.A.67, 3G27.A.97, 2G6Q.A.400, 2G9T.A.999, 3GA3.A.1, 1GDC.A.73, 1GDC.A.74, 2GFO.A.1200, 3GI1.A.501, 4GIZ.C.201, 2GQJ.A.300, 4GVE.A.601, 3HON.A.201, 4H12.A.1801, 4H12.A.1803, 1H7V.A.61, 3H8V.A.401, 3H99.A.601, 1HC7.A.490, 3HCS.A.303, 2HDP.A.492, 2HDP.A.493, 3HNA.A.502, 3HNA.A.503, 2HNC.A.263, 2HOC.A.263, 2HRV.A.143, 4HSU.A.904, 1HTD.A.401, 4I1F.A.503, 4I1F.A.508, 3I2D.A.1, 2I50.A.336, 2I50.A.337, 2I50.A.338, 4I51.A.3005, 1I8Z.A.262, 1IBI.A.195, 1IBI.A.196, 3IBN.A.262, 2IBI.A.1, 2IDA.A.104, 1IF9.A.262, 4II1.A.901, 1IML.A.78, 3IMI.A.201, 3IO2.A.202, 2IQJ.A.301, 1IRN.A.55, 3IRB.A.201, 3IUF.A.1, 4IUM.A.501, 1IYM.A.182, 1IYM.A.183, 1J2O.A.115, 2JMO.A.201, 2JM1.A.2, 2JM3.A.92, 2JQ5.A.129, 2JR7.A.85, 2JRJ.A.62, 2JRJ.A.63, 2JTG.A.88, 3JUE.A.999, 2JUN.A.220, 2JVX.A.29, 2JVN.A.400, 2JWO.A.488, 2JW6.A.601, 3JXP.A.320, 2K0A.A.108, 2K0A.A.109, 2K0A.A.110, 2K1P.A.96, 2K16.A.940, 2K16.A.941, 2K2C.A.138, 2K2C.A.139, 2K2C.A.141, 2K2C.A.142, 2K2C.A.143, 2K2D.A.80, 3K35.C.317, 2K4X.A.56, 3K5K.A.1197, 3K5K.B.1195, 3K5K.B.1196, 3K7H.B.1001, 2K7R.A.129, 4K7D.A.502, 1K81.A.144, 2K9H.A.101, 2K9H.A.102, 2KAK.A.170, 2KDX.A.120, 2KGG.A.53, 2KGG.A.54, 2KGO.A.109, 2KJE.A.501, 2KJE.A.502, 2KKH.A.201, 2KKR.A.500, 1KLR.A.31, 1KLS.A.31, 3KNV.A.201, 2KPI.A.150, 2KQ9.A.113, 2KQB.A.1001, 2KR1.A.65, 2KU3.A.63, 2KU3.A.64, 2KWJ.A.501, 2KWJ.A.601, 2KWJ.A.701, 2KWJ.A.801, 2LOZ.A.486, 3L11.A.601, 2L5U.A.62, 2L5U.A.63, 2L6Y.A.239, 2L6Z.B.37, 2L6M.A.201, 2L7X.A.106, 2L7X.A.107, 2L75.A.155, 2L75.A.156, 4L7X.A.101, 2L80.A.124, 2L9Z.A.403, 2LAU.A.82, 2LBM.A.1, 3LCZ.A.54, 2LCE.A.200, 2LGV.A.109, 2LGG.A.381, 2LHN.A.502, 2LHN.A.503, 2LI9.A.18, 2LJX.A.200, 2LJZ.A.201, 4LJO.A.1102, 4LJP.A.1101, 4LK9.A.401, 4LMY.A.202, 2LNO.A.401, 2LNO.A.601, 2LNO.A.701, 2LO2.A.101, 2LO3.A.101, 2LO4.A.300, 4LO9.A.401, 3LQH.A.1002, 2LRI.C.101, 2LRI.C.102, 2LUA.A.101, 2LUA.A.102, 2LUA.A.103, 2LV2.A.101, 2LV2.A.102, 2LV9.A.201, 2LV9.A.202, 2LWW.A.501, 2LWW.A.502, 2LZU.A.201, 2LZU.A.202, 2M0F.A.101, 4M0W.A.401, 2M13.A.601, 1M2K.A.999, 1M2O.A.800, 1M3V.A.123, 2M48.A.503, 2M6M.A.201, 2M7Q.A.102, 2M85.A.801, 2M85.A.802, 2M9Y.A.401, 2M9A.A.101, 2M9A.A.103, 2M9A.A.102, 2MA5.A.101, 2MA5.A.102, 2MA6.A.101, 2MA6.A.102, 2MD7.B.101, 2MD7.B.102, 2MDG.A.101, 2MDG.A.102, 1MEA.A.29, 3MEK.A.500, 3MEK.A.501, 3MEK.A.502, 3MHS.A.477, 4MI5.A.803, 4MI5.A.807, 2MKD.A.301, 1ML9.A.2, 1MM3.A.62, 1MM3.A.63, 2MNY.A.401, 2MNY.A.402, 3MNU.A.262, 1MR1.C.601, 2MRE.B.301, 4MSG.A.1401, 4MSX.A.501, 2MUM.A.301, 2MUM.A.302, 4NON.A.501, 1NOZ.A.46, 3N3K.A.1, 1NEE.A.136, 3NHE.A.1, 3NIS.A.2, 1NKU.A.188, 3NKM.A.1001

, 4NL4.H.803, 4NOS.A.3000, 4NQY.A.501, 2NYT.A.2000, 2O10.A.86, 2O10.A.87, 2O13.A
.190, 2O13.A.191, 3O36.A.2, 4O62.A.1001, 3O70.A.501, 3O7A.A.501, 4ODR.A.202, 4OD
R.B.202, 3OJ6.A.150, 2OM1.B.801, 3OOI.A.232, 1OQJ.A.183, 1OVX.A.61, 2OWA.A.201,
1OZB.I.50, 3P44.A.261, 3P55.A.261, 1P7A.A.38, 1PEG.A.4, 4PHT.B.601, 2PLI.D.702,
3PN3.A.1001, 2PRS.A.501, 3PT9.A.1, 3PT9.A.2, 1PTR.A.1, 2PUY.A.356, 1Q08.A.401, 1
Q08.A.402, 1Q08.B.403, 1Q08.B.404, 1Q68.A.201, 1Q69.A.207, 3Q87.A.126, 1QBH.A.36
4, 1QF8.A.216, 4QF3.A.2002, 2QKD.A.501, 4QQ4.A.2001, 3QU1.B.502, 1QYB.A.401, 1QY
P.A.58, 3R17.B.262, 4R2Y.A.102, 4R2Y.B.102, 2R3A.A.300, 2R3A.A.301, 2R3A.A.303,
3R6F.A.132, 2RGV.A.146, 1RGO.A.221, 1RGO.A.222, 2RHK.C.502, 2RI7.A.502, 3RIY.A.1
001, 3RMQ.A.114, 2RO1.A.201, 2RO1.A.301, 2RR4.A.501, 2RT9.A.701, 2RT9.A.702, 4RV
9.A.501, 1RXR.A.213, 1RXR.A.214, 3RZV.A.2, 3S2Q.B.501, 3S8P.A.400, 1SEO.A.201, 3
SOU.A.7, 3SP4.A.601, 1SRK.A.36, 1SU3.A.913, 1SVM.A.700, 3T6P.A.1001, 3T6P.A.1002
, 3T6P.A.1003, 3T6R.A.1, 3T6R.A.3, 3T7L.A.1, 3T85.A.261, 3T92.A.122, 3TGN.A.147,
 1TJL.A.200, 4TWJ.A.301, 1U2N.A.441, 3U31.A.276, 1U5K.A.300, 3U52.A.514, 1U85.A.
34, 1U86.A.36, 3U9G.A.229, 3UDZ.B.800, 3UEY.A.4, 3UEJ.A.302, 4UFO.A.2269, 3UFF.A
.1, 3UGD.A.2, 1UL4.A.139, 3UNG.C.903, 2UVL.A.1336, 3UW4.A.401, 3UX8.A.1001, 2UZG
.A.132, 2VOC.A.1815, 4V2Y.A.150, 1V54.F.99, 1V9X.A.200, 3VHS.A.51, 2VNF.A.1247,
2VRW.B.1565, 2VRD.A.1062, 2WOD.A.1269, 2W5Z.A.4970, 1W8P.B.1030, 1WFK.A.200, 3WI
D.A.1001, 1WJ2.A.470, 1WWD.A.57, 3WWL.A.102, 1XOT.A.150, 4X2Z.A.400, 1X4I.A.401,
 1X4J.A.201, 1X4L.A.401, 1X4S.A.201, 2X5R.A.1126, 1X63.A.201, 1X63.A.401, 2X7T.A
.1263, 2X7U.A.1261, 2X7M.A.1175, 2XCM.E.1222, 1XER.A.106, 1XF7.A.30, 2XIG.A.1151
, 1XJH.A.63, 1XOX.A.999, 2XOC.A.993, 2XOC.A.994, 2XOC.A.995, 1XPA.A.220, 1XPG.A.
1887, 1XQO.A.262, 1XRU.A.501, 2Y1N.A.1437, 2Y43.A.1097, 2Y43.A.1098, 2YHO.A.1002
, 1YOP.A.84, 2YRD.A.200, 2YRE.A.401, 2YRE.A.701, 2YRG.A.201, 2YSJ.A.201, 2YSJ.A.
401, 2YSV.A.201, 2YSL.A.401, 2YSM.A.901, 2YTO.A.201, 2YU4.A.201, 2YUC.A.201, 2YU
C.A.401, 1Z3A.A.301, 1Z84.A.604, 1Z8R.A.151, 2ZCO.B.408, 1ZFD.A.71, 3ZFJ.A.1159,
 1ZH1.A.199, 3ZME.A.313, 3ZNF.A.31, 3ZNI.A.1429, 1ZNM.A.29, 5ZNF.A.31, 7ZNF.A.31
, 2ZNR.A.1, 3ZPC.B.401, 1ZRP.A.54, 1ZSB.A.262, 1ZSC.A.262, 1ZU1.A.129, 1ZU1.A.13
0, 3ZVS.A.1160, 1ZW8.A.66, 1ZW8.A.67, 1ZY7.A.801

Table S3. 4-ligand Zn, combined group

	size	largest_angle*	middle_1*	middle_2*	middle_3*	middle_4*	smallest_opposite_angle*	Tetrahedral
1	"700"	"118.3+/-2.6"	"99.6+/-2.7"	"104.4+/-2.3"	"108.3+/-2.5"	"112.8+/-2.7"	"112.9+/-2.7"	"0.728"
2	"56"	"131.6+/-9.5"	"65.3+/-11.3"	"85.2+/-10"	"103.4+/-11.2"	"115.3+/-11.8"	"81+/-14.9"	"0"
3	"644"	"114.1+/-2.2"	"105.4+/-2"	"107.6+/-1.4"	"109.4+/-1.3"	"111.4+/-1.5"	"108.6+/-2.5"	"0.929"
4	"707"	"118.2+/-2.2"	"101.1+/-3.4"	"107.3+/-2.6"	"111.4+/-2"	"114.4+/-1.9"	"102.7+/-3.3"	"0.67"
5	"173"	"129.9+/-5.9"	"93.4+/-4.6"	"98.6+/-3.5"	"103.9+/-3.3"	"111.7+/-4.5"	"119.8+/-5.2"	"0.24"
6	"220"	"122.3+/-4"	"88.6+/-6.5"	"101.8+/-5.3"	"111.7+/-3.6"	"117.4+/-2.9"	"111.4+/-4.7"	"0.278"
7	"366"	"127+/-3.6"	"98.4+/-3"	"103+/-2.6"	"107.2+/-2.8"			
8	"140"	"141.4+/-5.6"	"89.6+/-6"	"96.9+/-4.4"	"104.4+/-4.3"			
9	"94"	"164.5+/-6.7"	"82.6+/-5.7"	"88.8+/-5"	"94+/-5"			
10	"75"	"149.2+/-10.9"	"60.3+/-8.2"	"84.1+/-8.3"	"95.8+/-7.4"			
11	"223"	"125.3+/-4.8"	"97.2+/-5.3"	"104.8+/-4.6"	"112.2+/-3.9"			

7	"114+/-3.9"	"104.1+/-3.7"	"0.369"
8	"114.2+/-7.2"	"99.9+/-7.6"	"0.013"
9	"100.4+/-6.9"	"103.1+/-12.3"	"0"
10	"108.3+/-10.5"	"117.1+/-13.4"	"0"
11	"117.8+/-3.8"	"92.2+/-5.4"	"0.187"

	TrigonalBipyramidalVA	TrigonalBipyramidalVP	SquarePyramidalV
1	"0.005"	"0"	"0"
2	"0.021"	"0.001"	"0"
3	"0.004"	"0"	"0"
4	"0.027"	"0"	"0"
5	"0.002"	"0.004"	"0.001"
6	"0.051"	"0"	"0"
7	"0.039"	"0"	"0"
8	"0.077"	"0.001"	"0.015"
9	"0.001"	"0.18"	"0.194"
10	"0"	"0.016"	"0.001"
11	"0.106"	"0"	"0"

	SquarePlanar
1	"0"
2	"0"
3	"0"
4	"0"
5	"0"
6	"0"
7	"0"
8	"0"
9	"0"
10	"0.003"
11	"0"

Table S4. Cluster members of 4-ligand Zn, combined group

[1] "Cluster 1"
2C7A.A.1641, 4CIS.A.300, 1CYQ.A.601, 2DRP.A.171, 4ESJ.A.301, 1F4S.P.64, 1F4S.P.6
5, 2FF0.A.1001, 1G2F.C.303, 2HAN.A.351, 2HAN.A.352, 2HGH.A.191, 4HN6.A.602, 4HP3
.C.201, 4HP3.C.202, 1HWT.D.138, 2I13.A.503, 2I13.A.506, 1I3J.A.100, 2IVH.A.1577,
1LAT.A.1515, 4LJ0.A.501, 1LLM.C.301, 1LLM.C.302, 1LO1.A.195, 3LRR.A.1, 3M9E.A.2
08, 1MEY.C.89, 2NLL.A.250, 3O9X.A.132, 3OD8.A.200, 4OLN.A.101, 4OND.A.102, 1P47.
A.203, 4PZI.A.1101, 4PZI.A.1102, 4QEN.A.802, 4QEN.A.803, 4QEN.A.804, 3QMD.A.300,
3QMD.A.301, 1TDZ.A.1001, 4TNT.A.701, 4TNT.A.702, 3TS2.A.1, 2XQC.A.1138, 2YKG.A.
927, 1ZGW.A.500, 3A1B.A.3, 4A2C.A.1349, 4A46.A.65, 1A5T.A.501, 2A5H.A.421, 2A6H.
D.7412, 1A71.A.402, 1A7T.A.251, 2AFW.A.996, 2AFX.A.996, 4AI5.A.200, 3ALR.A.601,
1AM6.A.262, 2APS.A.400, 2AQP.A.201, 4ARE.A.1790, 4AU7.A.1248, 3AVR.A.1502, 3AXS.
A.401, 4AY8.A.600, 1AZM.A.261, 3B4F.A.262, 2B5W.A.800, 4B6D.A.1341, 2B8T.A.1218,
4BF1.A.270, 4BF6.A.1262, 3BL0.A.262, 3BL1.A.262, 1BN1.A.262, 1BN3.A.262, 1BN4.A
.262, 1BNM.A.262, 1BNT.A.262, 1BNU.A.262, 1BNW.A.262, 3BO5.A.301, 3BOL.A.701, 1B
TK.A.1, 1C2G.A.409, 4C3E.A.201, 4C3T.A.300, 4C4O.B.600, 3C5K.A.202, 3C63.A.107,
3C63.B.107, 3C63.C.107, 2C6A.A.336, 3C7P.A.262, 2C7N.A.499, 1C8T.A.260, 4C8E.A.1
162, 3CA2.A.264, 4CA1.A.284, 1CCT.A.262, 4CCG.X.1374, 1CDO.B.377, 2CDC.A.1372, 2
CEX.C.1306, 1CG2.A.502, 1CIM.A.262, 2CKL.B.1115, 1CLC.A.653, 1CNG.A.1, 1CNI.A.1,
1CNJ.A.1, 1CNW.A.262, 1CNX.A.262, 1CNY.A.262, 3COS.A.502, 4COI.A.652, 3CQZ.L.30

05, 4CQ0.A.1262, 2CQE.A.822, 2CRW.A.401, 2CSZ.A.401, 2CS7.A.201, 2CT2.A.401, 2CTD.A.201, 2CTD.A.401, 2CTU.A.201, 2CUP.A.401, 3CXK.A.201, 3CXL.A.500, 4CYK.A.42, 1CZM.A.261, 1D1S.B.376, 4D1N.A.900, 2D5B.A.501, 2D8Y.A.401, 2D8Q.A.201, 2D8R.A.401, 2D9H.A.401, 3DAZ.A.262, 2DAR.A.401, 2DAS.A.101, 2DB6.A.201, 2DB6.A.401, 4DB3.A.401, 1DCQ.A.600, 3DD8.A.262, 1DDZ.A.2, 3DDT.A.46, 4DF9.A.503, 2DFV.A.1001, 4DLA.A.402, 3DMO.A.131, 2DMD.A.191, 2DMD.A.241, 2DPH.A.1001, 2DQ4.A.502, 1DVP.A.401, 1DVP.A.402, 3DWD.A.501, 3E1W.A.230, 4E2X.A.501, 1E3J.A.901, 2E6R.A.401, 2E6I.A.201, 2EA5.A.201, 2EBT.A.200, 2EBT.A.300, 3EB5.A.1001, 2ECJ.A.201, 2ECV.A.401, 2ECY.A.201, 2ECL.A.401, 2ECN.A.201, 2EER.A.501, 1EE2.A.1301, 1EE8.A.501, 3EED.A.194, 2EE8.A.701, 4EEZ.A.401, 1EF4.A.56, 3EFT.A.262, 2EGM.A.200, 2EGM.A.300, 3EH1.A.1269, 2ELN.A.181, 2ELI.A.201, 2EMJ.A.201, 2EM1.A.201, 2EMA.A.201, 2EME.A.201, 2EMH.A.201, 2EN1.A.201, 2EOS.A.201, 2EOZ.A.201, 2EOG.A.201, 2EOL.A.201, 2EOP.A.201, 2EPR.A.201, 2EPU.A.201, 2EPW.A.201, 2EPY.A.201, 2EPO.A.201, 2EPC.A.201, 2EQW.A.201, 2EQ2.A.201, 4ETS.A.302, 4EVB.A.204, 4EYL.A.303, 4EYU.A.1702, 1EZM.A.302, 3FOD.A.163, 1F18.A.155, 2F14.A.1262, 1F4T.A.369, 1F8F.A.373, 1F9X.A.999, 4F9V.A.401, 1FAQ.A.2, 4FAI.A.401, 2FEA.A.1302, 3FFP.X.262, 3FID.A.298, 3FL2.A.1002, 3FLO.B.2, 4FRC.A.302, 2FZW.A.375, 3G1P.A.300, 4G26.A.1001, 1G47.A.999, 1G48.A.262, 2G45.A.401, 4G7A.A.301, 2G84.A.506, 2GAG.D.101, 2GAH.D.101, 2GFJ.B.401, 2GFE.A.869, 3GJ3.B.300, 3GJ5.B.300, 3GJ7.D.300, 3GJ8.B.300, 4GRO.A.301, 4GR8.A.301, 2GVI.A.301, 3GZK.A.539, 1H2B.A.1362, 4H3O.A.301, 3H5A.B.360, 3H5N.A.500, 2H6E.A.500, 4H84.A.301, 4H9D.A.201, 3HCI.A.1000, 3HCJ.A.1000, 1HDY.A.376, 2HD6.A.262, 4HDH.A.1002, 4HEY.A.301, 2HF1.A.102, 4HF3.A.301, 4HI8.B.101, 4HI8.B.102, 2HJH.A.800, 3HKU.A.261, 2HL4.A.262, 3HNA.A.501, 3HNA.A.504, 2HQH.E.1500, 1HSO.A.1376, 4HTO.A.301, 4HT2.A.301, 4HTB.A.401, 1HUG.A.261, 4HU1.A.301, 2HZ8.A.117, 2IOO.A.579, 4I1H.A.507, 2I3H.A.1001, 3I4C.A.500, 1I6P.A.301, 2I9W.A.201, 1IA6.A.1264, 3IBI.A.262, 3IBL.A.262, 3IBU.A.262, 4IBY.A.301, 3IFJ.A.201, 3IJF.X.147, 1INN.B.167, 1IQ8.A.600, 3IR9.B.501, 4IXJ.A.301, 2IYB.E.1422, 2J1Y.A.1290, 2J6A.A.1138, 1J98.A.300, 2J9R.A.1194, 1JAZ.A.401, 2JA1.A.1192, 4JBG.A.401, 4JEA.A.202, 4JIV.D.101, 1JJE.B.251, 1JJT.B.251, 4JLW.A.401, 2JMO.A.401, 4JMY.A.201, 2JMD.A.65, 2JOX.A.110, 4JPA.A.301, 1JTK.A.137, 1JVB.A.400, 1JVB.A.500, 3JV7.A.501, 3JV7.A.502, 3JVH.A.163, 4JXE.A.501, 1JY8.A.300, 2JZ8.A.150, 3K2F.A.262, 3K5K.A.1194, 2K5C.A.96, 3K6J.A.800, 4K7D.A.501, 4K7D.A.505, 4K7D.A.507, 4K7W.B.101, 3KB1.A.302, 2KDP.A.1, 3KE1.A.163, 2KFN.A.1, 2KN9.A.82, 1KOL.A.1002, 4KUJ.A.301, 4KUY.A.301, 3KV5.A.490, 1KWG.A.806, 4KXQ.A.601, 2KZY.A.63, 3LOO.A.180, 3L11.A.602, 4L56.A.401, 4L58.A.102, 2L6L.A.201, 1LBU.A.214, 2LBM.A.3, 2LCE.A.300, 2LCQ.A.162, 2LGG.A.380, 2LGG.A.382, 1LIQ.A.28, 4LIM.A.401, 3LJU.X.401, 4LJO.A.1104, 3LKM.A.904, 2LKO.A.32, 1LPV.A.53, 4LQG.A.802, 3LSC.A.458, 3LT8.A.80, 4LU3.A.301, 1LV3.A.66, 2LWW.A.503, 4LXL.A.403, 3M04.A.501, 2M13.A.602, 3M2X.A.500, 3M2Y.A.500, 3M4C.B.108, 1M6H.A.1376, 2M6M.A.202, 3MBG.A.3, 1MBX.B.211, 3MEQ.A.501, 4ME3.A.301, 1MGO.A.376, 3MHC.A.262, 3MHH.E.97, 3MHS.A.474, 3MHS.A.475, 3MHS.A.476, 4MHQ.A.501, 4MHY.A.400, 4MI5.A.802, 4MI5.A.804, 4MI5.A.805, 2MIU.A.302, 4MJ7.A.201, 1ML9.A.1, 1ML9.A.3, 3ML5.A.263, 1MNC.A.282, 1MVH.A.501, 2MWX.A.201, 1MZ8.B.600, 1MZB.A.202, 4NOL.A.401, 4NON.A.503, 4N4F.A.1402, 1N5N.A.401, 1N8K.A.376, 1NCS.A.61, 3NIS.A.1, 3NIS.A.3, 3NIT.A.2, 3NI5.A.262, 4NJ5.A.803, 4NS5.A.401, 1NTO.A.500, 3NY1.A.4, 3NY1.A.5, 3NY1.A.6, 3NY3.A.2, 3NY3.A.3, 1NZJ.A.700, 2O03.A.202, 2O3K.A.401, 2O4Z.A.262, 3O4N.A.2002, 4O64.A.2001, 4O64.A.2002, 4O6I.A.601, 3O70.A.500, 3O7A.A.500, 4OAQ.A.403, 3OCA.A.300, 2OGW.A.500, 4OGE.A.1201, 4OIF.A.701, 1OKL.A.262, 1OKM.A.262, 3OOI.A.234, 2OSM.A.262, 2OSF.A.262, 2OUI.A.361, 1OX7.A.402, 1OXN.A.1001, 3OXF.A.440, 1OYW.A.801, 2OZU.A.800, 1P0F.A.2502, 2P09.A.200, 3P1V.A.427, 1P42.A.502, 2P57.A.201, 1P6O.A.401, 3P8B.A.101, 3PB4.X.400, 3PB7.X.400, 3PB9.X.400, 1PCX.A.950, 2PG3.A.300, 3PII.A.340, 3PJN.A.187, 3PJN.A.188, 1PL8.A.402, 3PN3.A.1002, 2POU.A.262, 2POW.A.262, 2POI.A.100, 2PPT.A.300, 1PQ4.A.1002, 2PQ8.A.501, 4PQT.A.501, 3PTM.A.1001, 2PVX.A.901, 2PVE.A.301, 3PYK.A.262, 3PZC.B.1000, 1PZW.A.100, 4Q09.A.301, 3Q1D.A.201, 3Q7C.A.1, 4Q7R.A.301, 4Q7R.A.302, 4Q7R.B.302, 4QF2.A.1802, 2QIC.A

.300, 2QIC.A.400, 3QL9.A.1, 3QL9.A.3, 4QN1.A.1501, 2QOA.A.262, 2QP6.A.262, 4QSI.A.301, 3QU1.A.501, 3QU1.A.503, 3QWP.A.500, 3QWP.A.501, 4R1X.B.501, 3R2N.A.135, 4R2Y.A.101, 4R2Y.A.103, 1R79.A.201, 1R79.A.401, 3RBU.A.1752, 2RI7.A.501, 1RJQ.A.601, 1RJW.A.401, 4RM5.D.300, 1RNI.A.256, 2ROW.A.601, 2RPC.A.201, 4RQT.A.401, 3RSN.A.200, 2RSH.A.101, 2RSI.A.101, 2RSI.A.102, 2RSJ.A.101, 1RUT.X.603, 3RZ0.B.262, 3RZ5.A.1, 3S2E.A.500, 3S2E.A.501, 1S3G.A.219, 3S73.B.262, 3S75.B.262, 3SAP.A.262, 3SD9.A.2, 3SI2.A.601, 3SJG.A.1752, 3SUB.A.161, 1SX1.A.23, 1T4W.A.201, 3T82.A.261, 1TAF.A.2003, 1TEQ.X.262, 3TIO.B.185, 3TIO.D.185, 3TIO.E.185, 3TMJ.A.262, 1TOT.A.53, 1TOT.A.54, 3TTC.A.1, 3TTC.A.2, 3TWO.A.349, 4TZC.A.501, 4TZU.A.503, 1U0A.A.5005, 3U1L.A.241, 1U3T.A.375, 1U3U.A.375, 1U3W.A.375, 1U5S.B.138, 3U9G.A.227, 3U9G.A.228, 1UAQ.A.200, 3UCJ.A.228, 3UCM.A.228, 3UCN.A.228, 3UEH.A.143, 3UEI.A.143, 3UEJ.A.301, 3UKO.A.400, 4UOV.A.298, 4UP0.A.1384, 2USN.A.257, 4UTV.A.1299, 1UUF.A.401, 3UVC.A.301, 3UWA.A.200, 1UX1.A.1132, 2UZG.A.133, 1V0D.A.401, 2V0C.A.1816, 4V0R.A.1001, 3V1F.B.703, 4V2W.A.502, 1V6G.A.401, 1V9E.B.260, 2V9E.B.1276, 2V9K.A.1533, 3VDP.A.201, 1VFY.A.301, 2VF7.A.1845, 1VGN.A.302, 3VGL.A.322, 1VHH.A.400, 3VHS.B.51, 3VHT.B.401, 1VJ0.A.400, 1VJE.A.167, 2VKR.A.106, 2VM5.A.1245, 2VO9.B.501, 2VPG.A.1400, 1VQ0.A.300, 2VQM.A.1412, 2VQG.C.1091, 2VRW.B.1566, 1VSR.A.201, 3VTH.A.801, 3VUW.E.801, 2VVB.X.1268, 2W0T.A.125, 1W4R.A.400, 1W5O.A.1339, 3W5K.B.504, 4W6Z.A.402, 1WAA.A.1090, 2WB0.X.601, 2WB0.X.602, 2WCB.A.100, 2WD2.A.1262, 2WD3.A.1263, 4WD8.B.303, 4WD8.C.303, 1WE9.A.401, 1WEE.A.401, 1WEW.A.401, 2WEH.A.1262, 1WFE.A.201, 1WFE.A.401, 1WFF.A.201, 1WFH.A.201, 1WFH.A.401, 1WFL.A.201, 1WFZ.A.201, 1WGE.A.201, 1WIR.A.201, 2WJV.A.1, 1WJP.A.701, 1WKQ.B.202, 1WN5.A.1001, 1WO4.A.26, 3WS6.C.201, 1WUQ.A.1001, 2WVJ.A.1193, 2WWO.A.1162, 1WYS.A.401, 1X31.D.1006, 1X4U.A.201, 1X4U.A.401, 1X4V.A.401, 1X4W.A.201, 1X4W.A.401, 1X5W.A.201, 1X61.A.401, 1X64.A.201, 1X6M.A.200, 2X7S.A.1265, 1XAF.A.501, 1XB0.A.403, 2XB4.A.1224, 2XCM.E.1223, 2XEU.A.1065, 1XEG.A.262, 2XOC.B.992, 1XTM.B.501, 1Y02.A.162, 2Y1N.A.1436, 1Y8Q.B.642, 1YO7.A.201, 1YQD.A.2000, 2YQM.A.201, 2YQM.A.401, 2YRK.A.201, 2YSM.A.501, 2YTG.A.201, 2YTS.A.201, 2YT9.A.201, 2YT9.A.203, 2YTA.A.201, 2YTE.A.201, 2YTI.A.201, 2YTJ.A.201, 2YUU.A.201, 2YVR.A.1001, 2YVR.A.1002, 2YWW.A.504, 2YZ3.A.301, 2YZ5.B.1501, 2Z2S.B.204, 2Z3J.A.2001, 2Z45.B.1004, 1Z5H.A.2001, 1Z6U.A.2, 1Z9Y.A.300, 2Z94.A.901, 2Z9L.A.701, 1ZE8.A.263, 2ZEF.A.391, 2ZEH.A.391, 2ZEL.A.391, 2ZEN.A.391, 2ZEO.A.391, 3ZFK.A.401, 1ZFQ.A.600, 1ZGF.A.400, 3ZGO.A.400, 1ZNF.A.27, 3ZNI.A.1428, 3ZTG.A.1336, 3ZTG.A.1337, 1ZVX.A.999, 3ZYQ.A.1223, 2ZZF.A.754

[1] "Cluster 2"

4LMG.B.202, 3P57.P.122, 3A1Z.C.226, 1ALH.B.450, 2ANH.A.452, 2ANU.B.505, 1BON.A.1002, 2CIH.A.213, 2DI2.A.30, 2E1W.A.400, 2E84.A.558, 2EC7.A.50, 1F30.H.201, 2F4L.A.1400, 4FC5.E.305, 2FZ6.A.201, 1GLC.F.169, 2H6H.B.1001, 2H6F.B.1001, 3H90.A.291, 4HDT.A.400, 4I0Z.A.504, 3ISO.B.220, 1KBE.A.2, 4KEQ.A.301, 1KHN.A.452, 3KVE.C.489, 2KVH.A.84, 2L1U.A.144, 3M7P.A.953, 2MQ1.A.103, 1N4P.H.378, 2OGJ.A.418, 2OX8.A.5, 2OX8.B.5, 1P4Q.B.302, 2POJ.A.265, 1Q9U.B.402, 2QQ4.I.139, 1R87.A.905, 3SOW.A.7, 3U24.A.594, 4UNI.C.1697, 2V8V.B.1455, 2VW4.A.503, 2VZ5.A.1131, 2W88.C.107, 2W9M.A.1565, 2WC0.B.3012, 2WKN.A.412, 2X3C.A.1342, 2X4H.A.1140, 1YEW.G.662, 2Z3I.A.2001, 3ZTV.A.1599, 1ZZM.A.403

[1] "Cluster 3"

1A1I.A.201, 1A73.A.202, 4AA6.A.253, 2C7A.A.1642, 3CBB.A.1001, 1CYQ.A.602, 2DRP.A.172, 1DSZ.A.1121, 1DSZ.A.1122, 1DSZ.B.1222, 3EQT.A.1, 1FFY.A.1001, 2FF0.A.1002, 3G9M.A.526, 2GAT.A.67, 4GAT.A.67, 6GAT.A.67, 3GOX.A.301, 4GZN.C.203, 2HAN.B.353, 2HAN.B.354, 4HC9.A.401, 2HGH.A.192, 2HGH.A.193, 4HN5.A.601, 2IHX.A.236, 2JP9.A.132, 2JZW.A.56, 1K82.A.450, 2KAE.A.175, 1KB2.A.151, 2KMK.A.83, 2KMK.A.84, 2KMK.A.85, 3KMP.A.2, 1LAT.A.1514, 4LJ0.A.502, 4LJ0.A.503, 2LT7.A.701, 2LT7.A.702, 2LT7.A.703, 3M7K.A.143, 3M7K.A.144, 4M8O.A.1303, 3M9E.B.209, 4M9E.A.505, 4M9V.C.201, 3NCU.A.1, 4NDH.A.402, 2NLL.B.450, 2NLL.B.451, 2O6M.A.601, 4OLN.A.102, 4OND.A.101, 4OOR.A.102, 4QEN.A.805, 4R2A.A.505, 4R2S.A.501, 1R4O.A.526, 1TF3.A.102, 1TF3

.A.3, 1UBD.C.501, 1UBD.C.502, 1UBD.C.503, 1UBD.C.504, 3UK3.C.968, 1YUI.A.64, 1ZA A.C.203, 1ZNS.A.1500, 2A1K.A.1, 3A1B.A.1, 3A1B.A.2, 4A24.A.601, 4A24.A.602, 4A2V .A.1000, 3A43.A.701, 2A51.A.54, 2A51.A.55, 2A5H.B.421, 2A6H.D.7458, 2A8D.A.1230, 3AII.A.1001, 2AKL.A.117, 3ASL.A.3, 4AUQ.B.1299, 4AUQ.B.1300, 2AYJ.A.57, 4AYC.A. 1484, 2B00.E.698, 2B44.A.400, 2B9D.A.1002, 1BBO.A.60, 4BM9.A.1466, 4BM9.A.1469, 1BNV.A.262, 3BO5.A.302, 3BO5.A.303, 3BO5.A.304, 3BOF.A.701, 3BQ5.A.800, 4BS9.A.1 782, 4BUE.A.2162, 2BYO.A.1209, 1BZM.A.261, 2BZ1.A.1174, 4C5W.A.403, 3C63.D.107, 3C6W.A.2, 1C9Q.A.999, 4CA1.A.283, 3CG7.A.299, 3CHQ.A.701, 3CHV.A.301, 2CJS.C.201 , 2CJS.C.202, 2CKL.A.1104, 2CKL.A.1105, 2CKL.B.1116, 4CPD.A.1300, 2CSV.A.200, 2C SV.A.400, 2CS2.A.200, 2CS3.A.200, 2CS8.A.401, 2CS8.A.601, 2CSH.A.200, 1CXX.A.1, 1D0C.A.900, 1D1T.A.375, 3D2Q.A.303, 3D2Q.A.304, 2D8Z.A.401, 2D9G.A.201, 2D9H.A.2 01, 2D9K.A.401, 3DBH.B.1, 1DFE.A.38, 3DGD.C.128, 2DLK.A.201, 2DLK.A.401, 1DL6.A. 60, 2DLO.A.401, 2DLQ.A.200, 2DLQ.A.400, 2DLQ.A.500, 2DMI.A.200, 2DMI.A.300, 2DMJ .A.200, 2DS7.A.100, 1E0E.A.147, 2E2Z.A.101, 1E4U.A.79, 1E4U.A.80, 2E5R.A.201, 2E 5S.A.201, 2E5S.A.401, 2E6R.A.201, 2E6S.A.201, 2E6S.A.401, 2E6S.A.601, 1E7L.A.116 5, 2EBL.A.241, 2EBT.A.100, 2EBV.A.201, 3EBE.A.500, 2EBQ.A.201, 2EBR.A.201, 2ECJ. A.401, 3EFO.A.766, 2EGQ.A.200, 2EGP.A.200, 2EGP.A.400, 2EHE.A.200, 2EHE.A.300, 2 ELY.A.200, 2ELZ.A.200, 2EL4.A.200, 2EL5.A.200, 2EL6.A.200, 2EM5.A.201, 2EM8.A.20 1, 2EMP.A.201, 2ENT.A.200, 2ENV.A.300, 1EN7.A.401, 2ENZ.A.300, 2ENZ.A.400, 2EOD. A.300, 2EOD.A.400, 2EPS.A.201, 2EPA.A.300, 2EPP.A.201, 2EQE.A.201, 2EQF.A.201, 2 EQG.A.201, 1ESK.A.55, 2ESL.A.4, 1EXK.A.80, 1EXK.A.81, 2F3B.A.341, 2F4M.A.501, 1F 62.A.52, 1F62.A.53, 1F81.A.88, 2F9I.B.601, 4FBE.A.403, 2FC6.A.201, 2FC7.A.201, 2 FE3.A.201, 2FGY.A.621, 4FKD.A.102, 3FL2.A.1001, 1FPO.A.90, 3FQM.A.901, 1FU9.A.37 , 1FWQ.A.124, 4FWE.A.902, 4FYY.B.201, 1G25.A.66, 1G25.A.67, 3G27.A.97, 2G6Q.A.40 0, 2G9T.A.999, 3GA3.A.1, 1GDC.A.73, 1GDC.A.74, 2GFO.A.1200, 3GI1.A.501, 4GIZ.C.2 01, 2GQJ.A.300, 4GVE.A.601, 3H0N.A.201, 4H12.A.1801, 4H12.A.1803, 1H7V.A.61, 3H8 V.A.401, 3H99.A.601, 1HC7.A.490, 3HCS.A.303, 2HDP.A.492, 2HDP.A.493, 3HNA.A.502, 3HNA.A.503, 3HNI.A.107, 2HNC.A.263, 2HOC.A.263, 2HRV.A.143, 4HSU.A.904, 1HTD.A. 401, 4I1F.A.503, 4I1F.A.508, 3I2D.A.1, 2I50.A.336, 2I50.A.337, 2I50.A.338, 4I51. A.3005, 1I8Z.A.262, 1IBI.A.195, 1IBI.A.196, 3IBN.A.262, 2IBI.A.1, 2IDA.A.104, 1I F9.A.262, 4II1.A.901, 3IMI.A.201, 3IO2.A.202, 2IQJ.A.301, 1IRN.A.55, 3IRB.A.201, 3IUF.A.1, 4IUM.A.501, 1IYM.A.182, 1IYM.A.183, 1J2O.A.115, 4JEA.B.202, 2JMO.A.20 1, 2JM1.A.2, 2JM3.A.92, 2JQ5.A.129, 2JR7.A.85, 2JRJ.A.62, 2JRJ.A.63, 2JTG.A.88, 3JUE.A.999, 2JUN.A.220, 2JVX.A.29, 2JVN.A.400, 2JWO.A.488, 2JW6.A.601, 3JXP.A.32 0, 2K0A.A.108, 2K0A.A.109, 2K0A.A.110, 2K1P.A.96, 2K16.A.940, 2K16.A.941, 2K2C.A .138, 2K2C.A.139, 2K2C.A.141, 2K2C.A.142, 2K2C.A.143, 2K2D.A.80, 3K35.C.317, 2K4 X.A.56, 3K5K.A.1197, 3K5K.B.1195, 3K5K.B.1196, 3K7H.B.1001, 2K7R.A.129, 4K7D.A.5 02, 1K81.A.144, 2K9H.A.101, 2K9H.A.102, 2KAK.A.170, 2KDX.A.120, 2KGG.A.53, 2KGG. A.54, 2KGO.A.109, 2KI7.B.124, 2KJE.A.501, 2KJE.A.502, 2KKH.A.201, 2KKR.A.500, 1K LR.A.31, 1KLS.A.31, 3KNV.A.201, 2KPI.A.150, 3KQI.A.71, 2KQ9.A.113, 2KQB.A.1001, 2KR1.A.65, 2KU3.A.63, 2KU3.A.64, 2KWJ.A.501, 2KWJ.A.601, 2KWJ.A.701, 2KWJ.A.801, 2L0Z.A.486, 3L11.A.601, 2L5U.A.62, 2L5U.A.63, 2L6Y.A.239, 2L6Z.B.37, 2L6M.A.201 , 2L7X.A.106, 2L7X.A.107, 2L75.A.155, 2L75.A.156, 4L7X.A.101, 2L80.A.124, 2L9Z.A .403, 2LAU.A.82, 2LBM.A.1, 3LCZ.A.54, 2LCE.A.200, 2LGV.A.109, 2LGG.A.381, 2LHN.A .502, 2LHN.A.503, 2LI9.A.18, 2LJX.A.200, 2LJZ.A.201, 4LJO.A.1101, 4LJO.A.1102, 4 LJP.A.1101, 4LK9.A.401, 4LMY.A.202, 2LNO.A.401, 2LNO.A.601, 2LNO.A.701, 2LO2.A.1 01, 2LO3.A.101, 2LO4.A.300, 4LO9.A.401, 3LQH.A.1002, 2LRI.C.101, 2LRI.C.102, 2LU A.A.101, 2LUA.A.102, 2LUA.A.103, 2LV2.A.101, 2LV2.A.102, 2LV9.A.201, 2LV9.A.202, 2LWW.A.501, 2LWW.A.502, 2LZU.A.201, 2LZU.A.202, 2M0F.A.101, 4M0W.A.401, 2M13.A. 601, 1M2K.A.999, 1M2O.A.800, 1M3V.A.123, 2M48.A.503, 2M6M.A.201, 2M7Q.A.102, 2M8 5.A.801, 2M85.A.802, 2M9Y.A.401, 2M9A.A.101, 2M9A.A.103, 2M9A.A.102, 2MA5.A.101, 2MA5.A.102, 2MA6.A.101, 2MA6.A.102, 2MD7.B.101, 2MD7.B.102, 2MDG.A.101, 2MDG.A. 102, 1MEA.A.29, 3MEK.A.500, 3MEK.A.501, 3MEK.A.502, 3MHS.A.477, 4MI5.A.803, 4MI5 .A.807, 2MKD.A.301, 1ML9.A.2, 1MM3.A.62, 1MM3.A.63, 2MNY.A.401, 2MNY.A.402, 3MNU

.A.262, 1MR1.C.601, 2MRE.B.301, 4MSG.A.1401, 4MSX.A.501, 2MUM.A.301, 2MUM.A.302, 4NON.A.501, 1NOZ.A.46, 3N3K.A.1, 1NEE.A.136, 3NHE.A.1, 3NIS.A.2, 1NKU.A.188, 3NKM.A.1001, 4NL4.H.803, 4NOS.A.3000, 4NQY.A.501, 2NYT.A.2000, 2O10.A.86, 2O10.A.87, 2O13.A.190, 2O13.A.191, 3O36.A.2, 4O62.A.1001, 4O64.A.2004, 3O70.A.501, 3O7A.A.501, 4ODR.A.202, 4ODR.B.202, 3OJ6.A.150, 2OM1.B.801, 3OOI.A.232, 1OQJ.A.183, 1OVX.A.61, 2OWA.A.201, 1OZB.I.50, 3P2A.C.151, 3P44.A.261, 3P55.A.261, 1P7A.A.38, 1PEG.A.4, 1PG5.B.500, 4PHT.B.601, 2PLI.D.702, 3PN3.A.1001, 2PRS.A.501, 3PT9.A.1, 3PT9.A.2, 1PTR.A.1, 2PUY.A.356, 1Q08.A.401, 1Q08.A.402, 1Q08.B.403, 1Q08.B.404, 1Q68.A.201, 1Q69.A.207, 3Q87.A.126, 1QBH.A.364, 1QF8.A.216, 4QF3.A.2002, 2QKD.A.501, 4QQ4.A.2001, 3QU1.B.502, 1QYB.A.401, 1QYP.A.58, 3R17.B.262, 4R2Y.A.102, 4R2Y.B.102, 2R3A.A.300, 2R3A.A.301, 2R3A.A.303, 3R6F.A.132, 2RGV.A.146, 1RGO.A.221, 1RGO.A.222, 2RHK.C.502, 2RI7.A.502, 3RIY.A.1001, 3RMQ.A.114, 2RO1.A.201, 2RO1.A.301, 2RR4.A.501, 2RT9.A.701, 2RT9.A.702, 4RV9.A.501, 1RXR.A.213, 1RXR.A.214, 3RZV.A.2, 3S2Q.B.501, 3S8P.A.400, 1SE0.A.201, 3SOU.A.7, 3SP4.A.601, 1SRK.A.36, 1SU3.A.913, 1SVM.A.700, 3T6P.A.1001, 3T6P.A.1002, 3T6P.A.1003, 3T6R.A.1, 3T6R.A.3, 3T7L.A.1, 3T85.A.261, 3T92.A.122, 3TGN.A.147, 3TG4.A.436, 1TJL.A.200, 4TWJ.A.301, 1U2N.A.441, 3U31.A.276, 4U4L.A.302, 1U5K.A.300, 3U52.A.514, 1U85.A.34, 1U86.A.36, 3U9G.A.229, 3UDZ.B.800, 3UEY.A.4, 3UEJ.A.302, 4UF0.A.2269, 3UFF.A.1, 3UGD.A.2, 1UL4.A.139, 3UNG.C.903, 2UVL.A.1336, 3UW4.A.401, 3UX8.A.1001, 2UZG.A.132, 2VOC.A.1815, 4V2Y.A.150, 1V54.F.99, 1V9X.A.200, 3VHS.A.51, 2VNF.A.1247, 3VOV.A.401, 2VRW.B.1565, 2VRD.A.1062, 3VTH.A.802, 1VZY.A.1291, 2W0D.A.1269, 2W5Z.A.4970, 1W8P.B.1030, 1WFK.A.200, 3WID.A.1001, 1WJ2.A.470, 1WWD.A.57, 3WWL.A.102, 1WWR.D.204, 1X0T.A.150, 4X2Z.A.400, 1X4I.A.401, 1X4J.A.201, 1X4L.A.401, 1X4S.A.201, 2X5R.A.1126, 1X63.A.201, 1X63.A.401, 1X64.A.401, 2X7T.A.1263, 2X7U.A.1261, 2X7M.A.1175, 2XCM.E.1222, 1XER.A.106, 1XF7.A.30, 4XIW.A.401, 2XIG.A.1151, 1XJH.A.63, 1XOX.A.999, 2XOC.A.993, 2XOC.A.994, 2XOC.A.995, 1XPA.A.220, 1XPG.A.1887, 1XQ0.A.262, 1XRU.A.501, 2Y1N.A.1437, 2Y43.A.1097, 2Y43.A.1098, 2YHO.A.1001, 2YHO.A.1002, 1YOP.A.84, 2YRD.A.200, 2YRE.A.401, 2YRE.A.701, 2YRG.A.201, 2YSJ.A.201, 2YSJ.A.401, 2YSV.A.201, 2YSL.A.401, 2YSM.A.901, 2YTO.A.201, 2YU4.A.201, 2YUC.A.201, 2YUC.A.401, 1Z3A.A.301, 1Z84.A.604, 1Z8R.A.151, 2ZC0.B.408, 1ZFD.A.71, 3ZFJ.A.1159, 1ZH1.A.199, 3ZME.A.313, 3ZNF.A.31, 3ZNI.A.1429, 1ZNM.A.29, 5ZNF.A.31, 7ZNF.A.31, 2ZNR.A.1, 3ZPC.B.401, 1ZRP.A.54, 1ZSB.A.262, 1ZSC.A.262, 1ZU1.A.129, 1ZU1.A.130, 3ZVS.A.1160, 1ZW8.A.66, 1ZW8.A.67, 1ZY7.A.801, 3ZYQ.A.1222
[1] "Cluster 4"
2A66.A.401, 2B3J.A.2001, 3EPH.A.1, 1F2I.G.1202, 1G2D.C.303, 3GOX.A.302, 4HC9.A.402, 2I13.A.502, 2I13.B.507, 2JP9.A.131, 2JP9.A.134, 1K3X.A.501, 1KB2.A.150, 3KDE.C.78, 1LO1.A.196, 4M9E.A.503, 4M9E.A.504, 1MEY.C.90, 3MLN.A.501, 2OFI.A.302, 3OYM.A.393, 1OZJ.A.145, 4R2A.A.503, 4R2A.A.504, 4R2Q.A.503, 1TF3.A.2, 3TS2.A.2, 3U6P.A.300, 3VD6.C.501, 2XQC.D.1141, 258L.A.500, 3A6G.A.301, 2AC3.A.531, 4ADN.A.1223, 1AF2.A.296, 2AFZ.A.391, 2AFM.A.391, 4AIA.A.200, 1AJB.A.451, 4AJX.H.1030, 3ALR.A.602, 2AP1.A.304, 4AR9.B.1731, 2ASH.A.400, 2AW1.A.262, 2AYD.A.369, 4AYC.A.1485, 2B3Z.A.1360, 1BBO.A.61, 1BCD.A.262, 3BET.A.262, 2BE7.D.1108, 3BJI.B.2, 2BL6.A.1059, 1BNN.A.262, 1BNQ.A.262, 2BNM.A.1199, 2BP0.A.1341, 1BUD.A.800, 1BYF.A.302, 4C09.A.351, 4C1F.A.502, 2C36.A.1311, 3C5K.A.201, 3C5K.A.203, 2C6C.A.1752, 1CAI.A.262, 1CAK.A.262, 2CBD.A.262, 4CCG.X.1375, 4CDG.A.1643, 3CE1.A.202, 2CFU.A.1002, 1CG2.C.502, 1CGL.A.302, 3CHS.A.701, 1CIL.A.262, 1CIN.A.262, 2CKI.A.999, 3CMR.A.450, 1CNH.A.1, 2CO8.A.401, 2CON.A.201, 2COR.A.401, 4CPD.A.1200, 2CR8.A.401, 2CRC.A.401, 2CSY.A.401, 2CS3.A.400, 2CSH.A.300, 2CSH.A.400, 2CT0.A.401, 2CT7.A.401, 4CVR.A.1158, 3CZV.A.262, 2D0W.B.1207, 1D0Q.B.151, 4D0Y.A.1239, 1D1T.A.376, 3D2N.A.101, 4D6S.A.1338, 3D7F.A.1752, 3D7V.A.2, 2D8X.A.201, 2D8X.A.401, 2D8Y.A.201, 2D8Z.A.201, 3D8W.A.262, 2D8U.A.201, 2D9K.A.601, 2D9L.A.401, 3DCC.A.262, 1DD6.A.503, 3DD0.A.262, 3DFM.A.402, 3DI4.B.286, 2DID.A.201, 2DIP.A.201, 2DIP.A.401, 2DJ7.A.201, 2DJ8.A.201, 2DKT.A.191, 2DKT.A.241, 2DKT.A.291, 2DKT.A.441, 2DLO.A.201, 2DLQ.A.300, 2DOO.A.502, 4DZ7.A.301, 1E3J.A.902, 2E5R.A.401, 3E6U.B.503, 2E7Y.A.1

301, 2E73.A.201, 2E73.A.401, 1E9P.B.153, 1E9Q.B.153, 2EA6.A.201, 2EBL.A.191, 2EC
T.A.401, 2ECY.A.401, 2ECG.A.201, 2ECL.A.201, 2ECM.A.401, 2ECN.A.401, 1ED9.A.451,
3EDI.A.210, 1EE2.A.1300, 2EE8.A.301, 2EGQ.A.300, 4EGU.A.202, 3EH2.A.800, 1EI6.A
.409, 2EJ4.A.401, 1EKJ.C.4003, 2ELO.A.181, 2ELR.A.181, 2ELT.A.181, 2ELX.A.181, 2
ELM.A.181, 2EMI.A.201, 2EM0.A.200, 2EM2.A.201, 2EM6.A.201, 2EM7.A.201, 2EM9.A.20
1, 2EMB.A.201, 2EMC.A.201, 2EMK.A.201, 2EML.A.201, 2EMM.A.201, 2ENV.A.200, 2EN2.
A.201, 2EN6.A.181, 2EN7.A.181, 2EN8.A.181, 2EN9.A.181, 2ENA.A.181, 2ENC.A.181, 2
ENE.A.181, 2ENF.A.181, 2ENN.A.300, 2ENN.A.400, 2EOR.A.201, 2EOU.A.201, 2EOV.A.20
1, 2EOW.A.201, 2EOX.A.201, 2EOY.A.201, 1EOU.A.300, 2EO4.A.201, 2EOK.A.201, 2EON.
A.201, 2EOO.A.201, 2EPT.A.201, 2EPV.A.201, 2EPZ.A.201, 2EP1.A.201, 2EP2.A.201, 2
EP3.A.201, 2EP4.A.200, 2EP4.A.300, 2EPA.A.400, 3EPZ.A.701, 2EPQ.A.201, 2EQ1.A.20
1, 2EQ4.A.201, 1EU3.B.402, 2EU3.A.262, 3EWF.A.400, 2EXU.A.501, 3EYX.A.1, 1F2W.A.
262, 4F3W.A.201, 3F7B.B.301, 3F7L.A.203, 3F7U.A.260, 3F9O.A.309, 1FAQ.A.1, 2FC7.
A.401, 2FGY.A.620, 2FHX.A.317, 3FID.A.299, 3FLO.B.1, 4FMN.B.902, 1FN9.A.1001, 2F
OQ.A.262, 2FOS.A.262, 2FOU.A.262, 2FOV.A.262, 2FOY.A.301, 4FO9.A.501, 3FPC.A.353
, 3FPL.A.352, 1FQL.A.262, 1FQM.A.262, 2FR5.A.147, 2FR6.A.147, 4FU5.A.302, 4FVD.A
.201, 4FVN.A.302, 4FVO.A.302, 4FVY.A.805, 3FW3.A.300, 4FWE.A.901, 4FWU.A.401, 2F
YG.A.302, 2G0D.A.416, 2G2N.A.1001, 1G52.A.262, 1G54.A.262, 1G5C.A.1001, 1G71.A.3
48, 1G9K.A.600, 3GC9.B.603, 2GD8.A.262, 4GGJ.A.301, 3GJ4.D.300, 4GNE.A.1501, 4GN
E.A.1502, 4GNE.A.1503, 4GNE.A.1504, 2GQJ.A.200, 4GR3.A.301, 2GSN.A.1000, 1GUP.A.
350, 3GV4.A.203, 1GVY.A.1425, 1GZH.A.1293, 3GZE.B.2, 3H0L.E.902, 4H0F.A.401, 2H1
5.A.262, 4H2K.B.1001, 2H39.A.352, 2H4N.A.262, 3H7H.A.119, 3HB2.P.486, 2HBA.A.101
, 1HCP.A.99, 4HCG.A.202, 3HI2.A.121, 2HJN.A.315, 3HKO.A.701, 3HKQ.A.261, 3HKT.A.
261, 3HLJ.A.272, 3HNJ.B.107, 3HS4.A.301, 2HU9.A.132, 1HXP.A.350, 1HXR.A.200, 1HY
7.A.302, 4I1F.A.504, 3I4C.A.400, 1I50.C.3002, 2I50.A.336, 1I73.A.998, 1IA9.A.200
1, 3IGP.A.262, 2IGI.A.1001, 4IJD.A.501, 4IJD.A.502, 4ILO.A.301, 4ILX.A.301, 2IMZ
.A.501, 2IMZ.B.502, 2INN.B.514, 2IOI.A.3001, 4ITO.A.301, 4ITP.A.302, 2IUC.B.1007
, 3IXE.B.302, 2IYB.E.1423, 4J1V.A.301, 1J2O.A.116, 2J7U.A.1884, 2J7J.A.1088, 2J9
U.B.1162, 2JBG.B.1577, 1JD0.A.901, 4JEA.C.202, 4JEA.D.202, 4JEB.B.202, 4JIU.A.20
1, 1JJD.A.104, 2JLP.A.226, 1JN7.A.37, 2JOX.A.109, 1JOC.A.300, 1JQ5.A.371, 4JSR.A
.401, 4JSZ.A.301, 2JTN.A.184, 2JUN.A.222, 3JWP.A.2001, 4JXE.A.502, 4JZ0.A.802, 1
K2Y.X.500, 4K2H.B.201, 3K34.A.1002, 1K7I.A.486, 4K7D.A.503, 4K7D.A.506, 2KAK.A.1
30, 2KAK.A.150, 3KEE.A.2000, 1KH7.A.451, 1KHK.A.451, 3KHI.A.301, 2KKT.A.500, 3KN
E.A.500, 3KNV.A.202, 4KNI.A.301, 4KNJ.A.301, 4KNM.A.301, 1KOL.A.1001, 4KP5.A.301
, 4KP8.A.301, 3KQI.A.72, 4KUV.A.301, 4KUW.A.301, 3KV4.A.448, 3KV5.A.489, 4KV0.A.
301, 4L05.A.203, 3L14.A.262, 4L3J.A.402, 1L6J.A.500, 4L6O.A.801, 4L6T.A.301, 2L7
P.A.201, 3L8H.A.901, 4LBU.A.402, 2LHN.A.501, 4LHI.A.301, 2LI8.A.187, 2LK5.A.37,
2LNO.A.501, 4LOF.A.401, 4LP6.A.310, 3LRQ.C.100, 4LW9.A.203, 4LW9.C.202, 4LW9.K.2
03, 3LXE.A.261, 2LXD.A.201, 2LXD.A.202, 3LYR.A.1, 3M0A.D.401, 2M1S.A.100, 3M14.A
.501, 3M1D.A.1000, 3M1W.A.500, 4M2R.A.301, 4M2V.A.301, 2M3Z.A.101, 2M3Z.A.102, 2
M3L.A.201, 4M3P.A.701, 3M40.A.262, 2M48.A.501, 3M5E.A.262, 3M5S.A.500, 3M67.A.26
3, 2M7Q.A.101, 2M7A.A.100, 2M9Y.A.402, 3M96.A.262, 3M98.A.262, 3MBG.A.1, 3MDZ.A.
263, 4MDM.A.301, 4MG3.A.201, 3MHI.A.262, 3MHL.A.262, 3MHM.A.262, 3MHS.A.472, 3MH
S.A.473, 4MHN.A.400, 4MI5.A.806, 3MJH.B.70, 3ML2.A.262, 3MMF.A.262, 3MNA.A.262,
3MND.A.153, 4MO8.A.301, 3MPZ.A.300, 3MP2.A.1, 1MS7.B.3001, 3MTW.A.2, 4MTY.A.301,
2MUQ.A.101, 1MXG.A.437, 3MYQ.A.262, 3MZC.A.262, 4N0X.B.301, 3N2P.A.262, 3N3J.A.
262, 3N4B.A.262, 3N67.A.900, 1N8K.A.375, 3NA7.A.300, 3NB5.A.261, 4NJ5.A.801, 2NM
X.A.301, 2NNV.A.262, 2NN1.A.301, 2NN7.A.301, 4NN2.A.401, 4NN2.A.402, 4NN2.A.403,
2NNG.A.262, 2NNO.A.262, 3NQ5.A.503, 3NQY.B.520, 4NQ4.A.302, 4NQ6.A.301, 4NTM.A.
201, 4NTN.A.201, 2NUP.B.1100, 3NY3.A.1, 4NZG.A.201, 3O0M.A.151, 3O36.A.1, 4O64.A
.2003, 4OBI.A.201, 3OCQ.A.184, 2OFK.A.201, 3OIL.A.262, 2OIK.B.201, 3OJ3.J.902, 3
OJ7.A.114, 3OOI.A.233, 1OQ5.A.600, 3OQ6.A.375, 2ORW.A.401, 3ORU.A.250, 2OU2.A.49
0, 3OY0.A.262, 3OYQ.A.262, 3OYS.A.262, 3P3H.A.261, 3P3J.A.261, 1P42.A.501, 3P58.
A.262, 3PB6.X.400, 3PB8.X.400, 3PBE.A.391, 1PFT.A.51, 3PLW.A.188, 3PN3.A.1010, 3

PO6.A.1, 4PQ7.A.301, 3PSQ.B.321, 4PTB.A.901, 4PTB.A.902, 2PUY.A.355, 3PU7.A.155, 3PUQ.A.2, 3PUR.A.3, 1PXE.A.64, 4PYX.A.301, 4PZH.A.302, 3Q01.A.1, 1Q0E.A.153, 3Q1D.A.202, 2Q1Q.A.262, 1Q3A.A.465, 4Q6D.A.301, 4Q6E.A.301, 4QF2.A.1801, 4QF3.A.2001, 3QGV.A.500, 2QO8.A.262, 4QSJ.A.301, 1QWY.A.400, 3QYK.A.262, 3R16.A.262, 1R9P.A.135, 1RAY.A.262, 3RF4.B.119, 2RHQ.A.1, 3RJ7.A.300, 4RL0.A.301, 1RM8.A.501, 1RMD.A.119, 1RMD.A.120, 4RN4.A.301, 2RPC.A.601, 2RPP.A.201, 3RQD.A.390, 2RSI.A.103, 2RSJ.A.102, 1RUT.X.604, 3RYM.A.106, 3RYV.B.262, 3RYX.B.262, 3RYY.A.1, 3RYZ.A.1, 3RYJ.B.262, 3RZ1.B.262, 3RZ7.A.1, 3RZ8.A.1, 3S71.B.262, 3S72.B.262, 3S76.A.1, 3S77.B.262, 3S9T.A.262, 3SAX.A.262, 3SBH.A.262, 3SBI.A.262, 3SEY.C.373, 1SLM.A.257, 1SML.A.271, 3SV6.A.4, 3T5U.A.262, 3T7L.A.2, 1T8H.A.275, 3T80.D.301, 3T84.A.261, 3T90.A.502, 1T9H.A.411, 3T92.A.124, 1TFI.A.51, 3TG4.A.435, 1TT5.B.1014, 3U5N.A.1, 3U5N.A.2, 3U9G.A.226, 3UCK.A.228, 3UCO.A.228, 1UD9.C.509, 3UEE.A.143, 3UNT.A.400, 4UP0.A.1383, 1USN.A.257, 3UVC.B.303, 3UVI.A.387, 1UW0.A.1118, 3V1C.A.101, 3V1E.A.102, 4V1T.A.776, 2V29.A.1276, 3V3G.B.301, 1V47.A.601, 3V4K.A.2, 1V51.A.602, 3V5G.A.262, 3V7M.A.509, 3V7X.A.2001, 2V9I.B.1275, 3VBD.A.2001, 1VDD.D.233, 1VFY.A.300, 2VJE.B.1491, 3VPB.E.100, 2VP7.A.1399, 2VPD.A.1399, 2VPG.A.1401, 1VQ2.A.701, 2VRS.A.1328, 3VRK.A.301, 2VR6.A.1156, 1VYK.A.1150, 1VYX.A.1062, 2W3N.B.1234, 2W3Q.A.1231, 2W4L.A.1170, 2W5X.A.1378, 4WAJ.A.301, 3WBH.B.505, 2WEJ.A.1262, 1WEM.A.201, 1WEO.A.401, 1WEW.A.201, 2WEO.A.1262, 3WF8.A.401, 2WGX.A.1300, 1WIG.A.201, 1WII.A.201, 2WJV.A.3, 1WJP.A.301, 1WJV.A.401, 3WLF.A.402, 3WNQ.A.501, 1WO3.A.26, 2WOJ.A.1353, 3WRG.A.702, 1X3Z.A.999, 1X4I.A.201, 1X4J.A.401, 1X4K.A.201, 1X4L.A.201, 1X4S.A.401, 2XBL.A.1197, 2XBL.C.1196, 1XC3.A.302, 1XCR.A.1001, 4XIX.B.401, 1XKI.A.1003, 2XQV.A.401, 1XRT.A.1423, 1XSO.A.152, 2XS4.A.998, 1Y02.A.161, 2Y6D.A.1267, 1Y93.A.265, 1YBO.B.160, 2YB5.A.1213, 2YHW.A.1719, 2YHY.A.2000, 2YIK.A.1533, 1YQD.A.1000, 2YQP.A.201, 2YRJ.A.200, 2YRT.A.201, 2YRT.A.401, 2YRC.A.200, 2YRE.A.501, 2YRE.A.601, 2YRG.A.401, 2YRH.A.200, 2YRM.A.201, 2YS2.A.201, 2YSA.A.181, 2YSM.A.301, 2YSM.A.701, 2YSO.A.181, 2YTH.A.201, 2YTP.A.181, 2YTQ.A.201, 2YTT.A.181, 2YT5.A.201, 2YT5.A.401, 2YTB.A.301, 2YTD.A.201, 2YTF.A.201, 2YTK.A.201, 2YTM.A.181, 2YU8.A.201, 2YYR.A.401, 2YYR.A.402, 1Z05.A.406, 2Z2Y.A.2001, 2Z3H.A.2001, 2Z3G.A.2001, 1Z60.A.2, 2Z9J.A.902, 2ZED.A.391, 2ZEE.A.391, 2ZEG.A.391, 2ZEM.A.391, 1ZFK.A.1300, 1ZFO.A.31, 1ZGE.A.1000, 1ZIN.A.219, 1ZNB.A.1, 1ZP5.A.999, 3ZP9.A.1009, 3ZQ6.A.1323, 1ZR9.A.117, 2ZTX.A.501, 3ZVS.A.1159, 3ZXH.A.301, 1ZZU.A.900, 2ZZE.A.753
[1] "Cluster 5"
3COQ.A.1002, 3F2D.A.5, 2KKF.A.2002, 3S14.A.1735, 3UK3.C.967, 3A6F.C.301, 3A6J.A.301, 3A6J.D.301, 1A7I.A.82, 1A7I.A.83, 1ADB.A.375, 1ADN.A.93, 2AF2.B.154, 2AS9.A.666, 2AS9.B.222, 1B4E.A.405, 1B8T.A.196, 4BHW.A.4, 4BLB.B.910, 4BOL.A.1261, 1BP3.A.500, 4C4O.D.500, 4CBY.A.2035, 2CJL.A.1217, 1CRA.A.262, 3D2Z.A.262, 4D9W.A.408, 2DGE.A.1001, 1DMY.A.280, 1DYO.A.401, 3E24.A.230, 2E26.A.603, 1E3I.A.381, 3E3F.A.230, 3E50.A.1, 2EA5.A.401, 3EBI.A.1, 2ECI.A.401, 4EEX.B.402, 2ELW.A.181, 1ELX.A.451, 1ELZ.A.451, 1EPW.A.1291, 1ESP.A.323, 3F5L.A.1001, 1FBX.A.3316, 3FCQ.A.322, 3FIE.A.428, 3FJU.A.507, 3FLF.A.2004, 2FNF.X.1, 1FR7.A.262, 3FV4.A.321, 3FVP.A.321, 3FXP.A.3000, 3G42.D.500, 1G4K.A.301, 1GI4.A.409, 3GIQ.A.482, 2GSU.A.1001, 3H2P.B.154, 4H57.A.405, 1H7N.A.1342, 1HCP.A.98, 4HEW.A.301, 1HP7.A.401, 1HY7.B.801, 1HYI.A.66, 4I28.A.602, 2I2X.A.524, 1I6N.A.401, 1I8J.B.400, 2I9W.A.200, 2IT4.A.561, 1JJ9.A.999, 1JM7.A.123, 2JOX.A.108, 4JOM.A.1004, 2JSD.A.275, 2JTN.A.183, 4K1R.A.502, 1K24.A.401, 1K83.I.3003, 1KHL.A.451, 4KJG.B.1001, 1KTO.A.405, 3KVE.B.489, 3KWO.C.161, 2L8E.A.829, 3LAT.A.215, 1LG5.A.262, 1LG6.A.262, 4LJP.A.1103, 3LUB.A.302, 2LUL.A.201, 2M3H.A.102, 1M65.A.300, 3MBM.A.163, 2MIU.A.303, 4MLT.A.301, 1MPO.A.376, 3MS3.A.401, 4MTW.E.401, 4MWP.E.401, 4MXJ.E.401, 4MZN.E.401, 4N0G.A.401, 4N4E.E.401, 4N5P.E.405, 4N66.E.501, 1NJ1.A.513, 1NJG.A.401, 3NQZ.B.1, 4OCM.E.401, 1OS0.A.600, 2OVX.A.444, 2OVZ.A.444, 1P6B.A.406, 3PBJ.D.31, 1PE8.A.317, 4Q7R.A.303, 3Q94.A.301, 4QEF.A.301, 1QF2.A.320, 3QH5.A.321, 3QHD.A.163, 2QL1.A.1, 4R9G.A.505, 3RCM.A.288, 2RSD.A.901, 1RUR.L.601, 3SSB.A.999, 3SU6.A.5, 1SW1.A.4

01, 3T02.A.501, 3T5Z.A.262, 3T73.A.410, 3T74.A.410, 3T8G.A.411, 3T8F.A.411, 3T87
.A.326, 3T8C.A.326, 3T8D.A.325, 3T8H.A.326, 1TBN.A.1, 1THL.A.324, 3TIO.A.1, 2TMN
.E.321, 6TMN.E.322, 3TTY.A.676, 1U3L.A.701, 4U4L.A.301, 3U7L.A.350, 4UA4.A.302,
1UD9.B.508, 3UKO.A.401, 2UX1.K.1173, 2VXX.A.201, 1WAA.E.1089, 4WK7.A.501, 2WWO.A
.1165, 1XLL.A.399, 1XUJ.A.246, 2Y28.B.1181, 1Y3G.E.321, 2Y4Y.C.1172, 1Z9G.E.1005
, 1ZDP.E.1005, 2ZNE.B.993, 3ZPG.A.1358, 1ZS0.A.999, 1ZUD.1.501, 2ZXG.A.900
[1] "Cluster 6"
1F2I.G.1201, 1G2F.C.301, 4HCC.A.504, 2I13.B.510, 4IQR.A.402, 2OPF.A.501, 1A42.A.
262, 2ADR.A.162, 2ADR.A.163, 4AF1.A.500, 4AIG.A.999, 1ANI.A.451, 4AR1.A.1722, 3A
SK.A.501, 3B6P.A.800, 2B83.C.3353, 4BHW.A.1, 1BIO.A.291, 3BKN.A.201, 3BKN.A.202,
1BNL.A.179, 2BNN.A.1200, 4C1E.A.502, 4C1G.B.301, 3C37.A.301, 3CHV.A.302, 1CO4.A
.43, 2CQF.A.330, 2CRR.A.401, 2CSY.A.201, 2CSZ.A.201, 3CSK.A.712, 2CT1.A.201, 2CT
1.A.401, 2CT5.A.201, 2CT7.A.201, 2CTT.A.201, 2CU8.A.201, 2CUP.A.201, 2CUP.A.601,
2CUQ.A.201, 2CUQ.A.401, 2CUR.A.201, 2CUR.A.401, 3CX3.A.314, 1CXV.A.3, 1D1T.A.40
1, 2D8S.A.201, 2D8S.A.401, 2D8U.A.401, 2D9N.A.256, 2DID.A.401, 2DJB.A.201, 2DJB.
A.401, 1DMX.A.280, 1DVB.A.194, 3E2I.A.200, 3E2U.E.102, 3E3I.A.230, 1E46.S.999, 2
E47.A.172, 2E72.A.201, 2ECI.A.201, 4EFS.A.301, 2ELQ.A.181, 1ELY.A.451, 2EMX.A.20
1, 2EMY.A.201, 2EMZ.A.201, 2EN4.A.201, 2EOI.A.201, 2EPX.A.201, 2F0Y.B.501, 3F4X.
A.262, 4F7O.B.301, 4F9C.B.401, 3FJU.A.502, 1FKW.A.400, 3FTN.D.357, 4GER.A.401, 3
GIQ.A.483, 4GQT.B.502, 3GRB.A.129, 3GTT.A.155, 4H12.A.1802, 4HVL.A.504, 2IOO.A.5
81, 3I9F.B.3, 1IB5.A.201, 1IBB.A.201, 1IF5.A.262, 1IML.A.77, 1IS8.B.3109, 3IXE.B
.301, 1J2T.A.302, 1J9Y.A.1004, 1JD5.A.501, 1JJD.A.102, 4JSA.A.301, 2JUN.A.221, 2
JUN.A.223, 2JWO.A.489, 1JW9.B.250, 2KBX.B.298, 1KEV.B.353, 1KFI.A.700, 2KIK.B.50
, 2KIZ.A.70, 2KIZ.A.71, 1KWQ.A.262, 2KZM.A.1, 4L6H.A.801, 2LI8.A.188, 3LPE.B.60,
2LUY.A.301, 2LXH.C.900, 3M15.A.107, 3M15.B.107, 4M3O.A.300, 1M4M.A.502, 2M48.A.
502, 1MC5.A.376, 3MEN.D.400, 2MIU.A.301, 4N4F.A.1401, 1N92.A.375, 4NQ5.A.302, 1N
UI.A.501, 4O1K.A.301, 2O4H.B.401, 3O7U.A.428, 1OHL.A.400, 2OH3.A.300, 1P9R.A.601
, 1PGU.A.616, 3PNU.A.336, 4Q0L.A.301, 1Q1A.A.701, 2Q1B.A.400, 1Q3K.A.301, 3Q6V.A
.2, 2QQ4.A.139, 3QU1.A.505, 1R22.A.502, 2R3A.A.302, 1R4V.A.202, 3RC6.A.1, 3RHG.A
.368, 1RJ6.A.601, 1RJW.A.402, 2RJP.B.1, 1RMD.A.117, 2RMN.A.1, 2ROW.A.602, 2RPP.A
.401, 4RQU.B.402, 3RUI.A.1, 1S4I.A.802, 3SOU.B.8, 2SRT.A.257, 3T7E.A.252, 3TGN.A
.148, 3TG4.A.437, 1U3T.A.376, 1U5S.B.139, 3UJZ.A.1, 1UZF.A.701, 2V08.A.1090, 1V5
N.A.201, 1V5R.A.201, 1V87.A.201, 1V87.A.401, 1VGN.A.301, 2VJE.B.1492, 1VK9.A.143
, 2VXX.B.202, 3W0F.A.301, 1WAA.D.1090, 1WE9.A.201, 1WEE.A.201, 1WEM.A.401, 1WEN.
A.201, 1WEO.A.201, 1WEP.A.401, 1WEQ.A.201, 1WEQ.A.401, 1WEV.A.201, 1WEV.A.401, 1
WG2.A.400, 4WH6.A.1203, 1WIL.A.201, 1WIM.A.401, 3WIE.B.1001, 1WJA.A.56, 3WL3.A.3
01, 1WUR.A.1001, 3WXC.A.302, 1WYH.A.401, 1WYS.A.201, 3X17.A.601, 1X3C.A.201, 1X3
H.A.201, 2X4H.B.1141, 1X4V.A.201, 1X6E.A.401, 1X6F.A.201, 2XOC.B.991, 1XUF.A.246
, 2XXH.B.1303, 1XYD.A.94, 1XYD.B.94, 2Y20.C.1331, 2Y4Y.A.1172, 2Y6C.A.1267, 1YM3
.A.301, 1YOU.A.301, 1YSB.A.501, 2Z2Y.D.2004, 1Z83.B.642, 1ZTQ.A.550
[1] "Cluster 7"
1A6Y.A.550, 2ER8.A.104, 1G2D.C.302, 1G2F.C.302, 3HAX.C.201, 2IHX.A.235, 2JP9.A.1
33, 2KKF.A.2001, 4LMG.A.201, 4M9V.C.202, 1MEY.C.88, 4MTD.A.201, 2NQ9.A.401, 4O6A
.A.601, 3PIH.A.917, 3QSV.A.1, 3A32.A.708, 2A5V.A.401, 1A71.A.401, 4A7K.A.950, 3A
9J.C.1, 3A9K.C.1, 2AA4.A.1001, 2AFU.A.391, 1ARE.A.1, 4ARF.A.1722, 3AUK.A.390, 2A
U3.A.501, 4AWY.B.3228, 3AX1.A.601, 4AXO.B.3228, 4AX1.B.3228, 4AXD.A.700, 2BOP.A.
400, 4B29.A.1205, 3B4N.B.712, 3B5Q.A.500, 3B7R.L.701, 1B8Y.A.301, 3B92.A.502, 3B
HX.A.1752, 3BIO.A.1752, 3BI1.A.1752, 2BL6.A.1060, 3BQ6.A.800, 1BS4.A.2001, 4BT7.
A.301, 1BV3.A.262, 3C10.A.102, 4C1D.A.501, 4C1E.A.501, 4C1F.A.501, 4C1G.A.300, 3
C2S.A.448, 4C2P.A.701, 3C52.B.401, 1C7K.A.133, 3C8Z.A.413, 4C81.A.1240, 2C9S.A.1
155, 1CAQ.A.301, 1CL4.A.81, 2CLT.A.1202, 3CNG.A.508, 2COT.A.401, 3COS.A.501, 3CQ
Z.I.3004, 3CSQ.A.335, 1CTT.A.296, 1CTU.A.296, 1CVE.A.262, 3CXL.A.501, 2D74.A.100
1, 1D9D.A.1, 3DBK.A.302, 1DCA.A.262, 1DD6.A.502, 2DH3.A.601, 3DH1.A.201, 2DKT.A.
341, 2DKT.A.391, 2DMD.A.291, 2DOO.A.501, 2DQ6.A.900, 4DR8.A.201, 1DSV.A.171, 2DS

N.B.2002, 2DW2.A.700, 4DYG.B.307, 3E2C.A.200, 1E3I.A.380, 1E3L.A.380, 1E4B.S.999
, 3E6U.C.502, 2E9H.A.301, 2EA6.A.401, 2ECW.A.401, 2ECG.A.401, 2ECL.A.601, 2ECM.A
.201, 3EER.A.2004, 2EE8.A.501, 4EEZ.A.402, 3EFO.B.1034, 2EG4.A.301, 2EG4.B.302,
3EHX.A.264, 1EI6.B.408, 1EKJ.A.4001, 1EKM.A.701, 2ELU.A.181, 2ELV.A.181, 2EM4.A.
201, 2EMF.A.201, 2EMG.A.201, 2ENH.A.181, 2EOE.A.201, 2EOF.A.201, 3EQN.B.757, 2EQ
0.A.201, 2EQ3.A.201, 4EXS.A.302, 3F0F.A.165, 1F1G.A.4002, 2FEJ.A.1, 4FGL.A.301,
2FIF.B.901, 4FKB.A.401, 4FKE.A.1024, 4FKK.A.1025, 1FLJ.A.262, 4FMP.A.400, 1FR2.B
.301, 2FSA.A.501, 3FTW.A.701, 3FUN.A.701, 2FU8.A.401, 2FU9.A.401, 3FVZ.A.821, 2F
ZW.B.376, 4G3M.B.401, 2GMN.A.805, 2GMW.A.300, 1GO8.P.1486, 4GU1.A.905, 4GUA.A.17
19, 1H19.A.701, 4H3S.A.901, 2HB9.A.401, 3HFF.A.154, 1HK8.A.1589, 3HKN.A.261, 3HN
I.G.107, 3HNI.H.107, 3HNJ.A.107, 3HNJ.C.107, 3HNJ.D.107, 4HNO.A.301, 3HPH.A.220,
 3HUG.D.109, 3I1U.A.401, 3I3T.A.700, 1I50.A.3006, 1I76.A.999, 4I7C.A.601, 4ICR.A
.501, 2IDA.A.103, 3IFJ.B.202, 2IGI.A.1004, 1IML.A.78, 2IMR.A.500, 2J21.A.1289, 4
J3D.B.302, 4J4M.A.301, 1JAO.A.999, 4JD1.B.202, 4JE6.A.200, 2JHG.A.401, 4JLX.A.50
1, 4JSW.A.301, 2JTN.A.186, 1JVO.A.261, 1JZS.A.1101, 4K2H.A.201, 3K6I.A.202, 1K7H
.A.478, 4K7D.A.504, 4K7D.A.508, 3KBF.A.159, 3KED.A.875, 1KH4.A.451, 1KK1.A.411,
1KOQ.A.301, 1KUO.A.701, 4KX8.A.1001, 4KXB.A.1001, 3KYC.B.641, 3L0V.A.1, 3L22.A.1
, 4L5O.A.303, 1L7O.B.301, 3LAS.A.167, 4LAO.A.401, 3LE9.B.2, 3LEA.A.485, 4LEF.A.3
02, 4LEV.A.601, 2LFD.A.400, 2LGV.A.111, 4LGJ.A.301, 4LJQ.B.1105, 3LMI.B.1002, 4L
OE.C.401, 1LPV.A.54, 3LQB.A.201, 3LQH.A.1001, 4LR2.A.505, 3LS1.A.1, 3LS9.A.457,
2LUY.A.300, 2LVU.A.101, 2LVT.A.101, 4LW9.D.201, 4LY4.D.301, 2MOD.A.101, 2MOE.A.1
01, 3M2N.A.263, 3M3X.A.262, 3M6I.A.402, 3M79.D.107, 4MB7.A.301, 3MEQ.A.401, 3MF1
.A.1000, 3MHS.E.97, 3MI9.C.88, 3MKG.A.155, 3MKV.B.426, 1MMR.A.1, 1MOO.A.262, 1MQ
0.B.147, 4MT2.A.67, 4MT2.A.68, 3MWM.A.140, 3MWM.A.142, 1ND1.A.400, 3NGJ.A.250, 3
NKQ.A.1001, 4NL4.H.802, 3NNQ.A.201, 1NO5.B.571, 4NQ5.A.301, 4NQ6.A.302, 4NQ7.A.3
01, 2OO3.A.201, 1OAL.A.152, 2OD1.A.902, 1ONW.A.801, 4OTE.A.304, 2OW9.B.606, 4OWF
.G.401, 2OXZ.A.264, 2OX8.B.3, 1P1V.A.201, 1P5D.X.500, 3P5A.A.262, 3P5L.A.262, 2P
53.A.401, 4P9C.B.201, 1PBO.A.1301, 1PBO.A.1303, 3PBB.A.391, 3PLW.A.187, 3PN3.A.1
009, 3PN3.B.1011, 3PZC.A.1000, 1Q2L.A.963, 2Q2L.A.1001, 3Q43.A.1, 3Q44.A.1, 1Q5W
.A.32, 2Q6E.A.501, 4QBG.B.301, 2QDT.A.401, 1QJI.A.1201, 3QNA.A.122, 2QNO.A.431,
4QP5.A.401, 1QUA.A.999, 3QWO.C.500, 3ROD.A.428, 3R3L.A.585, 1R5T.A.150, 2R59.A.7
01, 4RF1.A.1901, 1RMD.A.118, 4RQT.A.402, 4RUW.A.501, 3RZV.A.1, 1S1G.A.152, 3S2E.
G.500, 1S4B.P.1, 1SRP.A.920, 3SWR.A.3, 3T01.A.502, 1T3A.A.422, 3TBG.A.601, 3TGO.
B.505, 1TKF.A.901, 1TTM.A.262, 4TYT.A.302, 1U05.A.500, 1U10.A.601, 1U1H.A.766, 1
U4G.A.9800, 4UA4.A.301, 4UA4.B.303, 3UN6.A.325, 1UUF.A.402, 2UYV.B.1276, 1V13.B.
200, 3V1F.A.704, 1V4P.A.1001, 1V7Z.A.301, 2V9E.A.1276, 2VF7.B.1844, 2VL6.A.1266,
 1VQ2.A.702, 3VQZ.A.301, 2VUT.I.1713, 2VXI.B.201, 1VYX.A.1061, 1WAA.B.1090, 4WAI
.A.101, 2WCB.B.100, 2WHG.A.1263, 1WJB.A.56, 4WKE.A.501, 2WO8.C.1268, 1WY2.B.407,
 2X3B.A.1341, 1X62.A.401, 2X8Y.A.1616, 2X96.A.1617, 1XPZ.A.262, 1XUC.A.1261, 1Y2
3.A.1001, 1Y8F.A.702, 1YC5.A.1001, 1YE3.A.375, 2YQL.A.401, 2YQQ.A.201, 2YQQ.A.40
1, 2YSP.A.181, 2YTR.A.201, 2YTN.A.201, 2YUU.A.401, 2YXO.A.501, 2Z45.A.1001, 1Z6R
.A.501, 1Z6U.A.1, 1Z84.A.603, 1Z9N.A.201, 2ZEP.A.391, 2ZNC.A.1, 2ZNR.A.2, 1ZXV.B
.9002
[1] "Cluster 8"
3AUO.B.579, 4GNX.C.701, 4IFD.J.1106, 4L8H.R.105, 4MTD.B.201, 1ODH.A.1171, 1QUM.A
.301, 3A3O.B.65, 2A97.B.2437, 1AAF.A.57, 4AA1.A.1615, 1ARD.A.1, 1ARF.A.1, 2AYK.A
.171, 1B8T.A.194, 1BAW.A.107, 2BA1.A.201, 3BL5.A.300, 4BLD.D.910, 1BTG.B.902, 3C
0Y.A.401, 2C20.A.601, 2CEA.B.1606, 3D2N.A.102, 1D8M.B.801, 1DDZ.A.1, 1DE6.A.450,
 1DGS.B.2701, 2DJ7.A.401, 2DKD.B.922, 4DLA.A.401, 4DLF.A.404, 1DPM.A.801, 1DTH.A
.901, 3E4Z.B.2, 1E67.A.129, 2EIM.C.262, 1EU4.A.400, 1EYW.A.402, 1F5F.A.252, 1F8F
.A.372, 3FDK.A.402, 4FVL.A.501, 2G54.A.1100, 2GA3.A.451, 3GAY.B.328, 3GJN.B.600,
 1GKR.A.1452, 1GYT.J.600, 3GZE.B.14, 4H01.A.602, 3H8F.B.501, 4H82.B.300, 3H90.A.
293, 2HSI.A.283, 1HU8.A.501, 1IAU.A.504, 3ID7.A.402, 3IEW.B.801, 3ISI.X.3000, 1I
TU.A.401, 2IWE.A.1129, 4JIJ.A.302, 2JIG.A.1253, 1JT1.A.400, 4K5N.A.1101, 2K8D.A.

155, 2K08.C.54, 3KWO.B.161, 4KXC.A.1001, 1L10.F.2, 1LD3.A.500, 2LGV.A.110, 2LVR.
A.101, 2LXH.C.901, 3LZE.A.201, 3MON.A.201, 1MVH.A.502, 1MVH.A.503, 1NDV.A.400, 2
O36.A.690, 2O4Q.A.2402, 2O53.B.314, 4OIW.F.501, 1OJ7.B.1389, 1OKN.A.262, 2OO4.A.
6001, 1OS2.B.369, 1OS2.F.769, 2OW2.A.444, 2OXW.A.264, 1P91.B.2301, 4P9C.A.201, 1
PAA.A.160, 3PJN.A.186, 3PJN.A.189, 3PJN.B.186, 1PS7.A.331, 4Q7R.A.306, 2QJS.A.20
02, 3QJ5.A.376, 3QVY.A.500, 3QVZ.D.500, 1QX1.A.2004, 4R7M.D.1001, 3RAM.D.998, 2R
PR.A.201, 2RPC.A.401, 3RSM.A.500, 3SFH.A.403, 3SPU.D.1004, 3SZY.A.501, 1TF9.A.90
1, 1THJ.A.214, 1THJ.B.214, 1TKH.A.901, 3U7K.A.350, 3UW2.A.474, 2V2A.A.1275, 1V9P
.B.2701, 2W57.B.201, 4WD6.A.302, 1WNU.A.1001, 2X4H.B.1142, 2X5C.A.1128, 2X7M.A.1
174, 2XAA.C.1346, 2XAM.B.700, 1XEM.A.401, 1XTG.A.426, 1XWH.A.356, 2XY9.A.1628, 1
Y0J.A.244, 1Y7W.B.285, 1YEJ.L.605, 1YHC.A.601, 2YPU.A.1998, 2Z26.A.400, 2Z9K.A.9
01, 1ZXZ.A.198
[1] "Cluster 9"
3IE1.D.442, 1V15.A.1132, 3AF5.A.665, 2ANH.A.451, 2ANU.B.405, 2APO.B.501, 3B1B.A.
378, 1BH5.A.201, 1BQQ.M.289, 3C0Z.A.101, 2CBN.A.402, 3CE9.B.400, 2CG3.Z.1, 2CIH.
A.212, 3CQJ.B.285, 4CWM.B.433, 3D3X.A.428, 1DSQ.A.144, 1DXW.A.301, 4EGE.A.411, 3
EII.D.301, 1ENQ.C.238, 3EYV.L.220, 4F0R.A.501, 3FGG.A.161, 4FUK.A.401, 4H00.A.60
1, 2H42.A.501, 3H66.B.500, 3H90.D.5, 3H90.D.6, 3HDB.A.620, 2HD1.B.101, 4IGM.A.40
1, 4IGM.F.401, 3II1.A.571, 3ITM.A.1, 1JDI.A.301, 1JM7.B.143, 1JOE.A.205, 2K2G.A.
2, 1KAR.A.501, 1KAR.B.502, 2L0Z.A.487, 3L8Y.A.301, 1L9Y.A.2, 2M7Y.A.101, 3MKV.A.
425, 4MLX.A.301, 2MQ1.A.101, 4MZ7.A.701, 3N2C.D.426, 4NRZ.B.301, 1NYR.A.1002, 3O
90.A.192, 4O98.A.401, 2OC7.A.901, 1OLP.A.1373, 1OLP.A.1375, 1OLP.D.1374, 1P5X.A.
247, 2PJT.D.302, 2PTW.A.500, 3Q31.A.1, 1Q74.D.304, 2QFP.A.434, 3R2J.A.301, 1RO9.
A.529, 3S6L.B.185, 3SFW.A.502, 1SMP.A.472, 1T0A.B.760, 3T02.A.502, 4TQT.A.501, 3
TVX.A.900, 3UBF.A.7, 1UR6.B.79, 1UXA.C.1367, 3V94.E.702, 2VXX.D.201, 3WI2.B.801,
 4X2T.D.701, 4X2T.D.702, 2X4H.A.1141, 2XR1.A.1638, 2XR1.A.1639, 1YIX.A.601, 1YIX
.B.603, 1Z3J.A.264, 1ZKN.C.601, 3ZNS.A.101, 2ZNE.B.992, 1ZSW.A.315, 3ZTV.A.1598
[1] "Cluster 10"
1AJD.A.450, 1ANI.A.450, 2AQ2.B.1001, 2AXR.A.501, 3B0Z.A.22, 2B5L.C.3001, 3BYW.D.
1, 4C98.A.1266, 3CHP.A.701, 3CPA.A.308, 1D1T.B.406, 3D68.A.501, 3DLJ.A.2001, 3DL
J.B.2002, 3DNG.B.998, 2EC7.A.51, 4EGE.A.412, 1F30.I.201, 2FKM.X.500, 4FXO.A.301,
 4G1P.A.501, 2GLQ.A.2002, 3H90.A.292, 4HGX.B.301, 1HOV.A.165, 4IGN.A.401, 2IMC.A
.600, 2IUC.B.1006, 4J3D.A.301, 2JMD.A.66, 2JRP.A.150, 4JSS.A.301, 4K6T.B.403, 4K
7S.B.101, 1KH5.B.950, 1KQ0.A.479, 2KV1.A.125, 2KVG.A.85, 3LSF.H.2, 4LW9.E.204, 3
MO2.D.5, 2MQ1.A.102, 1MWO.A.438, 4N07.A.309, 4N07.B.306, 3NQ5.A.508, 2O1Q.B.145,
 2O3Z.A.501, 4PVT.A.404, 3PW3.C.406, 3PW3.D.406, 1PYT.B.350, 4Q7R.A.305, 3QU6.A.
114, 3QZC.A.2, 1R5X.A.122, 1TM6.A.23, 4U9D.A.205, 3U94.A.259, 3UBF.A.6, 1UD9.B.5
10, 1UDV.A.101, 1UUP.D.5222, 2VQH.B.1089, 3VUV.A.501, 3WC5.A.404, 4WD7.C.302, 1X
AF.A.503, 2Y2E.A.1180, 2Z2D.A.264, 2Z45.A.1003, 1Z5R.C.600, 1ZKX.A.423, 3ZNR.B.1
01, 3ZUQ.A.1440
[1] "Cluster 11"
1A1G.A.201, 1A6Y.A.551, 2ER8.A.105, 4GLX.A.601, 4IQR.A.403, 4NM6.A.2002, 3VD0.A.
401, 1A1R.A.901, 1A72.A.376, 1AAF.A.56, 2AB3.A.30, 2AMT.B.2900, 1ANJ.B.451, 1AXG
.A.401, 3AY2.A.1001, 2AZH.A.150, 4B6D.A.1340, 1B8T.A.193, 1B8T.A.195, 3BJI.A.1,
3BOC.A.1001, 3BTO.C.375, 3BYR.A.501, 2C1I.A.1465, 4C1D.A.502, 4C1G.A.301, 4C1Q.A
.493, 2C2F.A.1211, 1C3I.B.260, 1CDO.B.376, 3CJP.A.301, 2COT.A.201, 2CO8.A.201, 2
COR.A.201, 1CQR.B.2301, 3CQZ.B.3007, 2CQE.A.622, 2CT0.A.201, 2CT2.A.201, 2CTT.A.
401, 2CU8.A.401, 2D74.B.1002, 2D8T.A.201, 2D8T.A.401, 2D8V.A.201, 2D9M.A.1085, 2
D9N.A.456, 2DAN.A.201, 2DAR.A.201, 1DEH.A.376, 3DHA.A.256, 2DJ8.A.401, 2DJA.A.20
1, 2DJA.A.401, 1DK4.A.290, 1DMT.A.755, 1DO5.A.28, 3E1Z.A.111, 3E6U.A.501, 3E73.A
.501, 2ECT.A.201, 2ECV.A.201, 2ECW.A.201, 4EEX.A.402, 2ELS.A.181, 2ELI.A.401, 2E
OJ.A.201, 2EOQ.A.201, 2EOH.A.201, 2EOM.A.201, 2EWB.A.489, 1F35.A.306, 4FMN.B.901
, 2FNF.X.2, 2FPR.A.502, 3FTN.A.354, 2G9Y.B.451, 2GMN.A.801, 4GRI.A.501, 4GUT.A.9
04, 2GZL.A.900, 4H1Q.A.302, 1H9Q.A.262, 2HCS.A.1, 2HCS.A.2, 3HJT.A.1, 4HMA.A.301

, 1HSZ.A.1376, 1HT0.A.1376, 1HZ5.B.105, 1I73.A.999, 1IAG.A.999, 3IFU.A.181, 4IOU .D.1001, 4IR0.A.201, 2IUC.A.1002, 4IUQ.A.301, 4IUW.A.701, 4JH2.A.201, 4JH2.B.201 , 2JIG.B.1252, 1JJD.A.103, 2JTN.A.185, 1K07.A.1, 2KEM.A.195, 1KH5.A.451, 2KVF.A. 83, 1L0Y.B.706, 2L0B.A.143, 2L0B.A.161, 3L9Y.A.155, 1LDE.A.375, 1LDY.A.375, 1LI5 .B.964, 4LJQ.A.1101, 1LLU.B.343, 1M3V.A.124, 2M48.A.504, 1M6W.A.1376, 3M8T.A.300 , 3M8T.A.301, 1M9O.A.78, 3MA2.A.295, 3MDW.A.455, 1MG0.A.375, 1MG0.B.375, 3MI9.C. 87, 3MWM.A.141, 3N2C.E.425, 4NQ7.A.302, 2ODX.A.156, 2OHX.A.401, 2OI0.A.2, 1P1R.A .375, 1P42.A.503, 1P4Q.B.301, 2PLI.B.709, 1PV9.A.402, 2QDT.A.402, 3QE3.A.356, 2Q IN.A.2002, 2QL0.A.53, 2QSW.A.201, 1QTW.A.301, 1QV6.A.375, 1QV7.A.375, 1R1H.A.100 1, 1R37.A.500, 1R3N.B.501, 2RPC.A.801, 3S2F.E.500, 3SP1.A.481, 1SRD.B.156, 3SXK. B.3, 1T3K.A.201, 3T33.A.411, 1T9R.A.1, 1TBN.A.2, 3TEN.A.205, 1TKF.A.902, 1TWF.J. 3001, 1U0L.A.298, 1U10.A.400, 4U10.A.401, 1U3U.A.376, 1U3V.A.376, 1U40.A.160, 3U IK.A.341, 2UZG.A.131, 2V1X.A.1595, 1V5N.A.401, 1V6G.A.201, 2V8G.C.500, 3V96.B.30 2, 1VA1.A.100, 1VA2.A.100, 1VA3.A.100, 2VES.A.1297, 2VES.C.1302, 3VOW.A.201, 3VP E.A.301, 1VSH.A.281, 2W5V.B.1377, 3W5K.B.502, 3W5K.B.503, 4W6Z.A.401, 2WBT.A.113 0, 2WBT.A.1131, 1WEN.A.401, 1WEP.A.201, 1WES.A.201, 1WES.A.401, 1WFF.A.401, 1WFL .A.401, 1WFP.A.401, 1WG2.A.200, 1WIG.A.401, 1WIL.A.401, 1WIM.A.201, 1WJP.A.501, 1WJV.A.201, 3WLE.A.402, 4WOK.A.401, 1WYH.A.201, 1X3H.A.401, 1X4K.A.401, 1X61.A.2 01, 1X62.A.201, 1X6H.A.401, 2X8Z.A.1616, 2X95.A.1615, 1XB8.A.1001, 2XML.A.1349, 1XWY.A.401, 1Y8J.A.800, 1YC2.A.402, 1YC2.C.406, 1YLK.A.401, 2YQL.A.201, 1ZLH.A.5 55, 3ZNC.A.1, 3ZUK.A.1664, 2ZU2.A.5517

Table S5. 5-ligand Zn, normal group

	size	largest_angle*	middle_1*	middle_2	middle_3*	middle_4
1	"46"	"147.5+/-5.6"	"70.7+/-4.1"	"79.5+/-4.7"	"88.7+/-4.7"	"95.4+/-3.3"
2	"75"	"163.5+/-4.5"	"71.5+/-3.7"	"84.4+/-4.8"	"89.5+/-3.2"	"94.7+/-3.1"
3	"82"	"166.4+/-4.4"	"80.1+/-4.2"	"86.1+/-2.7"	"89.8+/-2.6"	"92.1+/-2.5"
4	"109"	"169.9+/-5.2"	"80.9+/-3.5"	"85.8+/-2.5"	"88.5+/-2.3"	"91.9+/-2.3"
5	"45"	"168.4+/-5"	"72+/-3.5"	"82.1+/-6.3"	"87.8+/-5.5"	"95.3+/-3.6"
6	"78"	"173.1+/-4"	"81.7+/-3.3"	"86.1+/-2.5"	"88.7+/-2"	"91.2+/-1.7"
7	"104"	"154.5+/-3.4"	"74.8+/-3.7"	"83.8+/-3.1"	"90.5+/-3.2"	"94.9+/-3"
8	"45"	"171.8+/-5.6"	"79.8+/-5.7"	"85.4+/-3.5"	"88.8+/-2.4"	"91.9+/-3.2"

	middle_5*	middle_6	middle_7	middle_8*
1	"101.7+/-3.4"	"108.9+/-5.2"	"119.1+/-6"	"135.1+/-4.5"
2	"98.6+/-2.7"	"103.5+/-3.8"	"117.2+/-6.7"	"135.3+/-4.1"
3	"95.6+/-2.6"	"100.4+/-3.6"	"109.1+/-5.3"	"151.4+/-4.3"
4	"95+/-2.4"	"99.5+/-3.7"	"116.6+/-5.9"	"136.5+/-4.5"
5	"99+/-4.1"	"103.9+/-5.1"	"117.2+/-3.3"	"124.9+/-4.7"
6	"94.1+/-2.4"	"98.3+/-4.2"	"119.3+/-2.3"	"125.8+/-3.3"
7	"99+/-2.6"	"105.6+/-3.2"	"112.3+/-5"	"145.6+/-3.8"
8	"94.6+/-2.9"	"98.2+/-4.1"	"105.2+/-7.3"	"162.8+/-5.9"

	smallest_opposite_angle*	TrigonalBipyramidal	SquarePyramidal
1	"104+/-5.3"	"0.032"	"0"
2	"101.5+/-5.1"	"0.109"	"0.001"
3	"97.8+/-4"	"0.059"	"0.114"
4	"105.4+/-3.8"	"0.32"	"0.008"
5	"109.6+/-3.6"	"0.126"	"0"
6	"113.4+/-3.2"	"0.604"	"0"
7	"99.9+/-4.6"	"0.027"	"0.008"
8	"87.7+/-6.6"	"0.004"	"0.336"

```
  TrigonalPrismaticV
1 "0.138"
2 "0.08"
3 "0.039"
4 "0.041"
5 "0.024"
6 "0.065"
7 "0.127"
8 "0.008"
```

Table S6. Cluster members of 5-ligand Zn, normal group

[1] "Cluster 1"
4A7B.A.1276, 2A8H.A.486, 1AF0.A.472, 4AR8.A.1731, 4BLB.A.910, 2BNN.B.1200, 1BQO.B.301, 1BS8.A.2001, 3C0Z.B.101, 4C6L.A.2823, 1CXV.A.1, 4DR9.A.201, 3DYC.A.451, 3E0L.A.1452, 3E2D.A.602, 3E4A.B.2000, 1EZ2.A.402, 1FLS.A.166, 1G05.B.801, 1G49.B.801, 4G9L.B.302, 4H49.A.301, 4H82.A.301, 2J83.A.999, 1JJE.A.252, 2JSD.A.276, 2JT5.A.256, 3LJZ.A.999, 4LV4.A.401, 3MK1.A.902, 1MMB.A.999, 3N2V.A.264, 3OD4.A.1350, 3PN4.A.1001, 3Q9F.B.344, 4QA0.A.401, 1RJ5.A.601, 3SPU.C.1010, 4TLN.A.321, 5TLN.A.321, 4TMN.E.322, 3U04.A.200, 3V77.A.302, 1W22.A.1375, 2Z3I.C.2003, 3ZXH.A.300
[1] "Cluster 2"
4A3W.A.1159, 4ASQ.A.1615, 4ASR.A.1615, 1B57.A.360, 3B7S.A.701, 3B7U.X.701, 4BT4.A.301, 4BT5.A.301, 4BXK.A.1620, 4BZR.A.1630, 3C52.A.401, 4CA5.A.1001, 4CA7.A.1616, 4CA8.A.1620, 3CZS.A.1102, 4DEF.A.401, 4DPE.A.301, 3EBH.A.1, 1EI6.B.409, 3ELF.A.352, 1FKX.A.400, 4FW3.B.300, 4FW4.C.301, 4FW5.A.301, 4FW7.A.301, 4FYT.A.1012, 1GKR.A.1453, 4GK8.A.302, 1GVF.A.288, 4H1Q.A.301, 1H48.C.900, 4H76.A.301, 2HC9.A.702, 3HK5.A.430, 3HK8.A.430, 2HPT.A.950, 3ID7.A.401, 2ILP.A.500, 4JP4.A.301, 2JT6.A.256, 3KR5.G.1004, 3M4C.D.109, 3MAX.A.379, 4MCA.B.1000, 4NTK.A.201, 3NXQ.A.650, 2OC2.A.701, 4OPN.A.201, 3P3C.A.401, 1P6E.A.248, 3P76.A.274, 1PV9.A.401, 3Q2H.A.401, 2QPJ.A.1, 1R43.A.500, 3T00.A.502, 1U7J.B.150, 1U7M.A.54, 1U7M.B.154, 3UHM.A.300, 1URA.A.451, 2USH.A.602, 2V5W.A.1380, 2V9G.A.1276, 2V9M.A.1275, 1WY2.A.406, 2XHM.A.1616, 1XXW.A.201, 2XYD.A.1620, 2Y6D.A.1268, 1Y7W.A.283, 2Z72.A.402, 2ZBM.A.402, 3ZU0.A.1595, 3ZU0.A.1596
[1] "Cluster 3"
4A7K.A.952, 3ASE.A.156, 4AWY.B.3229, 3B8Z.A.901, 2BNO.A.1201, 3BUD.A.1048, 3C10.A.101, 4C5W.A.402, 2CEA.A.1603, 2CFU.A.1001, 2DDY.A.177, 4DV8.A.801, 3DWB.A.771, 1E4C.S.999, 4E5V.B.401, 3F16.A.264, 1FA5.A.1200, 2FV5.A.3, 2FV9.B.4, 2FYV.A.2003, 3G4K.A.801, 2GC3.A.402, 1GKP.B.1460, 2GMN.A.802, 3H8F.E.501, 3HC4.L.401, 4HGX.A.301, 3HR1.A.1, 1HS6.A.701, 1HTY.A.1102, 3HYG.A.901, 2ICS.A.400, 1ITU.A.402, 1JAP.A.999, 2JNE.A.200, 1JWQ.A.1001, 3K5X.A.402, 1K9Z.A.402, 1LCP.A.489, 2LQ6.A.402, 3LS6.A.303, 4N27.A.201, 4N27.C.201, 3N9R.A.308, 3OHL.A.5, 1OS9.A.901, 4OUI.A.501, 2OW6.A.3001, 2OW7.A.5001, 2PAJ.A.493, 4PKT.A.802, 4PKW.A.801, 1PMI.A.445, 1PTW.A.501, 1PVW.A.401, 1Q74.A.304, 3Q9B.A.345, 3QAY.A.180, 1QH5.A.262, 1QIP.A.902, 1QIP.D.903, 4R76.A.1001, 3S2J.A.402, 3S2L.A.402, 3S2M.A.402, 3S2M.A.403, 3S2N.A.402, 3S2N.A.403, 1SML.A.270, 1T64.A.388, 1TQS.A.1401, 1TQT.A.1301, 1TQU.A.1400, 1TQV.A.1300, 1TQW.A.1400, 3VH9.A.301, 2VQM.A.1411, 2W3Z.A.1312, 2WM1.A.1333, 4X2T.B.1002, 1XP3.A.301, 1Y93.A.264
[1] "Cluster 4"
2L1G.A.88, 4QCL.A.1303, 1QUM.A.302, 1A7T.A.252, 4A7Y.A.951, 1AH7.A.246, 2AIO.A.315, 1AST.A.999, 4AX0.B.3229, 4AX1.B.3229, 2BH3.A.1002, 4BIN.A.500, 2BMI.A.272, 4BZ3.B.502, 4C24.A.301, 1CAM.A.262, 2CHI.A.212, 3D0N.A.262, 4DEL.A.402, 3DHA.A.25

5, 4DLM.A.401, 2DVT.A.1501, 2DVX.A.1501, 4DZH.A.504, 2E2D.A.500, 3E38.A.1, 2EG6.
A.400, 3EWD.A.371, 1F0J.A.1101, 1FA5.A.1201, 3FDK.A.401, 4FUA.A.216, 4GBD.A.502,
1GYT.G.600, 1GYT.G.601, 4H2E.A.301, 4H2G.A.601, 1H9N.A.262, 2HBV.A.401, 3HC8.A.
864, 3HKA.A.430, 1HZY.B.401, 4IE0.A.601, 4IE6.A.601, 4IG2.A.401, 4ISM.A.201, 3IT
C.A.501, 3ITC.A.502, 4J5F.A.301, 4J5H.A.302, 1J79.B.400, 2J9A.A.1493, 1JCZ.A.901
, 4JD1.A.201, 2JIG.A.1252, 1JK3.A.400, 1K4P.A.1004, 1KAE.B.1102, 1KEQ.A.280, 4KE
Q.A.302, 3KM8.A.400, 1LAM.A.488, 1LAM.A.489, 4LCQ.A.601, 4LE6.A.405, 4LE6.A.406,
3MDU.A.454, 3MKV.B.425, 3MVI.A.901, 2O4Q.A.2401, 2OB3.A.901, 2OW1.A.444, 1P5X.A
.248, 1P6D.A.246, 1P6D.A.248, 2PLM.A.407, 4PVO.A.402, 3Q4R.A.201, 2Q5B.A.107, 2Q
5B.C.107, 4QA5.A.401, 4QA6.A.401, 4RL2.B.302, 3SEY.E.373, 3SNG.A.402, 4TYT.A.301
, 3U79.D.110, 3U79.E.110, 1UEA.C.1, 2USH.A.601, 1UXA.B.1367, 2UX1.A.1174, 2UX1.C
.1173, 3V96.B.301, 3VPE.A.302, 3VTG.A.301, 2VUN.A.401, 4WB7.A.502, 1WPP.A.602, 2
XL9.B.1269, 2Z72.A.401, 2ZBM.A.401, 1ZNB.A.2, 2ZNE.B.991, 2ZO4.A.319, 3ZU0.B.158
8, 3ZU0.B.1589, 2ZWR.A.208, 1ZZM.A.401
[1] "Cluster 5"
2JZW.A.57, 4LJ0.B.502, 4OND.E.101, 2A7M.A.252, 1BIW.B.801, 1BLL.E.488, 4C8I.B.11
61, 3EWC.A.372, 4EYF.A.303, 2FGN.A.248, 2GSO.A.1000, 1H4N.A.262, 2HUC.A.248, 2JN
E.A.150, 1K07.B.3, 2K2C.A.140, 4KAP.A.301, 3KRY.A.1999, 3L6N.A.301, 3MA2.D.294,
3MA2.A.294, 3N9S.A.309, 3NJ9.A.262, 2NQH.A.301, 2NZE.A.401, 3O2X.A.1999, 3OHL.A.
4, 1PL6.A.402, 3PNU.A.337, 3Q6X.B.271, 2QVV.A.403, 1QXL.A.400, 2R2D.A.278, 3SXK.
A.4, 3TOM.B.108, 3V1E.A.101, 3VQZ.A.302, 3WAX.A.912, 3WAY.A.911, 2WXU.A.1375, 2W
XT.A.1371, 1XM8.A.700, 1Z60.A.1, 1ZED.A.904, 2ZWR.A.209
[1] "Cluster 6"
1AH7.A.248, 3BJC.A.876, 4BJH.A.423, 4C6L.A.2824, 3CKI.A.501, 3CQJ.A.285, 4CWM.A.
433, 4CXO.A.453, 4CXV.A.433, 1D5J.A.301, 2DVU.A.1501, 4DYK.A.501, 2EG7.A.401, 4E
NL.A.438, 6ENL.A.438, 2EWB.A.488, 1EYW.A.401, 4EYF.A.302, 1GKP.A.1461, 4H01.A.60
1, 2HBM.A.1001, 2HC9.A.701, 3HK9.A.431, 3HWP.A.295, 4ILW.D.301, 2J0T.A.1267, 4J4
K.A.402, 1J79.A.400, 4JH8.A.201, 4JH8.B.201, 1JIW.P.481, 4JS6.A.301, 3K2G.A.400,
1KMG.A.154, 3KNS.A.228, 4KTX.A.501, 4LEF.A.301, 2LFF.A.500, 3LGG.B.512, 3MJM.B.
401, 3MTW.A.1, 3N2C.A.425, 2NQH.A.302, 4O98.B.401, 1ONW.A.800, 1P6B.A.401, 1PB0.
A.1302, 2Q02.C.300, 3Q6X.A.3, 3QDF.A.266, 1QF0.A.320, 1QF1.A.320, 1QTW.A.303, 1R
3N.A.500, 3RHG.A.367, 4RL2.A.301, 1SHN.B.482, 3SNG.A.401, 1UIO.A.400, 1VFL.A.501
, 3W52.A.311, 2XF4.A.1211, 2XS3.A.999, 2XS4.A.999, 2Z24.A.400, 2Z24.B.400, 2Z25.
A.400, 2Z25.B.400, 2Z26.A.401, 2Z27.A.400, 2Z27.B.400, 2Z28.A.400, 2Z28.B.400, 2
Z29.A.400, 2Z29.B.400, 2Z2A.A.400, 2Z2B.A.338, 1ZZM.A.402
[1] "Cluster 7"
830C.A.272, 456C.A.272, 4A7Z.A.950, 1A85.A.999, 1A86.A.999, 2AB7.A.30, 1B3D.B.30
1, 2BIB.A.1549, 3BKQ.X.500, 4BT6.A.1257, 3BUB.A.1047, 3BUI.A.1046, 3BVT.A.1048,
3BVU.A.1048, 3BVV.A.1047, 3BVW.A.1046, 3BVX.A.1046, 4BZ5.A.500, 1C3R.A.501, 1C3S
.A.951, 2CA2.A.264, 3CV5.A.1047, 3CZN.A.1102, 2D1N.A.270, 2D1O.A.257, 1D7X.B.801
, 4DD8.A.1002, 2DKD.A.921, 1DQS.A.402, 2DW0.A.700, 1E48.S.999, 3EBG.A.1, 3EDZ.B.
2, 2ERP.A.700, 3F15.A.264, 3F17.A.264, 3F18.A.264, 3F19.A.264, 3F1A.A.264, 4FL7.
A.301, 2FVM.D.601, 2GO4.A.601, 4H3X.A.301, 1HFC.A.275, 1HJK.A.451, 1HOV.A.166, 4
HWO.A.701, 4HWP.A.701, 4HWR.A.701, 4HWS.A.701, 3HY7.A.901, 3HY9.A.901, 1HZY.A.40
2, 2I47.C.804, 4IE4.A.601, 1JAQ.A.999, 4JE7.A.202, 2JIH.B.1554, 1JJT.A.252, 2K4W
.A.156, 4K5P.A.1101, 1KBC.A.999, 3KDS.E.996, 1KYS.A.301, 2LFF.A.502, 3LJT.A.901,
3LK8.A.264, 2LQ6.A.401, 1MNC.A.281, 3N2U.A.264, 3NX7.A.264, 3O90.B.192, 3OHO.A.
5, 2OKL.A.601, 4OPN.B.201, 2PJT.A.302, 1PS6.A.330, 3Q2G.A.401, 4QA1.A.403, 4QA2.
A.403, 4QA4.A.502, 1QIN.A.401, 1QIN.B.301, 3QIZ.A.431, 1QJJ.A.250, 2RJQ.A.1, 1RM
8.A.500, 1RMZ.A.264, 3RTS.A.264, 3RTT.A.264, 2TCL.A.170, 3UWB.A.200, 2V5X.A.1377
, 3V77.A.301, 2VES.A.1295, 2W0D.A.1264, 2W15.A.1203, 4WD6.B.302, 2WO9.B.1268, 1X
BU.A.901, 1XBU.A.902, 2YD0.A.1946, 1YQY.A.781, 1ZXC.A.201
[1] "Cluster 8"
3ADR.A.263, 3AYK.A.170, 2BCN.A.295, 2BIB.A.1550, 2DI3.A.1002, 3EII.A.301, 2EK9.A

.1004, 2GC2.A.401, 2HD1.A.101, 4HTZ.B.1001, 4ICQ.A.501, 4IGN.B.401, 2J0E.B.1265, 4JAA.A.501, 2K78.A.151, 4KBP.A.439, 3KR5.E.1001, 4KYH.A.202, 3LL8.A.505, 2LZE.A.101, 1ML2.A.296, 4N7K.L.301, 4N7K.L.307, 4N7K.M.401, 4N7K.M.402, 4N7L.M.402, 3NO5.C.275, 3O64.A.1, 4OK2.A.801, 2OUN.B.404, 1Q3A.B.469, 1Q3K.B.300, 2QFR.A.434, 4R6T.A.1003, 4R6T.D.1001, 4R7M.C.1001, 4R7M.J.1003, 3SFW.A.501, 3V93.D.701, 1VKG.B.400, 3VUS.A.401, 2WEY.A.1772, 4X2T.A.702, 1XJS.A.150, 1Y13.A.174

Table S7. 5-ligand Zn, compressed group

	size	largest_angle*	middle_1*	middle_2	middle_3*	middle_4
1	"83"	"137.2+/-4.7"	"55+/-3.9"	"82.9+/-6"	"91.3+/-4.6"	"96.4+/-5.2"
2	"61"	"156.3+/-5.5"	"57.8+/-3.5"	"86.6+/-6.3"	"92.9+/-4.5"	"98+/-3.1"
3	"128"	"148.5+/-4.6"	"56.5+/-2.8"	"87.6+/-5.1"	"93.1+/-3.2"	"96.7+/-3.3"
4	"124"	"151.2+/-4.2"	"55.3+/-2.5"	"85.8+/-4.8"	"91.8+/-3.4"	"97.1+/-2.9"
5	"61"	"164.2+/-5.7"	"56.8+/-4.7"	"80.1+/-6.4"	"87.3+/-4.1"	"97.9+/-4"
6	"44"	"143.6+/-5.1"	"55.2+/-3"	"80.9+/-7.2"	"91.6+/-6.8"	"97.1+/-6"
7	"17"	"148.7+/-13.4"	"78.9+/-9.5"	"85.7+/-6.3"	"90.3+/-5.7"	"95.9+/-5.2"

	middle_5*	middle_6	middle_7	middle_8*
1	"103.1+/-4.7"	"112.8+/-4.3"	"120.1+/-4.3"	"127.4+/-3.8"
2	"101.6+/-3"	"105.4+/-3.7"	"110.2+/-5.1"	"144.8+/-5.8"
3	"101.1+/-3.4"	"106.5+/-4.5"	"114.5+/-4.6"	"134.6+/-3.8"
4	"102.1+/-3.2"	"108.3+/-4.3"	"117.3+/-3.2"	"122.7+/-3"
5	"104.5+/-3.6"	"109.8+/-3.6"	"115.6+/-4.1"	"123.6+/-5.9"
6	"103.6+/-5"	"111.1+/-5.9"	"121.8+/-5.2"	"133.8+/-4.5"
7	"101.7+/-6.9"	"106.5+/-8.8"	"112.7+/-10.6"	"141+/-10.9"

	smallest_opposite_angle*	TrigonalBipyramidal	SquarePyramidal
1	"105.2+/-4.8"	"0.054"	"0"
2	"94.2+/-6.1"	"0.028"	"0.058"
3	"102.3+/-3.2"	"0.091"	"0.002"
4	"107.5+/-4.3"	"0.132"	"0"
5	"103.8+/-5.7"	"0.038"	"0.001"
6	"88.3+/-7"	"0.012"	"0.001"
7	"54.7+/-4.1"	"0.012"	"0.119"

	TrigonalPrismaticV
1	"0.171"
2	"0.079"
3	"0.112"
4	"0.099"
5	"0.022"
6	"0.133"
7	"0.068"

Table S8. Cluster members of 5-ligand Zn, compressed group

[1] "Cluster 1"
4LMG.D.202, 2A7G.E.401, 3AHT.A.1001, 1ALK.A.451, 3B2Z.A.1, 3BKN.B.202, 3BOO.A.500, 4BP0.A.1314, 4C1H.A.351, 1CBX.A.309, 2CEA.E.1603, 1D8D.A.1001, 1DCE.B.900, 3DID.A.130, 3DSX.B.332, 3E37.B.1001, 1EC5.C.50, 2EG8.A.400, 3F28.A.321, 3F2P.A.200

5, 3FGD.A.321, 4FU4.A.502, 4G9L.B.301, 3GIP.A.484, 2GVI.A.302, 1HR7.B.501, 1HYT.A.805, 4I11.A.502, 3I9F.A.1, 3IQ6.B.201, 1J9Y.A.1003, 4JJJ.A.718, 1JML.A.102, 1KAP.P.613, 1KR6.A.405, 1KS7.A.405, 3KWO.C.162, 3LPE.D.60, 3LUB.B.302, 3M79.B.107, 3M79.C.107, 3MDJ.A.1000, 1MMP.A.2, 1N4Q.B.378, 1OEZ.W.1154, 4OKO.A.401, 4ONG.H.302, 1PE5.A.317, 1PE7.A.317, 2PLI.C.701, 1PSZ.A.1000, 4PUC.A.602, 1QBQ.B.1000, 3QW0.A.500, 3QW0.B.500, 3QW0.D.500, 3QW1.C.500, 1R43.A.501, 1RK6.A.601, 3S9C.A.503, 1TLP.E.322, 1TMN.E.322, 1TN6.B.1001, 1TN7.B.1001, 1TNB.B.378, 1TNO.B.378, 1TNU.B.378, 1TNY.B.378, 1TNZ.B.378, 3TOL.B.107, 3TT4.A.302, 3TVC.A.501, 2VXI.A.201, 3W5K.B.501, 1WAA.A.1091, 1WAA.F.1091, 4WD8.A.301, 2X4H.A.1142, 2X98.A.1476, 1XGE.A.400, 2Y28.A.1181, 2YJP.C.1270, 1ZG9.A.400

[1] "Cluster 2"

3B35.A.292, 3B3C.A.501, 3BHX.A.1751, 3BI0.A.1751, 3BI1.A.1751, 2C6P.A.1751, 3CSQ.B.335, 4DHL.B.501, 1DK4.B.591, 4DYO.A.502, 1DZW.P.999, 4EJ5.A.501, 2EK9.A.1002, 1FUA.A.216, 2GA3.A.450, 1GE7.B.200, 3H90.B.294, 1HR6.B.501, 4ICQ.B.502, 4ICR.A.502, 4IE7.A.601, 2IQ6.A.293, 1J2U.A.301, 4JBS.B.1009, 4JX5.D.1103, 4K5M.A.1101, 4K5O.A.1103, 1KBE.A.1, 4KFT.C.303, 2KIK.A.50, 3KNS.A.229, 3LOT.B.2, 1LFW.A.1001, 4LP6.A.302, 3LQ0.A.999, 3M3B.A.156, 1MH2.A.201, 4NAQ.A.1027, 4O3A.B.302, 4ON1.A.400, 4ONX.B.201, 2OR4.A.1751, 2PVW.A.1751, 4Q7R.A.307, 2QLA.B.502, 2QLA.D.503, 3SJX.A.1751, 3SZZ.A.502, 3T00.A.501, 1TKH.A.902, 1TKJ.A.901, 3TOL.D.107, 3U93.B.259, 2V29.A.1274, 2V29.A.1275, 3VAT.A.501, 1VKG.A.400, 2WO8.A.1268, 1XJO.A.902, 2XPY.A.1673, 1YHC.B.602

[1] "Cluster 3"

4MTD.A.202, 3AIG.A.999, 1ANJ.B.450, 2ANP.A.501, 2ANP.A.502, 4AR9.A.1731, 1ATL.A.401, 4AWZ.A.3230, 3AYU.A.415, 1BON.A.1001, 2B13.B.401, 2BH3.A.1001, 3BKK.A.701, 3BKL.A.701, 2C20.B.601, 2C6C.A.1751, 1CP7.A.901, 1CP7.A.902, 4CVR.A.1159, 4D0Y.B.1239, 1D1T.C.407, 4DPR.A.701, 2DSN.A.2001, 3E33.B.1001, 2EG7.A.400, 3EHY.A.264, 2EK8.A.1002, 3ELM.A.300, 1EW9.A.450, 3EWJ.B.2, 3FH4.A.301, 3FJU.A.999, 3FU1.B.201, 3FUK.A.701, 4FYQ.A.1012, 4FYR.A.1013, 3G42.A.500, 4GTM.B.501, 4GTP.B.501, 4GTQ.B.501, 4GTR.B.501, 1HDU.E.1308, 1HEE.E.1308, 2HIH.A.601, 2IEJ.B.939, 1IGB.A.502, 3IQ6.C.201, 2J13.A.1236, 4J5H.A.301, 2JAZ.B.600, 4JBS.A.1008, 1JI3.A.402, 4JYW.A.801, 4JZ0.A.801, 4K5L.A.1101, 4KAY.A.601, 1KH5.A.450, 4L2L.A.701, 4LCQ.A.602, 4LQG.A.801, 2M30.A.201, 3M4C.D.108, 3M79.A.107, 1MMP.A.1, 3NKQ.A.1002, 3NKR.A.1002, 3NKN.A.1002, 3NKO.A.1002, 4O2I.A.401, 4O3A.B.303, 2OY2.A.999, 3P24.A.999, 4P9C.J.201, 2PIY.A.400, 2PJ0.A.400, 2PJ3.A.400, 2PJ5.A.400, 2PJ7.A.400, 2PJ8.B.500, 2PJA.B.500, 2PJB.A.400, 2PJC.A.400, 3PZ4.B.1, 3Q4R.A.202, 3Q75.B.521, 3QBU.A.294, 1R42.A.804, 2RFH.A.1308, 2RJP.A.1, 1ROS.A.400, 1RTQ.A.702, 3RYM.B.107, 1S0E.A.1291, 3SKS.A.568, 1SQM.A.1001, 1TF9.A.902, 3TG0.A.502, 1TKJ.A.902, 3TS4.A.301, 3U24.A.595, 3V0A.A.1297, 2V77.A.1042, 2V9E.A.1275, 3VH9.A.302, 2VJ8.A.1611, 2VQQ.A.1411, 2VXX.C.202, 3WAW.A.913, 4WD8.A.302, 3WE7.A.301, 2WO9.C.1269, 2WOA.A.1270, 1WUO.A.301, 3WV3.A.301, 1WY2.A.405, 2X91.A.1619, 2X92.A.1615, 2X93.A.1615, 2X94.A.1616, 2X98.A.1475, 1XAF.B.507, 2XIG.C.1153, 2XR9.A.1867, 2Y2B.B.1180, 2YJP.A.1272, 2Z25.B.401, 2Z2D.A.265, 2ZTG.A.902

[1] "Cluster 4"

4A37.A.376, 3A52.A.1001, 4A69.A.500, 3B4N.A.711, 3B4N.B.715, 1B57.A.361, 4B6Z.C.385, 2BO9.A.999, 2BP8.B.1340, 1BYF.B.302, 4C2N.A.701, 4C2O.A.1629, 2C6P.A.1752, 1CG2.A.500, 1CG2.A.501, 3CHO.A.701, 2CLB.A.1175, 4COG.A.401, 1CPX.A.308, 5CPA.A.308, 4CVT.A.1159, 3E30.B.1001, 3E32.B.1001, 3E34.B.1001, 1ED9.A.450, 3EER.A.2001, 3EQN.A.757, 2EV6.B.2151, 1F57.A.310, 3FB4.A.217, 1FT7.A.501, 3FVL.A.1309, 4GM5.A.401, 2GSN.A.1001, 4GTO.B.501, 4GTV.B.401, 4H2K.A.1001, 1HFS.A.257, 2HH5.A.702, 1HI9.A.300, 1HLK.B.1004, 2I3C.A.314, 4IAV.A.401, 4IHM.A.402, 3IT7.A.183, 2IXD.A.1234, 1IY7.A.308, 2JBJ.A.1752, 1JCQ.B.1001, 1JCS.B.1001, 4JJI.A.402, 4K90.A.701, 1KH4.A.450, 1KRO.A.405, 3L3N.A.701, 4L9P.B.601, 1LD7.B.1001, 1LD8.B.1001, 1LGD.A.262, 1LND.E.800, 4LNB.B.602, 3LTV.A.1001, 2LVH.A.101, 4LW9.I.201, 1M4L.A.1308, 1M5E.A.1705, 4MBG.B.602, 4MKH.A.301, 4MKT.A.701, 4MRQ.A.501, 3MWM.B.142, 1MXD

.A.727, 1MZC.B.1001, 4N07.B.305, 3N21.A.401, 1N4Q.L.378, 1NW2.F.6006, 4NYY.B.501
, 1O1S.B.1001, 4O3A.A.303, 1O86.A.701, 1O8A.A.701, 4OJA.A.202, 4OTE.B.302, 4OXD.
C.301, 2PIZ.A.400, 4PPZ.A.601, 4PQA.A.403, 4PQA.A.404, 3PSQ.A.326, 3PZ1.B.332, 3
Q78.B.521, 3Q7A.B.521, 3RF4.A.118, 2RH6.A.1, 1S63.B.1001, 1S64.B.378, 1SA4.B.439
, 1SA5.B.438, 3SFX.B.521, 3SFY.B.521, 3TG0.A.501, 1TN8.B.1001, 3U9W.A.2001, 1UUP
.A.2222, 1UZE.A.701, 2VXI.A.202, 3W6H.A.302, 3WBH.A.505, 4WCM.A.538, 3WOJ.A.805,
 2X90.A.1618, 4XIX.A.401, 2XIG.B.1150, 2XX0.B.1340, 2XX7.B.1264, 2XXG.A.1339, 2Y
3D.B.149, 2Z2Y.C.2003, 1ZG7.A.400, 2ZIR.B.901, 2ZIS.B.901, 2ZKW.A.401, 3ZXO.A.15
79

[1] "Cluster 5"

1B8J.A.451, 1BA9.A.154, 1BOR.A.58, 4BZS.A.701, 4CA6.A.1001, 4COQ.A.299, 4CVT.A.1
158, 1DO5.B.29, 1ELX.B.451, 3F3Q.A.104, 4F7O.A.301, 2GFK.A.401, 2GFJ.A.401, 3GZE
.A.1, 2HSE.B.954, 3HTR.B.118, 2IOO.A.580, 1IF6.A.262, 1JJE.A.251, 1JJT.A.251, 2J
LP.B.226, 1JPU.A.371, 4K7W.A.102, 2KBX.B.299, 3KS3.A.262, 1L1O.C.1, 4LQY.A.506,
4LW9.A.201, 4LW9.R.201, 3M2Z.A.500, 2M30.B.201, 3MO2.D.6, 4MSM.C.501, 4NGE.A.110
1, 4NGE.E.101, 3NKN.A.1001, 2NQH.A.303, 1NW2.D.6004, 2P2L.A.901, 2PVV.A.1752, 4P
XY.A.302, 1Q3K.B.301, 1R3N.A.501, 1SHN.A.485, 3SIO.A.362, 1T0A.A.661, 1T3A.B.422
, 3TGN.B.147, 1U7J.A.50, 3UCT.B.102, 2V9I.A.1277, 2W5V.A.1377, 2W5X.B.1378, 2W57
.B.202, 4WD6.A.301, 4WNC.O.402, 3WS9.B.801, 3WXC.A.301, 2X97.A.1616, 1XP3.A.302,
 1ZG8.A.400

[1] "Cluster 6"

3A1Z.A.226, 4A94.B.501, 3AAK.A.992, 2AIG.P.999, 4B52.A.401, 3BKN.B.201, 1CGL.A.3
01, 6CPA.A.308, 7CPA.A.308, 8CPA.A.308, 3D10.A.95, 1D1S.B.406, 3D4U.A.309, 3DFM.
A.401, 4DWZ.A.302, 1E49.S.999, 2E46.A.172, 1EC5.B.50, 3FFZ.A.1300, 4FMP.B.400, 3
H90.C.2, 1HA5.C.4221, 1HZ5.A.103, 4I2F.A.603, 4IXN.A.401, 1JK9.C.302, 2JT5.A.257
, 1K53.A.1003, 4KA7.A.801, 1L0Y.B.702, 4LTE.A.1101, 3M52.B.116, 1NPC.A.323, 2OW0
.A.444, 3PSQ.B.328, 3PW3.A.406, 2QLA.A.500, 2QLA.C.501, 1QMU.A.999, 2R2D.A.277,
3U94.B.259, 3UBF.A.1, 3V94.D.702, 2WKN.B.412

[1] "Cluster 7"

4BLB.D.910, 4CPA.I.308, 1F30.B.201, 1F30.E.201, 4FUK.B.402, 4IOD.A.502, 4K0D.B.2
03, 4KJG.A.1001, 3M4B.B.108, 4MCS.A.814, 2MLS.A.302, 1NL4.B.500, 3OOF.A.302, 1P6
D.A.247, 1RLY.A.61, 2V8V.A.1456, 1ZEB.A.901

Table S9. 5-ligand Zn, combined group

	size	largest_angle*	middle_1*	middle_2	middle_3*
1	"200"	"141.2+/-5.5"	"56.3+/-4.4"	"83.8+/-6.2"	"91.8+/-4.2"
2	"103"	"169.4+/-5.5"	"80.2+/-5.4"	"85.9+/-3.2"	"89.3+/-2.5"
3	"23"	"149.1+/-11.6"	"74.6+/-11.8"	"82.8+/-9.3"	"89.3+/-6.1"
4	"221"	"171.3+/-4.5"	"79.3+/-5"	"85.2+/-3.6"	"88.6+/-2.8"
5	"180"	"157+/-6.5"	"56.6+/-4.4"	"83.8+/-6.3"	"90.2+/-4.6"
6	"144"	"151.5+/-5.2"	"57.2+/-4.1"	"86.5+/-6.1"	"93.1+/-4.3"
7	"232"	"158.3+/-6.3"	"74.4+/-4.7"	"83.8+/-4.1"	"89.5+/-3.5"

	middle_4	middle_5*	middle_6	middle_7	middle_8*
1	"96.3+/-4.5"	"102.3+/-4.3"	"110.9+/-5"	"118.9+/-4.5"	"129.2+/-5.2"
2	"91.8+/-2.6"	"95.1+/-2.8"	"99.1+/-3.9"	"107+/-6.2"	"157.4+/-6.9"
3	"95.7+/-6"	"101.8+/-6.8"	"106.4+/-8.5"	"115.2+/-10.6"	"141.4+/-10.1"
4	"92.3+/-2.8"	"95.3+/-3.1"	"99.9+/-4.6"	"118.1+/-4.6"	"130.3+/-6.2"
5	"97.6+/-3.2"	"102.9+/-3.6"	"108+/-4.2"	"116.4+/-4"	"123.5+/-4.7"
6	"97.5+/-3.3"	"101.8+/-3.4"	"106.3+/-4.4"	"113.6+/-6.5"	"139.7+/-5.9"
7	"94.4+/-3.2"	"98.7+/-3.1"	"104.7+/-4.3"	"113.8+/-6.2"	"141.9+/-5.8"

	smallest_opposite_angle*	TrigonalBipyramidal	SquarePyramidal	TrigonalPrismaticV
1	"104+/-5.4"	"0.004"	"0"	"0.063"
2	"92.8+/-6.6"	"0.024"	"0.226"	"0.019"
3	"58.7+/-9"	"0"	"0.012"	"0.019"
4	"108.7+/-5.3"	"0.387"	"0.003"	"0.047"
5	"106.1+/-5"	"0.009"	"0"	"0.023"
6	"95.5+/-6.9"	"0.001"	"0.002"	"0.027"
7	"101.4+/-4.7"	"0.07"	"0.011"	"0.108"

Table S10. Cluster members of 5-ligand Zn, combined group

[1] "Cluster 1"

4LMG.D.202, 4MTD.A.202, 2A7G.E.401, 4A7B.A.1276, 3AHT.A.1001, 3AIG.A.999, 1ALK.A
.451, 1ANJ.B.450, 4AR8.A.1731, 4AR9.A.1731, 1B0N.A.1001, 2B13.B.401, 3B2Z.A.1, 4
B52.A.401, 2BH3.A.1001, 3BKN.B.201, 3BKN.B.202, 3BOO.A.500, 4BP0.A.1314, 4C1H.A.
351, 2C20.B.601, 2C6P.A.1752, 1CBX.A.309, 2CEA.E.1603, 4CVT.A.1159, 1D1T.C.407,
1D8D.A.1001, 1DCE.B.900, 3DID.A.130, 3DSX.B.332, 3DYC.A.451, 3E2D.A.602, 3E30.B.
1001, 3E33.B.1001, 3E34.B.1001, 3E37.B.1001, 1EC5.B.50, 1EC5.C.50, 2EG8.A.400, 3
ELM.A.300, 1EW9.A.450, 1EZ2.A.402, 3F28.A.321, 3F2P.A.2005, 3FFZ.A.1300, 3FGD.A.
321, 4FMP.B.400, 1FT7.A.501, 4FU4.A.502, 1G05.B.801, 1G49.B.801, 3G42.A.500, 4G9
L.B.301, 3GIP.A.484, 4GTM.B.501, 4GTP.B.501, 4GTQ.B.501, 4GTR.B.501, 4GTV.B.401,
 2GVI.A.302, 4H2K.A.1001, 1HA5.C.4221, 1HR7.B.501, 1HYT.A.805, 1HZ5.A.103, 4I11.
A.502, 4I2F.A.603, 3I9F.A.1, 2IEJ.B.939, 1IGB.A.502, 3IQ6.B.201, 3IQ6.C.201, 2IX
D.A.1234, 1IY7.A.308, 2J13.A.1236, 4J5H.A.301, 1J9Y.A.1003, 4JBS.A.1008, 1JCS.B.
1001, 1JI3.A.402, 4JJJ.A.718, 1JML.A.102, 1K53.A.1003, 1KAP.P.613, 1KR6.A.405, 1
KRO.A.405, 1KS7.A.405, 3KWO.C.162, 4L9P.B.601, 4LCQ.A.602, 3LJZ.A.999, 3LPE.D.60
, 3LUB.B.302, 2LVH.A.101, 2M30.A.201, 3M4C.D.108, 1M5E.A.1705, 3M79.A.107, 3M79.
B.107, 3M79.C.107, 3MDJ.A.1000, 1MMP.A.2, 1N4Q.B.378, 1N4Q.L.378, 3NKR.A.1002, 3
NKN.A.1002, 1O1S.B.1001, 4O3A.B.303, 1OEZ.W.1154, 4OKO.A.401, 4ONG.H.302, 4OTE.B
.302, 2OW0.A.444, 2OY2.A.999, 3P24.A.999, 4P9C.J.201, 1PE5.A.317, 1PE7.A.317, 2P
IY.A.400, 2PIZ.A.400, 2PJ0.A.400, 2PJ7.A.400, 2PJA.B.500, 2PJB.A.400, 2PLI.C.701
, 4PPZ.A.601, 4PQA.A.404, 1PSZ.A.1000, 3PSQ.B.328, 4PUC.A.602, 3PZ1.B.332, 3PZ4.
B.1, 3Q75.B.521, 3Q78.B.521, 1QBQ.B.1000, 2QLA.C.501, 1QMU.A.999, 3QW0.A.500, 3Q
W0.B.500, 3QW0.D.500, 3QW1.C.500, 2R2D.A.277, 1R43.A.501, 2RFH.A.1308, 1RK6.A.60
1, 3S9C.A.503, 3SKS.A.568, 3TG0.A.501, 1TKJ.A.902, 4TLN.A.321, 1TLP.E.322, 1TMN.
E.322, 4TMN.E.322, 1TN6.B.1001, 1TN7.B.1001, 1TN8.B.1001, 1TNB.B.378, 1TNO.B.378
, 1TNU.B.378, 1TNY.B.378, 1TNZ.B.378, 3TOL.B.107, 3TS4.A.301, 3TT4.A.302, 3TVC.A
.501, 3UBF.A.1, 3V0A.A.1297, 3V77.A.302, 2V77.A.1042, 2V9E.A.1275, 3VH9.A.302, 2
VXI.A.201, 2VXX.C.202, 1W22.A.1375, 3W5K.B.501, 1WAA.A.1091, 1WAA.F.1091, 4WD8.A
.301, 4WD8.A.302, 3WOJ.A.805, 2WO9.C.1269, 2WOA.A.1270, 3WV3.A.301, 2X4H.A.1142,
 2X90.A.1618, 2X98.A.1476, 1XAF.B.507, 1XGE.A.400, 2XIG.C.1153, 2XR9.A.1867, 2XX
7.B.1264, 2Y28.A.1181, 2Y2B.B.1180, 2YJP.C.1270, 2Z2Y.C.2003, 2Z25.B.401, 2Z2D.A
.265, 1ZG9.A.400, 2ZIR.B.901, 2ZTG.A.902

[1] "Cluster 2"
4A7K.A.952, 3ADR.A.263, 3ASE.A.156, 3AYK.A.170, 3B8Z.A.901, 2BCN.A.295, 2BIB.A.1550, 3BUD.A.1048, 3C10.A.101, 2CEA.A.1603, 2CFU.A.1001, 4DHL.B.501, 2DI3.A.1002, 4DV8.A.801, 1E4C.S.999, 4E5V.B.401, 3EII.A.301, 2EK9.A.1004, 2FV9.B.4, 3G4K.A.801, 2GC2.A.401, 2GC3.A.402, 1GKP.B.1460, 2GMN.A.802, 3H8F.E.501, 3HC4.L.401, 2HD1.A.101, 4HGX.A.301, 3HR1.A.1, 1HTY.A.1102, 4HTZ.B.1001, 2ICS.A.400, 4ICQ.A.501, 4IGN.B.401, 1ITU.A.402, 2J0E.B.1265, 4JAA.A.501, 2JNE.A.200, 1JWQ.A.1001, 4JX5.D.1103, 3K5X.A.402, 2K78.A.151, 1K9Z.A.402, 4KBP.A.439, 3KR5.E.1001, 4KYH.A.202, 1LCP.A.489, 3LL8.A.505, 2LQ6.A.402, 3LS6.A.303, 2LZE.A.101, 1ML2.A.296, 4N27.A.201, 4N27.C.201, 4N7K.L.301, 4N7K.L.307, 4N7K.M.401, 4N7K.M.402, 4N7L.M.402, 3N9R.A.308, 3NO5.C.275, 3O64.A.1, 4OK2.A.801, 1OS9.A.901, 2OUN.B.404, 2OW6.A.3001, 2OW7.A.5001, 4PKT.A.802, 4PKW.A.801, 1Q3K.B.300, 1Q74.A.304, 3Q9B.A.345, 3QAY.A.180, 2QFR.A.434, 1QIP.A.902, 1QIP.D.903, 4R6T.A.1003, 4R6T.D.1001, 4R76.A.1001, 4R7M.C.1001, 4R7M.J.1003, 3S2J.A.402, 3S2L.A.402, 3S2M.A.402, 3S2M.A.403, 3S2N.A.402, 3S2N.A.403, 3SFW.A.501, 1T64.A.388, 1TQU.A.1400, 1TQW.A.1400, 3V93.D.701, 3VH9.A.301, 1VKG.B.400, 2VQM.A.1411, 3VUS.A.401, 2W3Z.A.1312, 2WEY.A.1772, 4X2T.A.702, 1XJS.A.150, 1XP3.A.301, 1Y13.A.174, 1Y93.A.264
[1] "Cluster 3"
4BLB.D.910, 4CPA.I.308, 1F30.B.201, 1F30.E.201, 4FUK.B.402, 2GA6.D.998, 3H90.C.2, 4IOD.A.502, 4KOD.B.203, 4KJG.A.1001, 2LQ6.A.401, 3M4B.B.108, 4MCS.A.814, 2MLS.A.302, 1NL4.B.500, 3OOF.A.302, 1P6D.A.247, 1Q3A.B.469, 1RLY.A.61, 3U94.B.259, 2V8V.A.1456, 3V94.D.702, 1ZEB.A.901
[1] "Cluster 4"
2JZW.A.57, 2L1G.A.88, 4LJ0.B.502, 4OND.E.101, 4QCL.A.1303, 1QUM.A.302, 1A7T.A.252, 2A7M.A.252, 4A7Y.A.951, 1AH7.A.246, 1AH7.A.248, 2AIO.A.315, 1AST.A.999, 4ASQ.A.1615, 4ASR.A.1615, 1B8J.A.451, 1BIW.B.801, 4BIN.A.500, 3BJC.A.876, 4BJH.A.423, 4BXK.A.1620, 4C24.A.301, 3C52.A.401, 4C6L.A.2824, 4C8I.B.1161, 2CHI.A.212, 3CKI.A.501, 3CQJ.A.285, 4CWM.A.433, 4CXO.A.453, 4CXV.A.433, 3D0N.A.262, 1D5J.A.301, 4DEL.A.402, 3DHA.A.255, 4DLM.A.401, 2DVT.A.1501, 2DVU.A.1501, 2DVX.A.1501, 4DYK.A.501, 4DZH.A.504, 2E2D.A.500, 3E38.A.1, 3EBH.A.1, 2EG6.A.400, 2EG7.A.401, 4ENL.A.438, 6ENL.A.438, 3EWC.A.372, 3EWD.A.371, 2EWB.A.488, 1EYW.A.401, 4EYF.A.302, 4EYF.A.303, 1F0J.A.1101, 1FA5.A.1201, 3FDK.A.401, 2FGN.A.248, 1FKX.A.400, 4GBD.A.502, 1GKP.A.1461, 2GSO.A.1000, 1GVF.A.288, 1GYT.G.600, 1GYT.G.601, 4H01.A.601, 4H1Q.A.301, 4H2E.A.301, 4H2G.A.601, 1H4N.A.262, 1H9N.A.262, 2HBV.A.401, 2HBM.A.1001, 3HC8.A.864, 2HC9.A.701, 3HK9.A.431, 3HKA.A.430, 2HUC.A.248, 3HWP.A.295, 1HZY.B.401, 4IE0.A.601, 4IE6.A.601, 4IG2.A.401, 4ILW.D.301, 4ISM.A.201, 3ITC.A.501, 3ITC.A.502, 2J0T.A.1267, 4J4K.A.402, 4J5H.A.302, 1J79.A.400, 1J79.B.400, 2J9A.A.1493, 1JCZ.A.901, 4JD1.A.201, 4JH8.A.201, 4JH8.B.201, 1JIW.P.481, 2JIG.A.1252, 2JNE.A.150, 4JS6.A.301, 1K07.B.3, 3K2G.A.400, 1K4P.A.1004, 1KAE.B.1102, 4KAP.A.301, 1KEQ.A.280, 4KEQ.A.302, 1KMG.A.154, 3KM8.A.400, 3KNS.A.228, 3KRY.A.1999, 4KTX.A.501, 3L6N.A.301, 1LAM.A.488, 4LCQ.A.601, 4LE6.A.405, 4LE6.A.406, 4LEF.A.301, 2LFF.A.500, 3LGG.B.512, 3M4C.D.109, 3MA2.D.294, 3MA2.A.294, 3MDU.A.454, 3MJM.B.401, 3MKV.B.425, 3MTW.A.1, 3MVI.A.901, 3N2C.A.425, 3N9S.A.309, 2NQH.A.302, 2NZE.A.401, 3O2X.A.1999, 2O4Q.A.2401, 4O98.B.401, 2OB3.A.901, 1ONW.A.800, 2OW1.A.444, 1P5X.A.248, 1P6B.A.401, 1P6D.A.246, 1P6D.A.248, 1PBO.A.1302, 1PL6.A.402, 3PNU.A.337, 1PV9.A.401, 4PVO.A.402, 2Q02.C.300, 3Q4R.A.201, 2Q5B.C.107, 3Q6X.A.3, 3Q6X.B.271, 4QA5.A.401, 4QA6.A.401, 3QDF.A.266, 1QF0.A.320, 1QF1.A.320, 1QTW.A.303, 2QVV.A.403, 1QXL.A.400, 2R2D.A.278, 1R3N.A.500, 3RHG.A.367, 4RL2.A.301, 4RL2.B.302, 3SEY.E.373, 1SHN.B.482, 3SNG.A.401, 3SNG.A.402, 3SXK.A.4, 3TOM.B.108, 4TYT.A.301, 3U79.D.110, 1UEA.C.1, 1UIO.A.400, 2USH.A.601, 2USH.A.602, 1UXA.B.1367, 2UX1.A.1174, 3V96.B.301, 1VFL.A.501, 3VQZ.A.302, 3VTG.A.301, 3W52.A.311, 3WAX.A.912, 3WAY.A.911, 4WB7.A.502, 1WPP.A.602, 2WXU.A.1375, 2WXT.A.1371, 1WY2.A.406, 2XF4.A.1211, 2XL9.B.1269, 1XM8.A.700, 2XS3.A.999, 2XS4.A.999, 2Z24.A.400, 2Z24.B.400, 2Z25.A.400, 2Z25.B.400, 2Z26.A.401, 2Z27.A.400, 2Z27.B.400, 2Z28.A.400, 2Z28.B.

400, 2Z29.A.400, 2Z29.B.400, 2Z2A.A.400, 2Z2B.A.338, 1Z60.A.1, 2Z72.A.401, 2ZBM.A.401, 2ZNE.B.991, 2ZO4.A.319, 3ZU0.A.1596, 3ZU0.B.1589, 2ZWR.A.208, 2ZWR.A.209, 1ZZM.A.401, 1ZZM.A.402
[1] "Cluster 5"
4A37.A.376, 3A52.A.1001, 4A69.A.500, 4AWZ.A.3230, 3B4N.A.711, 3B4N.B.715, 1B57.A.361, 4B6Z.C.385, 3B7S.A.701, 1BA9.A.154, 3BKK.A.701, 3BKL.A.701, 1BLL.E.488, 4BLB.A.910, 1BOR.A.58, 2BO9.A.999, 2BP8.B.1340, 1BYF.B.302, 4BZS.A.701, 4C2N.A.701, 4C2O.A.1629, 1CG2.A.500, 1CG2.A.501, 3CHO.A.701, 2CLB.A.1175, 4COG.A.401, 4COQ.A.299, 1CPX.A.308, 5CPA.A.308, 4CVR.A.1159, 4CVT.A.1158, 1DO5.B.29, 3E32.B.1001, 1ED9.A.450, 3EER.A.2001, 1ELX.B.451, 3EQN.A.757, 2EV6.B.2151, 3F3Q.A.104, 1F57.A.310, 4F7O.A.301, 3FB4.A.217, 3FU1.B.201, 3FVL.A.1309, 2GFK.A.401, 2GFJ.A.401, 4GM5.A.401, 2GSN.A.1001, 4GTO.B.501, 3GZE.A.1, 4H49.A.301, 1HFS.A.257, 2HH5.A.702, 1HI9.A.300, 1HLK.B.1004, 2HSE.B.954, 3HTR.B.118, 2I0O.A.580, 2I3C.A.314, 4IAV.A.401, 1IF6.A.262, 4IHM.A.402, 3IT7.A.183, 2JBJ.A.1752, 1JCQ.B.1001, 1JJE.A.251, 1JJT.A.251, 4JJI.A.402, 2JLP.B.226, 2K2C.A.140, 4K7W.A.102, 4K90.A.701, 2KBX.B.299, 1KH4.A.450, 3KS3.A.262, 1L1O.C.1, 4L2L.A.701, 3L3N.A.701, 1LD7.B.1001, 1LD8.B.1001, 1LGD.A.262, 1LND.E.800, 4LNB.B.602, 4LQY.A.506, 3LTV.A.1001, 4LW9.A.201, 4LW9.I.201, 4LW9.R.201, 3M2Z.A.500, 2M30.B.201, 1M4L.A.1308, 4MBG.B.602, 3MK1.A.902, 4MKH.A.301, 4MKT.A.701, 3MO2.D.6, 4MRQ.A.501, 4MSM.C.501, 3MWM.B.142, 1MXD.A.727, 1MZC.B.1001, 4N07.B.305, 3N21.A.401, 4NGE.A.1101, 4NGE.E.101, 3NJ9.A.262, 3NKQ.A.1002, 3NKN.A.1001, 2NQH.A.301, 2NQH.A.303, 4NTK.A.201, 1NW2.D.6004, 1NW2.F.6006, 4NYY.B.501, 4O3A.A.303, 1O86.A.701, 1O8A.A.701, 3OHL.A.4, 4OJA.A.202, 4OXD.C.301, 2P2L.A.901, 2PJC.A.400, 4PQA.A.403, 3PSQ.A.326, 2PVV.A.1752, 4PXY.A.302, 3Q7A.B.521, 2QPJ.A.1, 1R3N.A.501, 3RF4.A.118, 2RH6.A.1, 1S63.B.1001, 1S64.B.378, 1SA4.B.439, 1SA5.B.438, 3SFX.B.521, 3SFY.B.521, 1SHN.A.485, 3SIO.A.362, 1T0A.A.661, 1T3A.B.422, 3TGN.B.147, 3U24.A.595, 1U7J.A.50, 1U7M.A.54, 3U9W.A.2001, 3UCT.B.102, 1UUP.A.2222, 1UZE.A.701, 3V1E.A.101, 2V9I.A.1277, 2VJ8.A.1611, 2VXI.A.202, 2W5V.A.1377, 2W5X.B.1378, 2W57.B.202, 3W6H.A.302, 3WBH.A.505, 4WCM.A.538, 4WD6.A.301, 4WNC.O.402, 3WS9.B.801, 3WXC.A.301, 1WY2.A.405, 2X91.A.1619, 2X92.A.1615, 2X94.A.1616, 2X97.A.1616, 4XIX.A.401, 2XIG.B.1150, 1XP3.A.302, 2XX0.B.1340, 2XXG.A.1339, 2Y3D.B.149, 1ZED.A.904, 1ZG7.A.400, 1ZG8.A.400, 2ZIS.B.901, 2ZKW.A.401, 3ZXO.A.1579
[1] "Cluster 6"
3A1Z.A.226, 4A94.B.501, 3AAK.A.992, 2AIG.P.999, 2ANP.A.501, 2ANP.A.502, 1ATL.A.401, 3AYU.A.415, 3B35.A.292, 3B3C.A.501, 3BHX.A.1751, 3BIO.A.1751, 3BI1.A.1751, 1BQO.B.301, 3C0Z.B.101, 2C6P.A.1751, 2C6C.A.1751, 1CGL.A.301, 1CP7.A.901, 1CP7.A.902, 6CPA.A.308, 7CPA.A.308, 8CPA.A.308, 3CSQ.B.335, 4D0Y.B.1239, 3D10.A.95, 1D1S.B.406, 3D4U.A.309, 3DFM.A.401, 1DK4.B.591, 2DKD.A.921, 4DPR.A.701, 4DR9.A.201, 2DSN.A.2001, 4DWZ.A.302, 4DYO.A.502, 1DZW.P.999, 1E48.S.999, 1E49.S.999, 2E46.A.172, 2EG7.A.400, 3EHY.A.264, 4EJ5.A.501, 2EK8.A.1002, 2EK9.A.1002, 3EWJ.B.2, 3FH4.A.301, 3FJU.A.999, 1FUA.A.216, 3FUK.A.701, 4FYQ.A.1012, 4FYR.A.1013, 2GA3.A.450, 1GE7.B.200, 4H82.A.301, 3H90.B.294, 1HDU.E.1308, 1HEE.E.1308, 2HIH.A.601, 1HR6.B.501, 4ICQ.B.502, 4ICR.A.502, 4IE7.A.601, 2IQ6.A.293, 4IXN.A.401, 1J2U.A.301, 2JAZ.B.600, 4JBS.B.1009, 1JK9.C.302, 2JT5.A.257, 4JYW.A.801, 4JZO.A.801, 4K5L.A.1101, 4K5M.A.1101, 4K5O.A.1103, 4K5P.A.1101, 4KA7.A.801, 4KAY.A.601, 1KBE.A.1, 4KFT.C.303, 1KH5.A.450, 2KIK.A.50, 3KNS.A.229, 1L0Y.B.702, 3L0T.B.2, 1LFW.A.1001, 2LFF.A.502, 3LQ0.A.999, 4LQG.A.801, 4LTE.A.1101, 3M3B.A.156, 3M52.B.116, 1MMP.A.1, 4NAQ.A.1027, 3NKO.A.1002, 1NPC.A.323, 4O2I.A.401, 4O3A.B.302, 4ON1.A.400, 4ONX.B.201, 2OR4.A.1751, 2PJ3.A.400, 2PJ5.A.400, 2PJ8.B.500, 2PVW.A.1751, 3PW3.A.406, 3Q4R.A.202, 4Q7R.A.307, 3Q9F.B.344, 3QBU.A.294, 2QLA.A.500, 2QLA.B.502, 2QLA.D.503, 1R42.A.804, 2RJP.A.1, 1ROS.A.400, 1RTQ.A.702, 3RYM.B.107, 1S0E.A.1291, 3SJX.A.1751, 1SQM.A.1001, 3SZZ.A.502, 1TF9.A.902, 3TG0.A.502, 1TKH.A.902, 1TKJ.A.901, 3TOL.D.107, 3U93.B.259, 2V29.A.1274, 2V29.A.1275, 3VAT.A.501, 1VKG.A.400, 2VQQ.A.1411, 3WAW.A.913, 3WE7.A.301, 2WKN.B.412, 2WO8.A.1268, 1WUO.A.301, 2X93.

A.1615, 2X98.A.1475, 1XJO.A.902, 2XPY.A.1673, 1YHC.B.602, 2YJP.A.1272
[1] "Cluster 7"
830C.A.272, 456C.A.272, 4A3W.A.1159, 4A7Z.A.950, 1A85.A.999, 1A86.A.999, 2A8H.A.
486, 2AB7.A.30, 1AF0.A.472, 4AWY.B.3229, 4AX0.B.3229, 4AX1.B.3229, 1B3D.B.301, 1
B57.A.360, 3B7U.X.701, 2BH3.A.1002, 2BIB.A.1549, 3BKQ.X.500, 2BMI.A.272, 2BNN.B.
1200, 2BNO.A.1201, 1BS8.A.2001, 4BT4.A.301, 4BT5.A.301, 4BT6.A.1257, 3BUB.A.1047
, 3BUI.A.1046, 3BVT.A.1048, 3BVU.A.1048, 3BVV.A.1047, 3BVW.A.1046, 3BVX.A.1046,
4BZ3.B.502, 4BZ5.A.500, 4BZR.A.1630, 1C3R.A.501, 1C3S.A.951, 4C5W.A.402, 4C6L.A.
2823, 1CAM.A.262, 2CA2.A.264, 4CA5.A.1001, 4CA6.A.1001, 4CA7.A.1616, 4CA8.A.1620
, 3CV5.A.1047, 1CXV.A.1, 3CZN.A.1102, 3CZS.A.1102, 2D1N.A.270, 2D1O.A.257, 1D7X.
B.801, 2DDY.A.177, 4DD8.A.1002, 4DEF.A.401, 4DPE.A.301, 1DQS.A.402, 3DWB.A.771,
2DW0.A.700, 3E0L.A.1452, 3E4A.B.2000, 3EBG.A.1, 3EDZ.B.2, 1EI6.B.409, 3ELF.A.352
, 2ERP.A.700, 3F15.A.264, 3F16.A.264, 3F17.A.264, 3F18.A.264, 3F19.A.264, 3F1A.A
.264, 1FA5.A.1200, 1FLS.A.166, 4FL7.A.301, 4FUA.A.216, 2FV5.A.3, 2FVM.D.601, 4FW
3.B.300, 4FW4.C.301, 4FW5.A.301, 4FW7.A.301, 2FYV.A.2003, 4FYT.A.1012, 4G9L.B.30
2, 1GKR.A.1453, 4GK8.A.302, 2GO4.A.601, 4H3X.A.301, 1H48.C.900, 4H76.A.301, 2HC9
.A.702, 1HFC.A.275, 1HJK.A.451, 3HK5.A.430, 3HK8.A.430, 1HOV.A.166, 2HPT.A.950,
1HS6.A.701, 4HWO.A.701, 4HWP.A.701, 4HWR.A.701, 4HWS.A.701, 3HY7.A.901, 3HY9.A.9
01, 3HYG.A.901, 1HZY.A.402, 2I47.C.804, 3ID7.A.401, 4IE4.A.601, 2ILP.A.500, 4J5F
.A.301, 2J83.A.999, 1JAP.A.999, 1JAQ.A.999, 4JE7.A.202, 2JIH.B.1554, 1JJE.A.252,
1JJT.A.252, 1JK3.A.400, 4JP4.A.301, 1JPU.A.371, 2JSD.A.276, 2JT5.A.256, 2JT6.A.
256, 2K4W.A.156, 1KBC.A.999, 3KDS.E.996, 3KR5.G.1004, 1KYS.A.301, 1LAM.A.489, 3L
JT.A.901, 3LK8.A.264, 4LP6.A.302, 4LV4.A.401, 3MAX.A.379, 4MCA.B.1000, 1MH2.A.20
1, 1MMB.A.999, 1MNC.A.281, 3N2U.A.264, 3N2V.A.264, 3NX7.A.264, 3NXQ.A.650, 3O90.
B.192, 2OC2.A.701, 3OD4.A.1350, 3OHL.A.5, 3OHO.A.5, 2OKL.A.601, 4OPN.A.201, 4OPN
.B.201, 4OUI.A.501, 3P3C.A.401, 1P6E.A.248, 3P76.A.274, 2PAJ.A.493, 2PJT.A.302,
2PLM.A.407, 1PMI.A.445, 3PN4.A.1001, 1PS6.A.330, 1PTW.A.501, 1PVW.A.401, 3Q2G.A.
401, 3Q2H.A.401, 1Q3K.B.301, 2Q5B.A.107, 4QA0.A.401, 4QA1.A.403, 4QA2.A.403, 4QA
4.A.502, 1QH5.A.262, 1QIN.A.401, 1QIN.B.301, 3QIZ.A.431, 1QJJ.A.250, 1R43.A.500,
1RJ5.A.601, 2RJQ.A.1, 1RM8.A.500, 1RMZ.A.264, 3RTS.A.264, 3RTT.A.264, 1SML.A.27
0, 3SPU.C.1010, 3T00.A.501, 3T00.A.502, 2TCL.A.170, 5TLN.A.321, 1TQS.A.1401, 1TQ
T.A.1301, 1TQV.A.1300, 3U04.A.200, 1U7J.B.150, 1U7M.B.154, 3U79.E.110, 3UHM.A.30
0, 1URA.A.451, 3UWB.A.200, 2UX1.C.1173, 2V5W.A.1380, 2V5X.A.1377, 3V77.A.301, 2V
9G.A.1276, 2V9M.A.1275, 2VES.A.1295, 3VPE.A.302, 2VUN.A.401, 2W0D.A.1264, 2W15.A
.1203, 4WD6.B.302, 2WM1.A.1333, 2WO9.B.1268, 4X2T.B.1002, 1XBU.A.901, 1XBU.A.902
, 2XHM.A.1616, 1XXW.A.201, 2XYD.A.1620, 2Y6D.A.1268, 1Y7W.A.283, 2YD0.A.1946, 1Y
QY.A.781, 2Z3I.C.2003, 2Z72.A.402, 2ZBM.A.402, 1ZNB.A.2, 3ZU0.A.1595, 3ZU0.B.158
8, 1ZXC.A.201, 3ZXH.A.300

Table S11. 6-ligand Zn, normal group

	size	largest_angle*	middle_1*	middle_2	middle_3	middle_4
1	"22"	"175.3+/-2.5"	"76.6+/-2.7"	"81.1+/-2.7"	"84.1+/-2.2"	"87.2+/-2.1"
2	"31"	"170+/-3"	"74.6+/-2.9"	"81.2+/-3.4"	"84.5+/-2.7"	"87.2+/-1.7"
3	"27"	"176.3+/-2.1"	"81.4+/-3"	"85.2+/-1.8"	"86.9+/-1.5"	"88.3+/-1"
4	"14"	"165.8+/-3.7"	"70.4+/-4.2"	"77.3+/-6.3"	"83.4+/-4.7"	"85.9+/-2.9"
5	"27"	"175.7+/-1.5"	"83.4+/-2.5"	"85.2+/-2.2"	"86.8+/-2"	"88.3+/-1.4"
6	"29"	"170.2+/-3.4"	"82.8+/-2"	"84.9+/-1.6"	"86.5+/-1.5"	"88+/-1.4"

	middle_5*	middle_6	middle_7	middle_8	middle_9*
1	"89.4+/-1.4"	"91.6+/-1.8"	"93.8+/-1.8"	"95+/-1.8"	"97.2+/-1.9"
2	"89.5+/-1.5"	"90.8+/-1.8"	"92.6+/-1.6"	"94.2+/-2"	"96.6+/-2.1"

3	"89.3+/-1.1"	"90+/-1.1"	"91.9+/-0.9"	"93.3+/-1.1"	"94.4+/-1.4"
4	"87.5+/-2.7"	"90.7+/-2.9"	"93.3+/-2.6"	"96.5+/-2.6"	"101+/-5.8"
5	"89.7+/-1.3"	"91+/-1.1"	"92.6+/-1.4"	"93.7+/-1.5"	"94.8+/-1.6"
6	"89.5+/-1.5"	"91+/-1.4"	"92+/-1.3"	"93.3+/-1.3"	"94.5+/-1.4"

	middle_10	middle_11	middle_12	middle_13*
1	"101.1+/-2.8"	"106.9+/-6.4"	"155.3+/-5.4"	"164.6+/-3.6"
2	"99.7+/-2.9"	"104+/-4.5"	"160.8+/-4.4"	"165.9+/-2.5"
3	"96.1+/-2.2"	"98.5+/-2.9"	"169.4+/-4.1"	"173+/-2.2"
4	"105+/-5.5"	"112.5+/-8.3"	"149.8+/-8.9"	"159.2+/-4.7"
5	"96.7+/-1.9"	"99.4+/-2.9"	"165.5+/-4.8"	"172.9+/-2.2"
6	"98.2+/-2.4"	"105+/-4.3"	"160.3+/-3.6"	"166.1+/-3.1"

	smallest_opposite_angle*	Octahedral	TrigonalPrismatic
1	"73+/-3.7"	"0.127"	"0"
2	"81.4+/-2.4"	"0.192"	"0"
3	"85.1+/-1.7"	"0.564"	"0"
4	"73.1+/-3.3"	"0.004"	"0.002"
5	"78+/-2.7"	"0.271"	"0"
6	"72.1+/-2.5"	"0.083"	"0"

	PentagonalBipyramidalVA	PentagonalBipyramidalVP
1	"0"	"0.013"
2	"0"	"0.006"
3	"0"	"0.001"
4	"0"	"0.053"
5	"0"	"0.001"
6	"0"	"0.002"

Table S12. Cluster members of 6-ligand Zn, normal group

[1] "Cluster 1"
1BLL.E.489, 1DE5.A.450, 3E8R.B.2, 3IBM.A.200, 4IE5.A.601, 3ISI.X.3001, 3IVT.A.50
0, 3KMC.B.2, 3KME.B.2, 3KR5.A.1001, 3LGP.A.1, 4NZ3.A.501, 3O64.B.485, 4OJV.A.404
, 2OQL.A.401, 3QAY.D.180, 1R55.A.201, 4R6T.B.1003, 4R6T.F.1001, 4R7M.C.1003, 1SN
N.A.402, 3W0T.B.201
[1] "Cluster 2"
2ALW.A.5001, 4BBP.A.1316, 2BNO.B.1201, 3DDG.A.3001, 2DDF.A.1, 4DOO.A.502, 3DX2.A
.1046, 3EJP.A.1047, 3EJQ.A.1047, 3EJR.A.1047, 3EJU.A.1047, 2F7O.A.5001, 2F7Q.A.5
001, 2F92.F.1003, 2FQP.B.100, 4H1S.A.603, 3HPS.A.701, 1IM5.A.400, 1KQ3.A.401, 3L
X3.A.201, 3O93.A.192, 2OB3.B.904, 1PTM.A.330, 4QGE.A.602, 4R76.E.1001, 4TO8.A.30
1, 2V9N.A.1275, 3W0T.A.201, 2WHG.B.1263, 4X2T.G.702, 2Y33.A.900
[1] "Cluster 3"
2EK8.A.1004, 1ENQ.A.238, 2F92.F.1002, 2F94.F.1001, 2F94.F.1003, 4GQT.A.501, 3HDZ
.A.864, 1HQA.B.452, 3NO5.A.275, 4NT9.A.301, 3O91.A.192, 2OU3.A.161, 2OUP.B.777,
2P2L.A.201, 2PTZ.A.501, 2PU1.A.501, 2QYM.A.1, 2QYK.A.1, 1T9S.A.1, 1TB7.A.1001, 1
TBF.A.1, 1U74.A.1001, 3V93.A.701, 2WTA.A.1215, 1XM6.A.1001, 1Y2K.A.1001, 1ZKL.A.
501
[1] "Cluster 4"
3D4Y.A.1047, 1DK4.B.590, 3E49.B.500, 2I57.D.507, 4JDG.A.401, 4M6R.B.301, 4OJX.A.
404, 1OS9.F.926, 1QH3.A.262, 4R76.A.1003, 4RL0.B.302, 3V77.D.301, 4X2T.A.701, 2Z
ZW.A.362
[1] "Cluster 5"
3DZA.C.501, 3DZA.C.505, 2F92.F.1001, 2F9K.F.1001, 2F9K.F.1002, 2FUQ.A.1, 4GBD.A.

503, 1GT7.A.275, 2H44.A.501, 3LLX.A.376, 1M60.A.105, 4NPW.A.1001, 3O2G.A.388, 4O
K4.A.800, 2OUV.A.777, 4OV9.A.401, 2PTY.A.501, 2PTZ.A.500, 2PW3.A.501, 2R2V.C.35,
 1RRM.B.387, 3SL3.B.9, 1TB5.A.1001, 3U43.B.135, 3UUO.B.772, 1XOR.A.1001, 1Y9Q.A.
202
[1] "Cluster 6"
3BLB.A.1047, 3D4Z.A.1046, 3D52.A.1046, 3D51.A.1046, 3DDF.A.3001, 3DX1.A.1048, 3D
X3.A.1047, 3DX4.A.1047, 3E38.A.2, 2F18.A.1805, 2F1A.A.1805, 2F1B.A.1804, 2F7P.A.
5001, 2F7R.A.5001, 3GWT.A.504, 3ITU.A.1, 1KAE.A.1101, 1KRM.A.501, 1LCP.A.488, 4N
UR.A.701, 3OOJ.A.1, 4OJV.A.403, 4OJX.A.403, 2P18.A.301, 1PTM.B.331, 2PU1.A.500,
1R33.A.1163, 3RCQ.A.1, 1SR9.B.703

Table S13. 6-ligand Zn, combined group

	size	largest_angle*	middle_1*	middle_2	middle_3	middle_4
1	"50"	"172+/-3.8"	"81.1+/-3.5"	"83.9+/-2.6"	"85.8+/-1.9"	"87.5+/-1.9"
2	"49"	"171.8+/-3.2"	"80.4+/-3.2"	"84.5+/-2.1"	"87+/-2"	"88.6+/-1.6"
3	"55"	"176.1+/-1.9"	"81.8+/-3"	"85+/-2.1"	"86.8+/-1.8"	"88.2+/-1.3"
4	"55"	"164.2+/-4.1"	"70.8+/-6"	"79.6+/-5.3"	"84.7+/-4.3"	"88.1+/-3.6"
5	"44"	"169.3+/-3.7"	"72.4+/-4.3"	"80+/-4"	"84.1+/-3.3"	"87.1+/-2"
6	"21"	"150.2+/-9.4"	"55.1+/-6.5"	"73.5+/-11.9"	"81.9+/-7.1"	"87.1+/-5.7"
7	"24"	"160.4+/-5.9"	"56.9+/-3.2"	"70.9+/-8.4"	"83+/-4.8"	"88.2+/-3.5"

	middle_5*	middle_6	middle_7	middle_8	middle_9*
1	"89.3+/-1.7"	"91.2+/-1.6"	"92.6+/-1.7"	"93.9+/-1.6"	"95.7+/-2.4"
2	"90.2+/-1.8"	"92.2+/-1.6"	"94.1+/-1.8"	"96.2+/-2.2"	"98.2+/-2.5"
3	"89.4+/-1.2"	"90.6+/-1.2"	"92.2+/-1.2"	"93.6+/-1.3"	"94.7+/-1.6"
4	"90.5+/-2.9"	"92.9+/-3.2"	"95.9+/-2.6"	"98.4+/-3.2"	"100.9+/-3.6"
5	"89.1+/-1.8"	"90.9+/-2"	"92.9+/-1.9"	"94.8+/-2.3"	"97.4+/-3"
6	"90.2+/-4.2"	"93.9+/-5.1"	"97.4+/-5.5"	"102.6+/-4.3"	"110+/-6.1"
7	"90.3+/-3.2"	"93.6+/-3.4"	"97.3+/-3.8"	"101.4+/-4.4"	"103.8+/-4.7"

	middle_10	middle_11	middle_12	middle_13*
1	"99.4+/-3.1"	"105.4+/-5.5"	"158.5+/-5.1"	"166+/-3.6"
2	"100.6+/-2.4"	"109.3+/-5.3"	"150+/-5.6"	"157.8+/-4.6"
3	"96.5+/-2.1"	"99.1+/-2.9"	"167.4+/-4.8"	"172.6+/-2.6"
4	"104.2+/-4.5"	"114.4+/-8.3"	"143.5+/-7.4"	"153.5+/-4.9"
5	"101.1+/-3.7"	"106.3+/-5.5"	"157.6+/-7.6"	"164.8+/-2.9"
6	"115.9+/-6.6"	"120.9+/-6.8"	"133.1+/-6.2"	"140.9+/-6.1"
7	"107.8+/-6.3"	"113.2+/-9.1"	"145.3+/-8.6"	"153.4+/-5.6"

	smallest_opposite_angle*	Octahedral	TrigonalPrismatic
1	"71.9+/-2.8"	"0.086"	"0"
2	"58.2+/-3.2"	"0.024"	"0"
3	"82+/-3.6"	"0.439"	"0"
4	"57.7+/-4.2"	"0.006"	"0.004"
5	"79.4+/-4.1"	"0.125"	"0"
6	"56.8+/-8.3"	"0"	"0.116"
7	"79.3+/-5.8"	"0.008"	"0.003"

	PentagonalBipyramidalVA	PentagonalBipyramidalVP
1	"0"	"0.013"
2	"0"	"0.002"
3	"0"	"0.001"
4	"0.005"	"0.068"
5	"0"	"0.008"

6 "0" "0.004"
7 "0.003" "0.01"

Table S14. Cluster members of 6-ligand Zn, combined group

[1] "Cluster 1"
3BLB.A.1047, 1BLL.E.489, 3D4Z.A.1046, 3D52.A.1046, 3D51.A.1046, 3DDF.A.3001, 1DE5.A.450, 3DX1.A.1048, 3DX3.A.1047, 3DX4.A.1047, 3E38.A.2, 2F18.A.1805, 2F1A.A.1805, 2F1B.A.1804, 2F7P.A.5001, 2F7R.A.5001, 3GWT.A.504, 3IBM.A.200, 3ISI.X.3001, 3ITU.A.1, 3IVT.A.500, 1KAE.A.1101, 3KMC.B.2, 3KME.B.2, 3KR5.A.1001, 1KRM.A.501, 3LGP.A.1, 4NPW.A.1001, 4NUR.A.701, 3OOJ.A.1, 3O64.B.485, 4OJV.A.403, 4OJV.A.404, 4OJX.A.403, 2OQL.A.401, 2P18.A.301, 1PTM.B.331, 2PTZ.A.500, 2PU1.A.500, 3QAY.D.180, 2QF7.A.1157, 1R33.A.1163, 1R55.A.201, 4R6T.B.1003, 4R7M.C.1003, 3RCQ.A.1, 3SL3.B.9, 1SNN.A.402, 1SR9.B.703, 4X2T.A.701
[1] "Cluster 2"
3B3C.A.502, 2C1G.A.1465, 3C88.A.450, 3C8A.A.450, 3C8B.A.450, 2CB8.B.1090, 4CWM.A.432, 4CXV.A.432, 3DDA.A.450, 3DFK.A.300, 1EB6.A.178, 4ELC.A.501, 1FT7.A.502, 4GSZ.A.402, 1GW6.A.1615, 4ICQ.A.502, 2IWO.A.1255, 3K5X.A.403, 1LCP.A.488, 1LFW.A.1002, 3MK1.A.901, 4MTU.A.201, 1MZB.A.201, 4NGM.A.818, 4NGQ.A.817, 4NGT.A.811, 4NY2.A.501, 4O5O.A.301, 2OUN.A.402, 2OUQ.A.402, 1P5X.A.246, 2PTY.A.500, 4Q3J.A.401, 3QIY.A.431, 3QM3.A.355, 3QW7.A.501, 3QW8.A.501, 1R1J.A.1001, 1ROR.A.601, 3S2L.A.403, 1SDX.A.677, 3T3W.A.301, 2V9L.A.1275, 3VPB.A.503, 4WB7.A.503, 3WT4.A.502, 1XVX.A.315, 1ZED.A.903, 2ZXC.A.647
[1] "Cluster 3"
4BBP.A.1316, 3DZA.C.501, 3DZA.C.505, 2EK8.A.1004, 1ENQ.A.238, 2F92.F.1001, 2F92.F.1002, 2F94.F.1001, 2F94.F.1003, 2F9K.F.1001, 2F9K.F.1002, 2FUQ.A.1, 4GBD.A.503, 4GQT.A.501, 1GT7.A.275, 2H44.A.501, 3HDZ.A.864, 1HQA.B.452, 4IE5.A.601, 3LLX.A.376, 3LX3.A.201, 1M60.A.105, 3NO5.A.275, 4NT9.A.301, 3O2G.A.388, 3O91.A.192, 3O93.A.192, 4OK4.A.800, 2OUV.A.777, 2OU3.A.161, 2OUP.B.777, 4OV9.A.401, 2P2L.A.201, 2PTY.A.501, 2PTZ.A.501, 2PU1.A.501, 2PW3.A.501, 2QYM.A.1, 2QYK.A.1, 2R2V.C.35, 1RRM.B.387, 1T9S.A.1, 1TB5.A.1001, 1TB7.A.1001, 1TBF.A.1, 3U43.B.135, 1U74.A.1001, 3UUO.B.772, 3V93.A.701, 2WTA.A.1215, 1XM6.A.1001, 1XOR.A.1001, 1Y2K.A.1001, 1Y9Q.A.202, 1ZKL.A.501
[1] "Cluster 4"
3BON.A.500, 3BWI.A.450, 4CVR.A.1160, 4CVT.A.1160, 2DEA.A.402, 4DJ4.B.403, 1DK4.B.590, 3EZT.B.401, 4FW5.D.301, 4FW6.A.301, 4FW7.D.301, 3GBO.A.302, 3GJ9.B.127, 3GSH.A.101, 2GSO.A.1001, 4GTW.A.1010, 4GTW.A.1011, 2GYQ.A.407, 1H48.A.900, 2IMA.A.500, 2JT2.A.336, 1KH9.B.450, 3KR5.B.1004, 1LOY.D.704, 4L3T.B.1101, 4LCF.A.311, 4LCG.A.301, 4LCH.A.301, 3LE9.A.1, 1LOK.A.901, 1LOK.A.902, 4MCP.A.801, 4MCQ.A.801, 4MCR.A.801, 3MN8.A.999, 4OC1.A.814, 4OME.A.815, 3P3E.A.400, 3P3G.A.301, 2PLI.B.707, 4PPZ.A.602, 3PS1.A.301, 3PS2.A.301, 3PS3.A.301, 3QJ0.A.431, 1QTW.A.302, 1R1I.A.1001, 2RH6.A.2, 1SHN.B.481, 1TXR.A.501, 3U1Y.A.400, 3U79.H.110, 3V77.D.301, 3V94.A.702, 2ZZW.A.362
[1] "Cluster 5"
2ALW.A.5001, 2BNO.B.1201, 3D4Y.A.1047, 3DDG.A.3001, 2DDF.A.1, 4DOO.A.502, 3DX2.A.1046, 3E49.B.500, 3E8R.B.2, 3EJP.A.1047, 3EJQ.A.1047, 3EJR.A.1047, 3EJU.A.1047, 2F7O.A.5001, 2F7Q.A.5001, 2F92.F.1003, 2FQP.B.100, 4H1S.A.603, 3HPS.A.701, 2I57.D.507, 1IM5.A.400, 4JDG.A.401, 1KQ3.A.401, 4M6R.B.301, 4NZ3.A.501, 2OB3.B.904, 4OJX.A.404, 4OP4.A.302, 1PTM.A.330, 4QGE.A.602, 1QH3.A.262, 1QIP.B.901, 4R6T.F.1001, 4R76.A.1003, 4R76.E.1001, 4RLO.B.302, 4TO8.A.301, 2V9N.A.1275, 3WOT.A.201, 3WOT.B.201, 3WOU.A.201, 2WHG.B.1263, 4X2T.G.702, 2Y33.A.900

[1] "Cluster 6"
4BLL.A.1322, 4COQ.B.299, 1DK4.A.291, 4EWL.A.403, 1H8L.A.999, 1JJE.B.261, 3LMS.A.309, 1LNF.E.800, 4LQY.A.507, 4ONX.A.201, 2OOT.A.1751, 2OX8.A.4, 3PN6.B.202, 3R8B.B.122, 3R8B.D.122, 3R8B.F.122, 3R8B.P.122, 1UXB.A.1367, 2VME.A.501, 4X2T.E.701, 1YH8.A.501
[1] "Cluster 7"
1ANJ.A.450, 3BXM.A.1751, 4CBY.A.2034, 3DFF.A.274, 4DYO.A.501, 3E4A.A.2000, 4FW3.A.301, 1G12.A.200, 2G9Y.B.450, 2GO4.B.602, 1K07.A.2, 4L3T.A.1101, 4MN6.A.401, 1OS9.F.926, 3Q9B.E.345, 3RBU.A.1751, 1RTQ.A.701, 3SJG.A.1751, 1TXR.A.502, 2VQG.C.1092, 4WD8.C.302, 3WT4.A.501, 1XJO.A.901, 1YGD.A.142

Table S15. all-ligand-number Zn, normal group

	size	largest_angle*	middle_1*	middle_2*	middle_3*
1	"665"	"123.7+/-5.2"	"94.6+/-4.9"	"101.5+/-3.8"	"107.5+/-4.1"
2	"309"	"134.7+/-5.9"	"91+/-7.6"	"99.4+/-5.5"	"107.4+/-5.2"
3	"216"	"171.8+/-5.1"	"79.2+/-5.2"	"89.2+/-2"	"95.6+/-3.1"
4	"917"	"120.7+/-3.7"	"99.9+/-3.9"	"106.4+/-3.2"	"111.2+/-2.8"
5	"491"	"162.8+/-9.4"	"76.4+/-5.6"	"89.2+/-3.5"	"97.4+/-3.8"
6	"167"	"163.5+/-9.1"	"81.8+/-6.9"	"88.7+/-5.4"	"95.4+/-5.5"
7	"1269"	"115.8+/-2.8"	"103.2+/-3.2"	"106.6+/-2.1"	"109.2+/-1.9"

	middle_4*	smallest_opposite_angle*	Tetrahedral	TrigonalBipyramidal
1	"114.1+/-4"	"114.4+/-5.1"	"0.426"	"0"
2	"116.1+/-6.3"	"97.6+/-8.3"	"0.063"	"0"
3	"165.6+/-6.3"	"81.3+/-8.1"	"0.001"	"0.005"
4	"115+/-2.7"	"100.7+/-4.9"	"0.518"	"0"
5	"138+/-8.3"	"103.6+/-5.9"	"0.023"	"0.171"
6	"106.6+/-10.2"	"108.8+/-12.9"	"0.037"	"0.123"
7	"112+/-2.1"	"109.6+/-3.3"	"0.855"	"0"

	TrigonalBipyramidalVA	TrigonalBipyramidalVP	Octahedral	SquarePyramidal
1	"0.016"	"0"	"0"	"0"
2	"0.09"	"0"	"0"	"0"
3	"0.003"	"0.213"	"0.156"	"0.281"
4	"0.045"	"0"	"0"	"0"
5	"0.212"	"0.282"	"0"	"0.015"
6	"0.134"	"0.236"	"0"	"0"
7	"0.005"	"0"	"0"	"0"

	SquarePyramidalV	SquarePlanar	TrigonalPrismatic	TrigonalPrismaticV
1	"0"	"0"	"0"	"0"
2	"0.004"	"0"	"0"	"0"
3	"0.34"	"0.323"	"0"	"0.01"
4	"0"	"0"	"0"	"0"
5	"0.21"	"0.041"	"0"	"0.075"
6	"0.14"	"0.002"	"0"	"0.015"
7	"0"	"0"	"0"	"0"

	PentagonalBipyramidal	PentagonalBipyramidalVA	PentagonalBipyramidalVP
1	"0"	"0"	"0"
2	"0"	"0"	"0"
3	"0"	"0"	"0.006"
4	"0"	"0"	"0"
5	"0"	"0"	"0"

```
6 "0"                    "0"                       "0"
7 "0"                    "0"                       "0"
  SquareAntiprismatic SquareAntiprismaticV HexagonalBipyramidal
1 "0"                 "0"                  "0"
2 "0"                 "0"                  "0"
3 "0"                 "0"                  "0"
4 "0"                 "0"                  "0"
5 "0"                 "0"                  "0"
6 "0"                 "0"                  "0"
7 "0"                 "0"                  "0"
  HexagonalBipyramidalVA HexagonalBipyramidalVP
1 "0"                    "0"
2 "0"                    "0"
3 "0"                    "0"
4 "0"                    "0"
5 "0"                    "0"
6 "0"                    "0"
7 "0"                    "0"
```

Table S16. Cluster members of all-ligand-number Zn, normal group

```
[1] "Cluster 1"
3COQ.A.1002, 1F2I.G.1201, 3F2D.A.5, 1F4S.P.65, 4HCC.A.504, 4HP3.C.201, 1HWT.D.13
8, 2I13.A.503, 2I13.A.506, 2I13.B.510, 4IQR.A.402, 2IVH.A.1577, 2JP9.A.133, 2KKF
.A.2001, 2KKF.A.2002, 1LLM.C.302, 4LMG.A.201, 2NLL.A.250, 3O9X.A.132, 2OPF.A.501
, 4QEN.A.804, 3S14.A.1735, 3UK3.C.967, 1ZGW.A.500, 1A42.A.262, 4A46.A.65, 1A5T.A
.501, 3A6F.C.301, 3A6J.A.301, 3A6J.D.301, 1A7I.A.82, 1A7I.A.83, 2AA4.A.1001, 2AD
R.A.162, 1ADB.A.375, 1ADN.A.93, 2AF2.B.154, 4AI5.A.200, 4AIG.A.999, 1ANI.A.451,
2AQP.A.201, 3ASK.A.501, 2AS9.A.666, 2AS9.B.222, 3AUK.A.390, 4B29.A.1205, 1B4E.A.
405, 3B4F.A.262, 2B5W.A.800, 3B5Q.A.500, 1B8T.A.196, 2B83.C.3353, 2B8T.A.1218, 4
BHW.A.1, 4BHW.A.4, 1BI0.A.291, 3BKN.A.202, 3BL0.A.262, 4BLB.B.910, 1BNM.A.262, 3
BOL.A.701, 4BOL.A.1261, 1BP3.A.500, 1BTK.A.1, 3C10.A.102, 4C1D.A.501, 4C1E.A.501
, 4C1E.A.502, 4C1G.A.300, 4C2P.A.701, 3C37.A.301, 4C4O.D.500, 2C6A.A.336, 1C8T.A
.260, 3C8Z.A.413, 4C8E.A.1162, 1CAQ.A.301, 4CBY.A.2035, 4CCG.X.1374, 2CJL.A.1217
, 1CL4.A.81, 1CLC.A.653, 1CNI.A.1, 1CNW.A.262, 1CNX.A.262, 1CNY.A.262, 1CO4.A.43
, 4COI.A.652, 3CQZ.L.3005, 4CQ0.A.1262, 2CQE.A.822, 2CQF.A.330, 1CRA.A.262, 2CRR
.A.401, 2CSY.A.201, 2CSZ.A.401, 3CSK.A.712, 1CTU.A.296, 2CT1.A.201, 2CT2.A.401,
2CT7.A.201, 2CTD.A.201, 2CTD.A.401, 2CTT.A.201, 2CTU.A.201, 2CU8.A.201, 2CUP.A.2
01, 2CUP.A.601, 2CUQ.A.201, 2CUQ.A.401, 2CUR.A.401, 3CX3.A.314, 3CXL.A.501, 1CXV
.A.3, 4CYK.A.42, 1CZM.A.261, 1D1S.B.376, 1D1T.A.401, 4D1N.A.900, 3D2Z.A.262, 2D5
B.A.501, 2D8Q.A.201, 2D8S.A.201, 2D8S.A.401, 2D8U.A.401, 1D9D.A.1, 2D9N.A.256, 4
D9W.A.408, 2DAR.A.401, 2DB6.A.401, 3DD8.A.262, 3DDT.A.46, 4DF9.A.503, 2DGE.A.100
1, 2DID.A.401, 2DJB.A.201, 2DJB.A.401, 1DMX.A.280, 1DMY.A.280, 2DMD.A.191, 2DQ4.
A.502, 1DSV.A.171, 1DVB.A.194, 3DWD.A.501, 1DY0.A.401, 3E1W.A.230, 3E24.A.230, 3
E2I.A.200, 2E26.A.603, 3E2U.E.102, 4E2X.A.501, 1E3I.A.380, 1E3I.A.381, 3E3F.A.23
0, 3E3I.A.230, 1E4B.S.999, 2E47.A.172, 2E6I.A.201, 2E72.A.201, 2EA5.A.201, 2EA5.
A.401, 3EBI.A.1, 2ECJ.A.201, 2ECI.A.201, 2ECI.A.401, 2ECY.A.201, 2ECG.A.401, 2EC
L.A.401, 2EER.A.501, 4EEX.B.402, 3EFT.A.262, 4EFS.A.301, 3EH1.A.1269, 3EHX.A.264
, 2ELQ.A.181, 2ELW.A.181, 1ELX.A.451, 1ELY.A.451, 2EMX.A.201, 2EMY.A.201, 2EM1.A
.201, 2EMF.A.201, 2EMG.A.201, 2EMH.A.201, 2EN1.A.201, 2EOI.A.201, 2EOP.A.201, 2E
PU.A.201, 2EPX.A.201, 2EPY.A.201, 1EPW.A.1291, 2EQW.A.201, 2EQ0.A.201, 2EQ2.A.20
```

1, 2EQ3.A.201, 1ESP.A.323, 4EVB.A.204, 4EXS.A.302, 4EYL.A.303, 4EYU.A.1702, 2F0Y
.B.501, 3F0D.A.163, 3F0F.A.165, 1F4T.A.369, 3F4X.A.262, 3F5L.A.1001, 4F9C.B.401,
1FAQ.A.2, 1FBX.A.3316, 3FCQ.A.322, 3FFP.X.262, 3FIE.A.428, 2FIF.B.901, 3FJU.A.5
02, 3FJU.A.507, 1FKW.A.400, 4FKB.A.401, 3FLF.A.2004, 2FNF.X.1, 1FR7.A.262, 3FTN.
D.357, 2FU9.A.401, 3FV4.A.321, 3FVP.A.321, 3FXP.A.3000, 2FZW.B.376, 1G47.A.999,
3G42.D.500, 1G4K.A.301, 2GAH.D.101, 4GER.A.401, 2GFJ.B.401, 2GFE.A.869, 1GI4.A.4
09, 3GIQ.A.482, 3GIQ.A.483, 3GJ7.D.300, 4GQT.B.502, 3GRB.A.129, 4GR0.A.301, 4GR8
.A.301, 2GSU.A.1001, 3GTT.A.155, 2GVI.A.301, 1H19.A.701, 4H12.A.1802, 1H2B.A.136
2, 3H2P.B.154, 4H30.A.301, 3H5A.B.360, 3H5N.A.500, 4H57.A.405, 1H7N.A.1342, 4H84
.A.301, 2HB9.A.401, 1HCP.A.98, 1HDY.A.376, 4HDH.A.1002, 4HEW.A.301, 4HEY.A.301,
4HF3.A.301, 3HKN.A.261, 4HT0.A.301, 4HTB.A.401, 4HVL.A.504, 1HY7.B.801, 1HYI.A.6
6, 2HZ8.A.117, 2I0O.A.579, 4I28.A.602, 2I2X.A.524, 3I3T.A.700, 1I6N.A.401, 1I6P.
A.301, 1I76.A.999, 1I8J.B.400, 3I9F.B.3, 2I9W.A.200, 1IB5.A.201, 1IBB.A.201, 3IB
I.A.262, 3IFJ.A.201, 3IFJ.B.202, 3IJF.X.147, 1IML.A.77, 1IQ8.A.600, 2IT4.A.561,
3IXE.B.301, 4IXJ.A.301, 2J1Y.A.1290, 2J21.A.1289, 1J9Y.A.1004, 2J9R.A.1194, 2JA1
.A.1192, 1JD5.A.501, 4JIV.D.101, 1JJ9.A.999, 1JJD.A.102, 1JM7.A.123, 2JOX.A.108,
4JOM.A.1004, 4JPA.A.301, 2JSD.A.275, 2JTN.A.183, 2JTN.A.186, 2JUN.A.221, 2JUN.A
.223, 1JVB.A.500, 3JV7.A.501, 3JV7.A.502, 2JWO.A.489, 1JW9.B.250, 2JZ8.A.150, 4K
1R.A.502, 1K24.A.401, 2K5C.A.96, 3K6J.A.800, 4K7D.A.508, 1K83.I.3003, 3KB1.A.302
, 3KED.A.875, 1KEV.B.353, 1KFI.A.700, 2KFN.A.1, 1KH4.A.451, 1KHL.A.451, 2KIZ.A.7
0, 4KJG.B.1001, 1KK1.A.411, 2KN9.A.82, 1KOL.A.1002, 1KOQ.A.301, 1KTO.A.405, 4KUJ
.A.301, 3KVE.B.489, 1KWG.A.806, 1KWQ.A.262, 3KWO.C.161, 2KZM.A.1, 3L0V.A.1, 3L22
.A.1, 4L5O.A.303, 4L6H.A.801, 2L8E.A.829, 3LAT.A.215, 1LBU.A.214, 1LG5.A.262, 2L
GG.A.380, 4LGJ.A.301, 2LI8.A.188, 4LIM.A.401, 4LJP.A.1103, 4LOE.C.401, 3LPE.B.60
, 1LPV.A.53, 4LQG.A.802, 3LS1.A.1, 3LSC.A.458, 3LT8.A.80, 2LUY.A.301, 3LUB.A.302
, 2LUL.A.201, 2LVU.A.101, 4LW9.D.201, 2M3H.A.102, 4M3O.A.300, 2M48.A.502, 1M65.A
.300, 1M6H.A.1376, 3M6I.A.402, 3MBG.A.3, 3MBM.A.163, 1MC5.A.376, 3MHS.A.475, 2MI
U.A.301, 2MIU.A.303, 1ML9.A.3, 4MLT.A.301, 1MOO.A.262, 1MP0.A.376, 1MQ0.B.147, 3
MS3.A.401, 4MT2.A.67, 4MTW.E.401, 1MVH.A.501, 2MWX.A.201, 4MWP.E.401, 4MXJ.E.401
, 1MZB.A.202, 4MZN.E.401, 4N0G.A.401, 4N0L.A.401, 4N4E.E.401, 4N4F.A.1401, 4N5P.
E.405, 4N66.E.501, 1N92.A.375, 3NIT.A.2, 3NI5.A.262, 1NJ1.A.513, 1NJG.A.401, 4NJ
5.A.803, 4NL4.H.802, 3NQZ.B.1, 4NQ5.A.301, 4NQ5.A.302, 4NQ6.A.302, 4NS5.A.401, 1
NTO.A.500, 4O1K.A.301, 2O4H.B.401, 4O6I.A.601, 1OAL.A.152, 4OAQ.A.403, 4OCM.E.40
1, 2OD1.A.902, 1OHL.A.400, 2OH3.A.300, 1OS0.A.600, 2OSF.A.262, 4OTE.A.304, 2OUI.
A.361, 2OVX.A.444, 2OVZ.A.444, 1OX7.A.402, 3OXF.A.440, 1OYW.A.801, 3P1V.A.427, 1
P42.A.502, 1P6B.A.406, 1P9R.A.601, 4P9C.B.201, 3PBB.A.391, 3PBJ.D.31, 1PCX.A.950
, 1PE8.A.317, 1PGU.A.616, 2PG3.A.300, 3PII.A.340, 1PL8.A.402, 3PN3.A.1002, 3PN3.
A.1009, 3PN3.B.1011, 3PNU.A.336, 3PZC.B.1000, 4Q0L.A.301, 1Q1A.A.701, 3Q1D.A.201
, 2Q1B.A.400, 3Q6V.A.2, 2Q6E.A.501, 4Q7R.A.301, 4Q7R.A.302, 4Q7R.A.303, 3Q94.A.3
01, 2QDT.A.401, 4QEF.A.301, 1QF2.A.320, 3QH5.A.321, 3QHD.A.163, 2QIC.A.300, 2QL1
.A.1, 2QN0.A.431, 2QQ4.A.139, 1QUA.A.999, 3QU1.A.505, 3R0D.A.428, 1R22.A.502, 3R
2N.A.135, 4R2Y.A.103, 3R3L.A.585, 1R4V.A.202, 2R59.A.701, 4R9G.A.505, 3RCM.A.288
, 3RHG.A.368, 1RJ6.A.601, 1RJQ.A.601, 1RJW.A.402, 2RJP.B.1, 1RMD.A.117, 4RM5.D.3
00, 2RMN.A.1, 2ROW.A.601, 2ROW.A.602, 2RPC.A.201, 2RPP.A.401, 4RQU.B.402, 2RSD.A
.901, 2RSH.A.101, 2RSI.A.101, 3RUI.A.1, 1RUR.L.601, 3RZ5.A.1, 3S2E.A.500, 3SD9.A
.2, 3SOU.B.8, 3SSB.A.999, 3SU6.A.5, 1SW1.A.401, 1SX1.A.23, 3T02.A.501, 1T3A.A.42
2, 3T5Z.A.262, 3T73.A.410, 3T74.A.410, 3T7E.A.252, 3T8G.A.411, 3T8F.A.411, 3T87.
A.326, 3T8C.A.326, 3T8D.A.325, 3T8H.A.326, 1TBN.A.1, 3TGN.A.148, 1THL.A.324, 3TI
O.A.1, 2TMN.E.321, 6TMN.E.322, 3TMJ.A.262, 3TTY.A.676, 3TWO.A.349, 4TZC.A.501, 1
U3L.A.701, 1U3T.A.376, 1U4G.A.9800, 4U4L.A.301, 4UA4.A.302, 4UA4.B.303, 3UCM.A.2
28, 3UCN.A.228, 1UD9.B.508, 3UJZ.A.1, 3UKO.A.400, 3UKO.A.401, 4UOV.A.298, 2USN.A
.257, 1UUF.A.401, 3UVC.A.301, 3UWA.A.200, 1UX1.A.1132, 2UX1.K.1173, 1V0D.A.401,
4V0R.A.1001, 4V2W.A.502, 1V4P.A.1001, 1V5R.A.201, 1V87.A.201, 1V87.A.401, 1VGN.A
.301, 1VGN.A.302, 2VJE.B.1492, 2VM5.A.1245, 3VTH.A.801, 2VXI.B.201, 2VXX.A.201,

2VXX.B.202, 3W0F.A.301, 1W50.A.1339, 1WAA.A.1090, 1WAA.D.1090, 1WAA.E.1089, 4WAI .A.101, 2WCB.A.100, 4WD8.B.303, 1WE9.A.201, 1WEE.A.401, 1WEM.A.401, 1WEN.A.201, 1WEO.A.201, 1WEP.A.401, 1WEQ.A.201, 1WEQ.A.401, 1WEV.A.201, 1WEV.A.401, 1WEW.A.4 01, 1WFE.A.201, 1WFE.A.401, 1WFF.A.201, 1WFH.A.401, 1WFL.A.201, 1WG2.A.400, 4WH6 .A.1203, 2WHG.A.1263, 1WIL.A.201, 1WIM.A.401, 1WIR.A.201, 3WIE.B.1001, 2WJV.A.1, 1WJA.A.56, 1WJB.A.56, 1WJP.A.701, 4WK7.A.501, 4WKE.A.501, 3WL3.A.301, 2WO8.C.12 68, 3WS6.C.201, 1WUR.A.1001, 2WWO.A.1162, 3WXC.A.302, 1WYH.A.401, 1WYS.A.201, 3X 17.A.601, 1X3C.A.201, 1X3H.A.201, 2X4H.B.1141, 1X4U.A.201, 1X4U.A.401, 1X4V.A.20 1, 1X4V.A.401, 1X4W.A.201, 1X5W.A.201, 1X6E.A.401, 1X6F.A.201, 2XB4.A.1224, 2XEU .A.1065, 1XLL.A.399, 2XOC.B.991, 2XOC.B.992, 1XPZ.A.262, 1XUJ.A.246, 2XXH.B.1303 , 1XYD.A.94, 1XYD.B.94, 1Y23.A.1001, 2Y28.B.1181, 1Y3G.E.321, 2Y4Y.A.1172, 2Y4Y. C.1172, 1Y8F.A.702, 1YM3.A.301, 1YO7.A.201, 2YQQ.A.201, 2YRK.A.201, 1YSB.A.501, 2YSP.A.181, 2YT9.A.203, 2YTA.A.201, 2YTN.A.201, 2YUU.A.201, 2YUU.A.401, 2YVR.A.1 002, 2YXO.A.501, 2YZ3.A.301, 2Z2S.B.204, 2Z2Y.D.2004, 1Z5H.A.2001, 1Z6U.A.1, 1Z8 3.B.642, 1Z9G.E.1005, 1Z9N.A.201, 1Z9Y.A.300, 2Z94.A.901, 2Z9L.A.701, 1ZDP.E.100 5, 2ZEH.A.391, 2ZEN.A.391, 3ZFK.A.401, 2ZNE.B.993, 3ZPG.A.1358, 1ZSO.A.999, 1ZTQ .A.550, 1ZUD.1.501, 1ZVX.A.999, 2ZXG.A.900
[1] "Cluster 2"
1A6Y.A.551, 3AUO.B.579, 2ER8.A.105, 1G2F.C.301, 4GNX.C.701, 4IFD.J.1106, 4MTD.B. 201, 2NQ9.A.401, 4O6A.A.601, 1ODH.A.1171, 1QUM.A.301, 3VDO.A.401, 1A72.A.376, 2A 97.B.2437, 1AAF.A.57, 4AA1.A.1615, 2AB3.A.30, 2ADR.A.163, 2AFU.A.391, 2AMT.B.290 0, 2ANH.A.452, 1ARD.A.1, 1ARE.A.1, 1ARF.A.1, 4AR1.A.1722, 4ARF.A.1722, 1AXG.A.40 1, 2AYK.A.171, 3AY2.A.1001, 2AZH.A.150, 2B0P.A.400, 3B4N.B.712, 3B6P.A.800, 3B7R .L.701, 1B8T.A.193, 1B8T.A.194, 1B8T.A.195, 2BA1.A.201, 3BKN.A.201, 3BL5.A.300, 1BNL.A.179, 2BNN.A.1200, 1BTG.B.902, 2C20.A.601, 3C2S.A.448, 3C52.B.401, 3CHV.A. 302, 2COT.A.201, 2COR.A.201, 3CQZ.B.3007, 3CQZ.I.3004, 1CVE.A.262, 3D2N.A.102, 2 D74.A.1001, 2D74.B.1002, 2DAR.A.201, 1DDZ.A.1, 1DE6.A.450, 1DGS.B.2701, 2DJ7.A.4 01, 2DKD.B.922, 4DLA.A.401, 4DLF.A.404, 1DPM.A.801, 2DQ6.A.900, 2DSN.B.2002, 1DT H.A.901, 4DYG.B.307, 3E1Z.A.111, 2E1W.A.400, 1E46.S.999, 3E4Z.B.2, 1E67.A.129, 4 EEX.A.402, 4EEZ.A.402, 2EG4.A.301, 2EG4.B.302, 2ELU.A.181, 2ELV.A.181, 2EOM.A.20 1, 3EQN.B.757, 1EU4.A.400, 1EYW.A.402, 1F35.A.306, 1F5F.A.252, 1F8F.A.372, 2FEJ. A.1, 4FKK.A.1025, 4FMN.B.901, 4FMP.A.400, 2FNF.X.2, 3FVZ.A.821, 4FVL.A.501, 4G3M .B.401, 2G54.A.1100, 2GA3.A.451, 3GAY.B.328, 1GKR.A.1452, 1GLC.F.169, 2GMN.A.801 , 4GRI.A.501, 4GU1.A.905, 1GYT.J.600, 3GZE.B.14, 3H90.A.291, 3H90.A.293, 2HCS.A. 2, 4HMA.A.301, 3HNI.H.107, 3HPH.A.220, 2HSI.A.283, 1HU8.A.501, 2IOO.A.581, 1I50. A.3006, 4I7C.A.601, 1IAG.A.999, 1IAU.A.504, 3ID7.A.402, 3IEW.B.801, 4IOU.D.1001, 3ISI.X.3000, 1ITU.A.401, 4IUQ.A.301, 2IWE.A.1129, 4J3D.B.302, 4J4M.A.301, 4JD1. B.202, 4JIJ.A.302, 2JIG.A.1253, 4JSW.A.301, 1JT1.A.400, 4K2H.A.201, 4K5N.A.1101, 2K8D.A.155, 1KBE.A.2, 1KHN.A.452, 2KIK.B.50, 2KIZ.A.71, 2KO8.C.54, 2KVF.A.83, 2 KVH.A.84, 3KWO.B.161, 4KXC.A.1001, 1L1O.F.2, 1L7O.B.301, 4LAO.A.401, 1LD3.A.500, 1LDE.A.375, 1LDY.A.375, 3LE9.B.2, 4LEV.A.601, 2LGV.A.110, 4LJQ.A.1101, 2LVR.A.1 01, 2LVT.A.101, 2LXH.C.900, 2LXH.C.901, 3LZE.A.201, 3M0N.A.201, 2M0D.A.101, 3M15 .A.107, 3M15.B.107, 1M4M.A.502, 3M8T.A.301, 3MDW.A.455, 3MEN.D.400, 3MI9.C.88, 3 MKV.B.426, 1MMR.A.1, 2MQ1.A.103, 1MVH.A.502, 1MVH.A.503, 3MWM.A.142, 3N2C.E.425, 1ND1.A.400, 3NNQ.A.201, 1NUI.A.501, 2O36.A.690, 2O4Q.A.2402, 2O53.B.314, 2OI0.A .2, 4OIW.F.501, 1OJ7.B.1389, 1OKN.A.262, 1ONW.A.801, 2OO4.A.6001, 1OS2.B.369, 1O S2.F.769, 2OW2.A.444, 2OW9.B.606, 2OX8.B.3, 1P1R.A.375, 1P42.A.503, 1P5D.X.500, 2P53.A.401, 1P91.B.2301, 4P9C.A.201, 1PAA.A.160, 3PJN.A.186, 3PJN.B.186, 2PLI.B. 709, 2POJ.A.265, 1PS7.A.331, 3PZC.A.1000, 1Q3K.A.301, 3Q43.A.1, 3Q44.A.1, 4Q7R.A .306, 4QBG.B.301, 3QE3.A.356, 2QJS.A.2002, 3QJ5.A.376, 3QNA.A.122, 3QVY.A.500, 3 QVZ.D.500, 3QW0.C.500, 1QX1.A.2004, 1R1H.A.1001, 1R37.A.500, 1R3N.B.501, 2RPR.A. 201, 2RPC.A.401, 4RQT.A.402, 3RSM.A.500, 1S1G.A.152, 3S2F.E.500, 1S4B.P.1, 3SFH. A.403, 3SPU.D.1004, 3SP1.A.481, 2SRT.A.257, 3SZY.A.501, 3T01.A.502, 1T3K.A.201, 1T9R.A.1, 1TBN.A.2, 3TBG.A.601, 1TF9.A.901, 3TG0.B.505, 1THJ.A.214, 1THJ.B.214,

1TKF.A.901, 1TKH.A.901, 1U05.A.500, 1U0L.A.298, 1U10.A.601, 4U10.A.401, 1U3U.A.3
76, 1U3V.A.376, 3U7K.A.350, 3U7L.A.350, 3UIK.A.341, 4UNI.C.1697, 3UW2.A.474, 2UY
V.B.1276, 1UZF.A.701, 2V2A.A.1275, 1V5N.A.401, 2V8G.C.500, 2V8V.B.1455, 1V9P.B.2
701, 1VA1.A.100, 1VA2.A.100, 3VQZ.A.301, 1VSH.A.281, 1VYX.A.1061, 3W5K.B.503, 2W
57.B.201, 4W6Z.A.401, 2WBT.A.1131, 4WD6.A.302, 1WG2.A.200, 1WIL.A.401, 1WNU.A.10
01, 4WOK.A.401, 2X4H.A.1140, 2X4H.B.1142, 2X5C.A.1128, 1X61.A.201, 2X7M.A.1174,
2X8Y.A.1616, 2X96.A.1617, 2XAA.C.1346, 2XAM.B.700, 1XB8.A.1001, 1XEM.A.401, 1XTG
.A.426, 1XUC.A.1261, 1XUF.A.246, 1XWH.A.356, 1Y0J.A.244, 2Y20.C.1331, 2Y6C.A.126
7, 1Y7W.B.285, 1Y8J.A.800, 1YC2.A.402, 1YE3.A.375, 1YEJ.L.605, 1YHC.A.601, 1YOU.
A.301, 2YPU.A.1998, 2Z26.A.400, 2Z3I.A.2001, 2Z9K.A.901, 1ZLH.A.555, 3ZNC.A.1, 2
ZNC.A.1, 3ZTV.A.1599, 1ZXZ.A.198
[1] "Cluster 3"
4A7K.A.952, 3ADR.A.263, 3ASE.A.156, 3AYK.A.170, 2BCN.A.295, 2BIB.A.1550, 2DI3.A.
1002, 4E5V.B.401, 3EII.A.301, 2EK9.A.1004, 2FV9.B.4, 3G4K.A.801, 2GC2.A.401, 2GC
3.A.402, 2HD1.A.101, 4HGX.A.301, 4HTZ.B.1001, 2ICS.A.400, 4ICQ.A.501, 4IGN.B.401
, 2J0E.B.1265, 4JAA.A.501, 2K78.A.151, 4KBP.A.439, 3KR5.E.1001, 4KYH.A.202, 3LL8
.A.505, 2LQ6.A.401, 2LQ6.A.402, 2LZE.A.101, 1ML2.A.296, 4N27.C.201, 4N7K.L.301,
4N7K.L.307, 4N7K.M.401, 4N7K.M.402, 4N7L.M.402, 3N9R.A.308, 3NO5.C.275, 3O64.A.1
, 4OK2.A.801, 2OUN.B.404, 4PKT.A.802, 4PKW.A.801, 1Q3A.B.469, 1Q3K.B.300, 1Q74.A
.304, 3Q9B.A.345, 2QFR.A.434, 1QIP.A.902, 4R6T.A.1003, 4R6T.D.1001, 4R7M.C.1001,
 4R7M.J.1003, 3S2M.A.403, 3S2N.A.403, 3SFW.A.501, 3V93.D.701, 3VH9.A.301, 1VKG.B
.400, 3VUS.A.401, 2WEY.A.1772, 4X2T.A.702, 1XJS.A.150, 1XP3.A.301, 1Y13.A.174, 2
ALW.A.5001, 4BBP.A.1316, 3BLB.A.1047, 1BLL.E.489, 2BNO.B.1201, 3D4Y.A.1047, 3D4Z
.A.1046, 3D52.A.1046, 3D51.A.1046, 3DDF.A.3001, 3DDG.A.3001, 2DDF.A.1, 1DE5.A.45
0, 1DK4.B.590, 4DO0.A.502, 3DX1.A.1048, 3DX2.A.1046, 3DX3.A.1047, 3DX4.A.1047, 3
DZA.C.501, 3DZA.C.505, 3E38.A.2, 3E49.B.500, 3E8R.B.2, 3EJP.A.1047, 3EJQ.A.1047,
 3EJR.A.1047, 3EJU.A.1047, 2EK8.A.1004, 1ENQ.A.238, 2F18.A.1805, 2F1A.A.1805, 2F
1B.A.1804, 2F7O.A.5001, 2F7P.A.5001, 2F7Q.A.5001, 2F7R.A.5001, 2F92.F.1001, 2F92
.F.1002, 2F92.F.1003, 2F94.F.1001, 2F94.F.1003, 2F9K.F.1001, 2F9K.F.1002, 2FQP.B
.100, 2FUQ.A.1, 4GBD.A.503, 4GQT.A.501, 1GT7.A.275, 3GWT.A.504, 4H1S.A.603, 2H44
.A.501, 3HDZ.A.864, 3HPS.A.701, 1HQA.B.452, 2I57.D.507, 3IBM.A.200, 4IE5.A.601,
1IM5.A.400, 3ISI.X.3001, 3ITU.A.1, 3IVT.A.500, 4JDG.A.401, 1KAE.A.1101, 3KMC.B.2
, 3KME.B.2, 1KQ3.A.401, 3KR5.A.1001, 1KRM.A.501, 1LCP.A.488, 3LGP.A.1, 3LLX.A.37
6, 3LX3.A.201, 1M60.A.105, 4M6R.B.301, 3NO5.A.275, 4NPW.A.1001, 4NT9.A.301, 4NUR
.A.701, 4NZ3.A.501, 3O0J.A.1, 3O2G.A.388, 3O64.B.485, 3O91.A.192, 3O93.A.192, 2O
B3.B.904, 4OJV.A.403, 4OJV.A.404, 4OJX.A.403, 4OJX.A.404, 4OK4.A.800, 2OQL.A.401
, 1OS9.F.926, 2OUV.A.777, 2OU3.A.161, 2OUP.B.777, 4OV9.A.401, 2P18.A.301, 2P2L.A
.201, 1PTM.A.330, 1PTM.B.331, 2PTY.A.501, 2PTZ.A.500, 2PTZ.A.501, 2PU1.A.500, 2P
U1.A.501, 2PW3.A.501, 3QAY.D.180, 4QGE.A.602, 1QH3.A.262, 2QYM.A.1, 2QYK.A.1, 2R
2V.C.35, 1R33.A.1163, 1R55.A.201, 4R6T.B.1003, 4R6T.F.1001, 4R76.A.1003, 4R76.E.
1001, 4R7M.C.1003, 3RCQ.A.1, 4RL0.B.302, 1RRM.B.387, 3SL3.B.9, 1SNN.A.402, 1SR9.
B.703, 1T9S.A.1, 1TB5.A.1001, 1TB7.A.1001, 1TBF.A.1, 4TO8.A.301, 3U43.B.135, 1U7
4.A.1001, 3UUO.B.772, 3V77.D.301, 3V93.A.701, 2V9N.A.1275, 3W0T.A.201, 3W0T.B.20
1, 2WHG.B.1263, 2WTA.A.1215, 4X2T.A.701, 4X2T.G.702, 1XM6.A.1001, 1XOR.A.1001, 1
Y2K.A.1001, 2Y33.A.900, 1Y9Q.A.202, 1ZKL.A.501, 2ZZW.A.362
[1] "Cluster 4"
1A1G.A.201, 1A6Y.A.550, 2A66.A.401, 3EPH.A.1, 2ER8.A.104, 1F2I.G.1202, 1G2D.C.30
2, 1G2D.C.303, 1G2F.C.302, 4GLX.A.601, 3GOX.A.302, 3HAX.C.201, 2I13.A.502, 2I13.
B.507, 1I3J.A.100, 2IHX.A.235, 4IQR.A.403, 2JP9.A.131, 2JP9.A.134, 1K3X.A.501, 1
KB2.A.150, 1L01.A.196, 4M9E.A.503, 4M9E.A.504, 4M9V.C.202, 1MEY.C.88, 1MEY.C.90,
 3MLN.A.501, 4MTD.A.201, 4NM6.A.2002, 2OFI.A.302, 3OYM.A.393, 1OZJ.A.145, 3PIH.A
.917, 3QSV.A.1, 4R2A.A.504, 4R2Q.A.503, 2XQC.D.1141, 258L.A.500, 1A1R.A.901, 3A3
2.A.708, 2A5V.A.401, 3A6G.A.301, 1A71.A.401, 4A7K.A.950, 3A9J.C.1, 3A9K.C.1, 1AA
F.A.56, 2AC3.A.531, 4ADN.A.1223, 1AF2.A.296, 2AFM.A.391, 4AIA.A.200, 1AJB.A.451,

4AJX.H.1030, 3ALR.A.602, 1ANJ.B.451, 2AP1.A.304, 4AR9.B.1731, 2AU3.A.501, 2AW1.A.262, 4AWY.B.3228, 3AX1.A.601, 4AX0.B.3228, 4AX1.B.3228, 4AXD.A.700, 4AYC.A.1485, 2B3Z.A.1360, 4B6D.A.1340, 1B8Y.A.301, 3B92.A.502, 1BBO.A.61, 1BCD.A.262, 3BET.A.262, 2BE7.D.1108, 3BHX.A.1752, 3BI0.A.1752, 3BI1.A.1752, 3BJI.A.1, 3BJI.B.2, 2BL6.A.1060, 1BNQ.A.262, 2BNM.A.1199, 3BOC.A.1001, 2BP0.A.1341, 1BS4.A.2001, 3BTO.C.375, 4BT7.A.301, 1BUD.A.800, 1BV3.A.262, 1BYF.A.302, 3BYR.A.501, 2C1I.A.1465, 4C1D.A.502, 4C1F.A.501, 4C1F.A.502, 4C1G.A.301, 4C1G.B.301, 4C1Q.A.493, 2C2F.A.1211, 1C3I.B.260, 2C36.A.1311, 3C5K.A.203, 2C6C.A.1752, 1C7K.A.133, 4C81.A.1240, 2C9S.A.1155, 1CAI.A.262, 1CAK.A.262, 4CA1.A.284, 2CBD.A.262, 4CCG.X.1375, 1CDO.B.376, 4CDG.A.1643, 3CE1.A.202, 2CFU.A.1002, 1CG2.C.502, 1CGL.A.302, 1CIL.A.262, 3CJP.A.301, 2CKI.A.999, 2CLT.A.1202, 3CMR.A.450, 3CNG.A.508, 2COT.A.401, 3COS.A.501, 2CO8.A.201, 2CO8.A.401, 2CON.A.201, 2COR.A.401, 4CPD.A.1200, 1CQR.B.2301, 2CQE.A.622, 2CR8.A.401, 2CSY.A.401, 2CSZ.A.201, 3CSQ.A.335, 2CS3.A.400, 2CSH.A.300, 1CTT.A.296, 2CT0.A.201, 2CT0.A.401, 2CT1.A.401, 2CT2.A.201, 2CT5.A.201, 2CT7.A.401, 2CTT.A.401, 2CU8.A.401, 2CUR.A.201, 4CVR.A.1158, 3CZV.A.262, 2D0W.B.1207, 1D0Q.B.151, 4D0Y.A.1239, 1D1T.A.376, 3D2N.A.101, 4D6S.A.1338, 3D7V.A.2, 2D8X.A.201, 2D8Y.A.201, 2D8Z.A.201, 3D8W.A.262, 2D8T.A.201, 2D8T.A.401, 2D8U.A.201, 2D8V.A.201, 2D9M.A.1085, 2D9N.A.456, 2DAN.A.201, 3DBK.A.302, 1DCA.A.262, 1DD6.A.503, 1DEH.A.376, 3DFM.A.402, 2DH3.A.601, 3DH1.A.201, 3DHA.A.256, 3DI4.B.286, 2DID.A.201, 2DIP.A.401, 2DJ7.A.201, 2DJ8.A.201, 2DJ8.A.401, 2DJA.A.201, 2DJA.A.401, 1DK4.A.290, 2DKT.A.191, 2DKT.A.241, 2DKT.A.291, 2DKT.A.341, 2DKT.A.391, 2DKT.A.441, 2DLO.A.201, 2DLQ.A.300, 1DMT.A.755, 2DMD.A.291, 1DO5.A.28, 2DOO.A.501, 2DOO.A.502, 4DR8.A.201, 2DW2.A.700, 3E2C.A.200, 1E3J.A.902, 1E3L.A.380, 3E6U.A.501, 3E6U.C.502, 3E6U.B.503, 2E7Y.A.1301, 3E73.A.501, 2E73.A.201, 2E73.A.401, 2E9H.A.301, 1E9P.B.153, 1E9Q.B.153, 2EA6.A.201, 2EA6.A.401, 2EBL.A.191, 2ECT.A.201, 2ECT.A.401, 2ECV.A.201, 2ECW.A.201, 2ECW.A.401, 2ECY.A.401, 2ECG.A.201, 2ECL.A.201, 2ECL.A.601, 2ECM.A.201, 2ECM.A.401, 2ECN.A.401, 1ED9.A.451, 3EDI.A.210, 1EE2.A.1300, 3EER.A.2004, 2EE8.A.301, 2EE8.A.501, 3EFO.B.1034, 4EGU.A.202, 1EI6.A.409, 1EI6.B.408, 2EJ4.A.401, 1EKJ.A.4001, 1EKJ.C.4003, 1EKM.A.701, 2ELO.A.181, 2ELR.A.181, 2ELS.A.181, 2ELT.A.181, 2ELX.A.181, 2ELI.A.401, 2ELM.A.181, 2EMI.A.201, 2EMZ.A.201, 2EM0.A.200, 2EM2.A.201, 2EM4.A.201, 2EM6.A.201, 2EM9.A.201, 2EMB.A.201, 2EMC.A.201, 2EMK.A.201, 2EML.A.201, 2EMM.A.201, 2EN2.A.201, 2EN4.A.201, 2EN6.A.181, 2EN9.A.181, 2ENA.A.181, 2ENC.A.181, 2ENE.A.181, 2ENF.A.181, 2ENH.A.181, 2ENN.A.300, 2ENN.A.400, 2EOJ.A.201, 2EOQ.A.201, 2EOU.A.201, 2EOV.A.201, 2EOW.A.201, 2EOX.A.201, 2EOY.A.201, 1EOU.A.300, 2EO4.A.201, 2EOE.A.201, 2EOF.A.201, 2EOH.A.201, 2EOK.A.201, 2EON.A.201, 2EOO.A.201, 2EPT.A.201, 2EPV.A.201, 2EPZ.A.201, 2EP1.A.201, 2EP2.A.201, 2EP3.A.201, 2EP4.A.300, 3EPZ.A.701, 2EPQ.A.201, 2EQ1.A.201, 2EQ4.A.201, 1EU3.B.402, 2EU3.A.262, 3EWF.A.400, 2EWB.A.489, 2EXU.A.501, 3EYX.A.1, 1F1G.A.4002, 1F2W.A.262, 3F7B.B.301, 3F7L.A.203, 3F7U.A.260, 4F7O.B.301, 3F9O.A.309, 1FAQ.A.1, 4FGL.A.301, 3FID.A.299, 1FLJ.A.262, 4FMN.B.902, 2FOQ.A.262, 2FOS.A.262, 2FOU.A.262, 2FOV.A.262, 4FO9.A.501, 2FPR.A.502, 3FPC.A.353, 3FPL.A.352, 1FQL.A.262, 1FQM.A.262, 1FR2.B.301, 2FR6.A.147, 2FSA.A.501, 3FTN.A.354, 3FTW.A.701, 3FUN.A.701, 2FU8.A.401, 4FVN.A.302, 4FVO.A.302, 4FVY.A.805, 4FWU.A.401, 2FYG.A.302, 2G0D.A.416, 2G2N.A.1001, 1G5C.A.1001, 2G9Y.B.451, 1G9K.A.600, 3GC9.B.603, 2GD8.A.262, 3GJ4.D.300, 2GMN.A.805, 2GMW.A.300, 4GNE.A.1501, 4GNE.A.1503, 4GNE.A.1504, 1GO8.P.1486, 4GR3.A.301, 2GSN.A.1000, 4GUA.A.1719, 4GUT.A.904, 3GV4.A.203, 1GVY.A.1425, 3GZE.B.2, 2GZL.A.900, 3H0L.E.902, 2H15.A.262, 4H1Q.A.302, 4H2K.B.1001, 2H39.A.352, 4H3S.A.901, 2H4N.A.262, 1H9Q.A.262, 3HB2.P.486, 2HCS.A.1, 4HCG.A.202, 3HFF.A.154, 2HJN.A.315, 3HJT.A.1, 1HK8.A.1589, 3HKO.A.701, 3HKT.A.261, 3HLJ.A.272, 3HNI.G.107, 3HNJ.A.107, 3HNJ.B.107, 3HNJ.C.107, 3HNJ.D.107, 4HNO.A.301, 1HSZ.A.1376, 1HTO.A.1376, 3HUG.D.109, 1HXR.A.200, 1HY7.A.302, 1HZ5.B.105, 3I1U.A.401, 1I50.C.3002, 2I50.A.336, 1I73.A.998, 1I73.A.999, 1IA9.A.2001, 4ICR.A.501, 2IDA.A.103, 1IF5.A.262, 3IFU.A.181, 3IGP.A.262, 2IGI.A.1001, 2IGI.A.1004, 4IJD.A.501, 4IJD.A.502, 4ILO.A.301, 4ILX.A.301, 2IMZ.A.501, 2IMZ.B.502, 2IMR.A.

500, 2INN.B.514, 4IR0.A.201, 1IS8.B.3109, 4IT0.A.301, 4ITP.A.302, 2IUC.A.1002, 2IUC.B.1007, 4IUW.A.701, 3IXE.B.302, 4J1V.A.301, 1J2O.A.116, 1J2T.A.302, 2J7U.A.1884, 2J7J.A.1088, 1JAO.A.999, 2JBG.B.1577, 1JD0.A.901, 4JE6.A.200, 4JEB.B.202, 2JHG.A.401, 4JH2.A.201, 4JH2.B.201, 4JIU.A.201, 2JIG.B.1252, 1JJD.A.103, 4JLX.A.501, 2JLP.A.226, 1JN7.A.37, 2JOX.A.109, 1JQ5.A.371, 4JSA.A.301, 4JSR.A.401, 4JSZ.A.301, 2JTN.A.184, 2JTN.A.185, 2JUN.A.222, 1JV0.A.261, 3JWP.A.2001, 4JXE.A.502, 1JZS.A.1101, 4JZ0.A.802, 1K07.A.1, 1K2Y.X.500, 4K2H.B.201, 3K34.A.1002, 3K6I.A.202, 1K7H.A.478, 1K7I.A.486, 4K7D.A.503, 4K7D.A.504, 4K7D.A.506, 2KAK.A.150, 3KBF.A.159, 2KDP.A.1, 3KEE.A.2000, 2KEM.A.195, 1KH5.A.451, 1KH7.A.451, 3KHI.A.301, 3KNE.A.500, 4KNI.A.301, 4KNJ.A.301, 4KNM.A.301, 1KOL.A.1001, 4KP5.A.301, 4KP8.A.301, 3KQI.A.72, 1KU0.A.701, 4KUV.A.301, 4KUW.A.301, 3KV4.A.448, 4KX8.A.1001, 4KXB.A.1001, 3KYC.B.641, 1L0Y.B.706, 2L0B.A.143, 2L0B.A.161, 4L05.A.203, 3L14.A.262, 4L6O.A.801, 4L6T.A.301, 3L8H.A.901, 3L9Y.A.155, 3LAS.A.167, 4LBU.A.402, 3LEA.A.485, 4LEF.A.302, 2LFD.A.400, 2LGV.A.111, 1LI5.B.964, 2LI8.A.187, 4LJQ.B.1105, 2LK5.A.37, 1LLU.B.343, 3LMI.B.1002, 4LOF.A.401, 4LP6.A.310, 3LQB.A.201, 3LQH.A.1001, 4LR2.A.505, 3LS9.A.457, 2LUY.A.300, 4LW9.A.203, 4LW9.C.202, 4LW9.K.203, 3LXE.A.261, 2LXD.A.202, 3LYR.A.1, 4LY4.D.301, 3M0A.D.401, 2M0E.A.101, 3M14.A.501, 3M1W.A.500, 3M2N.A.263, 2M3Z.A.101, 2M3Z.A.102, 1M3V.A.124, 3M3X.A.262, 2M3L.A.201, 4M3P.A.701, 3M40.A.262, 2M48.A.501, 2M48.A.504, 3M5E.A.262, 3M5S.A.500, 3M67.A.263, 1M6W.A.1376, 2M7Q.A.101, 3M79.D.107, 2M7A.A.100, 3M8T.A.300, 2M9Y.A.402, 3M96.A.262, 3M98.A.262, 1M9O.A.78, 3MA2.A.295, 3MBG.A.1, 4MB7.A.301, 4MDM.A.301, 3MEQ.A.401, 3MF1.A.1000, 1MG0.A.375, 1MG0.B.375, 3MHI.A.262, 3MHL.A.262, 3MHM.A.262, 3MHS.A.473, 3MHS.E.97, 4MHN.A.400, 3MI9.C.87, 4MI5.A.806, 3MJH.B.70, 3MKG.A.155, 3ML2.A.262, 3MMF.A.262, 3MNA.A.262, 3MND.A.153, 3MP2.A.1, 1MS7.B.3001, 3MTW.A.2, 4MT2.A.68, 4MTY.A.301, 2MUQ.A.101, 3MWM.A.140, 3MWM.A.141, 1MXG.A.437, 3MYQ.A.262, 3MZC.A.262, 4N0X.B.301, 3N2P.A.262, 3N3J.A.262, 3N4B.A.262, 1N8K.A.375, 3NA7.A.300, 3NGJ.A.250, 4NJ5.A.801, 3NKQ.A.1001, 2NMX.A.301, 2NNV.A.262, 2NN7.A.301, 4NN2.A.401, 4NN2.A.402, 2NNG.A.262, 2NNO.A.262, 1NO5.B.571, 3NQ5.A.503, 3NQY.B.520, 4NQ4.A.302, 4NQ7.A.302, 4NTM.A.201, 4NTN.A.201, 2NUP.B.1100, 3NY3.A.1, 4NZG.A.201, 3O0M.A.151, 2O03.A.201, 3O36.A.1, 4O64.A.2003, 3O7U.A.428, 4OBI.A.201, 3OCQ.A.184, 2ODX.A.156, 2OFK.A.201, 2OHX.A.401, 3OIL.A.262, 2OIK.B.201, 3OJ3.J.902, 3OJ7.A.114, 3OOI.A.233, 1OQ5.A.600, 3OQ6.A.375, 2ORW.A.401, 3ORU.A.250, 2OU2.A.490, 4OWF.G.401, 2OXZ.A.264, 3OY0.A.262, 3OYS.A.262, 1P1V.A.201, 3P3H.A.261, 3P3J.A.261, 1P42.A.501, 1P4Q.B.301, 3P58.A.262, 3P5A.A.262, 3P5L.A.262, 1PB0.A.1301, 1PB0.A.1303, 3PB6.X.400, 3PB8.X.400, 1PFT.A.51, 3PLW.A.187, 3PN3.A.1010, 3PO6.A.1, 4PQ7.A.301, 3PSQ.B.321, 4PTB.A.901, 4PTB.A.902, 2PUY.A.355, 3PU7.A.155, 3PUR.A.3, 1PV9.A.402, 1PXE.A.64, 4PYX.A.301, 4PZH.A.302, 1Q0E.A.153, 3Q1D.A.202, 2Q1Q.A.262, 1Q2L.A.963, 2Q2L.A.1001, 1Q5W.A.32, 4Q6D.A.301, 4Q6E.A.301, 2QDT.A.402, 4QF2.A.1801, 4QF3.A.2001, 2QIN.A.2002, 1QJI.A.1201, 2QL0.A.53, 2QO8.A.262, 4QP5.A.401, 2QSW.A.201, 4QSJ.A.301, 1QTW.A.301, 1QV6.A.375, 1QV7.A.375, 3QYK.A.262, 3R16.A.262, 4R1X.B.501, 2R3A.A.302, 1R5T.A.150, 1R9P.A.135, 1RAY.A.262, 3RF4.B.119, 4RF1.A.1901, 2RHQ.A.1, 3RJ7.A.300, 4RL0.A.301, 1RM8.A.501, 1RMD.A.118, 1RMD.A.119, 1RMD.A.120, 2RPC.A.601, 2RPC.A.801, 2RPP.A.201, 3RQD.A.390, 2RSI.A.103, 2RSJ.A.102, 1RUT.X.604, 4RUW.A.501, 3RYM.A.106, 3RYV.B.262, 3RYX.B.262, 3RYY.A.1, 3RYZ.A.1, 3RYJ.B.262, 3RZV.A.1, 3RZ1.B.262, 3RZ7.A.1, 3S2E.G.500, 1S4I.A.802, 3S71.B.262, 3S77.B.262, 3S9T.A.262, 3SAX.A.262, 3SAP.A.262, 3SBI.A.262, 3SEY.C.373, 1SML.A.271, 1SRD.B.156, 1SRP.A.920, 3SV6.A.4, 3SWR.A.3, 3SXK.B.3, 3T33.A.411, 3T5U.A.262, 1T8H.A.275, 3T80.D.301, 3T90.A.502, 3TEN.A.205, 1TFI.A.51, 3TG4.A.435, 3TG4.A.437, 1TKF.A.902, 1TT5.B.1014, 1TTM.A.262, 1TWF.J.3001, 1U10.A.400, 1U1H.A.766, 1U40.A.160, 1U5S.B.139, 3U5N.A.1, 4UA4.A.301, 3UCK.A.228, 1UD9.C.509, 3UNT.A.400, 3UN6.A.325, 4UP0.A.1383, 1USN.A.257, 1UUF.A.402, 3UVC.B.303, 1UW0.A.1118, 2UZG.A.131, 2V08.A.1090, 2V1X.A.1595, 1V13.B.200, 3V1C.A.101, 3V1E.A.102, 3V1F.A.704, 4V1T.A.776, 2V29.A.1276, 1V47.A.601, 1V51.A.602, 1V5N.A.201, 3V5G.A.262, 1V6G.A.201, 3V7M.A.509, 1V7Z.A.301, 2V9I.B.1275, 3V96.B.302, 2V9E.A

.1276, 1VA3.A.100, 3VBD.A.2001, 1VDD.D.233, 2VES.A.1297, 2VES.C.1302, 2VF7.B.1844, 1VK9.A.143, 2VL6.A.1266, 3VOW.A.201, 3VPB.E.100, 3VPE.A.301, 2VP7.A.1399, 2VPD.A.1399, 2VPG.A.1401, 1VQ2.A.701, 1VQ2.A.702, 3VRK.A.301, 2VR6.A.1156, 2VUT.I.1713, 1VYK.A.1150, 1VYX.A.1062, 2W3N.B.1234, 2W3Q.A.1231, 2W4L.A.1170, 2W5V.B.1377, 2W5X.A.1378, 3W5K.B.502, 1WAA.B.1090, 4WAJ.A.301, 2WBT.A.1130, 3WBH.B.505, 1WEM.A.201, 1WEN.A.401, 1WEO.A.401, 1WEP.A.201, 1WES.A.201, 1WES.A.401, 1WFF.A.401, 1WFL.A.401, 3WF8.A.401, 1WFP.A.401, 1WIG.A.201, 1WIG.A.401, 1WII.A.201, 1WIM.A.201, 2WJV.A.3, 1WJP.A.301, 1WJP.A.501, 1WJV.A.201, 1WJV.A.401, 3WLE.A.402, 3WLF.A.402, 2WOJ.A.1353, 3WRG.A.702, 1WUQ.A.1001, 1WY2.B.407, 1WYH.A.201, 1X3H.A.401, 1X3Z.A.999, 2X3B.A.1341, 1X4I.A.201, 1X4J.A.401, 1X4K.A.201, 1X4K.A.401, 1X4S.A.401, 1X62.A.201, 1X6H.A.401, 2X8Z.A.1616, 2X95.A.1615, 2XBL.A.1197, 2XBL.C.1196, 1XC3.A.302, 1XCR.A.1001, 4XIX.B.401, 1XKI.A.1003, 2XML.A.1349, 2XQV.A.401, 1XRT.A.1423, 1XSO.A.152, 2XS4.A.998, 1XWY.A.401, 1Y02.A.161, 2Y6D.A.1267, 1Y93.A.265, 1YBO.B.160, 1YC2.C.406, 1YC5.A.1001, 2YHW.A.1719, 2YHY.A.2000, 1YLK.A.401, 1YQD.A.1000, 2YQL.A.201, 2YQL.A.401, 2YQP.A.201, 2YQQ.A.401, 2YRT.A.401, 2YRC.A.200, 2YRE.A.601, 2YRG.A.401, 2YRM.A.201, 2YS2.A.201, 2YSA.A.181, 2YSO.A.181, 2YTH.A.201, 2YTP.A.181, 2YTQ.A.201, 2YTR.A.201, 2YTT.A.181, 2YT5.A.201, 2YT5.A.401, 2YTB.A.301, 2YTD.A.201, 2YTJ.A.201, 2YTK.A.201, 2YTM.A.181, 2YU8.A.201, 2YVR.A.1001, 2YYR.A.401, 2YYR.A.402, 1Z05.A.406, 2Z3H.A.2001, 2Z45.A.1001, 1Z60.A.2, 1Z6R.A.501, 1Z84.A.603, 2Z9J.A.902, 2ZEP.A.391, 2ZED.A.391, 2ZEG.A.391, 1ZFK.A.1300, 1ZFO.A.31, 1ZGE.A.1000, 1ZIN.A.219, 2ZNR.A.2, 1ZP5.A.999, 3ZP9.A.1009, 3ZQ6.A.1323, 2ZTX.A.501, 3ZUK.A.1664, 2ZU2.A.5517, 3ZVS.A.1159, 3ZXH.A.301, 1ZXV.B.9002, 1ZZU.A.900, 2ZZE.A.753

[1] "Cluster 5"

2L1G.A.88, 4QCL.A.1303, 1QUM.A.302, 830C.A.272, 456C.A.272, 4A3W.A.1159, 1A7T.A.252, 2A7M.A.252, 4A7B.A.1276, 4A7Y.A.951, 4A7Z.A.950, 1A85.A.999, 1A86.A.999, 2A8H.A.486, 2AB7.A.30, 1AFO.A.472, 1AH7.A.246, 1AH7.A.248, 2AIO.A.315, 4AR8.A.1731, 1AST.A.999, 4ASQ.A.1615, 4ASR.A.1615, 4AWY.B.3229, 4AX0.B.3229, 4AX1.B.3229, 1B3D.B.301, 1B57.A.360, 3B7S.A.701, 3B7U.X.701, 3B8Z.A.901, 2BH3.A.1002, 1BIW.B.801, 2BIB.A.1549, 4BIN.A.500, 3BJC.A.876, 4BJH.A.423, 3BKQ.X.500, 1BLL.E.488, 4BLB.A.910, 2BMI.A.272, 2BNN.B.1200, 2BNO.A.1201, 1BQO.B.301, 1BS8.A.2001, 4BT4.A.301, 4BT5.A.301, 4BT6.A.1257, 3BUB.A.1047, 3BUD.A.1048, 3BUI.A.1046, 3BVT.A.1048, 3BVU.A.1048, 3BVV.A.1047, 3BVW.A.1046, 3BVX.A.1046, 4BXK.A.1620, 4BZ3.B.502, 4BZ5.A.500, 4BZR.A.1630, 3C0Z.B.101, 3C10.A.101, 4C24.A.301, 1C3R.A.501, 1C3S.A.951, 3C52.A.401, 4C5W.A.402, 4C6L.A.2823, 4C6L.A.2824, 4C8I.B.1161, 1CAM.A.262, 2CA2.A.264, 4CA5.A.1001, 4CA7.A.1616, 4CA8.A.1620, 2CEA.A.1603, 2CFU.A.1001, 2CHI.A.212, 3CKI.A.501, 3CV5.A.1047, 4CWM.A.433, 1CXV.A.1, 3CZN.A.1102, 3CZS.A.1102, 3D0N.A.262, 2D1N.A.270, 2D1O.A.257, 1D5J.A.301, 1D7X.B.801, 2DDY.A.177, 4DD8.A.1002, 4DEF.A.401, 4DEL.A.402, 3DHA.A.255, 2DKD.A.921, 4DLM.A.401, 4DPE.A.301, 1DQS.A.402, 4DR9.A.201, 2DVT.A.1501, 2DVX.A.1501, 4DV8.A.801, 3DWB.A.771, 2DW0.A.700, 3DYC.A.451, 4DYK.A.501, 4DZH.A.504, 3E0L.A.1452, 3E2D.A.602, 2E2D.A.500, 3E38.A.1, 1E48.S.999, 1E4C.S.999, 3E4A.B.2000, 3EBG.A.1, 3EBH.A.1, 3EDZ.B.2, 2EG6.A.400, 1EI6.B.409, 3ELF.A.352, 6ENL.A.438, 2ERP.A.700, 3EWC.A.372, 3EWD.A.371, 4EYF.A.303, 1EZ2.A.402, 1F0J.A.1101, 3F15.A.264, 3F16.A.264, 3F17.A.264, 3F18.A.264, 3F19.A.264, 3F1A.A.264, 1FA5.A.1200, 1FA5.A.1201, 3FDK.A.401, 2FGN.A.248, 1FKX.A.400, 1FLS.A.166, 4FL7.A.301, 4FUA.A.216, 2FV5.A.3, 2FVM.D.601, 4FW3.B.300, 4FW4.C.301, 4FW5.A.301, 4FW7.A.301, 2FYV.A.2003, 4FYT.A.1012, 1G05.B.801, 1G49.B.801, 4G9L.B.302, 4GBD.A.502, 1GKP.A.1461, 1GKP.B.1460, 1GKR.A.1453, 4GK8.A.302, 2GMN.A.802, 2GO4.A.601, 2GSO.A.1000, 1GVF.A.288, 1GYT.G.600, 1GYT.G.601, 4H01.A.601, 4H1Q.A.301, 4H2E.A.301, 4H2G.A.601, 4H3X.A.301, 1H48.C.900, 1H4N.A.262, 4H49.A.301, 4H76.A.301, 3H8F.E.501, 4H82.A.301, 1H9N.A.262, 2HBV.A.401, 2HBM.A.1001, 3HC4.L.401, 3HC8.A.864, 2HC9.A.701, 2HC9.A.702, 1HFC.A.275, 1HJK.A.451, 3HK5.A.430, 3HK8.A.430, 3HK9.A.431, 3HKA.A.430, 1HOV.A.166, 2HPT.A.950, 3HR1.A.1, 1HS6.A.701, 1HTY.A.1102, 2HUC.A.248, 3HWP.A.295, 4HWO.A.701, 4HWP.A.701, 4HWR.A.701,

4HWS.A.701, 3HY7.A.901, 3HY9.A.901, 3HYG.A.901, 1HZY.A.402, 1HZY.B.401, 2I47.C.804, 3ID7.A.401, 4IE0.A.601, 4IE4.A.601, 4IE6.A.601, 4IG2.A.401, 2ILP.A.500, 4ILW.D.301, 4ISM.A.201, 3ITC.A.501, 3ITC.A.502, 1ITU.A.402, 2J0T.A.1267, 4J4K.A.402, 4J5F.A.301, 4J5H.A.302, 1J79.A.400, 1J79.B.400, 2J83.A.999, 2J9A.A.1493, 1JAP.A.999, 1JAQ.A.999, 1JCZ.A.901, 4JD1.A.201, 4JE7.A.202, 4JH8.A.201, 4JH8.B.201, 1JIW.P.481, 2JIG.A.1252, 2JIH.B.1554, 1JJE.A.252, 1JJT.A.252, 1JK3.A.400, 2JNE.A.200, 4JP4.A.301, 2JSD.A.276, 2JT5.A.256, 2JT6.A.256, 1JWQ.A.1001, 1K07.B.3, 3K2G.A.400, 2K4W.A.156, 1K4P.A.1004, 3K5X.A.402, 4K5P.A.1101, 1K9Z.A.402, 1KAE.B.1102, 4KAP.A.301, 1KBC.A.999, 3KDS.E.996, 1KEQ.A.280, 4KEQ.A.302, 3KM8.A.400, 3KR5.G.1004, 3KRY.A.1999, 4KTX.A.501, 1KYS.A.301, 1LAM.A.488, 1LAM.A.489, 1LCP.A.489, 4LCQ.A.601, 4LE6.A.405, 4LE6.A.406, 4LEF.A.301, 2LFF.A.502, 3LGG.B.512, 3LJT.A.901, 3LJZ.A.999, 3LK8.A.264, 3LS6.A.303, 4LV4.A.401, 3M4C.D.109, 3MA2.D.294, 3MA2.A.294, 3MAX.A.379, 4MCA.B.1000, 3MDU.A.454, 3MJM.B.401, 3MK1.A.902, 3MKV.B.425, 1MMB.A.999, 1MNC.A.281, 3MTW.A.1, 3MVI.A.901, 3N2U.A.264, 3N2V.A.264, 4N27.A.201, 3N2C.A.425, 3N9S.A.309, 2NQH.A.301, 2NQH.A.302, 4NTK.A.201, 3NX7.A.264, 3NXQ.A.650, 2NZE.A.401, 3O2X.A.1999, 2O4Q.A.2401, 3O90.B.192, 4O98.B.401, 2OB3.A.901, 2OC2.A.701, 3OD4.A.1350, 3OHL.A.4, 3OHL.A.5, 3OHO.A.5, 2OKL.A.601, 1ONW.A.800, 4OPN.A.201, 4OPN.B.201, 1OS9.A.901, 4OUI.A.501, 2OW1.A.444, 2OW6.A.3001, 2OW7.A.5001, 3P3C.A.401, 1P5X.A.248, 1P6D.A.246, 1P6D.A.248, 1P6E.A.248, 3P76.A.274, 2PAJ.A.493, 1PB0.A.1302, 2PJT.A.302, 2PLM.A.407, 1PMI.A.445, 3PN4.A.1001, 3PNU.A.337, 1PS6.A.330, 1PTW.A.501, 1PV9.A.401, 1PVW.A.401, 4PVO.A.402, 2Q02.C.300, 3Q2G.A.401, 3Q2H.A.401, 3Q4R.A.201, 2Q5B.A.107, 2Q5B.C.107, 3Q6X.A.3, 3Q6X.B.271, 3Q9F.B.344, 3QAY.A.180, 4QA0.A.401, 4QA1.A.403, 4QA2.A.403, 4QA4.A.502, 4QA5.A.401, 4QA6.A.401, 1QF1.A.320, 1QH5.A.262, 1QIN.A.401, 1QIN.B.301, 1QIP.D.903, 3QIZ.A.431, 1QJJ.A.250, 2QPJ.A.1, 1QTW.A.303, 1QXL.A.400, 2R2D.A.278, 1R3N.A.500, 1R43.A.500, 4R76.A.1001, 1RJ5.A.601, 2RJQ.A.1, 4RL2.A.301, 4RL2.B.302, 1RM8.A.500, 1RMZ.A.264, 3RTS.A.264, 3RTT.A.264, 3S2J.A.402, 3S2L.A.402, 3S2M.A.402, 3S2N.A.402, 3SEY.E.373, 1SHN.B.482, 1SML.A.270, 3SNG.A.401, 3SNG.A.402, 3SPU.C.1010, 3T00.A.502, 1T64.A.388, 2TCL.A.170, 4TLN.A.321, 5TLN.A.321, 4TMN.E.322, 3TOM.B.108, 1TQS.A.1401, 1TQT.A.1301, 1TQU.A.1400, 1TQV.A.1300, 1TQW.A.1400, 4TYT.A.301, 3U04.A.200, 1U7J.B.150, 1U7M.A.54, 1U7M.B.154, 3U79.D.110, 3U79.E.110, 1UEA.C.1, 3UHM.A.300, 1UIO.A.400, 1URA.A.451, 2USH.A.601, 2USH.A.602, 3UWB.A.200, 1UXA.B.1367, 2UX1.A.1174, 2UX1.C.1173, 3V1E.A.101, 2V5W.A.1380, 2V5X.A.1377, 3V77.A.301, 3V77.A.302, 3V96.B.301, 2V9G.A.1276, 2V9M.A.1275, 2VES.A.1295, 1VFL.A.501, 3VPE.A.302, 2VQM.A.1411, 3VQZ.A.302, 3VTG.A.301, 2VUN.A.401, 2W0D.A.1264, 2W15.A.1203, 1W22.A.1375, 2W3Z.A.1312, 3WAX.A.912, 3WAY.A.911, 4WB7.A.502, 4WD6.B.302, 2WM1.A.1333, 2WO9.B.1268, 1WPP.A.602, 2WXU.A.1375, 2WXT.A.1371, 1WY2.A.406, 4X2T.B.1002, 1XBU.A.901, 1XBU.A.902, 2XHM.A.1616, 2XL9.B.1269, 1XM8.A.700, 2XS3.A.999, 2XS4.A.999, 1XXW.A.201, 2XYD.A.1620, 2Y6D.A.1268, 1Y7W.A.283, 1Y93.A.264, 2YD0.A.1946, 1YQY.A.781, 2Z24.B.400, 2Z25.B.400, 2Z27.B.400, 2Z28.B.400, 2Z29.B.400, 2Z3I.C.2003, 1Z60.A.1, 2Z72.A.401, 2Z72.A.402, 2ZBM.A.401, 2ZBM.A.402, 1ZED.A.904, 1ZNB.A.2, 2ZNE.B.991, 2ZO4.A.319, 3ZU0.A.1595, 3ZU0.A.1596, 3ZU0.B.1588, 3ZU0.B.1589, 2ZWR.A.208, 2ZWR.A.209, 1ZXC.A.201, 3ZXH.A.300, 1ZZM.A.401, 4BLD.D.910, 4FC5.E.305, 2FZ6.A.201, 4H82.B.300, 2JMD.A.66, 2KV1.A.125, 4LW9.E.204, 2MQ1.A.102, 1NDV.A.400, 2X4H.A.1141, 2Z45.A.1003

[1] "Cluster 6"

2JZW.A.57, 4LJ0.B.502, 4OND.E.101, 3CQJ.A.285, 4CXO.A.453, 4CXV.A.433, 2DVU.A.1501, 2EG7.A.401, 4ENL.A.438, 2EWB.A.488, 1EYW.A.401, 4EYF.A.302, 2JNE.A.150, 4JS6.A.301, 2K2C.A.140, 1KMG.A.154, 3KNS.A.228, 3L6N.A.301, 2LFF.A.500, 3NJ9.A.262, 1P6B.A.401, 1PL6.A.402, 3QDF.A.266, 1QF0.A.320, 2QVV.A.403, 3RHG.A.367, 3SXK.A.4, 3W52.A.311, 2XF4.A.1211, 2Z24.A.400, 2Z25.A.400, 2Z26.A.401, 2Z27.A.400, 2Z28.A.400, 2Z29.A.400, 2Z2A.A.400, 2Z2B.A.338, 1ZZM.A.402, 3IE1.D.442, 4L8H.R.105, 1V15.A.1132, 3A3O.B.65, 3AF5.A.665, 2ANH.A.451, 2ANU.B.405, 2APO.B.501, 3B1B.A.378, 2B5L.C.3001, 1BAW.A.107, 1BH5.A.201, 1BQQ.M.289, 3C0Y.A.401, 3C0Z.A.101, 2CBN

.A.402, 3CE9.B.400, 2CEA.B.1606, 2CG3.Z.1, 2CIH.A.212, 3CQJ.B.285, 4CWM.B.433, 3D3X.A.428, 1D8M.B.801, 1DSQ.A.144, 1DXW.A.301, 3E50.A.1, 2EC7.A.51, 4EGE.A.411, 3EII.D.301, 2EIM.C.262, 1ELZ.A.451, 1ENQ.C.238, 3EYV.L.220, 4F0R.A.501, 3FDK.A.402, 3FGG.A.161, 4FUK.A.401, 3GJN.B.600, 2GLQ.A.2002, 4H00.A.601, 4H01.A.602, 2H42.A.501, 3H66.B.500, 3H8F.B.501, 3H90.D.5, 3H90.D.6, 3HDB.A.620, 2HD1.B.101, 4HGX.B.301, 1HOV.A.165, 1HP7.A.401, 4IGM.A.401, 4IGM.F.401, 4IGN.A.401, 3II1.A.571, 3ITM.A.1, 4J3D.A.301, 1JDI.A.301, 1JM7.B.143, 1JOE.A.205, 2JRP.A.150, 4JSS.A.301, 2K2G.A.2, 4K6T.B.403, 1KAR.A.501, 1KAR.B.502, 2KVG.A.85, 2L0Z.A.487, 3L8Y.A.301, 1L9Y.A.2, 1LG6.A.262, 2M7Y.A.101, 3MKV.A.425, 4MLX.A.301, 3MO2.D.5, 2MQ1.A.101, 4MZ7.A.701, 3N2C.D.426, 4NRZ.B.301, 1NYR.A.1002, 3O90.A.192, 4O98.A.401, 2OC7.A.901, 1OLP.A.1373, 1OLP.A.1375, 1OLP.D.1374, 2OXW.A.264, 1P5X.A.247, 2PJT.D.302, 3PJN.A.189, 2PTW.A.500, 3Q31.A.1, 1Q74.D.304, 2QFP.A.434, 3R2J.A.301, 4R7M.D.1001, 3RAM.D.998, 1RO9.A.529, 3S6L.B.185, 3SFW.A.502, 1SMP.A.472, 1T0A.B.760, 3T02.A.502, 1TM6.A.23, 4TQT.A.501, 3TVX.A.900, 3UBF.A.7, 1UR6.B.79, 1UXA.C.1367, 3V94.E.702, 3VUV.A.501, 2VXX.D.201, 3WI2.B.801, 2WWO.A.1165, 4X2T.D.701, 4X2T.D.702, 2XR1.A.1638, 2XR1.A.1639, 2XY9.A.1628, 1YIX.A.601, 1YIX.B.603, 2Z2D.A.264, 1Z3J.A.264, 1ZKN.C.601, 3ZNS.A.101, 2ZNE.B.992, 1ZSW.A.315, 3ZTV.A.1598
[1] "Cluster 7"
1A1I.A.201, 1A73.A.202, 4AA6.A.253, 2B3J.A.2001, 2C7A.A.1641, 2C7A.A.1642, 3CBB.A.1001, 4CIS.A.300, 1CYQ.A.601, 1CYQ.A.602, 2DRP.A.171, 2DRP.A.172, 1DSZ.A.1121, 1DSZ.A.1122, 1DSZ.B.1222, 3EQT.A.1, 4ESJ.A.301, 1F4S.P.64, 1FFY.A.1001, 2FF0.A.1001, 2FF0.A.1002, 1G2F.C.303, 3G9M.A.526, 2GAT.A.67, 4GAT.A.67, 6GAT.A.67, 3GOX.A.301, 4GZN.C.203, 2HAN.A.351, 2HAN.A.352, 2HAN.B.353, 2HAN.B.354, 4HC9.A.401, 4HC9.A.402, 2HGH.A.191, 2HGH.A.192, 2HGH.A.193, 4HN5.A.601, 4HN6.A.602, 4HP3.C.202, 2IHX.A.236, 2JP9.A.132, 2JZW.A.56, 1K82.A.450, 2KAE.A.175, 1KB2.A.151, 3KDE.C.78, 2KMK.A.83, 2KMK.A.84, 2KMK.A.85, 3KMP.A.2, 1LAT.A.1514, 1LAT.A.1515, 4LJ0.A.501, 4LJ0.A.502, 4LJ0.A.503, 1LLM.C.301, 1LO1.A.195, 3LRR.A.1, 2LT7.A.701, 2LT7.A.702, 2LT7.A.703, 3M7K.A.143, 3M7K.A.144, 4M8O.A.1303, 3M9E.A.208, 3M9E.B.209, 4M9E.A.505, 4M9V.C.201, 1MEY.C.89, 3NCU.A.1, 4NDH.A.402, 2NLL.B.450, 2NLL.B.451, 2O6M.A.601, 3OD8.A.200, 4OLN.A.101, 4OLN.A.102, 4OND.A.101, 4OND.A.102, 4OOR.A.102, 1P47.A.203, 4PZI.A.1101, 4PZI.A.1102, 4QEN.A.802, 4QEN.A.803, 4QEN.A.805, 3QMD.A.300, 3QMD.A.301, 4R2A.A.503, 4R2A.A.505, 4R2S.A.501, 1R4O.A.526, 1TDZ.A.1001, 1TF3.A.102, 1TF3.A.2, 1TF3.A.3, 4TNT.A.701, 4TNT.A.702, 3TS2.A.1, 3TS2.A.2, 3U6P.A.300, 1UBD.C.501, 1UBD.C.502, 1UBD.C.503, 1UBD.C.504, 3UK3.C.968, 3VD6.C.501, 2XQC.A.1138, 2YKG.A.927, 1YUI.A.64, 1ZAA.C.203, 1ZNS.A.1500, 2A1K.A.1, 3A1B.A.1, 3A1B.A.2, 3A1B.A.3, 4A24.A.601, 4A24.A.602, 4A2C.A.1349, 4A2V.A.1000, 3A43.A.701, 2A51.A.54, 2A51.A.55, 2A5H.A.421, 2A5H.B.421, 2A6H.D.7458, 2A6H.D.7412, 1A71.A.402, 1A7T.A.251, 2A8D.A.1230, 2AFW.A.996, 2AFX.A.996, 2AFZ.A.391, 3AII.A.1001, 2AKL.A.117, 3ALR.A.601, 1AM6.A.262, 2APS.A.400, 4ARE.A.1790, 3ASL.A.3, 2ASH.A.400, 4AU7.A.1248, 4AUQ.B.1299, 4AUQ.B.1300, 3AVR.A.1502, 3AXS.A.401, 2AYD.A.369, 2AYJ.A.57, 4AY8.A.600, 4AYC.A.1484, 1AZM.A.261, 2B0O.E.698, 2B44.A.400, 4B6D.A.1341, 2B9D.A.1002, 1BBO.A.60, 4BF1.A.270, 4BF6.A.1262, 3BL1.A.262, 2BL6.A.1059, 4BM9.A.1466, 4BM9.A.1469, 1BN1.A.262, 1BN3.A.262, 1BN4.A.262, 1BNN.A.262, 1BNT.A.262, 1BNU.A.262, 1BNV.A.262, 1BNW.A.262, 3BO5.A.301, 3BO5.A.302, 3BO5.A.303, 3BO5.A.304, 3BOF.A.701, 3BQ5.A.800, 3BQ6.A.800, 4BS9.A.1782, 4BUE.A.2162, 2BYO.A.1209, 1BZM.A.261, 2BZ1.A.1174, 4C09.A.351, 1C2G.A.409, 4C3E.A.201, 4C3T.A.300, 4C4O.B.600, 3C5K.A.201, 3C5K.A.202, 4C5W.A.403, 3C63.A.107, 3C63.B.107, 3C63.C.107, 3C63.D.107, 3C6W.A.2, 3C7P.A.262, 2C7N.A.499, 1C9Q.A.999, 3CA2.A.264, 4CA1.A.283, 1CCT.A.262, 1CDO.B.377, 2CDC.A.1372, 2CEX.C.1306, 1CG2.A.502, 3CG7.A.299, 3CHQ.A.701, 3CHS.A.701, 3CHV.A.301, 1CIM.A.262, 1CIN.A.262, 2CJS.C.201, 2CJS.C.202, 2CKL.A.1104, 2CKL.A.1105, 2CKL.B.1115, 2CKL.B.1116, 1CNG.A.1, 1CNH.A.1, 1CNJ.A.1, 3COS.A.502, 4CPD.A.1300, 2CRW.A.401, 2CRC.A.401, 2CSV.A.200, 2CSV.A.400, 2CS2.A.200, 2CS3.A.200, 2CS7.A.201, 2CS8.A.401, 2CS8.A.601, 2CSH.A.200, 2CSH.A.400, 2CUP.A.401, 3CXK.A.201, 3CXL.A.500, 1CXX.A.1, 1D0C.A.900, 1D1T.A.375, 3D2Q

.A.303, 3D2Q.A.304, 3D7F.A.1752, 2D8X.A.401, 2D8Y.A.401, 2D8Z.A.401, 2D8R.A.401, 2D9G.A.201, 2D9H.A.201, 2D9H.A.401, 2D9K.A.401, 2D9K.A.601, 2D9L.A.401, 3DAZ.A.262, 2DAS.A.101, 3DBH.B.1, 2DB6.A.201, 4DB3.A.401, 3DCC.A.262, 1DCQ.A.600, 1DD6.A.502, 3DD0.A.262, 1DDZ.A.2, 1DFE.A.38, 2DFV.A.1001, 3DGD.C.128, 2DIP.A.201, 2DLK.A.201, 2DLK.A.401, 1DL6.A.60, 2DLO.A.401, 2DLQ.A.200, 2DLQ.A.400, 2DLQ.A.500, 4DLA.A.402, 3DMO.A.131, 2DMD.A.241, 2DMI.A.200, 2DMI.A.300, 2DMJ.A.200, 2DPH.A.1001, 2DS7.A.100, 1DVP.A.401, 1DVP.A.402, 4DZ7.A.301, 1E0E.A.147, 2E2Z.A.101, 1E3J.A.901, 1E4U.A.79, 1E4U.A.80, 2E5R.A.201, 2E5R.A.401, 2E5S.A.201, 2E5S.A.401, 2E6R.A.201, 2E6R.A.401, 2E6S.A.201, 2E6S.A.401, 2E6S.A.601, 1E7L.A.1165, 2EBL.A.241, 2EBT.A.100, 2EBT.A.200, 2EBT.A.300, 2EBV.A.201, 3EB5.A.1001, 3EBE.A.500, 2EBQ.A.201, 2EBR.A.201, 2ECJ.A.401, 2ECV.A.401, 2ECN.A.201, 1EE2.A.1301, 1EE8.A.501, 3EED.A.194, 2EE8.A.701, 4EEZ.A.401, 1EF4.A.56, 3EFO.A.766, 2EGQ.A.200, 2EGQ.A.300, 2EGM.A.200, 2EGM.A.300, 2EGP.A.200, 2EGP.A.400, 3EH2.A.800, 2EHE.A.200, 2EHE.A.300, 2ELN.A.181, 2ELY.A.200, 2ELZ.A.200, 2EL4.A.200, 2EL5.A.200, 2EL6.A.200, 2ELI.A.201, 2EMJ.A.201, 2EM5.A.201, 2EM7.A.201, 2EM8.A.201, 2EMA.A.201, 2EME.A.201, 2EMP.A.201, 2ENT.A.200, 2ENV.A.200, 2ENV.A.300, 1EN7.A.401, 2ENZ.A.300, 2ENZ.A.400, 2EN7.A.181, 2EN8.A.181, 2EOR.A.201, 2EOS.A.201, 2EOZ.A.201, 2EOD.A.300, 2EOD.A.400, 2EOG.A.201, 2EOL.A.201, 2EPR.A.201, 2EPS.A.201, 2EPW.A.201, 2EP0.A.201, 2EP4.A.200, 2EPA.A.300, 2EPA.A.400, 2EPC.A.201, 2EPP.A.201, 2EQE.A.201, 2EQF.A.201, 2EQG.A.201, 1ESK.A.55, 2ESL.A.4, 4ETS.A.302, 1EXK.A.80, 1EXK.A.81, 1EZM.A.302, 1F18.A.155, 2F14.A.1262, 2F3B.A.341, 4F3W.A.201, 2F4M.A.501, 1F62.A.52, 1F62.A.53, 1F81.A.88, 1F8F.A.373, 1F9X.A.999, 2F9I.B.601, 4F9V.A.401, 4FAI.A.401, 4FBE.A.403, 2FC6.A.201, 2FC7.A.201, 2FC7.A.401, 2FE3.A.201, 2FEA.A.1302, 2FGY.A.620, 2FGY.A.621, 2FHX.A.317, 3FID.A.298, 4FKD.A.102, 4FKE.A.1024, 3FL2.A.1001, 3FL2.A.1002, 3FLO.B.1, 3FLO.B.2, 1FN9.A.1001, 2FOY.A.301, 1FP0.A.90, 3FQM.A.901, 2FR5.A.147, 4FRC.A.302, 1FU9.A.37, 4FU5.A.302, 4FVD.A.201, 3FW3.A.300, 1FWQ.A.124, 4FWE.A.901, 4FWE.A.902, 4FYY.B.201, 2FZW.A.375, 3G1P.A.300, 1G25.A.66, 1G25.A.67, 3G27.A.97, 4G26.A.1001, 1G48.A.262, 2G45.A.401, 1G52.A.262, 1G54.A.262, 2G6Q.A.400, 1G71.A.348, 4G7A.A.301, 2G84.A.506, 2G9T.A.999, 3GA3.A.1, 2GAG.D.101, 1GDC.A.73, 1GDC.A.74, 2GFO.A.1200, 4GGJ.A.301, 3GI1.A.501, 4GIZ.C.201, 3GJ3.B.300, 3GJ5.B.300, 3GJ8.B.300, 4GNE.A.1502, 2GQJ.A.200, 2GQJ.A.300, 1GUP.A.350, 4GVE.A.601, 1GZH.A.1293, 3GZK.A.539, 3H0N.A.201, 4H0F.A.401, 4H12.A.1801, 4H12.A.1803, 2H6E.A.500, 3H7H.A.119, 1H7V.A.61, 3H8V.A.401, 3H99.A.601, 4H9D.A.201, 2HBA.A.101, 1HC7.A.490, 3HCI.A.1000, 1HCP.A.99, 3HCJ.A.1000, 3HCS.A.303, 2HD6.A.262, 2HDP.A.492, 2HDP.A.493, 2HF1.A.102, 3HI2.A.121, 4HI8.B.101, 4HI8.B.102, 2HJH.A.800, 3HKQ.A.261, 3HKU.A.261, 2HL4.A.262, 3HNA.A.501, 3HNA.A.502, 3HNA.A.503, 3HNA.A.504, 3HNI.A.107, 2HNC.A.263, 2HOC.A.263, 2HQH.E.1500, 2HRV.A.143, 3HS4.A.301, 1HSO.A.1376, 4HSU.A.904, 1HTD.A.401, 4HT2.A.301, 1HUG.A.261, 4HU1.A.301, 2HU9.A.132, 1HXP.A.350, 4I1F.A.503, 4I1F.A.504, 4I1F.A.508, 4I1H.A.507, 3I2D.A.1, 2I3H.A.1001, 3I4C.A.400, 3I4C.A.500, 2I50.A.336, 2I50.A.337, 2I50.A.338, 4I51.A.3005, 1I8Z.A.262, 2I9W.A.201, 1IA6.A.1264, 1IBI.A.195, 1IBI.A.196, 3IBL.A.262, 3IBN.A.262, 3IBU.A.262, 2IBI.A.1, 4IBY.A.301, 2IDA.A.104, 1IF9.A.262, 4II1.A.901, 1IML.A.78, 3IMI.A.201, 1INN.B.167, 3IO2.A.202, 2IOI.A.3001, 2IQJ.A.301, 1IRN.A.55, 3IR9.B.501, 3IRB.A.201, 3IUF.A.1, 4IUM.A.501, 1IYM.A.182, 1IYM.A.183, 2IYB.E.1422, 2IYB.E.1423, 1J2O.A.115, 2J6A.A.1138, 1J98.A.300, 2J9U.B.1162, 1JAZ.A.401, 4JBG.A.401, 4JEA.A.202, 4JEA.B.202, 4JEA.C.202, 4JEA.D.202, 1JJD.A.104, 1JJE.B.251, 1JJT.B.251, 4JLW.A.401, 2JMO.A.201, 2JMO.A.401, 2JM1.A.2, 2JM3.A.92, 4JMY.A.201, 2JMD.A.65, 2JOX.A.110, 1JOC.A.300, 2JQ5.A.129, 2JR7.A.85, 2JRJ.A.62, 2JRJ.A.63, 2JTG.A.88, 1JTK.A.137, 3JUE.A.999, 2JUN.A.220, 2JVX.A.29, 1JVB.A.400, 3JVH.A.163, 2JVN.A.400, 2JWO.A.488, 2JW6.A.601, 3JXP.A.320, 4JXE.A.501, 1JY8.A.300, 2K0A.A.108, 2K0A.A.109, 2K0A.A.110, 2K1P.A.96, 2K16.A.940, 2K16.A.941, 3K2F.A.262, 2K2C.A.138, 2K2C.A.139, 2K2C.A.141, 2K2C.A.142, 2K2C.A.143, 2K2D.A.80, 3K35.C.317, 2K4X.A.56, 3K5K.A.1194, 3K5K.A.1197, 3K5K.B.1195, 3K5K.B.1196, 3K7H.B.1001, 2K7R.A.129, 4K7D.A.501, 4K7D.A.502, 4K7D.A.505, 4K7D.A.507, 4K7W.B.101, 1K81.A.14

4, 2K9H.A.101, 2K9H.A.102, 2KAK.A.130, 2KAK.A.170, 2KDX.A.120, 3KE1.A.163, 2KGG.A.53, 2KGG.A.54, 2KGO.A.109, 1KHK.A.451, 2KI7.B.124, 2KJE.A.501, 2KJE.A.502, 2KKT.A.500, 2KKH.A.201, 2KKR.A.500, 1KLR.A.31, 1KLS.A.31, 3KNV.A.201, 3KNV.A.202, 2KPI.A.150, 3KQI.A.71, 2KQ9.A.113, 2KQB.A.1001, 2KR1.A.65, 2KU3.A.63, 2KU3.A.64, 4KUY.A.301, 3KV5.A.489, 3KV5.A.490, 4KV0.A.301, 2KWJ.A.501, 2KWJ.A.601, 2KWJ.A.701, 2KWJ.A.801, 4KXQ.A.601, 2KZY.A.63, 2L0Z.A.486, 3L00.A.180, 3L11.A.601, 3L11.A.602, 4L3J.A.402, 2L5U.A.62, 2L5U.A.63, 4L56.A.401, 4L58.A.102, 2L6Y.A.239, 2L6Z.B.37, 1L6J.A.500, 2L6L.A.201, 2L6M.A.201, 2L7X.A.106, 2L7X.A.107, 2L75.A.155, 2L75.A.156, 2L7P.A.201, 4L7X.A.101, 2L80.A.124, 2L9Z.A.403, 2LAU.A.82, 2LBM.A.1, 2LBM.A.3, 3LCZ.A.54, 2LCE.A.200, 2LCE.A.300, 2LCQ.A.162, 2LGV.A.109, 2LGG.A.381, 2LGG.A.382, 2LHN.A.501, 2LHN.A.502, 2LHN.A.503, 4LHI.A.301, 1LIQ.A.28, 2LI9.A.18, 2LJX.A.200, 2LJZ.A.201, 3LJU.X.401, 4LJO.A.1101, 4LJO.A.1102, 4LJO.A.1104, 4LJP.A.1101, 3LKM.A.904, 2LKO.A.32, 4LK9.A.401, 4LMY.A.202, 2LN0.A.401, 2LN0.A.501, 2LN0.A.601, 2LN0.A.701, 2LO2.A.101, 2LO3.A.101, 2LO4.A.300, 4LO9.A.401, 1LPV.A.54, 3LQH.A.1002, 3LRQ.C.100, 2LRI.C.101, 2LRI.C.102, 2LUA.A.101, 2LUA.A.102, 2LUA.A.103, 4LU3.A.301, 1LV3.A.66, 2LV2.A.101, 2LV2.A.102, 2LV9.A.201, 2LV9.A.202, 2LWW.A.501, 2LWW.A.502, 2LWW.A.503, 2LXD.A.201, 4LXL.A.403, 2LZU.A.201, 2LZU.A.202, 3M04.A.501, 2M0F.A.101, 4M0W.A.401, 2M1S.A.100, 3M1D.A.1000, 2M13.A.601, 2M13.A.602, 1M2K.A.999, 1M2O.A.800, 3M2X.A.500, 3M2Y.A.500, 4M2R.A.301, 4M2V.A.301, 1M3V.A.123, 3M4C.B.108, 2M48.A.503, 2M6M.A.201, 2M6M.A.202, 2M7Q.A.102, 2M85.A.801, 2M85.A.802, 2M9Y.A.401, 2M9A.A.101, 2M9A.A.103, 2M9A.A.102, 2MA5.A.101, 2MA5.A.102, 2MA6.A.101, 2MA6.A.102, 1MBX.B.211, 3MDZ.A.263, 2MD7.B.101, 2MD7.B.102, 2MDG.A.101, 2MDG.A.102, 1MEA.A.29, 3MEK.A.500, 3MEK.A.501, 3MEK.A.502, 3MEQ.A.501, 4ME3.A.301, 1MGO.A.376, 4MG3.A.201, 3MHC.A.262, 3MHH.E.97, 3MHS.A.472, 3MHS.A.474, 3MHS.A.476, 3MHS.A.477, 4MHQ.A.501, 4MHY.A.400, 4MI5.A.802, 4MI5.A.803, 4MI5.A.804, 4MI5.A.805, 4MI5.A.807, 2MIU.A.302, 4MJ7.A.201, 2MKD.A.301, 1ML9.A.1, 1ML9.A.2, 3ML5.A.263, 1MM3.A.62, 1MM3.A.63, 2MNY.A.401, 2MNY.A.402, 1MNC.A.282, 3MNU.A.262, 4MO8.A.301, 3MPZ.A.300, 1MR1.C.601, 2MRE.B.301, 4MSG.A.1401, 4MSX.A.501, 2MUM.A.301, 2MUM.A.302, 1MZ8.B.600, 4N0N.A.501, 4N0N.A.503, 1N0Z.A.46, 3N3K.A.1, 4N4F.A.1402, 1N5N.A.401, 3N67.A.900, 1N8K.A.376, 3NB5.A.261, 1NCS.A.61, 1NEE.A.136, 3NHE.A.1, 3NIS.A.1, 3NIS.A.2, 3NIS.A.3, 1NKU.A.188, 3NKM.A.1001, 4NL4.H.803, 2NN1.A.301, 4NN2.A.403, 4NOS.A.3000, 4NQ6.A.301, 4NQ7.A.301, 4NQY.A.501, 2NYT.A.2000, 3NY1.A.4, 3NY1.A.5, 3NY1.A.6, 3NY3.A.2, 3NY3.A.3, 1NZJ.A.700, 2O03.A.202, 2O10.A.86, 2O10.A.87, 2O13.A.190, 2O13.A.191, 3O36.A.2, 2O3K.A.401, 2O4Z.A.262, 3O4N.A.2002, 4O62.A.1001, 4O64.A.2001, 4O64.A.2002, 4O64.A.2004, 3O70.A.500, 3O70.A.501, 3O7A.A.500, 3O7A.A.501, 3OCA.A.300, 4ODR.A.202, 4ODR.B.202, 2OGW.A.500, 4OGE.A.1201, 4OIF.A.701, 3OJ6.A.150, 1OKL.A.262, 1OKM.A.262, 2OM1.B.801, 3OOI.A.232, 3OOI.A.234, 1OQJ.A.183, 2OSM.A.262, 1OVX.A.61, 2OWA.A.201, 1OXN.A.1001, 3OYQ.A.262, 1OZB.I.50, 2OZU.A.800, 1P0F.A.2502, 2P09.A.200, 3P2A.C.151, 3P44.A.261, 3P55.A.261, 2P57.A.201, 1P6O.A.401, 1P7A.A.38, 3P8B.A.101, 3PB4.X.400, 3PB7.X.400, 3PB9.X.400, 3PBE.A.391, 1PEG.A.4, 1PG5.B.500, 4PHT.B.601, 3PJN.A.187, 3PJN.A.188, 3PLW.A.188, 2PLI.D.702, 3PN3.A.1001, 2POU.A.262, 2POW.A.262, 2POI.A.100, 2PPT.A.300, 1PQ4.A.1002, 2PQ8.A.501, 4PQT.A.501, 2PRS.A.501, 3PT9.A.1, 3PT9.A.2, 3PTM.A.1001, 1PTR.A.1, 2PUY.A.356, 3PUQ.A.2, 2PVX.A.901, 2PVE.A.301, 3PYK.A.262, 1PZW.A.100, 1Q08.A.401, 1Q08.A.402, 1Q08.B.403, 1Q08.B.404, 3Q01.A.1, 4Q09.A.301, 1Q3A.A.465, 1Q68.A.201, 1Q69.A.207, 3Q7C.A.1, 4Q7R.B.302, 3Q87.A.126, 1QBH.A.364, 1QF8.A.216, 4QF2.A.1802, 4QF3.A.2002, 3QGV.A.500, 2QIC.A.400, 2QKD.A.501, 3QL9.A.1, 3QL9.A.3, 4QN1.A.1501, 2QOA.A.262, 2QP6.A.262, 4QQ4.A.2001, 4QSI.A.301, 3QU1.A.501, 3QU1.A.503, 3QU1.B.502, 1QWY.A.400, 3QWP.A.500, 3QWP.A.501, 1QYB.A.401, 1QYP.A.58, 3R17.B.262, 4R2Y.A.101, 4R2Y.A.102, 4R2Y.B.102, 2R3A.A.300, 2R3A.A.301, 2R3A.A.303, 3R6F.A.132, 1R79.A.201, 1R79.A.401, 3RBU.A.1752, 2RGV.A.146, 1RGO.A.221, 1RGO.A.222, 2RHK.C.502, 2RI7.A.501, 2RI7.A.502, 3RIY.A.1001, 1RJW.A.401, 3RMQ.A.114, 1RNI.A.256, 4RN4.A.301, 2RO1.A.201, 2RO1.A.301, 4RQT.A.401, 2RR4.A.501, 3RSN.A.200, 2RSI.A.102, 2RSJ.A.101, 2RT9.A.701, 2RT9.A.702,

1RUT.X.603, 4RV9.A.501, 1RXR.A.213, 1RXR.A.214, 3RZV.A.2, 3RZ0.B.262, 3RZ8.A.1, 3S2Q.B.501, 3S2E.A.501, 1S3G.A.219, 3S72.B.262, 3S73.B.262, 3S75.B.262, 3S76.A.1, 3S8P.A.400, 3SBH.A.262, 1SE0.A.201, 3SI2.A.601, 3SJG.A.1752, 1SLM.A.257, 3SOU.A.7, 3SP4.A.601, 1SRK.A.36, 1SU3.A.913, 3SUB.A.161, 1SVM.A.700, 1T4W.A.201, 3T6P.A.1001, 3T6P.A.1002, 3T6P.A.1003, 3T6R.A.1, 3T6R.A.3, 3T7L.A.1, 3T7L.A.2, 3T82.A.261, 3T84.A.261, 3T85.A.261, 1T9H.A.411, 3T92.A.122, 3T92.A.124, 1TAF.A.2003, 1TEQ.X.262, 3TGN.A.147, 3TG4.A.436, 3TIO.B.185, 3TIO.D.185, 3TIO.E.185, 1TJL.A.200, 1TOT.A.53, 1TOT.A.54, 3TTC.A.1, 3TTC.A.2, 4TWJ.A.301, 4TYT.A.302, 4TZU.A.503, 1U0A.A.5005, 3U1L.A.241, 1U2N.A.441, 1U3T.A.375, 1U3U.A.375, 1U3W.A.375, 3U31.A.276, 4U4L.A.302, 1U5K.A.300, 1U5S.B.138, 3U52.A.514, 3U5N.A.2, 1U85.A.34, 1U86.A.36, 3U9G.A.226, 3U9G.A.227, 3U9G.A.228, 3U9G.A.229, 1UAQ.A.200, 3UCJ.A.228, 3UCO.A.228, 3UDZ.B.800, 3UEH.A.143, 3UEY.A.4, 3UEE.A.143, 3UEI.A.143, 3UEJ.A.301, 3UEJ.A.302, 4UF0.A.2269, 3UFF.A.1, 3UGD.A.2, 1UL4.A.139, 3UNG.C.903, 4UP0.A.1384, 4UTV.A.1299, 3UVI.A.387, 2UVL.A.1336, 3UW4.A.401, 3UX8.A.1001, 2UZG.A.132, 2UZG.A.133, 2V0C.A.1815, 2V0C.A.1816, 3V1F.B.703, 4V2Y.A.150, 3V3G.B.301, 3V4K.A.2, 1V54.F.99, 1V6G.A.401, 3V7X.A.2001, 1V9E.B.260, 1V9X.A.200, 2V9E.B.1276, 2V9K.A.1533, 3VDP.A.201, 1VFY.A.300, 1VFY.A.301, 2VF7.A.1845, 3VGL.A.322, 1VHH.A.400, 3VHS.A.51, 3VHS.B.51, 3VHT.B.401, 1VJ0.A.400, 1VJE.A.167, 2VJE.B.1491, 2VKR.A.106, 2VNF.A.1247, 3VOV.A.401, 2VO9.B.501, 2VPG.A.1400, 1VQ0.A.300, 2VQM.A.1412, 2VQG.C.1091, 2VRS.A.1328, 2VRW.B.1565, 2VRW.B.1566, 2VRD.A.1062, 1VSR.A.201, 3VTH.A.802, 3VUW.E.801, 2VVB.X.1268, 1VZY.A.1291, 2W0T.A.125, 2W0D.A.1269, 1W4R.A.400, 2W5Z.A.4970, 3W5K.B.504, 4W6Z.A.402, 1W8P.B.1030, 2WB0.X.601, 2WB0.X.602, 2WCB.B.100, 2WD2.A.1262, 2WD3.A.1263, 4WD8.C.303, 2WEJ.A.1262, 1WE9.A.401, 1WEE.A.201, 1WEW.A.201, 2WEH.A.1262, 2WEO.A.1262, 1WFH.A.201, 1WFK.A.200, 1WFZ.A.201, 2WGX.A.1300, 1WGE.A.201, 3WID.A.1001, 1WJ2.A.470, 1WKQ.B.202, 1WN5.A.1001, 3WNQ.A.501, 1WO3.A.26, 1WO4.A.26, 2WVJ.A.1193, 1WWD.A.57, 3WWL.A.102, 1WWR.D.204, 1WYS.A.401, 1X0T.A.150, 4X2Z.A.400, 1X31.D.1006, 1X4I.A.401, 1X4J.A.201, 1X4L.A.201, 1X4L.A.401, 1X4S.A.201, 1X4W.A.401, 2X5R.A.1126, 1X61.A.401, 1X62.A.401, 1X63.A.201, 1X63.A.401, 1X64.A.201, 1X64.A.401, 1X6M.A.200, 2X7S.A.1265, 2X7T.A.1263, 2X7U.A.1261, 2X7M.A.1175, 1XAF.A.501, 1XB0.A.403, 2XCM.E.1222, 2XCM.E.1223, 1XEG.A.262, 1XER.A.106, 1XF7.A.30, 4XIW.A.401, 2XIG.A.1151, 1XJH.A.63, 1XOX.A.999, 2XOC.A.993, 2XOC.A.994, 2XOC.A.995, 1XPA.A.220, 1XPG.A.1887, 1XQ0.A.262, 1XRU.A.501, 1XTM.B.501, 1Y02.A.162, 2Y1N.A.1436, 2Y1N.A.1437, 2Y43.A.1097, 2Y43.A.1098, 1Y8Q.B.642, 2YB5.A.1213, 2YHO.A.1001, 2YHO.A.1002, 2YIK.A.1533, 1YOP.A.84, 1YQD.A.2000, 2YQM.A.201, 2YQM.A.401, 2YRJ.A.200, 2YRT.A.201, 2YRD.A.200, 2YRE.A.401, 2YRE.A.501, 2YRE.A.701, 2YRG.A.201, 2YRH.A.200, 2YSJ.A.201, 2YSJ.A.401, 2YSV.A.201, 2YSL.A.401, 2YSM.A.301, 2YSM.A.501, 2YSM.A.701, 2YSM.A.901, 2YTG.A.201, 2YTS.A.201, 2YT9.A.201, 2YTE.A.201, 2YTF.A.201, 2YTI.A.201, 2YTO.A.201, 2YU4.A.201, 2YUC.A.201, 2YUC.A.401, 2YWW.A.504, 2YZ5.B.1501, 2Z2Y.A.2001, 2Z3G.A.2001, 1Z3A.A.301, 2Z3J.A.2001, 2Z45.B.1004, 1Z6U.A.2, 1Z84.A.604, 1Z8R.A.151, 2ZC0.B.408, 1ZE8.A.263, 2ZEE.A.391, 2ZEF.A.391, 2ZEL.A.391, 2ZEM.A.391, 2ZEO.A.391, 1ZFD.A.71, 3ZFJ.A.1159, 1ZFQ.A.600, 1ZGF.A.400, 3ZGO.A.400, 1ZH1.A.199, 3ZME.A.313, 1ZNB.A.1, 1ZNF.A.27, 3ZNF.A.31, 3ZNI.A.1428, 3ZNI.A.1429, 1ZNM.A.29, 5ZNF.A.31, 7ZNF.A.31, 2ZNR.A.1, 3ZPC.B.401, 1ZR9.A.117, 1ZRP.A.54, 1ZSB.A.262, 1ZSC.A.262, 3ZTG.A.1336, 3ZTG.A.1337, 1ZU1.A.129, 1ZU1.A.130, 3ZVS.A.1160, 1ZW8.A.66, 1ZW8.A.67, 1ZY7.A.801, 3ZYQ.A.1222, 3ZYQ.A.1223, 2ZZF.A.754

Table S17. all-ligand-number Zn, compressed group

	size	largest_angle*	middle_1*	middle_2*	middle_3*
1	"60"	"144.4+/-5"	"55.5+/-3.1"	"90.5+/-6.7"	"104.8+/-6.6"

2	"24"	"137.1+/-10.6"	"75.3+/-10"	"89.4+/-5.7"	"106.6+/-6.4"
3	"41"	"160.8+/-5.6"	"57.7+/-3.7"	"89.8+/-4.5"	"103.1+/-4.4"
4	"103"	"167.8+/-5.4"	"76.8+/-6.3"	"90.2+/-2.4"	"99.2+/-3.1"
5	"148"	"150.7+/-4.2"	"55.4+/-2.5"	"91.9+/-3.8"	"101.8+/-3.2"
6	"33"	"130.8+/-9.6"	"56.1+/-3.7"	"82.5+/-8.7"	"96.8+/-9"
7	"32"	"147.9+/-8"	"56.6+/-3.1"	"86.1+/-5.3"	"96.4+/-5"
8	"103"	"137.3+/-5.1"	"55.3+/-3.9"	"92.2+/-5.1"	"103.1+/-5.3"
9	"130"	"151.8+/-5.2"	"57.1+/-3.1"	"93.6+/-3.5"	"100.8+/-2.8"
10	"67"	"164.1+/-5.6"	"56.8+/-4.5"	"86.3+/-5.1"	"104.7+/-3.6"

	middle_4*	smallest_opposite_angle*	Tetrahedral	TrigonalBipyramidal
1	"133.3+/-4.7"	"90.4+/-7"	"0.089"	"0.018"
2	"126.5+/-11.9"	"53.9+/-3.5"	"0.087"	"0.018"
3	"152.5+/-6.9"	"80.5+/-8.6"	"0.004"	"0.012"
4	"155.8+/-5.2"	"57.6+/-3.1"	"0.015"	"0.045"
5	"123.5+/-4.3"	"107.1+/-4.4"	"0.289"	"0.13"
6	"107.8+/-8.6"	"92+/-8.8"	"0.002"	"0"
7	"104.4+/-5.4"	"121.5+/-12.2"	"0"	"0"
8	"128.3+/-5"	"105.3+/-5"	"0.219"	"0.051"
9	"138.6+/-5"	"100.2+/-4.2"	"0.059"	"0.07"
10	"122+/-7"	"104.8+/-5.9"	"0.31"	"0.035"

	TrigonalBipyramidalVA	TrigonalBipyramidalVP	Octahedral	SquarePyramidal
1	"0.067"	"0.078"	"0"	"0.001"
2	"0.086"	"0.088"	"0.001"	"0"
3	"0.021"	"0.144"	"0.045"	"0.114"
4	"0.047"	"0.214"	"0.091"	"0.177"
5	"0.167"	"0.093"	"0"	"0"
6	"0.058"	"0.028"	"0"	"0"
7	"0.001"	"0.081"	"0"	"0"
8	"0.12"	"0.133"	"0"	"0"
9	"0.067"	"0.082"	"0"	"0.011"
10	"0.096"	"0.052"	"0"	"0.001"

	SquarePyramidalV	SquarePlanar	TrigonalPrismatic	TrigonalPrismaticV
1	"0.028"	"0.002"	"0.009"	"0.125"
2	"0.053"	"0.002"	"0.08"	"0.172"
3	"0.177"	"0.137"	"0.004"	"0.06"
4	"0.227"	"0.182"	"0.002"	"0.069"
5	"0.016"	"0"	"0"	"0.104"
6	"0.06"	"0"	"0"	"0"
7	"0.038"	"0"	"0"	"0"
8	"0.024"	"0.001"	"0"	"0.157"
9	"0.082"	"0.007"	"0"	"0.098"
10	"0.039"	"0.001"	"0"	"0.02"

	PentagonalBipyramidal	PentagonalBipyramidalVA	PentagonalBipyramidalVP
1	"0"	"0"	"0"
2	"0"	"0"	"0.002"
3	"0"	"0.001"	"0.033"
4	"0"	"0.002"	"0.125"
5	"0"	"0"	"0"
6	"0"	"0"	"0"
7	"0"	"0"	"0"
8	"0"	"0"	"0"
9	"0"	"0"	"0"
10	"0"	"0"	"0"

	SquareAntiprismatic	SquareAntiprismaticV	HexagonalBipyramidal

1	"0"	"0"	"0"
2	"0"	"0"	"0"
3	"0"	"0"	"0"
4	"0"	"0.008"	"0"
5	"0"	"0"	"0"
6	"0"	"0"	"0"
7	"0"	"0"	"0"
8	"0"	"0"	"0"
9	"0"	"0"	"0"
10	"0"	"0"	"0"

	HexagonalBipyramidalVA	HexagonalBipyramidalVP
1	"0"	"0"
2	"0"	"0"
3	"0"	"0"
4	"0"	"0"
5	"0"	"0"
6	"0"	"0"
7	"0"	"0"
8	"0"	"0"
9	"0"	"0"
10	"0"	"0"

Table S18. Cluster members of all-ligand-number Zn, compressed group

[1] "Cluster 1"
3A1Z.A.226, 4A94.B.501, 3AAK.A.992, 2AIG.P.999, 4B52.A.401, 3BKN.B.201, 1CGL.A.3
01, 6CPA.A.308, 7CPA.A.308, 8CPA.A.308, 3D10.A.95, 1D1S.B.406, 1D1T.C.407, 3D4U.
A.309, 3DFM.A.401, 4DWZ.A.302, 2E46.A.172, 1EC5.B.50, 1EW9.A.450, 3FFZ.A.1300, 4
FMP.B.400, 1HA5.C.4221, 1HDU.E.1308, 1HEE.E.1308, 1HZ5.A.103, 4I2F.A.603, 4IXN.A
.401, 1JK9.C.302, 2JT5.A.257, 1K53.A.1003, 4KA7.A.801, 1L0Y.B.702, 1LGD.A.262, 4
LTE.A.1101, 2M30.A.201, 3M52.B.116, 3M79.A.107, 1NPC.A.323, 4ONX.B.201, 2OW0.A.4
44, 4P9C.J.201, 3PSQ.B.328, 3PW3.A.406, 2QLA.A.500, 2QLA.C.501, 1QMU.A.999, 2R2D
.A.277, 3TOL.D.107, 3U94.B.259, 3UBF.A.1, 3V94.D.702, 2V9E.A.1275, 2WKN.B.412, 3
WS9.B.801, 1WUO.A.301, 2XIG.C.1153, 2ZTG.A.902, 2OX8.A.5, 2OX8.B.5, 3R8B.F.122
[1] "Cluster 2"
4BLB.D.910, 4CPA.I.308, 1F30.B.201, 1F30.E.201, 4IOD.A.502, 4K0D.B.203, 4KJG.A.1
001, 3M4B.B.108, 1NL4.B.500, 1RLY.A.61, 2V8V.A.1456, 2ANU.B.505, 4HDT.A.400, 3IS
O.B.220, 3M7P.A.953, 1N4P.H.378, 2W9M.A.1565, 1YEW.G.662, 1ZZM.A.403, 2OX8.A.4,
3R8B.B.122, 3R8B.D.122, 3R8B.P.122, 2VME.A.501
[1] "Cluster 3"
3CSQ.B.335, 4DHL.B.501, 1DK4.B.591, 3H90.C.2, 1HR6.B.501, 4ICQ.B.502, 4JBS.B.100
9, 4JX5.D.1103, 1KBE.A.1, 3M3B.A.156, 1MH2.A.201, 2QLA.D.503, 3SZZ.A.502, 1VKG.A
.400, 2XPY.A.1673, 1ANJ.A.450, 3BXM.A.1751, 4CBY.A.2034, 3DFF.A.274, 1DK4.A.291,
4DYO.A.501, 3E4A.A.2000, 4FW3.A.301, 1G12.A.200, 2G9Y.B.450, 2GO4.B.602, 1H48.A
.900, 1K07.A.2, 4L3T.A.1101, 2OOT.A.1751, 4OP4.A.302, 3Q9B.E.345, 1QIP.B.901, 3R
BU.A.1751, 1RTQ.A.701, 3SJG.A.1751, 1TXR.A.502, 3W0U.A.201, 4WD8.C.302, 1XJO.A.9
01, 1YGD.A.142
[1] "Cluster 4"
4FUK.B.402, 4MCS.A.814, 2MLS.A.302, 3OOF.A.302, 1P6D.A.247, 1ZEB.A.901, 3B3C.A.5
02, 3BON.A.500, 2C1G.A.1465, 3C88.A.450, 3C8A.A.450, 3C8B.A.450, 2CB8.B.1090, 4C
VR.A.1160, 4CVT.A.1160, 4CWM.A.432, 4CXV.A.432, 3DDA.A.450, 2DEA.A.402, 3DFK.A.3

00, 4DJ4.B.403, 1EB6.A.178, 4ELC.A.501, 3EZT.B.401, 1FT7.A.502, 4FW5.D.301, 4FW6
.A.301, 4FW7.D.301, 3GBO.A.302, 3GJ9.B.127, 2GSO.A.1001, 4GSZ.A.402, 4GTW.A.1010
, 4GTW.A.1011, 1GW6.A.1615, 2GYQ.A.407, 4ICQ.A.502, 2IMA.A.500, 2IWO.A.1255, 2JT
2.A.336, 3K5X.A.403, 3KR5.B.1004, 4L3T.B.1101, 4LCF.A.311, 4LCG.A.301, 4LCH.A.30
1, 3LE9.A.1, 1LFW.A.1002, 1LOK.A.901, 1LOK.A.902, 4MCP.A.801, 4MCQ.A.801, 4MCR.A
.801, 3MK1.A.901, 3MN8.A.999, 4MTU.A.201, 1MZB.A.201, 4NGM.A.818, 4NGQ.A.817, 4N
GT.A.811, 4NY2.A.501, 4O5O.A.301, 4OC1.A.814, 4OME.A.815, 2OUN.A.402, 2OUQ.A.402
, 3P3E.A.400, 3P3G.A.301, 1P5X.A.246, 2PLI.B.707, 4PPZ.A.602, 3PS1.A.301, 3PS2.A
.301, 3PS3.A.301, 2PTY.A.500, 4Q3J.A.401, 2QF7.A.1157, 3QIY.A.431, 3QJ0.A.431, 3
QM3.A.355, 1QTW.A.302, 3QW7.A.501, 3QW8.A.501, 1R1I.A.1001, 1R1J.A.1001, 2RH6.A.
2, 1ROR.A.601, 3S2L.A.403, 1SDX.A.677, 1SHN.B.481, 3T3W.A.301, 1TXR.A.501, 3U1Y.
A.400, 3U79.H.110, 3V94.A.702, 2V9L.A.1275, 3VPB.A.503, 4WB7.A.503, 3WT4.A.502,
1XVX.A.315, 1ZED.A.903, 2ZXC.A.647, 4X2T.L.701
[1] "Cluster 5"
4A37.A.376, 3A52.A.1001, 4A69.A.500, 4AWZ.A.3230, 2B13.B.401, 3B4N.A.711, 3B4N.B
.715, 4B6Z.C.385, 2BO9.A.999, 2BP8.B.1340, 1BYF.B.302, 4C2N.A.701, 4C2O.A.1629,
2C6P.A.1752, 1CG2.A.500, 1CG2.A.501, 3CHO.A.701, 2CLB.A.1175, 4COG.A.401, 1CPX.A
.308, 5CPA.A.308, 4CVT.A.1159, 3E30.B.1001, 3E32.B.1001, 3E33.B.1001, 3E34.B.100
1, 1ED9.A.450, 3EER.A.2001, 3EQN.A.757, 2EV6.B.2151, 1F57.A.310, 3FB4.A.217, 1FT
7.A.501, 3FU1.B.201, 3FVL.A.1309, 4GM5.A.401, 2GSN.A.1001, 4GTO.B.501, 4GTV.B.40
1, 4H2K.A.1001, 1HFS.A.257, 2HH5.A.702, 1HI9.A.300, 1HLK.B.1004, 2I3C.A.314, 4IA
V.A.401, 2IEJ.B.939, 4IHM.A.402, 3IQ6.C.201, 3IT7.A.183, 2IXD.A.1234, 1IY7.A.308
, 2JBJ.A.1752, 1JCQ.B.1001, 1JCS.B.1001, 1JI3.A.402, 4JJI.A.402, 1JML.A.102, 4K9
0.A.701, 1KH4.A.450, 1KRO.A.405, 3L3N.A.701, 4L9P.B.601, 1LD7.B.1001, 1LD8.B.100
1, 1LND.E.800, 4LNB.B.602, 3LTV.A.1001, 2LVH.A.101, 4LW9.I.201, 1M4L.A.1308, 1M5
E.A.1705, 4MBG.B.602, 4MKH.A.301, 4MKT.A.701, 4MRQ.A.501, 3MWM.B.142, 1MXD.A.727
, 1MZC.B.1001, 4N07.B.305, 3N21.A.401, 1N4Q.L.378, 3NKQ.A.1002, 3NKR.A.1002, 1NW
2.F.6006, 4NYY.B.501, 1O1S.B.1001, 4O3A.A.303, 4O3A.B.303, 1O86.A.701, 1O8A.A.70
1, 4OJA.A.202, 4OTE.B.302, 4OXD.C.301, 2PIZ.A.400, 4PPZ.A.601, 4PQA.A.403, 4PQA.
A.404, 3PSQ.A.326, 3PZ1.B.332, 3PZ4.B.1, 3Q78.B.521, 3Q7A.B.521, 2RFH.A.1308, 3R
F4.A.118, 2RH6.A.1, 1S63.B.1001, 1S64.B.378, 1SA4.B.439, 1SA5.B.438, 3SFX.B.521,
3SFY.B.521, 3TG0.A.501, 1TN8.B.1001, 3TS4.A.301, 3U9W.A.2001, 1UUP.A.2222, 1UZE
.A.701, 3VH9.A.302, 2VJ8.A.1611, 2VXI.A.202, 3W6H.A.302, 3WBH.A.505, 4WCM.A.538,
3WOJ.A.805, 3WV3.A.301, 1WY2.A.405, 2X90.A.1618, 2X91.A.1619, 2X92.A.1615, 4XIX
.A.401, 2XIG.B.1150, 2XX0.B.1340, 2XX7.B.1264, 2XXG.A.1339, 2Y3D.B.149, 2Z2Y.C.2
003, 2Z2D.A.265, 1ZG7.A.400, 2ZIR.B.901, 2ZIS.B.901, 2ZKW.A.401, 3ZXO.A.1579, 2A
Q2.B.1001, 2IUC.B.1006, 4N07.A.309, 1UD9.B.510, 4WD7.C.302
[1] "Cluster 6"
4LMG.B.202, 3A1Z.C.226, 1AJD.A.450, 1ALH.B.450, 1BON.A.1002, 4C98.A.1266, 2CIH.A
.213, 2E84.A.558, 4EGE.A.412, 1F30.H.201, 1F30.I.201, 2F4L.A.1400, 2H6H.B.1001,
2H6F.B.1001, 4IOZ.A.504, 4KEQ.A.301, 1KH5.B.950, 3KVE.C.489, 2L1U.A.144, 3NQ5.A.
508, 2O3Z.A.501, 2OGJ.A.418, 1P4Q.B.302, 1Q9U.B.402, 2QQ4.I.139, 3SOW.A.7, 3U24.
A.594, 2VZ5.A.1131, 2W88.C.107, 2WC0.B.3012, 2WKN.A.412, 2X3C.A.1342, 1ZKX.A.423
[1] "Cluster 7"
1ANI.A.450, 2AXR.A.501, 3BYW.D.1, 3CHP.A.701, 3CPA.A.308, 1D1T.B.406, 3D68.A.501
, 3DLJ.A.2001, 3DLJ.B.2002, 4G1P.A.501, 3H90.A.292, 2IMC.A.600, 4K7S.B.101, 1KQ0
.A.479, 3LSF.H.2, 1MWO.A.438, 4N07.B.306, 2O1Q.B.145, 4PVT.A.404, 3PW3.C.406, 3P
W3.D.406, 1PYT.B.350, 4Q7R.A.305, 3QU6.A.114, 4U9D.A.205, 3U94.A.259, 2VQH.B.108
9, 3WC5.A.404, 1XAF.A.503, 2Y2E.A.1180, 1Z5R.C.600, 3ZUQ.A.1440
[1] "Cluster 8"
4LMG.D.202, 2A7G.E.401, 3AHT.A.1001, 1ALK.A.451, 4AR9.A.1731, 3B2Z.A.1, 3BKN.B.2
02, 3BOO.A.500, 4BP0.A.1314, 4C1H.A.351, 2C20.B.601, 1CBX.A.309, 2CEA.E.1603, 1D
8D.A.1001, 1DCE.B.900, 3DID.A.130, 3DSX.B.332, 3E37.B.1001, 1EC5.C.50, 2EG8.A.40
0, 3ELM.A.300, 3F28.A.321, 3F2P.A.2005, 3FGD.A.321, 4FU4.A.502, 3G42.A.500, 4G9L

.B.301, 3GIP.A.484, 4GTM.B.501, 4GTQ.B.501, 4GTR.B.501, 2GVI.A.302, 1HR7.B.501, 1HYT.A.805, 4I11.A.502, 3I9F.A.1, 3IQ6.B.201, 2J13.A.1236, 1J9Y.A.1003, 4JJJ.A.7 18, 1KAP.P.613, 1KR6.A.405, 1KS7.A.405, 3KWO.C.162, 3LPE.D.60, 3LUB.B.302, 3M4C. D.108, 3M79.B.107, 3M79.C.107, 3MDJ.A.1000, 1MMP.A.2, 1N4Q.B.378, 1OEZ.W.1154, 4 OKO.A.401, 4ONG.H.302, 1PE5.A.317, 1PE7.A.317, 2PIY.A.400, 2PJB.A.400, 2PLI.C.70 1, 1PSZ.A.1000, 4PUC.A.602, 3Q75.B.521, 1QBQ.B.1000, 3QWO.A.500, 3QWO.B.500, 3QW O.D.500, 3QW1.C.500, 1R43.A.501, 1RK6.A.601, 3S9C.A.503, 3SKS.A.568, 1TLP.E.322, 1TMN.E.322, 1TN6.B.1001, 1TN7.B.1001, 1TNB.B.378, 1TNO.B.378, 1TNU.B.378, 1TNY. B.378, 1TNZ.B.378, 3TOL.B.107, 3TT4.A.302, 3TVC.A.501, 3V0A.A.1297, 2VXI.A.201, 3W5K.B.501, 1WAA.A.1091, 1WAA.F.1091, 4WD8.A.301, 2WOA.A.1270, 2X4H.A.1142, 2X98 .A.1476, 1XGE.A.400, 2Y28.A.1181, 2YJP.C.1270, 2Z25.B.401, 1ZG9.A.400, 4AF1.A.50 0, 2KBX.B.298, 1R87.A.905, 3RC6.A.1, 2VW4.A.503

[1] "Cluster 9"

4MTD.A.202, 3AIG.A.999, 1ANJ.B.450, 2ANP.A.501, 2ANP.A.502, 1ATL.A.401, 3AYU.A.4 15, 1BON.A.1001, 3B35.A.292, 3B3C.A.501, 2BH3.A.1001, 3BHX.A.1751, 3BIO.A.1751, 3BI1.A.1751, 3BKK.A.701, 3BKL.A.701, 2C6P.A.1751, 2C6C.A.1751, 1CP7.A.901, 1CP7. A.902, 4CVR.A.1159, 4D0Y.B.1239, 4DPR.A.701, 2DSN.A.2001, 4DYO.A.502, 1DZW.P.999 , 1E49.S.999, 2EG7.A.400, 3EHY.A.264, 4EJ5.A.501, 2EK8.A.1002, 2EK9.A.1002, 3EWJ .B.2, 3FH4.A.301, 3FJU.A.999, 1FUA.A.216, 3FUK.A.701, 4FYQ.A.1012, 4FYR.A.1013, 2GA3.A.450, 1GE7.B.200, 4GTP.B.501, 3H90.B.294, 2HIH.A.601, 4ICR.A.502, 4IE7.A.6 01, 1IGB.A.502, 2IQ6.A.293, 1J2U.A.301, 4J5H.A.301, 2JAZ.B.600, 4JBS.A.1008, 1JP U.A.371, 4JYW.A.801, 4JZO.A.801, 4K5L.A.1101, 4K5M.A.1101, 4K5O.A.1103, 4KAY.A.6 01, 4KFT.C.303, 1KH5.A.450, 2KIK.A.50, 3KNS.A.229, 3LOT.B.2, 4L2L.A.701, 4LCQ.A. 602, 1LFW.A.1001, 4LP6.A.302, 3LQO.A.999, 4LQG.A.801, 1MMP.A.1, 4NAQ.A.1027, 3NK N.A.1002, 3NKO.A.1002, 4O2I.A.401, 4O3A.B.302, 4ON1.A.400, 2OR4.A.1751, 2OY2.A.9 99, 3P24.A.999, 2PJ0.A.400, 2PJ3.A.400, 2PJ5.A.400, 2PJ7.A.400, 2PJ8.B.500, 2PJA .B.500, 2PJC.A.400, 2PVW.A.1751, 3Q4R.A.202, 4Q7R.A.307, 3QBU.A.294, 2QLA.B.502, 1R42.A.804, 2RJP.A.1, 1ROS.A.400, 1RTQ.A.702, 3RYM.B.107, 1S0E.A.1291, 3SJX.A.1 751, 1SQM.A.1001, 3T00.A.501, 1T3A.B.422, 1TF9.A.902, 3TGO.A.502, 1TKH.A.902, 1T KJ.A.901, 1TKJ.A.902, 3U24.A.595, 3U93.B.259, 2V29.A.1274, 2V29.A.1275, 2V77.A.1 042, 3VAT.A.501, 2VQQ.A.1411, 2VXX.C.202, 3WAW.A.913, 4WD8.A.302, 3WE7.A.301, 2W O8.A.1268, 2WO9.C.1269, 2X93.A.1615, 2X94.A.1616, 2X98.A.1475, 1XAF.B.507, 1XJO. A.902, 2XR9.A.1867, 2Y2B.B.1180, 1YHC.B.602, 2YJP.A.1272, 4FXO.A.301

[1] "Cluster 10"

1B57.A.361, 1B8J.A.451, 1BA9.A.154, 1BOR.A.58, 4BZS.A.701, 4CA6.A.1001, 4COQ.A.2 99, 4CVT.A.1158, 1DO5.B.29, 1ELX.B.451, 3F3Q.A.104, 4F7O.A.301, 2GFK.A.401, 2GFJ .A.401, 3GZE.A.1, 2HSE.B.954, 3HTR.B.118, 2IOO.A.580, 1IF6.A.262, 1JJE.A.251, 1J JT.A.251, 2JLP.B.226, 4K7W.A.102, 2KBX.B.299, 3KS3.A.262, 1L1O.C.1, 4LQY.A.506, 4LW9.A.201, 4LW9.R.201, 3M2Z.A.500, 2M30.B.201, 3MO2.D.6, 4MSM.C.501, 4NGE.A.110 1, 4NGE.E.101, 3NKN.A.1001, 2NQH.A.303, 1NW2.D.6004, 2P2L.A.901, 2PVV.A.1752, 4P XY.A.302, 1Q3K.B.301, 1R3N.A.501, 1SHN.A.485, 3SIO.A.362, 1T0A.A.661, 3TGN.B.147 , 1U7J.A.50, 3UCT.B.102, 2V9I.A.1277, 2W5V.A.1377, 2W5X.B.1378, 2W57.B.202, 4WD6 .A.301, 4WNC.O.402, 3WXC.A.301, 2X97.A.1616, 1XP3.A.302, 1ZG8.A.400, 3DNG.B.998, 2FKM.X.500, 3QZC.A.2, 1R5X.A.122, 3UBF.A.6, 1UDV.A.101, 1UUP.D.5222, 3ZNR.B.101

Table S19. all-ligand-number Zn, combined group

	size	largest_angle*	middle_1*	middle_2*	middle_3*
1	"541"	"147.3+/-9.3"	"56.8+/-4.8"	"91.1+/-5.6"	"102.2+/-4.8"
2	"509"	"165+/-8"	"76.7+/-6.1"	"89.2+/-3.2"	"97+/-3.5"
3	"170"	"159.7+/-11.1"	"74+/-12.2"	"86.9+/-6"	"95.3+/-6.3"

4	"728"	"122.8+/-4.1"	"98.9+/-4.3"	"105.7+/-3.4"	"111+/-3.3"
5	"92"	"147.8+/-13"	"62.8+/-11.5"	"89+/-7.2"	"105.8+/-7.9"
6	"252"	"137.7+/-6.7"	"90.2+/-7"	"98.3+/-4.8"	"105.6+/-4.9"
7	"1450"	"115.9+/-2.8"	"103.1+/-3.2"	"106.9+/-2.1"	"109.7+/-2"
8	"740"	"122.8+/-4.7"	"95.3+/-5"	"101.9+/-3.7"	"107.6+/-3.8"
9	"318"	"170.4+/-5.6"	"77.5+/-7"	"89.5+/-2.1"	"97+/-3.6"

	middle_4*	smallest_opposite_angle*	Tetrahedral	TrigonalBipyramidal
1	"128.8+/-8.6"	"102.9+/-7.1"	"0.115"	"0.004"
2	"137.9+/-9.3"	"103.9+/-6.4"	"0.024"	"0.199"
3	"103.2+/-8.4"	"109.7+/-14.4"	"0.042"	"0.017"
4	"115.6+/-3.2"	"98.9+/-5.4"	"0.419"	"0"
5	"136.8+/-13.1"	"64.4+/-12.1"	"0.014"	"0.004"
6	"114.8+/-6.9"	"100.2+/-8.7"	"0.04"	"0"
7	"112.4+/-2.1"	"108.3+/-3.6"	"0.83"	"0"
8	"113.9+/-3.8"	"114.4+/-4.9"	"0.464"	"0"
9	"162.8+/-7.1"	"73.7+/-12.3"	"0.001"	"0.01"

	TrigonalBipyramidalVA	TrigonalBipyramidalVP	Octahedral	SquarePyramidal
1	"0.061"	"0.012"	"0"	"0"
2	"0.235"	"0.316"	"0"	"0.021"
3	"0.023"	"0.13"	"0"	"0"
4	"0.064"	"0"	"0"	"0"
5	"0.023"	"0.033"	"0"	"0.003"
6	"0.077"	"0.001"	"0"	"0"
7	"0.007"	"0"	"0"	"0"
8	"0.015"	"0"	"0"	"0"
9	"0.01"	"0.179"	"0.112"	"0.205"

	SquarePyramidalV	SquarePlanar	TrigonalPrismatic	TrigonalPrismaticV
1	"0.01"	"0"	"0"	"0.038"
2	"0.224"	"0.048"	"0"	"0.07"
3	"0.11"	"0.002"	"0"	"0.004"
4	"0"	"0"	"0"	"0"
5	"0.041"	"0.009"	"0.028"	"0.064"
6	"0.01"	"0"	"0"	"0"
7	"0"	"0"	"0"	"0"
8	"0"	"0"	"0"	"0"
9	"0.285"	"0.253"	"0.001"	"0.018"

	PentagonalBipyramidal	PentagonalBipyramidalVA	PentagonalBipyramidalVP
1	"0"	"0"	"0"
2	"0"	"0"	"0"
3	"0"	"0"	"0"
4	"0"	"0"	"0"
5	"0"	"0.001"	"0.013"
6	"0"	"0"	"0"
7	"0"	"0"	"0"
8	"0"	"0"	"0"
9	"0"	"0.001"	"0.013"

	SquareAntiprismatic	SquareAntiprismaticV	HexagonalBipyramidal
1	"0"	"0"	"0"
2	"0"	"0"	"0"
3	"0"	"0"	"0"
4	"0"	"0"	"0"
5	"0"	"0"	"0"
6	"0"	"0"	"0"
7	"0"	"0"	"0"

```
8 "0"                "0"                  "0"
9 "0"                "0.002"              "0"
  HexagonalBipyramidalVA HexagonalBipyramidalVP
1 "0"                    "0"
2 "0"                    "0"
3 "0"                    "0"
4 "0"                    "0"
5 "0"                    "0"
6 "0"                    "0"
7 "0"                    "0"
8 "0"                    "0"
9 "0"                    "0"
```

Table S20. Cluster members of all-ligand-number Zn, combined group

```
[1] "Cluster 1"
4LMG.D.202, 4MTD.A.202, 3A1Z.A.226, 4A37.A.376, 3A52.A.1001, 4A69.A.500, 2A7G.E.
401, 4A7B.A.1276, 4A94.B.501, 3AHT.A.1001, 3AIG.A.999, 2AIG.P.999, 1ALK.A.451, 1
ANJ.B.450, 2ANP.A.501, 2ANP.A.502, 4AR8.A.1731, 4AR9.A.1731, 1ATL.A.401, 4AWZ.A.
3230, 3AYU.A.415, 1B0N.A.1001, 2B13.B.401, 3B2Z.A.1, 3B35.A.292, 3B3C.A.501, 3B4
N.A.711, 3B4N.B.715, 1B57.A.361, 4B52.A.401, 4B6Z.C.385, 1BA9.A.154, 2BH3.A.1001
, 3BHX.A.1751, 3BI0.A.1751, 3BI1.A.1751, 3BKK.A.701, 3BKL.A.701, 3BKN.B.201, 3BK
N.B.202, 4BLB.A.910, 2BNN.B.1200, 3BOO.A.500, 2BO9.A.999, 4BP0.A.1314, 2BP8.B.13
40, 1BQO.B.301, 1BYF.B.302, 4BZS.A.701, 3C0Z.B.101, 4C1H.A.351, 2C20.B.601, 4C2N
.A.701, 4C2O.A.1629, 2C6P.A.1751, 2C6P.A.1752, 2C6C.A.1751, 1CBX.A.309, 2CEA.E.1
603, 1CG2.A.500, 1CG2.A.501, 1CGL.A.301, 3CHO.A.701, 2CLB.A.1175, 4COG.A.401, 4C
OQ.A.299, 1CP7.A.901, 1CP7.A.902, 1CPX.A.308, 5CPA.A.308, 6CPA.A.308, 7CPA.A.308
, 8CPA.A.308, 4CVR.A.1159, 4CVT.A.1158, 4CVT.A.1159, 1CXV.A.1, 4D0Y.B.1239, 3D10
.A.95, 1D1T.C.407, 3D4U.A.309, 1D8D.A.1001, 1DCE.B.900, 3DFM.A.401, 3DID.A.130,
1DO5.B.29, 4DPR.A.701, 4DR9.A.201, 3DSX.B.332, 2DSN.A.2001, 3DYC.A.451, 4DYO.A.5
02, 1DZW.P.999, 3E2D.A.602, 3E30.B.1001, 3E32.B.1001, 3E33.B.1001, 3E34.B.1001,
3E37.B.1001, 1E49.S.999, 2E46.A.172, 1EC5.B.50, 1EC5.C.50, 1ED9.A.450, 3EER.A.20
01, 2EG7.A.400, 2EG8.A.400, 3EHY.A.264, 4EJ5.A.501, 2EK8.A.1002, 2EK9.A.1002, 1E
LX.B.451, 3ELM.A.300, 3EQN.A.757, 2EV6.B.2151, 1EW9.A.450, 3EWJ.B.2, 1EZ2.A.402,
 3F28.A.321, 3F2P.A.2005, 3F3Q.A.104, 1F57.A.310, 4F7O.A.301, 3FB4.A.217, 3FFZ.A
.1300, 3FGD.A.321, 3FH4.A.301, 3FJU.A.999, 1FLS.A.166, 4FMP.B.400, 1FT7.A.501, 3
FU1.B.201, 1FUA.A.216, 3FUK.A.701, 4FU4.A.502, 3FVL.A.1309, 4FYQ.A.1012, 4FYR.A.
1013, 1G05.B.801, 1G49.B.801, 3G42.A.500, 4G9L.B.301, 4G9L.B.302, 2GA3.A.450, 1G
E7.B.200, 2GFK.A.401, 2GFJ.A.401, 3GIP.A.484, 4GM5.A.401, 2GSN.A.1001, 4GTM.B.50
1, 4GTO.B.501, 4GTP.B.501, 4GTQ.B.501, 4GTR.B.501, 4GTV.B.401, 2GVI.A.302, 3GZE.
A.1, 4H2K.A.1001, 4H49.A.301, 3H90.B.294, 1HA5.C.4221, 1HDU.E.1308, 1HEE.E.1308,
 1HFS.A.257, 2HH5.A.702, 1HI9.A.300, 2HIH.A.601, 1HLK.B.1004, 1HR7.B.501, 2HSE.B
.954, 3HTR.B.118, 1HYT.A.805, 1HZ5.A.103, 4I11.A.502, 4I2F.A.603, 2I3C.A.314, 3I
9F.A.1, 4IAV.A.401, 4ICR.A.502, 2IEJ.B.939, 4IE7.A.601, 1IGB.A.502, 4IHM.A.402,
3IQ6.B.201, 3IQ6.C.201, 2IQ6.A.293, 3IT7.A.183, 2IXD.A.1234, 4IXN.A.401, 1IY7.A.
308, 2J13.A.1236, 4J5H.A.301, 1J9Y.A.1003, 2JAZ.B.600, 4JBS.A.1008, 2JBJ.A.1752,
 1JCQ.B.1001, 1JCS.B.1001, 1JI3.A.402, 1JJE.A.251, 1JJT.A.251, 4JJI.A.402, 4JJJ.
A.718, 1JK9.C.302, 2JLP.B.226, 1JML.A.102, 2JSD.A.276, 2JT5.A.256, 2JT5.A.257, 4
JYW.A.801, 4JZ0.A.801, 1K53.A.1003, 4K5L.A.1101, 4K5M.A.1101, 4K5O.A.1103, 4K5P.
A.1101, 4K7W.A.102, 4K90.A.701, 1KAP.P.613, 4KA7.A.801, 4KAY.A.601, 2KBX.B.299,
4KFT.C.303, 1KH4.A.450, 1KH5.A.450, 2KIK.A.50, 3KNS.A.229, 1KR6.A.405, 1KRO.A.40
```

5, 1KS7.A.405, 3KWO.C.162, 3LOT.B.2, 1L1O.C.1, 4L2L.A.701, 3L3N.A.701, 4L9P.B.60
1, 4LCQ.A.602, 1LD7.B.1001, 1LD8.B.1001, 1LFW.A.1001, 2LFF.A.502, 1LGD.A.262, 3L
JZ.A.999, 1LND.E.800, 4LNB.B.602, 3LPE.D.60, 3LQ0.A.999, 4LQG.A.801, 4LQY.A.506,
3LTV.A.1001, 3LUB.B.302, 2LVH.A.101, 4LW9.A.201, 4LW9.I.201, 4LW9.R.201, 3M2Z.A
.500, 2M30.A.201, 2M30.B.201, 3M4C.D.108, 1M4L.A.1308, 1M5E.A.1705, 3M52.B.116,
3M79.A.107, 3M79.B.107, 3M79.C.107, 4MBG.B.602, 3MDJ.A.1000, 3MK1.A.902, 4MKH.A.
301, 4MKT.A.701, 1MMB.A.999, 1MMP.A.1, 1MMP.A.2, 4MRQ.A.501, 4MSM.C.501, 3MWM.B.
142, 1MXD.A.727, 1MZC.B.1001, 4N07.B.305, 3N21.A.401, 1N4Q.B.378, 1N4Q.L.378, 4N
AQ.A.1027, 4NGE.A.1101, 3NKQ.A.1002, 3NKR.A.1002, 3NKN.A.1001, 3NKN.A.1002, 3NKO
.A.1002, 1NPC.A.323, 2NQH.A.303, 1NW2.D.6004, 1NW2.F.6006, 4NYY.B.501, 1O1S.B.10
01, 4O2I.A.401, 4O3A.A.303, 4O3A.B.302, 4O3A.B.303, 1O86.A.701, 1O8A.A.701, 1OEZ
.W.1154, 4OJA.A.202, 4OKO.A.401, 4ON1.A.400, 4ONG.H.302, 4ONX.B.201, 4OTE.B.302,
2OWO.A.444, 4OXD.C.301, 2OY2.A.999, 3P24.A.999, 2P2L.A.901, 4P9C.J.201, 1PE5.A.
317, 1PE7.A.317, 2PIY.A.400, 2PIZ.A.400, 2PJ0.A.400, 2PJ3.A.400, 2PJ5.A.400, 2PJ
7.A.400, 2PJ8.B.500, 2PJA.B.500, 2PJB.A.400, 2PJC.A.400, 2PLI.C.701, 3PN4.A.1001
, 4PPZ.A.601, 4PQA.A.403, 4PQA.A.404, 1PSZ.A.1000, 3PSQ.A.326, 3PSQ.B.328, 4PUC.
A.602, 2PVV.A.1752, 2PVW.A.1751, 3PW3.A.406, 3PZ1.B.332, 3PZ4.B.1, 3Q4R.A.202, 3
Q75.B.521, 3Q78.B.521, 3Q7A.B.521, 4Q7R.A.307, 3Q9F.B.344, 4QA0.A.401, 1QBQ.B.10
00, 3QBU.A.294, 2QLA.B.502, 2QLA.C.501, 2QLA.D.503, 1QMU.A.999, 3QW0.A.500, 3QW0
.B.500, 3QW0.D.500, 3QW1.C.500, 2R2D.A.277, 1R3N.A.501, 1R42.A.804, 1R43.A.501,
2RFH.A.1308, 3RF4.A.118, 2RH6.A.1, 2RJP.A.1, 1RK6.A.601, 1ROS.A.400, 1RTQ.A.702,
3RYM.B.107, 1S0E.A.1291, 1S63.B.1001, 1S64.B.378, 3S9C.A.503, 1SA4.B.439, 1SA5.
B.438, 3SFX.B.521, 3SFY.B.521, 1SHN.A.485, 3SI0.A.362, 3SKS.A.568, 1SQM.A.1001,
1T0A.A.661, 1T3A.B.422, 1TF9.A.902, 3TGN.B.147, 3TGO.A.501, 3TGO.A.502, 1TKH.A.9
02, 1TKJ.A.901, 1TKJ.A.902, 4TLN.A.321, 1TLP.E.322, 5TLN.A.321, 1TMN.E.322, 4TMN
.E.322, 1TN6.B.1001, 1TN7.B.1001, 1TN8.B.1001, 1TNB.B.378, 1TNO.B.378, 1TNU.B.37
8, 1TNY.B.378, 1TNZ.B.378, 3TOL.D.107, 3TOL.B.107, 3TS4.A.301, 3TT4.A.302, 3TVC.
A.501, 3U24.A.595, 3U9W.A.2001, 3U93.B.259, 3UBF.A.1, 1UUP.A.2222, 1UZE.A.701, 3
V0A.A.1297, 3V1E.A.101, 2V29.A.1274, 2V29.A.1275, 3V77.A.302, 2V77.A.1042, 2V9I.
A.1277, 2V9E.A.1275, 3VAT.A.501, 3VH9.A.302, 2VJ8.A.1611, 2VQQ.A.1411, 2VXI.A.20
1, 2VXI.A.202, 2VXX.C.202, 1W22.A.1375, 2W5V.A.1377, 2W5X.B.1378, 3W5K.B.501, 2W
57.B.202, 3W6H.A.302, 1WAA.A.1091, 1WAA.F.1091, 3WAW.A.913, 3WBH.A.505, 4WCM.A.5
38, 4WD8.A.301, 4WD8.A.302, 3WE7.A.301, 2WKN.B.412, 4WNC.O.402, 3WOJ.A.805, 2WO8
.A.1268, 2WO9.C.1269, 2WOA.A.1270, 3WS9.B.801, 1WUO.A.301, 3WV3.A.301, 1WY2.A.40
5, 2X4H.A.1142, 2X90.A.1618, 2X91.A.1619, 2X92.A.1615, 2X93.A.1615, 2X94.A.1616,
2X97.A.1616, 2X98.A.1475, 2X98.A.1476, 1XAF.B.507, 1XGE.A.400, 4XIX.A.401, 2XIG
.B.1150, 2XIG.C.1153, 1XJO.A.902, 2XR9.A.1867, 2XX0.B.1340, 2XX7.B.1264, 2XXG.A.
1339, 2Y28.A.1181, 2Y2B.B.1180, 2Y3D.B.149, 1YHC.B.602, 2YJP.A.1272, 2YJP.C.1270
, 2Z2Y.C.2003, 2Z25.B.401, 2Z2D.A.265, 1ZED.A.904, 1ZG7.A.400, 1ZG8.A.400, 1ZG9.
A.400, 2ZIR.B.901, 2ZIS.B.901, 2ZKW.A.401, 2ZTG.A.902, 3ZXO.A.1579, 3A1Z.C.226,
1ALH.B.450, 2AQ2.B.1001, 1BON.A.1002, 3BOZ.A.22, 4BLD.D.910, 4C98.A.1266, 2E84.A
.558, 1F30.H.201, 2FKM.X.500, 4FXO.A.301, 2H6H.B.1001, 2H6F.B.1001, 4H82.B.300,
3H90.A.292, 2IUC.B.1006, 2JMD.A.66, 2KBX.B.298, 4KEQ.A.301, 1KH5.B.950, 3KVE.C.4
89, 2KV1.A.125, 2KVG.A.85, 2KVH.A.84, 2L1U.A.144, 4LW9.E.204, 4N07.A.309, 1NDV.A
.400, 2OGJ.A.418, 2OX8.B.5, 1P4Q.B.302, 4PVT.A.404, 3PW3.C.406, 1Q9U.B.402, 2QQ4
.I.139, 1R87.A.905, 3RC6.A.1, 3SOW.A.7, 3U24.A.594, 4U9D.A.205, 1UD9.B.510, 2VW4
.A.503, 2VZ5.A.1131, 2W88.C.107, 4WD7.C.302, 3WIE.B.1001, 2WKN.A.412, 2X3C.A.134
2, 2X4H.A.1140, 2Z45.A.1003
[1] "Cluster 2"
2L1G.A.88, 4QCL.A.1303, 1QUM.A.302, 830C.A.272, 456C.A.272, 4A3W.A.1159, 1A7T.A.
252, 2A7M.A.252, 4A7K.A.952, 4A7Y.A.951, 4A7Z.A.950, 1A85.A.999, 1A86.A.999, 2A8
H.A.486, 2AB7.A.30, 1AF0.A.472, 1AH7.A.246, 1AH7.A.248, 2AIO.A.315, 1AST.A.999,
4ASQ.A.1615, 4ASR.A.1615, 4AWY.B.3229, 4AX0.B.3229, 4AX1.B.3229, 1B3D.B.301, 1B5
7.A.360, 3B7S.A.701, 3B7U.X.701, 1B8J.A.451, 3B8Z.A.901, 2BH3.A.1002, 1BIW.B.801

, 2BIB.A.1549, 4BIN.A.500, 3BJC.A.876, 4BJH.A.423, 3BKQ.X.500, 1BLL.E.488, 2BMI.A.272, 2BNO.A.1201, 1BS8.A.2001, 4BT4.A.301, 4BT5.A.301, 4BT6.A.1257, 3BUB.A.1047, 3BUD.A.1048, 3BUI.A.1046, 3BVT.A.1048, 3BVU.A.1048, 3BVV.A.1047, 3BVW.A.1046, 3BVX.A.1046, 4BXK.A.1620, 4BZ3.B.502, 4BZ5.A.500, 4BZR.A.1630, 3C10.A.101, 4C24.A.301, 1C3R.A.501, 1C3S.A.951, 3C52.A.401, 4C5W.A.402, 4C6L.A.2823, 4C6L.A.2824, 4C8I.B.1161, 1CAM.A.262, 2CA2.A.264, 4CA5.A.1001, 4CA6.A.1001, 4CA7.A.1616, 4CA8.A.1620, 2CEA.A.1603, 2CFU.A.1001, 2CHI.A.212, 3CKI.A.501, 3CQJ.A.285, 3CV5.A.1047, 4CWM.A.433, 4CXO.A.453, 3CZN.A.1102, 3CZS.A.1102, 3D0N.A.262, 2D1N.A.270, 2D1O.A.257, 1D5J.A.301, 1D7X.B.801, 2DDY.A.177, 4DD8.A.1002, 4DEF.A.401, 4DEL.A.402, 4DHL.B.501, 3DHA.A.255, 1DK4.B.591, 2DKD.A.921, 4DLM.A.401, 4DPE.A.301, 1DQS.A.402, 2DVT.A.1501, 2DVU.A.1501, 2DVX.A.1501, 4DV8.A.801, 3DWB.A.771, 2DW0.A.700, 4DYK.A.501, 4DZH.A.504, 3E0L.A.1452, 2E2D.A.500, 3E38.A.1, 1E48.S.999, 1E4C.S.999, 3E4A.B.2000, 3EBG.A.1, 3EBH.A.1, 3EDZ.B.2, 2EG6.A.400, 2EG7.A.401, 1EI6.B.409, 3ELF.A.352, 4ENL.A.438, 6ENL.A.438, 2ERP.A.700, 3EWC.A.372, 3EWD.A.371, 2EWB.A.488, 1EYW.A.401, 4EYF.A.302, 4EYF.A.303, 1F0J.A.1101, 3F15.A.264, 3F16.A.264, 3F17.A.264, 3F18.A.264, 3F19.A.264, 3F1A.A.264, 1FA5.A.1200, 1FA5.A.1201, 3FDK.A.401, 2FGN.A.248, 1FKX.A.400, 4FL7.A.301, 4FUA.A.216, 2FV5.A.3, 2FV9.B.4, 2FVM.D.601, 4FW3.B.300, 4FW4.C.301, 4FW5.A.301, 4FW7.A.301, 2FYV.A.2003, 4FYT.A.1012, 3G4K.A.801, 4GBD.A.502, 2GC3.A.402, 1GKP.A.1461, 1GKP.B.1460, 1GKR.A.1453, 4GK8.A.302, 2GMN.A.802, 2GO4.A.601, 2GSO.A.1000, 1GVF.A.288, 1GYT.G.600, 1GYT.G.601, 4H01.A.601, 4H1Q.A.301, 4H2E.A.301, 4H2G.A.601, 4H3X.A.301, 1H48.C.900, 1H4N.A.262, 4H76.A.301, 3H8F.E.501, 1H9N.A.262, 2HBV.A.401, 2HBM.A.1001, 3HC4.L.401, 3HC8.A.864, 2HC9.A.701, 2HC9.A.702, 1HFC.A.275, 1HJK.A.451, 3HK5.A.430, 3HK8.A.430, 3HK9.A.431, 3HKA.A.430, 1HOV.A.166, 2HPT.A.950, 3HR1.A.1, 1HS6.A.701, 1HTY.A.1102, 2HUC.A.248, 3HWP.A.295, 4HWO.A.701, 4HWP.A.701, 4HWR.A.701, 4HWS.A.701, 3HY7.A.901, 3HY9.A.901, 3HYG.A.901, 1HZY.A.402, 1HZY.B.401, 2I0O.A.580, 2I47.C.804, 2ICS.A.400, 3ID7.A.401, 4IE0.A.601, 4IE4.A.601, 4IE6.A.601, 1IF6.A.262, 4IG2.A.401, 2ILP.A.500, 4ILW.D.301, 4ISM.A.201, 3ITC.A.501, 3ITC.A.502, 1ITU.A.402, 2JOT.A.1267, 1J2U.A.301, 4J4K.A.402, 4J5F.A.301, 4J5H.A.302, 1J79.A.400, 1J79.B.400, 2J83.A.999, 2J9A.A.1493, 1JAP.A.999, 1JAQ.A.999, 1JCZ.A.901, 4JD1.A.201, 4JE7.A.202, 4JH8.A.201, 4JH8.B.201, 1JIW.P.481, 2JIG.A.1252, 2JIH.B.1554, 1JJE.A.252, 1JJT.A.252, 1JK3.A.400, 2JNE.A.150, 2JNE.A.200, 4JP4.A.301, 1JPU.A.371, 2JT6.A.256, 1JWQ.A.1001, 1K07.B.3, 3K2G.A.400, 2K4W.A.156, 1K4P.A.1004, 3K5X.A.402, 1K9Z.A.402, 1KAE.B.1102, 4KAP.A.301, 1KBC.A.999, 1KBE.A.1, 3KDS.E.996, 1KEQ.A.280, 4KEQ.A.302, 3KM8.A.400, 3KR5.G.1004, 3KRY.A.1999, 4KTX.A.501, 1KYS.A.301, 1LAM.A.488, 1LAM.A.489, 1LCP.A.489, 4LCQ.A.601, 4LE6.A.405, 4LE6.A.406, 4LEF.A.301, 2LFF.A.500, 3LGG.B.512, 3LJT.A.901, 3LK8.A.264, 4LP6.A.302, 2LQ6.A.402, 3LS6.A.303, 4LV4.A.401, 3M4C.D.109, 3MA2.D.294, 3MA2.A.294, 3MAX.A.379, 4MCA.B.1000, 3MDU.A.454, 1MH2.A.201, 3MJM.B.401, 3MKV.B.425, 1MNC.A.281, 3MO2.D.6, 3MTW.A.1, 3MVI.A.901, 3N2U.A.264, 3N2V.A.264, 4N27.A.201, 3N2C.A.425, 3N9S.A.309, 4NGE.E.101, 2NQH.A.301, 2NQH.A.302, 4NTK.A.201, 3NX7.A.264, 3NXQ.A.650, 2NZE.A.401, 3O2X.A.1999, 2O4Q.A.2401, 3O90.B.192, 4O98.B.401, 2OB3.A.901, 2OC2.A.701, 3OD4.A.1350, 3OHL.A.4, 3OHL.A.5, 3OHO.A.5, 2OKL.A.601, 1ONW.A.800, 4OPN.A.201, 4OPN.B.201, 2OR4.A.1751, 1OS9.A.901, 4OUI.A.501, 2OW1.A.444, 2OW6.A.3001, 2OW7.A.5001, 3P3C.A.401, 1P5X.A.248, 1P6B.A.401, 1P6D.A.246, 1P6D.A.248, 1P6E.A.248, 3P76.A.274, 2PAJ.A.493, 1PBO.A.1302, 2PJT.A.302, 1PL6.A.402, 2PLM.A.407, 1PMI.A.445, 3PNU.A.337, 1PS6.A.330, 1PTW.A.501, 1PV9.A.401, 1PVW.A.401, 4PVO.A.402, 4PXY.A.302, 2Q02.C.300, 3Q2G.A.401, 3Q2H.A.401, 1Q3K.B.301, 3Q4R.A.201, 2Q5B.A.107, 2Q5B.C.107, 3Q6X.A.3, 3Q6X.B.271, 3Q9B.A.345, 3QAY.A.180, 4QA1.A.403, 4QA2.A.403, 4QA4.A.502, 4QA5.A.401, 4QA6.A.401, 3QDF.A.266, 1QF1.A.320, 1QH5.A.262, 1QIN.A.401, 1QIN.B.301, 1QIP.A.902, 1QIP.D.903, 3QIZ.A.431, 1QJJ.A.250, 2QPJ.A.1, 1QTW.A.303, 1QXL.A.400, 2R2D.A.278, 1R3N.A.500, 1R43.A.500, 4R76.A.1001, 3RHG.A.367, 1RJ5.A.601, 2RJQ.A.1, 4RL2.A.301, 4RL2.B.302, 1RM8.A.500, 1RMZ.A.264, 3RTS.A.264, 3RTT.A.264, 3S2J.A.402, 3S2L.A.402, 3S2M.A.402, 3S2M.A.403, 3S2N.A.402, 3S2N.A.403, 3SEY.E.

373, 1SHN.B.482, 3SJX.A.1751, 1SML.A.270, 3SNG.A.401, 3SNG.A.402, 3SPU.C.1010, 3
SZZ.A.502, 3T00.A.501, 3T00.A.502, 1T64.A.388, 2TCL.A.170, 3TOM.B.108, 1TQS.A.14
01, 1TQT.A.1301, 1TQU.A.1400, 1TQV.A.1300, 1TQW.A.1400, 4TYT.A.301, 3U04.A.200,
1U7J.A.50, 1U7J.B.150, 1U7M.A.54, 1U7M.B.154, 3U79.D.110, 3U79.E.110, 1UEA.C.1,
3UHM.A.300, 1UIO.A.400, 1URA.A.451, 2USH.A.601, 2USH.A.602, 3UWB.A.200, 1UXA.B.1
367, 2UX1.A.1174, 2UX1.C.1173, 2V5W.A.1380, 2V5X.A.1377, 3V77.A.301, 3V96.B.301,
2V9G.A.1276, 2V9M.A.1275, 2VES.A.1295, 1VFL.A.501, 3VPE.A.302, 2VQM.A.1411, 3VQ
Z.A.302, 3VTG.A.301, 2VUN.A.401, 2WOD.A.1264, 2W15.A.1203, 2W3Z.A.1312, 3W52.A.3
11, 3WAX.A.912, 3WAY.A.911, 4WB7.A.502, 4WD6.A.301, 4WD6.B.302, 2WM1.A.1333, 2WO
9.B.1268, 1WPP.A.602, 2WXU.A.1375, 2WXT.A.1371, 1WY2.A.406, 4X2T.B.1002, 1XBU.A.
901, 1XBU.A.902, 2XF4.A.1211, 2XHM.A.1616, 2XL9.B.1269, 1XM8.A.700, 1XP3.A.301,
1XP3.A.302, 2XPY.A.1673, 2XS3.A.999, 2XS4.A.999, 1XXW.A.201, 2XYD.A.1620, 2Y6D.A
.1268, 1Y7W.A.283, 1Y93.A.264, 2YDO.A.1946, 1YQY.A.781, 2Z24.A.400, 2Z24.B.400,
2Z25.A.400, 2Z25.B.400, 2Z26.A.401, 2Z27.A.400, 2Z27.B.400, 2Z28.A.400, 2Z28.B.4
00, 2Z29.A.400, 2Z29.B.400, 2Z2A.A.400, 2Z2B.A.338, 2Z3I.C.2003, 1Z60.A.1, 2Z72.
A.401, 2Z72.A.402, 2ZBM.A.401, 2ZBM.A.402, 1ZNB.A.2, 2ZNE.B.991, 2ZO4.A.319, 3ZU
0.A.1595, 3ZU0.A.1596, 3ZU0.B.1588, 3ZU0.B.1589, 2ZWR.A.208, 2ZWR.A.209, 1ZXC.A.
201, 3ZXH.A.300, 1ZZM.A.401, 1ZZM.A.402, 2FZ6.A.201, 3H90.D.6, 2MQ1.A.102, 2X4H.
A.1141
[1] "Cluster 3"
2JZW.A.57, 4LJ0.B.502, 4OND.E.101, 1BOR.A.58, 4CXV.A.433, 4JS6.A.301, 2K2C.A.140
, 1KMG.A.154, 3KNS.A.228, 3KS3.A.262, 3L6N.A.301, 3NJ9.A.262, 1QFO.A.320, 2QVV.A
.403, 3SXK.A.4, 3UCT.B.102, 3WXC.A.301, 3IE1.D.442, 4L8H.R.105, 3AF5.A.665, 1AJD
.A.450, 1ANI.A.450, 2ANH.A.451, 2ANU.B.405, 2APO.B.501, 2AXR.A.501, 3B1B.A.378,
2B5L.C.3001, 1BH5.A.201, 1BQQ.M.289, 3BYW.D.1, 3C0Z.A.101, 2CBN.A.402, 3CE9.B.40
0, 2CG3.Z.1, 3CHP.A.701, 2CIH.A.212, 2CIH.A.213, 3CPA.A.308, 3CQJ.B.285, 4CWM.B.
433, 1D1T.B.406, 3D3X.A.428, 3D68.A.501, 3DLJ.A.2001, 3DLJ.B.2002, 3DNG.B.998, 1
DSQ.A.144, 1DXW.A.301, 3E50.A.1, 2EC7.A.50, 2EC7.A.51, 4EGE.A.411, 4EGE.A.412, 3
EII.D.301, 2EIM.C.262, 1ENQ.C.238, 3EYV.L.220, 4F0R.A.501, 1F30.I.201, 2F4L.A.14
00, 3FGG.A.161, 4FUK.A.401, 4G1P.A.501, 2GLQ.A.2002, 4H00.A.601, 2H42.A.501, 3H9
0.D.5, 3HDB.A.620, 2HD1.B.101, 4HGX.B.301, 1HOV.A.165, 1HP7.A.401, 4IGM.A.401, 4
IGM.F.401, 4IGN.A.401, 3II1.A.571, 2IMC.A.600, 3ITM.A.1, 4J3D.A.301, 1JDI.A.301,
1JM7.B.143, 1JOE.A.205, 2JRP.A.150, 4JSS.A.301, 4K6T.B.403, 4K7S.B.101, 1KAR.A.
501, 1KAR.B.502, 1KQ0.A.479, 2L0Z.A.487, 1L9Y.A.2, 1LG6.A.262, 3LSF.H.2, 2M7Y.A.
101, 3MKV.A.425, 4MLX.A.301, 3MO2.D.5, 2MQ1.A.101, 1MWO.A.438, 4MZ7.A.701, 4N07.
B.306, 3N2C.D.426, 3NQ5.A.508, 4NRZ.B.301, 1NYR.A.1002, 2O1Q.B.145, 2O3Z.A.501,
3O90.A.192, 4O98.A.401, 2OC7.A.901, 1OLP.A.1373, 1OLP.A.1375, 1P5X.A.247, 2PJT.D
.302, 2PTW.A.500, 3PW3.D.406, 1PYT.B.350, 3Q31.A.1, 1Q74.D.304, 4Q7R.A.305, 2QFP
.A.434, 3QU6.A.114, 3QZC.A.2, 3R2J.A.301, 1R5X.A.122, 4R7M.D.1001, 1RO9.A.529, 3
S6L.B.185, 3SFW.A.502, 1SMP.A.472, 1T0A.B.760, 3T02.A.502, 1TM6.A.23, 4TQT.A.501
, 3TVX.A.900, 3U94.A.259, 3UBF.A.6, 3UBF.A.7, 1UDV.A.101, 1UR6.B.79, 1UUP.D.5222
, 1UXA.C.1367, 3V94.E.702, 2VQH.B.1089, 3VUV.A.501, 2VXX.D.201, 3WC5.A.404, 2WCO
.B.3012, 3WI2.B.801, 2WWO.A.1165, 4X2T.D.701, 4X2T.D.702, 1XAF.A.503, 2XR1.A.163
8, 2XR1.A.1639, 2Y2E.A.1180, 1YIX.A.601, 1YIX.B.603, 2Z2D.A.264, 1Z3J.A.264, 1Z5
R.C.600, 1ZKN.C.601, 1ZKX.A.423, 3ZNR.B.101, 3ZNS.A.101, 2ZNE.B.992, 1ZSW.A.315,
3ZTV.A.1598, 3ZUQ.A.1440
[1] "Cluster 4"
1A1G.A.201, 1A6Y.A.550, 1A6Y.A.551, 2A66.A.401, 3EPH.A.1, 2ER8.A.104, 1F2I.G.120
2, 1G2D.C.302, 1G2F.C.301, 1G2F.C.302, 4GLX.A.601, 3HAX.C.201, 2I13.B.507, 4IQR.
A.403, 2JP9.A.131, 2JP9.A.134, 1K3X.A.501, 1KB2.A.150, 4M9E.A.503, 4M9E.A.504, 4
M9V.C.202, 4NM6.A.2002, 2NQ9.A.401, 4O6A.A.601, 2OFI.A.302, 3PIH.A.917, 3QSV.A.1
, 4R2Q.A.503, 258L.A.500, 1A1R.A.901, 3A32.A.708, 2A5V.A.401, 3A6G.A.301, 1A71.A
.401, 1A72.A.376, 4A7K.A.950, 3A9J.C.1, 3A9K.C.1, 1AAF.A.56, 2AC3.A.531, 4ADN.A.
1223, 1AF2.A.296, 2AFU.A.391, 2AFM.A.391, 1AJB.A.451, 2AMT.B.2900, 1ANJ.B.451, 4

AR9.B.1731, 3AUK.A.390, 2AU3.A.501, 4AWY.B.3228, 3AX1.A.601, 4AX0.B.3228, 4AX1.B .3228, 4AXD.A.700, 4B29.A.1205, 2B3Z.A.1360, 3B4N.B.712, 4B6D.A.1340, 1B8Y.A.301 , 3B92.A.502, 1BBO.A.61, 3BET.A.262, 2BE7.D.1108, 3BHX.A.1752, 3BI1.A.1752, 3BJI .A.1, 3BJI.B.2, 2BL6.A.1060, 1BNL.A.179, 1BNQ.A.262, 2BNM.A.1199, 3BOC.A.1001, 2 BP0.A.1341, 1BS4.A.2001, 3BTO.C.375, 4BT7.A.301, 1BUD.A.800, 1BV3.A.262, 1BYF.A. 302, 3BYR.A.501, 2C1I.A.1465, 4C1D.A.502, 4C1F.A.501, 4C1F.A.502, 4C1G.A.301, 4C 1G.B.301, 4C1Q.A.493, 3C2S.A.448, 2C2F.A.1211, 1C3I.B.260, 2C36.A.1311, 3C52.B.4 01, 4C81.A.1240, 2C9S.A.1155, 1CAK.A.262, 1CDO.B.376, 3CE1.A.202, 2CFU.A.1002, 1 CGL.A.302, 3CJP.A.301, 2CKI.A.999, 2CLT.A.1202, 3CMR.A.450, 3CNG.A.508, 2COT.A.2 01, 2COT.A.401, 3COS.A.501, 2CO8.A.201, 2CO8.A.401, 2CON.A.201, 2COR.A.201, 2COR .A.401, 1CQR.B.2301, 2CQE.A.622, 2CSY.A.401, 3CSQ.A.335, 1CTT.A.296, 2CTO.A.201, 2CTO.A.401, 2CT2.A.201, 2CT5.A.201, 2CT7.A.401, 2CTT.A.401, 2CU8.A.401, 2CUR.A. 201, 4CVR.A.1158, 2D0W.B.1207, 4D0Y.A.1239, 4D6S.A.1338, 3D7V.A.2, 2D74.A.1001, 2D74.B.1002, 2D8T.A.201, 2D8T.A.401, 2D8U.A.201, 2D8V.A.201, 2D9M.A.1085, 2D9N.A .456, 2DAN.A.201, 2DAR.A.201, 3DBK.A.302, 1DCA.A.262, 1DD6.A.503, 1DEH.A.376, 3D FM.A.402, 2DH3.A.601, 3DH1.A.201, 3DHA.A.256, 2DID.A.201, 2DJ7.A.201, 2DJ8.A.401 , 2DJA.A.201, 2DJA.A.401, 1DK4.A.290, 2DKT.A.191, 2DKT.A.241, 2DKT.A.291, 2DKT.A .341, 2DKT.A.391, 2DKT.A.441, 1DMT.A.755, 2DMD.A.291, 1DO5.A.28, 2DOO.A.501, 2DO O.A.502, 4DR8.A.201, 2DW2.A.700, 3E1Z.A.111, 3E2C.A.200, 1E3J.A.902, 1E3L.A.380, 3E6U.A.501, 3E6U.C.502, 3E6U.B.503, 2E7Y.A.1301, 3E73.A.501, 2E73.A.401, 2E9H.A .301, 1E9P.B.153, 1E9Q.B.153, 2EA6.A.201, 2EA6.A.401, 2ECT.A.201, 2ECT.A.401, 2E CV.A.201, 2ECW.A.201, 2ECW.A.401, 2ECY.A.401, 2ECG.A.201, 2ECL.A.601, 2ECM.A.201 , 2ECM.A.401, 1ED9.A.451, 3EDI.A.210, 1EE2.A.1300, 2EE8.A.501, 4EEX.A.402, 4EEZ. A.402, 3EFO.B.1034, 1EI6.A.409, 1EI6.B.408, 1EKJ.A.4001, 1EKJ.C.4003, 1EKM.A.701 , 2ELO.A.181, 2ELR.A.181, 2ELS.A.181, 2ELT.A.181, 2ELU.A.181, 2ELV.A.181, 2ELX.A .181, 2ELI.A.401, 2ELM.A.181, 2EMZ.A.201, 2EM2.A.201, 2EM4.A.201, 2EM6.A.201, 2E M9.A.201, 2EMB.A.201, 2EMC.A.201, 2EML.A.201, 2EN2.A.201, 2EN6.A.181, 2EN9.A.181 , 2ENA.A.181, 2ENC.A.181, 2ENE.A.181, 2ENH.A.181, 2EOJ.A.201, 2EOQ.A.201, 2EOU.A .201, 2EOV.A.201, 2EOW.A.201, 2EOX.A.201, 2EOE.A.201, 2EOF.A.201, 2EOH.A.201, 2E OK.A.201, 2EOM.A.201, 2EON.A.201, 2EOO.A.201, 2EPZ.A.201, 2EP2.A.201, 2EP4.A.300 , 3EPZ.A.701, 2EPQ.A.201, 2EQ1.A.201, 1EU3.B.402, 2EU3.A.262, 3EWF.A.400, 2EWB.A .489, 2EXU.A.501, 3EYX.A.1, 1F1G.A.4002, 1F2W.A.262, 3F7B.B.301, 4F7O.B.301, 3F9 O.A.309, 1FAQ.A.1, 4FGL.A.301, 4FKK.A.1025, 1FLJ.A.262, 4FMN.B.901, 4FMN.B.902, 4FMP.A.400, 2FNF.X.2, 4FO9.A.501, 2FPR.A.502, 3FPC.A.353, 3FPL.A.352, 1FQM.A.262 , 1FR2.B.301, 2FSA.A.501, 3FTN.A.354, 3FTW.A.701, 3FUN.A.701, 2FU8.A.401, 4FVY.A .805, 4FWU.A.401, 2G0D.A.416, 4G3M.B.401, 1G5C.A.1001, 2G9Y.B.451, 3GC9.B.603, 2 GMN.A.805, 4GNE.A.1501, 4GNE.A.1503, 1GO8.P.1486, 4GR3.A.301, 4GRI.A.501, 2GSN.A .1000, 4GU1.A.905, 4GUA.A.1719, 4GUT.A.904, 3GV4.A.203, 1GVY.A.1425, 2GZL.A.900, 4H1Q.A.302, 4H2K.B.1001, 2H39.A.352, 4H3S.A.901, 2H4N.A.262, 1H9Q.A.262, 3HB2.P .486, 2HCS.A.1, 2HCS.A.2, 4HCG.A.202, 3HFF.A.154, 3HJT.A.1, 1HK8.A.1589, 3HKT.A. 261, 3HLJ.A.272, 4HMA.A.301, 3HNI.G.107, 3HNJ.A.107, 3HNJ.B.107, 3HNJ.C.107, 3HN J.D.107, 4HNO.A.301, 3HPH.A.220, 1HSZ.A.1376, 1HTO.A.1376, 3HUG.D.109, 1HZ5.B.10 5, 3I1U.A.401, 1I50.A.3006, 1I73.A.998, 1I73.A.999, 1IA9.A.2001, 1IBB.A.201, 2ID A.A.103, 1IF5.A.262, 3IFU.A.181, 2IGI.A.1001, 2IGI.A.1004, 4IJD.A.501, 4IJD.A.50 2, 2IMZ.A.501, 2IMZ.B.502, 2IMR.A.500, 4IR0.A.201, 1IS8.B.3109, 2IUC.A.1002, 2IU C.B.1007, 4IUW.A.701, 4J1V.A.301, 1J2O.A.116, 4J4M.A.301, 2J7U.A.1884, 2J7J.A.10 88, 1JAO.A.999, 2JBG.B.1577, 4JEB.B.202, 2JHG.A.401, 4JH2.A.201, 4JH2.B.201, 2JI G.B.1252, 1JJD.A.103, 4JLX.A.501, 2JLP.A.226, 2JOX.A.109, 1JQ5.A.371, 4JSA.A.301 , 4JSW.A.301, 4JSZ.A.301, 2JTN.A.184, 2JTN.A.185, 2JUN.A.222, 1JV0.A.261, 3JWP.A .2001, 4JXE.A.502, 1JZS.A.1101, 1K07.A.1, 4K2H.A.201, 4K2H.B.201, 1K7H.A.478, 4K 7D.A.503, 4K7D.A.504, 4K7D.A.506, 3KBF.A.159, 2KEM.A.195, 1KH5.A.451, 1KH7.A.451 , 3KHI.A.301, 1KOL.A.1001, 4KP5.A.301, 4KP8.A.301, 1KU0.A.701, 2KVF.A.83, 3KV4.A .448, 4KX8.A.1001, 4KXB.A.1001, 3KYC.B.641, 1L0Y.B.706, 2L0B.A.143, 2L0B.A.161, 4L05.A.203, 4L6O.A.801, 4L6T.A.301, 3L8H.A.901, 3L9Y.A.155, 1LDY.A.375, 3LEA.A.4

85, 4LEF.A.302, 4LEV.A.601, 2LFD.A.400, 2LGV.A.111, 1LI5.B.964, 4LJQ.B.1105, 4LJ Q.A.1101, 2LK5.A.37, 1LLU.B.343, 3LPE.B.60, 4LP6.A.310, 3LQB.A.201, 3LQH.A.1001, 4LR2.A.505, 3LS9.A.457, 2LUY.A.300, 4LW9.K.203, 2LXD.A.202, 4LY4.D.301, 3M0A.D. 401, 2M0E.A.101, 3M15.A.107, 3M1W.A.500, 3M2N.A.263, 2M3Z.A.101, 1M3V.A.124, 3M3 X.A.262, 3M40.A.262, 2M48.A.501, 2M48.A.504, 3M5S.A.500, 3M67.A.263, 1M6W.A.1376 , 3M8T.A.300, 3M98.A.262, 1M9O.A.78, 3MA2.A.295, 3MBG.A.1, 4MB7.A.301, 3MDW.A.45 5, 4MDM.A.301, 3MEQ.A.401, 3MF1.A.1000, 1MGO.A.375, 1MGO.B.375, 3MHI.A.262, 3MHL .A.262, 3MHM.A.262, 3MHS.A.473, 3MHS.E.97, 4MHN.A.400, 3MI9.C.87, 3MI9.C.88, 3MJ H.B.70, 3MKG.A.155, 3ML2.A.262, 3MTW.A.2, 4MT2.A.68, 3MWM.A.141, 3MYQ.A.262, 3N2 C.E.425, 3N2P.A.262, 1N8K.A.375, 3NGJ.A.250, 3NKQ.A.1001, 4NN2.A.401, 4NN2.A.402 , 1NO5.B.571, 3NQY.B.520, 4NQ4.A.302, 4NQ7.A.302, 4NTM.A.201, 4NTN.A.201, 2NUP.B .1100, 2O03.A.201, 4O64.A.2003, 3O7U.A.428, 4OBI.A.201, 2ODX.A.156, 2OHX.A.401, 3OJ3.J.902, 3OQ6.A.375, 2ORW.A.401, 2OU2.A.490, 2OW9.B.606, 2OXZ.A.264, 2OX8.B.3 , 1P1R.A.375, 1P1V.A.201, 1P42.A.501, 1P42.A.503, 1P4Q.B.301, 3P58.A.262, 3P5A.A .262, 3P5L.A.262, 1PB0.A.1301, 1PB0.A.1303, 3PB6.X.400, 3PLW.A.187, 2PLI.B.709, 3PN3.A.1010, 3PO6.A.1, 3PSQ.B.321, 4PTB.A.901, 2PUY.A.355, 3PU7.A.155, 1PV9.A.40 2, 1PXE.A.64, 3PZC.A.1000, 1Q0E.A.153, 3Q1D.A.202, 2Q1Q.A.262, 2Q2L.A.1001, 1Q3K .A.301, 3Q43.A.1, 1Q5W.A.32, 4Q6D.A.301, 2QDT.A.401, 2QDT.A.402, 3QE3.A.356, 4QF 2.A.1801, 4QF3.A.2001, 2QIN.A.2002, 1QJI.A.1201, 2QL0.A.53, 4QP5.A.401, 2QSW.A.2 01, 4QSJ.A.301, 1QTW.A.301, 1QUA.A.999, 1QV6.A.375, 1QV7.A.375, 1R1H.A.1001, 1R3 7.A.500, 1R3N.B.501, 2R3A.A.302, 1RAY.A.262, 3RF4.B.119, 4RF1.A.1901, 4RL0.A.301 , 1RM8.A.501, 1RMD.A.119, 1RMD.A.120, 2RPC.A.601, 2RPC.A.801, 2RPP.A.201, 2RSI.A .103, 2RSJ.A.102, 1RUT.X.604, 3RYZ.A.1, 3RZV.A.1, 3S2E.G.500, 3S2F.E.500, 1S4B.P .1, 1S4I.A.802, 3S77.B.262, 3SEY.C.373, 1SML.A.271, 1SRD.B.156, 1SRP.A.920, 3SV6 .A.4, 3SWR.A.3, 3SXK.B.3, 1T3K.A.201, 3T33.A.411, 3T80.D.301, 3T9O.A.502, 1T9R.A .1, 3TBG.A.601, 3TEN.A.205, 1TKF.A.901, 1TKF.A.902, 1TTM.A.262, 1TWF.J.3001, 1U0 5.A.500, 1U10.A.400, 1U1H.A.766, 1U3U.A.376, 1U3V.A.376, 1U40.A.160, 1U5S.B.139, 3U5N.A.1, 4UA4.A.301, 3UCK.A.228, 1UD9.C.509, 3UN6.A.325, 1USN.A.257, 1UUF.A.40 2, 3UVC.B.303, 1UW0.A.1118, 2UZG.A.131, 2V08.A.1090, 2V1X.A.1595, 1V13.B.200, 3V 1C.A.101, 3V1E.A.102, 2V29.A.1276, 1V5N.A.201, 1V5N.A.401, 1V6G.A.201, 3V7M.A.50 9, 1V7Z.A.301, 2V9I.B.1275, 3V96.B.302, 2V9E.A.1276, 1VA1.A.100, 1VA2.A.100, 1VA 3.A.100, 2VES.A.1297, 2VES.C.1302, 2VF7.B.1844, 1VK9.A.143, 2VL6.A.1266, 3VOW.A. 201, 3VPE.A.301, 2VP7.A.1399, 1VQ2.A.702, 3VQZ.A.301, 3VRK.A.301, 2VUT.I.1713, 1 VYK.A.1150, 1VYX.A.1062, 2W3Q.A.1231, 2W5V.B.1377, 2W5X.A.1378, 3W5K.B.502, 1WAA .B.1090, 2WBT.A.1130, 2WBT.A.1131, 3WBH.B.505, 1WEM.A.201, 1WEN.A.401, 1WEO.A.40 1, 1WEP.A.201, 1WES.A.201, 1WES.A.401, 1WFF.A.401, 1WFL.A.401, 3WF8.A.401, 1WFP. A.401, 1WG2.A.200, 1WIG.A.201, 1WIG.A.401, 1WII.A.201, 1WIL.A.401, 1WIM.A.201, 2 WJV.A.3, 1WJP.A.301, 1WJP.A.501, 1WJV.A.201, 3WLE.A.402, 3WLF.A.402, 2WOJ.A.1353 , 4WOK.A.401, 3WRG.A.702, 1WY2.B.407, 1WYH.A.201, 1X3H.A.401, 1X3Z.A.999, 2X3B.A .1341, 1X4K.A.201, 1X4K.A.401, 1X61.A.201, 1X62.A.201, 1X6H.A.401, 2X8Y.A.1616, 2X8Z.A.1616, 2X95.A.1615, 2X96.A.1617, 2XBL.C.1196, 1XC3.A.302, 4XIX.B.401, 2XML .A.1349, 2XQV.A.401, 1XRT.A.1423, 1XSO.A.152, 2XS4.A.998, 1XWY.A.401, 2Y6C.A.126 7, 1Y8J.A.800, 1Y93.A.265, 1YB0.B.160, 1YC2.C.406, 1YC5.A.1001, 1YE3.A.375, 1YLK .A.401, 1YOU.A.301, 1YQD.A.1000, 2YQL.A.201, 2YQL.A.401, 2YQP.A.201, 2YQQ.A.401, 2YRT.A.401, 2YRG.A.401, 2YRM.A.201, 2YS2.A.201, 2YSA.A.181, 2YSO.A.181, 2YTH.A. 201, 2YTQ.A.201, 2YTR.A.201, 2YTT.A.181, 2YT5.A.201, 2YT5.A.401, 2YTB.A.301, 2YT D.A.201, 2YTK.A.201, 2Z45.A.1001, 1Z6R.A.501, 1Z84.A.603, 2ZEP.A.391, 2ZED.A.391 , 1ZFO.A.31, 3ZNC.A.1, 2ZNC.A.1, 3ZP9.A.1009, 3ZQ6.A.1323, 2ZTX.A.501, 3ZUK.A.16 64, 2ZU2.A.5517, 3ZVS.A.1159, 3ZXH.A.301, 1ZXV.B.9002, 1ZZU.A.900

[1] "Cluster 5"

3AAK.A.992, 4BLB.D.910, 4CPA.I.308, 3CSQ.B.335, 1D1S.B.406, 4DWZ.A.302, 1F30.B.2 01, 1F30.E.201, 2GA6.D.998, 4H82.A.301, 3H90.C.2, 1HR6.B.501, 4IOD.A.502, 4JBS.B .1009, 4K0D.B.203, 4KJG.A.1001, 1LOY.B.702, 4LTE.A.1101, 3M3B.A.156, 3M4B.B.108, 1NL4.B.500, 2QLA.A.500, 1RLY.A.61, 3U94.B.259, 2V8V.A.1456, 3V94.D.702, 1VKG.A.

400, 4LMG.B.202, 3P57.P.122, 2ANU.B.505, 2DI2.A.30, 4FC5.E.305, 1GLC.F.169, 3H66
.B.500, 4HDT.A.400, 4I0Z.A.504, 3ISO.B.220, 1KHN.A.452, 3M7P.A.953, 2MQ1.A.103,
1N4P.H.378, 2OX8.A.5, 2POJ.A.265, 4UNI.C.1697, 2V8V.B.1455, 2W9M.A.1565, 1YEW.G.
662, 1ZZM.A.403, 1ANJ.A.450, 4BLL.A.1322, 3BWI.A.450, 4COQ.B.299, 4CVR.A.1160, 4
CVT.A.1160, 1DK4.A.291, 4DYO.A.501, 4EWL.A.403, 4FW3.A.301, 1G12.A.200, 2GO4.B.6
02, 3GSH.A.101, 1H48.A.900, 1H8L.A.999, 1JJE.B.261, 2JT2.A.336, 1KH9.B.450, 1L0Y
.D.704, 4L3T.A.1101, 4L3T.B.1101, 3LMS.A.309, 1LNF.E.800, 4LQY.A.507, 3MN8.A.999
, 4MN6.A.401, 4ONX.A.201, 2OOT.A.1751, 2OX8.A.4, 3PN6.B.202, 3R8B.B.122, 3R8B.D.
122, 3R8B.F.122, 3R8B.P.122, 3RBU.A.1751, 3U1Y.A.400, 1UXB.A.1367, 2VME.A.501, 2
VQG.C.1092, 4WD8.C.302, 3WT4.A.501, 4X2T.E.701, 1XJO.A.901, 1YH8.A.501
[1] "Cluster 6"
3AUO.B.579, 2ER8.A.105, 3F2D.A.5, 4GNX.C.701, 4IFD.J.1106, 4MTD.B.201, 1ODH.A.11
71, 1QUM.A.301, 1V15.A.1132, 3VD0.A.401, 3A3O.B.65, 2A97.B.2437, 1AAF.A.57, 4AA1
.A.1615, 2AB3.A.30, 2ADR.A.163, 2AF2.B.154, 2ANH.A.452, 1ARD.A.1, 1ARE.A.1, 1ARF
.A.1, 4AR1.A.1722, 4ARF.A.1722, 1AXG.A.401, 2AYK.A.171, 3AY2.A.1001, 2AZH.A.150,
 2B0P.A.400, 3B6P.A.800, 3B7R.L.701, 1B8T.A.193, 1B8T.A.194, 1B8T.A.195, 1B8T.A.
196, 1BAW.A.107, 2BA1.A.201, 3BKN.A.201, 3BL5.A.300, 2BNN.A.1200, 1BTG.B.902, 3C
0Y.A.401, 2C20.A.601, 2CEA.B.1606, 3CHV.A.302, 3CQZ.B.3007, 3CQZ.I.3004, 1CVE.A.
262, 3D2N.A.102, 1D8M.B.801, 1D9D.A.1, 1DDZ.A.1, 1DE6.A.450, 1DGS.B.2701, 2DJ7.A
.401, 2DKD.B.922, 4DLA.A.401, 4DLF.A.404, 1DPM.A.801, 2DQ6.A.900, 2DSN.B.2002, 1
DTH.A.901, 4DYG.B.307, 2E1W.A.400, 1E46.S.999, 3E4Z.B.2, 1E67.A.129, 3EBI.A.1, 2
EG4.A.301, 2EG4.B.302, 1ELZ.A.451, 3EQN.B.757, 1EU4.A.400, 4EXS.A.302, 1EYW.A.40
2, 1F35.A.306, 1F5F.A.252, 1F8F.A.372, 3FDK.A.402, 2FEJ.A.1, 3FVZ.A.821, 4FVL.A.
501, 3G42.D.500, 2G54.A.1100, 2GA3.A.451, 3GAY.B.328, 3GJN.B.600, 1GKR.A.1452, 2
GMN.A.801, 1GYT.J.600, 3GZE.B.14, 4H01.A.602, 3H8F.B.501, 3H90.A.291, 3H90.A.293
, 3HNI.H.107, 2HSI.A.283, 1HU8.A.501, 2IOO.A.581, 4I28.A.602, 4I7C.A.601, 1IAG.A
.999, 1IAU.A.504, 3ID7.A.402, 3IEW.B.801, 4IOU.D.1001, 3ISI.X.3000, 1ITU.A.401,
4IUQ.A.301, 2IWE.A.1129, 4J3D.B.302, 4JD1.B.202, 4JIJ.A.302, 2JIG.A.1253, 1JM7.A
.123, 1JT1.A.400, 2K2G.A.2, 4K5N.A.1101, 2K8D.A.155, 1KBE.A.2, 2KIK.B.50, 2KIZ.A
.71, 2KO8.C.54, 3KWO.B.161, 4KXC.A.1001, 1L1O.F.2, 1L7O.B.301, 3L8Y.A.301, 4LAO.
A.401, 1LD3.A.500, 1LDE.A.375, 3LE9.B.2, 2LGV.A.110, 2LUL.A.201, 2LVR.A.101, 2LV
T.A.101, 2LXH.C.900, 2LXH.C.901, 3LZE.A.201, 3M0N.A.201, 2M0D.A.101, 3M15.B.107,
 1M4M.A.502, 3M8T.A.301, 3MEN.D.400, 3MKV.B.426, 1MMR.A.1, 1MVH.A.502, 1MVH.A.50
3, 3MWM.A.142, 1ND1.A.400, 3NNQ.A.201, 1NUI.A.501, 2O36.A.690, 2O4Q.A.2402, 2O53
.B.314, 2OI0.A.2, 4OIW.F.501, 1OJ7.B.1389, 1OKN.A.262, 1OLP.D.1374, 1ONW.A.801,
2OO4.A.6001, 1OS2.B.369, 1OS2.F.769, 2OW2.A.444, 2OXW.A.264, 1P5D.X.500, 2P53.A.
401, 1P91.B.2301, 4P9C.A.201, 1PAA.A.160, 3PJN.A.186, 3PJN.A.189, 3PJN.B.186, 1P
S7.A.331, 3Q44.A.1, 4Q7R.A.306, 4QBG.B.301, 2QJS.A.2002, 3QJ5.A.376, 2QL1.A.1, 3
QNA.A.122, 3QVY.A.500, 3QVZ.D.500, 3QWO.C.500, 1QX1.A.2004, 1R4V.A.202, 3RAM.D.9
98, 2RPR.A.201, 2RPC.A.401, 4RQT.A.402, 3RSM.A.500, 1RUR.L.601, 1S1G.A.152, 3SFH
.A.403, 3SPU.D.1004, 3SP1.A.481, 2SRT.A.257, 1SW1.A.401, 3SZY.A.501, 3T01.A.502,
 1TBN.A.2, 1TF9.A.901, 3TGO.B.505, 1THJ.A.214, 1THJ.B.214, 1TKH.A.901, 1U0L.A.29
8, 1U10.A.601, 4U10.A.401, 4U4L.A.301, 3U7K.A.350, 3U7L.A.350, 3UIK.A.341, 3UW2.
A.474, 2UYV.B.1276, 1UZF.A.701, 2V2A.A.1275, 2V8G.C.500, 1V9P.B.2701, 1VSH.A.281
, 1VYX.A.1061, 3W5K.B.503, 2W57.B.201, 4W6Z.A.401, 4WD6.A.302, 1WNU.A.1001, 2X4H
.B.1142, 2X5C.A.1128, 2X7M.A.1174, 2XAA.C.1346, 2XAM.B.700, 1XB8.A.1001, 1XEM.A.
401, 1XTG.A.426, 1XUC.A.1261, 1XUF.A.246, 1XWH.A.356, 2XY9.A.1628, 1Y0J.A.244, 2
Y20.C.1331, 1Y7W.B.285, 1YC2.A.402, 1YEJ.L.605, 1YHC.A.601, 2YPU.A.1998, 2Z26.A.
400, 2Z3I.A.2001, 2Z9K.A.901, 1ZLH.A.555, 3ZTV.A.1599, 1ZXZ.A.198
[1] "Cluster 7"
1A1I.A.201, 1A73.A.202, 4AA6.A.253, 2B3J.A.2001, 2C7A.A.1641, 2C7A.A.1642, 3CBB.
A.1001, 4CIS.A.300, 1CYQ.A.601, 1CYQ.A.602, 2DRP.A.171, 2DRP.A.172, 1DSZ.A.1121,
 1DSZ.A.1122, 1DSZ.B.1222, 3EQT.A.1, 4ESJ.A.301, 1F4S.P.64, 1FFY.A.1001, 2FF0.A.
1002, 1G2D.C.303, 1G2F.C.303, 3G9M.A.526, 2GAT.A.67, 4GAT.A.67, 6GAT.A.67, 3GOX.

A.301, 3GOX.A.302, 4GZN.C.203, 2HAN.A.351, 2HAN.A.352, 2HAN.B.353, 2HAN.B.354, 4
HC9.A.401, 4HC9.A.402, 2HGH.A.191, 2HGH.A.192, 2HGH.A.193, 4HN5.A.601, 4HN6.A.60
2, 2I13.A.502, 1I3J.A.100, 2IHX.A.235, 2IHX.A.236, 2JP9.A.132, 2JZW.A.56, 1K82.A
.450, 2KAE.A.175, 1KB2.A.151, 3KDE.C.78, 2KMK.A.83, 2KMK.A.84, 2KMK.A.85, 3KMP.A
.2, 1LAT.A.1514, 1LAT.A.1515, 4LJO.A.501, 4LJO.A.502, 4LJO.A.503, 1LLM.C.301, 1L
O1.A.195, 1LO1.A.196, 3LRR.A.1, 2LT7.A.701, 2LT7.A.702, 2LT7.A.703, 3M7K.A.143,
3M7K.A.144, 4M8O.A.1303, 3M9E.A.208, 3M9E.B.209, 4M9E.A.505, 4M9V.C.201, 1MEY.C.
89, 1MEY.C.90, 3MLN.A.501, 4MTD.A.201, 3NCU.A.1, 4NDH.A.402, 2NLL.B.450, 2NLL.B.
451, 2O6M.A.601, 3OD8.A.200, 4OLN.A.101, 4OLN.A.102, 4OND.A.101, 4OND.A.102, 4OO
R.A.102, 3OYM.A.393, 1OZJ.A.145, 1P47.A.203, 4QEN.A.802, 4QEN.A.803, 4QEN.A.805,
 4R2A.A.503, 4R2A.A.504, 4R2A.A.505, 4R2S.A.501, 1R4O.A.526, 1TDZ.A.1001, 1TF3.A
.102, 1TF3.A.2, 1TF3.A.3, 4TNT.A.701, 4TNT.A.702, 3TS2.A.1, 3TS2.A.2, 3U6P.A.300
, 1UBD.C.501, 1UBD.C.502, 1UBD.C.503, 1UBD.C.504, 3UK3.C.968, 3VD6.C.501, 2XQC.A
.1138, 2XQC.D.1141, 2YKG.A.927, 1YUI.A.64, 1ZAA.C.203, 1ZNS.A.1500, 2A1K.A.1, 3A
1B.A.1, 3A1B.A.2, 3A1B.A.3, 4A24.A.601, 4A24.A.602, 4A2C.A.1349, 4A2V.A.1000, 3A
43.A.701, 2A51.A.54, 2A51.A.55, 2A5H.A.421, 2A5H.B.421, 2A6H.D.7458, 2A6H.D.7412
, 1A71.A.402, 1A7T.A.251, 2A8D.A.1230, 2AFW.A.996, 2AFX.A.996, 2AFZ.A.391, 3AII.
A.1001, 4AIA.A.200, 4AJX.H.1030, 2AKL.A.117, 3ALR.A.601, 3ALR.A.602, 1AM6.A.262,
 2AP1.A.304, 2APS.A.400, 3ASL.A.3, 2ASH.A.400, 4AU7.A.1248, 4AUQ.B.1299, 4AUQ.B.
1300, 3AVR.A.1502, 2AW1.A.262, 3AXS.A.401, 2AYD.A.369, 2AYJ.A.57, 4AY8.A.600, 4A
YC.A.1484, 4AYC.A.1485, 1AZM.A.261, 2BOO.E.698, 2B44.A.400, 4B6D.A.1341, 2B9D.A.
1002, 1BBO.A.60, 1BCD.A.262, 4BF1.A.270, 4BF6.A.1262, 2BL6.A.1059, 4BM9.A.1466,
4BM9.A.1469, 1BN1.A.262, 1BN3.A.262, 1BN4.A.262, 1BNN.A.262, 1BNT.A.262, 1BNU.A.
262, 1BNV.A.262, 1BNW.A.262, 3BO5.A.302, 3BO5.A.303, 3BO5.A.304, 3BOF.A.701, 3BQ
5.A.800, 3BQ6.A.800, 4BS9.A.1782, 4BUE.A.2162, 2BYO.A.1209, 1BZM.A.261, 2BZ1.A.1
174, 4C09.A.351, 1C2G.A.409, 4C3E.A.201, 4C3T.A.300, 4C4O.B.600, 3C5K.A.201, 3C5
K.A.202, 3C5K.A.203, 4C5W.A.403, 3C63.A.107, 3C63.B.107, 3C63.C.107, 3C63.D.107,
 3C6W.A.2, 2C6C.A.1752, 1C7K.A.133, 3C7P.A.262, 2C7N.A.499, 1C9Q.A.999, 1CAI.A.2
62, 4CA1.A.283, 4CA1.A.284, 2CBD.A.262, 4CCG.X.1375, 1CDO.B.377, 2CDC.A.1372, 4C
DG.A.1643, 1CG2.A.502, 1CG2.C.502, 3CG7.A.299, 3CHQ.A.701, 3CHS.A.701, 3CHV.A.30
1, 1CIL.A.262, 1CIM.A.262, 1CIN.A.262, 2CJS.C.201, 2CJS.C.202, 2CKL.A.1104, 2CKL
.A.1105, 2CKL.B.1115, 2CKL.B.1116, 1CNG.A.1, 1CNH.A.1, 1CNJ.A.1, 3COS.A.502, 4CP
D.A.1200, 4CPD.A.1300, 2CR8.A.401, 2CRC.A.401, 2CSV.A.200, 2CSV.A.400, 2CS2.A.20
0, 2CS3.A.200, 2CS3.A.400, 2CS7.A.201, 2CS8.A.401, 2CS8.A.601, 2CSH.A.200, 2CSH.
A.300, 2CSH.A.400, 3CXK.A.201, 3CXL.A.500, 1CXX.A.1, 3CZV.A.262, 1D0C.A.900, 1D0
Q.B.151, 1D1T.A.375, 1D1T.A.376, 3D2N.A.101, 3D2Q.A.303, 3D2Q.A.304, 3D7F.A.1752
, 2D8X.A.201, 2D8X.A.401, 2D8Y.A.201, 2D8Y.A.401, 2D8Z.A.201, 2D8Z.A.401, 3D8W.A
.262, 2D8R.A.401, 2D9G.A.201, 2D9H.A.201, 2D9H.A.401, 2D9K.A.401, 2D9K.A.601, 2D
9L.A.401, 3DBH.B.1, 4DB3.A.401, 3DCC.A.262, 1DCQ.A.600, 1DD6.A.502, 3DDO.A.262,
1DDZ.A.2, 1DFE.A.38, 3DGD.C.128, 3DI4.B.286, 2DIP.A.201, 2DIP.A.401, 2DJ8.A.201,
 2DLK.A.201, 2DLK.A.401, 1DL6.A.60, 2DLO.A.201, 2DLO.A.401, 2DLQ.A.200, 2DLQ.A.3
00, 2DLQ.A.400, 2DLQ.A.500, 4DLA.A.402, 3DMO.A.131, 2DMD.A.241, 2DMI.A.200, 2DMI
.A.300, 2DMJ.A.200, 2DPH.A.1001, 2DS7.A.100, 1DVP.A.401, 1DVP.A.402, 4DZ7.A.301,
 1EOE.A.147, 2E2Z.A.101, 1E3J.A.901, 1E4U.A.79, 1E4U.A.80, 2E5R.A.201, 2E5R.A.40
1, 2E5S.A.201, 2E5S.A.401, 2E6R.A.201, 2E6R.A.401, 2E6S.A.201, 2E6S.A.401, 2E6S.
A.601, 1E7L.A.1165, 2E73.A.201, 2EBL.A.191, 2EBL.A.241, 2EBT.A.100, 2EBT.A.200,
2EBT.A.300, 2EBV.A.201, 3EB5.A.1001, 3EBE.A.500, 2EBQ.A.201, 2EBR.A.201, 2ECJ.A.
401, 2ECV.A.401, 2ECL.A.201, 2ECN.A.201, 2ECN.A.401, 1EE8.A.501, 3EED.A.194, 3EE
R.A.2004, 2EE8.A.301, 2EE8.A.701, 4EEZ.A.401, 1EF4.A.56, 3EFO.A.766, 2EGQ.A.200,
 2EGQ.A.300, 2EGM.A.200, 2EGM.A.300, 2EGP.A.200, 2EGP.A.400, 4EGU.A.202, 3EH2.A.
800, 2EHE.A.200, 2EHE.A.300, 2EJ4.A.401, 2ELN.A.181, 2ELY.A.200, 2ELZ.A.200, 2EL
4.A.200, 2EL5.A.200, 2EL6.A.200, 2EMJ.A.201, 2EMI.A.201, 2EMO.A.200, 2EM5.A.201,
 2EM7.A.201, 2EM8.A.201, 2EMK.A.201, 2EMM.A.201, 2EMP.A.201, 2ENT.A.200, 2ENV.A.
200, 2ENV.A.300, 1EN7.A.401, 2ENZ.A.300, 2ENZ.A.400, 2EN7.A.181, 2EN8.A.181, 2EN

F.A.181, 2ENN.A.300, 2ENN.A.400, 2EOR.A.201, 2EOS.A.201, 2EOY.A.201, 2EOZ.A.201,
1EOU.A.300, 2EO4.A.201, 2EOD.A.300, 2EOD.A.400, 2EOG.A.201, 2EOL.A.201, 2EPR.A.
201, 2EPS.A.201, 2EPT.A.201, 2EPV.A.201, 2EPW.A.201, 2EP0.A.201, 2EP1.A.201, 2EP
3.A.201, 2EP4.A.200, 2EPA.A.300, 2EPA.A.400, 2EPP.A.201, 2EQ4.A.201, 2EQE.A.201,
2EQF.A.201, 2EQG.A.201, 1ESK.A.55, 2ESL.A.4, 4ETS.A.302, 1EXK.A.80, 1EXK.A.81,
1EZM.A.302, 1F18.A.155, 2F14.A.1262, 2F3B.A.341, 4F3W.A.201, 2F4M.A.501, 1F62.A.
52, 1F62.A.53, 3F7L.A.203, 3F7U.A.260, 1F81.A.88, 1F9X.A.999, 2F9I.B.601, 4F9V.A
.401, 4FBE.A.403, 2FC6.A.201, 2FC7.A.201, 2FC7.A.401, 2FE3.A.201, 2FEA.A.1302, 2
FGY.A.620, 2FGY.A.621, 2FHX.A.317, 3FID.A.298, 3FID.A.299, 4FKD.A.102, 4FKE.A.10
24, 3FL2.A.1001, 3FLO.B.1, 3FLO.B.2, 1FN9.A.1001, 2FOQ.A.262, 2FOS.A.262, 2FOU.A
.262, 2FOV.A.262, 2FOY.A.301, 1FP0.A.90, 1FQL.A.262, 3FQM.A.901, 2FR5.A.147, 2FR
6.A.147, 4FRC.A.302, 1FU9.A.37, 4FU5.A.302, 4FVD.A.201, 4FVN.A.302, 4FVO.A.302,
3FW3.A.300, 1FWQ.A.124, 4FWE.A.901, 4FWE.A.902, 2FYG.A.302, 4FYY.B.201, 3G1P.A.3
00, 1G25.A.66, 1G25.A.67, 3G27.A.97, 4G26.A.1001, 2G2N.A.1001, 2G45.A.401, 1G52.
A.262, 1G54.A.262, 2G6Q.A.400, 1G71.A.348, 4G7A.A.301, 2G84.A.506, 2G9T.A.999, 1
G9K.A.600, 3GA3.A.1, 1GDC.A.73, 1GDC.A.74, 2GD8.A.262, 2GFO.A.1200, 4GGJ.A.301,
3GI1.A.501, 4GIZ.C.201, 3GJ3.B.300, 3GJ4.D.300, 3GJ5.B.300, 3GJ8.B.300, 2GMW.A.3
00, 4GNE.A.1502, 4GNE.A.1504, 2GQJ.A.200, 2GQJ.A.300, 1GUP.A.350, 4GVE.A.601, 1G
ZH.A.1293, 3GZE.B.2, 3GZK.A.539, 3H0L.E.902, 3H0N.A.201, 4H0F.A.401, 2H15.A.262,
4H12.A.1801, 4H12.A.1803, 2H6E.A.500, 3H7H.A.119, 1H7V.A.61, 3H8V.A.401, 3H99.A
.601, 4H9D.A.201, 2HBA.A.101, 1HC7.A.490, 3HCI.A.1000, 1HCP.A.99, 3HCJ.A.1000, 3
HCS.A.303, 2HD6.A.262, 2HDP.A.492, 2HDP.A.493, 2HF1.A.102, 3HI2.A.121, 4HI8.B.10
2, 2HJN.A.315, 2HJH.A.800, 3HKO.A.701, 3HKQ.A.261, 3HKU.A.261, 2HL4.A.262, 3HNA.
A.502, 3HNA.A.503, 3HNA.A.504, 3HNI.A.107, 2HNC.A.263, 2HOC.A.263, 2HQH.E.1500,
2HRV.A.143, 3HS4.A.301, 1HSO.A.1376, 4HSU.A.904, 1HTD.A.401, 4HT2.A.301, 1HUG.A.
261, 4HU1.A.301, 2HU9.A.132, 1HXP.A.350, 1HXR.A.200, 1HY7.A.302, 4I1F.A.503, 4I1
F.A.504, 4I1F.A.508, 4I1H.A.507, 3I2D.A.1, 3I4C.A.400, 3I4C.A.500, 1I50.C.3002,
2I50.A.336, 2I50.A.336, 2I50.A.337, 2I50.A.338, 4I51.A.3005, 1I8Z.A.262, 2I9W.A.
201, 1IA6.A.1264, 1IBI.A.195, 1IBI.A.196, 3IBL.A.262, 3IBN.A.262, 3IBU.A.262, 2I
BI.A.1, 4IBY.A.301, 2IDA.A.104, 1IF9.A.262, 3IGP.A.262, 4II1.A.901, 4ILO.A.301,
4ILX.A.301, 1IML.A.78, 3IMI.A.201, 1INN.B.167, 2INN.B.514, 3IO2.A.202, 2IOI.A.30
01, 2IQJ.A.301, 1IRN.A.55, 3IR9.B.501, 3IRB.A.201, 4ITO.A.301, 4ITP.A.302, 3IUF.
A.1, 4IUM.A.501, 3IXE.B.302, 1IYM.A.182, 1IYM.A.183, 2IYB.E.1422, 2IYB.E.1423, 1
J2O.A.115, 2J6A.A.1138, 1J98.A.300, 2J9U.B.1162, 1JAZ.A.401, 1JD0.A.901, 4JEA.A.
202, 4JEA.B.202, 4JEA.C.202, 4JEA.D.202, 4JIU.A.201, 1JJD.A.104, 1JJT.B.251, 2JM
0.A.201, 2JM0.A.401, 2JM1.A.2, 2JM3.A.92, 4JMY.A.201, 2JMD.A.65, 1JN7.A.37, 2JOX
.A.110, 1JOC.A.300, 2JQ5.A.129, 2JR7.A.85, 2JRJ.A.62, 2JRJ.A.63, 4JSR.A.401, 2JT
G.A.88, 3JUE.A.999, 2JUN.A.220, 2JVX.A.29, 1JVB.A.400, 3JVH.A.163, 2JVN.A.400, 2
JWO.A.488, 2JW6.A.601, 3JXP.A.320, 1JY8.A.300, 4JZO.A.802, 2K0A.A.108, 2K0A.A.10
9, 2K0A.A.110, 2K1P.A.96, 2K16.A.940, 2K16.A.941, 1K2Y.X.500, 2K2C.A.138, 2K2C.A
.139, 2K2C.A.141, 2K2C.A.142, 2K2C.A.143, 2K2D.A.80, 3K34.A.1002, 3K35.C.317, 2K
4X.A.56, 3K5K.A.1194, 3K5K.A.1197, 3K5K.B.1195, 3K5K.B.1196, 1K7I.A.486, 3K7H.B.
1001, 2K7R.A.129, 4K7D.A.501, 4K7D.A.502, 4K7D.A.505, 4K7D.A.507, 4K7W.B.101, 1K
81.A.144, 2K9H.A.101, 2K9H.A.102, 2KAK.A.130, 2KAK.A.150, 2KAK.A.170, 2KDX.A.120
, 3KE1.A.163, 3KEE.A.2000, 2KGG.A.53, 2KGG.A.54, 2KGO.A.109, 1KHK.A.451, 2KI7.B.
124, 2KJE.A.501, 2KJE.A.502, 2KKT.A.500, 2KKH.A.201, 2KKR.A.500, 1KLR.A.31, 1KLS
.A.31, 3KNE.A.500, 3KNV.A.201, 3KNV.A.202, 4KNI.A.301, 4KNJ.A.301, 4KNM.A.301, 2
KPI.A.150, 3KQI.A.71, 3KQI.A.72, 2KQ9.A.113, 2KQB.A.1001, 2KR1.A.65, 2KU3.A.63,
2KU3.A.64, 4KUV.A.301, 4KUW.A.301, 4KUY.A.301, 3KV5.A.489, 4KV0.A.301, 2KWJ.A.50
1, 2KWJ.A.601, 2KWJ.A.701, 2KWJ.A.801, 4KXQ.A.601, 2KZY.A.63, 2L0Z.A.486, 3L00.A
.180, 3L11.A.601, 3L11.A.602, 3L14.A.262, 4L3J.A.402, 2L5U.A.62, 2L5U.A.63, 4L58
.A.102, 2L6Y.A.239, 2L6Z.B.37, 1L6J.A.500, 2L6L.A.201, 2L6M.A.201, 2L7X.A.106, 2
L7X.A.107, 2L75.A.155, 2L75.A.156, 2L7P.A.201, 4L7X.A.101, 2L80.A.124, 2L9Z.A.40
3, 3LAS.A.167, 2LAU.A.82, 2LBM.A.1, 2LBM.A.3, 4LBU.A.402, 3LCZ.A.54, 2LCE.A.200,

2LCE.A.300, 2LCQ.A.162, 2LGV.A.109, 2LGG.A.381, 2LGG.A.382, 2LHN.A.501, 2LHN.A.502, 2LHN.A.503, 4LHI.A.301, 2LI8.A.187, 2LI9.A.18, 2LJX.A.200, 2LJZ.A.201, 3LJU.X.401, 4LJO.A.1101, 4LJO.A.1102, 4LJO.A.1104, 4LJP.A.1101, 3LKM.A.904, 2LK0.A.32, 4LK9.A.401, 4LMY.A.202, 2LN0.A.401, 2LN0.A.501, 2LN0.A.601, 2LN0.A.701, 2LO2.A.101, 2LO3.A.101, 2LO4.A.300, 4LO9.A.401, 4LOF.A.401, 1LPV.A.54, 3LQH.A.1002, 3LRQ.C.100, 2LRI.C.101, 2LRI.C.102, 2LUA.A.101, 2LUA.A.102, 2LUA.A.103, 1LV3.A.66, 2LV2.A.101, 2LV2.A.102, 2LV9.A.201, 2LV9.A.202, 2LWW.A.501, 2LWW.A.502, 2LWW.A.503, 4LW9.A.203, 4LW9.C.202, 3LXE.A.261, 2LXD.A.201, 3LYR.A.1, 2LZU.A.201, 2LZU.A.202, 3M04.A.501, 2M0F.A.101, 4M0W.A.401, 2M1S.A.100, 3M14.A.501, 3M1D.A.1000, 2M13.A.601, 2M13.A.602, 1M2K.A.999, 1M2O.A.800, 3M2X.A.500, 4M2R.A.301, 4M2V.A.301, 2M3Z.A.102, 1M3V.A.123, 2M3L.A.201, 4M3P.A.701, 2M48.A.503, 3M5E.A.262, 2M6M.A.201, 2M6M.A.202, 2M7Q.A.101, 2M7Q.A.102, 3M79.D.107, 2M7A.A.100, 2M85.A.801, 2M85.A.802, 2M9Y.A.401, 2M9Y.A.402, 3M96.A.262, 2M9A.A.101, 2M9A.A.103, 2M9A.A.102, 2MA5.A.101, 2MA5.A.102, 2MA6.A.101, 2MA6.A.102, 1MBX.B.211, 3MDZ.A.263, 2MD7.B.101, 2MD7.B.102, 2MDG.A.101, 2MDG.A.102, 1MEA.A.29, 3MEK.A.500, 3MEK.A.501, 3MEK.A.502, 3MEQ.A.501, 4ME3.A.301, 4MG3.A.201, 3MHC.A.262, 3MHH.E.97, 3MHS.A.472, 3MHS.A.474, 3MHS.A.477, 4MHQ.A.501, 4MHY.A.400, 4MI5.A.802, 4MI5.A.803, 4MI5.A.805, 4MI5.A.806, 4MI5.A.807, 4MJ7.A.201, 2MKD.A.301, 1ML9.A.1, 1ML9.A.2, 1MM3.A.62, 1MM3.A.63, 3MMF.A.262, 2MNY.A.401, 2MNY.A.402, 3MNA.A.262, 3MND.A.153, 1MNC.A.282, 3MNU.A.262, 4MO8.A.301, 3MPZ.A.300, 3MP2.A.1, 1MR1.C.601, 2MRE.B.301, 1MS7.B.3001, 4MSG.A.1401, 4MSX.A.501, 4MTY.A.301, 2MUM.A.301, 2MUM.A.302, 2MUQ.A.101, 3MWM.A.140, 1MXG.A.437, 1MZ8.B.600, 3MZC.A.262, 4N0N.A.501, 4N0N.A.503, 1N0Z.A.46, 4N0X.B.301, 3N3J.A.262, 3N3K.A.1, 4N4F.A.1402, 3N4B.A.262, 3N67.A.900, 1N8K.A.376, 3NA7.A.300, 3NB5.A.261, 1NCS.A.61, 1NEE.A.136, 3NHE.A.1, 3NIS.A.2, 3NIS.A.3, 4NJ5.A.801, 1NKU.A.188, 3NKM.A.1001, 4NL4.H.803, 2NMX.A.301, 2NNV.A.262, 2NN1.A.301, 2NN7.A.301, 4NN2.A.403, 2NNG.A.262, 2NNO.A.262, 4NOS.A.3000, 3NQ5.A.503, 4NQ6.A.301, 4NQ7.A.301, 4NQY.A.501, 2NYT.A.2000, 3NY1.A.4, 3NY1.A.5, 3NY1.A.6, 3NY3.A.1, 3NY3.A.2, 3NY3.A.3, 1NZJ.A.700, 4NZG.A.201, 300M.A.151, 2O03.A.202, 2O10.A.86, 2O10.A.87, 2O13.A.190, 2O13.A.191, 3O36.A.1, 3O36.A.2, 2O3K.A.401, 2O4Z.A.262, 3O4N.A.2002, 4O62.A.1001, 4O64.A.2001, 4O64.A.2004, 3O70.A.500, 3O70.A.501, 3O7A.A.500, 3O7A.A.501, 3OCA.A.300, 3OCQ.A.184, 4ODR.A.202, 4ODR.B.202, 2OFK.A.201, 2OGW.A.500, 4OGE.A.1201, 3OIL.A.262, 2OIK.B.201, 4OIF.A.701, 3OJ6.A.150, 3OJ7.A.114, 1OKL.A.262, 1OKM.A.262, 2OM1.B.801, 3OOI.A.232, 3OOI.A.233, 3OOI.A.234, 1OQ5.A.600, 1OQJ.A.183, 3ORU.A.250, 2OSM.A.262, 1OVX.A.61, 2OWA.A.201, 3OYO.A.262, 3OYQ.A.262, 3OYS.A.262, 1OZB.I.50, 2OZU.A.800, 2P09.A.200, 3P2A.C.151, 3P3H.A.261, 3P3J.A.261, 3P44.A.261, 3P55.A.261, 2P57.A.201, 1P6O.A.401, 1P7A.A.38, 3P8B.A.101, 3PB4.X.400, 3PB7.X.400, 3PB8.X.400, 3PB9.X.400, 3PBE.A.391, 1PEG.A.4, 1PFT.A.51, 1PG5.B.500, 4PHT.B.601, 3PJN.A.187, 3PJN.A.188, 3PLW.A.188, 2PLI.D.702, 3PN3.A.1001, 2POU.A.262, 2POW.A.262, 2POI.A.100, 2PPT.A.300, 1PQ4.A.1002, 4PQ7.A.301, 2PQ8.A.501, 4PQT.A.501, 2PRS.A.501, 3PT9.A.1, 3PT9.A.2, 3PTM.A.1001, 1PTR.A.1, 4PTB.A.902, 2PUY.A.356, 3PUQ.A.2, 3PUR.A.3, 2PVX.A.901, 2PVE.A.301, 4PYX.A.301, 1PZW.A.100, 4PZH.A.302, 1Q08.A.401, 1Q08.A.402, 1Q08.B.403, 1Q08.B.404, 3Q01.A.1, 1Q2L.A.963, 1Q3A.A.465, 1Q68.A.201, 1Q69.A.207, 4Q6E.A.301, 3Q7C.A.1, 4Q7R.B.302, 3Q87.A.126, 1QBH.A.364, 1QF8.A.216, 4QF2.A.1802, 4QF3.A.2002, 3QGV.A.500, 2QIC.A.400, 2QKD.A.501, 3QL9.A.1, 3QL9.A.3, 4QN1.A.1501, 2QO8.A.262, 2QOA.A.262, 2QP6.A.262, 4QQ4.A.2001, 4QSI.A.301, 3QU1.A.503, 3QU1.B.502, 1QWY.A.400, 3QWP.A.500, 3QWP.A.501, 1QYB.A.401, 1QYP.A.58, 3QYK.A.262, 3R16.A.262, 3R17.B.262, 4R1X.B.501, 4R2Y.A.101, 4R2Y.A.102, 4R2Y.B.102, 2R3A.A.300, 2R3A.A.301, 2R3A.A.303, 1R5T.A.150, 3R6F.A.132, 1R79.A.401, 1R9P.A.135, 3RBU.A.1752, 2RGV.A.146, 1RGO.A.221, 1RGO.A.222, 2RHQ.A.1, 2RHK.C.502, 2RI7.A.501, 2RI7.A.502, 3RIY.A.1001, 3RJ7.A.300, 1RJW.A.401, 1RMD.A.118, 3RMQ.A.114, 1RNI.A.256, 4RN4.A.301, 2RO1.A.201, 2RO1.A.301, 3RQD.A.390, 2RR4.A.501, 2RSI.A.102, 2RSJ.A.101, 2RT9.A.701, 2RT9.A.702, 1RUT.X.603, 4RUW.A.501, 4RV9.A.501, 1RXR.A.213, 1RXR.A.214, 3RYM.A.106, 3RYV.B.262, 3RYX.B.262, 3RYY.A.1, 3RYJ.B.262, 3RZV.A.2, 3RZ1.B.262,

3RZ7.A.1, 3RZ8.A.1, 3S2Q.B.501, 3S2E.A.501, 3S71.B.262, 3S72.B.262, 3S73.B.262, 3S75.B.262, 3S76.A.1, 3S8P.A.400, 3S9T.A.262, 3SAX.A.262, 3SAP.A.262, 3SBH.A.262 , 3SBI.A.262, 1SE0.A.201, 3SI2.A.601, 3SJG.A.1752, 1SLM.A.257, 3SOU.A.7, 3SP4.A.601, 1SRK.A.36, 1SU3.A.913, 3SUB.A.161, 1SVM.A.700, 1T4W.A.201, 3T5U.A.262, 3T6P .A.1001, 3T6P.A.1002, 3T6P.A.1003, 3T6R.A.1, 3T6R.A.3, 3T7L.A.1, 3T7L.A.2, 1T8H. A.275, 3T82.A.261, 3T84.A.261, 3T85.A.261, 1T9H.A.411, 3T92.A.122, 3T92.A.124, 1 TAF.A.2003, 1TEQ.X.262, 1TFI.A.51, 3TGN.A.147, 3TG4.A.435, 3TG4.A.436, 3TIO.B.18 5, 3TIO.D.185, 3TIO.E.185, 1TJL.A.200, 1TOT.A.53, 1TOT.A.54, 1TT5.B.1014, 3TTC.A .1, 3TTC.A.2, 4TWJ.A.301, 4TYT.A.302, 1U0A.A.5005, 3U1L.A.241, 1U2N.A.441, 1U3T. A.375, 3U31.A.276, 4U4L.A.302, 1U5K.A.300, 1U5S.B.138, 3U52.A.514, 3U5N.A.2, 1U8 5.A.34, 1U86.A.36, 3U9G.A.226, 3U9G.A.227, 3U9G.A.228, 3U9G.A.229, 1UAQ.A.200, 3 UCJ.A.228, 3UCO.A.228, 3UDZ.B.800, 3UEH.A.143, 3UEY.A.4, 3UEE.A.143, 3UEI.A.143, 3UEJ.A.301, 3UEJ.A.302, 4UFO.A.2269, 3UFF.A.1, 3UGD.A.2, 1UL4.A.139, 3UNG.C.903 , 3UNT.A.400, 4UPO.A.1383, 4UPO.A.1384, 4UTV.A.1299, 3UVI.A.387, 2UVL.A.1336, 3U W4.A.401, 3UX8.A.1001, 2UZG.A.132, 2UZG.A.133, 2V0C.A.1815, 2V0C.A.1816, 3V1F.A. 704, 3V1F.B.703, 4V1T.A.776, 4V2Y.A.150, 3V3G.B.301, 1V47.A.601, 3V4K.A.2, 1V51. A.602, 1V54.F.99, 3V5G.A.262, 1V6G.A.401, 3V7X.A.2001, 1V9X.A.200, 2V9E.B.1276, 2V9K.A.1533, 3VBD.A.2001, 1VDD.D.233, 3VDP.A.201, 1VFY.A.300, 1VFY.A.301, 2VF7.A .1845, 3VGL.A.322, 1VHH.A.400, 3VHS.A.51, 3VHS.B.51, 1VJ0.A.400, 1VJE.A.167, 2VJ E.B.1491, 2VKR.A.106, 2VNF.A.1247, 3VOV.A.401, 2VO9.B.501, 3VPB.E.100, 2VPD.A.13 99, 2VPG.A.1400, 2VPG.A.1401, 1VQ0.A.300, 2VQM.A.1412, 1VQ2.A.701, 2VQG.C.1091, 2VRS.A.1328, 2VRW.B.1565, 2VRW.B.1566, 2VR6.A.1156, 2VRD.A.1062, 1VSR.A.201, 3VT H.A.802, 3VUW.E.801, 2VVB.X.1268, 1VZY.A.1291, 2W0D.A.1269, 2W3N.B.1234, 1W4R.A. 400, 2W4L.A.1170, 2W5Z.A.4970, 3W5K.B.504, 1W8P.B.1030, 4WAJ.A.301, 2WB0.X.601, 2WB0.X.602, 2WCB.B.100, 2WD2.A.1262, 2WD3.A.1263, 4WD8.C.303, 2WEJ.A.1262, 1WE9. A.401, 1WEE.A.201, 1WEW.A.201, 2WEH.A.1262, 2WEO.A.1262, 1WFH.A.201, 1WFK.A.200, 1WFZ.A.201, 2WGX.A.1300, 1WGE.A.201, 3WID.A.1001, 1WJ2.A.470, 1WJV.A.401, 1WKQ. B.202, 1WN5.A.1001, 3WNQ.A.501, 1WO3.A.26, 1WO4.A.26, 2WVJ.A.1193, 1WWD.A.57, 3W WL.A.102, 1WWR.D.204, 1WYS.A.401, 1X0T.A.150, 4X2Z.A.400, 1X31.D.1006, 1X4I.A.20 1, 1X4I.A.401, 1X4J.A.201, 1X4J.A.401, 1X4L.A.201, 1X4L.A.401, 1X4S.A.201, 1X4S. A.401, 2X5R.A.1126, 1X61.A.401, 1X62.A.401, 1X63.A.201, 1X63.A.401, 1X64.A.201, 1X64.A.401, 1X6M.A.200, 2X7S.A.1265, 2X7T.A.1263, 2X7U.A.1261, 2X7M.A.1175, 1XAF .A.501, 1XB0.A.403, 2XBL.A.1197, 1XCR.A.1001, 2XCM.E.1222, 1XEG.A.262, 1XER.A.10 6, 1XF7.A.30, 4XIW.A.401, 2XIG.A.1151, 1XJH.A.63, 1XKI.A.1003, 1XOX.A.999, 2XOC. A.993, 2XOC.A.994, 2XOC.A.995, 1XPA.A.220, 1XPG.A.1887, 1XQ0.A.262, 1XRU.A.501, 1XTM.B.501, 1Y02.A.161, 2Y1N.A.1436, 2Y1N.A.1437, 2Y43.A.1097, 2Y43.A.1098, 2Y6D .A.1267, 1Y8Q.B.642, 2YB5.A.1213, 2YHO.A.1001, 2YHO.A.1002, 2YHW.A.1719, 2YHY.A. 2000, 2YIK.A.1533, 1YOP.A.84, 2YQM.A.201, 2YQM.A.401, 2YRJ.A.200, 2YRT.A.201, 2Y RC.A.200, 2YRD.A.200, 2YRE.A.401, 2YRE.A.501, 2YRE.A.601, 2YRE.A.701, 2YRG.A.201 , 2YRH.A.200, 2YSJ.A.201, 2YSJ.A.401, 2YSV.A.201, 2YSL.A.401, 2YSM.A.301, 2YSM.A .501, 2YSM.A.701, 2YSM.A.901, 2YTP.A.181, 2YTS.A.201, 2YT9.A.201, 2YTE.A.201, 2Y TF.A.201, 2YTI.A.201, 2YTM.A.181, 2YTO.A.201, 2YU4.A.201, 2YU8.A.201, 2YUC.A.201 , 2YUC.A.401, 2YWW.A.504, 2YYR.A.401, 2YYR.A.402, 1Z05.A.406, 2Z2Y.A.2001, 2Z3H. A.2001, 2Z3G.A.2001, 1Z3A.A.301, 2Z3J.A.2001, 2Z45.B.1004, 1Z60.A.2, 1Z6U.A.2, 1 Z84.A.604, 1Z8R.A.151, 2Z9J.A.902, 2ZC0.B.408, 1ZE8.A.263, 2ZEE.A.391, 2ZEF.A.39 1, 2ZEG.A.391, 2ZEM.A.391, 2ZEO.A.391, 1ZFD.A.71, 1ZFK.A.1300, 3ZFJ.A.1159, 1ZFQ .A.600, 1ZGE.A.1000, 1ZGF.A.400, 3ZGO.A.400, 1ZH1.A.199, 1ZIN.A.219, 3ZME.A.313, 1ZNB.A.1, 1ZNF.A.27, 3ZNF.A.31, 3ZNI.A.1428, 3ZNI.A.1429, 1ZNM.A.29, 5ZNF.A.31, 7ZNF.A.31, 2ZNR.A.1, 2ZNR.A.2, 1ZP5.A.999, 3ZPC.B.401, 1ZR9.A.117, 1ZRP.A.54, 1 ZSB.A.262, 1ZSC.A.262, 3ZTG.A.1337, 1ZU1.A.129, 1ZU1.A.130, 3ZVS.A.1160, 1ZW8.A. 66, 1ZW8.A.67, 1ZY7.A.801, 3ZYQ.A.1222, 2ZZE.A.753

[1] "Cluster 8"

3COQ.A.1002, 1F2I.G.1201, 1F4S.P.65, 2FF0.A.1001, 4HCC.A.504, 4HP3.C.201, 4HP3.C .202, 1HWT.D.138, 2I13.A.503, 2I13.A.506, 2I13.B.510, 4IQR.A.402, 2IVH.A.1577, 2

JP9.A.133, 2KKF.A.2001, 2KKF.A.2002, 1LLM.C.302, 4LMG.A.201, 1MEY.C.88, 2NLL.A.2
50, 3O9X.A.132, 2OPF.A.501, 4PZI.A.1101, 4PZI.A.1102, 4QEN.A.804, 3QMD.A.300, 3Q
MD.A.301, 3S14.A.1735, 3UK3.C.967, 1ZGW.A.500, 1A42.A.262, 4A46.A.65, 1A5T.A.501
, 3A6F.C.301, 3A6J.A.301, 3A6J.D.301, 1A7I.A.82, 1A7I.A.83, 2AA4.A.1001, 2ADR.A.
162, 1ADB.A.375, 1ADN.A.93, 4AF1.A.500, 4AI5.A.200, 4AIG.A.999, 1ANI.A.451, 2AQP
.A.201, 4ARE.A.1790, 3ASK.A.501, 2AS9.A.666, 2AS9.B.222, 1B4E.A.405, 3B4F.A.262,
 2B5W.A.800, 3B5Q.A.500, 2B83.C.3353, 2B8T.A.1218, 4BHW.A.1, 4BHW.A.4, 1BIO.A.29
1, 3BIO.A.1752, 3BKN.A.202, 3BL0.A.262, 3BL1.A.262, 4BLB.B.910, 1BNM.A.262, 3BO5
.A.301, 3BOL.A.701, 4BOL.A.1261, 1BP3.A.500, 1BTK.A.1, 3C10.A.102, 4C1D.A.501, 4
C1E.A.501, 4C1E.A.502, 4C1G.A.300, 4C2P.A.701, 3C37.A.301, 4C4O.D.500, 2C6A.A.33
6, 1C8T.A.260, 3C8Z.A.413, 4C8E.A.1162, 3CA2.A.264, 1CAQ.A.301, 4CBY.A.2035, 1CC
T.A.262, 4CCG.X.1374, 2CEX.C.1306, 2CJL.A.1217, 1CL4.A.81, 1CLC.A.653, 1CNI.A.1,
 1CNW.A.262, 1CNX.A.262, 1CNY.A.262, 1CO4.A.43, 4COI.A.652, 3CQZ.L.3005, 4CQ0.A.
1262, 2CQE.A.822, 2CQF.A.330, 2CRW.A.401, 1CRA.A.262, 2CRR.A.401, 2CSY.A.201, 2C
SZ.A.201, 2CSZ.A.401, 3CSK.A.712, 1CTU.A.296, 2CT1.A.201, 2CT1.A.401, 2CT2.A.401
, 2CT7.A.201, 2CTD.A.201, 2CTD.A.401, 2CTT.A.201, 2CTU.A.201, 2CU8.A.201, 2CUP.A
.201, 2CUP.A.401, 2CUP.A.601, 2CUQ.A.201, 2CUQ.A.401, 2CUR.A.401, 3CX3.A.314, 3C
XL.A.501, 1CXV.A.3, 4CYK.A.42, 1CZM.A.261, 1D1S.B.376, 1D1T.A.401, 4D1N.A.900, 3
D2Z.A.262, 2D5B.A.501, 2D8Q.A.201, 2D8S.A.201, 2D8S.A.401, 2D8U.A.401, 2D9N.A.25
6, 4D9W.A.408, 3DAZ.A.262, 2DAR.A.401, 2DAS.A.101, 2DB6.A.201, 2DB6.A.401, 3DD8.
A.262, 3DDT.A.46, 4DF9.A.503, 2DFV.A.1001, 2DGE.A.1001, 2DID.A.401, 2DJB.A.201,
 2DJB.A.401, 1DMX.A.280, 1DMY.A.280, 2DMD.A.191, 2DQ4.A.502, 1DSV.A.171, 1DVB.A.1
94, 3DWD.A.501, 1DY0.A.401, 3E1W.A.230, 3E24.A.230, 3E2I.A.200, 2E26.A.603, 3E2U
.E.102, 4E2X.A.501, 1E3I.A.380, 1E3I.A.381, 3E3F.A.230, 3E3I.A.230, 1E4B.S.999,
 2E47.A.172, 2E6I.A.201, 2E72.A.201, 2EA5.A.201, 2EA5.A.401, 2ECJ.A.201, 2ECI.A.2
01, 2ECI.A.401, 2ECY.A.201, 2ECG.A.401, 2ECL.A.401, 2EER.A.501, 1EE2.A.1301, 4EE
X.B.402, 3EFT.A.262, 4EFS.A.301, 3EH1.A.1269, 3EHX.A.264, 2ELQ.A.181, 2ELW.A.181
, 1ELX.A.451, 1ELY.A.451, 2ELI.A.201, 2EMX.A.201, 2EMY.A.201, 2EM1.A.201, 2EMA.A
.201, 2EME.A.201, 2EMF.A.201, 2EMG.A.201, 2EMH.A.201, 2EN1.A.201, 2EN4.A.201, 2E
OI.A.201, 2EOP.A.201, 2EPU.A.201, 2EPX.A.201, 2EPY.A.201, 1EPW.A.1291, 2EPC.A.20
1, 2EQW.A.201, 2EQ0.A.201, 2EQ2.A.201, 2EQ3.A.201, 1ESP.A.323, 4EVB.A.204, 4EYL.
A.303, 4EYU.A.1702, 2F0Y.B.501, 3F0D.A.163, 3F0F.A.165, 1F4T.A.369, 3F4X.A.262,
 3F5L.A.1001, 1F8F.A.373, 4F9C.B.401, 1FAQ.A.2, 4FAI.A.401, 1FBX.A.3316, 3FCQ.A.3
22, 3FFP.X.262, 3FIE.A.428, 2FIF.B.901, 3FJU.A.502, 3FJU.A.507, 1FKW.A.400, 4FKB
.A.401, 3FL2.A.1002, 3FLF.A.2004, 2FNF.X.1, 1FR7.A.262, 3FTN.D.357, 2FU9.A.401,
 3FV4.A.321, 3FVP.A.321, 3FXP.A.3000, 2FZW.A.375, 2FZW.B.376, 1G47.A.999, 1G48.A.
262, 1G4K.A.301, 2GAG.D.101, 2GAH.D.101, 4GER.A.401, 2GFJ.B.401, 2GFE.A.869, 1GI
4.A.409, 3GIQ.A.482, 3GIQ.A.483, 3GJ7.D.300, 4GQT.B.502, 3GRB.A.129, 4GR0.A.301,
 4GR8.A.301, 2GSU.A.1001, 3GTT.A.155, 2GVI.A.301, 1H19.A.701, 4H12.A.1802, 1H2B.
A.1362, 3H2P.B.154, 4H30.A.301, 3H5A.B.360, 3H5N.A.500, 4H57.A.405, 1H7N.A.1342,
 4H84.A.301, 2HB9.A.401, 1HCP.A.98, 1HDY.A.376, 4HDH.A.1002, 4HEW.A.301, 4HEY.A.
301, 4HF3.A.301, 4HI8.B.101, 3HKN.A.261, 3HNA.A.501, 4HT0.A.301, 4HTB.A.401, 4HV
L.A.504, 1HY7.B.801, 1HYI.A.66, 2HZ8.A.117, 2IOO.A.579, 2I2X.A.524, 2I3H.A.1001,
 3I3T.A.700, 1I6N.A.401, 1I6P.A.301, 1I76.A.999, 1I8J.B.400, 3I9F.B.3, 2I9W.A.20
0, 1IB5.A.201, 3IBI.A.262, 4ICR.A.501, 3IFJ.A.201, 3IFJ.B.202, 3IJF.X.147, 1IML.
A.77, 1IQ8.A.600, 2IT4.A.561, 3IXE.B.301, 4IXJ.A.301, 2J1Y.A.1290, 1J2T.A.302, 2
J21.A.1289, 1J9Y.A.1004, 2J9R.A.1194, 2JA1.A.1192, 4JBG.A.401, 1JD5.A.501, 4JE6.
A.200, 4JIV.D.101, 1JJ9.A.999, 1JJD.A.102, 1JJE.B.251, 4JLW.A.401, 2JOX.A.108, 4
JOM.A.1004, 4JPA.A.301, 2JSD.A.275, 1JTK.A.137, 2JTN.A.183, 2JTN.A.186, 2JUN.A.2
21, 2JUN.A.223, 1JVB.A.500, 3JV7.A.501, 3JV7.A.502, 2JWO.A.489, 1JW9.B.250, 4JXE
.A.501, 2JZ8.A.150, 4K1R.A.502, 1K24.A.401, 3K2F.A.262, 2K5C.A.96, 3K6I.A.202, 3
K6J.A.800, 4K7D.A.508, 1K83.I.3003, 3KB1.A.302, 2KDP.A.1, 3KED.A.875, 1KEV.B.353
, 1KFI.A.700, 2KFN.A.1, 1KH4.A.451, 1KHL.A.451, 2KIZ.A.70, 4KJG.B.1001, 1KK1.A.4
11, 2KN9.A.82, 1KOL.A.1002, 1KOQ.A.301, 1KTO.A.405, 4KUJ.A.301, 3KV5.A.490, 3KVE

.B.489, 1KWG.A.806, 1KWQ.A.262, 3KWO.C.161, 2KZM.A.1, 3L0V.A.1, 3L22.A.1, 4L56.A .401, 4L5O.A.303, 4L6H.A.801, 2L8E.A.829, 3LAT.A.215, 1LBU.A.214, 1LG5.A.262, 2L GG.A.380, 4LGJ.A.301, 1LIQ.A.28, 2LI8.A.188, 4LIM.A.401, 4LJP.A.1103, 3LMI.B.100 2, 4LOE.C.401, 1LPV.A.53, 4LQG.A.802, 3LS1.A.1, 3LSC.A.458, 3LT8.A.80, 2LUY.A.30 1, 3LUB.A.302, 4LU3.A.301, 2LVU.A.101, 4LW9.D.201, 4LXL.A.403, 3M2Y.A.500, 2M3H. A.102, 4M3O.A.300, 3M4C.B.108, 2M48.A.502, 1M65.A.300, 1M6H.A.1376, 3M6I.A.402, 3MBG.A.3, 3MBM.A.163, 1MC5.A.376, 1MGO.A.376, 3MHS.A.475, 3MHS.A.476, 4MI5.A.804 , 2MIU.A.301, 2MIU.A.302, 2MIU.A.303, 1ML9.A.3, 3ML5.A.263, 4MLT.A.301, 1MOO.A.2 62, 1MP0.A.376, 1MQ0.B.147, 3MS3.A.401, 4MT2.A.67, 4MTW.E.401, 1MVH.A.501, 2MWX. A.201, 4MWP.E.401, 4MXJ.E.401, 1MZB.A.202, 4MZN.E.401, 4N0G.A.401, 4N0L.A.401, 4 N4E.E.401, 4N4F.A.1401, 1N5N.A.401, 4N5P.E.405, 4N66.E.501, 1N92.A.375, 3NIS.A.1 , 3NIT.A.2, 3NI5.A.262, 1NJ1.A.513, 1NJG.A.401, 4NJ5.A.803, 4NL4.H.802, 3NQZ.B.1 , 4NQ5.A.301, 4NQ5.A.302, 4NQ6.A.302, 4NS5.A.401, 1NTO.A.500, 4O1K.A.301, 2O4H.B .401, 4O64.A.2002, 4O6I.A.601, 1OAL.A.152, 4OAQ.A.403, 4OCM.E.401, 2OD1.A.902, 1 OHL.A.400, 2OH3.A.300, 1OS0.A.600, 2OSF.A.262, 4OTE.A.304, 2OUI.A.361, 2OVX.A.44 4, 2OVZ.A.444, 4OWF.G.401, 1OX7.A.402, 1OXN.A.1001, 3OXF.A.440, 1OYW.A.801, 1P0F .A.2502, 3P1V.A.427, 1P42.A.502, 1P6B.A.406, 1P9R.A.601, 4P9C.B.201, 3PBB.A.391, 3PBJ.D.31, 1PCX.A.950, 1PE8.A.317, 1PGU.A.616, 2PG3.A.300, 3PII.A.340, 1PL8.A.4 02, 3PN3.A.1002, 3PN3.A.1009, 3PN3.B.1011, 3PNU.A.336, 3PYK.A.262, 3PZC.B.1000, 4Q09.A.301, 4Q0L.A.301, 1Q1A.A.701, 3Q1D.A.201, 2Q1B.A.400, 3Q6V.A.2, 2Q6E.A.501 , 4Q7R.A.301, 4Q7R.A.302, 4Q7R.A.303, 3Q94.A.301, 4QEF.A.301, 1QF2.A.320, 3QH5.A .321, 3QHD.A.163, 2QIC.A.300, 2QN0.A.431, 2QQ4.A.139, 3QU1.A.501, 3QU1.A.505, 3R 0D.A.428, 1R22.A.502, 3R2N.A.135, 4R2Y.A.103, 3R3L.A.585, 2R59.A.701, 1R79.A.201 , 4R9G.A.505, 3RCM.A.288, 3RHG.A.368, 1RJ6.A.601, 1RJQ.A.601, 1RJW.A.402, 2RJP.B .1, 1RMD.A.117, 4RM5.D.300, 2RMN.A.1, 2ROW.A.601, 2ROW.A.602, 2RPC.A.201, 2RPP.A .401, 4RQT.A.401, 4RQU.B.402, 3RSN.A.200, 2RSD.A.901, 2RSH.A.101, 2RSI.A.101, 3R UI.A.1, 3RZ0.B.262, 3RZ5.A.1, 3S2E.A.500, 1S3G.A.219, 3SD9.A.2, 3SOU.B.8, 3SSB.A .999, 3SU6.A.5, 1SX1.A.23, 3T02.A.501, 1T3A.A.422, 3T5Z.A.262, 3T73.A.410, 3T74. A.410, 3T7E.A.252, 3T8G.A.411, 3T8F.A.411, 3T87.A.326, 3T8C.A.326, 3T8D.A.325, 3 T8H.A.326, 1TBN.A.1, 3TGN.A.148, 3TG4.A.437, 1THL.A.324, 3TIO.A.1, 2TMN.E.321, 6 TMN.E.322, 3TMJ.A.262, 3TTY.A.676, 3TWO.A.349, 4TZC.A.501, 4TZU.A.503, 1U3L.A.70 1, 1U3T.A.376, 1U3U.A.375, 1U3W.A.375, 1U4G.A.9800, 4UA4.A.302, 4UA4.B.303, 3UCM .A.228, 3UCN.A.228, 1UD9.B.508, 3UJZ.A.1, 3UKO.A.400, 3UKO.A.401, 4UOV.A.298, 2U SN.A.257, 1UUF.A.401, 3UVC.A.301, 3UWA.A.200, 1UX1.A.1132, 2UX1.K.1173, 1V0D.A.4 01, 4V0R.A.1001, 4V2W.A.502, 1V4P.A.1001, 1V5R.A.201, 1V87.A.201, 1V87.A.401, 1V 9E.B.260, 1VGN.A.301, 1VGN.A.302, 3VHT.B.401, 2VJE.B.1492, 2VM5.A.1245, 3VTH.A.8 01, 2VXI.B.201, 2VXX.A.201, 2VXX.B.202, 2W0T.A.125, 3W0F.A.301, 1W5O.A.1339, 4W6 Z.A.402, 1WAA.A.1090, 1WAA.D.1090, 1WAA.E.1089, 4WAI.A.101, 2WCB.A.100, 4WD8.B.3 03, 1WE9.A.201, 1WEE.A.401, 1WEM.A.401, 1WEN.A.201, 1WEO.A.201, 1WEP.A.401, 1WEQ .A.201, 1WEQ.A.401, 1WEV.A.201, 1WEV.A.401, 1WEW.A.401, 1WFE.A.201, 1WFE.A.401, 1WFF.A.201, 1WFH.A.401, 1WFL.A.201, 1WG2.A.400, 4WH6.A.1203, 2WHG.A.1263, 1WIL.A .201, 1WIM.A.401, 1WIR.A.201, 2WJV.A.1, 1WJA.A.56, 1WJB.A.56, 1WJP.A.701, 4WK7.A .501, 4WKE.A.501, 3WL3.A.301, 2WO8.C.1268, 3WS6.C.201, 1WUQ.A.1001, 1WUR.A.1001, 2WWO.A.1162, 3WXC.A.302, 1WYH.A.401, 1WYS.A.201, 3X17.A.601, 1X3C.A.201, 1X3H.A .201, 2X4H.B.1141, 1X4U.A.201, 1X4U.A.401, 1X4V.A.201, 1X4V.A.401, 1X4W.A.201, 1 X4W.A.401, 1X5W.A.201, 1X6E.A.401, 1X6F.A.201, 2XB4.A.1224, 2XCM.E.1223, 2XEU.A. 1065, 1XLL.A.399, 2XOC.B.991, 2XOC.B.992, 1XPZ.A.262, 1XUJ.A.246, 2XXH.B.1303, 1 XYD.A.94, 1XYD.B.94, 1Y02.A.162, 1Y23.A.1001, 2Y28.B.1181, 1Y3G.E.321, 2Y4Y.A.11 72, 2Y4Y.C.1172, 1Y8F.A.702, 1YM3.A.301, 1YO7.A.201, 1YQD.A.2000, 2YQQ.A.201, 2Y RK.A.201, 1YSB.A.501, 2YSP.A.181, 2YTG.A.201, 2YT9.A.203, 2YTA.A.201, 2YTJ.A.201 , 2YTN.A.201, 2YUU.A.201, 2YUU.A.401, 2YVR.A.1001, 2YVR.A.1002, 2YXO.A.501, 2YZ3 .A.301, 2YZ5.B.1501, 2Z2S.B.204, 2Z2Y.D.2004, 1Z5H.A.2001, 1Z6U.A.1, 1Z83.B.642, 1Z9G.E.1005, 1Z9N.A.201, 1Z9Y.A.300, 2Z94.A.901, 2Z9L.A.701, 1ZDP.E.1005, 2ZEH. A.391, 2ZEL.A.391, 2ZEN.A.391, 3ZFK.A.401, 2ZNE.B.993, 3ZPG.A.1358, 1ZS0.A.999,

3ZTG.A.1336, 1ZTQ.A.550, 1ZUD.1.501, 1ZVX.A.999, 2ZXG.A.900, 3ZYQ.A.1223, 2ZZF.A
.754
[1] "Cluster 9"
3ADR.A.263, 3ASE.A.156, 3AYK.A.170, 2BCN.A.295, 2BIB.A.1550, 2DI3.A.1002, 4E5V.B
.401, 3EII.A.301, 2EK9.A.1004, 4FUK.B.402, 2GC2.A.401, 2HD1.A.101, 4HGX.A.301, 4
HTZ.B.1001, 4ICQ.A.501, 4ICQ.B.502, 4IGN.B.401, 2J0E.B.1265, 4JAA.A.501, 4JX5.D.
1103, 2K78.A.151, 4KBP.A.439, 3KR5.E.1001, 4KYH.A.202, 3LL8.A.505, 2LQ6.A.401, 2
LZE.A.101, 4MCS.A.814, 1ML2.A.296, 2MLS.A.302, 4N27.C.201, 4N7K.L.301, 4N7K.L.30
7, 4N7K.M.401, 4N7K.M.402, 4N7L.M.402, 3N9R.A.308, 3NO5.C.275, 3OOF.A.302, 3O64.
A.1, 4OK2.A.801, 2OUN.B.404, 1P6D.A.247, 4PKT.A.802, 4PKW.A.801, 1Q3A.B.469, 1Q3
K.B.300, 1Q74.A.304, 2QFR.A.434, 4R6T.A.1003, 4R6T.D.1001, 4R7M.C.1001, 4R7M.J.1
003, 3SFW.A.501, 3V93.D.701, 3VH9.A.301, 1VKG.B.400, 3VUS.A.401, 2WEY.A.1772, 4X
2T.A.702, 1XJS.A.150, 1Y13.A.174, 1ZEB.A.901, 2ALW.A.5001, 3B3C.A.502, 4BBP.A.13
16, 3BLB.A.1047, 1BLL.E.489, 2BNO.B.1201, 3BON.A.500, 3BXM.A.1751, 2C1G.A.1465,
3C88.A.450, 3C8A.A.450, 3C8B.A.450, 2CB8.B.1090, 4CBY.A.2034, 4CWM.A.432, 4CXV.A
.432, 3D4Y.A.1047, 3D4Z.A.1046, 3D52.A.1046, 3D51.A.1046, 3DDA.A.450, 3DDF.A.300
1, 3DDG.A.3001, 2DDF.A.1, 1DE5.A.450, 2DEA.A.402, 3DFF.A.274, 3DFK.A.300, 4DJ4.B
.403, 1DK4.B.590, 4DOO.A.502, 3DX1.A.1048, 3DX2.A.1046, 3DX3.A.1047, 3DX4.A.1047
, 3DZA.C.501, 3DZA.C.505, 3E38.A.2, 3E49.B.500, 3E4A.A.2000, 3E8R.B.2, 1EB6.A.17
8, 3EJP.A.1047, 3EJQ.A.1047, 3EJR.A.1047, 3EJU.A.1047, 2EK8.A.1004, 4ELC.A.501,
1ENQ.A.238, 3EZT.B.401, 2F18.A.1805, 2F1A.A.1805, 2F1B.A.1804, 2F7O.A.5001, 2F7P
.A.5001, 2F7Q.A.5001, 2F7R.A.5001, 2F92.F.1001, 2F92.F.1002, 2F92.F.1003, 2F94.F
.1001, 2F94.F.1003, 2F9K.F.1001, 2F9K.F.1002, 2FQP.B.100, 1FT7.A.502, 2FUQ.A.1,
4FW5.D.301, 4FW6.A.301, 4FW7.D.301, 2G9Y.B.450, 3GBO.A.302, 4GBD.A.503, 3GJ9.B.1
27, 4GQT.A.501, 2GSO.A.1001, 4GSZ.A.402, 1GT7.A.275, 4GTW.A.1010, 4GTW.A.1011, 1
GW6.A.1615, 3GWT.A.504, 2GYQ.A.407, 4H1S.A.603, 2H44.A.501, 3HDZ.A.864, 3HPS.A.7
01, 1HQA.B.452, 2I57.D.507, 3IBM.A.200, 4ICQ.A.502, 4IE5.A.601, 1IM5.A.400, 2IMA
.A.500, 3ISI.X.3001, 3ITU.A.1, 3IVT.A.500, 2IW0.A.1255, 4JDG.A.401, 1K07.A.2, 3K
5X.A.403, 1KAE.A.1101, 3KMC.B.2, 3KME.B.2, 1KQ3.A.401, 3KR5.A.1001, 3KR5.B.1004,
1KRM.A.501, 1LCP.A.488, 4LCF.A.311, 4LCG.A.301, 4LCH.A.301, 3LE9.A.1, 1LFW.A.10
02, 3LGP.A.1, 3LLX.A.376, 1LOK.A.901, 1LOK.A.902, 3LX3.A.201, 1M60.A.105, 4M6R.B
.301, 4MCP.A.801, 4MCQ.A.801, 4MCR.A.801, 3MK1.A.901, 4MTU.A.201, 1MZB.A.201, 4N
GM.A.818, 4NGQ.A.817, 4NGT.A.811, 3NO5.A.275, 4NPW.A.1001, 4NT9.A.301, 4NUR.A.70
1, 4NY2.A.501, 4NZ3.A.501, 3OOJ.A.1, 3O2G.A.388, 4O5O.A.301, 3O64.B.485, 3O91.A.
192, 3O93.A.192, 2OB3.B.904, 4OC1.A.814, 4OJV.A.403, 4OJV.A.404, 4OJX.A.403, 4OJ
X.A.404, 4OK4.A.800, 4OME.A.815, 4OP4.A.302, 2OQL.A.401, 1OS9.F.926, 2OUV.A.777,
2OU3.A.161, 2OUN.A.402, 2OUP.B.777, 2OUQ.A.402, 4OV9.A.401, 2P18.A.301, 2P2L.A.
201, 3P3E.A.400, 3P3G.A.301, 1P5X.A.246, 2PLI.B.707, 4PPZ.A.602, 3PS1.A.301, 3PS
2.A.301, 3PS3.A.301, 1PTM.A.330, 1PTM.B.331, 2PTY.A.500, 2PTY.A.501, 2PTZ.A.500,
2PTZ.A.501, 2PU1.A.500, 2PU1.A.501, 2PW3.A.501, 4Q3J.A.401, 3Q9B.E.345, 3QAY.D.
180, 2QF7.A.1157, 4QGE.A.602, 1QH3.A.262, 1QIP.B.901, 3QIY.A.431, 3QJ0.A.431, 3Q
M3.A.355, 1QTW.A.302, 3QW7.A.501, 3QW8.A.501, 2QYM.A.1, 2QYK.A.1, 1R1I.A.1001, 1
R1J.A.1001, 2R2V.C.35, 1R33.A.1163, 1R55.A.201, 4R6T.B.1003, 4R6T.F.1001, 4R76.A
.1003, 4R76.E.1001, 4R7M.C.1003, 3RCQ.A.1, 2RH6.A.2, 4RL0.B.302, 1ROR.A.601, 1RR
M.B.387, 1RTQ.A.701, 3S2L.A.403, 1SDX.A.677, 1SHN.B.481, 3SJG.A.1751, 3SL3.B.9,
1SNN.A.402, 1SR9.B.703, 3T3W.A.301, 1T9S.A.1, 1TB5.A.1001, 1TB7.A.1001, 1TBF.A.1
, 4TO8.A.301, 1TXR.A.501, 1TXR.A.502, 3U43.B.135, 1U74.A.1001, 3U79.H.110, 3UUO.
B.772, 3V77.D.301, 3V93.A.701, 3V94.A.702, 2V9L.A.1275, 2V9N.A.1275, 3VPB.A.503,
3W0T.A.201, 3W0T.B.201, 3W0U.A.201, 4WB7.A.503, 2WHG.B.1263, 3WT4.A.502, 2WTA.A
.1215, 4X2T.A.701, 4X2T.G.702, 1XM6.A.1001, 1XOR.A.1001, 1XVX.A.315, 1Y2K.A.1001
, 2Y33.A.900, 1Y9Q.A.202, 1YGD.A.142, 1ZED.A.903, 1ZKL.A.501, 2ZXC.A.647, 2ZZW.A
.362, 4X2T.L.701

Table S21. 4-ligand Mg, normal group

	size	largest_angle*	middle_1*	middle_2*	middle_3*	middle_4*	smallest_opposite_angle*	Tetrahedral	TrigonalBipyramidalVA	TrigonalBipyramidalVP	SquarePyramidalV	SquarePlanar
1	"34"	"161.2+/-8.1"	"77.1+/-6.1"	"84.6+/-5.3"	"92.2+/-5.7"	"103+/-9.1"	"149.5+/-10.2"	"0"	"0"	"0.002"	"0"	"0.092"
2	"34"	"129+/-7.7"	"91.7+/-6.4"	"100+/-4.9"	"108.8+/-5.3"	"116.2+/-5.8"	"105.8+/-10.9"	"0.052"	"0.005"	"0"	"0"	"0"
3	"67"	"167.2+/-5.7"	"79.9+/-4.5"	"85.9+/-4"	"90.5+/-4"	"95.8+/-4.6"	"88.2+/-8.2"	"0"	"0"	"0.004"	"0.199"	"0"
4	"41"	"151.5+/-11.6"	"80.5+/-8.4"	"88+/-8.5"	"101+/-7.8"	"117.7+/-8.7"	"85.3+/-7.2"	"0"	"0.005"	"0"	"0.004"	"0"
5	"43"	"148.6+/-8"	"71.4+/-4.2"	"77.3+/-3.8"	"82.4+/-4.3"	"91.3+/-7.1"	"76.3+/-7.4"	"0"	"0"	"0"	"0.002"	"0"
6	"14"	"137.9+/-9.4"	"70.2+/-4.5"	"76.9+/-4.8"	"82.6+/-4.2"	"93.3+/-14.4"	"121.3+/-9.5"	"0"	"0"	"0"	"0"	"0"
7	"47"	"157.4+/-9"	"82.2+/-6.2"	"90.4+/-5.2"	"96.8+/-4.9"	"107.1+/-6.6"	"109.5+/-8.9"	"0"	"0"	"0.019"	"0.003"	"0"

Table S22. Cluster members of 4-ligand Mg, normal group

[1] "Cluster 1"
4IRK.A.402, 2OTL.A.8066, 521P.A.168, 3AJP.A.183, 1AM4.D.679, 1CEE.A.180, 4D0L.B.
2001, 4DVG.A.201, 3EQB.A.9002, 3GFT.E.202, 3GOL.A.580, 3GT8.D.14, 4GZM.A.1001, 2
HAW.A.1002, 4HYP.C.302, 4I40.A.301, 4JVJ.F.403, 4JVJ.F.404, 3KRP.D.903, 1MAB.A.6
02, 2O1X.C.2003, 2P8E.A.306, 1Q3H.A.674, 1R0Z.D.674, 4R9U.D.302, 4RAB.C.303, 3T5
P.H.301, 4UOR.K.699, 3VHX.G.185, 1W1W.A.2001, 1W85.E.1368, 3WIG.A.402, 3WNW.J.20
1, 1YM0.A.402
[1] "Cluster 2"
2FLC.A.248, 4FLW.A.802, 1YJW.7.8044, 3ARA.A.166, 3C15.A.29, 1CLK.A.780, 2D32.B.2
524, 2E8A.A.501, 3EN9.A.600, 1G8G.A.521, 2H5N.B.1001, 4HJH.A.552, 4HN2.A.404, 1I
V4.D.1564, 2J4E.A.1002, 3KGX.A.503, 3LD0.J.54, 3M42.A.1, 4NNN.N.202, 2NOG.A.9220
, 2O4G.B.800, 4O6I.B.602, 4OKE.B.203, 4OKK.A.204, 3OPK.C.401, 4QDG.A.402, 1T9Z.A
.273, 3TAV.A.269, 1WOH.A.1001, 1XLB.A.399, 2XRI.A.1337, 2YFD.B.1145, 1YQ2.E.7005
, 1YYZ.A.341
[1] "Cluster 3"
3E54.A.702, 2AKZ.A.440, 2AQX.A.600, 1BR2.A.997, 3BU5.A.301, 3CNX.C.170, 2CW6.A.4
01, 4CYI.B.1000, 2DEJ.A.402, 2DH4.A.341, 2E92.B.1304, 2E95.A.1301, 3F74.C.1, 3FA
4.A.401, 3G2F.B.901, 2G74.A.185, 2GHQ.B.257, 2GWC.A.1, 3H8A.C.1431, 3HB0.A.701,
3HQP.M.502, 2HT6.B.444, 2IK2.A.290, 4IL6.C.505, 4IL6.c.506, 1IV2.F.1574, 4J99.D.
803, 2JK1.A.1144, 3KA9.A.189, 1LO0.A.602, 4LCZ.A.316, 4LRT.A.403, 1LVH.A.801, 4M

1W.A.201, 4MKS.A.502, 1NEL.A.438, 3NIZ.A.312, 1NV3.A.2341, 2O1U.A.302, 2O56.F.20
06, 2OI5.B.5000, 4OVN.B.202, 4POV.A.404, 1P7L.A.388, 4PRV.A.402, 2Q58.A.4, 1Q8Y.
A.809, 4QJB.A.301, 4QXD.B.302, 2R5T.A.600, 2RIO.A.1102, 1RLT.C.807, 3RYW.B.2003,
4S17.D.501, 3SY8.A.401, 4UON.B.401, 3UGJ.A.2006, 3VI4.B.502, 3VTI.C.401, 3VYT.C
.601, 1W9L.A.1748, 1WQA.A.456, 1YXO.A.5001, 1ZCW.A.501, 2ZCQ.A.453, 2ZEV.A.1302,
2ZRW.D.702
[1] "Cluster 4"
3ICE.D.1001, 4IR1.A.902, 1Q81.4.8078, 2W9C.A.1344, 2W9C.B.1342, 2A19.B.1642, 3AL
N.A.406, 4AVQ.C.902, 3CR3.B.1213, 4DPG.F.604, 3DYF.B.4002, 2FDR.A.1001, 3FYY.B.4
02, 1G9X.B.1301, 1GRV.A.490, 1HBN.A.1558, 4I1O.A.201, 2I5R.B.302, 2I07.B.5004, 3
JZM.A.701, 1KK8.B.998, 4MFE.D.1105, 2O1V.A.755, 2O1X.B.2002, 2PUN.B.401, 1PYX.A.
1002, 4QLQ.V.301, 3QU2.C.225, 3QU9.A.227, 4QXD.B.304, 4R3A.A.400, 1RC5.D.764, 3S
Z5.A.220, 4UM8.B.2001, 2VHQ.A.1328, 2WCJ.A.1144, 3WEJ.A.402, 3WGU.C.2003, 3WKA.A
.601, 1ZH4.A.201, 1ZYK.A.702
[1] "Cluster 5"
4DQP.A.903, 3S14.A.2001, 3ZC0.B.197, 3AQC.D.327, 2BM1.A.1690, 3CRL.B.2001, 4CW7.
C.1002, 4CYU.A.171, 1D1C.A.998, 2D33.C.2525, 3D7M.A.356, 3DLS.C.11, 4DPM.D.401,
1E1Q.A.601, 1E1Q.F.601, 2E6B.A.301, 2E92.A.1302, 1F4H.D.3001, 3FR8.B.1, 3G9D.B.2
99, 2GHT.B.257, 4HV6.B.201, 2I19.B.4004, 3IJQ.A.386, 3M1Y.D.300, 3MQT.A.1243, 3N
9V.A.61, 3OAC.D.3002, 3OHM.A.402, 4P9D.C.202, 4PFQ.C.400, 2Q80.A.401, 3RBM.B.100
2, 3SRD.C.603, 3SRF.D.533, 4TQ3.A.402, 3TW6.D.2002, 3U87.A.402, 2V0N.B.502, 2VKQ
.A.1288, 1WQ1.R.168, 1WUU.C.394, 2ZEV.B.1304
[1] "Cluster 6"
1AR1.A.560, 1BPM.A.488, 2DW7.L.2012, 3FPA.B.901, 3HYT.A.802, 2I07.A.5001, 2IOA.A
.5001, 3KZ1.E.550, 3LAW.B.1401, 4MPO.G.204, 3NC0.C.218, 1PKG.A.1481, 1SVW.B.301,
1XD2.A.167
[1] "Cluster 7"
4DLG.A.903, 3ICE.B.1001, 2PYJ.A.9004, 1RVB.B.302, 4UN4.B.2367, 1YJ9.0.8067, 3ALN
.B.406, 3BPD.G.126, 3CIK.A.690, 3D19.D.301, 3D19.E.301, 1D2E.D.504, 3E4O.A.501,
2E8W.B.1204, 4EOP.D.501, 4FMA.F.402, 1GQ9.B.1242, 4HNS.A.201, 3IAP.A.3001, 4IL6.
C.512, 4IL6.b.616, 3MG8.G.241, 4MPO.G.203, 3MWC.A.400, 1N5K.B.413, 4NNN.K.302, 2
O1X.D.2004, 2OPM.A.908, 3OPS.D.501, 2OQY.C.402, 4QVY.K.302, 1RK2.C.320, 4U3W.A.5
03, 4UUX.A.401, 2VOS.A.1491, 2VWI.B.1293, 3W9T.B.511, 2WCJ.A.1146, 3WEG.A.402, 2
WHE.A.1222, 3WU2.B.616, 3WU2.C.511, 3WU2.C.513, 1XMJ.A.2, 2Z2P.A.1001, 1Z5B.B.20
03, 2ZRY.D.702

Table S23. 4-ligand Mg, combined group

	size	largest_angle*	middle_1*	middle_2*	middle_3*
1	"14"	"133.2+/-10.5"	"67.5+/-5.5"	"75.6+/-3.8"	"81.1+/-4.5"
2	"23"	"151.4+/-6.5"	"82.7+/-6.4"	"91.6+/-5.6"	"99.7+/-3.8"
3	"25"	"167.1+/-6.4"	"80.3+/-5.6"	"87.6+/-3.8"	"92.3+/-3.8"
4	"18"	"132.7+/-6.8"	"62.3+/-4"	"70+/-5.2"	"77.6+/-5.1"
5	"20"	"162.5+/-9.6"	"64.1+/-7.7"	"78.1+/-6.9"	"104.1+/-9.6"
6	"21"	"153.1+/-10.2"	"65.6+/-7.5"	"79.9+/-7.5"	"93.6+/-11.5"
7	"12"	"133.3+/-8.7"	"62.1+/-7.3"	"73.9+/-6.5"	"88.2+/-8.6"
8	"27"	"162.9+/-7.2"	"78.5+/-5.8"	"85.9+/-2.9"	"91.3+/-3.8"
9	"24"	"126.4+/-6.9"	"91.9+/-6.8"	"100.1+/-5.1"	"108.9+/-4.4"
10	"38"	"152+/-5.3"	"72.1+/-4.4"	"78.1+/-3.6"	"82.8+/-4.3"
11	"33"	"154.1+/-5.9"	"86.4+/-5.8"	"93.9+/-5.1"	"99.4+/-4.7"
12	"55"	"167.3+/-5.6"	"79.7+/-4.1"	"85.5+/-3.9"	"90.1+/-3.7"

```
13 "16" "134.8+/-5.1"  "86.3+/-6.4" "93.5+/-7.7"  "107+/-8.6"
   middle_4*      smallest_opposite_angle* Tetrahedral
1  "89.3+/-8.1"   "114+/-7.9"              "0"
2  "109.7+/-6.1"  "112.1+/-7.5"            "0"
3  "100.5+/-5.1"  "107.8+/-8.7"            "0"
4  "91.8+/-8.7"   "71.9+/-8.6"             "0"
5  "120.2+/-11.8" "86.8+/-12"              "0"
6  "116.8+/-10.2" "135.6+/-7.3"            "0"
7  "115.1+/-12.1" "80.8+/-12.4"            "0"
8  "98.6+/-6.8"   "152.5+/-9.1"            "0"
9  "115.1+/-4.2"  "111.1+/-7.2"            "0.073"
10 "91.1+/-6.7"   "76+/-7.4"               "0"
11 "110.8+/-6.1"  "89.8+/-7.6"             "0"
12 "95.4+/-4.7"   "86.2+/-6.6"             "0"
13 "123.5+/-5.9"  "85.5+/-11.6"            "0"
   TrigonalBipyramidalVA TrigonalBipyramidalVP SquarePyramidalV
1  "0"                   "0"                   "0"
2  "0"                   "0.001"               "0.001"
3  "0"                   "0.043"               "0.031"
4  "0"                   "0"                   "0"
5  "0"                   "0"                   "0.001"
6  "0"                   "0.003"               "0"
7  "0"                   "0"                   "0"
8  "0"                   "0"                   "0"
9  "0"                   "0"                   "0"
10 "0"                   "0"                   "0.003"
11 "0"                   "0"                   "0.007"
12 "0"                   "0.001"               "0.229"
13 "0.022"               "0"                   "0"
   SquarePlanar
1  "0"
2  "0"
3  "0"
4  "0"
5  "0"
6  "0"
7  "0"
8  "0.116"
9  "0"
10 "0"
11 "0"
12 "0.001"
13 "0"
```

Table S24. Cluster members of 4-ligand Mg, combined group

```
[1] "Cluster 1"
1AZT.B.406, 1BPM.A.488, 2DW7.L.2012, 4F38.A.202, 2I19.B.4004, 2IO7.A.5001, 2IOA.
A.5001, 3KZ1.E.550, 3LAW.B.1401, 4MPO.G.204, 3NCO.C.218, 1PKG.A.1481, 1SVW.B.301
, 1ZOT.A.901
[1] "Cluster 2"
```

4DLG.A.903, 1RVB.B.302, 4UN4.B.2367, 1YJ9.0.8067, 3ALN.B.406, 3CIK.A.690, 3D19.E
.301, 1D2E.D.504, 1GQ9.B.1242, 4HNS.A.201, 3IAP.A.3001, 4IL6.C.512, 3MG8.G.241,
4NNN.K.302, 2O1X.D.2004, 2OPM.A.908, 1RK2.C.320, 4U3W.A.503, 2WCJ.A.1146, 3WEG.A
.402, 2WHE.A.1222, 3WU2.B.616, 3WU2.C.513
[1] "Cluster 3"
2PYJ.A.9004, 3BPD.G.126, 1BR2.A.997, 3D19.D.301, 3E4O.A.501, 2E8W.B.1204, 4EOP.D
.501, 3F74.C.1, 4FMA.F.402, 3H8A.C.1431, 2IK2.A.290, 4IL6.b.616, 2JK1.A.1144, 4L
CZ.A.316, 4MPO.G.203, 3OPS.D.501, 2OQY.C.402, 4PRV.A.402, 3RYW.B.2003, 4UUX.A.40
1, 2VWI.B.1293, 1XMJ.A.2, 1YXO.A.5001, 2Z2P.A.1001, 2ZRY.D.702
[1] "Cluster 4"
2NVQ.A.2001, 3AQC.D.327, 4CYU.A.171, 3D7M.A.356, 1DOA.A.199, 3EPS.A.1606, 3EQL.N
.1528, 1H8H.F.601, 4I1O.C.201, 1JFG.B.703, 3LCB.B.579, 3N9V.A.61, 4OAU.C.803, 3O
HM.A.402, 1S4E.B.1600, 2UXR.B.1398, 1WUU.C.394, 3ZXT.A.1278
[1] "Cluster 5"
3ICE.D.1001, 1Q81.4.8078, 1V14.C.1134, 2AEK.A.804, 3ALN.A.406, 1CUL.A.1007, 2E8W
.B.1203, 2I5R.B.302, 2ICJ.A.400, 3LGH.A.150, 1MXB.A.411, 2O1V.A.755, 4QXD.B.304,
1SO2.C.475, 3U87.B.403, 2VOS.A.1491, 3WEJ.A.402, 2Z2P.A.1003, 1ZH4.A.201, 1ZYK.
A.702
[1] "Cluster 6"
4IRK.A.402, 2OTL.A.8066, 1VQ7.0.8066, 4AG5.B.1588, 1AR1.A.560, 1CEE.A.180, 4D0L.
B.2001, 3GT8.D.14, 2HAW.A.1001, 3HYT.A.802, 4HYP.C.302, 1IOV.A.330, 4JVJ.F.404,
4K6T.B.411, 3KRP.D.903, 4QEH.A.401, 1SVT.E.601, 4UOR.K.699, 3VN9.A.402, 3WNW.J.2
01, 1Z6K.A.275
[1] "Cluster 7"
3DYF.B.4002, 1EQR.B.902, 1F4H.D.3001, 2G5H.B.501, 2G83.B.358, 1G9X.B.1301, 4I1O.
A.201, 3KRF.A.904, 3O7L.B.352, 3SRF.D.533, 1WBQ.C.1441, 1YXI.A.342
[1] "Cluster 8"
3HO1.X.22, 521P.A.168, 3AJP.A.183, 1AM4.D.679, 4DVG.A.201, 3EQB.A.9002, 3FPA.B.9
01, 3GFT.E.202, 3GOL.A.580, 4GZM.A.1001, 2HAW.A.1002, 4I4O.A.301, 4JVJ.F.403, 1M
AB.A.602, 2O1X.C.2003, 2P8E.A.306, 1Q3H.A.674, 1ROZ.D.674, 4R9U.D.302, 4RAB.C.30
3, 3T5P.H.301, 3VHX.G.185, 1W1W.A.2001, 1W85.E.1368, 3WIG.A.402, 1XD2.A.167, 1YM
0.A.402
[1] "Cluster 9"
2FLC.A.248, 4FLW.A.802, 1YJW.7.8044, 3ARA.A.166, 1CLK.A.780, 2E8A.A.501, 1G8G.A.
521, 2H5N.B.1001, 4HJH.A.552, 4HN2.A.404, 1IV4.D.1564, 2J4E.A.1002, 3LD0.J.54, 2
NOG.A.9220, 2O4G.B.800, 4O6I.B.602, 4OKE.B.203, 4OKK.A.204, 3OPK.C.401, 1T9Z.A.2
73, 3TAV.A.269, 1WOH.A.1001, 2XRI.A.1337, 1YQ2.E.7005
[1] "Cluster 10"
4DQP.A.903, 4P0P.A.601, 3S14.A.2001, 3ZC0.B.197, 2AQX.A.601, 2BM1.A.1690, 3CRL.B
.2001, 4CW7.C.1002, 1D1C.A.998, 2D33.C.2525, 3DLS.C.11, 4DPM.D.401, 1E1Q.A.601,
1E1Q.F.601, 2E6B.A.301, 2E92.A.1302, 3FR8.B.1, 3G9D.B.299, 2GHT.B.257, 4HV6.B.20
1, 3IG8.A.697, 3IJQ.A.386, 3M1Y.D.300, 3MQT.A.1243, 3OAC.D.3002, 4P9D.C.202, 4PF
Q.C.400, 2Q80.A.401, 3RBM.B.1002, 3SRD.C.603, 4TQ3.A.402, 3TW6.D.2002, 3U87.A.40
2, 2VON.B.502, 2VKQ.A.1288, 3VYT.C.601, 1WQ1.R.168, 2ZEV.B.1304
[1] "Cluster 11"
3ICE.B.1001, 2W9C.A.1344, 2W9C.B.1342, 4AVQ.C.902, 3CR3.B.1213, 4DPG.F.604, 2FDR
.A.1001, 3FYY.B.402, 1HBN.A.1558, 4IL6.c.506, 1KK8.B.998, 4MFE.D.1105, 3MWC.A.40
0, 1N5K.B.413, 2PUN.B.401, 4QLQ.V.301, 3QU2.C.225, 3QU9.A.227, 4QVY.K.302, 4R3A.
A.400, 1RC5.D.764, 3SY8.A.401, 3SZ5.A.220, 4UM8.B.2001, 2VHQ.A.1328, 3W9T.B.511,
2WCJ.A.1144, 3WGU.C.2003, 3WKA.A.601, 3WU2.C.511, 1XLB.A.399, 2YFD.B.1145, 1Z5B
.B.2003
[1] "Cluster 12"
3E54.A.702, 2AKZ.A.440, 2AQX.A.600, 3BU5.A.301, 3CNX.C.170, 2CW6.A.401, 4CYI.B.1
000, 2DEJ.A.402, 2DH4.A.341, 2E92.B.1304, 2E95.A.1301, 3FA4.A.401, 3G2F.B.901, 2

G74.A.185, 2GHQ.B.257, 2GWC.A.1, 3HBO.A.701, 3HQP.M.502, 2HT6.B.444, 4IL6.C.505,
1IV2.F.1574, 4J99.D.803, 3KA9.A.189, 1LOO.A.602, 4LRT.A.403, 1LVH.A.801, 4M1W.A
.201, 4MKS.A.502, 1NEL.A.438, 3NIZ.A.312, 1NV3.A.2341, 2O1U.A.302, 2O56.F.2006,
2OI5.B.5000, 4OVN.B.202, 4P0V.A.404, 1P7L.A.388, 2Q58.A.4, 1Q8Y.A.809, 4QJB.A.30
1, 4QXD.B.302, 2R5T.A.600, 2RIO.A.1102, 1RLT.C.807, 4S17.D.501, 4U0N.B.401, 3UGJ
.A.2006, 3VI4.B.502, 3VTI.C.401, 1W9L.A.1748, 1WQA.A.456, 1ZCW.A.501, 2ZCQ.A.453
, 2ZEV.A.1302, 2ZRW.D.702
[1] "Cluster 13"
4IR1.A.902, 2A19.B.1642, 3C15.A.29, 2D32.B.2524, 3EN9.A.600, 1GRV.A.490, 2IO7.B.
5004, 3JZM.A.701, 4K6T.E.412, 3KGX.A.503, 3M42.A.1, 4NNN.N.202, 2O1X.B.2002, 1PY
X.A.1002, 4QDG.A.402, 1YYZ.A.341

Table S25. 5-ligand Mg, normal group

	size	largest_angle*	middle_1*	middle_2	middle_3*	middle_4
1	"160"	"174.4+/-3.3"	"83.9+/-3.3"	"86.4+/-2.4"	"88.7+/-1.5"	"90.3+/-1.6"
2	"67"	"154.4+/-6.9"	"71.7+/-4.3"	"75.9+/-3.6"	"79.2+/-3.7"	"82.7+/-4.3"
3	"113"	"168.5+/-5.1"	"76.7+/-4.7"	"81.2+/-4"	"85.5+/-2.9"	"89.4+/-3.6"
4	"55"	"162.2+/-9.7"	"74+/-6.9"	"81+/-6.2"	"86.5+/-5.4"	"92.8+/-3.9"
5	"135"	"163+/-5.5"	"82.4+/-4.5"	"86.4+/-2.7"	"88.6+/-2.3"	"90.6+/-2.5"

	middle_5*	middle_6	middle_7	middle_8*
1	"92.1+/-1.8"	"95+/-3.2"	"98.8+/-4.6"	"170.3+/-4"
2	"87.4+/-5.1"	"93.4+/-8.6"	"107.1+/-13.4"	"145.3+/-6.5"
3	"93.5+/-4.9"	"97.5+/-5.9"	"106.6+/-10.2"	"157.8+/-5.3"
4	"98.8+/-5"	"106.5+/-8.2"	"118.6+/-6.7"	"135.6+/-7.5"
5	"95.1+/-3.4"	"102.2+/-3.6"	"108.3+/-5.4"	"156.2+/-5"

	smallest_opposite_angle*	TrigonalBipyramidal	SquarePyramidal
1	"86.6+/-4.4"	"0"	"0.437"
2	"72.6+/-5.5"	"0"	"0"
3	"78.4+/-5.4"	"0"	"0.055"
4	"101.3+/-7.8"	"0.045"	"0"
5	"96.3+/-4"	"0"	"0.011"

	TrigonalPrismaticV
1	"0"
2	"0"
3	"0"
4	"0.01"
5	"0"

Table S26. Cluster members of 5-ligand Mg, normal group

[1] "Cluster 1"
3A4K.C.301, 2IHM.A.700, 3A0U.A.201, 3A10.A.201, 4ACF.D.1480, 2AKZ.A.441, 1ALK.A.
452, 3B05.A.1001, 4BBJ.A.750, 2BHW.A.601, 2BHW.A.602, 2BHW.A.603, 2BHW.A.604, 2B
HW.A.609, 2BHW.A.614, 2BHW.B.605, 3BH7.A.1, 4BJU.A.998, 4BX2.A.301, 4BYF.A.1000,
4C5A.A.331, 3C9U.A.309, 1D0X.A.998, 1D0Y.A.998, 1D0Z.A.998, 1D1A.A.998, 1D1B.A.
998, 4DBQ.A.903, 2DCN.B.4006, 3DHF.A.502, 4DL8.A.304, 4DL8.A.305, 3DNT.B.442, 1D
XR.L.400, 3DYH.A.3002, 1E1R.F.601, 1E79.A.601, 2E8W.A.1201, 2E8X.A.1301, 3EF0.A.

1, 2FKW.A.1501, 2FKW.B.1601, 3G5A.C.307, 1G67.A.2007, 2G77.B.503, 1GFI.A.356, 2G J8.A.602, 3GL9.A.123, 2GLQ.A.2003, 3H1E.A.202, 1HE1.C.202, 2HEG.A.300, 2HF7.A.70 0, 4HGQ.C.201, 3HWX.1.602, 3HZH.A.202, 2I7D.A.728, 3ICM.A.403, 3ICN.A.402, 4IDO. A.503, 4IF4.A.300, 4IGA.A.200, 1IV2.B.1572, 2J7N.A.3374, 2J8C.L.1288, 1J97.A.220 , 4JA2.A.201, 1JB0.A.1107, 1JB0.A.1110, 1JB0.A.1118, 1JB0.A.1122, 1JB0.A.1129, 1 JB0.A.1130, 1JB0.A.1134, 1JB0.X.1701, 2JCS.B.1211, 4KEM.A.401, 1L3R.E.392, 1L5Y. A.701, 1L7N.A.221, 4LCZ.A.306, 4LCZ.A.307, 4LCZ.C.316, 4LEO.A.201, 1LGH.A.57, 1L GH.A.58, 1LGH.B.59, 4LRS.A.404, 1LVK.A.998, 1MPS.M.801, 1MXO.E.501, 1N24.B.706, 3N5K.A.2001, 1N6K.A.201, 2NGR.A.199, 4NM5.A.406, 3NNN.A.401, 4NST.A.1103, 1NVV.Q .1002, 4NVO.A.402, 2ODE.A.3001, 2OGX.A.291, 3OLV.A.130, 4OVN.A.201, 2OZE.A.299, 2PAN.A.851, 3PDE.B.310, 3PL9.A.602, 3PL9.A.603, 3PL9.A.609, 3PL9.A.610, 3PL9.A.6 12, 3PL9.A.613, 3PL9.A.614, 2PL1.A.204, 2PLS.I.606, 1Q3H.D.674, 3QHW.A.298, 1ROX .D.14, 2R25.B.1, 1RLO.A.801, 4RUR.W.301, 4S1H.A.303, 3SQS.A.450, 1SVK.A.356, 3T2 S.A.401, 3T34.A.1002, 3T6D.L.401, 3T6D.M.400, 3T6E.L.400, 1T91.A.1301, 3T9E.A.60 2, 3TCS.B.368, 3THU.A.500, 1TX4.B.681, 4UB6.C.507, 1UPB.A.601, 2VB6.A.1000, 3W6P .A.803, 1W7J.A.1793, 1W9I.A.1755, 3W9S.A.202, 2WF7.A.1220, 2WJN.M.1325, 3WK4.A.6 01, 1WQA.B.456, 2WZB.A.1417, 2X2E.A.1746, 1XBX.A.601, 1XHF.A.1001, 1Y9D.A.2601, 1YXO.A.5000, 1YZQ.A.901, 2Z4Z.A.1301, 1Z5B.A.2001, 2Z7I.B.1302, 1ZES.A.302, 1ZH4 .B.202, 1ZXN.A.900, 2ZXE.A.2002
[1] "Cluster 2"
2BPF.A.339, 4DPV.N.12, 4M30.B.302, 4W9M.E.902, 1A49.B.1134, 1A49.C.1734, 4AN9.A. 1383, 3AQC.D.328, 1AZT.A.403, 2BBO.A.2, 3C5P.D.302, 3CNX.B.170, 1CQP.A.310, 4CYI .D.1000, 4CYU.A.170, 2D33.A.525, 3DUF.C.1368, 3DVO.A.1368, 3DYF.A.3002, 3DYF.A.3 004, 4E1E.A.401, 3EA5.C.221, 1ELY.A.452, 3GFT.F.202, 1GIM.A.435, 3GT8.C.13, 1GY3 .A.1298, 2GZD.A.950, 4HYP.B.302, 3IAF.B.572, 2I07.A.5002, 4IYN.A.804, 4JLZ.B.502 , 1JM6.A.4601, 4JVJ.F.405, 4K81.B.203, 4LF2.D.601, 1MFO.A.1453, 4MGH.A.1302, 4NE H.A.1102, 1NUZ.A.2342, 2O1X.A.2001, 4O1P.C.903, 3OAB.A.904, 4OAU.C.802, 2OI2.A.4 00, 2POC.B.5, 4POV.A.403, 2PSN.A.701, 1Q19.A.504, 3RBM.A.1003, 4RJK.B.602, 3RUW. A.544, 4S17.C.502, 3SBE.A.501, 1SOJ.C.2127, 4TVU.B.601, 3TW6.B.2002, 4TXZ.B.502, 2WOO.A.1887, 2WB4.B.502, 3WQP.J.501, 2X9H.A.3001, 1XF9.B.11, 1Y8P.A.501, 1Y9I.B .602, 1ZCA.B.1383
[1] "Cluster 3"
3AUO.A.577, 4BDZ.A.1380, 4D6O.A.1186, 4D6O.A.1187, 4DLE.A.901, 2HVI.D.878, 3ICE. E.502, 4J9O.A.502, 3MDA.A.577, 1N56.A.401, 4NLK.A.402, 4NM1.A.401, 1TFW.B.1601, 2A5G.A.231, 4A7Y.A.952, 3ALO.A.1, 1AQF.A.534, 2AUU.A.201, 3B03.D.1001, 2B8W.A.59 5, 2B9J.A.600, 2BKK.A.1266, 1BS1.A.901, 2BVN.A.1395, 4BYG.A.996, 3C14.C.403, 2C4 3.A.1317, 3CBT.A.301, 3CK5.D.400, 3CRL.A.2000, 1CUL.A.1006, 3CX7.A.378, 3DKL.A.5 02, 4DLC.A.303, 1E1R.A.601, 4E1E.A.402, 2E8T.B.1303, 2E8X.A.1302, 1E9I.D.1433, 2 E91.B.1303, 2E92.B.1303, 3EA4.A.699, 3EF1.A.1, 3EFQ.B.4003, 3EHB.A.562, 4EKD.A.4 07, 2EWG.B.4003, 3EYA.A.613, 4FFR.A.406, 3FPA.A.901, 1FQJ.A.352, 2G07.A.601, 2G0 8.A.500, 3G8D.B.1002, 4GA3.A.1003, 2GTP.A.401, 4HEO.A.402, 4HEO.A.403, 4HGR.B.20 1, 3HIY.A.401, 2HO4.B.301, 3HU2.D.801, 3HYL.A.675, 3IOO.A.502, 2I19.A.3002, 2IK2 .A.289, 2IOA.A.5002, 4J5I.F.402, 2JCM.A.1490, 4KCV.B.1001, 1KK8.A.997, 3KRO.A.30 03, 3L0C.A.257, 3LMG.A.202, 4LNI.D.503, 4LNI.D.505, 4LRZ.A.302, 3LUZ.A.264, 3MCO .A.427, 3MCO.B.426, 3MGA.B.405, 3MLE.C.222, 4N0G.B.402, 4NCJ.A.903, 4NDN.A.401, 4NNN.Z.301, 2O56.A.2001, 3O61.A.202, 1PPW.A.401, 2PUL.A.400, 3PUW.A.1501, 3Q1O.D .400, 2Q58.A.3, 3Q7P.B.257, 4QXD.A.304, 3RBM.B.1001, 1RLT.A.805, 3RRA.A.406, 3SS 8.A.302, 3T5P.F.301, 3T9E.A.603, 4TQ4.C.402, 4UON.A.401, 3U2E.A.2, 2V54.B.1205, 3VAD.A.402, 1W5T.A.701, 3WBZ.F.403, 3WQM.A.403, 1XBT.D.4194, 1XZ8.B.180, 1ZCA.A. 383, 2ZRW.A.702
[1] "Cluster 4"
3EPH.B.2, 2G8F.A.301, 4IEM.A.502, 2OTJ.0.8044, 1QVG.0.8067, 1AJD.B.952, 4AZW.A.1 452, 2BJI.A.2278, 3CTL.A.601, 3D19.A.301, 2D32.A.1524, 3DGB.A.2001, 2EB6.A.1001, 7ENL.A.438, 3F5U.A.297, 2F9R.B.602, 3FSY.A.334, 3FXG.B.501, 3FYY.A.401, 4G61.A.

301, 3GIE.A.1, 3GQ7.A.692, 2GQ3.A.1002, 2GQ3.B.1003, 4GX6.A.402, 4IAC.A.402, 4IL
8.A.501, 1J7U.A.301, 1KJI.B.394, 3KR4.A.1004, 1L2O.B.902, 4LNI.A.503, 1LP4.A.342
, 2M32.A.401, 1NOW.A.401, 1N8W.A.900, 4NHO.A.1403, 1NUY.A.2342, 4NZN.A.404, 3Q2O
.B.384, 2QB8.B.401, 2QVU.A.340, 4QYI.E.203, 1SJA.B.701, 1SJB.A.1001, 3SRF.G.533,
 4UOM.B.503, 4UOR.A.699, 3V4S.A.402, 2VBI.G.1000, 2VPQ.B.1451, 1W88.A.1368, 3WNW
.A.201, 2XTI.A.1551, 2ZDR.A.1001
[1] "Cluster 5"
4DFM.A.903, 2PYJ.B.9002, 3Q8P.B.423, 3SNN.A.906, 3TFR.A.340, 3AU9.B.602, 3AUA.A.
601, 2BBT.A.3, 4BCL.A.367, 4BCL.A.368, 4BCL.A.369, 4BCL.A.370, 4BCL.A.371, 2BHW.
A.612, 1BHO.1.901, 4BJR.A.1517, 2BOZ.M.1304, 2BW7.D.2201, 1DAM.A.901, 4DFX.E.403
, 1E14.M.1303, 1E6D.M.1303, 1E9I.B.1431, 3ENI.A.375, 3ENI.A.378, 3EOJ.A.375, 2FK
A.A.9001, 1FMW.A.800, 1G65.G.301, 4GVE.A.602, 3I6E.B.386, 4IL6.j.102, 4IN7.M.411
, 1JB0.A.1011, 1JB0.A.1106, 1JB0.A.1112, 1JB0.A.1117, 1JB0.A.1121, 1JB0.A.1126,
1JB0.A.1128, 1JB0.A.1131, 1JB0.A.1132, 1JB0.A.1133, 1JB0.A.1136, 1JB0.B.1201, 1J
B0.B.1202, 1JB0.B.1203, 1JB0.B.1204, 1JB0.B.1206, 1JB0.B.1207, 1JB0.B.1211, 1JB0
.B.1214, 1JB0.B.1215, 1JB0.B.1221, 1JB0.B.1223, 1JB0.B.1224, 1JB0.B.1225, 1JB0.B
.1231, 1JB0.B.1234, 1JB0.B.1235, 1JB0.B.1236, 1JB0.B.1239, 1JB0.L.1501, 1JB0.L.1
502, 1JYX.A.3002, 1KJ8.B.394, 3KRF.D.901, 3L8F.A.401, 4LCZ.A.314, 4LRJ.A.302, 4L
YJ.A.201, 4MFE.C.1104, 4MKU.A.209, 1MNZ.A.389, 1N22.B.706, 4NHO.B.1403, 3NNS.A.4
01, 3O5T.A.299, 4OEC.A.401, 2OQY.B.402, 4PTK.A.302, 1Q9L.C.218, 3QQV.A.381, 1QSH
.B.147, 4QTD.A.426, 4QVP.V.302, 1R03.A.301, 4R02.Y.302, 4R17.K.302, 4R7O.E.402,
3RLG.A.286, 1RWT.A.614, 1RZH.L.854, 1RZH.M.851, 1RZH.M.853, 3TAV.A.266, 4UB6.B.6
03, 4UB6.B.607, 4UB6.B.610, 4UB6.B.616, 4UB6.C.504, 4UB6.C.506, 4UB6.C.509, 4UB6
.C.510, 4UB6.C.512, 4UB6.C.513, 4UB6.C.514, 4UB6.D.402, 4UM8.D.2001, 2UXR.A.1405
, 3V3Z.L.302, 2VP0.B.1209, 3VTH.A.805, 3WQP.H.501, 3WU2.A.405, 3WU2.A.410, 3WU2.
B.604, 3WU2.B.605, 3WU2.B.606, 3WU2.B.609, 3WU2.B.612, 3WU2.B.617, 3WU2.C.502, 3
WU2.C.510, 3WU2.D.402, 3WU2.b.615, 3WU2.b.616, 3WU2.b.617, 3WU2.c.902, 2YBE.A.14
17, 1YF6.M.853, 1YF6.M.856, 1YQ7.A.908, 1Z2P.X.1295, 1ZM7.D.400

Table S27. 5-ligand Mg, combined group

	size	largest_angle*	middle_1*	middle_2	middle_3*	middle_4
1	"49"	"162.7+/-2.4"	"83.9+/-2.8"	"87.7+/-1.8"	"89.2+/-1.5"	"90.7+/-1.8"
2	"14"	"145.8+/-5"	"62.7+/-5.6"	"77.4+/-6.7"	"87.3+/-6.8"	"93.4+/-7.3"
3	"40"	"169.6+/-4.2"	"80.9+/-2.6"	"83.3+/-2.5"	"85.9+/-2.1"	"88.2+/-2.4"
4	"10"	"150.4+/-7.9"	"73.3+/-3.2"	"78.4+/-5"	"85+/-4.2"	"92.9+/-4.6"
5	"18"	"170.4+/-3.5"	"81.5+/-3"	"84.8+/-2.1"	"88.3+/-2.2"	"90.5+/-2.8"
6	"22"	"162.6+/-5.2"	"71.2+/-4.3"	"81.7+/-5.1"	"87.3+/-5.6"	"92.2+/-4.7"
7	"24"	"143.1+/-7.6"	"63+/-4.6"	"69.5+/-4.9"	"75.1+/-5.7"	"79+/-6.8"
8	"24"	"172.4+/-2.6"	"72.3+/-3.8"	"78.9+/-4.5"	"84+/-3"	"91.7+/-3.6"
9	"18"	"162.8+/-5.5"	"62.7+/-5.4"	"77.5+/-6.6"	"85.6+/-7.5"	"96+/-5.9"
10	"17"	"161.1+/-6.2"	"56.9+/-6"	"79.5+/-5.4"	"85.7+/-4.5"	"90+/-5.5"
11	"24"	"162.6+/-5.4"	"73.1+/-5"	"78.4+/-4.1"	"83.4+/-4.1"	"87+/-4.8"
12	"60"	"177+/-1.4"	"85.6+/-2"	"87.6+/-1.6"	"89+/-1.2"	"90.5+/-1"
13	"24"	"173.9+/-3.1"	"76.7+/-2.4"	"83.6+/-2.7"	"88.2+/-1.8"	"91+/-2.3"
14	"28"	"170.6+/-3.7"	"82.1+/-3.1"	"85.7+/-2.4"	"88.5+/-2.3"	"91.2+/-2.6"
15	"37"	"157.2+/-2.1"	"84.1+/-2.5"	"86.4+/-1.3"	"88.5+/-1.1"	"90.3+/-2.2"
16	"51"	"173.6+/-2.7"	"83.5+/-2.1"	"85.7+/-2.2"	"88.4+/-1.5"	"90.1+/-1.7"
17	"26"	"163.5+/-4"	"73.9+/-4.5"	"80.3+/-4.8"	"86.2+/-3.7"	"89.7+/-4.2"
18	"36"	"151.9+/-4.6"	"69.5+/-4"	"74.7+/-3.5"	"77.8+/-3.2"	"81.9+/-3.8"
19	"32"	"160.7+/-3.8"	"76.2+/-2.2"	"79.7+/-2.7"	"82.1+/-2.7"	"84.8+/-2.4"

20	"36"	"170+/-3.3"	"84.6+/-2.4"	"87.5+/-2"	"89+/-1.7"	"90.5+/-1.7"
21	"18"	"171.3+/-4.4"	"81.4+/-3.4"	"85.2+/-2.7"	"87.6+/-1.5"	"91.9+/-2.8"

	middle_5*	middle_6	middle_7	middle_8*
1	"94.4+/-2.7"	"101.6+/-2.4"	"106.2+/-4.1"	"159.7+/-2.3"
2	"98.8+/-7.3"	"113.5+/-8.3"	"123.8+/-5.1"	"136.5+/-4.3"
3	"90.8+/-3"	"94.2+/-4"	"101.1+/-8.1"	"161.8+/-3.4"
4	"98.6+/-4.7"	"111.4+/-6.8"	"119.6+/-2.9"	"130.8+/-7.1"
5	"93.3+/-3.5"	"96.9+/-3.9"	"107.9+/-15"	"158.9+/-4.6"
6	"99.7+/-4.5"	"105.8+/-4.5"	"112.3+/-7"	"148.3+/-4.7"
7	"85.7+/-7.2"	"95.8+/-13.1"	"104.2+/-15.3"	"129.2+/-6.9"
8	"96.7+/-3.9"	"102.3+/-4.4"	"111.2+/-8.9"	"153.1+/-5.7"
9	"103+/-5.5"	"109.8+/-6.4"	"119.4+/-6.9"	"131+/-5.3"
10	"98.9+/-6.7"	"106.5+/-8.3"	"120.2+/-9.5"	"146.9+/-6.3"
11	"92.3+/-6.5"	"99.3+/-11"	"110.3+/-13.3"	"140.7+/-8.7"
12	"91.6+/-1.3"	"93.4+/-1.9"	"95.8+/-2.4"	"174.2+/-2.1"
13	"94.1+/-2.2"	"98.3+/-3.7"	"104.1+/-4"	"165.1+/-4.1"
14	"94.2+/-2.7"	"99.5+/-4"	"112.5+/-9.2"	"149.9+/-5.9"
15	"95.7+/-3.8"	"104.6+/-2.4"	"109.3+/-4.2"	"154.3+/-2.7"
16	"92.4+/-1.7"	"95+/-2.9"	"99.3+/-4.8"	"169.5+/-2"
17	"96.5+/-4.3"	"100.9+/-5.6"	"110.1+/-7.5"	"158.6+/-3.8"
18	"86.3+/-4.2"	"92.5+/-6.9"	"109.1+/-15"	"145.1+/-4.2"
19	"87.6+/-2.6"	"90.9+/-3.7"	"101.7+/-8.1"	"153.9+/-3.8"
20	"92.5+/-2.4"	"97.8+/-3.4"	"102.8+/-4.3"	"165.6+/-2.8"
21	"96.1+/-3.2"	"98.7+/-3"	"119.2+/-4.6"	"133.9+/-5.5"

	smallest_opposite_angle*	TrigonalBipyramidal	SquarePyramidal
1	"95.6+/-2.9"	"0"	"0.006"
2	"87+/-7.6"	"0"	"0"
3	"78.7+/-3.1"	"0"	"0.18"
4	"107.3+/-5.7"	"0"	"0"
5	"62.5+/-5.6"	"0"	"0.001"
6	"96+/-5.5"	"0"	"0.001"
7	"60.7+/-5.9"	"0"	"0"
8	"78.6+/-4.2"	"0"	"0.005"
9	"97.4+/-6.7"	"0"	"0"
10	"78.5+/-5.7"	"0"	"0"
11	"61.2+/-5.9"	"0"	"0"
12	"87.7+/-3.1"	"0"	"0.57"
13	"85+/-3.2"	"0"	"0.124"
14	"96+/-5.2"	"0.002"	"0.007"
15	"98.9+/-2.8"	"0"	"0"
16	"83.6+/-2.8"	"0"	"0.506"
17	"83.9+/-4.5"	"0"	"0.01"
18	"73.2+/-5.2"	"0"	"0"
19	"74.2+/-3.7"	"0"	"0.004"
20	"92.4+/-3.2"	"0"	"0.18"
21	"105.5+/-4.8"	"0.138"	"0"

	TrigonalPrismaticV
1	"0"
2	"0.04"
3	"0"
4	"0"
5	"0"
6	"0"
7	"0"

8 "0"
9 "0.001"
10 "0"
11 "0.001"
12 "0"
13 "0"
14 "0"
15 "0"
16 "0"
17 "0"
18 "0"
19 "0"
20 "0"
21 "0"

Table S28. Cluster members of 5-ligand Mg, combined group

[1] "Cluster 1"
2PYJ.B.9002, 2BBT.A.3, 4BCL.A.368, 1E6D.M.1303, 3ENI.A.375, 3ENI.A.378, 3EOJ.A.375, 1FMW.A.800, 1JB0.A.1106, 1JB0.A.1112, 1JB0.A.1117, 1JB0.A.1133, 1JB0.B.1201, 1JB0.B.1211, 1JB0.B.1215, 1JB0.B.1221, 1JB0.B.1223, 1JB0.B.1231, 1JB0.B.1234, 1JB0.L.1502, 1JYX.A.3002, 3L8F.A.401, 4LCZ.A.314, 1MNZ.A.389, 4QTD.A.426, 1R03.A.301, 3RLG.A.286, 1RZH.L.854, 1RZH.M.851, 1RZH.M.853, 4UB6.B.603, 4UB6.B.607, 4UB6.B.610, 4UB6.B.616, 4UB6.C.504, 4UB6.C.509, 4UB6.C.510, 4UB6.C.512, 4UB6.C.513, 4UB6.C.514, 4UB6.D.402, 3WU2.A.410, 3WU2.B.604, 3WU2.C.502, 3WU2.C.510, 3WU2.b.615, 3WU2.c.902, 1YF6.M.853, 1YF6.M.856
[1] "Cluster 2"
2G8F.A.301, 4ACF.B.1481, 1AJD.B.952, 4EO0.A.301, 2F1I.A.501, 2GQ3.B.1003, 1IV4.E.1565, 2M32.A.401, 4ML9.B.301, 4O1P.C.903, 4PV4.A.501, 3RUW.A.544, 3T80.B.160, 1ZCA.B.1383
[1] "Cluster 3"
3AUO.A.577, 4D6O.A.1186, 3ICE.E.502, 3MDA.A.577, 3ALO.A.1, 2AUU.A.201, 2B8W.A.595, 2BVN.A.1395, 4BYF.A.1000, 3CBT.A.301, 3CK5.D.400, 3CRL.A.2000, 3CX7.A.378, 1E1R.A.601, 1E1R.F.601, 2E8W.A.1201, 2E91.B.1303, 3EA4.A.699, 2G07.A.601, 2G08.A.500, 2GTP.A.401, 4HGQ.C.201, 4HGR.B.201, 2I19.A.3002, 2IOA.A.5002, 2JCM.A.1490, 1KK8.A.997, 4LRZ.A.302, 3MLE.C.222, 1N24.B.706, 4NNN.Z.301, 2PUL.A.400, 3PUW.A.1501, 4QXD.A.304, 1RLT.A.805, 3SS8.A.302, 4TQ4.C.402, 1Z5B.A.2001, 1ZCA.A.383, 2ZRW.A.702
[1] "Cluster 4"
4IEM.A.502, 3CTL.A.601, 3D19.A.301, 1NUY.A.2342, 3Q2O.B.384, 4QYI.E.203, 1SJB.A.1001, 3V4S.A.402, 3WNW.A.201, 2XTI.A.1551
[1] "Cluster 5"
4C5A.B.330, 4DLC.A.303, 1E4E.A.360, 3EHB.A.562, 4EKD.A.407, 2EWG.B.4003, 3FPA.A.901, 2IK2.A.289, 1L3R.E.391, 3LMG.A.202, 4N57.B.402, 4OVN.A.203, 4OVN.B.203, 4QXD.A.302, 3T9E.A.601, 4UAK.A.502, 1XBT.D.4194, 1YYZ.A.340
[1] "Cluster 6"
3AU9.B.602, 1BHO.1.901, 2BJI.A.2278, 4BJR.A.1517, 2BW7.D.2201, 4G61.A.301, 2GQ3.A.1002, 4GX6.A.402, 4HEO.A.402, 4IL6.j.102, 4IL8.A.501, 3KR4.A.1004, 3KRF.D.901, 4LNI.A.503, 4MFE.C.1104, 1NOW.A.401, 4NHO.B.1403, 1Q9L.C.218, 1SJA.B.701, 3SRF.G.533, 4UOM.B.503, 3WQP.H.501
[1] "Cluster 7"

3KK2.A.601, 2O5I.D.8001, 1QVG.0.8010, 1TTT.B.407, 2CN8.A.1504, 2DW7.A.2001, 4F0P
.A.501, 4FMC.B.203, 1GXB.C.1346, 4I1O.E.201, 2I07.A.5002, 3LZ9.A.852, 4N0G.A.405
, 1N8W.B.1900, 4NU1.A.404, 3OAB.A.904, 3OB8.D.3001, 3RBM.C.1002, 1S6P.A.601, 3SB
E.A.501, 4U81.A.501, 3UJ2.H.430, 2V0N.A.502, 2X9H.A.3001
[1] "Cluster 8"
2HVI.D.878, 4J9O.A.502, 4NLK.A.402, 1AQF.A.534, 3B03.D.1001, 2BKK.A.1266, 4BYG.A
.996, 3C14.C.403, 2C43.A.1317, 2E8T.B.1303, 4FFR.A.406, 3G8D.B.1002, 4HE0.A.403,
 3HU2.D.801, 3HYL.A.675, 4KCV.B.1001, 3MCO.B.426, 4NDN.A.401, 3RBM.B.1001, 3RRA.
A.406, 3T9E.A.603, 4UON.A.401, 3WQM.A.403, 1XZ8.B.180
[1] "Cluster 9"
3EPH.B.2, 2OTJ.0.8044, 1QVG.0.8067, 3ATT.A.379, 3B9T.A.485, 1DXI.A.390, 3F5U.A.2
97, 3GIE.A.1, 4GWZ.A.402, 1IV2.B.1562, 1L2O.B.902, 3LD0.8.54, 4NH0.A.1403, 4OTP.
A.502, 3U2E.A.3, 4UOR.A.699, 2VBI.G.1000, 2ZDR.A.1001
[1] "Cluster 10"
4IFD.J.1105, 4N41.E.101, 1N56.A.401, 2BVC.A.503, 3CWH.A.391, 2D32.A.1523, 1DIE.A
.398, 2DW7.B.2002, 3DYF.A.3004, 4FHY.A.402, 3GT8.C.13, 3HB0.B.702, 4I40.B.301, 4
LNI.D.504, 1MXA.A.411, 1SBJ.A.163, 4U03.A.504
[1] "Cluster 11"
2BPF.A.339, 2D33.B.1524, 4E1E.A.401, 3HQP.A.502, 4HYP.B.302, 3IAF.A.572, 3IG8.A.
696, 2I07.B.5003, 1JM6.A.4601, 4K10.A.405, 4LF2.D.601, 4LNI.D.505, 1MF0.A.1453,
3MQT.W.395, 1OL5.A.1389, 4P0V.A.403, 4P9D.B.202, 3PUG.A.601, 2PUI.A.401, 4PYK.A.
302, 3QHR.A.298, 4R9U.C.302, 4RJK.B.602, 4UOR.C.699
[1] "Cluster 12"
2IHM.A.700, 3A0U.A.201, 3A10.A.201, 3BH7.A.1, 4BX2.A.301, 1D0Z.A.998, 3DNT.B.442
, 3G5A.C.307, 2G77.B.503, 1GFI.A.356, 2GJ8.A.602, 3GL9.A.123, 1HE1.C.202, 2HEG.A
.300, 2HF7.A.700, 2I7D.A.728, 1IV2.B.1572, 4JA2.A.201, 1JB0.A.1118, 4KEM.A.401,
1L3R.E.392, 1L5Y.A.701, 1L7N.A.221, 4LCZ.A.307, 4LCZ.C.316, 1LGH.A.57, 1LGH.A.58
, 1LGH.B.59, 4LRS.A.404, 1LVK.A.998, 1MX0.E.501, 3N5K.A.2001, 4NV0.A.402, 2OGX.A
.291, 4OVN.A.201, 3PL9.A.602, 3PL9.A.603, 3PL9.A.609, 3PL9.A.610, 3PL9.A.612, 3P
L9.A.613, 3PL9.A.614, 2PL1.A.204, 2R25.B.1, 3T2S.A.401, 3T6D.L.401, 3T6D.M.400,
3T6E.L.400, 3T9E.A.602, 1TX4.B.681, 1W7J.A.1793, 3W9S.A.202, 2WF7.A.1220, 1WQA.B
.456, 2WZB.A.1417, 2X2E.A.1746, 1XBX.A.601, 1YZQ.A.901, 1ZES.A.302, 2ZXE.A.2002
[1] "Cluster 13"
3A4K.C.301, 4ACF.D.1480, 1ALK.A.452, 4BBJ.A.750, 2BHW.A.614, 4BJU.A.998, 3DHF.A.
502, 2E8X.A.1301, 3EFQ.B.4003, 1FQJ.A.352, 1G67.A.2007, 2H04.B.301, 3HWX.1.602,
2JCS.B.1211, 3MCO.A.427, 4NM5.A.406, 3PDE.B.310, 1PPW.A.401, 2Q58.A.3, 3SQS.A.45
0, 3T34.A.1002, 3U2E.A.2, 2V54.B.1205, 1W5T.A.701
[1] "Cluster 14"
3Q8P.B.423, 3AUA.A.601, 2D32.A.1524, 1DAM.A.901, 4DFX.E.403, 2FKA.A.9001, 3FXG.B
.501, 1G65.G.301, 3I6E.B.386, 1KJ8.B.394, 4LRJ.A.302, 4LYJ.A.201, 3MGA.B.405, 4M
KU.A.209, 4OEC.A.401, 2OQY.B.402, 4PTK.A.302, 3QQV.A.381, 4QVP.V.302, 4R02.Y.302
, 4R17.K.302, 3TAV.A.266, 4UM8.D.2001, 2VP0.B.1209, 3VTH.A.805, 2YBE.A.1417, 1Z2
P.X.1295, 1ZM7.D.400
[1] "Cluster 15"
4DFM.A.903, 3SNN.A.906, 4BCL.A.367, 4BCL.A.369, 4BCL.A.370, 4BCL.A.371, 2BOZ.M.1
304, 1E14.M.1303, 4IN7.M.411, 1JB0.A.1011, 1JB0.A.1121, 1JB0.A.1126, 1JB0.A.1128
, 1JB0.A.1131, 1JB0.A.1132, 1JB0.A.1136, 1JB0.B.1203, 1JB0.B.1206, 1JB0.B.1207,
1JB0.B.1214, 1JB0.B.1224, 1JB0.B.1225, 1JB0.B.1236, 1JB0.B.1239, 1JB0.L.1501, 1Q
SH.B.147, 4R7O.E.402, 3V3Z.L.302, 3WU2.A.405, 3WU2.B.605, 3WU2.B.606, 3WU2.B.609
, 3WU2.B.612, 3WU2.B.617, 3WU2.D.402, 3WU2.b.616, 3WU2.b.617
[1] "Cluster 16"
2AKZ.A.441, 3B05.A.1001, 2BHW.A.601, 2BHW.A.603, 2BHW.B.605, 4C5A.A.331, 3C9U.A.
309, 1D0X.A.998, 1D0Y.A.998, 1D1A.A.998, 1D1B.A.998, 4DBQ.A.903, 2DCN.B.4006, 4D
L8.A.304, 3DYH.A.3002, 1E79.A.601, 2FKW.A.1501, 3H1E.A.202, 3ICM.A.403, 3ICN.A.4

02, 4IDO.A.503, 4IF4.A.300, 4IGA.A.200, 2J7N.A.3374, 1J97.A.220, 1JB0.X.1701, 4L
CZ.A.306, 4LE0.A.201, 1N6K.A.201, 2NGR.A.199, 3NNN.A.401, 4NST.A.1103, 1NVV.Q.10
02, 2ODE.A.3001, 2PLS.I.606, 1Q3H.D.674, 3QHW.A.298, 1R0X.D.14, 1RLO.A.801, 4RUR
.W.301, 4S1H.A.303, 1SVK.A.356, 1T91.A.1301, 3TCS.B.368, 3W6P.A.803, 1W9I.A.1755
, 3WK4.A.601, 1YXO.A.5000, 2Z4Z.A.1301, 2Z7I.B.1302, 1ZH4.B.202
[1] "Cluster 17"
4BDZ.A.1380, 4D6O.A.1187, 4DLE.A.901, 3TFR.A.340, 1TFW.B.1601, 4A7Y.A.952, 2B9J.
A.600, 1BS1.A.901, 1CUL.A.1006, 3DKL.A.502, 1E9I.B.1431, 3EF1.A.1, 3EYA.A.613, 3
FDG.B.356, 3IOO.A.502, 4J5I.F.402, 1JB0.B.1202, 3KRO.A.3003, 3L0C.A.257, 4LNI.D.
503, 3O5T.A.299, 2O56.A.2001, 3Q1O.D.400, 3T5P.F.301, 2UXR.A.1405, 1YQ7.A.908
[1] "Cluster 18"
3A6P.H.1178, 4DPV.N.12, 4W9M.E.902, 1A49.B.1134, 1A49.C.1734, 4AN9.A.1383, 3AQC.
D.328, 1AZT.A.403, 2BBO.A.2, 3C5P.D.302, 3CNX.B.170, 1CQP.A.310, 4CYU.A.170, 3DY
F.A.3002, 3EA5.C.221, 1ELY.A.452, 1GIM.A.435, 1GY3.A.1298, 3IAF.B.572, 4IYN.A.80
4, 4JVJ.F.405, 4NEH.A.1102, 1NUZ.A.2342, 4OAU.C.802, 2P0C.B.5, 1Q19.A.504, 3RBM.
A.1003, 2RD5.C.1001, 4S17.C.502, 3TW6.B.2002, 2WB4.B.502, 3WQP.J.501, 1XF9.B.11,
1Y8P.A.501, 1Y9I.B.602, 1YHM.A.401
[1] "Cluster 19"
4M30.B.302, 4NM1.A.401, 2A5G.A.231, 4CYI.D.1000, 2D33.A.525, 3DUF.C.1368, 3DV0.A
.1368, 4E1E.A.402, 2E8X.A.1302, 1E9I.D.1433, 2E92.B.1303, 4GA3.A.1003, 3GFT.F.20
2, 2GZD.A.950, 3HIY.A.401, 4JLZ.B.502, 4K81.B.203, 3LUZ.A.264, 4MGH.A.1302, 4N0G
.B.402, 4NCJ.A.903, 2O1X.A.2001, 3O61.A.202, 2OI2.A.400, 2PSN.A.701, 3Q7P.B.257,
1SOJ.C.2127, 4TVU.B.601, 4TXZ.B.502, 3VAD.A.402, 2W00.A.1887, 3WBZ.F.403
[1] "Cluster 20"
2BHW.A.602, 2BHW.A.604, 2BHW.A.609, 2BHW.A.612, 4DL8.A.305, 1DXR.L.400, 3EF0.A.1
, 2FKW.B.1601, 2GLQ.A.2003, 4GVE.A.602, 3HZH.A.202, 2J8C.L.1288, 1JB0.A.1107, 1J
B0.A.1110, 1JB0.A.1122, 1JB0.A.1129, 1JB0.A.1130, 1JB0.A.1134, 1JB0.B.1204, 1JB0
.B.1235, 1MPS.M.801, 1N22.B.706, 3NNS.A.401, 3OLV.A.130, 2OZE.A.299, 2PAN.A.851,
1RWT.A.614, 3THU.A.500, 4UB6.C.506, 4UB6.C.507, 1UPB.A.601, 2VB6.A.1000, 2WJN.M
.1325, 1XHF.A.1001, 1Y9D.A.2601, 1ZXN.A.900
[1] "Cluster 21"
4AZW.A.1452, 3DGB.A.2001, 2EB6.A.1001, 7ENL.A.438, 2F9R.B.602, 3FSY.A.334, 3FYY.
A.401, 3GQ7.A.692, 4IAC.A.402, 1J7U.A.301, 1KJI.B.394, 1LP4.A.342, 1N8W.A.900, 4
NZN.A.404, 2QB8.B.401, 2QVU.A.340, 2VPQ.B.1451, 1W88.A.1368

Table S29. 6-ligand Mg, normal group

	size	largest_angle*	middle_1*	middle_2	middle_3	middle_4
1	"588"	"176.7+/-1.6"	"84.4+/-2"	"86.2+/-1.5"	"87.4+/-1.2"	"88.5+/-1"
2	"245"	"170.6+/-3.3"	"74.2+/-3.8"	"80.4+/-3.2"	"83.8+/-2.5"	"86.1+/-2.4"
3	"92"	"162.6+/-6.3"	"71.2+/-4.1"	"75.4+/-4.1"	"78.9+/-4"	"81.9+/-3.8"
4	"179"	"172.5+/-3.6"	"80.1+/-4"	"83.6+/-2.9"	"85.6+/-2.2"	"87.6+/-2"
5	"561"	"173.9+/-2.5"	"81+/-2.7"	"84+/-2.1"	"86+/-1.8"	"87.5+/-1.6"

	middle_5*	middle_6	middle_7	middle_8	middle_9*
1	"89.4+/-0.9"	"90.3+/-0.9"	"91.2+/-1"	"92.1+/-1"	"93.3+/-1.3"
2	"88.3+/-2.2"	"90.5+/-2.1"	"92.6+/-2.1"	"95.2+/-2.3"	"98.2+/-2.4"
3	"85+/-3.1"	"88.5+/-3.6"	"92.4+/-3.5"	"96.4+/-3.5"	"102.1+/-5"
4	"89.3+/-1.6"	"91+/-1.8"	"92.8+/-1.8"	"94.8+/-2.2"	"96.7+/-2.5"
5	"88.9+/-1.5"	"90.4+/-1.4"	"91.8+/-1.5"	"93.4+/-1.6"	"95.3+/-1.7"

	middle_10	middle_11	middle_12	middle_13*
1	"94.9+/-1.7"	"97.6+/-2.6"	"170.9+/-2.8"	"174.2+/-1.9"

```
2 "101.8+/-3.1" "107.2+/-4.9" "158.2+/-5.2" "164.5+/-3.6"
3 "108.4+/-6"   "118+/-8"     "147.1+/-7.2" "154+/-5.5"
4 "99.8+/-3.2"  "106.9+/-7.1" "158.8+/-5.8" "165.2+/-3.8"
5 "97.9+/-2.3"  "102.1+/-3.7" "165.2+/-3.9" "170.2+/-2.2"
  smallest_opposite_angle* Octahedral TrigonalPrismatic
1 "84.7+/-2.3"             "0.794"    "0"
2 "79.8+/-3.2"             "0.056"    "0"
3 "72.6+/-4.3"             "0"        "0.009"
4 "71.3+/-3.8"             "0.124"    "0"
5 "81.4+/-2.9"             "0.42"     "0"
  PentagonalBipyramidalVA PentagonalBipyramidalVP
1 "0"                     "0"
2 "0"                     "0.001"
3 "0"                     "0.015"
4 "0"                     "0.003"
5 "0"                     "0"
```

Table S30. Cluster members of 6-ligand Mg, normal group

[1] "Cluster 1"
4BE1.A.1381, 1BPY.A.339, 4BWJ.A.1834, 4DFK.A.902, 4DFM.A.902, 4DOC.A.401, 4DQP.D.902, 4ELT.A.902, 4ELU.A.902, 4F5O.A.402, 4FO6.A.601, 4GZ2.B.402, 2JOS.A.1412, 1JJ2.0.8010, 3JPQ.A.339, 3K58.A.1001, 3K59.A.1001, 3KD5.E.916, 4KLI.A.401, 4M04.A.702, 4M04.A.703, 4M9L.A.404, 4MDE.A.1002, 3MFI.A.515, 4MFC.A.401, 4O3O.A.502, 4O3Q.A.502, 3OJS.A.7, 4P4M.A.402, 3PNC.A.576, 4PUQ.B.401, 4Q8E.A.502, 4QM6.A.1002, 4R65.A.402, 3RJH.A.403, 3RTV.A.833, 3SNN.A.905, 3SPY.A.903, 3TFR.A.339, 3TFS.A.339, 3TIO.D.2, 1W7A.A.1802, 1XSN.A.576, 3ZVM.A.1526, 121P.A.168, 3A0T.A.800, 4A01.A.1767, 3A4L.A.401, 2A5Z.A.701, 2A5D.A.1231, 4A6X.A.350, 4ACF.A.1482, 4ACI.A.1187, 3AHC.A.826, 3AHD.A.826, 3AHE.A.826, 3AHG.A.826, 2AL1.B.439, 1AOX.A.400, 2AUU.A.203, 2AUU.A.204, 2AUT.A.601, 3AYX.A.701, 2B0T.A.800, 1B25.A.800, 4B2P.A.1351, 2B56.A.488, 3B7L.A.908, 3B7L.A.909, 2B82.A.1013, 2B9H.A.700, 4BAS.A.1183, 3BB1.A.282, 2BBS.A.3, 3BC1.A.194, 2BEK.A.501, 2BME.A.1184, 3BN3.A.1, 1BOF.A.800, 2BON.A.1302, 2BVC.A.504, 3BWV.A.300, 4BW9.A.501, 4BWR.A.1468, 4BX0.A.1291, 1BYQ.A.1001, 1C1Y.A.171, 3C4Z.A.563, 3C5H.A.302, 2C5L.A.1168, 4C5B.A.1314, 2C77.A.1407, 2C78.A.1407, 2CBZ.A.1872, 1CHN.A.200, 1CIP.A.356, 2CJE.A.1267, 2CK3.A.601, 2CK3.F.601, 2CN5.A.1506, 4COK.A.601, 1CTQ.A.168, 3CUR.H.553, 3CUS.Q.553, 3CX8.A.378, 3D2R.A.500, 4D6P.A.1352, 2D7C.A.1002, 4D7M.A.223, 1D8C.A.3001, 4DBR.A.810, 3DDC.A.600, 3DDH.B.232, 2DDT.A.311, 3DGT.A.800, 3DKC.A.2, 4DN5.A.1001, 4DSO.A.202, 4DUX.A.3001, 4DWG.A.401, 4DWO.A.301, 3DYH.A.3004, 3DYP.A.3001, 2DY1.A.701, 4DYK.A.502, 4DZH.A.501, 4E01.A.402, 1E2Q.A.401, 3E2D.A.603, 1E3D.B.901, 3E5H.A.200, 3E81.A.165, 3E8M.A.165, 2E9S.A.603, 1E9A.A.401, 4EEN.A.301, 4EFM.A.202, 3EHG.A.371, 1EK0.A.601, 2ERX.A.403, 3ET4.A.301, 3ET5.A.255, 4EX6.A.301, 4EX7.A.301, 2EZT.A.1510, 2EZU.A.1610, 3EZ3.A.1104, 2EZ4.A.1610, 2EZ8.A.1510, 4F1J.A.301, 2F2A.B.601, 1F5N.A.595, 3F61.A.310, 1F9H.A.161, 1F9H.A.162, 4FE3.A.304, 2FFQ.A.356, 2FH5.B.270, 4FI1.A.401, 4FK9.A.401, 2FOZ.A.348, 4FP1.A.401, 1FSG.A.302, 3FSY.A.333, 2FUE.A.500, 4FYP.A.301, 3FZN.A.605, 2G0W.A.501, 2G1T.A.1501, 1G17.A.301, 3G15.A.602, 1G4C.B.362, 1G4T.A.2005, 3G5A.D.307, 1G5T.A.998, 3G6K.A.307, 2G6B.A.301, 1G97.A.460, 4G9B.A.301, 3GAI.A.189, 2GCN.A.2001, 2GCP.A.2001, 2GHT.A.257, 2GIL.A.1201, 3GON.A.600, 4GOJ.A.202, 4GP2.A.402, 1GSA.A.319, 2GSM.A.3006, 4GT8.A.402, 3GYB.A.1, 4H1Z.D.401, 1H2A.L.1005, 1H2R.L.1005, 2H57.A.202, 3H70.A.342, 3H7V.A.331, 3H80.A.214, 4H81.A.402, 4H8E.A.301, 4HAT.A.302, 4HDO.B.200, 2HF8.A.301, 2HF9.A.

301, 4HGN.A.200, 2HNE.A.601, 4HOR.X.101, 1HQ2.A.162, 3HQJ.A.145, 3HRZ.A.628, 3HRZ.D.742, 3HSD.B.162, 1HTW.A.561, 3HW5.A.999, 1I0L.A.902, 2I1Q.A.501, 2I34.A.301, 2I5R.A.301, 3I76.A.1001, 4I94.A.402, 3IBA.A.403, 3ICM.A.401, 3ICZ.A.403, 4IDN.A.502, 4IDP.A.502, 2IHT.A.601, 1IH8.A.4002, 1IHU.A.592, 2IHP.A.287, 3IJL.A.386, 2IK4.B.287, 2IK4.B.289, 2IK6.B.287, 2IOR.A.2000, 2IO8.A.7002, 3IP0.A.161, 4ITR.D.203, 4IUC.L.702, 4IUD.L.1002, 1IV4.A.1571, 4IVG.A.803, 2IYW.A.202, 2J0V.A.1180, 2J7P.A.1401, 2J7P.D.1401, 1J9J.A.301, 2JC9.A.1491, 2JCB.A.1192, 4JDP.A.301, 2JD4.A.4061, 1JPM.A.1003, 4JS0.A.202, 3JTC.C.34, 3JYY.A.301, 3JZ0.A.303, 1K77.A.300, 3K8K.A.700, 3KA3.A.176, 3KAL.A.503, 3KB9.A.702, 3KC2.A.355, 1KCZ.A.901, 1KK1.A.413, 3KK0.A.180, 1KMQ.A.401, 4KO8.A.801, 1KQP.A.5001, 1KQP.A.5002, 4KQW.A.404, 1KSH.A.202, 1KTG.A.502, 1KTG.A.504, 1KTG.A.505, 3KTA.A.184, 3KUC.A.171, 4KUX.A.703, 4KVA.A.501, 4KVG.A.202, 4KWD.A.404, 4KXW.A.1001, 1KY2.A.401, 4L57.B.201, 3L8H.A.801, 4LFG.A.303, 4LFG.B.303, 4LGY.A.1302, 4LHW.A.301, 4LJ9.A.902, 3LLU.A.502, 4LPM.A.208, 3LUF.A.300, 3LX5.A.301, 3LXX.A.402, 4LYK.A.401, 4LZ0.A.403, 3M1I.A.1178, 4M53.A.527, 1M7B.A.550, 4M9Q.A.302, 4MDB.A.403, 4MGG.A.404, 3MHY.A.115, 1MJN.A.1001, 3MJH.A.201, 1MMG.A.998, 1MNE.A.998, 4MPO.B.205, 4MRT.A.301, 4MUM.A.301, 1MXG.A.439, 3MX3.A.601, 3MYH.X.997, 3MYK.X.998, 3MYL.X.998, 4N0D.A.402, 1N1Z.A.703, 1N20.A.702, 3N2N.A.1, 3N45.F.2, 3N45.F.3, 1N6I.A.201, 1N6L.A.201, 1N6N.A.201, 1N6O.A.201, 1N6P.A.201, 1N6R.A.201, 1NB0.A.201, 4NBS.A.502, 4ND0.A.302, 1NFZ.A.401, 3NKV.A.500, 1NN5.A.401, 1NRJ.B.1, 2NSY.A.305, 4NWI.A.401, 1O08.A.2800, 1O3Y.A.1002, 4O7I.A.401, 1OBW.A.176, 3OCV.A.264, 3OCW.A.263, 3OCY.A.264, 3OCZ.A.263, 4ODJ.A.502, 3OE1.A.601, 2OGD.A.3002, 2OGD.A.3004, 3OIW.A.170, 2OI6.B.6000, 4OI4.A.502, 1OKK.A.1002, 2OLR.A.543, 3OM2.A.486, 4OMF.A.503, 3OP2.B.500, 2ORW.A.501, 1OXV.A.1102, 1OXV.D.1101, 3OYX.A.601, 3OZX.A.613, 3OZX.A.614, 2P27.A.307, 4P31.A.402, 4P32.A.402, 1P4M.A.201, 1P5Z.B.401, 3P5P.A.901, 3P96.A.412, 4PFY.B.601, 3PGL.A.1, 1PHP.A.395, 4PHH.A.202, 3PIT.A.180, 2PKE.B.300, 3PNL.B.1212, 2PNQ.A.502, 1PPV.A.401, 4PQ9.A.301, 2PS2.A.401, 2PS5.B.701, 1PVF.A.401, 1PVG.A.903, 2PYW.A.501, 2PZ8.A.4001, 2PZE.A.3, 2Q28.A.1001, 1Q3H.C.674, 3Q3J.B.201, 2Q3F.A.301, 3Q46.A.307, 4Q4C.A.404, 3Q5V.B.599, 3Q60.A.603, 2Q66.A.602, 1Q92.A.1003, 4QC2.A.302, 3QF7.A.854, 1QGU.B.3002, 1QGU.D.3006, 2QG8.A.201, 4QHZ.A.302, 2QIS.A.907, 2QIS.A.908, 2QIS.A.909, 1QK5.A.303, 3QNM.A.400, 2QTY.A.348, 2QT0.A.1001, 3QUQ.A.225, 3QXC.A.222, 3QXJ.A.224, 3QXS.A.223, 2QX0.A.161, 3QYY.A.505, 1R2Q.A.300, 3R3S.A.296, 2R6O.A.801, 3R7W.A.600, 2R8E.A.201, 2RAH.A.354, 2RAR.A.501, 2RAV.A.701, 3RAP.R.200, 2RB5.A.701, 2RBK.A.501, 3REF.A.192, 1RKQ.A.1273, 4RKE.A.202, 4RKF.A.202, 3RLF.A.1501, 1RMT.A.1413, 3RO6.A.400, 4ROQ.A.401, 1RQI.A.604, 1RQJ.A.909, 3RUS.A.544, 3RV3.A.1004, 3RWM.B.1, 3RYE.A.908, 3RYE.A.909, 1RYA.A.1001, 1RYH.A.539, 4S1H.B.303, 3S4J.A.907, 3S4J.A.908, 3S4J.A.909, 3SAE.A.820, 3SF0.A.263, 3SHQ.A.321, 1SHT.X.219, 3SL2.A.701, 1SVM.A.750, 1SVS.A.356, 1T0P.A.901, 3T10.A.401, 3T1K.A.401, 3T2S.B.401, 3T2B.A.409, 3T2D.A.409, 3T7A.A.602, 1TC6.A.501, 2TCT.A.223, 3TEP.A.1, 3TG0.A.503, 1TMM.A.162, 4TMT.A.902, 4TMV.A.902, 4TMX.A.903, 4TN1.A.902, 3TS0.A.200, 4TY0.A.502, 4U5X.A.202, 4U82.A.301, 1U8Y.A.301, 4UAK.A.503, 4UAS.A.302, 4UAV.A.401, 2UAG.A.1001, 1UBK.L.1005, 3UCW.A.100, 4UCX.Q.1553, 4UE3.L.603, 3UJR.A.502, 3UJR.B.502, 3UJS.A.601, 3UJS.B.601, 1UMG.A.404, 4UM7.A.175, 3UPY.A.446, 1UPT.A.1183, 3UPL.A.447, 3UQY.L.603, 4UQL.Q.1552, 4URH.Q.1552, 4V0L.A.601, 4V1T.A.1777, 1V54.A.3518, 2V7Q.A.1512, 2VBU.A.1134, 3VC1.A.301, 3VC1.I.301, 3VCC.A.401, 1VG8.A.1401, 2VG3.A.1297, 2VK1.A.601, 2VK8.A.1565, 1VOM.A.997, 1VZM.B.1046, 1VZM.B.1047, 1W2Y.A.1231, 3W40.A.201, 2W4J.A.1280, 2W5V.A.1378, 2W5X.A.1379, 1W78.A.1422, 1W78.A.1423, 1WA5.A.1178, 3WBZ.A.404, 1WF3.A.401, 4WH2.A.402, 4WH3.A.402, 2WIC.A.1266, 3WJP.A.403, 3WJP.A.404, 2WKQ.A.1724, 3WNZ.A.502, 3WRY.C.1202, 1WUH.L.1005, 1WUK.L.1005, 3WXM.A.502, 1X06.A.900, 2X13.A.1418, 2X14.A.1418, 1X3S.A.200, 2X98.A.1477, 1XBY.A.601, 2XCW.A.1498, 1XFI.A.400, 2XH2.B.1439, 2XIS.A.392, 2XJB.A.1494, 2XJD.A.1497, 2XTN.A.1232, 1XX1.A.9001, 4XXP.A.301, 2Y8E.A.1177, 2YCH.A.501, 1YHL.A.1403, 1YMV.A.200, 1YNS.A.1258, 1YQ9.H.540, 1YQT.A.591, 1YQT.A.592, 1YRQ.H.553, 1YS7.A.1002, 1YU4.A.2002, 2YVO.A.1002, 2YVP.A.184, 1YV

D.A.850, 1YZL.A.401, 1YZN.A.301, 1YZT.A.700, 1Z06.A.203, 1Z07.A.300, 1Z08.A.1300
, 1Z08.C.3300, 1Z0J.A.400, 1Z2N.X.1295, 1Z4J.A.1001, 1Z4K.A.229, 1Z4L.A.2001, 2Z
4V.A.1502, 2Z4X.A.1201, 2Z4X.A.1202, 1Z59.A.1001, 1Z5G.A.601, 1Z5G.D.604, 2Z52.A
.1301, 2Z52.A.1302, 1Z88.A.601, 1ZC3.A.500, 1ZED.A.905, 3ZFD.A.500, 3ZIA.A.601,
1ZJJ.A.1001, 1ZS9.A.1257, 3ZVL.A.1524, 3ZX5.A.260
[1] "Cluster 2"
3A4K.A.301, 4AAB.B.1157, 2AQ4.A.302, 2BCV.A.576, 4BDY.A.1381, 4D6N.F.1196, 4DF4.
A.901, 4DLG.A.902, 4EEY.A.502, 4F5P.A.401, 3GDX.A.347, 3GQC.B.203, 4IR9.F.402, 4
IRD.F.903, 2ISP.A.339, 4K98.A.602, 3LK9.A.339, 4M30.A.502, 4M47.A.402, 4MFF.A.40
1, 3MQY.A.500, 3OS0.A.394, 3OYA.A.396, 3OYC.A.396, 3OYC.A.397, 3OYE.A.396, 3OYF.
A.397, 3OYG.A.397, 3OYH.A.396, 3OYH.A.397, 4PGQ.A.400, 4QCL.A.1302, 1QSY.A.1001,
3RJK.A.340, 3S3M.A.396, 1SKR.A.4001, 3SPY.A.904, 3SV3.A.836, 1TK8.A.901, 4TUQ.A
.402, 3UQ2.A.1, 2W35.A.1224, 1ZBL.B.204, 3A06.A.500, 4A01.A.1770, 4A01.A.1771, 1
A49.H.5334, 3A7D.A.300, 4AC0.A.1205, 3AJ0.A.183, 3AXK.A.478, 1AZS.C.403, 3B1X.A.
301, 1B7T.A.836, 3BNY.D.701, 4CE0.A.1251, 1CH8.A.434, 3CP6.A.501, 1CUL.C.396, 3C
WH.A.392, 1DAK.A.901, 4DBH.A.401, 3DFY.A.401, 3DHD.A.502, 1DIE.A.399, 1DQN.A.451
, 3DVA.A.1368, 2DW6.A.2001, 2DW6.D.2004, 4DWB.A.508, 4DXJ.A.401, 4DXJ.A.402, 3DY
G.A.3004, 4E1E.A.403, 1E9I.A.1431, 4EA0.A.303, 1EBG.A.438, 1EC9.A.498, 1ECB.A.50
7, 1ECQ.A.498, 3EFQ.A.3003, 3EFQ.B.4002, 3EQI.A.3, 3EYA.H.613, 3FD5.A.395, 3FD6.
B.395, 4FFL.A.904, 2FG5.A.301, 4FI4.A.501, 2FN0.A.701, 3FQI.A.1000, 1FTN.A.300,
1G3B.A.501, 2G4J.A.392, 4G61.A.302, 2G9Y.A.452, 2G9Z.A.704, 4GA3.A.1004, 4GIS.A.
405, 2GQS.A.240, 2GQ3.A.1000, 1H1D.A.300, 4H1Z.A.401, 3H4L.A.701, 3HDG.B.201, 4H
E1.A.404, 4HE2.A.405, 4HGR.A.201, 3HQD.A.501, 3HQP.B.502, 2HWG.A.901, 4I2B.A.602
, 4I3Y.A.302, 3I6E.A.386, 3IBA.A.402, 3ICZ.A.401, 2IDX.A.603, 4IEE.A.501, 1II0.A
.593, 2IK2.B.287, 2IK2.B.290, 2IOA.B.5004, 4IT1.A.501, 2IUC.B.1008, 4IX4.A.602,
2JCS.A.1211, 2JI6.A.1567, 2JI8.A.1567, 1JP4.A.701, 3JUK.C.307, 1K9Y.A.403, 4K9N.
A.601, 4KCV.A.1001, 1KEK.A.2237, 1KHK.A.452, 4KMQ.A.1102, 1KO5.A.1001, 1KP8.A.55
0, 3KRO.D.3002, 4KUX.A.701, 4KX3.A.302, 4LA7.B.601, 3LVV.A.695, 3LVV.A.697, 1M1B
.B.999, 4M69.A.403, 1MC1.A.603, 4MIT.A.202, 4MPO.B.204, 4MPO.E.201, 3MQT.H.626,
4NOG.A.403, 1N1Z.A.702, 1N20.A.703, 1N24.A.703, 3N3T.A.802, 4NEH.B.703, 1NHT.A.4
35, 4NM3.A.405, 3NO1.A.397, 1NUW.A.2497, 1NUY.A.2343, 2O1O.A.501, 4O4D.A.401, 1O
AD.A.392, 3OES.A.202, 1OFH.B.453, 4OKZ.A.901, 1ORK.A.223, 2OX4.A.402, 3OYZ.A.500
, 1OZF.A.699, 3P0X.A.430, 1P7T.A.1000, 1P9B.A.1600, 4PAL.A.110, 1PFK.A.325, 4PFK
.A.327, 2PLS.J.604, 2PP3.A.901, 6Q21.D.173, 3Q30.A.601, 3Q46.A.305, 1QC5.A.601,
1QC5.B.602, 4QEH.A.402, 1QF4.A.433, 4QPM.A.1502, 3QQV.A.382, 1QS0.A.501, 3QU4.A.
225, 2R9V.A.504, 3RBM.D.1003, 2RDX.A.378, 3RIM.A.1001, 4ROP.A.504, 1RQI.A.605, 1
S1C.A.300, 3S9I.A.743, 3SAD.A.801, 3SB0.A.801, 3SE1.A.182, 3SH6.A.176, 3SOP.A.40
1, 3SSN.A.501, 3T0Z.A.401, 3T8O.A.564, 1TND.A.352, 4TQ4.A.401, 3TTE.A.361, 3TTE.
B.361, 3UJ2.A.431, 3UXL.A.360, 4V1T.A.1778, 2V3W.A.1528, 2V5K.A.301, 1VA6.A.524,
2VBI.A.1000, 3VD3.A.3001, 2VDM.B.2001, 3VMM.A.501, 2VP0.A.1209, 2W00.B.1894, 3W
2W.A.904, 1W5T.B.701, 1W88.C.1368, 1WDD.A.1476, 4WK4.B.501, 4WRR.A.401, 2WX5.L.1
282, 2X5Z.A.602, 2XH4.A.1439, 5XIM.A.395, 5XIN.A.395, 1XIN.A.395, 8XIM.A.395, 1X
LC.A.399, 1XZ8.A.180, 1YVE.I.602, 1Z0K.A.1201, 2Z4W.A.1302, 2Z4Y.B.1301, 3ZCB.A.
301, 1ZVW.A.4001, 3ZYC.A.1750
[1] "Cluster 3"
2AGQ.A.4002, 4D6N.F.1197, 4DF8.A.903, 4DFP.A.901, 3LK9.A.340, 4M2Z.A.501, 2OZM.A
.904, 1QTM.A.1001, 3R7P.A.317, 3SI6.A.905, 2XCP.A.1004, 2XCA.A.3000, 2XCA.A.3001
, 1YVP.A.1001, 1ZBL.A.202, 3A58.B.401, 2AG1.B.611, 3B8I.A.289, 2BW7.A.2201, 4BYF
.C.1000, 1CG0.A.435, 4CTA.B.401, 4CZK.A.1335, 4D2I.A.1478, 3DYG.A.3002, 2E0A.A.5
00, 1E2A.C.106, 1EFL.B.1604, 3EG5.A.180, 2EGH.A.900, 1ELX.B.452, 1F1Z.A.2002, 3F
FU.A.156, 3FFU.B.155, 4FFL.A.905, 4FFO.A.904, 3FHY.B.404, 4FHX.A.402, 3G9D.A.298
, 1H65.A.282, 3HWX.A.602, 4HYV.A.1001, 3I3O.A.306, 4IOK.A.604, 2IUT.A.1724, 2J5X
.A.200, 1KH7.A.452, 3KR4.C.1004, 4KS0.A.1001, 3KUD.A.171, 4L2X.F.403, 4L2X.F.405
, 4LF1.A.801, 4LNI.E.505, 3M1Y.A.300, 1MB9.B.601, 1MBZ.A.603, 1MBZ.A.604, 2NOM.A

.401, 2NOM.A.402, 1NUW.A.2498, 1NV7.A.3341, 4OGE.A.1204, 2OPM.A.907, 3OPS.A.500, 2OQY.A.401, 1OVM.A.601, 2PLS.G.603, 3PP1.A.410, 2Q58.A.5, 1QF5.A.433, 2QTV.B.210, 4RAD.D.302, 1RC5.A.761, 4RNH.A.1501, 1SOJ.A.2123, 1T5S.A.1005, 3TXA.A.801, 4UOM.A.503, 3UGV.A.500, 2VQD.A.1449, 2VWT.A.301, 3VYT.C.602, 3W7F.A.303, 4WK2.B.501, 1WL6.A.801, 2XAM.B.1030, 2Y4M.A.400, 1Y9I.A.601, 1YHY.A.699, 2YWF.A.701, 3ZDY.B.2001

[1] "Cluster 4"

4AAB.B.1156, 4AQX.D.1526, 4BE2.A.1381, 4C2U.A.1666, 1CW0.N.202, 1CW0.A.203, 4D6O.D.1196, 4D6O.D.1197, 1G9Z.C.902, 1G9Z.D.901, 1G9Z.F.903, 4LOX.A.401, 4M30.A.501, 3MAQ.A.1001, 1MOW.D.374, 4NCB.A.702, 4NCB.B.702, 4NCB.B.703, 3OYA.A.397, 4PQU.A.602, 3S3O.A.397, 3S3M.A.397, 1T7P.A.4001, 1TK0.A.991, 2VBN.E.1026, 1ZBI.A.302, 1A49.A.534, 4ACF.A.1480, 2AE8.C.1009, 1AJB.A.452, 3BGA.A.1, 2BJI.A.2277, 3BM4.B.304, 1BWV.C.490, 3BWY.A.300, 4C5C.A.1314, 4C7X.A.700, 1CG1.A.435, 1CG4.A.435, 1CIB.A.434, 3CRR.A.324, 3CT2.A.401, 4CWB.A.1159, 3CXO.A.500, 3CXO.B.500, 4CYM.A.1199, 3D46.A.501, 3D47.A.501, 1DAY.A.341, 2DGN.A.1454, 4DH5.A.402, 3DUF.A.1368, 4DWB.A.507, 3DYH.B.4002, 3DYS.A.902, 2E8W.A.1202, 4EA0.A.301, 1EBH.A.438, 1ELZ.A.452, 1EXM.A.407, 3EZ3.B.1104, 4F0Q.A.501, 3F78.C.1, 4F71.A.301, 3FD5.A.396, 4FFL.A.906, 3FLK.A.401, 3FLK.A.405, 3FPA.C.901, 3FPB.A.1000, 3FTQ.A.371, 3FYY.A.402, 4GOK.B.202, 2GT4.B.401, 4GT3.A.403, 3GY1.A.500, 4HE1.A.403, 3HJN.A.501, 3HPF.A.402, 2HXU.A.601, 4I3Y.A.304, 1I6I.A.501, 1IGW.A.441, 2IK2.B.289, 2IK7.A.287, 1IV4.A.1572, 4J7L.A.402, 1JP4.A.702, 3JVT.B.502, 4K33.A.802, 1K9Y.A.401, 4KCU.A.1001, 4KCW.A.1001, 3KDN.A.500, 3KEU.A.400, 3KHQ.A.1, 4KI8.E.602, 1KKR.A.501, 4KQX.A.406, 3KRO.D.3001, 3LOY.A.257, 4L9Y.B.403, 3M00.A.550, 4M6U.A.401, 1MNS.A.360, 4MPO.A.206, 4MPO.C.205, 1MRS.A.300, 1MXO.A.501, 1NUY.A.2341, 4NZO.A.404, 2O1O.A.503, 1OIX.A.301, 4OKM.A.902, 3OP2.A.500, 3OPS.A.501, 4ORK.A.501, 2OUN.A.403, 4OVN.A.204, 1OW2.A.401, 1OZH.A.1405, 3OZM.D.390, 3P3B.A.393, 3P41.A.297, 3P93.A.406, 2PLS.H.602, 2PMQ.A.902, 1PUN.A.130, 4PU5.A.502, 1PYD.A.559, 1PYM.A.1003, 2PZA.A.6242, 2Q1A.X.294, 2Q1D.X.294, 4Q1V.A.803, 3Q30.A.600, 3Q46.A.306, 1Q6O.A.7300, 1Q6Q.A.7300, 4QE5.A.401, 1QMZ.A.383, 3QPE.A.393, 3QPE.B.393, 4QPM.A.1503, 3R1M.A.402, 3R1M.A.403, 3R25.A.402, 4RJJ.A.602, 4RJK.H.602, 3RLH.A.286, 4RN3.A.301, 1RQJ.A.907, 3SBF.A.402, 1SHQ.A.479, 3T6C.A.501, 1TE6.A.640, 4TQD.A.502, 3TW6.C.2002, 3TZF.A.279, 3UJR.A.501, 4USJ.C.302, 3V4B.A.403, 1V5G.A.1603, 2VBV.A.1136, 3VMK.A.402, 3WBZ.A.403, 3WDL.B.902, 2WEF.A.402, 4WKO.B.501, 2X3J.A.1590, 2XCL.A.480, 2XIM.A.395, 2XZW.A.202, 1YHM.B.1401, 1YVE.I.601, 1Z5C.A.2001, 2Z7H.A.1301, 2ZVJ.A.300, 3ZXW.A.476

[1] "Cluster 5"

4AQX.D.1525, 4BDY.A.1380, 4BE0.A.1380, 4BE2.A.1380, 4CEI.A.2234, 4CEI.B.2162, 4DFJ.A.902, 4DL4.A.501, 4DO9.A.401, 4DOA.A.401, 4DOB.A.401, 2DPI.A.871, 4DQI.A.901, 4DQI.D.901, 2EZ6.A.501, 3F2B.A.5, 1FIU.I.2222, 1FIU.A.5555, 2FMS.A.340, 3G6Y.A.871, 3GDX.A.348, 3GPL.A.800, 3IEV.A.400, 2ISO.A.339, 2IS4.A.1001, 3JPN.A.339, 3JPR.A.339, 3JPS.A.339, 3JPT.A.339, 4JWM.A.403, 3K57.A.1001, 4K97.A.603, 4K99.A.602, 3M8R.A.2, 3M8S.A.2, 4M8O.A.1302, 3MBY.A.339, 3MR5.A.435, 4O3N.A.503, 4O3O.A.503, 4O3Q.A.503, 4O5K.A.401, 3OHA.A.518, 3OYB.A.396, 3OYD.A.396, 3OYF.A.396, 3OYG.A.396, 2OZS.A.904, 2PFP.A.750, 2PFN.A.950, 3PML.A.2, 4R8U.B.402, 3RJF.A.340, 3RJK.A.339, 4RNN.A.503, 3SI8.A.451, 3SM4.B.227, 1SUZ.A.402, 3TFS.A.340, 3TIO.A.1, 3TWH.A.401, 4UAY.A.402, 4UB3.A.401, 3V6H.A.402, 1ZJN.A.339, 4A01.A.1769, 3A1U.A.5, 3A1U.A.6, 1A2B.A.550, 1A82.A.901, 3A99.A.401, 4ABZ.A.1210, 2AFK.E.1291, 2AGO.A.601, 1ALK.B.452, 4ANB.A.1384, 1AOR.A.609, 1AS0.A.356, 4AS2.A.1328, 2AUT.D.604, 4AUX.A.223, 3B05.D.1001, 3B1V.A.301, 1B4N.A.623, 3B7L.A.907, 1B8C.A.308, 1B8J.A.452, 2BKU.A.221, 3BRB.A.10, 2BU2.A.1388, 2BVN.B.1395, 4BX3.A.301, 1BZY.A.901, 2C18.A.1338, 2C3U.A.2238, 2C31.A.1553, 2C3P.A.2237, 2C42.A.3238, 2C4N.A.1251, 4C5A.B.331, 3C9U.B.312, 3CB3.A.501, 3CFX.A.704, 2CFS.A.1296, 3CG4.A.201, 2CHE.A.130, 1CJT.C.403, 1CJU.A.582, 2CJE.A.1268, 3CK5.A.400, 2CL5.A.1216, 2CLS.A.550, 3CMR.A.453, 3CP6.A.502, 3CP6.A.503, 3CR3.A.1212, 3CV2.A.1, 4CW7.A.1002, 3CZJ.B.3001

, 2DOO.A.1005, 1D2N.A.99, 3D36.A.478, 4DBF.A.401, 2DCN.A.4001, 4DCK.B.201, 2DEI.A.402, 4DEM.F.402, 4DEM.F.403, 4DEM.F.404, 4DFD.B.301, 4DN1.A.401, 4DN1.B.401, 3DOE.A.193, 4DSN.A.202, 1DTW.A.401, 2DUA.A.292, 1DXE.B.901, 4DXJ.A.403, 3DYH.A.3003, 3DYM.A.3001, 1E4E.A.365, 3E84.A.701, 4E8G.A.402, 2E91.A.1301, 2E91.A.1302, 2E95.A.1302, 3EA5.A.221, 4EA0.A.302, 1EBG.A.439, 2EB1.A.502, 2EB5.A.1001, 1EC7.A.498, 3EFQ.B.4004, 3EGT.A.3002, 3EGT.A.3003, 3EGT.A.3004, 3EKG.A.601, 3EQC.A.3, 3ES8.A.393, 3ETJ.A.401, 4EUK.A.1001, 2EW1.A.701, 3EZ3.B.1102, 2EZ9.A.1510, 1F2U.A.902, 1F8I.A.451, 2F9M.A.1201, 4F9A.A.602, 3FA5.A.282, 3FD5.A.397, 3FD5.B.397, 3FD6.B.397, 3FE4.B.902, 4FEG.A.707, 4FFR.A.403, 3FIU.A.5001, 3FIU.A.5002, 3FKQ.A.501, 2FOZ.A.349, 2FPR.A.503, 2FRV.B.540, 3FV9.A.501, 4FVR.A.902, 2G09.A.901, 3G2F.A.901, 1G4P.A.2003, 3G5A.B.307, 2G80.A.500, 2G9Z.B.701, 1GAG.A.201, 2GCQ.A.435, 2GGE.A.400, 4GIU.A.402, 2GL5.A.699, 4GME.C.501, 2GO7.A.207, 4GP2.A.401, 2GQS.A.241, 1GSI.A.1209, 1GUA.A.171, 4GYI.A.402, 4H19.A.405, 3H3X.Q.553, 2HCF.A.300, 2HCJ.A.998, 4HCH.A.405, 4HCL.A.401, 3HDG.A.202, 3HFW.A.361, 2HGS.A.502, 4HGQ.A.201, 4HHL.A.402, 3HIY.B.402, 2HJP.A.292, 4HNC.A.401, 4HNL.A.401, 4HPT.E.402, 4HQO.A.301, 3HVH.A.265, 3HVI.A.1, 3HVJ.A.265, 3HVK.A.1, 3HW3.A.999, 3HW4.A.999, 3HXX.A.445, 4I2B.A.604, 2I33.A.602, 4I3Z.A.302, 2I6K.A.302, 4IAD.A.402, 3IBA.A.401, 3ICK.A.401, 3ICK.A.402, 3ICK.A.403, 3ICM.A.402, 3ICZ.A.402, 4IEG.A.1001, 4IFW.A.502, 1IG5.A.78, 2IHU.A.601, 4IHC.A.501, 1II9.A.593, 3IIE.A.501, 3IJR.D.300, 4IJQ.A.304, 2IK4.A.287, 2IK4.B.288, 1IOW.A.331, 4IP4.A.503, 4IP5.A.502, 1IR3.A.301, 1ITZ.A.1001, 2IUC.A.1003, 1IV2.A.1571, 4IWH.A.401, 2IXE.A.2, 2IYN.C.1123, 1IZC.A.1001, 1J34.C.501, 1J7L.A.301, 2J7N.B.3374, 1J9J.B.301, 1JAH.A.168, 1JBW.A.998, 2JD4.B.4062, 1JGT.A.902, 2JI7.A.1567, 4JND.A.501, 1JSC.A.699, 1JUY.A.435, 3JVA.A.356, 3JVA.B.358, 4JX0.A.402, 3JYS.A.1, 3JYY.B.302, 1JZ7.A.3001, 3JZ0.A.300, 3K1S.H.107, 4K1W.A.501, 3K4Z.A.290, 4K6R.A.505, 3K9L.A.168, 1K9Y.A.402, 4K9Q.A.601, 1KA2.A.501, 3KB9.A.701, 4KCT.A.1001, 4KFU.A.307, 4KGD.A.702, 1KHZ.B.301, 1KHZ.B.310, 3KMW.A.501, 4KQX.A.405, 3KS6.C.251, 1KTG.A.503, 3KWS.B.401, 4KWD.A.403, 4KX5.A.314, 3L12.A.313, 4L2X.F.404, 1L8A.A.888, 4L80.A.403, 4L9W.A.202, 4L9Z.A.403, 4LA6.A.501, 2LCF.A.246, 4LFG.A.304, 1LNY.A.1453, 1LON.A.1454, 3LUF.A.301, 3LVO.A.264, 4LZ3.A.406, 3M07.A.595, 1MOW.A.502, 3M7I.A.901, 1MC1.A.601, 1MDL.A.360, 1MEZ.A.1453, 4MFG.A.201, 1MH1.A.201, 3MK2.A.903, 1MMA.A.998, 1MMN.A.998, 4MNE.A.902, 1MQ4.A.2088, 4MYO.A.301, 4MZU.C.404, 1N0H.A.699, 1N0H.B.1699, 3N07.A.200, 1N1Z.A.701, 1N20.A.701, 1N24.A.701, 1N24.A.702, 3N45.F.355, 3N4F.A.502, 1N8I.A.900, 4NFI.F.402, 4NFI.F.403, 4NFI.F.404, 3NJL.A.501, 3NOJ.A.239, 3NRJ.A.190, 1NSF.A.859, 1NSY.A.6241, 1NUX.A.2342, 2NXW.A.4002, 3NZG.A.507, 2O1S.A.1001, 3O61.B.202, 1O6Y.A.1280, 3O6Z.B.201, 2O7O.A.223, 4OAV.B.802, 3OCU.A.263, 3OCX.A.264, 2OCB.A.202, 4OCP.A.403, 2ODP.A.901, 2ODB.A.205, 3OE5.A.222, 2OEM.A.911, 2OEM.B.912, 2OFX.A.301, 2OGD.A.3003, 4OHY.A.502, 1OIW.A.1175, 1OKK.D.1002, 4OKM.A.901, 4OKM.A.903, 4OKZ.A.902, 4OKZ.A.903, 3OUZ.B.459, 3OZF.A.235, 3OZY.B.390, 2P3N.A.1758, 3P41.A.298, 3P5R.A.901, 3P93.C.406, 2PA4.A.325, 3PDE.A.311, 3PDE.A.312, 1PFK.A.327, 3PFF.A.831, 2PGN.A.610, 4PHG.A.201, 3PK7.A.406, 2PKO.A.502, 1POX.A.610, 1PT6.A.500, 3PUV.A.1501, 2PZA.A.6243, 3Q1O.A.400, 2Q5Q.A.4002, 1Q6L.A.5300, 1Q6R.A.7300, 3Q85.A.284, 1Q9S.A.201, 3Q9L.A.700, 4QEA.A.301, 2QGY.A.701, 2QJJ.C.1003, 3QKE.A.407, 3QKT.A.902, 2QME.A.179, 3QN3.A.601, 2QQ0.A.450, 2QQ0.B.452, 1QRA.A.168, 2QRZ.A.190, 2QTY.A.349, 2QTC.A.888, 3QUT.A.225, 3QVQ.C.310, 3QXH.A.223, 3QXX.A.224, 2QXO.A.162, 1R0X.A.13, 3R0U.A.380, 3R1M.A.404, 4R39.A.401, 3R6T.A.301, 3RBM.A.1001, 3RBM.A.1002, 3REG.A.550, 1RKU.A.301, 1RKV.A.401, 1RP7.A.890, 1RQI.A.603, 1RQJ.A.908, 3RUV.A.544, 1RVK.A.999, 3RYE.A.907, 3S9Z.A.802, 3SAZ.A.802, 1SAW.A.225, 3SBD.A.501, 3SDT.A.819, 3SDT.A.821, 3SEA.B.178, 3SJN.A.374, 3SLS.A.401, 3SN1.A.408, 3SN4.A.408, 1SO4.A.2300, 3STP.A.391, 3ST8.A.496, 3T1Q.A.198, 3T2D.A.408, 3T2E.A.409, 1T8Q.B.1602, 1T9B.A.1699, 1T9B.B.699, 1T9C.B.699, 3TCS.A.368, 3TDV.A.501, 1TE6.A.641, 3TJI.A.601, 3TKL.A.300, 4TMW.A.903, 3TMO.A.266, 4TSK.A.403, 1TW1.A.1, 3TWA.A.420, 3TWB.A.420, 3TYZ.A.281, 1TZZ.A.3501, 1U02.A.240, 3U2E.B.1, 4UAT.A.302, 3UCY.A.101, 3UIE.A.403, 1UMD.A.1401, 1UMG.A.403, 4UMF.A.1175, 4USI

.A.1151, 3UXK.A.360, 3UZR.A.300, 3V1V.A.501, 3V1X.A.501, 4V1T.A.1776, 3V2U.C.521
, 2V26.A.1801, 3V3W.A.403, 1V5F.A.1603, 1V8K.A.501, 1VA6.A.522, 3VC2.J.301, 3VCC
.A.402, 3VCN.A.501, 2VDO.B.2001, 2VDR.B.2001, 2VDL.B.2001, 2VDN.B.2001, 3VKB.A.7
01, 2VK4.A.601, 3VMK.B.402, 2VPR.A.1207, 3VPB.A.502, 3VR6.B.602, 3VVH.A.701, 3VX
4.A.802, 2VZB.A.1001, 1W2Y.A.1232, 1W6T.A.435, 3W6N.A.803, 3W6O.A.802, 1W7K.A.14
23, 3WBH.A.501, 1WC1.A.1501, 1WC1.A.1502, 3WEK.A.401, 2WEF.A.401, 4WF7.A.601, 3W
OO.A.502, 2WVG.A.601, 2WW8.A.1000, 1WZC.A.300, 1X07.A.900, 1X84.B.401, 2XB5.A.22
3, 1XEF.A.801, 1XEX.A.1002, 1XG3.A.2101, 2XH7.A.1441, 4XIA.A.399, 6XIM.A.395, 2X
JC.A.1499, 2XJE.A.1493, 2XSX.A.500, 2XTZ.A.1381, 2XUU.A.1307, 2Y6P.A.1234, 1Y8A.
A.501, 1Y8Q.B.641, 1Y9D.D.2901, 1YHL.A.1401, 1YHL.A.1402, 1YIO.A.212, 2YVO.A.100
1, 2YVP.A.183, 2YVM.A.1001, 2YXH.A.502, 1YYQ.B.701, 1YYQ.B.702, 1Z2O.X.1295, 1Z4
O.A.800, 1Z4P.X.1001, 1Z4Q.A.2001, 2Z4V.A.1501, 2Z7I.A.1301, 2ZCR.A.669, 2ZDH.A.
812, 2ZKJ.A.500, 3ZMC.A.1296, 3ZO9.A.1592, 3ZOU.A.1295, 1ZPD.A.601, 2ZPU.A.360,
3ZX4.B.260, 1ZXN.B.902

Table S31. 6-ligand Mg, compressed group

	size	largest_angle*	middle_1*	middle_2	middle_3	middle_4
1	"25"	"165.8+/-2.6"	"77.5+/-4.5"	"81.4+/-3.9"	"84.1+/-3.4"	"87.3+/-3.6"
2	"18"	"154.9+/-5.2"	"57.1+/-3.2"	"71.8+/-7.1"	"77.9+/-5.4"	"83.1+/-4.4"
3	"18"	"166.4+/-4.7"	"58.8+/-4.2"	"77.2+/-6.5"	"81.1+/-4.5"	"84.7+/-3.8"
4	"13"	"166.8+/-4.5"	"61.2+/-5.8"	"74.5+/-5.9"	"78.9+/-5.5"	"84.2+/-3.3"
5	"32"	"172.6+/-2.9"	"82.5+/-2.7"	"84.8+/-2.1"	"86.9+/-1.6"	"88+/-1.6"
6	"18"	"168.4+/-5.6"	"78.1+/-4.1"	"82+/-3.2"	"84.3+/-3.5"	"86.5+/-3.3"
7	"8"	"144.5+/-10.4"	"61.4+/-3.4"	"66.2+/-2.6"	"71+/-6.5"	"74.2+/-7.3"
8	"41"	"175.6+/-2.2"	"84.3+/-2.7"	"86.4+/-1.8"	"87.7+/-1.3"	"89.3+/-1.3"

	middle_5*	middle_6	middle_7	middle_8	middle_9*
1	"88.8+/-3.6"	"90.9+/-3"	"93.8+/-3.1"	"96.4+/-3"	"101.2+/-2.6"
2	"88.9+/-5.3"	"90.6+/-6"	"94.6+/-4.3"	"99.1+/-4.7"	"104.3+/-5.5"
3	"87.9+/-3"	"90.9+/-3.3"	"95.3+/-4.9"	"98.8+/-5.1"	"101.9+/-5"
4	"87.1+/-3.7"	"91.5+/-3.7"	"94.4+/-4"	"97.4+/-4.4"	"104+/-5.8"
5	"89.3+/-1.3"	"90.7+/-1.4"	"92.8+/-1.5"	"94.4+/-1.9"	"97.5+/-2.2"
6	"89.1+/-2.4"	"90.8+/-2.6"	"92.8+/-2.9"	"95.2+/-2.7"	"97.3+/-3.5"
7	"79+/-7.8"	"87.6+/-11.2"	"91.8+/-11.4"	"98+/-9.4"	"108.6+/-6.2"
8	"90.2+/-1.2"	"91.2+/-1.1"	"92.7+/-1.7"	"94+/-1.7"	"96.6+/-2.1"

	middle_10	middle_11	middle_12	middle_13*
1	"104.2+/-3.9"	"110.9+/-9"	"148.4+/-9.7"	"158.8+/-2.4"
2	"113.3+/-5.8"	"120.5+/-8.4"	"140.3+/-6.2"	"148.6+/-4.5"
3	"106.4+/-4.1"	"116.7+/-9.2"	"142.7+/-11"	"161.1+/-6.1"
4	"110.9+/-6.7"	"121.1+/-10.8"	"140.2+/-9.5"	"155.5+/-6.1"
5	"102+/-3.1"	"110.9+/-7.2"	"150.1+/-8.2"	"166.5+/-2.8"
6	"102.3+/-5.4"	"120.7+/-7.4"	"142.3+/-7.2"	"148.5+/-4"
7	"122+/-9"	"127.7+/-7.3"	"130+/-7.4"	"136.6+/-8.6"
8	"100.5+/-2.7"	"107.9+/-4.3"	"151.2+/-4.4"	"158.7+/-2.9"

	smallest_opposite_angle*	Octahedral	TrigonalPrismatic
1	"57.3+/-3.7"	"0.098"	"0"
2	"75.5+/-7.4"	"0"	"0"
3	"79.6+/-3.9"	"0.039"	"0"
4	"63+/-4.4"	"0"	"0"
5	"59.3+/-2.3"	"0.343"	"0"
6	"55.2+/-3"	"0.011"	"0"

```
7 "62.5+/-6.7"               "0"        "0"
8 "58.8+/-1.9"               "0.342"    "0"
  PentagonalBipyramidalVA PentagonalBipyramidalVP
1 "0"                     "0.016"
2 "0"                     "0.006"
3 "0"                     "0.014"
4 "0"                     "0"
5 "0"                     "0.075"
6 "0"                     "0.06"
7 "0"                     "0"
8 "0"                     "0.166"
```

Table S32. Cluster members of 6-ligand Mg, compressed group

```
[1] "Cluster 1"
4BDZ.A.1381, 4IRC.A.402, 3OYJ.A.397, 3S3N.A.397, 3SM4.E.15, 3A7E.A.215, 3BZN.A.5
01, 1DAW.A.342, 1DAY.A.342, 4DHP.A.303, 1EFK.A.604, 3ETH.A.401, 3ETJ.A.402, 3HWO
.A.1702, 3I4K.A.385, 4K10.D.404, 1KJI.A.393, 2PYW.A.500, 1PYX.A.1003, 1T5T.A.100
5, 4TY0.A.501, 4UOP.A.1612, 2VPQ.A.1450, 3WNZ.A.503, 3WQQ.A.502
[1] "Cluster 2"
2G8H.A.301, 2VBL.C.1026, 1AO0.A.469, 3G15.A.605, 4GMJ.B.302, 4GYZ.I.402, 2IOA.B.
5003, 1KFS.B.2, 4KMQ.A.1103, 2LVJ.A.101, 3MLE.A.222, 3P0W.A.471, 3RUW.D.544, 4S1
7.A.501, 3SH1.A.222, 4UM9.B.2001, 2W8D.A.1636, 3WQS.B.502
[1] "Cluster 3"
4BE1.A.1382, 4D6N.A.1188, 4E4F.A.504, 3FDG.A.357, 4GME.A.501, 3IJQ.B.386, 1JCT.A
.498, 3K5H.A.401, 3K5H.A.402, 4ORK.A.502, 3PMG.A.562, 3Q8U.E.159, 3U2E.B.4, 3U7F
.B.1, 2V0N.A.601, 4WB8.A.402, 1WC6.C.2202, 3WO0.A.503
[1] "Cluster 4"
4IEM.C.401, 2A9F.A.801, 3C41.J.603, 3CVJ.C.243, 3M00.A.551, 3N3T.B.803, 4P9D.A.2
02, 3PCR.B.1231, 1POW.B.610, 2PUI.B.401, 4RUB.B.491, 1VA6.A.523, 3WBH.B.503
[1] "Cluster 5"
4BE0.A.1381, 4NCB.C.101, 4O3S.A.503, 3OYB.A.397, 3OYD.A.397, 3OYE.A.397, 3OYL.A.
397, 3OYN.A.397, 1RVC.A.401, 1YTU.A.428, 4C5A.A.330, 4C5B.A.1313, 4C5C.A.1313, 3
DG6.A.2001, 2FN1.A.504, 4H2H.A.401, 1IOW.A.330, 2IO8.A.7001, 4IZG.A.414, 4J1O.A.
401, 1J7L.A.302, 4M3A.A.402, 3NA5.A.547, 1NFS.A.401, 4OAV.B.803, 3OLP.A.547, 3PF
R.A.456, 4PFY.A.602, 4QXD.B.301, 3R75.A.700, 3T2D.A.411, 1UMG.A.401
[1] "Cluster 6"
1MOW.A.373, 1MUH.A.479, 4ACF.A.1481, 3AU9.A.601, 4AZW.A.1453, 4EOM.A.302, 2FUV.A
.901, 4IAC.A.401, 2J0L.A.1688, 1KJI.A.394, 1KJJ.A.394, 4NZN.A.403, 4OHF.B.503, 2
ONS.A.702, 4OVN.A.202, 3V4S.B.402, 1W7V.A.1441, 3WQR.A.502
[1] "Cluster 7"
2ATX.A.201, 2EWG.A.3002, 1GUS.A.1069, 4GWS.A.402, 2J1L.A.1195, 3LDO.5.54, 4NH0.A
.1402, 3PLS.A.1
[1] "Cluster 8"
2AL1.B.438, 3CFX.A.703, 4DFX.E.404, 1EC8.A.498, 3ETH.A.402, 1EYZ.A.401, 1EYZ.A.4
02, 3GN6.A.321, 3GQ8.A.692, 2HGS.A.501, 3HPF.A.401, 4IAD.A.401, 3KAL.A.502, 1KJ8
.A.393, 1KJ8.A.394, 1KJ9.A.393, 1LP4.A.341, 3LVV.A.696, 4MDB.A.402, 4O4D.A.402,
1P43.A.438, 4PU5.A.501, 4Q4C.A.403, 2QVH.A.401, 3T7A.A.601, 3TDW.A.502, 3TM0.A.2
65, 3UJR.B.501, 3UJS.A.600, 3UJS.B.600, 3VA8.A.425, 3VAT.A.502, 3VC6.A.501, 2VPQ
.A.1451, 1WOH.A.1000, 4WB8.A.403, 4WH2.A.403, 4WH3.A.403, 2XH0.A.1439, 1Z2O.X.12
96, 2Z4X.B.1206
```

Table S33. 6-ligand Mg, combined group

	size	largest_angle*	middle_1*	middle_2	middle_3	middle_4
1	"295"	"173.5+/-3"	"81.7+/-2.7"	"84.3+/-2.1"	"86.1+/-1.9"	"87.8+/-1.8"
2	"66"	"156.1+/-8.8"	"62.4+/-6.4"	"71.3+/-6"	"76+/-5.3"	"80.2+/-5.3"
3	"193"	"167.8+/-4.8"	"71.3+/-5.5"	"78.2+/-3.8"	"81.9+/-3.3"	"84.6+/-2.9"
4	"362"	"172.2+/-2.9"	"77.2+/-3.6"	"82.1+/-2.7"	"84.9+/-2.1"	"86.9+/-1.8"
5	"778"	"176.2+/-2"	"83.9+/-2.1"	"85.8+/-1.6"	"87.2+/-1.3"	"88.3+/-1.1"
6	"149"	"171.7+/-4.7"	"80.6+/-5"	"84+/-3.3"	"86+/-2.8"	"88+/-2.4"

	middle_5*	middle_6	middle_7	middle_8	middle_9*
1	"89.3+/-1.5"	"90.7+/-1.4"	"92.3+/-1.6"	"93.8+/-1.7"	"95.7+/-2"
2	"84.5+/-5.7"	"88.4+/-5.9"	"92.7+/-5.7"	"98.3+/-5.7"	"105.5+/-6.7"
3	"87.4+/-2.7"	"90.3+/-2.9"	"93.2+/-2.9"	"96.3+/-3.2"	"100+/-3.4"
4	"88.5+/-1.7"	"90.4+/-1.7"	"92.2+/-1.8"	"94.3+/-1.8"	"96.7+/-2"
5	"89.3+/-1"	"90.3+/-1"	"91.2+/-1.1"	"92.3+/-1.2"	"93.7+/-1.5"
6	"89.5+/-2.1"	"91.2+/-2"	"93.2+/-2.2"	"95.1+/-2.4"	"98.1+/-2.8"

	middle_10	middle_11	middle_12	middle_13*
1	"98.5+/-2.8"	"104+/-5.3"	"162.5+/-4.7"	"168.5+/-3.2"
2	"114.3+/-7.3"	"123.2+/-7.8"	"138.6+/-7.9"	"147+/-6.8"
3	"104.7+/-3.8"	"112.5+/-7.1"	"151.9+/-6.9"	"159.7+/-3.9"
4	"99.7+/-2.8"	"104.3+/-4.4"	"162.4+/-4.6"	"168+/-2.8"
5	"95.4+/-1.9"	"98.3+/-3"	"170+/-3.3"	"173.5+/-2.2"
6	"101.8+/-3.7"	"110.9+/-8.5"	"150.1+/-8.2"	"160+/-5.9"

	smallest_opposite_angle*	Octahedral	TrigonalPrismatic
1	"75.8+/-3.4"	"0.277"	"0"
2	"70.3+/-8.1"	"0"	"0.012"
3	"75+/-5"	"0.004"	"0"
4	"82.1+/-2.4"	"0.223"	"0"
5	"84.2+/-2.3"	"0.738"	"0"
6	"59.7+/-4.4"	"0.006"	"0"

	PentagonalBipyramidalVA	PentagonalBipyramidalVP
1	"0"	"0.001"
2	"0"	"0.012"
3	"0"	"0.006"
4	"0"	"0"
5	"0"	"0"
6	"0"	"0.004"

Table S34. Cluster members of 6-ligand Mg, combined group

[1] "Cluster 1"
4AQX.D.1526, 4BE2.A.1381, 4C2U.A.1666, 4CEI.B.2162, 1CWO.N.202, 1CWO.A.203, 4DL4
.A.501, 4DLG.A.902, 2DPI.A.871, 4DQI.A.901, 2EZ6.A.501, 1FIU.I.2222, 1G9Z.C.902,
 1G9Z.F.903, 2ISO.A.339, 3JPN.A.339, 4LOX.A.401, 3M8S.A.2, 3MBY.A.339, 1MOW.D.37
4, 4NCB.B.703, 4O5K.A.401, 3OYA.A.397, 3OYC.A.396, 3OYH.A.396, 4PQU.A.602, 3RJF.
A.340, 3S3M.A.397, 3SI8.A.451, 1T7P.A.4001, 3TFS.A.340, 1TK0.A.991, 4UB3.A.401,
4A01.A.1769, 3A1U.A.6, 4AC0.A.1205, 2AE8.C.1009, 2AG0.A.601, 1AJB.A.452, 1ALK.B.

452, 4AUX.A.223, 3B05.D.1001, 3BGA.A.1, 2BJI.A.2277, 3BM4.B.304, 3BRB.A.10, 2BVN
.B.1395, 3BWY.A.300, 2C31.A.1553, 4C5C.A.1314, 4C7X.A.700, 1CG1.A.435, 1CG4.A.43
5, 3CG4.A.201, 1CIB.A.434, 1CJT.C.403, 1CJU.A.582, 3CK5.A.400, 2CL5.A.1216, 3CMR
.A.453, 3CP6.A.503, 3CRR.A.324, 3CT2.A.401, 3CV2.A.1, 4CW7.A.1002, 3CXO.A.500, 3
CXO.B.500, 4CYM.A.1199, 3CZJ.B.3001, 3D46.A.501, 3D47.A.501, 2DGN.A.1454, 4DH5.A
.402, 3DUF.A.1368, 2DUA.A.292, 3DYH.B.4002, 3DYS.A.902, 1E4E.A.365, 3E84.A.701,
4E8G.A.402, 2E91.A.1302, 2E95.A.1302, 4EA0.A.301, 1EBH.A.438, 2EB5.A.1001, 3EGT.
A.3002, 3EGT.A.3004, 3EKG.A.601, 1ELZ.A.452, 3ETJ.A.401, 1EXM.A.407, 3EZ3.B.1102
, 3EZ3.B.1104, 1F2U.A.902, 4F71.A.301, 2F9M.A.1201, 4F9A.A.602, 3FE4.B.902, 4FFL
.A.906, 3FIU.A.5002, 3FPA.C.901, 3FV9.A.501, 3FYY.A.402, 1G4P.A.2003, 3G5A.B.307
, 2G9Z.B.701, 4GME.C.501, 2GT4.B.401, 4GT3.A.403, 3GY1.A.500, 4GYI.A.402, 3H3X.Q
.553, 4HCL.A.401, 4HE1.A.403, 2HGS.A.502, 3HJN.A.501, 2HJP.A.292, 4HNC.A.401, 3H
PF.A.402, 3HVH.A.265, 3HVI.A.1, 3HVJ.A.265, 3HVK.A.1, 3HW3.A.999, 2HXU.A.601, 4I
3Y.A.304, 1I6I.A.501, 2I6K.A.302, 3ICK.A.403, 3ICM.A.402, 3ICZ.A.402, 4IEG.A.100
1, 4IHC.A.501, 1II9.A.593, 3IJR.D.300, 1IR3.A.301, 1IV4.A.1572, 1J34.C.501, 1J7L
.A.301, 2JD4.B.4062, 2JI7.A.1567, 1JP4.A.701, 1JP4.A.702, 1JSC.A.699, 3JVT.B.502
, 4K33.A.802, 3K9L.A.168, 4KCT.A.1001, 4KCU.A.1001, 3KDN.A.500, 3KEU.A.400, 1KHZ
.B.301, 3KHQ.A.1, 1KKR.A.501, 4KQX.A.406, 3LOY.A.257, 4L80.A.403, 4L9W.A.202, 4L
9Y.B.403, 1LNY.A.1453, 4M6U.A.401, 3M7I.A.901, 1MEZ.A.1453, 1MMA.A.998, 1MMN.A.9
98, 1MNS.A.360, 4MPO.A.206, 4MPO.C.205, 1MX0.A.501, 4MYO.A.301, 4MZU.C.404, 1NOH
.A.699, 1NOH.B.1699, 1N20.A.701, 1N24.A.701, 3N45.F.355, 1NSY.A.6241, 3NZG.A.507
, 4NZO.A.404, 2O1O.A.503, 2OCB.A.202, 3OE5.A.222, 2OEM.B.912, 1OIX.A.301, 4OKM.A
.901, 4OKM.A.902, 4OKZ.A.902, 1ORK.A.223, 2OUN.A.403, 1OW2.A.401, 1OZH.A.1405, 3
OZM.D.390, 3OZY.B.390, 3P3B.A.393, 3P41.A.297, 3P93.A.406, 2PLS.H.602, 2PMQ.A.90
2, 1PT6.A.500, 1PUN.A.130, 4PU5.A.502, 1PYD.A.559, 1PYM.A.1003, 3Q1O.A.400, 2Q1D
.X.294, 4Q1V.A.803, 3Q30.A.600, 3Q30.A.601, 1Q6L.A.5300, 1Q6O.A.7300, 1Q6Q.A.730
0, 1Q6R.A.7300, 3Q85.A.284, 1Q9S.A.201, 4QEA.A.301, 1QF4.A.433, 2QJJ.C.1003, 3QK
E.A.407, 3QKT.A.902, 1QMZ.A.383, 3QPE.A.393, 3QPE.B.393, 2QTY.A.349, 3QXH.A.223,
2QX0.A.162, 3R1M.A.402, 3R1M.A.403, 3R1M.A.404, 3R25.A.402, 3R6T.A.301, 2RDX.A.
378, 4RJK.H.602, 1RKU.A.301, 4RN3.A.301, 1RQJ.A.907, 3RUV.A.544, 3S9Z.A.802, 3SA
Z.A.802, 3SBD.A.501, 3SBF.A.402, 3SDT.A.819, 3SEA.B.178, 3SN1.A.408, 3T6C.A.501,
1T9B.B.699, 1TE6.A.640, 4TQD.A.502, 4TSK.A.403, 1TW1.A.1, 3TW6.C.2002, 3TWB.A.4
20, 3TZF.A.279, 1TZZ.A.3501, 1UMD.A.1401, 4USJ.C.302, 3UZR.A.300, 3V1V.A.501, 3V
4B.A.403, 1V5F.A.1603, 1V5G.A.1603, 1V8K.A.501, 2VBV.A.1136, 2VDO.B.2001, 2VDR.B
.2001, 2VDL.B.2001, 3VKB.A.701, 2VK4.A.601, 3VMK.A.402, 3VMK.B.402, 2VPR.A.1207,
3VX4.A.802, 1W6T.A.435, 3WBZ.A.403, 3WDL.B.902, 3WEK.A.401, 2WEF.A.402, 4WK0.B.
501, 2XCL.A.480, 1XEF.A.801, 1XG3.A.2101, 4XIA.A.399, 6XIM.A.395, 2XIM.A.395, 2X
JE.A.1493, 2XZW.A.202, 1Y8Q.B.641, 2YVP.A.183, 1YVE.I.601, 1Z2O.X.1295, 1Z5C.A.2
001, 2Z7H.A.1301, 2ZDH.A.812, 2ZKJ.A.500, 1ZPD.A.601, 1ZXN.B.902
[1] "Cluster 2"
4D6N.F.1197, 4G7O.D.2003, 2G8H.A.301, 3LK9.A.340, 4M2Z.A.501, 3R7P.A.317, 2VBL.C
.1026, 3A58.B.401, 1AO0.A.469, 2ATX.A.201, 4AZW.A.1453, 3C41.J.603, 4CZK.A.1335,
3DYG.A.3002, 1E2A.C.106, 2EWG.A.3002, 3FFU.B.155, 3FHY.B.404, 4FHX.A.402, 2FOL.
A.202, 3G15.A.605, 3G9D.A.298, 4GMJ.B.302, 1GUS.A.1069, 4GWS.A.402, 4GYZ.I.402,
1H65.A.282, 3HDG.E.204, 3HWX.A.602, 4IFW.A.503, 3IJQ.B.386, 2IOA.B.5003, 2IUT.A.
1724, 2J1L.A.1195, 1KFS.B.2, 4KMQ.A.1103, 3KUD.A.171, 3LD0.5.54, 2LVJ.A.101, 1MB
Z.A.604, 3MLE.A.222, 3N3T.B.803, 4NHO.A.1402, 2NOM.A.401, 1NV7.A.3341, 4OGE.A.12
04, 2OPM.A.907, 3P0W.A.471, 4P9D.A.202, 3PCR.B.1231, 3PLS.A.1, 3PP1.A.410, 2PUI.
B.401, 3RUW.D.544, 4RUB.B.491, 4S17.A.501, 3SH1.A.222, 1T5S.A.1005, 3TXA.A.801,
3U7F.B.1, 3UGV.A.500, 4UM9.B.2001, 2W8D.A.1636, 3WBH.B.503, 3WQS.B.502, 2Y4M.A.4
00
[1] "Cluster 3"
4AAB.B.1157, 2AGQ.A.4002, 4BDY.A.1381, 4BE1.A.1382, 4D6N.A.1188, 4D6O.D.1196, 4D
F8.A.903, 4DFP.A.901, 4F5P.A.401, 4IRD.F.903, 3LK9.A.339, 4M30.A.501, 4M30.A.502

, 4MFF.A.401, 4NCB.A.702, 3OYC.A.397, 3OYE.A.396, 2OZM.A.904, 1QTM.A.1001, 3S3M.A.396, 3SI6.A.905, 1SKR.A.4001, 3SPY.A.904, 1TK8.A.901, 2XCP.A.1004, 2XCA.A.3000, 2XCA.A.3001, 1YVP.A.1001, 1ZBL.A.202, 1ZBL.B.204, 3A06.A.500, 4A01.A.1770, 2A9F.A.801, 2AG1.B.611, 3AJO.A.183, 3AXK.A.478, 1AZS.C.403, 1B7T.A.836, 3B8I.A.289, 3BNY.D.701, 1BWV.C.490, 2BW7.A.2201, 4BYF.C.1000, 4CE0.A.1251, 1CG0.A.435, 4CTA.B.401, 3CWH.A.392, 4D2I.A.1478, 3DVA.A.1368, 2DW6.D.2004, 2E0A.A.500, 4E4F.A.504, 2E8W.A.1202, 1ECQ.A.498, 1EFL.B.1604, 3EG5.A.180, 2EGH.A.900, 3EYA.H.613, 1F1Z.A.2002, 3F78.C.1, 3FD5.A.396, 3FDG.A.357, 3FFU.A.156, 4FFL.A.904, 4FFL.A.905, 4FFO.A.904, 2FG5.A.301, 3FLK.A.405, 3FPB.A.1000, 3FQI.A.1000, 3FTQ.A.371, 1G3B.A.501, 2G4J.A.392, 2G9Z.A.704, 4GA3.A.1004, 4GME.A.501, 4GOK.B.202, 3HDG.B.201, 3HQP.B.502, 4HYV.A.1001, 4I2B.A.602, 3I3O.A.306, 1IGW.A.441, 2IK2.B.289, 2IK7.A.287, 2IOA.B.5004, 4IOK.A.604, 4IX4.A.602, 2J5X.A.200, 1JCT.A.498, 2JI8.A.1567, 3K5H.A.401, 3K5H.A.402, 4KCW.A.1001, 1KH7.A.452, 1KHK.A.452, 4KI8.E.602, 1KP8.A.550, 3KR4.C.1004, 3KRO.D.3002, 4KS0.A.1001, 4L2X.F.403, 4L2X.F.405, 4LF1.A.801, 4LNI.E.505, 3M00.A.550, 3M00.A.551, 1M1B.B.999, 3M1Y.A.300, 1MB9.B.601, 1MBZ.A.603, 1MC1.A.603, 3MQT.H.626, 1MRS.A.300, 4N0G.A.403, 1N20.A.703, 1NHT.A.435, 2NOM.A.402, 1NUW.A.2498, 2O1O.A.501, 3OES.A.202, 4OKZ.A.901, 3OPS.A.500, 3OPS.A.501, 2OQY.A.401, 4ORK.A.502, 1OVM.A.601, 4OVN.A.204, 3OYZ.A.500, 3P0X.A.430, 1PFK.A.325, 2PLS.G.603, 3PMG.A.562, 1POW.B.610, 6Q21.D.173, 2Q58.A.5, 3Q8U.E.159, 1QC5.A.601, 1QF5.A.433, 3QQV.A.382, 1QS0.A.501, 2QTV.B.210, 3QU4.A.225, 4RAD.D.302, 1RC5.A.761, 3RIM.A.1001, 4RJJ.A.602, 4RNH.A.1501, 4ROP.A.504, 1S1C.A.300, 3SE1.A.182, 3SH6.A.176, 1SOJ.A.2123, 3SSN.A.501, 3T0Z.A.401, 4TQ4.A.401, 4U0M.A.503, 3U2E.B.4, 3UJ2.A.431, 3UXL.A.360, 2V0N.A.601, 2V3W.A.1528, 1VA6.A.523, 2VBI.A.1000, 2VDM.B.2001, 3VMM.A.501, 2VQD.A.1449, 2VWT.A.301, 3VYT.C.602, 3W7F.A.303, 1W88.C.1368, 1WC6.C.2202, 4WK2.B.501, 4WK4.B.501, 1WL6.A.801, 3WO0.A.503, 2WX5.L.1282, 2X5Z.A.602, 2XAM.B.1030, 2XH4.A.1439, 5XIM.A.395, 1XLC.A.399, 1XZ8.A.180, 1Y9I.A.601, 1YHM.B.1401, 1YHY.A.699, 2YWF.A.701, 1Z0K.A.1201, 2Z4W.A.1302, 2Z4Y.B.1301, 3ZDY.B.2001, 2ZVJ.A.300, 3ZXW.A.476
[1] "Cluster 4"
3A4K.A.301, 2AQ4.A.302, 4AQX.D.1525, 2BCV.A.576, 4BDY.A.1380, 4CEI.A.2234, 4D6N.F.1196, 4DF4.A.901, 4DFJ.A.902, 4DOA.A.401, 4DQI.D.901, 4EEY.A.502, 3F2B.A.5, 1FIU.A.5555, 2FMS.A.340, 3GDX.A.347, 3GDX.A.348, 3GPL.A.800, 3GQC.B.203, 4IR9.F.402, 2ISP.A.339, 2IS4.A.1001, 3JPR.A.339, 3JPT.A.339, 4JWM.A.403, 3K57.A.1001, 4K97.A.603, 4K98.A.602, 4K99.A.602, 4M47.A.402, 3M8R.A.2, 3MQY.A.500, 3MR5.A.435, 4O3N.A.503, 4O3Q.A.503, 3OS0.A.394, 3OYA.A.396, 3OYB.A.396, 3OYD.A.396, 3OYF.A.396, 3OYF.A.397, 3OYG.A.397, 3OYH.A.397, 2OZS.A.904, 2PFN.A.950, 4PGQ.A.400, 3PML.A.2, 4QCL.A.1302, 1QSY.A.1001, 4R8U.B.402, 3RJK.A.339, 3RJK.A.340, 4RNN.A.503, 3SV3.A.836, 3TI0.A.1, 4TUQ.A.402, 3TWH.A.401, 3UQ2.A.1, 3V6H.A.402, 2W35.A.1224, 4A01.A.1771, 3A1U.A.5, 1A49.H.5334, 3A7D.A.300, 1A82.A.901, 4ABZ.A.1210, 2AUT.D.604, 3B1X.A.301, 1B4N.A.623, 1B8C.A.308, 2BKU.A.221, 4BX3.A.301, 1BZY.A.901, 2C3P.A.2237, 2C42.A.3238, 2C4N.A.1251, 4C5A.B.331, 3CB3.A.501, 3CFX.A.704, 1CH8.A.434, 2CJE.A.1268, 2CLS.A.550, 3CP6.A.501, 1CUL.C.396, 2D0O.A.1005, 1DAK.A.901, 4DBH.A.401, 2DCN.A.4001, 2DEI.A.402, 3DFY.A.401, 4DFD.B.301, 3DHD.A.502, 1DIE.A.399, 4DN1.A.401, 4DN1.B.401, 3DOE.A.193, 1DQN.A.451, 1DTW.A.401, 2DW6.A.2001, 4DWB.A.508, 1DXE.B.901, 4DXJ.A.401, 4DXJ.A.402, 4DXJ.A.403, 3DYG.A.3004, 3DYH.A.3003, 4E1E.A.403, 1E9I.A.1431, 2E91.A.1301, 4EA0.A.302, 4EA0.A.303, 1EBG.A.438, 1EBG.A.439, 2EB1.A.502, 1EC7.A.498, 1EC9.A.498, 1ECB.A.507, 3EFQ.A.3003, 3EFQ.B.4002, 3EFQ.B.4004, 3EGT.A.3003, 3EQI.A.3, 3ES8.A.393, 1F8I.A.451, 3FA5.A.282, 3FD5.A.395, 3FD5.A.397, 3FD5.B.397, 3FD6.B.395, 3FD6.B.397, 4FFR.A.403, 4FI4.A.501, 2FN0.A.701, 2FPR.A.503, 2FRV.B.540, 1FTN.A.300, 4FVR.A.902, 4G61.A.302, 2G9Y.A.452, 1GAG.A.201, 2GCQ.A.435, 2GGE.A.400, 4GIS.A.405, 4GIU.A.402, 2GO7.A.207, 4GP2.A.401, 2GQS.A.240, 2GQS.A.241, 2GQ3.A.1000, 1H1D.A.300, 4H19.A.405, 4H1Z.A.401, 3H4L.A.701, 2HCJ.A.998, 4HCH.A.405, 4HE1.A.404, 4HE2.A.405, 4HGQ.A.201, 4HGR.A.201, 4HHL.A.402, 3HIY.B.402, 3HQD.A.501, 4HQO.A.301, 2HWG.A.901, 3HXX.A.445, 2I33.

A.602, 4I3Y.A.302, 4I3Z.A.302, 3I6E.A.386, 3IBA.A.401, 3IBA.A.402, 3ICK.A.401, 3ICK.A.402, 3ICZ.A.401, 2IDX.A.603, 4IEE.A.501, 4IFW.A.502, 2IHU.A.601, 1II0.A.593, 3IIE.A.501, 4IJQ.A.304, 2IK2.B.287, 2IK2.B.290, 4IP5.A.502, 4IT1.A.501, 2IUC.B.1008, 4IWH.A.401, 2IXE.A.2, 1J9J.B.301, 1JAH.A.168, 2JCS.A.1211, 1JGT.A.902, 2JI6.A.1567, 4JND.A.501, 3JUK.C.307, 1JUY.A.435, 3JVA.A.356, 4JX0.A.402, 3JYS.A.1, 4K1W.A.501, 3K4Z.A.290, 1K9Y.A.403, 1K9Y.A.402, 4K9N.A.601, 1KA2.A.501, 4KCV.A.1001, 1KEK.A.2237, 4KFU.A.307, 1KHZ.B.310, 4KMQ.A.1102, 1KO5.A.1001, 4KQX.A.405, 3KS6.C.251, 4KUX.A.701, 3KWS.B.401, 4KWD.A.403, 4KX3.A.302, 4KX5.A.314, 4L2X.F.404, 1L8A.A.888, 4LA6.A.501, 4LA7.B.601, 1LON.A.1454, 3LV0.A.264, 3LVV.A.695, 3LVV.A.697, 4LZ3.A.406, 1M0W.A.502, 4M69.A.403, 1MC1.A.601, 1MDL.A.360, 4MFG.A.201, 1MH1.A.201, 4MIT.A.202, 4MPO.B.204, 4MPO.E.201, 1N1Z.A.702, 1N24.A.703, 3N3T.A.802, 3N4F.A.502, 1N8I.A.900, 4NEH.B.703, 4NFI.F.404, 3NJL.A.501, 4NM3.A.405, 3NO1.A.397, 1NUW.A.2497, 1NUX.A.2342, 1NUY.A.2343, 2O1S.A.1001, 4O4D.A.401, 3O61.B.202, 2O7O.A.223, 1OAD.A.392, 4OAV.B.802, 2ODB.A.205, 2OEM.A.911, 1OFH.B.453, 3OUZ.B.459, 2OX4.A.402, 1OZF.A.699, 3OZF.A.235, 2P3N.A.1758, 3P41.A.298, 3P5R.A.901, 1P7T.A.1000, 1P9B.A.1600, 2PA4.A.325, 4PAL.A.110, 3PDE.A.311, 1PFK.A.327, 4PFK.A.327, 2PGN.A.610, 3PK7.A.406, 2PK0.A.502, 2PLS.J.604, 2PP3.A.901, 3PUV.A.1501, 3Q46.A.305, 2Q5Q.A.4002, 3Q9L.A.700, 1QC5.B.602, 4QEH.A.402, 2QGY.A.701, 3QN3.A.601, 4QPM.A.1502, 2QQ0.A.450, 2QQ0.B.452, 2QTC.A.888, 1R0X.A.13, 3R0U.A.380, 4R39.A.401, 2R9V.A.504, 3RBM.A.1001, 3RBM.A.1002, 3RBM.D.1003, 3REG.A.550, 1RKV.A.401, 1RQI.A.603, 1RQI.A.605, 1RQJ.A.908, 3S9I.A.743, 3SAD.A.801, 3SB0.A.801, 3SOP.A.401, 3ST8.A.496, 3T1Q.A.198, 3T2E.A.409, 3T8O.A.564, 1T8Q.B.1602, 1T9B.A.1699, 1T9C.B.699, 1TND.A.352, 3TTE.A.361, 3TTE.B.361, 3U2E.B.1, 3UIE.A.403, 4UMF.A.1175, 4USI.A.1151, 3UXK.A.360, 4V1T.A.1776, 4V1T.A.1778, 2V5K.A.301, 1VA6.A.522, 1VA6.A.524, 3VD3.A.3001, 3VPB.A.502, 2VP0.A.1209, 3VR6.B.602, 2VZB.A.1001, 2W00.B.1894, 1W2Y.A.1232, 3W2W.A.904, 1W5T.B.701, 1W7K.A.1423, 4WB8.A.402, 1WC1.A.1502, 1WDD.A.1476, 2WEF.A.401, 4WF7.A.601, 3WO0.A.502, 4WRR.A.401, 2WW8.A.1000, 1WZC.A.300, 1X07.A.900, 5XIN.A.395, 1XIN.A.395, 8XIM.A.395, 2XJC.A.1499, 2XTZ.A.1381, 1YHL.A.1401, 1YIO.A.212, 1YVE.I.602, 2YXH.A.502, 1YYQ.B.701, 3ZCB.A.301, 2ZCR.A.669, 3ZO9.A.1592, 3ZOU.A.1295, 2ZPU.A.360, 1ZVW.A.4001, 3ZYC.A.1750
[1] "Cluster 5"
4BE0.A.1380, 4BE1.A.1381, 4BE2.A.1380, 1BPY.A.339, 4BWJ.A.1834, 4DFK.A.902, 4DFM.A.902, 4DO9.A.401, 4DOB.A.401, 4DOC.A.401, 4DQP.D.902, 4ELT.A.902, 4ELU.A.902, 4F5O.A.402, 4FO6.A.601, 3G6Y.A.871, 4GZ2.B.402, 3IEV.A.400, 2JOS.A.1412, 1JJ2.0.8010, 3JPQ.A.339, 3JPS.A.339, 3K58.A.1001, 3K59.A.1001, 3KD5.E.916, 4KLI.A.401, 4MO4.A.702, 4MO4.A.703, 4M8O.A.1302, 4M9L.A.404, 4MDE.A.1002, 3MFI.A.515, 4MFC.A.401, 4O3O.A.502, 4O3O.A.503, 4O3Q.A.502, 3OHA.A.518, 3OJS.A.7, 3OYG.A.396, 4P4M.A.402, 2PFP.A.750, 3PNC.A.576, 4PUQ.B.401, 4Q8E.A.502, 4QM6.A.1002, 4R65.A.402, 3RJH.A.403, 3RTV.A.833, 3SM4.B.227, 3SNN.A.905, 3SPY.A.903, 1SUZ.A.402, 3TFR.A.339, 3TFS.A.339, 3TIO.D.2, 4UAY.A.402, 1W7A.A.1802, 1XSN.A.576, 1ZJN.A.339, 3ZVM.A.1526, 121P.A.168, 3A0T.A.800, 4A01.A.1767, 1A2B.A.550, 3A4L.A.401, 2A5Z.A.701, 2A5D.A.1231, 4A6X.A.350, 3A99.A.401, 4ACF.A.1482, 4ACI.A.1187, 2AFK.E.1291, 3AHC.A.826, 3AHD.A.826, 3AHE.A.826, 3AHG.A.826, 2AL1.B.439, 4ANB.A.1384, 1AOR.A.609, 1AOX.A.400, 1ASO.A.356, 4AS2.A.1328, 2AUU.A.203, 2AUU.A.204, 2AUT.A.601, 3AYX.A.701, 2B0T.A.800, 3B1V.A.301, 1B25.A.800, 4B2P.A.1351, 2B56.A.488, 3B7L.A.907, 3B7L.A.908, 3B7L.A.909, 1B8J.A.452, 2B82.A.1013, 2B9H.A.700, 4BAS.A.1183, 3BB1.A.282, 2BBS.A.3, 3BC1.A.194, 2BEK.A.501, 2BME.A.1184, 3BN3.A.1, 1BOF.A.800, 2BON.A.1302, 2BU2.A.1388, 2BVC.A.504, 3BWV.A.300, 4BW9.A.501, 4BWR.A.1468, 4BX0.A.1291, 1BYQ.A.1001, 1C1Y.A.171, 2C18.A.1338, 2C3U.A.2238, 3C4Z.A.563, 3C5H.A.302, 2C5L.A.1168, 4C5B.A.1314, 2C77.A.1407, 2C78.A.1407, 3C9U.B.312, 2CBZ.A.1872, 2CFS.A.1296, 1CHN.A.200, 2CHE.A.130, 1CIP.A.356, 2CJE.A.1267, 2CK3.A.601, 2CK3.F.601, 2CN5.A.1506, 4COK.A.601, 3CP6.A.502, 3CR3.A.1212, 1CTQ.A.168, 3CUR.H.553, 3CUS.Q.553, 3CX8.A.378, 1D2N.A.99, 3D2R.A.500, 3D36.A.478, 4D6P.A.1352, 2D7C.A.1002, 4D7M.A.223, 1D8C.A.3001, 4DBF.A.401, 4DBR.A.810, 4DCK.B.201, 3DDC.A.600, 3DDH.B

.232, 2DDT.A.311, 4DEM.F.402, 4DEM.F.403, 4DEM.F.404, 3DGT.A.800, 3DKC.A.2, 4DN5
.A.1001, 4DSN.A.202, 4DSO.A.202, 4DUX.A.3001, 4DWG.A.401, 4DWO.A.301, 3DYH.A.300
4, 3DYM.A.3001, 3DYP.A.3001, 2DY1.A.701, 4DYK.A.502, 4DZH.A.501, 4E01.A.402, 1E2
Q.A.401, 3E2D.A.603, 1E3D.B.901, 3E5H.A.200, 3E81.A.165, 3E8M.A.165, 2E9S.A.603,
1E9A.A.401, 3EA5.A.221, 4EEN.A.301, 4EFM.A.202, 3EHG.A.371, 1EK0.A.601, 3EQC.A.
3, 2ERX.A.403, 3ET4.A.301, 3ET5.A.255, 4EUK.A.1001, 2EW1.A.701, 4EX6.A.301, 4EX7
.A.301, 2EZT.A.1510, 2EZU.A.1610, 3EZ3.A.1104, 2EZ4.A.1610, 2EZ8.A.1510, 2EZ9.A.
1510, 4F1J.A.301, 2F2A.B.601, 1F5N.A.595, 3F61.A.310, 1F9H.A.161, 1F9H.A.162, 4F
E3.A.304, 4FEG.A.707, 2FFQ.A.356, 2FH5.B.270, 3FIU.A.5001, 4FI1.A.401, 3FKQ.A.50
1, 4FK9.A.401, 2FOZ.A.348, 2FOZ.A.349, 4FP1.A.401, 1FSG.A.302, 3FSY.A.333, 2FUE.
A.500, 4FYP.A.301, 3FZN.A.605, 2G0W.A.501, 2G09.A.901, 2G1T.A.1501, 1G17.A.301,
3G15.A.602, 3G2F.A.901, 1G4C.B.362, 1G4T.A.2005, 3G5A.D.307, 1G5T.A.998, 3G6K.A.
307, 2G6B.A.301, 2G80.A.500, 1G97.A.460, 4G9B.A.301, 3GAI.A.189, 2GCN.A.2001, 2G
CP.A.2001, 2GHT.A.257, 2GIL.A.1201, 2GL5.A.699, 3GON.A.600, 4GOJ.A.202, 4GP2.A.4
02, 1GSA.A.319, 1GSI.A.1209, 2GSM.A.3006, 4GT8.A.402, 1GUA.A.171, 3GYB.A.1, 4H1Z
.D.401, 1H2A.L.1005, 1H2R.L.1005, 2H57.A.202, 3H70.A.342, 3H7V.A.331, 3H80.A.214
, 4H81.A.402, 4H8E.A.301, 4HAT.A.302, 2HCF.A.300, 3HDG.A.202, 4HDO.B.200, 3HFW.A
.361, 2HF8.A.301, 2HF9.A.301, 4HGN.A.200, 2HNE.A.601, 4HNL.A.401, 4HOR.X.101, 4H
PT.E.402, 1HQ2.A.162, 3HQJ.A.145, 3HRZ.A.628, 3HRZ.D.742, 3HSD.B.162, 1HTW.A.561
, 3HW4.A.999, 3HW5.A.999, 1I0L.A.902, 2I1Q.A.501, 4I2B.A.604, 2I34.A.301, 2I5R.A
.301, 3I76.A.1001, 4I94.A.402, 4IAD.A.402, 3IBA.A.403, 3ICM.A.401, 3ICZ.A.403, 4
IDN.A.502, 4IDP.A.502, 1IG5.A.78, 2IHT.A.601, 1IH8.A.4002, 1IHU.A.592, 2IHP.A.28
7, 3IJL.A.386, 2IK4.A.287, 2IK4.B.287, 2IK4.B.288, 2IK4.B.289, 2IK6.B.287, 2IOR.
A.2000, 1IOW.A.331, 2IO8.A.7002, 4IP4.A.503, 3IPO.A.161, 1ITZ.A.1001, 4ITR.D.203
, 2IUC.A.1003, 4IUC.L.702, 4IUD.L.1002, 1IV2.A.1571, 1IV4.A.1571, 4IVG.A.803, 2I
YW.A.202, 2IYN.C.1123, 1IZC.A.1001, 2J0V.A.1180, 2J7P.A.1401, 2J7P.D.1401, 2J7N.
B.3374, 1J9J.A.301, 1JBW.A.998, 2JC9.A.1491, 2JCB.A.1192, 4JDP.A.301, 2JD4.A.406
1, 1JPM.A.1003, 4JS0.A.202, 3JTC.C.34, 3JVA.B.358, 3JYY.A.301, 3JYY.B.302, 1JZ7.
A.3001, 3JZ0.A.300, 3JZ0.A.303, 3K1S.H.107, 4K6R.A.505, 1K77.A.300, 3K8K.A.700,
4K9Q.A.601, 3KA3.A.176, 3KAL.A.503, 3KB9.A.701, 3KB9.A.702, 3KC2.A.355, 1KCZ.A.9
01, 4KGD.A.702, 1KK1.A.413, 3KKO.A.180, 1KMQ.A.401, 3KMW.A.501, 4KO8.A.801, 1KQP
.A.5001, 1KQP.A.5002, 4KQW.A.404, 1KSH.A.202, 1KTG.A.502, 1KTG.A.503, 1KTG.A.504
, 1KTG.A.505, 3KTA.A.184, 3KUC.A.171, 4KUX.A.703, 4KVA.A.501, 4KVG.A.202, 4KWD.A
.404, 4KXW.A.1001, 1KY2.A.401, 3L12.A.313, 4L57.B.201, 3L8H.A.801, 4L9Z.A.403, 2
LCF.A.246, 4LFG.A.303, 4LFG.A.304, 4LFG.B.303, 4LGY.A.1302, 4LHW.A.301, 4LJ9.A.9
02, 3LLU.A.502, 4LPM.A.208, 3LUF.A.300, 3LUF.A.301, 3LX5.A.301, 3LXX.A.402, 4LYK
.A.401, 4LZ0.A.403, 3M07.A.595, 3M1I.A.1178, 4M53.A.527, 1M7B.A.550, 4M9Q.A.302,
4MDB.A.403, 4MGG.A.404, 3MHY.A.115, 1MJN.A.1001, 3MJH.A.201, 3MK2.A.903, 1MMG.A
.998, 1MNE.A.998, 4MNE.A.902, 4MPO.B.205, 1MQ4.A.2088, 4MRT.A.301, 4MUM.A.301, 1
MXG.A.439, 3MX3.A.601, 3MYH.X.997, 3MYK.X.998, 3MYL.X.998, 4N0D.A.402, 3N07.A.20
0, 1N1Z.A.701, 1N1Z.A.703, 1N20.A.702, 1N24.A.702, 3N2N.A.1, 3N45.F.2, 3N45.F.3,
1N6I.A.201, 1N6L.A.201, 1N6N.A.201, 1N6O.A.201, 1N6P.A.201, 1N6R.A.201, 1NB0.A.
201, 4NBS.A.502, 4NDO.A.302, 4NFI.F.402, 4NFI.F.403, 1NFZ.A.401, 3NKV.A.500, 1NN
5.A.401, 3NOJ.A.239, 1NRJ.B.1, 3NRJ.A.190, 2NSY.A.305, 1NSF.A.859, 4NWI.A.401, 2
NXW.A.4002, 1O08.A.2800, 1O3Y.A.1002, 1O6Y.A.1280, 3O6Z.B.201, 4O7I.A.401, 1OBW.
A.176, 3OCU.A.263, 3OCV.A.264, 3OCW.A.263, 3OCX.A.264, 3OCY.A.264, 3OCZ.A.263, 4
OCP.A.403, 2ODP.A.901, 4ODJ.A.502, 3OE1.A.601, 2OFX.A.301, 2OGD.A.3002, 2OGD.A.3
003, 2OGD.A.3004, 4OHY.A.502, 1OIW.A.1175, 3OIW.A.170, 2OI6.B.6000, 4OI4.A.502,
1OKK.A.1002, 1OKK.D.1002, 4OKM.A.903, 4OKZ.A.903, 2OLR.A.543, 3OM2.A.486, 4OMF.A
.503, 3OP2.B.500, 2ORW.A.501, 1OXV.A.1102, 1OXV.D.1101, 3OYX.A.601, 3OZX.A.613,
3OZX.A.614, 2P27.A.307, 4P31.A.402, 4P32.A.402, 1P4M.A.201, 1P5Z.B.401, 3P5P.A.9
01, 3P93.C.406, 3P96.A.412, 3PDE.A.312, 3PFF.A.831, 4PFY.B.601, 3PGL.A.1, 1PHP.A
.395, 4PHG.A.201, 4PHH.A.202, 3PIT.A.180, 2PKE.B.300, 3PNL.B.1212, 2PNQ.A.502, 1
POX.A.610, 1PPV.A.401, 4PQ9.A.301, 2PS2.A.401, 2PS5.B.701, 1PVF.A.401, 1PVG.A.90

3, 2PYW.A.501, 2PZ8.A.4001, 2PZA.A.6243, 2PZE.A.3, 2Q28.A.1001, 1Q3H.C.674, 3Q3J.B.201, 2Q3F.A.301, 3Q46.A.307, 4Q4C.A.404, 3Q5V.B.599, 3Q60.A.603, 2Q66.A.602, 1Q92.A.1003, 4QC2.A.302, 3QF7.A.854, 1QGU.B.3002, 1QGU.D.3006, 2QG8.A.201, 4QHZ.A.302, 2QIS.A.907, 2QIS.A.908, 2QIS.A.909, 1QK5.A.303, 2QME.A.179, 3QNM.A.400, 1QRA.A.168, 2QRZ.A.190, 2QTY.A.348, 2QTO.A.1001, 3QUQ.A.225, 3QUT.A.225, 3QVQ.C.310, 3QXC.A.222, 3QXJ.A.224, 3QXS.A.223, 3QXX.A.224, 2QXO.A.161, 3QYY.A.505, 1R2Q.A.300, 3R3S.A.296, 2R6O.A.801, 3R7W.A.600, 2R8E.A.201, 2RAH.A.354, 2RAR.A.501, 2RAV.A.701, 3RAP.R.200, 2RB5.A.701, 2RBK.A.501, 3REF.A.192, 1RKQ.A.1273, 4RKE.A.202, 4RKF.A.202, 3RLF.A.1501, 1RMT.A.1413, 3RO6.A.400, 4ROQ.A.401, 1RP7.A.890, 1RQI.A.604, 1RQJ.A.909, 3RUS.A.544, 1RVK.A.999, 3RV3.A.1004, 3RWM.B.1, 3RYE.A.907, 3RYE.A.908, 3RYE.A.909, 1RYA.A.1001, 1RYH.A.539, 4S1H.B.303, 3S4J.A.907, 3S4J.A.908, 3S4J.A.909, 1SAW.A.225, 3SAE.A.820, 3SDT.A.821, 3SFO.A.263, 3SHQ.A.321, 1SHT.X.219, 3SJN.A.374, 3SLS.A.401, 3SL2.A.701, 3SN4.A.408, 1SO4.A.2300, 3STP.A.391, 1SVM.A.750, 1SVS.A.356, 1TOP.A.901, 3T10.A.401, 3T1K.A.401, 3T2S.B.401, 3T2B.A.409, 3T2D.A.408, 3T2D.A.409, 3T7A.A.602, 1TC6.A.501, 2TCT.A.223, 3TCS.A.368, 3TDV.A.501, 1TE6.A.641, 3TEP.A.1, 3TGO.A.503, 3TJI.A.601, 3TKL.A.300, 1TMM.A.162, 4TMT.A.902, 4TMV.A.902, 4TMW.A.903, 4TMX.A.903, 3TMO.A.266, 4TN1.A.902, 3TSO.A.200, 3TWA.A.420, 3TYZ.A.281, 4TYO.A.502, 1UO2.A.240, 4U5X.A.202, 4U82.A.301, 1U8Y.A.301, 4UAK.A.503, 4UAS.A.302, 4UAT.A.302, 4UAV.A.401, 2UAG.A.1001, 1UBK.L.1005, 3UCW.A.100, 3UCY.A.101, 4UCX.Q.1553, 4UE3.L.603, 3UJR.A.502, 3UJR.B.502, 3UJS.A.601, 3UJS.B.601, 1UMG.A.403, 1UMG.A.404, 4UM7.A.175, 3UPY.A.446, 1UPT.A.1183, 3UPL.A.447, 3UQY.L.603, 4UQL.Q.1552, 4URH.Q.1552, 4V0L.A.601, 3V1X.A.501, 4V1T.A.1777, 3V2U.C.521, 2V26.A.1801, 3V3W.A.403, 1V54.A.3518, 2V7Q.A.1512, 2VBU.A.1134, 3VC1.A.301, 3VC1.I.301, 3VC2.J.301, 3VCC.A.401, 3VCC.A.402, 3VCN.A.501, 2VDN.B.2001, 1VG8.A.1401, 2VG3.A.1297, 2VK1.A.601, 2VK8.A.1565, 1VOM.A.997, 3VVH.A.701, 1VZM.B.1046, 1VZM.B.1047, 1W2Y.A.1231, 3W40.A.201, 2W4J.A.1280, 2W5V.A.1378, 2W5X.A.1379, 3W6N.A.803, 3W6O.A.802, 1W78.A.1422, 1W78.A.1423, 1WA5.A.1178, 3WBH.A.501, 3WBZ.A.404, 1WC1.A.1501, 1WF3.A.401, 4WH2.A.402, 4WH3.A.402, 2WIC.A.1266, 3WJP.A.403, 3WJP.A.404, 2WKQ.A.1724, 3WNZ.A.502, 3WRY.C.1202, 1WUH.L.1005, 1WUK.L.1005, 2WVG.A.601, 3WXM.A.502, 1X06.A.900, 2X13.A.1418, 2X14.A.1418, 1X3S.A.200, 1X84.B.401, 2X98.A.1477, 1XBY.A.601, 2XB5.A.223, 2XCW.A.1498, 1XEX.A.1002, 1XFI.A.400, 2XH2.B.1439, 2XH7.A.1441, 2XIS.A.392, 2XJB.A.1494, 2XJD.A.1497, 2XSX.A.500, 2XTN.A.1232, 2XUU.A.1307, 1XX1.A.9001, 4XXP.A.301, 2Y6P.A.1234, 1Y8A.A.501, 2Y8E.A.1177, 1Y9D.D.2901, 2YCH.A.501, 1YHL.A.1402, 1YHL.A.1403, 1YMV.A.200, 1YNS.A.1258, 1YQ9.H.540, 1YQT.A.591, 1YQT.A.592, 1YRQ.H.553, 1YS7.A.1002, 1YU4.A.2002, 2YVO.A.1001, 2YVO.A.1002, 2YVP.A.184, 1YVD.A.850, 2YVM.A.1001, 1YYQ.B.702, 1YZL.A.401, 1YZN.A.301, 1YZT.A.700, 1Z06.A.203, 1Z07.A.300, 1Z08.A.1300, 1Z08.C.3300, 1Z0J.A.400, 1Z2N.X.1295, 1Z4J.A.1001, 1Z4K.A.229, 1Z4L.A.2001, 1Z4O.A.800, 1Z4P.X.1001, 1Z4Q.A.2001, 2Z4V.A.1501, 2Z4V.A.1502, 2Z4X.A.1201, 2Z4X.A.1202, 1Z59.A.1001, 1Z5G.A.601, 1Z5G.D.604, 2Z52.A.1301, 2Z52.A.1302, 2Z7I.A.1301, 1Z88.A.601, 1ZC3.A.500, 1ZED.A.905, 3ZFD.A.500, 3ZIA.A.601, 1ZJJ.A.1001, 3ZMC.A.1296, 1ZS9.A.1257, 3ZVL.A.1524, 3ZX4.B.260, 3ZX5.A.260

[1] "Cluster 6"

4AAB.B.1156, 4BDZ.A.1381, 4BEO.A.1381, 4D6O.D.1197, 1G9Z.D.901, 4IEM.C.401, 4IRC.A.402, 3MAQ.A.1001, 1MOW.A.373, 1MUH.A.479, 4NCB.B.702, 4NCB.C.101, 4O3S.A.503, 3OYB.A.397, 3OYD.A.397, 3OYE.A.397, 3OYJ.A.397, 3OYL.A.397, 3OYN.A.397, 1RVC.A.401, 3S3O.A.397, 3S3N.A.397, 3SM4.E.15, 2VBN.E.1026, 1YTU.A.428, 1ZBI.A.302, 1A49.A.534, 3A7E.A.215, 4ACF.A.1480, 4ACF.A.1481, 2AL1.B.438, 3AU9.A.601, 3BZN.A.501, 4C5A.A.330, 4C5B.A.1313, 4C5C.A.1313, 3CFX.A.703, 3CVJ.C.243, 4CWB.A.1159, 1DAW.A.342, 1DAY.A.341, 1DAY.A.342, 4DFX.E.404, 3DG6.A.2001, 4DHP.A.303, 4DWB.A.507, 1EC8.A.498, 1EFK.A.604, 1ELX.B.452, 4EOM.A.302, 3ETH.A.401, 3ETH.A.402, 3ETJ.A.402, 1EYZ.A.401, 1EYZ.A.402, 4FOQ.A.501, 3FLK.A.401, 2FN1.A.504, 2FUV.A.901, 3GN6.A.321, 3GQ8.A.692, 4H2H.A.401, 2HGS.A.501, 3HPF.A.401, 3HWO.A.1702, 3I4K.A.385, 4IAC.A.401, 4IAD.A.401, 1IOW.A.330, 2IO8.A.7001, 4IZG.A.414, 2J0L.A.1688, 4J

1O.A.401, 1J7L.A.302, 4J7L.A.402, 4K10.D.404, 1K9Y.A.401, 3KAL.A.502, 1KJ8.A.393, 1KJ8.A.394, 1KJ9.A.393, 1KJI.A.393, 1KJI.A.394, 1KJJ.A.394, 3KRO.D.3001, 1LP4.A.341, 3LVV.A.696, 4M3A.A.402, 4MDB.A.402, 3NA5.A.547, 1NFS.A.401, 1NUY.A.2341, 4NZN.A.403, 4O4D.A.402, 4OAV.B.803, 4OHF.B.503, 3OLP.A.547, 2ONS.A.702, 3OP2.A.500, 4ORK.A.501, 4OVN.A.202, 1P43.A.438, 3PFR.A.456, 4PFY.A.602, 4PU5.A.501, 2PYW.A.500, 1PYX.A.1003, 2PZA.A.6242, 2Q1A.X.294, 3Q46.A.306, 4Q4C.A.403, 4QE5.A.401, 4QPM.A.1503, 2QVH.A.401, 4QXD.B.301, 3R75.A.700, 3RLH.A.286, 1SHQ.A.479, 3T12.A.198, 3T2D.A.411, 1T5T.A.1005, 3T7A.A.601, 3TDW.A.502, 3TM0.A.265, 4TY0.A.501, 3UJR.A.501, 3UJR.B.501, 3UJS.A.600, 3UJS.B.600, 1UMG.A.401, 4UOP.A.1612, 3V4S.B.402, 3VA8.A.425, 3VAT.A.502, 3VC6.A.501, 2VPQ.A.1450, 2VPQ.A.1451, 1WOH.A.1000, 1W7V.A.1441, 4WB8.A.403, 4WH2.A.403, 4WH3.A.403, 3WNZ.A.503, 3WQQ.A.502, 3WQR.A.502, 2X3J.A.1590, 2XHO.A.1439, 1Z2O.X.1296, 2Z4X.B.1206

Table S35. all-ligand-number Mg, normal group

	size	largest_angle*	middle_1*	middle_2*	middle_3*
1	"86"	"152.8+/-8.4"	"70.5+/-4.6"	"79.4+/-4.6"	"92.5+/-10.2"
2	"136"	"160.1+/-10.7"	"77+/-6.5"	"83.4+/-6.4"	"89.2+/-7.1"
3	"380"	"174.9+/-2.4"	"81.9+/-2.7"	"89.3+/-1.3"	"95+/-1.8"
4	"163"	"172.4+/-3.7"	"79.8+/-4.2"	"89.2+/-1.9"	"96.5+/-3.1"
5	"77"	"139.8+/-12.2"	"87.4+/-7.6"	"95.8+/-6.5"	"104.2+/-6.4"
6	"161"	"162.9+/-6.5"	"80.6+/-6"	"88.2+/-3.1"	"96.1+/-4.1"
7	"349"	"172.4+/-2.8"	"81.3+/-2.9"	"88.5+/-1.6"	"94.6+/-2.2"
8	"164"	"164.8+/-5.1"	"73.2+/-4.4"	"85.2+/-3"	"98.1+/-6.3"
9	"604"	"176.8+/-1.5"	"84.5+/-2"	"89.4+/-0.9"	"93.1+/-1.4"
10	"60"	"157.8+/-13.4"	"76.2+/-6.5"	"83.7+/-5.9"	"90.9+/-7"
11	"297"	"170.6+/-3.4"	"74.9+/-3.9"	"88+/-2.2"	"97.3+/-2.9"

	middle_4*	smallest_opposite_angle*	Tetrahedral	TrigonalBipyramidal
1	"141.1+/-8.1"	"74.3+/-7.7"	"0"	"0"
2	"96.7+/-8.2"	"85.4+/-10.6"	"0"	"0"
3	"170.7+/-2.4"	"79.1+/-2.5"	"0"	"0"
4	"164.1+/-3.7"	"70.8+/-3.9"	"0"	"0.001"
5	"114.4+/-7.1"	"102.9+/-11.5"	"0.024"	"0"
6	"150+/-9.8"	"98.3+/-5"	"0"	"0.015"
7	"169.1+/-2.7"	"85.2+/-3.4"	"0"	"0"
8	"155.4+/-4.3"	"74.6+/-4.6"	"0"	"0"
9	"174.3+/-1.9"	"85.2+/-2.3"	"0"	"0"
10	"101.5+/-11.1"	"137.2+/-16.7"	"0"	"0.002"
11	"164.4+/-3.7"	"80.6+/-3.3"	"0"	"0"

	TrigonalBipyramidalVA	TrigonalBipyramidalVP	Octahedral	SquarePyramidal
1	"0.009"	"0.001"	"0"	"0"
2	"0"	"0.002"	"0"	"0"
3	"0"	"0.172"	"0.411"	"0.568"
4	"0.001"	"0.171"	"0.077"	"0.236"
5	"0.005"	"0"	"0"	"0"
6	"0.014"	"0.061"	"0"	"0.006"
7	"0"	"0.119"	"0.355"	"0.529"
8	"0.001"	"0.024"	"0"	"0.006"
9	"0"	"0.099"	"0.707"	"0.807"
10	"0.001"	"0.021"	"0"	"0"
11	"0"	"0.102"	"0.063"	"0.184"

	SquarePyramidalV	SquarePlanar	TrigonalPrismatic	TrigonalPrismaticV
1	"0.01"	"0"	"0.009"	"0.034"
2	"0.1"	"0"	"0"	"0"
3	"0.685"	"0.66"	"0"	"0"
4	"0.468"	"0.378"	"0"	"0"
5	"0.001"	"0"	"0"	"0"
6	"0.065"	"0.042"	"0"	"0.001"
7	"0.621"	"0.631"	"0"	"0"
8	"0.08"	"0.031"	"0"	"0.001"
9	"0.85"	"0.826"	"0"	"0"
10	"0"	"0.052"	"0"	"0"
11	"0.37"	"0.285"	"0"	"0"

	PentagonalBipyramidal	PentagonalBipyramidalVA	PentagonalBipyramidalVP
1	"0"	"0"	"0.004"
2	"0"	"0"	"0"
3	"0"	"0"	"0"
4	"0"	"0"	"0.003"
5	"0"	"0"	"0"
6	"0"	"0"	"0"
7	"0"	"0"	"0"
8	"0"	"0"	"0.007"
9	"0"	"0"	"0"
10	"0"	"0"	"0"
11	"0"	"0"	"0"

	SquareAntiprismatic	SquareAntiprismaticV	HexagonalBipyramidal
1	"0"	"0"	"0"
2	"0"	"0"	"0"
3	"0"	"0"	"0"
4	"0"	"0"	"0"
5	"0"	"0"	"0"
6	"0"	"0"	"0"
7	"0"	"0"	"0"
8	"0"	"0"	"0"
9	"0"	"0"	"0"
10	"0"	"0"	"0"
11	"0"	"0"	"0"

	HexagonalBipyramidalVA	HexagonalBipyramidalVP
1	"0"	"0"
2	"0"	"0"
3	"0"	"0"
4	"0"	"0"
5	"0"	"0"
6	"0"	"0"
7	"0"	"0"
8	"0"	"0"
9	"0"	"0"
10	"0"	"0"
11	"0"	"0"

Table S36. Cluster members of all-ligand-number Mg, normal group

[1] "Cluster 1"
4CZK.A.1335, 3DYG.A.3002, 4FHX.A.402, 2IUT.A.1724, 3KUD.A.171, 2NOM.A.401, 3PP1.
A.410, 1T5S.A.1005, 3UGV.A.500, 2Y4M.A.400, 4FFO.A.903, 4LTZ.A.404, 2BPF.A.339,
4DPV.N.12, 4M30.B.302, 4W9M.E.902, 1A49.B.1134, 1A49.C.1734, 4AN9.A.1383, 3AQC.D
.328, 1AZT.A.403, 2BBO.A.2, 3C5P.D.302, 3CNX.B.170, 1CQP.A.310, 4CYU.A.170, 3DUF
.C.1368, 3DV0.A.1368, 3DYF.A.3002, 3DYF.A.3004, 4E1E.A.401, 3EA5.C.221, 1ELY.A.4
52, 3F5U.A.297, 1GIM.A.435, 2GQ3.B.1003, 3GT8.C.13, 1GY3.A.1298, 2GZD.A.950, 4HY
P.B.302, 3IAF.B.572, 2IO7.A.5002, 4IYN.A.804, 1JM6.A.4601, 4JVJ.F.405, 4K81.B.20
3, 3KR4.A.1004, 1L2O.B.902, 4LF2.D.601, 2M32.A.401, 1MFO.A.1453, 4NEH.A.1102, 4N
HO.A.1403, 1NUZ.A.2342, 2O1X.A.2001, 4O1P.C.903, 3OAB.A.904, 4OAU.C.802, 2OI2.A.
400, 2POC.B.5, 4P0V.A.403, 1Q19.A.504, 3RBM.A.1003, 4RJK.B.602, 3RUW.A.544, 4S17
.C.502, 3SBE.A.501, 3TW6.B.2002, 2VBI.G.1000, 2WB4.B.502, 3WQP.J.501, 2X9H.A.300
1, 1XF9.B.11, 1Y8P.A.501, 1Y9I.B.602, 1ZCA.B.1383, 3ICE.D.1001, 3ALN.A.406, 3DYF
.B.4002, 1G9X.B.1301, 4I1O.A.201, 3JZM.A.701, 2O1V.A.755, 2O1X.B.2002, 3QU2.C.22
5, 1RC5.D.764
[1] "Cluster 2"
4DQP.A.903, 3E54.A.702, 2PYJ.A.9004, 1Q81.4.8078, 3S14.A.2001, 2W9C.B.1342, 3ZC0
.B.197, 2AKZ.A.440, 2AQX.A.600, 3AQC.D.327, 4AVQ.C.902, 2BM1.A.1690, 3BPD.G.126,
 1BPM.A.488, 1BR2.A.997, 3BU5.A.301, 3CNX.C.170, 3CRL.B.2001, 2CW6.A.401, 4CW7.C
.1002, 4CYI.B.1000, 4CYU.A.171, 1D1C.A.998, 2D33.C.2525, 3D7M.A.356, 2DEJ.A.402,
 2DH4.A.341, 3DLS.C.11, 4DPM.D.401, 1E1Q.A.601, 1E1Q.F.601, 3E4O.A.501, 2E6B.A.3
01, 2E92.A.1302, 2E92.B.1304, 2E95.A.1301, 1F4H.D.3001, 3F74.C.1, 3FA4.A.401, 3F
R8.B.1, 3FYY.B.402, 3G2F.B.901, 2G74.A.185, 3G9D.B.299, 2GHT.B.257, 2GHQ.B.257,
2GWC.A.1, 3H8A.C.1431, 3HBO.A.701, 1HBN.A.1558, 3HQP.M.502, 2HT6.B.444, 4HV6.B.2
01, 2I19.B.4004, 2I5R.B.302, 3IJQ.A.386, 2IK2.A.290, 4IL6.C.505, 4IL6.c.506, 1IV
2.F.1574, 4J99.D.803, 2JK1.A.1144, 3KA9.A.189, 1KK8.B.998, 1LOO.A.602, 4LCZ.A.31
6, 4LRT.A.403, 1LVH.A.801, 3M1Y.D.300, 4M1W.A.201, 4MKS.A.502, 4MPO.G.203, 4MPO.
G.204, 3MQT.A.1243, 1N5K.B.413, 3N9V.A.61, 1NEL.A.438, 3NIZ.A.312, 1NV3.A.2341,
2O1U.A.302, 2O56.F.2006, 3OAC.D.3002, 3OHM.A.402, 2OI5.B.5000, 4OVN.B.202, 4P0V.
A.404, 1P7L.A.388, 4P9D.C.202, 4PFQ.C.400, 4PRV.A.402, 2Q58.A.4, 1Q8Y.A.809, 2Q8
0.A.401, 4QJB.A.301, 4QVY.K.302, 4QXD.B.302, 4QXD.B.304, 4R3A.A.400, 2R5T.A.600,
 3RBM.B.1002, 2RIO.A.1102, 1RLT.C.807, 3RYW.B.2003, 4S17.D.501, 3SRD.C.603, 3SRF
.D.533, 3SY8.A.401, 3SZ5.A.220, 4TQ3.A.402, 3TW6.D.2002, 4U0N.B.401, 3U87.A.402,
 3UGJ.A.2006, 4UUX.A.401, 2V0N.B.502, 2VHQ.A.1328, 3VI4.B.502, 2VKQ.A.1288, 3VTI
.C.401, 3VYT.C.601, 1W9L.A.1748, 3WEJ.A.402, 1WQ1.R.168, 1WQA.A.456, 3WU2.C.511,
 1WUU.C.394, 1XMJ.A.2, 1YXO.A.5001, 2Z2P.A.1001, 1ZCW.A.501, 2ZCQ.A.453, 2ZEV.A.
1302, 2ZEV.B.1304, 1ZH4.A.201, 2ZRW.D.702, 1ZYK.A.702
[1] "Cluster 3"
4AQX.D.1525, 4BDY.A.1380, 4BE0.A.1380, 4BE1.A.1381, 4CEI.B.2162, 4DL4.A.501, 4DL
G.A.902, 4DO9.A.401, 2DPI.A.871, 4DQI.A.901, 4DQI.D.901, 2EZ6.A.501, 1FIU.I.2222
, 3G6Y.A.871, 2IS4.A.1001, 3JPN.A.339, 3JPQ.A.339, 3JPS.A.339, 3K57.A.1001, 4KLI
.A.401, 3M8S.A.2, 4M8O.A.1302, 4MDE.A.1002, 3MR5.A.435, 4O5K.A.401, 3OYB.A.396,
3OYD.A.396, 2OZS.A.904, 3PML.A.2, 3PNC.A.576, 4R8U.B.402, 3RJF.A.340, 3SI8.A.451
, 3SM4.B.227, 1T7P.A.4001, 3TFS.A.340, 3TIO.A.1, 3TIO.D.2, 3TWH.A.401, 4UAY.A.40
2, 4UB3.A.401, 4A01.A.1767, 4A01.A.1769, 3A1U.A.5, 3A1U.A.6, 3A99.A.401, 4ABZ.A.
1210, 2AFK.E.1291, 2AGO.A.601, 1ALK.B.452, 4AS2.A.1328, 4AUX.A.223, 3B05.D.1001,
 1B4N.A.623, 3B7L.A.907, 1B8J.A.452, 2BEK.A.501, 3BGA.A.1, 3BRB.A.10, 2BU2.A.138
8, 2BVN.B.1395, 2C18.A.1338, 2C3U.A.2238, 2C31.A.1553, 4C5C.A.1314, 4C7X.A.700,
3C9U.B.312, 2CFS.A.1296, 1CG4.A.435, 3CG4.A.201, 2CHE.A.130, 1CJT.C.403, 1CJU.A.
582, 3CK5.A.400, 2CL5.A.1216, 3CMR.A.453, 3CP6.A.502, 3CP6.A.503, 3CRR.A.324, 3C
V2.A.1, 4CW7.A.1002, 3CZJ.B.3001, 1D2N.A.99, 2DCN.A.4001, 4DCK.B.201, 4DEM.F.402
, 4DEM.F.403, 4DN1.B.401, 4DSN.A.202, 2DUA.A.292, 3DYH.B.4002, 1E4E.A.365, 3E84.
A.701, 4E8G.A.402, 2E91.A.1302, 2E95.A.1302, 3EA5.A.221, 1EBH.A.438, 2EB5.A.1001
, 3EGT.A.3002, 3EGT.A.3004, 3EKG.A.601, 3EQC.A.3, 3ETJ.A.401, 4EUK.A.1001, 2EZT.

A.1510, 3EZ3.B.1102, 2EZ4.A.1610, 1F2U.A.902, 2F9M.A.1201, 4F9A.A.602, 3FD5.A.39
7, 3FD6.B.397, 3FE4.B.902, 3FIU.A.5002, 3FKQ.A.501, 3FV9.A.501, 4FVR.A.902, 1G17
.A.301, 1G4P.A.2003, 3G5A.B.307, 2G9Z.B.701, 4G9B.A.301, 1GAG.A.201, 2GHT.A.257,
2GL5.A.699, 4GME.C.501, 4GP2.A.401, 2GQS.A.241, 1GUA.A.171, 3GY1.A.500, 4GYI.A.
402, 3H3X.Q.553, 4H81.A.402, 4HCL.A.401, 3HDG.A.202, 2HGS.A.502, 4HGQ.A.201, 2HJ
P.A.292, 4HNC.A.401, 4HPT.E.402, 1HQ2.A.162, 3HVH.A.265, 3HVI.A.1, 3HVJ.A.265, 3
HVK.A.1, 3HW3.A.999, 3HW4.A.999, 4I3Z.A.302, 2I6K.A.302, 3ICK.A.402, 3ICK.A.403,
3ICM.A.402, 3ICZ.A.402, 4IEG.A.1001, 4IFW.A.502, 4IHC.A.501, 1II9.A.593, 1IR3.A
.301, 2IUC.A.1003, 1IV2.A.1571, 1IV4.A.1571, 1IV4.A.1572, 2IYN.C.1123, 1IZC.A.10
01, 1J34.C.501, 1J7L.A.301, 1J9J.B.301, 1JBW.A.998, 2JD4.B.4062, 2JI7.A.1567, 1J
PM.A.1003, 1JUY.A.435, 3JVA.B.358, 3JVT.B.502, 3JYY.B.302, 1JZ7.A.3001, 3JZ0.A.3
00, 3K1S.H.107, 4K6R.A.505, 3K9L.A.168, 3KAL.A.503, 4KCT.A.1001, 1KHZ.B.301, 4KV
A.A.501, 4L2X.F.404, 4L80.A.403, 4L9W.A.202, 4L9Z.A.403, 2LCF.A.246, 1LNY.A.1453
, 1M7B.A.550, 3M7I.A.901, 1MC1.A.601, 1MDL.A.360, 1MEZ.A.1453, 1MMA.A.998, 1MMN.
A.998, 4MNE.A.902, 4MPO.B.205, 4MPO.C.205, 1MX0.A.501, 4MYO.A.301, 4MZU.C.404, 1
NOH.A.699, 1NOH.B.1699, 1N1Z.A.703, 1N20.A.701, 1N20.A.702, 1N24.A.701, 3N45.F.3
55, 4NFI.F.402, 4NFI.F.403, 4NFI.F.404, 3NOJ.A.239, 3NRJ.A.190, 1NSY.A.6241, 1O6
Y.A.1280, 3O6Z.B.201, 2OCB.A.202, 4OCP.A.403, 3OE5.A.222, 2OEM.A.911, 2OEM.B.912
, 2OFX.A.301, 1OIW.A.1175, 1OIX.A.301, 1OKK.D.1002, 4OKM.A.901, 4OKM.A.903, 4OKZ
.A.902, 4OKZ.A.903, 3OM2.A.486, 1OW2.A.401, 3OZX.A.614, 3OZY.B.390, 3P41.A.297,
3P41.A.298, 3PFF.A.831, 1PHP.A.395, 2PK0.A.502, 2PLS.H.602, 2PS2.A.401, 1PT6.A.5
00, 1PVG.A.903, 1PYM.A.1003, 3Q1O.A.400, 2Q1D.X.294, 1Q6L.A.5300, 1Q6O.A.7300, 1
Q6Q.A.7300, 1Q6R.A.7300, 3Q85.A.284, 1Q9S.A.201, 4QEA.A.301, 2QJJ.C.1003, 3QKE.A
.407, 3QKT.A.902, 2QRZ.A.190, 2QTY.A.349, 3QVQ.C.310, 3QXH.A.223, 3QXX.A.224, 2Q
X0.A.162, 1R0X.A.13, 3R1M.A.403, 3R1M.A.404, 3R25.A.402, 3R6T.A.301, 3RBM.A.1001
, 1RKU.A.301, 4RKE.A.202, 1RP7.A.890, 1RQI.A.603, 1RQJ.A.907, 3RUV.A.544, 3RWM.B
.1, 1RYH.A.539, 3S9Z.A.802, 3SAZ.A.802, 3SBD.A.501, 3SBF.A.402, 3SDT.A.819, 3SEA
.B.178, 3SHQ.A.321, 3SN1.A.408, 3SN4.A.408, 1SO4.A.2300, 3T1Q.A.198, 3T2D.A.408,
3T2E.A.409, 1T9B.B.699, 3TDV.A.501, 1TE6.A.641, 3TJI.A.601, 3TKL.A.300, 3TMO.A.
266, 4TSK.A.403, 1TW1.A.1, 3TWB.A.420, 3TYZ.A.281, 1TZZ.A.3501, 1U02.A.240, 4UAS
.A.302, 3UCY.A.101, 1UMD.A.1401, 1UMG.A.403, 4UMF.A.1175, 3UXK.A.360, 3UZR.A.300
, 3V1V.A.501, 4V1T.A.1776, 3V2U.C.521, 3V4B.A.403, 1V5F.A.1603, 1VA6.A.522, 3VCC
.A.402, 3VCN.A.501, 2VDO.B.2001, 2VDR.B.2001, 2VDL.B.2001, 2VDN.B.2001, 3VKB.A.7
01, 2VK4.A.601, 3VMK.B.402, 2VPR.A.1207, 3VX4.A.802, 1W6T.A.435, 3W6N.A.803, 3W6
O.A.802, 3WBH.A.501, 3WBZ.A.404, 1WC1.A.1501, 3WEK.A.401, 2WIC.A.1266, 2WVG.A.60
1, 2XB5.A.223, 1XEF.A.801, 1XG3.A.2101, 2XIS.A.392, 4XIA.A.399, 6XIM.A.395, 2XJC
.A.1499, 2XJE.A.1493, 2XSX.A.500, 2XUU.A.1307, 1Y8Q.B.641, 1Y9D.D.2901, 1YHL.A.1
402, 1YIO.A.212, 2YVP.A.183, 1YVE.I.601, 2YVM.A.1001, 1YYQ.B.702, 1Z2O.X.1295, 1
Z4O.A.800, 1Z5C.A.2001, 2Z52.A.1301, 2ZCR.A.669, 2ZDH.A.812, 2ZKJ.A.500, 3ZMC.A.
1296, 1ZPD.A.601, 2ZPU.A.360, 1ZS9.A.1257, 1ZXN.B.902, 1ALK.A.452, 3B05.A.1001,
4DBQ.A.903, 2FKW.A.1501, 3ICM.A.403, 2NGR.A.199, 3NNN.A.401, 4NST.A.1103, 3PUW.A
.1501, 1Q3H.D.674, 1YXO.A.5000, 2Z4Z.A.1301, 1Z5B.A.2001, 1ZH4.B.202
[1] "Cluster 4"
4AAB.B.1156, 4AQX.D.1526, 4BE2.A.1381, 4C2U.A.1666, 1CW0.A.203, 4D6O.D.1196, 4D6
O.D.1197, 4F5P.A.401, 1G9Z.C.902, 1G9Z.D.901, 1G9Z.F.903, 4LOX.A.401, 4M30.A.501
, 3MAQ.A.1001, 1MOW.D.374, 4NCB.A.702, 4NCB.B.702, 4NCB.B.703, 3OYA.A.397, 4PQU.
A.602, 3S3O.A.397, 3S3M.A.397, 1TK0.A.991, 2VBN.E.1026, 1ZBI.A.302, 1A49.A.534,
4ACF.A.1480, 2AE8.C.1009, 1AJB.A.452, 2BJI.A.2277, 3BM4.B.304, 1BWV.C.490, 3BWY.
A.300, 1CG1.A.435, 3CT2.A.401, 4CWB.A.1159, 3CXO.A.500, 3CXO.B.500, 4CYM.A.1199,
3D46.A.501, 3D47.A.501, 1DAY.A.341, 2DGN.A.1454, 4DH5.A.402, 3DUF.A.1368, 4DWB.
A.507, 3DYS.A.902, 2E8W.A.1202, 4EA0.A.301, 1ELZ.A.452, 1EXM.A.407, 3EZ3.B.1104,
4FOQ.A.501, 3F78.C.1, 4F71.A.301, 3FD5.A.396, 4FFL.A.906, 3FLK.A.401, 3FLK.A.40
5, 3FPA.C.901, 3FPB.A.1000, 3FYY.A.402, 2G4J.A.392, 4GOK.B.202, 2GT4.B.401, 4GT3
.A.403, 4HE1.A.403, 3HJN.A.501, 3HPF.A.402, 2HXU.A.601, 4I3Y.A.304, 1I6I.A.501,

1IGW.A.441, 2IK2.B.289, 2IK7.A.287, 4J7L.A.402, 1JP4.A.702, 4K33.A.802, 1K9Y.A.4
01, 4KCU.A.1001, 3KDN.A.500, 3KEU.A.400, 3KHQ.A.1, 4KI8.E.602, 1KKR.A.501, 4KQX.
A.406, 3KRO.D.3001, 3LOY.A.257, 4L9Y.B.403, 3M00.A.550, 4M6U.A.401, 1MNS.A.360,
4MPO.A.206, 1NUY.A.2341, 4NZO.A.404, 2O1O.A.503, 4OKM.A.902, 3OP2.A.500, 3OPS.A.
501, 4ORK.A.501, 2OUN.A.403, 4OVN.A.204, 1OZH.A.1405, 3OZM.D.390, 3P3B.A.393, 3P
93.A.406, 2PMQ.A.902, 1PUN.A.130, 4PU5.A.502, 1PYD.A.559, 2PZA.A.6242, 2Q1A.X.29
4, 4Q1V.A.803, 3Q30.A.600, 3Q46.A.306, 4QE5.A.401, 1QMZ.A.383, 3QPE.A.393, 3QPE.
B.393, 4QPM.A.1503, 3R1M.A.402, 4RJJ.A.602, 4RJK.H.602, 3RLH.A.286, 4RN3.A.301,
1SHQ.A.479, 3T6C.A.501, 1TE6.A.640, 4TQD.A.502, 3TW6.C.2002, 3UJR.A.501, 1V5G.A.
1603, 2VBV.A.1136, 3VMK.A.402, 3WBZ.A.403, 3WDL.B.902, 2WEF.A.402, 4WK0.B.501, 2
X3J.A.1590, 2XCL.A.480, 2XIM.A.395, 2XZW.A.202, 1XZ8.A.180, 1YHM.B.1401, 2Z7H.A.
1301, 2ZVJ.A.300, 3ZXW.A.476, 3AUO.A.577, 4D6O.A.1186, 2BVN.A.1395, 4DLC.A.303,
3EHB.A.562, 4EKD.A.407, 2EWG.B.4003, 2G07.A.601, 2I19.A.3002, 2IK2.A.289, 2JCM.A
.1490, 1KK8.A.997, 3LMG.A.202, 2PUL.A.400, 4QXD.A.304, 4TQ4.C.402
[1] "Cluster 5"
4IEM.A.502, 1SJB.A.1001, 2XTI.A.1551, 4DLG.A.903, 2FLC.A.248, 4FLW.A.802, 3ICE.B
.1001, 4IR1.A.902, 1RVB.B.302, 4UN4.B.2367, 2W9C.A.1344, 1YJW.7.8044, 2A19.B.164
2, 3ARA.A.166, 3C15.A.29, 3CIK.A.690, 1CLK.A.780, 3CR3.B.1213, 3D19.E.301, 1D2E.
D.504, 2D32.B.2524, 4DPG.F.604, 2E8A.A.501, 3EN9.A.600, 2FDR.A.1001, 1G8G.A.521,
1GRV.A.490, 2H5N.B.1001, 4HJH.A.552, 4HN2.A.404, 4HNS.A.201, 3IAP.A.3001, 4IL6.
C.512, 4IL6.b.616, 2I07.B.5004, 1IV4.D.1564, 2J4E.A.1002, 3KGX.A.503, 3LD0.J.54,
3M42.A.1, 4MFE.D.1105, 3MG8.G.241, 3MWC.A.400, 4NNN.K.302, 4NNN.N.202, 2NOG.A.9
220, 2O1X.D.2004, 2O4G.B.800, 4O6I.B.602, 4OKE.B.203, 4OKK.A.204, 2OPM.A.908, 3O
PK.C.401, 2PUN.B.401, 1PYX.A.1002, 4QDG.A.402, 4QLQ.V.301, 3QU9.A.227, 1RK2.C.32
0, 1T9Z.A.273, 3TAV.A.269, 4U3W.A.503, 4UM8.B.2001, 1W0H.A.1001, 3W9T.B.511, 3WE
G.A.402, 3WGU.C.2003, 2WHE.A.1222, 3WKA.A.601, 3WU2.B.616, 3WU2.C.513, 1XLB.A.39
9, 2XRI.A.1337, 2YFD.B.1145, 1YQ2.E.7005, 1YYZ.A.341, 1Z5B.B.2003
[1] "Cluster 6"
4DFM.A.903, 3EPH.B.2, 2G8F.A.301, 2OTJ.0.8044, 3Q8P.B.423, 1QVG.0.8067, 3SNN.A.9
06, 1AJD.B.952, 3AU9.B.602, 3AUA.A.601, 4AZW.A.1452, 2BBT.A.3, 4BCL.A.367, 4BCL.
A.368, 4BCL.A.369, 4BCL.A.370, 4BCL.A.371, 1BHO.1.901, 2BJI.A.2278, 4BJR.A.1517,
2BOZ.M.1304, 2BW7.D.2201, 3CTL.A.601, 3D19.A.301, 2D32.A.1524, 1DAM.A.901, 4DFX
.E.403, 3DGB.A.2001, 1E14.M.1303, 1E6D.M.1303, 2EB6.A.1001, 3ENI.A.375, 3ENI.A.3
78, 7ENL.A.438, 3EOJ.A.375, 2F9R.B.602, 2FKA.A.9001, 1FMW.A.800, 3FXG.B.501, 3FY
Y.A.401, 1G65.G.301, 4G61.A.301, 3GIE.A.1, 3GQ7.A.692, 2GQ3.A.1002, 4GVE.A.602,
4GX6.A.402, 3I6E.B.386, 4IAC.A.402, 4IL6.j.102, 4IL8.A.501, 4IN7.M.411, 1J7U.A.3
01, 1JB0.A.1011, 1JB0.A.1106, 1JB0.A.1112, 1JB0.A.1121, 1JB0.A.1126, 1JB0.A.1128
, 1JB0.A.1131, 1JB0.A.1132, 1JB0.A.1136, 1JB0.B.1201, 1JB0.B.1203, 1JB0.B.1206,
1JB0.B.1207, 1JB0.B.1211, 1JB0.B.1214, 1JB0.B.1215, 1JB0.B.1221, 1JB0.B.1223, 1J
B0.B.1224, 1JB0.B.1225, 1JB0.B.1234, 1JB0.B.1236, 1JB0.B.1239, 1JB0.L.1501, 1JB0
.L.1502, 1JYX.A.3002, 1KJ8.B.394, 1KJI.B.394, 3KRF.D.901, 3L8F.A.401, 4LCZ.A.314
, 4LNI.A.503, 1LP4.A.342, 4LRJ.A.302, 4LYJ.A.201, 4MFE.C.1104, 3MGA.B.405, 4MKU.
A.209, 1MNZ.A.389, 1NOW.A.401, 1N8W.A.900, 4NHO.B.1403, 4NZN.A.404, 4OEC.A.401,
2OQY.B.402, 3Q2O.B.384, 1Q9L.C.218, 2QB8.B.401, 3QQV.A.381, 1QSH.B.147, 4QTD.A.4
26, 2QVU.A.340, 4QVP.V.302, 4QYI.E.203, 1R03.A.301, 4R02.Y.302, 4R17.K.302, 4R7O
.E.402, 3RLG.A.286, 1RZH.L.854, 1RZH.M.851, 1RZH.M.853, 1SJA.B.701, 3SRF.G.533,
3TAV.A.266, 4U0M.B.503, 4UB6.B.603, 4UB6.B.607, 4UB6.B.610, 4UB6.B.616, 4UB6.C.5
04, 4UB6.C.509, 4UB6.C.512, 4UB6.C.513, 4UB6.D.402, 4UM8.D.2001, 4UOR.A.699, 3V3
Z.L.302, 3V4S.A.402, 2VP0.B.1209, 2VPQ.B.1451, 3VTH.A.805, 1W88.A.1368, 3WNW.A.2
01, 3WQP.H.501, 3WU2.A.405, 3WU2.B.604, 3WU2.B.605, 3WU2.B.606, 3WU2.B.609, 3WU2
.B.612, 3WU2.B.617, 3WU2.C.502, 3WU2.C.510, 3WU2.D.402, 3WU2.b.615, 3WU2.b.616,
3WU2.b.617, 3WU2.c.902, 2YBE.A.1417, 1YF6.M.853, 1YF6.M.856, 1YQ7.A.908, 1Z2P.X.
1295, 2ZDR.A.1001, 1ZM7.D.400, 2VOS.A.1491, 2WCJ.A.1144
[1] "Cluster 7"

4BE2.A.1380, 4DFJ.A.902, 4DOA.A.401, 4DOB.A.401, 3F2B.A.5, 1FIU.A.5555, 2FMS.A.3 40, 3GDX.A.348, 3IEV.A.400, 3JPR.A.339, 3JPT.A.339, 4JWM.A.403, 4K97.A.603, 3M8R .A.2, 4M9L.A.404, 3MBY.A.339, 4MFC.A.401, 4O3N.A.503, 4O3O.A.503, 4O3Q.A.503, 3O HA.A.518, 3OYF.A.396, 3OYG.A.396, 2PFP.A.750, 2PFN.A.950, 3RJK.A.339, 4RNN.A.503 , 1SUZ.A.402, 3V6H.A.402, 1ZJN.A.339, 121P.A.168, 1A2B.A.550, 1A82.A.901, 1AOR.A .609, 2AUT.D.604, 3B1V.A.301, 1B8C.A.308, 4BX3.A.301, 2C3P.A.2237, 2C42.A.3238, 2C4N.A.1251, 4C5A.B.331, 2C77.A.1407, 2C78.A.1407, 3CB3.A.501, 3CFX.A.704, 2CLS. A.550, 3CR3.A.1212, 2DOO.A.1005, 4D7M.A.223, 4DBF.A.401, 2DEI.A.402, 4DEM.F.404, 4DFD.B.301, 4DN1.A.401, 3DOE.A.193, 1DTW.A.401, 1DXE.B.901, 4DXJ.A.403, 3DYH.A. 3003, 3DYM.A.3001, 2E91.A.1301, 4EA0.A.302, 1EBG.A.439, 2EB1.A.502, 1EC7.A.498, 3EFQ.B.4004, 2EW1.A.701, 2EZ9.A.1510, 1F8I.A.451, 3FA5.A.282, 3FD5.B.397, 4FE3.A .304, 4FEG.A.707, 4FFR.A.403, 3FIU.A.5001, 2FOZ.A.349, 2FPR.A.503, 2FRV.B.540, 2 G09.A.901, 3G2F.A.901, 2G80.A.500, 2GCQ.A.435, 2GGE.A.400, 4GIU.A.402, 2GO7.A.20 7, 1GSI.A.1209, 4H19.A.405, 2HCF.A.300, 4HCH.A.405, 3HFW.A.361, 4HHL.A.402, 3HIY .B.402, 4HNL.A.401, 4HQO.A.301, 3HXX.A.445, 4I2B.A.604, 2I33.A.602, 4IAD.A.402, 3ICK.A.401, 1IG5.A.78, 2IHU.A.601, 3IIE.A.501, 3IJR.D.300, 2IK4.A.287, 2IK4.B.28 8, 1IOW.A.331, 2IO8.A.7002, 4IP4.A.503, 4IP5.A.502, 1ITZ.A.1001, 4IWH.A.401, 2IX E.A.2, 2J7N.B.3374, 1JAH.A.168, 1JGT.A.902, 4JND.A.501, 1JSC.A.699, 3JVA.A.356, 4JXO.A.402, 3JYS.A.1, 4K1W.A.501, 3K4Z.A.290, 1K9Y.A.402, 4K9Q.A.601, 1KA2.A.501 , 3KB9.A.701, 4KFU.A.307, 4KGD.A.702, 1KHZ.B.310, 3KMW.A.501, 3KS6.C.251, 1KTG.A .503, 1KTG.A.505, 3KWS.B.401, 4KWD.A.403, 4KX5.A.314, 1KY2.A.401, 3L12.A.313, 1L 8A.A.888, 4LA6.A.501, 4LFG.A.304, 1LON.A.1454, 3LUF.A.301, 3LVO.A.264, 4LZ3.A.40 6, 3MO7.A.595, 1MOW.A.502, 4MFG.A.201, 1MH1.A.201, 3MK2.A.903, 1MQ4.A.2088, 3NO7 .A.200, 1N1Z.A.701, 1N24.A.702, 3N2N.A.1, 3N4F.A.502, 1N8I.A.900, 1NFZ.A.401, 3N JL.A.501, 1NSF.A.859, 1NUX.A.2342, 2NXW.A.4002, 2O1S.A.1001, 3O61.B.202, 2O7O.A. 223, 4OAV.B.802, 3OCU.A.263, 3OCV.A.264, 3OCX.A.264, 3OCZ.A.263, 2ODP.A.901, 2OD B.A.205, 2OGD.A.3003, 4OHY.A.502, 3OUZ.B.459, 3OZF.A.235, 2P3N.A.1758, 3P93.C.40 6, 2PA4.A.325, 3PDE.A.312, 1PFK.A.327, 2PGN.A.610, 4PHG.A.201, 3PK7.A.406, 1POX. A.610, 2PZA.A.6243, 2Q5Q.A.4002, 3Q9L.A.700, 2QGY.A.701, 2QIS.A.907, 2QME.A.179, 3QN3.A.601, 2QQO.A.450, 2QQO.B.452, 1QRA.A.168, 2QTC.A.888, 3QUT.A.225, 3ROU.A. 380, 4R39.A.401, 3RBM.A.1002, 3REG.A.550, 1RKV.A.401, 1RQJ.A.908, 1RVK.A.999, 3R YE.A.907, 3S4J.A.907, 1SAW.A.225, 3SDT.A.821, 3SJN.A.374, 3SLS.A.401, 3STP.A.391 , 3T2D.A.409, 1T8Q.B.1602, 1T9C.B.699, 3TCS.A.368, 4TMW.A.903, 3TWA.A.420, 3U2E. B.1, 4U5X.A.202, 4UAT.A.302, 3UIE.A.403, 1UMG.A.404, 4USI.A.1151, 3V1X.A.501, 2V 26.A.1801, 3V3W.A.403, 3VC2.J.301, 3VPB.A.502, 3VR6.B.602, 3VVH.A.701, 2VZB.A.10 01, 1W2Y.A.1232, 1W7K.A.1423, 1WC1.A.1502, 2WEF.A.401, 4WF7.A.601, 3WOO.A.502, 2 WW8.A.1000, 1WZC.A.300, 1X07.A.900, 1XEX.A.1002, 2XH7.A.1441, 2XTZ.A.1381, 4XXP. A.301, 2Y6P.A.1234, 1Y8A.A.501, 1YHL.A.1401, 2YVO.A.1001, 2YXH.A.502, 1YYQ.B.701 , 1Z4P.X.1001, 1Z4Q.A.2001, 2Z4V.A.1501, 2Z7I.A.1301, 1ZED.A.905, 3ZO9.A.1592, 3 ZOU.A.1295, 3ZX4.B.260, 2PYJ.B.9002, 4ACF.D.1480, 4BBJ.A.750, 2BHW.A.601, 2BHW.A .602, 2BHW.A.603, 2BHW.A.604, 2BHW.A.609, 2BHW.A.612, 2BHW.A.614, 2BHW.B.605, 4B YF.A.1000, 4C5A.A.331, 3C9U.A.309, 1D0X.A.998, 1D0Y.A.998, 1D1B.A.998, 2DCN.B.40 06, 3DHF.A.502, 4DL8.A.304, 4DL8.A.305, 1DXR.L.400, 3DYH.A.3002, 1E1R.F.601, 1E7 9.A.601, 2E8W.A.1201, 2E8X.A.1301, 3EFO.A.1, 2FKW.B.1601, 1G67.A.2007, 2GLQ.A.20 03, 4HGQ.C.201, 3HWX.1.602, 3HZH.A.202, 3ICN.A.402, 4IDO.A.503, 4IGA.A.200, 2IOA .A.5002, 1IV2.B.1572, 2J7N.A.3374, 2J8C.L.1288, 1JB0.A.1107, 1JB0.A.1110, 1JB0.A .1117, 1JB0.A.1122, 1JB0.A.1129, 1JB0.A.1130, 1JB0.A.1133, 1JB0.A.1134, 1JB0.B.1 204, 1JB0.B.1231, 1JB0.B.1235, 1JB0.X.1701, 2JCS.B.1211, 4LCZ.A.306, 4LEO.A.201, 3MLE.C.222, 1N22.B.706, 1N24.B.706, 3NNS.A.401, 4NNN.Z.301, 1NVV.Q.1002, 2ODE.A .3001, 3OLV.A.130, 2OZE.A.299, 2PAN.A.851, 2PLS.I.606, 4PTK.A.302, 1ROX.D.14, 4R UR.W.301, 1RWT.A.614, 3SQS.A.450, 1SVK.A.356, 1T91.A.1301, 3THU.A.500, 4UB6.C.50 6, 4UB6.C.507, 4UB6.C.510, 4UB6.C.514, 1UPB.A.601, 2VB6.A.1000, 3W6P.A.803, 2WJN .M.1325, 3WK4.A.601, 3WU2.A.410, 2X2E.A.1746, 1XHF.A.1001, 1Y9D.A.2601, 2Z7I.B.1 302, 1ZXN.A.900

[1] "Cluster 8"
2AGQ.A.4002, 4D6N.F.1197, 4DF8.A.903, 4DFP.A.901, 4IRD.F.903, 3LK9.A.340, 4M2Z.A
.501, 2OZM.A.904, 1QTM.A.1001, 3R7P.A.317, 3SI6.A.905, 2XCP.A.1004, 2XCA.A.3000,
 2XCA.A.3001, 1YVP.A.1001, 1ZBL.A.202, 3A06.A.500, 3A58.B.401, 2AG1.B.611, 3B8I.
A.289, 2BW7.A.2201, 4BYF.C.1000, 4CE0.A.1251, 1CG0.A.435, 4CTA.B.401, 4D2I.A.147
8, 2DW6.D.2004, 2E0A.A.500, 1E2A.C.106, 1ECQ.A.498, 1EFL.B.1604, 3EG5.A.180, 2EG
H.A.900, 1ELX.B.452, 1F1Z.A.2002, 3FFU.A.156, 3FFU.B.155, 4FFL.A.905, 4FFO.A.904
, 3FHY.B.404, 3FTQ.A.371, 2G9Z.A.704, 3G9D.A.298, 1H65.A.282, 3HWX.A.602, 4HYV.A
.1001, 3I3O.A.306, 4IOK.A.604, 2J5X.A.200, 4KCW.A.1001, 1KH7.A.452, 3KR4.C.1004,
 3KRO.D.3002, 4KS0.A.1001, 4L2X.F.403, 4L2X.F.405, 4LF1.A.801, 4LNI.E.505, 1M1B.
B.999, 3M1Y.A.300, 1MB9.B.601, 1MBZ.A.603, 1MBZ.A.604, 1MRS.A.300, 2NOM.A.402, 1
NUW.A.2498, 1NV7.A.3341, 4OGE.A.1204, 4OKZ.A.901, 2OPM.A.907, 3OPS.A.500, 2OQY.A
.401, 1OVM.A.601, 3P0X.A.430, 1PFK.A.325, 2PLS.G.603, 2Q58.A.5, 1QF5.A.433, 3QQV
.A.382, 2QTV.B.210, 4RAD.D.302, 1RC5.A.761, 3RIM.A.1001, 4RNH.A.1501, 1S1C.A.300
, 3SE1.A.182, 1SOJ.A.2123, 3SSN.A.501, 3TXA.A.801, 4U0M.A.503, 3UJ2.A.431, 2V3W.
A.1528, 2VQD.A.1449, 2VWT.A.301, 3VYT.C.602, 3W7F.A.303, 4WK2.B.501, 1WL6.A.801,
 2XAM.B.1030, 1Y9I.A.601, 1YHY.A.699, 2YWF.A.701, 1Z0K.A.1201, 3ZDY.B.2001, 4D6O
.A.1187, 4DLE.A.901, 2HVI.D.878, 1N56.A.401, 4NLK.A.402, 4NM1.A.401, 2A5G.A.231,
 4A7Y.A.952, 1AQF.A.534, 3B03.D.1001, 2B9J.A.600, 2BKK.A.1266, 4BYG.A.996, 3C14.
C.403, 3CK5.D.400, 1CUL.A.1006, 4CYI.D.1000, 2D33.A.525, 4E1E.A.402, 2E8X.A.1302
, 1E9I.D.1433, 2E92.B.1303, 3EA4.A.699, 3FPA.A.901, 4GA3.A.1003, 3GFT.F.202, 4HE
0.A.403, 4HGR.B.201, 3HIY.A.401, 3HU2.D.801, 3HYL.A.675, 3I0O.A.502, 4J5I.F.402,
 1JB0.B.1202, 4JLZ.B.502, 4KCV.B.1001, 4LNI.D.503, 4LNI.D.505, 3LUZ.A.264, 4MGH.
A.1302, 4N0G.B.402, 4NCJ.A.903, 4NDN.A.401, 2O56.A.2001, 3O61.A.202, 2PSN.A.701,
 3Q1O.D.400, 3Q7P.B.257, 1SOJ.C.2127, 3T9E.A.603, 4TVU.B.601, 4TXZ.B.502, 4U0N.A
.401, 3VAD.A.402, 2W00.A.1887, 3WBZ.F.403, 3WQM.A.403, 1XBT.D.4194, 1XZ8.B.180,
1ZCA.A.383
[1] "Cluster 9"
1BPY.A.339, 4BWJ.A.1834, 4DFK.A.902, 4DFM.A.902, 4DOC.A.401, 4DQP.D.902, 4ELT.A.
902, 4ELU.A.902, 4F5O.A.402, 4FO6.A.601, 4GZ2.B.402, 2J0S.A.1412, 1JJ2.0.8010, 3
K58.A.1001, 3K59.A.1001, 3KD5.E.916, 4M04.A.702, 4M04.A.703, 3MFI.A.515, 4O3O.A.
502, 4O3Q.A.502, 3OJS.A.7, 4P4M.A.402, 4PUQ.B.401, 4Q8E.A.502, 4QM6.A.1002, 4R65
.A.402, 3RJH.A.403, 3RTV.A.833, 3SNN.A.905, 3SPY.A.903, 3TFR.A.339, 3TFS.A.339,
1W7A.A.1802, 1XSN.A.576, 3ZVM.A.1526, 3A0T.A.800, 3A4L.A.401, 2A5Z.A.701, 2A5D.A
.1231, 4A6X.A.350, 4ACF.A.1482, 4ACI.A.1187, 3AHC.A.826, 3AHD.A.826, 3AHE.A.826,
 3AHG.A.826, 2AL1.B.439, 4ANB.A.1384, 1AOX.A.400, 1AS0.A.356, 2AUU.A.203, 2AUU.A
.204, 2AUT.A.601, 3AYX.A.701, 2B0T.A.800, 1B25.A.800, 4B2P.A.1351, 2B56.A.488, 3
B7L.A.908, 3B7L.A.909, 2B82.A.1013, 2B9H.A.700, 4BAS.A.1183, 3BB1.A.282, 2BBS.A.
3, 3BC1.A.194, 2BME.A.1184, 3BN3.A.1, 1BOF.A.800, 2BON.A.1302, 2BVC.A.504, 3BWV.
A.300, 4BW9.A.501, 4BWR.A.1468, 4BX0.A.1291, 1BYQ.A.1001, 1C1Y.A.171, 3C4Z.A.563
, 3C5H.A.302, 2C5L.A.1168, 4C5B.A.1314, 2CBZ.A.1872, 1CHN.A.200, 1CIP.A.356, 2CJ
E.A.1267, 2CK3.A.601, 2CK3.F.601, 2CN5.A.1506, 4COK.A.601, 1CTQ.A.168, 3CUR.H.55
3, 3CUS.Q.553, 3CX8.A.378, 3D2R.A.500, 3D36.A.478, 4D6P.A.1352, 2D7C.A.1002, 1D8
C.A.3001, 4DBR.A.810, 3DDC.A.600, 3DDH.B.232, 2DDT.A.311, 3DGT.A.800, 3DKC.A.2,
4DN5.A.1001, 4DSO.A.202, 4DUX.A.3001, 4DWG.A.401, 4DWO.A.301, 3DYH.A.3004, 3DYP.
A.3001, 2DY1.A.701, 4DYK.A.502, 4DZH.A.501, 4E01.A.402, 1E2Q.A.401, 3E2D.A.603,
1E3D.B.901, 3E5H.A.200, 3E81.A.165, 3E8M.A.165, 2E9S.A.603, 1E9A.A.401, 4EEN.A.3
01, 4EFM.A.202, 3EHG.A.371, 1EK0.A.601, 2ERX.A.403, 3ET4.A.301, 3ET5.A.255, 4EX6
.A.301, 4EX7.A.301, 2EZU.A.1610, 3EZ3.A.1104, 2EZ8.A.1510, 4F1J.A.301, 2F2A.B.60
1, 1F5N.A.595, 3F61.A.310, 1F9H.A.161, 1F9H.A.162, 2FFQ.A.356, 2FH5.B.270, 4FI1.
A.401, 4FK9.A.401, 2FOZ.A.348, 4FP1.A.401, 1FSG.A.302, 3FSY.A.333, 2FUE.A.500, 4
FYP.A.301, 3FZN.A.605, 2G0W.A.501, 2G1T.A.1501, 3G15.A.602, 1G4C.B.362, 1G4T.A.2
005, 3G5A.D.307, 1G5T.A.998, 3G6K.A.307, 2G6B.A.301, 1G97.A.460, 3GAI.A.189, 2GC
N.A.2001, 2GCP.A.2001, 2GIL.A.1201, 3GON.A.600, 4GOJ.A.202, 4GP2.A.402, 1GSA.A.3

19, 2GSM.A.3006, 4GT8.A.402, 3GYB.A.1, 4H1Z.D.401, 1H2A.L.1005, 1H2R.L.1005, 2H5 7.A.202, 3H70.A.342, 3H7V.A.331, 3H80.A.214, 4H8E.A.301, 4HAT.A.302, 4HDO.B.200, 2HF8.A.301, 2HF9.A.301, 4HGN.A.200, 2HNE.A.601, 4HOR.X.101, 3HQJ.A.145, 3HRZ.A. 628, 3HRZ.D.742, 3HSD.B.162, 1HTW.A.561, 3HW5.A.999, 1I0L.A.902, 2I1Q.A.501, 2I3 4.A.301, 2I5R.A.301, 3I76.A.1001, 4I94.A.402, 3IBA.A.403, 3ICM.A.401, 3ICZ.A.403 , 4IDN.A.502, 4IDP.A.502, 2IHT.A.601, 1IH8.A.4002, 1IHU.A.592, 2IHP.A.287, 3IJL. A.386, 2IK4.B.287, 2IK4.B.289, 2IK6.B.287, 2IOR.A.2000, 3IP0.A.161, 4ITR.D.203, 4IUC.L.702, 4IUD.L.1002, 4IVG.A.803, 2IYW.A.202, 2J0V.A.1180, 2J7P.A.1401, 2J7P. D.1401, 1J9J.A.301, 2JC9.A.1491, 2JCB.A.1192, 4JDP.A.301, 2JD4.A.4061, 4JS0.A.20 2, 3JTC.C.34, 3JYY.A.301, 3JZ0.A.303, 1K77.A.300, 3K8K.A.700, 3KA3.A.176, 3KB9.A .702, 3KC2.A.355, 1KCZ.A.901, 1KK1.A.413, 3KKO.A.180, 1KMQ.A.401, 4KO8.A.801, 1K QP.A.5001, 1KQP.A.5002, 4KQW.A.404, 1KSH.A.202, 1KTG.A.502, 1KTG.A.504, 3KTA.A.1 84, 3KUC.A.171, 4KUX.A.703, 4KVG.A.202, 4KWD.A.404, 4KXW.A.1001, 4L57.B.201, 3L8 H.A.801, 4LFG.A.303, 4LFG.B.303, 4LGY.A.1302, 4LHW.A.301, 4LJ9.A.902, 3LLU.A.502 , 4LPM.A.208, 3LUF.A.300, 3LX5.A.301, 3LXX.A.402, 4LYK.A.401, 4LZO.A.403, 3M1I.A .1178, 4M53.A.527, 4M9Q.A.302, 4MDB.A.403, 4MGG.A.404, 3MHY.A.115, 1MJN.A.1001, 3MJH.A.201, 1MMG.A.998, 1MNE.A.998, 4MRT.A.301, 4MUM.A.301, 1MXG.A.439, 3MX3.A.6 01, 3MYH.X.997, 3MYK.X.998, 3MYL.X.998, 4N0D.A.402, 3N45.F.2, 3N45.F.3, 1N6I.A.2 01, 1N6L.A.201, 1N6N.A.201, 1N6O.A.201, 1N6P.A.201, 1N6R.A.201, 1NBO.A.201, 4NBS .A.502, 4NDO.A.302, 3NKV.A.500, 1NN5.A.401, 1NRJ.B.1, 2NSY.A.305, 4NWI.A.401, 1O 08.A.2800, 1O3Y.A.1002, 4O7I.A.401, 1OBW.A.176, 3OCW.A.263, 3OCY.A.264, 4ODJ.A.5 02, 3OE1.A.601, 2OGD.A.3002, 2OGD.A.3004, 3OIW.A.170, 2OI6.B.6000, 4OI4.A.502, 1 OKK.A.1002, 2OLR.A.543, 4OMF.A.503, 3OP2.B.500, 2ORW.A.501, 1OXV.A.1102, 1OXV.D. 1101, 3OYX.A.601, 3OZX.A.613, 2P27.A.307, 4P31.A.402, 4P32.A.402, 1P4M.A.201, 1P 5Z.B.401, 3P5P.A.901, 3P96.A.412, 4PFY.B.601, 3PGL.A.1, 4PHH.A.202, 3PIT.A.180, 2PKE.B.300, 3PNL.B.1212, 2PNQ.A.502, 1PPV.A.401, 4PQ9.A.301, 2PS5.B.701, 1PVF.A. 401, 2PYW.A.501, 2PZ8.A.4001, 2PZE.A.3, 2Q28.A.1001, 1Q3H.C.674, 3Q3J.B.201, 2Q3 F.A.301, 3Q46.A.307, 4Q4C.A.404, 3Q5V.B.599, 3Q60.A.603, 2Q66.A.602, 1Q92.A.1003 , 4QC2.A.302, 3QF7.A.854, 1QGU.B.3002, 1QGU.D.3006, 2QG8.A.201, 4QHZ.A.302, 2QIS .A.908, 2QIS.A.909, 1QK5.A.303, 3QNM.A.400, 2QTY.A.348, 2QTO.A.1001, 3QUQ.A.225, 3QXC.A.222, 3QXJ.A.224, 3QXS.A.223, 2QXO.A.161, 3QYY.A.505, 1R2Q.A.300, 3R3S.A. 296, 2R6O.A.801, 3R7W.A.600, 2R8E.A.201, 2RAH.A.354, 2RAR.A.501, 2RAV.A.701, 3RA P.R.200, 2RB5.A.701, 2RBK.A.501, 3REF.A.192, 1RKQ.A.1273, 4RKF.A.202, 3RLF.A.150 1, 1RMT.A.1413, 3RO6.A.400, 4ROQ.A.401, 1RQI.A.604, 1RQJ.A.909, 3RUS.A.544, 3RV3 .A.1004, 3RYE.A.908, 3RYE.A.909, 1RYA.A.1001, 4S1H.B.303, 3S4J.A.908, 3S4J.A.909 , 3SAE.A.820, 3SF0.A.263, 1SHT.X.219, 3SL2.A.701, 1SVM.A.750, 1SVS.A.356, 1T0P.A .901, 3T10.A.401, 3T1K.A.401, 3T2S.B.401, 3T2B.A.409, 3T7A.A.602, 1TC6.A.501, 2T CT.A.223, 3TEP.A.1, 3TG0.A.503, 1TMM.A.162, 4TMT.A.902, 4TMV.A.902, 4TMX.A.903, 4TN1.A.902, 3TSO.A.200, 4TY0.A.502, 4U82.A.301, 1U8Y.A.301, 4UAK.A.503, 4UAV.A.4 01, 2UAG.A.1001, 1UBK.L.1005, 3UCW.A.100, 4UCX.Q.1553, 4UE3.L.603, 3UJR.A.502, 3 UJR.B.502, 3UJS.A.601, 3UJS.B.601, 4UM7.A.175, 3UPY.A.446, 1UPT.A.1183, 3UPL.A.4 47, 3UQY.L.603, 4UQL.Q.1552, 4URH.Q.1552, 4V0L.A.601, 4V1T.A.1777, 1V54.A.3518, 2V7Q.A.1512, 2VBU.A.1134, 3VC1.A.301, 3VC1.I.301, 3VCC.A.401, 1VG8.A.1401, 2VG3. A.1297, 2VK1.A.601, 2VK8.A.1565, 1VOM.A.997, 1VZM.B.1046, 1VZM.B.1047, 1W2Y.A.12 31, 3W40.A.201, 2W4J.A.1280, 2W5V.A.1378, 2W5X.A.1379, 1W78.A.1422, 1W78.A.1423, 1WA5.A.1178, 1WF3.A.401, 4WH2.A.402, 4WH3.A.402, 3WJP.A.403, 3WJP.A.404, 2WKQ.A .1724, 3WNZ.A.502, 3WRY.C.1202, 1WUH.L.1005, 1WUK.L.1005, 3WXM.A.502, 1X06.A.900 , 2X13.A.1418, 2X14.A.1418, 1X3S.A.200, 1X84.B.401, 2X98.A.1477, 1XBY.A.601, 2XC W.A.1498, 1XFI.A.400, 2XH2.B.1439, 2XJB.A.1494, 2XJD.A.1497, 2XTN.A.1232, 1XX1.A .9001, 2Y8E.A.1177, 2YCH.A.501, 1YHL.A.1403, 1YMV.A.200, 1YNS.A.1258, 1YQ9.H.540 , 1YQT.A.591, 1YQT.A.592, 1YRQ.H.553, 1YS7.A.1002, 1YU4.A.2002, 2YVO.A.1002, 2YV P.A.184, 1YVD.A.850, 1YZL.A.401, 1YZN.A.301, 1YZT.A.700, 1Z06.A.203, 1Z07.A.300, 1Z08.A.1300, 1Z08.C.3300, 1Z0J.A.400, 1Z2N.X.1295, 1Z4J.A.1001, 1Z4K.A.229, 1Z4 L.A.2001, 2Z4V.A.1502, 2Z4X.A.1201, 2Z4X.A.1202, 1Z59.A.1001, 1Z5G.A.601, 1Z5G.D

.604, 2Z52.A.1302, 1Z88.A.601, 1ZC3.A.500, 3ZFD.A.500, 3ZIA.A.601, 1ZJJ.A.1001, 3ZVL.A.1524, 3ZX5.A.260, 2IHM.A.700, 3A0U.A.201, 3A10.A.201, 2AKZ.A.441, 3BH7.A.1, 4BX2.A.301, 1D0Z.A.998, 1D1A.A.998, 3DNT.B.442, 3G5A.C.307, 2G77.B.503, 1GFI.A.356, 2GJ8.A.602, 3GL9.A.123, 3H1E.A.202, 1HE1.C.202, 2HEG.A.300, 2HF7.A.700, 2I7D.A.728, 4IF4.A.300, 1J97.A.220, 4JA2.A.201, 1JB0.A.1118, 4KEM.A.401, 1L3R.E.392, 1L5Y.A.701, 1L7N.A.221, 4LCZ.A.307, 4LCZ.C.316, 1LGH.A.57, 1LGH.A.58, 1LGH.B.59, 4LRS.A.404, 1LVK.A.998, 1MPS.M.801, 1MX0.E.501, 3N5K.A.2001, 1N6K.A.201, 4NV0.A.402, 2OGX.A.291, 4OVN.A.201, 3PDE.B.310, 3PL9.A.602, 3PL9.A.603, 3PL9.A.609, 3PL9.A.610, 3PL9.A.612, 3PL9.A.613, 3PL9.A.614, 2PL1.A.204, 3QHW.A.298, 2R25.B.1, 1RL0.A.801, 4S1H.A.303, 3T2S.A.401, 3T6D.L.401, 3T6D.M.400, 3T6E.L.400, 3T9E.A.602, 3TCS.B.368, 1TX4.B.681, 1W7J.A.1793, 1W9I.A.1755, 3W9S.A.202, 2WF7.A.1220, 1WQA.B.456, 2WZB.A.1417, 1XBX.A.601, 1YZQ.A.901, 1ZES.A.302, 2ZXE.A.2002

[1] "Cluster 10"

3FSY.A.334, 1NUY.A.2342, 4IRK.A.402, 2OTL.A.8066, 1YJ9.0.8067, 521P.A.168, 3AJP.A.183, 3ALN.B.406, 1AM4.D.679, 1AR1.A.560, 1CEE.A.180, 4D0L.B.2001, 3D19.D.301, 4DVG.A.201, 2DW7.L.2012, 2E8W.B.1204, 4EOP.D.501, 3EQB.A.9002, 4FMA.F.402, 3FPA.B.901, 3GFT.E.202, 3GOL.A.580, 1GQ9.B.1242, 3GT8.D.14, 4GZM.A.1001, 2HAW.A.1002, 3HYT.A.802, 4HYP.C.302, 4I40.A.301, 2I07.A.5001, 2IOA.A.5001, 4JVJ.F.403, 4JVJ.F.404, 3KRP.D.903, 3KZ1.E.550, 3LAW.B.1401, 1MAB.A.602, 3NC0.C.218, 2O1X.C.2003, 3OPS.D.501, 2OQY.C.402, 2P8E.A.306, 1PKG.A.1481, 1Q3H.A.674, 1R0Z.D.674, 4R9U.D.302, 4RAB.C.303, 1SVW.B.301, 3T5P.H.301, 4UOR.K.699, 3VHX.G.185, 2VWI.B.1293, 1W1W.A.2001, 1W85.E.1368, 2WCJ.A.1146, 3WIG.A.402, 3WNW.J.201, 1XD2.A.167, 1YM0.A.402, 2ZRY.D.702

[1] "Cluster 11"

3A4K.A.301, 4AAB.B.1157, 2AQ4.A.302, 2BCV.A.576, 4BDY.A.1381, 4CEI.A.2234, 1CW0.N.202, 4D6N.F.1196, 4DF4.A.901, 4EEY.A.502, 3GDX.A.347, 3GPL.A.800, 3GQC.B.203, 4IR9.F.402, 2IS0.A.339, 2ISP.A.339, 4K98.A.602, 4K99.A.602, 3LK9.A.339, 4M30.A.502, 4M47.A.402, 4MFF.A.401, 3MQY.A.500, 3OS0.A.394, 3OYA.A.396, 3OYC.A.396, 3OYC.A.397, 3OYE.A.396, 3OYF.A.397, 3OYG.A.397, 3OYH.A.396, 3OYH.A.397, 4PGQ.A.400, 4QCL.A.1302, 1QSY.A.1001, 3RJK.A.340, 3S3M.A.396, 1SKR.A.4001, 3SPY.A.904, 3SV3.A.836, 1TK8.A.901, 4TUQ.A.402, 3UQ2.A.1, 2W35.A.1224, 1ZBL.B.204, 4A01.A.1770, 4A01.A.1771, 1A49.H.5334, 3A7D.A.300, 4AC0.A.1205, 3AJO.A.183, 3AXK.A.478, 1AZS.C.403, 3B1X.A.301, 1B7T.A.836, 2BKU.A.221, 3BNY.D.701, 1BZY.A.901, 1CH8.A.434, 1CIB.A.434, 2CJE.A.1268, 3CP6.A.501, 1CUL.C.396, 3CWH.A.392, 1DAK.A.901, 4DBH.A.401, 3DFY.A.401, 3DHD.A.502, 1DIE.A.399, 1DQN.A.451, 3DVA.A.1368, 2DW6.A.2001, 4DWB.A.508, 4DXJ.A.401, 4DXJ.A.402, 3DYG.A.3004, 4E1E.A.403, 1E9I.A.1431, 4EA0.A.303, 1EBG.A.438, 1EC9.A.498, 1ECB.A.507, 3EFQ.A.3003, 3EFQ.B.4002, 3EGT.A.3003, 3EQI.A.3, 3ES8.A.393, 3EYA.H.613, 3FD5.A.395, 3FD6.B.395, 4FFL.A.904, 2FG5.A.301, 4FI4.A.501, 2FN0.A.701, 3FQI.A.1000, 1FTN.A.300, 1G3B.A.501, 4G61.A.302, 2G9Y.A.452, 4GA3.A.1004, 4GIS.A.405, 2GQS.A.240, 2GQ3.A.1000, 1H1D.A.300, 4H1Z.A.401, 3H4L.A.701, 2HCJ.A.998, 3HDG.B.201, 4HE1.A.404, 4HE2.A.405, 4HGR.A.201, 3HQD.A.501, 3HQP.B.502, 2HWG.A.901, 4I2B.A.602, 4I3Y.A.302, 3I6E.A.386, 3IBA.A.401, 3IBA.A.402, 3ICZ.A.401, 2IDX.A.603, 4IEE.A.501, 1II0.A.593, 4IJQ.A.304, 2IK2.B.287, 2IK2.B.290, 2IOA.B.5004, 4IT1.A.501, 2IUC.B.1008, 4IX4.A.602, 2JCS.A.1211, 2JI6.A.1567, 2JI8.A.1567, 1JP4.A.701, 3JUK.C.307, 1K9Y.A.403, 4K9N.A.601, 4KCV.A.1001, 1KEK.A.2237, 1KHK.A.452, 4KMQ.A.1102, 1KO5.A.1001, 1KP8.A.550, 4KQX.A.405, 4KUX.A.701, 4KX3.A.302, 4LA7.B.601, 3LVV.A.695, 3LVV.A.697, 4M69.A.403, 1MC1.A.603, 4MIT.A.202, 4MPO.B.204, 4MPO.E.201, 3MQT.H.626, 4N0G.A.403, 1N1Z.A.702, 1N20.A.703, 1N24.A.703, 3N3T.A.802, 4NEH.B.703, 1NHT.A.435, 4NM3.A.405, 3NO1.A.397, 1NUW.A.2497, 1NUY.A.2343, 3NZG.A.507, 2O1O.A.501, 4O4D.A.401, 1OAD.A.392, 3OES.A.202, 1OFH.B.453, 1ORK.A.223, 2OX4.A.402, 3OYZ.A.500, 1OZF.A.699, 3P5R.A.901, 1P7T.A.1000, 1P9B.A.1600, 4PAL.A.110, 3PDE.A.311, 4PFK.A.327, 2PLS.J.604, 2PP3.A.901, 3PUV.A.1501, 6Q21.D.173, 3Q30.A.601, 3Q46.A.305, 1QC5.A.601, 1QC5.B.602, 4QEH.A.402, 1QF4.A.433, 4QPM.A.1502, 1QS0.A.501, 3QU4.A.225, 2R9V.A.504, 3RBM.D.1003, 2

RDX.A.378, 4ROP.A.504, 1RQI.A.605, 3S9I.A.743, 3SAD.A.801, 3SB0.A.801, 3SH6.A.176, 3SOP.A.401, 3ST8.A.496, 3T0Z.A.401, 3T8O.A.564, 1T9B.A.1699, 1TND.A.352, 4TQ4.A.401, 3TTE.A.361, 3TTE.B.361, 3TZF.A.279, 4USJ.C.302, 3UXL.A.360, 4V1T.A.1778, 2V5K.A.301, 1V8K.A.501, 1VA6.A.524, 2VBI.A.1000, 3VD3.A.3001, 2VDM.B.2001, 3VMM.A.501, 2VP0.A.1209, 2W00.B.1894, 3W2W.A.904, 1W5T.B.701, 1W88.C.1368, 1WDD.A.1476, 4WK4.B.501, 4WRR.A.401, 2WX5.L.1282, 2X5Z.A.602, 2XH4.A.1439, 5XIM.A.395, 5XIN.A.395, 1XIN.A.395, 8XIM.A.395, 1XLC.A.399, 1YVE.I.602, 2Z4W.A.1302, 2Z4Y.B.1301, 3ZCB.A.301, 1ZVW.A.4001, 3ZYC.A.1750, 3A4K.C.301, 4BDZ.A.1380, 3ICE.E.502, 4J9O.A.502, 3MDA.A.577, 3TFR.A.340, 1TFW.B.1601, 3ALO.A.1, 2AUU.A.201, 2B8W.A.595, 4BJU.A.998, 1BS1.A.901, 2C43.A.1317, 3CBT.A.301, 3CRL.A.2000, 3CX7.A.378, 3DKL.A.502, 1E1R.A.601, 2E8T.B.1303, 1E9I.B.1431, 2E91.B.1303, 3EF1.A.1, 3EFQ.B.4003, 3EYA.A.613, 4FFR.A.406, 1FQJ.A.352, 2G08.A.500, 3G8D.B.1002, 2GTP.A.401, 4HEO.A.402, 2HO4.B.301, 3KRO.A.3003, 3LOC.A.257, 4LRZ.A.302, 3MCO.A.427, 3MCO.B.426, 4NM5.A.406, 3O5T.A.299, 1PPW.A.401, 2Q58.A.3, 3RBM.B.1001, 1RLT.A.805, 3RRA.A.406, 3SS8.A.302, 3T34.A.1002, 3T5P.F.301, 3U2E.A.2, 2UXR.A.1405, 2V54.B.1205, 1W5T.A.701, 2ZRW.A.702

Table S37. all-ligand-number Mg, compressed group

	size	largest_angle*	middle_1*	middle_2*	middle_3*
1	"24"	"135.4+/-11.8"	"60+/-4.5"	"71.4+/-6.7"	"81.3+/-8.7"
2	"68"	"158.6+/-8.6"	"57.2+/-3.9"	"86.9+/-5.2"	"102.5+/-7.3"
3	"38"	"148.5+/-10.8"	"65.2+/-6.3"	"77.5+/-6.3"	"92+/-10.4"
4	"57"	"167.2+/-5.9"	"66.5+/-6.1"	"86+/-3.4"	"101.8+/-4.8"
5	"114"	"172.1+/-4.3"	"82.4+/-3.1"	"89.7+/-1.8"	"97+/-2.9"
6	"18"	"159.6+/-10.6"	"58.1+/-3.6"	"82.5+/-8.5"	"101.2+/-10.9"

	middle_4*	smallest_opposite_angle*	Tetrahedral	TrigonalBipyramidal
1	"96.5+/-11.6"	"82.1+/-15.8"	"0"	"0"
2	"149.5+/-9.7"	"78.9+/-8.3"	"0"	"0.003"
3	"131.1+/-9.4"	"61.4+/-7.3"	"0"	"0"
4	"157.7+/-7.1"	"57.7+/-6.2"	"0.004"	"0.001"
5	"159.6+/-6.5"	"58.5+/-2.6"	"0"	"0.008"
6	"121.4+/-8.9"	"119.7+/-18.9"	"0.002"	"0.001"

	TrigonalBipyramidalVA	TrigonalBipyramidalVP	Octahedral	SquarePyramidal
1	"0"	"0"	"0"	"0"
2	"0.008"	"0.084"	"0.01"	"0.041"
3	"0.002"	"0.02"	"0"	"0"
4	"0.011"	"0.08"	"0.004"	"0.05"
5	"0.006"	"0.406"	"0.243"	"0.403"
6	"0.009"	"0.005"	"0"	"0"

	SquarePyramidalV	SquarePlanar	TrigonalPrismatic	TrigonalPrismaticV
1	"0"	"0"	"0"	"0"
2	"0.148"	"0.097"	"0"	"0.03"
3	"0.015"	"0"	"0"	"0.006"
4	"0.154"	"0.101"	"0"	"0.008"
5	"0.524"	"0.437"	"0"	"0.002"
6	"0"	"0.002"	"0"	"0"

	PentagonalBipyramidal	PentagonalBipyramidalVA	PentagonalBipyramidalVP
1	"0"	"0"	"0"
2	"0"	"0.002"	"0.008"
3	"0"	"0"	"0"

4	"0.002"	"0.01"	"0.009"
5	"0"	"0"	"0.093"
6	"0"	"0"	"0"

	SquareAntiprismatic	SquareAntiprismaticV	HexagonalBipyramidal
1	"0"	"0"	"0"
2	"0.004"	"0.004"	"0"
3	"0"	"0"	"0"
4	"0"	"0.002"	"0"
5	"0"	"0"	"0"
6	"0"	"0"	"0"

	HexagonalBipyramidalVA	HexagonalBipyramidalVP
1	"0"	"0"
2	"0"	"0"
3	"0"	"0"
4	"0"	"0"
5	"0"	"0"
6	"0"	"0"

Table S38. Cluster members of all-ligand-number Mg, compressed group

[1] "Cluster 1"
2NVQ.A.2001, 4P0P.A.601, 2AEK.A.804, 2AQX.A.601, 1AZT.B.406, 1DOA.A.199, 3EPS.A.1606, 3EQL.N.1528, 4F38.A.202, 2G5H.B.501, 1H8H.F.601, 4I1O.C.201, 3IG8.A.697, 1JFG.B.703, 3KRF.A.904, 3O7L.B.352, 4OAU.C.803, 1S4E.B.1600, 3U87.B.403, 2UXR.B.1398, 1WBQ.C.1441, 1YXI.A.342, 1ZOT.A.901, 3ZXT.A.1278

[1] "Cluster 2"
4BE1.A.1382, 4D6N.A.1188, 2G8H.A.301, 2VBL.C.1026, 1AO0.A.469, 2ATX.A.201, 4E4F.A.504, 3FDG.A.357, 3G15.A.605, 4GMJ.B.302, 4GYZ.I.402, 3IJQ.B.386, 2IOA.B.5003, 1JCT.A.498, 3K5H.A.401, 3K5H.A.402, 1KFS.B.2, 4KMQ.A.1103, 3LD0.5.54, 2LVJ.A.101, 3MLE.A.222, 3N3T.B.803, 4ORK.A.502, 3POW.A.471, 3PMG.A.562, 3Q8U.E.159, 3RUW.D.544, 4S17.A.501, 3SH1.A.222, 3U2E.B.4, 3U7F.B.1, 4UM9.B.2001, 2V0N.A.601, 2W8D.A.1636, 4WB8.A.402, 1WC6.C.2202, 3WO0.A.503, 3WQS.B.502, 4KI8.C.602, 4FFR.A.404, 3G9D.A.299, 1JR4.A.300, 4N41.E.101, 4ACF.B.1481, 3ATT.A.379, 3B9T.A.485, 2BVC.A.503, 3CWH.A.391, 2D32.A.1523, 1DIE.A.398, 2DW7.B.2002, 4EO0.A.301, 3FDG.B.356, 4FHY.A.402, 3HB0.B.702, 4I40.B.301, 1IV4.E.1565, 4LNI.D.504, 4ML9.B.301, 1MXA.A.411, 4PV4.A.501, 1SBJ.A.163, 3T80.B.160, 4U03.A.504, 3U2E.A.3, 1CUL.A.1007, 1SO2.C.475, 2Z2P.A.1003

[1] "Cluster 3"
2EWG.A.3002, 1GUS.A.1069, 4GWS.A.402, 2J1L.A.1195, 4NH0.A.1402, 3PLS.A.1, 3A6P.H.1178, 1QVG.0.8010, 1TTT.B.407, 2CN8.A.1504, 2D33.B.1524, 2DW7.A.2001, 4F0P.A.501, 2F1I.A.501, 4FMC.B.203, 1GXB.C.1346, 3HQP.A.502, 3IG8.A.696, 4K10.A.405, 3LZ9.A.852, 3MQT.W.395, 4N0G.A.405, 4NU1.A.404, 3OB8.D.3001, 4P9D.B.202, 4PYK.A.302, 3RBM.C.1002, 2RD5.C.1001, 1S6P.A.601, 4U81.A.501, 3UJ2.H.430, 4UOR.C.699, 2V0N.A.502, 1YHM.A.401, 1V14.C.1134, 1EQR.B.902, 2ICJ.A.400, 4K6T.E.412

[1] "Cluster 4"
4IEM.C.401, 4IRC.A.402, 1MUH.A.479, 3A7E.A.215, 2A9F.A.801, 4AZW.A.1453, 3C41.J.603, 3CVJ.C.243, 4DHP.A.303, 1EFK.A.604, 4GME.A.501, 2J0L.A.1688, 4K10.D.404, 3M00.A.551, 4OHF.B.503, 2ONS.A.702, 4P9D.A.202, 3PCR.B.1231, 1POW.B.610, 2PUI.B.401, 4RUB.B.491, 1T5T.A.1005, 4UOP.A.1612, 1VA6.A.523, 2VPQ.A.1450, 3WBH.B.503, 3WQQ.A.502, 4GZ2.A.402, 2HVI.A.878, 1MOW.A.371, 2OTJ.3.8078, 3V6J.A.403, 2A9F.B.800, 4CS3.A.1464, 4DCK.B.203, 1ECB.D.507, 3EW9.A.501, 4FVQ.A.902, 4FZL.B.302, 4GA3

.A.1002, 4GIR.B.401, 4GX4.B.402, 4GX6.B.402, 1HJK.B.452, 4KI8.A.602, 3MJX.A.901,
 3OTB.A.401, 3TLM.A.1005, 2VBI.B.1000, 1Z2P.X.1296, 2Z4W.B.1303, 4IFD.J.1105, 3I
AF.A.572, 3PUG.A.601, 2PUI.A.401, 3QHR.A.298, 4R9U.C.302
[1] "Cluster 5"
4BDZ.A.1381, 4BEO.A.1381, 1MOW.A.373, 4NCB.C.101, 4O3S.A.503, 3OYB.A.397, 3OYD.A
.397, 3OYE.A.397, 3OYJ.A.397, 3OYL.A.397, 3OYN.A.397, 1RVC.A.401, 3S3N.A.397, 3S
M4.E.15, 1YTU.A.428, 4ACF.A.1481, 2AL1.B.438, 3AU9.A.601, 3BZN.A.501, 4C5A.A.330
, 4C5B.A.1313, 4C5C.A.1313, 3CFX.A.703, 1DAW.A.342, 1DAY.A.342, 4DFX.E.404, 3DG6
.A.2001, 1EC8.A.498, 4EOM.A.302, 3ETH.A.401, 3ETH.A.402, 3ETJ.A.402, 1EYZ.A.401,
 1EYZ.A.402, 2FN1.A.504, 2FUV.A.901, 3GN6.A.321, 3GQ8.A.692, 4H2H.A.401, 2HGS.A.
501, 3HPF.A.401, 3HWO.A.1702, 3I4K.A.385, 4IAC.A.401, 4IAD.A.401, 1IOW.A.330, 2I
O8.A.7001, 4IZG.A.414, 4J1O.A.401, 1J7L.A.302, 3KAL.A.502, 1KJ8.A.393, 1KJ8.A.39
4, 1KJ9.A.393, 1KJI.A.393, 1KJI.A.394, 1KJJ.A.394, 1LP4.A.341, 3LVV.A.696, 4M3A.
A.402, 4MDB.A.402, 3NA5.A.547, 1NFS.A.401, 4NZN.A.403, 4O4D.A.402, 4OAV.B.803, 3
OLP.A.547, 4OVN.A.202, 1P43.A.438, 3PFR.A.456, 4PFY.A.602, 4PU5.A.501, 2PYW.A.50
0, 1PYX.A.1003, 4Q4C.A.403, 2QVH.A.401, 4QXD.B.301, 3R75.A.700, 3T2D.A.411, 3T7A
.A.601, 3TDW.A.502, 3TMO.A.265, 4TYO.A.501, 3UJR.B.501, 3UJS.A.600, 3UJS.B.600,
1UMG.A.401, 3V4S.B.402, 3VA8.A.425, 3VAT.A.502, 3VC6.A.501, 2VPQ.A.1451, 1WOH.A.
1000, 1W7V.A.1441, 4WB8.A.403, 4WH2.A.403, 4WH3.A.403, 3WNZ.A.503, 3WQR.A.502, 2
XHO.A.1439, 1Z2O.X.1296, 2Z4X.B.1206, 4C5A.B.330, 1E4E.A.360, 2IO7.B.5003, 1L3R.
E.391, 4N57.B.402, 1OL5.A.1389, 4OVN.A.203, 4OVN.B.203, 4QXD.A.302, 3T9E.A.601,
4UAK.A.502, 1YYZ.A.340
[1] "Cluster 6"
1DXI.A.390, 4GWZ.A.402, 1IV2.B.1562, 3LD0.8.54, 4OTP.A.502, 3HO1.X.22, 1VQ7.0.80
66, 4AG5.B.1588, 2E8W.B.1203, 2HAW.A.1001, 1IOV.A.330, 4K6T.B.411, 3LGH.A.150, 1
MXB.A.411, 4QEH.A.401, 1SVT.E.601, 3VN9.A.402, 1Z6K.A.275

Table S39. all-ligand-number Mg, combined group

	size	largest_angle*	middle_1*	middle_2*	middle_3*
1	"185"	"163.2+/-6.4"	"79.8+/-7.1"	"88.2+/-3.3"	"96+/-4.3"
2	"213"	"153.2+/-10.4"	"64.9+/-7.7"	"81.3+/-6.6"	"96.8+/-11"
3	"248"	"170.8+/-5"	"77.6+/-7.9"	"88.7+/-2.7"	"98.2+/-4"
4	"182"	"155.5+/-13.6"	"76.6+/-9.5"	"83.7+/-8.8"	"90.4+/-9.2"
5	"1255"	"175.4+/-2.5"	"83.2+/-2.8"	"89.2+/-1.2"	"93.9+/-1.9"
6	"129"	"149+/-16.3"	"78.3+/-11.2"	"87.9+/-9.2"	"96.8+/-10"
7	"601"	"169.7+/-4.4"	"75.5+/-5.1"	"87.6+/-2.6"	"97+/-4"

	middle_4*	smallest_opposite_angle*	Tetrahedral	TrigonalBipyramidal
1	"150.3+/-10.3"	"97.6+/-5.3"	"0"	"0.013"
2	"141.2+/-9.8"	"71.7+/-10.2"	"0"	"0"
3	"160.2+/-6.3"	"60.6+/-5.6"	"0"	"0.004"
4	"99.1+/-10.3"	"84.6+/-10.6"	"0"	"0"
5	"172.3+/-2.9"	"83.5+/-3.8"	"0"	"0"
6	"108.2+/-11.8"	"124.3+/-18.6"	"0.014"	"0.001"
7	"163.1+/-4.9"	"78.5+/-4.3"	"0"	"0"

	TrigonalBipyramidalVA	TrigonalBipyramidalVP	Octahedral	SquarePyramidal
1	"0.013"	"0.054"	"0"	"0.008"
2	"0.005"	"0.002"	"0"	"0"
3	"0.003"	"0.192"	"0.014"	"0.121"
4	"0.001"	"0.002"	"0"	"0"
5	"0"	"0.127"	"0.558"	"0.694"

6	"0.002"	"0.01"	"0"	"0"
7	"0"	"0.095"	"0.06"	"0.167"

	SquarePyramidalV	SquarePlanar	TrigonalPrismatic	TrigonalPrismaticV
1	"0.061"	"0.05"	"0"	"0.001"
2	"0.012"	"0"	"0.004"	"0.018"
3	"0.366"	"0.241"	"0"	"0"
4	"0.075"	"0"	"0"	"0"
5	"0.768"	"0.752"	"0"	"0"
6	"0"	"0.024"	"0"	"0"
7	"0.328"	"0.259"	"0"	"0"

	PentagonalBipyramidal	PentagonalBipyramidalVA	PentagonalBipyramidalVP
1	"0"	"0"	"0"
2	"0.001"	"0.004"	"0.008"
3	"0"	"0"	"0.005"
4	"0"	"0"	"0"
5	"0"	"0"	"0"
6	"0"	"0"	"0"
7	"0"	"0"	"0.001"

	SquareAntiprismatic	SquareAntiprismaticV	HexagonalBipyramidal
1	"0"	"0"	"0"
2	"0.001"	"0.001"	"0"
3	"0"	"0"	"0"
4	"0"	"0"	"0"
5	"0"	"0"	"0"
6	"0"	"0"	"0"
7	"0"	"0"	"0"

	HexagonalBipyramidalVA	HexagonalBipyramidalVP
1	"0"	"0"
2	"0"	"0"
3	"0"	"0"
4	"0"	"0"
5	"0"	"0"
6	"0"	"0"
7	"0"	"0"

Table S40. Cluster members of all-ligand-number Mg, combined group

[1] "Cluster 1"
4D6O.A.1187, 4DFM.A.903, 3EPH.B.2, 2G8F.A.301, 2OTJ.0.8044, 2PYJ.B.9002, 3Q8P.B.
423, 1QVG.0.8067, 3SNN.A.906, 1AJD.B.952, 3ATT.A.379, 3AU9.B.602, 3AUA.A.601, 4A
ZW.A.1452, 3B9T.A.485, 2BBT.A.3, 4BCL.A.367, 4BCL.A.368, 4BCL.A.369, 4BCL.A.370,
 4BCL.A.371, 1BHO.1.901, 2BJI.A.2278, 4BJR.A.1517, 2BOZ.M.1304, 2BW7.D.2201, 3CT
L.A.601, 3D19.A.301, 2D32.A.1524, 1DAM.A.901, 4DFX.E.403, 3DGB.A.2001, 1DXI.A.39
0, 1E14.M.1303, 1E6D.M.1303, 2EB6.A.1001, 3ENI.A.375, 3ENI.A.378, 7ENL.A.438, 3E
OJ.A.375, 2F9R.B.602, 2FKA.A.9001, 1FMW.A.800, 3FXG.B.501, 3FYY.A.401, 1G65.G.30
1, 4G61.A.301, 3GIE.A.1, 3GQ7.A.692, 2GQ3.A.1002, 4GVE.A.602, 4GWZ.A.402, 4GX6.A
.402, 3I6E.B.386, 4IAC.A.402, 4IL6.j.102, 4IL8.A.501, 4IN7.M.411, 1J7U.A.301, 1J
B0.A.1011, 1JB0.A.1106, 1JB0.A.1112, 1JB0.A.1117, 1JB0.A.1121, 1JB0.A.1126, 1JB0
.A.1128, 1JB0.A.1131, 1JB0.A.1132, 1JB0.A.1133, 1JB0.A.1136, 1JB0.B.1201, 1JB0.B
.1202, 1JB0.B.1203, 1JB0.B.1204, 1JB0.B.1206, 1JB0.B.1207, 1JB0.B.1211, 1JB0.B.1
214, 1JB0.B.1215, 1JB0.B.1221, 1JB0.B.1223, 1JB0.B.1224, 1JB0.B.1225, 1JB0.B.123

1, 1JB0.B.1234, 1JB0.B.1236, 1JB0.B.1239, 1JB0.L.1501, 1JB0.L.1502, 1JYX.A.3002, 1KJ8.B.394, 1KJI.B.394, 3KR4.A.1004, 3KRF.D.901, 3L8F.A.401, 4LCZ.A.314, 4LNI.A.503, 1LP4.A.342, 4LRJ.A.302, 4LYJ.A.201, 4MFE.C.1104, 3MGA.B.405, 4MKU.A.209, 1MNZ.A.389, 1N0W.A.401, 1N22.B.706, 1N8W.A.900, 4NH0.B.1403, 4NZN.A.404, 3O5T.A.299, 4OEC.A.401, 2OQY.B.402, 4PTK.A.302, 3Q2O.B.384, 1Q9L.C.218, 2QB8.B.401, 3QQV.A.381, 1QSH.B.147, 4QTD.A.426, 2QVU.A.340, 4QVP.V.302, 4QYI.E.203, 1R03.A.301, 4R02.Y.302, 4R17.K.302, 4R7O.E.402, 3RLG.A.286, 1RWT.A.614, 1RZH.L.854, 1RZH.M.851, 1RZH.M.853, 1SJA.B.701, 3SRF.G.533, 3T9E.A.603, 3TAV.A.266, 4U0M.B.503, 3U2E.A.3, 4UB6.B.603, 4UB6.B.607, 4UB6.B.610, 4UB6.B.616, 4UB6.C.504, 4UB6.C.506, 4UB6.C.509, 4UB6.C.510, 4UB6.C.512, 4UB6.C.513, 4UB6.C.514, 4UB6.D.402, 4UM8.D.2001, 4UOR.A.699, 2UXR.A.1405, 3V3Z.L.302, 3V4S.A.402, 2VP0.B.1209, 2VPQ.B.1451, 3VTH.A.805, 1W88.A.1368, 3WNW.A.201, 3WQP.H.501, 3WU2.A.405, 3WU2.A.410, 3WU2.B.604, 3WU2.B.605, 3WU2.B.606, 3WU2.B.609, 3WU2.B.612, 3WU2.B.617, 3WU2.C.502, 3WU2.C.510, 3WU2.D.402, 3WU2.b.615, 3WU2.b.616, 3WU2.b.617, 3WU2.c.902, 2YBE.A.1417, 1YF6.M.853, 1YF6.M.856, 1YQ7.A.908, 1Z2P.X.1295, 2ZDR.A.1001, 1ZM7.D.400, 1MXB.A.411, 2VOS.A.1491, 2WCJ.A.1144

[1] "Cluster 2"

4D6N.F.1197, 4G7O.D.2003, 2G8H.A.301, 3LK9.A.340, 4M2Z.A.501, 3R7P.A.317, 2VBL.C.1026, 3A58.B.401, 1AO0.A.469, 2ATX.A.201, 4AZW.A.1453, 3C41.J.603, 4CZK.A.1335, 3DYG.A.3002, 1E2A.C.106, 4E4F.A.504, 2EWG.A.3002, 3FFU.B.155, 4FHX.A.402, 2FOL.A.202, 3G15.A.605, 4GMJ.B.302, 1GUS.A.1069, 4GWS.A.402, 4GYZ.I.402, 1H65.A.282, 3HDG.E.204, 3HWX.A.602, 4IFW.A.503, 3IJQ.B.386, 2IOA.B.5003, 2IUT.A.1724, 2J1L.A.1195, 1KFS.B.2, 4KMQ.A.1103, 3KUD.A.171, 3LD0.5.54, 2LVJ.A.101, 1MBZ.A.604, 3MLE.A.222, 3N3T.B.803, 4NH0.A.1402, 2NOM.A.401, 4OGE.A.1204, 2OPM.A.907, 3P0W.A.471, 4P9D.A.202, 3PCR.B.1231, 3PLS.A.1, 3PMG.A.562, 3PP1.A.410, 2PUI.B.401, 3RUW.D.544, 4S17.A.501, 3SH1.A.222, 1T5S.A.1005, 3TXA.A.801, 3U7F.B.1, 3UGV.A.500, 4UM9.B.2001, 2W8D.A.1636, 3WBH.B.503, 3WQS.B.502, 2Y4M.A.400, 2W9C.B.1343, 4KI8.C.602, 4FFO.A.903, 4FFR.A.404, 3G9D.A.299, 1JR4.A.300, 4KI8.A.602, 4LTZ.A.404, 3MJX.A.901, 3A6P.H.1178, 2BPF.A.339, 4DPV.N.12, 4IFD.J.1105, 3KK2.A.601, 4M30.B.302, 1N56.A.401, 2O5I.D.8001, 1QVG.0.8010, 1TTT.B.407, 4W9M.E.902, 1A49.B.1134, 1A49.C.1734, 4ACF.B.1481, 4AN9.A.1383, 3AQC.D.328, 1AZT.A.403, 2BBO.A.2, 2BVC.A.503, 3C5P.D.302, 2CN8.A.1504, 3CNX.B.170, 1CQP.A.310, 3CWH.A.391, 4CYU.A.170, 2D32.A.1523, 2D33.A.525, 2D33.B.1524, 1DIE.A.398, 3DUF.C.1368, 3DV0.A.1368, 2DW7.A.2001, 2DW7.B.2002, 3DYF.A.3002, 3DYF.A.3004, 4E1E.A.401, 3EA5.C.221, 1ELY.A.452, 4EOO.A.301, 4F0P.A.501, 2F1I.A.501, 3F5U.A.297, 4FHY.A.402, 4FMC.B.203, 1GIM.A.435, 2GQ3.B.1003, 3GT8.C.13, 1GXB.C.1346, 1GY3.A.1298, 2GZD.A.950, 3HB0.B.702, 3HQP.A.502, 3HYL.A.675, 4I1O.E.201, 4I40.B.301, 3IAF.A.572, 3IAF.B.572, 3IG8.A.696, 2IO7.A.5002, 1IV4.E.1565, 4IYN.A.804, 1JM6.A.4601, 4JVJ.F.405, 4K10.A.405, 4K81.B.203, 1L2O.B.902, 3LD0.8.54, 4LF2.D.601, 4LNI.D.504, 3LZ9.A.852, 2M32.A.401, 1MFO.A.1453, 4ML9.B.301, 3MQT.W.395, 1MXA.A.411, 4N0G.A.405, 1N8W.B.1900, 4NEH.A.1102, 4NH0.A.1403, 1NUZ.A.2342, 4NU1.A.404, 2O1X.A.2001, 4O1P.C.903, 3OAB.A.904, 4OAU.C.802, 3OB8.D.3001, 2OI2.A.400, 4OTP.A.502, 2P0C.B.5, 4P0V.A.403, 4P9D.B.202, 3PUG.A.601, 4PV4.A.501, 4PYK.A.302, 1Q19.A.504, 3RBM.A.1003, 3RBM.C.1002, 2RD5.C.1001, 4RJK.B.602, 3RUW.A.544, 4S17.C.502, 1S6P.A.601, 1SBJ.A.163, 3SBE.A.501, 3T8O.B.160, 4TVU.B.601, 3TW6.B.2002, 4U03.A.504, 4U0N.A.401, 4U81.A.501, 3UJ2.H.430, 4UOR.C.699, 2VON.A.502, 2VBI.G.1000, 2WB4.B.502, 3WQP.J.501, 2X9H.A.3001, 1XF9.B.11, 1Y8P.A.501, 1Y9I.B.602, 1YHM.A.401, 1ZCA.B.1383, 4IR1.A.902, 2A19.B.1642, 3ALN.A.406, 1CUL.A.1007, 3DYF.B.4002, 1EQR.B.902, 2G83.B.358, 1G9X.B.1301, 4I1O.A.201, 3JZM.A.701, 3LCB.B.579, 2O1V.A.755, 2O1X.B.2002, 3O7L.B.352, 3QU2.C.225, 1RC5.D.764, 1SO2.C.475, 2Z2P.A.1003

[1] "Cluster 3"

4AAB.B.1156, 4AQX.D.1526, 4BDZ.A.1381, 4BE0.A.1381, 4BE2.A.1381, 1CW0.A.203, 4D6O.D.1197, 4DFP.A.901, 1G9Z.D.901, 4IEM.C.401, 4IRC.A.402, 3MAQ.A.1001, 1MOW.A.373, 1MUH.A.479, 4NCB.A.702, 4NCB.B.702, 4NCB.C.101, 4O3S.A.503, 3OYA.A.397, 3OYB.

A.397, 3OYD.A.397, 3OYE.A.397, 3OYJ.A.397, 3OYL.A.397, 3OYN.A.397, 1RVC.A.401, 3S3O.A.397, 3S3M.A.397, 3S3N.A.397, 3SM4.E.15, 2VBN.E.1026, 1YTU.A.428, 1ZBI.A.302, 1A49.A.534, 3A7E.A.215, 2A9F.A.801, 4ACF.A.1480, 4ACF.A.1481, 2AG1.B.611, 2AL1.B.438, 3AU9.A.601, 3B8I.A.289, 3BM4.B.304, 3BZN.A.501, 4C5A.A.330, 4C5B.A.1313, 4C5C.A.1313, 3CFX.A.703, 3CT2.A.401, 3CVJ.C.243, 4CWB.A.1159, 4D2I.A.1478, 1DAW.A.342, 1DAY.A.341, 1DAY.A.342, 4DFX.E.404, 3DG6.A.2001, 4DHP.A.303, 4DWB.A.507, 3DYS.A.902, 1EC8.A.498, 1EFK.A.604, 2EGH.A.900, 1ELX.B.452, 4EOM.A.302, 3ETH.A.401, 3ETH.A.402, 3ETJ.A.402, 1EYZ.A.401, 1EYZ.A.402, 4F0Q.A.501, 1F1Z.A.2002, 3FFU.A.156, 4FFL.A.905, 3FHY.B.404, 3FLK.A.401, 2FN1.A.504, 2FUV.A.901, 3GN6.A.321, 4GOK.B.202, 3GQ8.A.692, 4H2H.A.401, 4HE1.A.403, 2HGS.A.501, 3HPF.A.401, 3HWO.A.1702, 2HXU.A.601, 4I3Y.A.304, 3I4K.A.385, 4IAC.A.401, 4IAD.A.401, 1IOW.A.330, 2IO8.A.7001, 4IOK.A.604, 4IZG.A.414, 2J0L.A.1688, 4J1O.A.401, 1J7L.A.302, 4J7L.A.402, 1JP4.A.702, 4K10.D.404, 1K9Y.A.401, 3KAL.A.502, 4KCW.A.1001, 3KEU.A.400, 4KI8.E.602, 1KJ8.A.393, 1KJ8.A.394, 1KJ9.A.393, 1KJI.A.393, 1KJI.A.394, 1KJJ.A.394, 3KRO.D.3001, 3L0Y.A.257, 1LP4.A.341, 3LVV.A.696, 3M00.A.551, 4M3A.A.402, 4MDB.A.402, 4MPO.A.206, 3NA5.A.547, 1NFS.A.401, 2NOM.A.402, 1NUW.A.2498, 1NUY.A.2341, 4NZN.A.403, 4O4D.A.402, 4OAV.B.803, 4OHF.B.503, 3OLP.A.547, 2ONS.A.702, 3OP2.A.500, 4ORK.A.501, 2OUN.A.403, 4OVN.A.202, 4OVN.A.204, 1P43.A.438, 3PFR.A.456, 4PFY.A.602, 1POW.B.610, 4PU5.A.501, 4PU5.A.502, 2PYW.A.500, 1PYX.A.1003, 2PZA.A.6242, 2Q1A.X.294, 3Q46.A.306, 4Q4C.A.403, 4QE5.A.401, 1QF5.A.433, 4QPM.A.1503, 2QVH.A.401, 4QXD.B.301, 3R75.A.700, 4RAD.D.302, 1RC5.A.761, 4RJJ.A.602, 3RLH.A.286, 4RUB.B.491, 1SHQ.A.479, 3T12.A.198, 3T2D.A.411, 1T5T.A.1005, 3T7A.A.601, 3TDW.A.502, 1TE6.A.640, 3TMO.A.265, 4TY0.A.501, 3UJR.A.501, 3UJR.B.501, 3UJS.A.600, 3UJS.B.600, 1UMG.A.401, 4UOP.A.1612, 3V4S.B.402, 1VA6.A.523, 3VA8.A.425, 3VAT.A.502, 2VBV.A.1136, 3VC6.A.501, 2VPQ.A.1450, 2VPQ.A.1451, 1W0H.A.1000, 1W7V.A.1441, 4WB8.A.403, 4WH2.A.403, 4WH3.A.403, 4WK2.B.501, 3WNZ.A.503, 3WQQ.A.502, 3WQR.A.502, 2X3J.A.1590, 2XH0.A.1439, 1Z2O.X.1296, 2Z4X.B.1206, 3ZXW.A.476, 3GV7.B.871, 4GZ2.A.402, 2HVI.A.878, 4M2Z.B.301, 1MOW.A.371, 4OIN.D.2003, 2OTJ.3.8078, 3V6J.A.403, 2A9F.B.800, 4CS3.A.1464, 4DCK.B.203, 1ECB.D.507, 3EW9.A.501, 4FVQ.A.902, 4FZL.B.302, 4GA3.A.1002, 4GIR.B.401, 4GX4.B.402, 4GX6.B.402, 1HJK.B.452, 4RUB.A.491, 3TLM.A.1005, 2VBI.B.1000, 2XGZ.B.1439, 1Z2P.X.1296, 2Z4W.B.1303, 4C5A.B.330, 4DLC.A.303, 4E1E.A.402, 1E4E.A.360, 3EHB.A.562, 4EKD.A.407, 2EWG.B.4003, 3FPA.A.901, 4HYP.B.302, 2IK2.A.289, 2I07.B.5003, 1L3R.E.391, 3LMG.A.202, 4LNI.D.505, 4N57.B.402, 1OL5.A.1389, 4OVN.A.203, 4OVN.B.203, 2PSN.A.701, 2PUI.A.401, 3QHR.A.298, 4QXD.A.302, 4R9U.C.302, 3T9E.A.601, 4UAK.A.502, 1YYZ.A.340

[1] "Cluster 4"

4DQP.A.903, 3E54.A.702, 3ICE.B.1001, 3ICE.D.1001, 2NVQ.A.2001, 4P0P.A.601, 2PYJ.A.9004, 1Q81.4.8078, 3S14.A.2001, 1V14.C.1134, 2W9C.A.1344, 2W9C.B.1342, 3ZC0.B.197, 2AEK.A.804, 2AKZ.A.440, 2AQX.A.600, 2AQX.A.601, 3AQC.D.327, 4AVQ.C.902, 2BM1.A.1690, 3BPD.G.126, 1BR2.A.997, 3BU5.A.301, 3C15.A.29, 3CNX.C.170, 3CR3.B.1213, 3CRL.B.2001, 2CW6.A.401, 4CW7.C.1002, 4CYI.B.1000, 4CYU.A.171, 1D1C.A.998, 2D33.C.2525, 3D7M.A.356, 2DEJ.A.402, 2DH4.A.341, 3DLS.C.11, 1DOA.A.199, 4DPG.F.604, 4DPM.D.401, 1E1Q.A.601, 1E1Q.F.601, 3E4O.A.501, 2E6B.A.301, 2E92.A.1302, 2E92.B.1304, 2E95.A.1301, 3EPS.A.1606, 3EQL.N.1528, 4F38.A.202, 1F4H.D.3001, 3F74.C.1, 3FA4.A.401, 2FDR.A.1001, 3FR8.B.1, 3FYY.B.402, 3G2F.B.901, 2G5H.B.501, 2G74.A.185, 3G9D.B.299, 2GHT.B.257, 2GHQ.B.257, 1GRV.A.490, 2GWC.A.1, 3H8A.C.1431, 1H8H.F.601, 3HBO.A.701, 1HBN.A.1558, 3HQP.M.502, 2HT6.B.444, 4HV6.B.201, 2I19.B.4004, 4I1O.C.201, 2I5R.B.302, 2ICJ.A.400, 3IG8.A.697, 3IJQ.A.386, 2IK2.A.290, 4IL6.C.505, 4IL6.c.506, 2I07.B.5004, 1IV2.F.1574, 4J99.D.803, 1JFG.B.703, 2JK1.A.1144, 4K6T.E.412, 3KA9.A.189, 1KK8.B.998, 3KRF.A.904, 1L0O.A.602, 4LCZ.A.316, 4LRT.A.403, 1LVH.A.801, 3M1Y.D.300, 4M1W.A.201, 3M42.A.1, 4MFE.D.1105, 4MKS.A.502, 3MQT.A.1243, 3MWC.A.400, 1N5K.B.413, 3N9V.A.61, 1NEL.A.438, 3NIZ.A.312, 1NV3.A.2341, 2O1U.A.302, 2O56.F.2006, 3OAC.D.3002, 4OAU.C.803, 3OHM.A.402, 2OI5.B.5000, 4OVN.B.202, 4POV.A.404, 1P7L.A.388, 4P9D.C.202, 4PFQ.C.400, 4PRV.A.402, 2PUN.B.401, 1

PYX.A.1002, 2Q58.A.4, 1Q8Y.A.809, 2Q80.A.401, 4QDG.A.402, 4QJB.A.301, 4QLQ.V.301
, 3QU9.A.227, 4QVY.K.302, 4QXD.B.302, 4QXD.B.304, 4R3A.A.400, 2R5T.A.600, 3RBM.B
.1002, 2RIO.A.1102, 1RLT.C.807, 3RYW.B.2003, 4S17.D.501, 1S4E.B.1600, 3SRD.C.603
, 3SRF.D.533, 3SY8.A.401, 3SZ5.A.220, 4TQ3.A.402, 3TW6.D.2002, 4U0N.B.401, 3U87.
A.402, 3U87.B.403, 3UGJ.A.2006, 4UM8.B.2001, 4UUX.A.401, 2UXR.B.1398, 2V0N.B.502
, 2VHQ.A.1328, 3VI4.B.502, 2VKQ.A.1288, 3VTI.C.401, 3VYT.C.601, 1W9L.A.1748, 3W9
T.B.511, 1WBQ.C.1441, 3WEJ.A.402, 3WGU.C.2003, 3WKA.A.601, 1WQ1.R.168, 1WQA.A.45
6, 3WU2.C.511, 1WUU.C.394, 1XLB.A.399, 1XMJ.A.2, 2YFD.B.1145, 1YXI.A.342, 1YXO.A
.5001, 1YYZ.A.341, 2Z2P.A.1001, 1Z5B.B.2003, 1ZCW.A.501, 2ZCQ.A.453, 2ZEV.A.1302
, 2ZEV.B.1304, 1ZH4.A.201, 2ZRW.D.702, 3ZXT.A.1278, 1ZYK.A.702
[1] "Cluster 5"
4AQX.D.1525, 4BDY.A.1380, 4BE0.A.1380, 4BE1.A.1381, 4BE2.A.1380, 1BPY.A.339, 4BW
J.A.1834, 4CEI.B.2162, 4DFJ.A.902, 4DFK.A.902, 4DFM.A.902, 4DL4.A.501, 4DO9.A.40
1, 4DOB.A.401, 4DOC.A.401, 2DPI.A.871, 4DQI.A.901, 4DQI.D.901, 4DQP.D.902, 4ELT.
A.902, 4ELU.A.902, 2EZ6.A.501, 4F5O.A.402, 1FIU.I.2222, 1FIU.A.5555, 2FMS.A.340,
 4FO6.A.601, 3G6Y.A.871, 1G9Z.C.902, 1G9Z.F.903, 3GDX.A.348, 4GZ2.B.402, 3IEV.A.
400, 2JOS.A.1412, 1JJ2.0.8010, 3JPQ.A.339, 3JPS.A.339, 4JWM.A.403, 3K58.A.1001,
3K59.A.1001, 4K97.A.603, 3KD5.E.916, 4KLI.A.401, 4M04.A.702, 4M04.A.703, 3M8S.A.
2, 4M8O.A.1302, 4M9L.A.404, 3MBY.A.339, 4MDE.A.1002, 3MFI.A.515, 4MFC.A.401, 3MR
5.A.435, 4O3N.A.503, 4O3O.A.502, 4O3O.A.503, 4O3Q.A.502, 4O3Q.A.503, 4O5K.A.401,
 3OHA.A.518, 3OJS.A.7, 3OYB.A.396, 3OYD.A.396, 3OYG.A.396, 2OZS.A.904, 4P4M.A.40
2, 2PFP.A.750, 2PFN.A.950, 3PML.A.2, 3PNC.A.576, 4PUQ.B.401, 4Q8E.A.502, 4QM6.A.
1002, 4R65.A.402, 4R8U.B.402, 3RJH.A.403, 3RJK.A.339, 4RNN.A.503, 3RTV.A.833, 3S
I8.A.451, 3SM4.B.227, 3SNN.A.905, 3SPY.A.903, 1SUZ.A.402, 3TFR.A.339, 3TFS.A.339
, 3TIO.A.1, 3TIO.D.2, 4UAY.A.402, 3V6H.A.402, 1W7A.A.1802, 1XSN.A.576, 1ZJN.A.33
9, 3ZVM.A.1526, 121P.A.168, 3A0T.A.800, 4A01.A.1767, 4A01.A.1769, 3A1U.A.5, 3A1U
.A.6, 1A2B.A.550, 3A4L.A.401, 2A5Z.A.701, 2A5D.A.1231, 4A6X.A.350, 1A82.A.901, 3
A99.A.401, 4ACF.A.1482, 4ACI.A.1187, 2AFK.E.1291, 2AG0.A.601, 3AHC.A.826, 3AHD.A
.826, 3AHE.A.826, 3AHG.A.826, 1ALK.B.452, 2AL1.B.439, 4ANB.A.1384, 1AOR.A.609, 1
AOX.A.400, 1AS0.A.356, 4AS2.A.1328, 2AUU.A.203, 2AUU.A.204, 2AUT.A.601, 2AUT.D.6
04, 4AUX.A.223, 3AYX.A.701, 2B0T.A.800, 3B05.D.1001, 3B1V.A.301, 1B25.A.800, 4B2
P.A.1351, 1B4N.A.623, 2B56.A.488, 3B7L.A.907, 3B7L.A.908, 3B7L.A.909, 1B8C.A.308
, 1B8J.A.452, 2B82.A.1013, 2B9H.A.700, 4BAS.A.1183, 3BB1.A.282, 2BBS.A.3, 3BC1.A
.194, 2BEK.A.501, 3BGA.A.1, 2BME.A.1184, 3BN3.A.1, 1BOF.A.800, 2BON.A.1302, 3BRB
.A.10, 2BU2.A.1388, 2BVC.A.504, 2BVN.B.1395, 3BWV.A.300, 4BW9.A.501, 4BWR.A.1468
, 4BX0.A.1291, 4BX3.A.301, 1BYQ.A.1001, 1C1Y.A.171, 2C18.A.1338, 2C3U.A.2238, 2C
31.A.1553, 2C3P.A.2237, 2C42.A.3238, 3C4Z.A.563, 2C4N.A.1251, 3C5H.A.302, 2C5L.A
.1168, 4C5A.B.331, 4C5B.A.1314, 4C5C.A.1314, 2C77.A.1407, 2C78.A.1407, 4C7X.A.70
0, 3C9U.B.312, 2CBZ.A.1872, 3CB3.A.501, 3CFX.A.704, 2CFS.A.1296, 3CG4.A.201, 1CH
N.A.200, 2CHE.A.130, 1CIP.A.356, 1CJT.C.403, 1CJU.A.582, 2CJE.A.1267, 3CK5.A.400
, 2CK3.A.601, 2CK3.F.601, 2CL5.A.1216, 2CLS.A.550, 3CMR.A.453, 2CN5.A.1506, 4COK
.A.601, 3CP6.A.502, 3CP6.A.503, 3CR3.A.1212, 3CRR.A.324, 1CTQ.A.168, 3CUR.H.553,
 3CUS.Q.553, 3CV2.A.1, 3CX8.A.378, 3CXO.A.500, 3CZJ.B.3001, 2D0O.A.1005, 1D2N.A.
99, 3D2R.A.500, 3D36.A.478, 4D6P.A.1352, 2D7C.A.1002, 4D7M.A.223, 1D8C.A.3001, 4
DBF.A.401, 4DBR.A.810, 2DCN.A.4001, 4DCK.B.201, 3DDC.A.600, 3DDH.B.232, 2DDT.A.3
11, 2DEI.A.402, 4DEM.F.402, 4DEM.F.403, 4DEM.F.404, 4DFD.B.301, 3DGT.A.800, 3DKC
.A.2, 4DN1.A.401, 4DN1.B.401, 4DN5.A.1001, 4DSN.A.202, 4DSO.A.202, 1DTW.A.401, 2
DUA.A.292, 4DUX.A.3001, 4DWG.A.401, 4DWO.A.301, 1DXE.B.901, 4DXJ.A.403, 3DYH.A.3
003, 3DYH.A.3004, 3DYH.B.4002, 3DYM.A.3001, 3DYP.A.3001, 2DY1.A.701, 4DYK.A.502,
 4DZH.A.501, 4E01.A.402, 1E2Q.A.401, 3E2D.A.603, 1E3D.B.901, 3E5H.A.200, 3E81.A.
165, 3E84.A.701, 3E8M.A.165, 4E8G.A.402, 2E9S.A.603, 1E9A.A.401, 2E91.A.1301, 2E
95.A.1302, 3EA5.A.221, 4EA0.A.302, 1EBG.A.439, 2EB1.A.502, 2EB5.A.1001, 1EC7.A.4
98, 4EEN.A.301, 3EFQ.B.4004, 4EFM.A.202, 3EGT.A.3002, 3EGT.A.3004, 3EHG.A.371, 1
EKO.A.601, 3EKG.A.601, 3EQC.A.3, 2ERX.A.403, 3ET4.A.301, 3ET5.A.255, 3ETJ.A.401,

4EUK.A.1001, 2EW1.A.701, 4EX6.A.301, 4EX7.A.301, 2EZT.A.1510, 2EZU.A.1610, 3EZ3.A.1104, 3EZ3.B.1102, 2EZ4.A.1610, 2EZ8.A.1510, 2EZ9.A.1510, 4F1J.A.301, 1F2U.A.902, 2F2A.B.601, 1F5N.A.595, 3F61.A.310, 1F8I.A.451, 1F9H.A.161, 1F9H.A.162, 2F9M.A.1201, 4F9A.A.602, 3FD5.A.397, 3FD6.B.397, 4FE3.A.304, 4FEG.A.707, 2FFQ.A.356, 2FH5.B.270, 3FIU.A.5001, 3FIU.A.5002, 4FI1.A.401, 3FKQ.A.501, 4FK9.A.401, 2FOZ.A.348, 2FOZ.A.349, 2FPR.A.503, 4FP1.A.401, 2FRV.B.540, 1FSG.A.302, 3FSY.A.333, 2FUE.A.500, 3FV9.A.501, 4FVR.A.902, 4FYP.A.301, 3FZN.A.605, 2G0W.A.501, 2G09.A.901, 2G1T.A.1501, 1G17.A.301, 3G15.A.602, 3G2F.A.901, 1G4C.B.362, 1G4P.A.2003, 1G4T.A.2005, 3G5A.B.307, 3G5A.D.307, 1G5T.A.998, 3G6K.A.307, 2G6B.A.301, 2G80.A.500, 1G97.A.460, 4G9B.A.301, 3GAI.A.189, 2GCN.A.2001, 2GCP.A.2001, 2GCQ.A.435, 2GGE.A.400, 2GHT.A.257, 2GIL.A.1201, 4GIU.A.402, 2GL5.A.699, 4GME.C.501, 3GON.A.600, 2GO7.A.207, 4GOJ.A.202, 4GP2.A.401, 4GP2.A.402, 2GQS.A.241, 1GSA.A.319, 1GSI.A.1209, 2GSM.A.3006, 4GT8.A.402, 1GUA.A.171, 3GY1.A.500, 3GYB.A.1, 4H19.A.405, 4H1Z.D.401, 1H2A.L.1005, 1H2R.L.1005, 3H3X.Q.553, 2H57.A.202, 3H70.A.342, 3H7V.A.331, 3H80.A.214, 4H81.A.402, 4H8E.A.301, 4HAT.A.302, 2HCF.A.300, 4HCH.A.405, 4HCL.A.401, 3HDG.A.202, 4HDO.B.200, 3HFW.A.361, 2HF8.A.301, 2HF9.A.301, 2HGS.A.502, 4HGN.A.200, 4HGQ.A.201, 3HIY.B.402, 2HJP.A.292, 2HNE.A.601, 4HNC.A.401, 4HNL.A.401, 4HOR.X.101, 4HPT.E.402, 1HQ2.A.162, 3HQJ.A.145, 4HQO.A.301, 3HRZ.A.628, 3HRZ.D.742, 3HSD.B.162, 1HTW.A.561, 3HVH.A.265, 3HVI.A.1, 3HVJ.A.265, 3HVK.A.1, 3HW3.A.999, 3HW4.A.999, 3HW5.A.999, 3HXX.A.445, 1IOL.A.902, 2I1Q.A.501, 4I2B.A.604, 2I33.A.602, 2I34.A.301, 4I3Z.A.302, 2I5R.A.301, 2I6K.A.302, 3I76.A.1001, 4I94.A.402, 4IAD.A.402, 3IBA.A.403, 3ICK.A.401, 3ICK.A.402, 3ICK.A.403, 3ICM.A.401, 3ICM.A.402, 3ICZ.A.402, 3ICZ.A.403, 4IDN.A.502, 4IDP.A.502, 4IEG.A.1001, 4IFW.A.502, 1IG5.A.78, 2IHT.A.601, 2IHU.A.601, 1IH8.A.4002, 1IHU.A.592, 2IHP.A.287, 4IHC.A.501, 3IJL.A.386, 3IJR.D.300, 2IK4.A.287, 2IK4.B.287, 2IK4.B.288, 2IK4.B.289, 2IK6.B.287, 2IOR.A.2000, 1IOW.A.331, 2IO8.A.7002, 4IP4.A.503, 4IP5.A.502, 3IPO.A.161, 1IR3.A.301, 1ITZ.A.1001, 4ITR.D.203, 2IUC.A.1003, 4IUC.L.702, 4IUD.L.1002, 1IV2.A.1571, 1IV4.A.1571, 1IV4.A.1572, 4IVG.A.803, 4IWH.A.401, 2IXE.A.2, 2IYW.A.202, 2IYN.C.1123, 1IZC.A.1001, 2J0V.A.1180, 2J7P.A.1401, 2J7P.D.1401, 1J7L.A.301, 2J7N.B.3374, 1J9J.A.301, 1J9J.B.301, 1JBW.A.998, 2JC9.A.1491, 2JCB.A.1192, 4JDP.A.301, 2JD4.A.4061, 2JD4.B.4062, 1JGT.A.902, 2JI7.A.1567, 4JND.A.501, 1JPM.A.1003, 1JSC.A.699, 4JSO.A.202, 3JTC.C.34, 1JUY.A.435, 3JVA.A.356, 3JVA.B.358, 3JVT.B.502, 4JXO.A.402, 3JYS.A.1, 3JYY.A.301, 3JYY.B.302, 1JZ7.A.3001, 3JZO.A.300, 3JZO.A.303, 3K1S.H.107, 4K1W.A.501, 3K4Z.A.290, 4K6R.A.505, 1K77.A.300, 3K8K.A.700, 3K9L.A.168, 4K9Q.A.601, 1KA2.A.501, 3KA3.A.176, 3KAL.A.503, 3KB9.A.701, 3KB9.A.702, 3KC2.A.355, 1KCZ.A.901, 4KFU.A.307, 4KGD.A.702, 1KHZ.B.301, 1KK1.A.413, 3KKO.A.180, 1KMQ.A.401, 3KMW.A.501, 4KO8.A.801, 1KQP.A.5001, 1KQP.A.5002, 4KQW.A.404, 3KS6.C.251, 1KSH.A.202, 1KTG.A.502, 1KTG.A.503, 1KTG.A.504, 1KTG.A.505, 3KTA.A.184, 3KUC.A.171, 4KUX.A.703, 4KVA.A.501, 4KVG.A.202, 3KWS.B.401, 4KWD.A.404, 4KX5.A.314, 4KXW.A.1001, 1KY2.A.401, 3L12.A.313, 4L2X.F.404, 4L57.B.201, 3L8H.A.801, 4L80.A.403, 4L9W.A.202, 4L9Z.A.403, 4LA6.A.501, 2LCF.A.246, 4LFG.A.303, 4LFG.A.304, 4LFG.B.303, 4LGY.A.1302, 4LHW.A.301, 4LJ9.A.902, 3LLU.A.502, 1LNY.A.1453, 4LPM.A.208, 3LUF.A.300, 3LUF.A.301, 3LVO.A.264, 3LX5.A.301, 3LXX.A.402, 4LYK.A.401, 4LZO.A.403, 4LZ3.A.406, 3M07.A.595, 1M0W.A.502, 3M1I.A.1178, 4M53.A.527, 1M7B.A.550, 3M7I.A.901, 4M9Q.A.302, 1MC1.A.601, 1MDL.A.360, 4MDB.A.403, 4MFG.A.201, 4MGG.A.404, 1MH1.A.201, 3MHY.A.115, 1MJN.A.1001, 3MJH.A.201, 3MK2.A.903, 1MMA.A.998, 1MMG.A.998, 1MMN.A.998, 1MNE.A.998, 4MNE.A.902, 4MPO.B.205, 1MQ4.A.2088, 4MRT.A.301, 4MUM.A.301, 1MXO.A.501, 1MXG.A.439, 3MX3.A.601, 3MYH.X.997, 4MYO.A.301, 3MYK.X.998, 3MYL.X.998, 4MZU.C.404, 1N0H.A.699, 1N0H.B.1699, 4N0D.A.402, 3N07.A.200, 1N1Z.A.701, 1N1Z.A.703, 1N20.A.701, 1N20.A.702, 1N24.A.701, 1N24.A.702, 3N2N.A.1, 3N45.F.355, 3N45.F.2, 3N45.F.3, 3N4F.A.502, 1N6I.A.201, 1N6L.A.201, 1N6N.A.201, 1N6O.A.201, 1N6P.A.201, 1N6R.A.201, 1N8I.A.900, 1NBO.A.201, 4NBS.A.502, 4NDO.A.302, 4NFI.F.402, 4NFI.F.403, 4NFI.F.404, 1NFZ.A.401, 3NJL.A.501, 3NKV.A.500, 1NN5.A.401, 3NOJ.A.239, 1NRJ.B.1, 3NRJ.A.190, 2NSY.A.305, 1NSF.A.859, 1

NSY.A.6241, 4NWI.A.401, 2NXW.A.4002, 1O08.A.2800, 2O1S.A.1001, 1O3Y.A.1002, 3O61.B.202, 1O6Y.A.1280, 3O6Z.B.201, 2O7O.A.223, 4O7I.A.401, 4OAV.B.802, 1OBW.A.176, 3OCU.A.263, 3OCV.A.264, 3OCW.A.263, 3OCX.A.264, 3OCY.A.264, 3OCZ.A.263, 2OCB.A.202, 4OCP.A.403, 2ODP.A.901, 2ODB.A.205, 4ODJ.A.502, 3OE1.A.601, 3OE5.A.222, 2OEM.A.911, 2OEM.B.912, 2OFX.A.301, 2OGD.A.3002, 2OGD.A.3003, 2OGD.A.3004, 4OHY.A.502, 1OIW.A.1175, 1OIX.A.301, 3OIW.A.170, 2OI6.B.6000, 4OI4.A.502, 1OKK.A.1002, 1OKK.D.1002, 4OKM.A.901, 4OKM.A.902, 4OKM.A.903, 4OKZ.A.902, 4OKZ.A.903, 2OLR.A.543, 3OM2.A.486, 4OMF.A.503, 3OP2.B.500, 2ORW.A.501, 3OUZ.B.459, 1OXV.A.1102, 1OXV.D.1101, 3OYX.A.601, 3OZF.A.235, 3OZX.A.613, 3OZX.A.614, 2P27.A.307, 4P31.A.402, 4P32.A.402, 2P3N.A.1758, 3P41.A.297, 3P41.A.298, 1P4M.A.201, 1P5Z.B.401, 3P5P.A.901, 3P93.C.406, 3P96.A.412, 2PA4.A.325, 3PDE.A.312, 1PFK.A.327, 3PFF.A.831, 4PFY.B.601, 3PGL.A.1, 2PGN.A.610, 1PHP.A.395, 4PHG.A.201, 4PHH.A.202, 3PIT.A.180, 3PK7.A.406, 2PK0.A.502, 2PKE.B.300, 2PLS.H.602, 2PMQ.A.902, 3PNL.B.1212, 2PNQ.A.502, 1POX.A.610, 1PPV.A.401, 4PQ9.A.301, 2PS2.A.401, 2PS5.B.701, 1PT6.A.500, 1PVF.A.401, 1PVG.A.903, 2PYW.A.501, 2PZ8.A.4001, 2PZA.A.6243, 2PZE.A.3, 3Q10.A.400, 2Q1D.X.294, 2Q28.A.1001, 1Q3H.C.674, 3Q3J.B.201, 2Q3F.A.301, 3Q46.A.307, 4Q4C.A.404, 3Q5V.B.599, 2Q5Q.A.4002, 3Q60.A.603, 1Q6L.A.5300, 1Q6O.A.7300, 1Q6Q.A.7300, 1Q6R.A.7300, 2Q66.A.602, 3Q85.A.284, 1Q92.A.1003, 4QC2.A.302, 4QEA.A.301, 3QF7.A.854, 2QGY.A.701, 1QGU.B.3002, 1QGU.D.3006, 2QG8.A.201, 4QHZ.A.302, 2QIS.A.907, 2QIS.A.908, 2QIS.A.909, 2QJJ.C.1003, 1QK5.A.303, 3QKE.A.407, 3QKT.A.902, 2QME.A.179, 3QN3.A.601, 3QNM.A.400, 2QQ0.A.450, 2QQ0.B.452, 1QRA.A.168, 2QRZ.A.190, 2QTY.A.348, 2QTY.A.349, 2QT0.A.1001, 2QTC.A.888, 3QUQ.A.225, 3QUT.A.225, 3QVQ.C.310, 3QXC.A.222, 3QXH.A.223, 3QXJ.A.224, 3QXS.A.223, 3QXX.A.224, 2QXO.A.161, 3QYY.A.505, 1ROX.A.13, 3ROU.A.380, 3R1M.A.403, 3R1M.A.404, 1R2Q.A.300, 3R3S.A.296, 4R39.A.401, 3R6T.A.301, 2R6O.A.801, 3R7W.A.600, 2R8E.A.201, 2RAH.A.354, 2RAR.A.501, 2RAV.A.701, 3RAP.R.200, 3RBM.A.1001, 3RBM.A.1002, 2RB5.A.701, 2RBK.A.501, 3REF.A.192, 3REG.A.550, 1RKQ.A.1273, 1RKU.A.301, 1RKV.A.401, 4RKE.A.202, 4RKF.A.202, 3RLF.A.1501, 1RMT.A.1413, 3RO6.A.400, 4ROQ.A.401, 1RP7.A.890, 1RQI.A.603, 1RQI.A.604, 1RQJ.A.907, 1RQJ.A.908, 1RQJ.A.909, 3RUS.A.544, 1RVK.A.999, 3RV3.A.1004, 3RWM.B.1, 3RYE.A.907, 3RYE.A.908, 3RYE.A.909, 1RYA.A.1001, 1RYH.A.539, 4S1H.B.303, 3S4J.A.907, 3S4J.A.908, 3S4J.A.909, 3S9Z.A.802, 1SAW.A.225, 3SAE.A.820, 3SBD.A.501, 3SDT.A.819, 3SDT.A.821, 3SEA.B.178, 3SF0.A.263, 3SHQ.A.321, 1SHT.X.219, 3SJN.A.374, 3SLS.A.401, 3SL2.A.701, 3SN1.A.408, 3SN4.A.408, 1SO4.A.2300, 3STP.A.391, 1SVM.A.750, 1SVS.A.356, 1T0P.A.901, 3T10.A.401, 3T1K.A.401, 3T2S.B.401, 3T2B.A.409, 3T2D.A.408, 3T2D.A.409, 3T2E.A.409, 3T7A.A.602, 1T8Q.B.1602, 1T9B.B.699, 1T9C.B.699, 1TC6.A.501, 2TCT.A.223, 3TCS.A.368, 3TDV.A.501, 1TE6.A.641, 3TEP.A.1, 3TG0.A.503, 3TJI.A.601, 3TKL.A.300, 1TMM.A.162, 4TMT.A.902, 4TMV.A.902, 4TMW.A.903, 4TMX.A.903, 3TMO.A.266, 4TN1.A.902, 4TQD.A.502, 3TSO.A.200, 4TSK.A.403, 1TW1.A.1, 3TWA.A.420, 3TWB.A.420, 3TYZ.A.281, 4TYO.A.502, 1TZZ.A.3501, 1U02.A.240, 4U5X.A.202, 4U82.A.301, 1U8Y.A.301, 4UAK.A.503, 4UAS.A.302, 4UAT.A.302, 4UAV.A.401, 2UAG.A.1001, 1UBK.L.1005, 3UCW.A.100, 3UCY.A.101, 4UCX.Q.1553, 4UE3.L.603, 3UIE.A.403, 3UJR.A.502, 3UJR.B.502, 3UJS.A.601, 3UJS.B.601, 1UMD.A.1401, 1UMG.A.403, 1UMG.A.404, 4UM7.A.175, 4UMF.A.1175, 3UPY.A.446, 1UPT.A.1183, 3UPL.A.447, 3UQY.L.603, 4UQL.Q.1552, 4URH.Q.1552, 4USI.A.1151, 3UXK.A.360, 3UZR.A.300, 4V0L.A.601, 3V1V.A.501, 3V1X.A.501, 4V1T.A.1777, 3V2U.C.521, 2V26.A.1801, 3V3W.A.403, 3V4B.A.403, 1V54.A.3518, 1V5F.A.1603, 2V7Q.A.1512, 2VBU.A.1134, 3VC1.A.301, 3VC1.I.301, 3VC2.J.301, 3VCC.A.401, 3VCC.A.402, 3VCN.A.501, 2VD0.B.2001, 2VDR.B.2001, 2VDL.B.2001, 2VDN.B.2001, 1VG8.A.1401, 2VG3.A.1297, 3VKB.A.701, 2VK1.A.601, 2VK4.A.601, 2VK8.A.1565, 3VMK.A.402, 3VMK.B.402, 1VOM.A.997, 3VPB.A.502, 3VVH.A.701, 3VX4.A.802, 1VZM.B.1046, 1VZM.B.1047, 1W2Y.A.1231, 1W2Y.A.1232, 3W40.A.201, 2W4J.A.1280, 2W5V.A.1378, 2W5X.A.1379, 1W6T.A.435, 3W6N.A.803, 3W6O.A.802, 1W78.A.1422, 1W78.A.1423, 1W7K.A.1423, 1WA5.A.1178, 3WBH.A.501, 3WBZ.A.403, 3WBZ.A.404, 1WC1.A.1501, 1WC1.A.1502, 3WEK.A.401, 2WEF.A.401, 1WF3.A.401, 4WH2.A.402, 4WH3.A.402, 2WIC.A.1266, 3WJP.A.403, 3WJP.A.404, 2WKQ.A.1724, 3WNZ.A.502, 3WRY.C.

1202, 1WUH.L.1005, 1WUK.L.1005, 2WVG.A.601, 2WW8.A.1000, 3WXM.A.502, 1WZC.A.300, 1X06.A.900, 1X07.A.900, 2X13.A.1418, 2X14.A.1418, 1X3S.A.200, 1X84.B.401, 2X98.A.1477, 1XBY.A.601, 2XB5.A.223, 2XCW.A.1498, 1XEX.A.1002, 1XFI.A.400, 1XG3.A.2101, 2XH2.B.1439, 2XH7.A.1441, 2XIS.A.392, 4XIA.A.399, 2XJB.A.1494, 2XJC.A.1499, 2XJD.A.1497, 2XJE.A.1493, 2XSX.A.500, 2XTZ.A.1381, 2XTN.A.1232, 2XUU.A.1307, 1XX1.A.9001, 4XXP.A.301, 2Y6P.A.1234, 1Y8A.A.501, 1Y8Q.B.641, 2Y8E.A.1177, 1Y9D.D.2901, 2YCH.A.501, 1YHL.A.1401, 1YHL.A.1402, 1YHL.A.1403, 1YIO.A.212, 1YMV.A.200, 1YNS.A.1258, 1YQ9.H.540, 1YQT.A.591, 1YQT.A.592, 1YRQ.H.553, 1YS7.A.1002, 1YU4.A.2002, 2YVO.A.1001, 2YVO.A.1002, 2YVP.A.183, 2YVP.A.184, 1YVD.A.850, 1YVE.I.601, 2YVM.A.1001, 2YXH.A.502, 1YYQ.B.702, 1YZL.A.401, 1YZN.A.301, 1YZT.A.700, 1Z06.A.203, 1Z07.A.300, 1Z08.A.1300, 1Z08.C.3300, 1Z0J.A.400, 1Z2N.X.1295, 1Z2O.X.1295, 1Z4J.A.1001, 1Z4K.A.229, 1Z4L.A.2001, 1Z4O.A.800, 1Z4P.X.1001, 1Z4Q.A.2001, 2Z4V.A.1501, 2Z4V.A.1502, 2Z4X.A.1201, 2Z4X.A.1202, 1Z59.A.1001, 1Z5C.A.2001, 1Z5G.A.601, 1Z5G.D.604, 2Z52.A.1301, 2Z52.A.1302, 2Z7I.A.1301, 1Z88.A.601, 1ZC3.A.500, 2ZCR.A.669, 2ZDH.A.812, 1ZED.A.905, 3ZFD.A.500, 3ZIA.A.601, 1ZJJ.A.1001, 2ZKJ.A.500, 3ZMC.A.1296, 3ZO9.A.1592, 3ZOU.A.1295, 1ZPD.A.601, 2ZPU.A.360, 1ZS9.A.1257, 3ZVL.A.1524, 3ZX4.B.260, 3ZX5.A.260, 1ZXN.B.902, 2IHM.A.700, 3A0U.A.201, 3A10.A.201, 2AKZ.A.441, 1ALK.A.452, 3B05.A.1001, 4BBJ.A.750, 2BHW.A.601, 2BHW.A.602, 2BHW.A.603, 2BHW.A.604, 2BHW.A.609, 2BHW.A.612, 2BHW.A.614, 2BHW.B.605, 3BH7.A.1, 4BX2.A.301, 4C5A.A.331, 3C9U.A.309, 1D0X.A.998, 1D0Y.A.998, 1D0Z.A.998, 1D1A.A.998, 1D1B.A.998, 4DBQ.A.903, 2DCN.B.4006, 3DHF.A.502, 4DL8.A.304, 4DL8.A.305, 3DNT.B.442, 1DXR.L.400, 3DYH.A.3002, 1E1R.F.601, 1E79.A.601, 2E8W.A.1201, 2E8X.A.1301, 3EF0.A.1, 2FKW.A.1501, 2FKW.B.1601, 3G5A.C.307, 1G67.A.2007, 2G77.B.503, 1GFI.A.356, 2GJ8.A.602, 3GL9.A.123, 2GLQ.A.2003, 3H1E.A.202, 1HE1.C.202, 2HEG.A.300, 2HF7.A.700, 3HWX.1.602, 2I7D.A.728, 3ICM.A.403, 3ICN.A.402, 4IDO.A.503, 4IF4.A.300, 4IGA.A.200, 1IV2.B.1572, 2J7N.A.3374, 2J8C.L.1288, 1J97.A.220, 4JA2.A.201, 1JB0.A.1107, 1JB0.A.1110, 1JB0.A.1118, 1JB0.A.1122, 1JB0.A.1129, 1JB0.A.1130, 1JB0.A.1134, 1JB0.B.1235, 1JB0.X.1701, 2JCS.B.1211, 4KEM.A.401, 1L3R.E.392, 1L5Y.A.701, 1L7N.A.221, 4LCZ.A.306, 4LCZ.A.307, 4LCZ.C.316, 4LEO.A.201, 1LGH.A.57, 1LGH.A.58, 1LGH.B.59, 4LRS.A.404, 1LVK.A.998, 1MPS.M.801, 1MXO.E.501, 1N24.B.706, 3N5K.A.2001, 1N6K.A.201, 2NGR.A.199, 3NNN.A.401, 4NST.A.1103, 1NVV.Q.1002, 4NVO.A.402, 2ODE.A.3001, 2OGX.A.291, 3OLV.A.130, 4OVN.A.201, 2OZE.A.299, 2PAN.A.851, 3PDE.B.310, 3PL9.A.602, 3PL9.A.603, 3PL9.A.609, 3PL9.A.610, 3PL9.A.612, 3PL9.A.613, 3PL9.A.614, 2PL1.A.204, 2PLS.I.606, 1Q3H.D.674, 3QHW.A.298, 4QXD.A.304, 1ROX.D.14, 2R25.B.1, 1RLO.A.801, 4RUR.W.301, 4S1H.A.303, 1SVK.A.356, 3T2S.A.401, 3T6D.L.401, 3T6D.M.400, 3T6E.L.400, 1T91.A.1301, 3T9E.A.602, 3TCS.B.368, 3THU.A.500, 1TX4.B.681, 4UB6.C.507, 1UPB.A.601, 2VB6.A.1000, 3W6P.A.803, 1W7J.A.1793, 1W9I.A.1755, 3W9S.A.202, 2WF7.A.1220, 2WJN.M.1325, 3WK4.A.601, 1WQA.B.456, 2WZB.A.1417, 2X2E.A.1746, 1XBX.A.601, 1XHF.A.1001, 1Y9D.A.2601, 1YXO.A.5000, 1YZQ.A.901, 2Z4Z.A.1301, 1Z5B.A.2001, 2Z7I.B.1302, 1ZES.A.302, 1ZH4.B.202, 1ZXN.A.900, 2ZXE.A.2002

[1] "Cluster 6"

4IEM.A.502, 3FSY.A.334, 1IV2.B.1562, 1NUY.A.2342, 1SJB.A.1001, 2XTI.A.1551, 4DLG.A.903, 2FLC.A.248, 4FLW.A.802, 3HO1.X.22, 4IRK.A.402, 2OTL.A.8066, 1RVB.B.302, 4UN4.B.2367, 1VQ7.0.8066, 1YJ9.0.8067, 1YJW.7.8044, 521P.A.168, 4AG5.B.1588, 3AJP.A.183, 3ALN.B.406, 1AM4.D.679, 1AR1.A.560, 3ARA.A.166, 1AZT.B.406, 1BPM.A.488, 1CEE.A.180, 3CIK.A.690, 1CLK.A.780, 4DOL.B.2001, 3D19.D.301, 3D19.E.301, 1D2E.D.504, 2D32.B.2524, 4DVG.A.201, 2DW7.L.2012, 2E8W.B.1203, 2E8W.B.1204, 2E8A.A.501, 3EN9.A.600, 4EOP.D.501, 3EQB.A.9002, 4FMA.F.402, 3FPA.B.901, 1G8G.A.521, 3GFT.E.202, 3GOL.A.580, 1GQ9.B.1242, 3GT8.D.14, 4GZM.A.1001, 2H5N.B.1001, 2HAW.A.1001, 2HAW.A.1002, 4HJH.A.552, 4HN2.A.404, 4HNS.A.201, 3HYT.A.802, 4HYP.C.302, 4I40.A.301, 3IAP.A.3001, 4IL6.C.512, 4IL6.b.616, 1IOV.A.330, 2IO7.A.5001, 2IOA.A.5001, 1IV4.D.1564, 2J4E.A.1002, 4JVJ.F.403, 4JVJ.F.404, 4K6T.B.411, 3KGX.A.503, 3KRP.D.903, 3KZ1.E.550, 3LAW.B.1401, 3LDO.J.54, 3LGH.A.150, 1MAB.A.602, 3MG8.G.241,

4MPO.G.203, 4MPO.G.204, 3NC0.C.218, 4NNN.K.302, 4NNN.N.202, 2NOG.A.9220, 2O1X.C.
2003, 2O1X.D.2004, 2O4G.B.800, 4O6I.B.602, 4OKE.B.203, 4OKK.A.204, 2OPM.A.908, 3
OPK.C.401, 3OPS.D.501, 2OQY.C.402, 2P8E.A.306, 1PKG.A.1481, 1Q3H.A.674, 4QEH.A.4
01, 1ROZ.D.674, 4R9U.D.302, 4RAB.C.303, 1RK2.C.320, 1SVT.E.601, 1SVW.B.301, 3T5P
.H.301, 1T9Z.A.273, 3TAV.A.269, 4U3W.A.503, 4UOR.K.699, 3VHX.G.185, 3VN9.A.402,
2VWI.B.1293, 1W0H.A.1001, 1W1W.A.2001, 1W85.E.1368, 2WCJ.A.1146, 3WEG.A.402, 2WH
E.A.1222, 3WIG.A.402, 3WNW.J.201, 3WU2.B.616, 3WU2.C.513, 1XD2.A.167, 2XRI.A.133
7, 1YM0.A.402, 1YQ2.E.7005, 1Z6K.A.275, 1ZOT.A.901, 2ZRY.D.702
[1] "Cluster 7"
3A4K.A.301, 4AAB.B.1157, 2AGQ.A.4002, 2AQ4.A.302, 2BCV.A.576, 4BDY.A.1381, 4BE1.
A.1382, 4C2U.A.1666, 4CEI.A.2234, 1CW0.N.202, 4D6N.A.1188, 4D6N.F.1196, 4D6O.D.1
196, 4DF4.A.901, 4DF8.A.903, 4DLG.A.902, 4DOA.A.401, 4EEY.A.502, 3F2B.A.5, 4F5P.
A.401, 3GDX.A.347, 3GPL.A.800, 3GQC.B.203, 4IR9.F.402, 4IRD.F.903, 2ISO.A.339, 2
ISP.A.339, 2IS4.A.1001, 3JPN.A.339, 3JPR.A.339, 3JPT.A.339, 3K57.A.1001, 4K98.A.
602, 4K99.A.602, 3LK9.A.339, 4LOX.A.401, 4M30.A.501, 4M30.A.502, 4M47.A.402, 3M8
R.A.2, 4MFF.A.401, 1MOW.D.374, 3MQY.A.500, 4NCB.B.703, 3OS0.A.394, 3OYA.A.396, 3
OYC.A.396, 3OYC.A.397, 3OYE.A.396, 3OYF.A.396, 3OYF.A.397, 3OYG.A.397, 3OYH.A.39
6, 3OYH.A.397, 2OZM.A.904, 4PGQ.A.400, 4PQU.A.602, 4QCL.A.1302, 1QSY.A.1001, 1QT
M.A.1001, 3RJF.A.340, 3RJK.A.340, 3S3M.A.396, 3SI6.A.905, 1SKR.A.4001, 3SPY.A.90
4, 3SV3.A.836, 1T7P.A.4001, 3TFS.A.340, 1TK0.A.991, 1TK8.A.901, 4TUQ.A.402, 3TWH
.A.401, 4UB3.A.401, 3UQ2.A.1, 2W35.A.1224, 2XCP.A.1004, 2XCA.A.3000, 2XCA.A.3001
, 1YVP.A.1001, 1ZBL.A.202, 1ZBL.B.204, 3A06.A.500, 4A01.A.1770, 4A01.A.1771, 1A4
9.H.5334, 3A7D.A.300, 4ABZ.A.1210, 4AC0.A.1205, 2AE8.C.1009, 1AJB.A.452, 3AJO.A.
183, 3AXK.A.478, 1AZS.C.403, 3B1X.A.301, 1B7T.A.836, 2BJI.A.2277, 2BKU.A.221, 3B
NY.D.701, 1BWV.C.490, 2BW7.A.2201, 3BWY.A.300, 4BYF.C.1000, 1BZY.A.901, 4CE0.A.1
251, 1CG0.A.435, 1CG1.A.435, 1CG4.A.435, 1CH8.A.434, 1CIB.A.434, 2CJE.A.1268, 3C
P6.A.501, 4CTA.B.401, 1CUL.C.396, 3CWH.A.392, 4CW7.A.1002, 3CXO.B.500, 4CYM.A.11
99, 3D46.A.501, 3D47.A.501, 1DAK.A.901, 4DBH.A.401, 3DFY.A.401, 2DGN.A.1454, 4DH
5.A.402, 3DHD.A.502, 1DIE.A.399, 3DOE.A.193, 1DQN.A.451, 3DUF.A.1368, 3DVA.A.136
8, 2DW6.A.2001, 2DW6.D.2004, 4DWB.A.508, 4DXJ.A.401, 4DXJ.A.402, 3DYG.A.3004, 2E
0A.A.500, 4E1E.A.403, 1E4E.A.365, 2E8W.A.1202, 1E9I.A.1431, 2E91.A.1302, 4EA0.A.
301, 4EA0.A.303, 1EBG.A.438, 1EBH.A.438, 1EC9.A.498, 1ECB.A.507, 1ECQ.A.498, 1EF
L.B.1604, 3EFQ.A.3003, 3EFQ.B.4002, 3EG5.A.180, 3EGT.A.3003, 1ELZ.A.452, 3EQI.A.
3, 3ES8.A.393, 1EXM.A.407, 3EYA.H.613, 3EZ3.B.1104, 3F78.C.1, 4F71.A.301, 3FA5.A
.282, 3FD5.A.395, 3FD5.A.396, 3FD5.B.397, 3FD6.B.395, 3FDG.A.357, 3FE4.B.902, 4F
FL.A.904, 4FFL.A.906, 4FFO.A.904, 4FFR.A.403, 2FG5.A.301, 4FI4.A.501, 3FLK.A.405
, 2FN0.A.701, 3FPA.C.901, 3FPB.A.1000, 3FQI.A.1000, 1FTN.A.300, 3FTQ.A.371, 3FYY
.A.402, 1G3B.A.501, 2G4J.A.392, 4G61.A.302, 2G9Y.A.452, 2G9Z.A.704, 2G9Z.B.701,
3G9D.A.298, 1GAG.A.201, 4GA3.A.1004, 4GIS.A.405, 4GME.A.501, 2GQS.A.240, 2GQ3.A.
1000, 2GT4.B.401, 4GT3.A.403, 4GYI.A.402, 1H1D.A.300, 4H1Z.A.401, 3H4L.A.701, 2H
CJ.A.998, 3HDG.B.201, 4HE1.A.404, 4HE2.A.405, 4HGR.A.201, 4HHL.A.402, 3HJN.A.501
, 3HPF.A.402, 3HQD.A.501, 3HQP.B.502, 2HWG.A.901, 4HYV.A.1001, 4I2B.A.602, 3I3O.
A.306, 4I3Y.A.302, 1I6I.A.501, 3I6E.A.386, 3IBA.A.401, 3IBA.A.402, 3ICZ.A.401, 2
IDX.A.603, 4IEE.A.501, 1IGW.A.441, 1II0.A.593, 1II9.A.593, 3IIE.A.501, 4IJQ.A.30
4, 2IK2.B.287, 2IK2.B.289, 2IK2.B.290, 2IK7.A.287, 2IOA.B.5004, 4IT1.A.501, 2IUC
.B.1008, 4IX4.A.602, 1J34.C.501, 2J5X.A.200, 1JAH.A.168, 1JCT.A.498, 2JCS.A.1211
, 2JI6.A.1567, 2JI8.A.1567, 1JP4.A.701, 3JUK.C.307, 4K33.A.802, 3K5H.A.401, 3K5H
.A.402, 1K9Y.A.403, 1K9Y.A.402, 4K9N.A.601, 4KCT.A.1001, 4KCU.A.1001, 4KCV.A.100
1, 3KDN.A.500, 1KEK.A.2237, 1KH7.A.452, 1KHK.A.452, 1KHZ.B.310, 3KHQ.A.1, 1KKR.A
.501, 4KMQ.A.1102, 1KO5.A.1001, 1KP8.A.550, 4KQX.A.405, 4KQX.A.406, 3KR4.C.1004,
3KRO.D.3002, 4KS0.A.1001, 4KUX.A.701, 4KWD.A.403, 4KX3.A.302, 4L2X.F.403, 4L2X.
F.405, 1L8A.A.888, 4L9Y.B.403, 4LA7.B.601, 4LF1.A.801, 4LNI.E.505, 1LON.A.1454,
3LVV.A.695, 3LVV.A.697, 3M00.A.550, 1M1B.B.999, 3M1Y.A.300, 4M69.A.403, 4M6U.A.4
01, 1MB9.B.601, 1MBZ.A.603, 1MC1.A.603, 1MEZ.A.1453, 4MIT.A.202, 1MNS.A.360, 4MP

O.B.204, 4MPO.C.205, 4MPO.E.201, 3MQT.H.626, 1MRS.A.300, 4NOG.A.403, 1N1Z.A.702, 1N2O.A.703, 1N24.A.703, 3N3T.A.802, 4NEH.B.703, 1NHT.A.435, 4NM3.A.405, 3NO1.A.397, 1NUW.A.2497, 1NUX.A.2342, 1NUY.A.2343, 1NV7.A.3341, 3NZG.A.507, 4NZO.A.404, 2O1O.A.501, 2O1O.A.503, 4O4D.A.401, 1OAD.A.392, 3OES.A.202, 1OFH.B.453, 4OKZ.A.901, 3OPS.A.500, 3OPS.A.501, 2OQY.A.401, 1ORK.A.223, 4ORK.A.502, 1OVM.A.601, 1OW2.A.401, 2OX4.A.402, 3OYZ.A.500, 1OZF.A.699, 1OZH.A.1405, 3OZM.D.390, 3OZY.B.390, 3P0X.A.430, 3P3B.A.393, 3P5R.A.901, 1P7T.A.1000, 1P9B.A.1600, 3P93.A.406, 4PAL.A.110, 3PDE.A.311, 1PFK.A.325, 4PFK.A.327, 2PLS.G.603, 2PLS.J.604, 2PP3.A.901, 1PUN.A.130, 3PUV.A.1501, 1PYD.A.559, 1PYM.A.1003, 4Q1V.A.803, 6Q21.D.173, 3Q30.A.600, 3Q30.A.601, 3Q46.A.305, 2Q58.A.5, 3Q8U.E.159, 1Q9S.A.201, 3Q9L.A.700, 1QC5.A.601, 1QC5.B.602, 4QEH.A.402, 1QF4.A.433, 1QMZ.A.383, 3QPE.A.393, 3QPE.B.393, 4QPM.A.1502, 3QQV.A.382, 1QSO.A.501, 2QTV.B.210, 3QU4.A.225, 2QXO.A.162, 3R1M.A.402, 3R25.A.402, 2R9V.A.504, 3RBM.D.1003, 2RDX.A.378, 3RIM.A.1001, 4RJK.H.602, 4RN3.A.301, 4RNH.A.1501, 4ROP.A.504, 1RQI.A.605, 3RUV.A.544, 1S1C.A.300, 3S9I.A.743, 3SAZ.A.802, 3SAD.A.801, 3SBO.A.801, 3SBF.A.402, 3SE1.A.182, 3SH6.A.176, 3SOP.A.401, 1SOJ.A.2123, 3SSN.A.501, 3ST8.A.496, 3T0Z.A.401, 3T1Q.A.198, 3T6C.A.501, 3T8O.A.564, 1T9B.A.1699, 1TND.A.352, 4TQ4.A.401, 3TTE.A.361, 3TTE.B.361, 3TW6.C.2002, 3TZF.A.279, 4U0M.A.503, 3U2E.B.1, 3U2E.B.4, 3UJ2.A.431, 4USJ.C.302, 3UXL.A.360, 2VON.A.601, 4V1T.A.1776, 4V1T.A.1778, 2V3W.A.1528, 1V5G.A.1603, 2V5K.A.301, 1V8K.A.501, 1VA6.A.522, 1VA6.A.524, 2VBI.A.1000, 3VD3.A.3001, 2VDM.B.2001, 3VMM.A.501, 2VPR.A.1207, 2VPO.A.1209, 2VQD.A.1449, 3VR6.B.602, 2VWT.A.301, 3VYT.C.602, 2VZB.A.1001, 2W00.B.1894, 3W2W.A.904, 1W5T.B.701, 3W7F.A.303, 1W88.C.1368, 4WB8.A.402, 1WC6.C.2202, 1WDD.A.1476, 3WDL.B.902, 2WEF.A.402, 4WF7.A.601, 4WK0.B.501, 4WK4.B.501, 1WL6.A.801, 3WO0.A.502, 3WO0.A.503, 4WRR.A.401, 2WX5.L.1282, 2X5Z.A.602, 2XAM.B.1030, 2XCL.A.480, 1XEF.A.801, 2XH4.A.1439, 5XIM.A.395, 5XIN.A.395, 1XIN.A.395, 6XIM.A.395, 8XIM.A.395, 2XIM.A.395, 1XLC.A.399, 2XZW.A.202, 1XZ8.A.180, 1Y9I.A.601, 1YHM.B.1401, 1YHY.A.699, 1YVE.I.602, 2YWF.A.701, 1YYQ.B.701, 1Z0K.A.1201, 2Z4W.A.1302, 2Z4Y.B.1301, 2Z7H.A.1301, 3ZCB.A.301, 3ZDY.B.2001, 1ZVW.A.4001, 2ZVJ.A.300, 3ZYC.A.1750, 3OTB.A.401, 3A4K.C.301, 3AUO.A.577, 4BDZ.A.1380, 4D6O.A.1186, 4DLE.A.901, 2HVI.D.878, 3ICE.E.502, 4J9O.A.502, 3MDA.A.577, 4N41.E.101, 4NLK.A.402, 4NM1.A.401, 3TFR.A.340, 1TFW.B.1601, 2A5G.A.231, 4A7Y.A.952, 4ACF.D.1480, 3ALO.A.1, 1AQF.A.534, 2AUU.A.201, 3B03.D.1001, 2B8W.A.595, 2B9J.A.600, 4BJU.A.998, 2BKK.A.1266, 1BS1.A.901, 2BVN.A.1395, 4BYF.A.1000, 4BYG.A.996, 3C14.C.403, 2C43.A.1317, 3CBT.A.301, 3CK5.D.400, 3CRL.A.2000, 1CUL.A.1006, 3CX7.A.378, 4CYI.D.1000, 3DKL.A.502, 1E1R.A.601, 2E8T.B.1303, 2E8X.A.1302, 1E9I.B.1431, 1E9I.D.1433, 2E91.B.1303, 2E92.B.1303, 3EA4.A.699, 3EF1.A.1, 3EFQ.B.4003, 3EYA.A.613, 3FDG.B.356, 4FFR.A.406, 1FQJ.A.352, 2G07.A.601, 2G08.A.500, 3G8D.B.1002, 4GA3.A.1003, 3GFT.F.202, 2GTP.A.401, 4HEO.A.402, 4HEO.A.403, 4HGQ.C.201, 4HGR.B.201, 3HIY.A.401, 2HO4.B.301, 3HU2.D.801, 3HZH.A.202, 3I0O.A.502, 2I19.A.3002, 2IOA.A.5002, 4J5I.F.402, 2JCM.A.1490, 4JLZ.B.502, 4KCV.B.1001, 1KK8.A.997, 3KRO.A.3003, 3L0C.A.257, 4LNI.D.503, 4LRZ.A.302, 3LUZ.A.264, 3MCO.A.427, 3MCO.B.426, 4MGH.A.1302, 3MLE.C.222, 4NOG.B.402, 4NCJ.A.903, 4NDN.A.401, 4NM5.A.406, 3NNS.A.401, 4NNN.Z.301, 2O56.A.2001, 3O61.A.202, 1PPW.A.401, 2PUL.A.400, 3PUW.A.1501, 3Q1O.D.400, 2Q58.A.3, 3Q7P.B.257, 3RBM.B.1001, 1RLT.A.805, 3RRA.A.406, 1SOJ.C.2127, 3SQS.A.450, 3SS8.A.302, 3T34.A.1002, 3T5P.F.301, 4TQ4.C.402, 4TXZ.B.502, 3U2E.A.2, 2V54.B.1205, 3VAD.A.402, 2W00.A.1887, 1W5T.A.701, 3WBZ.F.403, 3WQM.A.403, 1XBT.D.4194, 1XZ8.B.180, 1ZCA.A.383, 2ZRW.A.702

Table S41. 4-ligand Ca, normal group

size	largest_angle*	middle_1*	middle_2*	middle_3*

```
1 "33"  "139.1+/-10.8" "68.5+/-4.4" "74+/-4.9"   "79.6+/-5.3"
2 "51"  "158.3+/-10.8" "76.2+/-7.6" "85.2+/-7.2" "95.6+/-7.5"
3 "107" "164.9+/-6.2"  "79.3+/-5.4" "86.3+/-4.9" "92.1+/-5.9"
4 "53"  "147.3+/-9.7"  "77.5+/-7.4" "85.5+/-7.1" "97.1+/-11.3"
5 "49"  "162+/-10.8"   "76.4+/-6.5" "83.4+/-5.3" "92.4+/-6"
  middle_4*      smallest_opposite_angle* Tetrahedral
1 "91.2+/-10.8"  "84+/-14.7"              "0.002"
2 "107.1+/-11.2" "110.9+/-7.2"            "0"
3 "99.8+/-7.3"   "85.7+/-8.4"             "0"
4 "121.3+/-8.3"  "84.5+/-10.2"            "0.001"
5 "107.5+/-11.9" "143+/-12.4"             "0"
  TrigonalBipyramidalVA TrigonalBipyramidalVP SquarePyramidalV
1 "0.003"               "0.001"               "0.027"
2 "0.003"               "0.145"               "0.103"
3 "0.006"               "0.048"               "0.276"
4 "0.018"               "0.013"               "0.013"
5 "0"                   "0.069"               "0.004"
  SquarePlanar
1 "0"
2 "0"
3 "0"
4 "0"
5 "0.081"
```

Table S42. Cluster members of 4-ligand Ca, normal group

```
[1] "Cluster 1"
2AU0.H.1415, 3GIJ.B.1415, 3KHL.B.1417, 3NHG.A.909, 3B2Z.C.3, 4BTX.A.1764, 4BWE.A
.503, 2CLT.A.1102, 1CVM.A.802, 1FBL.A.994, 1FZA.C.1, 1G5N.A.403, 1GU6.A.1480, 4I
9F.A.401, 3IAE.B.572, 2J0T.C.1268, 2JF9.P.1014, 3KQA.C.420, 3M0W.G.202, 1MTS.A.4
80, 3N9V.A.64, 4NUP.C.304, 3P1O.A.234, 3Q2N.B.304, 1UG9.A.2002, 4WFE.A.306, 4WIW
.C.701, 2WJS.A.5002, 3WMW.A.401, 2WWO.E.800, 1XJL.A.342, 2XSG.A.1772, 2YGM.B.141
8
[1] "Cluster 2"
2B0D.A.502, 4EBC.A.504, 4G3I.A.401, 4K4I.E.603, 3OOR.A.236, 4Q0W.B.1001, 4AAH.A.
702, 4AG4.A.5002, 1ALA.A.401, 1AXK.A.395, 2B00.A.252, 1BK9.A.200, 1BRW.A.3001, 1
DM5.E.1135, 3E1I.B.503, 4EJ7.A.404, 1FBL.A.993, 4G1M.B.2001, 3GG1.A.503, 3GG1.B.
503, 1HOV.A.168, 1IJL.A.203, 4ILW.F.304, 1IVG.B.470, 1KVY.A.124, 2LMV.A.151, 1LW
U.K.4, 1MWN.A.100, 1NGS.B.681, 3NSJ.A.702, 2OVZ.B.449, 2OZR.F.4030, 4PIB.B.203,
4POQ.G.401, 4POR.E.401, 2Q04.C.211, 1SEL.B.277, 1TFX.A.1007, 1UCN.A.1162, 4UM9.B
.2003, 3UMJ.A.902, 2V5C.A.1625, 3V96.B.305, 2VME.E.500, 2W1W.B.1134, 3W9T.B.509,
 1XJL.A.344, 1YAX.A.1002, 2YDP.B.502, 2Z2D.A.267, 2Z2D.A.268
[1] "Cluster 3"
4BXO.A.2050, 2C28.A.1344, 4EFJ.C.101, 4EFJ.A.401, 2GIH.B.401, 4K4H.M.605, 4K4I.E
.606, 4KHW.A.1005, 1M5X.C.801, 2NOF.A.328, 3OOR.A.237, 3SQ2.A.906, 2WTF.A.1512,
1A85.A.996, 1AEI.D.317, 1AEI.A.317, 2AZ1.D.202, 4B7U.B.401, 3B90.B.702, 3BCF.A.7
05, 4BCU.A.201, 3BS6.A.1, 4CAG.A.606, 2CDP.C.1140, 2COL.B.801, 3D4G.A.484, 3D6E.
B.202, 1D8F.A.305, 3DF0.A.717, 3DF0.B.601, 2EAA.C.904, 4EMU.B.401, 1ESL.A.164, 2
EXJ.D.2004, 2FH3.A.1001, 4GEJ.A.201, 4GGB.A.402, 4GH8.B.203, 3GRI.B.700, 2GXS.B.
604, 2H0K.A.402, 2H0K.A.408, 1H71.P.500, 3HDB.A.657, 2HTY.A.991, 2IAP.A.402, 3IJ
E.B.4002, 3IRH.A.457, 2J1G.F.1291, 2J60.C.1277, 4JP8.A.706, 3K39.F.1000, 3K8Y.A.
```

167, 1KVO.D.192, 4LN6.G.403, 4M7K.H.301, 3M83.B.408, 4MBE.D.201, 2MG9.A.101, 1N2
8.B.127, 4N25.A.705, 4N2D.A.705, 4N96.B.401, 4NAS.C.501, 2NPO.A.1293, 4NUP.C.301
, 4NVR.C.401, 1OLP.A.1372, 1OS2.A.872, 2OVX.B.447, 2OVX.B.450, 2P5W.B.701, 1Q3A.
A.467, 4Q4Y.1.5006, 1Q7B.B.9003, 1QDO.C.245, 1QMD.A.404, 4QN3.A.501, 3QWU.B.602,
 2R1D.I.3000, 2R8Y.I.209, 2RHP.A.16, 1RJV.A.112, 4RSR.A.404, 1SOE.A.1292, 3SH5.A
.196, 3SHI.M.305, 3SNZ.A.97, 1SPJ.A.300, 1SRR.C.532, 1UBN.A.277, 3UBH.A.855, 1UE
A.A.4, 2VB6.B.1148, 1VE5.B.2003, 2VVD.A.1329, 1W1X.A.1479, 2WII.A.1643, 3WNX.A.5
02, 1X36.A.269, 2XTJ.A.1423, 1Y6P.A.217, 1Y6P.B.218, 2ZUX.A.629, 2ZUX.A.637, 2ZU
Y.A.621, 2ZWP.B.401

[1] "Cluster 4"

2C2R.A.1344, 4DTJ.A.1003, 4DTU.A.1003, 4DU4.A.1003, 2GIH.A.402, 3M9M.B.354, 3QER
.A.906, 3QEV.A.906, 3RBD.A.415, 4RI8.A.1101, 3V20.A.302, 4WUZ.B.301, 3ATS.A.380,
 1B1G.A.77, 1B90.A.930, 3D4G.A.485, 1D8M.A.304, 3DEM.B.6001, 4DLK.B.402, 4EJ7.A.
402, 4EM6.B.604, 1EN7.A.403, 3FHA.A.705, 2HF4.A.902, 2HYW.A.506, 2II1.C.404, 2IW
V.A.1284, 2K0J.A.503, 4KS3.A.502, 1KTW.A.5, 4L06.A.501, 4LIN.A.1301, 3MDO.A.391,
 3O82.B.544, 4OO2.C.601, 2P5V.B.1008, 2P5V.C.1001, 4P99.B.540, 1PZ8.C.703, 1QD6.
C.2, 1QHD.A.603, 2QVF.B.703, 4R5O.A.509, 3R6Q.H.469, 1RFN.A.500, 3S5U.F.220, 1SB
H.A.291, 4TQO.H.701, 2UX1.K.174, 2WG8.C.201, 2WJS.A.5001, 1Y1A.A.501, 1ZH2.A.202

[1] "Cluster 5"

3KHG.A.415, 4KHU.A.1003, 2NOL.A.328, 4QWD.A.702, 3RBD.B.1415, 4AC8.B.1311, 1AWB.
B.280, 2BD4.A.260, 2BZ6.H.1260, 3C14.A.29, 1C9P.A.501, 1D8M.B.804, 4FVL.A.506, 4
H82.C.305, 1HKB.A.923, 3HQ8.B.402, 1HVD.A.600, 2II1.D.401, 2IO4.B.701, 3IS5.F.1,
 1ITC.A.1500, 1J24.A.1001, 1JDC.A.452, 4KW7.A.402, 4L41.B.201, 4MC7.A.503, 4MIX.
A.2501, 4N2I.A.707, 1NMB.N.478, 4NUY.A.1001, 3OHO.A.1, 4OKH.C.903, 3OXQ.D.516, 4
P99.A.533, 2PC6.B.303, 1PEX.A.502, 2PR3.A.901, 4Q4X.1.5007, 1QMD.B.405, 3R4I.D.3
42, 2RJP.D.2, 3RMK.B.308, 3VEQ.B.301, 3VOB.A.401, 2W0Q.B.803, 3W9T.B.510, 1Y7O.B
.1004, 2YA9.A.1303, 2YN3.D.6355

Table S43. 4-ligand Ca, combined group

	size	largest_angle*	middle_1*	middle_2*	middle_3*	middle_4*	smallest_opposite_angle*	Tetrahedral	TrigonalBipyramidalVA	TrigonalBipyramidalVP	SquarePyramidalV	SquarePlanar
1	"69"	"148.2+/-11.1"	"67.9+/-13"	"82.7+/-9.3"	"100.6+/-12.7"	"127.5+/-11"	"90.7+/-14.5"	"0.001"	"0.01"	"0.007"	"0.006"	"0"
2	"67"	"159.2+/-12.3"	"72.9+/-10.4"	"83.3+/-5.7"	"93.8+/-6.9"	"109.9+/-12.6"	"138.9+/-13.4"	"0"	"0"	"0.054"	"0.004"	"0.059"
3	"74"	"127.4+/-13"	"57.3+/-8.5"	"72.3+/-7.9"	"80.2+/-7.5"	"94.8+/-11.6"	"84+/-16.2"	"0.002"	"0.004"	"0.001"	"0.013"	
4	"61"	"161.1+/-10"	"75.7+/-6.1"	"82.9+/-6.7"	"91+/-7.3"	"101.2+/-9.4"	"69.1+/-11"	"0"	"0.001"	"0.01"	"0.14"	
5	"120"	"163.4+/-7.5"	"79.5+/-6.4"	"87.1+/-5.2"	"93.2+/-6.4"	"101.7+/-8"	"95.5+/-9.8"	"0"	"0.008"	"0.1"	"0.223"	

3 "0"
4 "0"
5 "0"

Table S44. Cluster members of 4-ligand Ca, combined group

[1] "Cluster 1"
4DTJ.A.1003, 4DTU.A.1003, 4DU4.A.1003, 4EBC.A.504, 2GIH.A.402, 3QER.A.906, 3QEV.A.906, 4WUZ.B.301, 3ATS.A.380, 1B1G.A.77, 1B90.A.930, 1BRW.A.3001, 4CP1.A.1297, 1D8M.A.304, 4DLK.B.402, 3E1I.B.502, 3EDD.A.701, 1EN7.A.403, 1EUB.A.277, 3FHA.A.705, 3GG1.A.503, 2HF4.A.902, 2HYW.A.506, 2II1.C.404, 1IJL.A.203, 2IWV.A.1284, 1JHN.A.900, 1JYI.D.408, 4K6T.G.408, 4KS3.A.502, 4KS4.A.502, 1KTW.A.496, 1KTW.A.5, 1KTW.A.9, 4L06.A.501, 4L76.E.402, 4LIN.A.1301, 1LPK.B.1, 1LRW.A.702, 1M1J.B.503, 2M29.A.401, 3O82.B.544, 4OO2.C.601, 2P5V.C.1001, 4P99.B.540, 1PZ8.C.703, 2Q04.C.211, 1QD6.C.2, 1QHD.A.603, 4QNP.A.506, 2QPS.A.501, 4R5O.A.509, 3R6Q.H.469, 1RFN.A.500, 1RK9.A.112, 3S5U.F.220, 1SBH.A.291, 4TQO.H.701, 1TRQ.A.106, 1UCN.A.1162, 4UM9.D.2002, 2UX1.K.174, 1W52.X.602, 3WIU.B.1004, 4WK4.B.502, 1XZO.A.1014, 1XZO.B.1018, 1Y1A.A.501, 1ZH2.A.202
[1] "Cluster 2"
2B0D.A.502, 3KHG.A.415, 4KHU.A.1003, 2NOL.A.328, 4Q0W.A.1001, 4QWD.A.702, 3QZ7.A.363, 3RBD.B.1415, 4AAH.A.702, 4AC8.B.1311, 2AER.L.3005, 1AWB.B.280, 1AXK.A.395, 2BD4.A.260, 2BZ6.H.1260, 3C14.A.29, 1C9P.A.501, 1D8M.B.804, 4FVL.A.506, 4H82.C.305, 1HKB.A.923, 3HQ8.B.402, 3HR4.H.203, 1HVD.A.600, 2II1.D.401, 2IO4.B.701, 3IS5.F.1, 1ITC.A.1500, 1J24.A.1001, 1JDC.A.452, 1K6S.A.302, 1K6S.B.301, 4KW7.A.402, 4L41.B.201, 4MC7.A.503, 4MIX.A.2501, 4N2I.A.707, 1N41.A.410, 1NMB.N.478, 4NUY.A.1001, 3OHO.A.1, 4OKH.C.903, 2OVZ.B.449, 3OXQ.D.516, 4P99.A.533, 2PC6.B.303, 1PEX.A.502, 4PIB.B.203, 4POQ.G.401, 4POR.E.401, 2PR3.A.901, 4Q4X.1.5007, 1QMD.B.405, 3R4I.D.342, 2RJP.D.2, 3RMK.B.308, 3RRV.C.255, 2TBV.A.388, 3VEQ.B.301, 2VME.E.500, 3VOB.A.401, 2W0Q.B.803, 3W9T.B.510, 1Y7O.B.1004, 2YA9.A.1303, 2YN3.D.6355, 2Z2D.A.268
[1] "Cluster 3"
2AUO.H.1415, 3GIJ.B.1415, 4J2A.A.1006, 3KHL.B.1417, 3NHG.A.909, 4RIC.B.1101, 1AFB.2.3, 3B2Z.C.3, 2B30.A.302, 4BTX.A.1764, 4BY6.A.3080, 2C11.C.1739, 2CLT.A.1102, 2EOX.B.701, 2E6V.E.12, 3EAD.C.1001, 1FBL.A.994, 2FH3.A.1003, 1FZA.C.1, 1G5N.A.403, 3GHG.L.601, 1GU6.A.1480, 4I9F.A.401, 3IAE.B.572, 2IEZ.A.219, 4IEF.H.704, 1IT4.A.200, 2J0T.C.1268, 2J3G.A.1289, 4JBE.A.503, 1JED.B.535, 2K0J.A.503, 4K1C.B.504, 2KBM.B.101, 3KQA.C.420, 4L76.B.402, 4L76.D.402, 1LGC.C.301, 4LLF.O.401, 1LWU.C.5, 3MOW.G.202, 4M8D.B.305, 2M98.A.201, 4MDV.A.404, 1MTS.A.480, 2NWH.A.402, 4OVG.A.404, 4POS.B.401, 3Q2N.B.304, 1QDO.D.247, 1QLK.A.93, 3RBX.A.601, 2RHP.A.15, 3RRY.A.202, 3S55.D.280, 1S6I.A.192, 1SAV.A.321, 1SUI.B.306, 1SUS.C.307, 1TCF.A.164, 2TEC.E.344, 1UG9.A.2002, 2W67.B.1590, 1WD9.A.902, 4WFE.A.306, 4WIW.C.701, 3WMW.A.401, 2WM4.A.1430, 2WWO.E.800, 2XJO.B.1175, 1XJL.A.342, 2XSG.A.1772, 1YN8.B.1008, 1Z3J.A.268
[1] "Cluster 4"
2C2R.A.1344, 2C28.A.1344, 4K4I.E.606, 3RBD.A.415, 4RI8.A.1101, 3SQ2.A.906, 1AEI.D.317, 1AEI.A.317, 3AKB.A.173, 2AZ1.D.202, 3BOW.B.404, 4BWE.A.503, 1CVM.A.802, 3D4G.A.484, 3D4G.A.485, 3DF0.B.601, 2DW2.B.711, 2E3X.A.801, 2EAA.C.904, 3GE4.A.167, 1GQM.A.1090, 1GQM.C.1088, 3GRI.B.700, 2GXS.B.604, 2HOK.A.408, 2HOL.A.1015, 2HTY.A.991, 2IAP.A.402, 3IJE.B.4002, 3IRH.A.457, 2JF9.P.1014, 4JWS.A.502, 3LMW.A.9, 4LN6.G.403, 3M83.B.408, 3MDO.A.391, 3N9V.A.64, 4NAS.C.501, 4NUP.C.301, 4NUP.C.304, 1OLP.A.1372, 3P1O.A.234, 3QWU.B.602, 2R1D.I.3000, 4RSR.A.404, 3SH5.A.196, 1

SPJ.A.300, 1T9H.A.414, 3U24.A.602, 1UBN.A.277, 1UEA.A.4, 2VB6.B.1148, 2VVD.A.132
9, 1WOO.A.1781, 3WCV.B.203, 2WII.A.1643, 2WJS.A.5001, 2WJS.A.5002, 3WNX.A.501, 2
XTJ.A.1423, 2YGM.B.1418
[1] "Cluster 5"
4BXO.A.2050, 4EFJ.C.101, 4EFJ.A.401, 4G3I.A.401, 2GIH.B.401, 4K4H.M.605, 4K4I.E.
603, 4KHW.A.1005, 1M5X.C.801, 3M9M.B.354, 2NOF.A.328, 3OOR.A.236, 3OOR.A.237, 4Q
OW.B.1001, 3V20.A.302, 2WTF.A.1512, 1A85.A.996, 4AG4.A.5002, 1ALA.A.401, 2BOO.A.
252, 4B7U.B.401, 3B90.B.702, 3BCF.A.705, 4BCU.A.201, 1BK9.A.200, 3BS6.A.1, 4CAG.
A.606, 2CDP.C.1140, 2COL.B.801, 3D6E.B.202, 1D8F.A.305, 3DEM.B.6001, 3DFO.A.717,
1DM5.E.1135, 3E1I.B.503, 4EJ7.A.402, 4EJ7.A.404, 4EM6.B.604, 4EMU.B.401, 1ESL.A
.164, 2EXJ.D.2004, 1FBL.A.993, 2FH3.A.1001, 4G1M.B.2001, 4GEJ.A.201, 3GG1.B.503,
4GGB.A.402, 4GH8.B.203, 2H0K.A.402, 1H71.P.500, 3HDB.A.657, 1HOV.A.168, 4ILW.F.
304, 1IVG.B.470, 2J1G.F.1291, 2J60.C.1277, 4JP8.A.706, 1JX6.A.401, 3K39.F.1000,
3K8Y.A.167, 1KVO.D.192, 1KVY.A.124, 2LMV.A.151, 1LWU.K.4, 4M7K.H.301, 4MBE.D.201
, 2MG9.A.101, 1MWN.A.100, 1N28.B.127, 4N25.A.705, 4N2D.A.705, 4N96.B.401, 1NGS.B
.681, 2NPO.A.1293, 3NSJ.A.702, 4NVR.C.401, 1OS2.A.872, 2OVX.B.447, 2OVX.B.450, 2
OZR.F.4030, 2P5V.B.1008, 2P5W.B.701, 1Q3A.A.467, 4Q4Y.1.5006, 1Q7B.B.9003, 1QDO.
C.245, 1QMD.A.404, 4QN3.A.501, 2QVF.B.703, 2R8Y.I.209, 2RHP.A.16, 1RJV.A.112, 1S
OE.A.1292, 1SEL.B.277, 3SHI.M.305, 3SNZ.A.97, 1SRR.C.532, 1TFX.A.1007, 3UBH.A.85
5, 4UM9.B.2003, 3UMJ.A.902, 2V5C.A.1625, 3V96.B.305, 1VE5.B.2003, 2W1W.B.1134, 1
W1X.A.1479, 3W9T.B.509, 2WG8.C.201, 3WNX.A.502, 1X36.A.269, 1XJL.A.344, 1Y6P.A.2
17, 1Y6P.B.218, 1YAX.A.1002, 2YDP.B.502, 2Z2D.A.267, 2ZUX.A.629, 2ZUX.A.637, 2ZU
Y.A.621, 2ZWP.B.401

Table S45. 5-ligand Ca, normal group

	size	largest_angle*	middle_1*	middle_2	middle_3*	middle_4
1	"89"	"172+/-3.7"	"80.3+/-3.6"	"84.4+/-2.3"	"87.5+/-2.1"	"90.1+/-2.2"
2	"20"	"168.6+/-6.3"	"78.4+/-5.5"	"84.2+/-2.7"	"88.1+/-3.1"	"91.6+/-2.6"
3	"79"	"167.9+/-5.1"	"75.2+/-4.8"	"81.1+/-3.7"	"86.5+/-3.1"	"90+/-3.6"
4	"34"	"152.4+/-8.2"	"67.8+/-3.1"	"71.8+/-3"	"76.8+/-3.6"	"82.8+/-5.6"
5	"54"	"164.5+/-6.5"	"77.4+/-4.8"	"84.1+/-3.2"	"88.3+/-2.6"	"92+/-3.1"
6	"59"	"161.3+/-5.8"	"73.1+/-4.5"	"77.5+/-4.1"	"81.6+/-3.4"	"85.9+/-4.1"
7	"34"	"156.5+/-8.2"	"69.5+/-5.4"	"77.7+/-5.4"	"82.2+/-5.6"	"90.3+/-6"

	middle_5*	middle_6	middle_7	middle_8*
1	"92.6+/-2.8"	"96.6+/-3.6"	"102.2+/-5"	"165.7+/-3.8"
2	"95.4+/-3.1"	"102.7+/-5.8"	"118.3+/-5.2"	"132.5+/-5.4"
3	"94.5+/-4.9"	"99.9+/-5.7"	"109.5+/-9"	"156.7+/-3.9"
4	"89.6+/-7.8"	"99.5+/-10.7"	"116.3+/-13.6"	"135+/-6.3"
5	"96.8+/-3.6"	"102.6+/-4.3"	"109.9+/-5.1"	"152.9+/-5"
6	"90.6+/-5.1"	"99.5+/-8.5"	"114+/-12.9"	"147.2+/-5.2"
7	"99.5+/-5.3"	"110.6+/-7"	"125.7+/-7.2"	"140.1+/-5.7"

	smallest_opposite_angle*	TrigonalBipyramidal	SquarePyramidal
1	"84.9+/-4.7"	"0.008"	"0.383"
2	"107.1+/-5.5"	"0.255"	"0.023"
3	"77.3+/-4.8"	"0.008"	"0.128"
4	"66.7+/-3.9"	"0.008"	"0.011"
5	"95.7+/-4.3"	"0.062"	"0.118"
6	"72.9+/-5.7"	"0.007"	"0.058"
7	"90.6+/-6.3"	"0.009"	"0.005"

TrigonalPrismaticV

1 "0.034"
2 "0.158"
3 "0.043"
4 "0.069"
5 "0.093"
6 "0.101"
7 "0.066"

Table S46. Cluster members of 5-ligand Ca, normal group

[1] "Cluster 1"
4BXO.B.1216, 4G3I.B.401, 3GII.A.415, 4K4H.M.604, 4K4I.A.603, 4LQO.A.401, 1R7M.A.304, 1TW8.C.803, 2WTF.B.1509, 4AG4.A.5001, 4B7M.B.1471, 2BV2.A.1085, 2BW7.A.2202, 3BYC.A.901, 2CDO.B.1140, 2CHI.A.218, 5CHY.A.401, 4CPN.A.500, 4CPO.A.1466, 1CXV.B.6, 2D00.B.1002, 1D7X.B.805, 3DPE.A.997, 1DYK.A.4001, 1E35.B.260, 3EKI.A.602, 2ERQ.B.702, 1EZS.C.650, 1F2N.A.1002, 3FRP.A.628, 4FVL.B.506, 4GDI.C.507, 2GNT.A.254, 4GN7.B.301, 2HTV.A.995, 4ILW.D.304, 1IME.B.278, 1INW.A.501, 1J0Y.D.701, 1JAO.A.996, 2JG9.B.1224, 1JI3.A.401, 4JUC.B.601, 3K37.A.467, 1L6J.A.502, 1L9N.A.703, 3LNF.B.305, 1N28.A.128, 1NGO.A.1002, 1NGO.C.1001, 2NVO.A.533, 2NW6.A.613, 1NXO.A.902, 2OO4.B.5006, 2OW1.B.447, 3Q2L.A.703, 4Q4X.1.5005, 1QI5.A.452, 2R1D.B.1000, 2R8Z.J.210, 1R8L.B.902, 2RHP.A.10, 2RHP.A.20, 2RJP.A.2, 2RJP.A.3, 2RJQ.A.6, 1ROS.B.503, 1RU4.A.2, 3SHI.G.305, 3SJS.A.222, 1STB.A.150, 1T6B.X.800, 4U32.A.301, 1ULV.A.2005, 1UYX.B.1135, 3V03.B.585, 1V7V.A.1001, 3VV3.A.404, 1W3M.C.3014, 2WG8.A.201, 3WIU.B.1002, 3WNK.A.812, 1Y4A.E.1001, 1Z4V.A.600, 2ZJ6.A.625, 1ZTQ.A.561, 2ZUX.B.638, 2ZUY.A.627, 2ZWO.A.400
[1] "Cluster 2"
2VA2.B.1343, 1AWB.B.279, 4B7U.D.401, 1CIZ.A.305, 2DSN.A.2011, 3ELM.A.303, 2EXH.C.2003, 3G20.A.201, 2J7A.D.1007, 4LM8.A.812, 1OLP.B.1372, 3QHQ.A.230, 1RM8.A.504, 3SOO.A.98, 3SOO.B.97, 3TRQ.A.359, 1UG9.A.2003, 2W1W.A.1132, 1WY9.A.148, 2ZUY.A.629
[1] "Cluster 3"
2FKC.A.248, 4KHN.A.1002, 4KHW.A.1003, 1A25.A.292, 1A25.B.292, 4AIO.A.1890, 4B4F.B.607, 2BAT.A.601, 2BD3.A.260, 3BOW.A.718, 1CJY.A.951, 3CK7.B.720, 3D4G.H.484, 2DEW.X.903, 1DM5.A.1131, 1DM5.C.1133, 1DQ1.A.238, 3E9T.A.6, 2EA7.B.452, 1EE6.A.300, 3F19.A.266, 2F2O.B.1001, 1GQM.E.1089, 4GZS.A.501, 4H2E.B.304, 1HFZ.C.124, 4I5K.A.501, 1JIW.P.489, 1JRF.A.48, 3K1A.B.524, 4K3Y.D.604, 3K9X.D.249, 3KF9.A.303, 3KL6.A.3, 4KNA.A.504, 1KVO.A.192, 4KVK.A.712, 1KWH.A.800, 4LMF.B.302, 4LXF.A.701, 4MDV.A.402, 3MIN.D.524, 1MMP.B.3, 1MTV.A.480, 4NRE.A.716, 4NRE.A.717, 4NUP.A.301, 3O9J.A.995, 2OO4.B.5004, 3OX6.E.502, 2P5V.C.1002, 4PHK.A.304, 1PK8.F.817, 3PRT.A.404, 3QNI.B.400, 3QQZ.A.324, 1QUO.B.702, 4R83.D.501, 2RHP.A.18, 2RLD.C.121, 3SOB.B.1, 1SRR.A.531, 1TAD.A.352, 3TH3.L.145, 1TLD.A.480, 3U1R.A.700, 3UBH.A.852, 3V03.A.584, 3W9T.A.1006, 2YFS.A.1711, 2YGM.A.1417, 2YN5.A.6365, 1YO8.A.1188, 1YO8.A.1197, 2Z8S.B.641, 2ZID.A.882, 2ZKT.A.413, 1ZTQ.B.565, 2ZWO.B.402
[1] "Cluster 4"
4K4G.I.602, 1AFD.2.2, 1AR1.A.561, 1AVX.A.700, 1B09.C.302, 3BJU.C.608, 2CLT.A.1101, 1EAK.D.998, 4ELF.F.201, 4FU4.A.505, 3GCW.E.1, 4GKX.B.302, 1IXX.D.124, 4KZW.B.400, 4L06.B.501, 4L06.D.501, 3LND.A.210, 1LWJ.A.883, 3MOW.H.202, 3MW3.A.302, 2OKX.A.4004, 3QWU.A.601, 2RHP.A.25, 2RJQ.A.5, 3S5U.A.220, 4SBV.A.261, 1TN3.A.183, 4TVU.B.600, 1UZJ.C.3648, 2W2O.E.1333, 2WWO.C.800, 2WWO.F.800, 1YO8.A.1184, 1ZZH.A.401
[1] "Cluster 5"

4QWE.A.703, 3UIQ.A.905, 1AF4.A.276, 3AFG.B.541, 1ALA.A.402, 3AYU.A.419, 4B7R.B.5
02, 1B9V.A.500, 4CBX.G.1151, 4CCE.A.4001, 2CDP.B.1140, 3CKC.B.700, 2DDF.A.475, 4
DWW.A.301, 1E5J.A.402, 1EA7.A.315, 1F5R.A.800, 1FZD.F.1, 1GMM.A.1132, 4H1Q.A.304
, 4HZY.A.507, 3K5T.A.802, 4KS2.A.501, 1MR8.A.102, 1N28.A.126, 4N2F.A.703, 1NGS.A
.681, 1NKQ.A.260, 4NPK.A.803, 1OT5.A.602, 2OVX.B.449, 4PKH.J.1201, 1PZ7.B.702, 1
Q3A.B.472, 3Q3K.A.262, 1QL9.A.480, 4QN3.B.501, 2R8Y.C.203, 2RHP.A.22, 1S0E.A.129
3, 1S1D.B.1002, 1SEL.A.277, 1SMP.A.478, 1SPU.A.802, 2TCL.A.174, 1TFX.B.4007, 1V0
Z.A.1477, 1V6C.A.505, 3VI4.C.2004, 3VRQ.A.401, 1WVM.A.604, 2Z8S.B.647, 2ZRQ.A.7,
2ZWO.A.403
[1] "Cluster 6"
4K4I.I.604, 3QEP.A.906, 3SQ1.A.907, 4AQ8.D.1238, 4BTX.A.1763, 2CLT.B.1301, 2DEW.
X.904, 3E3R.B.195, 1E8U.A.1002, 1EGZ.B.300, 1ESP.A.320, 2EXK.D.2004, 1F2N.C.1001
, 3FCS.D.2002, 4FGC.C.203, 3FWO.A.823, 4GGB.A.401, 1HFY.A.124, 3HLH.D.315, 4HSZ.
B.101, 2HTY.G.997, 1HVD.A.602, 1KLJ.H.900, 3KQA.A.420, 1KX1.C.301, 4L06.C.501, 1
L7L.A.201, 3L95.X.2001, 3LCP.D.159, 4LIN.E.1301, 1LQD.B.1, 4LT6.A.602, 4O1Q.A.40
1, 1OS2.C.473, 3OSH.A.121, 3PK0.D.280, 4PLS.A.305, 1PYT.C.650, 1R1Z.B.315, 1RE3.
C.408, 2RHP.A.2, 2RHP.A.24, 2RHP.A.28, 1ROS.A.404, 3S5U.D.220, 4SBV.C.261, 1TD7.
A.1001, 1TYE.B.1402, 4UB6.O.301, 2V3T.A.1264, 1VFP.B.995, 2VME.A.500, 3VOT.A.504
, 3W9T.C.1010, 3W9T.B.508, 1WD9.A.901, 2WVX.C.801, 2X0G.B.1149, 3ZHG.B.1323
[1] "Cluster 7"
4K4G.M.603, 3KHH.A.416, 3KHH.B.1417, 3KHL.B.1415, 3MQ6.A.340, 3NDK.A.906, 3OOL.A
.237, 3D4G.B.485, 1EGZ.C.300, 7EST.E.11, 3FHA.B.704, 2FYC.B.404, 1G5N.A.404, 2GA
9.D.480, 3HQ8.A.402, 2HTY.C.993, 2HT5.A.995, 2HYW.A.502, 4JQG.A.305, 4MIV.C.601,
3NHH.A.151, 4NIY.A.301, 2OVZ.A.449, 2Q1F.B.2002, 3Q3L.B.437, 4Q4X.1.5006, 3SON.
A.201, 1TK2.A.1276, 1V3J.B.689, 1VFO.B.1002, 1W2M.E.1442, 1WD9.A.900, 2YC2.B.201
, 2YN3.C.6370

Table S47. 5-ligand Ca, compressed group

	size	largest_angle*	middle_1*	middle_2	middle_3*	middle_4
1	"20"	"155.2+/-11.7"	"72.9+/-5"	"80.2+/-4.6"	"84.9+/-4.5"	"89.8+/-5.9"
2	"27"	"147.8+/-9.8"	"51.9+/-4.2"	"77.8+/-6.8"	"87.9+/-4.7"	"95.4+/-5.3"
3	"14"	"144+/-8.4"	"56.1+/-6.9"	"69.7+/-7.5"	"74.2+/-5.9"	"78.8+/-5.4"
4	"22"	"161.8+/-6.3"	"53+/-3.7"	"70.4+/-5"	"78.7+/-5.6"	"93+/-9.1"
5	"39"	"162.6+/-8"	"71.4+/-4.8"	"77.6+/-4.5"	"81.9+/-4.6"	"85.8+/-4.8"
6	"59"	"160.2+/-5.8"	"51.8+/-3"	"75.3+/-5.6"	"81.8+/-4.7"	"86.4+/-4.7"

	middle_5*	middle_6	middle_7	middle_8*
1	"100.8+/-7.6"	"109.8+/-10.1"	"129.4+/-15.1"	"146.1+/-9.6"
2	"105.6+/-6"	"112.6+/-7.6"	"121.8+/-6.1"	"135.9+/-7.2"
3	"86+/-7"	"97.3+/-10.1"	"114.1+/-12.5"	"126.7+/-7.6"
4	"107.7+/-8.4"	"115.2+/-9.8"	"124.8+/-9.9"	"142.7+/-8.4"
5	"91.3+/-5.3"	"100.7+/-8"	"108.2+/-8.5"	"123.5+/-6.6"
6	"91.1+/-4.5"	"103.6+/-8.2"	"123.1+/-8.7"	"144.5+/-5.4"

	smallest_opposite_angle*	TrigonalBipyramidal	SquarePyramidal
1	"52.7+/-3.8"	"0.01"	"0.034"
2	"92.2+/-9"	"0.037"	"0.001"
3	"62.6+/-8.5"	"0.001"	"0.008"
4	"73.5+/-5.3"	"0.005"	"0.002"
5	"51.2+/-3.9"	"0.022"	"0.068"
6	"79.6+/-7.3"	"0.01"	"0.007"

TrigonalPrismaticV

1 "0.077"
2 "0.031"
3 "0.017"
4 "0.099"
5 "0.085"
6 "0.083"

Table S48. Cluster members of 5-ligand Ca, compressed group

[1] "Cluster 1"
2ASJ.B.1415, 4K4H.E.604, 2BTW.B.400, 6CGT.A.685, 2DDY.A.175, 2DPK.A.4001, 1DTH.A
.903, 3GE4.B.167, 4GQR.A.502, 1JKU.B.2272, 1JKU.E.5272, 3K7L.A.701, 3KOO.H.302,
2LP2.A.202, 2MLS.A.304, 3NP5.A.732, 4P99.A.505, 4POQ.C.401, 1TNQ.A.91, 1ULV.A.20
06
[1] "Cluster 2"
2EX5.X.802, 1G9Y.C.803, 4Q10.A.1001, 1RYS.B.803, 1B09.C.301, 2BTW.A.400, 3C5I.A.
371, 1DJZ.A.2, 2E30.A.300, 4G64.B.301, 1IJL.B.202, 2JAL.B.1447, 4KPP.B.501, 3L9I
.C.1151, 1LGC.E.301, 1M63.B.500, 2ML2.A.206, 1NW1.A.430, 4OVY.A.409, 2OYH.B.2, 3
POW.A.900, 3RGO.A.1, 2TBV.C.388, 1V1G.A.1209, 1WDA.A.902, 3WIV.B.1004, 1Z3U.B.49
7
[1] "Cluster 3"
1BCZ.A.410, 4EJ7.C.403, 1EXZ.C.805, 3FAX.A.1223, 1G1Q.C.803, 3HR4.H.202, 3IJE.A.
4004, 3IJE.A.4008, 2K2F.A.94, 4MDV.B.404, 2MLR.A.305, 2OXE.B.600, 2RHP.A.27, 4WF
F.A.304
[1] "Cluster 4"
1BSS.A.433, 1SON.A.403, 1AEI.A.320, 1CTR.A.150, 1F4M.E.105, 3FLP.L.302, 4GOD.A.5
03, 4G9L.B.306, 1IHO.A.2, 1IVE.A.470, 2IWV.B.1284, 1K90.D.801, 1LU1.A.301, 4MBE.
A.202, 2RJP.B.4, 3UJO.D.304, 1VAH.A.500, 4WK2.B.502, 1WMZ.B.203, 1YAX.C.1003, 1Z
3J.A.266, 2ZWO.A.401
[1] "Cluster 5"
2I3P.B.1, 3RBE.A.415, 4RIC.A.1101, 3ALA.E.1764, 2BBM.A.182, 3BJU.C.606, 2C10.C.1
773, 2CHO.A.1717, 1CTR.A.149, 3E1I.C.502, 2FH2.B.2003, 2FH3.B.2001, 1FIH.B.3, 3I
JE.A.4007, 3IKQ.A.403, 4JWU.A.502, 2KOE.A.153, 1K9I.A.407, 1LT9.B.1, 4OMD.F.604,
 2OX9.B.803, 4PJO.A.601, 4POQ.E.401, 2PRK.A.281, 4Q6O.A.501, 1QDO.B.243, 1QNI.D.
903, 2QVF.B.704, 2RGB.A.601, 2RHP.A.11, 1RTM.2.3, 1S1E.A.225, 2SNI.E.276, 4UM8.B
.2003, 3VI3.B.2001, 2VRO.D.1006, 1WOP.A.1781, 3W9T.G.506, 1WT9.A.1001
[1] "Cluster 6"
3KHL.A.415, 3A7Q.A.4001, 2AAO.B.299, 1AEI.A.318, 1ALA.A.400, 1AXK.B.395, 3BRX.A.
328, 3BXK.A.152, 2D3P.A.237, 2D7F.A.240, 3E3R.B.193, 2E6V.D.11, 4EJ7.C.402, 1ETH
.A.449, 2FH3.A.1002, 2FHC.A.2405, 3FLT.B.302, 1FZC.C.1, 1FZD.G.1, 1GYK.A.1206, 2
HOK.A.401, 4ILW.F.305, 2J3U.C.1292, 2J64.C.1222, 1J84.A.366, 4JDZ.B.704, 4JDZ.A.
702, 1JN2.P.238, 3JQL.A.121, 2K0J.A.501, 4KTP.B.804, 1LGN.A.302, 1M1J.C.501, 3M8
3.E.407, 3M8D.A.710, 2ML3.A.206, 2MLS.A.305, 1N29.A.125, 1NXO.A.900, 4OJQ.B.1002
, 4OMC.E.607, 1OTN.A.250, 3OX5.B.502, 4P99.D.512, 1Q3A.C.478, 1QOO.A.302, 3RYD.C
.267, 1SOC.A.1879, 3TRP.A.360, 4UM8.A.2004, 3VU1.A.1001, 1W2M.A.1442, 4W4Q.A.401
, 3WYN.B.402, 2WZE.A.1552, 1XFE.A.100, 2ZOC.A.501, 2ZOC.A.502, 2ZOC.A.504

Table S49. 5-ligand Ca, combined group

	size	largest_angle*	middle_1*	middle_2	middle_3*	middle_4
1	"164"	"170.4+/-4.7"	"78.8+/-4.3"	"83.5+/-3"	"87.2+/-2.4"	"90.4+/-2.9"
2	"120"	"157.4+/-9.2"	"52.3+/-4.4"	"74+/-7.5"	"82.1+/-5.9"	"89.5+/-7.2"
3	"78"	"161.2+/-8.9"	"73.8+/-7.6"	"81.6+/-5.6"	"86.4+/-5.1"	"91.5+/-4.2"
4	"112"	"162+/-6.6"	"72.7+/-4.6"	"78.2+/-4.4"	"83.1+/-4.3"	"87.6+/-5.1"
5	"101"	"153.9+/-12.9"	"65.6+/-9.1"	"72.6+/-7.9"	"78.3+/-6.4"	"83.3+/-6.4"

	middle_5*	middle_6	middle_7	middle_8*
1	"93.8+/-3.8"	"98.5+/-4.9"	"105.8+/-7.6"	"161.8+/-5.8"
2	"98.3+/-9.9"	"108.6+/-10.5"	"123.7+/-8.9"	"142+/-7.2"
3	"97.4+/-4.4"	"105.8+/-6.4"	"118+/-8.3"	"140.8+/-8.9"
4	"93.3+/-6.6"	"101+/-8.8"	"114.6+/-14.4"	"149.7+/-6.2"
5	"89.8+/-7.7"	"99.6+/-9.8"	"112+/-11.4"	"126.5+/-8.9"

	smallest_opposite_angle*	TrigonalBipyramidal	SquarePyramidal
1	"84.4+/-6.4"	"0.011"	"0.272"
2	"80.7+/-8.5"	"0.003"	"0.001"
3	"99.1+/-7.5"	"0.109"	"0.046"
4	"71.2+/-8.4"	"0.005"	"0.058"
5	"57.2+/-7.6"	"0.007"	"0.016"

	TrigonalPrismaticV
1	"0.041"
2	"0.044"
3	"0.123"
4	"0.07"
5	"0.041"

Table S50. Cluster members of 5-ligand Ca, combined group

[1] "Cluster 1"

4BXO.B.1216, 2FKC.A.248, 4G3I.B.401, 3GII.A.415, 4K4H.M.604, 4K4I.A.603, 4KHW.A.1003, 4LQ0.A.401, 4QWE.A.703, 1R7M.A.304, 1TW8.C.803, 3UIQ.A.905, 2WTF.B.1509, 1A25.B.292, 4AG4.A.5001, 4AIO.A.1890, 4B4F.B.607, 4B7M.B.1471, 2BAT.A.601, 2BD3.A.260, 3BOW.A.718, 2BV2.A.1085, 2BW7.A.2202, 3BYC.A.901, 4CCE.A.4001, 2CDO.B.1140, 2CDP.B.1140, 2CHI.A.218, 5CHY.A.401, 1CJY.A.951, 3CKC.B.700, 4CPN.A.500, 4CPO.A.1466, 1CXV.B.6, 2D00.B.1002, 1D7X.B.805, 2DEW.X.903, 1DM5.A.1131, 3DPE.A.997, 1DQ1.A.238, 4DWW.A.301, 1DYK.A.4001, 1E35.B.260, 1E5J.A.402, 2EA7.B.452, 3EKI.A.602, 2ERQ.B.702, 1EZS.C.650, 3F19.A.266, 1F2N.A.1002, 3FRP.A.628, 4FVL.B.506, 4GDI.C.507, 2GNT.A.254, 4GN7.B.301, 1GQM.E.1089, 1HFZ.C.124, 2HTV.A.995, 4I5K.A.501, 4ILW.D.304, 1IME.B.278, 1INW.A.501, 1J0Y.D.701, 1JAO.A.996, 2JG9.B.1224, 1JI3.A.401, 1JRF.A.48, 4JUC.B.601, 3K37.A.467, 4K3Y.D.604, 3K5T.A.802, 3KF9.A.303, 4KNA.A.504, 4KS2.A.501, 1KVO.A.192, 1KWH.A.800, 1L6J.A.502, 1L9N.A.703, 3LNF.B.305, 4LXF.A.701, 4MDV.A.402, 3MIN.D.524, 1MR8.A.102, 1MTV.A.480, 1N28.A.128, 4N2F.A.703, 1NG0.A.1002, 1NG0.C.1001, 1NKQ.A.260, 4NRE.A.717, 4NUP.A.301, 2NVO.A.533, 2NW6.A.613, 1NX0.A.902, 3O9J.A.995, 2O04.B.5004, 2O04.B.5006, 2OW1.B.447, 3OX6.E.502, 2P5V.C.1002, 4PHK.A.304, 1PK8.F.817, 4PKH.J.1201, 3PRT.A.404, 3Q2L.A.703, 3Q3K.A.262, 4Q4X.1.5005, 1QI5.A.452, 1QL9.A.480, 3QNI.B.400, 4QN3.B.501, 1QU0.B.702, 2R1D.B.1000, 2R8Y.C.203, 2R8Z.J.210, 1R8L.B.902, 4R83.D.501, 2RHP.A.10, 2RHP.A.18, 2RHP.A.20, 2RJP.A.2, 2RJP.A.3, 2RJQ.A.6, 1ROS.B.503, 1RU4.A.2, 1S0E.A.1293, 1S1D.B.1002, 3SHI.G.305, 3SJS.A.222, 1SMP.A.478, 3SOB.B.1, 1STB.A.150, 1T6B.X.800, 1TLD.A.480, 3U1R.A.700, 4U32.A.301, 1ULV.A.2005, 1UYX.B.1135, 3V03.A.584, 3V03.B.585, 1V0Z.A.1477, 1V7V.A.1001, 3VRQ.A.401, 3VV3.A.404, 1W3M.C.3014, 3W9

T.A.1006, 2WG8.A.201, 3WIU.B.1002, 3WNK.A.812, 1WVM.A.604, 1Y4A.E.1001, 2YN5.A.6
365, 1YO8.A.1197, 1Z4V.A.600, 2Z8S.B.641, 2Z8S.B.647, 2ZID.A.882, 2ZJ6.A.625, 2Z
RQ.A.7, 1ZTQ.A.561, 2ZUX.B.638, 2ZUY.A.627, 2ZWO.A.400, 2ZWO.A.403
[1] "Cluster 2"
1BSS.A.433, 1G9Y.C.803, 3GIK.A.415, 3KHH.B.1417, 3OOL.A.237, 4Q10.A.1001, 1RYS.B
.803, 1SON.A.403, 3S9H.A.906, 3A7Q.A.4001, 2AAO.B.299, 1AEI.A.318, 1AEI.A.320, 1
ALA.A.400, 1AXK.B.395, 1B09.C.301, 3BRX.A.328, 2BTW.A.400, 3BXK.A.152, 3C5I.A.37
1, 1CTR.A.150, 2D3P.A.237, 3D4G.B.485, 2D7F.A.240, 2E30.A.300, 3E3R.B.193, 2E6V.
D.11, 4EJ7.C.402, 4EJ7.C.403, 1ETH.A.449, 1F4M.E.105, 2FH3.A.1002, 2FHC.A.2405,
3FLP.L.302, 3FLT.B.302, 3FW0.A.823, 1FZC.C.1, 1FZD.G.1, 4G0D.A.503, 4G64.B.301,
4G9L.B.306, 2GA9.D.480, 1GYK.A.1206, 2HOK.A.401, 3HQ8.A.402, 1IHO.A.2, 1IJL.B.20
2, 4ILW.F.305, 2IWV.B.1284, 2J3U.C.1292, 2J64.C.1222, 1J84.A.366, 2JAL.B.1447, 4
JDZ.B.704, 4JDZ.A.702, 1JN2.P.238, 3JQL.A.121, 2K0J.A.501, 1K90.D.801, 1K90.E.80
2, 4KPP.B.501, 4KTP.B.804, 1KX1.C.301, 4L76.A.402, 3L9I.C.1151, 1LGC.E.301, 1LGN
.A.302, 1LU1.A.301, 1LWU.B.1, 1M1J.C.501, 3M83.E.407, 3M8D.A.710, 4MBE.A.202, 2M
L2.A.206, 2ML3.A.206, 2MLR.A.305, 2MLS.A.305, 1N29.A.125, 1NW1.A.430, 1NX0.A.900
, 1NX1.A.4, 4OJQ.B.1002, 4OMC.E.607, 1OTN.A.250, 2OXE.B.600, 2OYH.B.2, 4P99.D.51
2, 3POG.B.1, 3POW.A.900, 1Q3A.C.478, 1QOO.A.302, 3RG0.A.1, 2RJP.B.4, 3RMK.E.313,
 3RYD.C.267, 1SOC.A.1879, 2TBV.C.388, 3TRP.A.360, 3UJO.D.304, 4UM8.A.2004, 1V1G.
A.1209, 1VAH.A.500, 3VU1.A.1001, 1W2M.A.1442, 4W4Q.A.401, 1WDA.A.902, 3WIV.B.100
4, 4WK2.B.502, 1WMZ.B.203, 3WYN.B.402, 2WZE.A.1552, 1XFE.A.100, 1YAX.C.1003, 2YN
3.C.6370, 1Z3J.A.266, 1Z3U.B.497, 2ZOC.A.501, 2ZOC.A.502, 2ZOC.A.504, 2ZWO.A.401
[1] "Cluster 3"
2EX5.X.802, 4K4G.M.603, 3KHH.A.416, 3KHL.B.1415, 3MQ6.A.340, 3NDK.A.906, 2VA2.B.
1343, 1AF4.A.276, 3AFG.B.541, 1ALA.A.402, 1AWB.B.279, 3AYU.A.419, 4B7R.B.502, 4B
7U.D.401, 1B9V.A.500, 4CBX.G.1151, 1CIZ.A.305, 2DDF.A.475, 1DJZ.A.2, 2DSN.A.2011
, 1EA7.A.315, 1EGZ.C.300, 3ELM.A.303, 7EST.E.11, 2EXH.C.2003, 1F5R.A.800, 3FHA.B
.704, 2FYC.B.404, 1FZD.F.1, 3G20.A.201, 1G5N.A.404, 1GMM.A.1132, 4H1Q.A.304, 2HT
5.A.995, 2HYW.A.502, 4HZY.A.507, 2J7A.D.1007, 4JQG.A.305, 4LM8.A.812, 1M63.B.500
, 4MIV.C.601, 1N28.A.126, 1NGS.A.681, 3NHH.A.151, 4NIY.A.301, 4NPK.A.803, 1OLP.B
.1372, 1OT5.A.602, 2OVX.B.449, 2OVZ.A.449, 4OVY.A.409, 3P2P.A.125, 1PZ7.B.702, 2
Q1F.B.2002, 1Q3A.B.472, 4Q4X.1.5006, 3QHQ.A.230, 2RHP.A.22, 1RM8.A.504, 1SEL.A.2
77, 3SO0.A.98, 3SO0.B.97, 3SON.A.201, 1SPU.A.802, 2TCL.A.174, 1TFX.B.4007, 1TK2.
A.1276, 3TRQ.A.359, 1UG9.A.2003, 1V3J.B.689, 1V6C.A.505, 3VI4.C.2004, 2W1W.A.113
2, 1W2M.E.1442, 1WD9.A.900, 1WY9.A.148, 2YC2.B.201, 2ZUY.A.629
[1] "Cluster 4"
2ASJ.B.1415, 4K4H.E.604, 4K4I.I.604, 3KHL.A.415, 4KHN.A.1002, 3QEP.A.906, 3SQ1.A
.907, 1A25.A.292, 4AQ8.D.1238, 3BJU.C.608, 4BTX.A.1763, 6CGT.A.685, 3CK7.B.720,
2CLT.A.1101, 2CLT.B.1301, 3D4G.H.484, 2DEW.X.904, 1DM5.C.1133, 2DPK.A.4001, 1DTH
.A.903, 3E3R.B.195, 1E8U.A.1002, 3E9T.A.6, 1EE6.A.300, 1EGZ.B.300, 1ESP.A.320, 2
EXK.D.2004, 2F2O.B.1001, 1F2N.C.1001, 3FCS.D.2002, 4FGC.C.203, 3GE4.B.167, 4GGB.
A.401, 4GQR.A.502, 4GZS.A.501, 4H2E.B.304, 1HFY.A.124, 3HLH.D.315, 4HSZ.B.101, 2
HTY.C.993, 2HTY.G.997, 1HVD.A.602, 1JIW.P.489, 1JKU.B.2272, 1JKU.E.5272, 3K1A.B.
524, 3K7L.A.701, 3K9X.D.249, 3KL6.A.3, 1KLJ.H.900, 3KQA.A.420, 4KVK.A.712, 4L06.
C.501, 1L7L.A.201, 3L95.X.2001, 3LCP.D.159, 4LIN.E.1301, 4LMF.B.302, 3LND.A.210,
 2LP2.A.202, 1LQD.B.1, 4LT6.A.602, 1MMP.B.3, 4NRE.A.716, 4O1Q.A.401, 1OS2.C.473,
 3OSH.A.121, 3PK0.D.280, 4PLS.A.305, 1PYT.C.650, 3Q3L.B.437, 3QQZ.A.324, 1R1Z.B.
315, 1RE3.C.408, 2RHP.A.2, 2RHP.A.24, 2RHP.A.28, 2RJQ.A.5, 2RLD.C.121, 1ROS.A.40
4, 3S5U.A.220, 3S5U.D.220, 4SBV.C.261, 1SRR.A.531, 1TAD.A.352, 1TD7.A.1001, 3TH3
.L.145, 1TN3.A.183, 1TYE.B.1402, 4UB6.O.301, 3UBH.A.852, 1ULV.A.2006, 1UZJ.C.364
8, 2V3T.A.1264, 1VFO.B.1002, 1VFP.B.995, 2VME.A.500, 3VOT.A.504, 2W2O.E.1333, 3W
9T.C.1010, 3W9T.B.508, 1WD9.A.901, 2WVX.C.801, 2X0G.B.1149, 2YFS.A.1711, 2YGM.A.
1417, 1YO8.A.1184, 1YO8.A.1188, 3ZHG.B.1323, 2ZKT.A.413, 1ZTQ.B.565, 2ZWO.B.402
[1] "Cluster 5"

4EFJ.A.402, 2I3P.B.1, 4K4G.I.602, 3RBE.A.415, 4RIC.A.1101, 1AFD.2.2, 3ALA.E.1764, 1AR1.A.561, 1AVX.A.700, 1B09.C.302, 2BBM.A.182, 1BCZ.A.410, 3BJU.C.606, 2BTW.B.400, 2C10.C.1773, 2C11.A.1738, 4CAG.A.601, 2CHO.A.1717, 1CTR.A.149, 2DDY.A.175, 3DF0.A.720, 3E1I.C.502, 1EAK.D.998, 4ELF.F.201, 1EXZ.C.805, 3FAX.A.1223, 2FH2.B.2003, 2FH3.B.2001, 1FIH.B.3, 4FU4.A.505, 1G1Q.C.803, 3GCW.E.1, 4GKX.B.302, 3HR4.H.202, 3IJE.A.4004, 3IJE.A.4007, 3IJE.A.4008, 3IKQ.A.403, 1IVE.A.470, 1IXX.D.124, 4JP8.A.704, 4JWU.A.502, 2K0E.A.153, 2K2F.A.94, 1K9I.A.407, 3KOO.H.302, 4KZW.B.400, 4L06.B.501, 4L06.D.501, 2LAN.A.301, 1LT9.B.1, 1LWJ.A.883, 1LWU.F.6, 3MOW.H.202, 2MOK.A.202, 2MOK.A.203, 4MDV.B.404, 2MLS.A.304, 1MPR.A.428, 3MW3.A.302, 3NP5.A.732, 2OKX.A.4004, 4OMD.F.604, 3OX5.B.502, 3OX5.C.501, 2OX9.B.803, 4P99.A.505, 4PJO.A.601, 4POQ.C.401, 4POQ.E.401, 2PRK.A.281, 4Q6O.A.501, 1QDO.B.243, 1QLK.B.93, 1QNI.D.903, 2QVF.B.704, 3QWU.A.601, 2RGB.A.601, 2RHP.A.11, 2RHP.A.25, 2RHP.A.27, 1RTM.2.3, 1S1E.A.225, 4SBV.A.261, 1SDD.A.2184, 2SNI.E.276, 1SW8.A.81, 1SZB.B.1004, 1TNQ.A.91, 4TVU.B.600, 4UM8.B.2003, 3VI3.B.2001, 2VR0.D.1006, 1WOP.A.1781, 3W9T.G.506, 4WFF.A.304, 3WHU.A.501, 1WT9.A.1001, 2WW0.C.800, 2WW0.F.800, 1ZZH.A.401

Table S51. 6-ligand Ca, normal group

	size	largest_angle*	middle_1*	middle_2	middle_3	middle_4
1	"148"	"167+/-3.7"	"75.4+/-4.4"	"79.4+/-2.9"	"82.1+/-2.5"	"84.1+/-2.3"
2	"64"	"155.6+/-5.7"	"69.5+/-4.8"	"72.9+/-4.8"	"76.4+/-3.7"	"79.1+/-3.3"
3	"230"	"172+/-2.8"	"79.5+/-3.2"	"82.8+/-2.1"	"84.7+/-1.7"	"86.5+/-1.7"
4	"81"	"168.4+/-5.5"	"71.6+/-4.4"	"75.5+/-3.6"	"79.4+/-3.5"	"82.7+/-3.2"
5	"159"	"175.7+/-1.9"	"81.7+/-2.8"	"84.4+/-2"	"86.3+/-1.6"	"87.8+/-1.3"
6	"94"	"172+/-3.8"	"77.2+/-4.2"	"81.3+/-3.1"	"84.2+/-2.5"	"86.4+/-2.3"

	middle_5*	middle_6	middle_7	middle_8	middle_9*
1	"86.2+/-2.1"	"88.8+/-2.7"	"91.9+/-3"	"95.5+/-3.4"	"101.5+/-3.8"
2	"82.3+/-3.6"	"85.2+/-3.9"	"89+/-4.8"	"94.2+/-5.6"	"107.7+/-7.4"
3	"88.1+/-1.8"	"89.8+/-1.8"	"91.7+/-1.8"	"93.9+/-2.1"	"96.6+/-2.1"
4	"85.5+/-3.1"	"88.4+/-3.2"	"91.6+/-3.3"	"96.1+/-3.8"	"100.1+/-4.5"
5	"89.2+/-1.3"	"90.3+/-1.3"	"91.6+/-1.2"	"93.2+/-1.4"	"94.8+/-1.8"
6	"88.7+/-2.3"	"90.9+/-2.3"	"93+/-2.3"	"95.6+/-2.7"	"98.5+/-2.8"

	middle_10	middle_11	middle_12	middle_13*
1	"106.3+/-4.1"	"113.9+/-6.2"	"154.4+/-5.9"	"160.5+/-3.2"
2	"119.2+/-9.6"	"130.9+/-5.5"	"141+/-4.9"	"147.9+/-4.5"
3	"100.4+/-3.1"	"106.6+/-4.6"	"162+/-4"	"167+/-2.3"
4	"107.3+/-6.8"	"128.6+/-9.7"	"144.1+/-5.4"	"151+/-4.3"
5	"97+/-2.1"	"101+/-3.5"	"166.4+/-4"	"171.9+/-2.2"
6	"102+/-3.5"	"112+/-8.7"	"155.3+/-6.9"	"164.2+/-4.2"

	smallest_opposite_angle*	Octahedral	TrigonalPrismatic
1	"78.7+/-2.9"	"0.151"	"0.081"
2	"70.7+/-5.1"	"0.006"	"0.045"
3	"80.2+/-2.8"	"0.472"	"0.051"
4	"69.3+/-4.5"	"0.019"	"0.07"
5	"82.8+/-2.6"	"0.667"	"0.02"
6	"70.4+/-3.8"	"0.179"	"0.045"

	PentagonalBipyramidalVA	PentagonalBipyramidalVP
1	"0"	"0.116"
2	"0.057"	"0.076"
3	"0"	"0.096"

4 "0.001" "0.199"
5 "0" "0.04"
6 "0" "0.112"

Table S52. Cluster members of 6-ligand Ca, normal group

[1] "Cluster 1"
2AOQ.A.232, 2ASD.A.415, 3BQ1.A.4001, 3COW.A.304, 3EH8.A.302, 1FOO.B.761, 4F4W.B.
403, 2GIJ.A.401, 2IBK.A.401, 2JEJ.A.1344, 1JX4.A.4001, 4K4H.A.607, 4KLD.A.402, 4
LQO.A.402, 3M9O.B.4003, 3MXB.B.173, 3MXB.R.175, 3ODH.A.195, 1R7M.B.536, 3RAX.A.4
15, 1SOO.A.401, 1S9F.A.4001, 2A8K.B.404, 2AER.L.3008, 1AFO.A.486, 3AMR.A.910, 1A
WB.A.1, 3BOI.A.124, 3BC9.A.702, 3BC9.A.705, 3BH4.B.1, 1BLI.A.600, 4C9F.B.401, 3C
KC.A.600, 2DOO.D.1001, 3D4G.B.484, 2DEW.X.901, 2DF7.C.5904, 4DOU.A.1001, 2DSN.B.
2012, 4EJ7.B.402, 4EPU.A.601, 2EXH.D.2004, 2F3C.E.242, 2FIB.A.412, 2FPS.A.503, 3
FP8.E.601, 1G5N.A.402, 4G6O.A.302, 1G87.A.615, 2GGM.B.402, 2GJP.A.1486, 4GQ7.A.3
01, 4GZT.B.510, 2HOK.B.410, 1H3G.A.701, 1H5V.A.306, 1H71.P.502, 1HL5.D.156, 4HOW
.A.704, 4HPN.A.401, 2HYU.A.502, 2I4B.A.454, 4I8H.A.301, 3IOX.A.903, 4JCM.A.706,
2JKH.A.1245, 4JZX.A.403, 1KAP.P.617, 1KAP.P.620, 3KM5.A.2011, 2L51.A.207, 1L9M.A
.702, 3L9I.C.1148, 4LJ3.B.403, 4LLS.A.303, 4LLT.A.303, 3LNH.A.303, 4LQR.A.202, 4
LVN.A.704, 2MOP.A.1201, 3M1H.C.2001, 3MBR.X.300, 4MKM.A.403, 2ML1.A.204, 2ML2.A.
201, 2ML3.A.203, 2ML3.A.204, 3MVS.A.214, 4N2G.A.705, 4N2I.A.705, 4NAM.A.801, 1NB
W.A.650, 1NRW.A.903, 1NX1.A.3, 1OAH.B.1526, 1OBO.A.501, 3OTJ.E.1000, 3P4G.A.402,
3P4G.A.403, 3P4G.A.404, 3P4G.A.405, 3P4G.A.406, 3P4G.A.407, 3P4G.A.408, 3P4G.A.
409, 3P4G.A.410, 3P4G.A.412, 3P4G.B.411, 3PKO.A.280, 1PZ7.A.701, 2PZO.A.501, 3Q8
F.A.736, 4QD2.E.302, 3QGV.A.504, 4QN7.A.501, 2QUB.A.616, 2QUB.A.618, 2RA3.A.1, 2
RA3.B.1, 3S6J.A.4, 1SBH.A.290, 1SCB.A.276, 3SOO.D.97, 3SVL.A.201, 1TCM.B.687, 3T
EW.A.801, 3UBF.A.754, 1ULV.A.2002, 4UM9.B.2002, 1UYX.A.1133, 1V0Z.B.1477, 3V96.B
.304, 1VCL.B.1002, 2VDR.B.2002, 2VL8.A.1545, 3VTO.A.302, 2W1W.B.1135, 1WPC.A.502
, 2XSG.B.1772, 1Y6W.A.149, 1YO8.A.1201, 1YO8.A.1203, 1YO8.A.1206, 2Z2X.A.1007, 2
Z8X.A.624, 2Z8S.B.643, 2ZQO.A.901
[1] "Cluster 2"
3FSP.A.501, 3KHH.B.1416, 1M5X.A.802, 3NE6.A.905, 4PTF.A.1202, 2WTF.B.1510, 2A8K.
C.403, 3B1T.A.900, 4BY5.C.1187, 1CGU.A.686, 3CGT.A.686, 1CRU.A.503, 1D7F.A.5003,
3DAS.A.351, 4DRZ.A.202, 1E8U.B.1003, 3EHJ.A.1, 3FCU.B.2002, 1FHF.A.501, 4FU4.B.
505, 1HDF.B.1101, 3HLI.D.315, 4I5N.B.601, 3JTX.B.396, 2KAY.A.185, 4KVJ.A.714, 1K
X1.E.502, 1KX1.F.601, 1L6R.A.901, 4LMF.A.303, 1LPG.B.1, 1MAC.B.389, 2ML2.A.202,
4NHF.B.301, 3NN7.A.503, 3O5S.A.243, 2PF2.A.174, 4PLM.A.504, 2PPL.A.481, 1Q7B.A.9
002, 2RDZ.B.1502, 3SO1.B.97, 3TSK.A.304, 3TUY.C.157, 1ULV.A.2003, 1UMS.A.3, 1UZJ
.A.1648, 1V2I.A.1001, 1VFO.A.1001, 2VKH.A.1543, 2VZP.B.1129, 2W2N.E.1334, 2W3J.A
.1139, 3W9T.A.1008, 3W9T.D.503, 3W9T.D.504, 4WBQ.B.602, 3WH3.A.501, 2WO9.B.1272,
2WVX.B.801, 2WVZ.B.800, 2WWO.A.800, 2XQX.B.1949, 1YO8.A.1211
[1] "Cluster 3"
2AOR.A.402, 2ASD.A.416, 3AVX.A.3001, 1DMU.A.300, 4DTP.A.1002, 4ELV.A.908, 4F4W.A
.402, 4FJ9.A.1002, 4FJK.A.1002, 4FJL.A.1002, 4FJM.A.1002, 3IAY.A.1, 4J2A.A.1002,
4J2B.A.1002, 1JXL.A.1402, 4K4H.E.602, 4K4I.E.602, 3KHR.A.416, 4KHQ.A.1001, 3LZJ
.A.905, 4M3Z.A.1002, 3M9N.B.4003, 3M9O.B.4001, 1N3E.F.491, 3O3G.A.1, 3QET.A.905,
4QWB.A.402, 4QWB.A.403, 2RDJ.A.353, 3SLP.B.227, 3SUN.A.897, 1TW8.A.801, 3A7Q.B.
5001, 2AEP.A.601, 1AFO.A.484, 4AIE.A.1540, 3AJ7.A.602, 4AQ1.A.1925, 4AQ1.A.1926,
4AQE.A.1208, 4AR9.A.1732, 4ARF.A.1723, 1AVA.A.502, 3AYU.A.418, 3B1U.A.901, 1B4N
.A.620, 1B8L.A.110, 3B8Z.A.904, 1B9T.A.500, 2BU3.A.1242, 2BV2.B.1085, 4BZ4.A.123
3, 2CDP.A.1140, 1CLC.A.650, 1CLC.A.652, 4CPY.A.1466, 4CU9.A.2999, 4CUB.A.2645, 2

CYY.A.2002, 2DCJ.A.1003, 2DDU.A.1, 2DDR.A.1324, 2DIE.A.779, 4DK4.A.302, 4DKB.A.3
02, 1DPO.A.246, 2EA7.A.450, 3EF2.A.304, 3EF2.A.305, 1EGZ.A.300, 1EX9.A.286, 4F8Z
.A.409, 1FBL.A.996, 3FSJ.X.600, 3FU1.A.301, 2FWN.A.532, 3G4E.A.1, 4G62.A.302, 4G
DJ.A.507, 3GG1.A.502, 4GG1.A.602, 2GKO.A.611, 3GK2.A.92, 4GN7.A.301, 4GW3.A.401,
4H1Q.A.303, 1H71.P.501, 1H9H.E.1246, 3HB2.P.482, 1HDF.A.1101, 3HI7.A.802, 3HJR.
A.603, 1HM9.A.1901, 1HVX.A.517, 1HY7.A.305, 1HYO.A.1006, 2HYV.A.608, 1I76.A.996,
3IBZ.A.192, 4IHM.A.403, 1IOD.G.503, 2IUF.E.1697, 4IU2.A.301, 4IU3.A.301, 2IXT.A
.1311, 1J0H.A.601, 1J11.A.701, 1J35.C.501, 4J7M.A.403, 1J9K.A.301, 1JI3.B.403, 1
JK3.A.403, 4JZB.A.402, 4JZE.H.302, 3K37.B.467, 4K3K.B.401, 1K7I.A.483, 1K7I.A.48
7, 1K7Q.A.485, 4K70.B.1002, 4K89.A.408, 4K9P.A.601, 1KA1.A.401, 1KAP.P.618, 3KCG
.H.500, 4KTY.A.802, 4KXY.A.707, 3LNI.A.303, 3LPD.A.342, 4MB1.A.602, 1MCT.A.246,
3MHF.A.328, 2ML1.A.202, 3MMZ.A.500, 4MPR.A.601, 3MVS.A.211, 3MVS.A.215, 4MWL.A.5
12, 3MW3.A.301, 4N20.A.705, 4N2E.A.705, 1N7V.A.601, 4NAS.A.503, 3NIF.D.2002, 3NJ
H.B.502, 3NKQ.A.1003, 1NKG.A.800, 4NOT.A.302, 1NSC.A.468, 4NUQ.A.301, 4NUZ.A.100
1, 2O72.A.403, 3OJY.A.555, 1OM6.A.701, 1OM8.A.705, 3OM6.B.1, 3P95.A.1, 4PHN.A.30
2, 2PNY.A.228, 3POJ.B.1, 3PPE.A.401, 2Q16.A.200, 3Q4W.A.224, 1QCO.A.1002, 3QU7.A
.230, 3QU7.B.225, 2QUB.A.614, 3QXG.B.230, 4R12.A.809, 2R1B.A.1001, 2R8Y.A.201, 3
R8Y.A.242, 1RQ5.A.819, 3RQ0.A.301, 1SAT.A.476, 3T3P.B.2003, 1T5S.A.1004, 3TEW.A.
800, 1TO2.E.450, 4TSH.B.1502, 3U1R.A.703, 3U1R.A.704, 3U1R.A.705, 3U1R.A.706, 3U
8D.A.203, 1ULV.A.2001, 4USU.A.1471, 1UTM.A.247, 1UX6.B.2002, 1UX6.B.2003, 1UX6.B
.2004, 1UX6.B.2007, 1UX6.B.2010, 1UYX.A.1134, 4UZU.A.1484, 1VCL.A.1004, 1VCL.B.1
001, 1VL9.A.125, 2VNG.B.1214, 3VOC.A.501, 3VV3.A.401, 4WA3.A.503, 2WFK.A.1250, 1
WMD.A.1003, 2WW3.A.800, 1WZA.A.601, 2XR9.A.1869, 2XVT.F.1137, 2Y6D.A.1266, 1Y7B.
A.3001, 1Y9Z.A.604, 1Y9Z.B.605, 2YAY.A.1267, 2YEQ.A.1526, 2YGL.A.1413, 1YI7.A.30
01, 1YO8.A.1185, 1YO8.A.1193, 1YO8.A.1199, 1YO8.A.1209, 1YS6.A.1001, 1Z6O.A.5302
, 1Z70.X.3001, 2Z8X.A.620, 2Z8X.A.622, 2Z8X.A.623, 2ZUX.A.630, 2ZUY.A.626, 3ZWH.
A.501, 3ZXH.A.304
[1] "Cluster 4"
3C0W.A.301, 1DMU.A.302, 1N3E.C.492, 3QEX.A.907, 1R7M.A.306, 2RDJ.B.353, 4A6S.C.1
122, 1AFA.2.2, 1AWB.A.278, 4AWD.A.1321, 3AZX.B.301, 3B00.B.301, 4B7R.A.502, 3BCD
.A.708, 3BJU.A.608, 4CAJ.B.1325, 2CDP.B.1139, 3CK7.D.730, 1CR8.A.45, 4CRR.A.1386
, 1CYG.A.682, 1DGL.B.301, 4DLK.A.403, 3EAD.A.1003, 3EDD.A.700, 3EHJ.B.1, 3ESQ.A.
213, 1F6S.E.205, 4G62.A.301, 1G9K.A.700, 3GN4.B.204, 3GQF.D.154, 2GSM.A.3007, 4H
3X.A.304, 1HFZ.A.124, 1HQV.A.999, 1I22.A.198, 3I4I.A.1001, 3I57.A.186, 4JDZ.B.70
2, 3JXS.A.302, 4K5W.A.201, 3KF9.C.304, 1KMB.1.2, 3KQA.B.421, 3LCP.B.279, 3LHM.A.
131, 1LOC.E.688, 4M5I.A.203, 1MDU.A.403, 4N25.A.707, 4N2I.A.704, 3N4E.A.500, 4N8
5.A.502, 3N8G.A.1002, 2OKX.B.4002, 2QT6.A.3713, 4QU6.A.904, 1R1Z.C.415, 3RRW.A.2
71, 3SLE.B.401, 3SO0.F.98, 1TT2.A.502, 1UF3.D.913, 1UH2.A.1002, 1UH3.A.1001, 1UH
3.A.1002, 1UKS.A.688, 1UPS.A.501, 3V03.A.585, 1V3J.A.687, 1V3L.B.689, 2VJ3.A.153
3, 2VVE.B.1338, 2VXJ.G.200, 4WF7.D.600, 2WQS.A.2415, 2YDP.A.502, 1YO8.A.1195, 2Z
49.A.1002, 3ZHG.D.1323
[1] "Cluster 5"
3DPG.B.501, 4ECQ.A.501, 4FJ8.A.1002, 4K4H.A.605, 1N3F.C.498, 2OAA.A.601, 2ODI.A.
701, 2Q10.A.701, 3AFG.A.540, 2AFH.D.2490, 3AHW.A.122, 3AI7.B.901, 1AJJ.A.73, 3AK
B.A.2, 4APX.B.1239, 3AUK.A.391, 4AWN.A.300, 4AXN.A.1329, 3AYU.A.417, 3B7E.A.1005
, 3BCD.A.707, 2BL0.B.1146, 4BNR.A.600, 2BQ4.A.1119, 2C10.A.1771, 3C9I.A.1, 3CKZ.
A.1, 4CPL.A.500, 4CUA.A.2644, 1CVL.A.320, 3D3I.A.1001, 3D7K.A.571, 4E5U.B.302, 3
EDY.A.1, 2EEK.A.401, 3F5V.A.223, 2FCW.B.3001, 2FCW.B.3002, 2FGZ.A.1192, 3FG1.A.1
501, 2FHF.A.2404, 3FVQ.A.360, 1G9K.A.703, 1G9K.A.706, 1GA6.A.374, 4GDI.A.509, 2G
KO.A.612, 1GTT.A.1430, 2HOB.A.1000, 4H1Q.A.305, 3H81.A.279, 3H81.C.279, 3H81.C.2
80, 1HFC.A.277, 3HGN.A.250, 4HJF.A.601, 4HS9.A.401, 1HT6.A.502, 1HV5.A.5503, 4HZ
W.A.507, 4I35.A.513, 1I76.A.997, 2I8U.A.202, 2I8T.A.402, 3I9G.L.301, 3I9G.L.302,
4IHM.A.404, 2IXT.A.1310, 1J8E.A.201, 2JKP.A.1728, 4JZB.A.401, 4JZB.A.403, 4JZX.
A.404, 4JZX.A.405, 4K1K.A.501, 4K70.A.1002, 1KAP.P.616, 1KAP.P.619, 4L74.A.401,

1LQV.C.35, 4LVN.A.703, 4M5I.A.201, 3M6L.A.801, 3MA2.A.293, 3MA2.A.296, 4MEW.A.50
2, 1MNC.A.283, 3MOS.A.1, 4MWV.A.512, 3N1U.A.200, 4N2P.A.201, 4N4E.E.405, 1N9E.A.
802, 1NPC.A.320, 1OAC.A.802, 4OCI.A.201, 4OKH.B.904, 3OOY.A.621, 1OU9.A.131, 3OY
R.B.337, 3OYR.B.338, 3P2P.B.126, 2P3U.B.501, 3P4G.A.401, 2PHI.B.125, 4PMX.A.401,
 1Q3A.A.466, 4Q8K.A.501, 1QCN.A.1001, 4QN6.A.501, 3QRB.A.301, 2QUB.A.615, 2R5N.A
.2000, 1R6V.A.1, 2R8O.A.670, 2R8Z.A.201, 2R8P.A.670, 1RM8.A.502, 3RRX.A.901, 3RU
P.A.1004, 3RVV.A.225, 3RVW.A.223, 3S4Y.B.1303, 3SAL.A.601, 1SIO.A.601, 1T1E.A.70
0, 3TI4.A.601, 1TRK.A.681, 3U8I.A.201, 3UPT.A.691, 1UR4.A.1398, 1UX6.B.2012, 1UX
6.B.2016, 3V5U.A.705, 3V96.B.303, 2VOV.A.1338, 3VV3.B.404, 1W7C.A.802, 3W7T.A.10
01, 1WAD.A.116, 4WIW.A.702, 4WK0.B.502, 4WK0.B.503, 4WK7.A.504, 2WNV.B.1225, 2WO
Y.A.2414, 1Y93.A.266, 1Y93.A.268, 2YN3.A.6362, 2YN5.A.6362, 2YN5.A.6363, 1YO8.A.
1191, 2YOA.A.1003, 1YS1.X.400, 2Z2X.A.1004, 2Z2X.A.1005, 2Z30.A.1006, 2Z8X.A.626
, 2ZWO.B.400
[1] "Cluster 6"
4ABT.B.1287, 2AOR.A.401, 3DVO.A.340, 3GV5.B.424, 2IBK.A.402, 2IMW.P.406, 4KYW.A.
302, 1N3E.D.493, 1N3F.D.499, 1N3F.F.497, 1OUP.A.300, 1Q9Y.A.939, 1RYS.A.801, 3SP
Z.A.905, 3A09.A.601, 3A13.E.445, 1AG9.A.200, 1AG9.B.1000, 3AMR.A.909, 3ASI.A.200
1, 3B4N.A.702, 4B4F.A.607, 1C3H.D.8003, 1C3H.F.8001, 4CAG.A.602, 1CGE.A.305, 5CN
A.C.240, 3D6E.A.202, 4DOU.A.1002, 2E9B.A.741, 3EHB.A.563, 1ELT.A.300, 5ENL.A.438
, 4G60.A.301, 4GI6.A.601, 3GN9.A.201, 1GVK.B.1246, 3HGP.A.250, 3I98.A.627, 1J1N.
B.493, 2J1T.A.1154, 1J9L.B.1303, 1JE5.B.502, 2JKE.A.1728, 2KAY.B.187, 4KKF.A.703
, 3KMV.A.163, 4KS1.A.501, 1KU0.A.703, 3KZP.A.240, 2MIN.B.525, 2ML1.A.201, 4N20.A
.706, 4N2B.A.705, 4NEH.B.701, 1NNL.B.2002, 1NUD.A.703, 2NXP.A.600, 3OJY.B.538, 3
OM5.B.1, 4OUL.B.1201, 3PGB.A.903, 4PLS.A.301, 2POO.A.805, 1PVY.B.603, 1PW9.A.404
, 2Q1C.X.294, 3Q2L.B.703, 3Q5I.A.528, 2QIM.A.158, 1QLB.A.1658, 1RXO.B.477, 1SOB.
A.1292, 3S5U.E.221, 2SAS.A.187, 1TU5.A.902, 3U1R.A.707, 4UP4.A.501, 4UP4.A.502,
2UWF.A.1369, 2VCC.A.1917, 1WC5.C.2100, 3WN6.A.502, 2WOB.E.1161, 1WRZ.A.154, 3X17
.A.602, 1XKD.A.1005, 2Y09.A.1242, 1YO8.A.1183, 1YO8.A.1205, 1YO8.A.1208, 2ZE0.A.
552, 2ZPR.A.2001, 2ZWP.A.402

Table S53. 6-ligand Ca, compressed group

	size	largest_angle*	middle_1*	middle_2	middle_3	middle_4
1	"64"	"172+/-4.2"	"74.3+/-4.6"	"78.2+/-3.9"	"82.3+/-2.8"	"85.2+/-2.9"
2	"44"	"157.6+/-5"	"51.9+/-3.2"	"71.2+/-4.3"	"75.1+/-3.6"	"77.9+/-3.7"
3	"75"	"161.3+/-4"	"72.9+/-3.7"	"76.5+/-2.6"	"78.9+/-2.2"	"81.8+/-3.2"
4	"45"	"169+/-5.2"	"51+/-3.1"	"72.9+/-4.3"	"77.9+/-4"	"81.4+/-3.4"
5	"67"	"158.3+/-6.2"	"53.2+/-3"	"70.8+/-4.8"	"75.8+/-4.5"	"78.5+/-3.9"
6	"29"	"164+/-5.1"	"72.8+/-5.5"	"76.6+/-4.7"	"79.7+/-4.6"	"83.3+/-4.4"
7	"47"	"169.8+/-3.5"	"68.4+/-4.1"	"73.9+/-3.4"	"77.5+/-3.7"	"80.6+/-3.5"
8	"30"	"153.9+/-7.1"	"68.4+/-4.6"	"72.5+/-4.7"	"76.1+/-4.6"	"79.9+/-4.8"

	middle_5*	middle_6	middle_7	middle_8	middle_9*
1	"87.5+/-2.8"	"89.5+/-2.4"	"91.4+/-2.5"	"94.6+/-3"	"97.4+/-3.9"
2	"80.4+/-3.8"	"82.8+/-4.1"	"85.4+/-3.8"	"90.7+/-4.7"	"96.1+/-4.7"
3	"84.1+/-3.1"	"86.8+/-3.4"	"90.5+/-4"	"96.8+/-5.2"	"104.7+/-5.6"
4	"84.6+/-3.7"	"88.2+/-3.3"	"91.5+/-3.7"	"95.8+/-3.7"	"102.2+/-4.9"
5	"82.7+/-4"	"86.3+/-4.7"	"91.4+/-5.2"	"98+/-7.6"	"111.6+/-6"
6	"85.5+/-4.4"	"87.5+/-3.8"	"90.9+/-4"	"93.8+/-3.7"	"98+/-4.8"
7	"83.8+/-3.1"	"88.2+/-4.3"	"92.5+/-3.9"	"97.4+/-3.5"	"101.9+/-4.2"
8	"83+/-3.7"	"87.3+/-5.7"	"91.5+/-7.2"	"100+/-7.5"	"109.9+/-6.2"

	middle_10	middle_11	middle_12	middle_13*

1	"104.1+/-5.5"	"123.4+/-9"	"142.6+/-9.5"	"156+/-4.7"
2	"117.2+/-9.3"	"127.5+/-7.2"	"142.4+/-7.1"	"148.7+/-6.5"
3	"115.1+/-7.3"	"125.3+/-5.7"	"145.4+/-9.1"	"155.2+/-2.8"
4	"112.5+/-7.1"	"123.1+/-7.2"	"139.6+/-6.5"	"159+/-5.9"
5	"121.5+/-6.7"	"129.7+/-6.6"	"141.8+/-6.1"	"147.5+/-4.1"
6	"108.3+/-7.6"	"127.1+/-6.2"	"136.3+/-4.7"	"143.9+/-5"
7	"107.7+/-5.8"	"117.8+/-6.2"	"136.5+/-11.7"	"163+/-4.6"
8	"117.4+/-5.8"	"127.4+/-6.4"	"134.1+/-6.9"	"144+/-5.7"

	smallest_opposite_angle*	Octahedral	TrigonalPrismatic
1	"51.5+/-3"	"0.066"	"0.072"
2	"68.9+/-5.1"	"0"	"0.019"
3	"51.3+/-2.7"	"0.01"	"0.071"
4	"73.5+/-6.5"	"0.012"	"0.032"
5	"73.3+/-6"	"0.002"	"0.045"
6	"51.5+/-2.9"	"0.06"	"0.087"
7	"50.9+/-2.9"	"0.061"	"0.048"
8	"52.6+/-3.3"	"0.008"	"0.084"

	PentagonalBipyramidalVA	PentagonalBipyramidalVP
1	"0.001"	"0.194"
2	"0.124"	"0.014"
3	"0.063"	"0.072"
4	"0.004"	"0.067"
5	"0.145"	"0.036"
6	"0"	"0.195"
7	"0.01"	"0.072"
8	"0.12"	"0.077"

Table S54. Cluster members of 6-ligand Ca, compressed group

[1] "Cluster 1"
3EH8.D.303, 2FLD.A.601, 2I3P.A.154, 2I3Q.B.81, 3LDY.A.145, 3PR4.A.343, 2A3W.R.33
6, 1ANW.A.351, 4AQA.A.1210, 1AUI.B.502, 2BB4.A.260, 3BOW.A.717, 2C10.A.1773, 4CA
G.A.608, 4CAG.A.609, 1DAN.L.155, 2DBX.B.701, 4DD8.B.1001, 1DJX.A.2, 2DW2.A.703,
2FH2.B.2001, 1FZC.B.2, 3GIN.A.2, 1GU6.A.1479, 1H5V.A.305, 2HYW.B.514, 2II1.B.400
, 1J1N.A.493, 2J7A.B.1007, 4JDZ.B.703, 3K5S.A.218, 3K7L.A.702, 3K7N.A.702, 3K7N.
A.703, 4KDV.A.202, 1KTW.B.8, 4KTR.D.814, 4N2I.A.706, 1NKQ.F.560, 1NX2.A.3, 2OA8.
C.302, 4OKH.A.901, 4OMD.D.605, 1OT5.A.601, 2OXE.A.600, 2P37.C.239, 4P99.D.508, 2
Q16.B.200, 2Q17.E.315, 2QT7.B.201, 3RHT.A.257, 1SL6.A.3, 1U94.A.701, 3UBR.B.476,
3V03.A.586, 2VY0.A.1298, 3WN6.B.501, 2XVT.C.1137, 2Z57.A.1006, 2Z8Z.A.618, 2Z8S
.A.646, 2ZJ7.A.619, 2ZUX.A.638, 2ZUY.A.628
[1] "Cluster 2"
3A7Q.A.4002, 1AFD.3.2, 1AMY.A.502, 1ANW.B.354, 1ATN.D.264, 1CFF.A.150, 2E3X.A.80
3, 2E6W.A.300, 3ECQ.A.2000, 1FDK.A.124, 4FL4.C.402, 1GQM.A.1089, 4GTW.A.1012, 2H
YW.A.505, 2I08.A.200, 3IA7.A.402, 3IA7.B.402, 3IGO.A.601, 1IZJ.A.1002, 4JBE.A.50
2, 1KTW.A.3, 1KX1.A.222, 4LMF.A.304, 1LWJ.B.10, 1M34.B.2299, 1M8T.C.1003, 1NIW.A
.1002, 1ODB.F.1092, 3OX6.A.501, 4P5W.A.1001, 1Q8H.A.72, 1QNI.E.903, 2RHP.A.1176,
3RV2.A.405, 1THL.A.3233, 1UG9.A.2006, 3USU.H.272, 3W9T.A.1005, 3WHU.B.502, 1WMD
.A.1001, 3WU2.O.301, 2Z8S.B.642, 2ZBA.A.461, 2ZKT.B.413
[1] "Cluster 3"
1AZO.B.283, 1A25.A.290, 3A9Q.G.214, 3AKA.A.173, 1AVA.A.501, 3BBY.A.215, 2BLO.C.1
155, 2CDP.C.1139, 3CGT.A.685, 2CHN.A.1717, 3CK9.A.710, 3DOY.A.93, 2D3L.A.503, 3E

1I.C.501, 2E6V.C.10, 1E8A.B.1090, 3ESR.A.213, 1F9O.A.2000, 3FAX.A.4, 2FHF.A.2405 , 3FLP.B.302, 1G5N.A.407, 4G9L.A.304, 1GCY.A.529, 2GDF.B.302, 3GK2.A.93, 1HFZ.D. 124, 2HIH.A.603, 3HTL.X.1, 3IJ8.A.497, 4ILW.D.305, 2JBK.A.803, 2JHL.F.1298, 2K0F .A.153, 3K5T.A.803, 1K9I.F.903, 2KAY.A.186, 3L2Y.A.302, 3LCP.D.158, 1LGN.B.302, 1LN8.A.201, 2LV6.A.203, 1M56.A.1007, 4MVF.A.603, 4MVF.A.604, 4NDD.B.401, 1NIW.A. 1003, 1OBR.A.404, 2OO4.A.5003, 2OZN.B.401, 4P99.A.501, 4P99.B.509, 2PMY.A.103, 4 POS.E.401, 3PVN.B.5003, 4Q6P.A.509, 1R1Z.C.410, 2R28.A.1003, 2RHP.A.8, 2SEC.E.27 6, 1SU3.B.907, 1SVN.A.276, 3UNX.A.280, 3VI3.A.2003, 3VI3.A.2004, 4WBQ.B.601, 3WI U.A.1004, 2WL3.A.1290, 1WMY.B.203, 1Y4D.E.1001, 1Z3U.A.497, 2Z8S.A.648, 2Z8S.B.6 46, 2ZEY.B.152, 2ZWP.A.401

[1] "Cluster 4"

2AGO.A.403, 4DTS.A.1002, 4K4I.A.606, 1ANN.A.319, 4CAG.A.605, 3CHK.A.502, 1DCY.A. 198, 2DDY.A.174, 2DPK.A.2001, 3E3R.B.194, 3EAD.B.1004, 4ELF.C.201, 2ERO.B.702, 4 FGC.B.204, 3FLP.B.301, 3FLT.A.302, 4FL4.C.401, 4GKX.B.301, 1IME.A.278, 4K1C.A.50 6, 3K8L.B.800, 2KAY.B.188, 1LGC.A.301, 1LHV.A.401, 4MDV.B.403, 2ML1.A.206, 3NOM. B.263, 1O9I.C.269, 4P99.A.512, 2PQY.A.500, 4Q4W.1.905, 2RHP.A.26, 3S5U.C.220, 1T EC.E.343, 3TRQ.A.358, 1TYE.A.1406, 1U7W.A.501, 3UJQ.D.305, 2V3T.B.1264, 1V3J.B.6 90, 2VB6.B.1151, 1W2M.C.1441, 3W9T.C.1009, 2Z8S.A.644, 2ZUY.A.624

[1] "Cluster 5"

3CFR.A.910, 2G8K.A.401, 1N56.B.403, 1A2X.A.160, 1A2X.A.161, 3AAJ.A.991, 1AEI.A.3 19, 5AER.A.201, 1AJ4.A.163, 1BAG.A.431, 3BOW.A.719, 3BOW.B.403, 1C9U.B.1002, 3CG A.A.102, 4E52.A.403, 2E6W.A.301, 3E9T.A.1, 3EAD.B.1002, 4ELG.F.202, 1ESP.A.322, 2EXI.D.3004, 1GOH.A.290, 2H0K.A.407, 1H4B.A.1085, 1H4B.A.1086, 2HQ8.B.303, 1IXX. B.124, 2J1G.D.1289, 1JBA.A.501, 4JEO.A.401, 1K96.A.92, 3KF9.A.302, 4LMH.D.811, 2 LV6.A.204, 2LVK.A.102, 2M5E.A.2001, 2ML3.A.205, 4N25.A.706, 1NAE.A.900, 4NDD.B.4 02, 4NEN.A.1115, 1NIW.A.1001, 1NIW.C.1005, 1NUB.A.301, 1NUB.A.302, 1NX2.A.4, 3OX 6.A.502, 1PK8.A.817, 3PM8.B.514, 1PTK.A.281, 3QRX.A.170, 3QRX.A.173, 2SAS.A.186, 1TCF.A.162, 3TRQ.A.360, 1TTX.A.110, 1TYE.A.1405, 1TYE.A.1407, 4UM8.C.2004, 1UZJ .C.3649, 1V1G.A.1211, 2WO9.D.1270, 1WRL.D.104, 1YUU.B.199, 2Z2D.A.266, 2Z8S.A.64 7, 2ZJ7.A.627

[1] "Cluster 6"

3MIS.A.2, 3ODH.A.196, 3A7Q.A.4003, 3B1T.A.903, 1B47.A.351, 3D94.A.2, 1DE4.C.801, 2DIE.A.778, 4EJ7.A.403, 1ESV.S.395, 4FDI.A.603, 2IWV.B.1281, 2J7A.E.1006, 1K7Q. A.481, 3K7N.A.701, 4KTR.G.806, 4LP7.A.301, 4M7Z.B.409, 4MHX.A.601, 4MIV.E.600, 1 N2K.A.600, 4NHD.B.403, 1NT0.A.3001, 3OUU.B.455, 1P8J.G.3014, 4PIB.C.204, 3PVN.B. 5004, 4UM9.C.2002, 1VFP.A.995

[1] "Cluster 7"

2BQR.A.2002, 3GIM.A.415, 4NLG.A.401, 3A7Q.A.4004, 1ATN.D.265, 3B2Z.D.3, 1BAG.A.4 32, 1BCJ.2.3, 4BQ3.B.1803, 2BW7.B.2202, 3CGA.B.102, 1DSY.A.501, 3E9T.B.3, 1FZD.E .1, 4GOD.B.503, 1G7Y.F.254, 2H0K.A.410, 4IMM.B.402, 2J7A.A.1007, 1JXN.B.246, 4JX 1.A.504, 3K7L.A.703, 1KWV.B.604, 1L9N.B.704, 3LPD.A.341, 1M63.F.502, 1N47.A.252, 1NUD.A.701, 3NWK.B.238, 3OM7.B.1, 2OX9.A.803, 4PKG.A.403, 2PPL.A.482, 3Q3L.A.43 6, 3QQZ.A.326, 2RJP.B.3, 3RRD.A.239, 3RTT.A.267, 1S2N.A.1291, 4TVU.A.600, 4U2A.A .301, 3USU.A.264, 3VI3.A.2001, 1WBL.D.303, 3WCT.B.203, 1Y3N.A.493, 1YO8.A.1202

[1] "Cluster 8"

4DU3.A.1003, 2COO.B.1507, 2D3P.C.241, 1DJY.A.2, 3E78.A.601, 2E85.B.1004, 4ELG.A. 202, 3G5C.B.802, 2GJR.A.1488, 4IAV.A.420, 1JBA.A.500, 3JQ5.A.201, 1KB0.A.801, 1K V9.A.802, 2LMT.A.149, 2ML2.A.205, 3MXW.A.402, 1NHE.A.805, 2NQA.B.902, 2SAS.A.188 , 1ULV.A.2004, 1V3D.B.2001, 1XJL.B.340, 2YN3.B.6361, 1YUU.A.197, 2Z8S.A.642, 1ZF S.A.104, 1ZIV.A.1, 2ZN9.A.902, 2ZN9.B.901

Table S55. 6-ligand Ca, combined group

	size	largest_angle*	middle_1*	middle_2	middle_3	middle_4
1	"214"	"167.3+/-6.2"	"71.6+/-5.5"	"76.2+/-3.9"	"79.6+/-3.7"	"82.9+/-3.6"
2	"324"	"174.1+/-2.7"	"80.6+/-3.3"	"83.7+/-2.1"	"85.6+/-1.8"	"87.2+/-1.5"
3	"128"	"172.1+/-3.6"	"77.8+/-4"	"81.6+/-3.1"	"84.3+/-2.5"	"86.4+/-2.4"
4	"118"	"163.6+/-7.1"	"72.2+/-4.3"	"75.6+/-3.8"	"78.7+/-3.5"	"81.6+/-3.2"
5	"86"	"157.2+/-6.7"	"55.9+/-6.6"	"67.9+/-6.5"	"74.3+/-5"	"77.9+/-3.9"
6	"184"	"167.6+/-3.6"	"75.9+/-4.7"	"79.8+/-3"	"82.5+/-2.5"	"84.5+/-2.2"
7	"105"	"163.4+/-7.1"	"51.2+/-3.1"	"71.6+/-5.7"	"76.5+/-4.5"	"80.2+/-4.2"
8	"82"	"155.8+/-7.5"	"60.2+/-8.6"	"68.1+/-7.2"	"73+/-6.2"	"78+/-6.3"

	middle_5*	middle_6	middle_7	middle_8	middle_9*
1	"85.3+/-3.3"	"88.2+/-3.5"	"91.5+/-3.6"	"96+/-4.2"	"101.1+/-5.6"
2	"88.7+/-1.6"	"90.1+/-1.5"	"91.7+/-1.5"	"93.6+/-1.8"	"95.6+/-2"
3	"88.7+/-2.3"	"90.8+/-2.2"	"93+/-2.3"	"95.3+/-2.6"	"98.1+/-2.9"
4	"84.6+/-3.2"	"87.3+/-3.5"	"90.5+/-3.2"	"95+/-4"	"101.8+/-5.5"
5	"82.4+/-4.7"	"86.7+/-5.7"	"92.5+/-7"	"99.6+/-9.1"	"114.4+/-5.8"
6	"86.4+/-2.1"	"88.9+/-2.5"	"91.9+/-2.8"	"95.2+/-3.3"	"100.7+/-3.7"
7	"83.4+/-4"	"86.5+/-4.5"	"89.6+/-5"	"94.5+/-5.2"	"100.5+/-5.7"
8	"81.8+/-6.2"	"86.2+/-7"	"90.7+/-8"	"97.2+/-8.7"	"105.2+/-8.4"

	middle_10	middle_11	middle_12	middle_13*
1	"109.1+/-7.6"	"123.1+/-7.6"	"141.4+/-10.2"	"156.3+/-6.1"
2	"98.5+/-2.8"	"103.4+/-4.5"	"164.6+/-4.3"	"169.8+/-2.9"
3	"101.6+/-3.6"	"111.5+/-8.4"	"155.9+/-7"	"164.4+/-4.2"
4	"110.8+/-8.9"	"130.4+/-7.6"	"143+/-5.2"	"149.6+/-4.1"
5	"123.4+/-6.6"	"130.6+/-6.4"	"140.1+/-6.1"	"146.4+/-5.2"
6	"105.4+/-4"	"112.6+/-5.8"	"155.6+/-6"	"161.5+/-3.3"
7	"115.6+/-8.4"	"126.2+/-7.6"	"142+/-7"	"153.8+/-6.6"
8	"114.3+/-8.4"	"124.7+/-8.1"	"134.2+/-8.3"	"143.4+/-7.8"

	smallest_opposite_angle*	Octahedral	TrigonalPrismatic
1	"51.4+/-3.1"	"0.029"	"0.048"
2	"81.8+/-2.7"	"0.576"	"0.031"
3	"71.5+/-4.4"	"0.225"	"0.048"
4	"69.6+/-4.8"	"0.014"	"0.071"
5	"72.6+/-6.5"	"0.002"	"0.043"
6	"79.1+/-2.8"	"0.209"	"0.083"
7	"72.1+/-6.8"	"0.003"	"0.018"
8	"53.9+/-5.1"	"0.003"	"0.034"

	PentagonalBipyramidalVA	PentagonalBipyramidalVP
1	"0.026"	"0.111"
2	"0"	"0.061"
3	"0"	"0.124"
4	"0.017"	"0.17"
5	"0.113"	"0.033"
6	"0"	"0.118"
7	"0.075"	"0.035"
8	"0.047"	"0.044"

Table S56. Cluster members of 6-ligand Ca, combined group

[1] "Cluster 1"

1AZO.B.283, 2BQR.A.2002, 3C0W.A.301, 3EH8.D.303, 2FLD.A.601, 3GIM.A.415, 2I3P.A.
154, 2I3Q.B.81, 3MIS.A.2, 4NLG.A.401, 3PR4.A.343, 1A25.A.290, 2A3W.R.336, 4A6S.C
.1122, 3A7Q.A.4003, 3A7Q.A.4004, 3A9Q.G.214, 3AKA.A.173, 1ANW.A.351, 4AQA.A.1210
, 1ATN.D.265, 1AUI.B.502, 1AVA.A.501, 3B1T.A.903, 3B2Z.D.3, 1BAG.A.432, 2BB4.A.2
60, 3BBY.A.215, 1BCJ.2.3, 2BL0.C.1155, 3BOW.A.717, 4BQ3.B.1803, 2BW7.B.2202, 2C1
0.A.1773, 4CAG.A.608, 4CAG.A.609, 2CDP.C.1139, 1CFF.A.152, 3CGA.B.102, 2CHN.A.17
17, 3CK9.A.710, 1CR8.A.45, 3D0Y.A.93, 2D3L.A.503, 3D94.A.2, 1DAN.L.155, 2DBX.B.7
01, 4DD8.B.1001, 1DE4.C.801, 1DGL.B.301, 1DJX.A.2, 1DSY.A.501, 2DW2.A.703, 3E1I.
C.501, 2E6V.C.10, 1E8A.B.1090, 3E9T.B.3, 4ECG.A.502, 4EJ7.A.403, 1ESV.S.395, 3ES
R.A.213, 1F9O.A.2000, 3FAX.A.4, 2FH2.B.2001, 2FHF.A.2405, 3FLP.B.302, 1FZC.B.2,
1FZD.E.1, 4G0D.B.503, 1G5N.A.407, 1G7Y.F.254, 4G9L.A.304, 1GCY.A.529, 2GDF.B.302
, 3GIN.A.2, 3GK2.A.93, 1GU6.A.1479, 2H0K.A.410, 1H5V.A.305, 1HFZ.D.124, 3HGP.A.2
50, 2HIH.A.603, 2HTV.B.993, 3HTL.X.1, 2HYW.B.514, 2II1.B.400, 3IJ8.A.497, 4ILW.D
.305, 4IMM.B.402, 1J1N.A.493, 2J7A.B.1007, 2J7A.E.1006, 2JBK.A.803, 4JDZ.B.703,
2JHL.F.1298, 1JXN.B.246, 4JX1.A.504, 2K0F.A.153, 3K5S.A.218, 3K5T.A.803, 1K7Q.A.
481, 3K7L.A.702, 3K7L.A.703, 3K7N.A.701, 3K7N.A.702, 3K7N.A.703, 1K9I.F.903, 4KD
V.A.202, 1KTW.B.8, 4KTR.D.814, 4KTR.G.806, 1KWV.B.604, 3L2Y.A.302, 1L8S.B.314, 1
L9N.B.704, 3LCP.D.158, 1LGN.B.302, 1LN8.A.201, 3LPD.A.341, 4LP7.A.301, 2LV6.A.20
3, 1M56.A.1007, 4M5I.A.203, 1M63.F.502, 4MHX.A.601, 4MIV.E.600, 4MVF.A.603, 4MVF
.A.604, 1N2K.A.600, 4N2I.A.706, 1N47.A.252, 4NDD.B.401, 4NHD.B.403, 1NIW.A.1003,
1NKQ.F.560, 1NT0.A.3001, 1NUD.A.701, 3NWK.B.238, 1NX2.A.3, 2OA8.C.302, 1OBR.A.4
04, 4OKH.A.901, 3OM7.B.1, 4OMD.D.605, 2OO4.A.5003, 1OT5.A.601, 3OUU.B.455, 2OX9.
A.803, 2OXE.A.600, 2OZN.B.401, 2P37.C.239, 4P99.A.501, 4P99.B.509, 4P99.D.508, 4
PIB.C.204, 4PKG.A.403, 2PMY.A.103, 4POS.E.401, 2PPL.A.482, 3PVN.B.5003, 3PVN.B.5
004, 2Q16.B.200, 2Q17.E.315, 3Q3L.A.436, 4Q6P.A.509, 3QQZ.A.326, 2QT7.B.201, 1R1
Z.C.410, 2R28.A.1003, 3RHT.A.257, 2RHP.A.8, 2RJP.B.3, 3RRD.A.239, 3RTT.A.267, 1S
2N.A.1291, 2SEC.E.276, 1SL6.A.3, 1SU3.B.907, 1SVN.A.276, 4TVU.A.600, 4U2A.A.301,
1U94.A.701, 3UBR.B.476, 3UNX.A.280, 3USU.A.264, 3V03.A.586, 1VFP.A.995, 3VI3.A.
2001, 3VI3.A.2003, 3VI3.A.2004, 2VY0.A.1298, 1W0P.A.1780, 1WBL.D.303, 4WBQ.B.601
, 3WCT.B.203, 3WIU.A.1004, 1WMY.B.203, 3WN6.B.501, 1XJL.B.342, 2XVT.C.1137, 1Y3N
.A.493, 1Y4D.E.1001, 1YO8.A.1192, 1YO8.A.1202, 1Z3U.A.497, 2Z57.A.1006, 2Z8Z.A.6
18, 2Z8S.A.646, 2Z8S.A.648, 2Z8S.B.646, 2ZEY.B.152, 2ZJ7.A.619, 2ZUY.A.628, 2ZWP
.A.401
[1] "Cluster 2"
2A0R.A.402, 2ASD.A.416, 3AVX.A.3001, 3DPG.B.501, 4DTP.A.1002, 4ECQ.A.501, 4ELV.A
.908, 4F4W.A.402, 4FJ8.A.1002, 4FJ9.A.1002, 4FJM.A.1002, 3IAY.A.1, 4J2A.A.1002,
1JXL.A.1402, 4K4H.A.605, 4K4H.E.602, 4K4I.E.602, 4KHQ.A.1001, 4M3Z.A.1002, 3M9N.
B.4003, 1N3E.F.491, 1N3F.C.498, 2OAA.A.601, 2ODI.A.701, 2Q10.A.701, 4QWB.A.402,
2RDJ.A.353, 1TW8.A.801, 3A7Q.B.5001, 2AEP.A.601, 1AF0.A.484, 3AFG.A.540, 2AFH.D.
2490, 3AHW.A.122, 3AI7.B.901, 1AJJ.A.73, 3AJ7.A.602, 3AKB.A.2, 4APX.B.1239, 4AQ1
.A.1926, 4AQE.A.1208, 4ARF.A.1723, 3AUK.A.391, 1AVA.A.502, 4AWN.A.300, 4AXN.A.13
29, 3AYU.A.417, 3AYU.A.418, 1B4N.A.620, 3B7E.A.1005, 1B8L.A.110, 3B8Z.A.904, 3BC
D.A.707, 2BL0.B.1146, 4BNR.A.600, 2BQ4.A.1119, 2BU3.A.1242, 2C10.A.1771, 3C9I.A.
1, 2CDP.A.1140, 3CKZ.A.1, 1CLC.A.650, 4CPL.A.500, 4CPY.A.1466, 4CU9.A.2999, 4CUA
.A.2644, 4CUB.A.2645, 1CVL.A.320, 2CYY.A.2002, 3D3I.A.1001, 3D7K.A.571, 2DCJ.A.1
003, 2DDU.A.1, 2DDR.A.1324, 2DIE.A.779, 4DK4.A.302, 1DPO.A.246, 4E5U.B.302, 2EA7
.A.450, 3EDY.A.1, 2EEK.A.401, 3EF2.A.304, 3EF2.A.305, 1EGZ.A.300, 1EX9.A.286, 3F
5V.A.223, 4F8Z.A.409, 2FCW.B.3001, 2FCW.B.3002, 2FGZ.A.1192, 3FG1.A.1501, 2FHF.A
.2404, 3FSJ.X.600, 3FVQ.A.360, 2FWN.A.532, 1G9K.A.703, 1G9K.A.706, 1GA6.A.374, 4
GDI.A.509, 4GDJ.A.507, 4GG1.A.602, 2GKO.A.612, 3GK2.A.92, 4GN7.A.301, 1GTT.A.143
0, 4GW3.A.401, 2H0B.A.1000, 4H1Q.A.303, 4H1Q.A.305, 1H71.P.501, 3H81.A.279, 3H81
.C.279, 3H81.C.280, 1H9H.E.1246, 3HB2.P.482, 1HDF.A.1101, 1HFC.A.277, 3HGN.A.250
, 3HI7.A.802, 3HJR.A.603, 4HJF.A.601, 1HM9.A.1901, 4HS9.A.401, 1HT6.A.502, 1HV5.
A.5503, 1HY7.A.305, 1HYO.A.1006, 4HZW.A.507, 4I35.A.513, 1I76.A.996, 1I76.A.997,

2I8U.A.202, 2I8T.A.402, 3I9G.L.301, 3I9G.L.302, 3IBZ.A.192, 4IHM.A.404, 1IOD.G.
503, 4IU2.A.301, 4IU3.A.301, 2IXT.A.1310, 2IXT.A.1311, 1J11.A.701, 4J7M.A.403, 1
J8E.A.201, 1JI3.B.403, 1JK3.A.403, 2JKP.A.1728, 4JZB.A.401, 4JZB.A.403, 4JZX.A.4
04, 4JZX.A.405, 4K1K.A.501, 3K37.B.467, 4K3K.B.401, 1K7I.A.487, 4K70.A.1002, 4K8
9.A.408, 4K9P.A.601, 1KAP.P.616, 1KAP.P.618, 1KAP.P.619, 3KCG.H.500, 4KTY.A.802,
4KXY.A.707, 4L74.A.401, 3LNI.A.303, 1LQV.C.35, 4LVN.A.703, 4M5I.A.201, 3M6L.A.8
01, 3MA2.A.293, 3MA2.A.296, 4MB1.A.602, 1MCT.A.246, 4MEW.A.502, 2ML1.A.202, 1MNC
.A.283, 3MOS.A.1, 4MPR.A.601, 3MVS.A.211, 4MWL.A.512, 3MW3.A.301, 4MWV.A.512, 3N
1U.A.200, 4N20.A.705, 4N2E.A.705, 4N2P.A.201, 4N4E.E.405, 1N7V.A.601, 1N9E.A.802
, 3NIF.D.2002, 3NJH.B.502, 3NKQ.A.1003, 1NKG.A.800, 4NOT.A.302, 1NPC.A.320, 1NSC
.A.468, 2O72.A.403, 1OAC.A.802, 4OCI.A.201, 3OJY.A.555, 4OKH.B.904, 1OM6.A.701,
1OM8.A.705, 3OOY.A.621, 1OU9.A.131, 3OYR.B.337, 3OYR.B.338, 3P2P.B.126, 2P3U.B.5
01, 3P4G.A.401, 3P95.A.1, 2PHI.B.125, 4PMX.A.401, 2PNY.A.228, 3POJ.B.1, 3PPE.A.4
01, 2Q16.A.200, 1Q3A.A.466, 3Q4W.A.224, 4Q8K.A.501, 1QCN.A.1001, 4QN6.A.501, 3QR
B.A.301, 3QU7.A.230, 3QU7.B.225, 2QUB.A.614, 2QUB.A.615, 4R12.A.809, 2R1B.A.1001
, 2R5N.A.2000, 1R6V.A.1, 2R8O.A.670, 2R8Y.A.201, 2R8Z.A.201, 3R8Y.A.242, 2R8P.A.
670, 1RM8.A.502, 1RQ5.A.819, 3RRX.A.901, 3RUP.A.1004, 3RVV.A.225, 3RVW.A.223, 3S
4Y.B.1303, 1SAT.A.476, 3SAL.A.601, 1SIO.A.601, 1T1E.A.700, 3T3P.B.2003, 3TEW.A.8
00, 3TI4.A.601, 1TO2.E.450, 1TRK.A.681, 4TSH.B.1502, 3U1R.A.703, 3U1R.A.706, 3U8
D.A.203, 3U8I.A.201, 1ULV.A.2001, 3UPT.A.691, 1UR4.A.1398, 4USU.A.1471, 1UTM.A.2
47, 1UX6.B.2002, 1UX6.B.2003, 1UX6.B.2004, 1UX6.B.2010, 1UX6.B.2012, 1UX6.B.2016
, 1UYX.A.1134, 4UZU.A.1484, 3V5U.A.705, 3V96.B.303, 1VCL.A.1004, 1VL9.A.125, 2VN
G.B.1214, 2VOV.A.1338, 3VV3.A.401, 3VV3.B.404, 1W7C.A.802, 3W7T.A.1001, 1WAD.A.1
16, 4WA3.A.503, 2WFK.A.1250, 4WIW.A.702, 4WKO.B.502, 4WKO.B.503, 4WK7.A.504, 1WM
D.A.1003, 2WNV.B.1225, 2WOY.A.2414, 2WW3.A.800, 1WZA.A.601, 2XVT.F.1137, 2Y6D.A.
1266, 1Y93.A.266, 1Y93.A.268, 1Y9Z.A.604, 2YAY.A.1267, 2YEQ.A.1526, 2YGL.A.1413,
2YN3.A.6362, 2YN5.A.6362, 2YN5.A.6363, 1YO8.A.1185, 1YO8.A.1191, 1YO8.A.1193, 1
YO8.A.1199, 2YOA.A.1003, 1YS1.X.400, 1YS6.A.1001, 2Z2X.A.1004, 2Z2X.A.1005, 2Z30
.A.1006, 1Z6O.A.5302, 1Z70.X.3001, 2Z8X.A.620, 2Z8X.A.626, 2ZUX.A.630, 2ZUY.A.62
6, 3ZWH.A.501, 2ZWO.B.400, 3ZXH.A.304
[1] "Cluster 3"
4ABT.B.1287, 2AOR.A.401, 1DMU.A.300, 3DVO.A.340, 3GV5.B.424, 2IBK.A.402, 2IMW.P.
406, 4J2B.A.1002, 4KYW.A.302, 3LDY.A.145, 3M9O.B.4001, 1N3E.D.493, 1N3F.D.499, 1
N3F.F.497, 3O3G.A.1, 1OUP.A.300, 1Q9Y.A.939, 3QET.A.905, 1RYS.A.801, 3SPZ.A.905,
3A09.A.601, 3A13.E.445, 1AG9.A.200, 1AG9.B.1000, 4AIE.A.1540, 3AMR.A.909, 4AQ1.
A.1925, 3ASI.A.2001, 3B4N.A.702, 4B4F.A.607, 1B9T.A.500, 3BCD.A.708, 2BV2.B.1085
, 4BZ4.A.1233, 1C3H.D.8003, 1C3H.F.8001, 4CAG.A.602, 1CGE.A.305, 1CLC.A.652, 5CN
A.C.240, 3D6E.A.202, 4DLK.A.403, 4DOU.A.1002, 2E9B.A.741, 3EHB.A.563, 1ELT.A.300
, 5ENL.A.438, 1FBL.A.996, 3G4E.A.1, 4G60.A.301, 3GG1.A.502, 4GI6.A.601, 3GN9.A.2
01, 1GVK.B.1246, 1HVX.A.517, 3I98.A.627, 4IHM.A.403, 2IUF.E.1697, 1JOH.A.601, 1J
1N.B.493, 2J1T.A.1154, 1J35.C.501, 2J7A.A.1007, 1J9L.B.1303, 1JE5.B.502, 2JKE.A.
1728, 4JZB.A.402, 1K7Q.A.485, 4K70.B.1002, 2KAY.B.187, 4KKF.A.703, 3KMV.A.163, 4
KS1.A.501, 1KUO.A.703, 3KZP.A.240, 1LOC.E.688, 2MIN.B.525, 2ML1.A.201, 3MMZ.A.50
0, 4N20.A.706, 4N2B.A.705, 4NEH.B.701, 1NNL.B.2002, 1NUD.A.703, 4NUZ.A.1001, 2NX
P.A.600, 3OJY.B.538, 3OM5.B.1, 4OUL.B.1201, 3PGB.A.903, 4PLS.A.301, 2POO.A.805,
1PVY.B.603, 1PW9.A.404, 2Q1C.X.294, 3Q2L.B.703, 3Q5I.A.528, 2QIM.A.158, 1QLB.A.1
658, 4QU6.A.904, 1RXO.B.477, 1SOB.A.1292, 3S5U.E.221, 2SAS.A.187, 1T5S.A.1004, 1
TU5.A.902, 3U1R.A.705, 3U1R.A.707, 4UP4.A.501, 4UP4.A.502, 2UWF.A.1369, 1UX6.B.2
007, 2VCC.A.1917, 1WC5.C.2100, 3WN6.A.502, 2WOB.E.1161, 1WRZ.A.154, 3X17.A.602,
1XKD.A.1005, 2Y09.A.1242, 1YO8.A.1183, 1YO8.A.1205, 1YO8.A.1208, 1YO8.A.1209, 2Z
E0.A.552, 2ZPR.A.2001, 2ZUX.A.638, 2ZWP.A.402
[1] "Cluster 4"
3FSP.A.501, 3KHH.B.1416, 1M5X.A.802, 1N3E.C.492, 4PTF.A.1202, 3QEX.A.907, 1R7M.A
.306, 2RDJ.B.353, 2WTF.B.1510, 2A8K.C.403, 1AFA.2.2, 1AWB.A.278, 4AWD.A.1321, 3A

ZX.B.301, 3B00.B.301, 4B7R.A.502, 3BJU.A.608, 4BY5.C.1187, 4CAJ.B.1325, 2CDP.B.1139, 1CGU.A.686, 3CGT.A.686, 3CK7.D.730, 1CRU.A.503, 4CRR.A.1386, 1CYG.A.682, 3DAS.A.351, 4DRZ.A.202, 3EAD.A.1003, 3EDD.A.700, 3ESQ.A.213, 1F6S.E.205, 3FCU.B.2002, 1FHF.A.501, 4G62.A.301, 1G9K.A.700, 3GN4.B.204, 3GQF.D.154, 2GSM.A.3007, 4H3X.A.304, 1HDF.B.1101, 1HFZ.A.124, 1HL5.D.156, 1HQV.A.999, 1I22.A.198, 3I4I.A.1001, 3I57.A.186, 2IWV.B.1281, 4JDZ.B.702, 3JTX.B.396, 3JXS.A.302, 4K5W.A.201, 2KAY.A.186, 3KF9.C.304, 1KMB.1.2, 3KQA.B.421, 4KVJ.A.714, 1KX1.E.502, 1KX1.F.601, 1L6R.A.901, 3LCP.B.279, 3LHM.A.131, 1MAC.B.389, 1MDU.A.403, 4N25.A.707, 4N2I.A.704, 4N85.A.502, 3N8G.A.1002, 4NAM.A.801, 4NHF.B.301, 3NN7.A.503, 3O5S.A.243, 2OKX.B.4002, 1P8J.G.3014, 2PPL.A.481, 1Q7B.A.9002, 2QT6.A.3713, 1R1Z.C.415, 2RDZ.B.1502, 3RRW.A.271, 3SLE.B.401, 3SO0.F.98, 3TEW.A.801, 3TSK.A.304, 1TT2.A.502, 3TUY.C.157, 1UF3.D.913, 1UH2.A.1002, 1UH3.A.1001, 1UH3.A.1002, 1UKS.A.688, 1ULV.A.2003, 1UPS.A.501, 3V03.A.585, 1V2I.A.1001, 1V3D.B.2001, 1V3J.A.687, 1V3L.B.689, 2VDR.B.2002, 1VFO.A.1001, 2VJ3.A.1533, 2VVE.B.1338, 2VXJ.G.200, 2VZP.B.1129, 2W2N.E.1334, 2W3J.A.1139, 3W9T.A.1008, 3W9T.D.504, 4WBQ.B.602, 4WF7.D.600, 3WH3.A.501, 2WO9.B.1272, 2WQS.A.2415, 2XQX.B.1949, 2YDP.A.502, 1YO8.A.1195, 2Z49.A.1002, 3ZHG.D.1323

[1] "Cluster 5"

3CFR.A.910, 2G8K.A.401, 1N56.B.403, 3NE6.A.905, 1A2X.A.160, 1A2X.A.161, 1AEI.A.319, 5AER.A.201, 5AER.A.202, 1AJ4.A.163, 3B1T.A.900, 1BAG.A.431, 3BOW.A.719, 3BOW.B.403, 4BY5.D.1185, 1C9U.B.1002, 3CGA.A.102, 1D7F.A.5003, 2E6W.A.301, 1E8U.B.1003, 3EAD.B.1002, 4ELG.F.202, 2EQD.A.701, 1ESP.A.322, 2EXI.D.3004, 1FI5.A.162, 4FU4.B.505, 1GOH.A.290, 2HOK.A.407, 3HLI.D.315, 4I5N.B.601, 2IWV.D.1286, 1IXX.B.124, 2J1G.D.1289, 1JBA.A.501, 1JC2.A.3, 4JEO.A.401, 2K7O.A.103, 1K90.F.804, 1K96.A.92, 2KAY.A.185, 3KF9.A.302, 2KZ2.A.149, 1LBX.A.290, 4LMF.A.303, 4LMH.D.811, 1LPG.B.1, 2LV6.A.204, 2LVK.A.102, 2M5E.A.2001, 4MIV.D.601, 2ML3.A.205, 4N25.A.706, 4NDD.B.402, 4NEN.A.1115, 1NIW.A.1001, 1NIW.C.1005, 1NUB.A.301, 1NUB.A.302, 3OX6.A.502, 2PF2.A.174, 4PLM.A.504, 3PM8.B.514, 1PTK.A.281, 3QRX.A.170, 2SAS.A.186, 1SCV.A.162, 3SO1.B.97, 1TCF.A.162, 3TRQ.A.360, 1TTX.A.110, 1TTX.A.111, 4UM8.C.2004, 1UMS.A.3, 1UZJ.A.1648, 1UZJ.C.3649, 1V1G.A.1211, 2VKH.A.1543, 2WVX.B.801, 2WVZ.B.800, 1YCM.A.266, 1YO8.A.1211, 1YUU.B.199, 2Z2D.A.266, 2Z8S.A.647, 2ZJ7.A.627

[1] "Cluster 6"

2AOQ.A.232, 2ASD.A.415, 3BQ1.A.4001, 3COW.A.304, 1DMU.A.302, 3EH8.A.302, 1FOO.B.761, 4F4W.B.403, 4FJK.A.1002, 4FJL.A.1002, 2GIJ.A.401, 2IBK.A.401, 2JEJ.A.1344, 1JX4.A.4001, 4K4H.A.607, 3KHR.A.416, 4KLD.A.402, 4LQ0.A.402, 3LZJ.A.905, 3M9O.B.4003, 3MXB.B.173, 3MXB.R.175, 3ODH.A.195, 4QWB.A.403, 1R7M.B.536, 3RAX.A.415, 1SOO.A.401, 1S9F.A.4001, 3SLP.B.227, 3SUN.A.897, 2A8K.B.404, 2AER.L.3008, 1AFO.A.486, 3AMR.A.910, 4AR9.A.1732, 1AWB.A.1, 3B0I.A.124, 3B1U.A.901, 3BC9.A.702, 3BC9.A.705, 3BH4.B.1, 1BLI.A.600, 4C9F.B.401, 3CKC.A.600, 2D00.D.1001, 3D4G.B.484, 2DEW.X.901, 2DF7.C.5904, 4DKB.A.302, 4DOU.A.1001, 2DSN.B.2012, 4EJ7.B.402, 4EPU.A.601, 2EXH.D.2004, 2F3C.E.242, 2FIB.A.412, 2FPS.A.503, 3FP8.E.601, 3FU1.A.301, 1G5N.A.402, 4G60.A.302, 4G62.A.302, 1G87.A.615, 2GGM.B.402, 2GJP.A.1486, 2GKO.A.611, 4GQ7.A.301, 4GZT.B.510, 2HOK.B.410, 1H3G.A.701, 1H5V.A.306, 1H71.P.502, 4HOW.A.704, 4HPN.A.401, 2HYU.A.502, 2HYV.A.608, 2I4B.A.454, 4I8H.A.301, 3IOX.A.903, 1J9K.A.301, 4JCM.A.706, 2JKH.A.1245, 4JZE.H.302, 4JZX.A.403, 1K7I.A.483, 1KA1.A.401, 2KAY.B.188, 1KAP.P.617, 1KAP.P.620, 3KM5.A.2011, 2L51.A.207, 1L9M.A.702, 3L9I.C.1148, 4LJ3.B.403, 4LLS.A.303, 4LLT.A.303, 3LNH.A.303, 3LPD.A.342, 4LQR.A.202, 4LVN.A.704, 2M0P.A.1201, 3M1H.C.2001, 3MBR.X.300, 3MHF.A.328, 4MKM.A.403, 2ML1.A.204, 2ML2.A.201, 2ML3.A.203, 2ML3.A.204, 3MVS.A.214, 3MVS.A.215, 4N2G.A.705, 4N2I.A.705, 3N4E.A.500, 4NAS.A.503, 1NBW.A.650, 1NRW.A.903, 4NUQ.A.301, 1NX1.A.3, 1OAH.B.1526, 1OB0.A.501, 3OM6.B.1, 3OTJ.E.1000, 3P4G.A.402, 3P4G.A.403, 3P4G.A.404, 3P4G.A.405, 3P4G.A.406, 3P4G.A.407, 3P4G.A.408, 3P4G.A.409, 3P4G.A.410, 3P4G.A.412, 3P4G.B.411, 4PHN.A.302, 3PKO.A.280, 1PZ7.A.701, 2PZO.A.501, 3Q8F.A.736, 1QCO.A.1002, 4QD2.E.302, 3QGV.A.504, 4QN7.A.501, 2QUB.A.616, 2QUB.A.618, 3QXG.

B.230, 2RA3.A.1, 2RA3.B.1, 3RQ0.A.301, 3S6J.A.4, 1SBH.A.290, 1SCB.A.276, 3SO0.D.97, 3SVL.A.201, 1TCM.B.687, 3U1R.A.704, 3UBF.A.754, 1ULV.A.2002, 4UM9.B.2002, 1UYX.A.1133, 1V0Z.B.1477, 3V96.B.304, 1VCL.B.1001, 1VCL.B.1002, 2VL8.A.1545, 3VOC.A.501, 3VTO.A.302, 2W1W.B.1135, 1WPC.A.502, 2XR9.A.1869, 2XSG.B.1772, 1Y6W.A.149, 1Y7B.A.3001, 1Y9Z.B.605, 1YI7.A.3001, 1YO8.A.1201, 1YO8.A.1203, 1YO8.A.1206, 2Z2X.A.1007, 2Z8X.A.622, 2Z8X.A.623, 2Z8X.A.624, 2Z8S.B.643, 2ZQ0.A.901

[1] "Cluster 7"

2AGO.A.403, 4DTS.A.1002, 4K4I.A.606, 3A7Q.A.4002, 3AAJ.A.991, 1AFD.3.2, 1AMY.A.502, 1ANN.A.319, 1ANW.B.354, 4CAG.A.605, 3CHK.A.502, 1D2L.A.46, 1DCY.A.198, 2DDY.A.174, 2DPK.A.2001, 2E3X.A.803, 3E3R.B.194, 4E52.A.403, 3E9T.A.1, 3EAD.B.1004, 3ECQ.A.2000, 3EHJ.B.1, 4ELF.C.201, 2ERO.B.702, 1FDK.A.124, 4FGC.B.204, 3FLP.B.301, 3FLT.A.302, 4FL4.C.401, 4FL4.C.402, 3FVI.D.125, 3GIN.A.1, 4GKX.B.301, 1GQM.A.1089, 1H4B.A.1085, 1H4B.A.1086, 2HQ8.B.303, 2HYW.A.505, 2I08.A.200, 3IA7.A.402, 3IA7.B.402, 3IGO.A.601, 1IME.A.278, 1IZJ.A.1002, 4JBE.A.502, 1JL5.A.2004, 4K1C.A.506, 3K8L.B.800, 1KIT.A.803, 4KPP.A.501, 1KTW.A.3, 1KX1.A.222, 1LGC.A.301, 1LHV.A.401, 4LMF.A.304, 2LP3.A.201, 1M34.B.2299, 1M8T.C.1003, 4MBZ.H.401, 4MDV.B.403, 2ML1.A.206, 1NAE.A.900, 1NIW.A.1002, 3NOM.B.263, 1NX2.A.4, 1O9I.C.269, 1ODB.F.1092, 3OX6.A.501, 4P5W.A.1001, 4P99.A.512, 1PK8.A.817, 2PQY.A.500, 4Q4W.1.905, 1Q8H.A.72, 1QNI.E.903, 3QRX.A.173, 2RHP.A.1176, 2RHP.A.26, 3RV2.A.405, 3S5U.C.220, 1TEC.E.343, 1THL.A.3233, 3TRQ.A.358, 1TYE.A.1405, 1TYE.A.1406, 1TYE.A.1407, 1U7W.A.501, 1UG9.A.2006, 3UJQ.D.305, 3USU.H.272, 2V3T.B.1264, 1V3J.B.690, 2VB6.B.1151, 1W2M.C.1441, 3W9T.A.1005, 3W9T.C.1009, 1WMD.A.1001, 2WO9.D.1270, 1WRL.D.104, 3WU2.O.301, 2Z8S.A.644, 2Z8S.B.642, 2ZBA.A.461, 2ZKT.B.413, 2ZUY.A.624

[1] "Cluster 8"

4DU3.A.1003, 3GIL.B.1417, 4K4H.I.602, 4LQ0.A.403, 3ODH.A.196, 1ATN.D.264, 1B47.A.351, 4BTW.B.1764, 2COO.B.1507, 1CFF.A.149, 1CFF.A.150, 3CGT.A.685, 2D3P.C.241, 3DF0.A.715, 3DF0.A.716, 3DF0.B.604, 2DIE.A.778, 1DJY.A.2, 2E6W.A.300, 3E78.A.601, 2E85.B.1004, 3EHJ.A.1, 4ELG.A.202, 1EUB.A.278, 4FDI.A.603, 3G5C.B.802, 2GJR.A.1488, 4GTW.A.1012, 4IAV.A.420, 1JBA.A.500, 3JQ5.A.201, 1JSA.A.500, 1JSA.A.501, 4K1C.A.509, 1KB0.A.801, 1KV9.A.802, 1KX1.E.501, 2KYF.A.110, 1LMJ.A.101, 1LMJ.A.102, 2LMT.A.149, 2LMT.A.150, 2LP2.A.203, 1LTJ.B.2, 1LWJ.B.10, 2MOK.A.201, 1M63.B.502, 4M7Z.B.409, 2ML2.A.202, 2ML2.A.205, 3MXW.A.402, 1N28.B.125, 1NHE.A.805, 2NQA.B.902, 1NUD.A.702, 1NX3.A.4, 2P0R.B.1004, 2POJ.A.266, 4POQ.A.401, 4R9X.A.302, 3RBX.A.600, 2SAS.A.188, 1ULV.A.2004, 4UM9.C.2001, 4UM9.C.2002, 3VI3.A.2002, 3W9T.D.503, 3WHU.B.502, 2WL3.A.1290, 2WW0.A.800, 2WYS.A.1554, 1XJL.A.346, 1XJL.B.340, 2XRM.A.405, 2YN3.B.6361, 1YUU.A.197, 1Z6C.A.247, 2Z8S.A.642, 1ZFS.A.104, 1ZIV.A.1, 2ZN9.A.902, 2ZN9.B.901

Table S57. 7-ligand Ca, normal group

	size	largest_angle*	middle_1*	middle_2	middle_3	middle_4
1	"46"	"157.1+/-3.1"	"69.7+/-2.7"	"72.4+/-2.6"	"74.2+/-1.8"	"76+/-1.7"
2	"34"	"153.3+/-3.4"	"69.8+/-3.2"	"72.1+/-3"	"73.9+/-2.4"	"75.6+/-1.8"
3	"49"	"165.4+/-2.4"	"70.4+/-1.2"	"72.3+/-1.4"	"74.5+/-1.3"	"76.6+/-1.5"
4	"49"	"173.8+/-2.4"	"70.8+/-1.3"	"72.2+/-1.1"	"73.9+/-1.4"	"76.2+/-1.7"
5	"38"	"172.5+/-3"	"68.6+/-2.5"	"72.7+/-2"	"75.3+/-2.1"	"78.9+/-1.8"
6	"31"	"165.8+/-3.7"	"71+/-2.9"	"73.3+/-2.1"	"74.8+/-1.3"	"76.5+/-1.5"
7	"32"	"165.8+/-3.3"	"69.1+/-2.4"	"72.6+/-2.3"	"74.5+/-2"	"76.8+/-2.4"
8	"56"	"161.7+/-2.5"	"71.1+/-2.5"	"73.9+/-1.8"	"75.6+/-1.8"	"77.3+/-1.1"

	middle_5	middle_6	middle_7*	middle_8	middle_9
1	"77.4+/-1.6"	"79+/-1.8"	"80.7+/-1.6"	"82.7+/-2"	"85.4+/-2.4"

2	"76.8+/-1.7"	"78.2+/-2"	"79.4+/-2"	"80.7+/-2"	"82.9+/-1.9"
3	"79.2+/-2"	"81.5+/-1.7"	"83.5+/-1.4"	"85.4+/-1.6"	"87.8+/-1.8"
4	"80.9+/-2.1"	"83+/-1.7"	"84.8+/-1.8"	"86.6+/-1.8"	"88.5+/-1.5"
5	"81.2+/-1.8"	"83.6+/-2"	"85.3+/-1.9"	"86.9+/-1.6"	"88.7+/-1.5"
6	"78.2+/-1.4"	"79.8+/-1.5"	"81.3+/-1.6"	"83.3+/-1.9"	"85+/-1.9"
7	"78.5+/-2"	"80.4+/-1.5"	"82.5+/-2.1"	"84.7+/-2.4"	"87.5+/-1.9"
8	"78.5+/-1.1"	"79.6+/-1.1"	"81+/-1.1"	"82.8+/-1.3"	"85.3+/-1.6"

	middle_10	middle_11	middle_12	middle_13*	middle_14
1	"88.1+/-2.8"	"91.8+/-3.7"	"98.3+/-4.8"	"105.8+/-3.6"	"118.4+/-7"
2	"85.8+/-2.4"	"88.9+/-3.8"	"99+/-5.7"	"116.6+/-3.4"	"123.2+/-4.2"
3	"89.3+/-1.8"	"91.8+/-2.6"	"95.4+/-2.3"	"99.7+/-2.5"	"108.2+/-4.9"
4	"90.5+/-1.4"	"92.4+/-1.7"	"95+/-2.2"	"97.6+/-2.9"	"101.5+/-3.6"
5	"90+/-1.6"	"91.7+/-1.9"	"94.6+/-2"	"97.9+/-2.1"	"102.2+/-4.5"
6	"88.1+/-2.5"	"91.7+/-3.4"	"96.9+/-3.8"	"109+/-2.9"	"113.4+/-5.2"
7	"90.3+/-1.8"	"92.3+/-2.1"	"98.2+/-5.4"	"105.8+/-3.2"	"113.3+/-4.6"
8	"86.8+/-2"	"89.5+/-2.3"	"100.7+/-4.2"	"113.9+/-2.2"	"116.8+/-2.5"

	middle_15	middle_16	middle_17	middle_18	middle_19*
1	"131.9+/-4.7"	"136.6+/-2.9"	"140.3+/-3.3"	"145.4+/-3.5"	"150.1+/-3"
2	"130.1+/-4.3"	"134.7+/-3.6"	"139.2+/-3"	"145.1+/-3.2"	"149+/-2.7"
3	"136.3+/-3.9"	"139.5+/-2"	"142.6+/-1.8"	"145.9+/-2.3"	"148.6+/-2.3"
4	"137.9+/-2.6"	"140.6+/-1.7"	"142.8+/-1.6"	"145.4+/-2"	"147.9+/-1.9"
5	"132.4+/-3.8"	"136.9+/-2.1"	"142.6+/-2.8"	"149.8+/-2.4"	"154.1+/-2.6"
6	"133.3+/-3.8"	"137.8+/-3.1"	"140.1+/-3"	"144.2+/-2.7"	"147.5+/-2.6"
7	"124.4+/-6.5"	"132.2+/-3.6"	"138.7+/-4.3"	"150.6+/-4.6"	"157.9+/-3.4"
8	"122+/-4.2"	"131+/-3.2"	"136.8+/-3.5"	"150.3+/-3.7"	"157.8+/-2.7"

	smallest_opposite_angle*	PentagonalBipyramidal	SquareAntiprismaticV
1	"67.4+/-3.2"	"0.125"	"0.162"
2	"70.1+/-2.8"	"0.187"	"0.329"
3	"68.1+/-2"	"0.249"	"0.222"
4	"68.7+/-1.6"	"0.15"	"0.091"
5	"64.5+/-2.1"	"0.254"	"0.19"
6	"68.6+/-3"	"0.231"	"0.22"
7	"66.1+/-2.9"	"0.303"	"0.404"
8	"67.8+/-3.1"	"0.095"	"0.191"

	HexagonalBipyramidalVA	HexagonalBipyramidalVP
1	"0"	"0.038"
2	"0.001"	"0.039"
3	"0"	"0.087"
4	"0"	"0.047"
5	"0"	"0.14"
6	"0.001"	"0.073"
7	"0.002"	"0.178"
8	"0.001"	"0.045"

Table S58. Cluster members of 7-ligand Ca, normal group

[1] "Cluster 1"
4A5G.A.1308, 4A6O.A.2346, 4A6S.A.1122, 3AFG.A.541, 4AL9.A.1122, 1BJ3.B.124, 3BPS
.E.1, 1BYF.A.201, 1CGT.A.686, 4CPB.A.1122, 4CPB.D.1123, 1D2V.A.600, 2DCK.A.1002,
3DED.B.506, 1DV8.A.1002, 2FF3.A.701, 1FHF.A.502, 4FHP.A.402, 1FIF.B.2, 3INM.A.5
21, 1J34.C.504, 3K8L.A.700, 4LHK.A.303, 3LI3.A.402, 4LJH.A.201, 1LQV.C.42, 4M65.

A.404, 1MN1.A.371, 1NL1.A.204, 1NZI.A.1001, 1PA2.A.307, 3PAQ.A.300, 3PAR.A.300, 1PJX.A.491, 1SCH.A.302, 1SH7.A.1292, 1SZB.A.1001, 3TH4.L.148, 2VZR.B.1132, 2W2M.E.1334, 3WU2.A.401, 2WZS.F.800, 1XO5.A.1, 2Z48.A.1007, 1ZH2.B.201, 3ZYH.A.1123
[1] "Cluster 2"
2B6N.A.300, 4B9F.A.153, 1BCJ.2.2, 2BO2.A.155, 2BO2.A.156, 4D0E.A.1531, 3DBZ.A.401, 3DED.A.504, 3DEM.B.4001, 2DSO.A.1001, 4FVL.A.505, 1G1Q.A.801, 2GGX.C.401, 2GVU.A.500, 4HHR.A.703, 3HLH.B.315, 2IAW.A.401, 2IAX.A.401, 2IAO.A.401, 3IQT.A.1, 4JSD.A.603, 1KWZ.A.504, 3LI4.A.316, 4MZA.A.612, 4N4E.E.404, 4N7A.A.605, 1NPC.A.322, 3P7F.D.1, 3Q9K.A.606, 3R5Q.A.1001, 1USR.B.1573, 2VZP.A.1129, 3WH3.A.500, 3WHD.A.501
[1] "Cluster 3"
4A3Z.A.2344, 3A4U.A.287, 4A41.A.2494, 1ALC.A.200, 3ALU.A.201, 3BOK.A.201, 3BOX.A.578, 1B9O.A.124, 4B96.A.1155, 1CPM.A.215, 3EDF.A.603, 2EJN.A.1003, 3FMU.A.351, 3HDL.A.307, 1I22.B.197, 4IAU.A.800, 2IWV.D.1283, 2J1A.A.1769, 4JCL.A.701, 2JDA.A.1146, 1JI1.A.2002, 1JUG.A.126, 1KZM.A.501, 1LYC.B.9002, 1MAC.A.388, 1O4Y.A.700, 3OWF.A.151, 1PAM.A.687, 1PJ9.A.890, 4Q1U.A.402, 1QGJ.A.2001, 1R1Z.A.286, 3SRE.A.1357, 1SU4.A.996, 3TBD.A.401, 1TDQ.B.127, 1TLG.A.201, 1UX6.B.2014, 2V72.A.1139, 2VZQ.A.1130, 2W1Q.A.1947, 2W1S.A.1946, 1WZL.A.1601, 2YFU.A.1141, 2Z48.A.1103, 2Z48.A.1104, 2Z48.A.1205, 2Z49.A.1004, 3ZUC.A.1154
[1] "Cluster 4"
4A4A.A.1925, 4ASM.B.1359, 4ATE.A.1275, 4AWD.B.1321, 3AXD.A.3002, 1B80.B.351, 4B9C.A.1151, 2BIB.A.1551, 4BLK.A.400, 3BMV.A.685, 4BM1.A.401, 1CPN.A.209, 4CZN.A.1370, 2DEW.X.900, 2E39.A.501, 2E8Y.A.741, 1F6S.A.201, 2FHF.A.2401, 3HOO.A.401, 2HD9.A.2001, 1HFX.A.124, 3HR9.A.401, 3ILF.A.278, 4J3V.A.920, 4J3W.A.907, 4JGL.A.202, 1LLP.A.351, 4LPL.A.1101, 4LQR.A.201, 1LY8.A.9001, 3M5Q.A.372, 1MVE.A.400, 4N2B.A.707, 4N2G.A.703, 4N2L.A.704, 4N6F.A.302, 3NNG.A.402, 4ODG.A.202, 2OKX.A.4001, 3OMI.A.613, 3Q3U.A.340, 4QF4.A.202, 1SNC.A.150, 1UPS.B.501, 1URX.A.1300, 3WDH.A.801, 2WOY.A.2415, 2WZA.A.2415, 1YRO.A.124
[1] "Cluster 5"
4K4G.A.602, 4K4H.A.602, 4K4I.A.605, 4UAW.A.403, 4A42.A.1690, 4A5G.A.1309, 4AQ1.A.1924, 3ATG.A.301, 3AZY.A.301, 1BGP.A.501, 3CK7.A.710, 4CRQ.A.260, 4CUO.A.1326, 1D3C.A.687, 2EXH.A.2001, 1GW2.A.502, 1GWT.A.502, 1GWU.A.1308, 1GX2.A.1310, 2H2N.B.1001, 3HB3.A.563, 3HDL.A.306, 2HYK.A.477, 2JD9.A.1146, 2JKA.A.1727, 3MMZ.A.501, 1PA2.A.308, 3PGV.A.267, 1QGJ.A.2002, 4R83.B.501, 1SCH.A.301, 3SO1.A.97, 1TE2.A.702, 1U0A.A.5004, 1W3M.E.3013, 2YLJ.A.1308, 2Z30.A.1002, 2Z49.A.1005
[1] "Cluster 6"
1T9I.C.801, 1T9I.C.802, 3ALT.A.201, 2BF6.A.1693, 1CIU.A.684, 4D0E.A.1533, 4DZT.A.302, 1G5N.A.408, 4GER.A.404, 3GIS.Z.1003, 1GWU.A.1307, 1JI1.A.2001, 2JKX.A.1641, 3K8K.A.710, 1L6R.B.903, 4LHN.A.302, 1LPZ.B.1, 3S18.A.229, 3TO5.A.131, 1UX7.A.1134, 3V6N.A.229, 3W57.A.202, 3W5N.A.1201, 2W86.A.1148, 2W86.A.1149, 2WQ8.A.1641, 2XFD.A.1111, 2XFE.A.1113, 2Z48.A.1102, 2Z49.A.1001, 3ZO9.A.1589
[1] "Cluster 7"
4A3X.A.1268, 4AFA.A.1267, 4AFB.A.1267, 4AOC.A.1129, 4AOC.E.1129, 4ASL.A.1268, 1BIW.B.803, 4CI7.A.1505, 4CP0.A.1294, 4CP1.A.1294, 3CQO.A.4004, 1DED.B.5004, 2ERV.A.195, 4G01.A.300, 4GWI.A.204, 1H3G.A.700, 1HFZ.B.124, 4I5L.B.601, 4IAI.A.402, 2J22.A.1150, 1K12.A.160, 3K8L.A.710, 2ML2.A.203, 2ML3.A.201, 2NZM.A.406, 1OUX.A.402, 4PIB.C.201, 3SRG.A.1357, 2VUD.C.1118, 4WF7.A.600, 2WR9.A.1131, 2Z49.A.1003
[1] "Cluster 8"
3RAX.B.1416, 4AFC.A.1267, 3AUJ.A.1603, 2BOI.A.300, 2BOI.B.600, 2BOJ.A.1116, 2BOJ.C.1117, 2BP6.A.802, 2BP6.C.805, 1BQB.A.353, 2BV4.A.300, 2BV4.B.300, 4CE8.A.998, 4CE8.B.998, 4COU.A.1270, 4COV.A.1269, 4COY.A.1270, 3CQO.A.4001, 3DCQ.A.116, 3DCQ.B.116, 3EHU.A.500, 3EIF.A.1, 1ESL.A.163, 2EWE.A.703, 2FPW.A.503, 1GEN.A.302, 2GVV.A.500, 2H2T.B.322, 3HLI.B.315, 2JDY.A.1116, 2JDY.B.1117, 2JDM.B.1115, 2JDM.C.1115, 2JDN.A.881, 2JDN.B.881, 2JDN.C.881, 4KVL.A.703, 1OUX.B.404, 1OVS.A.402, 1

0VS.B.404, 1PAM.A.688, 1SNN.B.503, 3SO0.A.97, 1SU3.A.904, 1V3E.A.4001, 2VNV.A.302, 2VNV.B.302, 2VUC.B.990, 2VUC.C.991, 3W5M.A.1201, 1WDC.C.501, 2WR9.D.1132, 1YDY.A.904, 3ZDV.A.200, 3ZDV.B.200, 1ZJA.A.7001

Table S59. 7-ligand Ca, compressed group

	size	largest_angle*	middle_1*	middle_2	middle_3
1	"65"	"172.2+/-2.6"	"69.5+/-3.5"	"73.8+/-2.9"	"76.5+/-2.9"
2	"105"	"174.7+/-2.3"	"70.9+/-2.8"	"75.2+/-2.1"	"78.2+/-2"
3	"136"	"166.9+/-2.3"	"69.3+/-3.5"	"74.6+/-2.7"	"77.2+/-2.7"
4	"117"	"161.3+/-2.5"	"73.3+/-3.3"	"75.7+/-2.3"	"77.2+/-2"
5	"27"	"156.4+/-4.9"	"67.3+/-4.2"	"71.2+/-3.7"	"73.6+/-3.4"
6	"132"	"169.1+/-2.7"	"72.5+/-3"	"76+/-2.3"	"78.1+/-1.9"
7	"184"	"172.8+/-2.6"	"73.5+/-2.1"	"75.8+/-1.7"	"77.8+/-1.3"
8	"169"	"164.3+/-2.6"	"73.8+/-2.4"	"75.9+/-2"	"77.7+/-1.6"
9	"67"	"167+/-4.6"	"51+/-2.2"	"71.4+/-4.1"	"75.4+/-3.4"
10	"75"	"159.3+/-4.3"	"51.7+/-2.6"	"70.7+/-5"	"74.1+/-3.7"

	middle_4	middle_5	middle_6	middle_7*	middle_8
1	"78.6+/-2.7"	"81+/-2.6"	"83+/-2.6"	"84.8+/-2.3"	"87.3+/-2"
2	"80.9+/-1.8"	"83+/-1.4"	"84.6+/-1.4"	"86.2+/-1.6"	"87.7+/-1.5"
3	"79.4+/-2.5"	"81.5+/-2.2"	"83.4+/-2"	"85.6+/-1.8"	"87.3+/-2"
4	"78.7+/-1.7"	"80.3+/-1.5"	"81.6+/-1.6"	"83+/-1.5"	"84.6+/-1.7"
5	"75.4+/-3.2"	"77.1+/-2.6"	"78.9+/-2.8"	"80.8+/-2.4"	"82.9+/-3.1"
6	"79.5+/-1.7"	"80.7+/-1.6"	"82.1+/-1.5"	"83.5+/-1.6"	"85.1+/-1.6"
7	"79.7+/-1.5"	"81.5+/-1.6"	"83.3+/-1.7"	"84.8+/-1.6"	"86.5+/-1.7"
8	"79.3+/-1.5"	"80.8+/-1.5"	"82.2+/-1.5"	"83.9+/-1.6"	"85.6+/-1.9"
9	"77.8+/-2.7"	"80.3+/-2.3"	"82.5+/-2.4"	"84.3+/-2.1"	"86.3+/-2.2"
10	"76.5+/-3.1"	"78.4+/-2.7"	"80+/-2.7"	"81.8+/-2.6"	"83.6+/-2.8"

	middle_9	middle_10	middle_11	middle_12	middle_13*
1	"89.1+/-2"	"91.2+/-2.5"	"93.9+/-3.2"	"98.4+/-3.2"	"105+/-3.1"
2	"89.2+/-1.4"	"90.9+/-1.2"	"92.6+/-1.5"	"94.1+/-2"	"96.4+/-2.2"
3	"89+/-1.9"	"90.9+/-1.9"	"93+/-2.1"	"96.4+/-2.5"	"99.4+/-2.3"
4	"86.1+/-1.9"	"88.2+/-2.5"	"91.2+/-3"	"97.3+/-4"	"107.4+/-2.8"
5	"84.6+/-3.4"	"86.5+/-3.4"	"91.4+/-4.8"	"97.5+/-5.3"	"112.6+/-6.1"
6	"87.1+/-1.9"	"89.6+/-2.3"	"92.6+/-3.2"	"98.2+/-3.9"	"105.3+/-2.5"
7	"88.5+/-1.6"	"90.2+/-1.6"	"92.4+/-2"	"95.4+/-2.1"	"98.6+/-2.3"
8	"87.4+/-2.1"	"89.5+/-2.2"	"92+/-2.5"	"96.7+/-2.7"	"100.3+/-2.1"
9	"88.3+/-2.4"	"91.3+/-2.9"	"94.1+/-3.2"	"98+/-3.6"	"103.9+/-3.8"
10	"86.1+/-3.1"	"89.3+/-3.6"	"93.6+/-4.4"	"99.3+/-4.7"	"109.3+/-5.4"

	middle_14	middle_15	middle_16	middle_17	middle_18
1	"108+/-3.3"	"113.5+/-3.2"	"119.7+/-6.3"	"144.7+/-7.7"	"157.7+/-4.3"
2	"100.5+/-4.5"	"122.7+/-4.3"	"129.2+/-5.3"	"146.6+/-3.8"	"154.9+/-4.4"
3	"105.9+/-5.3"	"120.3+/-5.1"	"127.5+/-5.6"	"145.9+/-5.5"	"155.4+/-4.9"
4	"113.6+/-4"	"123+/-4.5"	"127.5+/-4.8"	"146.5+/-5.7"	"151.9+/-4"
5	"120.6+/-5.8"	"126.5+/-4.7"	"131.1+/-4.5"	"139.4+/-4.3"	"144.7+/-4.7"
6	"109.3+/-3.3"	"119.5+/-4.2"	"125.9+/-5.1"	"146+/-5"	"153.9+/-4.9"
7	"103.2+/-3.8"	"125.8+/-3.4"	"130.8+/-3.4"	"148+/-3.4"	"152.1+/-2.8"
8	"109+/-5.2"	"124.6+/-3"	"129.3+/-3.9"	"148.6+/-4.1"	"153+/-3.1"
9	"110.5+/-5.6"	"120.9+/-5.1"	"130.2+/-5.5"	"140.3+/-4.4"	"152.5+/-6.1"
10	"116.7+/-5.1"	"123.5+/-4.7"	"129.8+/-4.9"	"142.3+/-5.7"	"149.2+/-5.4"

	middle_19*	smallest_opposite_angle*	PentagonalBipyramidal

1	"167.7+/-2.9"	"51+/-2.6"	"0.048"
2	"161.6+/-2.7"	"51.4+/-1.8"	"0.124"
3	"162.3+/-2.5"	"51+/-2.1"	"0.121"
4	"156.2+/-2.9"	"51.6+/-2"	"0.15"
5	"148.4+/-4.4"	"50.2+/-2"	"0.047"
6	"159.8+/-2.7"	"51.1+/-1.9"	"0.104"
7	"155.7+/-2.4"	"52+/-1.9"	"0.215"
8	"156.2+/-2.6"	"51.8+/-1.8"	"0.172"
9	"160.7+/-4.3"	"70.3+/-4.7"	"0.071"
10	"153.6+/-4.4"	"71.7+/-4.5"	"0.074"

	SquareAntiprismaticV	HexagonalBipyramidalVA	HexagonalBipyramidalVP
1	"0.19"	"0"	"0.108"
2	"0.081"	"0"	"0.067"
3	"0.132"	"0"	"0.078"
4	"0.233"	"0"	"0.046"
5	"0.085"	"0"	"0.006"
6	"0.167"	"0.001"	"0.062"
7	"0.158"	"0"	"0.081"
8	"0.196"	"0"	"0.055"
9	"0.129"	"0.001"	"0.046"
10	"0.173"	"0.001"	"0.029"

Table S60. Cluster members of 7-ligand Ca, compressed group

[1] "Cluster 1"
2ATL.B.1415, 4J2D.A.1002, 4KHU.A.1002, 2ODI.B.702, 2Q10.B.702, 3QEP.A.905, 3QER.A.905, 1ANW.B.353, 1AX0.A.290, 1BTU.A.260, 2C0T.A.1507, 1C8T.B.264, 3CLN.A.152, 1D2S.A.401, 1DB5.A.198, 1DBN.A.301, 2EIG.A.1102, 3EU3.A.1, 3FCS.A.2004, 1FIB.A.500, 2FMD.A.301, 1FX5.A.251, 4FZM.A.301, 1G7Y.A.254, 2GKO.A.610, 1GO8.P.1482, 1GSL.A.251, 1H3G.B.701, 1H6X.A.1162, 1HQL.A.302, 3HR4.D.202, 4I35.A.514, 2JE7.A.1241, 2JEC.A.240, 1JX9.B.601, 1LEN.A.184, 1LHW.A.401, 3LNP.A.472, 1LOC.A.228, 2LTN.A.191, 2ML2.A.204, 2ML3.A.202, 1MVQ.A.238, 3N35.A.290, 1NIW.G.1014, 1NLS.A.240, 2OVU.A.238, 2P2K.A.239, 1QNW.A.302, 1QPK.A.451, 1QX2.A.1005, 1R1Z.A.285, 1RLW.A.401, 4U36.A.302, 3U4X.A.237, 1UKG.A.1262, 1UP8.A.599, 2UWP.A.1246, 1UX6.B.2009, 1VCL.A.1002, 1WC5.A.2100, 3WCS.A.1003, 2YFS.A.1712, 2ZVD.C.620, 3ZYR.A.401
[1] "Cluster 2"
3AVW.A.3001, 2VBO.A.1155, 2VS7.A.1183, 3ACH.A.204, 2AEF.A.602, 4AQA.A.1209, 4AQE.A.1207, 4AQO.A.1881, 1AVA.A.500, 4AVS.A.207, 4BQ2.A.1797, 2C4X.A.1252, 2CBL.A.352, 2CFT.A.1297, 2CM5.A.1679, 2CM6.A.1679, 2CM6.A.1680, 3DEM.A.5001, 3DNZ.A.403, 2E26.A.605, 3EAD.B.1003, 3ECQ.A.2001, 1EDM.B.2, 2ERO.A.702, 2EXH.B.2002, 2FI1.A.191, 1FNY.A.500, 1G8K.A.5008, 1GCA.A.351, 4GER.A.403, 1GPL.A.500, 1HDF.A.1102, 4HHO.A.401, 1HPL.A.960, 3HX4.A.602, 2HYV.A.607, 3IBZ.A.193, 3IK2.A.514, 2J7A.A.1006, 1J83.A.4001, 4JDZ.B.701, 1KQU.A.301, 3KQR.A.205, 1KVO.A.191, 3KWU.A.901, 4LOR.A.301, 4LOS.A.401, 1LPB.B.453, 1LQV.C.34, 4LXO.A.1601, 4LXO.B.1601, 3M1H.A.2002, 4MGQ.A.601, 3N5A.A.101, 1NPC.A.321, 2NXP.D.603, 1OAC.A.803, 1OAH.A.1525, 3OEA.A.200, 3OM6.A.1, 3OM7.A.1, 4OMC.A.605, 4OY7.G.302, 1P8J.A.3001, 3PDD.A.193, 3PGB.A.902, 3POJ.A.1, 2PQX.A.500, 2Q17.A.315, 3QR0.A.1000, 2QUB.A.617, 1R64.A.701, 1RLW.A.400, 3S5U.A.221, 1SH7.A.1291, 1SL8.A.670, 3SRE.A.1356, 1SU4.A.995, 3T3P.B.2002, 1TU5.A.903, 1UX6.B.2008, 1UX6.B.2011, 1V0A.A.1177, 2V0B.A.1241, 3V9M.A.205, 3VLV.A.503, 2VPT.A.1235, 3VRR.A.401, 2W08.A.206, 3WCT.F.203, 2WN3.A.400, 2WN3.C.400, 2WZE.B.1552, 1YO8.A.1189, 1Z70.X.3002, 2Z8X.A.618, 2Z8X.A.621, 2Z8X.A.6

25, 2ZEX.A.406, 2ZKM.X.800, 2ZUX.A.635, 2ZUX.A.636, 2ZUY.A.625, 2ZYH.A.700, 2ZZJ .A.239
[1] "Cluster 3"
4E3S.A.1002, 3EH8.A.301, 4J2E.A.1002, 3KHR.B.1416, 3MX9.A.363, 3MXB.A.175, 3NCI. A.905, 3NDK.A.905, 3NE6.A.904, 3NGI.A.905, 3QNO.A.903, 4UB4.A.401, 4UB4.A.402, 2 A3Y.A.601, 1AJP.B.558, 1AYP.A.301, 2AYH.A.417, 4AYU.D.205, 2AZZ.A.125, 3B2Z.A.2, 3B4N.B.701, 1BF2.A.751, 2BWR.A.500, 3BYK.A.474, 2C10.B.1777, 4CAG.A.603, 4CBU.G .1151, 2CCM.A.1194, 3CIO.K.402, 1D0L.A.400, 4D0E.A.1532, 2DBX.D.702, 1DBI.A.703, 4DIR.A.101, 1DJX.B.2, 4DKB.A.301, 2E26.A.601, 2E26.A.602, 3E9T.B.4, 3ETO.A.2001 , 3EXM.A.301, 3FLP.A.301, 3FLP.A.302, 1G8K.C.5108, 1G9K.A.705, 1GK9.B.1579, 1GKF .B.1571, 1GO7.P.1482, 3GPE.A.501, 2GSK.A.1, 4H2B.A.604, 2H61.D.817, 3HB2.P.481, 4I2Y.A.503, 4IHM.A.406, 2II1.C.400, 4ITC.A.1201, 4IU3.B.301, 1J1A.A.201, 1J9L.A. 1301, 4JX1.E.505, 1K9I.I.1201, 1K9J.A.403, 4K90.A.702, 1KAP.P.621, 3KM5.B.2011, 1KVW.A.124, 3KWU.A.902, 2KYF.A.109, 3L2Y.A.301, 4LMH.A.811, 3LPC.A.360, 3LPC.A.3 62, 4LP7.B.301, 1LVU.A.8001, 1LVU.D.8002, 3M1H.A.2001, 4M5E.A.505, 1MCX.A.352, 1 MKU.A.124, 1MKV.A.124, 3MVS.A.216, 4N2L.A.707, 1N47.B.252, 4NEH.A.1101, 2NXP.C.6 02, 2NZM.A.405, 2O72.A.402, 1O8F.A.1353, 1O8P.A.1149, 3OM2.A.1, 3OM4.A.1, 3OM5.A .1, 4OMC.A.606, 4OMC.A.607, 1OYG.A.500, 5P2P.A.125, 4P99.B.517, 2PEL.A.237, 4PHJ .A.302, 4PKI.A.403, 2PMY.B.104, 1PT2.A.500, 3PVN.M.5026, 1QI3.A.451, 2QUB.A.621, 3R5V.B.201, 2R8Z.N.214, 1RU4.A.1, 1S01.A.295, 1S1E.A.226, 1S6C.A.218, 1SL4.A.40 7, 1THM.A.302, 3TI7.A.354, 3TI9.A.354, 1TM7.E.470, 4TNC.A.164, 3UJO.B.304, 4UM9. A.2001, 1UNE.A.124, 1UWW.A.1192, 3VV3.A.402, 1WBF.A.303, 1WC0.A.2100, 4WJK.B.502 , 4WK7.A.502, 2WW8.A.1002, 1Y1X.A.201, 1Y6O.A.207, 2YAY.A.1266, 2YOA.A.1002, 1YX H.A.1001, 2ZUX.A.632, 2ZUY.A.622, 2ZVD.A.628
[1] "Cluster 4"
4DTM.A.1002, 2FLD.B.602, 3RAX.A.416, 2WTF.A.1511, 2AAO.A.295, 2AAO.A.296, 3AIE.A .4001, 4APX.B.1240, 1AVS.A.94, 3BOO.A.124, 1B8R.A.109, 3BFM.A.235, 2CCL.B.1061, 1CJY.A.950, 4CPV.A.109, 2CT9.A.301, 2CT9.A.302, 4CT3.A.1170, 3CZT.X.93, 3DBK.A.3 03, 2DIE.A.780, 3DO1.A.401, 1DTL.A.203, 1DVI.A.273, 2E3X.A.802, 1EXR.A.1000, 4F0 Z.B.201, 4F0Z.B.204, 1FAT.A.255, 1FZD.A.1, 1G8I.A.1599, 3GDC.A.401, 1GGZ.A.149, 2GGM.A.401, 4GGF.A.101, 4GM5.A.404, 2GSK.A.2, 2GXS.A.601, 4H2A.A.805, 2HQ8.A.202 , 3HZ3.A.1, 2I7A.A.2, 4ICB.A.77, 4IK8.A.502, 4IU3.B.302, 2J1G.B.1290, 1JC9.A.301 , 4JWQ.A.202, 4K70.B.1003, 1K9K.A.400, 4KKK.A.701, 3KLK.A.1, 3KLL.A.1, 3KQR.C.20 6, 4KWU.A.1109, 3LI6.A.149, 3LI6.D.150, 3LNI.A.302, 4LVN.A.702, 4M7H.A.501, 1MDW .B.4, 3MHZ.A.736, 2ML1.A.205, 4MNO.A.302, 3MVS.A.212, 4N5X.A.201, 4N5X.A.205, 2O 04.A.402, 3OOV.A.1, 2O5G.A.401, 2O5G.A.402, 2O5G.A.403, 2OBH.A.1001, 1OHZ.B.1058 , 2OLG.A.2001, 2OZN.B.402, 3P4G.A.413, 4P5X.A.1001, 5PAL.A.110, 4PET.A.403, 2PVB .A.111, 2Q1F.A.2001, 3Q5I.A.525, 3QRX.A.171, 1QTX.A.152, 2R9F.A.366, 2RHP.A.29, 3RUP.A.1006, 1RWY.A.422, 1SBF.A.601, 1SL7.A.301, 1SRA.A.302, 1TCF.A.163, 1TN4.A. 162, 3U1R.A.702, 3UBG.A.902, 3UCP.A.912, 3UL4.B.66, 4UZU.A.1483, 2VN6.B.1067, 2W 46.A.1148, 2W87.A.1149, 3WA5.A.504, 3WH2.A.302, 3WHT.B.501, 2WNP.F.1298, 1WP6.A. 502, 2Y3N.B.1068, 2YA2.A.1691, 1YO8.A.1186, 1YO8.A.1187, 1YO8.A.1190, 1Z3J.A.267 , 2Z30.A.1004, 1ZCM.A.1001, 2ZFD.A.229, 2ZWP.B.402
[1] "Cluster 5"
4DU1.A.1003, 4DU3.A.1002, 4BY5.A.1184, 3D1M.A.2, 3ECQ.B.2000, 3FZO.A.400, 4GER.A .402, 3GIS.Y.1002, 1HVX.A.516, 2HYW.A.501, 4I75.A.401, 3IJ9.A.497, 1IOD.A.501, 4 K70.A.1003, 3KF9.C.302, 1KIC.B.328, 1KXO.C.703, 4LMF.D.303, 3N1F.A.6, 3N1G.B.190 , 4NHF.F.301, 1S2N.A.1290, 3S5U.D.221, 2VUD.D.1118, 3WNP.A.801, 1YO8.A.1204, 2ZW O.A.402
[1] "Cluster 6"
4DTU.A.1002, 4ED0.A.503, 4FK0.A.1002, 4K4G.A.603, 3QEV.A.905, 4QWA.A.403, 1SOM.A .402, 4UAW.A.402, 1A25.B.290, 3A4U.A.286, 1A75.A.109, 1A8B.A.407, 4AE2.A.246, 5A ER.A.200, 4AQA.A.1208, 4AQE.A.1209, 3BOX.A.579, 3B8Z.A.903, 2BIB.A.1552, 4BJ0.A. 1167, 1BU3.A.109, 3C1Q.A.2, 4CFQ.A.501, 2CM5.A.1678, 4DIR.A.102, 1DTL.A.201, 2DU

R.A.1, 4DZ3.A.202, 4DZ3.B.202, 2E4T.A.701, 1E8A.A.1089, 2E85.A.1002, 1EDH.A.302, 2EGD.A.301, 2EWE.A.701, 2FH1.A.2001, 1FS7.A.651, 1FXH.B.1002, 1G9K.A.704, 4GGF.C.204, 2GJP.A.1488, 4GUK.A.207, 1GZC.A.290, 1H2G.B.1558, 3HB2.P.484, 3HJR.A.602, 4HPH.A.701, 1HT6.A.500, 1HT9.A.1001, 1HVX.A.518, 3HX4.A.603, 2HYW.A.503, 4I35.A.515, 3I57.B.186, 4IAI.A.401, 4ICB.A.76, 3IPV.A.252, 4IT5.D.202, 2J1V.A.1152, 2J5Z.A.1277, 4JA8.A.503, 1JIA.A.134, 1JIW.P.485, 4JJJ.A.704, 1JK3.A.404, 3JXS.A.301, 1K7I.A.480, 1K94.A.998, 4KTY.A.801, 4KTY.A.803, 3L2Y.C.302, 1L9N.A.700, 1L9N.A.702, 3LCP.C.159, 4M00.A.801, 1MCX.A.349, 3MVS.A.213, 4N1G.A.204, 3N5A.A.102, 1NIW.A.1004, 3NOL.A.263, 3NQZ.B.520, 3OOW.A.377, 1OFL.A.528, 4OKH.A.904, 4OKH.B.901, 3OX6.B.502, 3P4G.A.411, 3P4G.B.405, 4P99.A.514, 4PEO.A.102, 4PEU.A.401, 3PPE.A.402, 3PPE.A.403, 1Q3A.A.468, 3Q5I.A.524, 2QNG.A.201, 3QRB.A.302, 3QRX.A.172, 2QUB.A.619, 2QUB.G.616, 2QV6.A.302, 1RP8.A.500, 1RRO.A.109, 1S6B.B.402, 3TI9.A.355, 4UM9.A.2003, 3USU.C.266, 1UX6.B.2001, 1UX6.B.2005, 1UX7.A.1135, 1UY4.A.1147, 1UYZ.A.1132, 4UZU.A.1485, 4V29.B.1179, 2VN5.B.102, 2WBX.A.1103, 2WD6.A.1765, 1WKY.A.503, 4WK7.A.503, 3WN6.A.503, 2WZ8.A.1149, 2XOM.A.1169, 1Y1X.A.202, 2Y3N.B.1067, 2Y5I.A.101, 1YO8.A.1198, 1YUT.A.197, 2Z2X.A.1003, 2Z8X.A.619, 2Z8X.A.627, 2Z8Z.A.623

[1] "Cluster 7"

4AQU.A.1154, 4AQU.A.1156, 1G9Y.B.802, 3ODH.B.195, 2VBJ.A.1154, 2VBJ.A.1155, 2VBO.A.1154, 1A75.A.110, 1A8A.A.407, 3AMR.A.905, 3AMR.A.907, 4APX.B.1238, 1AVS.A.93, 2B96.A.124, 3BEU.A.249, 3BI1.A.1753, 3BNG.A.508, 1BQB.A.352, 1BSW.A.900, 2C26.A.302, 3C3Y.A.238, 4CAG.A.604, 4CAG.A.610, 1CB8.A.3000, 4CBU.G.1150, 2CDO.A.1139, 3CHJ.A.338, 3CHK.A.503, 3CIP.G.201, 2CKI.A.997, 2CN3.A.1778, 4CTE.B.280, 1CVR.A.504, 1DBI.A.701, 1DBI.A.704, 3DEM.A.8001, 4DUQ.A.101, 1DVI.A.271, 2DWO.A.701, 2DWO.A.703, 2E26.A.604, 2EHB.A.1001, 1EXR.A.1004, 4F0Z.B.202, 4F0Z.B.203, 3FED.A.1755, 2FMJ.A.338, 3FO3.A.527, 2FVY.A.311, 3G5C.A.801, 1G5N.A.401, 1G9G.A.630, 1G9K.A.701, 3GBO.A.301, 1GCY.A.528, 1GGZ.A.150, 1GGZ.A.151, 3GPE.A.502, 1H6Y.A.1161, 1H80.A.1493, 3HDB.A.621, 3HI7.A.803, 1HQV.A.998, 2HQ8.A.201, 3HX4.A.601, 1HY7.A.304, 2HYV.A.605, 4I35.A.511, 2ID4.A.906, 3IGO.A.602, 3IKW.A.1, 4IMM.A.407, 2IPL.A.502, 4ITC.A.1202, 4JGU.A.901, 1JI1.A.2003, 3JQ1.B.1, 3K21.A.192, 1K96.A.91, 1KAP.P.614, 3KCP.A.701, 3KHE.A.192, 3KHE.A.195, 3KM5.A.2012, 4LDC.A.502, 1LE6.A.461, 4LJ3.A.402, 4LM8.A.811, 3LPA.A.361, 4M2P.A.301, 4M5E.A.506, 1M8T.A.1001, 4MEW.A.501, 4MNO.A.303, 1MPX.A.638, 3MSE.B.180, 4N1G.A.202, 4N2B.A.709, 4N2G.A.704, 4N2N.A.704, 4N5X.A.203, 1N9E.A.803, 1NNL.A.2001, 3NQX.A.511, 4NUQ.A.303, 2O72.A.401, 2O9Q.A.501, 1OAH.A.1526, 1OF3.A.1174, 1OH4.A.1186, 1OHZ.B.1057, 2OW9.B.610, 3OX6.B.501, 3P6B.A.205, 4P99.C.505, 3PF2.A.1, 4PHN.B.303, 4PKG.G.1201, 4PLS.B.301, 3PM8.A.1, 1PMH.X.300, 1POA.A.201, 1POC.A.501, 2PPL.A.478, 1PVA.A.111, 3Q2N.A.303, 3Q5I.A.1, 3QGM.A.503, 2QQM.A.1, 3QRB.A.303, 2QUB.A.620, 1R55.A.515, 2RAN.A.324, 2RDZ.A.1501, 2RF7.A.1501, 1RRO.A.110, 1S3P.A.210, 1S6B.A.401, 2SCP.A.192, 1SGT.A.246, 3SIB.A.221, 3SJG.A.1753, 1SL6.D.3, 1SL6.E.3, 1SL8.A.669, 1SL8.A.671, 3SNY.A.97, 3SXQ.A.526, 1T44.G.702, 1TF4.A.3002, 3TOY.A.361, 4TSH.B.1501, 3TZ1.A.1, 3U1R.A.701, 3UBH.A.857, 3UXF.A.2, 4V29.B.1178, 2VDQ.B.2002, 1VL9.A.124, 2VVE.A.1336, 1WON.A.1132, 3W57.A.201, 3W57.A.203, 1W7C.A.803, 3WLC.A.501, 2WPH.S.1246, 3WU2.c.901, 2WW8.A.1001, 4XDQ.A.301, 2XFG.B.1619, 2XFD.A.1112, 1XJO.A.905, 1XVJ.A.422, 1Y4J.A.1001, 2Y6L.A.1168, 2YIH.A.1520, 2YKK.A.1520, 2YN5.A.6364, 1YO8.A.1200, 2YOA.A.1001, 2ZEZ.A.200, 2ZFD.A.227, 2ZFD.A.228, 2ZP4.A.124

[1] "Cluster 8"

1G9Y.A.801, 4KLD.A.403, 3MQ6.A.1, 3QNN.A.903, 3A24.A.1268, 1A2Q.A.295, 3A8R.A.401, 2AAO.A.293, 1AF0.A.476, 1AK9.A.295, 3AKB.A.171, 3AKB.A.172, 3AKB.A.175, 3AMR.A.908, 4AQ1.A.1923, 4AQ1.C.1923, 4AQJ.A.1097, 1AXN.A.353, 3BC9.A.701, 1BH6.A.501, 1BJF.A.402, 1BLI.A.500, 1BQB.A.351, 1BU3.A.110, 4BY5.A.1183, 4BY5.A.1185, 3C1Q.B.1, 2C4X.A.1253, 2C4F.L.1143, 1C9N.A.277, 2CCL.B.1060, 2CCM.A.1192, 4CCD.A.3669, 2CDP.A.1139, 4CFQ.A.502, 4CFY.A.301, 1CP9.B.554, 4CPV.A.110, 1CRU.A.501, 4DA2.A.301, 2DCK.A.1001, 4DUQ.A.102, 2DWO.A.702, 1E43.A.502, 1E8A.A.1090, 3EDF.A.602

, 2EGD.A.302, 3ETO.A.2003, 1EXR.A.1002, 2FH1.A.2002, 3FIA.A.201, 1G8I.B.1600, 1G
9K.A.702, 4GGF.L.204, 2GJP.A.1487, 4H1Q.B.304, 3HDB.A.622, 3HJR.A.601, 2HQ8.A.20
3, 3HX4.A.604, 3HX6.A.1, 4I2Y.A.501, 1I82.A.192, 2ID4.A.901, 4IEF.B.702, 3IGO.A.
603, 1J55.A.102, 3JQW.A.1001, 3K21.A.193, 3K21.A.194, 1K94.A.999, 1K9U.A.1001, 1
K9U.A.1002, 1KAP.P.615, 4KDW.A.201, 3KHE.A.193, 3KHE.A.194, 3LND.B.208, 3LPC.A.3
61, 2LRP.A.202, 1MCX.A.351, 4MSP.A.201, 1MXE.A.506, 4N1G.A.203, 3N4E.B.500, 4N5X
.A.204, 4NEH.A.1103, 1NIW.C.1007, 1NPC.A.319, 3O4Y.A.198, 2O5G.A.404, 2O8O.A.100
9, 1OBO.A.502, 4OKH.A.902, 2OO4.A.5001, 2OPO.A.301, 2OPO.A.302, 2OX9.A.804, 2POR
.A.1002, 5PAL.A.111, 4PEO.X.103, 4PHJ.A.303, 4PHJ.A.304, 3PM8.A.514, 3PM8.B.1, 1
PMJ.X.300, 1POB.A.801, 1POE.A.801, 1PVA.A.110, 3PVN.E.5009, 2PVB.A.110, 3Q2L.A.7
01, 3Q5I.A.526, 1Q5P.A.271, 4Q6O.A.502, 2QQO.A.1, 1QTX.A.153, 1QTX.A.154, 1QX2.A
.1001, 1QX2.B.1006, 1ROR.E.302, 2R2I.A.500, 2R2I.A.501, 2R2I.A.502, 1RFJ.A.1001,
1RFJ.A.1003, 1RFJ.A.1004, 3RM1.A.102, 1RWY.A.421, 1SO2.A.276, 1S6C.A.217, 2SCP.
A.190, 2SCP.A.191, 3SIB.A.222, 1ST3.A.270, 1SUD.A.295, 1TCF.A.160, 1THM.A.301, 3
TI7.A.353, 3TI7.A.355, 3TI9.A.353, 1TKF.A.905, 5TNC.A.163, 3TTQ.A.2867, 3UL4.B.6
7, 1UXX.X.1130, 1VCL.A.1001, 1VCL.A.1005, 2VVD.A.1328, 3VYV.A.303, 2VZP.A.1128,
2W47.A.1137, 3WFD.B.806, 3WHI.A.401, 2WND.A.102, 2WNO.A.1251, 1WPC.A.501, 1X1J.A
.2000, 3X17.A.603, 1Y93.A.267, 1Y9Z.A.603, 1YU6.A.401, 1YUT.B.199, 2Z2X.A.1002,
2Z30.A.1005, 2ZN9.A.901, 2ZUX.A.631, 2ZWP.A.404, 3ZYP.A.1220
[1] "Cluster 9"
3FD2.A.374, 4K4G.I.603, 4K4H.A.604, 1N48.A.501, 3NAE.A.906, 3S9H.A.905, 3SCX.A.9
06, 3SQ2.A.904, 2AAO.A.294, 1AFD.1.2, 3AMR.A.901, 1ANN.A.320, 4ANR.A.400, 3B2Z.B
.2, 1BJF.A.403, 2COT.B.1507, 3C7F.A.803, 1CGE.A.304, 1DB4.A.198, 1E43.A.504, 3EK
I.A.601, 4ELG.B.202, 4ELG.C.202, 2ERO.A.701, 3FCS.A.2008, 2FH1.A.2003, 3FLT.A.30
1, 4GON.A.202, 4GKY.A.302, 1HDH.A.1528, 4I2Y.B.504, 4IRZ.A.2006, 1J55.A.101, 3KE
Z.B.501, 4KHO.A.1001, 1KP4.A.200, 2MLR.A.303, 3NOK.A.257, 1O9I.A.269, 1O9I.E.269
, 2P69.A.305, 4P99.A.515, 3PDD.A.194, 4RDQ.A.504, 4RDQ.B.502, 4RGP.B.302, 1SRA.A
.301, 3T3P.A.2005, 3T3P.A.2006, 3T3P.A.2007, 1TMQ.A.1001, 4U65.E.302, 1UX6.B.200
6, 2VNG.A.1213, 1W0Y.H.1259, 2WG7.A.1126, 4WKO.A.502, 4WKO.A.504, 2Y8K.A.1526, 2
YHG.A.1936, 1YO8.A.1194, 1YO8.A.1207, 2ZFD.A.230, 2ZUX.A.633, 2ZUX.A.634, 2ZUY.A
.623, 2ZZV.A.501
[1] "Cluster 10"
3NHG.A.908, 3QEW.A.905, 3QEX.A.905, 1B1G.A.76, 1BJF.A.404, 3BJU.A.606, 1BLI.A.70
0, 2BPE.A.1245, 4COK.A.1615, 4CAJ.C.1323, 2CCM.A.1193, 4CGT.A.685, 2CHN.B.1716,
3CIO.K.401, 1CXE.A.690, 4DH2.B.101, 4DH2.B.102, 2DIJ.A.689, 4DLK.A.401, 3DSL.A.2
, 1DTL.A.202, 1EO7.A.689, 1ESP.A.319, 3ETO.A.2002, 1EXR.A.1001, 3FAW.A.4, 1FIF.C
.2, 1GOH.A.291, 1GGZ.A.152, 4HEX.A.204, 1HQV.A.997, 1HT6.A.501, 3IUC.A.2, 2IWA.A
.501, 4JRF.A.601, 3KCP.A.702, 3KF9.A.301, 1KXT.A.4001, 4L03.B.502, 3LND.A.209, 4
MNO.A.301, 4MSP.A.202, 3NJH.C.503, 4NUQ.A.302, 2OO4.A.5002, 4P5F.B.501, 1PVY.A.5
03, 4QB2.A.202, 1R17.A.599, 4R1D.A.601, 3TRP.A.358, 4U6B.A.501, 3U8D.A.202, 3UBG
.A.901, 3UBH.A.856, 1UKT.B.690, 4UM9.A.2002, 4UM9.A.2004, 1UZJ.B.2648, 4V29.A.11
78, 1V3J.A.688, 2VN5.B.101, 2VN6.B.1066, 1WOP.A.1779, 3WHT.B.502, 4WJK.A.503, 3W
NO.A.801, 1WPC.A.503, 2WVZ.A.800, 1X2T.A.603, 1XO5.A.5, 2Y5I.A.102, 2YEQ.A.1525,
1YO8.A.1210, 3ZHG.A.1323

Table S61. 7-ligand Ca, combined group

	size	largest_angle*	middle_1*	middle_2	middle_3
1	"64"	"157.6+/-5.9"	"52.5+/-3.7"	"64.3+/-5.9"	"69+/-4.3"
2	"159"	"168.9+/-2.9"	"72.4+/-2.8"	"75.8+/-2.2"	"78+/-1.9"
3	"250"	"164.7+/-2.6"	"73.3+/-2.5"	"75.8+/-1.9"	"77.7+/-1.7"

4	"124"	"160.2+/-3.7"	"71.7+/-4.1"	"74.6+/-3.1"	"76.2+/-2.8"
5	"115"	"172.1+/-3.5"	"70+/-2.1"	"72.5+/-1.6"	"74.6+/-1.7"
6	"241"	"173.6+/-2.6"	"73.1+/-2.3"	"75.8+/-1.7"	"77.9+/-1.4"
7	"64"	"168.4+/-4.9"	"53.8+/-4.8"	"66.8+/-7.8"	"71.6+/-6.5"
8	"129"	"163.9+/-5.5"	"51.2+/-2.1"	"70.5+/-6.2"	"75.2+/-3.5"
9	"117"	"158.3+/-4.9"	"70.4+/-3"	"73+/-2.6"	"74.8+/-2.1"
10	"150"	"171.2+/-3.5"	"68.6+/-3.4"	"74.3+/-2.8"	"77.3+/-2.9"
11	"105"	"163.2+/-3.8"	"70+/-2.4"	"72.6+/-2.2"	"74.5+/-1.6"

	middle_4	middle_5	middle_6	middle_7*	middle_8
1	"72.1+/-4.1"	"75.1+/-3.8"	"77.4+/-3.6"	"80.3+/-3.6"	"83.4+/-4.1"
2	"79.5+/-1.6"	"80.9+/-1.7"	"82.4+/-1.5"	"83.9+/-1.6"	"85.6+/-1.7"
3	"79.4+/-1.5"	"80.9+/-1.4"	"82.4+/-1.5"	"84.1+/-1.6"	"85.8+/-1.8"
4	"77.9+/-2.5"	"79.5+/-2.2"	"81+/-2.2"	"82.6+/-1.8"	"84.3+/-2.4"
5	"77.2+/-2.1"	"80.7+/-2.1"	"82.9+/-2.1"	"84.7+/-1.9"	"86.5+/-1.8"
6	"80+/-1.7"	"81.9+/-1.6"	"83.7+/-1.7"	"85.1+/-1.7"	"86.8+/-1.7"
7	"74.4+/-6.1"	"77+/-5.2"	"79.4+/-5.4"	"82.3+/-4.6"	"85.4+/-4.1"
8	"77.7+/-2.5"	"79.8+/-2.4"	"81.6+/-2.5"	"83.3+/-2.4"	"85.3+/-2.6"
9	"76.5+/-1.7"	"77.7+/-1.6"	"79+/-1.7"	"80.4+/-1.7"	"82+/-1.9"
10	"80+/-2.6"	"82.2+/-2.2"	"84.1+/-1.8"	"86.1+/-1.8"	"87.7+/-1.9"
11	"76.6+/-1.8"	"78.3+/-1.7"	"80.2+/-1.8"	"82.1+/-1.9"	"84.1+/-2.1"

	middle_9	middle_10	middle_11	middle_12	middle_13*
1	"86.4+/-5.2"	"91+/-6"	"95+/-6.3"	"102.7+/-7.6"	"114.4+/-5.7"
2	"87.3+/-1.9"	"89.6+/-2.2"	"92.2+/-2.8"	"97.9+/-3.5"	"105.7+/-2.6"
3	"87.7+/-2"	"89.6+/-2.1"	"92.1+/-2.5"	"96.5+/-2.6"	"100.4+/-2.4"
4	"85.8+/-2.5"	"87.9+/-2.9"	"91.6+/-3.5"	"97.6+/-4.3"	"108.3+/-3.8"
5	"88.5+/-1.6"	"90.2+/-1.7"	"92.1+/-2.1"	"95.2+/-2.5"	"98.2+/-3"
6	"88.6+/-1.5"	"90.4+/-1.5"	"92.5+/-2.1"	"95.1+/-2.3"	"97.9+/-2.6"
7	"88.6+/-3.8"	"91.9+/-4.2"	"96.6+/-5.3"	"100.9+/-5.2"	"105.1+/-5.3"
8	"87.5+/-2.7"	"90.3+/-3"	"93.6+/-3.4"	"98.4+/-3.9"	"105.9+/-5"
9	"84.4+/-2"	"86.7+/-2.3"	"89.8+/-3.1"	"99.7+/-5.2"	"113.8+/-3.6"
10	"89.3+/-1.8"	"91.2+/-1.9"	"93.4+/-2.3"	"96.3+/-2.9"	"99.5+/-3.3"
11	"86.6+/-2.2"	"89.1+/-2.4"	"92.1+/-3.1"	"96.9+/-3.8"	"104.4+/-3.7"

	middle_14	middle_15	middle_16	middle_17	middle_18
1	"120.1+/-6"	"125.3+/-4.9"	"131.1+/-5.1"	"137.6+/-5"	"143.5+/-5.3"
2	"109.7+/-3.2"	"118.1+/-4.4"	"124.8+/-6"	"146.3+/-5.1"	"154.8+/-4.9"
3	"108.5+/-5.2"	"124+/-3.4"	"129+/-4.2"	"148.1+/-4"	"153.4+/-3.3"
4	"114.9+/-4.9"	"124.1+/-4.7"	"128.6+/-4.9"	"144.5+/-6.3"	"150+/-4.8"
5	"102.9+/-4.6"	"135.3+/-4.8"	"139+/-2.7"	"142.6+/-2.3"	"147+/-3.2"
6	"102.1+/-4"	"125+/-3.7"	"130.2+/-3.9"	"148+/-3.4"	"152.9+/-3.3"
7	"109.8+/-6.1"	"119.7+/-6.1"	"127.5+/-6.4"	"136.5+/-7.6"	"147.9+/-8.1"
8	"113.1+/-5.9"	"122.2+/-4.9"	"129.3+/-5.2"	"141.5+/-5.5"	"151.5+/-5.5"
9	"119.9+/-4.7"	"126+/-5.8"	"133.2+/-4"	"138+/-3.6"	"147.5+/-4.5"
10	"104.6+/-5.1"	"118.4+/-5.8"	"125.5+/-7"	"145.3+/-5.8"	"156.2+/-5.2"
11	"112.6+/-5.4"	"132+/-6.2"	"136.5+/-4"	"140.8+/-3.4"	"146.9+/-3.9"

	middle_19*	smallest_opposite_angle*	PentagonalBipyramidal
1	"148+/-4.2"	"57.6+/-7.6"	"0.017"
2	"161.4+/-3.4"	"51.2+/-2.2"	"0.097"
3	"157.4+/-2.8"	"51.6+/-1.7"	"0.174"
4	"153.8+/-4.2"	"51.5+/-2.2"	"0.159"
5	"150.1+/-3.7"	"66.9+/-3"	"0.21"
6	"157+/-2.8"	"51.7+/-1.8"	"0.204"
7	"160.3+/-5.7"	"51.8+/-4.6"	"0.031"
8	"158.2+/-4.6"	"72.1+/-4.1"	"0.089"
9	"153.4+/-5.2"	"68.6+/-3.4"	"0.123"

10	"165.2+/-3"	"50.9+/-2.2"	"0.073"
11	"151.3+/-4.9"	"67.4+/-2.6"	"0.243"

	SquareAntiprismaticV	HexagonalBipyramidalVA	HexagonalBipyramidalVP
1	"0.106"	"0.006"	"0.012"
2	"0.173"	"0.001"	"0.08"
3	"0.178"	"0"	"0.07"
4	"0.217"	"0.001"	"0.056"
5	"0.154"	"0"	"0.095"
6	"0.136"	"0"	"0.098"
7	"0.095"	"0"	"0.034"
8	"0.163"	"0.003"	"0.056"
9	"0.219"	"0.001"	"0.04"
10	"0.093"	"0"	"0.082"
11	"0.272"	"0.001"	"0.095"

Table S62. Cluster members of 7-ligand Ca, combined group

[1] "Cluster 1"
4K4I.I.603, 3SQ1.A.903, 1AJ4.A.164, 3AR2.A.1001, 4CAJ.C.1323, 3CI0.K.401, 1CKK.A.151, 2CL8.B.1245, 2DIJ.A.689, 1DJW.B.2, 1E07.A.689, 2EV5.A.1150, 1FI6.A.100, 1FWX.D.4903, 3GIS.Y.1002, 1HQV.A.997, 3HR4.F.202, 4I5K.A.502, 4IEF.F.703, 4IRZ.A.2005, 4IT5.B.201, 1KIE.B.328, 1KWW.B.602, 1KXT.A.4001, 4LJ3.A.403, 4LMF.D.303, 2LMV.A.150, 2M55.A.301, 4M5H.A.305, 1M63.B.501, 2MTE.A.101, 2MTE.A.102, 1MXD.A.731, 1NIW.E.1010, 3NJH.C.503, 1NL0.G.905, 1NL2.A.205, 1NYA.A.501, 4O6N.B.401, 4OV2.A.201, 2OW2.B.446, 4PHK.B.305, 1PVY.A.503, 3QED.A.348, 1QNI.C.901, 1RF1.E.462, 1S2N.A.1290, 3SBR.A.703, 1TF4.A.3001, 3TRP.A.358, 4TV8.A.503, 3UBG.A.901, 1UZJ.B.2648, 4V29.A.1178, 1V3J.A.688, 3VI3.C.2001, 2WR9.B.1129, 2WVZ.A.800, 1X2T.A.603, 1XYD.A.93, 2Y4Q.A.800, 2YEQ.A.1525, 1YUU.B.200, 1Z6C.A.246
[1] "Cluster 2"
4DTU.A.1002, 4E3S.A.1002, 4ED0.A.503, 4FK0.A.1002, 4J2E.A.1002, 4K4G.A.603, 3NDK.A.905, 3NE6.A.904, 3NGI.A.905, 2ODI.B.702, 3QER.A.905, 3QEV.A.905, 4QWA.A.403, 4UAW.A.402, 1A25.B.290, 3A4U.A.286, 1A75.A.109, 1A8B.A.407, 4AE2.A.246, 5AER.A.200, 1ANW.B.353, 4AQA.A.1208, 4AQE.A.1209, 1AX0.A.290, 3B8Z.A.903, 3BFM.A.235, 1BIW.B.803, 2BIB.A.1552, 4BJ0.A.1167, 1BU3.A.109, 2C0T.A.1507, 3C1Q.A.2, 2CCL.B.1061, 4CFQ.A.501, 3CLN.A.152, 2CM5.A.1678, 1DB5.A.198, 4DIR.A.102, 2DUR.A.1, 4DZ3.A.202, 4DZ3.B.202, 1E8A.A.1089, 2E85.A.1002, 1EDH.A.302, 2EGD.A.301, 2EIG.A.1102, 2FH1.A.2001, 1FIB.A.500, 1FXH.B.1002, 1G7Y.A.254, 4GGF.C.204, 2GJP.A.1488, 2GSK.A.1, 1GSL.A.251, 1GZC.A.290, 3HB2.P.484, 3HJR.A.602, 4HPH.A.701, 1HT6.A.500, 1HT9.A.1001, 1HVX.A.518, 3HX4.A.603, 2HYW.A.503, 4I35.A.515, 3I57.B.186, 2I7A.A.2, 4IAI.A.401, 3IPV.A.252, 4IT5.D.202, 2J1V.A.1152, 2J5Z.A.1277, 2JE7.A.1241, 2JEC.A.240, 1JIA.A.134, 4JJJ.A.704, 1JK3.A.404, 3JXS.A.301, 1K94.A.998, 4KTY.A.801, 4KTY.A.803, 4KWU.A.1109, 3L2Y.C.302, 1L9N.A.700, 1L9N.A.702, 1LEN.A.184, 4M00.A.801, 1MCX.A.349, 3MVS.A.212, 3MVS.A.213, 1MVQ.A.238, 4N1G.A.204, 4N2L.A.707, 3N5A.A.102, 1NIW.A.1004, 1NIW.G.1014, 1NLS.A.240, 3NOL.A.263, 3NQZ.B.520, 2NXP.C.602, 3O0W.A.377, 1OFL.A.528, 1OHZ.B.1058, 4OKH.A.904, 4OKH.B.901, 1OUX.A.402, 2OVU.A.238, 3OX6.B.502, 2P2K.A.239, 3P4G.A.411, 3P4G.B.405, 4P99.A.514, 5PAL.A.110, 4PE0.A.102, 4PEU.A.401, 4PHJ.A.302, 3PPE.A.402, 3PPE.A.403, 1Q3A.A.468, 3Q5I.A.524, 2QNG.A.201, 1QPK.A.451, 3QRB.A.302, 3QRX.A.172, 2QUB.A.619, 2QUB.G.616, 2QV6.A.302, 1R1Z.A.285, 1RLW.A.401, 1RP8.A.500, 1RR0.A.109, 1RU4.A.1, 1S1E.A.226, 1S6B.B.402, 3UBG.A.902, 3USU.C.266, 1UX6.B.2005, 1UX6.B.2009, 1UX7.A.1135, 4UZU.A.1485, 4V29.B.1179, 2VN5.B.102, 2WD6.A.1765, 1WKY.A.503, 4WK7.A.503, 3WN6.A.503,

2WZ8.A.1149, 2XOM.A.1169, 1Y1X.A.202, 2Y3N.B.1067, 2Y5I.A.101, 2YFS.A.1712, 1YO8.A.1198, 1YUT.A.197, 1YXH.A.1001, 2Z2X.A.1003, 2Z8X.A.619, 2Z8X.A.627, 2Z8Z.A.623, 2ZVD.C.620
[1] "Cluster 3"
1G9Y.A.801, 4KLD.A.403, 3MQ6.A.1, 3MX9.A.363, 3QNN.A.903, 3QNO.A.903, 4UB4.A.401, 1A2Q.A.295, 2A3Y.A.601, 3A8R.A.401, 2AAO.A.293, 1AFO.A.476, 1AK9.A.295, 3AKB.A.171, 3AKB.A.172, 3AKB.A.175, 3AMR.A.907, 3AMR.A.908, 4AQ1.A.1923, 4AQ1.C.1923, 4AQJ.A.1097, 1AVS.A.93, 1AVS.A.94, 1AXN.A.353, 2AYH.A.417, 4AYU.D.205, 2AZZ.A.125, 3BC9.A.701, 1BF2.A.751, 1BH6.A.501, 1BJF.A.402, 1BLI.A.500, 1BQB.A.351, 1BU3.A.110, 3BYK.A.474, 4BY5.A.1183, 4BY5.A.1185, 2C10.B.1777, 3C1Q.B.1, 3C3Y.A.238, 2C4X.A.1253, 2C4F.L.1143, 1C9N.A.277, 4CAG.A.603, 4CBU.G.1151, 2CCL.B.1060, 2CCM.A.1192, 2CCM.A.1194, 4CCD.A.3669, 2CDP.A.1139, 4CFQ.A.502, 4CFY.A.301, 1CP9.B.554, 4CPV.A.110, 1CRU.A.501, 3CZT.X.93, 4DA2.A.301, 2DCK.A.1001, 4DIR.A.101, 3DO1.A.401, 1DTL.A.201, 1DTL.A.203, 4DUQ.A.102, 1DVI.A.271, 2DWO.A.701, 2DWO.A.702, 2E26.A.601, 2E4T.A.701, 1E43.A.502, 1E8A.A.1090, 3EDF.A.602, 2EGD.A.302, 3ETO.A.2003, 2EWE.A.701, 1EXR.A.1000, 1EXR.A.1002, 4F0Z.B.204, 2FH1.A.2002, 3FIA.A.201, 3FLP.A.301, 3FLP.A.302, 3G5C.A.801, 1G8I.B.1600, 1G9K.A.702, 4GGF.L.204, 2GJP.A.1487, 4GUK.A.207, 4H1Q.B.304, 1H2G.B.1558, 3HB2.P.481, 3HDB.A.622, 3HJR.A.601, 1HQV.A.998, 2HQ8.A.203, 3HX4.A.604, 3HX6.A.1, 2HYV.A.605, 4I2Y.A.501, 4I2Y.A.503, 1I82.A.192, 2ID4.A.901, 4IEF.B.702, 3IGO.A.603, 2II1.C.400, 4ITC.A.1202, 1J1A.A.201, 1J55.A.102, 3JQW.A.1001, 4JX1.E.505, 3K21.A.193, 3K21.A.194, 1K7I.A.480, 1K94.A.999, 1K9J.A.403, 1K9U.A.1001, 1K9U.A.1002, 4K90.A.702, 1KAP.P.615, 4KDW.A.201, 3KHE.A.193, 3KHE.A.194, 3KLL.A.1, 1KVW.A.124, 3L2Y.A.301, 3LND.B.208, 3LNI.A.302, 3LPC.A.361, 2LRP.A.202, 4M5E.A.505, 1MCX.A.351, 1MCX.A.352, 1MKV.A.124, 2ML1.A.205, 4MSP.A.201, 1MXE.A.506, 4N1G.A.203, 3N4E.B.500, 4N5X.A.204, 4NEH.A.1101, 4NEH.A.1103, 1NIW.C.1007, 1NPC.A.319, 2NZM.A.405, 3O4Y.A.198, 2O5G.A.404, 2O72.A.402, 2O8O.A.1009, 1OBO.A.502, 4OKH.A.902, 2OLG.A.2001, 4OMC.A.607, 2OO4.A.5001, 2OPO.A.301, 2OPO.A.302, 2OX9.A.804, 1OYG.A.500, 2P0R.A.1002, 5P2P.A.125, 5PAL.A.111, 4PEO.X.103, 4PHJ.A.303, 4PHJ.A.304, 4PKI.A.403, 2PMY.B.104, 3PM8.A.514, 3PM8.B.1, 1PMJ.X.300, 1POE.A.801, 1PT2.A.500, 1PVA.A.110, 3PVN.E.5009, 3PVN.M.5026, 2PVB.A.110, 3Q2L.A.701, 3Q5I.A.526, 1Q5P.A.271, 4Q6O.A.502, 1QI3.A.451, 2QQO.A.1, 3QRX.A.171, 1QTX.A.153, 1QTX.A.154, 1QX2.A.1001, 1QX2.B.1006, 1ROR.E.302, 2R2I.A.500, 2R2I.A.501, 2R2I.A.502, 1RFJ.A.1001, 1RFJ.A.1003, 1RFJ.A.1004, 3RM1.A.102, 3RUP.A.1006, 1RWY.A.421, 1S01.A.295, 1S02.A.276, 1S6C.A.217, 2SCP.A.190, 2SCP.A.191, 3SIB.A.222, 1SL6.E.3, 1ST3.A.270, 1SUD.A.295, 1TCF.A.160, 1THM.A.301, 1THM.A.302, 3TI7.A.353, 3TI7.A.355, 3TI9.A.353, 3TI9.A.355, 1TKF.A.905, 4TNC.A.164, 5TNC.A.163, 3TTQ.A.2867, 3UJO.B.304, 3UL4.B.67, 4UM9.A.2001, 1UX6.B.2001, 1UXX.X.1130, 1UY4.A.1147, 1UYZ.A.1132, 1VCL.A.1001, 1VCL.A.1005, 2VN6.B.1067, 2VVD.A.1328, 3VYV.A.303, 2VZP.A.1128, 2W47.A.1137, 2WBX.A.1103, 3WFD.B.806, 3WHI.A.401, 3WHT.B.501, 4WK7.A.502, 2WND.A.102, 2WNO.A.1251, 1WP6.A.502, 1WPC.A.501, 1X1J.A.2000, 3X17.A.603, 1Y93.A.267, 1Y9Z.A.603, 2YOA.A.1002, 1YU6.A.401, 2Z2X.A.1002, 2Z30.A.1005, 2ZFD.A.229, 2ZUX.A.631, 2ZUX.A.632, 2ZUY.A.622, 3ZYP.A.1220
[1] "Cluster 4"
4DTM.A.1002, 4DU1.A.1003, 4DU3.A.1002, 2FLD.B.602, 3RAX.A.416, 2WTF.A.1511, 3A24.A.1268, 2AAO.A.295, 2AAO.A.296, 3AIE.A.4001, 4APX.B.1240, 3BOO.A.124, 1B8R.A.109, 4BY5.A.1184, 1CJY.A.950, 4CPV.A.109, 2CT9.A.301, 2CT9.A.302, 4CT3.A.1170, 3D1M.A.2, 3DBK.A.303, 2DIE.A.780, 1DVI.A.273, 2E3X.A.802, 3ECQ.B.2000, 4F0Z.B.201, 1FAT.A.255, 3FZO.A.400, 1FZD.A.1, 1G8I.A.1599, 3GDC.A.401, 4GER.A.402, 1GGZ.A.149, 2GGM.A.401, 4GGF.A.101, 4GM5.A.404, 2GSK.A.2, 2GXS.A.601, 4H2A.A.805, 2HQ8.A.202, 1HVX.A.516, 2HYW.A.501, 3HZ3.A.1, 4I75.A.401, 4ICB.A.77, 3IJ9.A.497, 4IK8.A.502, 1IOD.A.501, 4IU3.B.302, 2J1G.B.1290, 1JC9.A.301, 1JIW.P.485, 4JWQ.A.202, 4K70.A.1003, 4K70.B.1003, 1K9K.A.400, 3KF9.C.302, 1KIC.B.328, 4KKK.A.701, 3KLK.A.1, 3KQR.C.206, 1KXO.C.703, 3LI6.A.149, 3LI6.D.150, 4LVN.A.702, 4M7H.A.501, 1MDW.

B.4, 3MHZ.A.736, 4MN0.A.302, 3N1F.A.6, 3N1G.B.190, 4N5X.A.201, 4N5X.A.205, 4NHF.F.301, 2O04.A.402, 3O0V.A.1, 2O5G.A.401, 2O5G.A.402, 2O5G.A.403, 2OBH.A.1001, 2OZN.B.402, 3P4G.A.413, 4P5X.A.1001, 4PET.A.403, 1P0B.A.801, 2PVB.A.111, 2Q1F.A.2001, 3Q5I.A.525, 1QTX.A.152, 2R9F.A.366, 2RHP.A.29, 1RWY.A.422, 3S5U.D.221, 1SBF.A.601, 1SL7.A.301, 1SRA.A.302, 1TCF.A.163, 1TN4.A.162, 3U1R.A.702, 3UCP.A.912, 3UL4.B.66, 4UM9.A.2003, 4UZU.A.1483, 2VUD.D.1118, 2W46.A.1148, 2W87.A.1149, 3WA5.A.504, 3WH2.A.302, 3WNP.A.801, 2WNP.F.1298, 2Y3N.B.1068, 2YA2.A.1691, 1YO8.A.1186, 1YO8.A.1187, 1YO8.A.1190, 1YO8.A.1204, 1YUT.B.199, 1Z3J.A.267, 2Z30.A.1004, 1ZCM.A.1001, 2ZN9.A.901, 2ZWO.A.402, 2ZWP.A.404, 2ZWP.B.402

[1] "Cluster 5"

4K4G.A.602, 4K4H.A.602, 4K4I.A.605, 4UAW.A.403, 4A3Z.A.2344, 3A4U.A.287, 4A41.A.2494, 4A42.A.1690, 4A4A.A.1925, 4A5G.A.1309, 3ALU.A.201, 4AOC.A.1129, 4AQ1.A.1924, 4ASM.B.1359, 3ATG.A.301, 4ATE.A.1275, 4AWD.B.1321, 3AXD.A.3002, 3AZY.A.301, 3B0K.A.201, 1B80.B.351, 1B9O.A.124, 4B96.A.1155, 4B9C.A.1151, 1BGP.A.501, 2BIB.A.1551, 4BLK.A.400, 3BMV.A.685, 4BM1.A.401, 3CK7.A.710, 1CPN.A.209, 4CRQ.A.260, 4CUO.A.1326, 4CZN.A.1370, 1D3C.A.687, 2DEW.X.900, 2E39.A.501, 2E8Y.A.741, 2EXH.A.2001, 1F6S.A.201, 2FHF.A.2401, 3FMU.A.351, 1GW2.A.502, 1GWT.A.502, 1GWU.A.1308, 1GX2.A.1310, 3H0O.A.401, 2H2N.B.1001, 3HB3.A.563, 3HDL.A.306, 2HD9.A.2001, 1HFX.A.124, 3HR9.A.401, 2HYK.A.477, 1I22.B.197, 4IAU.A.800, 3ILF.A.278, 2IWV.D.1283, 2J1A.A.1769, 4J3V.A.920, 4J3W.A.907, 4JCL.A.701, 2JD9.A.1146, 2JDA.A.1146, 4JGL.A.202, 1JI1.A.2002, 2JKA.A.1727, 1JUG.A.126, 1L6R.B.903, 1LLP.A.351, 4LPL.A.1101, 4LQR.A.201, 1LY8.A.9001, 3M5Q.A.372, 3MMZ.A.501, 1MVE.A.400, 4N2B.A.707, 4N2G.A.703, 4N2L.A.704, 4N6F.A.302, 3NNG.A.402, 1O4Y.A.700, 4ODG.A.202, 2OKX.A.4001, 3OMI.A.613, 3OWF.A.151, 1PA2.A.308, 3PGV.A.267, 4PLS.B.301, 3Q3U.A.340, 4QF4.A.202, 1QGJ.A.2002, 1R1Z.A.286, 4R83.B.501, 3S18.A.229, 1SCH.A.301, 3SNY.A.97, 1SNC.A.150, 3SO1.A.97, 1TE2.A.702, 1U0A.A.5004, 1UPS.B.501, 1URX.A.1300, 1UX6.B.2014, 3V6N.A.229, 2VDQ.B.2002, 1W3M.E.3013, 3WDH.A.801, 2WOY.A.2415, 2WZA.A.2415, 2YLJ.A.1308, 1YRO.A.124, 2Z30.A.1002, 2Z49.A.1004, 2Z49.A.1005

[1] "Cluster 6"

4AQU.A.1154, 4AQU.A.1156, 1G9Y.B.802, 3ODH.B.195, 1S0M.A.402, 2VBJ.A.1154, 2VBJ.A.1155, 2VBO.A.1154, 2VBO.A.1155, 2VS7.A.1183, 1A75.A.110, 1A8A.A.407, 3AMR.A.905, 4APX.B.1238, 4AQA.A.1209, 4AQE.A.1207, 1AVA.A.500, 3B2Z.A.2, 2B96.A.124, 3BEU.A.249, 3BI1.A.1753, 3BNG.A.508, 1BQB.A.352, 4BQ2.A.1797, 1BSW.A.900, 2C26.A.302, 2C4X.A.1252, 4CAG.A.604, 4CAG.A.610, 1CB8.A.3000, 2CBL.A.352, 4CBU.G.1150, 2CDO.A.1139, 2CFT.A.1297, 3CHJ.A.338, 3CHK.A.503, 3CI0.K.402, 3CIP.G.201, 2CKI.A.997, 2CM5.A.1679, 2CM6.A.1680, 2CN3.A.1778, 4CTE.B.280, 1CVR.A.504, 1DBI.A.701, 1DBI.A.703, 1DBI.A.704, 3DEM.A.8001, 3DNZ.A.403, 4DUQ.A.101, 2DW0.A.703, 2E26.A.604, 2E26.A.605, 3ECQ.A.2001, 1EDM.B.2, 2EHB.A.1001, 2ERO.A.702, 1EXR.A.1004, 4F0Z.B.202, 4F0Z.B.203, 3FED.A.1755, 2FI1.A.191, 2FMJ.A.338, 3FO3.A.527, 1FS7.A.651, 2FVY.A.311, 1G5N.A.401, 1G9G.A.630, 1G9K.A.701, 3GBO.A.301, 1GCA.A.351, 1GCY.A.528, 4GER.A.403, 1GGZ.A.150, 1GGZ.A.151, 1GPL.A.500, 3GPE.A.502, 1H6Y.A.1161, 1H80.A.1493, 3HDB.A.621, 3HI7.A.803, 2HQ8.A.201, 3HX4.A.601, 3HX4.A.602, 1HY7.A.304, 2HYV.A.607, 4I35.A.511, 3IBZ.A.193, 2ID4.A.906, 3IGO.A.602, 3IKW.A.1, 3IK2.A.514, 4IMM.A.407, 2IPL.A.502, 1J83.A.4001, 4JDZ.B.701, 4JGU.A.901, 1JI1.A.2003, 3JQ1.B.1, 3K21.A.192, 1K96.A.91, 1KAP.P.614, 3KCP.A.701, 3KHE.A.192, 3KHE.A.195, 3KM5.A.2012, 1KQU.A.301, 3KWU.A.901, 3LCP.C.159, 4LDC.A.502, 1LE6.A.461, 4LJ3.A.402, 4LM8.A.811, 4LOS.A.401, 3LPA.A.361, 1LQV.C.34, 4LXO.A.1601, 4LXO.B.1601, 3M1H.A.2002, 4M2P.A.301, 4M5E.A.506, 1M8T.A.1001, 4MEW.A.501, 4MN0.A.303, 1MPX.A.638, 3MSE.B.180, 4N1G.A.202, 4N2B.A.709, 4N2G.A.704, 4N2N.A.704, 4N5X.A.203, 3N5A.A.101, 1N9E.A.803, 1NNL.A.2001, 3NQX.A.511, 4NUQ.A.303, 2NXP.D.603, 2O72.A.401, 2O9Q.A.501, 1OAH.A.1525, 1OAH.A.1526, 1OF3.A.1174, 1OH4.A.1186, 1OHZ.B.1057, 4OMC.A.605, 2OW9.B.610, 3OX6.B.501, 3P6B.A.205, 1P8J.A.3001, 4P99.C.505, 3PDD.A.193, 3PF2.A.1, 3PGB.A.902, 4PHN.B.303, 4PKG.G.1201, 3PM8.A.1, 1PMH.X.300, 1POA.A.201, 1POC.A.501, 3POJ.A.1, 2PPL.A.478, 2PQX.A.500, 1PVA.A.111, 2Q17.A.315, 3Q2N.A

.303, 3Q5I.A.1, 3QGM.A.503, 2QQM.A.1, 3QRB.A.303, 2QUB.A.617, 2QUB.A.620, 1R55.A
.515, 3R5V.B.201, 1R64.A.701, 2RAN.A.324, 2RDZ.A.1501, 2RF7.A.1501, 1RLW.A.400,
1RRO.A.110, 1S3P.A.210, 3S5U.A.221, 1S6B.A.401, 2SCP.A.192, 1SGT.A.246, 1SH7.A.1
291, 3SIB.A.221, 3SJG.A.1753, 1SL6.D.3, 1SL8.A.669, 1SL8.A.670, 1SL8.A.671, 3SXQ
.A.526, 3T3P.B.2002, 1T44.G.702, 1TF4.A.3002, 3TOY.A.361, 4TSH.B.1501, 3TZ1.A.1,
3U1R.A.701, 3UBH.A.857, 3UXF.A.2, 1V0A.A.1177, 2V0B.A.1241, 4V29.B.1178, 1VL9.A
.124, 2VPT.A.1235, 3VRR.A.401, 2VVE.A.1336, 1WON.A.1132, 3W57.A.201, 3W57.A.203,
1W7C.A.803, 3WCT.F.203, 3WLC.A.501, 2WN3.A.400, 2WN3.C.400, 2WPH.S.1246, 3WU2.c
.901, 2WW8.A.1001, 2WZE.B.1552, 4XDQ.A.301, 2XFG.B.1619, 2XFD.A.1112, 1XJO.A.905
, 1XVJ.A.422, 1Y4J.A.1001, 2Y6L.A.1168, 2YIH.A.1520, 2YKK.A.1520, 2YN5.A.6364, 1
YO8.A.1200, 2YOA.A.1001, 1Z70.X.3002, 2Z8X.A.618, 2ZEZ.A.200, 2ZFD.A.227, 2ZFD.A
.228, 2ZP4.A.124, 2ZUX.A.635, 2ZUX.A.636, 2ZUY.A.625
[1] "Cluster 7"
2AGQ.A.4001, 2ATL.B.1415, 3EH8.A.301, 3FD2.A.375, 4K4G.I.603, 3KHR.B.1416, 3RB4.
A.415, 1AP4.A.90, 2ARY.B.404, 4AR1.A.1723, 3B2Z.B.2, 3B4N.B.701, 3BSG.A.501, 1C8
T.B.264, 1CKK.A.153, 1D2J.A.1, 1DJY.B.2, 4DLK.B.401, 2EHB.A.1004, 1EMN.A.2225, 4
ENZ.A.1110, 1FPW.A.502, 4FZM.A.301, 4G1M.A.2008, 1G9K.A.704, 3H2W.A.538, 4I5L.E.
702, 1I8A.A.192, 4IEF.D.704, 4IRZ.A.2007, 4IT5.C.201, 4JA8.A.503, 1JKU.A.1272, 2
JT6.A.258, 2JU0.A.500, 1KX1.F.602, 2KYF.A.109, 4L73.A.403, 2LUX.A.202, 3MOW.I.20
2, 4MO0.A.803, 4MO2.A.701, 2M28.A.301, 1M63.B.503, 1NIW.E.1009, 4NPK.A.802, 4O6N
.A.401, 1O8P.A.1149, 1O9I.E.269, 3QC6.X.1, 2QUB.K.614, 2RJP.C.3, 1SZB.A.1003, 1T
DQ.B.128, 4TVU.C.600, 4U65.E.301, 4V0C.C.201, 2VN7.A.650, 1WC0.A.2100, 3WLC.A.50
3, 1YO8.A.1194, 1YO8.A.1207, 1ZED.A.906, 1ZIV.A.2
[1] "Cluster 8"
3FD2.A.374, 4K4H.A.604, 1N48.A.501, 3NAE.A.906, 3NHG.A.908, 3QEW.A.905, 3QEX.A.9
05, 3S9H.A.905, 3SCX.A.906, 3SQ2.A.904, 2AAO.A.294, 1AFD.1.2, 3AMR.A.901, 3AMR.A
.906, 1ANN.A.320, 4ANR.A.400, 1B1G.A.76, 1BJF.A.403, 1BJF.A.404, 3BJU.A.606, 1BL
I.A.700, 2BPE.A.1245, 2C0T.B.1507, 4C0K.A.1615, 3C7F.A.803, 2CCM.A.1193, 1CGE.A.
304, 2CHN.B.1716, 1CXE.A.690, 1DB4.A.198, 2DEW.X.902, 4DH2.B.101, 4DH2.B.102, 4D
LK.A.401, 3DSL.A.2, 1DTL.A.202, 1E43.A.504, 3EKI.A.601, 4ELG.B.202, 4ELG.C.202,
2ERO.A.701, 1ESP.A.319, 3ETO.A.2002, 1EXR.A.1001, 3FAW.A.4, 3FCS.A.2008, 2FH1.A.
2003, 1FIF.C.2, 3FLT.A.301, 1GOH.A.291, 4GON.A.202, 1GGZ.A.152, 4GKY.A.302, 3GQ8
.A.1, 1HDH.A.1528, 4HEX.A.204, 1HT6.A.501, 4I2Y.B.504, 4IRZ.A.2006, 3IUC.A.2, 2I
WA.A.501, 1J1D.A.202, 1J55.A.101, 4JRF.A.601, 3KCP.A.702, 3KEZ.B.501, 3KF9.A.301
, 3KF9.A.304, 4KHO.A.1001, 2KPN.A.762, 1KP4.A.200, 4L03.B.502, 4L73.B.402, 3LND.
A.209, 2MLR.A.303, 4MNO.A.301, 4MSP.A.202, 3NOK.A.257, 4NUQ.A.302, 1O9I.A.269, 2
OO4.A.5002, 4P5F.B.501, 2P69.A.305, 4P99.A.515, 3PDD.A.194, 4QB2.A.202, 1R17.A.5
99, 4R1D.A.601, 4RDQ.A.504, 4RDQ.B.502, 4RGP.B.302, 1SRA.A.301, 3T3P.A.2005, 3T3
P.A.2006, 3T3P.A.2007, 1TMQ.A.1001, 4U65.E.302, 4U6B.A.501, 3U8D.A.202, 3UBH.A.8
56, 1UKT.B.690, 4UM9.A.2002, 4UM9.A.2004, 1UX6.B.2006, 2VN5.B.101, 2VN6.B.1066,
2VNG.A.1213, 1WOP.A.1779, 1WOY.H.1259, 2WG7.A.1126, 3WHT.B.502, 4WJK.A.503, 4WKO
.A.502, 4WKO.A.504, 3WNO.A.801, 1WPC.A.503, 1XO5.A.5, 2Y5I.A.102, 2Y8K.A.1526, 2
YHG.A.1936, 1YO8.A.1196, 1YO8.A.1210, 1YUT.A.198, 2ZFD.A.230, 3ZHG.A.1323, 2ZUX.
A.633, 2ZUX.A.634, 2ZUY.A.623, 2ZZV.A.501
[1] "Cluster 9"
3RAX.B.1416, 4AFC.A.1267, 3AUJ.A.1603, 2B6N.A.300, 4B9F.A.153, 1BCJ.2.2, 2BO2.A.
155, 2BO2.A.156, 2BOI.A.300, 2BOI.B.600, 2BOJ.A.1116, 2BOJ.C.1117, 2BP6.A.802, 2
BP6.C.805, 1BQB.A.353, 2BV4.A.300, 2BV4.B.300, 4CE8.A.998, 4CE8.B.998, 4CGT.A.68
5, 1CIU.A.684, 4COU.A.1270, 4COV.A.1269, 4COY.A.1270, 4CPB.A.1122, 3CQO.A.4001,
4D0E.A.1531, 4D0E.A.1533, 3DBZ.A.401, 3DCQ.A.116, 3DCQ.B.116, 2DCK.A.1002, 3DED.
A.504, 3DEM.B.4001, 2DSO.A.1001, 3EHU.A.500, 3EIF.A.1, 1ESL.A.163, 2EWE.A.703, 4
FHP.A.402, 2FPW.A.503, 4FVL.A.505, 1G1Q.A.801, 1GEN.A.302, 4GER.A.404, 2GGX.C.40
1, 3GIS.Z.1003, 2GVU.A.500, 2GVV.A.500, 2H2T.B.322, 4HHR.A.703, 3HLH.B.315, 3HLI
.B.315, 2IAW.A.401, 2IAX.A.401, 2IAO.A.401, 3INM.A.521, 3IQT.A.1, 1J34.C.504, 2J

DY.A.1116, 2JDY.B.1117, 2JDM.B.1115, 2JDM.C.1115, 2JDN.A.881, 2JDN.B.881, 2JDN.C.881, 4JSD.A.603, 4KVL.A.703, 1KWZ.A.504, 3LI3.A.402, 3LI4.A.316, 1LQV.C.42, 4M65.A.404, 1MN1.A.371, 4MZA.A.612, 4N4E.E.404, 4N7A.A.605, 1NL1.A.204, 1NPC.A.322, 1NZI.A.1001, 2NZM.A.406, 1OUX.B.404, 1OVS.A.402, 1OVS.B.404, 3P7F.D.1, 1PAM.A.688, 3PAQ.A.300, 1PJX.A.491, 3Q9K.A.606, 3R5Q.A.1001, 1SNN.B.503, 3SO0.A.97, 1SU3.A.904, 1SZB.A.1001, 3TH4.L.148, 1USR.B.1573, 1V3E.A.4001, 2VNV.A.302, 2VNV.B.302, 2VUC.B.990, 2VUC.C.991, 2VZP.A.1129, 2VZR.B.1132, 3W5M.A.1201, 3W5N.A.1201, 2W86.A.1149, 1WDC.C.501, 3WH3.A.500, 3WHD.A.501, 2WR9.D.1132, 3WU2.A.401, 1YDY.A.904, 3ZDV.A.200, 3ZDV.B.200, 1ZH2.B.201, 1ZJA.A.7001, 3ZO9.A.1589

[1] "Cluster 10"

3AVW.A.3001, 4J2D.A.1002, 4KHU.A.1002, 3MXB.A.175, 3NCI.A.905, 2Q10.B.702, 3QEP.A.905, 4UB4.A.402, 3ACH.A.204, 2AEF.A.602, 1AJP.B.558, 4AQO.A.1881, 4AVS.A.207, 1AYP.A.301, 3B0X.A.579, 1BTU.A.260, 2BWR.A.500, 2CM6.A.1679, 1D0L.A.400, 4D0E.A.1532, 1D2S.A.401, 2DBX.D.702, 1DBN.A.301, 4DD8.A.1001, 3DEM.A.5001, 1DJX.B.2, 4DKB.A.301, 2DPK.A.3001, 2E26.A.602, 3E9T.B.4, 3EAD.B.1003, 3ETO.A.2001, 3EU3.A.1, 2EXH.B.2002, 3EXM.A.301, 3FCS.A.2004, 2FMD.A.301, 1FNY.A.500, 1FX5.A.251, 1G8K.A.5008, 1G8K.C.5108, 1G9K.A.705, 2GKO.A.610, 1GK9.B.1579, 1GKF.B.1571, 1GO7.P.1482, 1GO8.P.1482, 3GPE.A.501, 4H2B.A.604, 1H3G.B.701, 1H6X.A.1162, 2H61.D.817, 1HDF.A.1102, 4HHO.A.401, 4HHQ.A.401, 1HPL.A.960, 1HQL.A.302, 3HR4.D.202, 4I35.A.514, 4ICB.A.76, 4IHM.A.406, 4ITC.A.1201, 4IU3.B.301, 2J7A.A.1006, 1J9L.A.1301, 1JX9.B.601, 1K9I.I.1201, 1KAP.P.621, 3KM5.B.2011, 3KQR.A.205, 1KVO.A.191, 3KWU.A.902, 1LHW.A.401, 4LMH.A.811, 3LNF.A.302, 3LNP.A.472, 1LOC.A.228, 4LOR.A.301, 1LPB.B.453, 3LPC.A.360, 3LPC.A.362, 4LP7.B.301, 2LTN.A.191, 1LVU.A.8001, 1LVU.D.8002, 3M1H.A.2001, 4MGQ.A.601, 1MKU.A.124, 2ML2.A.204, 2ML3.A.202, 3MVS.A.216, 3N35.A.290, 1N47.B.252, 1NPC.A.321, 1O8F.A.1353, 1OAC.A.803, 3OEA.A.200, 3OM2.A.1, 3OM4.A.1, 3OM5.A.1, 3OM6.A.1, 3OM7.A.1, 4OMC.A.606, 4OY7.G.302, 4P99.B.517, 2PEL.A.237, 1QNW.A.302, 3QR0.A.1000, 2QUB.A.621, 1QX2.A.1005, 2R8Z.N.214, 1S6C.A.218, 1SL4.A.407, 3SRE.A.1356, 1SU4.A.995, 3TI7.A.354, 3TI9.A.354, 1TM7.E.470, 1TU5.A.903, 4U36.A.302, 3U4X.A.237, 1UKG.A.1262, 1UNE.A.124, 1UP8.A.599, 2UWP.A.1246, 1UWW.A.1192, 1UX6.B.2008, 1UX6.B.2011, 3V9M.A.205, 1VCL.A.1002, 3VLV.A.503, 3VV3.A.402, 2W08.A.206, 1WBF.A.303, 1WC5.A.2100, 3WCS.A.1003, 4WJK.B.502, 2WW8.A.1002, 1Y1X.A.201, 1Y6O.A.207, 2YAY.A.1266, 1YO8.A.1189, 2Z8X.A.621, 2Z8X.A.625, 2ZEX.A.406, 2ZKM.X.800, 2ZVD.A.628, 3ZYR.A.401, 2ZYH.A.700, 2ZZJ.A.239

[1] "Cluster 11"

1T9I.C.801, 1T9I.C.802, 4A3X.A.1268, 4A5G.A.1308, 4A6O.A.2346, 4A6S.A.1122, 3AFG.A.541, 4AFA.A.1267, 4AFB.A.1267, 1ALC.A.200, 3ALT.A.201, 4AL9.A.1122, 4AOC.E.1129, 4ASL.A.1268, 3B0X.A.578, 2BF6.A.1693, 1BJ3.B.124, 3BPS.E.1, 1BYF.A.201, 1CGT.A.686, 4CI7.A.1505, 1CPM.A.215, 4CP0.A.1294, 4CP1.A.1294, 4CPB.D.1123, 3CQ0.A.4004, 1D2V.A.600, 1DED.B.5004, 3DED.B.506, 1DV8.A.1002, 4DZT.A.302, 3EDF.A.603, 2EJN.A.1003, 2ERV.A.195, 2FF3.A.701, 1FHF.A.502, 1FIF.B.2, 4G01.A.300, 1G5N.A.408, 1GWU.A.1307, 4GWI.A.204, 1H3G.A.700, 3HDL.A.307, 1HFZ.B.124, 4I5L.B.601, 4IAI.A.402, 2J22.A.1150, 1JI1.A.2001, 2JKX.A.1641, 1K12.A.160, 3K8K.A.710, 3K8L.A.700, 3K8L.A.710, 1KZM.A.501, 4LHK.A.303, 4LHN.A.302, 4LJH.A.201, 1LPZ.B.1, 1LYC.B.9002, 1MAC.A.388, 2ML2.A.203, 2ML3.A.201, 1PA2.A.307, 1PAM.A.687, 3PAR.A.300, 4PIB.C.201, 1PJ9.A.890, 4Q1U.A.402, 1QGJ.A.2001, 1SCH.A.302, 1SH7.A.1292, 3SRE.A.1357, 3SRG.A.1357, 1SU4.A.996, 3TBD.A.401, 1TDQ.B.127, 1TLG.A.201, 3TO5.A.131, 1UX7.A.1134, 2V72.A.1139, 2VUD.C.1118, 2VZQ.A.1130, 2W1Q.A.1947, 2W1S.A.1946, 2W2M.E.1334, 3W57.A.202, 2W86.A.1148, 4WF7.A.600, 2WQ8.A.1641, 2WR9.A.1131, 1WZL.A.1601, 2WZS.F.800, 2XFD.A.1111, 2XFE.A.1113, 1XO5.A.1, 2YFU.A.1141, 2Z48.A.1007, 2Z48.A.1102, 2Z48.A.1103, 2Z48.A.1104, 2Z48.A.1205, 2Z49.A.1001, 2Z49.A.1003, 3ZUC.A.1154, 3ZYH.A.1123

Table S63. 8-ligand Ca, compressed group

	size	largest_angle*	middle_1*	middle_2	middle_3	middle_4
1	"43"	"155.9+/-4.2"	"51.5+/-2.1"	"69+/-2.3"	"70.9+/-1.6"	"72.3+/-1.6"
2	"15"	"148.8+/-4.1"	"66.6+/-3.1"	"69.9+/-1"	"70.8+/-0.6"	"71.7+/-1"
3	"43"	"157.6+/-2.9"	"51.2+/-2.3"	"69.5+/-2.1"	"71.4+/-1.4"	"72.6+/-1.2"
4	"22"	"159.5+/-5.3"	"64.4+/-2.6"	"68.2+/-2.4"	"70.4+/-2"	"71.7+/-2"

	middle_5	middle_6	middle_7	middle_8	middle_9*
1	"73.8+/-1.7"	"74.9+/-1.5"	"76+/-1.5"	"77.1+/-1.4"	"78.4+/-1.4"
2	"72.6+/-1.2"	"73.6+/-1.2"	"74.9+/-1.5"	"76.4+/-1.5"	"77.4+/-2.1"
3	"74.2+/-1.4"	"75.3+/-1.2"	"76.4+/-1.2"	"77.6+/-1.2"	"78.8+/-1.2"
4	"73.1+/-1.9"	"74.3+/-1.3"	"76.3+/-1.4"	"77.9+/-1.5"	"79.4+/-1.9"

	middle_10	middle_11	middle_12	middle_13	middle_14
1	"79.8+/-1.5"	"81+/-1.4"	"82.6+/-1.9"	"84.1+/-1.8"	"87.5+/-2.3"
2	"78.6+/-1.9"	"80.9+/-1.9"	"82.1+/-2"	"84+/-2.1"	"86+/-2.4"
3	"80.1+/-1.4"	"81.3+/-1.3"	"83.2+/-1.8"	"85.3+/-1.9"	"87.6+/-1.8"
4	"80.9+/-1.6"	"82+/-1.6"	"83.9+/-1.9"	"85.6+/-2.4"	"88.7+/-2.1"

	middle_15	middle_16	middle_17*	middle_18	middle_19
1	"90.9+/-2.8"	"96.7+/-5"	"114.1+/-3.9"	"117.4+/-3.5"	"121.3+/-3.6"
2	"90.8+/-2"	"95.3+/-4.5"	"116.3+/-6.6"	"124.2+/-4.3"	"127+/-2.8"
3	"92.1+/-2.9"	"96+/-3.3"	"102.7+/-2.2"	"116.9+/-3.6"	"123+/-2.8"
4	"92.8+/-4.4"	"97.4+/-4.7"	"103.7+/-4.5"	"117.6+/-3.9"	"123+/-4.7"

	middle_20	middle_21	middle_22	middle_23	middle_24
1	"126.5+/-3.7"	"131.6+/-3.4"	"135.9+/-2.3"	"139.6+/-2.7"	"143.5+/-2.1"
2	"131.8+/-1.7"	"135.9+/-1.3"	"137.6+/-1.6"	"139.4+/-1.7"	"141.3+/-1.3"
3	"126.2+/-3"	"131.8+/-3.5"	"138+/-2.9"	"140.7+/-2.1"	"145+/-2.3"
4	"126.8+/-5.3"	"131.3+/-5"	"135.3+/-3.3"	"139+/-3"	"143.5+/-2.9"

	middle_25	middle_26*	smallest_opposite_angle*	SquareAntiprismatic
1	"147.3+/-3"	"151.9+/-3.5"	"66+/-2.8"	"0.022"
2	"143.4+/-2.1"	"145+/-2.1"	"51+/-1.5"	"0"
3	"147.7+/-1.9"	"151.7+/-2.7"	"67.6+/-2.5"	"0.018"
4	"148.6+/-4.2"	"154.2+/-4.4"	"50.8+/-2.6"	"0.101"

	HexagonalBipyramidal
1	"0"
2	"0"
3	"0"
4	"0"

Table S64. Cluster members of 8-ligand Ca, compressed group

[1] "Cluster 1"
2A40.B.1273, 4AW7.A.1599, 3B9X.A.400, 4B9P.A.1167, 3BMV.A.684, 1CGV.A.692, 1CGY.A.692, 1CXL.A.689, 1D3C.A.688, 3DED.C.508, 3DHP.A.497, 1DTU.A.688, 1EO5.A.689, 2FF2.A.601, 1G43.A.200, 1G94.A.800, 2GUY.A.601, 2HYV.A.601, 1I75.A.1688, 4I71.A.402, 1IOD.G.506, 1JAE.A.500, 1KCK.A.691, 1KCL.A.1692, 1KWT.A.502, 1KXH.A.800, 4KZW.A.302, 2MAS.A.316, 1MCX.A.347, 1NBC.B.156, 1OT1.A.1693, 1PEZ.A.891, 1PJ9.A.891, 1SH7.A.1290, 3TEC.E.344, 1UA7.A.601, 3VM7.A.501, 2WHK.A.1339, 1WMD.A.1002, 3WMS.A.801, 2WNX.A.1163, 2WZS.A.800, 1YO8.A.1182
[1] "Cluster 2"
1B9A.A.110, 3B9G.A.318, 2C40.A.1311, 3EPW.A.1001, 3EPX.A.1001, 4I70.A.401, 4I72.

A.401, 1J34.A.511, 3MKM.A.501, 3MKN.A.501, 1Q8F.A.2001, 3S82.B.405, 1UZK.A.2512, 3WMT.A.606, 1YOE.A.1001
[1] "Cluster 3"
2ASP.A.600, 4AWN.A.301, 4B97.A.1152, 4BM1.A.402, 2BOQ.A.1352, 1CLC.A.651, 4CZN.A.1369, 4DLK.A.402, 1DV8.A.1001, 2E39.A.502, 3ER9.B.901, 2EWE.A.702, 4EW9.A.203, 2F3Y.A.1174, 4FCS.A.403, 3FM1.A.352, 3FM4.A.352, 3FMU.A.352, 1G87.A.616, 1H3J.A.1346, 1HUP.A.302, 1HX0.A.500, 1IA6.A.1263, 1J34.B.512, 1K72.A.779, 1LLP.A.352, 3M5Q.A.371, 1MN2.A.371, 2NZM.A.407, 3PDD.A.192, 3PF2.A.2, 3POE.A.2, 2PWA.A.1280, 3Q3U.A.341, 3QEE.A.21, 1SNN.A.403, 1UOV.A.1419, 2VMH.A.3050, 2VMI.A.3050, 1W3H.B.1348, 1XKD.A.1006, 2ZIC.A.944, 3ZQX.A.1146
[1] "Cluster 4"
1ANX.B.321, 4AOC.B.1129, 4AYU.A.205, 1C0G.S.129, 1CGU.A.685, 1CGW.A.692, 3DEM.A.3001, 3DR2.A.707, 1DX5.I.1001, 3FZ0.B.400, 3HR6.A.1, 2JBH.A.1228, 1KXQ.A.4001, 2LRO.A.202, 4QB6.A.203, 3T8I.C.400, 3TH2.L.152, 4U6D.A.501, 4U6D.B.502, 4X9Y.A.502, 1YCM.A.267, 1Z32.X.497

Table S65. 8-ligand Ca, combined group

	size	largest_angle*	middle_1*	middle_2	middle_3	middle_4
1	"17"	"148.5+/-3.3"	"60.7+/-7.1"	"67.6+/-3.9"	"70.2+/-1.9"	"71.4+/-1.6"
2	"44"	"160.8+/-3"	"51.2+/-2.8"	"66.6+/-6.6"	"70.8+/-4.2"	"73.6+/-3.3"
3	"29"	"156.6+/-4.2"	"64.6+/-3.2"	"68.4+/-2.8"	"70.3+/-2.5"	"71.6+/-2.4"
4	"18"	"171.6+/-3.7"	"51.1+/-3.5"	"62.2+/-6.3"	"67.4+/-4.1"	"70.2+/-4.1"
5	"44"	"158+/-2.9"	"50.9+/-2.2"	"68.8+/-4.1"	"71.2+/-2.2"	"72.5+/-1.6"
6	"46"	"158.3+/-3.1"	"51.1+/-1.2"	"68.7+/-4.6"	"72.6+/-1.6"	"74.4+/-1.3"
7	"60"	"149+/-2.5"	"66.3+/-2.2"	"69.8+/-1.3"	"71.1+/-1.2"	"72.1+/-1"
8	"29"	"162.8+/-3.6"	"52.5+/-3.4"	"66.1+/-5.8"	"69.5+/-5.2"	"71.3+/-5"
9	"22"	"152.3+/-3.7"	"64+/-2.6"	"68.7+/-2.2"	"70.3+/-1.9"	"71.4+/-1.5"
10	"41"	"156.5+/-3.8"	"51.2+/-1.8"	"68.8+/-3.6"	"70.9+/-1.6"	"72.5+/-1.8"

	middle_5	middle_6	middle_7	middle_8	middle_9*
1	"72.2+/-1.2"	"73.4+/-1.2"	"74.8+/-1.5"	"76.3+/-1.8"	"77.2+/-2"
2	"75.1+/-2.5"	"76.7+/-2"	"77.9+/-1.5"	"79.4+/-1.3"	"80.9+/-1.7"
3	"73+/-2.4"	"74.4+/-2.3"	"76.2+/-1.6"	"77.5+/-1.7"	"79+/-1.9"
4	"72.1+/-3.5"	"74.8+/-3.4"	"77.6+/-2.7"	"79.6+/-2.6"	"81.7+/-2.4"
5	"74.1+/-1.7"	"75.2+/-1.4"	"76.5+/-1.3"	"77.6+/-1.2"	"78.8+/-1.3"
6	"75.7+/-1.1"	"76.9+/-1"	"78+/-1.1"	"79.1+/-1.3"	"80.1+/-1.3"
7	"72.9+/-1"	"73.6+/-1.1"	"74.6+/-0.9"	"75.4+/-1.1"	"76.3+/-1.1"
8	"73+/-4.6"	"74.5+/-4"	"75.6+/-3.7"	"76.9+/-3.3"	"78.1+/-2.8"
9	"72.4+/-1.4"	"74+/-1.3"	"75+/-1.4"	"76.2+/-1.6"	"77.5+/-1.5"
10	"74+/-1.7"	"75.1+/-1.4"	"76.2+/-1.4"	"77.3+/-1.3"	"78.5+/-1.2"

	middle_10	middle_11	middle_12	middle_13	middle_14
1	"79.1+/-2.2"	"81.4+/-2"	"82.8+/-2.2"	"84.4+/-2.1"	"86.3+/-2.5"
2	"82.6+/-2.2"	"83.9+/-2.2"	"85.7+/-2.2"	"87.2+/-2.3"	"88.9+/-2.7"
3	"80.5+/-1.8"	"81.8+/-2"	"83.5+/-2.4"	"85.5+/-2.9"	"88.4+/-3.1"
4	"83.5+/-2.9"	"86.2+/-3"	"88.7+/-3.5"	"90.5+/-2.7"	"92.7+/-3"
5	"80.2+/-1.5"	"81.5+/-1.8"	"83.4+/-2.2"	"85.4+/-2.2"	"87.8+/-2.5"
6	"81.3+/-1.2"	"82.5+/-1.2"	"83.9+/-1.1"	"85.7+/-1.9"	"87.3+/-2.3"
7	"77.6+/-1.3"	"79+/-1.5"	"81.3+/-1.9"	"83.1+/-2.1"	"85.9+/-1.9"
8	"79.8+/-2"	"81.8+/-1.9"	"83.8+/-2"	"86.6+/-2.7"	"90.5+/-4"
9	"78.6+/-1.6"	"80.1+/-2"	"81.9+/-2.1"	"84.3+/-2.6"	"89.5+/-2.5"
10	"79.8+/-1.4"	"81.1+/-1.4"	"82.5+/-1.9"	"84.1+/-1.9"	"87.6+/-2.3"

	middle_15	middle_16	middle_17*	middle_18	middle_19
1	"92.2+/-4"	"95.9+/-5"	"120.5+/-4.2"	"124.3+/-3.4"	"126.7+/-2.9"
2	"91.7+/-2.8"	"96.7+/-3"	"101.1+/-2.9"	"111.8+/-6"	"118+/-6.2"
3	"91.9+/-3.7"	"97+/-4.7"	"105.3+/-4.5"	"119.2+/-4.8"	"124.8+/-3.9"
4	"95.1+/-3.7"	"98+/-4.5"	"101.9+/-3.9"	"110.1+/-5.4"	"114.4+/-4.6"
5	"92.3+/-3.1"	"96.2+/-3.3"	"102.6+/-2.3"	"117.2+/-3.5"	"123+/-2.9"
6	"90+/-3.1"	"97.7+/-4.2"	"107.2+/-2.6"	"115.5+/-5.4"	"120.6+/-4.2"
7	"89+/-2"	"94+/-3.3"	"113.9+/-3.3"	"119.7+/-2.8"	"127.8+/-2.8"
8	"95.7+/-5.1"	"101.3+/-5.2"	"112.4+/-3.2"	"115.2+/-3.1"	"118.6+/-3.1"
9	"93.5+/-3"	"97.2+/-3.3"	"101.6+/-3.2"	"121.7+/-6.1"	"126.8+/-3.8"
10	"91.1+/-2.7"	"96.7+/-4.7"	"113.6+/-3.3"	"117.2+/-3.1"	"121+/-3"

	middle_20	middle_21	middle_22	middle_23	middle_24
1	"131.1+/-2.3"	"135+/-1.9"	"136.3+/-2.4"	"138.1+/-2.5"	"140.2+/-2.5"
2	"123.3+/-4.2"	"127.8+/-3.4"	"132.5+/-4.5"	"142.5+/-4.2"	"148.1+/-3.6"
3	"128+/-4.5"	"132.5+/-3.8"	"135.8+/-2.6"	"139.2+/-2.5"	"142.9+/-2.6"
4	"119.6+/-5.9"	"125.4+/-4.9"	"134+/-5.6"	"139.9+/-5.6"	"146.9+/-5.4"
5	"126.1+/-3"	"131.4+/-3.4"	"137.7+/-3.1"	"140.6+/-2.2"	"144.7+/-2.7"
6	"126+/-3.4"	"129.7+/-3"	"134.4+/-4.1"	"141.8+/-3.9"	"146+/-3.3"
7	"132.6+/-2.2"	"135.6+/-2"	"138.9+/-1.5"	"140.8+/-1.5"	"142.5+/-1.3"
8	"122+/-3.3"	"125.3+/-4.3"	"130.9+/-3.9"	"140.4+/-5.1"	"145.2+/-4.8"
9	"130.4+/-3.4"	"134.8+/-2.7"	"137.2+/-2.5"	"140.2+/-2.5"	"143.3+/-2.6"
10	"126.3+/-3.5"	"131.2+/-3.4"	"135.7+/-2.4"	"139.8+/-2.8"	"143.8+/-2"

	middle_25	middle_26*	smallest_opposite_angle*
1	"143+/-1.9"	"144.8+/-1.7"	"53.2+/-4.3"
2	"152.4+/-2.6"	"156+/-2.4"	"50.4+/-2"
3	"146.9+/-3"	"151.8+/-4.4"	"51.1+/-2.6"
4	"150.8+/-6.1"	"161.6+/-4.6"	"50.6+/-3.7"
5	"147.7+/-2.5"	"151.8+/-2.7"	"67.6+/-2.4"
6	"149.1+/-3.4"	"151.8+/-2.5"	"52+/-1.6"
7	"144.3+/-1.3"	"146.5+/-1.5"	"67.9+/-1.8"
8	"150+/-4.6"	"156.9+/-3.8"	"51.3+/-2.7"
9	"146.2+/-2.1"	"149.3+/-2.7"	"64.7+/-3.6"
10	"147.5+/-2.9"	"152.3+/-3.1"	"66.1+/-2.7"

	SquareAntiprismatic	HexagonalBipyramidal
1	"0.077"	"0"
2	"0.017"	"0"
3	"0.118"	"0.005"
4	"0.134"	"0.009"
5	"0.017"	"0"
6	"0.022"	"0"
7	"0.033"	"0"
8	"0.105"	"0"
9	"0.045"	"0"
10	"0.023"	"0"

Table S66. Cluster members of 8-ligand Ca, combined group

[1] "Cluster 1"
3B9G.A.318, 1DV8.A.1003, 3EPW.A.1001, 3EPX.A.1001, 3G5I.A.501, 4I71.A.402, 4I72.A.401, 4I73.A.401, 2M3S.A.202, 2MAS.A.316, 3MKM.A.501, 3MKN.A.501, 1Q8F.A.2001, 3S82.B.405, 3WMT.A.606, 1YOE.A.1001, 1YUT.B.200

[1] "Cluster 2"
1AZ0.A.284, 1A25.A.291, 1A25.B.291, 4AQ8.D.1239, 4AYU.A.205, 1CVR.A.503, 3D34.A.228, 3DED.A.505, 1DSY.A.502, 3EF2.A.303, 3EF2.B.303, 4EW9.A.204, 3GZK.A.538, 3HR4.B.201, 1I82.A.193, 1KSC.A.500, 1KWZ.A.503, 1LQV.C.45, 3N1G.A.192, 4N4E.E.402, 4NPK.A.801, 2NQI.A.401, 2NXP.B.601, 1OUX.A.401, 2OX9.B.804, 4PE0.A.103, 4PHJ.A.301, 2POR.A.303, 3Q13.A.601, 3Q4W.A.225, 1QNI.A.901, 1RDL.1.227, 1RP8.A.501, 3SG4.A.453, 3SWB.A.91, 1T44.G.701, 3TH4.L.149, 1TLQ.A.190, 1UV4.A.1294, 1V0A.A.1176, 3WC3.A.501, 4WK0.A.501, 1Y9I.A.501, 2ZWP.B.403
[1] "Cluster 3"
4RN0.A.502, 1ANX.B.321, 4AOC.B.1129, 1B9A.A.110, 1C0G.S.129, 2C40.A.1311, 1CGW.A.692, 3DEM.A.3001, 1DX5.I.1001, 3FZ0.B.400, 3HR6.A.1, 4I5N.E.701, 4I70.A.401, 1J34.A.511, 2JBH.A.1228, 1KXQ.A.4001, 2LR0.A.202, 4QB1.A.202, 4QB6.A.203, 1SMD.A.497, 3T8I.C.400, 3TH2.L.152, 4U6B.C.501, 4U6D.A.501, 4U6D.B.502, 1UZK.A.2512, 4X9Y.A.502, 1YCM.A.267, 1Z32.X.497
[1] "Cluster 4"
1N48.A.502, 4QWD.A.703, 1S0O.A.402, 1AMY.A.501, 3B8Z.A.902, 3DR2.A.707, 4I5L.E.701, 4IT5.A.301, 2J5W.A.3042, 2KXV.A.201, 1LGN.A.301, 3LJT.A.902, 3LND.A.208, 3ONR.A.72, 2POR.A.1003, 3T3P.A.2004, 1UOV.A.1420, 4WKE.A.502
[1] "Cluster 5"
2ASP.A.600, 4AWN.A.301, 4B97.A.1152, 4BM1.A.402, 2BOQ.A.1352, 1CLC.A.651, 4CZN.A.1369, 4DLK.A.402, 1DV8.A.1001, 2E39.A.502, 3ER9.B.901, 2EWE.A.702, 4EW9.A.203, 2F3Y.A.1174, 4FCS.A.403, 3FM1.A.352, 3FM4.A.352, 3FMU.A.352, 1G87.A.616, 4GNC.A.301, 1H3J.A.1346, 1HUP.A.302, 1HX0.A.500, 1IA6.A.1263, 1J34.B.512, 1K72.A.779, 1LLP.A.352, 3M5Q.A.371, 1MN2.A.371, 2NZM.A.407, 3PDD.A.192, 3PF2.A.2, 3POE.A.2, 2PWA.A.1280, 3Q3U.A.341, 3QEE.A.21, 1SNN.A.403, 1UOV.A.1419, 2VMH.A.3050, 2VMI.A.3050, 3VV3.A.403, 1W3H.B.1348, 2ZIC.A.944, 3ZQX.A.1146
[1] "Cluster 6"
1AFB.1.227, 3BJE.B.402, 2BOI.A.200, 2BOJ.A.1117, 2BP6.A.801, 2BPE.B.1245, 2BV4.A.200, 4CE8.A.997, 3D1M.A.190, 3DCQ.A.115, 3DE8.A.109, 4EW9.B.203, 1FIF.A.1, 1FWX.B.4903, 4HPK.A.1102, 1HUP.A.301, 2IE7.A.401, 2IWK.A.1607, 1J34.C.505, 2JDM.B.1116, 2JDN.B.882, 3JQW.A.1002, 1K9J.A.401, 2KRD.C.90, 4KZV.A.303, 2LR0.A.201, 1MDW.A.1, 2MSB.A.1, 1MXG.A.438, 3N1F.A.5, 2O8O.A.1010, 1OUR.A.401, 1OVP.A.116, 4PHN.A.304, 1PW9.B.402, 2QVM.A.1001, 3SBQ.A.703, 1TN3.A.182, 1UQX.A.1115, 2VNV.A.301, 2VUC.B.991, 1WMZ.A.201, 3WN6.A.501, 2WR9.A.1132, 2XR6.A.1390, 1Y6W.A.150
[1] "Cluster 7"
4AK8.A.1326, 4AYO.A.500, 4AYP.A.500, 4AYQ.A.500, 4AYR.A.500, 2CKI.A.998, 2DG1.A.3001, 4E52.B.401, 1FO3.A.700, 1G1T.A.160, 3G81.A.401, 2GGU.A.401, 2GGX.A.401, 3IKP.B.401, 3IKR.A.401, 1K9I.B.502, 1K9J.A.402, 1KWU.A.503, 1KWV.A.503, 1KWW.A.503, 1KWX.A.503, 1KWZ.B.604, 1KX0.A.504, 1KZA.1.502, 1KZD.1.502, 4KZV.A.302, 2MSB.B.2, 4N32.A.402, 4N33.A.404, 4N34.A.402, 4N35.A.404, 4N36.A.402, 4N37.A.402, 4N38.A.403, 2ORJ.A.401, 2OS9.A.401, 3P5G.A.500, 3P5H.A.500, 3P5I.A.500, 3P7G.A.1, 3P7H.A.1, 1PW9.A.401, 1PWB.B.401, 1RDI.1.2, 1RDJ.1.2, 1RDK.1.2, 1RDL.1.2, 1RDN.1.2, 1RDO.1.2, 3RQI.A.181, 1RTM.1.2, 1SL5.A.402, 1SL6.A.2, 2VUV.A.200, 2VUZ.A.1130, 3VYK.A.1007, 3WH2.A.301, 1WMY.A.202, 2WZS.A.800, 2XR6.A.1391
[1] "Cluster 8"
1BSU.A.301, 2A3Y.A.600, 4AOC.A.1130, 4AVS.A.206, 4AYU.A.206, 3BVH.B.1, 1CGT.A.685, 1CGU.A.685, 1CGX.A.692, 4DOE.A.502, 1DVI.A.274, 4I5L.B.602, 4IEF.B.703, 4IEF.H.703, 4IT5.D.201, 4JA8.B.502, 3KQR.A.206, 1KXR.A.1, 2KZ2.A.150, 2M3S.A.203, 4NEH.A.1104, 2OX9.A.801, 2R9F.A.365, 1TF4.B.3003, 4TV9.A.503, 4V0C.D.201, 2W08.A.205, 2XFG.A.1447, 1ZCM.A.1002
[1] "Cluster 9"
1AFA.1.2, 1AFB.1.2, 1BCH.1.2, 1BCH.2.2, 2DSO.B.1002, 1FIF.A.2, 1FIH.A.2, 1H80.A.1492, 1JZN.A.1138, 3KMB.1.2, 4KZO.A.501, 4KZO.B.501, 1LQV.D.47, 1MUQ.B.202, 4NVR.A.401, 2OX9.A.802, 3PAK.A.300, 1WOY.L.1156, 2W87.A.1148, 1WMZ.A.202, 1WMZ.B.202

, 1XKD.A.1006
[1] "Cluster 10"
2A40.B.1273, 4AW7.A.1599, 3B9X.A.400, 4B9P.A.1167, 3BMV.A.684, 1CGV.A.692, 1CGY.A.692, 1CXL.A.689, 1D3C.A.688, 3DED.C.508, 3DHP.A.497, 1DTU.A.688, 1EO5.A.689, 2FF2.A.601, 1G43.A.200, 1G94.A.800, 2GUY.A.601, 1GYK.A.1205, 2HYV.A.601, 1I75.A.1688, 1IOD.G.506, 1JAE.A.500, 1KCK.A.691, 1KCL.A.1692, 1KWT.A.502, 1KXH.A.800, 4KZW.A.302, 1MCX.A.347, 1NBC.B.156, 1OT1.A.1693, 1PEZ.A.891, 1PJ9.A.891, 1SH7.A.1290, 3TEC.E.344, 1UA7.A.601, 3VM7.A.501, 2WHK.A.1339, 1WMD.A.1002, 3WMS.A.801, 2WNX.A.1163, 1YO8.A.1182

Table S67. all-ligand-number Ca, normal group

	size	largest_angle*	middle_1*	middle_2*	middle_3*
1	"704"	"172+/-4"	"79.4+/-3.9"	"88.1+/-2.1"	"96.3+/-3.5"
2	"196"	"158.2+/-12.1"	"77.4+/-6.9"	"84.3+/-7.1"	"90.9+/-8.4"
3	"114"	"158.2+/-10.8"	"74.5+/-6.6"	"85.5+/-5.6"	"96.7+/-6.1"
4	"327"	"155.3+/-6.3"	"68.9+/-3.7"	"79.8+/-3.1"	"109.6+/-7.6"
5	"439"	"167.4+/-5.9"	"71.6+/-4.1"	"84.3+/-3.4"	"98.2+/-5.9"
6	"73"	"158.8+/-13.5"	"75.8+/-7.1"	"83.6+/-6.6"	"93.6+/-7.7"

	middle_4*	smallest_opposite_angle*	Tetrahedral	TrigonalBipyramidal
1	"166.4+/-4.7"	"80.6+/-5.2"	"0.001"	"0.036"
2	"101.8+/-10.4"	"87+/-11.5"	"0"	"0"
3	"140+/-10"	"96+/-8.9"	"0.014"	"0.074"
4	"149.3+/-6.1"	"68.7+/-4.1"	"0.007"	"0.013"
5	"151.4+/-6.5"	"70+/-5.2"	"0.005"	"0.028"
6	"106.6+/-12.4"	"134.6+/-16"	"0.001"	"0"

	TrigonalBipyramidalVA	TrigonalBipyramidalVP	Octahedral	SquarePyramidal
1	"0.023"	"0.359"	"0.353"	"0.477"
2	"0.008"	"0.048"	"0"	"0"
3	"0.089"	"0.152"	"0"	"0.04"
4	"0.024"	"0.067"	"0.012"	"0.037"
5	"0.028"	"0.169"	"0.034"	"0.123"
6	"0.001"	"0.099"	"0"	"0"

	SquarePyramidalV	SquarePlanar	TrigonalPrismatic	TrigonalPrismaticV
1	"0.527"	"0.496"	"0.037"	"0.09"
2	"0.176"	"0"	"0"	"0"
3	"0.165"	"0.07"	"0"	"0.101"
4	"0.089"	"0.064"	"0.112"	"0.141"
5	"0.215"	"0.171"	"0.094"	"0.169"
6	"0.027"	"0.054"	"0"	"0"

	PentagonalBipyramidal	PentagonalBipyramidalVA	PentagonalBipyramidalVP
1	"0"	"0"	"0.066"
2	"0"	"0"	"0"
3	"0"	"0"	"0"
4	"0.074"	"0.106"	"0.112"
5	"0.095"	"0.105"	"0.172"
6	"0"	"0"	"0"

	SquareAntiprismatic	SquareAntiprismaticV	HexagonalBipyramidal
1	"0"	"0"	"0"
2	"0"	"0"	"0"
3	"0"	"0"	"0"

4	"0.009"	"0.112"	"0"
5	"0"	"0.086"	"0"
6	"0"	"0"	"0"

	HexagonalBipyramidalVA	HexagonalBipyramidalVP
1	"0"	"0"
2	"0"	"0"
3	"0"	"0"
4	"0"	"0.021"
5	"0"	"0.043"
6	"0"	"0"

Table S68. Cluster members of all-ligand-number Ca, normal group

[1] "Cluster 1"
4BXO.B.1216, 2FKC.A.248, 4G3I.B.401, 3GII.A.415, 4K4H.M.604, 4K4I.A.603, 4KHW.A.
1003, 4LQ0.A.401, 1R7M.A.304, 1TW8.C.803, 3UIQ.A.905, 2WTF.B.1509, 4AG4.A.5001,
4AIO.A.1890, 4B7M.B.1471, 2BAT.A.601, 2BD3.A.260, 2BV2.A.1085, 2BW7.A.2202, 3BYC
.A.901, 4CCE.A.4001, 2CDO.B.1140, 2CDP.B.1140, 2CHI.A.218, 5CHY.A.401, 1CJY.A.95
1, 3CKC.B.700, 4CPN.A.500, 4CPO.A.1466, 1CXV.B.6, 2D00.B.1002, 1D7X.B.805, 2DEW.
X.903, 3DPE.A.997, 1DYK.A.4001, 1E35.B.260, 3EKI.A.602, 2ERQ.B.702, 1EZS.C.650,
3F19.A.266, 1F2N.A.1002, 3FRP.A.628, 4FVL.B.506, 4GDI.C.507, 2GNT.A.254, 4GN7.B.
301, 1GQM.E.1089, 2HTV.A.995, 4ILW.D.304, 1IME.B.278, 1INW.A.501, 1J0Y.D.701, 1J
AO.A.996, 2JG9.B.1224, 1JI3.A.401, 1JRF.A.48, 4JUC.B.601, 3K37.A.467, 4K3Y.D.604
, 3K5T.A.802, 3KF9.A.303, 1KVO.A.192, 1KWH.A.800, 1L6J.A.502, 1L9N.A.703, 3LNF.B
.305, 4MDV.A.402, 3MIN.D.524, 1MR8.A.102, 1MTV.A.480, 1N28.A.128, 4N2F.A.703, 1N
G0.A.1002, 1NG0.C.1001, 1NKQ.A.260, 4NRE.A.716, 4NRE.A.717, 4NUP.A.301, 2NVO.A.5
33, 2NW6.A.613, 1NXO.A.902, 3O9J.A.995, 2OO4.B.5004, 2OO4.B.5006, 2OW1.B.447, 3O
X6.E.502, 2P5V.C.1002, 4PHK.A.304, 1PK8.F.817, 4PKH.J.1201, 3Q2L.A.703, 3Q3K.A.2
62, 4Q4X.1.5005, 1QI5.A.452, 1QL9.A.480, 3QNI.B.400, 4QN3.B.501, 1QUO.B.702, 2R1
D.B.1000, 2R8Y.C.203, 2R8Z.J.210, 1R8L.B.902, 2RHP.A.10, 2RHP.A.20, 2RJP.A.2, 2R
JP.A.3, 2RJQ.A.6, 1ROS.B.503, 1RU4.A.2, 1S0E.A.1293, 3SHI.G.305, 3SJS.A.222, 1ST
B.A.150, 1T6B.X.800, 1TLD.A.480, 3U1R.A.700, 4U32.A.301, 1ULV.A.2005, 1UYX.B.113
5, 3V03.A.584, 3V03.B.585, 1V0Z.A.1477, 1V7V.A.1001, 3VV3.A.404, 1W3M.C.3014, 3W
9T.A.1006, 2WG8.A.201, 3WIU.B.1002, 3WNK.A.812, 1WVM.A.604, 1Y4A.E.1001, 2YN5.A.
6365, 1YO8.A.1197, 1Z4V.A.600, 2ZID.A.882, 2ZJ6.A.625, 2ZRQ.A.7, 1ZTQ.A.561, 2ZU
X.B.638, 2ZUY.A.627, 2ZWO.A.400, 4ABT.B.1287, 2AOR.A.401, 2AOR.A.402, 2ASD.A.415
, 2ASD.A.416, 3AVX.A.3001, 3C0W.A.304, 1DMU.A.300, 3DPG.B.501, 4DTP.A.1002, 3DVO
.A.340, 4ECQ.A.501, 4ELV.A.908, 1FOO.B.761, 4F4W.A.402, 4F4W.B.403, 4FJ8.A.1002,
 4FJ9.A.1002, 4FJK.A.1002, 4FJL.A.1002, 4FJM.A.1002, 2GIJ.A.401, 3IAY.A.1, 2IBK.
A.402, 2IMW.P.406, 4J2A.A.1002, 4J2B.A.1002, 2JEJ.A.1344, 1JXL.A.1402, 4K4H.A.60
5, 4K4H.A.607, 4K4H.E.602, 4K4I.E.602, 3KHR.A.416, 4KHQ.A.1001, 4KLD.A.402, 4KYW
.A.302, 4LQ0.A.402, 3LZJ.A.905, 4M3Z.A.1002, 3M9N.B.4003, 3M9O.B.4001, 3M9O.B.40
03, 3MXB.B.173, 3MXB.R.175, 1N3E.D.493, 1N3E.F.491, 1N3F.C.498, 1N3F.D.499, 1N3F
.F.497, 3O3G.A.1, 2OAA.A.601, 3ODH.A.195, 2ODI.A.701, 1OUP.A.300, 2Q10.A.701, 1Q
9Y.A.939, 3QET.A.905, 4QWB.A.402, 4QWB.A.403, 1R7M.B.536, 3RAX.A.415, 2RDJ.A.353
, 1RYS.A.801, 1SOO.A.401, 3SLP.B.227, 3SPZ.A.905, 3SUN.A.897, 1TW8.A.801, 3A09.A
.601, 3A7Q.B.5001, 2A8K.B.404, 2AEP.A.601, 1AF0.A.484, 1AF0.A.486, 3AFG.A.540, 2
AFH.D.2490, 1AG9.A.200, 1AG9.B.1000, 3AHW.A.122, 3AI7.B.901, 4AIE.A.1540, 1AJJ.A
.73, 3AJ7.A.602, 3AKB.A.2, 3AMR.A.909, 3AMR.A.910, 4APX.B.1239, 4AQ1.A.1925, 4AQ
1.A.1926, 4AQE.A.1208, 4AR9.A.1732, 4ARF.A.1723, 3ASI.A.2001, 3AUK.A.391, 1AVA.A
.502, 1AWB.A.1, 4AWN.A.300, 4AXN.A.1329, 3AYU.A.417, 3AYU.A.418, 3B1U.A.901, 1B4

N.A.620, 3B4N.A.702, 4B4F.A.607, 3B7E.A.1005, 1B8L.A.110, 3B8Z.A.904, 1B9T.A.500, 3BC9.A.702, 3BC9.A.705, 3BCD.A.707, 3BH4.B.1, 1BLI.A.600, 2BL0.B.1146, 4BNR.A.600, 2BQ4.A.1119, 2BU3.A.1242, 2BV2.B.1085, 4BZ4.A.1233, 2C10.A.1771, 1C3H.D.8003, 1C3H.F.8001, 3C9I.A.1, 4C9F.B.401, 4CAG.A.602, 2CDP.A.1140, 1CGE.A.305, 3CKZ.A.1, 1CLC.A.650, 1CLC.A.652, 4CPL.A.500, 4CPY.A.1466, 4CU9.A.2999, 4CUA.A.2644, 4CUB.A.2645, 1CVL.A.320, 2CYY.A.2002, 2D00.D.1001, 3D3I.A.1001, 3D7K.A.571, 2DCJ.A.1003, 2DDU.A.1, 2DDR.A.1324, 2DEW.X.901, 2DF7.C.5904, 2DIE.A.779, 4DK4.A.302, 4DKB.A.302, 4DOU.A.1001, 4DOU.A.1002, 1DPO.A.246, 4E5U.B.302, 2EA7.A.450, 3EDY.A.1, 2EEK.A.401, 3EF2.A.304, 3EF2.A.305, 1EGZ.A.300, 4EJ7.B.402, 1ELT.A.300, 5ENL.A.438, 4EPU.A.601, 1EX9.A.286, 2F3C.E.242, 3F5V.A.223, 4F8Z.A.409, 1FBL.A.996, 2FCW.B.3001, 2FCW.B.3002, 2FGZ.A.1192, 3FG1.A.1501, 2FHF.A.2404, 3FP8.E.601, 3FSJ.X.600, 3FU1.A.301, 3FVQ.A.360, 2FWN.A.532, 3G4E.A.1, 1G5N.A.402, 4G60.A.302, 4G62.A.302, 1G9K.A.703, 1G9K.A.706, 1GA6.A.374, 4GDI.A.509, 4GDJ.A.507, 3GG1.A.502, 4GG1.A.602, 2GJP.A.1486, 2GKO.A.611, 2GKO.A.612, 3GK2.A.92, 3GN9.A.201, 4GN7.A.301, 1GTT.A.1430, 1GVK.B.1246, 4GW3.A.401, 4GZT.B.510, 2H0K.B.410, 2H0B.A.1000, 4H1Q.A.303, 4H1Q.A.305, 1H3G.A.701, 1H71.P.501, 1H71.P.502, 3H81.A.279, 3H81.C.279, 3H81.C.280, 1H9H.E.1246, 3HB2.P.482, 1HDF.A.1101, 1HFC.A.277, 3HGN.A.250, 3HI7.A.802, 3HJR.A.603, 4HJF.A.601, 1HM9.A.1901, 4HOW.A.704, 4HPN.A.401, 4HS9.A.401, 1HT6.A.502, 1HV5.A.5503, 1HVX.A.517, 1HY7.A.305, 1HYO.A.1006, 2HYU.A.502, 2HYV.A.608, 4HZW.A.507, 4I35.A.513, 1I76.A.996, 1I76.A.997, 2I8U.A.202, 4I8H.A.301, 2I8T.A.402, 3I98.A.627, 3I9G.L.301, 3I9G.L.302, 3IBZ.A.192, 4IHM.A.403, 4IHM.A.404, 1IOD.G.503, 3IOX.A.903, 2IUF.E.1697, 4IU2.A.301, 4IU3.A.301, 2IXT.A.1310, 2IXT.A.1311, 1J0H.A.601, 1J11.A.701, 1J1N.B.493, 1J35.C.501, 4J7M.A.403, 1J8E.A.201, 1J9K.A.301, 1JE5.B.502, 1JI3.B.403, 1JK3.A.403, 2JKP.A.1728, 2JKE.A.1728, 2JKH.A.1245, 4JZB.A.401, 4JZB.A.402, 4JZB.A.403, 4JZE.H.302, 4JZX.A.403, 4JZX.A.404, 4JZX.A.405, 4K1K.A.501, 3K37.B.467, 4K3K.B.401, 1K7I.A.483, 1K7I.A.487, 1K7Q.A.485, 4K70.A.1002, 4K70.B.1002, 4K89.A.408, 4K9P.A.601, 1KA1.A.401, 1KAP.P.616, 1KAP.P.617, 1KAP.P.618, 1KAP.P.619, 1KAP.P.620, 3KCG.H.500, 4KKF.A.703, 3KM5.A.2011, 3KMV.A.163, 4KS1.A.501, 4KTY.A.802, 1KU0.A.703, 4KXY.A.707, 4L74.A.401, 4LLS.A.303, 4LLT.A.303, 3LNH.A.303, 3LNI.A.303, 3LPD.A.342, 1LQV.C.35, 4LQR.A.202, 4LVN.A.703, 3M1H.C.2001, 4M5I.A.201, 3M6L.A.801, 3MA2.A.293, 3MA2.A.296, 3MBR.X.300, 4MB1.A.602, 1MCT.A.246, 4MEW.A.502, 3MHF.A.328, 2MIN.B.525, 4MKM.A.403, 2ML1.A.201, 2ML1.A.202, 2ML1.A.204, 2ML2.A.201, 2ML3.A.203, 2ML3.A.204, 3MMZ.A.500, 1MNC.A.283, 3MOS.A.1, 4MPR.A.601, 3MVS.A.211, 3MVS.A.215, 4MWL.A.512, 3MW3.A.301, 4MWV.A.512, 3N1U.A.200, 4N20.A.705, 4N20.A.706, 4N2B.A.705, 4N2E.A.705, 4N2G.A.705, 4N2I.A.705, 4N2P.A.201, 4N4E.E.405, 1N7V.A.601, 1N9E.A.802, 4NAS.A.503, 1NBW.A.650, 4NEH.B.701, 3NIF.D.2002, 3NJH.B.502, 3NKQ.A.1003, 1NKG.A.800, 1NNL.B.2002, 4NOT.A.302, 1NPC.A.320, 1NRW.A.903, 1NSC.A.468, 1NUD.A.703, 4NUQ.A.301, 4NUZ.A.1001, 1NX1.A.3, 2O72.A.403, 1OAC.A.802, 1OAH.B.1526, 1OBO.A.501, 4OCI.A.201, 3OJY.A.555, 3OJY.B.538, 4OKH.B.904, 1OM6.A.701, 1OM8.A.705, 3OM5.B.1, 3OM6.B.1, 3OOY.A.621, 3OTJ.E.1000, 1OU9.A.131, 4OUL.B.1201, 3OYR.B.337, 3OYR.B.338, 3P2P.B.126, 2P3U.B.501, 3P4G.A.401, 3P4G.A.402, 3P4G.A.406, 3P4G.A.408, 3P4G.A.409, 3P4G.A.410, 3P4G.B.411, 3P95.A.1, 3PGB.A.903, 2PHI.B.125, 4PHN.A.302, 3PKO.A.280, 4PMX.A.401, 2PNY.A.228, 3POJ.B.1, 2POO.A.805, 3PPE.A.401, 1PW9.A.404, 1PZ7.A.701, 2PZ0.A.501, 2Q16.A.200, 3Q2L.B.703, 1Q3A.A.466, 3Q4W.A.224, 4Q8K.A.501, 1QCN.A.1001, 1QCO.A.1002, 4QD2.E.302, 2QIM.A.158, 1QLB.A.1658, 4QN6.A.501, 4QN7.A.501, 3QRB.A.301, 3QU7.A.230, 3QU7.B.225, 2QUB.A.614, 2QUB.A.615, 2QUB.A.616, 2QUB.A.618, 3QXG.B.230, 4R12.A.809, 2R1B.A.1001, 2R5N.A.2000, 1R6V.A.1, 2R8O.A.670, 2R8Y.A.201, 2R8Z.A.201, 3R8Y.A.242, 2R8P.A.670, 2RA3.A.1, 2RA3.B.1, 1RM8.A.502, 1RQ5.A.819, 3RQ0.A.301, 3RRX.A.901, 3RUP.A.1004, 3RVV.A.225, 3RVW.A.223, 1RXO.B.477, 1S0B.A.1292, 3S4Y.B.1303, 3S5U.E.221, 1SAT.A.476, 2SAS.A.187, 3SAL.A.601, 1SBH.A.290, 1SCB.A.276, 1SIO.A.601, 3SO0.D.97, 1T1E.A.700, 3T3P.B.2003, 1T5S.A.1004, 3TEW.A.800, 3TI4.A.601, 1TO2.E.450, 1TRK.A.681, 4TSH.B.1502, 1TU5.A.902, 3U1R.A.703, 3U1R.A.704, 3U1R.A.705, 3U1R.A.706, 3U1R.A.707, 3U8D.A.203, 3U8I.A.2

01, 1ULV.A.2001, 1ULV.A.2002, 4UM9.B.2002, 3UPT.A.691, 4UP4.A.501, 4UP4.A.502, 1UR4.A.1398, 4USU.A.1471, 1UTM.A.247, 2UWF.A.1369, 1UX6.B.2002, 1UX6.B.2003, 1UX6.B.2004, 1UX6.B.2007, 1UX6.B.2010, 1UX6.B.2012, 1UX6.B.2016, 1UYX.A.1133, 1UYX.A.1134, 4UZU.A.1484, 1V0Z.B.1477, 3V5U.A.705, 3V96.B.303, 3V96.B.304, 1VCL.A.1004, 1VCL.B.1001, 1VCL.B.1002, 1VL9.A.125, 2VNG.B.1214, 2VOV.A.1338, 3VOC.A.501, 3VTO.A.302, 3VV3.A.401, 3VV3.B.404, 2W1W.B.1135, 1W7C.A.802, 3W7T.A.1001, 1WAD.A.116, 4WA3.A.503, 2WFK.A.1250, 4WIW.A.702, 4WK0.B.502, 4WK0.B.503, 4WK7.A.504, 1WMD.A.1003, 2WNV.B.1225, 3WN6.A.502, 2WOY.A.2414, 2WOB.E.1161, 1WPC.A.502, 1WRZ.A.154, 2WW3.A.800, 1WZA.A.601, 3X17.A.602, 1XKD.A.1005, 2XR9.A.1869, 2XSG.B.1772, 2XVT.F.1137, 2Y09.A.1242, 1Y6W.A.149, 2Y6D.A.1266, 1Y7B.A.3001, 1Y93.A.266, 1Y93.A.268, 1Y9Z.A.604, 1Y9Z.B.605, 2YAY.A.1267, 2YEQ.A.1526, 2YGL.A.1413, 1YI7.A.3001, 2YN3.A.6362, 2YN5.A.6362, 2YN5.A.6363, 1YO8.A.1183, 1YO8.A.1185, 1YO8.A.1191, 1YO8.A.1193, 1YO8.A.1199, 1YO8.A.1205, 1YO8.A.1206, 1YO8.A.1209, 2YOA.A.1003, 1YS1.X.400, 1YS6.A.1001, 2Z2X.A.1004, 2Z2X.A.1005, 2Z2X.A.1007, 2Z30.A.1006, 1Z6O.A.5302, 1Z70.X.3001, 2Z8X.A.620, 2Z8X.A.622, 2Z8X.A.623, 2Z8X.A.624, 2Z8X.A.626, 2Z8S.B.643, 2ZE0.A.552, 2ZPR.A.2001, 2ZUX.A.630, 2ZUY.A.626, 3ZWH.A.501, 2ZWO.B.400, 3ZXH.A.304
[1] "Cluster 2"
4KZW.B.400, 2AU0.H.1415, 4BXO.A.2050, 2C2R.A.1344, 2C28.A.1344, 4DU4.A.1003, 4EFJ.C.101, 4EFJ.A.401, 3GIJ.B.1415, 2GIH.B.401, 4K4H.M.605, 4K4I.E.606, 3KHL.B.1417, 4KHW.A.1005, 1M5X.C.801, 3M9M.B.354, 3NHG.A.909, 2NOF.A.328, 3OOR.A.237, 4QOW.B.1001, 3QER.A.906, 3RBD.A.415, 4RI8.A.1101, 3SQ2.A.906, 3V20.A.302, 2WTF.A.1512, 4WUZ.B.301, 1A85.A.996, 1AEI.D.317, 1AEI.A.317, 4AG4.A.5002, 3ATS.A.380, 2AZ1.D.202, 2B00.A.252, 1B1G.A.77, 3B2Z.C.3, 4B7U.B.401, 1B90.A.930, 3B90.B.702, 3BCF.A.705, 4BCU.A.201, 1BK9.A.200, 3BS6.A.1, 4BTX.A.1764, 4BWE.A.503, 4CAG.A.606, 2CDP.C.1140, 2COL.B.801, 1CVM.A.802, 3D4G.A.484, 3D4G.A.485, 3D6E.B.202, 1D8F.A.305, 3DEM.B.6001, 3DF0.A.717, 3DF0.B.601, 4DLK.B.402, 1DM5.E.1135, 3E1I.B.503, 2EAA.C.904, 4EJ7.A.402, 4EJ7.A.404, 4EM6.B.604, 4EMU.B.401, 1ESL.A.164, 2EXJ.D.2004, 2FH3.A.1001, 1FZA.C.1, 4G1M.B.2001, 1G5N.A.403, 4GEJ.A.201, 3GG1.B.503, 4GGB.A.402, 4GH8.B.203, 3GRI.B.700, 1GU6.A.1480, 2GXS.B.604, 2HOK.A.402, 2HOK.A.408, 1H71.P.500, 3HDB.A.657, 2HF4.A.902, 1HOV.A.168, 2HTY.A.991, 4I9F.A.401, 2IAP.A.402, 3IAE.B.572, 3IJE.B.4002, 4ILW.F.304, 3IRH.A.457, 1IVG.B.470, 2J0T.C.1268, 2J1G.F.1291, 2J60.C.1277, 2JF9.P.1014, 4JP8.A.706, 2K0J.A.503, 3K39.F.1000, 3K8Y.A.167, 3KQA.C.420, 4KS3.A.502, 1KTW.A.5, 1KVO.D.192, 1KVY.A.124, 4LIN.A.1301, 4LN6.G.403, 1LWU.K.4, 3M0W.G.202, 4M7K.H.301, 3M83.B.408, 4MBE.D.201, 3MDO.A.391, 2MG9.A.101, 1MTS.A.480, 1MWN.A.100, 1N28.B.127, 4N25.A.705, 4N2D.A.705, 3N9V.A.64, 4N96.B.401, 4NAS.C.501, 1NGS.B.681, 2NP0.A.1293, 4NUP.C.301, 4NUP.C.304, 4NVR.C.401, 1OLP.A.1372, 1OS2.A.872, 2OVX.B.447, 2OVX.B.450, 3P1O.A.234, 2P5V.B.1008, 2P5V.C.1001, 2P5W.B.701, 1PZ8.C.703, 3Q2N.B.304, 1Q3A.A.467, 4Q4Y.1.5006, 1Q7B.B.9003, 1QDO.C.245, 1QHD.A.603, 1QMD.A.404, 4QN3.A.501, 2QVF.B.703, 3QWU.B.602, 2R1D.I.3000, 4R5O.A.509, 2R8Y.I.209, 1RFN.A.500, 2RHP.A.16, 1RJV.A.112, 4RSR.A.404, 1S0E.A.1292, 3S5U.F.220, 1SEL.B.277, 3SH5.A.196, 3SHI.M.305, 3SNZ.A.97, 1SPJ.A.300, 1SRR.C.532, 1UBN.A.277, 3UBH.A.855, 1UEA.A.4, 1UG9.A.2002, 4UM9.B.2003, 3UMJ.A.902, 2V5C.A.1625, 2VB6.B.1148, 1VE5.B.2003, 2VVD.A.1329, 2W1W.B.1134, 1W1X.A.1479, 3W9T.B.509, 4WFE.A.306, 2WG8.C.201, 2WII.A.1643, 4WIW.C.701, 2WJS.A.5001, 2WJS.A.5002, 3WMW.A.401, 3WNX.A.502, 2WWO.E.800, 1X36.A.269, 1XJL.A.342, 1XJL.A.344, 2XSG.A.1772, 2XTJ.A.1423, 1Y6P.A.217, 1Y6P.B.218, 2YDP.B.502, 2YGM.B.1418, 2Z2D.A.267, 2ZUX.A.629, 2ZUX.A.637, 2ZUY.A.621, 2ZWP.B.401
[1] "Cluster 3"
4K4G.M.603, 3KHH.A.416, 3KHH.B.1417, 3KHL.B.1415, 3MQ6.A.340, 3NDK.A.906, 4QWE.A.703, 2VA2.B.1343, 1AF4.A.276, 3AFG.B.541, 1ALA.A.402, 1AWB.B.279, 3AYU.A.419, 4B7R.B.502, 4B7U.D.401, 1B9V.A.500, 4CBX.G.1151, 1CIZ.A.305, 3D4G.B.485, 2DDF.A.475, 2DSN.A.2011, 4DWW.A.301, 1E5J.A.402, 1E8U.A.1002, 1EA7.A.315, 1EGZ.C.300, 3ELM.A.303, 7EST.E.11, 2EXH.C.2003, 2EXK.D.2004, 1F5R.A.800, 3FHA.B.704, 2FYC.B.40

4, 1FZD.F.1, 3G20.A.201, 1G5N.A.404, 1GMM.A.1132, 4H1Q.A.304, 3HLH.D.315, 3HQ8.A
.402, 2HTY.C.993, 2HT5.A.995, 2HYW.A.502, 4HZY.A.507, 2J7A.D.1007, 4JQG.A.305, 4
KS2.A.501, 1KX1.C.301, 4LM8.A.812, 4MIV.C.601, 1N28.A.126, 1NGS.A.681, 3NHH.A.15
1, 4NIY.A.301, 4NPK.A.803, 1OLP.B.1372, 3OSH.A.121, 1OT5.A.602, 2OVX.B.449, 2OVZ
.A.449, 1PZ7.B.702, 2Q1F.B.2002, 1Q3A.B.472, 3Q3L.B.437, 4Q4X.1.5006, 3QHQ.A.230
, 2RHP.A.22, 1RM8.A.504, 1S1D.B.1002, 1SEL.A.277, 1SMP.A.478, 3SO0.A.98, 3SO0.B.
97, 3SON.A.201, 1SPU.A.802, 2TCL.A.174, 1TFX.B.4007, 1TK2.A.1276, 1UG9.A.2003, 1
V3J.B.689, 1V6C.A.505, 3VI4.C.2004, 3VRQ.A.401, 2W1W.A.1132, 1W2M.E.1442, 1WD9.A
.900, 1WY9.A.148, 2YC2.B.201, 2Z8S.B.647, 2ZUY.A.629, 2ZWO.A.403, 4DTJ.A.1003, 4
DTU.A.1003, 4EBC.A.504, 2GIH.A.402, 3QEV.A.906, 1D8M.A.304, 1EN7.A.403, 3FHA.A.7
05, 3GG1.A.503, 2HYW.A.506, 2II1.C.404, 1IJL.A.203, 3O82.B.544, 4OO2.C.601, 4P99
.B.540, 2Q04.C.211, 1QD6.C.2, 1SBH.A.291, 4TQO.H.701, 1UCN.A.1162, 2UX1.K.174, 1
Y1A.A.501, 1ZH2.A.202
[1] "Cluster 4"
4K4I.I.604, 3OOL.A.237, 1AFD.2.2, 1AR1.A.561, 1B09.C.302, 3BJU.C.608, 3GCW.E.1,
4L06.B.501, 4L06.D.501, 4LT6.A.602, 1LWJ.A.883, 3MW3.A.302, 2OKX.A.4004, 3QQZ.A.
324, 3QWU.A.601, 3S5U.A.220, 4SBV.A.261, 1TN3.A.183, 2W2O.E.1333, 2WW0.F.800, 2Y
N3.C.6370, 3FSP.A.501, 3KHH.B.1416, 1M5X.A.802, 3NE6.A.905, 4PTF.A.1202, 1S9F.A.
4001, 2WTF.B.1510, 2A8K.C.403, 3B1T.A.900, 4BY5.C.1187, 3CGT.A.686, 1D7F.A.5003,
 3DAS.A.351, 4DRZ.A.202, 1E8U.B.1003, 3EHJ.A.1, 3FCU.B.2002, 1FHF.A.501, 4FU4.B.
505, 1HDF.B.1101, 3HLI.D.315, 4I5N.B.601, 4JCM.A.706, 3JTX.B.396, 2KAY.A.185, 4K
VJ.A.714, 1KX1.E.502, 1KX1.F.601, 1L6R.A.901, 4LMF.A.303, 1LPG.B.1, 2ML2.A.202,
4NHF.B.301, 3NN7.A.503, 3O5S.A.243, 2PF2.A.174, 4PLM.A.504, 2PPL.A.481, 1Q7B.A.9
002, 2RDZ.B.1502, 3SO1.B.97, 3TSK.A.304, 3TUY.C.157, 1ULV.A.2003, 1UMS.A.3, 1UZJ
.A.1648, 1V2I.A.1001, 1VFO.A.1001, 2VKH.A.1543, 2VZP.B.1129, 2W2N.E.1334, 2W3J.A
.1139, 3W9T.A.1008, 3W9T.D.503, 4WBQ.B.602, 3WH3.A.501, 2WO9.B.1272, 2WVX.B.801,
 2WVZ.B.800, 2WW0.A.800, 2XQX.B.1949, 1YO8.A.1211, 1AFA.1.2, 1AFB.1.2, 4AK8.A.13
26, 4AYO.A.500, 4AYP.A.500, 4AYQ.A.500, 4AYR.A.500, 1BCH.1.2, 1BCH.2.2, 2CKI.A.9
98, 2DG1.A.3001, 2DSO.B.1002, 4E52.B.401, 1FIF.A.2, 1FIH.A.2, 1FO3.A.700, 1G1T.A
.160, 3G81.A.401, 2GGU.A.401, 2GGX.A.401, 1H80.A.1492, 3IKP.B.401, 3IKR.A.401, 1
JZN.A.1138, 1K9I.B.502, 1K9J.A.402, 3KMB.1.2, 1KWU.A.503, 1KWV.A.503, 1KWW.A.503
, 1KWX.A.503, 1KWZ.B.604, 1KX0.A.504, 1KZA.1.502, 1KZD.1.502, 4KZO.A.501, 4KZO.B
.501, 4KZV.A.302, 1LQV.D.47, 2MSB.B.2, 1MUQ.B.202, 4N32.A.402, 4N33.A.404, 4N34.
A.402, 4N35.A.404, 4N36.A.402, 4N37.A.402, 4N38.A.403, 4NVR.A.401, 2ORJ.A.401, 2
OS9.A.401, 2OX9.A.802, 3P5G.A.500, 3P5H.A.500, 3P5I.A.500, 3P7G.A.1, 3P7H.A.1, 3
PAK.A.300, 1PW9.A.401, 1PWB.B.401, 1RDI.1.2, 1RDJ.1.2, 1RDK.1.2, 1RDL.1.2, 1RDN.
1.2, 1RDO.1.2, 3RQI.A.181, 1RTM.1.2, 1SL5.A.402, 1SL6.A.2, 2VUV.A.200, 2VUZ.A.11
30, 3VYK.A.1007, 1W0Y.L.1156, 2W87.A.1148, 3WH2.A.301, 1WMY.A.202, 1WMZ.A.202, 1
WMZ.B.202, 2XR6.A.1391, 3RAX.B.1416, 1T9I.C.801, 1T9I.C.802, 4A5G.A.1308, 4A6O.A
.2346, 4A6S.A.1122, 3AFG.A.541, 4AFA.A.1267, 4AFC.A.1267, 3ALT.A.201, 3AUJ.A.160
3, 2B6N.A.300, 4B9F.A.153, 1BCJ.2.2, 1BJ3.B.124, 2BO2.A.155, 2BO2.A.156, 2BOI.A.
300, 2BOI.B.600, 2BOJ.A.1116, 2BOJ.C.1117, 3BPS.E.1, 2BP6.A.802, 2BP6.C.805, 1BQ
B.A.353, 2BV4.A.300, 2BV4.B.300, 1BYF.A.201, 4CE8.A.998, 4CE8.B.998, 1CGT.A.686,
 1CIU.A.684, 4COU.A.1270, 4COV.A.1269, 4COY.A.1270, 4CPB.A.1122, 4CPB.D.1123, 3C
QO.A.4001, 4D0E.A.1531, 4D0E.A.1533, 1D2V.A.600, 3DBZ.A.401, 3DCQ.A.116, 3DCQ.B.
116, 2DCK.A.1002, 3DED.A.504, 3DED.B.506, 3DEM.B.4001, 2DSO.A.1001, 1DV8.A.1002,
 4DZT.A.302, 3EHU.A.500, 3EIF.A.1, 2ERV.A.195, 1ESL.A.163, 2EWE.A.703, 2FF3.A.70
1, 1FHF.A.502, 4FHP.A.402, 1FIF.B.2, 2FPW.A.503, 4FVL.A.505, 1G1Q.A.801, 1G5N.A.
408, 1GEN.A.302, 4GER.A.404, 2GGX.C.401, 3GIS.Z.1003, 2GVU.A.500, 2GVV.A.500, 1G
WU.A.1307, 2H2T.B.322, 1HFZ.B.124, 4HHR.A.703, 3HLH.B.315, 3HLI.B.315, 2IAW.A.40
1, 2IAX.A.401, 2IAO.A.401, 3INM.A.521, 3IQT.A.1, 1J34.C.504, 2JDY.A.1116, 2JDY.B
.1117, 2JDM.B.1115, 2JDM.C.1115, 2JDN.A.881, 2JDN.B.881, 2JDN.C.881, 2JKX.A.1641
, 4JSD.A.603, 3K8L.A.700, 4KVL.A.703, 1KWZ.A.504, 4LHK.A.303, 4LHN.A.302, 3LI3.A
.402, 3LI4.A.316, 4LJH.A.201, 1LPZ.B.1, 1LQV.C.42, 4M65.A.404, 1MN1.A.371, 4MZA.

A.612, 4N4E.E.404, 4N7A.A.605, 1NL1.A.204, 1NPC.A.322, 1NZI.A.1001, 2NZM.A.406, 1OUX.B.404, 1OVS.A.402, 1OVS.B.404, 3P7F.D.1, 1PA2.A.307, 1PAM.A.688, 3PAQ.A.300 , 3PAR.A.300, 1PJX.A.491, 3Q9K.A.606, 3R5Q.A.1001, 1SCH.A.302, 1SNN.B.503, 3SO0.A.97, 1SU3.A.904, 1SZB.A.1001, 1TDQ.B.127, 3TH4.L.148, 1USR.B.1573, 1UX7.A.1134, 1V3E.A.4001, 2VNV.A.302, 2VNV.B.302, 2VUC.B.990, 2VUC.C.991, 2VUD.C.1118, 2VZP.A.1129, 2VZR.B.1132, 2W2M.E.1334, 3W57.A.202, 3W5M.A.1201, 3W5N.A.1201, 2W86.A.1149, 1WDC.C.501, 3WH3.A.500, 3WHD.A.501, 2WR9.D.1132, 3WU2.A.401, 2WZS.F.800, 2XFD.A.1111, 2XFE.A.1113, 1XO5.A.1, 1YDY.A.904, 2Z48.A.1007, 2Z49.A.1001, 3ZDV.A.200, 3ZDV.B.200, 1ZH2.B.201, 1ZJA.A.7001, 3ZO9.A.1589, 3ZYH.A.1123, 2IWV.A.1284, 4L06.A.501, 3R6Q.H.469
[1] "Cluster 5"
4K4G.I.602, 4KHN.A.1002, 3QEP.A.906, 3SQ1.A.907, 1A25.A.292, 1A25.B.292, 4AQ8.D.1238, 1AVX.A.700, 4B4F.B.607, 3BOW.A.718, 4BTX.A.1763, 3CK7.B.720, 2CLT.A.1101, 2CLT.B.1301, 3D4G.H.484, 2DEW.X.904, 1DM5.A.1131, 1DM5.C.1133, 1DQ1.A.238, 3E3R.B.195, 3E9T.A.6, 1EAK.D.998, 2EA7.B.452, 1EE6.A.300, 1EGZ.B.300, 4ELF.F.201, 1ESP.A.320, 2F2O.B.1001, 1F2N.C.1001, 3FCS.D.2002, 4FGC.C.203, 4FU4.A.505, 3FW0.A.823, 2GA9.D.480, 4GGB.A.401, 4GKX.B.302, 4GZS.A.501, 4H2E.B.304, 1HFY.A.124, 1HFZ.C.124, 4HSZ.B.101, 2HTY.G.997, 1HVD.A.602, 4I5K.A.501, 1IXX.D.124, 1JIW.P.489, 3K1A.B.524, 3K9X.D.249, 3KL6.A.3, 1KLJ.H.900, 4KNA.A.504, 3KQA.A.420, 4KVK.A.712 , 4L06.C.501, 1L7L.A.201, 3L95.X.2001, 3LCP.D.159, 4LIN.E.1301, 4LMF.B.302, 3LND.A.210, 1LQD.B.1, 4LXF.A.701, 3MOW.H.202, 1MMP.B.3, 4O1Q.A.401, 1OS2.C.473, 3PK0.D.280, 4PLS.A.305, 3PRT.A.404, 1PYT.C.650, 1R1Z.B.315, 4R83.D.501, 1RE3.C.408, 2RHP.A.2, 2RHP.A.18, 2RHP.A.24, 2RHP.A.25, 2RHP.A.28, 2RJQ.A.5, 2RLD.C.121, 1ROS.A.404, 3S5U.D.220, 4SBV.C.261, 3SOB.B.1, 1SRR.A.531, 1TAD.A.352, 1TD7.A.1001, 3TH3.L.145, 4TVU.B.600, 1TYE.B.1402, 4UB6.O.301, 3UBH.A.852, 1UZJ.C.3648, 2V3T.A.1264, 1VFO.B.1002, 1VFP.B.995, 2VME.A.500, 3VOT.A.504, 3W9T.C.1010, 3W9T.B.508, 1WD9.A.901, 2WVX.C.801, 2WW0.C.800, 2X0G.B.1149, 2YFS.A.1711, 2YGM.A.1417, 1YO8.A.1184, 1YO8.A.1188, 2Z8S.B.641, 3ZHG.B.1323, 2ZKT.A.413, 1ZTQ.B.565, 2ZWO.B.402 , 1ZZH.A.401, 2AOQ.A.232, 3BQ1.A.4001, 3COW.A.301, 1DMU.A.302, 3EH8.A.302, 3GV5.B.424, 2IBK.A.401, 1JX4.A.4001, 1N3E.C.492, 3QEX.A.907, 1R7M.A.306, 2RDJ.B.353, 3A13.E.445, 4A6S.C.1122, 2AER.L.3008, 1AFA.2.2, 1AWB.A.278, 4AWD.A.1321, 3AZX.B.301, 3BOO.B.301, 3BOI.A.124, 4B7R.A.502, 3BCD.A.708, 3BJU.A.608, 4CAJ.B.1325, 2CDP.B.1139, 1CGU.A.686, 3CK7.D.730, 3CKC.A.600, 5CNA.C.240, 1CR8.A.45, 1CRU.A.503 , 4CRR.A.1386, 1CYG.A.682, 3D4G.B.484, 3D6E.A.202, 1DGL.B.301, 4DLK.A.403, 2DSN.B.2012, 2E9B.A.741, 3EAD.A.1003, 3EDD.A.700, 3EHB.A.563, 3EHJ.B.1, 3ESQ.A.213, 2EXH.D.2004, 1F6S.E.205, 2FIB.A.412, 2FPS.A.503, 4G60.A.301, 4G62.A.301, 1G87.A.615, 1G9K.A.700, 2GGM.B.402, 4GI6.A.601, 3GN4.B.204, 3GQF.D.154, 4GQ7.A.301, 2GSM.A.3007, 4H3X.A.304, 1H5V.A.306, 1HFZ.A.124, 3HGP.A.250, 1HL5.D.156, 1HQV.A.999, 1I22.A.198, 3I4I.A.1001, 2I4B.A.454, 3I57.A.186, 2J1T.A.1154, 1J9L.B.1303, 4JDZ.B.702, 3JXS.A.302, 4K5W.A.201, 2KAY.B.187, 3KF9.C.304, 1KMB.1.2, 3KQA.B.421, 3KZP.A.240, 2L51.A.207, 1L9M.A.702, 3L9I.C.1148, 3LCP.B.279, 3LHM.A.131, 4LJ3.B.403, 1LOC.E.688, 4LVN.A.704, 2MOP.A.1201, 4M5I.A.203, 1MAC.B.389, 1MDU.A.403, 3MVS.A.214, 4N25.A.707, 4N2I.A.704, 3N4E.A.500, 4N85.A.502, 3N8G.A.1002, 4NAM.A.801, 2NXP.A.600, 2OKX.B.4002, 3P4G.A.403, 3P4G.A.404, 3P4G.A.405, 3P4G.A.407, 3P4G.A.412, 4PLS.A.301, 1PVY.B.603, 2Q1C.X.294, 3Q5I.A.528, 3Q8F.A.736, 3QGV.A.504, 2QT6.A.3713, 4QU6.A.904, 1R1Z.C.415, 3RRW.A.271, 3S6J.A.4, 3SLE.B.401, 3SO0.F.98, 3SVL.A.201, 1TCM.B.687, 3TEW.A.801, 1TT2.A.502, 3UBF.A.754, 1UF3.D.913, 1UH2.A.1002, 1UH3.A.1001, 1UH3.A.1002, 1UKS.A.688, 1UPS.A.501, 3V03.A.585, 1V3J.A.687, 1V3L.B.689, 2VCC.A.1917, 2VDR.B.2002, 2VJ3.A.1533, 2VL8.A.1545, 2VVE.B.1338, 2VXJ.G.200, 3W9T.D.504, 1WC5.C.2100, 4WF7.D.600, 2WQS.A.2415, 2YDP.A.502, 1YO8.A.1195, 1YO8.A.1201, 1YO8.A.1203, 1YO8.A.1208, 2Z49.A.1002, 3ZHG.D.1323, 2ZQ0.A.901, 2ZWP.A.402, 4K4G.A.602, 4K4H.A.602, 4K4I.A.605, 4UAW.A.403, 4A3X.A.1268, 4A3Z.A.2344, 3A4U.A.287, 4A41.A.2494, 4A42.A.1690, 4A4A.A.1925, 4A5G.A.1309, 4AFB.A.1267, 1ALC.A.200, 3ALU.A.201, 4AL9.A.1122, 4AOC.A.1129, 4AOC.E.1129, 4AQ1.A.1924, 4

ASL.A.1268, 4ASM.B.1359, 3ATG.A.301, 4ATE.A.1275, 4AWD.B.1321, 3AXD.A.3002, 3AZY
.A.301, 3B0K.A.201, 3B0X.A.578, 1B80.B.351, 1B90.A.124, 4B96.A.1155, 4B9C.A.1151
, 2BF6.A.1693, 1BGP.A.501, 1BIW.B.803, 2BIB.A.1551, 4BLK.A.400, 3BMV.A.685, 4BM1
.A.401, 4CI7.A.1505, 3CK7.A.710, 1CPM.A.215, 1CPN.A.209, 4CP0.A.1294, 4CP1.A.129
4, 3CQ0.A.4004, 4CRQ.A.260, 4CU0.A.1326, 4CZN.A.1370, 1D3C.A.687, 2DEW.X.900, 1D
ED.B.5004, 2E39.A.501, 2E8Y.A.741, 3EDF.A.603, 2EJN.A.1003, 2EXH.A.2001, 1F6S.A.
201, 2FHF.A.2401, 3FMU.A.351, 4G01.A.300, 1GW2.A.502, 1GWT.A.502, 1GWU.A.1308, 4
GWI.A.204, 1GX2.A.1310, 3H00.A.401, 2H2N.B.1001, 1H3G.A.700, 3HB3.A.563, 3HDL.A.
306, 3HDL.A.307, 2HD9.A.2001, 1HFX.A.124, 3HR9.A.401, 2HYK.A.477, 1I22.B.197, 4I
5L.B.601, 4IAI.A.402, 4IAU.A.800, 3ILF.A.278, 2IWV.D.1283, 2J1A.A.1769, 2J22.A.1
150, 4J3V.A.920, 4J3W.A.907, 4JCL.A.701, 2JD9.A.1146, 2JDA.A.1146, 4JGL.A.202, 1
JI1.A.2001, 1JI1.A.2002, 2JKA.A.1727, 1JUG.A.126, 1K12.A.160, 3K8K.A.710, 3K8L.A
.710, 1KZM.A.501, 1L6R.B.903, 1LLP.A.351, 4LPL.A.1101, 4LQR.A.201, 1LY8.A.9001,
1LYC.B.9002, 3M5Q.A.372, 1MAC.A.388, 2ML2.A.203, 2ML3.A.201, 3MMZ.A.501, 1MVE.A.
400, 4N2B.A.707, 4N2G.A.703, 4N2L.A.704, 4N6F.A.302, 3NNG.A.402, 1O4Y.A.700, 4OD
G.A.202, 2OKX.A.4001, 3OMI.A.613, 1OUX.A.402, 3OWF.A.151, 1PA2.A.308, 1PAM.A.687
, 3PGV.A.267, 4PIB.C.201, 1PJ9.A.890, 4Q1U.A.402, 3Q3U.A.340, 4QF4.A.202, 1QGJ.A
.2001, 1QGJ.A.2002, 1R1Z.A.286, 4R83.B.501, 3S18.A.229, 1SCH.A.301, 1SH7.A.1292,
1SNC.A.150, 3SO1.A.97, 3SRE.A.1357, 3SRG.A.1357, 1SU4.A.996, 3TBD.A.401, 1TE2.A
.702, 1TLG.A.201, 3TO5.A.131, 1U0A.A.5004, 1UPS.B.501, 1URX.A.1300, 1UX6.B.2014,
3V6N.A.229, 2V72.A.1139, 2VZQ.A.1130, 2W1Q.A.1947, 2W1S.A.1946, 1W3M.E.3013, 2W
86.A.1148, 3WDH.A.801, 4WF7.A.600, 2WOY.A.2415, 2WQ8.A.1641, 2WR9.A.1131, 1WZL.A
.1601, 2WZA.A.2415, 2YFU.A.1141, 2YLJ.A.1308, 1YRO.A.124, 2Z30.A.1002, 2Z48.A.11
02, 2Z48.A.1103, 2Z48.A.1104, 2Z48.A.1205, 2Z49.A.1003, 2Z49.A.1004, 2Z49.A.1005
, 3ZUC.A.1154
[1] "Cluster 6"
3TRQ.A.359, 2B0D.A.502, 4G3I.A.401, 4K4I.E.603, 3KHG.A.415, 4KHU.A.1003, 2NOL.A.
328, 3OOR.A.236, 4QWD.A.702, 3RBD.B.1415, 4AAH.A.702, 4AC8.B.1311, 1ALA.A.401, 1
AWB.B.280, 1AXK.A.395, 2BD4.A.260, 1BRW.A.3001, 2BZ6.H.1260, 3C14.A.29, 1C9P.A.5
01, 2CLT.A.1102, 1D8M.B.804, 1FBL.A.993, 1FBL.A.994, 4FVL.A.506, 4H82.C.305, 1HK
B.A.923, 3HQ8.B.402, 1HVD.A.600, 2II1.D.401, 2IO4.B.701, 3IS5.F.1, 1ITC.A.1500,
1J24.A.1001, 1JDC.A.452, 4KW7.A.402, 4L41.B.201, 2LMV.A.151, 4MC7.A.503, 4MIX.A.
2501, 4N2I.A.707, 1NMB.N.478, 3NSJ.A.702, 4NUY.A.1001, 3OHO.A.1, 4OKH.C.903, 2OV
Z.B.449, 3OXQ.D.516, 2OZR.F.4030, 4P99.A.533, 2PC6.B.303, 1PEX.A.502, 4PIB.B.203
, 4POQ.G.401, 4POR.E.401, 2PR3.A.901, 4Q4X.1.5007, 1QMD.B.405, 3R4I.D.342, 2RJP.
D.2, 3RMK.B.308, 1TFX.A.1007, 3V96.B.305, 3VEQ.B.301, 2VME.E.500, 3VOB.A.401, 2W
0Q.B.803, 3W9T.B.510, 1Y7O.B.1004, 1YAX.A.1002, 2YA9.A.1303, 2YN3.D.6355, 2Z2D.A
.268

Table S69. all-ligand-number Ca, compressed group

	size	largest_angle*	middle_1*	middle_2*	middle_3*
1	"341"	"173.4+/-2.8"	"72.9+/-3.1"	"85.5+/-2.1"	"98+/-3"
2	"322"	"164.2+/-3.2"	"73.3+/-3.2"	"84.4+/-2.5"	"100+/-3"
3	"214"	"162.6+/-3.9"	"71.9+/-4.2"	"82.7+/-2.1"	"108.1+/-3.4"
4	"214"	"158.1+/-5.9"	"52.2+/-2.8"	"80.8+/-3.7"	"111.7+/-5.3"
5	"68"	"159.4+/-12.1"	"69.3+/-8.3"	"80.5+/-6.8"	"91.6+/-6.6"
6	"83"	"151.7+/-7"	"68.6+/-5.9"	"81.8+/-4.5"	"109.7+/-8.2"
7	"263"	"162.4+/-6.6"	"51.4+/-2.8"	"82.7+/-4.2"	"98.6+/-6.1"
8	"45"	"149.6+/-12.9"	"52.4+/-3.6"	"83.6+/-7.8"	"100.5+/-10.5"
9	"40"	"126+/-11.6"	"51.5+/-3.3"	"73.8+/-7.1"	"82.1+/-7.9"

```
10 "280" "170+/-3.2"    "69.5+/-4.1" "85+/-2.3"   "102.1+/-3.9"
   middle_4*      smallest_opposite_angle* Tetrahedral TrigonalBipyramidal
1  "157.2+/-3.5"  "51.6+/-2.1"             "0.023"     "0.19"
2  "155.6+/-4.3"  "51.7+/-2.2"             "0.025"     "0.135"
3  "156.3+/-3.3"  "51.5+/-2.3"             "0.032"     "0.103"
4  "151.1+/-5.9"  "70+/-5.4"               "0.023"     "0.03"
5  "119.5+/-10.9" "53.2+/-6"               "0.012"     "0.013"
6  "143.8+/-5.3"  "51.6+/-3"               "0.014"     "0.033"
7  "151.9+/-8.3"  "73.8+/-7"               "0.018"     "0.037"
8  "129.2+/-10.6" "105.8+/-17.3"           "0.027"     "0.022"
9  "96.9+/-10.9"  "85.3+/-14.3"            "0.004"     "0"
10 "164.4+/-3.4"  "51.1+/-2.6"             "0.034"     "0.102"
   TrigonalBipyramidalVA TrigonalBipyramidalVP Octahedral SquarePyramidal
1  "0.195"               "0.282"               "0.111"    "0.257"
2  "0.177"               "0.239"               "0.081"    "0.211"
3  "0.156"               "0.215"               "0.066"    "0.176"
4  "0.088"               "0.121"               "0.023"    "0.065"
5  "0.03"                "0.091"               "0"        "0.039"
6  "0.079"               "0.109"               "0.013"    "0.052"
7  "0.096"               "0.13"                "0.022"    "0.074"
8  "0.046"               "0.035"               "0"        "0.001"
9  "0.026"               "0.053"               "0"        "0"
10 "0.124"               "0.282"               "0.168"    "0.277"
   SquarePyramidalV SquarePlanar TrigonalPrismatic TrigonalPrismaticV
1  "0.3"            "0.28"       "0.078"           "0.192"
2  "0.271"          "0.258"      "0.12"            "0.211"
3  "0.246"          "0.216"      "0.155"           "0.222"
4  "0.139"          "0.093"      "0.063"           "0.153"
5  "0.078"          "0.079"      "0"               "0.049"
6  "0.132"          "0.05"       "0.063"           "0.131"
7  "0.171"          "0.098"      "0.044"           "0.12"
8  "0.014"          "0.001"      "0"               "0.018"
9  "0.064"          "0"          "0"               "0"
10 "0.316"          "0.308"      "0.087"           "0.17"
   PentagonalBipyramidal PentagonalBipyramidalVA PentagonalBipyramidalVP
1  "0.15"                "0.148"                 "0.233"
2  "0.129"               "0.135"                 "0.207"
3  "0.112"               "0.128"                 "0.181"
4  "0.024"               "0.075"                 "0.058"
5  "0"                   "0"                     "0"
6  "0.015"               "0.062"                 "0.056"
7  "0.025"               "0.047"                 "0.057"
8  "0"                   "0"                     "0"
9  "0"                   "0"                     "0"
10 "0.063"               "0.068"                 "0.179"
   SquareAntiprismatic SquareAntiprismaticV HexagonalBipyramidal
1  "0"                 "0.109"              "0"
2  "0.003"             "0.14"               "0"
3  "0.005"             "0.17"               "0"
4  "0.004"             "0.054"              "0"
5  "0"                 "0"                  "0"
6  "0.003"             "0.027"              "0"
7  "0.003"             "0.046"              "0"
8  "0"                 "0"                  "0"
```

```
9  "0"                "0"                "0"
10 "0"                "0.122"            "0"
   HexagonalBipyramidalVA HexagonalBipyramidalVP
1  "0"                   "0.063"
2  "0"                   "0.044"
3  "0.001"               "0.04"
4  "0"                   "0.01"
5  "0"                   "0"
6  "0"                   "0.006"
7  "0"                   "0.013"
8  "0"                   "0"
9  "0"                   "0"
10 "0"                   "0.065"
```

Table S70. Cluster members of all-ligand-number Ca, compressed group

[1] "Cluster 1"
2DPK.A.4001, 3EH8.D.303, 2FLD.A.601, 2I3P.A.154, 2I3Q.B.81, 3LDY.A.145, 3PR4.A.3
43, 2A3W.R.336, 3A7Q.A.4003, 1ANW.A.351, 4AQA.A.1210, 2BB4.A.260, 1BCJ.2.3, 2C10
.A.1773, 4DD8.B.1001, 1DJX.A.2, 1DSY.A.501, 2DW2.A.703, 2FH2.B.2001, 1FZC.B.2, 3
GIN.A.2, 1GU6.A.1479, 1H5V.A.305, 2HYW.B.514, 2II1.B.400, 1J1N.A.493, 2J7A.E.100
6, 4JDZ.B.703, 4JX1.A.504, 3K5S.A.218, 3K7L.A.702, 4KDV.A.202, 1KTW.B.8, 4KTR.D.
814, 4N2I.A.706, 1N47.A.252, 1NKQ.F.560, 1NX2.A.3, 4OMD.D.605, 1OT5.A.601, 2OXE.
A.600, 2P37.C.239, 4P99.D.508, 2Q16.B.200, 2Q17.E.315, 2QT7.B.201, 3RHT.A.257, 1
SL6.A.3, 1U94.A.701, 3UBR.B.476, 3V03.A.586, 3VI3.A.2001, 2VY0.A.1298, 3WN6.B.50
1, 2XVT.C.1137, 1Y3N.A.493, 2Z8Z.A.618, 2Z8S.A.646, 2ZJ7.A.619, 2ZUX.A.638, 2ZUY
.A.628, 3DR2.A.707, 4AQU.A.1154, 4AQU.A.1156, 3EH8.A.301, 1G9Y.B.802, 3MXB.A.175
, 3ODH.B.195, 1SOM.A.402, 2VBJ.A.1154, 2VBJ.A.1155, 2VBO.A.1154, 2VBO.A.1155, 2V
S7.A.1183, 1A75.A.110, 1A8A.A.407, 3AMR.A.905, 4APX.B.1238, 4AQA.A.1208, 4AQA.A.
1209, 4AQE.A.1207, 4AQO.A.1881, 1AVA.A.500, 4AVS.A.207, 2AYH.A.417, 2AZZ.A.125,
3B2Z.A.2, 2B96.A.124, 3BEU.A.249, 3BI1.A.1753, 4BJ0.A.1167, 3BNG.A.508, 1BQB.A.3
52, 4BQ2.A.1797, 1BSW.A.900, 2C26.A.302, 2C4X.A.1252, 4CAG.A.604, 4CAG.A.610, 1C
B8.A.3000, 2CBL.A.352, 4CBU.G.1150, 2CDO.A.1139, 2CFT.A.1297, 3CHJ.A.338, 3CHK.A
.503, 3CIP.G.201, 2CKI.A.997, 2CM5.A.1679, 2CM6.A.1680, 2CN3.A.1778, 4CTE.B.280,
 1CVR.A.504, 1DBI.A.701, 1DBI.A.703, 1DBI.A.704, 3DEM.A.8001, 4DIR.A.102, 3DNZ.A
.403, 4DUQ.A.101, 1DVI.A.271, 2DWO.A.703, 4DZ3.B.202, 2E26.A.604, 2E26.A.605, 2E
85.A.1002, 3ECQ.A.2001, 1EDM.B.2, 1EXR.A.1004, 4F0Z.B.202, 4F0Z.B.203, 3FED.A.17
55, 2FI1.A.191, 2FMJ.A.338, 3FO3.A.527, 1FS7.A.651, 2FVY.A.311, 1G5N.A.401, 1G9G
.A.630, 1G9K.A.701, 3GBO.A.301, 1GCA.A.351, 1GCY.A.528, 4GER.A.403, 1GGZ.A.150,
1GGZ.A.151, 1GPL.A.500, 3GPE.A.502, 1H6Y.A.1161, 1H80.A.1493, 3HB2.P.481, 1HDF.A
.1102, 3HDB.A.621, 3HI7.A.803, 3HJR.A.602, 1HPL.A.960, 2HQ8.A.201, 1HT9.A.1001,
3HX4.A.601, 3HX4.A.602, 1HY7.A.304, 2HYV.A.607, 4I35.A.511, 3IBZ.A.193, 2ID4.A.9
06, 3IGO.A.602, 3IKW.A.1, 3IK2.A.514, 4IMM.A.407, 2IPL.A.502, 2J7A.A.1006, 1J83.
A.4001, 4JDZ.B.701, 4JGU.A.901, 1JI1.A.2003, 1JIA.A.134, 3JQ1.B.1, 3JXS.A.301, 4
JX1.E.505, 3K21.A.192, 1K96.A.91, 4K90.A.702, 1KAP.P.614, 3KCP.A.701, 3KHE.A.192
, 3KHE.A.195, 3KM5.A.2012, 1KQU.A.301, 3KQR.A.205, 3KWU.A.901, 3LCP.C.159, 4LDC.
A.502, 1LE6.A.461, 4LJ3.A.402, 4LM8.A.811, 4LOS.A.401, 1LPB.B.453, 3LPA.A.361, 1
LQV.C.34, 4LXO.A.1601, 4LXO.B.1601, 3M1H.A.2002, 4M2P.A.301, 4M5E.A.506, 1M8T.A.
1001, 1MCX.A.352, 4MEW.A.501, 4MNO.A.303, 1MPX.A.638, 3MSE.B.180, 4N1G.A.202, 4N
1G.A.204, 4N2B.A.709, 4N2G.A.704, 4N2N.A.704, 4N5X.A.203, 3N5A.A.101, 1N9E.A.803
, 1NIW.A.1004, 1NNL.A.2001, 1NPC.A.321, 3NQX.A.511, 4NUQ.A.303, 2NXP.D.603, 2NZM

.A.405, 2O72.A.401, 2O9Q.A.501, 1OAC.A.803, 1OAH.A.1525, 1OAH.A.1526, 1OF3.A.117
4, 1OH4.A.1186, 1OHZ.B.1057, 4OMC.A.605, 2OW9.B.610, 3OX6.B.501, 4OY7.G.302, 3P6
B.A.205, 1P8J.A.3001, 4P99.C.505, 3PDD.A.193, 3PF2.A.1, 3PGB.A.902, 4PHN.B.303,
4PKG.G.1201, 3PM8.A.1, 1PMH.X.300, 1POA.A.201, 1POC.A.501, 3POJ.A.1, 2PPL.A.478,
2PQX.A.500, 2Q17.A.315, 3Q2N.A.303, 3Q5I.A.1, 3Q5I.A.524, 3QGM.A.503, 2QQM.A.1,
3QRO.A.1000, 3QRB.A.303, 2QUB.A.617, 2QUB.A.620, 1R55.A.515, 3R5V.B.201, 1R64.A
.701, 2RAN.A.324, 2RDZ.A.1501, 2RF7.A.1501, 1RLW.A.400, 1RRO.A.110, 1S3P.A.210,
3S5U.A.221, 1S6B.A.401, 2SCP.A.192, 1SGT.A.246, 1SH7.A.1291, 3SIB.A.221, 3SJG.A.
1753, 1SL6.D.3, 1SL8.A.669, 1SL8.A.670, 1SL8.A.671, 3SNY.A.97, 3SRE.A.1356, 3SXQ
.A.526, 3T3P.B.2002, 1T44.G.702, 1TF4.A.3002, 3TOY.A.361, 4TSH.B.1501, 3TZ1.A.1,
3U1R.A.701, 3UBH.A.857, 1UX6.B.2001, 1UX6.B.2008, 1UX7.A.1135, 3UXF.A.2, 1V0A.A
.1177, 2V0B.A.1241, 4V29.B.1178, 3V9M.A.205, 1VL9.A.124, 2VPT.A.1235, 3VRR.A.401
, 2VVE.A.1336, 1WON.A.1132, 3W57.A.201, 1W7C.A.803, 3WCT.F.203, 4WK7.A.502, 3WLC
.A.501, 2WN3.A.400, 2WN3.C.400, 2WPH.S.1246, 3WU2.c.901, 2WW8.A.1001, 2WZE.B.155
2, 4XDQ.A.301, 2XFG.B.1619, 2XFD.A.1112, 1XJO.A.905, 2XOM.A.1169, 1XVJ.A.422, 1Y
4J.A.1001, 2Y6L.A.1168, 2YIH.A.1520, 2YKK.A.1520, 2YN5.A.6364, 1YO8.A.1198, 1YO8
.A.1200, 2YOA.A.1001, 1Z70.X.3002, 2Z8X.A.618, 2Z8X.A.621, 2ZEZ.A.200, 2ZFD.A.22
7, 2ZFD.A.228, 2ZP4.A.124, 2ZUX.A.635, 2ZUX.A.636, 2ZUY.A.625
[1] "Cluster 2"
2ASJ.B.1415, 4K4H.E.604, 1DTH.A.903, 3GE4.B.167, 1JKU.B.2272, 1JKU.E.5272, 3MIS.
A.2, 1AUI.B.502, 1AVA.A.501, 3B1T.A.903, 3BOW.A.717, 4BQ3.B.1803, 4CAG.A.608, 4C
AG.A.609, 2CDP.C.1139, 2CHN.A.1717, 3CK9.A.710, 3D94.A.2, 1DAN.L.155, 2DBX.B.701
, 1DE4.C.801, 2DIE.A.778, 1E8A.B.1090, 4EJ7.A.403, 1F9O.A.2000, 3FAX.A.4, 2FHF.A
.2405, 3FLP.B.302, 4G9L.A.304, 1GCY.A.529, 2HIH.A.603, 3HTL.X.1, 4ILW.D.305, 2IW
V.B.1281, 2JBK.A.803, 3K5T.A.803, 1K7Q.A.481, 3K7N.A.701, 3K7N.A.702, 3K7N.A.703
, 1K9I.F.903, 4KTR.G.806, 3L2Y.A.302, 1L9N.B.704, 4LP7.A.301, 1M56.A.1007, 4MHX.
A.601, 4MIV.E.600, 4MVF.A.604, 1N2K.A.600, 4NDD.B.401, 4NHD.B.403, 1NTO.A.3001,
2OA8.C.302, 1OBR.A.404, 4OKH.A.901, 2OO4.A.5003, 3OUU.B.455, 4P99.A.501, 4P99.B.
509, 4PIB.C.204, 2PMY.A.103, 3PVN.B.5003, 3PVN.B.5004, 4Q6P.A.509, 1S2N.A.1291,
2SEC.E.276, 1SU3.B.907, 1SVN.A.276, 4UM9.C.2002, 3UNX.A.280, 1VFP.A.995, 3VI3.A.
2004, 2WL3.A.1290, 1WMY.B.203, 1Y4D.E.1001, 2Z57.A.1006, 2Z8S.A.648, 2Z8S.B.646,
2ZEY.B.152, 3HR6.A.1, 2JBH.A.1228, 4U6D.A.501, 4U6D.B.502, 1YCM.A.267, 1G9Y.A.8
01, 4KLD.A.403, 3MQ6.A.1, 3MX9.A.363, 3QNN.A.903, 3QNO.A.903, 4UB4.A.401, 3A24.A
.1268, 1A2Q.A.295, 3A8R.A.401, 2AAO.A.293, 1AFO.A.476, 1AK9.A.295, 3AKB.A.171, 3
AKB.A.172, 3AKB.A.175, 3AMR.A.907, 3AMR.A.908, 4AQ1.A.1923, 4AQ1.C.1923, 4AQJ.A.
1097, 1AVS.A.93, 1AXN.A.353, 4AYU.D.205, 3BC9.A.701, 1BF2.A.751, 1BH6.A.501, 1BJ
F.A.402, 1BLI.A.500, 1BQB.A.351, 1BU3.A.109, 1BU3.A.110, 3BYK.A.474, 4BY5.A.1183
, 4BY5.A.1185, 3C1Q.B.1, 3C3Y.A.238, 2C4X.A.1253, 2C4F.L.1143, 1C9N.A.277, 4CAG.
A.603, 4CBU.G.1151, 2CCL.B.1060, 2CCM.A.1192, 2CCM.A.1194, 4CCD.A.3669, 2CDP.A.1
139, 4CFQ.A.502, 4CFY.A.301, 1CJY.A.950, 1CP9.B.554, 4CPV.A.110, 1CRU.A.501, 2CT
9.A.301, 3CZT.X.93, 4DA2.A.301, 2DCK.A.1001, 3DO1.A.401, 1DTL.A.201, 4DUQ.A.102,
2DWO.A.701, 2DWO.A.702, 2E26.A.601, 2E4T.A.701, 1E43.A.502, 1E8A.A.1090, 1EDH.A
.302, 3EDF.A.602, 2EGD.A.302, 2EHB.A.1001, 3ETO.A.2003, 2EWE.A.701, 1EXR.A.1002,
2FH1.A.2002, 3FIA.A.201, 3FLP.A.301, 3G5C.A.801, 1G8I.B.1600, 1G9K.A.702, 1GGZ.
A.149, 4GGF.C.204, 4GGF.L.204, 2GJP.A.1487, 4GUK.A.207, 4H1Q.B.304, 1H2G.B.1558,
3HDB.A.622, 3HJR.A.601, 1HQV.A.998, 2HQ8.A.203, 1HT6.A.500, 3HX4.A.604, 3HX6.A.
1, 2HYV.A.605, 2HYW.A.503, 4I2Y.A.501, 4I2Y.A.503, 1I82.A.192, 2ID4.A.901, 4IEF.
B.702, 3IGO.A.603, 2II1.C.400, 4ITC.A.1202, 1J1A.A.201, 1J55.A.102, 1JK3.A.404,
3JQW.A.1001, 3K21.A.193, 3K21.A.194, 1K7I.A.480, 1K94.A.999, 1K9U.A.1001, 1K9U.A
.1002, 1KAP.P.615, 4KDW.A.201, 3KHE.A.193, 3KHE.A.194, 1KVW.A.124, 3L2Y.A.301, 3
LND.B.208, 3LNI.A.302, 3LPC.A.361, 2LRP.A.202, 4M5E.A.505, 1MCX.A.351, 1MKV.A.12
4, 4MSP.A.201, 1MXE.A.506, 4N1G.A.203, 3N4E.B.500, 4N5X.A.204, 4NEH.A.1101, 4NEH
.A.1103, 1NIW.C.1007, 1NPC.A.319, 3O4Y.A.198, 2O5G.A.404, 2O8O.A.1009, 1OBO.A.50
2, 4OKH.A.902, 4OMC.A.607, 2OO4.A.5001, 2OPO.A.301, 2OPO.A.302, 2OX9.A.804, 1OYG

.A.500, 2POR.A.1002, 5PAL.A.111, 4PEO.X.103, 4PHJ.A.303, 4PHJ.A.304, 4PLS.B.301, 2PMY.B.104, 3PM8.A.514, 3PM8.B.1, 1PMJ.X.300, 1POB.A.801, 1POE.A.801, 1PVA.A.110, 1PVA.A.111, 3PVN.E.5009, 3PVN.M.5026, 2PVB.A.110, 3Q2L.A.701, 3Q5I.A.526, 1Q5P.A.271, 4Q6O.A.502, 1QI3.A.451, 2QQO.A.1, 1QTX.A.153, 1QTX.A.154, 1QX2.A.1001, 1QX2.B.1006, 1ROR.E.302, 2R2I.A.500, 2R2I.A.501, 2R2I.A.502, 1RFJ.A.1001, 1RFJ.A.1003, 1RFJ.A.1004, 3RM1.A.102, 1RWY.A.421, 1S01.A.295, 1S02.A.276, 1S6C.A.217, 2SCP.A.190, 2SCP.A.191, 3SIB.A.222, 1SL6.E.3, 1ST3.A.270, 1SUD.A.295, 1TCF.A.160, 1THM.A.301, 3TI7.A.353, 3TI7.A.355, 3TI9.A.353, 3TI9.A.355, 1TKF.A.905, 4TNC.A.164, 5TNC.A.163, 3UJO.B.304, 3UL4.B.67, 4UM9.A.2001, 1UXX.X.1130, 1UY4.A.1147, 1UYZ.A.1132, 1VCL.A.1001, 1VCL.A.1005, 2VDQ.B.2002, 2VVD.A.1328, 3VYV.A.303, 2VZP.A.1128, 2W47.A.1137, 3W57.A.203, 2WBX.A.1103, 2WD6.A.1765, 3WFD.B.806, 3WHI.A.401, 3WHT.B.501, 4WK7.A.503, 2WND.A.102, 2WNO.A.1251, 1WPC.A.501, 1X1J.A.2000, 3X17.A.603, 1Y93.A.267, 1Y9Z.A.603, 2YOA.A.1002, 1YU6.A.401, 2Z2X.A.1002, 2Z30.A.1005, 2ZN9.A.901, 2ZUX.A.631, 2ZUX.A.632, 2ZUY.A.622, 2ZWP.A.404, 3ZYP.A.1220

[1] "Cluster 3"

6CGT.A.685, 4GQR.A.502, 3K7L.A.701, 1ULV.A.2006, 1AZO.B.283, 3A9Q.G.214, 3AKA.A.173, 3BBY.A.215, 2BLO.C.1155, 3CGT.A.685, 3D0Y.A.93, 2D3L.A.503, 3E1I.C.501, 2E6V.C.10, 1ESV.S.395, 3ESR.A.213, 1FZD.E.1, 1G5N.A.407, 2GDF.B.302, 3GK2.A.93, 1HFZ.D.124, 3IJ8.A.497, 1JBA.A.500, 2JHL.F.1298, 1JXN.B.246, 2K0F.A.153, 2KAY.A.186, 3LCP.D.158, 1LGN.B.302, 2LMT.A.149, 1LN8.A.201, 2LV6.A.203, 4MVF.A.603, 1NIW.A.1003, 2OZN.B.401, 4POS.E.401, 3Q3L.A.436, 1R1Z.C.410, 2R28.A.1003, 2RHP.A.8, 1V3D.B.2001, 3VI3.A.2003, 4WBQ.B.601, 3WIU.A.1004, 1Z3U.A.497, 2ZWP.A.401, 4AOC.B.1129, 1CGW.A.692, 1DX5.I.1001, 1KXQ.A.4001, 2LRO.A.202, 3TH2.L.152, 4X9Y.A.502, 1Z32.X.497, 4DTM.A.1002, 4DU1.A.1003, 4DU3.A.1002, 4FKO.A.1002, 2FLD.B.602, 4QWA.A.403, 3RAX.A.416, 2WTF.A.1511, 1A25.B.290, 3A4U.A.286, 1A75.A.109, 2AAO.A.295, 2AAO.A.296, 5AER.A.200, 3AIE.A.4001, 4APX.B.1240, 1AVS.A.94, 3BOO.A.124, 1B8R.A.109, 3B8Z.A.903, 3BFM.A.235, 2CCL.B.1061, 4CPV.A.109, 2CT9.A.302, 4CT3.A.1170, 3DBK.A.303, 2DIE.A.780, 1DTL.A.203, 1DVI.A.273, 2E3X.A.802, 3ECQ.B.2000, 1EXR.A.1000, 4F0Z.B.201, 4F0Z.B.204, 1FAT.A.255, 3FZO.A.400, 1FZD.A.1, 1G8I.A.1599, 1G9K.A.704, 3GDC.A.401, 2GGM.A.401, 4GGF.A.101, 2GJP.A.1488, 4GM5.A.404, 2GSK.A.2, 2GXS.A.601, 4H2A.A.805, 3HB2.P.484, 2HQ8.A.202, 1HVX.A.516, 1HVX.A.518, 3HZ3.A.1, 4I35.A.515, 2I7A.A.2, 4IAI.A.401, 4ICB.A.77, 3IJ9.A.497, 4IK8.A.502, 4IU3.B.302, 2J1V.A.1152, 2J1G.B.1290, 4JA8.A.503, 1JC9.A.301, 1JIW.P.485, 4JWQ.A.202, 4K70.B.1003, 1K9K.A.400, 4KKK.A.701, 3KLK.A.1, 3KLL.A.1, 3KQR.C.206, 4KTY.A.801, 4KWU.A.1109, 3LI6.A.149, 3LI6.D.150, 4LVN.A.702, 4M00.A.801, 4M7H.A.501, 1MDW.B.4, 3MHZ.A.736, 2ML1.A.205, 4MNO.A.302, 3MVS.A.212, 4N5X.A.201, 4N5X.A.205, 2O04.A.402, 3O0V.A.1, 3O0W.A.377, 2O5G.A.401, 2O5G.A.402, 2O5G.A.403, 2OBH.A.1001, 1OHZ.B.1058, 2OLG.A.2001, 3OX6.B.502, 2OZN.B.402, 5P2P.A.125, 3P4G.A.411, 3P4G.A.413, 3P4G.B.405, 4P5X.A.1001, 4P99.A.514, 5PAL.A.110, 4PEO.A.102, 4PET.A.403, 2PVB.A.111, 2Q1F.A.2001, 1Q3A.A.468, 3Q5I.A.525, 2QNG.A.201, 3QRB.A.302, 3QRX.A.171, 1QTX.A.152, 2R9F.A.366, 2RHP.A.29, 1RRO.A.109, 1RU4.A.1, 3RUP.A.1006, 1RWY.A.422, 1S2N.A.1290, 1SBF.A.601, 1SL7.A.301, 1SRA.A.302, 1TCF.A.163, 1TN4.A.162, 3TTQ.A.2867, 3U1R.A.702, 3UBG.A.902, 3UCP.A.912, 3UL4.B.66, 4UM9.A.2003, 3USU.C.266, 1UX6.B.2005, 4UZU.A.1483, 4V29.B.1179, 2VN5.B.102, 2VN6.B.1067, 2VUD.D.1118, 2W46.A.1148, 2W87.A.1149, 3WA5.A.504, 3WN6.A.503, 2WNP.F.1298, 1WP6.A.502, 2WZ8.A.1149, 2Y3N.B.1067, 2Y3N.B.1068, 2Y5I.A.101, 2YA2.A.1691, 1YO8.A.1186, 1YO8.A.1187, 1YO8.A.1190, 1YUT.A.197, 1Z3J.A.267, 2Z30.A.1004, 2Z8X.A.627, 1ZCM.A.1001, 2ZFD.A.229, 2ZWO.A.402, 2ZWP.B.402

[1] "Cluster 4"

1BSS.A.433, 1SON.A.403, 1B09.C.301, 1F4M.E.105, 3FLP.L.302, 1IHO.A.2, 2IWV.B.1284, 2JAL.B.1447, 1K90.D.801, 2OYH.B.2, 1VAH.A.500, 1WDA.A.902, 4WK2.B.502, 1Z3J.A.266, 2ZWO.A.401, 3CFR.A.910, 2G8K.A.401, 1N56.B.403, 1A2X.A.160, 1A2X.A.161, 1AEI.A.319, 5AER.A.201, 1AJ4.A.163, 1BAG.A.431, 3BOW.A.719, 3BOW.B.403, 1C9U.B.1002, 3CGA.A.102, 2DDY.A.174, 2E6W.A.301, 3EAD.B.1002, 4ELG.F.202, 1ESP.A.322, 2EXI

.D.3004, 1G0H.A.290, 4GKX.B.301, 1GQM.A.1089, 2H0K.A.407, 1H4B.A.1085, 1H4B.A.10
86, 2HQ8.B.303, 1IXX.B.124, 2J1G.D.1289, 1JBA.A.501, 4JE0.A.401, 4K1C.A.506, 1K9
6.A.92, 3KF9.A.302, 4LMH.D.811, 2LV6.A.204, 2LVK.A.102, 2M5E.A.2001, 2ML3.A.205,
4N25.A.706, 4NEN.A.1115, 1NIW.A.1001, 1NIW.A.1002, 1NIW.C.1005, 1NUB.A.301, 1NU
B.A.302, 1NX2.A.4, 4P5W.A.1001, 3PM8.B.514, 1PTK.A.281, 3QRX.A.170, 3QRX.A.173,
2SAS.A.186, 1TCF.A.162, 1TTX.A.110, 4UM8.C.2004, 1UZJ.C.3649, 1V1G.A.1211, 2VB6.
B.1151, 3W9T.C.1009, 3WHU.B.502, 1WRL.D.104, 1YUU.B.199, 2Z2D.A.266, 2Z8S.A.647,
2ZJ7.A.627, 2A40.B.1273, 4AW7.A.1599, 3B9X.A.400, 4B97.A.1152, 4B9P.A.1167, 3BM
V.A.684, 4BM1.A.402, 2BOQ.A.1352, 1CGV.A.692, 1CGY.A.692, 1CLC.A.651, 1CXL.A.689
, 4CZN.A.1369, 1D3C.A.688, 3DED.C.508, 3DHP.A.497, 1DTU.A.688, 1EO5.A.689, 3ER9.
B.901, 2FF2.A.601, 1G43.A.200, 1G94.A.800, 2GUY.A.601, 1HX0.A.500, 2HYV.A.601, 1
I75.A.1688, 4I71.A.402, 1IA6.A.1263, 1IOD.G.506, 1JAE.A.500, 1KCK.A.691, 1KCL.A.
1692, 1KWT.A.502, 1KXH.A.800, 4KZW.A.302, 3M5Q.A.371, 2MAS.A.316, 1MCX.A.347, 1M
N2.A.371, 1NBC.B.156, 2NZM.A.407, 1OT1.A.1693, 1PEZ.A.891, 1PJ9.A.891, 3POE.A.2,
2PWA.A.1280, 3QEE.A.21, 1SH7.A.1290, 1SNN.A.403, 3TEC.E.344, 1UA7.A.601, 1UOV.A
.1419, 3VM7.A.501, 2VMH.A.3050, 1W3H.B.1348, 2WHK.A.1339, 1WMD.A.1002, 3WMS.A.80
1, 2WNX.A.1163, 2WZS.A.800, 1XKD.A.1006, 1YO8.A.1182, 3ZQX.A.1146, 4K4G.I.603, 3
NHG.A.908, 3QEW.A.905, 3QEX.A.905, 3S9H.A.905, 3SCX.A.906, 4ANR.A.400, 1BJF.A.40
4, 2BPE.A.1245, 4C0K.A.1615, 4CAJ.C.1323, 2CCM.A.1193, 1CGE.A.304, 4CGT.A.685, 2
CHN.B.1716, 3CIO.K.401, 1CXE.A.690, 1DB4.A.198, 4DH2.B.102, 2DIJ.A.689, 4DLK.A.4
01, 3DSL.A.2, 1DTL.A.202, 3EKI.A.601, 1EO7.A.689, 1ESP.A.319, 1FIF.C.2, 3FLT.A.3
01, 1GOH.A.291, 1HQV.A.997, 1HT6.A.501, 4IRZ.A.2006, 3IUC.A.2, 2IWA.A.501, 3KCP.
A.702, 3KF9.A.301, 1KXT.A.4001, 4L03.B.502, 3LND.A.209, 4MNO.A.301, 4MSP.A.202,
3NJH.C.503, 4P5F.B.501, 1PVY.A.503, 4QB2.A.202, 3T3P.A.2005, 1TMQ.A.1001, 3TRP.A
.358, 4U6B.A.501, 3U8D.A.202, 3UBG.A.901, 1UKT.B.690, 4UM9.A.2002, 1UZJ.B.2648,
4V29.A.1178, 1V3J.A.688, 2VN6.B.1066, 1WOP.A.1779, 3WHT.B.502, 4WKO.A.502, 3WNO.
A.801, 1WPC.A.503, 2WVZ.A.800, 1X2T.A.603, 1XO5.A.5, 2YEQ.A.1525, 3ZHG.A.1323, 3
E1I.B.502, 1KTW.A.496, 1KTW.A.9, 4QNP.A.506
[1] "Cluster 5"
2I3P.B.1, 3RBE.A.415, 4RIC.A.1101, 3ALA.E.1764, 2BBM.A.182, 3BJU.C.606, 2C10.C.1
773, 2CHO.A.1717, 1CTR.A.149, 3E1I.C.502, 1EXZ.C.805, 3FAX.A.1223, 2FH2.B.2003,
2FH3.B.2001, 1FIH.B.3, 1G1Q.C.803, 3HR4.H.202, 3IJE.A.4004, 3IJE.A.4007, 3IJE.A.
4008, 3IKQ.A.403, 1IVE.A.470, 4JWU.A.502, 2K0E.A.153, 2K2F.A.94, 1K9I.A.407, 1LT
9.B.1, 4MDV.B.404, 4OMD.F.604, 2OX9.B.803, 4PJ0.A.601, 4POQ.E.401, 2PRK.A.281, 4
Q6O.A.501, 1QDO.B.243, 1QNI.D.903, 2QVF.B.704, 2RGB.A.601, 2RHP.A.11, 2RHP.A.27,
1RTM.2.3, 1S1E.A.225, 2SNI.E.276, 4UM8.B.2003, 3VI3.B.2001, 2VRO.D.1006, 1WOP.A
.1781, 3W9T.G.506, 4WFF.A.304, 1WT9.A.1001, 1ATN.D.264, 4M7Z.B.409, 3AKB.A.173,
3BOW.B.404, 2DW2.B.711, 2E3X.A.801, 3GE4.A.167, 1GQM.A.1090, 1GQM.C.1088, 2HOL.A
.1015, 4JWS.A.502, 3LMW.A.9, 2M29.A.401, 2NWH.A.402, 1T9H.A.414, 3U24.A.602, 1WO
O.A.1781, 3WCV.B.203
[1] "Cluster 6"
2BTW.B.400, 2DDY.A.175, 3KOO.H.302, 2MLS.A.304, 3NP5.A.732, 4P99.A.505, 4POQ.C.4
01, 1TNQ.A.91, 4DU3.A.1003, 3ODH.A.196, 1A25.A.290, 1B47.A.351, 2COO.B.1507, 1CF
F.A.150, 2D3P.C.241, 1DJY.A.2, 3E78.A.601, 2E85.B.1004, 4ELG.A.202, 4FDI.A.603,
3G5C.B.802, 2GJR.A.1488, 4IAV.A.420, 3JQ5.A.201, 1KBO.A.801, 1KV9.A.802, 2ML2.A.
205, 3MXW.A.402, 1NHE.A.805, 2NQA.B.902, 1P8J.G.3014, 2SAS.A.188, 1ULV.A.2004, 1
XJL.B.340, 2YN3.B.6361, 1YUU.A.197, 2Z8S.A.642, 1ZFS.A.104, 1ZIV.A.1, 2ZN9.A.902
, 2ZN9.B.901, 1ANX.B.321, 1B9A.A.110, 3B9G.A.318, 1COG.S.129, 2C40.A.1311, 3DEM.
A.3001, 3EPW.A.1001, 3EPX.A.1001, 3FZO.B.400, 4I70.A.401, 4I72.A.401, 1J34.A.511
, 3MKM.A.501, 3MKN.A.501, 1Q8F.A.2001, 4QB6.A.203, 3S82.B.405, 3T8I.C.400, 1UZK.
A.2512, 3WMT.A.606, 1YOE.A.1001, 4BY5.A.1184, 3D1M.A.2, 4GER.A.402, 3GIS.Y.1002,
2HYW.A.501, 4I75.A.401, 1IOD.A.501, 4K70.A.1003, 3KF9.C.302, 1KIC.B.328, 1KXO.C
.703, 4LMF.D.303, 3N1F.A.6, 3N1G.B.190, 4NHF.F.301, 3S5U.D.221, 3WH2.A.302, 3WNP
.A.801, 1YO8.A.1204, 1YUT.B.199, 1LRW.A.702

[1] "Cluster 7"
1G9Y.C.803, 3KHL.A.415, 3A7Q.A.4001, 2AAO.B.299, 1AEI.A.318, 1AEI.A.320, 1ALA.A.400, 1AXK.B.395, 3BRX.A.328, 3BXK.A.152, 1CTR.A.150, 2D3P.A.237, 2D7F.A.240, 3E3R.B.193, 2E6V.D.11, 4EJ7.C.402, 4EJ7.C.403, 1ETH.A.449, 2FH3.A.1002, 2FHC.A.2405, 1FZC.C.1, 1FZD.G.1, 4G0D.A.503, 4G9L.B.306, 1GYK.A.1206, 2H0K.A.401, 4ILW.F.305, 2J3U.C.1292, 2J64.C.1222, 1J84.A.366, 4JDZ.B.704, 4JDZ.A.702, 1JN2.P.238, 3JQL.A.121, 4KTP.B.804, 1LGC.E.301, 1LGN.A.302, 1LU1.A.301, 1M1J.C.501, 3M83.E.407, 3M8D.A.710, 4MBE.A.202, 2ML2.A.206, 2ML3.A.206, 2MLR.A.305, 2MLS.A.305, 1N29.A.125, 1NX0.A.900, 4OJQ.B.1002, 4OMC.E.607, 1OTN.A.250, 3OX5.B.502, 2OXE.B.600, 4P99.D.512, 1Q3A.C.478, 1QOO.A.302, 2RJP.B.4, 3RYD.C.267, 1S0C.A.1879, 3TRP.A.360, 3UJO.D.304, 4UM8.A.2004, 3VU1.A.1001, 1W2M.A.1442, 4W4Q.A.401, 1WMZ.B.203, 3WYN.B.402, 2WZE.A.1552, 1XFE.A.100, 1YAX.C.1003, 1Z3U.B.497, 2ZOC.A.501, 2ZOC.A.502, 2ZOC.A.504, 4DTS.A.1002, 4K4I.A.606, 3A7Q.A.4002, 3AAJ.A.991, 1AFD.3.2, 1AMY.A.502, 1ANN.A.319, 1ANW.B.354, 4CAG.A.605, 3CHK.A.502, 1DCY.A.198, 2DPK.A.2001, 2E3X.A.803, 3E3R.B.194, 4E52.A.403, 2E6W.A.300, 3E9T.A.1, 3EAD.B.1004, 3ECQ.A.2000, 4ELF.C.201, 2ERO.B.702, 1FDK.A.124, 4FGC.B.204, 3FLP.B.301, 3FLT.A.302, 4FL4.C.401, 4FL4.C.402, 4GTW.A.1012, 2HYW.A.505, 2I08.A.200, 3IA7.A.402, 3IA7.B.402, 3IGO.A.601, 1IME.A.278, 1IZJ.A.1002, 4JBE.A.502, 3K8L.B.800, 2KAY.B.188, 1KTW.A.3, 1KX1.A.222, 1LGC.A.301, 1LHV.A.401, 4LMF.A.304, 1LWJ.B.10, 1M34.B.2299, 1M8T.C.1003, 4MDV.B.403, 2ML1.A.206, 1NAE.A.900, 4NDD.B.402, 3NOM.B.263, 1O9I.C.269, 1ODB.F.1092, 3OX6.A.501, 3OX6.A.502, 4P99.A.512, 1PK8.A.817, 2PQY.A.500, 4Q4W.1.905, 1Q8H.A.72, 1QNI.E.903, 2RHP.A.1176, 2RHP.A.26, 3RV2.A.405, 3S5U.C.220, 1TEC.E.343, 1THL.A.3233, 3TRQ.A.358, 3TRQ.A.360, 1TYE.A.1405, 1TYE.A.1406, 1TYE.A.1407, 1U7W.A.501, 1UG9.A.2006, 3UJQ.D.305, 3USU.H.272, 2V3T.B.1264, 1V3J.B.690, 1W2M.C.1441, 3W9T.A.1005, 1WMD.A.1001, 2WO9.D.1270, 3WU2.O.301, 2Z8S.A.644, 2Z8S.B.642, 2ZBA.A.461, 2ZKT.B.413, 2ZUY.A.624, 2ASP.A.600, 4AWN.A.301, 4DLK.A.402, 1DV8.A.1001, 2E39.A.502, 2EWE.A.702, 4EW9.A.203, 2F3Y.A.1174, 4FCS.A.403, 3FM1.A.352, 3FM4.A.352, 3FMU.A.352, 1G87.A.616, 1H3J.A.1346, 1HUP.A.302, 1J34.B.512, 1K72.A.779, 1LLP.A.352, 3PDD.A.192, 3PF2.A.2, 3Q3U.A.341, 2VMI.A.3050, 2ZIC.A.944, 3FD2.A.374, 4K4H.A.604, 1N48.A.501, 3NAE.A.906, 3SQ2.A.904, 2AAO.A.294, 1AFD.1.2, 3AMR.A.901, 1ANN.A.320, 1B1G.A.76, 3B2Z.B.2, 1BJF.A.403, 3BJU.A.606, 1BLI.A.700, 2COT.B.1507, 3C7F.A.803, 4DH2.B.101, 1E43.A.504, 4ELG.B.202, 4ELG.C.202, 2ERO.A.701, 3ETO.A.2002, 1EXR.A.1001, 3FAW.A.4, 3FCS.A.2008, 2FH1.A.2003, 4GON.A.202, 1GGZ.A.152, 4GKY.A.302, 1HDH.A.1528, 4HEX.A.204, 4I2Y.B.504, 1J55.A.101, 4JRF.A.601, 3KEZ.B.501, 4KHO.A.1001, 1KP4.A.200, 2MLR.A.303, 3NOK.A.257, 4NUQ.A.302, 1O9I.A.269, 1O9I.E.269, 2OO4.A.5002, 2P69.A.305, 4P99.A.515, 1R17.A.599, 4R1D.A.601, 4RDQ.A.504, 4RDQ.B.502, 4RGP.B.302, 1SRA.A.301, 3T3P.A.2006, 3T3P.A.2007, 4U65.E.302, 3UBH.A.856, 4UM9.A.2004, 1UX6.B.2006, 2VN5.B.101, 2VNG.A.1213, 1WOY.H.1259, 2WG7.A.1126, 4WJK.A.503, 4WKO.A.504, 2Y5I.A.102, 2Y8K.A.1526, 2YHG.A.1936, 1YO8.A.1194, 1YO8.A.1207, 1YO8.A.1210, 2ZFD.A.230, 2ZUX.A.633, 2ZUX.A.634, 2ZUY.A.623, 2ZZV.A.501, 1EUB.A.277, 2QPS.A.501, 1W52.X.602, 1XZO.B.1018
[1] "Cluster 8"
2EX5.X.802, 4Q10.A.1001, 1RYS.B.803, 2BTW.A.400, 3C5I.A.371, 1DJZ.A.2, 2E30.A.300, 3FLT.B.302, 4G64.B.301, 1IJL.B.202, 2K0J.A.501, 4KPP.B.501, 3L9I.C.1151, 1M63.B.500, 1NW1.A.430, 4OVY.A.409, 3POW.A.900, 3RGO.A.1, 2TBV.C.388, 1V1G.A.1209, 3WIV.B.1004, 4QOW.A.1001, 3QZ7.A.363, 2AER.L.3005, 4CP1.A.1297, 3EDD.A.701, 3HR4.H.203, 1JHN.A.900, 1JYI.D.408, 1K6S.A.302, 1K6S.B.301, 4KS4.A.502, 4L76.E.402, 1LPK.B.1, 1M1J.B.503, 1N41.A.410, 1RK9.A.112, 3RRV.C.255, 2TBV.A.388, 1TRQ.A.106, 4UM9.D.2002, 3WIU.B.1004, 4WK4.B.502, 1XZO.A.1014, 1Z3J.A.268
[1] "Cluster 9"
1BCZ.A.410, 4J2A.A.1006, 4RIC.B.1101, 1AFB.2.3, 4BY6.A.3080, 2E0X.B.701, 2E6V.E.12, 3EAD.C.1001, 2FH3.A.1003, 3GHG.L.601, 2IEZ.A.219, 1IT4.A.200, 2J3G.A.1289, 4JBE.A.503, 1JED.B.535, 1JX6.A.401, 2KBM.B.101, 4L76.B.402, 4L76.D.402, 1LGC.C.301, 4LLF.O.401, 1LWU.C.5, 4M8D.B.305, 4MDV.A.404, 4OVG.A.404, 4POS.B.401, 1QDO.D.

247, 3RBX.A.601, 2RHP.A.15, 3RRY.A.202, 3S55.D.280, 1SAV.A.321, 1SUI.B.306, 1TCF
.A.164, 2TEC.E.344, 2W67.B.1590, 1WD9.A.902, 2WM4.A.1430, 2XJO.B.1175, 1YN8.B.10
08
[1] "Cluster 10"
2LP2.A.202, 2AGO.A.403, 2BQR.A.2002, 3GIM.A.415, 4NLG.A.401, 3A7Q.A.4004, 1ATN.D
.265, 3B2Z.D.3, 1BAG.A.432, 2BW7.B.2202, 3CGA.B.102, 3E9T.B.3, 4GOD.B.503, 1G7Y.
F.254, 2HOK.A.410, 4IMM.B.402, 2J7A.A.1007, 2J7A.B.1007, 3K7L.A.703, 1KWV.B.604,
3LPD.A.341, 1M63.F.502, 1NUD.A.701, 3NWK.B.238, 3OM7.B.1, 2OX9.A.803, 4PKG.A.40
3, 2PPL.A.482, 3QQZ.A.326, 2RJP.B.3, 3RRD.A.239, 3RTT.A.267, 4TVU.A.600, 4U2A.A.
301, 3USU.A.264, 1WBL.D.303, 3WCT.B.203, 1YO8.A.1202, 4AYU.A.205, 1CGU.A.685, 2A
TL.B.1415, 3AVW.A.3001, 4DTU.A.1002, 4E3S.A.1002, 4EDO.A.503, 4J2D.A.1002, 4J2E.
A.1002, 4K4G.A.603, 3KHR.B.1416, 4KHU.A.1002, 3NCI.A.905, 3NDK.A.905, 3NE6.A.904
, 3NGI.A.905, 2ODI.B.702, 2Q10.B.702, 3QEP.A.905, 3QER.A.905, 3QEV.A.905, 4UAW.A
.402, 4UB4.A.402, 2A3Y.A.601, 1A8B.A.407, 3ACH.A.204, 2AEF.A.602, 4AE2.A.246, 1A
JP.B.558, 1ANW.B.353, 4AQE.A.1209, 1AXO.A.290, 1AYP.A.301, 3BOX.A.579, 3B4N.B.70
1, 2BIB.A.1552, 1BTU.A.260, 2BWR.A.500, 2COT.A.1507, 2C10.B.1777, 3C1Q.A.2, 1C8T
.B.264, 4CFQ.A.501, 3CIO.K.402, 3CLN.A.152, 2CM5.A.1678, 2CM6.A.1679, 1D0L.A.400
, 4D0E.A.1532, 1D2S.A.401, 1DB5.A.198, 2DBX.D.702, 1DBN.A.301, 3DEM.A.5001, 4DIR
.A.101, 1DJX.B.2, 4DKB.A.301, 2DUR.A.1, 4DZ3.A.202, 2E26.A.602, 1E8A.A.1089, 3E9
T.B.4, 3EAD.B.1003, 2EGD.A.301, 2EIG.A.1102, 2ERO.A.702, 3ETO.A.2001, 3EU3.A.1,
2EXH.B.2002, 3EXM.A.301, 3FCS.A.2004, 2FH1.A.2001, 1FIB.A.500, 3FLP.A.302, 2FMD.
A.301, 1FNY.A.500, 1FX5.A.251, 1FXH.B.1002, 4FZM.A.301, 1G7Y.A.254, 1G8K.A.5008,
1G8K.C.5108, 1G9K.A.705, 2GKO.A.610, 1GK9.B.1579, 1GKF.B.1571, 1GO7.P.1482, 1GO
8.P.1482, 3GPE.A.501, 2GSK.A.1, 1GSL.A.251, 1GZC.A.290, 4H2B.A.604, 1H3G.B.701,
1H6X.A.1162, 2H61.D.817, 4HHO.A.401, 4HPH.A.701, 1HQL.A.302, 3HR4.D.202, 3HX4.A.
603, 4I35.A.514, 3I57.B.186, 4ICB.A.76, 4IHM.A.406, 3IPV.A.252, 4IT5.D.202, 4ITC
.A.1201, 4IU3.B.301, 2J5Z.A.1277, 1J9L.A.1301, 2JE7.A.1241, 2JEC.A.240, 4JJJ.A.7
04, 1JX9.B.601, 1K94.A.998, 1K9I.I.1201, 1K9J.A.403, 1KAP.P.621, 3KM5.B.2011, 4K
TY.A.803, 1KVO.A.191, 3KWU.A.902, 2KYF.A.109, 3L2Y.C.302, 1L9N.A.700, 1L9N.A.702
, 1LEN.A.184, 1LHW.A.401, 4LMH.A.811, 3LNP.A.472, 1LOC.A.228, 4LOR.A.301, 3LPC.A
.360, 3LPC.A.362, 4LP7.B.301, 2LTN.A.191, 1LVU.A.8001, 1LVU.D.8002, 3M1H.A.2001,
1MCX.A.349, 4MGQ.A.601, 1MKU.A.124, 2ML2.A.204, 2ML3.A.202, 3MVS.A.213, 3MVS.A.
216, 1MVQ.A.238, 4N2L.A.707, 3N35.A.290, 1N47.B.252, 3N5A.A.102, 1NIW.G.1014, 1N
LS.A.240, 3NOL.A.263, 3NQZ.B.520, 2NXP.C.602, 2O72.A.402, 1O8F.A.1353, 1O8P.A.11
49, 3OEA.A.200, 1OFL.A.528, 4OKH.A.904, 4OKH.B.901, 3OM2.A.1, 3OM4.A.1, 3OM5.A.1
, 3OM6.A.1, 3OM7.A.1, 4OMC.A.606, 2OVU.A.238, 2P2K.A.239, 4P99.B.517, 3PDD.A.194
, 2PEL.A.237, 4PEU.A.401, 4PHJ.A.302, 4PKI.A.403, 3PPE.A.402, 3PPE.A.403, 1PT2.A
.500, 1QNW.A.302, 1QPK.A.451, 3QRX.A.172, 2QUB.A.619, 2QUB.A.621, 2QUB.G.616, 2Q
V6.A.302, 1QX2.A.1005, 1R1Z.A.285, 2R8Z.N.214, 1RLW.A.401, 1RP8.A.500, 1S1E.A.22
6, 1S6B.B.402, 1S6C.A.218, 1SL4.A.407, 1SU4.A.995, 1THM.A.302, 3TI7.A.354, 3TI9.
A.354, 1TM7.E.470, 1TU5.A.903, 4U36.A.302, 3U4X.A.237, 1UKG.A.1262, 1UNE.A.124,
1UP8.A.599, 2UWP.A.1246, 1UWW.A.1192, 1UX6.B.2009, 1UX6.B.2011, 4UZU.A.1485, 1VC
L.A.1002, 3VLV.A.503, 3VV3.A.402, 2W08.A.206, 1WBF.A.303, 1WCO.A.2100, 1WC5.A.21
00, 3WCS.A.1003, 4WJK.B.502, 1WKY.A.503, 2WW8.A.1002, 1Y1X.A.201, 1Y1X.A.202, 1Y
6O.A.207, 2YAY.A.1266, 2YFS.A.1712, 1YO8.A.1189, 1YXH.A.1001, 2Z2X.A.1003, 2Z8X.
A.619, 2Z8X.A.625, 2Z8Z.A.623, 2ZEX.A.406, 2ZKM.X.800, 2ZVD.A.628, 2ZVD.C.620, 3
ZYR.A.401, 2ZYH.A.700, 2ZZJ.A.239

Table S71. all-ligand-number Ca, combined group

size largest_angle* middle_1* middle_2* middle_3*

1	"81"	"127.7+/-13.1"	"57.1+/-8.5"	"72.1+/-7.5"	"80.5+/-8"
2	"138"	"154.9+/-12"	"66.3+/-9.1"	"78.9+/-6.8"	"90.5+/-7.2"
3	"458"	"171.7+/-3.7"	"73.3+/-2.9"	"85.3+/-2.1"	"98.4+/-3.1"
4	"74"	"159.4+/-12"	"73.4+/-9.8"	"83.6+/-5.8"	"93.8+/-6.8"
5	"170"	"161.6+/-8.9"	"79+/-6.4"	"86.3+/-5.8"	"93.2+/-7.3"
6	"354"	"170.1+/-3.7"	"67.8+/-5.8"	"85+/-2.4"	"101.9+/-4.1"
7	"498"	"166.3+/-5.9"	"71.5+/-3.9"	"84+/-3.2"	"100.2+/-6.3"
8	"445"	"161.3+/-6.2"	"51.3+/-2.9"	"82.1+/-4.4"	"104.2+/-8.1"
9	"281"	"152.2+/-5.9"	"66+/-6.5"	"79.2+/-3.3"	"113.7+/-6.6"
10	"721"	"171.9+/-4.1"	"79.3+/-3.8"	"88+/-2.1"	"96.2+/-3.6"
11	"138"	"156.3+/-11.1"	"67.3+/-11.4"	"84.9+/-6.5"	"97.7+/-7.2"
12	"283"	"161.1+/-5.5"	"52.2+/-3.5"	"80.6+/-3.8"	"107.8+/-6.1"
13	"439"	"161.7+/-4"	"72.6+/-3.7"	"83.3+/-2.5"	"104.8+/-4.9"

	middle_4*	smallest_opposite_angle*	Tetrahedral	TrigonalBipyramidal
1	"95.9+/-12.1"	"83+/-16.2"	"0.002"	"0"
2	"124.4+/-11.3"	"58.1+/-8.6"	"0.007"	"0.006"
3	"156.8+/-3.8"	"51.8+/-2.4"	"0.004"	"0.049"
4	"108.8+/-12.5"	"136.6+/-14.6"	"0"	"0"
5	"102.7+/-9.2"	"89.6+/-10.8"	"0"	"0"
6	"164+/-3.7"	"51.5+/-3.3"	"0.011"	"0.052"
7	"151.7+/-5.3"	"70.3+/-5"	"0.005"	"0.025"
8	"151.8+/-7.2"	"72.9+/-6.2"	"0.009"	"0.016"
9	"147.4+/-5.5"	"66.8+/-6.6"	"0.007"	"0.009"
10	"166.1+/-5.1"	"80.9+/-5.4"	"0.001"	"0.036"
11	"137.4+/-8.7"	"97.1+/-9.6"	"0.017"	"0.06"
12	"153.3+/-5.9"	"52.7+/-4.3"	"0.014"	"0.025"
13	"154.8+/-4.8"	"51.8+/-2.8"	"0.005"	"0.025"

	TrigonalBipyramidalVA	TrigonalBipyramidalVP	Octahedral	SquarePyramidal
1	"0.004"	"0.001"	"0"	"0"
2	"0.013"	"0.06"	"0"	"0.013"
3	"0.035"	"0.261"	"0.09"	"0.203"
4	"0"	"0.088"	"0"	"0"
5	"0.009"	"0.057"	"0"	"0"
6	"0.044"	"0.228"	"0.141"	"0.235"
7	"0.028"	"0.156"	"0.029"	"0.109"
8	"0.028"	"0.074"	"0.017"	"0.053"
9	"0.022"	"0.048"	"0.01"	"0.024"
10	"0.023"	"0.352"	"0.343"	"0.465"
11	"0.083"	"0.114"	"0"	"0.022"
12	"0.044"	"0.077"	"0.017"	"0.041"
13	"0.029"	"0.169"	"0.053"	"0.124"

	SquarePyramidalV	SquarePlanar	TrigonalPrismatic	TrigonalPrismaticV
1	"0.013"	"0"	"0"	"0"
2	"0.05"	"0.003"	"0"	"0.035"
3	"0.284"	"0.246"	"0.083"	"0.177"
4	"0.022"	"0.054"	"0"	"0"
5	"0.2"	"0"	"0"	"0"
6	"0.284"	"0.272"	"0.074"	"0.144"
7	"0.203"	"0.159"	"0.106"	"0.173"
8	"0.116"	"0.067"	"0.047"	"0.12"
9	"0.054"	"0.037"	"0.092"	"0.124"
10	"0.516"	"0.484"	"0.036"	"0.089"
11	"0.123"	"0.042"	"0"	"0.079"
12	"0.078"	"0.05"	"0.065"	"0.115"

13	"0.201"	"0.169"	"0.122"	"0.187"

	PentagonalBipyramidal	PentagonalBipyramidalVA	PentagonalBipyramidalVP
1	"0"	"0"	"0"
2	"0"	"0.002"	"0"
3	"0.157"	"0.157"	"0.242"
4	"0"	"0"	"0"
5	"0"	"0"	"0"
6	"0.074"	"0.083"	"0.184"
7	"0.098"	"0.113"	"0.174"
8	"0.025"	"0.064"	"0.062"
9	"0.049"	"0.081"	"0.079"
10	"0"	"0"	"0.064"
11	"0"	"0"	"0"
12	"0.035"	"0.046"	"0.077"
13	"0.111"	"0.132"	"0.176"

	SquareAntiprismatic	SquareAntiprismaticV	HexagonalBipyramidal
1	"0"	"0"	"0"
2	"0"	"0"	"0"
3	"0"	"0.113"	"0"
4	"0"	"0"	"0"
5	"0"	"0"	"0"
6	"0.002"	"0.114"	"0"
7	"0"	"0.097"	"0"
8	"0.002"	"0.047"	"0"
9	"0.014"	"0.087"	"0"
10	"0"	"0"	"0"
11	"0"	"0"	"0"
12	"0.035"	"0.093"	"0.002"
13	"0.005"	"0.141"	"0"

	HexagonalBipyramidalVA	HexagonalBipyramidalVP
1	"0"	"0"
2	"0"	"0"
3	"0"	"0.072"
4	"0"	"0"
5	"0"	"0"
6	"0.001"	"0.076"
7	"0"	"0.04"
8	"0.001"	"0.015"
9	"0.001"	"0.014"
10	"0"	"0"
11	"0"	"0"
12	"0.007"	"0.022"
13	"0"	"0.041"

Table S72. Cluster members of all-ligand-number Ca, combined group

[1] "Cluster 1"
1BCZ.A.410, 3DF0.A.720, 2M0K.A.202, 1MPR.A.428, 1QLK.B.93, 2AU0.H.1415, 3GIJ.B.1415, 4J2A.A.1006, 3KHL.B.1417, 3NHG.A.909, 4RIC.B.1101, 1AFB.2.3, 3B2Z.C.3, 2B30.A.302, 4BTX.A.1764, 4BY6.A.3080, 2C11.C.1739, 2CLT.A.1102, 2E0X.B.701, 2E6V.E.12, 3EAD.C.1001, 1FBL.A.994, 2FH3.A.1003, 1FZA.C.1, 1G5N.A.403, 3GHG.L.601, 1GU6.

A.1480, 4I9F.A.401, 3IAE.B.572, 2IEZ.A.219, 4IEF.H.704, 1IT4.A.200, 2J0T.C.1268, 2J3G.A.1289, 4JBE.A.503, 1JED.B.535, 4K1C.B.504, 2KBM.B.101, 3KQA.C.420, 4L76.B.402, 4L76.D.402, 1LGC.C.301, 4LLF.O.401, 1LWU.C.5, 3MOW.G.202, 4M8D.B.305, 2M98.A.201, 4MDV.A.404, 1MTS.A.480, 3N9V.A.64, 4NUP.C.304, 4OVG.A.404, 4POS.B.401, 3Q2N.B.304, 1QDO.D.247, 1QLK.A.93, 3RBX.A.601, 2RHP.A.15, 3RRY.A.202, 3S55.D.280, 1S6I.A.192, 1SAV.A.321, 1SUI.B.306, 1SUS.C.307, 1TCF.A.164, 2TEC.E.344, 1UG9.A.2002, 2W67.B.1590, 1WD9.A.902, 4WFE.A.306, 4WIW.C.701, 2WJS.A.5002, 3WMW.A.401, 2WM4.A.1430, 2WW0.E.800, 2XJO.B.1175, 1XJL.A.342, 2XSG.A.1772, 1XZO.A.1014, 1YN8.B.1008, 1Z3J.A.268

[1] "Cluster 2"

4EFJ.A.402, 3GIK.A.415, 4K4G.I.602, 3RBE.A.415, 4RIC.A.1101, 1AFD.2.2, 3ALA.E.1764, 1AR1.A.561, 1AVX.A.700, 1B09.C.302, 2BBM.A.182, 3BJU.C.606, 2C10.C.1773, 2C11.A.1738, 4CAG.A.601, 2CHO.A.1717, 1CTR.A.149, 3E1I.C.502, 1EAK.D.998, 4EJ7.C.403, 4ELF.F.201, 1EXZ.C.805, 3FAX.A.1223, 2FH2.B.2003, 2FH3.B.2001, 1FIH.B.3, 4FU4.A.505, 1G1Q.C.803, 3GCW.E.1, 4GKX.B.302, 3HR4.H.202, 3IJE.A.4004, 3IJE.A.4007, 3IJE.A.4008, 3IKQ.A.403, 1IVE.A.470, 1IXX.D.124, 4JP8.A.704, 4JWU.A.502, 2K0E.A.153, 2K2F.A.94, 1K9I.A.407, 3KOO.H.302, 3KQA.A.420, 4KZW.B.400, 4L06.B.501, 2LAN.A.301, 3LCP.D.159, 1LT9.B.1, 1LWJ.A.883, 1LWU.F.6, 3MOW.H.202, 2MOK.A.203, 4MDV.B.404, 2MLR.A.305, 2MLS.A.304, 3MW3.A.302, 3NP5.A.732, 1NX1.A.4, 2OKX.A.4004, 4OMD.F.604, 3OX5.B.502, 3OX5.C.501, 2OX9.B.803, 4P99.A.505, 4PJO.A.601, 4POQ.C.401, 4POQ.E.401, 2PRK.A.281, 4Q6O.A.501, 1QDO.B.243, 1QNI.D.903, 2QVF.B.704, 3QWU.A.601, 2RGB.A.601, 2RHP.A.11, 2RHP.A.25, 2RHP.A.27, 2RJQ.A.5, 1RTM.2.3, 1S1E.A.225, 3S5U.A.220, 4SBV.A.261, 1SDD.A.2184, 2SNI.E.276, 1SW8.A.81, 1TNQ.A.91, 4TVU.B.600, 4UM8.B.2003, 1UZJ.C.3648, 3VI3.B.2001, 2VR0.D.1006, 1WOP.A.1781, 3W9T.G.506, 4WFF.A.304, 3WHU.A.501, 1WT9.A.1001, 2WW0.C.800, 2WW0.F.800, 1ZZH.A.401, 3GIL.B.1417, 3ODH.A.196, 1ATN.D.264, 1CFF.A.150, 3DF0.A.715, 2E6W.A.300, 4ELG.A.202, 4K1C.A.509, 2MOK.A.201, 4M7Z.B.409, 1NUD.A.702, 2C2R.A.1344, 4DTJ.A.1003, 4DTU.A.1003, 4RI8.A.1101, 3AKB.A.173, 1B1G.A.77, 3BOW.B.404, 3D4G.A.485, 2DW2.B.711, 2E3X.A.801, 3GE4.A.167, 1GQM.A.1090, 1GQM.C.1088, 2HOL.A.1015, 3IRH.A.457, 2IWV.A.1284, 4JWS.A.502, 2K0J.A.503, 3LMW.A.9, 2M29.A.401, 2NWH.A.402, 1T9H.A.414, 3U24.A.602, 1WOO.A.1781, 3WCV.B.203, 2WJS.A.5001, 3WNX.A.501

[1] "Cluster 3"

2I3P.B.1, 2DPK.A.4001, 1JKU.B.2272, 3COW.A.301, 3EH8.D.303, 2FLD.A.601, 2I3P.A.154, 2I3Q.B.81, 3LDY.A.145, 3MIS.A.2, 1N3E.D.493, 3PR4.A.343, 2A3W.R.336, 3A7Q.A.4003, 1ANW.A.351, 4AQA.A.1210, 1AUI.B.502, 3B1T.A.903, 2BB4.A.260, 1BCJ.2.3, 3BOW.A.717, 2C10.A.1773, 4CAG.A.608, 4CAG.A.609, 3D94.A.2, 1DAN.L.155, 2DBX.B.701, 4DD8.B.1001, 1DJX.A.2, 1DSY.A.501, 2DW2.A.703, 2FH2.B.2001, 1FZC.B.2, 3GIN.A.2, 1GU6.A.1479, 1H5V.A.305, 2HYW.B.514, 2II1.B.400, 1J1N.A.493, 2J7A.B.1007, 2J7A.E.1006, 4JDZ.B.703, 3K5S.A.218, 1K7Q.A.481, 3K7L.A.702, 3K7N.A.702, 3K7N.A.703, 2KAY.B.187, 4KDV.A.202, 1KTW.B.8, 4KTR.D.814, 4KTR.G.806, 1L9N.B.704, 4LP7.A.301, 4N2I.A.706, 1N47.A.252, 1NKQ.F.560, 1NX2.A.3, 4OKH.A.901, 4OMD.D.605, 1OT5.A.601, 2OXE.A.600, 2P37.C.239, 4P99.D.508, 2Q16.B.200, 2Q17.E.315, 2Q1C.X.294, 2QT7.B.201, 3RHT.A.257, 1SL6.A.3, 1U94.A.701, 3UBR.B.476, 3V03.A.586, 3VI3.A.2001, 3VI3.A.2004, 2VY0.A.1298, 3WN6.B.501, 2XVT.C.1137, 1Y3N.A.493, 2Z57.A.1006, 2Z8Z.A.618, 2Z8S.A.646, 2ZJ7.A.619, 2ZUX.A.638, 2ZUY.A.628, 4AQU.A.1154, 4AQU.A.1156, 3EH8.A.301, 1G9Y.A.801, 1G9Y.B.802, 3MXB.A.175, 3ODH.B.195, 1SOM.A.402, 2VBJ.A.1154, 2VBJ.A.1155, 2VBO.A.1154, 2VBO.A.1155, 2VS7.A.1183, 1A75.A.110, 1A8A.A.407, 1AFO.A.476, 3AKB.A.171, 3AMR.A.905, 3AMR.A.907, 4APX.B.1238, 4AQ1.A.1923, 4AQ1.C.1923, 4AQA.A.1208, 4AQA.A.1209, 4AQE.A.1207, 4AQO.A.1881, 1AVA.A.500, 1AVS.A.93, 4AVS.A.207, 2AYH.A.417, 2AZZ.A.125, 3B2Z.A.2, 2B96.A.124, 3BEU.A.249, 1BF2.A.751, 3BI1.A.1753, 4BJO.A.1167, 1BLI.A.500, 3BNG.A.508, 1BQB.A.352, 4BQ2.A.1797, 1BSW.A.900, 1BU3.A.109, 3C1Q.B.1, 2C26.A.302, 3C3Y.A.238, 2C4X.A.1252, 2C4X.A.1253, 4CAG.A.604, 4CAG.A.610, 1CB8.A.3000, 2CBL.A.352, 4CBU.G.1150, 4CBU.G.1151, 2CCM.A.1194, 4CCD.A.3669, 2CDO.A.1139, 2CDP.A.1139, 2CFT.A.1297, 4CFY.A.301, 3CHJ

.A.338, 3CHK.A.503, 3CIP.G.201, 2CKI.A.997, 2CM5.A.1679, 2CM6.A.1680, 2CN3.A.177
8, 1CP9.B.554, 1CRU.A.501, 4CTE.B.280, 1CVR.A.504, 1DBI.A.701, 1DBI.A.703, 1DBI.
A.704, 2DCK.A.1001, 3DEM.A.8001, 4DIR.A.102, 3DNZ.A.403, 4DUQ.A.101, 1DVI.A.271,
 2DW0.A.701, 2DW0.A.703, 4DZ3.B.202, 2E26.A.601, 2E26.A.604, 2E26.A.605, 2E85.A.
1002, 3ECQ.A.2001, 1EDH.A.302, 1EDM.B.2, 2EHB.A.1001, 1EXR.A.1002, 1EXR.A.1004,
4F0Z.B.202, 4F0Z.B.203, 3FED.A.1755, 3FIA.A.201, 2FI1.A.191, 2FMJ.A.338, 3FO3.A.
527, 1FS7.A.651, 2FVY.A.311, 3G5C.A.801, 1G5N.A.401, 1G9G.A.630, 1G9K.A.701, 3GB
O.A.301, 1GCA.A.351, 1GCY.A.528, 4GER.A.403, 1GGZ.A.150, 1GGZ.A.151, 1GPL.A.500,
 3GPE.A.502, 4GUK.A.207, 4H1Q.B.304, 1H6Y.A.1161, 1H80.A.1493, 3HB2.P.481, 3HB3.
A.563, 3HDB.A.621, 3HDB.A.622, 3HI7.A.803, 3HJR.A.602, 1HPL.A.960, 1HQV.A.998, 2
HQ8.A.201, 1HT6.A.500, 1HT9.A.1001, 3HX4.A.601, 3HX4.A.602, 1HY7.A.304, 2HYV.A.6
05, 2HYV.A.607, 2HYW.A.503, 4I2Y.A.503, 4I35.A.511, 1I82.A.192, 3IBZ.A.193, 4ICB
.A.76, 2ID4.A.901, 2ID4.A.906, 3IGO.A.602, 3IGO.A.603, 2II1.C.400, 3IKW.A.1, 3IK
2.A.514, 4IMM.A.407, 2IPL.A.502, 4ITC.A.1202, 1J1A.A.201, 1J83.A.4001, 4JDZ.B.70
1, 4JGU.A.901, 1JI1.A.2003, 1JIA.A.134, 2JKA.A.1727, 3JQ1.B.1, 3JQW.A.1001, 3JXS
.A.301, 4JX1.E.505, 3K21.A.192, 3K21.A.193, 1K7I.A.480, 1K94.A.998, 1K96.A.91, 1
K9U.A.1001, 1K9U.A.1002, 4K90.A.702, 1KAP.P.614, 1KAP.P.615, 3KCP.A.701, 3KHE.A.
192, 3KHE.A.195, 3KM5.A.2012, 1KQU.A.301, 3KQR.A.205, 3KWU.A.901, 3L2Y.A.301, 3L
CP.C.159, 4LDC.A.502, 1LE6.A.461, 4LJ3.A.402, 4LM8.A.811, 4LOS.A.401, 1LPB.B.453
, 3LPA.A.361, 3LPC.A.361, 1LQV.C.34, 2LRP.A.202, 4LXO.A.1601, 4LXO.B.1601, 4M00.
A.801, 3M1H.A.2002, 4M2P.A.301, 4M5E.A.506, 1M8T.A.1001, 1MCX.A.352, 4MEW.A.501,
 4MNO.A.303, 1MPX.A.638, 3MSE.B.180, 4MSP.A.201, 4N1G.A.202, 4N1G.A.204, 4N2B.A.
709, 4N2G.A.704, 4N2N.A.704, 4N5X.A.203, 3N5A.A.101, 3N5A.A.102, 1N9E.A.803, 4NE
H.A.1101, 1NIW.A.1004, 1NIW.C.1007, 1NNL.A.2001, 3NQX.A.511, 4NUQ.A.303, 2NXP.D.
603, 2NZM.A.405, 2O72.A.401, 2O8O.A.1009, 2O9Q.A.501, 1OAH.A.1525, 1OAH.A.1526,
3OEA.A.200, 1OF3.A.1174, 1OH4.A.1186, 1OHZ.B.1057, 4OKH.A.902, 4OKH.A.904, 4OMC.
A.605, 4OMC.A.607, 2OO4.A.5001, 2OPO.A.301, 2OW9.B.610, 3OX6.B.501, 2OX9.A.804,
1OYG.A.500, 2P0R.A.1002, 3P6B.A.205, 1P8J.A.3001, 4P99.C.505, 3PDD.A.193, 3PF2.A
.1, 3PGB.A.902, 4PHN.B.303, 4PKG.G.1201, 4PKI.A.403, 4PLS.B.301, 2PMY.B.104, 3PM
8.A.1, 3PM8.A.514, 1PMH.X.300, 1PMJ.X.300, 1POA.A.201, 1POC.A.501, 3POJ.A.1, 2PP
L.A.478, 2PQX.A.500, 1PVA.A.111, 3PVN.M.5026, 2Q17.A.315, 3Q2N.A.303, 3Q5I.A.1,
3Q5I.A.524, 3Q5I.A.526, 4Q6O.A.502, 3QGM.A.503, 2QNG.A.201, 2QQM.A.1, 2QQO.A.1,
3QRB.A.303, 2QUB.A.617, 2QUB.A.620, 1QX2.B.1006, 1R55.A.515, 3R5V.B.201, 1R64.A.
701, 2RAN.A.324, 2RDZ.A.1501, 1RFJ.A.1003, 2RF7.A.1501, 1RLW.A.400, 1RP8.A.500,
1RRO.A.110, 1RWY.A.421, 1S3P.A.210, 3S5U.A.221, 1S6B.A.401, 2SCP.A.190, 2SCP.A.1
92, 1SGT.A.246, 1SH7.A.1291, 3SIB.A.221, 3SJG.A.1753, 1SL6.D.3, 1SL6.E.3, 1SL8.A
.669, 1SL8.A.670, 1SL8.A.671, 3SNY.A.97, 3SXQ.A.526, 3T3P.B.2002, 1T44.G.702, 1T
F4.A.3002, 1THM.A.301, 3TI9.A.353, 3TI9.A.355, 3TOY.A.361, 4TSH.B.1501, 3TZ1.A.1
, 3U1R.A.701, 3UBH.A.857, 3UL4.B.67, 1UX6.B.2001, 1UX7.A.1135, 3UXF.A.2, 1UY4.A.
1147, 1V0A.A.1177, 2V0B.A.1241, 4V29.B.1178, 3V9M.A.205, 1VCL.A.1005, 2VDQ.B.200
2, 1VL9.A.124, 2VPT.A.1235, 3VRR.A.401, 2VVE.A.1336, 1W0N.A.1132, 2W47.A.1137, 3
W57.A.201, 3W57.A.203, 1W7C.A.803, 3WCT.F.203, 2WD6.A.1765, 4WK7.A.502, 4WK7.A.5
03, 3WLC.A.501, 2WN3.A.400, 2WN3.C.400, 2WNO.A.1251, 2WPH.S.1246, 3WU2.c.901, 2W
W8.A.1001, 2WZE.B.1552, 4XDQ.A.301, 2XFG.B.1619, 2XFD.A.1112, 1XJO.A.905, 2XOM.A
.1169, 1XVJ.A.422, 1Y4J.A.1001, 2Y6L.A.1168, 2YIH.A.1520, 2YKK.A.1520, 2YN5.A.63
64, 1YO8.A.1198, 1YO8.A.1200, 2YOA.A.1001, 2Z2X.A.1002, 2Z30.A.1005, 1Z70.X.3002
, 2Z8X.A.618, 2Z8X.A.621, 2ZEZ.A.200, 2ZFD.A.227, 2ZFD.A.228, 2ZP4.A.124, 2ZUX.A
.631, 2ZUX.A.635, 2ZUX.A.636, 2ZUY.A.625
[1] "Cluster 4"
3TRQ.A.359, 2B0D.A.502, 4G3I.A.401, 4K4I.E.603, 3KHG.A.415, 4KHU.A.1003, 2NOL.A.
328, 4Q0W.A.1001, 4QWD.A.702, 3QZ7.A.363, 3RBD.B.1415, 4AAH.A.702, 4AC8.B.1311,
1AWB.B.280, 1AXK.A.395, 2BD4.A.260, 2BZ6.H.1260, 3C14.A.29, 1C9P.A.501, 1D8M.B.8
04, 1FBL.A.993, 4FVL.A.506, 4H82.C.305, 1HKB.A.923, 3HQ8.B.402, 3HR4.H.203, 1HVD
.A.600, 2II1.D.401, 2IO4.B.701, 3IS5.F.1, 1ITC.A.1500, 1J24.A.1001, 1JDC.A.452,

1K6S.A.302, 1K6S.B.301, 4KW7.A.402, 4L41.B.201, 2LMV.A.151, 4MC7.A.503, 4MIX.A.2
501, 4N2I.A.707, 1N41.A.410, 1NMB.N.478, 4NUY.A.1001, 3OHO.A.1, 4OKH.C.903, 2OVZ
.B.449, 3OXQ.D.516, 4P99.A.533, 2PC6.B.303, 1PEX.A.502, 4PIB.B.203, 4POQ.G.401,
4POR.E.401, 2PR3.A.901, 4Q4X.1.5007, 1QMD.B.405, 3R4I.D.342, 2RJP.D.2, 3RMK.B.30
8, 3RRV.C.255, 2TBV.A.388, 1TFX.A.1007, 3V96.B.305, 3VEQ.B.301, 2VME.E.500, 3VOB
.A.401, 2WOQ.B.803, 3W9T.B.510, 1Y7O.B.1004, 1YAX.A.1002, 2YA9.A.1303, 2YN3.D.63
55, 2Z2D.A.268
[1] "Cluster 5"
4BXO.A.2050, 2C28.A.1344, 4DU4.A.1003, 4EFJ.C.101, 4EFJ.A.401, 2GIH.B.401, 4K4H.
M.605, 4K4I.E.606, 4KHW.A.1005, 1M5X.C.801, 3M9M.B.354, 2NOF.A.328, 3OOR.A.236,
3OOR.A.237, 4Q0W.B.1001, 3QER.A.906, 3RBD.A.415, 3SQ2.A.906, 3V20.A.302, 2WTF.A.
1512, 4WUZ.B.301, 1A85.A.996, 1AEI.D.317, 1AEI.A.317, 4AG4.A.5002, 1ALA.A.401, 3
ATS.A.380, 2AZ1.D.202, 2B00.A.252, 4B7U.B.401, 1B90.A.930, 3B90.B.702, 3BCF.A.70
5, 4BCU.A.201, 1BK9.A.200, 1BRW.A.3001, 3BS6.A.1, 4BWE.A.503, 4CAG.A.606, 2CDP.C
.1140, 2COL.B.801, 1CVM.A.802, 3D4G.A.484, 3D6E.B.202, 1D8F.A.305, 3DEM.B.6001,
3DF0.A.717, 3DF0.B.601, 4DLK.B.402, 1DM5.E.1135, 3E1I.B.503, 2EAA.C.904, 4EJ7.A.
402, 4EJ7.A.404, 4EM6.B.604, 4EMU.B.401, 1ESL.A.164, 2EXJ.D.2004, 2FH3.A.1001, 4
G1M.B.2001, 4GEJ.A.201, 3GG1.B.503, 4GGB.A.402, 4GH8.B.203, 3GRI.B.700, 2GXS.B.6
04, 2HOK.A.402, 2HOK.A.408, 1H71.P.500, 3HDB.A.657, 2HF4.A.902, 1HOV.A.168, 2HTY
.A.991, 2IAP.A.402, 3IJE.B.4002, 4ILW.F.304, 1IVG.B.470, 2J1G.F.1291, 2J60.C.127
7, 2JF9.P.1014, 4JP8.A.706, 1JX6.A.401, 3K39.F.1000, 3K8Y.A.167, 4KS3.A.502, 1KT
W.A.5, 1KVO.D.192, 1KVY.A.124, 4L76.E.402, 4LIN.A.1301, 4LN6.G.403, 1LWU.K.4, 4M
7K.H.301, 3M83.B.408, 4MBE.D.201, 3MDO.A.391, 2MG9.A.101, 1MWN.A.100, 1N28.B.127
, 4N25.A.705, 4N2D.A.705, 4N96.B.401, 4NAS.C.501, 1NGS.B.681, 2NPO.A.1293, 3NSJ.
A.702, 4NUP.C.301, 4NVR.C.401, 1OLP.A.1372, 1OS2.A.872, 2OVX.B.447, 2OVX.B.450,
2OZR.F.4030, 3P1O.A.234, 2P5V.B.1008, 2P5V.C.1001, 2P5W.B.701, 1PZ8.C.703, 1Q3A.
A.467, 4Q4Y.1.5006, 1Q7B.B.9003, 1QDO.C.245, 1QHD.A.603, 1QMD.A.404, 4QN3.A.501,
2QVF.B.703, 3QWU.B.602, 2R1D.I.3000, 4R5O.A.509, 2R8Y.I.209, 1RFN.A.500, 2RHP.A
.16, 1RJV.A.112, 4RSR.A.404, 1S0E.A.1292, 3S5U.F.220, 1SBH.A.291, 1SEL.B.277, 3S
H5.A.196, 3SHI.M.305, 3SNZ.A.97, 1SPJ.A.300, 1SRR.C.532, 1UBN.A.277, 3UBH.A.855,
1UEA.A.4, 4UM9.B.2003, 3UMJ.A.902, 2V5C.A.1625, 2VB6.B.1148, 1VE5.B.2003, 2VVD.
A.1329, 2W1W.B.1134, 1W1X.A.1479, 3W9T.B.509, 2WG8.C.201, 2WII.A.1643, 3WNX.A.50
2, 1X36.A.269, 1XJL.A.344, 2XTJ.A.1423, 1Y6P.A.217, 1Y6P.B.218, 2YDP.B.502, 2YGM
.B.1418, 2Z2D.A.267, 2ZUX.A.629, 2ZUX.A.637, 2ZUY.A.621, 2ZWP.B.401
[1] "Cluster 6"
3GE4.B.167, 2LP2.A.202, 2AGO.A.403, 2BQR.A.2002, 3GIM.A.415, 4NLG.A.401, 1Q9Y.A.
939, 3A7Q.A.4004, 1ATN.D.265, 3B2Z.D.3, 1BAG.A.432, 4BQ3.B.1803, 2BW7.B.2202, 3C
GA.B.102, 1CR8.A.45, 1DGL.B.301, 3E9T.B.3, 4ECG.A.502, 1FZD.E.1, 4GOD.B.503, 1G7
Y.F.254, 2HOK.A.410, 3HGP.A.250, 4IMM.B.402, 2J7A.A.1007, 1JXN.B.246, 4JX1.A.504
, 3K7L.A.703, 1KWV.B.604, 3LPD.A.341, 1M63.F.502, 1NUD.A.701, 3NWK.B.238, 3OM7.B
.1, 2OX9.A.803, 4PKG.A.403, 2PPL.A.482, 3Q3L.A.436, 3QQZ.A.326, 2RJP.B.3, 3RRD.A
.239, 3RTT.A.267, 1S2N.A.1291, 4TVU.A.600, 4U2A.A.301, 3USU.A.264, 1WOP.A.1780,
1WBL.D.303, 1WC5.C.2100, 3WCT.B.203, 1XJL.B.342, 1YO8.A.1192, 1YO8.A.1202, 4QWD.
A.703, 4AYU.A.205, 1CGU.A.685, 3DR2.A.707, 4I5L.E.701, 3LND.A.208, 3ONR.A.72, 2A
GQ.A.4001, 2ATL.B.1415, 3AVW.A.3001, 4DTU.A.1002, 4E3S.A.1002, 4EDO.A.503, 4J2D.
A.1002, 4J2E.A.1002, 4K4G.A.603, 4K4H.A.602, 4K4I.A.605, 3KHR.B.1416, 4KHU.A.100
2, 3MX9.A.363, 3NCI.A.905, 3NDK.A.905, 3NE6.A.904, 3NGI.A.905, 2ODI.B.702, 2Q10.
B.702, 3QEP.A.905, 3QER.A.905, 3QEV.A.905, 3QNO.A.903, 4UAW.A.402, 4UB4.A.401, 4
UB4.A.402, 2A3Y.A.601, 1A8B.A.407, 3ACH.A.204, 2AEF.A.602, 4AE2.A.246, 1AJP.B.55
8, 1ANW.B.353, 4AQE.A.1209, 1AXO.A.290, 1AYP.A.301, 4AYU.D.205, 3B0X.A.579, 3B4N
.B.701, 1BIW.B.803, 2BIB.A.1552, 1BTU.A.260, 2BWR.A.500, 3BYK.A.474, 2C0T.A.1507
, 2C10.B.1777, 3C1Q.A.2, 1C8T.B.264, 4CFQ.A.501, 3CIO.K.402, 3CLN.A.152, 2CM5.A.
1678, 2CM6.A.1679, 1D0L.A.400, 4D0E.A.1532, 1D2J.A.1, 1D2S.A.401, 1DB5.A.198, 2D
BX.D.702, 1DBN.A.301, 4DD8.A.1001, 3DEM.A.5001, 4DIR.A.101, 1DJX.B.2, 1DJY.B.2,

4DKB.A.301, 4DLK.B.401, 2DPK.A.3001, 2DUR.A.1, 4DZ3.A.202, 2E26.A.602, 1E8A.A.10
89, 3E9T.B.4, 3EAD.B.1003, 2EGD.A.301, 2EIG.A.1102, 2ERO.A.702, 3ETO.A.2001, 3EU
3.A.1, 2EXH.B.2002, 3EXM.A.301, 3FCS.A.2004, 2FH1.A.2001, 1FIB.A.500, 3FLP.A.301
, 3FLP.A.302, 2FMD.A.301, 1FNY.A.500, 1FX5.A.251, 1FXH.B.1002, 4FZM.A.301, 4G1M.
A.2008, 1G7Y.A.254, 1G8K.A.5008, 1G8K.C.5108, 1G9K.A.704, 1G9K.A.705, 2GKO.A.610
, 1GK9.B.1579, 1GKF.B.1571, 1GO7.P.1482, 1GO8.P.1482, 3GPE.A.501, 2GSK.A.1, 1GSL
.A.251, 1GZC.A.290, 4H2B.A.604, 1H3G.B.701, 1H6X.A.1162, 2H61.D.817, 3HB2.P.484,
1HDF.A.1102, 4HHO.A.401, 4HHQ.A.401, 4HPH.A.701, 1HQL.A.302, 3HR4.D.202, 3HX4.A
.603, 4I35.A.514, 4I35.A.515, 3I57.B.186, 4IHM.A.406, 3IPV.A.252, 4IT5.D.202, 4I
TC.A.1201, 4IU3.B.301, 2J5Z.A.1277, 2J7A.A.1006, 1J9L.A.1301, 4JA8.A.503, 2JE7.A
.1241, 2JEC.A.240, 4JJJ.A.704, 1JKU.A.1272, 1JX9.B.601, 1K9I.I.1201, 1K9J.A.403,
1KAP.P.621, 3KM5.B.2011, 4KTY.A.803, 1KVO.A.191, 3KWU.A.902, 2KYF.A.109, 3L2Y.C
.302, 1L9N.A.700, 1L9N.A.702, 1LEN.A.184, 1LHW.A.401, 4LMH.A.811, 3LNF.A.302, 3L
NP.A.472, 1LOC.A.228, 4LOR.A.301, 3LPC.A.360, 3LPC.A.362, 4LP7.B.301, 2LTN.A.191
, 1LVU.A.8001, 1LVU.D.8002, 3M0W.I.202, 4M00.A.803, 4M02.A.701, 3M1H.A.2001, 4M5
E.A.505, 1MCX.A.349, 4MGQ.A.601, 1MKU.A.124, 1MKV.A.124, 2ML2.A.203, 2ML2.A.204,
2ML3.A.202, 3MVS.A.213, 3MVS.A.216, 1MVQ.A.238, 4N2L.A.707, 3N35.A.290, 1N47.B.
252, 1NIW.G.1014, 1NLS.A.240, 3NOL.A.263, 1NPC.A.321, 3NQZ.B.520, 2NXP.C.602, 2O
72.A.402, 1O8F.A.1353, 1O8P.A.1149, 1OAC.A.803, 1OFL.A.528, 4OKH.B.901, 3OM2.A.1
, 3OM4.A.1, 3OM5.A.1, 3OM6.A.1, 3OM7.A.1, 4OMC.A.606, 2OVU.A.238, 3OX6.B.502, 4O
Y7.G.302, 2P2K.A.239, 5P2P.A.125, 3P4G.A.411, 3P4G.B.405, 4P99.B.517, 3PDD.A.194
, 2PEL.A.237, 4PEU.A.401, 3PGV.A.267, 4PHJ.A.302, 3PPE.A.402, 3PPE.A.403, 1PT2.A
.500, 3QC6.X.1, 1QI3.A.451, 1QNW.A.302, 1QPK.A.451, 3QRO.A.1000, 3QRX.A.172, 2QU
B.A.619, 2QUB.A.621, 2QUB.G.616, 2QUB.K.614, 2QV6.A.302, 1QX2.A.1005, 1R1Z.A.285
, 2R8Z.N.214, 2RJP.C.3, 1RLW.A.401, 1RU4.A.1, 1S1E.A.226, 1S6B.B.402, 1S6C.A.218
, 1SL4.A.407, 3SRE.A.1356, 1SU4.A.995, 1THM.A.302, 3TI7.A.354, 3TI9.A.354, 1TM7.
E.470, 4TNC.A.164, 1TU5.A.903, 4TVU.C.600, 4U36.A.302, 3U4X.A.237, 4U65.E.301, 1
UKG.A.1262, 4UM9.A.2001, 1UNE.A.124, 1UP8.A.599, 3USU.C.266, 2UWP.A.1246, 1UWW.A
.1192, 1UX6.B.2005, 1UX6.B.2008, 1UX6.B.2009, 1UX6.B.2011, 4UZU.A.1485, 1VCL.A.1
002, 3VLV.A.503, 2VN7.A.650, 2VUD.C.1118, 3VV3.A.402, 2W08.A.206, 1WBF.A.303, 1W
CO.A.2100, 1WC5.A.2100, 3WCS.A.1003, 4WJK.B.502, 1WKY.A.503, 3WLC.A.503, 2WW8.A.
1002, 1Y1X.A.201, 1Y1X.A.202, 1Y6O.A.207, 2YAY.A.1266, 2YFS.A.1712, 1YO8.A.1189,
1YO8.A.1207, 2YOA.A.1002, 1YUT.A.197, 1YXH.A.1001, 2Z2X.A.1003, 2Z8X.A.619, 2Z8
X.A.625, 2Z8Z.A.623, 1ZED.A.906, 2ZEX.A.406, 2ZKM.X.800, 2ZUY.A.622, 2ZVD.A.628,
2ZVD.C.620, 3ZYR.A.401, 2ZYH.A.700, 2ZZJ.A.239
[1] "Cluster 7"
2ASJ.B.1415, 4K4I.I.604, 3KHL.A.415, 4KHN.A.1002, 3QEP.A.906, 3SQ1.A.907, 1A25.A
.292, 4AQ8.D.1238, 4B4F.B.607, 3BOW.A.718, 4BTX.A.1763, 3CK7.B.720, 2CLT.A.1101,
2CLT.B.1301, 3D4G.H.484, 2DEW.X.904, 1DM5.A.1131, 1DM5.C.1133, 3E3R.B.195, 1E8U
.A.1002, 3E9T.A.6, 1EE6.A.300, 1EGZ.B.300, 1ESP.A.320, 2EXK.D.2004, 2F2O.B.1001,
1F2N.C.1001, 3FCS.D.2002, 4FGC.C.203, 3FHA.B.704, 3FWO.A.823, 2GA9.D.480, 4GGB.
A.401, 4GZS.A.501, 4H2E.B.304, 1HFY.A.124, 1HFZ.C.124, 4HSZ.B.101, 2HTY.C.993, 2
HTY.G.997, 1HVD.A.602, 4I5K.A.501, 1JIW.P.489, 3K1A.B.524, 3K9X.D.249, 1KLJ.H.90
0, 4KNA.A.504, 4KVK.A.712, 4L06.C.501, 1L7L.A.201, 3L95.X.2001, 4LIN.E.1301, 4LM
F.B.302, 3LND.A.210, 1LQD.B.1, 4LT6.A.602, 4LXF.A.701, 1MMP.B.3, 4O1Q.A.401, 1OS
2.C.473, 3PKO.D.280, 4PLS.A.305, 1PYT.C.650, 3QQZ.A.324, 1R1Z.B.315, 1RE3.C.408,
2RHP.A.2, 2RHP.A.24, 2RHP.A.28, 2RLD.C.121, 1ROS.A.404, 3S5U.D.220, 4SBV.C.261,
3SOB.B.1, 1SRR.A.531, 1TAD.A.352, 1TD7.A.1001, 3TH3.L.145, 1TYE.B.1402, 4UB6.O.
301, 3UBH.A.852, 2V3T.A.1264, 1VFO.B.1002, 1VFP.B.995, 2VME.A.500, 3VOT.A.504, 3
W9T.C.1010, 3W9T.B.508, 1WD9.A.901, 2WVX.C.801, 2X0G.B.1149, 2YFS.A.1711, 2YGM.A
.1417, 1YO8.A.1184, 1YO8.A.1188, 2Z8S.B.641, 3ZHG.B.1323, 2ZKT.A.413, 1ZTQ.B.565
, 2ZWO.B.402, 2AOQ.A.232, 3BQ1.A.4001, 1DMU.A.302, 3EH8.A.302, 3FSP.A.501, 3GV5.
B.424, 2IBK.A.401, 1JX4.A.4001, 3KHH.B.1416, 1M5X.A.802, 1N3E.C.492, 4PTF.A.1202
, 3QEX.A.907, 1R7M.A.306, 2RDJ.B.353, 1S9F.A.4001, 2WTF.B.1510, 3A13.E.445, 2A8K

.C.403, 2AER.L.3008, 1AFA.2.2, 4AWD.A.1321, 3AZX.B.301, 3B00.B.301, 3B0I.A.124, 4B7R.A.502, 3BCD.A.708, 3BJU.A.608, 4CAJ.B.1325, 2CDP.B.1139, 1CGU.A.686, 3CGT.A .686, 3CK7.D.730, 3CKC.A.600, 5CNA.C.240, 1CRU.A.503, 4CRR.A.1386, 1CYG.A.682, 3 D4G.B.484, 3DAS.A.351, 4DLK.A.403, 4DRZ.A.202, 2DSN.B.2012, 3EAD.A.1003, 3EDD.A. 700, 3EHB.A.563, 3EHJ.B.1, 4EJ7.B.402, 3ESQ.A.213, 2EXH.D.2004, 1F6S.E.205, 3FCU .B.2002, 2FIB.A.412, 2FPS.A.503, 4G60.A.301, 4G62.A.301, 1G87.A.615, 1G9K.A.700, 2GGM.B.402, 4GI6.A.601, 3GN4.B.204, 3GQF.D.154, 4GQ7.A.301, 2GSM.A.3007, 4H3X.A .304, 1H5V.A.306, 1HDF.B.1101, 1HFZ.A.124, 1HL5.D.156, 1HQV.A.999, 1I22.A.198, 3 I4I.A.1001, 2I4B.A.454, 3I57.A.186, 2IWV.B.1281, 2J1T.A.1154, 1J9L.B.1303, 4JCM. A.706, 4JDZ.B.702, 3JTX.B.396, 3JXS.A.302, 4K5W.A.201, 3KF9.C.304, 1KMB.1.2, 3KQ A.B.421, 4KVJ.A.714, 1KX1.E.502, 1KX1.F.601, 3KZP.A.240, 2L51.A.207, 1L9M.A.702, 3L9I.C.1148, 3LCP.B.279, 3LHM.A.131, 4LJ3.B.403, 1LOC.E.688, 4LVN.A.704, 2MOP.A .1201, 4M5I.A.203, 1MAC.B.389, 1MDU.A.403, 3MVS.A.214, 4N25.A.707, 4N2I.A.704, 3 N4E.A.500, 4N85.A.502, 3N8G.A.1002, 4NAM.A.801, 4NHF.B.301, 3NN7.A.503, 2NXP.A.6 00, 3O5S.A.243, 2OKX.B.4002, 3P4G.A.403, 3P4G.A.404, 3P4G.A.405, 3P4G.A.407, 3P4 G.A.412, 4PLS.A.301, 1PVY.B.603, 3Q5I.A.528, 3Q8F.A.736, 4QD2.E.302, 3QGV.A.504, 2QT6.A.3713, 4QU6.A.904, 1R1Z.C.415, 3RRW.A.271, 3S6J.A.4, 3SLE.B.401, 3SO0.F.9 8, 3SVL.A.201, 1TCM.B.687, 3TEW.A.801, 1TT2.A.502, 3UBF.A.754, 1UF3.D.913, 1UH2. A.1002, 1UH3.A.1001, 1UH3.A.1002, 1UKS.A.688, 1ULV.A.2003, 4UM9.B.2002, 1UPS.A.5 01, 1UYX.A.1133, 3V03.A.585, 1V3J.A.687, 1V3L.B.689, 2VCC.A.1917, 2VDR.B.2002, 2 VJ3.A.1533, 2VL8.A.1545, 2VVE.B.1338, 2VXJ.G.200, 3W9T.A.1008, 3W9T.D.504, 4WBQ. B.602, 4WF7.D.600, 3WH3.A.501, 2WO9.B.1272, 2WQS.A.2415, 2XQX.B.1949, 2YDP.A.502 , 1YO8.A.1195, 1YO8.A.1201, 1YO8.A.1203, 1YO8.A.1208, 2Z49.A.1002, 3ZHG.D.1323, 2ZQ0.A.901, 2ZWP.A.402, 4KZO.A.501, 4KZO.B.501, 4K4G.A.602, 1T9I.C.801, 1T9I.C.8 02, 4UAW.A.403, 4A3X.A.1268, 4A3Z.A.2344, 3A4U.A.287, 4A41.A.2494, 4A42.A.1690, 4A4A.A.1925, 4A5G.A.1308, 4A5G.A.1309, 4A6O.A.2346, 4A6S.A.1122, 4AFA.A.1267, 4A FB.A.1267, 4AFC.A.1267, 1ALC.A.200, 3ALT.A.201, 3ALU.A.201, 4AL9.A.1122, 4AOC.A. 1129, 4AOC.E.1129, 4AQ1.A.1924, 4ASL.A.1268, 4ASM.B.1359, 3ATG.A.301, 4ATE.A.127 5, 4AWD.B.1321, 3AXD.A.3002, 3AZY.A.301, 3B0K.A.201, 3B0X.A.578, 1B80.B.351, 1B9 O.A.124, 4B96.A.1155, 4B9C.A.1151, 2BF6.A.1693, 1BGP.A.501, 2BIB.A.1551, 1BJ3.B. 124, 4BLK.A.400, 3BMV.A.685, 4BM1.A.401, 2BOI.A.300, 2BOI.B.600, 1BQB.A.353, 2BV 4.A.300, 2BV4.B.300, 1BYF.A.201, 1CGT.A.686, 1CIU.A.684, 4CI7.A.1505, 3CK7.A.710 , 4COU.A.1270, 4COV.A.1269, 1CPM.A.215, 1CPN.A.209, 4CP0.A.1294, 4CP1.A.1294, 4C PB.D.1123, 3CQO.A.4001, 3CQO.A.4004, 4CRQ.A.260, 4CUO.A.1326, 4CZN.A.1370, 1D3C. A.687, 2DEW.X.900, 1DED.B.5004, 3DED.B.506, 1DV8.A.1002, 4DZT.A.302, 2E39.A.501, 2E8Y.A.741, 3EDF.A.603, 2EJN.A.1003, 2ERV.A.195, 2EWE.A.703, 2EXH.A.2001, 1F6S. A.201, 2FHF.A.2401, 1FIF.B.2, 3FMU.A.351, 4G01.A.300, 1G5N.A.408, 1GW2.A.502, 1G WT.A.502, 1GWU.A.1307, 1GWU.A.1308, 4GWI.A.204, 1GX2.A.1310, 3HOO.A.401, 2H2N.B. 1001, 1H3G.A.700, 3HDL.A.306, 3HDL.A.307, 2HD9.A.2001, 1HFX.A.124, 1HFZ.B.124, 3 HR9.A.401, 2HYK.A.477, 1I22.B.197, 4IAI.A.402, 4IAU.A.800, 3ILF.A.278, 2IWV.D.12 83, 2J1A.A.1769, 2J22.A.1150, 4J3V.A.920, 4J3W.A.907, 4JCL.A.701, 2JDY.A.1116, 2 JDY.B.1117, 2JD9.A.1146, 2JDA.A.1146, 2JDM.C.1115, 2JDN.A.881, 4JGL.A.202, 1JI1. A.2001, 1JI1.A.2002, 2JKX.A.1641, 1JUG.A.126, 1K12.A.160, 3K8K.A.710, 3K8L.A.700 , 3K8L.A.710, 1KZM.A.501, 1L6R.B.903, 4LHK.A.303, 4LHN.A.302, 4LJH.A.201, 1LLP.A .351, 4LPL.A.1101, 4LQR.A.201, 1LY8.A.9001, 1LYC.B.9002, 3M5Q.A.372, 1MAC.A.388, 2ML3.A.201, 3MMZ.A.501, 1MVE.A.400, 4N2B.A.707, 4N2G.A.703, 4N2L.A.704, 4N6F.A. 302, 3NNG.A.402, 2NZM.A.406, 1O4Y.A.700, 4ODG.A.202, 2OKX.A.4001, 3OMI.A.613, 1O UX.B.404, 3OWF.A.151, 1PA2.A.307, 1PA2.A.308, 1PAM.A.687, 1PAM.A.688, 3PAR.A.300 , 4PIB.C.201, 1PJ9.A.890, 4Q1U.A.402, 3Q3U.A.340, 4QF4.A.202, 1QGJ.A.2001, 1QGJ. A.2002, 1R1Z.A.286, 4R83.B.501, 3S18.A.229, 1SCH.A.301, 1SCH.A.302, 1SH7.A.1292, 1SNC.A.150, 3SO1.A.97, 3SRE.A.1357, 3SRG.A.1357, 1SU4.A.996, 3TBD.A.401, 1TDQ.B .127, 1TE2.A.702, 1TLG.A.201, 3TO5.A.131, 1UOA.A.5004, 1UPS.B.501, 1URX.A.1300, 1UX6.B.2014, 1UX7.A.1134, 3V6N.A.229, 2V72.A.1139, 2VNV.A.302, 2VNV.B.302, 2VZQ. A.1130, 2W1Q.A.1947, 2W1S.A.1946, 1W3M.E.3013, 3W57.A.202, 3W5N.A.1201, 2W86.A.1

148, 3WDH.A.801, 4WF7.A.600, 2WOY.A.2415, 2WQ8.A.1641, 2WR9.A.1131, 2WR9.D.1132, 1WZL.A.1601, 2WZA.A.2415, 2XFD.A.1111, 2XFE.A.1113, 1XO5.A.1, 2YFU.A.1141, 2YLJ .A.1308, 1YRO.A.124, 2Z30.A.1002, 2Z48.A.1007, 2Z48.A.1102, 2Z48.A.1103, 2Z48.A. 1104, 2Z48.A.1205, 2Z49.A.1001, 2Z49.A.1003, 2Z49.A.1004, 2Z49.A.1005, 1ZJA.A.70 01, 3ZO9.A.1589, 3ZUC.A.1154, 3ZYH.A.1123
[1] "Cluster 8"
1BSS.A.433, 3S9H.A.906, 3A7Q.A.4001, 2AAO.B.299, 1ALA.A.400, 1AXK.B.395, 1B09.C. 301, 3BRX.A.328, 3BXK.A.152, 1CTR.A.150, 2D3P.A.237, 2D7F.A.240, 3E3R.B.193, 2E6 V.D.11, 1ETH.A.449, 1F4M.E.105, 2FH3.A.1002, 2FHC.A.2405, 3FLP.L.302, 1FZC.C.1, 1FZD.G.1, 4G0D.A.503, 4G9L.B.306, 1GYK.A.1206, 2H0K.A.401, 1IHO.A.2, 2IWV.B.1284 , 2J3U.C.1292, 2J64.C.1222, 1J84.A.366, 4JDZ.B.704, 4JDZ.A.702, 1JN2.P.238, 1K90 .E.802, 4L76.A.402, 1LGC.E.301, 1LGN.A.302, 1LU1.A.301, 1LWU.B.1, 1M1J.C.501, 3M 83.E.407, 3M8D.A.710, 4MBE.A.202, 2ML3.A.206, 2MLS.A.305, 1N29.A.125, 1NXO.A.900 , 4OJQ.B.1002, 4OMC.E.607, 1OTN.A.250, 2OXE.B.600, 2OYH.B.2, 4P99.D.512, 3POG.B. 1, 1Q3A.C.478, 1QOO.A.302, 2RJP.B.4, 3RMK.E.313, 3RYD.C.267, 1SOC.A.1879, 2TBV.C .388, 3UJO.D.304, 4UM8.A.2004, 1VAH.A.500, 3VU1.A.1001, 1W2M.A.1442, 4W4Q.A.401, 1WDA.A.902, 4WK2.B.502, 1WMZ.B.203, 3WYN.B.402, 2WZE.A.1552, 1XFE.A.100, 1YAX.C .1003, 2YN3.C.6370, 1Z3U.B.497, 2ZOC.A.501, 2ZOC.A.502, 2ZWO.A.401, 3CFR.A.910, 4DTS.A.1002, 4K4I.A.606, 1N56.B.403, 3A7Q.A.4002, 3AAJ.A.991, 5AER.A.201, 1AFD.3 .2, 1AJ4.A.163, 1AMY.A.502, 1ANN.A.319, 1ANW.B.354, 1BAG.A.431, 3BOW.A.719, 3BOW .B.403, 4BY5.D.1185, 1C9U.B.1002, 4CAG.A.605, 3CGA.A.102, 3CHK.A.502, 1D2L.A.46, 1DCY.A.198, 2DDY.A.174, 2DPK.A.2001, 2E3X.A.803, 3E3R.B.194, 4E52.A.403, 2E6W.A .301, 3E9T.A.1, 3EAD.B.1002, 3EAD.B.1004, 3ECQ.A.2000, 4ELF.C.201, 4ELG.F.202, 2 EQD.A.701, 2ERO.B.702, 1FDK.A.124, 4FGC.B.204, 1FI5.A.162, 3FLP.B.301, 3FLT.A.30 2, 4FL4.C.401, 4FL4.C.402, 3FVI.D.125, 1GOH.A.290, 4GKX.B.301, 1GQM.A.1089, 4GTW .A.1012, 2H0K.A.407, 1H4B.A.1085, 1H4B.A.1086, 2HQ8.B.303, 2HYW.A.505, 2I08.A.20 0, 4I5N.B.601, 3IA7.A.402, 3IA7.B.402, 3IGO.A.601, 1IME.A.278, 2IWV.D.1286, 1IXX .B.124, 1IZJ.A.1002, 2J1G.D.1289, 1JBA.A.501, 4JBE.A.502, 4JEO.A.401, 1JL5.A.200 4, 4K1C.A.506, 3K8L.B.800, 1K90.F.804, 1K96.A.92, 2KAY.A.185, 3KF9.A.302, 1KIT.A .803, 4KPP.A.501, 1KTW.A.3, 1KX1.A.222, 1LBX.A.290, 1LGC.A.301, 1LHV.A.401, 4LMF .A.304, 4LMH.D.811, 2LP3.A.201, 2LV6.A.204, 2LVK.A.102, 1LWJ.B.10, 1M34.B.2299, 1M8T.C.1003, 4MDV.B.403, 4MIV.D.601, 2ML1.A.206, 2ML3.A.205, 4N25.A.706, 1NAE.A. 900, 4NDD.B.402, 4NEN.A.1115, 1NIW.A.1002, 1NIW.C.1005, 3NOM.B.263, 1NUB.A.301, 1NUB.A.302, 1NX2.A.4, 1O9I.C.269, 1ODB.F.1092, 3OX6.A.501, 3OX6.A.502, 4P5W.A.10 01, 4P99.A.512, 1PK8.A.817, 3PM8.B.514, 2PQY.A.500, 1PTK.A.281, 4Q4W.1.905, 1Q8H .A.72, 1QNI.E.903, 3QRX.A.173, 2RHP.A.1176, 2RHP.A.26, 3RV2.A.405, 3S5U.C.220, 2 SAS.A.186, 1SCV.A.162, 1TCF.A.162, 1TEC.E.343, 1THL.A.3233, 3TRQ.A.358, 3TRQ.A.3 60, 1TTX.A.110, 1TTX.A.111, 1TYE.A.1405, 1TYE.A.1406, 1TYE.A.1407, 1U7W.A.501, 1 UG9.A.2006, 4UM8.C.2004, 3USU.H.272, 1V1G.A.1211, 2V3T.B.1264, 1V3J.B.690, 2VB6. B.1151, 1W2M.C.1441, 3W9T.A.1005, 3WHU.B.502, 1WMD.A.1001, 2WO9.D.1270, 3WU2.O.3 01, 1YUU.B.199, 2Z2D.A.266, 2Z8S.A.644, 2Z8S.B.642, 2ZBA.A.461, 2ZJ7.A.627, 2ZKT .B.413, 2ZUY.A.624, 2A40.B.1273, 1AFB.1.2, 2ASP.A.600, 4AW7.A.1599, 4AWN.A.301, 4B97.A.1152, 4B9P.A.1167, 3BMV.A.684, 4BM1.A.402, 2BOQ.A.1352, 1CGV.A.692, 1CLC. A.651, 1CXL.A.689, 4CZN.A.1369, 1D3C.A.688, 3DED.C.508, 3DHP.A.497, 4DLK.A.402, 1DTU.A.688, 1DV8.A.1001, 2E39.A.502, 3ER9.B.901, 2EWE.A.702, 4EW9.A.203, 2F3Y.A. 1174, 4FCS.A.403, 3FM1.A.352, 3FM4.A.352, 3FMU.A.352, 1G87.A.616, 1G94.A.800, 2G UY.A.601, 1GYK.A.1205, 1H3J.A.1346, 1HUP.A.302, 2HYV.A.601, 1I75.A.1688, 1IA6.A. 1263, 1IOD.G.506, 1J34.B.512, 1JAE.A.500, 1K72.A.779, 1KXH.A.800, 1LLP.A.352, 3M 5Q.A.371, 1MN2.A.371, 1NBC.B.156, 2NZM.A.407, 1OT1.A.1693, 3PDD.A.192, 3PF2.A.2, 3POE.A.2, 2PWA.A.1280, 3Q3U.A.341, 3QEE.A.21, 1SH7.A.1290, 1UA7.A.601, 1UOV.A.1 419, 3VM7.A.501, 2VMH.A.3050, 2VMI.A.3050, 3VV3.A.403, 1W3H.B.1348, 2WHK.A.1339, 1WMD.A.1002, 2WNX.A.1163, 2ZIC.A.944, 3ZQX.A.1146, 3FD2.A.374, 4K4H.A.604, 1N48 .A.501, 3NAE.A.906, 3NHG.A.908, 3QEW.A.905, 3QEX.A.905, 3S9H.A.905, 3SCX.A.906, 3SQ2.A.904, 2AAO.A.294, 1AFD.1.2, 3AMR.A.901, 3AMR.A.906, 1ANN.A.320, 4ANR.A.400

, 1B1G.A.76, 1BJF.A.403, 1BJF.A.404, 3BJU.A.606, 1BLI.A.700, 2BPE.A.1245, 2COT.B
.1507, 4COK.A.1615, 3C7F.A.803, 4CAJ.C.1323, 2CCM.A.1193, 1CGE.A.304, 2CHN.B.171
6, 3CIO.K.401, 1CXE.A.690, 1DB4.A.198, 2DEW.X.902, 4DH2.B.101, 4DH2.B.102, 4DLK.
A.401, 3DSL.A.2, 1DTL.A.202, 1E43.A.504, 3EKI.A.601, 4ELG.B.202, 4ELG.C.202, 1EO
7.A.689, 2ERO.A.701, 1ESP.A.319, 3ETO.A.2002, 1EXR.A.1001, 3FAW.A.4, 3FCS.A.2008
, 2FH1.A.2003, 1FIF.C.2, 3FLT.A.301, 4GON.A.202, 1GGZ.A.152, 4GKY.A.302, 3GQ8.A.
1, 1HDH.A.1528, 4HEX.A.204, 1HQV.A.997, 1HT6.A.501, 4I2Y.B.504, 4I5K.A.502, 3IUC
.A.2, 2IWA.A.501, 1J1D.A.202, 1J55.A.101, 4JRF.A.601, 3KCP.A.702, 3KEZ.B.501, 3K
F9.A.301, 3KF9.A.304, 4KHO.A.1001, 2KPN.A.762, 1KP4.A.200, 4L03.B.502, 4L73.B.40
2, 3LND.A.209, 2MLR.A.303, 4MNO.A.301, 4MSP.A.202, 2MTE.A.101, 3NJH.C.503, 3NOK.
A.257, 4NUQ.A.302, 1O9I.A.269, 2OO4.A.5002, 4OV2.A.201, 4P5F.B.501, 2P69.A.305,
4P99.A.515, 4QB2.A.202, 1R17.A.599, 4R1D.A.601, 4RDQ.A.504, 4RDQ.B.502, 4RGP.B.3
02, 1SRA.A.301, 3T3P.A.2005, 3T3P.A.2006, 3T3P.A.2007, 1TMQ.A.1001, 3TRP.A.358,
4U65.E.302, 4U6B.A.501, 3U8D.A.202, 3UBH.A.856, 1UKT.B.690, 4UM9.A.2002, 4UM9.A.
2004, 1UX6.B.2006, 4V29.A.1178, 1V3J.A.688, 2VN5.B.101, 2VN6.B.1066, 2VNG.A.1213
, 1WOP.A.1779, 1WOY.H.1259, 2WG7.A.1126, 3WHT.B.502, 4WJK.A.503, 4WKO.A.502, 4WK
O.A.504, 3WNO.A.801, 1WPC.A.503, 2WVZ.A.800, 1XO5.A.5, 2Y5I.A.102, 2Y8K.A.1526,
2YHG.A.1936, 1YO8.A.1196, 1YO8.A.1210, 1YUT.A.198, 2ZFD.A.230, 3ZHG.A.1323, 2ZUX
.A.633, 2ZUX.A.634, 2ZUY.A.623, 2ZZV.A.501, 3E1I.B.502, 1EUB.A.277, 4K6T.G.408,
4QNP.A.506, 2QPS.A.501, 1W52.X.602
[1] "Cluster 9"
1SON.A.403, 3BJU.C.608, 2DDY.A.175, 1IJL.B.202, 2JAL.B.1447, 4L06.D.501, 1TN3.A.
183, 2W2O.E.1333, 1Z3J.A.266, 2G8K.A.401, 4K4H.I.602, 3NE6.A.905, 1A2X.A.160, 1A
EI.A.319, 5AER.A.202, 3B1T.A.900, 4BY5.C.1187, 1D7F.A.5003, 3E78.A.601, 1E8U.B.1
003, 3EHJ.A.1, 1ESP.A.322, 2EXI.D.3004, 4FU4.B.505, 3HLI.D.315, 1JC2.A.3, 2K7O.A
.103, 1KBO.A.801, 1KV9.A.802, 2KZ2.A.149, 1L6R.A.901, 4LMF.A.303, 1LPG.B.1, 2M5E
.A.2001, 1M63.B.502, 2ML2.A.202, 2ML2.A.205, 3MXW.A.402, 1NHE.A.805, 1NIW.A.1001
, 2PF2.A.174, 4PLM.A.504, 2POJ.A.266, 2PPL.A.481, 1Q7B.A.9002, 3QRX.A.170, 2RDZ.
B.1502, 3SO1.B.97, 3TSK.A.304, 3TUY.C.157, 1ULV.A.2004, 1UZJ.A.1648, 1UZJ.C.3649
, 1V2I.A.1001, 1VFO.A.1001, 2VKH.A.1543, 2VZP.B.1129, 2W2N.E.1334, 2W3J.A.1139,
2WVX.B.801, 2WVZ.B.800, 2WWO.A.800, 1XJL.B.340, 1YCM.A.266, 1YO8.A.1211, 2Z8S.A.
647, 1ZFS.A.104, 1AFA.1.2, 4AK8.A.1326, 4AYO.A.500, 4AYP.A.500, 4AYQ.A.500, 4AYR
.A.500, 3B9G.A.318, 3B9X.A.400, 1BCH.1.2, 1BCH.2.2, 2CKI.A.998, 2DG1.A.3001, 2DS
O.B.1002, 4E52.B.401, 1EO5.A.689, 3EPW.A.1001, 3EPX.A.1001, 2FF2.A.601, 1FIF.A.2
, 1FIH.A.2, 1FO3.A.700, 1G1T.A.160, 3G5I.A.501, 3G81.A.401, 2GGU.A.401, 2GGX.A.4
01, 1H8O.A.1492, 4I5N.E.701, 4I71.A.402, 4I72.A.401, 4I73.A.401, 3IKP.B.401, 3IK
R.A.401, 1J34.A.511, 1JZN.A.1138, 1K9I.B.502, 1K9J.A.402, 3KMB.1.2, 1KWT.A.502,
1KWU.A.503, 1KWV.A.503, 1KWW.A.503, 1KWX.A.503, 1KWZ.B.604, 1KXO.A.504, 1KZA.1.5
02, 1KZD.1.502, 4KZV.A.302, 4KZW.A.302, 1LQV.D.47, 2MAS.A.316, 3MKM.A.501, 3MKN.
A.501, 2MSB.B.2, 1MUQ.B.202, 4N32.A.402, 4N33.A.404, 4N34.A.402, 4N35.A.404, 4N3
6.A.402, 4N37.A.402, 4N38.A.403, 4NVR.A.401, 2ORJ.A.401, 2OS9.A.401, 2OX9.A.802,
3P5G.A.500, 3P5H.A.500, 3P5I.A.500, 3P7G.A.1, 3P7H.A.1, 3PAK.A.300, 1PW9.A.401,
1PWB.B.401, 1Q8F.A.2001, 1RDI.1.2, 1RDJ.1.2, 1RDK.1.2, 1RDL.1.2, 1RDN.1.2, 1RDO
.1.2, 3RQI.A.181, 1RTM.1.2, 3S82.B.405, 1SL5.A.402, 1SL6.A.2, 2VUV.A.200, 2VUZ.A
.1130, 3VYK.A.1007, 1WOY.L.1156, 3WH2.A.301, 3WMS.A.801, 3WMT.A.606, 1WMY.A.202,
1WMZ.A.202, 1WMZ.B.202, 2WZS.A.800, 1XKD.A.1006, 2XR6.A.1391, 1YOE.A.1001, 3RAX
.B.1416, 3AFG.A.541, 3AUJ.A.1603, 2B6N.A.300, 4B9F.A.153, 1BCJ.2.2, 2BO2.A.155,
2BO2.A.156, 2BOJ.A.1116, 2BOJ.C.1117, 3BPS.E.1, 2BP6.A.802, 2BP6.C.805, 4CE8.A.9
98, 4CGT.A.685, 4COY.A.1270, 4CPB.A.1122, 4D0E.A.1531, 4D0E.A.1533, 1D2V.A.600,
3DBZ.A.401, 3DCQ.A.116, 3DCQ.B.116, 2DCK.A.1002, 3DED.A.504, 3DEM.B.4001, 2DIJ.A
.689, 2DSO.A.1001, 3EHU.A.500, 3EIF.A.1, 1ESL.A.163, 2FF3.A.701, 1FHF.A.502, 4FH
P.A.402, 1FI6.A.100, 4FVL.A.505, 1G1Q.A.801, 1GEN.A.302, 4GER.A.402, 4GER.A.404,
2GGX.C.401, 3GIS.Z.1003, 2GVU.A.500, 2GVV.A.500, 2H2T.B.322, 4HHR.A.703, 3HLH.B
.315, 3HLI.B.315, 2IAW.A.401, 2IAX.A.401, 2IAO.A.401, 3INM.A.521, 1IOD.A.501, 3I

QT.A.1, 4IT5.B.201, 1J34.C.504, 2JDM.B.1115, 4JSD.A.603, 4KVL.A.703, 1KWZ.A.504, 3LI3.A.402, 3LI4.A.316, 4LMF.D.303, 1LPZ.B.1, 1LQV.C.42, 4M65.A.404, 1MN1.A.371 , 2MTE.A.102, 4MZA.A.612, 4N4E.E.404, 4N7A.A.605, 1NL1.A.204, 1NPC.A.322, 1NZI.A .1001, 1OVS.A.402, 1OVS.B.404, 3P7F.D.1, 3PAQ.A.300, 1PJX.A.491, 1PVY.A.503, 3Q9 K.A.606, 3R5Q.A.1001, 3SO0.A.97, 1SU3.A.904, 1SZB.A.1001, 3TH4.L.148, 1USR.B.157 3, 1UZJ.B.2648, 1V3E.A.4001, 2VUC.B.990, 2VUC.C.991, 2VZP.A.1129, 2VZR.B.1132, 2 W2M.E.1334, 3W5M.A.1201, 2W86.A.1149, 1WDC.C.501, 3WH3.A.500, 3WHD.A.501, 3WU2.A .401, 2WZS.F.800, 1X2T.A.603, 2Y4Q.A.800, 1YDY.A.904, 2YEQ.A.1525, 3ZDV.A.200, 3 ZDV.B.200, 1ZH2.B.201, 1JHN.A.900, 1KTW.A.496, 1KTW.A.9, 4L06.A.501, 1LRW.A.702, 3R6Q.H.469

[1] "Cluster 10"

4BXO.B.1216, 2FKC.A.248, 4G3I.B.401, 3GII.A.415, 4K4H.M.604, 4K4I.A.603, 4KHW.A. 1003, 4LQ0.A.401, 4QWE.A.703, 1R7M.A.304, 1TW8.C.803, 3UIQ.A.905, 2WTF.B.1509, 1 A25.B.292, 4AG4.A.5001, 4AIO.A.1890, 1ALA.A.402, 4B7M.B.1471, 2BAT.A.601, 2BD3.A .260, 2BV2.A.1085, 2BW7.A.2202, 3BYC.A.901, 4CCE.A.4001, 2CDO.B.1140, 2CDP.B.114 0, 2CHI.A.218, 5CHY.A.401, 1CJY.A.951, 3CKC.B.700, 4CPN.A.500, 4CPO.A.1466, 1CXV .B.6, 2D00.B.1002, 1D7X.B.805, 2DEW.X.903, 3DPE.A.997, 1DQ1.A.238, 4DWW.A.301, 1 DYK.A.4001, 1E35.B.260, 1E5J.A.402, 2EA7.B.452, 3EKI.A.602, 2ERQ.B.702, 1EZS.C.6 50, 3F19.A.266, 1F2N.A.1002, 3FRP.A.628, 4FVL.B.506, 4GDI.C.507, 2GNT.A.254, 4GN 7.B.301, 1GQM.E.1089, 2HTV.A.995, 4HZY.A.507, 4ILW.D.304, 1IME.B.278, 1INW.A.501 , 1J0Y.D.701, 1JAO.A.996, 2JG9.B.1224, 1JI3.A.401, 1JRF.A.48, 4JUC.B.601, 3K37.A .467, 4K3Y.D.604, 3K5T.A.802, 3KF9.A.303, 3KL6.A.3, 4KS2.A.501, 1KVO.A.192, 1KWH .A.800, 1L6J.A.502, 1L9N.A.703, 3LNF.B.305, 4MDV.A.402, 3MIN.D.524, 1MR8.A.102, 1MTV.A.480, 1N28.A.126, 1N28.A.128, 4N2F.A.703, 1NG0.A.1002, 1NG0.C.1001, 1NKQ.A .260, 4NRE.A.716, 4NRE.A.717, 4NUP.A.301, 2NVO.A.533, 2NW6.A.613, 1NX0.A.902, 3O 9J.A.995, 2O04.B.5004, 2O04.B.5006, 2OW1.B.447, 3OX6.E.502, 2P5V.C.1002, 4PHK.A. 304, 1PK8.F.817, 4PKH.J.1201, 3PRT.A.404, 3Q2L.A.703, 1Q3A.B.472, 3Q3K.A.262, 4Q 4X.1.5005, 1QI5.A.452, 1QL9.A.480, 3QNI.B.400, 4QN3.B.501, 1QU0.B.702, 2R1D.B.10 00, 2R8Y.C.203, 2R8Z.J.210, 1R8L.B.902, 4R83.D.501, 2RHP.A.10, 2RHP.A.18, 2RHP.A .20, 2RJP.A.2, 2RJP.A.3, 2RJQ.A.6, 1ROS.B.503, 1RU4.A.2, 1S0E.A.1293, 1S1D.B.100 2, 3SHI.G.305, 3SJS.A.222, 1SMP.A.478, 1STB.A.150, 1T6B.X.800, 1TLD.A.480, 3U1R. A.700, 4U32.A.301, 1ULV.A.2005, 1UYX.B.1135, 3V03.A.584, 3V03.B.585, 1V0Z.A.1477 , 1V7V.A.1001, 3VRQ.A.401, 3VV3.A.404, 1W3M.C.3014, 3W9T.A.1006, 2WG8.A.201, 3WI U.B.1002, 3WNK.A.812, 1WVM.A.604, 1Y4A.E.1001, 2YN5.A.6365, 1YO8.A.1197, 1Z4V.A. 600, 2Z8S.B.647, 2ZID.A.882, 2ZJ6.A.625, 2ZRQ.A.7, 1ZTQ.A.561, 2ZUX.B.638, 2ZUY. A.627, 2ZWO.A.400, 2ZWO.A.403, 4ABT.B.1287, 2AOR.A.401, 2AOR.A.402, 2ASD.A.415, 2ASD.A.416, 3AVX.A.3001, 3C0W.A.304, 1DMU.A.300, 3DPG.B.501, 4DTP.A.1002, 3DVO.A .340, 4ECQ.A.501, 4ELV.A.908, 1FOO.B.761, 4F4W.A.402, 4F4W.B.403, 4FJ8.A.1002, 4 FJ9.A.1002, 4FJK.A.1002, 4FJL.A.1002, 4FJM.A.1002, 2GIJ.A.401, 3IAY.A.1, 2IBK.A. 402, 2IMW.P.406, 4J2A.A.1002, 4J2B.A.1002, 2JEJ.A.1344, 1JXL.A.1402, 4K4H.A.605, 4K4H.A.607, 4K4H.E.602, 4K4I.E.602, 3KHR.A.416, 4KHQ.A.1001, 4KLD.A.402, 4KYW.A .302, 4LQ0.A.402, 3LZJ.A.905, 4M3Z.A.1002, 3M9N.B.4003, 3M9O.B.4001, 3M9O.B.4003 , 3MXB.B.173, 3MXB.R.175, 1N3E.F.491, 1N3F.C.498, 1N3F.D.499, 1N3F.F.497, 3O3G.A .1, 2OAA.A.601, 3ODH.A.195, 2ODI.A.701, 1OUP.A.300, 2Q10.A.701, 3QET.A.905, 4QWB .A.402, 4QWB.A.403, 1R7M.B.536, 3RAX.A.415, 2RDJ.A.353, 1RYS.A.801, 1SO0.A.401, 3SLP.B.227, 3SPZ.A.905, 3SUN.A.897, 1TW8.A.801, 3A09.A.601, 3A7Q.B.5001, 2A8K.B. 404, 2AEP.A.601, 1AF0.A.484, 1AF0.A.486, 3AFG.A.540, 2AFH.D.2490, 1AG9.A.200, 1A G9.B.1000, 3AHW.A.122, 3AI7.B.901, 4AIE.A.1540, 1AJJ.A.73, 3AJ7.A.602, 3AKB.A.2, 3AMR.A.909, 3AMR.A.910, 4APX.B.1239, 4AQ1.A.1925, 4AQ1.A.1926, 4AQE.A.1208, 4AR 9.A.1732, 4ARF.A.1723, 3ASI.A.2001, 3AUK.A.391, 1AVA.A.502, 1AWB.A.1, 4AWN.A.300 , 4AXN.A.1329, 3AYU.A.417, 3AYU.A.418, 3B1U.A.901, 1B4N.A.620, 3B4N.A.702, 4B4F. A.607, 3B7E.A.1005, 1B8L.A.110, 3B8Z.A.904, 1B9T.A.500, 3BC9.A.702, 3BC9.A.705, 3BCD.A.707, 3BH4.B.1, 1BLI.A.600, 2BLO.B.1146, 4BNR.A.600, 2BQ4.A.1119, 2BU3.A.1 242, 2BV2.B.1085, 4BZ4.A.1233, 2C10.A.1771, 1C3H.D.8003, 1C3H.F.8001, 3C9I.A.1,

4C9F.B.401, 4CAG.A.602, 2CDP.A.1140, 1CGE.A.305, 3CKZ.A.1, 1CLC.A.650, 1CLC.A.652, 4CPL.A.500, 4CPY.A.1466, 4CU9.A.2999, 4CUA.A.2644, 4CUB.A.2645, 1CVL.A.320, 2CYY.A.2002, 2D00.D.1001, 3D3I.A.1001, 3D6E.A.202, 3D7K.A.571, 2DCJ.A.1003, 2DDU.A.1, 2DDR.A.1324, 2DEW.X.901, 2DF7.C.5904, 2DIE.A.779, 4DK4.A.302, 4DKB.A.302, 4DOU.A.1001, 4DOU.A.1002, 1DPO.A.246, 4E5U.B.302, 2E9B.A.741, 2EA7.A.450, 3EDY.A.1, 2EEK.A.401, 3EF2.A.304, 3EF2.A.305, 1EGZ.A.300, 1ELT.A.300, 5ENL.A.438, 4EPU.A.601, 1EX9.A.286, 2F3C.E.242, 3F5V.A.223, 4F8Z.A.409, 1FBL.A.996, 2FCW.B.3001, 2FCW.B.3002, 2FGZ.A.1192, 3FG1.A.1501, 2FHF.A.2404, 3FP8.E.601, 3FSJ.X.600, 3FU1.A.301, 3FVQ.A.360, 2FWN.A.532, 3G4E.A.1, 1G5N.A.402, 4G60.A.302, 4G62.A.302, 1G9K.A.703, 1G9K.A.706, 1GA6.A.374, 4GDI.A.509, 4GDJ.A.507, 3GG1.A.502, 4GG1.A.602, 2GJP.A.1486, 2GKO.A.611, 2GKO.A.612, 3GK2.A.92, 3GN9.A.201, 4GN7.A.301, 1GTT.A.1430, 1GVK.B.1246, 4GW3.A.401, 4GZT.B.510, 2HOK.B.410, 2HOB.A.1000, 4H1Q.A.303, 4H1Q.A.305, 1H3G.A.701, 1H71.P.501, 1H71.P.502, 3H81.A.279, 3H81.C.279, 3H81.C.280, 1H9H.E.1246, 3HB2.P.482, 1HDF.A.1101, 1HFC.A.277, 3HGN.A.250, 3HI7.A.802, 3HJR.A.603, 4HJF.A.601, 1HM9.A.1901, 4HOW.A.704, 4HPN.A.401, 4HS9.A.401, 1HT6.A.502, 1HV5.A.5503, 1HVX.A.517, 1HY7.A.305, 1HYO.A.1006, 2HYU.A.502, 2HYV.A.608, 4HZW.A.507, 4I35.A.513, 1I76.A.996, 1I76.A.997, 2I8U.A.202, 4I8H.A.301, 2I8T.A.402, 3I98.A.627, 3I9G.L.301, 3I9G.L.302, 3IBZ.A.192, 4IHM.A.403, 4IHM.A.404, 1IOD.G.503, 3IOX.A.903, 2IUF.E.1697, 4IU2.A.301, 4IU3.A.301, 2IXT.A.1310, 2IXT.A.1311, 1JOH.A.601, 1J11.A.701, 1J1N.B.493, 1J35.C.501, 4J7M.A.403, 1J8E.A.201, 1J9K.A.301, 1JE5.B.502, 1JI3.B.403, 1JK3.A.403, 2JKP.A.1728, 2JKE.A.1728, 2JKH.A.1245, 4JZB.A.401, 4JZB.A.402, 4JZB.A.403, 4JZE.H.302, 4JZX.A.403, 4JZX.A.404, 4JZX.A.405, 4K1K.A.501, 3K37.B.467, 4K3K.B.401, 1K7I.A.483, 1K7I.A.487, 1K7Q.A.485, 4K70.A.1002, 4K70.B.1002, 4K89.A.408, 4K9P.A.601, 1KA1.A.401, 2KAY.B.188, 1KAP.P.616, 1KAP.P.617, 1KAP.P.618, 1KAP.P.619, 1KAP.P.620, 3KCG.H.500, 4KKF.A.703, 3KM5.A.2011, 3KMV.A.163, 4KS1.A.501, 4KTY.A.802, 1KUO.A.703, 4KXY.A.707, 4L74.A.401, 4LLS.A.303, 4LLT.A.303, 3LNH.A.303, 3LNI.A.303, 3LPD.A.342, 1LQV.C.35, 4LQR.A.202, 4LVN.A.703, 3M1H.C.2001, 4M5I.A.201, 3M6L.A.801, 3MA2.A.293, 3MA2.A.296, 3MBR.X.300, 4MB1.A.602, 1MCT.A.246, 4MEW.A.502, 3MHF.A.328, 2MIN.B.525, 4MKM.A.403, 2ML1.A.201, 2ML1.A.202, 2ML1.A.204, 2ML2.A.201, 2ML3.A.203, 2ML3.A.204, 3MMZ.A.500, 1MNC.A.283, 3MOS.A.1, 4MPR.A.601, 3MVS.A.211, 3MVS.A.215, 4MWL.A.512, 3MW3.A.301, 4MWV.A.512, 3N1U.A.200, 4N20.A.705, 4N20.A.706, 4N2B.A.705, 4N2E.A.705, 4N2G.A.705, 4N2I.A.705, 4N2P.A.201, 4N4E.E.405, 1N7V.A.601, 1N9E.A.802, 4NAS.A.503, 1NBW.A.650, 4NEH.B.701, 3NIF.D.2002, 3NJH.B.502, 3NKQ.A.1003, 1NKG.A.800, 1NNL.B.2002, 4NOT.A.302, 1NPC.A.320, 1NRW.A.903, 1NSC.A.468, 1NUD.A.703, 4NUQ.A.301, 4NUZ.A.1001, 1NX1.A.3, 2O72.A.403, 1OAC.A.802, 1OAH.B.1526, 1OBO.A.501, 4OCI.A.201, 3OJY.A.555, 3OJY.B.538, 4OKH.B.904, 1OM6.A.701, 1OM8.A.705, 3OM5.B.1, 3OM6.B.1, 3OOY.A.621, 3OTJ.E.1000, 1OU9.A.131, 4OUL.B.1201, 3OYR.B.337, 3OYR.B.338, 3P2P.B.126, 2P3U.B.501, 3P4G.A.401, 3P4G.A.402, 3P4G.A.406, 3P4G.A.408, 3P4G.A.409, 3P4G.A.410, 3P4G.B.411, 3P95.A.1, 3PGB.A.903, 2PHI.B.125, 4PHN.A.302, 3PKO.A.280, 4PMX.A.401, 2PNY.A.228, 3POJ.B.1, 2POO.A.805, 3PPE.A.401, 1PW9.A.404, 1PZ7.A.701, 2PZO.A.501, 2Q16.A.200, 3Q2L.B.703, 1Q3A.A.466, 3Q4W.A.224, 4Q8K.A.501, 1QCN.A.1001, 1QCO.A.1002, 2QIM.A.158, 1QLB.A.1658, 4QN6.A.501, 4QN7.A.501, 3QRB.A.301, 3QU7.A.230, 3QU7.B.225, 2QUB.A.614, 2QUB.A.615, 2QUB.A.616, 2QUB.A.618, 3QXG.B.230, 4R12.A.809, 2R1B.A.1001, 2R5N.A.2000, 1R6V.A.1, 2R8O.A.670, 2R8Y.A.201, 2R8Z.A.201, 3R8Y.A.242, 2R8P.A.670, 2RA3.A.1, 2RA3.B.1, 1RM8.A.502, 1RQ5.A.819, 3RQO.A.301, 3RRX.A.901, 3RUP.A.1004, 3RVV.A.225, 3RVW.A.223, 1RXO.B.477, 1SOB.A.1292, 3S4Y.B.1303, 3S5U.E.221, 1SAT.A.476, 2SAS.A.187, 3SAL.A.601, 1SBH.A.290, 1SCB.A.276, 1SIO.A.601, 3SOO.D.97, 1T1E.A.700, 3T3P.B.2003, 1T5S.A.1004, 3TEW.A.800, 3TI4.A.601, 1TO2.E.450, 1TRK.A.681, 4TSH.B.1502, 1TU5.A.902, 3U1R.A.703, 3U1R.A.704, 3U1R.A.705, 3U1R.A.706, 3U1R.A.707, 3U8D.A.203, 3U8I.A.201, 1ULV.A.2001, 1ULV.A.2002, 3UPT.A.691, 4UP4.A.501, 4UP4.A.502, 1UR4.A.1398, 4USU.A.1471, 1UTM.A.247, 2UWF.A.1369, 1UX6.B.2002, 1UX6.B.2003, 1UX6.B.2004, 1UX6.B.2007, 1UX6.B.2010, 1UX6.B.2012, 1UX6.B.2016, 1UYX.A.1134, 4UZU.A.1484, 1V0Z.B.1477, 3V5U.A.

705, 3V96.B.303, 3V96.B.304, 1VCL.A.1004, 1VCL.B.1001, 1VCL.B.1002, 1VL9.A.125, 2VNG.B.1214, 2VOV.A.1338, 3VOC.A.501, 3VTO.A.302, 3VV3.A.401, 3VV3.B.404, 2W1W.B.1135, 1W7C.A.802, 3W7T.A.1001, 1WAD.A.116, 4WA3.A.503, 2WFK.A.1250, 4WIW.A.702, 4WK0.B.502, 4WK0.B.503, 4WK7.A.504, 1WMD.A.1003, 2WNV.B.1225, 3WN6.A.502, 2WOY.A.2414, 2WOB.E.1161, 1WPC.A.502, 1WRZ.A.154, 2WW3.A.800, 1WZA.A.601, 3X17.A.602, 1XKD.A.1005, 2XR9.A.1869, 2XSG.B.1772, 2XVT.F.1137, 2Y09.A.1242, 1Y6W.A.149, 2Y6D.A.1266, 1Y7B.A.3001, 1Y93.A.266, 1Y93.A.268, 1Y9Z.A.604, 1Y9Z.B.605, 2YAY.A.1267, 2YEQ.A.1526, 2YGL.A.1413, 1YI7.A.3001, 2YN3.A.6362, 2YN5.A.6362, 2YN5.A.6363, 1YO8.A.1183, 1YO8.A.1185, 1YO8.A.1191, 1YO8.A.1193, 1YO8.A.1199, 1YO8.A.1205, 1YO8.A.1206, 1YO8.A.1209, 2YOA.A.1003, 1YS1.X.400, 1YS6.A.1001, 2Z2X.A.1004, 2Z2X.A.1005, 2Z2X.A.1007, 2Z30.A.1006, 1Z6O.A.5302, 1Z70.X.3001, 2Z8X.A.620, 2Z8X.A.622, 2Z8X.A.623, 2Z8X.A.624, 2Z8X.A.626, 2Z8S.B.643, 2ZE0.A.552, 2ZPR.A.2001, 2ZUX.A.630, 2ZUY.A.626, 3ZWH.A.501, 2ZWO.B.400, 3ZXH.A.304

[1] "Cluster 11"

2EX5.X.802, 1G9Y.C.803, 4K4G.M.603, 3KHH.A.416, 3KHH.B.1417, 3KHL.B.1415, 3MQ6.A.340, 3NDK.A.906, 3OOL.A.237, 4Q10.A.1001, 1RYS.B.803, 2VA2.B.1343, 1AEI.A.318, 1AEI.A.320, 1AF4.A.276, 3AFG.B.541, 1AWB.B.279, 3AYU.A.419, 4B7R.B.502, 4B7U.D.401, 1B9V.A.500, 2BTW.A.400, 3C5I.A.371, 4CBX.G.1151, 1CIZ.A.305, 3D4G.B.485, 2DDF.A.475, 1DJZ.A.2, 2DSN.A.2011, 2E30.A.300, 1EA7.A.315, 1EGZ.C.300, 4EJ7.C.402, 3ELM.A.303, 7EST.E.11, 2EXH.C.2003, 1F5R.A.800, 3FLT.B.302, 2FYC.B.404, 1FZD.F.1, 3G20.A.201, 1G5N.A.404, 4G64.B.301, 1GMM.A.1132, 4H1Q.A.304, 3HLH.D.315, 3HQ8.A.402, 2HT5.A.995, 2HYW.A.502, 4ILW.F.305, 2J7A.D.1007, 3JQL.A.121, 4JQG.A.305, 2K0J.A.501, 4KPP.B.501, 4KTP.B.804, 1KX1.C.301, 3L9I.C.1151, 4LM8.A.812, 1M63.B.500, 4MIV.C.601, 2ML2.A.206, 1NGS.A.681, 3NHH.A.151, 4NIY.A.301, 4NPK.A.803, 1NW1.A.430, 1OLP.B.1372, 3OSH.A.121, 1OT5.A.602, 2OVX.B.449, 2OVZ.A.449, 4OVY.A.409, 3P2P.A.125, 3POW.A.900, 1PZ7.B.702, 2Q1F.B.2002, 3Q3L.B.437, 4Q4X.1.5006, 3QHQ.A.230, 3RG0.A.1, 2RHP.A.22, 1RM8.A.504, 1SEL.A.277, 3SO0.A.98, 3SO0.B.97, 3SON.A.201, 1SPU.A.802, 2TCL.A.174, 1TFX.B.4007, 1TK2.A.1276, 3TRP.A.360, 1UG9.A.2003, 1V1G.A.1209, 1V3J.B.689, 1V6C.A.505, 3VI4.C.2004, 2W1W.A.1132, 1W2M.E.1442, 1WD9.A.900, 3WIV.B.1004, 1WY9.A.148, 2YC2.B.201, 2ZOC.A.504, 2ZUY.A.629, 4EBC.A.504, 2GIH.A.402, 3QEV.A.906, 2AER.L.3005, 4CP1.A.1297, 1D8M.A.304, 3EDD.A.701, 1EN7.A.403, 3FHA.A.705, 3GG1.A.503, 2HYW.A.506, 2II1.C.404, 1IJL.A.203, 1JYI.D.408, 4KS4.A.502, 1LPK.B.1, 1M1J.B.503, 3O82.B.544, 4OO2.C.601, 4P99.B.540, 2Q04.C.211, 1QD6.C.2, 1RK9.A.112, 4TQO.H.701, 1TRQ.A.106, 1UCN.A.1162, 4UM9.D.2002, 2UX1.K.174, 3WIU.B.1004, 4WK4.B.502, 1XZO.B.1018, 1Y1A.A.501, 1ZH2.A.202

[1] "Cluster 12"

1K90.D.801, 1SZB.B.1004, 4LQ0.A.403, 1A2X.A.161, 4BTW.B.1764, 2C0O.B.1507, 1CFF.A.149, 1CFF.A.152, 3CGT.A.685, 3DF0.A.716, 3DF0.B.604, 1EUB.A.278, 3G5C.B.802, 3GIN.A.1, 2HTV.B.993, 1JBA.A.500, 1JSA.A.500, 1JSA.A.501, 1KX1.E.501, 2KYF.A.110, 1L8S.B.314, 1LMJ.A.101, 1LMJ.A.102, 2LMT.A.150, 2LP2.A.203, 1LTJ.B.2, 4MBZ.H.401, 1N28.B.125, 1NX3.A.4, 2P0R.B.1004, 4POQ.A.401, 4R9X.A.302, 3RBX.A.600, 3UJQ.D.305, 4UM9.C.2001, 1UMS.A.3, 3VI3.A.2002, 3W9T.C.1009, 1WRL.D.104, 2WYS.A.1554, 1XJL.A.346, 2XRM.A.405, 1Z6C.A.247, 2ZN9.B.901, 4BYA.A.77, 1J35.C.505, 2M3S.A.201, 2M3S.A.204, 3ZDY.C.2004, 1AZO.A.284, 1BSU.A.301, 1N48.A.502, 4RNO.A.502, 1SOO.A.402, 1A25.A.291, 1A25.B.291, 2A3Y.A.600, 1AFB.1.227, 1AMY.A.501, 4AOC.A.1130, 4AQ8.D.1239, 4AVS.A.206, 4AYU.A.206, 3B8Z.A.902, 3BJE.B.402, 2BOI.A.200, 2BOJ.A.1117, 2BP6.A.801, 2BPE.B.1245, 3BVH.B.1, 2BV4.A.200, 1C0G.S.129, 4CE8.A.997, 1CGT.A.685, 1CGW.A.692, 1CGX.A.692, 1CGY.A.692, 1CVR.A.503, 3D1M.A.190, 3D34.A.228, 3DCQ.A.115, 3DE8.A.109, 3DED.A.505, 4DOE.A.502, 1DSY.A.502, 1DV8.A.1003, 1DVI.A.274, 3EF2.A.303, 3EF2.B.303, 4EW9.A.204, 4EW9.B.203, 1FIF.A.1, 1FWX.B.4903, 3FZO.B.400, 1G43.A.200, 4GNC.A.301, 3GZK.A.538, 4HPK.A.1102, 3HR4.B.201, 1HUP.A.301, 1HXO.A.500, 4I5L.B.602, 1I82.A.193, 2IE7.A.401, 4IEF.B.703, 4IEF.H.703, 4IT5.A.301, 4IT5.D.201, 2IWK.A.1607, 1J34.C.505, 2J5W.A.3042, 4JA8.B.502, 2JDM.B.1116, 2JDN.B.882, 3JQW.A.1002, 1K9J.A.401, 1KCK.A.691, 1KCL.A.1692, 3KQR.A.206, 2KRD

.C.90, 1KSC.A.500, 1KWZ.A.503, 2KXV.A.201, 1KXR.A.1, 2KZ2.A.150, 4KZV.A.303, 1LG
N.A.301, 3LJT.A.902, 1LQV.C.45, 2LRO.A.201, 2M3S.A.202, 2M3S.A.203, 1MCX.A.347,
1MDW.A.1, 2MSB.A.1, 1MXG.A.438, 3N1F.A.5, 3N1G.A.192, 4N4E.E.402, 4NEH.A.1104, 4
NPK.A.801, 2NQI.A.401, 2NXP.B.601, 2O8O.A.1010, 1OUR.A.401, 1OUX.A.401, 1OVP.A.1
16, 2OX9.A.801, 2OX9.B.804, 2P0R.A.1003, 1PEZ.A.891, 4PEO.A.103, 4PHJ.A.301, 4PH
N.A.304, 1PJ9.A.891, 2POR.A.303, 1PW9.B.402, 3Q13.A.601, 3Q4W.A.225, 4QB1.A.202,
1QNI.A.901, 2QVM.A.1001, 2R9F.A.365, 1RDL.1.227, 1RP8.A.501, 3SBQ.A.703, 3SG4.A
.453, 1SMD.A.497, 1SNN.A.403, 3SWB.A.91, 3T3P.A.2004, 1T44.G.701, 3TEC.E.344, 1T
F4.B.3003, 3TH4.L.149, 1TLQ.A.190, 1TN3.A.182, 4TV9.A.503, 4U6B.C.501, 1UOV.A.14
20, 1UQX.A.1115, 1UV4.A.1294, 1V0A.A.1176, 4V0C.D.201, 2VNV.A.301, 2VUC.B.991, 2
W08.A.205, 3WC3.A.501, 4WK0.A.501, 4WKE.A.502, 1WMZ.A.201, 3WN6.A.501, 2WR9.A.11
32, 2XFG.A.1447, 2XR6.A.1390, 1Y6W.A.150, 1Y9I.A.501, 1YO8.A.1182, 1YUT.B.200, 1
ZCM.A.1002, 2ZWP.B.403, 3FD2.A.375, 4K4G.I.603, 4K4I.I.603, 3RB4.A.415, 3SQ1.A.9
03, 1AJ4.A.164, 1AP4.A.90, 2ARY.B.404, 3AR2.A.1001, 4AR1.A.1723, 3B2Z.B.2, 3BSG.
A.501, 1CKK.A.151, 1CKK.A.153, 2CL8.B.1245, 1DJW.B.2, 2EHB.A.1004, 1EMN.A.2225,
4ENZ.A.1110, 2EV5.A.1150, 1FPW.A.502, 1FWX.D.4903, 1G0H.A.291, 3GIS.Y.1002, 3H2W
.A.538, 3HR4.F.202, 4I5L.E.702, 1I8A.A.192, 4IEF.D.704, 4IEF.F.703, 3IJ9.A.497,
4IRZ.A.2005, 4IRZ.A.2006, 4IRZ.A.2007, 4IT5.C.201, 2JT6.A.258, 2JU0.A.500, 3KF9.
C.302, 1KIE.B.328, 1KWW.B.602, 1KX1.F.602, 1KXT.A.4001, 4L73.A.403, 4LJ3.A.403,
2LMV.A.150, 2LUX.A.202, 2M28.A.301, 2M55.A.301, 4M5H.A.305, 1M63.B.501, 1M63.B.5
03, 1MXD.A.731, 4NHF.F.301, 1NIW.E.1009, 1NIW.E.1010, 1NL0.G.905, 1NL2.A.205, 4N
PK.A.802, 1NYA.A.501, 4O6N.A.401, 4O6N.B.401, 1O9I.E.269, 2OW2.B.446, 4PHK.B.305
, 3QED.A.348, 1QNI.C.901, 1RF1.E.462, 1S2N.A.1290, 3SBR.A.703, 1SZB.A.1003, 1TDQ
.B.128, 1TF4.A.3001, 4TV8.A.503, 3UBG.A.901, 4V0C.C.201, 3VI3.C.2001, 2WR9.B.112
9, 1XYD.A.93, 1YO8.A.1194, 1YUU.B.200, 1Z6C.A.246, 1ZIV.A.2
[1] "Cluster 13"
4K4H.E.604, 2BTW.B.400, 6CGT.A.685, 1DTH.A.903, 4GQR.A.502, 1JKU.E.5272, 3K7L.A.
701, 1ULV.A.2006, 1AZO.B.283, 4DU3.A.1003, 1A25.A.290, 4A6S.C.1122, 3A9Q.G.214,
3AKA.A.173, 1AVA.A.501, 1AWB.A.278, 1B47.A.351, 3BBY.A.215, 2BL0.C.1155, 2CDP.C.
1139, 2CHN.A.1717, 3CK9.A.710, 3D0Y.A.93, 2D3P.C.241, 2D3L.A.503, 1DE4.C.801, 2D
IE.A.778, 1DJY.A.2, 3E1I.C.501, 2E6V.C.10, 1E8A.B.1090, 2E85.B.1004, 4EJ7.A.403,
1ESV.S.395, 3ESR.A.213, 1F9O.A.2000, 3FAX.A.4, 4FDI.A.603, 1FHF.A.501, 2FHF.A.2
405, 3FLP.B.302, 1G5N.A.407, 4G9L.A.304, 1GCY.A.529, 2GDF.B.302, 2GJR.A.1488, 3G
K2.A.93, 1HFZ.D.124, 2HIH.A.603, 3HTL.X.1, 4IAV.A.420, 3IJ8.A.497, 4ILW.D.305, 2
JBK.A.803, 2JHL.F.1298, 3JQ5.A.201, 2K0F.A.153, 3K5T.A.803, 3K7N.A.701, 1K9I.F.9
03, 2KAY.A.186, 3L2Y.A.302, 3LCP.D.158, 1LGN.B.302, 2LMT.A.149, 1LN8.A.201, 2LV6
.A.203, 1M56.A.1007, 4MHX.A.601, 4MIV.E.600, 4MVF.A.603, 4MVF.A.604, 1N2K.A.600,
4NDD.B.401, 4NHD.B.403, 1NIW.A.1003, 2NQA.B.902, 1NT0.A.3001, 2OA8.C.302, 1OBR.
A.404, 2O04.A.5003, 3OUU.B.455, 2OZN.B.401, 1P8J.G.3014, 4P99.A.501, 4P99.B.509,
4PIB.C.204, 2PMY.A.103, 4POS.E.401, 3PVN.B.5003, 3PVN.B.5004, 4Q6P.A.509, 1R1Z.
C.410, 2R28.A.1003, 2RHP.A.8, 2SAS.A.188, 2SEC.E.276, 1SU3.B.907, 1SVN.A.276, 4U
M9.C.2002, 3UNX.A.280, 1V3D.B.2001, 1VFP.A.995, 3VI3.A.2003, 3W9T.D.503, 4WBQ.B.
601, 3WIU.A.1004, 2WL3.A.1290, 1WMY.B.203, 1Y4D.E.1001, 2YN3.B.6361, 1YUU.A.197,
1Z3U.A.497, 2Z8S.A.642, 2Z8S.A.648, 2Z8S.B.646, 2ZEY.B.152, 1ZIV.A.1, 2ZN9.A.90
2, 2ZWP.A.401, 1ANX.B.321, 4AOC.B.1129, 1B9A.A.110, 2C40.A.1311, 3DEM.A.3001, 1D
X5.I.1001, 3HR6.A.1, 4I70.A.401, 2JBH.A.1228, 1KXQ.A.4001, 2LRO.A.202, 4QB6.A.20
3, 3T8I.C.400, 3TH2.L.152, 4U6D.A.501, 4U6D.B.502, 1UZK.A.2512, 2W87.A.1148, 4X9
Y.A.502, 1YCM.A.267, 1Z32.X.497, 4DTM.A.1002, 4DU1.A.1003, 4DU3.A.1002, 4FK0.A.1
002, 2FLD.B.602, 4KLD.A.403, 3MQ6.A.1, 3QNN.A.903, 4QWA.A.403, 3RAX.A.416, 2WTF.
A.1511, 1A25.B.290, 3A24.A.1268, 1A2Q.A.295, 3A4U.A.286, 1A75.A.109, 3A8R.A.401,
2AAO.A.293, 2AAO.A.295, 2AAO.A.296, 5AER.A.200, 3AIE.A.4001, 1AK9.A.295, 3AKB.A
.172, 3AKB.A.175, 3AMR.A.908, 4APX.B.1240, 4AQJ.A.1097, 1AVS.A.94, 1AXN.A.353, 3
B0O.A.124, 1B8R.A.109, 3B8Z.A.903, 3BC9.A.701, 3BFM.A.235, 1BH6.A.501, 1BJF.A.40
2, 1BQB.A.351, 1BU3.A.110, 4BY5.A.1183, 4BY5.A.1184, 4BY5.A.1185, 2C4F.L.1143, 1

C9N.A.277, 4CAG.A.603, 2CCL.B.1060, 2CCL.B.1061, 2CCM.A.1192, 4CE8.B.998, 4CFQ.A.502, 1CJY.A.950, 4CPV.A.109, 4CPV.A.110, 2CT9.A.301, 2CT9.A.302, 4CT3.A.1170, 3CZT.X.93, 3D1M.A.2, 4DA2.A.301, 3DBK.A.303, 2DIE.A.780, 3DO1.A.401, 1DTL.A.201, 1DTL.A.203, 4DUQ.A.102, 1DVI.A.273, 2DW0.A.702, 2E3X.A.802, 2E4T.A.701, 1E43.A.502, 1E8A.A.1090, 3ECQ.B.2000, 3EDF.A.602, 2EGD.A.302, 3ETO.A.2003, 2EWE.A.701, 1EXR.A.1000, 4F0Z.B.201, 4F0Z.B.204, 1FAT.A.255, 2FH1.A.2002, 2FPW.A.503, 3FZ0.A.400, 1FZD.A.1, 1G8I.A.1599, 1G8I.B.1600, 1G9K.A.702, 3GDC.A.401, 1GGZ.A.149, 2GGM.A.401, 4GGF.A.101, 4GGF.C.204, 4GGF.L.204, 2GJP.A.1487, 2GJP.A.1488, 4GM5.A.404, 2GSK.A.2, 2GXS.A.601, 1H2G.B.1558, 4H2A.A.805, 3HJR.A.601, 2HQ8.A.202, 2HQ8.A.203, 1HVX.A.516, 1HVX.A.518, 3HX4.A.604, 3HX6.A.1, 2HYW.A.501, 3HZ3.A.1, 4I2Y.A.501, 4I5L.B.601, 2I7A.A.2, 4I75.A.401, 4IAI.A.401, 4ICB.A.77, 4IEF.B.702, 4IK8.A.502, 4IU3.B.302, 2J1V.A.1152, 2J1G.B.1290, 1J55.A.102, 1JC9.A.301, 2JDN.B.881, 2JDN.C.881, 1JIW.P.485, 1JK3.A.404, 4JWQ.A.202, 3K21.A.194, 4K70.A.1003, 4K70.B.1003, 1K94.A.999, 1K9K.A.400, 4KDW.A.201, 3KHE.A.193, 3KHE.A.194, 1KIC.B.328, 4KKK.A.701, 3KLK.A.1, 3KLL.A.1, 3KQR.C.206, 4KTY.A.801, 1KVW.A.124, 4KWU.A.1109, 1KXO.C.703, 3LI6.A.149, 3LI6.D.150, 3LND.B.208, 3LNI.A.302, 4LVN.A.702, 4M7H.A.501, 1MCX.A.351, 1MDW.B.4, 3MHZ.A.736, 2ML1.A.205, 4MN0.A.302, 3MVS.A.212, 1MXE.A.506, 4N1G.A.203, 3N1F.A.6, 3N1G.B.190, 3N4E.B.500, 4N5X.A.201, 4N5X.A.204, 4N5X.A.205, 4NEH.A.1103, 1NPC.A.319, 2O04.A.402, 3O0V.A.1, 3O0W.A.377, 3O4Y.A.198, 2O5G.A.401, 2O5G.A.402, 2O5G.A.403, 2O5G.A.404, 1OB0.A.502, 2OBH.A.1001, 1OHZ.B.1058, 2OLG.A.2001, 2OPO.A.302, 1OUX.A.402, 2OZN.B.402, 3P4G.A.413, 4P5X.A.1001, 4P99.A.514, 5PAL.A.110, 5PAL.A.111, 4PE0.X.103, 4PE0.A.102, 4PET.A.403, 4PHJ.A.303, 4PHJ.A.304, 3PM8.B.1, 1POB.A.801, 1POE.A.801, 1PVA.A.110, 3PVN.E.5009, 2PVB.A.110, 2PVB.A.111, 2Q1F.A.2001, 3Q2L.A.701, 1Q3A.A.468, 3Q5I.A.525, 1Q5P.A.271, 3QRB.A.302, 3QRX.A.171, 1QTX.A.152, 1QTX.A.153, 1QTX.A.154, 1QX2.A.1001, 1R0R.E.302, 2R2I.A.500, 2R2I.A.501, 2R2I.A.502, 2R9F.A.366, 1RFJ.A.1001, 1RFJ.A.1004, 2RHP.A.29, 3RM1.A.102, 1RRO.A.109, 3RUP.A.1006, 1RWY.A.422, 1S01.A.295, 1S02.A.276, 3S5U.D.221, 1S6C.A.217, 1SBF.A.601, 2SCP.A.191, 3SIB.A.222, 1SL7.A.301, 1SNN.B.503, 1SRA.A.302, 1ST3.A.270, 1SUD.A.295, 1TCF.A.160, 1TCF.A.163, 3TI7.A.353, 3TI7.A.355, 1TKF.A.905, 1TN4.A.162, 5TNC.A.163, 3TTQ.A.2867, 3U1R.A.702, 3UBG.A.902, 3UCP.A.912, 3UJO.B.304, 3UL4.B.66, 4UM9.A.2003, 1UXX.X.1130, 1UYZ.A.1132, 4UZU.A.1483, 4V29.B.1179, 1VCL.A.1001, 2VN5.B.102, 2VN6.B.1067, 2VUD.D.1118, 2VVD.A.1328, 3VYV.A.303, 2VZP.A.1128, 2W46.A.1148, 2W87.A.1149, 3WA5.A.504, 2WBX.A.1103, 3WFD.B.806, 3WH2.A.302, 3WHI.A.401, 3WHT.B.501, 3WN6.A.503, 3WNP.A.801, 2WND.A.102, 2WNP.F.1298, 1WP6.A.502, 1WPC.A.501, 2WZ8.A.1149, 1X1J.A.2000, 3X17.A.603, 2Y3N.B.1067, 2Y3N.B.1068, 2Y5I.A.101, 1Y93.A.267, 1Y9Z.A.603, 2YA2.A.1691, 1YO8.A.1186, 1YO8.A.1187, 1YO8.A.1190, 1YO8.A.1204, 1YU6.A.401, 1YUT.B.199, 1Z3J.A.267, 2Z30.A.1004, 2Z8X.A.627, 1ZCM.A.1001, 2ZFD.A.229, 2ZN9.A.901, 2ZUX.A.632, 2ZWO.A.402, 2ZWP.A.404, 2ZWP.B.402, 3ZYP.A.1220

Table S73. 4-ligand Fe, normal group

	size	largest_angle*	middle_1*	middle_2*	middle_3*
1	"57"	"124.2+/-5.7"	"96.9+/-5"	"102.6+/-3.8"	"108.4+/-3.5"
2	"56"	"168.1+/-6.1"	"80.4+/-5.4"	"87.2+/-5"	"93.8+/-3.7"
3	"15"	"170.5+/-6.7"	"80.1+/-4.3"	"87.5+/-1.4"	"91.6+/-2.8"
4	"30"	"134.1+/-6.9"	"85.6+/-5.9"	"94.1+/-7.3"	"106.5+/-6.1"
5	"26"	"143.9+/-7.4"	"87.2+/-6.8"	"95+/-3.9"	"101+/-4.1"

	middle_4*	smallest_opposite_angle*	Tetrahedral	TrigonalBipyramidalVA
1	"114.1+/-3.7"	"108.7+/-7.3"	"0.014"	"0"
2	"98.4+/-5.7"	"95.3+/-11.9"	"0"	"0"

3 "98.5+/-5.1" "157.7+/-9.5"   "0"   "0"
4 "119.1+/-4.3" "90.8+/-7.1"   "0"   "0.001"
5 "113.8+/-6.5" "108.3+/-8.5"   "0"   "0"
  TrigonalBipyramidalVP SquarePyramidalV SquarePlanar
1 "0"   "0"   "0"
2 "0.009"   "0.017"   "0"
3 "0"   "0"   "0"
4 "0"   "0"   "0"
5 "0"   "0"   "0"

Table S74. Cluster members of 4-ligand Fe, normal group

[1] "Cluster 1"
4AM5.A.1162, 1B2O.B.55, 2B5H.A.501, 1B71.A.192, 1BE7.A.55, 1BFY.A.55, 1BOU.B.501
, 1CJX.B.629, 3D3L.A.801, 2DE6.B.501, 3E2T.A.1, 1E3D.A.269, 4EB5.D.201, 1FZ0.A.5
002, 2GBX.E.455, 3GKE.A.501, 3GL0.A.501, 3GL2.A.501, 2HMK.A.451, 4HSL.A.202, 1JI
G.A.402, 1JIG.B.401, 2JI3.A.1127, 1JQK.B.801, 4K9F.A.101, 4KU0.D.101, 4KWL.A.301
, 4KX6.N.301, 1LNB.E.900, 1N1Q.A.516, 3N9Y.D.151, 3NA0.C.150, 3NA0.D.150, 4NBA.A
.501, 4NBF.A.501, 4NBG.A.501, 1NF6.A.200, 2OHJ.A.502, 2OHJ.A.511, 4QLW.B.201, 3Q
VD.G.173, 1R2F.A.400, 1R9X.A.501, 4REU.B.202, 1SHR.B.801, 1T9O.A.255, 3V7P.A.429
, 1VCX.A.54, 3VMH.C.501, 2W3S.E.1464, 2WLB.B.619, 1WQL.A.502, 2XSO.G.901, 2YFI.A
.901, 2YFI.G.900, 1YK5.A.54, 1YUX.A.303
[1] "Cluster 2"
2BI4.A.1384, 4CMY.A.1164, 4D8F.A.401, 1DGG.A.2000, 1DT0.A.1601, 3FMR.B.401, 1H2A
.L.1004, 1HJF.A.1310, 1HJG.A.1307, 1I4Y.B.602, 1I4Z.D.604, 1I4Z.G.607, 2IBN.A.70
4, 3ICF.A.602, 1IDS.C.208, 2INP.A.3, 2ITB.B.501, 1KBP.A.438, 4KBP.A.438, 3KCY.A.
1350, 4KEV.D.401, 1LGT.A.500, 1LKD.A.500, 3LKT.M.600, 3LMX.O.600, 1MOJ.B.301, 1N
NF.A.401, 1NX8.A.300, 2OHH.B.1501, 2OHH.E.3501, 1OQ9.A.365, 1OS7.C.302, 3PCB.O.6
00, 3PCJ.M.600, 1PIY.A.376, 3PM5.A.1002, 3Q1G.A.1001, 3Q1G.B.1001, 1QFC.A.402, 2
QJE.A.692, 2R2F.B.320, 4RC5.A.1003, 1SQ3.B.906, 1SQ3.C.907, 4TOA.A.206, 4TOA.B.2
07, 3U52.B.512, 3U9M.E.205, 2VHL.A.1398, 3VTI.B.803, 3WHN.B.201, 2XRX.G.1461, 2X
SO.K.900, 1XZW.B.929, 1YKM.J.600, 1ZZ9.C.199
[1] "Cluster 3"
4BMT.B.1323, 3DHG.A.501, 1FZH.B.5004, 3I4V.A.281, 2ITB.A.501, 4IWK.F.201, 1N7X.A
.339, 3NJZ.A.369, 2P6B.C.513, 3PCA.N.600, 3PCL.R.600, 3R2M.A.155, 1T47.A.431, 2V
C7.D.1315, 2Z4G.A.503
[1] "Cluster 4"
4AC8.B.500, 1BIQ.A.377, 2BKB.C.1193, 2BOY.E.1255, 3CF4.A.808, 3DBY.A.306, 1E2U.A
.701, 3FG1.B.1500, 4FWI.B.401, 1GNL.A.1545, 2GPC.B.195, 1GUP.C.351, 3I01.B.800,
2JD7.O.203, 1JRO.G.3001, 1NO3.A.858, 2PT2.A.400, 2PUZ.A.500, 2Q0J.B.997, 3Q36.A.
458, 4QDD.A.401, 3QFM.A.264, 1QGH.H.157, 1RA5.A.500, 3T81.B.606, 3U9M.A.202, 1W9
M.A.1555, 1Y4T.A.650, 1YKP.F.2600, 2ZZI.A.208
[1] "Cluster 5"
1AOR.A.606, 4C4U.B.201, 4C4U.I.201, 2CKF.C.501, 3D19.F.302, 4DHL.A.502, 4F1E.P.2
01, 2FKZ.A.1600, 2GBX.A.456, 3GC1.A.605, 2GYQ.A.402, 1I4Z.E.605, 4ILT.A.301, 2J2
F.E.371, 1JI5.A.152, 1JI5.B.151, 1N1Q.B.515, 1OQU.A.1001, 3PCE.M.600, 3PCK.Q.600
, 2PCD.M.600, 1RSR.A.1004, 1ULI.A.600, 3USS.B.212, 1W69.A.1349, 2ZQX.A.501

Table S75. 4-ligand Fe, combined group

	size	largest_angle*	middle_1*	middle_2*	middle_3*
1	"58"	"167.6+/-6.5"	"81+/-5.4"	"87.7+/-5.1"	"94.1+/-3.8"
2	"64"	"125.4+/-6.6"	"96.2+/-5.4"	"101.9+/-4.5"	"108+/-3.8"
3	"35"	"161.9+/-10.9"	"69.4+/-11.5"	"87.2+/-4.1"	"93.6+/-4.8"
4	"21"	"149.4+/-10.6"	"60.2+/-5.1"	"87.2+/-6.8"	"100.2+/-8.9"
5	"44"	"137.3+/-7.1"	"85.8+/-6"	"94.6+/-6.1"	"104.5+/-6.1"

	middle_4*	smallest_opposite_angle*	Tetrahedral
1	"98.7+/-5.8"	"95.1+/-11.2"	"0"
2	"114.2+/-3.9"	"109.3+/-7.9"	"0.013"
3	"108.7+/-11.9"	"148.5+/-12.5"	"0"
4	"111.7+/-12.8"	"94.3+/-10"	"0"
5	"117.7+/-5.4"	"95.5+/-9.2"	"0"

	TrigonalBipyramidalVA	TrigonalBipyramidalVP	SquarePyramidalV
1	"0"	"0.008"	"0.016"
2	"0"	"0"	"0"
3	"0"	"0"	"0"
4	"0"	"0"	"0"
5	"0.001"	"0"	"0"

	SquarePlanar
1	"0"
2	"0"
3	"0"
4	"0"
5	"0"

Table S76. Cluster members of 4-ligand Fe, combined group

[1] "Cluster 1"
2BI4.A.1384, 4CMY.A.1164, 4D8F.A.401, 1DGG.A.2000, 1DT0.A.1601, 3FMR.B.401, 1H2A.L.1004, 1HJF.A.1310, 1HJG.A.1307, 1I4Y.B.602, 1I4Z.D.604, 1I4Z.G.607, 2IBN.A.704, 3ICF.A.602, 1IDS.C.208, 2INP.A.3, 2ITB.B.501, 2J2F.E.371, 1KBP.A.438, 4KBP.A.438, 3KCY.A.1350, 4KEV.D.401, 1LGT.A.500, 1LKD.A.500, 3LKT.M.600, 3LMX.O.600, 1MOJ.B.301, 1NNF.A.401, 1NX8.A.300, 2OHH.B.1501, 2OHH.E.3501, 1OQ9.A.365, 1OS7.C.302, 3PCB.O.600, 3PCJ.M.600, 1PIY.A.376, 3PM5.A.1002, 3Q1G.A.1001, 3Q1G.B.1001, 1QFC.A.402, 2QJE.A.692, 2R2F.B.320, 4RC5.A.1003, 1SQ3.B.906, 1SQ3.C.907, 4TOA.A.206, 4TOA.B.207, 3U52.B.512, 3U9M.E.205, 2VHL.A.1398, 3VTI.B.803, 1W69.A.1349, 3WHN.B.201, 2XRX.G.1461, 1XZW.B.929, 1YKM.J.600, 2ZQX.A.501, 1ZZ9.C.199
[1] "Cluster 2"
4AM5.A.1162, 1AOR.A.606, 1B2O.B.55, 2B5H.A.501, 1B71.A.192, 1BE7.A.55, 1BFY.A.55, 1BOU.B.501, 1CJX.B.629, 3D3L.A.801, 2DE6.B.501, 3E2T.A.1, 1E3D.A.269, 4EB5.D.201, 4F1E.P.201, 1FZ0.A.5002, 2GBX.E.455, 3GKE.A.501, 3GL0.A.501, 3GL2.A.501, 2HMK.A.451, 4HSL.A.202, 1JI5.A.152, 1JI5.B.151, 1JIG.A.402, 1JIG.B.401, 2JI3.A.1127, 1JQK.B.801, 4K9F.A.101, 4KU0.D.101, 4KWL.A.301, 4KX6.N.301, 1LNB.E.900, 1N1Q.A.516, 1N1Q.B.515, 3N9Y.D.151, 3NA0.C.150, 3NA0.D.150, 4NBA.A.501, 4NBF.A.501, 4NBG.A.501, 1NF6.A.200, 2OHJ.A.502, 2OHJ.A.511, 4QDD.A.401, 4QLW.B.201, 3QVD.G.173, 1R2F.A.400, 1R9X.A.501, 4REU.B.202, 1RSR.A.1004, 1SHR.B.801, 1T9O.A.255, 3V7P.A.429, 1VCX.A.54, 3VMH.C.501, 2W3S.E.1464, 2WLB.B.619, 1WQL.A.502, 2XSO.G.901, 2YFI.A.901, 2YFI.G.900, 1YK5.A.54, 1YUX.A.303
[1] "Cluster 3"

4QQW.G.1002, 1AFR.B.454, 4BMT.B.1323, 4BMT.B.1324, 4CVP.A.1155, 3DHG.A.501, 3E1N.D.301, 3E1N.H.300, 2FKZ.A.1601, 1FZH.B.5004, 1GUP.A.351, 3I4V.A.281, 3IS8.N.162, 2ITB.A.501, 4IWK.F.201, 1JYB.A.600, 1N7X.A.339, 1NFV.A.201, 1NFV.M.200, 3NJZ.A.369, 1OQ4.B.364, 2P6B.C.513, 3PCA.N.600, 3PCL.R.600, 1PFR.A.502, 1PIU.A.401, 3R2M.A.155, 1T47.A.431, 4TOA.B.203, 4TOE.A.203, 3USS.B.212, 2VC7.D.1315, 1W2N.A.312, 2XSO.K.900, 2Z4G.A.503
[1] "Cluster 4"
4CMY.B.1165, 3FE5.A.1, 2FLO.B.1605, 3FM3.A.452, 1FRF.L.565, 1GUP.D.351, 3GZY.A.701, 1MOJ.A.302, 1O7P.A.1453, 2OHJ.A.501, 1OQU.A.1001, 1OVB.A.300, 2PHD.C.370, 1PIY.A.377, 1RSR.A.1003, 1SQ3.C.908, 3T81.B.607, 1ULJ.E.600, 3VV9.C.502, 2XRX.Q.1461, 2XSO.O.900
[1] "Cluster 5"
4AC8.B.500, 1BIQ.A.377, 2BKB.C.1193, 2BOY.E.1255, 4C4U.B.201, 4C4U.I.201, 3CF4.A.808, 2CKF.C.501, 3D19.F.302, 3DBY.A.306, 4DHL.A.502, 1E2U.A.701, 3FG1.B.1500, 2FKZ.A.1600, 4FWI.B.401, 2GBX.A.456, 3GC1.A.605, 1GNL.A.1545, 2GPC.B.195, 1GUP.C.351, 2GYQ.A.402, 3IO1.B.800, 1I4Z.E.605, 4ILT.A.301, 2JD7.O.203, 1JRO.G.3001, 1NO3.A.858, 3PCE.M.600, 3PCK.Q.600, 2PCD.M.600, 2PT2.A.400, 2PUZ.A.500, 2Q0J.B.997, 3Q36.A.458, 3QFM.A.264, 1QGH.H.157, 1RA5.A.500, 3T81.B.606, 3U9M.A.202, 1ULI.A.600, 1W9M.A.1555, 1Y4T.A.650, 1YKP.F.2600, 2ZZI.A.208

Table S77. 5-ligand Fe, normal group

	size	largest_angle*	middle_1*	middle_2	middle_3*	middle_4
1	"192"	"177.8+/-1.5"	"87+/-2.2"	"88.7+/-1"	"89.5+/-0.8"	"90.5+/-0.6"
2	"36"	"161.4+/-6.7"	"79+/-4.5"	"84.3+/-3"	"88.8+/-3.5"	"91.9+/-3.8"
3	"107"	"170.6+/-2.7"	"86.2+/-2.7"	"88.2+/-1.4"	"89.5+/-1.1"	"90.9+/-1.2"
4	"58"	"174.5+/-2.7"	"81.8+/-3.3"	"85.9+/-2.5"	"88.3+/-1.8"	"90.5+/-1.9"
5	"56"	"172+/-4.6"	"83+/-3.9"	"86.1+/-2.5"	"88.3+/-2.1"	"90.7+/-1.9"
6	"48"	"164.3+/-3.1"	"86.3+/-2.3"	"87.9+/-1"	"89.1+/-1"	"90.4+/-1.6"
7	"36"	"166+/-6.4"	"75+/-3.8"	"84.1+/-3.9"	"88.3+/-3.9"	"91.9+/-3.8"

	middle_5*	middle_6	middle_7	middle_8*
1	"91.3+/-1"	"92.8+/-1.7"	"95+/-2.7"	"176.4+/-1.9"
2	"97+/-3.1"	"103.1+/-3.8"	"114.5+/-7.4"	"147.4+/-5.1"
3	"92.7+/-1.9"	"97.4+/-2.6"	"100.7+/-3"	"167.7+/-2.3"
4	"93+/-2.2"	"95.8+/-3"	"99.9+/-4.7"	"170.1+/-3.9"
5	"93.9+/-3.3"	"98.2+/-4.5"	"119.3+/-4.8"	"132.1+/-6.4"
6	"95.5+/-2.2"	"99.9+/-2.2"	"103.2+/-2.8"	"160.6+/-3"
7	"96.1+/-4"	"100.2+/-4.7"	"107.6+/-6.9"	"157.4+/-4.1"

	smallest_opposite_angle*	TrigonalBipyramidal	SquarePyramidal
1	"88.5+/-2.4"	"0"	"0.234"
2	"97+/-4.7"	"0"	"0"
3	"92.1+/-2.4"	"0"	"0.07"
4	"83+/-4.4"	"0"	"0.09"
5	"107.3+/-6.2"	"0.013"	"0"
6	"96.5+/-2.7"	"0"	"0.004"
7	"84.2+/-6.1"	"0"	"0.001"

	TrigonalPrismaticV
1	"0"
2	"0"
3	"0"
4	"0"

5 "0"
6 "0"
7 "0"

Table S78. Cluster members of 5-ligand Fe, normal group

[1] "Cluster 1"
3A15.A.354, 4AJ9.A.1715, 2AKJ.A.564, 3ARJ.A.153, 3AT6.A.142, 2AUO.A.153, 2AUQ.A.147, 2AVO.A.147, 4AVD.A.144, 3AYF.A.802, 1B0B.A.144, 3BA2.A.158, 1BCF.A.200, 3BK9.A.401, 2C1D.H.1158, 1C6S.A.88, 4C9L.A.1418, 1CG8.B.142, 1CH4.A.147, 2CMM.A.155, 1CRC.A.105, 4CZC.A.1337, 3D1K.A.200, 3D1K.B.400, 2D5X.B.147, 3DAM.A.600, 3DE8.D.150, 1DGF.A.3000, 2DKK.A.430, 1DLY.A.144, 4DVQ.A.601, 1DW1.A.113, 1E2R.A.602, 2E39.A.401, 3E4W.A.501, 2E84.A.1315, 4EGO.A.501, 1EQD.A.185, 4ESA.A.202, 4ESA.B.202, 2FDU.A.500, 1FHF.A.350, 1FHJ.B.147, 4FVC.A.201, 2G3H.A.154, 3G46.A.147, 3GAS.A.1290, 1GEJ.A.501, 2GGN.X.251, 1GJQ.A.602, 2GKM.A.144, 4GQS.B.501, 4H0K.A.200, 1H1X.A.1154, 1H97.A.148, 1HJ4.B.601, 4HRR.A.201, 4HRR.B.201, 2HYS.A.201, 1HZU.A.601, 1I3D.A.147, 4I8V.A.601, 2I96.A.129, 3IBD.A.500, 2IG3.A.700, 1IOP.A.154, 3IQB.A.500, 1IRD.B.347, 1IWH.A.142, 1IX4.A.300, 2J7A.D.1001, 1JEB.D.147, 4JET.A.201, 2JXM.B.250, 2KII.A.182, 4L1Y.A.300, 4L1Z.A.300, 4L2M.A.201, 4L3H.A.402, 2L4D.A.107, 4L54.A.501, 1LGA.A.396, 1LHT.A.155, 2LHB.A.151, 1M54.F.1620, 1M56.A.1002, 1M7S.D.600, 3MM3.A.501, 3MMB.A.580, 3MMO.A.1004, 1MN1.A.396, 1MYF.A.154, 3MYM.A.139, 3MYN.A.139, 4N4M.A.616, 4N4N.C.601, 3N8Y.B.601, 3NA0.A.601, 4NK2.A.700, 3NN2.A.239, 2NNJ.A.500, 2NP1.A.350, 1NR6.A.500, 3NTG.D.601, 3O5C.B.402, 1OAE.A.1125, 3OCD.C.401, 3OCD.D.401, 1OCZ.A.515, 1ODO.A.1407, 3OFU.A.417, 1OG5.A.501, 2OIF.A.163, 1OR4.A.180, 1OZW.B.300, 3P3X.A.501, 2P85.A.500, 3PM0.A.900, 3PT8.A.500, 3PT8.B.500, 2Q8P.A.300, 3QGP.A.200, 3QQR.B.163, 2QRW.A.700, 2QSS.A.142, 4R21.A.600, 3R9B.A.501, 2RF7.D.1, 3RIW.A.305, 4RKM.K.809, 4RKM.L.813, 3RUR.A.200, 1S13.A.300, 1S1F.A.430, 1S61.A.144, 1S69.A.125, 3S66.A.142, 3S79.A.600, 1SCH.A.300, 1SI8.A.501, 1SPG.B.148, 3TBG.A.800, 3TM8.A.903, 1TWN.A.300, 3TYW.A.501, 1U5U.A.999, 4U9D.D.201, 3UA1.A.508, 4UBS.A.501, 3UHB.A.147, 3UHD.B.147, 1UMO.A.1172, 3UOI.I.200, 3UT2.A.1500, 1UX8.A.700, 1V4U.A.144, 1V4U.B.147, 1V9Z.B.1140, 3VED.A.401, 2VHD.A.401, 3VNO.A.501, 3VOL.A.401, 1VRE.A.148, 3VRF.B.201, 2VZW.A.1206, 3W08.A.501, 1W92.A.1149, 3WCU.A.200, 3WCU.C.200, 3WCU.B.201, 3WCU.D.201, 3WFB.B.802, 4WG2.A.603, 3WNU.A.801, 1WOW.A.300, 3WXO.A.801, 2WY4.A.150, 2X66.A.1359, 1X8V.A.470, 1X9F.A.160, 1X9F.B.160, 1X9F.C.160, 1X9F.D.160, 2XQ1.B.1503, 2Y4F.A.389, 2YGX.D.450, 2YL1.A.128, 1YMC.A.154, 2Z6N.A.150, 2Z6N.B.150, 3ZK5.A.1407, 2ZVU.A.300

[1] "Cluster 2"
2AWC.A.137, 2BIW.C.1492, 1BIQ.A.376, 4BMT.A.1323, 1EO2.B.600, 1EYS.C.609, 1EYS.C.611, 3FG4.A.1500, 3HHX.A.281, 3HHY.A.281, 3HJ8.A.281, 3HJS.A.281, 1HLM.A.159, 2HMQ.C.115, 3I4Y.A.281, 3I51.A.281, 3IVD.B.601, 4J6C.A.502, 4KVQ.A.301, 1KW6.B.301, 4L7Y.A.201, 1NFT.A.333, 3NKT.A.369, 1NX4.A.300, 3O5U.A.300, 1ODN.A.1326, 4P1B.D.501, 3PCE.N.600, 2PCD.N.600, 2Q0J.A.998, 1QFC.A.401, 3T4V.A.300, 3UFK.A.920, 1YCH.A.501, 1YFX.A.300, 2ZZI.B.209

[1] "Cluster 3"
3A51.C.412, 3ABB.A.1430, 3AK3.C.215, 1B7V.A.93, 2BCN.B.109, 1BGP.A.400, 2BMO.A.1441, 3BUJ.A.398, 4C50.A.1741, 3C6G.A.601, 4CAB.A.537, 2CCY.A.129, 1CGN.A.128, 2CJ1.A.1300, 1CPQ.A.130, 4D3O.A.901, 3DAX.A.601, 3DBG.A.500, 4DWU.A.201, 3E5L.A.1408, 1ECD.A.137, 3EJ8.A.1901, 3EJD.B.405, 4ENU.A.801, 1EUP.A.410, 2FDG.A.300, 3G1Q.A.480, 4G2C.A.501, 4G45.A.401, 2GB8.A.295, 1GW2.A.350, 1GWS.A.615, 1GWU.A.1306, 4H8Q.A.201, 3HF2.A.482, 1HJ5.B.601, 2HMQ.B.115, 3HX9.A.300, 4IAM.A.501, 2IIZ.A

.400, 2ISA.A.486, 1IT2.A.147, 1IZO.C.501, 4J1X.A.201, 4JS9.A.501, 3K9V.A.520, 4K
FO.A.501, 3KX4.A.999, 2L8M.A.416, 4M26.C.401, 4M71.B.403, 1M85.A.1001, 1MBA.A.14
8, 2MHR.A.119, 3MM6.B.570, 4MMO.A.401, 3MOL.B.185, 1MQV.A.150, 3N3R.A.1500, 1N97
.B.603, 4NKW.A.600, 2NOX.C.500, 1OIK.A.1302, 4OQR.A.501, 2OYY.A.201, 1P3T.A.300,
 2PMS.A.347, 3Q14.A.501, 1Q5E.A.440, 3Q9K.A.605, 2Q9F.A.602, 1QGJ.A.1350, 1QJS.A
.500, 3QPI.A.1001, 1QWL.B.550, 3QY8.A.252, 3QZM.B.201, 3QZX.A.200, 2R1H.D.148, 2
R79.A.500, 2RCL.B.600, 2RDN.A.1, 2RFB.A.410, 3SIK.A.154, 1SOG.A.296, 1SPG.A.144,
 1T85.A.417, 1TMX.A.861, 3TM8.A.902, 2TOH.A.501, 4TRI.A.501, 3TTW.A.760, 3TTX.B.
760, 3U9J.A.200, 1UC3.A.150, 1V54.A.516, 3W4U.A.201, 3W8M.A.201, 2WIV.A.1553, 4W
XO.B.301, 2XBK.A.1398, 2XF2.A.690, 2XMO.A.1128, 2XN8.A.1434, 2Z3U.A.500, 2ZDO.A.
200, 2ZFO.D.200
[1] "Cluster 4"
4AC8.D.500, 1AHJ.A.208, 1BT8.B.202, 3BXD.A.302, 2D0Q.A.300, 3DHG.D.507, 3EAH.A.8
61, 3GE3.A.502, 4GEP.A.580, 3GPH.A.500, 2GYQ.A.401, 1H2L.A.1350, 2HBT.A.900, 3HC
1.A.305, 3HC1.A.306, 1HDS.A.142, 3HF4.F.147, 1HV4.A.151, 1I4Y.E.605, 2IBN.B.706,
 4IGO.A.1000, 2INN.A.513, 2J0P.A.1342, 1J1L.A.1001, 1L0L.D.242, 1LC1.A.105, 1LH2
.A.154, 1LH6.A.154, 1LH7.A.154, 2LH2.A.154, 3LL8.A.506, 3LMX.M.600, 3MZS.A.500,
3NNF.A.600, 3NNL.A.600, 3O0F.A.304, 2O68.A.401, 2OGI.A.301, 4OZ5.A.201, 3PCJ.R.6
00, 2PQ7.A.221, 1QHW.A.433, 3QY6.A.264, 2RI4.A.142, 1S05.A.130, 1SMJ.C.472, 1SQ3
.A.903, 1T47.B.430, 3U9M.A.201, 1W2A.X.1302, 1WRA.A.401, 2XMO.B.556, 2Y0I.A.1350
, 1Y01.B.142, 2Z36.A.450, 3ZKY.A.1332, 2ZPG.A.300, 2ZZS.2.220
[1] "Cluster 5"
4QQZ.C.1001, 4AIQ.A.1746, 1B06.A.322, 2BKB.A.1193, 1BS3.A.202, 2BUT.B.1541, 2BUU
.B.1541, 2BUV.B.1541, 2BUW.B.600, 2BUM.B.1541, 2BUQ.B.1541, 3CEI.A.500, 2CW2.A.4
02, 2CW3.A.524, 4DVH.A.301, 3E13.X.326, 3ESF.A.198, 4F2N.B.300, 4FFK.A.301, 3FG3
.D.1500, 4G2D.A.402, 1GN6.A.999, 2GOJ.A.198, 2GPC.A.195, 3H1S.A.1001, 3HJQ.A.281
, 3HKP.A.281, 1HMD.A.115, 4IEZ.A.501, 1ISA.A.193, 3IS8.A.163, 4KEZ.A.401, 4L2B.A
.201, 4L2C.A.201, 3LIO.A.5000, 1MY6.B.200, 3PCN.P.600, 2QFR.A.433, 3QFM.B.264, 1
QNN.A.201, 2R1K.A.800, 2R1N.A.800, 3R2R.A.155, 4REU.A.201, 1SQ3.A.901, 1TDW.A.42
5, 3TQJ.A.1001, 1UNF.X.1239, 2VHL.A.1397, 2VP1.A.1347, 2W7W.A.1195, 1WB7.A.212,
1WB8.A.212, 1XM8.A.701, 1Z6O.M.300, 1ZA5.B.393
[1] "Cluster 6"
2AA1.B.400, 4AUL.A.754, 1BBH.A.132, 4BLY.A.500, 4BM1.A.500, 1CG5.A.142, 1CGO.A.1
28, 1D06.A.501, 1D0C.A.500, 1D2V.A.605, 3E65.A.901, 3EH5.A.801, 4ENP.A.801, 1EYS
.C.612, 4FB2.B.501, 1FT5.A.214, 1GBU.B.148, 1GCV.A.141, 1GCV.B.137, 1GVH.A.1398,
 1HRM.A.154, 1IBE.A.143, 3ICF.A.601, 1ITH.A.143, 2J2M.A.501, 1JAF.A.130, 4L7Y.B.
201, 1MGN.A.154, 1MXR.A.1003, 3NU1.A.302, 1OM4.A.750, 1OUT.A.143, 1OUT.B.148, 4S
1C.A.801, 3SCF.C.203, 3TKT.A.431, 3TTV.A.760, 3UHK.A.147, 2VE3.B.1444, 1VHB.A.15
0, 2VV6.C.1259, 3WAQ.A.201, 1XZ5.A.142, 1Y5F.B.147, 1Y5J.B.147, 1YFW.A.300, 2Z6F
.A.3747, 2ZYQ.A.301
[1] "Cluster 7"
4ANP.A.1426, 4B2O.A.1266, 1B7Z.A.690, 1DRT.A.325, 1EOB.B.600, 4FAG.A.401, 1FRV.B
.537, 1FZ1.B.5003, 1GY9.A.300, 1H2K.A.1350, 4M26.B.401, 1N04.A.688, 3N1Y.A.503,
3N1Y.A.504, 4N71.A.201, 3O32.A.300, 3O6J.A.300, 4OJ8.B.301, 3PCA.M.600, 3PCJ.N.6
00, 3PCK.M.600, 2PHD.B.370, 3Q3O.A.502, 2R2F.A.320, 1SP8.A.500, 3UF9.B.315, 1UOF
.A.1311, 1UTE.A.501, 3VER.A.601, 3VSI.B.401, 1VZ4.A.1299, 1WZD.A.901, 1XVE.A.117
0, 2YDE.A.501, 2YU1.A.600, 1ZJ9.B.1569

Table S79. 5-ligand Fe, compressed group

size largest_angle* middle_1* middle_2 middle_3* middle_4

1	"41"	"151.1+/-8.1"	"58+/-3.5"	"82.7+/-6.5"	"91.1+/-3.6"	"96.9+/-3.5"
2	"14"	"172.3+/-5.1"	"79.8+/-5.2"	"84.4+/-5.3"	"87.9+/-3"	"91.3+/-2.1"
3	"22"	"147.4+/-4.5"	"59.2+/-6.1"	"80.6+/-6.8"	"87.2+/-6"	"92.3+/-4.4"
4	"34"	"164+/-5.9"	"60.3+/-3.8"	"85.6+/-4.9"	"89.9+/-3.9"	"93.4+/-4.3"

	middle_5*	middle_6	middle_7	middle_8*
1	"101.9+/-3.7"	"107.5+/-4.9"	"117.7+/-6.7"	"134.2+/-7.7"
2	"93.7+/-1.6"	"97+/-4.1"	"105.4+/-13.1"	"153.1+/-11.2"
3	"96+/-4.3"	"104.1+/-9.2"	"123.7+/-6.5"	"141+/-4.7"
4	"97.6+/-4"	"102.5+/-4.1"	"110.1+/-6.2"	"151.1+/-7.7"

	smallest_opposite_angle*	TrigonalBipyramidal	SquarePyramidal
1	"100.4+/-6.8"	"0.008"	"0"
2	"60.2+/-3"	"0"	"0.043"
3	"78.9+/-8.5"	"0"	"0"
4	"86.5+/-6"	"0"	"0.015"

	TrigonalPrismaticV
1	"0.004"
2	"0"
3	"0"
4	"0"

Table S80. Cluster members of 5-ligand Fe, compressed group

[1] "Cluster 1"
4AM4.A.1161, 1CJX.A.629, 2CKF.A.501, 4D8F.B.402, 2DE6.A.501, 1DLM.A.400, 3E1N.C.
300, 3E1Q.A.301, 4ELR.A.401, 2FKZ.C.1600, 3FM3.B.552, 1FZ0.B.5004, 4GAM.F.601, 3
GHQ.K.300, 1GUQ.A.351, 4HJL.A.502, 2HMN.A.450, 1HSE.A.400, 2HTN.A.301, 4HVR.A.20
1, 4JPY.A.301, 4NB9.A.501, 4NBC.A.501, 4NBG.C.501, 4NBH.A.501, 3O6R.A.300, 1OQU.
B.1004, 2OWT.A.324, 4P9G.A.401, 1PIZ.A.376, 3R2R.A.156, 4RC6.A.302, 4REU.A.202,
1RSV.A.1004, 1SI0.A.321, 4TOH.A.202, 4V06.A.1491, 1XIK.B.377, 2XRX.M.1461, 2XSH.
G.900, 2YFJ.C.901
[1] "Cluster 2"
4QQZ.G.1002, 3AK9.J.168, 4AQ2.B.800, 4AQ6.F.837, 2BJJ.X.694, 1EH3.A.400, 1GVC.A.
1253, 3MPS.D.1, 2O84.X.500, 1OQH.A.500, 3PCC.M.600, 3TOD.A.694, 3V83.A.703, 3W54
.B.501
[1] "Cluster 3"
1B1X.A.691, 1BIQ.B.376, 3CI8.A.6, 3E1M.B.301, 1F9B.A.691, 2FKZ.C.1601, 2FL0.A.16
02, 3FVB.A.164, 3HH8.A.1, 3IS8.A.161, 3IS8.E.162, 1LKO.A.601, 1LKP.A.601, 4N71.A
.202, 1NFV.A.200, 1PFR.B.503, 3QVD.B.173, 3QVD.C.175, 4RC6.A.301, 1SQ3.A.902, 2V
ZB.B.6204, 1YV1.B.301
[1] "Cluster 4"
4AC8.A.500, 1B7Z.A.691, 3CHH.A.501, 3EE4.A.317, 1F9B.A.690, 1FYZ.B.5004, 3GCF.D.
501, 1HDS.B.146, 2ITB.A.502, 2IW4.B.1311, 2J2F.A.371, 1JI5.D.153, 1LTV.A.900, 3M
PS.D.172, 4NB8.B.501, 3OWO.B.384, 3PCC.N.600, 3PCD.M.600, 3PCF.N.600, 3PCH.R.600
, 4PG0.A.302, 1PRC.M.607, 1Q0C.B.500, 3Q1G.B.1002, 3QHB.A.182, 1RNR.A.402, 1TKP.
D.302, 2UW1.A.1359, 3W54.A.501, 3W54.B.502, 4WWZ.B.301, 2XSH.C.901, 1XZW.A.429,
1YUZ.A.302

Table S81. 5-ligand Fe, combined group

	size	largest_angle*	middle_1*	middle_2	middle_3*
1	"40"	"150.5+/-8.2"	"58.2+/-3.9"	"82.8+/-6.5"	"90.8+/-4"
2	"191"	"177.8+/-1.5"	"87+/-2.2"	"88.7+/-1"	"89.5+/-0.7"
3	"57"	"174.7+/-2.7"	"82+/-3.2"	"86+/-2.4"	"88.4+/-1.7"
4	"46"	"154.8+/-8.5"	"58.9+/-4.3"	"82.5+/-6.3"	"88+/-5.5"
5	"43"	"167.6+/-5"	"71.5+/-5.9"	"84.5+/-4.2"	"89.2+/-3.6"
6	"19"	"168.9+/-9.6"	"79.9+/-5.2"	"84.4+/-4.9"	"87.5+/-3.1"
7	"55"	"171.9+/-4.6"	"83.1+/-3.8"	"86.2+/-2.4"	"88.3+/-2.1"
8	"107"	"170.6+/-2.7"	"86.2+/-2.7"	"88.2+/-1.4"	"89.5+/-1.1"
9	"49"	"164+/-3.2"	"86.4+/-2.1"	"87.9+/-1"	"89+/-1"
10	"37"	"162.9+/-5.8"	"78.5+/-4.7"	"84.7+/-2.9"	"89.5+/-2.7"

	middle_4	middle_5*	middle_6	middle_7	middle_8*
1	"96.6+/-3.6"	"101.9+/-3.7"	"107.8+/-5"	"118.3+/-6.7"	"134+/-7.7"
2	"90.5+/-0.6"	"91.3+/-1"	"92.8+/-1.7"	"95+/-2.7"	"176.5+/-1.9"
3	"90.5+/-1.9"	"92.9+/-2.2"	"95.7+/-3"	"99.9+/-4.8"	"170.3+/-3.8"
4	"92.3+/-5.3"	"96.6+/-4.7"	"103.3+/-7.4"	"117+/-8.9"	"144.1+/-5.7"
5	"92.7+/-3"	"96.9+/-3.5"	"101+/-3.8"	"107.8+/-6.2"	"158.5+/-3.9"
6	"91.2+/-2.6"	"93.7+/-2.1"	"97.1+/-4.5"	"105.5+/-13.2"	"153+/-11"
7	"90.6+/-1.8"	"93.8+/-3.3"	"98.1+/-4.5"	"119.3+/-4.8"	"132+/-6.4"
8	"90.9+/-1.2"	"92.7+/-1.9"	"97.4+/-2.6"	"100.7+/-3"	"167.7+/-2.3"
9	"90.4+/-1.6"	"95.5+/-2.2"	"100.1+/-2.5"	"103.6+/-3.4"	"160.4+/-3.1"
10	"92.7+/-3.5"	"97.1+/-3"	"102.5+/-3.1"	"113.8+/-7"	"147.5+/-5.2"

	smallest_opposite_angle*	TrigonalBipyramidal	SquarePyramidal
1	"100.6+/-6.8"	"0"	"0"
2	"88.5+/-2.4"	"0"	"0.235"
3	"83.2+/-4.1"	"0"	"0.091"
4	"82.8+/-7.2"	"0"	"0"
5	"86.1+/-5.2"	"0"	"0.001"
6	"62.7+/-6.1"	"0"	"0"
7	"107.6+/-5.9"	"0.013"	"0"
8	"92.1+/-2.4"	"0"	"0.07"
9	"96.4+/-3"	"0"	"0.004"
10	"97.1+/-4.7"	"0"	"0"

	TrigonalPrismaticV
1	"0.001"
2	"0"
3	"0"
4	"0"
5	"0"
6	"0"
7	"0"
8	"0"
9	"0"
10	"0"

Table S82. Cluster members of 5-ligand Fe, combined group

[1] "Cluster 1"
4AM4.A.1161, 4BMT.A.1323, 1CJX.A.629, 2CKF.A.501, 4D8F.B.402, 2DE6.A.501, 1DLM.A
.400, 3E1N.C.300, 3E1Q.A.301, 4ELR.A.401, 2FKZ.C.1600, 3FM3.B.552, 1FZ0.B.5004,

4GAM.F.601, 3GHQ.K.300, 1GUQ.A.351, 4HJL.A.502, 2HMN.A.450, 1HSE.A.400, 2HTN.A.3
01, 4JPY.A.301, 4NB9.A.501, 4NBC.A.501, 4NBG.C.501, 4NBH.A.501, 1OQU.B.1004, 2OW
T.A.324, 4P9G.A.401, 1PIZ.A.376, 3R2R.A.156, 4RC6.A.302, 4REU.A.202, 1RSV.A.1004
, 1SIO.A.321, 4TOH.A.202, 4V06.A.1491, 1XIK.B.377, 2XRX.M.1461, 2XSH.G.900, 2YFJ
.C.901
[1] "Cluster 2"
3A15.A.354, 4AJ9.A.1715, 2AKJ.A.564, 3ARJ.A.153, 3AT6.A.142, 2AUO.A.153, 2AUQ.A.
147, 2AV0.A.147, 4AVD.A.144, 3AYF.A.802, 1BOB.A.144, 3BA2.A.158, 1BCF.A.200, 3BK
9.A.401, 2C1D.H.1158, 1C6S.A.88, 4C9L.A.1418, 1CG8.B.142, 1CH4.A.147, 2CMM.A.155
, 1CRC.A.105, 4CZC.A.1337, 3D1K.A.200, 3D1K.B.400, 2D5X.B.147, 3DAM.A.600, 3DE8.
D.150, 1DGF.A.3000, 2DKK.A.430, 1DLY.A.144, 4DVQ.A.601, 1DW1.A.113, 1E2R.A.602,
2E39.A.401, 3E4W.A.501, 2E84.A.1315, 4EGO.A.501, 1EQD.A.185, 4ESA.A.202, 4ESA.B.
202, 2FDU.A.500, 1FHF.A.350, 1FHJ.B.147, 4FVC.A.201, 2G3H.A.154, 3G46.A.147, 3GA
S.A.1290, 1GEJ.A.501, 2GGN.X.251, 1GJQ.A.602, 2GKM.A.144, 4GQS.B.501, 4HOK.A.200
, 1H1X.A.1154, 1H97.A.148, 1HJ4.B.601, 4HRR.A.201, 4HRR.B.201, 2HYS.A.201, 1HZU.
A.601, 1I3D.A.147, 4I8V.A.601, 2I96.A.129, 3IBD.A.500, 2IG3.A.700, 1IOP.A.154, 3
IQB.A.500, 1IRD.B.347, 1IWH.A.142, 1IX4.A.300, 2J7A.D.1001, 1JEB.D.147, 4JET.A.2
01, 2JXM.B.250, 2KII.A.182, 4L1Y.A.300, 4L1Z.A.300, 4L2M.A.201, 4L3H.A.402, 2L4D
.A.107, 4L54.A.501, 1LGA.A.396, 1LHT.A.155, 2LHB.A.151, 1M54.F.1620, 1M56.A.1002
, 1M7S.D.600, 3MM3.A.501, 3MMO.A.1004, 1MN1.A.396, 1MYF.A.154, 3MYM.A.139, 3MYN.
A.139, 4N4M.A.616, 4N4N.C.601, 3N8Y.B.601, 3NA0.A.601, 4NK2.A.700, 3NN2.A.239, 2
NNJ.A.500, 2NP1.A.350, 1NR6.A.500, 3NTG.D.601, 3O5C.B.402, 1OAE.A.1125, 3OCD.C.4
01, 3OCD.D.401, 1OCZ.A.515, 1ODO.A.1407, 3OFU.A.417, 1OG5.A.501, 2OIF.A.163, 1OR
4.A.180, 1OZW.B.300, 3P3X.A.501, 2P85.A.500, 3PM0.A.900, 3PT8.A.500, 3PT8.B.500,
2Q8P.A.300, 3QGP.A.200, 3QQR.B.163, 2QRW.A.700, 2QSS.A.142, 4R21.A.600, 3R9B.A.
501, 2RF7.D.1, 3RIW.A.305, 4RKM.K.809, 4RKM.L.813, 3RUR.A.200, 1S13.A.300, 1S1F.
A.430, 1S61.A.144, 1S69.A.125, 3S66.A.142, 3S79.A.600, 1SCH.A.300, 1SI8.A.501, 1
SPG.B.148, 3TBG.A.800, 3TM8.A.903, 1TWN.A.300, 3TYW.A.501, 1U5U.A.999, 4U9D.D.20
1, 3UA1.A.508, 4UBS.A.501, 3UHB.A.147, 3UHD.B.147, 1UMO.A.1172, 3UOI.I.200, 3UT2
.A.1500, 1UX8.A.700, 1V4U.A.144, 1V4U.B.147, 1V9Z.B.1140, 3VED.A.401, 2VHD.A.401
, 3VNO.A.501, 3VOL.A.401, 1VRE.A.148, 3VRF.B.201, 2VZW.A.1206, 3W08.A.501, 1W92.
A.1149, 3WCU.A.200, 3WCU.C.200, 3WCU.B.201, 3WCU.D.201, 3WFB.B.802, 4WG2.A.603,
3WNU.A.801, 1WOW.A.300, 3WXO.A.801, 2WY4.A.150, 2X66.A.1359, 1X8V.A.470, 1X9F.A.
160, 1X9F.B.160, 1X9F.C.160, 1X9F.D.160, 2XQ1.B.1503, 2Y4F.A.389, 2YGX.D.450, 2Y
L1.A.128, 1YMC.A.154, 2Z6N.A.150, 2Z6N.B.150, 3ZK5.A.1407, 2ZVU.A.300
[1] "Cluster 3"
4AC8.D.500, 1AHJ.A.208, 1BT8.B.202, 2D0Q.A.300, 3DHG.D.507, 3EAH.A.861, 3GE3.A.5
02, 4GEP.A.580, 3GPH.A.500, 2GYQ.A.401, 1H2L.A.1350, 2HBT.A.900, 3HC1.A.305, 3HC
1.A.306, 1HDS.A.142, 3HF4.F.147, 1HV4.A.151, 1I4Y.E.605, 2IBN.B.706, 4IGO.A.1000
, 2INN.A.513, 2J0P.A.1342, 1J1L.A.1001, 1L0L.D.242, 1LC1.A.105, 1LH2.A.154, 1LH6
.A.154, 1LH7.A.154, 2LH2.A.154, 3LL8.A.506, 3LMX.M.600, 3MMB.A.580, 3MZS.A.500,
3NNF.A.600, 3NNL.A.600, 3OOF.A.304, 2O68.A.401, 2OGI.A.301, 4OZ5.A.201, 3PCJ.R.6
00, 2PQ7.A.221, 1QHW.A.433, 3QY6.A.264, 2RI4.A.142, 1S05.A.130, 1SMJ.C.472, 1SQ3
.A.903, 1T47.B.430, 3U9M.A.201, 1WRA.A.401, 2XMO.B.556, 2Y0I.A.1350, 1Y01.B.142,
2Z36.A.450, 3ZKY.A.1332, 2ZPG.A.300, 2ZZS.2.220
[1] "Cluster 4"
4AC8.A.500, 1B1X.A.691, 1BIQ.B.376, 3CI8.A.6, 3E1M.B.301, 1F9B.A.691, 2FKZ.C.160
1, 2FLO.A.1602, 3FVB.A.164, 1FYZ.B.5004, 4HVR.A.201, 3IS8.A.161, 3IS8.E.162, 2IT
B.A.502, 2IW4.B.1311, 2J2F.A.371, 1JI5.D.153, 1LKO.A.601, 1LKP.A.601, 1LTV.A.900
, 3MPS.D.172, 4N71.A.202, 4NB8.B.501, 1NFV.A.200, 3PCD.M.600, 3PCF.N.600, 3PCH.R
.600, 1PFR.B.503, 4PGO.A.302, 1PRC.M.607, 3Q1G.B.1002, 3QHB.A.182, 3QVD.B.173, 3
QVD.C.175, 4RC6.A.301, 1SQ3.A.902, 1TKP.D.302, 2UW1.A.1359, 2VZB.B.6204, 3W54.A.
501, 2XSH.C.901, 1XVE.A.1170, 1YCH.A.501, 1YUZ.A.302, 2YU1.A.600, 1YV1.B.301
[1] "Cluster 5"

4ANP.A.1426, 4B2O.A.1266, 1B7Z.A.690, 1B7Z.A.691, 3BXD.A.302, 3CHH.A.501, 1DRT.A
.325, 3EE4.A.317, 1EOB.B.600, 1F9B.A.690, 1FRV.B.537, 1FZ1.B.5003, 3GCF.D.501, 1
GY9.A.300, 1H2K.A.1350, 1HDS.B.146, 4M26.B.401, 1N04.A.688, 3N1Y.A.503, 3N1Y.A.5
04, 3O32.A.300, 3O6J.A.300, 4OJ8.B.301, 3OWO.B.384, 3PCA.M.600, 3PCC.N.600, 3PCJ
.N.600, 3PCK.M.600, 2PHD.B.370, 2R2F.A.320, 1RNR.A.402, 1SP8.A.500, 3UF9.B.315,
1UOF.A.1311, 1UTE.A.501, 3VER.A.601, 3VSI.B.401, 1VZ4.A.1299, 3W54.B.502, 4WWZ.B
.301, 1WZD.A.901, 1XZW.A.429, 2YDE.A.501
[1] "Cluster 6"
4QQZ.G.1002, 3AK9.J.168, 4AQ2.B.800, 4AQ6.F.837, 2BJJ.X.694, 1EH3.A.400, 1GVC.A.
1253, 3HH8.A.1, 3MPS.D.1, 4N71.A.201, 2O84.X.500, 1OQH.A.500, 3PCC.M.600, 3Q3O.A
.502, 3TOD.A.694, 3V83.A.703, 1W2A.X.1302, 3W54.B.501, 1ZJ9.B.1569
[1] "Cluster 7"
4QQZ.C.1001, 4AIQ.A.1746, 1B06.A.322, 2BKB.A.1193, 1BS3.A.202, 2BUT.B.1541, 2BUU
.B.1541, 2BUV.B.1541, 2BUW.B.600, 2BUM.B.1541, 2BUQ.B.1541, 3CEI.A.500, 2CW2.A.4
02, 2CW3.A.524, 4DVH.A.301, 3E13.X.326, 3ESF.A.198, 4F2N.B.300, 4FFK.A.301, 3FG3
.D.1500, 4G2D.A.402, 1GN6.A.999, 2GOJ.A.198, 2GPC.A.195, 3H1S.A.1001, 3HKP.A.281
, 1HMD.A.115, 4IEZ.A.501, 1ISA.A.193, 3IS8.A.163, 4KEZ.A.401, 4L2B.A.201, 4L2C.A
.201, 3LIO.A.5000, 1MY6.B.200, 3PCN.P.600, 2QFR.A.433, 3QFM.B.264, 1QNN.A.201, 2
R1K.A.800, 2R1N.A.800, 3R2R.A.155, 4REU.A.201, 1SQ3.A.901, 1TDW.A.425, 3TQJ.A.10
01, 1UNF.X.1239, 2VHL.A.1397, 2VP1.A.1347, 2W7W.A.1195, 1WB7.A.212, 1WB8.A.212,
1XM8.A.701, 1Z6O.M.300, 1ZA5.B.393
[1] "Cluster 8"
3A51.C.412, 3ABB.A.1430, 3AK3.C.215, 1B7V.A.93, 2BCN.B.109, 1BGP.A.400, 2BMO.A.1
441, 3BUJ.A.398, 4C50.A.1741, 3C6G.A.601, 4CAB.A.537, 2CCY.A.129, 1CGN.A.128, 2C
J1.A.1300, 1CPQ.A.130, 4D3O.A.901, 3DAX.A.601, 3DBG.A.500, 4DWU.A.201, 3E5L.A.14
08, 1ECD.A.137, 3EJ8.A.1901, 3EJD.B.405, 4ENU.A.801, 1EUP.A.410, 2FDG.A.300, 3G1
Q.A.480, 4G2C.A.501, 4G45.A.401, 2GB8.A.295, 1GW2.A.350, 1GWS.A.615, 1GWU.A.1306
, 4H8Q.A.201, 3HF2.A.482, 1HJ5.B.601, 2HMQ.B.115, 3HX9.A.300, 4IAM.A.501, 2IIZ.A
.400, 2ISA.A.486, 1IT2.A.147, 1IZO.C.501, 4J1X.A.201, 4JS9.A.501, 3K9V.A.520, 4K
FO.A.501, 3KX4.A.999, 2L8M.A.416, 4M26.C.401, 4M71.B.403, 1M85.A.1001, 1MBA.A.14
8, 2MHR.A.119, 3MM6.B.570, 4MMO.A.401, 3MOL.B.185, 1MQV.A.150, 3N3R.A.1500, 1N97
.B.603, 4NKW.A.600, 2NOX.C.500, 1OIK.A.1302, 4OQR.A.501, 2OYY.A.201, 1P3T.A.300,
2PMS.A.347, 3Q14.A.501, 1Q5E.A.440, 3Q9K.A.605, 2Q9F.A.602, 1QGJ.A.1350, 1QJS.A
.500, 3QPI.A.1001, 1QWL.B.550, 3QY8.A.252, 3QZM.B.201, 3QZX.A.200, 2R1H.D.148, 2
R79.A.500, 2RCL.B.600, 2RDN.A.1, 2RFB.A.410, 3SIK.A.154, 1SOG.A.296, 1SPG.A.144,
1T85.A.417, 1TMX.A.861, 3TM8.A.902, 2TOH.A.501, 4TRI.A.501, 3TTW.A.760, 3TTX.B.
760, 3U9J.A.200, 1UC3.A.150, 1V54.A.516, 3W4U.A.201, 3W8M.A.201, 2WIV.A.1553, 4W
XO.B.301, 2XBK.A.1398, 2XF2.A.690, 2XMO.A.1128, 2XN8.A.1434, 2Z3U.A.500, 2ZDO.A.
200, 2ZFO.D.200
[1] "Cluster 9"
2AA1.B.400, 4AUL.A.754, 1BBH.A.132, 4BLY.A.500, 4BM1.A.500, 1CG5.A.142, 1CGO.A.1
28, 1D06.A.501, 1D0C.A.500, 1D2V.A.605, 3E65.A.901, 3EH5.A.801, 4ENP.A.801, 1EYS
.C.612, 4FAG.A.401, 4FB2.B.501, 1FT5.A.214, 1GBU.B.148, 1GCV.A.141, 1GCV.B.137,
1GVH.A.1398, 1HRM.A.154, 1IBE.A.143, 3ICF.A.601, 1ITH.A.143, 2J2M.A.501, 1JAF.A.
130, 4L7Y.A.201, 4L7Y.B.201, 1MGN.A.154, 1MXR.A.1003, 3NU1.A.302, 1OM4.A.750, 1O
UT.A.143, 1OUT.B.148, 4S1C.A.801, 3SCF.C.203, 3TKT.A.431, 3TTV.A.760, 3UHK.A.147
, 2VE3.B.1444, 1VHB.A.150, 2VV6.C.1259, 3WAQ.A.201, 1XZ5.A.142, 1Y5F.B.147, 1Y5J
.B.147, 2Z6F.A.3747, 2ZYQ.A.301
[1] "Cluster 10"
2AWC.A.137, 2BIW.C.1492, 1BIQ.A.376, 1EO2.B.600, 1EYS.C.609, 1EYS.C.611, 3FG4.A.
1500, 3HHX.A.281, 3HHY.A.281, 3HJ8.A.281, 3HJQ.A.281, 3HJS.A.281, 1HLM.A.159, 2H
MQ.C.115, 3I4Y.A.281, 3I51.A.281, 3IVD.B.601, 4J6C.A.502, 4KVQ.A.301, 1KW6.B.301
, 1NFT.A.333, 3NKT.A.369, 1NX4.A.300, 3O5U.A.300, 3O6R.A.300, 1ODN.A.1326, 4P1B.
D.501, 3PCE.N.600, 2PCD.N.600, 1Q0C.B.500, 2Q0J.A.998, 1QFC.A.401, 3T4V.A.300, 3

UFK.A.920, 1YFW.A.300, 1YFX.A.300, 2ZZI.B.209

Table S83. 6-ligand Fe, normal group

	size	largest_angle*	middle_1*	middle_2	middle_3	middle_4
1	"176"	"178+/-1.2"	"82.3+/-2.1"	"86.1+/-1.7"	"87.9+/-1.3"	"88.8+/-1"
2	"58"	"173+/-3.2"	"79.9+/-3.3"	"84.1+/-3"	"86.5+/-2.2"	"88.2+/-2"
3	"308"	"178+/-1.2"	"86.2+/-1.2"	"87.6+/-1.1"	"88.5+/-0.9"	"89.1+/-0.7"
4	"52"	"171+/-3.3"	"73.9+/-3.1"	"80.6+/-3.5"	"83.3+/-2.8"	"85.7+/-2.4"
5	"502"	"178.9+/-0.7"	"87.3+/-1.2"	"88.3+/-0.8"	"89+/-0.6"	"89.4+/-0.5"
6	"123"	"176.8+/-1.8"	"82.9+/-2.4"	"85.8+/-1.9"	"87.4+/-1.5"	"88.7+/-1.2"
7	"130"	"174.3+/-2.2"	"80.3+/-2.6"	"84.2+/-2"	"86.4+/-1.5"	"88+/-1.2"

	middle_5*	middle_6	middle_7	middle_8	middle_9*
1	"89.7+/-0.6"	"90.4+/-0.6"	"91.1+/-0.7"	"91.8+/-1"	"93.1+/-1.4"
2	"89.8+/-1.7"	"91.2+/-1.4"	"92.7+/-1.8"	"94.1+/-1.8"	"95.9+/-1.9"
3	"89.7+/-0.6"	"90.3+/-0.6"	"90.9+/-0.7"	"91.5+/-0.8"	"92.3+/-1"
4	"87.8+/-1.7"	"89.8+/-1.8"	"92.1+/-2.3"	"94.5+/-2.5"	"98.8+/-2.6"
5	"89.8+/-0.4"	"90.2+/-0.3"	"90.5+/-0.4"	"90.9+/-0.5"	"91.4+/-0.6"
6	"89.7+/-1.2"	"90.9+/-1"	"91.8+/-1.1"	"92.9+/-1.3"	"94.3+/-1.6"
7	"89.1+/-1.1"	"90.1+/-1"	"91.2+/-1.1"	"92.7+/-1.5"	"94.3+/-1.7"

	middle_10	middle_11	middle_12	middle_13*
1	"94.6+/-1.6"	"97.5+/-2.4"	"172+/-3.5"	"176.4+/-1.6"
2	"99.2+/-2.8"	"103.7+/-4.6"	"161.6+/-4.4"	"167.2+/-3.5"
3	"93.7+/-1.3"	"95.9+/-2"	"173.4+/-2.4"	"176.7+/-1.6"
4	"103+/-3.5"	"108.5+/-5.3"	"156.7+/-6.9"	"164.3+/-4.2"
5	"92.2+/-1"	"93.4+/-1.4"	"176.4+/-1.6"	"178.1+/-0.9"
6	"96.4+/-2.4"	"99.4+/-3.3"	"168.6+/-4.1"	"174.7+/-2.2"
7	"97.3+/-2.2"	"100.3+/-3.3"	"168.2+/-3.3"	"171.1+/-2"

	smallest_opposite_angle*	Octahedral	TrigonalPrismatic
1	"86.6+/-1.7"	"0.286"	"0"
2	"74+/-3.1"	"0.007"	"0"
3	"84.4+/-1.5"	"0.269"	"0"
4	"80.6+/-4.2"	"0"	"0"
5	"87.8+/-1"	"0.333"	"0"
6	"79.8+/-2.4"	"0.167"	"0"
7	"85.2+/-2.5"	"0.07"	"0"

	PentagonalBipyramidalVA	PentagonalBipyramidalVP
1	"0"	"0"
2	"0"	"0"
3	"0"	"0"
4	"0"	"0.001"
5	"0"	"0"
6	"0"	"0"
7	"0"	"0"

Table S84. Cluster members of 6-ligand Fe, normal group

[1] "Cluster 1"

3A15.B.354, 2A9E.A.550, 2AIU.A.200, 1AKK.A.105, 4APY.A.1418, 3AT5.B.147, 2B0Z.B.109, 2BGV.X.1121, 3BK9.B.401, 2BPN.A.110, 2C1V.A.401, 2C1D.A.1292, 2C1D.B.1158, 1C2N.A.117, 1C52.A.200, 1CH1.A.154, 1CH2.A.154, 2CN4.A.1175, 2CN4.B.1175, 1CPT.A.430, 3CSL.A.866, 2CVC.A.1012, 2CVC.A.1014, 2CVC.A.1016, 1D4D.A.604, 1DP8.A.719, 1DTI.A.154, 1E39.A.801, 3E5K.A.1408, 2E84.A.1302, 2E84.A.1306, 2E84.A.1307, 2E84.A.1310, 2E84.A.1316, 1EGY.A.410, 3EHB.A.559, 2FAM.A.148, 1FCD.C.901, 4FDH.A.601, 1FT9.B.300, 1G09.B.147, 4G7Q.A.602, 4G7S.A.602, 3GEO.A.580, 3GQP.C.143, 1GWS.A.603, 1GWS.A.606, 1GWS.A.610, 1GWS.A.616, 1H21.A.1248, 1H29.A.1107, 1H29.A.1108, 1H29.A.1114, 1H32.A.1263, 3HDL.A.305, 3HF4.A.142, 1HGB.D.147, 1HJ5.A.601, 3HQ6.A.400, 3HQ7.A.401, 3HYU.B.147, 1IB7.A.95, 2IUF.A.1691, 2IVF.C.1217, 2J7A.A.1003, 2JJP.A.412, 2K3V.A.278, 3K3O.A.1, 2KMY.A.233, 2KSU.A.282, 3L1M.A.150, 3L1T.A.479, 1L2K.A.154, 3LD6.A.601, 3LGN.A.200, 4LM8.A.806, 4LMH.A.804, 4LMH.A.806, 4LMH.A.810, 4M2F.B.401, 4M4A.B.201, 1MLW.A.403, 1MNI.B.154, 3MOM.A.186, 1MQF.A.501, 3N3N.B.1500, 4N4K.A.610, 4N4N.A.607, 4N4O.A.608, 4N8T.B.201, 4NFG.B.201, 4NK1.B.201, 1NS6.A.142, 2NSR.A.154, 4O1W.A.101, 4O4Z.A.201, 4O6U.A.203, 4O7G.A.301, 3O89.A.2154, 1OAH.A.1522, 1OAH.A.1524, 3OMA.A.1, 1OQU.C.1008, 3OUQ.A.604, 3OZW.A.404, 1P2E.A.801, 1P2E.A.803, 1P2H.A.803, 1P2Y.A.430, 3P3L.A.501, 1PA2.A.306, 1PBY.A.991, 3QNS.A.351, 1QO8.A.602, 1QO8.A.604, 1QPA.A.350, 1QPU.A.107, 1QQ3.A.107, 3QU8.A.500, 2RF7.A.2, 3RGS.A.1, 4RKM.B.808, 4RKM.D.807, 4RKN.A.903, 1RSE.A.154, 3RUK.B.600, 3S8G.A.800, 3SDN.A.160, 1SE6.A.430, 3SLE.A.402, 1SUO.A.500, 3T3Z.A.500, 3TF0.A.500, 3TIK.A.482, 3TJS.A.508, 4TT5.A.401, 1U13.A.460, 3UBR.A.473, 3UBR.A.474, 3UBR.A.475, 4UQH.A.1450, 2UUQ.A.1405, 4UVR.A.1450, 1V0H.X.251, 2V08.A.1087, 2V0M.A.1499, 3V2V.A.154, 4V3W.A.750, 3V5X.A.201, 2V7K.A.1360, 1V8X.A.901, 1VB6.A.1140, 2VEB.A.200, 3VHB.A.150, 2VHB.B.150, 3VM9.A.154, 3VP5.A.201, 3VXJ.A.501, 3W9C.A.501, 3WCT.C.200, 3WCT.D.201, 2WJN.C.1333, 1WMU.A.201, 1WOX.A.300, 2WX2.A.1450, 1XVB.A.1171, 2YEV.A.1015, 2YEV.B.587, 1YWD.A.185, 1Z1N.X.607, 1Z1N.X.612, 1Z1N.X.614, 2ZCF.A.206, 3ZE6.B.502, 3ZOX.A.1082, 1ZZH.A.803

[1] "Cluster 2"

1A2F.A.1, 1A7E.A.119, 4B7G.A.3000, 3BFJ.M.1388, 1BKA.A.694, 2BQ8.X.1305, 2BQ8.X.1306, 2BV0.B.600, 4CHL.A.501, 1DRY.A.332, 3DXU.A.360, 3E6S.B.200, 1FCD.C.902, 1FSL.A.144, 4G51.B.202, 4GHF.B.401, 3GM6.A.1004, 4GP4.A.602, 4GP5.A.602, 2H0V.A.501, 2HBU.A.900, 1HMO.B.115, 2HUO.A.301, 3IXF.A.139, 3KT7.A.701, 3LXV.M.600, 4M25.A.401, 3MDT.A.505, 3MOO.A.911, 3N2O.A.506, 3NC3.A.406, 2NOX.A.500, 4OJ8.C.301, 1OQU.C.1009, 3OUH.A.600, 1PIU.A.402, 2Q0J.B.998, 3Q1G.A.1002, 3QFO.A.264, 2QPP.A.300, 3QY8.A.253, 4RC8.A.303, 3RNC.A.500, 3RNF.A.500, 1SYY.A.1319, 1SYY.A.1320, 1TOQ.A.500, 3TMZ.A.501, 2VUN.A.402, 3WEC.A.501, 2X9P.A.1398, 1XK3.A.300, 1XU5.A.1175, 1XVF.B.1175, 1XVG.A.529, 1YGF.B.147, 3ZLI.A.4001, 3ZPI.A.1407

[1] "Cluster 3"

2A3F.X.201, 1A4E.A.503, 3A8G.A.301, 3A8L.A.300, 4AAN.A.400, 4AAN.A.401, 4AM5.A.1160, 1AQE.A.121, 3AWM.A.501, 1AWP.A.201, 2B10.D.909, 4B2N.A.700, 4B4Y.A.1155, 3B6H.B.600, 1B80.A.350, 1B82.A.350, 1B85.A.350, 3B99.A.600, 2BDM.A.500, 2BH4.X.1123, 1BJE.A.154, 4BJK.A.1450, 2BLF.B.1582, 3BNG.A.513, 2BPN.A.109, 2BQ4.A.1115, 2BQ4.A.1116, 2BQ4.A.1117, 2BS4.C.1255, 1BVY.A.1000, 4C0C.A.1450, 2C1U.A.401, 1C2R.A.120, 4C28.A.1450, 1C40.A.150, 3C62.A.150, 3C63.A.150, 1C6R.A.90, 4C9M.A.1418, 2CDV.A.109, 2CDV.A.111, 1CH5.A.154, 1CH7.A.154, 1CH9.A.154, 1CI3.M.254, 4CK8.A.1480, 1CO6.A.108, 1COT.A.130, 4COH.A.1450, 4COO.A.1549, 3CQV.A.601, 2CVC.A.1001, 2CVC.A.1007, 2CVC.A.1011, 3CX5.C.4001, 2CY3.A.120, 2CY3.A.121, 2CZS.A.500, 2CZS.A.501, 1CZJ.A.119, 1CZJ.A.121, 1CZJ.A.122, 3CZY.A.300, 2D0W.A.200, 2D0S.A.80, 4D30.B.750, 4D34.A.500, 4D35.A.500, 4D36.A.500, 4D37.A.500, 4D3A.A.500, 1D7B.A.401, 2DGE.A.200, 1DJ5.A.1, 3DMI.A.146, 3DR0.A.94, 4DTZ.A.500, 1DUW.A.300, 1DUW.A.301, 1DW0.A.113, 4DXY.A.501, 4DY9.A.201, 1E08.E.80, 1E39.A.802, 1E39.A.803, 3E5J.A.1408, 2E84.A.1301, 2E84.A.1304, 2E84.A.1305, 2E84.A.1308, 2E84.A.1309, 2E84.A.1313, 4EID.A.101, 2EKT.A.154, 1ETP.A.200, 1EUE.A.201, 2EXV.A.83, 1EZV.C.401, 1F1C.A.200, 1F1F.A.200, 4FA7.A.603, 4FAS.A.602, 4FAS.A.605, 4FAS.A.607, 2FBZ.X.901,

4FEF.A.403, 1FGJ.A.552, 1FGJ.A.553, 1FI7.A.110, 2FMY.A.300, 1FOP.A.500, 3FOO.A.1
50, 1FS7.A.510, 4G3J.A.501, 4GED.B.201, 1GKS.A.0, 2GNV.A.166, 1GQ1.A.602, 4GQS.A
.501, 1GWF.A.504, 1H21.A.1249, 1H21.B.1249, 1H29.A.1102, 4H2L.B.201, 4H44.A.302,
 4H6O.A.501, 2H88.C.142, 4HB6.A.72, 4HB8.A.72, 4HC3.A.72, 3HF4.B.147, 4HHR.A.701
, 4HHS.A.701, 1HJ5.B.602, 1HLB.A.158, 3HNJ.A.150, 3HNK.A.150, 3HQ9.A.401, 1HRC.A
.105, 3HSP.A.750, 4HSW.A.201, 3HYU.A.142, 1I3E.A.147, 1I8P.A.115, 2IBN.A.703, 1I
CC.A.201, 2IJ3.B.999, 1IO7.A.1401, 1IQC.A.402, 3IQ5.A.150, 1IWO.A.901, 1J02.A.30
0, 1J77.A.300, 2J7A.A.1004, 2J7A.A.1005, 2J7A.C.1004, 1JDL.A.500, 1JDR.A.296, 4J
E9.A.201, 1JNI.A.126, 2K3V.A.218, 4K8F.B.300, 4KF2.B.501, 4KIB.A.403, 2KMY.A.251
, 1KQG.C.809, 4KVK.A.701, 4KVL.A.701, 3L61.A.420, 2LDO.A.154, 4LJI.A.301, 1LM3.B
.200, 1LMS.A.118, 4LM8.A.801, 4LMH.A.807, 4LMH.A.808, 1LS9.A.92, 1LSX.A.719, 2LZ
Z.A.102, 3M15.A.150, 4M4A.A.201, 4M72.A.403, 4M73.A.403, 1MDV.A.110, 1MDV.A.112,
 4MLN.A.201, 1MNY.A.95, 4MQJ.B.201, 1MXR.A.1004, 4N4N.A.601, 4N4N.A.602, 4N4N.A.
604, 4N4N.A.605, 3NMI.A.150, 3NN1.A.239, 4NP1.A.185, 2NRL.A.148, 3NWV.A.105, 3NX
U.A.508, 3O1A.A.385, 3O5C.A.401, 3O5C.D.504, 1OAH.A.1521, 3OA8.A.401, 3OA8.B.401
, 1OFW.A.1295, 3OFT.A.417, 3OO4.A.142, 3ORV.B.600, 2ORR.A.600, 3OUE.A.612, 3OUQ.
A.602, 3OUQ.A.605, 3OUQ.A.606, 2OZY.A.203, 3OZV.A.404, 1P2E.A.804, 1PBY.A.992, 2
PEG.B.400, 4PK5.A.501, 1PP9.C.501, 2PQ7.A.220, 4PXH.A.501, 1QHU.A.500, 2QJY.A.50
1, 1QN2.B.101, 2QSP.A.142, 1QYZ.A.200, 1ROQ.A.200, 4R20.A.601, 2R50.A.166, 2R80.
A.150, 3R9C.A.450, 2RAO.B.147, 2RDZ.A.5, 3RJ6.A.154, 4RKM.A.808, 4RKN.A.906, 1RW
J.A.91, 1RZ5.A.401, 3S1J.A.140, 3S8F.A.800, 1SP3.A.801, 1SP3.A.804, 1SP3.A.805,
 1SP3.A.806, 1SP3.A.808, 3SWZ.B.600, 3SXQ.A.1006, 3SXQ.A.1007, 3SXQ.A.1001, 3T3R.
A.500, 1T68.X.201, 3T6D.C.401, 3T6D.C.403, 3TDA.A.800, 3TGA.A.185, 1TH2.D.2003,
 3TMC.A.309, 4TOB.C.201, 4TUV.A.401, 3U8P.A.347, 4U9D.A.201, 3UBR.A.472, 3UCP.A.9
02, 3UCP.A.905, 3UOI.B.200, 1URV.A.1172, 2V07.A.1102, 4V3Z.B.750, 1V54.A.515, 1V
75.B.201, 2V7L.A.1360, 3V8D.A.601, 2VHD.A.402, 3VKP.A.601, 3VKS.A.601, 3VRD.A.20
2, 3VRG.A.201, 3VRG.B.201, 2VXH.A.1001, 2W0B.A.470, 2WDQ.C.1129, 3WFD.B.801, 3WF
X.A.201, 2WJN.C.1335, 4WPD.A.402, 3WU2.F.101, 2XFH.A.1412, 2XKR.A.1400, 1XQ5.B.1
48, 1XQ5.C.143, 2XTS.B.500, 2Y5N.A.450, 2YIU.A.501, 2YK3.A.200, 1Z1N.X.604, 1Z1N
.X.608, 1Z1N.X.610, 2Z47.A.1004, 3ZBY.A.1402, 2ZBO.A.111, 3ZG2.A.1480, 3ZH0.A.20
0, 3ZHW.A.1163, 3ZJO.A.200, 2ZPB.A.300, 1ZZH.A.802
[1] "Cluster 4"
2A1X.A.450, 1B1X.A.690, 4B2O.A.1267, 4BGL.A.1001, 1BKA.A.693, 2CAG.A.485, 1D9Y.A
.310, 3DHG.A.502, 1EO9.B.600, 2FR7.A.501, 1FZ1.A.5001, 1FZ3.A.5002, 1FZ7.B.5003,
 2G1M.A.600, 1GGF.B.760, 1I4Z.A.601, 4I4G.A.601, 4I4H.A.601, 1JNF.A.702, 1JNF.A.
703, 4K0F.A.601, 4K9T.A.601, 4K9U.A.601, 4K9V.A.601, 4K9W.A.601, 4K9X.A.601, 1KW
9.B.301, 4ME4.A.402, 1MMO.D.3, 1MMO.D.4, 3MZS.C.500, 1NX4.B.300, 1OCZ.A.516, 1OQ
U.C.1010, 3P3N.A.350, 3PCJ.O.600, 3PER.A.1001, 1PHG.A.417, 3Q3M.A.509, 3QFN.A.26
5, 3QY6.A.263, 3QY7.A.263, 1R1N.C.400, 2RDB.A.499, 3RNC.A.499, 3RNF.A.501, 1TOQ.
A.499, 1U74.B.1101, 1WRA.B.402, 1XVB.A.1170, 1XVF.B.1174, 3ZK3.A.1311
[1] "Cluster 5"
19HC.A.301, 19HC.A.302, 19HC.A.303, 19HC.A.304, 19HC.A.305, 19HC.A.306, 19HC.A.3
07, 19HC.A.308, 19HC.A.309, 2A3M.A.501, 2A3M.A.502, 2A3M.A.503, 2A3M.A.504, 1A56
.A.82, 3A9F.A.207, 4AAL.A.423, 4AAO.A.400, 2AI5.A.81, 1AOF.A.602, 1AOF.B.601, 1A
OM.A.602, 1AOM.B.601, 1AOM.B.602, 1AQA.A.95, 1AQE.A.122, 3AQ5.A.144, 3AQ9.A.144,
 3AT5.A.142, 2AT3.X.185, 2B4Z.A.500, 3B42.A.199, 3B42.B.399, 3B47.A.199, 1BFR.B.
200, 4BJA.A.300, 2BK9.A.1154, 4BMM.A.1450, 3BNJ.A.513, 3BNJ.A.514, 3BNJ.A.515, 3
BNJ.A.516, 3BNJ.A.517, 2BPN.A.108, 2BPN.A.111, 2BQ4.A.1118, 2BS2.C.1255, 2BS2.C.
1256, 2BS3.C.1255, 3BXU.A.72, 3BXU.A.73, 3BXU.A.74, 4C0N.A.1157, 2C1V.A.402, 3C2
C.A.113, 4C27.A.1450, 4C44.A.1151, 1C75.A.93, 3C76.X.185, 3C78.X.185, 2C8S.A.117
3, 3CAO.A.104, 3CAO.A.105, 3CAO.A.106, 3CAO.A.107, 1CCH.A.83, 1CCR.A.112, 4CDP.A
.402, 2CEO.A.1102, 1CNO.A.200, 1COR.A.83, 3CP5.A.202, 2CTH.A.109, 2CTH.A.110, 2C
TH.A.111, 2CTH.A.112, 3CU4.A.199, 2CVC.A.1002, 2CVC.A.1003, 2CVC.A.1004, 2CVC.A.
1005, 2CVC.A.1006, 2CVC.A.1008, 2CVC.A.1009, 2CVC.A.1010, 3CX5.C.4002, 3CX5.D.40

03, 3CX5.W.4026, 1CXA.A.126, 1CXC.A.125, 3CXH.W.4026, 1CXY.A.204, 1CYI.A.200, 3C
YR.A.203, 3CYR.A.204, 2CY3.A.119, 2CY3.A.122, 5CYT.R.105, 1CZJ.A.120, 2D0T.A.404
, 3D7O.A.143, 2DC3.A.191, 3DE8.A.150, 3DHR.A.142, 1DK0.A.200, 2DN1.A.142, 2DN1.B
.147, 1DUW.A.293, 1DUW.A.297, 1DW2.A.113, 1DWL.B.80, 1DY7.B.601, 2DY5.A.300, 1E2
9.A.136, 1E2R.B.601, 1E2W.A.900, 1E39.A.804, 2E3A.A.401, 1E8E.A.125, 2E80.A.1508
, 2E84.A.1314, 1EB7.A.401, 3EGW.C.806, 3EGW.C.807, 1EHJ.A.1030, 1EHJ.A.1053, 1EH
J.A.1066, 4EIE.A.101, 4EIF.A.101, 2EKU.A.154, 1ETP.A.199, 2EU7.X.201, 2EWK.A.100
1, 2EWK.A.1003, 2EWU.A.1001, 2EWU.A.1003, 1EWH.A.253, 2EWI.A.1004, 2EWI.A.1002,
2EWI.A.1001, 2EWI.A.1003, 1EZV.C.402, 1F03.A.201, 1F24.A.501, 4F6I.A.201, 4FA7.A
.602, 4FAS.A.601, 4FAS.A.603, 4FAS.A.604, 4FAS.A.606, 2FFN.A.1003, 1FGJ.A.548, 1
FI3.A.83, 1FJ0.A.115, 3FLL.A.185, 3FO3.A.1004, 3FO3.A.1005, 3FO3.A.1006, 3FO3.A.
1007, 3FO3.A.1008, 3FO3.A.1002, 3FO3.A.1003, 3FO3.A.1001, 2FRF.A.154, 1FS7.A.509
, 1FS8.A.508, 1FT5.A.213, 1FT5.A.215, 1FT5.A.216, 1FT9.A.300, 2FWT.A.803, 2FWT.A
.805, 2FWL.A.132, 2FYU.D.242, 4G1V.A.401, 3G5N.A.500, 4G7L.A.301, 3GAS.D.1294, 2
GC4.D.200, 1GDV.A.101, 1GQ1.A.601, 2GSM.A.2001, 2GTF.X.201, 3GW9.A.480, 1GYO.A.1
11, 1GYO.A.112, 1GYO.A.113, 1GYO.A.114, 4GYD.A.200, 4H0K.B.200, 1H1O.A.1184, 1H1
O.A.1185, 1H21.B.1248, 1H29.A.1104, 1H32.A.1264, 1H32.B.1139, 3H33.A.75, 3H33.A.
76, 3H33.A.77, 3H34.A.72, 3H34.A.73, 3H34.A.74, 3H4N.A.72, 3H4N.A.73, 3H4N.A.74,
1H55.A.350, 1H57.A.350, 3H8T.A.301, 1HBI.A.153, 4HB6.A.73, 4HB8.A.73, 4HBF.A.72
, 1HCZ.A.253, 1HJ3.B.602, 2HJI.A.180, 4HPA.A.201, 4HPB.A.201, 4HPD.A.201, 3HQ9.A
.400, 2I5N.C.404, 1I5U.A.201, 1I77.A.108, 1I77.A.109, 1I77.A.110, 1I77.A.111, 4I
7Z.A.302, 3I8R.A.901, 1I8O.A.115, 2I8F.A.83, 3I9T.A.300, 3I9U.A.300, 1IDR.A.144,
1IKE.A.185, 1IT1.A.201, 1IT1.A.202, 1IT1.A.203, 1IT1.A.204, 1IVJ.A.300, 1IYN.A.
296, 1J0P.A.1002, 1J0Q.A.201, 2J1M.A.1456, 4J20.A.107, 1J3S.A.105, 2J5M.A.1300,
2J7A.C.1003, 4JEB.A.201, 4JJ0.A.501, 4JJ0.A.502, 1JMX.A.1001, 1JMX.A.1002, 1JNI.
A.125, 1KB0.A.802, 1KBI.A.760, 4KMG.A.101, 1KOK.A.296, 2KSU.A.305, 1KV9.A.901, 4
LM8.A.802, 4LM8.A.803, 4LM8.A.804, 4LM8.A.805, 4LM8.A.807, 4LM8.A.808, 4LM8.A.80
9, 4LM8.A.810, 4LMH.A.801, 4LMH.A.803, 4LMH.A.805, 4LMH.A.809, 1LQX.A.201, 1LR6.
A.201, 1M1P.A.802, 1M1Q.A.804, 1M1Q.A.803, 1M1R.A.801, 1M2I.A.201, 3M4C.A.150, 1
M59.A.201, 1M70.A.199, 1M70.A.200, 3MDM.A.505, 1MJ4.A.502, 3ML1.B.1128, 3ML1.B.1
129, 1ML7.A.185, 4MPM.A.201, 3MUS.A.201, 3MVC.A.500, 3MVF.A.185, 1MWB.A.125, 1MZ
4.A.151, 1N45.A.300, 4N4J.A.609, 4N4J.A.610, 4N4J.A.611, 4N4J.A.612, 4N4J.A.613,
4N4J.A.614, 4N4J.A.615, 4N4J.A.616, 4N4L.A.616, 4N4N.A.603, 4N4N.A.606, 1NAZ.A.
200, 1NIR.A.601, 1NIR.A.602, 1NML.A.401, 1NNO.A.602, 1NOS.A.901, 1NS9.B.147, 4O6
Q.A.202, 4O7G.A.302, 1OFW.A.1294, 1OFW.A.1296, 1OFW.A.1297, 1OFW.A.1298, 1OFW.A.
1299, 1OFW.A.1300, 1OFW.A.1301, 1OFW.A.1302, 3OMI.A.607, 3OO4.B.147, 2ORT.A.600,
2ORO.A.600, 2ORP.A.600, 2ORQ.A.600, 2ORS.A.600, 2OT4.A.1004, 2OT4.A.1007, 3OUE.
A.609, 3OUE.A.610, 3OUE.A.611, 3OUQ.A.603, 2OZY.A.202, 2OZY.A.204, 2OZY.A.205, 3
OZU.A.404, 3PC3.A.701, 2PEG.A.200, 3PH2.B.1087, 3PI2.B.500, 1PL3.A.401, 1PM1.X.1
80, 1PP9.C.502, 1PP9.D.501, 3PXW.A.500, 1Q16.C.806, 1Q16.C.807, 2Q8Q.A.300, 3Q99
.B.750, 1QDB.A.516, 1QDB.A.517, 1QDB.A.518, 1QDB.A.519, 2QJY.A.502, 2QJY.B.301,
1QKS.A.601, 1QKS.A.602, 1QNO.A.113, 1QNO.A.114, 1QNO.A.115, 1QNO.A.116, 3QQQ.A.1
63, 3QWO.A.150, 2R80.B.150, 2RAO.C.142, 2RDZ.A.2, 2RDZ.A.3, 2RDZ.A.4, 4RKN.A.902
, 4RKN.A.905, 4RKN.A.907, 4RKN.A.908, 4RKN.A.909, 1RWJ.A.90, 1RWJ.A.92, 3SEL.X.7
3, 3SEL.X.74, 1SH4.A.201, 3SJ0.X.73, 3SJ0.X.74, 3SJ1.X.73, 3SJ1.X.74, 3SJ4.X.73,
3SJ4.X.74, 3SJ5.A.500, 3SJL.A.500, 3SJL.A.600, 1SOX.A.502, 1SP3.A.803, 3SXQ.A.1
005, 3SXQ.A.1008, 3SXQ.A.1002, 3SXQ.A.1003, 1SY2.A.185, 3T6D.C.404, 3T6E.C.401,
3T6E.C.402, 3T6E.C.403, 3TGU.C.501, 3TGU.C.502, 3TGU.D.501, 3TGM.A.300, 1TKW.B.2
53, 3TOL.A.150, 1TU2.B.255, 1U4H.A.500, 1U7R.A.154, 1U9M.A.90, 1U9U.A.90, 3U99.A
.500, 3U99.A.700, 3UBC.A.201, 3UCP.A.901, 3UCP.A.903, 3UCP.A.904, 3UCP.A.906, 3U
CP.A.907, 3UCP.A.908, 3UCP.A.909, 3UCP.A.910, 3UCP.A.911, 1UP9.A.201, 1UP9.A.202
, 1UP9.A.203, 1UP9.A.204, 4V2K.A.601, 4V3V.A.750, 4V3X.A.750, 1V9Y.A.1140, 3VAU.
A.201, 1VGI.A.300, 3VRD.A.201, 1VYD.A.1117, 2VYW.A.149, 1W2L.A.1100, 2W31.A.200,
2W3G.A.500, 1W7O.A.1119, 1W7O.A.1120, 1W7O.A.1121, 1W7O.A.1122, 1WAD.A.117, 1WA

D.A.113, 1WAD.A.114, 1WAD.A.115, 3WAH.A.201, 3WC8.A.201, 3WCT.A.200, 3WCT.B.201, 1WE1.A.300, 3WFD.C.201, 2WJM.C.1334, 2WJN.C.1336, 1WOV.A.300, 4WQ8.A.1002, 4WQ9.A.1001, 4WQ9.A.1002, 4WQC.A.1002, 4WQD.A.1002, 2WTG.A.180, 1WVE.C.699, 1WVP.A.154, 3X15.A.200, 3X15.J.200, 1X3X.B.202, 1X46.A.151, 4XDI.A.201, 2XKI.A.1110, 2XSJ.B.503, 2XYK.A.700, 1Y0P.A.801, 1Y0P.A.802, 1Y0P.A.803, 1Y0P.A.804, 1Y5I.C.806, 1Y5I.C.807, 1Y5L.C.806, 2YIU.A.500, 1YIQ.A.901, 2YL7.A.128, 2YXC.A.1001, 2YYW.A.1001, 2YYW.A.1003, 2YYX.A.1004, 2YYX.A.1001, 1Z1N.X.616, 2Z47.B.3003, 2Z6S.A.201, 2Z6T.A.201, 1Z8O.A.410, 1Z9N.A.1001, 1Z9N.C.2001, 3ZIY.A.600, 3ZJQ.A.200, 3ZOO.A.105, 2ZS0.A.200, 2ZS0.B.200, 2ZS0.C.200, 2ZS0.D.200, 2ZXY.A.200, 2ZZS.1.220

[1] "Cluster 6"

3A17.A.354, 1A2S.A.90, 1ASH.A.301, 3B0H.B.601, 2B2R.A.1500, 3BDZ.A.450, 1BIN.B.144, 2BMM.A.1157, 1CC5.A.1, 1CED.A.90, 4CK9.A.1480, 2CVC.A.1013, 2CZ1.A.300, 4D02.A.603, 4D38.A.500, 1D4D.A.601, 1D4D.A.602, 1D4D.A.603, 1DCC.A.296, 1DD7.A.600, 3DHH.A.501, 1DP9.A.719, 4DTW.A.500, 2E3B.A.401, 2E84.A.1303, 2E84.A.1311, 2E84.A.1312, 3EHB.A.560, 3EJ6.A.4000, 4EJI.A.501, 4EP6.A.601, 1F4U.A.410, 4G70.A.601, 4G71.A.602, 7GEP.A.580, 4GP8.A.602, 1GWS.A.601, 1GWS.A.605, 1GWS.A.611, 1GWS.A.612, 1GWS.A.613, 2H0V.B.500, 3HB3.A.559, 3HB3.A.560, 2HUO.A.302, 3I63.A.501, 3I63.A.502, 1IQC.A.401, 2IUW.A.500, 2J7A.A.1002, 2J7A.C.1002, 1JEX.A.95, 1JIP.A.410, 3K1O.A.488, 4K8F.A.300, 4KIC.A.403, 4KJT.A.201, 1KQG.C.810, 2KSC.A.125, 4L0F.A.501, 3L4D.A.481, 1LFK.A.430, 4LXJ.A.601, 3M3A.A.155, 4M71.A.403, 5MBA.A.148, 3MDR.A.505, 4ME4.A.401, 4MLM.A.201, 4MLN.B.201, 3MM9.A.580, 4N4N.A.608, 4N4Y.A.602, 3N8Y.A.601, 1N9C.A.93, 3NA1.A.601, 1NML.A.402, 1NPF.A.154, 4O6J.A.302, 1OAH.A.1523, 3OUI.A.1, 3OUQ.A.601, 1P2H.A.804, 2PAC.A.83, 5PAH.A.425, 3PER.A.1002, 3PF7.A.1001, 4PG0.A.301, 4PH9.A.602, 3PT7.B.500, 2QDY.A.300, 1QO8.A.601, 1QO8.A.603, 3QQR.A.163, 2QU0.A.142, 2R6S.A.501, 3RI7.A.494, 3RMZ.A.500, 3S8F.A.801, 1SQ3.D.912, 3TK3.A.500, 3TOR.A.3, 3UBR.A.471, 1ULI.B.700, 1VB6.B.1140, 4VHB.A.150, 2VHD.B.401, 3VR8.C.201, 3VTH.A.807, 1W0G.A.1501, 1W4W.A.1307, 3WFC.B.802, 3WFE.B.802, 2WU5.C.305, 4WWJ.B.301, 2X2N.A.1479, 4X8B.A.508, 1XK1.A.300, 2YIU.B.500, 1Z1N.X.606, 1Z1N.X.613, 1Z8U.B.201, 1Z0Y.C.1305

[1] "Cluster 7"

3A0G.A.201, 3A16.B.354, 2A1O.A.417, 3A51.B.412, 3ABM.A.516, 4AUM.A.900, 2AVK.A.201, 3AYF.A.801, 2B11.D.1301, 2B2O.B.1500, 4B2N.A.701, 4B8N.A.201, 2BC5.A.150, 1BEP.A.296, 1BJ9.A.296, 3BOM.A.143, 3BOM.B.148, 2BUZ.B.1541, 2C1D.A.1291, 1CH3.A.154, 2CJ2.A.1300, 4CKA.A.1480, 1CLS.A.142, 4CVJ.A.1295, 2CYP.A.295, 4D02.A.602, 2D2M.D.200, 3DHI.A.600, 1DLW.A.144, 1DM1.A.148, 4DTY.A.500, 3E2O.A.296, 3EH5.A.800, 1EHE.A.501, 4ENH.A.601, 4ENU.B.801, 1EOC.B.600, 4F4O.B.201, 4FIA.A.600, 4G7G.A.501, 2GEP.A.580, 3GE3.A.501, 2GJ1.A.605, 2GSM.A.2002, 1GWS.A.609, 1GWT.A.350, 4H44.A.301, 1HBZ.A.504, 2HBD.A.142, 1HRO.A.107, 4HR0.A.402, 4I7Z.A.301, 4I7Z.C.301, 2IJ4.A.471, 2INC.A.502, 4IPS.A.401, 1IZO.A.501, 4J14.A.601, 2JJN.A.412, 2JTI.B.104, 2K3V.A.238, 2K3V.A.261, 4KIG.A.502, 1LA6.B.147, 2LDO.A.130, 2LDO.A.168, 4LMH.A.802, 2LZZ.A.101, 1M7S.A.600, 4M73.B.403, 4MBA.A.148, 3MGX.A.397, 3MKB.B.137, 4MLN.B.202, 1MRP.A.310, 1MTY.D.4, 4N6W.A.202, 3N9Q.A.1, 2NNB.A.472, 1NS9.A.142, 3NYH.A.605, 4NZ2.A.501, 3OOR.B.802, 3O72.A.500, 1OAF.A.1251, 1OAH.A.1520, 3OJT.D.500, 2OZY.A.201, 1P2H.A.801, 3PAH.A.425, 4PAH.A.425, 2PCC.A.296, 3PF7.A.1002, 3PUQ.A.1, 3PUR.A.1, 3Q14.A.502, 3Q3N.A.509, 1Q5D.A.440, 3QPI.B.1001, 3QY7.A.264, 3RIV.A.305, 3RMK.A.494, 1RYO.A.329, 4S1B.A.802, 1S73.A.296, 1SMI.A.472, 1SP3.A.807, 1STQ.A.600, 3TMC.A.310, 1TQN.A.508, 3TTX.A.760, 4UAX.A.501, 1UED.A.1430, 2V7I.A.1362, 2VZW.B.1209, 2WM5.A.450, 4WNV.A.601, 2WU2.C.1130, 2XC3.A.1433, 1XU5.A.1174, 1XVG.A.528, 1XVX.A.313, 2Y69.A.516, 1Y8W.A.142, 1YMA.A.154, 1YYG.A.396, 1Z1N.X.602, 1Z1N.X.603, 1Z1N.X.605, 3ZG3.A.490

Table S85. 6-ligand Fe, compressed group

	size	largest_angle*	middle_1*	middle_2	middle_3	middle_4	middle_5*	middle_6	middle_7	middle_8	middle_9*	middle_10	middle_11	middle_12	middle_13*	smallest_opposite_angle*	Octahedral	TrigonalPrismatic	PentagonalBipyramidalVA	PentagonalBipyramidalVP
1	"42"	"170.4+/-4.6"	"77.1+/-5.3"	"82+/-3.5"	"84.4+/-2.7"	"87.2+/-2.1"	"89.7+/-2.1"	"91.6+/-1.9"	"93.9+/-2.4"	"96.9+/-2.7"	"99.2+/-3.1"	"103+/-4.9"	"115.9+/-7.1"	"146.3+/-5.2"	"151.8+/-4.1"	"58.4+/-2.4"	"0.014"	"0"	"0"	"0.037"
2	"54"	"172.9+/-3.6"	"81.3+/-3.5"	"84.9+/-1.9"	"87.1+/-1.7"	"88.7+/-1.7"	"90.2+/-1.7"	"92+/-1.7"	"93.4+/-1.4"	"95.1+/-1.6"	"97.4+/-2.2"	"100.8+/-3.4"	"108.5+/-5.2"	"153.2+/-5.8"	"164.6+/-4.6"	"60.6+/-3.5"	"0.025"	"0"	"0"	"0.004"
3	"32"	"170.6+/-4.7"	"63.4+/-3"	"80.7+/-3.7"	"84.9+/-2.4"	"87.5+/-1.9"	"89.3+/-2"	"91.3+/-2"	"93.2+/-2.5"	"95.7+/-2"	"97.9+/-2.7"	"102.9+/-3.6"	"113.1+/-6.4"	"151.9+/-5.6"	"165.3+/-5.1"	"80.9+/-4.6"	"0.013"	"0"	"0"	"0"
4	"21"	"162.4+/-8"	"59.8+/-3.2"	"74.8+/-8.4"	"80.5+/-6"	"85.2+/-4"	"87.8+/-3"	"92.3+/-4"	"95.8+/-5.2"	"99.2+/-4.9"	"104.8+/-4.6"	"110.6+/-6.5"	"119.4+/-8.4"	"140.9+/-7.6"	"155+/-7.9"	"70.2+/-6.8"	"0"	"0"	"0"	"0.001"

Table S86. Cluster members of 6-ligand Fe, compressed group

[1] "Cluster 1"
4QQZ.A.1002, 4AM5.A.1161, 4AQ6.D.837, 1B3E.A.400, 1DLT.A.400, 1DSN.A.400, 3E1M.A
.301, 3E1M.C.300, 3E1N.B.300, 1EYS.M.607, 1FZ6.B.5004, 3GCF.A.501, 4H99.M.402, 4
H9L.M.402, 3IS8.B.162, 1J30.A.401, 1J30.B.404, 2J8C.M.1307, 4JO0.A.601, 1LKO.A.6
00, 4NB8.A.501, 1NF4.A.200, 2OHJ.D.2501, 3PWF.A.201, 3PWF.A.202, 3PWF.B.201, 3PW
F.B.202, 3PZA.A.173, 3PZA.B.173, 4QDF.A.402, 3QVD.H.173, 1R2F.A.401, 1RVJ.M.857,
1RZH.M.857, 1SQI.A.1450, 2UW1.B.1359, 1UZR.A.1293, 3VEO.A.602, 1VRN.M.500, 3VVA
.A.501, 1VZ4.D.1301, 2VZB.A.6204
[1] "Cluster 2"
1A8E.A.339, 3AK9.A.168, 3AK9.C.168, 4AQ2.E.800, 1B0L.A.694, 1C7M.A.101, 2CSG.A.5
04, 2D09.A.430, 1D3K.A.339, 3DU3.M.500, 3DUQ.M.500, 3EMR.A.400, 3FGS.A.402, 1FQE
.A.500, 4G51.A.202, 3GVY.A.162, 2GYQ.B.404, 1H43.A.1315, 1H44.A.1326, 1H76.A.702
, 4HBH.M.402, 3HF8.A.400, 4HR4.A.402, 1IEJ.A.333, 1JQF.A.500, 4KF1.A.402, 4KVR.A
.302, 4M26.D.401, 3MPS.F.173, 3MPS.G.172, 1N7W.A.339, 2OOC.A.500, 1OQG.A.500, 3Q
3N.A.510, 2QED.A.252, 3QHB.B.182, 3R0G.A.200, 3R1J.A.299, 4RC8.A.302, 3RI7.A.1,
3RMK.D.495, 1SQY.A.701, 1TH3.D.2003, 1TKP.A.304, 1TKP.B.303, 1UMX.M.1306, 2UW1.A
.1360, 2V27.A.1268, 3V83.F.703, 3VEZ.A.601, 1VFE.A.400, 1W69.A.1350, 2YAV.A.402,
1YUX.A.302
[1] "Cluster 3"
2B1X.E.502, 1BLF.A.700, 1BLF.A.701, 2BOY.G.1255, 2BUR.B.600, 1CE2.A.690, 1CE2.A.
691, 4H0W.A.701, 1H76.A.703, 1HAB.A.200, 1HAC.B.200, 3HGI.A.281, 3IB0.A.999, 1LC

T.A.400, 1LFG.A.693, 1LFG.A.694, 1LKM.A.601, 4M1I.A.402, 1MTY.D.3, 2O7U.A.500, 1OVT.A.689, 3PCC.O.600, 3PCF.M.600, 3QYT.A.680, 1S9A.A.300, 1TFD.A.950, 3UF9.A.315, 1VFD.A.400, 3VMG.B.501, 3WFD.B.803, 1YUZ.A.301, 3ZK4.A.800

[1] "Cluster 4"

4BMT.A.1324, 3DHI.A.601, 4E2P.A.401, 1FZ6.B.5003, 4GAM.F.602, 3GJB.A.320, 2HMO.A.450, 4KVQ.A.302, 4N71.D.201, 1NNT.A.333, 3OOR.B.803, 4P1B.D.502, 3PCH.M.600, 3QJV.A.801, 2RDB.A.500, 1SP8.B.500, 1SQD.A.500, 3VE1.B.702, 3W54.A.502, 3WFC.B.803, 2ZI8.A.701

Table S87. 6-ligand Fe, combined group

	size	largest_angle*	middle_1*	middle_2	middle_3	middle_4
1	"88"	"173.3+/-3.2"	"80.2+/-3.4"	"84.4+/-2.8"	"86.6+/-2.1"	"88.3+/-2"
2	"669"	"178.7+/-0.8"	"86.9+/-1.5"	"88.2+/-1"	"88.9+/-0.7"	"89.3+/-0.6"
3	"73"	"171.6+/-4.3"	"79.9+/-4.2"	"83.6+/-3"	"85.9+/-2.4"	"88+/-2"
4	"28"	"164.5+/-8.2"	"59.9+/-7.1"	"76+/-7.8"	"81.1+/-5.5"	"85.7+/-4"
5	"204"	"174.8+/-2.4"	"80.1+/-2.7"	"84.3+/-2.2"	"86.5+/-1.6"	"88+/-1.4"
6	"371"	"177.8+/-1.3"	"84.4+/-2.1"	"86.7+/-1.5"	"88.1+/-1.1"	"88.9+/-0.9"
7	"70"	"170.1+/-4.5"	"67.6+/-5.7"	"79.6+/-4.6"	"83.3+/-3.6"	"86.4+/-2.4"

	middle_5*	middle_6	middle_7	middle_8	middle_9*
1	"89.7+/-1.6"	"91.1+/-1.4"	"92.6+/-1.6"	"94.1+/-1.7"	"96+/-1.9"
2	"89.8+/-0.4"	"90.2+/-0.4"	"90.6+/-0.4"	"91+/-0.6"	"91.5+/-0.7"
3	"90.2+/-1.9"	"92.1+/-1.9"	"93.8+/-1.9"	"96+/-2"	"98.2+/-2.5"
4	"88.6+/-3.1"	"92.1+/-3.4"	"96.1+/-4.9"	"99.3+/-4.6"	"104.7+/-5"
5	"89.2+/-1.2"	"90.3+/-1.1"	"91.5+/-1.2"	"92.9+/-1.5"	"94.5+/-1.9"
6	"89.6+/-0.7"	"90.5+/-0.8"	"91.1+/-0.9"	"91.9+/-1.1"	"93.1+/-1.3"
7	"88.4+/-2"	"90.7+/-2.4"	"92.9+/-2.9"	"95.6+/-2.9"	"99+/-3.3"

	middle_10	middle_11	middle_12	middle_13*
1	"99.3+/-3"	"105.2+/-5.8"	"160.7+/-6.3"	"167.9+/-4"
2	"92.4+/-1.1"	"93.8+/-1.6"	"175.9+/-1.9"	"177.8+/-1.2"
3	"101.8+/-4.2"	"111.7+/-7.2"	"149.7+/-6"	"157.3+/-6.4"
4	"109.2+/-6.6"	"119.4+/-8.1"	"140.5+/-7.8"	"153.5+/-9.1"
5	"97.2+/-2.4"	"100.5+/-3.4"	"167.7+/-4"	"171.7+/-2.6"
6	"94.7+/-1.8"	"97.3+/-2.4"	"171.7+/-3.5"	"176.3+/-1.7"
7	"104.1+/-4.1"	"111.8+/-6.2"	"152.5+/-7.1"	"163.6+/-4.9"

	smallest_opposite_angle*	Octahedral	TrigonalPrismatic
1	"72.3+/-5"	"0.005"	"0"
2	"87.4+/-1.3"	"0.322"	"0"
3	"58.7+/-2.6"	"0"	"0"
4	"63.4+/-8.3"	"0"	"0"
5	"84.2+/-3.1"	"0.092"	"0"
6	"83.6+/-2.5"	"0.259"	"0"
7	"80.6+/-4.5"	"0"	"0"

	PentagonalBipyramidalVA	PentagonalBipyramidalVP
1	"0"	"0"
2	"0"	"0"
3	"0"	"0"
4	"0"	"0"
5	"0"	"0"
6	"0"	"0"
7	"0"	"0.001"

Table S88. Cluster members of 6-ligand Fe, combined group

[1] "Cluster 1"
1A2F.A.1, 1A7E.A.119, 1A8E.A.339, 1B0L.A.694, 3B0H.B.601, 4B7G.A.3000, 3BFJ.M.13
88, 1BKA.A.693, 1BKA.A.694, 2BQ8.X.1305, 2BQ8.X.1306, 2BV0.B.600, 1C7M.A.101, 4C
HL.A.501, 2D09.A.430, 1DD7.A.600, 3DHG.A.502, 1DRY.A.332, 4DTW.A.500, 3DXU.A.360
, 3E6S.B.200, 1FCD.C.902, 1FQE.A.500, 1FSL.A.144, 1FZ1.A.5001, 1FZ3.A.5002, 4G51
.A.202, 4G51.B.202, 4GHF.B.401, 3GM6.A.1004, 4GP4.A.602, 4GP5.A.602, 2H0V.A.501,
 1H76.A.702, 2HBU.A.900, 3HB3.A.560, 2HUO.A.301, 1I4Z.A.601, 3IXF.A.139, 3KT7.A.
701, 3LXV.M.600, 4M25.A.401, 3MDT.A.505, 3MOO.A.911, 3MPS.F.173, 3MPS.G.172, 3NC
3.A.406, 2NOX.A.500, 4OJ8.C.301, 1OQG.A.500, 1OQU.C.1009, 1OQU.C.1010, 3OUH.A.60
0, 3OUI.A.1, 1PIU.A.402, 2Q0J.B.998, 3Q1G.A.1002, 3Q3N.A.510, 2QED.A.252, 3QFO.A
.264, 2QPP.A.300, 3QY6.A.263, 3QY8.A.253, 3R0G.A.200, 3R1J.A.299, 4RC8.A.303, 3R
I7.A.494, 3RMK.D.495, 3RNC.A.500, 3RNF.A.500, 1SYY.A.1319, 1SYY.A.1320, 1T0Q.A.5
00, 3TMZ.A.501, 3UBR.A.471, 3V83.F.703, 1VFE.A.400, 2VHD.B.401, 2VUN.A.402, 3WEC
.A.501, 2X9P.A.1398, 1XK3.A.300, 1XU5.A.1175, 1XVF.B.1175, 1XVG.A.529, 1YGF.B.14
7, 3ZLI.A.4001, 3ZPI.A.1407
[1] "Cluster 2"
19HC.A.301, 19HC.A.302, 19HC.A.303, 19HC.A.304, 19HC.A.305, 19HC.A.306, 19HC.A.3
07, 19HC.A.308, 19HC.A.309, 2A3M.A.501, 2A3M.A.502, 2A3M.A.503, 2A3M.A.504, 1A56
.A.82, 3A9F.A.207, 4AAL.A.423, 4AAN.A.401, 4AAO.A.400, 2AI5.A.81, 1AOF.A.602, 1A
OF.B.601, 1AOM.A.602, 1AOM.B.601, 1AOM.B.602, 1AQA.A.95, 1AQE.A.121, 1AQE.A.122,
 3AQ5.A.144, 3AQ9.A.144, 3AT5.A.142, 3AT5.B.147, 2AT3.X.185, 2B4Z.A.500, 3B42.A.
199, 3B42.B.399, 3B47.A.199, 1B80.A.350, 1B82.A.350, 1B85.A.350, 1BFR.B.200, 2BG
V.X.1121, 4BJA.A.300, 2BK9.A.1154, 2BLF.B.1582, 4BMM.A.1450, 3BNG.A.513, 3BNJ.A.
513, 3BNJ.A.514, 3BNJ.A.515, 3BNJ.A.516, 3BNJ.A.517, 2BPN.A.108, 2BPN.A.109, 2BP
N.A.110, 2BPN.A.111, 2BQ4.A.1117, 2BQ4.A.1118, 2BS2.C.1255, 2BS2.C.1256, 2BS3.C.
1255, 2BS4.C.1255, 3BXU.A.72, 3BXU.A.73, 3BXU.A.74, 4C0C.A.1450, 4C0N.A.1157, 2C
1U.A.401, 2C1V.A.401, 2C1V.A.402, 2C1D.A.1292, 3C2C.A.113, 1C2R.A.120, 4C27.A.14
50, 4C44.A.1151, 1C52.A.200, 1C6R.A.90, 1C75.A.93, 3C76.X.185, 3C78.X.185, 2C8S.
A.1173, 3CAO.A.104, 3CAO.A.105, 3CAO.A.106, 3CAO.A.107, 1CCH.A.83, 1CCR.A.112, 2
CDV.A.109, 2CDV.A.111, 4CDP.A.402, 2CE0.A.1102, 1CH1.A.154, 1CH2.A.154, 1CH5.A.1
54, 1CH9.A.154, 1CNO.A.200, 2CN4.A.1175, 1CO6.A.108, 1COR.A.83, 3CP5.A.202, 3CQV
.A.601, 2CTH.A.109, 2CTH.A.110, 2CTH.A.111, 2CTH.A.112, 3CU4.A.199, 2CVC.A.1002,
 2CVC.A.1003, 2CVC.A.1004, 2CVC.A.1005, 2CVC.A.1006, 2CVC.A.1008, 2CVC.A.1009, 2
CVC.A.1010, 2CVC.A.1014, 2CVC.A.1016, 3CX5.C.4001, 3CX5.C.4002, 3CX5.D.4003, 3CX
5.W.4026, 1CXA.A.126, 1CXC.A.125, 3CXH.W.4026, 1CXY.A.204, 1CYI.A.200, 3CYR.A.20
3, 3CYR.A.204, 2CY3.A.119, 2CY3.A.120, 2CY3.A.121, 2CY3.A.122, 5CYT.R.105, 2CZS.
A.501, 1CZJ.A.120, 1CZJ.A.122, 3CZY.A.300, 2D0S.A.80, 2D0T.A.404, 4D30.B.750, 3D
7O.A.143, 2DC3.A.191, 3DE8.A.150, 3DHR.A.142, 1DK0.A.200, 2DN1.A.142, 2DN1.B.147
, 1DTI.A.154, 4DTZ.A.500, 1DUW.A.293, 1DUW.A.297, 1DUW.A.300, 1DW0.A.113, 1DW2.A
.113, 1DWL.B.80, 1DY7.B.601, 2DY5.A.300, 1E29.A.136, 1E2R.B.601, 1E2W.A.900, 1E3
9.A.804, 2E3A.A.401, 1E8E.A.125, 2E80.A.1508, 2E84.A.1305, 2E84.A.1310, 2E84.A.1
314, 1EB7.A.401, 3EGW.C.806, 3EGW.C.807, 1EHJ.A.1030, 1EHJ.A.1053, 1EHJ.A.1066,
4EID.A.101, 4EIE.A.101, 4EIF.A.101, 2EKU.A.154, 1ETP.A.199, 1ETP.A.200, 2EU7.X.2
01, 2EWK.A.1001, 2EWK.A.1003, 2EWU.A.1001, 2EWU.A.1003, 1EWH.A.253, 2EWI.A.1004,
 2EWI.A.1002, 2EWI.A.1001, 2EWI.A.1003, 1EZV.C.401, 1EZV.C.402, 1F03.A.201, 1F1C
.A.200, 1F1F.A.200, 1F24.A.501, 4F6I.A.201, 4FA7.A.602, 4FAS.A.601, 4FAS.A.603,
4FAS.A.604, 4FAS.A.605, 4FAS.A.606, 4FAS.A.607, 1FCD.C.901, 4FEF.A.403, 2FFN.A.1
003, 1FGJ.A.548, 1FGJ.A.552, 1FI3.A.83, 1FI7.A.110, 1FJ0.A.115, 3FLL.A.185, 2FMY

.A.300, 3F03.A.1004, 3F03.A.1005, 3F03.A.1006, 3F03.A.1007, 3F03.A.1008, 3F03.A.1002, 3F03.A.1003, 3F03.A.1001, 2FRF.A.154, 1FS7.A.509, 1FS8.A.508, 1FT5.A.213, 1FT5.A.215, 1FT5.A.216, 1FT9.A.300, 1FT9.B.300, 2FWT.A.803, 2FWT.A.805, 2FWL.A.132, 2FYU.D.242, 4G1V.A.401, 3G5N.A.500, 4G7L.A.301, 3GAS.D.1294, 2GC4.D.200, 1GDV.A.101, 1GKS.A.0, 2GNV.A.166, 1GQ1.A.601, 1GQ1.A.602, 2GSM.A.2001, 2GTF.X.201, 3GW9.A.480, 1GYO.A.111, 1GYO.A.112, 1GYO.A.113, 1GYO.A.114, 4GYD.A.200, 4H0K.B.200, 1H1O.A.1184, 1H1O.A.1185, 1H21.A.1248, 1H21.B.1248, 1H29.A.1104, 1H32.A.1263, 1H32.A.1264, 1H32.B.1139, 3H33.A.75, 3H33.A.76, 3H33.A.77, 3H34.A.72, 3H34.A.73, 3H34.A.74, 3H4N.A.72, 3H4N.A.73, 3H4N.A.74, 1H55.A.350, 1H57.A.350, 3H8T.A.301, 1HBI.A.153, 4HB6.A.72, 4HB6.A.73, 4HB8.A.72, 4HB8.A.73, 4HBF.A.72, 1HCZ.A.253, 3HDL.A.305, 4HHR.A.701, 1HJ3.B.602, 1HJ5.A.601, 1HJ5.B.602, 2HJI.A.180, 3HNJ.A.150, 4HPA.A.201, 4HPB.A.201, 4HPD.A.201, 3HQ9.A.400, 3HQ9.A.401, 1HRC.A.105, 3HYU.B.147, 2I5N.C.404, 1I5U.A.201, 1I77.A.108, 1I77.A.109, 1I77.A.110, 1I77.A.111, 4I7Z.A.302, 3I8R.A.901, 1I8O.A.115, 1I8P.A.115, 2I8F.A.83, 3I9T.A.300, 3I9U.A.300, 1IDR.A.144, 1IKE.A.185, 1IQC.A.402, 1IT1.A.201, 1IT1.A.202, 1IT1.A.203, 1IT1.A.204, 2IUF.A.1691, 1IVJ.A.300, 1IYN.A.296, 1J02.A.300, 1J0P.A.1002, 1J0Q.A.201, 2J1M.A.1456, 4J20.A.107, 1J3S.A.105, 2J5M.A.1300, 2J7A.A.1004, 2J7A.C.1003, 1JDL.A.500, 4JE9.A.201, 4JEB.A.201, 4JJ0.A.501, 4JJ0.A.502, 2JJP.A.412, 1JMX.A.1001, 1JMX.A.1002, 1JNI.A.125, 1KB0.A.802, 1KBI.A.760, 2KMY.A.251, 4KMG.A.101, 1KOK.A.296, 2KSU.A.282, 2KSU.A.305, 1KV9.A.901, 4KVK.A.701, 4KVL.A.701, 1L2K.A.154, 3L61.A.420, 2LDO.A.154, 1LM3.B.200, 4LM8.A.801, 4LM8.A.802, 4LM8.A.803, 4LM8.A.804, 4LM8.A.805, 4LM8.A.807, 4LM8.A.808, 4LM8.A.809, 4LM8.A.810, 4LMH.A.801, 4LMH.A.803, 4LMH.A.804, 4LMH.A.805, 4LMH.A.806, 4LMH.A.808, 4LMH.A.809, 4LMH.A.810, 1LQX.A.201, 1LR6.A.201, 1LS9.A.92, 1M1P.A.802, 1M1Q.A.804, 1M1Q.A.803, 1M1R.A.801, 1M2I.A.201, 3M4C.A.150, 1M59.A.201, 1M70.A.199, 1M70.A.200, 4M72.A.403, 3MDM.A.505, 1MJ4.A.502, 3ML1.B.1128, 3ML1.B.1129, 1ML7.A.185, 1MNI.B.154, 4MPM.A.201, 3MUS.A.201, 3MVC.A.500, 3MVF.A.185, 1MWB.A.125, 1MZ4.A.151, 1N45.A.300, 4N4J.A.609, 4N4J.A.610, 4N4J.A.611, 4N4J.A.612, 4N4J.A.613, 4N4J.A.614, 4N4J.A.615, 4N4J.A.616, 4N4L.A.616, 4N4N.A.601, 4N4N.A.603, 4N4N.A.604, 4N4N.A.605, 4N4N.A.606, 4N4N.A.607, 4N8T.B.201, 1NAZ.A.200, 1NIR.A.601, 1NIR.A.602, 3NMI.A.150, 1NML.A.401, 3NN1.A.239, 1NNO.A.602, 1NOS.A.901, 1NS6.A.142, 1NS9.B.147, 3NWV.A.105, 3NXU.A.508, 4O4Z.A.201, 3O5C.A.401, 4O6Q.A.202, 4O6U.A.203, 4O7G.A.302, 3O89.A.2154, 1OFW.A.1294, 1OFW.A.1295, 1OFW.A.1296, 1OFW.A.1297, 1OFW.A.1298, 1OFW.A.1299, 1OFW.A.1300, 1OFW.A.1301, 1OFW.A.1302, 3OMI.A.607, 3OO4.B.147, 2ORT.A.600, 2ORO.A.600, 2ORP.A.600, 2ORQ.A.600, 2ORS.A.600, 2OT4.A.1004, 2OT4.A.1007, 3OUE.A.609, 3OUE.A.610, 3OUE.A.611, 3OUE.A.612, 3OUQ.A.602, 3OUQ.A.603, 2OZY.A.202, 2OZY.A.203, 2OZY.A.204, 2OZY.A.205, 3OZU.A.404, 1P2E.A.801, 1PBY.A.991, 3PC3.A.701, 2PEG.A.200, 2PEG.B.400, 3PH2.B.1087, 3PI2.B.500, 1PL3.A.401, 1PM1.X.180, 1PP9.C.502, 1PP9.D.501, 3PXW.A.500, 1Q16.C.806, 1Q16.C.807, 2Q8Q.A.300, 3Q99.B.750, 1QDB.A.516, 1QDB.A.517, 1QDB.A.518, 1QDB.A.519, 1QHU.A.500, 2QJY.A.502, 2QJY.B.301, 1QKS.A.601, 1QKS.A.602, 1QNO.A.113, 1QNO.A.114, 1QNO.A.115, 1QNO.A.116, 1QN2.B.101, 1QPA.A.350, 3QQQ.A.163, 3QWO.A.150, 1QYZ.A.200, 2R80.A.150, 2R80.B.150, 2RAO.B.147, 2RAO.C.142, 2RDZ.A.2, 2RDZ.A.3, 2RDZ.A.4, 3RJ6.A.154, 4RKM.A.808, 4RKM.D.807, 4RKN.A.902, 4RKN.A.905, 4RKN.A.906, 4RKN.A.907, 4RKN.A.908, 4RKN.A.909, 1RSE.A.154, 3RUK.B.600, 1RWJ.A.90, 1RWJ.A.91, 1RWJ.A.92, 3SEL.X.73, 3SEL.X.74, 1SH4.A.201, 3SJ0.X.73, 3SJ0.X.74, 3SJ1.X.73, 3SJ1.X.74, 3SJ4.X.73, 3SJ4.X.74, 3SJ5.A.500, 3SJL.A.500, 3SJL.A.600, 3SLE.A.402, 1SOX.A.502, 1SP3.A.801, 1SP3.A.803, 1SP3.A.804, 1SP3.A.805, 1SP3.A.806, 1SUO.A.500, 3SXQ.A.1005, 3SXQ.A.1006, 3SXQ.A.1007, 3SXQ.A.1008, 3SXQ.A.1002, 3SXQ.A.1003, 3SXQ.A.1001, 1SY2.A.185, 3T6D.C.403, 3T6D.C.404, 3T6E.C.401, 3T6E.C.402, 3T6E.C.403, 3TGU.C.501, 3TGU.C.502, 3TGU.D.501, 3TGM.A.300, 1TKW.B.253, 3TOL.A.150, 1TU2.B.255, 1U4H.A.500, 1U7R.A.154, 3U8P.A.347, 1U9M.A.90, 1U9U.A.90, 3U99.A.500, 3U99.A.700, 3UBR.A.475, 3UBC.A.201, 3UCP.A.901, 3UCP.A.903, 3UCP.A.904, 3UCP.A.906, 3UCP.A.907, 3UCP.A.908, 3UCP.A.909, 3UCP.A.910, 3UCP.A.911, 1UP9.A.201, 1UP9.A.202, 1UP9.A.203, 1UP9.A.204, 2UUQ.

A.1405, 4UVR.A.1450, 2V07.A.1102, 4V2K.A.601, 4V3V.A.750, 4V3W.A.750, 4V3X.A.750
, 1V54.A.515, 2V7K.A.1360, 1V8X.A.901, 1V9Y.A.1140, 3VAU.A.201, 1VGI.A.300, 3VKP
.A.601, 3VKS.A.601, 3VM9.A.154, 3VP5.A.201, 3VRD.A.201, 3VRD.A.202, 3VRG.A.201,
3VXJ.A.501, 1VYD.A.1117, 2VYW.A.149, 2W0B.A.470, 1W2L.A.1100, 2W31.A.200, 2W3G.A
.500, 1W7O.A.1119, 1W7O.A.1120, 1W7O.A.1121, 1W7O.A.1122, 1WAD.A.117, 1WAD.A.113
, 1WAD.A.114, 1WAD.A.115, 3WAH.A.201, 3WC8.A.201, 3WCT.A.200, 3WCT.B.201, 1WE1.A
.300, 3WFD.C.201, 2WJM.C.1334, 2WJN.C.1336, 1WOV.A.300, 1WOX.A.300, 4WQ8.A.1002,
4WQ9.A.1001, 4WQ9.A.1002, 4WQC.A.1002, 4WQD.A.1002, 2WTG.A.180, 3WU2.F.101, 1WV
E.C.699, 1WVP.A.154, 3X15.A.200, 3X15.J.200, 1X3X.B.202, 1X46.A.151, 4XDI.A.201,
2XKI.A.1110, 2XSJ.B.503, 2XTS.B.500, 2XYK.A.700, 1Y0P.A.801, 1Y0P.A.802, 1Y0P.A
.803, 1Y0P.A.804, 1Y5I.C.806, 1Y5I.C.807, 1Y5L.C.806, 2Y5N.A.450, 2YIU.A.500, 1Y
IQ.A.901, 2YL7.A.128, 1YWD.A.185, 2YXC.A.1001, 2YYW.A.1001, 2YYW.A.1003, 2YYX.A.
1004, 2YYX.A.1001, 1Z1N.X.616, 2Z47.A.1004, 2Z47.B.3003, 2Z6S.A.201, 2Z6T.A.201,
1Z8O.A.410, 1Z9N.A.1001, 1Z9N.C.2001, 2ZBO.A.111, 3ZHO.A.200, 3ZHW.A.1163, 3ZIY
.A.600, 3ZJQ.A.200, 3ZOO.A.105, 2ZS0.A.200, 2ZS0.B.200, 2ZS0.C.200, 2ZS0.D.200,
2ZXY.A.200, 2ZZS.1.220
[1] "Cluster 3"
4QQZ.A.1002, 3AK9.A.168, 3AK9.C.168, 4AM5.A.1161, 4AQ2.E.800, 4AQ6.D.837, 1B3E.A
.400, 2CSG.A.504, 1D3K.A.339, 1DLT.A.400, 1DSN.A.400, 3DU3.M.500, 3DUQ.M.500, 3E
1M.A.301, 3E1M.C.300, 3E1N.B.300, 3EMR.A.400, 1EYS.M.607, 3FGS.A.402, 3GCF.A.501
, 3GVY.A.162, 2GYQ.B.404, 1H43.A.1315, 1H44.A.1326, 4H99.M.402, 4H9L.M.402, 4HBH
.M.402, 3HF8.A.400, 4HR4.A.402, 1IEJ.A.333, 1J30.A.401, 1J30.B.404, 2J8C.M.1307,
4JOO.A.601, 1JQF.A.500, 4KF1.A.402, 4KVR.A.302, 1LKO.A.600, 4M26.D.401, 1N7W.A.
339, 1NF4.A.200, 2OOC.A.500, 2OHJ.D.2501, 3PWF.A.202, 3PWF.B.202, 3PZA.A.173, 3P
ZA.B.173, 3QHB.B.182, 3QVD.H.173, 1R2F.A.401, 4RC8.A.302, 3RI7.A.1, 1RVJ.M.857,
1RZH.M.857, 1SQI.A.1450, 1SQY.A.701, 1TH3.D.2003, 1TKP.A.304, 1TKP.B.303, 1UMX.M
.1306, 2UW1.A.1360, 2UW1.B.1359, 1UZR.A.1293, 2V27.A.1268, 3VEO.A.602, 3VEZ.A.60
1, 1VRN.M.500, 3VVA.A.501, 1VZ4.D.1301, 2VZB.A.6204, 1W69.A.1350, 2YAV.A.402, 1Y
UX.A.302
[1] "Cluster 4"
4BMT.A.1324, 8CAT.A.507, 3DHI.A.601, 1DMH.A.400, 4E2P.A.401, 1FZ6.B.5003, 1FZ6.B
.5004, 4GAM.F.602, 3GJB.A.320, 3IS8.B.162, 4KVQ.A.302, 3N9T.A.292, 4NB8.A.501, 1
NNT.A.333, 3OOR.B.803, 4P1B.D.502, 3PCH.M.600, 3PWF.A.201, 3PWF.B.201, 3Q1G.D.10
02, 4QDF.A.402, 3QJV.A.801, 1SP8.B.500, 1SQD.A.500, 3VVA.B.502, 3W54.A.502, 3WFC
.B.803, 2ZI8.A.701
[1] "Cluster 5"
3A0G.A.201, 2A1X.A.450, 3A16.B.354, 2A1O.A.417, 1A2S.A.90, 3A51.B.412, 3ABM.A.51
6, 4AUM.A.900, 2AVK.A.201, 3AYF.A.801, 2B11.D.1301, 2B2O.B.1500, 4B2N.A.701, 4B8
N.A.201, 2BC5.A.150, 3BDZ.A.450, 1BEP.A.296, 4BGL.A.1001, 1BIN.B.144, 1BJ9.A.296
, 2BMM.A.1157, 3BOM.A.143, 3BOM.B.148, 2BUZ.B.1541, 2C1D.A.1291, 1C2N.A.117, 1CC
5.A.1, 1CH3.A.154, 2CJ2.A.1300, 4CKA.A.1480, 1CLS.A.142, 4CVJ.A.1295, 2CYP.A.295
, 2CZ1.A.300, 4D02.A.602, 4D02.A.603, 2D2M.D.200, 1DCC.A.296, 1DLW.A.144, 1DM1.A
.148, 4DTY.A.500, 3E2O.A.296, 3E5K.A.1408, 3EH5.A.800, 3EHB.A.560, 1EHE.A.501, 4
ENH.A.601, 4ENU.B.801, 1EO9.B.600, 1EOC.B.600, 1F4U.A.410, 4F4O.B.201, 4FIA.A.60
0, 1FZ7.B.5003, 1G09.B.147, 4G71.A.602, 4G7G.A.501, 4G7Q.A.602, 2GEP.A.580, 3GE3
.A.501, 7GEP.A.580, 2GJ1.A.605, 4GP8.A.602, 2GSM.A.2002, 1GWS.A.609, 1GWS.A.616,
1GWT.A.350, 2H0V.B.500, 1H29.A.1108, 1H29.A.1114, 4H44.A.301, 3HB3.A.559, 1HBZ.
A.504, 2HBD.A.142, 1HMO.B.115, 3HQ7.A.401, 1HRO.A.107, 4HRO.A.402, 3I63.A.501, 4
I7Z.A.301, 4I7Z.C.301, 2IJ4.A.471, 2INC.A.502, 4IPS.A.401, 2IVF.C.1217, 1IZO.A.5
01, 4J14.A.601, 2JJN.A.412, 2JTI.B.104, 4K0F.A.601, 3K1O.A.488, 2K3V.A.238, 2K3V
.A.261, 3K3O.A.1, 4K8F.A.300, 4KIG.A.502, 3L1T.A.479, 1LA6.B.147, 2LDO.A.168, 4L
MH.A.802, 4LXJ.A.601, 2LZZ.A.101, 4M4A.B.201, 1M7S.A.600, 4M71.A.403, 4M73.B.403
, 4MBA.A.148, 3MDR.A.505, 4ME4.A.401, 3MGX.A.397, 3MKB.B.137, 4MLN.B.202, 1MRP.A
.310, 1MTY.D.4, 3N2O.A.506, 3N3N.B.1500, 4N4K.A.610, 4N6W.A.202, 3N9Q.A.1, 2NNB.

A.472, 2NSR.A.154, 1NS9.A.142, 3NYH.A.605, 4NZ2.A.501, 3O0R.B.802, 4O6J.A.302, 3O72.A.500, 1OAF.A.1251, 1OAH.A.1520, 1OAH.A.1523, 1OCZ.A.516, 3OJT.D.500, 1OQU.C.1008, 3OZW.A.404, 1P2H.A.801, 1PA2.A.306, 3PAH.A.425, 4PAH.A.425, 5PAH.A.425, 3PCJ.O.600, 2PCC.A.296, 3PER.A.1002, 3PF7.A.1002, 4PG0.A.301, 1PHG.A.417, 3PUQ.A.1, 3PUR.A.1, 3Q14.A.502, 3Q3N.A.509, 1Q5D.A.440, 2QDY.A.300, 1QO8.A.601, 1QO8.A.604, 3QPI.B.1001, 3QU8.A.500, 2QUO.A.142, 3QY7.A.264, 2R6S.A.501, 3RIV.A.305, 3RMK.A.494, 3RMZ.A.500, 3RNF.A.501, 1RYO.A.329, 4S1B.A.802, 1S73.A.296, 3S8F.A.801, 1SMI.A.472, 1SP3.A.807, 1SQ3.D.912, 1STQ.A.600, 3TMC.A.310, 1TQN.A.508, 3TTX.A.760, 1U74.B.1101, 4UAX.A.501, 3UBR.A.473, 1UED.A.1430, 1ULI.B.700, 3V5X.A.201, 2V7I.A.1362, 2VEB.A.200, 2VZW.B.1209, 1W4W.A.1307, 2WM5.A.450, 4WNV.A.601, 2WU2.C.1130, 4WWJ.B.301, 2XC3.A.1433, 1XU5.A.1174, 1XVB.A.1170, 1XVB.A.1171, 1XVG.A.528, 1XVX.A.313, 2Y69.A.516, 1Y8W.A.142, 2YIU.B.500, 1YMA.A.154, 1YYG.A.396, 1Z1N.X.602, 1Z1N.X.603, 1Z1N.X.605, 1Z1N.X.606, 1Z1N.X.607, 3ZG3.A.490

[1] "Cluster 6"

3A15.B.354, 3A17.A.354, 2A3F.X.201, 1A4E.A.503, 3A8G.A.301, 3A8L.A.300, 2A9E.A.550, 4AAN.A.400, 2AIU.A.200, 1AKK.A.105, 4AM5.A.1160, 4APY.A.1418, 1ASH.A.301, 3AWM.A.501, 1AWP.A.201, 2B0Z.B.109, 2B10.D.909, 2B2R.A.1500, 4B2N.A.700, 4B4Y.A.1155, 3B6H.B.600, 3B99.A.600, 2BDM.A.500, 2BH4.X.1123, 1BJE.A.154, 4BJK.A.1450, 3BK9.B.401, 2BQ4.A.1115, 2BQ4.A.1116, 1BVY.A.1000, 2C1D.B.1158, 4C28.A.1450, 1C40.A.150, 3C62.A.150, 3C63.A.150, 4C9M.A.1418, 1CED.A.90, 1CH7.A.154, 1CI3.M.254, 4CK8.A.1480, 4CK9.A.1480, 2CN4.B.1175, 1COT.A.130, 4COH.A.1450, 4COO.A.1549, 1CPT.A.430, 3CSL.A.866, 2CVC.A.1001, 2CVC.A.1007, 2CVC.A.1011, 2CVC.A.1012, 2CVC.A.1013, 2CZS.A.500, 1CZJ.A.119, 1CZJ.A.121, 2D0W.A.200, 4D34.A.500, 4D35.A.500, 4D36.A.500, 4D37.A.500, 4D38.A.500, 4D3A.A.500, 1D4D.A.601, 1D4D.A.602, 1D4D.A.603, 1D4D.A.604, 1D7B.A.401, 2DGE.A.200, 3DHH.A.501, 3DHI.A.600, 1DJ5.A.1, 3DMI.A.146, 1DP8.A.719, 1DP9.A.719, 3DR0.A.94, 1DUW.A.301, 4DXY.A.501, 4DY9.A.201, 1E08.E.80, 1E39.A.801, 1E39.A.802, 1E39.A.803, 2E3B.A.401, 3E5J.A.1408, 2E84.A.1301, 2E84.A.1302, 2E84.A.1303, 2E84.A.1304, 2E84.A.1306, 2E84.A.1307, 2E84.A.1308, 2E84.A.1309, 2E84.A.1311, 2E84.A.1312, 2E84.A.1313, 2E84.A.1316, 1EGY.A.410, 3EHB.A.559, 3EJ6.A.4000, 4EJI.A.501, 2EKT.A.154, 4EP6.A.601, 1EUE.A.201, 2EXV.A.83, 4FA7.A.603, 2FAM.A.148, 4FAS.A.602, 2FBZ.X.901, 4FDH.A.601, 1FGJ.A.553, 1FOP.A.500, 3FOO.A.150, 1FS7.A.510, 4G3J.A.501, 4G70.A.601, 4G7S.A.602, 3GEO.A.580, 4GED.B.201, 3GQP.C.143, 4GQS.A.501, 1GWF.A.504, 1GWS.A.601, 1GWS.A.603, 1GWS.A.605, 1GWS.A.606, 1GWS.A.610, 1GWS.A.611, 1GWS.A.612, 1GWS.A.613, 1H21.A.1249, 1H21.B.1249, 1H29.A.1102, 1H29.A.1107, 4H2L.B.201, 4H44.A.302, 4H6O.A.501, 2H88.C.142, 4HC3.A.72, 3HF4.A.142, 3HF4.B.147, 1HGB.D.147, 4HHS.A.701, 1HLB.A.158, 3HNK.A.150, 3HQ6.A.400, 3HSP.A.750, 4HSW.A.201, 2HUO.A.302, 3HYU.A.142, 1I3E.A.147, 3I63.A.502, 2IBN.A.703, 1IB7.A.95, 1ICC.A.201, 2IJ3.B.999, 1IO7.A.1401, 1IQC.A.401, 3IQ5.A.150, 2IUW.A.500, 1IWO.A.901, 1J77.A.300, 2J7A.A.1002, 2J7A.A.1003, 2J7A.A.1005, 2J7A.C.1002, 2J7A.C.1004, 1JDR.A.296, 1JEX.A.95, 1JIP.A.410, 1JNI.A.126, 2K3V.A.218, 2K3V.A.278, 4K8F.B.300, 4KF2.B.501, 4KIB.A.403, 4KIC.A.403, 4KJT.A.201, 2KMY.A.233, 1KQG.C.809, 1KQG.C.810, 2KSC.A.125, 4L0F.A.501, 3L1M.A.150, 3L4D.A.481, 3LD6.A.601, 2LDO.A.130, 1LFK.A.430, 3LGN.A.200, 4LJI.A.301, 1LMS.A.118, 4LM8.A.806, 4LMH.A.807, 1LSX.A.719, 2LZZ.A.102, 3M15.A.150, 4M2F.B.401, 3M3A.A.155, 4M4A.A.201, 4M73.A.403, 5MBA.A.148, 1MDV.A.110, 1MDV.A.112, 1MLW.A.403, 4MLM.A.201, 4MLN.A.201, 4MLN.B.201, 3MM9.A.580, 1MNY.A.95, 3MOM.A.186, 1MQF.A.501, 4MQJ.B.201, 1MXR.A.1004, 4N4N.A.602, 4N4N.A.608, 4N4O.A.608, 4N4Y.A.602, 3N8Y.A.601, 1N9C.A.93, 3NA1.A.601, 4NFG.B.201, 4NK1.B.201, 1NML.A.402, 1NPF.A.154, 4NP1.A.185, 2NRL.A.148, 3O1A.A.385, 4O1W.A.101, 3O5C.D.504, 4O7G.A.301, 1OAH.A.1521, 1OAH.A.1522, 1OAH.A.1524, 3OA8.A.401, 3OA8.B.401, 3OFT.A.417, 3OMA.A.1, 3OO4.A.142, 3ORV.B.600, 2ORR.A.600, 3OUQ.A.601, 3OUQ.A.604, 3OUQ.A.605, 3OUQ.A.606, 2OZY.A.201, 3OZV.A.404, 1P2E.A.803, 1P2E.A.804, 1P2H.A.803, 1P2H.A.804, 1P2Y.A.430, 3P3L.A.501, 2PAC.A.83, 1PBY.A.992, 3PF7.A.1001, 4PH9.A.602, 4PK5.A.501, 1PP9.C.501, 2PQ7.A.220, 3PT7.B.500, 4PXH.A.501, 2QJY.A.501, 3QNS.A.351, 1QO8.A.602, 1QO8.A.

603, 1QPU.A.107, 1QQ3.A.107, 3QQR.A.163, 2QSP.A.142, 1R0Q.A.200, 4R20.A.601, 2R5
0.A.166, 3R9C.A.450, 2RDZ.A.5, 2RF7.A.2, 3RGS.A.1, 4RKM.B.808, 4RKN.A.903, 1RZ5.
A.401, 3S1J.A.140, 3S8F.A.800, 3S8G.A.800, 3SDN.A.160, 1SE6.A.430, 1SP3.A.808, 3
SWZ.B.600, 3T3R.A.500, 3T3Z.A.500, 1T68.X.201, 3T6D.C.401, 3TDA.A.800, 3TF0.A.50
0, 3TGA.A.185, 1TH2.D.2003, 3TIK.A.482, 3TJS.A.508, 3TK3.A.500, 3TMC.A.309, 3TOR
.A.3, 4TOB.C.201, 4TT5.A.401, 4TUV.A.401, 1U13.A.460, 4U9D.A.201, 3UBR.A.472, 3U
BR.A.474, 3UCP.A.902, 3UCP.A.905, 3UOI.B.200, 4UQH.A.1450, 1URV.A.1172, 1V0H.X.2
51, 2V08.A.1087, 2V0M.A.1499, 3V2V.A.154, 4V3Z.B.750, 1V75.B.201, 2V7L.A.1360, 3
V8D.A.601, 1VB6.A.1140, 1VB6.B.1140, 3VHB.A.150, 4VHB.A.150, 2VHB.B.150, 2VHD.A.
402, 3VR8.C.201, 3VRG.B.201, 3VTH.A.807, 2VXH.A.1001, 1W0G.A.1501, 3W9C.A.501, 3
WCT.C.200, 3WCT.D.201, 2WDQ.C.1129, 3WFC.B.802, 3WFD.B.801, 3WFE.B.802, 3WFX.A.2
01, 2WJN.C.1333, 2WJN.C.1335, 1WMU.A.201, 4WPD.A.402, 2WU5.C.305, 2WX2.A.1450, 2
X2N.A.1479, 4X8B.A.508, 2XFH.A.1412, 1XK1.A.300, 2XKR.A.1400, 1XQ5.B.148, 1XQ5.C
.143, 2YEV.A.1015, 2YEV.B.587, 2YIU.A.501, 2YK3.A.200, 1Z1N.X.604, 1Z1N.X.608, 1
Z1N.X.610, 1Z1N.X.612, 1Z1N.X.613, 1Z1N.X.614, 1Z8U.B.201, 3ZBY.A.1402, 2ZCF.A.2
06, 3ZE6.B.502, 3ZG2.A.1480, 3ZJO.A.200, 1ZOY.C.1305, 3ZOX.A.1082, 2ZPB.A.300, 1
ZZH.A.802, 1ZZH.A.803
[1] "Cluster 7"
1B1X.A.690, 2B1X.E.502, 4B2O.A.1267, 1BLF.A.700, 1BLF.A.701, 2BOY.G.1255, 2BUR.B
.600, 2CAG.A.485, 1CE2.A.690, 1CE2.A.691, 1D9Y.A.310, 2FR7.A.501, 2G1M.A.600, 1G
GF.B.760, 4H0W.A.701, 1H76.A.703, 1HAB.A.200, 1HAC.B.200, 3HGI.A.281, 2HMO.A.450
, 4I4G.A.601, 4I4H.A.601, 3IB0.A.999, 1JNF.A.702, 1JNF.A.703, 4K9T.A.601, 4K9U.A
.601, 4K9V.A.601, 4K9W.A.601, 4K9X.A.601, 1KW9.B.301, 1LCT.A.400, 1LFG.A.693, 1L
FG.A.694, 1LKM.A.601, 4M1I.A.402, 4ME4.A.402, 1MMO.D.3, 1MMO.D.4, 1MTY.D.3, 3MZS
.C.500, 4N71.D.201, 1NX4.B.300, 2O7U.A.500, 1OVT.A.689, 3P3N.A.350, 3PCC.O.600,
3PCF.M.600, 3PER.A.1001, 3Q3M.A.509, 3QFN.A.265, 3QY7.A.263, 3QYT.A.680, 1R1N.C.
400, 2RDB.A.499, 2RDB.A.500, 3RNC.A.499, 1S9A.A.300, 1T0Q.A.499, 1TFD.A.950, 3UF
9.A.315, 3VE1.B.702, 1VFD.A.400, 3VMG.B.501, 3WFD.B.803, 1WRA.B.402, 1XVF.B.1174
, 1YUZ.A.301, 3ZK3.A.1311, 3ZK4.A.800

Table S89. all-ligand-number Fe, normal group

	size	largest_angle*	middle_1*	middle_2*	middle_3*
1	"105"	"129.7+/-8.5"	"92.3+/-7.5"	"99.1+/-6.2"	"106.9+/-4.8"
2	"157"	"170.7+/-4.6"	"76.8+/-4.2"	"88.4+/-2.4"	"96.7+/-3.2"
3	"75"	"169.2+/-7.5"	"81.8+/-4.5"	"88.3+/-2.6"	"94.7+/-3.6"
4	"78"	"167.3+/-7.4"	"80.7+/-5.3"	"87.7+/-4.6"	"93.6+/-3.7"
5	"1035"	"178.4+/-1.2"	"86.7+/-1.7"	"89.7+/-0.5"	"91.7+/-0.9"
6	"455"	"176.3+/-2.1"	"82.2+/-2.9"	"89.4+/-1.2"	"93.7+/-1.6"
7	"161"	"167.1+/-4.6"	"84.8+/-4.2"	"89.5+/-1.5"	"94.4+/-2.7"

	middle_4*	smallest_opposite_angle*	Tetrahedral	TrigonalBipyramidal
1	"115.6+/-4.9"	"103.6+/-10.7"	"0.008"	"0"
2	"164.3+/-5.1"	"78.9+/-5.3"	"0"	"0"
3	"134.8+/-7.8"	"105.3+/-6.9"	"0"	"0.01"
4	"99.5+/-6.5"	"108.3+/-27"	"0"	"0"
5	"177.4+/-1.6"	"87.2+/-2.1"	"0"	"0"
6	"173.7+/-2.8"	"83.4+/-3.6"	"0"	"0"
7	"162.7+/-5.8"	"93.9+/-3.5"	"0"	"0"

	TrigonalBipyramidalVA	TrigonalBipyramidalVP	Octahedral	SquarePyramidal
1	"0"	"0"	"0"	"0"
2	"0"	"0.02"	"0.003"	"0.03"

3	"0.013"	"0.036"	"0"	"0"
4	"0"	"0.006"	"0"	"0"
5	"0"	"0.002"	"0.253"	"0.357"
6	"0"	"0.014"	"0.149"	"0.278"
7	"0"	"0.003"	"0"	"0.02"

	SquarePyramidalV	SquarePlanar	TrigonalPrismatic	TrigonalPrismaticV
1	"0"	"0"	"0"	"0"
2	"0.097"	"0.057"	"0"	"0"
3	"0.012"	"0"	"0"	"0"
4	"0.012"	"0"	"0"	"0"
5	"0.37"	"0.375"	"0"	"0"
6	"0.323"	"0.323"	"0"	"0"
7	"0.051"	"0.055"	"0"	"0"

	PentagonalBipyramidal	PentagonalBipyramidalVA	PentagonalBipyramidalVP
1	"0"	"0"	"0"
2	"0"	"0"	"0"
3	"0"	"0"	"0"
4	"0"	"0"	"0"
5	"0"	"0"	"0"
6	"0"	"0"	"0"
7	"0"	"0"	"0"

	SquareAntiprismatic	SquareAntiprismaticV	HexagonalBipyramidal
1	"0"	"0"	"0"
2	"0"	"0"	"0"
3	"0"	"0"	"0"
4	"0"	"0"	"0"
5	"0"	"0"	"0"
6	"0"	"0"	"0"
7	"0"	"0"	"0"

	HexagonalBipyramidalVA	HexagonalBipyramidalVP
1	"0"	"0"
2	"0"	"0"
3	"0"	"0"
4	"0"	"0"
5	"0"	"0"
6	"0"	"0"
7	"0"	"0"

Table S90. Cluster members of all-ligand-number Fe, normal group

[1] "Cluster 1"

4AC8.B.500, 4AM5.A.1162, 1AOR.A.606, 1B2O.B.55, 2B5H.A.501, 1B71.A.192, 1BE7.A.5
5, 1BFY.A.55, 1BIQ.A.377, 2BKB.C.1193, 2BOY.E.1255, 1BOU.B.501, 4C4U.B.201, 4C4U
.I.201, 3CF4.A.808, 1CJX.B.629, 2CKF.C.501, 3D19.F.302, 3D3L.A.801, 3DBY.A.306,
2DE6.B.501, 4DHL.A.502, 3E2T.A.1, 1E3D.A.269, 4EB5.D.201, 4F1E.P.201, 3FG1.B.150
0, 2FKZ.A.1600, 4FWI.B.401, 1FZO.A.5002, 2GBX.E.455, 3GC1.A.605, 3GKE.A.501, 3GL
0.A.501, 3GL2.A.501, 1GNL.A.1545, 2GPC.B.195, 1GUP.C.351, 2GYQ.A.402, 2HMK.A.451
, 4HSL.A.202, 3I01.B.800, 1I4Z.E.605, 4ILT.A.301, 2JD7.0.203, 1JI5.A.152, 1JI5.B
.151, 1JIG.A.402, 1JIG.B.401, 2JI3.A.1127, 1JQK.B.801, 1JRO.G.3001, 4K9F.A.101,
4KU0.D.101, 4KWL.A.301, 4KX6.N.301, 1LNB.E.900, 1N1Q.A.516, 1N1Q.B.515, 3N9Y.D.1
51, 3NA0.C.150, 3NA0.D.150, 4NBA.A.501, 4NBF.A.501, 4NBG.A.501, 1NF6.A.200, 1NO3

.A.858, 2OHJ.A.502, 2OHJ.A.511, 3PCK.Q.600, 2PCD.M.600, 2PT2.A.400, 2PUZ.A.500, 2Q0J.B.997, 3Q36.A.458, 4QDD.A.401, 3QFM.A.264, 1QGH.H.157, 4QLW.B.201, 3QVD.G.1 73, 1R2F.A.400, 1R9X.A.501, 1RA5.A.500, 4REU.B.202, 1RSR.A.1004, 1SHR.B.801, 3T8 1.B.606, 1T9O.A.255, 3U9M.A.202, 1ULI.A.600, 3V7P.A.429, 1VCX.A.54, 3VMH.C.501, 2W3S.E.1464, 1W9M.A.1555, 2WLB.B.619, 1WQL.A.502, 2XSO.G.901, 1Y4T.A.650, 2YFI.A .901, 2YFI.G.900, 1YK5.A.54, 1YKP.F.2600, 1YUX.A.303, 2ZZI.A.208
[1] "Cluster 2"
2A1X.A.450, 1A7E.A.119, 3ABM.A.516, 1B1X.A.690, 2B2O.B.1500, 4B2O.A.1267, 4B7G.A .3000, 3BFJ.M.1388, 4BGL.A.1001, 1BKA.A.693, 1BKA.A.694, 2BQ8.X.1305, 2BQ8.X.130 6, 2BUZ.B.1541, 2BV0.B.600, 2CAG.A.485, 4CHL.A.501, 1D9Y.A.310, 3DHG.A.502, 1DRY .A.332, 3DXU.A.360, 3E6S.B.200, 1EO9.B.600, 1FCD.C.902, 2FR7.A.501, 1FSL.A.144, 1FZ1.A.5001, 1FZ3.A.5002, 1FZ7.B.5003, 2G1M.A.600, 4G51.B.202, 3GE3.A.501, 1GGF. B.760, 4GHF.B.401, 3GM6.A.1004, 4GP5.A.602, 2HOV.A.501, 2HUO.A.301, 1I4Z.A.601, 4I4G.A.601, 4I4H.A.601, 2INC.A.502, 3IXF.A.139, 1IZO.A.501, 1JNF.A.702, 1JNF.A.7 03, 4K0F.A.601, 4K9T.A.601, 4K9U.A.601, 4K9V.A.601, 4K9W.A.601, 4K9X.A.601, 3KT7 .A.701, 1KW9.B.301, 3LXV.M.600, 4M25.A.401, 4M73.B.403, 3MDT.A.505, 4ME4.A.402, 1MMO.D.3, 1MMO.D.4, 3MOO.A.911, 3MZS.C.500, 3N20.A.506, 2NOX.A.500, 1NX4.B.300, 3OOR.B.802, 1OCZ.A.516, 4OJ8.C.301, 1OQU.C.1009, 1OQU.C.1010, 3OUH.A.600, 3P3N.A .350, 3PCJ.O.600, 2PCC.A.296, 3PER.A.1001, 1PHG.A.417, 1PIU.A.402, 3PUQ.A.1, 2Q0 J.B.998, 3Q14.A.502, 3Q1G.A.1002, 3Q3M.A.509, 3QFN.A.265, 3QFO.A.264, 2QPP.A.300 , 3QY6.A.263, 3QY7.A.263, 3QY8.A.253, 1R1N.C.400, 4RC8.A.303, 2RDB.A.499, 3RNC.A .499, 3RNC.A.500, 3RNF.A.500, 3RNF.A.501, 1RYO.A.329, 1SYY.A.1319, 1SYY.A.1320, 1TOQ.A.499, 1TOQ.A.500, 3TMZ.A.501, 3TTX.A.760, 1U74.B.1101, 2VUN.A.402, 3WEC.A. 501, 1WRA.B.402, 2X9P.A.1398, 1XU5.A.1175, 1XVB.A.1170, 1XVF.B.1174, 1XVF.B.1175 , 1XVG.A.529, 1Y8W.A.142, 1YGF.B.147, 1YMA.A.154, 1Z1N.X.602, 3ZK3.A.1311, 3ZLI. A.4001, 4AC8.D.500, 1AHJ.A.208, 4ANP.A.1426, 1B7Z.A.690, 3BXD.A.302, 1FRV.B.537, 1FZ1.B.5003, 1H2K.A.1350, 1H2L.A.1350, 4IGO.A.1000, 4M26.B.401, 1N04.A.688, 3N1 Y.A.504, 4N71.A.201, 3OOF.A.304, 3O32.A.300, 3O6J.A.300, 2OGI.A.301, 3PCA.M.600, 3PCJ.N.600, 3PCK.M.600, 2PQ7.A.221, 3Q3O.A.502, 1QHW.A.433, 2R2F.A.320, 1SP8.A. 500, 3UF9.B.315, 1UOF.A.1311, 1UTE.A.501, 1VZ4.A.1299, 1W2A.X.1302, 1WZD.A.901, 2XMO.B.556, 1XVE.A.1170, 2Y0I.A.1350, 2YDE.A.501, 2YU1.A.600, 1ZJ9.B.1569
[1] "Cluster 3"
4QQZ.C.1001, 4AIQ.A.1746, 1B06.A.322, 1BIQ.A.376, 2BKB.A.1193, 4BMT.A.1323, 1BS3 .A.202, 2BUT.B.1541, 2BUU.B.1541, 2BUV.B.1541, 2BUW.B.600, 2BUM.B.1541, 2BUQ.B.1 541, 3CEI.A.500, 2CW2.A.402, 2CW3.A.524, 4DVH.A.301, 3E13.X.326, 1EO2.B.600, 3ES F.A.198, 1EYS.C.609, 4F2N.B.300, 4FFK.A.301, 3FG3.D.1500, 3FG4.A.1500, 4G2D.A.40 2, 1GN6.A.999, 2GOJ.A.198, 2GPC.A.195, 3H1S.A.1001, 3HHY.A.281, 3HJQ.A.281, 3HJS .A.281, 3HKP.A.281, 1HMD.A.115, 3I4Y.A.281, 3I51.A.281, 4IEZ.A.501, 1ISA.A.193, 3IS8.A.163, 4J6C.A.502, 4KEZ.A.401, 1KW6.B.301, 4L2B.A.201, 4L2C.A.201, 3LIO.A.5 000, 1MY6.B.200, 1NFT.A.333, 3O5U.A.300, 4P1B.D.501, 3PCE.N.600, 3PCN.P.600, 2PC D.N.600, 2QFR.A.433, 3QFM.B.264, 1QNN.A.201, 2R1K.A.800, 2R1N.A.800, 3R2R.A.155, 4REU.A.201, 1SQ3.A.901, 1TDW.A.425, 3TQJ.A.1001, 1UNF.X.1239, 2VHL.A.1397, 2VP1 .A.1347, 2W7W.A.1195, 1WB7.A.212, 1WB8.A.212, 1XM8.A.701, 1YCH.A.501, 1YFX.A.300 , 1Z6O.M.300, 1ZA5.B.393, 1OQU.A.1001
[1] "Cluster 4"
2BI4.A.1384, 4BMT.B.1323, 4CMY.A.1164, 4D8F.A.401, 1DGG.A.2000, 3DHG.A.501, 1DTO .A.1601, 1E2U.A.701, 3FMR.B.401, 1FZH.B.5004, 2GBX.A.456, 1H2A.L.1004, 1HJF.A.13 10, 1HJG.A.1307, 1I4Y.B.602, 1I4Z.D.604, 1I4Z.G.607, 3I4V.A.281, 2IBN.A.704, 3IC F.A.602, 1IDS.C.208, 2INP.A.3, 2ITB.A.501, 2ITB.B.501, 4IWK.F.201, 2J2F.E.371, 1 KBP.A.438, 4KBP.A.438, 3KCY.A.1350, 4KEV.D.401, 1LGT.A.500, 1LKD.A.500, 3LKT.M.6 00, 3LMX.O.600, 1MOJ.B.301, 1N7X.A.339, 3NJZ.A.369, 1NNF.A.401, 1NX8.A.300, 2OHH .B.1501, 2OHH.E.3501, 1OQ9.A.365, 1OS7.C.302, 2P6B.C.513, 3PCA.N.600, 3PCB.O.600 , 3PCE.M.600, 3PCJ.M.600, 3PCL.R.600, 1PIY.A.376, 3PM5.A.1002, 3Q1G.A.1001, 3Q1G .B.1001, 1QFC.A.402, 2QJE.A.692, 3R2M.A.155, 2R2F.B.320, 4RC5.A.1003, 1SQ3.B.906

, 1SQ3.C.907, 1T47.A.431, 4TOA.A.206, 4TOA.B.207, 3U52.B.512, 3U9M.E.205, 3USS.B
.212, 2VC7.D.1315, 2VHL.A.1398, 3VTI.B.803, 1W69.A.1349, 3WHN.B.201, 2XRX.G.1461
, 2XSO.K.900, 1XZW.B.929, 1YKM.J.600, 2Z4G.A.503, 2ZQX.A.501, 1ZZ9.C.199
[1] "Cluster 5"
19HC.A.301, 19HC.A.302, 19HC.A.303, 19HC.A.304, 19HC.A.305, 19HC.A.306, 19HC.A.3
07, 19HC.A.308, 19HC.A.309, 3A15.B.354, 2A3M.A.501, 2A3M.A.502, 2A3M.A.503, 2A3M
.A.504, 1A4E.A.503, 1A56.A.82, 3A8G.A.301, 3A9F.A.207, 2A9E.A.550, 4AAL.A.423, 4
AAN.A.400, 4AAN.A.401, 4AAO.A.400, 2AIU.A.200, 2AI5.A.81, 4AM5.A.1160, 1AOF.A.60
2, 1AOF.B.601, 1AOM.A.602, 1AOM.B.601, 1AOM.B.602, 1AQA.A.95, 1AQE.A.121, 1AQE.A
.122, 3AQ5.A.144, 3AQ9.A.144, 3AT5.A.142, 3AT5.B.147, 2AT3.X.185, 1AWP.A.201, 4B
2N.A.700, 2B4Z.A.500, 3B42.A.199, 3B42.B.399, 3B47.A.199, 4B4Y.A.1155, 1B80.A.35
0, 1B82.A.350, 1B85.A.350, 3B99.A.600, 2BDM.A.500, 1BFR.B.200, 2BGV.X.1121, 2BH4
.X.1123, 1BJE.A.154, 4BJA.A.300, 3BK9.B.401, 2BK9.A.1154, 2BLF.B.1582, 4BMM.A.14
50, 3BNG.A.513, 3BNJ.A.513, 3BNJ.A.514, 3BNJ.A.515, 3BNJ.A.516, 3BNJ.A.517, 2BPN
.A.108, 2BPN.A.109, 2BPN.A.110, 2BPN.A.111, 2BQ4.A.1115, 2BQ4.A.1117, 2BQ4.A.111
8, 2BS2.C.1255, 2BS2.C.1256, 2BS3.C.1255, 2BS4.C.1255, 1BVY.A.1000, 3BXU.A.72, 3
BXU.A.73, 3BXU.A.74, 4C0C.A.1450, 4C0N.A.1157, 2C1U.A.401, 2C1V.A.401, 2C1V.A.40
2, 2C1D.A.1292, 3C2C.A.113, 1C2R.A.120, 4C27.A.1450, 4C28.A.1450, 4C44.A.1151, 1
C52.A.200, 3C63.A.150, 1C6R.A.90, 1C75.A.93, 3C76.X.185, 3C78.X.185, 2C8S.A.1173
, 3CAO.A.104, 3CAO.A.105, 3CAO.A.106, 3CAO.A.107, 1CCH.A.83, 1CCR.A.112, 2CDV.A.
109, 2CDV.A.111, 4CDP.A.402, 2CEO.A.1102, 1CH1.A.154, 1CH2.A.154, 1CH5.A.154, 1C
H9.A.154, 1CI3.M.254, 4CK8.A.1480, 1CNO.A.200, 2CN4.A.1175, 1CO6.A.108, 1COR.A.8
3, 1COT.A.130, 4COH.A.1450, 4COO.A.1549, 3CP5.A.202, 3CQV.A.601, 3CSL.A.866, 2CT
H.A.109, 2CTH.A.110, 2CTH.A.111, 2CTH.A.112, 3CU4.A.199, 2CVC.A.1001, 2CVC.A.100
2, 2CVC.A.1003, 2CVC.A.1004, 2CVC.A.1005, 2CVC.A.1006, 2CVC.A.1008, 2CVC.A.1009,
2CVC.A.1010, 2CVC.A.1014, 2CVC.A.1016, 3CX5.C.4001, 3CX5.C.4002, 3CX5.D.4003, 3
CX5.W.4026, 1CXA.A.126, 1CXC.A.125, 3CXH.W.4026, 1CXY.A.204, 1CYI.A.200, 3CYR.A.
203, 3CYR.A.204, 2CY3.A.119, 2CY3.A.120, 2CY3.A.121, 2CY3.A.122, 5CYT.R.105, 2CZ
S.A.500, 2CZS.A.501, 1CZJ.A.119, 1CZJ.A.120, 1CZJ.A.121, 1CZJ.A.122, 3CZY.A.300,
2D0S.A.80, 2D0T.A.404, 4D30.B.750, 4D34.A.500, 4D35.A.500, 1D7B.A.401, 3D7O.A.1
43, 2DC3.A.191, 3DE8.A.150, 2DGE.A.200, 3DHR.A.142, 1DKO.A.200, 3DMI.A.146, 2DN1
.A.142, 2DN1.B.147, 3DRO.A.94, 1DTI.A.154, 4DTZ.A.500, 1DUW.A.293, 1DUW.A.297, 1
DUW.A.300, 1DUW.A.301, 1DW0.A.113, 1DW2.A.113, 1DWL.B.80, 1DY7.B.601, 2DY5.A.300
, 4DY9.A.201, 1E08.E.80, 1E29.A.136, 1E2R.B.601, 1E2W.A.900, 1E39.A.803, 1E39.A.
804, 2E3A.A.401, 3E5J.A.1408, 1E8E.A.125, 2E80.A.1508, 2E84.A.1301, 2E84.A.1305,
2E84.A.1307, 2E84.A.1308, 2E84.A.1309, 2E84.A.1310, 2E84.A.1313, 2E84.A.1314, 1
EB7.A.401, 3EGW.C.806, 3EGW.C.807, 3EHB.A.559, 1EHJ.A.1030, 1EHJ.A.1053, 1EHJ.A.
1066, 4EID.A.101, 4EIE.A.101, 4EIF.A.101, 2EKT.A.154, 2EKU.A.154, 1ETP.A.199, 1E
TP.A.200, 1EUE.A.201, 2EU7.X.201, 2EWK.A.1001, 2EWK.A.1003, 2EWU.A.1001, 2EWU.A.
1003, 1EWH.A.253, 2EWI.A.1004, 2EWI.A.1002, 2EWI.A.1001, 2EWI.A.1003, 2EXV.A.83,
1EZV.C.401, 1EZV.C.402, 1F03.A.201, 1F1C.A.200, 1F1F.A.200, 1F24.A.501, 4F6I.A.
201, 4FA7.A.602, 4FA7.A.603, 4FAS.A.601, 4FAS.A.602, 4FAS.A.603, 4FAS.A.604, 4FA
S.A.605, 4FAS.A.606, 4FAS.A.607, 2FBZ.X.901, 1FCD.C.901, 4FEF.A.403, 2FFN.A.1003
, 1FGJ.A.548, 1FGJ.A.552, 1FGJ.A.553, 1FI3.A.83, 1FI7.A.110, 1FJ0.A.115, 3FLL.A.
185, 2FMY.A.300, 3FO3.A.1004, 3FO3.A.1005, 3FO3.A.1006, 3FO3.A.1007, 3FO3.A.1008
, 3FO3.A.1002, 3FO3.A.1003, 3FO3.A.1001, 1FOP.A.500, 3FOO.A.150, 2FRF.A.154, 1FS
7.A.509, 1FS7.A.510, 1FS8.A.508, 1FT5.A.213, 1FT5.A.215, 1FT5.A.216, 1FT9.A.300,
1FT9.B.300, 2FWT.A.803, 2FWT.A.805, 2FWL.A.132, 2FYU.D.242, 4G1V.A.401, 3G5N.A.
500, 4G7L.A.301, 3GAS.D.1294, 2GC4.D.200, 1GDV.A.101, 4GED.B.201, 1GKS.A.0, 2GNV
.A.166, 1GQ1.A.601, 1GQ1.A.602, 3GQP.C.143, 2GSM.A.2001, 2GTF.X.201, 1GWF.A.504,
3GW9.A.480, 1GWS.A.603, 1GYO.A.111, 1GYO.A.112, 1GYO.A.113, 1GYO.A.114, 4GYD.A.
200, 4H0K.B.200, 1H1O.A.1184, 1H1O.A.1185, 1H21.A.1248, 1H21.A.1249, 1H21.B.1248
, 1H29.A.1102, 1H29.A.1104, 4H2L.B.201, 1H32.A.1263, 1H32.A.1264, 1H32.B.1139, 3
H33.A.75, 3H33.A.76, 3H33.A.77, 3H34.A.72, 3H34.A.73, 3H34.A.74, 3H4N.A.72, 3H4N

.A.73, 3H4N.A.74, 4H44.A.302, 1H55.A.350, 1H57.A.350, 3H8T.A.301, 2H88.C.142, 1HBI.A.153, 4HB6.A.72, 4HB6.A.73, 4HB8.A.72, 4HB8.A.73, 4HBF.A.72, 1HCZ.A.253, 4HC3.A.72, 3HDL.A.305, 3HF4.A.142, 1HGB.D.147, 4HHR.A.701, 4HHS.A.701, 1HJ3.B.602, 1HJ5.A.601, 1HJ5.B.602, 2HJI.A.180, 1HLB.A.158, 3HNJ.A.150, 3HNK.A.150, 4HPA.A.201, 4HPB.A.201, 4HPD.A.201, 3HQ9.A.400, 3HQ9.A.401, 1HRC.A.105, 3HYU.A.142, 3HYU.B.147, 1I3E.A.147, 2I5N.C.404, 1I5U.A.201, 1I77.A.108, 1I77.A.109, 1I77.A.110, 1I77.A.111, 4I7Z.A.302, 3I8R.A.901, 1I8O.A.115, 1I8P.A.115, 2I8F.A.83, 3I9T.A.300, 3I9U.A.300, 1ICC.A.201, 1IDR.A.144, 2IJ3.B.999, 1IKE.A.185, 1IQC.A.402, 3IQ5.A.150, 1IT1.A.201, 1IT1.A.202, 1IT1.A.203, 1IT1.A.204, 2IUF.A.1691, 1IVJ.A.300, 1IWO.A.901, 1IYN.A.296, 1J02.A.300, 1J0P.A.1002, 1J0Q.A.201, 2J1M.A.1456, 4J20.A.107, 1J3S.A.105, 2J5M.A.1300, 2J7A.A.1004, 2J7A.A.1005, 2J7A.C.1003, 1JDL.A.500, 4JE9.A.201, 4JEB.A.201, 4JJ0.A.501, 4JJ0.A.502, 2JJP.A.412, 1JMX.A.1001, 1JMX.A.1002, 1JNI.A.125, 1JNI.A.126, 2K3V.A.218, 1KB0.A.802, 1KBI.A.760, 4KF2.B.501, 2KMY.A.251, 4KMG.A.101, 1KOK.A.296, 2KSU.A.282, 2KSU.A.305, 1KV9.A.901, 4KVK.A.701, 4KVL.A.701, 3L1M.A.150, 1L2K.A.154, 3L61.A.420, 3LD6.A.601, 2LDO.A.154, 4LJI.A.301, 1LM3.B.200, 1LMS.A.118, 4LM8.A.801, 4LM8.A.802, 4LM8.A.803, 4LM8.A.804, 4LM8.A.805, 4LM8.A.807, 4LM8.A.808, 4LM8.A.809, 4LM8.A.810, 4LMH.A.801, 4LMH.A.803, 4LMH.A.804, 4LMH.A.805, 4LMH.A.806, 4LMH.A.807, 4LMH.A.808, 4LMH.A.809, 4LMH.A.810, 1LQX.A.201, 1LR6.A.201, 1LS9.A.92, 1LSX.A.719, 1M1P.A.802, 1M1Q.A.804, 1M1Q.A.803, 1M1R.A.801, 1M2I.A.201, 3M4C.A.150, 4M4A.A.201, 1M59.A.201, 1M70.A.199, 1M70.A.200, 4M72.A.403, 4M73.A.403, 3MDM.A.505, 1MDV.A.112, 1MJ4.A.502, 3ML1.B.1128, 3ML1.B.1129, 1ML7.A.185, 1MNI.B.154, 4MPM.A.201, 4MQJ.B.201, 3MUS.A.201, 3MVC.A.500, 3MVF.A.185, 1MWB.A.125, 1MZ4.A.151, 1N45.A.300, 4N4J.A.609, 4N4J.A.610, 4N4J.A.611, 4N4J.A.612, 4N4J.A.613, 4N4J.A.614, 4N4J.A.615, 4N4J.A.616, 4N4L.A.616, 4N4N.A.601, 4N4N.A.602, 4N4N.A.603, 4N4N.A.604, 4N4N.A.605, 4N4N.A.606, 4N4N.A.607, 4N4O.A.608, 4N8T.B.201, 1NAZ.A.200, 4NFG.B.201, 1NIR.A.601, 1NIR.A.602, 4NK1.B.201, 3NMI.A.150, 1NML.A.401, 3NN1.A.239, 1NNO.A.602, 1NOS.A.901, 4NP1.A.185, 2NRL.A.148, 1NS6.A.142, 1NS9.B.147, 3NWV.A.105, 3NXU.A.508, 3O1A.A.385, 4O1W.A.101, 4O4Z.A.201, 3O5C.A.401, 3O5C.D.504, 4O6Q.A.202, 4O6U.A.203, 4O7G.A.302, 3O89.A.2154, 3OA8.A.401, 3OA8.B.401, 1OFW.A.1294, 1OFW.A.1295, 1OFW.A.1296, 1OFW.A.1297, 1OFW.A.1298, 1OFW.A.1299, 1OFW.A.1300, 1OFW.A.1301, 1OFW.A.1302, 3OFT.A.417, 3OMA.A.1, 3OMI.A.607, 3OO4.A.142, 3OO4.B.147, 2ORT.A.600, 3ORV.B.600, 2ORO.A.600, 2ORP.A.600, 2ORQ.A.600, 2ORR.A.600, 2ORS.A.600, 2OT4.A.1004, 2OT4.A.1007, 3OUE.A.609, 3OUE.A.610, 3OUE.A.611, 3OUE.A.612, 3OUQ.A.602, 3OUQ.A.603, 3OUQ.A.605, 3OUQ.A.606, 2OZY.A.202, 2OZY.A.203, 2OZY.A.204, 2OZY.A.205, 3OZU.A.404, 1P2E.A.801, 1P2E.A.803, 1P2E.A.804, 3P3L.A.501, 1PBY.A.991, 1PBY.A.992, 3PC3.A.701, 2PEG.A.200, 2PEG.B.400, 3PH2.B.1087, 3PI2.B.500, 4PK5.A.501, 1PL3.A.401, 1PM1.X.180, 1PP9.C.501, 1PP9.C.502, 1PP9.D.501, 2PQ7.A.220, 3PXW.A.500, 4PXH.A.501, 1Q16.C.806, 1Q16.C.807, 2Q8Q.A.300, 3Q99.B.750, 1QDB.A.516, 1QDB.A.517, 1QDB.A.518, 1QDB.A.519, 1QHU.A.500, 2QJY.A.501, 2QJY.A.502, 2QJY.B.301, 1QKS.A.601, 1QKS.A.602, 1QNO.A.113, 1QNO.A.114, 1QNO.A.115, 1QNO.A.116, 1QN2.B.101, 3QNS.A.351, 1QPA.A.350, 3QQQ.A.163, 2QSP.A.142, 3QWO.A.150, 1QYZ.A.200, 1R0Q.A.200, 4R20.A.601, 2R50.A.166, 2R80.A.150, 2R80.B.150, 3R9C.A.450, 2RAO.B.147, 2RAO.C.142, 2RDZ.A.2, 2RDZ.A.3, 2RDZ.A.4, 2RDZ.A.5, 3RJ6.A.154, 4RKM.A.808, 4RKM.D.807, 4RKN.A.902, 4RKN.A.903, 4RKN.A.905, 4RKN.A.906, 4RKN.A.907, 4RKN.A.908, 4RKN.A.909, 1RSE.A.154, 3RUK.B.600, 1RWJ.A.90, 1RWJ.A.91, 1RWJ.A.92, 3S8G.A.800, 1SE6.A.430, 3SEL.X.73, 3SEL.X.74, 1SH4.A.201, 3SJ0.X.73, 3SJ0.X.74, 3SJ1.X.73, 3SJ1.X.74, 3SJ4.X.73, 3SJ4.X.74, 3SJ5.A.500, 3SJL.A.500, 3SJL.A.600, 3SLE.A.402, 1SOX.A.502, 1SP3.A.801, 1SP3.A.803, 1SP3.A.804, 1SP3.A.805, 1SP3.A.806, 1SP3.A.808, 1SUO.A.500, 3SXQ.A.1005, 3SXQ.A.1006, 3SXQ.A.1007, 3SXQ.A.1008, 3SXQ.A.1002, 3SXQ.A.1003, 3SXQ.A.1001, 1SY2.A.185, 3T3R.A.500, 1T68.X.201, 3T6D.C.401, 3T6D.C.403, 3T6D.C.404, 3T6E.C.401, 3T6E.C.402, 3T6E.C.403, 3TDA.A.800, 3TGU.C.501, 3TGU.C.502, 3TGU.D.501, 3TGA.A.185, 3TGM.A.300, 3TIK.A.482, 1TKW.B.253, 3TMC.A.309, 4TOB.C.201, 3TOL.A.150, 4TT5.A.401, 1TU2.B.255, 1U4H.A.500, 1U7R.A.154, 3U8P.A.347, 1U9M

.A.90, 4U9D.A.201, 1U9U.A.90, 3U99.A.500, 3U99.A.700, 3UBR.A.472, 3UBR.A.475, 3U
BC.A.201, 3UCP.A.901, 3UCP.A.902, 3UCP.A.903, 3UCP.A.904, 3UCP.A.905, 3UCP.A.906
, 3UCP.A.907, 3UCP.A.908, 3UCP.A.909, 3UCP.A.910, 3UCP.A.911, 1UP9.A.201, 1UP9.A
.202, 1UP9.A.203, 1UP9.A.204, 2UUQ.A.1405, 4UVR.A.1450, 1V0H.X.251, 2V07.A.1102,
 4V2K.A.601, 4V3V.A.750, 4V3W.A.750, 4V3X.A.750, 1V54.A.515, 1V75.B.201, 2V7K.A.
1360, 2V7L.A.1360, 3V8D.A.601, 1V8X.A.901, 1V9Y.A.1140, 3VAU.A.201, 1VB6.A.1140,
 1VGI.A.300, 3VHB.A.150, 2VHD.A.402, 3VKP.A.601, 3VKS.A.601, 3VM9.A.154, 3VP5.A.
201, 3VRD.A.201, 3VRD.A.202, 3VRG.A.201, 3VRG.B.201, 3VXJ.A.501, 2VXH.A.1001, 1V
YD.A.1117, 2VYW.A.149, 2W0B.A.470, 1W2L.A.1100, 2W31.A.200, 2W3G.A.500, 1W7O.A.1
119, 1W7O.A.1120, 1W7O.A.1121, 1W7O.A.1122, 3W9C.A.501, 1WAD.A.117, 1WAD.A.113,
1WAD.A.114, 1WAD.A.115, 3WAH.A.201, 3WC8.A.201, 3WCT.A.200, 3WCT.B.201, 3WCT.C.2
00, 1WE1.A.300, 3WFD.C.201, 3WFX.A.201, 2WJM.C.1334, 2WJN.C.1333, 2WJN.C.1335, 2
WJN.C.1336, 1WMU.A.201, 1WOV.A.300, 1WOX.A.300, 4WPD.A.402, 4WQ8.A.1002, 4WQ9.A.
1001, 4WQ9.A.1002, 4WQC.A.1002, 4WQD.A.1002, 2WTG.A.180, 3WU2.F.101, 1WVE.C.699,
 1WVP.A.154, 3X15.A.200, 3X15.J.200, 1X3X.B.202, 1X46.A.151, 4XDI.A.201, 2XFH.A.
1412, 2XKR.A.1400, 2XKI.A.1110, 2XSJ.B.503, 2XTS.B.500, 2XYK.A.700, 1Y0P.A.801,
1Y0P.A.802, 1Y0P.A.803, 1Y0P.A.804, 1Y5I.C.806, 1Y5I.C.807, 1Y5L.C.806, 2Y5N.A.4
50, 2YEV.B.587, 2YIU.A.500, 2YIU.A.501, 1YIQ.A.901, 2YK3.A.200, 2YL7.A.128, 1YWD
.A.185, 2YXC.A.1001, 2YYW.A.1001, 2YYW.A.1003, 2YYX.A.1004, 2YYX.A.1001, 1Z1N.X.
608, 1Z1N.X.610, 1Z1N.X.616, 2Z47.A.1004, 2Z47.B.3003, 2Z6S.A.201, 2Z6T.A.201, 1
Z8O.A.410, 1Z9N.A.1001, 1Z9N.C.2001, 2ZBO.A.111, 3ZG2.A.1480, 3ZHO.A.200, 3ZHW.A
.1163, 3ZIY.A.600, 3ZJQ.A.200, 3ZOO.A.105, 3ZOX.A.1082, 2ZS0.A.200, 2ZS0.B.200,
2ZS0.C.200, 2ZS0.D.200, 2ZXY.A.200, 2ZZS.1.220, 1ZZH.A.802, 3A15.A.354, 4AJ9.A.1
715, 2AKJ.A.564, 3ARJ.A.153, 3AT6.A.142, 2AUO.A.153, 2AUQ.A.147, 2AVO.A.147, 4AV
D.A.144, 3AYF.A.802, 1B0B.A.144, 3BA2.A.158, 1BCF.A.200, 3BK9.A.401, 3BUJ.A.398,
 2C1D.H.1158, 3C6G.A.601, 4C9L.A.1418, 1CG8.B.142, 1CH4.A.147, 2CMM.A.155, 1CRC.
A.105, 4CZC.A.1337, 3D1K.A.200, 3D1K.B.400, 2D5X.B.147, 3DAM.A.600, 3DE8.D.150,
1DGF.A.3000, 2DKK.A.430, 1DLY.A.144, 4DVQ.A.601, 1DW1.A.113, 1E2R.A.602, 2E39.A.
401, 3E4W.A.501, 2E84.A.1315, 4ENU.A.801, 1EQD.A.185, 4ESA.A.202, 4ESA.B.202, 2F
DU.A.500, 1FHF.A.350, 1FHJ.B.147, 4FVC.A.201, 2G3H.A.154, 3G46.A.147, 4G45.A.401
, 3GAS.A.1290, 1GEJ.A.501, 2GGN.X.251, 1GJQ.A.602, 2GKM.A.144, 4GQS.B.501, 4H0K.
A.200, 1H1X.A.1154, 1H97.A.148, 1HJ4.B.601, 1HJ5.B.601, 4HRR.A.201, 4HRR.B.201,
2HYS.A.201, 1HZU.A.601, 1I3D.A.147, 4I8V.A.601, 2I96.A.129, 4IAM.A.501, 3IBD.A.5
00, 2IG3.A.700, 1IOP.A.154, 3IQB.A.500, 1IRD.B.347, 1IWH.A.142, 1IX4.A.300, 2J7A
.D.1001, 1JEB.D.147, 4JET.A.201, 2JXM.B.250, 2KII.A.182, 4L1Y.A.300, 4L1Z.A.300,
 4L2M.A.201, 4L3H.A.402, 2L4D.A.107, 4L54.A.501, 1LGA.A.396, 1LHT.A.155, 2LHB.A.
151, 1M54.F.1620, 1M56.A.1002, 1M7S.D.600, 3MM3.A.501, 3MMO.A.1004, 4MMO.A.401,
1MN1.A.396, 1MYF.A.154, 3MYM.A.139, 3MYN.A.139, 4N4M.A.616, 4N4N.C.601, 3N8Y.B.6
01, 3NAO.A.601, 4NK2.A.700, 3NN2.A.239, 2NNJ.A.500, 2NP1.A.350, 1NR6.A.500, 3NTG
.D.601, 3O5C.B.402, 1OAE.A.1125, 3OCD.C.401, 3OCD.D.401, 1OCZ.A.515, 1ODO.A.1407
, 3OFU.A.417, 1OG5.A.501, 2OIF.A.163, 1OR4.A.180, 1OZW.B.300, 3P3X.A.501, 2P85.A
.500, 3PMO.A.900, 3PT8.A.500, 3PT8.B.500, 2Q8P.A.300, 3QGP.A.200, 3QQR.B.163, 2Q
RW.A.700, 2QSS.A.142, 4R21.A.600, 3R9B.A.501, 2RF7.D.1, 3RIW.A.305, 4RKM.K.809,
4RKM.L.813, 3RUR.A.200, 1S13.A.300, 1S1F.A.430, 1S61.A.144, 1S69.A.125, 3S66.A.1
42, 3S79.A.600, 1SCH.A.300, 1SI8.A.501, 1SPG.B.148, 1T85.A.417, 3TBG.A.800, 3TM8
.A.903, 3TTX.B.760, 1TWN.A.300, 1U5U.A.999, 4U9D.D.201, 3UA1.A.508, 4UBS.A.501,
1UC3.A.150, 3UHB.A.147, 3UHD.B.147, 1UMO.A.1172, 3UOI.I.200, 3UT2.A.1500, 1UX8.A
.700, 1V4U.B.147, 1V9Z.B.1140, 3VED.A.401, 2VHD.A.401, 3VNO.A.501, 1VRE.A.148, 3
VRF.B.201, 2VZW.A.1206, 3W08.A.501, 3W4U.A.201, 1W92.A.1149, 3WCU.A.200, 3WCU.C.
200, 3WFB.B.802, 4WG2.A.603, 3WNU.A.801, 1WOW.A.300, 3WXO.A.801, 2WY4.A.150, 2X6
6.A.1359, 1X8V.A.470, 1X9F.A.160, 1X9F.B.160, 1X9F.C.160, 1X9F.D.160, 2XBK.A.139
8, 2XN8.A.1434, 2XQ1.B.1503, 2Y4F.A.389, 2YGX.D.450, 2YL1.A.128, 1YMC.A.154, 2Z6
N.A.150, 2Z6N.B.150, 2ZFO.D.200, 3ZK5.A.1407, 2ZVU.A.300
[1] "Cluster 6"

3A0G.A.201, 3A16.B.354, 3A17.A.354, 2A1O.A.417, 1A2F.A.1, 1A2S.A.90, 2A3F.X.201, 3A51.B.412, 3A8L.A.300, 1AKK.A.105, 4APY.A.1418, 1ASH.A.301, 4AUM.A.900, 2AVK.A.201, 3AWM.A.501, 3AYF.A.801, 2B0Z.B.109, 3B0H.B.601, 2B10.D.909, 2B11.D.1301, 2B2R.A.1500, 4B2N.A.701, 3B6H.B.600, 4B8N.A.201, 2BC5.A.150, 3BDZ.A.450, 1BEP.A.296, 1BIN.B.144, 1BJ9.A.296, 4BJK.A.1450, 2BMM.A.1157, 3BOM.A.143, 3BOM.B.148, 2BQ4.A.1116, 2C1D.A.1291, 2C1D.B.1158, 1C2N.A.117, 1C40.A.150, 3C62.A.150, 4C9M.A.1418, 1CC5.A.1, 1CED.A.90, 1CH3.A.154, 1CH7.A.154, 2CJ2.A.1300, 4CK9.A.1480, 4CKA.A.1480, 1CLS.A.142, 2CN4.B.1175, 1CPT.A.430, 2CVC.A.1007, 2CVC.A.1011, 2CVC.A.1012, 2CVC.A.1013, 4CVJ.A.1295, 2CYP.A.295, 2CZ1.A.300, 2D0W.A.200, 4D02.A.602, 4D02.A.603, 2D2M.D.200, 4D36.A.500, 4D37.A.500, 4D38.A.500, 4D3A.A.500, 1D4D.A.601, 1D4D.A.602, 1D4D.A.603, 1D4D.A.604, 1DCC.A.296, 1DD7.A.600, 3DHH.A.501, 3DHI.A.600, 1DJ5.A.1, 1DLW.A.144, 1DM1.A.148, 1DP8.A.719, 1DP9.A.719, 4DTW.A.500, 4DTY.A.500, 4DXY.A.501, 3E2O.A.296, 1E39.A.801, 1E39.A.802, 2E3B.A.401, 3E5K.A.1408, 2E84.A.1302, 2E84.A.1303, 2E84.A.1304, 2E84.A.1306, 2E84.A.1311, 2E84.A.1312, 2E84.A.1316, 1EGY.A.410, 3EH5.A.800, 3EHB.A.560, 1EHE.A.501, 3EJ6.A.4000, 4EJI.A.501, 4ENH.A.601, 4ENU.B.801, 1EOC.B.600, 4EP6.A.601, 1F4U.A.410, 4F4O.B.201, 2FAM.A.148, 4FDH.A.601, 4FIA.A.600, 1G09.B.147, 4G3J.A.501, 4G70.A.601, 4G71.A.602, 4G7G.A.501, 4G7Q.A.602, 4G7S.A.602, 2GEP.A.580, 3GEO.A.580, 7GEP.A.580, 2GJ1.A.605, 4GP4.A.602, 4GP8.A.602, 4GQS.A.501, 2GSM.A.2002, 1GWS.A.601, 1GWS.A.605, 1GWS.A.606, 1GWS.A.609, 1GWS.A.610, 1GWS.A.611, 1GWS.A.612, 1GWS.A.613, 1GWS.A.616, 1GWT.A.350, 2H0V.B.500, 1H21.B.1249, 1H29.A.1107, 1H29.A.1108, 1H29.A.1114, 4H44.A.301, 4H6O.A.501, 2HBU.A.900, 3HB3.A.559, 3HB3.A.560, 1HBZ.A.504, 2HBD.A.142, 3HF4.B.147, 1HMO.B.115, 3HQ6.A.400, 3HQ7.A.401, 1HRO.A.107, 4HRO.A.402, 3HSP.A.750, 4HSW.A.201, 2HUO.A.302, 3I63.A.501, 3I63.A.502, 4I7Z.A.301, 4I7Z.C.301, 2IBN.A.703, 1IB7.A.95, 2IJ4.A.471, 1IO7.A.1401, 4IPS.A.401, 1IQC.A.401, 2IUW.A.500, 2IVF.C.1217, 4J14.A.601, 1J77.A.300, 2J7A.A.1002, 2J7A.A.1003, 2J7A.C.1002, 2J7A.C.1004, 1JDR.A.296, 1JEX.A.95, 1JIP.A.410, 2JJN.A.412, 2JTI.B.104, 3K1O.A.488, 2K3V.A.238, 2K3V.A.261, 2K3V.A.278, 3K3O.A.1, 4K8F.A.300, 4K8F.B.300, 4KIB.A.403, 4KIC.A.403, 4KIG.A.502, 4KJT.A.201, 2KMY.A.233, 1KQG.C.809, 1KQG.C.810, 2KSC.A.125, 4L0F.A.501, 3L1T.A.479, 3L4D.A.481, 1LA6.B.147, 2LDO.A.130, 2LDO.A.168, 1LFK.A.430, 3LGN.A.200, 4LM8.A.806, 4LMH.A.802, 4LXJ.A.601, 2LZZ.A.101, 2LZZ.A.102, 3M15.A.150, 4M2F.B.401, 3M3A.A.155, 4M4A.B.201, 1M7S.A.600, 4M71.A.403, 4MBA.A.148, 5MBA.A.148, 1MDV.A.110, 3MDR.A.505, 4ME4.A.401, 3MGX.A.397, 3MKB.B.137, 1MLW.A.403, 4MLM.A.201, 4MLN.A.201, 4MLN.B.201, 4MLN.B.202, 3MM9.A.580, 1MNY.A.95, 3MOM.A.186, 1MQF.A.501, 1MRP.A.310, 1MTY.D.4, 1MXR.A.1004, 3N3N.B.1500, 4N4K.A.610, 4N4N.A.608, 4N4Y.A.602, 4N6W.A.202, 3N8Y.A.601, 1N9C.A.93, 3N9Q.A.1, 3NA1.A.601, 3NC3.A.406, 1NML.A.402, 2NNB.A.472, 1NPF.A.154, 2NSR.A.154, 1NS9.A.142, 3NYH.A.605, 4NZ2.A.501, 4O6J.A.302, 3O72.A.500, 4O7G.A.301, 1OAF.A.1251, 1OAH.A.1520, 1OAH.A.1521, 1OAH.A.1522, 1OAH.A.1523, 1OAH.A.1524, 3OJT.D.500, 1OQU.C.1008, 3OUI.A.1, 3OUQ.A.601, 3OUQ.A.604, 2OZY.A.201, 3OZV.A.404, 3OZW.A.404, 1P2H.A.801, 1P2H.A.803, 1P2H.A.804, 1P2Y.A.430, 1PA2.A.306, 3PAH.A.425, 2PAC.A.83, 4PAH.A.425, 5PAH.A.425, 3PER.A.1002, 3PF7.A.1001, 3PF7.A.1002, 4PGO.A.301, 4PH9.A.602, 3PT7.B.500, 3PUR.A.1, 3Q3N.A.509, 1Q5D.A.440, 2QDY.A.300, 1QO8.A.601, 1QO8.A.602, 1QO8.A.603, 1QO8.A.604, 3QPI.B.1001, 1QPU.A.107, 1QQ3.A.107, 3QQR.A.163, 3QU8.A.500, 2QUO.A.142, 3QY7.A.264, 2R6S.A.501, 2RF7.A.2, 3RGS.A.1, 3RI7.A.494, 3RIV.A.305, 4RKM.B.808, 3RMK.A.494, 3RMZ.A.500, 1RZ5.A.401, 4S1B.A.802, 3S1J.A.140, 1S73.A.296, 3S8F.A.800, 3S8F.A.801, 3SDN.A.160, 1SMI.A.472, 1SP3.A.807, 1SQ3.D.912, 1STQ.A.600, 3SWZ.B.600, 3T3Z.A.500, 3TF0.A.500, 1TH2.D.2003, 3TJS.A.508, 3TK3.A.500, 3TMC.A.310, 3TOR.A.3, 1TQN.A.508, 4TUV.A.401, 1U13.A.460, 4UAX.A.501, 3UBR.A.471, 3UBR.A.473, 3UBR.A.474, 1UED.A.1430, 1ULI.B.700, 3UOI.B.200, 4UQH.A.1450, 1URV.A.1172, 2V08.A.1087, 2V0M.A.1499, 3V2V.A.154, 4V3Z.B.750, 3V5X.A.201, 2V7I.A.1362, 1VB6.B.1140, 2VEB.A.200, 4VHB.A.150, 2VHB.B.150, 2VHD.B.401, 3VR8.C.201, 3VTH.A.807, 2VZW.B.1209, 1WOG.A.1501, 1W4W.A.1307, 3WCT.D.201, 2WDQ.C.1129, 3WFC.B.802, 3WFD.B.801, 3WFE.B.802, 2WM5.A.450, 4WNV.A.601, 2WU2.C.1130, 2

WU5.C.305, 4WWJ.B.301, 2WX2.A.1450, 2X2N.A.1479, 4X8B.A.508, 2XC3.A.1433, 1XK1.A.300, 1XK3.A.300, 1XQ5.B.148, 1XQ5.C.143, 1XU5.A.1174, 1XVB.A.1171, 1XVG.A.528, 1XVX.A.313, 2Y69.A.516, 2YEV.A.1015, 2YIU.B.500, 1YYG.A.396, 1Z1N.X.603, 1Z1N.X.604, 1Z1N.X.605, 1Z1N.X.606, 1Z1N.X.607, 1Z1N.X.612, 1Z1N.X.613, 1Z1N.X.614, 1Z8U.B.201, 3ZBY.A.1402, 2ZCF.A.206, 3ZE6.B.502, 3ZG3.A.490, 3ZJO.A.200, 1ZOY.C.1305, 3ZPI.A.1407, 2ZPB.A.300, 1ZZH.A.803, 3A51.C.412, 1BT8.B.202, 1C6S.A.88, 2D0Q.A.300, 3DBG.A.500, 3DHG.D.507, 3E5L.A.1408, 3EAH.A.861, 4EGO.A.501, 3GE3.A.502, 4GEP.A.580, 3GPH.A.500, 2GYQ.A.401, 2HBT.A.900, 3HC1.A.305, 3HC1.A.306, 1HDS.A.142, 3HF4.F.147, 1HV4.A.151, 1I4Y.E.605, 2IBN.B.706, 2INN.A.513, 2JOP.A.1342, 1J1L.A.1001, 4JS9.A.501, 1L0L.D.242, 1LC1.A.105, 1LH2.A.154, 1LH6.A.154, 1LH7.A.154, 2LH2.A.154, 3LL8.A.506, 3LMX.M.600, 1M85.A.1001, 3MMB.A.580, 3MZS.A.500, 3NNF.A.600, 3NNL.A.600, 2O68.A.401, 4OZ5.A.201, 3PCJ.R.600, 2PMS.A.347, 3Q9K.A.605, 3QY6.A.264, 2RI4.A.142, 1SO5.A.130, 1SMJ.C.472, 1SQ3.A.903, 1T47.B.430, 2TOH.A.501, 3TYW.A.501, 3U9M.A.201, 1V4U.A.144, 3VOL.A.401, 3WCU.B.201, 3WCU.D.201, 1WRA.A.401, 1Y01.B.142, 2Z36.A.450, 3ZKY.A.1332, 2ZPG.A.300, 2ZZS.2.220
[1] "Cluster 7"
2AA1.B.400, 3ABB.A.1430, 3AK3.C.215, 4AUL.A.754, 2AWC.A.137, 4B2O.A.1266, 1B7V.A.93, 1BBH.A.132, 2BCN.B.109, 1BGP.A.400, 2BIW.C.1492, 4BLY.A.500, 4BM1.A.500, 2BMO.A.1441, 4C50.A.1741, 4CAB.A.537, 2CCY.A.129, 1CG5.A.142, 1CGN.A.128, 1CGO.A.128, 2CJ1.A.1300, 1CPQ.A.130, 1D06.A.501, 1D0C.A.500, 1D2V.A.605, 4D3O.A.901, 3DAX.A.601, 1DRT.A.325, 4DWU.A.201, 3E65.A.901, 1ECD.A.137, 3EH5.A.801, 3EJ8.A.1901, 3EJD.B.405, 4ENP.A.801, 1EOB.B.600, 1EUP.A.410, 1EYS.C.611, 1EYS.C.612, 4FAG.A.401, 4FB2.B.501, 2FDG.A.300, 1FT5.A.214, 3G1Q.A.480, 4G2C.A.501, 1GBU.B.148, 2GB8.A.295, 1GCV.A.141, 1GCV.B.137, 1GVH.A.1398, 1GW2.A.350, 1GWS.A.615, 1GWU.A.1306, 1GY9.A.300, 4H8Q.A.201, 3HF2.A.482, 3HHX.A.281, 3HJ8.A.281, 1HLM.A.159, 2HMQ.B.115, 2HMQ.C.115, 1HRM.A.154, 3HX9.A.300, 1IBE.A.143, 3ICF.A.601, 2IIZ.A.400, 2ISA.A.486, 1IT2.A.147, 1ITH.A.143, 3IVD.B.601, 1IZO.C.501, 4J1X.A.201, 2J2M.A.501, 1JAF.A.130, 3K9V.A.520, 4KFO.A.501, 4KVQ.A.301, 3KX4.A.999, 4L7Y.A.201, 4L7Y.B.201, 2L8M.A.416, 4M26.C.401, 4M71.B.403, 1MBA.A.148, 1MGN.A.154, 2MHR.A.119, 3MM6.B.570, 3MOL.B.185, 1MQV.A.150, 1MXR.A.1003, 3N1Y.A.503, 3N3R.A.1500, 1N97.B.603, 3NKT.A.369, 4NKW.A.600, 2NOX.C.500, 3NU1.A.302, 1NX4.A.300, 1ODN.A.1326, 1OIK.A.1302, 4OJ8.B.301, 1OM4.A.750, 4OQR.A.501, 1OUT.A.143, 1OUT.B.148, 2OYY.A.201, 1P3T.A.300, 2PHD.B.370, 2Q0J.A.998, 3Q14.A.501, 1Q5E.A.440, 2Q9F.A.602, 1QFC.A.401, 1QGJ.A.1350, 1QJS.A.500, 3QPI.A.1001, 1QWL.B.550, 3QY8.A.252, 3QZM.B.201, 3QZX.A.200, 2R1H.D.148, 2R79.A.500, 2RCL.B.600, 2RDN.A.1, 2RFB.A.410, 4S1C.A.801, 3SCF.C.203, 3SIK.A.154, 1SOG.A.296, 1SPG.A.144, 3T4V.A.300, 3TKT.A.431, 1TMX.A.861, 3TM8.A.902, 4TRI.A.501, 3TTV.A.760, 3TTW.A.760, 3U9J.A.200, 3UFK.A.920, 3UHK.A.147, 1V54.A.516, 3VER.A.601, 2VE3.B.1444, 1VHB.A.150, 3VSI.B.401, 2VV6.C.1259, 3W8M.A.201, 3WAQ.A.201, 2WIV.A.1553, 4WXO.B.301, 2XF2.A.690, 2XMO.A.1128, 1XZ5.A.142, 1Y5F.B.147, 1Y5J.B.147, 1YFW.A.300, 2Z3U.A.500, 2Z6F.A.3747, 2ZDO.A.200, 2ZYQ.A.301, 2ZZI.B.209

Table S91. all-ligand-number Fe, compressed group

	size	largest_angle*	middle_1*	middle_2*	middle_3*
1	"70"	"154+/-7.9"	"58.7+/-3.3"	"89.1+/-5.1"	"99.6+/-5.4"
2	"75"	"172.8+/-3.7"	"81.4+/-3.3"	"90.1+/-1.7"	"96.9+/-2.7"
3	"18"	"154.8+/-9.1"	"59.4+/-5"	"87.3+/-5.5"	"95.3+/-5.7"
4	"40"	"167.5+/-8.4"	"74.5+/-6.1"	"89.1+/-2.7"	"99.3+/-3.4"
5	"34"	"148.2+/-9.6"	"58+/-3.8"	"89.3+/-5.7"	"100.8+/-7.2"
6	"61"	"168.6+/-5.2"	"62+/-3.6"	"89.1+/-2.8"	"99.1+/-4.4"

	middle_4*	smallest_opposite_angle*	Tetrahedral	TrigonalBipyramidal
1	"141.8+/-6.3"	"88.3+/-9.4"	"0"	"0"
2	"162.5+/-5.5"	"60.5+/-3.2"	"0"	"0"
3	"117.3+/-9.5"	"143.5+/-8"	"0"	"0"
4	"149.2+/-6.9"	"58.2+/-2.7"	"0"	"0"
5	"117.3+/-12.5"	"99.2+/-10.4"	"0.013"	"0.01"
6	"161.4+/-6.8"	"79.9+/-6.9"	"0"	"0"

	TrigonalBipyramidalVA	TrigonalBipyramidalVP	Octahedral	SquarePyramidal
1	"0.001"	"0.006"	"0"	"0.006"
2	"0"	"0.185"	"0.026"	"0.169"
3	"0"	"0"	"0"	"0"
4	"0"	"0.035"	"0"	"0.013"
5	"0.017"	"0.023"	"0"	"0"
6	"0"	"0.144"	"0.007"	"0.089"

	SquarePyramidalV	SquarePlanar	TrigonalPrismatic	TrigonalPrismaticV
1	"0.024"	"0"	"0"	"0.001"
2	"0.232"	"0.21"	"0"	"0"
3	"0"	"0"	"0"	"0"
4	"0.091"	"0.037"	"0"	"0"
5	"0.008"	"0"	"0"	"0.004"
6	"0.185"	"0.117"	"0"	"0"

	PentagonalBipyramidal	PentagonalBipyramidalVA	PentagonalBipyramidalVP
1	"0"	"0"	"0"
2	"0"	"0"	"0.022"
3	"0"	"0"	"0"
4	"0"	"0"	"0.003"
5	"0"	"0"	"0"
6	"0"	"0"	"0"

	SquareAntiprismatic	SquareAntiprismaticV	HexagonalBipyramidal
1	"0"	"0"	"0"
2	"0"	"0"	"0"
3	"0"	"0"	"0"
4	"0"	"0"	"0"
5	"0"	"0"	"0"
6	"0"	"0"	"0"

	HexagonalBipyramidalVA	HexagonalBipyramidalVP
1	"0"	"0"
2	"0"	"0"
3	"0"	"0"
4	"0"	"0"
5	"0"	"0"
6	"0"	"0"

Table S92. Cluster members of all-ligand-number Fe, compressed group

[1] "Cluster 1"
4GAM.F.602, 4KVQ.A.302, 1NNT.A.333, 2ZI8.A.701, 4AC8.A.500, 1B1X.A.691, 1BIQ.B.376, 3CI8.A.6, 2CKF.A.501, 4D8F.B.402, 2DE6.A.501, 1DLM.A.400, 3E1M.B.301, 3E1N.C.300, 2FKZ.C.1600, 2FKZ.C.1601, 2FLO.A.1602, 3FVB.A.164, 1FYZ.B.5004, 1FZ0.B.5004, 4GAM.F.601, 3GCF.D.501, 3GHQ.K.300, 1GUQ.A.351, 1HSE.A.400, 4HVR.A.201, 3IS8.A.161, 3IS8.E.162, 2ITB.A.502, 2IW4.B.1311, 2J2F.A.371, 1JI5.D.153, 4JPY.A.301,

1LKO.A.601, 1LKP.A.601, 3MPS.D.172, 4N71.A.202, 4NB8.B.501, 4NBC.A.501, 4NBG.C.501, 1NFV.A.200, 3O6R.A.300, 1OQU.B.1004, 3PCD.M.600, 3PCF.N.600, 3PCH.R.600, 1PFR.B.503, 1PIZ.A.376, 1PRC.M.607, 1Q0C.B.500, 3QHB.A.182, 3QVD.B.173, 3QVD.C.175, 3R2R.A.156, 4RC6.A.301, 4RC6.A.302, 1RSV.A.1004, 1SQ3.A.902, 4TOH.A.202, 2UW1.A.1359, 2VZB.B.6204, 3W54.A.501, 2XRX.M.1461, 2XSH.C.901, 2XSH.G.900, 2YFJ.C.901, 1YUZ.A.302, 1YV1.B.301, 2FL0.B.1605, 2XRX.Q.1461
[1] "Cluster 2"
1A8E.A.339, 3AK9.A.168, 3AK9.C.168, 4AQ2.E.800, 4AQ6.D.837, 1B0L.A.694, 1B3E.A.400, 1C7M.A.101, 2CSG.A.504, 2D09.A.430, 1D3K.A.339, 3DU3.M.500, 3DUQ.M.500, 3EMR.A.400, 3FGS.A.402, 1FQE.A.500, 4G51.A.202, 3GCF.A.501, 3GVY.A.162, 2GYQ.B.404, 1H43.A.1315, 1H44.A.1326, 1H76.A.702, 4H99.M.402, 4H9L.M.402, 4HBH.M.402, 3HF8.A.400, 4HR4.A.402, 1IEJ.A.333, 2J8C.M.1307, 4JO0.A.601, 1JQF.A.500, 4KF1.A.402, 4KVR.A.302, 4M26.D.401, 3MPS.F.173, 3MPS.G.172, 1N7W.A.339, 2O0C.A.500, 1OQG.A.500, 3Q3N.A.510, 2QED.A.252, 3QHB.B.182, 3ROG.A.200, 3R1J.A.299, 4RC8.A.302, 3RI7.A.1, 3RMK.D.495, 1RVJ.M.857, 1SQI.A.1450, 1SQY.A.701, 1TH3.D.2003, 1TKP.A.304, 1TKP.B.303, 1UMX.M.1306, 2UW1.A.1360, 2UW1.B.1359, 2V27.A.1268, 3V83.F.703, 3VEO.A.602, 3VEZ.A.601, 1VFE.A.400, 1VRN.M.500, 1W69.A.1350, 2YAV.A.402, 1YUX.A.302, 3AK9.J.168, 4AQ2.B.800, 4AQ6.F.837, 2BJJ.X.694, 1EH3.A.400, 2O84.X.500, 1OQH.A.500, 3TOD.A.694, 3V83.A.703
[1] "Cluster 3"
4QQW.G.1002, 1AFR.B.454, 4BMT.B.1324, 4CVP.A.1155, 3E1N.D.301, 3E1N.H.300, 2FKZ.A.1601, 1GUP.A.351, 3IS8.N.162, 1JYB.A.600, 1NFV.A.201, 1NFV.M.200, 1OQ4.B.364, 1PFR.A.502, 1PIU.A.401, 4TOA.B.203, 4TOE.A.203, 1W2N.A.312
[1] "Cluster 4"
4QQZ.A.1002, 4AM5.A.1161, 4BMT.A.1324, 1DLT.A.400, 1DSN.A.400, 3E1M.A.301, 3E1M.C.300, 3E1N.B.300, 1EYS.M.607, 1FZ6.B.5003, 1FZ6.B.5004, 3IS8.B.162, 1J30.A.401, 1J30.B.404, 1LKO.A.600, 4NB8.A.501, 1NF4.A.200, 2OHJ.D.2501, 3PWF.A.201, 3PWF.A.202, 3PWF.B.201, 3PWF.B.202, 3PZA.A.173, 3PZA.B.173, 4QDF.A.402, 3QVD.H.173, 1R2F.A.401, 1RZH.M.857, 1SP8.B.500, 1UZR.A.1293, 3VVA.A.501, 1VZ4.D.1301, 2VZB.A.6204, 4QQZ.G.1002, 1F9B.A.691, 1GVC.A.1253, 3HH8.A.1, 3MPS.D.1, 3PCC.M.600, 3W54.B.501
[1] "Cluster 5"
4AM4.A.1161, 1CJX.A.629, 3E1Q.A.301, 4ELR.A.401, 3FM3.B.552, 4HJL.A.502, 2HMN.A.450, 2HTN.A.301, 4NB9.A.501, 4NBH.A.501, 2OWT.A.324, 4P9G.A.401, 4REU.A.202, 1SIO.A.321, 4V06.A.1491, 1XIK.B.377, 4CMY.B.1165, 3FE5.A.1, 3FM3.A.452, 1FRF.L.565, 1GUP.D.351, 3GZY.A.701, 1MOJ.A.302, 1O7P.A.1453, 2OHJ.A.501, 1OVB.A.300, 2PHD.C.370, 1PIY.A.377, 1RSR.A.1003, 1SQ3.C.908, 3T81.B.607, 1ULJ.E.600, 3VV9.C.502, 2XSO.O.900
[1] "Cluster 6"
2B1X.E.502, 1BLF.A.700, 1BLF.A.701, 2BOY.G.1255, 2BUR.B.600, 1CE2.A.690, 1CE2.A.691, 3DHI.A.601, 4E2P.A.401, 3GJB.A.320, 4HOW.A.701, 1H76.A.703, 1HAB.A.200, 1HAC.B.200, 3HGI.A.281, 2HMO.A.450, 3IBO.A.999, 1LCT.A.400, 1LFG.A.693, 1LFG.A.694, 1LKM.A.601, 4M1I.A.402, 1MTY.D.3, 4N71.D.201, 3OOR.B.803, 2O7U.A.500, 1OVT.A.689, 4P1B.D.502, 3PCC.O.600, 3PCF.M.600, 3PCH.M.600, 3QJV.A.801, 3QYT.A.680, 2RDB.A.500, 1S9A.A.300, 1SQD.A.500, 1TFD.A.950, 3UF9.A.315, 3VE1.B.702, 1VFD.A.400, 3VMG.B.501, 3W54.A.502, 3WFC.B.803, 3WFD.B.803, 1YUZ.A.301, 3ZK4.A.800, 1B7Z.A.691, 3CHH.A.501, 3EE4.A.317, 1F9B.A.690, 1HDS.B.146, 1LTV.A.900, 3OWO.B.384, 3PCC.N.600, 4PGO.A.302, 3Q1G.B.1002, 1RNR.A.402, 1TKP.D.302, 3W54.B.502, 4WWZ.B.301, 1XZW.A.429

Table S93. all-ligand-number Fe, combined group

	size	largest_angle*	middle_1*	middle_2*	middle_3*
1	"73"	"164.5+/-9"	"77.5+/-9.7"	"88+/-5.7"	"94.7+/-4.2"
2	"131"	"168+/-5.4"	"67.5+/-7.2"	"88.6+/-2.9"	"98.7+/-4.3"
3	"35"	"161.9+/-10.9"	"69.4+/-11.5"	"87.2+/-4.1"	"93.6+/-4.8"
4	"70"	"170.2+/-6.4"	"82.3+/-4"	"88.6+/-2.2"	"94.4+/-3.4"
5	"128"	"170.9+/-6.2"	"78.2+/-7.2"	"89.7+/-2.1"	"97.7+/-3.4"
6	"100"	"151.3+/-8.9"	"60.3+/-6"	"89.3+/-5.2"	"100.2+/-6"
7	"416"	"175.1+/-2.5"	"81.1+/-3.4"	"89.3+/-1.4"	"94.4+/-2.1"
8	"157"	"167.4+/-4.2"	"85.1+/-3.7"	"89.3+/-1.4"	"94.3+/-2.7"
9	"1160"	"178.3+/-1.3"	"86.4+/-2"	"89.7+/-0.5"	"91.8+/-1.1"
10	"100"	"129+/-8.1"	"93.1+/-6.7"	"99.3+/-6.3"	"107.3+/-4.6"

	middle_4*	smallest_opposite_angle*	Tetrahedral	TrigonalBipyramidal
1	"99.9+/-6.5"	"95.1+/-11"	"0"	"0"
2	"160.2+/-5.7"	"80.4+/-6.7"	"0"	"0"
3	"108.7+/-11.9"	"148.5+/-12.5"	"0"	"0"
4	"133.9+/-7.8"	"106.3+/-6.6"	"0"	"0.011"
5	"158+/-8.7"	"60.2+/-4.7"	"0"	"0"
6	"137.1+/-9.5"	"92+/-10.7"	"0.002"	"0"
7	"171.9+/-3.4"	"82.1+/-4.5"	"0"	"0"
8	"163+/-5.7"	"94+/-3.5"	"0"	"0"
9	"177.2+/-1.7"	"86.9+/-2.3"	"0"	"0"
10	"115.5+/-4.7"	"104.1+/-10.6"	"0.008"	"0"

	TrigonalBipyramidalVA	TrigonalBipyramidalVP	Octahedral	SquarePyramidal
1	"0"	"0.007"	"0"	"0"
2	"0.001"	"0.03"	"0"	"0.021"
3	"0"	"0"	"0"	"0"
4	"0.013"	"0.039"	"0"	"0"
5	"0.001"	"0.049"	"0.001"	"0.02"
6	"0"	"0"	"0"	"0"
7	"0"	"0.019"	"0.086"	"0.207"
8	"0"	"0.003"	"0"	"0.021"
9	"0"	"0.003"	"0.254"	"0.358"
10	"0"	"0"	"0"	"0"

	SquarePyramidalV	SquarePlanar	TrigonalPrismatic	TrigonalPrismaticV
1	"0.013"	"0"	"0"	"0"
2	"0.067"	"0.028"	"0"	"0"
3	"0"	"0"	"0"	"0"
4	"0.013"	"0"	"0"	"0"
5	"0.121"	"0.052"	"0"	"0"
6	"0"	"0"	"0"	"0"
7	"0.266"	"0.254"	"0"	"0"
8	"0.053"	"0.059"	"0"	"0"
9	"0.373"	"0.379"	"0"	"0"
10	"0"	"0"	"0"	"0"

	PentagonalBipyramidal	PentagonalBipyramidalVA	PentagonalBipyramidalVP
1	"0"	"0"	"0"
2	"0"	"0"	"0"
3	"0"	"0"	"0"
4	"0"	"0"	"0"
5	"0"	"0"	"0"
6	"0"	"0"	"0"
7	"0"	"0"	"0"
8	"0"	"0"	"0"

```
9  "0"                   "0"                      "0"
10 "0"                   "0"                      "0"
   SquareAntiprismatic SquareAntiprismaticV HexagonalBipyramidal
1  "0"                 "0"                  "0"
2  "0"                 "0"                  "0"
3  "0"                 "0"                  "0"
4  "0"                 "0"                  "0"
5  "0"                 "0"                  "0"
6  "0"                 "0"                  "0"
7  "0"                 "0"                  "0"
8  "0"                 "0"                  "0"
9  "0"                 "0"                  "0"
10 "0"                 "0"                  "0"
   HexagonalBipyramidalVA HexagonalBipyramidalVP
1  "0"                    "0"
2  "0"                    "0"
3  "0"                    "0"
4  "0"                    "0"
5  "0"                    "0"
6  "0"                    "0"
7  "0"                    "0"
8  "0"                    "0"
9  "0"                    "0"
10 "0"                    "0"
```

Table S94. Cluster members of all-ligand-number Fe, combined group

[1] "Cluster 1"
4AC8.B.500, 2BI4.A.1384, 4C4U.B.201, 4CMY.A.1164, 4CMY.B.1165, 4D8F.A.401, 1DGG.A.2000, 1DT0.A.1601, 1E2U.A.701, 3FE5.A.1, 3FM3.A.452, 3FMR.B.401, 3GZY.A.701, 1H2A.L.1004, 1HJF.A.1310, 1HJG.A.1307, 1I4Y.B.602, 1I4Z.D.604, 1I4Z.G.607, 2IBN.A.704, 3ICF.A.602, 1IDS.C.208, 2INP.A.3, 2ITB.B.501, 2J2F.E.371, 1KBP.A.438, 4KBP.A.438, 3KCY.A.1350, 4KEV.D.401, 1LGT.A.500, 1LKD.A.500, 3LKT.M.600, 3LMX.O.600, 1MOJ.A.302, 1MOJ.B.301, 1NNF.A.401, 1NX8.A.300, 1O7P.A.1453, 2OHH.B.1501, 2OHH.E.3501, 1OQ9.A.365, 1OS7.C.302, 3PCB.O.600, 3PCE.M.600, 3PCJ.M.600, 2PHD.C.370, 1PIY.A.376, 1PIY.A.377, 3PM5.A.1002, 3Q1G.A.1001, 3Q1G.B.1001, 1QFC.A.402, 2QJE.A.692, 2R2F.B.320, 4RC5.A.1003, 1RSR.A.1003, 1SQ3.B.906, 1SQ3.C.907, 1SQ3.C.908, 4TOA.A.206, 4TOA.B.207, 3U52.B.512, 3U9M.E.205, 1ULJ.E.600, 2VHL.A.1398, 3VTI.B.803, 1W69.A.1349, 3WHN.B.201, 2XRX.G.1461, 1XZW.B.929, 1YKM.J.600, 2ZQX.A.501, 1ZZ9.C.199
[1] "Cluster 2"
1B1X.A.690, 2B1X.E.502, 4B2O.A.1267, 1BLF.A.700, 1BLF.A.701, 2BOY.G.1255, 2BUR.B.600, 2CAG.A.485, 1CE2.A.690, 1CE2.A.691, 1D9Y.A.310, 3DHI.A.601, 1DMH.A.400, 4E2P.A.401, 2FR7.A.501, 1FZ1.A.5001, 1FZ3.A.5002, 2G1M.A.600, 1GGF.B.760, 3GJB.A.320, 4HOW.A.701, 1H76.A.703, 3HGI.A.281, 2HMO.A.450, 1I4Z.A.601, 4I4G.A.601, 4I4H.A.601, 3IBO.A.999, 1JNF.A.702, 1JNF.A.703, 4K9T.A.601, 4K9U.A.601, 4K9V.A.601, 4K9X.A.601, 1KW9.B.301, 1LCT.A.400, 1LFG.A.693, 1LFG.A.694, 1LKM.A.601, 4M1I.A.402, 4ME4.A.402, 1MMO.D.3, 1MMO.D.4, 1MTY.D.3, 3MZS.C.500, 4N71.D.201, 1NX4.B.300, 3OOR.B.803, 2O7U.A.500, 4OJ8.C.301, 1OVT.A.689, 4P1B.D.502, 3P3N.A.350, 3PCC.O.600, 3PCF.M.600, 3PCH.M.600, 3Q3M.A.509, 3QFN.A.265, 3QJV.A.801, 3QY6.A.263, 3QYT.A.680, 1R1N.C.400, 2RDB.A.499, 2RDB.A.500, 3RNC.A.499, 1S9A.A.300, 1SQD.A.500

, 1TOQ.A.499, 1TFD.A.950, 3UF9.A.315, 3VE1.B.702, 1VFD.A.400, 3VMG.B.501, 3VVA.B .502, 3W54.A.502, 3WFC.B.803, 3WFD.B.803, 1WRA.B.402, 1XVF.B.1174, 1XVG.A.529, 1 YGF.B.147, 1YUZ.A.301, 2ZI8.A.701, 3ZK3.A.1311, 3ZK4.A.800, 4ANP.A.1426, 1B7Z.A. 690, 1B7Z.A.691, 3CHH.A.501, 3EE4.A.317, 1F9B.A.690, 1FRV.B.537, 1FZ1.B.5003, 3G CF.D.501, 1H2K.A.1350, 1HDS.B.146, 1LTV.A.900, 4M26.B.401, 3MPS.D.172, 1N04.A.68 8, 3N1Y.A.504, 4N71.A.201, 3O32.A.300, 3O6J.A.300, 4OJ8.B.301, 3OWO.B.384, 3PCA. M.600, 3PCC.N.600, 3PCJ.N.600, 3PCK.M.600, 4PGO.A.302, 2PHD.B.370, 3Q1G.B.1002, 1QFC.A.401, 2R2F.A.320, 1RNR.A.402, 1SP8.A.500, 1TKP.D.302, 3UF9.B.315, 1UOF.A.1 311, 1UTE.A.501, 2UW1.A.1359, 3VER.A.601, 1VZ4.A.1299, 3W54.B.502, 4WWZ.B.301, 1 WZD.A.901, 1XVE.A.1170, 1XZW.A.429, 2YDE.A.501, 1ZJ9.B.1569

[1] "Cluster 3"

4QQW.G.1002, 1AFR.B.454, 4BMT.B.1323, 4BMT.B.1324, 4CVP.A.1155, 3DHG.A.501, 3E1N .D.301, 3E1N.H.300, 2FKZ.A.1601, 1FZH.B.5004, 1GUP.A.351, 3I4V.A.281, 3IS8.N.162 , 2ITB.A.501, 4IWK.F.201, 1JYB.A.600, 1N7X.A.339, 1NFV.A.201, 1NFV.M.200, 3NJZ.A .369, 1OQ4.B.364, 2P6B.C.513, 3PCA.N.600, 3PCL.R.600, 1PFR.A.502, 1PIU.A.401, 3R 2M.A.155, 1T47.A.431, 4TOA.B.203, 4TOE.A.203, 3USS.B.212, 2VC7.D.1315, 1W2N.A.31 2, 2XSO.K.900, 2Z4G.A.503

[1] "Cluster 4"

4QQZ.C.1001, 4AIQ.A.1746, 1B06.A.322, 2BKB.A.1193, 1BS3.A.202, 2BUT.B.1541, 2BUU .B.1541, 2BUV.B.1541, 2BUW.B.600, 2BUM.B.1541, 2BUQ.B.1541, 3CEI.A.500, 2CW2.A.4 02, 2CW3.A.524, 4DVH.A.301, 3E13.X.326, 1EO2.B.600, 3ESF.A.198, 4F2N.B.300, 4FFK .A.301, 3FG3.D.1500, 3FG4.A.1500, 4G2D.A.402, 1GN6.A.999, 2GOJ.A.198, 2GPC.A.195 , 3H1S.A.1001, 3HHY.A.281, 3HJQ.A.281, 3HKP.A.281, 1HMD.A.115, 3I4Y.A.281, 3I51. A.281, 4IEZ.A.501, 1ISA.A.193, 3IS8.A.163, 4J6C.A.502, 4KEZ.A.401, 1KW6.B.301, 4 L2B.A.201, 4L2C.A.201, 3LIO.A.5000, 1MY6.B.200, 3O5U.A.300, 4P1B.D.501, 3PCE.N.6 00, 3PCN.P.600, 2PCD.N.600, 2QFR.A.433, 3QFM.B.264, 1QNN.A.201, 2R1K.A.800, 2R1N .A.800, 3R2R.A.155, 4REU.A.201, 1SQ3.A.901, 1TDW.A.425, 3TQJ.A.1001, 1UNF.X.1239 , 2VHL.A.1397, 2VP1.A.1347, 2W7W.A.1195, 1WB7.A.212, 1WB8.A.212, 1XM8.A.701, 1YF X.A.300, 1Z6O.M.300, 1ZA5.B.393, 2GBX.A.456, 1OQU.A.1001

[1] "Cluster 5"

4QQZ.A.1002, 1A8E.A.339, 3AK9.A.168, 3AK9.C.168, 4AM5.A.1161, 4AQ2.E.800, 4AQ6.D .837, 1B0L.A.694, 1B3E.A.400, 3BFJ.M.1388, 4BMT.A.1324, 2BVO.B.600, 1C7M.A.101, 8CAT.A.507, 2CSG.A.504, 1D3K.A.339, 1DLT.A.400, 1DSN.A.400, 3DU3.M.500, 3DUQ.M.5 00, 3E1M.A.301, 3E1M.C.300, 3E1N.B.300, 3EMR.A.400, 1EYS.M.607, 3FGS.A.402, 1FQE .A.500, 1FSL.A.144, 1FZ6.B.5003, 1FZ6.B.5004, 4G51.A.202, 3GCF.A.501, 3GVY.A.162 , 2GYQ.B.404, 1H43.A.1315, 1H44.A.1326, 1H76.A.702, 4H99.M.402, 4H9L.M.402, 4HBH .M.402, 3HF8.A.400, 4HR4.A.402, 2HUO.A.301, 1IEJ.A.333, 3IS8.B.162, 1J30.A.401, 1J30.B.404, 2J8C.M.1307, 4JOO.A.601, 1JQF.A.500, 4KF1.A.402, 4KVR.A.302, 1LKO.A. 600, 4M26.D.401, 3MPS.F.173, 3MPS.G.172, 1N7W.A.339, 3N9T.A.292, 4NB8.A.501, 1NF 4.A.200, 2OOC.A.500, 2OHJ.D.2501, 1OQG.A.500, 3PWF.A.201, 3PWF.A.202, 3PWF.B.201 , 3PWF.B.202, 3PZA.A.173, 3PZA.B.173, 2QOJ.B.998, 3Q1G.D.1002, 4QDF.A.402, 2QED. A.252, 3QFO.A.264, 3QHB.B.182, 3QVD.H.173, 3QY8.A.253, 3R0G.A.200, 3R1J.A.299, 1 R2F.A.401, 4RC8.A.302, 3RI7.A.1, 3RMK.D.495, 1RVJ.M.857, 1RZH.M.857, 1SP8.B.500, 1SQI.A.1450, 1SQY.A.701, 1SYY.A.1319, 1TH3.D.2003, 1TKP.A.304, 1TKP.B.303, 1UMX .M.1306, 2UW1.A.1360, 2UW1.B.1359, 1UZR.A.1293, 2V27.A.1268, 3V83.F.703, 3VEO.A. 602, 3VEZ.A.601, 1VFE.A.400, 1VRN.M.500, 2VUN.A.402, 3VVA.A.501, 1VZ4.D.1301, 2V ZB.A.6204, 1W69.A.1350, 2YAV.A.402, 1YUX.A.302, 4QQZ.G.1002, 3AK9.J.168, 4AQ2.B. 800, 4AQ6.F.837, 2BJJ.X.694, 1EH3.A.400, 1F9B.A.691, 1GVC.A.1253, 3HH8.A.1, 3MPS .D.1, 2O84.X.500, 1OQH.A.500, 3PCC.M.600, 3Q3O.A.502, 3TOD.A.694, 3V83.A.703, 1W 2A.X.1302, 3W54.B.501, 3E6S.A.200

[1] "Cluster 6"

4GAM.F.602, 4KVQ.A.302, 1NNT.A.333, 4AC8.A.500, 4AM4.A.1161, 1B1X.A.691, 1BIQ.A. 376, 1BIQ.B.376, 4BMT.A.1323, 3CI8.A.6, 1CJX.A.629, 2CKF.A.501, 4D8F.B.402, 2DE6 .A.501, 1DLM.A.400, 3E1M.B.301, 3E1N.C.300, 3E1Q.A.301, 4ELR.A.401, 2FKZ.C.1600,

2FKZ.C.1601, 2FL0.A.1602, 3FM3.B.552, 3FVB.A.164, 1FYZ.B.5004, 1FZ0.B.5004, 4GA
M.F.601, 3GHQ.K.300, 1GUQ.A.351, 3HJ8.A.281, 3HJS.A.281, 4HJL.A.502, 2HMN.A.450,
1HSE.A.400, 2HTN.A.301, 4HVR.A.201, 3IS8.A.161, 3IS8.E.162, 2ITB.A.502, 2IW4.B.
1311, 2J2F.A.371, 1JI5.D.153, 4JPY.A.301, 4KVQ.A.301, 1LKO.A.601, 1LKP.A.601, 4N
71.A.202, 4NB8.B.501, 4NB9.A.501, 4NBC.A.501, 4NBG.C.501, 4NBH.A.501, 1NFT.A.333
, 1NFV.A.200, 3O6R.A.300, 1OQU.B.1004, 2OWT.A.324, 4P9G.A.401, 3PCD.M.600, 3PCF.
N.600, 3PCH.R.600, 1PFR.B.503, 1PIZ.A.376, 1PRC.M.607, 1Q0C.B.500, 3QHB.A.182, 3
QVD.B.173, 3QVD.C.175, 3R2R.A.156, 4RC6.A.301, 4RC6.A.302, 4REU.A.202, 1RSV.A.10
04, 1SI0.A.321, 1SQ3.A.902, 4TOH.A.202, 4V06.A.1491, 2VZB.B.6204, 3W54.A.501, 1X
IK.B.377, 2XRX.M.1461, 2XSH.C.901, 2XSH.G.900, 1YCH.A.501, 2YFJ.C.901, 1YUZ.A.30
2, 2YU1.A.600, 1YV1.B.301, 2FKZ.A.1600, 2FL0.B.1605, 1FRF.L.565, 1GUP.D.351, 2OH
J.A.501, 1OVB.A.300, 3QFM.A.264, 3T81.B.607, 3VV9.C.502, 2XRX.Q.1461, 2XSO.O.900
, 1YKP.F.2600
[1] "Cluster 7"
3A0G.A.201, 2A1X.A.450, 3A16.B.354, 3A17.A.354, 2A1O.A.417, 1A2F.A.1, 1A2S.A.90,
2A3F.X.201, 3A51.B.412, 1A7E.A.119, 3ABM.A.516, 1ASH.A.301, 4AUM.A.900, 2AVK.A.
201, 3AYF.A.801, 3B0H.B.601, 2B11.D.1301, 2B2O.B.1500, 2B2R.A.1500, 4B2N.A.701,
4B7G.A.3000, 4B8N.A.201, 2BC5.A.150, 3BDZ.A.450, 1BEP.A.296, 4BGL.A.1001, 1BIN.B
.144, 1BJ9.A.296, 1BKA.A.693, 1BKA.A.694, 2BMM.A.1157, 3BOM.A.143, 3BOM.B.148, 2
BQ8.X.1305, 2BQ8.X.1306, 2BUZ.B.1541, 2C1D.A.1291, 1C2N.A.117, 1CC5.A.1, 1CED.A.
90, 1CH3.A.154, 4CHL.A.501, 2CJ2.A.1300, 4CK9.A.1480, 4CKA.A.1480, 1CLS.A.142, 2
CVC.A.1013, 4CVJ.A.1295, 2CYP.A.295, 2CZ1.A.300, 2D09.A.430, 4D02.A.602, 4D02.A.
603, 2D2M.D.200, 4D38.A.500, 1D4D.A.601, 1D4D.A.602, 1DCC.A.296, 1DD7.A.600, 3DH
G.A.502, 3DHI.A.600, 1DLW.A.144, 1DM1.A.148, 1DP8.A.719, 1DP9.A.719, 1DRY.A.332,
4DTW.A.500, 4DTY.A.500, 3DXU.A.360, 3E2O.A.296, 2E3B.A.401, 3E6S.B.200, 2E84.A.
1303, 2E84.A.1311, 3EH5.A.800, 3EHB.A.560, 1EHE.A.501, 3EJ6.A.4000, 4EJI.A.501,
4ENH.A.601, 4ENU.B.801, 1EO9.B.600, 1EOC.B.600, 4EP6.A.601, 1F4U.A.410, 4F4O.B.2
01, 2FAM.A.148, 1FCD.C.902, 4FIA.A.600, 1FZ7.B.5003, 1G09.B.147, 4G51.B.202, 4G7
0.A.601, 4G71.A.602, 4G7G.A.501, 4G7Q.A.602, 2GEP.A.580, 3GE3.A.501, 3GEO.A.580,
7GEP.A.580, 4GHF.B.401, 2GJ1.A.605, 3GM6.A.1004, 4GP4.A.602, 4GP5.A.602, 4GP8.A
.602, 2GSM.A.2002, 1GWS.A.601, 1GWS.A.605, 1GWS.A.609, 1GWS.A.610, 1GWS.A.611, 1
GWS.A.612, 1GWS.A.613, 1GWS.A.616, 1GWT.A.350, 2H0V.A.501, 2H0V.B.500, 1H29.A.11
08, 1H29.A.1114, 4H44.A.301, 1HAB.A.200, 1HAC.B.200, 2HBU.A.900, 3HB3.A.559, 3HB
3.A.560, 1HBZ.A.504, 2HBD.A.142, 1HMO.B.115, 3HQ6.A.400, 3HQ7.A.401, 1HRO.A.107,
4HRO.A.402, 2HUO.A.302, 3I63.A.501, 3I63.A.502, 4I7Z.A.301, 4I7Z.C.301, 2IBN.A.
703, 2IJ4.A.471, 2INC.A.502, 1IO7.A.1401, 4IPS.A.401, 1IQC.A.401, 2IUW.A.500, 2I
VF.C.1217, 3IXF.A.139, 1IZO.A.501, 4J14.A.601, 1J77.A.300, 2J7A.A.1002, 2J7A.C.1
002, 2J7A.C.1004, 1JEX.A.95, 1JIP.A.410, 2JJN.A.412, 2JTI.B.104, 4K0F.A.601, 3K1
O.A.488, 2K3V.A.238, 2K3V.A.261, 3K3O.A.1, 4K8F.A.300, 4K9W.A.601, 4KIB.A.403, 4
KIC.A.403, 4KIG.A.502, 4KJT.A.201, 1KQG.C.810, 2KSC.A.125, 3KT7.A.701, 4L0F.A.50
1, 3L1T.A.479, 3L4D.A.481, 1LA6.B.147, 2LDO.A.130, 2LDO.A.168, 1LFK.A.430, 4LMH.
A.802, 3LXV.M.600, 4LXJ.A.601, 2LZZ.A.101, 2LZZ.A.102, 4M25.A.401, 4M2F.B.401, 3
M3A.A.155, 4M4A.B.201, 1M7S.A.600, 4M71.A.403, 4M73.B.403, 4MBA.A.148, 5MBA.A.14
8, 3MDR.A.505, 3MDT.A.505, 4ME4.A.401, 3MGX.A.397, 3MKB.B.137, 1MLW.A.403, 4MLN.
B.201, 4MLN.B.202, 3MOO.A.911, 1MRP.A.310, 1MTY.D.4, 3N20.A.506, 3N3N.B.1500, 4N
4K.A.610, 4N4N.A.608, 4N4Y.A.602, 4N6W.A.202, 3N8Y.A.601, 1N9C.A.93, 3N9Q.A.1, 3
NA1.A.601, 3NC3.A.406, 2NNB.A.472, 2NOX.A.500, 1NPF.A.154, 2NSR.A.154, 1NS9.A.14
2, 3NYH.A.605, 4NZ2.A.501, 4O6J.A.302, 3O72.A.500, 1OAF.A.1251, 1OAH.A.1520, 1OA
H.A.1522, 1OAH.A.1523, 1OCZ.A.516, 3OJT.D.500, 1OQU.C.1008, 1OQU.C.1009, 1OQU.C.
1010, 3OUH.A.600, 3OUI.A.1, 2OZY.A.201, 3OZW.A.404, 1P2H.A.801, 1P2H.A.803, 1P2H
.A.804, 3PAH.A.425, 2PAC.A.83, 4PAH.A.425, 5PAH.A.425, 3PCJ.O.600, 2PCC.A.296, 3
PER.A.1001, 3PER.A.1002, 3PF7.A.1001, 3PF7.A.1002, 4PG0.A.301, 1PHG.A.417, 4PH9.
A.602, 1PIU.A.402, 3PT7.B.500, 3PUQ.A.1, 3PUR.A.1, 3Q14.A.502, 3Q1G.A.1002, 3Q3N
.A.509, 3Q3N.A.510, 1Q5D.A.440, 2QDY.A.300, 1QO8.A.601, 1QO8.A.602, 1QO8.A.604,

2QPP.A.300, 3QPI.B.1001, 1QPU.A.107, 3QU8.A.500, 2QU0.A.142, 3QY7.A.263, 3QY7.A.
264, 2R6S.A.501, 4RC8.A.303, 3RI7.A.494, 3RIV.A.305, 3RMK.A.494, 3RMZ.A.500, 3RN
C.A.500, 3RNF.A.500, 3RNF.A.501, 1RYO.A.329, 4S1B.A.802, 1S73.A.296, 3S8F.A.801,
3SDN.A.160, 1SMI.A.472, 1SP3.A.807, 1SQ3.D.912, 1STQ.A.600, 1SYY.A.1320, 1TOQ.A
.500, 3TK3.A.500, 3TMC.A.310, 3TMZ.A.501, 3TOR.A.3, 1TQN.A.508, 3TTX.A.760, 4TUV
.A.401, 1U74.B.1101, 4UAX.A.501, 3UBR.A.471, 3UBR.A.473, 1UED.A.1430, 1ULI.B.700
, 4V3Z.B.750, 3V5X.A.201, 2V7I.A.1362, 2VHD.B.401, 3VR8.C.201, 3VTH.A.807, 2VZW.
B.1209, 1W0G.A.1501, 1W4W.A.1307, 3WEC.A.501, 3WFC.B.802, 3WFE.B.802, 2WM5.A.450
, 4WNV.A.601, 2WU2.C.1130, 2WU5.C.305, 4WWJ.B.301, 2X2N.A.1479, 4X8B.A.508, 2X9P
.A.1398, 2XC3.A.1433, 1XK1.A.300, 1XK3.A.300, 1XU5.A.1174, 1XU5.A.1175, 1XVB.A.1
170, 1XVB.A.1171, 1XVF.B.1175, 1XVG.A.528, 1XVX.A.313, 2Y69.A.516, 1Y8W.A.142, 2
YIU.B.500, 1YMA.A.154, 1YYG.A.396, 1Z1N.X.602, 1Z1N.X.603, 1Z1N.X.604, 1Z1N.X.60
5, 1Z1N.X.606, 1Z1N.X.613, 1Z1N.X.614, 1Z8U.B.201, 2ZCF.A.206, 3ZG3.A.490, 3ZLI.
A.4001, 1ZOY.C.1305, 3ZPI.A.1407, 4AC8.D.500, 1AHJ.A.208, 1BT8.B.202, 3BXD.A.302
, 2D0Q.A.300, 3DBG.A.500, 3DHG.D.507, 3E5L.A.1408, 3EAH.A.861, 3GE3.A.502, 4GEP.
A.580, 3GPH.A.500, 2GYQ.A.401, 1H2L.A.1350, 2HBT.A.900, 3HC1.A.305, 3HC1.A.306,
1HDS.A.142, 3HF4.F.147, 1HV4.A.151, 1I4Y.E.605, 2IBN.B.706, 4IGO.A.1000, 2INN.A.
513, 2JOP.A.1342, 1J1L.A.1001, 1L0L.D.242, 1LC1.A.105, 1LH2.A.154, 1LH6.A.154, 1
LH7.A.154, 2LH2.A.154, 3LL8.A.506, 3LMX.M.600, 1M85.A.1001, 3MZS.A.500, 3NNF.A.6
00, 3NNL.A.600, 3OOF.A.304, 2O68.A.401, 2OGI.A.301, 4OZ5.A.201, 3PCJ.R.600, 2PMS
.A.347, 2PQ7.A.221, 1QHW.A.433, 3QY6.A.264, 2RI4.A.142, 1S05.A.130, 1SMJ.C.472,
1SQ3.A.903, 1T47.B.430, 1TMX.A.861, 2TOH.A.501, 3U9M.A.201, 1WRA.A.401, 2XMO.B.5
56, 2YOI.A.1350, 1Y01.B.142, 2Z36.A.450, 3ZKY.A.1332, 2ZPG.A.300, 2ZZS.2.220
[1] "Cluster 8"
3OOR.B.802, 2AA1.B.400, 3ABB.A.1430, 3AK3.C.215, 4AUL.A.754, 2AWC.A.137, 4B2O.A.
1266, 1B7V.A.93, 1BBH.A.132, 2BCN.B.109, 1BGP.A.400, 2BIW.C.1492, 4BLY.A.500, 4B
M1.A.500, 2BMO.A.1441, 4C50.A.1741, 4CAB.A.537, 2CCY.A.129, 1CG5.A.142, 1CGN.A.1
28, 1CGO.A.128, 2CJ1.A.1300, 1CPQ.A.130, 1D06.A.501, 1DOC.A.500, 1D2V.A.605, 4D3
O.A.901, 3DAX.A.601, 1DRT.A.325, 4DWU.A.201, 3E65.A.901, 1ECD.A.137, 3EH5.A.801,
3EJ8.A.1901, 3EJD.B.405, 4ENP.A.801, 1EOB.B.600, 1EUP.A.410, 1EYS.C.609, 1EYS.C
.611, 1EYS.C.612, 4FAG.A.401, 4FB2.B.501, 2FDG.A.300, 1FT5.A.214, 3G1Q.A.480, 4G
2C.A.501, 1GBU.B.148, 2GB8.A.295, 1GCV.A.141, 1GCV.B.137, 1GVH.A.1398, 1GW2.A.35
0, 1GWS.A.615, 1GWU.A.1306, 1GY9.A.300, 4H8Q.A.201, 3HF2.A.482, 3HHX.A.281, 1HLM
.A.159, 2HMQ.B.115, 2HMQ.C.115, 1HRM.A.154, 3HX9.A.300, 1IBE.A.143, 3ICF.A.601,
2IIZ.A.400, 2ISA.A.486, 1IT2.A.147, 1ITH.A.143, 3IVD.B.601, 1IZO.C.501, 4J1X.A.2
01, 2J2M.A.501, 1JAF.A.130, 4JS9.A.501, 3K9V.A.520, 4KFO.A.501, 3KX4.A.999, 4L7Y
.A.201, 4L7Y.B.201, 2L8M.A.416, 4M26.C.401, 4M71.B.403, 1MBA.A.148, 1MGN.A.154,
2MHR.A.119, 3MM6.B.570, 3MOL.B.185, 1MQV.A.150, 1MXR.A.1003, 3N1Y.A.503, 3N3R.A.
1500, 1N97.B.603, 3NKT.A.369, 4NKW.A.600, 2NOX.C.500, 3NU1.A.302, 1NX4.A.300, 1O
DN.A.1326, 1OIK.A.1302, 1OM4.A.750, 4OQR.A.501, 1OUT.A.143, 1OUT.B.148, 2OYY.A.2
01, 1P3T.A.300, 2Q0J.A.998, 3Q14.A.501, 1Q5E.A.440, 2Q9F.A.602, 1QGJ.A.1350, 1QJ
S.A.500, 3QPI.A.1001, 1QWL.B.550, 3QY8.A.252, 3QZM.B.201, 3QZX.A.200, 2R1H.D.148
, 2R79.A.500, 2RCL.B.600, 2RDN.A.1, 2RFB.A.410, 4S1C.A.801, 3SCF.C.203, 3SIK.A.1
54, 1SOG.A.296, 1SPG.A.144, 3T4V.A.300, 3TKT.A.431, 3TM8.A.902, 4TRI.A.501, 3TTV
.A.760, 3TTW.A.760, 3U9J.A.200, 3UFK.A.920, 3UHK.A.147, 1V54.A.516, 2VE3.B.1444,
1VHB.A.150, 3VSI.B.401, 2VV6.C.1259, 3W8M.A.201, 3WAQ.A.201, 2WIV.A.1553, 4WXO.
B.301, 2XF2.A.690, 2XM0.A.1128, 1XZ5.A.142, 1Y5F.B.147, 1Y5J.B.147, 1YFW.A.300,
2Z3U.A.500, 2Z6F.A.3747, 2ZDO.A.200, 2ZYQ.A.301, 2ZZI.B.209
[1] "Cluster 9"
19HC.A.301, 19HC.A.302, 19HC.A.303, 19HC.A.304, 19HC.A.305, 19HC.A.306, 19HC.A.3
07, 19HC.A.308, 19HC.A.309, 3A15.B.354, 2A3M.A.501, 2A3M.A.502, 2A3M.A.503, 2A3M
.A.504, 1A4E.A.503, 1A56.A.82, 3A8G.A.301, 3A8L.A.300, 3A9F.A.207, 2A9E.A.550, 4
AAL.A.423, 4AAN.A.400, 4AAN.A.401, 4AAO.A.400, 2AIU.A.200, 2AI5.A.81, 1AKK.A.105
, 4AM5.A.1160, 1AOF.A.602, 1AOF.B.601, 1AOM.A.602, 1AOM.B.601, 1AOM.B.602, 4APY.

A.1418, 1AQA.A.95, 1AQE.A.121, 1AQE.A.122, 3AQ5.A.144, 3AQ9.A.144, 3AT5.A.142, 3
AT5.B.147, 2AT3.X.185, 3AWM.A.501, 1AWP.A.201, 2B0Z.B.109, 2B10.D.909, 4B2N.A.70
0, 2B4Z.A.500, 3B42.A.199, 3B42.B.399, 3B47.A.199, 4B4Y.A.1155, 3B6H.B.600, 1B80
.A.350, 1B82.A.350, 1B85.A.350, 3B99.A.600, 2BDM.A.500, 1BFR.B.200, 2BGV.X.1121,
2BH4.X.1123, 1BJE.A.154, 4BJA.A.300, 4BJK.A.1450, 3BK9.B.401, 2BK9.A.1154, 2BLF
.B.1582, 4BMM.A.1450, 3BNG.A.513, 3BNJ.A.513, 3BNJ.A.514, 3BNJ.A.515, 3BNJ.A.516
, 3BNJ.A.517, 2BPN.A.108, 2BPN.A.109, 2BPN.A.110, 2BPN.A.111, 2BQ4.A.1115, 2BQ4.
A.1116, 2BQ4.A.1117, 2BQ4.A.1118, 2BS2.C.1255, 2BS2.C.1256, 2BS3.C.1255, 2BS4.C.
1255, 1BVY.A.1000, 3BXU.A.72, 3BXU.A.73, 3BXU.A.74, 4C0C.A.1450, 4C0N.A.1157, 2C
1U.A.401, 2C1V.A.401, 2C1V.A.402, 2C1D.A.1292, 2C1D.B.1158, 3C2C.A.113, 1C2R.A.1
20, 4C27.A.1450, 4C28.A.1450, 1C40.A.150, 4C44.A.1151, 1C52.A.200, 3C62.A.150, 3
C63.A.150, 1C6R.A.90, 1C75.A.93, 3C76.X.185, 3C78.X.185, 2C8S.A.1173, 4C9M.A.141
8, 3CAO.A.104, 3CAO.A.105, 3CAO.A.106, 3CAO.A.107, 1CCH.A.83, 1CCR.A.112, 2CDV.A
.109, 2CDV.A.111, 4CDP.A.402, 2CE0.A.1102, 1CH1.A.154, 1CH2.A.154, 1CH5.A.154, 1
CH7.A.154, 1CH9.A.154, 1CI3.M.254, 4CK8.A.1480, 1CNO.A.200, 2CN4.A.1175, 2CN4.B.
1175, 1CO6.A.108, 1COR.A.83, 1COT.A.130, 4COH.A.1450, 4COO.A.1549, 3CP5.A.202, 1
CPT.A.430, 3CQV.A.601, 3CSL.A.866, 2CTH.A.109, 2CTH.A.110, 2CTH.A.111, 2CTH.A.11
2, 3CU4.A.199, 2CVC.A.1001, 2CVC.A.1002, 2CVC.A.1003, 2CVC.A.1004, 2CVC.A.1005,
2CVC.A.1006, 2CVC.A.1007, 2CVC.A.1008, 2CVC.A.1009, 2CVC.A.1010, 2CVC.A.1011, 2C
VC.A.1012, 2CVC.A.1014, 2CVC.A.1016, 3CX5.C.4001, 3CX5.C.4002, 3CX5.D.4003, 3CX5
.W.4026, 1CXA.A.126, 1CXC.A.125, 3CXH.W.4026, 1CXY.A.204, 1CYI.A.200, 3CYR.A.203
, 3CYR.A.204, 2CY3.A.119, 2CY3.A.120, 2CY3.A.121, 2CY3.A.122, 5CYT.R.105, 2CZS.A
.500, 2CZS.A.501, 1CZJ.A.119, 1CZJ.A.120, 1CZJ.A.121, 1CZJ.A.122, 3CZY.A.300, 2D
0W.A.200, 2D0S.A.80, 2D0T.A.404, 4D30.B.750, 4D34.A.500, 4D35.A.500, 4D36.A.500,
4D37.A.500, 4D3A.A.500, 1D4D.A.603, 1D4D.A.604, 1D7B.A.401, 3D7O.A.143, 2DC3.A.
191, 3DE8.A.150, 2DGE.A.200, 3DHH.A.501, 3DHR.A.142, 1DJ5.A.1, 1DK0.A.200, 3DMI.
A.146, 2DN1.A.142, 2DN1.B.147, 3DR0.A.94, 1DTI.A.154, 4DTZ.A.500, 1DUW.A.293, 1D
UW.A.297, 1DUW.A.300, 1DUW.A.301, 1DW0.A.113, 1DW2.A.113, 1DWL.B.80, 4DXY.A.501,
1DY7.B.601, 2DY5.A.300, 4DY9.A.201, 1E08.E.80, 1E29.A.136, 1E2R.B.601, 1E2W.A.9
00, 1E39.A.801, 1E39.A.802, 1E39.A.803, 1E39.A.804, 2E3A.A.401, 3E5J.A.1408, 3E5
K.A.1408, 1E8E.A.125, 2E80.A.1508, 2E84.A.1301, 2E84.A.1302, 2E84.A.1304, 2E84.A
.1305, 2E84.A.1306, 2E84.A.1307, 2E84.A.1308, 2E84.A.1309, 2E84.A.1310, 2E84.A.1
312, 2E84.A.1313, 2E84.A.1314, 2E84.A.1316, 1EB7.A.401, 1EGY.A.410, 3EGW.C.806,
3EGW.C.807, 3EHB.A.559, 1EHJ.A.1030, 1EHJ.A.1053, 1EHJ.A.1066, 4EID.A.101, 4EIE.
A.101, 4EIF.A.101, 2EKT.A.154, 2EKU.A.154, 1ETP.A.199, 1ETP.A.200, 1EUE.A.201, 2
EU7.X.201, 2EWK.A.1001, 2EWK.A.1003, 2EWU.A.1001, 2EWU.A.1003, 1EWH.A.253, 2EWI.
A.1004, 2EWI.A.1002, 2EWI.A.1001, 2EWI.A.1003, 2EXV.A.83, 1EZV.C.401, 1EZV.C.402
, 1F03.A.201, 1F1C.A.200, 1F1F.A.200, 1F24.A.501, 4F6I.A.201, 4FA7.A.602, 4FA7.A
.603, 4FAS.A.601, 4FAS.A.602, 4FAS.A.603, 4FAS.A.604, 4FAS.A.605, 4FAS.A.606, 4F
AS.A.607, 2FBZ.X.901, 1FCD.C.901, 4FDH.A.601, 4FEF.A.403, 2FFN.A.1003, 1FGJ.A.54
8, 1FGJ.A.552, 1FGJ.A.553, 1FI3.A.83, 1FI7.A.110, 1FJ0.A.115, 3FLL.A.185, 2FMY.A
.300, 3FO3.A.1004, 3FO3.A.1005, 3FO3.A.1006, 3FO3.A.1007, 3FO3.A.1008, 3FO3.A.10
02, 3FO3.A.1003, 3FO3.A.1001, 1FOP.A.500, 3FOO.A.150, 2FRF.A.154, 1FS7.A.509, 1F
S7.A.510, 1FS8.A.508, 1FT5.A.213, 1FT5.A.215, 1FT5.A.216, 1FT9.A.300, 1FT9.B.300
, 2FWT.A.803, 2FWT.A.805, 2FWL.A.132, 2FYU.D.242, 4G1V.A.401, 4G3J.A.501, 3G5N.A
.500, 4G7L.A.301, 4G7S.A.602, 3GAS.D.1294, 2GC4.D.200, 1GDV.A.101, 4GED.B.201, 1
GKS.A.0, 2GNV.A.166, 1GQ1.A.601, 1GQ1.A.602, 3GQP.C.143, 4GQS.A.501, 2GSM.A.2001
, 2GTF.X.201, 1GWF.A.504, 3GW9.A.480, 1GWS.A.603, 1GWS.A.606, 1GYO.A.111, 1GYO.A
.112, 1GYO.A.113, 1GYO.A.114, 4GYD.A.200, 4H0K.B.200, 1H1O.A.1184, 1H1O.A.1185,
1H21.A.1248, 1H21.A.1249, 1H21.B.1248, 1H21.B.1249, 1H29.A.1102, 1H29.A.1104, 1H
29.A.1107, 4H2L.B.201, 1H32.A.1263, 1H32.A.1264, 1H32.B.1139, 3H33.A.75, 3H33.A.
76, 3H33.A.77, 3H34.A.72, 3H34.A.73, 3H34.A.74, 3H4N.A.72, 3H4N.A.73, 3H4N.A.74,
4H44.A.302, 1H55.A.350, 1H57.A.350, 4H6O.A.501, 3H8T.A.301, 2H88.C.142, 1HBI.A.
153, 4HB6.A.72, 4HB6.A.73, 4HB8.A.72, 4HB8.A.73, 4HBF.A.72, 1HCZ.A.253, 4HC3.A.7

2, 3HDL.A.305, 3HF4.A.142, 3HF4.B.147, 1HGB.D.147, 4HHR.A.701, 4HHS.A.701, 1HJ3.
B.602, 1HJ5.A.601, 1HJ5.B.602, 2HJI.A.180, 1HLB.A.158, 3HNJ.A.150, 3HNK.A.150, 4
HPA.A.201, 4HPB.A.201, 4HPD.A.201, 3HQ9.A.400, 3HQ9.A.401, 1HRC.A.105, 3HSP.A.75
0, 4HSW.A.201, 3HYU.A.142, 3HYU.B.147, 1I3E.A.147, 2I5N.C.404, 1I5U.A.201, 1I77.
A.108, 1I77.A.109, 1I77.A.110, 1I77.A.111, 4I7Z.A.302, 3I8R.A.901, 1I8O.A.115, 1
I8P.A.115, 2I8F.A.83, 3I9T.A.300, 3I9U.A.300, 1IB7.A.95, 1ICC.A.201, 1IDR.A.144,
2IJ3.B.999, 1IKE.A.185, 1IQC.A.402, 3IQ5.A.150, 1IT1.A.201, 1IT1.A.202, 1IT1.A.
203, 1IT1.A.204, 2IUF.A.1691, 1IVJ.A.300, 1IW0.A.901, 1IYN.A.296, 1J02.A.300, 1J
0P.A.1002, 1J0Q.A.201, 2J1M.A.1456, 4J20.A.107, 1J3S.A.105, 2J5M.A.1300, 2J7A.A.
1003, 2J7A.A.1004, 2J7A.A.1005, 2J7A.C.1003, 1JDL.A.500, 1JDR.A.296, 4JE9.A.201,
4JEB.A.201, 4JJ0.A.501, 4JJ0.A.502, 2JJP.A.412, 1JMX.A.1001, 1JMX.A.1002, 1JNI.
A.125, 1JNI.A.126, 2K3V.A.218, 2K3V.A.278, 4K8F.B.300, 1KB0.A.802, 1KBI.A.760, 4
KF2.B.501, 2KMY.A.233, 2KMY.A.251, 4KMG.A.101, 1KOK.A.296, 1KQG.C.809, 2KSU.A.28
2, 2KSU.A.305, 1KV9.A.901, 4KVK.A.701, 4KVL.A.701, 3L1M.A.150, 1L2K.A.154, 3L61.
A.420, 3LD6.A.601, 2LDO.A.154, 3LGN.A.200, 4LJI.A.301, 1LM3.B.200, 1LMS.A.118, 4
LM8.A.801, 4LM8.A.802, 4LM8.A.803, 4LM8.A.804, 4LM8.A.805, 4LM8.A.806, 4LM8.A.80
7, 4LM8.A.808, 4LM8.A.809, 4LM8.A.810, 4LMH.A.801, 4LMH.A.803, 4LMH.A.804, 4LMH.
A.805, 4LMH.A.806, 4LMH.A.807, 4LMH.A.808, 4LMH.A.809, 4LMH.A.810, 1LQX.A.201, 1
LR6.A.201, 1LS9.A.92, 1LSX.A.719, 3M15.A.150, 1M1P.A.802, 1M1Q.A.804, 1M1Q.A.803
, 1M1R.A.801, 1M2I.A.201, 3M4C.A.150, 4M4A.A.201, 1M59.A.201, 1M70.A.199, 1M70.A
.200, 4M72.A.403, 4M73.A.403, 3MDM.A.505, 1MDV.A.110, 1MDV.A.112, 1MJ4.A.502, 3M
L1.B.1128, 3ML1.B.1129, 1ML7.A.185, 4MLM.A.201, 4MLN.A.201, 3MM9.A.580, 1MNI.B.1
54, 1MNY.A.95, 3MOM.A.186, 4MPM.A.201, 1MQF.A.501, 4MQJ.B.201, 3MUS.A.201, 3MVC.
A.500, 3MVF.A.185, 1MWB.A.125, 1MXR.A.1004, 1MZ4.A.151, 1N45.A.300, 4N4J.A.609,
4N4J.A.610, 4N4J.A.611, 4N4J.A.612, 4N4J.A.613, 4N4J.A.614, 4N4J.A.615, 4N4J.A.6
16, 4N4L.A.616, 4N4N.A.601, 4N4N.A.602, 4N4N.A.603, 4N4N.A.604, 4N4N.A.605, 4N4N
.A.606, 4N4N.A.607, 4N4O.A.608, 4N8T.B.201, 1NAZ.A.200, 4NFG.B.201, 1NIR.A.601,
1NIR.A.602, 4NK1.B.201, 3NMI.A.150, 1NML.A.401, 1NML.A.402, 3NN1.A.239, 1NNO.A.6
02, 1NOS.A.901, 4NP1.A.185, 2NRL.A.148, 1NS6.A.142, 1NS9.B.147, 3NWV.A.105, 3NXU
.A.508, 3O1A.A.385, 4O1W.A.101, 4O4Z.A.201, 3O5C.A.401, 3O5C.D.504, 4O6Q.A.202,
4O6U.A.203, 4O7G.A.301, 4O7G.A.302, 3O89.A.2154, 1OAH.A.1521, 1OAH.A.1524, 3OA8.
A.401, 3OA8.B.401, 1OFW.A.1294, 1OFW.A.1295, 1OFW.A.1296, 1OFW.A.1297, 1OFW.A.12
98, 1OFW.A.1299, 1OFW.A.1300, 1OFW.A.1301, 1OFW.A.1302, 3OFT.A.417, 3OMA.A.1, 3O
MI.A.607, 3OO4.A.142, 3OO4.B.147, 2ORT.A.600, 3ORV.B.600, 2ORO.A.600, 2ORP.A.600
, 2ORQ.A.600, 2ORR.A.600, 2ORS.A.600, 2OT4.A.1004, 2OT4.A.1007, 3OUE.A.609, 3OUE
.A.610, 3OUE.A.611, 3OUE.A.612, 3OUQ.A.601, 3OUQ.A.602, 3OUQ.A.603, 3OUQ.A.604,
3OUQ.A.605, 3OUQ.A.606, 2OZY.A.202, 2OZY.A.203, 2OZY.A.204, 2OZY.A.205, 3OZU.A.4
04, 3OZV.A.404, 1P2E.A.801, 1P2E.A.803, 1P2E.A.804, 1P2Y.A.430, 3P3L.A.501, 1PA2
.A.306, 1PBY.A.991, 1PBY.A.992, 3PC3.A.701, 2PEG.A.200, 2PEG.B.400, 3PH2.B.1087,
3PI2.B.500, 4PK5.A.501, 1PL3.A.401, 1PM1.X.180, 1PP9.C.501, 1PP9.C.502, 1PP9.D.
501, 2PQ7.A.220, 3PXW.A.500, 4PXH.A.501, 1Q16.C.806, 1Q16.C.807, 2Q8Q.A.300, 3Q9
9.B.750, 1QDB.A.516, 1QDB.A.517, 1QDB.A.518, 1QDB.A.519, 1QHU.A.500, 2QJY.A.501,
2QJY.A.502, 2QJY.B.301, 1QKS.A.601, 1QKS.A.602, 1QNO.A.113, 1QNO.A.114, 1QNO.A.
115, 1QNO.A.116, 1QN2.B.101, 3QNS.A.351, 1QO8.A.603, 1QPA.A.350, 1QQ3.A.107, 3QQ
Q.A.163, 3QQR.A.163, 2QSP.A.142, 3QWO.A.150, 1QYZ.A.200, 1R0Q.A.200, 4R20.A.601,
2R50.A.166, 2R80.A.150, 2R80.B.150, 3R9C.A.450, 2RAO.B.147, 2RAO.C.142, 2RDZ.A.
2, 2RDZ.A.3, 2RDZ.A.4, 2RDZ.A.5, 2RF7.A.2, 3RGS.A.1, 3RJ6.A.154, 4RKM.A.808, 4RK
M.B.808, 4RKM.D.807, 4RKN.A.902, 4RKN.A.903, 4RKN.A.905, 4RKN.A.906, 4RKN.A.907,
4RKN.A.908, 4RKN.A.909, 1RSE.A.154, 3RUK.B.600, 1RWJ.A.90, 1RWJ.A.91, 1RWJ.A.92
, 1RZ5.A.401, 3S1J.A.140, 3S8F.A.800, 3S8G.A.800, 1SE6.A.430, 3SEL.X.73, 3SEL.X.
74, 1SH4.A.201, 3SJ0.X.73, 3SJ0.X.74, 3SJ1.X.73, 3SJ1.X.74, 3SJ4.X.73, 3SJ4.X.74
, 3SJ5.A.500, 3SJL.A.500, 3SJL.A.600, 3SLE.A.402, 1SOX.A.502, 1SP3.A.801, 1SP3.A
.803, 1SP3.A.804, 1SP3.A.805, 1SP3.A.806, 1SP3.A.808, 1SUO.A.500, 3SWZ.B.600, 3S
XQ.A.1005, 3SXQ.A.1006, 3SXQ.A.1007, 3SXQ.A.1008, 3SXQ.A.1002, 3SXQ.A.1003, 3SXQ

.A.1001, 1SY2.A.185, 3T3R.A.500, 3T3Z.A.500, 1T68.X.201, 3T6D.C.401, 3T6D.C.403, 3T6D.C.404, 3T6E.C.401, 3T6E.C.402, 3T6E.C.403, 3TDA.A.800, 3TF0.A.500, 3TGU.C.501, 3TGU.C.502, 3TGU.D.501, 3TGA.A.185, 3TGM.A.300, 1TH2.D.2003, 3TIK.A.482, 3TJS.A.508, 1TKW.B.253, 3TMC.A.309, 4TOB.C.201, 3TOL.A.150, 4TT5.A.401, 1TU2.B.255, 1U13.A.460, 1U4H.A.500, 1U7R.A.154, 3U8P.A.347, 1U9M.A.90, 4U9D.A.201, 1U9U.A.90, 3U99.A.500, 3U99.A.700, 3UBR.A.472, 3UBR.A.474, 3UBR.A.475, 3UBC.A.201, 3UCP.A.901, 3UCP.A.902, 3UCP.A.903, 3UCP.A.904, 3UCP.A.905, 3UCP.A.906, 3UCP.A.907, 3UCP.A.908, 3UCP.A.909, 3UCP.A.910, 3UCP.A.911, 3UOI.B.200, 1UP9.A.201, 1UP9.A.202, 1UP9.A.203, 1UP9.A.204, 4UQH.A.1450, 1URV.A.1172, 2UUQ.A.1405, 4UVR.A.1450, 1V0H.X.251, 2V07.A.1102, 2V08.A.1087, 2V0M.A.1499, 3V2V.A.154, 4V2K.A.601, 4V3V.A.750, 4V3W.A.750, 4V3X.A.750, 1V54.A.515, 1V75.B.201, 2V7K.A.1360, 2V7L.A.1360, 3V8D.A.601, 1V8X.A.901, 1V9Y.A.1140, 3VAU.A.201, 1VB6.A.1140, 1VB6.B.1140, 2VEB.A.200, 1VGI.A.300, 3VHB.A.150, 4VHB.A.150, 2VHB.B.150, 2VHD.A.402, 3VKP.A.601, 3VKS.A.601, 3VM9.A.154, 3VP5.A.201, 3VRD.A.201, 3VRD.A.202, 3VRG.A.201, 3VRG.B.201, 3VXJ.A.501, 2VXH.A.1001, 1VYD.A.1117, 2VYW.A.149, 2W0B.A.470, 1W2L.A.1100, 2W31.A.200, 2W3G.A.500, 1W7O.A.1119, 1W7O.A.1120, 1W7O.A.1121, 1W7O.A.1122, 3W9C.A.501, 1WAD.A.117, 1WAD.A.113, 1WAD.A.114, 1WAD.A.115, 3WAH.A.201, 3WC8.A.201, 3WCT.A.200, 3WCT.B.201, 3WCT.C.200, 3WCT.D.201, 2WDQ.C.1129, 1WE1.A.300, 3WFD.B.801, 3WFD.C.201, 3WFX.A.201, 2WJM.C.1334, 2WJN.C.1333, 2WJN.C.1335, 2WJN.C.1336, 1WMU.A.201, 1WOV.A.300, 1WOX.A.300, 4WPD.A.402, 4WQ8.A.1002, 4WQ9.A.1001, 4WQ9.A.1002, 4WQC.A.1002, 4WQD.A.1002, 2WTG.A.180, 3WU2.F.101, 1WVE.C.699, 1WVP.A.154, 2WX2.A.1450, 3X15.A.200, 3X15.J.200, 1X3X.B.202, 1X46.A.151, 4XDI.A.201, 2XFH.A.1412, 2XKR.A.1400, 2XKI.A.1110, 1XQ5.B.148, 1XQ5.C.143, 2XSJ.B.503, 2XTS.B.500, 2XYK.A.700, 1Y0P.A.801, 1Y0P.A.802, 1Y0P.A.803, 1Y0P.A.804, 1Y5I.C.806, 1Y5I.C.807, 1Y5L.C.806, 2Y5N.A.450, 2YEV.A.1015, 2YEV.B.587, 2YIU.A.500, 2YIU.A.501, 1YIQ.A.901, 2YK3.A.200, 2YL7.A.128, 1YWD.A.185, 2YXC.A.1001, 2YYW.A.1001, 2YYW.A.1003, 2YYX.A.1004, 2YYX.A.1001, 1Z1N.X.607, 1Z1N.X.608, 1Z1N.X.610, 1Z1N.X.612, 1Z1N.X.616, 2Z47.A.1004, 2Z47.B.3003, 2Z6S.A.201, 2Z6T.A.201, 1Z8O.A.410, 1Z9N.A.1001, 1Z9N.C.2001, 3ZBY.A.1402, 2ZBO.A.111, 3ZE6.B.502, 3ZG2.A.1480, 3ZHO.A.200, 3ZHW.A.1163, 3ZIY.A.600, 3ZJO.A.200, 3ZJQ.A.200, 3ZOO.A.105, 3ZOX.A.1082, 2ZPB.A.300, 2ZS0.A.200, 2ZS0.B.200, 2ZS0.C.200, 2ZS0.D.200, 2ZXY.A.200, 2ZZS.1.220, 1ZZH.A.802, 1ZZH.A.803, 3A15.A.354, 3A51.C.412, 4AJ9.A.1715, 2AKJ.A.564, 3ARJ.A.153, 3AT6.A.142, 2AUO.A.153, 2AUQ.A.147, 2AV0.A.147, 4AVD.A.144, 3AYF.A.802, 1B0B.A.144, 3BA2.A.158, 1BCF.A.200, 3BK9.A.401, 3BUJ.A.398, 2C1D.H.1158, 3C6G.A.601, 1C6S.A.88, 4C9L.A.1418, 1CG8.B.142, 1CH4.A.147, 2CMM.A.155, 1CRC.A.105, 4CZC.A.1337, 3D1K.A.200, 3D1K.B.400, 2D5X.B.147, 3DAM.A.600, 3DE8.D.150, 1DGF.A.3000, 2DKK.A.430, 1DLY.A.144, 4DVQ.A.601, 1DW1.A.113, 1E2R.A.602, 2E39.A.401, 3E4W.A.501, 2E84.A.1315, 4EGO.A.501, 4ENU.A.801, 1EQD.A.185, 4ESA.A.202, 4ESA.B.202, 2FDU.A.500, 1FHF.A.350, 1FHJ.B.147, 4FVC.A.201, 2G3H.A.154, 3G46.A.147, 4G45.A.401, 3GAS.A.1290, 1GEJ.A.501, 2GGN.X.251, 1GJQ.A.602, 2GKM.A.144, 4GQS.B.501, 4H0K.A.200, 1H1X.A.1154, 1H97.A.148, 1HJ4.B.601, 1HJ5.B.601, 4HRR.A.201, 4HRR.B.201, 2HYS.A.201, 1HZU.A.601, 1I3D.A.147, 4I8V.A.601, 2I96.A.129, 4IAM.A.501, 3IBD.A.500, 2IG3.A.700, 1IOP.A.154, 3IQB.A.500, 1IRD.B.347, 1IWH.A.142, 1IX4.A.300, 2J7A.D.1001, 1JEB.D.147, 4JET.A.201, 2JXM.B.250, 2KII.A.182, 4L1Y.A.300, 4L1Z.A.300, 4L2M.A.201, 4L3H.A.402, 2L4D.A.107, 4L54.A.501, 1LGA.A.396, 1LHT.A.155, 2LHB.A.151, 1M54.F.1620, 1M56.A.1002, 1M7S.D.600, 3MM3.A.501, 3MMB.A.580, 3MMO.A.1004, 4MMO.A.401, 1MN1.A.396, 1MYF.A.154, 3MYM.A.139, 3MYN.A.139, 4N4M.A.616, 4N4N.C.601, 3N8Y.B.601, 3NA0.A.601, 4NK2.A.700, 3NN2.A.239, 2NNJ.A.500, 2NP1.A.350, 1NR6.A.500, 3NTG.D.601, 3O5C.B.402, 1OAE.A.1125, 3OCD.C.401, 3OCD.D.401, 1OCZ.A.515, 1ODO.A.1407, 3OFU.A.417, 1OG5.A.501, 2OIF.A.163, 1OR4.A.180, 1OZW.B.300, 3P3X.A.501, 2P85.A.500, 3PM0.A.900, 3PT8.A.500, 3PT8.B.500, 2Q8P.A.300, 3Q9K.A.605, 3QGP.A.200, 3QQR.B.163, 2QRW.A.700, 2QSS.A.142, 4R21.A.600, 3R9B.A.501, 2RF7.D.1, 3RIW.A.305, 4RKM.K.809, 4RKM.L.813, 3RUR.A.200, 1S13.A.300, 1S1F.A.430, 1S61.A.144, 1S69.A.125, 3S66.A.142, 3S79.A.600, 1SCH.A.300, 1SI8.A.501, 1SPG.B.148, 1T85.A

.417, 3TBG.A.800, 3TM8.A.903, 3TTX.B.760, 1TWN.A.300, 3TYW.A.501, 1U5U.A.999, 4U
9D.D.201, 3UA1.A.508, 4UBS.A.501, 1UC3.A.150, 3UHB.A.147, 3UHD.B.147, 1UMO.A.117
2, 3UOI.I.200, 3UT2.A.1500, 1UX8.A.700, 1V4U.A.144, 1V4U.B.147, 1V9Z.B.1140, 3VE
D.A.401, 2VHD.A.401, 3VNO.A.501, 3VOL.A.401, 1VRE.A.148, 3VRF.B.201, 2VZW.A.1206
, 3W08.A.501, 3W4U.A.201, 1W92.A.1149, 3WCU.A.200, 3WCU.C.200, 3WCU.B.201, 3WCU.
D.201, 3WFB.B.802, 4WG2.A.603, 3WNU.A.801, 1WOW.A.300, 3WXO.A.801, 2WY4.A.150, 2
X66.A.1359, 1X8V.A.470, 1X9F.A.160, 1X9F.B.160, 1X9F.C.160, 1X9F.D.160, 2XBK.A.1
398, 2XN8.A.1434, 2XQ1.B.1503, 2Y4F.A.389, 2YGX.D.450, 2YL1.A.128, 1YMC.A.154, 2
Z6N.A.150, 2Z6N.B.150, 2ZFO.D.200, 3ZK5.A.1407, 2ZVU.A.300
[1] "Cluster 10"
4AM5.A.1162, 1AOR.A.606, 1B2O.B.55, 2B5H.A.501, 1B71.A.192, 1BE7.A.55, 1BFY.A.55
, 1BIQ.A.377, 2BKB.C.1193, 2BOY.E.1255, 1BOU.B.501, 4C4U.I.201, 3CF4.A.808, 1CJX
.B.629, 2CKF.C.501, 3D19.F.302, 3D3L.A.801, 3DBY.A.306, 2DE6.B.501, 4DHL.A.502,
3E2T.A.1, 1E3D.A.269, 4EB5.D.201, 4F1E.P.201, 3FG1.B.1500, 4FWI.B.401, 1FZO.A.50
02, 2GBX.E.455, 3GC1.A.605, 3GKE.A.501, 3GL0.A.501, 3GL2.A.501, 1GNL.A.1545, 2GP
C.B.195, 1GUP.C.351, 2GYQ.A.402, 2HMK.A.451, 4HSL.A.202, 3I01.B.800, 1I4Z.E.605,
4ILT.A.301, 2JD7.0.203, 1JI5.A.152, 1JI5.B.151, 1JIG.A.402, 1JIG.B.401, 2JI3.A.
1127, 1JQK.B.801, 1JRO.G.3001, 4K9F.A.101, 4KUO.D.101, 4KWL.A.301, 4KX6.N.301, 1
LNB.E.900, 1N1Q.A.516, 1N1Q.B.515, 3N9Y.D.151, 3NAO.C.150, 3NAO.D.150, 4NBA.A.50
1, 4NBF.A.501, 4NBG.A.501, 1NF6.A.200, 1NO3.A.858, 2OHJ.A.502, 2OHJ.A.511, 3PCK.
Q.600, 2PCD.M.600, 2PT2.A.400, 2PUZ.A.500, 2Q0J.B.997, 3Q36.A.458, 4QDD.A.401, 1
QGH.H.157, 4QLW.B.201, 3QVD.G.173, 1R2F.A.400, 1R9X.A.501, 1RA5.A.500, 4REU.B.20
2, 1RSR.A.1004, 1SHR.B.801, 3T81.B.606, 1T9O.A.255, 3U9M.A.202, 1ULI.A.600, 3V7P
.A.429, 1VCX.A.54, 3VMH.C.501, 2W3S.E.1464, 1W9M.A.1555, 2WLB.B.619, 1WQL.A.502,
2XSO.G.901, 1Y4T.A.650, 2YFI.A.901, 2YFI.G.900, 1YK5.A.54, 1YUX.A.303, 2ZZI.A.2
08

Table S95. 4-ligand Na, normal group

	size	largest_angle*	middle_1*	middle_2*	middle_3*
1	"13"	"133.7+/-7.2"	"80.9+/-6.6"	"99+/-5.3"	"112.2+/-4.5"
2	"27"	"153.6+/-5.3"	"84.5+/-5.1"	"90.3+/-4.9"	"99.2+/-5.4"
3	"14"	"150.4+/-6.9"	"71.8+/-6.2"	"81.5+/-7.7"	"97.9+/-13"
4	"14"	"154.8+/-11.2"	"78+/-5.2"	"81.9+/-3.8"	"87.8+/-3.8"
5	"34"	"166.8+/-5.7"	"78.5+/-6.1"	"87.3+/-5.8"	"95.2+/-5.1"
6	"21"	"166.8+/-6.9"	"79+/-4.3"	"85.8+/-3.8"	"91.4+/-4.3"
7	"22"	"154.8+/-6.7"	"72.1+/-5.5"	"79.3+/-4.7"	"84.9+/-4.1"
8	"37"	"166.7+/-4.5"	"81.6+/-5.4"	"87.4+/-4.8"	"92.4+/-3.4"
9	"15"	"132+/-6.8"	"85.5+/-7.4"	"95.9+/-6"	"107.6+/-7.1"
10	"15"	"143.4+/-8.7"	"74.1+/-6.7"	"82.9+/-6"	"91.7+/-6.9"

	middle_4*	smallest_opposite_angle*	Tetrahedral
1	"118.7+/-3.8"	"85.5+/-7.8"	"0.022"
2	"112+/-4.4"	"97.8+/-7.2"	"0.002"
3	"121.3+/-9.4"	"125.5+/-10.2"	"0.001"
4	"93.9+/-6.6"	"130.6+/-6.7"	"0.001"
5	"103.8+/-6.5"	"81.4+/-7.5"	"0"
6	"101.9+/-5.3"	"152.5+/-7.5"	"0"
7	"95.3+/-7.3"	"83.6+/-6.9"	"0"
8	"99.6+/-4.4"	"102.4+/-7.6"	"0"
9	"116.8+/-6"	"111.3+/-8.1"	"0.056"
10	"128.8+/-8.7"	"83.7+/-9.4"	"0.001"

	TrigonalBipyramidalVA	TrigonalBipyramidalVP	SquarePyramidalV
1	"0.286"	"0.003"	"0.007"
2	"0.056"	"0.086"	"0.248"
3	"0.004"	"0.077"	"0.003"
4	"0.001"	"0.465"	"0.031"
5	"0.005"	"0.04"	"0.498"
6	"0"	"0.139"	"0"
7	"0.003"	"0.026"	"0.308"
8	"0.003"	"0.402"	"0.504"
9	"0.148"	"0.002"	"0.004"
10	"0.069"	"0.015"	"0.023"

	SquarePlanar
1	"0"
2	"0"
3	"0"
4	"0.062"
5	"0"
6	"0.472"
7	"0"
8	"0"
9	"0"
10	"0"

Table S96. Cluster members of 4-ligand Na, normal group

[1] "Cluster 1"
4FZY.A.201, 4FZY.B.201, 1QVG.L.8347, 3EPZ.A.1, 3FFZ.B.1302, 4FLK.A.504, 4FO2.E.101, 4IQZ.E.305, 2NQL.A.401, 2OCC.A.519, 3T34.A.1003, 3UA6.B.2, 3ZPR.B.1
[1] "Cluster 2"
8ICP.A.341, 2IHM.A.900, 3JPS.A.340, 3KNT.A.208, 4A22.C.1346, 1A5S.B.2000, 3ASP.A.701, 3BX1.B.284, 3C17.B.326, 2C9R.A.1103, 3GVF.A.178, 3IAQ.C.3101, 4J1I.A.502, 4J2H.A.306, 4JDO.D.301, 4JVL.A.703, 4O4W.A.301, 4OBO.A.402, 3PYM.B.504, 1Q1Z.A.313, 3RNO.A.402, 1S81.A.2, 1T64.B.1392, 3VD7.A.3101, 3VDA.A.3101, 3W6P.A.804, 4XOO.B.401
[1] "Cluster 3"
4DOC.A.402, 1Q81.M.8380, 3AR8.A.1000, 1DI4.A.501, 2FQE.A.901, 4HCH.A.407, 4HXV.A.403, 3I3D.C.3101, 1MXO.D.901, 3OB8.A.3006, 3P0J.A.711, 3T2Q.D.3101, 1UD2.A.1003, 3VD3.C.3101
[1] "Cluster 4"
4M47.A.403, 3AXG.A.3005, 3DR3.A.336, 2E7U.A.1002, 1EBU.A.901, 3HVU.D.182, 3K13.A.647, 4ODI.A.301, 1QJS.A.512, 1QJS.A.523, 3UA7.A.145, 1XC6.A.8001, 1YCE.A.201, 1YCE.C.201
[1] "Cluster 5"
4NLN.A.402, 3AST.A.701, 4D1I.C.600, 4D1I.F.600, 3D32.A.120, 2DDA.C.303, 2EPF.C.307, 3EUW.B.343, 4FEW.B.303, 3GCD.A.215, 4GY9.A.206, 4HMM.B.302, 3IAQ.B.3104, 3IJ6.B.313, 4J0Y.A.501, 4JHG.A.205, 1JZN.D.4139, 4L73.A.404, 4LH7.A.404, 3N83.A.701, 4NAW.N.304, 1OA8.A.1690, 3OB8.B.3003, 2OSW.B.602, 3OTK.C.587, 1Q1Q.A.351, 4Q4X.1.5004, 2WDO.A.601, 2WG8.B.202, 2WWH.B.212, 3WXO.A.808, 4WXG.A.502, 2Y00.A.1359, 4ZNB.A.3
[1] "Cluster 6"
3E45.A.260, 2OTJ.R.8537, 3C17.A.324, 4CH8.D.1580, 1CM5.A.1056, 3DYQ.A.902, 4ENZ.

A.1112, 1F7T.A.472, 1F7T.C.474, 4FOI.A.1005, 4FOI.A.1006, 3H1V.X.600, 3IGQ.F.801
, 2J5W.A.3043, 3NRB.C.287, 1QJS.A.513, 4R3W.B.402, 1R4P.F.4004, 3V6N.A.232, 3W0L
.A.502, 1XAR.B.200
[1] "Cluster 7"
3EPG.A.421, 4KHS.A.1009, 4KHU.A.1006, 1YIT.0.8538, 3C9F.B.603, 3G1N.A.5001, 4G1K
.A.301, 4HKT.B.410, 3I4Q.A.177, 4IJK.B.301, 1KNR.A.542, 4L3F.H.401, 4NWH.A.201,
3OO2.B.383, 4R6C.A.213, 3T2P.B.3102, 3TXJ.A.1138, 2VDX.A.1385, 3VD5.D.3102, 2WCF
.B.1091, 2WOI.B.1491, 3ZLY.A.1384
[1] "Cluster 8"
4DOC.A.404, 2ISP.A.340, 2ISP.A.342, 2A2A.C.3158, 4B1L.A.1679, 2C9A.A.1289, 4D9U.
A.901, 3DKI.B.324, 4FMT.A.301, 3GA5.A.700, 3HVY.C.427, 4I2B.A.601, 4JRX.D.301, 1
JYX.B.3103, 4K7V.A.407, 3L27.B.3, 4LDZ.A.205, 1O4Z.B.1005, 2O5W.C.171, 2OSY.B.60
2, 3P8O.A.187, 3PJ0.C.367, 2POC.D.5004, 1PX4.A.3104, 2QF2.A.700, 4QFE.G.304, 2QZ
7.A.195, 2QZ7.B.196, 3S30.A.383, 1VIZ.B.239, 1W90.A.1154, 2WOI.D.1488, 3WV2.A.30
5, 3WXO.A.807, 2X7J.D.1581, 2ZHJ.A.320, 2ZXK.A.1
[1] "Cluster 9"
2AA0.A.2003, 2EHS.A.201, 4FHA.A.402, 1G8I.B.1595, 2GG2.A.703, 1GW1.A.1423, 1IP3.
A.999, 1MAU.A.499, 4NT8.A.206, 1ODZ.A.1427, 4OVZ.B.903, 4TMV.A.903, 1W7Z.A.1032,
2WHM.A.1425, 2X2E.A.1747
[1] "Cluster 10"
4KHU.A.1008, 3UXP.A.339, 4C0K.A.1614, 4CBY.C.2037, 3IWK.B.504, 4JCO.D.406, 4KAF.
A.404, 4MVJ.D.402, 1PX3.A.3101, 4Q4B.A.530, 3Q9E.L.343, 3UA6.A.147, 3VD5.C.3101,
1YAO.A.501, 3ZX2.B.521

Table S97. 4-ligand Na, combined group

	size	largest_angle*	middle_1*	middle_2*	middle_3*
1	"32"	"163.5+/-8.8"	"79.2+/-3.9"	"84.8+/-3.9"	"90.3+/-4.4"
2	"59"	"163.8+/-6.5"	"81.9+/-5.7"	"87.9+/-5.2"	"93.8+/-4.6"
3	"12"	"123.8+/-12.7"	"55.5+/-9.6"	"74.3+/-10.1"	"81.7+/-9.2"
4	"23"	"148.6+/-10"	"68.3+/-11.6"	"83.5+/-7.9"	"97.5+/-11.3"
5	"45"	"136.4+/-8.7"	"81+/-9.1"	"94.2+/-7.6"	"105.9+/-8.5"
6	"13"	"153.4+/-11.4"	"58+/-9.7"	"72.7+/-11.5"	"106.1+/-15.9"
7	"56"	"161.2+/-8"	"76.6+/-7.2"	"84.9+/-5.7"	"91.5+/-6.7"

	middle_4*	smallest_opposite_angle*	Tetrahedral
1	"99.4+/-6.8"	"145.8+/-11.5"	"0"
2	"103+/-7"	"101.4+/-7.8"	"0.001"
3	"100.3+/-11"	"80.8+/-15.8"	"0.001"
4	"118.5+/-13.6"	"122+/-10.7"	"0.004"
5	"118.6+/-6.7"	"93.5+/-13.7"	"0.024"
6	"134.8+/-11"	"72.2+/-15.4"	"0"
7	"102+/-8.6"	"81.2+/-7.9"	"0"

	TrigonalBipyramidalVA	TrigonalBipyramidalVP	SquarePyramidalV
1	"0"	"0.266"	"0.007"
2	"0.014"	"0.31"	"0.445"
3	"0.01"	"0.017"	"0.035"
4	"0.003"	"0.053"	"0.004"
5	"0.17"	"0.009"	"0.016"
6	"0"	"0"	"0.001"
7	"0.009"	"0.028"	"0.407"

SquarePlanar

1 "0.337"
2 "0"
3 "0"
4 "0"
5 "0"
6 "0"
7 "0"

Table S98. Cluster members of 4-ligand Na, combined group

[1] "Cluster 1"
3E45.A.260, 2OTJ.R.8537, 3AXG.A.3005, 3C17.A.324, 4CH8.D.1580, 1CM5.A.1056, 3DR3.A.336, 3DYQ.A.902, 1EBU.A.901, 4ENZ.A.1112, 1F7T.A.472, 1F7T.C.474, 4FOI.A.1005, 4FOI.A.1006, 3H1V.X.600, 3HVU.D.182, 3IGQ.F.801, 2J5W.A.3043, 3K13.A.647, 3NRB.C.287, 4ODI.A.301, 1QJS.A.513, 1QJS.A.523, 4R3W.B.402, 1R4P.F.4004, 3UA7.A.145, 3V6N.A.232, 3WOL.A.502, 1XAR.B.200, 1XC6.A.8001, 1YCE.A.201, 1YCE.C.201
[1] "Cluster 2"
4DOC.A.404, 2IHM.A.900, 2ISP.A.340, 2ISP.A.342, 3JPS.A.340, 3KNT.A.208, 2A2A.C.3158, 4B1L.A.1679, 2C9R.A.1103, 2C9A.A.1289, 4D9U.A.901, 3DKI.B.324, 2E7U.A.1002, 3EUW.B.343, 4FMT.A.301, 3G1N.A.5001, 3GA5.A.700, 3GVF.A.178, 3HVY.C.427, 4I2B.A.601, 3IAQ.C.3101, 4J1I.A.502, 4J2H.A.306, 4JDO.D.301, 4JRX.D.301, 1JYX.B.3103, 1JZN.D.4139, 4K7V.A.407, 3L27.B.3, 4LDZ.A.205, 1O4Z.B.1005, 4O4W.A.301, 2O5W.C.171, 4OBO.A.402, 2OSY.B.602, 3P8O.A.187, 3PJ0.C.367, 2POC.D.5004, 1PX4.A.3104, 3PYM.B.504, 1Q1Z.A.313, 4Q4X.1.5004, 2QF2.A.700, 4QFE.G.304, 2QZ7.A.195, 2QZ7.B.196, 3RNO.A.402, 3S30.A.383, 3VD7.A.3101, 3VDA.A.3101, 1VIZ.B.239, 1W90.A.1154, 2WOI.D.1488, 3WV2.A.305, 3WXO.A.807, 4WXG.A.502, 2X7J.D.1581, 2ZHJ.A.320, 2ZXK.A.1
[1] "Cluster 3"
1U8R.D.4104, 3BFT.A.1007, 4D1I.H.600, 2D4E.C.1905, 1G8G.A.522, 1G8G.A.527, 1G8H.A.526, 4L73.A.406, 2NWH.A.404, 4R6C.A.213, 3T2P.B.3102, 1YAO.A.501
[1] "Cluster 4"
4DOC.A.402, 4KHN.A.1010, 4M47.A.403, 1Q81.M.8380, 1YIT.0.8517, 3AR8.A.1000, 1DI4.A.501, 3DYQ.A.901, 3FKR.A.409, 2FQE.A.901, 2GG2.A.703, 4HCH.A.407, 4HXV.A.403, 3I3D.C.3101, 1MXO.D.901, 4NT8.A.206, 3OB8.A.3006, 3P0J.A.711, 1QJS.A.512, 3T2Q.D.3101, 1UD2.A.1003, 3VD3.C.3101, 2X1Z.M.1163
[1] "Cluster 5"
4FZY.A.201, 4FZY.B.201, 8ICP.A.341, 4KHU.A.1008, 1QVG.L.8347, 3UXP.A.339, 4A22.C.1346, 2AAO.A.2003, 3BX1.B.284, 4CBY.C.2037, 2EHS.A.201, 3EPZ.A.1, 3FFZ.B.1302, 4FHA.A.402, 4FLK.A.504, 4FO2.E.101, 1G8G.A.525, 1G8I.B.1595, 1GW1.A.1423, 1IP3.A.999, 4IQZ.E.305, 1JED.A.525, 4JVL.A.703, 4K70.B.1011, 1MAU.A.499, 4MVJ.D.402, 2NQL.A.401, 2OCC.A.519, 1ODZ.A.1427, 4OVZ.B.903, 1PX3.A.3101, 3Q9E.L.343, 1S81.A.2, 3T34.A.1003, 1T64.B.1392, 4TMV.A.903, 3UA6.A.147, 3UA6.B.2, 3W6P.A.804, 1W7Z.A.1032, 2WHM.A.1425, 4XOO.B.401, 2X2E.A.1747, 3ZPR.B.1, 3ZX2.B.521
[1] "Cluster 6"
4COK.A.1614, 3DYQ.B.901, 1G8H.A.523, 3GCD.B.215, 3IAP.D.3103, 3IO1.A.501, 4JCO.D.406, 4KAF.A.404, 4L73.B.405, 4ODN.A.207, 4Q4B.A.530, 1QY1.A.204, 3ZDU.A.353
[1] "Cluster 7"
3EPG.A.421, 4KHS.A.1009, 4KHU.A.1006, 4NLN.A.402, 1YIT.0.8538, 1A5S.B.2000, 3ASP.A.701, 3AST.A.701, 3C17.B.326, 3C9F.B.603, 4D1I.C.600, 4D1I.F.600, 3D32.A.120, 2DDA.C.303, 2EPF.C.307, 4FEW.B.303, 3FGH.A.180, 4G1K.A.301, 3GCD.A.215, 4GY9.A.206, 4HKT.B.410, 4HMM.B.302, 3I4Q.A.177, 3IAQ.B.3104, 3IJ6.B.313, 4IJK.B.301, 3IWK.B.504, 4JOY.A.501, 4JHG.A.205, 1KNR.A.542, 4L3F.H.401, 4L73.A.404, 4LH7.A.404,

3N83.A.701, 4NAW.N.304, 4NWH.A.201, 1OA8.A.1690, 3OB8.B.3003, 3OO2.B.383, 2OSW.B.602, 3OTK.C.587, 1Q1Q.A.351, 3TXJ.A.1138, 2VDX.A.1385, 3VD5.C.3101, 3VD5.D.3102, 2WCF.B.1091, 2WD0.A.601, 2WG8.B.202, 2WO0.B.1728, 2WOI.B.1491, 2WWH.B.212, 3WXO.A.808, 2YO0.A.1359, 3ZLY.A.1384, 4ZNB.A.3

Table S99. 5-ligand Na, normal group

	size	largest_angle*	middle_1*	middle_2	middle_3*	middle_4
1	"22"	"164.3+/-5.4"	"67.1+/-5.2"	"78.8+/-7"	"89.4+/-3.8"	"93.9+/-2.4"
2	"46"	"168.4+/-5.3"	"81.5+/-3.5"	"85.4+/-2.6"	"88.5+/-2.4"	"90.9+/-2.2"
3	"40"	"174.4+/-2.7"	"82.3+/-3"	"86.4+/-1.9"	"88.6+/-2.1"	"90.2+/-1.7"
4	"33"	"169.1+/-4.5"	"81.8+/-3.2"	"84.9+/-2.6"	"88+/-2.1"	"90.6+/-1.8"
5	"28"	"163+/-6"	"73.6+/-5"	"80+/-3.9"	"84.8+/-4"	"89.5+/-4.3"
6	"46"	"170.6+/-4.5"	"77.3+/-5"	"83+/-3"	"86.7+/-3"	"89.7+/-3.2"
7	"14"	"147.6+/-8.3"	"70.3+/-3.1"	"74.2+/-2.8"	"79.5+/-4.3"	"85.6+/-5.6"
8	"37"	"151.5+/-5.6"	"73.7+/-4.7"	"80.5+/-2.6"	"84.6+/-2.9"	"89.9+/-4.7"
9	"50"	"163.6+/-3.7"	"77.4+/-4.8"	"82.8+/-4.2"	"87.1+/-3.8"	"91.2+/-3.3"
10	"44"	"151+/-5"	"75.1+/-5.8"	"82.9+/-3.7"	"86.7+/-3.7"	"91.2+/-4.3"

	middle_5*	middle_6	middle_7	middle_8*
1	"100.2+/-3.4"	"108.1+/-5.6"	"121.6+/-7"	"133.2+/-5"
2	"94.5+/-2.6"	"100.6+/-5.2"	"118.3+/-8.1"	"143.5+/-5"
3	"93.2+/-2.3"	"97.1+/-2.8"	"101.5+/-4.7"	"167.1+/-4.8"
4	"94+/-2.8"	"101.1+/-4.8"	"120.7+/-3.4"	"129+/-4.5"
5	"94.9+/-3.9"	"101.5+/-6.4"	"116.3+/-12.9"	"149.5+/-5.4"
6	"94.6+/-3.9"	"98.9+/-4.4"	"106.4+/-7.1"	"162.6+/-3.8"
7	"92.7+/-8"	"110.8+/-15.2"	"125.8+/-12.2"	"139.6+/-6.5"
8	"95.2+/-5.3"	"112.3+/-5.2"	"121.5+/-5.3"	"131.4+/-4.6"
9	"98+/-4.8"	"103.5+/-4.4"	"111.2+/-6.1"	"155.6+/-4.4"
10	"99+/-4.6"	"108.8+/-5.9"	"120.9+/-9"	"142.7+/-4.7"

	smallest_opposite_angle*	TrigonalBipyramidal	SquarePyramidal
1	"96.3+/-6.1"	"0.169"	"0.028"
2	"97.8+/-5.7"	"0.357"	"0.13"
3	"89.2+/-5.1"	"0.026"	"0.845"
4	"109.2+/-4.5"	"0.739"	"0.009"
5	"74.9+/-5"	"0.011"	"0.137"
6	"78.9+/-4.7"	"0.005"	"0.531"
7	"72.1+/-6.6"	"0.001"	"0.005"
8	"108.1+/-3.9"	"0.203"	"0.007"
9	"91.6+/-4.5"	"0.05"	"0.314"
10	"96.1+/-6.1"	"0.101"	"0.031"

	TrigonalPrismaticV
1	"0.217"
2	"0.243"
3	"0.04"
4	"0.269"
5	"0.135"
6	"0.071"
7	"0.24"
8	"0.424"
9	"0.166"
10	"0.408"

Table S100. Cluster members of 5-ligand Na, normal group

[1] "Cluster 1"
1JJ2.A.8345, 1SUZ.A.403, 1A7T.A.255, 4A87.A.1162, 4CFY.A.302, 4FEV.B.303, 4FLL.A.504, 4FO2.Q.101, 3KEU.A.402, 3KWM.A.225, 4LDE.A.1402, 3MX6.A.261, 1O4Z.A.1004, 1O68.E.274, 4OMC.A.608, 4QKU.A.501, 2QWL.A.589, 3WA2.X.702, 2WOI.A.1491, 2WPC.C.1492, 2WX5.H.1252, 3ZYV.A.2337
[1] "Cluster 2"
2BCV.A.579, 2GIJ.F.403, 4ORJ.A.305, 2A65.A.751, 2ABS.A.1003, 2B2N.B.1001, 2DDB.B.303, 4EXR.A.301, 4FMT.D.301, 1GVF.A.289, 4GVO.A.702, 4H7O.C.302, 4H83.E.401, 3IMX.A.467, 2JBW.D.1367, 4JEX.A.512, 2JLN.A.1471, 1JZ8.D.3104, 1KA0.A.501, 4LHL.A.301, 3LJQ.A.597, 4M9U.A.402, 3MAX.A.381, 4MM7.A.601, 4MMB.A.601, 4MMF.A.601, 3MPN.A.751, 3MPQ.A.751, 4O1G.A.401, 1OAF.A.1252, 4OUA.A.403, 2PFL.A.2001, 3Q9B.A.344, 3QS4.A.751, 3QS5.A.751, 1RW9.A.900, 3S9J.A.401, 1SK4.A.342, 1TO2.E.451, 3VD3.A.3101, 3VDA.B.3101, 2Z5D.B.180, 1ZH8.A.329, 3ZK1.A.90, 3ZK1.B.90, 3ZQS.B.1295
[1] "Cluster 3"
2FMS.A.342, 4GXK.A.405, 3JPP.A.340, 2PFQ.A.1, 4TUR.A.404, 4ADB.A.1405, 3AGB.A.1, 3AST.B.701, 4AYO.A.502, 4BDR.A.902, 3C7E.A.489, 4DF9.A.501, 4G1K.B.301, 2GEZ.A.401, 1GEN.A.304, 1GUU.A.1090, 1GV2.A.1192, 2HU3.A.9002, 1HXN.A.2, 4I2F.A.602, 3IFV.C.408, 2IY6.B.1540, 2J5W.A.3044, 2J5W.A.3045, 2JHN.A.1298, 4KXW.A.1013, 4KZV.A.304, 3LP5.A.251, 2O34.B.502, 4PFI.A.401, 4PUV.A.405, 1QHU.A.437, 3STH.A.501, 1SU3.A.911, 3U21.A.500, 3VS8.A.501, 1W16.A.1002, 1W9W.A.900, 2XZK.B.507, 1ZDN.A.157
[1] "Cluster 4"
2GIG.A.502, 4ATF.C.500, 3B1N.A.403, 4BEM.A.201, 4BEM.B.201, 4BEM.J.201, 2BL2.A.1157, 4D77.A.1543, 2DKB.A.436, 3EPR.A.266, 4FHA.B.402, 3G0T.B.437, 1H80.A.1497, 2HIG.B.488, 4JNQ.A.402, 4MB4.A.604, 3MS8.A.401, 3MUI.A.401, 1MVO.A.202, 1N82.A.401, 1OA8.C.1691, 2PPL.A.480, 3PZJ.A.301, 2Q8X.A.401, 4R7U.A.504, 3RGA.A.312, 3T8J.A.401, 1V54.A.3519, 3VDG.A.502, 1W9S.A.1142, 2WGM.A.201, 2WGM.B.201, 3WZ1.A.401
[1] "Cluster 5"
3PML.B.7, 2A7L.A.201, 2A9Y.A.2002, 4D9T.A.901, 1EZ1.B.1002, 3FZQ.B.274, 3G1N.B.5002, 4GKI.F.303, 4GRX.A.501, 3IAQ.A.3103, 3IC9.A.491, 2JBW.A.1368, 1MGV.A.501, 4NPJ.B.701, 4NRH.C.401, 4OOC.B.402, 2ONP.G.707, 4OUC.A.801, 4QVS.A.502, 1S5C.A.241, 1SOO.A.547, 2WWG.B.213, 1XAR.A.100, 4XEL.A.201, 1YQ2.A.7501, 2ZN8.A.995, 3ZPQ.A.1360, 3ZX0.C.522
[1] "Cluster 6"
3HW8.A.580, 1ORP.A.224, 1Q81.K.8346, 4BR6.A.401, 4BVN.A.1360, 4C7A.B.1159, 4CZN.A.1371, 4D1I.A.600, 4D1J.E.604, 4F3Y.A.301, 4FEW.D.304, 1G5I.C.902, 4GDK.E.301, 2GTW.E.3006, 4GY9.A.207, 3HYS.A.267, 4I0W.D.602, 3I2W.A.304, 4I2R.C.602, 3I44.A.477, 3JS4.A.208, 4KA8.A.806, 3KED.A.951, 4L3H.A.404, 4M4V.A.505, 4MJD.A.203, 4MM9.A.602, 4MPT.A.402, 3OEC.A.300, 3OTK.A.586, 4PV3.A.201, 4QFE.B.305, 1TQY.H.1094, 1UD8.A.1001, 2V4B.B.1562, 1VI6.A.208, 3VS8.C.501, 3WNM.A.802, 1WPG.D.1300, 2WU2.A.1590, 2WV7.B.401, 1XOG.C.1001, 1X7D.A.1501, 2X7J.A.1581, 1XDF.A.401, 3ZQ5.A.1530
[1] "Cluster 7"
4KHY.A.1013, 2A5G.B.242, 4B6C.A.1257, 4C1P.A.1728, 4CBY.B.2036, 1D7U.A.435, 3G8Q.D.302, 4H41.B.405, 3KZW.D.498, 4MVJ.A.401, 4NLQ.A.910, 4NT1.A.301, 2P6Z.A.402, 3WGU.A.2005
[1] "Cluster 8"
1TX3.A.801, 3C7X.A.1001, 3CB8.A.820, 4CD5.A.1420, 3CZJ.A.3101, 4D1J.A.601, 4DUW.

A.3101, 4DUX.A.3101, 3DYM.A.3101, 1EBU.B.902, 1EJA.A.246, 3GVK.A.916, 3I3B.A.3101, 4IQZ.A.317, 3IRD.A.301, 4J4B.B.303, 4JPA.A.305, 3JU4.A.7, 1JZ2.A.3101, 1JZ3.A.3101, 1JZ6.A.3101, 1JZ7.A.3101, 1JZ8.A.3101, 3LKB.A.394, 4MPY.A.503, 3MUZ.1.3101, 3MV1.1.3101, 3PNX.C.169, 1PX3.B.3101, 3QS6.A.751, 3T09.A.3101, 4TMW.A.902, 3UNX.A.281, 3VD7.B.3101, 1VI6.C.208, 1XFF.A.5301, 1ZNB.A.3

[1] "Cluster 9"

3HW8.A.577, 1VQ7.Q.9148, 3ASQ.B.701, 4CSH.A.1169, 3CZJ.A.3102, 3DR3.A.335, 2DV1.A.1000, 3EEB.A.211, 4EZE.A.302, 3H7K.A.387, 2HZY.A.1201, 3IJP.A.301, 3IWK.D.504, 1IYN.A.298, 2J5A.A.1109, 4JDO.G.301, 1JZ7.C.3103, 3K6A.E.178, 4KA5.A.801, 1L2T.A.1502, 1LZS.A.131, 3MC1.A.301, 1MUQ.A.206, 3MUZ.1.3102, 4N3M.A.403, 3N3O.A.1000, 2O34.A.503, 4O4V.A.301, 4ODI.B.301, 2OKQ.A.119, 2OYC.A.305, 4PMO.A.310, 3Q94.A.310, 1QOP.B.501, 4QTO.A.501, 4QTO.B.501, 2QZ7.A.193, 3TXF.A.1138, 3VW7.A.2012, 3WGU.C.2007, 1WQR.A.131, 2WUW.E.1277, 3WXO.A.805, 1X0G.A.1002, 1X7U.A.1000, 2Y8K.A.1527, 1YAP.A.501, 2Z2F.A.2001, 2ZJ9.A.1, 2ZND.A.195

[1] "Cluster 10"

1DIZ.A.825, 8ICK.A.341, 1VQ8.M.9147, 1YJW.A.8545, 4CD5.A.1421, 3CKI.A.502, 3CTP.A.402, 3DYO.A.3101, 3DYP.A.3101, 1F6D.A.1378, 2FBL.A.304, 4FXZ.A.603, 3GBV.B.1, 2GKO.A.614, 1GNY.A.1244, 1HXN.A.4, 3I3D.A.3101, 3IMM.C.3, 1JYN.A.3101, 1JYV.A.3101, 1JYW.A.3101, 3MVO.1.3101, 3N3R.A.1000, 4OFI.A.801, 3ONF.A.507, 2OZ3.B.2001, 2P3Z.B.501, 3PNX.E.168, 4PV3.A.202, 1PX4.A.3101, 1Q20.A.313, 1QHU.A.436, 3QST.A.253, 1T64.A.391, 3TAV.A.267, 3TYP.A.155, 3V5U.A.701, 3V5U.A.703, 3VD4.A.3101, 3VD5.A.3101, 3VD9.A.3101, 3VDC.A.3101, 3WOL.C.502, 2WWH.C.213

Table S101. 5-ligand Na, combined group

	size	largest_angle*	middle_1*	middle_2	middle_3*	middle_4
1	"30"	"160.3+/-11.6"	"54.6+/-8.2"	"77.4+/-7.8"	"88.4+/-6.8"	"95.1+/-5.5"
2	"49"	"160.5+/-8.6"	"70.4+/-8.8"	"78.5+/-3.9"	"83.3+/-4.6"	"87.8+/-4.8"
3	"84"	"172.1+/-4.3"	"79.9+/-4.4"	"84.7+/-3"	"87.8+/-2.6"	"90.2+/-2.5"
4	"49"	"169+/-4.3"	"80.8+/-4.2"	"84.9+/-3.4"	"88.3+/-2"	"90.8+/-2.1"
5	"91"	"164.8+/-6.2"	"79+/-4.9"	"83.8+/-3.9"	"87.7+/-3.4"	"91.4+/-2.9"
6	"23"	"156.2+/-11.5"	"61.1+/-10.3"	"76.3+/-6.4"	"83.4+/-7.2"	"88.4+/-7.4"
7	"80"	"151.6+/-5.9"	"74.3+/-5"	"81.4+/-3.5"	"85.7+/-3.5"	"90.6+/-4.5"

	middle_5*	middle_6	middle_7	middle_8*
1	"101.4+/-4.6"	"112.8+/-7.5"	"124.1+/-8.4"	"135+/-7.2"
2	"93.7+/-5.4"	"103.6+/-10.7"	"116.7+/-13.2"	"149.7+/-7.5"
3	"94.4+/-3.6"	"98.4+/-3.9"	"104.4+/-6.5"	"164.9+/-4.7"
4	"94.3+/-3.1"	"101.2+/-4.9"	"121.2+/-4.9"	"131.5+/-5.6"
5	"96.6+/-4.3"	"102.5+/-5"	"114.2+/-8.4"	"150.8+/-6.1"
6	"96+/-8.7"	"109.2+/-9.8"	"119.2+/-11.2"	"133+/-9.3"
7	"97.3+/-5.3"	"110.3+/-5.9"	"121.6+/-7.3"	"136.2+/-6.4"

	smallest_opposite_angle*	TrigonalBipyramidal	SquarePyramidal
1	"91.6+/-7.7"	"0.022"	"0.023"
2	"72.8+/-6.7"	"0.003"	"0.111"
3	"83.8+/-6.6"	"0.014"	"0.69"
4	"106+/-6.4"	"0.658"	"0.022"
5	"93.9+/-5.9"	"0.143"	"0.228"
6	"50.1+/-7.6"	"0"	"0.003"
7	"102.3+/-7.5"	"0.17"	"0.014"

	TrigonalPrismaticV
1	"0.111"

2 "0.12"
3 "0.064"
4 "0.254"
5 "0.218"
6 "0.032"
7 "0.42"

Table S102. Cluster members of 5-ligand Na, combined group

[1] "Cluster 1"
4FZZ.A.201, 1JJ2.A.8345, 4KHY.A.1006, 4NLK.A.403, 2BBH.A.301, 1BUN.A.121, 4FEV.B
.303, 3FFZ.A.1302, 4FLL.A.504, 4FO2.Q.101, 2GG8.A.503, 1GVH.A.1399, 4H83.A.402,
4J4B.A.902, 1JJU.C.996, 3KEU.A.402, 3KWM.A.225, 4MAT.A.501, 3MX6.A.261, 1NSX.A.1
401, 1O68.E.274, 2QWL.A.589, 1SU4.A.997, 1TC8.A.121, 1U4J.B.1001, 3VD3.D.3101, 3
VD5.A.3101, 2WPC.C.1492, 2WWH.C.213, 2WX5.H.1252
[1] "Cluster 2"
4KHY.A.1013, 3PML.B.7, 2A5G.B.242, 2A7L.A.201, 2A9Y.A.2002, 4B6C.A.1257, 4BR6.A.
401, 4C1P.A.1728, 4CBY.B.2036, 4CVU.A.1998, 4D1J.E.604, 1D7U.A.435, 4D9T.A.901,
4DD8.A.1005, 1EZ1.B.1002, 3G1N.B.5002, 3G8Q.D.302, 4G8T.B.502, 4GKI.F.303, 4GRX.
A.501, 3I2W.A.304, 3IAQ.A.3103, 3IC9.A.491, 2JBW.A.1368, 4LLH.B.602, 1MGV.A.501,
4MVJ.A.401, 4NLQ.A.910, 4NPJ.B.701, 4NRH.C.401, 4NT1.A.301, 1NZA.A.104, 4OOC.B.
402, 4O54.A.302, 2ONP.G.707, 4OUC.A.801, 2P6Z.A.402, 4QVS.A.502, 1S5C.A.241, 1SO
O.A.547, 2WD0.C.601, 2WWG.B.213, 1XAR.A.100, 4XEL.A.201, 1ZEL.A.401, 2ZN8.A.995,
3ZPQ.A.1360, 3ZQ5.A.1530, 3ZX0.C.522
[1] "Cluster 3"
2FMS.A.342, 4GXK.A.405, 3HW8.A.580, 3JPP.A.340, 1ORP.A.224, 2PFQ.A.1, 1Q81.K.834
6, 4TUR.A.404, 3AGB.A.1, 3AST.B.701, 4BDR.A.902, 4BVN.A.1360, 3C7E.A.489, 4C7A.B
.1159, 4CZN.A.1371, 4D1I.A.600, 4DF9.A.501, 3DR3.A.335, 4F3Y.A.301, 4FEW.D.304,
4G1K.B.301, 1G5I.C.902, 4GDK.E.301, 2GEZ.A.401, 1GEN.A.304, 2GTW.E.3006, 1GUU.A.
1090, 1GV2.A.1192, 4GY9.A.207, 2HU3.A.9002, 1HXN.A.2, 3HYS.A.267, 4I0W.D.602, 4I
2F.A.602, 4I2R.C.602, 3I44.A.477, 3IFV.C.408, 2IY6.B.1540, 2J5W.A.3044, 2J5W.A.3
045, 2JHN.A.1298, 3JS4.A.208, 4KA8.A.806, 3KED.A.951, 4KXW.A.1013, 4KZV.A.304, 4
L3H.A.404, 3LP5.A.251, 1LZS.A.131, 4M4V.A.505, 4MJD.A.203, 4MM9.A.602, 4MPT.A.40
2, 2O34.B.502, 3OEC.A.300, 2OKQ.A.119, 3OTK.A.586, 4PFI.A.401, 4PUV.A.405, 4PV3.
A.201, 4QFE.B.305, 1QHU.A.437, 3STH.A.501, 1SU3.A.911, 1TQY.H.1094, 3U21.A.500,
1UD8.A.1001, 2V4B.B.1562, 1VI6.A.208, 3VS8.A.501, 3VS8.C.501, 1W16.A.1002, 1W9W.
A.900, 3WNM.A.802, 1WPG.D.1300, 2WU2.A.1590, 2WV7.B.401, 1X0G.C.1001, 1X7D.A.150
1, 1X7U.A.1000, 2X7J.A.1581, 1XDF.A.401, 2XZK.B.507, 1ZDN.A.157
[1] "Cluster 4"
2GIG.A.502, 1SUZ.A.403, 1A7T.A.255, 4A87.A.1162, 2ABS.A.1003, 4ATF.C.500, 3B1N.A
.403, 2B2N.B.1001, 4BEM.A.201, 4BEM.B.201, 4BEM.J.201, 2BL2.A.1157, 4D77.A.1543,
2DKB.A.436, 3EPR.A.266, 4EXR.A.301, 4FHA.B.402, 3G0T.B.437, 1H80.A.1497, 2HIG.B
.488, 2JBW.D.1367, 4JNQ.A.402, 4LDE.A.1402, 3MAX.A.381, 4MB4.A.604, 4MMB.A.601,
4MMF.A.601, 3MS8.A.401, 3MUI.A.401, 1MVO.A.202, 1N82.A.401, 1OA8.C.1691, 1OAF.A.
1252, 2PFL.A.2001, 2PPL.A.480, 3PZJ.A.301, 2Q8X.A.401, 3QS4.A.751, 4R7U.A.504, 3
RGA.A.312, 1SK4.A.342, 3T8J.A.401, 1V54.A.3519, 3VDG.A.502, 1W9S.A.1142, 2WGM.A.
201, 2WGM.B.201, 3WZ1.A.401, 2Z5D.B.180
[1] "Cluster 5"
2BCV.A.579, 3HW8.A.577, 4ORJ.A.305, 1VQ7.Q.9148, 2A65.A.751, 4ADB.A.1405, 3ASQ.B
.701, 4AYO.A.502, 4CSH.A.1169, 3CTP.A.402, 3CZJ.A.3102, 2DDB.B.303, 2DV1.A.1000,
3DYP.A.3101, 3EEB.A.211, 4EZE.A.302, 4FMT.D.301, 4FXZ.A.603, 3FZQ.B.274, 1GVF.A

.289, 3H7K.A.387, 4H7O.C.302, 4H83.E.401, 2HZY.A.1201, 3IJP.A.301, 3IMX.A.467, 3IWK.D.504, 1IYN.A.298, 2J5A.A.1109, 4JDO.G.301, 4JEX.A.512, 2JLN.A.1471, 1JYV.A.3101, 1JZ7.C.3103, 1JZ8.D.3104, 3K6A.E.178, 1KA0.A.501, 4KA5.A.801, 1L2T.A.1502, 4LHL.A.301, 3LJQ.A.597, 4M9U.A.402, 3MC1.A.301, 4MM7.A.601, 3MPN.A.751, 3MPQ.A.751, 1MUQ.A.206, 3MUZ.1.3102, 3N3R.A.1000, 4N3M.A.403, 3N3O.A.1000, 4O1G.A.401, 2O34.A.503, 4O4V.A.301, 4ODI.B.301, 4OFI.A.801, 4OUA.A.403, 2OYC.A.305, 4PMO.A.310, 3Q94.A.310, 3Q9B.A.344, 1QOP.B.501, 3QS5.A.751, 3QST.A.253, 4QTO.A.501, 4QTO.B.501, 2QZ7.A.193, 1RW9.A.900, 3S9J.A.401, 1T64.A.391, 3TAV.A.267, 1TO2.E.451, 3TXF.A.1138, 3VD3.A.3101, 3VDA.B.3101, 3VW7.A.2012, 3WGU.C.2007, 1WQR.A.131, 2WUW.E.1277, 3WXO.A.805, 1X0G.A.1002, 2Y8K.A.1527, 1YAP.A.501, 1YQ2.A.7501, 2Z2F.A.2001, 1ZH8.A.329, 2ZJ9.A.1, 3ZK1.A.90, 3ZK1.B.90, 2ZND.A.195, 3ZQS.B.1295

[1] "Cluster 6"

4DOA.A.402, 4DOB.A.402, 2FMQ.A.340, 3JPT.A.340, 3PNC.A.2, 2PXI.A.340, 1U8R.A.1101, 1VQ9.C.9104, 2BWU.A.1441, 3CBT.A.420, 3FKR.B.408, 4H41.B.405, 3INJ.C.601, 3KZW.D.498, 4LCZ.B.320, 4M5P.A.505, 1MMX.A.349, 4NTL.A.311, 4NYP.A.1001, 3R2H.A.156, 1SVY.A.2, 1YCE.B.201, 1YCE.a.201

[1] "Cluster 7"

1DIZ.A.825, 2GIJ.F.403, 8ICK.A.341, 1TX3.A.801, 1VQ8.M.9147, 1YJW.A.8545, 3C7X.A.1001, 3CB8.A.820, 4CD5.A.1420, 4CD5.A.1421, 4CFY.A.302, 3CKI.A.502, 3CZJ.A.3101, 4D1J.A.601, 4DUW.A.3101, 4DUX.A.3101, 3DYM.A.3101, 3DYO.A.3101, 1EBU.B.902, 1EJA.A.246, 1F6D.A.1378, 2FBL.A.304, 3GBV.B.1, 2GKO.A.614, 1GNY.A.1244, 3GVK.A.916, 4GVO.A.702, 1HXN.A.4, 3I3B.A.3101, 3I3D.A.3101, 3IMM.C.3, 4IQZ.A.317, 3IRD.A.301, 4J4B.B.303, 4JPA.A.305, 3JU4.A.7, 1JYN.A.3101, 1JYW.A.3101, 1JZ2.A.3101, 1JZ3.A.3101, 1JZ6.A.3101, 1JZ7.A.3101, 1JZ8.A.3101, 3LKB.A.394, 4MPY.A.503, 3MUZ.1.3101, 3MV0.1.3101, 3MV1.1.3101, 1O4Z.A.1004, 4OMC.A.608, 3ONF.A.507, 2OZ3.B.2001, 2P3Z.B.501, 3PNX.C.169, 3PNX.E.168, 4PV3.A.202, 1PX3.B.3101, 1PX4.A.3101, 1Q20.A.313, 1QHU.A.436, 4QKU.A.501, 3QS6.A.751, 3T09.A.3101, 4TMW.A.902, 3TYP.A.155, 3UNX.A.281, 3V5U.A.701, 3V5U.A.703, 3VD4.A.3101, 3VD7.B.3101, 3VD9.A.3101, 3VDC.A.3101, 1VI6.C.208, 3W0L.C.502, 3WA2.X.702, 3WGU.A.2005, 2WOI.A.1491, 1XFF.A.5301, 1ZNB.A.3, 3ZYV.A.2337

Table S103. 6-ligand Na, normal group

	size	largest_angle*	middle_1*	middle_2	middle_3	middle_4
1	"34"	"171.2+/-3.3"	"65.6+/-3.3"	"79.2+/-4.9"	"82.5+/-3.6"	"85.6+/-3.4"
2	"37"	"163.9+/-3.5"	"76.3+/-3.2"	"79.1+/-2.9"	"82.3+/-2.8"	"84.4+/-2.3"
3	"108"	"175+/-2.3"	"80.1+/-3.8"	"83.4+/-2.2"	"85.5+/-2.2"	"87.4+/-1.7"
4	"36"	"158.2+/-6.2"	"65.3+/-4.9"	"73.7+/-3.9"	"78.5+/-3.5"	"82.5+/-4"
5	"81"	"170+/-3.6"	"76.3+/-2.9"	"80.6+/-2.4"	"83.3+/-2"	"85.3+/-2.1"
6	"66"	"172.6+/-3.2"	"79+/-3.9"	"82.7+/-2.6"	"85.1+/-2.3"	"87.1+/-2.1"

	middle_5*	middle_6	middle_7	middle_8	middle_9*
1	"88.5+/-2.6"	"91.2+/-2.3"	"94.4+/-2.8"	"97.2+/-3.3"	"100.8+/-3.8"
2	"87.3+/-2.4"	"89.6+/-2.6"	"91.9+/-3"	"95.1+/-3.1"	"101.2+/-4.7"
3	"88.8+/-1.6"	"90.6+/-1.7"	"92+/-1.8"	"93.8+/-1.9"	"95.7+/-1.9"
4	"86.4+/-4"	"90+/-4.6"	"95.1+/-4.5"	"99.3+/-5.1"	"106.6+/-6.4"
5	"87.4+/-2"	"89.6+/-1.9"	"91.9+/-2.2"	"94.6+/-2.3"	"99.2+/-2.7"
6	"89+/-1.7"	"91+/-1.9"	"93.1+/-2.1"	"95.1+/-2.2"	"97.4+/-2.2"

	middle_10	middle_11	middle_12	middle_13*
1	"105.7+/-4.9"	"113.7+/-6.4"	"148.7+/-9.7"	"165+/-5.4"
2	"107.8+/-5.7"	"118+/-8.3"	"150.8+/-5.1"	"156.5+/-4.6"
3	"98.3+/-2.8"	"102.5+/-4.5"	"164.3+/-5.4"	"171.7+/-2.8"

```
4 "115.4+/-8.4" "124.7+/-8.9" "138.7+/-6.6" "149.2+/-5.5"
5 "103.8+/-3.7" "109.1+/-4.6" "158+/-4.2"   "164+/-2.9"
6 "100.7+/-2.9" "109.4+/-6.1" "154.9+/-6.6" "165.4+/-3.9"
  smallest_opposite_angle* Octahedral TrigonalPrismatic
1 "77+/-6"                  "0.24"     "0.076"
2 "71.6+/-3.8"              "0.184"    "0.248"
3 "82+/-3.1"                "0.868"    "0.016"
4 "68.2+/-6.9"              "0.02"     "0.216"
5 "80.3+/-2.5"              "0.522"    "0.077"
6 "70.5+/-4.5"              "0.519"    "0.051"
  PentagonalBipyramidalVA PentagonalBipyramidalVP
1 "0"                     "0.225"
2 "0"                     "0.359"
3 "0"                     "0.063"
4 "0.033"                 "0.261"
5 "0"                     "0.154"
6 "0"                     "0.202"
```

Table S104. Cluster members of 6-ligand Na, normal group

[1] "Cluster 1"
2AHR.C.1259, 4AMJ.A.1360, 3BIA.X.117, 3BIB.X.117, 3CRN.A.131, 4D7C.A.1544, 2DDB.C.302, 2E5X.A.303, 2E54.A.1004, 4E6P.A.301, 4EAE.A.302, 4FEX.A.303, 4G8T.C.502, 4GIB.A.301, 2GJU.A.2001, 1H16.A.9001, 3I2W.B.304, 4J07.A.201, 1JTP.A.501, 1JZN.A.1139, 3MYV.A.502, 3N83.G.707, 1O04.A.6601, 2OYN.A.201, 1Q3X.A.800, 2QSV.A.222, 1S82.A.4, 1T8U.B.701, 1TQY.B.1091, 3VGL.A.323, 2VNZ.X.9252, 2X7J.B.1581, 2X8J.A.1317, 1ZUD.1.701
[1] "Cluster 2"
2BCR.A.604, 4KYW.A.303, 1Q81.A.8378, 1VQ8.Q.9148, 2BER.A.1649, 3C17.A.323, 4DOU.A.1004, 3GZA.A.471, 2HZG.A.1101, 3I01.M.730, 3IC9.B.491, 3IWJ.A.505, 2J80.A.1134, 4JN7.A.401, 3K1U.A.412, 3L7X.A.142, 3LG1.A.530, 4LL2.A.301, 3N0U.A.208, 4O57.A.301, 4O5H.A.605, 1O68.A.274, 3ON4.C.189, 3OND.A.509, 4PD6.A.502, 4PMO.A.312, 1Q6X.A.1002, 2QR7.A.1000, 1S0A.A.1501, 1T64.A.392, 3TDQ.A.117, 4US3.A.701, 4USW.A.1468, 1V0H.X.252, 1W9S.A.1141, 2ZJ9.B.2, 1ZOR.A.1001
[1] "Cluster 3"
4EJY.A.301, 1EWN.A.501, 3I0W.A.296, 4KLI.A.404, 4KLI.A.405, 4M04.A.707, 1ORN.A.224, 3OSN.A.423, 4P4M.A.403, 1RZT.A.2001, 4TUP.A.402, 2A5F.B.1326, 3AGC.A.1, 3AJN.A.136, 4AK1.A.1702, 2AU7.A.206, 4B1M.A.1680, 3BGA.A.6, 4C1O.A.1731, 4C3X.A.561, 3C7F.A.804, 4CCY.A.1298, 4CIT.A.1454, 3CYM.A.501, 3D9R.A.135, 2DDA.A.301, 4DW8.A.304, 4E6P.D.301, 2E7U.A.1003, 3E9L.A.1, 3ELF.A.351, 2FPR.B.505, 1G5I.A.901, 3GED.A.251, 3GIR.A.373, 4GNJ.B.302, 4GRX.B.501, 2GTW.B.3005, 1GV5.A.1142, 4H83.A.401, 4HUR.A.316, 4I29.A.601, 4I2A.A.602, 3ICF.A.515, 3IFV.A.402, 4IIB.A.944, 3IJP.B.301, 3IMM.B.2, 4IQZ.A.316, 3IRS.A.290, 4JEX.B.511, 2JHJ.B.1296, 4JVL.B.304, 1JZ7.A.3104, 4KA7.A.805, 3KRS.A.301, 1L5B.A.302, 4LG8.A.601, 4LIZ.A.202, 1M4Y.A.252, 3M9Y.B.254, 4M9B.A.201, 3MJ6.A.503, 4MMB.A.602, 4MMF.A.602, 3MQG.A.193, 4NAW.B.303, 3NNB.A.401, 4NSJ.A.222, 3OLJ.A.1, 2OPL.A.187, 4P33.A.401, 4PCG.C.304, 3PGO.A.147, 1PYF.A.315, 3PZR.A.373, 3PZS.A.287, 4Q5K.A.302, 1QNJ.A.280, 3QXT.A.133, 3R0L.D.124, 4R3N.B.401, 4R6K.A.501, 2RGI.B.98, 1S5D.A.241, 3SJL.A.402, 3SSB.A.995, 4TKX.L.706, 4U99.A.203, 1UD2.A.1002, 3UWP.A.424, 2V3U.A.1263, 2V79.A.1116, 1VMF.A.134, 1W15.A.2002, 3W5N.A.1210, 3WAY.A.914, 3WC3.A.502, 4WFX.A.504, 3WNO.A.802, 2WOF.A.1728, 2WW2.A.800, 4XCZ.A.405, 4XCZ.A.406, 1XKN.A.700, 1Y7W.B.282, 2

ZQ3.A.160, 3ZX3.C.522
[1] "Cluster 4"
2OTL.A.8545, 1TW8.B.902, 3UXP.A.338, 1VQ9.M.9147, 1A5U.G.4732, 2AB8.A.2003, 4B52
.A.1305, 1BGP.A.502, 4BY5.B.1188, 4CBY.A.2036, 2D4E.A.1901, 4FMT.B.301, 2GFH.A.2
49, 1HBN.D.1561, 3HSC.A.491, 2IM2.A.3001, 1JZ4.A.3101, 4KZW.A.304, 3ME4.B.2, 4NL
V.A.911, 4OMG.A.402, 2P6Z.B.401, 2P6Z.B.403, 4PCG.A.302, 4Q92.C.502, 3SIS.B.3001
, 3SIT.A.3000, 3TAY.A.1, 4TMX.A.902, 2W4M.A.1245, 3WFA.A.801, 4WFZ.A.501, 2WPC.A
.1491, 2YDG.A.1130, 2YFO.A.1743, 1Z45.A.702
[1] "Cluster 5"
4P4P.A.401, 4AFK.A.1507, 2AMF.A.850, 3AR7.A.1000, 3B34.A.951, 3BLJ.B.701, 3BOS.B
.302, 4BVO.A.1396, 4CCY.A.1297, 2CD7.A.1132, 4D1J.E.602, 4D1J.G.603, 4DF9.B.501,
 3E85.A.163, 1EAS.A.5, 4EEK.A.302, 4EEL.A.302, 3EIF.A.1033, 3EIY.A.177, 3EUW.A.3
43, 3F3C.A.752, 2FV7.A.403, 4GAF.B.505, 3GOD.B.327, 1GV2.A.1191, 3HON.A.204, 3H1
2.A.500, 3HVI.A.265, 1HX6.A.705, 4IOW.B.603, 3IAN.A.1, 3IC3.C.100, 2JBW.C.1367,
4JB3.A.301, 4JDO.A.301, 2JHN.B.1299, 1JMM.A.3001, 1JTP.L.503, 4JTE.C.301, 4JTF.C
.303, 4JTG.C.302, 4JTH.C.303, 4JTJ.C.302, 1K2X.A.801, 1KSU.A.810, 1LLA.A.631, 4N
9S.A.402, 4N9V.A.404, 3NGJ.A.249, 3NMB.A.1, 4NPI.A.602, 4OOC.A.401, 2O34.A.501,
4O47.A.401, 3OEC.C.300, 1P0Z.A.1633, 4PMO.A.311, 2POC.A.5001, 2QKF.C.283, 1R4P.A
.4003, 2R85.A.600, 3RU5.A.133, 1RWH.A.900, 1S5E.A.241, 3SIB.A.223, 1T3M.A.801, 4
TKX.L.705, 1UD2.A.1001, 3USL.A.752, 2V4V.A.3052, 2V4B.A.1566, 2WCB.A.101, 2WGE.A
.1426, 1WKY.A.504, 2XNA.C.1216, 1YOP.A.1810, 2Y5F.A.1245, 2YNQ.A.1392, 2ZND.A.19
6, 1ZOD.A.435, 3ZWF.B.1365
[1] "Cluster 6"
2BCU.A.577, 2BCQ.A.1, 1CZ0.C.606, 4ED3.A.502, 1JJ2.0.8338, 1VQ8.J.9146, 2A5D.B.3
26, 4A6U.B.1460, 3AR4.A.1000, 2AUT.D.605, 1B57.A.364, 2BS2.A.1658, 2DDB.A.301, 4
DD8.D.1005, 4DEL.A.401, 3E85.A.162, 2EHQ.A.1540, 4FDZ.B.301, 4FET.A.301, 2FM1.D.
344, 1G3K.A.500, 1G5H.A.901, 3GA5.B.701, 4GKI.D.303, 3HON.A.203, 1HBN.A.1561, 3H
IJ.B.295, 2HIO.B.240, 3HSS.A.268, 3IGZ.B.601, 2IJA.A.401, 3IPO.A.436, 3IPP.A.438
, 1IYN.A.297, 2IZV.A.1430, 1JAY.A.215, 3JS4.D.208, 4JTK.C.302, 1JZ7.A.3102, 4LGN
.A.827, 4M48.A.701, 4M4U.A.501, 4M60.A.502, 3MQD.A.500, 3MZG.B.211, 4NRH.A.401,
4OF8.A.301, 3OPK.A.301, 4PSR.A.619, 4PYJ.A.301, 4Q69.A.501, 2QJY.R.2001, 2QV6.A.
303, 4QXK.A.402, 1U7H.B.912, 3UES.B.503, 3UF4.A.601, 2VPB.A.1398, 2WDQ.A.1590, 2
WOI.D.1489, 2WWF.B.214, 4X9K.A.401, 2XZI.A.502, 1YYA.A.1001, 2ZHJ.A.321, 1ZOR.B.
1002

Table S105. 6-ligand Na, combined group

	size	largest_angle*	middle_1*	middle_2	middle_3	middle_4
1	"48"	"159.9+/-4.5"	"71.2+/-6"	"76.3+/-4"	"80.1+/-3.4"	"83.2+/-3.1"
2	"46"	"166.2+/-5.6"	"68.6+/-7.4"	"76.6+/-5.8"	"80.5+/-4.9"	"84.6+/-4"
3	"28"	"154.4+/-10.8"	"59+/-10.4"	"72.9+/-6.9"	"79.3+/-6.7"	"84.3+/-6.1"
4	"44"	"165.3+/-6.1"	"50.2+/-6"	"73.2+/-8.5"	"80.4+/-4.9"	"84.1+/-4.3"
5	"85"	"171.5+/-3.8"	"78.5+/-3.8"	"82.2+/-2.5"	"84.7+/-2.3"	"86.7+/-2.1"
6	"85"	"169.7+/-3.8"	"70.9+/-6.1"	"79.5+/-3.8"	"82.8+/-2.7"	"85.1+/-2.6"
7	"135"	"174.4+/-2.6"	"79.9+/-3.6"	"83.2+/-2.2"	"85.2+/-2.2"	"87.2+/-1.7"

	middle_5*	middle_6	middle_7	middle_8	middle_9*
1	"86.5+/-3.5"	"89.2+/-3.7"	"92.9+/-4.2"	"96.3+/-3.8"	"103.7+/-5.6"
2	"88.1+/-3.6"	"90.7+/-3.3"	"94+/-4.5"	"97.9+/-4.4"	"103.3+/-4.8"
3	"88.8+/-4.3"	"92.8+/-3.9"	"97.1+/-4.3"	"102+/-5.7"	"109.9+/-7.3"
4	"87.6+/-3.4"	"90.8+/-3.9"	"94.5+/-5.2"	"98.9+/-5.5"	"105.4+/-5.5"
5	"88.7+/-1.9"	"90.9+/-2"	"93.1+/-2.3"	"95.1+/-2.4"	"97.7+/-2.5"

6	"87.7+/-2.5"	"90.1+/-2.3"	"92.9+/-2.5"	"95.7+/-2.6"	"100.2+/-3.3"
7	"88.6+/-1.7"	"90.4+/-1.7"	"92+/-1.8"	"93.8+/-1.9"	"96.2+/-2.1"

	middle_10	middle_11	middle_12	middle_13*
1	"112.6+/-7.4"	"122.9+/-8.5"	"144.5+/-6.3"	"151.8+/-5.2"
2	"109.1+/-5.6"	"118.5+/-6.9"	"135.2+/-8.7"	"158.2+/-6"
3	"119.1+/-5.7"	"124.8+/-5.2"	"131.8+/-5.8"	"139.1+/-5.3"
4	"111.9+/-5.9"	"121.7+/-7.3"	"137.6+/-7.5"	"153.2+/-6.2"
5	"101.5+/-3.4"	"110.2+/-6.7"	"154.9+/-5.9"	"164+/-4.4"
6	"105.3+/-4.5"	"111.9+/-5.7"	"153.6+/-8.6"	"164.1+/-3.5"
7	"98.9+/-3"	"103.2+/-4.6"	"163.4+/-5.3"	"170.8+/-3.3"

	smallest_opposite_angle*	Octahedral	TrigonalPrismatic
1	"70.8+/-5.5"	"0.06"	"0.28"
2	"50.2+/-5.3"	"0.051"	"0.048"
3	"53+/-7.3"	"0.008"	"0.09"
4	"70.7+/-9.5"	"0.005"	"0.049"
5	"70.8+/-4.9"	"0.465"	"0.067"
6	"79.7+/-3.7"	"0.342"	"0.085"
7	"81.6+/-3.2"	"0.85"	"0.022"

	PentagonalBipyramidalVA	PentagonalBipyramidalVP
1	"0.024"	"0.354"
2	"0.01"	"0.186"
3	"0.002"	"0.067"
4	"0.015"	"0.074"
5	"0"	"0.223"
6	"0"	"0.172"
7	"0"	"0.074"

Table S106. Cluster members of 6-ligand Na, combined group

[1] "Cluster 1"
3UXP.A.338, 1VQ8.Q.9148, 1VQ9.M.9147, 1A5U.G.4732, 2AB8.A.2003, 4B52.A.1305, 2BER.A.1649, 1BGP.A.502, 4BY5.B.1188, 3C17.A.323, 4CBY.A.2036, 2D4E.A.1901, 2GFH.A.249, 1HBN.D.1561, 2HZG.A.1101, 3I01.M.730, 3IC9.B.491, 2IM2.A.3001, 3IWJ.A.505, 2J80.A.1134, 4JN7.A.401, 1JZ4.A.3101, 4LL2.A.301, 3ME4.B.2, 3N0U.A.208, 4NLV.A.911, 4O57.A.301, 4O5H.A.605, 1O68.A.274, 3ON4.C.189, 3OND.A.509, 2P6Z.B.403, 4PCG.A.302, 1Q6X.A.1002, 1S0A.A.1501, 3SIS.B.3001, 3SIT.A.3000, 1T64.A.392, 3TAY.A.1, 4US3.A.701, 4USW.A.1468, 1V0H.X.252, 2W4M.A.1245, 4WFZ.A.501, 2WPC.A.1491, 2YDG.A.1130, 1Z45.A.702, 2ZJ9.B.2
[1] "Cluster 2"
1BPZ.A.341, 3C5G.A.803, 2FMP.A.1340, 4FZX.B.101, 4FZX.A.101, 4FZY.C.101, 4FZY.D.101, 2ISO.A.340, 3JPO.A.340, 3JPQ.A.340, 3JPR.A.340, 4M9L.A.403, 1SA3.A.401, 1VQ8.0.9117, 3A6V.A.1005, 3A6V.B.1006, 3ATU.A.6267, 1AVT.A.301, 3B8X.B.668, 1BA0.A.490, 4DCC.A.302, 2DDA.B.302, 4DD8.B.1005, 3E3T.A.243, 3ED4.A.519, 4FMT.B.301, 3HSC.A.490, 3HSC.A.491, 1HVX.A.519, 3MZG.B.211, 4O52.A.301, 1QHU.A.438, 2QSV.A.222, 1QUS.A.400, 3R0L.D.125, 4UZU.A.1491, 1V7T.A.406, 3VDC.A.3103, 2WCP.A.501, 4WED.A.601, 3WFA.A.801, 3WNK.A.813, 3WXO.A.806, 3WZ1.A.402, 1YQ2.C.7503, 1ZDN.B.157
[1] "Cluster 3"
4FZX.C.201, 4KLI.A.403, 3MBY.A.340, 2OTL.A.8545, 1TW8.B.902, 4UAY.A.403, 1YIJ.0.8519, 1YJ9.0.8517, 3B1Q.C.332, 2GA4.A.713, 3GQ9.A.1, 3GQ9.A.692, 3HLT.A.269, 4IQL.A.407, 4J43.A.902, 3KZW.A.497, 3OQ8.A.460, 3OQ8.C.460, 2P6Z.B.401, 3Q9C.A.344, 4Q92.C.502, 2QZ7.A.194, 3R2H.A.157, 1VK1.A.302, 3WGV.C.2005, 2WGM.u.201, 2YXU.B

.2406, 3ZK2.L.90
[1] "Cluster 4"
3C0W.A.303, 3C5G.B.808, 2FLD.C.603, 1Q81.N.8347, 1VQ5.A.9145, 4ATF.A.500, 3BC9.A
.704, 3BH4.A.489, 4D1I.B.600, 2DIE.A.781, 4DMI.A.202, 4F4R.A.501, 4FUS.A.828, 2G
JP.A.1489, 4H83.B.402, 2HEU.A.4001, 1HN0.A.1800, 2HZG.B.1102, 2ID4.A.907, 2JBA.A
.1127, 4JQR.A.301, 3KQB.A.303, 4KZW.A.304, 4MPY.C.503, 3MV1.3.3101, 1O04.A.6601,
 1OB0.A.504, 4OMG.A.402, 2P3Z.A.501, 3Q2H.A.701, 4TMW.B.902, 4TMX.A.902, 3USZ.A.
902, 1V8Z.A.389, 3VD5.B.3101, 3VHS.B.52, 3W5N.A.1211, 2WOF.A.1727, 1WPC.A.504, 1
WX5.C.282, 1XAR.B.201, 2YFO.A.1743, 3ZK1.D.90, 1ZUD.1.701
[1] "Cluster 5"
2BCU.A.577, 2BCQ.A.1, 2BCR.A.604, 1CZ0.C.606, 4ED3.A.502, 1JJ2.0.8338, 4KYW.A.30
3, 4P4P.A.401, 1VQ8.J.9146, 2A5D.B.326, 4A6U.B.1460, 3AR4.A.1000, 2AUT.D.605, 1B
57.A.364, 3BIB.X.117, 2BS2.A.1658, 4D1J.G.603, 2DDB.A.301, 4DD8.D.1005, 4DEL.A.4
01, 4DF9.B.501, 4DOU.A.1004, 2E5X.A.303, 3E85.A.162, 2EHQ.A.1540, 4FDZ.B.301, 4F
ET.A.301, 2FM1.D.344, 1G5H.A.901, 4GKI.D.303, 3GZA.A.471, 3H0N.A.203, 1HBN.A.156
1, 3HIJ.B.295, 2HI0.B.240, 3HSS.A.268, 4HUC.A.509, 4I0W.B.603, 3IGZ.B.601, 2IJA.
A.401, 3IPP.A.438, 1IYN.A.297, 2IZV.A.1430, 1JAY.A.215, 3JS4.D.208, 4JTH.C.303,
4JTK.C.302, 1JZ7.A.3102, 3K1U.A.412, 3L7X.A.142, 3LG1.A.530, 4LGN.A.827, 4M48.A.
701, 4M4U.A.501, 4M60.A.502, 3MQD.A.500, 4NPI.A.602, 4NRH.A.401, 4OF8.A.301, 3OP
K.A.301, 4PD6.A.502, 4PMO.A.312, 4PSR.A.619, 4Q69.A.501, 2QJY.R.2001, 2QR7.A.100
0, 2QV6.A.303, 4QXK.A.402, 3SLZ.B.130, 3TDQ.A.117, 1U7H.B.912, 3UES.B.503, 3UF4.
A.601, 2VPB.A.1398, 2WDQ.A.1590, 2WOI.D.1489, 2WWF.B.214, 4X9K.A.401, 2XNA.C.121
6, 2XZI.A.502, 1YYA.A.1001, 2ZHJ.A.321, 2ZND.A.196, 1ZOR.A.1001, 1ZOR.B.1002
[1] "Cluster 6"
1Q81.A.8378, 4AFK.A.1507, 2AHR.C.1259, 4AMJ.A.1360, 3AR7.A.1000, 3BIA.X.117, 3BL
J.B.701, 3BOS.B.302, 4BVO.A.1396, 2CD7.A.1132, 3CRN.A.131, 4D1J.E.602, 4D7C.A.15
44, 2DDB.C.302, 2E54.A.1004, 4E6P.A.301, 3E85.A.163, 1EAS.A.5, 4EAE.A.302, 4EEK.
A.302, 4EEL.A.302, 3EIF.A.1033, 3EIY.A.177, 3EUW.A.343, 4FEX.A.303, 4G8T.C.502,
4GIB.A.301, 2GJU.A.2001, 3GOD.B.327, 1H16.A.9001, 3HVI.A.265, 3I2W.B.304, 4J07.A
.201, 2JBW.C.1367, 4JB3.A.301, 4JDO.A.301, 2JHN.B.1299, 1JMM.A.3001, 1JTP.A.501,
 1JTP.L.503, 4JTE.C.301, 4JTF.C.303, 4JTG.C.302, 1JZN.A.1139, 1K2X.A.801, 1LLA.A
.631, 4M48.A.702, 3MYV.A.502, 3N83.G.707, 4N9S.A.402, 4N9V.A.404, 3NMB.A.1, 4O0C
.A.401, 2O34.A.501, 4O47.A.401, 3OEC.C.300, 2OYN.A.201, 1P0Z.A.1633, 2POC.A.5001
, 1Q3X.A.800, 2QKF.C.283, 1R4P.A.4003, 2R85.A.600, 1RWH.A.900, 1S82.A.4, 1T8U.B.
701, 1TQY.B.1091, 3TSH.A.604, 3USL.A.752, 2V4V.A.3052, 2V4B.A.1566, 3VGL.A.323,
2VNZ.X.9252, 1W9S.A.1141, 2WCB.A.101, 3WG7.N.605, 1WKY.A.504, 2WQK.A.254, 2X7J.B
.1581, 2X8J.A.1317, 1Y0P.A.1810, 2Y5F.A.1245, 2YNQ.A.1392, 1ZOD.A.435, 3ZWF.B.13
65
[1] "Cluster 7"
4EJY.A.301, 1EWN.A.501, 3I0W.A.296, 4KLI.A.404, 4KLI.A.405, 4M04.A.707, 1ORN.A.2
24, 3OSN.A.423, 4P4M.A.403, 1RZT.A.2001, 4TUP.A.402, 2A5F.B.1326, 3AGC.A.1, 3AJN
.A.136, 4AK1.A.1702, 2AMF.A.850, 2AU7.A.206, 4B1M.A.1680, 3B34.A.951, 3BGA.A.6,
4C1O.A.1731, 4C3X.A.561, 3C7F.A.804, 4CCY.A.1297, 4CCY.A.1298, 4CIT.A.1454, 3CYM
.A.501, 3D9R.A.135, 2DDA.A.301, 4DW8.A.304, 4E6P.D.301, 2E7U.A.1003, 3E9L.A.1, 3
ELF.A.351, 3F3C.A.752, 2FPR.B.505, 2FV7.A.403, 1G3K.A.500, 1G5I.A.901, 3GA5.B.70
1, 4GAF.B.505, 3GED.A.251, 3GIR.A.373, 4GNJ.B.302, 4GRX.B.501, 2GTW.B.3005, 1GV2
.A.1191, 1GV5.A.1142, 3H0N.A.204, 3H12.A.500, 4H83.A.401, 4HUR.A.316, 1HX6.A.705
, 4I29.A.601, 4I2A.A.602, 3IAN.A.1, 3IC3.C.100, 3ICF.A.515, 3IFV.A.402, 4IIB.A.9
44, 3IJP.B.301, 3IMM.B.2, 3IPO.A.436, 4IQZ.A.316, 3IRS.A.290, 4JEX.B.511, 2JHJ.B
.1296, 4JTJ.C.302, 4JVL.B.304, 1JZ7.A.3104, 4KA7.A.805, 3KRS.A.301, 1KSU.A.810,
1L5B.A.302, 4LG8.A.601, 4LIZ.A.202, 1M4Y.A.252, 3M9Y.B.254, 4M9B.A.201, 3MJ6.A.5
03, 4MMB.A.602, 4MMF.A.602, 3MQG.A.193, 4NAW.B.303, 3NGJ.A.249, 3NNB.A.401, 4NSJ
.A.222, 3OLJ.A.1, 2OPL.A.187, 4P33.A.401, 4PCG.C.304, 3PGO.A.147, 4PMO.A.311, 1P
YF.A.315, 4PYJ.A.301, 3PZR.A.373, 3PZS.A.287, 4Q5K.A.302, 1QNJ.A.280, 3QXT.A.133

, 3R0L.D.124, 4R3N.B.401, 4R6K.A.501, 2RGI.B.98, 3RU5.A.133, 1S5D.A.241, 1S5E.A.241, 3SIB.A.223, 3SJL.A.402, 3SSB.A.995, 1T3M.A.801, 4TKX.L.705, 4TKX.L.706, 4U99.A.203, 1UD2.A.1001, 1UD2.A.1002, 3UWP.A.424, 2V3U.A.1263, 2V79.A.1116, 1VMF.A.134, 1W15.A.2002, 3W5N.A.1210, 3WAY.A.914, 3WC3.A.502, 4WFX.A.504, 2WGE.A.1426, 3WNO.A.802, 2WOF.A.1728, 2WW2.A.800, 4XCZ.A.405, 4XCZ.A.406, 1XKN.A.700, 1Y7W.B.282, 2ZQ3.A.160, 3ZX3.C.522

Table S107. all-ligand-number Na, normal group

	size	largest_angle*	middle_1*	middle_2*	middle_3*
1	"210"	"158.6+/-11.2"	"76.7+/-6.6"	"87.1+/-4.2"	"97+/-6.1"
2	"148"	"161+/-8.2"	"70.3+/-6.2"	"85.7+/-4.4"	"100.3+/-7.2"
3	"397"	"172+/-4.3"	"78.2+/-5"	"88.2+/-2.3"	"96.9+/-3.5"
4	"140"	"157.1+/-12.7"	"80+/-7.1"	"88+/-7.4"	"95.4+/-8.4"
5	"51"	"158.5+/-12.2"	"78.1+/-6.3"	"84.8+/-6"	"91.5+/-6.6"

	middle_4*	smallest_opposite_angle*	Tetrahedral	TrigonalBipyramidal
1	"137+/-8.7"	"101.4+/-8"	"0.046"	"0.272"
2	"151.1+/-7.8"	"71.6+/-7.4"	"0.014"	"0.057"
3	"166.2+/-5.4"	"80.6+/-6.8"	"0.003"	"0.062"
4	"105+/-9.1"	"91+/-11.6"	"0.006"	"0"
5	"103.9+/-11.2"	"138.1+/-14.5"	"0.001"	"0"

	TrigonalBipyramidalVA	TrigonalBipyramidalVP	Octahedral	SquarePyramidal
1	"0.348"	"0.434"	"0"	"0.051"
2	"0.076"	"0.308"	"0.051"	"0.171"
3	"0.042"	"0.622"	"0.451"	"0.715"
4	"0.047"	"0.127"	"0"	"0"
5	"0.001"	"0.238"	"0"	"0"

	SquarePyramidalV	SquarePlanar	TrigonalPrismatic	TrigonalPrismaticV
1	"0.358"	"0.142"	"0"	"0.286"
2	"0.385"	"0.275"	"0.123"	"0.281"
3	"0.817"	"0.797"	"0.036"	"0.13"
4	"0.35"	"0"	"0"	"0"
5	"0.018"	"0.211"	"0"	"0"

	PentagonalBipyramidal	PentagonalBipyramidalVA	PentagonalBipyramidalVP
1	"0"	"0"	"0"
2	"0.023"	"0.033"	"0.193"
3	"0"	"0"	"0.098"
4	"0"	"0"	"0"
5	"0"	"0"	"0"

	SquareAntiprismatic	SquareAntiprismaticV	HexagonalBipyramidal
1	"0"	"0"	"0"
2	"0"	"0.017"	"0"
3	"0"	"0"	"0"
4	"0"	"0"	"0"
5	"0"	"0"	"0"

	HexagonalBipyramidalVA	HexagonalBipyramidalVP
1	"0"	"0"
2	"0"	"0.008"
3	"0"	"0.001"
4	"0"	"0"
5	"0"	"0"

Table S108. Cluster members of all-ligand-number Na, normal group

[1] "Cluster 1"
4DOC.A.402, 4FZY.A.201, 4KHU.A.1008, 3AR8.A.1000, 4CBY.C.2037, 1G8I.B.1595, 1IP3
.A.999, 4KAF.A.404, 1MAU.A.499, 4MVJ.D.402, 2NQL.A.401, 4NT8.A.206, 4OVZ.B.903,
1PX3.A.3101, 4Q4B.A.530, 4TMV.A.903, 3UA6.A.147, 3VD3.C.3101, 3VDA.A.3101, 2BCV.
A.579, 1DIZ.A.825, 2GIG.A.502, 2GIJ.F.403, 3HW8.A.577, 8ICK.A.341, 1JJ2.A.8345,
4ORJ.A.305, 1SUZ.A.403, 1TX3.A.801, 1VQ8.M.9147, 1YJW.A.8545, 2A65.A.751, 1A7T.A
.255, 4A87.A.1162, 2ABS.A.1003, 4ATF.C.500, 3B1N.A.403, 2B2N.B.1001, 4BEM.A.201,
4BEM.B.201, 4BEM.J.201, 2BL2.A.1157, 3C7X.A.1001, 3CB8.A.820, 4CD5.A.1420, 4CD5
.A.1421, 4CFY.A.302, 3CKI.A.502, 3CTP.A.402, 3CZJ.A.3101, 4D1J.A.601, 4D77.A.154
3, 2DDB.B.303, 2DKB.A.436, 4DUW.A.3101, 4DUX.A.3101, 3DYM.A.3101, 3DYO.A.3101, 3
DYP.A.3101, 1EBU.B.902, 3EEB.A.211, 1EJA.A.246, 3EPR.A.266, 4EXR.A.301, 4EZE.A.3
02, 1F6D.A.1378, 2FBL.A.304, 4FEV.B.303, 4FHA.B.402, 4FLL.A.504, 4FMT.D.301, 4FO
2.Q.101, 4FXZ.A.603, 3G0T.B.437, 3GBV.B.1, 2GKO.A.614, 1GNY.A.1244, 1GVF.A.289,
3GVK.A.916, 4GVO.A.702, 3H7K.A.387, 4H7O.C.302, 1H80.A.1497, 4H83.E.401, 2HIG.B.
488, 1HXN.A.4, 2HZY.A.1201, 3I3B.A.3101, 3I3D.A.3101, 3IMM.C.3, 3IMX.A.467, 4IQZ
.A.317, 3IRD.A.301, 4J4B.B.303, 2J5A.A.1109, 2JBW.D.1367, 4JEX.A.512, 2JLN.A.147
1, 4JNQ.A.402, 4JPA.A.305, 3JU4.A.7, 1JYN.A.3101, 1JYW.A.3101, 1JZ2.A.3101, 1JZ3
.A.3101, 1JZ6.A.3101, 1JZ7.A.3101, 1JZ8.A.3101, 1JZ8.D.3104, 1KAO.A.501, 3KEU.A.
402, 3KWM.A.225, 4LDE.A.1402, 4LHL.A.301, 3LJQ.A.597, 3LKB.A.394, 4M9U.A.402, 3M
AX.A.381, 4MB4.A.604, 4MM7.A.601, 4MMB.A.601, 4MMF.A.601, 3MPN.A.751, 3MPQ.A.751
, 4MPY.A.503, 3MS8.A.401, 3MUI.A.401, 1MUQ.A.206, 3MUZ.1.3101, 3MUZ.1.3102, 1MVO
.A.202, 3MV0.1.3101, 3MV1.1.3101, 3MX6.A.261, 3N3R.A.1000, 1N82.A.401, 1O4Z.A.10
04, 4O4V.A.301, 1O68.E.274, 1OA8.C.1691, 1OAF.A.1252, 4OFI.A.801, 4OMC.A.608, 3O
NF.A.507, 4OUA.A.403, 2OZ3.B.2001, 2P3Z.B.501, 2PFL.A.2001, 3PNX.C.169, 3PNX.E.1
68, 2PPL.A.480, 4PV3.A.202, 1PX3.B.3101, 1PX4.A.3101, 3PZJ.A.301, 1Q20.A.313, 2Q
8X.A.401, 3Q9B.A.344, 1QHU.A.436, 4QKU.A.501, 3QS4.A.751, 3QS5.A.751, 3QS6.A.751
, 3QST.A.253, 4QTO.A.501, 4QTO.B.501, 2QWL.A.589, 4R7U.A.504, 3RGA.A.312, 3S9J.A
.401, 1SK4.A.342, 3T09.A.3101, 1T64.A.391, 3T8J.A.401, 3TAV.A.267, 4TMW.A.902, 1
TO2.E.451, 3TXF.A.1138, 3TYP.A.155, 3UNX.A.281, 1V54.A.3519, 3V5U.A.701, 3V5U.A.
703, 3VD3.A.3101, 3VD4.A.3101, 3VD7.B.3101, 3VD9.A.3101, 3VDA.B.3101, 3VDC.A.310
1, 3VDG.A.502, 1VI6.C.208, 3W0L.C.502, 1W9S.A.1142, 3WA2.X.702, 3WGU.C.2007, 2WG
M.A.201, 2WGM.B.201, 2WOI.A.1491, 2WPC.C.1492, 2WUW.E.1277, 2WWH.C.213, 3WZ1.A.4
01, 1X0G.A.1002, 1XFF.A.5301, 2Z5D.B.180, 3ZK1.A.90, 3ZK1.B.90, 1ZNB.A.3, 3ZQS.B
.1295, 3ZYV.A.2337
[1] "Cluster 2"
2GIG.A.501, 1Q81.C.8345, 1VQ9.Q.9148, 1GOF.A.702, 4IIL.A.402, 3MMD.A.410, 3Q2G.A
.701, 3U0F.A.410, 1W8O.A.1654, 4C0K.A.1614, 4JCO.D.406, 2B2N.A.345, 4B6C.B.1257,
4KHY.A.1013, 3PML.B.7, 2A5G.B.242, 2A7L.A.201, 2A9Y.A.2002, 4B6C.A.1257, 4C1P.A
.1728, 4CBY.B.2036, 1D7U.A.435, 4D9T.A.901, 1EZ1.B.1002, 3FZQ.B.274, 3G1N.B.5002
, 3G8Q.D.302, 4GKI.F.303, 4GRX.A.501, 4H41.B.405, 3IAQ.A.3103, 3IC9.A.491, 3IWK.
D.504, 2JBW.A.1368, 4JDO.G.301, 1JYV.A.3101, 3KZW.D.498, 1MGV.A.501, 4MVJ.A.401,
4NLQ.A.910, 4NPJ.B.701, 4NRH.C.401, 4NT1.A.301, 4OOC.B.402, 2O34.A.503, 2ONP.G.
707, 4OUC.A.801, 2OYC.A.305, 2P6Z.A.402, 4QVS.A.502, 1S5C.A.241, 1SOO.A.547, 3VD
5.A.3101, 3VW7.A.2012, 3WGU.A.2005, 1WQR.A.131, 2WWG.B.213, 2WX5.H.1252, 1XAR.A.
100, 4XEL.A.201, 1YQ2.A.7501, 2ZJ9.A.1, 2ZN8.A.995, 3ZPQ.A.1360, 3ZQ5.A.1530, 3Z
XO.C.522, 2BCR.A.604, 1JJ2.0.8338, 2OTL.A.8545, 1Q81.A.8378, 1TW8.B.902, 3UXP.A.
338, 1VQ8.Q.9148, 1VQ9.M.9147, 1A5U.G.4732, 2AB8.A.2003, 3AR4.A.1000, 4B52.A.130
5, 2BER.A.1649, 1BGP.A.502, 4BY5.B.1188, 3C17.A.323, 4CBY.A.2036, 2D4E.A.1901, 2

E5X.A.303, 4FMT.B.301, 2GFH.A.249, 1H16.A.9001, 1HBN.D.1561, 3HSC.A.491, 2HZG.A.1101, 3I01.M.730, 3I2W.B.304, 3IC9.B.491, 2IM2.A.3001, 3IWJ.A.505, 2J80.A.1134, 4JN7.A.401, 1JZ4.A.3101, 3K1U.A.412, 4KZW.A.304, 3L7X.A.142, 3LG1.A.530, 4LL2.A.301, 3ME4.B.2, 3MZG.B.211, 3N0U.A.208, 3N83.G.707, 4NLV.A.911, 1O04.A.6601, 4O57.A.301, 4O5H.A.605, 1O68.A.274, 4OMG.A.402, 3ON4.C.189, 3OND.A.509, 2P6Z.B.401, 2P6Z.B.403, 4PCG.A.302, 4PMO.A.312, 1Q3X.A.800, 1Q6X.A.1002, 4Q92.C.502, 2QR7.A.1000, 2QSV.A.222, 1S0A.A.1501, 3SIS.B.3001, 3SIT.A.3000, 1T64.A.392, 1T8U.B.701, 3TAY.A.1, 4TMX.A.902, 4US3.A.701, 4USW.A.1468, 1V0H.X.252, 3VGL.A.323, 2W4M.A.1245, 3WFA.A.801, 4WFZ.A.501, 2WPC.A.1491, 2X8J.A.1317, 2YDG.A.1130, 2YFO.A.1743, 1Z45.A.702, 2ZHJ.A.321, 2ZJ9.B.2, 1ZOR.A.1001, 1ZUD.1.701

[1] "Cluster 3"

4KQ7.B.502, 2FMS.A.342, 4GXK.A.405, 3HW8.A.580, 3JPP.A.340, 1ORP.A.224, 2PFQ.A.1, 1Q81.K.8346, 4TUR.A.404, 1VQ7.Q.9148, 4ADB.A.1405, 3AGB.A.1, 3ASQ.B.701, 3AST.B.701, 4AYO.A.502, 4BDR.A.902, 4BR6.A.401, 4BVN.A.1360, 3C7E.A.489, 4C7A.B.1159, 4CSH.A.1169, 3CZJ.A.3102, 4CZN.A.1371, 4D1I.A.600, 4D1J.E.604, 4DF9.A.501, 3DR3.A.335, 2DV1.A.1000, 4F3Y.A.301, 4FEW.D.304, 4G1K.B.301, 1G5I.C.902, 4GDK.E.301, 2GEZ.A.401, 1GEN.A.304, 2GTW.E.3006, 1GUU.A.1090, 1GV2.A.1192, 4GY9.A.207, 2HU3.A.9002, 1HXN.A.2, 3HYS.A.267, 4I0W.D.602, 3I2W.A.304, 4I2F.A.602, 4I2R.C.602, 3I44.A.477, 3IFV.C.408, 3IJP.A.301, 1IYN.A.298, 2IY6.B.1540, 2J5W.A.3044, 2J5W.A.3045, 2JHN.A.1298, 3JS4.A.208, 1JZ7.C.3103, 3K6A.E.178, 4KA5.A.801, 4KA8.A.806, 3KED.A.951, 4KXW.A.1013, 4KZV.A.304, 1L2T.A.1502, 4L3H.A.404, 3LP5.A.251, 1LZS.A.131, 4M4V.A.505, 3MC1.A.301, 4MJD.A.203, 4MM9.A.602, 4MPT.A.402, 4N3M.A.403, 3N3O.A.1000, 4O1G.A.401, 2O34.B.502, 4ODI.B.301, 3OEC.A.300, 2OKQ.A.119, 3OTK.A.586, 4PFI.A.401, 4PMO.A.310, 4PUV.A.405, 4PV3.A.201, 3Q94.A.310, 4QFE.B.305, 1QHU.A.437, 1QOP.B.501, 2QZ7.A.193, 1RW9.A.900, 3STH.A.501, 1SU3.A.911, 1TQY.H.1094, 3U21.A.500, 1UD8.A.1001, 2V4B.B.1562, 1VI6.A.208, 3VS8.A.501, 3VS8.C.501, 1W16.A.1002, 1W9W.A.900, 3WNM.A.802, 1WPG.D.1300, 2WU2.A.1590, 2WV7.B.401, 3WXO.A.805, 1X0G.C.1001, 1X7D.A.1501, 1X7U.A.1000, 2X7J.A.1581, 1XDF.A.401, 2XZK.B.507, 2Y8K.A.1527, 1YAP.A.501, 2Z2F.A.2001, 1ZDN.A.157, 1ZH8.A.329, 2ZND.A.195, 2BCU.A.577, 2BCQ.A.1, 1CZO.C.606, 4ED3.A.502, 4EJY.A.301, 1EWN.A.501, 3I0W.A.296, 4KLI.A.404, 4KLI.A.405, 4KYW.A.303, 4M04.A.707, 1ORN.A.224, 3OSN.A.423, 4P4M.A.403, 4P4P.A.401, 1RZT.A.2001, 4TUP.A.402, 1VQ8.J.9146, 2A5F.B.1326, 2A5D.B.326, 4A6U.B.1460, 4AFK.A.1507, 3AGC.A.1, 2AHR.C.1259, 3AJN.A.136, 4AK1.A.1702, 2AMF.A.850, 4AMJ.A.1360, 3AR7.A.1000, 2AU7.A.206, 2AUT.D.605, 4B1M.A.1680, 3B34.A.951, 1B57.A.364, 3BGA.A.6, 3BIA.X.117, 3BIB.X.117, 3BLJ.B.701, 3BOS.B.302, 2BS2.A.1658, 4BVO.A.1396, 4C1O.A.1731, 4C3X.A.561, 3C7F.A.804, 4CCY.A.1297, 4CCY.A.1298, 2CD7.A.1132, 4CIT.A.1454, 3CRN.A.131, 3CYM.A.501, 4D1J.E.602, 4D1J.G.603, 4D7C.A.1544, 3D9R.A.135, 2DDA.A.301, 2DDB.A.301, 2DDB.C.302, 4DD8.D.1005, 4DEL.A.401, 4DF9.B.501, 4DOU.A.1004, 4DW8.A.304, 2E54.A.1004, 4E6P.A.301, 4E6P.D.301, 2E7U.A.1003, 3E85.A.162, 3E85.A.163, 3E9L.A.1, 1EAS.A.5, 4EAE.A.302, 4EEK.A.302, 4EEL.A.302, 2EHQ.A.1540, 3EIF.A.1033, 3EIY.A.177, 3ELF.A.351, 3EUW.A.343, 3F3C.A.752, 4FDZ.B.301, 4FET.A.301, 4FEX.A.303, 2FM1.D.344, 2FPR.B.505, 2FV7.A.403, 1G3K.A.500, 1G5H.A.901, 1G5I.A.901, 4G8T.C.502, 3GA5.B.701, 4GAF.B.505, 3GED.A.251, 3GIR.A.373, 4GIB.A.301, 2GJU.A.2001, 4GKI.D.303, 4GNJ.B.302, 3GOD.B.327, 4GRX.B.501, 2GTW.B.3005, 1GV2.A.1191, 1GV5.A.1142, 3GZA.A.471, 3H0N.A.203, 3H0N.A.204, 3H12.A.500, 4H83.A.401, 1HBN.A.1561, 3HIJ.B.295, 2HI0.B.240, 3HSS.A.268, 4HUR.A.316, 3HVI.A.265, 1HX6.A.705, 4I0W.B.603, 4I29.A.601, 4I2A.A.602, 3IAN.A.1, 3IC3.C.100, 3ICF.A.515, 3IFV.A.402, 3IGZ.B.601, 4IIB.A.944, 3IJP.B.301, 2IJA.A.401, 3IMM.B.2, 3IPO.A.436, 3IPP.A.438, 4IQZ.A.316, 3IRS.A.290, 1IYN.A.297, 2IZV.A.1430, 4J07.A.201, 1JAY.A.215, 2JBW.C.1367, 4JB3.A.301, 4JDO.A.301, 4JEX.B.511, 2JHJ.B.1296, 2JHN.B.1299, 1JMM.A.3001, 3JS4.D.208, 1JTP.A.501, 1JTP.L.503, 4JTE.C.301, 4JTF.C.303, 4JTG.C.302, 4JTH.C.303, 4JTJ.C.302, 4JTK.C.302, 4JVL.B.304, 1JZ7.A.3102, 1JZ7.A.3104, 1JZN.A.1139, 1K2X.A.801, 4KA7.A.805, 3KRS.A.301, 1KSU.A.810, 1L5B.A.302, 4LG8.A.601, 4LGN.A.827, 4LIZ.A.202, 1LLA.A.631, 1M4Y.A.252, 4M48.A.701, 4M4U.A

.501, 4M60.A.502, 3M9Y.B.254, 4M9B.A.201, 3MJ6.A.503, 4MMB.A.602, 4MMF.A.602, 3M
QD.A.500, 3MQG.A.193, 3MYV.A.502, 4N9S.A.402, 4N9V.A.404, 4NAW.B.303, 3NGJ.A.249
, 3NMB.A.1, 3NNB.A.401, 4NPI.A.602, 4NRH.A.401, 4NSJ.A.222, 4O0C.A.401, 2O34.A.5
01, 4O47.A.401, 3OEC.C.300, 4OF8.A.301, 3OLJ.A.1, 2OPL.A.187, 3OPK.A.301, 2OYN.A
.201, 1P0Z.A.1633, 4P33.A.401, 4PCG.C.304, 4PD6.A.502, 3PG0.A.147, 4PMO.A.311, 2
POC.A.5001, 4PSR.A.619, 1PYF.A.315, 4PYJ.A.301, 3PZR.A.373, 3PZS.A.287, 4Q5K.A.3
02, 4Q69.A.501, 2QJY.R.2001, 2QKF.C.283, 1QNJ.A.280, 2QV6.A.303, 3QXT.A.133, 4QX
K.A.402, 3R0L.D.124, 4R3N.B.401, 1R4P.A.4003, 4R6K.A.501, 2R85.A.600, 2RGI.B.98,
3RU5.A.133, 1RWH.A.900, 1S5D.A.241, 1S5E.A.241, 1S82.A.4, 3SIB.A.223, 3SJL.A.40
2, 3SSB.A.995, 1T3M.A.801, 3TDQ.A.117, 4TKX.L.705, 4TKX.L.706, 1TQY.B.1091, 1U7H
.B.912, 4U99.A.203, 1UD2.A.1001, 1UD2.A.1002, 3UES.B.503, 3UF4.A.601, 3USL.A.752
, 3UWP.A.424, 2V3U.A.1263, 2V4V.A.3052, 2V4B.A.1566, 2V79.A.1116, 1VMF.A.134, 2V
NZ.X.9252, 2VPB.A.1398, 1W15.A.2002, 3W5N.A.1210, 1W9S.A.1141, 3WAY.A.914, 3WC3.
A.502, 2WCB.A.101, 2WDQ.A.1590, 4WFX.A.504, 2WGE.A.1426, 1WKY.A.504, 3WNO.A.802,
2WOF.A.1728, 2WOI.D.1489, 2WW2.A.800, 2WWF.B.214, 2X7J.B.1581, 4X9K.A.401, 4XCZ
.A.405, 4XCZ.A.406, 1XKN.A.700, 2XNA.C.1216, 2XZI.A.502, 1Y0P.A.1810, 2Y5F.A.124
5, 1Y7W.B.282, 2YNQ.A.1392, 1YYA.A.1001, 2ZND.A.196, 1ZOD.A.435, 1ZOR.B.1002, 2Z
Q3.A.160, 3ZWF.B.1365, 3ZX3.C.522
[1] "Cluster 4"
4DOC.A.404, 3EPG.A.421, 4FZY.B.201, 8ICP.A.341, 2IHM.A.900, 2ISP.A.340, 2ISP.A.3
42, 3JPS.A.340, 4KHS.A.1009, 4KHU.A.1006, 3KNT.A.208, 4NLN.A.402, 1QVG.L.8347, 3
UXP.A.339, 1YIT.0.8538, 2A2A.C.3158, 4A22.C.1346, 1A5S.B.2000, 2AA0.A.2003, 3ASP
.A.701, 3AST.A.701, 4B1L.A.1679, 3BX1.B.284, 3C17.B.326, 2C9R.A.1103, 3C9F.B.603
, 2C9A.A.1289, 4D1I.C.600, 4D1I.F.600, 3D32.A.120, 4D9U.A.901, 2DDA.C.303, 3DKI.
B.324, 3EPZ.A.1, 2EPF.C.307, 3EUW.B.343, 4FEW.B.303, 3FFZ.B.1302, 4FHA.A.402, 4F
LK.A.504, 4FMT.A.301, 4FO2.E.101, 3G1N.A.5001, 4G1K.A.301, 3GA5.A.700, 3GCD.A.21
5, 3GVF.A.178, 1GW1.A.1423, 4GY9.A.206, 4HKT.B.410, 4HMM.B.302, 3HVY.C.427, 4I2B
.A.601, 3I4Q.A.177, 3IAQ.B.3104, 3IAQ.C.3101, 3IJ6.B.313, 4IJK.B.301, 4IQZ.E.305
, 3IWK.B.504, 4J0Y.A.501, 4J1I.A.502, 4J2H.A.306, 4JDO.D.301, 4JHG.A.205, 4JRX.D
.301, 4JVL.A.703, 1JYX.B.3103, 1JZN.D.4139, 4K7V.A.407, 1KNR.A.542, 4L3F.H.401,
4L73.A.404, 4LDZ.A.205, 4LH7.A.404, 3N83.A.701, 4NAW.N.304, 4NWH.A.201, 1O4Z.B.1
005, 2O5W.C.171, 1OA8.A.1690, 3OB8.B.3003, 4OBO.A.402, 2OCC.A.519, 1ODZ.A.1427,
3OO2.B.383, 2OSW.B.602, 2OSY.B.602, 3OTK.C.587, 3P8O.A.187, 3PJ0.C.367, 2POC.D.5
004, 1PX4.A.3104, 3PYM.B.504, 1Q1Q.A.351, 1Q1Z.A.313, 4Q4X.1.5004, 3Q9E.L.343, 2
QF2.A.700, 4QFE.G.304, 2QZ7.B.196, 4R6C.A.213, 3RN0.A.402, 3S30.A.383, 1S81.A.2,
3T2P.B.3102, 3T34.A.1003, 1T64.B.1392, 3TXJ.A.1138, 3UA6.B.2, 2VDX.A.1385, 3VD5
.C.3101, 3VD5.D.3102, 3VD7.A.3101, 1VIZ.B.239, 3W6P.A.804, 1W7Z.A.1032, 1W90.A.1
154, 2WCF.B.1091, 2WD0.A.601, 2WG8.B.202, 2WHM.A.1425, 2WOI.B.1491, 2WOI.D.1488,
3WV2.A.305, 2WWH.B.212, 3WXO.A.807, 3WXO.A.808, 4WXG.A.502, 4X0O.B.401, 2X2E.A.
1747, 2X7J.D.1581, 2Y00.A.1359, 1YAO.A.501, 2ZHJ.A.320, 3ZLY.A.1384, 4ZNB.A.3, 3
ZPR.B.1, 2ZXK.A.1, 3ZX2.B.521
[1] "Cluster 5"
3E45.A.260, 4M47.A.403, 2OTJ.R.8537, 1Q81.M.8380, 3AXG.A.3005, 3C17.A.324, 4CH8.
D.1580, 1CM5.A.1056, 1DI4.A.501, 3DR3.A.336, 3DYQ.A.902, 2E7U.A.1002, 1EBU.A.901
, 2EHS.A.201, 4ENZ.A.1112, 1F7T.A.472, 1F7T.C.474, 4FOI.A.1005, 4FOI.A.1006, 2FQ
E.A.901, 2GG2.A.703, 3H1V.X.600, 4HCH.A.407, 3HVU.D.182, 4HXV.A.403, 3I3D.C.3101
, 3IGQ.F.801, 2J5W.A.3043, 3K13.A.647, 3L27.B.3, 1MXO.D.901, 3NRB.C.287, 4O4W.A.
301, 3OB8.A.3006, 4ODI.A.301, 3P0J.A.711, 1QJS.A.512, 1QJS.A.513, 1QJS.A.523, 2Q
Z7.A.195, 4R3W.B.402, 1R4P.F.4004, 3T2Q.D.3101, 3UA7.A.145, 1UD2.A.1003, 3V6N.A.
232, 3W0L.A.502, 1XAR.B.200, 1XC6.A.8001, 1YCE.A.201, 1YCE.C.201

Table S109. all-ligand-number Na, compressed group

	size	largest_angle*	middle_1*	middle_2*	middle_3*
1	"24"	"162.3+/-8.1"	"48.7+/-5"	"83.6+/-7.8"	"106.5+/-10.9"
2	"23"	"153+/-11.2"	"71.1+/-6.9"	"85.3+/-7.9"	"101.8+/-9"
3	"24"	"160.8+/-3.7"	"66.7+/-3.9"	"84.7+/-3.7"	"107.1+/-5.4"
4	"19"	"171.4+/-4"	"68.5+/-4.9"	"87.1+/-2.6"	"99.8+/-4.1"
5	"23"	"168+/-4.1"	"76.3+/-4.2"	"88.8+/-3"	"100.7+/-4.5"
6	"8"	"120.3+/-10.3"	"50.8+/-3.9"	"72.1+/-7.9"	"79.1+/-8.6"
7	"15"	"146.9+/-13"	"50.8+/-4.5"	"90.1+/-8.5"	"101.5+/-5.5"
8	"37"	"166.3+/-5.9"	"50+/-4.6"	"86.5+/-4.3"	"102.7+/-6.5"

	middle_4*	smallest_opposite_angle*	Tetrahedral	TrigonalBipyramidal
1	"141.3+/-7.3"	"80.7+/-10.7"	"0.044"	"0.057"
2	"133.4+/-9.1"	"48.3+/-3.9"	"0.047"	"0.092"
3	"154.5+/-4.7"	"48.5+/-3.4"	"0.096"	"0.226"
4	"164.1+/-4.4"	"47+/-3"	"0.049"	"0.258"
5	"158.1+/-5.8"	"53.2+/-3"	"0.082"	"0.34"
6	"100.3+/-8.7"	"86.8+/-15.2"	"0.023"	"0"
7	"123.7+/-12.8"	"97.6+/-12.9"	"0.183"	"0.079"
8	"157+/-6.3"	"72.4+/-6.3"	"0.065"	"0.181"

	TrigonalBipyramidalVA	TrigonalBipyramidalVP	Octahedral	SquarePyramidal
1	"0.155"	"0.201"	"0.003"	"0.035"
2	"0.172"	"0.26"	"0.014"	"0.077"
3	"0.357"	"0.599"	"0.129"	"0.298"
4	"0.241"	"0.558"	"0.255"	"0.471"
5	"0.342"	"0.66"	"0.322"	"0.57"
6	"0.079"	"0.127"	"0"	"0"
7	"0.255"	"0.19"	"0"	"0.038"
8	"0.286"	"0.504"	"0.111"	"0.309"

	SquarePyramidalV	SquarePlanar	TrigonalPrismatic	TrigonalPrismaticV
1	"0.19"	"0.054"	"0.052"	"0.183"
2	"0.222"	"0.125"	"0.053"	"0.246"
3	"0.585"	"0.53"	"0.257"	"0.531"
4	"0.596"	"0.558"	"0.057"	"0.257"
5	"0.721"	"0.717"	"0.154"	"0.423"
6	"0.16"	"0"	"0"	"0"
7	"0.137"	"0.012"	"0"	"0.158"
8	"0.513"	"0.468"	"0.103"	"0.354"

	PentagonalBipyramidal	PentagonalBipyramidalVA	PentagonalBipyramidalVP
1	"0"	"0"	"0.04"
2	"0"	"0"	"0.044"
3	"0.156"	"0.171"	"0.401"
4	"0.117"	"0.109"	"0.316"
5	"0.041"	"0.048"	"0.465"
6	"0"	"0"	"0"
7	"0"	"0"	"0"
8	"0.028"	"0.047"	"0.162"

	SquareAntiprismatic	SquareAntiprismaticV	HexagonalBipyramidal
1	"0"	"0"	"0"
2	"0"	"0"	"0"
3	"0.041"	"0.255"	"0"
4	"0"	"0.117"	"0"
5	"0"	"0.042"	"0"
6	"0"	"0"	"0"

```
7 "0"                 "0"                 "0"
8 "0"                 "0.074"             "0"
  HexagonalBipyramidalVA HexagonalBipyramidalVP
1 "0"                    "0"
2 "0"                    "0"
3 "0.001"                "0.05"
4 "0"                    "0.095"
5 "0"                    "0.01"
6 "0"                    "0"
7 "0"                    "0"
8 "0"                    "0.01"
```

Table S110. Cluster members of all-ligand-number Na, compressed group

[1] "Cluster 1"
3DYQ.B.901, 3GCD.B.215, 3I01.A.501, 1QY1.A.204, 3ZDU.A.353, 4NLK.A.403, 1BUN.A.121, 3FFZ.A.1302, 1GVH.A.1399, 4H83.A.402, 4M5P.A.505, 4MAT.A.501, 4O54.A.302, 1SU4.A.997, 1TC8.A.121, 3VD3.D.3101, 3C5G.B.808, 2HZG.B.1102, 4IQL.A.407, 4J43.A.902, 2JBA.A.1127, 4JQR.A.301, 4MPY.C.503, 3USZ.A.902
[1] "Cluster 2"
1G8G.A.525, 1G8H.A.523, 4L73.B.405, 2WO0.B.1728, 4DOA.A.402, 4DOB.A.402, 2FMQ.A.340, 3JPT.A.340, 3PNC.A.2, 2PXI.A.340, 3CBT.A.420, 3INJ.C.601, 4LCZ.B.320, 4NTL.A.311, 1SVY.A.2, 4KLI.A.403, 3MBY.A.340, 2GA4.A.713, 3GQ9.A.1, 3HLT.A.269, 3Q9C.A.344, 2QZ7.A.194, 1VK1.A.302
[1] "Cluster 3"
2HZL.A.500, 4LUG.A.301, 1O68.D.274, 4O7J.A.201, 3PFV.A.1, 3UT0.A.902, 3V7Z.A.404, 1W9W.A.901, 4X26.A.402, 3RWK.X.521, 1BPZ.A.341, 3C5G.A.803, 2FMP.A.1340, 2ISO.A.340, 3JPO.A.340, 3JPQ.A.340, 4M9L.A.403, 1SA3.A.401, 2DDA.B.302, 3ED4.A.519, 3R2H.A.157, 4WED.A.601, 1YQ2.C.7503, 1ZDN.B.157
[1] "Cluster 4"
1WTE.A.1001, 1XSP.A.576, 4DKA.A.201, 4DNL.A.300, 4JP4.A.305, 3MNC.A.280, 3NUQ.A.283, 2VX5.A.1421, 2XRM.A.401, 2YEQ.A.582, 3JPR.A.340, 3ATU.A.6267, 1BA0.A.490, 4DD8.B.1005, 1HVX.A.519, 1QHU.A.438, 2WCP.A.501, 3WNK.A.813, 3WZ1.A.402
[1] "Cluster 5"
4DMI.C.203, 4IB0.A.402, 3UES.A.503, 4CVU.A.1998, 4LLH.B.602, 1NZA.A.104, 4FZX.B.101, 4FZX.A.101, 4FZY.C.101, 4FZY.D.101, 1VQ8.0.9117, 3A6V.A.1005, 3A6V.B.1006, 1AVT.A.301, 3B8X.B.668, 4DCC.A.302, 4HUC.A.509, 4O52.A.301, 1QUS.A.400, 3SLZ.B.130, 4UZU.A.1491, 1V7T.A.406, 3WXO.A.806
[1] "Cluster 6"
1U8R.D.4104, 3BFT.A.1007, 4D1I.H.600, 1G8G.A.522, 1G8G.A.527, 1G8H.A.526, 4L73.A.406, 2NWH.A.404
[1] "Cluster 7"
4KHN.A.1010, 1YIT.0.8517, 3DYQ.A.901, 3FGH.A.180, 3FKR.A.409, 1JED.A.525, 4K70.B.1011, 2X1Z.M.1163, 4FZZ.A.201, 2BBH.A.301, 2GG8.A.503, 4J4B.A.902, 1JJU.C.996, 1NSX.A.1401, 1U4J.B.1001
[1] "Cluster 8"
4NLZ.A.402, 2E5X.A.302, 4IB0.A.401, 3N80.B.602, 2PKC.A.280, 1WX5.A.282, 1U8R.A.1101, 4DD8.A.1005, 4G8T.B.502, 2WD0.C.601, 1ZEL.A.401, 3C0W.A.303, 1VQ5.A.9145, 4ATF.A.500, 3BC9.A.704, 3BH4.A.489, 2DIE.A.781, 4DMI.A.202, 2GJP.A.1489, 2HEU.A.4001, 2ID4.A.907, 3KQB.A.303, 4M48.A.702, 3MV1.3.3101, 1OB0.A.504, 2P3Z.A.501, 3Q2H.A.701, 3TSH.A.604, 1V8Z.A.389, 3VD5.B.3101, 3VHS.B.52, 3W5N.A.1211, 3WG7.N.60

5, 1WPC.A.504, 2WQK.A.254, 1WX5.C.282, 1XAR.B.201

Table S111. all-ligand-number Na, combined group

	size	largest_angle*	middle_1*	middle_2*	middle_3*
1	"217"	"167.4+/-5.2"	"74.3+/-5.8"	"87.2+/-3.1"	"98.7+/-4.7"
2	"125"	"157.5+/-9.8"	"57.1+/-10"	"84.2+/-7.4"	"103.6+/-9.7"
3	"61"	"133.8+/-10.7"	"73.7+/-15"	"90.8+/-10.9"	"101.9+/-12.1"
4	"147"	"165.5+/-7.7"	"63+/-10.2"	"86.3+/-4.1"	"102.6+/-5.9"
5	"48"	"159.9+/-10.8"	"78.2+/-6.3"	"84.4+/-5.3"	"90.5+/-5.6"
6	"210"	"159.7+/-9.4"	"75.9+/-8"	"87+/-4"	"96.5+/-5.3"
7	"108"	"162.6+/-7.6"	"78.8+/-7.7"	"86.1+/-6.2"	"92.6+/-6.1"
8	"268"	"173.4+/-3.6"	"79.5+/-4.6"	"88.4+/-2"	"96.1+/-3"

	middle_4*	smallest_opposite_angle*	Tetrahedral	TrigonalBipyramidal
1	"159.2+/-6"	"75.7+/-6.9"	"0.003"	"0.079"
2	"144+/-10.4"	"71.1+/-10.6"	"0.014"	"0.035"
3	"115.1+/-8.6"	"93.7+/-16.2"	"0.027"	"0.001"
4	"155.1+/-10.4"	"50.8+/-6"	"0.029"	"0.109"
5	"103.4+/-11.3"	"139.1+/-14.2"	"0.001"	"0"
6	"137.3+/-8.6"	"101.2+/-7.8"	"0.048"	"0.273"
7	"101.5+/-7.3"	"90.1+/-12.1"	"0"	"0"
8	"168.2+/-4.7"	"82.5+/-6.1"	"0.003"	"0.06"

	TrigonalBipyramidalVA	TrigonalBipyramidalVP	Octahedral	SquarePyramidal
1	"0.07"	"0.478"	"0.198"	"0.411"
2	"0.119"	"0.192"	"0.009"	"0.051"
3	"0.139"	"0.015"	"0"	"0"
4	"0.127"	"0.407"	"0.126"	"0.25"
5	"0"	"0.25"	"0"	"0"
6	"0.339"	"0.44"	"0"	"0.051"
7	"0.01"	"0.155"	"0"	"0"
8	"0.036"	"0.666"	"0.534"	"0.815"

	SquarePyramidalV	SquarePlanar	TrigonalPrismatic	TrigonalPrismaticV
1	"0.626"	"0.563"	"0.087"	"0.212"
2	"0.201"	"0.106"	"0.088"	"0.246"
3	"0.022"	"0"	"0"	"0.007"
4	"0.452"	"0.373"	"0.078"	"0.231"
5	"0.019"	"0.225"	"0"	"0"
6	"0.359"	"0.143"	"0"	"0.291"
7	"0.44"	"0"	"0"	"0"
8	"0.881"	"0.863"	"0.023"	"0.104"

	PentagonalBipyramidal	PentagonalBipyramidalVA	PentagonalBipyramidalVP
1	"0.009"	"0.009"	"0.169"
2	"0.009"	"0.027"	"0.099"
3	"0"	"0"	"0"
4	"0.059"	"0.076"	"0.216"
5	"0"	"0"	"0"
6	"0"	"0"	"0"
7	"0"	"0"	"0"
8	"0"	"0"	"0.072"

	SquareAntiprismatic	SquareAntiprismaticV	HexagonalBipyramidal
1	"0"	"0.006"	"0"

```
2 "0"                 "0.018"              "0"
3 "0"                 "0"                  "0"
4 "0.012"             "0.084"              "0"
5 "0"                 "0"                  "0"
6 "0"                 "0"                  "0"
7 "0"                 "0"                  "0"
8 "0"                 "0"                  "0"
  HexagonalBipyramidalVA HexagonalBipyramidalVP
1 "0"                    "0.003"
2 "0"                    "0.002"
3 "0"                    "0"
4 "0"                    "0.033"
5 "0"                    "0"
6 "0"                    "0"
7 "0"                    "0"
8 "0"                    "0"
```

Table S112. Cluster members of all-ligand-number Na, combined group

[1] "Cluster 1"
3MMD.A.410, 1W8O.A.1654, 3HW8.A.580, 3PML.B.7, 1Q81.K.8346, 1VQ7.Q.9148, 2A7L.A.201, 2A9Y.A.2002, 4BR6.A.401, 3CZJ.A.3102, 4D1J.E.604, 4D9T.A.901, 1EZ1.B.1002, 3G1N.B.5002, 1G5I.C.902, 4GKI.F.303, 4GRX.A.501, 3HYS.A.267, 3I2W.A.304, 3IAQ.A.3103, 3IWK.D.504, 1IYN.A.298, 4JDO.G.301, 1JZ7.C.3103, 4KA5.A.801, 3KED.A.951, 4LLH.B.602, 3MC1.A.301, 1MGV.A.501, 4MJD.A.203, 4MPT.A.402, 3N3O.A.1000, 4NPJ.B.701, 4NRH.C.401, 4O1G.A.401, 2O34.A.503, 4ODI.B.301, 3OEC.A.300, 2OKQ.A.119, 2ONP.G.707, 4OUC.A.801, 2OYC.A.305, 4QVS.A.502, 1RW9.A.900, 1S5C.A.241, 1SOO.A.547, 1TQY.H.1094, 1UD8.A.1001, 1VI6.A.208, 3VW7.A.2012, 3WNM.A.802, 1WQR.A.131, 2WU2.A.1590, 1XOG.A.1002, 1XOG.C.1001, 1X7D.A.1501, 2X7J.A.1581, 1XAR.A.100, 4XEL.A.201, 1YAP.A.501, 1YQ2.A.7501, 1ZH8.A.329, 2ZJ9.A.1, 2ZN8.A.995, 2ZND.A.195, 3ZPQ.A.1360, 3ZQ5.A.1530, 3ZXO.C.522, 2BCR.A.604, 1JJ2.0.8338, 4KYW.A.303, 1Q81.A.8378, 1VQ5.A.9145, 1VQ8.J.9146, 1VQ8.Q.9148, 2AB8.A.2003, 4AFK.A.1507, 2AHR.C.1259, 3AR4.A.1000, 3AR7.A.1000, 2BER.A.1649, 3BIA.X.117, 3BIB.X.117, 3BLJ.B.701, 3BOS.B.302, 3C17.A.323, 2CD7.A.1132, 3CRN.A.131, 4D1J.E.602, 4D1J.G.603, 4D7C.A.1544, 2DDB.A.301, 4DD8.D.1005, 4DF9.B.501, 4DOU.A.1004, 2E5X.A.303, 2E54.A.1004, 3E85.A.162, 3E85.A.163, 1EAS.A.5, 4EAE.A.302, 4EEK.A.302, 4EEL.A.302, 2EHQ.A.1540, 3EIF.A.1033, 3EUW.A.343, 4FDZ.B.301, 4FET.A.301, 1G5H.A.901, 4G8T.C.502, 4GKI.D.303, 3GZA.A.471, 3HON.A.203, 1H16.A.9001, 1HBN.A.1561, 3HIJ.B.295, 2HIO.B.240, 3HSS.A.268, 4HUC.A.509, 3HVI.A.265, 2HZG.A.1101, 3I01.M.730, 4I0W.B.603, 3I2W.B.304, 3IC9.B.491, 2IJA.A.401, 3IPP.A.438, 3IWJ.A.505, 1IYN.A.297, 2IZV.A.1430, 4J07.A.201, 2J80.A.1134, 2JBW.C.1367, 4JDO.A.301, 2JHN.B.1299, 1JMM.A.3001, 4JN7.A.401, 1JTP.L.503, 4JTE.C.301, 1JZ7.A.3102, 3K1U.A.412, 3L7X.A.142, 3LG1.A.530, 4LGN.A.827, 1LLA.A.631, 4LL2.A.301, 4M48.A.701, 4M48.A.702, 4M60.A.502, 3ME4.B.2, 3MQD.A.500, 3MYV.A.502, 3NOU.A.208, 3N83.G.707, 4N9S.A.402, 4N9V.A.404, 4NRH.A.401, 1O04.A.6601, 4O0C.A.401, 4O47.A.401, 4O57.A.301, 4O5H.A.605, 1O68.A.274, 3OEC.C.300, 4OF8.A.301, 3ON4.C.189, 3OND.A.509, 3OPK.A.301, 2OYN.A.201, 1P0Z.A.1633, 4PD6.A.502, 4PMO.A.312, 2POC.A.5001, 1Q3X.A.800, 1Q6X.A.1002, 2QJY.R.2001, 2QR7.A.1000, 1R4P.A.4003, 2R85.A.600, 1RWH.A.900, 1S0A.A.1501, 1S82.A.4, 3SLZ.B.130, 1T64.A.392, 1T8U.B.701, 3TDQ.A.117, 3TSH.A.604, 1U7H.B.912, 3UES.B.503, 3UF4.A.601, 4US3.A.701, 4USW.A.1468, 3USL.A.752, 1V0H.X.252, 2V4V.A.3052, 3VGL.A.323, 2VNZ.X.9252, 2VPB.A.1398, 1W9S.A.1141, 2WCB.A.101, 2WDQ.A.1590, 3WG7.N.605, 1WKY

.A.504, 2WWF.B.214, 2X7J.B.1581, 2X8J.A.1317, 4X9K.A.401, 1XAR.B.201, 2XNA.C.121
6, 2Y5F.A.1245, 2ZHJ.A.321, 2ZJ9.B.2, 1ZOD.A.435, 1ZOR.A.1001, 1ZOR.B.1002, 1ZUD
.1.701, 3ZWF.B.1365
[1] "Cluster 2"
1Q81.A.8353, 1VQ9.Q.9148, 2E5X.A.302, 1GOF.A.702, 4IBO.A.401, 1WX5.A.282, 4COK.A
.1614, 3DYQ.B.901, 1G8H.A.523, 3GCD.B.215, 3IAP.D.3103, 3IO1.A.501, 4JCO.D.406,
4L73.B.405, 4ODN.A.207, 1QY1.A.204, 3ZDU.A.353, 4B6C.B.1257, 4KHY.A.1013, 4NLK.A
.403, 1U8R.A.1101, 1VQ9.C.9104, 2A5G.B.242, 4B6C.A.1257, 1BUN.A.121, 2BWU.A.1441
, 4C1P.A.1728, 4CBY.B.2036, 1D7U.A.435, 4DD8.A.1005, 3FFZ.A.1302, 3FKR.B.408, 4F
O2.Q.101, 3G8Q.D.302, 4G8T.B.502, 1GVH.A.1399, 4H41.B.405, 4H83.A.402, 3IC9.A.49
1, 2JBW.A.1368, 1JJU.C.996, 3KZW.D.498, 4LCZ.B.320, 4M5P.A.505, 4MVJ.A.401, 4NLQ
.A.910, 4NT1.A.301, 4NYP.A.1001, 4OOC.B.402, 4O54.A.302, 2P6Z.A.402, 3R2H.A.156,
1SU4.A.997, 1TC8.A.121, 1U4J.B.1001, 3VD3.D.3101, 3VD5.A.3101, 2WDO.C.601, 3WGU
.A.2005, 2WWG.B.213, 2WX5.H.1252, 1ZEL.A.401, 3COW.A.303, 3C5G.B.808, 2FLD.C.603
, 4FZX.C.201, 4KLI.A.403, 2OTL.A.8545, 1TW8.B.902, 1VQ9.M.9147, 1YJ9.O.8517, 1A5
U.G.4732, 4ATF.A.500, 4B52.A.1305, 3BC9.A.704, 1BGP.A.502, 3BH4.A.489, 4BY5.B.11
88, 4CBY.A.2036, 2D4E.A.1901, 2DIE.A.781, 4DMI.A.202, 2GFH.A.249, 2GJP.A.1489, 1
HBN.D.1561, 2HEU.A.4001, 1HNO.A.1800, 2HZG.B.1102, 2ID4.A.907, 2IM2.A.3001, 4IQL
.A.407, 4J43.A.902, 2JBA.A.1127, 4JQR.A.301, 1JZ4.A.3101, 3KQB.A.303, 3KZW.A.497
, 4KZW.A.304, 4MPY.C.503, 3MV1.3.3101, 4NLV.A.911, 1OBO.A.504, 4OMG.A.402, 3OQ8.
A.460, 3OQ8.C.460, 2P3Z.A.501, 2P6Z.B.401, 2P6Z.B.403, 4PCG.A.302, 3Q2H.A.701, 4
Q92.C.502, 3SIS.B.3001, 3USZ.A.902, 1V8Z.A.389, 3VD5.B.3101, 3VHS.B.52, 2W4M.A.1
245, 3W5N.A.1211, 4WFZ.A.501, 3WGV.C.2005, 1WPC.A.504, 1WX5.C.282, 2YDG.A.1130,
2YFO.A.1743, 1Z45.A.702
[1] "Cluster 3"
4FZY.A.201, 4FZY.B.201, 8ICP.A.341, 1QVG.L.8347, 1U8R.D.4104, 3UXP.A.339, 1YIT.O
.8517, 4A22.C.1346, 2AAO.A.2003, 3BFT.A.1007, 3BX1.B.284, 4D1I.H.600, 2D4E.C.190
5, 2EHS.A.201, 3EPZ.A.1, 3FFZ.B.1302, 4FHA.A.402, 4FLK.A.504, 4FO2.E.101, 1G8G.A
.522, 1G8G.A.525, 1G8G.A.527, 1G8H.A.526, 1G8I.B.1595, 3GVF.A.178, 1GW1.A.1423,
4HCH.A.407, 3I3D.C.3101, 1IP3.A.999, 4IQZ.E.305, 3IWK.B.504, 1JED.A.525, 4JVL.A.
703, 4K70.B.1011, 4L73.A.406, 1MAU.A.499, 4MVJ.D.402, 2NQL.A.401, 4NT8.A.206, 2N
WH.A.404, 2OCC.A.519, 1ODZ.A.1427, 4OVZ.B.903, 3Q9E.L.343, 1S81.A.2, 3T34.A.1003
, 1T64.B.1392, 4TMV.A.903, 3UA6.B.2, 3W6P.A.804, 1W7Z.A.1032, 2WHM.A.1425, 4XOO.
B.401, 2X1Z.M.1163, 2X2E.A.1747, 1YAO.A.501, 3ZPR.B.1, 3ZX2.B.521, 4FZZ.A.201, 3
DYM.A.3101, 4J4B.A.902
[1] "Cluster 4"
2GIG.A.501, 4NLZ.A.402, 1Q81.C.8345, 1QVG.O.8317, 4TUP.A.401, 1WTE.A.1001, 1XSP.
A.576, 4D1I.D.602, 4DKA.A.201, 4DMI.C.203, 4DNL.A.300, 4DXK.A.502, 2FBB.A.131, 4
G8T.D.502, 3HIJ.A.294, 2HZL.A.500, 4IBO.A.402, 4IIL.A.402, 4JP4.A.305, 1JZ3.B.31
02, 3KQC.A.303, 4KQ7.B.502, 1LTM.A.400, 4LUG.A.301, 4MM7.A.602, 3MNC.A.280, 3N8O
.B.602, 3NUQ.A.283, 1O68.D.274, 4O7J.A.201, 3OPO.A.2, 3PDG.A.99, 3PFV.A.1, 2PKC.
A.280, 3Q2G.A.701, 3UOF.A.410, 3U27.A.306, 3UCY.A.100, 3UES.A.503, 3UTO.A.902, 3
V7Z.A.404, 2VX5.A.1421, 3W5D.A.1001, 1W9W.A.901, 3WGV.A.2005, 4X26.A.402, 2XRM.A
.401, 2Y8K.A.1528, 2YEQ.A.582, 2ZHI.A.321, 2ZND.A.194, 1AG9.A.400, 1AG9.A.401, 2
B2N.A.345, 4D1I.G.602, 1GOH.A.702, 3RWK.X.521, 1Y4A.E.1002, 4DOA.A.402, 4DOB.A.4
02, 2FMQ.A.340, 3JPT.A.340, 3PNC.A.2, 2PXI.A.340, 3CBT.A.420, 4CVU.A.1998, 3INJ.
C.601, 1MMX.A.349, 4NTL.A.311, 1NZA.A.104, 1SVY.A.2, 1YCE.B.201, 1YCE.a.201, 1BP
Z.A.341, 3C5G.A.803, 2FMP.A.1340, 4FZX.B.101, 4FZX.A.101, 4FZY.C.101, 4FZY.D.101
, 2ISO.A.340, 3JPO.A.340, 3JPQ.A.340, 3JPR.A.340, 4M9L.A.403, 3MBY.A.340, 1Q81.N
.8347, 1SA3.A.401, 4UAY.A.403, 3UXP.A.338, 1VQ8.O.9117, 1YIJ.O.8519, 3A6V.A.1005
, 3A6V.B.1006, 3ATU.A.6267, 1AVT.A.301, 3B1Q.C.332, 3B8X.B.668, 1BAO.A.490, 4D1I
.B.600, 4DCC.A.302, 2DDA.B.302, 4DD8.B.1005, 3E3T.A.243, 3ED4.A.519, 4F4R.A.501,
4FMT.B.301, 4FUS.A.828, 2GA4.A.713, 3GQ9.A.1, 3GQ9.A.692, 4H83.B.402, 3HLT.A.26
9, 3HSC.A.490, 3HSC.A.491, 1HVX.A.519, 3MZG.B.211, 4O52.A.301, 3Q9C.A.344, 1QHU.

A.438, 2QSV.A.222, 1QUS.A.400, 2QZ7.A.194, 3R0L.D.125, 3R2H.A.157, 3SIT.A.3000, 3TAY.A.1, 4TMW.B.902, 4TMX.A.902, 4UZU.A.1491, 1V7T.A.406, 3VDC.A.3103, 1VK1.A.3 02, 2WCP.A.501, 4WED.A.601, 3WFA.A.801, 2WGM.u.201, 3WNK.A.813, 2WOF.A.1727, 2WP C.A.1491, 3WXO.A.806, 3WZ1.A.402, 1YQ2.C.7503, 2YXU.B.2406, 1ZDN.B.157, 3ZK1.D.9 0, 3ZK2.L.90
[1] "Cluster 5"
3E45.A.260, 4M47.A.403, 2OTJ.R.8537, 1Q81.M.8380, 3AXG.A.3005, 3C17.A.324, 4CH8. D.1580, 1CM5.A.1056, 1DI4.A.501, 3DR3.A.336, 3DYQ.A.902, 2E7U.A.1002, 1EBU.A.901 , 4ENZ.A.1112, 1F7T.A.472, 1F7T.C.474, 4FOI.A.1005, 4FOI.A.1006, 2FQE.A.901, 2GG 2.A.703, 3H1V.X.600, 3HVU.D.182, 4HXV.A.403, 3IGQ.F.801, 2J5W.A.3043, 3K13.A.647 , 3L27.B.3, 1MXO.D.901, 3NRB.C.287, 4O4W.A.301, 3OB8.A.3006, 4ODI.A.301, 3P0J.A. 711, 1QJS.A.512, 1QJS.A.513, 1QJS.A.523, 2QZ7.A.195, 4R3W.B.402, 1R4P.F.4004, 3T 2Q.D.3101, 3UA7.A.145, 1UD2.A.1003, 3V6N.A.232, 3W0L.A.502, 1XAR.B.200, 1XC6.A.8 001, 1YCE.A.201, 1YCE.C.201
[1] "Cluster 6"
4DOC.A.402, 4KHN.A.1010, 4KHU.A.1008, 3AR8.A.1000, 4CBY.C.2037, 3DYQ.A.901, 3IAQ .C.3101, 4KAF.A.404, 1PX3.A.3101, 4Q4B.A.530, 3UA6.A.147, 3VD3.C.3101, 3VD5.C.31 01, 3VD7.A.3101, 3VDA.A.3101, 2BCV.A.579, 1DIZ.A.825, 2GIG.A.502, 2GIJ.F.403, 3H W8.A.577, 8ICK.A.341, 1JJ2.A.8345, 4KHY.A.1006, 4ORJ.A.305, 1SUZ.A.403, 1TX3.A.8 01, 1VQ8.M.9147, 1YJW.A.8545, 2A65.A.751, 1A7T.A.255, 4A87.A.1162, 2ABS.A.1003, 4ATF.C.500, 3B1N.A.403, 2B2N.B.1001, 2BBH.A.301, 4BEM.A.201, 4BEM.B.201, 4BEM.J. 201, 2BL2.A.1157, 3C7X.A.1001, 3CB8.A.820, 4CD5.A.1420, 4CD5.A.1421, 4CFY.A.302, 3CKI.A.502, 3CTP.A.402, 3CZJ.A.3101, 4D1J.A.601, 4D77.A.1543, 2DDB.B.303, 2DKB. A.436, 4DUW.A.3101, 4DUX.A.3101, 3DYO.A.3101, 3DYP.A.3101, 1EBU.B.902, 3EEB.A.21 1, 1EJA.A.246, 3EPR.A.266, 4EXR.A.301, 4EZE.A.302, 1F6D.A.1378, 2FBL.A.304, 4FEV .B.303, 4FHA.B.402, 4FLL.A.504, 4FMT.D.301, 4FXZ.A.603, 3FZQ.B.274, 3G0T.B.437, 3GBV.B.1, 2GG8.A.503, 2GKO.A.614, 1GNY.A.1244, 1GVF.A.289, 3GVK.A.916, 4GVO.A.70 2, 3H7K.A.387, 4H7O.C.302, 1H80.A.1497, 4H83.E.401, 2HIG.B.488, 1HXN.A.4, 2HZY.A .1201, 3I3B.A.3101, 3I3D.A.3101, 3IMM.C.3, 3IMX.A.467, 4IQZ.A.317, 3IRD.A.301, 4 J4B.B.303, 2J5A.A.1109, 2JBW.D.1367, 4JEX.A.512, 2JLN.A.1471, 4JNQ.A.402, 4JPA.A .305, 3JU4.A.7, 1JYN.A.3101, 1JYV.A.3101, 1JYW.A.3101, 1JZ2.A.3101, 1JZ3.A.3101, 1JZ6.A.3101, 1JZ7.A.3101, 1JZ8.A.3101, 1JZ8.D.3104, 1KA0.A.501, 3KEU.A.402, 3KW M.A.225, 4LDE.A.1402, 4LHL.A.301, 3LJQ.A.597, 3LKB.A.394, 4M9U.A.402, 3MAX.A.381 , 4MAT.A.501, 4MB4.A.604, 4MM7.A.601, 4MMB.A.601, 4MMF.A.601, 3MPN.A.751, 3MPQ.A .751, 4MPY.A.503, 3MS8.A.401, 3MUI.A.401, 1MUQ.A.206, 3MUZ.1.3101, 3MUZ.1.3102, 1MVO.A.202, 3MV0.1.3101, 3MV1.1.3101, 3MX6.A.261, 3N3R.A.1000, 1N82.A.401, 1NSX. A.1401, 1O4Z.A.1004, 4O4V.A.301, 1O68.E.274, 1OA8.C.1691, 1OAF.A.1252, 4OFI.A.80 1, 4OMC.A.608, 3ONF.A.507, 4OUA.A.403, 2OZ3.B.2001, 2P3Z.B.501, 2PFL.A.2001, 3PN X.C.169, 3PNX.E.168, 2PPL.A.480, 4PV3.A.202, 1PX3.B.3101, 1PX4.A.3101, 3PZJ.A.30 1, 1Q20.A.313, 2Q8X.A.401, 3Q9B.A.344, 1QHU.A.436, 4QKU.A.501, 3QS4.A.751, 3QS5. A.751, 3QS6.A.751, 3QST.A.253, 4QTO.A.501, 4QTO.B.501, 2QWL.A.589, 4R7U.A.504, 3 RGA.A.312, 3S9J.A.401, 1SK4.A.342, 3T09.A.3101, 1T64.A.391, 3T8J.A.401, 3TAV.A.2 67, 4TMW.A.902, 1TO2.E.451, 3TXF.A.1138, 3TYP.A.155, 3UNX.A.281, 1V54.A.3519, 3V 5U.A.701, 3V5U.A.703, 3VD3.A.3101, 3VD4.A.3101, 3VD7.B.3101, 3VD9.A.3101, 3VDA.B .3101, 3VDC.A.3101, 3VDG.A.502, 1VI6.C.208, 3W0L.C.502, 1W9S.A.1142, 3WA2.X.702, 3WGU.C.2007, 2WGM.A.201, 2WGM.B.201, 2WOI.A.1491, 2WPC.C.1492, 2WUW.E.1277, 2WW H.C.213, 3WZ1.A.401, 1XFF.A.5301, 2Z5D.B.180, 3ZK1.A.90, 3ZK1.B.90, 1ZNB.A.3, 3Z QS.B.1295, 3ZYV.A.2337
[1] "Cluster 7"
4DOC.A.404, 3EPG.A.421, 2IHM.A.900, 2ISP.A.340, 2ISP.A.342, 3JPS.A.340, 4KHS.A.1 009, 4KHU.A.1006, 3KNT.A.208, 4NLN.A.402, 1YIT.0.8538, 2A2A.C.3158, 1A5S.B.2000, 3ASP.A.701, 3AST.A.701, 4B1L.A.1679, 3C17.B.326, 2C9R.A.1103, 3C9F.B.603, 2C9A. A.1289, 4D1I.C.600, 4D1I.F.600, 3D32.A.120, 4D9U.A.901, 2DDA.C.303, 3DKI.B.324, 2EPF.C.307, 3EUW.B.343, 4FEW.B.303, 3FGH.A.180, 3FKR.A.409, 4FMT.A.301, 3G1N.A.5

001, 4G1K.A.301, 3GA5.A.700, 3GCD.A.215, 4GY9.A.206, 4HKT.B.410, 4HMM.B.302, 3HV
Y.C.427, 4I2B.A.601, 3I4Q.A.177, 3IAQ.B.3104, 3IJ6.B.313, 4IJK.B.301, 4J0Y.A.501
, 4J1I.A.502, 4J2H.A.306, 4JD0.D.301, 4JHG.A.205, 4JRX.D.301, 1JYX.B.3103, 1JZN.
D.4139, 4K7V.A.407, 1KNR.A.542, 4L3F.H.401, 4L73.A.404, 4LDZ.A.205, 4LH7.A.404,
3N83.A.701, 4NAW.N.304, 4NWH.A.201, 1O4Z.B.1005, 2O5W.C.171, 1OA8.A.1690, 3OB8.B
.3003, 4OBO.A.402, 3OO2.B.383, 2OSW.B.602, 2OSY.B.602, 3OTK.C.587, 3P8O.A.187, 3
PJ0.C.367, 2POC.D.5004, 1PX4.A.3104, 3PYM.B.504, 1Q1Q.A.351, 1Q1Z.A.313, 4Q4X.1.
5004, 2QF2.A.700, 4QFE.G.304, 2QZ7.B.196, 4R6C.A.213, 3RN0.A.402, 3S30.A.383, 3T
2P.B.3102, 3TXJ.A.1138, 2VDX.A.1385, 3VD5.D.3102, 1VIZ.B.239, 1W90.A.1154, 2WCF.
B.1091, 2WD0.A.601, 2WG8.B.202, 2WOO.B.1728, 2WOI.B.1491, 2WOI.D.1488, 3WV2.A.30
5, 2WWH.B.212, 3WXO.A.807, 3WXO.A.808, 4WXG.A.502, 2X7J.D.1581, 2YOO.A.1359, 2ZH
J.A.320, 3ZLY.A.1384, 4ZNB.A.3, 2ZXK.A.1
[1] "Cluster 8"
2FMS.A.342, 4GXK.A.405, 3JPP.A.340, 1ORP.A.224, 2PFQ.A.1, 4TUR.A.404, 4ADB.A.140
5, 3AGB.A.1, 3ASQ.B.701, 3AST.B.701, 4AYO.A.502, 4BDR.A.902, 4BVN.A.1360, 3C7E.A
.489, 4C7A.B.1159, 4CSH.A.1169, 4CZN.A.1371, 4D1I.A.600, 4DF9.A.501, 3DR3.A.335,
2DV1.A.1000, 4F3Y.A.301, 4FEW.D.304, 4G1K.B.301, 4GDK.E.301, 2GEZ.A.401, 1GEN.A
.304, 2GTW.E.3006, 1GUU.A.1090, 1GV2.A.1192, 4GY9.A.207, 2HU3.A.9002, 1HXN.A.2,
4IOW.D.602, 4I2F.A.602, 4I2R.C.602, 3I44.A.477, 3IFV.C.408, 3IJP.A.301, 2IY6.B.1
540, 2J5W.A.3044, 2J5W.A.3045, 2JHN.A.1298, 3JS4.A.208, 3K6A.E.178, 4KA8.A.806,
4KXW.A.1013, 4KZV.A.304, 1L2T.A.1502, 4L3H.A.404, 3LP5.A.251, 1LZS.A.131, 4M4V.A
.505, 4MM9.A.602, 4N3M.A.403, 2O34.B.502, 3OTK.A.586, 4PFI.A.401, 4PMO.A.310, 4P
UV.A.405, 4PV3.A.201, 3Q94.A.310, 4QFE.B.305, 1QHU.A.437, 1QOP.B.501, 2QZ7.A.193
, 3STH.A.501, 1SU3.A.911, 3U21.A.500, 2V4B.B.1562, 3VS8.A.501, 3VS8.C.501, 1W16.
A.1002, 1W9W.A.900, 1WPG.D.1300, 2WV7.B.401, 3WXO.A.805, 1X7U.A.1000, 1XDF.A.401
, 2XZK.B.507, 2Y8K.A.1527, 2Z2F.A.2001, 1ZDN.A.157, 2BCU.A.577, 2BCQ.A.1, 1CZ0.C
.606, 4ED3.A.502, 4EJY.A.301, 1EWN.A.501, 3IOW.A.296, 4KLI.A.404, 4KLI.A.405, 4M
O4.A.707, 1ORN.A.224, 3OSN.A.423, 4P4M.A.403, 4P4P.A.401, 1RZT.A.2001, 4TUP.A.40
2, 2A5F.B.1326, 2A5D.B.326, 4A6U.B.1460, 3AGC.A.1, 3AJN.A.136, 4AK1.A.1702, 2AMF
.A.850, 4AMJ.A.1360, 2AU7.A.206, 2AUT.D.605, 4B1M.A.1680, 3B34.A.951, 1B57.A.364
, 3BGA.A.6, 2BS2.A.1658, 4BVO.A.1396, 4C1O.A.1731, 4C3X.A.561, 3C7F.A.804, 4CCY.
A.1297, 4CCY.A.1298, 4CIT.A.1454, 3CYM.A.501, 3D9R.A.135, 2DDA.A.301, 2DDB.C.302
, 4DEL.A.401, 4DW8.A.304, 4E6P.A.301, 4E6P.D.301, 2E7U.A.1003, 3E9L.A.1, 3EIY.A.
177, 3ELF.A.351, 3F3C.A.752, 4FEX.A.303, 2FM1.D.344, 2FPR.B.505, 2FV7.A.403, 1G3
K.A.500, 1G5I.A.901, 3GA5.B.701, 4GAF.B.505, 3GED.A.251, 3GIR.A.373, 4GIB.A.301,
2GJU.A.2001, 4GNJ.B.302, 3GOD.B.327, 4GRX.B.501, 2GTW.B.3005, 1GV2.A.1191, 1GV5
.A.1142, 3HON.A.204, 3H12.A.500, 4H83.A.401, 4HUR.A.316, 1HX6.A.705, 4I29.A.601,
4I2A.A.602, 3IAN.A.1, 3IC3.C.100, 3ICF.A.515, 3IFV.A.402, 3IGZ.B.601, 4IIB.A.94
4, 3IJP.B.301, 3IMM.B.2, 3IPO.A.436, 4IQZ.A.316, 3IRS.A.290, 1JAY.A.215, 4JB3.A.
301, 4JEX.B.511, 2JHJ.B.1296, 3JS4.D.208, 1JTP.A.501, 4JTF.C.303, 4JTG.C.302, 4J
TH.C.303, 4JTJ.C.302, 4JTK.C.302, 4JVL.B.304, 1JZ7.A.3104, 1JZN.A.1139, 1K2X.A.8
01, 4KA7.A.805, 3KRS.A.301, 1KSU.A.810, 1L5B.A.302, 4LG8.A.601, 4LIZ.A.202, 1M4Y
.A.252, 4M4U.A.501, 3M9Y.B.254, 4M9B.A.201, 3MJ6.A.503, 4MMB.A.602, 4MMF.A.602,
3MQG.A.193, 4NAW.B.303, 3NGJ.A.249, 3NMB.A.1, 3NNB.A.401, 4NPI.A.602, 4NSJ.A.222
, 2O34.A.501, 3OLJ.A.1, 2OPL.A.187, 4P33.A.401, 4PCG.C.304, 3PGO.A.147, 4PMO.A.3
11, 4PSR.A.619, 1PYF.A.315, 4PYJ.A.301, 3PZR.A.373, 3PZS.A.287, 4Q5K.A.302, 4Q69
.A.501, 2QKF.C.283, 1QNJ.A.280, 2QV6.A.303, 3QXT.A.133, 4QXK.A.402, 3ROL.D.124,
4R3N.B.401, 4R6K.A.501, 2RGI.B.98, 3RU5.A.133, 1S5D.A.241, 1S5E.A.241, 3SIB.A.22
3, 3SJL.A.402, 3SSB.A.995, 1T3M.A.801, 4TKX.L.705, 4TKX.L.706, 1TQY.B.1091, 4U99
.A.203, 1UD2.A.1001, 1UD2.A.1002, 3UWP.A.424, 2V3U.A.1263, 2V4B.A.1566, 2V79.A.1
116, 1VMF.A.134, 1W15.A.2002, 3W5N.A.1210, 3WAY.A.914, 3WC3.A.502, 4WFX.A.504, 2
WGE.A.1426, 3WNO.A.802, 2WOF.A.1728, 2WOI.D.1489, 2WQK.A.254, 2WW2.A.800, 4XCZ.A
.405, 4XCZ.A.406, 1XKN.A.700, 2XZI.A.502, 1YOP.A.1810, 1Y7W.B.282, 2YNQ.A.1392,
1YYA.A.1001, 2ZND.A.196, 2ZQ3.A.160, 3ZX3.C.522

Table S113. 4-ligand combined metal, normal group

	size	largest_angle*	middle_1*	middle_2*	middle_3*	middle_4*	smallest_opposite_angle*	Tetrahedral	TrigonalBipyramidalVA	TrigonalBipyramidalVP	SquarePyramidalV	SquarePlanar
1	"705"	"125.1+/-5.3"	"97.6+/-5.3"	"104.5+/-4.2"	"110.6+/-3.9"	"116.1+/-3.9"	"97.3+/-5.8"	"0.303"	"0.078"	"0"	"0"	"0"
2	"350"	"146.9+/-8.7"	"81.9+/-8"	"90.4+/-7.8"	"100.8+/-8.2"	"116.7+/-9"	"95.2+/-11.9"	"0.001"	"0.036"	"0.013"	"0.038"	"0"
3	"1814"	"116.6+/-2.9"	"102.3+/-3.6"	"106.6+/-2.4"	"109.7+/-2.2"	"112.6+/-2.2"	"108.4+/-4.2"	"0.791"	"0.008"	"0"	"0"	"0"
4	"209"	"160.7+/-11.6"	"77.4+/-6.8"	"84.6+/-5.7"	"91.7+/-6"	"103+/-10.6"	"138.4+/-15.5"	"0.001"	"0"	"0.127"	"0.014"	"0.087"
5	"699"	"126.2+/-6.3"	"93.3+/-5.8"	"100.5+/-4.1"	"107.1+/-4.3"	"114.3+/-4.5"	"113.7+/-5.8"	"0.31"	"0.021"	"0.001"	"0.001"	"0"
6	"492"	"162.5+/-10.4"	"77.8+/-6.5"	"84.6+/-6.1"	"90.5+/-6.6"	"97.9+/-7.6"	"89.8+/-12.4"	"0"	"0.002"	"0.072"	"0.214"	"0"

Table S114. Cluster members of 4-ligand combined metal, normal group

[1] "Cluster 1"
1A1G.A.201, 1A6Y.A.551, 3EPH.A.1, 2ER8.A.104, 2ER8.A.105, 1G2D.C.302, 1G2F.C.301
, 4GLX.A.601, 3HAX.C.201, 2I13.B.507, 4IFD.J.1106, 4IQR.A.403, 2JP9.A.131, 4M9E.
A.503, 4M9V.C.202, 4NM6.A.2002, 2NQ9.A.401, 4O6A.A.601, 1ODH.A.1171, 3PIH.A.917,
3QSV.A.1, 4R2Q.A.503, 1A1R.A.901, 3A32.A.708, 2A5V.A.401, 1A71.A.401, 1A72.A.37
6, 4A7K.A.950, 3A9J.C.1, 3A9K.C.1, 1AAF.A.56, 4AA1.A.1615, 2AB3.A.30, 2AC3.A.531
, 2ADR.A.163, 2AFU.A.391, 2AMT.B.2900, 1ANJ.B.451, 1ARE.A.1, 4ARF.A.1722, 2AU3.A
.501, 4AWY.B.3228, 3AX1.A.601, 1AXG.A.401, 4AX0.B.3228, 4AX1.B.3228, 4AXD.A.700,
3AY2.A.1001, 2AZH.A.150, 4B29.A.1205, 3B4N.B.712, 4B6D.A.1340, 1B8T.A.193, 1B8T
.A.195, 1B8Y.A.301, 3B92.A.502, 3BHX.A.1752, 3BJI.A.1, 3BJI.B.2, 3BL5.A.300, 2BL
6.A.1060, 1BNL.A.179, 1BNQ.A.262, 2BNM.A.1199, 2BNN.A.1200, 3BOC.A.1001, 1BS4.A.
2001, 3BTO.C.375, 4BT7.A.301, 1BUD.A.800, 1BV3.A.262, 1BYF.A.302, 3BYR.A.501, 2C
1I.A.1465, 4C1D.A.502, 4C1F.A.502, 4C1G.A.301, 4C1G.B.301, 4C1Q.A.493, 3C2S.A.44
8, 2C2F.A.1211, 1C3I.B.260, 2C36.A.1311, 3C52.B.401, 4C81.A.1240, 2C9S.A.1155, 1
CAK.A.262, 1CDO.B.376, 3CE1.A.202, 2CFU.A.1002, 1CGL.A.302, 3CJP.A.301, 2CLT.A.1
202, 3CMR.A.450, 2COT.A.201, 2COT.A.401, 3COS.A.501, 2CO8.A.201, 2CON.A.201, 2CO
R.A.201, 1CQR.B.2301, 3CQZ.B.3007, 2CQE.A.622, 2CSY.A.401, 3CSQ.A.335, 2CT0.A.20
1, 2CT2.A.201, 2CT7.A.401, 2CTT.A.401, 2CU8.A.401, 2CUR.A.201, 2D0W.B.1207, 4D0Y
.A.1239, 3D7V.A.2, 2D74.A.1001, 2D74.B.1002, 2D8T.A.201, 2D8T.A.401, 2D8V.A.201,
2D9M.A.1085, 2D9N.A.456, 2DAN.A.201, 2DAR.A.201, 1DCA.A.262, 1DEH.A.376, 3DFM.A
.402, 2DH3.A.601, 3DH1.A.201, 3DHA.A.256, 2DJ7.A.401, 2DJ8.A.401, 2DJA.A.201, 2D

JA.A.401, 1DK4.A.290, 2DKT.A.191, 2DKT.A.241, 2DKT.A.291, 2DKT.A.341, 2DKT.A.391
, 2DKT.A.441, 2DKD.B.922, 1DMT.A.755, 2DMD.A.291, 1DO5.A.28, 2DOO.A.501, 2DQ6.A.
900, 4DR8.A.201, 2DSN.B.2002, 2DW2.A.700, 3E1Z.A.111, 3E2C.A.200, 1E3L.A.380, 1E
67.A.129, 3E6U.A.501, 3E6U.C.502, 2E7Y.A.1301, 3E73.A.501, 2E9H.A.301, 1E9Q.B.15
3, 2EA6.A.201, 2ECT.A.201, 2ECT.A.401, 2ECV.A.201, 2ECW.A.201, 2ECW.A.401, 2ECY.
A.401, 2ECG.A.201, 2ECL.A.601, 2ECM.A.201, 2ECM.A.401, 1ED9.A.451, 3EDI.A.210, 1
EE2.A.1300, 2EE8.A.501, 4EEX.A.402, 4EEZ.A.402, 3EFO.B.1034, 1EI6.A.409, 1EI6.B.
408, 1EKJ.A.4001, 1EKJ.C.4003, 1EKM.A.701, 2ELO.A.181, 2ELR.A.181, 2ELS.A.181, 2
ELU.A.181, 2ELV.A.181, 2ELX.A.181, 2ELI.A.401, 2EMZ.A.201, 2EM2.A.201, 2EM4.A.20
1, 2EM6.A.201, 2EM9.A.201, 2EMB.A.201, 2EMC.A.201, 2EML.A.201, 2EN2.A.201, 2EN9.
A.181, 2ENA.A.181, 2ENC.A.181, 2ENE.A.181, 2ENH.A.181, 2EOJ.A.201, 2EOQ.A.201, 2
EOW.A.201, 2EOE.A.201, 2EOH.A.201, 2EOM.A.201, 2EON.A.201, 2EOO.A.201, 3EPZ.A.70
1, 2EPQ.A.201, 1EU4.A.400, 3EWF.A.400, 2EWB.A.489, 3EYX.A.1, 1F2W.A.262, 1F35.A.
306, 4F7O.B.301, 3F9O.A.309, 1FAQ.A.1, 4FGL.A.301, 4FKK.A.1025, 1FLJ.A.262, 4FMN
.B.901, 4FMN.B.902, 4FMP.A.400, 2FNF.X.2, 4FO9.A.501, 2FPR.A.502, 3FPC.A.353, 3F
PL.A.352, 1FR2.B.301, 3FTN.A.354, 3FTW.A.701, 3FUN.A.701, 2FU8.A.401, 4FVY.A.805
, 4FWU.A.401, 2G0D.A.416, 4G3M.B.401, 1G5C.A.1001, 2G9Y.B.451, 3GAY.B.328, 3GC9.
B.603, 2GMN.A.801, 2GMN.A.805, 4GNE.A.1501, 4GNE.A.1503, 1GO8.P.1486, 4GR3.A.301
, 4GRI.A.501, 2GSN.A.1000, 4GU1.A.905, 4GUA.A.1719, 4GUT.A.904, 1GVY.A.1425, 1GY
T.J.600, 2GZL.A.900, 4H1Q.A.302, 4H2K.B.1001, 2H39.A.352, 2H4N.A.262, 1H9Q.A.262
, 3HB2.P.486, 2HCS.A.1, 2HCS.A.2, 3HFF.A.154, 3HJT.A.1, 1HK8.A.1589, 4HMA.A.301,
3HNI.G.107, 3HNI.H.107, 3HNJ.A.107, 3HNJ.B.107, 3HNJ.C.107, 3HNJ.D.107, 4HNO.A.
301, 3HPH.A.220, 1HSZ.A.1376, 1HTO.A.1376, 1HU8.A.501, 3HUG.D.109, 1HZ5.B.105, 3
I1U.A.401, 1I5O.A.3006, 1I73.A.998, 1I73.A.999, 4I7C.A.601, 1IA9.A.2001, 1IAG.A.
999, 1IAU.A.504, 2IDA.A.103, 1IF5.A.262, 3IFU.A.181, 4IJD.A.501, 4IJD.A.502, 2IM
R.A.500, 4IOU.D.1001, 4IR0.A.201, 1IS8.B.3109, 2IUC.A.1002, 2IUC.B.1007, 4IUQ.A.
301, 4IUW.A.701, 2IWE.A.1129, 1J2O.A.116, 4J3D.B.302, 4J4M.A.301, 2J7U.A.1884, 4
JD1.B.202, 4JEB.B.202, 2JHG.A.401, 4JH2.A.201, 4JH2.B.201, 2JIG.A.1253, 2JIG.B.1
252, 1JJD.A.103, 4JLX.A.501, 2JLP.A.226, 2JOX.A.109, 1JQ5.A.371, 4JSA.A.301, 4JS
W.A.301, 4JSZ.A.301, 1JT1.A.400, 2JTN.A.184, 2JTN.A.185, 2JUN.A.222, 1JV0.A.261,
1JZS.A.1101, 1K07.A.1, 4K2H.A.201, 1K7H.A.478, 4K7D.A.503, 4K7D.A.504, 4K7D.A.5
06, 3KBF.A.159, 2KEM.A.195, 1KH5.A.451, 1KH7.A.451, 3KHI.A.301, 1KOL.A.1001, 4KP
5.A.301, 1KU0.A.701, 2KVF.A.83, 3KWO.B.161, 4KX8.A.1001, 4KXB.A.1001, 3KYC.B.641
, 1L0Y.B.706, 2L0B.A.143, 2L0B.A.161, 4L6O.A.801, 1L7O.B.301, 3L8H.A.901, 3L9Y.A
.155, 4LAO.A.401, 1LDE.A.375, 1LDY.A.375, 3LEA.A.485, 4LEV.A.601, 2LFD.A.400, 2L
GV.A.111, 1LI5.B.964, 4LJQ.B.1105, 4LJQ.A.1101, 1LLU.B.343, 4LP6.A.310, 3LQB.A.2
01, 3LQH.A.1001, 4LR2.A.505, 3LS9.A.457, 2LUY.A.300, 2LXD.A.202, 4LY4.D.301, 3M0
A.D.401, 3M15.A.107, 2M3Z.A.101, 1M3V.A.124, 3M3X.A.262, 2M48.A.501, 2M48.A.504,
3M5S.A.500, 1M6W.A.1376, 3M8T.A.300, 3M8T.A.301, 1M9O.A.78, 3MA2.A.295, 4MB7.A.
301, 3MDW.A.455, 3MEQ.A.401, 3MF1.A.1000, 1MGO.A.375, 1MGO.B.375, 3MHS.A.473, 3M
HS.E.97, 3MI9.C.87, 3MI9.C.88, 3MJH.B.70, 3MKG.A.155, 3MKV.B.426, 3ML2.A.262, 3M
TW.A.2, 3MWM.A.141, 3N2C.E.425, 1N8K.A.375, 1ND1.A.400, 3NGJ.A.250, 3NKQ.A.1001,
3NNQ.A.201, 1NO5.B.571, 3NQY.B.520, 4NQ7.A.302, 4NTM.A.201, 4NTN.A.201, 2NUP.B.
1100, 2O03.A.201, 2O53.B.314, 4O64.A.2003, 3O7U.A.428, 4OBI.A.201, 2ODX.A.156, 2
OHX.A.401, 2OI0.A.2, 3OJ3.J.902, 2ORW.A.401, 2OW9.B.606, 2OXZ.A.264, 2OX8.B.3, 1
P1R.A.375, 1P1V.A.201, 1P42.A.501, 1P42.A.503, 1P4Q.B.301, 3P5A.A.262, 3P5L.A.26
2, 2P53.A.401, 1PAA.A.160, 1PBO.A.1303, 3PB6.X.400, 3PLW.A.187, 2PLI.B.709, 3PN3
.A.1010, 3PSQ.B.321, 4PTB.A.901, 2PUY.A.355, 1PV9.A.402, 1PXE.A.64, 3PZC.A.1000,
1Q3K.A.301, 3Q43.A.1, 3Q44.A.1, 1Q5W.A.32, 4Q7R.A.306, 2QDT.A.401, 2QDT.A.402,
3QE3.A.356, 2QIN.A.2002, 1QJI.A.1201, 2QLO.A.53, 3QNA.A.122, 4QP5.A.401, 2QSW.A.
201, 1QTW.A.301, 1QV6.A.375, 1QV7.A.375, 3QVY.A.500, 1R1H.A.1001, 1R37.A.500, 1R
3N.B.501, 2R3A.A.302, 3RF4.B.119, 4RF1.A.1901, 1RM8.A.501, 1RMD.A.119, 1RMD.A.12
0, 2RPC.A.401, 2RPC.A.601, 2RPC.A.801, 2RPP.A.201, 3RSM.A.500, 2RSJ.A.102, 3RZV.
A.1, 1S1G.A.152, 3S2E.G.500, 3S2F.E.500, 1S4B.P.1, 1S4I.A.802, 3SEY.C.373, 3SFH.

A.403, 1SML.A.271, 3SP1.A.481, 1SRD.B.156, 1SRP.A.920, 3SV6.A.4, 3SWR.A.3, 3SXK.B.3, 3T01.A.502, 1T3K.A.201, 3T33.A.411, 1T9R.A.1, 1TBN.A.2, 3TBG.A.601, 3TEN.A.205, 1TKF.A.901, 1TKF.A.902, 1TKH.A.901, 1TTM.A.262, 1TWF.J.3001, 1U05.A.500, 1U0L.A.298, 1U10.A.400, 1U10.A.601, 4U10.A.401, 1U1H.A.766, 1U3U.A.376, 1U3V.A.376, 1U40.A.160, 1U5S.B.139, 3U5N.A.1, 4UA4.A.301, 3UCK.A.228, 3UIK.A.341, 3UN6.A.325, 1UUF.A.402, 1UW0.A.1118, 2UZG.A.131, 2V1X.A.1595, 1V13.B.200, 3V1C.A.101, 1V5N.A.201, 1V5N.A.401, 1V6G.A.201, 3V7M.A.509, 1V7Z.A.301, 2V8G.C.500, 2V9I.B.1275, 3V96.B.302, 1VA1.A.100, 1VA2.A.100, 1VA3.A.100, 2VES.A.1297, 2VES.C.1302, 2VF7.B.1844, 1VK9.A.143, 2VL6.A.1266, 3VOW.A.201, 3VPE.A.301, 1VQ2.A.702, 3VQZ.A.301, 3VRK.A.301, 1VSH.A.281, 2VUT.I.1713, 1VYK.A.1150, 1VYX.A.1061, 1VYX.A.1062, 2W3Q.A.1231, 2W5V.B.1377, 3W5K.B.502, 3W5K.B.503, 4W6Z.A.401, 1WAA.B.1090, 2WBT.A.1130, 2WBT.A.1131, 3WBH.B.505, 1WEM.A.201, 1WEN.A.401, 1WEO.A.401, 1WEP.A.201, 1WES.A.201, 1WES.A.401, 1WFF.A.401, 1WFL.A.401, 1WFP.A.401, 1WG2.A.200, 1WIG.A.201, 1WIG.A.401, 1WII.A.201, 1WIL.A.401, 1WIM.A.201, 2WJV.A.3, 1WJP.A.301, 1WJP.A.501, 1WJV.A.201, 3WLE.A.402, 3WLF.A.402, 2WOJ.A.1353, 4WOK.A.401, 1WY2.B.407, 1WYH.A.201, 1X3H.A.401, 2X3B.A.1341, 1X4K.A.201, 1X4K.A.401, 1X61.A.201, 1X62.A.201, 1X6H.A.401, 2X7M.A.1174, 2X8Y.A.1616, 2X8Z.A.1616, 2X95.A.1615, 2X96.A.1617, 2XAA.C.1346, 1XB8.A.1001, 2XBL.C.1196, 1XC3.A.302, 4XIX.B.401, 2XML.A.1349, 2XQV.A.401, 1XRT.A.1423, 2XS4.A.998, 1XUC.A.1261, 1XWY.A.401, 2Y6C.A.1267, 1Y8J.A.800, 1Y93.A.265, 1YB0.B.160, 1YC2.A.402, 1YC2.C.406, 1YE3.A.375, 1YLK.A.401, 1YOU.A.301, 2YPU.A.1998, 1YQD.A.1000, 2YQL.A.201, 2YQL.A.401, 2YQQ.A.401, 2YRT.A.401, 2YRM.A.201, 2YSA.A.181, 2YTH.A.201, 2YTQ.A.201, 2YTT.A.181, 2YT5.A.201, 2YTB.A.301, 2Z3I.A.2001, 2Z45.A.1001, 1Z6R.A.501, 1Z84.A.603, 2Z9K.A.901, 2ZEP.A.391, 1ZFO.A.31, 1ZLH.A.555, 3ZNC.A.1, 2ZNC.A.1, 3ZQ6.A.1323, 2ZTX.A.501, 3ZUK.A.1664, 2ZU2.A.5517, 3ZVS.A.1159, 3ZXH.A.301, 1ZXV.B.9002, 1ZXZ.A.198, 1ZZU.A.900, 2A19.B.1642, 3ARA.A.166, 3C15.A.29, 2D32.B.2524, 1G8G.A.521, 3M42.A.1, 4NNN.N.202, 2O4G.B.800, 4QDG.A.402, 2YFD.B.1145, 1YYZ.A.341, 4R5O.A.509, 4AM5.A.1162, 2B5H.A.501, 1BIQ.A.377, 2BKB.C.1193, 2BOY.E.1255, 3CF4.A.808, 3DBY.A.306, 4FWI.B.401, 2GPC.B.195, 2HMK.A.451, 1I4Z.E.605, 1JRO.G.3001, 4KWL.A.301, 4KX6.N.301, 4NBG.A.501, 1NF6.A.200, 2OHJ.A.502, 2Q0J.B.997, 4QDD.A.401, 1QGH.H.157, 1R9X.A.501, 4REU.B.202, 3U9M.A.202, 3V7P.A.429, 3VMH.C.501, 2W3S.E.1464, 1WQL.A.502, 2YFI.A.901, 2YFI.G.900, 2ZZI.A.208, 4FZY.A.201, 4FZY.B.201, 3FFZ.B.1302, 4FLK.A.504, 4FO2.E.101, 4IQZ.E.305, 2OCC.A.519
[1] "Cluster 2"
3AUO.B.579, 4GNX.C.701, 3IE1.D.442, 4L8H.R.105, 4MTD.B.201, 1V15.A.1132, 3VDO.A.401, 2A97.B.2437, 1AAF.A.57, 2ANH.A.451, 2ANH.A.452, 1ARD.A.1, 1ARF.A.1, 2AYK.A.171, 1B8T.A.194, 1BAW.A.107, 1BH5.A.201, 4BLD.D.910, 1BQQ.M.289, 1BTG.B.902, 3C0Y.A.401, 3C0Z.A.101, 2C20.A.601, 2CEA.B.1606, 1D8M.B.801, 1DDZ.A.1, 1DE6.A.450, 1DGS.B.2701, 4DLF.A.404, 1DSQ.A.144, 2E1W.A.400, 3E4Z.B.2, 2EIM.C.262, 1F5F.A.252, 4FC5.E.305, 3FDK.A.402, 2FZ6.A.201, 2G54.A.1100, 2GA3.A.451, 3GJN.B.600, 1GKR.A.1452, 1GLC.F.169, 3GZE.B.14, 4H01.A.602, 3H8F.B.501, 4H82.B.300, 3H90.A.291, 3H90.D.6, 3ID7.A.402, 3IEW.B.801, 3II1.A.571, 3ISI.X.3000, 1ITU.A.401, 4J3D.A.301, 2JMD.A.66, 4JSS.A.301, 4K5N.A.1101, 2K8D.A.155, 1KBE.A.2, 1KHN.A.452, 2KIZ.A.71, 2KO8.C.54, 2KV1.A.125, 2KVG.A.85, 2KVH.A.84, 4KXC.A.1001, 2L0Z.A.487, 1L1O.F.2, 3L8Y.A.301, 1LD3.A.500, 2LGV.A.110, 2LVR.A.101, 4LW9.E.204, 2LXH.C.901, 3LZE.A.201, 4MLX.A.301, 2MQ1.A.102, 2MQ1.A.103, 1MVH.A.502, 1MVH.A.503, 1NDV.A.400, 1NYR.A.1002, 2O36.A.690, 4OIW.F.501, 1OJ7.B.1389, 1OLP.D.1374, 2OO4.A.6001, 1OS2.B.369, 1OS2.F.769, 2OW2.A.444, 2OXW.A.264, 1P5X.A.247, 1P91.B.2301, 4P9C.A.201, 3PJN.A.186, 3PJN.A.189, 3PJN.B.186, 2POJ.A.265, 1PS7.A.331, 1QX1.A.2004, 4R7M.D.1001, 3RAM.D.998, 2RPR.A.201, 3SPU.D.1004, 3SZY.A.501, 1T0A.B.760, 3T02.A.502, 1TF9.A.901, 1THJ.A.214, 1THJ.B.214, 3U7K.A.350, 4UNI.C.1697, 3UW2.A.474, 2V2A.A.1275, 2V8V.B.1455, 1V9P.B.2701, 2W57.B.201, 4WD6.A.302, 2X4H.A.1140, 2X4H.A.1141, 2X5C.A.1128, 2XAM.B.700, 1XTG.A.426, 2XY9.A.1628, 1Y0J.A.244, 1Y7W.B.285, 2Z45.A.1003, 2ZNE.B.992, 3ZTV.A.1599, 4DLG.A.903, 3ICE.B.1001, 3ICE.D.1001, 4IR1.A.9

02, 1Q81.4.8078, 1RVB.B.302, 4UN4.B.2367, 2W9C.A.1344, 2W9C.B.1342, 3ALN.A.406, 4AVQ.C.902, 3CR3.B.1213, 1D2E.D.504, 4DPG.F.604, 3DYF.B.4002, 3EN9.A.600, 2FDR.A.1001, 3FYY.B.402, 1G9X.B.1301, 1GRV.A.490, 4HN2.A.404, 4HNS.A.201, 4I10.A.201, 3IAP.A.3001, 4IL6.C.512, 2I07.B.5004, 3JZM.A.701, 3KGX.A.503, 1KK8.B.998, 4MFE.D.1105, 3MG8.G.241, 3MWC.A.400, 1N5K.B.413, 2O1V.A.755, 2O1X.B.2002, 2O1X.D.2004, 2OPM.A.908, 2PUN.B.401, 1PYX.A.1002, 4QLQ.V.301, 3QU2.C.225, 3QU9.A.227, 4QVY.K.302, 4R3A.A.400, 1RC5.D.764, 1RK2.C.320, 3SZ5.A.220, 4U3W.A.503, 4UM8.B.2001, 2VOS.A.1491, 3W9T.B.511, 2WCJ.A.1144, 3WEG.A.402, 3WGU.C.2003, 3WKA.A.601, 3WU2.B.616, 3WU2.C.513, 1XLB.A.399, 1Z5B.B.2003, 4DTJ.A.1003, 4DTU.A.1003, 4DU4.A.1003, 4EBC.A.504, 4G3I.A.401, 2GIH.A.402, 3M9M.B.354, 3QER.A.906, 3QEV.A.906, 3RBD.A.415, 4RI8.A.1101, 3V20.A.302, 4WUZ.B.301, 2B00.A.252, 1B1G.A.77, 1B90.A.930, 1BK9.A.200, 3D4G.A.485, 1D8M.A.304, 3DEM.B.6001, 3DF0.A.717, 4DLK.B.402, 3E1I.B.503, 4EJ7.A.402, 4EM6.B.604, 1EN7.A.403, 3FHA.A.705, 3GG1.A.503, 1GU6.A.1480, 2HF4.A.902, 2HYW.A.506, 2II1.C.404, 1IJL.A.203, 2IWV.A.1284, 2K0J.A.503, 4KS3.A.502, 1KTW.A.5, 1KVY.A.124, 4L06.A.501, 4LIN.A.1301, 3MOW.G.202, 4N25.A.705, 1NGS.B.681, 3NSJ.A.702, 3O82.B.544, 4OO2.C.601, 2P5V.B.1008, 2P5V.C.1001, 4P99.B.540, 4P0R.E.401, 1PZ8.C.703, 2Q04.C.211, 1QD6.C.2, 1QHD.A.603, 2QVF.B.703, 3R6Q.H.469, 1RFN.A.500, 1RJV.A.112, 1S0E.A.1292, 3S5U.F.220, 1SBH.A.291, 1SEL.B.277, 4TQ0.H.701, 1UCN.A.1162, 2UX1.K.174, 2W1W.B.1134, 1W1X.A.1479, 2WG8.C.201, 2WW0.E.800, 1Y1A.A.501, 2Z2D.A.267, 1ZH2.A.202, 4AC8.B.500, 4C4U.B.201, 4C4U.I.201, 2CKF.C.501, 3D19.F.302, 4DHL.A.502, 1E2U.A.701, 3FG1.B.1500, 2FKZ.A.1600, 2GBX.A.456, 3GC1.A.605, 1GNL.A.1545, 1GUP.C.351, 2GYQ.A.402, 3I01.B.800, 4ILT.A.301, 2J2F.E.371, 2JD7.0.203, 1OQU.A.1001, 3PCE.M.600, 3PCK.Q.600, 2PCD.M.600, 2PT2.A.400, 2PUZ.A.500, 3Q36.A.458, 3QFM.A.264, 1RA5.A.500, 3T81.B.606, 1ULI.A.600, 1W69.A.1349, 1W9M.A.1555, 1Y4T.A.650, 1YKP.F.2600, 2ZQX.A.501, 4DOC.A.402, 8ICP.A.341, 2IHM.A.900, 3JPS.A.340, 4KHU.A.1008, 3KNT.A.208, 1QVG.L.8347, 3UXP.A.339, 4A22.C.1346, 1A5S.B.2000, 3AR8.A.1000, 3ASP.A.701, 3BX1.B.284, 4C0K.A.1614, 3C17.B.326, 2C9R.A.1103, 4CBY.C.2037, 3EPZ.A.1, 3GVF.A.178, 4HCH.A.407, 3I3D.C.3101, 3IAQ.C.3101, 3IWK.B.504, 4J1I.A.502, 4J2H.A.306, 4JC0.D.406, 4JD0.D.301, 4JVL.A.703, 4KAF.A.404, 4MVJ.D.402, 2NQL.A.401, 4O4W.A.301, 4OB0.A.402, 1PX3.A.3101, 3PYM.B.504, 1Q1Z.A.313, 4Q4B.A.530, 3Q9E.L.343, 3RN0.A.402, 1S81.A.2, 3T34.A.1003, 1T64.B.1392, 4TMV.A.903, 3UA6.A.147, 3UA6.B.2, 3VD3.C.3101, 3VD5.C.3101, 3VD7.A.3101, 3VDA.A.3101, 3W6P.A.804, 2WG8.B.202, 4X00.B.401, 2X2E.A.1747, 1YA0.A.501, 3ZPR.B.1, 3ZX2.B.521

[1] "Cluster 3"

1A1I.A.201, 1A6Y.A.550, 2A66.A.401, 1A73.A.202, 4AA6.A.253, 2B3J.A.2001, 2C7A.A.1641, 2C7A.A.1642, 3CBB.A.1001, 4CIS.A.300, 1CYQ.A.601, 1CYQ.A.602, 2DRP.A.171, 2DRP.A.172, 1DSZ.A.1121, 1DSZ.A.1122, 1DSZ.B.1222, 3EQT.A.1, 4ESJ.A.301, 1F2I.G.1202, 1F4S.P.64, 1FFY.A.1001, 2FF0.A.1001, 2FF0.A.1002, 1G2D.C.303, 1G2F.C.303, 3G9M.A.526, 2GAT.A.67, 4GAT.A.67, 6GAT.A.67, 3GOX.A.301, 3GOX.A.302, 4GZN.C.203, 2HAN.A.351, 2HAN.A.352, 2HAN.B.353, 2HAN.B.354, 4HC9.A.401, 4HC9.A.402, 2HGH.A.191, 2HGH.A.192, 2HGH.A.193, 4HN5.A.601, 4HN6.A.602, 4HP3.C.202, 2I13.A.502, 2I13.A.503, 1I3J.A.100, 2IHX.A.235, 2IHX.A.236, 2JP9.A.132, 2JP9.A.134, 2JZW.A.56, 1K3X.A.501, 1K82.A.450, 2KAE.A.175, 1KB2.A.150, 1KB2.A.151, 3KDE.C.78, 2KMK.A.83, 2KMK.A.84, 2KMK.A.85, 3KMP.A.2, 1LAT.A.1514, 1LAT.A.1515, 4LJ0.A.501, 4LJ0.A.502, 4LJ0.A.503, 1LLM.C.301, 1LLM.C.302, 1LO1.A.195, 1LO1.A.196, 3LRR.A.1, 2LT7.A.701, 2LT7.A.702, 2LT7.A.703, 3M7K.A.143, 3M7K.A.144, 4M80.A.1303, 3M9E.A.208, 3M9E.B.209, 4M9E.A.504, 4M9E.A.505, 4M9V.C.201, 1MEY.C.89, 1MEY.C.90, 3MLN.A.501, 4MTD.A.201, 3NCU.A.1, 4NDH.A.402, 2NLL.A.250, 2NLL.B.450, 2NLL.B.451, 2O6M.A.601, 3O9X.A.132, 3OD8.A.200, 2OFI.A.302, 4OLN.A.101, 4OLN.A.102, 4OND.A.101, 4OND.A.102, 4OOR.A.102, 3OYM.A.393, 1OZJ.A.145, 1P47.A.203, 4PZI.A.1101, 4PZI.A.1102, 4QEN.A.802, 4QEN.A.803, 4QEN.A.804, 4QEN.A.805, 3QMD.A.300, 3QMD.A.301, 4R2A.A.503, 4R2A.A.504, 4R2A.A.505, 4R2S.A.501, 1R4O.A.526, 1TDZ.A.1001, 1TF3.A.102, 1TF3.A.2, 1TF3.A.3, 4TNT.A.701, 4TNT.A.702, 3TS2.A.1, 3TS2.A.2, 3U6P.A.300, 1UBD.C

.501, 1UBD.C.502, 1UBD.C.503, 1UBD.C.504, 3UK3.C.968, 3VD6.C.501, 2XQC.A.1138, 2XQC.D.1141, 2YKG.A.927, 1YUI.A.64, 1ZAA.C.203, 1ZNS.A.1500, 258L.A.500, 2A1K.A.1, 3A1B.A.1, 3A1B.A.2, 3A1B.A.3, 4A24.A.601, 4A24.A.602, 4A2C.A.1349, 4A2V.A.1000, 3A43.A.701, 2A51.A.54, 2A51.A.55, 2A5H.A.421, 2A5H.B.421, 3A6G.A.301, 2A6H.D.7458, 2A6H.D.7412, 1A71.A.402, 1A7T.A.251, 2A8D.A.1230, 4ADN.A.1223, 1AF2.A.296, 2AFW.A.996, 2AFX.A.996, 2AFZ.A.391, 2AFM.A.391, 3AII.A.1001, 4AI5.A.200, 4AIA.A.200, 1AJB.A.451, 4AJX.H.1030, 2AKL.A.117, 3ALR.A.601, 3ALR.A.602, 1AM6.A.262, 2AP1.A.304, 2APS.A.400, 4AR9.B.1731, 4ARE.A.1790, 3ASL.A.3, 2ASH.A.400, 4AU7.A.1248, 4AUQ.B.1299, 4AUQ.B.1300, 3AVR.A.1502, 2AW1.A.262, 3AXS.A.401, 2AYD.A.369, 2AYJ.A.57, 4AY8.A.600, 4AYC.A.1484, 4AYC.A.1485, 1AZM.A.261, 2B0O.E.698, 2B3Z.A.1360, 2B44.A.400, 4B6D.A.1341, 2B8T.A.1218, 2B9D.A.1002, 1BBO.A.60, 1BBO.A.61, 1BCD.A.262, 3BET.A.262, 2BE7.D.1108, 4BF1.A.270, 4BF6.A.1262, 3BI1.A.1752, 3BL1.A.262, 2BL6.A.1059, 4BM9.A.1466, 4BM9.A.1469, 1BN1.A.262, 1BN3.A.262, 1BN4.A.262, 1BNN.A.262, 1BNT.A.262, 1BNU.A.262, 1BNV.A.262, 1BNW.A.262, 3BO5.A.301, 3BO5.A.302, 3BO5.A.303, 3BO5.A.304, 3BOF.A.701, 3BOL.A.701, 2BP0.A.1341, 3BQ5.A.800, 3BQ6.A.800, 4BS9.A.1782, 4BUE.A.2162, 2BYO.A.1209, 1BZM.A.261, 2BZ1.A.1174, 4C09.A.351, 1C2G.A.409, 4C3E.A.201, 4C3T.A.300, 4C4O.B.600, 3C5K.A.201, 3C5K.A.202, 3C5K.A.203, 4C5W.A.403, 3C63.A.107, 3C63.B.107, 3C63.C.107, 3C63.D.107, 3C6W.A.2, 2C6A.A.336, 2C6C.A.1752, 1C7K.A.133, 3C7P.A.262, 2C7N.A.499, 1C9Q.A.999, 3CA2.A.264, 1CAI.A.262, 4CA1.A.283, 4CA1.A.284, 2CBD.A.262, 1CCT.A.262, 4CCG.X.1375, 1CDO.B.377, 2CDC.A.1372, 4CDG.A.1643, 2CEX.C.1306, 1CG2.A.502, 1CG2.C.502, 3CG7.A.299, 3CHQ.A.701, 3CHS.A.701, 3CHV.A.301, 1CIL.A.262, 1CIM.A.262, 1CIN.A.262, 2CJS.C.201, 2CJS.C.202, 2CKL.A.1104, 2CKL.A.1105, 2CKL.B.1115, 2CKL.B.1116, 2CKI.A.999, 1CNG.A.1, 1CNH.A.1, 1CNI.A.1, 1CNJ.A.1, 3CNG.A.508, 1CNX.A.262, 1CNY.A.262, 3COS.A.502, 2CO8.A.401, 2COR.A.401, 4CPD.A.1200, 4CPD.A.1300, 2CRW.A.401, 2CR8.A.401, 2CRC.A.401, 2CSV.A.200, 2CSV.A.400, 2CSZ.A.201, 2CSZ.A.401, 2CS2.A.200, 2CS3.A.200, 2CS3.A.400, 2CS7.A.201, 2CS8.A.401, 2CS8.A.601, 2CSH.A.200, 2CSH.A.300, 2CSH.A.400, 1CTT.A.296, 2CT0.A.401, 2CT5.A.201, 2CTD.A.201, 2CTU.A.201, 2CUP.A.401, 4CVR.A.1158, 3CXK.A.201, 3CXL.A.500, 1CXX.A.1, 1CZM.A.261, 3CZV.A.262, 1D0C.A.900, 1D0Q.B.151, 1D1S.B.376, 1D1T.A.375, 1D1T.A.376, 4D1N.A.900, 3D2N.A.101, 3D2Q.A.303, 3D2Q.A.304, 2D5B.A.501, 4D6S.A.1338, 3D7F.A.1752, 2D8X.A.201, 2D8X.A.401, 2D8Y.A.201, 2D8Y.A.401, 2D8Z.A.201, 2D8Z.A.401, 3D8W.A.262, 2D8R.A.401, 2D8U.A.201, 2D9G.A.201, 2D9H.A.201, 2D9H.A.401, 2D9K.A.401, 2D9K.A.601, 2D9L.A.401, 3DAZ.A.262, 2DAS.A.101, 3DBH.B.1, 3DBK.A.302, 2DB6.A.201, 4DB3.A.401, 3DCC.A.262, 1DCQ.A.600, 1DD6.A.502, 1DD6.A.503, 3DDO.A.262, 1DDZ.A.2, 1DFE.A.38, 2DFV.A.1001, 3DGD.C.128, 3DI4.B.286, 2DID.A.201, 2DIP.A.201, 2DIP.A.401, 2DJ7.A.201, 2DJ8.A.201, 2DLK.A.201, 2DLK.A.401, 1DL6.A.60, 2DLO.A.201, 2DLO.A.401, 2DLQ.A.200, 2DLQ.A.300, 2DLQ.A.400, 2DLQ.A.500, 4DLA.A.402, 3DMO.A.131, 2DMD.A.191, 2DMD.A.241, 2DMI.A.200, 2DMI.A.300, 2DMJ.A.200, 2DOO.A.502, 2DPH.A.1001, 2DS7.A.100, 1DVP.A.401, 1DVP.A.402, 3DWD.A.501, 4DZ7.A.301, 1E0E.A.147, 3E1W.A.230, 2E2Z.A.101, 4E2X.A.501, 1E3J.A.901, 1E3J.A.902, 1E4U.A.79, 1E4U.A.80, 2E5R.A.201, 2E5R.A.401, 2E5S.A.201, 2E5S.A.401, 2E6R.A.201, 2E6R.A.401, 2E6S.A.201, 2E6S.A.401, 2E6S.A.601, 3E6U.B.503, 2E6I.A.201, 1E7L.A.1165, 2E73.A.201, 2E73.A.401, 1E9P.B.153, 2EA6.A.401, 2EBL.A.191, 2EBL.A.241, 2EBT.A.100, 2EBT.A.200, 2EBT.A.300, 2EBV.A.201, 3EB5.A.1001, 3EBE.A.500, 2EBQ.A.201, 2EBR.A.201, 2ECJ.A.401, 2ECV.A.401, 2ECY.A.201, 2ECL.A.201, 2ECN.A.201, 2ECN.A.401, 2EER.A.501, 1EE2.A.1301, 1EE8.A.501, 3EED.A.194, 3EER.A.2004, 2EE8.A.301, 2EE8.A.701, 4EEZ.A.401, 1EF4.A.56, 3EFO.A.766, 3EFT.A.262, 2EGQ.A.200, 2EGQ.A.300, 2EGM.A.200, 2EGM.A.300, 2EGP.A.200, 2EGP.A.400, 4EGU.A.202, 3EH2.A.800, 2EHE.A.200, 2EHE.A.300, 2EJ4.A.401, 2ELN.A.181, 2ELQ.A.181, 2ELT.A.181, 2ELY.A.200, 2ELZ.A.200, 2EL4.A.200, 2EL5.A.200, 2EL6.A.200, 2ELI.A.201, 2ELM.A.181, 2EMJ.A.201, 2EMI.A.201, 2EM0.A.200, 2EM1.A.201, 2EM5.A.201, 2EM7.A.201, 2EM8.A.201, 2EMA.A.201, 2EME.A.201, 2EMK.A.201, 2EMM.A.201, 2EMP.A.201, 2ENT.A.200, 2ENV.A.200, 2ENV.A.300, 1EN7.A.401, 2ENZ.A.300, 2ENZ.A.400, 2EN4.A.201, 2EN6.A.181, 2EN7.A.181, 2EN8.A.181, 2ENF.A.181, 2ENN.A.300, 2

ENN.A.400, 2EOR.A.201, 2EOS.A.201, 2EOU.A.201, 2EOV.A.201, 2EOX.A.201, 2EOY.A.20
1, 2EOZ.A.201, 1EOU.A.300, 2EO4.A.201, 2EOD.A.300, 2EOD.A.400, 2EOF.A.201, 2EOG.
A.201, 2EOK.A.201, 2EOL.A.201, 2EOP.A.201, 2EPR.A.201, 2EPS.A.201, 2EPT.A.201, 2
EPV.A.201, 2EPW.A.201, 2EPY.A.201, 2EPZ.A.201, 2EP0.A.201, 2EP1.A.201, 2EP2.A.20
1, 2EP3.A.201, 2EP4.A.200, 2EP4.A.300, 2EPA.A.300, 2EPA.A.400, 2EPC.A.201, 2EPP.
A.201, 2EQW.A.201, 2EQ1.A.201, 2EQ4.A.201, 2EQE.A.201, 2EQF.A.201, 2EQG.A.201, 1
ESK.A.55, 2ESL.A.4, 4ETS.A.302, 1EU3.B.402, 2EU3.A.262, 4EVB.A.204, 2EXU.A.501,
1EXK.A.80, 1EXK.A.81, 4EYL.A.303, 1EZM.A.302, 1F18.A.155, 1F1G.A.4002, 2F14.A.12
62, 2F3B.A.341, 4F3W.A.201, 1F4T.A.369, 2F4M.A.501, 1F62.A.52, 1F62.A.53, 3F7B.B
.301, 3F7L.A.203, 3F7U.A.260, 1F81.A.88, 1F8F.A.373, 1F9X.A.999, 2F9I.B.601, 4F9
V.A.401, 4FAI.A.401, 4FBE.A.403, 2FC6.A.201, 2FC7.A.201, 2FC7.A.401, 2FE3.A.201,
2FEA.A.1302, 2FGY.A.620, 2FGY.A.621, 2FHX.A.317, 3FID.A.298, 3FID.A.299, 2FIF.B
.901, 4FKD.A.102, 4FKE.A.1024, 3FL2.A.1001, 3FL2.A.1002, 3FLO.B.1, 3FLO.B.2, 1FN
9.A.1001, 2FOQ.A.262, 2FOS.A.262, 2FOU.A.262, 2FOV.A.262, 2FOY.A.301, 1FP0.A.90,
1FQL.A.262, 1FQM.A.262, 3FQM.A.901, 2FR5.A.147, 2FR6.A.147, 4FRC.A.302, 2FSA.A.
501, 1FU9.A.37, 4FU5.A.302, 4FVD.A.201, 4FVN.A.302, 4FVO.A.302, 3FW3.A.300, 1FWQ
.A.124, 4FWE.A.901, 4FWE.A.902, 2FYG.A.302, 4FYY.B.201, 2FZW.A.375, 3G1P.A.300,
1G25.A.66, 1G25.A.67, 3G27.A.97, 4G26.A.1001, 2G2N.A.1001, 1G48.A.262, 2G45.A.40
1, 1G52.A.262, 1G54.A.262, 2G6Q.A.400, 1G71.A.348, 4G7A.A.301, 2G84.A.506, 2G9T.
A.999, 1G9K.A.600, 3GA3.A.1, 2GAG.D.101, 2GAH.D.101, 1GDC.A.73, 1GDC.A.74, 2GD8.
A.262, 2GFJ.B.401, 2GFO.A.1200, 4GGJ.A.301, 3GI1.A.501, 4GIZ.C.201, 3GJ3.B.300,
3GJ4.D.300, 3GJ5.B.300, 3GJ7.D.300, 3GJ8.B.300, 2GMW.A.300, 4GNE.A.1502, 4GNE.A.
1504, 2GQJ.A.200, 2GQJ.A.300, 1GUP.A.350, 3GV4.A.203, 2GVI.A.301, 4GVE.A.601, 1G
ZH.A.1293, 3GZE.B.2, 3GZK.A.539, 3H0L.E.902, 3H0N.A.201, 4H0F.A.401, 2H15.A.262,
4H12.A.1801, 4H12.A.1803, 1H2B.A.1362, 4H3S.A.901, 2H6E.A.500, 3H7H.A.119, 1H7V
.A.61, 3H8V.A.401, 3H99.A.601, 4H9D.A.201, 2HBA.A.101, 1HC7.A.490, 3HCI.A.1000,
1HCP.A.99, 3HCJ.A.1000, 3HCS.A.303, 4HCG.A.202, 1HDY.A.376, 2HD6.A.262, 2HDP.A.4
92, 2HDP.A.493, 2HF1.A.102, 3HI2.A.121, 4HI8.B.101, 4HI8.B.102, 2HJN.A.315, 2HJH
.A.800, 3HKO.A.701, 3HKQ.A.261, 3HKT.A.261, 3HKU.A.261, 3HLJ.A.272, 2HL4.A.262,
3HNA.A.501, 3HNA.A.502, 3HNA.A.503, 3HNA.A.504, 3HNI.A.107, 2HNC.A.263, 2HOC.A.2
63, 2HQH.E.1500, 2HRV.A.143, 3HS4.A.301, 1HSO.A.1376, 4HSU.A.904, 1HTD.A.401, 4H
T0.A.301, 4HT2.A.301, 4HTB.A.401, 1HUG.A.261, 4HU1.A.301, 2HU9.A.132, 1HXP.A.350
, 1HXR.A.200, 1HY7.A.302, 2I00.A.579, 4I1F.A.503, 4I1F.A.504, 4I1F.A.508, 4I1H.A
.507, 3I2D.A.1, 2I3H.A.1001, 3I4C.A.400, 3I4C.A.500, 1I50.C.3002, 2I50.A.336, 2I
50.A.336, 2I50.A.337, 2I50.A.338, 4I51.A.3005, 1I8Z.A.262, 2I9W.A.201, 1IA6.A.12
64, 1IBI.A.195, 1IBI.A.196, 3IBI.A.262, 3IBL.A.262, 3IBN.A.262, 3IBU.A.262, 2IBI
.A.1, 4IBY.A.301, 2IDA.A.104, 1IF9.A.262, 3IFJ.A.201, 3IFJ.B.202, 3IGP.A.262, 2I
GI.A.1001, 2IGI.A.1004, 4II1.A.901, 3IJF.X.147, 4ILO.A.301, 4ILX.A.301, 2IMZ.A.5
01, 2IMZ.B.502, 1IML.A.78, 3IMI.A.201, 1INN.B.167, 2INN.B.514, 3IO2.A.202, 2IOI.
A.3001, 1IQ8.A.600, 2IQJ.A.301, 1IRN.A.55, 3IR9.B.501, 3IRB.A.201, 4ITO.A.301, 4
ITP.A.302, 3IUF.A.1, 4IUM.A.501, 3IXE.B.301, 3IXE.B.302, 1IYM.A.182, 1IYM.A.183,
2IYB.E.1422, 2IYB.E.1423, 2J1Y.A.1290, 4J1V.A.301, 1J2O.A.115, 1J2T.A.302, 2J6A
.A.1138, 2J7J.A.1088, 1J98.A.300, 2J9U.B.1162, 1JAO.A.999, 1JAZ.A.401, 4JBG.A.40
1, 2JBG.B.1577, 1JD0.A.901, 1JD5.A.501, 4JE6.A.200, 4JEA.A.202, 4JEA.B.202, 4JEA
.C.202, 4JEA.D.202, 4JIU.A.201, 4JIV.D.101, 1JJD.A.104, 1JJE.B.251, 1JJT.B.251,
4JLW.A.401, 2JM0.A.201, 2JM0.A.401, 2JM1.A.2, 2JM3.A.92, 4JMY.A.201, 2JMD.A.65,
1JN7.A.37, 2JOX.A.110, 1JOC.A.300, 2JQ5.A.129, 2JR7.A.85, 2JRJ.A.62, 2JRJ.A.63,
4JSR.A.401, 2JTG.A.88, 1JTK.A.137, 3JUE.A.999, 2JUN.A.220, 2JVX.A.29, 1JVB.A.400
, 3JV7.A.501, 3JVH.A.163, 2JVN.A.400, 2JWO.A.488, 2JWO.A.489, 3JWP.A.2001, 2JW6.
A.601, 3JXP.A.320, 4JXE.A.501, 4JXE.A.502, 1JY8.A.300, 4JZ0.A.802, 2K0A.A.108, 2
K0A.A.109, 2K0A.A.110, 2K1P.A.96, 2K16.A.940, 2K16.A.941, 3K2F.A.262, 1K2Y.X.500
, 2K2C.A.138, 2K2C.A.139, 2K2C.A.141, 2K2C.A.142, 2K2C.A.143, 2K2D.A.80, 4K2H.B.
201, 3K34.A.1002, 3K35.C.317, 2K4X.A.56, 3K5K.A.1194, 3K5K.A.1197, 3K5K.B.1195,
3K5K.B.1196, 1K7I.A.486, 3K7H.B.1001, 2K7R.A.129, 4K7D.A.501, 4K7D.A.502, 4K7D.A

.505, 4K7D.A.507, 4K7W.B.101, 1K81.A.144, 2K9H.A.101, 2K9H.A.102, 2KAK.A.130, 2KAK.A.150, 2KAK.A.170, 2KDP.A.1, 2KDX.A.120, 3KE1.A.163, 3KEE.A.2000, 2KGG.A.53, 2KGG.A.54, 2KGO.A.109, 1KHK.A.451, 2KI7.B.124, 2KJE.A.501, 2KJE.A.502, 2KKT.A.500, 2KKH.A.201, 2KKR.A.500, 1KLR.A.31, 1KLS.A.31, 3KNE.A.500, 3KNV.A.201, 3KNV.A.202, 4KNI.A.301, 4KNJ.A.301, 4KNM.A.301, 1KOL.A.1002, 2KPI.A.150, 4KP8.A.301, 3KQI.A.71, 3KQI.A.72, 2KQ9.A.113, 2KQB.A.1001, 2KR1.A.65, 2KU3.A.63, 2KU3.A.64, 4KUV.A.301, 4KUW.A.301, 4KUY.A.301, 3KV4.A.448, 3KV5.A.489, 3KV5.A.490, 4KVO.A.301, 1KWG.A.806, 2KWJ.A.501, 2KWJ.A.601, 2KWJ.A.701, 2KWJ.A.801, 4KXQ.A.601, 2KZY.A.63, 2L0Z.A.486, 3L00.A.180, 4L05.A.203, 3L11.A.601, 3L11.A.602, 3L14.A.262, 4L3J.A.402, 2L5U.A.62, 2L5U.A.63, 4L56.A.401, 4L58.A.102, 2L6Y.A.239, 2L6Z.B.37, 1L6J.A.500, 2L6L.A.201, 2L6M.A.201, 4L6T.A.301, 2L7X.A.106, 2L7X.A.107, 2L75.A.155, 2L75.A.156, 2L7P.A.201, 4L7X.A.101, 2L80.A.124, 2L9Z.A.403, 3LAS.A.167, 2LAU.A.82, 2LBM.A.1, 2LBM.A.3, 4LBU.A.402, 3LCZ.A.54, 2LCE.A.200, 2LCE.A.300, 2LCQ.A.162, 2LGV.A.109, 2LGG.A.381, 2LGG.A.382, 2LHN.A.501, 2LHN.A.502, 2LHN.A.503, 4LHI.A.301, 1LIQ.A.28, 2LI8.A.187, 2LI9.A.18, 2LJX.A.200, 2LJZ.A.201, 3LJU.X.401, 4LJO.A.1101, 4LJO.A.1102, 4LJO.A.1104, 4LJP.A.1101, 3LKM.A.904, 2LKO.A.32, 2LK5.A.37, 4LK9.A.401, 3LMI.B.1002, 4LMY.A.202, 2LN0.A.401, 2LN0.A.501, 2LN0.A.601, 2LN0.A.701, 2LO2.A.101, 2LO3.A.101, 2LO4.A.300, 4LO9.A.401, 4LOF.A.401, 1LPV.A.54, 3LQH.A.1002, 4LQG.A.802, 3LRQ.C.100, 2LRI.C.101, 2LRI.C.102, 3LSC.A.458, 3LT8.A.80, 2LUA.A.101, 2LUA.A.102, 2LUA.A.103, 4LU3.A.301, 1LV3.A.66, 2LV2.A.101, 2LV2.A.102, 2LV9.A.201, 2LV9.A.202, 2LWW.A.501, 2LWW.A.502, 2LWW.A.503, 4LW9.A.203, 4LW9.C.202, 4LW9.K.203, 3LXE.A.261, 2LXD.A.201, 4LXL.A.403, 3LYR.A.1, 2LZU.A.201, 2LZU.A.202, 3M04.A.501, 2M0E.A.101, 2M0F.A.101, 4M0W.A.401, 2M1S.A.100, 3M14.A.501, 3M1D.A.1000, 2M13.A.601, 2M13.A.602, 3M1W.A.500, 1M2K.A.999, 1M2O.A.800, 3M2N.A.263, 3M2X.A.500, 3M2Y.A.500, 4M2R.A.301, 4M2V.A.301, 2M3Z.A.102, 1M3V.A.123, 2M3L.A.201, 4M3P.A.701, 3M40.A.262, 3M4C.B.108, 2M48.A.503, 3M5E.A.262, 3M67.A.263, 2M6M.A.201, 2M6M.A.202, 2M7Q.A.101, 2M7Q.A.102, 3M79.D.107, 2M7A.A.100, 2M85.A.801, 2M85.A.802, 2M9Y.A.401, 2M9Y.A.402, 3M96.A.262, 3M98.A.262, 2M9A.A.101, 2M9A.A.103, 2M9A.A.102, 2MA5.A.101, 2MA5.A.102, 2MA6.A.101, 2MA6.A.102, 3MBG.A.3, 3MBG.A.1, 1MBX.B.211, 3MDZ.A.263, 2MD7.B.101, 2MD7.B.102, 2MDG.A.101, 2MDG.A.102, 4MDM.A.301, 1MEA.A.29, 3MEK.A.500, 3MEK.A.501, 3MEK.A.502, 3MEQ.A.501, 4ME3.A.301, 1MGO.A.376, 4MG3.A.201, 3MHC.A.262, 3MHH.E.97, 3MHI.A.262, 3MHL.A.262, 3MHM.A.262, 3MHS.A.472, 3MHS.A.474, 3MHS.A.475, 3MHS.A.476, 3MHS.A.477, 4MHN.A.400, 4MHQ.A.501, 4MHY.A.400, 4MI5.A.802, 4MI5.A.803, 4MI5.A.804, 4MI5.A.805, 4MI5.A.806, 4MI5.A.807, 2MIU.A.302, 4MJ7.A.201, 2MKD.A.301, 1ML9.A.1, 1ML9.A.2, 1ML9.A.3, 3ML5.A.263, 1MM3.A.62, 1MM3.A.63, 3MMF.A.262, 2MNY.A.401, 2MNY.A.402, 3MNA.A.262, 3MND.A.153, 1MNC.A.282, 3MNU.A.262, 4MO8.A.301, 3MPZ.A.300, 3MP2.A.1, 1MR1.C.601, 2MRE.B.301, 1MS7.B.3001, 4MSG.A.1401, 4MSX.A.501, 4MT2.A.68, 4MTY.A.301, 2MUM.A.301, 2MUM.A.302, 2MUQ.A.101, 1MVH.A.501, 3MWM.A.140, 2MWX.A.201, 1MXG.A.437, 3MYQ.A.262, 1MZ8.B.600, 3MZC.A.262, 4N0N.A.501, 4N0N.A.503, 1N0Z.A.46, 4N0X.B.301, 3N2P.A.262, 3N3J.A.262, 3N3K.A.1, 4N4F.A.1402, 3N4B.A.262, 1N5N.A.401, 3N67.A.900, 1N8K.A.376, 3NA7.A.300, 3NB5.A.261, 1NCS.A.61, 1NEE.A.136, 3NHE.A.1, 3NIS.A.1, 3NIS.A.2, 3NIS.A.3, 4NJ5.A.801, 1NKU.A.188, 3NKM.A.1001, 4NL4.H.803, 2NMX.A.301, 2NNV.A.262, 2NN1.A.301, 2NN7.A.301, 4NN2.A.401, 4NN2.A.402, 4NN2.A.403, 2NNG.A.262, 2NNO.A.262, 4NOS.A.3000, 3NQ5.A.503, 4NQ4.A.302, 4NQ6.A.301, 4NQ7.A.301, 4NQY.A.501, 4NS5.A.401, 2NYT.A.2000, 3NY1.A.4, 3NY1.A.5, 3NY1.A.6, 3NY3.A.1, 3NY3.A.2, 3NY3.A.3, 1NZJ.A.700, 4NZG.A.201, 3O0M.A.151, 2O03.A.202, 2O10.A.86, 2O10.A.87, 2O13.A.190, 2O13.A.191, 3O36.A.1, 3O36.A.2, 2O3K.A.401, 2O4Z.A.262, 3O4N.A.2002, 4O62.A.1001, 4O64.A.2001, 4O64.A.2002, 4O64.A.2004, 4O6I.A.601, 3O70.A.500, 3O70.A.501, 3O7A.A.500, 3O7A.A.501, 3OCA.A.300, 3OCQ.A.184, 4ODR.A.202, 4ODR.B.202, 2OFK.A.201, 2OGW.A.500, 4OGE.A.1201, 3OIL.A.262, 2OIK.B.201, 4OIF.A.701, 3OJ6.A.150, 3OJ7.A.114, 1OKL.A.262, 1OKM.A.262, 2OM1.B.801, 3OOI.A.232, 3OOI.A.233, 3OOI.A.234, 1OQ5.A.600, 3OQ6.A.375, 1OQJ.A.183, 3ORU.A.250, 2OSM.A.262, 2OSF.A.262, 2OU2.A.490, 1OVX.A.61, 2OWA.A.201, 1OX7.A.402, 1OXN.A.1001, 3

OXF.A.440, 3OY0.A.262, 1OYW.A.801, 3OYQ.A.262, 3OYS.A.262, 1OZB.I.50, 2OZU.A.800 , 1P0F.A.2502, 2P09.A.200, 3P1V.A.427, 3P2A.C.151, 3P3H.A.261, 3P3J.A.261, 3P44. A.261, 3P55.A.261, 3P58.A.262, 2P57.A.201, 1P6O.A.401, 1P7A.A.38, 3P8B.A.101, 1P 9R.A.601, 1PB0.A.1301, 3PB4.X.400, 3PB7.X.400, 3PB8.X.400, 3PB9.X.400, 3PBE.A.39 1, 1PEG.A.4, 1PFT.A.51, 1PG5.B.500, 4PHT.B.601, 3PJN.A.187, 3PJN.A.188, 3PLW.A.1 88, 2PLI.D.702, 3PN3.A.1001, 2POU.A.262, 2POW.A.262, 3PO6.A.1, 2POI.A.100, 2PPT. A.300, 1PQ4.A.1002, 4PQ7.A.301, 2PQ8.A.501, 4PQT.A.501, 2PRS.A.501, 3PT9.A.1, 3P T9.A.2, 3PTM.A.1001, 1PTR.A.1, 4PTB.A.902, 2PUY.A.356, 3PU7.A.155, 3PUQ.A.2, 3PU R.A.3, 2PVX.A.901, 2PVE.A.301, 3PYK.A.262, 4PYX.A.301, 1PZW.A.100, 4PZH.A.302, 1 Q08.A.401, 1Q08.A.402, 1Q08.B.403, 1Q08.B.404, 3Q01.A.1, 1Q0E.A.153, 4Q09.A.301, 3Q1D.A.201, 3Q1D.A.202, 2Q1Q.A.262, 1Q2L.A.963, 2Q2L.A.1001, 1Q3A.A.465, 1Q68.A .201, 1Q69.A.207, 4Q6D.A.301, 4Q6E.A.301, 3Q7C.A.1, 4Q7R.B.302, 3Q87.A.126, 1QBH .A.364, 1QF8.A.216, 4QF2.A.1801, 4QF2.A.1802, 4QF3.A.2001, 4QF3.A.2002, 3QGV.A.5 00, 2QIC.A.400, 2QKD.A.501, 3QL9.A.1, 3QL9.A.3, 4QN1.A.1501, 2QO8.A.262, 2QOA.A. 262, 2QP6.A.262, 4QQ4.A.2001, 4QSI.A.301, 4QSJ.A.301, 3QU1.A.501, 3QU1.A.503, 3Q U1.B.502, 1QWY.A.400, 3QWP.A.500, 3QWP.A.501, 1QYB.A.401, 1QYP.A.58, 3QYK.A.262, 3R16.A.262, 3R17.B.262, 4R1X.B.501, 3R2N.A.135, 4R2Y.A.101, 4R2Y.A.102, 4R2Y.A. 103, 4R2Y.B.102, 2R3A.A.300, 2R3A.A.301, 2R3A.A.303, 1R5T.A.150, 3R6F.A.132, 1R7 9.A.201, 1R79.A.401, 1R9P.A.135, 1RAY.A.262, 3RBU.A.1752, 2RGV.A.146, 1RGO.A.221 , 1RGO.A.222, 2RHQ.A.1, 2RHK.C.502, 2RI7.A.501, 2RI7.A.502, 3RIY.A.1001, 3RJ7.A. 300, 1RJW.A.401, 4RL0.A.301, 1RMD.A.118, 3RMQ.A.114, 4RM5.D.300, 1RNI.A.256, 4RN 4.A.301, 2RO1.A.201, 2RO1.A.301, 2ROW.A.601, 3RQD.A.390, 4RQT.A.401, 2RR4.A.501, 3RSN.A.200, 2RSH.A.101, 2RSI.A.102, 2RSI.A.103, 2RSJ.A.101, 2RT9.A.701, 2RT9.A. 702, 1RUT.X.603, 1RUT.X.604, 4RUW.A.501, 4RV9.A.501, 1RXR.A.213, 1RXR.A.214, 3RY M.A.106, 3RYV.B.262, 3RYX.B.262, 3RYY.A.1, 3RYZ.A.1, 3RYJ.B.262, 3RZV.A.2, 3RZ0. B.262, 3RZ1.B.262, 3RZ7.A.1, 3RZ8.A.1, 3S2Q.B.501, 3S2E.A.500, 3S2E.A.501, 1S3G. A.219, 3S71.B.262, 3S72.B.262, 3S73.B.262, 3S75.B.262, 3S76.A.1, 3S77.B.262, 3S8 P.A.400, 3S9T.A.262, 3SAX.A.262, 3SAP.A.262, 3SBH.A.262, 3SBI.A.262, 1SE0.A.201, 3SI2.A.601, 3SJG.A.1752, 1SLM.A.257, 3SOU.A.7, 3SP4.A.601, 1SRK.A.36, 1SU3.A.91 3, 3SUB.A.161, 1SVM.A.700, 1SX1.A.23, 1T4W.A.201, 3T5U.A.262, 3T6P.A.1001, 3T6P. A.1002, 3T6P.A.1003, 3T6R.A.1, 3T6R.A.3, 3T7L.A.1, 3T7L.A.2, 1T8H.A.275, 3T80.D. 301, 3T82.A.261, 3T84.A.261, 3T85.A.261, 3T9O.A.502, 1T9H.A.411, 3T92.A.122, 3T9 2.A.124, 1TAF.A.2003, 1TEQ.X.262, 1TFI.A.51, 3TGN.A.147, 3TG4.A.435, 3TG4.A.436, 3TG4.A.437, 3TIO.B.185, 3TIO.D.185, 3TIO.E.185, 1TJL.A.200, 3TMJ.A.262, 1TOT.A. 53, 1TOT.A.54, 1TT5.B.1014, 3TTC.A.1, 3TTC.A.2, 4TWJ.A.301, 4TYT.A.302, 4TZU.A.5 03, 1U0A.A.5005, 3U1L.A.241, 1U2N.A.441, 1U3T.A.375, 1U3U.A.375, 1U3W.A.375, 3U3 1.A.276, 4U4L.A.302, 1U5K.A.300, 1U5S.B.138, 3U52.A.514, 3U5N.A.2, 1U85.A.34, 1U 86.A.36, 3U9G.A.226, 3U9G.A.227, 3U9G.A.228, 3U9G.A.229, 1UAQ.A.200, 3UCJ.A.228, 3UCM.A.228, 3UCO.A.228, 1UD9.C.509, 3UDZ.B.800, 3UEH.A.143, 3UEY.A.4, 3UEE.A.14 3, 3UEI.A.143, 3UEJ.A.301, 3UEJ.A.302, 4UF0.A.2269, 3UFF.A.1, 3UGD.A.2, 3UKO.A.4 00, 1UL4.A.139, 3UNG.C.903, 3UNT.A.400, 4UOV.A.298, 4UP0.A.1383, 4UP0.A.1384, 1U SN.A.257, 2USN.A.257, 4UTV.A.1299, 1UUF.A.401, 3UVC.A.301, 3UVC.B.303, 3UVI.A.38 7, 2UVL.A.1336, 3UW4.A.401, 1UX1.A.1132, 3UX8.A.1001, 2UZG.A.132, 2UZG.A.133, 2V 08.A.1090, 2V0C.A.1815, 2V0C.A.1816, 3V1E.A.102, 3V1F.A.704, 3V1F.B.703, 4V1T.A. 776, 2V29.A.1276, 4V2W.A.502, 4V2Y.A.150, 3V3G.B.301, 1V47.A.601, 3V4K.A.2, 1V4P .A.1001, 1V51.A.602, 1V54.F.99, 3V5G.A.262, 1V6G.A.401, 3V7X.A.2001, 1V9E.B.260, 1V9X.A.200, 2V9E.A.1276, 2V9E.B.1276, 2V9K.A.1533, 3VBD.A.2001, 1VDD.D.233, 3VD P.A.201, 1VFY.A.300, 1VFY.A.301, 2VF7.A.1845, 3VGL.A.322, 1VHH.A.400, 3VHS.A.51, 3VHS.B.51, 3VHT.B.401, 1VJ0.A.400, 1VJE.A.167, 2VJE.B.1491, 2VKR.A.106, 2VM5.A. 1245, 2VNF.A.1247, 3VOV.A.401, 2VO9.B.501, 3VPB.E.100, 2VP7.A.1399, 2VPD.A.1399, 2VPG.A.1400, 2VPG.A.1401, 1VQ0.A.300, 2VQM.A.1412, 1VQ2.A.701, 2VQG.C.1091, 2VR S.A.1328, 2VRW.B.1565, 2VRW.B.1566, 2VR6.A.1156, 2VRD.A.1062, 1VSR.A.201, 3VTH.A .802, 3VUW.E.801, 2VVB.X.1268, 1VZY.A.1291, 2W0T.A.125, 2W0D.A.1269, 2W3N.B.1234 , 1W4R.A.400, 2W4L.A.1170, 2W5X.A.1378, 2W5Z.A.4970, 1W5O.A.1339, 3W5K.B.504, 4W

6Z.A.402, 1W8P.B.1030, 1WAA.A.1090, 4WAJ.A.301, 2WB0.X.601, 2WB0.X.602, 2WCB.B.1 00, 2WD2.A.1262, 2WD3.A.1263, 4WD8.C.303, 2WEJ.A.1262, 1WE9.A.401, 1WEE.A.201, 1 WEW.A.201, 2WEH.A.1262, 2WEO.A.1262, 1WFE.A.201, 1WFE.A.401, 1WFH.A.201, 1WFK.A. 200, 1WFL.A.201, 3WF8.A.401, 1WFZ.A.201, 2WGX.A.1300, 1WGE.A.201, 3WID.A.1001, 1 WIR.A.201, 1WJ2.A.470, 2WJV.A.1, 1WJV.A.401, 1WKQ.B.202, 1WN5.A.1001, 3WNQ.A.501 , 1WO3.A.26, 1WO4.A.26, 3WRG.A.702, 1WUQ.A.1001, 2WVJ.A.1193, 1WWD.A.57, 3WWL.A. 102, 1WWR.D.204, 1WYS.A.401, 1X0T.A.150, 4X2Z.A.400, 1X31.D.1006, 1X3Z.A.999, 1X 4I.A.201, 1X4I.A.401, 1X4J.A.201, 1X4J.A.401, 1X4L.A.201, 1X4L.A.401, 1X4S.A.201 , 1X4S.A.401, 1X4U.A.201, 1X4V.A.401, 1X4W.A.401, 2X5R.A.1126, 1X61.A.401, 1X62. A.401, 1X63.A.201, 1X63.A.401, 1X64.A.201, 1X64.A.401, 1X6M.A.200, 2X7S.A.1265, 2X7T.A.1263, 2X7U.A.1261, 2X7M.A.1175, 1XAF.A.501, 1XB0.A.403, 2XB4.A.1224, 2XBL .A.1197, 1XCR.A.1001, 2XCM.E.1222, 2XCM.E.1223, 2XEU.A.1065, 1XEG.A.262, 1XER.A. 106, 1XF7.A.30, 4XIW.A.401, 2XIG.A.1151, 1XJH.A.63, 1XKI.A.1003, 1XOX.A.999, 2XO C.A.993, 2XOC.A.994, 2XOC.A.995, 1XPA.A.220, 1XPG.A.1887, 1XQ0.A.262, 1XRU.A.501 , 1XSO.A.152, 1XTM.B.501, 1Y02.A.161, 1Y02.A.162, 2Y1N.A.1436, 2Y1N.A.1437, 2Y43 .A.1097, 2Y43.A.1098, 2Y6D.A.1267, 1Y8Q.B.642, 2YB5.A.1213, 1YC5.A.1001, 2YHO.A. 1001, 2YHO.A.1002, 2YHW.A.1719, 2YHY.A.2000, 2YIK.A.1533, 1YOP.A.84, 1YQD.A.2000 , 2YQM.A.201, 2YQM.A.401, 2YQP.A.201, 2YRJ.A.200, 2YRT.A.201, 2YRC.A.200, 2YRD.A .200, 2YRE.A.401, 2YRE.A.501, 2YRE.A.601, 2YRE.A.701, 2YRG.A.201, 2YRG.A.401, 2Y RH.A.200, 2YRK.A.201, 2YSJ.A.201, 2YSJ.A.401, 2YSV.A.201, 2YS2.A.201, 2YSL.A.401 , 2YSM.A.301, 2YSM.A.501, 2YSM.A.701, 2YSM.A.901, 2YSO.A.181, 2YTG.A.201, 2YTP.A .181, 2YTR.A.201, 2YTS.A.201, 2YT5.A.401, 2YT9.A.201, 2YT9.A.203, 2YTD.A.201, 2Y TE.A.201, 2YTF.A.201, 2YTI.A.201, 2YTJ.A.201, 2YTK.A.201, 2YTM.A.181, 2YTO.A.201 , 2YUU.A.201, 2YU4.A.201, 2YU8.A.201, 2YUC.A.201, 2YUC.A.401, 2YVR.A.1001, 2YVR. A.1002, 2YWW.A.504, 2YYR.A.401, 2YYR.A.402, 2YZ5.B.1501, 1Z05.A.406, 2Z2Y.A.2001 , 2Z3H.A.2001, 2Z3G.A.2001, 1Z3A.A.301, 2Z3J.A.2001, 2Z45.B.1004, 1Z5H.A.2001, 1 Z60.A.2, 1Z6U.A.2, 1Z84.A.604, 1Z8R.A.151, 2Z9J.A.902, 1Z9Y.A.300, 2ZC0.B.408, 1 ZE8.A.263, 2ZED.A.391, 2ZEE.A.391, 2ZEF.A.391, 2ZEG.A.391, 2ZEL.A.391, 2ZEM.A.39 1, 2ZEN.A.391, 2ZEO.A.391, 1ZFD.A.71, 1ZFK.A.1300, 3ZFJ.A.1159, 3ZFK.A.401, 1ZFQ .A.600, 1ZGE.A.1000, 1ZGF.A.400, 3ZGO.A.400, 1ZH1.A.199, 1ZIN.A.219, 3ZME.A.313, 1ZNB.A.1, 1ZNF.A.27, 3ZNF.A.31, 3ZNI.A.1428, 3ZNI.A.1429, 1ZNM.A.29, 5ZNF.A.31, 7ZNF.A.31, 2ZNR.A.1, 2ZNR.A.2, 1ZP5.A.999, 3ZP9.A.1009, 3ZPC.B.401, 1ZR9.A.117, 1ZRP.A.54, 1ZSB.A.262, 1ZSC.A.262, 3ZTG.A.1336, 3ZTG.A.1337, 1ZU1.A.129, 1ZU1.A .130, 3ZVS.A.1160, 1ZW8.A.66, 1ZW8.A.67, 1ZY7.A.801, 3ZYQ.A.1222, 3ZYQ.A.1223, 2 ZZE.A.753, 2ZZF.A.754, 4FLW.A.802, 1YJW.7.8044, 4OKK.A.204, 3OPK.C.401, 1B2O.B.5 5, 1B71.A.192, 1BE7.A.55, 3D3L.A.801, 3E2T.A.1, 2JI3.A.1127, 4KU0.D.101, 1SHR.B. 801, 1T9O.A.255, 1VCX.A.54, 2WLB.B.619, 1YK5.A.54

[1] "Cluster 4"

1A7I.A.82, 3B1B.A.378, 2B5L.C.3001, 2CIH.A.212, 3CQJ.B.285, 4CWM.B.433, 3E50.A.1 , 2EC7.A.51, 4EGE.A.411, 4FUK.A.401, 2GLQ.A.2002, 4HGX.B.301, 1HOV.A.165, 1HP7.A .401, 4IGN.A.401, 2JRP.A.150, 4K6T.B.403, 1KAR.B.502, 4KJG.B.1001, 3MO2.D.5, 3N2 C.D.426, 4O98.A.401, 2OC7.A.901, 3Q31.A.1, 1TM6.A.23, 3UBF.A.7, 3VUV.A.501, 3WI2 .B.801, 2WWO.A.1165, 1YIX.B.603, 2Z2D.A.264, 3ZTV.A.1598, 4IRK.A.402, 2OTL.A.806 6, 1YJ9.0.8067, 521P.A.168, 3AJP.A.183, 3ALN.B.406, 1AM4.D.679, 1AR1.A.560, 1CEE .A.180, 4D0L.B.2001, 4DVG.A.201, 2DW7.L.2012, 2E8W.B.1204, 4EOP.D.501, 3EQB.A.90 02, 4FMA.F.402, 3FPA.B.901, 3GFT.E.202, 3GOL.A.580, 1GQ9.B.1242, 3GT8.D.14, 4GZM .A.1001, 2HAW.A.1002, 3HYT.A.802, 4HYP.C.302, 4I40.A.301, 4JVJ.F.403, 4JVJ.F.404 , 3KRP.D.903, 3KZ1.E.550, 3LAW.B.1401, 1MAB.A.602, 3NCO.C.218, 2O1X.C.2003, 2OQY .C.402, 2P8E.A.306, 1Q3H.A.674, 1ROZ.D.674, 4R9U.D.302, 4RAB.C.303, 3T5P.H.301, 4UOR.K.699, 3VHX.G.185, 2VWI.B.1293, 1W1W.A.2001, 1W85.E.1368, 2WCJ.A.1146, 3WIG .A.402, 3WNW.J.201, 1XD2.A.167, 1YMO.A.402, 4K4I.E.603, 4KHU.A.1003, 2NOL.A.328, 3OOR.A.236, 4QWD.A.702, 3RBD.B.1415, 4AC8.B.1311, 1AWB.B.280, 1AXK.A.395, 2BD4. A.260, 2BZ6.H.1260, 3C14.A.29, 1C9P.A.501, 2CLT.A.1102, 1D8M.B.804, 1FBL.A.993, 1FBL.A.994, 4FVL.A.506, 4H82.C.305, 1HKB.A.923, 3HQ8.B.402, 1HVD.A.600, 2II1.D.4

01, 2I04.B.701, 3IS5.F.1, 1ITC.A.1500, 1J24.A.1001, 1JDC.A.452, 4KW7.A.402, 4L41
.B.201, 4MC7.A.503, 4MIX.A.2501, 4N2I.A.707, 1NMB.N.478, 4NUY.A.1001, 3OHO.A.1,
4OKH.C.903, 3OXQ.D.516, 4P99.A.533, 2PC6.B.303, 1PEX.A.502, 4PIB.B.203, 4POQ.G.4
01, 2PR3.A.901, 4Q4X.1.5007, 1QMD.B.405, 3R4I.D.342, 2RJP.D.2, 3RMK.B.308, 1TFX.
A.1007, 3V96.B.305, 3VEQ.B.301, 2VME.E.500, 3VOB.A.401, 2W0Q.B.803, 3W9T.B.510,
1Y7O.B.1004, 2YA9.A.1303, 2YN3.D.6355, 2Z2D.A.268, 4BMT.B.1323, 3DHG.A.501, 1DTO
.A.1601, 1FZH.B.5004, 3I4V.A.281, 2ITB.A.501, 4IWK.F.201, 4KEV.D.401, 1MOJ.B.301
, 1N7X.A.339, 3NJZ.A.369, 2P6B.C.513, 3PCA.N.600, 3PCL.R.600, 3R2M.A.155, 1T47.A
.431, 3USS.B.212, 2VC7.D.1315, 2XSO.K.900, 2Z4G.A.503, 3E45.A.260, 4M47.A.403, 2
OTJ.R.8537, 1Q81.M.8380, 3AXG.A.3005, 3C17.A.324, 4CH8.D.1580, 1CM5.A.1056, 1DI4
.A.501, 3DR3.A.336, 3DYQ.A.902, 2E7U.A.1002, 1EBU.A.901, 4ENZ.A.1112, 1F7T.A.472
, 1F7T.C.474, 4FOI.A.1005, 4FOI.A.1006, 2FQE.A.901, 3H1V.X.600, 3HVU.D.182, 4HXV
.A.403, 3IGQ.F.801, 2J5W.A.3043, 3K13.A.647, 3L27.B.3, 1MXO.D.901, 3NRB.C.287, 3
OB8.A.3006, 4ODI.A.301, 3P0J.A.711, 1QJS.A.512, 1QJS.A.513, 1QJS.A.523, 2QZ7.A.1
95, 4R3W.B.402, 1R4P.F.4004, 3T2Q.D.3101, 3UA7.A.145, 1UD2.A.1003, 3V6N.A.232, 3
WOL.A.502, 1XAR.B.200, 1XC6.A.8001, 1YCE.A.201, 1YCE.C.201
[1] "Cluster 5"
3COQ.A.1002, 1F2I.G.1201, 3F2D.A.5, 1F4S.P.65, 1G2F.C.302, 4HCC.A.504, 4HP3.C.20
1, 1HWT.D.138, 2I13.A.506, 2I13.B.510, 4IQR.A.402, 2IVH.A.1577, 2JP9.A.133, 2KKF
.A.2001, 2KKF.A.2002, 4LMG.A.201, 1MEY.C.88, 2OPF.A.501, 1QUM.A.301, 3S14.A.1735
, 3UK3.C.967, 1ZGW.A.500, 3A3O.B.65, 1A42.A.262, 4A46.A.65, 1A5T.A.501, 3A6F.C.3
01, 3A6J.A.301, 3A6J.D.301, 1A7I.A.83, 2AA4.A.1001, 2ADR.A.162, 1ADB.A.375, 1ADN
.A.93, 2AF2.B.154, 4AIG.A.999, 1ANI.A.451, 2AQP.A.201, 4AR1.A.1722, 3ASK.A.501,
2AS9.A.666, 2AS9.B.222, 3AUK.A.390, 2B0P.A.400, 1B4E.A.405, 3B4F.A.262, 2B5W.A.8
00, 3B5Q.A.500, 3B6P.A.800, 3B7R.L.701, 1B8T.A.196, 2B83.C.3353, 2BA1.A.201, 4BH
W.A.1, 4BHW.A.4, 1BIO.A.291, 3BIO.A.1752, 3BKN.A.201, 3BKN.A.202, 3BL0.A.262, 4B
LB.B.910, 1BNM.A.262, 4BOL.A.1261, 1BP3.A.500, 1BTK.A.1, 3C10.A.102, 4C1D.A.501,
4C1E.A.501, 4C1E.A.502, 4C1F.A.501, 4C1G.A.300, 4C2P.A.701, 3C37.A.301, 4C4O.D.
500, 1C8T.A.260, 3C8Z.A.413, 4C8E.A.1162, 1CAQ.A.301, 4CBY.A.2035, 4CCG.X.1374,
3CHV.A.302, 2CJL.A.1217, 1CL4.A.81, 1CLC.A.653, 1CNW.A.262, 1CO4.A.43, 4COI.A.65
2, 3CQZ.I.3004, 3CQZ.L.3005, 4CQ0.A.1262, 2CQE.A.822, 2CQF.A.330, 1CRA.A.262, 2C
RR.A.401, 2CSY.A.201, 3CSK.A.712, 1CTU.A.296, 2CT1.A.201, 2CT1.A.401, 2CT2.A.401
, 2CT7.A.201, 2CTD.A.401, 2CTT.A.201, 2CU8.A.201, 2CUP.A.201, 2CUP.A.601, 2CUQ.A
.201, 2CUQ.A.401, 2CUR.A.401, 1CVE.A.262, 3CX3.A.314, 3CXL.A.501, 1CXV.A.3, 4CYK
.A.42, 1D1T.A.401, 3D2N.A.102, 3D2Z.A.262, 2D8Q.A.201, 2D8S.A.201, 2D8S.A.401, 2
D8U.A.401, 1D9D.A.1, 2D9N.A.256, 4D9W.A.408, 2DAR.A.401, 2DB6.A.401, 3DD8.A.262,
3DDT.A.46, 4DF9.A.503, 2DGE.A.1001, 2DID.A.401, 2DJB.A.201, 2DJB.A.401, 4DLA.A.
401, 1DMX.A.280, 1DMY.A.280, 1DPM.A.801, 2DQ4.A.502, 1DSV.A.171, 1DTH.A.901, 1DV
B.A.194, 1DYO.A.401, 4DYG.B.307, 3E24.A.230, 3E2I.A.200, 2E26.A.603, 3E2U.E.102,
1E3I.A.380, 1E3I.A.381, 3E3F.A.230, 3E3I.A.230, 1E46.S.999, 1E4B.S.999, 2E47.A.
172, 2E72.A.201, 2EA5.A.201, 2EA5.A.401, 3EBI.A.1, 2ECJ.A.201, 2ECI.A.201, 2ECI.
A.401, 2ECG.A.401, 2ECL.A.401, 4EEX.B.402, 4EFS.A.301, 2EG4.A.301, 2EG4.B.302, 3
EH1.A.1269, 3EHX.A.264, 2ELW.A.181, 1ELX.A.451, 1ELY.A.451, 1ELZ.A.451, 2EMX.A.2
01, 2EMY.A.201, 2EMF.A.201, 2EMG.A.201, 2EMH.A.201, 2EN1.A.201, 2EOI.A.201, 2EPU
.A.201, 2EPX.A.201, 1EPW.A.1291, 3EQN.B.757, 2EQ0.A.201, 2EQ2.A.201, 2EQ3.A.201,
1ESP.A.323, 4EXS.A.302, 1EYW.A.402, 4EYU.A.1702, 2F0Y.B.501, 3F0D.A.163, 3F0F.A
.165, 3F4X.A.262, 3F5L.A.1001, 1F8F.A.372, 4F9C.B.401, 1FAQ.A.2, 1FBX.A.3316, 3F
CQ.A.322, 2FEJ.A.1, 3FFP.X.262, 3FIE.A.428, 3FJU.A.502, 3FJU.A.507, 1FKW.A.400,
4FKB.A.401, 3FLF.A.2004, 2FNF.X.1, 1FR7.A.262, 3FTN.D.357, 2FU9.A.401, 3FV4.A.32
1, 3FVP.A.321, 3FVZ.A.821, 4FVL.A.501, 3FXP.A.3000, 2FZW.B.376, 1G47.A.999, 3G42
.D.500, 1G4K.A.301, 4GER.A.401, 2GFE.A.869, 1GI4.A.409, 3GIQ.A.482, 3GIQ.A.483,
4GQT.B.502, 3GRB.A.129, 4GR0.A.301, 4GR8.A.301, 2GSU.A.1001, 3GTT.A.155, 1H19.A.
701, 4H12.A.1802, 3H2P.B.154, 4H30.A.301, 3H5A.B.360, 3H5N.A.500, 4H57.A.405, 1H
7N.A.1342, 4H84.A.301, 3H90.A.293, 2HB9.A.401, 1HCP.A.98, 4HDH.A.1002, 4HEW.A.30

1, 4HEY.A.301, 4HF3.A.301, 3HKN.A.261, 2HSI.A.283, 4HVL.A.504, 1HY7.B.801, 1HYI.
A.66, 2HZ8.A.117, 2IOO.A.581, 4I28.A.602, 2I2X.A.524, 3I3T.A.700, 1I6N.A.401, 1I
6P.A.301, 1I76.A.999, 1I8J.B.400, 3I9F.B.3, 2I9W.A.200, 1IB5.A.201, 1IBB.A.201,
4ICR.A.501, 1IML.A.77, 2IT4.A.561, 4IXJ.A.301, 2J21.A.1289, 1J9Y.A.1004, 2J9R.A.
1194, 2JA1.A.1192, 4JIJ.A.302, 1JJ9.A.999, 1JJD.A.102, 1JM7.A.123, 2JOX.A.108, 4
JOM.A.1004, 4JPA.A.301, 2JSD.A.275, 2JTN.A.183, 2JTN.A.186, 2JUN.A.221, 2JUN.A.2
23, 1JVB.A.500, 3JV7.A.502, 1JW9.B.250, 2JZ8.A.150, 4K1R.A.502, 1K24.A.401, 2K5C
.A.96, 3K6I.A.202, 3K6J.A.800, 4K7D.A.508, 1K83.I.3003, 3KB1.A.302, 3KED.A.875,
1KEV.B.353, 1KFI.A.700, 2KFN.A.1, 1KH4.A.451, 1KHL.A.451, 2KIK.B.50, 2KIZ.A.70,
1KK1.A.411, 2KN9.A.82, 1KOQ.A.301, 1KTO.A.405, 4KUJ.A.301, 3KVE.B.489, 1KWQ.A.26
2, 3KWO.C.161, 2KZM.A.1, 3L0V.A.1, 3L22.A.1, 4L5O.A.303, 4L6H.A.801, 2L8E.A.829,
3LAT.A.215, 1LBU.A.214, 3LE9.B.2, 4LEF.A.302, 1LG5.A.262, 1LG6.A.262, 2LGG.A.38
0, 4LGJ.A.301, 2LI8.A.188, 4LIM.A.401, 4LJP.A.1103, 4LOE.C.401, 3LPE.B.60, 1LPV.
A.53, 3LS1.A.1, 2LUY.A.301, 3LUB.A.302, 2LUL.A.201, 2LVU.A.101, 2LVT.A.101, 4LW9
.D.201, 2LXH.C.900, 3M0N.A.201, 2M0D.A.101, 3M15.B.107, 2M3H.A.102, 4M3O.A.300,
1M4M.A.502, 2M48.A.502, 1M65.A.300, 1M6H.A.1376, 3M6I.A.402, 3MBM.A.163, 1MC5.A.
376, 3MEN.D.400, 2MIU.A.301, 2MIU.A.303, 4MLT.A.301, 1MMR.A.1, 1MOO.A.262, 1MPO.
A.376, 1MQO.B.147, 3MS3.A.401, 4MT2.A.67, 4MTW.E.401, 3MWM.A.142, 4MWP.E.401, 4M
XJ.E.401, 1MZB.A.202, 4MZN.E.401, 4N0G.A.401, 4N0L.A.401, 4N4E.E.401, 4N4F.A.140
1, 4N5P.E.405, 4N66.E.501, 1N92.A.375, 3NIT.A.2, 3NI5.A.262, 1NJ1.A.513, 1NJG.A.
401, 4NJ5.A.803, 4NL4.H.802, 3NQZ.B.1, 4NQ5.A.301, 4NQ5.A.302, 4NQ6.A.302, 1NTO.
A.500, 1NUI.A.501, 4O1K.A.301, 2O4H.B.401, 2O4Q.A.2402, 1OAL.A.152, 4OAQ.A.403,
4OCM.E.401, 2OD1.A.902, 1OHL.A.400, 2OH3.A.300, 1OKN.A.262, 1ONW.A.801, 1OS0.A.6
00, 4OTE.A.304, 2OUI.A.361, 2OVX.A.444, 2OVZ.A.444, 4OWF.G.401, 1P42.A.502, 1P5D
.X.500, 1P6B.A.406, 4P9C.B.201, 3PBB.A.391, 3PBJ.D.31, 1PCX.A.950, 1PE8.A.317, 1
PGU.A.616, 2PG3.A.300, 3PII.A.340, 1PL8.A.402, 3PN3.A.1002, 3PN3.A.1009, 3PN3.B.
1011, 3PNU.A.336, 3PZC.B.1000, 4Q0L.A.301, 1Q1A.A.701, 2Q1B.A.400, 3Q6V.A.2, 2Q6
E.A.501, 4Q7R.A.301, 4Q7R.A.302, 4Q7R.A.303, 3Q94.A.301, 4QBG.B.301, 4QEF.A.301,
1QF2.A.320, 3QH5.A.321, 3QHD.A.163, 2QIC.A.300, 2QJS.A.2002, 3QJ5.A.376, 2QL1.A
.1, 2QNO.A.431, 2QQ4.A.139, 1QUA.A.999, 3QU1.A.505, 3QVZ.D.500, 3QWO.C.500, 3ROD
.A.428, 1R22.A.502, 3R3L.A.585, 1R4V.A.202, 2R59.A.701, 4R9G.A.505, 3RCM.A.288,
3RHG.A.368, 1RJ6.A.601, 1RJQ.A.601, 1RJW.A.402, 2RJP.B.1, 1RMD.A.117, 2RMN.A.1,
2ROW.A.602, 2RPC.A.201, 2RPP.A.401, 4RQT.A.402, 4RQU.B.402, 2RSD.A.901, 2RSI.A.1
01, 3RUI.A.1, 1RUR.L.601, 3RZ5.A.1, 3SD9.A.2, 3SOU.B.8, 2SRT.A.257, 3SSB.A.999,
3SU6.A.5, 1SW1.A.401, 3T02.A.501, 1T3A.A.422, 3T5Z.A.262, 3T73.A.410, 3T74.A.410
, 3T7E.A.252, 3T8G.A.411, 3T8F.A.411, 3T87.A.326, 3T8C.A.326, 3T8D.A.325, 3T8H.A
.326, 1TBN.A.1, 3TGN.A.148, 3TGO.B.505, 1THL.A.324, 3TIO.A.1, 2TMN.E.321, 6TMN.E
.322, 3TTY.A.676, 3TWO.A.349, 4TZC.A.501, 1U3L.A.701, 1U3T.A.376, 1U4G.A.9800, 4
U4L.A.301, 3U7L.A.350, 4UA4.A.302, 4UA4.B.303, 3UCN.A.228, 1UD9.B.508, 3UJZ.A.1,
3UKO.A.401, 3UWA.A.200, 2UX1.K.1173, 2UYV.B.1276, 1UZF.A.701, 1V0D.A.401, 4V0R.
A.1001, 1V5R.A.201, 1V87.A.201, 1V87.A.401, 1VGN.A.301, 1VGN.A.302, 2VJE.B.1492,
3VTH.A.801, 2VXI.B.201, 2VXX.A.201, 2VXX.B.202, 3W0F.A.301, 1WAA.D.1090, 1WAA.E
.1089, 4WAI.A.101, 2WCB.A.100, 4WD8.B.303, 1WE9.A.201, 1WEE.A.401, 1WEM.A.401, 1
WEN.A.201, 1WEO.A.201, 1WEP.A.401, 1WEQ.A.201, 1WEQ.A.401, 1WEV.A.201, 1WEV.A.40
1, 1WEW.A.401, 1WFF.A.201, 1WFH.A.401, 1WG2.A.400, 4WH6.A.1203, 2WHG.A.1263, 1WI
L.A.201, 1WIM.A.401, 3WIE.B.1001, 1WJA.A.56, 1WJB.A.56, 1WJP.A.701, 4WK7.A.501,
4WKE.A.501, 3WL3.A.301, 1WNU.A.1001, 2WO8.C.1268, 3WS6.C.201, 1WUR.A.1001, 2WWO.
A.1162, 3WXC.A.302, 1WYH.A.401, 1WYS.A.201, 3X17.A.601, 1X3C.A.201, 1X3H.A.201,
2X4H.B.1141, 2X4H.B.1142, 1X4U.A.401, 1X4V.A.201, 1X4W.A.201, 1X5W.A.201, 1X6E.A
.401, 1X6F.A.201, 1XEM.A.401, 1XLL.A.399, 2XOC.B.991, 2XOC.B.992, 1XPZ.A.262, 1X
UF.A.246, 1XUJ.A.246, 1XWH.A.356, 2XXH.B.1303, 1XYD.A.94, 1XYD.B.94, 1Y23.A.1001
, 2Y20.C.1331, 2Y28.B.1181, 1Y3G.E.321, 2Y4Y.A.1172, 2Y4Y.C.1172, 1Y8F.A.702, 1Y
EJ.L.605, 1YHC.A.601, 1YM3.A.301, 1YO7.A.201, 2YQQ.A.201, 1YSB.A.501, 2YSP.A.181
, 2YTA.A.201, 2YTN.A.201, 2YUU.A.401, 2YXO.A.501, 2YZ3.A.301, 2Z2S.B.204, 2Z2Y.D

.2004, 2Z26.A.400, 1Z6U.A.1, 1Z83.B.642, 1Z9G.E.1005, 1Z9N.A.201, 2Z94.A.901, 2Z
9L.A.701, 1ZDP.E.1005, 2ZEH.A.391, 2ZNE.B.993, 3ZPG.A.1358, 1ZS0.A.999, 1ZTQ.A.5
50, 1ZUD.1.501, 1ZVX.A.999, 2ZXG.A.900, 2FLC.A.248, 3CIK.A.690, 1CLK.A.780, 3D19
.E.301, 2E8A.A.501, 2H5N.B.1001, 4HJH.A.552, 1IV4.D.1564, 2J4E.A.1002, 3LD0.J.54
, 4NNN.K.302, 2NOG.A.9220, 4O6I.B.602, 4OKE.B.203, 1T9Z.A.273, 3TAV.A.269, 1W0H.
A.1001, 2WHE.A.1222, 2XRI.A.1337, 1YQ2.E.7005, 2B0D.A.502, 3KHG.A.415, 4AAH.A.70
2, 1BRW.A.3001, 2OVZ.B.449, 1AOR.A.606, 1BFY.A.55, 1BOU.B.501, 1CJX.B.629, 2DE6.
B.501, 1E3D.A.269, 4EB5.D.201, 4F1E.P.201, 1FZ0.A.5002, 2GBX.E.455, 3GKE.A.501,
3GL0.A.501, 3GL2.A.501, 4HSL.A.202, 1JI5.A.152, 1JI5.B.151, 1JIG.A.402, 1JIG.B.4
01, 1JQK.B.801, 4K9F.A.101, 1LNB.E.900, 1N1Q.A.516, 1N1Q.B.515, 3N9Y.D.151, 3NA0
.C.150, 3NA0.D.150, 4NBA.A.501, 4NBF.A.501, 1NO3.A.858, 2OHJ.A.511, 4QLW.B.201,
3QVD.G.173, 1R2F.A.400, 1RSR.A.1004, 2XSO.G.901, 1YUX.A.303, 2AA0.A.2003, 2EHS.A
.201, 4FHA.A.402, 1G8I.B.1595, 2GG2.A.703, 1GW1.A.1423, 1IP3.A.999, 1MAU.A.499,
4NT8.A.206, 1ODZ.A.1427, 4OVZ.B.903, 1W7Z.A.1032, 2WHM.A.1425
[1] "Cluster 6"
3AF5.A.665, 2ANU.B.405, 2APO.B.501, 2CBN.A.402, 3CE9.B.400, 2CG3.Z.1, 3D3X.A.428
, 1DXW.A.301, 3EII.D.301, 1ENQ.C.238, 3EYV.L.220, 4F0R.A.501, 3FGG.A.161, 4H00.A
.601, 2H42.A.501, 3H66.B.500, 3H90.D.5, 3HDB.A.620, 2HD1.B.101, 4IGM.A.401, 4IGM
.F.401, 3ITM.A.1, 1JDI.A.301, 1JM7.B.143, 1JOE.A.205, 2K2G.A.2, 1KAR.A.501, 1L9Y
.A.2, 2M7Y.A.101, 3MKV.A.425, 2MQ1.A.101, 4MZ7.A.701, 4NRZ.B.301, 3O90.A.192, 1O
LP.A.1373, 1OLP.A.1375, 2PJT.D.302, 2PTW.A.500, 1Q74.D.304, 2QFP.A.434, 3R2J.A.3
01, 1RO9.A.529, 3S6L.B.185, 3SFW.A.502, 1SMP.A.472, 4TQT.A.501, 3TVX.A.900, 1UR6
.B.79, 1UXA.C.1367, 3V94.E.702, 2VXX.D.201, 4X2T.D.701, 4X2T.D.702, 2XR1.A.1638,
 2XR1.A.1639, 1YIX.A.601, 1Z3J.A.264, 1ZKN.C.601, 3ZNS.A.101, 1ZSW.A.315, 4DQP.A
.903, 3E54.A.702, 2PYJ.A.9004, 3S14.A.2001, 3ZC0.B.197, 2AKZ.A.440, 2AQX.A.600,
3AQC.D.327, 2BM1.A.1690, 3BPD.G.126, 1BPM.A.488, 1BR2.A.997, 3BU5.A.301, 3CNX.C.
170, 3CRL.B.2001, 2CW6.A.401, 4CW7.C.1002, 4CYI.B.1000, 4CYU.A.171, 1D1C.A.998,
3D19.D.301, 2D33.C.2525, 3D7M.A.356, 2DEJ.A.402, 2DH4.A.341, 3DLS.C.11, 4DPM.D.4
01, 1E1Q.A.601, 1E1Q.F.601, 3E4O.A.501, 2E6B.A.301, 2E92.A.1302, 2E92.B.1304, 2E
95.A.1301, 1F4H.D.3001, 3F74.C.1, 3FA4.A.401, 3FR8.B.1, 3G2F.B.901, 2G74.A.185,
3G9D.B.299, 2GHT.B.257, 2GHQ.B.257, 2GWC.A.1, 3H8A.C.1431, 3HB0.A.701, 1HBN.A.15
58, 3HQP.M.502, 2HT6.B.444, 4HV6.B.201, 2I19.B.4004, 2I5R.B.302, 3IJQ.A.386, 2IK
2.A.290, 4IL6.C.505, 4IL6.b.616, 4IL6.c.506, 2IO7.A.5001, 2IOA.A.5001, 1IV2.F.15
74, 4J99.D.803, 2JK1.A.1144, 3KA9.A.189, 1LO0.A.602, 4LCZ.A.316, 4LRT.A.403, 1LV
H.A.801, 3M1Y.D.300, 4M1W.A.201, 4MKS.A.502, 4MPO.G.203, 4MPO.G.204, 3MQT.A.1243
, 3N9V.A.61, 1NEL.A.438, 3NIZ.A.312, 1NV3.A.2341, 2O1U.A.302, 2O56.F.2006, 3OAC.
D.3002, 3OHM.A.402, 2OI5.B.5000, 3OPS.D.501, 4OVN.B.202, 4P0V.A.404, 1P7L.A.388,
 4P9D.C.202, 4PFQ.C.400, 1PKG.A.1481, 4PRV.A.402, 2Q58.A.4, 1Q8Y.A.809, 2Q80.A.4
01, 4QJB.A.301, 4QXD.B.302, 4QXD.B.304, 2R5T.A.600, 3RBM.B.1002, 2RIO.A.1102, 1R
LT.C.807, 3RYW.B.2003, 4S17.D.501, 3SRD.C.603, 3SRF.D.533, 1SVW.B.301, 3SY8.A.40
1, 4TQ3.A.402, 3TW6.D.2002, 4UON.B.401, 3U87.A.402, 3UGJ.A.2006, 4UUX.A.401, 2V0
N.B.502, 2VHQ.A.1328, 3VI4.B.502, 2VKQ.A.1288, 3VTI.C.401, 3VYT.C.601, 1W9L.A.17
48, 3WEJ.A.402, 1WQ1.R.168, 1WQA.A.456, 3WU2.C.511, 1WUU.C.394, 1XMJ.A.2, 1YXO.A
.5001, 2Z2P.A.1001, 1ZCW.A.501, 2ZCQ.A.453, 2ZEV.A.1302, 2ZEV.B.1304, 1ZH4.A.201
, 2ZRW.D.702, 2ZRY.D.702, 1ZYK.A.702, 2AU0.H.1415, 4BXO.A.2050, 2C2R.A.1344, 2C2
8.A.1344, 4EFJ.C.101, 4EFJ.A.401, 3GIJ.B.1415, 2GIH.B.401, 4K4H.M.605, 4K4I.E.60
6, 3KHL.B.1417, 4KHW.A.1005, 1M5X.C.801, 3NHG.A.909, 2NOF.A.328, 3OOR.A.237, 4Q0
W.B.1001, 3SQ2.A.906, 2WTF.A.1512, 1A85.A.996, 1AEI.D.317, 1AEI.A.317, 4AG4.A.50
02, 1ALA.A.401, 3ATS.A.380, 2AZ1.D.202, 3B2Z.C.3, 4B7U.B.401, 3B90.B.702, 3BCF.A
.705, 4BCU.A.201, 3BS6.A.1, 4BTX.A.1764, 4BWE.A.503, 4CAG.A.606, 2CDP.C.1140, 2C
OL.B.801, 1CVM.A.802, 3D4G.A.484, 3D6E.B.202, 1D8F.A.305, 3DF0.B.601, 1DM5.E.113
5, 2EAA.C.904, 4EJ7.A.404, 4EMU.B.401, 1ESL.A.164, 2EXJ.D.2004, 2FH3.A.1001, 1FZ
A.C.1, 4G1M.B.2001, 1G5N.A.403, 4GEJ.A.201, 3GG1.B.503, 4GGB.A.402, 4GH8.B.203,
3GRI.B.700, 2GXS.B.604, 2H0K.A.402, 2H0K.A.408, 1H71.P.500, 3HDB.A.657, 1HOV.A.1

68, 2HTY.A.991, 4I9F.A.401, 2IAP.A.402, 3IAE.B.572, 3IJE.B.4002, 4ILW.F.304, 3IR
H.A.457, 1IVG.B.470, 2J0T.C.1268, 2J1G.F.1291, 2J60.C.1277, 2JF9.P.1014, 4JP8.A.
706, 3K39.F.1000, 3K8Y.A.167, 3KQA.C.420, 1KVO.D.192, 2LMV.A.151, 4LN6.G.403, 1L
WU.K.4, 4M7K.H.301, 3M83.B.408, 4MBE.D.201, 3MDO.A.391, 2MG9.A.101, 1MTS.A.480,
1MWN.A.100, 1N28.B.127, 4N2D.A.705, 3N9V.A.64, 4N96.B.401, 4NAS.C.501, 2NP0.A.12
93, 4NUP.C.301, 4NUP.C.304, 4NVR.C.401, 1OLP.A.1372, 1OS2.A.872, 2OVX.B.447, 2OV
X.B.450, 2OZR.F.4030, 3P1O.A.234, 2P5W.B.701, 3Q2N.B.304, 1Q3A.A.467, 4Q4Y.1.500
6, 1Q7B.B.9003, 1QDO.C.245, 1QMD.A.404, 4QN3.A.501, 3QWU.B.602, 2R1D.I.3000, 2R8
Y.I.209, 2RHP.A.16, 4RSR.A.404, 3SH5.A.196, 3SHI.M.305, 3SNZ.A.97, 1SPJ.A.300, 1
SRR.C.532, 1UBN.A.277, 3UBH.A.855, 1UEA.A.4, 1UG9.A.2002, 4UM9.B.2003, 3UMJ.A.90
2, 2V5C.A.1625, 2VB6.B.1148, 1VE5.B.2003, 2VVD.A.1329, 3W9T.B.509, 4WFE.A.306, 2
WII.A.1643, 4WIW.C.701, 2WJS.A.5001, 2WJS.A.5002, 3WMW.A.401, 3WNX.A.502, 1X36.A
.269, 1XJL.A.342, 1XJL.A.344, 2XSG.A.1772, 2XTJ.A.1423, 1Y6P.A.217, 1Y6P.B.218,
1YAX.A.1002, 2YDP.B.502, 2YGM.B.1418, 2ZUX.A.629, 2ZUX.A.637, 2ZUY.A.621, 2ZWP.B
.401, 2BI4.A.1384, 4CMY.A.1164, 4D8F.A.401, 1DGG.A.2000, 3FMR.B.401, 1H2A.L.1004
, 1HJF.A.1310, 1HJG.A.1307, 1I4Y.B.602, 1I4Z.D.604, 1I4Z.G.607, 2IBN.A.704, 3ICF
.A.602, 1IDS.C.208, 2INP.A.3, 2ITB.B.501, 1KBP.A.438, 4KBP.A.438, 3KCY.A.1350, 1
LGT.A.500, 1LKD.A.500, 3LKT.M.600, 3LMX.O.600, 1NNF.A.401, 1NX8.A.300, 2OHH.B.15
01, 2OHH.E.3501, 1OQ9.A.365, 1OS7.C.302, 3PCB.O.600, 3PCJ.M.600, 1PIY.A.376, 3PM
5.A.1002, 3Q1G.A.1001, 3Q1G.B.1001, 1QFC.A.402, 2QJE.A.692, 2R2F.B.320, 4RC5.A.1
003, 1SQ3.B.906, 1SQ3.C.907, 4TOA.A.206, 4TOA.B.207, 3U52.B.512, 3U9M.E.205, 2VH
L.A.1398, 3VTI.B.803, 3WHN.B.201, 2XRX.G.1461, 1XZW.B.929, 1YKM.J.600, 1ZZ9.C.19
9, 4DOC.A.404, 3EPG.A.421, 2ISP.A.340, 2ISP.A.342, 4KHS.A.1009, 4KHU.A.1006, 4NL
N.A.402, 1YIT.0.8538, 2A2A.C.3158, 3AST.A.701, 4B1L.A.1679, 3C9F.B.603, 2C9A.A.1
289, 4D1I.C.600, 4D1I.F.600, 3D32.A.120, 4D9U.A.901, 2DDA.C.303, 3DKI.B.324, 2EP
F.C.307, 3EUW.B.343, 4FEW.B.303, 4FMT.A.301, 3G1N.A.5001, 4G1K.A.301, 3GA5.A.700
, 3GCD.A.215, 4GY9.A.206, 4HKT.B.410, 4HMM.B.302, 3HVY.C.427, 4I2B.A.601, 3I4Q.A
.177, 3IAQ.B.3104, 3IJ6.B.313, 4IJK.B.301, 4J0Y.A.501, 4JHG.A.205, 4JRX.D.301, 1
JYX.B.3103, 1JZN.D.4139, 4K7V.A.407, 1KNR.A.542, 4L3F.H.401, 4L73.A.404, 4LDZ.A.
205, 4LH7.A.404, 3N83.A.701, 4NAW.N.304, 4NWH.A.201, 1O4Z.B.1005, 2O5W.C.171, 1O
A8.A.1690, 3OB8.B.3003, 3OO2.B.383, 2OSW.B.602, 2OSY.B.602, 3OTK.C.587, 3P8O.A.1
87, 3PJO.C.367, 2POC.D.5004, 1PX4.A.3104, 1Q1Q.A.351, 4Q4X.1.5004, 2QF2.A.700, 4
QFE.G.304, 2QZ7.B.196, 4R6C.A.213, 3S30.A.383, 3T2P.B.3102, 3TXJ.A.1138, 2VDX.A.
1385, 3VD5.D.3102, 1VIZ.B.239, 1W90.A.1154, 2WCF.B.1091, 2WDO.A.601, 2WOI.B.1491
, 2WOI.D.1488, 3WV2.A.305, 2WWH.B.212, 3WXO.A.807, 3WXO.A.808, 4WXG.A.502, 2X7J.
D.1581, 2Y0O.A.1359, 2ZHJ.A.320, 3ZLY.A.1384, 4ZNB.A.3, 2ZXK.A.1

Table S115. 4-ligand combined metal, compressed group

	size	largest_angle*	middle_1*	middle_2*	middle_3*
1	"79"	"142.5+/-12.1"	"56+/-3.7"	"87.6+/-8.1"	"99.1+/-7.4"
2	"41"	"155.4+/-10.8"	"54.9+/-5.6"	"79.2+/-9.1"	"107.3+/-13.4"
3	"79"	"126.7+/-10.8"	"54.3+/-5.2"	"73+/-6.7"	"82.4+/-8.5"
4	"32"	"148.4+/-19.3"	"77.6+/-8.4"	"84.6+/-8.6"	"98.8+/-10.9"
5	"58"	"154.5+/-10.5"	"57.5+/-4.5"	"84.6+/-6.9"	"96.9+/-8.1"

	middle_4*	smallest_opposite_angle*	Tetrahedral
1	"107+/-8"	"101.3+/-11"	"0.027"
2	"134.5+/-10.8"	"90.5+/-15.5"	"0"
3	"99+/-11.6"	"84.8+/-13.9"	"0.004"
4	"109.3+/-12.2"	"53.3+/-2.9"	"0.008"
5	"116.7+/-11.1"	"134.5+/-11.3"	"0.004"

	TrigonalBipyramidalVA	TrigonalBipyramidalVP	SquarePyramidalV
1	"0.034"	"0.024"	"0.048"
2	"0.013"	"0"	"0.001"
3	"0.026"	"0.044"	"0.057"
4	"0.031"	"0.023"	"0.023"
5	"0"	"0.031"	"0"

	SquarePlanar
1	"0"
2	"0"
3	"0"
4	"0.026"
5	"0.001"

Table S116. Cluster members of 4-ligand combined metal, compressed group

[1] "Cluster 1"
4AF1.A.500, 1AJD.A.450, 1ALH.B.450, 1ANI.A.450, 1BON.A.1002, 3BYW.D.1, 3CHP.A.70 1, 2CIH.A.213, 3CPA.A.308, 3D68.A.501, 3DLJ.A.2001, 3DLJ.B.2002, 2E84.A.558, 4EG E.A.412, 4G1P.A.501, 2H6H.B.1001, 2H6F.B.1001, 2IMC.A.600, 2IUC.B.1006, 4K7S.B.1 01, 2KBX.B.298, 1KH5.B.950, 1KQ0.A.479, 3KVE.C.489, 1MWO.A.438, 4N07.A.309, 4N07 .B.306, 2O3Z.A.501, 4PVT.A.404, 1Q9U.B.402, 2QQ4.I.139, 1R87.A.905, 3RC6.A.1, 3S OW.A.7, 3U24.A.594, 4U9D.A.205, 3UBF.A.6, 1UD9.B.510, 1UDV.A.101, 1UUP.D.5222, 2 VQH.B.1089, 2VW4.A.503, 2VZ5.A.1131, 2WC0.B.3012, 4WD7.C.302, 2WKN.A.412, 1Z5R.C .600, 1ZKX.A.423, 3ZNR.B.101, 3ZUQ.A.1440, 2AEK.A.804, 3U87.B.403, 1WBQ.C.1441, 1JX6.A.401, 2KBM.B.101, 4L76.E.402, 3RRV.C.255, 1SAV.A.321, 3WIU.B.1004, 1XZO.A. 1014, 1Z3J.A.268, 4CMY.B.1165, 3FE5.A.1, 3FM3.A.452, 1GUP.D.351, 3GZY.A.701, 1MO J.A.302, 1O7P.A.1453, 2OHJ.A.501, 2PHD.C.370, 1PIY.A.377, 1ULJ.E.600, 2XSO.O.900 , 3FGH.A.180, 3FKR.A.409, 1G8H.A.526, 1JED.A.525, 4K70.B.1011, 2X1Z.M.1163
[1] "Cluster 2"
3DNG.B.998, 2FKM.X.500, 4FXO.A.301, 2OX8.A.5, 2OX8.B.5, 1CUL.A.1007, 2E8W.B.1203 , 2ICJ.A.400, 3LGH.A.150, 1MXB.A.411, 1SO2.C.475, 2Z2P.A.1003, 4CP1.A.1297, 3E1I .B.502, 1EUB.A.277, 1JHN.A.900, 1JYI.D.408, 1KTW.A.496, 1KTW.A.9, 1M1J.B.503, 2M 29.A.401, 4QNP.A.506, 2QPS.A.501, 1RK9.A.112, 1TRQ.A.106, 4UM9.D.2002, 1W52.X.60 2, 1XZO.B.1018, 2FLO.B.1605, 1OVB.A.300, 1RSR.A.1003, 3T81.B.607, 3VV9.C.502, 2X RX.Q.1461, 4KHN.A.1010, 3DYQ.A.901, 3DYQ.B.901, 3GCD.B.215, 3IO1.A.501, 1QY1.A.2 04, 3ZDU.A.353
[1] "Cluster 3"
4LMG.B.202, 3A1Z.C.226, 4C98.A.1266, 1F30.H.201, 1F30.I.201, 2F4L.A.1400, 4IOZ.A .504, 4KEQ.A.301, 2L1U.A.144, 3NQ5.A.508, 2OGJ.A.418, 1P4Q.B.302, 2W88.C.107, 2X 3C.A.1342, 2NVQ.A.2001, 4P0P.A.601, 2AQX.A.601, 1AZT.B.406, 1DOA.A.199, 3EPS.A.1 606, 3EQL.N.1528, 4F38.A.202, 2G5H.B.501, 1H8H.F.601, 4I1O.C.201, 1JFG.B.703, 3K RF.A.904, 3O7L.B.352, 4OAU.C.803, 1S4E.B.1600, 2UXR.B.1398, 1YXI.A.342, 1ZOT.A.9 01, 3ZXT.A.1278, 4J2A.A.1006, 4RIC.B.1101, 1AFB.2.3, 4BY6.A.3080, 2E0X.B.701, 2E 6V.E.12, 3EAD.C.1001, 2FH3.A.1003, 3GHG.L.601, 2IEZ.A.219, 1IT4.A.200, 2J3G.A.12 89, 4JBE.A.503, 1JED.B.535, 4L76.B.402, 4L76.D.402, 1LGC.C.301, 4LLF.O.401, 1LWU .C.5, 4M8D.B.305, 4MDV.A.404, 4OVG.A.404, 4POS.B.401, 1QDO.D.247, 3RBX.A.601, 2R HP.A.15, 3RRY.A.202, 3S55.D.280, 1SUI.B.306, 1TCF.A.164, 2TEC.E.344, 2W67.B.1590 , 1WD9.A.902, 2WM4.A.1430, 2XJO.B.1175, 1YN8.B.1008, 1FRF.L.565, 1SQ3.C.908, 1U8 R.D.4104, 3BFT.A.1007, 4D1I.H.600, 1G8G.A.522, 1G8G.A.527, 4L73.A.406, 2NWH.A.40 4
[1] "Cluster 4"

2ANU.B.505, 4HDT.A.400, 3ISO.B.220, 3M7P.A.953, 1N4P.H.378, 2W9M.A.1565, 1YEW.G.662, 1ZZM.A.403, 1V14.C.1134, 1EQR.B.902, 3IG8.A.697, 4K6T.E.412, 3AKB.A.173, 3BOW.B.404, 2DW2.B.711, 2E3X.A.801, 3GE4.A.167, 1GQM.A.1090, 1GQM.C.1088, 2HOL.A.1015, 4JWS.A.502, 3LMW.A.9, 1LRW.A.702, 2NWH.A.402, 1T9H.A.414, 3U24.A.602, 1WOO.A.1781, 3WCV.B.203, 1G8G.A.525, 1G8H.A.523, 4L73.B.405, 2WO0.B.1728

[1] "Cluster 5"

2AQ2.B.1001, 2AXR.A.501, 1D1T.B.406, 3H90.A.292, 3LSF.H.2, 2O1Q.B.145, 3PW3.C.406, 3PW3.D.406, 1PYT.B.350, 4Q7R.A.305, 3QU6.A.114, 3QZC.A.2, 1R5X.A.122, 3U94.A.259, 3WC5.A.404, 1XAF.A.503, 2Y2E.A.1180, 3HO1.X.22, 1VQ7.0.8066, 4AG5.B.1588, 2HAW.A.1001, 1IOV.A.330, 4K6T.B.411, 4QEH.A.401, 1SVT.E.601, 3VN9.A.402, 1Z6K.A.275, 4Q0W.A.1001, 3QZ7.A.363, 2AER.L.3005, 3EDD.A.701, 3HR4.H.203, 1K6S.A.302, 1K6S.B.301, 4KS4.A.502, 1LPK.B.1, 1N41.A.410, 2TBV.A.388, 4WK4.B.502, 4QQW.G.1002, 1AFR.B.454, 4BMT.B.1324, 4CVP.A.1155, 3E1N.D.301, 3E1N.H.300, 2FKZ.A.1601, 1GUP.A.351, 3IS8.N.162, 1JYB.A.600, 1NFV.A.201, 1NFV.M.200, 1OQ4.B.364, 1PFR.A.502, 1PIU.A.401, 4TOA.B.203, 4TOE.A.203, 1W2N.A.312, 1YIT.0.8517

Table S117. 4-ligand combined metal, combined group

	size	largest_angle*	middle_1*	middle_2*	middle_3*
1	"1980"	"116.9+/-3.1"	"101.8+/-3.9"	"106.3+/-2.6"	"109.6+/-2.3"
2	"576"	"163.6+/-7.4"	"79.8+/-7.6"	"87.4+/-5.7"	"93.6+/-5.9"
3	"697"	"125.4+/-5.6"	"97.4+/-5.4"	"104.4+/-4.3"	"110.6+/-4"
4	"236"	"144.4+/-11.1"	"69.7+/-11.7"	"85.2+/-9.1"	"102.5+/-10.7"
5	"194"	"133.4+/-13.5"	"60.8+/-8.8"	"74+/-7.8"	"82.7+/-8.4"
6	"263"	"158.6+/-11"	"71.7+/-10.5"	"84.5+/-5.9"	"93.3+/-6.7"
7	"631"	"129.5+/-7.6"	"91.8+/-6.4"	"99.4+/-4.4"	"106.2+/-4.6"

	middle_4*	smallest_opposite_angle*	Tetrahedral
1	"112.7+/-2.4"	"108.7+/-4.3"	"0.772"
2	"101+/-7.4"	"92.4+/-13"	"0"
3	"116.2+/-4"	"96.8+/-6.7"	"0.292"
4	"124.3+/-10.1"	"89.6+/-14.7"	"0"
5	"96.3+/-11.2"	"82.3+/-16.9"	"0.001"
6	"106.7+/-11.4"	"137.8+/-14.8"	"0"
7	"114.2+/-5.1"	"112.9+/-6.9"	"0.212"

	TrigonalBipyramidalVA	TrigonalBipyramidalVP	SquarePyramidalV
1	"0.009"	"0"	"0"
2	"0.004"	"0.079"	"0.204"
3	"0.079"	"0"	"0"
4	"0.023"	"0.005"	"0.006"
5	"0.003"	"0.003"	"0.01"
6	"0"	"0.074"	"0.003"
7	"0.028"	"0.001"	"0.002"

	SquarePlanar
1	"0"
2	"0"
3	"0"
4	"0"
5	"0"
6	"0.069"
7	"0"

Table S118. Cluster members of 4-ligand combined metal, combined group

[1] "Cluster 1"
1A1I.A.201, 1A6Y.A.550, 2A66.A.401, 1A73.A.202, 4AA6.A.253, 2B3J.A.2001, 2C7A.A.1641, 2C7A.A.1642, 3CBB.A.1001, 4CIS.A.300, 1CYQ.A.601, 1CYQ.A.602, 2DRP.A.171, 2DRP.A.172, 1DSZ.A.1121, 1DSZ.A.1122, 1DSZ.B.1222, 3EQT.A.1, 4ESJ.A.301, 1F2I.G.1202, 1F4S.P.64, 1F4S.P.65, 1FFY.A.1001, 2FF0.A.1001, 2FF0.A.1002, 1G2D.C.303, 1G2F.C.303, 3G9M.A.526, 2GAT.A.67, 4GAT.A.67, 6GAT.A.67, 3GOX.A.301, 3GOX.A.302, 4GZN.C.203, 2HAN.A.351, 2HAN.A.352, 2HAN.B.353, 2HAN.B.354, 4HC9.A.401, 4HC9.A.402, 2HGH.A.191, 2HGH.A.192, 2HGH.A.193, 4HN5.A.601, 4HN6.A.602, 4HP3.C.202, 1HWT.D.138, 2I13.A.502, 2I13.A.503, 2I13.A.506, 1I3J.A.100, 2IHX.A.235, 2IHX.A.236, 2IVH.A.1577, 2JP9.A.132, 2JP9.A.133, 2JP9.A.134, 2JZW.A.56, 1K3X.A.501, 1K82.A.450, 2KAE.A.175, 1KB2.A.150, 1KB2.A.151, 3KDE.C.78, 2KMK.A.83, 2KMK.A.84, 2KMK.A.85, 3KMP.A.2, 1LAT.A.1514, 1LAT.A.1515, 4LJ0.A.501, 4LJ0.A.502, 4LJ0.A.503, 1LLM.C.301, 1LLM.C.302, 1LO1.A.195, 1LO1.A.196, 3LRR.A.1, 2LT7.A.701, 2LT7.A.702, 2LT7.A.703, 3M7K.A.143, 3M7K.A.144, 4M8O.A.1303, 3M9E.A.208, 3M9E.B.209, 4M9E.A.504, 4M9E.A.505, 4M9V.C.201, 1MEY.C.88, 1MEY.C.89, 1MEY.C.90, 3MLN.A.501, 4MTD.A.201, 3NCU.A.1, 4NDH.A.402, 2NLL.A.250, 2NLL.B.450, 2NLL.B.451, 2O6M.A.601, 3O9X.A.132, 3OD8.A.200, 2OFI.A.302, 4OLN.A.101, 4OLN.A.102, 4OND.A.101, 4OND.A.102, 4OOR.A.102, 2OPF.A.501, 3OYM.A.393, 1OZJ.A.145, 1P47.A.203, 4PZI.A.1101, 4PZI.A.1102, 4QEN.A.802, 4QEN.A.803, 4QEN.A.804, 4QEN.A.805, 3QMD.A.300, 3QMD.A.301, 4R2A.A.503, 4R2A.A.504, 4R2A.A.505, 4R2Q.A.503, 4R2S.A.501, 1R4O.A.526, 1TDZ.A.1001, 1TF3.A.102, 1TF3.A.2, 1TF3.A.3, 4TNT.A.701, 4TNT.A.702, 3TS2.A.1, 3TS2.A.2, 3U6P.A.300, 1UBD.C.501, 1UBD.C.502, 1UBD.C.503, 1UBD.C.504, 3UK3.C.968, 3VD6.C.501, 2XQC.A.1138, 2XQC.D.1141, 2YKG.A.927, 1YUI.A.64, 1ZAA.C.203, 1ZGW.A.500, 1ZNS.A.1500, 258L.A.500, 2A1K.A.1, 3A1B.A.1, 3A1B.A.2, 3A1B.A.3, 4A24.A.601, 4A24.A.602, 4A2C.A.1349, 4A2V.A.1000, 3A43.A.701, 4A46.A.65, 2A51.A.54, 2A51.A.55, 2A5H.A.421, 2A5H.B.421, 3A6G.A.301, 2A6H.D.7458, 2A6H.D.7412, 1A71.A.402, 1A7T.A.251, 2A8D.A.1230, 4ADN.A.1223, 1AF2.A.296, 2AFW.A.996, 2AFX.A.996, 2AFZ.A.391, 2AFM.A.391, 3AII.A.1001, 4AI5.A.200, 4AIA.A.200, 1AJB.A.451, 4AJX.H.1030, 2AKL.A.117, 3ALR.A.601, 3ALR.A.602, 1AM6.A.262, 2AP1.A.304, 2APS.A.400, 2AQP.A.201, 4AR9.B.1731, 4ARE.A.1790, 3ASK.A.501, 3ASL.A.3, 2ASH.A.400, 4AU7.A.1248, 4AUQ.B.1299, 4AUQ.B.1300, 3AVR.A.1502, 2AW1.A.262, 3AXS.A.401, 2AYD.A.369, 2AYJ.A.57, 4AY8.A.600, 4AYC.A.1484, 4AYC.A.1485, 1AZM.A.261, 2B0O.E.698, 2B3Z.A.1360, 2B44.A.400, 2B5W.A.800, 4B6D.A.1341, 2B8T.A.1218, 2B9D.A.1002, 1BBO.A.60, 1BBO.A.61, 1BCD.A.262, 3BET.A.262, 2BE7.D.1108, 4BF1.A.270, 4BF6.A.1262, 3BI0.A.1752, 3BI1.A.1752, 3BKN.A.202, 3BL0.A.262, 3BL1.A.262, 2BL6.A.1059, 4BM9.A.1466, 4BM9.A.1469, 1BN1.A.262, 1BN3.A.262, 1BN4.A.262, 1BNM.A.262, 1BNN.A.262, 1BNT.A.262, 1BNU.A.262, 1BNV.A.262, 1BNW.A.262, 3BO5.A.301, 3BO5.A.302, 3BO5.A.303, 3BO5.A.304, 3BOF.A.701, 3BOL.A.701, 2BP0.A.1341, 3BQ5.A.800, 3BQ6.A.800, 4BS9.A.1782, 4BUE.A.2162, 2BYO.A.1209, 1BZM.A.261, 2BZ1.A.1174, 4C09.A.351, 1C2G.A.409, 3C37.A.301, 4C3E.A.201, 4C3T.A.300, 4C4O.B.600, 3C5K.A.201, 3C5K.A.202, 3C5K.A.203, 4C5W.A.403, 3C63.A.107, 3C63.B.107, 3C63.C.107, 3C63.D.107, 3C6W.A.2, 2C6A.A.336, 2C6C.A.1752, 1C7K.A.133, 3C7P.A.262, 2C7N.A.499, 4C8E.A.1162, 1C9Q.A.999, 3CA2.A.264, 1CAI.A.262, 4CA1.A.283, 4CA1.A.284, 2CBD.A.262, 1CCT.A.262, 4CCG.X.1374, 4CCG.X.1375, 1CDO.B.377, 2CDC.A.1372, 4CDG.A.1643, 2CEX.C.1306, 2CFU.A.1002, 1CG2.A.502, 1CG2.C.502, 3CG7.A.299, 3CHQ.A.701, 3CHS.A.701, 3CHV.A.301, 1CIL.A.262, 1CIM.A.262, 1CIN.A.262, 2CJS.C.201, 2CJS.C.202, 2CKL.A.1104, 2CKL.A.1105, 2CKL.B.1115, 2CKL.B.1116, 2CKI.A.999, 1CL4.A.81, 1CNG.A.1, 1CNH.A.1, 1CNI.A.1, 1CNJ.A.1, 3CNG.A.508, 1CNW.A.262, 1CNX.A.262, 1CNY.A.262, 3COS.A.502, 2CO8.A.401, 2COR.A.401, 4COI.A.652, 4CPD.A.1200, 4CPD.A.1300, 4CQ0.A.1262, 2CQE.A.822, 2CRW.A.401, 2CR8.A.401, 2

CRC.A.401, 2CRR.A.401, 2CSV.A.200, 2CSV.A.400, 2CSZ.A.201, 2CSZ.A.401, 2CS2.A.200, 2CS3.A.200, 2CS3.A.400, 2CS7.A.201, 2CS8.A.401, 2CS8.A.601, 2CSH.A.200, 2CSH.A.300, 2CSH.A.400, 1CTT.A.296, 2CT0.A.401, 2CT1.A.201, 2CT1.A.401, 2CT2.A.401, 2CT5.A.201, 2CT7.A.201, 2CTD.A.201, 2CTD.A.401, 2CTU.A.201, 2CUP.A.401, 2CUP.A.601, 2CUR.A.401, 4CVR.A.1158, 3CXK.A.201, 3CXL.A.500, 3CXL.A.501, 1CXX.A.1, 4CYK.A.42, 1CZM.A.261, 3CZV.A.262, 1D0C.A.900, 1D0Q.B.151, 1D1S.B.376, 1D1T.A.375, 1D1T.A.376, 4D1N.A.900, 3D2N.A.101, 3D2Q.A.303, 3D2Q.A.304, 2D5B.A.501, 4D6S.A.1338, 3D7F.A.1752, 2D8X.A.201, 2D8X.A.401, 2D8Y.A.201, 2D8Y.A.401, 2D8Z.A.201, 2D8Z.A.401, 3D8W.A.262, 2D8Q.A.201, 2D8R.A.401, 2D8S.A.201, 2D8S.A.401, 2D8U.A.201, 2D9G.A.201, 2D9H.A.201, 2D9H.A.401, 2D9K.A.401, 2D9K.A.601, 2D9L.A.401, 3DAZ.A.262, 2DAR.A.401, 2DAS.A.101, 3DBH.B.1, 3DBK.A.302, 2DB6.A.201, 4DB3.A.401, 3DCC.A.262, 1DCQ.A.600, 1DD6.A.502, 1DD6.A.503, 3DD0.A.262, 3DD8.A.262, 1DDZ.A.2, 3DDT.A.46, 1DFE.A.38, 4DF9.A.503, 2DFV.A.1001, 3DGD.C.128, 3DI4.B.286, 2DID.A.201, 2DIP.A.201, 2DIP.A.401, 2DJ7.A.201, 2DJ8.A.201, 2DKT.A.391, 2DLK.A.201, 2DLK.A.401, 1DL6.A.60, 2DLO.A.201, 2DLO.A.401, 2DLQ.A.200, 2DLQ.A.300, 2DLQ.A.400, 2DLQ.A.500, 4DLA.A.402, 3DMO.A.131, 2DMD.A.191, 2DMD.A.241, 2DMI.A.200, 2DMI.A.300, 2DMJ.A.200, 2DOO.A.502, 2DPH.A.1001, 2DS7.A.100, 1DVP.A.401, 1DVP.A.402, 3DWD.A.501, 4DZ7.A.301, 1E0E.A.147, 3E1W.A.230, 2E2Z.A.101, 3E2I.A.200, 3E2U.E.102, 4E2X.A.501, 1E3J.A.901, 1E3J.A.902, 1E4U.A.79, 1E4U.A.80, 2E5R.A.201, 2E5R.A.401, 2E5S.A.201, 2E5S.A.401, 2E6R.A.201, 2E6R.A.401, 2E6S.A.201, 2E6S.A.401, 2E6S.A.601, 3E6U.B.503, 2E6I.A.201, 1E7L.A.1165, 2E73.A.201, 2E73.A.401, 1E9P.B.153, 2EA5.A.201, 2EA6.A.401, 2EBL.A.191, 2EBL.A.241, 2EBT.A.100, 2EBT.A.200, 2EBT.A.300, 2EBV.A.201, 3EB5.A.1001, 3EBE.A.500, 2EBQ.A.201, 2EBR.A.201, 2ECJ.A.201, 2ECJ.A.401, 2ECV.A.401, 2ECY.A.201, 2ECL.A.201, 2ECL.A.401, 2ECN.A.201, 2ECN.A.401, 2EER.A.501, 1EE2.A.1301, 1EE8.A.501, 3EED.A.194, 3EER.A.2004, 2EE8.A.301, 2EE8.A.701, 4EEZ.A.401, 1EF4.A.56, 3EFO.A.766, 3EFT.A.262, 2EGQ.A.200, 2EGQ.A.300, 2EGM.A.200, 2EGM.A.300, 2EGP.A.200, 2EGP.A.400, 4EGU.A.202, 3EH1.A.1269, 3EH2.A.800, 2EHE.A.200, 2EHE.A.300, 2EJ4.A.401, 2ELN.A.181, 2ELO.A.181, 2ELQ.A.181, 2ELT.A.181, 2ELY.A.200, 2ELZ.A.200, 2EL4.A.200, 2EL5.A.200, 2EL6.A.200, 2ELI.A.201, 2ELM.A.181, 2EMJ.A.201, 2EMI.A.201, 2EMY.A.201, 2EMZ.A.201, 2EM0.A.200, 2EM1.A.201, 2EM5.A.201, 2EM7.A.201, 2EM8.A.201, 2EMA.A.201, 2EME.A.201, 2EMG.A.201, 2EMH.A.201, 2EMK.A.201, 2EMM.A.201, 2EMP.A.201, 2ENT.A.200, 2ENV.A.200, 2ENV.A.300, 1EN7.A.401, 2ENZ.A.300, 2ENZ.A.400, 2EN1.A.201, 2EN4.A.201, 2EN6.A.181, 2EN7.A.181, 2EN8.A.181, 2ENF.A.181, 2ENN.A.300, 2ENN.A.400, 2EOR.A.201, 2EOS.A.201, 2EOU.A.201, 2EOV.A.201, 2EOX.A.201, 2EOY.A.201, 2EOZ.A.201, 1EOU.A.300, 2EO4.A.201, 2EOD.A.300, 2EOD.A.400, 2EOF.A.201, 2EOG.A.201, 2EOK.A.201, 2EOL.A.201, 2EOP.A.201, 2EPR.A.201, 2EPS.A.201, 2EPT.A.201, 2EPV.A.201, 2EPW.A.201, 2EPY.A.201, 2EPZ.A.201, 2EP0.A.201, 2EP1.A.201, 2EP2.A.201, 2EP3.A.201, 2EP4.A.200, 2EP4.A.300, 2EPA.A.300, 2EPA.A.400, 2EPC.A.201, 2EPP.A.201, 2EQW.A.201, 2EQ0.A.201, 2EQ1.A.201, 2EQ4.A.201, 2EQE.A.201, 2EQF.A.201, 2EQG.A.201, 1ESK.A.55, 2ESL.A.4, 4ETS.A.302, 1EU3.B.402, 2EU3.A.262, 4EVB.A.204, 2EXU.A.501, 1EXK.A.80, 1EXK.A.81, 4EYL.A.303, 1EZM.A.302, 2F0Y.B.501, 3F0D.A.163, 1F18.A.155, 1F1G.A.4002, 2F14.A.1262, 1F2W.A.262, 2F3B.A.341, 4F3W.A.201, 1F4T.A.369, 2F4M.A.501, 1F62.A.52, 1F62.A.53, 3F7B.B.301, 3F7L.A.203, 3F7U.A.260, 1F81.A.88, 1F8F.A.373, 1F9X.A.999, 2F9I.B.601, 4F9V.A.401, 1FAQ.A.2, 4FAI.A.401, 4FBE.A.403, 2FC6.A.201, 2FC7.A.201, 2FC7.A.401, 2FE3.A.201, 2FEA.A.1302, 3FFP.X.262, 2FGY.A.620, 2FGY.A.621, 2FHX.A.317, 3FID.A.298, 3FID.A.299, 2FIF.B.901, 4FKD.A.102, 4FKE.A.1024, 3FL2.A.1001, 3FL2.A.1002, 3FLO.B.1, 3FLO.B.2, 1FN9.A.1001, 2FOQ.A.262, 2FOS.A.262, 2FOU.A.262, 2FOV.A.262, 2FOY.A.301, 1FP0.A.90, 1FQL.A.262, 1FQM.A.262, 3FQM.A.901, 2FR5.A.147, 2FR6.A.147, 4FRC.A.302, 2FSA.A.501, 1FU9.A.37, 2FU9.A.401, 4FU5.A.302, 4FVD.A.201, 4FVN.A.302, 4FVO.A.302, 3FW3.A.300, 1FWQ.A.124, 4FWE.A.901, 4FWE.A.902, 2FYG.A.302, 4FYY.B.201, 2FZW.A.375, 3G1P.A.300, 1G25.A.66, 1G25.A.67, 3G27.A.97, 4G26.A.1001, 2G2N.A.1001, 1G48.A.262, 2G45.A.401, 1G52.A.262, 1G54.A.262, 2G6Q.A.400, 1G71.A.348, 4G7A.A.301, 2G84.A.506, 2G9T.A.999, 1G9K.A.600, 3GA3.A.1, 2GAG.D.101, 2GAH

.D.101, 1GDC.A.73, 1GDC.A.74, 2GD8.A.262, 2GFJ.B.401, 2GFO.A.1200, 4GGJ.A.301, 3GI1.A.501, 3GIQ.A.483, 4GIZ.C.201, 3GJ3.B.300, 3GJ4.D.300, 3GJ5.B.300, 3GJ7.D.300, 3GJ8.B.300, 2GMW.A.300, 4GNE.A.1502, 4GNE.A.1503, 4GNE.A.1504, 2GQJ.A.200, 2GQJ.A.300, 1GUP.A.350, 3GV4.A.203, 2GVI.A.301, 4GVE.A.601, 1GZH.A.1293, 3GZE.B.2, 3GZK.A.539, 3H0L.E.902, 3H0N.A.201, 4H0F.A.401, 2H15.A.262, 4H12.A.1801, 4H12.A.1802, 4H12.A.1803, 1H2B.A.1362, 4H3S.A.901, 3H5A.B.360, 3H5N.A.500, 2H6E.A.500, 3H7H.A.119, 1H7V.A.61, 3H8V.A.401, 3H99.A.601, 4H9D.A.201, 2HBA.A.101, 1HC7.A.490, 3HCI.A.1000, 1HCP.A.99, 3HCJ.A.1000, 3HCS.A.303, 4HCG.A.202, 1HDY.A.376, 2HD6.A.262, 2HDP.A.492, 2HDP.A.493, 4HEY.A.301, 2HF1.A.102, 3HI2.A.121, 4HI8.B.101, 4HI8.B.102, 2HJN.A.315, 2HJH.A.800, 3HKN.A.261, 3HKO.A.701, 3HKQ.A.261, 3HKT.A.261, 3HKU.A.261, 3HLJ.A.272, 2HL4.A.262, 3HNA.A.501, 3HNA.A.502, 3HNA.A.503, 3HNA.A.504, 3HNI.A.107, 2HNC.A.263, 2HOC.A.263, 2HQH.E.1500, 2HRV.A.143, 3HS4.A.301, 1HSO.A.1376, 4HSU.A.904, 1HTD.A.401, 4HT0.A.301, 4HT2.A.301, 4HTB.A.401, 1HUG.A.261, 4HU1.A.301, 2HU9.A.132, 1HXP.A.350, 1HXR.A.200, 1HY7.A.302, 2HZ8.A.117, 2IOO.A.579, 4I1F.A.503, 4I1F.A.504, 4I1F.A.508, 4I1H.A.507, 3I2D.A.1, 2I3H.A.1001, 3I4C.A.400, 3I4C.A.500, 1I5O.C.3002, 2I5O.A.336, 2I5O.A.336, 2I5O.A.337, 2I5O.A.338, 4I51.A.3005, 1I6P.A.301, 1I8Z.A.262, 2I9W.A.201, 1IA6.A.1264, 1IA9.A.2001, 1IBI.A.195, 1IBI.A.196, 3IBI.A.262, 3IBL.A.262, 3IBN.A.262, 3IBU.A.262, 2IBI.A.1, 4IBY.A.301, 4ICR.A.501, 2IDA.A.104, 1IF9.A.262, 3IFJ.A.201, 3IFJ.B.202, 3IGP.A.262, 2IGI.A.1001, 2IGI.A.1004, 4II1.A.901, 3IJF.X.147, 4ILO.A.301, 4ILX.A.301, 2IMZ.A.501, 2IMZ.B.502, 1IML.A.78, 3IMI.A.201, 1INN.B.167, 2INN.B.514, 3IO2.A.202, 2IOI.A.3001, 1IQ8.A.600, 2IQJ.A.301, 1IRN.A.55, 3IR9.B.501, 3IRB.A.201, 4ITO.A.301, 4ITP.A.302, 3IUF.A.1, 4IUM.A.501, 3IXE.B.301, 3IXE.B.302, 1IYM.A.182, 1IYM.A.183, 2IYB.E.1422, 2IYB.E.1423, 2J1Y.A.1290, 4J1V.A.301, 1J2O.A.115, 1J2T.A.302, 2J21.A.1289, 2J6A.A.1138, 2J7J.A.1088, 1J98.A.300, 2J9R.A.1194, 2J9U.B.1162, 1JAO.A.999, 1JAZ.A.401, 4JBG.A.401, 2JBG.B.1577, 1JD0.A.901, 1JD5.A.501, 4JE6.A.200, 4JEA.A.202, 4JEA.B.202, 4JEA.C.202, 4JEA.D.202, 4JIU.A.201, 4JIV.D.101, 1JJD.A.104, 1JJE.B.251, 1JJT.B.251, 4JLW.A.401, 2JM0.A.201, 2JM0.A.401, 2JM1.A.2, 2JM3.A.92, 4JMY.A.201, 2JMD.A.65, 1JN7.A.37, 2JOX.A.109, 2JOX.A.110, 1JOC.A.300, 2JQ5.A.129, 2JR7.A.85, 2JRJ.A.62, 2JRJ.A.63, 4JSR.A.401, 2JTG.A.88, 1JTK.A.137, 3JUE.A.999, 2JUN.A.220, 2JVX.A.29, 1JVB.A.400, 1JVB.A.500, 3JV7.A.501, 3JV7.A.502, 3JVH.A.163, 2JVN.A.400, 2JWO.A.488, 2JWO.A.489, 3JWP.A.2001, 2JW6.A.601, 3JXP.A.320, 4JXE.A.501, 4JXE.A.502, 1JY8.A.300, 1JZS.A.1101, 2JZ8.A.150, 4JZ0.A.802, 2K0A.A.108, 2K0A.A.109, 2K0A.A.110, 2K1P.A.96, 2K16.A.940, 2K16.A.941, 3K2F.A.262, 1K2Y.X.500, 2K2C.A.138, 2K2C.A.139, 2K2C.A.141, 2K2C.A.142, 2K2C.A.143, 2K2D.A.80, 4K2H.B.201, 3K34.A.1002, 3K35.C.317, 2K4X.A.56, 3K5K.A.1194, 3K5K.A.1197, 3K5K.B.1195, 3K5K.B.1196, 2K5C.A.96, 3K6I.A.202, 1K7I.A.486, 3K7H.B.1001, 2K7R.A.129, 4K7D.A.501, 4K7D.A.502, 4K7D.A.505, 4K7D.A.507, 4K7D.A.508, 4K7W.B.101, 1K81.A.144, 2K9H.A.101, 2K9H.A.102, 2KAK.A.130, 2KAK.A.150, 2KAK.A.170, 3KB1.A.302, 3KBF.A.159, 2KDP.A.1, 2KDX.A.120, 3KE1.A.163, 3KEE.A.2000, 2KFN.A.1, 2KGG.A.53, 2KGG.A.54, 2KGO.A.109, 1KHK.A.451, 3KHI.A.301, 2KI7.B.124, 2KJE.A.501, 2KJE.A.502, 2KKT.A.500, 2KKH.A.201, 2KKR.A.500, 1KLR.A.31, 1KLS.A.31, 3KNE.A.500, 3KNV.A.201, 3KNV.A.202, 4KNI.A.301, 4KNJ.A.301, 4KNM.A.301, 1KOL.A.1002, 2KPI.A.150, 4KP8.A.301, 3KQI.A.71, 3KQI.A.72, 2KQ9.A.113, 2KQB.A.1001, 2KR1.A.65, 2KU3.A.63, 2KU3.A.64, 4KUJ.A.301, 4KUV.A.301, 4KUW.A.301, 4KUY.A.301, 3KV4.A.448, 3KV5.A.489, 3KV5.A.490, 4KV0.A.301, 1KWG.A.806, 1KWQ.A.262, 2KWJ.A.501, 2KWJ.A.601, 2KWJ.A.701, 2KWJ.A.801, 4KXQ.A.601, 2KZY.A.63, 2L0Z.A.486, 3L00.A.180, 4L05.A.203, 3L11.A.601, 3L11.A.602, 3L14.A.262, 3L22.A.1, 4L3J.A.402, 2L5U.A.62, 2L5U.A.63, 4L56.A.401, 4L58.A.102, 2L6Y.A.239, 2L6Z.B.37, 1L6J.A.500, 2L6L.A.201, 2L6M.A.201, 4L6H.A.801, 4L6T.A.301, 2L7X.A.106, 2L7X.A.107, 2L75.A.155, 2L75.A.156, 2L7P.A.201, 4L7X.A.101, 2L80.A.124, 2L9Z.A.403, 3LAS.A.167, 2LAU.A.82, 2LBM.A.1, 2LBM.A.3, 4LBU.A.402, 3LCZ.A.54, 2LCE.A.200, 2LCE.A.300, 2LCQ.A.162, 4LEF.A.302, 2LGV.A.109, 2LGG.A.380, 2LGG.A.381, 2LGG.A.382, 2LHN.A.501, 2LHN.A.502, 2LHN.A.503, 4LHI.A.301, 1LIQ.A.28, 2LI8.A.187, 2LI8.A.188, 2LI9.A.18, 4LIM.A.401, 2LJX.A

.200, 2LJZ.A.201, 3LJU.X.401, 4LJO.A.1101, 4LJO.A.1102, 4LJO.A.1104, 4LJP.A.1101
, 3LKM.A.904, 2LKO.A.32, 2LK5.A.37, 4LK9.A.401, 3LMI.B.1002, 4LMY.A.202, 2LNO.A.
401, 2LNO.A.501, 2LNO.A.601, 2LNO.A.701, 2LO2.A.101, 2LO3.A.101, 2LO4.A.300, 4LO
9.A.401, 4LOF.A.401, 1LPV.A.54, 3LQH.A.1002, 4LQG.A.802, 3LRQ.C.100, 2LRI.C.101,
 2LRI.C.102, 3LSC.A.458, 3LT8.A.80, 2LUA.A.101, 2LUA.A.102, 2LUA.A.103, 4LU3.A.3
01, 1LV3.A.66, 2LV2.A.101, 2LV2.A.102, 2LV9.A.201, 2LV9.A.202, 2LWW.A.501, 2LWW.
A.502, 2LWW.A.503, 4LW9.A.203, 4LW9.C.202, 4LW9.K.203, 3LXE.A.261, 2LXD.A.201, 2
LXD.A.202, 4LXL.A.403, 3LYR.A.1, 2LZU.A.201, 2LZU.A.202, 3M04.A.501, 2M0E.A.101,
 2M0F.A.101, 4M0W.A.401, 2M1S.A.100, 3M14.A.501, 3M1D.A.1000, 2M13.A.601, 2M13.A
.602, 3M1W.A.500, 1M2K.A.999, 1M2O.A.800, 3M2N.A.263, 3M2X.A.500, 3M2Y.A.500, 4M
2R.A.301, 4M2V.A.301, 2M3Z.A.102, 1M3V.A.123, 3M3X.A.262, 2M3L.A.201, 4M3O.A.300
, 4M3P.A.701, 3M40.A.262, 3M4C.B.108, 2M48.A.501, 2M48.A.503, 3M5E.A.262, 3M67.A
.263, 3M6I.A.402, 2M6M.A.201, 2M6M.A.202, 2M7Q.A.101, 2M7Q.A.102, 3M79.D.107, 2M
7A.A.100, 2M85.A.801, 2M85.A.802, 2M9Y.A.401, 2M9Y.A.402, 3M96.A.262, 3M98.A.262
, 2M9A.A.101, 2M9A.A.103, 2M9A.A.102, 2MA5.A.101, 2MA5.A.102, 2MA6.A.101, 2MA6.A
.102, 3MBG.A.3, 3MBG.A.1, 1MBX.B.211, 3MDZ.A.263, 2MD7.B.101, 2MD7.B.102, 2MDG.A
.101, 2MDG.A.102, 4MDM.A.301, 1MEA.A.29, 3MEK.A.500, 3MEK.A.501, 3MEK.A.502, 3ME
Q.A.501, 4ME3.A.301, 1MGO.A.376, 4MG3.A.201, 3MHC.A.262, 3MHH.E.97, 3MHI.A.262,
3MHL.A.262, 3MHM.A.262, 3MHS.A.472, 3MHS.A.473, 3MHS.A.474, 3MHS.A.475, 3MHS.A.4
76, 3MHS.A.477, 4MHN.A.400, 4MHQ.A.501, 4MHY.A.400, 4MI5.A.802, 4MI5.A.803, 4MI5
.A.804, 4MI5.A.805, 4MI5.A.806, 4MI5.A.807, 2MIU.A.302, 3MJH.B.70, 4MJ7.A.201, 2
MKD.A.301, 1ML9.A.1, 1ML9.A.2, 1ML9.A.3, 3ML5.A.263, 1MM3.A.62, 1MM3.A.63, 3MMF.
A.262, 2MNY.A.401, 2MNY.A.402, 3MNA.A.262, 3MND.A.153, 1MNC.A.282, 3MNU.A.262, 4
MO8.A.301, 3MPZ.A.300, 3MP2.A.1, 1MR1.C.601, 2MRE.B.301, 1MS7.B.3001, 4MSG.A.140
1, 4MSX.A.501, 4MT2.A.67, 4MT2.A.68, 4MTY.A.301, 2MUM.A.301, 2MUM.A.302, 2MUQ.A.
101, 1MVH.A.501, 3MWM.A.140, 2MWX.A.201, 1MXG.A.437, 3MYQ.A.262, 1MZ8.B.600, 3MZ
C.A.262, 4N0N.A.501, 4N0N.A.503, 1N0Z.A.46, 4N0X.B.301, 3N2P.A.262, 3N3J.A.262,
3N3K.A.1, 4N4F.A.1402, 3N4B.A.262, 1N5N.A.401, 3N67.A.900, 1N8K.A.376, 3NA7.A.30
0, 3NB5.A.261, 1NCS.A.61, 1NEE.A.136, 3NHE.A.1, 3NIS.A.1, 3NIS.A.2, 3NIS.A.3, 3N
IT.A.2, 4NJ5.A.801, 1NKU.A.188, 3NKM.A.1001, 4NL4.H.803, 2NMX.A.301, 2NNV.A.262,
 2NN1.A.301, 2NN7.A.301, 4NN2.A.401, 4NN2.A.402, 4NN2.A.403, 2NNG.A.262, 2NNO.A.
262, 4NOS.A.3000, 3NQ5.A.503, 4NQ4.A.302, 4NQ5.A.302, 4NQ6.A.301, 4NQ7.A.301, 4N
QY.A.501, 4NS5.A.401, 1NTO.A.500, 2NYT.A.2000, 3NY1.A.4, 3NY1.A.5, 3NY1.A.6, 3NY
3.A.1, 3NY3.A.2, 3NY3.A.3, 1NZJ.A.700, 4NZG.A.201, 3O0M.A.151, 2O03.A.202, 2O10.
A.86, 2O10.A.87, 2O13.A.190, 2O13.A.191, 3O36.A.1, 3O36.A.2, 2O3K.A.401, 2O4Z.A.
262, 3O4N.A.2002, 4O62.A.1001, 4O64.A.2001, 4O64.A.2002, 4O64.A.2004, 4O6I.A.601
, 3O70.A.500, 3O70.A.501, 3O7A.A.500, 3O7A.A.501, 4OAQ.A.403, 3OCA.A.300, 3OCQ.A
.184, 4ODR.A.202, 4ODR.B.202, 2OFK.A.201, 2OGW.A.500, 4OGE.A.1201, 1OHL.A.400, 3
OIL.A.262, 2OIK.B.201, 4OIF.A.701, 3OJ6.A.150, 3OJ7.A.114, 1OKL.A.262, 1OKM.A.26
2, 2OM1.B.801, 3OOI.A.232, 3OOI.A.233, 3OOI.A.234, 1OQ5.A.600, 3OQ6.A.375, 1OQJ.
A.183, 3ORU.A.250, 2OSM.A.262, 2OSF.A.262, 2OU2.A.490, 2OUI.A.361, 1OVX.A.61, 2O
WA.A.201, 4OWF.G.401, 1OX7.A.402, 1OXN.A.1001, 3OXF.A.440, 3OY0.A.262, 1OYW.A.80
1, 3OYQ.A.262, 3OYS.A.262, 1OZB.I.50, 2OZU.A.800, 1P0F.A.2502, 2P09.A.200, 3P1V.
A.427, 3P2A.C.151, 3P3H.A.261, 3P3J.A.261, 3P44.A.261, 3P55.A.261, 3P58.A.262, 2
P57.A.201, 1P6O.A.401, 1P7A.A.38, 3P8B.A.101, 1P9R.A.601, 1PBO.A.1301, 3PB4.X.40
0, 3PB7.X.400, 3PB8.X.400, 3PB9.X.400, 3PBE.A.391, 1PCX.A.950, 1PEG.A.4, 1PFT.A.
51, 1PG5.B.500, 4PHT.B.601, 3PII.A.340, 3PJN.A.187, 3PJN.A.188, 3PLW.A.187, 3PLW
.A.188, 2PLI.D.702, 3PN3.A.1001, 2POU.A.262, 2POW.A.262, 3PO6.A.1, 2POI.A.100, 2
PPT.A.300, 1PQ4.A.1002, 4PQ7.A.301, 2PQ8.A.501, 4PQT.A.501, 2PRS.A.501, 3PT9.A.1
, 3PT9.A.2, 3PTM.A.1001, 1PTR.A.1, 4PTB.A.902, 2PUY.A.356, 3PU7.A.155, 3PUQ.A.2,
 3PUR.A.3, 2PVX.A.901, 2PVE.A.301, 3PYK.A.262, 4PYX.A.301, 1PZW.A.100, 4PZH.A.30
2, 1Q08.A.401, 1Q08.A.402, 1Q08.B.403, 1Q08.B.404, 3Q01.A.1, 1Q0E.A.153, 4Q09.A.
301, 3Q1D.A.201, 3Q1D.A.202, 2Q1Q.A.262, 1Q2L.A.963, 2Q2L.A.1001, 1Q3A.A.465, 1Q
68.A.201, 1Q69.A.207, 4Q6D.A.301, 4Q6E.A.301, 3Q7C.A.1, 4Q7R.A.302, 4Q7R.B.302,

3Q87.A.126, 1QBH.A.364, 1QF8.A.216, 4QF2.A.1801, 4QF2.A.1802, 4QF3.A.2001, 4QF3.A.2002, 3QGV.A.500, 2QIC.A.300, 2QIC.A.400, 2QKD.A.501, 3QL9.A.1, 3QL9.A.3, 2QN0.A.431, 4QN1.A.1501, 2QO8.A.262, 2QOA.A.262, 2QP6.A.262, 4QQ4.A.2001, 4QSI.A.301, 4QSJ.A.301, 3QU1.A.501, 3QU1.A.503, 3QU1.A.505, 3QU1.B.502, 1QWY.A.400, 3QWP.A.500, 3QWP.A.501, 1QYB.A.401, 1QYP.A.58, 3QYK.A.262, 3R0D.A.428, 3R16.A.262, 3R17.B.262, 4R1X.B.501, 3R2N.A.135, 4R2Y.A.101, 4R2Y.A.102, 4R2Y.A.103, 4R2Y.B.102, 2R3A.A.300, 2R3A.A.301, 2R3A.A.303, 1R5T.A.150, 3R6F.A.132, 1R79.A.201, 1R79.A.401, 1R9P.A.135, 1RAY.A.262, 3RBU.A.1752, 2RGV.A.146, 1RGO.A.221, 1RGO.A.222, 2RHQ.A.1, 2RHK.C.502, 2RI7.A.501, 2RI7.A.502, 3RIY.A.1001, 3RJ7.A.300, 1RJW.A.401, 4RL0.A.301, 1RMD.A.117, 1RMD.A.118, 3RMQ.A.114, 4RM5.D.300, 1RNI.A.256, 4RN4.A.301, 2RO1.A.201, 2RO1.A.301, 2ROW.A.601, 2ROW.A.602, 2RPP.A.401, 3RQD.A.390, 4RQT.A.401, 2RR4.A.501, 3RSN.A.200, 2RSH.A.101, 2RSI.A.102, 2RSI.A.103, 2RSJ.A.101, 2RT9.A.701, 2RT9.A.702, 3RUI.A.1, 1RUT.X.603, 1RUT.X.604, 4RUW.A.501, 4RV9.A.501, 1RXR.A.213, 1RXR.A.214, 3RYM.A.106, 3RYV.B.262, 3RYX.B.262, 3RYY.A.1, 3RYZ.A.1, 3RYJ.B.262, 3RZV.A.2, 3RZ0.B.262, 3RZ1.B.262, 3RZ5.A.1, 3RZ7.A.1, 3RZ8.A.1, 3S2Q.B.501, 3S2E.A.500, 3S2E.A.501, 1S3G.A.219, 3S71.B.262, 3S72.B.262, 3S73.B.262, 3S75.B.262, 3S76.A.1, 3S77.B.262, 3S8P.A.400, 3S9T.A.262, 3SAX.A.262, 3SAP.A.262, 3SBH.A.262, 3SBI.A.262, 3SD9.A.2, 1SE0.A.201, 3SI2.A.601, 3SJG.A.1752, 1SLM.A.257, 3SOU.A.7, 3SOU.B.8, 3SP4.A.601, 1SRK.A.36, 1SU3.A.913, 3SUB.A.161, 1SVM.A.700, 3SWR.A.3, 1SX1.A.23, 1T4W.A.201, 3T5U.A.262, 3T6P.A.1001, 3T6P.A.1002, 3T6P.A.1003, 3T6R.A.1, 3T6R.A.3, 3T7E.A.252, 3T7L.A.1, 3T7L.A.2, 1T8H.A.275, 3T80.D.301, 3T82.A.261, 3T84.A.261, 3T85.A.261, 3T90.A.502, 1T9H.A.411, 3T92.A.122, 3T92.A.124, 1TAF.A.2003, 1TEQ.X.262, 1TFI.A.51, 3TGN.A.147, 3TG4.A.435, 3TG4.A.436, 3TG4.A.437, 3TIO.B.185, 3TIO.D.185, 3TIO.E.185, 1TJL.A.200, 3TMJ.A.262, 1TOT.A.53, 1TOT.A.54, 1TT5.B.1014, 3TTC.A.1, 3TTC.A.2, 4TWJ.A.301, 3TWO.A.349, 4TYT.A.302, 4TZU.A.503, 1U0A.A.5005, 3U1L.A.241, 1U2N.A.441, 1U3T.A.375, 1U3U.A.375, 1U3W.A.375, 3U31.A.276, 4U4L.A.302, 1U5K.A.300, 1U5S.B.138, 3U52.A.514, 3U5N.A.2, 1U85.A.34, 1U86.A.36, 3U9G.A.226, 3U9G.A.227, 3U9G.A.228, 3U9G.A.229, 1UAQ.A.200, 3UCJ.A.228, 3UCM.A.228, 3UCO.A.228, 1UD9.C.509, 3UDZ.B.800, 3UEH.A.143, 3UEY.A.4, 3UEE.A.143, 3UEI.A.143, 3UEJ.A.301, 3UEJ.A.302, 4UF0.A.2269, 3UFF.A.1, 3UGD.A.2, 3UK0.A.400, 1UL4.A.139, 3UNG.C.903, 3UNT.A.400, 4UOV.A.298, 4UP0.A.1383, 4UP0.A.1384, 1USN.A.257, 2USN.A.257, 4UTV.A.1299, 1UUF.A.401, 3UVC.A.301, 3UVC.B.303, 3UVI.A.387, 2UVL.A.1336, 3UW4.A.401, 3UWA.A.200, 1UX1.A.1132, 3UX8.A.1001, 2UZG.A.132, 2UZG.A.133, 2V08.A.1090, 2V0C.A.1815, 2V0C.A.1816, 4V0R.A.1001, 3V1E.A.102, 3V1F.A.704, 3V1F.B.703, 4V1T.A.776, 2V29.A.1276, 4V2W.A.502, 4V2Y.A.150, 3V3G.B.301, 1V47.A.601, 3V4K.A.2, 1V4P.A.1001, 1V51.A.602, 1V54.F.99, 1V5R.A.201, 3V5G.A.262, 1V6G.A.401, 3V7X.A.2001, 1V9E.B.260, 1V9X.A.200, 2V9E.A.1276, 2V9E.B.1276, 2V9K.A.1533, 3VBD.A.2001, 1VDD.D.233, 3VDP.A.201, 1VFY.A.300, 1VFY.A.301, 2VF7.A.1845, 1VGN.A.302, 3VGL.A.322, 1VHH.A.400, 3VHS.A.51, 3VHS.B.51, 3VHT.B.401, 1VJ0.A.400, 1VJE.A.167, 2VJE.B.1491, 2VKR.A.106, 2VM5.A.1245, 2VNF.A.1247, 3VOV.A.401, 2VO9.B.501, 3VPB.E.100, 2VP7.A.1399, 2VPD.A.1399, 2VPG.A.1400, 2VPG.A.1401, 1VQ0.A.300, 2VQM.A.1412, 1VQ2.A.701, 2VQG.C.1091, 2VRS.A.1328, 2VRW.B.1565, 2VRW.B.1566, 2VR6.A.1156, 2VRD.A.1062, 1VSR.A.201, 3VTH.A.801, 3VTH.A.802, 3VUW.E.801, 2VVB.X.1268, 1VZY.A.1291, 2W0T.A.125, 2W0D.A.1269, 2W3N.B.1234, 1W4R.A.400, 2W4L.A.1170, 2W5X.A.1378, 2W5Z.A.4970, 1W5O.A.1339, 3W5K.B.504, 4W6Z.A.402, 1W8P.B.1030, 1WAA.A.1090, 4WAJ.A.301, 2WB0.X.601, 2WB0.X.602, 2WCB.A.100, 2WCB.B.100, 2WD2.A.1262, 2WD3.A.1263, 4WD8.B.303, 4WD8.C.303, 2WEJ.A.1262, 1WE9.A.401, 1WEE.A.201, 1WEE.A.401, 1WEQ.A.201, 1WEW.A.201, 1WEW.A.401, 2WEH.A.1262, 2WEO.A.1262, 1WFE.A.201, 1WFE.A.401, 1WFF.A.201, 1WFH.A.201, 1WFH.A.401, 1WFK.A.200, 1WFL.A.201, 3WF8.A.401, 1WFZ.A.201, 2WGX.A.1300, 1WGE.A.201, 3WID.A.1001, 1WIR.A.201, 1WJ2.A.470, 2WJV.A.1, 1WJP.A.701, 1WJV.A.401, 1WKQ.B.202, 1WN5.A.1001, 3WNQ.A.501, 1WO3.A.26, 1WO4.A.26, 3WRG.A.702, 3WS6.C.201, 1WUQ.A.1001, 2WVJ.A.1193, 1WWD.A.57, 3WWL.A.102, 1WWR.D.204, 1WYS.A.401, 1X0T.A.150, 4X2Z.A.400, 1X31.D.1006, 1X3Z.A.999, 1X4I.A.201, 1X4I.A.401, 1X4J.A.201, 1X4J.A.401, 1X4L.A.201,

1X4L.A.401, 1X4S.A.201, 1X4S.A.401, 1X4U.A.201, 1X4V.A.401, 1X4W.A.201, 1X4W.A.401, 1X5W.A.201, 2X5R.A.1126, 1X61.A.401, 1X62.A.401, 1X63.A.201, 1X63.A.401, 1X64.A.201, 1X64.A.401, 1X6F.A.201, 1X6M.A.200, 2X7S.A.1265, 2X7T.A.1263, 2X7U.A.1261, 2X7M.A.1175, 1XAF.A.501, 1XBO.A.403, 2XB4.A.1224, 2XBL.A.1197, 1XCR.A.1001, 2XCM.E.1222, 2XCM.E.1223, 2XEU.A.1065, 1XEG.A.262, 1XER.A.106, 1XF7.A.30, 4XIW.A.401, 2XIG.A.1151, 1XJH.A.63, 1XKI.A.1003, 1XOX.A.999, 2XOC.A.993, 2XOC.A.994, 2XOC.A.995, 2XOC.B.992, 1XPA.A.220, 1XPG.A.1887, 1XQ0.A.262, 1XRU.A.501, 1XSO.A.152, 1XTM.B.501, 1Y02.A.161, 1Y02.A.162, 2Y1N.A.1436, 2Y1N.A.1437, 2Y43.A.1097, 2Y43.A.1098, 2Y6D.A.1267, 1Y8Q.B.642, 2YB5.A.1213, 1YC5.A.1001, 2YHO.A.1001, 2YHO.A.1002, 2YHW.A.1719, 2YHY.A.2000, 2YIK.A.1533, 1YOP.A.84, 1YQD.A.2000, 2YQL.A.401, 2YQM.A.201, 2YQM.A.401, 2YQP.A.201, 2YQQ.A.201, 2YRJ.A.200, 2YRT.A.201, 2YRT.A.401, 2YRC.A.200, 2YRD.A.200, 2YRE.A.401, 2YRE.A.501, 2YRE.A.601, 2YRE.A.701, 2YRG.A.201, 2YRG.A.401, 2YRH.A.200, 2YRK.A.201, 2YSJ.A.201, 2YSJ.A.401, 2YSV.A.201, 2YS2.A.201, 2YSL.A.401, 2YSM.A.301, 2YSM.A.501, 2YSM.A.701, 2YSM.A.901, 2YSO.A.181, 2YTH.A.201, 2YTG.A.201, 2YTP.A.181, 2YTR.A.201, 2YTS.A.201, 2YT5.A.401, 2YT9.A.201, 2YT9.A.203, 2YTD.A.201, 2YTE.A.201, 2YTF.A.201, 2YTI.A.201, 2YTJ.A.201, 2YTK.A.201, 2YTM.A.181, 2YTN.A.201, 2YTO.A.201, 2YUU.A.201, 2YU4.A.201, 2YU8.A.201, 2YUC.A.201, 2YUC.A.401, 2YVR.A.1001, 2YVR.A.1002, 2YWW.A.504, 2YYR.A.401, 2YYR.A.402, 2YZ3.A.301, 2YZ5.B.1501, 1Z05.A.406, 2Z2S.B.204, 2Z2Y.A.2001, 2Z3H.A.2001, 2Z3G.A.2001, 1Z3A.A.301, 2Z3J.A.2001, 2Z45.B.1004, 1Z5H.A.2001, 1Z60.A.2, 1Z6U.A.2, 1Z83.B.642, 1Z84.A.604, 1Z8R.A.151, 2Z9J.A.902, 1Z9Y.A.300, 2Z94.A.901, 2Z9L.A.701, 2ZCO.B.408, 1ZE8.A.263, 2ZED.A.391, 2ZEE.A.391, 2ZEF.A.391, 2ZEG.A.391, 2ZEH.A.391, 2ZEL.A.391, 2ZEM.A.391, 2ZEN.A.391, 2ZEO.A.391, 1ZFD.A.71, 1ZFK.A.1300, 3ZFJ.A.1159, 3ZFK.A.401, 1ZFQ.A.600, 1ZGE.A.1000, 1ZGF.A.400, 3ZGO.A.400, 1ZH1.A.199, 1ZIN.A.219, 3ZME.A.313, 1ZNB.A.1, 1ZNF.A.27, 3ZNF.A.31, 3ZNI.A.1428, 3ZNI.A.1429, 1ZNM.A.29, 5ZNF.A.31, 7ZNF.A.31, 2ZNR.A.1, 2ZNR.A.2, 1ZP5.A.999, 3ZP9.A.1009, 3ZPC.B.401, 1ZR9.A.117, 1ZRP.A.54, 1ZSB.A.262, 1ZSC.A.262, 3ZTG.A.1336, 3ZTG.A.1337, 1ZU1.A.129, 1ZU1.A.130, 1ZVX.A.999, 3ZVS.A.1160, 1ZW8.A.66, 1ZW8.A.67, 1ZY7.A.801, 3ZYQ.A.1222, 3ZYQ.A.1223, 2ZZE.A.753, 2ZZF.A.754, 2FLC.A.248, 4FLW.A.802, 1YJW.7.8044, 4OKK.A.204, 3OPK.C.401, 1B2O.B.55, 1B71.A.192, 1BE7.A.55, 3D3L.A.801, 3E2T.A.1, 1FZO.A.5002, 3GKE.A.501, 2JI3.A.1127, 4K9F.A.101, 4KUO.D.101, 3N9Y.D.151, 3NAO.D.150, 2OHJ.A.511, 1SHR.B.801, 1T9O.A.255, 1VCX.A.54, 2WLB.B.619, 1YK5.A.54
[1] "Cluster 2"
3IE1.D.442, 1V15.A.1132, 2A97.B.2437, 3AF5.A.665, 1ANI.A.450, 2ANH.A.451, 2ANU.B.405, 2APO.B.501, 3B1B.A.378, 1BAW.A.107, 1BH5.A.201, 1BQQ.M.289, 3COZ.A.101, 2CBN.A.402, 3CE9.B.400, 2CEA.B.1606, 2CG3.Z.1, 3CHP.A.701, 2CIH.A.212, 3D3X.A.428, 1DSQ.A.144, 1DXW.A.301, 4EGE.A.412, 3EII.D.301, 2EIM.C.262, 1ENQ.C.238, 3EYV.L.220, 4FOR.A.501, 1F5F.A.252, 3FGG.A.161, 3GJN.B.600, 4H00.A.601, 2H42.A.501, 3H66.B.500, 3H90.D.5, 3HDB.A.620, 2HD1.B.101, 4IGM.A.401, 4IGM.F.401, 3II1.A.571, 2IMC.A.600, 3ITM.A.1, 1JDI.A.301, 1JM7.B.143, 1JOE.A.205, 2K2G.A.2, 1KAR.A.501, 1KAR.B.502, 2KO8.C.54, 1KQ0.A.479, 2LOZ.A.487, 3L8Y.A.301, 1L9Y.A.2, 3LZE.A.201, 2M7Y.A.101, 3MKV.A.425, 4MLX.A.301, 2MQ1.A.101, 4MZ7.A.701, 4N07.B.306, 4NRZ.B.301, 1NYR.A.1002, 2O36.A.690, 3O90.A.192, 1OLP.A.1373, 1OLP.A.1375, 1OLP.D.1374, 2OXW.A.264, 1P5X.A.247, 2PJT.D.302, 3PJN.A.189, 2PTW.A.500, 3Q31.A.1, 1Q74.D.304, 2QFP.A.434, 1QX1.A.2004, 3R2J.A.301, 3RAM.D.998, 1RO9.A.529, 3S6L.B.185, 3SFW.A.502, 1SMP.A.472, 1T0A.B.760, 3T02.A.502, 1TF9.A.901, 4TQT.A.501, 3TVX.A.900, 3UBF.A.6, 1UDV.A.101, 1UR6.B.79, 1UUP.D.5222, 1UXA.C.1367, 3V94.E.702, 2VXX.D.201, 3WI2.B.801, 4X2T.D.701, 4X2T.D.702, 2XR1.A.1638, 2XR1.A.1639, 2XY9.A.1628, 1YIX.A.601, 1Z3J.A.264, 1ZKN.C.601, 3ZNR.B.101, 3ZNS.A.101, 2ZNE.B.992, 1ZSW.A.315, 3E54.A.702, 3ICE.B.1001, 2PYJ.A.9004, 3S14.A.2001, 1V14.C.1134, 2W9C.B.1342, 2AEK.A.804, 2AKZ.A.440, 2AQX.A.600, 4AVQ.C.902, 2BM1.A.1690, 3BPD.G.126, 1BR2.A.997, 3BU5.A.301, 3CNX.C.170, 3CR3.B.1213, 2CW6.A.401, 4CYI.B.1000, 1D1C.A.998, 3D19.D.301, 2D33.C.2525, 2DEJ.A.402, 2DH4.A.341, 3DLS.C.11, 3E4O.A.501, 2E6B.A.301,

2E8W.B.1203, 2E92.B.1304, 2E95.A.1301, 3F74.C.1, 3FA4.A.401, 2FDR.A.1001, 4FMA.F.402, 3FYY.B.402, 3G2F.B.901, 2G74.A.185, 3G9D.B.299, 2GHT.B.257, 2GHQ.B.257, 2GWC.A.1, 3H8A.C.1431, 3HBO.A.701, 1HBN.A.1558, 4HNS.A.201, 3HQP.M.502, 2HT6.B.444, 2I5R.B.302, 3IAP.A.3001, 2ICJ.A.400, 2IK2.A.290, 4IL6.C.505, 4IL6.C.512, 4IL6.b.616, 4IL6.c.506, 1IV2.F.1574, 4J99.D.803, 2JK1.A.1144, 3KA9.A.189, 1KK8.B.998, 1LOO.A.602, 4LCZ.A.316, 4LRT.A.403, 1LVH.A.801, 3M1Y.D.300, 4M1W.A.201, 4MFE.D.1105, 3MG8.G.241, 4MKS.A.502, 4MPO.G.203, 4MPO.G.204, 3MWC.A.400, 1N5K.B.413, 1NEL.A.438, 3NIZ.A.312, 1NV3.A.2341, 2O1U.A.302, 2O56.F.2006, 2OI5.B.5000, 3OPS.D.501, 4OVN.B.202, 4POV.A.404, 1P7L.A.388, 4PFQ.C.400, 4PRV.A.402, 2PUN.B.401, 2Q58.A.4, 1Q8Y.A.809, 4QJB.A.301, 3QU9.A.227, 4QVY.K.302, 4QXD.B.302, 4QXD.B.304, 4R3A.A.400, 2R5T.A.600, 2RIO.A.1102, 1RLT.C.807, 3RYW.B.2003, 4S17.D.501, 3SY8.A.401, 3SZ5.A.220, 3TW6.D.2002, 4UON.B.401, 3UGJ.A.2006, 4UM8.B.2001, 4UUX.A.401, 2VON.B.502, 2VHQ.A.1328, 3VI4.B.502, 3VTI.C.401, 3VYT.C.601, 1W9L.A.1748, 3W9T.B.511, 3WEG.A.402, 3WEJ.A.402, 3WGU.C.2003, 1WQA.A.456, 3WU2.B.616, 3WU2.C.511, 3WU2.C.513, 1XMJ.A.2, 1YXO.A.5001, 2Z2P.A.1001, 1Z5B.B.2003, 1ZCW.A.501, 2ZCQ.A.453, 2ZEV.A.1302, 2ZEV.B.1304, 1ZH4.A.201, 2ZRW.D.702, 2ZRY.D.702, 1ZYK.A.702, 4BXO.A.2050, 2C28.A.1344, 4EFJ.C.101, 4EFJ.A.401, 2GIH.B.401, 4K4H.M.605, 4K4I.E.603, 4K4I.E.606, 4KHW.A.1005, 1M5X.C.801, 3M9M.B.354, 2NOF.A.328, 3OOR.A.236, 3OOR.A.237, 4QOW.B.1001, 3RBD.A.415, 3SQ2.A.906, 3V20.A.302, 2WTF.A.1512, 1A85.A.996, 1AEI.D.317, 1AEI.A.317, 4AG4.A.5002, 1ALA.A.401, 2AZ1.D.202, 2B00.A.252, 4B7U.B.401, 3B90.B.702, 3BCF.A.705, 4BCU.A.201, 1BK9.A.200, 3BOW.B.404, 3BS6.A.1, 4CAG.A.606, 2CDP.C.1140, 2COL.B.801, 1CVM.A.802, 3D4G.A.484, 3D6E.B.202, 1D8F.A.305, 3DEM.B.6001, 3DFO.A.717, 3DFO.B.601, 1DM5.E.1135, 2DW2.B.711, 3E1I.B.503, 2EAA.C.904, 4EJ7.A.402, 4EJ7.A.404, 4EM6.B.604, 4EMU.B.401, 1ESL.A.164, 2EXJ.D.2004, 2FH3.A.1001, 4G1M.B.2001, 3GE4.A.167, 4GEJ.A.201, 3GG1.B.503, 4GGB.A.402, 4GH8.B.203, 1GQM.A.1090, 1GQM.C.1088, 3GRI.B.700, 2GXS.B.604, 2HOK.A.402, 2HOK.A.408, 2HOL.A.1015, 1H71.P.500, 3HDB.A.657, 1HOV.A.168, 2HTY.A.991, 2IAP.A.402, 3IJE.B.4002, 4ILW.F.304, 3IRH.A.457, 1IVG.B.470, 2J1G.F.1291, 2J60.C.1277, 2JF9.P.1014, 4JP8.A.706, 4JWS.A.502, 3K39.F.1000, 3K8Y.A.167, 4KS3.A.502, 1KVO.D.192, 1KVY.A.124, 3LMW.A.9, 2LMV.A.151, 4LN6.G.403, 1LWU.K.4, 4M7K.H.301, 3M83.B.408, 4MBE.D.201, 3MDO.A.391, 2MG9.A.101, 1MWN.A.100, 1N28.B.127, 4N25.A.705, 4N2D.A.705, 4N96.B.401, 4NAS.C.501, 1NGS.B.681, 2NPO.A.1293, 4NUP.C.301, 4NVR.C.401, 1OLP.A.1372, 1OS2.A.872, 2OVX.B.447, 2OVX.B.450, 2OZR.F.4030, 2P5V.B.1008, 2P5W.B.701, 1Q3A.A.467, 4Q4Y.1.5006, 1Q7B.B.9003, 1QDO.C.245, 1QMD.A.404, 4QN3.A.501, 2QVF.B.703, 3QWU.B.602, 2R1D.I.3000, 2R8Y.I.209, 2RHP.A.16, 1RJV.A.112, 4RSR.A.404, 1SOE.A.1292, 1SEL.B.277, 3SH5.A.196, 3SHI.M.305, 3SNZ.A.97, 1SPJ.A.300, 1SRR.C.532, 1TFX.A.1007, 3U24.A.602, 1UBN.A.277, 3UBH.A.855, 1UEA.A.4, 4UM9.B.2003, 3UMJ.A.902, 2V5C.A.1625, 3V96.B.305, 2VB6.B.1148, 1VE5.B.2003, 2VVD.A.1329, 1WOO.A.1781, 2W1W.B.1134, 1W1X.A.1479, 3W9T.B.509, 3WCV.B.203, 2WG8.C.201, 2WII.A.1643, 3WNX.A.502, 1X36.A.269, 1XJL.A.344, 2XTJ.A.1423, 1Y6P.A.217, 1Y6P.B.218, 1YAX.A.1002, 2YDP.B.502, 2Z2D.A.267, 2ZUX.A.629, 2ZUX.A.637, 2ZUY.A.621, 2ZWP.B.401, 2BI4.A.1384, 4C4U.B.201, 4CMY.A.1164, 4D8F.A.401, 1DGG.A.2000, 1E2U.A.701, 3FE5.A.1, 3FMR.B.401, 3GZY.A.701, 1H2A.L.1004, 1HJF.A.1310, 1HJG.A.1307, 1I4Y.B.602, 1I4Z.D.604, 1I4Z.G.607, 2IBN.A.704, 3ICF.A.602, 1IDS.C.208, 2INP.A.3, 2ITB.B.501, 2J2F.E.371, 1KBP.A.438, 4KBP.A.438, 3KCY.A.1350, 4KEV.D.401, 1LGT.A.500, 1LKD.A.500, 3LKT.M.600, 3LMX.O.600, 1MOJ.A.302, 1MOJ.B.301, 1NNF.A.401, 1NX8.A.300, 1O7P.A.1453, 2OHH.B.1501, 2OHH.E.3501, 1OQ9.A.365, 1OS7.C.302, 3PCB.O.600, 3PCJ.M.600, 2PHD.C.370, 1PIY.A.376, 1PIY.A.377, 3PM5.A.1002, 3Q1G.A.1001, 3Q1G.B.1001, 1QFC.A.402, 2QJE.A.692, 2R2F.B.320, 4RC5.A.1003, 1RSR.A.1003, 1SQ3.B.906, 1SQ3.C.907, 4TOA.A.206, 4TOA.B.207, 3U52.B.512, 3U9M.E.205, 1ULJ.E.600, 2VHL.A.1398, 3VTI.B.803, 1W69.A.1349, 3WHN.B.201, 2XRX.G.1461, 1XZW.B.929, 1YKM.J.600, 2ZQX.A.501, 1ZZ9.C.199, 4DOC.A.404, 3EPG.A.421, 2IHM.A.900, 2ISP.A.340, 2ISP.A.342, 3JPS.A.340, 4KHS.A.1009, 3KNT.A.208, 4NLN.A.402, 1YIT.0.8538, 2A2A.C.3158, 1A5S.B.2000, 3ASP.A.701, 3AST.A.701, 4B1L.A.1679, 3BX1.B.284, 3C17.B.326, 2C9R.A.1103, 2C9

A.A.1289, 4D1I.C.600, 4D1I.F.600, 3D32.A.120, 4D9U.A.901, 2DDA.C.303, 3DKI.B.324
, 2EPF.C.307, 3EUW.B.343, 4FEW.B.303, 3FKR.A.409, 4FMT.A.301, 3G1N.A.5001, 4G1K.
A.301, 3GA5.A.700, 3GCD.A.215, 4GY9.A.206, 4HKT.B.410, 4HMM.B.302, 3HVY.C.427, 4
I2B.A.601, 3I4Q.A.177, 3IAQ.B.3104, 3IAQ.C.3101, 3IJ6.B.313, 4IJK.B.301, 4J0Y.A.
501, 4J1I.A.502, 4J2H.A.306, 4JDO.D.301, 4JHG.A.205, 4JRX.D.301, 4JVL.A.703, 1JY
X.B.3103, 1JZN.D.4139, 4K7V.A.407, 3L27.B.3, 4L3F.H.401, 4L73.A.404, 4LDZ.A.205,
4LH7.A.404, 3N83.A.701, 4NAW.N.304, 4NWH.A.201, 1O4Z.B.1005, 4O4W.A.301, 2O5W.C
.171, 1OA8.A.1690, 3OB8.B.3003, 4OBO.A.402, 2OSW.B.602, 2OSY.B.602, 3OTK.C.587,
3P8O.A.187, 3PJ0.C.367, 2POC.D.5004, 1PX4.A.3104, 3PYM.B.504, 1Q1Q.A.351, 1Q1Z.A
.313, 4Q4X.1.5004, 2QF2.A.700, 4QFE.G.304, 2QZ7.A.195, 2QZ7.B.196, 3RN0.A.402, 3
S30.A.383, 3TXJ.A.1138, 2VDX.A.1385, 3VD5.D.3102, 3VD7.A.3101, 1VIZ.B.239, 1W90.
A.1154, 2WCF.B.1091, 2WD0.A.601, 2WG8.B.202, 2WOI.B.1491, 2WOI.D.1488, 3WV2.A.30
5, 2WWH.B.212, 3WXO.A.807, 3WXO.A.808, 4WXG.A.502, 2X7J.D.1581, 2Y00.A.1359, 2ZH
J.A.320, 3ZLY.A.1384, 4ZNB.A.3, 2ZXK.A.1
[1] "Cluster 3"
1A1G.A.201, 1A6Y.A.551, 3AUO.B.579, 3EPH.A.1, 2ER8.A.104, 2ER8.A.105, 1G2D.C.302
, 1G2F.C.301, 1G2F.C.302, 4GLX.A.601, 3HAX.C.201, 2I13.B.507, 4IFD.J.1106, 4IQR.
A.403, 2JP9.A.131, 4M9E.A.503, 4M9V.C.202, 4NM6.A.2002, 2NQ9.A.401, 4O6A.A.601,
1ODH.A.1171, 3PIH.A.917, 3QSV.A.1, 3VD0.A.401, 1A1R.A.901, 3A32.A.708, 2A5V.A.40
1, 1A71.A.401, 1A72.A.376, 4A7K.A.950, 3A9J.C.1, 3A9K.C.1, 1AAF.A.56, 1AAF.A.57,
4AA1.A.1615, 2AB3.A.30, 2AC3.A.531, 2AFU.A.391, 2AMT.B.2900, 1ANJ.B.451, 1ARE.A
.1, 3AUK.A.390, 2AU3.A.501, 4AWY.B.3228, 3AX1.A.601, 1AXG.A.401, 4AX0.B.3228, 4A
X1.B.3228, 4AXD.A.700, 3AY2.A.1001, 2AZH.A.150, 4B29.A.1205, 3B4N.B.712, 4B6D.A.
1340, 1B8T.A.193, 1B8T.A.195, 1B8Y.A.301, 3B92.A.502, 3BHX.A.1752, 3BJI.A.1, 3BJ
I.B.2, 3BL5.A.300, 2BL6.A.1060, 1BNL.A.179, 1BNQ.A.262, 2BNM.A.1199, 2BNN.A.1200
, 3BOC.A.1001, 1BS4.A.2001, 3BTO.C.375, 4BT7.A.301, 1BUD.A.800, 1BV3.A.262, 1BYF
.A.302, 3BYR.A.501, 3C10.A.102, 2C1I.A.1465, 4C1D.A.502, 4C1F.A.501, 4C1F.A.502,
4C1G.A.301, 4C1G.B.301, 4C1Q.A.493, 2C20.A.601, 3C2S.A.448, 2C2F.A.1211, 1C3I.B
.260, 2C36.A.1311, 3C52.B.401, 4C81.A.1240, 2C9S.A.1155, 1CAK.A.262, 1CDO.B.376,
3CE1.A.202, 1CGL.A.302, 3CJP.A.301, 2CLT.A.1202, 3CMR.A.450, 2COT.A.201, 2COT.A
.401, 3COS.A.501, 2CO8.A.201, 2CON.A.201, 2COR.A.201, 1CQR.B.2301, 3CQZ.B.3007,
2CQE.A.622, 2CSY.A.401, 3CSQ.A.335, 2CT0.A.201, 2CT2.A.201, 2CT7.A.401, 2CTT.A.4
01, 2CU8.A.401, 2CUR.A.201, 2D0W.B.1207, 4D0Y.A.1239, 3D7V.A.2, 2D74.A.1001, 2D7
4.B.1002, 2D8T.A.201, 2D8T.A.401, 2D8V.A.201, 2D9M.A.1085, 2D9N.A.456, 2DAN.A.20
1, 2DAR.A.201, 1DCA.A.262, 1DEH.A.376, 3DFM.A.402, 2DH3.A.601, 3DH1.A.201, 3DHA.
A.256, 2DJ7.A.401, 2DJ8.A.401, 2DJA.A.201, 2DJA.A.401, 1DK4.A.290, 2DKT.A.191, 2
DKT.A.241, 2DKT.A.291, 2DKT.A.341, 2DKT.A.441, 2DKD.B.922, 1DMT.A.755, 2DMD.A.29
1, 1DO5.A.28, 2DO0.A.501, 2DQ6.A.900, 4DR8.A.201, 2DW2.A.700, 3E1Z.A.111, 3E2C.A
.200, 1E3L.A.380, 1E67.A.129, 3E6U.A.501, 3E6U.C.502, 2E7Y.A.1301, 3E73.A.501, 2
E9H.A.301, 1E9Q.B.153, 2EA6.A.201, 2ECT.A.201, 2ECT.A.401, 2ECV.A.201, 2ECW.A.20
1, 2ECW.A.401, 2ECY.A.401, 2ECG.A.201, 2ECL.A.601, 2ECM.A.201, 2ECM.A.401, 1ED9.
A.451, 3EDI.A.210, 1EE2.A.1300, 2EE8.A.501, 4EEX.A.402, 4EEZ.A.402, 3EFO.B.1034,
1EI6.A.409, 1EI6.B.408, 1EKJ.A.4001, 1EKJ.C.4003, 1EKM.A.701, 2ELR.A.181, 2ELS.
A.181, 2ELU.A.181, 2ELV.A.181, 2ELX.A.181, 2ELI.A.401, 2EM2.A.201, 2EM4.A.201, 2
EM6.A.201, 2EM9.A.201, 2EMB.A.201, 2EMC.A.201, 2EML.A.201, 2EN2.A.201, 2EN9.A.18
1, 2ENA.A.181, 2ENC.A.181, 2ENE.A.181, 2ENH.A.181, 2EOJ.A.201, 2EOQ.A.201, 2EOW.
A.201, 2EOE.A.201, 2EOH.A.201, 2EOM.A.201, 2EON.A.201, 2EOO.A.201, 3EPZ.A.701, 2
EPQ.A.201, 3EWF.A.400, 2EWB.A.489, 3EYX.A.1, 1F35.A.306, 4F70.B.301, 3F90.A.309,
1FAQ.A.1, 4FGL.A.301, 4FKK.A.1025, 1FLJ.A.262, 4FMN.B.901, 4FMN.B.902, 4FMP.A.4
00, 2FNF.X.2, 4FO9.A.501, 2FPR.A.502, 3FPC.A.353, 3FPL.A.352, 1FR2.B.301, 3FTN.A
.354, 3FTW.A.701, 3FUN.A.701, 2FU8.A.401, 4FVY.A.805, 4FWU.A.401, 2G0D.A.416, 4G
3M.B.401, 1G5C.A.1001, 2G9Y.B.451, 2GA3.A.451, 3GAY.B.328, 3GC9.B.603, 2GMN.A.80
1, 2GMN.A.805, 4GNE.A.1501, 1GO8.P.1486, 4GR3.A.301, 4GRI.A.501, 2GSN.A.1000, 4G
U1.A.905, 4GUA.A.1719, 4GUT.A.904, 1GVY.A.1425, 3GZE.B.14, 2GZL.A.900, 4H1Q.A.30

2, 4H2K.B.1001, 2H39.A.352, 2H4N.A.262, 1H9Q.A.262, 3HB2.P.486, 2HCS.A.1, 2HCS.A.2, 3HFF.A.154, 3HJT.A.1, 1HK8.A.1589, 4HMA.A.301, 3HNI.G.107, 3HNI.H.107, 3HNJ.A.107, 3HNJ.B.107, 3HNJ.C.107, 3HNJ.D.107, 4HNO.A.301, 3HPH.A.220, 1HSZ.A.1376, 1HTO.A.1376, 3HUG.D.109, 1HZ5.B.105, 3I1U.A.401, 1I50.A.3006, 1I73.A.998, 1I73.A.999, 4I7C.A.601, 1IAG.A.999, 1IAU.A.504, 1IBB.A.201, 2IDA.A.103, 3IEW.B.801, 1IF5.A.262, 3IFU.A.181, 4IJD.A.501, 4IJD.A.502, 2IMR.A.500, 4IOU.D.1001, 4IR0.A.201, 1IS8.B.3109, 2IUC.A.1002, 2IUC.B.1007, 4IUQ.A.301, 4IUW.A.701, 1J2O.A.116, 4J3D.B.302, 4J4M.A.301, 2J7U.A.1884, 4JEB.B.202, 2JHG.A.401, 4JH2.A.201, 4JH2.B.201, 2JIG.A.1253, 2JIG.B.1252, 1JJD.A.103, 4JLX.A.501, 2JLP.A.226, 1JQ5.A.371, 4JSA.A.301, 4JSW.A.301, 4JSZ.A.301, 1JT1.A.400, 2JTN.A.184, 2JTN.A.185, 2JUN.A.222, 1JV0.A.261, 1K07.A.1, 4K2H.A.201, 1K7H.A.478, 4K7D.A.503, 4K7D.A.504, 4K7D.A.506, 2K8D.A.155, 2KEM.A.195, 1KH5.A.451, 1KH7.A.451, 1KOL.A.1001, 4KP5.A.301, 1KU0.A.701, 2KVF.A.83, 3KWO.B.161, 4KX8.A.1001, 4KXB.A.1001, 3KYC.B.641, 1LOY.B.706, 2L0B.A.143, 2L0B.A.161, 1L1O.F.2, 4L6O.A.801, 1L7O.B.301, 3L8H.A.901, 3L9Y.A.155, 4LAO.A.401, 1LD3.A.500, 1LDE.A.375, 1LDY.A.375, 3LEA.A.485, 4LEV.A.601, 2LFD.A.400, 2LGV.A.111, 1LI5.B.964, 4LJQ.B.1105, 4LJQ.A.1101, 1LLU.B.343, 3LPE.B.60, 4LP6.A.310, 3LQB.A.201, 3LQH.A.1001, 4LR2.A.505, 3LS9.A.457, 2LUY.A.300, 4LY4.D.301, 3M0A.D.401, 3M15.A.107, 2M3Z.A.101, 1M3V.A.124, 2M48.A.504, 3M5S.A.500, 1M6W.A.1376, 3M8T.A.300, 3M8T.A.301, 1M9O.A.78, 3MA2.A.295, 4MB7.A.301, 3MDW.A.455, 3MEQ.A.401, 3MF1.A.1000, 1MGO.A.375, 1MGO.B.375, 3MHS.E.97, 3MI9.C.87, 3MI9.C.88, 3MKG.A.155, 3MKV.B.426, 3ML2.A.262, 3MTW.A.2, 3MWM.A.141, 3N2C.E.425, 1N8K.A.375, 1ND1.A.400, 3NGJ.A.250, 3NKQ.A.1001, 3NNQ.A.201, 1NO5.B.571, 3NQY.B.520, 4NQ7.A.302, 4NTM.A.201, 4NTN.A.201, 2NUP.B.1100, 2O03.A.201, 2O53.B.314, 4O64.A.2003, 3O7U.A.428, 4OBI.A.201, 2ODX.A.156, 2OD1.A.902, 2OHX.A.401, 2OI0.A.2, 3OJ3.J.902, 2ORW.A.401, 2OW9.B.606, 2OXZ.A.264, 2OX8.B.3, 1P1R.A.375, 1P1V.A.201, 1P42.A.501, 1P42.A.503, 1P4Q.B.301, 3P5A.A.262, 3P5L.A.262, 2P53.A.401, 1PB0.A.1303, 3PB6.X.400, 2PLI.B.709, 3PN3.A.1010, 1PS7.A.331, 3PSQ.B.321, 4PTB.A.901, 2PUY.A.355, 1PV9.A.402, 1PXE.A.64, 3PZC.A.1000, 1Q3K.A.301, 3Q43.A.1, 3Q44.A.1, 1Q5W.A.32, 2QDT.A.401, 2QDT.A.402, 3QE3.A.356, 2QIN.A.2002, 1QJI.A.1201, 2QL0.A.53, 3QNA.A.122, 4QP5.A.401, 2QSW.A.201, 1QTW.A.301, 1QV6.A.375, 1QV7.A.375, 3QVY.A.500, 1R1H.A.1001, 1R37.A.500, 1R3N.B.501, 2R3A.A.302, 3RF4.B.119, 4RF1.A.1901, 1RM8.A.501, 1RMD.A.119, 1RMD.A.120, 2RPC.A.401, 2RPC.A.601, 2RPC.A.801, 2RPP.A.201, 3RSM.A.500, 2RSJ.A.102, 3RZV.A.1, 1S1G.A.152, 3S2E.G.500, 3S2F.E.500, 1S4I.A.802, 3SEY.C.373, 3SFH.A.403, 1SML.A.271, 3SP1.A.481, 1SRD.B.156, 1SRP.A.920, 3SV6.A.4, 3SXK.B.3, 1T3A.A.422, 1T3K.A.201, 3T33.A.411, 1T9R.A.1, 1TBN.A.2, 3TBG.A.601, 3TEN.A.205, 1TKF.A.901, 1TKF.A.902, 1TTM.A.262, 1TWF.J.3001, 1U05.A.500, 1U0L.A.298, 1U10.A.400, 1U10.A.601, 4U10.A.401, 1U1H.A.766, 1U3U.A.376, 1U3V.A.376, 1U40.A.160, 1U5S.B.139, 3U5N.A.1, 4UA4.A.301, 3UCK.A.228, 3UIK.A.341, 3UN6.A.325, 1UUF.A.402, 1UW0.A.1118, 3UW2.A.474, 2UZG.A.131, 2V1X.A.1595, 1V13.B.200, 3V1C.A.101, 1V5N.A.201, 1V5N.A.401, 1V6G.A.201, 3V7M.A.509, 1V7Z.A.301, 2V8G.C.500, 2V9I.B.1275, 3V96.B.302, 1VA1.A.100, 1VA2.A.100, 1VA3.A.100, 2VES.A.1297, 2VES.C.1302, 2VF7.B.1844, 1VK9.A.143, 2VL6.A.1266, 3VOW.A.201, 3VPE.A.301, 1VQ2.A.702, 3VQZ.A.301, 3VRK.A.301, 1VSH.A.281, 2VUT.I.1713, 1VYK.A.1150, 1VYX.A.1062, 2W3Q.A.1231, 2W5V.B.1377, 3W5K.B.502, 3W5K.B.503, 4W6Z.A.401, 1WAA.B.1090, 2WBT.A.1130, 2WBT.A.1131, 3WBH.B.505, 1WEM.A.201, 1WEN.A.401, 1WEO.A.401, 1WEP.A.201, 1WES.A.201, 1WES.A.401, 1WFF.A.401, 1WFL.A.401, 1WFP.A.401, 1WG2.A.200, 1WIG.A.201, 1WIG.A.401, 1WII.A.201, 1WIL.A.401, 1WIM.A.201, 2WJV.A.3, 1WJP.A.301, 1WJP.A.501, 1WJV.A.201, 3WLE.A.402, 3WLF.A.402, 2WOJ.A.1353, 4WOK.A.401, 1WY2.B.407, 1WYH.A.201, 1X3H.A.401, 2X3B.A.1341, 1X4K.A.201, 1X4K.A.401, 1X61.A.201, 1X62.A.201, 1X6H.A.401, 2X7M.A.1174, 2X8Y.A.1616, 2X8Z.A.1616, 2X95.A.1615, 2X96.A.1617, 2XAA.C.1346, 1XB8.A.1001, 2XBL.C.1196, 1XC3.A.302, 4XIX.B.401, 2XML.A.1349, 2XQV.A.401, 1XRT.A.1423, 2XS4.A.998, 1XUC.A.1261, 1XWY.A.401, 1Y0J.A.244, 2Y6C.A.1267, 1Y8J.A.800, 1Y93.A.265, 1YB0.B.160, 1YC2.A.402, 1YC2.C.406, 1YE3.A.375, 1YEW.G.662, 1YLK.A.401, 1YOU.A.301, 2YPU.A.1998, 1YQD.A.1000, 2YQL.A.201, 2YQQ.A.401, 2Y

RM.A.201, 2YSA.A.181, 2YTQ.A.201, 2YTT.A.181, 2YT5.A.201, 2YTB.A.301, 2Z3I.A.2001, 2Z45.A.1001, 1Z6R.A.501, 1Z84.A.603, 2Z9K.A.901, 2ZEP.A.391, 1ZFO.A.31, 1ZLH.A.555, 3ZNC.A.1, 2ZNC.A.1, 3ZQ6.A.1323, 2ZTX.A.501, 3ZUK.A.1664, 2ZU2.A.5517, 3ZVS.A.1159, 3ZXH.A.301, 1ZXV.B.9002, 1ZXZ.A.198, 1ZZU.A.900, 2A19.B.1642, 3ARA.A.166, 3C15.A.29, 2D32.B.2524, 1G8G.A.521, 3M42.A.1, 4NNN.N.202, 2O4G.B.800, 4QDG.A.402, 1XLB.A.399, 2YFD.B.1145, 1YYZ.A.341, 1LRW.A.702, 4R5O.A.509, 4AM5.A.1162, 2B5H.A.501, 2BKB.C.1193, 2BOY.E.1255, 3CF4.A.808, 1CJX.B.629, 3DBY.A.306, 4FWI.B.401, 2GPC.B.195, 1GUP.C.351, 2HMK.A.451, 1I4Z.E.605, 1JRO.G.3001, 4KWL.A.301, 4KX6.N.301, 4NBG.A.501, 1NF6.A.200, 2OHJ.A.502, 2Q0J.B.997, 3Q36.A.458, 4QDD.A.401, 1QGH.H.157, 1R9X.A.501, 4REU.B.202, 3U9M.A.202, 3V7P.A.429, 3VMH.C.501, 2W3S.E.1464, 1WQL.A.502, 1Y4T.A.650, 2YFI.A.901, 2YFI.G.900, 2ZZI.A.208, 4FZY.A.201, 4FZY.B.201, 3FFZ.B.1302, 4FLK.A.504, 4FO2.E.101, 1G8G.A.525, 4IQZ.E.305, 2OCC.A.519
[1] "Cluster 4"
4GNX.C.701, 4L8H.R.105, 1ALH.B.450, 2ANH.A.452, 2ANU.B.505, 2AQ2.B.1001, 1ARD.A.1, 2AYK.A.171, 1BON.A.1002, 4BLD.D.910, 1BTG.B.902, 1DGS.B.2701, 2DI2.A.30, 4DLF.A.404, 2E1W.A.400, 2E84.A.558, 4FC5.E.305, 2FKM.X.500, 4FXO.A.301, 2FZ6.A.201, 1GLC.F.169, 2H6H.B.1001, 2H6F.B.1001, 4H82.B.300, 3H90.A.291, 3H90.D.6, 4HDT.A.400, 3ID7.A.402, 3ISO.B.220, 2IUC.B.1006, 4J3D.A.301, 2JMD.A.66, 1KBE.A.2, 1KH5.B.950, 1KHN.A.452, 3KVE.C.489, 2KV1.A.125, 2KVH.A.84, 2L1U.A.144, 4LW9.E.204, 3M7P.A.953, 2MQ1.A.102, 2MQ1.A.103, 1MVH.A.502, 4N07.A.309, 1NDV.A.400, 2O3Z.A.501, 1OS2.F.769, 2OW2.A.444, 2OX8.A.5, 2OX8.B.5, 1P91.B.2301, 2POJ.A.265, 4PVT.A.404, 1Q9U.B.402, 2QQ4.I.139, 1R87.A.905, 3SOW.A.7, 3SPU.D.1004, 3U24.A.594, 3U7K.A.350, 1UD9.B.510, 4UNI.C.1697, 2V8V.B.1455, 1V9P.B.2701, 2VW4.A.503, 2VZ5.A.1131, 4WD7.C.302, 2WKN.A.412, 2X4H.A.1140, 2X4H.A.1141, 2X5C.A.1128, 2XAM.B.700, 2Z45.A.1003, 3ZTV.A.1599, 1ZZM.A.403, 3ICE.D.1001, 4IR1.A.902, 1Q81.4.8078, 4UN4.B.2367, 2W9C.A.1344, 3ALN.A.406, 1CUL.A.1007, 1D2E.D.504, 3DYF.B.4002, 3EN9.A.600, 1EQR.B.902, 1G9X.B.1301, 1GRV.A.490, 4I1O.A.201, 2IO7.B.5004, 3JZM.A.701, 4K6T.E.412, 3LGH.A.150, 1MXB.A.411, 2O1V.A.755, 2O1X.B.2002, 1PYX.A.1002, 4QLQ.V.301, 3QU2.C.225, 1RC5.D.764, 1SO2.C.475, 3VN9.A.402, 2VOS.A.1491, 2WCJ.A.1144, 3WKA.A.601, 2Z2P.A.1003, 2C2R.A.1344, 4DTJ.A.1003, 4DTU.A.1003, 4DU4.A.1003, 4EBC.A.504, 2GIH.A.402, 3QER.A.906, 3QEV.A.906, 4RI8.A.1101, 4WUZ.B.301, 3AKB.A.173, 3ATS.A.380, 1B1G.A.77, 1B90.A.930, 4CP1.A.1297, 1D8M.A.304, 4DLK.B.402, 3E1I.B.502, 1EN7.A.403, 1EUB.A.277, 3FHA.A.705, 3GG1.A.503, 2HF4.A.902, 2HYW.A.506, 2II1.C.404, 1IJL.A.203, 2IWV.A.1284, 1JHN.A.900, 1JYI.D.408, 4K6T.G.408, 1KTW.A.496, 1KTW.A.5, 1KTW.A.9, 4L06.A.501, 4L76.E.402, 1M1J.B.503, 2M29.A.401, 3O82.B.544, 4OO2.C.601, 2P5V.C.1001, 4P99.B.540, 1PZ8.C.703, 2Q04.C.211, 1QD6.C.2, 4QNP.A.506, 2QPS.A.501, 3R6Q.H.469, 1RFN.A.500, 1RK9.A.112, 3S5U.F.220, 1T9H.A.414, 4TQO.H.701, 1TRQ.A.106, 1UCN.A.1162, 4UM9.D.2002, 2UX1.K.174, 1W52.X.602, 3WIU.B.1004, 4WK4.B.502, 1XZO.A.1014, 1XZO.B.1018, 1Y1A.A.501, 1ZH2.A.202, 4AC8.B.500, 3FG1.B.1500, 2FKZ.A.1600, 2FL0.B.1605, 1FRF.L.565, 1GNL.A.1545, 1GUP.D.351, 2JD7.0.203, 2OHJ.A.501, 1OQU.A.1001, 1OVB.A.300, 3PCE.M.600, 2PT2.A.400, 2PUZ.A.500, 3QFM.A.264, 1RA5.A.500, 3T81.B.606, 3T81.B.607, 3VV9.C.502, 1W9M.A.1555, 2XRX.Q.1461, 2XSO.O.900, 1YKP.F.2600, 4DOC.A.402, 8ICP.A.341, 4KHN.A.1010, 4KHU.A.1008, 1QVG.L.8347, 3UXP.A.339, 4A22.C.1346, 3AR8.A.1000, 4COK.A.1614, 4CBY.C.2037, 3DYQ.A.901, 3DYQ.B.901, 3EPZ.A.1, 1G8H.A.523, 3GCD.B.215, 3GVF.A.178, 3I01.A.501, 3IWK.B.504, 4JCO.D.406, 1JED.A.525, 4K70.B.1011, 4KAF.A.404, 4L73.B.405, 4MVJ.D.402, 2NQL.A.401, 1PX3.A.3101, 4Q4B.A.530, 3Q9E.L.343, 1QY1.A.204, 1S81.A.2, 3T34.A.1003, 1T64.B.1392, 3UA6.A.147, 3UA6.B.2, 3VD3.C.3101, 3VD5.C.3101, 3VDA.A.3101, 3W6P.A.804, 4XOO.B.401, 2X1Z.M.1163, 3ZDU.A.353, 3ZPR.B.1, 3ZX2.B.521
[1] "Cluster 5"
4LMG.B.202, 3P57.P.122, 3A1Z.C.226, 1AJD.A.450, 3B0Z.A.22, 4C98.A.1266, 2CIH.A.213, 3D68.A.501, 2EC7.A.50, 1F30.H.201, 1F30.I.201, 2F4L.A.1400, 4IOZ.A.504, 4KEQ.A.301, 1N4P.H.378, 3NQ5.A.508, 2OGJ.A.418, 1P4Q.B.302, 2W88.C.107, 2W9M.A.1565,

2WC0.B.3012, 2X3C.A.1342, 1ZKX.A.423, 4DQP.A.903, 2NVQ.A.2001, 4P0P.A.601, 3ZC0
.B.197, 2AQX.A.601, 3AQC.D.327, 1AZT.B.406, 1BPM.A.488, 3CRL.B.2001, 4CW7.C.1002
, 4CYU.A.171, 3D7M.A.356, 1D0A.A.199, 4DPM.D.401, 2DW7.L.2012, 1E1Q.A.601, 1E1Q.
F.601, 2E92.A.1302, 3EPS.A.1606, 3EQL.N.1528, 4F38.A.202, 1F4H.D.3001, 3FR8.B.1,
2G5H.B.501, 2G83.B.358, 1H8H.F.601, 4HV6.B.201, 2I19.B.4004, 4I10.C.201, 3IG8.A
.697, 3IJQ.A.386, 2IOA.A.5001, 1JFG.B.703, 3KRF.A.904, 3LCB.B.579, 3MQT.A.1243,
3N9V.A.61, 3NC0.C.218, 3O7L.B.352, 3OAC.D.3002, 4OAU.C.803, 3OHM.A.402, 4P9D.C.2
02, 1PKG.A.1481, 2Q80.A.401, 3RBM.B.1002, 1S4E.B.1600, 3SRD.C.603, 3SRF.D.533, 1
SVW.B.301, 4TQ3.A.402, 3U87.A.402, 3U87.B.403, 2UXR.B.1398, 2VKQ.A.1288, 1WBQ.C.
1441, 1WQ1.R.168, 1WUU.C.394, 1YXI.A.342, 1ZOT.A.901, 3ZXT.A.1278, 2AU0.H.1415,
3GIJ.B.1415, 4J2A.A.1006, 3KHL.B.1417, 3NHG.A.909, 4RIC.B.1101, 1AFB.2.3, 3B2Z.C
.3, 2B30.A.302, 4BTX.A.1764, 4BWE.A.503, 4BY6.A.3080, 2C11.C.1739, 2CLT.A.1102,
3D4G.A.485, 2E0X.B.701, 2E3X.A.801, 2E6V.E.12, 3EAD.C.1001, 1FBL.A.994, 2FH3.A.1
003, 1FZA.C.1, 1G5N.A.403, 3GHG.L.601, 1GU6.A.1480, 4I9F.A.401, 3IAE.B.572, 2IEZ
.A.219, 4IEF.H.704, 1IT4.A.200, 2J0T.C.1268, 2J3G.A.1289, 4JBE.A.503, 1JED.B.535
, 1JX6.A.401, 2K0J.A.503, 4K1C.B.504, 2KBM.B.101, 3KQA.C.420, 4L76.B.402, 4L76.D
.402, 1LGC.C.301, 4LIN.A.1301, 4LLF.O.401, 1LWU.C.5, 3M0W.G.202, 4M8D.B.305, 2M9
8.A.201, 4MDV.A.404, 1MTS.A.480, 3N9V.A.64, 4NUP.C.304, 2NWH.A.402, 4OVG.A.404,
3P1O.A.234, 4POS.B.401, 3Q2N.B.304, 1QD0.D.247, 1QLK.A.93, 3RBX.A.601, 2RHP.A.15
, 3RRY.A.202, 3S55.D.280, 1S6I.A.192, 1SAV.A.321, 1SUI.B.306, 1SUS.C.307, 1TCF.A
.164, 2TEC.E.344, 1UG9.A.2002, 2W67.B.1590, 1WD9.A.902, 4WFE.A.306, 4WIW.C.701,
2WJS.A.5001, 2WJS.A.5002, 3WMW.A.401, 2WM4.A.1430, 3WNX.A.501, 2WW0.E.800, 2XJO.
B.1175, 1XJL.A.342, 2XSG.A.1772, 2YGM.B.1418, 1YN8.B.1008, 1Z3J.A.268, 4CMY.B.11
65, 3FM3.A.452, 1SQ3.C.908, 4KHU.A.1006, 4M47.A.403, 1U8R.D.4104, 3BFT.A.1007, 3
C9F.B.603, 4D1I.H.600, 2D4E.C.1905, 3FGH.A.180, 1G8G.A.522, 1G8G.A.527, 1G8H.A.5
26, 3IAP.D.3103, 1KNR.A.542, 4L73.A.406, 2NWH.A.404, 4ODN.A.207, 3OO2.B.383, 4R6
C.A.213, 3T2P.B.3102, 2WO0.B.1728, 1YAO.A.501
[1] "Cluster 6"
2AXR.A.501, 2B5L.C.3001, 3BYW.D.1, 3CPA.A.308, 3CQJ.B.285, 4CWM.B.433, 1D1T.B.40
6, 3DLJ.A.2001, 3DLJ.B.2002, 3DNG.B.998, 3E50.A.1, 2EC7.A.51, 4EGE.A.411, 4FUK.A
.401, 4G1P.A.501, 2GLQ.A.2002, 3H90.A.292, 4HGX.B.301, 1HOV.A.165, 4IGN.A.401, 2
JRP.A.150, 4JSS.A.301, 4K6T.B.403, 4K7S.B.101, 4KJG.B.1001, 3LSF.H.2, 3MO2.D.5,
1MWO.A.438, 2O1Q.B.145, 4O98.A.401, 2OC7.A.901, 3PW3.C.406, 3PW3.D.406, 1PYT.B.3
50, 4Q7R.A.305, 3QU6.A.114, 3QZC.A.2, 1R5X.A.122, 1TM6.A.23, 4U9D.A.205, 3U94.A.
259, 3UBF.A.7, 2VQH.B.1089, 3VUV.A.501, 3WC5.A.404, 2WWO.A.1165, 1XAF.A.503, 2Y2
E.A.1180, 1YIX.B.603, 2Z2D.A.264, 1Z5R.C.600, 3ZTV.A.1598, 3ZUQ.A.1440, 3HO1.X.2
2, 4IRK.A.402, 2OTL.A.8066, 1VQ7.0.8066, 1YJ9.0.8067, 521P.A.168, 4AG5.B.1588, 3
AJP.A.183, 3ALN.B.406, 1AM4.D.679, 1AR1.A.560, 1CEE.A.180, 4D0L.B.2001, 4DVG.A.2
01, 2E8W.B.1204, 4EOP.D.501, 3EQB.A.9002, 3FPA.B.901, 3GFT.E.202, 3GOL.A.580, 1G
Q9.B.1242, 3GT8.D.14, 4GZM.A.1001, 2HAW.A.1001, 2HAW.A.1002, 3HYT.A.802, 4HYP.C.
302, 4I40.A.301, 1IOV.A.330, 2I07.A.5001, 4JVJ.F.403, 4JVJ.F.404, 4K6T.B.411, 3K
RP.D.903, 3KZ1.E.550, 3LAW.B.1401, 1MAB.A.602, 2O1X.C.2003, 2OPM.A.908, 2OQY.C.4
02, 2P8E.A.306, 1Q3H.A.674, 4QEH.A.401, 1R0Z.D.674, 4R9U.D.302, 4RAB.C.303, 1SVT
.E.601, 3T5P.H.301, 4UOR.K.699, 3VHX.G.185, 2VWI.B.1293, 1W1W.A.2001, 1W85.E.136
8, 2WCJ.A.1146, 3WIG.A.402, 3WNW.J.201, 1XD2.A.167, 1YM0.A.402, 1Z6K.A.275, 4G3I
.A.401, 3KHG.A.415, 4KHU.A.1003, 2NOL.A.328, 4Q0W.A.1001, 4QWD.A.702, 3QZ7.A.363
, 3RBD.B.1415, 4AC8.B.1311, 2AER.L.3005, 1AWB.B.280, 1AXK.A.395, 2BD4.A.260, 2BZ
6.H.1260, 3C14.A.29, 1C9P.A.501, 1D8M.B.804, 3EDD.A.701, 1FBL.A.993, 4FVL.A.506,
4H82.C.305, 1HKB.A.923, 3HQ8.B.402, 3HR4.H.203, 1HVD.A.600, 2II1.D.401, 2I04.B.
701, 3IS5.F.1, 1ITC.A.1500, 1J24.A.1001, 1JDC.A.452, 1K6S.A.302, 1K6S.B.301, 4KS
4.A.502, 4KW7.A.402, 4L41.B.201, 1LPK.B.1, 4MC7.A.503, 4MIX.A.2501, 4N2I.A.707,
1N41.A.410, 1NMB.N.478, 4NUY.A.1001, 3OHO.A.1, 4OKH.C.903, 3OXQ.D.516, 4P99.A.53
3, 2PC6.B.303, 1PEX.A.502, 4PIB.B.203, 4POQ.G.401, 2PR3.A.901, 4Q4X.1.5007, 1QMD
.B.405, 3R4I.D.342, 2RJP.D.2, 3RMK.B.308, 3RRV.C.255, 2TBV.A.388, 3VEQ.B.301, 2V

ME.E.500, 3VOB.A.401, 2WOQ.B.803, 3W9T.B.510, 1Y7O.B.1004, 2YA9.A.1303, 2YN3.D.6
355, 2Z2D.A.268, 4QQW.G.1002, 1AFR.B.454, 4BMT.B.1323, 4BMT.B.1324, 4CVP.A.1155,
3DHG.A.501, 1DTO.A.1601, 3E1N.D.301, 3E1N.H.300, 2FKZ.A.1601, 1FZH.B.5004, 2GBX
.A.456, 1GUP.A.351, 3I4V.A.281, 3IS8.N.162, 2ITB.A.501, 4IWK.F.201, 1JYB.A.600,
1N7X.A.339, 1NFV.A.201, 1NFV.M.200, 3NJZ.A.369, 1OQ4.B.364, 2P6B.C.513, 3PCA.N.6
00, 3PCL.R.600, 1PFR.A.502, 1PIU.A.401, 3R2M.A.155, 1T47.A.431, 4TOA.B.203, 4TOE
.A.203, 3USS.B.212, 2VC7.D.1315, 1W2N.A.312, 2XSO.K.900, 2Z4G.A.503, 3E45.A.260,
2OTJ.R.8537, 1Q81.M.8380, 1YIT.O.8517, 3AXG.A.3005, 3C17.A.324, 4CH8.D.1580, 1C
M5.A.1056, 1DI4.A.501, 3DR3.A.336, 3DYQ.A.902, 2E7U.A.1002, 1EBU.A.901, 4ENZ.A.1
112, 1F7T.A.472, 1F7T.C.474, 4FOI.A.1005, 4FOI.A.1006, 2FQE.A.901, 3H1V.X.600, 4
HCH.A.407, 3HVU.D.182, 4HXV.A.403, 3I3D.C.3101, 3IGQ.F.801, 2J5W.A.3043, 3K13.A.
647, 1MXO.D.901, 3NRB.C.287, 3OB8.A.3006, 4ODI.A.301, 3P0J.A.711, 1QJS.A.512, 1Q
JS.A.513, 1QJS.A.523, 4R3W.B.402, 1R4P.F.4004, 3T2Q.D.3101, 3UA7.A.145, 1UD2.A.1
003, 3V6N.A.232, 3W0L.A.502, 1XAR.B.200, 1XC6.A.8001, 1YCE.A.201, 1YCE.C.201
[1] "Cluster 7"
3COQ.A.1002, 1F2I.G.1201, 3F2D.A.5, 4HCC.A.504, 4HP3.C.201, 2I13.B.510, 4IQR.A.4
02, 2KKF.A.2001, 2KKF.A.2002, 4LMG.A.201, 4MTD.B.201, 1QUM.A.301, 3S14.A.1735, 3
UK3.C.967, 3A3O.B.65, 1A42.A.262, 1A5T.A.501, 3A6F.C.301, 3A6J.A.301, 3A6J.D.301
, 1A7I.A.82, 1A7I.A.83, 2AA4.A.1001, 2ADR.A.162, 2ADR.A.163, 1ADB.A.375, 1ADN.A.
93, 2AF2.B.154, 4AF1.A.500, 4AIG.A.999, 1ANI.A.451, 1ARF.A.1, 4AR1.A.1722, 4ARF.
A.1722, 2AS9.A.666, 2AS9.B.222, 2B0P.A.400, 1B4E.A.405, 3B4F.A.262, 3B5Q.A.500,
3B6P.A.800, 3B7R.L.701, 1B8T.A.194, 1B8T.A.196, 2B83.C.3353, 2BA1.A.201, 4BHW.A.
1, 4BHW.A.4, 1BIO.A.291, 3BKN.A.201, 4BLB.B.910, 4BOL.A.1261, 1BP3.A.500, 1BTK.A
.1, 3C0Y.A.401, 4C1D.A.501, 4C1E.A.501, 4C1E.A.502, 4C1G.A.300, 4C2P.A.701, 4C4O
.D.500, 1C8T.A.260, 3C8Z.A.413, 1CAQ.A.301, 4CBY.A.2035, 3CHV.A.302, 2CJL.A.1217
, 1CLC.A.653, 1CO4.A.43, 3CQZ.I.3004, 3CQZ.L.3005, 2CQF.A.330, 1CRA.A.262, 2CSY.
A.201, 3CSK.A.712, 1CTU.A.296, 2CTT.A.201, 2CU8.A.201, 2CUP.A.201, 2CUQ.A.201, 2
CUQ.A.401, 1CVE.A.262, 3CX3.A.314, 1CXV.A.3, 1D1T.A.401, 3D2N.A.102, 3D2Z.A.262,
1D8M.B.801, 2D8U.A.401, 1D9D.A.1, 2D9N.A.256, 4D9W.A.408, 2DB6.A.401, 1DDZ.A.1,
1DE6.A.450, 2DGE.A.1001, 2DID.A.401, 2DJB.A.201, 2DJB.A.401, 4DLA.A.401, 1DMX.A
.280, 1DMY.A.280, 1DPM.A.801, 2DQ4.A.502, 1DSV.A.171, 2DSN.B.2002, 1DTH.A.901, 1
DVB.A.194, 1DY0.A.401, 4DYG.B.307, 3E24.A.230, 2E26.A.603, 1E3I.A.380, 1E3I.A.38
1, 3E3F.A.230, 3E3I.A.230, 1E46.S.999, 1E4B.S.999, 3E4Z.B.2, 2E47.A.172, 2E72.A.
201, 2EA5.A.401, 3EBI.A.1, 2ECI.A.201, 2ECI.A.401, 2ECG.A.401, 4EEX.B.402, 4EFS.
A.301, 2EG4.A.301, 2EG4.B.302, 3EHX.A.264, 2ELW.A.181, 1ELX.A.451, 1ELY.A.451, 1
ELZ.A.451, 2EMX.A.201, 2EMF.A.201, 2EOI.A.201, 2EPU.A.201, 2EPX.A.201, 1EPW.A.12
91, 3EQN.B.757, 2EQ2.A.201, 2EQ3.A.201, 1ESP.A.323, 1EU4.A.400, 4EXS.A.302, 1EYW
.A.402, 4EYU.A.1702, 3F0F.A.165, 3F4X.A.262, 3F5L.A.1001, 1F8F.A.372, 4F9C.B.401
, 1FBX.A.3316, 3FCQ.A.322, 3FDK.A.402, 2FEJ.A.1, 3FIE.A.428, 3FJU.A.502, 3FJU.A.
507, 1FKW.A.400, 4FKB.A.401, 3FLF.A.2004, 2FNF.X.1, 1FR7.A.262, 3FTN.D.357, 3FV4
.A.321, 3FVP.A.321, 3FVZ.A.821, 4FVL.A.501, 3FXP.A.3000, 2FZW.B.376, 1G47.A.999,
3G42.D.500, 1G4K.A.301, 2G54.A.1100, 4GER.A.401, 2GFE.A.869, 1GI4.A.409, 3GIQ.A
.482, 1GKR.A.1452, 4GQT.B.502, 3GRB.A.129, 4GR0.A.301, 4GR8.A.301, 2GSU.A.1001,
3GTT.A.155, 1GYT.J.600, 4H01.A.602, 1H19.A.701, 3H2P.B.154, 4H30.A.301, 4H57.A.4
05, 1H7N.A.1342, 3H8F.B.501, 4H84.A.301, 3H90.A.293, 2HB9.A.401, 1HCP.A.98, 4HDH
.A.1002, 4HEW.A.301, 4HF3.A.301, 1HP7.A.401, 2HSI.A.283, 1HU8.A.501, 4HVL.A.504,
1HY7.B.801, 1HYI.A.66, 2I0O.A.581, 4I28.A.602, 2I2X.A.524, 3I3T.A.700, 1I6N.A.4
01, 1I76.A.999, 1I8J.B.400, 3I9F.B.3, 2I9W.A.200, 1IB5.A.201, 1IML.A.77, 3ISI.X.
3000, 1ITU.A.401, 2IT4.A.561, 2IWE.A.1129, 4IXJ.A.301, 1J9Y.A.1004, 2JA1.A.1192,
4JD1.B.202, 4JIJ.A.302, 1JJ9.A.999, 1JJD.A.102, 1JM7.A.123, 2JOX.A.108, 4JOM.A.
1004, 4JPA.A.301, 2JSD.A.275, 2JTN.A.183, 2JTN.A.186, 2JUN.A.221, 2JUN.A.223, 1J
W9.B.250, 4K1R.A.502, 1K24.A.401, 4K5N.A.1101, 3K6J.A.800, 1K83.I.3003, 2KBX.B.2
98, 3KED.A.875, 1KEV.B.353, 1KFI.A.700, 1KH4.A.451, 1KHL.A.451, 2KIK.B.50, 2KIZ.
A.70, 2KIZ.A.71, 1KK1.A.411, 2KN9.A.82, 1KOQ.A.301, 1KTO.A.405, 3KVE.B.489, 2KVG

.A.85, 3KWO.C.161, 4KXC.A.1001, 2KZM.A.1, 3L0V.A.1, 4L5O.A.303, 2L8E.A.829, 3LAT .A.215, 1LBU.A.214, 3LE9.B.2, 2LGV.A.110, 1LG5.A.262, 1LG6.A.262, 4LGJ.A.301, 4L JP.A.1103, 4LOE.C.401, 1LPV.A.53, 3LS1.A.1, 2LUY.A.301, 3LUB.A.302, 2LUL.A.201, 2LVU.A.101, 2LVR.A.101, 2LVT.A.101, 4LW9.D.201, 2LXH.C.900, 2LXH.C.901, 3M0N.A.2 01, 2M0D.A.101, 3M15.B.107, 2M3H.A.102, 1M4M.A.502, 2M48.A.502, 1M65.A.300, 1M6H .A.1376, 3MBM.A.163, 1MC5.A.376, 3MEN.D.400, 2MIU.A.301, 2MIU.A.303, 4MLT.A.301, 1MMR.A.1, 1MOO.A.262, 1MP0.A.376, 1MQ0.B.147, 3MS3.A.401, 4MTW.E.401, 1MVH.A.50 3, 3MWM.A.142, 4MWP.E.401, 4MXJ.E.401, 1MZB.A.202, 4MZN.E.401, 4NOG.A.401, 4NOL. A.401, 3N2C.D.426, 4N4E.E.401, 4N4F.A.1401, 4N5P.E.405, 4N66.E.501, 1N92.A.375, 3NI5.A.262, 1NJ1.A.513, 1NJG.A.401, 4NJ5.A.803, 4NL4.H.802, 3NQZ.B.1, 4NQ5.A.301 , 4NQ6.A.302, 1NUI.A.501, 4O1K.A.301, 2O4H.B.401, 2O4Q.A.2402, 1OAL.A.152, 4OCM. E.401, 2OH3.A.300, 4OIW.F.501, 1OJ7.B.1389, 1OKN.A.262, 1ONW.A.801, 2OO4.A.6001, 1OSO.A.600, 1OS2.B.369, 4OTE.A.304, 2OVX.A.444, 2OVZ.A.444, 1P42.A.502, 1P5D.X. 500, 1P6B.A.406, 4P9C.A.201, 4P9C.B.201, 1PAA.A.160, 3PBB.A.391, 3PBJ.D.31, 1PE8 .A.317, 1PGU.A.616, 2PG3.A.300, 3PJN.A.186, 3PJN.B.186, 1PL8.A.402, 3PN3.A.1002, 3PN3.A.1009, 3PN3.B.1011, 3PNU.A.336, 3PZC.B.1000, 4Q0L.A.301, 1Q1A.A.701, 2Q1B .A.400, 3Q6V.A.2, 2Q6E.A.501, 4Q7R.A.301, 4Q7R.A.303, 4Q7R.A.306, 3Q94.A.301, 4Q BG.B.301, 4QEF.A.301, 1QF2.A.320, 3QH5.A.321, 3QHD.A.163, 2QJS.A.2002, 3QJ5.A.37 6, 2QL1.A.1, 2QQ4.A.139, 1QUA.A.999, 3QVZ.D.500, 3QW0.C.500, 1R22.A.502, 3R3L.A. 585, 1R4V.A.202, 2R59.A.701, 4R7M.D.1001, 4R9G.A.505, 3RC6.A.1, 3RCM.A.288, 3RHG .A.368, 1RJ6.A.601, 1RJQ.A.601, 1RJW.A.402, 2RJP.B.1, 2RMN.A.1, 2RPR.A.201, 2RPC .A.201, 4RQT.A.402, 4RQU.B.402, 2RSD.A.901, 2RSI.A.101, 1RUR.L.601, 1S4B.P.1, 2S RT.A.257, 3SSB.A.999, 3SU6.A.5, 1SW1.A.401, 3SZY.A.501, 3T01.A.502, 3T02.A.501, 3T5Z.A.262, 3T73.A.410, 3T74.A.410, 3T8G.A.411, 3T8F.A.411, 3T87.A.326, 3T8C.A.3 26, 3T8D.A.325, 3T8H.A.326, 1TBN.A.1, 3TGN.A.148, 3TGO.B.505, 1THJ.A.214, 1THJ.B .214, 1THL.A.324, 3TIO.A.1, 1TKH.A.901, 2TMN.E.321, 6TMN.E.322, 3TTY.A.676, 4TZC .A.501, 1U3L.A.701, 1U3T.A.376, 1U4G.A.9800, 4U4L.A.301, 3U7L.A.350, 4UA4.A.302, 4UA4.B.303, 3UCN.A.228, 1UD9.B.508, 3UJZ.A.1, 3UKO.A.401, 2UX1.K.1173, 2UYV.B.1 276, 1UZF.A.701, 1V0D.A.401, 2V2A.A.1275, 1V87.A.201, 1V87.A.401, 1VGN.A.301, 2V JE.B.1492, 2VXI.B.201, 2VXX.A.201, 2VXX.B.202, 1VYX.A.1061, 3W0F.A.301, 2W57.B.2 01, 1WAA.D.1090, 1WAA.E.1089, 4WAI.A.101, 4WD6.A.302, 1WE9.A.201, 1WEM.A.401, 1W EN.A.201, 1WEO.A.201, 1WEP.A.401, 1WEQ.A.401, 1WEV.A.201, 1WEV.A.401, 1WG2.A.400 , 4WH6.A.1203, 2WHG.A.1263, 1WIL.A.201, 1WIM.A.401, 3WIE.B.1001, 1WJA.A.56, 1WJB .A.56, 4WK7.A.501, 4WKE.A.501, 3WL3.A.301, 1WNU.A.1001, 2WO8.C.1268, 1WUR.A.1001 , 2WWO.A.1162, 3WXC.A.302, 1WYH.A.401, 1WYS.A.201, 3X17.A.601, 1X3C.A.201, 1X3H. A.201, 2X4H.B.1141, 2X4H.B.1142, 1X4U.A.401, 1X4V.A.201, 1X6E.A.401, 1XEM.A.401, 1XLL.A.399, 2XOC.B.991, 1XPZ.A.262, 1XTG.A.426, 1XUF.A.246, 1XUJ.A.246, 1XWH.A. 356, 2XXH.B.1303, 1XYD.A.94, 1XYD.B.94, 1Y23.A.1001, 2Y20.C.1331, 2Y28.B.1181, 1 Y3G.E.321, 2Y4Y.A.1172, 2Y4Y.C.1172, 1Y7W.B.285, 1Y8F.A.702, 1YEJ.L.605, 1YHC.A. 601, 1YM3.A.301, 1YO7.A.201, 1YSB.A.501, 2YSP.A.181, 2YTA.A.201, 2YUU.A.401, 2YX O.A.501, 2Z2Y.D.2004, 2Z26.A.400, 1Z6U.A.1, 1Z9G.E.1005, 1Z9N.A.201, 1ZDP.E.1005 , 2ZNE.B.993, 3ZPG.A.1358, 1ZSO.A.999, 1ZTQ.A.550, 1ZUD.1.501, 2ZXG.A.900, 4DLG. A.903, 1RVB.B.302, 3CIK.A.690, 1CLK.A.780, 3D19.E.301, 4DPG.F.604, 2E8A.A.501, 2 H5N.B.1001, 4HJH.A.552, 4HN2.A.404, 1IV4.D.1564, 2J4E.A.1002, 3KGX.A.503, 3LD0.J .54, 4NNN.K.302, 2NOG.A.9220, 2O1X.D.2004, 4O6I.B.602, 4OKE.B.203, 1RK2.C.320, 1 T9Z.A.273, 3TAV.A.269, 4U3W.A.503, 1WOH.A.1001, 2WHE.A.1222, 2XRI.A.1337, 1YQ2.E .7005, 2B0D.A.502, 4AAH.A.702, 1BRW.A.3001, 3NSJ.A.702, 2OVZ.B.449, 4POR.E.401, 1QHD.A.603, 1SBH.A.291, 1AOR.A.606, 1BFY.A.55, 1BIQ.A.377, 1BOU.B.501, 4C4U.I.20 1, 2CKF.C.501, 3D19.F.302, 2DE6.B.501, 4DHL.A.502, 1E3D.A.269, 4EB5.D.201, 4F1E. P.201, 2GBX.E.455, 3GC1.A.605, 3GL0.A.501, 3GL2.A.501, 2GYQ.A.402, 4HSL.A.202, 3 I01.B.800, 4ILT.A.301, 1JI5.A.152, 1JI5.B.151, 1JIG.A.402, 1JIG.B.401, 1JQK.B.80 1, 1LNB.E.900, 1N1Q.A.516, 1N1Q.B.515, 3NAO.C.150, 4NBA.A.501, 4NBF.A.501, 1NO3. A.858, 3PCK.Q.600, 2PCD.M.600, 4QLW.B.201, 3QVD.G.173, 1R2F.A.400, 1RSR.A.1004, 1ULI.A.600, 2XSO.G.901, 1YUX.A.303, 2AA0.A.2003, 2EHS.A.201, 4FHA.A.402, 1G8I.B.

1595, 2GG2.A.703, 1GW1.A.1423, 1IP3.A.999, 1MAU.A.499, 4NT8.A.206, 1ODZ.A.1427, 4OVZ.B.903, 4TMV.A.903, 1W7Z.A.1032, 2WHM.A.1425, 2X2E.A.1747

Table S119. 5-ligand combined metal, normal group

	size	largest_angle*	middle_1*	middle_2	middle_3*	middle_4
1	"298"	"172+/-3.7"	"79.8+/-4"	"84.4+/-2.7"	"87.5+/-2.2"	"90.1+/-2.5"
2	"144"	"153.9+/-7.9"	"70.1+/-4.2"	"74.7+/-4"	"78.9+/-3.9"	"83.5+/-5"
3	"333"	"166+/-4.7"	"83+/-4.3"	"86.8+/-2.6"	"89+/-2.2"	"91.1+/-2.3"
4	"387"	"176.5+/-2.5"	"86.1+/-2.5"	"88.1+/-1.7"	"89.3+/-1.1"	"90.5+/-1"
5	"260"	"171.4+/-4.4"	"80.2+/-5.1"	"85.3+/-3.6"	"88.4+/-2.8"	"91.8+/-2.7"
6	"254"	"165.6+/-5.7"	"74.9+/-4.5"	"80.4+/-4"	"85.2+/-3.7"	"89.4+/-4.3"
7	"217"	"154.2+/-7.3"	"71.8+/-5.1"	"81.1+/-5.4"	"87.2+/-5"	"93.2+/-4.4"
8	"238"	"156.3+/-5.1"	"74.9+/-5.5"	"83.5+/-4"	"89.1+/-4"	"93.2+/-3.7"
9	"245"	"169.3+/-4.4"	"79.6+/-4.9"	"84.8+/-3.4"	"88.5+/-2.7"	"91.9+/-2.7"

	middle_5*	middle_6	middle_7	middle_8*
1	"93.2+/-3"	"97+/-4.2"	"103.2+/-6"	"165.3+/-4"
2	"89.2+/-6.4"	"99.5+/-11.9"	"113.6+/-14.2"	"141.4+/-7"
3	"94.8+/-3"	"100.1+/-3.4"	"105.1+/-4.4"	"160.1+/-4.3"
4	"91.6+/-1.4"	"93.9+/-2.4"	"96.4+/-3.3"	"174+/-3.5"
5	"95+/-3.4"	"99.6+/-4.7"	"118.9+/-3.6"	"128.2+/-4.8"
6	"94.5+/-5.3"	"99.5+/-6.3"	"110.8+/-10.9"	"154.3+/-4.9"
7	"98.9+/-4.5"	"108.5+/-6.2"	"119.7+/-6.1"	"134.1+/-5"
8	"98.8+/-3.8"	"105.7+/-3.7"	"113.4+/-7.6"	"147.7+/-4.3"
9	"95.6+/-3"	"101+/-5"	"116.4+/-7.4"	"141.7+/-5.5"

	smallest_opposite_angle*	TrigonalBipyramidal	SquarePyramidal
1	"82.8+/-4.7"	"0.004"	"0.318"
2	"71.5+/-6.3"	"0.003"	"0.008"
3	"94.7+/-3.5"	"0.011"	"0.111"
4	"88.5+/-3.4"	"0.001"	"0.335"
5	"110.3+/-4.2"	"0.392"	"0.002"
6	"77.1+/-5.5"	"0.005"	"0.085"
7	"103.3+/-5.9"	"0.1"	"0.004"
8	"97.5+/-5.2"	"0.036"	"0.033"
9	"99.3+/-5.7"	"0.166"	"0.04"

	TrigonalPrismaticV
1	"0.021"
2	"0.062"
3	"0.023"
4	"0.002"
5	"0.071"
6	"0.047"
7	"0.192"
8	"0.131"
9	"0.072"

Table S120. Cluster members of 5-ligand combined metal, normal group

[1] "Cluster 1"
2BIB.A.1550, 3EII.A.301, 2GC2.A.401, 2HD1.A.101, 4ICQ.A.501, 2K78.A.151, 3KR5.E.1001, 4KYH.A.202, 4N7K.L.307, 3NO5.C.275, 4OK2.A.801, 1Q3K.B.300, 4R6T.D.1001, 4R7M.C.1001, 3V93.D.701, 1VKG.B.400, 4X2T.A.702, 3A4K.C.301, 4BDZ.A.1380, 4D6O.A.1186, 3ICE.E.502, 3MDA.A.577, 4ACF.D.1480, 1ALK.A.452, 2AUU.A.201, 3B05.A.1001, 2B8W.A.595, 4BBJ.A.750, 2BHW.A.601, 2BHW.A.602, 2BHW.A.603, 2BHW.A.614, 2BHW.B.605, 4BJU.A.998, 2BVN.A.1395, 4BYF.A.1000, 3C9U.A.309, 3CBT.A.301, 3CRL.A.2000, 3CX7.A.378, 1D0X.A.998, 1D0Y.A.998, 1D1B.A.998, 4DBQ.A.903, 2DCN.B.4006, 3DHF.A.502, 3DKL.A.502, 4DL8.A.304, 4DLC.A.303, 3DYH.A.3002, 1E1R.A.601, 1E1R.F.601, 1E79.A.601, 2E8W.A.1201, 2E8X.A.1301, 2E91.B.1303, 3EF1.A.1, 3EFQ.B.4003, 2FKW.A.1501, 1FQJ.A.352, 2G07.A.601, 2G08.A.500, 1G67.A.2007, 3G8D.B.1002, 2GTP.A.401, 4HGQ.C.201, 2HO4.B.301, 3HWX.1.602, 3HZH.A.202, 2I19.A.3002, 3ICN.A.402, 4IDO.A.503, 4IGA.A.200, 2IK2.A.289, 2IOA.A.5002, 2J7N.A.3374, 2JCM.A.1490, 2JCS.B.1211, 1KK8.A.997, 4LCZ.A.306, 3MCO.A.427, 3MLE.C.222, 1N24.B.706, 2NGR.A.199, 4NM5.A.406, 3NNN.A.401, 4NST.A.1103, 1NVV.Q.1002, 2ODE.A.3001, 3OLV.A.130, 2PLS.I.606, 1PPW.A.401, 2PUL.A.400, 3PUW.A.1501, 1Q3H.D.674, 2Q58.A.3, 4QXD.A.304, 1ROX.D.14, 1RLT.A.805, 3SQS.A.450, 3SS8.A.302, 1SVK.A.356, 3T34.A.1002, 3T5P.F.301, 1T91.A.1301, 4TQ4.C.402, 3U2E.A.2, 1UPB.A.601, 2V54.B.1205, 1W5T.A.701, 3W6P.A.803, 3WK4.A.601, 1YXO.A.5000, 2Z4Z.A.1301, 1Z5B.A.2001, 2Z7I.B.1302, 1ZH4.B.202, 2ZRW.A.702, 4BXO.B.1216, 4G3I.B.401, 3GII.A.415, 4K4I.A.603, 4LQ0.A.401, 1R7M.A.304, 1TW8.C.803, 4AG4.A.5001, 4B7M.B.1471, 2BAT.A.601, 2BD3.A.260, 2BV2.A.1085, 2BW7.A.2202, 3BYC.A.901, 2CDO.B.1140, 2CHI.A.218, 5CHY.A.401, 1CJY.A.951, 4CPN.A.500, 4CPO.A.1466, 1CXV.B.6, 2D00.B.1002, 2DEW.X.903, 1DYK.A.4001, 1E35.B.260, 3EKI.A.602, 2ERQ.B.702, 1EZS.C.650, 1F2N.A.1002, 3FRP.A.628, 4GDI.C.507, 2HTV.A.995, 4ILW.D.304, 1IME.B.278, 1INW.A.501, 1J0Y.D.701, 1JI3.A.401, 4JUC.B.601, 3K37.A.467, 1L6J.A.502, 1L9N.A.703, 3LNF.B.305, 1MTV.A.480, 1NG0.A.1002, 1NG0.C.1001, 1NKQ.A.260, 2NVO.A.533, 1NXO.A.902, 3O9J.A.995, 2OO4.B.5006, 2OW1.B.447, 2P5V.C.1002, 1PK8.F.817, 3Q2L.A.703, 4Q4X.1.5005, 3QNI.B.400, 2R1D.B.1000, 2R8Z.J.210, 1R8L.B.902, 2RHP.A.10, 2RHP.A.20, 2RJP.A.2, 2RJP.A.3, 2RJQ.A.6, 3SHI.G.305, 3SJS.A.222, 1STB.A.150, 1T6B.X.800, 3V03.A.584, 3V03.B.585, 1V7V.A.1001, 1W3M.C.3014, 2WG8.A.201, 3WIU.B.1002, 3WNK.A.812, 1YO8.A.1197, 1Z4V.A.600, 2ZJ6.A.625, 2ZUX.B.638, 2ZWO.A.400, 4AC8.D.500, 1AHJ.A.208, 3AK3.C.215, 1BT8.B.202, 3BXD.A.302, 4CAB.A.537, 3DHG.D.507, 1FZ1.B.5003, 1H2K.A.1350, 1H2L.A.1350, 3HC1.A.305, 1HDS.A.142, 3HF4.F.147, 1HV4.A.151, 4IGO.A.1000, 2INN.A.513, 1J1L.A.1001, 1LC1.A.105, 1LH2.A.154, 1LH6.A.154, 3LMX.M.600, 4M26.C.401, 4M71.B.403, 3MZS.A.500, 3N1Y.A.503, 3N1Y.A.504, 3OOF.A.304, 2O68.A.401, 2OGI.A.301, 2OYY.A.201, 4OZ5.A.201, 3PCA.M.600, 3PCJ.R.600, 2PQ7.A.221, 1QHW.A.433, 3QY6.A.264, 2RDN.A.1, 2RFB.A.410, 1S05.A.130, 1SMJ.C.472, 1TMX.A.861, 3U9J.A.200, 3U9M.A.201, 3UF9.B.315, 1UTE.A.501, 3VSI.B.401, 1W2A.X.1302, 1WRA.A.401, 2XMO.B.556, 2Y0I.A.1350, 2Z36.A.450, 2ZPG.A.300, 3JPP.A.340, 1ORP.A.224, 3AGB.A.1, 4BDR.A.902, 4BVN.A.1360, 4C7A.B.1159, 4CZN.A.1371, 4D1I.A.600, 4DF9.A.501, 4F3Y.A.301, 4FEW.D.304, 4GDK.E.301, 2GEZ.A.401, 2GTW.E.3006, 1GV2.A.1192, 4GY9.A.207, 4I0W.D.602, 4I2F.A.602, 4I2R.C.602, 3I44.A.477, 3IFV.C.408, 2J5W.A.3044, 3JS4.A.208, 4KA8.A.806, 4L3H.A.404, 3LP5.A.251, 4M4V.A.505, 4MM9.A.602, 4MPT.A.402, 2O34.B.502, 2OKQ.A.119, 3OTK.A.586, 4PUV.A.405, 4PV3.A.201, 4QFE.B.305, 1QHU.A.437, 1TQY.H.1094, 2V4B.B.1562, 1VI6.A.208, 3VS8.A.501, 3VS8.C.501, 3WNM.A.802, 1WPG.D.1300, 2WU2.A.1590, 2WV7.B.401, 1X7D.A.1501, 1XDF.A.401, 2XZK.B.507
[1] "Cluster 2"
2BPF.A.339, 4DPV.N.12, 4M30.B.302, 4W9M.E.902, 1A49.B.1134, 1A49.C.1734, 4AN9.A.1383, 3AQC.D.328, 1AZT.A.403, 2BBO.A.2, 3C5P.D.302, 3CNX.B.170, 1CQP.A.310, 4CYU.A.170, 3DUF.C.1368, 3DV0.A.1368, 3DYF.A.3002, 3DYF.A.3004, 4E1E.A.401, 3EA5.C.221, 1ELY.A.452, 1GIM.A.435, 1GY3.A.1298, 2GZD.A.950, 4HYP.B.302, 3IAF.B.572, 2IO7.A.5002, 4IYN.A.804, 1JM6.A.4601, 4JVJ.F.405, 4K81.B.203, 4LF2.D.601, 1MF0.A.1453, 4NEH.A.1102, 1NUZ.A.2342, 2O1X.A.2001, 4O1P.C.903, 3OAB.A.904, 4OAU.C.802, 2

OI2.A.400, 2POC.B.5, 4POV.A.403, 1Q19.A.504, 3RBM.A.1003, 4RJK.B.602, 3RUW.A.544
, 4S17.C.502, 3SBE.A.501, 3TW6.B.2002, 2WB4.B.502, 3WQP.J.501, 2X9H.A.3001, 1XF9
.B.11, 1Y8P.A.501, 1Y9I.B.602, 1ZCA.B.1383, 4K4G.I.602, 3NDK.A.906, 3OOL.A.237,
3SQ1.A.907, 1AFD.2.2, 1AR1.A.561, 1AVX.A.700, 1B09.C.302, 3BJU.C.608, 2CLT.A.110
1, 3D4G.B.485, 1EAK.D.998, 1EGZ.B.300, 4ELF.F.201, 1ESP.A.320, 3FCS.D.2002, 4FU4
.A.505, 3FW0.A.823, 3GCW.E.1, 4GKX.B.302, 3HLH.D.315, 2HTY.G.997, 1HVD.A.602, 1I
XX.D.124, 3KQA.A.420, 1KX1.C.301, 4KZW.B.400, 4L06.B.501, 4L06.C.501, 4L06.D.501
, 1L7L.A.201, 3L95.X.2001, 3LCP.D.159, 4LIN.E.1301, 3LND.A.210, 1LWJ.A.883, 3MOW
.H.202, 3MW3.A.302, 2OKX.A.4004, 1OS2.C.473, 3OSH.A.121, 3QWU.A.601, 2RHP.A.24,
2RHP.A.25, 2RJQ.A.5, 3S5U.A.220, 3S5U.D.220, 4SBV.A.261, 4SBV.C.261, 1TN3.A.183,
4TVU.B.600, 1TYE.B.1402, 4UB6.O.301, 1UZJ.C.3648, 2V3T.A.1264, 1V3J.B.689, 3VOT
.A.504, 2W2O.E.1333, 1W2M.E.1442, 3W9T.C.1010, 3W9T.B.508, 2WVX.C.801, 2WW0.C.80
0, 2WW0.F.800, 2X0G.B.1149, 1YO8.A.1184, 1ZZH.A.401, 1XVE.A.1170, 2YU1.A.600, 4K
HY.A.1013, 2A5G.B.242, 4B6C.A.1257, 4C1P.A.1728, 4CBY.B.2036, 1D7U.A.435, 3G8Q.D
.302, 4GKI.F.303, 4H41.B.405, 3IC9.A.491, 3KZW.D.498, 4MVJ.A.401, 4NLQ.A.910, 4N
T1.A.301, 4OOC.B.402, 2P6Z.A.402, 3WGU.A.2005, 2WWG.B.213, 2WX5.H.1252
[1] "Cluster 3"
4A7K.A.952, 3ADR.A.263, 3ASE.A.156, 2BCN.A.295, 3BUD.A.1048, 2DI3.A.1002, 4E5V.B
.401, 2FV9.B.4, 3G4K.A.801, 2GC3.A.402, 1GKP.B.1460, 3H8F.E.501, 2ICS.A.400, 4IG
N.B.401, 1ITU.A.402, 2J0E.B.1265, 4JAA.A.501, 1K9Z.A.402, 4KBP.A.439, 1LCP.A.489
, 3LL8.A.505, 2LQ6.A.402, 4N27.C.201, 3O64.A.1, 2OUN.B.404, 2OW7.A.5001, 4PKT.A.
802, 4PKW.A.801, 1Q74.A.304, 3Q9B.A.345, 2QFR.A.434, 1QIP.A.902, 1QIP.D.903, 3S2
M.A.403, 3S2N.A.403, 3SFW.A.501, 1TQU.A.1400, 3VH9.A.301, 3VUS.A.401, 2W3Z.A.131
2, 1XP3.A.301, 1Y13.A.174, 2PYJ.B.9002, 3Q8P.B.423, 3SNN.A.906, 3TFR.A.340, 3AU9
.B.602, 2BBT.A.3, 4BCL.A.367, 4BCL.A.368, 4BCL.A.369, 4BCL.A.370, 2BHW.A.604, 2B
HW.A.612, 2BOZ.M.1304, 1BS1.A.901, 4DL8.A.305, 1DXR.L.400, 1E14.M.1303, 1E6D.M.1
303, 1E9I.B.1431, 3ENI.A.375, 3ENI.A.378, 3EOJ.A.375, 2FKW.B.1601, 2FKA.A.9001,
1FMW.A.800, 2GLQ.A.2003, 4GVE.A.602, 4HEO.A.402, 2J8C.L.1288, 1JB0.A.1011, 1JB0.
A.1106, 1JB0.A.1107, 1JB0.A.1112, 1JB0.A.1117, 1JB0.A.1121, 1JB0.A.1122, 1JB0.A.
1128, 1JB0.A.1129, 1JB0.A.1132, 1JB0.A.1133, 1JB0.A.1134, 1JB0.A.1136, 1JB0.B.12
01, 1JB0.B.1203, 1JB0.B.1204, 1JB0.B.1206, 1JB0.B.1207, 1JB0.B.1211, 1JB0.B.1214
, 1JB0.B.1215, 1JB0.B.1221, 1JB0.B.1223, 1JB0.B.1231, 1JB0.B.1234, 1JB0.B.1235,
1JB0.B.1239, 1JB0.L.1501, 1JB0.L.1502, 1JYX.A.3002, 3L8F.A.401, 4LCZ.A.314, 4LRJ
.A.302, 1MNZ.A.389, 1N22.B.706, 3NNS.A.401, 4NNN.Z.301, 3O5T.A.299, 4OEC.A.401,
2PAN.A.851, 4PTK.A.302, 3QQV.A.381, 1QSH.B.147, 4QTD.A.426, 1R03.A.301, 4R02.Y.3
02, 3RLG.A.286, 1RWT.A.614, 1RZH.L.854, 1RZH.M.851, 1RZH.M.853, 3TAV.A.266, 3THU
.A.500, 4UB6.B.603, 4UB6.B.607, 4UB6.B.610, 4UB6.B.616, 4UB6.C.504, 4UB6.C.506,
4UB6.C.507, 4UB6.C.509, 4UB6.C.510, 4UB6.C.512, 4UB6.C.513, 4UB6.C.514, 4UB6.D.4
02, 4UM8.D.2001, 2UXR.A.1405, 2VP0.B.1209, 3VTH.A.805, 3WU2.A.410, 3WU2.B.604, 3
WU2.C.502, 3WU2.C.510, 3WU2.D.402, 3WU2.b.615, 3WU2.b.616, 3WU2.b.617, 3WU2.c.90
2, 2YBE.A.1417, 1YF6.M.853, 1YF6.M.856, 1YQ7.A.908, 1Z2P.X.1295, 1ZM7.D.400, 1ZX
N.A.900, 2WTF.B.1509, 1ALA.A.402, 4CCE.A.4001, 2CDP.B.1140, 3CKC.B.700, 4DWW.A.3
01, 1E5J.A.402, 4HZY.A.507, 1JAO.A.996, 3K5T.A.802, 4KS2.A.501, 1MR8.A.102, 1N28
.A.126, 1N28.A.128, 4N2F.A.703, 4PKH.J.1201, 1Q3A.B.472, 3Q3K.A.262, 1QL9.A.480,
4QN3.B.501, 2R8Y.C.203, 1ROS.B.503, 1SOE.A.1293, 1SMP.A.478, 4U32.A.301, 1ULV.A
.2005, 1VOZ.A.1477, 1WVM.A.604, 1Y4A.E.1001, 2Z8S.B.647, 2ZUY.A.627, 2ZWO.A.403,
2AA1.B.400, 4AUL.A.754, 4B2O.A.1266, 1BBH.A.132, 2BCN.B.109, 1BGP.A.400, 4BLY.A
.500, 4BM1.A.500, 2BMO.A.1441, 2CCY.A.129, 1CG5.A.142, 1CGN.A.128, 1CGO.A.128, 2
CJ1.A.1300, 1CPQ.A.130, 1D06.A.501, 1DOC.A.500, 1D2V.A.605, 4D3O.A.901, 1DRT.A.3
25, 3E65.A.901, 1ECD.A.137, 3EH5.A.801, 3EJD.B.405, 4ENP.A.801, 1EOB.B.600, 1EUP
.A.410, 1EYS.C.612, 4FAG.A.401, 4FB2.B.501, 2FDG.A.300, 1FT5.A.214, 3G1Q.A.480,
4G2C.A.501, 1GBU.B.148, 2GB8.A.295, 1GCV.A.141, 1GCV.B.137, 1GVH.A.1398, 1GW2.A.
350, 1GWS.A.615, 1GWU.A.1306, 1GY9.A.300, 4H8Q.A.201, 3HF2.A.482, 1HRM.A.154, 3H
X9.A.300, 1IBE.A.143, 3ICF.A.601, 2IIZ.A.400, 2ISA.A.486, 1IT2.A.147, 1ITH.A.143

, 1IZO.C.501, 4J1X.A.201, 2J2M.A.501, 1JAF.A.130, 3K9V.A.520, 4L7Y.B.201, 2L8M.A.416, 1MBA.A.148, 1MGN.A.154, 3MOL.B.185, 1MQV.A.150, 1MXR.A.1003, 3N3R.A.1500, 4NKW.A.600, 2NOX.C.500, 3NU1.A.302, 3O6J.A.300, 4OJ8.B.301, 1OM4.A.750, 1OUT.A.143, 1OUT.B.148, 1P3T.A.300, 3Q14.A.501, 3QPI.A.1001, 1QWL.B.550, 3QY8.A.252, 3QZM.B.201, 2RCL.B.600, 4S1C.A.801, 3SCF.C.203, 3SIK.A.154, 1SOG.A.296, 3T4V.A.300, 3TKT.A.431, 4TRI.A.501, 3TTV.A.760, 3UFK.A.920, 3UHK.A.147, 1V54.A.516, 3VER.A.601, 2VE3.B.1444, 1VHB.A.150, 2VV6.C.1259, 3W8M.A.201, 3WAQ.A.201, 2WIV.A.1553, 2XF2.A.690, 2XMO.A.1128, 1XZ5.A.142, 1Y5F.B.147, 1Y5J.B.147, 1YFW.A.300, 2Z3U.A.500, 2Z6F.A.3747, 2ZDO.A.200, 2ZYQ.A.301, 1VQ7.Q.9148, 4ADB.A.1405, 3ASQ.B.701, 3AST.B.701, 4AYO.A.502, 3C7E.A.489, 4CSH.A.1169, 3CZJ.A.3102, 3DR3.A.335, 2DV1.A.1000, 3EEB.A.211, 3H7K.A.387, 3IJP.A.301, 1IYN.A.298, 2IY6.B.1540, 3K6A.E.178, 4KXW.A.1013, 4KZV.A.304, 1L2T.A.1502, 1LZS.A.131, 3MUZ.1.3102, 4N3M.A.403, 4ODI.B.301, 4PMO.A.310, 3Q94.A.310, 1QOP.B.501, 2QZ7.A.193, 3U21.A.500, 2WUW.E.1277, 3WXO.A.805, 1X0G.A.1002, 1X7U.A.1000, 2Y8K.A.1527, 2Z2F.A.2001, 2ZND.A.195
[1] "Cluster 4"
2EK9.A.1004, 4HTZ.B.1001, 2LZE.A.101, 1ML2.A.296, 4N7K.L.301, 4N7K.M.401, 4N7K.M.402, 4N7L.M.402, 2IHM.A.700, 3A0U.A.201, 3A10.A.201, 2AKZ.A.441, 2BHW.A.609, 3BH7.A.1, 4BX2.A.301, 4C5A.A.331, 1D0Z.A.998, 1D1A.A.998, 3DNT.B.442, 3EF0.A.1, 3G5A.C.307, 2G77.B.503, 1GFI.A.356, 2GJ8.A.602, 3GL9.A.123, 3H1E.A.202, 1HE1.C.202, 2HEG.A.300, 2HF7.A.700, 2I7D.A.728, 3ICM.A.403, 4IF4.A.300, 1IV2.B.1572, 1J97.A.220, 4JA2.A.201, 1JB0.A.1110, 1JB0.A.1118, 1JB0.A.1130, 1JB0.X.1701, 4KEM.A.401, 1L3R.E.392, 1L5Y.A.701, 1L7N.A.221, 4LCZ.A.307, 4LCZ.C.316, 4LEO.A.201, 1LGH.A.57, 1LGH.A.58, 1LGH.B.59, 4LRS.A.404, 1LVK.A.998, 1MPS.M.801, 1MXO.E.501, 3N5K.A.2001, 1N6K.A.201, 4NV0.A.402, 2OGX.A.291, 4OVN.A.201, 2OZE.A.299, 3PDE.B.310, 3PL9.A.602, 3PL9.A.603, 3PL9.A.609, 3PL9.A.610, 3PL9.A.612, 3PL9.A.613, 3PL9.A.614, 2PL1.A.204, 3QHW.A.298, 2R25.B.1, 1RLO.A.801, 4RUR.W.301, 4S1H.A.303, 3T2S.A.401, 3T6D.L.401, 3T6D.M.400, 3T6E.L.400, 3T9E.A.602, 3TCS.B.368, 1TX4.B.681, 2VB6.A.1000, 1W7J.A.1793, 1W9I.A.1755, 3W9S.A.202, 2WF7.A.1220, 2WJN.M.1325, 1WQA.B.456, 2WZB.A.1417, 2X2E.A.1746, 1XBX.A.601, 1XHF.A.1001, 1Y9D.A.2601, 1YZQ.A.901, 1ZES.A.302, 2ZXE.A.2002, 4K4H.M.604, 1D7X.B.805, 3DPE.A.997, 4FVL.B.506, 2GNT.A.254, 4GN7.B.301, 2JG9.B.1224, 2NW6.A.613, 1QI5.A.452, 1RU4.A.2, 1UYX.B.1135, 3VV3.A.404, 1ZTQ.A.561, 3A15.A.354, 3A51.C.412, 3ABB.A.1430, 4AJ9.A.1715, 2AKJ.A.564, 3ARJ.A.153, 3AT6.A.142, 2AUO.A.153, 2AUQ.A.147, 2AV0.A.147, 4AVD.A.144, 3AYF.A.802, 1B0B.A.144, 1B7V.A.93, 3BA2.A.158, 1BCF.A.200, 3BK9.A.401, 3BUJ.A.398, 2C1D.H.1158, 4C50.A.1741, 3C6G.A.601, 1C6S.A.88, 4C9L.A.1418, 1CG8.B.142, 1CH4.A.147, 2CMM.A.155, 1CRC.A.105, 4CZC.A.1337, 2D0Q.A.300, 3D1K.A.200, 3D1K.B.400, 2D5X.B.147, 3DAM.A.600, 3DAX.A.601, 3DBG.A.500, 3DE8.D.150, 1DGF.A.3000, 2DKK.A.430, 1DLY.A.144, 4DVQ.A.601, 1DW1.A.113, 4DWU.A.201, 1E2R.A.602, 2E39.A.401, 3E4W.A.501, 3E5L.A.1408, 2E84.A.1315, 3EAH.A.861, 4EGO.A.501, 3EJ8.A.1901, 4ENU.A.801, 1EQD.A.185, 4ESA.A.202, 4ESA.B.202, 2FDU.A.500, 1FHF.A.350, 1FHJ.B.147, 4FVC.A.201, 2G3H.A.154, 3G46.A.147, 4G45.A.401, 3GAS.A.1290, 3GE3.A.502, 1GEJ.A.501, 4GEP.A.580, 2GGN.X.251, 1GJQ.A.602, 2GKM.A.144, 3GPH.A.500, 4GQS.B.501, 2GYQ.A.401, 4H0K.A.200, 1H1X.A.1154, 1H97.A.148, 2HBT.A.900, 3HC1.A.306, 1HJ4.B.601, 1HJ5.B.601, 2HMQ.B.115, 4HRR.A.201, 4HRR.B.201, 2HYS.A.201, 1HZU.A.601, 1I3D.A.147, 1I4Y.E.605, 4I8V.A.601, 2I96.A.129, 4IAM.A.501, 2IBN.B.706, 3IBD.A.500, 2IG3.A.700, 1IOP.A.154, 3IQB.A.500, 1IRD.B.347, 1IWH.A.142, 1IX4.A.300, 2J0P.A.1342, 2J7A.D.1001, 1JEB.D.147, 4JET.A.201, 4JS9.A.501, 2JXM.B.250, 4KFO.A.501, 2KII.A.182, 3KX4.A.999, 1L0L.D.242, 4L1Y.A.300, 4L1Z.A.300, 4L2M.A.201, 4L3H.A.402, 2L4D.A.107, 4L54.A.501, 1LGA.A.396, 1LH7.A.154, 1LHT.A.155, 2LH2.A.154, 2LHB.A.151, 3LL8.A.506, 1M54.F.1620, 1M56.A.1002, 1M7S.D.600, 1M85.A.1001, 2MHR.A.119, 3MM3.A.501, 3MM6.B.570, 3MMB.A.580, 3MMO.A.1004, 4MMO.A.401, 1MN1.A.396, 1MYF.A.154, 3MYM.A.139, 3MYN.A.139, 4N4M.A.616, 4N4N.C.601, 3N8Y.B.601, 1N97.B.603, 3NAO.A.601, 4NK2.A.700, 3NN2.A.239, 3NNF.A.600, 3NNL.A.600, 2NNJ.A.500, 2NP1.A.350, 1NR6.A.500, 3NTG.D.601, 3O5C.B.402, 1OAE.A.1125, 3OCD.C.401, 3OCD.D.401, 1OCZ.A.515

, 1OD0.A.1407, 3OFU.A.417, 1OG5.A.501, 1OIK.A.1302, 2OIF.A.163, 4OQR.A.501, 1OR4
.A.180, 1OZW.B.300, 3P3X.A.501, 2P85.A.500, 3PM0.A.900, 2PMS.A.347, 3PT8.A.500,
3PT8.B.500, 1Q5E.A.440, 2Q8P.A.300, 3Q9K.A.605, 2Q9F.A.602, 1QGJ.A.1350, 3QGP.A.
200, 1QJS.A.500, 3QQR.B.163, 2QRW.A.700, 2QSS.A.142, 3QZX.A.200, 2R1H.D.148, 4R2
1.A.600, 2R79.A.500, 3R9B.A.501, 2RF7.D.1, 2RI4.A.142, 3RIW.A.305, 4RKM.K.809, 4
RKM.L.813, 3RUR.A.200, 1S13.A.300, 1S1F.A.430, 1S61.A.144, 1S69.A.125, 3S66.A.14
2, 3S79.A.600, 1SCH.A.300, 1SI8.A.501, 1SPG.A.144, 1SPG.B.148, 1SQ3.A.903, 1T47.
B.430, 1T85.A.417, 3TBG.A.800, 3TM8.A.902, 3TM8.A.903, 2TOH.A.501, 3TTW.A.760, 3
TTX.B.760, 1TWN.A.300, 3TYW.A.501, 1U5U.A.999, 4U9D.D.201, 3UA1.A.508, 4UBS.A.50
1, 1UC3.A.150, 3UHB.A.147, 3UHD.B.147, 1UMO.A.1172, 3UOI.I.200, 3UT2.A.1500, 1UX
8.A.700, 1V4U.A.144, 1V4U.B.147, 1V9Z.B.1140, 3VED.A.401, 2VHD.A.401, 3VNO.A.501
, 3VOL.A.401, 1VRE.A.148, 3VRF.B.201, 2VZW.A.1206, 3W08.A.501, 3W4U.A.201, 1W92.
A.1149, 3WCU.A.200, 3WCU.C.200, 3WCU.B.201, 3WCU.D.201, 3WFB.B.802, 4WG2.A.603,
3WNU.A.801, 1WOW.A.300, 3WXO.A.801, 4WX0.B.301, 2WY4.A.150, 2X66.A.1359, 1X8V.A.
470, 1X9F.A.160, 1X9F.B.160, 1X9F.C.160, 1X9F.D.160, 2XBK.A.1398, 2XN8.A.1434, 2
XQ1.B.1503, 1Y01.B.142, 2Y4F.A.389, 2YGX.D.450, 2YL1.A.128, 1YMC.A.154, 2Z6N.A.1
50, 2Z6N.B.150, 2ZFO.D.200, 3ZK5.A.1407, 3ZKY.A.1332, 2ZVU.A.300, 2ZZS.2.220, 2F
MS.A.342, 4GXK.A.405, 2PFQ.A.1, 4TUR.A.404, 4G1K.B.301, 1GEN.A.304, 1GUU.A.1090,
 2HU3.A.9002, 1HXN.A.2, 2J5W.A.3045, 2JHN.A.1298, 4PFI.A.401, 3STH.A.501, 1SU3.A
.911, 1W16.A.1002, 1W9W.A.900, 1ZDN.A.157
[1] "Cluster 5"
2JZW.A.57, 4LJ0.B.502, 4OND.E.101, 1QUM.A.302, 2A7M.A.252, 1AH7.A.246, 1AH7.A.24
8, 2AIO.A.315, 1AST.A.999, 3BJC.A.876, 4BJH.A.423, 4C24.A.301, 4C6L.A.2824, 4C8I
.B.1161, 2CHI.A.212, 3CKI.A.501, 3CQJ.A.285, 4CWM.A.433, 4CXO.A.453, 4CXV.A.433,
 3D0N.A.262, 1D5J.A.301, 4DEL.A.402, 3DHA.A.255, 4DLM.A.401, 2DVU.A.1501, 4DYK.A
.501, 2E2D.A.500, 2EG6.A.400, 2EG7.A.401, 4ENL.A.438, 6ENL.A.438, 3EWC.A.372, 3E
WD.A.371, 2EWB.A.488, 1EYW.A.401, 4EYF.A.302, 4EYF.A.303, 1F0J.A.1101, 3FDK.A.40
1, 2FGN.A.248, 4GBD.A.502, 1GKP.A.1461, 2GSO.A.1000, 1GVF.A.288, 1GYT.G.600, 1GY
T.G.601, 4H01.A.601, 4H2E.A.301, 1H4N.A.262, 1H9N.A.262, 2HBM.A.1001, 3HC8.A.864
, 2HC9.A.701, 3HK9.A.431, 3HKA.A.430, 2HUC.A.248, 3HWP.A.295, 1HZY.B.401, 4IG2.A
.401, 4ILW.D.301, 4ISM.A.201, 2J0T.A.1267, 4J4K.A.402, 4J5H.A.302, 1J79.A.400, 1
J79.B.400, 4JD1.A.201, 4JH8.A.201, 4JH8.B.201, 1JIW.P.481, 2JNE.A.150, 4JS6.A.30
1, 1K07.B.3, 3K2G.A.400, 2K2C.A.140, 4KAP.A.301, 4KEQ.A.302, 1KMG.A.154, 3KM8.A.
400, 3KNS.A.228, 3KRY.A.1999, 4KTX.A.501, 3L6N.A.301, 1LAM.A.488, 4LCQ.A.601, 4L
EF.A.301, 2LFF.A.500, 3LGG.B.512, 3M4C.D.109, 3MA2.D.294, 3MA2.A.294, 3MJM.B.401
, 3MKV.B.425, 3MTW.A.1, 3MVI.A.901, 3N2C.A.425, 3N9S.A.309, 3NJ9.A.262, 2NQH.A.3
01, 2NQH.A.302, 2NZE.A.401, 3O2X.A.1999, 4O98.B.401, 2OB3.A.901, 1ONW.A.800, 2OW
1.A.444, 1P6B.A.401, 1P6D.A.248, 1PBO.A.1302, 1PL6.A.402, 3PNU.A.337, 1PV9.A.401
, 4PVO.A.402, 2Q02.C.300, 3Q6X.A.3, 3Q6X.B.271, 3QDF.A.266, 1QF0.A.320, 1QF1.A.3
20, 1QTW.A.303, 2QVV.A.403, 1QXL.A.400, 2R2D.A.278, 1R3N.A.500, 3RHG.A.367, 4RL2
.A.301, 4RL2.B.302, 1SHN.B.482, 3SNG.A.401, 3SXK.A.4, 3TOM.B.108, 4TYT.A.301, 1U
IO.A.400, 2USH.A.601, 1UXA.B.1367, 3V96.B.301, 1VFL.A.501, 3VQZ.A.302, 3W52.A.31
1, 3WAX.A.912, 3WAY.A.911, 1WPP.A.602, 2WXU.A.1375, 2WXT.A.1371, 2XF4.A.1211, 1X
M8.A.700, 2XS3.A.999, 2XS4.A.999, 2Z24.A.400, 2Z24.B.400, 2Z25.A.400, 2Z25.B.400
, 2Z26.A.401, 2Z27.A.400, 2Z27.B.400, 2Z28.A.400, 2Z28.B.400, 2Z29.A.400, 2Z29.B
.400, 2Z2A.A.400, 2Z2B.A.338, 1Z60.A.1, 2Z04.A.319, 3ZU0.B.1589, 2ZWR.A.208, 2ZW
R.A.209, 1ZZM.A.401, 1ZZM.A.402, 4AZW.A.1452, 2EB6.A.1001, 3FSY.A.334, 3FYY.A.40
1, 3GQ7.A.692, 1J7U.A.301, 1LP4.A.342, 1NUY.A.2342, 2QVU.A.340, 2VPQ.B.1451, 2VA
2.B.1343, 1AWB.B.279, 4B7U.D.401, 1CIZ.A.305, 2DSN.A.2011, 3ELM.A.303, 2EXH.C.20
03, 2J7A.D.1007, 1OLP.B.1372, 3QHQ.A.230, 1RM8.A.504, 3TRQ.A.359, 2ZUY.A.629, 4A
IQ.A.1746, 1B06.A.322, 2BKB.A.1193, 2BUU.B.1541, 2BUW.B.600, 2BUQ.B.1541, 3CEI.A
.500, 2CW2.A.402, 2CW3.A.524, 4DVH.A.301, 3E13.X.326, 3ESF.A.198, 4F2N.B.300, 4F
FK.A.301, 2GOJ.A.198, 2GPC.A.195, 3H1S.A.1001, 1HMD.A.115, 4IEZ.A.501, 1ISA.A.19
3, 4KEZ.A.401, 4L2B.A.201, 4L2C.A.201, 3LIO.A.5000, 1MY6.B.200, 1QNN.A.201, 2R1K

.A.800, 4REU.A.201, 3TQJ.A.1001, 1UNF.X.1239, 2W7W.A.1195, 1WB7.A.212, 1WB8.A.21 2, 1Z60.M.300, 1ZA5.B.393, 2GIG.A.502, 4A87.A.1162, 4ATF.C.500, 3B1N.A.403, 4BEM .A.201, 4BEM.B.201, 4BEM.J.201, 2BL2.A.1157, 4D77.A.1543, 2DKB.A.436, 3EPR.A.266 , 3GOT.B.437, 1H80.A.1497, 2HIG.B.488, 4JNQ.A.402, 4MB4.A.604, 3MS8.A.401, 3MUI. A.401, 1MVO.A.202, 1N82.A.401, 1OA8.C.1691, 2PPL.A.480, 3PZJ.A.301, 2Q8X.A.401, 4R7U.A.504, 3RGA.A.312, 3T8J.A.401, 1V54.A.3519, 3VDG.A.502, 1W9S.A.1142, 2WGM.A .201, 2WGM.B.201, 3WZ1.A.401

[1] "Cluster 6"

3AYK.A.170, 4HGX.A.301, 2LQ6.A.401, 3N9R.A.308, 1Q3A.B.469, 4R6T.A.1003, 4R7M.J. 1003, 2WEY.A.1772, 1XJS.A.150, 3AUO.A.577, 4D6O.A.1187, 4DLE.A.901, 2HVI.D.878, 4J9O.A.502, 1N56.A.401, 4NLK.A.402, 4NM1.A.401, 1TFW.B.1601, 2A5G.A.231, 4A7Y.A. 952, 3ALO.A.1, 1AQF.A.534, 3B03.D.1001, 2B9J.A.600, 2BKK.A.1266, 4BYG.A.996, 3C1 4.C.403, 2C43.A.1317, 3CK5.D.400, 1CUL.A.1006, 4CYI.D.1000, 2D33.A.525, 4E1E.A.4 02, 2E8T.B.1303, 2E8X.A.1302, 1E9I.D.1433, 2E92.B.1303, 3EA4.A.699, 3EHB.A.562, 4EKD.A.407, 2EWG.B.4003, 3EYA.A.613, 4FFR.A.406, 3FPA.A.901, 4GA3.A.1003, 3GFT.F .202, 3GT8.C.13, 4HEO.A.403, 4HGR.B.201, 3HIY.A.401, 3HU2.D.801, 3HYL.A.675, 3I0 0.A.502, 4J5I.F.402, 1JBO.B.1202, 4JLZ.B.502, 4KCV.B.1001, 3KRO.A.3003, 3L0C.A.2 57, 3LMG.A.202, 4LNI.D.503, 4LNI.D.505, 4LRZ.A.302, 3LUZ.A.264, 3MCO.B.426, 3MGA .B.405, 4MGH.A.1302, 4N0G.B.402, 4NCJ.A.903, 4NDN.A.401, 2O56.A.2001, 3O61.A.202 , 2PSN.A.701, 3Q10.D.400, 3Q7P.B.257, 3RBM.B.1001, 3RRA.A.406, 1SOJ.C.2127, 4TVU .B.601, 4TXZ.B.502, 4U0N.A.401, 3VAD.A.402, 2W00.A.1887, 3WBZ.F.403, 3WQM.A.403, 1XBT.D.4194, 1XZ8.B.180, 1ZCA.A.383, 2FKC.A.248, 4K4I.I.604, 4KHN.A.1002, 4KHW. A.1003, 3QEP.A.906, 1A25.A.292, 1A25.B.292, 4AIO.A.1890, 4AQ8.D.1238, 4B4F.B.607 , 3BOW.A.718, 4BTX.A.1763, 3CK7.B.720, 2CLT.B.1301, 3D4G.H.484, 2DEW.X.904, 1DM5 .A.1131, 1DM5.C.1133, 1DQ1.A.238, 3E3R.B.195, 1E8U.A.1002, 3E9T.A.6, 2EA7.B.452, 1EE6.A.300, 2EXK.D.2004, 3F19.A.266, 2F2O.B.1001, 1F2N.C.1001, 4FGC.C.203, 2GA9 .D.480, 4GGB.A.401, 1GQM.E.1089, 4GZS.A.501, 4H2E.B.304, 1HFY.A.124, 1HFZ.C.124, 4HSZ.B.101, 2HTY.C.993, 4I5K.A.501, 1JIW.P.489, 1JRF.A.48, 3K1A.B.524, 4K3Y.D.6 04, 3K9X.D.249, 3KF9.A.303, 3KL6.A.3, 1KLJ.H.900, 4KNA.A.504, 1KVO.A.192, 4KVK.A .712, 1KWH.A.800, 4LMF.B.302, 1LQD.B.1, 4LT6.A.602, 4LXF.A.701, 4MDV.A.402, 3MIN .D.524, 1MMP.B.3, 4NRE.A.716, 4NRE.A.717, 4NUP.A.301, 4O1Q.A.401, 2OO4.B.5004, 3 OX6.E.502, 4PHK.A.304, 3PKO.D.280, 4PLS.A.305, 3PRT.A.404, 1PYT.C.650, 3QQZ.A.32 4, 1QUO.B.702, 1R1Z.B.315, 4R83.D.501, 1RE3.C.408, 2RHP.A.2, 2RHP.A.18, 2RHP.A.2 8, 2RLD.C.121, 1ROS.A.404, 3SOB.B.1, 1SRR.A.531, 1TAD.A.352, 1TD7.A.1001, 3TH3.L .145, 1TLD.A.480, 3U1R.A.700, 3UBH.A.852, 1VFO.B.1002, 1VFP.B.995, 2VME.A.500, 3 W9T.A.1006, 1WD9.A.901, 2YFS.A.1711, 2YGM.A.1417, 2YN5.A.6365, 1YO8.A.1188, 2Z8S .B.641, 3ZHG.B.1323, 2ZID.A.882, 2ZKT.A.413, 2ZRQ.A.7, 1ZTQ.B.565, 2ZWO.B.402, 4 ANP.A.1426, 1B7Z.A.690, 1FRV.B.537, 4M26.B.401, 1NO4.A.688, 4N71.A.201, 3O32.A.3 00, 3PCJ.N.600, 3PCK.M.600, 3Q3O.A.502, 2R2F.A.320, 1SP8.A.500, 1UOF.A.1311, 1VZ 4.A.1299, 1WZD.A.901, 2YDE.A.501, 1ZJ9.B.1569, 3HW8.A.580, 3PML.B.7, 1Q81.K.8346 , 2A7L.A.201, 2A9Y.A.2002, 4BR6.A.401, 4D1J.E.604, 4D9T.A.901, 1EZ1.B.1002, 3FZQ .B.274, 3G1N.B.5002, 1G5I.C.902, 4GRX.A.501, 3HYS.A.267, 3I2W.A.304, 3IAQ.A.3103 , 2JBW.A.1368, 4JDO.G.301, 4KA5.A.801, 3KED.A.951, 3MC1.A.301, 1MGV.A.501, 4MJD. A.203, 3N3O.A.1000, 4NPJ.B.701, 4NRH.C.401, 3OEC.A.300, 2ONP.G.707, 4OUC.A.801, 2OYC.A.305, 4QVS.A.502, 1S5C.A.241, 1SOO.A.547, 1UD8.A.1001, 3VW7.A.2012, 1X0G.C .1001, 2X7J.A.1581, 1XAR.A.100, 4XEL.A.201, 1YAP.A.501, 1YQ2.A.7501, 2ZJ9.A.1, 2 ZN8.A.995, 3ZPQ.A.1360, 3ZQ5.A.1530, 3ZX0.C.522

[1] "Cluster 7"

830C.A.272, 1A7T.A.252, 4A7B.A.1276, 1A86.A.999, 2A8H.A.486, 1AFO.A.472, 4AR8.A. 1731, 1B3D.B.301, 3B7S.A.701, 1BIW.B.801, 1BLL.E.488, 4BLB.A.910, 2BNN.B.1200, 1 BQO.B.301, 1BS8.A.2001, 4BT4.A.301, 4BT5.A.301, 4BXK.A.1620, 4BZ3.B.502, 4BZR.A. 1630, 3C0Z.B.101, 4C6L.A.2823, 1CAM.A.262, 4CA5.A.1001, 4CA7.A.1616, 4CA8.A.1620 , 1CXV.A.1, 3CZN.A.1102, 3CZS.A.1102, 4DD8.A.1002, 4DR9.A.201, 3DYC.A.451, 3E0L. A.1452, 3E2D.A.602, 3E4A.B.2000, 1EI6.B.409, 3ELF.A.352, 1EZ2.A.402, 1FLS.A.166,

4FW3.B.300, 4FW4.C.301, 4FW5.A.301, 4FW7.A.301, 4FYT.A.1012, 1G05.B.801, 1G49.B .801, 4G9L.B.302, 4GK8.A.302, 4H1Q.A.301, 4H49.A.301, 4H76.A.301, 3HK5.A.430, 3H K8.A.430, 1HZY.A.402, 2I47.C.804, 3ID7.A.401, 2ILP.A.500, 2J83.A.999, 4JE7.A.202 , 1JJE.A.252, 1JJT.A.252, 2JSD.A.276, 2JT5.A.256, 2JT6.A.256, 1KYS.A.301, 3LJZ.A .999, 4LV4.A.401, 4MCA.B.1000, 3MK1.A.902, 1MMB.A.999, 3N2V.A.264, 4NTK.A.201, 3 NXQ.A.650, 2OC2.A.701, 3OD4.A.1350, 3OHL.A.4, 2OKL.A.601, 4OPN.A.201, 3P3C.A.401 , 3P76.A.274, 3PN4.A.1001, 3Q2G.A.401, 3Q2H.A.401, 3Q9F.B.344, 4QA0.A.401, 2QPJ. A.1, 1R43.A.500, 1RJ5.A.601, 3SPU.C.1010, 4TLN.A.321, 5TLN.A.321, 4TMN.E.322, 3U 04.A.200, 1U7J.B.150, 1U7M.A.54, 1U7M.B.154, 3U79.E.110, 1URA.A.451, 2UX1.C.1173 , 3V1E.A.101, 2V5W.A.1380, 3V77.A.302, 2V9G.A.1276, 1W22.A.1375, 4WD6.B.302, 1WY 2.A.406, 2XHM.A.1616, 2Y6D.A.1268, 1Y7W.A.283, 2YD0.A.1946, 2Z3I.C.2003, 1ZED.A. 904, 3ZXH.A.300, 3EPH.B.2, 4IEM.A.502, 2OTJ.0.8044, 1QVG.0.8067, 3CTL.A.601, 3D1 9.A.301, 3F5U.A.297, 3GIE.A.1, 1L2O.B.902, 2M32.A.401, 4NH0.A.1403, 3Q2O.B.384, 4QYI.E.203, 1SJB.A.1001, 4UOR.A.699, 3V4S.A.402, 3WNW.A.201, 2XTI.A.1551, 2ZDR.A .1001, 3KHH.B.1417, 3KHL.B.1415, 2FYC.B.404, 4LM8.A.812, 4MIV.C.601, 4NIY.A.301, 2OVZ.A.449, 3SON.A.201, 1TK2.A.1276, 2W1W.A.1132, 1WD9.A.900, 2YC2.B.201, 4BMT. A.1323, 1NFT.A.333, 1SQ3.A.901, 1YCH.A.501, 1DIZ.A.825, 8ICK.A.341, 1JJ2.A.8345, 1TX3.A.801, 1VQ8.M.9147, 1A7T.A.255, 3C7X.A.1001, 3CB8.A.820, 4CD5.A.1420, 4CD5 .A.1421, 4CFY.A.302, 3CZJ.A.3101, 4D1J.A.601, 4DUW.A.3101, 4DUX.A.3101, 3DYM.A.3 101, 1EBU.B.902, 1EJA.A.246, 1F6D.A.1378, 2FBL.A.304, 4FEV.B.303, 4FHA.B.402, 4F LL.A.504, 3GBV.B.1, 3GVK.A.916, 3I3B.A.3101, 3I3D.A.3101, 4IQZ.A.317, 3IRD.A.301 , 4J4B.B.303, 4JPA.A.305, 3JU4.A.7, 1JZ2.A.3101, 1JZ3.A.3101, 1JZ6.A.3101, 1JZ7. A.3101, 1JZ8.A.3101, 3KEU.A.402, 3KWM.A.225, 3LKB.A.394, 4MPY.A.503, 3MUZ.1.3101 , 3MV0.1.3101, 3MV1.1.3101, 3MX6.A.261, 1O4Z.A.1004, 4OMC.A.608, 3ONF.A.507, 2OZ 3.B.2001, 3PNX.C.169, 3PNX.E.168, 4PV3.A.202, 1PX3.B.3101, 4QKU.A.501, 3QS6.A.75 1, 2QWL.A.589, 3T09.A.3101, 4TMW.A.902, 3UNX.A.281, 3VD7.B.3101, 3VD9.A.3101, 3V DC.A.3101, 1VI6.C.208, 3W0L.C.502, 3WA2.X.702, 2WOI.A.1491, 1XFF.A.5301, 1ZNB.A. 3, 3ZYV.A.2337

[1] "Cluster 8"

456C.A.272, 4A7Z.A.950, 1A85.A.999, 2AB7.A.30, 3B7U.X.701, 2BIB.A.1549, 3BKQ.X.5 00, 2BNO.A.1201, 4BT6.A.1257, 3BUB.A.1047, 3BUI.A.1046, 3BVT.A.1048, 3BVU.A.1048 , 3BVV.A.1047, 3BVW.A.1046, 3BVX.A.1046, 4BZ5.A.500, 3C10.A.101, 1C3R.A.501, 1C3 S.A.951, 4C5W.A.402, 2CA2.A.264, 3CV5.A.1047, 2D1N.A.270, 2D1O.A.257, 1D7X.B.801 , 2DDY.A.177, 2DKD.A.921, 1DQS.A.402, 4DV8.A.801, 3DWB.A.771, 2DW0.A.700, 1E48.S .999, 1E4C.S.999, 3EBG.A.1, 3EDZ.B.2, 2ERP.A.700, 3F15.A.264, 3F17.A.264, 3F18.A .264, 3F19.A.264, 3F1A.A.264, 4FL7.A.301, 2FV5.A.3, 2FVM.D.601, 2FYV.A.2003, 1GK R.A.1453, 2GO4.A.601, 4H3X.A.301, 4H82.A.301, 1HFC.A.275, 1HJK.A.451, 1HOV.A.166 , 4HWO.A.701, 4HWP.A.701, 4HWR.A.701, 4HWS.A.701, 3HY7.A.901, 3HY9.A.901, 3HYG.A .901, 4IE4.A.601, 1JAQ.A.999, 2JIH.B.1554, 2K4W.A.156, 4K5P.A.1101, 1KBC.A.999, 3KDS.E.996, 2LFF.A.502, 3LJT.A.901, 3LK8.A.264, 1MNC.A.281, 3N2U.A.264, 4N27.A.2 01, 3NX7.A.264, 3O90.B.192, 3OHO.A.5, 4OPN.B.201, 2OW6.A.3001, 1P6E.A.248, 2PJT. A.302, 1PS6.A.330, 4QA1.A.403, 4QA2.A.403, 4QA4.A.502, 1QIN.A.401, 1QIN.B.301, 3 QIZ.A.431, 2RJQ.A.1, 1RM8.A.500, 1RMZ.A.264, 3RTS.A.264, 3RTT.A.264, 1T64.A.388, 2TCL.A.170, 3UWB.A.200, 2V5X.A.1377, 3V77.A.301, 2V9M.A.1275, 2VES.A.1295, 2VQM .A.1411, 2W0D.A.1264, 2W15.A.1203, 2WO9.B.1268, 1XBU.A.901, 1XBU.A.902, 1Y93.A.2 64, 1YQY.A.781, 1ZXC.A.201, 4DFM.A.903, 2G8F.A.301, 1AJD.B.952, 4BCL.A.371, 1BHO .1.901, 2BJI.A.2278, 4BJR.A.1517, 2BW7.D.2201, 4G61.A.301, 2GQ3.B.1003, 4GX6.A.4 02, 4IL6.j.102, 4IL8.A.501, 4IN7.M.411, 1JB0.A.1126, 1JB0.A.1131, 1JB0.B.1224, 1 JB0.B.1225, 1JB0.B.1236, 3KRF.D.901, 4LNI.A.503, 1NOW.A.401, 4NHO.B.1403, 1Q9L.C .218, 4R7O.E.402, 1SJA.B.701, 3SRF.G.533, 4UOM.B.503, 3V3Z.L.302, 3WU2.A.405, 3W U2.B.605, 3WU2.B.606, 3WU2.B.609, 3WU2.B.612, 3WU2.B.617, 4K4G.M.603, 3MQ6.A.340 , 1AF4.A.276, 3AFG.B.541, 4B7R.B.502, 1B9V.A.500, 1EA7.A.315, 1FZD.F.1, 1G5N.A.4 04, 1GMM.A.1132, 3HQ8.A.402, 2HT5.A.995, 2HYW.A.502, 4JQG.A.305, 1NGS.A.681, 3NH H.A.151, 4NPK.A.803, 1OT5.A.602, 2OVX.B.449, 1PZ7.B.702, 2Q1F.B.2002, 3Q3L.B.437

, 4Q4X.1.5006, 2RHP.A.22, 1S1D.B.1002, 1SEL.A.277, 2TCL.A.174, 1UG9.A.2003, 1V6C .A.505, 3VI4.C.2004, 3VRQ.A.401, 2YN3.C.6370, 1BIQ.A.376, 1EYS.C.611, 3HHX.A.281 , 3HJ8.A.281, 3HJS.A.281, 1HLM.A.159, 3I51.A.281, 4KVQ.A.301, 1KW6.B.301, 4L7Y.A .201, 3NKT.A.369, 1NX4.A.300, 3O5U.A.300, 1ODN.A.1326, 1QFC.A.401, 2ZZI.B.209, 3 HW8.A.577, 1YJW.A.8545, 3CKI.A.502, 3CTP.A.402, 3DYO.A.3101, 3DYP.A.3101, 4EZE.A .302, 4FXZ.A.603, 2GKO.A.614, 1GNY.A.1244, 1GVF.A.289, 4GVO.A.702, 1HXN.A.4, 3IM M.C.3, 3IWK.D.504, 2J5A.A.1109, 4JEX.A.512, 1JYN.A.3101, 1JYV.A.3101, 1JYW.A.310 1, 1JZ7.C.3103, 1MUQ.A.206, 3N3R.A.1000, 2O34.A.503, 4O4V.A.301, 1O68.E.274, 4OF I.A.801, 2P3Z.B.501, 1PX4.A.3101, 1Q20.A.313, 1QHU.A.436, 3QST.A.253, 4QTO.A.501 , 4QTO.B.501, 1T64.A.391, 3TAV.A.267, 1TO2.E.451, 3TXF.A.1138, 3TYP.A.155, 3V5U. A.701, 3V5U.A.703, 3VD4.A.3101, 3VD5.A.3101, 3WGU.C.2007, 1WQR.A.131, 2WWH.C.213 , 3ZK1.B.90

[1] "Cluster 9"

2L1G.A.88, 4QCL.A.1303, 4A3W.A.1159, 4A7Y.A.951, 4ASQ.A.1615, 4ASR.A.1615, 4AWY. B.3229, 4AX0.B.3229, 4AX1.B.3229, 1B57.A.360, 3B8Z.A.901, 2BH3.A.1002, 4BIN.A.50 0, 2BMI.A.272, 3C52.A.401, 2CEA.A.1603, 2CFU.A.1001, 4DEF.A.401, 4DPE.A.301, 2DV T.A.1501, 2DVX.A.1501, 4DZH.A.504, 3E38.A.1, 3EBH.A.1, 3F16.A.264, 1FA5.A.1200, 1FA5.A.1201, 1FKX.A.400, 4FUA.A.216, 2GMN.A.802, 4H2G.A.601, 1H48.C.900, 2HBV.A. 401, 3HC4.L.401, 2HC9.A.702, 2HPT.A.950, 3HR1.A.1, 1HS6.A.701, 1HTY.A.1102, 4IE0 .A.601, 4IE6.A.601, 3ITC.A.501, 3ITC.A.502, 4J5F.A.301, 2J9A.A.1493, 1JAP.A.999, 1JCZ.A.901, 2JIG.A.1252, 1JK3.A.400, 2JNE.A.200, 4JP4.A.301, 1JWQ.A.1001, 1K4P. A.1004, 3K5X.A.402, 1KAE.B.1102, 1KEQ.A.280, 3KR5.G.1004, 1LAM.A.489, 4LE6.A.405 , 4LE6.A.406, 3LS6.A.303, 3MAX.A.379, 3MDU.A.454, 2O4Q.A.2401, 3OHL.A.5, 1OS9.A. 901, 4OUI.A.501, 1P5X.A.248, 1P6D.A.246, 2PAJ.A.493, 2PLM.A.407, 1PMI.A.445, 1PT W.A.501, 1PVW.A.401, 3Q4R.A.201, 2Q5B.A.107, 2Q5B.C.107, 3QAY.A.180, 4QA5.A.401, 4QA6.A.401, 1QH5.A.262, 1QJJ.A.250, 4R76.A.1001, 3S2J.A.402, 3S2L.A.402, 3S2M.A .402, 3S2N.A.402, 3SEY.E.373, 1SML.A.270, 3SNG.A.402, 3T00.A.502, 1TQS.A.1401, 1 TQT.A.1301, 1TQV.A.1300, 1TQW.A.1400, 3U79.D.110, 1UEA.C.1, 3UHM.A.300, 2USH.A.6 02, 2UX1.A.1174, 3VPE.A.302, 3VTG.A.301, 2VUN.A.401, 4WB7.A.502, 2WM1.A.1333, 4X 2T.B.1002, 2XL9.B.1269, 1XXW.A.201, 2XYD.A.1620, 2Z72.A.401, 2Z72.A.402, 2ZBM.A. 401, 2ZBM.A.402, 1ZNB.A.2, 2ZNE.B.991, 3ZU0.A.1595, 3ZU0.A.1596, 3ZU0.B.1588, 3A UA.A.601, 2D32.A.1524, 1DAM.A.901, 4DFX.E.403, 3DGB.A.2001, 7ENL.A.438, 2F9R.B.6 02, 3FXG.B.501, 1G65.G.301, 2GQ3.A.1002, 3I6E.B.386, 4IAC.A.402, 1KJ8.B.394, 1KJ I.B.394, 3KR4.A.1004, 4LYJ.A.201, 4MFE.C.1104, 4MKU.A.209, 1N8W.A.900, 4NZN.A.40 4, 2OQY.B.402, 2QB8.B.401, 4QVP.V.302, 4R17.K.302, 3T9E.A.603, 2VBI.G.1000, 1W88 .A.1368, 3WQP.H.501, 3KHH.A.416, 4QWE.A.703, 3UIQ.A.905, 3AYU.A.419, 4CBX.G.1151 , 2DDF.A.475, 1EGZ.C.300, 7EST.E.11, 1F5R.A.800, 3FHA.B.704, 3G20.A.201, 4H1Q.A. 304, 3SO0.A.98, 3SO0.B.97, 1SPU.A.802, 1TFX.B.4007, 1WY9.A.148, 4QQZ.C.1001, 2AW C.A.137, 2BIW.C.1492, 1BS3.A.202, 2BUT.B.1541, 2BUV.B.1541, 2BUM.B.1541, 1EO2.B. 600, 1EYS.C.609, 3FG3.D.1500, 3FG4.A.1500, 4G2D.A.402, 1GN6.A.999, 3HHY.A.281, 3 HJQ.A.281, 3HKP.A.281, 2HMQ.C.115, 3I4Y.A.281, 3IS8.A.163, 3IVD.B.601, 4J6C.A.50 2, 4P1B.D.501, 3PCE.N.600, 3PCN.P.600, 2PCD.N.600, 2PHD.B.370, 2Q0J.A.998, 2QFR. A.433, 3QFM.B.264, 2R1N.A.800, 3R2R.A.155, 1TDW.A.425, 2VHL.A.1397, 2VP1.A.1347, 1XM8.A.701, 1YFX.A.300, 2BCV.A.579, 2GIJ.F.403, 4ORJ.A.305, 1SUZ.A.403, 2A65.A. 751, 2ABS.A.1003, 2B2N.B.1001, 2DDB.B.303, 4EXR.A.301, 4FMT.D.301, 4FO2.Q.101, 4 H7O.C.302, 4H83.E.401, 2HZY.A.1201, 3IMX.A.467, 2JBW.D.1367, 2JLN.A.1471, 1JZ8.D .3104, 1KAO.A.501, 4LDE.A.1402, 4LHL.A.301, 3LJQ.A.597, 4M9U.A.402, 3MAX.A.381, 4MM7.A.601, 4MMB.A.601, 4MMF.A.601, 3MPN.A.751, 3MPQ.A.751, 4O1G.A.401, 1OAF.A.1 252, 4OUA.A.403, 2PFL.A.2001, 3Q9B.A.344, 3QS4.A.751, 3QS5.A.751, 1RW9.A.900, 3S 9J.A.401, 1SK4.A.342, 3VD3.A.3101, 3VDA.B.3101, 2WPC.C.1492, 2Z5D.B.180, 1ZH8.A. 329, 3ZK1.A.90, 3ZQS.B.1295

Table S121. 5-ligand combined metal, compressed group

	size	largest_angle*	middle_1*	middle_2	middle_3*	middle_4
1	"53"	"167.9+/-6.3"	"78.3+/-6.4"	"83.5+/-4.1"	"87.3+/-3.3"	"90.6+/-4"
2	"202"	"156.5+/-6.8"	"56+/-3.4"	"84+/-6.1"	"90.4+/-4.2"	"97.6+/-3.4"
3	"104"	"162.9+/-6.4"	"57+/-5.2"	"82.3+/-7.2"	"88.9+/-4.7"	"93.9+/-4.9"
4	"192"	"149.3+/-4.9"	"56.5+/-3.6"	"85.6+/-6.5"	"93.1+/-3.8"	"97.1+/-3.8"
5	"121"	"156.1+/-8"	"53.1+/-4.3"	"74.2+/-6.4"	"80.6+/-5.7"	"88+/-7.5"
6	"98"	"152.1+/-12"	"70.1+/-6.5"	"77.2+/-6.7"	"82.1+/-7"	"87.1+/-7.9"
7	"151"	"139.2+/-5.1"	"54.9+/-3.7"	"83.5+/-5.9"	"91.4+/-4.7"	"96.3+/-4.9"

	middle_5*	middle_6	middle_7	middle_8*
1	"96+/-5.8"	"101.5+/-9.4"	"113.1+/-17.7"	"153.6+/-8"
2	"102.8+/-3.5"	"108.4+/-4.1"	"116.3+/-3.9"	"124.1+/-4.9"
3	"98.9+/-6.2"	"104.8+/-6.4"	"115.5+/-10"	"148+/-7.7"
4	"101.7+/-3.5"	"107.5+/-5.7"	"116.2+/-6.8"	"136.9+/-4.8"
5	"95.5+/-9"	"106.1+/-9.9"	"122.6+/-8.6"	"141.6+/-7.2"
6	"93.5+/-8.8"	"102.4+/-10.4"	"111.4+/-12.5"	"128.6+/-8.3"
7	"103.1+/-5"	"112+/-5"	"119.3+/-4.5"	"128.2+/-5"

	smallest_opposite_angle*	TrigonalBipyramidal	SquarePyramidal
1	"57+/-4.6"	"0.02"	"0.09"
2	"105.7+/-5.1"	"0.095"	"0"
3	"88.9+/-6.8"	"0.006"	"0.045"
4	"96.7+/-5.9"	"0.049"	"0.007"
5	"75.2+/-6"	"0.006"	"0.006"
6	"53+/-5.3"	"0.011"	"0.034"
7	"104.4+/-6"	"0.069"	"0"

	TrigonalPrismaticV
1	"0.022"
2	"0.064"
3	"0.025"
4	"0.094"
5	"0.063"
6	"0.071"
7	"0.155"

Table S122. Cluster members of 5-ligand combined metal, compressed group

[1] "Cluster 1"
4FUK.B.402, 4MCS.A.814, 2MLS.A.302, 3OOF.A.302, 1P6D.A.247, 1ZEB.A.901, 4C5A.B.3
30, 1E4E.A.360, 3IAF.A.572, 2IO7.B.5003, 1L3R.E.391, 4N57.B.402, 1OL5.A.1389, 4O
VN.A.203, 4OVN.B.203, 3PUG.A.601, 2PUI.A.401, 3QHR.A.298, 4QXD.A.302, 4R9U.C.302
, 3T9E.A.601, 4UAK.A.502, 1YYZ.A.340, 2ASJ.B.1415, 2I3P.B.1, 4K4H.E.604, 3ALA.E.
1764, 6CGT.A.685, 2DPK.A.4001, 1DTH.A.903, 3GE4.B.167, 4GQR.A.502, 1JKU.B.2272,
1JKU.E.5272, 3K7L.A.701, 2LP2.A.202, 1ULV.A.2006, 4QQZ.G.1002, 3AK9.J.168, 4AQ2.
B.800, 4AQ6.F.837, 2BJJ.X.694, 1EH3.A.400, 1GVC.A.1253, 3MPS.D.1, 2O84.X.500, 1O
QH.A.500, 3PCC.M.600, 3TOD.A.694, 3V83.A.703, 4CVU.A.1998, 4LLH.B.602, 1NZA.A.10
4
[1] "Cluster 2"
4A37.A.376, 3A52.A.1001, 4A69.A.500, 4AWZ.A.3230, 2B13.B.401, 3B4N.A.711, 3B4N.B
.715, 1B57.A.361, 4B6Z.C.385, 1B8J.A.451, 1BA9.A.154, 3BKK.A.701, 3BKL.A.701, 1B

OR.A.58, 2BO9.A.999, 2BP8.B.1340, 1BYF.B.302, 4BZS.A.701, 4C2N.A.701, 4C2O.A.162
9, 4CA6.A.1001, 1CG2.A.500, 1CG2.A.501, 3CHO.A.701, 2CLB.A.1175, 4COG.A.401, 4CO
Q.A.299, 1CPX.A.308, 5CPA.A.308, 4CVR.A.1159, 4CVT.A.1158, 4CVT.A.1159, 1DO5.B.2
9, 3E30.B.1001, 3E32.B.1001, 3E34.B.1001, 1ED9.A.450, 3EER.A.2001, 3EQN.A.757, 2
EV6.B.2151, 3F3Q.A.104, 1F57.A.310, 4F7O.A.301, 3FB4.A.217, 1FT7.A.501, 3FU1.B.2
01, 3FVL.A.1309, 2GFK.A.401, 2GFJ.A.401, 4GM5.A.401, 2GSN.A.1001, 4GTO.B.501, 4G
TV.B.401, 3GZE.A.1, 1HFS.A.257, 2HH5.A.702, 1HI9.A.300, 1HLK.B.1004, 2HSE.B.954,
 3HTR.B.118, 2IOO.A.580, 2I3C.A.314, 4IAV.A.401, 2IEJ.B.939, 1IF6.A.262, 4IHM.A.
402, 3IT7.A.183, 1IY7.A.308, 2JBJ.A.1752, 1JCQ.B.1001, 1JJE.A.251, 1JJT.A.251, 4
JJI.A.402, 2JLP.B.226, 1JPU.A.371, 4K7W.A.102, 4K90.A.701, 2KBX.B.299, 1KH4.A.45
0, 3KS3.A.262, 1L1O.C.1, 4L2L.A.701, 3L3N.A.701, 1LD7.B.1001, 1LD8.B.1001, 1LGD.
A.262, 1LND.E.800, 4LNB.B.602, 4LQY.A.506, 3LTV.A.1001, 4LW9.A.201, 4LW9.I.201,
4LW9.R.201, 3M2Z.A.500, 2M30.B.201, 1M4L.A.1308, 4MBG.B.602, 4MKH.A.301, 4MKT.A.
701, 3MO2.D.6, 4MRQ.A.501, 4MSM.C.501, 3MWM.B.142, 1MXD.A.727, 1MZC.B.1001, 4N07
.B.305, 3N21.A.401, 4NGE.A.1101, 4NGE.E.101, 3NKQ.A.1002, 3NKN.A.1001, 2NQH.A.30
3, 1NW2.D.6004, 1NW2.F.6006, 4NYY.B.501, 4O3A.A.303, 1O86.A.701, 1O8A.A.701, 4OJ
A.A.202, 4OXD.C.301, 2P2L.A.901, 2PJC.A.400, 4PQA.A.403, 4PQA.A.404, 3PSQ.A.326,
 2PVV.A.1752, 4PXY.A.302, 3PZ1.B.332, 3PZ4.B.1, 3Q7A.B.521, 1R3N.A.501, 3RF4.A.1
18, 2RH6.A.1, 1S63.B.1001, 1S64.B.378, 1SA4.B.439, 1SA5.B.438, 3SFX.B.521, 3SFY.
B.521, 1SHN.A.485, 3SIO.A.362, 1T0A.A.661, 1T3A.B.422, 3TGN.B.147, 3U24.A.595, 1
U7J.A.50, 3U9W.A.2001, 3UCT.B.102, 1UUP.A.2222, 1UZE.A.701, 2V9I.A.1277, 2VJ8.A.
1611, 2VXI.A.202, 2W5V.A.1377, 2W5X.B.1378, 2W57.B.202, 3W6H.A.302, 3WBH.A.505,
4WCM.A.538, 4WD6.A.301, 4WNC.O.402, 3WS9.B.801, 3WXC.A.301, 1WY2.A.405, 2X90.A.1
618, 2X91.A.1619, 2X92.A.1615, 2X97.A.1616, 4XIX.A.401, 2XIG.B.1150, 1XP3.A.302,
 2XXO.B.1340, 2XXG.A.1339, 2Y3D.B.149, 1ZG7.A.400, 1ZG8.A.400, 2ZIR.B.901, 2ZIS.
B.901, 2ZKW.A.401, 3ZXO.A.1579, 1DXI.A.390, 4GWZ.A.402, 1IV2.B.1562, 3LDO.8.54,
4OTP.A.502, 1RYS.B.803, 1DJZ.A.2, 1M63.B.500, 1CJX.A.629, 3FM3.B.552, 3GHQ.K.300
, 1GUQ.A.351, 4HJL.A.502, 2HMN.A.450, 4NB9.A.501, 4NBH.A.501, 2OWT.A.324, 4P9G.A
.401, 4REU.A.202, 1SIO.A.321, 1BUN.A.121, 2GG8.A.503
[1] "Cluster 3"
3B35.A.292, 4DHL.B.501, 1DK4.B.591, 4DYO.A.502, 2EK9.A.1002, 1ELX.B.451, 2GA3.A.
450, 1GE7.B.200, 1HR6.B.501, 4ICQ.B.502, 1J2U.A.301, 4JX5.D.1103, 1KBE.A.1, 4KFT
.C.303, 3LOT.B.2, 4LP6.A.302, 1MH2.A.201, 4NAQ.A.1027, 2OR4.A.1751, 1Q3K.B.301,
2QLA.B.502, 2QLA.D.503, 3SJX.A.1751, 3SZZ.A.502, 3T00.A.501, 1VKG.A.400, 1XJO.A.
902, 2XPY.A.1673, 4N41.E.101, 3ATT.A.379, 3CWH.A.391, 2D32.A.1523, 1DIE.A.398, 3
FDG.B.356, 4I40.B.301, 4LNI.D.504, 1SBJ.A.163, 4U03.A.504, 3U2E.A.3, 1G9Y.C.803,
 3KHL.A.415, 1AEI.A.318, 1AEI.A.320, 1B09.C.301, 3BRX.A.328, 4EJ7.C.402, 3FLP.L.
302, 3FLT.B.302, 1GYK.A.1206, 4ILW.F.305, 4JDZ.A.702, 4KTP.B.804, 2ML2.A.206, 1N
29.A.125, 1NXO.A.900, 4OMC.E.607, 4P99.D.512, 1SOC.A.1879, 4UM8.A.2004, 1VAH.A.5
00, 4W4Q.A.401, 1Z3U.B.497, 2ZOC.A.504, 4AC8.A.500, 1B1X.A.691, 1B7Z.A.691, 3CHH
.A.501, 4D8F.B.402, 3EE4.A.317, 1F9B.A.690, 1FYZ.B.5004, 3GCF.D.501, 1HDS.B.146,
 1HSE.A.400, 2ITB.A.502, 2IW4.B.1311, 2J2F.A.371, 1JI5.D.153, 3MPS.D.172, 4NB8.B
.501, 3OWO.B.384, 3PCC.N.600, 3PCD.M.600, 3PCF.N.600, 4PGO.A.302, 1PRC.M.607, 1Q
OC.B.500, 3Q1G.B.1002, 3QHB.A.182, 1RNR.A.402, 1TKP.D.302, 2UW1.A.1359, 2VZB.B.6
204, 3W54.A.501, 3W54.B.502, 4WWZ.B.301, 2XSH.C.901, 1XZW.A.429, 3FFZ.A.1302, 1G
VH.A.1399, 4H83.A.402, 4MAT.A.501, 1SU4.A.997, 1ZEL.A.401
[1] "Cluster 4"
4MTD.A.202, 3A1Z.A.226, 4A94.B.501, 3AIG.A.999, 2AIG.P.999, 1ANJ.B.450, 2ANP.A.5
01, 2ANP.A.502, 1ATL.A.401, 3AYU.A.415, 3B3C.A.501, 2BH3.A.1001, 3BHX.A.1751, 3B
IO.A.1751, 3BI1.A.1751, 2C6P.A.1751, 2C6C.A.1751, 1CGL.A.301, 1CP7.A.901, 1CP7.A
.902, 6CPA.A.308, 7CPA.A.308, 8CPA.A.308, 4D0Y.B.1239, 3D10.A.95, 3D4U.A.309, 3D
FM.A.401, 4DPR.A.701, 2DSN.A.2001, 1DZW.P.999, 1E49.S.999, 1EC5.B.50, 2EG7.A.400
, 3EHY.A.264, 4EJ5.A.501, 2EK8.A.1002, 1EW9.A.450, 3EWJ.B.2, 3FFZ.A.1300, 3FH4.A
.301, 3FJU.A.999, 1FUA.A.216, 3FUK.A.701, 4FYQ.A.1012, 4FYR.A.1013, 4GTP.B.501,

3H90.B.294, 1HDU.E.1308, 1HEE.E.1308, 2HIH.A.601, 4I2F.A.603, 4ICR.A.502, 4IE7.A.601, 1IGB.A.502, 3IQ6.C.201, 2IQ6.A.293, 4IXN.A.401, 4J5H.A.301, 2JAZ.B.600, 4JBS.A.1008, 1JK9.C.302, 2JT5.A.257, 4JYW.A.801, 4JZ0.A.801, 4K5L.A.1101, 4K5M.A.1101, 4K5O.A.1103, 4KA7.A.801, 4KAY.A.601, 1KH5.A.450, 2KIK.A.50, 3KNS.A.229, 1L0Y.B.702, 4LCQ.A.602, 1LFW.A.1001, 3LQ0.A.999, 4LQG.A.801, 4LTE.A.1101, 2M30.A.201, 3M52.B.116, 3M79.A.107, 1MMP.A.1, 3NKN.A.1002, 3NKO.A.1002, 1NPC.A.323, 4O2I.A.401, 4O3A.B.302, 4ON1.A.400, 4ONX.B.201, 2OY2.A.999, 3P24.A.999, 4P9C.J.201, 2PJ0.A.400, 2PJ3.A.400, 2PJ5.A.400, 2PJ7.A.400, 2PJ8.B.500, 2PJA.B.500, 3PSQ.B.328, 2PVW.A.1751, 3PW3.A.406, 3Q4R.A.202, 4Q7R.A.307, 3QBU.A.294, 2QLA.A.500, 2QLA.C.501, 1R42.A.804, 2RJP.A.1, 1ROS.A.400, 1RTQ.A.702, 3RYM.B.107, 1S0E.A.1291, 1SQM.A.1001, 1TF9.A.902, 3TG0.A.502, 1TKH.A.902, 1TKJ.A.901, 1TKJ.A.902, 3TOL.D.107, 3TS4.A.301, 3U93.B.259, 2V29.A.1274, 2V29.A.1275, 2V77.A.1042, 2V9E.A.1275, 3VAT.A.501, 3VH9.A.302, 2VQQ.A.1411, 2VXX.C.202, 3WAW.A.913, 4WD8.A.302, 3WE7.A.301, 2WKN.B.412, 2WO8.A.1268, 2WO9.C.1269, 1WUO.A.301, 2X93.A.1615, 2X94.A.1616, 2X98.A.1475, 1XAF.B.507, 2XIG.C.1153, 2XR9.A.1867, 2Y2B.B.1180, 1YHC.B.602, 2YJP.A.1272, 2ZTG.A.902, 3B9T.A.485, 4EO0.A.301, 1IV4.E.1565, 4ML9.B.301, 4PV4.A.501, 3T80.B.160, 4Q10.A.1001, 2E30.A.300, 4G64.B.301, 4KPP.B.501, 3L9I.C.1151, 4OVY.A.409, 3POW.A.900, 2TBV.C.388, 1V1G.A.1209, 3WIV.B.1004, 1BIQ.B.376, 2CKF.A.501, 2DE6.A.501, 1DLM.A.400, 3E1N.C.300, 2FKZ.C.1600, 2FL0.A.1602, 3FVB.A.164, 1FZ0.B.5004, 4GAM.F.601, 4HVR.A.201, 3IS8.A.161, 4JPY.A.301, 4NBC.A.501, 4NBG.C.501, 1NFV.A.200, 3O6R.A.300, 1OQU.B.1004, 1PFR.B.503, 1PIZ.A.376, 3R2R.A.156, 4RC6.A.302, 1RSV.A.1004, 4TOH.A.202, 2XRX.M.1461, 2XSH.G.900, 2YFJ.C.901, 1JJU.C.996, 1NSX.A.1401, 1U4J.B.1001

[1] "Cluster 5"

3AAK.A.992, 3CSQ.B.335, 1D1S.B.406, 4DWZ.A.302, 2E46.A.172, 3H90.C.2, 4JBS.B.1009, 3M3B.A.156, 3U94.B.259, 3V94.D.702, 3A6P.H.1178, 4IFD.J.1105, 4ACF.B.1481, 2BVC.A.503, 2DW7.B.2002, 4FHY.A.402, 4FMC.B.203, 3HB0.B.702, 3LZ9.A.852, 1MXA.A.411, 4NOG.A.405, 2RD5.C.1001, 1YHM.A.401, 1BSS.A.433, 1SON.A.403, 3A7Q.A.4001, 2AAO.B.299, 1ALA.A.400, 1AXK.B.395, 2BBM.A.182, 3BXK.A.152, 1CTR.A.150, 2D3P.A.237, 2D7F.A.240, 3E3R.B.193, 2E6V.D.11, 4EJ7.C.403, 1ETH.A.449, 1F4M.E.105, 2FH3.A.1002, 2FHC.A.2405, 1FZC.C.1, 1FZD.G.1, 4G0D.A.503, 4G9L.B.306, 2HOK.A.401, 1IH0.A.2, 3IJE.A.4008, 1IVE.A.470, 2IWV.B.1284, 2J3U.C.1292, 2J64.C.1222, 1J84.A.366, 2JAL.B.1447, 4JDZ.B.704, 1JN2.P.238, 3JQL.A.121, 2K0J.A.501, 2K2F.A.94, 1K90.D.801, 1LGC.E.301, 1LGN.A.302, 1LU1.A.301, 1M1J.C.501, 3M83.E.407, 3M8D.A.710, 4MBE.A.202, 4MDV.B.404, 2ML3.A.206, 2MLR.A.305, 2MLS.A.305, 4OJQ.B.1002, 1OTN.A.250, 3OX5.B.502, 2OXE.B.600, 2OYH.B.2, 1Q3A.C.478, 1QOO.A.302, 3RG0.A.1, 2RJP.B.4, 3RYD.C.267, 3TRP.A.360, 3UJ0.D.304, 3VU1.A.1001, 1W2M.A.1442, 1WDA.A.902, 4WFF.A.304, 4WK2.B.502, 1WMZ.B.203, 3WYN.B.402, 2WZE.A.1552, 1XFE.A.100, 1YAX.C.1003, 1Z3J.A.266, 2ZOC.A.501, 2ZOC.A.502, 2ZWO.A.401, 3CI8.A.6, 3E1M.B.301, 2FKZ.C.1601, 3IS8.E.162, 1LKO.A.601, 1LKP.A.601, 1LTV.A.900, 4N71.A.202, 3PCH.R.600, 3QVD.B.173, 3QVD.C.175, 4RC6.A.301, 1SQ3.A.902, 1YUZ.A.302, 1YV1.B.301, 4NLK.A.403, 1U8R.A.1101, 4DD8.A.1005, 4G8T.B.502, 4M5P.A.505, 4O54.A.302, 1TC8.A.121, 3VD3.D.3101, 2WD0.C.601

[1] "Cluster 6"

4BLB.D.910, 4CPA.I.308, 1F30.B.201, 1F30.E.201, 4IOD.A.502, 4K0D.B.203, 4KJG.A.1001, 3M4B.B.108, 1NL4.B.500, 1RLY.A.61, 2V8V.A.1456, 1QVG.0.8010, 1TTT.B.407, 2CN8.A.1504, 2D33.B.1524, 2DW7.A.2001, 4FOP.A.501, 2F1I.A.501, 1GXB.C.1346, 3HQP.A.502, 3IG8.A.696, 4K10.A.405, 3MQT.W.395, 4NU1.A.404, 3OB8.D.3001, 4P9D.B.202, 4PYK.A.302, 3RBM.C.1002, 1S6P.A.601, 4U81.A.501, 3UJ2.H.430, 4UOR.C.699, 2V0N.A.502, 3RBE.A.415, 4RIC.A.1101, 1BCZ.A.410, 3BJU.C.606, 2BTW.B.400, 2C10.C.1773, 2CHO.A.1717, 1CTR.A.149, 2DDY.A.175, 3E1I.C.502, 1EXZ.C.805, 3FAX.A.1223, 2FH2.B.2003, 2FH3.B.2001, 1FIH.B.3, 1G1Q.C.803, 3HR4.H.202, 3IJE.A.4004, 3IJE.A.4007, 3IKQ.A.403, 4JWU.A.502, 2K0E.A.153, 1K9I.A.407, 3KO0.H.302, 1LT9.B.1, 2MLS.A.304, 3NP5.A.732, 4OMD.F.604, 2OX9.B.803, 4P99.A.505, 4PJ0.A.601, 4POQ.C.401, 4POQ.E.4

01, 2PRK.A.281, 4Q6O.A.501, 1QDO.B.243, 1QNI.D.903, 2QVF.B.704, 2RGB.A.601, 2RHP.A.11, 2RHP.A.27, 1RTM.2.3, 1S1E.A.225, 2SNI.E.276, 1TNQ.A.91, 4UM8.B.2003, 3VI3.B.2001, 2VR0.D.1006, 1WOP.A.1781, 3W9T.G.506, 1WT9.A.1001, 1F9B.A.691, 3HH8.A.1, 3W54.B.501, 4DOA.A.402, 4DOB.A.402, 2FMQ.A.340, 3JPT.A.340, 3PNC.A.2, 2PXI.A.340, 3CBT.A.420, 3INJ.C.601, 4LCZ.B.320, 4NTL.A.311, 1SVY.A.2

[1] "Cluster 7"

4LMG.D.202, 2A7G.E.401, 3AHT.A.1001, 1ALK.A.451, 4AR9.A.1731, 1B0N.A.1001, 3B2Z.A.1, 4B52.A.401, 3BKN.B.201, 3BKN.B.202, 3BOO.A.500, 4BPO.A.1314, 4C1H.A.351, 2C20.B.601, 2C6P.A.1752, 1CBX.A.309, 2CEA.E.1603, 1D1T.C.407, 1D8D.A.1001, 1DCE.B.900, 3DID.A.130, 3DSX.B.332, 3E33.B.1001, 3E37.B.1001, 1EC5.C.50, 2EG8.A.400, 3ELM.A.300, 3F28.A.321, 3F2P.A.2005, 3FGD.A.321, 4FMP.B.400, 4FU4.A.502, 3G42.A.500, 4G9L.B.301, 3GIP.A.484, 4GTM.B.501, 4GTQ.B.501, 4GTR.B.501, 2GVI.A.302, 4H2K.A.1001, 1HA5.C.4221, 1HR7.B.501, 1HYT.A.805, 1HZ5.A.103, 4I11.A.502, 3I9F.A.1, 3IQ6.B.201, 2IXD.A.1234, 2J13.A.1236, 1J9Y.A.1003, 1JCS.B.1001, 1JI3.A.402, 4JJJ.A.718, 1JML.A.102, 1K53.A.1003, 1KAP.P.613, 1KR6.A.405, 1KRO.A.405, 1KS7.A.405, 3KWO.C.162, 4L9P.B.601, 3LPE.D.60, 3LUB.B.302, 2LVH.A.101, 3M4C.D.108, 1M5E.A.1705, 3M79.B.107, 3M79.C.107, 3MDJ.A.1000, 1MMP.A.2, 1N4Q.B.378, 1N4Q.L.378, 3NKR.A.1002, 1O1S.B.1001, 4O3A.B.303, 1OEZ.W.1154, 4OKO.A.401, 4ONG.H.302, 4OTE.B.302, 2OWO.A.444, 1PE5.A.317, 1PE7.A.317, 2PIY.A.400, 2PIZ.A.400, 2PJB.A.400, 2PLI.C.701, 4PPZ.A.601, 1PSZ.A.1000, 4PUC.A.602, 3Q75.B.521, 3Q78.B.521, 1QBQ.B.1000, 1QMU.A.999, 3QWO.A.500, 3QWO.B.500, 3QWO.D.500, 3QW1.C.500, 2R2D.A.277, 1R43.A.501, 2RFH.A.1308, 1RK6.A.601, 3S9C.A.503, 3SKS.A.568, 3TGO.A.501, 1TLP.E.322, 1TMN.E.322, 1TN6.B.1001, 1TN7.B.1001, 1TN8.B.1001, 1TNB.B.378, 1TNO.B.378, 1TNU.B.378, 1TNY.B.378, 1TNZ.B.378, 3TOL.B.107, 3TT4.A.302, 3TVC.A.501, 3UBF.A.1, 3V0A.A.1297, 2VXI.A.201, 3W5K.B.501, 1WAA.A.1091, 1WAA.F.1091, 4WD8.A.301, 3WOJ.A.805, 2WOA.A.1270, 3WV3.A.301, 2X4H.A.1142, 2X98.A.1476, 1XGE.A.400, 2XX7.B.1264, 2Y28.A.1181, 2YJP.C.1270, 2Z2Y.C.2003, 2Z25.B.401, 2Z2D.A.265, 1ZG9.A.400, 2EX5.X.802, 2BTW.A.400, 3C5I.A.371, 1IJL.B.202, 1NW1.A.430, 4AM4.A.1161, 3E1Q.A.301, 4ELR.A.401, 2HTN.A.301, 4V06.A.1491, 1XIK.B.377, 4FZZ.A.201, 2BBH.A.301, 4J4B.A.902

Table S123. 5-ligand combined metal, combined group

	size	largest_angle*	middle_1*	middle_2	middle_3*	middle_4
1	"248"	"152.7+/-11.6"	"65.7+/-9.1"	"74+/-7.3"	"79.4+/-6.6"	"84.5+/-7.4"
2	"496"	"168+/-7"	"77.9+/-6.2"	"84.5+/-4.1"	"88.1+/-3.1"	"92.2+/-3.2"
3	"525"	"148+/-8.3"	"57.2+/-5.4"	"84.1+/-6.2"	"91.4+/-4.4"	"96.9+/-4"
4	"722"	"174.1+/-4.1"	"84.2+/-4"	"87+/-2.4"	"88.8+/-1.6"	"90.5+/-1.5"
5	"599"	"161.9+/-6.9"	"78.9+/-5.4"	"84.9+/-3.4"	"88.8+/-3.2"	"92+/-3.3"
6	"425"	"166.1+/-6.3"	"75.5+/-5.5"	"81.1+/-4.3"	"85.4+/-3.8"	"89.1+/-4.1"
7	"321"	"157.3+/-8.3"	"57.2+/-6.6"	"78.6+/-8.1"	"86.3+/-6.7"	"92.7+/-6.4"

	middle_5*	middle_6	middle_7	middle_8*
1	"91.1+/-8.5"	"100.9+/-11.1"	"113.1+/-13.5"	"132.7+/-10"
2	"96+/-3.9"	"101.7+/-5.8"	"118.6+/-5.2"	"131.3+/-6"
3	"102.3+/-4.1"	"109.4+/-5.2"	"117.3+/-5.3"	"129.2+/-6.8"
4	"92.4+/-2.1"	"95.5+/-3.5"	"99.2+/-5"	"170.2+/-5.4"
5	"96.8+/-3.6"	"103+/-4.7"	"111.2+/-7"	"150.7+/-6.6"
6	"93.7+/-5.1"	"98.9+/-6.9"	"109.5+/-11.4"	"155.4+/-6.1"
7	"99.5+/-7"	"107.6+/-8.4"	"120.8+/-9.5"	"142.2+/-7"

	smallest_opposite_angle*	TrigonalBipyramidal	SquarePyramidal
1	"60.1+/-9.1"	"0.004"	"0.008"
2	"107.1+/-5.7"	"0.287"	"0.005"

3 "103.6+/-5.7" "0.008" "0.001"
4 "88+/-4.7" "0.003" "0.303"
5 "97.2+/-4.9" "0.052" "0.067"
6 "75+/-7.9" "0.003" "0.098"
7 "86+/-7.9" "0.004" "0.003"
TrigonalPrismaticV
1 "0.036"
2 "0.092"
3 "0.052"
4 "0.007"
5 "0.094"
6 "0.038"
7 "0.044"

Table S124. Cluster members of 5-ligand combined metal, combined group

[1] "Cluster 1"
4BLB.D.910, 4CPA.I.308, 1F30.B.201, 1F30.E.201, 2GA6.D.998, 4I0D.A.502, 4K0D.B.203, 4KJG.A.1001, 3M4B.B.108, 1NL4.B.500, 1RLY.A.61, 2V8V.A.1456, 3V94.D.702, 3A6P.H.1178, 2BPF.A.339, 4DPV.N.12, 3KK2.A.601, 2O5I.D.8001, 1QVG.O.8010, 1TTT.B.407, 1A49.B.1134, 1A49.C.1734, 4AN9.A.1383, 3AQC.D.328, 2BBO.A.2, 3C5P.D.302, 2CN8.A.1504, 3CNX.B.170, 4CYU.A.170, 2D33.B.1524, 2DW7.A.2001, 3DYF.A.3002, 4E1E.A.401, 3EA5.C.221, 1ELY.A.452, 4F0P.A.501, 2F1I.A.501, 4FMC.B.203, 1GXB.C.1346, 3HQP.A.502, 4I1O.E.201, 3IG8.A.696, 2IO7.A.5002, 4JVJ.F.405, 4K10.A.405, 4LF2.D.601, 3LZ9.A.852, 3MQT.W.395, 4N0G.A.405, 1N8W.B.1900, 4NU1.A.404, 3OAB.A.904, 4OAU.C.802, 3OB8.D.3001, 4P9D.B.202, 4PYK.A.302, 4R9U.C.302, 3RBM.A.1003, 3RBM.C.1002, 1S6P.A.601, 3SBE.A.501, 3TW6.B.2002, 4U81.A.501, 3UJ2.H.430, 4UOR.C.699, 2V0N.A.502, 2X9H.A.3001, 1Y8P.A.501, 1Y9I.B.602, 1YHM.A.401, 2ASJ.B.1415, 4EFJ.A.402, 3GIK.A.415, 2I3P.B.1, 4K4G.I.602, 4K4H.E.604, 3RBE.A.415, 4RIC.A.1101, 1SON.A.403, 3SQ1.A.907, 1AFD.2.2, 3ALA.E.1764, 1AR1.A.561, 1AVX.A.700, 1B09.C.302, 2BBM.A.182, 1BCZ.A.410, 3BJU.C.606, 3BJU.C.608, 2BTW.B.400, 3BXK.A.152, 2C10.C.1773, 2C11.A.1738, 4CAG.A.601, 6CGT.A.685, 2CHO.A.1717, 2CLT.A.1101, 1CTR.A.149, 2DDY.A.175, 3DF0.A.720, 1DTH.A.903, 3E1I.C.502, 1EAK.D.998, 4EJ7.C.403, 4ELF.F.201, 1ESP.A.320, 1EXZ.C.805, 3FAX.A.1223, 2FH2.B.2003, 2FH3.B.2001, 1FIH.B.3, 4FU4.A.505, 1G1Q.C.803, 3GCW.E.1, 4GKX.B.302, 3HR4.H.202, 2HTY.G.997, 3IJE.A.4004, 3IJE.A.4007, 3IJE.A.4008, 3IKQ.A.403, 1IVE.A.470, 1IXX.D.124, 1JKU.E.5272, 4JP8.A.704, 4JWU.A.502, 2K0E.A.153, 2K2F.A.94, 3K7L.A.701, 1K9I.A.407, 3KO0.H.302, 3KQA.A.420, 4KZW.B.400, 4L06.B.501, 4L06.D.501, 2LAN.A.301, 3LCP.D.159, 1LGN.A.302, 4LIN.E.1301, 3LND.A.210, 1LT9.B.1, 1LU1.A.301, 1LWJ.A.883, 1LWU.F.6, 3M0W.H.202, 2M0K.A.202, 2M0K.A.203, 3M8D.A.710, 4MDV.B.404, 2MLR.A.305, 2MLS.A.304, 1MPR.A.428, 3MW3.A.302, 3NP5.A.732, 1NX1.A.4, 2OKX.A.4004, 4OMD.F.604, 3OX5.B.502, 3OX5.C.501, 2OX9.B.803, 2OXE.B.600, 4P99.A.505, 4PJ0.A.601, 3POG.B.1, 4POQ.C.401, 4POQ.E.401, 2PRK.A.281, 4Q6O.A.501, 1QDO.B.243, 1QLK.B.93, 1QNI.D.903, 1QOO.A.302, 2QVF.B.704, 3QWU.A.601, 2RGB.A.601, 2RHP.A.11, 2RHP.A.24, 2RHP.A.25, 2RHP.A.27, 2RJP.B.4, 2RJQ.A.5, 1RTM.2.3, 1S1E.A.225, 3S5U.A.220, 4SBV.A.261, 1SDD.A.2184, 2SNI.E.276, 1SW8.A.81, 1SZB.B.1004, 1TN3.A.183, 1TNQ.A.91, 4TVU.B.600, 1TYE.B.1402, 4UM8.B.2003, 1UZJ.C.3648, 3VI3.B.2001, 2VR0.D.1006, 1W0P.A.1781, 3W9T.G.506, 4WFF.A.304, 3WHU.A.501, 1WT9.A.1001, 2WW0.C.800, 2WW0.F.800, 2X0G.B.1149, 1XFE.A.100, 1YO8.A.1184, 1ZZH.A.401, 1F9B.A.691, 3HH8.A.1, 3IS8.E.162, 3MPS.D.1, 4RC6.A.301, 3W54.B.501, 2YU1.A.600, 4DOA.A.402, 4DOB.A.402, 2FMQ.A.340, 3JPT.A.340, 4KHY.A.1013, 3PNC.A.2, 2PXI.A.340, 1U8R.A.1101, 1VQ9.C.9104, 4B6C.A.1257, 2BWU.A.14

41, 4C1P.A.1728, 3CBT.A.420, 1D7U.A.435, 3FKR.B.408, 3G8Q.D.302, 4H41.B.405, 3IN J.C.601, 3KZW.D.498, 4LCZ.B.320, 4M5P.A.505, 1MMX.A.349, 4MVJ.A.401, 4NLQ.A.910, 4NT1.A.301, 4NTL.A.311, 4NYP.A.1001, 4OOC.B.402, 2P6Z.A.402, 3R2H.A.156, 1SVY.A .2, 1YCE.B.201, 1YCE.a.201

[1] "Cluster 2"

2JZW.A.57, 2L1G.A.88, 4LJO.B.502, 4OND.E.101, 4QCL.A.1303, 1QUM.A.302, 830C.A.27 2, 1A7T.A.252, 2A7M.A.252, 4A7Y.A.951, 2A8H.A.486, 1AH7.A.246, 1AH7.A.248, 2AIO. A.315, 1AST.A.999, 4ASQ.A.1615, 4ASR.A.1615, 1B57.A.360, 3B7S.A.701, 1B8J.A.451, 2BH3.A.1002, 1BIW.B.801, 4BIN.A.500, 3BJC.A.876, 4BJH.A.423, 1BLL.E.488, 1BOR.A .58, 4BT4.A.301, 4BT5.A.301, 4BXK.A.1620, 4BZ3.B.502, 4BZR.A.1630, 4C24.A.301, 3 C52.A.401, 4C6L.A.2823, 4C6L.A.2824, 4C8I.B.1161, 1CAM.A.262, 4CA5.A.1001, 4CA6. A.1001, 4CA7.A.1616, 4CA8.A.1620, 2CHI.A.212, 3CKI.A.501, 3CQJ.A.285, 4CWM.A.433 , 4CXO.A.453, 4CXV.A.433, 3CZS.A.1102, 3DON.A.262, 1D5J.A.301, 4DEF.A.401, 4DEL. A.402, 3DHA.A.255, 4DLM.A.401, 4DPE.A.301, 2DVT.A.1501, 2DVU.A.1501, 2DVX.A.1501 , 4DYK.A.501, 4DZH.A.504, 2E2D.A.500, 3E38.A.1, 3EBH.A.1, 2EG6.A.400, 2EG7.A.401 , 1EI6.B.409, 3ELF.A.352, 4ENL.A.438, 6ENL.A.438, 3EWC.A.372, 3EWD.A.371, 2EWB.A .488, 1EYW.A.401, 4EYF.A.302, 4EYF.A.303, 1F0J.A.1101, 1FA5.A.1201, 3FDK.A.401, 2FGN.A.248, 1FKX.A.400, 4FW3.B.300, 4FW5.A.301, 4FW7.A.301, 4FYT.A.1012, 4GBD.A. 502, 1GKP.A.1461, 4GK8.A.302, 2GSO.A.1000, 1GVF.A.288, 1GYT.G.600, 1GYT.G.601, 4 H01.A.601, 4H1Q.A.301, 4H2E.A.301, 4H2G.A.601, 1H48.C.900, 1H4N.A.262, 4H76.A.30 1, 1H9N.A.262, 2HBV.A.401, 2HBM.A.1001, 3HC8.A.864, 2HC9.A.701, 2HC9.A.702, 3HK5 .A.430, 3HK8.A.430, 3HK9.A.431, 3HKA.A.430, 2HPT.A.950, 2HUC.A.248, 3HWP.A.295, 1HZY.A.402, 1HZY.B.401, 3ID7.A.401, 4IEO.A.601, 4IE6.A.601, 1IF6.A.262, 4IG2.A.4 01, 2ILP.A.500, 4ILW.D.301, 4ISM.A.201, 3ITC.A.501, 2J0T.A.1267, 4J4K.A.402, 4J5 H.A.302, 1J79.A.400, 1J79.B.400, 2J9A.A.1493, 1JCZ.A.901, 4JD1.A.201, 4JH8.A.201 , 4JH8.B.201, 1JIW.P.481, 2JIG.A.1252, 1JK3.A.400, 2JNE.A.150, 4JP4.A.301, 1JPU. A.371, 4JS6.A.301, 2JT6.A.256, 1K07.B.3, 3K2G.A.400, 2K2C.A.140, 1K4P.A.1004, 4K AP.A.301, 1KEQ.A.280, 4KEQ.A.302, 1KMG.A.154, 3KM8.A.400, 3KNS.A.228, 3KRY.A.199 9, 3KS3.A.262, 4KTX.A.501, 1L1O.C.1, 3L6N.A.301, 1LAM.A.488, 1LAM.A.489, 4LCQ.A. 601, 4LE6.A.405, 4LE6.A.406, 4LEF.A.301, 2LFF.A.500, 3LGG.B.512, 4LV4.A.401, 3M4 C.D.109, 3MA2.D.294, 3MA2.A.294, 3MAX.A.379, 3MDU.A.454, 3MJM.B.401, 3MKV.B.425, 3MO2.D.6, 3MTW.A.1, 3MVI.A.901, 3N2C.A.425, 3N9S.A.309, 4NGE.E.101, 3NJ9.A.262, 2NQH.A.301, 2NQH.A.302, 4NTK.A.201, 3NXQ.A.650, 2NZE.A.401, 3O2X.A.1999, 2O4Q.A .2401, 4O98.B.401, 2OB3.A.901, 2OC2.A.701, 3OD4.A.1350, 3OHL.A.4, 1ONW.A.800, 4O PN.A.201, 2OW1.A.444, 3P3C.A.401, 1P5X.A.248, 1P6B.A.401, 1P6D.A.246, 1P6D.A.248 , 3P76.A.274, 1PBO.A.1302, 1PL6.A.402, 2PLM.A.407, 3PNU.A.337, 1PV9.A.401, 4PVO. A.402, 4PXY.A.302, 2Q02.C.300, 3Q2H.A.401, 3Q4R.A.201, 3Q6X.A.3, 3Q6X.B.271, 4QA 5.A.401, 4QA6.A.401, 3QDF.A.266, 1QF0.A.320, 1QF1.A.320, 2QPJ.A.1, 1QTW.A.303, 2 QVV.A.403, 1QXL.A.400, 2R2D.A.278, 1R3N.A.500, 1R43.A.500, 3RHG.A.367, 1RJ5.A.60 1, 4RL2.A.301, 4RL2.B.302, 3SEY.E.373, 1SHN.B.482, 3SNG.A.401, 3SNG.A.402, 3SPU. C.1010, 3SXK.A.4, 3T00.A.501, 3T00.A.502, 3TOM.B.108, 4TYT.A.301, 1U7J.A.50, 1U7 J.B.150, 1U7M.B.154, 3U79.D.110, 3U79.E.110, 3UCT.B.102, 1UEA.C.1, 3UHM.A.300, 1 UIO.A.400, 1URA.A.451, 2USH.A.601, 2USH.A.602, 1UXA.B.1367, 2UX1.A.1174, 2UX1.C. 1173, 2V5W.A.1380, 3V96.B.301, 1VFL.A.501, 3VQZ.A.302, 3VTG.A.301, 2VUN.A.401, 3 W52.A.311, 3WAX.A.912, 3WAY.A.911, 4WB7.A.502, 4WD6.A.301, 1WPP.A.602, 2WXU.A.13 75, 2WXT.A.1371, 1WY2.A.406, 2XF4.A.1211, 2XHM.A.1616, 2XL9.B.1269, 1XM8.A.700, 1XP3.A.302, 2XS3.A.999, 2XS4.A.999, 2XYD.A.1620, 2Y6D.A.1268, 1Y7W.A.283, 2Z24.A .400, 2Z24.B.400, 2Z25.A.400, 2Z25.B.400, 2Z26.A.401, 2Z27.A.400, 2Z27.B.400, 2Z 28.A.400, 2Z28.B.400, 2Z29.A.400, 2Z29.B.400, 2Z2A.A.400, 2Z2B.A.338, 2Z3I.C.200 3, 1Z60.A.1, 2Z72.A.401, 2Z72.A.402, 2ZBM.A.401, 2ZBM.A.402, 1ZNB.A.2, 2ZNE.B.99 1, 2ZO4.A.319, 3ZU0.A.1596, 3ZU0.B.1588, 3ZU0.B.1589, 2ZWR.A.208, 2ZWR.A.209, 1Z ZM.A.401, 1ZZM.A.402, 3EPH.B.2, 2OTJ.0.8044, 1QVG.0.8067, 4AZW.A.1452, 3CTL.A.60 1, 3D19.A.301, 2D32.A.1524, 3DGB.A.2001, 2EB6.A.1001, 7ENL.A.438, 2F9R.B.602, 3F SY.A.334, 3FYY.A.401, 3GIE.A.1, 3GQ7.A.692, 4IAC.A.402, 1J7U.A.301, 1KJI.B.394,

1LP4.A.342, 1N8W.A.900, 1NUY.A.2342, 4NZN.A.404, 3Q2O.B.384, 2QB8.B.401, 2QVU.A.
340, 1SJB.A.1001, 2VPQ.B.1451, 2ZDR.A.1001, 2VA2.B.1343, 1AWB.B.279, 4B7U.D.401,
1CIZ.A.305, 2DSN.A.2011, 3ELM.A.303, 2EXH.C.2003, 3G20.A.201, 2J7A.D.1007, 4LM8
.A.812, 1OLP.B.1372, 2OVZ.A.449, 3QHQ.A.230, 1RM8.A.504, 3SO0.A.98, 3SO0.B.97, 3
SON.A.201, 3TRQ.A.359, 2W1W.A.1132, 1WY9.A.148, 2YC2.B.201, 2ZUY.A.629, 4QQZ.C.1
001, 4AIQ.A.1746, 1B06.A.322, 2BKB.A.1193, 2BUT.B.1541, 2BUU.B.1541, 2BUV.B.1541
, 2BUW.B.600, 2BUM.B.1541, 2BUQ.B.1541, 3CEI.A.500, 2CW2.A.402, 2CW3.A.524, 4DVH
.A.301, 3E13.X.326, 3ESF.A.198, 4F2N.B.300, 4FFK.A.301, 3FG3.D.1500, 4G2D.A.402,
1GN6.A.999, 2GOJ.A.198, 2GPC.A.195, 3H1S.A.1001, 3HJQ.A.281, 3HKP.A.281, 1HMD.A
.115, 4IEZ.A.501, 1ISA.A.193, 3IS8.A.163, 4KEZ.A.401, 4L2B.A.201, 4L2C.A.201, 3L
IO.A.5000, 1MY6.B.200, 3PCN.P.600, 2QFR.A.433, 3QFM.B.264, 1QNN.A.201, 2R1K.A.80
0, 2R1N.A.800, 4REU.A.201, 1SQ3.A.901, 1TDW.A.425, 3TQJ.A.1001, 1UNF.X.1239, 2VH
L.A.1397, 2VP1.A.1347, 2W7W.A.1195, 1WB7.A.212, 1WB8.A.212, 1XM8.A.701, 1Z6O.M.3
00, 1ZA5.B.393, 2GIG.A.502, 2GIJ.F.403, 1SUZ.A.403, 1A7T.A.255, 4A87.A.1162, 2AB
S.A.1003, 4ATF.C.500, 3B1N.A.403, 2B2N.B.1001, 4BEM.A.201, 4BEM.B.201, 4BEM.J.20
1, 2BL2.A.1157, 3C7X.A.1001, 3CB8.A.820, 4CD5.A.1420, 4CFY.A.302, 3CZJ.A.3101, 4
D1J.A.601, 4D77.A.1543, 2DKB.A.436, 4DUW.A.3101, 4DUX.A.3101, 1EJA.A.246, 3EPR.A
.266, 4EXR.A.301, 4FHA.B.402, 3G0T.B.437, 3GVK.A.916, 1H80.A.1497, 2HIG.B.488, 3
I3B.A.3101, 4IQZ.A.317, 3IRD.A.301, 2JBW.D.1367, 4JNQ.A.402, 4JPA.A.305, 3JU4.A.
7, 1JZ2.A.3101, 1JZ6.A.3101, 1JZ7.A.3101, 3KWM.A.225, 4LDE.A.1402, 3LKB.A.394, 3
MAX.A.381, 4MB4.A.604, 4MMB.A.601, 4MMF.A.601, 4MPY.A.503, 3MS8.A.401, 3MUI.A.40
1, 3MUZ.1.3101, 1MVO.A.202, 3MV0.1.3101, 3MV1.1.3101, 3MX6.A.261, 1N82.A.401, 1O
4Z.A.1004, 1OA8.C.1691, 1OAF.A.1252, 2PFL.A.2001, 2PPL.A.480, 3PZJ.A.301, 2Q8X.A
.401, 4QKU.A.501, 3QS4.A.751, 3QS6.A.751, 4R7U.A.504, 3RGA.A.312, 1SK4.A.342, 3T
09.A.3101, 3T8J.A.401, 1V54.A.3519, 3VDG.A.502, 1VI6.C.208, 1W9S.A.1142, 3WA2.X.
702, 2WGM.A.201, 2WGM.B.201, 2WOI.A.1491, 3WZ1.A.401, 1XFF.A.5301, 2Z5D.B.180, 1
ZNB.A.3, 3ZYV.A.2337
[1] "Cluster 3"
4LMG.D.202, 4MTD.A.202, 3A1Z.A.226, 4A37.A.376, 3A52.A.1001, 4A69.A.500, 2A7G.E.
401, 4A7B.A.1276, 1A86.A.999, 3AHT.A.1001, 3AIG.A.999, 2AIG.P.999, 1ALK.A.451, 1
ANJ.B.450, 2ANP.A.501, 2ANP.A.502, 4AR8.A.1731, 4AR9.A.1731, 1ATL.A.401, 4AWZ.A.
3230, 3AYU.A.415, 1BON.A.1001, 2B13.B.401, 3B2Z.A.1, 3B3C.A.501, 3B4N.A.711, 3B4
N.B.715, 1B57.A.361, 4B52.A.401, 4B6Z.C.385, 1BA9.A.154, 2BH3.A.1001, 3BKK.A.701
, 3BKL.A.701, 3BKN.B.201, 3BKN.B.202, 4BLB.A.910, 2BNN.B.1200, 3BOO.A.500, 2BO9.
A.999, 4BPO.A.1314, 2BP8.B.1340, 1BQO.B.301, 1BS8.A.2001, 1BYF.B.302, 4BZS.A.701
, 3COZ.B.101, 4C1H.A.351, 2C20.B.601, 4C2N.A.701, 4C2O.A.1629, 2C6P.A.1752, 2C6C
.A.1751, 1CBX.A.309, 2CEA.E.1603, 1CG2.A.500, 1CG2.A.501, 1CGL.A.301, 3CHO.A.701
, 2CLB.A.1175, 4COG.A.401, 4COQ.A.299, 1CP7.A.901, 1CP7.A.902, 1CPX.A.308, 5CPA.
A.308, 6CPA.A.308, 4CVR.A.1159, 4CVT.A.1158, 4CVT.A.1159, 1CXV.A.1, 4D0Y.B.1239,
1D1T.C.407, 1D8D.A.1001, 1DCE.B.900, 3DFM.A.401, 3DID.A.130, 1DO5.B.29, 4DPR.A.
701, 4DR9.A.201, 3DSX.B.332, 2DSN.A.2001, 3DYC.A.451, 1DZW.P.999, 3E2D.A.602, 3E
30.B.1001, 3E32.B.1001, 3E33.B.1001, 3E34.B.1001, 3E37.B.1001, 3E4A.B.2000, 1EC5
.B.50, 1EC5.C.50, 1ED9.A.450, 3EER.A.2001, 2EG7.A.400, 2EG8.A.400, 3EHY.A.264, 4
EJ5.A.501, 2EK8.A.1002, 3ELM.A.300, 3EQN.A.757, 2EV6.B.2151, 1EW9.A.450, 3EWJ.B.
2, 1EZ2.A.402, 3F28.A.321, 3F2P.A.2005, 3F3Q.A.104, 1F57.A.310, 3FB4.A.217, 3FFZ
.A.1300, 3FGD.A.321, 3FH4.A.301, 3FJU.A.999, 4FMP.B.400, 1FT7.A.501, 3FU1.B.201,
3FUK.A.701, 4FU4.A.502, 3FVL.A.1309, 4FYQ.A.1012, 4FYR.A.1013, 1G05.B.801, 1G49
.B.801, 3G42.A.500, 4G9L.B.301, 4G9L.B.302, 2GFK.A.401, 2GFJ.A.401, 3GIP.A.484,
4GM5.A.401, 2GSN.A.1001, 4GTM.B.501, 4GTO.B.501, 4GTP.B.501, 4GTQ.B.501, 4GTR.B.
501, 4GTV.B.401, 2GVI.A.302, 3GZE.A.1, 4H2K.A.1001, 4H49.A.301, 3H90.B.294, 1HA5
.C.4221, 1HDU.E.1308, 1HEE.E.1308, 1HFS.A.257, 2HH5.A.702, 1HI9.A.300, 2HIH.A.60
1, 1HLK.B.1004, 1HR7.B.501, 2HSE.B.954, 1HYT.A.805, 1HZ5.A.103, 4I11.A.502, 4I2F
.A.603, 2I3C.A.314, 3I9F.A.1, 4IAV.A.401, 2IEJ.B.939, 1IGB.A.502, 4IHM.A.402, 3I
Q6.B.201, 3IQ6.C.201, 3IT7.A.183, 2IXD.A.1234, 1IY7.A.308, 2J13.A.1236, 4J5H.A.3

01, 1J9Y.A.1003, 2JAZ.B.600, 4JBS.A.1008, 2JBJ.A.1752, 1JCQ.B.1001, 1JCS.B.1001, 1JI3.A.402, 1JJE.A.251, 1JJT.A.251, 4JJI.A.402, 4JJJ.A.718, 1JK9.C.302, 2JLP.B.226, 1JML.A.102, 2JSD.A.276, 2JT5.A.256, 4JYW.A.801, 4JZ0.A.801, 1K53.A.1003, 4K5L.A.1101, 4K7W.A.102, 4K90.A.701, 1KAP.P.613, 4KAY.A.601, 2KBX.B.299, 1KH4.A.450, 2KIK.A.50, 1KR6.A.405, 1KR0.A.405, 1KS7.A.405, 3KW0.C.162, 4L2L.A.701, 3L3N.A.701, 4L9P.B.601, 4LCQ.A.602, 1LD7.B.1001, 1LD8.B.1001, 1LFW.A.1001, 2LFF.A.502, 1LGD.A.262, 3LJZ.A.999, 1LND.E.800, 4LNB.B.602, 3LPE.D.60, 3LQ0.A.999, 4LQG.A.801, 4LQY.A.506, 3LTV.A.1001, 3LUB.B.302, 2LVH.A.101, 4LW9.A.201, 4LW9.I.201, 4LW9.R.201, 3M2Z.A.500, 2M30.A.201, 2M30.B.201, 3M4C.D.108, 1M4L.A.1308, 1M5E.A.1705, 3M79.A.107, 3M79.B.107, 3M79.C.107, 4MBG.B.602, 3MDJ.A.1000, 3MK1.A.902, 4MKH.A.301, 4MKT.A.701, 1MMB.A.999, 1MMP.A.1, 1MMP.A.2, 4MRQ.A.501, 4MSM.C.501, 3MWM.B.142, 1MXD.A.727, 1MZC.B.1001, 4N07.B.305, 3N2V.A.264, 3N21.A.401, 1N4Q.B.378, 1N4Q.L.378, 4NGE.A.1101, 3NKQ.A.1002, 3NKR.A.1002, 3NKN.A.1001, 3NKN.A.1002, 3NKO.A.1002, 1NPC.A.323, 2NQH.A.303, 1NW2.D.6004, 1NW2.F.6006, 4NYY.B.501, 1O1S.B.1001, 4O2I.A.401, 4O3A.A.303, 4O3A.B.302, 4O3A.B.303, 1O86.A.701, 1O8A.A.701, 1OEZ.W.1154, 4OJA.A.202, 4OKO.A.401, 4ONG.H.302, 4OTE.B.302, 2OW0.A.444, 4OXD.C.301, 2OY2.A.999, 3P24.A.999, 2P2L.A.901, 4P9C.J.201, 1PE5.A.317, 1PE7.A.317, 2PIY.A.400, 2PIZ.A.400, 2PJ0.A.400, 2PJ3.A.400, 2PJ5.A.400, 2PJ7.A.400, 2PJ8.B.500, 2PJA.B.500, 2PJB.A.400, 2PJC.A.400, 2PLI.C.701, 3PN4.A.1001, 4PPZ.A.601, 4PQA.A.403, 4PQA.A.404, 1PSZ.A.1000, 3PSQ.A.326, 3PSQ.B.328, 4PUC.A.602, 2PVV.A.1752, 3PW3.A.406, 3PZ1.B.332, 3PZ4.B.1, 3Q4R.A.202, 3Q75.B.521, 3Q78.B.521, 3Q7A.B.521, 4Q7R.A.307, 4QA0.A.401, 1QBQ.B.1000, 3QBU.A.294, 2QLA.C.501, 1QMU.A.999, 3QW0.A.500, 3QW0.B.500, 3QW0.D.500, 3QW1.C.500, 2R2D.A.277, 1R3N.A.501, 1R42.A.804, 1R43.A.501, 2RFH.A.1308, 3RF4.A.118, 2RH6.A.1, 2RJP.A.1, 1RK6.A.601, 1ROS.A.400, 1RTQ.A.702, 3RYM.B.107, 1S0E.A.1291, 1S63.B.1001, 1S64.B.378, 3S9C.A.503, 1SA4.B.439, 1SA5.B.438, 3SFX.B.521, 3SFY.B.521, 1SHN.A.485, 3SI0.A.362, 3SKS.A.568, 1SQM.A.1001, 1T0A.A.661, 1T3A.B.422, 1TF9.A.902, 3TGN.B.147, 3TG0.A.501, 3TG0.A.502, 1TKJ.A.901, 1TKJ.A.902, 4TLN.A.321, 1TLP.E.322, 5TLN.A.321, 1TMN.E.322, 4TMN.E.322, 1TN6.B.1001, 1TN7.B.1001, 1TN8.B.1001, 1TNB.B.378, 1TNO.B.378, 1TNU.B.378, 1TNY.B.378, 1TNZ.B.378, 3TOL.B.107, 3TS4.A.301, 3TT4.A.302, 3TVC.A.501, 3U04.A.200, 3U24.A.595, 1U7M.A.54, 3U9W.A.2001, 3UBF.A.1, 1UUP.A.2222, 1UZE.A.701, 3V0A.A.1297, 3V1E.A.101, 2V29.A.1275, 3V77.A.302, 2V77.A.1042, 2V9I.A.1277, 2V9E.A.1275, 3VH9.A.302, 2VJ8.A.1611, 2VQQ.A.1411, 2VXI.A.201, 2VXI.A.202, 2VXX.C.202, 1W22.A.1375, 2W5V.A.1377, 2W5X.B.1378, 3W5K.B.501, 2W57.B.202, 3W6H.A.302, 1WAA.A.1091, 1WAA.F.1091, 3WAW.A.913, 3WBH.A.505, 4WCM.A.538, 4WD8.A.301, 4WD8.A.302, 3WE7.A.301, 4WNC.O.402, 3WOJ.A.805, 2WO8.A.1268, 2WO9.C.1269, 2WOA.A.1270, 1WUO.A.301, 3WV3.A.301, 3WXC.A.301, 1WY2.A.405, 2X4H.A.1142, 2X90.A.1618, 2X91.A.1619, 2X92.A.1615, 2X93.A.1615, 2X94.A.1616, 2X97.A.1616, 2X98.A.1475, 2X98.A.1476, 1XAF.B.507, 1XGE.A.400, 4XIX.A.401, 2XIG.B.1150, 2XIG.C.1153, 2XR9.A.1867, 2XX0.B.1340, 2XX7.B.1264, 2XXG.A.1339, 2Y28.A.1181, 2Y2B.B.1180, 2Y3D.B.149, 1YHC.B.602, 2YJP.A.1272, 2YJP.C.1270, 2Z2Y.C.2003, 2Z25.B.401, 2Z2D.A.265, 1ZED.A.904, 1ZG7.A.400, 1ZG8.A.400, 1ZG9.A.400, 2ZIR.B.901, 2ZIS.B.901, 2ZKW.A.401, 2ZTG.A.902, 3ZXO.A.1579, 2G8F.A.301, 4IEM.A.502, 4EOO.A.301, 4GWZ.A.402, 1IV2.B.1562, 1IV4.E.1565, 3LD0.8.54, 2M32.A.401, 4ML9.B.301, 4OTP.A.502, 4QYI.E.203, 4UOR.A.699, 3V4S.A.402, 3WNW.A.201, 2XTI.A.1551, 2EX5.X.802, 2BTW.A.400, 3C5I.A.371, 1DJZ.A.2, 2E30.A.300, 1IJL.B.202, 4KPP.B.501, 3L9I.C.1151, 1M63.B.500, 1NW1.A.430, 3WIV.B.1004, 4AM4.A.1161, 4BMT.A.1323, 1CJX.A.629, 2CKF.A.501, 2DE6.A.501, 1DLM.A.400, 3E1Q.A.301, 4ELR.A.401, 2FKZ.C.1600, 3FM3.B.552, 4GAM.F.601, 3GHQ.K.300, 1GUQ.A.351, 4HJL.A.502, 2HMN.A.450, 2HTN.A.301, 4NB9.A.501, 4NBG.C.501, 4NBH.A.501, 1OQU.B.1004, 2OWT.A.324, 4P9G.A.401, 1PIZ.A.376, 3R2R.A.156, 4RC6.A.302, 4REU.A.202, 1SI0.A.321, 4V06.A.1491, 1XIK.B.377, 2XSH.G.900, 1DIZ.A.825, 4FZZ.A.201, 8ICK.A.341, 1JJ2.A.8345, 4KHY.A.1006, 1TX3.A.801, 2BBH.A.301, 3DYM.A.3101, 1EBU.B.902, 1GNY.A.1244, 3I3D.A.3101, 4J4B.A.902, 4J4B.B.303, 1JZ3.A.3101, 1NSX.A.1401, 3ONF.A.507, 3PNX.C.169, 1PX3.B.3101, 2QWL.A.589, 4TMW.A.902, 1U4J.B.1001, 3VD7.

B.3101
[1] "Cluster 4"
3ADR.A.263, 2BIB.A.1550, 2DI3.A.1002, 3EII.A.301, 2EK9.A.1004, 3G4K.A.801, 2HD1.A.101, 4HTZ.B.1001, 4ICQ.A.501, 2K78.A.151, 4KBP.A.439, 3KR5.E.1001, 4KYH.A.202, 3LL8.A.505, 2LZE.A.101, 1ML2.A.296, 4N7K.L.301, 4N7K.L.307, 4N7K.M.401, 4N7K.M.402, 4N7L.M.402, 3NO5.C.275, 3O64.A.1, 4OK2.A.801, 2OUN.B.404, 1Q3K.B.300, 3V93.D.701, 1VKG.B.400, 4X2T.A.702, 3A4K.C.301, 2IHM.A.700, 2PYJ.B.9002, 3AOU.A.201, 3A10.A.201, 4ACF.D.1480, 2AKZ.A.441, 1ALK.A.452, 3B05.A.1001, 4BBJ.A.750, 2BHW.A.601, 2BHW.A.602, 2BHW.A.603, 2BHW.A.604, 2BHW.A.609, 2BHW.A.612, 2BHW.A.614, 2BHW.B.605, 3BH7.A.1, 4BJU.A.998, 4BX2.A.301, 4BYF.A.1000, 4C5A.A.331, 3C9U.A.309, 1D0X.A.998, 1D0Y.A.998, 1D0Z.A.998, 1D1A.A.998, 1D1B.A.998, 4DBQ.A.903, 2DCN.B.4006, 3DHF.A.502, 4DL8.A.304, 4DL8.A.305, 3DNT.B.442, 1DXR.L.400, 3DYH.A.3002, 1E1R.F.601, 1E79.A.601, 2E8W.A.1201, 2E8X.A.1301, 3EF0.A.1, 2FKW.A.1501, 2FKW.B.1601, 1FQJ.A.352, 3G5A.C.307, 1G67.A.2007, 2G77.B.503, 1GFI.A.356, 2GJ8.A.602, 3GL9.A.123, 2GLQ.A.2003, 4GVE.A.602, 3H1E.A.202, 1HE1.C.202, 2HEG.A.300, 2HF7.A.700, 4HGQ.C.201, 2HO4.B.301, 3HWX.1.602, 3HZH.A.202, 2I7D.A.728, 3ICM.A.403, 3ICN.A.402, 4IDO.A.503, 4IF4.A.300, 4IGA.A.200, 2IOA.A.5002, 1IV2.B.1572, 2J7N.A.3374, 2J8C.L.1288, 1J97.A.220, 4JA2.A.201, 1JB0.A.1107, 1JB0.A.1110, 1JB0.A.1117, 1JB0.A.1118, 1JB0.A.1122, 1JB0.A.1129, 1JB0.A.1130, 1JB0.A.1133, 1JB0.A.1134, 1JB0.B.1204, 1JB0.B.1231, 1JB0.B.1235, 1JB0.X.1701, 2JCS.B.1211, 4KEM.A.401, 1L3R.E.392, 1L5Y.A.701, 1L7N.A.221, 4LCZ.A.306, 4LCZ.A.307, 4LCZ.C.316, 4LEO.A.201, 1LGH.A.57, 1LGH.A.58, 1LGH.B.59, 4LRS.A.404, 1LVK.A.998, 3MCO.A.427, 3MLE.C.222, 1MPS.M.801, 1MXO.E.501, 1N22.B.706, 1N24.B.706, 3N5K.A.2001, 1N6K.A.201, 2NGR.A.199, 4NM5.A.406, 3NNN.A.401, 3NNS.A.401, 4NST.A.1103, 1NVV.Q.1002, 4NVO.A.402, 2ODE.A.3001, 2OGX.A.291, 3OLV.A.130, 4OVN.A.201, 2OZE.A.299, 2PAN.A.851, 3PDE.B.310, 3PL9.A.602, 3PL9.A.603, 3PL9.A.609, 3PL9.A.610, 3PL9.A.612, 3PL9.A.613, 3PL9.A.614, 2PL1.A.204, 2PLS.I.606, 1PPW.A.401, 4PTK.A.302, 3PUW.A.1501, 1Q3H.D.674, 2Q58.A.3, 3QHW.A.298, 4QXD.A.304, 1R0X.D.14, 2R25.B.1, 1RLO.A.801, 4RUR.W.301, 1RWT.A.614, 4S1H.A.303, 3SQS.A.450, 1SVK.A.356, 3T2S.A.401, 3T34.A.1002, 3T6D.L.401, 3T6D.M.400, 3T6E.L.400, 1T91.A.1301, 3T9E.A.602, 3TCS.B.368, 3THU.A.500, 1TX4.B.681, 3U2E.A.2, 4UB6.C.506, 4UB6.C.507, 4UB6.C.510, 1UPB.A.601, 2V54.B.1205, 2VB6.A.1000, 1W5T.A.701, 3W6P.A.803, 1W7J.A.1793, 1W9I.A.1755, 3W9S.A.202, 2WF7.A.1220, 2WJN.M.1325, 3WK4.A.601, 1WQA.B.456, 2WZB.A.1417, 2X2E.A.1746, 1XBX.A.601, 1XHF.A.1001, 1Y9D.A.2601, 1YXO.A.5000, 1YZQ.A.901, 2Z4Z.A.1301, 1Z5B.A.2001, 2Z7I.B.1302, 1ZES.A.302, 1ZH4.B.202, 1ZXN.A.900, 2ZXE.A.2002, 4BXO.B.1216, 4G3I.B.401, 3GII.A.415, 4K4H.M.604, 4LQ0.A.401, 2WTF.B.1509, 4AG4.A.5001, 4B7M.B.1471, 2BV2.A.1085, 2BW7.A.2202, 3BYC.A.901, 2CDO.B.1140, 4CPN.A.500, 4CPO.A.1466, 1CXV.B.6, 1D7X.B.805, 3DPE.A.997, 1DYK.A.4001, 1E35.B.260, 3EKI.A.602, 2ERQ.B.702, 1EZS.C.650, 1F2N.A.1002, 4FVL.B.506, 4GDI.C.507, 2GNT.A.254, 4GN7.B.301, 2HTV.A.995, 4ILW.D.304, 1IME.B.278, 1INW.A.501, 1JAO.A.996, 2JG9.B.1224, 1JI3.A.401, 4JUC.B.601, 3K37.A.467, 1L6J.A.502, 1L9N.A.703, 3LNF.B.305, 1N28.A.128, 1NG0.A.1002, 1NG0.C.1001, 2NVO.A.533, 2NW6.A.613, 2OO4.B.5006, 2OW1.B.447, 4Q4X.1.5005, 1QI5.A.452, 2R1D.B.1000, 2R8Z.J.210, 1R8L.B.902, 2RHP.A.10, 2RHP.A.20, 2RJP.A.2, 2RJQ.A.6, 1ROS.B.503, 1RU4.A.2, 3SJS.A.222, 1STB.A.150, 1T6B.X.800, 4U32.A.301, 1ULV.A.2005, 1UYX.B.1135, 3V03.B.585, 3VV3.A.404, 1W3M.C.3014, 2WG8.A.201, 3WIU.B.1002, 3WNK.A.812, 1Y4A.E.1001, 1Z4V.A.600, 2ZJ6.A.625, 1ZTQ.A.561, 2ZUX.B.638, 2ZUY.A.627, 2ZWO.A.400, 3A15.A.354, 3A51.C.412, 3ABB.A.1430, 1AHJ.A.208, 4AJ9.A.1715, 3AK3.C.215, 2AKJ.A.564, 3ARJ.A.153, 3AT6.A.142, 2AUO.A.153, 2AUQ.A.147, 2AVO.A.147, 4AVD.A.144, 3AYF.A.802, 1BOB.A.144, 1B7V.A.93, 3BA2.A.158, 1BBH.A.132, 1BCF.A.200, 2BCN.B.109, 1BGP.A.400, 3BK9.A.401, 2BMO.A.1441, 1BT8.B.202, 3BUJ.A.398, 3BXD.A.302, 2C1D.H.1158, 4C50.A.1741, 3C6G.A.601, 1C6S.A.88, 4C9L.A.1418, 4CAB.A.537, 2CCY.A.129, 1CG8.B.142, 1CGN.A.128, 1CH4.A.147, 2CJ1.A.1300, 2CMM.A.155, 1CPQ.A.130, 1CRC.A.105, 4CZC.A.1337, 2D0Q.A.300, 3D1K.A.200, 3D1K.B.400, 4D3O.A.901, 2D5X.B.147, 3DAM.A.600, 3DAX.A.601, 3DBG.A.500, 3DE8.D.150, 1DGF.A.300

0, 3DHG.D.507, 2DKK.A.430, 1DLY.A.144, 4DVQ.A.601, 1DW1.A.113, 4DWU.A.201, 1E2R.A.602, 2E39.A.401, 3E4W.A.501, 3E5L.A.1408, 2E84.A.1315, 3EAH.A.861, 1ECD.A.137, 4EGO.A.501, 3EH5.A.801, 3EJ8.A.1901, 3EJD.B.405, 4ENP.A.801, 4ENU.A.801, 1EQD.A.185, 4ESA.A.202, 4ESA.B.202, 1EUP.A.410, 1EYS.C.612, 4FB2.B.501, 2FDU.A.500, 2FDG.A.300, 1FHF.A.350, 1FHJ.B.147, 4FVC.A.201, 1FZ1.B.5003, 3G1Q.A.480, 4G2C.A.501, 2G3H.A.154, 3G46.A.147, 4G45.A.401, 3GAS.A.1290, 1GBU.B.148, 2GB8.A.295, 1GCV.A.141, 1GCV.B.137, 3GE3.A.502, 1GEJ.A.501, 4GEP.A.580, 2GGN.X.251, 1GJQ.A.602, 2GKM.A.144, 3GPH.A.500, 4GQS.B.501, 1GVH.A.1398, 1GW2.A.350, 1GWS.A.615, 1GWU.A.1306, 2GYQ.A.401, 4H0K.A.200, 1H1X.A.1154, 1H2K.A.1350, 1H2L.A.1350, 4H8Q.A.201, 1H97.A.148, 2HBT.A.900, 3HC1.A.305, 3HC1.A.306, 1HDS.A.142, 1HDS.B.146, 3HF2.A.482, 3HF4.F.147, 1HJ4.B.601, 1HJ5.B.601, 2HMQ.B.115, 4HRR.A.201, 4HRR.B.201, 1HV4.A.151, 3HX9.A.300, 2HYS.A.201, 1HZU.A.601, 1I3D.A.147, 1I4Y.E.605, 4I8V.A.601, 2I96.A.129, 4IAM.A.501, 2IBN.B.706, 3IBD.A.500, 2IG3.A.700, 4IGO.A.1000, 2IIZ.A.400, 2INN.A.513, 1IOP.A.154, 3IQB.A.500, 1IRD.B.347, 2ISA.A.486, 1IT2.A.147, 1IWH.A.142, 1IX4.A.300, 1IZO.C.501, 2J0P.A.1342, 1J1L.A.1001, 4J1X.A.201, 2J2M.A.501, 2J7A.D.1001, 1JAF.A.130, 1JEB.D.147, 4JET.A.201, 4JS9.A.501, 2JXM.B.250, 3K9V.A.520, 4KF0.A.501, 2KII.A.182, 3KX4.A.999, 1L0L.D.242, 4L1Y.A.300, 4L1Z.A.300, 4L2M.A.201, 4L3H.A.402, 2L4D.A.107, 4L54.A.501, 2L8M.A.416, 1LC1.A.105, 1LGA.A.396, 1LH2.A.154, 1LH6.A.154, 1LH7.A.154, 1LHT.A.155, 2LH2.A.154, 2LHB.A.151, 3LL8.A.506, 3LMX.M.600, 4M26.C.401, 1M54.F.1620, 1M56.A.1002, 1M7S.D.600, 4M71.B.403, 1M85.A.1001, 1MBA.A.148, 1MGN.A.154, 2MHR.A.119, 3MM3.A.501, 3MM6.B.570, 3MMB.A.580, 3MMO.A.1004, 4MMO.A.401, 1MN1.A.396, 3MOL.B.185, 1MQV.A.150, 1MYF.A.154, 3MYM.A.139, 3MYN.A.139, 3MZS.A.500, 3N1Y.A.503, 3N1Y.A.504, 3N3R.A.1500, 4N4M.A.616, 4N4N.C.601, 3N8Y.B.601, 1N97.B.603, 3NA0.A.601, 4NK2.A.700, 4NKW.A.600, 3NN2.A.239, 3NNF.A.600, 3NNL.A.600, 2NNJ.A.500, 2NOX.C.500, 2NP1.A.350, 1NR6.A.500, 3NTG.D.601, 3NU1.A.302, 3O0F.A.304, 3O5C.B.402, 2O68.A.401, 1OAE.A.1125, 3OCD.C.401, 3OCD.D.401, 1OCZ.A.515, 1ODO.A.1407, 3OFU.A.417, 1OG5.A.501, 2OGI.A.301, 1OIK.A.1302, 2OIF.A.163, 4OQR.A.501, 1OR4.A.180, 2OYY.A.201, 1OZW.B.300, 4OZ5.A.201, 1P3T.A.300, 3P3X.A.501, 2P85.A.500, 3PCJ.R.600, 3PM0.A.900, 2PMS.A.347, 3PT8.A.500, 3PT8.B.500, 3Q14.A.501, 1Q5E.A.440, 2Q8P.A.300, 3Q9K.A.605, 2Q9F.A.602, 1QGJ.A.1350, 3QGP.A.200, 1QHW.A.433, 1QJS.A.500, 3QPI.A.1001, 3QQR.B.163, 2QRW.A.700, 2QSS.A.142, 1QWL.B.550, 3QY6.A.264, 3QY8.A.252, 3QZM.B.201, 3QZX.A.200, 2R1H.D.148, 4R21.A.600, 2R79.A.500, 3R9B.A.501, 2RCL.B.600, 2RDN.A.1, 2RF7.D.1, 2RFB.A.410, 2RI4.A.142, 3RIW.A.305, 4RKM.K.809, 4RKM.L.813, 3RUR.A.200, 1S05.A.130, 1S13.A.300, 1S1F.A.430, 1S61.A.144, 1S69.A.125, 3S66.A.142, 3S79.A.600, 1SCH.A.300, 1SI8.A.501, 3SIK.A.154, 1SMJ.C.472, 1SOG.A.296, 1SPG.A.144, 1SPG.B.148, 1SQ3.A.903, 1T47.B.430, 1T85.A.417, 3TBG.A.800, 3TKT.A.431, 1TMX.A.861, 3TM8.A.902, 3TM8.A.903, 2TOH.A.501, 4TRI.A.501, 3TTV.A.760, 3TTW.A.760, 3TTX.B.760, 1TWN.A.300, 3TYW.A.501, 1U5U.A.999, 4U9D.D.201, 3U9J.A.200, 3U9M.A.201, 3UA1.A.508, 4UBS.A.501, 1UC3.A.150, 3UHB.A.147, 3UHD.B.147, 1UMO.A.1172, 3UOI.I.200, 3UT2.A.1500, 1UX8.A.700, 1V4U.A.144, 1V4U.B.147, 1V54.A.516, 1V9Z.B.1140, 3VED.A.401, 2VE3.B.1444, 2VHD.A.401, 3VNO.A.501, 3VOL.A.401, 1VRE.A.148, 3VRF.B.201, 3VSI.B.401, 2VZW.A.1206, 3W08.A.501, 3W4U.A.201, 3W8M.A.201, 1W92.A.1149, 3WCU.A.200, 3WCU.C.200, 3WCU.B.201, 3WCU.D.201, 3WFB.B.802, 4WG2.A.603, 2WIV.A.1553, 3WNU.A.801, 1WOW.A.300, 1WRA.A.401, 3WXO.A.801, 4WXO.B.301, 2WY4.A.150, 2X66.A.1359, 1X8V.A.470, 1X9F.A.160, 1X9F.B.160, 1X9F.C.160, 1X9F.D.160, 2XBK.A.1398, 2XF2.A.690, 2XMO.A.1128, 2XMO.B.556, 2XN8.A.1434, 2XQ1.B.1503, 1Y01.B.142, 2Y4F.A.389, 2YGX.D.450, 2YL1.A.128, 1YMC.A.154, 2Z3U.A.500, 2Z36.A.450, 2Z6F.A.3747, 2Z6N.A.150, 2Z6N.B.150, 2ZDO.A.200, 2ZFO.D.200, 3ZK5.A.1407, 3ZKY.A.1332, 2ZPG.A.300, 2ZVU.A.300, 2ZZS.2.220, 2FMS.A.342, 4GXK.A.405, 3JPP.A.340, 2PFQ.A.1, 4TUR.A.404, 3AGB.A.1, 3AST.B.701, 4AYO.A.502, 4BDR.A.902, 3C7E.A.489, 4C7A.B.1159, 4CZN.A.1371, 4DF9.A.501, 4F3Y.A.301, 4FEW.D.304, 4G1K.B.301, 4GDK.E.301, 2GEZ.A.401, 1GEN.A.304, 1GUU.A.1090, 1GV2.A.1192, 4GY9.A.207, 2HU3.A.9002, 1HXN.A.2, 4I2F.A.602, 3I44.A.477, 3IFV.C.408, 2IY6.B.1540, 2J5W.A.3044, 2J5W.A.3045, 2JHN.A.1298, 3JS4.A.

208, 4KA8.A.806, 4KXW.A.1013, 4KZV.A.304, 3LP5.A.251, 2O34.B.502, 3OTK.A.586, 4P
FI.A.401, 4PUV.A.405, 4PV3.A.201, 4QFE.B.305, 1QHU.A.437, 3STH.A.501, 1SU3.A.911
, 3U21.A.500, 2V4B.B.1562, 3VS8.A.501, 3VS8.C.501, 1W16.A.1002, 1W9W.A.900, 1WPG
.D.1300, 2WV7.B.401, 2XZK.B.507, 1ZDN.A.157
[1] "Cluster 5"
456C.A.272, 4A3W.A.1159, 4A7K.A.952, 4A7Z.A.950, 1A85.A.999, 2AB7.A.30, 1AF0.A.4
72, 3ASE.A.156, 4AWY.B.3229, 4AX0.B.3229, 4AX1.B.3229, 1B3D.B.301, 3B7U.X.701, 3
B8Z.A.901, 2BCN.A.295, 2BIB.A.1549, 3BKQ.X.500, 2BMI.A.272, 2BNO.A.1201, 4BT6.A.
1257, 3BUB.A.1047, 3BUD.A.1048, 3BUI.A.1046, 3BVT.A.1048, 3BVU.A.1048, 3BVV.A.10
47, 3BVW.A.1046, 3BVX.A.1046, 4BZ5.A.500, 3C10.A.101, 1C3R.A.501, 1C3S.A.951, 4C
5W.A.402, 2CA2.A.264, 2CEA.A.1603, 2CFU.A.1001, 3CV5.A.1047, 3CZN.A.1102, 2D1N.A
.270, 2D1O.A.257, 1D7X.B.801, 2DDY.A.177, 4DD8.A.1002, 4DHL.B.501, 1DQS.A.402, 4
DV8.A.801, 3DWB.A.771, 2DW0.A.700, 3E0L.A.1452, 1E4C.S.999, 4E5V.B.401, 3EBG.A.1
, 3EDZ.B.2, 2ERP.A.700, 3F15.A.264, 3F16.A.264, 3F17.A.264, 3F18.A.264, 3F19.A.2
64, 3F1A.A.264, 1FA5.A.1200, 4FL7.A.301, 4FUA.A.216, 2FV5.A.3, 2FV9.B.4, 2FVM.D.
601, 4FW4.C.301, 2FYV.A.2003, 2GC3.A.402, 1GKP.B.1460, 1GKR.A.1453, 2GMN.A.802,
2GO4.A.601, 4H3X.A.301, 3H8F.E.501, 3HC4.L.401, 1HFC.A.275, 4HGX.A.301, 1HJK.A.4
51, 1HOV.A.166, 3HR1.A.1, 1HS6.A.701, 1HTY.A.1102, 4HWO.A.701, 4HWP.A.701, 4HWR.
A.701, 4HWS.A.701, 3HY7.A.901, 3HY9.A.901, 3HYG.A.901, 2I47.C.804, 2ICS.A.400, 4
IE4.A.601, 4IGN.B.401, 3ITC.A.502, 1ITU.A.402, 2J0E.B.1265, 4J5F.A.301, 2J83.A.9
99, 4JAA.A.501, 1JAP.A.999, 1JAQ.A.999, 4JE7.A.202, 2JIH.B.1554, 1JJE.A.252, 1JJ
T.A.252, 2JNE.A.200, 1JWQ.A.1001, 2K4W.A.156, 3K5X.A.402, 1K9Z.A.402, 1KAE.B.110
2, 1KBC.A.999, 3KDS.E.996, 1KYS.A.301, 1LCP.A.489, 3LJT.A.901, 3LK8.A.264, 2LQ6.
A.402, 3LS6.A.303, 1MNC.A.281, 3N2U.A.264, 4N27.A.201, 4N27.C.201, 3N9R.A.308, 3
NX7.A.264, 3O90.B.192, 3OHL.A.5, 3OHO.A.5, 2OKL.A.601, 4OPN.B.201, 1OS9.A.901, 4
OUI.A.501, 2OW6.A.3001, 2OW7.A.5001, 1P6E.A.248, 2PAJ.A.493, 2PJT.A.302, 4PKT.A.
802, 4PKW.A.801, 1PMI.A.445, 1PS6.A.330, 1PTW.A.501, 1PVW.A.401, 3Q2G.A.401, 2Q5
B.A.107, 2Q5B.C.107, 1Q74.A.304, 3Q9B.A.345, 3QAY.A.180, 4QA1.A.403, 4QA2.A.403,
4QA4.A.502, 2QFR.A.434, 1QH5.A.262, 1QIN.A.401, 1QIN.B.301, 1QIP.A.902, 1QIP.D.
903, 3QIZ.A.431, 1QJJ.A.250, 4R76.A.1001, 2RJQ.A.1, 1RM8.A.500, 1RMZ.A.264, 3RTS
.A.264, 3RTT.A.264, 3S2J.A.402, 3S2L.A.402, 3S2M.A.402, 3S2M.A.403, 3S2N.A.402,
3S2N.A.403, 3SFW.A.501, 1SML.A.270, 1T64.A.388, 2TCL.A.170, 1TQS.A.1401, 1TQT.A.
1301, 1TQU.A.1400, 1TQV.A.1300, 1TQW.A.1400, 3UWB.A.200, 2V5X.A.1377, 3V77.A.301
, 2V9M.A.1275, 2VES.A.1295, 3VH9.A.301, 3VPE.A.302, 2VQM.A.1411, 3VUS.A.401, 2W0
D.A.1264, 2W15.A.1203, 2W3Z.A.1312, 4WD6.B.302, 2WM1.A.1333, 2WO9.B.1268, 4X2T.B
.1002, 1XBU.A.901, 1XBU.A.902, 1XP3.A.301, 1Y13.A.174, 1Y93.A.264, 2YD0.A.1946,
1YQY.A.781, 3ZU0.A.1595, 1ZXC.A.201, 3ZXH.A.300, 4D6O.A.1187, 4DFM.A.903, 3Q8P.B
.423, 3SNN.A.906, 3TFR.A.340, 1AJD.B.952, 3AU9.B.602, 3AUA.A.601, 2BBT.A.3, 4BCL
.A.367, 4BCL.A.368, 4BCL.A.369, 4BCL.A.370, 4BCL.A.371, 1BHO.1.901, 4BJR.A.1517,
2BOZ.M.1304, 1BS1.A.901, 2BW7.D.2201, 1DAM.A.901, 4DFX.E.403, 1E14.M.1303, 1E6D
.M.1303, 1E9I.B.1431, 3ENI.A.375, 3ENI.A.378, 3EOJ.A.375, 2FKA.A.9001, 1FMW.A.80
0, 3FXG.B.501, 1G65.G.301, 4G61.A.301, 2GQ3.A.1002, 4GX6.A.402, 4HEO.A.402, 3I6E
.B.386, 4IL6.j.102, 4IL8.A.501, 4IN7.M.411, 1JB0.A.1011, 1JB0.A.1106, 1JB0.A.111
2, 1JB0.A.1121, 1JB0.A.1126, 1JB0.A.1128, 1JB0.A.1131, 1JB0.A.1132, 1JB0.A.1136,
1JB0.B.1201, 1JB0.B.1203, 1JB0.B.1206, 1JB0.B.1207, 1JB0.B.1211, 1JB0.B.1214, 1
JB0.B.1215, 1JB0.B.1221, 1JB0.B.1223, 1JB0.B.1224, 1JB0.B.1225, 1JB0.B.1234, 1JB
0.B.1236, 1JB0.B.1239, 1JB0.L.1501, 1JB0.L.1502, 1JYX.A.3002, 1KJ8.B.394, 3KR4.A
.1004, 3KRF.D.901, 3L8F.A.401, 4LCZ.A.314, 4LNI.A.503, 4LRJ.A.302, 4LYJ.A.201, 4
MFE.C.1104, 4MKU.A.209, 1MNZ.A.389, 1NOW.A.401, 4NHO.B.1403, 3O5T.A.299, 4OEC.A.
401, 2OQY.B.402, 1Q9L.C.218, 3QQV.A.381, 1QSH.B.147, 4QTD.A.426, 4QVP.V.302, 1R0
3.A.301, 4R02.Y.302, 4R17.K.302, 4R7O.E.402, 3RLG.A.286, 1RZH.L.854, 1RZH.M.851,
1RZH.M.853, 1SJA.B.701, 3TAV.A.266, 4U0M.B.503, 4UB6.B.603, 4UB6.B.607, 4UB6.B.
610, 4UB6.B.616, 4UB6.C.504, 4UB6.C.509, 4UB6.C.512, 4UB6.C.513, 4UB6.C.514, 4UB
6.D.402, 4UM8.D.2001, 2UXR.A.1405, 3V3Z.L.302, 2VP0.B.1209, 3VTH.A.805, 1W88.A.1

368, 3WQP.H.501, 3WU2.A.405, 3WU2.A.410, 3WU2.B.604, 3WU2.B.605, 3WU2.B.606, 3WU 2.B.609, 3WU2.B.612, 3WU2.B.617, 3WU2.C.502, 3WU2.C.510, 3WU2.D.402, 3WU2.b.615, 3WU2.b.616, 3WU2.b.617, 3WU2.c.902, 2YBE.A.1417, 1YF6.M.853, 1YF6.M.856, 1YQ7.A .908, 1Z2P.X.1295, 1ZM7.D.400, 4K4G.M.603, 4QWE.A.703, 3UIQ.A.905, 1AF4.A.276, 3 AFG.B.541, 1ALA.A.402, 3AYU.A.419, 4B7R.B.502, 1B9V.A.500, 4CBX.G.1151, 4CCE.A.4 001, 2CDP.B.1140, 3CKC.B.700, 2DDF.A.475, 4DWW.A.301, 1E5J.A.402, 1EA7.A.315, 7E ST.E.11, 1F5R.A.800, 2FYC.B.404, 1FZD.F.1, 1G5N.A.404, 1GMM.A.1132, 4H1Q.A.304, 2HT5.A.995, 4HZY.A.507, 3K5T.A.802, 4KS2.A.501, 4MIV.C.601, 1MR8.A.102, 1N28.A.1 26, 4N2F.A.703, 1NGS.A.681, 1NKQ.A.260, 4NPK.A.803, 1OT5.A.602, 2OVX.B.449, 4PKH .J.1201, 1PZ7.B.702, 1Q3A.B.472, 3Q3K.A.262, 4Q4X.1.5006, 1QL9.A.480, 4QN3.B.501 , 2R8Y.C.203, 2RHP.A.22, 1S0E.A.1293, 1S1D.B.1002, 1SEL.A.277, 1SMP.A.478, 1SPU. A.802, 2TCL.A.174, 1TFX.B.4007, 1TK2.A.1276, 1UG9.A.2003, 1V0Z.A.1477, 1V6C.A.50 5, 3VI4.C.2004, 3VRQ.A.401, 1W2M.E.1442, 1WVM.A.604, 2Z8S.B.647, 2ZRQ.A.7, 2ZWO. A.403, 2AA1.B.400, 4AUL.A.754, 2AWC.A.137, 4B2O.A.1266, 2BIW.C.1492, 1BIQ.A.376, 4BLY.A.500, 4BM1.A.500, 1BS3.A.202, 1CG5.A.142, 1CGO.A.128, 3CHH.A.501, 1D06.A. 501, 1D0C.A.500, 1D2V.A.605, 1DRT.A.325, 3E65.A.901, 1EO2.B.600, 1EOB.B.600, 1EY S.C.609, 1EYS.C.611, 1F9B.A.690, 4FAG.A.401, 3FG4.A.1500, 1FT5.A.214, 1GY9.A.300 , 3HHX.A.281, 3HHY.A.281, 3HJ8.A.281, 3HJS.A.281, 1HLM.A.159, 2HMQ.C.115, 1HRM.A .154, 1HSE.A.400, 3I4Y.A.281, 3I51.A.281, 1IBE.A.143, 3ICF.A.601, 1ITH.A.143, 3I VD.B.601, 4J6C.A.502, 4KVQ.A.301, 1KW6.B.301, 4L7Y.A.201, 4L7Y.B.201, 1MXR.A.100 3, 1NFT.A.333, 3NKT.A.369, 1NX4.A.300, 3O5U.A.300, 3O6J.A.300, 3O6R.A.300, 1ODN. A.1326, 4OJ8.B.301, 1OM4.A.750, 1OUT.A.143, 1OUT.B.148, 4P1B.D.501, 3PCE.N.600, 2PCD.N.600, 2PHD.B.370, 1Q0C.B.500, 2Q0J.A.998, 1QFC.A.401, 3R2R.A.155, 4S1C.A.8 01, 3SCF.C.203, 1SP8.A.500, 3T4V.A.300, 3UFK.A.920, 3UHK.A.147, 3VER.A.601, 1VHB .A.150, 2VV6.C.1259, 3WAQ.A.201, 1XZ5.A.142, 1Y5F.B.147, 1Y5J.B.147, 1YFW.A.300, 1YFX.A.300, 2ZYQ.A.301, 2ZZI.B.209, 2BCV.A.579, 3HW8.A.577, 4ORJ.A.305, 1VQ7.Q. 9148, 1VQ8.M.9147, 2A65.A.751, 4ADB.A.1405, 3ASQ.B.701, 4CD5.A.1421, 4CSH.A.1169 , 3CTP.A.402, 3CZJ.A.3102, 2DDB.B.303, 3DR3.A.335, 2DV1.A.1000, 3DYO.A.3101, 3DY P.A.3101, 3EEB.A.211, 4EZE.A.302, 2FBL.A.304, 4FMT.D.301, 4FXZ.A.603, 3FZQ.B.274 , 3GBV.B.1, 2GKO.A.614, 1GVF.A.289, 4GVO.A.702, 3H7K.A.387, 4H7O.C.302, 4H83.E.4 01, 1HXN.A.4, 2HZY.A.1201, 3IJP.A.301, 3IMM.C.3, 3IMX.A.467, 1IYN.A.298, 2J5A.A. 1109, 4JEX.A.512, 2JLN.A.1471, 1JYN.A.3101, 1JYV.A.3101, 1JYW.A.3101, 1JZ7.C.310 3, 1JZ8.A.3101, 1JZ8.D.3104, 3K6A.E.178, 1KA0.A.501, 4KA5.A.801, 1L2T.A.1502, 4L HL.A.301, 3LJQ.A.597, 1LZS.A.131, 4M9U.A.402, 4MM7.A.601, 3MPN.A.751, 3MPQ.A.751 , 1MUQ.A.206, 3MUZ.1.3102, 3N3R.A.1000, 4N3M.A.403, 4O1G.A.401, 2O34.A.503, 4O4V .A.301, 4ODI.B.301, 4OFI.A.801, 4OUA.A.403, 2P3Z.B.501, 4PMO.A.310, 3PNX.E.168, 1PX4.A.3101, 1Q20.A.313, 3Q94.A.310, 3Q9B.A.344, 1QHU.A.436, 1QOP.B.501, 3QS5.A. 751, 3QST.A.253, 4QTO.A.501, 4QTO.B.501, 2QZ7.A.193, 3S9J.A.401, 1T64.A.391, 3TA V.A.267, 1TO2.E.451, 3TXF.A.1138, 3TYP.A.155, 3UNX.A.281, 3V5U.A.701, 3V5U.A.703 , 3VD3.A.3101, 3VD4.A.3101, 3VD9.A.3101, 3VDA.B.3101, 3VDC.A.3101, 3W0L.C.502, 3 WGU.C.2007, 1WQR.A.131, 2WUW.E.1277, 3WXO.A.805, 1X0G.A.1002, 1X7U.A.1000, 2Y8K. A.1527, 1YAP.A.501, 2Z2F.A.2001, 1ZH8.A.329, 2ZJ9.A.1, 3ZK1.A.90, 3ZK1.B.90, 2ZN D.A.195, 3ZQS.B.1295

[1] "Cluster 6"

3AYK.A.170, 4FUK.B.402, 2GC2.A.401, 4JX5.D.1103, 2LQ6.A.401, 4MCS.A.814, 2MLS.A. 302, 3OOF.A.302, 1P6D.A.247, 1Q3A.B.469, 4R6T.A.1003, 4R6T.D.1001, 4R7M.C.1001, 4R7M.J.1003, 2WEY.A.1772, 1XJS.A.150, 1ZEB.A.901, 3AUO.A.577, 4BDZ.A.1380, 4D6O. A.1186, 4DLE.A.901, 2HVI.D.878, 3ICE.E.502, 4IFD.J.1105, 4J9O.A.502, 4M3O.B.302, 3MDA.A.577, 4N41.E.101, 4NLK.A.402, 4NM1.A.401, 1TFW.B.1601, 4W9M.E.902, 2A5G.A .231, 4A7Y.A.952, 3ALO.A.1, 1AQF.A.534, 2AUU.A.201, 1AZT.A.403, 3B03.D.1001, 2B8 W.A.595, 2B9J.A.600, 2BKK.A.1266, 2BVN.A.1395, 4BYG.A.996, 3C14.C.403, 2C43.A.13 17, 4C5A.B.330, 3CBT.A.301, 3CK5.D.400, 1CQP.A.310, 3CRL.A.2000, 1CUL.A.1006, 3C X7.A.378, 4CYI.D.1000, 2D33.A.525, 3DKL.A.502, 4DLC.A.303, 3DUF.C.1368, 3DV0.A.1 368, 1E1R.A.601, 4E1E.A.402, 1E4E.A.360, 2E8T.B.1303, 2E8X.A.1302, 1E9I.D.1433,

2E91.B.1303, 2E92.B.1303, 3EA4.A.699, 3EF1.A.1, 3EFQ.B.4003, 3EHB.A.562, 4EKD.A.407, 2EWG.B.4003, 3EYA.A.613, 3FDG.B.356, 4FFR.A.406, 3FPA.A.901, 2G07.A.601, 2G08.A.500, 3G8D.B.1002, 4GA3.A.1003, 3GFT.F.202, 1GIM.A.435, 2GTP.A.401, 2GZD.A.950, 4HE0.A.403, 4HGR.B.201, 3HIY.A.401, 3HU2.D.801, 3HYL.A.675, 4HYP.B.302, 3I00.A.502, 2I19.A.3002, 3IAF.A.572, 3IAF.B.572, 2IK2.A.289, 2I07.B.5003, 4IYN.A.804, 4J5I.F.402, 1JB0.B.1202, 2JCM.A.1490, 4JLZ.B.502, 1JM6.A.4601, 4K81.B.203, 4KCV.B.1001, 1KK8.A.997, 3KR0.A.3003, 3L0C.A.257, 1L3R.E.391, 3LMG.A.202, 4LNI.D.503, 4LNI.D.505, 4LRZ.A.302, 3LUZ.A.264, 3MC0.B.426, 1MF0.A.1453, 3MGA.B.405, 4MGH.A.1302, 4N0G.B.402, 4N57.B.402, 4NCJ.A.903, 4NDN.A.401, 4NEH.A.1102, 4NNN.Z.301, 1NUZ.A.2342, 2O1X.A.2001, 2O56.A.2001, 3O61.A.202, 2OI2.A.400, 1OL5.A.1389, 4OVN.A.203, 4OVN.B.203, 4P0V.A.403, 2PSN.A.701, 2PUL.A.400, 3PUG.A.601, 2PUI.A.401, 3Q1O.D.400, 3Q7P.B.257, 3QHR.A.298, 4QXD.A.302, 3RBM.B.1001, 4RJK.B.602, 1RLT.A.805, 3RRA.A.406, 4S17.C.502, 1SOJ.C.2127, 3SS8.A.302, 3T5P.F.301, 3T9E.A.601, 3T9E.A.603, 4TQ4.C.402, 4TVU.B.601, 4TXZ.B.502, 4U0N.A.401, 4UAK.A.502, 3VAD.A.402, 2W00.A.1887, 3WBZ.F.403, 3WQM.A.403, 3WQP.J.501, 1XBT.D.4194, 1XF9.B.11, 1XZ8.B.180, 1YYZ.A.340, 1ZCA.A.383, 2ZRW.A.702, 2FKC.A.248, 4K4I.A.603, 4K4I.I.604, 3KHL.A.415, 4KHN.A.1002, 4KHW.A.1003, 3QEP.A.906, 1R7M.A.304, 1TW8.C.803, 1A25.A.292, 1A25.B.292, 4AIO.A.1890, 4AQ8.D.1238, 4B4F.B.607, 2BAT.A.601, 2BD3.A.260, 3BOW.A.718, 4BTX.A.1763, 2CHI.A.218, 5CHY.A.401, 1CJY.A.951, 3CK7.B.720, 2CLT.B.1301, 2D00.B.1002, 3D4G.H.484, 2DEW.X.903, 2DEW.X.904, 1DM5.A.1131, 1DM5.C.1133, 2DPK.A.4001, 1DQ1.A.238, 3E3R.B.195, 1E8U.A.1002, 3E9T.A.6, 2EA7.B.452, 1EE6.A.300, 1EGZ.B.300, 2EXK.D.2004, 3F19.A.266, 2F20.B.1001, 1F2N.C.1001, 3FCS.D.2002, 4FGC.C.203, 3FHA.B.704, 3FRP.A.628, 3GE4.B.167, 4GGB.A.401, 1GQM.E.1089, 4GQR.A.502, 4GZS.A.501, 4H2E.B.304, 1HFY.A.124, 1HFZ.C.124, 3HLH.D.315, 4HSZ.B.101, 1HVD.A.602, 4I5K.A.501, 1J0Y.D.701, 1JIW.P.489, 1JKU.B.2272, 1JRF.A.48, 3K1A.B.524, 4K3Y.D.604, 3K9X.D.249, 3KF9.A.303, 3KL6.A.3, 1KLJ.H.900, 4KNA.A.504, 1KV0.A.192, 4KVK.A.712, 1KWH.A.800, 4L06.C.501, 1L7L.A.201, 3L95.X.2001, 4LMF.B.302, 2LP2.A.202, 1LQD.B.1, 4LT6.A.602, 4LXF.A.701, 4MDV.A.402, 3MIN.D.524, 1MMP.B.3, 1MTV.A.480, 4NRE.A.716, 4NRE.A.717, 4NUP.A.301, 1NX0.A.902, 4O1Q.A.401, 3O9J.A.995, 2O04.B.5004, 1OS2.C.473, 3OSH.A.121, 3OX6.E.502, 2P5V.C.1002, 4PHK.A.304, 3PK0.D.280, 1PK8.F.817, 4PLS.A.305, 3PRT.A.404, 1PYT.C.650, 3Q2L.A.703, 3QNI.B.400, 3QQZ.A.324, 1QU0.B.702, 1R1Z.B.315, 4R83.D.501, 1RE3.C.408, 2RHP.A.2, 2RHP.A.18, 2RHP.A.28, 2RJP.A.3, 2RLD.C.121, 1ROS.A.404, 3S5U.D.220, 4SBV.C.261, 3SHI.G.305, 3SOB.B.1, 1SRR.A.531, 1TAD.A.352, 1TD7.A.1001, 3TH3.L.145, 1TLD.A.480, 3U1R.A.700, 4UB6.O.301, 3UBH.A.852, 1ULV.A.2006, 3V03.A.584, 2V3T.A.1264, 1V7V.A.1001, 1VF0.B.1002, 1VFP.B.995, 2VME.A.500, 3VOT.A.504, 3W9T.A.1006, 3W9T.C.1010, 3W9T.B.508, 1WD9.A.901, 2WVX.C.801, 2YFS.A.1711, 2YGM.A.1417, 2YN5.A.6365, 1YO8.A.1188, 1YO8.A.1197, 2Z8S.B.641, 3ZHG.B.1323, 2ZID.A.882, 2ZKT.A.413, 1ZTQ.B.565, 2ZWO.B.402, 4QQZ.G.1002, 4AC8.D.500, 3AK9.J.168, 4ANP.A.1426, 4AQ2.B.800, 4AQ6.F.837, 1B7Z.A.690, 1B7Z.A.691, 2BJJ.X.694, 3EE4.A.317, 1EH3.A.400, 1FRV.B.537, 1GVC.A.1253, 4M26.B.401, 1N04.A.688, 4N71.A.201, 3O32.A.300, 2O84.X.500, 1OQH.A.500, 3OWO.B.384, 3PCA.M.600, 3PCC.M.600, 3PCJ.N.600, 3PCK.M.600, 2PQ7.A.221, 3Q3O.A.502, 2R2F.A.320, 3TOD.A.694, 3UF9.B.315, 1UOF.A.1311, 1UTE.A.501, 3V83.A.703, 1VZ4.A.1299, 1W2A.X.1302, 3W54.B.502, 4WWZ.B.301, 1WZD.A.901, 1XVE.A.1170, 1XZW.A.429, 2Y0I.A.1350, 2YDE.A.501, 1ZJ9.B.1569, 3HW8.A.580, 1ORP.A.224, 3PML.B.7, 1Q81.K.8346, 2A5G.B.242, 2A7L.A.201, 2A9Y.A.2002, 4BR6.A.401, 4BVN.A.1360, 4CVU.A.1998, 4D1I.A.600, 4D1J.E.604, 4D9T.A.901, 1EZ1.B.1002, 3G1N.B.5002, 1G5I.C.902, 4GKI.F.303, 4GRX.A.501, 2GTW.E.3006, 3HYS.A.267, 4IOW.D.602, 3I2W.A.304, 4I2R.C.602, 3IAQ.A.3103, 3IC9.A.491, 2JBW.A.1368, 4JDO.G.301, 3KED.A.951, 4L3H.A.404, 4LLH.B.602, 4M4V.A.505, 3MC1.A.301, 1MGV.A.501, 4MJD.A.203, 4MM9.A.602, 4MPT.A.402, 3N3O.A.1000, 4NPJ.B.701, 4NRH.C.401, 1NZA.A.104, 3OEC.A.300, 2OKQ.A.119, 2ONP.G.707, 4OUC.A.801, 2OYC.A.305, 4QVS.A.502, 1RW9.A.900, 1S5C.A.241, 1SOO.A.547, 1TQY.H.1094, 1UD8.A.1001, 1VI6.A.208, 3VW7.A.2012, 3WNM.A.802, 2WU2.A.1590, 2WWG.B.213, 1XOG.C.1001, 1X7D.A.1501, 2X7J.A.1581, 1XAR.A.100, 1XDF.A.401, 4XEL.A.201,

1YQ2.A.7501, 2ZN8.A.995, 3ZPQ.A.1360, 3ZQ5.A.1530, 3ZX0.C.522
[1] "Cluster 7"
4A94.B.501, 3AAK.A.992, 3B35.A.292, 3BHX.A.1751, 3BI0.A.1751, 3BI1.A.1751, 2C6P.
A.1751, 7CPA.A.308, 8CPA.A.308, 3CSQ.B.335, 3D10.A.95, 1D1S.B.406, 3D4U.A.309, 1
DK4.B.591, 2DKD.A.921, 4DWZ.A.302, 4DY0.A.502, 1E48.S.999, 1E49.S.999, 2E46.A.17
2, 2EK9.A.1002, 1ELX.B.451, 4F7O.A.301, 1FLS.A.166, 1FUA.A.216, 2GA3.A.450, 1GE7
.B.200, 4H82.A.301, 3H90.C.2, 1HR6.B.501, 3HTR.B.118, 2I0O.A.580, 4ICQ.B.502, 4I
CR.A.502, 4IE7.A.601, 2IQ6.A.293, 4IXN.A.401, 1J2U.A.301, 4JBS.B.1009, 2JT5.A.25
7, 4K5M.A.1101, 4K5O.A.1103, 4K5P.A.1101, 4KA7.A.801, 1KBE.A.1, 4KFT.C.303, 1KH5
.A.450, 3KNS.A.229, 3KR5.G.1004, 1L0Y.B.702, 3L0T.B.2, 4LP6.A.302, 4LTE.A.1101,
3M3B.A.156, 3M52.B.116, 4MCA.B.1000, 1MH2.A.201, 4NAQ.A.1027, 4ON1.A.400, 4ONX.B
.201, 2OR4.A.1751, 2PVW.A.1751, 1Q3K.B.301, 3Q9F.B.344, 2QLA.A.500, 2QLA.B.502,
2QLA.D.503, 3SJX.A.1751, 3SZZ.A.502, 1TKH.A.902, 3TOL.D.107, 3U93.B.259, 3U94.B.
259, 2V29.A.1274, 2V9G.A.1276, 3VAT.A.501, 1VKG.A.400, 2WKN.B.412, 3WS9.B.801, 1
XJO.A.902, 2XPY.A.1673, 1XXW.A.201, 1N56.A.401, 4ACF.B.1481, 3ATT.A.379, 3B9T.A.
485, 2BJI.A.2278, 2BVC.A.503, 3CWH.A.391, 2D32.A.1523, 1DIE.A.398, 2DW7.B.2002,
1DXI.A.390, 3DYF.A.3004, 3F5U.A.297, 4FHY.A.402, 2GQ3.B.1003, 3GT8.C.13, 1GY3.A.
1298, 3HB0.B.702, 4I40.B.301, 1L2O.B.902, 4LNI.D.504, 1MXA.A.411, 4NH0.A.1403, 4
O1P.C.903, 2P0C.B.5, 4PV4.A.501, 1Q19.A.504, 2RD5.C.1001, 3RUW.A.544, 1SBJ.A.163
, 3SRF.G.533, 3T80.B.160, 4U03.A.504, 3U2E.A.3, 2VBI.G.1000, 2WB4.B.502, 1ZCA.B.
1383, 1BSS.A.433, 1G9Y.C.803, 3KHH.A.416, 3KHH.B.1417, 3KHL.B.1415, 3MQ6.A.340,
3NDK.A.906, 3OOL.A.237, 4Q10.A.1001, 1RYS.B.803, 3S9H.A.906, 3A7Q.A.4001, 2AAO.B
.299, 1AEI.A.318, 1AEI.A.320, 1ALA.A.400, 1AXK.B.395, 1B09.C.301, 3BRX.A.328, 1C
TR.A.150, 2D3P.A.237, 3D4G.B.485, 2D7F.A.240, 3E3R.B.193, 2E6V.D.11, 1EGZ.C.300,
4EJ7.C.402, 1ETH.A.449, 1F4M.E.105, 2FH3.A.1002, 2FHC.A.2405, 3FLP.L.302, 3FLT.
B.302, 3FW0.A.823, 1FZC.C.1, 1FZD.G.1, 4G0D.A.503, 4G64.B.301, 4G9L.B.306, 2GA9.
D.480, 1GYK.A.1206, 2H0K.A.401, 3HQ8.A.402, 2HTY.C.993, 2HYW.A.502, 1IHO.A.2, 4I
LW.F.305, 2IWV.B.1284, 2J3U.C.1292, 2J64.C.1222, 1J84.A.366, 2JAL.B.1447, 4JDZ.B
.704, 4JDZ.A.702, 1JN2.P.238, 3JQL.A.121, 4JQG.A.305, 2K0J.A.501, 1K90.D.801, 1K
90.E.802, 4KTP.B.804, 1KX1.C.301, 4L76.A.402, 1LGC.E.301, 1LWU.B.1, 1M1J.C.501,
3M83.E.407, 4MBE.A.202, 2ML2.A.206, 2ML3.A.206, 2MLS.A.305, 1N29.A.125, 3NHH.A.1
51, 4NIY.A.301, 1NX0.A.900, 4OJQ.B.1002, 4OMC.E.607, 1OTN.A.250, 4OVY.A.409, 2OY
H.B.2, 3P2P.A.125, 4P99.D.512, 3POW.A.900, 2Q1F.B.2002, 1Q3A.C.478, 3Q3L.B.437,
3RG0.A.1, 3RMK.E.313, 3RYD.C.267, 1S0C.A.1879, 2TBV.C.388, 3TRP.A.360, 3UJ0.D.30
4, 4UM8.A.2004, 1V1G.A.1209, 1V3J.B.689, 1VAH.A.500, 3VU1.A.1001, 2W2O.E.1333, 1
W2M.A.1442, 4W4Q.A.401, 1WD9.A.900, 1WDA.A.902, 4WK2.B.502, 1WMZ.B.203, 3WYN.B.4
02, 2WZE.A.1552, 1YAX.C.1003, 2YN3.C.6370, 1Z3J.A.266, 1Z3U.B.497, 2ZOC.A.501, 2
ZOC.A.502, 2ZOC.A.504, 2ZWO.A.401, 4AC8.A.500, 1B1X.A.691, 1BIQ.B.376, 3CI8.A.6,
4D8F.B.402, 3E1M.B.301, 3E1N.C.300, 2FKZ.C.1601, 2FL0.A.1602, 3FVB.A.164, 1FYZ.
B.5004, 1FZ0.B.5004, 3GCF.D.501, 4HVR.A.201, 3IS8.A.161, 2ITB.A.502, 2IW4.B.1311
, 2J2F.A.371, 1JI5.D.153, 4JPY.A.301, 1LKO.A.601, 1LKP.A.601, 1LTV.A.900, 3MPS.D
.172, 4N71.A.202, 4NB8.B.501, 4NBC.A.501, 1NFV.A.200, 3PCC.N.600, 3PCD.M.600, 3P
CF.N.600, 3PCH.R.600, 1PFR.B.503, 4PG0.A.302, 1PRC.M.607, 3Q1G.B.1002, 3QHB.A.18
2, 3QVD.B.173, 3QVD.C.175, 1RNR.A.402, 1RSV.A.1004, 1SQ3.A.902, 1TKP.D.302, 4TOH
.A.202, 2UW1.A.1359, 2VZB.B.6204, 3W54.A.501, 2XRX.M.1461, 2XSH.C.901, 1YCH.A.50
1, 2YFJ.C.901, 1YUZ.A.302, 1YV1.B.301, 4NLK.A.403, 1YJW.A.8545, 1BUN.A.121, 4CBY
.B.2036, 3CKI.A.502, 4DD8.A.1005, 1F6D.A.1378, 4FEV.B.303, 3FFZ.A.1302, 4FLL.A.5
04, 4FO2.Q.101, 4G8T.B.502, 2GG8.A.503, 1GVH.A.1399, 4H83.A.402, 3IWK.D.504, 1JJ
U.C.996, 3KEU.A.402, 4MAT.A.501, 4O54.A.302, 1O68.E.274, 4OMC.A.608, 2OZ3.B.2001
, 4PV3.A.202, 1SU4.A.997, 1TC8.A.121, 3VD3.D.3101, 3VD5.A.3101, 2WD0.C.601, 3WGU
.A.2005, 2WPC.C.1492, 2WWH.C.213, 2WX5.H.1252, 1ZEL.A.401

Table S125. 6-ligand combined metal, normal group

	size	largest_angle*	middle_1*	middle_2	middle_3
1	"1260"	"175.3+/-2.1"	"82+/-2.6"	"84.7+/-1.9"	"86.5+/-1.6"
2	"1278"	"178.2+/-1.2"	"86+/-2"	"87.5+/-1.3"	"88.4+/-1"
3	"450"	"172.7+/-3.4"	"80.4+/-3.4"	"83.6+/-2.7"	"85.7+/-2.1"
4	"184"	"158.9+/-6.6"	"69.3+/-5.1"	"73.8+/-4.4"	"77.3+/-3.8"
5	"410"	"168.3+/-4.6"	"72.9+/-4.5"	"78.6+/-3.5"	"82+/-2.9"
6	"720"	"171.1+/-3.1"	"76.8+/-3.9"	"81.6+/-2.8"	"84.3+/-2.3"

	middle_4	middle_5*	middle_6	middle_7	middle_8
1	"88+/-1.4"	"89.2+/-1.3"	"90.4+/-1.3"	"91.6+/-1.3"	"93+/-1.4"
2	"89.1+/-0.7"	"89.7+/-0.6"	"90.2+/-0.6"	"90.8+/-0.7"	"91.4+/-0.8"
3	"87.5+/-2"	"89.3+/-1.7"	"91.1+/-1.8"	"92.8+/-1.9"	"94.6+/-2.1"
4	"80.6+/-3.8"	"83.9+/-3.7"	"87+/-4.1"	"90.9+/-4.6"	"95.8+/-5.1"
5	"84.5+/-2.8"	"87+/-2.6"	"89.8+/-2.7"	"92.8+/-2.9"	"96.2+/-3.2"
6	"86.4+/-2"	"88.2+/-1.9"	"90+/-1.9"	"92+/-2"	"94.3+/-2.2"

	middle_9*	middle_10	middle_11	middle_12	middle_13*
1	"94.6+/-1.6"	"96.9+/-2.1"	"100.4+/-3.4"	"167.3+/-3.9"	"172+/-2.3"
2	"92.1+/-1.1"	"93.3+/-1.6"	"95.2+/-2.4"	"174.2+/-2.9"	"176.9+/-1.6"
3	"96.6+/-2.4"	"99.7+/-3"	"106.7+/-6.5"	"159+/-6"	"166.3+/-3.7"
4	"105+/-7"	"114.7+/-8.8"	"127.8+/-8.2"	"141.6+/-6.1"	"149+/-4.9"
5	"100.4+/-3.7"	"105.4+/-4.5"	"115.1+/-8.4"	"151.6+/-6.3"	"158.8+/-4.1"
6	"97.5+/-2.5"	"101.2+/-3.3"	"106.5+/-4.7"	"160.5+/-5.2"	"166.5+/-2.8"

	smallest_opposite_angle*	Octahedral	TrigonalPrismatic
1	"82.6+/-2.9"	"0.516"	"0.004"
2	"86.3+/-2.1"	"0.438"	"0"
3	"72+/-3.9"	"0.219"	"0.015"
4	"69.9+/-5.3"	"0.008"	"0.077"
5	"74.6+/-4.7"	"0.078"	"0.063"
6	"81.1+/-2.7"	"0.282"	"0.027"

	PentagonalBipyramidalVA	PentagonalBipyramidalVP
1	"0"	"0.01"
2	"0"	"0"
3	"0"	"0.051"
4	"0.027"	"0.132"
5	"0"	"0.113"
6	"0"	"0.051"

Table S126. Cluster members of 6-ligand combined metal, normal group

[1] "Cluster 1"
3DZA.C.501, 3DZA.C.505, 1ENQ.A.238, 2F92.F.1001, 2F94.F.1001, 2F94.F.1003, 2F9K.F.1001, 2F9K.F.1002, 2FUQ.A.1, 4GBD.A.503, 4GQT.A.501, 1GT7.A.275, 2H44.A.501, 3HDZ.A.864, 4IE5.A.601, 3LLX.A.376, 1M60.A.105, 3NO5.A.275, 4NT9.A.301, 3O2G.A.388, 3O91.A.192, 4OK4.A.800, 2OUV.A.777, 2OU3.A.161, 2OUP.B.777, 4OV9.A.401, 2PTY.A.501, 2PW3.A.501, 2QYM.A.1, 2QYK.A.1, 2R2V.C.35, 1RRM.B.387, 1T9S.A.1, 1TB5.A.1001, 1TBF.A.1, 3U43.B.135, 1U74.A.1001, 3UUO.B.772, 3V93.A.701, 2WTA.A.1215, 1XOR.A.1001, 1Y9Q.A.202, 1ZKL.A.501, 4AQX.D.1525, 4BDY.A.1380, 4BE0.A.1380, 4BE1.A.1381, 4BE2.A.1380, 4CEI.B.2162, 4DL4.A.501, 4DO9.A.401, 4DOB.A.401, 2DPI.A.871, 4DQI.A.901, 4DQI.D.901, 4F5O.A.402, 1FIU.I.2222, 1FIU.A.5555, 3G6Y.A.871, 4GZ2.B

.402, 3IEV.A.400, 1JJ2.0.8010, 3JPQ.A.339, 3JPS.A.339, 4JWM.A.403, 3K58.A.1001, 3K59.A.1001, 4K97.A.603, 4KLI.A.401, 4M04.A.702, 3M8S.A.2, 4M8O.A.1302, 4M9L.A.404, 4MDE.A.1002, 4MFC.A.401, 3MR5.A.435, 4O3O.A.502, 4O3O.A.503, 4O3Q.A.502, 4O3Q.A.503, 4O5K.A.401, 3OHA.A.518, 3OJS.A.7, 3OYB.A.396, 3OYD.A.396, 3OYG.A.396, 2OZS.A.904, 2PFP.A.750, 3PML.A.2, 3PNC.A.576, 4PUQ.B.401, 4Q8E.A.502, 4R65.A.402, 3RJH.A.403, 3RJK.A.339, 4RNN.A.503, 3SI8.A.451, 3SM4.B.227, 3SNN.A.905, 1SUZ.A.402, 3TFR.A.339, 3TFS.A.339, 3TI0.A.1, 3TI0.D.2, 4UAY.A.402, 3V6H.A.402, 1XSN.A.576, 1ZJN.A.339, 121P.A.168, 4A01.A.1767, 4A01.A.1769, 3A1U.A.5, 3A1U.A.6, 1A2B.A.550, 2A5Z.A.701, 1A82.A.901, 3A99.A.401, 4ACI.A.1187, 2AFK.E.1291, 2AG0.A.601, 1ALK.B.452, 2AL1.B.439, 4ANB.A.1384, 1AOR.A.609, 1AS0.A.356, 4AS2.A.1328, 2AUU.A.203, 2AUT.D.604, 4AUX.A.223, 2B0T.A.800, 3B05.D.1001, 3B1V.A.301, 1B4N.A.623, 3B7L.A.907, 3B7L.A.908, 3B7L.A.909, 1B8J.A.452, 2B82.A.1013, 2BEK.A.501, 1BOF.A.800, 2BON.A.1302, 3BRB.A.10, 2BU2.A.1388, 2BVN.B.1395, 4BW9.A.501, 4BX3.A.301, 1C1Y.A.171, 2C18.A.1338, 2C3U.A.2238, 2C42.A.3238, 3C4Z.A.563, 2C4N.A.1251, 3C5H.A.302, 2C77.A.1407, 2C78.A.1407, 3C9U.B.312, 2CBZ.A.1872, 3CFX.A.704, 2CFS.A.1296, 3CG4.A.201, 2CHE.A.130, 1CJT.C.403, 1CJU.A.582, 2CJE.A.1267, 3CK5.A.400, 2CL5.A.1216, 2CLS.A.550, 3CMR.A.453, 3CP6.A.502, 3CP6.A.503, 3CR3.A.1212, 1CTQ.A.168, 3CV2.A.1, 3CX8.A.378, 3CZJ.B.3001, 2D0O.A.1005, 1D2N.A.99, 3D36.A.478, 4D6P.A.1352, 4D7M.A.223, 1D8C.A.3001, 4DBF.A.401, 4DBR.A.810, 4DCK.B.201, 3DDH.B.232, 2DDT.A.311, 2DEI.A.402, 4DEM.F.402, 4DEM.F.403, 4DEM.F.404, 3DKC.A.2, 4DN1.A.401, 4DN5.A.1001, 4DSN.A.202, 1DTW.A.401, 4DUX.A.3001, 4DWG.A.401, 4DXJ.A.403, 3DYH.A.3003, 3DYH.A.3004, 3DYM.A.3001, 3DYP.A.3001, 4DYK.A.502, 4DZH.A.501, 3E84.A.701, 3E8M.A.165, 4E8G.A.402, 2E9S.A.603, 1E9A.A.401, 2E91.A.1301, 2E95.A.1302, 3EA5.A.221, 2EB5.A.1001, 3EFQ.B.4004, 3EGT.A.3004, 3EQC.A.3, 2ERX.A.403, 3ET5.A.255, 3ETJ.A.401, 4EUK.A.1001, 2EW1.A.701, 4EX6.A.301, 2EZT.A.1510, 2EZU.A.1610, 3EZ3.A.1104, 2EZ4.A.1610, 2EZ8.A.1510, 2EZ9.A.1510, 4F1J.A.301, 2F2A.B.601, 1F9H.A.162, 2F9M.A.1201, 4F9A.A.602, 3FD5.A.397, 3FD6.B.397, 4FE3.A.304, 4FEG.A.707, 3FIU.A.5001, 3FIU.A.5002, 4FI1.A.401, 3FKQ.A.501, 4FK9.A.401, 2FOZ.A.348, 2FOZ.A.349, 4FP1.A.401, 2FRV.B.540, 3FV9.A.501, 4FVR.A.902, 4FYP.A.301, 3FZN.A.605, 2G09.A.901, 1G17.A.301, 3G2F.A.901, 1G5T.A.998, 2G6B.A.301, 2G80.A.500, 1G97.A.460, 4G9B.A.301, 3GAI.A.189, 2GCP.A.2001, 2GHT.A.257, 2GIL.A.1201, 2GL5.A.699, 4GME.C.501, 3GON.A.600, 2GO7.A.207, 4GP2.A.401, 4GP2.A.402, 1GSI.A.1209, 1GUA.A.171, 4H1Z.D.401, 3H3X.Q.553, 2H57.A.202, 3H70.A.342, 3H7V.A.331, 4H81.A.402, 2HCF.A.300, 4HCH.A.405, 4HCL.A.401, 3HDG.A.202, 3HFW.A.361, 4HGN.A.200, 4HGQ.A.201, 3HIY.B.402, 2HJP.A.292, 2HNE.A.601, 4HNL.A.401, 4HPT.E.402, 1HQ2.A.162, 4HQO.A.301, 3HRZ.A.628, 3HVJ.A.265, 3HW3.A.999, 3HW4.A.999, 3HW5.A.999, 1I0L.A.902, 2I1Q.A.501, 4I2B.A.604, 2I33.A.602, 2I6K.A.302, 4IAD.A.402, 3IBA.A.403, 3ICK.A.402, 3ICK.A.403, 3ICM.A.401, 3ICM.A.402, 4IDP.A.502, 4IEG.A.1001, 1IG5.A.78, 2IHT.A.601, 2IHU.A.601, 1IH8.A.4002, 1IHU.A.592, 4IHC.A.501, 3IJL.A.386, 3IJR.D.300, 2IK4.A.287, 2IK4.B.287, 2IK4.B.288, 1IOW.A.331, 2IO8.A.7002, 4IP4.A.503, 4IP5.A.502, 1IR3.A.301, 1ITZ.A.1001, 4ITR.D.203, 2IUC.A.1003, 1IV2.A.1571, 1IV4.A.1571, 4IVG.A.803, 4IWH.A.401, 2IYW.A.202, 2IYN.C.1123, 1IZC.A.1001, 2J7P.A.1401, 2J7N.B.3374, 1J9J.B.301, 1JBW.A.998, 2JCB.A.1192, 2JD4.A.4061, 2JD4.B.4062, 1JGT.A.902, 2JI7.A.1567, 4JND.A.501, 1JPM.A.1003, 1JSC.A.699, 4JSO.A.202, 1JUY.A.435, 3JVA.A.356, 3JVA.B.358, 4JX0.A.402, 3JYS.A.1, 3JYY.B.302, 1JZ7.A.3001, 3JZ0.A.300, 3JZ0.A.303, 3K1S.H.107, 3K4Z.A.290, 4K6R.A.505, 3K9L.A.168, 4K9Q.A.601, 3KA3.A.176, 3KAL.A.503, 3KB9.A.701, 3KB9.A.702, 3KC2.A.355, 4KFU.A.307, 4KGD.A.702, 1KHZ.B.301, 3KMW.A.501, 4KQW.A.404, 3KS6.C.251, 1KTG.A.502, 1KTG.A.503, 1KTG.A.505, 4KUX.A.703, 4KVA.A.501, 4KWD.A.404, 4KX5.A.314, 4KXW.A.1001, 1KY2.A.401, 3L12.A.313, 4L2X.F.404, 4L80.A.403, 4L9W.A.202, 4L9Z.A.403, 4LA6.A.501, 2LCF.A.246, 4LFG.A.304, 4LGY.A.1302, 4LJ9.A.902, 1LNY.A.1453, 3LUF.A.301, 3LX5.A.301, 4LYK.A.401, 4LZ0.A.403, 3M07.A.595, 1M0W.A.502, 4M53.A.527, 1M7B.A.550, 3M7I.A.901, 1MC1.A.601, 1MDL.A.360, 4MDB.A.403, 4MGG.A.404, 1MH1.A.201, 3MHY.A.115, 3MJH.A.201, 3MK2.A.903, 1MMN.A.998, 1MNE.A.998, 4MNE.A.902, 4MPO.B.205, 1MQ4.A.2088, 4MUM.A.301, 4MY

O.A.301, 3MYK.X.998, 3MYL.X.998, 4MZU.C.404, 1NOH.A.699, 1NOH.B.1699, 3N07.A.200
, 1N1Z.A.701, 1N1Z.A.703, 1N20.A.701, 1N20.A.702, 1N24.A.701, 1N24.A.702, 3N2N.A
.1, 3N45.F.355, 3N45.F.3, 3N4F.A.502, 1N6I.A.201, 1N6N.A.201, 1N6O.A.201, 1N6R.A
.201, 4NDO.A.302, 4NFI.F.402, 4NFI.F.403, 1NFZ.A.401, 3NJL.A.501, 3NKV.A.500, 3N
OJ.A.239, 3NRJ.A.190, 1NSF.A.859, 1NSY.A.6241, 4NWI.A.401, 2NXW.A.4002, 2O1S.A.1
001, 1O6Y.A.1280, 3O6Z.B.201, 2O7O.A.223, 4O7I.A.401, 4OAV.B.802, 3OCU.A.263, 3O
CV.A.264, 3OCW.A.263, 3OCX.A.264, 3OCZ.A.263, 2OCB.A.202, 4OCP.A.403, 2ODP.A.901
, 2OEM.A.911, 2OEM.B.912, 2OFX.A.301, 2OGD.A.3002, 2OGD.A.3003, 2OGD.A.3004, 4OH
Y.A.502, 1OIW.A.1175, 3OIW.A.170, 4OI4.A.502, 1OKK.D.1002, 4OKM.A.901, 4OKM.A.90
3, 4OKZ.A.903, 3OM2.A.486, 3OP2.B.500, 2ORW.A.501, 3OUZ.B.459, 3OYX.A.601, 3OZF.
A.235, 3OZX.A.614, 4P31.A.402, 3P41.A.298, 3P5P.A.901, 3P93.C.406, 2PA4.A.325, 3
PDE.A.312, 3PFF.A.831, 4PFY.B.601, 1PHP.A.395, 4PHG.A.201, 4PHH.A.202, 3PK7.A.40
6, 2PKO.A.502, 2PKE.B.300, 3PNL.B.1212, 1POX.A.610, 1PPV.A.401, 2PS2.A.401, 2PS5
.B.701, 1PVF.A.401, 1PVG.A.903, 2PYW.A.501, 2PZ8.A.4001, 2PZA.A.6243, 3Q1O.A.400
, 2Q28.A.1001, 1Q3H.C.674, 2Q3F.A.301, 4Q4C.A.404, 3Q5V.B.599, 2Q5Q.A.4002, 1Q6L
.A.5300, 1Q6R.A.7300, 2Q66.A.602, 3Q85.A.284, 4QC2.A.302, 4QEA.A.301, 3QF7.A.854
, 2QG8.A.201, 2QIS.A.907, 2QJJ.C.1003, 3QKT.A.902, 2QME.A.179, 3QN3.A.601, 2QQO.
A.450, 1QRA.A.168, 2QRZ.A.190, 2QTY.A.348, 2QTY.A.349, 2QTC.A.888, 3QUQ.A.225, 3
QUT.A.225, 3QVQ.C.310, 3QXC.A.222, 3QXH.A.223, 3QXJ.A.224, 3QXS.A.223, 3QXX.A.22
4, 2QXO.A.161, 3QYY.A.505, 1ROX.A.13, 3R1M.A.404, 3R6T.A.301, 3R7W.A.600, 2RAH.A
.354, 2RAR.A.501, 2RAV.A.701, 3RBM.A.1001, 2RB5.A.701, 2RBK.A.501, 3REF.A.192, 3
REG.A.550, 1RKQ.A.1273, 1RKU.A.301, 1RKV.A.401, 4RKE.A.202, 3RO6.A.400, 1RP7.A.8
90, 1RQI.A.603, 1RQI.A.604, 1RQJ.A.908, 1RQJ.A.909, 3RUS.A.544, 1RVK.A.999, 3RWM
.B.1, 3RYE.A.907, 3RYE.A.909, 1RYA.A.1001, 1RYH.A.539, 4S1H.B.303, 3S4J.A.907, 3
S4J.A.909, 3S9Z.A.802, 1SAW.A.225, 3SAE.A.820, 3SBD.A.501, 3SDT.A.819, 3SDT.A.82
1, 3SEA.B.178, 3SFO.A.263, 3SHQ.A.321, 3SJN.A.374, 3SLS.A.401, 3SN1.A.408, 3SN4.
A.408, 1SO4.A.2300, 3STP.A.391, 1SVS.A.356, 3T2D.A.408, 3T2D.A.409, 1T8Q.B.1602,
1T9B.B.699, 1T9C.B.699, 2TCT.A.223, 3TCS.A.368, 3TDV.A.501, 1TE6.A.641, 3TEP.A.
1, 3TJI.A.601, 3TKL.A.300, 1TMM.A.162, 4TMT.A.902, 4TMW.A.903, 4TMX.A.903, 3TMO.
A.266, 4TN1.A.902, 4TSK.A.403, 1TW1.A.1, 3TWA.A.420, 3TYZ.A.281, 4TYO.A.502, 1TZ
Z.A.3501, 1U02.A.240, 4U5X.A.202, 4UAK.A.503, 4UAS.A.302, 4UAT.A.302, 4UAV.A.401
, 3UCW.A.100, 3UCY.A.101, 3UIE.A.403, 3UJR.B.502, 1UMD.A.1401, 1UMG.A.403, 1UMG.
A.404, 4UM7.A.175, 4UMF.A.1175, 3UPY.A.446, 1UPT.A.1183, 3UXK.A.360, 3UZR.A.300,
3V1V.A.501, 3V1X.A.501, 4V1T.A.1777, 3V2U.C.521, 2V26.A.1801, 3V3W.A.403, 1V54.
A.3518, 3VC1.A.301, 3VC2.J.301, 3VCC.A.402, 3VCN.A.501, 2VDO.B.2001, 2VDL.B.2001
, 2VDN.B.2001, 3VKB.A.701, 2VK1.A.601, 2VK4.A.601, 3VPB.A.502, 3VVH.A.701, 3VX4.
A.802, 1W2Y.A.1231, 1W2Y.A.1232, 1W6T.A.435, 3W6N.A.803, 3W6O.A.802, 1W7K.A.1423
, 1WA5.A.1178, 3WBH.A.501, 3WBZ.A.404, 1WC1.A.1501, 1WC1.A.1502, 3WEK.A.401, 2WE
F.A.401, 1WF3.A.401, 4WH2.A.402, 4WH3.A.402, 2WIC.A.1266, 3WJP.A.403, 3WJP.A.404
, 3WNZ.A.502, 3WRY.C.1202, 2WVG.A.601, 2WW8.A.1000, 3WXM.A.502, 1WZC.A.300, 1X07
.A.900, 1X84.B.401, 1XBY.A.601, 2XB5.A.223, 2XCW.A.1498, 1XEX.A.1002, 2XH2.B.143
9, 2XH7.A.1441, 2XIS.A.392, 4XIA.A.399, 2XJB.A.1494, 2XJD.A.1497, 2XJE.A.1493, 2
XSX.A.500, 2XTZ.A.1381, 2XUU.A.1307, 1XX1.A.9001, 4XXP.A.301, 2Y6P.A.1234, 1Y8A.
A.501, 1Y8Q.B.641, 1Y9D.D.2901, 2YCH.A.501, 1YHL.A.1401, 1YHL.A.1402, 1YMV.A.200
, 1YNS.A.1258, 1YQ9.H.540, 1YQT.A.591, 1YU4.A.2002, 2YVO.A.1001, 2YVO.A.1002, 2Y
VP.A.183, 2YVP.A.184, 2YVM.A.1001, 2YXH.A.502, 1YYQ.B.702, 1YZN.A.301, 1Z06.A.20
3, 1Z07.A.300, 1Z08.C.3300, 1Z2O.X.1295, 1Z4L.A.2001, 1Z4O.A.800, 1Z4P.X.1001, 1
Z4Q.A.2001, 2Z4V.A.1501, 2Z4V.A.1502, 2Z4X.A.1201, 2Z4X.A.1202, 1Z59.A.1001, 2Z5
2.A.1301, 2Z7I.A.1301, 1Z88.A.601, 1ZC3.A.500, 2ZCR.A.669, 1ZED.A.905, 1ZJJ.A.10
01, 2ZKJ.A.500, 3ZMC.A.1296, 3ZO9.A.1592, 3ZOU.A.1295, 1ZPD.A.601, 2ZPU.A.360, 1
ZS9.A.1257, 3ZVL.A.1524, 3ZX4.B.260, 3ZX5.A.260, 1ZXN.B.902, 3DPG.B.501, 4ECQ.A.
501, 4FJ8.A.1002, 4K4H.A.605, 4K4I.E.602, 1N3F.C.498, 2OAA.A.601, 2ODI.A.701, 2Q
1O.A.701, 4QWB.A.402, 2AEP.A.601, 3AFG.A.540, 3AHW.A.122, 3AI7.B.901, 1AJJ.A.73,
3AJ7.A.602, 3AKB.A.2, 4APX.B.1239, 3AUK.A.391, 4AXN.A.1329, 3B7E.A.1005, 3B8Z.A

.904, 3BCD.A.707, 2BL0.B.1146, 4BNR.A.600, 2BQ4.A.1119, 2C10.A.1771, 3C9I.A.1, 3CKZ.A.1, 4CPL.A.500, 4CUA.A.2644, 1CVL.A.320, 3D3I.A.1001, 3D7K.A.571, 1DPO.A.246, 4E5U.B.302, 2EA7.A.450, 2EEK.A.401, 1EGZ.A.300, 3F5V.A.223, 2FCW.B.3001, 2FCW.B.3002, 3FG1.A.1501, 2FHF.A.2404, 3FSJ.X.600, 3FVQ.A.360, 2FWN.A.532, 1G9K.A.703, 1G9K.A.706, 1GA6.A.374, 4GDI.A.509, 4GG1.A.602, 2GKO.A.612, 1GTT.A.1430, 2HOB.A.1000, 4H1Q.A.303, 3H81.C.279, 3H81.C.280, 3HGN.A.250, 3HJR.A.603, 4HJF.A.601, 4HS9.A.401, 1HY7.A.305, 4HZW.A.507, 4I35.A.513, 2I8U.A.202, 2I8T.A.402, 3I9G.L.301, 3I9G.L.302, 4IHM.A.404, 4IU2.A.301, 4IU3.A.301, 2IXT.A.1310, 1J8E.A.201, 1JK3.A.403, 2JKP.A.1728, 4JZB.A.401, 4JZB.A.403, 4JZX.A.404, 4JZX.A.405, 4K1K.A.501, 1K7I.A.487, 4K70.A.1002, 1KAP.P.616, 1KAP.P.619, 4L74.A.401, 1LQV.C.35, 4LVN.A.703, 4M5I.A.201, 3M6L.A.801, 3MA2.A.293, 3MA2.A.296, 4MEW.A.502, 1MNC.A.283, 3MOS.A.1, 4MPR.A.601, 4MWV.A.512, 3N1U.A.200, 4N2E.A.705, 4N2P.A.201, 4N4E.E.405, 1N9E.A.802, 1NPC.A.320, 2O72.A.403, 1OAC.A.802, 4OCI.A.201, 4OKH.B.904, 3OOY.A.621, 1OU9.A.131, 3OYR.B.337, 3OYR.B.338, 3P2P.B.126, 3P4G.A.401, 2PHI.B.125, 4PMX.A.401, 1Q3A.A.466, 3Q4W.A.224, 4Q8K.A.501, 1QCN.A.1001, 4QN6.A.501, 3QRB.A.301, 2QUB.A.615, 2R5N.A.2000, 1R6V.A.1, 2R8O.A.670, 2R8Z.A.201, 2R8P.A.670, 1RQ5.A.819, 3RRX.A.901, 3RUP.A.1004, 3RVV.A.225, 3RVW.A.223, 3S4Y.B.1303, 1SAT.A.476, 3SAL.A.601, 1SIO.A.601, 1T1E.A.700, 3TEW.A.800, 3TI4.A.601, 1TRK.A.681, 3U1R.A.703, 3U8D.A.203, 3U8I.A.201, 3UPT.A.691, 1UR4.A.1398, 4USU.A.1471, 1UX6.B.2002, 1UX6.B.2004, 1UX6.B.2012, 1UX6.B.2016, 4UZU.A.1484, 3V5U.A.705, 2VOV.A.1338, 3VV3.A.401, 3VV3.B.404, 1W7C.A.802, 3W7T.A.1001, 1WAD.A.116, 4WIW.A.702, 4WKO.B.502, 4WK7.A.504, 2WNV.B.1225, 2WOY.A.2414, 1WZA.A.601, 1Y93.A.266, 2YEQ.A.1526, 2YN3.A.6362, 2YN5.A.6362, 2YN5.A.6363, 1YO8.A.1191, 2YOA.A.1003, 1YS1.X.400, 2Z2X.A.1004, 2Z2X.A.1005, 2Z30.A.1006, 2Z8X.A.626, 3ZXH.A.304, 3A0G.A.201, 3A16.B.354, 3A17.A.354, 2A1O.A.417, 1A2S.A.90, 2A3F.X.201, 3A51.B.412, 1ASH.A.301, 4AUM.A.900, 2AVK.A.201, 3AYF.A.801, 3B0H.B.601, 2B11.D.1301, 2B2R.A.1500, 4B2N.A.701, 4B8N.A.201, 2BC5.A.150, 3BDZ.A.450, 1BEP.A.296, 1BIN.B.144, 1BJ9.A.296, 2BMM.A.1157, 3BOM.A.143, 3BOM.B.148, 2C1D.A.1291, 1C2N.A.117, 1CC5.A.1, 1CED.A.90, 1CH3.A.154, 2CJ2.A.1300, 4CK9.A.1480, 4CKA.A.1480, 1CLS.A.142, 2CVC.A.1013, 4CVJ.A.1295, 2CYP.A.295, 2CZ1.A.300, 4D02.A.602, 4D02.A.603, 2D2M.D.200, 4D38.A.500, 1D4D.A.601, 1D4D.A.602, 1DCC.A.296, 1DD7.A.600, 3DHI.A.600, 1DLW.A.144, 1DM1.A.148, 1DP9.A.719, 4DTY.A.500, 3E2O.A.296, 2E3B.A.401, 2E84.A.1303, 3EH5.A.800, 3EHB.A.560, 1EHE.A.501, 4ENH.A.601, 4ENU.B.801, 1EOC.B.600, 4EP6.A.601, 1F4U.A.410, 4F4O.B.201, 2FAM.A.148, 4FIA.A.600, 1G09.B.147, 4G70.A.601, 4G71.A.602, 4G7G.A.501, 4G7Q.A.602, 2GEP.A.580, 3GEO.A.580, 7GEP.A.580, 4GP8.A.602, 2GSM.A.2002, 1GWS.A.601, 1GWS.A.605, 1GWS.A.609, 1GWS.A.610, 1GWS.A.611, 1GWS.A.612, 1GWS.A.613, 1GWS.A.616, 1GWT.A.350, 2HOV.B.500, 1H29.A.1108, 1H29.A.1114, 4H44.A.301, 3HB3.A.559, 3HB3.A.560, 1HBZ.A.504, 2HBD.A.142, 1HMO.B.115, 3HQ6.A.400, 3HQ7.A.401, 1HRO.A.107, 4HRO.A.402, 2HUO.A.302, 3I63.A.501, 3I63.A.502, 4I7Z.A.301, 4I7Z.C.301, 2IBN.A.703, 2IJ4.A.471, 1IO7.A.1401, 4IPS.A.401, 1IQC.A.401, 2IUW.A.500, 4J14.A.601, 1J77.A.300, 2J7A.A.1002, 2J7A.C.1002, 2J7A.C.1004, 1JEX.A.95, 1JIP.A.410, 2JJN.A.412, 2JTI.B.104, 3K1O.A.488, 2K3V.A.238, 2K3V.A.261, 3K3O.A.1, 4K8F.A.300, 4KIB.A.403, 4KIC.A.403, 4KIG.A.502, 4KJT.A.201, 1KQG.C.810, 2KSC.A.125, 4L0F.A.501, 3L1T.A.479, 3L4D.A.481, 1LA6.B.147, 2LDO.A.130, 2LDO.A.168, 1LFK.A.430, 4LMH.A.802, 4LXJ.A.601, 2LZZ.A.101, 2LZZ.A.102, 4M2F.B.401, 3M3A.A.155, 4M4A.B.201, 1M7S.A.600, 4M71.A.403, 4MBA.A.148, 5MBA.A.148, 3MDR.A.505, 4ME4.A.401, 3MGX.A.397, 3MKB.B.137, 1MLW.A.403, 4MLN.B.201, 4MLN.B.202, 1MRP.A.310, 1MTY.D.4, 3N3N.B.1500, 4N4K.A.610, 4N4N.A.608, 4N4Y.A.602, 4N6W.A.202, 1N9C.A.93, 3N9Q.A.1, 3NA1.A.601, 2NNB.A.472, 1NPF.A.154, 2NSR.A.154, 1NS9.A.142, 3NYH.A.605, 4NZ2.A.501, 4O6J.A.302, 3O72.A.500, 1OAF.A.1251, 1OAH.A.1520, 1OAH.A.1522, 1OAH.A.1523, 3OJT.D.500, 1OQU.C.1008, 3OUI.A.1, 2OZY.A.201, 3OZW.A.404, 1P2H.A.804, 3PAH.A.425, 2PAC.A.83, 4PAH.A.425, 5PAH.A.425, 3PER.A.1002, 3PF7.A.1001, 3PF7.A.1002, 4PGO.A.301, 4PH9.A.602, 3PT7.B.500, 3PUR.A.1, 3Q3N.A.509, 1Q5D.A.440, 2QDY.A.300, 1QO8.A.601, 1QO8.A.602, 1QO8.A.604, 3QPI.B.1001, 1QPU.A.107, 2QUO.A.142, 3QY7.A.264, 2R6

S.A.501, 3RI7.A.494, 3RIV.A.305, 3RMK.A.494, 3RMZ.A.500, 3RNF.A.500, 4S1B.A.802, 1S73.A.296, 3S8F.A.801, 3SDN.A.160, 1SMI.A.472, 1SP3.A.807, 1SQ3.D.912, 1STQ.A.600, 1TH2.D.2003, 3TK3.A.500, 3TMC.A.310, 3TOR.A.3, 1TQN.A.508, 4TUV.A.401, 4UAX.A.501, 3UBR.A.473, 1UED.A.1430, 1ULI.B.700, 4V3Z.B.750, 3V5X.A.201, 2V7I.A.1362, 2VHD.B.401, 3VR8.C.201, 3VTH.A.807, 2VZW.B.1209, 1W0G.A.1501, 1W4W.A.1307, 3WFC.B.802, 2WM5.A.450, 4WNV.A.601, 2WU2.C.1130, 2WU5.C.305, 4WWJ.B.301, 2X2N.A.1479, 4X8B.A.508, 2XC3.A.1433, 1XU5.A.1174, 1XVB.A.1171, 1XVG.A.528, 1XVX.A.313, 2Y69.A.516, 2YIU.B.500, 1YMA.A.154, 1YYG.A.396, 1Z1N.X.603, 1Z1N.X.604, 1Z1N.X.605, 1Z1N.X.606, 1Z1N.X.613, 1Z1N.X.614, 1Z8U.B.201, 2ZCF.A.206, 3ZG3.A.490, 3ZLI.A.4001, 1ZOY.C.1305, 3ZPI.A.1407, 4EJY.A.301, 1EWN.A.501, 3I0W.A.296, 4KLI.A.405, 1ORN.A.224, 3OSN.A.423, 4P4M.A.403, 4TUP.A.402, 2A5F.B.1326, 3AGC.A.1, 3AJN.A.136, 4AK1.A.1702, 2AU7.A.206, 4C1O.A.1731, 3C7F.A.804, 4CIT.A.1454, 3E9L.A.1, 3ELF.A.351, 2FPR.B.505, 3GED.A.251, 4GNJ.B.302, 2GTW.B.3005, 1GV5.A.1142, 4H83.A.401, 4HUR.A.316, 4I29.A.601, 4I2A.A.602, 4IIB.A.944, 3IJP.B.301, 3IMM.B.2, 4IQZ.A.316, 3IRS.A.290, 1JZ7.A.3104, 4KA7.A.805, 3KRS.A.301, 4LG8.A.601, 4LIZ.A.202, 3M9Y.B.254, 4MMB.A.602, 4NAW.B.303, 3NNB.A.401, 4NSJ.A.222, 3OLJ.A.1, 2OPL.A.187, 4PCG.C.304, 3PG0.A.147, 1PYF.A.315, 3PZR.A.373, 3PZS.A.287, 4Q5K.A.302, 1QNJ.A.280, 3QXT.A.133, 3R0L.D.124, 4R3N.B.401, 2RGI.B.98, 3SJL.A.402, 4TKX.L.706, 4U99.A.203, 1UD2.A.1002, 3UWP.A.424, 2V3U.A.1263, 2V79.A.1116, 1VMF.A.134, 1W15.A.2002, 3W5N.A.1210, 3WNO.A.802, 2WW2.A.800, 4XCZ.A.405, 4XCZ.A.406, 1XKN.A.700, 1Y7W.B.282, 3ZX3.C.522
[1] "Cluster 2"
2EK8.A.1004, 2F92.F.1002, 1HQA.B.452, 2P2L.A.201, 2PTZ.A.501, 2PU1.A.501, 1TB7.A.1001, 1Y2K.A.1001, 1BPY.A.339, 4BWJ.A.1834, 4DFK.A.902, 4DFM.A.902, 4DOC.A.401, 4DQP.D.902, 4ELT.A.902, 4ELU.A.902, 4F06.A.601, 2J0S.A.1412, 3KD5.E.916, 4M04.A.703, 3MFI.A.515, 4P4M.A.402, 4QM6.A.1002, 3RTV.A.833, 3SPY.A.903, 1W7A.A.1802, 3ZVM.A.1526, 3A0T.A.800, 3A4L.A.401, 2A5D.A.1231, 4A6X.A.350, 4ACF.A.1482, 3AHC.A.826, 3AHD.A.826, 3AHE.A.826, 3AHG.A.826, 1AOX.A.400, 2AUU.A.204, 2AUT.A.601, 3AYX.A.701, 1B25.A.800, 4B2P.A.1351, 2B56.A.488, 2B9H.A.700, 4BAS.A.1183, 3BB1.A.282, 2BBS.A.3, 3BC1.A.194, 2BME.A.1184, 3BN3.A.1, 2BVC.A.504, 3BWV.A.300, 4BWR.A.1468, 4BX0.A.1291, 1BYQ.A.1001, 2C5L.A.1168, 4C5B.A.1314, 1CHN.A.200, 1CIP.A.356, 2CK3.A.601, 2CK3.F.601, 2CN5.A.1506, 4COK.A.601, 3CUR.H.553, 3CUS.Q.553, 3D2R.A.500, 2D7C.A.1002, 3DDC.A.600, 3DGT.A.800, 4DSO.A.202, 4DWO.A.301, 2DY1.A.701, 4E01.A.402, 1E2Q.A.401, 3E2D.A.603, 1E3D.B.901, 3E5H.A.200, 3E81.A.165, 4EEN.A.301, 4EFM.A.202, 3EHG.A.371, 1EK0.A.601, 3ET4.A.301, 4EX7.A.301, 1F5N.A.595, 3F61.A.310, 1F9H.A.161, 2FFQ.A.356, 2FH5.B.270, 1FSG.A.302, 3FSY.A.333, 2FUE.A.500, 2G0W.A.501, 2G1T.A.1501, 3G15.A.602, 1G4C.B.362, 1G4T.A.2005, 3G5A.D.307, 3G6K.A.307, 2GCN.A.2001, 4GOJ.A.202, 1GSA.A.319, 2GSM.A.3006, 4GT8.A.402, 3GYB.A.1, 1H2A.L.1005, 1H2R.L.1005, 3H80.A.214, 4H8E.A.301, 4HAT.A.302, 4HDO.B.200, 2HF8.A.301, 2HF9.A.301, 4HOR.X.101, 3HQJ.A.145, 3HRZ.D.742, 3HSD.B.162, 1HTW.A.561, 2I34.A.301, 2I5R.A.301, 3I76.A.1001, 4I94.A.402, 3ICZ.A.403, 4IDN.A.502, 2IHP.A.287, 2IK4.B.289, 2IK6.B.287, 2IOR.A.2000, 3IP0.A.161, 4IUC.L.702, 4IUD.L.1002, 2J0V.A.1180, 2J7P.D.1401, 1J9J.A.301, 2JC9.A.1491, 4JDP.A.301, 3JTC.C.34, 3JYY.A.301, 1K77.A.300, 3K8K.A.700, 1KCZ.A.901, 1KK1.A.413, 3KKO.A.180, 1KMQ.A.401, 4KO8.A.801, 1KQP.A.5001, 1KQP.A.5002, 1KSH.A.202, 1KTG.A.504, 3KTA.A.184, 3KUC.A.171, 4KVG.A.202, 4L57.B.201, 3L8H.A.801, 4LFG.A.303, 4LFG.B.303, 4LHW.A.301, 3LLU.A.502, 4LPM.A.208, 3LUF.A.300, 3LXX.A.402, 3M1I.A.1178, 4M9Q.A.302, 1MJN.A.1001, 1MMG.A.998, 4MRT.A.301, 1MXG.A.439, 3MX3.A.601, 3MYH.X.997, 4N0D.A.402, 3N45.F.2, 1N6L.A.201, 1N6P.A.201, 1NB0.A.201, 4NBS.A.502, 1NN5.A.401, 1NRJ.B.1, 2NSY.A.305, 1O08.A.2800, 1O3Y.A.1002, 1OBW.A.176, 3OCY.A.264, 4ODJ.A.502, 3OE1.A.601, 2OI6.B.6000, 1OKK.A.1002, 2OLR.A.543, 4OMF.A.503, 1OXV.A.1102, 1OXV.D.1101, 3OZX.A.613, 2P27.A.307, 4P32.A.402, 1P4M.A.201, 1P5Z.B.401, 3P96.A.412, 3PGL.A.1, 3PIT.A.180, 2PNQ.A.502, 4PQ9.A.301, 2PZE.A.3, 3Q3J.B.201, 3Q46.A.307, 3Q60.A.603, 1Q92.A.1003, 1QGU.B.3002, 1QGU.D.3006, 4QHZ.A.302, 2QIS.A.908, 2QIS.A.909, 1QK5.A.30

3, 3QNM.A.400, 2QT0.A.1001, 1R2Q.A.300, 3R3S.A.296, 2R6O.A.801, 2R8E.A.201, 3RAP .R.200, 4RKF.A.202, 3RLF.A.1501, 1RMT.A.1413, 4ROQ.A.401, 3RV3.A.1004, 3RYE.A.90 8, 3S4J.A.908, 1SHT.X.219, 3SL2.A.701, 1SVM.A.750, 1T0P.A.901, 3T10.A.401, 3T1K. A.401, 3T2S.B.401, 3T2B.A.409, 3T7A.A.602, 1TC6.A.501, 3TG0.A.503, 4TMV.A.902, 3 TSO.A.200, 4U82.A.301, 1U8Y.A.301, 2UAG.A.1001, 1UBK.L.1005, 4UCX.Q.1553, 4UE3.L .603, 3UJR.A.502, 3UJS.A.601, 3UJS.B.601, 3UPL.A.447, 3UQY.L.603, 4UQL.Q.1552, 4 URH.Q.1552, 4V0L.A.601, 2V7Q.A.1512, 2VBU.A.1134, 3VC1.I.301, 3VCC.A.401, 1VG8.A .1401, 2VG3.A.1297, 2VK8.A.1565, 1VOM.A.997, 1VZM.B.1046, 1VZM.B.1047, 3W40.A.20 1, 2W4J.A.1280, 2W5V.A.1378, 2W5X.A.1379, 1W78.A.1422, 1W78.A.1423, 2WKQ.A.1724, 1WUH.L.1005, 1WUK.L.1005, 1X06.A.900, 2X13.A.1418, 2X14.A.1418, 1X3S.A.200, 2X9 8.A.1477, 1XFI.A.400, 2XTN.A.1232, 2Y8E.A.1177, 1YHL.A.1403, 1YQT.A.592, 1YRQ.H. 553, 1YS7.A.1002, 1YVD.A.850, 1YZL.A.401, 1YZT.A.700, 1Z08.A.1300, 1Z0J.A.400, 1 Z2N.X.1295, 1Z4J.A.1001, 1Z4K.A.229, 1Z5G.A.601, 1Z5G.D.604, 2Z52.A.1302, 3ZFD.A .500, 3ZIA.A.601, 2AFH.D.2490, 4AWN.A.300, 3AYU.A.417, 3EDY.A.1, 2FGZ.A.1192, 4H 1Q.A.305, 3H81.A.279, 1HFC.A.277, 1HV5.A.5503, 1I76.A.997, 1RM8.A.502, 3V96.B.30 3, 1Y93.A.268, 2ZWO.B.400, 19HC.A.301, 19HC.A.302, 19HC.A.303, 19HC.A.304, 19HC. A.305, 19HC.A.306, 19HC.A.307, 19HC.A.308, 19HC.A.309, 3A15.B.354, 2A3M.A.501, 2 A3M.A.502, 2A3M.A.503, 2A3M.A.504, 1A4E.A.503, 1A56.A.82, 3A8G.A.301, 3A8L.A.300 , 3A9F.A.207, 2A9E.A.550, 4AAL.A.423, 4AAN.A.400, 4AAN.A.401, 4AAO.A.400, 2AIU.A .200, 2AI5.A.81, 1AKK.A.105, 4AM5.A.1160, 1AOF.A.602, 1AOF.B.601, 1AOM.A.602, 1A OM.B.601, 1AOM.B.602, 4APY.A.1418, 1AQA.A.95, 1AQE.A.121, 1AQE.A.122, 3AQ5.A.144 , 3AQ9.A.144, 3AT5.A.142, 3AT5.B.147, 2AT3.X.185, 3AWM.A.501, 1AWP.A.201, 2B0Z.B .109, 2B10.D.909, 4B2N.A.700, 2B4Z.A.500, 3B42.A.199, 3B42.B.399, 3B47.A.199, 4B 4Y.A.1155, 3B6H.B.600, 1B80.A.350, 1B82.A.350, 1B85.A.350, 3B99.A.600, 2BDM.A.50 0, 1BFR.B.200, 2BGV.X.1121, 2BH4.X.1123, 1BJE.A.154, 4BJA.A.300, 4BJK.A.1450, 3B K9.B.401, 2BK9.A.1154, 2BLF.B.1582, 4BMM.A.1450, 3BNG.A.513, 3BNJ.A.513, 3BNJ.A. 514, 3BNJ.A.515, 3BNJ.A.516, 3BNJ.A.517, 2BPN.A.108, 2BPN.A.109, 2BPN.A.110, 2BP N.A.111, 2BQ4.A.1115, 2BQ4.A.1116, 2BQ4.A.1117, 2BQ4.A.1118, 2BS2.C.1255, 2BS2.C .1256, 2BS3.C.1255, 2BS4.C.1255, 1BVY.A.1000, 3BXU.A.72, 3BXU.A.73, 3BXU.A.74, 4 C0C.A.1450, 4C0N.A.1157, 2C1U.A.401, 2C1V.A.401, 2C1V.A.402, 2C1D.A.1292, 2C1D.B .1158, 3C2C.A.113, 1C2R.A.120, 4C27.A.1450, 4C28.A.1450, 1C40.A.150, 4C44.A.1151 , 1C52.A.200, 3C62.A.150, 3C63.A.150, 1C6R.A.90, 1C75.A.93, 3C76.X.185, 3C78.X.1 85, 2C8S.A.1173, 4C9M.A.1418, 3CAO.A.104, 3CAO.A.105, 3CAO.A.106, 3CAO.A.107, 1C CH.A.83, 1CCR.A.112, 2CDV.A.109, 2CDV.A.111, 4CDP.A.402, 2CE0.A.1102, 1CH1.A.154 , 1CH2.A.154, 1CH5.A.154, 1CH7.A.154, 1CH9.A.154, 1CI3.M.254, 4CK8.A.1480, 1CNO. A.200, 2CN4.A.1175, 2CN4.B.1175, 1CO6.A.108, 1COR.A.83, 1COT.A.130, 4COH.A.1450, 4COO.A.1549, 3CP5.A.202, 1CPT.A.430, 3CQV.A.601, 3CSL.A.866, 2CTH.A.109, 2CTH.A .110, 2CTH.A.111, 2CTH.A.112, 3CU4.A.199, 2CVC.A.1001, 2CVC.A.1002, 2CVC.A.1003, 2CVC.A.1004, 2CVC.A.1005, 2CVC.A.1006, 2CVC.A.1007, 2CVC.A.1008, 2CVC.A.1009, 2 CVC.A.1010, 2CVC.A.1011, 2CVC.A.1012, 2CVC.A.1014, 2CVC.A.1016, 3CX5.C.4001, 3CX 5.C.4002, 3CX5.D.4003, 3CX5.W.4026, 1CXA.A.126, 1CXC.A.125, 3CXH.W.4026, 1CXY.A. 204, 1CYI.A.200, 3CYR.A.203, 3CYR.A.204, 2CY3.A.119, 2CY3.A.120, 2CY3.A.121, 2CY 3.A.122, 5CYT.R.105, 2CZS.A.500, 2CZS.A.501, 1CZJ.A.119, 1CZJ.A.120, 1CZJ.A.121, 1CZJ.A.122, 3CZY.A.300, 2D0W.A.200, 2D0S.A.80, 2D0T.A.404, 4D30.B.750, 4D34.A.5 00, 4D35.A.500, 4D36.A.500, 4D37.A.500, 4D3A.A.500, 1D4D.A.603, 1D4D.A.604, 1D7B .A.401, 3D7O.A.143, 2DC3.A.191, 3DE8.A.150, 2DGE.A.200, 3DHH.A.501, 3DHR.A.142, 1DJ5.A.1, 1DK0.A.200, 3DMI.A.146, 2DN1.A.142, 2DN1.B.147, 1DP8.A.719, 3DR0.A.94, 1DTI.A.154, 4DTZ.A.500, 1DUW.A.293, 1DUW.A.297, 1DUW.A.300, 1DUW.A.301, 1DW0.A. 113, 1DW2.A.113, 1DWL.B.80, 4DXY.A.501, 1DY7.B.601, 2DY5.A.300, 4DY9.A.201, 1E08 .E.80, 1E29.A.136, 1E2R.B.601, 1E2W.A.900, 1E39.A.801, 1E39.A.802, 1E39.A.803, 1 E39.A.804, 2E3A.A.401, 3E5J.A.1408, 3E5K.A.1408, 1E8E.A.125, 2E80.A.1508, 2E84.A .1301, 2E84.A.1302, 2E84.A.1304, 2E84.A.1305, 2E84.A.1306, 2E84.A.1307, 2E84.A.1 308, 2E84.A.1309, 2E84.A.1310, 2E84.A.1311, 2E84.A.1312, 2E84.A.1313, 2E84.A.131 4, 2E84.A.1316, 1EB7.A.401, 1EGY.A.410, 3EGW.C.806, 3EGW.C.807, 3EHB.A.559, 1EHJ

.A.1030, 1EHJ.A.1053, 1EHJ.A.1066, 4EID.A.101, 4EIE.A.101, 4EIF.A.101, 3EJ6.A.40
00, 4EJI.A.501, 2EKT.A.154, 2EKU.A.154, 1ETP.A.199, 1ETP.A.200, 1EUE.A.201, 2EU7
.X.201, 2EWK.A.1001, 2EWK.A.1003, 2EWU.A.1001, 2EWU.A.1003, 1EWH.A.253, 2EWI.A.1
004, 2EWI.A.1002, 2EWI.A.1001, 2EWI.A.1003, 2EXV.A.83, 1EZV.C.401, 1EZV.C.402, 1
F03.A.201, 1F1C.A.200, 1F1F.A.200, 1F24.A.501, 4F6I.A.201, 4FA7.A.602, 4FA7.A.60
3, 4FAS.A.601, 4FAS.A.602, 4FAS.A.603, 4FAS.A.604, 4FAS.A.605, 4FAS.A.606, 4FAS.
A.607, 2FBZ.X.901, 1FCD.C.901, 4FDH.A.601, 4FEF.A.403, 2FFN.A.1003, 1FGJ.A.548,
1FGJ.A.552, 1FGJ.A.553, 1FI3.A.83, 1FI7.A.110, 1FJ0.A.115, 3FLL.A.185, 2FMY.A.30
0, 3FO3.A.1004, 3FO3.A.1005, 3FO3.A.1006, 3FO3.A.1007, 3FO3.A.1008, 3FO3.A.1002,
 3FO3.A.1003, 3FO3.A.1001, 1FOP.A.500, 3FOO.A.150, 2FRF.A.154, 1FS7.A.509, 1FS7.
A.510, 1FS8.A.508, 1FT5.A.213, 1FT5.A.215, 1FT5.A.216, 1FT9.A.300, 1FT9.B.300, 2
FWT.A.803, 2FWT.A.805, 2FWL.A.132, 2FYU.D.242, 4G1V.A.401, 4G3J.A.501, 3G5N.A.50
0, 4G7L.A.301, 4G7S.A.602, 3GAS.D.1294, 2GC4.D.200, 1GDV.A.101, 4GED.B.201, 1GKS
.A.0, 2GNV.A.166, 1GQ1.A.601, 1GQ1.A.602, 3GQP.C.143, 4GQS.A.501, 2GSM.A.2001, 2
GTF.X.201, 1GWF.A.504, 3GW9.A.480, 1GWS.A.603, 1GWS.A.606, 1GYO.A.111, 1GYO.A.11
2, 1GYO.A.113, 1GYO.A.114, 4GYD.A.200, 4H0K.B.200, 1H1O.A.1184, 1H1O.A.1185, 1H2
1.A.1248, 1H21.A.1249, 1H21.B.1248, 1H21.B.1249, 1H29.A.1102, 1H29.A.1104, 1H29.
A.1107, 4H2L.B.201, 1H32.A.1263, 1H32.A.1264, 1H32.B.1139, 3H33.A.75, 3H33.A.76,
 3H33.A.77, 3H34.A.72, 3H34.A.73, 3H34.A.74, 3H4N.A.72, 3H4N.A.73, 3H4N.A.74, 4H
44.A.302, 1H55.A.350, 1H57.A.350, 4H6O.A.501, 3H8T.A.301, 2H88.C.142, 1HBI.A.153
, 4HB6.A.72, 4HB6.A.73, 4HB8.A.72, 4HB8.A.73, 4HBF.A.72, 1HCZ.A.253, 4HC3.A.72,
3HDL.A.305, 3HF4.A.142, 3HF4.B.147, 1HGB.D.147, 4HHR.A.701, 4HHS.A.701, 1HJ3.B.6
02, 1HJ5.A.601, 1HJ5.B.602, 2HJI.A.180, 1HLB.A.158, 3HNJ.A.150, 3HNK.A.150, 4HPA
.A.201, 4HPB.A.201, 4HPD.A.201, 3HQ9.A.400, 3HQ9.A.401, 1HRC.A.105, 3HSP.A.750,
4HSW.A.201, 3HYU.A.142, 3HYU.B.147, 1I3E.A.147, 2I5N.C.404, 1I5U.A.201, 1I77.A.1
08, 1I77.A.109, 1I77.A.110, 1I77.A.111, 4I7Z.A.302, 3I8R.A.901, 1I8O.A.115, 1I8P
.A.115, 2I8F.A.83, 3I9T.A.300, 3I9U.A.300, 1IB7.A.95, 1ICC.A.201, 1IDR.A.144, 2I
J3.B.999, 1IKE.A.185, 1IQC.A.402, 3IQ5.A.150, 1IT1.A.201, 1IT1.A.202, 1IT1.A.203
, 1IT1.A.204, 2IUF.A.1691, 1IVJ.A.300, 2IVF.C.1217, 1IW0.A.901, 1IYN.A.296, 1J02
.A.300, 1J0P.A.1002, 1J0Q.A.201, 2J1M.A.1456, 4J20.A.107, 1J3S.A.105, 2J5M.A.130
0, 2J7A.A.1003, 2J7A.A.1004, 2J7A.A.1005, 2J7A.C.1003, 1JDL.A.500, 1JDR.A.296, 4
JE9.A.201, 4JEB.A.201, 4JJ0.A.501, 4JJ0.A.502, 2JJP.A.412, 1JMX.A.1001, 1JMX.A.1
002, 1JNI.A.125, 1JNI.A.126, 2K3V.A.218, 2K3V.A.278, 4K8F.B.300, 1KB0.A.802, 1KB
I.A.760, 4KF2.B.501, 2KMY.A.233, 2KMY.A.251, 4KMG.A.101, 1KOK.A.296, 1KQG.C.809,
 2KSU.A.282, 2KSU.A.305, 1KV9.A.901, 4KVK.A.701, 4KVL.A.701, 3L1M.A.150, 1L2K.A.
154, 3L61.A.420, 3LD6.A.601, 2LDO.A.154, 3LGN.A.200, 4LJI.A.301, 1LM3.B.200, 1LM
S.A.118, 4LM8.A.801, 4LM8.A.802, 4LM8.A.803, 4LM8.A.804, 4LM8.A.805, 4LM8.A.806,
 4LM8.A.807, 4LM8.A.808, 4LM8.A.809, 4LM8.A.810, 4LMH.A.801, 4LMH.A.803, 4LMH.A.
804, 4LMH.A.805, 4LMH.A.806, 4LMH.A.807, 4LMH.A.808, 4LMH.A.809, 4LMH.A.810, 1LQ
X.A.201, 1LR6.A.201, 1LS9.A.92, 1LSX.A.719, 3M15.A.150, 1M1P.A.802, 1M1Q.A.804,
1M1Q.A.803, 1M1R.A.801, 1M2I.A.201, 3M4C.A.150, 4M4A.A.201, 1M59.A.201, 1M70.A.1
99, 1M70.A.200, 4M72.A.403, 4M73.A.403, 3MDM.A.505, 1MDV.A.110, 1MDV.A.112, 1MJ4
.A.502, 3ML1.B.1128, 3ML1.B.1129, 1ML7.A.185, 4MLM.A.201, 4MLN.A.201, 3MM9.A.580
, 1MNI.B.154, 1MNY.A.95, 3MOM.A.186, 4MPM.A.201, 1MQF.A.501, 4MQJ.B.201, 3MUS.A.
201, 3MVC.A.500, 3MVF.A.185, 1MWB.A.125, 1MXR.A.1004, 1MZ4.A.151, 1N45.A.300, 4N
4J.A.609, 4N4J.A.610, 4N4J.A.611, 4N4J.A.612, 4N4J.A.613, 4N4J.A.614, 4N4J.A.615
, 4N4J.A.616, 4N4L.A.616, 4N4N.A.601, 4N4N.A.602, 4N4N.A.603, 4N4N.A.604, 4N4N.A
.605, 4N4N.A.606, 4N4N.A.607, 4N4O.A.608, 4N8T.B.201, 3N8Y.A.601, 1NAZ.A.200, 4N
FG.B.201, 1NIR.A.601, 1NIR.A.602, 4NK1.B.201, 3NMI.A.150, 1NML.A.401, 1NML.A.402
, 3NN1.A.239, 1NNO.A.602, 1NOS.A.901, 4NP1.A.185, 2NRL.A.148, 1NS6.A.142, 1NS9.B
.147, 3NWV.A.105, 3NXU.A.508, 3O1A.A.385, 4O1W.A.101, 4O4Z.A.201, 3O5C.A.401, 3O
5C.D.504, 4O6Q.A.202, 4O6U.A.203, 4O7G.A.301, 4O7G.A.302, 3O89.A.2154, 1OAH.A.15
21, 1OAH.A.1524, 3OA8.A.401, 3OA8.B.401, 1OFW.A.1294, 1OFW.A.1295, 1OFW.A.1296,
1OFW.A.1297, 1OFW.A.1298, 1OFW.A.1299, 1OFW.A.1300, 1OFW.A.1301, 1OFW.A.1302, 3O

FT.A.417, 3OMA.A.1, 3OMI.A.607, 3OO4.A.142, 3OO4.B.147, 2ORT.A.600, 3ORV.B.600, 2ORO.A.600, 2ORP.A.600, 2ORQ.A.600, 2ORR.A.600, 2ORS.A.600, 2OT4.A.1004, 2OT4.A.1007, 3OUE.A.609, 3OUE.A.610, 3OUE.A.611, 3OUE.A.612, 3OUQ.A.601, 3OUQ.A.602, 3OUQ.A.603, 3OUQ.A.604, 3OUQ.A.605, 3OUQ.A.606, 2OZY.A.202, 2OZY.A.203, 2OZY.A.204, 2OZY.A.205, 3OZU.A.404, 3OZV.A.404, 1P2E.A.801, 1P2E.A.803, 1P2E.A.804, 1P2H.A.803, 1P2Y.A.430, 3P3L.A.501, 1PA2.A.306, 1PBY.A.991, 1PBY.A.992, 3PC3.A.701, 2PEG.A.200, 2PEG.B.400, 3PH2.B.1087, 3PI2.B.500, 4PK5.A.501, 1PL3.A.401, 1PM1.X.180, 1PP9.C.501, 1PP9.C.502, 1PP9.D.501, 2PQ7.A.220, 3PXW.A.500, 4PXH.A.501, 1Q16.C.806, 1Q16.C.807, 2Q8Q.A.300, 3Q99.B.750, 1QDB.A.516, 1QDB.A.517, 1QDB.A.518, 1QDB.A.519, 1QHU.A.500, 2QJY.A.501, 2QJY.A.502, 2QJY.B.301, 1QKS.A.601, 1QKS.A.602, 1QNO.A.113, 1QNO.A.114, 1QNO.A.115, 1QNO.A.116, 1QN2.B.101, 3QNS.A.351, 1QO8.A.603, 1QPA.A.350, 1QQ3.A.107, 3QQQ.A.163, 3QQR.A.163, 2QSP.A.142, 3QU8.A.500, 3QWO.A.150, 1QYZ.A.200, 1R0Q.A.200, 4R20.A.601, 2R50.A.166, 2R80.A.150, 2R80.B.150, 3R9C.A.450, 2RAO.B.147, 2RAO.C.142, 2RDZ.A.2, 2RDZ.A.3, 2RDZ.A.4, 2RDZ.A.5, 2RF7.A.2, 3RGS.A.1, 3RJ6.A.154, 4RKM.A.808, 4RKM.B.808, 4RKM.D.807, 4RKN.A.902, 4RKN.A.903, 4RKN.A.905, 4RKN.A.906, 4RKN.A.907, 4RKN.A.908, 4RKN.A.909, 1RSE.A.154, 3RUK.B.600, 1RWJ.A.90, 1RWJ.A.91, 1RWJ.A.92, 1RZ5.A.401, 3S1J.A.140, 3S8F.A.800, 3S8G.A.800, 1SE6.A.430, 3SEL.X.73, 3SEL.X.74, 1SH4.A.201, 3SJ0.X.73, 3SJ0.X.74, 3SJ1.X.73, 3SJ1.X.74, 3SJ4.X.73, 3SJ4.X.74, 3SJ5.A.500, 3SJL.A.500, 3SJL.A.600, 3SLE.A.402, 1SOX.A.502, 1SP3.A.801, 1SP3.A.803, 1SP3.A.804, 1SP3.A.805, 1SP3.A.806, 1SP3.A.808, 1SUO.A.500, 3SWZ.B.600, 3SXQ.A.1005, 3SXQ.A.1006, 3SXQ.A.1007, 3SXQ.A.1008, 3SXQ.A.1002, 3SXQ.A.1003, 3SXQ.A.1001, 1SY2.A.185, 3T3R.A.500, 3T3Z.A.500, 1T68.X.201, 3T6D.C.401, 3T6D.C.403, 3T6D.C.404, 3T6E.C.401, 3T6E.C.402, 3T6E.C.403, 3TDA.A.800, 3TFO.A.500, 3TGU.C.501, 3TGU.C.502, 3TGU.D.501, 3TGA.A.185, 3TGM.A.300, 3TIK.A.482, 3TJS.A.508, 1TKW.B.253, 3TMC.A.309, 4TOB.C.201, 3TOL.A.150, 4TT5.A.401, 1TU2.B.255, 1U13.A.460, 1U4H.A.500, 1U7R.A.154, 3U8P.A.347, 1U9M.A.90, 4U9D.A.201, 1U9U.A.90, 3U99.A.500, 3U99.A.700, 3UBR.A.472, 3UBR.A.474, 3UBR.A.475, 3UBC.A.201, 3UCP.A.901, 3UCP.A.902, 3UCP.A.903, 3UCP.A.904, 3UCP.A.905, 3UCP.A.906, 3UCP.A.907, 3UCP.A.908, 3UCP.A.909, 3UCP.A.910, 3UCP.A.911, 3UOI.B.200, 1UP9.A.201, 1UP9.A.202, 1UP9.A.203, 1UP9.A.204, 4UQH.A.1450, 1URV.A.1172, 2UUQ.A.1405, 4UVR.A.1450, 1V0H.X.251, 2V07.A.1102, 2V08.A.1087, 2V0M.A.1499, 3V2V.A.154, 4V2K.A.601, 4V3V.A.750, 4V3W.A.750, 4V3X.A.750, 1V54.A.515, 1V75.B.201, 2V7K.A.1360, 2V7L.A.1360, 3V8D.A.601, 1V8X.A.901, 1V9Y.A.1140, 3VAU.A.201, 1VB6.A.1140, 1VB6.B.1140, 2VEB.A.200, 1VGI.A.300, 3VHB.A.150, 4VHB.A.150, 2VHB.B.150, 2VHD.A.402, 3VKP.A.601, 3VKS.A.601, 3VM9.A.154, 3VP5.A.201, 3VRD.A.201, 3VRD.A.202, 3VRG.A.201, 3VRG.B.201, 3VXJ.A.501, 2VXH.A.1001, 1VYD.A.1117, 2VYW.A.149, 2WOB.A.470, 1W2L.A.1100, 2W31.A.200, 2W3G.A.500, 1W7O.A.1119, 1W7O.A.1120, 1W7O.A.1121, 1W7O.A.1122, 3W9C.A.501, 1WAD.A.117, 1WAD.A.113, 1WAD.A.114, 1WAD.A.115, 3WAH.A.201, 3WC8.A.201, 3WCT.A.200, 3WCT.B.201, 3WCT.C.200, 3WCT.D.201, 2WDQ.C.1129, 1WE1.A.300, 3WFD.B.801, 3WFD.C.201, 3WFE.B.802, 3WFX.A.201, 2WJM.C.1334, 2WJN.C.1333, 2WJN.C.1335, 2WJN.C.1336, 1WMU.A.201, 1WOV.A.300, 1WOX.A.300, 4WPD.A.402, 4WQ8.A.1002, 4WQ9.A.1001, 4WQ9.A.1002, 4WQC.A.1002, 4WQD.A.1002, 2WTG.A.180, 3WU2.F.101, 1WVE.C.699, 1WVP.A.154, 2WX2.A.1450, 3X15.A.200, 3X15.J.200, 1X3X.B.202, 1X46.A.151, 4XDI.A.201, 2XFH.A.1412, 1XK1.A.300, 2XKR.A.1400, 2XKI.A.1110, 1XQ5.B.148, 1XQ5.C.143, 2XSJ.B.503, 2XTS.B.500, 2XYK.A.700, 1Y0P.A.801, 1Y0P.A.802, 1Y0P.A.803, 1Y0P.A.804, 1Y5I.C.806, 1Y5I.C.807, 1Y5L.C.806, 2Y5N.A.450, 2YEV.A.1015, 2YEV.B.587, 2YIU.A.500, 2YIU.A.501, 1YIQ.A.901, 2YK3.A.200, 2YL7.A.128, 1YWD.A.185, 2YXC.A.1001, 2YYW.A.1001, 2YYW.A.1003, 2YYX.A.1004, 2YYX.A.1001, 1Z1N.X.607, 1Z1N.X.608, 1Z1N.X.610, 1Z1N.X.612, 1Z1N.X.616, 2Z47.A.1004, 2Z47.B.3003, 2Z6S.A.201, 2Z6T.A.201, 1Z8O.A.410, 1Z9N.A.1001, 1Z9N.C.2001, 3ZBY.A.1402, 2ZBO.A.111, 3ZE6.B.502, 3ZG2.A.1480, 3ZHO.A.200, 3ZHW.A.1163, 3ZIY.A.600, 3ZJO.A.200, 3ZJQ.A.200, 3ZOO.A.105, 3ZOX.A.1082, 2ZPB.A.300, 2ZSO.A.200, 2ZSO.B.200, 2ZSO.C.200, 2ZSO.D.200, 2ZXY.A.200, 2ZZS.1.220, 1ZZH.A.802, 1ZZH.A.803, 4KLI.A.404, 4M04.A.707, 1RZT.A.2001, 4C3X.A.561, 4JVL.B.304, 3MJ6.A.503, 3MQG.A.

193
[1] "Cluster 3"
3BLB.A.1047, 1BLL.E.489, 3D4Z.A.1046, 3D52.A.1046, 3D51.A.1046, 3DDF.A.3001, 1DE
5.A.450, 3DX1.A.1048, 3DX3.A.1047, 3DX4.A.1047, 3E38.A.2, 2F18.A.1805, 2F1A.A.18
05, 2F1B.A.1804, 2F7P.A.5001, 2F7R.A.5001, 3GWT.A.504, 3IBM.A.200, 3ITU.A.1, 3IV
T.A.500, 1KAE.A.1101, 3KMC.B.2, 3KME.B.2, 3KR5.A.1001, 1KRM.A.501, 1LCP.A.488, 3
LGP.A.1, 4NPW.A.1001, 4NUR.A.701, 3OOJ.A.1, 3O64.B.485, 4OJV.A.403, 4OJX.A.403,
2OQL.A.401, 2P18.A.301, 1PTM.B.331, 2PTZ.A.500, 2PU1.A.500, 3QAY.D.180, 1R33.A.1
163, 1R55.A.201, 4R6T.B.1003, 4R7M.C.1003, 3RCQ.A.1, 3SL3.B.9, 1SNN.A.402, 1SR9.
B.703, 4AAB.B.1156, 4AQX.D.1526, 4BE2.A.1381, 4C2U.A.1666, 1CW0.N.202, 1CW0.A.20
3, 4DLG.A.902, 2EZ6.A.501, 1G9Z.C.902, 1G9Z.D.901, 1G9Z.F.903, 4LOX.A.401, 3MAQ.
A.1001, 1MOW.D.374, 4NCB.B.702, 4NCB.B.703, 3OYA.A.397, 4PQU.A.602, 3RJF.A.340,
3S3O.A.397, 3S3M.A.397, 1T7P.A.4001, 1TK0.A.991, 4UB3.A.401, 2VBN.E.1026, 1ZBI.A
.302, 1A49.A.534, 4ACF.A.1480, 2AE8.C.1009, 1AJB.A.452, 3BGA.A.1, 2BJI.A.2277, 3
BM4.B.304, 3BWY.A.300, 2C31.A.1553, 4C5C.A.1314, 4C7X.A.700, 1CG1.A.435, 1CG4.A.
435, 3CRR.A.324, 3CT2.A.401, 4CW7.A.1002, 4CWB.A.1159, 3CXO.A.500, 3CXO.B.500, 4
CYM.A.1199, 3D46.A.501, 3D47.A.501, 1DAY.A.341, 2DGN.A.1454, 4DH5.A.402, 3DUF.A.
1368, 2DUA.A.292, 4DWB.A.507, 3DYH.B.4002, 3DYS.A.902, 4EA0.A.301, 1EBH.A.438, 3
EGT.A.3002, 3EKG.A.601, 1ELZ.A.452, 1EXM.A.407, 3EZ3.B.1102, 3EZ3.B.1104, 4F0Q.A
.501, 4F71.A.301, 3FD5.A.396, 4FFL.A.906, 3FLK.A.401, 3FLK.A.405, 3FPA.C.901, 3F
YY.A.402, 1G4P.A.2003, 3G5A.B.307, 2G9Z.B.701, 2GT4.B.401, 4GT3.A.403, 3GY1.A.50
0, 4HE1.A.403, 2HGS.A.502, 3HJN.A.501, 4HNC.A.401, 3HPF.A.402, 3HVI.A.1, 3HVK.A.
1, 2HXU.A.601, 4I3Y.A.304, 1I6I.A.501, 3ICZ.A.402, 2IK2.B.289, 1IV4.A.1572, 1J7L
.A.301, 4J7L.A.402, 1JP4.A.702, 3JVT.B.502, 4K33.A.802, 1K9Y.A.401, 4KCU.A.1001,
3KDN.A.500, 3KEU.A.400, 3KHQ.A.1, 1KKR.A.501, 4KQX.A.406, 3KRO.D.3001, 3LOY.A.2
57, 4L9Y.B.403, 4M6U.A.401, 1MEZ.A.1453, 1MMA.A.998, 1MNS.A.360, 4MPO.A.206, 4MP
O.C.205, 1MXO.A.501, 1NUY.A.2341, 4NZO.A.404, 2O1O.A.503, 3OE5.A.222, 1OIX.A.301
, 4OKM.A.902, 4OKZ.A.902, 3OP2.A.500, 3OPS.A.501, 4ORK.A.501, 2OUN.A.403, 1OZH.A
.1405, 3OZM.D.390, 3OZY.B.390, 3P3B.A.393, 3P41.A.297, 3P93.A.406, 2PLS.H.602, 2
PMQ.A.902, 1PT6.A.500, 1PUN.A.130, 4PU5.A.502, 1PYD.A.559, 1PYM.A.1003, 2PZA.A.6
242, 2Q1A.X.294, 2Q1D.X.294, 4Q1V.A.803, 3Q30.A.600, 3Q30.A.601, 3Q46.A.306, 1Q6
O.A.7300, 1Q6Q.A.7300, 4QE5.A.401, 3QKE.A.407, 1QMZ.A.383, 3QPE.A.393, 3QPE.B.39
3, 4QPM.A.1503, 3R1M.A.402, 3R1M.A.403, 3R25.A.402, 4RJJ.A.602, 4RJK.H.602, 3RLH
.A.286, 4RN3.A.301, 1RQJ.A.907, 3RUV.A.544, 3SAZ.A.802, 3SBF.A.402, 1SHQ.A.479,
3T6C.A.501, 1TE6.A.640, 4TQD.A.502, 3TW6.C.2002, 3TWB.A.420, 3UJR.A.501, 4USJ.C.
302, 3V4B.A.403, 1V5F.A.1603, 1V5G.A.1603, 2VBV.A.1136, 2VDR.B.2001, 3VMK.A.402,
3VMK.B.402, 2VPR.A.1207, 3WBZ.A.403, 1WDD.A.1476, 3WDL.B.902, 2WEF.A.402, 4WKO.
B.501, 2X3J.A.1590, 2XCL.A.480, 1XG3.A.2101, 2XIM.A.395, 2XZW.A.202, 1YVE.I.601,
1Z5C.A.2001, 2Z7H.A.1301, 2ZDH.A.812, 2ZVJ.A.300, 4ABT.B.1287, 1DMU.A.300, 2IBK
.A.402, 4KHQ.A.1001, 4KYW.A.302, 3M9O.B.4001, 1N3E.D.493, 1N3F.D.499, 1N3F.F.497
, 3O3G.A.1, 1OUP.A.300, 1Q9Y.A.939, 2RDJ.A.353, 1RYS.A.801, 3SPZ.A.905, 3A09.A.6
01, 1AG9.A.200, 1AG9.B.1000, 4AIE.A.1540, 3AMR.A.909, 4AQ1.A.1925, 4AQE.A.1208,
3ASI.A.2001, 1AVA.A.502, 3B4N.A.702, 4B4F.A.607, 1B9T.A.500, 2BV2.B.1085, 1C3H.D
.8003, 4CAG.A.602, 1CLC.A.652, 4CUB.A.2645, 4DOU.A.1002, 2E9B.A.741, 1ELT.A.300,
5ENL.A.438, 1FBL.A.996, 3GG1.A.502, 1GVK.B.1246, 1HVX.A.517, 3I98.A.627, 2IUF.E
.1697, 1JOH.A.601, 1J1N.B.493, 1J35.C.501, 1JE5.B.502, 1JI3.B.403, 2JKE.A.1728,
4JZB.A.402, 1K7Q.A.485, 2KAY.B.187, 4KKF.A.703, 3KMV.A.163, 1KUO.A.703, 3KZP.A.2
40, 2MIN.B.525, 2ML1.A.201, 4N20.A.705, 4N20.A.706, 4N2B.A.705, 4NEH.B.701, 3NJH
.B.502, 4NUZ.A.1001, 3OJY.B.538, 3OM5.B.1, 4OUL.B.1201, 3PGB.A.903, 4PLS.A.301,
2POO.A.805, 1PW9.A.404, 2Q1C.X.294, 3Q2L.B.703, 2QIM.A.158, 1QLB.A.1658, 1RXO.B.
477, 1SOB.A.1292, 3S5U.E.221, 2SAS.A.187, 1TO2.E.450, 3U1R.A.706, 3U1R.A.707, 4U
P4.A.501, 4UP4.A.502, 1UTM.A.247, 2UWF.A.1369, 1UX6.B.2003, 1UX6.B.2007, 2VCC.A.
1917, 3WN6.A.502, 3X17.A.602, 1XKD.A.1005, 1YO8.A.1183, 1YO8.A.1205, 2ZEO.A.552,
2ZPR.A.2001, 3ZWH.A.501, 2ZWP.A.402, 1A2F.A.1, 4B7G.A.3000, 3BFJ.M.1388, 1BKA.A

.694, 2BQ8.X.1305, 2BV0.B.600, 4CHL.A.501, 1DRY.A.332, 4DTW.A.500, 3DXU.A.360, 3
E6S.B.200, 1FSL.A.144, 4G51.B.202, 4GHF.B.401, 3GM6.A.1004, 4GP4.A.602, 4GP5.A.6
02, 2HBU.A.900, 2HUO.A.301, 3IXF.A.139, 3LXV.M.600, 4M25.A.401, 3MDT.A.505, 3MOO
.A.911, 3NC3.A.406, 2NOX.A.500, 4OJ8.C.301, 1OQU.C.1009, 3OUH.A.600, 1PIU.A.402,
 2Q0J.B.998, 3Q1G.A.1002, 3QY8.A.253, 4RC8.A.303, 3RNC.A.500, 1SYY.A.1319, 1SYY.
A.1320, 1TOQ.A.500, 3TMZ.A.501, 3UBR.A.471, 2VUN.A.402, 3WEC.A.501, 2X9P.A.1398,
 1XK3.A.300, 1XVG.A.529, 1YGF.B.147, 2BCU.A.577, 2BCQ.A.1, 1CZO.C.606, 4ED3.A.50
2, 1JJ2.0.8338, 4P4P.A.401, 1VQ8.J.9146, 2A5D.B.326, 4A6U.B.1460, 2AUT.D.605, 1B
57.A.364, 2BS2.A.1658, 3CYM.A.501, 2DDB.A.301, 4DD8.D.1005, 4DEL.A.401, 3E85.A.1
62, 2EHQ.A.1540, 4FDZ.B.301, 4FET.A.301, 4FEX.A.303, 2FM1.D.344, 1G3K.A.500, 1G5
H.A.901, 3GA5.B.701, 4GKI.D.303, 3HON.A.203, 1HBN.A.1561, 2HI0.B.240, 3HSS.A.268
, 3IFV.A.402, 3IGZ.B.601, 2IJA.A.401, 3IPO.A.436, 3IPP.A.438, 1IYN.A.297, 2IZV.A
.1430, 1JAY.A.215, 3JS4.D.208, 4JTK.C.302, 1JZ7.A.3102, 3L7X.A.142, 4LGN.A.827,
4M4U.A.501, 4M60.A.502, 3MQD.A.500, 3MZG.B.211, 4NRH.A.401, 3OPK.A.301, 4PSR.A.6
19, 4PYJ.A.301, 4Q69.A.501, 2QJY.R.2001, 2QV6.A.303, 4QXK.A.402, 1U7H.B.912, 2VP
B.A.1398, 2WDQ.A.1590, 2WOI.D.1489, 2WWF.B.214, 4X9K.A.401, 2XNA.C.1216, 2XZI.A.
502, 1YYA.A.1001, 2ZHJ.A.321, 1ZOR.B.1002
[1] "Cluster 4"
1DK4.B.590, 2ZZW.A.362, 4D6N.F.1197, 4DFP.A.901, 3LK9.A.340, 4M2Z.A.501, 3R7P.A.
317, 3A58.B.401, 4CZK.A.1335, 3DYG.A.3002, 1E2A.C.106, 1EFL.B.1604, 2EGH.A.900,
3FFU.A.156, 3FFU.B.155, 3FHY.B.404, 4FHX.A.402, 3G9D.A.298, 1H65.A.282, 3HWX.A.6
02, 2IUT.A.1724, 3KUD.A.171, 4L2X.F.403, 1MBZ.A.604, 2NOM.A.401, 1NV7.A.3341, 4O
GE.A.1204, 2OPM.A.907, 3PP1.A.410, 1RC5.A.761, 4RNH.A.1501, 1SOJ.A.2123, 1T5S.A.
1005, 3TXA.A.801, 3UGV.A.500, 2VQD.A.1449, 4WK2.B.501, 2Y4M.A.400, 2YWF.A.701, 3
FSP.A.501, 3KHH.B.1416, 1M5X.A.802, 3NE6.A.905, 4PTF.A.1202, 3QEX.A.907, 2RDJ.B.
353, 1S9F.A.4001, 2WTF.B.1510, 4A6S.C.1122, 2A8K.C.403, 1AFA.2.2, 1AWB.A.278, 3B
1T.A.900, 4B7R.A.502, 3BJU.A.608, 4BY5.C.1187, 4CAJ.B.1325, 2CDP.B.1139, 1CGU.A.
686, 3CGT.A.686, 1CRU.A.503, 1CYG.A.682, 1D7F.A.5003, 3DAS.A.351, 4DRZ.A.202, 1E
8U.B.1003, 3EDD.A.700, 3EHJ.A.1, 1F6S.E.205, 3FCU.B.2002, 1FHF.A.501, 4FU4.B.505
, 3GQF.D.154, 1HDF.B.1101, 1HFZ.A.124, 1HL5.D.156, 3HLI.D.315, 1HQV.A.999, 1I22.
A.198, 4I5N.B.601, 4JDZ.B.702, 3JTX.B.396, 4K5W.A.201, 2KAY.A.185, 1KMB.1.2, 4KV
J.A.714, 1KX1.E.502, 1KX1.F.601, 1L6R.A.901, 3LCP.B.279, 3LHM.A.131, 4LMF.A.303,
 1LPG.B.1, 1MAC.B.389, 1MDU.A.403, 2ML2.A.202, 4N85.A.502, 3N8G.A.1002, 4NAM.A.8
01, 4NHF.B.301, 3NN7.A.503, 3O5S.A.243, 2OKX.B.4002, 2PF2.A.174, 4PLM.A.504, 2PP
L.A.481, 1Q7B.A.9002, 2QT6.A.3713, 1R1Z.C.415, 2RDZ.B.1502, 3SO0.F.98, 3SO1.B.97
, 3TSK.A.304, 3TUY.C.157, 1UH2.A.1002, 1UH3.A.1001, 1ULV.A.2003, 1UMS.A.3, 1UZJ.
A.1648, 3V03.A.585, 1V2I.A.1001, 1V3J.A.687, 1V3L.B.689, 1VFO.A.1001, 2VJ3.A.153
3, 2VKH.A.1543, 2VZP.B.1129, 2W2N.E.1334, 2W3J.A.1139, 3W9T.A.1008, 3W9T.D.503,
3W9T.D.504, 4WBQ.B.602, 4WF7.D.600, 3WH3.A.501, 2WO9.B.1272, 2WVX.B.801, 2WVZ.B.
800, 2WW0.A.800, 2XQX.B.1949, 1YO8.A.1195, 1YO8.A.1211, 2OTL.A.8545, 1TW8.B.902,
 3UXP.A.338, 1VQ9.M.9147, 1A5U.G.4732, 2AB8.A.2003, 4B52.A.1305, 2BER.A.1649, 1B
GP.A.502, 4BY5.B.1188, 4CBY.A.2036, 2D4E.A.1901, 4FMT.B.301, 2GFH.A.249, 1HBN.D.
1561, 3HSC.A.491, 2IM2.A.3001, 3IWJ.A.505, 1JZ4.A.3101, 4KZW.A.304, 4LL2.A.301,
3ME4.B.2, 4NLV.A.911, 4OMG.A.402, 2P6Z.B.401, 2P6Z.B.403, 4PCG.A.302, 4Q92.C.502
, 3SIS.B.3001, 3SIT.A.3000, 1T64.A.392, 3TAY.A.1, 4TMX.A.902, 4US3.A.701, 4USW.A
.1468, 2W4M.A.1245, 3WFA.A.801, 4WFZ.A.501, 2WPC.A.1491, 2YDG.A.1130, 2YFO.A.174
3, 1Z45.A.702
[1] "Cluster 5"
2BNO.B.1201, 3D4Y.A.1047, 3E49.B.500, 3E8R.B.2, 2I57.D.507, 3ISI.X.3001, 4JDG.A.
401, 4M6R.B.301, 4OJV.A.404, 4OJX.A.404, 1OS9.F.926, 1QH3.A.262, 4R76.A.1003, 4R
LO.B.302, 3V77.D.301, 2WHG.B.1263, 4X2T.A.701, 4AAB.B.1157, 2AGQ.A.4002, 4BDY.A.
1381, 4D6O.D.1196, 4D6O.D.1197, 4DF8.A.903, 4F5P.A.401, 4IRD.F.903, 3LK9.A.339,
4M3O.A.501, 4M3O.A.502, 4MFF.A.401, 4NCB.A.702, 3OYC.A.397, 3OYE.A.396, 2OZM.A.9
04, 1QTM.A.1001, 3SI6.A.905, 3SPY.A.904, 2XCP.A.1004, 2XCA.A.3000, 2XCA.A.3001,

1YVP.A.1001, 1ZBL.A.202, 1ZBL.B.204, 3A06.A.500, 4A01.A.1770, 2AG1.B.611, 3AJO.A
.183, 3AXK.A.478, 1B7T.A.836, 3B8I.A.289, 1BWV.C.490, 2BW7.A.2201, 4BYF.C.1000,
4CEO.A.1251, 1CG0.A.435, 4CTA.B.401, 4D2I.A.1478, 3DVA.A.1368, 2DW6.D.2004, 2E0A
.A.500, 2E8W.A.1202, 1ECQ.A.498, 3EG5.A.180, 1ELX.B.452, 3EYA.H.613, 1F1Z.A.2002
, 3F78.C.1, 4FFL.A.904, 4FFL.A.905, 4FFO.A.904, 2FG5.A.301, 3FPB.A.1000, 3FTQ.A.
371, 1G3B.A.501, 2G4J.A.392, 2G9Z.A.704, 4GA3.A.1004, 4GOK.B.202, 4HYV.A.1001, 4
I2B.A.602, 3I3O.A.306, 1IGW.A.441, 2IK7.A.287, 2IOA.B.5004, 4IOK.A.604, 4IX4.A.6
02, 2J5X.A.200, 2JI8.A.1567, 4KCW.A.1001, 1KH7.A.452, 1KHK.A.452, 4KI8.E.602, 1K
P8.A.550, 3KR4.C.1004, 3KRO.D.3002, 4KS0.A.1001, 4L2X.F.405, 4LF1.A.801, 4LNI.E.
505, 3M00.A.550, 1M1B.B.999, 3M1Y.A.300, 1MB9.B.601, 1MBZ.A.603, 3MQT.H.626, 1MR
S.A.300, 1N20.A.703, 1NHT.A.435, 2NOM.A.402, 1NUW.A.2498, 3OES.A.202, 4OKZ.A.901
, 3OPS.A.500, 2OQY.A.401, 1OVM.A.601, 4OVN.A.204, 3OYZ.A.500, 3P0X.A.430, 1PFK.A
.325, 2PLS.G.603, 6Q21.D.173, 2Q58.A.5, 1QF5.A.433, 3QQV.A.382, 1QS0.A.501, 2QTV
.B.210, 3QU4.A.225, 4RAD.D.302, 3RIM.A.1001, 1S1C.A.300, 3SE1.A.182, 3SH6.A.176,
3SSN.A.501, 3T0Z.A.401, 4TQ4.A.401, 4U0M.A.503, 3UJ2.A.431, 3UXL.A.360, 2V3W.A.
1528, 2VBI.A.1000, 3VMM.A.501, 2VWT.A.301, 3VYT.C.602, 3W7F.A.303, 1W88.C.1368,
4WK4.B.501, 1WL6.A.801, 2X5Z.A.602, 2XAM.B.1030, 2XH4.A.1439, 5XIM.A.395, 1XLC.A
.399, 1XZ8.A.180, 1Y9I.A.601, 1YHM.B.1401, 1YHY.A.699, 1Z0K.A.1201, 2Z4W.A.1302,
2Z4Y.B.1301, 3ZDY.B.2001, 3ZXW.A.476, 2AOQ.A.232, 2AOR.A.401, 2ASD.A.415, 3BQ1.
A.4001, 3COW.A.301, 1DMU.A.302, 3DVO.A.340, 3EH8.A.302, 2GIJ.A.401, 3GV5.B.424,
2IBK.A.401, 2IMW.P.406, 1JX4.A.4001, 4KLD.A.402, 3MXB.B.173, 3MXB.R.175, 1N3E.C.
492, 3ODH.A.195, 1R7M.A.306, 1R7M.B.536, 3A13.E.445, 2A8K.B.404, 2AER.L.3008, 1A
WB.A.1, 4AWD.A.1321, 3AZX.B.301, 3B00.B.301, 3B0I.A.124, 3BCD.A.708, 1BLI.A.600,
1C3H.F.8001, 4C9F.B.401, 1CGE.A.305, 3CK7.D.730, 3CKC.A.600, 5CNA.C.240, 1CR8.A
.45, 4CRR.A.1386, 3D4G.B.484, 3D6E.A.202, 2DF7.C.5904, 1DGL.B.301, 4DLK.A.403, 2
DSN.B.2012, 3EAD.A.1003, 3EHB.A.563, 3EHJ.B.1, 4EJ7.B.402, 4EPU.A.601, 3ESQ.A.21
3, 2EXH.D.2004, 2FIB.A.412, 2FPS.A.503, 1G5N.A.402, 4G60.A.301, 4G60.A.302, 4G62
.A.301, 1G87.A.615, 1G9K.A.700, 2GGM.B.402, 4GI6.A.601, 3GN4.B.204, 4GQ7.A.301,
2GSM.A.3007, 4GZT.B.510, 1H3G.A.701, 4H3X.A.304, 1H5V.A.306, 3HGP.A.250, 4HOW.A.
704, 4HPN.A.401, 2HYU.A.502, 3I4I.A.1001, 2I4B.A.454, 3I57.A.186, 4I8H.A.301, 3I
OX.A.903, 2J1T.A.1154, 1J9L.B.1303, 4JCM.A.706, 3JXS.A.302, 4JZX.A.403, 1KAP.P.6
20, 3KF9.C.304, 3KQA.B.421, 4KS1.A.501, 2L51.A.207, 1L9M.A.702, 3L9I.C.1148, 4LJ
3.B.403, 4LLT.A.303, 3LNH.A.303, 1LOC.E.688, 4LVN.A.704, 2M0P.A.1201, 4M5I.A.203
, 4MKM.A.403, 2ML2.A.201, 2ML3.A.203, 3MVS.A.214, 4N25.A.707, 4N2G.A.705, 4N2I.A
.704, 3N4E.A.500, 1NBW.A.650, 1NNL.B.2002, 1NUD.A.703, 2NXP.A.600, 1OAH.B.1526,
1OBO.A.501, 3OTJ.E.1000, 3P4G.A.402, 3P4G.A.403, 3P4G.A.404, 3P4G.A.405, 3P4G.A.
407, 3P4G.A.408, 3P4G.A.409, 3P4G.A.412, 3P4G.B.411, 3PKO.A.280, 1PVY.B.603, 3Q5
I.A.528, 3Q8F.A.736, 4QD2.E.302, 3QGV.A.504, 4QU6.A.904, 3RRW.A.271, 3S6J.A.4, 1
SCB.A.276, 3SLE.B.401, 3SO0.D.97, 3SVL.A.201, 1TCM.B.687, 3TEW.A.801, 1TT2.A.502
, 1TU5.A.902, 3UBF.A.754, 1UF3.D.913, 1UH3.A.1002, 1UKS.A.688, 4UM9.B.2002, 1UPS
.A.501, 1UYX.A.1133, 3V96.B.304, 2VDR.B.2002, 2VL8.A.1545, 2VVE.B.1338, 2VXJ.G.2
00, 2W1W.B.1135, 1WC5.C.2100, 2WOB.E.1161, 1WPC.A.502, 2WQS.A.2415, 2XSG.B.1772,
2Y09.A.1242, 1Y6W.A.149, 2YDP.A.502, 1YO8.A.1201, 1YO8.A.1203, 1YO8.A.1206, 1YO
8.A.1208, 2Z2X.A.1007, 2Z49.A.1002, 3ZHG.D.1323, 2ZQ0.A.901, 1FZ3.A.5002, 1JNF.A
.703, 4ME4.A.402, 1MMO.D.3, 1MMO.D.4, 1NX4.B.300, 3QFN.A.265, 3QFO.A.264, 1R1N.C
.400, 2RDB.A.499, 1T0Q.A.499, 1XVF.B.1174, 3ZK3.A.1311, 2BCR.A.604, 4KYW.A.303,
1Q81.A.8378, 1VQ8.Q.9148, 4AFK.A.1507, 2AHR.C.1259, 3AR4.A.1000, 3BIA.X.117, 3BI
B.X.117, 3BOS.B.302, 3C17.A.323, 2CD7.A.1132, 3CRN.A.131, 4DOU.A.1004, 2E5X.A.30
3, 1EAS.A.5, 4EAE.A.302, 3EUW.A.343, 4G8T.C.502, 3GZA.A.471, 1H16.A.9001, 3HIJ.B
.295, 2HZG.A.1101, 3I01.M.730, 3I2W.B.304, 3IC9.B.491, 2J80.A.1134, 2JBW.C.1367,
2JHN.B.1299, 4JN7.A.401, 4JTE.C.301, 3K1U.A.412, 3LG1.A.530, 4M48.A.701, 3N0U.A
.208, 3N83.G.707, 4N9S.A.402, 4N9V.A.404, 1O04.A.6601, 4O57.A.301, 4O5H.A.605, 1
O68.A.274, 3OEC.C.300, 4OF8.A.301, 3ON4.C.189, 3OND.A.509, 2OYN.A.201, 4PD6.A.50
2, 4PMO.A.312, 1Q3X.A.800, 1Q6X.A.1002, 2QR7.A.1000, 2QSV.A.222, 1R4P.A.4003, 2R

85.A.600, 1RWH.A.900, 1S0A.A.1501, 1T8U.B.701, 3TDQ.A.117, 3UES.B.503, 3UF4.A.60
1, 1V0H.X.252, 2V4V.A.3052, 3VGL.A.323, 2VNZ.X.9252, 1W9S.A.1141, 2X8J.A.1317, 2
ZJ9.B.2, 1Z0D.A.435, 1Z0R.A.1001, 1ZUD.1.701
[1] "Cluster 6"
2ALW.A.5001, 4BBP.A.1316, 3DDG.A.3001, 2DDF.A.1, 4D00.A.502, 3DX2.A.1046, 3EJP.A
.1047, 3EJQ.A.1047, 3EJR.A.1047, 3EJU.A.1047, 2F70.A.5001, 2F7Q.A.5001, 2F92.F.1
003, 2FQP.B.100, 4H1S.A.603, 3HPS.A.701, 1IM5.A.400, 1KQ3.A.401, 3LX3.A.201, 4NZ
3.A.501, 3O93.A.192, 2OB3.B.904, 1PTM.A.330, 4QGE.A.602, 4R6T.F.1001, 4R76.E.100
1, 4T08.A.301, 2V9N.A.1275, 3W0T.A.201, 3W0T.B.201, 4X2T.G.702, 1XM6.A.1001, 2Y3
3.A.900, 3A4K.A.301, 2AQ4.A.302, 2BCV.A.576, 4CEI.A.2234, 4D6N.F.1196, 4DF4.A.90
1, 4DFJ.A.902, 4DOA.A.401, 4EEY.A.502, 3F2B.A.5, 2FMS.A.340, 3GDX.A.347, 3GDX.A.
348, 3GPL.A.800, 3GQC.B.203, 4IR9.F.402, 2ISO.A.339, 2ISP.A.339, 2IS4.A.1001, 3J
PN.A.339, 3JPR.A.339, 3JPT.A.339, 3K57.A.1001, 4K98.A.602, 4K99.A.602, 4M47.A.40
2, 3M8R.A.2, 3MBY.A.339, 3MQY.A.500, 4O3N.A.503, 3OS0.A.394, 3OYA.A.396, 3OYC.A.
396, 3OYF.A.396, 3OYF.A.397, 3OYG.A.397, 3OYH.A.396, 3OYH.A.397, 2PFN.A.950, 4PG
Q.A.400, 4QCL.A.1302, 1QSY.A.1001, 4R8U.B.402, 3RJK.A.340, 3S3M.A.396, 1SKR.A.40
01, 3SV3.A.836, 3TFS.A.340, 1TK8.A.901, 4TUQ.A.402, 3TWH.A.401, 3UQ2.A.1, 2W35.A
.1224, 4A01.A.1771, 1A49.H.5334, 3A7D.A.300, 4ABZ.A.1210, 4AC0.A.1205, 1AZS.C.40
3, 3B1X.A.301, 1B8C.A.308, 2BKU.A.221, 3BNY.D.701, 1BZY.A.901, 2C3P.A.2237, 4C5A
.B.331, 3CB3.A.501, 1CH8.A.434, 1CIB.A.434, 2CJE.A.1268, 3CP6.A.501, 1CUL.C.396,
3CWH.A.392, 1DAK.A.901, 4DBH.A.401, 2DCN.A.4001, 3DFY.A.401, 4DFD.B.301, 3DHD.A
.502, 1DIE.A.399, 4DN1.B.401, 3DOE.A.193, 1DQN.A.451, 2DW6.A.2001, 4DWB.A.508, 1
DXE.B.901, 4DXJ.A.401, 4DXJ.A.402, 3DYG.A.3004, 4E1E.A.403, 1E4E.A.365, 1E9I.A.1
431, 2E91.A.1302, 4EA0.A.302, 4EA0.A.303, 1EBG.A.438, 1EBG.A.439, 2EB1.A.502, 1E
C7.A.498, 1EC9.A.498, 1ECB.A.507, 3EFQ.A.3003, 3EFQ.B.4002, 3EGT.A.3003, 3EQI.A.
3, 3ES8.A.393, 1F2U.A.902, 1F8I.A.451, 3FA5.A.282, 3FD5.A.395, 3FD5.B.397, 3FD6.
B.395, 3FE4.B.902, 4FFR.A.403, 4FI4.A.501, 2FN0.A.701, 2FPR.A.503, 3FQI.A.1000,
1FTN.A.300, 4G61.A.302, 2G9Y.A.452, 1GAG.A.201, 2GCQ.A.435, 2GGE.A.400, 4GIS.A.4
05, 4GIU.A.402, 2GQS.A.240, 2GQS.A.241, 2GQ3.A.1000, 4GYI.A.402, 1H1D.A.300, 4H1
9.A.405, 4H1Z.A.401, 3H4L.A.701, 2HCJ.A.998, 3HDG.B.201, 4HE1.A.404, 4HE2.A.405,
4HGR.A.201, 4HHL.A.402, 3HQD.A.501, 3HQP.B.502, 3HVH.A.265, 2HWG.A.901, 3HXX.A.
445, 4I3Y.A.302, 4I3Z.A.302, 3I6E.A.386, 3IBA.A.401, 3IBA.A.402, 3ICK.A.401, 3IC
Z.A.401, 2IDX.A.603, 4IEE.A.501, 4IFW.A.502, 1II0.A.593, 1II9.A.593, 3IIE.A.501,
4IJQ.A.304, 2IK2.B.287, 2IK2.B.290, 4IT1.A.501, 2IUC.B.1008, 2IXE.A.2, 1J34.C.5
01, 1JAH.A.168, 2JCS.A.1211, 2JI6.A.1567, 1JP4.A.701, 3JUK.C.307, 4K1W.A.501, 1K
9Y.A.403, 1K9Y.A.402, 4K9N.A.601, 1KA2.A.501, 4KCT.A.1001, 4KCV.A.1001, 1KEK.A.2
237, 1KHZ.B.310, 4KMQ.A.1102, 1KO5.A.1001, 4KQX.A.405, 4KUX.A.701, 3KWS.B.401, 4
KWD.A.403, 4KX3.A.302, 1L8A.A.888, 4LA7.B.601, 1LON.A.1454, 3LV0.A.264, 3LVV.A.6
95, 3LVV.A.697, 4LZ3.A.406, 4M69.A.403, 1MC1.A.603, 4MFG.A.201, 4MIT.A.202, 4MPO
.B.204, 4MPO.E.201, 4N0G.A.403, 1N1Z.A.702, 1N24.A.703, 3N3T.A.802, 1N8I.A.900,
4NEH.B.703, 4NFI.F.404, 4NM3.A.405, 3NO1.A.397, 1NUW.A.2497, 1NUX.A.2342, 1NUY.A
.2343, 3NZG.A.507, 2O10.A.501, 4O4D.A.401, 3O61.B.202, 1OAD.A.392, 2ODB.A.205, 1
OFH.B.453, 1ORK.A.223, 1OW2.A.401, 2OX4.A.402, 1OZF.A.699, 2P3N.A.1758, 3P5R.A.9
01, 1P7T.A.1000, 1P9B.A.1600, 4PAL.A.110, 3PDE.A.311, 1PFK.A.327, 4PFK.A.327, 2P
GN.A.610, 2PLS.J.604, 2PP3.A.901, 3PUV.A.1501, 3Q46.A.305, 1Q9S.A.201, 3Q9L.A.70
0, 1QC5.A.601, 1QC5.B.602, 4QEH.A.402, 1QF4.A.433, 2QGY.A.701, 4QPM.A.1502, 2QQ0
.B.452, 2QX0.A.162, 3R0U.A.380, 4R39.A.401, 2R9V.A.504, 3RBM.A.1002, 3RBM.D.1003
, 2RDX.A.378, 4ROP.A.504, 1RQI.A.605, 3S9I.A.743, 3SAD.A.801, 3SB0.A.801, 3SOP.A
.401, 3ST8.A.496, 3T1Q.A.198, 3T2E.A.409, 3T8O.A.564, 1T9B.A.1699, 1TND.A.352, 3
TTE.A.361, 3TTE.B.361, 3TZF.A.279, 3U2E.B.1, 4USI.A.1151, 4V1T.A.1776, 4V1T.A.17
78, 2V5K.A.301, 1V8K.A.501, 1VA6.A.522, 1VA6.A.524, 3VD3.A.3001, 2VDM.B.2001, 2V
P0.A.1209, 3VR6.B.602, 2VZB.A.1001, 2W00.B.1894, 3W2W.A.904, 1W5T.B.701, 4WF7.A.
601, 3WO0.A.502, 4WRR.A.401, 2WX5.L.1282, 1XEF.A.801, 5XIN.A.395, 1XIN.A.395, 6X
IM.A.395, 8XIM.A.395, 2XJC.A.1499, 1YI0.A.212, 1YVE.I.602, 1YYQ.B.701, 3ZCB.A.30

1, 1ZVW.A.4001, 3ZYC.A.1750, 2AOR.A.402, 2ASD.A.416, 3AVX.A.3001, 3COW.A.304, 4DTP.A.1002, 4ELV.A.908, 1FOO.B.761, 4F4W.A.402, 4F4W.B.403, 4FJ9.A.1002, 4FJK.A.1002, 4FJL.A.1002, 4FJM.A.1002, 3IAY.A.1, 4J2A.A.1002, 4J2B.A.1002, 2JEJ.A.1344, 1JXL.A.1402, 4K4H.A.607, 4K4H.E.602, 3KHR.A.416, 4LQO.A.402, 3LZJ.A.905, 4M3Z.A.1002, 3M9N.B.4003, 3M9O.B.4003, 1N3E.F.491, 3QET.A.905, 4QWB.A.403, 3RAX.A.415, 1SOO.A.401, 3SLP.B.227, 3SUN.A.897, 1TW8.A.801, 3A7Q.B.5001, 1AF0.A.484, 1AF0.A.486, 3AMR.A.910, 4AQ1.A.1926, 4AR9.A.1732, 4ARF.A.1723, 3AYU.A.418, 3B1U.A.901, 1B4N.A.620, 1B8L.A.110, 3BC9.A.702, 3BC9.A.705, 3BH4.B.1, 2BU3.A.1242, 4BZ4.A.1233, 2CDP.A.1140, 1CLC.A.650, 4CPY.A.1466, 4CU9.A.2999, 2CYY.A.2002, 2D00.D.1001, 2DCJ.A.1003, 2DDU.A.1, 2DDR.A.1324, 2DEW.X.901, 2DIE.A.779, 4DK4.A.302, 4DKB.A.302, 4DOU.A.1001, 3EF2.A.304, 3EF2.A.305, 1EX9.A.286, 2F3C.E.242, 4F8Z.A.409, 3FP8.E.601, 3FU1.A.301, 3G4E.A.1, 4G62.A.302, 4GDJ.A.507, 2GJP.A.1486, 2GKO.A.611, 3GK2.A.92, 3GN9.A.201, 4GN7.A.301, 4GW3.A.401, 2HOK.B.410, 1H71.P.501, 1H71.P.502, 1H9H.E.1246, 3HB2.P.482, 1HDF.A.1101, 3HI7.A.802, 1HM9.A.1901, 1HT6.A.502, 1HYO.A.1006, 2HYV.A.608, 1I76.A.996, 3IBZ.A.192, 4IHM.A.403, 1IOD.G.503, 2IXT.A.1311, 1J11.A.701, 4J7M.A.403, 1J9K.A.301, 2JKH.A.1245, 4JZE.H.302, 3K37.B.467, 4K3K.B.401, 1K7I.A.483, 4K70.B.1002, 4K89.A.408, 4K9P.A.601, 1KA1.A.401, 1KAP.P.617, 1KAP.P.618, 3KCG.H.500, 3KM5.A.2011, 4KTY.A.802, 4KXY.A.707, 4LLS.A.303, 3LNI.A.303, 3LPD.A.342, 4LQR.A.202, 3M1H.C.2001, 3MBR.X.300, 4MB1.A.602, 1MCT.A.246, 3MHF.A.328, 2ML1.A.202, 2ML1.A.204, 2ML3.A.204, 3MMZ.A.500, 3MVS.A.211, 3MVS.A.215, 4MWL.A.512, 3MW3.A.301, 4N2I.A.705, 1N7V.A.601, 4NAS.A.503, 3NIF.D.2002, 3NKQ.A.1003, 1NKG.A.800, 4NOT.A.302, 1NRW.A.903, 1NSC.A.468, 4NUQ.A.301, 1NX1.A.3, 3OJY.A.555, 1OM6.A.701, 1OM8.A.705, 3OM6.B.1, 2P3U.B.501, 3P4G.A.406, 3P4G.A.410, 3P95.A.1, 4PHN.A.302, 2PNY.A.228, 3POJ.B.1, 3PPE.A.401, 1PZ7.A.701, 2PZ0.A.501, 2Q16.A.200, 1QCO.A.1002, 4QN7.A.501, 3QU7.A.230, 3QU7.B.225, 2QUB.A.614, 2QUB.A.616, 2QUB.A.618, 3QXG.B.230, 4R12.A.809, 2R1B.A.1001, 2R8Y.A.201, 3R8Y.A.242, 2RA3.A.1, 2RA3.B.1, 3RQ0.A.301, 1SBH.A.290, 3T3P.B.2003, 1T5S.A.1004, 4TSH.B.1502, 3U1R.A.704, 3U1R.A.705, 1ULV.A.2001, 1ULV.A.2002, 1UX6.B.2010, 1UYX.A.1134, 1V0Z.B.1477, 1VCL.A.1004, 1VCL.B.1001, 1VCL.B.1002, 1VL9.A.125, 2VNG.B.1214, 3VOC.A.501, 3VTO.A.302, 4WA3.A.503, 2WFK.A.1250, 4WKO.B.503, 1WMD.A.1003, 1WRZ.A.154, 2WW3.A.800, 2XR9.A.1869, 2XVT.F.1137, 2Y6D.A.1266, 1Y7B.A.3001, 1Y9Z.A.604, 1Y9Z.B.605, 2YAY.A.1267, 2YGL.A.1413, 1YI7.A.3001, 1YO8.A.1185, 1YO8.A.1193, 1YO8.A.1199, 1YO8.A.1209, 1YS6.A.1001, 1Z6O.A.5302, 1Z70.X.3001, 2Z8X.A.620, 2Z8X.A.622, 2Z8X.A.623, 2Z8X.A.624, 2Z8S.B.643, 2ZUX.A.630, 2ZUY.A.626, 2A1X.A.450, 1A7E.A.119, 3ABM.A.516, 1B1X.A.690, 2B2O.B.1500, 4B2O.A.1267, 4BGL.A.1001, 1BKA.A.693, 2BQ8.X.1306, 2BUZ.B.1541, 2CAG.A.485, 1D9Y.A.310, 3DHG.A.502, 1EO9.B.600, 1FCD.C.902, 2FR7.A.501, 1FZ1.A.5001, 1FZ7.B.5003, 2G1M.A.600, 3GE3.A.501, 1GGF.B.760, 2GJ1.A.605, 2HOV.A.501, 1I4Z.A.601, 4I4G.A.601, 4I4H.A.601, 2INC.A.502, 1IZO.A.501, 1JNF.A.702, 4KOF.A.601, 4K9T.A.601, 4K9U.A.601, 4K9V.A.601, 4K9W.A.601, 4K9X.A.601, 3KT7.A.701, 1KW9.B.301, 4M73.B.403, 3MZS.C.500, 3N2O.A.506, 3OOR.B.802, 1OCZ.A.516, 1OQU.C.1010, 1P2H.A.801, 3P3N.A.350, 3PCJ.O.600, 2PCC.A.296, 3PER.A.1001, 1PHG.A.417, 3PUQ.A.1, 3Q14.A.502, 3Q3M.A.509, 2QPP.A.300, 3QY6.A.263, 3QY7.A.263, 3RNC.A.499, 3RNF.A.501, 1RYO.A.329, 3TTX.A.760, 1U74.B.1101, 1WRA.B.402, 1XU5.A.1175, 1XVB.A.1170, 1XVF.B.1175, 1Y8W.A.142, 1Z1N.X.602, 2AMF.A.850, 4AMJ.A.1360, 3AR7.A.1000, 4B1M.A.1680, 3B34.A.951, 3BGA.A.6, 3BLJ.B.701, 4BVO.A.1396, 4CCY.A.1297, 4CCY.A.1298, 4D1J.E.602, 4D1J.G.603, 4D7C.A.1544, 3D9R.A.135, 2DDA.A.301, 2DDB.C.302, 4DF9.B.501, 4DW8.A.304, 2E54.A.1004, 4E6P.A.301, 4E6P.D.301, 2E7U.A.1003, 3E85.A.163, 4EEK.A.302, 4EEL.A.302, 3EIF.A.1033, 3EIY.A.177, 3F3C.A.752, 2FV7.A.403, 1G5I.A.901, 4GAF.B.505, 3GIR.A.373, 4GIB.A.301, 2GJU.A.2001, 3GOD.B.327, 4GRX.B.501, 1GV2.A.1191, 3HON.A.204, 3H12.A.500, 3HVI.A.265, 1HX6.A.705, 4IOW.B.603, 3IAN.A.1, 3IC3.C.100, 3ICF.A.515, 4J07.A.201, 4JB3.A.301, 4JDO.A.301, 4JEX.B.511, 2JHJ.B.1296, 1JMM.A.3001, 1JTP.A.501, 1JTP.L.503, 4JTF.C.303, 4JTG.C.302, 4JTH.C.303, 4JTJ.C.302, 1JZN.A.1139, 1K2X.A.801, 1KSU.A.810, 1L5B.A.302, 1LLA.A.631, 1M4Y.A.252, 4M9B.A.201, 4MMF.A.602, 3MYV.A.502, 3NGJ.A.24

9, 3NMB.A.1, 4NPI.A.602, 4O0C.A.401, 2O34.A.501, 4O47.A.401, 1P0Z.A.1633, 4P33.A.401, 4PM0.A.311, 2P0C.A.5001, 2QKF.C.283, 4R6K.A.501, 3RU5.A.133, 1S5D.A.241, 1S5E.A.241, 1S82.A.4, 3SIB.A.223, 3SSB.A.995, 1T3M.A.801, 4TKX.L.705, 1TQY.B.1091, 1UD2.A.1001, 3USL.A.752, 2V4B.A.1566, 3WAY.A.914, 3WC3.A.502, 2WCB.A.101, 4WFX.A.504, 2WGE.A.1426, 1WKY.A.504, 2WOF.A.1728, 2X7J.B.1581, 1Y0P.A.1810, 2Y5F.A.1245, 2YNQ.A.1392, 2ZND.A.196, 2ZQ3.A.160, 3ZWF.B.1365

Table S127. 6-ligand combined metal, compressed group

	size	largest_angle*	middle_1*	middle_2	middle_3
1	"98"	"165.1+/-3.3"	"75.3+/-4.4"	"81.1+/-4"	"84.7+/-3.3"
2	"107"	"174.3+/-3.1"	"82.3+/-3.2"	"85+/-2.6"	"86.9+/-2.1"
3	"77"	"170.1+/-4.5"	"75+/-4.6"	"80.1+/-4.1"	"82.7+/-3.5"
4	"89"	"172.3+/-3.1"	"82+/-2.9"	"84.9+/-2.1"	"86.8+/-1.7"
5	"69"	"168.6+/-5.7"	"60.2+/-5"	"79+/-5"	"83.4+/-4.1"
6	"84"	"170.8+/-3.4"	"69.1+/-3.9"	"74.8+/-3.7"	"79.3+/-4.2"
7	"29"	"145.2+/-7.6"	"63.2+/-5.4"	"70.6+/-6.4"	"75.3+/-6.5"
8	"76"	"156.4+/-5.4"	"53.4+/-3.9"	"70.3+/-5.7"	"76.6+/-5.2"
9	"74"	"157.6+/-4.3"	"53.3+/-4.3"	"72.5+/-4.7"	"76.9+/-4.9"
10	"88"	"168.3+/-4.3"	"52.7+/-4.8"	"73.3+/-6.9"	"78.9+/-4.9"
11	"93"	"161.4+/-3.9"	"71.2+/-4.6"	"76.1+/-3.7"	"79+/-3.3"
12	"49"	"158.8+/-4.7"	"70.2+/-4.8"	"74.9+/-4.3"	"78+/-4.6"

	middle_4	middle_5*	middle_6	middle_7	middle_8
1	"87.8+/-2.8"	"90.2+/-2.5"	"92.2+/-2.6"	"94.8+/-2.7"	"97.4+/-2.5"
2	"88.6+/-1.8"	"90.2+/-1.8"	"91.7+/-1.6"	"93.3+/-1.8"	"95.2+/-2.1"
3	"86.1+/-3"	"87.9+/-2.6"	"89.9+/-2.6"	"92.4+/-2.9"	"95.3+/-3.5"
4	"88.3+/-1.7"	"89.8+/-1.4"	"91.3+/-1.6"	"93.2+/-1.5"	"94.8+/-1.8"
5	"86.8+/-2.6"	"88.9+/-2.2"	"91.2+/-2.5"	"93.7+/-2.8"	"96.7+/-2.9"
6	"82.7+/-4.1"	"85.5+/-3.5"	"89.1+/-3.6"	"92.3+/-3.5"	"96+/-3.6"
7	"79.7+/-6.6"	"83.6+/-6.1"	"90+/-8.6"	"94.6+/-9.7"	"102.3+/-7.3"
8	"80.6+/-5.3"	"84.8+/-5.2"	"88.3+/-5.6"	"92.8+/-5.7"	"99+/-7.2"
9	"79.9+/-4.9"	"83+/-5.4"	"85.6+/-5.7"	"88.3+/-5.7"	"93.1+/-5.6"
10	"82.4+/-4.4"	"85.8+/-4"	"89.3+/-4.1"	"93+/-4.4"	"97.4+/-4.9"
11	"82+/-3.6"	"84.5+/-3.4"	"87.5+/-3.8"	"91.3+/-4.5"	"97.9+/-5.6"
12	"81.3+/-4.4"	"84.1+/-4.6"	"87.3+/-4.4"	"91+/-4.9"	"96.2+/-6"

	middle_9*	middle_10	middle_11	middle_12	middle_13*
1	"100.5+/-2.7"	"104.5+/-4.3"	"113.5+/-8.7"	"147+/-7"	"156.8+/-3.2"
2	"97.2+/-2.4"	"100.3+/-2.7"	"109.2+/-5.5"	"150.6+/-5.7"	"157.5+/-2.9"
3	"98.3+/-4.1"	"103.8+/-5.1"	"124+/-6.5"	"139.8+/-6.3"	"147.6+/-4.1"
4	"97.4+/-2.4"	"101.5+/-3.7"	"110.1+/-7.2"	"150.9+/-8.4"	"166.4+/-3.1"
5	"99.2+/-3.7"	"104.5+/-4.6"	"114.8+/-8.2"	"148.2+/-8.8"	"164+/-5.2"
6	"100.2+/-4.5"	"106.9+/-6"	"119.1+/-7.4"	"138.5+/-11.6"	"161+/-4.9"
7	"111+/-6.7"	"120.1+/-6.5"	"126.7+/-6.2"	"130.7+/-6.4"	"138.4+/-6.2"
8	"111.4+/-6.1"	"120.2+/-7.4"	"127.2+/-8.3"	"142.1+/-6.2"	"147.4+/-4.6"
9	"98.5+/-5.3"	"115.4+/-8.8"	"125.9+/-8.8"	"142.4+/-6.6"	"149.6+/-5.5"
10	"104.9+/-4.9"	"112.6+/-6.1"	"121.8+/-7.5"	"137.8+/-6.9"	"155.6+/-6.5"
11	"105.7+/-5.5"	"114.1+/-7"	"124.2+/-6.6"	"143.2+/-9.4"	"155.9+/-3.1"
12	"103.1+/-6.4"	"114.2+/-7.6"	"126.4+/-6.3"	"136.3+/-7.3"	"144.5+/-5.1"

	smallest_opposite_angle*	Octahedral	TrigonalPrismatic
1	"57+/-3.7"	"0.113"	"0.039"
2	"58.4+/-2.9"	"0.177"	"0.003"

3	"54.3+/-3.9"	"0.043"	"0.042"
4	"59.7+/-3.7"	"0.175"	"0.001"
5	"81.2+/-4.4"	"0.062"	"0.008"
6	"50.4+/-2.9"	"0.054"	"0.047"
7	"54.9+/-6.4"	"0.003"	"0.093"
8	"76.1+/-6.3"	"0.002"	"0.059"
9	"69.2+/-4.8"	"0.003"	"0.022"
10	"72+/-5.7"	"0.015"	"0.03"
11	"50.9+/-3.3"	"0.025"	"0.066"
12	"52.6+/-3.8"	"0.024"	"0.09"

	PentagonalBipyramidalVA	PentagonalBipyramidalVP
1	"0"	"0.166"
2	"0"	"0.11"
3	"0.002"	"0.144"
4	"0"	"0.056"
5	"0"	"0.036"
6	"0.006"	"0.109"
7	"0.027"	"0.033"
8	"0.118"	"0.038"
9	"0.093"	"0.025"
10	"0.003"	"0.048"
11	"0.053"	"0.084"
12	"0.059"	"0.155"

Table S128. Cluster members of 6-ligand combined metal, compressed group

[1] "Cluster 1"
3BON.A.500, 4CWM.A.432, 4CXV.A.432, 3DDA.A.450, 3DFK.A.300, 4ELC.A.501, 4FW5.D.301, 3GBO.A.302, 3GJ9.B.127, 2GSO.A.1001, 4GSZ.A.402, 4GTW.A.1011, 1GW6.A.1615, 2GYQ.A.407, 2IMA.A.500, 4LCF.A.311, 4LCG.A.301, 4LCH.A.301, 3LE9.A.1, 1LOK.A.901, 4MCP.A.801, 4MCQ.A.801, 4MCR.A.801, 4NGM.A.818, 4NGT.A.811, 4OC1.A.814, 4OME.A.815, 3P3E.A.400, 3P3G.A.301, 2PLI.B.707, 4PPZ.A.602, 3PS1.A.301, 3PS2.A.301, 3PS3.A.301, 3QIY.A.431, 3QJ0.A.431, 1QTW.A.302, 2RH6.A.2, 3VPB.A.503, 2ZXC.A.647, 4BDZ.A.1381, 4IEM.C.401, 4IRC.A.402, 3OYJ.A.397, 3S3N.A.397, 3SM4.E.15, 3AU9.A.601, 3BZN.A.501, 1DAW.A.342, 1DAY.A.342, 1EFK.A.604, 3ETH.A.401, 3HWO.A.1702, 4IAC.A.401, 1PYX.A.1003, 4TY0.A.501, 4UOP.A.1612, 1VA6.A.523, 2VPQ.A.1450, 1W7V.A.1441, 3WNZ.A.503, 3WQQ.A.502, 3PR4.A.343, 3BOW.A.717, 4CAG.A.609, 1DAN.L.155, 1DJX.A.2, 4G9L.A.304, 2HIH.A.603, 3K7N.A.703, 2KAY.A.186, 1M56.A.1007, 4MVF.A.604, 2OA8.C.302, 4OKH.A.901, 1OT5.A.601, 2SEC.E.276, 3UNX.A.280, 2Z57.A.1006, 1DLT.A.400, 1DSN.A.400, 1FZ6.B.5003, 1FZ6.B.5004, 4NB8.A.501, 1R2F.A.401, 1SP8.B.500, 1SQI.A.1450, 1TH3.D.2003, 1UZR.A.1293, 3VVA.A.501, 2VZB.A.6204, 4FZX.B.101, 4FZX.A.101, 4FZY.C.101, 4FZY.D.101, 1VQ8.0.9117, 4UZU.A.1491, 1V7T.A.406
[1] "Cluster 2"
3B3C.A.502, 3C88.A.450, 3C8A.A.450, 3C8B.A.450, 1EB6.A.178, 4ICQ.A.502, 3K5X.A.403, 1LFW.A.1002, 3MK1.A.901, 4MTU.A.201, 4NGQ.A.817, 4O5O.A.301, 1P5X.A.246, 2PTY.A.500, 3QM3.A.355, 3QW7.A.501, 3QW8.A.501, 1ROR.A.601, 3S2L.A.403, 2V9L.A.1275, 4WB7.A.503, 3WT4.A.502, 1XVX.A.315, 1ZED.A.903, 2AL1.B.438, 3CFX.A.703, 4DFX.E.404, 1EC8.A.498, 3ETH.A.402, 3ETJ.A.402, 1EYZ.A.401, 1EYZ.A.402, 3GN6.A.321, 3GQ8.A.692, 2HGS.A.501, 3HPF.A.401, 3I4K.A.385, 4IAD.A.401, 3KAL.A.502, 1KJ8.A.393, 1KJ8.A.394, 1KJ9.A.393, 1KJI.A.393, 1KJI.A.394, 1KJJ.A.394, 1LP4.A.341, 3LVV.A.696, 4MDB.A.402, 4NZN.A.403, 4O4D.A.402, 4PU5.A.501, 4Q4C.A.403, 2QVH.A.401, 3T

7A.A.601, 3TDW.A.502, 3TM0.A.265, 3UJR.B.501, 3UJS.A.600, 3UJS.B.600, 3V4S.B.402
, 3VA8.A.425, 3VAT.A.502, 3VC6.A.501, 2VPQ.A.1451, 1W0H.A.1000, 4WB8.A.403, 4WH2
.A.403, 4WH3.A.403, 2XH0.A.1439, 1Z2O.X.1296, 2Z4X.B.1206, 1ANW.A.351, 1GU6.A.14
79, 4JDZ.B.703, 2QT7.B.201, 3RHT.A.257, 2ZUY.A.628, 4AM5.A.1161, 4AQ6.D.837, 1B3
E.A.400, 2CSG.A.504, 3DUQ.M.500, 3FGS.A.402, 3GCF.A.501, 2GYQ.B.404, 4H99.M.402,
4H9L.M.402, 4HBH.M.402, 2J8C.M.1307, 4J00.A.601, 1JQF.A.500, 4M26.D.401, 2OHJ.D
.2501, 3QHB.B.182, 1RVJ.M.857, 1RZH.M.857, 1TKP.A.304, 1TKP.B.303, 1UMX.M.1306,
2UW1.B.1359, 2V27.A.1268, 3VEO.A.602, 3VEZ.A.601, 1VRN.M.500, 2YAV.A.402, 1YUX.A
.302, 3SLZ.B.130
[1] "Cluster 3"
4CVT.A.1160, 2DEA.A.402, 1FT7.A.502, 4FW6.A.301, 4FW7.D.301, 3KR5.B.1004, 1LOK.A
.902, 1MZB.A.201, 1R1I.A.1001, 1R1J.A.1001, 1TXR.A.501, 3U1Y.A.400, 3U79.H.110,
3V94.A.702, 1MOW.A.373, 1MUH.A.479, 4ACF.A.1481, 4EOM.A.302, 2FUV.A.901, 4OHF.B.
503, 2ONS.A.702, 4OVN.A.202, 3WQR.A.502, 2FLD.A.601, 2I3P.A.154, 2I3Q.B.81, 3MIS
.A.2, 3A7Q.A.4003, 1AUI.B.502, 3B1T.A.903, 4CAG.A.608, 3D94.A.2, 1ESV.S.395, 1H5
V.A.305, 2HYW.B.514, 2II1.B.400, 2IWV.B.1281, 2J7A.E.1006, 1K7Q.A.481, 3K7N.A.70
1, 3K7N.A.702, 4KTR.G.806, 4LP7.A.301, 1N2K.A.600, 1NT0.A.3001, 4OMD.D.605, 3OUU
.B.455, 2OXE.A.600, 2Q16.B.200, 2Q17.E.315, 2VY0.A.1298, 3WN6.B.501, 2XVT.C.1137
, 2Z8Z.A.618, 2Z8S.A.646, 4QQZ.A.1002, 3E1M.A.301, 3E1M.C.300, 3E1N.B.300, 1EYS.
M.607, 3IS8.B.162, 1J30.A.401, 1J30.B.404, 1LKO.A.600, 1NF4.A.200, 3PWF.A.201, 3
PWF.A.202, 3PWF.B.201, 3PWF.B.202, 3PZA.A.173, 3PZA.B.173, 4QDF.A.402, 3QVD.H.17
3, 1VZ4.D.1301, 3A6V.A.1005, 3A6V.B.1006, 3HLT.A.269
[1] "Cluster 4"
2C1G.A.1465, 2CB8.B.1090, 2IW0.A.1255, 4NY2.A.501, 2OUN.A.402, 2OUQ.A.402, 4Q3J.
A.401, 2QF7.A.1157, 1SDX.A.677, 3T3W.A.301, 4BE0.A.1381, 4NCB.C.101, 4O3S.A.503,
3OYB.A.397, 3OYD.A.397, 3OYE.A.397, 3OYL.A.397, 3OYN.A.397, 1RVC.A.401, 1YTU.A.
428, 4C5A.A.330, 4C5B.A.1313, 4C5C.A.1313, 3DG6.A.2001, 2FN1.A.504, 4H2H.A.401,
1IOW.A.330, 2IO8.A.7001, 4IZG.A.414, 4J1O.A.401, 1J7L.A.302, 4M3A.A.402, 3NA5.A.
547, 1NFS.A.401, 4OAV.B.803, 3OLP.A.547, 1P43.A.438, 3PFR.A.456, 4PFY.A.602, 2PY
W.A.500, 4QXD.B.301, 3R75.A.700, 3T2D.A.411, 1UMG.A.401, 3LDY.A.145, 2J7A.A.1007
, 2J7A.B.1007, 4P99.D.508, 2ZUX.A.638, 1A8E.A.339, 3AK9.A.168, 3AK9.C.168, 4AQ2.
E.800, 1B0L.A.694, 1C7M.A.101, 2D09.A.430, 1D3K.A.339, 3DU3.M.500, 3EMR.A.400, 1
FQE.A.500, 4G51.A.202, 3GVY.A.162, 1H43.A.1315, 1H44.A.1326, 1H76.A.702, 3HF8.A.
400, 1IEJ.A.333, 4KF1.A.402, 4KVR.A.302, 3MPS.F.173, 3MPS.G.172, 1N7W.A.339, 2OO
C.A.500, 1OQG.A.500, 3Q3N.A.510, 2QED.A.252, 3R0G.A.200, 3R1J.A.299, 4RC8.A.302,
3RI7.A.1, 3RMK.D.495, 1SQY.A.701, 2UW1.A.1360, 3V83.F.703, 1VFE.A.400, 1W69.A.1
350, 4DCC.A.302, 4HUC.A.509, 1HVX.A.519
[1] "Cluster 5"
3BXM.A.1751, 3DFF.A.274, 3E4A.A.2000, 4OP4.A.302, 1QIP.B.901, 1RTQ.A.701, 3SJG.A
.1751, 3WOU.A.201, 1YGD.A.142, 4BE1.A.1382, 4D6N.A.1188, 4E4F.A.504, 3FDG.A.357,
4GME.A.501, 1JCT.A.498, 3K5H.A.401, 3K5H.A.402, 4ORK.A.502, 3Q8U.E.159, 3U2E.B.
4, 2VON.A.601, 4WB8.A.402, 3WO0.A.503, 4K4I.A.606, 1IME.A.278, 2KAY.B.188, 1O9I.
C.269, 2PQY.A.500, 3S5U.C.220, 1TYE.A.1406, 2B1X.E.502, 1BLF.A.700, 1BLF.A.701,
2BOY.G.1255, 2BUR.B.600, 1CE2.A.690, 1CE2.A.691, 4HOW.A.701, 1H76.A.703, 1HAB.A.
200, 1HAC.B.200, 3HGI.A.281, 3IB0.A.999, 1LCT.A.400, 1LFG.A.693, 1LFG.A.694, 1LK
M.A.601, 4M1I.A.402, 1MTY.D.3, 4N71.D.201, 2O7U.A.500, 1OVT.A.689, 3PCC.O.600, 3
PCF.M.600, 3QYT.A.680, 2RDB.A.500, 1S9A.A.300, 1TFD.A.950, 3UF9.A.315, 3VE1.B.70
2, 1VFD.A.400, 3VMG.B.501, 3WFD.B.803, 1YUZ.A.301, 3ZK4.A.800, 4M48.A.702, 3TSH.
A.604, 3WG7.N.605, 2WQK.A.254
[1] "Cluster 6"
4GTW.A.1010, 1SHN.B.481, 3CVJ.C.243, 4DHP.A.303, 2BQR.A.2002, 3EH8.D.303, 3GIM.A
.415, 4NLG.A.401, 2A3W.R.336, 3A7Q.A.4004, 4AQA.A.1210, 1ATN.D.265, 3B2Z.D.3, 1B
AG.A.432, 2BB4.A.260, 1BCJ.2.3, 4BQ3.B.1803, 2BW7.B.2202, 2C10.A.1773, 3CGA.B.10
2, 2DBX.B.701, 4DD8.B.1001, 1DSY.A.501, 2DW2.A.703, 3E9T.B.3, 2FH2.B.2001, 1FZC.

B.2, 4GOD.B.503, 1G7Y.F.254, 3GIN.A.2, 2H0K.A.410, 4IMM.B.402, 1J1N.A.493, 4JX1.A.504, 3K5S.A.218, 3K7L.A.702, 3K7L.A.703, 4KDV.A.202, 1KTW.B.8, 4KTR.D.814, 1KWV.B.604, 1L9N.B.704, 3LPD.A.341, 1M63.F.502, 4N2I.A.706, 1N47.A.252, 1NKQ.F.560, 1NUD.A.701, 3NWK.B.238, 1NX2.A.3, 3OM7.B.1, 2OX9.A.803, 2P37.C.239, 4PKG.A.403, 2PPL.A.482, 3QQZ.A.326, 2RJP.B.3, 3RRD.A.239, 3RTT.A.267, 1S2N.A.1291, 1SL6.A.3, 4TVU.A.600, 4U2A.A.301, 1U94.A.701, 3UBR.B.476, 3USU.A.264, 3V03.A.586, 3VI3.A.2001, 1WBL.D.303, 3WCT.B.203, 1Y3N.A.493, 1YO8.A.1202, 2ZJ7.A.619, 4HR4.A.402, 3JPR.A.340, 3ATU.A.6267, 3B8X.B.668, 1BA0.A.490, 4DD8.B.1005, 4O52.A.301, 1QHU.A.438, 2WCP.A.501, 3WNK.A.813, 3WZ1.A.402

[1] "Cluster 7"

1DK4.A.291, 2OX8.A.4, 3R8B.B.122, 3R8B.D.122, 3R8B.P.122, 2VME.A.501, 2EWG.A.3002, 1GUS.A.1069, 4GWS.A.402, 2J1L.A.1195, 3LD0.5.54, 4NHO.A.1402, 3PLS.A.1, 1CFF.A.150, 3E78.A.601, 1ESP.A.322, 3JQ5.A.201, 1KB0.A.801, 1KV9.A.802, 2ML2.A.205, 3MXW.A.402, 1XJL.B.340, 1ZFS.A.104, 2ZN9.B.901, 4GAM.F.602, 4KLI.A.403, 3MBY.A.340, 3R2H.A.157, 1VK1.A.302

[1] "Cluster 8"

1ANJ.A.450, 4DYO.A.501, 4L3T.A.1101, 3R8B.F.122, 4WD8.C.302, 1XJO.A.901, 2G8H.A.301, 2VBL.C.1026, 2ATX.A.201, 4GMJ.B.302, 1KFS.B.2, 2LVJ.A.101, 3RUW.D.544, 3SH1.A.222, 4UM9.B.2001, 3WQS.B.502, 2G8K.A.401, 1N56.B.403, 1A2X.A.160, 3AAJ.A.991, 1AEI.A.319, 5AER.A.201, 1AJ4.A.163, 1BAG.A.431, 3BOW.A.719, 3BOW.B.403, 1C9U.B.1002, 3CGA.A.102, 3EAD.B.1002, 4ELG.F.202, 2H0K.A.407, 1H4B.A.1085, 1H4B.A.1086, 1IXX.B.124, 2J1G.D.1289, 1JBA.A.501, 4JEO.A.401, 1K96.A.92, 3KF9.A.302, 2LV6.A.204, 2M5E.A.2001, 2ML3.A.205, 4N25.A.706, 1NAE.A.900, 4NDD.B.402, 4NEN.A.1115, 1NIW.A.1001, 1NIW.C.1005, 1NUB.A.301, 1NUB.A.302, 3OX6.A.502, 1PK8.A.817, 3PM8.B.514, 1PTK.A.281, 3QRX.A.170, 2SAS.A.186, 1TCF.A.162, 3TRQ.A.360, 1TTX.A.110, 4UM8.C.2004, 1UZJ.C.3649, 1V1G.A.1211, 2WO9.D.1270, 1YUU.B.199, 2Z2D.A.266, 2Z8S.A.647, 2ZJ7.A.627, 4KVQ.A.302, 2ZI8.A.701, 3C5G.B.808, 2HZG.B.1102, 4J43.A.902, 2JBA.A.1127, 4MPY.C.503, 3USZ.A.902, 3VHS.B.52

[1] "Cluster 9"

4FW3.A.301, 1G12.A.200, 2GO4.B.602, 1H48.A.900, 2OOT.A.1751, 3RBU.A.1751, 1AO0.A.469, 3G15.A.605, 4GYZ.I.402, 2IOA.B.5003, 3MLE.A.222, 3POW.A.471, 4S17.A.501, 2W8D.A.1636, 3WBH.B.503, 3CFR.A.910, 3A7Q.A.4002, 1AFD.3.2, 1AMY.A.502, 1ANW.B.354, 1ATN.D.264, 3CHK.A.502, 2E3X.A.803, 2E6W.A.300, 2E6W.A.301, 3ECQ.A.2000, 2EXI.D.3004, 1FDK.A.124, 4FL4.C.402, 1GQM.A.1089, 4GTW.A.1012, 2HQ8.B.303, 2HYW.A.505, 2I08.A.200, 3IA7.A.402, 3IA7.B.402, 3IGO.A.601, 1IZJ.A.1002, 4JBE.A.502, 1KTW.A.3, 1KX1.A.222, 4LMF.A.304, 2LVK.A.102, 1LWJ.B.10, 1M34.B.2299, 1M8T.C.1003, 1NIW.A.1002, 1ODB.F.1092, 3OX6.A.501, 4P5W.A.1001, 1Q8H.A.72, 1QNI.E.903, 3QRX.A.173, 2RHP.A.1176, 3RV2.A.405, 1THL.A.3233, 1TYE.A.1405, 1UG9.A.2006, 3USU.H.272, 3W9T.A.1005, 3WHU.B.502, 1WMD.A.1001, 3WU2.O.301, 2Z8S.B.642, 2ZBA.A.461, 2ZKT.B.413, 1NNT.A.333, 3OOR.B.803, 1SQD.A.500, 4DMI.A.202, 2HEU.A.4001, 2ID4.A.907, 4IQL.A.407, 3VD5.B.3101

[1] "Cluster 10"

4CBY.A.2034, 2G9Y.B.450, 1K07.A.2, 3Q9B.E.345, 1TXR.A.502, 2A9F.A.801, 3C41.J.603, 3IJQ.B.386, 4KMQ.A.1103, 3MOO.A.551, 3N3T.B.803, 3PMG.A.562, 1POW.B.610, 2PUI.B.401, 3U7F.B.1, 1WC6.C.2202, 2AGO.A.403, 4DTS.A.1002, 1A2X.A.161, 1ANN.A.319, 4CAG.A.605, 1DCY.A.198, 2DDY.A.174, 2DPK.A.2001, 3E3R.B.194, 4E52.A.403, 3E9T.A.1, 3EAD.B.1004, 4ELF.C.201, 2ERO.B.702, 4FGC.B.204, 3FLP.B.301, 3FLT.A.302, 4FL4.C.401, 1GOH.A.290, 4GKX.B.301, 4K1C.A.506, 3K8L.B.800, 1LGC.A.301, 1LHV.A.401, 4LMH.D.811, 4MDV.B.403, 2ML1.A.206, 3NOM.B.263, 1NX2.A.4, 4P99.A.512, 4Q4W.1.905, 2RHP.A.26, 1TEC.E.343, 3TRQ.A.358, 1TYE.A.1407, 1U7W.A.501, 3UJQ.D.305, 2V3T.B.1264, 1V3J.B.690, 2VB6.B.1151, 1W2M.C.1441, 3W9T.C.1009, 1WRL.D.104, 2Z8S.A.644, 2ZUY.A.624, 3DHI.A.601, 4E2P.A.401, 3GJB.A.320, 2HMO.A.450, 4P1B.D.502, 3PCH.M.600, 3QJV.A.801, 3W54.A.502, 3WFC.B.803, 3COW.A.303, 1VQ5.A.9145, 4ATF.A.500, 3BC9.A.704, 3BH4.A.489, 2DIE.A.781, 2GJP.A.1489, 4JQR.A.301, 3KQB.A.303, 3MV1.3.3

101, 1OB0.A.504, 2P3Z.A.501, 3Q2H.A.701, 1V8Z.A.389, 3W5N.A.1211, 1WPC.A.504, 1WX5.C.282, 1XAR.B.201

[1] "Cluster 11"

4DJ4.B.403, 3EZT.B.401, 2JT2.A.336, 4L3T.B.1101, 3A7E.A.215, 4K10.D.404, 4RUB.B.491, 1T5T.A.1005, 1AZO.B.283, 3A9Q.G.214, 3AKA.A.173, 1AVA.A.501, 3BBY.A.215, 2BL0.C.1155, 2CDP.C.1139, 3CGT.A.685, 2CHN.A.1717, 3CK9.A.710, 3D0Y.A.93, 2D3L.A.503, 3E1I.C.501, 2E6V.C.10, 1E8A.B.1090, 3ESR.A.213, 1F9O.A.2000, 3FAX.A.4, 3FLP.B.302, 1FZD.E.1, 1G5N.A.407, 1GCY.A.529, 2GDF.B.302, 3GK2.A.93, 1HFZ.D.124, 3HTL.X.1, 3IJ8.A.497, 4ILW.D.305, 2JBK.A.803, 1JBA.A.500, 2JHL.F.1298, 1JXN.B.246, 2K0F.A.153, 3K5T.A.803, 1K9I.F.903, 3L2Y.A.302, 3LCP.D.158, 1LGN.B.302, 1LN8.A.201, 2LV6.A.203, 4MVF.A.603, 4NDD.B.401, 1NIW.A.1003, 1OBR.A.404, 2OZN.B.401, 4P99.A.501, 4P99.B.509, 2PMY.A.103, 4POS.E.401, 3PVN.B.5003, 3Q3L.A.436, 4Q6P.A.509, 1R1Z.C.410, 2R28.A.1003, 2RHP.A.8, 2SAS.A.188, 1SU3.B.907, 1SVN.A.276, 3VI3.A.2003, 3VI3.A.2004, 4WBQ.B.601, 3WIU.A.1004, 1WMY.B.203, 1Y4D.E.1001, 1Z3U.A.497, 2Z8S.A.648, 2Z8S.B.646, 2ZEY.B.152, 2ZN9.A.902, 2ZWP.A.401, 4BMT.A.1324, 1BPZ.A.341, 3C5G.A.803, 2FMP.A.1340, 2ISO.A.340, 3JPO.A.340, 3JPQ.A.340, 1SA3.A.401, 1AVT.A.301, 2DDA.B.302, 3ED4.A.519, 1QUS.A.400, 3WXO.A.806, 1YQ2.C.7503, 1ZDN.B.157

[1] "Cluster 12"

4CVR.A.1160, 3MN8.A.999, 4AZW.A.1453, 2J0L.A.1688, 4P9D.A.202, 3PCR.B.1231, 4DU3.A.1003, 3ODH.A.196, 1A25.A.290, 1B47.A.351, 2C0O.B.1507, 2D3P.C.241, 1DE4.C.801, 2DIE.A.778, 1DJY.A.2, 2E85.B.1004, 4EJ7.A.403, 4ELG.A.202, 4FDI.A.603, 2FHF.A.2405, 3G5C.B.802, 2GJR.A.1488, 4IAV.A.420, 2LMT.A.149, 4M7Z.B.409, 4MHX.A.601, 4MIV.E.600, 1NHE.A.805, 4NHD.B.403, 2NQA.B.902, 2OO4.A.5003, 1P8J.G.3014, 4PIB.C.204, 3PVN.B.5004, 1ULV.A.2004, 4UM9.C.2002, 1V3D.B.2001, 1VFP.A.995, 2WL3.A.1290, 2YN3.B.6361, 1YUU.A.197, 2Z8S.A.642, 1ZIV.A.1, 4M9L.A.403, 2GA4.A.713, 3GQ9.A.1, 3Q9C.A.344, 2QZ7.A.194, 4WED.A.601

Table S129. 6-ligand combined metal, combined group

	size	largest_angle*	middle_1*	middle_2	middle_3
1	"360"	"165.8+/-5.8"	"70.3+/-6.5"	"76.8+/-4.9"	"80.5+/-4.5"
2	"308"	"172.5+/-3.9"	"80.6+/-4"	"84+/-2.9"	"86.2+/-2.4"
3	"1344"	"174.9+/-2.3"	"81.6+/-2.7"	"84.4+/-2"	"86.3+/-1.7"
4	"644"	"171.9+/-3.3"	"80.2+/-2.9"	"83.2+/-2.5"	"85.2+/-2.1"
5	"1341"	"178.2+/-1.2"	"85.9+/-2"	"87.5+/-1.4"	"88.4+/-1"
6	"316"	"164.5+/-5.6"	"72.4+/-4.4"	"76.4+/-4"	"79.9+/-3.3"
7	"290"	"162.4+/-6.7"	"53+/-4.8"	"71.4+/-7.4"	"77.8+/-5.5"
8	"172"	"152.4+/-8.2"	"60.2+/-7.9"	"69.3+/-7.3"	"74.8+/-6.7"
9	"581"	"170.4+/-3.7"	"72.7+/-5"	"80.2+/-3.3"	"83.6+/-2.7"

	middle_4	middle_5*	middle_6	middle_7	middle_8
1	"84+/-4.2"	"86.8+/-4"	"89.5+/-4"	"92.9+/-4.1"	"96.9+/-4.3"
2	"88+/-2.1"	"89.8+/-1.9"	"91.6+/-1.8"	"93.4+/-1.8"	"95.5+/-2.3"
3	"87.8+/-1.4"	"89.1+/-1.4"	"90.4+/-1.3"	"91.6+/-1.3"	"93.1+/-1.5"
4	"87+/-2"	"88.7+/-1.8"	"90.4+/-1.8"	"92.3+/-2"	"94.1+/-2.2"
5	"89.1+/-0.8"	"89.7+/-0.6"	"90.3+/-0.6"	"90.8+/-0.7"	"91.4+/-0.9"
6	"82.8+/-3.2"	"85.5+/-2.9"	"88.5+/-3.2"	"92+/-3.5"	"96.1+/-3.8"
7	"82.1+/-4.9"	"85.8+/-4.7"	"89.1+/-5.3"	"92.9+/-5.8"	"97.8+/-6.4"
8	"79.2+/-6.8"	"82.9+/-6.3"	"87.4+/-6.9"	"92.2+/-7.9"	"98.5+/-8.4"
9	"86+/-2.4"	"88.2+/-2.2"	"90.4+/-2.2"	"92.7+/-2.3"	"95.3+/-2.5"

	middle_9*	middle_10	middle_11	middle_12	middle_13*

1	"101.7+/-5"	"108.7+/-7.1"	"121.4+/-8.2"	"140.7+/-9.5"	"154.7+/-6.5"
2	"97.8+/-2.7"	"101.3+/-3.4"	"110.7+/-7.8"	"150.5+/-7.3"	"160.7+/-5.4"
3	"94.9+/-1.7"	"97.2+/-2.3"	"100.9+/-3.6"	"166.8+/-4"	"171.5+/-2.4"
4	"96.7+/-2.4"	"100.3+/-3.2"	"107.1+/-6.1"	"159.9+/-5.1"	"166.1+/-3.5"
5	"92.2+/-1.2"	"93.4+/-1.6"	"95.3+/-2.4"	"174+/-3"	"176.8+/-1.7"
6	"102.1+/-5.1"	"109.1+/-6.8"	"122.3+/-9.6"	"146.8+/-6.6"	"153.8+/-5"
7	"104.7+/-6.9"	"114.5+/-8.1"	"123.5+/-8.9"	"140.8+/-7.5"	"152.4+/-6.6"
8	"109+/-8.7"	"118.2+/-8.9"	"126.4+/-7.9"	"134.4+/-7.1"	"141.6+/-6.4"
9	"98.8+/-2.9"	"102.8+/-3.7"	"108.7+/-5.5"	"156.8+/-6.5"	"164.7+/-3.8"

	smallest_opposite_angle*	Octahedral	TrigonalPrismatic
1	"52.6+/-4.4"	"0.022"	"0.037"
2	"59.6+/-4.1"	"0.033"	"0.007"
3	"82.6+/-2.9"	"0.493"	"0.005"
4	"74.8+/-3.7"	"0.258"	"0.026"
5	"86.1+/-2.2"	"0.448"	"0"
6	"72.2+/-4.4"	"0.035"	"0.08"
7	"72.9+/-7.7"	"0.002"	"0.022"
8	"59.3+/-8.9"	"0.003"	"0.065"
9	"80.3+/-3.5"	"0.168"	"0.028"

	PentagonalBipyramidalVA	PentagonalBipyramidalVP
1	"0.02"	"0.095"
2	"0"	"0.038"
3	"0"	"0.012"
4	"0"	"0.062"
5	"0"	"0"
6	"0.007"	"0.137"
7	"0.054"	"0.034"
8	"0.039"	"0.054"
9	"0"	"0.049"

Table S130. Cluster members of 6-ligand combined metal, combined group

[1] "Cluster 1"
3BON.A.500, 3BWI.A.450, 4COQ.B.299, 4CVR.A.1160, 4CVT.A.1160, 4CWM.A.432, 2DEA.A
.402, 4DJ4.B.403, 3EZT.B.401, 4FW5.D.301, 4FW6.A.301, 4FW7.D.301, 3GBO.A.302, 3G
SH.A.101, 2GSO.A.1001, 4GTW.A.1010, 4GTW.A.1011, 1GW6.A.1615, 2IMA.A.500, 2JT2.A
.336, 1KH9.B.450, 1L0Y.D.704, 4L3T.B.1101, 4LCF.A.311, 4LCG.A.301, 4LCH.A.301, 1
LOK.A.901, 1LOK.A.902, 4MCP.A.801, 4MCQ.A.801, 4MCR.A.801, 3MN8.A.999, 4OC1.A.81
4, 4OME.A.815, 3P3E.A.400, 3P3G.A.301, 2PLI.B.707, 4PPZ.A.602, 3PS1.A.301, 3PS2.
A.301, 3PS3.A.301, 3QJ0.A.431, 1QTW.A.302, 1R1I.A.1001, 1R1J.A.1001, 2RH6.A.2, 1
SHN.B.481, 1TXR.A.501, 3U1Y.A.400, 3U79.H.110, 3V94.A.702, 4IEM.C.401, 4IRC.A.40
2, 1MUH.A.479, 3A7E.A.215, 3AU9.A.601, 4AZW.A.1453, 3CVJ.C.243, 4DHP.A.303, 1EFK
.A.604, 4EOM.A.302, 2FUV.A.901, 4IAC.A.401, 2J0L.A.1688, 4K10.D.404, 4OHF.B.503,
 2ONS.A.702, 4OVN.A.202, 3PCR.B.1231, 1POW.B.610, 4RUB.B.491, 3T12.A.198, 1T5T.A
.1005, 1VA6.A.523, 2VPQ.A.1450, 3WQQ.A.502, 3WQR.A.502, 1AZ0.B.283, 3EH8.D.303,
2FLD.A.601, 3GIM.A.415, 2I3P.A.154, 2I3Q.B.81, 4LQ0.A.403, 3MIS.A.2, 4NLG.A.401,
 3PR4.A.343, 1A25.A.290, 2A3W.R.336, 4A6S.C.1122, 3A7Q.A.4003, 3A7Q.A.4004, 3A9Q
.G.214, 3AKA.A.173, 4AQA.A.1210, 1ATN.D.265, 1AUI.B.502, 1AVA.A.501, 1AWB.A.278,
 3B1T.A.903, 3B2Z.D.3, 1B47.A.351, 1BAG.A.432, 2BB4.A.260, 3BBY.A.215, 1BCJ.2.3,
 2BL0.C.1155, 3BOW.A.717, 4BQ3.B.1803, 2BW7.B.2202, 2C10.A.1773, 4CAG.A.608, 4CA
G.A.609, 2CDP.C.1139, 1CFF.A.152, 3CGA.B.102, 3CGT.A.685, 2CHN.A.1717, 3CK9.A.71

0, 1CR8.A.45, 3D0Y.A.93, 2D3L.A.503, 3D94.A.2, 1DAN.L.155, 2DBX.B.701, 1DE4.C.80
1, 1DGL.B.301, 2DIE.A.778, 1DSY.A.501, 3E1I.C.501, 2E6V.C.10, 1E8A.B.1090, 3E9T.
B.3, 4ECG.A.502, 4EJ7.A.403, 1ESV.S.395, 3ESR.A.213, 1F9O.A.2000, 3FAX.A.4, 4FDI
.A.603, 1FHF.A.501, 2FH2.B.2001, 2FHF.A.2405, 3FLP.B.302, 1FZC.B.2, 1FZD.E.1, 4G
0D.B.503, 1G5N.A.407, 1G7Y.F.254, 4G9L.A.304, 1GCY.A.529, 2GDF.B.302, 3GIN.A.2,
3GK2.A.93, 2H0K.A.410, 1HFZ.D.124, 3HGP.A.250, 2HIH.A.603, 2HTV.B.993, 3HTL.X.1,
2HYW.B.514, 2II1.B.400, 3IJ8.A.497, 4ILW.D.305, 4IMM.B.402, 2IWV.B.1281, 1J1N.A
.493, 2J7A.E.1006, 2JBK.A.803, 2JHL.F.1298, 1JXN.B.246, 4JX1.A.504, 2K0F.A.153,
3K5S.A.218, 3K5T.A.803, 4K5W.A.201, 1K7Q.A.481, 3K7L.A.702, 3K7L.A.703, 3K7N.A.7
01, 3K7N.A.702, 3K7N.A.703, 1K9I.F.903, 2KAY.A.186, 4KDV.A.202, 4KTR.D.814, 4KTR
.G.806, 1KWV.B.604, 1KX1.E.501, 3L2Y.A.302, 1L8S.B.314, 1L9N.B.704, 3LCP.D.158,
1LGN.B.302, 1LMJ.A.102, 2LMT.A.149, 1LN8.A.201, 3LPD.A.341, 4LP7.A.301, 2LV6.A.2
03, 1M56.A.1007, 4M5I.A.203, 1M63.F.502, 4MHX.A.601, 4MIV.E.600, 4MVF.A.603, 4MV
F.A.604, 1N2K.A.600, 4N2I.A.706, 1N47.A.252, 4NDD.B.401, 4NHD.B.403, 1NIW.A.1003
, 1NT0.A.3001, 1NUD.A.701, 1NX2.A.3, 2OA8.C.302, 1OBR.A.404, 4OKH.A.901, 3OM7.B.
1, 4OMD.D.605, 2OO4.A.5003, 1OT5.A.601, 3OUU.B.455, 2OX9.A.803, 2OXE.A.600, 2OZN
.B.401, 2P37.C.239, 1P8J.G.3014, 4P99.A.501, 4P99.B.509, 4PIB.C.204, 4PKG.A.403,
2PMY.A.103, 4POS.E.401, 2PPL.A.482, 3PVN.B.5003, 3PVN.B.5004, 2Q16.B.200, 2Q17.
E.315, 3Q3L.A.436, 4Q6P.A.509, 3QQZ.A.326, 1R1Z.C.410, 2R28.A.1003, 2RHP.A.8, 2R
JP.B.3, 3RRD.A.239, 3RTT.A.267, 1S2N.A.1291, 2SEC.E.276, 1SL6.A.3, 1SU3.B.907, 1
SVN.A.276, 4TVU.A.600, 4U2A.A.301, 1U94.A.701, 3UBR.B.476, 4UM9.C.2001, 4UM9.C.2
002, 3UNX.A.280, 3USU.A.264, 3V03.A.586, 1V3D.B.2001, 1VFP.A.995, 3VI3.A.2001, 3
VI3.A.2003, 3VI3.A.2004, 1W0P.A.1780, 1WBL.D.303, 4WBQ.B.601, 3WCT.B.203, 3WIU.A
.1004, 2WL3.A.1290, 1WMY.B.203, 3WN6.B.501, 1XJL.B.342, 2XVT.C.1137, 1Y3N.A.493,
1Y4D.E.1001, 2YN3.B.6361, 1Y08.A.1192, 1Y08.A.1202, 1Z3U.A.497, 2Z8S.A.646, 2Z8
S.A.648, 2Z8S.B.646, 2ZEY.B.152, 2ZN9.A.902, 2ZWP.A.401, 4QQZ.A.1002, 4BMT.A.132
4, 8CAT.A.507, 1DLT.A.400, 1DSN.A.400, 3E1M.A.301, 3E1M.C.300, 3E1N.B.300, 3IS8.
B.162, 1J30.B.404, 4NB8.A.501, 1NF4.A.200, 3PWF.A.201, 3PWF.A.202, 3PWF.B.201, 3
PWF.B.202, 3Q1G.D.1002, 4QDF.A.402, 1SP8.B.500, 1UZR.A.1293, 3VVA.A.501, 1BPZ.A.
341, 3C5G.A.803, 2FMP.A.1340, 4FZX.B.101, 4FZX.A.101, 4FZY.C.101, 4FZY.D.101, 2I
SO.A.340, 3JPO.A.340, 3JPQ.A.340, 3JPR.A.340, 4M9L.A.403, 1SA3.A.401, 3UXP.A.338
, 3A6V.A.1005, 3A6V.B.1006, 3ATU.A.6267, 1AVT.A.301, 3B8X.B.668, 1BA0.A.490, 2DD
A.B.302, 4DD8.B.1005, 3E3T.A.243, 3ED4.A.519, 4FMT.B.301, 2GA4.A.713, 3GQ9.A.1,
3HLT.A.269, 3HSC.A.490, 3HSC.A.491, 4O52.A.301, 1QHU.A.438, 2QSV.A.222, 1QUS.A.4
00, 2QZ7.A.194, 3R0L.D.125, 3TAY.A.1, 1V7T.A.406, 3VDC.A.3103, 2WCP.A.501, 4WED.
A.601, 3WFA.A.801, 3WNK.A.813, 2WPC.A.1491, 3WXO.A.806, 3WZ1.A.402, 1YQ2.C.7503,
1ZDN.B.157, 3ZK2.L.90
[1] "Cluster 2"
3B3C.A.502, 2C1G.A.1465, 3C88.A.450, 3C8A.A.450, 3C8B.A.450, 2CB8.B.1090, 4CXV.A
.432, 3DDA.A.450, 1DE5.A.450, 3DFK.A.300, 1EB6.A.178, 4ELC.A.501, 1FT7.A.502, 4G
SZ.A.402, 2GYQ.A.407, 4ICQ.A.502, 2IWO.A.1255, 3K5X.A.403, 1LCP.A.488, 3LE9.A.1,
1LFW.A.1002, 3MK1.A.901, 4MTU.A.201, 1MZB.A.201, 4NGM.A.818, 4NGQ.A.817, 4NGT.A
.811, 4NY2.A.501, 4O5O.A.301, 2OQL.A.401, 2OUN.A.402, 2OUQ.A.402, 1P5X.A.246, 2P
TY.A.500, 4Q3J.A.401, 3QAY.D.180, 2QF7.A.1157, 3QIY.A.431, 3QM3.A.355, 3QW7.A.50
1, 3QW8.A.501, 1ROR.A.601, 3S2L.A.403, 1SDX.A.677, 3T3W.A.301, 2V9L.A.1275, 3VPB
.A.503, 4WB7.A.503, 3WT4.A.502, 1XVX.A.315, 1ZED.A.903, 2ZXC.A.647, 4AAB.B.1156,
4BDZ.A.1381, 4BEO.A.1381, 1G9Z.D.901, 3MAQ.A.1001, 1MOW.A.373, 4NCB.A.702, 4NCB
.B.702, 4NCB.C.101, 4O3S.A.503, 3OYA.A.397, 3OYB.A.397, 3OYD.A.397, 3OYE.A.397,
3OYJ.A.397, 3OYL.A.397, 3OYN.A.397, 1RVC.A.401, 3S3O.A.397, 3S3N.A.397, 3SM4.E.1
5, 2VBN.E.1026, 1YTU.A.428, 1ZBI.A.302, 1A49.A.534, 4ACF.A.1480, 4ACF.A.1481, 2A
L1.B.438, 3BZN.A.501, 4C5A.A.330, 4C5B.A.1313, 4C5C.A.1313, 3CFX.A.703, 4CWB.A.1
159, 1DAW.A.342, 1DAY.A.341, 1DAY.A.342, 4DFX.E.404, 3DG6.A.2001, 4DWB.A.507, 1E
C8.A.498, 1ELX.B.452, 3ETH.A.401, 3ETH.A.402, 3ETJ.A.402, 1EYZ.A.401, 1EYZ.A.402
, 4FOQ.A.501, 3FLK.A.401, 2FN1.A.504, 3GN6.A.321, 3GQ8.A.692, 4H2H.A.401, 2HGS.A

.501, 3HPF.A.401, 3HWO.A.1702, 3I4K.A.385, 4IAD.A.401, 1IOW.A.330, 2IO8.A.7001, 4IZG.A.414, 4J1O.A.401, 1J7L.A.302, 4J7L.A.402, 3KAL.A.502, 4KI8.E.602, 1KJ8.A.393, 1KJ8.A.394, 1KJ9.A.393, 1KJI.A.393, 1KJI.A.394, 1KJJ.A.394, 3KRO.D.3001, 1LP4.A.341, 3LVV.A.696, 4M3A.A.402, 4MDB.A.402, 3NA5.A.547, 1NFS.A.401, 1NUY.A.2341, 4NZN.A.403, 4O4D.A.402, 4OAV.B.803, 3OLP.A.547, 4ORK.A.501, 1P43.A.438, 3PFR.A.456, 4PFY.A.602, 4PU5.A.501, 2PYW.A.500, 1PYX.A.1003, 2PZA.A.6242, 2Q1A.X.294, 4Q4C.A.403, 4QPM.A.1503, 2QVH.A.401, 4QXD.B.301, 3R75.A.700, 3RLH.A.286, 3T2D.A.411, 3T7A.A.601, 3TDW.A.502, 3TMO.A.265, 4TYO.A.501, 3UJR.A.501, 3UJR.B.501, 3UJS.A.600, 3UJS.B.600, 1UMG.A.401, 4UOP.A.1612, 3V4S.B.402, 3VA8.A.425, 3VAT.A.502, 3VC6.A.501, 2VPQ.A.1451, 1WOH.A.1000, 1W7V.A.1441, 4WB8.A.403, 4WH2.A.403, 4WH3.A.403, 3WNZ.A.503, 2X3J.A.1590, 2XHO.A.1439, 1Z2O.X.1296, 2Z4X.B.1206, 2BQR.A.2002, 3COW.A.301, 3LDY.A.145, 1N3E.D.493, 1N3F.D.499, 1Q9Y.A.939, 3A13.E.445, 1ANW.A.351, 3ASI.A.2001, 4DD8.B.1001, 1DJX.A.2, 2DW2.A.703, 4G60.A.301, 1GU6.A.1479, 1H5V.A.305, 2J7A.A.1007, 2J7A.B.1007, 4JDZ.B.703, 2KAY.B.187, 1KTW.B.8, 1KUO.A.703, 4N20.A.706, 1NKQ.F.560, 3NWK.B.238, 4P99.D.508, 3PGB.A.903, 4PLS.A.301, 2Q1C.X.294, 2QT7.B.201, 3RHT.A.257, 2VCC.A.1917, 2VYO.A.1298, 1WC5.C.2100, 2Z57.A.1006, 2Z8Z.A.618, 2ZJ7.A.619, 2ZUX.A.638, 2ZUY.A.628, 2ZWP.A.402, 1A8E.A.339, 3AK9.A.168, 3AK9.C.168, 4AM5.A.1161, 4AQ2.E.800, 4AQ6.D.837, 1BOL.A.694, 1B3E.A.400, 2CSG.A.504, 1D3K.A.339, 3DU3.M.500, 3DUQ.M.500, 3EMR.A.400, 1EYS.M.607, 3FGS.A.402, 1FQE.A.500, 3GCF.A.501, 3GVY.A.162, 2GYQ.B.404, 1H43.A.1315, 1H44.A.1326, 1H76.A.702, 4H99.M.402, 4H9L.M.402, 4HBH.M.402, 3HF8.A.400, 4HR4.A.402, 1IEJ.A.333, 1J30.A.401, 2J8C.M.1307, 4JOO.A.601, 1JQF.A.500, 4KF1.A.402, 4KVR.A.302, 1LKO.A.600, 4M26.D.401, 3MPS.F.173, 3MPS.G.172, 1N7W.A.339, 2OOC.A.500, 2OHJ.D.2501, 3PZA.A.173, 3PZA.B.173, 2QED.A.252, 3QHB.B.182, 3QVD.H.173, 3ROG.A.200, 3R1J.A.299, 4RC8.A.302, 3RI7.A.1, 3RMK.D.495, 1RVJ.M.857, 1RZH.M.857, 1SQI.A.1450, 1SQY.A.701, 1TH3.D.2003, 1TKP.A.304, 1TKP.B.303, 1UMX.M.1306, 2UW1.A.1360, 2UW1.B.1359, 2V27.A.1268, 3V83.F.703, 3VEO.A.602, 3VEZ.A.601, 1VFE.A.400, 1VRN.M.500, 1VZ4.D.1301, 2VZB.A.6204, 1W69.A.1350, 2YAV.A.402, 1YUX.A.302, 1CZO.C.606, 1JJ2.0.8338, 1VQ8.0.9117, 3AR4.A.1000, 4DCC.A.302, 4DD8.D.1005, 3HON.A.203, 2HIO.B.240, 4HUC.A.509, 1HVX.A.519, 1JZ7.A.3102, 3L7X.A.142, 3MZG.B.211, 4O57.A.301, 3OPK.A.301, 3SLZ.B.130, 4UZU.A.1491, 2VPB.A.1398, 2WDQ.A.1590, 2ZHJ.A.321

[1] "Cluster 3"

4BBP.A.1316, 3DZA.C.501, 3DZA.C.505, 1ENQ.A.238, 2F92.F.1001, 2F94.F.1001, 2F94.F.1003, 2F9K.F.1001, 2F9K.F.1002, 2FUQ.A.1, 4GBD.A.503, 4GQT.A.501, 1GT7.A.275, 2H44.A.501, 3HDZ.A.864, 4IE5.A.601, 3LLX.A.376, 3LX3.A.201, 1M60.A.105, 3NO5.A.275, 4NT9.A.301, 3O2G.A.388, 3O91.A.192, 3O93.A.192, 4OK4.A.800, 2OUV.A.777, 2OU3.A.161, 2OUP.B.777, 4OV9.A.401, 2PTY.A.501, 2PW3.A.501, 4QGE.A.602, 2QYM.A.1, 2QYK.A.1, 2R2V.C.35, 1RRM.B.387, 1T9S.A.1, 1TB5.A.1001, 1TBF.A.1, 3U43.B.135, 1U74.A.1001, 3UUO.B.772, 3V93.A.701, 2WTA.A.1215, 1XM6.A.1001, 1XOR.A.1001, 1Y9Q.A.202, 1ZKL.A.501, 4AQX.D.1525, 4BDY.A.1380, 4BEO.A.1380, 4BE1.A.1381, 4BE2.A.1380, 4CEI.A.2234, 4CEI.B.2162, 4DFJ.A.902, 4DL4.A.501, 4DO9.A.401, 4DOA.A.401, 4DOB.A.401, 2DPI.A.871, 4DQI.A.901, 4DQI.D.901, 3F2B.A.5, 4F5O.A.402, 1FIU.I.2222, 1FIU.A.5555, 2FMS.A.340, 3G6Y.A.871, 3GDX.A.348, 4GZ2.B.402, 3IEV.A.400, 1JJ2.0.8010, 3JPQ.A.339, 3JPR.A.339, 3JPS.A.339, 4JWM.A.403, 3K58.A.1001, 3K59.A.1001, 4K97.A.603, 4KLI.A.401, 4MO4.A.702, 3M8R.A.2, 3M8S.A.2, 4M8O.A.1302, 4M9L.A.404, 3MBY.A.339, 4MDE.A.1002, 4MFC.A.401, 3MR5.A.435, 4O3N.A.503, 4O3O.A.502, 4O3O.A.503, 4O3Q.A.502, 4O3Q.A.503, 4O5K.A.401, 3OHA.A.518, 3OJS.A.7, 3OYB.A.396, 3OYD.A.396, 3OYF.A.396, 3OYG.A.396, 2OZS.A.904, 2PFP.A.750, 2PFN.A.950, 3PML.A.2, 3PNC.A.576, 4PUQ.B.401, 4Q8E.A.502, 4R65.A.402, 4R8U.B.402, 3RJH.A.403, 3RJK.A.339, 4RNN.A.503, 3SI8.A.451, 3SM4.B.227, 3SNN.A.905, 1SUZ.A.402, 3TFR.A.339, 3TFS.A.339, 3TIO.A.1, 3TIO.D.2, 4UAY.A.402, 3V6H.A.402, 1XSN.A.576, 1ZJN.A.339, 121P.A.168, 4A01.A.1767, 4A01.A.1769, 3A1U.A.5, 3A1U.A.6, 1A2B.A.550, 2A5Z.A.701, 1A82.A.901, 3A99.A.401, 4ACI.A.1187, 2AFK.E.1291, 2AGO.A.601, 1ALK.B.452, 2AL1.B.439, 4ANB.A.1384, 1AOR.A.609, 1ASO.A.356, 4AS2.A.1328, 2AUU.A.203, 2AUT.D.604, 4AUX.A

.223, 2B0T.A.800, 3B05.D.1001, 3B1V.A.301, 1B4N.A.623, 3B7L.A.907, 3B7L.A.909, 1B8C.A.308, 1B8J.A.452, 2B82.A.1013, 2BEK.A.501, 2BKU.A.221, 1BOF.A.800, 2BON.A.1302, 2BU2.A.1388, 2BVN.B.1395, 4BW9.A.501, 4BX3.A.301, 1C1Y.A.171, 2C18.A.1338, 2C3U.A.2238, 2C3P.A.2237, 2C42.A.3238, 3C4Z.A.563, 2C4N.A.1251, 3C5H.A.302, 4C5A.B.331, 2C77.A.1407, 2C78.A.1407, 3C9U.B.312, 2CBZ.A.1872, 3CB3.A.501, 3CFX.A.704, 2CFS.A.1296, 3CG4.A.201, 2CHE.A.130, 1CJT.C.403, 1CJU.A.582, 2CJE.A.1267, 3CK5.A.400, 2CLS.A.550, 3CMR.A.453, 3CP6.A.502, 3CP6.A.503, 3CR3.A.1212, 1CTQ.A.168, 3CV2.A.1, 3CX8.A.378, 3CZJ.B.3001, 2D0O.A.1005, 1D2N.A.99, 3D36.A.478, 4D6P.A.1352, 4D7M.A.223, 1D8C.A.3001, 4DBF.A.401, 4DBR.A.810, 2DCN.A.4001, 4DCK.B.201, 3DDH.B.232, 2DDT.A.311, 2DEI.A.402, 4DEM.F.402, 4DEM.F.403, 4DEM.F.404, 4DFD.B.301, 4DN1.A.401, 3DOE.A.193, 4DSN.A.202, 1DTW.A.401, 4DUX.A.3001, 4DWG.A.401, 1DXE.B.901, 4DXJ.A.403, 3DYH.A.3003, 3DYH.A.3004, 3DYM.A.3001, 4DZH.A.501, 3E84.A.701, 3E8M.A.165, 4E8G.A.402, 1E9A.A.401, 2E91.A.1301, 2E95.A.1302, 3EA5.A.221, 4EA0.A.302, 1EBG.A.439, 2EB1.A.502, 2EB5.A.1001, 1EC7.A.498, 3EFQ.B.4004, 3EGT.A.3004, 3EQC.A.3, 2ERX.A.403, 3ET5.A.255, 3ETJ.A.401, 4EUK.A.1001, 2EW1.A.701, 4EX6.A.301, 2EZT.A.1510, 2EZU.A.1610, 2EZ4.A.1610, 2EZ8.A.1510, 2EZ9.A.1510, 2F2A.B.601, 1F8I.A.451, 1F9H.A.162, 2F9M.A.1201, 4F9A.A.602, 3FA5.A.282, 3FD5.A.397, 3FD5.B.397, 3FD6.B.397, 4FE3.A.304, 4FEG.A.707, 3FIU.A.5001, 3FIU.A.5002, 4FI1.A.401, 3FKQ.A.501, 4FK9.A.401, 2FOZ.A.348, 2FOZ.A.349, 2FPR.A.503, 4FP1.A.401, 2FRV.B.540, 3FV9.A.501, 4FVR.A.902, 4FYP.A.301, 3FZN.A.605, 2G09.A.901, 1G17.A.301, 3G2F.A.901, 1G5T.A.998, 2G6B.A.301, 2G80.A.500, 1G97.A.460, 4G9B.A.301, 3GAI.A.189, 2GCP.A.2001, 2GCQ.A.435, 2GGE.A.400, 2GHT.A.257, 2GIL.A.1201, 4GIU.A.402, 2GL5.A.699, 4GME.C.501, 3GON.A.600, 2GO7.A.207, 4GP2.A.401, 4GP2.A.402, 2GQS.A.241, 1GSI.A.1209, 1GUA.A.171, 4H19.A.405, 4H1Z.D.401, 3H3X.Q.553, 2H57.A.202, 3H7V.A.331, 4H81.A.402, 2HCF.A.300, 4HCH.A.405, 4HCL.A.401, 3HDG.A.202, 3HFW.A.361, 4HGN.A.200, 4HGQ.A.201, 4HHL.A.402, 3HIY.B.402, 2HJP.A.292, 2HNE.A.601, 4HNL.A.401, 4HPT.E.402, 1HQ2.A.162, 4HQO.A.301, 3HRZ.A.628, 3HVJ.A.265, 3HW3.A.999, 3HW4.A.999, 3HW5.A.999, 3HXX.A.445, 1I0L.A.902, 2I1Q.A.501, 4I2B.A.604, 2I33.A.602, 4I3Z.A.302, 2I6K.A.302, 4IAD.A.402, 3IBA.A.403, 3ICK.A.401, 3ICK.A.402, 3ICK.A.403, 3ICM.A.402, 4IDP.A.502, 4IEG.A.1001, 1IG5.A.78, 2IHT.A.601, 2IHU.A.601, 1IH8.A.4002, 1IHU.A.592, 4IHC.A.501, 3IIE.A.501, 3IJL.A.386, 3IJR.D.300, 2IK4.A.287, 2IK4.B.287, 2IK4.B.288, 1IOW.A.331, 2IO8.A.7002, 4IP4.A.503, 4IP5.A.502, 1IR3.A.301, 1ITZ.A.1001, 4ITR.D.203, 2IUC.A.1003, 1IV2.A.1571, 1IV4.A.1571, 4IVG.A.803, 4IWH.A.401, 2IXE.A.2, 2IYW.A.202, 2IYN.C.1123, 1IZC.A.1001, 2J7N.B.3374, 1J9J.B.301, 1JBW.A.998, 2JCB.A.1192, 2JD4.A.4061, 2JD4.B.4062, 1JGT.A.902, 2JI7.A.1567, 4JND.A.501, 1JPM.A.1003, 1JSC.A.699, 4JS0.A.202, 1JUY.A.435, 3JVA.A.356, 3JVA.B.358, 4JX0.A.402, 3JYS.A.1, 3JYY.B.302, 1JZ7.A.3001, 3JZ0.A.300, 3JZ0.A.303, 3K1S.H.107, 4K1W.A.501, 3K4Z.A.290, 4K6R.A.505, 3K9L.A.168, 1K9Y.A.402, 4K9Q.A.601, 1KA2.A.501, 3KA3.A.176, 3KAL.A.503, 3KB9.A.701, 3KB9.A.702, 3KC2.A.355, 4KFU.A.307, 4KGD.A.702, 1KHZ.B.301, 1KHZ.B.310, 3KMW.A.501, 3KS6.C.251, 1KTG.A.502, 1KTG.A.503, 1KTG.A.505, 4KUX.A.703, 4KVA.A.501, 3KWS.B.401, 4KWD.A.403, 4KWD.A.404, 4KX5.A.314, 4KXW.A.1001, 1KY2.A.401, 3L12.A.313, 4L2X.F.404, 4L80.A.403, 4L9W.A.202, 4L9Z.A.403, 4LA6.A.501, 2LCF.A.246, 4LFG.A.304, 4LGY.A.1302, 4LJ9.A.902, 1LNY.A.1453, 1LON.A.1454, 3LUF.A.301, 3LV0.A.264, 3LX5.A.301, 4LYK.A.401, 4LZ0.A.403, 4LZ3.A.406, 3M07.A.595, 1M0W.A.502, 4M53.A.527, 1M7B.A.550, 3M7I.A.901, 1MC1.A.601, 1MDL.A.360, 4MFG.A.201, 4MGG.A.404, 1MH1.A.201, 3MHY.A.115, 3MJH.A.201, 3MK2.A.903, 1MMN.A.998, 1MNE.A.998, 4MNE.A.902, 4MPO.B.205, 1MQ4.A.2088, 4MYO.A.301, 3MYK.X.998, 3MYL.X.998, 4MZU.C.404, 1N0H.A.699, 1N0H.B.1699, 3N07.A.200, 1N1Z.A.701, 1N1Z.A.703, 1N20.A.701, 1N20.A.702, 1N24.A.702, 3N2N.A.1, 3N45.F.355, 3N45.F.3, 3N4F.A.502, 1N6I.A.201, 1N6O.A.201, 1N8I.A.900, 4NDO.A.302, 4NFI.F.402, 4NFI.F.403, 4NFI.F.404, 1NFZ.A.401, 3NJL.A.501, 3NKV.A.500, 3NOJ.A.239, 3NRJ.A.190, 1NSF.A.859, 1NSY.A.6241, 4NWI.A.401, 2NXW.A.4002, 2O1S.A.1001, 3O61.B.202, 1O6Y.A.1280, 3O6Z.B.201, 2O7O.A.223, 4O7I.A.401, 4OAV.B.802, 3OCU.A.263, 3OCV.A.264, 3OCW.A.263, 3OCX.A.264, 3OCZ.A.263, 2OCB.A.202, 4OCP.A.403, 2ODP.A.901,

2ODB.A.205, 2OEM.A.911, 2OEM.B.912, 2OFX.A.301, 2OGD.A.3003, 2OGD.A.3004, 4OHY.A.502, 1OIW.A.1175, 3OIW.A.170, 4OI4.A.502, 1OKK.D.1002, 4OKM.A.901, 4OKM.A.903, 4OKZ.A.903, 3OM2.A.486, 3OP2.B.500, 2ORW.A.501, 3OUZ.B.459, 3OZF.A.235, 3OZX.A.614, 4P31.A.402, 2P3N.A.1758, 3P41.A.298, 3P5R.A.901, 3P93.C.406, 2PA4.A.325, 3PDE.A.312, 1PFK.A.327, 3PFF.A.831, 4PFY.B.601, 2PGN.A.610, 1PHP.A.395, 4PHG.A.201, 4PHH.A.202, 3PK7.A.406, 2PK0.A.502, 2PKE.B.300, 3PNL.B.1212, 1POX.A.610, 1PPV.A.401, 2PS2.A.401, 2PS5.B.701, 1PVG.A.903, 2PYW.A.501, 2PZ8.A.4001, 2PZA.A.6243, 3Q1O.A.400, 2Q28.A.1001, 1Q3H.C.674, 2Q3F.A.301, 4Q4C.A.404, 3Q5V.B.599, 2Q5Q.A.4002, 1Q6L.A.5300, 1Q6R.A.7300, 2Q66.A.602, 3Q85.A.284, 3Q9L.A.700, 4QC2.A.302, 4QEA.A.301, 3QF7.A.854, 2QGY.A.701, 2QG8.A.201, 2QIS.A.907, 2QJJ.C.1003, 3QKT.A.902, 2QME.A.179, 3QN3.A.601, 2QQ0.A.450, 2QQ0.B.452, 1QRA.A.168, 2QRZ.A.190, 2QTY.A.348, 2QTY.A.349, 2QTC.A.888, 3QUQ.A.225, 3QUT.A.225, 3QVQ.C.310, 3QXC.A.222, 3QXH.A.223, 3QXJ.A.224, 3QXS.A.223, 3QXX.A.224, 2QX0.A.161, 3QYY.A.505, 1ROX.A.13, 3ROU.A.380, 3R1M.A.404, 4R39.A.401, 2RAH.A.354, 2RAR.A.501, 2RAV.A.701, 3RBM.A.1001, 3RBM.A.1002, 2RB5.A.701, 2RBK.A.501, 3REF.A.192, 3REG.A.550, 1RKQ.A.1273, 1RKU.A.301, 1RKV.A.401, 4RKE.A.202, 3RO6.A.400, 1RP7.A.890, 1RQI.A.603, 1RQI.A.604, 1RQJ.A.908, 1RQJ.A.909, 3RUS.A.544, 1RVK.A.999, 3RWM.B.1, 3RYE.A.907, 3RYE.A.909, 1RYA.A.1001, 1RYH.A.539, 4S1H.B.303, 3S4J.A.907, 3S4J.A.909, 3S9Z.A.802, 1SAW.A.225, 3SDT.A.819, 3SDT.A.821, 3SEA.B.178, 3SF0.A.263, 3SHQ.A.321, 3SJN.A.374, 3SLS.A.401, 3SN1.A.408, 3SN4.A.408, 1SO4.A.2300, 3STP.A.391, 1SVS.A.356, 3T1Q.A.198, 3T2D.A.408, 3T2D.A.409, 3T2E.A.409, 1T8Q.B.1602, 1T9B.B.699, 1T9C.B.699, 2TCT.A.223, 3TCS.A.368, 3TDV.A.501, 1TE6.A.641, 3TEP.A.1, 3TJI.A.601, 3TKL.A.300, 1TMM.A.162, 4TMT.A.902, 4TMW.A.903, 4TMX.A.903, 3TM0.A.266, 4TN1.A.902, 4TSK.A.403, 1TW1.A.1, 3TWA.A.420, 3TYZ.A.281, 4TY0.A.502, 1TZZ.A.3501, 1U02.A.240, 3U2E.B.1, 4U5X.A.202, 4UAK.A.503, 4UAS.A.302, 4UAT.A.302, 4UAV.A.401, 3UCW.A.100, 3UCY.A.101, 3UIE.A.403, 1UMD.A.1401, 1UMG.A.403, 1UMG.A.404, 4UM7.A.175, 4UMF.A.1175, 3UPY.A.446, 1UPT.A.1183, 4USI.A.1151, 3UXK.A.360, 3UZR.A.300, 3V1V.A.501, 3V1X.A.501, 4V1T.A.1777, 3V2U.C.521, 2V26.A.1801, 3V3W.A.403, 3VC1.A.301, 3VC2.J.301, 3VCC.A.402, 3VCN.A.501, 2VDO.B.2001, 2VDL.B.2001, 2VDN.B.2001, 2VK1.A.601, 2VK4.A.601, 3VPB.A.502, 3VR6.B.602, 3VVH.A.701, 3VX4.A.802, 1W2Y.A.1231, 1W2Y.A.1232, 1W6T.A.435, 3W6N.A.803, 3W6O.A.802, 1W7K.A.1423, 1WA5.A.1178, 3WBH.A.501, 3WBZ.A.404, 1WC1.A.1501, 1WC1.A.1502, 3WEK.A.401, 2WEF.A.401, 1WF3.A.401, 4WF7.A.601, 4WH2.A.402, 4WH3.A.402, 2WIC.A.1266, 3WJP.A.403, 3WNZ.A.502, 3WRY.C.1202, 2WVG.A.601, 2WW8.A.1000, 3WXM.A.502, 1WZC.A.300, 1X07.A.900, 1X84.B.401, 2XB5.A.223, 2XCW.A.1498, 1XEX.A.1002, 2XH2.B.1439, 2XH7.A.1441, 2XIS.A.392, 4XIA.A.399, 2XJB.A.1494, 2XJC.A.1499, 2XJD.A.1497, 2XJE.A.1493, 2XSX.A.500, 2XTZ.A.1381, 2XUU.A.1307, 1XX1.A.9001, 4XXP.A.301, 2Y6P.A.1234, 1Y8A.A.501, 1Y8Q.B.641, 1Y9D.D.2901, 2YCH.A.501, 1YHL.A.1401, 1YHL.A.1402, 1YIO.A.212, 1YNS.A.1258, 1YQ9.H.540, 1YQT.A.591, 1YU4.A.2002, 2YVO.A.1001, 2YVO.A.1002, 2YVP.A.183, 2YVP.A.184, 2YVM.A.1001, 2YXH.A.502, 1YYQ.B.702, 1YZN.A.301, 1Z06.A.203, 1Z07.A.300, 1Z08.C.3300, 1Z2O.X.1295, 1Z4L.A.2001, 1Z4O.A.800, 1Z4P.X.1001, 1Z4Q.A.2001, 2Z4V.A.1501, 2Z4X.A.1201, 2Z4X.A.1202, 1Z59.A.1001, 2Z52.A.1301, 2Z7I.A.1301, 1ZC3.A.500, 2ZCR.A.669, 1ZED.A.905, 1ZJJ.A.1001, 2ZKJ.A.500, 3ZMC.A.1296, 3ZO9.A.1592, 3ZOU.A.1295, 1ZPD.A.601, 2ZPU.A.360, 1ZS9.A.1257, 3ZVL.A.1524, 3ZX4.B.260, 3ZX5.A.260, 1ZXN.B.902, 3DPG.B.501, 4ECQ.A.501, 4F4W.A.402, 4FJ8.A.1002, 4FJM.A.1002, 4K4H.A.605, 4K4I.E.602, 1N3F.C.498, 2OAA.A.601, 2ODI.A.701, 2Q10.A.701, 4QWB.A.402, 2AEP.A.601, 3AFG.A.540, 3AHW.A.122, 3AI7.B.901, 1AJJ.A.73, 3AJ7.A.602, 3AKB.A.2, 4APX.B.1239, 3AUK.A.391, 4AXN.A.1329, 3AYU.A.418, 3B7E.A.1005, 1B8L.A.110, 3B8Z.A.904, 3BCD.A.707, 2BL0.B.1146, 4BNR.A.600, 2BQ4.A.1119, 2C10.A.1771, 3C9I.A.1, 3CKZ.A.1, 4CPL.A.500, 4CPY.A.1466, 4CU9.A.2999, 4CUA.A.2644, 1CVL.A.320, 2CYY.A.2002, 3D3I.A.1001, 3D7K.A.571, 2DDR.A.1324, 1DPO.A.246, 4E5U.B.302, 2EA7.A.450, 2EEK.A.401, 1EGZ.A.300, 1EX9.A.286, 3F5V.A.223, 2FCW.B.3001, 2FCW.B.3002, 3FG1.A.1501, 2FHF.A.2404, 3FSJ.X.600, 3FVQ.A.360, 2FWN.A.532, 1G9K.A.703, 1G9K.A.706, 1GA6.A.374, 4GDI.A.509, 4GG1.A.602, 3GK2.A.92, 4GN7.A.301, 1GTT.A.1430, 2HOB.A.

1000, 4H1Q.A.303, 1H71.P.501, 3H81.C.279, 3H81.C.280, 3HB2.P.482, 3HGN.A.250, 3HJR.A.603, 4HJF.A.601, 4HS9.A.401, 1HT6.A.502, 1HY7.A.305, 4HZW.A.507, 4I35.A.513, 1I76.A.996, 2I8U.A.202, 3I9G.L.301, 3I9G.L.302, 3IBZ.A.192, 4IHM.A.404, 1IOD.G.503, 4IU2.A.301, 4IU3.A.301, 2IXT.A.1311, 1J8E.A.201, 1JK3.A.403, 2JKP.A.1728, 4JZB.A.401, 4JZB.A.403, 4JZX.A.404, 4JZX.A.405, 4K1K.A.501, 3K37.B.467, 1K7I.A.487, 4K70.A.1002, 1KAP.P.616, 1KAP.P.618, 1KAP.P.619, 3LNI.A.303, 4LVN.A.703, 4M5I.A.201, 3M6L.A.801, 3MA2.A.293, 3MA2.A.296, 4MEW.A.502, 2ML1.A.202, 1MNC.A.283, 3MOS.A.1, 4MPR.A.601, 4MWL.A.512, 4MWV.A.512, 3N1U.A.200, 4N2E.A.705, 4N2P.A.201, 4N4E.E.405, 1N9E.A.802, 3NKQ.A.1003, 1NPC.A.320, 2O72.A.403, 1OAC.A.802, 4OCI.A.201, 4OKH.B.904, 3OOY.A.621, 1OU9.A.131, 3OYR.B.337, 3OYR.B.338, 3P2P.B.126, 2P3U.B.501, 3P4G.A.401, 2PHI.B.125, 4PMX.A.401, 2PNY.A.228, 1Q3A.A.466, 3Q4W.A.224, 4Q8K.A.501, 1QCN.A.1001, 4QN6.A.501, 3QRB.A.301, 3QU7.A.230, 3QU7.B.225, 2QUB.A.614, 2QUB.A.615, 2R1B.A.1001, 2R5N.A.2000, 1R6V.A.1, 2R8O.A.670, 2R8Z.A.201, 3R8Y.A.242, 1RQ5.A.819, 3RRX.A.901, 3RUP.A.1004, 3RVV.A.225, 3RVW.A.223, 3S4Y.B.1303, 1SAT.A.476, 3SAL.A.601, 1SIO.A.601, 1T1E.A.700, 3TEW.A.800, 3TI4.A.601, 1TRK.A.681, 3U1R.A.703, 3U8D.A.203, 3U8I.A.201, 3UPT.A.691, 1UR4.A.1398, 4USU.A.1471, 1UX6.B.2002, 1UX6.B.2004, 1UX6.B.2010, 1UX6.B.2012, 1UX6.B.2016, 4UZU.A.1484, 3V5U.A.705, 1VCL.A.1004, 1VL9.A.125, 2VOV.A.1338, 3VV3.A.401, 3VV3.B.404, 1W7C.A.802, 3W7T.A.1001, 1WAD.A.116, 4WIW.A.702, 4WKO.B.502, 4WKO.B.503, 4WK7.A.504, 1WMD.A.1003, 2WNV.B.1225, 2WOY.A.2414, 1WZA.A.601, 2Y6D.A.1266, 1Y93.A.266, 2YAY.A.1267, 2YGL.A.1413, 2YN3.A.6362, 2YN5.A.6362, 2YN5.A.6363, 1YO8.A.1191, 2YOA.A.1003, 1YS1.X.400, 1YS6.A.1001, 2Z2X.A.1004, 2Z30.A.1006, 1Z6O.A.5302, 2Z8X.A.626, 2ZUX.A.630, 2ZUY.A.626, 3ZXH.A.304, 3A0G.A.201, 3A16.B.354, 3A17.A.354, 2A1O.A.417, 1A2S.A.90, 2A3F.X.201, 3A51.B.412, 3ABM.A.516, 1ASH.A.301, 4AUM.A.900, 2AVK.A.201, 3AYF.A.801, 3B0H.B.601, 2B11.D.1301, 2B2O.B.1500, 2B2R.A.1500, 4B2N.A.701, 4B8N.A.201, 2BC5.A.150, 3BDZ.A.450, 1BEP.A.296, 1BIN.B.144, 1BJ9.A.296, 2BMM.A.1157, 3BOM.A.143, 3BOM.B.148, 2BUZ.B.1541, 2C1D.A.1291, 1C2N.A.117, 1CC5.A.1, 1CED.A.90, 1CH3.A.154, 2CJ2.A.1300, 4CK9.A.1480, 4CKA.A.1480, 1CLS.A.142, 2CVC.A.1013, 4CVJ.A.1295, 2CYP.A.295, 2CZ1.A.300, 4D02.A.602, 4D02.A.603, 2D2M.D.200, 4D38.A.500, 1D4D.A.602, 1DCC.A.296, 1DD7.A.600, 3DHI.A.600, 1DLW.A.144, 1DM1.A.148, 4DTW.A.500, 4DTY.A.500, 3E2O.A.296, 2E3B.A.401, 2E84.A.1303, 3EH5.A.800, 3EHB.A.560, 1EHE.A.501, 4ENH.A.601, 4ENU.B.801, 1EOC.B.600, 1F4U.A.410, 4F4O.B.201, 2FAM.A.148, 4FIA.A.600, 4G71.A.602, 4G7G.A.501, 4G7Q.A.602, 2GEP.A.580, 3GE3.A.501, 3GEO.A.580, 7GEP.A.580, 2GJ1.A.605, 4GP8.A.602, 2GSM.A.2002, 1GWS.A.601, 1GWS.A.605, 1GWS.A.609, 1GWS.A.610, 1GWS.A.612, 1GWS.A.613, 1GWS.A.616, 1GWT.A.350, 2H0V.B.500, 1H29.A.1108, 4H44.A.301, 3HB3.A.559, 3HB3.A.560, 1HBZ.A.504, 2HBD.A.142, 1HMO.B.115, 3HQ7.A.401, 1HRO.A.107, 4HRO.A.402, 2HUO.A.302, 3I63.A.501, 3I63.A.502, 4I7Z.A.301, 4I7Z.C.301, 2IBN.A.703, 2IJ4.A.471, 2INC.A.502, 1IO7.A.1401, 4IPS.A.401, 1IQC.A.401, 2IUW.A.500, 1IZO.A.501, 4J14.A.601, 1J77.A.300, 2J7A.A.1002, 2J7A.C.1002, 1JEX.A.95, 1JIP.A.410, 2JJN.A.412, 2JTI.B.104, 3K1O.A.488, 2K3V.A.238, 2K3V.A.261, 3K3O.A.1, 4K8F.A.300, 4KIB.A.403, 4KIG.A.502, 4KJT.A.201, 1KQG.C.810, 2KSC.A.125, 4L0F.A.501, 3L1T.A.479, 3L4D.A.481, 1LA6.B.147, 2LDO.A.130, 2LDO.A.168, 1LFK.A.430, 4LMH.A.802, 4LXJ.A.601, 2LZZ.A.101, 4M4A.B.201, 1M7S.A.600, 4M71.A.403, 4M73.B.403, 4MBA.A.148, 5MBA.A.148, 3MDR.A.505, 4ME4.A.401, 3MGX.A.397, 3MKB.B.137, 1MLW.A.403, 4MLN.B.201, 4MLN.B.202, 1MRP.A.310, 1MTY.D.4, 3N3N.B.1500, 4N4N.A.608, 4N4Y.A.602, 4N6W.A.202, 1N9C.A.93, 3N9Q.A.1, 3NA1.A.601, 2NNB.A.472, 1NPF.A.154, 2NSR.A.154, 1NS9.A.142, 3NYH.A.605, 4NZ2.A.501, 4O6J.A.302, 3O72.A.500, 1OAF.A.1251, 1OAH.A.1520, 1OAH.A.1522, 1OAH.A.1523, 1OCZ.A.516, 3OJT.D.500, 1OQU.C.1008, 3OUI.A.1, 2OZY.A.201, 1P2H.A.801, 1P2H.A.804, 3PAH.A.425, 4PAH.A.425, 5PAH.A.425, 2PCC.A.296, 3PER.A.1002, 3PF7.A.1001, 3PF7.A.1002, 4PG0.A.301, 4PH9.A.602, 3PT7.B.500, 3PUQ.A.1, 3PUR.A.1, 3Q14.A.502, 3Q3N.A.509, 1Q5D.A.440, 2QDY.A.300, 1QO8.A.601, 1QO8.A.602, 1QO8.A.604, 3QPI.B.1001, 1QPU.A.107, 2QUO.A.142, 3QY7.A.264, 2R6S.A.501, 3RI7.A.494, 3RIV.A.305, 3RMK.A.494, 3RMZ.A.500, 1RYO.A.329, 4S1B.A.802, 1S73.A.296, 3S8F.A.801, 3SDN.A.160, 1S

MI.A.472, 1SP3.A.807, 1SQ3.D.912, 1STQ.A.600, 3TK3.A.500, 3TMC.A.310, 1TQN.A.508
, 4TUV.A.401, 4UAX.A.501, 3UBR.A.473, 1UED.A.1430, 1ULI.B.700, 4V3Z.B.750, 3V5X.
A.201, 2V7I.A.1362, 2VHD.B.401, 3VTH.A.807, 2VZW.B.1209, 1W0G.A.1501, 1W4W.A.130
7, 3WFC.B.802, 2WM5.A.450, 4WNV.A.601, 2WU2.C.1130, 2WU5.C.305, 4WWJ.B.301, 2X2N
.A.1479, 4X8B.A.508, 2XC3.A.1433, 1XU5.A.1174, 1XVB.A.1171, 1XVG.A.528, 1XVX.A.3
13, 2Y69.A.516, 1Y8W.A.142, 2YIU.B.500, 1YMA.A.154, 1YYG.A.396, 1Z1N.X.602, 1Z1N
.X.603, 1Z1N.X.605, 1Z1N.X.606, 1Z1N.X.613, 1Z8U.B.201, 2ZCF.A.206, 3ZG3.A.490,
1ZOY.C.1305, 3ZPI.A.1407, 4EJY.A.301, 1EWN.A.501, 3IOW.A.296, 4KLI.A.405, 1ORN.A
.224, 3OSN.A.423, 4P4M.A.403, 2A5F.B.1326, 3AGC.A.1, 3AJN.A.136, 4AK1.A.1702, 2A
U7.A.206, 3BGA.A.6, 4C1O.A.1731, 3C7F.A.804, 4CCY.A.1298, 4CIT.A.1454, 3D9R.A.13
5, 2DDA.A.301, 2E7U.A.1003, 3ELF.A.351, 3GED.A.251, 3GIR.A.373, 4GRX.B.501, 2GTW
.B.3005, 1GV5.A.1142, 4H83.A.401, 4HUR.A.316, 4I29.A.601, 3IC3.C.100, 3ICF.A.515
, 4IIB.A.944, 3IJP.B.301, 3IMM.B.2, 4IQZ.A.316, 3IRS.A.290, 2JHJ.B.1296, 1JZ7.A.
3104, 4KA7.A.805, 3KRS.A.301, 4LG8.A.601, 4LIZ.A.202, 1M4Y.A.252, 3M9Y.B.254, 4M
9B.A.201, 4MMB.A.602, 4MMF.A.602, 4NAW.B.303, 3NNB.A.401, 4NSJ.A.222, 3OLJ.A.1,
2OPL.A.187, 4P33.A.401, 4PCG.C.304, 3PG0.A.147, 1PYF.A.315, 3PZR.A.373, 3PZS.A.2
87, 4Q5K.A.302, 1QNJ.A.280, 3QXT.A.133, 3R0L.D.124, 4R3N.B.401, 2RGI.B.98, 1S5D.
A.241, 3SJL.A.402, 4TKX.L.706, 4U99.A.203, 3UWP.A.424, 2V3U.A.1263, 2V79.A.1116,
 1VMF.A.134, 1W15.A.2002, 3W5N.A.1210, 3WAY.A.914, 3WC3.A.502, 3WNO.A.802, 2WW2.
A.800, 4XCZ.A.405, 4XCZ.A.406, 1XKN.A.700, 1Y7W.B.282, 2ZQ3.A.160, 3ZX3.C.522
[1] "Cluster 4"
3BLB.A.1047, 1BLL.E.489, 3D4Z.A.1046, 3D52.A.1046, 3D51.A.1046, 3DDF.A.3001, 2DD
F.A.1, 3DX1.A.1048, 3DX3.A.1047, 3DX4.A.1047, 3E38.A.2, 2F18.A.1805, 2F1A.A.1805
, 2F1B.A.1804, 2F7P.A.5001, 2F7R.A.5001, 3GWT.A.504, 3IBM.A.200, 3ISI.X.3001, 3I
TU.A.1, 3IVT.A.500, 1KAE.A.1101, 3KMC.B.2, 3KME.B.2, 3KR5.A.1001, 1KRM.A.501, 3L
GP.A.1, 4NPW.A.1001, 4NUR.A.701, 3O0J.A.1, 3O64.B.485, 4OJV.A.403, 4OJV.A.404, 4
OJX.A.403, 2P18.A.301, 1PTM.B.331, 2PTZ.A.500, 2PU1.A.500, 1R33.A.1163, 1R55.A.2
01, 4R6T.B.1003, 4R7M.C.1003, 3RCQ.A.1, 3SL3.B.9, 1SNN.A.402, 1SR9.B.703, 4AQX.D
.1526, 4BE2.A.1381, 4C2U.A.1666, 1CW0.N.202, 1CW0.A.203, 4D6O.D.1196, 4D6O.D.119
7, 4DLG.A.902, 2EZ6.A.501, 1G9Z.C.902, 1G9Z.F.903, 3GDX.A.347, 3GPL.A.800, 2ISO.
A.339, 2ISP.A.339, 3JPN.A.339, 3JPT.A.339, 4K99.A.602, 4LOX.A.401, 4M30.A.501, 1
MOW.D.374, 4NCB.B.703, 3OYC.A.396, 3OYH.A.396, 4PQU.A.602, 3RJF.A.340, 3RJK.A.34
0, 3S3M.A.397, 1SKR.A.4001, 1T7P.A.4001, 3TFS.A.340, 1TK0.A.991, 3TWH.A.401, 4UB
3.A.401, 4ABZ.A.1210, 4AC0.A.1205, 2AE8.C.1009, 1AJB.A.452, 3AXK.A.478, 3BGA.A.1
, 2BJI.A.2277, 3BM4.B.304, 3BRB.A.10, 3BWY.A.300, 1BZY.A.901, 2C31.A.1553, 4C5C.
A.1314, 4C7X.A.700, 4CE0.A.1251, 1CG1.A.435, 1CG4.A.435, 1CIB.A.434, 2CJE.A.1268
, 2CL5.A.1216, 3CRR.A.324, 3CT2.A.401, 3CWH.A.392, 4CW7.A.1002, 3CXO.A.500, 3CXO
.B.500, 4CYM.A.1199, 3D46.A.501, 3D47.A.501, 3DFY.A.401, 2DGN.A.1454, 4DH5.A.402
, 4DN1.B.401, 3DUF.A.1368, 2DUA.A.292, 2DW6.A.2001, 4DXJ.A.401, 3DYH.B.4002, 3DY
S.A.902, 1E4E.A.365, 2E8W.A.1202, 2E91.A.1302, 4EA0.A.301, 4EA0.A.303, 1EBH.A.43
8, 3EGT.A.3002, 3EGT.A.3003, 3EKG.A.601, 1ELZ.A.452, 3ES8.A.393, 1EXM.A.407, 3EZ
3.B.1102, 3EZ3.B.1104, 1F2U.A.902, 4F71.A.301, 3FD5.A.396, 3FE4.B.902, 4FFL.A.90
6, 4FFR.A.403, 3FLK.A.405, 3FPA.C.901, 3FPB.A.1000, 3FTQ.A.371, 3FYY.A.402, 1G4P
.A.2003, 3G5A.B.307, 2G9Z.B.701, 1GAG.A.201, 4GIS.A.405, 2GT4.B.401, 4GT3.A.403,
 3GY1.A.500, 4GYI.A.402, 2HCJ.A.998, 4HE1.A.403, 2HGS.A.502, 3HJN.A.501, 4HNC.A.
401, 3HPF.A.402, 3HQP.B.502, 3HVH.A.265, 3HVI.A.1, 3HVK.A.1, 2HXU.A.601, 4I3Y.A.
304, 1I6I.A.501, 3IBA.A.401, 3ICZ.A.402, 4IFW.A.502, 1IGW.A.441, 1II9.A.593, 4IJ
Q.A.304, 2IK2.B.289, 2IK7.A.287, 4IT1.A.501, 2IUC.B.1008, 1IV4.A.1572, 1J34.C.50
1, 1J7L.A.301, 1JP4.A.701, 1JP4.A.702, 3JUK.C.307, 3JVT.B.502, 4K33.A.802, 1K9Y.
A.401, 4KCT.A.1001, 4KCU.A.1001, 3KDN.A.500, 3KEU.A.400, 3KHQ.A.1, 1KKR.A.501, 4
KMQ.A.1102, 4KQX.A.405, 4KQX.A.406, 4KUX.A.701, 3L0Y.A.257, 1L8A.A.888, 4L9Y.B.4
03, 3M0O.A.550, 1M1B.B.999, 4M6U.A.401, 1MEZ.A.1453, 1MMA.A.998, 1MNS.A.360, 4MP
O.A.206, 4MPO.C.205, 1MXO.A.501, 1N1Z.A.702, 1N24.A.701, 3N3T.A.802, 1NUX.A.2342
, 3NZG.A.507, 4NZO.A.404, 2O1O.A.503, 4O4D.A.401, 3OE5.A.222, 1OIX.A.301, 4OKM.A

.902, 4OKZ.A.902, 3OP2.A.500, 3OPS.A.501, 1ORK.A.223, 2OUN.A.403, 1OW2.A.401, 2O X4.A.402, 1OZH.A.1405, 3OZM.D.390, 3OZY.B.390, 3P3B.A.393, 3P41.A.297, 3P93.A.40 6, 4PAL.A.110, 2PLS.H.602, 2PMQ.A.902, 1PT6.A.500, 1PUN.A.130, 3PUV.A.1501, 4PU5 .A.502, 1PYD.A.559, 1PYM.A.1003, 2Q1D.X.294, 4Q1V.A.803, 3Q30.A.600, 3Q30.A.601, 3Q46.A.306, 1Q6O.A.7300, 1Q6Q.A.7300, 1Q9S.A.201, 4QE5.A.401, 1QF4.A.433, 3QKE. A.407, 1QMZ.A.383, 3QPE.A.393, 3QPE.B.393, 2QX0.A.162, 3R1M.A.402, 3R1M.A.403, 3 R25.A.402, 3R6T.A.301, 2RDX.A.378, 4RJJ.A.602, 4RJK.H.602, 4RN3.A.301, 1RQJ.A.90 7, 3RUV.A.544, 1S1C.A.300, 3SAZ.A.802, 3SBD.A.501, 3SBF.A.402, 1SHQ.A.479, 3SOP. A.401, 3T6C.A.501, 1T9B.A.1699, 1TE6.A.640, 1TND.A.352, 4TQD.A.502, 3TTE.A.361, 3TW6.C.2002, 3TWB.A.420, 3TZF.A.279, 4USJ.C.302, 4V1T.A.1776, 3V4B.A.403, 1V5F.A .1603, 1V5G.A.1603, 1V8K.A.501, 1VA6.A.522, 2VBV.A.1136, 2VDR.B.2001, 3VKB.A.701 , 3VMK.A.402, 3VMK.B.402, 2VPR.A.1207, 2VZB.A.1001, 1W5T.B.701, 3WBZ.A.403, 3WDL .B.902, 2WEF.A.402, 4WK0.B.501, 3WOO.A.502, 2XCL.A.480, 1XEF.A.801, 1XG3.A.2101, 1XIN.A.395, 6XIM.A.395, 2XIM.A.395, 2XZW.A.202, 1YHM.B.1401, 1YVE.I.601, 1YYQ.B .701, 1Z5C.A.2001, 2Z7H.A.1301, 2ZDH.A.812, 2ZVJ.A.300, 3ZYC.A.1750, 4ABT.B.1287 , 2AOR.A.402, 2ASD.A.415, 3AVX.A.3001, 3COW.A.304, 1DMU.A.300, 3DVO.A.340, 4ELV. A.908, 4FJ9.A.1002, 4FJK.A.1002, 4FJL.A.1002, 3IAY.A.1, 2IBK.A.402, 2IMW.P.406, 4J2A.A.1002, 4J2B.A.1002, 2JEJ.A.1344, 1JXL.A.1402, 3KHR.A.416, 4KHQ.A.1001, 4KL D.A.402, 4KYW.A.302, 3LZJ.A.905, 3M9O.B.4001, 1N3F.F.497, 3O3G.A.1, 1OUP.A.300, 3QET.A.905, 4QWB.A.403, 3RAX.A.415, 2RDJ.A.353, 1RYS.A.801, 3SLP.B.227, 3SPZ.A.9 05, 1TW8.A.801, 3A09.A.601, 3A7Q.B.5001, 1AF0.A.484, 1AG9.A.200, 1AG9.B.1000, 4A IE.A.1540, 3AMR.A.909, 3AMR.A.910, 4AQ1.A.1925, 4AQ1.A.1926, 4AQE.A.1208, 4ARF.A .1723, 1AVA.A.502, 3B4N.A.702, 4B4F.A.607, 1B9T.A.500, 2BV2.B.1085, 4BZ4.A.1233, 1C3H.D.8003, 4CAG.A.602, 2CDP.A.1140, 1CGE.A.305, 1CLC.A.650, 1CLC.A.652, 4CUB. A.2645, 3D6E.A.202, 2DDU.A.1, 2DF7.C.5904, 2DIE.A.779, 4DK4.A.302, 4DOU.A.1001, 4DOU.A.1002, 2E9B.A.741, 1ELT.A.300, 5ENL.A.438, 1FBL.A.996, 3FU1.A.301, 3G4E.A. 1, 4GDJ.A.507, 3GG1.A.502, 2GKO.A.611, 1GVK.B.1246, 4GW3.A.401, 1H9H.E.1246, 1HD F.A.1101, 1HVX.A.517, 2HYU.A.502, 4I8H.A.301, 3I98.A.627, 4IHM.A.403, 2IUF.E.169 7, 1J0H.A.601, 1J1N.B.493, 1J35.C.501, 4J7M.A.403, 1JE5.B.502, 1JI3.B.403, 2JKE. A.1728, 4JZB.A.402, 4JZE.H.302, 4K3K.B.401, 1K7I.A.483, 1K7Q.A.485, 4K70.B.1002, 3KCG.H.500, 4KKF.A.703, 3KM5.A.2011, 3KMV.A.163, 4KTY.A.802, 4KXY.A.707, 3KZP.A .240, 4LLS.A.303, 3LNH.A.303, 3LPD.A.342, 3MBR.X.300, 4MB1.A.602, 2MIN.B.525, 4M KM.A.403, 2ML1.A.201, 3MMZ.A.500, 3MVS.A.211, 3MVS.A.215, 3MW3.A.301, 4N20.A.705 , 4N2B.A.705, 4NEH.B.701, 3NIF.D.2002, 3NJH.B.502, 1NNL.B.2002, 1NSC.A.468, 4NUZ .A.1001, 1OAH.B.1526, 3OJY.A.555, 3OJY.B.538, 1OM6.A.701, 1OM8.A.705, 3OM5.B.1, 3OM6.B.1, 4OUL.B.1201, 3P95.A.1, 4PHN.A.302, 3POJ.B.1, 2POO.A.805, 3PPE.A.401, 1 PW9.A.404, 2PZ0.A.501, 3Q2L.B.703, 2QIM.A.158, 1QLB.A.1658, 2QUB.A.616, 2QUB.A.6 18, 2R8Y.A.201, 1RXO.B.477, 1SOB.A.1292, 3S5U.E.221, 2SAS.A.187, 1T5S.A.1004, 1T O2.E.450, 3U1R.A.705, 3U1R.A.706, 3U1R.A.707, 4UP4.A.501, 4UP4.A.502, 1UTM.A.247 , 2UWF.A.1369, 1UX6.B.2003, 1UX6.B.2007, 1UYX.A.1134, 1VCL.B.1001, 3VTO.A.302, 2 W1W.B.1135, 4WA3.A.503, 2WFK.A.1250, 3WN6.A.502, 2WOB.E.1161, 3X17.A.602, 1XKD.A .1005, 2Y09.A.1242, 1Y7B.A.3001, 1Y9Z.A.604, 2YEQ.A.1526, 1YI7.A.3001, 1YO8.A.11 83, 1YO8.A.1185, 1YO8.A.1193, 1YO8.A.1199, 1YO8.A.1205, 1YO8.A.1206, 2Z8X.A.620, 2Z8X.A.622, 2Z8X.A.623, 2ZEO.A.552, 2ZPR.A.2001, 3ZWH.A.501, 1A2F.A.1, 1A7E.A.1 19, 4B7G.A.3000, 3BFJ.M.1388, 1BKA.A.694, 2BQ8.X.1305, 2BQ8.X.1306, 2BVO.B.600, 1C7M.A.101, 4CHL.A.501, 2D09.A.430, 3DHG.A.502, 3DXU.A.360, 3E6S.B.200, 1FCD.C.9 02, 1FSL.A.144, 1FZ3.A.5002, 4G51.A.202, 4G51.B.202, 4GHF.B.401, 3GM6.A.1004, 4G P4.A.602, 4GP5.A.602, 2HOV.A.501, 2HBU.A.900, 2HUO.A.301, 3IXF.A.139, 3KT7.A.701 , 3LXV.M.600, 4M25.A.401, 3MDT.A.505, 3MOO.A.911, 3N20.A.506, 3NC3.A.406, 2NOX.A .500, 4OJ8.C.301, 1OQG.A.500, 1OQU.C.1009, 3OUH.A.600, 1PIU.A.402, 2Q0J.B.998, 3 Q1G.A.1002, 3Q3N.A.510, 3QFO.A.264, 2QPP.A.300, 3QY8.A.253, 4RC8.A.303, 3RNC.A.5 00, 3RNF.A.500, 1SYY.A.1319, 1SYY.A.1320, 1TOQ.A.500, 3TMZ.A.501, 3UBR.A.471, 2V UN.A.402, 3WEC.A.501, 2X9P.A.1398, 1XK3.A.300, 1XU5.A.1175, 1XVF.B.1175, 1XVG.A. 529, 1YGF.B.147, 3ZLI.A.4001, 2BCU.A.577, 2BCQ.A.1, 4ED3.A.502, 4KYW.A.303, 4P4P

.A.401, 1VQ8.J.9146, 2A5D.B.326, 4A6U.B.1460, 2AMF.A.850, 2AUT.D.605, 4B1M.A.168
0, 1B57.A.364, 2BS2.A.1658, 3CYM.A.501, 4D1J.G.603, 2DDB.A.301, 4DEL.A.401, 4DF9
.B.501, 4DW8.A.304, 3E85.A.162, 2EHQ.A.1540, 4FDZ.B.301, 4FET.A.301, 2FM1.D.344,
 2FV7.A.403, 1G3K.A.500, 1G5H.A.901, 3GA5.B.701, 4GAF.B.505, 4GKI.D.303, 1GV2.A.
1191, 3GZA.A.471, 3H12.A.500, 1HBN.A.1561, 3HSS.A.268, 1HX6.A.705, 4I0W.B.603, 3
IFV.A.402, 3IGZ.B.601, 2IJA.A.401, 3IPO.A.436, 3IPP.A.438, 1IYN.A.297, 2IZV.A.14
30, 1JAY.A.215, 3JS4.D.208, 4JTH.C.303, 4JTJ.C.302, 4JTK.C.302, 4LGN.A.827, 4M48
.A.701, 4M4U.A.501, 4M60.A.502, 3MQD.A.500, 4NPI.A.602, 4NRH.A.401, 4PSR.A.619,
4PYJ.A.301, 4Q69.A.501, 2QJY.R.2001, 2QV6.A.303, 4QXK.A.402, 3SIB.A.223, 1T3M.A.
801, 3TDQ.A.117, 1U7H.B.912, 1UD2.A.1001, 3UES.B.503, 3UF4.A.601, 2WOI.D.1489, 2
WWF.B.214, 4X9K.A.401, 2XNA.C.1216, 2XZI.A.502, 1YYA.A.1001, 2ZND.A.196, 1ZOR.B.
1002
[1] "Cluster 5"
2EK8.A.1004, 2F92.F.1002, 1HQA.B.452, 2P2L.A.201, 2PTZ.A.501, 2PU1.A.501, 1TB7.A
.1001, 1Y2K.A.1001, 1BPY.A.339, 4BWJ.A.1834, 4DFK.A.902, 4DFM.A.902, 4DOC.A.401,
 4DQP.D.902, 4ELT.A.902, 4ELU.A.902, 4FO6.A.601, 2J0S.A.1412, 3KD5.E.916, 4M04.A
.703, 3MFI.A.515, 4P4M.A.402, 4QM6.A.1002, 3RTV.A.833, 3SPY.A.903, 1W7A.A.1802,
3ZVM.A.1526, 3AOT.A.800, 3A4L.A.401, 2A5D.A.1231, 4A6X.A.350, 4ACF.A.1482, 3AHC.
A.826, 3AHD.A.826, 3AHE.A.826, 3AHG.A.826, 1AOX.A.400, 2AUU.A.204, 2AUT.A.601, 3
AYX.A.701, 1B25.A.800, 4B2P.A.1351, 2B56.A.488, 3B7L.A.908, 2B9H.A.700, 4BAS.A.1
183, 3BB1.A.282, 2BBS.A.3, 3BC1.A.194, 2BME.A.1184, 3BN3.A.1, 2BVC.A.504, 3BWV.A
.300, 4BWR.A.1468, 4BXO.A.1291, 1BYQ.A.1001, 2C5L.A.1168, 4C5B.A.1314, 1CHN.A.20
0, 1CIP.A.356, 2CK3.A.601, 2CK3.F.601, 2CN5.A.1506, 4COK.A.601, 3CUR.H.553, 3CUS
.Q.553, 3D2R.A.500, 2D7C.A.1002, 3DDC.A.600, 3DGT.A.800, 3DKC.A.2, 4DN5.A.1001,
4DSO.A.202, 4DWO.A.301, 3DYP.A.3001, 2DY1.A.701, 4DYK.A.502, 4E01.A.402, 1E2Q.A.
401, 3E2D.A.603, 1E3D.B.901, 3E5H.A.200, 3E81.A.165, 2E9S.A.603, 4EEN.A.301, 4EF
M.A.202, 3EHG.A.371, 1EK0.A.601, 3ET4.A.301, 4EX7.A.301, 3EZ3.A.1104, 4F1J.A.301
, 1F5N.A.595, 3F61.A.310, 1F9H.A.161, 2FFQ.A.356, 2FH5.B.270, 1FSG.A.302, 3FSY.A
.333, 2FUE.A.500, 2G0W.A.501, 2G1T.A.1501, 3G15.A.602, 1G4C.B.362, 1G4T.A.2005,
3G5A.D.307, 3G6K.A.307, 2GCN.A.2001, 4GOJ.A.202, 1GSA.A.319, 2GSM.A.3006, 4GT8.A
.402, 3GYB.A.1, 1H2A.L.1005, 1H2R.L.1005, 3H7O.A.342, 3H80.A.214, 4H8E.A.301, 4H
AT.A.302, 4HDO.B.200, 2HF8.A.301, 2HF9.A.301, 4HOR.X.101, 3HQJ.A.145, 3HRZ.D.742
, 3HSD.B.162, 1HTW.A.561, 2I34.A.301, 2I5R.A.301, 3I76.A.1001, 4I94.A.402, 3ICM.
A.401, 3ICZ.A.403, 4IDN.A.502, 2IHP.A.287, 2IK4.B.289, 2IK6.B.287, 2IOR.A.2000,
3IPO.A.161, 4IUC.L.702, 4IUD.L.1002, 2J0V.A.1180, 2J7P.A.1401, 2J7P.D.1401, 1J9J
.A.301, 2JC9.A.1491, 4JDP.A.301, 3JTC.C.34, 3JYY.A.301, 1K77.A.300, 3K8K.A.700,
1KCZ.A.901, 1KK1.A.413, 3KKO.A.180, 1KMQ.A.401, 4KO8.A.801, 1KQP.A.5001, 1KQP.A.
5002, 4KQW.A.404, 1KSH.A.202, 1KTG.A.504, 3KTA.A.184, 3KUC.A.171, 4KVG.A.202, 4L
57.B.201, 3L8H.A.801, 4LFG.A.303, 4LFG.B.303, 4LHW.A.301, 3LLU.A.502, 4LPM.A.208
, 3LUF.A.300, 3LXX.A.402, 3M1I.A.1178, 4M9Q.A.302, 4MDB.A.403, 1MJN.A.1001, 1MMG
.A.998, 4MRT.A.301, 4MUM.A.301, 1MXG.A.439, 3MX3.A.601, 3MYH.X.997, 4NOD.A.402,
3N45.F.2, 1N6L.A.201, 1N6N.A.201, 1N6P.A.201, 1N6R.A.201, 1NBO.A.201, 4NBS.A.502
, 1NN5.A.401, 1NRJ.B.1, 2NSY.A.305, 1O08.A.2800, 1O3Y.A.1002, 1OBW.A.176, 3OCY.A
.264, 4ODJ.A.502, 3OE1.A.601, 2OGD.A.3002, 2OI6.B.6000, 1OKK.A.1002, 2OLR.A.543,
 4OMF.A.503, 1OXV.A.1102, 1OXV.D.1101, 3OYX.A.601, 3OZX.A.613, 2P27.A.307, 4P32.
A.402, 1P4M.A.201, 1P5Z.B.401, 3P5P.A.901, 3P96.A.412, 3PGL.A.1, 3PIT.A.180, 2PN
Q.A.502, 4PQ9.A.301, 1PVF.A.401, 2PZE.A.3, 3Q3J.B.201, 3Q46.A.307, 3Q60.A.603, 1
Q92.A.1003, 1QGU.B.3002, 1QGU.D.3006, 4QHZ.A.302, 2QIS.A.908, 2QIS.A.909, 1QK5.A
.303, 3QNM.A.400, 2QTO.A.1001, 1R2Q.A.300, 3R3S.A.296, 2R6O.A.801, 3R7W.A.600, 2
R8E.A.201, 3RAP.R.200, 4RKF.A.202, 3RLF.A.1501, 1RMT.A.1413, 4ROQ.A.401, 3RV3.A.
1004, 3RYE.A.908, 3S4J.A.908, 3SAE.A.820, 1SHT.X.219, 3SL2.A.701, 1SVM.A.750, 1T
0P.A.901, 3T10.A.401, 3T1K.A.401, 3T2S.B.401, 3T2B.A.409, 3T7A.A.602, 1TC6.A.501
, 3TG0.A.503, 4TMV.A.902, 3TSO.A.200, 4U82.A.301, 1U8Y.A.301, 2UAG.A.1001, 1UBK.
L.1005, 4UCX.Q.1553, 4UE3.L.603, 3UJR.A.502, 3UJR.B.502, 3UJS.A.601, 3UJS.B.601,

3UPL.A.447, 3UQY.L.603, 4UQL.Q.1552, 4URH.Q.1552, 4V0L.A.601, 1V54.A.3518, 2V7Q.A.1512, 2VBU.A.1134, 3VC1.I.301, 3VCC.A.401, 1VG8.A.1401, 2VG3.A.1297, 2VK8.A.1565, 1VOM.A.997, 1VZM.B.1046, 1VZM.B.1047, 3W40.A.201, 2W4J.A.1280, 2W5V.A.1378, 2W5X.A.1379, 1W78.A.1422, 1W78.A.1423, 3WJP.A.404, 2WKQ.A.1724, 1WUH.L.1005, 1WUK.L.1005, 1X06.A.900, 2X13.A.1418, 2X14.A.1418, 1X3S.A.200, 2X98.A.1477, 1XBY.A.601, 1XFI.A.400, 2XTN.A.1232, 2Y8E.A.1177, 1YHL.A.1403, 1YMV.A.200, 1YQT.A.592, 1YRQ.H.553, 1YS7.A.1002, 1YVD.A.850, 1YZL.A.401, 1YZT.A.700, 1Z08.A.1300, 1Z0J.A.400, 1Z2N.X.1295, 1Z4J.A.1001, 1Z4K.A.229, 2Z4V.A.1502, 1Z5G.A.601, 1Z5G.D.604, 2Z52.A.1302, 1Z88.A.601, 3ZFD.A.500, 3ZIA.A.601, 2AFH.D.2490, 4AWN.A.300, 3AYU.A.417, 3EDY.A.1, 2FGZ.A.1192, 2GKO.A.612, 4H1Q.A.305, 3H81.A.279, 1HFC.A.277, 1HV5.A.5503, 1I76.A.997, 2I8T.A.402, 2IXT.A.1310, 4L74.A.401, 1LQV.C.35, 2R8P.A.670, 1RM8.A.502, 3V96.B.303, 1Y93.A.268, 2Z2X.A.1005, 2ZWO.B.400, 19HC.A.301, 19HC.A.302, 19HC.A.303, 19HC.A.304, 19HC.A.305, 19HC.A.306, 19HC.A.307, 19HC.A.308, 19HC.A.309, 3A15.B.354, 2A3M.A.501, 2A3M.A.502, 2A3M.A.503, 2A3M.A.504, 1A4E.A.503, 1A56.A.82, 3A8G.A.301, 3A8L.A.300, 3A9F.A.207, 2A9E.A.550, 4AAL.A.423, 4AAN.A.400, 4AAN.A.401, 4AAO.A.400, 2AIU.A.200, 2AI5.A.81, 1AKK.A.105, 4AM5.A.1160, 1AOF.A.602, 1AOF.B.601, 1AOM.A.602, 1AOM.B.601, 1AOM.B.602, 4APY.A.1418, 1AQA.A.95, 1AQE.A.121, 1AQE.A.122, 3AQ5.A.144, 3AQ9.A.144, 3AT5.A.142, 3AT5.B.147, 2AT3.X.185, 3AWM.A.501, 1AWP.A.201, 2B0Z.B.109, 2B10.D.909, 4B2N.A.700, 2B4Z.A.500, 3B42.A.199, 3B42.B.399, 3B47.A.199, 4B4Y.A.1155, 3B6H.B.600, 1B80.A.350, 1B82.A.350, 1B85.A.350, 3B99.A.600, 2BDM.A.500, 1BFR.B.200, 2BGV.X.1121, 2BH4.X.1123, 1BJE.A.154, 4BJA.A.300, 4BJK.A.1450, 3BK9.B.401, 2BK9.A.1154, 2BLF.B.1582, 4BMM.A.1450, 3BNG.A.513, 3BNJ.A.513, 3BNJ.A.514, 3BNJ.A.515, 3BNJ.A.516, 3BNJ.A.517, 2BPN.A.108, 2BPN.A.109, 2BPN.A.110, 2BPN.A.111, 2BQ4.A.1115, 2BQ4.A.1116, 2BQ4.A.1117, 2BQ4.A.1118, 2BS2.C.1255, 2BS2.C.1256, 2BS3.C.1255, 2BS4.C.1255, 1BVY.A.1000, 3BXU.A.72, 3BXU.A.73, 3BXU.A.74, 4C0C.A.1450, 4C0N.A.1157, 2C1U.A.401, 2C1V.A.401, 2C1V.A.402, 2C1D.A.1292, 2C1D.B.1158, 3C2C.A.113, 1C2R.A.120, 4C27.A.1450, 4C28.A.1450, 1C40.A.150, 4C44.A.1151, 1C52.A.200, 3C62.A.150, 3C63.A.150, 1C6R.A.90, 1C75.A.93, 3C76.X.185, 3C78.X.185, 2C8S.A.1173, 4C9M.A.1418, 3CAO.A.104, 3CAO.A.105, 3CAO.A.106, 3CAO.A.107, 1CCH.A.83, 1CCR.A.112, 2CDV.A.109, 2CDV.A.111, 4CDP.A.402, 2CE0.A.1102, 1CH1.A.154, 1CH2.A.154, 1CH5.A.154, 1CH7.A.154, 1CH9.A.154, 1CI3.M.254, 4CK8.A.1480, 1CNO.A.200, 2CN4.A.1175, 2CN4.B.1175, 1CO6.A.108, 1COR.A.83, 1COT.A.130, 4COH.A.1450, 4COO.A.1549, 3CP5.A.202, 1CPT.A.430, 3CQV.A.601, 3CSL.A.866, 2CTH.A.109, 2CTH.A.110, 2CTH.A.111, 2CTH.A.112, 3CU4.A.199, 2CVC.A.1001, 2CVC.A.1002, 2CVC.A.1003, 2CVC.A.1004, 2CVC.A.1005, 2CVC.A.1006, 2CVC.A.1007, 2CVC.A.1008, 2CVC.A.1009, 2CVC.A.1010, 2CVC.A.1011, 2CVC.A.1012, 2CVC.A.1014, 2CVC.A.1016, 3CX5.C.4001, 3CX5.C.4002, 3CX5.D.4003, 3CX5.W.4026, 1CXA.A.126, 1CXC.A.125, 3CXH.W.4026, 1CXY.A.204, 1CYI.A.200, 3CYR.A.203, 3CYR.A.204, 2CY3.A.119, 2CY3.A.120, 2CY3.A.121, 2CY3.A.122, 5CYT.R.105, 2CZS.A.500, 2CZS.A.501, 1CZJ.A.119, 1CZJ.A.120, 1CZJ.A.121, 1CZJ.A.122, 3CZY.A.300, 2D0W.A.200, 2D0S.A.80, 2D0T.A.404, 4D30.B.750, 4D34.A.500, 4D35.A.500, 4D36.A.500, 4D37.A.500, 4D3A.A.500, 1D4D.A.601, 1D4D.A.603, 1D4D.A.604, 1D7B.A.401, 3D7O.A.143, 2DC3.A.191, 3DE8.A.150, 2DGE.A.200, 3DHH.A.501, 3DHR.A.142, 1DJ5.A.1, 1DK0.A.200, 3DMI.A.146, 2DN1.A.142, 2DN1.B.147, 1DP8.A.719, 1DP9.A.719, 3DR0.A.94, 1DTI.A.154, 4DTZ.A.500, 1DUW.A.293, 1DUW.A.297, 1DUW.A.300, 1DUW.A.301, 1DW0.A.113, 1DW2.A.113, 1DWL.B.80, 4DXY.A.501, 1DY7.B.601, 2DY5.A.300, 4DY9.A.201, 1E08.E.80, 1E29.A.136, 1E2R.B.601, 1E2W.A.900, 1E39.A.801, 1E39.A.802, 1E39.A.803, 1E39.A.804, 2E3A.A.401, 3E5J.A.1408, 3E5K.A.1408, 1E8E.A.125, 2E80.A.1508, 2E84.A.1301, 2E84.A.1302, 2E84.A.1304, 2E84.A.1305, 2E84.A.1306, 2E84.A.1307, 2E84.A.1308, 2E84.A.1309, 2E84.A.1310, 2E84.A.1311, 2E84.A.1312, 2E84.A.1313, 2E84.A.1314, 2E84.A.1316, 1EB7.A.401, 1EGY.A.410, 3EGW.C.806, 3EGW.C.807, 3EHB.A.559, 1EHJ.A.1030, 1EHJ.A.1053, 1EHJ.A.1066, 4EID.A.101, 4EIE.A.101, 4EIF.A.101, 3EJ6.A.4000, 4EJI.A.501, 2EKT.A.154, 2EKU.A.154, 4EP6.A.601, 1ETP.A.199, 1ETP.A.200, 1EUE.A.201, 2EU7.X.201, 2EWK.A.1001, 2EWK.A.1003, 2EWU.A.1001, 2EWU.A.1003, 1EWH.A.253, 2EWI.A.1004, 2

EWI.A.1002, 2EWI.A.1001, 2EWI.A.1003, 2EXV.A.83, 1EZV.C.401, 1EZV.C.402, 1F03.A.201, 1F1C.A.200, 1F1F.A.200, 1F24.A.501, 4F6I.A.201, 4FA7.A.602, 4FA7.A.603, 4FAS.A.601, 4FAS.A.602, 4FAS.A.603, 4FAS.A.604, 4FAS.A.605, 4FAS.A.606, 4FAS.A.607, 2FBZ.X.901, 1FCD.C.901, 4FDH.A.601, 4FEF.A.403, 2FFN.A.1003, 1FGJ.A.548, 1FGJ.A.552, 1FGJ.A.553, 1FI3.A.83, 1FI7.A.110, 1FJ0.A.115, 3FLL.A.185, 2FMY.A.300, 3FO3.A.1004, 3FO3.A.1005, 3FO3.A.1006, 3FO3.A.1007, 3FO3.A.1008, 3FO3.A.1002, 3FO3.A.1003, 3FO3.A.1001, 1FOP.A.500, 3FOO.A.150, 2FRF.A.154, 1FS7.A.509, 1FS7.A.510, 1FS8.A.508, 1FT5.A.213, 1FT5.A.215, 1FT5.A.216, 1FT9.A.300, 1FT9.B.300, 2FWT.A.803, 2FWT.A.805, 2FWL.A.132, 2FYU.D.242, 1G09.B.147, 4G1V.A.401, 4G3J.A.501, 3G5N.A.500, 4G70.A.601, 4G7L.A.301, 4G7S.A.602, 3GAS.D.1294, 2GC4.D.200, 1GDV.A.101, 4GED.B.201, 1GKS.A.0, 2GNV.A.166, 1GQ1.A.601, 1GQ1.A.602, 3GQP.C.143, 4GQS.A.501, 2GSM.A.2001, 2GTF.X.201, 1GWF.A.504, 3GW9.A.480, 1GWS.A.603, 1GWS.A.606, 1GWS.A.611, 1GYO.A.111, 1GYO.A.112, 1GYO.A.113, 1GYO.A.114, 4GYD.A.200, 4HOK.B.200, 1H1O.A.1184, 1H1O.A.1185, 1H21.A.1248, 1H21.A.1249, 1H21.B.1248, 1H21.B.1249, 1H29.A.1102, 1H29.A.1104, 1H29.A.1107, 1H29.A.1114, 4H2L.B.201, 1H32.A.1263, 1H32.A.1264, 1H32.B.1139, 3H33.A.75, 3H33.A.76, 3H33.A.77, 3H34.A.72, 3H34.A.73, 3H34.A.74, 3H4N.A.72, 3H4N.A.73, 3H4N.A.74, 4H44.A.302, 1H55.A.350, 1H57.A.350, 4H6O.A.501, 3H8T.A.301, 2H88.C.142, 1HBI.A.153, 4HB6.A.72, 4HB6.A.73, 4HB8.A.72, 4HB8.A.73, 4HBF.A.72, 1HCZ.A.253, 4HC3.A.72, 3HDL.A.305, 3HF4.A.142, 3HF4.B.147, 1HGB.D.147, 4HHR.A.701, 4HHS.A.701, 1HJ3.B.602, 1HJ5.A.601, 1HJ5.B.602, 2HJI.A.180, 1HLB.A.158, 3HNJ.A.150, 3HNK.A.150, 4HPA.A.201, 4HPB.A.201, 4HPD.A.201, 3HQ6.A.400, 3HQ9.A.400, 3HQ9.A.401, 1HRC.A.105, 3HSP.A.750, 4HSW.A.201, 3HYU.A.142, 3HYU.B.147, 1I3E.A.147, 2I5N.C.404, 1I5U.A.201, 1I77.A.108, 1I77.A.109, 1I77.A.110, 1I77.A.111, 4I7Z.A.302, 3I8R.A.901, 1I8O.A.115, 1I8P.A.115, 2I8F.A.83, 3I9T.A.300, 3I9U.A.300, 1IB7.A.95, 1ICC.A.201, 1IDR.A.144, 2IJ3.B.999, 1IKE.A.185, 1IQC.A.402, 3IQ5.A.150, 1IT1.A.201, 1IT1.A.202, 1IT1.A.203, 1IT1.A.204, 2IUF.A.1691, 1IVJ.A.300, 2IVF.C.1217, 1IW0.A.901, 1IYN.A.296, 1J02.A.300, 1J0P.A.1002, 1J0Q.A.201, 2J1M.A.1456, 4J20.A.107, 1J3S.A.105, 2J5M.A.1300, 2J7A.A.1003, 2J7A.A.1004, 2J7A.A.1005, 2J7A.C.1003, 2J7A.C.1004, 1JDL.A.500, 1JDR.A.296, 4JE9.A.201, 4JEB.A.201, 4JJ0.A.501, 4JJ0.A.502, 2JJP.A.412, 1JMX.A.1001, 1JMX.A.1002, 1JNI.A.125, 1JNI.A.126, 2K3V.A.218, 2K3V.A.278, 4K8F.B.300, 1KB0.A.802, 1KBI.A.760, 4KF2.B.501, 4KIC.A.403, 2KMY.A.233, 2KMY.A.251, 4KMG.A.101, 1KOK.A.296, 1KQG.C.809, 2KSU.A.282, 2KSU.A.305, 1KV9.A.901, 4KVK.A.701, 4KVL.A.701, 3L1M.A.150, 1L2K.A.154, 3L61.A.420, 3LD6.A.601, 2LDO.A.154, 3LGN.A.200, 4LJI.A.301, 1LM3.B.200, 1LMS.A.118, 4LM8.A.801, 4LM8.A.802, 4LM8.A.803, 4LM8.A.804, 4LM8.A.805, 4LM8.A.806, 4LM8.A.807, 4LM8.A.808, 4LM8.A.809, 4LM8.A.810, 4LMH.A.801, 4LMH.A.803, 4LMH.A.804, 4LMH.A.805, 4LMH.A.806, 4LMH.A.807, 4LMH.A.808, 4LMH.A.809, 4LMH.A.810, 1LQX.A.201, 1LR6.A.201, 1LS9.A.92, 1LSX.A.719, 2LZZ.A.102, 3M15.A.150, 1M1P.A.802, 1M1Q.A.804, 1M1Q.A.803, 1M1R.A.801, 1M2I.A.201, 4M2F.B.401, 3M3A.A.155, 3M4C.A.150, 4M4A.A.201, 1M59.A.201, 1M70.A.199, 1M70.A.200, 4M72.A.403, 4M73.A.403, 3MDM.A.505, 1MDV.A.110, 1MDV.A.112, 1MJ4.A.502, 3ML1.B.1128, 3ML1.B.1129, 1ML7.A.185, 4MLM.A.201, 4MLN.A.201, 3MM9.A.580, 1MNI.B.154, 1MNY.A.95, 3MOM.A.186, 4MPM.A.201, 1MQF.A.501, 4MQJ.B.201, 3MUS.A.201, 3MVC.A.500, 3MVF.A.185, 1MWB.A.125, 1MXR.A.1004, 1MZ4.A.151, 1N45.A.300, 4N4J.A.609, 4N4J.A.610, 4N4J.A.611, 4N4J.A.612, 4N4J.A.613, 4N4J.A.614, 4N4J.A.615, 4N4J.A.616, 4N4K.A.610, 4N4L.A.616, 4N4N.A.601, 4N4N.A.602, 4N4N.A.603, 4N4N.A.604, 4N4N.A.605, 4N4N.A.606, 4N4N.A.607, 4N4O.A.608, 4N8T.B.201, 3N8Y.A.601, 1NAZ.A.200, 4NFG.B.201, 1NIR.A.601, 1NIR.A.602, 4NK1.B.201, 3NMI.A.150, 1NML.A.401, 1NML.A.402, 3NN1.A.239, 1NNO.A.602, 1NOS.A.901, 4NP1.A.185, 2NRL.A.148, 1NS6.A.142, 1NS9.B.147, 3NWV.A.105, 3NXU.A.508, 3O1A.A.385, 4O1W.A.101, 4O4Z.A.201, 3O5C.A.401, 3O5C.D.504, 4O6Q.A.202, 4O6U.A.203, 4O7G.A.301, 4O7G.A.302, 3O89.A.2154, 1OAH.A.1521, 1OAH.A.1524, 3OA8.A.401, 3OA8.B.401, 1OFW.A.1294, 1OFW.A.1295, 1OFW.A.1296, 1OFW.A.1297, 1OFW.A.1298, 1OFW.A.1299, 1OFW.A.1300, 1OFW.A.1301, 1OFW.A.1302, 3OFT.A.417, 3OMA.A.1, 3OMI.A.607, 3OO4.A.142, 3OO4.B.147, 2ORT.A.600, 3ORV.B.600, 2ORO.A.600, 2ORP.A.600, 2ORQ.A.6

00, 2ORR.A.600, 2ORS.A.600, 2OT4.A.1004, 2OT4.A.1007, 3OUE.A.609, 3OUE.A.610, 3O
UE.A.611, 3OUE.A.612, 3OUQ.A.601, 3OUQ.A.602, 3OUQ.A.603, 3OUQ.A.604, 3OUQ.A.605
, 3OUQ.A.606, 2OZY.A.202, 2OZY.A.203, 2OZY.A.204, 2OZY.A.205, 3OZU.A.404, 3OZV.A
.404, 3OZW.A.404, 1P2E.A.801, 1P2E.A.803, 1P2E.A.804, 1P2H.A.803, 1P2Y.A.430, 3P
3L.A.501, 1PA2.A.306, 2PAC.A.83, 1PBY.A.991, 1PBY.A.992, 3PC3.A.701, 2PEG.A.200,
2PEG.B.400, 3PH2.B.1087, 3PI2.B.500, 4PK5.A.501, 1PL3.A.401, 1PM1.X.180, 1PP9.C
.501, 1PP9.C.502, 1PP9.D.501, 2PQ7.A.220, 3PXW.A.500, 4PXH.A.501, 1Q16.C.806, 1Q
16.C.807, 2Q8Q.A.300, 3Q99.B.750, 1QDB.A.516, 1QDB.A.517, 1QDB.A.518, 1QDB.A.519
, 1QHU.A.500, 2QJY.A.501, 2QJY.A.502, 2QJY.B.301, 1QKS.A.601, 1QKS.A.602, 1QN0.A
.113, 1QN0.A.114, 1QN0.A.115, 1QN0.A.116, 1QN2.B.101, 3QNS.A.351, 1QO8.A.603, 1Q
PA.A.350, 1QQ3.A.107, 3QQQ.A.163, 3QQR.A.163, 2QSP.A.142, 3QU8.A.500, 3QW0.A.150
, 1QYZ.A.200, 1R0Q.A.200, 4R20.A.601, 2R50.A.166, 2R80.A.150, 2R80.B.150, 3R9C.A
.450, 2RAO.B.147, 2RAO.C.142, 2RDZ.A.2, 2RDZ.A.3, 2RDZ.A.4, 2RDZ.A.5, 2RF7.A.2,
3RGS.A.1, 3RJ6.A.154, 4RKM.A.808, 4RKM.B.808, 4RKM.D.807, 4RKN.A.902, 4RKN.A.903
, 4RKN.A.905, 4RKN.A.906, 4RKN.A.907, 4RKN.A.908, 4RKN.A.909, 1RSE.A.154, 3RUK.B
.600, 1RWJ.A.90, 1RWJ.A.91, 1RWJ.A.92, 1RZ5.A.401, 3S1J.A.140, 3S8F.A.800, 3S8G.
A.800, 1SE6.A.430, 3SEL.X.73, 3SEL.X.74, 1SH4.A.201, 3SJ0.X.73, 3SJ0.X.74, 3SJ1.
X.73, 3SJ1.X.74, 3SJ4.X.73, 3SJ4.X.74, 3SJ5.A.500, 3SJL.A.500, 3SJL.A.600, 3SLE.
A.402, 1SOX.A.502, 1SP3.A.801, 1SP3.A.803, 1SP3.A.804, 1SP3.A.805, 1SP3.A.806, 1
SP3.A.808, 1SUO.A.500, 3SWZ.B.600, 3SXQ.A.1005, 3SXQ.A.1006, 3SXQ.A.1007, 3SXQ.A
.1008, 3SXQ.A.1002, 3SXQ.A.1003, 3SXQ.A.1001, 1SY2.A.185, 3T3R.A.500, 3T3Z.A.500
, 1T68.X.201, 3T6D.C.401, 3T6D.C.403, 3T6D.C.404, 3T6E.C.401, 3T6E.C.402, 3T6E.C
.403, 3TDA.A.800, 3TF0.A.500, 3TGU.C.501, 3TGU.C.502, 3TGU.D.501, 3TGA.A.185, 3T
GM.A.300, 1TH2.D.2003, 3TIK.A.482, 3TJS.A.508, 1TKW.B.253, 3TMC.A.309, 3TOR.A.3,
4TOB.C.201, 3TOL.A.150, 4TT5.A.401, 1TU2.B.255, 1U13.A.460, 1U4H.A.500, 1U7R.A.
154, 3U8P.A.347, 1U9M.A.90, 4U9D.A.201, 1U9U.A.90, 3U99.A.500, 3U99.A.700, 3UBR.
A.472, 3UBR.A.474, 3UBR.A.475, 3UBC.A.201, 3UCP.A.901, 3UCP.A.902, 3UCP.A.903, 3
UCP.A.904, 3UCP.A.905, 3UCP.A.906, 3UCP.A.907, 3UCP.A.908, 3UCP.A.909, 3UCP.A.91
0, 3UCP.A.911, 3UOI.B.200, 1UP9.A.201, 1UP9.A.202, 1UP9.A.203, 1UP9.A.204, 4UQH.
A.1450, 1URV.A.1172, 2UUQ.A.1405, 4UVR.A.1450, 1V0H.X.251, 2V07.A.1102, 2V08.A.1
087, 2V0M.A.1499, 3V2V.A.154, 4V2K.A.601, 4V3V.A.750, 4V3W.A.750, 4V3X.A.750, 1V
54.A.515, 1V75.B.201, 2V7K.A.1360, 2V7L.A.1360, 3V8D.A.601, 1V8X.A.901, 1V9Y.A.1
140, 3VAU.A.201, 1VB6.A.1140, 1VB6.B.1140, 2VEB.A.200, 1VGI.A.300, 3VHB.A.150, 4
VHB.A.150, 2VHB.B.150, 2VHD.A.402, 3VKP.A.601, 3VKS.A.601, 3VM9.A.154, 3VP5.A.20
1, 3VR8.C.201, 3VRD.A.201, 3VRD.A.202, 3VRG.A.201, 3VRG.B.201, 3VXJ.A.501, 2VXH.
A.1001, 1VYD.A.1117, 2VYW.A.149, 2W0B.A.470, 1W2L.A.1100, 2W31.A.200, 2W3G.A.500
, 1W7O.A.1119, 1W7O.A.1120, 1W7O.A.1121, 1W7O.A.1122, 3W9C.A.501, 1WAD.A.117, 1W
AD.A.113, 1WAD.A.114, 1WAD.A.115, 3WAH.A.201, 3WC8.A.201, 3WCT.A.200, 3WCT.B.201
, 3WCT.C.200, 3WCT.D.201, 2WDQ.C.1129, 1WE1.A.300, 3WFD.B.801, 3WFD.C.201, 3WFE.
B.802, 3WFX.A.201, 2WJM.C.1334, 2WJN.C.1333, 2WJN.C.1335, 2WJN.C.1336, 1WMU.A.20
1, 1WOV.A.300, 1WOX.A.300, 4WPD.A.402, 4WQ8.A.1002, 4WQ9.A.1001, 4WQ9.A.1002, 4W
QC.A.1002, 4WQD.A.1002, 2WTG.A.180, 3WU2.F.101, 1WVE.C.699, 1WVP.A.154, 2WX2.A.1
450, 3X15.A.200, 3X15.J.200, 1X3X.B.202, 1X46.A.151, 4XDI.A.201, 2XFH.A.1412, 1X
K1.A.300, 2XKR.A.1400, 2XKI.A.1110, 1XQ5.B.148, 1XQ5.C.143, 2XSJ.B.503, 2XTS.B.5
00, 2XYK.A.700, 1Y0P.A.801, 1Y0P.A.802, 1Y0P.A.803, 1Y0P.A.804, 1Y5I.C.806, 1Y5I
.C.807, 1Y5L.C.806, 2Y5N.A.450, 2YEV.A.1015, 2YEV.B.587, 2YIU.A.500, 2YIU.A.501,
1YIQ.A.901, 2YK3.A.200, 2YL7.A.128, 1YWD.A.185, 2YXC.A.1001, 2YYW.A.1001, 2YYW.
A.1003, 2YYX.A.1004, 2YYX.A.1001, 1Z1N.X.604, 1Z1N.X.607, 1Z1N.X.608, 1Z1N.X.610
, 1Z1N.X.612, 1Z1N.X.614, 1Z1N.X.616, 2Z47.A.1004, 2Z47.B.3003, 2Z6S.A.201, 2Z6T
.A.201, 1Z8O.A.410, 1Z9N.A.1001, 1Z9N.C.2001, 3ZBY.A.1402, 2ZBO.A.111, 3ZE6.B.50
2, 3ZG2.A.1480, 3ZH0.A.200, 3ZHW.A.1163, 3ZIY.A.600, 3ZJO.A.200, 3ZJQ.A.200, 3ZO
O.A.105, 3ZOX.A.1082, 2ZPB.A.300, 2ZS0.A.200, 2ZS0.B.200, 2ZS0.C.200, 2ZS0.D.200
, 2ZXY.A.200, 2ZZS.1.220, 1ZZH.A.802, 1ZZH.A.803, 4KLI.A.404, 4M04.A.707, 1RZT.A
.2001, 4TUP.A.402, 4C3X.A.561, 3E9L.A.1, 2FPR.B.505, 4GNJ.B.302, 4I2A.A.602, 4JV

L.B.304, 3MJ6.A.503, 3MQG.A.193, 1UD2.A.1002
[1] "Cluster 6"
1DK4.B.590, 3GJ9.B.127, 1K07.A.2, 3KR5.B.1004, 4M6R.B.301, 4OJX.A.404, 1OS9.F.92
6, 4R76.A.1003, 3V77.D.301, 4X2T.A.701, 2ZZW.A.362, 2AGQ.A.4002, 4D6N.F.1197, 4D
F8.A.903, 4DFP.A.901, 3LK9.A.340, 4M2Z.A.501, 2OZM.A.904, 1QTM.A.1001, 3R7P.A.31
7, 3SI6.A.905, 2XCP.A.1004, 2XCA.A.3000, 2XCA.A.3001, 1YVP.A.1001, 1ZBL.A.202, 3
A58.B.401, 2AG1.B.611, 3B8I.A.289, 2BW7.A.2201, 4BYF.C.1000, 1CG0.A.435, 4CTA.B.
401, 4D2I.A.1478, 3DYG.A.3002, 2E0A.A.500, 1E2A.C.106, 1EFL.B.1604, 3EG5.A.180,
2EGH.A.900, 1F1Z.A.2002, 3FFU.A.156, 3FFU.B.155, 4FFL.A.905, 4FFO.A.904, 3FHY.B.
404, 3G9D.A.298, 1H65.A.282, 3HWX.A.602, 4HYV.A.1001, 3I3O.A.306, 4IOK.A.604, 2J
5X.A.200, 4KCW.A.1001, 1KH7.A.452, 3KR4.C.1004, 4KS0.A.1001, 4L2X.F.403, 4L2X.F.
405, 4LF1.A.801, 4LNI.E.505, 3M1Y.A.300, 1MB9.B.601, 1MBZ.A.603, 1MBZ.A.604, 1MR
S.A.300, 2NOM.A.402, 1NUW.A.2498, 1NV7.A.3341, 4OGE.A.1204, 2OPM.A.907, 3OPS.A.5
00, 2OQY.A.401, 1OVM.A.601, 4OVN.A.204, 1PFK.A.325, 2PLS.G.603, 2Q58.A.5, 1QF5.A
.433, 2QTV.B.210, 4RAD.D.302, 1RC5.A.761, 3RIM.A.1001, 4RNH.A.1501, 1SOJ.A.2123,
 3SSN.A.501, 1T5S.A.1005, 3TXA.A.801, 4UOM.A.503, 2VQD.A.1449, 2VWT.A.301, 3VYT.
C.602, 3W7F.A.303, 1WC6.C.2202, 4WK2.B.501, 1WL6.A.801, 2XAM.B.1030, 2Y4M.A.400,
 1Y9I.A.601, 1YHY.A.699, 2YWF.A.701, 3ZDY.B.2001, 3ZXW.A.476, 2AOQ.A.232, 3BQ1.A
.4001, 1DMU.A.302, 3EH8.A.302, 3FSP.A.501, 2IBK.A.401, 1JX4.A.4001, 3KHH.B.1416,
 1M5X.A.802, 1N3E.C.492, 4PTF.A.1202, 3QEX.A.907, 1R7M.A.306, 2RDJ.B.353, 1S9F.A
.4001, 2WTF.B.1510, 2A8K.C.403, 2AER.L.3008, 1AFA.2.2, 4AWD.A.1321, 3AZX.B.301,
3B00.B.301, 3B0I.A.124, 4B7R.A.502, 3BCD.A.708, 3BJU.A.608, 4CAJ.B.1325, 2CDP.B.
1139, 1CGU.A.686, 3CK7.D.730, 5CNA.C.240, 1CRU.A.503, 4CRR.A.1386, 1CYG.A.682, 3
DAS.A.351, 4DLK.A.403, 4DRZ.A.202, 2DSN.B.2012, 3EAD.A.1003, 3EDD.A.700, 3EHB.A.
563, 3ESQ.A.213, 1F6S.E.205, 3FCU.B.2002, 2FPS.A.503, 4G62.A.301, 1G87.A.615, 1G
9K.A.700, 2GGM.B.402, 4GI6.A.601, 3GN4.B.204, 3GQF.D.154, 4GQ7.A.301, 2GSM.A.300
7, 4H3X.A.304, 1H5V.A.306, 1HDF.B.1101, 1HFZ.A.124, 1HL5.D.156, 1HQV.A.999, 1I22
.A.198, 3I4I.A.1001, 2I4B.A.454, 3I57.A.186, 2J1T.A.1154, 1J9L.B.1303, 4JCM.A.70
6, 4JDZ.B.702, 3JTX.B.396, 3JXS.A.302, 3KF9.C.304, 1KMB.1.2, 3KQA.B.421, 4KVJ.A.
714, 1KX1.E.502, 1KX1.F.601, 2L51.A.207, 1L6R.A.901, 1L9M.A.702, 3L9I.C.1148, 3L
CP.B.279, 3LHM.A.131, 1LPG.B.1, 4LVN.A.704, 2M0P.A.1201, 1MAC.B.389, 1MDU.A.403,
 3MVS.A.214, 4N25.A.707, 4N2I.A.704, 3N4E.A.500, 4N85.A.502, 3N8G.A.1002, 4NAM.A
.801, 4NHF.B.301, 3NN7.A.503, 2NXP.A.600, 3O5S.A.243, 1OB0.A.501, 2OKX.B.4002, 3
P4G.A.403, 3P4G.A.404, 3P4G.A.405, 3P4G.A.407, 3P4G.A.412, 4PLM.A.504, 3Q5I.A.52
8, 1Q7B.A.9002, 3Q8F.A.736, 4QD2.E.302, 2QT6.A.3713, 4QU6.A.904, 1R1Z.C.415, 2RD
Z.B.1502, 3RRW.A.271, 3SLE.B.401, 3SO0.F.98, 3SO1.B.97, 3SVL.A.201, 3TEW.A.801,
3TSK.A.304, 1TT2.A.502, 3UBF.A.754, 1UF3.D.913, 1UH2.A.1002, 1UH3.A.1001, 1UH3.A
.1002, 1UKS.A.688, 1ULV.A.2003, 1UPS.A.501, 1UYX.A.1133, 1V3J.A.687, 1V3L.B.689,
 2VDR.B.2002, 2VJ3.A.1533, 2VL8.A.1545, 2VVE.B.1338, 2VXJ.G.200, 2W3J.A.1139, 3W
9T.A.1008, 3W9T.D.504, 4WBQ.B.602, 4WF7.D.600, 3WH3.A.501, 2WO9.B.1272, 2WQS.A.2
415, 2XQX.B.1949, 1Y6W.A.149, 2YDP.A.502, 1YO8.A.1195, 1YO8.A.1208, 2Z49.A.1002,
 3ZHG.D.1323, 2ZQ0.A.901, 1FZ6.B.5003, 1FZ6.B.5004, 4ME4.A.402, 1MMO.D.3, 3QFN.A
.265, 1R1N.C.400, 1R2F.A.401, 1XVF.B.1174, 2BCR.A.604, 1Q81.A.8378, 1VQ8.Q.9148,
 1A5U.G.4732, 2AB8.A.2003, 2BER.A.1649, 4BY5.B.1188, 3C17.A.323, 4CBY.A.2036, 2D
4E.A.1901, 4DOU.A.1004, 2E5X.A.303, 1HBN.D.1561, 2HZG.A.1101, 3I01.M.730, 3I2W.B
.304, 3IC9.B.491, 2IM2.A.3001, 3IWJ.A.505, 2J80.A.1134, 4JN7.A.401, 1JZ4.A.3101,
 3K1U.A.412, 3LG1.A.530, 4LL2.A.301, 3ME4.B.2, 3N0U.A.208, 4NLV.A.911, 4O5H.A.60
5, 1O68.A.274, 3ON4.C.189, 3OND.A.509, 4PCG.A.302, 4PD6.A.502, 4PMO.A.312, 1Q3X.
A.800, 1Q6X.A.1002, 2QR7.A.1000, 1SOA.A.1501, 1T64.A.392, 4US3.A.701, 4USW.A.146
8, 1VOH.X.252, 3VGL.A.323, 2W4M.A.1245, 1W9S.A.1141, 4WFZ.A.501, 2X8J.A.1317, 2Z
J9.B.2, 1ZOR.A.1001, 1ZUD.1.701
[1] "Cluster 7"
1ANJ.A.450, 3BXM.A.1751, 4CBY.A.2034, 3DFF.A.274, 4DYO.A.501, 3E4A.A.2000, 4FW3.
A.301, 1G12.A.200, 2G9Y.B.450, 2GO4.B.602, 1H48.A.900, 4L3T.A.1101, 4MN6.A.401,

2OOT.A.1751, 3Q9B.E.345, 3RBU.A.1751, 1RTQ.A.701, 3SJG.A.1751, 1TXR.A.502, 2VQG.C.1092, 4WD8.C.302, 3WT4.A.501, 4X2T.E.701, 1XJO.A.901, 2G8H.A.301, 2VBL.C.1026, 2A9F.A.801, 1AO0.A.469, 3C41.J.603, 4E4F.A.504, 3G15.A.605, 4GMJ.B.302, 4GYZ.I.402, 3HDG.E.204, 3IJQ.B.386, 2IOA.B.5003, 1JCT.A.498, 3K5H.A.401, 3K5H.A.402, 1KFS.B.2, 4KMQ.A.1103, 2LVJ.A.101, 3MO0.A.551, 3MLE.A.222, 3N3T.B.803, 4ORK.A.502, 3POW.A.471, 3PMG.A.562, 2PUI.B.401, 3Q8U.E.159, 3RUW.D.544, 3SH1.A.222, 3U2E.B.4, 3U7F.B.1, 4UM9.B.2001, 2VON.A.601, 2W8D.A.1636, 3WBH.B.503, 3WQS.B.502, 2AGO.A.403, 4DTS.A.1002, 1N56.B.403, 1A2X.A.161, 3A7Q.A.4002, 3AAJ.A.991, 5AER.A.201, 1AFD.3.2, 1AJ4.A.163, 1AMY.A.502, 1ANN.A.319, 1ANW.B.354, 1BAG.A.431, 3BOW.A.719, 3BOW.B.403, 4BY5.D.1185, 1C9U.B.1002, 4CAG.A.605, 3CGA.A.102, 3CHK.A.502, 1D2L.A.46, 1DCY.A.198, 2DDY.A.174, 2DPK.A.2001, 2E3X.A.803, 3E3R.B.194, 4E52.A.403, 2E6W.A.301, 3E9T.A.1, 3EAD.B.1002, 3EAD.B.1004, 3ECQ.A.2000, 3EHJ.B.1, 4ELF.C.201, 4ELG.F.202, 2EQD.A.701, 2ERO.B.702, 2EXI.D.3004, 1FDK.A.124, 4FGC.B.204, 1FI5.A.162, 3FLP.B.301, 3FLT.A.302, 4FL4.C.401, 4FL4.C.402, 3FVI.D.125, 1GOH.A.290, 3GIN.A.1, 4GKX.B.301, 1GQM.A.1089, 2HOK.A.407, 1H4B.A.1085, 1H4B.A.1086, 2HQ8.B.303, 2HYW.A.505, 2I08.A.200, 3IA7.A.402, 3IA7.B.402, 3IGO.A.601, 1IME.A.278, 2IWV.D.1286, 1IXX.B.124, 1IZJ.A.1002, 2J1G.D.1289, 1JBA.A.501, 4JBE.A.502, 4JEO.A.401, 1JL5.A.2004, 4K1C.A.506, 3K8L.B.800, 1K90.F.804, 1K96.A.92, 2KAY.A.185, 3KF9.A.302, 1KIT.A.803, 4KPP.A.501, 1KTW.A.3, 1KX1.A.222, 1LBX.A.290, 1LGC.A.301, 1LHV.A.401, 1LMJ.A.101, 4LMF.A.304, 2LMT.A.150, 4LMH.D.811, 2LP3.A.201, 2LV6.A.204, 2LVK.A.102, 1M34.B.2299, 1M8T.C.1003, 4MBZ.H.401, 4MDV.B.403, 4MIV.D.601, 2ML1.A.206, 2ML3.A.205, 1N28.B.125, 4N25.A.706, 1NAE.A.900, 4NDD.B.402, 4NEN.A.1115, 1NIW.A.1002, 1NIW.C.1005, 3NOM.B.263, 1NUB.A.301, 1NUB.A.302, 1NX2.A.4, 1O9I.C.269, 1ODB.F.1092, 3OX6.A.501, 3OX6.A.502, 4P5W.A.1001, 4P99.A.512, 1PK8.A.817, 3PM8.B.514, 2PQY.A.500, 1PTK.A.281, 4Q4W.1.905, 1Q8H.A.72, 1QNI.E.903, 3QRX.A.173, 2RHP.A.1176, 2RHP.A.26, 3RV2.A.405, 3S5U.C.220, 2SAS.A.186, 1SCV.A.162, 1TCF.A.162, 1TEC.E.343, 1THL.A.3233, 3TRQ.A.358, 3TRQ.A.360, 1TTX.A.110, 1TTX.A.111, 1TYE.A.1405, 1TYE.A.1406, 1TYE.A.1407, 1U7W.A.501, 1UG9.A.2006, 3UJQ.D.305, 1UMS.A.3, 3USU.H.272, 3V03.A.585, 2V3T.B.1264, 1V3J.B.690, 2VB6.B.1151, 1W2M.C.1441, 3W9T.A.1005, 3W9T.C.1009, 1WMD.A.1001, 2WO9.D.1270, 1WRL.D.104, 3WU2.O.301, 2WYS.A.1554, 1YUU.B.199, 2Z2D.A.266, 2Z8S.A.644, 2Z8S.B.642, 2ZBA.A.461, 2ZJ7.A.627, 2ZKT.B.413, 2ZUY.A.624, 2B1X.E.502, 3DHI.A.601, 1DMH.A.400, 4E2P.A.401, 3GJB.A.320, 2HMO.A.450, 4KVQ.A.302, 4N71.D.201, 3OOR.B.803, 4P1B.D.502, 3PCH.M.600, 3QJV.A.801, 3QYT.A.680, 2RDB.A.500, 1SQD.A.500, 3VE1.B.702, 3VVA.B.502, 3W54.A.502, 3WFC.B.803, 3WFD.B.803, 2ZI8.A.701, 3COW.A.303, 3C5G.B.808, 2FLD.C.603, 1Q81.N.8347, 1VQ5.A.9145, 4ATF.A.500, 3BC9.A.704, 3BH4.A.489, 4D1I.B.600, 2DIE.A.781, 4DMI.A.202, 4F4R.A.501, 4FUS.A.828, 2GFH.A.249, 2GJP.A.1489, 4H83.B.402, 2HEU.A.4001, 1HNO.A.1800, 2HZG.B.1102, 2ID4.A.907, 2JBA.A.1127, 4JQR.A.301, 3KQB.A.303, 4KZW.A.304, 4M48.A.702, 4MPY.C.503, 3MV1.3.3101, 1O04.A.6601, 1OBO.A.504, 4OMG.A.402, 2P3Z.A.501, 2P6Z.B.403, 3Q2H.A.701, 3SIS.B.3001, 4TMW.B.902, 4TMX.A.902, 3USZ.A.902, 1V8Z.A.389, 3VD5.B.3101, 3VHS.B.52, 3W5N.A.1211, 2WOF.A.1727, 1WPC.A.504, 1WX5.C.282, 1XAR.B.201, 2YDG.A.1130, 2YFO.A.1743, 1Z45.A.702, 3ZK1.D.90

[1] "Cluster 8"

4BLL.A.1322, 1DK4.A.291, 4EWL.A.403, 1H8L.A.999, 1JJE.B.261, 3LMS.A.309, 1LNF.E.800, 4LQY.A.507, 4ONX.A.201, 2OX8.A.4, 3PN6.B.202, 3R8B.B.122, 3R8B.D.122, 3R8B.F.122, 3R8B.P.122, 1UXB.A.1367, 2VME.A.501, 1YH8.A.501, 4G7O.D.2003, 2ATX.A.201, 4CZK.A.1335, 2EWG.A.3002, 4FHX.A.402, 2FOL.A.202, 1GUS.A.1069, 4GWS.A.402, 4IFW.A.503, 2IUT.A.1724, 2J1L.A.1195, 3KUD.A.171, 3LDO.5.54, 4NHO.A.1402, 2NOM.A.401, 4P9D.A.202, 3PLS.A.1, 3PP1.A.410, 4S17.A.501, 3UGV.A.500, 3CFR.A.910, 4DU3.A.1003, 2G8K.A.401, 3GIL.B.1417, 4K4H.I.602, 3NE6.A.905, 3ODH.A.196, 1A2X.A.160, 1AEI.A.319, 5AER.A.202, 1ATN.D.264, 3B1T.A.900, 4BTW.B.1764, 4BY5.C.1187, 2COO.B.1507, 1CFF.A.149, 1CFF.A.150, 3CGT.A.686, 2D3P.C.241, 1D7F.A.5003, 3DFO.A.715, 3DFO.A.716, 3DFO.B.604, 1DJY.A.2, 2E6W.A.300, 3E78.A.601, 1E8U.B.1003, 2E85.B.1004, 3EHJ.A.1, 4ELG.A.202, 1ESP.A.322, 1EUB.A.278, 4FU4.B.505, 3G5C.B.802, 2GJR.A.1

488, 4GTW.A.1012, 3HLI.D.315, 4I5N.B.601, 4IAV.A.420, 1JBA.A.500, 1JC2.A.3, 3JQ5
.A.201, 1JSA.A.500, 1JSA.A.501, 4K1C.A.509, 2K7O.A.103, 1KBO.A.801, 1KV9.A.802,
2KYF.A.110, 2KZ2.A.149, 4LMF.A.303, 2LP2.A.203, 1LTJ.B.2, 1LWJ.B.10, 2MOK.A.201,
 2M5E.A.2001, 1M63.B.502, 4M7Z.B.409, 2ML2.A.202, 2ML2.A.205, 3MXW.A.402, 1NHE.A
.805, 1NIW.A.1001, 2NQA.B.902, 1NUD.A.702, 1NX3.A.4, 2P0R.B.1004, 2PF2.A.174, 2P
OJ.A.266, 4POQ.A.401, 2PPL.A.481, 3QRX.A.170, 4R9X.A.302, 3RBX.A.600, 2SAS.A.188
, 3TUY.C.157, 1ULV.A.2004, 4UM8.C.2004, 1UZJ.A.1648, 1UZJ.C.3649, 1V1G.A.1211, 1
V2I.A.1001, 1VFO.A.1001, 3VI3.A.2002, 2VKH.A.1543, 2VZP.B.1129, 2W2N.E.1334, 3W9
T.D.503, 3WHU.B.502, 2WVX.B.801, 2WVZ.B.800, 2WW0.A.800, 1XJL.A.346, 1XJL.B.340,
 2XRM.A.405, 1YCM.A.266, 1YO8.A.1211, 1YUU.A.197, 1Z6C.A.247, 2Z8S.A.642, 2Z8S.A
.647, 1ZFS.A.104, 1ZIV.A.1, 2ZN9.B.901, 4GAM.F.602, 3N9T.A.292, 1NNT.A.333, 4FZX
.C.201, 4KLI.A.403, 3MBY.A.340, 2OTL.A.8545, 1TW8.B.902, 4UAY.A.403, 1VQ9.M.9147
, 1YIJ.0.8519, 1YJ9.0.8517, 3B1Q.C.332, 4B52.A.1305, 1BGP.A.502, 3GQ9.A.692, 4IQ
L.A.407, 4J43.A.902, 3KZW.A.497, 3OQ8.A.460, 3OQ8.C.460, 2P6Z.B.401, 3Q9C.A.344,
 4Q92.C.502, 3R2H.A.157, 3SIT.A.3000, 1VK1.A.302, 3WGV.C.2005, 2WGM.u.201, 2YXU.
B.2406
[1] "Cluster 9"
2ALW.A.5001, 2BNO.B.1201, 3D4Y.A.1047, 3DDG.A.3001, 4DO0.A.502, 3DX2.A.1046, 3E4
9.B.500, 3E8R.B.2, 3EJP.A.1047, 3EJQ.A.1047, 3EJR.A.1047, 3EJU.A.1047, 2F7O.A.50
01, 2F7Q.A.5001, 2F92.F.1003, 2FQP.B.100, 4H1S.A.603, 3HPS.A.701, 2I57.D.507, 1I
M5.A.400, 4JDG.A.401, 1KQ3.A.401, 4NZ3.A.501, 2OB3.B.904, 4OP4.A.302, 1PTM.A.330
, 1QH3.A.262, 1QIP.B.901, 4R6T.F.1001, 4R76.E.1001, 4RLO.B.302, 4TO8.A.301, 2V9N
.A.1275, 3WOT.A.201, 3WOT.B.201, 3WOU.A.201, 2WHG.B.1263, 4X2T.G.702, 2Y33.A.900
, 1YGD.A.142, 3A4K.A.301, 4AAB.B.1157, 2AQ4.A.302, 2BCV.A.576, 4BDY.A.1381, 4BE1
.A.1382, 4D6N.A.1188, 4D6N.F.1196, 4DF4.A.901, 4EEY.A.502, 4F5P.A.401, 3GQC.B.20
3, 4IR9.F.402, 4IRD.F.903, 2IS4.A.1001, 3K57.A.1001, 4K98.A.602, 3LK9.A.339, 4M3
0.A.502, 4M47.A.402, 4MFF.A.401, 3MQY.A.500, 3OS0.A.394, 3OYA.A.396, 3OYC.A.397,
 3OYE.A.396, 3OYF.A.397, 3OYG.A.397, 3OYH.A.397, 4PGQ.A.400, 4QCL.A.1302, 1QSY.A
.1001, 3S3M.A.396, 3SPY.A.904, 3SV3.A.836, 1TK8.A.901, 4TUQ.A.402, 3UQ2.A.1, 2W3
5.A.1224, 1ZBL.B.204, 3A06.A.500, 4A01.A.1770, 4A01.A.1771, 1A49.H.5334, 3A7D.A.
300, 3AJO.A.183, 1AZS.C.403, 3B1X.A.301, 1B7T.A.836, 3BNY.D.701, 1BWV.C.490, 1CH
8.A.434, 3CP6.A.501, 1CUL.C.396, 1DAK.A.901, 4DBH.A.401, 3DHD.A.502, 1DIE.A.399,
 1DQN.A.451, 3DVA.A.1368, 2DW6.D.2004, 4DWB.A.508, 4DXJ.A.402, 3DYG.A.3004, 4E1E
.A.403, 1E9I.A.1431, 1EBG.A.438, 1EC9.A.498, 1ECB.A.507, 1ECQ.A.498, 3EFQ.A.3003
, 3EFQ.B.4002, 3EQI.A.3, 3EYA.H.613, 3F78.C.1, 3FD5.A.395, 3FD6.B.395, 3FDG.A.35
7, 4FFL.A.904, 2FG5.A.301, 4FI4.A.501, 2FNO.A.701, 3FQI.A.1000, 1FTN.A.300, 1G3B
.A.501, 2G4J.A.392, 4G61.A.302, 2G9Y.A.452, 2G9Z.A.704, 4GA3.A.1004, 4GME.A.501,
 4GOK.B.202, 2GQS.A.240, 2GQ3.A.1000, 1H1D.A.300, 4H1Z.A.401, 3H4L.A.701, 3HDG.B
.201, 4HE1.A.404, 4HE2.A.405, 4HGR.A.201, 3HQD.A.501, 2HWG.A.901, 4I2B.A.602, 4I
3Y.A.302, 3I6E.A.386, 3IBA.A.402, 3ICZ.A.401, 2IDX.A.603, 4IEE.A.501, 1II0.A.593
, 2IK2.B.287, 2IK2.B.290, 2IOA.B.5004, 4IX4.A.602, 1JAH.A.168, 2JCS.A.1211, 2JI6
.A.1567, 2JI8.A.1567, 1K9Y.A.403, 4K9N.A.601, 4KCV.A.1001, 1KEK.A.2237, 1KHK.A.4
52, 1KO5.A.1001, 1KP8.A.550, 3KRO.D.3002, 4KX3.A.302, 4LA7.B.601, 3LVV.A.695, 3L
VV.A.697, 4M69.A.403, 1MC1.A.603, 4MIT.A.202, 4MPO.B.204, 4MPO.E.201, 3MQT.H.626
, 4N0G.A.403, 1N20.A.703, 1N24.A.703, 4NEH.B.703, 1NHT.A.435, 4NM3.A.405, 3NO1.A
.397, 1NUW.A.2497, 1NUY.A.2343, 2O1O.A.501, 1OAD.A.392, 3OES.A.202, 1OFH.B.453,
4OKZ.A.901, 3OYZ.A.500, 1OZF.A.699, 3POX.A.430, 1P7T.A.1000, 1P9B.A.1600, 3PDE.A
.311, 4PFK.A.327, 2PLS.J.604, 2PP3.A.901, 6Q21.D.173, 3Q46.A.305, 1QC5.A.601, 1Q
C5.B.602, 4QEH.A.402, 4QPM.A.1502, 3QQV.A.382, 1QS0.A.501, 3QU4.A.225, 2R9V.A.50
4, 3RBM.D.1003, 4ROP.A.504, 1RQI.A.605, 3S9I.A.743, 3SAD.A.801, 3SB0.A.801, 3SE1
.A.182, 3SH6.A.176, 3ST8.A.496, 3TOZ.A.401, 3T8O.A.564, 4TQ4.A.401, 3TTE.B.361,
3UJ2.A.431, 3UXL.A.360, 4V1T.A.1778, 2V3W.A.1528, 2V5K.A.301, 1VA6.A.524, 2VBI.A
.1000, 3VD3.A.3001, 2VDM.B.2001, 3VMM.A.501, 2VPO.A.1209, 2W00.B.1894, 3W2W.A.90
4, 1W88.C.1368, 4WB8.A.402, 1WDD.A.1476, 4WK4.B.501, 3WO0.A.503, 4WRR.A.401, 2WX

5.L.1282, 2X5Z.A.602, 2XH4.A.1439, 5XIM.A.395, 5XIN.A.395, 8XIM.A.395, 1XLC.A.39 9, 1XZ8.A.180, 1YVE.I.602, 1Z0K.A.1201, 2Z4W.A.1302, 2Z4Y.B.1301, 3ZCB.A.301, 1Z VW.A.4001, 2A0R.A.401, 2ASD.A.416, 4DTP.A.1002, 1F00.B.761, 4F4W.B.403, 2GIJ.A.4 01, 3GV5.B.424, 4K4H.A.607, 4K4H.E.602, 4K4I.A.606, 4LQ0.A.402, 4M3Z.A.1002, 3M9 N.B.4003, 3M9O.B.4003, 3MXB.B.173, 3MXB.R.175, 1N3E.F.491, 3ODH.A.195, 1R7M.B.53 6, 1S00.A.401, 3SUN.A.897, 2A8K.B.404, 1AF0.A.486, 4AR9.A.1732, 1AWB.A.1, 3B1U.A .901, 1B4N.A.620, 3BC9.A.702, 3BC9.A.705, 3BH4.B.1, 1BLI.A.600, 2BU3.A.1242, 1C3 H.F.8001, 4C9F.B.401, 3CKC.A.600, 2D00.D.1001, 3D4G.B.484, 2DCJ.A.1003, 2DEW.X.9 01, 4DKB.A.302, 3EF2.A.304, 3EF2.A.305, 4EJ7.B.402, 4EPU.A.601, 2EXH.D.2004, 2F3 C.E.242, 4F8Z.A.409, 2FIB.A.412, 3FP8.E.601, 1G5N.A.402, 4G60.A.302, 4G62.A.302, 2GJP.A.1486, 3GN9.A.201, 4GZT.B.510, 2HOK.B.410, 1H3G.A.701, 1H71.P.502, 3HI7.A .802, 1HM9.A.1901, 4HOW.A.704, 4HPN.A.401, 1HYO.A.1006, 2HYV.A.608, 3IOX.A.903, 1J11.A.701, 1J9K.A.301, 2JKH.A.1245, 4JZX.A.403, 4K89.A.408, 4K9P.A.601, 1KA1.A. 401, 2KAY.B.188, 1KAP.P.617, 1KAP.P.620, 4KS1.A.501, 4LJ3.B.403, 4LLT.A.303, 1LO C.E.688, 4LQR.A.202, 3M1H.C.2001, 1MCT.A.246, 3MHF.A.328, 2ML1.A.204, 2ML2.A.201 , 2ML3.A.203, 2ML3.A.204, 4N2G.A.705, 4N2I.A.705, 1N7V.A.601, 4NAS.A.503, 1NBW.A .650, 1NKG.A.800, 4NOT.A.302, 1NRW.A.903, 1NUD.A.703, 4NUQ.A.301, 1NX1.A.3, 3OTJ .E.1000, 3P4G.A.402, 3P4G.A.406, 3P4G.A.408, 3P4G.A.409, 3P4G.A.410, 3P4G.B.411, 3PK0.A.280, 1PVY.B.603, 1PZ7.A.701, 2Q16.A.200, 1QCO.A.1002, 3QGV.A.504, 4QN7.A .501, 3QXG.B.230, 4R12.A.809, 2RA3.A.1, 2RA3.B.1, 3RQ0.A.301, 3S6J.A.4, 1SBH.A.2 90, 1SCB.A.276, 3SO0.D.97, 3T3P.B.2003, 1TCM.B.687, 4TSH.B.1502, 1TU5.A.902, 3U1 R.A.704, 1ULV.A.2001, 1ULV.A.2002, 4UM9.B.2002, 1V0Z.B.1477, 3V96.B.304, 1VCL.B. 1002, 2VNG.B.1214, 3VOC.A.501, 1WPC.A.502, 1WRZ.A.154, 2WW3.A.800, 2XR9.A.1869, 2XSG.B.1772, 2XVT.F.1137, 1Y9Z.B.605, 1YO8.A.1201, 1YO8.A.1203, 1YO8.A.1209, 2Z2 X.A.1007, 1Z70.X.3001, 2Z8X.A.624, 2Z8S.B.643, 2A1X.A.450, 1B1X.A.690, 4B2O.A.12 67, 4BGL.A.1001, 1BKA.A.693, 1BLF.A.700, 1BLF.A.701, 2BOY.G.1255, 2BUR.B.600, 2C AG.A.485, 1CE2.A.690, 1CE2.A.691, 1D9Y.A.310, 1DRY.A.332, 1E09.B.600, 2FR7.A.501 , 1FZ1.A.5001, 1FZ7.B.5003, 2G1M.A.600, 1GGF.B.760, 4HOW.A.701, 1H76.A.703, 1HAB .A.200, 1HAC.B.200, 3HGI.A.281, 1I4Z.A.601, 4I4G.A.601, 4I4H.A.601, 3IB0.A.999, 1JNF.A.702, 1JNF.A.703, 4K0F.A.601, 4K9T.A.601, 4K9U.A.601, 4K9V.A.601, 4K9W.A.6 01, 4K9X.A.601, 1KW9.B.301, 1LCT.A.400, 1LFG.A.693, 1LFG.A.694, 1LKM.A.601, 4M1I .A.402, 1MMO.D.4, 1MTY.D.3, 3MZS.C.500, 1NX4.B.300, 3OOR.B.802, 2O7U.A.500, 1OQU .C.1010, 1OVT.A.689, 3P3N.A.350, 3PCC.O.600, 3PCF.M.600, 3PCJ.O.600, 3PER.A.1001 , 1PHG.A.417, 3Q3M.A.509, 3QY6.A.263, 3QY7.A.263, 2RDB.A.499, 3RNC.A.499, 3RNF.A .501, 1S9A.A.300, 1T0Q.A.499, 1TFD.A.950, 3TTX.A.760, 1U74.B.1101, 3UF9.A.315, 1 VFD.A.400, 3VMG.B.501, 1WRA.B.402, 1XVB.A.1170, 1YUZ.A.301, 3ZK3.A.1311, 3ZK4.A. 800, 4AFK.A.1507, 2AHR.C.1259, 4AMJ.A.1360, 3AR7.A.1000, 3B34.A.951, 3BIA.X.117, 3BIB.X.117, 3BLJ.B.701, 3BOS.B.302, 4BVO.A.1396, 4CCY.A.1297, 2CD7.A.1132, 3CRN .A.131, 4D1J.E.602, 4D7C.A.1544, 2DDB.C.302, 2E54.A.1004, 4E6P.A.301, 4E6P.D.301 , 3E85.A.163, 1EAS.A.5, 4EAE.A.302, 4EEK.A.302, 4EEL.A.302, 3EIF.A.1033, 3EIY.A. 177, 3EUW.A.343, 3F3C.A.752, 4FEX.A.303, 1G5I.A.901, 4G8T.C.502, 4GIB.A.301, 2GJ U.A.2001, 3GOD.B.327, 3HON.A.204, 1H16.A.9001, 3HIJ.B.295, 3HVI.A.265, 3IAN.A.1, 4J07.A.201, 2JBW.C.1367, 4JB3.A.301, 4JDO.A.301, 4JEX.B.511, 2JHN.B.1299, 1JMM. A.3001, 1JTP.A.501, 1JTP.L.503, 4JTE.C.301, 4JTF.C.303, 4JTG.C.302, 1JZN.A.1139, 1K2X.A.801, 1KSU.A.810, 1L5B.A.302, 1LLA.A.631, 3MYV.A.502, 3N83.G.707, 4N9S.A. 402, 4N9V.A.404, 3NGJ.A.249, 3NMB.A.1, 4O0C.A.401, 2O34.A.501, 4O47.A.401, 3OEC. C.300, 4OF8.A.301, 2OYN.A.201, 1P0Z.A.1633, 4PMO.A.311, 2POC.A.5001, 2QKF.C.283, 1R4P.A.4003, 4R6K.A.501, 2R85.A.600, 3RU5.A.133, 1RWH.A.900, 1S5E.A.241, 1S82.A .4, 3SSB.A.995, 1T8U.B.701, 4TKX.L.705, 1TQY.B.1091, 3TSH.A.604, 3USL.A.752, 2V4 V.A.3052, 2V4B.A.1566, 2VNZ.X.9252, 2WCB.A.101, 4WFX.A.504, 3WG7.N.605, 2WGE.A.1 426, 1WKY.A.504, 2WOF.A.1728, 2WQK.A.254, 2X7J.B.1581, 1YOP.A.1810, 2Y5F.A.1245, 2YNQ.A.1392, 1ZOD.A.435, 3ZWF.B.1365

Table S131. 7-ligand combined metal, normal group

	size	largest_angle*	middle_1*	middle_2	middle_3	middle_4
1	"45"	"163.2+/-2.6"	"71.8+/-1.7"	"73.9+/-1.8"	"75.6+/-1.6"	"77.4+/-1"
2	"44"	"173.8+/-2.4"	"71+/-1.1"	"72.3+/-1"	"73.9+/-1.4"	"76+/-1.6"
3	"37"	"172.9+/-2.9"	"69.2+/-2"	"72.5+/-1.8"	"74.9+/-2"	"78.7+/-1.8"
4	"46"	"166+/-2.3"	"70.4+/-1.3"	"72.4+/-1.4"	"74.5+/-1.4"	"76.7+/-1.7"
5	"26"	"151.9+/-2.8"	"69.1+/-3.6"	"71.6+/-3.2"	"73.7+/-2.6"	"75.4+/-2"
6	"17"	"170.5+/-4.6"	"66.2+/-2.9"	"72+/-3.6"	"75+/-2.4"	"77.1+/-2.5"
7	"38"	"160.7+/-3.2"	"69.1+/-2.1"	"72.2+/-2.4"	"74.2+/-2.2"	"76.7+/-2.2"
8	"26"	"159.9+/-2.8"	"69+/-2.9"	"73.1+/-2.4"	"75.3+/-2"	"77.1+/-1.3"
9	"31"	"166.5+/-3.4"	"71.1+/-2.6"	"73.6+/-1.6"	"74.8+/-1.2"	"76.5+/-1.5"
10	"37"	"156.5+/-3"	"70.3+/-2.8"	"72.2+/-2.8"	"73.8+/-2"	"75.7+/-1.7"

	middle_5	middle_6	middle_7*	middle_8	middle_9
1	"78.5+/-1"	"79.8+/-1"	"81.2+/-1.3"	"83.2+/-1.4"	"86+/-1.5"
2	"80.8+/-2.2"	"82.8+/-1.8"	"84.7+/-1.8"	"86.5+/-1.8"	"88.5+/-1.5"
3	"81.3+/-1.9"	"83.8+/-1.9"	"85.5+/-1.9"	"87+/-1.6"	"88.8+/-1.4"
4	"79.4+/-2"	"81.7+/-1.8"	"83.6+/-1.5"	"85.6+/-1.7"	"87.9+/-1.8"
5	"76.6+/-1.9"	"78+/-2.1"	"79+/-2.2"	"80.4+/-2"	"82.9+/-2"
6	"79.1+/-1.9"	"81.4+/-2.4"	"84.3+/-2.1"	"86.9+/-1.9"	"89.4+/-1.9"
7	"78.4+/-2"	"80.2+/-1.7"	"82.1+/-1.9"	"84.1+/-1.9"	"87.1+/-1.7"
8	"78.4+/-1.3"	"79.6+/-1.2"	"80.8+/-1.1"	"82.3+/-1.5"	"84.5+/-1.8"
9	"78.2+/-1.4"	"79.9+/-1.4"	"81.3+/-1.6"	"83.3+/-2"	"85.1+/-2"
10	"77+/-1.5"	"78.6+/-1.9"	"80.5+/-1.6"	"82.1+/-1.9"	"84.1+/-2"

	middle_10	middle_11	middle_12	middle_13*	middle_14
1	"87.8+/-2.1"	"89.8+/-2.3"	"99.7+/-4.2"	"113+/-2.7"	"115.5+/-2.1"
2	"90.6+/-1.4"	"92.5+/-1.7"	"95+/-2.2"	"97.7+/-2.9"	"101.6+/-3.7"
3	"90.1+/-1.5"	"91.6+/-1.7"	"94.4+/-1.7"	"97.3+/-1.9"	"101.7+/-4.2"
4	"89.3+/-1.9"	"91.9+/-2.7"	"95.3+/-2.3"	"99.4+/-2.4"	"107.5+/-5"
5	"85.7+/-2.6"	"89+/-4.2"	"98.8+/-4.7"	"117.8+/-3.1"	"124.1+/-3.5"
6	"91.4+/-1.9"	"93.8+/-2.6"	"97.5+/-4.5"	"102.4+/-3.4"	"108.5+/-5.9"
7	"89.7+/-2.1"	"92.4+/-3.1"	"96.8+/-3.3"	"102.7+/-2.1"	"113.1+/-6.1"
8	"85.9+/-1.8"	"89.4+/-2.3"	"102.3+/-5.2"	"113+/-2.7"	"118.7+/-3.3"
9	"88.3+/-2.5"	"91.9+/-3.3"	"97.3+/-4.1"	"108.2+/-2.7"	"112.5+/-4.3"
10	"87.2+/-2.5"	"91.3+/-3.4"	"98.4+/-5.8"	"109.2+/-3.2"	"121.1+/-5.4"

	middle_15	middle_16	middle_17	middle_18	middle_19*
1	"121.7+/-5"	"131.2+/-3.2"	"137.1+/-3.3"	"150.5+/-3.4"	"158.8+/-2.1"
2	"138.3+/-2.5"	"140.7+/-1.7"	"142.8+/-1.5"	"145.2+/-1.8"	"147.4+/-1.7"
3	"133.6+/-3"	"137.5+/-2.2"	"142.8+/-2.7"	"149+/-2.4"	"153.2+/-2"
4	"136.2+/-3.9"	"139.6+/-2"	"142.7+/-1.6"	"145.9+/-2.1"	"148.5+/-2.3"
5	"130+/-4.5"	"134.5+/-3.2"	"138.7+/-3.1"	"144.9+/-3.4"	"149.1+/-2.7"
6	"122.8+/-5.8"	"132.4+/-4"	"139.6+/-5.2"	"152.9+/-5.6"	"160.3+/-3.5"
7	"131.8+/-4.2"	"135.7+/-3.1"	"141+/-3.3"	"148+/-3.4"	"153+/-3.1"
8	"124.3+/-3.1"	"130.6+/-2.8"	"136.5+/-3.8"	"149.7+/-3.8"	"155.4+/-2.8"
9	"132.7+/-5.8"	"137.7+/-3.4"	"140.4+/-2.8"	"144.5+/-3.1"	"147.8+/-3.1"
10	"131.2+/-4.7"	"136.9+/-3.2"	"140.4+/-3.2"	"144.4+/-3.1"	"148.5+/-2.5"

	smallest_opposite_angle*	PentagonalBipyramidal	SquareAntiprismaticV
1	"66.1+/-1.8"	"0.129"	"0.188"
2	"69+/-1.5"	"0.19"	"0.118"
3	"65.2+/-2"	"0.21"	"0.161"
4	"67.9+/-2.2"	"0.24"	"0.204"
5	"70.4+/-3.2"	"0.191"	"0.356"

6	"63.1+/-2.9"	"0.269"	"0.273"
7	"66.5+/-2.6"	"0.278"	"0.338"
8	"71.3+/-2"	"0.118"	"0.261"
9	"68.6+/-2.5"	"0.282"	"0.287"
10	"67.6+/-3.7"	"0.055"	"0.077"

	HexagonalBipyramidalVA	HexagonalBipyramidalVP
1	"0.001"	"0.066"
2	"0"	"0.059"
3	"0"	"0.108"
4	"0"	"0.083"
5	"0.001"	"0.031"
6	"0"	"0.198"
7	"0.001"	"0.12"
8	"0.001"	"0.049"
9	"0.001"	"0.099"
10	"0"	"0.013"

Table S132. Cluster members of 7-ligand combined metal, normal group

[1] "Cluster 1"
4A3X.A.1268, 4AFA.A.1267, 4AFC.A.1267, 4ASL.A.1268, 2BOI.A.300, 2BOI.B.600, 2BOJ.A.1116, 2BOJ.C.1117, 2BP6.A.802, 2BP6.C.805, 1BQB.A.353, 2BV4.A.300, 2BV4.B.300, 4CE8.A.998, 4CE8.B.998, 4COU.A.1270, 4COV.A.1269, 4COY.A.1270, 4CPO.A.1294, 4CP1.A.1294, 3CQO.A.4001, 3DCQ.B.116, 3EIF.A.1, 2EWE.A.703, 2FPW.A.503, 4IAI.A.402, 2JDY.A.1116, 2JDY.B.1117, 2JDM.C.1115, 2JDN.A.881, 2JDN.B.881, 2JDN.C.881, 1OUX.A.402, 1OVS.A.402, 1PAM.A.688, 1SNN.B.503, 2VNV.A.302, 2VNV.B.302, 2VUC.B.990, 2VUC.C.991, 3W5M.A.1201, 2WR9.A.1131, 2WR9.D.1132, 1YDY.A.904, 3ZDV.A.200
[1] "Cluster 2"
4A4A.A.1925, 4ASM.B.1359, 4ATE.A.1275, 4AWD.B.1321, 3AXD.A.3002, 1B8O.B.351, 4B9C.A.1151, 2BIB.A.1551, 4BLK.A.400, 3BMV.A.685, 4BM1.A.401, 1CPN.A.209, 2DEW.X.900, 2E39.A.501, 2E8Y.A.741, 2FHF.A.2401, 3HOO.A.401, 2HD9.A.2001, 1HFX.A.124, 3HR9.A.401, 3ILF.A.278, 4J3V.A.920, 4J3W.A.907, 4JGL.A.202, 1LLP.A.351, 4LPL.A.1101, 4LQR.A.201, 1LY8.A.9001, 1MVE.A.400, 4N2B.A.707, 4N2G.A.703, 4N2L.A.704, 4N6F.A.302, 3NNG.A.402, 3OMI.A.613, 3Q3U.A.340, 4QF4.A.202, 1UPS.B.501, 1URX.A.1300, 3WDH.A.801, 2WOY.A.2415, 2WZA.A.2415, 1YRO.A.124, 1W8O.A.1654
[1] "Cluster 3"
4K4G.A.602, 4UAW.A.403, 4A42.A.1690, 4A5G.A.1309, 4AQ1.A.1924, 3ATG.A.301, 3AZY.A.301, 1BGP.A.501, 3CK7.A.710, 4CRQ.A.260, 4CUO.A.1326, 4CZN.A.1370, 1D3C.A.687, 2EXH.A.2001, 1F6S.A.201, 1GW2.A.502, 1GWT.A.502, 1GWU.A.1308, 1GX2.A.1310, 2H2N.B.1001, 3HB3.A.563, 2HYK.A.477, 2JKA.A.1727, 3M5Q.A.372, 3MMZ.A.501, 4ODG.A.202, 2OKX.A.4001, 1PA2.A.308, 1QGJ.A.2002, 4R83.B.501, 1SCH.A.301, 3SO1.A.97, 1TE2.A.702, 1U0A.A.5004, 1W3M.E.3013, 2YLJ.A.1308, 2Z49.A.1005
[1] "Cluster 4"
4A3Z.A.2344, 3A4U.A.287, 4A41.A.2494, 1ALC.A.200, 3ALU.A.201, 3B0K.A.201, 3BOX.A.578, 1B9O.A.124, 4B96.A.1155, 1CPM.A.215, 2EJN.A.1003, 3FMU.A.351, 3HDL.A.307, 1I22.B.197, 4IAU.A.800, 2IWV.D.1283, 2J1A.A.1769, 4JCL.A.701, 2JD9.A.1146, 2JDA.A.1146, 1JI1.A.2002, 1JUG.A.126, 1LYC.B.9002, 1MAC.A.388, 1O4Y.A.700, 3OWF.A.151, 1PJ9.A.890, 4Q1U.A.402, 1QGJ.A.2001, 1R1Z.A.286, 1SNC.A.150, 3SRE.A.1357, 1SU4.A.996, 1TDQ.B.127, 1TLG.A.201, 1UX6.B.2014, 2V72.A.1139, 2VZQ.A.1130, 2W1Q.A.1947, 2W1S.A.1946, 2YFU.A.1141, 2Z48.A.1104, 2Z48.A.1205, 2Z49.A.1004, 3ZUC.A.1154, 3MMD.A.410

[1] "Cluster 5"
4FFO.A.903, 1BCJ.2.2, 2BO2.A.155, 2BO2.A.156, 4D0E.A.1531, 3DBZ.A.401, 3DED.A.50
4, 2DSO.A.1001, 4FVL.A.505, 1G1Q.A.801, 2GGX.C.401, 2GVU.A.500, 4HHR.A.703, 2IAW
.A.401, 2IAO.A.401, 1KWZ.A.504, 3LI4.A.316, 4N4E.E.404, 4N7A.A.605, 1NPC.A.322,
3P7F.D.1, 3Q9K.A.606, 3R5Q.A.1001, 1USR.B.1573, 3WH3.A.500, 3WHD.A.501
[1] "Cluster 6"
4K4H.A.602, 4K4I.A.605, 4AOC.A.1129, 1BIW.B.803, 4CI7.A.1505, 1DED.B.5004, 4G01.
A.300, 3HDL.A.306, 2ML2.A.203, 3PGV.A.267, 2VUD.C.1118, 2Z30.A.1002, 2Z49.A.1003
, 2GIG.A.501, 4KQ7.B.502, 3Q2G.A.701, 3UOF.A.410
[1] "Cluster 7"
4A6O.A.2346, 4A6S.A.1122, 3AFG.A.541, 4AFB.A.1267, 4AL9.A.1122, 1BJ3.B.124, 1CGT
.A.686, 4CPB.D.1123, 3CQO.A.4004, 1DV8.A.1002, 3EDF.A.603, 2ERV.A.195, 2FF3.A.70
1, 1FHF.A.502, 1FIF.B.2, 4GWI.A.204, 1H3G.A.700, 1HFZ.B.124, 4I5L.B.601, 2J22.A.
1150, 1K12.A.160, 3K8L.A.700, 1KZM.A.501, 4LHK.A.303, 2ML3.A.201, 1PA2.A.307, 4P
IB.C.201, 1SCH.A.302, 1SH7.A.1292, 3SRG.A.1357, 3TBD.A.401, 1WZL.A.1601, 1XO5.A.
1, 2Z48.A.1103, 3ZYH.A.1123, 1VQ9.Q.9148, 1GOF.A.702, 4IIL.A.402
[1] "Cluster 8"
3RAX.B.1416, 3AUJ.A.1603, 1CIU.A.684, 3DCQ.A.116, 3EHU.A.500, 1ESL.A.163, 1GEN.A
.302, 2GVV.A.500, 2H2T.B.322, 3HLH.B.315, 3HLI.B.315, 2JDM.B.1115, 4KVL.A.703, 4
M65.A.404, 4MZA.A.612, 2NZM.A.406, 1OUX.B.404, 1OVS.B.404, 3PAR.A.300, 3SO0.A.97
, 1SU3.A.904, 1V3E.A.4001, 1WDC.C.501, 3ZDV.B.200, 1ZH2.B.201, 1ZJA.A.7001
[1] "Cluster 9"
1T9I.C.801, 1T9I.C.802, 3ALT.A.201, 4AOC.E.1129, 2BF6.A.1693, 4DZT.A.302, 1G5N.A
.408, 1GWU.A.1307, 1JI1.A.2001, 2JKX.A.1641, 3K8K.A.710, 3K8L.A.710, 1L6R.B.903,
 4LHN.A.302, 1LPZ.B.1, 1PAM.A.687, 3S18.A.229, 3TO5.A.131, 1UX7.A.1134, 3V6N.A.2
29, 3W57.A.202, 3W5N.A.1201, 2W86.A.1148, 2W86.A.1149, 4WF7.A.600, 2WQ8.A.1641,
2XFD.A.1111, 2XFE.A.1113, 2Z48.A.1102, 2Z49.A.1001, 3ZO9.A.1589
[1] "Cluster 10"
4LTZ.A.404, 4A5G.A.1308, 2B6N.A.300, 4B9F.A.153, 3BPS.E.1, 1BYF.A.201, 4CPB.A.11
22, 4D0E.A.1533, 1D2V.A.600, 2DCK.A.1002, 3DED.B.506, 3DEM.B.4001, 4FHP.A.402, 4
GER.A.404, 3GIS.Z.1003, 2IAX.A.401, 3INM.A.521, 3IQT.A.1, 1J34.C.504, 4JSD.A.603
, 3LI3.A.402, 4LJH.A.201, 1LQV.C.42, 1MN1.A.371, 1NL1.A.204, 1NZI.A.1001, 3PAQ.A
.300, 1PJX.A.491, 1SZB.A.1001, 3TH4.L.148, 2VZP.A.1129, 2VZR.B.1132, 2W2M.E.1334
, 3WU2.A.401, 2WZS.F.800, 2Z48.A.1007, 1Q81.C.8345

Table S133. 7-ligand combined metal, compressed group

	size	largest_angle*	middle_1*	middle_2	middle_3
1	"85"	"161.2+/-2.6"	"74.4+/-2"	"76.2+/-1.7"	"77.4+/-1.6"
2	"80"	"159.3+/-4.2"	"51.7+/-2.6"	"70.8+/-5"	"74.1+/-3.6"
3	"72"	"172+/-2.8"	"69.2+/-3.6"	"73.4+/-3.2"	"76.3+/-2.9"
4	"115"	"162.6+/-1.8"	"74.4+/-1.9"	"76.2+/-1.6"	"77.7+/-1.4"
5	"132"	"166.9+/-2.1"	"70+/-3"	"74.9+/-2.4"	"77.4+/-2.4"
6	"60"	"163.4+/-3.7"	"65.6+/-2.7"	"71.8+/-4.4"	"74.9+/-4.1"
7	"73"	"174.5+/-2.6"	"68.9+/-3.1"	"74.5+/-2.7"	"78.2+/-2.2"
8	"134"	"168.5+/-2.1"	"73.4+/-2.1"	"75.8+/-1.8"	"77.8+/-1.6"
9	"167"	"174.3+/-2.2"	"73.2+/-2.2"	"75.8+/-1.9"	"78+/-1.5"
10	"120"	"169.7+/-2.7"	"72.9+/-2.4"	"76+/-2.1"	"78.1+/-1.9"
11	"73"	"167.2+/-4.7"	"51.3+/-2.9"	"70.9+/-4.3"	"75.3+/-3.4"
12	"22"	"154.6+/-4.5"	"67.4+/-4.1"	"71+/-3.7"	"73.2+/-3.5"

middle_4 middle_5 middle_6 middle_7* middle_8

1	"79+/-1.4"	"80.4+/-1.3"	"81.5+/-1.3"	"82.8+/-1.4"	"84.2+/-1.6"
2	"76.4+/-3"	"78.4+/-2.6"	"79.9+/-2.6"	"81.7+/-2.6"	"83.8+/-2.9"
3	"78.4+/-2.8"	"80.9+/-2.5"	"82.9+/-2.4"	"84.7+/-2.3"	"87.2+/-2"
4	"79.2+/-1.3"	"80.5+/-1.3"	"82+/-1.4"	"83.7+/-1.4"	"85.3+/-1.6"
5	"79.6+/-2.2"	"81.8+/-1.8"	"83.5+/-1.6"	"85.5+/-1.5"	"87.1+/-1.7"
6	"77.1+/-3.6"	"79.1+/-3"	"81.4+/-2.8"	"83.9+/-2.6"	"86.5+/-3.1"
7	"81.1+/-2"	"83.3+/-2.1"	"85.1+/-1.7"	"86.7+/-1.6"	"88+/-1.6"
8	"79.5+/-1.6"	"81.1+/-1.5"	"82.6+/-1.7"	"84.3+/-1.7"	"85.9+/-1.8"
9	"80+/-1.7"	"82+/-1.6"	"83.8+/-1.6"	"85.3+/-1.6"	"87+/-1.5"
10	"79.6+/-1.6"	"80.7+/-1.7"	"82+/-1.6"	"83.5+/-1.7"	"85.2+/-1.7"
11	"78.1+/-2.7"	"80.4+/-2.3"	"82.5+/-2.3"	"84.4+/-2"	"86.4+/-2.1"
12	"75+/-3"	"76.8+/-2.3"	"78.4+/-2.5"	"80.3+/-2.2"	"82.3+/-2.5"

	middle_9	middle_10	middle_11	middle_12	middle_13*
1	"85.7+/-1.8"	"87.6+/-2.3"	"90.8+/-2.9"	"97+/-4.4"	"108.4+/-2.8"
2	"86.3+/-3.2"	"89.6+/-3.7"	"93.8+/-4.4"	"99.3+/-4.7"	"109.1+/-5.3"
3	"89.1+/-2"	"91.3+/-2.5"	"94+/-3.3"	"98.7+/-3.3"	"105.4+/-2.7"
4	"86.9+/-1.9"	"89.2+/-2.1"	"91.9+/-2.5"	"96.9+/-2.8"	"101.2+/-2.4"
5	"88.7+/-1.7"	"90.8+/-1.8"	"92.9+/-2.1"	"96.2+/-2.5"	"99.2+/-2"
6	"88.8+/-2.9"	"90.9+/-3.3"	"94.5+/-3.4"	"99.4+/-3.8"	"105.1+/-3.3"
7	"89.3+/-1.5"	"91+/-1.3"	"92.6+/-1.5"	"94.3+/-2"	"96.5+/-2.1"
8	"87.9+/-2"	"89.7+/-2.1"	"91.9+/-2.5"	"96.2+/-2.6"	"100+/-2.2"
9	"88.8+/-1.3"	"90.5+/-1.4"	"92.6+/-1.8"	"94.9+/-2"	"97.4+/-2.3"
10	"87.1+/-1.9"	"89.7+/-2.3"	"92.7+/-3.3"	"97.9+/-3.6"	"105.1+/-2.5"
11	"88.5+/-2.5"	"91.4+/-2.9"	"94.1+/-3.2"	"98+/-3.4"	"103.7+/-3.8"
12	"83.9+/-2.9"	"86+/-3.3"	"91.1+/-4.8"	"97.5+/-5.4"	"113.5+/-6.1"

	middle_14	middle_15	middle_16	middle_17	middle_18
1	"113.9+/-3.9"	"122.9+/-4.4"	"126.8+/-4.4"	"147+/-5.6"	"152+/-3.9"
2	"116.3+/-5.2"	"123.5+/-4.7"	"130.1+/-5"	"142.3+/-5.5"	"149.2+/-5.3"
3	"108.5+/-3.2"	"113.7+/-3.6"	"119.7+/-6.5"	"144.1+/-8"	"157.4+/-4.4"
4	"111.2+/-4.8"	"123.6+/-3.2"	"128+/-3.8"	"149+/-4.1"	"153.4+/-3.6"
5	"105.9+/-5.1"	"120.1+/-4.8"	"127.3+/-5.4"	"146.2+/-4.9"	"155.9+/-4.3"
6	"110.8+/-5.7"	"122.1+/-6.1"	"130.3+/-6.1"	"142.1+/-5.5"	"150.1+/-5.1"
7	"101.6+/-5.4"	"120.6+/-5.7"	"128.3+/-6.1"	"145.6+/-4.2"	"155.5+/-5.3"
8	"106+/-4.3"	"125.9+/-2.9"	"131.2+/-3.3"	"147.6+/-3.7"	"151.6+/-2.9"
9	"101.3+/-3.6"	"125.1+/-3.4"	"130.1+/-4.1"	"148.4+/-3.4"	"153.1+/-3.1"
10	"108.9+/-3.1"	"119.4+/-3.9"	"125.8+/-5"	"146.3+/-4.8"	"154.1+/-4.6"
11	"110.2+/-5.6"	"121+/-5.2"	"130.1+/-5.5"	"140.1+/-4.5"	"152.5+/-6"
12	"122+/-5.9"	"127.3+/-4.6"	"131+/-4.6"	"139.2+/-4.6"	"144.7+/-4.5"

	middle_19*	smallest_opposite_angle*	PentagonalBipyramidal
1	"155.8+/-2.9"	"51.5+/-1.7"	"0.162"
2	"153.5+/-4.3"	"71.5+/-4.5"	"0.078"
3	"167.3+/-2.9"	"51.2+/-2.9"	"0.051"
4	"157.5+/-2.4"	"51.6+/-1.6"	"0.153"
5	"162.4+/-2.4"	"50.8+/-2"	"0.108"
6	"155.9+/-4.1"	"52.3+/-3.7"	"0.121"
7	"164.1+/-3.1"	"51+/-2.3"	"0.099"
8	"154.9+/-2.4"	"52.2+/-1.9"	"0.22"
9	"157.1+/-2.4"	"51.7+/-1.9"	"0.199"
10	"159.9+/-2.5"	"51.1+/-2.1"	"0.103"
11	"161+/-4.5"	"69.8+/-4.7"	"0.069"
12	"147.5+/-4.3"	"51.2+/-3.7"	"0.052"

	SquareAntiprismaticV	HexagonalBipyramidalVA	HexagonalBipyramidalVP
1	"0.255"	"0.001"	"0.046"
2	"0.177"	"0.001"	"0.029"

3	"0.193"	"0"	"0.109"
4	"0.205"	"0"	"0.047"
5	"0.119"	"0"	"0.073"
6	"0.202"	"0"	"0.057"
7	"0.066"	"0"	"0.066"
8	"0.187"	"0"	"0.069"
9	"0.14"	"0"	"0.087"
10	"0.163"	"0.001"	"0.057"
11	"0.14"	"0.001"	"0.045"
12	"0.081"	"0"	"0.006"

Table S134. Cluster members of 7-ligand combined metal, compressed group

[1] "Cluster 1"
2FLD.B.602, 3RAX.A.416, 2WTF.A.1511, 2AAO.A.296, 3AIE.A.4001, 4APX.B.1240, 3BOO.
A.124, 1B8R.A.109, 3BFM.A.235, 2CCL.B.1061, 4CPV.A.109, 2CT9.A.302, 4CT3.A.1170,
 3DBK.A.303, 2DIE.A.780, 1DTL.A.203, 1EXR.A.1000, 4F0Z.B.201, 1FZD.A.1, 1G8I.A.1
599, 3GDC.A.401, 1GGZ.A.149, 2GGM.A.401, 4GGF.A.101, 4H2A.A.805, 2HQ8.A.202, 1HV
X.A.516, 3HZ3.A.1, 4ICB.A.77, 4IK8.A.502, 4IU3.B.302, 2J1G.B.1290, 1JC9.A.301, 4
JWQ.A.202, 1K9K.A.400, 3KLK.A.1, 3KQR.C.206, 3LI6.A.149, 4LVN.A.702, 4M7H.A.501,
 3MHZ.A.736, 4MNO.A.302, 3MVS.A.212, 4N5X.A.201, 4N5X.A.205, 2O04.A.402, 2O5G.A.
401, 2O5G.A.402, 2O5G.A.403, 2OBH.A.1001, 1OHZ.B.1058, 2OZN.B.402, 3P4G.A.413, 4
P5X.A.1001, 5PAL.A.110, 2PVB.A.111, 3Q5I.A.525, 3QRX.A.171, 1QTX.A.152, 2R9F.A.3
66, 2RHP.A.29, 1RWY.A.422, 1SBF.A.601, 1SL7.A.301, 1SRA.A.302, 1TN4.A.162, 3U1R.
A.702, 3UBG.A.902, 3UCP.A.912, 3UL4.B.66, 4UZU.A.1483, 2VN6.B.1067, 2W46.A.1148,
 2W87.A.1149, 3WA5.A.504, 3WH2.A.302, 2WNP.F.1298, 2Y3N.B.1068, 2YA2.A.1691, 1YO
8.A.1190, 1Z3J.A.267, 2Z30.A.1004, 1ZCM.A.1001, 2ZWO.A.402, 1W9W.A.901
[1] "Cluster 2"
4FFR.A.404, 1JR4.A.300, 3NHG.A.908, 3QEW.A.905, 3QEX.A.905, 1B1G.A.76, 1BJF.A.40
4, 3BJU.A.606, 1BLI.A.700, 2BPE.A.1245, 4C0K.A.1615, 4CAJ.C.1323, 2CCM.A.1193, 4
CGT.A.685, 2CHN.B.1716, 3CIO.K.401, 1CXE.A.690, 4DH2.B.101, 4DH2.B.102, 2DIJ.A.6
89, 4DLK.A.401, 3DSL.A.2, 1DTL.A.202, 1E07.A.689, 1ESP.A.319, 3ETO.A.2002, 1EXR.
A.1001, 3FAW.A.4, 1FIF.C.2, 1G0H.A.291, 1GGZ.A.152, 4HEX.A.204, 1HQV.A.997, 1HT6
.A.501, 3IUC.A.2, 2IWA.A.501, 1J55.A.101, 4JRF.A.601, 3KCP.A.702, 3KF9.A.301, 1K
XT.A.4001, 4L03.B.502, 3LND.A.209, 4MNO.A.301, 4MSP.A.202, 3NJH.C.503, 4NUQ.A.30
2, 2O04.A.5002, 4P5F.B.501, 1PVY.A.503, 4QB2.A.202, 1R17.A.599, 4R1D.A.601, 3TRP
.A.358, 4U6B.A.501, 3U8D.A.202, 3UBG.A.901, 3UBH.A.856, 1UKT.B.690, 4UM9.A.2002,
 4UM9.A.2004, 1UZJ.B.2648, 4V29.A.1178, 1V3J.A.688, 2VN5.B.101, 2VN6.B.1066, 1WO
P.A.1779, 3WHT.B.502, 4WJK.A.503, 3WNO.A.801, 1WPC.A.503, 2WVZ.A.800, 1X2T.A.603
, 1XO5.A.5, 2Y5I.A.102, 2YEQ.A.1525, 1YO8.A.1210, 3ZHG.A.1323, 2E5X.A.302, 4IBO.
A.401
[1] "Cluster 3"
1MOW.A.371, 3V6J.A.403, 2A9F.B.800, 4GA3.A.1002, 3TLM.A.1005, 2ATL.B.1415, 4J2D.
A.1002, 4K4G.A.603, 4KHU.A.1002, 2ODI.B.702, 2Q10.B.702, 3QER.A.905, 1ANW.B.353,
 1AXO.A.290, 2C0T.A.1507, 1C8T.B.264, 3CLN.A.152, 1D2S.A.401, 1DB5.A.198, 1DBN.A
.301, 2DUR.A.1, 2EIG.A.1102, 3EU3.A.1, 3EXM.A.301, 1FIB.A.500, 2FMD.A.301, 1FX5.
A.251, 4FZM.A.301, 1G7Y.A.254, 2GKO.A.610, 1GO8.P.1482, 1GSL.A.251, 1H3G.B.701,
1H6X.A.1162, 1HQL.A.302, 3HR4.D.202, 4I35.A.514, 2JE7.A.1241, 2JEC.A.240, 1JX9.B
.601, 1LEN.A.184, 1LHW.A.401, 3LNP.A.472, 1LOC.A.228, 2LTN.A.191, 2ML2.A.204, 2M
L3.A.202, 1MVQ.A.238, 3N35.A.290, 1NIW.G.1014, 1NLS.A.240, 2OVU.A.238, 2P2K.A.23
9, 1QNW.A.302, 1QPK.A.451, 1QX2.A.1005, 1R1Z.A.285, 1RLW.A.401, 4U36.A.302, 3U4X

.A.237, 1UKG.A.1262, 2UWP.A.1246, 1UX6.B.2009, 1VCL.A.1002, 1WC5.A.2100, 3WCS.A.
1003, 2YFS.A.1712, 2ZVD.C.620, 3ZYR.A.401, 1XSP.A.576, 4DNL.A.300, 2XRM.A.401
[1] "Cluster 4"
4KLD.A.403, 3MQ6.A.1, 3QNN.A.903, 1A2Q.A.295, 3A8R.A.401, 2AAO.A.293, 1AK9.A.295
, 3AKB.A.172, 3AKB.A.175, 3AMR.A.908, 4AQJ.A.1097, 1AVS.A.94, 3BC9.A.701, 1BH6.A
.501, 1BJF.A.402, 1BLI.A.500, 1BQB.A.351, 1BU3.A.110, 4BY5.A.1183, 4BY5.A.1185,
2C4F.L.1143, 1C9N.A.277, 2CCM.A.1192, 4CFQ.A.502, 4CFY.A.301, 2CT9.A.301, 3CZT.X
.93, 4DA2.A.301, 3DO1.A.401, 4DUQ.A.102, 2DW0.A.702, 2E4T.A.701, 1E43.A.502, 1E8
A.A.1090, 3EDF.A.602, 2EGD.A.302, 1EXR.A.1002, 4F0Z.B.204, 2FH1.A.2002, 4GGF.L.2
04, 2GJP.A.1487, 3HJR.A.601, 3HX4.A.604, 3HX6.A.1, 4I2Y.A.501, 2I7A.A.2, 1I82.A.
192, 4IEF.B.702, 1J55.A.102, 1JK3.A.404, 1K94.A.999, 4KDW.A.201, 3KHE.A.193, 3KH
E.A.194, 3KLL.A.1, 4KWU.A.1109, 3LND.B.208, 3LNI.A.302, 2ML1.A.205, 1MXE.A.506,
4N5X.A.204, 1NPC.A.319, 3O4Y.A.198, 2O5G.A.404, 1OBO.A.502, 2OLG.A.2001, 2OPO.A.
302, 5PAL.A.111, 4PEO.X.103, 4PHJ.A.303, 4PHJ.A.304, 3PM8.B.1, 1POB.A.801, 1POE.
A.801, 1PVA.A.110, 3PVN.E.5009, 3Q2L.A.701, 1Q5P.A.271, 3QRB.A.302, 1QTX.A.153,
1QTX.A.154, 1QX2.A.1001, 1ROR.E.302, 2R2I.A.502, 1RFJ.A.1004, 3RM1.A.102, 3RUP.A
.1006, 1S01.A.295, 1S02.A.276, 1S6C.A.217, 2SCP.A.191, 3SIB.A.222, 1ST3.A.270, 1
SUD.A.295, 3TI7.A.353, 3TI7.A.355, 3TI9.A.353, 1TKF.A.905, 3TTQ.A.2867, 3VYV.A.3
03, 2VZP.A.1128, 2WBX.A.1103, 3WFD.B.806, 3WHT.B.501, 2WND.A.102, 1WP6.A.502, 1W
PC.A.501, 2WZ8.A.1149, 3X17.A.603, 1Y93.A.267, 1Y9Z.A.603, 1YU6.A.401, 2ZFD.A.22
9, 4O7J.A.201, 3V7Z.A.404
[1] "Cluster 5"
4GZ2.A.402, 2HVI.A.878, 4GIR.B.401, 1HJK.B.452, 2Z4W.B.1303, 4E3S.A.1002, 4J2E.A
.1002, 3MX9.A.363, 3MXB.A.175, 3NCI.A.905, 3NDK.A.905, 3NE6.A.904, 3NGI.A.905, 3
QNO.A.903, 4UB4.A.401, 4UB4.A.402, 2A3Y.A.601, 1AJP.B.558, 1AYP.A.301, 2AYH.A.41
7, 4AYU.D.205, 3B2Z.A.2, 1BF2.A.751, 2BWR.A.500, 3BYK.A.474, 2C10.B.1777, 4CBU.G
.1151, 2CCM.A.1194, 3CIO.K.402, 1D0L.A.400, 4D0E.A.1532, 2DBX.D.702, 1DBI.A.703,
 4DIR.A.101, 1DJX.B.2, 4DKB.A.301, 2E26.A.601, 3E9T.B.4, 3ETO.A.2001, 3FCS.A.200
4, 3FLP.A.301, 3FLP.A.302, 1G8I.B.1600, 1G8K.C.5108, 1G9K.A.705, 1GK9.B.1579, 1G
KF.B.1571, 1GO7.P.1482, 3GPE.A.501, 2GSK.A.1, 4H2B.A.604, 2H61.D.817, 3HB2.P.481
, 2HYV.A.607, 4I2Y.A.503, 4IHM.A.406, 2II1.C.400, 4ITC.A.1201, 4IU3.B.301, 1J1A.
A.201, 1J9L.A.1301, 4JX1.E.505, 1K9I.I.1201, 1K9J.A.403, 4K90.A.702, 1KAP.P.621,
 3KM5.B.2011, 3KWU.A.902, 3L2Y.A.301, 4LMH.A.811, 3LPC.A.360, 3LPC.A.362, 1LVU.A
.8001, 3M1H.A.2001, 4M5E.A.505, 1MCX.A.352, 1MKU.A.124, 1MKV.A.124, 3MVS.A.216,
4N2L.A.707, 1N47.B.252, 4NEH.A.1101, 2NXP.C.602, 2NZM.A.405, 2O72.A.402, 1O8F.A.
1353, 3OM2.A.1, 3OM4.A.1, 3OM5.A.1, 4OMC.A.606, 4OMC.A.607, 1OYG.A.500, 2POR.A.1
002, 4P99.B.517, 2PEL.A.237, 4PHJ.A.302, 4PKI.A.403, 2PMY.B.104, 1PT2.A.500, 3PV
N.M.5026, 1QI3.A.451, 2QUB.A.621, 3R5V.B.201, 1S1E.A.226, 1S6C.A.218, 1SL4.A.407
, 1THM.A.302, 3TI7.A.354, 3TI9.A.354, 1TM7.E.470, 4TNC.A.164, 4UM9.A.2001, 1UNE.
A.124, 1UP8.A.599, 1UWW.A.1192, 1UY4.A.1147, 3VLV.A.503, 2VVD.A.1328, 3VV3.A.402
, 4WJK.B.502, 4WK7.A.502, 1Y1X.A.201, 1Y6O.A.207, 2YAY.A.1266, 2YOA.A.1002, 1YXH
.A.1001, 2ZUX.A.631, 2ZUX.A.632, 2ZUY.A.622, 2ZVD.A.628, 4IBO.A.402, 2VX5.A.1421
[1] "Cluster 6"
4X2T.L.701, 3EW9.A.501, 4GX6.B.402, 3MJX.A.901, 4DTM.A.1002, 4DU1.A.1003, 4DU3.A
.1002, 3EH8.A.301, 3KHR.B.1416, 2AAO.A.295, 3B4N.B.701, 4CAG.A.603, 1CJY.A.950,
1DVI.A.273, 2E3X.A.802, 1FAT.A.255, 3FZO.A.400, 1G9K.A.704, 4GM5.A.404, 2GSK.A.2
, 2GXS.A.601, 2J1V.A.1152, 4JA8.A.503, 1JIW.P.485, 4K70.B.1003, 4KKK.A.701, 1KVW
.A.124, 2KYF.A.109, 3LI6.D.150, 1MDW.B.4, 4NEH.A.1103, 4NHF.F.301, 3OOV.A.1, 1O8
P.A.1149, 3OX6.B.502, 5P2P.A.125, 3P4G.B.405, 4PET.A.403, 2Q1F.A.2001, 1RU4.A.1,
 1S6B.B.402, 1TCF.A.160, 1TCF.A.163, 3UJO.B.304, 3USU.C.266, 1VCL.A.1001, 2VUD.D
.1118, 1WC0.A.2100, 1X1J.A.2000, 1Y08.A.1186, 1Y08.A.1187, 1YUT.B.199, 2ZN9.A.90
1, 2ZWP.B.402, 2HZL.A.500, 4LUG.A.301, 1O68.D.274, 3PFV.A.1, 3UTO.A.902, 4X26.A.
402
[1] "Cluster 7"

2OTJ.3.8078, 4DCK.B.203, 1ECB.D.507, 4FVQ.A.902, 3AVW.A.3001, 3QEP.A.905, 2VS7.A
.1183, 3ACH.A.204, 2AEF.A.602, 4AQO.A.1881, 4AVS.A.207, 1BTU.A.260, 2CFT.A.1297,
2CM6.A.1679, 3DEM.A.5001, 3DNZ.A.403, 2E26.A.602, 3EAD.B.1003, 2ERO.A.702, 2EXH
.B.2002, 2FI1.A.191, 1FNY.A.500, 1G8K.A.5008, 1GCA.A.351, 1HDF.A.1102, 4HHO.A.40
1, 1HPL.A.960, 3IBZ.A.193, 2J7A.A.1006, 1J83.A.4001, 4JDZ.B.701, 3KQR.A.205, 1KV
O.A.191, 4LOR.A.301, 1LPB.B.453, 4LP7.B.301, 1LVU.D.8002, 4MGQ.A.601, 1NPC.A.321
, 2NXP.D.603, 1OAC.A.803, 1OAH.A.1525, 3OEA.A.200, 3OM6.A.1, 3OM7.A.1, 4OY7.G.30
2, 1P8J.A.3001, 2Q17.A.315, 3QRO.A.1000, 1R64.A.701, 2R8Z.N.214, 1RLW.A.400, 3SR
E.A.1356, 1SU4.A.995, 1TU5.A.903, 1UX6.B.2008, 1UX6.B.2011, 2V0B.A.1241, 3V9M.A.
205, 3VRR.A.401, 2W08.A.206, 1WBF.A.303, 3WCT.F.203, 2WW8.A.1002, 1YO8.A.1189, 2
Z8X.A.621, 2Z8X.A.625, 2ZEX.A.406, 2ZKM.X.800, 2ZYH.A.700, 1WTE.A.1001, 3MNC.A.2
80, 2YEQ.A.582
[1] "Cluster 8"
1G9Y.A.801, 3A24.A.1268, 1A75.A.110, 1AFO.A.476, 3AKB.A.171, 3AMR.A.907, 4AQ1.A.
1923, 4AQ1.C.1923, 1AVS.A.93, 1AXN.A.353, 2AZZ.A.125, 3BEU.A.249, 3BI1.A.1753, 1
BQB.A.352, 3C1Q.B.1, 2C26.A.302, 3C3Y.A.238, 2C4X.A.1253, 1CB8.A.3000, 2CCL.B.10
60, 4CCD.A.3669, 2CDP.A.1139, 3CHJ.A.338, 2CKI.A.997, 1CP9.B.554, 4CPV.A.110, 1C
RU.A.501, 1DBI.A.701, 2DCK.A.1001, 1DVI.A.271, 2DW0.A.701, 2EHB.A.1001, 3ETO.A.2
003, 3FED.A.1755, 3FIA.A.201, 3G5C.A.801, 1G5N.A.401, 1G9K.A.702, 1GGZ.A.151, 4G
UK.A.207, 4H1Q.B.304, 3HDB.A.622, 3HI7.A.803, 1HQV.A.998, 2HQ8.A.201, 2HQ8.A.203
, 1HY7.A.304, 2HYV.A.605, 2HYW.A.503, 4I35.A.511, 2ID4.A.901, 3IGO.A.602, 3IGO.A
.603, 4ITC.A.1202, 3JQW.A.1001, 3K21.A.192, 3K21.A.193, 1K7I.A.480, 1K9U.A.1001,
1K9U.A.1002, 1KAP.P.615, 3KM5.A.2012, 4LDC.A.502, 4LM8.A.811, 3LPA.A.361, 3LPC.
A.361, 2LRP.A.202, 1MCX.A.351, 4MEW.A.501, 1MPX.A.638, 4MSP.A.201, 4N1G.A.203, 4
N2B.A.709, 4N2G.A.704, 3N4E.B.500, 1NIW.C.1007, 4NUQ.A.303, 2O8O.A.1009, 2O9Q.A.
501, 4OKH.A.902, 2OO4.A.5001, 2OPO.A.301, 2OW9.B.610, 2OX9.A.804, 4P99.C.505, 4P
HN.B.303, 4PLS.B.301, 3PM8.A.1, 3PM8.A.514, 1PMJ.X.300, 1PVA.A.111, 2PVB.A.110,
3Q2N.A.303, 3Q5I.A.526, 4Q6O.A.502, 2QQO.A.1, 3QRB.A.303, 1QX2.B.1006, 2R2I.A.50
0, 2R2I.A.501, 1RFJ.A.1001, 1RFJ.A.1003, 1RWY.A.421, 2SCP.A.190, 2SCP.A.192, 1SG
T.A.246, 3SIB.A.221, 1SL6.E.3, 1SL8.A.671, 3SNY.A.97, 1T44.G.702, 1THM.A.301, 5T
NC.A.163, 3TOY.A.361, 3UL4.B.67, 4UM9.A.2003, 3UXF.A.2, 1UXX.X.1130, 1VCL.A.1005
, 2VDQ.B.2002, 2W47.A.1137, 3W57.A.203, 3WHI.A.401, 3WLC.A.501, 2WNO.A.1251, 2WW
8.A.1001, 2Z2X.A.1002, 2Z30.A.1005, 2ZFD.A.227, 2ZFD.A.228, 2ZWP.A.404, 3ZYP.A.1
220, 4DMI.C.203, 3UES.A.503
[1] "Cluster 9"
4FZL.B.302, 1Z2P.X.1296, 4AQU.A.1154, 4AQU.A.1156, 1G9Y.B.802, 3ODH.B.195, 2VBJ.
A.1154, 2VBJ.A.1155, 2VBO.A.1154, 2VBO.A.1155, 1A8A.A.407, 3AMR.A.905, 4APX.B.12
38, 4AQA.A.1209, 4AQE.A.1207, 1AVA.A.500, 2B96.A.124, 3BNG.A.508, 4BQ2.A.1797, 1
BSW.A.900, 2C4X.A.1252, 4CAG.A.604, 4CAG.A.610, 2CBL.A.352, 4CBU.G.1150, 2CDO.A.
1139, 3CHK.A.503, 3CIP.G.201, 2CM5.A.1679, 2CM6.A.1680, 2CN3.A.1778, 4CTE.B.280,
1CVR.A.504, 1DBI.A.704, 3DEM.A.8001, 4DUQ.A.101, 2DW0.A.703, 2E26.A.604, 2E26.A
.605, 3ECQ.A.2001, 1EDM.B.2, 1EXR.A.1004, 4F0Z.B.202, 4F0Z.B.203, 2FMJ.A.338, 3F
O3.A.527, 2FVY.A.311, 1G9G.A.630, 1G9K.A.701, 3GBO.A.301, 4GER.A.403, 1GGZ.A.150
, 1GPL.A.500, 3GPE.A.502, 1H6Y.A.1161, 1H80.A.1493, 3HDB.A.621, 3HX4.A.601, 3HX4
.A.602, 2ID4.A.906, 3IKW.A.1, 3IK2.A.514, 4IMM.A.407, 2IPL.A.502, 4JGU.A.901, 1J
I1.A.2003, 3JQ1.B.1, 1K96.A.91, 1KAP.P.614, 3KCP.A.701, 3KHE.A.195, 1KQU.A.301,
3KWU.A.901, 1LE6.A.461, 4LJ3.A.402, 4LOS.A.401, 1LQV.C.34, 4LXO.A.1601, 4LXO.B.1
601, 3M1H.A.2002, 4M2P.A.301, 4M5E.A.506, 1M8T.A.1001, 4MNO.A.303, 3MSE.B.180, 4
N1G.A.202, 4N2N.A.704, 4N5X.A.203, 3N5A.A.101, 1N9E.A.803, 1NNL.A.2001, 3NQX.A.5
11, 2O72.A.401, 1OAH.A.1526, 1OF3.A.1174, 1OH4.A.1186, 1OHZ.B.1057, 4OMC.A.605,
3OX6.B.501, 3P6B.A.205, 3PDD.A.193, 3PF2.A.1, 3PGB.A.902, 4PKG.G.1201, 1PMH.X.30
0, 1POA.A.201, 1POC.A.501, 3POJ.A.1, 2PPL.A.478, 2PQX.A.500, 3Q5I.A.1, 3QGM.A.50
3, 2QQM.A.1, 2QUB.A.617, 2QUB.A.620, 1R55.A.515, 2RAN.A.324, 2RDZ.A.1501, 2RF7.A
.1501, 1RRO.A.110, 1S3P.A.210, 3S5U.A.221, 1S6B.A.401, 1SH7.A.1291, 3SJG.A.1753,

1SL6.D.3, 1SL8.A.669, 1SL8.A.670, 3SXQ.A.526, 3T3P.B.2002, 1TF4.A.3002, 4TSH.B.1501, 3TZ1.A.1, 3U1R.A.701, 3UBH.A.857, 1V0A.A.1177, 4V29.B.1178, 1VL9.A.124, 2VPT.A.1235, 2VVE.A.1336, 1W0N.A.1132, 3W57.A.201, 1W7C.A.803, 2WN3.A.400, 2WN3.C.400, 2WPH.S.1246, 3WU2.c.901, 2WZE.B.1552, 4XDQ.A.301, 2XFG.B.1619, 2XFD.A.1112, 1XJO.A.905, 1XVJ.A.422, 1Y4J.A.1001, 2YIH.A.1520, 2YKK.A.1520, 2YN5.A.6364, 1YO8.A.1200, 2YOA.A.1001, 1Z70.X.3002, 2Z8X.A.618, 2ZEZ.A.200, 2ZUX.A.635, 2ZUX.A.636, 2ZUY.A.625, 4DKA.A.201, 3NUQ.A.283
[1] "Cluster 10"
4GX4.B.402, 4DTU.A.1002, 4ED0.A.503, 4FK0.A.1002, 3QEV.A.905, 4QWA.A.403, 1S0M.A.402, 4UAW.A.402, 1A25.B.290, 3A4U.A.286, 1A75.A.109, 1A8B.A.407, 4AE2.A.246, 5AER.A.200, 4AQA.A.1208, 4AQE.A.1209, 3B0X.A.579, 3B8Z.A.903, 2BIB.A.1552, 4BJ0.A.1167, 1BU3.A.109, 3C1Q.A.2, 4CFQ.A.501, 2CM5.A.1678, 4DIR.A.102, 1DTL.A.201, 4DZ3.A.202, 4DZ3.B.202, 1E8A.A.1089, 2E85.A.1002, 1EDH.A.302, 2EGD.A.301, 2EWE.A.701, 2FH1.A.2001, 1FS7.A.651, 1FXH.B.1002, 1GCY.A.528, 4GGF.C.204, 2GJP.A.1488, 1GZC.A.290, 1H2G.B.1558, 3HB2.P.484, 3HJR.A.602, 4HPH.A.701, 1HT6.A.500, 1HT9.A.1001, 1HVX.A.518, 3HX4.A.603, 4I35.A.515, 3I57.B.186, 4IAI.A.401, 4ICB.A.76, 3IPV.A.252, 4IT5.D.202, 2J5Z.A.1277, 1JIA.A.134, 4JJJ.A.704, 3JXS.A.301, 3K21.A.194, 1K94.A.998, 3KHE.A.192, 4KTY.A.801, 4KTY.A.803, 3L2Y.C.302, 1L9N.A.700, 1L9N.A.702, 3LCP.C.159, 4M00.A.801, 1MCX.A.349, 3MVS.A.213, 4N1G.A.204, 3N5A.A.102, 1NIW.A.1004, 3NOL.A.263, 3NQZ.B.520, 3OOW.A.377, 1OFL.A.528, 4OKH.A.904, 4OKH.B.901, 3P4G.A.411, 4P99.A.514, 4PE0.A.102, 4PEU.A.401, 3PPE.A.402, 3PPE.A.403, 1Q3A.A.468, 3Q5I.A.524, 2QNG.A.201, 3QRX.A.172, 2QUB.A.619, 2QUB.G.616, 2QV6.A.302, 1RP8.A.500, 1RRO.A.109, 3TI9.A.355, 1UX6.B.2001, 1UX6.B.2005, 1UX7.A.1135, 1UYZ.A.1132, 4UZU.A.1485, 4V29.B.1179, 2VN5.B.102, 2WD6.A.1765, 1WKY.A.503, 4WK7.A.503, 3WN6.A.503, 2XOM.A.1169, 1Y1X.A.202, 2Y3N.B.1067, 2Y5I.A.101, 2Y6L.A.1168, 1YO8.A.1198, 1YUT.A.197, 2Z2X.A.1003, 2Z8X.A.619, 2Z8X.A.627, 2Z8Z.A.623, 2ZP4.A.124, 2ZZJ.A.239, 4JP4.A.305
[1] "Cluster 11"
4CS3.A.1464, 3OTB.A.401, 2VBI.B.1000, 3FD2.A.374, 4K4G.I.603, 4K4H.A.604, 1N48.A.501, 3NAE.A.906, 3S9H.A.905, 3SCX.A.906, 3SQ2.A.904, 2AAO.A.294, 1AFD.1.2, 3AMR.A.901, 1ANN.A.320, 4ANR.A.400, 3B2Z.B.2, 1BJF.A.403, 2C0T.B.1507, 3C7F.A.803, 1CGE.A.304, 1DB4.A.198, 1E43.A.504, 3EKI.A.601, 4ELG.B.202, 4ELG.C.202, 2ERO.A.701, 3FCS.A.2008, 2FH1.A.2003, 3FLT.A.301, 4GON.A.202, 4GKY.A.302, 1HDH.A.1528, 4I2Y.B.504, 4IRZ.A.2006, 3KEZ.B.501, 4KHO.A.1001, 1KP4.A.200, 2MLR.A.303, 3NOK.A.257, 1O9I.A.269, 1O9I.E.269, 2P69.A.305, 4P99.A.515, 3PDD.A.194, 4RDQ.A.504, 4RDQ.B.502, 4RGP.B.302, 1SRA.A.301, 3T3P.A.2005, 3T3P.A.2006, 3T3P.A.2007, 1TMQ.A.1001, 4U65.E.302, 1UX6.B.2006, 2VNG.A.1213, 1W0Y.H.1259, 2WG7.A.1126, 4WK0.A.502, 4WK0.A.504, 2Y8K.A.1526, 2YHG.A.1936, 1YO8.A.1194, 1YO8.A.1207, 2ZFD.A.230, 2ZUX.A.633, 2ZUX.A.634, 2ZUY.A.623, 2ZZV.A.501, 4NLZ.A.402, 3N80.B.602, 2PKC.A.280, 1WX5.A.282
[1] "Cluster 12"
3G9D.A.299, 4KI8.A.602, 4BY5.A.1184, 3D1M.A.2, 3ECQ.B.2000, 4GER.A.402, 3GIS.Y.1002, 2HYW.A.501, 4I75.A.401, 3IJ9.A.497, 1IOD.A.501, 4K70.A.1003, 3KF9.C.302, 1KIC.B.328, 1KX0.C.703, 4LMF.D.303, 3N1F.A.6, 3N1G.B.190, 1S2N.A.1290, 3S5U.D.221, 3WNP.A.801, 1YO8.A.1204

Table S135. 7-ligand combined metal, combined group

	size	largest_angle*	middle_1*	middle_2	middle_3
1	"152"	"171.8+/-3.7"	"68.9+/-3"	"74.1+/-2.8"	"77.4+/-2.8"
2	"121"	"172.1+/-3.4"	"70+/-2.1"	"72.4+/-1.7"	"74.5+/-1.7"

3	"108"	"163+/-3.8"	"69.8+/-2.5"	"72.5+/-2.3"	"74.4+/-2"
4	"68"	"162.3+/-5.7"	"52.7+/-3.5"	"63.3+/-6.5"	"68.6+/-5.3"
5	"281"	"164.8+/-2.6"	"73.3+/-2.7"	"75.8+/-2.1"	"77.7+/-1.7"
6	"158"	"169.4+/-2.8"	"71.4+/-3.4"	"75.3+/-2.7"	"77.6+/-2.3"
7	"128"	"160+/-3.9"	"71.5+/-4"	"74.4+/-3.2"	"76.1+/-2.9"
8	"120"	"158.1+/-5"	"70.2+/-3.2"	"72.8+/-2.8"	"74.7+/-2.4"
9	"44"	"157.2+/-5.9"	"51.8+/-3.1"	"64.3+/-6.2"	"69.7+/-4.3"
10	"244"	"173.4+/-2.6"	"73+/-2.4"	"75.8+/-1.9"	"77.9+/-1.5"
11	"62"	"171.2+/-4"	"54.5+/-5.3"	"70.1+/-6.8"	"76+/-4.6"
12	"127"	"164.1+/-5.3"	"51.5+/-2.4"	"70.8+/-6"	"75.5+/-3.1"

	middle_4	middle_5	middle_6	middle_7*	middle_8
1	"80+/-2.6"	"82.3+/-2.3"	"84.2+/-2"	"86.1+/-1.8"	"87.8+/-1.9"
2	"77.2+/-2.1"	"80.6+/-2.2"	"82.8+/-2.2"	"84.6+/-2"	"86.4+/-1.8"
3	"76.5+/-1.9"	"78.2+/-1.8"	"80.2+/-1.7"	"82.1+/-1.9"	"84.2+/-2.1"
4	"71.9+/-5.2"	"74.9+/-4.8"	"77.4+/-4.9"	"80.2+/-4.2"	"83.9+/-4.2"
5	"79.3+/-1.5"	"80.8+/-1.4"	"82.4+/-1.5"	"84.1+/-1.6"	"85.8+/-1.8"
6	"79.3+/-2.1"	"80.9+/-2"	"82.3+/-1.9"	"83.9+/-1.7"	"85.7+/-1.8"
7	"77.8+/-2.6"	"79.4+/-2.3"	"80.9+/-2.3"	"82.6+/-2"	"84.3+/-2.3"
8	"76.4+/-1.9"	"77.6+/-1.7"	"78.9+/-1.8"	"80.3+/-1.7"	"82+/-1.9"
9	"72.6+/-3.4"	"75.8+/-3.1"	"77.8+/-2.9"	"80.8+/-3.4"	"83.5+/-4"
10	"79.9+/-1.7"	"81.8+/-1.7"	"83.6+/-1.7"	"85.1+/-1.7"	"86.8+/-1.7"
11	"79+/-4.3"	"81.5+/-3.9"	"83.8+/-3.4"	"86+/-2.9"	"88.2+/-2.7"
12	"77.9+/-2.4"	"80+/-2.3"	"81.7+/-2.4"	"83.4+/-2.4"	"85.4+/-2.4"

	middle_9	middle_10	middle_11	middle_12	middle_13*
1	"89.4+/-1.8"	"91.4+/-1.8"	"93.3+/-2.2"	"96+/-2.8"	"98.8+/-3"
2	"88.5+/-1.7"	"90.3+/-1.8"	"92.3+/-2.5"	"95.3+/-2.8"	"98.3+/-3.2"
3	"86.7+/-2.2"	"89.2+/-2.4"	"92.1+/-3.2"	"97+/-3.8"	"104.3+/-3.7"
4	"86.9+/-4.7"	"90.8+/-5.2"	"95.9+/-6.1"	"102+/-6"	"109.2+/-5.4"
5	"87.6+/-2"	"89.6+/-2.1"	"92.1+/-2.6"	"96.8+/-2.8"	"100.8+/-2.6"
6	"87.6+/-2"	"89.9+/-2.3"	"92.6+/-2.9"	"98.2+/-3.7"	"105.9+/-2.6"
7	"85.9+/-2.5"	"88.1+/-3.2"	"91.7+/-3.6"	"97.6+/-4.4"	"108.5+/-4.1"
8	"84.3+/-2"	"86.7+/-2.4"	"89.9+/-3.2"	"99.7+/-5.1"	"113.8+/-3.6"
9	"87.1+/-5.1"	"91.1+/-5.9"	"95.4+/-6"	"102.4+/-7.6"	"114.6+/-5.6"
10	"88.6+/-1.6"	"90.4+/-1.6"	"92.5+/-2.1"	"95.2+/-2.2"	"98+/-2.6"
11	"90.5+/-2.3"	"92.8+/-2.9"	"95.6+/-3.6"	"98.2+/-4.1"	"101.6+/-4.7"
12	"87.6+/-2.6"	"90.4+/-3"	"93.6+/-3.4"	"98.3+/-3.8"	"105.5+/-4.7"

	middle_14	middle_15	middle_16	middle_17	middle_18
1	"103.9+/-5.1"	"118.4+/-5.7"	"126.1+/-6.8"	"145.5+/-5.1"	"156.3+/-4.8"
2	"103.2+/-4.7"	"134.9+/-5.4"	"138.8+/-3.1"	"142.6+/-2.3"	"147.1+/-3.2"
3	"112.4+/-5.4"	"132.1+/-5.9"	"136.6+/-3.9"	"140.8+/-3.2"	"146.9+/-3.5"
4	"114.8+/-6"	"122+/-5.2"	"128.5+/-5.8"	"136.4+/-7.5"	"145+/-6.6"
5	"108.7+/-5.1"	"123.6+/-3.6"	"128.7+/-4.3"	"148+/-4.1"	"153.5+/-3.4"
6	"109.6+/-3.2"	"117.4+/-4.2"	"124.1+/-6.2"	"145.6+/-5.8"	"155.2+/-5.1"
7	"115+/-5.3"	"124.1+/-4.8"	"128.6+/-5.2"	"144.2+/-6.4"	"149.8+/-4.9"
8	"120+/-4.7"	"126.1+/-5.7"	"133.2+/-3.9"	"138+/-3.6"	"147.4+/-4.5"
9	"119.8+/-6"	"125.2+/-4.8"	"131.7+/-4.9"	"137.7+/-4.4"	"143.9+/-4.8"
10	"102.2+/-4"	"125.1+/-3.7"	"130.3+/-4"	"148+/-3.4"	"152.7+/-3.2"
11	"106.4+/-5.3"	"116.6+/-8.2"	"126.9+/-7.1"	"138+/-7.2"	"154.6+/-9"
12	"112.9+/-5.9"	"122.2+/-4.8"	"129.5+/-5.2"	"141.7+/-5.3"	"151.5+/-5.3"

	middle_19*	smallest_opposite_angle*	PentagonalBipyramidal
1	"164.9+/-3"	"50.8+/-2.4"	"0.075"
2	"150.4+/-3.9"	"66.8+/-3"	"0.223"
3	"151.2+/-4.6"	"67.4+/-2.7"	"0.239"
4	"153.4+/-5"	"51.5+/-4"	"0.017"

5	"157.6+/-2.9"	"51.5+/-1.7"	"0.162"
6	"162.3+/-3.7"	"51.4+/-2.7"	"0.104"
7	"153.8+/-4.2"	"51.6+/-2.3"	"0.171"
8	"153.3+/-5.2"	"68.5+/-3.4"	"0.12"
9	"147.8+/-4.5"	"64.2+/-6"	"0.011"
10	"156.8+/-2.7"	"51.7+/-2"	"0.201"
11	"165.6+/-4.6"	"52.1+/-5.7"	"0.046"
12	"158.5+/-4.3"	"72.1+/-4.1"	"0.096"

	SquareAntiprismaticV	HexagonalBipyramidalVA	HexagonalBipyramidalVP
1	"0.081"	"0"	"0.081"
2	"0.162"	"0"	"0.099"
3	"0.268"	"0.001"	"0.092"
4	"0.127"	"0.006"	"0.015"
5	"0.175"	"0"	"0.066"
6	"0.189"	"0.001"	"0.091"
7	"0.228"	"0.001"	"0.061"
8	"0.214"	"0.001"	"0.039"
9	"0.049"	"0"	"0.002"
10	"0.134"	"0"	"0.094"
11	"0.082"	"0"	"0.054"
12	"0.176"	"0.003"	"0.059"

Table S136. Cluster members of 7-ligand combined metal, combined group

[1] "Cluster 1"
2HVI.A.878, 1MOW.A.371, 2OTJ.3.8078, 4DCK.B.203, 1ECB.D.507, 4FVQ.A.902, 4GA3.A.
1002, 1HJK.B.452, 3TLM.A.1005, 2Z4W.B.1303, 3AVW.A.3001, 4J2D.A.1002, 4KHU.A.100
2, 3MXB.A.175, 3NCI.A.905, 2Q10.B.702, 3QEP.A.905, 4UB4.A.402, 2VS7.A.1183, 3ACH
.A.204, 2AEF.A.602, 1AJP.B.558, 4AQO.A.1881, 4AVS.A.207, 1AYP.A.301, 1BTU.A.260,
2BWR.A.500, 2CFT.A.1297, 2CM6.A.1679, 4D0E.A.1532, 1D2S.A.401, 2DBX.D.702, 1DBN
.A.301, 3DEM.A.5001, 1DJX.B.2, 4DKB.A.301, 2E26.A.602, 3E9T.B.4, 3EAD.B.1003, 2E
RO.A.702, 3ETO.A.2001, 2EXH.B.2002, 3FCS.A.2004, 2FI1.A.191, 2FMD.A.301, 1FNY.A.
500, 1FX5.A.251, 1G8K.A.5008, 1G8K.C.5108, 2GKO.A.610, 1GK9.B.1579, 1GKF.B.1571,
1GO7.P.1482, 1GO8.P.1482, 3GPE.A.501, 4H2B.A.604, 1H3G.B.701, 1H6X.A.1162, 1HDF
.A.1102, 4HHO.A.401, 1HPL.A.960, 1HQL.A.302, 2HYV.A.607, 3IBZ.A.193, 4ICB.A.76,
4IHM.A.406, 4ITC.A.1201, 2J7A.A.1006, 1JX9.B.601, 1K9I.I.1201, 1KAP.P.621, 3KM5.
B.2011, 3KQR.A.205, 1KVO.A.191, 3KWU.A.902, 1LHW.A.401, 4LMH.A.811, 3LNP.A.472,
1LOC.A.228, 4LOR.A.301, 1LPB.B.453, 3LPC.A.360, 3LPC.A.362, 4LP7.B.301, 2LTN.A.1
91, 1LVU.A.8001, 1LVU.D.8002, 3M1H.A.2001, 4MGQ.A.601, 1MKU.A.124, 2ML3.A.202, 3
MVS.A.216, 1N47.B.252, 1NPC.A.321, 2NXP.D.603, 1OAC.A.803, 1OAH.A.1525, 3OEA.A.2
00, 3OM2.A.1, 3OM4.A.1, 3OM5.A.1, 3OM6.A.1, 3OM7.A.1, 4OMC.A.606, 4OY7.G.302, 4P
99.B.517, 2PEL.A.237, 3QR0.A.1000, 2QUB.A.621, 2R8Z.N.214, 1S6C.A.218, 1SL4.A.40
7, 3SRE.A.1356, 1SU4.A.995, 3TI7.A.354, 3TI9.A.354, 1TM7.E.470, 1TU5.A.903, 4U36
.A.302, 3U4X.A.237, 1UNE.A.124, 1UP8.A.599, 2UWP.A.1246, 1UWW.A.1192, 1UX6.B.200
8, 1UX6.B.2011, 3V9M.A.205, 1VCL.A.1002, 3VLV.A.503, 3VV3.A.402, 2W08.A.206, 1WB
F.A.303, 1WC5.A.2100, 4WJK.B.502, 2WW8.A.1002, 1Y1X.A.201, 1Y6O.A.207, 2YAY.A.12
66, 1YO8.A.1189, 2Z8X.A.621, 2Z8X.A.625, 2ZEX.A.406, 2ZKM.X.800, 2ZVD.A.628, 2ZY
H.A.700, 2ZZJ.A.239, 1WTE.A.1001, 4KQ7.B.502, 3MNC.A.280, 2VX5.A.1421, 2XRM.A.40
1, 2YEQ.A.582
[1] "Cluster 2"
4GX4.B.402, 4K4G.A.602, 4K4H.A.602, 4K4I.A.605, 4UAW.A.403, 4A3Z.A.2344, 3A4U.A.

287, 4A41.A.2494, 4A42.A.1690, 4A4A.A.1925, 4A5G.A.1309, 3ALU.A.201, 4AOC.A.1129, 4AQ1.A.1924, 4ASM.B.1359, 3ATG.A.301, 4ATE.A.1275, 4AWD.B.1321, 3AXD.A.3002, 3AZY.A.301, 3B0K.A.201, 1B80.B.351, 1B9O.A.124, 4B96.A.1155, 4B9C.A.1151, 1BGP.A.501, 2BIB.A.1551, 4BLK.A.400, 3BMV.A.685, 4BM1.A.401, 4CI7.A.1505, 3CK7.A.710, 1CPN.A.209, 4CRQ.A.260, 4CUO.A.1326, 4CZN.A.1370, 1D3C.A.687, 2DEW.X.900, 2E39.A.501, 2E8Y.A.741, 2EXH.A.2001, 1F6S.A.201, 2FHF.A.2401, 3FMU.A.351, 1GW2.A.502, 1GWT.A.502, 1GWU.A.1308, 1GX2.A.1310, 3HOO.A.401, 2H2N.B.1001, 3HB3.A.563, 3HDL.A.306, 2HD9.A.2001, 1HFX.A.124, 3HR9.A.401, 2HYK.A.477, 1I22.B.197, 4IAU.A.800, 3ILF.A.278, 2IWV.D.1283, 2J1A.A.1769, 4J3V.A.920, 4J3W.A.907, 4JCL.A.701, 2JD9.A.1146, 2JDA.A.1146, 4JGL.A.202, 1JI1.A.2002, 2JKA.A.1727, 1JUG.A.126, 1L6R.B.903, 1LLP.A.351, 4LPL.A.1101, 4LQR.A.201, 1LY8.A.9001, 3M5Q.A.372, 3MMZ.A.501, 1MVE.A.400, 4N2B.A.707, 4N2G.A.703, 4N2L.A.704, 4N6F.A.302, 3NNG.A.402, 1O4Y.A.700, 4ODG.A.202, 2OKX.A.4001, 3OMI.A.613, 3OWF.A.151, 1PA2.A.308, 3PGV.A.267, 4PLS.B.301, 3Q3U.A.340, 4QF4.A.202, 1QGJ.A.2002, 1R1Z.A.286, 4R83.B.501, 3S18.A.229, 1SCH.A.301, 3SNY.A.97, 1SNC.A.150, 3SO1.A.97, 1TE2.A.702, 1U0A.A.5004, 1UPS.B.501, 1URX.A.1300, 1UX6.B.2014, 3V6N.A.229, 2VDQ.B.2002, 1W3M.E.3013, 3WDH.A.801, 2WOY.A.2415, 2WZA.A.2415, 2YLJ.A.1308, 1YRO.A.124, 2Z30.A.1002, 2Z49.A.1004, 2Z49.A.1005, 2GIG.A.501, 3MMD.A.410, 3U0F.A.410, 1W8O.A.1654

[1] "Cluster 3"

4GX6.B.402, 3MJX.A.901, 1T9I.C.801, 1T9I.C.802, 4A3X.A.1268, 4A5G.A.1308, 4A6O.A.2346, 4A6S.A.1122, 3AFG.A.541, 4AFA.A.1267, 4AFB.A.1267, 1ALC.A.200, 3ALT.A.201, 4AL9.A.1122, 4AOC.E.1129, 4ASL.A.1268, 3BOX.A.578, 2BF6.A.1693, 1BJ3.B.124, 3BPS.E.1, 1BYF.A.201, 1CGT.A.686, 1CPM.A.215, 4CP0.A.1294, 4CP1.A.1294, 4CPB.D.1123, 3CQO.A.4004, 1D2V.A.600, 1DED.B.5004, 3DED.B.506, 1DV8.A.1002, 4DZT.A.302, 3EDF.A.603, 2EJN.A.1003, 2ERV.A.195, 2FF3.A.701, 1FHF.A.502, 1FIF.B.2, 4G01.A.300, 1G5N.A.408, 1GWU.A.1307, 4GWI.A.204, 1H3G.A.700, 3HDL.A.307, 1HFZ.B.124, 4I5L.B.601, 4IAI.A.402, 2J22.A.1150, 1JI1.A.2001, 2JKX.A.1641, 1K12.A.160, 3K8K.A.710, 3K8L.A.700, 3K8L.A.710, 1KZM.A.501, 4LHK.A.303, 4LHN.A.302, 4LJH.A.201, 1LPZ.B.1, 1LYC.B.9002, 1MAC.A.388, 2ML3.A.201, 1PA2.A.307, 1PAM.A.687, 3PAR.A.300, 4PIB.C.201, 1PJ9.A.890, 4Q1U.A.402, 1QGJ.A.2001, 1SCH.A.302, 1SH7.A.1292, 3SRE.A.1357, 3SRG.A.1357, 1SU4.A.996, 3TBD.A.401, 1TDQ.B.127, 1TLG.A.201, 3TO5.A.131, 1UX7.A.1134, 2V72.A.1139, 2VUD.C.1118, 2VZQ.A.1130, 2W1Q.A.1947, 2W1S.A.1946, 2W2M.E.1334, 3W57.A.202, 2W86.A.1148, 4WF7.A.600, 2WQ8.A.1641, 2WR9.A.1131, 1WZL.A.1601, 2WZS.F.800, 2XFD.A.1111, 2XFE.A.1113, 1XO5.A.1, 2YFU.A.1141, 2Z48.A.1007, 2Z48.A.1102, 2Z48.A.1103, 2Z48.A.1104, 2Z48.A.1205, 2Z49.A.1001, 2Z49.A.1003, 3ZUC.A.1154, 3ZYH.A.1123, 1VQ9.Q.9148, 1GOF.A.702, 4IIL.A.402

[1] "Cluster 4"

4M2Z.B.301, 4RUB.A.491, 3FD2.A.375, 4K4I.I.603, 1AJ4.A.164, 1AP4.A.90, 2ARY.B.404, 3AR2.A.1001, 3B2Z.B.2, 3BSG.A.501, 1CKK.A.151, 1CKK.A.153, 2CL8.B.1245, 2EHB.A.1004, 1EMN.A.2225, 4ENZ.A.1110, 2EV5.A.1150, 1FPW.A.502, 1FWX.D.4903, 1GOH.A.291, 3GIS.Y.1002, 3H2W.A.538, 3HR4.F.202, 4I5L.E.702, 1I8A.A.192, 4IEF.D.704, 4IEF.F.703, 4IRZ.A.2005, 4IRZ.A.2007, 4IT5.C.201, 2JU0.A.500, 1KWW.B.602, 1KX1.F.602, 2LMV.A.150, 2LUX.A.202, 2M28.A.301, 1M63.B.501, 1M63.B.503, 1MXD.A.731, 4NHF.F.301, 1NIW.E.1009, 1NIW.E.1010, 1NL2.A.205, 4NPK.A.802, 1NYA.A.501, 4O6N.A.401, 4O6N.B.401, 2OW2.B.446, 4PHK.B.305, 1QNI.C.901, 1S2N.A.1290, 3SBR.A.703, 1TDQ.B.128, 1TF4.A.3001, 4TV8.A.503, 4V0C.C.201, 3VI3.C.2001, 2WR9.B.1129, 1XYD.A.93, 1ZIV.A.2, 1QVG.O.8317, 3HIJ.A.294, 4MM7.A.602, 3U27.A.306, 3UT0.A.902, 3WGV.A.2005, 2Y8K.A.1528, 2ZND.A.194

[1] "Cluster 5"

4X2T.L.701, 1G9Y.A.801, 4J2E.A.1002, 4KLD.A.403, 3MQ6.A.1, 3MX9.A.363, 3NDK.A.905, 3NE6.A.904, 3QNN.A.903, 3QNO.A.903, 4UB4.A.401, 1A2Q.A.295, 2A3Y.A.601, 3A8R.A.401, 2AAO.A.293, 1AFO.A.476, 1AK9.A.295, 3AKB.A.171, 3AKB.A.172, 3AKB.A.175, 3AMR.A.908, 4AQ1.A.1923, 4AQ1.C.1923, 4AQJ.A.1097, 1AVS.A.93, 1AVS.A.94, 1AXN.A.353, 2AYH.A.417, 4AYU.D.205, 2AZZ.A.125, 3B8Z.A.903, 3BC9.A.701, 1BF2.A.751, 1BH6

.A.501, 1BJF.A.402, 1BLI.A.500, 1BQB.A.351, 1BU3.A.109, 1BU3.A.110, 3BYK.A.474, 4BY5.A.1183, 4BY5.A.1185, 2C10.B.1777, 3C1Q.B.1, 3C3Y.A.238, 2C4X.A.1253, 2C4F.L.1143, 1C9N.A.277, 4CAG.A.603, 4CBU.G.1151, 2CCL.B.1060, 2CCM.A.1192, 2CCM.A.1194, 4CCD.A.3669, 2CDP.A.1139, 4CFQ.A.502, 4CFY.A.301, 1CP9.B.554, 4CPV.A.110, 1CRU.A.501, 3CZT.X.93, 4DA2.A.301, 2DCK.A.1001, 2DIE.A.780, 4DIR.A.101, 3DO1.A.401, 1DTL.A.201, 1DTL.A.203, 4DUQ.A.102, 1DVI.A.271, 2DW0.A.701, 2DW0.A.702, 2E26.A.601, 2E4T.A.701, 1E43.A.502, 1E8A.A.1090, 3EDF.A.602, 2EGD.A.302, 3ETO.A.2003, 2EWE.A.701, 1EXR.A.1000, 1EXR.A.1002, 4F0Z.B.204, 2FH1.A.2002, 3FIA.A.201, 3FLP.A.301, 3FLP.A.302, 3G5C.A.801, 1G8I.B.1600, 1G9K.A.702, 1GGZ.A.149, 4GGF.C.204, 4GGF.L.204, 2GJP.A.1487, 4GUK.A.207, 4H1Q.B.304, 1H2G.B.1558, 3HB2.P.481, 3HDB.A.622, 3HJR.A.601, 2HQ8.A.203, 1HT6.A.500, 1HT9.A.1001, 3HX4.A.604, 3HX6.A.1, 2HYV.A.605, 2HYW.A.503, 4I2Y.A.501, 4I2Y.A.503, 2I7A.A.2, 1I82.A.192, 2ID4.A.901, 4IEF.B.702, 3IGO.A.603, 2II1.C.400, 4ITC.A.1202, 1J1A.A.201, 1J55.A.102, 1JK3.A.404, 3JQW.A.1001, 4JX1.E.505, 3K21.A.193, 3K21.A.194, 1K7I.A.480, 1K94.A.999, 1K9J.A.403, 1K9U.A.1001, 1K9U.A.1002, 4K90.A.702, 1KAP.P.615, 4KDW.A.201, 3KHE.A.193, 3KHE.A.194, 3KLL.A.1, 1KVW.A.124, 4KWU.A.1109, 3L2Y.A.301, 3LND.B.208, 3LNI.A.302, 3LPC.A.361, 2LRP.A.202, 4M5E.A.505, 1MCX.A.351, 1MCX.A.352, 1MKV.A.124, 2ML1.A.205, 4MSP.A.201, 1MXE.A.506, 4N1G.A.203, 3N4E.B.500, 4N5X.A.204, 4NEH.A.1101, 4NEH.A.1103, 1NIW.C.1007, 3NOL.A.263, 1NPC.A.319, 2NXP.C.602, 2NZM.A.405, 3OOW.A.377, 3O4Y.A.198, 2O5G.A.404, 2O72.A.402, 2O8O.A.1009, 1OB0.A.502, 4OKH.A.902, 2OLG.A.2001, 4OMC.A.607, 2OO4.A.5001, 2OPO.A.301, 2OPO.A.302, 2OX9.A.804, 1OYG.A.500, 2P0R.A.1002, 5P2P.A.125, 4P99.A.514, 5PAL.A.110, 5PAL.A.111, 4PEO.X.103, 4PHJ.A.302, 4PHJ.A.303, 4PHJ.A.304, 4PKI.A.403, 2PMY.B.104, 3PM8.A.514, 3PM8.B.1, 1PMJ.X.300, 1POE.A.801, 1PT2.A.500, 1PVA.A.110, 3PVN.E.5009, 3PVN.M.5026, 2PVB.A.110, 3Q2L.A.701, 3Q5I.A.524, 3Q5I.A.526, 1Q5P.A.271, 4Q6O.A.502, 1QI3.A.451, 2QQO.A.1, 3QRB.A.302, 3QRX.A.171, 1QTX.A.153, 1QTX.A.154, 1QX2.A.1001, 1QX2.B.1006, 1R0R.E.302, 2R2I.A.500, 2R2I.A.501, 2R2I.A.502, 1RFJ.A.1001, 1RFJ.A.1003, 1RFJ.A.1004, 2RHP.A.29, 3RM1.A.102, 3RUP.A.1006, 1RWY.A.421, 1S01.A.295, 1S02.A.276, 1S6C.A.217, 2SCP.A.190, 2SCP.A.191, 3SIB.A.222, 1SL6.E.3, 1ST3.A.270, 1SUD.A.295, 1TCF.A.160, 1THM.A.301, 1THM.A.302, 3TI7.A.353, 3TI7.A.355, 3TI9.A.353, 3TI9.A.355, 1TKF.A.905, 4TNC.A.164, 5TNC.A.163, 3TTQ.A.2867, 3UJO.B.304, 3UL4.B.67, 4UM9.A.2001, 1UX6.B.2001, 1UXX.X.1130, 1UY4.A.1147, 1UYZ.A.1132, 1VCL.A.1001, 1VCL.A.1005, 2VN5.B.102, 2VN6.B.1067, 2VVD.A.1328, 3VYV.A.303, 2VZP.A.1128, 2W47.A.1137, 2WBX.A.1103, 3WFD.B.806, 3WHI.A.401, 3WHT.B.501, 4WK7.A.502, 2WND.A.102, 2WNO.A.1251, 1WP6.A.502, 1WPC.A.501, 2WZ8.A.1149, 1X1J.A.2000, 3X17.A.603, 2Y3N.B.1067, 1Y93.A.267, 1Y9Z.A.603, 2YOA.A.1002, 1YU6.A.401, 2Z2X.A.1002, 2Z30.A.1005, 2Z8X.A.627, 2ZFD.A.229, 2ZUX.A.631, 2ZUX.A.632, 2ZUY.A.622, 3ZYP.A.1220, 4DMI.C.203, 4IB0.A.402, 4O7J.A.201, 3PFV.A.1, 4X26.A.402
[1] "Cluster 6"
3V6J.A.403, 2A9F.B.800, 3EW9.A.501, 4DTU.A.1002, 4E3S.A.1002, 4ED0.A.503, 4FK0.A.1002, 4K4G.A.603, 3NGI.A.905, 2ODI.B.702, 3QER.A.905, 3QEV.A.905, 4QWA.A.403, 4UAW.A.402, 1A25.B.290, 3A4U.A.286, 1A75.A.109, 1A8B.A.407, 4AE2.A.246, 5AER.A.200, 1ANW.B.353, 4AQA.A.1208, 4AQE.A.1209, 1AX0.A.290, 3B0X.A.579, 3BFM.A.235, 1BIW.B.803, 2BIB.A.1552, 4BJ0.A.1167, 2C0T.A.1507, 3C1Q.A.2, 1C8T.B.264, 2CCL.B.1061, 4CFQ.A.501, 3CLN.A.152, 2CM5.A.1678, 1D0L.A.400, 1DB5.A.198, 4DIR.A.102, 2DUR.A.1, 4DZ3.A.202, 4DZ3.B.202, 1E8A.A.1089, 2E85.A.1002, 1EDH.A.302, 2EGD.A.301, 2EIG.A.1102, 3EU3.A.1, 2FH1.A.2001, 1FIB.A.500, 1FXH.B.1002, 4FZM.A.301, 1G7Y.A.254, 1G9K.A.704, 1G9K.A.705, 2GJP.A.1488, 2GSK.A.1, 1GSL.A.251, 1GZC.A.290, 3HB2.P.484, 4HPH.A.701, 3HR4.D.202, 1HVX.A.518, 3HX4.A.603, 4I35.A.514, 4I35.A.515, 3I57.B.186, 4IAI.A.401, 3IPV.A.252, 4IT5.D.202, 4IU3.B.301, 2J1V.A.1152, 2J5Z.A.1277, 1J9L.A.1301, 4JA8.A.503, 2JE7.A.1241, 2JEC.A.240, 4JJJ.A.704, 1K94.A.998, 4KTY.A.801, 4KTY.A.803, 3L2Y.C.302, 1L9N.A.700, 1L9N.A.702, 1LEN.A.184, 4M00.A.801, 1MCX.A.349, 2ML2.A.203, 3MVS.A.212, 3MVS.A.213, 1MVQ.A.238, 4N1G.A.204, 4N2L.A.707, 3N35.A.290, 3N5A.A.102, 1NIW.A.1004, 1NIW.G.1014, 1NLS.A.240, 3NQZ.B.520

, 1O8F.A.1353, 1OFL.A.528, 1OHZ.B.1058, 4OKH.A.904, 4OKH.B.901, 1OUX.A.402, 2OVU.A.238, 3OX6.B.502, 2P2K.A.239, 3P4G.A.411, 3P4G.B.405, 4PE0.A.102, 4PEU.A.401, 3PPE.A.402, 3PPE.A.403, 1Q3A.A.468, 1QNW.A.302, 2QNG.A.201, 1QPK.A.451, 3QRX.A.172, 2QUB.A.619, 2QUB.G.616, 2QV6.A.302, 1QX2.A.1005, 1R1Z.A.285, 1RLW.A.401, 1RP8.A.500, 1RRO.A.109, 1RU4.A.1, 1S1E.A.226, 1S6B.B.402, 3UBG.A.902, 1UKG.A.1262, 3USU.C.266, 1UX6.B.2005, 1UX6.B.2009, 1UX7.A.1135, 4UZU.A.1485, 4V29.B.1179, 3WCS.A.1003, 2WD6.A.1765, 1WKY.A.503, 4WK7.A.503, 3WN6.A.503, 2XOM.A.1169, 1Y1X.A.202, 2Y5I.A.101, 2YFS.A.1712, 1YO8.A.1198, 1YUT.A.197, 1YXH.A.1001, 2Z2X.A.1003, 2Z8X.A.619, 2Z8Z.A.623, 2ZVD.C.620, 3ZYR.A.401, 1XSP.A.576, 4DNL.A.300, 4JP4.A.305

[1] "Cluster 7"

4KI8.A.602, 4DTM.A.1002, 4DU1.A.1003, 4DU3.A.1002, 2FLD.B.602, 3RAX.A.416, 2WTF.A.1511, 3A24.A.1268, 2AAO.A.295, 2AAO.A.296, 3AIE.A.4001, 4APX.B.1240, 3BOO.A.124, 1B8R.A.109, 4BY5.A.1184, 1CJY.A.950, 4CPV.A.109, 2CT9.A.301, 2CT9.A.302, 4CT3.A.1170, 3D1M.A.2, 3DBK.A.303, 1DVI.A.273, 2E3X.A.802, 3ECQ.B.2000, 4F0Z.B.201, 1FAT.A.255, 3FZO.A.400, 1FZD.A.1, 1G8I.A.1599, 3GDC.A.401, 4GER.A.402, 2GGM.A.401, 4GGF.A.101, 4GM5.A.404, 2GSK.A.2, 2GXS.A.601, 4H2A.A.805, 2HQ8.A.202, 1HVX.A.516, 2HYW.A.501, 3HZ3.A.1, 4I75.A.401, 4ICB.A.77, 3IJ9.A.497, 4IK8.A.502, 1IOD.A.501, 4IU3.B.302, 2J1G.B.1290, 1JC9.A.301, 1JIW.P.485, 4JWQ.A.202, 4K70.A.1003, 4K70.B.1003, 1K9K.A.400, 3KF9.C.302, 1KIC.B.328, 4KKK.A.701, 3KLK.A.1, 3KQR.C.206, 1KXO.C.703, 3LI6.A.149, 3LI6.D.150, 4LMF.D.303, 4LVN.A.702, 4M7H.A.501, 1MDW.B.4, 3MHZ.A.736, 4MNO.A.302, 3N1F.A.6, 3N1G.B.190, 4N5X.A.201, 4N5X.A.205, 2OO4.A.402, 3OOV.A.1, 2O5G.A.401, 2O5G.A.402, 2O5G.A.403, 2OBH.A.1001, 2OZN.B.402, 3P4G.A.413, 4P5X.A.1001, 4PET.A.403, 1POB.A.801, 2PVB.A.111, 2Q1F.A.2001, 3Q5I.A.525, 1QTX.A.152, 2R9F.A.366, 1RWY.A.422, 3S5U.D.221, 1SBF.A.601, 1SL7.A.301, 1SRA.A.302, 1TCF.A.163, 1TN4.A.162, 3U1R.A.702, 3UCP.A.912, 3UL4.B.66, 4UM9.A.2003, 4UZU.A.1483, 2VUD.D.1118, 2W46.A.1148, 2W87.A.1149, 3WA5.A.504, 3WH2.A.302, 3WNP.A.801, 2WNP.F.1298, 2Y3N.B.1068, 2YA2.A.1691, 1YO8.A.1186, 1YO8.A.1187, 1YO8.A.1190, 1YO8.A.1204, 1YUT.B.199, 1Z3J.A.267, 2Z30.A.1004, 1ZCM.A.1001, 2ZN9.A.901, 2ZWO.A.402, 2ZWP.A.404, 2ZWP.B.402, 1Q81.C.8345, 2HZL.A.500, 4LUG.A.301, 1O68.D.274, 3V7Z.A.404, 1W9W.A.901

[1] "Cluster 8"

4FFO.A.903, 3G9D.A.299, 4LTZ.A.404, 3RAX.B.1416, 4AFC.A.1267, 3AUJ.A.1603, 2B6N.A.300, 4B9F.A.153, 1BCJ.2.2, 2BO2.A.155, 2BO2.A.156, 2BOI.A.300, 2BOI.B.600, 2BOJ.A.1116, 2BOJ.C.1117, 2BP6.A.802, 2BP6.C.805, 1BQB.A.353, 2BV4.A.300, 2BV4.B.300, 4CE8.A.998, 4CE8.B.998, 4CGT.A.685, 1CIU.A.684, 4COU.A.1270, 4COV.A.1269, 4COY.A.1270, 4CPB.A.1122, 3CQO.A.4001, 4D0E.A.1531, 4D0E.A.1533, 3DBZ.A.401, 3DCQ.A.116, 3DCQ.B.116, 2DCK.A.1002, 3DED.A.504, 3DEM.B.4001, 2DSO.A.1001, 3EHU.A.500, 3EIF.A.1, 1ESL.A.163, 2EWE.A.703, 4FHP.A.402, 2FPW.A.503, 4FVL.A.505, 1G1Q.A.801, 1GEN.A.302, 4GER.A.404, 2GGX.C.401, 3GIS.Z.1003, 2GVU.A.500, 2GVV.A.500, 2H2T.B.322, 4HHR.A.703, 3HLH.B.315, 3HLI.B.315, 2IAW.A.401, 2IAX.A.401, 2IAO.A.401, 3INM.A.521, 3IQT.A.1, 1J34.C.504, 2JDY.A.1116, 2JDY.B.1117, 2JDM.B.1115, 2JDM.C.1115, 2JDN.A.881, 2JDN.B.881, 2JDN.C.881, 4JSD.A.603, 4KVL.A.703, 1KWZ.A.504, 3LI3.A.402, 3LI4.A.316, 1LQV.C.42, 4M65.A.404, 1MN1.A.371, 4MZA.A.612, 4N4E.E.404, 4N7A.A.605, 1NL1.A.204, 1NPC.A.322, 1NZI.A.1001, 2NZM.A.406, 1OUX.B.404, 1OVS.A.402, 1OVS.B.404, 3P7F.D.1, 1PAM.A.688, 3PAQ.A.300, 1PJX.A.491, 3Q9K.A.606, 3R5Q.A.1001, 1SNN.B.503, 3SOO.A.97, 1SU3.A.904, 1SZB.A.1001, 3TH4.L.148, 1USR.B.1573, 1V3E.A.4001, 2VNV.A.302, 2VNV.B.302, 2VUC.B.990, 2VUC.C.991, 2VZP.A.1129, 2VZR.B.1132, 3W5M.A.1201, 3W5N.A.1201, 2W86.A.1149, 1WDC.C.501, 3WH3.A.500, 3WHD.A.501, 2WR9.D.1132, 3WU2.A.401, 1YDY.A.904, 3ZDV.A.200, 3ZDV.B.200, 1ZH2.B.201, 1ZJA.A.7001, 3ZO9.A.1589

[1] "Cluster 9"

4FFR.A.404, 1JR4.A.300, 3SQ1.A.903, 4CAJ.C.1323, 3CIO.K.401, 1CXE.A.690, 2DIJ.A.689, 1DJW.B.2, 3DSL.A.2, 1EO7.A.689, 1FI6.A.100, 1FIF.C.2, 1HQV.A.997, 4I5K.A.50

2, 4IT5.B.201, 1J1D.A.202, 3KF9.A.304, 1KIE.B.328, 1KXT.A.4001, 4LJ3.A.403, 2M55
.A.301, 4M5H.A.305, 2MTE.A.101, 2MTE.A.102, 3NJH.C.503, 1NL0.G.905, 4OV2.A.201,
4P5F.B.501, 1PVY.A.503, 3QED.A.348, 1RF1.E.462, 3TRP.A.358, 3UBG.A.901, 1UZJ.B.2
648, 4V29.A.1178, 1V3J.A.688, 2WVZ.A.800, 1X2T.A.603, 2Y4Q.A.800, 2YEQ.A.1525, 1
YUU.B.200, 1Z6C.A.246, 1Q81.A.8353, 2E5X.A.302
[1] "Cluster 10"
4FZL.B.302, 1Z2P.X.1296, 4AQU.A.1154, 4AQU.A.1156, 3EH8.A.301, 1G9Y.B.802, 3ODH.
B.195, 1SOM.A.402, 2VBJ.A.1154, 2VBJ.A.1155, 2VBO.A.1154, 2VBO.A.1155, 1A75.A.11
0, 1A8A.A.407, 3AMR.A.905, 3AMR.A.907, 4APX.B.1238, 4AQA.A.1209, 4AQE.A.1207, 1A
VA.A.500, 3B2Z.A.2, 2B96.A.124, 3BEU.A.249, 3BI1.A.1753, 3BNG.A.508, 1BQB.A.352,
 4BQ2.A.1797, 1BSW.A.900, 2C26.A.302, 2C4X.A.1252, 4CAG.A.604, 4CAG.A.610, 1CB8.
A.3000, 2CBL.A.352, 4CBU.G.1150, 2CDO.A.1139, 3CHJ.A.338, 3CHK.A.503, 3CIO.K.402
, 3CIP.G.201, 2CKI.A.997, 2CM5.A.1679, 2CM6.A.1680, 2CN3.A.1778, 4CTE.B.280, 1CV
R.A.504, 1DBI.A.701, 1DBI.A.703, 1DBI.A.704, 3DEM.A.8001, 3DNZ.A.403, 4DUQ.A.101
, 2DWO.A.703, 2E26.A.604, 2E26.A.605, 3ECQ.A.2001, 1EDM.B.2, 2EHB.A.1001, 1EXR.A
.1004, 4F0Z.B.202, 4F0Z.B.203, 3FED.A.1755, 2FMJ.A.338, 3FO3.A.527, 1FS7.A.651,
2FVY.A.311, 1G5N.A.401, 1G9G.A.630, 1G9K.A.701, 3GBO.A.301, 1GCA.A.351, 1GCY.A.5
28, 4GER.A.403, 1GGZ.A.150, 1GGZ.A.151, 1GPL.A.500, 3GPE.A.502, 1H6Y.A.1161, 1H8
0.A.1493, 3HDB.A.621, 3HI7.A.803, 3HJR.A.602, 1HQV.A.998, 2HQ8.A.201, 3HX4.A.601
, 3HX4.A.602, 1HY7.A.304, 4I35.A.511, 2ID4.A.906, 3IGO.A.602, 3IKW.A.1, 3IK2.A.5
14, 4IMM.A.407, 2IPL.A.502, 1J83.A.4001, 4JDZ.B.701, 4JGU.A.901, 1JI1.A.2003, 1J
IA.A.134, 3JQ1.B.1, 3JXS.A.301, 3K21.A.192, 1K96.A.91, 1KAP.P.614, 3KCP.A.701, 3
KHE.A.192, 3KHE.A.195, 3KM5.A.2012, 1KQU.A.301, 3KWU.A.901, 3LCP.C.159, 4LDC.A.5
02, 1LE6.A.461, 4LJ3.A.402, 4LM8.A.811, 4LOS.A.401, 3LPA.A.361, 1LQV.C.34, 4LXO.
A.1601, 4LXO.B.1601, 3M1H.A.2002, 4M2P.A.301, 4M5E.A.506, 1M8T.A.1001, 4MEW.A.50
1, 4MNO.A.303, 1MPX.A.638, 3MSE.B.180, 4N1G.A.202, 4N2B.A.709, 4N2G.A.704, 4N2N.
A.704, 4N5X.A.203, 3N5A.A.101, 1N9E.A.803, 1NNL.A.2001, 3NQX.A.511, 4NUQ.A.303,
2O72.A.401, 2O9Q.A.501, 1OAH.A.1526, 1OF3.A.1174, 1OH4.A.1186, 1OHZ.B.1057, 4OMC
.A.605, 2OW9.B.610, 3OX6.B.501, 3P6B.A.205, 1P8J.A.3001, 4P99.C.505, 3PDD.A.193,
 3PF2.A.1, 3PGB.A.902, 4PHN.B.303, 4PKG.G.1201, 3PM8.A.1, 1PMH.X.300, 1POA.A.201
, 1POC.A.501, 3POJ.A.1, 2PPL.A.478, 2PQX.A.500, 1PVA.A.111, 2Q17.A.315, 3Q2N.A.3
03, 3Q5I.A.1, 3QGM.A.503, 2QQM.A.1, 3QRB.A.303, 2QUB.A.617, 2QUB.A.620, 1R55.A.5
15, 3R5V.B.201, 1R64.A.701, 2RAN.A.324, 2RDZ.A.1501, 2RF7.A.1501, 1RLW.A.400, 1R
RO.A.110, 1S3P.A.210, 3S5U.A.221, 1S6B.A.401, 2SCP.A.192, 1SGT.A.246, 1SH7.A.129
1, 3SIB.A.221, 3SJG.A.1753, 1SL6.D.3, 1SL8.A.669, 1SL8.A.670, 1SL8.A.671, 3SXQ.A
.526, 3T3P.B.2002, 1T44.G.702, 1TF4.A.3002, 3TOY.A.361, 4TSH.B.1501, 3TZ1.A.1, 3
U1R.A.701, 3UBH.A.857, 3UXF.A.2, 1V0A.A.1177, 2V0B.A.1241, 4V29.B.1178, 1VL9.A.1
24, 2VPT.A.1235, 3VRR.A.401, 2VVE.A.1336, 1WON.A.1132, 3W57.A.201, 3W57.A.203, 1
W7C.A.803, 3WCT.F.203, 3WLC.A.501, 2WN3.A.400, 2WN3.C.400, 2WPH.S.1246, 3WU2.c.9
01, 2WW8.A.1001, 2WZE.B.1552, 4XDQ.A.301, 2XFG.B.1619, 2XFD.A.1112, 1XJO.A.905,
1XVJ.A.422, 1Y4J.A.1001, 2Y6L.A.1168, 2YIH.A.1520, 2YKK.A.1520, 2YN5.A.6364, 1YO
8.A.1200, 2YOA.A.1001, 1Z70.X.3002, 2Z8X.A.618, 2ZEZ.A.200, 2ZFD.A.227, 2ZFD.A.2
28, 2ZP4.A.124, 2ZUX.A.635, 2ZUX.A.636, 2ZUY.A.625, 4DKA.A.201, 3NUQ.A.283, 3UES
.A.503
[1] "Cluster 11"
3GV7.B.871, 4GZ2.A.402, 4OIN.D.2003, 4CS3.A.1464, 4GIR.B.401, 2XGZ.B.1439, 2AGQ.
A.4001, 2ATL.B.1415, 4K4G.I.603, 3KHR.B.1416, 3RB4.A.415, 4AR1.A.1723, 3B4N.B.70
1, 1D2J.A.1, 4DD8.A.1001, 1DJY.B.2, 4DLK.B.401, 2DPK.A.3001, 3EXM.A.301, 4G1M.A.
2008, 2H61.D.817, 4HHQ.A.401, 1JKU.A.1272, 2JT6.A.258, 2KYF.A.109, 4L73.A.403, 3
LNF.A.302, 3MOW.I.202, 4MOO.A.803, 4MO2.A.701, 2ML2.A.204, 1O8P.A.1149, 3PDD.A.1
94, 3QC6.X.1, 2QUB.K.614, 2RJP.C.3, 1SZB.A.1003, 4TVU.C.600, 4U65.E.301, 2VN7.A.
650, 1WCO.A.2100, 3WLC.A.503, 1YO8.A.1194, 1YO8.A.1207, 1ZED.A.906, 3E6S.A.200,
4NLZ.A.402, 4TUP.A.401, 4D1I.D.602, 4DXK.A.502, 2FBB.A.131, 4G8T.D.502, 1JZ3.B.3
102, 3KQC.A.303, 1LTM.A.400, 3OP0.A.2, 3PDG.A.99, 2PKC.A.280, 3Q2G.A.701, 3UCY.A

.100, 3W5D.A.1001, 2ZHI.A.321
[1] "Cluster 12"
3OTB.A.401, 2VBI.B.1000, 3FD2.A.374, 4K4H.A.604, 1N48.A.501, 3NAE.A.906, 3NHG.A.908, 3QEW.A.905, 3QEX.A.905, 3S9H.A.905, 3SCX.A.906, 3SQ2.A.904, 2AAO.A.294, 1AFD.1.2, 3AMR.A.901, 3AMR.A.906, 1ANN.A.320, 4ANR.A.400, 1B1G.A.76, 1BJF.A.403, 1BJF.A.404, 3BJU.A.606, 1BLI.A.700, 2BPE.A.1245, 2COT.B.1507, 4COK.A.1615, 3C7F.A.803, 2CCM.A.1193, 1CGE.A.304, 2CHN.B.1716, 1DB4.A.198, 2DEW.X.902, 4DH2.B.101, 4DH2.B.102, 4DLK.A.401, 1DTL.A.202, 1E43.A.504, 3EKI.A.601, 4ELG.B.202, 4ELG.C.202, 2ERO.A.701, 1ESP.A.319, 3ETO.A.2002, 1EXR.A.1001, 3FAW.A.4, 3FCS.A.2008, 2FH1.A.2003, 3FLT.A.301, 4GON.A.202, 1GGZ.A.152, 4GKY.A.302, 3GQ8.A.1, 1HDH.A.1528, 4HEX.A.204, 1HT6.A.501, 4I2Y.B.504, 4IRZ.A.2006, 3IUC.A.2, 2IWA.A.501, 1J55.A.101, 4JRF.A.601, 3KCP.A.702, 3KEZ.B.501, 3KF9.A.301, 4KHO.A.1001, 2KPN.A.762, 1KP4.A.200, 4L03.B.502, 4L73.B.402, 3LND.A.209, 2MLR.A.303, 4MNO.A.301, 4MSP.A.202, 3NOK.A.257, 4NUQ.A.302, 1O9I.A.269, 1O9I.E.269, 2O04.A.5002, 2P69.A.305, 4P99.A.515, 4QB2.A.202, 1R17.A.599, 4R1D.A.601, 4RDQ.A.504, 4RDQ.B.502, 4RGP.B.302, 1SRA.A.301, 3T3P.A.2005, 3T3P.A.2006, 3T3P.A.2007, 1TMQ.A.1001, 4U65.E.302, 4U6B.A.501, 3U8D.A.202, 3UBH.A.856, 1UKT.B.690, 4UM9.A.2002, 4UM9.A.2004, 1UX6.B.2006, 2VN5.B.101, 2VN6.B.1066, 2VNG.A.1213, 1WOP.A.1779, 1WOY.H.1259, 2WG7.A.1126, 3WHT.B.502, 4WJK.A.503, 4WKO.A.502, 4WKO.A.504, 3WNO.A.801, 1WPC.A.503, 1XO5.A.5, 2Y5I.A.102, 2Y8K.A.1526, 2YHG.A.1936, 1YO8.A.1196, 1YO8.A.1210, 1YUT.A.198, 2ZFD.A.230, 3ZHG.A.1323, 2ZUX.A.633, 2ZUX.A.634, 2ZUY.A.623, 2ZZV.A.501, 4IBO.A.401, 3N8O.B.602, 1WX5.A.282

Table S137. 8-ligand combined metal, normal group

	size	largest_angle*	middle_1*	middle_2	middle_3	middle_4
1	"1"	"173.7+/-NA"	"59.5+/-NA"	"61+/-NA"	"61.7+/-NA"	"63.7+/-NA"
2	"12"	"153.2+/-2.4"	"64.5+/-1.8"	"68.9+/-1.6"	"70.6+/-1.7"	"72+/-1.3"
3	"22"	"148+/-1.6"	"67.5+/-1.4"	"70.1+/-1"	"71.4+/-1"	"72.2+/-0.9"
4	"12"	"148.5+/-1.4"	"65.5+/-1.8"	"69.4+/-1.6"	"70.6+/-1.6"	"71.3+/-1.5"
5	"17"	"153.3+/-3.6"	"63.9+/-2.6"	"69+/-2.1"	"70.7+/-1.6"	"71.7+/-1.5"
6	"18"	"147.9+/-1.2"	"66.5+/-1.9"	"69.8+/-1.3"	"71.1+/-1.4"	"72.2+/-0.9"

	middle_5	middle_6	middle_7	middle_8	middle_9*
1	"63.8+/-NA"	"70.5+/-NA"	"73.6+/-NA"	"82.7+/-NA"	"84.2+/-NA"
2	"72.5+/-1.4"	"73.6+/-1.7"	"75.2+/-1.1"	"76.8+/-1.4"	"77.8+/-1.2"
3	"73.1+/-0.9"	"73.7+/-0.8"	"74.4+/-0.7"	"75.1+/-0.7"	"76+/-0.8"
4	"72.3+/-1.5"	"73.7+/-1.3"	"74.7+/-0.9"	"75.2+/-0.6"	"76.1+/-0.8"
5	"72.6+/-1.2"	"73.8+/-1.5"	"74.9+/-1.7"	"75.9+/-1.6"	"77.3+/-1.4"
6	"73+/-0.9"	"73.7+/-0.9"	"74.5+/-1"	"75.2+/-1.2"	"76.1+/-0.9"

	middle_10	middle_11	middle_12	middle_13	middle_14
1	"87.5+/-NA"	"90.2+/-NA"	"90.3+/-NA"	"96.7+/-NA"	"97.4+/-NA"
2	"78.7+/-1.7"	"80.1+/-1.9"	"81.6+/-1.5"	"83.3+/-1.6"	"85.9+/-2.5"
3	"77.5+/-1.3"	"78.6+/-1.3"	"80.6+/-1.5"	"82.3+/-1.9"	"85.2+/-1.7"
4	"77.2+/-1.4"	"79.5+/-1.9"	"82.7+/-3.1"	"85.2+/-3.1"	"88.3+/-1.5"
5	"78.5+/-1.5"	"79.8+/-1.6"	"81.9+/-1.6"	"84+/-2.4"	"89.9+/-2.2"
6	"77.4+/-0.8"	"78.7+/-1.3"	"81+/-1.5"	"83.1+/-1.3"	"85.8+/-1.2"

	middle_15	middle_16	middle_17*	middle_18	middle_19
1	"99.3+/-NA"	"102+/-NA"	"105.2+/-NA"	"113.8+/-NA"	"114.8+/-NA"
2	"89.3+/-1.6"	"95.2+/-4.6"	"112.5+/-1.6"	"118.3+/-4"	"127.2+/-3.5"
3	"89.1+/-2.1"	"94.9+/-2.7"	"113.5+/-1.3"	"120.1+/-2.3"	"128.8+/-2.1"
4	"92.1+/-3"	"93.9+/-3.1"	"106.6+/-2.7"	"120.3+/-5.2"	"128+/-1.5"

5	"93.7+/-2.6"	"97.1+/-2.4"	"100.8+/-2.9"	"122.9+/-4.1"	"127.4+/-3.3"
6	"88.1+/-1.6"	"92.6+/-2.4"	"117.7+/-1.7"	"120.3+/-2"	"126.8+/-2.9"

	middle_20	middle_21	middle_22	middle_23	middle_24
1	"117.5+/-NA"	"119.2+/-NA"	"123.4+/-NA"	"124.3+/-NA"	"162.7+/-NA"
2	"132+/-2"	"133.9+/-2.3"	"138.4+/-1.9"	"140.2+/-2.4"	"142.2+/-1.7"
3	"133.3+/-1.8"	"136.3+/-1.6"	"139.1+/-1.5"	"140.7+/-1.3"	"142.4+/-1.6"
4	"132.1+/-2.2"	"136.9+/-2.1"	"140.1+/-1"	"141.9+/-1.1"	"142.9+/-0.9"
5	"130+/-3.7"	"134+/-2.3"	"136.3+/-2.3"	"139.8+/-2.3"	"143.1+/-2.9"
6	"132.6+/-1.5"	"135.3+/-1.6"	"138.4+/-1"	"140.6+/-1.3"	"142.8+/-0.9"

	middle_25	middle_26*	smallest_opposite_angle*	SquareAntiprismatic
1	"167.1+/-NA"	"170.8+/-NA"	"60.2+/-NA"	"0"
2	"144.6+/-2"	"147.3+/-2.2"	"66.3+/-2.6"	"0"
3	"144.3+/-1.3"	"146.2+/-1"	"68.3+/-1.2"	"0"
4	"144.3+/-1.1"	"146.7+/-1.2"	"67.1+/-2.7"	"0.083"
5	"146.4+/-2.3"	"149.9+/-2.5"	"64.5+/-3.5"	"0.059"
6	"144.4+/-0.9"	"146.2+/-1.3"	"68.1+/-1.4"	"0.056"

	HexagonalBipyramidal
1	"0"
2	"0"
3	"0"
4	"0"
5	"0"
6	"0"

Table S138. Cluster members of 8-ligand combined metal, normal group

[1] "Cluster 1"
2B2N.A.345
[1] "Cluster 2"
4AK8.A.1326, 2DG1.A.3001, 2DSO.B.1002, 1G1T.A.160, 2GGX.A.401, 4N38.A.403, 3P7H.A.1, 1RDO.1.2, 3RQI.A.181, 1RTM.1.2, 2VUZ.A.1130, 3WH2.A.301
[1] "Cluster 3"
4AYO.A.500, 2CKI.A.998, 4E52.B.401, 3G81.A.401, 3IKP.B.401, 1K9J.A.402, 1KWU.A.503, 1KWV.A.503, 1KWW.A.503, 1KWX.A.503, 1KWZ.B.604, 1KXO.A.504, 1KZA.1.502, 2MSB.B.2, 4N32.A.402, 4N37.A.402, 3P7G.A.1, 1RDI.1.2, 1RDJ.1.2, 1RDL.1.2, 1RDN.1.2, 1SL6.A.2
[1] "Cluster 4"
1BCH.1.2, 3KMB.1.2, 4N33.A.404, 4N35.A.404, 4N36.A.402, 3P5H.A.500, 3P5I.A.500, 3PAK.A.300, 1RDK.1.2, 3VYK.A.1007, 1WOY.L.1156, 1WMY.A.202
[1] "Cluster 5"
1AFA.1.2, 1AFB.1.2, 1BCH.2.2, 1FIF.A.2, 1FIH.A.2, 1H8O.A.1492, 1JZN.A.1138, 4KZO.A.501, 4KZO.B.501, 1LQV.D.47, 1MUQ.B.202, 4NVR.A.401, 2OX9.A.802, 2W87.A.1148, 1WMZ.A.202, 1WMZ.B.202, 4B6C.B.1257
[1] "Cluster 6"
4AYP.A.500, 4AYQ.A.500, 4AYR.A.500, 1FO3.A.700, 2GGU.A.401, 3IKR.A.401, 1K9I.B.502, 1KZD.1.502, 4KZV.A.302, 4N34.A.402, 2ORJ.A.401, 2OS9.A.401, 3P5G.A.500, 1PW9.A.401, 1PWB.B.401, 1SL5.A.402, 2VUV.A.200, 2XR6.A.1391

Table S139. 8-ligand combined metal, compressed group

	size	largest_angle*	middle_1*	middle_2	middle_3	middle_4
1	"44"	"157.5+/-3"	"51.4+/-2.5"	"69.5+/-2.1"	"71.3+/-1.4"	"72.6+/-1.3"
2	"22"	"159.5+/-5.3"	"64.4+/-2.6"	"68.2+/-2.4"	"70.4+/-2"	"71.7+/-2"
3	"43"	"155.9+/-4.2"	"51.5+/-2.1"	"69+/-2.3"	"70.9+/-1.6"	"72.3+/-1.6"
4	"16"	"149.4+/-4.6"	"66.7+/-3"	"69.9+/-0.9"	"70.8+/-0.6"	"71.7+/-0.9"

	middle_5	middle_6	middle_7	middle_8	middle_9*
1	"74.1+/-1.5"	"75.2+/-1.3"	"76.3+/-1.4"	"77.5+/-1.4"	"78.7+/-1.2"
2	"73.1+/-1.9"	"74.3+/-1.3"	"76.3+/-1.4"	"77.9+/-1.5"	"79.4+/-1.9"
3	"73.8+/-1.7"	"74.9+/-1.5"	"76+/-1.5"	"77.1+/-1.4"	"78.4+/-1.4"
4	"72.6+/-1.2"	"73.7+/-1.2"	"74.9+/-1.4"	"76.3+/-1.5"	"77.4+/-2"

	middle_10	middle_11	middle_12	middle_13	middle_14
1	"80.1+/-1.3"	"81.3+/-1.3"	"83.2+/-1.7"	"85.3+/-1.9"	"87.8+/-2.2"
2	"80.9+/-1.6"	"82+/-1.6"	"83.9+/-1.9"	"85.6+/-2.4"	"88.7+/-2.1"
3	"79.8+/-1.5"	"81+/-1.4"	"82.6+/-1.9"	"84.1+/-1.8"	"87.5+/-2.3"
4	"78.6+/-1.8"	"80.7+/-1.9"	"82+/-1.9"	"84+/-2"	"86+/-2.3"

	middle_15	middle_16	middle_17*	middle_18	middle_19
1	"92.3+/-3.1"	"96.1+/-3.3"	"102.7+/-2.2"	"117+/-3.6"	"123+/-2.7"
2	"92.8+/-4.4"	"97.4+/-4.7"	"103.7+/-4.5"	"117.6+/-3.9"	"123+/-4.7"
3	"90.9+/-2.8"	"96.7+/-5"	"114.1+/-3.9"	"117.4+/-3.5"	"121.3+/-3.6"
4	"90.7+/-2"	"95.2+/-4.4"	"116.7+/-6.6"	"124.1+/-4.2"	"126.9+/-2.8"

	middle_20	middle_21	middle_22	middle_23	middle_24
1	"126.3+/-3.1"	"131.8+/-3.5"	"137.9+/-2.9"	"140.7+/-2.1"	"144.9+/-2.3"
2	"126.8+/-5.3"	"131.3+/-5"	"135.3+/-3.3"	"139+/-3"	"143.5+/-2.9"
3	"126.5+/-3.7"	"131.6+/-3.4"	"135.9+/-2.3"	"139.6+/-2.7"	"143.5+/-2.1"
4	"131.8+/-1.6"	"135.6+/-1.6"	"137.3+/-2"	"139.3+/-1.7"	"141.2+/-1.3"

	middle_25	middle_26*	smallest_opposite_angle*	SquareAntiprismatic
1	"147.8+/-1.9"	"151.7+/-2.7"	"67.5+/-2.6"	"0.023"
2	"148.6+/-4.2"	"154.2+/-4.4"	"50.8+/-2.6"	"0.101"
3	"147.3+/-3"	"151.9+/-3.5"	"66+/-2.8"	"0.022"
4	"143.5+/-2.1"	"145.2+/-2.2"	"51.1+/-1.4"	"0.062"

	HexagonalBipyramidal
1	"0"
2	"0"
3	"0"
4	"0"

Table S140. Cluster members of 8-ligand combined metal, compressed group

[1] "Cluster 1"
4KI8.C.602, 2ASP.A.600, 4AWN.A.301, 4B97.A.1152, 4BM1.A.402, 2BOQ.A.1352, 1CLC.A.651, 4CZN.A.1369, 4DLK.A.402, 1DV8.A.1001, 2E39.A.502, 3ER9.B.901, 2EWE.A.702, 4EW9.A.203, 2F3Y.A.1174, 4FCS.A.403, 3FM1.A.352, 3FM4.A.352, 3FMU.A.352, 1G87.A.616, 1H3J.A.1346, 1HUP.A.302, 1HXO.A.500, 1IA6.A.1263, 1J34.B.512, 1K72.A.779, 1LLP.A.352, 3M5Q.A.371, 1MN2.A.371, 2NZM.A.407, 3PDD.A.192, 3PF2.A.2, 3POE.A.2, 2PWA.A.1280, 3Q3U.A.341, 3QEE.A.21, 1SNN.A.403, 1UOV.A.1419, 2VMH.A.3050, 2VMI.A.3050, 1W3H.B.1348, 1XKD.A.1006, 2ZIC.A.944, 3ZQX.A.1146
[1] "Cluster 2"
1ANX.B.321, 4AOC.B.1129, 4AYU.A.205, 1COG.S.129, 1CGU.A.685, 1CGW.A.692, 3DEM.A.3001, 3DR2.A.707, 1DX5.I.1001, 3FZO.B.400, 3HR6.A.1, 2JBH.A.1228, 1KXQ.A.4001, 2

LRO.A.202, 4QB6.A.203, 3T8I.C.400, 3TH2.L.152, 4U6D.A.501, 4U6D.B.502, 4X9Y.A.502, 1YCM.A.267, 1Z32.X.497
[1] "Cluster 3"
2A40.B.1273, 4AW7.A.1599, 3B9X.A.400, 4B9P.A.1167, 3BMV.A.684, 1CGV.A.692, 1CGY.A.692, 1CXL.A.689, 1D3C.A.688, 3DED.C.508, 3DHP.A.497, 1DTU.A.688, 1EO5.A.689, 2FF2.A.601, 1G43.A.200, 1G94.A.800, 2GUY.A.601, 2HYV.A.601, 1I75.A.1688, 4I71.A.402, 1IOD.G.506, 1JAE.A.500, 1KCK.A.691, 1KCL.A.1692, 1KWT.A.502, 1KXH.A.800, 4KZW.A.302, 2MAS.A.316, 1MCX.A.347, 1NBC.B.156, 1OT1.A.1693, 1PEZ.A.891, 1PJ9.A.891, 1SH7.A.1290, 3TEC.E.344, 1UA7.A.601, 3VM7.A.501, 2WHK.A.1339, 1WMD.A.1002, 3WMS.A.801, 2WNX.A.1163, 2WZS.A.800, 1YO8.A.1182
[1] "Cluster 4"
1B9A.A.110, 3B9G.A.318, 2C40.A.1311, 3EPW.A.1001, 3EPX.A.1001, 4I70.A.401, 4I72.A.401, 1J34.A.511, 3MKM.A.501, 3MKN.A.501, 1Q8F.A.2001, 3S82.B.405, 1UZK.A.2512, 3WMT.A.606, 1YOE.A.1001, 3RWK.X.521

Table S141. 8-ligand combined metal, combined group

	size	largest_angle*	middle_1*	middle_2	middle_3	middle_4
1	"60"	"149+/-2.5"	"66.3+/-2.2"	"69.8+/-1.3"	"71.1+/-1.2"	"72.1+/-1"
2	"24"	"152.4+/-3.6"	"63.7+/-2.8"	"68.6+/-2.2"	"70.2+/-1.8"	"71.2+/-1.6"
3	"44"	"158+/-2.9"	"50.9+/-2.2"	"68.8+/-4.1"	"71.2+/-2.2"	"72.5+/-1.6"
4	"22"	"172.1+/-3.6"	"50.9+/-4.3"	"61.4+/-6.5"	"66.5+/-4.9"	"69.4+/-4.8"
5	"49"	"158.3+/-3.3"	"50.9+/-1.6"	"68.4+/-4.7"	"72.2+/-2.4"	"74+/-2.4"
6	"17"	"149.2+/-4"	"61.6+/-7"	"67.8+/-3.9"	"70.2+/-1.9"	"71.3+/-1.6"
7	"42"	"156.3+/-4.2"	"51.2+/-1.8"	"68.7+/-3.5"	"70.9+/-1.6"	"72.5+/-1.7"
8	"29"	"162.8+/-3.6"	"52.5+/-3.4"	"66.1+/-5.8"	"69.5+/-5.2"	"71.3+/-5"
9	"29"	"156.6+/-4.2"	"64.6+/-3.2"	"68.4+/-2.8"	"70.3+/-2.5"	"71.6+/-2.4"
10	"44"	"160.8+/-3"	"51.2+/-2.8"	"66.6+/-6.6"	"70.8+/-4.2"	"73.6+/-3.3"

	middle_5	middle_6	middle_7	middle_8	middle_9*
1	"72.9+/-1"	"73.6+/-1.1"	"74.6+/-0.9"	"75.4+/-1.1"	"76.3+/-1.1"
2	"72.2+/-1.5"	"73.7+/-1.6"	"74.7+/-1.6"	"75.9+/-1.8"	"77.4+/-1.4"
3	"74.1+/-1.7"	"75.2+/-1.4"	"76.5+/-1.3"	"77.6+/-1.2"	"78.8+/-1.3"
4	"71.9+/-4"	"75.2+/-3.9"	"77.9+/-3.1"	"80+/-2.8"	"81.9+/-2.6"
5	"75.3+/-2.3"	"76.6+/-1.9"	"77.7+/-2"	"78.8+/-2"	"80+/-1.4"
6	"72.3+/-1.2"	"73.6+/-1.1"	"74.9+/-1.4"	"76.4+/-1.7"	"77.3+/-2"
7	"73.9+/-1.7"	"75+/-1.5"	"76.2+/-1.5"	"77.2+/-1.4"	"78.5+/-1.3"
8	"73+/-4.6"	"74.5+/-4"	"75.6+/-3.7"	"76.9+/-3.3"	"78.1+/-2.8"
9	"73+/-2.4"	"74.4+/-2.3"	"76.2+/-1.6"	"77.5+/-1.7"	"79+/-1.9"
10	"75.1+/-2.5"	"76.7+/-2"	"77.9+/-1.5"	"79.4+/-1.3"	"80.9+/-1.7"

	middle_10	middle_11	middle_12	middle_13	middle_14
1	"77.6+/-1.3"	"79+/-1.5"	"81.3+/-1.9"	"83.1+/-2.1"	"85.9+/-1.9"
2	"78.7+/-1.5"	"80.2+/-2"	"82.1+/-2.2"	"84.6+/-2.8"	"89.9+/-2.8"
3	"80.2+/-1.5"	"81.5+/-1.8"	"83.4+/-2.2"	"85.4+/-2.2"	"87.8+/-2.5"
4	"83.8+/-3.1"	"86.5+/-3.1"	"89+/-3.2"	"90.9+/-2.8"	"93.1+/-3"
5	"81.4+/-1.4"	"82.6+/-1.6"	"84.1+/-1.4"	"85.9+/-2.1"	"87.6+/-2.5"
6	"79.2+/-2"	"81.3+/-2.1"	"82.6+/-2.2"	"84.3+/-2.1"	"86.3+/-2.5"
7	"79.7+/-1.5"	"81.1+/-1.4"	"82.6+/-1.9"	"84.1+/-1.9"	"87.6+/-2.3"
8	"79.8+/-2"	"81.8+/-1.9"	"83.8+/-2"	"86.6+/-2.7"	"90.5+/-4"
9	"80.5+/-1.8"	"81.8+/-2"	"83.5+/-2.4"	"85.5+/-2.9"	"88.4+/-3.1"
10	"82.6+/-2.2"	"83.9+/-2.2"	"85.7+/-2.2"	"87.2+/-2.3"	"88.9+/-2.7"

	middle_15	middle_16	middle_17*	middle_18	middle_19

1	"89+/-2"	"94+/-3.3"	"113.9+/-3.3"	"119.7+/-2.8"	"127.8+/-2.8"
2	"93.8+/-3.1"	"97.4+/-3.3"	"101.8+/-3.2"	"121.6+/-5.9"	"126.6+/-3.7"
3	"92.3+/-3.1"	"96.2+/-3.3"	"102.6+/-2.3"	"117.2+/-3.5"	"123+/-2.9"
4	"95.3+/-3.5"	"98+/-4.2"	"101.6+/-3.8"	"109.3+/-5.7"	"113.6+/-4.7"
5	"90.3+/-3.3"	"98.2+/-4.6"	"107.3+/-2.7"	"115.4+/-5.3"	"120.4+/-4.4"
6	"91.9+/-4.1"	"95.7+/-5"	"120.7+/-4.2"	"124.3+/-3.4"	"126.3+/-2.7"
7	"91.2+/-2.7"	"96.7+/-4.6"	"113.8+/-3.4"	"117.3+/-3.2"	"121.2+/-3.4"
8	"95.7+/-5.1"	"101.3+/-5.2"	"112.4+/-3.2"	"115.2+/-3.1"	"118.6+/-3.1"
9	"91.9+/-3.7"	"97+/-4.7"	"105.3+/-4.5"	"119.2+/-4.8"	"124.8+/-3.9"
10	"91.7+/-2.8"	"96.7+/-3"	"101.1+/-2.9"	"111.8+/-6"	"118+/-6.2"

	middle_20	middle_21	middle_22	middle_23	middle_24
1	"132.6+/-2.2"	"135.6+/-2"	"138.9+/-1.5"	"140.8+/-1.5"	"142.5+/-1.3"
2	"130.4+/-3.3"	"134.6+/-2.7"	"137+/-2.5"	"140.1+/-2.4"	"143.2+/-2.5"
3	"126.1+/-3"	"131.4+/-3.4"	"137.7+/-3.1"	"140.6+/-2.2"	"144.7+/-2.7"
4	"118.6+/-5.9"	"125.4+/-5.1"	"133.5+/-5.6"	"139.1+/-6.4"	"148+/-7"
5	"126+/-3.4"	"129.8+/-3"	"134.5+/-4"	"141.5+/-4"	"145.7+/-3.6"
6	"131+/-2.3"	"134.8+/-2"	"136.1+/-2.5"	"138.1+/-2.5"	"140.2+/-2.4"
7	"126.5+/-3.7"	"131.3+/-3.4"	"135.8+/-2.3"	"139.7+/-2.7"	"143.7+/-2.1"
8	"122+/-3.3"	"125.3+/-4.3"	"130.9+/-3.9"	"140.4+/-5.1"	"145.2+/-4.8"
9	"128+/-4.5"	"132.5+/-3.8"	"135.8+/-2.6"	"139.2+/-2.5"	"142.9+/-2.6"
10	"123.3+/-4.2"	"127.8+/-3.4"	"132.5+/-4.5"	"142.5+/-4.2"	"148.1+/-3.6"

	middle_25	middle_26*	smallest_opposite_angle*
1	"144.3+/-1.3"	"146.5+/-1.5"	"67.9+/-1.8"
2	"146.3+/-2.2"	"149.3+/-2.6"	"64.5+/-3.5"
3	"147.7+/-2.5"	"151.8+/-2.7"	"67.6+/-2.4"
4	"152.7+/-7.7"	"162.1+/-5.4"	"50.6+/-5.3"
5	"148.9+/-3.7"	"151.5+/-3"	"52+/-1.5"
6	"143.2+/-1.9"	"145+/-1.9"	"52.5+/-3.3"
7	"147.4+/-3"	"152.2+/-3.2"	"66+/-2.7"
8	"150+/-4.6"	"156.9+/-3.8"	"51.3+/-2.7"
9	"146.9+/-3"	"151.8+/-4.4"	"51.1+/-2.6"
10	"152.4+/-2.6"	"156+/-2.4"	"50.4+/-2"

	SquareAntiprismatic	HexagonalBipyramidal
1	"0.033"	"0"
2	"0.046"	"0"
3	"0.017"	"0"
4	"0.11"	"0.007"
5	"0.038"	"0"
6	"0.133"	"0"
7	"0.022"	"0"
8	"0.105"	"0"
9	"0.118"	"0.005"
10	"0.017"	"0"

Table S142. Cluster members of 8-ligand combined metal, combined group

[1] "Cluster 1"
4AK8.A.1326, 4AYO.A.500, 4AYP.A.500, 4AYQ.A.500, 4AYR.A.500, 2CKI.A.998, 2DG1.A.3001, 4E52.B.401, 1FO3.A.700, 1G1T.A.160, 3G81.A.401, 2GGU.A.401, 2GGX.A.401, 3IKP.B.401, 3IKR.A.401, 1K9I.B.502, 1K9J.A.402, 1KWU.A.503, 1KWV.A.503, 1KWW.A.503, 1KWX.A.503, 1KWZ.B.604, 1KX0.A.504, 1KZA.1.502, 1KZD.1.502, 4KZV.A.302, 2MSB.B

.2, 4N32.A.402, 4N33.A.404, 4N34.A.402, 4N35.A.404, 4N36.A.402, 4N37.A.402, 4N38
.A.403, 2ORJ.A.401, 2OS9.A.401, 3P5G.A.500, 3P5H.A.500, 3P5I.A.500, 3P7G.A.1, 3P
7H.A.1, 1PW9.A.401, 1PWB.B.401, 1RDI.1.2, 1RDJ.1.2, 1RDK.1.2, 1RDL.1.2, 1RDN.1.2
, 1RDO.1.2, 3RQI.A.181, 1RTM.1.2, 1SL5.A.402, 1SL6.A.2, 2VUV.A.200, 2VUZ.A.1130,
 3VYK.A.1007, 3WH2.A.301, 1WMY.A.202, 2WZS.A.800, 2XR6.A.1391
[1] "Cluster 2"
4KI8.C.602, 1AFA.1.2, 1AFB.1.2, 1BCH.1.2, 1BCH.2.2, 2DSO.B.1002, 1FIF.A.2, 1FIH.
A.2, 1H8O.A.1492, 1JZN.A.1138, 3KMB.1.2, 4KZO.A.501, 4KZO.B.501, 1LQV.D.47, 1MUQ
.B.202, 4NVR.A.401, 2OX9.A.802, 3PAK.A.300, 1WOY.L.1156, 2W87.A.1148, 1WMZ.A.202
, 1WMZ.B.202, 1XKD.A.1006, 4B6C.B.1257
[1] "Cluster 3"
2ASP.A.600, 4AWN.A.301, 4B97.A.1152, 4BM1.A.402, 2BOQ.A.1352, 1CLC.A.651, 4CZN.A
.1369, 4DLK.A.402, 1DV8.A.1001, 2E39.A.502, 3ER9.B.901, 2EWE.A.702, 4EW9.A.203,
2F3Y.A.1174, 4FCS.A.403, 3FM1.A.352, 3FM4.A.352, 3FMU.A.352, 1G87.A.616, 4GNC.A.
301, 1H3J.A.1346, 1HUP.A.302, 1HXO.A.500, 1IA6.A.1263, 1J34.B.512, 1K72.A.779, 1
LLP.A.352, 3M5Q.A.371, 1MN2.A.371, 2NZM.A.407, 3PDD.A.192, 3PF2.A.2, 3POE.A.2, 2
PWA.A.1280, 3Q3U.A.341, 3QEE.A.21, 1SNN.A.403, 1UOV.A.1419, 2VMH.A.3050, 2VMI.A.
3050, 3VV3.A.403, 1W3H.B.1348, 2ZIC.A.944, 3ZQX.A.1146
[1] "Cluster 4"
1N48.A.502, 4QWD.A.703, 1SOO.A.402, 1AMY.A.501, 3B8Z.A.902, 3DR2.A.707, 4I5L.E.7
01, 4IT5.A.301, 2J5W.A.3042, 2KXV.A.201, 1LGN.A.301, 3LJT.A.902, 3LND.A.208, 3ON
R.A.72, 2POR.A.1003, 3T3P.A.2004, 1UOV.A.1420, 4WKE.A.502, 2B2N.A.345, 4D1I.G.60
2, 1GOH.A.702, 1Y4A.E.1002
[1] "Cluster 5"
2W9C.B.1343, 1AFB.1.227, 3BJE.B.402, 2BOI.A.200, 2BOJ.A.1117, 2BP6.A.801, 2BPE.B
.1245, 2BV4.A.200, 4CE8.A.997, 3D1M.A.190, 3DCQ.A.115, 3DE8.A.109, 4EW9.B.203, 1
FIF.A.1, 1FWX.B.4903, 4HPK.A.1102, 1HUP.A.301, 2IE7.A.401, 2IWK.A.1607, 1J34.C.5
05, 2JDM.B.1116, 2JDN.B.882, 3JQW.A.1002, 1K9J.A.401, 2KRD.C.90, 4KZV.A.303, 2LR
O.A.201, 1MDW.A.1, 2MSB.A.1, 1MXG.A.438, 3N1F.A.5, 2O8O.A.1010, 1OUR.A.401, 1OVP
.A.116, 4PHN.A.304, 1PW9.B.402, 2QVM.A.1001, 3SBQ.A.703, 1TN3.A.182, 1UQX.A.1115
, 2VNV.A.301, 2VUC.B.991, 1WMZ.A.201, 3WN6.A.501, 2WR9.A.1132, 2XR6.A.1390, 1Y6W
.A.150, 1AG9.A.400, 1AG9.A.401
[1] "Cluster 6"
3B9G.A.318, 1DV8.A.1003, 3EPW.A.1001, 3EPX.A.1001, 3G5I.A.501, 4I72.A.401, 4I73.
A.401, 2M3S.A.202, 2MAS.A.316, 3MKM.A.501, 3MKN.A.501, 1Q8F.A.2001, 3S82.B.405,
3WMT.A.606, 1YOE.A.1001, 1YUT.B.200, 3RWK.X.521
[1] "Cluster 7"
2A40.B.1273, 4AW7.A.1599, 3B9X.A.400, 4B9P.A.1167, 3BMV.A.684, 1CGV.A.692, 1CGY.
A.692, 1CXL.A.689, 1D3C.A.688, 3DED.C.508, 3DHP.A.497, 1DTU.A.688, 1EO5.A.689, 2
FF2.A.601, 1G43.A.200, 1G94.A.800, 2GUY.A.601, 1GYK.A.1205, 2HYV.A.601, 1I75.A.1
688, 4I71.A.402, 1IOD.G.506, 1JAE.A.500, 1KCK.A.691, 1KCL.A.1692, 1KWT.A.502, 1K
XH.A.800, 4KZW.A.302, 1MCX.A.347, 1NBC.B.156, 1OT1.A.1693, 1PEZ.A.891, 1PJ9.A.89
1, 1SH7.A.1290, 3TEC.E.344, 1UA7.A.601, 3VM7.A.501, 2WHK.A.1339, 1WMD.A.1002, 3W
MS.A.801, 2WNX.A.1163, 1YO8.A.1182
[1] "Cluster 8"
1BSU.A.301, 2A3Y.A.600, 4AOC.A.1130, 4AVS.A.206, 4AYU.A.206, 3BVH.B.1, 1CGT.A.68
5, 1CGU.A.685, 1CGX.A.692, 4DOE.A.502, 1DVI.A.274, 4I5L.B.602, 4IEF.B.703, 4IEF.
H.703, 4IT5.D.201, 4JA8.B.502, 3KQR.A.206, 1KXR.A.1, 2KZ2.A.150, 2M3S.A.203, 4NE
H.A.1104, 2OX9.A.801, 2R9F.A.365, 1TF4.B.3003, 4TV9.A.503, 4V0C.D.201, 2W08.A.20
5, 2XFG.A.1447, 1ZCM.A.1002
[1] "Cluster 9"
4RNO.A.502, 1ANX.B.321, 4AOC.B.1129, 1B9A.A.110, 1C0G.S.129, 2C40.A.1311, 1CGW.A
.692, 3DEM.A.3001, 1DX5.I.1001, 3FZO.B.400, 3HR6.A.1, 4I5N.E.701, 4I70.A.401, 1J
34.A.511, 2JBH.A.1228, 1KXQ.A.4001, 2LRO.A.202, 4QB1.A.202, 4QB6.A.203, 1SMD.A.4

97, 3T8I.C.400, 3TH2.L.152, 4U6B.C.501, 4U6D.A.501, 4U6D.B.502, 1UZK.A.2512, 4X9Y.A.502, 1YCM.A.267, 1Z32.X.497
[1] "Cluster 10"
1AZ0.A.284, 1A25.A.291, 1A25.B.291, 4AQ8.D.1239, 4AYU.A.205, 1CVR.A.503, 3D34.A.228, 3DED.A.505, 1DSY.A.502, 3EF2.A.303, 3EF2.B.303, 4EW9.A.204, 3GZK.A.538, 3HR4.B.201, 1I82.A.193, 1KSC.A.500, 1KWZ.A.503, 1LQV.C.45, 3N1G.A.192, 4N4E.E.402, 4NPK.A.801, 2NQI.A.401, 2NXP.B.601, 1OUX.A.401, 2OX9.B.804, 4PE0.A.103, 4PHJ.A.301, 2POR.A.303, 3Q13.A.601, 3Q4W.A.225, 1QNI.A.901, 1RDL.1.227, 1RP8.A.501, 3SG4.A.453, 3SWB.A.91, 1T44.G.701, 3TH4.L.149, 1TLQ.A.190, 1UV4.A.1294, 1V0A.A.1176, 3WC3.A.501, 4WK0.A.501, 1Y9I.A.501, 2ZWP.B.403

Table S143. all-ligand-number combined metal, normal group

	size	largest_angle*	middle_1*	middle_2*	middle_3*
1	"3251"	"120.7+/-6.5"	"99.2+/-6"	"104.7+/-4.1"	"109.3+/-3.4"
2	"676"	"158.7+/-11.4"	"79.5+/-7.2"	"86.6+/-7.1"	"93.2+/-7.8"
3	"1073"	"162.4+/-9.7"	"77.2+/-6.2"	"88.2+/-4"	"96.8+/-4.5"
4	"1019"	"160.5+/-8.7"	"70.3+/-4.4"	"82.5+/-4.3"	"101.9+/-9.3"
5	"2903"	"176.6+/-2.6"	"84.5+/-2.9"	"89.4+/-1"	"93+/-1.8"
6	"261"	"158+/-12.8"	"78.5+/-7.4"	"85.6+/-6.2"	"92.8+/-6.7"
7	"2193"	"170.8+/-4.3"	"77.9+/-4.6"	"88.1+/-2.3"	"96.7+/-3.5"

	middle_4*	smallest_opposite_angle*	Tetrahedral	TrigonalBipyramidal
1	"113.8+/-3.6"	"106.9+/-7.4"	"0.574"	"0"
2	"102.2+/-9.7"	"89.9+/-11.6"	"0"	"0"
3	"138.2+/-10"	"102.7+/-7"	"0.026"	"0.162"
4	"149.3+/-6.8"	"70.2+/-5.6"	"0.006"	"0.02"
5	"174.3+/-3.6"	"85.5+/-3.8"	"0"	"0.003"
6	"103.7+/-10.7"	"134.5+/-16.1"	"0.001"	"0"
7	"164.4+/-4.7"	"79.4+/-6.5"	"0.001"	"0.022"

	TrigonalBipyramidalVA	TrigonalBipyramidalVP	Octahedral	SquarePyramidal
1	"0.028"	"0"	"0"	"0"
2	"0.011"	"0.051"	"0"	"0"
3	"0.196"	"0.26"	"0"	"0.021"
4	"0.028"	"0.118"	"0.017"	"0.065"
5	"0.001"	"0.096"	"0.386"	"0.508"
6	"0.001"	"0.122"	"0"	"0"
7	"0.015"	"0.243"	"0.19"	"0.327"

	SquarePyramidalV	SquarePlanar	TrigonalPrismatic	TrigonalPrismaticV
1	"0"	"0"	"0"	"0"
2	"0.171"	"0"	"0"	"0"
3	"0.194"	"0.054"	"0"	"0.1"
4	"0.15"	"0.105"	"0.089"	"0.152"
5	"0.544"	"0.536"	"0.001"	"0.005"
6	"0.019"	"0.07"	"0"	"0"
7	"0.441"	"0.405"	"0.02"	"0.053"

	PentagonalBipyramidal	PentagonalBipyramidalVA	PentagonalBipyramidalVP
1	"0"	"0"	"0"
2	"0"	"0"	"0"
3	"0"	"0"	"0"
4	"0.068"	"0.084"	"0.126"
5	"0"	"0"	"0.002"

```
6 "0"                   "0"                      "0"
7 "0"                   "0"                      "0.043"
  SquareAntiprismatic SquareAntiprismaticV HexagonalBipyramidal
1 "0"                 "0"                  "0"
2 "0"                 "0"                  "0"
3 "0"                 "0"                  "0"
4 "0.003"             "0.076"              "0"
5 "0"                 "0"                  "0"
6 "0"                 "0"                  "0"
7 "0"                 "0"                  "0"
  HexagonalBipyramidalVA HexagonalBipyramidalVP
1 "0"                    "0"
2 "0"                    "0"
3 "0"                    "0"
4 "0"                    "0.027"
5 "0"                    "0"
6 "0"                    "0"
7 "0"                    "0"
```

Table S144. Cluster members of all-ligand-number combined metal, normal group

[1] "Cluster 1"
1A1G.A.201, 1A1I.A.201, 1A6Y.A.550, 1A6Y.A.551, 2A66.A.401, 1A73.A.202, 4AA6.A.2
53, 3AUO.B.579, 2B3J.A.2001, 2C7A.A.1641, 2C7A.A.1642, 3CBB.A.1001, 4CIS.A.300,
3COQ.A.1002, 1CYQ.A.601, 1CYQ.A.602, 2DRP.A.171, 2DRP.A.172, 1DSZ.A.1121, 1DSZ.A
.1122, 1DSZ.B.1222, 3EPH.A.1, 3EQT.A.1, 2ER8.A.104, 2ER8.A.105, 4ESJ.A.301, 1F2I
.G.1201, 1F2I.G.1202, 1F4S.P.64, 1F4S.P.65, 1FFY.A.1001, 2FF0.A.1001, 2FF0.A.100
2, 1G2D.C.302, 1G2D.C.303, 1G2F.C.301, 1G2F.C.302, 1G2F.C.303, 3G9M.A.526, 2GAT.
A.67, 4GAT.A.67, 6GAT.A.67, 4GLX.A.601, 3GOX.A.301, 3GOX.A.302, 4GZN.C.203, 3HAX
.C.201, 2HAN.A.351, 2HAN.A.352, 2HAN.B.353, 2HAN.B.354, 4HC9.A.401, 4HC9.A.402,
4HCC.A.504, 2HGH.A.191, 2HGH.A.192, 2HGH.A.193, 4HN5.A.601, 4HN6.A.602, 4HP3.C.2
01, 4HP3.C.202, 1HWT.D.138, 2I13.A.502, 2I13.A.503, 2I13.A.506, 2I13.B.507, 2I13
.B.510, 1I3J.A.100, 4IFD.J.1106, 2IHX.A.235, 2IHX.A.236, 4IQR.A.402, 4IQR.A.403,
 2IVH.A.1577, 2JP9.A.131, 2JP9.A.132, 2JP9.A.133, 2JP9.A.134, 2JZW.A.56, 1K3X.A.
501, 1K82.A.450, 2KAE.A.175, 1KB2.A.150, 1KB2.A.151, 3KDE.C.78, 2KKF.A.2001, 2KK
F.A.2002, 2KMK.A.83, 2KMK.A.84, 2KMK.A.85, 3KMP.A.2, 1LAT.A.1514, 1LAT.A.1515, 4
LJ0.A.501, 4LJ0.A.502, 4LJ0.A.503, 1LLM.C.301, 1LLM.C.302, 4LMG.A.201, 1LO1.A.19
5, 1LO1.A.196, 3LRR.A.1, 2LT7.A.701, 2LT7.A.702, 2LT7.A.703, 3M7K.A.143, 3M7K.A.
144, 4M8O.A.1303, 3M9E.A.208, 3M9E.B.209, 4M9E.A.503, 4M9E.A.504, 4M9E.A.505, 4M
9V.C.201, 4M9V.C.202, 1MEY.C.88, 1MEY.C.89, 1MEY.C.90, 3MLN.A.501, 4MTD.A.201, 3
NCU.A.1, 4NDH.A.402, 2NLL.A.250, 2NLL.B.450, 2NLL.B.451, 4NM6.A.2002, 2NQ9.A.401
, 2O6M.A.601, 4O6A.A.601, 3O9X.A.132, 1ODH.A.1171, 3OD8.A.200, 2OFI.A.302, 4OLN.
A.101, 4OLN.A.102, 4OND.A.101, 4OND.A.102, 4OOR.A.102, 2OPF.A.501, 3OYM.A.393, 1
OZJ.A.145, 1P47.A.203, 3PIH.A.917, 4PZI.A.1101, 4PZI.A.1102, 4QEN.A.802, 4QEN.A.
803, 4QEN.A.804, 4QEN.A.805, 3QMD.A.300, 3QMD.A.301, 3QSV.A.1, 1QUM.A.301, 4R2A.
A.503, 4R2A.A.504, 4R2A.A.505, 4R2Q.A.503, 4R2S.A.501, 1R4O.A.526, 3S14.A.1735,
1TDZ.A.1001, 1TF3.A.102, 1TF3.A.2, 1TF3.A.3, 4TNT.A.701, 4TNT.A.702, 3TS2.A.1, 3
TS2.A.2, 3U6P.A.300, 1UBD.C.501, 1UBD.C.502, 1UBD.C.503, 1UBD.C.504, 3UK3.C.967,
 3UK3.C.968, 3VD6.C.501, 2XQC.A.1138, 2XQC.D.1141, 2YKG.A.927, 1YUI.A.64, 1ZAA.C
.203, 1ZGW.A.500, 1ZNS.A.1500, 258L.A.500, 2A1K.A.1, 3A1B.A.1, 3A1B.A.2, 3A1B.A.
3, 1A1R.A.901, 4A24.A.601, 4A24.A.602, 4A2C.A.1349, 4A2V.A.1000, 3A32.A.708, 1A4

2.A.262, 3A43.A.701, 4A46.A.65, 2A5V.A.401, 1A5T.A.501, 2A51.A.54, 2A51.A.55, 2A5H.A.421, 2A5H.B.421, 3A6F.C.301, 3A6G.A.301, 3A6J.A.301, 3A6J.D.301, 2A6H.D.7458, 2A6H.D.7412, 1A71.A.401, 1A71.A.402, 1A72.A.376, 1A7T.A.251, 4A7K.A.950, 2A8D.A.1230, 3A9J.C.1, 3A9K.C.1, 1AAF.A.56, 2AA4.A.1001, 4AA1.A.1615, 2AB3.A.30, 2AC3.A.531, 2ADR.A.162, 2ADR.A.163, 1ADN.A.93, 4ADN.A.1223, 1AF2.A.296, 2AFU.A.391, 2AFW.A.996, 2AFX.A.996, 2AFZ.A.391, 2AFM.A.391, 3AII.A.1001, 4AI5.A.200, 4AIA.A.200, 4AIG.A.999, 1AJB.A.451, 4AJX.H.1030, 2AKL.A.117, 3ALR.A.601, 3ALR.A.602, 1AM6.A.262, 2AMT.B.2900, 1ANI.A.451, 1ANJ.B.451, 2AP1.A.304, 2APS.A.400, 2AQP.A.201, 1ARD.A.1, 1ARE.A.1, 1ARF.A.1, 4AR1.A.1722, 4AR9.B.1731, 4ARE.A.1790, 4ARF.A.1722, 3ASK.A.501, 3ASL.A.3, 2AS9.A.666, 2AS9.B.222, 2ASH.A.400, 3AUK.A.390, 2AU3.A.501, 4AU7.A.1248, 4AUQ.B.1299, 4AUQ.B.1300, 3AVR.A.1502, 2AW1.A.262, 4AWY.B.3228, 3AX1.A.601, 1AXG.A.401, 3AXS.A.401, 4AX0.B.3228, 4AX1.B.3228, 4AXD.A.700, 2AYK.A.171, 3AY2.A.1001, 2AYD.A.369, 2AYJ.A.57, 4AY8.A.600, 4AYC.A.1484, 4AYC.A.1485, 1AZM.A.261, 2AZH.A.150, 2B0O.E.698, 2B0P.A.400, 4B29.A.1205, 2B3Z.A.1360, 1B4E.A.405, 3B4F.A.262, 3B4N.B.712, 2B44.A.400, 2B5W.A.800, 3B5Q.A.500, 3B6P.A.800, 4B6D.A.1340, 4B6D.A.1341, 3B7R.L.701, 1B8T.A.193, 1B8T.A.194, 1B8T.A.195, 1B8Y.A.301, 2B83.C.3353, 2B8T.A.1218, 3B92.A.502, 2B9D.A.1002, 2BA1.A.201, 1BBO.A.60, 1BBO.A.61, 1BCD.A.262, 3BET.A.262, 2BE7.D.1108, 4BF1.A.270, 4BF6.A.1262, 3BHX.A.1752, 4BHW.A.1, 4BHW.A.4, 1BI0.A.291, 3BI0.A.1752, 3BI1.A.1752, 3BJI.A.1, 3BJI.B.2, 3BKN.A.201, 3BKN.A.202, 3BL0.A.262, 3BL1.A.262, 3BL5.A.300, 2BL6.A.1059, 2BL6.A.1060, 4BLB.B.910, 4BM9.A.1466, 4BM9.A.1469, 1BN1.A.262, 1BN3.A.262, 1BN4.A.262, 1BNL.A.179, 1BNM.A.262, 1BNN.A.262, 1BNQ.A.262, 1BNT.A.262, 1BNU.A.262, 1BNV.A.262, 1BNW.A.262, 2BNM.A.1199, 2BNN.A.1200, 3BO5.A.301, 3BO5.A.302, 3BO5.A.303, 3BO5.A.304, 3BOC.A.1001, 3BOF.A.701, 3BOL.A.701, 4BOL.A.1261, 1BP3.A.500, 2BP0.A.1341, 3BQ5.A.800, 3BQ6.A.800, 1BS4.A.2001, 4BS9.A.1782, 1BTK.A.1, 3BTO.C.375, 4BT7.A.301, 1BUD.A.800, 4BUE.A.2162, 1BV3.A.262, 2BYO.A.1209, 1BYF.A.302, 3BYR.A.501, 1BZM.A.261, 2BZ1.A.1174, 4C09.A.351, 3C10.A.102, 2C1I.A.1465, 4C1D.A.501, 4C1D.A.502, 4C1E.A.501, 4C1E.A.502, 4C1F.A.501, 4C1F.A.502, 4C1G.A.300, 4C1G.A.301, 4C1G.B.301, 4C1Q.A.493, 1C2G.A.409, 2C20.A.601, 3C2S.A.448, 2C2F.A.1211, 4C2P.A.701, 1C3I.B.260, 3C37.A.301, 2C36.A.1311, 4C3E.A.201, 4C3T.A.300, 4C40.B.600, 4C40.D.500, 3C52.B.401, 3C5K.A.201, 3C5K.A.202, 3C5K.A.203, 4C5W.A.403, 3C63.A.107, 3C63.B.107, 3C63.C.107, 3C63.D.107, 3C6W.A.2, 2C6A.A.336, 2C6C.A.1752, 1C7K.A.133, 3C7P.A.262, 2C7N.A.499, 1C8T.A.260, 3C8Z.A.413, 4C81.A.1240, 4C8E.A.1162, 2C9S.A.1155, 1C9Q.A.999, 3CA2.A.264, 1CAI.A.262, 1CAK.A.262, 1CAQ.A.301, 4CA1.A.283, 4CA1.A.284, 2CBD.A.262, 4CBY.A.2035, 1CCT.A.262, 4CCG.X.1374, 4CCG.X.1375, 1CDO.B.376, 1CDO.B.377, 2CDC.A.1372, 4CDG.A.1643, 2CEX.C.1306, 3CE1.A.202, 2CFU.A.1002, 1CG2.A.502, 1CG2.C.502, 3CG7.A.299, 1CGL.A.302, 3CHQ.A.701, 3CHS.A.701, 3CHV.A.301, 3CHV.A.302, 1CIL.A.262, 1CIM.A.262, 1CIN.A.262, 2CJL.A.1217, 2CJS.C.201, 2CJS.C.202, 3CJP.A.301, 2CKL.A.1104, 2CKL.A.1105, 2CKL.B.1115, 2CKL.B.1116, 2CKI.A.999, 1CL4.A.81, 1CLC.A.653, 2CLT.A.1202, 3CMR.A.450, 1CNG.A.1, 1CNH.A.1, 1CNI.A.1, 1CNJ.A.1, 3CNG.A.508, 1CNW.A.262, 1CNX.A.262, 1CNY.A.262, 2COT.A.201, 2COT.A.401, 1CO4.A.43, 3COS.A.501, 3COS.A.502, 2CO8.A.201, 2CO8.A.401, 2CON.A.201, 2COR.A.201, 2COR.A.401, 4COI.A.652, 4CPD.A.1200, 4CPD.A.1300, 1CQR.B.2301, 3CQZ.B.3007, 3CQZ.I.3004, 3CQZ.L.3005, 4CQ0.A.1262, 2CQE.A.622, 2CQE.A.822, 2CQF.A.330, 2CRW.A.401, 1CRA.A.262, 2CR8.A.401, 2CRC.A.401, 2CRR.A.401, 2CSV.A.200, 2CSV.A.400, 2CSY.A.201, 2CSY.A.401, 2CSZ.A.201, 2CSZ.A.401, 3CSK.A.712, 3CSQ.A.335, 2CS2.A.200, 2CS3.A.200, 2CS3.A.400, 2CS7.A.201, 2CS8.A.401, 2CS8.A.601, 2CSH.A.200, 2CSH.A.300, 2CSH.A.400, 1CTT.A.296, 1CTU.A.296, 2CT0.A.201, 2CT0.A.401, 2CT1.A.201, 2CT1.A.401, 2CT2.A.201, 2CT2.A.401, 2CT5.A.201, 2CT7.A.201, 2CT7.A.401, 2CTD.A.201, 2CTD.A.401, 2CTT.A.201, 2CTT.A.401, 2CTU.A.201, 2CU8.A.201, 2CU8.A.401, 2CUP.A.201, 2CUP.A.401, 2CUP.A.601, 2CUQ.A.201, 2CUQ.A.401, 2CUR.A.201, 2CUR.A.401, 1CVE.A.262, 4CVR.A.1158, 3CX3.A.314, 3CXK.A.201, 3CXL.A.500, 3CXL.A.501, 1CXV.A.3, 1CXX.A.1, 4CYK.A.42, 1CZM.A.261, 3CZV.A.262, 2D0W.B.1207, 1D0C.A.900, 1D0Q.B.151, 4D0Y.A.1239, 1D1S.B.376, 1D1T.A.375, 1D1T.A.376, 1D1T.A.4

01, 4D1N.A.900, 3D2N.A.101, 3D2N.A.102, 3D2Q.A.303, 3D2Q.A.304, 3D2Z.A.262, 2D5B.A.501, 4D6S.A.1338, 3D7F.A.1752, 3D7V.A.2, 2D74.A.1001, 2D74.B.1002, 2D8X.A.201, 2D8X.A.401, 2D8Y.A.201, 2D8Y.A.401, 2D8Z.A.201, 2D8Z.A.401, 3D8W.A.262, 2D8Q.A.201, 2D8R.A.401, 2D8S.A.201, 2D8S.A.401, 2D8T.A.201, 2D8T.A.401, 2D8U.A.201, 2D8U.A.401, 2D8V.A.201, 1D9D.A.1, 2D9G.A.201, 2D9H.A.201, 2D9H.A.401, 2D9K.A.401, 2D9K.A.601, 2D9L.A.401, 2D9M.A.1085, 2D9N.A.256, 2D9N.A.456, 4D9W.A.408, 3DAZ.A.262, 2DAN.A.201, 2DAR.A.201, 2DAR.A.401, 2DAS.A.101, 3DBH.B.1, 3DBK.A.302, 2DB6.A.201, 2DB6.A.401, 4DB3.A.401, 1DCA.A.262, 3DCC.A.262, 1DCQ.A.600, 1DD6.A.502, 1DD6.A.503, 3DD0.A.262, 3DD8.A.262, 1DDZ.A.2, 3DDT.A.46, 1DE6.A.450, 1DEH.A.376, 1DFE.A.38, 3DFM.A.402, 4DF9.A.503, 2DFV.A.1001, 3DGD.C.128, 1DGS.B.2701, 2DGE.A.1001, 2DH3.A.601, 3DH1.A.201, 3DHA.A.256, 3DI4.B.286, 2DID.A.201, 2DID.A.401, 2DIP.A.201, 2DIP.A.401, 2DJ7.A.201, 2DJ7.A.401, 2DJ8.A.201, 2DJ8.A.401, 2DJA.A.201, 2DJA.A.401, 2DJB.A.201, 2DJB.A.401, 1DK4.A.290, 2DKT.A.191, 2DKT.A.241, 2DKT.A.291, 2DKT.A.341, 2DKT.A.391, 2DKT.A.441, 2DKD.B.922, 2DLK.A.201, 2DLK.A.401, 1DL6.A.60, 2DLO.A.201, 2DLO.A.401, 2DLQ.A.200, 2DLQ.A.300, 2DLQ.A.400, 2DLQ.A.500, 4DLA.A.401, 4DLA.A.402, 1DMT.A.755, 1DMX.A.280, 1DMY.A.280, 3DMO.A.131, 2DMD.A.191, 2DMD.A.241, 2DMD.A.291, 2DMI.A.200, 2DMI.A.300, 2DMJ.A.200, 1DO5.A.28, 2DOO.A.501, 2DOO.A.502, 1DPM.A.801, 2DPH.A.1001, 2DQ4.A.502, 2DQ6.A.900, 4DR8.A.201, 1DSV.A.171, 2DS7.A.100, 2DSN.B.2002, 1DTH.A.901, 1DVB.A.194, 1DVP.A.401, 1DVP.A.402, 3DWD.A.501, 2DW2.A.700, 1DY0.A.401, 4DYG.B.307, 4DZ7.A.301, 1E0E.A.147, 3E1W.A.230, 3E1Z.A.111, 2E2Z.A.101, 3E24.A.230, 3E2C.A.200, 3E2I.A.200, 3E2U.E.102, 4E2X.A.501, 1E3I.A.380, 1E3I.A.381, 1E3J.A.901, 1E3J.A.902, 1E3L.A.380, 3E3F.A.230, 3E3I.A.230, 1E46.S.999, 1E4B.S.999, 1E4U.A.79, 1E4U.A.80, 3E4Z.B.2, 2E47.A.172, 2E5R.A.201, 2E5R.A.401, 2E5S.A.201, 2E5S.A.401, 2E6R.A.201, 2E6R.A.401, 2E6S.A.201, 2E6S.A.401, 2E6S.A.601, 1E67.A.129, 3E6U.A.501, 3E6U.C.502, 3E6U.B.503, 2E6I.A.201, 2E7Y.A.1301, 3E73.A.501, 1E7L.A.1165, 2E72.A.201, 2E73.A.201, 2E73.A.401, 2E9H.A.301, 1E9P.B.153, 1E9Q.B.153, 2EA5.A.201, 2EA5.A.401, 2EA6.A.201, 2EA6.A.401, 2EBL.A.191, 2EBL.A.241, 2EBT.A.100, 2EBT.A.200, 2EBT.A.300, 2EBV.A.201, 3EB5.A.1001, 3EBE.A.500, 3EBI.A.1, 2EBQ.A.201, 2EBR.A.201, 2ECJ.A.201, 2ECJ.A.401, 2ECI.A.201, 2ECI.A.401, 2ECT.A.201, 2ECT.A.401, 2ECV.A.201, 2ECV.A.401, 2ECW.A.201, 2ECW.A.401, 2ECY.A.201, 2ECY.A.401, 2ECG.A.201, 2ECG.A.401, 2ECL.A.201, 2ECL.A.401, 2ECL.A.601, 2ECM.A.201, 2ECM.A.401, 2ECN.A.201, 2ECN.A.401, 1ED9.A.451, 3EDI.A.210, 2EER.A.501, 1EE2.A.1300, 1EE2.A.1301, 1EE8.A.501, 3EED.A.194, 3EER.A.2004, 2EE8.A.301, 2EE8.A.501, 2EE8.A.701, 4EEX.A.402, 4EEX.B.402, 4EEZ.A.401, 4EEZ.A.402, 1EF4.A.56, 3EFO.A.766, 3EFO.B.1034, 3EFT.A.262, 4EFS.A.301, 2EGQ.A.200, 2EGQ.A.300, 2EG4.A.301, 2EG4.B.302, 2EGM.A.200, 2EGM.A.300, 2EGP.A.200, 2EGP.A.400, 4EGU.A.202, 3EH1.A.1269, 3EH2.A.800, 3EHX.A.264, 2EHE.A.200, 2EHE.A.300, 1EI6.A.409, 1EI6.B.408, 2EJ4.A.401, 1EKJ.A.4001, 1EKJ.C.4003, 1EKM.A.701, 2ELN.A.181, 2ELO.A.181, 2ELQ.A.181, 2ELR.A.181, 2ELS.A.181, 2ELT.A.181, 2ELU.A.181, 2ELV.A.181, 2ELW.A.181, 2ELX.A.181, 2ELY.A.200, 2ELZ.A.200, 1ELY.A.451, 2EL4.A.200, 2EL5.A.200, 2EL6.A.200, 2ELI.A.201, 2ELI.A.401, 2ELM.A.181, 2EMJ.A.201, 2EMI.A.201, 2EMX.A.201, 2EMY.A.201, 2EMZ.A.201, 2EM0.A.200, 2EM1.A.201, 2EM2.A.201, 2EM4.A.201, 2EM5.A.201, 2EM6.A.201, 2EM7.A.201, 2EM8.A.201, 2EM9.A.201, 2EMA.A.201, 2EMB.A.201, 2EMC.A.201, 2EME.A.201, 2EMF.A.201, 2EMG.A.201, 2EMH.A.201, 2EMK.A.201, 2EML.A.201, 2EMM.A.201, 2EMP.A.201, 2ENT.A.200, 2ENV.A.200, 2ENV.A.300, 1EN7.A.401, 2ENZ.A.300, 2ENZ.A.400, 2EN1.A.201, 2EN2.A.201, 2EN4.A.201, 2EN6.A.181, 2EN7.A.181, 2EN8.A.181, 2EN9.A.181, 2ENA.A.181, 2ENC.A.181, 2ENE.A.181, 2ENF.A.181, 2ENH.A.181, 2ENN.A.300, 2ENN.A.400, 2EOJ.A.201, 2EOI.A.201, 2EOQ.A.201, 2EOR.A.201, 2EOS.A.201, 2EOU.A.201, 2EOV.A.201, 2EOW.A.201, 2EOX.A.201, 2EOY.A.201, 2EOZ.A.201, 1EOU.A.300, 2EO4.A.201, 2EOD.A.300, 2EOD.A.400, 2EOE.A.201, 2EOF.A.201, 2EOG.A.201, 2EOH.A.201, 2EOK.A.201, 2EOL.A.201, 2EOM.A.201, 2EON.A.201, 2EOO.A.201, 2EOP.A.201, 2EPR.A.201, 2EPS.A.201, 2EPT.A.201, 2EPU.A.201, 2EPV.A.201, 2EPW.A.201, 2EPX.A.201, 2EPY.A.201, 2EPZ.A.201, 2EP0.A.201, 2EP1.A.201, 2EP2.A.201, 2EP3.A.201, 2EP4.A.200, 2EP4.A.300, 2EPA.A.300, 2EPA.A.400, 2EPC.A.

201, 3EPZ.A.701, 2EPP.A.201, 2EPQ.A.201, 2EQW.A.201, 3EQN.B.757, 2EQ0.A.201, 2EQ
1.A.201, 2EQ2.A.201, 2EQ3.A.201, 2EQ4.A.201, 2EQE.A.201, 2EQF.A.201, 2EQG.A.201,
1ESK.A.55, 1ESP.A.323, 2ESL.A.4, 4ETS.A.302, 1EU3.B.402, 1EU4.A.400, 2EU3.A.262
, 4EVB.A.204, 3EWF.A.400, 2EWB.A.489, 2EXU.A.501, 1EXK.A.80, 1EXK.A.81, 4EXS.A.3
02, 1EYW.A.402, 3EYX.A.1, 4EYL.A.303, 4EYU.A.1702, 1EZM.A.302, 2F0Y.B.501, 3F0D.
A.163, 3F0F.A.165, 1F18.A.155, 1F1G.A.4002, 2F14.A.1262, 1F2W.A.262, 1F35.A.306,
2F3B.A.341, 4F3W.A.201, 1F4T.A.369, 3F4X.A.262, 2F4M.A.501, 3F5L.A.1001, 1F62.A
.52, 1F62.A.53, 3F7B.B.301, 3F7L.A.203, 3F7U.A.260, 4F70.B.301, 1F81.A.88, 1F8F.
A.372, 1F8F.A.373, 1F9X.A.999, 3F90.A.309, 2F9I.B.601, 4F9C.B.401, 4F9V.A.401, 1
FAQ.A.1, 1FAQ.A.2, 4FAI.A.401, 1FBX.A.3316, 4FBE.A.403, 3FCQ.A.322, 2FC6.A.201,
2FC7.A.201, 2FC7.A.401, 2FE3.A.201, 2FEA.A.1302, 2FEJ.A.1, 3FFP.X.262, 2FGY.A.62
0, 2FGY.A.621, 4FGL.A.301, 2FHX.A.317, 3FID.A.298, 3FID.A.299, 3FIE.A.428, 2FIF.
B.901, 3FJU.A.502, 3FJU.A.507, 1FKW.A.400, 4FKB.A.401, 4FKD.A.102, 4FKE.A.1024,
4FKK.A.1025, 3FL2.A.1001, 3FL2.A.1002, 1FLJ.A.262, 3FLF.A.2004, 3FLO.B.1, 3FLO.B
.2, 4FMN.B.901, 4FMN.B.902, 4FMP.A.400, 1FN9.A.1001, 2FNF.X.1, 2FNF.X.2, 2FOQ.A.
262, 2FOS.A.262, 2FOU.A.262, 2FOV.A.262, 2FOY.A.301, 4FO9.A.501, 1FP0.A.90, 2FPR
.A.502, 3FPC.A.353, 3FPL.A.352, 1FQL.A.262, 1FQM.A.262, 3FQM.A.901, 1FR2.B.301,
1FR7.A.262, 2FR5.A.147, 2FR6.A.147, 4FRC.A.302, 2FSA.A.501, 3FTN.A.354, 3FTN.D.3
57, 3FTW.A.701, 1FU9.A.37, 3FUN.A.701, 2FU8.A.401, 2FU9.A.401, 4FU5.A.302, 3FV4.
A.321, 3FVP.A.321, 3FVZ.A.821, 4FVD.A.201, 4FVL.A.501, 4FVN.A.302, 4FVO.A.302, 4
FVY.A.805, 3FW3.A.300, 1FWQ.A.124, 4FWE.A.901, 4FWE.A.902, 4FWU.A.401, 3FXP.A.30
00, 2FYG.A.302, 4FYY.B.201, 2FZW.A.375, 2FZW.B.376, 2G0D.A.416, 3G1P.A.300, 1G25
.A.66, 1G25.A.67, 3G27.A.97, 4G26.A.1001, 2G2N.A.1001, 4G3M.B.401, 1G47.A.999, 1
G48.A.262, 3G42.D.500, 1G4K.A.301, 2G45.A.401, 1G52.A.262, 1G54.A.262, 1G5C.A.10
01, 2G54.A.1100, 2G6Q.A.400, 1G71.A.348, 4G7A.A.301, 2G84.A.506, 2G9T.A.999, 2G9
Y.B.451, 1G9K.A.600, 3GA3.A.1, 2GA3.A.451, 3GAY.B.328, 2GAG.D.101, 2GAH.D.101, 3
GC9.B.603, 1GDC.A.73, 1GDC.A.74, 2GD8.A.262, 4GER.A.401, 2GFJ.B.401, 2GFE.A.869,
2GFO.A.1200, 4GGJ.A.301, 1GI4.A.409, 3GI1.A.501, 3GIQ.A.482, 3GIQ.A.483, 4GIZ.C
.201, 3GJ3.B.300, 3GJ4.D.300, 3GJ5.B.300, 3GJ7.D.300, 3GJ8.B.300, 1GKR.A.1452, 2
GMN.A.801, 2GMN.A.805, 2GMW.A.300, 4GNE.A.1501, 4GNE.A.1502, 4GNE.A.1503, 4GNE.A
.1504, 1GO8.P.1486, 2GQJ.A.200, 2GQJ.A.300, 4GQT.B.502, 3GRB.A.129, 4GR0.A.301,
4GR3.A.301, 4GR8.A.301, 4GRI.A.501, 2GSN.A.1000, 3GTT.A.155, 1GUP.A.350, 4GU1.A.
905, 4GUA.A.1719, 4GUT.A.904, 3GV4.A.203, 1GVY.A.1425, 2GVI.A.301, 4GVE.A.601, 1
GYT.J.600, 1GZH.A.1293, 3GZE.B.2, 3GZK.A.539, 2GZL.A.900, 3H0L.E.902, 3H0N.A.201
, 4H0F.A.401, 1H19.A.701, 2H15.A.262, 4H12.A.1801, 4H12.A.1802, 4H12.A.1803, 4H1
Q.A.302, 1H2B.A.1362, 3H2P.B.154, 4H2K.B.1001, 2H39.A.352, 4H30.A.301, 4H3S.A.90
1, 2H4N.A.262, 3H5A.B.360, 3H5N.A.500, 4H57.A.405, 2H6E.A.500, 1H7N.A.1342, 3H7H
.A.119, 1H7V.A.61, 3H8V.A.401, 4H84.A.301, 3H90.A.293, 3H99.A.601, 1H9Q.A.262, 4
H9D.A.201, 3HB2.P.486, 2HB9.A.401, 2HBA.A.101, 1HC7.A.490, 3HCI.A.1000, 1HCP.A.9
8, 1HCP.A.99, 3HCJ.A.1000, 3HCS.A.303, 2HCS.A.1, 2HCS.A.2, 4HCG.A.202, 1HDY.A.37
6, 2HD6.A.262, 2HDP.A.492, 2HDP.A.493, 4HDH.A.1002, 4HEW.A.301, 4HEY.A.301, 3HFF
.A.154, 2HF1.A.102, 4HF3.A.301, 3HI2.A.121, 4HI8.B.101, 4HI8.B.102, 2HJN.A.315,
3HJT.A.1, 2HJH.A.800, 1HK8.A.1589, 3HKN.A.261, 3HKO.A.701, 3HKQ.A.261, 3HKT.A.26
1, 3HKU.A.261, 3HLJ.A.272, 2HL4.A.262, 4HMA.A.301, 3HNA.A.501, 3HNA.A.502, 3HNA.
A.503, 3HNA.A.504, 3HNI.A.107, 3HNI.G.107, 3HNI.H.107, 3HNJ.A.107, 3HNJ.B.107, 3
HNJ.C.107, 3HNJ.D.107, 2HNC.A.263, 4HNO.A.301, 2HOC.A.263, 3HPH.A.220, 2HQH.E.15
00, 2HRV.A.143, 3HS4.A.301, 1HSO.A.1376, 1HSZ.A.1376, 2HSI.A.283, 4HSU.A.904, 1H
T0.A.1376, 1HTD.A.401, 4HT0.A.301, 4HT2.A.301, 4HTB.A.401, 1HU8.A.501, 1HUG.A.26
1, 3HUG.D.109, 4HU1.A.301, 2HU9.A.132, 4HVL.A.504, 1HXP.A.350, 1HXR.A.200, 1HY7.
A.302, 1HY7.B.801, 1HZ5.B.105, 2HZ8.A.117, 2IO0.A.579, 2IO0.A.581, 3I1U.A.401, 4
I1F.A.503, 4I1F.A.504, 4I1F.A.508, 4I1H.A.507, 3I2D.A.1, 4I28.A.602, 2I2X.A.524,
2I3H.A.1001, 3I3T.A.700, 3I4C.A.400, 3I4C.A.500, 1I50.A.3006, 1I50.C.3002, 2I5O
.A.336, 2I50.A.336, 2I50.A.337, 2I50.A.338, 4I51.A.3005, 1I6N.A.401, 1I6P.A.301,
1I73.A.998, 1I73.A.999, 1I76.A.999, 4I7C.A.601, 1I8J.B.400, 1I8Z.A.262, 3I9F.B.

3, 2I9W.A.200, 2I9W.A.201, 1IA6.A.1264, 1IA9.A.2001, 1IAG.A.999, 1IAU.A.504, 1IB
5.A.201, 1IBB.A.201, 1IBI.A.195, 1IBI.A.196, 3IBI.A.262, 3IBL.A.262, 3IBN.A.262,
3IBU.A.262, 2IBI.A.1, 4IBY.A.301, 4ICR.A.501, 2IDA.A.103, 2IDA.A.104, 3IEW.B.80
1, 1IF5.A.262, 1IF9.A.262, 3IFJ.A.201, 3IFJ.B.202, 3IFU.A.181, 3IGP.A.262, 2IGI.
A.1001, 2IGI.A.1004, 4II1.A.901, 3IJF.X.147, 4IJD.A.501, 4IJD.A.502, 4ILO.A.301,
4ILX.A.301, 2IMZ.A.501, 2IMZ.B.502, 1IML.A.77, 1IML.A.78, 3IMI.A.201, 2IMR.A.50
0, 1INN.B.167, 2INN.B.514, 3IO2.A.202, 2IOI.A.3001, 4IOU.D.1001, 1IQ8.A.600, 2IQ
J.A.301, 4IR0.A.201, 1IRN.A.55, 3IR9.B.501, 3IRB.A.201, 1IS8.B.3109, 3ISI.X.3000
, 1ITU.A.401, 2IT4.A.561, 4ITO.A.301, 4ITP.A.302, 3IUF.A.1, 2IUC.A.1002, 2IUC.B.
1007, 4IUM.A.501, 4IUQ.A.301, 4IUW.A.701, 2IWE.A.1129, 3IXE.B.301, 3IXE.B.302, 4
IXJ.A.301, 1IYM.A.182, 1IYM.A.183, 2IYB.E.1422, 2IYB.E.1423, 2J1Y.A.1290, 4J1V.A
.301, 1J2O.A.115, 1J2O.A.116, 1J2T.A.302, 2J21.A.1289, 4J3D.B.302, 4J4M.A.301, 2
J6A.A.1138, 2J7U.A.1884, 2J7J.A.1088, 1J98.A.300, 1J9Y.A.1004, 2J9R.A.1194, 2J9U
.B.1162, 1JAO.A.999, 1JAZ.A.401, 2JA1.A.1192, 4JBG.A.401, 2JBG.B.1577, 1JD0.A.90
1, 1JD5.A.501, 4JD1.B.202, 4JE6.A.200, 4JEA.A.202, 4JEA.B.202, 4JEA.C.202, 4JEA.
D.202, 4JEB.B.202, 2JHG.A.401, 4JH2.A.201, 4JH2.B.201, 4JIJ.A.302, 4JIU.A.201, 4
JIV.D.101, 2JIG.A.1253, 2JIG.B.1252, 1JJ9.A.999, 1JJD.A.102, 1JJD.A.103, 1JJD.A.
104, 1JJE.B.251, 1JJT.B.251, 4JLW.A.401, 4JLX.A.501, 2JLP.A.226, 2JMO.A.201, 2JM
O.A.401, 2JM1.A.2, 2JM3.A.92, 4JMY.A.201, 2JMD.A.65, 1JN7.A.37, 2JOX.A.108, 2JOX
.A.109, 2JOX.A.110, 1JOC.A.300, 4JOM.A.1004, 4JPA.A.301, 1JQ5.A.371, 2JQ5.A.129,
2JR7.A.85, 2JRJ.A.62, 2JRJ.A.63, 2JSD.A.275, 4JSA.A.301, 4JSR.A.401, 4JSW.A.301
, 4JSZ.A.301, 2JTG.A.88, 1JT1.A.400, 1JTK.A.137, 2JTN.A.183, 2JTN.A.184, 2JTN.A.
185, 2JTN.A.186, 3JUE.A.999, 2JUN.A.220, 2JUN.A.221, 2JUN.A.222, 2JUN.A.223, 1JV
0.A.261, 2JVX.A.29, 1JVB.A.400, 1JVB.A.500, 3JV7.A.501, 3JV7.A.502, 3JVH.A.163,
2JVN.A.400, 2JWO.A.488, 2JWO.A.489, 1JW9.B.250, 3JWP.A.2001, 2JW6.A.601, 3JXP.A.
320, 4JXE.A.501, 4JXE.A.502, 1JY8.A.300, 1JZS.A.1101, 2JZ8.A.150, 4JZO.A.802, 1K
07.A.1, 2K0A.A.108, 2K0A.A.109, 2K0A.A.110, 2K1P.A.96, 2K16.A.940, 2K16.A.941, 4
K1R.A.502, 1K24.A.401, 3K2F.A.262, 1K2Y.X.500, 2K2C.A.138, 2K2C.A.139, 2K2C.A.14
1, 2K2C.A.142, 2K2C.A.143, 2K2D.A.80, 4K2H.A.201, 4K2H.B.201, 3K34.A.1002, 3K35.
C.317, 2K4X.A.56, 3K5K.A.1194, 3K5K.A.1197, 3K5K.B.1195, 3K5K.B.1196, 2K5C.A.96,
4K5N.A.1101, 3K6I.A.202, 3K6J.A.800, 1K7H.A.478, 1K7I.A.486, 3K7H.B.1001, 2K7R.
A.129, 4K7D.A.501, 4K7D.A.502, 4K7D.A.503, 4K7D.A.504, 4K7D.A.505, 4K7D.A.506, 4
K7D.A.507, 4K7D.A.508, 4K7W.B.101, 1K81.A.144, 1K83.I.3003, 2K8D.A.155, 2K9H.A.1
01, 2K9H.A.102, 2KAK.A.130, 2KAK.A.150, 2KAK.A.170, 1KBE.A.2, 3KB1.A.302, 3KBF.A
.159, 2KDP.A.1, 2KDX.A.120, 3KE1.A.163, 3KED.A.875, 3KEE.A.2000, 1KEV.B.353, 2KE
M.A.195, 1KFI.A.700, 2KFN.A.1, 2KGG.A.53, 2KGG.A.54, 2KGO.A.109, 1KH4.A.451, 1KH
5.A.451, 1KH7.A.451, 1KHK.A.451, 1KHL.A.451, 3KHI.A.301, 2KIK.B.50, 2KIZ.A.70, 2
KIZ.A.71, 2KI7.B.124, 2KJE.A.501, 2KJE.A.502, 1KK1.A.411, 2KKT.A.500, 2KKH.A.201
, 2KKR.A.500, 1KLR.A.31, 1KLS.A.31, 3KNE.A.500, 3KNV.A.201, 3KNV.A.202, 2KN9.A.8
2, 4KNI.A.301, 4KNJ.A.301, 4KNM.A.301, 1KOL.A.1001, 1KOL.A.1002, 1KOQ.A.301, 4KP
5.A.301, 2KPI.A.150, 4KP8.A.301, 3KQI.A.71, 3KQI.A.72, 2KQ9.A.113, 2KQB.A.1001,
2KR1.A.65, 1KTO.A.405, 1KU0.A.701, 2KU3.A.63, 2KU3.A.64, 4KUJ.A.301, 4KUV.A.301,
4KUW.A.301, 4KUY.A.301, 2KVF.A.83, 3KV4.A.448, 3KV5.A.489, 3KV5.A.490, 3KVE.B.4
89, 4KV0.A.301, 1KWG.A.806, 1KWQ.A.262, 3KWO.B.161, 3KWO.C.161, 2KWJ.A.501, 2KWJ
.A.601, 2KWJ.A.701, 2KWJ.A.801, 4KX8.A.1001, 4KXB.A.1001, 4KXC.A.1001, 4KXQ.A.60
1, 3KYC.B.641, 2KZY.A.63, 2KZM.A.1, 2L0Z.A.486, 3L00.A.180, 1L0Y.B.706, 3L0V.A.1
, 2L0B.A.143, 2L0B.A.161, 4L05.A.203, 3L11.A.601, 3L11.A.602, 3L14.A.262, 1L1O.F
.2, 3L22.A.1, 4L3J.A.402, 2L5U.A.62, 2L5U.A.63, 4L56.A.401, 4L58.A.102, 4L5O.A.3
03, 2L6Y.A.239, 2L6Z.B.37, 1L6J.A.500, 2L6L.A.201, 2L6M.A.201, 4L6H.A.801, 4L6O.
A.801, 4L6T.A.301, 2L7X.A.106, 2L7X.A.107, 1L7O.B.301, 2L75.A.155, 2L75.A.156, 2
L7P.A.201, 4L7X.A.101, 3L8H.A.901, 2L80.A.124, 2L8E.A.829, 2L9Z.A.403, 3L9Y.A.15
5, 3LAS.A.167, 3LAT.A.215, 2LAU.A.82, 4LAO.A.401, 1LBU.A.214, 2LBM.A.1, 2LBM.A.3
, 4LBU.A.402, 3LCZ.A.54, 2LCE.A.200, 2LCE.A.300, 2LCQ.A.162, 1LD3.A.500, 1LDE.A.
375, 1LDY.A.375, 3LE9.B.2, 3LEA.A.485, 4LEF.A.302, 4LEV.A.601, 2LFD.A.400, 2LGV.

A.109, 2LGV.A.110, 2LGV.A.111, 1LG5.A.262, 2LGG.A.380, 2LGG.A.381, 2LGG.A.382, 4LGJ.A.301, 2LHN.A.501, 2LHN.A.502, 2LHN.A.503, 4LHI.A.301, 1LI5.B.964, 1LIQ.A.28, 2LI8.A.187, 2LI8.A.188, 2LI9.A.18, 4LIM.A.401, 2LJX.A.200, 2LJZ.A.201, 3LJU.X.401, 4LJO.A.1101, 4LJO.A.1102, 4LJO.A.1104, 4LJP.A.1101, 4LJP.A.1103, 4LJQ.B.1105, 4LJQ.A.1101, 3LKM.A.904, 2LKO.A.32, 2LK5.A.37, 4LK9.A.401, 1LLU.B.343, 3LMI.B.1002, 4LMY.A.202, 2LNO.A.401, 2LNO.A.501, 2LNO.A.601, 2LNO.A.701, 2LO2.A.101, 2LO3.A.101, 2LO4.A.300, 4LO9.A.401, 4LOE.C.401, 4LOF.A.401, 3LPE.B.60, 1LPV.A.53, 1LPV.A.54, 4LP6.A.310, 3LQB.A.201, 3LQH.A.1001, 3LQH.A.1002, 4LQG.A.802, 3LRQ.C.100, 4LR2.A.505, 2LRI.C.101, 2LRI.C.102, 3LS1.A.1, 3LS9.A.457, 3LSC.A.458, 3LT8.A.80, 2LUY.A.300, 2LUY.A.301, 3LUB.A.302, 2LUA.A.101, 2LUA.A.102, 2LUA.A.103, 4LU3.A.301, 2LUL.A.201, 2LVU.A.101, 1LV3.A.66, 2LV2.A.101, 2LV2.A.102, 2LV9.A.201, 2LV9.A.202, 2LVR.A.101, 2LVT.A.101, 2LWW.A.501, 2LWW.A.502, 2LWW.A.503, 4LW9.A.203, 4LW9.C.202, 4LW9.D.201, 4LW9.K.203, 3LXE.A.261, 2LXD.A.201, 2LXD.A.202, 2LXH.C.900, 4LXL.A.403, 3LYR.A.1, 4LY4.D.301, 2LZU.A.201, 2LZU.A.202, 3M04.A.501, 3M0A.D.401, 3M0N.A.201, 2M0D.A.101, 2M0E.A.101, 2M0F.A.101, 4M0W.A.401, 2M1S.A.100, 3M14.A.501, 3M15.A.107, 3M15.B.107, 3M1D.A.1000, 2M13.A.601, 2M13.A.602, 3M1W.A.500, 1M2K.A.999, 1M2O.A.800, 3M2N.A.263, 3M2X.A.500, 3M2Y.A.500, 4M2R.A.301, 4M2V.A.301, 2M3Z.A.101, 2M3Z.A.102, 1M3V.A.123, 1M3V.A.124, 3M3X.A.262, 2M3H.A.102, 2M3L.A.201, 4M3O.A.300, 4M3P.A.701, 3M40.A.262, 3M4C.B.108, 1M4M.A.502, 2M48.A.501, 2M48.A.502, 2M48.A.503, 2M48.A.504, 3M5E.A.262, 3M5S.A.500, 1M65.A.300, 1M6H.A.1376, 3M67.A.263, 3M6I.A.402, 1M6W.A.1376, 2M6M.A.201, 2M6M.A.202, 2M7Q.A.101, 2M7Q.A.102, 3M79.D.107, 2M7A.A.100, 3M8T.A.300, 3M8T.A.301, 2M85.A.801, 2M85.A.802, 2M9Y.A.401, 2M9Y.A.402, 3M96.A.262, 3M98.A.262, 1M9O.A.78, 2M9A.A.101, 2M9A.A.103, 2M9A.A.102, 3MA2.A.295, 2MA5.A.101, 2MA5.A.102, 2MA6.A.101, 2MA6.A.102, 3MBG.A.3, 3MBG.A.1, 3MBM.A.163, 1MBX.B.211, 4MB7.A.301, 1MC5.A.376, 3MDW.A.455, 3MDZ.A.263, 2MD7.B.101, 2MD7.B.102, 2MDG.A.101, 2MDG.A.102, 4MDM.A.301, 1MEA.A.29, 3MEK.A.500, 3MEK.A.501, 3MEK.A.502, 3MEN.D.400, 3MEQ.A.401, 3MEQ.A.501, 4ME3.A.301, 3MF1.A.1000, 1MGO.A.375, 1MGO.A.376, 1MGO.B.375, 4MG3.A.201, 3MHC.A.262, 3MHH.E.97, 3MHI.A.262, 3MHL.A.262, 3MHM.A.262, 3MHS.A.472, 3MHS.A.473, 3MHS.A.474, 3MHS.A.475, 3MHS.A.476, 3MHS.A.477, 3MHS.E.97, 4MHN.A.400, 4MHQ.A.501, 4MHY.A.400, 3MI9.C.87, 3MI9.C.88, 4MI5.A.802, 4MI5.A.803, 4MI5.A.804, 4MI5.A.805, 4MI5.A.806, 4MI5.A.807, 2MIU.A.301, 2MIU.A.302, 2MIU.A.303, 3MJH.B.70, 4MJ7.A.201, 3MKG.A.155, 3MKV.B.426, 2MKD.A.301, 3ML2.A.262, 1ML9.A.1, 1ML9.A.2, 1ML9.A.3, 3ML5.A.263, 4MLT.A.301, 1MM3.A.62, 1MM3.A.63, 3MMF.A.262, 1MMR.A.1, 2MNY.A.401, 2MNY.A.402, 3MNA.A.262, 3MND.A.153, 1MNC.A.282, 3MNU.A.262, 1MOO.A.262, 4MO8.A.301, 1MPO.A.376, 3MPZ.A.300, 3MP2.A.1, 1MQ0.B.147, 1MR1.C.601, 2MRE.B.301, 3MS3.A.401, 1MS7.B.3001, 4MSG.A.1401, 4MSX.A.501, 3MTW.A.2, 4MT2.A.67, 4MT2.A.68, 4MTW.E.401, 4MTY.A.301, 2MUM.A.301, 2MUM.A.302, 2MUQ.A.101, 1MVH.A.501, 1MVH.A.502, 3MWM.A.140, 3MWM.A.141, 3MWM.A.142, 2MWX.A.201, 4MWP.E.401, 1MXG.A.437, 4MXJ.E.401, 3MYQ.A.262, 1MZ8.B.600, 1MZB.A.202, 4MZN.E.401, 3MZC.A.262, 4NOG.A.401, 4NOL.A.401, 4NON.A.501, 4NON.A.503, 1NOZ.A.46, 4NOX.B.301, 3N2C.E.425, 3N2P.A.262, 3N3J.A.262, 3N3K.A.1, 4N4E.E.401, 4N4F.A.1401, 4N4F.A.1402, 3N4B.A.262, 1N5N.A.401, 4N5P.E.405, 4N66.E.501, 3N67.A.900, 1N8K.A.375, 1N8K.A.376, 1N92.A.375, 3NA7.A.300, 3NB5.A.261, 1NCS.A.61, 1ND1.A.400, 1NEE.A.136, 3NGJ.A.250, 3NHE.A.1, 3NIS.A.1, 3NIS.A.2, 3NIS.A.3, 3NIT.A.2, 3NI5.A.262, 1NJ1.A.513, 1NJG.A.401, 4NJ5.A.801, 4NJ5.A.803, 3NKQ.A.1001, 1NKU.A.188, 3NKM.A.1001, 4NL4.H.802, 4NL4.H.803, 2NMX.A.301, 2NNV.A.262, 3NNQ.A.201, 2NN1.A.301, 2NN7.A.301, 4NN2.A.401, 4NN2.A.402, 4NN2.A.403, 2NNG.A.262, 2NNO.A.262, 1NO5.B.571, 4NOS.A.3000, 3NQ5.A.503, 3NQY.B.520, 3NQZ.B.1, 4NQ4.A.302, 4NQ5.A.301, 4NQ5.A.302, 4NQ6.A.301, 4NQ6.A.302, 4NQ7.A.301, 4NQ7.A.302, 4NQY.A.501, 4NS5.A.401, 1NTO.A.500, 4NTM.A.201, 4NTN.A.201, 2NUP.B.1100, 1NUI.A.501, 2NYT.A.2000, 3NY1.A.4, 3NY1.A.5, 3NY1.A.6, 3NY3.A.1, 3NY3.A.2, 3NY3.A.3, 1NZJ.A.700, 4NZG.A.201, 3O0M.A.151, 2O03.A.201, 2O03.A.202, 2O10.A.86, 2O10.A.87, 2O13.A.190, 2O13.A.191, 4O1K.A.301, 3O36.A.1, 3O36.A.2, 2O3K.A.401, 2O4Z.A.262, 3O4N.A.2002, 2O4H.B.401, 2O4Q.A.2402, 2O53.B.314, 4O62.A

.1001, 4O64.A.2001, 4O64.A.2002, 4O64.A.2003, 4O64.A.2004, 4O6I.A.601, 3O70.A.50
0, 3O70.A.501, 3O7A.A.500, 3O7A.A.501, 3O7U.A.428, 1OAL.A.152, 4OAQ.A.403, 4OBI.
A.201, 3OCA.A.300, 3OCQ.A.184, 4OCM.E.401, 2ODX.A.156, 2OD1.A.902, 4ODR.A.202, 4
ODR.B.202, 2OFK.A.201, 2OGW.A.500, 4OGE.A.1201, 2OHX.A.401, 1OHL.A.400, 2OH3.A.3
00, 3OIL.A.262, 2OI0.A.2, 2OIK.B.201, 4OIF.A.701, 4OIW.F.501, 1OJ7.B.1389, 3OJ3.
J.902, 3OJ6.A.150, 3OJ7.A.114, 1OKL.A.262, 1OKM.A.262, 1OKN.A.262, 2OM1.B.801, 1
ONW.A.801, 3OOI.A.232, 3OOI.A.233, 3OOI.A.234, 2OO4.A.6001, 1OQ5.A.600, 3OQ6.A.3
75, 1OQJ.A.183, 2ORW.A.401, 3ORU.A.250, 1OS0.A.600, 2OSM.A.262, 2OSF.A.262, 4OTE
.A.304, 2OU2.A.490, 2OUI.A.361, 2OVX.A.444, 2OVZ.A.444, 1OVX.A.61, 2OW9.B.606, 2
OWA.A.201, 4OWF.G.401, 1OX7.A.402, 2OXZ.A.264, 1OXN.A.1001, 3OXF.A.440, 2OX8.B.3
, 3OY0.A.262, 1OYW.A.801, 3OYQ.A.262, 3OYS.A.262, 1OZB.I.50, 2OZU.A.800, 1P0F.A.
2502, 2P09.A.200, 1P1R.A.375, 1P1V.A.201, 3P1V.A.427, 3P2A.C.151, 3P3H.A.261, 3P
3J.A.261, 1P42.A.501, 1P42.A.502, 1P42.A.503, 3P44.A.261, 1P4Q.B.301, 1P5D.X.500
, 3P55.A.261, 3P58.A.262, 3P5A.A.262, 3P5L.A.262, 2P53.A.401, 2P57.A.201, 1P6B.A
.406, 1P6O.A.401, 1P7A.A.38, 3P8B.A.101, 1P9R.A.601, 4P9C.A.201, 4P9C.B.201, 1PA
A.A.160, 1PB0.A.1301, 1PB0.A.1303, 3PB4.X.400, 3PB6.X.400, 3PB7.X.400, 3PB8.X.40
0, 3PB9.X.400, 3PBB.A.391, 3PBE.A.391, 3PBJ.D.31, 1PCX.A.950, 1PE8.A.317, 1PEG.A
.4, 1PFT.A.51, 1PG5.B.500, 1PGU.A.616, 2PG3.A.300, 4PHT.B.601, 3PII.A.340, 3PJN.
A.187, 3PJN.A.188, 1PL8.A.402, 3PLW.A.187, 3PLW.A.188, 2PLI.B.709, 2PLI.D.702, 3
PN3.A.1001, 3PN3.A.1002, 3PN3.A.1009, 3PN3.A.1010, 3PN3.B.1011, 3PNU.A.336, 2POU
.A.262, 2POW.A.262, 3PO6.A.1, 2POI.A.100, 2PPT.A.300, 1PQ4.A.1002, 4PQ7.A.301, 2
PQ8.A.501, 4PQT.A.501, 2PRS.A.501, 3PSQ.B.321, 3PT9.A.1, 3PT9.A.2, 3PTM.A.1001,
1PTR.A.1, 4PTB.A.901, 4PTB.A.902, 2PUY.A.355, 2PUY.A.356, 3PU7.A.155, 3PUQ.A.2,
3PUR.A.3, 1PV9.A.402, 2PVX.A.901, 2PVE.A.301, 1PXE.A.64, 3PYK.A.262, 4PYX.A.301,
3PZC.A.1000, 3PZC.B.1000, 1PZW.A.100, 4PZH.A.302, 1Q08.A.401, 1Q08.A.402, 1Q08.
B.403, 1Q08.B.404, 3Q01.A.1, 1Q0E.A.153, 4Q09.A.301, 4Q0L.A.301, 1Q1A.A.701, 3Q1
D.A.201, 3Q1D.A.202, 2Q1B.A.400, 2Q1Q.A.262, 1Q2L.A.963, 2Q2L.A.1001, 1Q3A.A.465
, 1Q3K.A.301, 3Q43.A.1, 3Q44.A.1, 1Q5W.A.32, 1Q68.A.201, 1Q69.A.207, 3Q6V.A.2, 2
Q6E.A.501, 4Q6D.A.301, 4Q6E.A.301, 3Q7C.A.1, 4Q7R.A.301, 4Q7R.A.302, 4Q7R.A.303,
4Q7R.A.306, 4Q7R.B.302, 3Q87.A.126, 3Q94.A.301, 1QBH.A.364, 4QBG.B.301, 2QDT.A.
401, 2QDT.A.402, 4QEF.A.301, 3QE3.A.356, 1QF2.A.320, 1QF8.A.216, 4QF2.A.1801, 4Q
F2.A.1802, 4QF3.A.2001, 4QF3.A.2002, 3QGV.A.500, 3QH5.A.321, 3QHD.A.163, 2QIC.A.
300, 2QIC.A.400, 2QIN.A.2002, 2QJS.A.2002, 3QJ5.A.376, 1QJI.A.1201, 2QKD.A.501,
3QL9.A.1, 3QL9.A.3, 2QL0.A.53, 2QL1.A.1, 3QNA.A.122, 2QN0.A.431, 4QN1.A.1501, 2Q
O8.A.262, 2QOA.A.262, 2QP6.A.262, 4QP5.A.401, 2QQ4.A.139, 4QQ4.A.2001, 2QSW.A.20
1, 4QSI.A.301, 4QSJ.A.301, 1QTW.A.301, 1QUA.A.999, 3QU1.A.501, 3QU1.A.503, 3QU1.
A.505, 3QU1.B.502, 1QV6.A.375, 1QV7.A.375, 3QVY.A.500, 3QVZ.D.500, 3QW0.C.500, 1
QWY.A.400, 3QWP.A.500, 3QWP.A.501, 1QYB.A.401, 1QYP.A.58, 3QYK.A.262, 3R0D.A.428
, 1R1H.A.1001, 3R16.A.262, 3R17.B.262, 4R1X.B.501, 1R22.A.502, 3R2N.A.135, 4R2Y.
A.101, 4R2Y.A.102, 4R2Y.A.103, 4R2Y.B.102, 1R37.A.500, 1R3N.B.501, 3R3L.A.585, 2
R3A.A.300, 2R3A.A.301, 2R3A.A.302, 2R3A.A.303, 1R4V.A.202, 1R5T.A.150, 2R59.A.70
1, 3R6F.A.132, 1R79.A.201, 1R79.A.401, 1R9P.A.135, 4R9G.A.505, 1RAY.A.262, 3RBU.
A.1752, 3RCM.A.288, 3RF4.B.119, 4RF1.A.1901, 2RGV.A.146, 1RGO.A.221, 1RGO.A.222,
2RHQ.A.1, 3RHG.A.368, 2RHK.C.502, 2RI7.A.501, 2RI7.A.502, 3RIY.A.1001, 1RJ6.A.6
01, 3RJ7.A.300, 1RJQ.A.601, 1RJW.A.401, 1RJW.A.402, 2RJP.B.1, 4RL0.A.301, 1RM8.A
.501, 1RMD.A.117, 1RMD.A.118, 1RMD.A.119, 1RMD.A.120, 3RMQ.A.114, 4RM5.D.300, 2R
MN.A.1, 1RNI.A.256, 4RN4.A.301, 2RO1.A.201, 2RO1.A.301, 2ROW.A.601, 2ROW.A.602,
2RPR.A.201, 2RPC.A.201, 2RPC.A.401, 2RPC.A.601, 2RPC.A.801, 2RPP.A.201, 2RPP.A.4
01, 3RQD.A.390, 4RQT.A.401, 4RQT.A.402, 4RQU.B.402, 2RR4.A.501, 3RSM.A.500, 3RSN
.A.200, 2RSD.A.901, 2RSH.A.101, 2RSI.A.101, 2RSI.A.102, 2RSI.A.103, 2RSJ.A.101,
2RSJ.A.102, 2RT9.A.701, 2RT9.A.702, 3RUI.A.1, 1RUR.L.601, 1RUT.X.603, 1RUT.X.604
, 4RUW.A.501, 4RV9.A.501, 1RXR.A.213, 1RXR.A.214, 3RYM.A.106, 3RYV.B.262, 3RYX.B
.262, 3RYY.A.1, 3RYZ.A.1, 3RYJ.B.262, 3RZV.A.1, 3RZV.A.2, 3RZ0.B.262, 3RZ1.B.262
, 3RZ5.A.1, 3RZ7.A.1, 3RZ8.A.1, 1S1G.A.152, 3S2Q.B.501, 3S2E.A.500, 3S2E.A.501,

3S2E.G.500, 3S2F.E.500, 1S3G.A.219, 1S4B.P.1, 1S4I.A.802, 3S71.B.262, 3S72.B.262
, 3S73.B.262, 3S75.B.262, 3S76.A.1, 3S77.B.262, 3S8P.A.400, 3S9T.A.262, 3SAX.A.2
62, 3SAP.A.262, 3SBH.A.262, 3SBI.A.262, 3SD9.A.2, 1SEO.A.201, 3SEY.C.373, 3SFH.A
.403, 3SI2.A.601, 3SJG.A.1752, 1SLM.A.257, 1SML.A.271, 3SOU.A.7, 3SOU.B.8, 3SP1.
A.481, 3SP4.A.601, 1SRD.B.156, 1SRK.A.36, 1SRP.A.920, 2SRT.A.257, 3SSB.A.999, 1S
U3.A.913, 3SU6.A.5, 3SUB.A.161, 1SVM.A.700, 3SV6.A.4, 1SW1.A.401, 3SWR.A.3, 1SX1
.A.23, 3SXK.B.3, 3SZY.A.501, 3T01.A.502, 3T02.A.501, 1T3A.A.422, 1T3K.A.201, 3T3
3.A.411, 1T4W.A.201, 3T5U.A.262, 3T5Z.A.262, 3T6P.A.1001, 3T6P.A.1002, 3T6P.A.10
03, 3T6R.A.1, 3T6R.A.3, 3T73.A.410, 3T74.A.410, 3T7E.A.252, 3T7L.A.1, 3T7L.A.2,
3T8G.A.411, 3T8F.A.411, 1T8H.A.275, 3T80.D.301, 3T82.A.261, 3T84.A.261, 3T85.A.2
61, 3T87.A.326, 3T8C.A.326, 3T8D.A.325, 3T8H.A.326, 3T9O.A.502, 1T9H.A.411, 1T9R
.A.1, 3T92.A.122, 3T92.A.124, 1TAF.A.2003, 1TBN.A.1, 1TBN.A.2, 3TBG.A.601, 1TEQ.
X.262, 3TEN.A.205, 1TFI.A.51, 3TGN.A.147, 3TGN.A.148, 3TGO.B.505, 3TG4.A.435, 3T
G4.A.436, 3TG4.A.437, 1THJ.A.214, 1THJ.B.214, 1THL.A.324, 3TIO.A.1, 3TIO.B.185,
3TIO.D.185, 3TIO.E.185, 1TJL.A.200, 1TKF.A.901, 1TKF.A.902, 1TKH.A.901, 2TMN.E.3
21, 6TMN.E.322, 3TMJ.A.262, 1TOT.A.53, 1TOT.A.54, 1TT5.B.1014, 3TTY.A.676, 1TTM.
A.262, 3TTC.A.1, 3TTC.A.2, 1TWF.J.3001, 4TWJ.A.301, 3TWO.A.349, 4TYT.A.302, 4TZC
.A.501, 4TZU.A.503, 1U05.A.500, 1U0A.A.5005, 1U0L.A.298, 1U10.A.400, 1U10.A.601,
4U10.A.401, 1U1H.A.766, 3U1L.A.241, 1U2N.A.441, 1U3L.A.701, 1U3T.A.375, 1U3T.A.
376, 1U3U.A.375, 1U3U.A.376, 1U3V.A.376, 1U3W.A.375, 3U31.A.276, 1U40.A.160, 1U4
G.A.9800, 4U4L.A.301, 4U4L.A.302, 1U5K.A.300, 1U5S.B.138, 1U5S.B.139, 3U52.A.514
, 3U5N.A.1, 3U5N.A.2, 3U7L.A.350, 1U85.A.34, 1U86.A.36, 3U9G.A.226, 3U9G.A.227,
3U9G.A.228, 3U9G.A.229, 4UA4.A.301, 4UA4.A.302, 4UA4.B.303, 1UAQ.A.200, 3UCJ.A.2
28, 3UCK.A.228, 3UCM.A.228, 3UCN.A.228, 3UCO.A.228, 1UD9.B.508, 1UD9.C.509, 3UDZ
.B.800, 3UEH.A.143, 3UEY.A.4, 3UEE.A.143, 3UEI.A.143, 3UEJ.A.301, 3UEJ.A.302, 4U
FO.A.2269, 3UFF.A.1, 3UGD.A.2, 3UIK.A.341, 3UJZ.A.1, 3UKO.A.400, 3UKO.A.401, 1UL
4.A.139, 3UNG.C.903, 3UNT.A.400, 3UN6.A.325, 4UOV.A.298, 4UPO.A.1383, 4UPO.A.138
4, 1USN.A.257, 2USN.A.257, 4UTV.A.1299, 1UUF.A.401, 1UUF.A.402, 3UVC.A.301, 3UVC
.B.303, 3UVI.A.387, 2UVL.A.1336, 1UWO.A.1118, 3UW2.A.474, 3UW4.A.401, 3UWA.A.200
, 1UX1.A.1132, 3UX8.A.1001, 2UX1.K.1173, 2UYV.B.1276, 1UZF.A.701, 2UZG.A.131, 2U
ZG.A.132, 2UZG.A.133, 1V0D.A.401, 2V08.A.1090, 2V0C.A.1815, 2V0C.A.1816, 4V0R.A.
1001, 2V1X.A.1595, 1V13.B.200, 3V1C.A.101, 3V1E.A.102, 3V1F.A.704, 3V1F.B.703, 4
V1T.A.776, 2V29.A.1276, 4V2W.A.502, 4V2Y.A.150, 3V3G.B.301, 1V47.A.601, 3V4K.A.2
, 1V4P.A.1001, 1V51.A.602, 1V54.F.99, 1V5N.A.201, 1V5N.A.401, 1V5R.A.201, 3V5G.A
.262, 1V6G.A.201, 1V6G.A.401, 3V7M.A.509, 1V7Z.A.301, 3V7X.A.2001, 2V8G.C.500, 1
V87.A.201, 1V87.A.401, 2V9I.B.1275, 1V9E.B.260, 3V96.B.302, 1V9X.A.200, 2V9E.A.1
276, 2V9E.B.1276, 2V9K.A.1533, 1VA1.A.100, 1VA2.A.100, 1VA3.A.100, 3VBD.A.2001,
1VDD.D.233, 3VDP.A.201, 2VES.A.1297, 2VES.C.1302, 1VFY.A.300, 1VFY.A.301, 2VF7.A
.1845, 2VF7.B.1844, 1VGN.A.301, 1VGN.A.302, 3VGL.A.322, 1VHH.A.400, 3VHS.A.51, 3
VHS.B.51, 3VHT.B.401, 1VJ0.A.400, 1VJE.A.167, 2VJE.B.1491, 2VJE.B.1492, 2VKR.A.1
06, 1VK9.A.143, 2VL6.A.1266, 2VM5.A.1245, 2VNF.A.1247, 3VOV.A.401, 3VOW.A.201, 2
VO9.B.501, 3VPB.E.100, 3VPE.A.301, 2VP7.A.1399, 2VPD.A.1399, 2VPG.A.1400, 2VPG.A
.1401, 1VQ0.A.300, 2VQM.A.1412, 1VQ2.A.701, 1VQ2.A.702, 3VQZ.A.301, 2VQG.C.1091,
2VRS.A.1328, 2VRW.B.1565, 2VRW.B.1566, 3VRK.A.301, 2VR6.A.1156, 2VRD.A.1062, 1V
SH.A.281, 1VSR.A.201, 3VTH.A.801, 3VTH.A.802, 3VUW.E.801, 2VUT.I.1713, 2VVB.X.12
68, 2VXI.B.201, 2VXX.A.201, 2VXX.B.202, 1VYK.A.1150, 1VYX.A.1061, 1VYX.A.1062, 1
VZY.A.1291, 2W0T.A.125, 3W0F.A.301, 2WOD.A.1269, 2W3N.B.1234, 2W3Q.A.1231, 1W4R.
A.400, 2W4L.A.1170, 2W5V.B.1377, 2W5X.A.1378, 2W5Z.A.4970, 1W5O.A.1339, 3W5K.B.5
02, 3W5K.B.503, 3W5K.B.504, 2W57.B.201, 4W6Z.A.401, 4W6Z.A.402, 1W8P.B.1030, 1WA
A.A.1090, 1WAA.B.1090, 1WAA.D.1090, 1WAA.E.1089, 4WAI.A.101, 4WAJ.A.301, 2WBT.A.
1130, 2WBT.A.1131, 3WBH.B.505, 2WBO.X.601, 2WBO.X.602, 2WCB.A.100, 2WCB.B.100, 2
WD2.A.1262, 2WD3.A.1263, 4WD6.A.302, 4WD8.B.303, 4WD8.C.303, 2WEJ.A.1262, 1WE9.A
.201, 1WE9.A.401, 1WEE.A.201, 1WEE.A.401, 1WEM.A.201, 1WEM.A.401, 1WEN.A.201, 1W
EN.A.401, 1WEO.A.201, 1WEO.A.401, 1WEP.A.201, 1WEP.A.401, 1WEQ.A.201, 1WEQ.A.401

, 1WES.A.201, 1WES.A.401, 1WEV.A.201, 1WEV.A.401, 1WEW.A.201, 1WEW.A.401, 2WEH.A.1262, 2WEO.A.1262, 1WFE.A.201, 1WFE.A.401, 1WFF.A.201, 1WFF.A.401, 1WFH.A.201, 1WFH.A.401, 1WFK.A.200, 1WFL.A.201, 1WFL.A.401, 3WF8.A.401, 1WFP.A.401, 1WFZ.A.201, 1WG2.A.200, 1WG2.A.400, 2WGX.A.1300, 1WGE.A.201, 4WH6.A.1203, 2WHG.A.1263, 1WIG.A.201, 1WIG.A.401, 1WII.A.201, 1WIL.A.201, 1WIL.A.401, 1WIM.A.201, 1WIM.A.401, 3WID.A.1001, 1WIR.A.201, 3WIE.B.1001, 1WJ2.A.470, 2WJV.A.1, 2WJV.A.3, 1WJA.A.56, 1WJB.A.56, 1WJP.A.301, 1WJP.A.501, 1WJP.A.701, 1WJV.A.201, 1WJV.A.401, 1WKQ.B.202, 4WK7.A.501, 4WKE.A.501, 3WL3.A.301, 3WLE.A.402, 3WLF.A.402, 1WN5.A.1001, 1WNU.A.1001, 3WNQ.A.501, 1WO3.A.26, 1WO4.A.26, 2WO8.C.1268, 2WOJ.A.1353, 4WOK.A.401, 3WRG.A.702, 3WS6.C.201, 1WUQ.A.1001, 1WUR.A.1001, 2WVJ.A.1193, 1WWD.A.57, 3WWL.A.102, 1WWR.D.204, 2WWO.A.1162, 3WXC.A.302, 1WY2.B.407, 1WYH.A.201, 1WYH.A.401, 1WYS.A.201, 1WYS.A.401, 1X0T.A.150, 3X17.A.601, 4X2Z.A.400, 1X31.D.1006, 1X3C.A.201, 1X3H.A.201, 1X3H.A.401, 1X3Z.A.999, 2X3B.A.1341, 2X4H.B.1141, 2X4H.B.1142, 1X4I.A.201, 1X4I.A.401, 1X4J.A.201, 1X4J.A.401, 1X4K.A.201, 1X4K.A.401, 1X4L.A.201, 1X4L.A.401, 1X4S.A.201, 1X4S.A.401, 1X4U.A.201, 1X4U.A.401, 1X4V.A.201, 1X4V.A.401, 1X4W.A.201, 1X4W.A.401, 1X5W.A.201, 2X5R.A.1126, 1X61.A.201, 1X61.A.401, 1X62.A.201, 1X62.A.401, 1X63.A.201, 1X63.A.401, 1X64.A.201, 1X64.A.401, 1X6E.A.401, 1X6F.A.201, 1X6H.A.401, 1X6M.A.200, 2X7S.A.1265, 2X7T.A.1263, 2X7U.A.1261, 2X7M.A.1174, 2X7M.A.1175, 2X8Y.A.1616, 2X8Z.A.1616, 2X95.A.1615, 2X96.A.1617, 1XAF.A.501, 2XAA.C.1346, 1XB0.A.403, 1XB8.A.1001, 2XB4.A.1224, 2XBL.A.1197, 2XBL.C.1196, 1XC3.A.302, 1XCR.A.1001, 2XCM.E.1222, 2XCM.E.1223, 2XEU.A.1065, 1XEG.A.262, 1XEM.A.401, 1XER.A.106, 1XF7.A.30, 4XIW.A.401, 4XIX.B.401, 2XIG.A.1151, 1XJH.A.63, 1XKI.A.1003, 1XLL.A.399, 2XML.A.1349, 1XOX.A.999, 2XOC.A.993, 2XOC.A.994, 2XOC.A.995, 2XOC.B.991, 2XOC.B.992, 1XPA.A.220, 1XPG.A.1887, 1XPZ.A.262, 1XQ0.A.262, 2XQV.A.401, 1XRT.A.1423, 1XRU.A.501, 1XSO.A.152, 2XS4.A.998, 1XTG.A.426, 1XTM.B.501, 1XUC.A.1261, 1XUF.A.246, 1XUJ.A.246, 1XWH.A.356, 1XWY.A.401, 2XXH.B.1303, 1XYD.A.94, 1XYD.B.94, 1Y02.A.161, 1Y02.A.162, 1Y0J.A.244, 2Y1N.A.1436, 2Y1N.A.1437, 1Y23.A.1001, 2Y20.C.1331, 2Y28.B.1181, 1Y3G.E.321, 2Y4Y.A.1172, 2Y4Y.C.1172, 2Y43.A.1097, 2Y43.A.1098, 2Y6C.A.1267, 2Y6D.A.1267, 1Y7W.B.285, 1Y8F.A.702, 1Y8J.A.800, 1Y8Q.B.642, 1Y93.A.265, 1YB0.B.160, 2YB5.A.1213, 1YC2.A.402, 1YC2.C.406, 1YC5.A.1001, 1YE3.A.375, 1YEJ.L.605, 2YHO.A.1001, 2YHO.A.1002, 1YHC.A.601, 2YHW.A.1719, 2YHY.A.2000, 2YIK.A.1533, 1YLK.A.401, 1YM3.A.301, 1YO7.A.201, 1YOP.A.84, 1YOU.A.301, 2YPU.A.1998, 1YQD.A.1000, 1YQD.A.2000, 2YQL.A.201, 2YQL.A.401, 2YQM.A.201, 2YQM.A.401, 2YQP.A.201, 2YQQ.A.201, 2YQQ.A.401, 2YRJ.A.200, 2YRT.A.201, 2YRT.A.401, 2YRC.A.200, 2YRD.A.200, 2YRE.A.401, 2YRE.A.501, 2YRE.A.601, 2YRE.A.701, 2YRG.A.201, 2YRG.A.401, 2YRH.A.200, 2YRK.A.201, 2YRM.A.201, 2YSJ.A.201, 2YSJ.A.401, 2YSV.A.201, 1YSB.A.501, 2YS2.A.201, 2YSA.A.181, 2YSL.A.401, 2YSM.A.301, 2YSM.A.501, 2YSM.A.701, 2YSM.A.901, 2YSO.A.181, 2YSP.A.181, 2YTH.A.201, 2YTG.A.201, 2YTP.A.181, 2YTQ.A.201, 2YTR.A.201, 2YTS.A.201, 2YTT.A.181, 2YT5.A.201, 2YT5.A.401, 2YT9.A.201, 2YT9.A.203, 2YTA.A.201, 2YTB.A.301, 2YTD.A.201, 2YTE.A.201, 2YTF.A.201, 2YTI.A.201, 2YTJ.A.201, 2YTK.A.201, 2YTM.A.181, 2YTN.A.201, 2YTO.A.201, 2YUU.A.201, 2YUU.A.401, 2YU4.A.201, 2YU8.A.201, 2YUC.A.201, 2YUC.A.401, 2YVR.A.1001, 2YVR.A.1002, 2YWW.A.504, 2YXO.A.501, 2YYR.A.401, 2YYR.A.402, 2YZ3.A.301, 2YZ5.B.1501, 1Z05.A.406, 2Z2S.B.204, 2Z2Y.A.2001, 2Z2Y.D.2004, 2Z26.A.400, 2Z3H.A.2001, 2Z3G.A.2001, 1Z3A.A.301, 2Z3I.A.2001, 2Z3J.A.2001, 2Z45.A.1001, 2Z45.B.1004, 1Z5H.A.2001, 1Z60.A.2, 1Z6R.A.501, 1Z6U.A.1, 1Z6U.A.2, 1Z83.B.642, 1Z84.A.603, 1Z84.A.604, 1Z8R.A.151, 2Z9J.A.902, 1Z9G.E.1005, 1Z9N.A.201, 1Z9Y.A.300, 2Z94.A.901, 2Z9K.A.901, 2Z9L.A.701, 2ZC0.B.408, 1ZDP.E.1005, 2ZEP.A.391, 1ZE8.A.263, 2ZED.A.391, 2ZEE.A.391, 2ZEF.A.391, 2ZEG.A.391, 2ZEH.A.391, 2ZEL.A.391, 2ZEM.A.391, 2ZEN.A.391, 2ZEO.A.391, 1ZFD.A.71, 1ZFK.A.1300, 3ZFJ.A.1159, 3ZFK.A.401, 1ZFO.A.31, 1ZFQ.A.600, 1ZGE.A.1000, 1ZGF.A.400, 3ZGO.A.400, 1ZH1.A.199, 1ZIN.A.219, 1ZLH.A.555, 3ZME.A.313, 1ZNB.A.1, 3ZNC.A.1, 1ZNF.A.27, 3ZNF.A.31, 3ZNI.A.1428, 3ZNI.A.1429, 1ZNM.A.29, 5ZNF.A.31, 7ZNF.A.31, 2ZNC.A.1, 2ZNE.B.993, 2ZNR.A.1, 2ZNR.A.2, 1ZP5.A.999, 3ZP9.A.1009, 3ZPC.B.401, 3ZPG.A.1358, 3ZQ6.A.1

323, 1ZR9.A.117, 1ZRP.A.54, 1ZS0.A.999, 1ZSB.A.262, 1ZSC.A.262, 2ZTX.A.501, 3ZTG
.A.1336, 3ZTG.A.1337, 1ZTQ.A.550, 1ZU1.A.129, 1ZU1.A.130, 1ZUD.1.501, 3ZUK.A.166
4, 2ZU2.A.5517, 1ZVX.A.999, 3ZVS.A.1159, 3ZVS.A.1160, 1ZW8.A.66, 1ZW8.A.67, 3ZXH
.A.301, 1ZXV.B.9002, 1ZXZ.A.198, 2ZXG.A.900, 1ZY7.A.801, 3ZYQ.A.1222, 3ZYQ.A.122
3, 1ZZU.A.900, 2ZZE.A.753, 2ZZF.A.754, 2FLC.A.248, 4FLW.A.802, 1YJW.7.8044, 2A19
.B.1642, 3ARA.A.166, 3C15.A.29, 1CLK.A.780, 2D32.B.2524, 2E8A.A.501, 1G8G.A.521,
2H5N.B.1001, 4HJH.A.552, 4HN2.A.404, 1IV4.D.1564, 2J4E.A.1002, 3KGX.A.503, 3LDO
.J.54, 3M42.A.1, 4NNN.N.202, 2NOG.A.9220, 2O4G.B.800, 4O6I.B.602, 4OKE.B.203, 4O
KK.A.204, 3OPK.C.401, 1PYX.A.1002, 4QDG.A.402, 1T9Z.A.273, 3TAV.A.269, 1WOH.A.10
01, 1XLB.A.399, 2XRI.A.1337, 2YFD.B.1145, 1YQ2.E.7005, 1YYZ.A.341, 2GIH.A.402, 1
BRW.A.3001, 4R5O.A.509, 1SBH.A.291, 4AM5.A.1162, 1AOR.A.606, 1B2O.B.55, 2B5H.A.5
01, 1B71.A.192, 1BE7.A.55, 1BFY.A.55, 1BIQ.A.377, 2BKB.C.1193, 2BOY.E.1255, 1BOU
.B.501, 4C4U.I.201, 3CF4.A.808, 1CJX.B.629, 2CKF.C.501, 3D19.F.302, 3D3L.A.801,
3DBY.A.306, 2DE6.B.501, 3E2T.A.1, 1E3D.A.269, 4EB5.D.201, 4F1E.P.201, 4FWI.B.401
, 1FZO.A.5002, 2GBX.E.455, 3GC1.A.605, 3GKE.A.501, 3GL0.A.501, 3GL2.A.501, 2GPC.
B.195, 1GUP.C.351, 2GYQ.A.402, 2HMK.A.451, 4HSL.A.202, 3I01.B.800, 1I4Z.E.605, 4
ILT.A.301, 1JI5.A.152, 1JI5.B.151, 1JIG.A.402, 1JIG.B.401, 2JI3.A.1127, 1JQK.B.8
01, 1JRO.G.3001, 4K9F.A.101, 4KU0.D.101, 4KWL.A.301, 4KX6.N.301, 1LNB.E.900, 1N1
Q.A.516, 1N1Q.B.515, 3N9Y.D.151, 3NA0.C.150, 3NA0.D.150, 4NBA.A.501, 4NBF.A.501,
4NBG.A.501, 1NF6.A.200, 1NO3.A.858, 2OHJ.A.502, 2OHJ.A.511, 2PUZ.A.500, 2Q0J.B.
997, 3Q36.A.458, 4QDD.A.401, 1QGH.H.157, 4QLW.B.201, 3QVD.G.173, 1R2F.A.400, 1R9
X.A.501, 4REU.B.202, 1SHR.B.801, 1T9O.A.255, 3U9M.A.202, 1ULI.A.600, 3V7P.A.429,
1VCX.A.54, 3VMH.C.501, 2W3S.E.1464, 2WLB.B.619, 1WQL.A.502, 2XSO.G.901, 2YFI.A.
901, 2YFI.G.900, 1YK5.A.54, 1YUX.A.303, 2ZZI.A.208, 4FZY.A.201, 4FZY.B.201, 2AA0
.A.2003, 2EHS.A.201, 3FFZ.B.1302, 4FHA.A.402, 4FLK.A.504, 4FO2.E.101, 1G8I.B.159
5, 1GW1.A.1423, 1IP3.A.999, 4IQZ.E.305, 1MAU.A.499, 4NT8.A.206, 2OCC.A.519, 1ODZ
.A.1427, 4OVZ.B.903, 1W7Z.A.1032, 2WHM.A.1425, 2X2E.A.1747
[1] "Cluster 2"
3IE1.D.442, 4MTD.B.201, 1V15.A.1132, 3VDO.A.401, 2A97.B.2437, 1AAF.A.57, 3AF5.A.
665, 2ANH.A.451, 2ANH.A.452, 2ANU.B.405, 2APO.B.501, 1BAW.A.107, 1BH5.A.201, 1BQ
Q.M.289, 1BTG.B.902, 3C0Y.A.401, 3C0Z.A.101, 2CBN.A.402, 3CE9.B.400, 2CEA.B.1606
, 2CG3.Z.1, 3D3X.A.428, 1DDZ.A.1, 4DLF.A.404, 1DSQ.A.144, 1DXW.A.301, 2E1W.A.400
, 3EII.D.301, 2EIM.C.262, 1ENQ.C.238, 3EYV.L.220, 4F0R.A.501, 1F5F.A.252, 3FDK.A
.402, 3FGG.A.161, 3GJN.B.600, 3GZE.B.14, 4H00.A.601, 4H01.A.602, 2H42.A.501, 3H6
6.B.500, 3H8F.B.501, 3H90.A.291, 3H90.D.5, 3HDB.A.620, 2HD1.B.101, 3ID7.A.402, 4
IGM.A.401, 4IGM.F.401, 3ITM.A.1, 4J3D.A.301, 1JDI.A.301, 1JM7.B.143, 1JOE.A.205,
2K2G.A.2, 1KAR.A.501, 1KHN.A.452, 2KO8.C.54, 2LOZ.A.487, 3L8Y.A.301, 1L9Y.A.2,
3LZE.A.201, 2M7Y.A.101, 3MKV.A.425, 4MLX.A.301, 2MQ1.A.101, 1MVH.A.503, 4MZ7.A.7
01, 4NRZ.B.301, 1NYR.A.1002, 2O36.A.690, 3O90.A.192, 1OLP.A.1373, 1OLP.A.1375, 1
OLP.D.1374, 1OS2.B.369, 2OW2.A.444, 2OXW.A.264, 1P5X.A.247, 1P91.B.2301, 2PJT.D.
302, 3PJN.A.186, 3PJN.A.189, 3PJN.B.186, 1PS7.A.331, 1Q74.D.304, 2QFP.A.434, 1QX
1.A.2004, 3R2J.A.301, 3RAM.D.998, 1RO9.A.529, 3S6L.B.185, 3SFW.A.502, 1SMP.A.472
, 3SPU.D.1004, 3T02.A.502, 1TF9.A.901, 4TQT.A.501, 3TVX.A.900, 4UNI.C.1697, 1UR6
.B.79, 1UXA.C.1367, 2V2A.A.1275, 2V8V.B.1455, 3V94.E.702, 1V9P.B.2701, 2VXX.D.20
1, 4X2T.D.701, 4X2T.D.702, 2X4H.A.1140, 2XR1.A.1638, 2XR1.A.1639, 2XY9.A.1628, 1
Z3J.A.264, 1ZKN.C.601, 3ZNS.A.101, 2ZNE.B.992, 3ZTV.A.1599, 4DQP.A.903, 3E54.A.7
02, 3ICE.B.1001, 3ICE.D.1001, 2PYJ.A.9004, 1Q81.4.8078, 1RVB.B.302, 3S14.A.2001,
2W9C.A.1344, 2W9C.B.1342, 3ZC0.B.197, 2AKZ.A.440, 2AQX.A.600, 3AQC.D.327, 4AVQ.
C.902, 2BM1.A.1690, 3BPD.G.126, 1BPM.A.488, 1BR2.A.997, 3BU5.A.301, 3CNX.C.170,
3CR3.B.1213, 3CRL.B.2001, 2CW6.A.401, 4CW7.C.1002, 4CYI.B.1000, 4CYU.A.171, 1D1C
.A.998, 3D19.D.301, 2D33.C.2525, 3D7M.A.356, 2DEJ.A.402, 2DH4.A.341, 3DLS.C.11,
4DPG.F.604, 4DPM.D.401, 1E1Q.A.601, 1E1Q.F.601, 3E4O.A.501, 2E6B.A.301, 2E92.A.1
302, 2E92.B.1304, 2E95.A.1301, 1F4H.D.3001, 3F74.C.1, 3FA4.A.401, 2FDR.A.1001, 3
FR8.B.1, 3FYY.B.402, 3G2F.B.901, 2G74.A.185, 3G9D.B.299, 2GHT.B.257, 2GHQ.B.257,

2GWC.A.1, 3H8A.C.1431, 3HB0.A.701, 1HBN.A.1558, 4HNS.A.201, 3HQP.M.502, 2HT6.B.
444, 4HV6.B.201, 2I19.B.4004, 4I1O.A.201, 2I5R.B.302, 3IAP.A.3001, 3IJQ.A.386, 2
IK2.A.290, 4IL6.C.505, 4IL6.b.616, 4IL6.c.506, 2IO7.B.5004, 1IV2.F.1574, 4J99.D.
803, 2JK1.A.1144, 3KA9.A.189, 1KK8.B.998, 1LOO.A.602, 4LCZ.A.316, 4LRT.A.403, 1L
VH.A.801, 3M1Y.D.300, 4M1W.A.201, 4MFE.D.1105, 3MG8.G.241, 4MKS.A.502, 4MPO.G.20
3, 4MPO.G.204, 3MQT.A.1243, 3MWC.A.400, 1N5K.B.413, 3N9V.A.61, 1NEL.A.438, 3NIZ.
A.312, 1NV3.A.2341, 2O1U.A.302, 2O1X.B.2002, 2O56.F.2006, 3OAC.D.3002, 3OHM.A.40
2, 2OI5.B.5000, 4OVN.B.202, 4P0V.A.404, 1P7L.A.388, 4P9D.C.202, 4PFQ.C.400, 4PRV
.A.402, 2PUN.B.401, 2Q58.A.4, 1Q8Y.A.809, 2Q80.A.401, 4QJB.A.301, 4QLQ.V.301, 3Q
U2.C.225, 3QU9.A.227, 4QVY.K.302, 4QXD.B.302, 4QXD.B.304, 4R3A.A.400, 2R5T.A.600
, 3RBM.B.1002, 1RC5.D.764, 2RIO.A.1102, 1RK2.C.320, 1RLT.C.807, 3RYW.B.2003, 4S1
7.D.501, 3SRD.C.603, 3SRF.D.533, 3SY8.A.401, 3SZ5.A.220, 4TQ3.A.402, 3TW6.D.2002
, 4U0N.B.401, 3U87.A.402, 3UGJ.A.2006, 4UM8.B.2001, 4UUX.A.401, 2V0N.B.502, 2VHQ
.A.1328, 3VI4.B.502, 2VKQ.A.1288, 3VTI.C.401, 3VYT.C.601, 1W9L.A.1748, 3W9T.B.51
1, 3WEG.A.402, 3WEJ.A.402, 3WGU.C.2003, 3WKA.A.601, 1WQ1.R.168, 1WQA.A.456, 3WU2
.B.616, 3WU2.C.511, 3WU2.C.513, 1WUU.C.394, 1XMJ.A.2, 1YXO.A.5001, 2Z2P.A.1001,
1Z5B.B.2003, 1ZCW.A.501, 2ZCQ.A.453, 2ZEV.A.1302, 2ZEV.B.1304, 1ZH4.A.201, 2ZRW.
D.702, 1ZYK.A.702, 2AU0.H.1415, 4BXO.A.2050, 2C2R.A.1344, 2C28.A.1344, 4DTJ.A.10
03, 4DU4.A.1003, 4EFJ.C.101, 4EFJ.A.401, 3GIJ.B.1415, 2GIH.B.401, 4K4H.M.605, 4K
4I.E.606, 3KHL.B.1417, 4KHW.A.1005, 1M5X.C.801, 3M9M.B.354, 3NHG.A.909, 2NOF.A.3
28, 3OOR.A.237, 4Q0W.B.1001, 3QER.A.906, 3RBD.A.415, 4RI8.A.1101, 3SQ2.A.906, 3V
20.A.302, 2WTF.A.1512, 4WUZ.B.301, 1A85.A.996, 1AEI.D.317, 1AEI.A.317, 4AG4.A.50
02, 1ALA.A.401, 3ATS.A.380, 2AZ1.D.202, 1B1G.A.77, 3B2Z.C.3, 4B7U.B.401, 1B90.A.
930, 3B90.B.702, 3BCF.A.705, 4BCU.A.201, 1BK9.A.200, 3BS6.A.1, 4BTX.A.1764, 4BWE
.A.503, 4CAG.A.606, 2CDP.C.1140, 2COL.B.801, 1CVM.A.802, 3D4G.A.484, 3D4G.A.485,
3D6E.B.202, 1D8F.A.305, 3DEM.B.6001, 3DF0.A.717, 3DF0.B.601, 4DLK.B.402, 1DM5.E
.1135, 3E1I.B.503, 2EAA.C.904, 4EJ7.A.402, 4EJ7.A.404, 4EM6.B.604, 4EMU.B.401, 1
ESL.A.164, 2EXJ.D.2004, 2FH3.A.1001, 1FZA.C.1, 4G1M.B.2001, 1G5N.A.403, 4GEJ.A.2
01, 3GG1.B.503, 4GGB.A.402, 4GH8.B.203, 3GRI.B.700, 1GU6.A.1480, 2GXS.B.604, 2H0
K.A.402, 2H0K.A.408, 1H71.P.500, 3HDB.A.657, 2HF4.A.902, 1HOV.A.168, 2HTY.A.991,
4I9F.A.401, 2IAP.A.402, 3IAE.B.572, 3IJE.B.4002, 4ILW.F.304, 3IRH.A.457, 1IVG.B
.470, 2J0T.C.1268, 2J1G.F.1291, 2J60.C.1277, 2JF9.P.1014, 4JP8.A.706, 2K0J.A.503
, 3K39.F.1000, 3K8Y.A.167, 3KQA.C.420, 4KS3.A.502, 1KTW.A.5, 1KVO.D.192, 1KVY.A.
124, 4LIN.A.1301, 4LN6.G.403, 1LWU.K.4, 3MOW.G.202, 4M7K.H.301, 3M83.B.408, 4MBE
.D.201, 3MDO.A.391, 2MG9.A.101, 1MTS.A.480, 1MWN.A.100, 1N28.B.127, 4N25.A.705,
4N2D.A.705, 3N9V.A.64, 4N96.B.401, 4NAS.C.501, 1NGS.B.681, 2NP0.A.1293, 3NSJ.A.7
02, 4NUP.C.301, 4NUP.C.304, 4NVR.C.401, 1OLP.A.1372, 1OS2.A.872, 2OVX.B.447, 2OV
X.B.450, 2OZR.F.4030, 3P1O.A.234, 2P5V.B.1008, 2P5V.C.1001, 2P5W.B.701, 1PZ8.C.7
03, 3Q2N.B.304, 1Q3A.A.467, 4Q4Y.1.5006, 1Q7B.B.9003, 1QDO.C.245, 1QHD.A.603, 1Q
MD.A.404, 4QN3.A.501, 2QVF.B.703, 3QWU.B.602, 2R1D.I.3000, 2R8Y.I.209, 1RFN.A.50
0, 2RHP.A.16, 1RJV.A.112, 4RSR.A.404, 1SOE.A.1292, 3S5U.F.220, 1SEL.B.277, 3SH5.
A.196, 3SHI.M.305, 3SNZ.A.97, 1SPJ.A.300, 1SRR.C.532, 4TQO.H.701, 1UBN.A.277, 3U
BH.A.855, 1UEA.A.4, 1UG9.A.2002, 4UM9.B.2003, 3UMJ.A.902, 2V5C.A.1625, 2VB6.B.11
48, 1VE5.B.2003, 2VVD.A.1329, 2W1W.B.1134, 1W1X.A.1479, 3W9T.B.509, 4WFE.A.306,
2WG8.C.201, 2WII.A.1643, 4WIW.C.701, 2WJS.A.5001, 2WJS.A.5002, 3WMW.A.401, 3WNX.
A.502, 2WWO.E.800, 1X36.A.269, 1XJL.A.342, 1XJL.A.344, 2XSG.A.1772, 2XTJ.A.1423,
1Y1A.A.501, 1Y6P.A.217, 1Y6P.B.218, 2YDP.B.502, 2YGM.B.1418, 2Z2D.A.267, 2ZUX.A
.629, 2ZUX.A.637, 2ZUY.A.621, 2ZWP.B.401, 4AC8.B.500, 4C4U.B.201, 4CMY.A.1164, 4
D8F.A.401, 1DGG.A.2000, 4DHL.A.502, 1E2U.A.701, 3FG1.B.1500, 3FMR.B.401, 1GNL.A.
1545, 1H2A.L.1004, 1HJF.A.1310, 1HJG.A.1307, 1I4Y.B.602, 1I4Z.D.604, 1I4Z.G.607,
2IBN.A.704, 3ICF.A.602, 1IDS.C.208, 2INP.A.3, 2ITB.B.501, 2J2F.E.371, 1KBP.A.43
8, 4KBP.A.438, 1LGT.A.500, 1LKD.A.500, 3LKT.M.600, 3LMX.O.600, 1NNF.A.401, 1NX8.
A.300, 2OHH.B.1501, 2OHH.E.3501, 1OQ9.A.365, 1OS7.C.302, 3PCB.O.600, 3PCE.M.600,
3PCJ.M.600, 3PCK.Q.600, 1PIY.A.376, 3PM5.A.1002, 2PT2.A.400, 3Q1G.A.1001, 3Q1G.

B.1001, 1QFC.A.402, 3QFM.A.264, 2QJE.A.692, 2R2F.B.320, 1RA5.A.500, 4RC5.A.1003, 1SQ3.B.906, 1SQ3.C.907, 3T81.B.606, 4TOA.A.206, 4TOA.B.207, 3U52.B.512, 3U9M.E.205, 2VHL.A.1398, 3VTI.B.803, 1W69.A.1349, 1W9M.A.1555, 3WHN.B.201, 2XRX.G.1461, 1XZW.B.929, 1Y4T.A.650, 1YKM.J.600, 2ZQX.A.501, 1ZZ9.C.199, 4DOC.A.404, 3EPG.A.421, 8ICP.A.341, 2IHM.A.900, 2ISP.A.340, 2ISP.A.342, 3JPS.A.340, 4KHS.A.1009, 4KHU.A.1006, 3KNT.A.208, 4NLN.A.402, 1QVG.L.8347, 3UXP.A.339, 1YIT.0.8538, 2A2A.C.3158, 4A22.C.1346, 1A5S.B.2000, 3ASP.A.701, 3AST.A.701, 4B1L.A.1679, 3BX1.B.284, 3C17.B.326, 2C9R.A.1103, 3C9F.B.603, 2C9A.A.1289, 4D1I.C.600, 4D1I.F.600, 3D32.A.120, 4D9U.A.901, 2DDA.C.303, 3DKI.B.324, 3EPZ.A.1, 2EPF.C.307, 3EUW.B.343, 4FEW.B.303, 4FMT.A.301, 3G1N.A.5001, 4G1K.A.301, 3GA5.A.700, 3GCD.A.215, 3GVF.A.178, 4GY9.A.206, 4HKT.B.410, 4HMM.B.302, 3HVY.C.427, 4I2B.A.601, 3I4Q.A.177, 3IAQ.B.3104, 3IAQ.C.3101, 3IJ6.B.313, 4IJK.B.301, 3IWK.B.504, 4J0Y.A.501, 4J1I.A.502, 4J2H.A.306, 4JDO.D.301, 4JHG.A.205, 4JVL.A.703, 1JYX.B.3103, 1JZN.D.4139, 4K7V.A.407, 1KNR.A.542, 4L3F.H.401, 4L73.A.404, 4LDZ.A.205, 4LH7.A.404, 3N83.A.701, 4NAW.N.304, 4NWH.A.201, 1O4Z.B.1005, 2O5W.C.171, 1OA8.A.1690, 3OB8.B.3003, 4OBO.A.402, 3OO2.B.383, 2OSW.B.602, 2OSY.B.602, 3OTK.C.587, 3P8O.A.187, 3PJO.C.367, 2POC.D.5004, 1PX4.A.3104, 3PYM.B.504, 1Q1Q.A.351, 1Q1Z.A.313, 4Q4X.1.5004, 3Q9E.L.343, 2QF2.A.700, 4QFE.G.304, 2QZ7.B.196, 4R6C.A.213, 3RNO.A.402, 3S30.A.383, 1S81.A.2, 3T2P.B.3102, 3T34.A.1003, 1T64.B.1392, 3TXJ.A.1138, 3UA6.B.2, 2VDX.A.1385, 3VD5.C.3101, 3VD5.D.3102, 1VIZ.B.239, 3W6P.A.804, 1W90.A.1154, 2WCF.B.1091, 2WD0.A.601, 2WG8.B.202, 2WOI.B.1491, 2WOI.D.1488, 3WV2.A.305, 2WWH.B.212, 3WXO.A.807, 3WXO.A.808, 4WXG.A.502, 4XOO.B.401, 2X7J.D.1581, 2Y00.A.1359, 1YAO.A.501, 2ZHJ.A.320, 3ZLY.A.1384, 4ZNB.A.3, 3ZPR.B.1, 2ZXK.A.1, 3ZX2.B.521
[1] "Cluster 3"
2JZW.A.57, 2L1G.A.88, 4LJ0.B.502, 4OND.E.101, 4QCL.A.1303, 1QUM.A.302, 830C.A.272, 456C.A.272, 4A3W.A.1159, 1A7T.A.252, 2A7M.A.252, 4A7B.A.1276, 4A7K.A.952, 4A7Y.A.951, 4A7Z.A.950, 1A85.A.999, 1A86.A.999, 2A8H.A.486, 2AB7.A.30, 1AFO.A.472, 1AH7.A.246, 1AH7.A.248, 2AIO.A.315, 4AR8.A.1731, 1AST.A.999, 4ASQ.A.1615, 4ASR.A.1615, 4AWY.B.3229, 4AX0.B.3229, 4AX1.B.3229, 1B3D.B.301, 1B57.A.360, 3B7S.A.701, 3B7U.X.701, 3B8Z.A.901, 2BH3.A.1002, 1BIW.B.801, 4BIN.A.500, 3BJC.A.876, 4BJH.A.423, 3BKQ.X.500, 1BLL.E.488, 4BLB.A.910, 2BMI.A.272, 2BNN.B.1200, 2BNO.A.1201, 1BQO.B.301, 1BS8.A.2001, 4BT4.A.301, 4BT5.A.301, 4BT6.A.1257, 3BUB.A.1047, 3BUD.A.1048, 3BUI.A.1046, 3BVT.A.1048, 3BVU.A.1048, 3BVV.A.1047, 3BVW.A.1046, 3BVX.A.1046, 4BXK.A.1620, 4BZ3.B.502, 4BZ5.A.500, 4BZR.A.1630, 3C0Z.B.101, 3C10.A.101, 4C24.A.301, 1C3R.A.501, 1C3S.A.951, 3C52.A.401, 4C5W.A.402, 4C6L.A.2823, 4C6L.A.2824, 4C8I.B.1161, 1CAM.A.262, 2CA2.A.264, 4CA5.A.1001, 4CA7.A.1616, 4CA8.A.1620, 2CEA.A.1603, 2CFU.A.1001, 2CHI.A.212, 3CKI.A.501, 3CQJ.A.285, 3CV5.A.1047, 4CWM.A.433, 1CXV.A.1, 4CXO.A.453, 4CXV.A.433, 3CZN.A.1102, 3CZS.A.1102, 3D0N.A.262, 2D1N.A.270, 2D1O.A.257, 1D5J.A.301, 1D7X.B.801, 2DDY.A.177, 4DD8.A.1002, 4DEF.A.401, 4DEL.A.402, 3DHA.A.255, 2DKD.A.921, 4DLM.A.401, 4DPE.A.301, 1DQS.A.402, 4DR9.A.201, 2DVT.A.1501, 2DVU.A.1501, 2DVX.A.1501, 4DV8.A.801, 3DWB.A.771, 2DW0.A.700, 3DYC.A.451, 4DYK.A.501, 4DZH.A.504, 3E0L.A.1452, 3E2D.A.602, 2E2D.A.500, 3E38.A.1, 1E48.S.999, 1E4C.S.999, 3E4A.B.2000, 3EBG.A.1, 3EBH.A.1, 3EDZ.B.2, 2EG6.A.400, 2EG7.A.401, 1EI6.B.409, 3ELF.A.352, 4ENL.A.438, 6ENL.A.438, 2ERP.A.700, 3EWC.A.372, 3EWD.A.371, 2EWB.A.488, 1EYW.A.401, 4EYF.A.302, 4EYF.A.303, 1EZ2.A.402, 1F0J.A.1101, 3F15.A.264, 3F16.A.264, 3F17.A.264, 3F18.A.264, 3F19.A.264, 3F1A.A.264, 1FA5.A.1200, 1FA5.A.1201, 3FDK.A.401, 2FGN.A.248, 1FKX.A.400, 1FLS.A.166, 4FL7.A.301, 4FUA.A.216, 2FV5.A.3, 2FVM.D.601, 4FW3.B.300, 4FW4.C.301, 4FW5.A.301, 4FW7.A.301, 2FYV.A.2003, 4FYT.A.1012, 1G05.B.801, 1G49.B.801, 4G9L.B.302, 4GBD.A.502, 1GKP.A.1461, 1GKP.B.1460, 1GKR.A.1453, 4GK8.A.302, 2GMN.A.802, 2GO4.A.601, 2GSO.A.1000, 1GVF.A.288, 1GYT.G.600, 1GYT.G.601, 4H01.A.601, 4H1Q.A.301, 4H2E.A.301, 4H2G.A.601, 4H3X.A.301, 1H48.C.900, 1H4N.A.262, 4H49.A.301, 4H76.A.301, 3H8F.E.501, 1H9N.A.262, 2HBV.A.401, 2HBM.A.1001, 3HC4.L.401, 3HC8.A.864, 2HC9.A.701, 2HC9.A.702, 1HFC.A.275, 1HJK.A.451, 3HK5.A.430, 3HK8.A.430, 3HK9.A.431,

3HKA.A.430, 1HOV.A.166, 2HPT.A.950, 3HR1.A.1, 1HS6.A.701, 1HTY.A.1102, 2HUC.A.24
8, 3HWP.A.295, 4HWO.A.701, 4HWP.A.701, 4HWR.A.701, 4HWS.A.701, 3HY7.A.901, 3HY9.
A.901, 3HYG.A.901, 1HZY.A.402, 1HZY.B.401, 2I47.C.804, 2ICS.A.400, 3ID7.A.401, 4
IE0.A.601, 4IE4.A.601, 4IE6.A.601, 4IG2.A.401, 2ILP.A.500, 4ILW.D.301, 4ISM.A.20
1, 3ITC.A.501, 3ITC.A.502, 1ITU.A.402, 2J0T.A.1267, 4J4K.A.402, 4J5F.A.301, 4J5H
.A.302, 1J79.A.400, 1J79.B.400, 2J83.A.999, 2J9A.A.1493, 1JAP.A.999, 1JAQ.A.999,
1JCZ.A.901, 4JD1.A.201, 4JE7.A.202, 4JH8.A.201, 4JH8.B.201, 1JIW.P.481, 2JIG.A.
1252, 2JIH.B.1554, 1JJE.A.252, 1JJT.A.252, 1JK3.A.400, 2JNE.A.150, 2JNE.A.200, 4
JP4.A.301, 2JSD.A.276, 4JS6.A.301, 2JT5.A.256, 2JT6.A.256, 1JWQ.A.1001, 1K07.B.3
, 3K2G.A.400, 2K2C.A.140, 2K4W.A.156, 1K4P.A.1004, 3K5X.A.402, 4K5P.A.1101, 1K9Z
.A.402, 1KAE.B.1102, 4KAP.A.301, 1KBC.A.999, 3KDS.E.996, 1KEQ.A.280, 4KEQ.A.302,
1KMG.A.154, 3KM8.A.400, 3KNS.A.228, 3KR5.G.1004, 3KRY.A.1999, 4KTX.A.501, 1KYS.
A.301, 3L6N.A.301, 1LAM.A.488, 1LAM.A.489, 1LCP.A.489, 4LCQ.A.601, 4LE6.A.405, 4
LE6.A.406, 4LEF.A.301, 2LFF.A.500, 2LFF.A.502, 3LGG.B.512, 3LJT.A.901, 3LJZ.A.99
9, 3LK8.A.264, 3LS6.A.303, 4LV4.A.401, 3M4C.D.109, 3MA2.D.294, 3MA2.A.294, 3MAX.
A.379, 4MCA.B.1000, 3MDU.A.454, 3MJM.B.401, 3MK1.A.902, 3MKV.B.425, 1MMB.A.999,
1MNC.A.281, 3MTW.A.1, 3MVI.A.901, 3N2U.A.264, 3N2V.A.264, 4N27.A.201, 3N2C.A.425
, 3N9S.A.309, 3NJ9.A.262, 2NQH.A.301, 2NQH.A.302, 4NTK.A.201, 3NX7.A.264, 3NXQ.A
.650, 2NZE.A.401, 3O2X.A.1999, 2O4Q.A.2401, 3O90.B.192, 4O98.B.401, 2OB3.A.901,
2OC2.A.701, 3OD4.A.1350, 3OHL.A.4, 3OHL.A.5, 3OHO.A.5, 2OKL.A.601, 1ONW.A.800, 4
OPN.A.201, 4OPN.B.201, 1OS9.A.901, 4OUI.A.501, 2OW1.A.444, 2OW6.A.3001, 2OW7.A.5
001, 3P3C.A.401, 1P5X.A.248, 1P6B.A.401, 1P6D.A.246, 1P6D.A.248, 1P6E.A.248, 3P7
6.A.274, 2PAJ.A.493, 1PB0.A.1302, 2PJT.A.302, 1PL6.A.402, 2PLM.A.407, 1PMI.A.445
, 3PN4.A.1001, 3PNU.A.337, 1PS6.A.330, 1PTW.A.501, 1PV9.A.401, 1PVW.A.401, 4PVO.
A.402, 2Q02.C.300, 3Q2G.A.401, 3Q2H.A.401, 3Q4R.A.201, 2Q5B.A.107, 2Q5B.C.107, 3
Q6X.A.3, 3Q6X.B.271, 3Q9F.B.344, 3QAY.A.180, 4QA0.A.401, 4QA1.A.403, 4QA2.A.403,
4QA4.A.502, 4QA5.A.401, 4QA6.A.401, 3QDF.A.266, 1QF0.A.320, 1QF1.A.320, 1QH5.A.
262, 1QIN.A.401, 1QIN.B.301, 1QIP.D.903, 3QIZ.A.431, 1QJJ.A.250, 2QPJ.A.1, 1QTW.
A.303, 2QVV.A.403, 1QXL.A.400, 2R2D.A.278, 1R3N.A.500, 1R43.A.500, 4R76.A.1001,
3RHG.A.367, 1RJ5.A.601, 2RJQ.A.1, 4RL2.A.301, 4RL2.B.302, 1RM8.A.500, 1RMZ.A.264
, 3RTS.A.264, 3RTT.A.264, 3S2J.A.402, 3S2L.A.402, 3S2M.A.402, 3S2N.A.402, 3S2N.A
.403, 3SEY.E.373, 1SHN.B.482, 1SML.A.270, 3SNG.A.401, 3SNG.A.402, 3SPU.C.1010, 3
SXK.A.4, 3T00.A.502, 1T64.A.388, 2TCL.A.170, 4TLN.A.321, 5TLN.A.321, 4TMN.E.322,
3TOM.B.108, 1TQS.A.1401, 1TQT.A.1301, 1TQU.A.1400, 1TQV.A.1300, 1TQW.A.1400, 4T
YT.A.301, 3U04.A.200, 1U7J.B.150, 1U7M.A.54, 1U7M.B.154, 3U79.D.110, 3U79.E.110,
1UEA.C.1, 3UHM.A.300, 1UIO.A.400, 1URA.A.451, 2USH.A.601, 2USH.A.602, 3UWB.A.20
0, 1UXA.B.1367, 2UX1.A.1174, 2UX1.C.1173, 3V1E.A.101, 2V5W.A.1380, 2V5X.A.1377,
3V77.A.301, 3V77.A.302, 3V96.B.301, 2V9G.A.1276, 2V9M.A.1275, 2VES.A.1295, 1VFL.
A.501, 3VPE.A.302, 2VQM.A.1411, 3VQZ.A.302, 3VTG.A.301, 2VUN.A.401, 2W0D.A.1264,
2W15.A.1203, 1W22.A.1375, 2W3Z.A.1312, 3W52.A.311, 3WAX.A.912, 3WAY.A.911, 4WB7
.A.502, 4WD6.B.302, 2WM1.A.1333, 2WO9.B.1268, 1WPP.A.602, 2WXU.A.1375, 2WXT.A.13
71, 1WY2.A.406, 4X2T.B.1002, 1XBU.A.901, 1XBU.A.902, 2XF4.A.1211, 2XHM.A.1616, 2
XL9.B.1269, 1XM8.A.700, 2XS3.A.999, 2XS4.A.999, 1XXW.A.201, 2XYD.A.1620, 2Y6D.A.
1268, 1Y7W.A.283, 1Y93.A.264, 2YDO.A.1946, 1YQY.A.781, 2Z24.A.400, 2Z24.B.400, 2
Z25.A.400, 2Z25.B.400, 2Z26.A.401, 2Z27.A.400, 2Z27.B.400, 2Z28.A.400, 2Z28.B.40
0, 2Z29.A.400, 2Z29.B.400, 2Z2A.A.400, 2Z2B.A.338, 2Z3I.C.2003, 1Z60.A.1, 2Z72.A
.401, 2Z72.A.402, 2ZBM.A.401, 2ZBM.A.402, 1ZED.A.904, 1ZNB.A.2, 2ZNE.B.991, 2ZO4
.A.319, 3ZU0.A.1595, 3ZU0.A.1596, 3ZU0.B.1588, 3ZU0.B.1589, 2ZWR.A.208, 2ZWR.A.2
09, 1ZXC.A.201, 3ZXH.A.300, 1ZZM.A.401, 1ZZM.A.402, 4GNX.C.701, 4L8H.R.105, 4BLD
.D.910, 4H82.B.300, 3H90.D.6, 2JMD.A.66, 2KV1.A.125, 2KVH.A.84, 4LW9.E.204, 2LXH
.C.901, 2MQ1.A.102, 1NDV.A.400, 1OS2.F.769, 3U7K.A.350, 2X4H.A.1141, 2X5C.A.1128
, 2XAM.B.700, 2Z45.A.1003, 4DFM.A.903, 3EPH.B.2, 2G8F.A.301, 4IEM.A.502, 2OTJ.0.
8044, 3Q8P.B.423, 1QVG.0.8067, 3SNN.A.906, 1AJD.B.952, 3AU9.B.602, 3AUA.A.601, 4
AZW.A.1452, 4BCL.A.367, 4BCL.A.368, 4BCL.A.369, 4BCL.A.370, 4BCL.A.371, 1BHO.1.9

01, 2BJI.A.2278, 4BJR.A.1517, 2BOZ.M.1304, 2BW7.D.2201, 3CTL.A.601, 3D19.A.301, 2D32.A.1524, 1DAM.A.901, 4DFX.E.403, 3DGB.A.2001, 1E14.M.1303, 2EB6.A.1001, 7ENL .A.438, 3F5U.A.297, 2F9R.B.602, 2FKA.A.9001, 3FSY.A.334, 3FXG.B.501, 3FYY.A.401, 1G65.G.301, 4G61.A.301, 3GIE.A.1, 3GQ7.A.692, 2GQ3.A.1002, 2GQ3.B.1003, 4GX6.A. 402, 3I6E.B.386, 4IAC.A.402, 4IL6.j.102, 4IL8.A.501, 4IN7.M.411, 1J7U.A.301, 1JB 0.A.1011, 1JB0.A.1121, 1JB0.A.1126, 1JB0.A.1128, 1JB0.A.1131, 1JB0.A.1132, 1JB0. A.1136, 1JB0.B.1201, 1JB0.B.1203, 1JB0.B.1206, 1JB0.B.1207, 1JB0.B.1211, 1JB0.B. 1224, 1JB0.B.1225, 1JB0.B.1234, 1JB0.B.1236, 1JB0.B.1239, 1JB0.L.1501, 1JYX.A.30 02, 1KJ8.B.394, 1KJI.B.394, 3KR4.A.1004, 1L2O.B.902, 4LCZ.A.314, 4LNI.A.503, 1LP 4.A.342, 4LYJ.A.201, 2M32.A.401, 4MFE.C.1104, 4MKU.A.209, 1N0W.A.401, 1N8W.A.900 , 4NH0.B.1403, 1NUY.A.2342, 4NZN.A.404, 4O1P.C.903, 2OQY.B.402, 3Q2O.B.384, 1Q9L .C.218, 2QB8.B.401, 1QSH.B.147, 2QVU.A.340, 4QVP.V.302, 4QYI.E.203, 4R02.Y.302, 4R17.K.302, 4R7O.E.402, 1RZH.L.854, 1RZH.M.853, 1SJA.B.701, 1SJB.A.1001, 3SRF.G. 533, 4U0M.B.503, 4UB6.B.607, 4UB6.C.509, 4UM8.D.2001, 4UOR.A.699, 3V3Z.L.302, 3V 4S.A.402, 2VBI.G.1000, 2VPQ.B.1451, 1W88.A.1368, 3WNW.A.201, 3WQP.H.501, 3WU2.A. 405, 3WU2.B.605, 3WU2.B.606, 3WU2.B.609, 3WU2.B.612, 3WU2.B.617, 3WU2.D.402, 3WU 2.b.616, 3WU2.b.617, 2XTI.A.1551, 1YF6.M.853, 2ZDR.A.1001, 1ZM7.D.400, 4DLG.A.90 3, 4IR1.A.902, 4UN4.B.2367, 1D2E.D.504, 3DYF.B.4002, 3EN9.A.600, 1GRV.A.490, 2O1 V.A.755, 2VOS.A.1491, 2WCJ.A.1144, 4K4G.M.603, 3KHH.A.416, 3KHH.B.1417, 3KHL.B.1 415, 4QWE.A.703, 2VA2.B.1343, 1AF4.A.276, 3AFG.B.541, 1ALA.A.402, 1AWB.B.279, 3A YU.A.419, 4B7R.B.502, 4B7U.D.401, 1B9V.A.500, 4CBX.G.1151, 1CIZ.A.305, 2DDF.A.47 5, 2DSN.A.2011, 1E5J.A.402, 1EA7.A.315, 1EGZ.C.300, 3ELM.A.303, 7EST.E.11, 2EXH. C.2003, 1F5R.A.800, 2FYC.B.404, 1FZD.F.1, 3G20.A.201, 1G5N.A.404, 1GMM.A.1132, 4 H1Q.A.304, 2HT5.A.995, 2HYW.A.502, 4HZY.A.507, 2J7A.D.1007, 4JQG.A.305, 4LM8.A.8 12, 4MIV.C.601, 1N28.A.126, 1NGS.A.681, 3NHH.A.151, 4NIY.A.301, 4NPK.A.803, 1OLP .B.1372, 1OT5.A.602, 2OVX.B.449, 2OVZ.A.449, 1PZ7.B.702, 2Q1F.B.2002, 1Q3A.B.472 , 4Q4X.1.5006, 3QHQ.A.230, 2RHP.A.22, 1RM8.A.504, 1SEL.A.277, 1SMP.A.478, 3SO0.A .98, 3SO0.B.97, 3SON.A.201, 1SPU.A.802, 2TCL.A.174, 1TFX.B.4007, 1TK2.A.1276, 3T RQ.A.359, 1UG9.A.2003, 1V6C.A.505, 3VI4.C.2004, 2W1W.A.1132, 1W2M.E.1442, 1WD9.A .900, 1WY9.A.148, 2YC2.B.201, 2Z8S.B.647, 2ZUY.A.629, 4EBC.A.504, 2B00.A.252, 1D 8M.A.304, 1EN7.A.403, 3FHA.A.705, 3GG1.A.503, 2HYW.A.506, 2II1.C.404, 1IJL.A.203 , 4P99.B.540, 4POR.E.401, 2Q04.C.211, 1QD6.C.2, 1UCN.A.1162, 2UX1.K.174, 1YAX.A. 1002, 1ZH2.A.202, 4QQZ.C.1001, 4AIQ.A.1746, 4AUL.A.754, 2AWC.A.137, 1B06.A.322, 2BIW.C.1492, 1BIQ.A.376, 2BKB.A.1193, 4BMT.A.1323, 1BS3.A.202, 2BUT.B.1541, 2BUU .B.1541, 2BUV.B.1541, 2BUW.B.600, 2BUM.B.1541, 2BUQ.B.1541, 3CEI.A.500, 2CW2.A.4 02, 2CW3.A.524, 4DVH.A.301, 3E13.X.326, 3E65.A.901, 1EO2.B.600, 3ESF.A.198, 1EYS .C.609, 1EYS.C.611, 4F2N.B.300, 4FFK.A.301, 3FG3.D.1500, 3FG4.A.1500, 4G2D.A.402 , 1GN6.A.999, 2GOJ.A.198, 2GPC.A.195, 3H1S.A.1001, 3HHX.A.281, 3HHY.A.281, 3HJ8. A.281, 3HJQ.A.281, 3HJS.A.281, 3HKP.A.281, 1HLM.A.159, 1HMD.A.115, 2HMQ.C.115, 1 HRM.A.154, 3I4Y.A.281, 3I51.A.281, 1IBE.A.143, 3ICF.A.601, 4IEZ.A.501, 1ISA.A.19 3, 3IS8.A.163, 3IVD.B.601, 4J6C.A.502, 4KEZ.A.401, 4KVQ.A.301, 1KW6.B.301, 4L2B. A.201, 4L2C.A.201, 4L7Y.A.201, 3LIO.A.5000, 1MY6.B.200, 1NFT.A.333, 3NKT.A.369, 1NX4.A.300, 3O5U.A.300, 1ODN.A.1326, 1OM4.A.750, 1OUT.B.148, 4P1B.D.501, 3PCE.N. 600, 3PCN.P.600, 2PCD.N.600, 2Q0J.A.998, 2QFR.A.433, 1QFC.A.401, 3QFM.B.264, 1QN N.A.201, 2R1K.A.800, 2R1N.A.800, 3R2R.A.155, 4REU.A.201, 4S1C.A.801, 1SQ3.A.901, 3T4V.A.300, 1TDW.A.425, 3TQJ.A.1001, 3UFK.A.920, 1UNF.X.1239, 2VHL.A.1397, 2VP1 .A.1347, 2W7W.A.1195, 1WB7.A.212, 1WB8.A.212, 1XM8.A.701, 1YCH.A.501, 1YFW.A.300 , 1YFX.A.300, 1Z6O.M.300, 1ZA5.B.393, 2ZYQ.A.301, 2ZZI.B.209, 2FKZ.A.1600, 2JD7. 0.203, 1OQU.A.1001, 2PCD.M.600, 1YKP.F.2600, 4DOC.A.402, 4KHU.A.1008, 3AR8.A.100 0, 4CBY.C.2037, 4KAF.A.404, 2NQL.A.401, 1PX3.A.3101, 4TMV.A.903, 3UA6.A.147, 3VD 3.C.3101, 3VD7.A.3101, 3VDA.A.3101, 2BCV.A.579, 1DIZ.A.825, 2GIG.A.502, 2GIJ.F.4 03, 3HW8.A.577, 8ICK.A.341, 1JJ2.A.8345, 4ORJ.A.305, 1SUZ.A.403, 1TX3.A.801, 1VQ 8.M.9147, 1YJW.A.8545, 2A65.A.751, 1A7T.A.255, 4A87.A.1162, 2ABS.A.1003, 4ATF.C. 500, 3B1N.A.403, 2B2N.B.1001, 4BEM.A.201, 4BEM.B.201, 4BEM.J.201, 2BL2.A.1157, 3

C7X.A.1001, 3CB8.A.820, 4CD5.A.1420, 4CD5.A.1421, 4CFY.A.302, 3CKI.A.502, 3CTP.A .402, 3CZJ.A.3101, 4D1J.A.601, 4D77.A.1543, 2DDB.B.303, 2DKB.A.436, 4DUW.A.3101, 4DUX.A.3101, 3DYM.A.3101, 3DYO.A.3101, 3DYP.A.3101, 1EBU.B.902, 1EJA.A.246, 3EP R.A.266, 4EXR.A.301, 4EZE.A.302, 1F6D.A.1378, 2FBL.A.304, 4FEV.B.303, 4FHA.B.402 , 4FLL.A.504, 4FMT.D.301, 4FO2.Q.101, 4FXZ.A.603, 3G0T.B.437, 3GBV.B.1, 2GKO.A.6 14, 1GNY.A.1244, 1GVF.A.289, 3GVK.A.916, 4GVO.A.702, 3H7K.A.387, 4H7O.C.302, 1H8 0.A.1497, 4H83.E.401, 2HIG.B.488, 1HXN.A.4, 2HZY.A.1201, 3I3B.A.3101, 3I3D.A.310 1, 3IMM.C.3, 3IMX.A.467, 4IQZ.A.317, 3IRD.A.301, 3IWK.D.504, 4J4B.B.303, 2J5A.A. 1109, 2JBW.D.1367, 4JEX.A.512, 2JLN.A.1471, 4JNQ.A.402, 4JPA.A.305, 3JU4.A.7, 1J YN.A.3101, 1JZ2.A.3101, 1JZ3.A.3101, 1JZ6.A.3101, 1JZ7.A.3101, 1JZ8.A.3101, 1JZ8 .D.3104, 1KA0.A.501, 3KEU.A.402, 3KWM.A.225, 4LDE.A.1402, 4LHL.A.301, 3LJQ.A.597 , 3LKB.A.394, 4M9U.A.402, 3MAX.A.381, 4MB4.A.604, 4MM7.A.601, 4MMB.A.601, 4MMF.A .601, 3MPN.A.751, 3MPQ.A.751, 4MPY.A.503, 3MS8.A.401, 3MUI.A.401, 1MUQ.A.206, 3M UZ.1.3101, 3MUZ.1.3102, 1MVO.A.202, 3MV0.1.3101, 3MV1.1.3101, 3MX6.A.261, 3N3R.A .1000, 1N82.A.401, 1O4Z.A.1004, 4O4V.A.301, 1O68.E.274, 1OA8.C.1691, 1OAF.A.1252 , 4OFI.A.801, 4OMC.A.608, 3ONF.A.507, 4OUA.A.403, 2OZ3.B.2001, 2P3Z.B.501, 2PFL. A.2001, 3PNX.C.169, 3PNX.E.168, 2PPL.A.480, 4PV3.A.202, 1PX3.B.3101, 3PZJ.A.301, 1Q20.A.313, 2Q8X.A.401, 3Q9B.A.344, 1QHU.A.436, 4QKU.A.501, 3QS4.A.751, 3QS5.A. 751, 3QS6.A.751, 3QST.A.253, 4QTO.A.501, 4QTO.B.501, 2QWL.A.589, 4R7U.A.504, 3RG A.A.312, 3S9J.A.401, 1SK4.A.342, 3T09.A.3101, 1T64.A.391, 3T8J.A.401, 3TAV.A.267 , 4TMW.A.902, 1TO2.E.451, 3TXF.A.1138, 3TYP.A.155, 3UNX.A.281, 1V54.A.3519, 3V5U .A.701, 3V5U.A.703, 3VD3.A.3101, 3VD4.A.3101, 3VD7.B.3101, 3VD9.A.3101, 3VDA.B.3 101, 3VDC.A.3101, 3VDG.A.502, 1VI6.C.208, 3W0L.C.502, 1W9S.A.1142, 3WA2.X.702, 3 WGU.C.2007, 2WGM.A.201, 2WGM.B.201, 2WOI.A.1491, 2WPC.C.1492, 2WUW.E.1277, 2WWH. C.213, 3WZ1.A.401, 1XFF.A.5301, 2Z5D.B.180, 1ZH8.A.329, 3ZK1.A.90, 3ZK1.B.90, 1Z NB.A.3, 3ZQS.B.1295, 3ZYV.A.2337
[1] "Cluster 4"
3AYK.A.170, 2BIB.A.1549, 4H82.A.301, 2LQ6.A.401, 1Q3A.B.469, 4FC5.E.305, 2FZ6.A. 201, 1GLC.F.169, 2MQ1.A.103, 2POJ.A.265, 1DK4.B.590, 4OJX.A.404, 1OS9.F.926, 3V7 7.D.301, 4X2T.A.701, 2ZZW.A.362, 4D6N.F.1197, 4DFP.A.901, 3LK9.A.340, 4M2Z.A.501 , 2OZM.A.904, 1QTM.A.1001, 3R7P.A.317, 2XCP.A.1004, 2XCA.A.3001, 1ZBL.A.202, 3A5 8.B.401, 2AG1.B.611, 3B8I.A.289, 2BW7.A.2201, 4BYF.C.1000, 1CG0.A.435, 4CTA.B.40 1, 4CZK.A.1335, 4D2I.A.1478, 3DYG.A.3002, 1E2A.C.106, 1EFL.B.1604, 3EG5.A.180, 2 EGH.A.900, 1ELX.B.452, 1F1Z.A.2002, 3FFU.A.156, 3FFU.B.155, 4FFL.A.905, 3FHY.B.4 04, 4FHX.A.402, 3G9D.A.298, 1H65.A.282, 3HWX.A.602, 4HYV.A.1001, 4IOK.A.604, 2IU T.A.1724, 1KH7.A.452, 3KR4.C.1004, 3KUD.A.171, 4L2X.F.403, 4LNI.E.505, 3M1Y.A.30 0, 1MBZ.A.603, 1MBZ.A.604, 2NOM.A.401, 2NOM.A.402, 1NUW.A.2498, 1NV7.A.3341, 4OG E.A.1204, 2OPM.A.907, 2OQY.A.401, 3PP1.A.410, 2Q58.A.5, 1QF5.A.433, 4RAD.D.302, 1RC5.A.761, 4RNH.A.1501, 1SOJ.A.2123, 1T5S.A.1005, 3TXA.A.801, 4UOM.A.503, 3UGV. A.500, 2VQD.A.1449, 3VYT.C.602, 3W7F.A.303, 4WK2.B.501, 1WL6.A.801, 2XAM.B.1030, 2Y4M.A.400, 2YWF.A.701, 3ZDY.B.2001, 4FFO.A.903, 4LTZ.A.404, 2BPF.A.339, 4DPV.N .12, 4M30.B.302, 1N56.A.401, 4NLK.A.402, 4W9M.E.902, 1A49.B.1134, 1A49.C.1734, 4 A7Y.A.952, 4AN9.A.1383, 1AQF.A.534, 3AQC.D.328, 1AZT.A.403, 2BBO.A.2, 2BKK.A.126 6, 4BYG.A.996, 3C5P.D.302, 3CNX.B.170, 1CQP.A.310, 1CUL.A.1006, 4CYI.D.1000, 4CY U.A.170, 2D33.A.525, 3DUF.C.1368, 3DV0.A.1368, 3DYF.A.3002, 3DYF.A.3004, 4E1E.A. 401, 4E1E.A.402, 1E9I.D.1433, 3EA5.C.221, 1ELY.A.452, 3FPA.A.901, 4GA3.A.1003, 3 GFT.F.202, 1GIM.A.435, 3GT8.C.13, 1GY3.A.1298, 2GZD.A.950, 4HEO.A.403, 3HYL.A.67 5, 4HYP.B.302, 3IAF.B.572, 2I07.A.5002, 4IYN.A.804, 1JBO.B.1202, 4JLZ.B.502, 1JM 6.A.4601, 4JVJ.F.405, 4K81.B.203, 4LF2.D.601, 4LNI.D.505, 1MFO.A.1453, 4MGH.A.13 02, 4NEH.A.1102, 4NHO.A.1403, 1NUZ.A.2342, 2O1X.A.2001, 3OAB.A.904, 4OAU.C.802, 2OI2.A.400, 2P0C.B.5, 4P0V.A.403, 2PSN.A.701, 1Q19.A.504, 3Q1O.D.400, 3RBM.A.100 3, 4RJK.B.602, 3RUW.A.544, 4S17.C.502, 3SBE.A.501, 1SOJ.C.2127, 3T9E.A.603, 4TVU .B.601, 3TW6.B.2002, 4TXZ.B.502, 4UON.A.401, 3VAD.A.402, 2W00.A.1887, 2WB4.B.502 , 3WQP.J.501, 2X9H.A.3001, 1XF9.B.11, 1Y8P.A.501, 1Y9I.B.602, 1ZCA.B.1383, 3ALN.

A.406, 1G9X.B.1301, 3JZM.A.701, 4K4G.I.602, 4K4I.I.604, 4KHN.A.1002, 3MQ6.A.340, 3NDK.A.906, 3OOL.A.237, 3QEP.A.906, 3SQ1.A.907, 1AFD.2.2, 4AQ8.D.1238, 1AR1.A.561, 1AVX.A.700, 1B09.C.302, 3BJU.C.608, 4BTX.A.1763, 3CK7.B.720, 2CLT.A.1101, 2CLT.B.1301, 3D4G.B.485, 3D4G.H.484, 2DEW.X.904, 1DM5.A.1131, 1DM5.C.1133, 3E3R.B.195, 1E8U.A.1002, 3E9T.A.6, 1EAK.D.998, 1EGZ.B.300, 4ELF.F.201, 1ESP.A.320, 2EXK.D.2004, 2F2O.B.1001, 3FCS.D.2002, 4FGC.C.203, 3FHA.B.704, 4FU4.A.505, 3FW0.A.823, 2GA9.D.480, 3GCW.E.1, 4GGB.A.401, 4GKX.B.302, 4H2E.B.304, 1HFY.A.124, 3HLH.D.315, 3HQ8.A.402, 4HSZ.B.101, 2HTY.C.993, 2HTY.G.997, 1HVD.A.602, 1IXX.D.124, 1KLJ.H.900, 3KQA.A.420, 1KX1.C.301, 4KZW.B.400, 4L06.B.501, 4L06.C.501, 4L06.D.501, 1L7L.A.201, 3L95.X.2001, 3LCP.D.159, 4LIN.E.1301, 4LMF.B.302, 3LND.A.210, 1LQD.B.1, 4LT6.A.602, 1LWJ.A.883, 3MOW.H.202, 1MMP.B.3, 3MW3.A.302, 4O1Q.A.401, 2OKX.A.4004, 1OS2.C.473, 3OSH.A.121, 3PKO.D.280, 4PLS.A.305, 1PYT.C.650, 3Q3L.B.437, 3QQZ.A.324, 3QWU.A.601, 1R1Z.B.315, 1RE3.C.408, 2RHP.A.2, 2RHP.A.24, 2RHP.A.25, 2RHP.A.28, 2RJQ.A.5, 2RLD.C.121, 1ROS.A.404, 3S5U.A.220, 3S5U.D.220, 4SBV.A.261, 4SBV.C.261, 1SRR.A.531, 1TD7.A.1001, 3TH3.L.145, 1TN3.A.183, 4TVU.B.600, 1TYE.B.1402, 4UB6.O.301, 3UBH.A.852, 1UZJ.C.3648, 2V3T.A.1264, 1V3J.B.689, 1VFO.B.1002, 1VFP.B.995, 2VME.A.500, 3VOT.A.504, 2W2O.E.1333, 3W9T.C.1010, 3W9T.B.508, 1WD9.A.901, 2WVX.C.801, 2WW0.C.800, 2WW0.F.800, 2X0G.B.1149, 2YFS.A.1711, 2YN3.C.6370, 1YO8.A.1184, 3ZHG.B.1323, 2ZWO.B.402, 1ZZH.A.401, 2AOQ.A.232, 3BQ1.A.4001, 3C0W.A.301, 3EH8.A.302, 3FSP.A.501, 1JX4.A.4001, 3KHH.B.1416, 1M5X.A.802, 1N3E.C.492, 3NE6.A.905, 4PTF.A.1202, 3QEX.A.907, 1R7M.A.306, 2RDJ.B.353, 1S9F.A.4001, 2WTF.B.1510, 4A6S.C.1122, 2A8K.C.403, 1AFA.2.2, 1AWB.A.278, 4AWD.A.1321, 3AZX.B.301, 3B1T.A.900, 4B7R.A.502, 3BCD.A.708, 3BJU.A.608, 4BY5.C.1187, 4CAJ.B.1325, 2CDP.B.1139, 1CGU.A.686, 3CGT.A.686, 3CK7.D.730, 1CR8.A.45, 1CRU.A.503, 4CRR.A.1386, 1CYG.A.682, 1D7F.A.5003, 3DAS.A.351, 1DGL.B.301, 4DRZ.A.202, 2DSN.B.2012, 1E8U.B.1003, 3EAD.A.1003, 3EDD.A.700, 3EHJ.A.1, 3EHJ.B.1, 3ESQ.A.213, 1F6S.E.205, 3FCU.B.2002, 1FHF.A.501, 2FPS.A.503, 4FU4.B.505, 4G62.A.301, 1G9K.A.700, 3GN4.B.204, 3GQF.D.154, 4GQ7.A.301, 2GSM.A.3007, 4H3X.A.304, 1HDF.B.1101, 1HFZ.A.124, 1HL5.D.156, 3HLI.D.315, 1HQV.A.999, 1I22.A.198, 3I4I.A.1001, 2I4B.A.454, 3I57.A.186, 4I5N.B.601, 2J1T.A.1154, 1J9L.B.1303, 4JCM.A.706, 4JDZ.B.702, 3JTX.B.396, 3JXS.A.302, 4K5W.A.201, 2KAY.A.185, 3KF9.C.304, 1KMB.1.2, 4KVJ.A.714, 1KX1.E.502, 1KX1.F.601, 2L51.A.207, 1L6R.A.901, 1L9M.A.702, 3LCP.B.279, 3LHM.A.131, 4LMF.A.303, 1LPG.B.1, 4M5I.A.203, 1MAC.B.389, 1MDU.A.403, 2ML2.A.202, 4N25.A.707, 4N2I.A.704, 3N4E.A.500, 4N85.A.502, 3N8G.A.1002, 4NAM.A.801, 4NHF.B.301, 3NN7.A.503, 3O5S.A.243, 2OKX.B.4002, 3P4G.A.403, 2PF2.A.174, 4PLM.A.504, 2PPL.A.481, 1Q7B.A.9002, 3Q8F.A.736, 2QT6.A.3713, 1R1Z.C.415, 2RDZ.B.1502, 3RRW.A.271, 3SLE.B.401, 3SO0.F.98, 3SO1.B.97, 3TEW.A.801, 3TSK.A.304, 1TT2.A.502, 3TUY.C.157, 3UBF.A.754, 1UF3.D.913, 1UH2.A.1002, 1UH3.A.1001, 1UH3.A.1002, 1UKS.A.688, 1ULV.A.2003, 1UMS.A.3, 1UPS.A.501, 1UZJ.A.1648, 3V03.A.585, 1V2I.A.1001, 1V3J.A.687, 1V3L.B.689, 2VDR.B.2002, 1VFO.A.1001, 2VJ3.A.1533, 2VKH.A.1543, 2VL8.A.1545, 2VVE.B.1338, 2VXJ.G.200, 2VZP.B.1129, 2W2N.E.1334, 2W3J.A.1139, 3W9T.A.1008, 3W9T.D.503, 3W9T.D.504, 4WBQ.B.602, 4WF7.D.600, 3WH3.A.501, 2WO9.B.1272, 2WQS.A.2415, 2WVX.B.801, 2WVZ.B.800, 2WW0.A.800, 2XQX.B.1949, 2YDP.A.502, 1YO8.A.1195, 1YO8.A.1208, 1YO8.A.1211, 2Z49.A.1002, 3ZHG.D.1323, 1AFA.1.2, 1AFB.1.2, 4AK8.A.1326, 4AYO.A.500, 4AYP.A.500, 4AYQ.A.500, 4AYR.A.500, 1BCH.1.2, 1BCH.2.2, 2CKI.A.998, 2DG1.A.3001, 2DSO.B.1002, 4E52.B.401, 1FIF.A.2, 1FIH.A.2, 1FO3.A.700, 1G1T.A.160, 3G81.A.401, 2GGU.A.401, 2GGX.A.401, 1H80.A.1492, 3IKP.B.401, 3IKR.A.401, 1JZN.A.1138, 1K9I.B.502, 1K9J.A.402, 3KMB.1.2, 1KWU.A.503, 1KWV.A.503, 1KWW.A.503, 1KWX.A.503, 1KWZ.B.604, 1KX0.A.504, 1KZA.1.502, 1KZD.1.502, 4KZO.A.501, 4KZO.B.501, 4KZV.A.302, 1LQV.D.47, 2MSB.B.2, 1MUQ.B.202, 4N32.A.402, 4N33.A.404, 4N34.A.402, 4N35.A.404, 4N36.A.402, 4N37.A.402, 4N38.A.403, 4NVR.A.401, 2ORJ.A.401, 2OS9.A.401, 2OX9.A.802, 3P5G.A.500, 3P5H.A.500, 3P5I.A.500, 3P7G.A.1, 3P7H.A.1, 3PAK.A.300, 1PW9.A.401, 1PWB.B.401, 1RDI.1.2, 1RDJ.1.2, 1RDK.1.2, 1RDL.1.2, 1RDN.1.2, 1RDO.1.2, 3RQI.A.181, 1RTM.1.2, 1SL5.A.402, 1SL6.A.2, 2VUV.A.200, 2VUZ.A.1130, 3VYK.A.1007, 1

W0Y.L.1156, 2W87.A.1148, 3WH2.A.301, 1WMY.A.202, 1WMZ.A.202, 1WMZ.B.202, 2XR6.A.1391, 4K4G.A.602, 4K4H.A.602, 4K4I.A.605, 3RAX.B.1416, 1T9I.C.801, 1T9I.C.802, 4UAW.A.403, 4A3X.A.1268, 4A3Z.A.2344, 3A4U.A.287, 4A41.A.2494, 4A42.A.1690, 4A4A.A.1925, 4A5G.A.1308, 4A5G.A.1309, 4A60.A.2346, 4A6S.A.1122, 3AFG.A.541, 4AFA.A.1267, 4AFB.A.1267, 4AFC.A.1267, 1ALC.A.200, 3ALT.A.201, 3ALU.A.201, 4AL9.A.1122, 4AOC.A.1129, 4AOC.E.1129, 4AQ1.A.1924, 4ASL.A.1268, 4ASM.B.1359, 3ATG.A.301, 4ATE.A.1275, 3AUJ.A.1603, 4AWD.B.1321, 3AXD.A.3002, 3AZY.A.301, 3B0K.A.201, 3B0X.A.578, 2B6N.A.300, 1B80.B.351, 1B90.A.124, 4B96.A.1155, 4B9C.A.1151, 4B9F.A.153, 1BCJ.2.2, 2BF6.A.1693, 1BGP.A.501, 1BIW.B.803, 2BIB.A.1551, 1BJ3.B.124, 4BLK.A.400, 3BMV.A.685, 4BM1.A.401, 2BO2.A.155, 2BO2.A.156, 2BOI.A.300, 2BOI.B.600, 2BOJ.A.1116, 2BOJ.C.1117, 3BPS.E.1, 2BP6.A.802, 2BP6.C.805, 1BQB.A.353, 2BV4.A.300, 2BV4.B.300, 1BYF.A.201, 4CE8.A.998, 4CE8.B.998, 1CGT.A.686, 1CIU.A.684, 4CI7.A.1505, 3CK7.A.710, 4COU.A.1270, 4COV.A.1269, 4COY.A.1270, 1CPM.A.215, 1CPN.A.209, 4CP0.A.1294, 4CP1.A.1294, 4CPB.A.1122, 4CPB.D.1123, 3CQO.A.4001, 3CQO.A.4004, 4CRQ.A.260, 4CUO.A.1326, 4CZN.A.1370, 4D0E.A.1531, 4D0E.A.1533, 1D2V.A.600, 1D3C.A.687, 3DBZ.A.401, 3DCQ.A.116, 3DCQ.B.116, 2DCK.A.1002, 2DEW.X.900, 1DED.B.5004, 3DED.A.504, 3DED.B.506, 3DEM.B.4001, 2DSO.A.1001, 1DV8.A.1002, 4DZT.A.302, 2E39.A.501, 2E8Y.A.741, 3EDF.A.603, 3EHU.A.500, 3EIF.A.1, 2EJN.A.1003, 2ERV.A.195, 1ESL.A.163, 2EWE.A.703, 2EXH.A.2001, 1F6S.A.201, 2FF3.A.701, 1FHF.A.502, 2FHF.A.2401, 4FHP.A.402, 1FIF.B.2, 3FMU.A.351, 2FPW.A.503, 4FVL.A.505, 4G01.A.300, 1G1Q.A.801, 1G5N.A.408, 1GEN.A.302, 4GER.A.404, 2GGX.C.401, 3GIS.Z.1003, 2GVU.A.500, 2GVV.A.500, 1GW2.A.502, 1GWT.A.502, 1GWU.A.1307, 1GWU.A.1308, 4GWI.A.204, 1GX2.A.1310, 3HOO.A.401, 2H2T.B.322, 2H2N.B.1001, 1H3G.A.700, 3HB3.A.563, 3HDL.A.306, 3HDL.A.307, 2HD9.A.2001, 1HFX.A.124, 1HFZ.B.124, 4HHR.A.703, 3HLH.B.315, 3HLI.B.315, 3HR9.A.401, 2HYK.A.477, 1I22.B.197, 4I5L.B.601, 2IAW.A.401, 2IAX.A.401, 2IAO.A.401, 4IAI.A.402, 4IAU.A.800, 3ILF.A.278, 3INM.A.521, 3IQT.A.1, 2IWV.D.1283, 2J1A.A.1769, 2J22.A.1150, 1J34.C.504, 4J3V.A.920, 4J3W.A.907, 4JCL.A.701, 2JDY.A.1116, 2JDY.B.1117, 2JD9.A.1146, 2JDA.A.1146, 2JDM.B.1115, 2JDM.C.1115, 2JDN.A.881, 2JDN.B.881, 2JDN.C.881, 4JGL.A.202, 1JI1.A.2001, 1JI1.A.2002, 2JKX.A.1641, 2JKA.A.1727, 4JSD.A.603, 1JUG.A.126, 1K12.A.160, 3K8K.A.710, 3K8L.A.700, 3K8L.A.710, 4KVL.A.703, 1KWZ.A.504, 1KZM.A.501, 1L6R.B.903, 4LHK.A.303, 4LHN.A.302, 3LI3.A.402, 3LI4.A.316, 4LJH.A.201, 1LLP.A.351, 1LPZ.B.1, 4LPL.A.1101, 1LQV.C.42, 4LQR.A.201, 1LY8.A.9001, 1LYC.B.9002, 3M5Q.A.372, 4M65.A.404, 1MAC.A.388, 2ML2.A.203, 2ML3.A.201, 3MMZ.A.501, 1MN1.A.371, 1MVE.A.400, 4MZA.A.612, 4N2B.A.707, 4N2G.A.703, 4N2L.A.704, 4N4E.E.404, 4N6F.A.302, 4N7A.A.605, 1NL1.A.204, 3NNG.A.402, 1NPC.A.322, 1NZI.A.1001, 2NZM.A.406, 1O4Y.A.700, 4ODG.A.202, 2OKX.A.4001, 3OMI.A.613, 1OUX.A.402, 1OUX.B.404, 1OVS.A.402, 1OVS.B.404, 3OWF.A.151, 3P7F.D.1, 1PA2.A.307, 1PA2.A.308, 1PAM.A.687, 1PAM.A.688, 3PAQ.A.300, 3PAR.A.300, 3PGV.A.267, 4PIB.C.201, 1PJ9.A.890, 1PJX.A.491, 4Q1U.A.402, 3Q3U.A.340, 3Q9K.A.606, 4QF4.A.202, 1QGJ.A.2001, 1QGJ.A.2002, 1R1Z.A.286, 3R5Q.A.1001, 4R83.B.501, 3S18.A.229, 1SCH.A.301, 1SCH.A.302, 1SH7.A.1292, 1SNC.A.150, 1SNN.B.503, 3SO0.A.97, 3SO1.A.97, 3SRE.A.1357, 3SRG.A.1357, 1SU3.A.904, 1SU4.A.996, 1SZB.A.1001, 3TBD.A.401, 1TDQ.B.127, 1TE2.A.702, 3TH4.L.148, 1TLG.A.201, 3TO5.A.131, 1U0A.A.5004, 1UPS.B.501, 1URX.A.1300, 1USR.B.1573, 1UX6.B.2014, 1UX7.A.1134, 1V3E.A.4001, 3V6N.A.229, 2V72.A.1139, 2VNV.A.302, 2VNV.B.302, 2VUC.B.990, 2VUC.C.991, 2VUD.C.1118, 2VZP.A.1129, 2VZQ.A.1130, 2VZR.B.1132, 2W1Q.A.1947, 2W1S.A.1946, 2W2M.E.1334, 1W3M.E.3013, 3W57.A.202, 3W5M.A.1201, 3W5N.A.1201, 2W86.A.1148, 2W86.A.1149, 1WDC.C.501, 3WDH.A.801, 4WF7.A.600, 3WH3.A.500, 3WHD.A.501, 2WOY.A.2415, 2WQ8.A.1641, 2WR9.A.1131, 2WR9.D.1132, 3WU2.A.401, 1WZL.A.1601, 2WZA.A.2415, 2WZS.F.800, 2XFD.A.1111, 2XFE.A.1113, 1XO5.A.1, 1YDY.A.904, 2YFU.A.1141, 2YLJ.A.1308, 1YRO.A.124, 2Z30.A.1002, 2Z48.A.1007, 2Z48.A.1102, 2Z48.A.1103, 2Z48.A.1104, 2Z48.A.1205, 2Z49.A.1001, 2Z49.A.1003, 2Z49.A.1004, 2Z49.A.1005, 3ZDV.A.200, 3ZDV.B.200, 1ZH2.B.201, 1ZJA.A.7001, 3ZO9.A.1589, 3ZUC.A.1154, 3ZYH.A.1123, 4DTU.A.1003, 3QEV.A.906, 2IWV.A.1284, 4L06.A.501, 3O82.B.544, 4OO2.C.601, 3R6Q.H.469, 4ME4.A.402, 1R1N.C.400

, 4ANP.A.1426, 4N71.A.201, 3PCK.M.600, 1XVE.A.1170, 2YU1.A.600, 2GIG.A.501, 1Q81
.C.8345, 1VQ9.Q.9148, 1GOF.A.702, 4IIL.A.402, 3MMD.A.410, 3Q2G.A.701, 3U0F.A.410
, 1W8O.A.1654, 4C0K.A.1614, 4JC0.D.406, 4MVJ.D.402, 4Q4B.A.530, 4B6C.B.1257, 4KH
Y.A.1013, 3PML.B.7, 2A5G.B.242, 2A7L.A.201, 2A9Y.A.2002, 4B6C.A.1257, 4C1P.A.172
8, 4CBY.B.2036, 1D7U.A.435, 4D9T.A.901, 1EZ1.B.1002, 3FZQ.B.274, 3G1N.B.5002, 3G
8Q.D.302, 4GKI.F.303, 4GRX.A.501, 4H41.B.405, 3IAQ.A.3103, 3IC9.A.491, 2JBW.A.13
68, 4JD0.G.301, 1JYV.A.3101, 1JYW.A.3101, 3KZW.D.498, 1MGV.A.501, 4MVJ.A.401, 4N
LQ.A.910, 4NT1.A.301, 4OOC.B.402, 2ONP.G.707, 4OUC.A.801, 2P6Z.A.402, 1PX4.A.310
1, 4QVS.A.502, 1S5C.A.241, 1SOO.A.547, 3VD5.A.3101, 3WGU.A.2005, 1WQR.A.131, 2WW
G.B.213, 2WX5.H.1252, 1XAR.A.100, 4XEL.A.201, 1YQ2.A.7501, 2ZJ9.A.1, 2ZN8.A.995,
 3ZPQ.A.1360, 3ZX0.C.522, 2OTL.A.8545, 1Q81.A.8378, 1TW8.B.902, 3UXP.A.338, 1VQ9
.M.9147, 1A5U.G.4732, 2AB8.A.2003, 4B52.A.1305, 2BER.A.1649, 1BGP.A.502, 4BY5.B.
1188, 3C17.A.323, 4CBY.A.2036, 2D4E.A.1901, 2E5X.A.303, 4FMT.B.301, 2GFH.A.249,
1HBN.D.1561, 3HSC.A.491, 2HZG.A.1101, 3I01.M.730, 3I2W.B.304, 3IC9.B.491, 2IM2.A
.3001, 3IWJ.A.505, 2J80.A.1134, 4JN7.A.401, 1JZ4.A.3101, 4KZW.A.304, 3LG1.A.530,
 4LL2.A.301, 3ME4.B.2, 3N0U.A.208, 4NLV.A.911, 1O04.A.6601, 4O57.A.301, 4O5H.A.6
05, 1O68.A.274, 4OMG.A.402, 3OND.A.509, 2P6Z.B.401, 2P6Z.B.403, 4PCG.A.302, 1Q6X
.A.1002, 4Q92.C.502, 2QR7.A.1000, 2QSV.A.222, 1S0A.A.1501, 3SIS.B.3001, 3SIT.A.3
000, 1T64.A.392, 3TAY.A.1, 4TMX.A.902, 4US3.A.701, 4USW.A.1468, 1V0H.X.252, 2W4M
.A.1245, 3WFA.A.801, 4WFZ.A.501, 2WPC.A.1491, 2X8J.A.1317, 2YDG.A.1130, 2YF0.A.1
743, 1Z45.A.702, 2ZJ9.B.2, 1ZUD.1.701
[1] "Cluster 5"
3ADR.A.263, 2DI3.A.1002, 2EK9.A.1004, 3G4K.A.801, 4HTZ.B.1001, 4KBP.A.439, 3LL8.
A.505, 2LQ6.A.402, 2LZE.A.101, 1ML2.A.296, 4N7K.L.301, 4N7K.M.401, 4N7K.M.402, 4
N7L.M.402, 4OK2.A.801, 1VKG.B.400, 4X2T.A.702, 3DZA.C.501, 3DZA.C.505, 2EK8.A.10
04, 1ENQ.A.238, 2F92.F.1002, 2F94.F.1001, 2F94.F.1003, 2F9K.F.1001, 2F9K.F.1002,
 4GBD.A.503, 4GQT.A.501, 2H44.A.501, 3HDZ.A.864, 1HQA.B.452, 3LLX.A.376, 1M60.A.
105, 3NO5.A.275, 4NT9.A.301, 3O2G.A.388, 3O91.A.192, 4OK4.A.800, 2OUV.A.777, 2OU
3.A.161, 2OUP.B.777, 4OV9.A.401, 2P2L.A.201, 2PTY.A.501, 2PTZ.A.500, 2PTZ.A.501,
 2PU1.A.501, 2PW3.A.501, 2QYM.A.1, 2QYK.A.1, 2R2V.C.35, 1RRM.B.387, 3SL3.B.9, 1T
9S.A.1, 1TB5.A.1001, 1TB7.A.1001, 1TBF.A.1, 1U74.A.1001, 3UUO.B.772, 3V93.A.701,
 2WTA.A.1215, 1XOR.A.1001, 1Y2K.A.1001, 1Y9Q.A.202, 1ZKL.A.501, 4BE0.A.1380, 4BE
1.A.1381, 4BE2.A.1380, 1BPY.A.339, 4BWJ.A.1834, 4CEI.B.2162, 4DFK.A.902, 4DFM.A.
902, 4DL4.A.501, 4DO9.A.401, 4DOB.A.401, 4DOC.A.401, 4DQI.A.901, 4DQP.D.902, 4EL
T.A.902, 4ELU.A.902, 4F5O.A.402, 1FIU.I.2222, 1FIU.A.5555, 4FO6.A.601, 3G6Y.A.87
1, 4GZ2.B.402, 3IEV.A.400, 2JOS.A.1412, 1JJ2.0.8010, 3JPQ.A.339, 3JPS.A.339, 4JW
M.A.403, 3K58.A.1001, 3K59.A.1001, 4K97.A.603, 3KD5.E.916, 4KLI.A.401, 4M04.A.70
2, 4M04.A.703, 3M8S.A.2, 4M8O.A.1302, 4M9L.A.404, 4MDE.A.1002, 3MFI.A.515, 4MFC.
A.401, 4O3O.A.502, 4O3O.A.503, 4O3Q.A.502, 4O3Q.A.503, 4O5K.A.401, 3OHA.A.518, 3
OJS.A.7, 3OYG.A.396, 4P4M.A.402, 2PFP.A.750, 3PNC.A.576, 4PUQ.B.401, 4Q8E.A.502,
 4QM6.A.1002, 4R65.A.402, 3RJH.A.403, 3RJK.A.339, 4RNN.A.503, 3RTV.A.833, 3SI8.A
.451, 3SM4.B.227, 3SNN.A.905, 3SPY.A.903, 1SUZ.A.402, 3TFR.A.339, 3TFS.A.339, 3T
IO.A.1, 3TIO.D.2, 4UAY.A.402, 1W7A.A.1802, 1XSN.A.576, 1ZJN.A.339, 3ZVM.A.1526,
121P.A.168, 3A0T.A.800, 4A01.A.1767, 4A01.A.1769, 3A1U.A.5, 3A1U.A.6, 1A2B.A.550
, 3A4L.A.401, 2A5Z.A.701, 2A5D.A.1231, 4A6X.A.350, 1A82.A.901, 3A99.A.401, 4ACF.
A.1482, 4ACI.A.1187, 2AFK.E.1291, 2AG0.A.601, 3AHC.A.826, 3AHD.A.826, 3AHE.A.826
, 3AHG.A.826, 2AL1.B.439, 4ANB.A.1384, 1AOR.A.609, 1AOX.A.400, 1ASO.A.356, 4AS2.
A.1328, 2AUU.A.203, 2AUU.A.204, 2AUT.A.601, 2AUT.D.604, 4AUX.A.223, 3AYX.A.701,
2B0T.A.800, 3B1V.A.301, 1B25.A.800, 4B2P.A.1351, 2B56.A.488, 3B7L.A.907, 3B7L.A.
908, 3B7L.A.909, 1B8J.A.452, 2B82.A.1013, 2B9H.A.700, 4BAS.A.1183, 3BB1.A.282, 2
BBS.A.3, 3BC1.A.194, 2BEK.A.501, 2BME.A.1184, 3BN3.A.1, 1BOF.A.800, 2BON.A.1302,
 2BU2.A.1388, 2BVC.A.504, 2BVN.B.1395, 3BWV.A.300, 4BW9.A.501, 4BWR.A.1468, 4BX0
.A.1291, 4BX3.A.301, 1BYQ.A.1001, 1C1Y.A.171, 2C18.A.1338, 2C3U.A.2238, 3C4Z.A.5
63, 2C4N.A.1251, 3C5H.A.302, 2C5L.A.1168, 4C5B.A.1314, 2C77.A.1407, 2C78.A.1407,

3C9U.B.312, 2CBZ.A.1872, 2CFS.A.1296, 3CG4.A.201, 1CHN.A.200, 2CHE.A.130, 1CIP.A.356, 1CJU.A.582, 2CJE.A.1267, 3CK5.A.400, 2CK3.A.601, 2CK3.F.601, 2CLS.A.550, 2CN5.A.1506, 4COK.A.601, 3CP6.A.502, 3CP6.A.503, 3CR3.A.1212, 1CTQ.A.168, 3CUR.H.553, 3CUS.Q.553, 3CV2.A.1, 3CX8.A.378, 2D0O.A.1005, 1D2N.A.99, 3D2R.A.500, 3D36.A.478, 4D6P.A.1352, 2D7C.A.1002, 4D7M.A.223, 1D8C.A.3001, 4DBF.A.401, 4DBR.A.810, 4DCK.B.201, 3DDC.A.600, 3DDH.B.232, 2DDT.A.311, 4DEM.F.402, 4DEM.F.403, 4DEM.F.404, 3DGT.A.800, 3DKC.A.2, 4DN1.A.401, 4DN5.A.1001, 4DSN.A.202, 4DSO.A.202, 1DTW.A.401, 4DUX.A.3001, 4DWG.A.401, 4DWO.A.301, 3DYH.A.3003, 3DYH.A.3004, 3DYM.A.3001, 3DYP.A.3001, 2DY1.A.701, 4DYK.A.502, 4DZH.A.501, 4E01.A.402, 1E2Q.A.401, 3E2D.A.603, 1E3D.B.901, 3E5H.A.200, 3E81.A.165, 3E84.A.701, 3E8M.A.165, 4E8G.A.402, 2E9S.A.603, 1E9A.A.401, 2E91.A.1301, 2E95.A.1302, 3EA5.A.221, 2EB5.A.1001, 4EEN.A.301, 3EFQ.B.4004, 4EFM.A.202, 3EGT.A.3004, 3EHG.A.371, 1EKO.A.601, 3EQC.A.3, 2ERX.A.403, 3ET4.A.301, 3ET5.A.255, 4EUK.A.1001, 2EW1.A.701, 4EX6.A.301, 4EX7.A.301, 2EZT.A.1510, 2EZU.A.1610, 3EZ3.A.1104, 2EZ4.A.1610, 2EZ8.A.1510, 2EZ9.A.1510, 4F1J.A.301, 2F2A.B.601, 1F5N.A.595, 3F61.A.310, 1F9H.A.161, 1F9H.A.162, 2F9M.A.1201, 4F9A.A.602, 3FD5.A.397, 4FE3.A.304, 4FEG.A.707, 2FFQ.A.356, 2FH5.B.270, 3FIU.A.5001, 3FIU.A.5002, 4FI1.A.401, 3FKQ.A.501, 4FK9.A.401, 2FOZ.A.348, 2FOZ.A.349, 4FP1.A.401, 2FRV.B.540, 1FSG.A.302, 3FSY.A.333, 2FUE.A.500, 4FYP.A.301, 3FZN.A.605, 2G0W.A.501, 2G09.A.901, 2G1T.A.1501, 1G17.A.301, 3G15.A.602, 3G2F.A.901, 1G4C.B.362, 1G4T.A.2005, 3G5A.D.307, 1G5T.A.998, 3G6K.A.307, 2G6B.A.301, 2G80.A.500, 1G97.A.460, 4G9B.A.301, 3GAI.A.189, 2GCN.A.2001, 2GCP.A.2001, 2GHT.A.257, 2GIL.A.1201, 2GL5.A.699, 3GON.A.600, 4GOJ.A.202, 4GP2.A.402, 1GSA.A.319, 1GSI.A.1209, 2GSM.A.3006, 4GT8.A.402, 1GUA.A.171, 3GYB.A.1, 4H1Z.D.401, 1H2A.L.1005, 1H2R.L.1005, 2H57.A.202, 3H70.A.342, 3H7V.A.331, 3H80.A.214, 4H81.A.402, 4H8E.A.301, 4HAT.A.302, 2HCF.A.300, 4HCH.A.405, 4HCL.A.401, 3HDG.A.202, 4HDO.B.200, 3HFW.A.361, 2HF8.A.301, 2HF9.A.301, 4HGN.A.200, 3HIY.B.402, 2HJP.A.292, 2HNE.A.601, 4HNL.A.401, 4HOR.X.101, 4HPT.E.402, 1HQ2.A.162, 3HQJ.A.145, 4HQO.A.301, 3HRZ.A.628, 3HRZ.D.742, 3HSD.B.162, 1HTW.A.561, 3HW4.A.999, 3HW5.A.999, 1IOL.A.902, 2I1Q.A.501, 4I2B.A.604, 2I34.A.301, 2I5R.A.301, 3I76.A.1001, 4I94.A.402, 4IAD.A.402, 3IBA.A.403, 3ICM.A.401, 3ICM.A.402, 3ICZ.A.403, 4IDN.A.502, 4IDP.A.502, 4IEG.A.1001, 1IG5.A.78, 2IHT.A.601, 2IHU.A.601, 1IH8.A.4002, 1IHU.A.592, 2IHP.A.287, 3IJL.A.386, 2IK4.A.287, 2IK4.B.287, 2IK4.B.288, 2IK4.B.289, 2IK6.B.287, 2IOR.A.2000, 1IOW.A.331, 2IO8.A.7002, 4IP4.A.503, 3IPO.A.161, 1ITZ.A.1001, 4ITR.D.203, 2IUC.A.1003, 4IUC.L.702, 4IUD.L.1002, 1IV2.A.1571, 1IV4.A.1571, 4IVG.A.803, 4IWH.A.401, 2IYW.A.202, 2IYN.C.1123, 1IZC.A.1001, 2J0V.A.1180, 2J7P.A.1401, 2J7P.D.1401, 2J7N.B.3374, 1J9J.A.301, 1J9J.B.301, 1JBW.A.998, 2JC9.A.1491, 2JCB.A.1192, 4JDP.A.301, 2JD4.A.4061, 1JPM.A.1003, 4JS0.A.202, 3JTC.C.34, 1JUY.A.435, 3JVA.A.356, 3JVA.B.358, 3JYY.A.301, 3JYY.B.302, 1JZ7.A.3001, 3JZ0.A.300, 3JZ0.A.303, 3K1S.H.107, 4K6R.A.505, 1K77.A.300, 3K8K.A.700, 3K9L.A.168, 4K9Q.A.601, 3KA3.A.176, 3KAL.A.503, 3KB9.A.701, 3KB9.A.702, 3KC2.A.355, 1KCZ.A.901, 4KGD.A.702, 1KK1.A.413, 3KKO.A.180, 1KMQ.A.401, 3KMW.A.501, 4KO8.A.801, 1KQP.A.5001, 1KQP.A.5002, 4KQW.A.404, 3KS6.C.251, 1KSH.A.202, 1KTG.A.502, 1KTG.A.503, 1KTG.A.504, 1KTG.A.505, 3KTA.A.184, 3KUC.A.171, 4KUX.A.703, 4KVA.A.501, 4KVG.A.202, 4KWD.A.404, 4KX5.A.314, 4KXW.A.1001, 1KY2.A.401, 3L12.A.313, 4L57.B.201, 3L8H.A.801, 4L80.A.403, 4L9W.A.202, 4L9Z.A.403, 4LA6.A.501, 2LCF.A.246, 4LFG.A.303, 4LFG.A.304, 4LFG.B.303, 4LGY.A.1302, 4LHW.A.301, 4LJ9.A.902, 3LLU.A.502, 4LPM.A.208, 3LUF.A.300, 3LUF.A.301, 3LX5.A.301, 3LXX.A.402, 4LYK.A.401, 4LZ0.A.403, 3M07.A.595, 3M1I.A.1178, 4M53.A.527, 1M7B.A.550, 3M7I.A.901, 4M9Q.A.302, 4MDB.A.403, 4MGG.A.404, 1MH1.A.201, 3MHY.A.115, 1MJN.A.1001, 3MJH.A.201, 3MK2.A.903, 1MMG.A.998, 1MMN.A.998, 1MNE.A.998, 4MNE.A.902, 4MPO.B.205, 1MQ4.A.2088, 4MRT.A.301, 4MUM.A.301, 1MXG.A.439, 3MX3.A.601, 3MYH.X.997, 4MYO.A.301, 3MYK.X.998, 3MYL.X.998, 4MZU.C.404, 1N0H.A.699, 4N0D.A.402, 3N07.A.200, 1N1Z.A.701, 1N1Z.A.703, 1N20.A.702, 1N24.A.702, 3N2N.A.1, 3N45.F.2, 3N45.F.3, 3N4F.A.502, 1N6I.A.201, 1N6L.A.201, 1N6N.A.201, 1N6O.A.201, 1N6P.A.201, 1N6R.A.201, 1NBO.A.201, 4NBS.A.502, 4NDO.A.302, 4NFI.F.40

2, 4NFI.F.403, 1NFZ.A.401, 3NKV.A.500, 1NN5.A.401, 3NOJ.A.239, 1NRJ.B.1, 3NRJ.A.190, 2NSY.A.305, 1NSF.A.859, 1NSY.A.6241, 4NWI.A.401, 2NXW.A.4002, 1O08.A.2800, 2O1S.A.1001, 1O3Y.A.1002, 1O6Y.A.1280, 3O6Z.B.201, 2O7O.A.223, 4O7I.A.401, 1OBW.A.176, 3OCU.A.263, 3OCV.A.264, 3OCW.A.263, 3OCX.A.264, 3OCY.A.264, 3OCZ.A.263, 4OCP.A.403, 2ODP.A.901, 4ODJ.A.502, 3OE1.A.601, 2OEM.B.912, 2OFX.A.301, 2OGD.A.3002, 2OGD.A.3003, 2OGD.A.3004, 4OHY.A.502, 1OIW.A.1175, 3OIW.A.170, 2OI6.B.6000, 4OI4.A.502, 1OKK.A.1002, 1OKK.D.1002, 4OKM.A.901, 4OKM.A.903, 4OKZ.A.903, 2OLR.A.543, 3OM2.A.486, 4OMF.A.503, 3OP2.B.500, 2ORW.A.501, 3OUZ.B.459, 1OXV.A.1102, 1OXV.D.1101, 3OYX.A.601, 3OZX.A.613, 3OZX.A.614, 2P27.A.307, 4P31.A.402, 4P32.A.402, 1P4M.A.201, 1P5Z.B.401, 3P5P.A.901, 3P93.C.406, 3P96.A.412, 2PA4.A.325, 3PDE.A.312, 3PFF.A.831, 4PFY.B.601, 3PGL.A.1, 1PHP.A.395, 4PHG.A.201, 4PHH.A.202, 3PIT.A.180, 3PK7.A.406, 2PKE.B.300, 3PNL.B.1212, 2PNQ.A.502, 1POX.A.610, 1PPV.A.401, 4PQ9.A.301, 2PS2.A.401, 2PS5.B.701, 1PVF.A.401, 1PVG.A.903, 2PYW.A.501, 2PZ8.A.4001, 2PZA.A.6243, 2PZE.A.3, 2Q28.A.1001, 1Q3H.C.674, 3Q3J.B.201, 2Q3F.A.301, 3Q46.A.307, 4Q4C.A.404, 3Q5V.B.599, 3Q60.A.603, 1Q6L.A.5300, 1Q6R.A.7300, 2Q66.A.602, 1Q92.A.1003, 4QC2.A.302, 4QEA.A.301, 3QF7.A.854, 1QGU.B.3002, 1QGU.D.3006, 2QG8.A.201, 4QHZ.A.302, 2QIS.A.907, 2QIS.A.908, 2QIS.A.909, 2QJJ.C.1003, 1QK5.A.303, 3QKE.A.407, 3QKT.A.902, 2QME.A.179, 3QNM.A.400, 2QQ0.A.450, 1QRA.A.168, 2QRZ.A.190, 2QTY.A.348, 2QTO.A.1001, 3QUQ.A.225, 3QUT.A.225, 3QVQ.C.310, 3QXC.A.222, 3QXH.A.223, 3QXJ.A.224, 3QXS.A.223, 3QXX.A.224, 2QXO.A.161, 3QYY.A.505, 1R2Q.A.300, 3R3S.A.296, 2R6O.A.801, 3R7W.A.600, 2R8E.A.201, 2RAH.A.354, 2RAR.A.501, 2RAV.A.701, 3RAP.R.200, 2RB5.A.701, 2RBK.A.501, 3REF.A.192, 3REG.A.550, 1RKQ.A.1273, 1RKU.A.301, 1RKV.A.401, 4RKE.A.202, 4RKF.A.202, 3RLF.A.1501, 1RMT.A.1413, 3RO6.A.400, 4ROQ.A.401, 1RP7.A.890, 1RQI.A.603, 1RQI.A.604, 1RQJ.A.908, 1RQJ.A.909, 3RUS.A.544, 1RVK.A.999, 3RV3.A.1004, 3RWM.B.1, 3RYE.A.907, 3RYE.A.908, 3RYE.A.909, 1RYA.A.1001, 1RYH.A.539, 4S1H.B.303, 3S4J.A.907, 3S4J.A.908, 3S4J.A.909, 3S9Z.A.802, 1SAW.A.225, 3SAE.A.820, 3SDT.A.819, 3SDT.A.821, 3SFO.A.263, 3SHQ.A.321, 1SHT.X.219, 3SJN.A.374, 3SLS.A.401, 3SL2.A.701, 3SN1.A.408, 3SN4.A.408, 1SO4.A.2300, 3STP.A.391, 1SVM.A.750, 1SVS.A.356, 1TOP.A.901, 3T10.A.401, 3T1K.A.401, 3T2S.B.401, 3T2B.A.409, 3T2D.A.408, 3T2D.A.409, 3T7A.A.602, 1TC6.A.501, 2TCT.A.223, 3TCS.A.368, 3TDV.A.501, 1TE6.A.641, 3TEP.A.1, 3TGO.A.503, 3TJI.A.601, 3TKL.A.300, 1TMM.A.162, 4TMT.A.902, 4TMV.A.902, 4TMW.A.903, 4TMX.A.903, 3TMO.A.266, 4TN1.A.902, 3TSO.A.200, 1TW1.A.1, 3TWA.A.420, 3TYZ.A.281, 4TY0.A.502, 1U02.A.240, 4U5X.A.202, 4U82.A.301, 1U8Y.A.301, 4UAK.A.503, 4UAS.A.302, 4UAT.A.302, 4UAV.A.401, 2UAG.A.1001, 1UBK.L.1005, 3UCW.A.100, 3UCY.A.101, 4UCX.Q.1553, 4UE3.L.603, 3UIE.A.403, 3UJR.A.502, 3UJR.B.502, 3UJS.A.601, 3UJS.B.601, 1UMD.A.1401, 1UMG.A.403, 1UMG.A.404, 4UM7.A.175, 3UPY.A.446, 1UPT.A.1183, 3UPL.A.447, 3UQY.L.603, 4UQL.Q.1552, 4URH.Q.1552, 3UXK.A.360, 3UZR.A.300, 4V0L.A.601, 3V1V.A.501, 3V1X.A.501, 4V1T.A.1777, 3V2U.C.521, 2V26.A.1801, 3V3W.A.403, 1V54.A.3518, 2V7Q.A.1512, 2VBU.A.1134, 3VC1.A.301, 3VC1.I.301, 3VC2.J.301, 3VCC.A.401, 3VCC.A.402, 3VCN.A.501, 2VDO.B.2001, 2VDN.B.2001, 1VG8.A.1401, 2VG3.A.1297, 2VK1.A.601, 2VK8.A.1565, 1VOM.A.997, 3VVH.A.701, 3VX4.A.802, 1VZM.B.1046, 1VZM.B.1047, 1W2Y.A.1231, 1W2Y.A.1232, 3W40.A.201, 2W4J.A.1280, 2W5V.A.1378, 2W5X.A.1379, 3W6N.A.803, 3W6O.A.802, 1W78.A.1422, 1W78.A.1423, 1WA5.A.1178, 3WBH.A.501, 3WBZ.A.404, 1WC1.A.1501, 1WC1.A.1502, 3WEK.A.401, 2WEF.A.401, 1WF3.A.401, 4WH2.A.402, 4WH3.A.402, 2WIC.A.1266, 3WJP.A.403, 3WJP.A.404, 2WKQ.A.1724, 3WNZ.A.502, 3WRY.C.1202, 1WUH.L.1005, 1WUK.L.1005, 2WVG.A.601, 3WXM.A.502, 1WZC.A.300, 1X06.A.900, 1X07.A.900, 2X13.A.1418, 2X14.A.1418, 1X3S.A.200, 1X84.B.401, 2X98.A.1477, 1XBY.A.601, 2XB5.A.223, 2XCW.A.1498, 1XEX.A.1002, 1XFI.A.400, 1XG3.A.2101, 2XH2.B.1439, 2XH7.A.1441, 2XIS.A.392, 4XIA.A.399, 2XJB.A.1494, 2XJD.A.1497, 2XJE.A.1493, 2XSX.A.500, 2XTN.A.1232, 2XUU.A.1307, 1XX1.A.9001, 4XXP.A.301, 2Y6P.A.1234, 1Y8A.A.501, 1Y8Q.B.641, 2Y8E.A.1177, 1Y9D.D.2901, 2YCH.A.501, 1YHL.A.1402, 1YHL.A.1403, 1YMV.A.200, 1YNS.A.1258, 1YQ9.H.540, 1YQT.A.591, 1YQT.A.592, 1YRQ.H.553, 1YS7.A.1002, 1YU4.A.2002, 2YVO.A.1001, 2YVO.A.1002, 2YVP.A.184, 1YVD.A.850, 2YVM.A.1001, 2YXH.A.502, 1Y

YQ.B.702, 1YZL.A.401, 1YZN.A.301, 1YZT.A.700, 1Z06.A.203, 1Z07.A.300, 1Z08.A.130
0, 1Z08.C.3300, 1Z0J.A.400, 1Z2N.X.1295, 1Z2O.X.1295, 1Z4J.A.1001, 1Z4K.A.229, 1
Z4L.A.2001, 1Z4O.A.800, 1Z4P.X.1001, 1Z4Q.A.2001, 2Z4V.A.1501, 2Z4V.A.1502, 2Z4X
.A.1201, 2Z4X.A.1202, 1Z59.A.1001, 1Z5C.A.2001, 1Z5G.A.601, 1Z5G.D.604, 2Z52.A.1
301, 2Z52.A.1302, 2Z7I.A.1301, 1Z88.A.601, 1ZC3.A.500, 1ZED.A.905, 3ZFD.A.500, 3
ZIA.A.601, 1ZJJ.A.1001, 3ZMC.A.1296, 3ZO9.A.1592, 3ZOU.A.1295, 1ZPD.A.601, 1ZS9.
A.1257, 3ZVL.A.1524, 3ZX4.B.260, 3ZX5.A.260, 2IHM.A.700, 3A0U.A.201, 3A10.A.201,
2AKZ.A.441, 2BHW.A.601, 2BHW.A.603, 2BHW.A.604, 2BHW.A.609, 2BHW.A.612, 3BH7.A.
1, 4BX2.A.301, 4C5A.A.331, 3C9U.A.309, 1D0X.A.998, 1D0Y.A.998, 1D0Z.A.998, 1D1A.
A.998, 1D1B.A.998, 2DCN.B.4006, 4DL8.A.304, 3DNT.B.442, 1DXR.L.400, 1E79.A.601,
2E8X.A.1301, 3EF0.A.1, 2FKW.A.1501, 2FKW.B.1601, 3G5A.C.307, 2G77.B.503, 1GFI.A.
356, 2GJ8.A.602, 3GL9.A.123, 2GLQ.A.2003, 4GVE.A.602, 3H1E.A.202, 1HE1.C.202, 2H
EG.A.300, 2HF7.A.700, 2I7D.A.728, 3ICM.A.403, 3ICN.A.402, 4IDO.A.503, 4IF4.A.300
, 1IV2.B.1572, 2J7N.A.3374, 2J8C.L.1288, 1J97.A.220, 4JA2.A.201, 1JB0.A.1107, 1J
B0.A.1110, 1JB0.A.1112, 1JB0.A.1118, 1JB0.A.1130, 1JB0.A.1134, 1JB0.B.1204, 1JB0
.B.1231, 1JB0.B.1235, 1JB0.X.1701, 4KEM.A.401, 1L3R.E.392, 1L5Y.A.701, 1L7N.A.22
1, 4LCZ.A.307, 4LCZ.C.316, 4LE0.A.201, 1LGH.A.57, 1LGH.A.58, 1LGH.B.59, 4LRS.A.4
04, 1LVK.A.998, 1MPS.M.801, 1MX0.E.501, 3N5K.A.2001, 1N6K.A.201, 1NVV.Q.1002, 4N
V0.A.402, 2ODE.A.3001, 2OGX.A.291, 3OLV.A.130, 4OVN.A.201, 2OZE.A.299, 3PDE.B.31
0, 3PL9.A.602, 3PL9.A.603, 3PL9.A.609, 3PL9.A.610, 3PL9.A.612, 3PL9.A.613, 3PL9.
A.614, 2PL1.A.204, 2PLS.I.606, 3QHW.A.298, 1R0X.D.14, 2R25.B.1, 1RLO.A.801, 4RUR
.W.301, 1RWT.A.614, 4S1H.A.303, 1SVK.A.356, 3T2S.A.401, 3T6D.L.401, 3T6D.M.400,
3T6E.L.400, 1T91.A.1301, 3T9E.A.602, 3TCS.B.368, 3THU.A.500, 1TX4.B.681, 1UPB.A.
601, 2VB6.A.1000, 3W6P.A.803, 1W7J.A.1793, 1W9I.A.1755, 3W9S.A.202, 2WF7.A.1220,
2WJN.M.1325, 1WQA.B.456, 2WZB.A.1417, 2X2E.A.1746, 1XBX.A.601, 1XHF.A.1001, 1Y9
D.A.2601, 1YZQ.A.901, 1Z2P.X.1295, 2Z4Z.A.1301, 2Z7I.B.1302, 1ZES.A.302, 1ZH4.B.
202, 1ZXN.A.900, 2ZXE.A.2002, 4G3I.B.401, 4K4H.M.604, 4LQ0.A.401, 4AG4.A.5001, 3
BYC.A.901, 2CD0.B.1140, 1D7X.B.805, 3DPE.A.997, 1DYK.A.4001, 3EKI.A.602, 4FVL.B.
506, 2GNT.A.254, 4GN7.B.301, 4ILW.D.304, 1INW.A.501, 2JG9.B.1224, 1L6J.A.502, 2N
W6.A.613, 1QI5.A.452, 2RJQ.A.6, 1RU4.A.2, 1T6B.X.800, 4U32.A.301, 1ULV.A.2005, 1
UYX.B.1135, 3V03.B.585, 3VV3.A.404, 3WNK.A.812, 1ZTQ.A.561, 3DPG.B.501, 4ECQ.A.5
01, 4K4H.A.605, 1N3F.C.498, 2OAA.A.601, 2ODI.A.701, 3AFG.A.540, 2AFH.D.2490, 3AH
W.A.122, 3AI7.B.901, 3AKB.A.2, 4AWN.A.300, 4AXN.A.1329, 3AYU.A.417, 3B7E.A.1005,
3BCD.A.707, 2BL0.B.1146, 4BNR.A.600, 2BQ4.A.1119, 2C10.A.1771, 3C9I.A.1, 3CKZ.A
.1, 4CPL.A.500, 4CUA.A.2644, 1CVL.A.320, 3D3I.A.1001, 3D7K.A.571, 4E5U.B.302, 3E
DY.A.1, 2EEK.A.401, 3F5V.A.223, 2FCW.B.3001, 2FCW.B.3002, 2FGZ.A.1192, 2FHF.A.24
04, 3FVQ.A.360, 1G9K.A.706, 1GA6.A.374, 4GDI.A.509, 2GKO.A.612, 1GTT.A.1430, 2H0
B.A.1000, 4H1Q.A.305, 3H81.A.279, 3H81.C.280, 1HFC.A.277, 4HJF.A.601, 4HS9.A.401
, 1HV5.A.5503, 4HZW.A.507, 1I76.A.997, 2I8U.A.202, 2I8T.A.402, 3I9G.L.301, 3I9G.
L.302, 4IHM.A.404, 2IXT.A.1310, 1J8E.A.201, 2JKP.A.1728, 4JZB.A.401, 4JZB.A.403,
4JZX.A.404, 4JZX.A.405, 4K1K.A.501, 4K70.A.1002, 1KAP.P.616, 1KAP.P.619, 4L74.A
.401, 1LQV.C.35, 4LVN.A.703, 4M5I.A.201, 3M6L.A.801, 3MA2.A.293, 3MA2.A.296, 4ME
W.A.502, 1MNC.A.283, 3MOS.A.1, 4N2P.A.201, 4N4E.E.405, 1NPC.A.320, 1OAC.A.802, 4
OCI.A.201, 4OKH.B.904, 3OOY.A.621, 1OU9.A.131, 3OYR.B.337, 3OYR.B.338, 3P2P.B.12
6, 2PHI.B.125, 1Q3A.A.466, 4Q8K.A.501, 1QCN.A.1001, 4QN6.A.501, 3QRB.A.301, 2QUB
.A.615, 2R5N.A.2000, 2R8O.A.670, 2R8Z.A.201, 2R8P.A.670, 1RM8.A.502, 3RRX.A.901,
3RUP.A.1004, 3RVW.A.223, 3SAL.A.601, 1SIO.A.601, 1T1E.A.700, 3TI4.A.601, 3U8I.A
.201, 3UPT.A.691, 1UR4.A.1398, 1UX6.B.2012, 1UX6.B.2016, 3V5U.A.705, 3V96.B.303,
2VOV.A.1338, 3VV3.B.404, 1W7C.A.802, 3W7T.A.1001, 1WAD.A.116, 4WIW.A.702, 2WNV.
B.1225, 2WOY.A.2414, 1Y93.A.266, 1Y93.A.268, 2YN3.A.6362, 2YN5.A.6362, 2YN5.A.63
63, 1YO8.A.1191, 2YOA.A.1003, 1YS1.X.400, 2Z2X.A.1004, 2Z2X.A.1005, 2Z30.A.1006,
2Z8X.A.626, 2ZWO.B.400, 19HC.A.301, 19HC.A.302, 19HC.A.303, 19HC.A.304, 19HC.A.
305, 19HC.A.306, 19HC.A.307, 19HC.A.308, 19HC.A.309, 3A0G.A.201, 3A15.B.354, 3A1
6.B.354, 3A17.A.354, 2A1O.A.417, 1A2S.A.90, 2A3F.X.201, 2A3M.A.501, 2A3M.A.502,

2A3M.A.503, 2A3M.A.504, 1A4E.A.503, 1A56.A.82, 3A51.B.412, 3A8G.A.301, 3A8L.A.300, 3A9F.A.207, 2A9E.A.550, 4AAL.A.423, 4AAN.A.400, 4AAN.A.401, 4AAO.A.400, 2AIU.A.200, 2AI5.A.81, 1AKK.A.105, 4AM5.A.1160, 1AOF.A.602, 1AOF.B.601, 1AOM.A.602, 1AOM.B.601, 1AOM.B.602, 4APY.A.1418, 1AQA.A.95, 1AQE.A.121, 1AQE.A.122, 3AQ5.A.144, 3AQ9.A.144, 1ASH.A.301, 3AT5.A.142, 3AT5.B.147, 2AT3.X.185, 4AUM.A.900, 2AVK.A.201, 3AWM.A.501, 1AWP.A.201, 3AYF.A.801, 2B0Z.B.109, 3B0H.B.601, 2B10.D.909, 2B11.D.1301, 2B2R.A.1500, 4B2N.A.700, 4B2N.A.701, 2B4Z.A.500, 3B42.A.199, 3B42.B.399, 3B47.A.199, 4B4Y.A.1155, 3B6H.B.600, 1B80.A.350, 1B82.A.350, 1B85.A.350, 4B8N.A.201, 3B99.A.600, 2BC5.A.150, 3BDZ.A.450, 2BDM.A.500, 1BFR.B.200, 2BGV.X.1121, 2BH4.X.1123, 1BIN.B.144, 1BJ9.A.296, 1BJE.A.154, 4BJA.A.300, 4BJK.A.1450, 3BK9.B.401, 2BK9.A.1154, 2BLF.B.1582, 4BMM.A.1450, 2BMM.A.1157, 3BNG.A.513, 3BNJ.A.513, 3BNJ.A.514, 3BNJ.A.515, 3BNJ.A.516, 3BNJ.A.517, 3BOM.A.143, 3BOM.B.148, 2BPN.A.108, 2BPN.A.109, 2BPN.A.110, 2BPN.A.111, 2BQ4.A.1115, 2BQ4.A.1116, 2BQ4.A.1117, 2BQ4.A.1118, 2BS2.C.1255, 2BS2.C.1256, 2BS3.C.1255, 2BS4.C.1255, 1BVY.A.1000, 3BXU.A.72, 3BXU.A.73, 3BXU.A.74, 4C0C.A.1450, 4C0N.A.1157, 2C1U.A.401, 2C1V.A.401, 2C1V.A.402, 2C1D.A.1291, 2C1D.A.1292, 2C1D.B.1158, 1C2N.A.117, 3C2C.A.113, 1C2R.A.120, 4C27.A.1450, 4C28.A.1450, 1C40.A.150, 4C44.A.1151, 1C52.A.200, 3C62.A.150, 3C63.A.150, 1C6R.A.90, 1C75.A.93, 3C76.X.185, 3C78.X.185, 2C8S.A.1173, 4C9M.A.1418, 3CAO.A.104, 3CAO.A.105, 3CAO.A.106, 3CAO.A.107, 1CC5.A.1, 1CCH.A.83, 1CCR.A.112, 2CDV.A.109, 2CDV.A.111, 4CDP.A.402, 1CED.A.90, 2CE0.A.1102, 1CH1.A.154, 1CH2.A.154, 1CH3.A.154, 1CH5.A.154, 1CH7.A.154, 1CH9.A.154, 1CI3.M.254, 2CJ2.A.1300, 4CK8.A.1480, 4CK9.A.1480, 4CKA.A.1480, 1CLS.A.142, 1CNO.A.200, 2CN4.A.1175, 2CN4.B.1175, 1CO6.A.108, 1COR.A.83, 1COT.A.130, 4COH.A.1450, 4COO.A.1549, 3CP5.A.202, 1CPT.A.430, 3CQV.A.601, 3CSL.A.866, 2CTH.A.109, 2CTH.A.110, 2CTH.A.111, 2CTH.A.112, 3CU4.A.199, 2CVC.A.1001, 2CVC.A.1002, 2CVC.A.1003, 2CVC.A.1004, 2CVC.A.1005, 2CVC.A.1006, 2CVC.A.1007, 2CVC.A.1008, 2CVC.A.1009, 2CVC.A.1010, 2CVC.A.1011, 2CVC.A.1012, 2CVC.A.1013, 2CVC.A.1014, 2CVC.A.1016, 4CVJ.A.1295, 3CX5.C.4001, 3CX5.C.4002, 3CX5.D.4003, 3CX5.W.4026, 1CXA.A.126, 1CXC.A.125, 3CXH.W.4026, 1CXY.A.204, 1CYI.A.200, 3CYR.A.203, 3CYR.A.204, 2CY3.A.119, 2CY3.A.120, 2CY3.A.121, 2CY3.A.122, 2CYP.A.295, 5CYT.R.105, 2CZS.A.500, 2CZS.A.501, 1CZJ.A.119, 1CZJ.A.120, 1CZJ.A.121, 1CZJ.A.122, 2CZ1.A.300, 3CZY.A.300, 2D0W.A.200, 4D02.A.602, 4D02.A.603, 2D0S.A.80, 2D0T.A.404, 2D2M.D.200, 4D30.B.750, 4D34.A.500, 4D35.A.500, 4D36.A.500, 4D37.A.500, 4D38.A.500, 4D3A.A.500, 1D4D.A.601, 1D4D.A.602, 1D4D.A.603, 1D4D.A.604, 1D7B.A.401, 3D7O.A.143, 1DCC.A.296, 2DC3.A.191, 1DD7.A.600, 3DE8.A.150, 2DGE.A.200, 3DHH.A.501, 3DHI.A.600, 3DHR.A.142, 1DJ5.A.1, 1DK0.A.200, 1DLW.A.144, 1DM1.A.148, 3DMI.A.146, 2DN1.A.142, 2DN1.B.147, 1DP8.A.719, 1DP9.A.719, 3DR0.A.94, 1DTI.A.154, 4DTW.A.500, 4DTZ.A.500, 1DUW.A.293, 1DUW.A.297, 1DUW.A.300, 1DUW.A.301, 1DW0.A.113, 1DW2.A.113, 1DWL.B.80, 4DXY.A.501, 1DY7.B.601, 2DY5.A.300, 4DY9.A.201, 1E08.E.80, 1E29.A.136, 1E2R.B.601, 1E2W.A.900, 3E2O.A.296, 1E39.A.801, 1E39.A.802, 1E39.A.803, 1E39.A.804, 2E3A.A.401, 2E3B.A.401, 3E5J.A.1408, 3E5K.A.1408, 1E8E.A.125, 2E80.A.1508, 2E84.A.1301, 2E84.A.1302, 2E84.A.1303, 2E84.A.1304, 2E84.A.1305, 2E84.A.1306, 2E84.A.1307, 2E84.A.1308, 2E84.A.1309, 2E84.A.1310, 2E84.A.1311, 2E84.A.1312, 2E84.A.1313, 2E84.A.1314, 2E84.A.1316, 1EB7.A.401, 1EGY.A.410, 3EGW.C.806, 3EGW.C.807, 3EH5.A.800, 3EHB.A.559, 3EHB.A.560, 1EHE.A.501, 1EHJ.A.1030, 1EHJ.A.1053, 1EHJ.A.1066, 4EID.A.101, 4EIE.A.101, 4EIF.A.101, 3EJ6.A.4000, 4EJI.A.501, 2EKT.A.154, 2EKU.A.154, 4ENH.A.601, 4ENU.B.801, 4EP6.A.601, 1ETP.A.199, 1ETP.A.200, 1EUE.A.201, 2EU7.X.201, 2EWK.A.1001, 2EWK.A.1003, 2EWU.A.1001, 2EWU.A.1003, 1EWH.A.253, 2EWI.A.1004, 2EWI.A.1002, 2EWI.A.1001, 2EWI.A.1003, 2EXV.A.83, 1EZV.C.401, 1EZV.C.402, 1F03.A.201, 1F1C.A.200, 1F1F.A.200, 1F24.A.501, 1F4U.A.410, 4F4O.B.201, 4F6I.A.201, 4FA7.A.602, 4FA7.A.603, 2FAM.A.148, 4FAS.A.601, 4FAS.A.602, 4FAS.A.603, 4FAS.A.604, 4FAS.A.605, 4FAS.A.606, 4FAS.A.607, 2FBZ.X.901, 1FCD.C.901, 4FDH.A.601, 4FEF.A.403, 2FFN.A.1003, 1FGJ.A.548, 1FGJ.A.552, 1FGJ.A.553, 1FI3.A.83, 1FI7.A.110, 4FIA.A.600, 1FJ0.A.115, 3FLL.A.185, 2FMY.A.300, 3FO3.A.1004, 3FO3.A.1005, 3FO3.A.1006, 3FO3.A.100

7, 3FO3.A.1008, 3FO3.A.1002, 3FO3.A.1003, 3FO3.A.1001, 1FOP.A.500, 3FOO.A.150, 2FRF.A.154, 1FS7.A.509, 1FS7.A.510, 1FS8.A.508, 1FT5.A.213, 1FT5.A.215, 1FT5.A.216, 1FT9.A.300, 1FT9.B.300, 2FWT.A.803, 2FWT.A.805, 2FWL.A.132, 2FYU.D.242, 1G09.B.147, 4G1V.A.401, 4G3J.A.501, 3G5N.A.500, 4G70.A.601, 4G71.A.602, 4G7G.A.501, 4G7L.A.301, 4G7Q.A.602, 4G7S.A.602, 3GAS.D.1294, 2GC4.D.200, 1GDV.A.101, 2GEP.A.580, 3GEO.A.580, 4GED.B.201, 7GEP.A.580, 1GKS.A.0, 2GNV.A.166, 4GP8.A.602, 1GQ1.A.601, 1GQ1.A.602, 3GQP.C.143, 4GQS.A.501, 2GSM.A.2001, 2GSM.A.2002, 2GTF.X.201, 1GWF.A.504, 3GW9.A.480, 1GWS.A.601, 1GWS.A.603, 1GWS.A.605, 1GWS.A.606, 1GWS.A.609, 1GWS.A.610, 1GWS.A.611, 1GWS.A.612, 1GWS.A.613, 1GWS.A.616, 1GWT.A.350, 1GYO.A.111, 1GYO.A.112, 1GYO.A.113, 1GYO.A.114, 4GYD.A.200, 2H0V.B.500, 4H0K.B.200, 1H10.A.1184, 1H10.A.1185, 1H21.A.1248, 1H21.A.1249, 1H21.B.1248, 1H21.B.1249, 1H29.A.1102, 1H29.A.1104, 1H29.A.1107, 1H29.A.1108, 1H29.A.1114, 4H2L.B.201, 1H32.A.1263, 1H32.A.1264, 1H32.B.1139, 3H33.A.75, 3H33.A.76, 3H33.A.77, 3H34.A.72, 3H34.A.73, 3H34.A.74, 3H4N.A.72, 3H4N.A.73, 3H4N.A.74, 4H44.A.301, 4H44.A.302, 1H55.A.350, 1H57.A.350, 4H6O.A.501, 3H8T.A.301, 2H88.C.142, 2HBU.A.900, 3HB3.A.559, 3HB3.A.560, 1HBI.A.153, 1HBZ.A.504, 2HBD.A.142, 4HB6.A.72, 4HB6.A.73, 4HB8.A.72, 4HB8.A.73, 4HBF.A.72, 1HCZ.A.253, 4HC3.A.72, 3HDL.A.305, 3HF4.A.142, 3HF4.B.147, 1HGB.D.147, 4HHR.A.701, 4HHS.A.701, 1HJ3.B.602, 1HJ5.A.601, 1HJ5.B.602, 2HJI.A.180, 1HLB.A.158, 3HNJ.A.150, 3HNK.A.150, 4HPA.A.201, 4HPB.A.201, 4HPD.A.201, 3HQ6.A.400, 3HQ7.A.401, 3HQ9.A.400, 3HQ9.A.401, 1HRC.A.105, 1HRO.A.107, 4HR0.A.402, 3HSP.A.750, 4HSW.A.201, 2HUO.A.302, 3HYU.A.142, 3HYU.B.147, 1I3E.A.147, 2I5N.C.404, 1I5U.A.201, 3I63.A.501, 3I63.A.502, 1I77.A.108, 1I77.A.109, 1I77.A.110, 1I77.A.111, 4I7Z.A.301, 4I7Z.A.302, 4I7Z.C.301, 3I8R.A.901, 1I8O.A.115, 1I8P.A.115, 2I8F.A.83, 3I9T.A.300, 3I9U.A.300, 2IBN.A.703, 1IB7.A.95, 1ICC.A.201, 1IDR.A.144, 2IJ3.B.999, 2IJ4.A.471, 1IKE.A.185, 1IO7.A.1401, 4IPS.A.401, 1IQC.A.401, 1IQC.A.402, 3IQ5.A.150, 1IT1.A.201, 1IT1.A.202, 1IT1.A.203, 1IT1.A.204, 2IUW.A.500, 2IUF.A.1691, 1IVJ.A.300, 2IVF.C.1217, 1IW0.A.901, 1IYN.A.296, 1J02.A.300, 1J0P.A.1002, 1J0Q.A.201, 2J1M.A.1456, 4J14.A.601, 4J20.A.107, 1J3S.A.105, 2J5M.A.1300, 1J77.A.300, 2J7A.A.1002, 2J7A.A.1003, 2J7A.A.1004, 2J7A.A.1005, 2J7A.C.1002, 2J7A.C.1003, 2J7A.C.1004, 1JDL.A.500, 1JDR.A.296, 4JE9.A.201, 4JEB.A.201, 1JEX.A.95, 1JIP.A.410, 4JJ0.A.501, 4JJ0.A.502, 2JJN.A.412, 2JJP.A.412, 1JMX.A.1001, 1JMX.A.1002, 1JNI.A.125, 1JNI.A.126, 2JTI.B.104, 3K1O.A.488, 2K3V.A.218, 2K3V.A.238, 2K3V.A.261, 2K3V.A.278, 3K3O.A.1, 4K8F.A.300, 4K8F.B.300, 1KB0.A.802, 1KBI.A.760, 4KF2.B.501, 4KIB.A.403, 4KIC.A.403, 4KIG.A.502, 4KJT.A.201, 2KMY.A.233, 2KMY.A.251, 4KMG.A.101, 1KOK.A.296, 1KQG.C.809, 1KQG.C.810, 2KSC.A.125, 2KSU.A.282, 2KSU.A.305, 1KV9.A.901, 4KVK.A.701, 4KVL.A.701, 4L0F.A.501, 3L1M.A.150, 3L1T.A.479, 1L2K.A.154, 3L4D.A.481, 3L61.A.420, 1LA6.B.147, 3LD6.A.601, 2LDO.A.130, 2LDO.A.154, 2LDO.A.168, 1LFK.A.430, 3LGN.A.200, 4LJI.A.301, 1LM3.B.200, 1LMS.A.118, 4LM8.A.801, 4LM8.A.802, 4LM8.A.803, 4LM8.A.804, 4LM8.A.805, 4LM8.A.806, 4LM8.A.807, 4LM8.A.808, 4LM8.A.809, 4LM8.A.810, 4LMH.A.801, 4LMH.A.802, 4LMH.A.803, 4LMH.A.804, 4LMH.A.805, 4LMH.A.806, 4LMH.A.807, 4LMH.A.808, 4LMH.A.809, 4LMH.A.810, 1LQX.A.201, 1LR6.A.201, 1LS9.A.92, 1LSX.A.719, 4LXJ.A.601, 2LZZ.A.101, 2LZZ.A.102, 3M15.A.150, 1M1P.A.802, 1M1Q.A.804, 1M1Q.A.803, 1M1R.A.801, 1M2I.A.201, 4M2F.B.401, 3M3A.A.155, 3M4C.A.150, 4M4A.A.201, 4M4A.B.201, 1M59.A.201, 1M70.A.199, 1M70.A.200, 1M7S.A.600, 4M71.A.403, 4M72.A.403, 4M73.A.403, 4MBA.A.148, 5MBA.A.148, 3MDM.A.505, 1MDV.A.110, 1MDV.A.112, 3MDR.A.505, 4ME4.A.401, 3MGX.A.397, 1MJ4.A.502, 3MKB.B.137, 3ML1.B.1128, 3ML1.B.1129, 1ML7.A.185, 1MLW.A.403, 4MLM.A.201, 4MLN.A.201, 4MLN.B.201, 4MLN.B.202, 3MM9.A.580, 1MNI.B.154, 1MNY.A.95, 3MOM.A.186, 4MPM.A.201, 1MQF.A.501, 4MQJ.B.201, 3MUS.A.201, 3MVC.A.500, 3MVF.A.185, 1MWB.A.125, 1MXR.A.1004, 1MZ4.A.151, 3N3N.B.1500, 1N45.A.300, 4N4J.A.609, 4N4J.A.610, 4N4J.A.611, 4N4J.A.612, 4N4J.A.613, 4N4J.A.614, 4N4J.A.615, 4N4J.A.616, 4N4K.A.610, 4N4L.A.616, 4N4N.A.601, 4N4N.A.602, 4N4N.A.603, 4N4N.A.604, 4N4N.A.605, 4N4N.A.606, 4N4N.A.607, 4N4N.A.608, 4N4O.A.608, 4N4Y.A.602, 4N6W.A.202, 4N8T.B.201, 3N8Y.A.601, 1N9C.A.93, 3N9Q.A.1, 1NAZ.A.200, 3NA1.A.601, 4NFG.B.201, 1NIR.A.6

01, 1NIR.A.602, 4NK1.B.201, 3NMI.A.150, 1NML.A.401, 1NML.A.402, 3NN1.A.239, 1NNO.A.602, 2NNB.A.472, 1NOS.A.901, 1NPF.A.154, 4NP1.A.185, 2NRL.A.148, 1NS6.A.142, 2NSR.A.154, 1NS9.A.142, 1NS9.B.147, 3NWV.A.105, 3NXU.A.508, 3NYH.A.605, 4NZ2.A.501, 3O1A.A.385, 4O1W.A.101, 4O4Z.A.201, 3O5C.A.401, 3O5C.D.504, 4O6J.A.302, 4O6Q.A.202, 4O6U.A.203, 3O72.A.500, 4O7G.A.301, 4O7G.A.302, 3O89.A.2154, 1OAF.A.1251, 1OAH.A.1520, 1OAH.A.1521, 1OAH.A.1522, 1OAH.A.1523, 1OAH.A.1524, 3OA8.A.401, 3OA8.B.401, 1OFW.A.1294, 1OFW.A.1295, 1OFW.A.1296, 1OFW.A.1297, 1OFW.A.1298, 1OFW.A.1299, 1OFW.A.1300, 1OFW.A.1301, 1OFW.A.1302, 3OFT.A.417, 3OJT.D.500, 3OMA.A.1, 3OMI.A.607, 3OO4.A.142, 3OO4.B.147, 1OQU.C.1008, 2ORT.A.600, 3ORV.B.600, 2ORO.A.600, 2ORP.A.600, 2ORQ.A.600, 2ORR.A.600, 2ORS.A.600, 2OT4.A.1004, 2OT4.A.1007, 3OUE.A.609, 3OUE.A.610, 3OUE.A.611, 3OUE.A.612, 3OUI.A.1, 3OUQ.A.601, 3OUQ.A.602, 3OUQ.A.603, 3OUQ.A.604, 3OUQ.A.605, 3OUQ.A.606, 2OZY.A.201, 2OZY.A.202, 2OZY.A.203, 2OZY.A.204, 2OZY.A.205, 3OZU.A.404, 3OZV.A.404, 3OZW.A.404, 1P2E.A.801, 1P2E.A.803, 1P2E.A.804, 1P2H.A.803, 1P2H.A.804, 1P2Y.A.430, 3P3L.A.501, 1PA2.A.306, 3PAH.A.425, 2PAC.A.83, 4PAH.A.425, 5PAH.A.425, 1PBY.A.991, 1PBY.A.992, 3PC3.A.701, 3PER.A.1002, 2PEG.A.200, 2PEG.B.400, 3PF7.A.1001, 3PF7.A.1002, 3PH2.B.1087, 4PH9.A.602, 3PI2.B.500, 4PK5.A.501, 1PL3.A.401, 1PM1.X.180, 1PP9.C.501, 1PP9.C.502, 1PP9.D.501, 2PQ7.A.220, 3PT7.B.500, 3PUR.A.1, 3PXW.A.500, 4PXH.A.501, 1Q16.C.806, 1Q16.C.807, 3Q3N.A.509, 1Q5D.A.440, 2Q8Q.A.300, 3Q99.B.750, 2QDY.A.300, 1QDB.A.516, 1QDB.A.517, 1QDB.A.518, 1QDB.A.519, 1QHU.A.500, 2QJY.A.501, 2QJY.A.502, 2QJY.B.301, 1QKS.A.601, 1QKS.A.602, 1QNO.A.113, 1QNO.A.114, 1QNO.A.115, 1QNO.A.116, 1QN2.B.101, 3QNS.A.351, 1QO8.A.601, 1QO8.A.602, 1QO8.A.603, 1QO8.A.604, 1QPA.A.350, 1QPU.A.107, 1QQ3.A.107, 3QQQ.A.163, 3QQR.A.163, 2QSP.A.142, 3QU8.A.500, 2QUO.A.142, 3QWO.A.150, 3QY7.A.264, 1QYZ.A.200, 1R0Q.A.200, 4R20.A.601, 2R50.A.166, 2R6S.A.501, 2R80.A.150, 2R80.B.150, 3R9C.A.450, 2RAO.B.147, 2RAO.C.142, 2RDZ.A.2, 2RDZ.A.3, 2RDZ.A.4, 2RDZ.A.5, 2RF7.A.2, 3RGS.A.1, 3RI7.A.494, 3RIV.A.305, 3RJ6.A.154, 4RKM.A.808, 4RKM.B.808, 4RKM.D.807, 4RKN.A.902, 4RKN.A.903, 4RKN.A.905, 4RKN.A.906, 4RKN.A.907, 4RKN.A.908, 4RKN.A.909, 3RMK.A.494, 3RMZ.A.500, 1RSE.A.154, 3RUK.B.600, 1RWJ.A.90, 1RWJ.A.91, 1RWJ.A.92, 1RZ5.A.401, 3S1J.A.140, 1S73.A.296, 3S8F.A.800, 3S8F.A.801, 3S8G.A.800, 3SDN.A.160, 1SE6.A.430, 3SEL.X.73, 3SEL.X.74, 1SH4.A.201, 3SJ0.X.73, 3SJ0.X.74, 3SJ1.X.73, 3SJ1.X.74, 3SJ4.X.73, 3SJ4.X.74, 3SJ5.A.500, 3SJL.A.500, 3SJL.A.600, 3SLE.A.402, 1SMI.A.472, 1SOX.A.502, 1SP3.A.801, 1SP3.A.803, 1SP3.A.804, 1SP3.A.805, 1SP3.A.806, 1SP3.A.807, 1SP3.A.808, 1SQ3.D.912, 1STQ.A.600, 1SUO.A.500, 3SWZ.B.600, 3SXQ.A.1005, 3SXQ.A.1006, 3SXQ.A.1007, 3SXQ.A.1008, 3SXQ.A.1002, 3SXQ.A.1003, 3SXQ.A.1001, 1SY2.A.185, 3T3R.A.500, 3T3Z.A.500, 1T68.X.201, 3T6D.C.401, 3T6D.C.403, 3T6D.C.404, 3T6E.C.401, 3T6E.C.402, 3T6E.C.403, 3TDA.A.800, 3TFO.A.500, 3TGU.C.501, 3TGU.C.502, 3TGU.D.501, 3TGA.A.185, 3TGM.A.300, 1TH2.D.2003, 3TIK.A.482, 3TJS.A.508, 1TKW.B.253, 3TK3.A.500, 3TMC.A.309, 3TMC.A.310, 3TOR.A.3, 4TOB.C.201, 3TOL.A.150, 1TQN.A.508, 4TT5.A.401, 1TU2.B.255, 4TUV.A.401, 1U13.A.460, 1U4H.A.500, 1U7R.A.154, 3U8P.A.347, 1U9M.A.90, 4U9D.A.201, 1U9U.A.90, 3U99.A.500, 3U99.A.700, 4UAX.A.501, 3UBR.A.471, 3UBR.A.472, 3UBR.A.473, 3UBR.A.474, 3UBR.A.475, 3UBC.A.201, 3UCP.A.901, 3UCP.A.902, 3UCP.A.903, 3UCP.A.904, 3UCP.A.905, 3UCP.A.906, 3UCP.A.907, 3UCP.A.908, 3UCP.A.909, 3UCP.A.910, 3UCP.A.911, 1UED.A.1430, 1ULI.B.700, 3UOI.B.200, 1UP9.A.201, 1UP9.A.202, 1UP9.A.203, 1UP9.A.204, 4UQH.A.1450, 1URV.A.1172, 2UUQ.A.1405, 4UVR.A.1450, 1V0H.X.251, 2V07.A.1102, 2V08.A.1087, 2V0M.A.1499, 3V2V.A.154, 4V2K.A.601, 4V3V.A.750, 4V3W.A.750, 4V3X.A.750, 4V3Z.B.750, 1V54.A.515, 3V5X.A.201, 1V75.B.201, 2V7I.A.1362, 2V7K.A.1360, 2V7L.A.1360, 3V8D.A.601, 1V8X.A.901, 1V9Y.A.1140, 3VAU.A.201, 1VB6.A.1140, 1VB6.B.1140, 2VEB.A.200, 1VGI.A.300, 3VHB.A.150, 4VHB.A.150, 2VHB.B.150, 2VHD.A.402, 2VHD.B.401, 3VKP.A.601, 3VKS.A.601, 3VM9.A.154, 3VP5.A.201, 3VR8.C.201, 3VRD.A.201, 3VRD.A.202, 3VRG.A.201, 3VRG.B.201, 3VTH.A.807, 3VXJ.A.501, 2VXH.A.1001, 1VYD.A.1117, 2VYW.A.149, 2VZW.B.1209, 1W0G.A.1501, 2W0B.A.470, 1W2L.A.1100, 2W31.A.200, 2W3G.A.500, 1W4W.A.1307, 1W7O.A.1119, 1W7O.A.1120, 1W7O.A.1121, 1W7O.A.1122, 3W9C.A.501, 1WAD.A.117, 1WAD.A.113,

1WAD.A.114, 1WAD.A.115, 3WAH.A.201, 3WC8.A.201, 3WCT.A.200, 3WCT.B.201, 3WCT.C.
200, 3WCT.D.201, 2WDQ.C.1129, 1WE1.A.300, 3WFC.B.802, 3WFD.B.801, 3WFD.C.201, 3W
FE.B.802, 3WFX.A.201, 2WJM.C.1334, 2WJN.C.1333, 2WJN.C.1335, 2WJN.C.1336, 1WMU.A
.201, 2WM5.A.450, 4WNV.A.601, 1WOV.A.300, 1WOX.A.300, 4WPD.A.402, 4WQ8.A.1002, 4
WQ9.A.1001, 4WQ9.A.1002, 4WQC.A.1002, 4WQD.A.1002, 2WTG.A.180, 3WU2.F.101, 2WU2.
C.1130, 2WU5.C.305, 1WVE.C.699, 1WVP.A.154, 4WWJ.B.301, 2WX2.A.1450, 3X15.A.200,
3X15.J.200, 2X2N.A.1479, 1X3X.B.202, 1X46.A.151, 4X8B.A.508, 2XC3.A.1433, 4XDI.
A.201, 2XFH.A.1412, 1XK1.A.300, 2XKR.A.1400, 2XKI.A.1110, 1XQ5.B.148, 1XQ5.C.143
, 2XSJ.B.503, 2XTS.B.500, 1XU5.A.1174, 1XVB.A.1171, 1XVG.A.528, 1XVX.A.313, 2XYK
.A.700, 1Y0P.A.801, 1Y0P.A.802, 1Y0P.A.803, 1Y0P.A.804, 1Y5I.C.806, 1Y5I.C.807,
1Y5L.C.806, 2Y5N.A.450, 2YEV.A.1015, 2YEV.B.587, 2YIU.A.500, 2YIU.A.501, 2YIU.B.
500, 1YIQ.A.901, 2YK3.A.200, 2YL7.A.128, 1YWD.A.185, 2YXC.A.1001, 2YYW.A.1001, 2
YYW.A.1003, 2YYX.A.1004, 2YYX.A.1001, 1YYG.A.396, 1Z1N.X.603, 1Z1N.X.604, 1Z1N.X
.605, 1Z1N.X.606, 1Z1N.X.607, 1Z1N.X.608, 1Z1N.X.610, 1Z1N.X.612, 1Z1N.X.613, 1Z
1N.X.614, 1Z1N.X.616, 2Z47.A.1004, 2Z47.B.3003, 2Z6S.A.201, 2Z6T.A.201, 1Z8O.A.4
10, 1Z8U.B.201, 1Z9N.A.1001, 1Z9N.C.2001, 3ZBY.A.1402, 2ZBO.A.111, 2ZCF.A.206, 3
ZE6.B.502, 3ZG2.A.1480, 3ZG3.A.490, 3ZH0.A.200, 3ZHW.A.1163, 3ZIY.A.600, 3ZJO.A.
200, 3ZJQ.A.200, 1ZOY.C.1305, 3ZOO.A.105, 3ZOX.A.1082, 2ZPB.A.300, 2ZS0.A.200, 2
ZS0.B.200, 2ZS0.C.200, 2ZS0.D.200, 2ZXY.A.200, 2ZZS.1.220, 1ZZH.A.802, 1ZZH.A.80
3, 3A15.A.354, 3A51.C.412, 2AA1.B.400, 3ABB.A.1430, 4AJ9.A.1715, 2AKJ.A.564, 3AR
J.A.153, 3AT6.A.142, 2AUO.A.153, 2AUQ.A.147, 2AV0.A.147, 4AVD.A.144, 3AYF.A.802,
1B0B.A.144, 1B7V.A.93, 3BA2.A.158, 1BBH.A.132, 1BCF.A.200, 2BCN.B.109, 1BGP.A.4
00, 3BK9.A.401, 1BT8.B.202, 3BUJ.A.398, 2C1D.H.1158, 4C50.A.1741, 3C6G.A.601, 1C
6S.A.88, 4C9L.A.1418, 4CAB.A.537, 2CCY.A.129, 1CG8.B.142, 1CGN.A.128, 1CH4.A.147
, 2CJ1.A.1300, 2CMM.A.155, 1CPQ.A.130, 1CRC.A.105, 4CZC.A.1337, 1D0C.A.500, 2D0Q
.A.300, 3D1K.A.200, 3D1K.B.400, 4D3O.A.901, 2D5X.B.147, 3DAM.A.600, 3DAX.A.601,
3DBG.A.500, 3DE8.D.150, 1DGF.A.3000, 2DKK.A.430, 1DLY.A.144, 4DVQ.A.601, 1DW1.A.
113, 4DWU.A.201, 1E2R.A.602, 2E39.A.401, 3E4W.A.501, 3E5L.A.1408, 2E84.A.1315, 3
EAH.A.861, 1ECD.A.137, 4EGO.A.501, 3EJ8.A.1901, 3EJD.B.405, 4ENP.A.801, 4ENU.A.8
01, 1EQD.A.185, 4ESA.A.202, 4ESA.B.202, 1EUP.A.410, 4FB2.B.501, 2FDU.A.500, 1FHF
.A.350, 1FHJ.B.147, 4FVC.A.201, 3G1Q.A.480, 4G2C.A.501, 2G3H.A.154, 3G46.A.147,
4G45.A.401, 3GAS.A.1290, 2GB8.A.295, 1GCV.A.141, 1GCV.B.137, 3GE3.A.502, 1GEJ.A.
501, 4GEP.A.580, 2GGN.X.251, 1GJQ.A.602, 2GKM.A.144, 3GPH.A.500, 4GQS.B.501, 1GV
H.A.1398, 1GW2.A.350, 1GWS.A.615, 1GWU.A.1306, 2GYQ.A.401, 4H0K.A.200, 1H1X.A.11
54, 4H8Q.A.201, 1H97.A.148, 2HBT.A.900, 3HC1.A.305, 3HC1.A.306, 1HDS.A.142, 3HF2
.A.482, 3HF4.F.147, 1HJ4.B.601, 1HJ5.B.601, 2HMQ.B.115, 4HRR.A.201, 4HRR.B.201,
1HV4.A.151, 3HX9.A.300, 2HYS.A.201, 1HZU.A.601, 1I3D.A.147, 1I4Y.E.605, 4I8V.A.6
01, 2I96.A.129, 4IAM.A.501, 2IBN.B.706, 3IBD.A.500, 2IG3.A.700, 2IIZ.A.400, 2INN
.A.513, 1IOP.A.154, 3IQB.A.500, 1IRD.B.347, 2ISA.A.486, 1IT2.A.147, 1ITH.A.143,
1IWH.A.142, 1IX4.A.300, 1IZO.C.501, 2J0P.A.1342, 1J1L.A.1001, 2J7A.D.1001, 1JAF.
A.130, 1JEB.D.147, 4JET.A.201, 4JS9.A.501, 2JXM.B.250, 4KF0.A.501, 2KII.A.182, 3
KX4.A.999, 1L0L.D.242, 4L1Y.A.300, 4L1Z.A.300, 4L2M.A.201, 4L3H.A.402, 2L4D.A.10
7, 4L54.A.501, 2L8M.A.416, 1LC1.A.105, 1LGA.A.396, 1LH2.A.154, 1LH6.A.154, 1LH7.
A.154, 1LHT.A.155, 2LH2.A.154, 2LHB.A.151, 3LL8.A.506, 4M26.C.401, 1M54.F.1620,
1M56.A.1002, 1M7S.D.600, 4M71.B.403, 1M85.A.1001, 1MBA.A.148, 1MGN.A.154, 2MHR.A
.119, 3MM3.A.501, 3MM6.B.570, 3MMB.A.580, 3MMO.A.1004, 4MMO.A.401, 1MN1.A.396, 3
MOL.B.185, 1MYF.A.154, 3MYM.A.139, 3MYN.A.139, 3N3R.A.1500, 4N4M.A.616, 4N4N.C.6
01, 3N8Y.B.601, 1N97.B.603, 3NA0.A.601, 4NK2.A.700, 4NKW.A.600, 3NN2.A.239, 3NNF
.A.600, 3NNL.A.600, 2NNJ.A.500, 2NOX.C.500, 2NP1.A.350, 1NR6.A.500, 3NTG.D.601,
3NU1.A.302, 3O5C.B.402, 2O68.A.401, 1OAE.A.1125, 3OCD.C.401, 3OCD.D.401, 1OCZ.A.
515, 1ODO.A.1407, 3OFU.A.417, 1OG5.A.501, 1OIK.A.1302, 2OIF.A.163, 4OQR.A.501, 1
OR4.A.180, 2OYY.A.201, 1OZW.B.300, 4OZ5.A.201, 3P3X.A.501, 2P85.A.500, 3PCJ.R.60
0, 3PM0.A.900, 2PMS.A.347, 3PT8.A.500, 3PT8.B.500, 3Q14.A.501, 1Q5E.A.440, 2Q8P.
A.300, 3Q9K.A.605, 2Q9F.A.602, 1QGJ.A.1350, 3QGP.A.200, 1QJS.A.500, 3QQR.B.163,

2QRW.A.700, 2QSS.A.142, 1QWL.B.550, 3QZM.B.201, 3QZX.A.200, 2R1H.D.148, 4R21.A.6
00, 2R79.A.500, 3R9B.A.501, 2RCL.B.600, 2RF7.D.1, 2RFB.A.410, 2RI4.A.142, 3RIW.A
.305, 4RKM.K.809, 4RKM.L.813, 3RUR.A.200, 1S05.A.130, 1S13.A.300, 1S1F.A.430, 1S
61.A.144, 1S69.A.125, 3S66.A.142, 3S79.A.600, 1SCH.A.300, 1SI8.A.501, 3SIK.A.154
, 1SOG.A.296, 1SPG.A.144, 1SPG.B.148, 1SQ3.A.903, 1T47.B.430, 1T85.A.417, 3TBG.A
.800, 1TMX.A.861, 3TM8.A.902, 3TM8.A.903, 2TOH.A.501, 4TRI.A.501, 3TTV.A.760, 3T
TW.A.760, 3TTX.B.760, 1TWN.A.300, 3TYW.A.501, 1U5U.A.999, 4U9D.D.201, 3U9J.A.200
, 3UA1.A.508, 4UBS.A.501, 1UC3.A.150, 3UHB.A.147, 3UHD.B.147, 3UHK.A.147, 1UMO.A
.1172, 3UOI.I.200, 3UT2.A.1500, 1UX8.A.700, 1V4U.A.144, 1V4U.B.147, 1V54.A.516,
1V9Z.B.1140, 3VED.A.401, 2VHD.A.401, 3VNO.A.501, 3VOL.A.401, 1VRE.A.148, 3VRF.B.
201, 2VZW.A.1206, 3W08.A.501, 3W4U.A.201, 1W92.A.1149, 3WCU.A.200, 3WCU.C.200, 3
WCU.B.201, 3WCU.D.201, 3WFB.B.802, 4WG2.A.603, 2WIV.A.1553, 3WNU.A.801, 1WOW.A.3
00, 1WRA.A.401, 3WXO.A.801, 4WXO.B.301, 2WY4.A.150, 2X66.A.1359, 1X8V.A.470, 1X9
F.A.160, 1X9F.B.160, 1X9F.C.160, 1X9F.D.160, 2XBK.A.1398, 2XF2.A.690, 2XMO.A.112
8, 2XN8.A.1434, 2XQ1.B.1503, 1Y01.B.142, 2Y4F.A.389, 1Y5F.B.147, 2YGX.D.450, 2YL
1.A.128, 1YMC.A.154, 2Z3U.A.500, 2Z36.A.450, 2Z6F.A.3747, 2Z6N.A.150, 2Z6N.B.150
, 2ZDO.A.200, 2ZFO.D.200, 3ZK5.A.1407, 3ZKY.A.1332, 2ZPG.A.300, 2ZVU.A.300, 2ZZS
.2.220, 2FMS.A.342, 4GXK.A.405, 3JPP.A.340, 2PFQ.A.1, 4TUR.A.404, 4ADB.A.1405, 4
AYO.A.502, 4BDR.A.902, 3C7E.A.489, 4DF9.A.501, 4G1K.B.301, 2GEZ.A.401, 1GEN.A.30
4, 1GUU.A.1090, 1GV2.A.1192, 2HU3.A.9002, 1HXN.A.2, 4I2F.A.602, 3IFV.C.408, 2IY6
.B.1540, 2J5W.A.3045, 2JHN.A.1298, 4KXW.A.1013, 4KZV.A.304, 3LP5.A.251, 4PFI.A.4
01, 1QHU.A.437, 3STH.A.501, 1SU3.A.911, 3U21.A.500, 1W16.A.1002, 1W9W.A.900, 1ZD
N.A.157, 4EJY.A.301, 1EWN.A.501, 3IOW.A.296, 4KLI.A.404, 4KLI.A.405, 4M04.A.707,
1ORN.A.224, 4P4M.A.403, 1RZT.A.2001, 4TUP.A.402, 3AGC.A.1, 4AK1.A.1702, 4C3X.A.
561, 3E9L.A.1, 3ELF.A.351, 2FPR.B.505, 3GED.A.251, 4GNJ.B.302, 2GTW.B.3005, 1GV5
.A.1142, 4H83.A.401, 4HUR.A.316, 4I2A.A.602, 4IIB.A.944, 3IMM.B.2, 3IRS.A.290, 4
JVL.B.304, 1JZ7.A.3104, 4KA7.A.805, 4LG8.A.601, 3MJ6.A.503, 4MMB.A.602, 3MQG.A.1
93, 4NAW.B.303, 3OLJ.A.1, 2OPL.A.187, 4PCG.C.304, 1PYF.A.315, 3PZR.A.373, 3PZS.A
.287, 1QNJ.A.280, 3QXT.A.133, 3R0L.D.124, 4R3N.B.401, 3SJL.A.402, 1UD2.A.1002, 3
UWP.A.424, 2V3U.A.1263, 1VMF.A.134, 3W5N.A.1210, 3WNO.A.802, 2WW2.A.800, 4XCZ.A.
405, 4XCZ.A.406, 1XKN.A.700, 1Y7W.B.282, 3ZX3.C.522
[1] "Cluster 6"
3F2D.A.5, 3A3O.B.65, 1A7I.A.82, 1A7I.A.83, 1ADB.A.375, 2AF2.B.154, 3B1B.A.378, 2
B5L.C.3001, 1B8T.A.196, 2CIH.A.212, 3CQJ.B.285, 4CWM.B.433, 1D8M.B.801, 2E26.A.6
03, 3E50.A.1, 2EC7.A.51, 4EGE.A.411, 1ELX.A.451, 1ELZ.A.451, 1EPW.A.1291, 4FUK.A
.401, 2GLQ.A.2002, 2GSU.A.1001, 4HGX.B.301, 1HOV.A.165, 1HP7.A.401, 1HYI.A.66, 4
IGN.A.401, 3II1.A.571, 1JM7.A.123, 2JRP.A.150, 4JSS.A.301, 4K6T.B.403, 1KAR.B.50
2, 4KJG.B.1001, 2KVG.A.85, 1LG6.A.262, 3MO2.D.5, 3N2C.D.426, 4O98.A.401, 2OC7.A.
901, 2PTW.A.500, 3Q31.A.1, 4R7M.D.1001, 1T0A.B.760, 1TM6.A.23, 3UBF.A.7, 3VUV.A.
501, 3WI2.B.801, 2WWO.A.1165, 1YIX.A.601, 1YIX.B.603, 2Z2D.A.264, 1ZSW.A.315, 3Z
TV.A.1598, 4IRK.A.402, 2OTL.A.8066, 1YJ9.0.8067, 521P.A.168, 3AJP.A.183, 3ALN.B.
406, 1AM4.D.679, 1AR1.A.560, 1CEE.A.180, 3CIK.A.690, 4D0L.B.2001, 3D19.E.301, 4D
VG.A.201, 2DW7.L.2012, 2E8W.B.1204, 4EOP.D.501, 3EQB.A.9002, 4FMA.F.402, 3FPA.B.
901, 3GFT.E.202, 3GOL.A.580, 1GQ9.B.1242, 3GT8.D.14, 4GZM.A.1001, 2HAW.A.1002, 3
HYT.A.802, 4HYP.C.302, 4I40.A.301, 4IL6.C.512, 2I07.A.5001, 2IOA.A.5001, 4JVJ.F.
403, 4JVJ.F.404, 3KRP.D.903, 3KZ1.E.550, 3LAW.B.1401, 1MAB.A.602, 3NCO.C.218, 4N
NN.K.302, 2O1X.C.2003, 2O1X.D.2004, 2OPM.A.908, 3OPS.D.501, 2OQY.C.402, 2P8E.A.3
06, 1PKG.A.1481, 1Q3H.A.674, 1R0Z.D.674, 4R9U.D.302, 4RAB.C.303, 1SVW.B.301, 3T5
P.H.301, 4U3W.A.503, 4UOR.K.699, 3VHX.G.185, 2VWI.B.1293, 1W1W.A.2001, 1W85.E.13
68, 2WCJ.A.1146, 2WHE.A.1222, 3WIG.A.402, 3WNW.J.201, 1XD2.A.167, 1YMO.A.402, 2Z
RY.D.702, 2B0D.A.502, 4G3I.A.401, 4K4I.E.603, 3KHG.A.415, 4KHU.A.1003, 2NOL.A.32
8, 3OOR.A.236, 4QWD.A.702, 3RBD.B.1415, 4AAH.A.702, 4AC8.B.1311, 1AWB.B.280, 1AX
K.A.395, 2BD4.A.260, 2BZ6.H.1260, 3C14.A.29, 1C9P.A.501, 2CLT.A.1102, 1D8M.B.804
, 1FBL.A.993, 1FBL.A.994, 4FVL.A.506, 4H82.C.305, 1HKB.A.923, 3HQ8.B.402, 1HVD.A

.600, 2II1.D.401, 2I04.B.701, 3IS5.F.1, 1ITC.A.1500, 1J24.A.1001, 1JDC.A.452, 4K
W7.A.402, 4L41.B.201, 2LMV.A.151, 4MC7.A.503, 4MIX.A.2501, 4N2I.A.707, 1NMB.N.47
8, 4NUY.A.1001, 3OHO.A.1, 4OKH.C.903, 2OVZ.B.449, 3OXQ.D.516, 4P99.A.533, 2PC6.B
.303, 1PEX.A.502, 4PIB.B.203, 4POQ.G.401, 2PR3.A.901, 4Q4X.1.5007, 1QMD.B.405, 3
R4I.D.342, 2RJP.D.2, 3RMK.B.308, 1TFX.A.1007, 3V96.B.305, 3VEQ.B.301, 2VME.E.500
, 3VOB.A.401, 2WOQ.B.803, 3W9T.B.510, 1Y7O.B.1004, 2YA9.A.1303, 2YN3.D.6355, 2Z2
D.A.268, 2BI4.A.1384, 4BMT.B.1323, 3DHG.A.501, 1DTO.A.1601, 1FZH.B.5004, 2GBX.A.
456, 3I4V.A.281, 2ITB.A.501, 4IWK.F.201, 3KCY.A.1350, 4KEV.D.401, 1MOJ.B.301, 1N
7X.A.339, 3NJZ.A.369, 2P6B.C.513, 3PCA.N.600, 3PCL.R.600, 3R2M.A.155, 1RSR.A.100
4, 1T47.A.431, 3USS.B.212, 2VC7.D.1315, 2XSO.K.900, 2Z4G.A.503, 3E45.A.260, 4M47
.A.403, 2OTJ.R.8537, 1Q81.M.8380, 3AXG.A.3005, 3C17.A.324, 4CH8.D.1580, 1CM5.A.1
056, 1DI4.A.501, 3DR3.A.336, 3DYQ.A.902, 2E7U.A.1002, 1EBU.A.901, 4ENZ.A.1112, 1
F7T.A.472, 1F7T.C.474, 4FOI.A.1005, 4FOI.A.1006, 2FQE.A.901, 2GG2.A.703, 3H1V.X.
600, 4HCH.A.407, 3HVU.D.182, 4HXV.A.403, 3I3D.C.3101, 3IGQ.F.801, 2J5W.A.3043, 4
JRX.D.301, 3K13.A.647, 3L27.B.3, 1MXO.D.901, 3NRB.C.287, 4O4W.A.301, 3OB8.A.3006
, 4ODI.A.301, 3P0J.A.711, 1QJS.A.512, 1QJS.A.513, 1QJS.A.523, 2QZ7.A.195, 4R3W.B
.402, 1R4P.F.4004, 3T2Q.D.3101, 3UA7.A.145, 1UD2.A.1003, 3V6N.A.232, 3WOL.A.502,
1XAR.B.200, 1XC6.A.8001, 1YCE.A.201, 1YCE.C.201
[1] "Cluster 7"
3ASE.A.156, 2BCN.A.295, 2BIB.A.1550, 4E5V.B.401, 3EII.A.301, 2FV9.B.4, 2GC2.A.40
1, 2GC3.A.402, 2HD1.A.101, 4HGX.A.301, 4ICQ.A.501, 4IGN.B.401, 2J0E.B.1265, 4JAA
.A.501, 2K78.A.151, 3KR5.E.1001, 4KYH.A.202, 4N27.C.201, 4N7K.L.307, 3N9R.A.308,
3NO5.C.275, 3O64.A.1, 2OUN.B.404, 4PKT.A.802, 4PKW.A.801, 1Q3K.B.300, 1Q74.A.30
4, 3Q9B.A.345, 2QFR.A.434, 1QIP.A.902, 4R6T.A.1003, 4R6T.D.1001, 4R7M.C.1001, 4R
7M.J.1003, 3S2M.A.403, 3SFW.A.501, 3V93.D.701, 3VH9.A.301, 3VUS.A.401, 2WEY.A.17
72, 1XJS.A.150, 1XP3.A.301, 1Y13.A.174, 2ALW.A.5001, 4BBP.A.1316, 3BLB.A.1047, 1
BLL.E.489, 2BNO.B.1201, 3D4Y.A.1047, 3D4Z.A.1046, 3D52.A.1046, 3D51.A.1046, 3DDF
.A.3001, 3DDG.A.3001, 2DDF.A.1, 1DE5.A.450, 4DO0.A.502, 3DX1.A.1048, 3DX2.A.1046
, 3DX3.A.1047, 3DX4.A.1047, 3E38.A.2, 3E49.B.500, 3E8R.B.2, 3EJP.A.1047, 3EJQ.A.
1047, 3EJR.A.1047, 3EJU.A.1047, 2F18.A.1805, 2F1A.A.1805, 2F1B.A.1804, 2F7O.A.50
01, 2F7P.A.5001, 2F7Q.A.5001, 2F7R.A.5001, 2F92.F.1001, 2F92.F.1003, 2FQP.B.100,
2FUQ.A.1, 1GT7.A.275, 3GWT.A.504, 4H1S.A.603, 3HPS.A.701, 2I57.D.507, 3IBM.A.20
0, 4IE5.A.601, 1IM5.A.400, 3ISI.X.3001, 3ITU.A.1, 3IVT.A.500, 4JDG.A.401, 1KAE.A
.1101, 3KMC.B.2, 3KME.B.2, 1KQ3.A.401, 3KR5.A.1001, 1KRM.A.501, 1LCP.A.488, 3LGP
.A.1, 3LX3.A.201, 4M6R.B.301, 4NPW.A.1001, 4NUR.A.701, 4NZ3.A.501, 3O0J.A.1, 3O6
4.B.485, 3O93.A.192, 2OB3.B.904, 4OJV.A.403, 4OJV.A.404, 4OJX.A.403, 2OQL.A.401,
2P18.A.301, 1PTM.A.330, 1PTM.B.331, 2PU1.A.500, 3QAY.D.180, 4QGE.A.602, 1QH3.A.
262, 1R33.A.1163, 1R55.A.201, 4R6T.B.1003, 4R6T.F.1001, 4R76.A.1003, 4R76.E.1001
, 4R7M.C.1003, 3RCQ.A.1, 4RLO.B.302, 1SNN.A.402, 1SR9.B.703, 4TO8.A.301, 3U43.B.
135, 2V9N.A.1275, 3WOT.A.201, 3WOT.B.201, 2WHG.B.1263, 4X2T.G.702, 1XM6.A.1001,
2Y33.A.900, 3A4K.A.301, 4AAB.B.1156, 4AAB.B.1157, 2AGQ.A.4002, 2AQ4.A.302, 4AQX.
D.1525, 4AQX.D.1526, 2BCV.A.576, 4BDY.A.1380, 4BDY.A.1381, 4BE2.A.1381, 4C2U.A.1
666, 4CEI.A.2234, 1CWO.N.202, 1CWO.A.203, 4D6N.F.1196, 4D6O.D.1196, 4D6O.D.1197,
4DF4.A.901, 4DF8.A.903, 4DFJ.A.902, 4DLG.A.902, 4DOA.A.401, 2DPI.A.871, 4DQI.D.
901, 4EEY.A.502, 2EZ6.A.501, 3F2B.A.5, 4F5P.A.401, 2FMS.A.340, 1G9Z.C.902, 1G9Z.
D.901, 1G9Z.F.903, 3GDX.A.347, 3GDX.A.348, 3GPL.A.800, 3GQC.B.203, 4IR9.F.402, 4
IRD.F.903, 2ISO.A.339, 2ISP.A.339, 2IS4.A.1001, 3JPN.A.339, 3JPR.A.339, 3JPT.A.3
39, 3K57.A.1001, 4K98.A.602, 4K99.A.602, 3LK9.A.339, 4LOX.A.401, 4M30.A.501, 4M3
0.A.502, 4M47.A.402, 3M8R.A.2, 3MAQ.A.1001, 3MBY.A.339, 4MFF.A.401, 1MOW.D.374,
3MQY.A.500, 3MR5.A.435, 4NCB.A.702, 4NCB.B.702, 4NCB.B.703, 4O3N.A.503, 3OSO.A.3
94, 3OYA.A.396, 3OYA.A.397, 3OYB.A.396, 3OYC.A.396, 3OYC.A.397, 3OYD.A.396, 3OYE
.A.396, 3OYF.A.396, 3OYF.A.397, 3OYG.A.397, 3OYH.A.396, 3OYH.A.397, 2OZS.A.904,
2PFN.A.950, 4PGQ.A.400, 3PML.A.2, 4PQU.A.602, 4QCL.A.1302, 1QSY.A.1001, 4R8U.B.4
02, 3RJF.A.340, 3RJK.A.340, 3S3O.A.397, 3S3M.A.396, 3S3M.A.397, 3SI6.A.905, 1SKR

.A.4001, 3SPY.A.904, 3SV3.A.836, 1T7P.A.4001, 3TFS.A.340, 1TK0.A.991, 1TK8.A.901, 4TUQ.A.402, 3TWH.A.401, 4UB3.A.401, 3UQ2.A.1, 3V6H.A.402, 2VBN.E.1026, 2W35.A.1224, 2XCA.A.3000, 1YVP.A.1001, 1ZBI.A.302, 1ZBL.B.204, 3A06.A.500, 4A01.A.1770, 4A01.A.1771, 1A49.A.534, 1A49.H.5334, 3A7D.A.300, 4ABZ.A.1210, 4AC0.A.1205, 4ACF.A.1480, 2AE8.C.1009, 1AJB.A.452, 3AJ0.A.183, 1ALK.B.452, 3AXK.A.478, 1AZS.C.403, 3B05.D.1001, 3B1X.A.301, 1B4N.A.623, 1B7T.A.836, 1B8C.A.308, 3BGA.A.1, 2BJI.A.2277, 2BKU.A.221, 3BM4.B.304, 3BNY.D.701, 3BRB.A.10, 1BWV.C.490, 3BWY.A.300, 1BZY.A.901, 2C31.A.1553, 2C3P.A.2237, 2C42.A.3238, 4C5A.B.331, 4C5C.A.1314, 4C7X.A.700, 3CB3.A.501, 4CE0.A.1251, 3CFX.A.704, 1CG1.A.435, 1CG4.A.435, 1CH8.A.434, 1CIB.A.434, 1CJT.C.403, 2CJE.A.1268, 2CL5.A.1216, 3CMR.A.453, 3CP6.A.501, 3CRR.A.324, 3CT2.A.401, 1CUL.C.396, 3CWH.A.392, 4CW7.A.1002, 4CWB.A.1159, 3CX0.A.500, 3CX0.B.500, 4CYM.A.1199, 3CZJ.B.3001, 3D46.A.501, 3D47.A.501, 1DAK.A.901, 1DAY.A.341, 4DBH.A.401, 2DCN.A.4001, 2DEI.A.402, 3DFY.A.401, 4DFD.B.301, 2DGN.A.1454, 4DH5.A.402, 3DHD.A.502, 1DIE.A.399, 4DN1.B.401, 3DOE.A.193, 1DQN.A.451, 3DUF.A.1368, 2DUA.A.292, 3DVA.A.1368, 2DW6.A.2001, 2DW6.D.2004, 4DWB.A.507, 4DWB.A.508, 1DXE.B.901, 4DXJ.A.401, 4DXJ.A.402, 4DXJ.A.403, 3DYG.A.3004, 3DYH.B.4002, 3DYS.A.902, 2E0A.A.500, 4E1E.A.403, 1E4E.A.365, 2E8W.A.1202, 1E9I.A.1431, 2E91.A.1302, 4EA0.A.301, 4EA0.A.302, 4EA0.A.303, 1EBG.A.438, 1EBG.A.439, 1EBH.A.438, 2EB1.A.502, 1EC7.A.498, 1EC9.A.498, 1ECB.A.507, 1ECQ.A.498, 3EFQ.A.3003, 3EFQ.B.4002, 3EGT.A.3002, 3EGT.A.3003, 3EKG.A.601, 1ELZ.A.452, 3EQI.A.3, 3ES8.A.393, 3ETJ.A.401, 1EXM.A.407, 3EYA.H.613, 3EZ3.B.1102, 3EZ3.B.1104, 4F0Q.A.501, 1F2U.A.902, 3F78.C.1, 4F71.A.301, 1F8I.A.451, 3FA5.A.282, 3FD5.A.395, 3FD5.A.396, 3FD5.B.397, 3FD6.B.395, 3FD6.B.397, 3FE4.B.902, 4FFL.A.904, 4FFL.A.906, 4FFO.A.904, 4FFR.A.403, 2FG5.A.301, 4FI4.A.501, 3FLK.A.401, 3FLK.A.405, 2FN0.A.701, 2FPR.A.503, 3FPA.C.901, 3FPB.A.1000, 3FQI.A.1000, 1FTN.A.300, 3FTQ.A.371, 3FV9.A.501, 4FVR.A.902, 3FYY.A.402, 1G3B.A.501, 1G4P.A.2003, 2G4J.A.392, 3G5A.B.307, 4G61.A.302, 2G9Y.A.452, 2G9Z.A.704, 2G9Z.B.701, 1GAG.A.201, 4GA3.A.1004, 2GCQ.A.435, 2GGE.A.400, 4GIS.A.405, 4GIU.A.402, 4GME.C.501, 2GO7.A.207, 4GOK.B.202, 4GP2.A.401, 2GQS.A.240, 2GQS.A.241, 2GQ3.A.1000, 2GT4.B.401, 4GT3.A.403, 3GY1.A.500, 4GYI.A.402, 1H1D.A.300, 4H19.A.405, 4H1Z.A.401, 3H3X.Q.553, 3H4L.A.701, 2HCJ.A.998, 3HDG.B.201, 4HE1.A.403, 4HE1.A.404, 4HE2.A.405, 2HGS.A.502, 4HGQ.A.201, 4HGR.A.201, 4HHL.A.402, 3HJN.A.501, 4HNC.A.401, 3HPF.A.402, 3HQD.A.501, 3HQP.B.502, 3HVH.A.265, 3HVI.A.1, 3HVJ.A.265, 3HVK.A.1, 3HW3.A.999, 2HWG.A.901, 2HXU.A.601, 3HXX.A.445, 4I2B.A.602, 3I3O.A.306, 2I33.A.602, 4I3Y.A.302, 4I3Y.A.304, 4I3Z.A.302, 1I6I.A.501, 3I6E.A.386, 2I6K.A.302, 3IBA.A.401, 3IBA.A.402, 3ICK.A.401, 3ICK.A.402, 3ICK.A.403, 3ICZ.A.401, 3ICZ.A.402, 2IDX.A.603, 4IEE.A.501, 4IFW.A.502, 1IGW.A.441, 4IHC.A.501, 1II0.A.593, 1II9.A.593, 3IIE.A.501, 3IJR.D.300, 4IJQ.A.304, 2IK2.B.287, 2IK2.B.289, 2IK2.B.290, 2IK7.A.287, 2IOA.B.5004, 4IP5.A.502, 1IR3.A.301, 4IT1.A.501, 2IUC.B.1008, 1IV4.A.1572, 4IX4.A.602, 2IXE.A.2, 1J34.C.501, 2J5X.A.200, 1J7L.A.301, 4J7L.A.402, 1JAH.A.168, 2JCS.A.1211, 2JD4.B.4062, 1JGT.A.902, 2JI6.A.1567, 2JI7.A.1567, 2JI8.A.1567, 4JND.A.501, 1JP4.A.701, 1JP4.A.702, 1JSC.A.699, 3JUK.C.307, 3JVT.B.502, 4JX0.A.402, 3JYS.A.1, 4K1W.A.501, 4K33.A.802, 3K4Z.A.290, 1K9Y.A.401, 1K9Y.A.403, 1K9Y.A.402, 4K9N.A.601, 1KA2.A.501, 4KCT.A.1001, 4KCU.A.1001, 4KCV.A.1001, 4KCW.A.1001, 3KDN.A.500, 1KEK.A.2237, 3KEU.A.400, 4KFU.A.307, 1KHK.A.452, 1KHZ.B.301, 1KHZ.B.310, 3KHQ.A.1, 4KI8.E.602, 1KKR.A.501, 4KMQ.A.1102, 1KO5.A.1001, 1KP8.A.550, 4KQX.A.405, 4KQX.A.406, 3KRO.D.3001, 3KRO.D.3002, 4KSO.A.1001, 4KUX.A.701, 3KWS.B.401, 4KWD.A.403, 4KX3.A.302, 3L0Y.A.257, 4L2X.F.404, 4L2X.F.405, 1L8A.A.888, 4L9Y.B.403, 4LA7.B.601, 4LF1.A.801, 1LNY.A.1453, 1LON.A.1454, 3LVO.A.264, 3LVV.A.695, 3LVV.A.697, 4LZ3.A.406, 3M00.A.550, 1M0W.A.502, 1M1B.B.999, 4M69.A.403, 4M6U.A.401, 1MB9.B.601, 1MC1.A.601, 1MC1.A.603, 1MDL.A.360, 1MEZ.A.1453, 4MFG.A.201, 4MIT.A.202, 1MMA.A.998, 1MNS.A.360, 4MPO.A.206, 4MPO.B.204, 4MPO.C.205, 4MPO.E.201, 3MQT.H.626, 1MRS.A.300, 1MX0.A.501, 1N0H.B.1699, 4N0G.A.403, 1N1Z.A.702, 1N20.A.701, 1N20.A.703, 1N24.A.701, 1N24.A.703, 3N3T.A.802, 3N45.F.355, 1N8I.A.900, 4NEH.B.703, 4NFI.F.404, 1NHT.A.435, 3NJL.A.501, 4

NM3.A.405, 3NO1.A.397, 1NUW.A.2497, 1NUX.A.2342, 1NUY.A.2341, 1NUY.A.2343, 3NZG.A.507, 4NZO.A.404, 2OIO.A.501, 2OIO.A.503, 4O4D.A.401, 3O61.B.202, 1OAD.A.392, 4OAV.B.802, 2OCB.A.202, 2ODB.A.205, 3OE5.A.222, 3OES.A.202, 2OEM.A.911, 1OFH.B.453, 1OIX.A.301, 4OKM.A.902, 4OKZ.A.901, 4OKZ.A.902, 3OP2.A.500, 3OPS.A.500, 3OPS.A.501, 1ORK.A.223, 4ORK.A.501, 2OUN.A.403, 1OVM.A.601, 4OVN.A.204, 1OW2.A.401, 2OX4.A.402, 3OYZ.A.500, 1OZF.A.699, 1OZH.A.1405, 3OZF.A.235, 3OZM.D.390, 3OZY.B.390, 3P0X.A.430, 3P3B.A.393, 2P3N.A.1758, 3P41.A.297, 3P41.A.298, 3P5R.A.901, 1P7T.A.1000, 1P9B.A.1600, 3P93.A.406, 4PAL.A.110, 3PDE.A.311, 1PFK.A.325, 1PFK.A.327, 4PFK.A.327, 2PGN.A.610, 2PK0.A.502, 2PLS.G.603, 2PLS.H.602, 2PLS.J.604, 2PMQ.A.902, 2PP3.A.901, 1PT6.A.500, 1PUN.A.130, 3PUV.A.1501, 4PU5.A.502, 1PYD.A.559, 1PYM.A.1003, 2PZA.A.6242, 3Q1O.A.400, 2Q1A.X.294, 2Q1D.X.294, 4Q1V.A.803, 6Q21.D.173, 3Q30.A.600, 3Q30.A.601, 3Q46.A.305, 3Q46.A.306, 2Q5Q.A.4002, 1Q6O.A.7300, 1Q6Q.A.7300, 3Q85.A.284, 1Q9S.A.201, 3Q9L.A.700, 1QC5.A.601, 1QC5.B.602, 4QE5.A.401, 4QEH.A.402, 1QF4.A.433, 2QGY.A.701, 1QMZ.A.383, 3QN3.A.601, 3QPE.A.393, 3QPE.B.393, 4QPM.A.1502, 4QPM.A.1503, 2QQ0.B.452, 3QQV.A.382, 1QS0.A.501, 2QTV.B.210, 2QTY.A.349, 2QTC.A.888, 3QU4.A.225, 2QX0.A.162, 1R0X.A.13, 3R0U.A.380, 3R1M.A.402, 3R1M.A.403, 3R1M.A.404, 3R25.A.402, 4R39.A.401, 3R6T.A.301, 2R9V.A.504, 3RBM.A.1001, 3RBM.A.1002, 3RBM.D.1003, 2RDX.A.378, 3RIM.A.1001, 4RJJ.A.602, 4RJK.H.602, 3RLH.A.286, 4RN3.A.301, 4ROP.A.504, 1RQI.A.605, 1RQJ.A.907, 3RUV.A.544, 1S1C.A.300, 3S9I.A.743, 3SAZ.A.802, 3SAD.A.801, 3SB0.A.801, 3SBD.A.501, 3SBF.A.402, 3SE1.A.182, 3SEA.B.178, 1SHQ.A.479, 3SH6.A.176, 3SOP.A.401, 3SSN.A.501, 3ST8.A.496, 3T0Z.A.401, 3T1Q.A.198, 3T2E.A.409, 3T6C.A.501, 3T8O.A.564, 1T8Q.B.1602, 1T9B.A.1699, 1T9B.B.699, 1T9C.B.699, 1TE6.A.640, 1TND.A.352, 4TQ4.A.401, 4TQD.A.502, 4TSK.A.403, 3TTE.A.361, 3TTE.B.361, 3TW6.C.2002, 3TWB.A.420, 3TZF.A.279, 1TZZ.A.3501, 3U2E.B.1, 3UJ2.A.431, 3UJR.A.501, 4UMF.A.1175, 4USI.A.1151, 4USJ.C.302, 3UXL.A.360, 4V1T.A.1776, 4V1T.A.1778, 2V3W.A.1528, 3V4B.A.403, 1V5F.A.1603, 1V5G.A.1603, 2V5K.A.301, 1V8K.A.501, 1VA6.A.522, 1VA6.A.524, 2VBI.A.1000, 2VBV.A.1136, 2VDR.B.2001, 3VD3.A.3001, 2VDL.B.2001, 2VDM.B.2001, 3VKB.A.701, 2VK4.A.601, 3VMK.A.402, 3VMK.B.402, 3VMM.A.501, 2VPR.A.1207, 3VPB.A.502, 2VPO.A.1209, 3VR6.B.602, 2VWT.A.301, 2VZB.A.1001, 2W00.B.1894, 3W2W.A.904, 1W5T.B.701, 1W6T.A.435, 1W7K.A.1423, 1W88.C.1368, 3WBZ.A.403, 1WDD.A.1476, 3WDL.B.902, 2WEF.A.402, 4WF7.A.601, 4WK0.B.501, 4WK4.B.501, 3WOO.A.502, 4WRR.A.401, 2WW8.A.1000, 2WX5.L.1282, 2X3J.A.1590, 2X5Z.A.602, 2XCL.A.480, 1XEF.A.801, 2XH4.A.1439, 5XIM.A.395, 5XIN.A.395, 1XIN.A.395, 6XIM.A.395, 8XIM.A.395, 2XIM.A.395, 2XJC.A.1499, 1XLC.A.399, 2XTZ.A.1381, 2XZW.A.202, 1XZ8.A.180, 1Y9I.A.601, 1YHL.A.1401, 1YHM.B.1401, 1YHY.A.699, 1YIO.A.212, 2YVP.A.183, 1YVE.I.601, 1YVE.I.602, 1YYQ.B.701, 1Z0K.A.1201, 2Z4W.A.1302, 2Z4Y.B.1301, 2Z7H.A.1301, 3ZCB.A.301, 2ZCR.A.669, 2ZDH.A.812, 2ZKJ.A.500, 2ZPU.A.360, 1ZVW.A.4001, 2ZVJ.A.300, 1ZXN.B.902, 3ZXW.A.476, 3ZYC.A.1750, 3A4K.C.301, 3AUO.A.577, 4BDZ.A.1380, 4D6O.A.1186, 4D6O.A.1187, 4DLE.A.901, 2HVI.D.878, 3ICE.E.502, 4J9O.A.502, 3MDA.A.577, 4NM1.A.401, 2PYJ.B.9002, 3TFR.A.340, 1TFW.B.1601, 2A5G.A.231, 4ACF.D.1480, 1ALK.A.452, 3ALO.A.1, 2AUU.A.201, 3B03.D.1001, 3B05.A.1001, 2B8W.A.595, 2B9J.A.600, 2BBT.A.3, 4BBJ.A.750, 2BHW.A.602, 2BHW.A.614, 2BHW.B.605, 4BJU.A.998, 1BS1.A.901, 2BVN.A.1395, 4BYF.A.1000, 3C14.C.403, 2C43.A.1317, 3CBT.A.301, 3CK5.D.400, 3CRL.A.2000, 3CX7.A.378, 4DBQ.A.903, 3DHF.A.502, 3DKL.A.502, 4DL8.A.305, 4DLC.A.303, 3DYH.A.3002, 1E1R.A.601, 1E1R.F.601, 1E6D.M.1303, 2E8T.B.1303, 2E8W.A.1201, 2E8X.A.1302, 1E9I.B.1431, 2E91.B.1303, 2E92.B.1303, 3EA4.A.699, 3EF1.A.1, 3EFQ.B.4003, 3EHB.A.562, 4EKD.A.407, 3ENI.A.375, 3ENI.A.378, 3EOJ.A.375, 2EWG.B.4003, 3EYA.A.613, 4FFR.A.406, 1FMW.A.800, 1FQJ.A.352, 2G07.A.601, 2G08.A.500, 1G67.A.2007, 3G8D.B.1002, 2GTP.A.401, 4HEO.A.402, 4HGQ.C.201, 4HGR.B.201, 3HIY.A.401, 2HO4.B.301, 3HU2.D.801, 3HWX.1.602, 3HZH.A.202, 3IOO.A.502, 2I19.A.3002, 4IGA.A.200, 2IK2.A.289, 2IOA.A.5002, 4J5I.F.402, 1JB0.A.1106, 1JB0.A.1117, 1JB0.A.1122, 1JB0.A.1129, 1JB0.A.1133, 1JB0.B.1214, 1JB0.B.1215, 1JB0.B.1221, 1JB0.B.1223, 1JB0.L.1502, 2JCM.A.1490, 2JCS.B.1211, 4KCV.B.1001, 1KK8.A.997, 3KRF.D.901, 3KRO.A.3003, 3L0C.A.257, 3L8F.A.401, 4LCZ.A.3

06, 3LMG.A.202, 4LNI.D.503, 4LRJ.A.302, 4LRZ.A.302, 3LUZ.A.264, 3MCO.A.427, 3MCO.B.426, 3MGA.B.405, 3MLE.C.222, 1MNZ.A.389, 4NOG.B.402, 1N22.B.706, 1N24.B.706, 4NCJ.A.903, 4NDN.A.401, 2NGR.A.199, 4NM5.A.406, 3NNN.A.401, 3NNS.A.401, 4NNN.Z.301, 4NST.A.1103, 3O5T.A.299, 2O56.A.2001, 3O61.A.202, 4OEC.A.401, 2PAN.A.851, 1PPW.A.401, 4PTK.A.302, 2PUL.A.400, 3PUW.A.1501, 1Q3H.D.674, 2Q58.A.3, 3Q7P.B.257, 3QQV.A.381, 4QTD.A.426, 4QXD.A.304, 1R03.A.301, 3RBM.B.1001, 3RLG.A.286, 1RLT.A.805, 3RRA.A.406, 1RZH.M.851, 3SQS.A.450, 3SS8.A.302, 3T34.A.1002, 3T5P.F.301, 3TAV.A.266, 4TQ4.C.402, 3U2E.A.2, 4UB6.B.603, 4UB6.B.610, 4UB6.B.616, 4UB6.C.504, 4UB6.C.506, 4UB6.C.507, 4UB6.C.510, 4UB6.C.512, 4UB6.C.513, 4UB6.C.514, 4UB6.D.402, 2UXR.A.1405, 2V54.B.1205, 2VPO.B.1209, 3VTH.A.805, 1W5T.A.701, 3WBZ.F.403, 3WK4.A.601, 3WQM.A.403, 3WU2.A.410, 3WU2.B.604, 3WU2.C.502, 3WU2.C.510, 3WU2.b.615, 3WU2.c.902, 1XBT.D.4194, 1XZ8.B.180, 2YBE.A.1417, 1YF6.M.856, 1YQ7.A.908, 1YXO.A.5000, 1Z5B.A.2001, 1ZCA.A.383, 2ZRW.A.702, 4BXO.B.1216, 2FKC.A.248, 3GII.A.415, 4K4I.A.603, 4KHW.A.1003, 1R7M.A.304, 1TW8.C.803, 3UIQ.A.905, 2WTF.B.1509, 1A25.A.292, 1A25.B.292, 4AIO.A.1890, 4B4F.B.607, 4B7M.B.1471, 2BAT.A.601, 2BD3.A.260, 3BOW.A.718, 2BV2.A.1085, 2BW7.A.2202, 4CCE.A.4001, 2CDP.B.1140, 2CHI.A.218, 5CHY.A.401, 1CJY.A.951, 3CKC.B.700, 4CPN.A.500, 4CPO.A.1466, 1CXV.B.6, 2DOO.B.1002, 2DEW.X.903, 1DQ1.A.238, 4DWW.A.301, 1E35.B.260, 2EA7.B.452, 1EE6.A.300, 2ERQ.B.702, 1EZS.C.650, 3F19.A.266, 1F2N.A.1002, 1F2N.C.1001, 3FRP.A.628, 4GDI.C.507, 1GQM.E.1089, 4GZS.A.501, 1HFZ.C.124, 2HTV.A.995, 4I5K.A.501, 1IME.B.278, 1JOY.D.701, 1JAO.A.996, 1JI3.A.401, 1JIW.P.489, 1JRF.A.48, 4JUC.B.601, 3K1A.B.524, 3K37.A.467, 4K3Y.D.604, 3K5T.A.802, 3K9X.D.249, 3KF9.A.303, 3KL6.A.3, 4KNA.A.504, 4KS2.A.501, 1KVO.A.192, 4KVK.A.712, 1KWH.A.800, 1L9N.A.703, 3LNF.B.305, 4LXF.A.701, 4MDV.A.402, 3MIN.D.524, 1MR8.A.102, 1MTV.A.480, 1N28.A.128, 4N2F.A.703, 1NG0.A.1002, 1NG0.C.1001, 1NKQ.A.260, 4NRE.A.716, 4NRE.A.717, 4NUP.A.301, 2NVO.A.533, 1NXO.A.902, 3O9J.A.995, 2OO4.B.5004, 2OO4.B.5006, 2OW1.B.447, 3OX6.E.502, 2P5V.C.1002, 4PHK.A.304, 1PK8.F.817, 4PKH.J.1201, 3PRT.A.404, 3Q2L.A.703, 3Q3K.A.262, 4Q4X.1.5005, 1QL9.A.480, 3QNI.B.400, 4QN3.B.501, 1QUO.B.702, 2R1D.B.1000, 2R8Y.C.203, 2R8Z.J.210, 1R8L.B.902, 4R83.D.501, 2RHP.A.10, 2RHP.A.18, 2RHP.A.20, 2RJP.A.2, 2RJP.A.3, 1ROS.B.503, 1SOE.A.1293, 1S1D.B.1002, 3SHI.G.305, 3SJS.A.222, 3SOB.B.1, 1STB.A.150, 1TAD.A.352, 1TLD.A.480, 3U1R.A.700, 3V03.A.584, 1VOZ.A.1477, 1V7V.A.1001, 3VRQ.A.401, 1W3M.C.3014, 3W9T.A.1006, 2WG8.A.201, 3WIU.B.1002, 1WVM.A.604, 1Y4A.E.1001, 2YGM.A.1417, 2YN5.A.6365, 1YO8.A.1188, 1YO8.A.1197, 1Z4V.A.600, 2Z8S.B.641, 2ZID.A.882, 2ZJ6.A.625, 2ZKT.A.413, 2ZRQ.A.7, 1ZTQ.B.565, 2ZUX.B.638, 2ZUY.A.627, 2ZWO.A.400, 2ZWO.A.403, 4ABT.B.1287, 2AOR.A.401, 2AOR.A.402, 2ASD.A.415, 2ASD.A.416, 3AVX.A.3001, 3COW.A.304, 1DMU.A.300, 1DMU.A.302, 4DTP.A.1002, 3DVO.A.340, 4ELV.A.908, 1FOO.B.761, 4F4W.A.402, 4F4W.B.403, 4FJ8.A.1002, 4FJ9.A.1002, 4FJK.A.1002, 4FJL.A.1002, 4FJM.A.1002, 2GIJ.A.401, 3GV5.B.424, 3IAY.A.1, 2IBK.A.401, 2IBK.A.402, 2IMW.P.406, 4J2A.A.1002, 4J2B.A.1002, 2JEJ.A.1344, 1JXL.A.1402, 4K4H.A.607, 4K4H.E.602, 4K4I.E.602, 3KHR.A.416, 4KHQ.A.1001, 4KLD.A.402, 4KYW.A.302, 4LQO.A.402, 3LZJ.A.905, 4M3Z.A.1002, 3M9N.B.4003, 3M9O.B.4001, 3M9O.B.4003, 3MXB.B.173, 3MXB.R.175, 1N3E.D.493, 1N3E.F.491, 1N3F.D.499, 1N3F.F.497, 3O3G.A.1, 3ODH.A.195, 1OUP.A.300, 2Q10.A.701, 1Q9Y.A.939, 3QET.A.905, 4QWB.A.402, 4QWB.A.403, 1R7M.B.536, 3RAX.A.415, 2RDJ.A.353, 1RYS.A.801, 1SOO.A.401, 3SLP.B.227, 3SPZ.A.905, 3SUN.A.897, 1TW8.A.801, 3A09.A.601, 3A13.E.445, 3A7Q.B.5001, 2A8K.B.404, 2AER.L.3008, 2AEP.A.601, 1AFO.A.484, 1AFO.A.486, 1AG9.A.200, 1AG9.B.1000, 4AIE.A.1540, 1AJJ.A.73, 3AJ7.A.602, 3AMR.A.909, 3AMR.A.910, 4APX.B.1239, 4AQ1.A.1925, 4AQ1.A.1926, 4AQE.A.1208, 4AR9.A.1732, 4ARF.A.1723, 3ASI.A.2001, 3AUK.A.391, 1AVA.A.502, 1AWB.A.1, 3AYU.A.418, 3B00.B.301, 3B0I.A.124, 3B1U.A.901, 1B4N.A.620, 3B4N.A.702, 4B4F.A.607, 1B8L.A.110, 3B8Z.A.904, 1B9T.A.500, 3BC9.A.702, 3BC9.A.705, 3BH4.B.1, 1BLI.A.600, 2BU3.A.1242, 2BV2.B.1085, 4BZ4.A.1233, 1C3H.D.8003, 1C3H.F.8001, 4C9F.B.401, 4CAG.A.602, 2CDP.A.1140, 1CGE.A.305, 3CKC.A.600, 1CLC.A.650, 1CLC.A.652, 5CNA.C.240, 4CPY.A.1466, 4CU9.A.2999, 4CUB.A.2645, 2CYY.A.2002, 2DOO.D.1001, 3D4G.B.484, 3D6E.A.202, 2DCJ.A.1003, 2DDU.A.1, 2

DDR.A.1324, 2DEW.X.901, 2DF7.C.5904, 2DIE.A.779, 4DK4.A.302, 4DKB.A.302, 4DLK.A.
403, 4DOU.A.1001, 4DOU.A.1002, 1DPO.A.246, 2E9B.A.741, 2EA7.A.450, 3EF2.A.304, 3
EF2.A.305, 1EGZ.A.300, 3EHB.A.563, 4EJ7.B.402, 1ELT.A.300, 5ENL.A.438, 4EPU.A.60
1, 2EXH.D.2004, 1EX9.A.286, 2F3C.E.242, 4F8Z.A.409, 1FBL.A.996, 3FG1.A.1501, 2FI
B.A.412, 3FP8.E.601, 3FSJ.X.600, 3FU1.A.301, 2FWN.A.532, 3G4E.A.1, 1G5N.A.402, 4
G60.A.301, 4G60.A.302, 4G62.A.302, 1G87.A.615, 1G9K.A.703, 4GDJ.A.507, 3GG1.A.50
2, 4GG1.A.602, 2GGM.B.402, 4GI6.A.601, 2GJP.A.1486, 2GKO.A.611, 3GK2.A.92, 3GN9.
A.201, 4GN7.A.301, 1GVK.B.1246, 4GW3.A.401, 4GZT.B.510, 2HOK.B.410, 4H1Q.A.303,
1H3G.A.701, 1H5V.A.306, 1H71.P.501, 1H71.P.502, 3H81.C.279, 1H9H.E.1246, 3HB2.P.
482, 1HDF.A.1101, 3HGN.A.250, 3HGP.A.250, 3HI7.A.802, 3HJR.A.603, 1HM9.A.1901, 4
HOW.A.704, 4HPN.A.401, 1HT6.A.502, 1HVX.A.517, 1HY7.A.305, 1HYO.A.1006, 2HYU.A.5
02, 2HYV.A.608, 4I35.A.513, 1I76.A.996, 4I8H.A.301, 3I98.A.627, 3IBZ.A.192, 4IHM
.A.403, 1IOD.G.503, 3IOX.A.903, 2IUF.E.1697, 4IU2.A.301, 4IU3.A.301, 2IXT.A.1311
, 1J0H.A.601, 1J11.A.701, 1J1N.B.493, 1J35.C.501, 4J7M.A.403, 1J9K.A.301, 1JE5.B
.502, 1JI3.B.403, 1JK3.A.403, 2JKE.A.1728, 2JKH.A.1245, 4JZB.A.402, 4JZE.H.302,
4JZX.A.403, 3K37.B.467, 4K3K.B.401, 1K7I.A.483, 1K7I.A.487, 1K7Q.A.485, 4K70.B.1
002, 4K89.A.408, 4K9P.A.601, 1KA1.A.401, 2KAY.B.187, 1KAP.P.617, 1KAP.P.618, 1KA
P.P.620, 3KCG.H.500, 4KKF.A.703, 3KM5.A.2011, 3KMV.A.163, 3KQA.B.421, 4KS1.A.501
, 4KTY.A.802, 1KUO.A.703, 4KXY.A.707, 3KZP.A.240, 3L9I.C.1148, 4LJ3.B.403, 4LLS.
A.303, 4LLT.A.303, 3LNH.A.303, 3LNI.A.303, 1LOC.E.688, 3LPD.A.342, 4LQR.A.202, 4
LVN.A.704, 2M0P.A.1201, 3M1H.C.2001, 3MBR.X.300, 4MB1.A.602, 1MCT.A.246, 3MHF.A.
328, 2MIN.B.525, 4MKM.A.403, 2ML1.A.201, 2ML1.A.202, 2ML1.A.204, 2ML2.A.201, 2ML
3.A.203, 2ML3.A.204, 3MMZ.A.500, 4MPR.A.601, 3MVS.A.211, 3MVS.A.214, 3MVS.A.215,
4MWL.A.512, 3MW3.A.301, 4MWV.A.512, 3N1U.A.200, 4N20.A.705, 4N20.A.706, 4N2B.A.
705, 4N2E.A.705, 4N2G.A.705, 4N2I.A.705, 1N7V.A.601, 1N9E.A.802, 4NAS.A.503, 1NB
W.A.650, 4NEH.B.701, 3NIF.D.2002, 3NJH.B.502, 3NKQ.A.1003, 1NKG.A.800, 1NNL.B.20
02, 4NOT.A.302, 1NRW.A.903, 1NSC.A.468, 1NUD.A.703, 4NUQ.A.301, 4NUZ.A.1001, 1NX
1.A.3, 2NXP.A.600, 2O72.A.403, 1OAH.B.1526, 1OBO.A.501, 3OJY.A.555, 3OJY.B.538,
1OM6.A.701, 1OM8.A.705, 3OM5.B.1, 3OM6.B.1, 3OTJ.E.1000, 4OUL.B.1201, 2P3U.B.501
, 3P4G.A.401, 3P4G.A.402, 3P4G.A.404, 3P4G.A.405, 3P4G.A.406, 3P4G.A.407, 3P4G.A
.408, 3P4G.A.409, 3P4G.A.410, 3P4G.A.412, 3P4G.B.411, 3P95.A.1, 3PGB.A.903, 4PHN
.A.302, 3PK0.A.280, 4PLS.A.301, 4PMX.A.401, 2PNY.A.228, 3POJ.B.1, 2POO.A.805, 3P
PE.A.401, 1PVY.B.603, 1PW9.A.404, 1PZ7.A.701, 2PZ0.A.501, 2Q16.A.200, 2Q1C.X.294
, 3Q2L.B.703, 3Q4W.A.224, 3Q5I.A.528, 1QCO.A.1002, 4QD2.E.302, 3QGV.A.504, 2QIM.
A.158, 1QLB.A.1658, 4QN7.A.501, 3QU7.A.230, 3QU7.B.225, 2QUB.A.614, 2QUB.A.616,
2QUB.A.618, 4QU6.A.904, 3QXG.B.230, 4R12.A.809, 2R1B.A.1001, 1R6V.A.1, 2R8Y.A.20
1, 3R8Y.A.242, 2RA3.A.1, 2RA3.B.1, 1RQ5.A.819, 3RQ0.A.301, 3RVV.A.225, 1RXO.B.47
7, 1S0B.A.1292, 3S4Y.B.1303, 3S5U.E.221, 3S6J.A.4, 1SAT.A.476, 2SAS.A.187, 1SBH.
A.290, 1SCB.A.276, 3SO0.D.97, 3SVL.A.201, 3T3P.B.2003, 1T5S.A.1004, 1TCM.B.687,
3TEW.A.800, 1TO2.E.450, 1TRK.A.681, 4TSH.B.1502, 1TU5.A.902, 3U1R.A.703, 3U1R.A.
704, 3U1R.A.705, 3U1R.A.706, 3U1R.A.707, 3U8D.A.203, 1ULV.A.2001, 1ULV.A.2002, 4
UM9.B.2002, 4UP4.A.501, 4UP4.A.502, 4USU.A.1471, 1UTM.A.247, 2UWF.A.1369, 1UX6.B
.2002, 1UX6.B.2003, 1UX6.B.2004, 1UX6.B.2007, 1UX6.B.2010, 1UYX.A.1133, 1UYX.A.1
134, 4UZU.A.1484, 1V0Z.B.1477, 3V96.B.304, 1VCL.A.1004, 1VCL.B.1001, 1VCL.B.1002
, 2VCC.A.1917, 1VL9.A.125, 2VNG.B.1214, 3VOC.A.501, 3VTO.A.302, 3VV3.A.401, 2W1W
.B.1135, 4WA3.A.503, 1WC5.C.2100, 2WFK.A.1250, 4WKO.B.502, 4WKO.B.503, 4WK7.A.50
4, 1WMD.A.1003, 3WN6.A.502, 2WOB.E.1161, 1WPC.A.502, 1WRZ.A.154, 2WW3.A.800, 1WZ
A.A.601, 3X17.A.602, 1XKD.A.1005, 2XR9.A.1869, 2XSG.B.1772, 2XVT.F.1137, 2Y09.A.
1242, 1Y6W.A.149, 2Y6D.A.1266, 1Y7B.A.3001, 1Y9Z.A.604, 1Y9Z.B.605, 2YAY.A.1267,
2YEQ.A.1526, 2YGL.A.1413, 1YI7.A.3001, 1YO8.A.1183, 1YO8.A.1185, 1YO8.A.1193, 1
YO8.A.1199, 1YO8.A.1201, 1YO8.A.1203, 1YO8.A.1205, 1YO8.A.1206, 1YO8.A.1209, 1YS
6.A.1001, 2Z2X.A.1007, 1Z6O.A.5302, 1Z70.X.3001, 2Z8X.A.620, 2Z8X.A.622, 2Z8X.A.
623, 2Z8X.A.624, 2Z8S.B.643, 2ZE0.A.552, 2ZPR.A.2001, 2ZQ0.A.901, 2ZUX.A.630, 2Z
UY.A.626, 3ZWH.A.501, 2ZWP.A.402, 3ZXH.A.304, 2A1X.A.450, 1A2F.A.1, 1A7E.A.119,

3ABM.A.516, 1B1X.A.690, 2B2O.B.1500, 4B2O.A.1267, 4B7G.A.3000, 1BEP.A.296, 3BFJ.M.1388, 4BGL.A.1001, 1BKA.A.693, 1BKA.A.694, 2BQ8.X.1305, 2BQ8.X.1306, 2BUZ.B.1541, 2BV0.B.600, 2CAG.A.485, 4CHL.A.501, 1D9Y.A.310, 3DHG.A.502, 1DRY.A.332, 4DTY.A.500, 3DXU.A.360, 3E6S.B.200, 1EO9.B.600, 1EOC.B.600, 1FCD.C.902, 2FR7.A.501, 1FSL.A.144, 1FZ1.A.5001, 1FZ3.A.5002, 1FZ7.B.5003, 2G1M.A.600, 4G51.B.202, 3GE3.A.501, 1GGF.B.760, 4GHF.B.401, 2GJ1.A.605, 3GM6.A.1004, 4GP4.A.602, 4GP5.A.602, 2HOV.A.501, 1HMO.B.115, 2HUO.A.301, 1I4Z.A.601, 4I4G.A.601, 4I4H.A.601, 2INC.A.502, 3IXF.A.139, 1IZO.A.501, 1JNF.A.702, 1JNF.A.703, 4K0F.A.601, 4K9T.A.601, 4K9U.A.601, 4K9V.A.601, 4K9W.A.601, 4K9X.A.601, 3KT7.A.701, 1KW9.B.301, 3LXV.M.600, 4M25.A.401, 4M73.B.403, 3MDT.A.505, 1MMO.D.3, 1MMO.D.4, 3MOO.A.911, 1MRP.A.310, 1MTY.D.4, 3MZS.C.500, 3N2O.A.506, 3NC3.A.406, 2NOX.A.500, 1NX4.B.300, 3OOR.B.802, 1OCZ.A.516, 4OJ8.C.301, 1OQU.C.1009, 1OQU.C.1010, 3OUH.A.600, 1P2H.A.801, 3P3N.A.350, 3PCJ.O.600, 2PCC.A.296, 3PER.A.1001, 4PGO.A.301, 1PHG.A.417, 1PIU.A.402, 3PUQ.A.1, 2Q0J.B.998, 3Q14.A.502, 3Q1G.A.1002, 3Q3M.A.509, 3QFN.A.265, 3QFO.A.264, 2QPP.A.300, 3QPI.B.1001, 3QY6.A.263, 3QY7.A.263, 3QY8.A.253, 4RC8.A.303, 2RDB.A.499, 3RNC.A.499, 3RNC.A.500, 3RNF.A.500, 3RNF.A.501, 1RYO.A.329, 4S1B.A.802, 1SYY.A.1319, 1SYY.A.1320, 1TOQ.A.499, 1TOQ.A.500, 3TMZ.A.501, 3TTX.A.760, 1U74.B.1101, 2VUN.A.402, 3WEC.A.501, 1WRA.B.402, 2X9P.A.1398, 1XK3.A.300, 1XU5.A.1175, 1XVB.A.1170, 1XVF.B.1174, 1XVF.B.1175, 1XVG.A.529, 2Y69.A.516, 1Y8W.A.142, 1YGF.B.147, 1YMA.A.154, 1Z1N.X.602, 3ZK3.A.1311, 3ZLI.A.4001, 3ZPI.A.1407, 4AC8.D.500, 1AHJ.A.208, 3AK3.C.215, 4B2O.A.1266, 1B7Z.A.690, 4BLY.A.500, 4BM1.A.500, 2BMO.A.1441, 3BXD.A.302, 1CG5.A.142, 1CGO.A.128, 1D06.A.501, 1D2V.A.605, 3DHG.D.507, 1DRT.A.325, 3EH5.A.801, 1EOB.B.600, 1EYS.C.612, 4FAG.A.401, 2FDG.A.300, 1FRV.B.537, 1FT5.A.214, 1FZ1.B.5003, 1GBU.B.148, 1GY9.A.300, 1H2K.A.1350, 1H2L.A.1350, 4IGO.A.1000, 4J1X.A.201, 2J2M.A.501, 3K9V.A.520, 4L7Y.B.201, 3LMX.M.600, 4M26.B.401, 1MQV.A.150, 1MXR.A.1003, 3MZS.A.500, 1N04.A.688, 3N1Y.A.503, 3N1Y.A.504, 3OOF.A.304, 3O32.A.300, 3O6J.A.300, 2OGI.A.301, 4OJ8.B.301, 1OUT.A.143, 1P3T.A.300, 3PCA.M.600, 3PCJ.N.600, 2PHD.B.370, 2PQ7.A.221, 3Q3O.A.502, 1QHW.A.433, 3QPI.A.1001, 3QY6.A.264, 3QY8.A.252, 2R2F.A.320, 2RDN.A.1, 3SCF.C.203, 1SMJ.C.472, 1SP8.A.500, 3TKT.A.431, 3U9M.A.201, 3UF9.B.315, 1UOF.A.1311, 1UTE.A.501, 3VER.A.601, 2VE3.B.1444, 1VHB.A.150, 3VSI.B.401, 2VV6.C.1259, 1VZ4.A.1299, 1W2A.X.1302, 3W8M.A.201, 3WAQ.A.201, 1WZD.A.901, 2XMO.B.556, 1XZ5.A.142, 2Y0I.A.1350, 1Y5J.B.147, 2YDE.A.501, 1ZJ9.B.1569, 4KQ7.B.502, 2B2N.A.345, 3HW8.A.580, 1ORP.A.224, 1Q81.K.8346, 1VQ7.Q.9148, 3AGB.A.1, 3ASQ.B.701, 3AST.B.701, 4BR6.A.401, 4BVN.A.1360, 4C7A.B.1159, 4CSH.A.1169, 3CZJ.A.3102, 4CZN.A.1371, 4D1I.A.600, 4D1J.E.604, 3DR3.A.335, 2DV1.A.1000, 3EEB.A.211, 4F3Y.A.301, 4FEW.D.304, 1G5I.C.902, 4GDK.E.301, 2GTW.E.3006, 4GY9.A.207, 3HYS.A.267, 4IOW.D.602, 3I2W.A.304, 4I2R.C.602, 3I44.A.477, 3IJP.A.301, 1IYN.A.298, 2J5W.A.3044, 3JS4.A.208, 1JZ7.C.3103, 3K6A.E.178, 4KA5.A.801, 4KA8.A.806, 3KED.A.951, 1L2T.A.1502, 4L3H.A.404, 1LZS.A.131, 4M4V.A.505, 3MC1.A.301, 4MJD.A.203, 4MM9.A.602, 4MPT.A.402, 4N3M.A.403, 3N3O.A.1000, 4NPJ.B.701, 4NRH.C.401, 4O1G.A.401, 2O34.A.503, 2O34.B.502, 4ODI.B.301, 3OEC.A.300, 2OKQ.A.119, 3OTK.A.586, 2OYC.A.305, 4PMO.A.310, 4PUV.A.405, 4PV3.A.201, 3Q94.A.310, 4QFE.B.305, 1QOP.B.501, 2QZ7.A.193, 1RW9.A.900, 1TQY.H.1094, 1UD8.A.1001, 2V4B.B.1562, 1VI6.A.208, 3VS8.A.501, 3VS8.C.501, 3VW7.A.2012, 3WNM.A.802, 1WPG.D.1300, 2WU2.A.1590, 2WV7.B.401, 3WXO.A.805, 1X0G.A.1002, 1X0G.C.1001, 1X7D.A.1501, 1X7U.A.1000, 2X7J.A.1581, 1XDF.A.401, 2XZK.B.507, 2Y8K.A.1527, 1YAP.A.501, 2Z2F.A.2001, 2ZND.A.195, 3ZQ5.A.1530, 2BCU.A.577, 2BCQ.A.1, 2BCR.A.604, 1CZO.C.606, 4ED3.A.502, 1JJ2.0.8338, 4KYW.A.303, 3OSN.A.423, 4P4P.A.401, 1VQ8.J.9146, 1VQ8.Q.9148, 2A5F.B.1326, 2A5D.B.326, 4A6U.B.1460, 4AFK.A.1507, 2AHR.C.1259, 3AJN.A.136, 2AMF.A.850, 4AMJ.A.1360, 3AR4.A.1000, 3AR7.A.1000, 2AU7.A.206, 2AUT.D.605, 4B1M.A.1680, 3B34.A.951, 1B57.A.364, 3BGA.A.6, 3BIA.X.117, 3BIB.X.117, 3BLJ.B.701, 3BOS.B.302, 2BS2.A.1658, 4BVO.A.1396, 4C1O.A.1731, 3C7F.A.804, 4CCY.A.1297, 4CCY.A.1298, 2CD7.A.1132, 4CIT.A.1454, 3CRN.A.131, 3CYM.A.501, 4D1J.E.602, 4D1J.G.603, 4D7C.A.1544, 3D9R.A.135, 2DDA.A.301, 2DDB.A.301, 2DDB.C.302, 4DD8.D.1005

, 4DEL.A.401, 4DF9.B.501, 4DOU.A.1004, 4DW8.A.304, 2E54.A.1004, 4E6P.A.301, 4E6P
.D.301, 2E7U.A.1003, 3E85.A.162, 3E85.A.163, 1EAS.A.5, 4EAE.A.302, 4EEK.A.302, 4
EEL.A.302, 2EHQ.A.1540, 3EIF.A.1033, 3EIY.A.177, 3EUW.A.343, 3F3C.A.752, 4FDZ.B.
301, 4FET.A.301, 4FEX.A.303, 2FM1.D.344, 2FV7.A.403, 1G3K.A.500, 1G5H.A.901, 1G5
I.A.901, 4G8T.C.502, 3GA5.B.701, 4GAF.B.505, 3GIR.A.373, 4GIB.A.301, 2GJU.A.2001
, 4GKI.D.303, 3GOD.B.327, 4GRX.B.501, 1GV2.A.1191, 3GZA.A.471, 3HON.A.203, 3HON.
A.204, 1H16.A.9001, 3H12.A.500, 1HBN.A.1561, 3HIJ.B.295, 2HIO.B.240, 3HSS.A.268,
 3HVI.A.265, 1HX6.A.705, 4IOW.B.603, 4I29.A.601, 3IAN.A.1, 3IC3.C.100, 3ICF.A.51
5, 3IFV.A.402, 3IGZ.B.601, 3IJP.B.301, 2IJA.A.401, 3IPO.A.436, 3IPP.A.438, 4IQZ.
A.316, 1IYN.A.297, 2IZV.A.1430, 4J07.A.201, 1JAY.A.215, 2JBW.C.1367, 4JB3.A.301,
 4JDO.A.301, 4JEX.B.511, 2JHJ.B.1296, 2JHN.B.1299, 1JMM.A.3001, 3JS4.D.208, 1JTP
.A.501, 1JTP.L.503, 4JTE.C.301, 4JTF.C.303, 4JTG.C.302, 4JTH.C.303, 4JTJ.C.302,
4JTK.C.302, 1JZ7.A.3102, 1JZN.A.1139, 3K1U.A.412, 1K2X.A.801, 3KRS.A.301, 1KSU.A
.810, 1L5B.A.302, 3L7X.A.142, 4LGN.A.827, 4LIZ.A.202, 1LLA.A.631, 1M4Y.A.252, 4M
48.A.701, 4M4U.A.501, 4M60.A.502, 3M9Y.B.254, 4M9B.A.201, 4MMF.A.602, 3MQD.A.500
, 3MYV.A.502, 3MZG.B.211, 3N83.G.707, 4N9S.A.402, 4N9V.A.404, 3NGJ.A.249, 3NMB.A
.1, 3NNB.A.401, 4NPI.A.602, 4NRH.A.401, 4NSJ.A.222, 4OOC.A.401, 2O34.A.501, 4O47
.A.401, 3OEC.C.300, 4OF8.A.301, 3ON4.C.189, 3OPK.A.301, 2OYN.A.201, 1POZ.A.1633,
 4P33.A.401, 4PD6.A.502, 3PGO.A.147, 4PMO.A.311, 4PMO.A.312, 2POC.A.5001, 4PSR.A
.619, 4PYJ.A.301, 1Q3X.A.800, 4Q5K.A.302, 4Q69.A.501, 2QJY.R.2001, 2QKF.C.283, 2
QV6.A.303, 4QXK.A.402, 1R4P.A.4003, 4R6K.A.501, 2R85.A.600, 2RGI.B.98, 3RU5.A.13
3, 1RWH.A.900, 1S5D.A.241, 1S5E.A.241, 1S82.A.4, 3SIB.A.223, 3SSB.A.995, 1T3M.A.
801, 1T8U.B.701, 3TDQ.A.117, 4TKX.L.705, 4TKX.L.706, 1TQY.B.1091, 1U7H.B.912, 4U
99.A.203, 1UD2.A.1001, 3UES.B.503, 3UF4.A.601, 3USL.A.752, 2V4V.A.3052, 2V4B.A.1
566, 2V79.A.1116, 3VGL.A.323, 2VNZ.X.9252, 2VPB.A.1398, 1W15.A.2002, 1W9S.A.1141
, 3WAY.A.914, 3WC3.A.502, 2WCB.A.101, 2WDQ.A.1590, 4WFX.A.504, 2WGE.A.1426, 1WKY
.A.504, 2WOF.A.1728, 2WOI.D.1489, 2WWF.B.214, 2X7J.B.1581, 4X9K.A.401, 2XNA.C.12
16, 2XZI.A.502, 1Y0P.A.1810, 2Y5F.A.1245, 2YNQ.A.1392, 1YYA.A.1001, 2ZHJ.A.321,
2ZND.A.196, 1ZOD.A.435, 1ZOR.A.1001, 1ZOR.B.1002, 2ZQ3.A.160, 3ZWF.B.1365

Table S145. all-ligand-number combined metal, compressed group

	size	largest_angle*	middle_1*	middle_2*	middle_3*
1	"705"	"161.5+/-7"	"53.5+/-4.8"	"83.7+/-5.1"	"103.7+/-8"
2	"171"	"151+/-13.5"	"69.4+/-8.8"	"81.9+/-7.6"	"96.3+/-10.2"
3	"108"	"129.7+/-12"	"54.9+/-5.2"	"75.5+/-8.2"	"86.3+/-10.8"
4	"479"	"149.1+/-9.4"	"56+/-3.9"	"90.9+/-5.7"	"102+/-5.7"
5	"1020"	"171.5+/-3.8"	"74.4+/-5.7"	"86.7+/-3"	"98.9+/-3.7"
6	"649"	"162.2+/-4.8"	"71.3+/-4.6"	"84+/-3.4"	"104.7+/-5.6"
7	"269"	"152.1+/-10.4"	"56.1+/-3.9"	"88.7+/-6.4"	"101+/-6.2"

	middle_4*	smallest_opposite_angle*	Tetrahedral	TrigonalBipyramidal
1	"152.7+/-7.7"	"73.6+/-7.3"	"0.019"	"0.035"
2	"126.5+/-11.5"	"54.6+/-6.5"	"0.022"	"0.017"
3	"100.5+/-11.2"	"86.2+/-13.3"	"0.004"	"0"
4	"134+/-7"	"97.8+/-7.2"	"0.11"	"0.046"
5	"159.8+/-5.5"	"53.7+/-4.4"	"0.022"	"0.119"
6	"154.4+/-5.4"	"51.8+/-3.1"	"0.028"	"0.099"
7	"118.1+/-8.7"	"114.4+/-12.6"	"0.189"	"0.066"

	TrigonalBipyramidalVA	TrigonalBipyramidalVP	Octahedral	SquarePyramidal
1	"0.083"	"0.144"	"0.026"	"0.085"
2	"0.051"	"0.092"	"0.002"	"0.027"

3	"0.031"	"0.039"	"0"	"0"
4	"0.082"	"0.081"	"0"	"0.005"
5	"0.127"	"0.284"	"0.134"	"0.267"
6	"0.148"	"0.222"	"0.066"	"0.171"
7	"0.092"	"0.066"	"0"	"0"

	SquarePyramidalV	SquarePlanar	TrigonalPrismatic	TrigonalPrismaticV
1	"0.178"	"0.121"	"0.043"	"0.12"
2	"0.08"	"0.049"	"0.017"	"0.082"
3	"0.064"	"0"	"0"	"0"
4	"0.045"	"0.004"	"0.001"	"0.088"
5	"0.326"	"0.299"	"0.061"	"0.14"
6	"0.249"	"0.215"	"0.119"	"0.203"
7	"0.018"	"0"	"0"	"0.055"

	PentagonalBipyramidal	PentagonalBipyramidalVA	PentagonalBipyramidalVP
1	"0.018"	"0.043"	"0.052"
2	"0"	"0.001"	"0.006"
3	"0"	"0"	"0"
4	"0"	"0"	"0"
5	"0.085"	"0.085"	"0.178"
6	"0.087"	"0.103"	"0.171"
7	"0"	"0"	"0"

	SquareAntiprismatic	SquareAntiprismaticV	HexagonalBipyramidal
1	"0.003"	"0.038"	"0"
2	"0"	"0"	"0"
3	"0"	"0"	"0"
4	"0"	"0"	"0"
5	"0"	"0.083"	"0"
6	"0.005"	"0.124"	"0"
7	"0"	"0"	"0"

	HexagonalBipyramidalVA	HexagonalBipyramidalVP
1	"0"	"0.008"
2	"0"	"0"
3	"0"	"0"
4	"0"	"0"
5	"0"	"0.046"
6	"0"	"0.03"
7	"0"	"0"

Table S146. Cluster members of all-ligand-number combined metal, compressed group

[1] "Cluster 1"
3CSQ.B.335, 4DHL.B.501, 4DWZ.A.302, 3H90.C.2, 1HR6.B.501, 4ICQ.B.502, 4JBS.B.100
9, 4JX5.D.1103, 3M3B.A.156, 1MH2.A.201, 2QLA.D.503, 3SZZ.A.502, 3U94.B.259, 1VKG
.A.400, 2XPY.A.1673, 1ANJ.A.450, 3BXM.A.1751, 4CBY.A.2034, 3DFF.A.274, 1DK4.A.29
1, 4DYO.A.501, 3E4A.A.2000, 4FW3.A.301, 1G12.A.200, 2G9Y.B.450, 2GO4.B.602, 1H48
.A.900, 1K07.A.2, 4L3T.A.1101, 2OOT.A.1751, 4OP4.A.302, 3Q9B.E.345, 1QIP.B.901,
3RBU.A.1751, 1RTQ.A.701, 3SJG.A.1751, 1TXR.A.502, 3WOU.A.201, 4WD8.C.302, 1XJO.A
.901, 1YGD.A.142, 4BE1.A.1382, 4D6N.A.1188, 2G8H.A.301, 2A9F.A.801, 1AO0.A.469,
2ATX.A.201, 3C41.J.603, 4E4F.A.504, 2EWG.A.3002, 3FDG.A.357, 3G15.A.605, 4GME.A.
501, 4GMJ.B.302, 4GYZ.I.402, 3IJQ.B.386, 2IOA.B.5003, 1JCT.A.498, 3K5H.A.401, 3K
5H.A.402, 1KFS.B.2, 4KMQ.A.1103, 3LDO.5.54, 2LVJ.A.101, 3MOO.A.551, 3MLE.A.222,

3N3T.B.803, 4ORK.A.502, 3P0W.A.471, 3PMG.A.562, 2PUI.B.401, 3Q8U.E.159, 3RUW.D.5 44, 4S17.A.501, 3U2E.B.4, 3U7F.B.1, 4UM9.B.2001, 2V0N.A.601, 2W8D.A.1636, 3WBH.B .503, 4WB8.A.402, 1WC6.C.2202, 3WO0.A.503, 3WQS.B.502, 4KI8.C.602, 4CS3.A.1464, 4FFR.A.404, 3G9D.A.299, 1JR4.A.300, 3OTB.A.401, 2VBI.B.1000, 4IFD.J.1105, 4N41.E .101, 3CWH.A.391, 2D32.A.1523, 1DIE.A.398, 2DW7.B.2002, 3FDG.B.356, 4FHY.A.402, 3HB0.B.702, 4I40.B.301, 4LNI.D.504, 1MXA.A.411, 1SBJ.A.163, 4U03.A.504, 1YHM.A.4 01, 1SO2.C.475, 1BSS.A.433, 3KHL.A.415, 1SON.A.403, 3A7Q.A.4001, 2AAO.B.299, 1AE I.A.320, 1ALA.A.400, 1AXK.B.395, 1B09.C.301, 3BRX.A.328, 3BXK.A.152, 1CTR.A.150, 2D3P.A.237, 2D7F.A.240, 3E3R.B.193, 2E6V.D.11, 1ETH.A.449, 1F4M.E.105, 2FH3.A.1 002, 2FHC.A.2405, 3FLP.L.302, 1FZC.C.1, 1FZD.G.1, 4G0D.A.503, 4G9L.B.306, 1GYK.A .1206, 2HOK.A.401, 1IHO.A.2, 2IWV.B.1284, 2J3U.C.1292, 2J64.C.1222, 1J84.A.366, 4JDZ.B.704, 4JDZ.A.702, 1JN2.P.238, 3JQL.A.121, 1K90.D.801, 1LGN.A.302, 1LU1.A.3 01, 1M1J.C.501, 3M83.E.407, 3M8D.A.710, 4MBE.A.202, 2ML3.A.206, 2MLS.A.305, 1N29 .A.125, 1NX0.A.900, 4OJQ.B.1002, 4OMC.E.607, 1OTN.A.250, 3OX5.B.502, 2OXE.B.600, 2OYH.B.2, 4P99.D.512, 1Q3A.C.478, 1QO0.A.302, 2RJP.B.4, 1S0C.A.1879, 3TRP.A.360 , 3UJO.D.304, 4UM8.A.2004, 1VAH.A.500, 3VU1.A.1001, 1W2M.A.1442, 4W4Q.A.401, 4WK 2.B.502, 1WMZ.B.203, 3WYN.B.402, 2WZE.A.1552, 1XFE.A.100, 1YAX.C.1003, 1Z3J.A.26 6, 2ZOC.A.501, 2ZOC.A.502, 2ZWO.A.401, 2AGO.A.403, 3CFR.A.910, 4DTS.A.1002, 2G8K .A.401, 4K4I.A.606, 1N56.B.403, 1A2X.A.160, 1A2X.A.161, 3A7Q.A.4002, 3AAJ.A.991, 1AEI.A.319, 5AER.A.201, 1AFD.3.2, 1AJ4.A.163, 1AMY.A.502, 1ANN.A.319, 1ANW.B.35 4, 1BAG.A.431, 3BOW.A.719, 3BOW.B.403, 1C9U.B.1002, 4CAG.A.605, 3CGA.A.102, 3CHK .A.502, 1DCY.A.198, 2DDY.A.174, 2DPK.A.2001, 2E3X.A.803, 3E3R.B.194, 4E52.A.403, 2E6W.A.301, 3E9T.A.1, 3EAD.B.1002, 3EAD.B.1004, 3ECQ.A.2000, 4ELF.C.201, 4ELG.F .202, 2ERO.B.702, 1ESP.A.322, 2EXI.D.3004, 1FDK.A.124, 4FGC.B.204, 3FLP.B.301, 3 FLT.A.302, 4FL4.C.401, 4FL4.C.402, 1GOH.A.290, 4GKX.B.301, 1GQM.A.1089, 4GTW.A.1 012, 2HOK.A.407, 1H4B.A.1085, 1H4B.A.1086, 2HQ8.B.303, 2HYW.A.505, 2I08.A.200, 3 IA7.A.402, 3IA7.B.402, 3IGO.A.601, 1IME.A.278, 1IXX.B.124, 1IZJ.A.1002, 2J1G.D.1 289, 1JBA.A.501, 4JBE.A.502, 4JEO.A.401, 4K1C.A.506, 3K8L.B.800, 1K96.A.92, 2KAY .B.188, 3KF9.A.302, 1KTW.A.3, 1KX1.A.222, 1LGC.A.301, 1LHV.A.401, 4LMF.A.304, 4L MH.D.811, 2LV6.A.204, 2LVK.A.102, 1LWJ.B.10, 1M34.B.2299, 2M5E.A.2001, 1M8T.C.10 03, 4MDV.B.403, 2ML1.A.206, 2ML3.A.205, 4N25.A.706, 1NAE.A.900, 4NDD.B.402, 4NEN .A.1115, 1NIW.A.1001, 1NIW.A.1002, 1NIW.C.1005, 3NOM.B.263, 1NUB.A.301, 1NUB.A.3 02, 1NX2.A.4, 1O9I.C.269, 1ODB.F.1092, 3OX6.A.501, 3OX6.A.502, 4P5W.A.1001, 4P99 .A.512, 1PK8.A.817, 3PM8.B.514, 2PQY.A.500, 1PTK.A.281, 4Q4W.1.905, 1Q8H.A.72, 1 QNI.E.903, 3QRX.A.170, 3QRX.A.173, 2RHP.A.1176, 2RHP.A.26, 3RV2.A.405, 3S5U.C.22 0, 2SAS.A.186, 1TCF.A.162, 1TEC.E.343, 1THL.A.3233, 3TRQ.A.358, 3TRQ.A.360, 1TTX .A.110, 1TYE.A.1405, 1TYE.A.1406, 1TYE.A.1407, 1U7W.A.501, 1UG9.A.2006, 3UJQ.D.3 05, 4UM8.C.2004, 3USU.H.272, 1UZJ.C.3649, 1V1G.A.1211, 2V3T.B.1264, 1V3J.B.690, 2VB6.B.1151, 1W2M.C.1441, 3W9T.A.1005, 3W9T.C.1009, 3WHU.B.502, 1WMD.A.1001, 2WO 9.D.1270, 1WRL.D.104, 3WU2.O.301, 1YUU.B.199, 2Z2D.A.266, 2Z8S.A.644, 2Z8S.A.647 , 2Z8S.B.642, 2ZBA.A.461, 2ZJ7.A.627, 2ZKT.B.413, 2ZUY.A.624, 2A40.B.1273, 2ASP. A.600, 4AW7.A.1599, 4AWN.A.301, 3B9X.A.400, 4B97.A.1152, 4B9P.A.1167, 3BMV.A.684 , 4BM1.A.402, 2BOQ.A.1352, 1CGV.A.692, 1CGY.A.692, 1CLC.A.651, 1CXL.A.689, 4CZN. A.1369, 1D3C.A.688, 3DED.C.508, 3DHP.A.497, 4DLK.A.402, 1DTU.A.688, 1DV8.A.1001, 2E39.A.502, 1E05.A.689, 3ER9.B.901, 2EWE.A.702, 4EW9.A.203, 2F3Y.A.1174, 4FCS.A .403, 2FF2.A.601, 3FM1.A.352, 3FM4.A.352, 3FMU.A.352, 1G43.A.200, 1G87.A.616, 1G 94.A.800, 2GUY.A.601, 1H3J.A.1346, 1HUP.A.302, 1HX0.A.500, 2HYV.A.601, 1I75.A.16 88, 4I71.A.402, 1IA6.A.1263, 1IOD.G.506, 1J34.B.512, 1JAE.A.500, 1K72.A.779, 1KC K.A.691, 1KCL.A.1692, 1KWT.A.502, 1KXH.A.800, 4KZW.A.302, 1LLP.A.352, 3M5Q.A.371 , 2MAS.A.316, 1MCX.A.347, 1MN2.A.371, 1NBC.B.156, 2NZM.A.407, 1OT1.A.1693, 3PDD. A.192, 1PEZ.A.891, 3PF2.A.2, 1PJ9.A.891, 3POE.A.2, 2PWA.A.1280, 3Q3U.A.341, 3QEE .A.21, 1SH7.A.1290, 1SNN.A.403, 3TEC.E.344, 1UA7.A.601, 1UOV.A.1419, 3VM7.A.501, 2VMH.A.3050, 2VMI.A.3050, 1W3H.B.1348, 2WHK.A.1339, 1WMD.A.1002, 3WMS.A.801, 2W NX.A.1163, 2WZS.A.800, 1XKD.A.1006, 1YO8.A.1182, 2ZIC.A.944, 3ZQX.A.1146, 3FD2.A

.374, 4K4G.I.603, 4K4H.A.604, 1N48.A.501, 3NAE.A.906, 3NHG.A.908, 3QEW.A.905, 3QEX.A.905, 3S9H.A.905, 3SCX.A.906, 3SQ2.A.904, 2AAO.A.294, 1AFD.1.2, 3AMR.A.901, 1ANN.A.320, 4ANR.A.400, 1B1G.A.76, 3B2Z.B.2, 1BJF.A.403, 1BJF.A.404, 3BJU.A.606, 1BLI.A.700, 2BPE.A.1245, 2C0T.B.1507, 4C0K.A.1615, 3C7F.A.803, 4CAJ.C.1323, 2CCM.A.1193, 1CGE.A.304, 4CGT.A.685, 2CHN.B.1716, 3CIO.K.401, 1CXE.A.690, 1DB4.A.198, 4DH2.B.101, 4DH2.B.102, 2DIJ.A.689, 4DLK.A.401, 3DSL.A.2, 1DTL.A.202, 1E43.A.504, 3EKI.A.601, 4ELG.B.202, 4ELG.C.202, 1E07.A.689, 2ERO.A.701, 1ESP.A.319, 3ETO.A.2002, 1EXR.A.1001, 3FAW.A.4, 3FCS.A.2008, 2FH1.A.2003, 1FIF.C.2, 3FLT.A.301, 1GOH.A.291, 4GON.A.202, 1GGZ.A.152, 4GKY.A.302, 1HDH.A.1528, 4HEX.A.204, 1HQV.A.997, 1HT6.A.501, 4I2Y.B.504, 4IRZ.A.2006, 3IUC.A.2, 2IWA.A.501, 1J55.A.101, 4JRF.A.601, 3KCP.A.702, 3KEZ.B.501, 3KF9.A.301, 4KHO.A.1001, 1KP4.A.200, 1KXT.A.4001, 4L03.B.502, 3LND.A.209, 2MLR.A.303, 4MNO.A.301, 4MSP.A.202, 3NJH.C.503, 3NOK.A.257, 4NUQ.A.302, 1O9I.A.269, 1O9I.E.269, 2OO4.A.5002, 4P5F.B.501, 2P69.A.305, 4P99.A.515, 3PDD.A.194, 1PVY.A.503, 4QB2.A.202, 1R17.A.599, 4R1D.A.601, 4RDQ.A.504, 4RDQ.B.502, 4RGP.B.302, 1SRA.A.301, 3T3P.A.2005, 3T3P.A.2006, 3T3P.A.2007, 1TMQ.A.1001, 3TRP.A.358, 4U65.E.302, 4U6B.A.501, 3U8D.A.202, 3UBG.A.901, 3UBH.A.856, 1UKT.B.690, 4UM9.A.2002, 4UM9.A.2004, 1UX6.B.2006, 1UZJ.B.2648, 4V29.A.1178, 1V3J.A.688, 2VN5.B.101, 2VN6.B.1066, 2VNG.A.1213, 1W0P.A.1779, 1W0Y.H.1259, 2WG7.A.1126, 3WHT.B.502, 4WJK.A.503, 4WKO.A.502, 4WKO.A.504, 3WNO.A.801, 1WPC.A.503, 2WVZ.A.800, 1X2T.A.603, 1X05.A.5, 2Y5I.A.102, 2Y8K.A.1526, 2YEQ.A.1525, 2YHG.A.1936, 1YO8.A.1194, 1YO8.A.1207, 1YO8.A.1210, 2ZFD.A.230, 3ZHG.A.1323, 2ZUX.A.633, 2ZUX.A.634, 2ZUY.A.623, 2ZZV.A.501, 3E1I.B.502, 1EUB.A.277, 1KTW.A.496, 1KTW.A.9, 2QPS.A.501, 1W52.X.602, 2B1X.E.502, 1BLF.A.700, 1BLF.A.701, 2BOY.G.1255, 2BUR.B.600, 1CE2.A.690, 1CE2.A.691, 3DHI.A.601, 4E2P.A.401, 3GJB.A.320, 4HOW.A.701, 1H76.A.703, 1HAB.A.200, 2HMO.A.450, 3IBO.A.999, 4KVQ.A.302, 1LCT.A.400, 1LFG.A.693, 1LFG.A.694, 1LKM.A.601, 4M1I.A.402, 1MTY.D.3, 4N71.D.201, 1NNT.A.333, 3OOR.B.803, 2O7U.A.500, 1OVT.A.689, 4P1B.D.502, 3PCC.O.600, 3PCF.M.600, 3PCH.M.600, 3QJV.A.801, 3QYT.A.680, 2RDB.A.500, 1SQD.A.500, 1TFD.A.950, 3UF9.A.315, 3VE1.B.702, 1VFD.A.400, 3VMG.B.501, 3W54.A.502, 3WFC.B.803, 3WFD.B.803, 1YUZ.A.301, 2ZI8.A.701, 3ZK4.A.800, 4AC8.A.500, 1B1X.A.691, 1B7Z.A.691, 3CHH.A.501, 3EE4.A.317, 1F9B.A.690, 3GCF.D.501, 1HDS.B.146, 3IS8.E.162, 1LKO.A.601, 1LKP.A.601, 1LTV.A.900, 3MPS.D.172, 4NB8.B.501, 3OWO.B.384, 3PCC.N.600, 3PCD.M.600, 3PCH.R.600, 4PGO.A.302, 1PRC.M.607, 3Q1G.B.1002, 3QHB.A.182, 3QVD.C.175, 4RC6.A.301, 1RNR.A.402, 1SQ3.A.902, 1TKP.D.302, 2UW1.A.1359, 2VZB.B.6204, 3W54.A.501, 3W54.B.502, 4WWZ.B.301, 1XZW.A.429, 1YUZ.A.302, 1YV1.B.301, 4NLZ.A.402, 2E5X.A.302, 4IBO.A.401, 3N80.B.602, 2PKC.A.280, 1WX5.A.282, 3DYQ.B.901, 3GCD.B.215, 3I01.A.501, 1QY1.A.204, 4NLK.A.403, 1U8R.A.1101, 4DD8.A.1005, 4G8T.B.502, 1GVH.A.1399, 4054.A.302, 1SU4.A.997, 1TC8.A.121, 3VD3.D.3101, 2WDO.C.601, 1ZEL.A.401, 3C0W.A.303, 3C5G.B.808, 1VQ5.A.9145, 4ATF.A.500, 3BC9.A.704, 3BH4.A.489, 2DIE.A.781, 4DMI.A.202, 2GJP.A.1489, 2HEU.A.4001, 2HZG.B.1102, 2ID4.A.907, 4IQL.A.407, 4J43.A.902, 2JBA.A.1127, 4JQR.A.301, 3KQB.A.303, 4M48.A.702, 4MPY.C.503, 3MV1.3.3101, 1OBO.A.504, 2P3Z.A.501, 3Q2H.A.701, 3TSH.A.604, 3USZ.A.902, 1V8Z.A.389, 3VD5.B.3101, 3VHS.B.52, 3W5N.A.1211, 3WG7.N.605, 1WPC.A.504, 2WQK.A.254, 1WX5.C.282, 1XAR.B.201

[1] "Cluster 2"

4BLB.D.910, 4CPA.I.308, 1F30.B.201, 1F30.E.201, 4IOD.A.502, 4K0D.B.203, 4KJG.A.1001, 3M4B.B.108, 1NL4.B.500, 1RLY.A.61, 2V8V.A.1456, 3V94.D.702, 2ANU.B.505, 4HDT.A.400, 3ISO.B.220, 3M7P.A.953, 1N4P.H.378, 2W9M.A.1565, 1YEW.G.662, 1ZZM.A.403, 2OX8.A.4, 3R8B.B.122, 3R8B.D.122, 3R8B.P.122, 2VME.A.501, 1GUS.A.1069, 4GWS.A.402, 2J1L.A.1195, 4NHO.A.1402, 3PLS.A.1, 3A6P.H.1178, 1QVG.0.8010, 1TTT.B.407, 2CN8.A.1504, 2D33.B.1524, 2DW7.A.2001, 4FOP.A.501, 2F1I.A.501, 4FMC.B.203, 1GXB.C.1346, 3HQP.A.502, 3IG8.A.696, 4K10.A.405, 3LZ9.A.852, 3MQT.W.395, 4NOG.A.405, 4NU1.A.404, 3OB8.D.3001, 4P9D.B.202, 4PYK.A.302, 3RBM.C.1002, 1S6P.A.601, 4U81.A.501, 3UJ2.H.430, 4UOR.C.699, 2VON.A.502, 1V14.C.1134, 1EQR.B.902, 4K6T.E.412, 2I3P.B.1, 3RBE.A.415, 4RIC.A.1101, 3ALA.E.1764, 2BBM.A.182, 3BJU.C.606, 2BTW.B.400

, 2C10.C.1773, 2CHO.A.1717, 1CTR.A.149, 2DDY.A.175, 3E1I.C.502, 4EJ7.C.403, 1EXZ .C.805, 3FAX.A.1223, 2FH2.B.2003, 2FH3.B.2001, 1FIH.B.3, 1G1Q.C.803, 3HR4.H.202, 3IJE.A.4004, 3IJE.A.4007, 3IJE.A.4008, 3IKQ.A.403, 1IVE.A.470, 4JWU.A.502, 2K0E .A.153, 2K2F.A.94, 1K9I.A.407, 3KOO.H.302, 1LT9.B.1, 4MDV.B.404, 2MLS.A.304, 3NP 5.A.732, 4OMD.F.604, 2OX9.B.803, 4P99.A.505, 4PJ0.A.601, 4POQ.C.401, 4POQ.E.401, 2PRK.A.281, 4Q6O.A.501, 1QDO.B.243, 1QNI.D.903, 2QVF.B.704, 2RGB.A.601, 2RHP.A. 11, 2RHP.A.27, 1RTM.2.3, 1S1E.A.225, 2SNI.E.276, 1TNQ.A.91, 4UM8.B.2003, 3VI3.B. 2001, 2VRO.D.1006, 1WOP.A.1781, 3W9T.G.506, 4WFF.A.304, 1WT9.A.1001, 3ODH.A.196, 1ATN.D.264, 1CFF.A.150, 2E6W.A.300, 4ELG.A.202, 4IAV.A.420, 1KBO.A.801, 1KV9.A. 802, 4M7Z.B.409, 2ML2.A.205, 1XJL.B.340, 1ZFS.A.104, 1J34.A.511, 3AKB.A.173, 3BO W.B.404, 2DW2.B.711, 2E3X.A.801, 3GE4.A.167, 1GQM.A.1090, 1GQM.C.1088, 2HOL.A.10 15, 4JWS.A.502, 3LMW.A.9, 1LRW.A.702, 2M29.A.401, 2NWH.A.402, 1T9H.A.414, 3U24.A .602, 1WOO.A.1781, 3WCV.B.203, 4GAM.F.602, 1F9B.A.691, 3HH8.A.1, 3W54.B.501, 1G8 G.A.525, 1G8H.A.523, 2WOO.B.1728, 4DOA.A.402, 4DOB.A.402, 2FMQ.A.340, 3JPT.A.340 , 3PNC.A.2, 2PXI.A.340, 3INJ.C.601, 4LCZ.B.320, 4M5P.A.505, 4NTL.A.311, 1SVY.A.2 , 4KLI.A.403, 3MBY.A.340, 3GQ9.A.1, 3Q9C.A.344, 1VK1.A.302

[1] "Cluster 3"

4LMG.B.202, 3A1Z.C.226, 1AJD.A.450, 1ALH.B.450, 1B0N.A.1002, 4C98.A.1266, 2CIH.A .213, 1F30.H.201, 1F30.I.201, 2F4L.A.1400, 4IOZ.A.504, 4KEQ.A.301, 1KH5.B.950, 3 KVE.C.489, 2L1U.A.144, 3NQ5.A.508, 2OGJ.A.418, 1P4Q.B.302, 1Q9U.B.402, 2QQ4.I.13 9, 3SOW.A.7, 2VZ5.A.1131, 2W88.C.107, 2WC0.B.3012, 2WKN.A.412, 2X3C.A.1342, 1ZKX .A.423, 2NVQ.A.2001, 4P0P.A.601, 2AEK.A.804, 2AQX.A.601, 1AZT.B.406, 1DOA.A.199, 3EPS.A.1606, 3EQL.N.1528, 4F38.A.202, 2G5H.B.501, 1H8H.F.601, 4I1O.C.201, 3IG8. A.697, 1JFG.B.703, 3KRF.A.904, 3O7L.B.352, 4OAU.C.803, 1S4E.B.1600, 3U87.B.403, 2UXR.B.1398, 1WBQ.C.1441, 1YXI.A.342, 1ZOT.A.901, 3ZXT.A.1278, 1BCZ.A.410, 4J2A. A.1006, 4RIC.B.1101, 1AFB.2.3, 4BY6.A.3080, 2E0X.B.701, 2E6V.E.12, 3EAD.C.1001, 2FH3.A.1003, 3GHG.L.601, 2IEZ.A.219, 1IT4.A.200, 2J3G.A.1289, 4JBE.A.503, 1JED.B .535, 2KBM.B.101, 4L76.B.402, 4L76.D.402, 4L76.E.402, 1LGC.C.301, 4LLF.O.401, 1L WU.C.5, 4M8D.B.305, 4MDV.A.404, 4OVG.A.404, 4POS.B.401, 1QDO.D.247, 3RBX.A.601, 2RHP.A.15, 3RRY.A.202, 3S55.D.280, 1SAV.A.321, 1SUI.B.306, 1TCF.A.164, 2TEC.E.34 4, 2W67.B.1590, 1WD9.A.902, 2WM4.A.1430, 2XJO.B.1175, 1XZO.A.1014, 1YN8.B.1008, 4CMY.B.1165, 1FRF.L.565, 1GUP.D.351, 2OHJ.A.501, 2PHD.C.370, 1SQ3.C.908, 1U8R.D. 4104, 3BFT.A.1007, 4D1I.H.600, 3FGH.A.180, 1G8G.A.522, 1G8G.A.527, 1G8H.A.526, 4 K70.B.1011, 4L73.A.406, 2NWH.A.404

[1] "Cluster 4"

4LMG.D.202, 4MTD.A.202, 3A1Z.A.226, 3A52.A.1001, 2A7G.E.401, 4A94.B.501, 3AAK.A. 992, 3AIG.A.999, 2AIG.P.999, 1ANJ.B.450, 2ANP.A.501, 2ANP.A.502, 4AR9.A.1731, 1A TL.A.401, 4AWZ.A.3230, 3AYU.A.415, 1B0N.A.1001, 2B13.B.401, 3B2Z.A.1, 3B35.A.292 , 3B3C.A.501, 3B4N.B.715, 4B52.A.401, 1B8J.A.451, 2BH3.A.1001, 3BHX.A.1751, 3BI0 .A.1751, 3BI1.A.1751, 3BKK.A.701, 3BKL.A.701, 3BKN.B.201, 3BOO.A.500, 4BP0.A.131 4, 1BYF.B.302, 4C1H.A.351, 2C20.B.601, 2C6P.A.1751, 2C6P.A.1752, 2C6C.A.1751, 4C A6.A.1001, 1CBX.A.309, 2CEA.E.1603, 1CGL.A.301, 1CP7.A.901, 1CP7.A.902, 6CPA.A.3 08, 7CPA.A.308, 8CPA.A.308, 4CVR.A.1159, 4CVT.A.1159, 4D0Y.B.1239, 3D10.A.95, 1D 1S.B.406, 1D1T.C.407, 3D4U.A.309, 1DCE.B.900, 3DFM.A.401, 1DK4.B.591, 4DPR.A.701 , 3DSX.B.332, 2DSN.A.2001, 4DYO.A.502, 1DZW.P.999, 3E30.B.1001, 3E32.B.1001, 3E3 3.B.1001, 3E34.B.1001, 3E37.B.1001, 1E49.S.999, 2E46.A.172, 1EC5.B.50, 1EC5.C.50 , 2EG7.A.400, 2EG8.A.400, 3EHY.A.264, 4EJ5.A.501, 2EK8.A.1002, 2EK9.A.1002, 1ELX .B.451, 3ELM.A.300, 2EV6.B.2151, 1EW9.A.450, 3EWJ.B.2, 3F28.A.321, 3F2P.A.2005, 4F7O.A.301, 3FFZ.A.1300, 3FGD.A.321, 3FH4.A.301, 3FJU.A.999, 4FMP.B.400, 3FU1.B. 201, 1FUA.A.216, 3FUK.A.701, 4FYQ.A.1012, 4FYR.A.1013, 3G42.A.500, 4G9L.B.301, 2 GA3.A.450, 1GE7.B.200, 3GIP.A.484, 4GTM.B.501, 4GTP.B.501, 4GTQ.B.501, 4GTR.B.50 1, 4GTV.B.401, 2GVI.A.302, 3GZE.A.1, 3H90.B.294, 1HA5.C.4221, 1HDU.E.1308, 1HEE. E.1308, 1HFS.A.257, 1HI9.A.300, 2HIH.A.601, 3HTR.B.118, 1HYT.A.805, 1HZ5.A.103, 2IOO.A.580, 4I11.A.502, 4I2F.A.603, 3I9F.A.1, 4ICR.A.502, 2IEJ.B.939, 4IE7.A.601

, 1IGB.A.502, 3IQ6.B.201, 3IQ6.C.201, 2IQ6.A.293, 3IT7.A.183, 4IXN.A.401, 2J13.A
.1236, 1J2U.A.301, 4J5H.A.301, 2JAZ.B.600, 4JBS.A.1008, 1JI3.A.402, 4JJI.A.402,
1JK9.C.302, 2JLP.B.226, 1JML.A.102, 1JPU.A.371, 2JT5.A.257, 4JYW.A.801, 4JZ0.A.8
01, 1K53.A.1003, 4K5L.A.1101, 4K5M.A.1101, 4K5O.A.1103, 1KAP.P.613, 4KA7.A.801,
4KAY.A.601, 2KBX.B.299, 1KBE.A.1, 4KFT.C.303, 1KH5.A.450, 2KIK.A.50, 3KNS.A.229,
1KR6.A.405, 1KS7.A.405, 3KW0.C.162, 1L0Y.B.702, 3L0T.B.2, 4L2L.A.701, 4L9P.B.60
1, 4LCQ.A.602, 1LD7.B.1001, 1LD8.B.1001, 1LFW.A.1001, 1LGD.A.262, 3LPE.D.60, 4LP
6.A.302, 3LQ0.A.999, 4LQG.A.801, 4LTE.A.1101, 2M30.A.201, 2M30.B.201, 3M4C.D.108
, 3M52.B.116, 3M79.A.107, 3M79.B.107, 3M79.C.107, 1MMP.A.1, 1MZC.B.1001, 4NAQ.A.
1027, 4NGE.A.1101, 3NKQ.A.1002, 3NKR.A.1002, 3NKN.A.1001, 3NKN.A.1002, 3NKO.A.10
02, 1NPC.A.323, 2NQH.A.303, 1NW2.D.6004, 1O1S.B.1001, 4O2I.A.401, 4O3A.B.302, 4O
3A.B.303, 1O8A.A.701, 1OEZ.W.1154, 4ON1.A.400, 4ONG.H.302, 4ONX.B.201, 2OR4.A.17
51, 2OW0.A.444, 4OXD.C.301, 2OY2.A.999, 3P24.A.999, 4P9C.J.201, 1PE5.A.317, 1PE7
.A.317, 2PIY.A.400, 2PJ0.A.400, 2PJ3.A.400, 2PJ5.A.400, 2PJ7.A.400, 2PJ8.B.500,
2PJA.B.500, 2PJB.A.400, 2PJC.A.400, 2PLI.C.701, 1PSZ.A.1000, 3PSQ.B.328, 4PUC.A.
602, 2PVV.A.1752, 2PVW.A.1751, 3PW3.A.406, 3PZ4.B.1, 1Q3K.B.301, 3Q4R.A.202, 3Q7
5.B.521, 4Q7R.A.307, 1QBQ.B.1000, 3QBU.A.294, 2QLA.A.500, 2QLA.B.502, 2QLA.C.501
, 1QMU.A.999, 3QW0.A.500, 3QW0.D.500, 3QW1.C.500, 2R2D.A.277, 1R3N.A.501, 1R42.A
.804, 1R43.A.501, 2RFH.A.1308, 3RF4.A.118, 2RJP.A.1, 1ROS.A.400, 1RTQ.A.702, 3RY
M.B.107, 1S0E.A.1291, 3S9C.A.503, 3SFY.B.521, 1SHN.A.485, 3SI0.A.362, 3SJX.A.175
1, 3SKS.A.568, 1SQM.A.1001, 3T00.A.501, 1T3A.B.422, 1TF9.A.902, 3TG0.A.502, 1TKH
.A.902, 1TKJ.A.901, 1TKJ.A.902, 1TLP.E.322, 1TMN.E.322, 1TN6.B.1001, 1TNB.B.378,
1TNU.B.378, 1TNY.B.378, 1TNZ.B.378, 3TOL.D.107, 3TOL.B.107, 3TS4.A.301, 3TVC.A.
501, 3U24.A.595, 3U93.B.259, 3UBF.A.1, 3V0A.A.1297, 2V29.A.1274, 2V29.A.1275, 2V
77.A.1042, 2V9E.A.1275, 3VAT.A.501, 3VH9.A.302, 2VJ8.A.1611, 2VQQ.A.1411, 2VXI.A
.201, 2VXX.C.202, 1WAA.A.1091, 1WAA.F.1091, 3WAW.A.913, 3WBH.A.505, 4WD8.A.301,
4WD8.A.302, 3WE7.A.301, 2WKN.B.412, 2WO8.A.1268, 2WO9.C.1269, 2WOA.A.1270, 3WS9.
B.801, 1WUO.A.301, 3WV3.A.301, 1WY2.A.405, 2X4H.A.1142, 2X90.A.1618, 2X91.A.1619
, 2X92.A.1615, 2X93.A.1615, 2X94.A.1616, 2X98.A.1475, 1XAF.B.507, 1XGE.A.400, 4X
IX.A.401, 2XIG.B.1150, 2XIG.C.1153, 1XJO.A.902, 2XR9.A.1867, 2XX7.B.1264, 2Y28.A
.1181, 2Y2B.B.1180, 1YHC.B.602, 2YJP.A.1272, 2Z25.B.401, 2Z2D.A.265, 1ZG8.A.400,
1ZG9.A.400, 2ZKW.A.401, 2ZTG.A.902, 2E84.A.558, 4FXO.A.301, 2H6H.B.1001, 2H6F.B
.1001, 2OX8.A.5, 2OX8.B.5, 1R87.A.905, 3U24.A.594, 3R8B.F.122, 2VBL.C.1026, 3SH1
.A.222, 4ACF.B.1481, 3ATT.A.379, 3B9T.A.485, 2BVC.A.503, 1DXI.A.390, 4EO0.A.301,
4GWZ.A.402, 1IV2.B.1562, 1IV4.E.1565, 3LD0.8.54, 4ML9.B.301, 4OTP.A.502, 4PV4.A
.501, 2RD5.C.1001, 3T80.B.160, 3U2E.A.3, 1CUL.A.1007, 2ICJ.A.400, 1MXB.A.411, 2Z
2P.A.1003, 2EX5.X.802, 1G9Y.C.803, 4Q10.A.1001, 1RYS.B.803, 1AEI.A.318, 2BTW.A.4
00, 3C5I.A.371, 1DJZ.A.2, 2E30.A.300, 4EJ7.C.402, 3FLT.B.302, 4G64.B.301, 1IJL.B
.202, 4ILW.F.305, 2JAL.B.1447, 2K0J.A.501, 4KPP.B.501, 4KTP.B.804, 3L9I.C.1151,
1LGC.E.301, 2ML2.A.206, 2MLR.A.305, 1NW1.A.430, 4OVY.A.409, 3POW.A.900, 3RGO.A.1
, 3RYD.C.267, 2TBV.C.388, 1V1G.A.1209, 1WDA.A.902, 3WIV.B.1004, 1Z3U.B.497, 2ZOC
.A.504, 4CP1.A.1297, 1JHN.A.900, 1JYI.D.408, 1M1J.B.503, 4QNP.A.506, 1RK9.A.112,
1TRQ.A.106, 4UM9.D.2002, 3WIU.B.1004, 1XZO.B.1018, 4AM4.A.1161, 1BIQ.B.376, 3CI
8.A.6, 2CKF.A.501, 4D8F.B.402, 2DE6.A.501, 1DLM.A.400, 3E1M.B.301, 3E1N.C.300, 2
FKZ.C.1600, 2FKZ.C.1601, 2FL0.A.1602, 3FM3.B.552, 3FVB.A.164, 1FYZ.B.5004, 1FZ0.
B.5004, 4GAM.F.601, 3GHQ.K.300, 1GUQ.A.351, 4HJL.A.502, 1HSE.A.400, 2HTN.A.301,
4HVR.A.201, 3IS8.A.161, 2ITB.A.502, 2IW4.B.1311, 2J2F.A.371, 1JI5.D.153, 4JPY.A.
301, 4N71.A.202, 4NB9.A.501, 4NBC.A.501, 4NBG.C.501, 4NBH.A.501, 1NFV.A.200, 3O6
R.A.300, 1OQU.B.1004, 3PCF.N.600, 1PFR.B.503, 1PIZ.A.376, 1Q0C.B.500, 3QVD.B.173
, 3R2R.A.156, 4RC6.A.302, 1RSV.A.1004, 1SI0.A.321, 4TOH.A.202, 1XIK.B.377, 2XRX.
M.1461, 2XSH.C.901, 2XSH.G.900, 2YFJ.C.901, 2FL0.B.1605, 1OVB.A.300, 1RSR.A.1003
, 3T81.B.607, 3VV9.C.502, 2XRX.Q.1461, 2XSO.O.900, 4KHN.A.1010, 3DYQ.A.901, 1JED
.A.525, 3ZDU.A.353, 4FZZ.A.201, 2BBH.A.301, 1BUN.A.121, 3FFZ.A.1302, 2GG8.A.503,
4H83.A.402, 4J4B.A.902, 1JJU.C.996, 4MAT.A.501, 1NSX.A.1401, 1U4J.B.1001

[1] "Cluster 5"
4FUK.B.402, 4MCS.A.814, 2MLS.A.302, 3OOF.A.302, 1ZEB.A.901, 3B3C.A.502, 2C1G.A.1
465, 3C88.A.450, 3C8A.A.450, 3C8B.A.450, 2CB8.B.1090, 4CWM.A.432, 4CXV.A.432, 3D
DA.A.450, 2DEA.A.402, 3DFK.A.300, 1EB6.A.178, 4ELC.A.501, 1FT7.A.502, 4GSZ.A.402
, 4GTW.A.1010, 4GTW.A.1011, 1GW6.A.1615, 2GYQ.A.407, 4ICQ.A.502, 2IMA.A.500, 2IW
0.A.1255, 3K5X.A.403, 3KR5.B.1004, 3LE9.A.1, 1LFW.A.1002, 1LOK.A.901, 1LOK.A.902
, 4MCP.A.801, 4MCR.A.801, 3MK1.A.901, 4MTU.A.201, 1MZB.A.201, 4NGM.A.818, 4NGQ.A
.817, 4NGT.A.811, 4NY2.A.501, 4O5O.A.301, 4OC1.A.814, 4OME.A.815, 2OUN.A.402, 2O
UQ.A.402, 3P3G.A.301, 1P5X.A.246, 3PS1.A.301, 2PTY.A.500, 4Q3J.A.401, 2QF7.A.115
7, 3QIY.A.431, 3QM3.A.355, 3QW7.A.501, 3QW8.A.501, 1R1I.A.1001, 1R1J.A.1001, 1RO
R.A.601, 3S2L.A.403, 1SDX.A.677, 3T3W.A.301, 3U79.H.110, 3V94.A.702, 2V9L.A.1275
, 3VPB.A.503, 4WB7.A.503, 3WT4.A.502, 1XVX.A.315, 1ZED.A.903, 2ZXC.A.647, 4BDZ.A
.1381, 4BEO.A.1381, 4IEM.C.401, 1MOW.A.373, 1MUH.A.479, 4NCB.C.101, 4O3S.A.503,
3OYB.A.397, 3OYD.A.397, 3OYE.A.397, 3OYJ.A.397, 3OYL.A.397, 3OYN.A.397, 1RVC.A.4
01, 3S3N.A.397, 3SM4.E.15, 1YTU.A.428, 4ACF.A.1481, 2AL1.B.438, 3AU9.A.601, 3BZN
.A.501, 4C5A.A.330, 4C5B.A.1313, 4C5C.A.1313, 3CFX.A.703, 3CVJ.C.243, 1DAW.A.342
, 1DAY.A.342, 4DFX.E.404, 3DG6.A.2001, 1EC8.A.498, 1EFK.A.604, 4EOM.A.302, 3ETH.
A.401, 3ETH.A.402, 3ETJ.A.402, 1EYZ.A.401, 1EYZ.A.402, 2FN1.A.504, 2FUV.A.901, 3
GN6.A.321, 3GQ8.A.692, 4H2H.A.401, 2HGS.A.501, 3HPF.A.401, 3HWO.A.1702, 3I4K.A.3
85, 4IAC.A.401, 4IAD.A.401, 1IOW.A.330, 2IO8.A.7001, 4IZG.A.414, 4J1O.A.401, 1J7
L.A.302, 3KAL.A.502, 1KJ8.A.393, 1KJ8.A.394, 1KJ9.A.393, 1KJI.A.393, 1KJI.A.394,
1KJJ.A.394, 1LP4.A.341, 3LVV.A.696, 4M3A.A.402, 4MDB.A.402, 3NA5.A.547, 1NFS.A.
401, 4NZN.A.403, 4O4D.A.402, 4OAV.B.803, 3OLP.A.547, 2ONS.A.702, 4OVN.A.202, 1P4
3.A.438, 3PFR.A.456, 4PFY.A.602, 1POW.B.610, 4PU5.A.501, 2PYW.A.500, 1PYX.A.1003
, 4Q4C.A.403, 2QVH.A.401, 4QXD.B.301, 3R75.A.700, 3T2D.A.411, 3T7A.A.601, 3TDW.A
.502, 3TMO.A.265, 4TYO.A.501, 3UJR.B.501, 3UJS.A.600, 3UJS.B.600, 1UMG.A.401, 4U
OP.A.1612, 3V4S.B.402, 1VA6.A.523, 3VA8.A.425, 3VAT.A.502, 3VC6.A.501, 2VPQ.A.14
51, 1WOH.A.1000, 1W7V.A.1441, 4WB8.A.403, 4WH2.A.403, 4WH3.A.403, 3WNZ.A.503, 2X
HO.A.1439, 1Z2O.X.1296, 2Z4X.B.1206, 2HVI.A.878, 1MOW.A.371, 2OTJ.3.8078, 3V6J.A
.403, 2A9F.B.800, 4DCK.B.203, 1ECB.D.507, 4FVQ.A.902, 4FZL.B.302, 4GA3.A.1002, 4
GIR.B.401, 4GX4.B.402, 3TLM.A.1005, 1Z2P.X.1296, 2Z4W.B.1303, 4C5A.B.330, 1E4E.A
.360, 2IO7.B.5003, 1L3R.E.391, 4N57.B.402, 1OL5.A.1389, 4OVN.A.203, 4OVN.B.203,
3QHR.A.298, 4QXD.A.302, 3T9E.A.601, 4UAK.A.502, 1YYZ.A.340, 2DPK.A.4001, 3GE4.B.
167, 1JKU.B.2272, 2LP2.A.202, 2BQR.A.2002, 3EH8.D.303, 2FLD.A.601, 3GIM.A.415, 2
I3P.A.154, 2I3Q.B.81, 3LDY.A.145, 4NLG.A.401, 3PR4.A.343, 2A3W.R.336, 3A7Q.A.400
4, 1ANW.A.351, 4AQA.A.1210, 1AUI.B.502, 1BAG.A.432, 2BB4.A.260, 1BCJ.2.3, 3BOW.A
.717, 4BQ3.B.1803, 2BW7.B.2202, 2C1O.A.1773, 4CAG.A.609, 1DAN.L.155, 2DBX.B.701,
4DD8.B.1001, 1DJX.A.2, 1DSY.A.501, 2DW2.A.703, 2FH2.B.2001, 1FZC.B.2, 1G7Y.F.25
4, 3GIN.A.2, 1GU6.A.1479, 1H5V.A.305, 2HYW.B.514, 2II1.B.400, 4IMM.B.402, 1J1N.A
.493, 2J7A.A.1007, 2J7A.B.1007, 2J7A.E.1006, 4JDZ.B.703, 4JX1.A.504, 3K5S.A.218,
1K7Q.A.481, 3K7L.A.702, 3K7L.A.703, 3K7N.A.702, 3K7N.A.703, 4KDV.A.202, 1KTW.B.
8, 4KTR.D.814, 4KTR.G.806, 1KWV.B.604, 1L9N.B.704, 3LPD.A.341, 1M63.F.502, 4MVF.
A.604, 4N2I.A.706, 1NKQ.F.560, 1NUD.A.701, 3NWK.B.238, 1NX2.A.3, 4OKH.A.901, 3OM
7.B.1, 4OMD.D.605, 1OT5.A.601, 2OX9.A.803, 2OXE.A.600, 2P37.C.239, 4P99.D.508, 4
PKG.A.403, 2PPL.A.482, 2Q16.B.200, 2Q17.E.315, 3QQZ.A.326, 2QT7.B.201, 1R1Z.C.41
0, 3RHT.A.257, 2RJP.B.3, 3RRD.A.239, 3RTT.A.267, 1SL6.A.3, 4TVU.A.600, 4U2A.A.30
1, 1U94.A.701, 3UBR.B.476, 3USU.A.264, 3V03.A.586, 3VI3.A.2001, 2VYO.A.1298, 3WC
T.B.203, 2XVT.C.1137, 1Y3N.A.493, 1YO8.A.1202, 2Z57.A.1006, 2Z8Z.A.618, 2Z8S.A.6
46, 2ZJ7.A.619, 2ZUX.A.638, 2ZUY.A.628, 3DR2.A.707, 4AQU.A.1154, 4AQU.A.1156, 2A
TL.B.1415, 3AVW.A.3001, 4DTU.A.1002, 4E3S.A.1002, 4EDO.A.503, 1G9Y.A.801, 1G9Y.B
.802, 4J2D.A.1002, 3KHR.B.1416, 4KHU.A.1002, 3MX9.A.363, 3MXB.A.175, 3NCI.A.905,
3NDK.A.905, 3NE6.A.904, 3NGI.A.905, 3ODH.B.195, 2ODI.B.702, 2Q10.B.702, 3QEP.A.
905, 3QER.A.905, 3QEV.A.905, 3QNO.A.903, 1SOM.A.402, 4UAW.A.402, 4UB4.A.401, 4UB
4.A.402, 2VBJ.A.1154, 2VBJ.A.1155, 2VBO.A.1154, 2VBO.A.1155, 2VS7.A.1183, 2A3Y.A

.601, 1A75.A.110, 1A8A.A.407, 1A8B.A.407, 3ACH.A.204, 2AEF.A.602, 4AE2.A.246, 1AJP.B.558, 3AKB.A.171, 3AMR.A.905, 3AMR.A.907, 4APX.B.1238, 4AQ1.A.1923, 4AQ1.C.1923, 4AQA.A.1208, 4AQA.A.1209, 4AQE.A.1207, 4AQE.A.1209, 4AQO.A.1881, 1AVA.A.500, 4AVS.A.207, 1AXO.A.290, 1AYP.A.301, 2AYH.A.417, 4AYU.D.205, 2AZZ.A.125, 3B0X.A.579, 3B2Z.A.2, 3B4N.B.701, 2B96.A.124, 3BEU.A.249, 1BF2.A.751, 3BI1.A.1753, 2BIB.A.1552, 4BJ0.A.1167, 1BLI.A.500, 3BNG.A.508, 1BQB.A.352, 4BQ2.A.1797, 1BSW.A.900, 1BTU.A.260, 2BWR.A.500, 2C0T.A.1507, 2C10.B.1777, 3C1Q.A.2, 3C1Q.B.1, 2C26.A.302, 3C3Y.A.238, 2C4X.A.1252, 4CAG.A.604, 4CAG.A.610, 1CB8.A.3000, 2CBL.A.352, 4CBU.G.1150, 4CBU.G.1151, 2CCM.A.1194, 2CDO.A.1139, 2CDP.A.1139, 2CFT.A.1297, 4CFQ.A.501, 4CFY.A.301, 3CHJ.A.338, 3CHK.A.503, 3CIO.K.402, 3CIP.G.201, 2CKI.A.997, 3CLN.A.152, 2CM5.A.1678, 2CM5.A.1679, 2CM6.A.1679, 2CM6.A.1680, 2CN3.A.1778, 1CP9.B.554, 1CRU.A.501, 4CTE.B.280, 1CVR.A.504, 1D0L.A.400, 4D0E.A.1532, 1D2S.A.401, 1DB5.A.198, 1DBI.A.701, 1DBI.A.703, 1DBI.A.704, 1DBN.A.301, 2DCK.A.1001, 3DEM.A.5001, 3DEM.A.8001, 4DIR.A.102, 1DJX.B.2, 4DKB.A.301, 3DNZ.A.403, 4DUQ.A.101, 1DVI.A.271, 2DW0.A.701, 2DW0.A.703, 4DZ3.A.202, 4DZ3.B.202, 2E26.A.601, 2E26.A.602, 2E26.A.604, 2E26.A.605, 1E8A.A.1089, 2E85.A.1002, 3E9T.B.4, 3EAD.B.1003, 3ECQ.A.2001, 1EDH.A.302, 1EDM.B.2, 2EGD.A.301, 2EIG.A.1102, 2ERO.A.702, 3ETO.A.2001, 3EU3.A.1, 2EXH.B.2002, 1EXR.A.1002, 1EXR.A.1004, 3EXM.A.301, 4F0Z.B.202, 4F0Z.B.203, 3FCS.A.2004, 3FED.A.1755, 2FH1.A.2001, 1FIB.A.500, 3FIA.A.201, 2FI1.A.191, 2FMJ.A.338, 2FMD.A.301, 1FNY.A.500, 3FO3.A.527, 1FS7.A.651, 2FVY.A.311, 1FX5.A.251, 1FXH.B.1002, 1G5N.A.401, 1G7Y.A.254, 1G8K.A.5008, 1G8K.C.5108, 1G9G.A.630, 1G9K.A.701, 3GB0.A.301, 1GCA.A.351, 1GCY.A.528, 4GER.A.403, 1GGZ.A.150, 1GGZ.A.151, 4GGF.C.204, 2GKO.A.610, 1GK9.B.1579, 1GKF.B.1571, 1GO7.P.1482, 1GO8.P.1482, 1GPL.A.500, 3GPE.A.501, 3GPE.A.502, 2GSK.A.1, 1GSL.A.251, 4GUK.A.207, 1GZC.A.290, 4H2B.A.604, 1H3G.B.701, 1H6X.A.1162, 1H6Y.A.1161, 2H61.D.817, 1H80.A.1493, 3HB2.P.481, 1HDF.A.1102, 3HDB.A.621, 3HDB.A.622, 4HHO.A.401, 3HI7.A.803, 3HJR.A.602, 1HPL.A.960, 4HPH.A.701, 1HQL.A.302, 1HQV.A.998, 2HQ8.A.201, 3HR4.D.202, 1HT6.A.500, 1HT9.A.1001, 3HX4.A.601, 3HX4.A.602, 1HY7.A.304, 2HYV.A.607, 4I2Y.A.503, 4I35.A.511, 4I35.A.514, 3I57.B.186, 1I82.A.192, 3IBZ.A.193, 4ICB.A.76, 2ID4.A.906, 3IGO.A.602, 3IGO.A.603, 4IHM.A.406, 2II1.C.400, 3IKW.A.1, 3IK2.A.514, 4IMM.A.407, 3IPV.A.252, 2IPL.A.502, 4IT5.D.202, 4ITC.A.1201, 4ITC.A.1202, 4IU3.B.301, 1J1A.A.201, 2J5Z.A.1277, 2J7A.A.1006, 1J83.A.4001, 1J9L.A.1301, 4JDZ.B.701, 2JE7.A.1241, 2JEC.A.240, 4JGU.A.901, 1JI1.A.2003, 1JIA.A.134, 4JJJ.A.704, 3JQ1.B.1, 1JX9.B.601, 3JXS.A.301, 4JX1.E.505, 3K21.A.192, 3K21.A.193, 1K7I.A.480, 1K94.A.998, 1K96.A.91, 1K9I.I.1201, 1K9J.A.403, 1K9U.A.1001, 4K90.A.702, 1KAP.P.614, 1KAP.P.621, 3KCP.A.701, 3KHE.A.192, 3KHE.A.195, 3KM5.A.2012, 3KM5.B.2011, 1KQU.A.301, 3KQR.A.205, 4KTY.A.801, 4KTY.A.803, 1KVO.A.191, 3KWU.A.901, 3KWU.A.902, 3L2Y.A.301, 3LCP.C.159, 4LDC.A.502, 1LE6.A.461, 1LEN.A.184, 1LHW.A.401, 4LJ3.A.402, 4LM8.A.811, 4LMH.A.811, 3LNP.A.472, 1LOC.A.228, 4LOR.A.301, 4LOS.A.401, 1LPB.B.453, 3LPA.A.361, 3LPC.A.360, 3LPC.A.361, 3LPC.A.362, 4LP7.B.301, 1LQV.C.34, 2LRP.A.202, 2LTN.A.191, 1LVU.A.8001, 1LVU.D.8002, 4LXO.A.1601, 4LXO.B.1601, 4M00.A.801, 3M1H.A.2001, 3M1H.A.2002, 4M2P.A.301, 4M5E.A.506, 1M8T.A.1001, 1MCX.A.349, 1MCX.A.352, 4MEW.A.501, 4MGQ.A.601, 1MKU.A.124, 1MKV.A.124, 2ML2.A.204, 2ML3.A.202, 4MNO.A.303, 1MPX.A.638, 3MSE.B.180, 4MSP.A.201, 3MVS.A.213, 3MVS.A.216, 1MVQ.A.238, 4N1G.A.202, 4N1G.A.204, 4N2B.A.709, 4N2G.A.704, 4N2N.A.704, 3N35.A.290, 1N47.B.252, 4N5X.A.203, 3N5A.A.101, 3N5A.A.102, 1N9E.A.803, 4NEH.A.1101, 1NIW.A.1004, 1NIW.C.1007, 1NIW.G.1014, 1NLS.A.240, 1NNL.A.2001, 3NOL.A.263, 1NPC.A.321, 3NQX.A.511, 3NQZ.B.520, 4NUQ.A.303, 2NXP.D.603, 2NZM.A.405, 2O72.A.401, 2O72.A.402, 2O8O.A.1009, 1O8F.A.1353, 2O9Q.A.501, 1OAC.A.803, 1OAH.A.1525, 1OAH.A.1526, 3OEA.A.200, 1OF3.A.1174, 1OFL.A.528, 1OH4.A.1186, 1OHZ.B.1057, 4OKH.A.902, 4OKH.A.904, 3OM2.A.1, 3OM4.A.1, 3OM5.A.1, 3OM6.A.1, 3OM7.A.1, 4OMC.A.605, 4OMC.A.606, 4OMC.A.607, 2OO4.A.5001, 2OPO.A.301, 2OVU.A.238, 2OW9.B.610, 3OX6.B.501, 2OX9.A.804, 1OYG.A.500, 4OY7.G.302, 2P2K.A.239, 3P6B.A.205, 1P8J.A.3001, 4P99.B.517, 4P99.C.505, 3PDD.A.193, 2PEL.A.237, 4PEU.A.401, 3PF2.A.1, 3PGB.A.902, 4PHN.B.303, 4

PKG.G.1201, 4PKI.A.403, 4PLS.B.301, 2PMY.B.104, 3PM8.A.1, 1PMH.X.300, 1PMJ.X.300
, 1POA.A.201, 1POC.A.501, 3POJ.A.1, 2PPL.A.478, 3PPE.A.402, 3PPE.A.403, 2PQX.A.5
00, 1PT2.A.500, 3PVN.M.5026, 2Q17.A.315, 3Q2N.A.303, 3Q5I.A.1, 3Q5I.A.524, 3Q5I.
A.526, 4Q6O.A.502, 3QGM.A.503, 1QI3.A.451, 1QNW.A.302, 2QNG.A.201, 1QPK.A.451, 2
QQM.A.1, 2QQO.A.1, 3QRO.A.1000, 3QRB.A.303, 2QUB.A.617, 2QUB.A.619, 2QUB.A.620,
2QUB.A.621, 2QUB.G.616, 2QV6.A.302, 1QX2.A.1005, 1R1Z.A.285, 1R55.A.515, 3R5V.B.
201, 1R64.A.701, 2R8Z.N.214, 2RAN.A.324, 2RDZ.A.1501, 2RF7.A.1501, 1RLW.A.400, 1
RLW.A.401, 1RP8.A.500, 1RRO.A.110, 1S1E.A.226, 1S3P.A.210, 3S5U.A.221, 1S6B.A.40
1, 1S6C.A.218, 2SCP.A.190, 2SCP.A.192, 1SGT.A.246, 1SH7.A.1291, 3SIB.A.221, 3SJG
.A.1753, 1SL4.A.407, 1SL6.D.3, 1SL6.E.3, 1SL8.A.669, 1SL8.A.670, 1SL8.A.671, 3SN
Y.A.97, 3SRE.A.1356, 1SU4.A.995, 3SXQ.A.526, 3T3P.B.2002, 1T44.G.702, 1TF4.A.300
2, 3TI7.A.354, 3TI9.A.353, 3TI9.A.354, 3TI9.A.355, 1TM7.E.470, 4TSH.B.1501, 1TU5
.A.903, 3TZ1.A.1, 3U1R.A.701, 4U36.A.302, 3U4X.A.237, 3UBH.A.857, 1UKG.A.1262, 3
UL4.B.67, 4UM9.A.2001, 1UNE.A.124, 1UP8.A.599, 2UWP.A.1246, 1UWW.A.1192, 1UX6.B.
2001, 1UX6.B.2008, 1UX6.B.2009, 1UX6.B.2011, 1UX7.A.1135, 3UXF.A.2, 1UY4.A.1147,
4UZU.A.1485, 1V0A.A.1177, 2V0B.A.1241, 4V29.B.1178, 3V9M.A.205, 1VCL.A.1002, 1V
CL.A.1005, 1VL9.A.124, 3VLV.A.503, 2VPT.A.1235, 3VRR.A.401, 2VVE.A.1336, 3VV3.A.
402, 1WON.A.1132, 2W08.A.206, 3W57.A.201, 1W7C.A.803, 1WBF.A.303, 1WC5.A.2100, 3
WCS.A.1003, 3WCT.F.203, 2WD6.A.1765, 4WJK.B.502, 1WKY.A.503, 4WK7.A.502, 4WK7.A.
503, 3WLC.A.501, 2WN3.A.400, 2WN3.C.400, 2WNO.A.1251, 2WPH.S.1246, 3WU2.c.901, 2
WW8.A.1001, 2WW8.A.1002, 2WZE.B.1552, 4XDQ.A.301, 2XFG.B.1619, 2XFD.A.1112, 1XJO
.A.905, 2XOM.A.1169, 1XVJ.A.422, 1Y1X.A.201, 1Y1X.A.202, 1Y4J.A.1001, 1Y6O.A.207
, 2Y6L.A.1168, 1Y93.A.267, 2YAY.A.1266, 2YFS.A.1712, 2YIH.A.1520, 2YKK.A.1520, 2
YN5.A.6364, 1YO8.A.1189, 1YO8.A.1198, 1YO8.A.1200, 2YOA.A.1001, 1YXH.A.1001, 2Z2
X.A.1002, 2Z2X.A.1003, 2Z30.A.1005, 1Z70.X.3002, 2Z8X.A.618, 2Z8X.A.619, 2Z8X.A.
621, 2Z8X.A.625, 2ZEZ.A.200, 2ZEX.A.406, 2ZFD.A.227, 2ZFD.A.228, 2ZKM.X.800, 2ZP
4.A.124, 2ZUX.A.631, 2ZUX.A.635, 2ZUX.A.636, 2ZUY.A.625, 2ZVD.A.628, 2ZVD.C.620,
3ZYR.A.401, 2ZYH.A.700, 2ZZJ.A.239, 4QQZ.A.1002, 1A8E.A.339, 3AK9.A.168, 3AK9.C
.168, 4AM5.A.1161, 4AQ2.E.800, 4AQ6.D.837, 1B0L.A.694, 1B3E.A.400, 1C7M.A.101, 2
CSG.A.504, 2D09.A.430, 1D3K.A.339, 1DLT.A.400, 3DU3.M.500, 3DUQ.M.500, 3E1M.C.30
0, 3EMR.A.400, 1EYS.M.607, 3FGS.A.402, 1FQE.A.500, 1FZ6.B.5004, 4G51.A.202, 3GCF
.A.501, 3GVY.A.162, 2GYQ.B.404, 1H43.A.1315, 1H44.A.1326, 1H76.A.702, 4H99.M.402
, 4H9L.M.402, 1HAC.B.200, 4HBH.M.402, 3HF8.A.400, 3HGI.A.281, 4HR4.A.402, 1IEJ.A
.333, 1J30.A.401, 1J30.B.404, 2J8C.M.1307, 4JO0.A.601, 1JQF.A.500, 4KF1.A.402, 4
KVR.A.302, 1LKO.A.600, 4M26.D.401, 3MPS.F.173, 3MPS.G.172, 1N7W.A.339, 1NF4.A.20
0, 2OOC.A.500, 2OHJ.D.2501, 1OQG.A.500, 3PZA.A.173, 3PZA.B.173, 3Q3N.A.510, 4QDF
.A.402, 2QED.A.252, 3QHB.B.182, 3QVD.H.173, 3R0G.A.200, 3R1J.A.299, 4RC8.A.302,
3RI7.A.1, 3RMK.D.495, 1RVJ.M.857, 1RZH.M.857, 1S9A.A.300, 1SQI.A.1450, 1SQY.A.70
1, 1TH3.D.2003, 1TKP.A.304, 1TKP.B.303, 1UMX.M.1306, 2UW1.A.1360, 2UW1.B.1359, 1
UZR.A.1293, 2V27.A.1268, 3V83.F.703, 3VEO.A.602, 3VEZ.A.601, 1VFE.A.400, 1VRN.M.
500, 3VVA.A.501, 1VZ4.D.1301, 2VZB.A.6204, 1W69.A.1350, 2YAV.A.402, 1YUX.A.302,
4QQZ.G.1002, 3AK9.J.168, 4AQ2.B.800, 4AQ6.F.837, 2BJJ.X.694, 1EH3.A.400, 1GVC.A.
1253, 3MPS.D.1, 2O84.X.500, 1OQH.A.500, 3PCC.M.600, 3TOD.A.694, 3V83.A.703, 1WTE
.A.1001, 1XSP.A.576, 4DKA.A.201, 4IBO.A.402, 4JP4.A.305, 3MNC.A.280, 3NUQ.A.283,
3UES.A.503, 2VX5.A.1421, 2XRM.A.401, 2YEQ.A.582, 4CVU.A.1998, 4LLH.B.602, 1NZA.
A.104, 4FZX.B.101, 4FZX.A.101, 4FZY.C.101, 4FZY.D.101, 1VQ8.0.9117, 3A6V.B.1006,
3ATU.A.6267, 3B8X.B.668, 1BA0.A.490, 4DCC.A.302, 4DD8.B.1005, 4HUC.A.509, 1HVX.
A.519, 4O52.A.301, 1QHU.A.438, 3SLZ.B.130, 4UZU.A.1491, 2WCP.A.501, 3WZ1.A.402
[1] "Cluster 6"
1P6D.A.247, 3BON.A.500, 4CVR.A.1160, 4CVT.A.1160, 4DJ4.B.403, 3EZT.B.401, 4FW5.D
.301, 4FW6.A.301, 4FW7.D.301, 3GBO.A.302, 3GJ9.B.127, 2GSO.A.1001, 2JT2.A.336, 4
L3T.B.1101, 4LCF.A.311, 4LCG.A.301, 4LCH.A.301, 4MCQ.A.801, 3MN8.A.999, 3P3E.A.4
00, 2PLI.B.707, 4PPZ.A.602, 3PS2.A.301, 3PS3.A.301, 3QJ0.A.431, 1QTW.A.302, 2RH6
.A.2, 1SHN.B.481, 1TXR.A.501, 3U1Y.A.400, 4X2T.L.701, 4IRC.A.402, 3A7E.A.215, 4A

ZW.A.1453, 4DHP.A.303, 2J0L.A.1688, 4K10.D.404, 4OHF.B.503, 4P9D.A.202, 3PCR.B.1231, 4RUB.B.491, 1T5T.A.1005, 2VPQ.A.1450, 3WQQ.A.502, 3WQR.A.502, 4GZ2.A.402, 3EW9.A.501, 4GX6.B.402, 1HJK.B.452, 4KI8.A.602, 3MJX.A.901, 3IAF.A.572, 3PUG.A.601, 2PUI.A.401, 4R9U.C.302, 2ASJ.B.1415, 4K4H.E.604, 6CGT.A.685, 1DTH.A.903, 4GQR.A.502, 1JKU.E.5272, 3K7L.A.701, 1ULV.A.2006, 1AZ0.B.283, 4DU3.A.1003, 3MIS.A.2, 1A25.A.290, 3A7Q.A.4003, 3A9Q.G.214, 3AKA.A.173, 1ATN.D.265, 1AVA.A.501, 3B1T.A.903, 3B2Z.D.3, 1B47.A.351, 3BBY.A.215, 2BL0.C.1155, 2C0O.B.1507, 4CAG.A.608, 2CDP.C.1139, 3CGA.B.102, 3CGT.A.685, 2CHN.A.1717, 3CK9.A.710, 3D0Y.A.93, 2D3P.C.241, 2D3L.A.503, 3D94.A.2, 1DE4.C.801, 2DIE.A.778, 1DJY.A.2, 3E1I.C.501, 2E6V.C.10, 3E78.A.601, 1E8A.B.1090, 2E85.B.1004, 3E9T.B.3, 4EJ7.A.403, 1ESV.S.395, 3ESR.A.213, 1F9O.A.2000, 3FAX.A.4, 4FDI.A.603, 2FHF.A.2405, 3FLP.B.302, 1FZD.E.1, 4G0D.B.503, 3G5C.B.802, 1G5N.A.407, 4G9L.A.304, 1GCY.A.529, 2GDF.B.302, 2GJR.A.1488, 3GK2.A.93, 2H0K.A.410, 1HFZ.D.124, 2HIH.A.603, 3HTL.X.1, 3IJ8.A.497, 4ILW.D.305, 2IWV.B.1281, 2JBK.A.803, 1JBA.A.500, 2JHL.F.1298, 3JQ5.A.201, 1JXN.B.246, 2K0F.A.153, 3K5T.A.803, 3K7N.A.701, 1K9I.F.903, 2KAY.A.186, 3L2Y.A.302, 3LCP.D.158, 1LGN.B.302, 2LMT.A.149, 1LN8.A.201, 4LP7.A.301, 2LV6.A.203, 1M56.A.1007, 4MHX.A.601, 4MIV.E.600, 4MVF.A.603, 3MXW.A.402, 1N2K.A.600, 1N47.A.252, 4NDD.B.401, 1NHE.A.805, 4NHD.B.403, 1NIW.A.1003, 2NQA.B.902, 1NT0.A.3001, 2OA8.C.302, 1OBR.A.404, 2OO4.A.5003, 3OUU.B.455, 2OZN.B.401, 1P8J.G.3014, 4P99.A.501, 4P99.B.509, 4PIB.C.204, 2PMY.A.103, 4POS.E.401, 3PVN.B.5003, 3PVN.B.5004, 3Q3L.A.436, 4Q6P.A.509, 2R28.A.1003, 2RHP.A.8, 1S2N.A.1291, 2SAS.A.188, 2SEC.E.276, 1SU3.B.907, 1SVN.A.276, 1ULV.A.2004, 4UM9.C.2002, 3UNX.A.280, 1V3D.B.2001, 1VFP.A.995, 3VI3.A.2003, 3VI3.A.2004, 1WBL.D.303, 4WBQ.B.601, 3WIU.A.1004, 2WL3.A.1290, 1WMY.B.203, 3WN6.B.501, 1Y4D.E.1001, 2YN3.B.6361, 1YUU.A.197, 1Z3U.A.497, 2Z8S.A.642, 2Z8S.A.648, 2Z8S.B.646, 2ZEY.B.152, 1ZIV.A.1, 2ZN9.A.902, 2ZN9.B.901, 2ZWP.A.401, 1ANX.B.321, 4AOC.B.1129, 4AYU.A.205, 1B9A.A.110, 3B9G.A.318, 1C0G.S.129, 2C40.A.1311, 1CGU.A.685, 1CGW.A.692, 3DEM.A.3001, 1DX5.I.1001, 3EPW.A.1001, 3EPX.A.1001, 3FZ0.B.400, 3HR6.A.1, 4I70.A.401, 4I72.A.401, 2JBH.A.1228, 1KXQ.A.4001, 2LRO.A.202, 3MKM.A.501, 3MKN.A.501, 1Q8F.A.2001, 4QB6.A.203, 3S82.B.405, 3T8I.C.400, 3TH2.L.152, 4U6D.A.501, 4U6D.B.502, 1UZK.A.2512, 3WMT.A.606, 4X9Y.A.502, 1YCM.A.267, 1YOE.A.1001, 1Z32.X.497, 4DTM.A.1002, 4DU1.A.1003, 4DU3.A.1002, 3EH8.A.301, 4FK0.A.1002, 2FLD.B.602, 4J2E.A.1002, 4K4G.A.603, 4KLD.A.403, 3MQ6.A.1, 3QNN.A.903, 4QWA.A.403, 3RAX.A.416, 2WTF.A.1511, 1A25.B.290, 3A24.A.1268, 1A2Q.A.295, 3A4U.A.286, 1A75.A.109, 3A8R.A.401, 2AAO.A.293, 2AAO.A.295, 2AAO.A.296, 5AER.A.200, 1AF0.A.476, 3AIE.A.4001, 1AK9.A.295, 3AKB.A.172, 3AKB.A.175, 3AMR.A.908, 1ANW.B.353, 4APX.B.1240, 4AQJ.A.1097, 1AVS.A.93, 1AVS.A.94, 1AXN.A.353, 3B0O.A.124, 1B8R.A.109, 3B8Z.A.903, 3BC9.A.701, 3BFM.A.235, 1BH6.A.501, 1BJF.A.402, 1BQB.A.351, 1BU3.A.109, 1BU3.A.110, 3BYK.A.474, 4BY5.A.1183, 4BY5.A.1184, 4BY5.A.1185, 2C4X.A.1253, 2C4F.L.1143, 1C8T.B.264, 1C9N.A.277, 4CAG.A.603, 2CCL.B.1060, 2CCL.B.1061, 2CCM.A.1192, 4CCD.A.3669, 4CFQ.A.502, 1CJY.A.950, 4CPV.A.109, 4CPV.A.110, 2CT9.A.301, 2CT9.A.302, 4CT3.A.1170, 3CZT.X.93, 3D1M.A.2, 4DA2.A.301, 2DBX.D.702, 3DBK.A.303, 2DIE.A.780, 4DIR.A.101, 3DO1.A.401, 1DTL.A.201, 1DTL.A.203, 2DUR.A.1, 4DUQ.A.102, 1DVI.A.273, 2DW0.A.702, 2E3X.A.802, 2E4T.A.701, 1E43.A.502, 1E8A.A.1090, 3ECQ.B.2000, 3EDF.A.602, 2EGD.A.302, 2EHB.A.1001, 3ET0.A.2003, 2EWE.A.701, 1EXR.A.1000, 4F0Z.B.201, 4F0Z.B.204, 1FAT.A.255, 2FH1.A.2002, 3FLP.A.301, 3FLP.A.302, 3FZ0.A.400, 1FZD.A.1, 4FZM.A.301, 3G5C.A.801, 1G8I.A.1599, 1G8I.B.1600, 1G9K.A.702, 1G9K.A.704, 1G9K.A.705, 3GDC.A.401, 4GER.A.402, 1GGZ.A.149, 2GGM.A.401, 4GGF.A.101, 4GGF.L.204, 3GIS.Y.1002, 2GJP.A.1487, 2GJP.A.1488, 4GM5.A.404, 2GSK.A.2, 2GXS.A.601, 4H1Q.B.304, 1H2G.B.1558, 4H2A.A.805, 3HB2.P.484, 3HJR.A.601, 2HQ8.A.202, 2HQ8.A.203, 1HVX.A.516, 1HVX.A.518, 3HX4.A.603, 3HX4.A.604, 3HX6.A.1, 2HYV.A.605, 2HYW.A.501, 2HYW.A.503, 3HZ3.A.1, 4I2Y.A.501, 4I35.A.515, 2I7A.A.2, 4I75.A.401, 4IAI.A.401, 4ICB.A.77, 2ID4.A.901, 4IEF.B.702, 3IJ9.A.497, 4IK8.A.502, 1IOD.A.501, 4IU3.B.302, 2J1V.A.1152, 2J1G.B.1290, 1J55.A.102, 4JA8.A.503, 1JC9.A.301, 1JIW.P.485, 1JK3.A.404, 3JQW.A.1001, 4JWQ.A.202, 3K21.A.194, 4K70.A.100

3, 4K70.B.1003, 1K94.A.999, 1K9K.A.400, 1K9U.A.1002, 1KAP.P.615, 4KDW.A.201, 3KF 9.C.302, 3KHE.A.193, 3KHE.A.194, 1KIC.B.328, 4KKK.A.701, 3KLK.A.1, 3KLL.A.1, 3KQ R.C.206, 1KVW.A.124, 4KWU.A.1109, 1KX0.C.703, 2KYF.A.109, 3L2Y.C.302, 1L9N.A.700 , 1L9N.A.702, 3LI6.A.149, 3LI6.D.150, 4LMF.D.303, 3LND.B.208, 3LNI.A.302, 4LVN.A .702, 4M5E.A.505, 4M7H.A.501, 1MCX.A.351, 1MDW.B.4, 3MHZ.A.736, 2ML1.A.205, 4MN0 .A.302, 3MVS.A.212, 1MXE.A.506, 4N1G.A.203, 3N1F.A.6, 3N1G.B.190, 4N2L.A.707, 3N 4E.B.500, 4N5X.A.201, 4N5X.A.204, 4N5X.A.205, 4NEH.A.1103, 4NHF.F.301, 1NPC.A.31 9, 2NXP.C.602, 2O04.A.402, 3O0V.A.1, 3O0W.A.377, 3O4Y.A.198, 2O5G.A.401, 2O5G.A. 402, 2O5G.A.403, 2O5G.A.404, 1O8P.A.1149, 1OB0.A.502, 2OBH.A.1001, 1OHZ.B.1058, 4OKH.B.901, 2OLG.A.2001, 2OPO.A.302, 3OX6.B.502, 2OZN.B.402, 2P0R.A.1002, 5P2P.A .125, 3P4G.A.411, 3P4G.A.413, 3P4G.B.405, 4P5X.A.1001, 4P99.A.514, 5PAL.A.110, 5 PAL.A.111, 4PE0.X.103, 4PE0.A.102, 4PET.A.403, 4PHJ.A.302, 4PHJ.A.303, 4PHJ.A.30 4, 3PM8.A.514, 3PM8.B.1, 1POB.A.801, 1POE.A.801, 1PVA.A.110, 1PVA.A.111, 3PVN.E. 5009, 2PVB.A.110, 2PVB.A.111, 2Q1F.A.2001, 3Q2L.A.701, 1Q3A.A.468, 3Q5I.A.525, 1 Q5P.A.271, 3QRB.A.302, 3QRX.A.171, 3QRX.A.172, 1QTX.A.152, 1QTX.A.153, 1QTX.A.15 4, 1QX2.A.1001, 1QX2.B.1006, 1R0R.E.302, 2R2I.A.500, 2R2I.A.501, 2R2I.A.502, 2R9 F.A.366, 1RFJ.A.1001, 1RFJ.A.1003, 1RFJ.A.1004, 2RHP.A.29, 3RM1.A.102, 1RRO.A.10 9, 1RU4.A.1, 3RUP.A.1006, 1RWY.A.421, 1RWY.A.422, 1S01.A.295, 1S02.A.276, 1S2N.A .1290, 3S5U.D.221, 1S6B.B.402, 1S6C.A.217, 1SBF.A.601, 2SCP.A.191, 3SIB.A.222, 1 SL7.A.301, 1SRA.A.302, 1ST3.A.270, 1SUD.A.295, 1TCF.A.160, 1TCF.A.163, 1THM.A.30 1, 1THM.A.302, 3TI7.A.353, 3TI7.A.355, 1TKF.A.905, 1TN4.A.162, 4TNC.A.164, 5TNC. A.163, 3TOY.A.361, 3TTQ.A.2867, 3U1R.A.702, 3UBG.A.902, 3UCP.A.912, 3UJ0.B.304, 3UL4.B.66, 4UM9.A.2003, 3USU.C.266, 1UX6.B.2005, 1UXX.X.1130, 1UYZ.A.1132, 4UZU. A.1483, 4V29.B.1179, 1VCL.A.1001, 2VDQ.B.2002, 2VN5.B.102, 2VN6.B.1067, 2VUD.D.1 118, 2VVD.A.1328, 3VYV.A.303, 2VZP.A.1128, 2W46.A.1148, 2W47.A.1137, 3W57.A.203, 2W87.A.1149, 3WA5.A.504, 2WBX.A.1103, 1WC0.A.2100, 3WFD.B.806, 3WH2.A.302, 3WHI .A.401, 3WHT.B.501, 3WN6.A.503, 3WNP.A.801, 2WND.A.102, 2WNP.F.1298, 1WP6.A.502, 1WPC.A.501, 2WZ8.A.1149, 1X1J.A.2000, 3X17.A.603, 2Y3N.B.1067, 2Y3N.B.1068, 2Y5 I.A.101, 1Y9Z.A.603, 2YA2.A.1691, 1YO8.A.1186, 1YO8.A.1187, 1YO8.A.1190, 1YO8.A. 1204, 2YOA.A.1002, 1YU6.A.401, 1YUT.A.197, 1YUT.B.199, 1Z3J.A.267, 2Z30.A.1004, 2Z8X.A.627, 2Z8Z.A.623, 1ZCM.A.1001, 2ZFD.A.229, 2ZN9.A.901, 2ZUX.A.632, 2ZUY.A. 622, 2ZWO.A.402, 2ZWP.A.404, 2ZWP.B.402, 3ZYP.A.1220, 4BMT.A.1324, 1DSN.A.400, 3 E1M.A.301, 3E1N.B.300, 1FZ6.B.5003, 3IS8.B.162, 4NB8.A.501, 3PWF.A.201, 3PWF.A.2 02, 3PWF.B.201, 3PWF.B.202, 1R2F.A.401, 1SP8.B.500, 4DMI.C.203, 4DNL.A.300, 2HZL .A.500, 4LUG.A.301, 1O68.D.274, 4O7J.A.201, 3PFV.A.1, 3UT0.A.902, 3V7Z.A.404, 1W 9W.A.901, 4X26.A.402, 4L73.B.405, 3RWK.X.521, 3CBT.A.420, 1BPZ.A.341, 3C5G.A.803 , 2FMP.A.1340, 2ISO.A.340, 3JPO.A.340, 3JPQ.A.340, 3JPR.A.340, 4M9L.A.403, 1SA3. A.401, 3A6V.A.1005, 1AVT.A.301, 2DDA.B.302, 3ED4.A.519, 2GA4.A.713, 3HLT.A.269, 1QUS.A.400, 2QZ7.A.194, 3R2H.A.157, 1V7T.A.406, 4WED.A.601, 3WNK.A.813, 3WXO.A.8 06, 1YQ2.C.7503, 1ZDN.B.157

[1] "Cluster 7"

4A37.A.376, 4A69.A.500, 3AHT.A.1001, 1ALK.A.451, 3B4N.A.711, 1B57.A.361, 4B6Z.C. 385, 1BA9.A.154, 3BKN.B.202, 1BOR.A.58, 2BO9.A.999, 2BP8.B.1340, 4BZS.A.701, 4C2 N.A.701, 4C2O.A.1629, 1CG2.A.500, 1CG2.A.501, 3CHO.A.701, 2CLB.A.1175, 4COG.A.40 1, 4COQ.A.299, 1CPX.A.308, 5CPA.A.308, 4CVT.A.1158, 1D8D.A.1001, 3DID.A.130, 1DO 5.B.29, 1ED9.A.450, 3EER.A.2001, 3EQN.A.757, 3F3Q.A.104, 1F57.A.310, 3FB4.A.217, 1FT7.A.501, 4FU4.A.502, 3FVL.A.1309, 2GFK.A.401, 2GFJ.A.401, 4GM5.A.401, 2GSN.A .1001, 4GTO.B.501, 4H2K.A.1001, 2HH5.A.702, 1HLK.B.1004, 1HR7.B.501, 2HSE.B.954, 2I3C.A.314, 4IAV.A.401, 1IF6.A.262, 4IHM.A.402, 2IXD.A.1234, 1IY7.A.308, 1J9Y.A .1003, 2JBJ.A.1752, 1JCQ.B.1001, 1JCS.B.1001, 1JJE.A.251, 1JJT.A.251, 4JJJ.A.718 , 4K7W.A.102, 4K90.A.701, 1KH4.A.450, 1KRO.A.405, 3KS3.A.262, 1L1O.C.1, 3L3N.A.7 01, 1LND.E.800, 4LNB.B.602, 4LQY.A.506, 3LTV.A.1001, 3LUB.B.302, 2LVH.A.101, 4LW 9.A.201, 4LW9.I.201, 4LW9.R.201, 3M2Z.A.500, 1M4L.A.1308, 1M5E.A.1705, 4MBG.B.60 2, 3MDJ.A.1000, 4MKH.A.301, 4MKT.A.701, 1MMP.A.2, 3MO2.D.6, 4MRQ.A.501, 4MSM.C.5

01, 3MWM.B.142, 1MXD.A.727, 4N07.B.305, 3N21.A.401, 1N4Q.B.378, 1N4Q.L.378, 4NGE .E.101, 1NW2.F.6006, 4NYY.B.501, 4O3A.A.303, 1O86.A.701, 4OJA.A.202, 4OKO.A.401, 4OTE.B.302, 2P2L.A.901, 2PIZ.A.400, 4PPZ.A.601, 4PQA.A.403, 4PQA.A.404, 3PSQ.A. 326, 4PXY.A.302, 3PZ1.B.332, 3Q78.B.521, 3Q7A.B.521, 3QWO.B.500, 2RH6.A.1, 1RK6. A.601, 1S63.B.1001, 1S64.B.378, 1SA4.B.439, 1SA5.B.438, 3SFX.B.521, 1TOA.A.661, 3TGN.B.147, 3TGO.A.501, 1TN7.B.1001, 1TN8.B.1001, 1TNO.B.378, 3TT4.A.302, 1U7J.A .50, 3U9W.A.2001, 3UCT.B.102, 1UUP.A.2222, 1UZE.A.701, 2V9I.A.1277, 2VXI.A.202, 2W5V.A.1377, 2W5X.B.1378, 3W5K.B.501, 2W57.B.202, 3W6H.A.302, 4WCM.A.538, 4WD6.A .301, 4WNC.O.402, 3WOJ.A.805, 3WXC.A.301, 2X97.A.1616, 2X98.A.1476, 1XP3.A.302, 2XXO.B.1340, 2XXG.A.1339, 2Y3D.B.149, 2YJP.C.1270, 2Z2Y.C.2003, 1ZG7.A.400, 2ZIR .B.901, 2ZIS.B.901, 3ZXO.A.1579, 4AF1.A.500, 1ANI.A.450, 2AQ2.B.1001, 2AXR.A.501 , 3BYW.D.1, 3CHP.A.701, 3CPA.A.308, 1D1T.B.406, 3D68.A.501, 3DLJ.A.2001, 3DLJ.B. 2002, 3DNG.B.998, 4EGE.A.412, 2FKM.X.500, 4G1P.A.501, 3H90.A.292, 2IMC.A.600, 2I UC.B.1006, 4K7S.B.101, 2KBX.B.298, 1KQO.A.479, 3LSF.H.2, 1MWO.A.438, 4N07.A.309, 4N07.B.306, 2O1Q.B.145, 2O3Z.A.501, 4PVT.A.404, 3PW3.C.406, 3PW3.D.406, 1PYT.B. 350, 4Q7R.A.305, 3QU6.A.114, 3QZC.A.2, 1R5X.A.122, 3RC6.A.1, 4U9D.A.205, 3U94.A. 259, 3UBF.A.6, 1UD9.B.510, 1UDV.A.101, 1UUP.D.5222, 2VQH.B.1089, 2VW4.A.503, 3WC 5.A.404, 4WD7.C.302, 1XAF.A.503, 2Y2E.A.1180, 1Z5R.C.600, 3ZNR.B.101, 3ZUQ.A.144 0, 3HO1.X.22, 1VQ7.0.8066, 4AG5.B.1588, 2E8W.B.1203, 2HAW.A.1001, 1IOV.A.330, 4K 6T.B.411, 3LGH.A.150, 4QEH.A.401, 1SVT.E.601, 3VN9.A.402, 1Z6K.A.275, 1M63.B.500 , 4QOW.A.1001, 3QZ7.A.363, 2AER.L.3005, 3EDD.A.701, 3HR4.H.203, 1JX6.A.401, 1K6S .A.302, 1K6S.B.301, 4KS4.A.502, 1LPK.B.1, 1N41.A.410, 3RRV.C.255, 2TBV.A.388, 4W K4.B.502, 1Z3J.A.268, 1CJX.A.629, 3E1Q.A.301, 4ELR.A.401, 2HMN.A.450, 2OWT.A.324 , 4P9G.A.401, 4REU.A.202, 4V06.A.1491, 4QQW.G.1002, 1AFR.B.454, 4BMT.B.1324, 4CV P.A.1155, 3E1N.D.301, 3E1N.H.300, 3FE5.A.1, 2FKZ.A.1601, 3FM3.A.452, 1GUP.A.351, 3GZY.A.701, 3IS8.N.162, 1JYB.A.600, 1MOJ.A.302, 1NFV.A.201, 1NFV.M.200, 1O7P.A. 1453, 1OQ4.B.364, 1PFR.A.502, 1PIU.A.401, 1PIY.A.377, 4TOA.B.203, 4TOE.A.203, 1U LJ.E.600, 1W2N.A.312, 1YIT.0.8517, 3FKR.A.409, 2X1Z.M.1163

Table S147. all-ligand-number combined metal, combined group

	size	largest_angle*	middle_1*	middle_2*	middle_3*
1	"3223"	"176.4+/-2.7"	"84.1+/-3.2"	"89.4+/-1.1"	"93.2+/-2"
2	"2322"	"169.2+/-5"	"76.7+/-5.8"	"87.8+/-2.7"	"97+/-4"
3	"1912"	"168.6+/-5.6"	"72.2+/-6.8"	"85.7+/-3.3"	"100.8+/-5"
4	"3249"	"120.7+/-6.5"	"99.2+/-5.9"	"104.8+/-4.1"	"109.3+/-3.4"
5	"751"	"152.2+/-16.7"	"73.7+/-11.8"	"83.7+/-9"	"91.3+/-9.1"
6	"1716"	"157.9+/-8.1"	"59.1+/-9"	"81.8+/-5.3"	"104.8+/-9.5"
7	"1553"	"156.1+/-11.3"	"67.4+/-11.2"	"89.1+/-5.3"	"99.6+/-5.6"
8	"521"	"160.6+/-12"	"77+/-10.2"	"86.8+/-5.6"	"94.1+/-6.1"

	middle_4*	smallest_opposite_angle*	Tetrahedral	TrigonalBipyramidal
1	"173.8+/-3.7"	"85+/-4.1"	"0"	"0.004"
2	"161.7+/-6.2"	"80.1+/-7.7"	"0.001"	"0.024"
3	"157.9+/-6.2"	"54+/-5.4"	"0.007"	"0.039"
4	"113.8+/-3.6"	"106.9+/-7.4"	"0.574"	"0"
5	"102.2+/-10.6"	"83.9+/-13.6"	"0.001"	"0"
6	"147.8+/-8.5"	"66+/-9.7"	"0.009"	"0.017"
7	"134.4+/-9.1"	"101.7+/-7.3"	"0.058"	"0.085"
8	"107.9+/-11.3"	"125.1+/-17"	"0.01"	"0.072"

	TrigonalBipyramidalVA	TrigonalBipyramidalVP	Octahedral	SquarePyramidal
1	"0.002"	"0.115"	"0.385"	"0.511"

2	"0.019"	"0.214"	"0.129"	"0.247"
3	"0.035"	"0.217"	"0.074"	"0.165"
4	"0.028"	"0"	"0"	"0"
5	"0.01"	"0.027"	"0"	"0.001"
6	"0.035"	"0.082"	"0.013"	"0.039"
7	"0.13"	"0.144"	"0"	"0.009"
8	"0.075"	"0.165"	"0"	"0"

	SquarePyramidalV	SquarePlanar	TrigonalPrismatic	TrigonalPrismaticV
1	"0.551"	"0.543"	"0.002"	"0.008"
2	"0.368"	"0.322"	"0.023"	"0.06"
3	"0.269"	"0.223"	"0.075"	"0.141"
4	"0"	"0"	"0"	"0"
5	"0.137"	"0"	"0"	"0.001"
6	"0.097"	"0.058"	"0.064"	"0.125"
7	"0.115"	"0.026"	"0"	"0.077"
8	"0.058"	"0.035"	"0"	"0.01"

	PentagonalBipyramidal	PentagonalBipyramidalVA	PentagonalBipyramidalVP
1	"0"	"0"	"0.005"
2	"0.001"	"0.001"	"0.045"
3	"0.092"	"0.099"	"0.166"
4	"0"	"0"	"0"
5	"0"	"0"	"0"
6	"0.037"	"0.061"	"0.077"
7	"0"	"0"	"0"
8	"0"	"0"	"0"

	SquareAntiprismatic	SquareAntiprismaticV	HexagonalBipyramidal
1	"0"	"0"	"0"
2	"0"	"0.001"	"0"
3	"0.002"	"0.097"	"0"
4	"0"	"0"	"0"
5	"0"	"0"	"0"
6	"0.009"	"0.06"	"0"
7	"0"	"0"	"0"
8	"0"	"0"	"0"

	HexagonalBipyramidalVA	HexagonalBipyramidalVP
1	"0"	"0"
2	"0"	"0"
3	"0"	"0.049"
4	"0"	"0"
5	"0"	"0"
6	"0.002"	"0.015"
7	"0"	"0"
8	"0"	"0"

Table S148. Cluster members of all-ligand-number combined metal, combined group

[1] "Cluster 1"
3ADR.A.263, 2DI3.A.1002, 2EK9.A.1004, 3G4K.A.801, 4HTZ.B.1001, 2K78.A.151, 4KBP.A.439, 3LL8.A.505, 2LQ6.A.402, 2LZE.A.101, 1ML2.A.296, 4N7K.L.301, 4N7K.L.307, 4N7K.M.401, 4N7K.M.402, 4N7L.M.402, 3NO5.C.275, 4OK2.A.801, 1VKG.B.400, 4X2T.A.702, 3DZA.C.501, 3DZA.C.505, 2EK8.A.1004, 1ENQ.A.238, 2F92.F.1001, 2F92.F.1002, 2F

94.F.1001, 2F94.F.1003, 2F9K.F.1001, 2F9K.F.1002, 2FUQ.A.1, 4GBD.A.503, 4GQT.A.5 01, 1GT7.A.275, 3GWT.A.504, 2H44.A.501, 3HDZ.A.864, 1HQA.B.452, 3LLX.A.376, 1M60 .A.105, 3NO5.A.275, 4NPW.A.1001, 4NT9.A.301, 3O2G.A.388, 3O91.A.192, 4OK4.A.800, 2OUV.A.777, 2OU3.A.161, 2OUP.B.777, 4OV9.A.401, 2P2L.A.201, 2PTY.A.501, 2PTZ.A. 500, 2PTZ.A.501, 2PU1.A.501, 2PW3.A.501, 2QYM.A.1, 2QYK.A.1, 2R2V.C.35, 1RRM.B.3 87, 3SL3.B.9, 1T9S.A.1, 1TB5.A.1001, 1TB7.A.1001, 1TBF.A.1, 3U43.B.135, 1U74.A.1 001, 3UUO.B.772, 3V93.A.701, 2WTA.A.1215, 1XM6.A.1001, 1XOR.A.1001, 1Y2K.A.1001, 1Y9Q.A.202, 1ZKL.A.501, 4BDY.A.1380, 4BE0.A.1380, 4BE1.A.1381, 4BE2.A.1380, 4BE 2.A.1381, 1BPY.A.339, 4BWJ.A.1834, 4CEI.B.2162, 4DFJ.A.902, 4DFK.A.902, 4DFM.A.9 02, 4DL4.A.501, 4DO9.A.401, 4DOB.A.401, 4DOC.A.401, 2DPI.A.871, 4DQI.A.901, 4DQI .D.901, 4DQP.D.902, 4ELT.A.902, 4ELU.A.902, 4F5O.A.402, 1FIU.I.2222, 1FIU.A.5555 , 4FO6.A.601, 3G6Y.A.871, 1G9Z.F.903, 4GZ2.B.402, 3IEV.A.400, 2JOS.A.1412, 1JJ2. 0.8010, 3JPQ.A.339, 3JPS.A.339, 4JWM.A.403, 3K58.A.1001, 3K59.A.1001, 4K97.A.603 , 3KD5.E.916, 4KLI.A.401, 4M04.A.702, 4M04.A.703, 3M8S.A.2, 4M8O.A.1302, 4M9L.A. 404, 4MDE.A.1002, 3MFI.A.515, 4MFC.A.401, 3MR5.A.435, 4O3O.A.502, 4O3O.A.503, 4O 3Q.A.502, 4O3Q.A.503, 4O5K.A.401, 3OHA.A.518, 3OJS.A.7, 3OYB.A.396, 3OYD.A.396, 3OYG.A.396, 2OZS.A.904, 4P4M.A.402, 2PFP.A.750, 3PML.A.2, 3PNC.A.576, 4PUQ.B.401 , 4Q8E.A.502, 4QM6.A.1002, 4R65.A.402, 3RJH.A.403, 3RJK.A.339, 4RNN.A.503, 3RTV. A.833, 3SI8.A.451, 3SM4.B.227, 3SNN.A.905, 3SPY.A.903, 1SUZ.A.402, 3TFR.A.339, 3 TFS.A.339, 3TIO.A.1, 3TIO.D.2, 4UAY.A.402, 3V6H.A.402, 1W7A.A.1802, 1XSN.A.576, 1ZJN.A.339, 3ZVM.A.1526, 121P.A.168, 3A0T.A.800, 4A01.A.1767, 4A01.A.1769, 3A1U. A.5, 3A1U.A.6, 1A2B.A.550, 3A4L.A.401, 2A5Z.A.701, 2A5D.A.1231, 4A6X.A.350, 1A82 .A.901, 3A99.A.401, 4ACF.A.1482, 4ACI.A.1187, 2AFK.E.1291, 2AGO.A.601, 3AHC.A.82 6, 3AHD.A.826, 3AHE.A.826, 3AHG.A.826, 1ALK.B.452, 2AL1.B.439, 4ANB.A.1384, 1AOR .A.609, 1AOX.A.400, 1ASO.A.356, 4AS2.A.1328, 2AUU.A.203, 2AUU.A.204, 2AUT.A.601, 2AUT.D.604, 4AUX.A.223, 3AYX.A.701, 2B0T.A.800, 3B05.D.1001, 3B1V.A.301, 1B25.A .800, 4B2P.A.1351, 1B4N.A.623, 2B56.A.488, 3B7L.A.907, 3B7L.A.908, 3B7L.A.909, 1 B8J.A.452, 2B82.A.1013, 2B9H.A.700, 4BAS.A.1183, 3BB1.A.282, 2BBS.A.3, 3BC1.A.19 4, 2BEK.A.501, 3BGA.A.1, 2BME.A.1184, 3BN3.A.1, 1BOF.A.800, 2BON.A.1302, 2BU2.A. 1388, 2BVC.A.504, 2BVN.B.1395, 3BWV.A.300, 4BW9.A.501, 4BWR.A.1468, 4BX0.A.1291, 4BX3.A.301, 1BYQ.A.1001, 1C1Y.A.171, 2C18.A.1338, 2C3U.A.2238, 2C42.A.3238, 3C4 Z.A.563, 2C4N.A.1251, 3C5H.A.302, 2C5L.A.1168, 4C5B.A.1314, 4C5C.A.1314, 2C77.A. 1407, 2C78.A.1407, 4C7X.A.700, 3C9U.B.312, 2CBZ.A.1872, 3CFX.A.704, 2CFS.A.1296, 3CG4.A.201, 1CHN.A.200, 2CHE.A.130, 1CIP.A.356, 1CJT.C.403, 1CJU.A.582, 2CJE.A. 1267, 3CK5.A.400, 2CK3.A.601, 2CK3.F.601, 2CL5.A.1216, 2CLS.A.550, 3CMR.A.453, 2 CN5.A.1506, 4COK.A.601, 3CP6.A.502, 3CP6.A.503, 3CR3.A.1212, 1CTQ.A.168, 3CUR.H. 553, 3CUS.Q.553, 3CV2.A.1, 3CX8.A.378, 3CZJ.B.3001, 2D0O.A.1005, 1D2N.A.99, 3D2R .A.500, 3D36.A.478, 4D6P.A.1352, 2D7C.A.1002, 4D7M.A.223, 1D8C.A.3001, 4DBF.A.40 1, 4DBR.A.810, 2DCN.A.4001, 4DCK.B.201, 3DDC.A.600, 3DDH.B.232, 2DDT.A.311, 2DEI .A.402, 4DEM.F.402, 4DEM.F.403, 4DEM.F.404, 3DGT.A.800, 3DKC.A.2, 4DN1.A.401, 4D N5.A.1001, 4DSN.A.202, 4DSO.A.202, 1DTW.A.401, 4DUX.A.3001, 4DWG.A.401, 4DWO.A.3 01, 1DXE.B.901, 4DXJ.A.403, 3DYH.A.3003, 3DYH.A.3004, 3DYM.A.3001, 3DYP.A.3001, 2DY1.A.701, 4DYK.A.502, 4DZH.A.501, 4E01.A.402, 1E2Q.A.401, 3E2D.A.603, 1E3D.B.9 01, 3E5H.A.200, 3E81.A.165, 3E84.A.701, 3E8M.A.165, 4E8G.A.402, 2E9S.A.603, 1E9A .A.401, 2E91.A.1301, 2E95.A.1302, 3EA5.A.221, 2EB1.A.502, 2EB5.A.1001, 4EEN.A.30 1, 3EFQ.B.4004, 4EFM.A.202, 3EGT.A.3002, 3EGT.A.3004, 3EHG.A.371, 1EKO.A.601, 3E QC.A.3, 2ERX.A.403, 3ET4.A.301, 3ET5.A.255, 3ETJ.A.401, 4EUK.A.1001, 2EW1.A.701, 4EX6.A.301, 4EX7.A.301, 2EZT.A.1510, 2EZU.A.1610, 3EZ3.A.1104, 3EZ3.B.1102, 2EZ 4.A.1610, 2EZ8.A.1510, 2EZ9.A.1510, 4F1J.A.301, 2F2A.B.601, 1F5N.A.595, 3F61.A.3 10, 1F9H.A.161, 1F9H.A.162, 2F9M.A.1201, 4F9A.A.602, 3FD5.A.397, 3FD6.B.397, 4FE 3.A.304, 4FEG.A.707, 2FFQ.A.356, 2FH5.B.270, 3FIU.A.5001, 3FIU.A.5002, 4FI1.A.40 1, 3FKQ.A.501, 4FK9.A.401, 2FOZ.A.348, 2FOZ.A.349, 4FP1.A.401, 2FRV.B.540, 1FSG. A.302, 3FSY.A.333, 2FUE.A.500, 3FV9.A.501, 4FVR.A.902, 4FYP.A.301, 3FZN.A.605, 2 G0W.A.501, 2G09.A.901, 2G1T.A.1501, 1G17.A.301, 3G15.A.602, 3G2F.A.901, 1G4C.B.3

62, 1G4P.A.2003, 1G4T.A.2005, 3G5A.D.307, 1G5T.A.998, 3G6K.A.307, 2G6B.A.301, 2G80.A.500, 1G97.A.460, 4G9B.A.301, 3GAI.A.189, 2GCN.A.2001, 2GCP.A.2001, 2GHT.A.257, 2GIL.A.1201, 2GL5.A.699, 4GME.C.501, 3GON.A.600, 2GO7.A.207, 4GOJ.A.202, 4GP2.A.401, 4GP2.A.402, 2GQS.A.241, 1GSA.A.319, 1GSI.A.1209, 2GSM.A.3006, 4GT8.A.402, 1GUA.A.171, 3GYB.A.1, 4H19.A.405, 4H1Z.D.401, 1H2A.L.1005, 1H2R.L.1005, 3H3X.Q.553, 2H57.A.202, 3H70.A.342, 3H7V.A.331, 3H80.A.214, 4H81.A.402, 4H8E.A.301, 4HAT.A.302, 2HCF.A.300, 4HCH.A.405, 4HCL.A.401, 3HDG.A.202, 4HDO.B.200, 3HFW.A.361, 2HF8.A.301, 2HF9.A.301, 2HGS.A.502, 4HGN.A.200, 4HGQ.A.201, 3HIY.B.402, 2HJP.A.292, 2HNE.A.601, 4HNC.A.401, 4HNL.A.401, 4HOR.X.101, 4HPT.E.402, 1HQ2.A.162, 3HQJ.A.145, 4HQO.A.301, 3HRZ.A.628, 3HRZ.D.742, 3HSD.B.162, 1HTW.A.561, 3HVJ.A.265, 3HW3.A.999, 3HW4.A.999, 3HW5.A.999, 1I0L.A.902, 2I1Q.A.501, 4I2B.A.604, 2I33.A.602, 2I34.A.301, 4I3Z.A.302, 2I5R.A.301, 2I6K.A.302, 3I76.A.1001, 4I94.A.402, 4IAD.A.402, 3IBA.A.403, 3ICK.A.401, 3ICK.A.402, 3ICK.A.403, 3ICM.A.401, 3ICM.A.402, 3ICZ.A.402, 3ICZ.A.403, 4IDN.A.502, 4IDP.A.502, 4IEG.A.1001, 1IG5.A.78, 2IHT.A.601, 2IHU.A.601, 1IH8.A.4002, 1IHU.A.592, 2IHP.A.287, 4IHC.A.501, 3IJL.A.386, 3IJR.D.300, 2IK4.A.287, 2IK4.B.287, 2IK4.B.288, 2IK4.B.289, 2IK6.B.287, 2IOR.A.2000, 1IOW.A.331, 2IO8.A.7002, 4IP4.A.503, 4IP5.A.502, 3IPO.A.161, 1IR3.A.301, 1ITZ.A.1001, 4ITR.D.203, 2IUC.A.1003, 4IUC.L.702, 4IUD.L.1002, 1IV2.A.1571, 1IV4.A.1571, 4IVG.A.803, 4IWH.A.401, 2IXE.A.2, 2IYW.A.202, 2IYN.C.1123, 1IZC.A.1001, 2J0V.A.1180, 2J7P.A.1401, 2J7P.D.1401, 1J7L.A.301, 2J7N.B.3374, 1J9J.A.301, 1J9J.B.301, 1JBW.A.998, 2JC9.A.1491, 2JCB.A.1192, 4JDP.A.301, 2JD4.A.4061, 2JD4.B.4062, 1JGT.A.902, 2JI7.A.1567, 4JND.A.501, 1JPM.A.1003, 1JSC.A.699, 4JSO.A.202, 3JTC.C.34, 1JUY.A.435, 3JVA.A.356, 3JVA.B.358, 3JVT.B.502, 4JXO.A.402, 3JYS.A.1, 3JYY.A.301, 3JYY.B.302, 1JZ7.A.3001, 3JZO.A.300, 3JZO.A.303, 3K1S.H.107, 4K1W.A.501, 3K4Z.A.290, 4K6R.A.505, 1K77.A.300, 3K8K.A.700, 3K9L.A.168, 4K9Q.A.601, 3KA3.A.176, 3KAL.A.503, 3KB9.A.701, 3KB9.A.702, 3KC2.A.355, 1KCZ.A.901, 4KFU.A.307, 4KGD.A.702, 1KHZ.B.301, 1KK1.A.413, 3KKO.A.180, 1KMQ.A.401, 3KMW.A.501, 4KO8.A.801, 1KQP.A.5001, 1KQP.A.5002, 4KQW.A.404, 3KS6.C.251, 1KSH.A.202, 1KTG.A.502, 1KTG.A.503, 1KTG.A.504, 1KTG.A.505, 3KTA.A.184, 3KUC.A.171, 4KUX.A.703, 4KVA.A.501, 4KVG.A.202, 4KWD.A.404, 4KX5.A.314, 4KXW.A.1001, 1KY2.A.401, 3L12.A.313, 4L2X.F.404, 4L57.B.201, 3L8H.A.801, 4L80.A.403, 4L9W.A.202, 4L9Z.A.403, 4LA6.A.501, 2LCF.A.246, 4LFG.A.303, 4LFG.A.304, 4LFG.B.303, 4LGY.A.1302, 4LHW.A.301, 4LJ9.A.902, 3LLU.A.502, 1LNY.A.1453, 4LPM.A.208, 3LUF.A.300, 3LUF.A.301, 3LX5.A.301, 3LXX.A.402, 4LYK.A.401, 4LZO.A.403, 3M07.A.595, 1M0W.A.502, 3M1I.A.1178, 4M53.A.527, 1M7B.A.550, 3M7I.A.901, 4M9Q.A.302, 1MC1.A.601, 1MDL.A.360, 4MDB.A.403, 4MGG.A.404, 1MH1.A.201, 3MHY.A.115, 1MJN.A.1001, 3MJH.A.201, 3MK2.A.903, 1MMG.A.998, 1MMN.A.998, 1MNE.A.998, 4MNE.A.902, 4MPO.B.205, 1MQ4.A.2088, 4MRT.A.301, 4MUM.A.301, 1MXO.A.501, 1MXG.A.439, 3MX3.A.601, 3MYH.X.997, 4MYO.A.301, 3MYK.X.998, 3MYL.X.998, 4MZU.C.404, 1N0H.A.699, 1N0H.B.1699, 4N0D.A.402, 3N07.A.200, 1N1Z.A.701, 1N1Z.A.703, 1N20.A.701, 1N20.A.702, 1N24.A.702, 3N2N.A.1, 3N45.F.355, 3N45.F.2, 3N45.F.3, 3N4F.A.502, 1N6I.A.201, 1N6L.A.201, 1N6N.A.201, 1N6O.A.201, 1N6P.A.201, 1N6R.A.201, 1NBO.A.201, 4NBS.A.502, 4NDO.A.302, 4NFI.F.402, 4NFI.F.403, 4NFI.F.404, 1NFZ.A.401, 3NKV.A.500, 1NN5.A.401, 3NOJ.A.239, 1NRJ.B.1, 3NRJ.A.190, 2NSY.A.305, 1NSF.A.859, 1NSY.A.6241, 4NWI.A.401, 2NXW.A.4002, 1O08.A.2800, 2O1S.A.1001, 1O3Y.A.1002, 3O61.B.202, 1O6Y.A.1280, 3O6Z.B.201, 2O70.A.223, 4O7I.A.401, 4OAV.B.802, 1OBW.A.176, 3OCU.A.263, 3OCV.A.264, 3OCW.A.263, 3OCX.A.264, 3OCY.A.264, 3OCZ.A.263, 2OCB.A.202, 4OCP.A.403, 2ODP.A.901, 4ODJ.A.502, 3OE1.A.601, 3OE5.A.222, 2OEM.A.911, 2OEM.B.912, 2OFX.A.301, 2OGD.A.3002, 2OGD.A.3003, 2OGD.A.3004, 4OHY.A.502, 1OIW.A.1175, 1OIX.A.301, 3OIW.A.170, 2OI6.B.6000, 4OI4.A.502, 1OKK.A.1002, 1OKK.D.1002, 4OKM.A.901, 4OKM.A.903, 4OKZ.A.903, 2OLR.A.543, 3OM2.A.486, 4OMF.A.503, 3OP2.B.500, 2ORW.A.501, 3OUZ.B.459, 1OXV.A.1102, 1OXV.D.1101, 3OYX.A.601, 3OZF.A.235, 3OZX.A.613, 3OZX.A.614, 2P27.A.307, 4P31.A.402, 4P32.A.402, 3P41.A.298, 1P4M.A.201, 1P5Z.B.401, 3P5P.A.901, 3P93.C.406, 3P96.A.412, 2PA4.A.325, 3PDE.A.312, 3PFF.A.831, 4PFY.B.601, 3PGL.A.1, 1PHP.A.395, 4PHG.A.201, 4PHH.A

.202, 3PIT.A.180, 3PK7.A.406, 2PK0.A.502, 2PKE.B.300, 2PLS.H.602, 2PMQ.A.902, 3PNL.B.1212, 2PNQ.A.502, 1POX.A.610, 1PPV.A.401, 4PQ9.A.301, 2PS2.A.401, 2PS5.B.701, 1PT6.A.500, 1PVF.A.401, 1PVG.A.903, 2PYW.A.501, 2PZ8.A.4001, 2PZA.A.6243, 2PZE.A.3, 3Q10.A.400, 2Q28.A.1001, 1Q3H.C.674, 3Q3J.B.201, 2Q3F.A.301, 3Q46.A.307, 4Q4C.A.404, 3Q5V.B.599, 2Q5Q.A.4002, 3Q60.A.603, 1Q6L.A.5300, 1Q6O.A.7300, 1Q6Q.A.7300, 1Q6R.A.7300, 2Q66.A.602, 3Q85.A.284, 1Q92.A.1003, 4QC2.A.302, 4QEA.A.301, 3QF7.A.854, 1QGU.B.3002, 1QGU.D.3006, 2QG8.A.201, 4QHZ.A.302, 2QIS.A.907, 2QIS.A.908, 2QIS.A.909, 2QJJ.C.1003, 1QK5.A.303, 3QKE.A.407, 3QKT.A.902, 2QME.A.179, 3QN3.A.601, 3QNM.A.400, 2QQ0.A.450, 1QRA.A.168, 2QRZ.A.190, 2QTY.A.348, 2QTY.A.349, 2QT0.A.1001, 2QTC.A.888, 3QUQ.A.225, 3QUT.A.225, 3QVQ.C.310, 3QXC.A.222, 3QXH.A.223, 3QXJ.A.224, 3QXS.A.223, 3QXX.A.224, 2QX0.A.161, 3QYY.A.505, 1R0X.A.13, 3R0U.A.380, 3R1M.A.403, 3R1M.A.404, 1R2Q.A.300, 3R3S.A.296, 4R39.A.401, 3R6T.A.301, 2R6O.A.801, 3R7W.A.600, 2R8E.A.201, 2RAH.A.354, 2RAR.A.501, 2RAV.A.701, 3RAP.R.200, 3RBM.A.1001, 2RB5.A.701, 2RBK.A.501, 3REF.A.192, 3REG.A.550, 1RKQ.A.1273, 1RKU.A.301, 1RKV.A.401, 4RKE.A.202, 4RKF.A.202, 3RLF.A.1501, 1RMT.A.1413, 3RO6.A.400, 4ROQ.A.401, 1RP7.A.890, 1RQI.A.603, 1RQI.A.604, 1RQJ.A.907, 1RQJ.A.908, 1RQJ.A.909, 3RUS.A.544, 1RVK.A.999, 3RV3.A.1004, 3RWM.B.1, 3RYE.A.907, 3RYE.A.908, 3RYE.A.909, 1RYA.A.1001, 1RYH.A.539, 4S1H.B.303, 3S4J.A.907, 3S4J.A.908, 3S4J.A.909, 3S9Z.A.802, 3SAZ.A.802, 1SAW.A.225, 3SAE.A.820, 3SBD.A.501, 3SDT.A.819, 3SDT.A.821, 3SEA.B.178, 3SF0.A.263, 3SHQ.A.321, 1SHT.X.219, 3SJN.A.374, 3SLS.A.401, 3SL2.A.701, 3SN1.A.408, 3SN4.A.408, 1SO4.A.2300, 3STP.A.391, 1SVM.A.750, 1SVS.A.356, 1T0P.A.901, 3T10.A.401, 3T1K.A.401, 3T2S.B.401, 3T2B.A.409, 3T2D.A.408, 3T2D.A.409, 3T7A.A.602, 1T8Q.B.1602, 1T9B.B.699, 1T9C.B.699, 1TC6.A.501, 2TCT.A.223, 3TCS.A.368, 3TDV.A.501, 1TE6.A.641, 3TEP.A.1, 3TG0.A.503, 3TJI.A.601, 3TKL.A.300, 1TMM.A.162, 4TMT.A.902, 4TMV.A.902, 4TMW.A.903, 4TMX.A.903, 3TMO.A.266, 4TN1.A.902, 4TQD.A.502, 3TSO.A.200, 4TSK.A.403, 1TW1.A.1, 3TWA.A.420, 3TWB.A.420, 3TYZ.A.281, 4TY0.A.502, 1TZZ.A.3501, 1U02.A.240, 4U5X.A.202, 4U82.A.301, 1U8Y.A.301, 4UAK.A.503, 4UAS.A.302, 4UAT.A.302, 4UAV.A.401, 2UAG.A.1001, 1UBK.L.1005, 3UCW.A.100, 3UCY.A.101, 4UCX.Q.1553, 4UE3.L.603, 3UIE.A.403, 3UJR.A.502, 3UJR.B.502, 3UJS.A.601, 3UJS.B.601, 1UMD.A.1401, 1UMG.A.403, 1UMG.A.404, 4UM7.A.175, 4UMF.A.1175, 3UPY.A.446, 1UPT.A.1183, 3UPL.A.447, 3UQY.L.603, 4UQL.Q.1552, 4URH.Q.1552, 3UXK.A.360, 3UZR.A.300, 4V0L.A.601, 3V1V.A.501, 3V1X.A.501, 4V1T.A.1777, 3V2U.C.521, 2V26.A.1801, 3V3W.A.403, 3V4B.A.403, 1V54.A.3518, 2V7Q.A.1512, 2VBU.A.1134, 3VC1.A.301, 3VC1.I.301, 3VC2.J.301, 3VCC.A.401, 3VCC.A.402, 3VCN.A.501, 2VDO.B.2001, 2VDR.B.2001, 2VDL.B.2001, 2VDN.B.2001, 1VG8.A.1401, 2VG3.A.1297, 2VK1.A.601, 2VK4.A.601, 2VK8.A.1565, 3VMK.A.402, 3VMK.B.402, 1VOM.A.997, 3VPB.A.502, 3VVH.A.701, 3VX4.A.802, 1VZM.B.1046, 1VZM.B.1047, 1W2Y.A.1231, 1W2Y.A.1232, 3W40.A.201, 2W4J.A.1280, 2W5V.A.1378, 2W5X.A.1379, 1W6T.A.435, 3W6N.A.803, 3W6O.A.802, 1W78.A.1422, 1W78.A.1423, 1W7K.A.1423, 1WA5.A.1178, 3WBH.A.501, 3WBZ.A.403, 3WBZ.A.404, 1WC1.A.1501, 1WC1.A.1502, 3WEK.A.401, 2WEF.A.401, 1WF3.A.401, 4WH2.A.402, 4WH3.A.402, 2WIC.A.1266, 3WJP.A.403, 3WJP.A.404, 2WKQ.A.1724, 3WNZ.A.502, 3WRY.C.1202, 1WUH.L.1005, 1WUK.L.1005, 2WVG.A.601, 2WW8.A.1000, 3WXM.A.502, 1WZC.A.300, 1X06.A.900, 1X07.A.900, 2X13.A.1418, 2X14.A.1418, 1X3S.A.200, 1X84.B.401, 2X98.A.1477, 1XBY.A.601, 2XB5.A.223, 2XCW.A.1498, 1XEX.A.1002, 1XFI.A.400, 1XG3.A.2101, 2XH2.B.1439, 2XH7.A.1441, 2XIS.A.392, 4XIA.A.399, 2XJB.A.1494, 2XJD.A.1497, 2XJE.A.1493, 2XSX.A.500, 2XTZ.A.1381, 2XTN.A.1232, 2XUU.A.1307, 1XX1.A.9001, 4XXP.A.301, 2Y6P.A.1234, 1Y8A.A.501, 1Y8Q.B.641, 2Y8E.A.1177, 1Y9D.D.2901, 2YCH.A.501, 1YHL.A.1401, 1YHL.A.1402, 1YHL.A.1403, 1YMV.A.200, 1YNS.A.1258, 1YQ9.H.540, 1YQT.A.591, 1YQT.A.592, 1YRQ.H.553, 1YS7.A.1002, 1YU4.A.2002, 2YVO.A.1001, 2YVO.A.1002, 2YVP.A.183, 2YVP.A.184, 1YVD.A.850, 1YVE.I.601, 2YVM.A.1001, 2YXH.A.502, 1YYQ.B.702, 1YZL.A.401, 1YZN.A.301, 1YZT.A.700, 1Z06.A.203, 1Z07.A.300, 1Z08.A.1300, 1Z08.C.3300, 1Z0J.A.400, 1Z2N.X.1295, 1Z2O.X.1295, 1Z4J.A.1001, 1Z4K.A.229, 1Z4L.A.2001, 1Z4O.A.800, 1Z4P.X.1001, 1Z4Q.A.2001, 2Z4V.A.1501, 2Z4V.A.1502, 2Z4X.A.1201, 2Z4X.A.1202, 1Z59.A.1001, 1Z5C.A.2001, 1Z5G.A.601, 1Z5G.D.6

04, 2Z52.A.1301, 2Z52.A.1302, 2Z7I.A.1301, 1Z88.A.601, 1ZC3.A.500, 2ZCR.A.669, 2ZDH.A.812, 1ZED.A.905, 3ZFD.A.500, 3ZIA.A.601, 1ZJJ.A.1001, 2ZKJ.A.500, 3ZMC.A.1296, 3ZO9.A.1592, 3ZOU.A.1295, 1ZPD.A.601, 2ZPU.A.360, 1ZS9.A.1257, 3ZVL.A.1524, 3ZX4.B.260, 3ZX5.A.260, 1ZXN.B.902, 2IHM.A.700, 3A0U.A.201, 3A10.A.201, 2AKZ.A.441, 1ALK.A.452, 3B05.A.1001, 4BBJ.A.750, 2BHW.A.601, 2BHW.A.602, 2BHW.A.603, 2BHW.A.604, 2BHW.A.609, 2BHW.A.612, 2BHW.A.614, 3BH7.A.1, 4BX2.A.301, 4C5A.A.331, 3C9U.A.309, 1D0X.A.998, 1D0Y.A.998, 1D0Z.A.998, 1D1A.A.998, 1D1B.A.998, 4DBQ.A.903, 2DCN.B.4006, 4DL8.A.304, 3DNT.B.442, 1DXR.L.400, 3DYH.A.3002, 1E79.A.601, 2E8X.A.1301, 3EF0.A.1, 2FKW.A.1501, 2FKW.B.1601, 3G5A.C.307, 1G67.A.2007, 2G77.B.503, 1GFI.A.356, 2GJ8.A.602, 3GL9.A.123, 2GLQ.A.2003, 4GVE.A.602, 3H1E.A.202, 1HE1.C.202, 2HEG.A.300, 2HF7.A.700, 3HWX.1.602, 2I7D.A.728, 3ICM.A.403, 3ICN.A.402, 4IDO.A.503, 4IF4.A.300, 4IGA.A.200, 1IV2.B.1572, 2J7N.A.3374, 2J8C.L.1288, 1J97.A.220, 4JA2.A.201, 1JB0.A.1107, 1JB0.A.1110, 1JB0.A.1112, 1JB0.A.1118, 1JB0.A.1129, 1JB0.A.1130, 1JB0.A.1134, 1JB0.B.1231, 1JB0.B.1235, 1JB0.X.1701, 4KEM.A.401, 1L3R.E.392, 1L5Y.A.701, 1L7N.A.221, 4LCZ.A.306, 4LCZ.A.307, 4LCZ.C.316, 4LE0.A.201, 1LGH.A.57, 1LGH.A.58, 1LGH.B.59, 4LRS.A.404, 1LVK.A.998, 1MPS.M.801, 1MX0.E.501, 3N5K.A.2001, 1N6K.A.201, 2NGR.A.199, 3NNN.A.401, 4NST.A.1103, 1NVV.Q.1002, 4NV0.A.402, 2ODE.A.3001, 2OGX.A.291, 3OLV.A.130, 4OVN.A.201, 2OZE.A.299, 3PDE.B.310, 3PL9.A.602, 3PL9.A.603, 3PL9.A.609, 3PL9.A.610, 3PL9.A.612, 3PL9.A.613, 3PL9.A.614, 2PL1.A.204, 2PLS.I.606, 1Q3H.D.674, 3QHW.A.298, 1R0X.D.14, 2R25.B.1, 1RLO.A.801, 4RUR.W.301, 1RWT.A.614, 4S1H.A.303, 1SVK.A.356, 3T2S.A.401, 3T6D.L.401, 3T6D.M.400, 3T6E.L.400, 1T91.A.1301, 3T9E.A.602, 3TCS.B.368, 3THU.A.500, 1TX4.B.681, 1UPB.A.601, 2VB6.A.1000, 3W6P.A.803, 1W7J.A.1793, 1W9I.A.1755, 3W9S.A.202, 2WF7.A.1220, 2WJN.M.1325, 3WK4.A.601, 1WQA.B.456, 2WZB.A.1417, 2X2E.A.1746, 1XBX.A.601, 1XHF.A.1001, 1Y9D.A.2601, 1YXO.A.5000, 1YZQ.A.901, 1Z2P.X.1295, 2Z4Z.A.1301, 2Z7I.B.1302, 1ZES.A.302, 1ZH4.B.202, 1ZXN.A.900, 2ZXE.A.2002, 4G3I.B.401, 4K4H.M.604, 4LQ0.A.401, 4AG4.A.5001, 2BV2.A.1085, 3BYC.A.901, 2CD0.B.1140, 4CPN.A.500, 1D7X.B.805, 3DPE.A.997, 1DYK.A.4001, 3EKI.A.602, 4FVL.B.506, 2GNT.A.254, 4GN7.B.301, 4ILW.D.304, 1INW.A.501, 2JG9.B.1224, 1L6J.A.502, 1L9N.A.703, 2NW6.A.613, 4Q4X.1.5005, 1QI5.A.452, 2R1D.B.1000, 2R8Z.J.210, 1R8L.B.902, 2RJQ.A.6, 1RU4.A.2, 1T6B.X.800, 4U32.A.301, 1ULV.A.2005, 1UYX.B.1135, 3V03.B.585, 3VV3.A.404, 3WNK.A.812, 1Z4V.A.600, 1ZTQ.A.561, 3DPG.B.501, 4ECQ.A.501, 4FJ8.A.1002, 4K4H.A.605, 4K4I.E.602, 4KHQ.A.1001, 1N3F.C.498, 2OAA.A.601, 2ODI.A.701, 2Q10.A.701, 4QWB.A.402, 2RDJ.A.353, 3AFG.A.540, 2AFH.D.2490, 3AHW.A.122, 3AI7.B.901, 1AJJ.A.73, 3AJ7.A.602, 3AKB.A.2, 4APX.B.1239, 4AQE.A.1208, 3AUK.A.391, 1AVA.A.502, 4AWN.A.300, 4AXN.A.1329, 3AYU.A.417, 3B7E.A.1005, 3B8Z.A.904, 3BCD.A.707, 2BL0.B.1146, 4BNR.A.600, 2BQ4.A.1119, 2C10.A.1771, 3C9I.A.1, 3CKZ.A.1, 4CPL.A.500, 4CU9.A.2999, 4CUA.A.2644, 1CVL.A.320, 3D3I.A.1001, 3D7K.A.571, 2DDR.A.1324, 1DPO.A.246, 4E5U.B.302, 2EA7.A.450, 3EDY.A.1, 2EEK.A.401, 1EGZ.A.300, 3F5V.A.223, 2FCW.B.3001, 2FCW.B.3002, 2FGZ.A.1192, 3FG1.A.1501, 2FHF.A.2404, 3FSJ.X.600, 3FVQ.A.360, 2FWN.A.532, 1G9K.A.703, 1G9K.A.706, 1GA6.A.374, 4GDI.A.509, 4GG1.A.602, 2GKO.A.612, 4GN7.A.301, 1GTT.A.1430, 2HOB.A.1000, 4H1Q.A.303, 4H1Q.A.305, 3H81.A.279, 3H81.C.279, 3H81.C.280, 1HFC.A.277, 3HGN.A.250, 3HJR.A.603, 4HJF.A.601, 4HS9.A.401, 1HT6.A.502, 1HV5.A.5503, 1HY7.A.305, 4HZW.A.507, 4I35.A.513, 1I76.A.997, 2I8U.A.202, 2I8T.A.402, 3I9G.L.301, 3I9G.L.302, 4IHM.A.404, 4IU2.A.301, 4IU3.A.301, 2IXT.A.1310, 1J8E.A.201, 1JK3.A.403, 2JKP.A.1728, 4JZB.A.401, 4JZB.A.403, 4JZX.A.404, 4JZX.A.405, 4K1K.A.501, 1K7I.A.487, 4K70.A.1002, 1KAP.P.616, 1KAP.P.619, 4L74.A.401, 1LQV.C.35, 4LVN.A.703, 4M5I.A.201, 3M6L.A.801, 3MA2.A.293, 3MA2.A.296, 4MEW.A.502, 1MNC.A.283, 3MOS.A.1, 4MPR.A.601, 4MWV.A.512, 3N1U.A.200, 4N2E.A.705, 4N2P.A.201, 4N4E.E.405, 1N9E.A.802, 1NPC.A.320, 2O72.A.403, 1OAC.A.802, 4OCI.A.201, 4OKH.B.904, 3OOY.A.621, 1OU9.A.131, 3OYR.B.337, 3OYR.B.338, 3P2P.B.126, 2P3U.B.501, 3P4G.A.401, 2PHI.B.125, 4PMX.A.401, 1Q3A.A.466, 3Q4W.A.224, 4Q8K.A.501, 1QCN.A.1001, 4QN6.A.501, 3QRB.A.301, 3QU7.A.230, 3QU7.B.225, 2QUB.A.615, 2R1B.A.1001, 2R5N.A.2000, 1R6V.A.1, 2R8O.A.670, 2R8Z.A.201, 2R8P.A.670, 1RM8.A.502

, 1RQ5.A.819, 3RRX.A.901, 3RUP.A.1004, 3RVV.A.225, 3RVW.A.223, 3S4Y.B.1303, 1SAT .A.476, 3SAL.A.601, 1SIO.A.601, 1T1E.A.700, 3TI4.A.601, 1TRK.A.681, 3U1R.A.703, 3U8D.A.203, 3U8I.A.201, 3UPT.A.691, 4UP4.A.501, 1UR4.A.1398, 4USU.A.1471, 1UX6.B .2002, 1UX6.B.2004, 1UX6.B.2012, 1UX6.B.2016, 4UZU.A.1484, 3V5U.A.705, 3V96.B.30 3, 2VOV.A.1338, 3VV3.B.404, 1W7C.A.802, 3W7T.A.1001, 1WAD.A.116, 4WIW.A.702, 4WK 0.B.502, 4WK0.B.503, 4WK7.A.504, 1WMD.A.1003, 2WNV.B.1225, 2WOY.A.2414, 1WZA.A.6 01, 1Y93.A.266, 1Y93.A.268, 2YN3.A.6362, 2YN5.A.6362, 2YN5.A.6363, 1YO8.A.1191, 2YOA.A.1003, 1YS1.X.400, 2Z2X.A.1004, 2Z2X.A.1005, 2Z30.A.1006, 2Z8X.A.626, 2ZWO .B.400, 3ZXH.A.304, 19HC.A.301, 19HC.A.302, 19HC.A.303, 19HC.A.304, 19HC.A.305, 19HC.A.306, 19HC.A.307, 19HC.A.308, 19HC.A.309, 3A0G.A.201, 3A15.B.354, 3A16.B.3 54, 3A17.A.354, 2A1O.A.417, 1A2F.A.1, 1A2S.A.90, 2A3F.X.201, 2A3M.A.501, 2A3M.A. 502, 2A3M.A.503, 2A3M.A.504, 1A4E.A.503, 1A56.A.82, 3A51.B.412, 3A8G.A.301, 3A8L .A.300, 3A9F.A.207, 2A9E.A.550, 4AAL.A.423, 4AAN.A.400, 4AAN.A.401, 4AAO.A.400, 2AIU.A.200, 2AI5.A.81, 1AKK.A.105, 4AM5.A.1160, 1AOF.A.602, 1AOF.B.601, 1AOM.A.6 02, 1AOM.B.601, 1AOM.B.602, 4APY.A.1418, 1AQA.A.95, 1AQE.A.121, 1AQE.A.122, 3AQ5 .A.144, 3AQ9.A.144, 1ASH.A.301, 3AT5.A.142, 3AT5.B.147, 2AT3.X.185, 4AUM.A.900, 2AVK.A.201, 3AWM.A.501, 1AWP.A.201, 3AYF.A.801, 2B0Z.B.109, 3B0H.B.601, 2B10.D.9 09, 2B11.D.1301, 2B2R.A.1500, 4B2N.A.700, 4B2N.A.701, 2B4Z.A.500, 3B42.A.199, 3B 42.B.399, 3B47.A.199, 4B4Y.A.1155, 3B6H.B.600, 1B80.A.350, 1B82.A.350, 1B85.A.35 0, 4B8N.A.201, 3B99.A.600, 2BC5.A.150, 3BDZ.A.450, 2BDM.A.500, 1BEP.A.296, 1BFR. B.200, 2BGV.X.1121, 2BH4.X.1123, 1BIN.B.144, 1BJ9.A.296, 1BJE.A.154, 4BJA.A.300, 4BJK.A.1450, 3BK9.B.401, 2BK9.A.1154, 2BLF.B.1582, 4BMM.A.1450, 2BMM.A.1157, 3B NG.A.513, 3BNJ.A.513, 3BNJ.A.514, 3BNJ.A.515, 3BNJ.A.516, 3BNJ.A.517, 3BOM.A.143 , 3BOM.B.148, 2BPN.A.108, 2BPN.A.109, 2BPN.A.110, 2BPN.A.111, 2BQ4.A.1115, 2BQ4. A.1116, 2BQ4.A.1117, 2BQ4.A.1118, 2BS2.C.1255, 2BS2.C.1256, 2BS3.C.1255, 2BS4.C. 1255, 1BVY.A.1000, 3BXU.A.72, 3BXU.A.73, 3BXU.A.74, 4C0C.A.1450, 4C0N.A.1157, 2C 1U.A.401, 2C1V.A.401, 2C1V.A.402, 2C1D.A.1291, 2C1D.A.1292, 2C1D.B.1158, 1C2N.A. 117, 3C2C.A.113, 1C2R.A.120, 4C27.A.1450, 4C28.A.1450, 1C40.A.150, 4C44.A.1151, 1C52.A.200, 3C62.A.150, 3C63.A.150, 1C6R.A.90, 1C75.A.93, 3C76.X.185, 3C78.X.185 , 2C8S.A.1173, 4C9M.A.1418, 3CAO.A.104, 3CAO.A.105, 3CAO.A.106, 3CAO.A.107, 1CC5 .A.1, 1CCH.A.83, 1CCR.A.112, 2CDV.A.109, 2CDV.A.111, 4CDP.A.402, 1CED.A.90, 2CE0 .A.1102, 1CH1.A.154, 1CH2.A.154, 1CH3.A.154, 1CH5.A.154, 1CH7.A.154, 1CH9.A.154, 1CI3.M.254, 2CJ2.A.1300, 4CK8.A.1480, 4CK9.A.1480, 4CKA.A.1480, 1CLS.A.142, 1CN O.A.200, 2CN4.A.1175, 2CN4.B.1175, 1CO6.A.108, 1COR.A.83, 1COT.A.130, 4COH.A.145 0, 4COO.A.1549, 3CP5.A.202, 1CPT.A.430, 3CQV.A.601, 3CSL.A.866, 2CTH.A.109, 2CTH .A.110, 2CTH.A.111, 2CTH.A.112, 3CU4.A.199, 2CVC.A.1001, 2CVC.A.1002, 2CVC.A.100 3, 2CVC.A.1004, 2CVC.A.1005, 2CVC.A.1006, 2CVC.A.1007, 2CVC.A.1008, 2CVC.A.1009, 2CVC.A.1010, 2CVC.A.1011, 2CVC.A.1012, 2CVC.A.1013, 2CVC.A.1014, 2CVC.A.1016, 4 CVJ.A.1295, 3CX5.C.4001, 3CX5.C.4002, 3CX5.D.4003, 3CX5.W.4026, 1CXA.A.126, 1CXC .A.125, 3CXH.W.4026, 1CXY.A.204, 1CYI.A.200, 3CYR.A.203, 3CYR.A.204, 2CY3.A.119, 2CY3.A.120, 2CY3.A.121, 2CY3.A.122, 2CYP.A.295, 5CYT.R.105, 2CZS.A.500, 2CZS.A. 501, 1CZJ.A.119, 1CZJ.A.120, 1CZJ.A.121, 1CZJ.A.122, 2CZ1.A.300, 3CZY.A.300, 2D0 W.A.200, 2D09.A.430, 4D02.A.602, 4D02.A.603, 2D0S.A.80, 2D0T.A.404, 2D2M.D.200, 4D30.B.750, 4D34.A.500, 4D35.A.500, 4D36.A.500, 4D37.A.500, 4D38.A.500, 4D3A.A.5 00, 1D4D.A.601, 1D4D.A.602, 1D4D.A.603, 1D4D.A.604, 1D7B.A.401, 3D7O.A.143, 1DCC .A.296, 2DC3.A.191, 1DD7.A.600, 3DE8.A.150, 2DGE.A.200, 3DHH.A.501, 3DHI.A.600, 3DHR.A.142, 1DJ5.A.1, 1DK0.A.200, 1DLW.A.144, 1DM1.A.148, 3DMI.A.146, 2DN1.A.142 , 2DN1.B.147, 1DP8.A.719, 1DP9.A.719, 3DR0.A.94, 1DTI.A.154, 4DTW.A.500, 4DTY.A. 500, 4DTZ.A.500, 1DUW.A.293, 1DUW.A.297, 1DUW.A.300, 1DUW.A.301, 1DWO.A.113, 1DW 2.A.113, 1DWL.B.80, 4DXY.A.501, 1DY7.B.601, 2DY5.A.300, 4DY9.A.201, 1E08.E.80, 1 E29.A.136, 1E2R.B.601, 1E2W.A.900, 3E2O.A.296, 1E39.A.801, 1E39.A.802, 1E39.A.80 3, 1E39.A.804, 2E3A.A.401, 2E3B.A.401, 3E5J.A.1408, 3E5K.A.1408, 1E8E.A.125, 2E8 0.A.1508, 2E84.A.1301, 2E84.A.1302, 2E84.A.1303, 2E84.A.1304, 2E84.A.1305, 2E84. A.1306, 2E84.A.1307, 2E84.A.1308, 2E84.A.1309, 2E84.A.1310, 2E84.A.1311, 2E84.A.

1312, 2E84.A.1313, 2E84.A.1314, 2E84.A.1316, 1EB7.A.401, 1EGY.A.410, 3EGW.C.806, 3EGW.C.807, 3EH5.A.800, 3EHB.A.559, 3EHB.A.560, 1EHE.A.501, 1EHJ.A.1030, 1EHJ.A.1053, 1EHJ.A.1066, 4EID.A.101, 4EIE.A.101, 4EIF.A.101, 3EJ6.A.4000, 4EJI.A.501, 2EKT.A.154, 2EKU.A.154, 4ENH.A.601, 4ENU.B.801, 1EOC.B.600, 4EP6.A.601, 1ETP.A.199, 1ETP.A.200, 1EUE.A.201, 2EU7.X.201, 2EWK.A.1001, 2EWK.A.1003, 2EWU.A.1001, 2EWU.A.1003, 1EWH.A.253, 2EWI.A.1004, 2EWI.A.1002, 2EWI.A.1001, 2EWI.A.1003, 2EXV.A.83, 1EZV.C.401, 1EZV.C.402, 1F03.A.201, 1F1C.A.200, 1F1F.A.200, 1F24.A.501, 1F4U.A.410, 4F4O.B.201, 4F6I.A.201, 4FA7.A.602, 4FA7.A.603, 2FAM.A.148, 4FAS.A.601, 4FAS.A.602, 4FAS.A.603, 4FAS.A.604, 4FAS.A.605, 4FAS.A.606, 4FAS.A.607, 2FBZ.X.901, 1FCD.C.901, 4FDH.A.601, 4FEF.A.403, 2FFN.A.1003, 1FGJ.A.548, 1FGJ.A.552, 1FGJ.A.553, 1FI3.A.83, 1FI7.A.110, 4FIA.A.600, 1FJ0.A.115, 3FLL.A.185, 2FMY.A.300, 3FO3.A.1004, 3FO3.A.1005, 3FO3.A.1006, 3FO3.A.1007, 3FO3.A.1008, 3FO3.A.1002, 3FO3.A.1003, 3FO3.A.1001, 1FOP.A.500, 3FOO.A.150, 2FRF.A.154, 1FS7.A.509, 1FS7.A.510, 1FS8.A.508, 1FT5.A.213, 1FT5.A.215, 1FT5.A.216, 1FT9.A.300, 1FT9.B.300, 2FWT.A.803, 2FWT.A.805, 2FWL.A.132, 2FYU.D.242, 1G09.B.147, 4G1V.A.401, 4G3J.A.501, 3G5N.A.500, 4G70.A.601, 4G71.A.602, 4G7G.A.501, 4G7L.A.301, 4G7Q.A.602, 4G7S.A.602, 3GAS.D.1294, 2GC4.D.200, 1GDV.A.101, 2GEP.A.580, 3GEO.A.580, 4GED.B.201, 7GEP.A.580, 2GJ1.A.605, 1GKS.A.0, 3GM6.A.1004, 2GNV.A.166, 4GP4.A.602, 4GP8.A.602, 1GQ1.A.601, 1GQ1.A.602, 3GQP.C.143, 4GQS.A.501, 2GSM.A.2001, 2GSM.A.2002, 2GTF.X.201, 1GWF.A.504, 3GW9.A.480, 1GWS.A.601, 1GWS.A.603, 1GWS.A.605, 1GWS.A.606, 1GWS.A.609, 1GWS.A.610, 1GWS.A.611, 1GWS.A.612, 1GWS.A.613, 1GWS.A.616, 1GWT.A.350, 1GYO.A.111, 1GYO.A.112, 1GYO.A.113, 1GYO.A.114, 4GYD.A.200, 2H0V.B.500, 4H0K.B.200, 1H1O.A.1184, 1H1O.A.1185, 1H21.A.1248, 1H21.A.1249, 1H21.B.1248, 1H21.B.1249, 1H29.A.1102, 1H29.A.1104, 1H29.A.1107, 1H29.A.1108, 1H29.A.1114, 4H2L.B.201, 1H32.A.1263, 1H32.A.1264, 1H32.B.1139, 3H33.A.75, 3H33.A.76, 3H33.A.77, 3H34.A.72, 3H34.A.73, 3H34.A.74, 3H4N.A.72, 3H4N.A.73, 3H4N.A.74, 4H44.A.301, 4H44.A.302, 1H55.A.350, 1H57.A.350, 4H6O.A.501, 3H8T.A.301, 2H88.C.142, 1HAB.A.200, 2HBU.A.900, 3HB3.A.559, 3HB3.A.560, 1HBI.A.153, 1HBZ.A.504, 2HBD.A.142, 4HB6.A.72, 4HB6.A.73, 4HB8.A.72, 4HB8.A.73, 4HBF.A.72, 1HCZ.A.253, 4HC3.A.72, 3HDL.A.305, 3HF4.A.142, 3HF4.B.147, 1HGB.D.147, 4HHR.A.701, 4HHS.A.701, 1HJ3.B.602, 1HJ5.A.601, 1HJ5.B.602, 2HJI.A.180, 1HLB.A.158, 1HMO.B.115, 3HNJ.A.150, 3HNK.A.150, 4HPA.A.201, 4HPB.A.201, 4HPD.A.201, 3HQ6.A.400, 3HQ7.A.401, 3HQ9.A.400, 3HQ9.A.401, 1HRC.A.105, 1HRO.A.107, 4HRO.A.402, 3HSP.A.750, 4HSW.A.201, 2HUO.A.302, 3HYU.A.142, 3HYU.B.147, 1I3E.A.147, 2I5N.C.404, 1I5U.A.201, 3I63.A.501, 3I63.A.502, 1I77.A.108, 1I77.A.109, 1I77.A.110, 1I77.A.111, 4I7Z.A.301, 4I7Z.A.302, 4I7Z.C.301, 3I8R.A.901, 1I8O.A.115, 1I8P.A.115, 2I8F.A.83, 3I9T.A.300, 3I9U.A.300, 2IBN.A.703, 1IB7.A.95, 1ICC.A.201, 1IDR.A.144, 2IJ3.B.999, 2IJ4.A.471, 1IKE.A.185, 1IO7.A.1401, 4IPS.A.401, 1IQC.A.401, 1IQC.A.402, 3IQ5.A.150, 1IT1.A.201, 1IT1.A.202, 1IT1.A.203, 1IT1.A.204, 2IUW.A.500, 2IUF.A.1691, 1IVJ.A.300, 2IVF.C.1217, 1IW0.A.901, 1IYN.A.296, 1J02.A.300, 1J0P.A.1002, 1J0Q.A.201, 2J1M.A.1456, 4J14.A.601, 4J20.A.107, 1J3S.A.105, 2J5M.A.1300, 1J77.A.300, 2J7A.A.1002, 2J7A.A.1003, 2J7A.A.1004, 2J7A.A.1005, 2J7A.C.1002, 2J7A.C.1003, 2J7A.C.1004, 1JDL.A.500, 1JDR.A.296, 4JE9.A.201, 4JEB.A.201, 1JEX.A.95, 1JIP.A.410, 4JJ0.A.501, 4JJ0.A.502, 2JJN.A.412, 2JJP.A.412, 1JMX.A.1001, 1JMX.A.1002, 1JNI.A.125, 1JNI.A.126, 2JTI.B.104, 3K1O.A.488, 2K3V.A.218, 2K3V.A.238, 2K3V.A.261, 2K3V.A.278, 3K3O.A.1, 4K8F.A.300, 4K8F.B.300, 1KB0.A.802, 1KBI.A.760, 4KF2.B.501, 4KIB.A.403, 4KIC.A.403, 4KIG.A.502, 4KJT.A.201, 2KMY.A.233, 2KMY.A.251, 4KMG.A.101, 1KOK.A.296, 1KQG.C.809, 1KQG.C.810, 2KSC.A.125, 2KSU.A.282, 2KSU.A.305, 1KV9.A.901, 4KVK.A.701, 4KVL.A.701, 4L0F.A.501, 3L1M.A.150, 3L1T.A.479, 1L2K.A.154, 3L4D.A.481, 3L61.A.420, 1LA6.B.147, 3LD6.A.601, 2LDO.A.130, 2LDO.A.154, 2LDO.A.168, 1LFK.A.430, 3LGN.A.200, 4LJI.A.301, 1LM3.B.200, 1LMS.A.118, 4LM8.A.801, 4LM8.A.802, 4LM8.A.803, 4LM8.A.804, 4LM8.A.805, 4LM8.A.806, 4LM8.A.807, 4LM8.A.808, 4LM8.A.809, 4LM8.A.810, 4LMH.A.801, 4LMH.A.802, 4LMH.A.803, 4LMH.A.804, 4LMH.A.805, 4LMH.A.806, 4LMH.A.807, 4LMH.A.808, 4LMH.A.809, 4LMH.A.810, 1LQX.A.201, 1LR6.A.201, 1LS9.A.92, 1LSX.A.719

, 4LXJ.A.601, 2LZZ.A.101, 2LZZ.A.102, 3M15.A.150, 1M1P.A.802, 1M1Q.A.804, 1M1Q.A
.803, 1M1R.A.801, 1M2I.A.201, 4M25.A.401, 4M2F.B.401, 3M3A.A.155, 3M4C.A.150, 4M
4A.A.201, 4M4A.B.201, 1M59.A.201, 1M70.A.199, 1M70.A.200, 1M7S.A.600, 4M71.A.403
, 4M72.A.403, 4M73.A.403, 4M73.B.403, 4MBA.A.148, 5MBA.A.148, 3MDM.A.505, 1MDV.A
.110, 1MDV.A.112, 3MDR.A.505, 4ME4.A.401, 3MGX.A.397, 1MJ4.A.502, 3MKB.B.137, 3M
L1.B.1128, 3ML1.B.1129, 1ML7.A.185, 1MLW.A.403, 4MLM.A.201, 4MLN.A.201, 4MLN.B.2
01, 4MLN.B.202, 3MM9.A.580, 1MNI.B.154, 1MNY.A.95, 3MOM.A.186, 3MOO.A.911, 4MPM.
A.201, 1MQF.A.501, 4MQJ.B.201, 1MRP.A.310, 1MTY.D.4, 3MUS.A.201, 3MVC.A.500, 3MV
F.A.185, 1MWB.A.125, 1MXR.A.1004, 1MZ4.A.151, 3N3N.B.1500, 1N45.A.300, 4N4J.A.60
9, 4N4J.A.610, 4N4J.A.611, 4N4J.A.612, 4N4J.A.613, 4N4J.A.614, 4N4J.A.615, 4N4J.
A.616, 4N4K.A.610, 4N4L.A.616, 4N4N.A.601, 4N4N.A.602, 4N4N.A.603, 4N4N.A.604, 4
N4N.A.605, 4N4N.A.606, 4N4N.A.607, 4N4N.A.608, 4N4O.A.608, 4N4Y.A.602, 4N6W.A.20
2, 4N8T.B.201, 3N8Y.A.601, 1N9C.A.93, 3N9Q.A.1, 1NAZ.A.200, 3NA1.A.601, 3NC3.A.4
06, 4NFG.B.201, 1NIR.A.601, 1NIR.A.602, 4NK1.B.201, 3NMI.A.150, 1NML.A.401, 1NML
.A.402, 3NN1.A.239, 1NNO.A.602, 2NNB.A.472, 1NOS.A.901, 1NPF.A.154, 4NP1.A.185,
2NRL.A.148, 1NS6.A.142, 2NSR.A.154, 1NS9.A.142, 1NS9.B.147, 3NWV.A.105, 3NXU.A.5
08, 3NYH.A.605, 4NZ2.A.501, 3O1A.A.385, 4O1W.A.101, 4O4Z.A.201, 3O5C.A.401, 3O5C
.D.504, 4O6J.A.302, 4O6Q.A.202, 4O6U.A.203, 3O72.A.500, 4O7G.A.301, 4O7G.A.302,
3O89.A.2154, 1OAF.A.1251, 1OAH.A.1520, 1OAH.A.1521, 1OAH.A.1522, 1OAH.A.1523, 1O
AH.A.1524, 3OA8.A.401, 3OA8.B.401, 1OFW.A.1294, 1OFW.A.1295, 1OFW.A.1296, 1OFW.A
.1297, 1OFW.A.1298, 1OFW.A.1299, 1OFW.A.1300, 1OFW.A.1301, 1OFW.A.1302, 3OFT.A.4
17, 3OJT.D.500, 3OMA.A.1, 3OMI.A.607, 3OO4.A.142, 3OO4.B.147, 1OQU.C.1008, 2ORT.
A.600, 3ORV.B.600, 2ORO.A.600, 2ORP.A.600, 2ORQ.A.600, 2ORR.A.600, 2ORS.A.600, 2
OT4.A.1004, 2OT4.A.1007, 3OUE.A.609, 3OUE.A.610, 3OUE.A.611, 3OUE.A.612, 3OUH.A.
600, 3OUI.A.1, 3OUQ.A.601, 3OUQ.A.602, 3OUQ.A.603, 3OUQ.A.604, 3OUQ.A.605, 3OUQ.
A.606, 2OZY.A.201, 2OZY.A.202, 2OZY.A.203, 2OZY.A.204, 2OZY.A.205, 3OZU.A.404, 3
OZV.A.404, 3OZW.A.404, 1P2E.A.801, 1P2E.A.803, 1P2E.A.804, 1P2H.A.801, 1P2H.A.80
3, 1P2H.A.804, 1P2Y.A.430, 3P3L.A.501, 1PA2.A.306, 3PAH.A.425, 2PAC.A.83, 4PAH.A
.425, 5PAH.A.425, 1PBY.A.991, 1PBY.A.992, 3PC3.A.701, 3PER.A.1002, 2PEG.A.200, 2
PEG.B.400, 3PF7.A.1001, 3PF7.A.1002, 4PGO.A.301, 3PH2.B.1087, 4PH9.A.602, 3PI2.B
.500, 4PK5.A.501, 1PL3.A.401, 1PM1.X.180, 1PP9.C.501, 1PP9.C.502, 1PP9.D.501, 2P
Q7.A.220, 3PT7.B.500, 3PUQ.A.1, 3PUR.A.1, 3PXW.A.500, 4PXH.A.501, 1Q16.C.806, 1Q
16.C.807, 3Q3N.A.509, 3Q3N.A.510, 1Q5D.A.440, 2Q8Q.A.300, 3Q99.B.750, 2QDY.A.300
, 1QDB.A.516, 1QDB.A.517, 1QDB.A.518, 1QDB.A.519, 1QHU.A.500, 2QJY.A.501, 2QJY.A
.502, 2QJY.B.301, 1QKS.A.601, 1QKS.A.602, 1QNO.A.113, 1QNO.A.114, 1QNO.A.115, 1Q
NO.A.116, 1QN2.B.101, 3QNS.A.351, 1QO8.A.601, 1QO8.A.602, 1QO8.A.603, 1QO8.A.604
, 1QPA.A.350, 3QPI.B.1001, 1QPU.A.107, 1QQ3.A.107, 3QQQ.A.163, 3QQR.A.163, 2QSP.
A.142, 3QU8.A.500, 2QUO.A.142, 3QWO.A.150, 3QY7.A.264, 1QYZ.A.200, 1ROQ.A.200, 4
R20.A.601, 2R50.A.166, 2R6S.A.501, 2R80.A.150, 2R80.B.150, 3R9C.A.450, 2RAO.B.14
7, 2RAO.C.142, 2RDZ.A.2, 2RDZ.A.3, 2RDZ.A.4, 2RDZ.A.5, 2RF7.A.2, 3RGS.A.1, 3RI7.
A.494, 3RIV.A.305, 3RJ6.A.154, 4RKM.A.808, 4RKM.B.808, 4RKM.D.807, 4RKN.A.902, 4
RKN.A.903, 4RKN.A.905, 4RKN.A.906, 4RKN.A.907, 4RKN.A.908, 4RKN.A.909, 3RMK.A.49
4, 3RMZ.A.500, 1RSE.A.154, 3RUK.B.600, 1RWJ.A.90, 1RWJ.A.91, 1RWJ.A.92, 1RZ5.A.4
01, 4S1B.A.802, 3S1J.A.140, 1S73.A.296, 3S8F.A.800, 3S8F.A.801, 3S8G.A.800, 3SDN
.A.160, 1SE6.A.430, 3SEL.X.73, 3SEL.X.74, 1SH4.A.201, 3SJ0.X.73, 3SJ0.X.74, 3SJ1
.X.73, 3SJ1.X.74, 3SJ4.X.73, 3SJ4.X.74, 3SJ5.A.500, 3SJL.A.500, 3SJL.A.600, 3SLE
.A.402, 1SMI.A.472, 1SOX.A.502, 1SP3.A.801, 1SP3.A.803, 1SP3.A.804, 1SP3.A.805,
1SP3.A.806, 1SP3.A.807, 1SP3.A.808, 1SQ3.D.912, 1STQ.A.600, 1SUO.A.500, 3SWZ.B.6
00, 3SXQ.A.1005, 3SXQ.A.1006, 3SXQ.A.1007, 3SXQ.A.1008, 3SXQ.A.1002, 3SXQ.A.1003
, 3SXQ.A.1001, 1SY2.A.185, 3T3R.A.500, 3T3Z.A.500, 1T68.X.201, 3T6D.C.401, 3T6D.
C.403, 3T6D.C.404, 3T6E.C.401, 3T6E.C.402, 3T6E.C.403, 3TDA.A.800, 3TFO.A.500, 3
TGU.C.501, 3TGU.C.502, 3TGU.D.501, 3TGA.A.185, 3TGM.A.300, 1TH2.D.2003, 3TIK.A.4
82, 3TJS.A.508, 1TKW.B.253, 3TK3.A.500, 3TMC.A.309, 3TMC.A.310, 3TOR.A.3, 4TOB.C
.201, 3TOL.A.150, 1TQN.A.508, 4TT5.A.401, 1TU2.B.255, 4TUV.A.401, 1U13.A.460, 1U

4H.A.500, 1U7R.A.154, 3U8P.A.347, 1U9M.A.90, 4U9D.A.201, 1U9U.A.90, 3U99.A.500, 3U99.A.700, 4UAX.A.501, 3UBR.A.471, 3UBR.A.472, 3UBR.A.473, 3UBR.A.474, 3UBR.A.475, 3UBC.A.201, 3UCP.A.901, 3UCP.A.902, 3UCP.A.903, 3UCP.A.904, 3UCP.A.905, 3UCP.A.906, 3UCP.A.907, 3UCP.A.908, 3UCP.A.909, 3UCP.A.910, 3UCP.A.911, 1UED.A.1430, 1ULI.B.700, 3UOI.B.200, 1UP9.A.201, 1UP9.A.202, 1UP9.A.203, 1UP9.A.204, 4UQH.A.1450, 1URV.A.1172, 2UUQ.A.1405, 4UVR.A.1450, 1V0H.X.251, 2V07.A.1102, 2V08.A.1087, 2V0M.A.1499, 3V2V.A.154, 4V2K.A.601, 4V3V.A.750, 4V3W.A.750, 4V3X.A.750, 4V3Z.B.750, 1V54.A.515, 3V5X.A.201, 1V75.B.201, 2V7I.A.1362, 2V7K.A.1360, 2V7L.A.1360, 3V8D.A.601, 1V8X.A.901, 1V9Y.A.1140, 3VAU.A.201, 1VB6.A.1140, 1VB6.B.1140, 2VEB.A.200, 1VGI.A.300, 3VHB.A.150, 4VHB.A.150, 2VHB.B.150, 2VHD.A.402, 2VHD.B.401, 3VKP.A.601, 3VKS.A.601, 3VM9.A.154, 3VP5.A.201, 3VR8.C.201, 3VRD.A.201, 3VRD.A.202, 3VRG.A.201, 3VRG.B.201, 3VTH.A.807, 3VXJ.A.501, 2VXH.A.1001, 1VYD.A.1117, 2VYW.A.149, 2VZW.B.1209, 1W0G.A.1501, 2W0B.A.470, 1W2L.A.1100, 2W31.A.200, 2W3G.A.500, 1W4W.A.1307, 1W7O.A.1119, 1W7O.A.1120, 1W7O.A.1121, 1W7O.A.1122, 3W9C.A.501, 1WAD.A.117, 1WAD.A.113, 1WAD.A.114, 1WAD.A.115, 3WAH.A.201, 3WC8.A.201, 3WCT.A.200, 3WCT.B.201, 3WCT.C.200, 3WCT.D.201, 2WDQ.C.1129, 1WE1.A.300, 3WFC.B.802, 3WFD.B.801, 3WFD.C.201, 3WFE.B.802, 3WFX.A.201, 2WJM.C.1334, 2WJN.C.1333, 2WJN.C.1335, 2WJN.C.1336, 1WMU.A.201, 2WM5.A.450, 4WNV.A.601, 1WOV.A.300, 1WOX.A.300, 4WPD.A.402, 4WQ8.A.1002, 4WQ9.A.1001, 4WQ9.A.1002, 4WQC.A.1002, 4WQD.A.1002, 2WTG.A.180, 3WU2.F.101, 2WU2.C.1130, 2WU5.C.305, 1WVE.C.699, 1WVP.A.154, 4WWJ.B.301, 2WX2.A.1450, 3X15.A.200, 3X15.J.200, 2X2N.A.1479, 1X3X.B.202, 1X46.A.151, 4X8B.A.508, 2XC3.A.1433, 4XDI.A.201, 2XFH.A.1412, 1XK1.A.300, 1XK3.A.300, 2XKR.A.1400, 2XKI.A.1110, 1XQ5.B.148, 1XQ5.C.143, 2XSJ.B.503, 2XTS.B.500, 1XU5.A.1174, 1XVB.A.1171, 1XVG.A.528, 1XVX.A.313, 2XYK.A.700, 1Y0P.A.801, 1Y0P.A.802, 1Y0P.A.803, 1Y0P.A.804, 1Y5I.C.806, 1Y5I.C.807, 1Y5L.C.806, 2Y5N.A.450, 2Y69.A.516, 2YEV.A.1015, 2YEV.B.587, 2YIU.A.500, 2YIU.A.501, 2YIU.B.500, 1YIQ.A.901, 2YK3.A.200, 2YL7.A.128, 1YMA.A.154, 1YWD.A.185, 2YXC.A.1001, 2YYW.A.1001, 2YYW.A.1003, 2YYX.A.1004, 2YYX.A.1001, 1YYG.A.396, 1Z1N.X.602, 1Z1N.X.603, 1Z1N.X.604, 1Z1N.X.605, 1Z1N.X.606, 1Z1N.X.607, 1Z1N.X.608, 1Z1N.X.610, 1Z1N.X.612, 1Z1N.X.613, 1Z1N.X.614, 1Z1N.X.616, 2Z47.A.1004, 2Z47.B.3003, 2Z6S.A.201, 2Z6T.A.201, 1Z8O.A.410, 1Z8U.B.201, 1Z9N.A.1001, 1Z9N.C.2001, 3ZBY.A.1402, 2ZBO.A.111, 2ZCF.A.206, 3ZE6.B.502, 3ZG2.A.1480, 3ZG3.A.490, 3ZHO.A.200, 3ZHW.A.1163, 3ZIY.A.600, 3ZJO.A.200, 3ZJQ.A.200, 3ZLI.A.4001, 1ZOY.C.1305, 3ZOO.A.105, 3ZOX.A.1082, 3ZPI.A.1407, 2ZPB.A.300, 2ZS0.A.200, 2ZS0.B.200, 2ZS0.C.200, 2ZS0.D.200, 2ZXY.A.200, 2ZZS.1.220, 1ZZH.A.802, 1ZZH.A.803, 3A15.A.354, 3A51.C.412, 2AA1.B.400, 3ABB.A.1430, 4AJ9.A.1715, 3AK3.C.215, 2AKJ.A.564, 3ARJ.A.153, 3AT6.A.142, 2AUO.A.153, 2AUQ.A.147, 2AV0.A.147, 4AVD.A.144, 3AYF.A.802, 1B0B.A.144, 1B7V.A.93, 3BA2.A.158, 1BBH.A.132, 1BCF.A.200, 2BCN.B.109, 1BGP.A.400, 3BK9.A.401, 2BMO.A.1441, 1BT8.B.202, 3BUJ.A.398, 2C1D.H.1158, 4C50.A.1741, 3C6G.A.601, 1C6S.A.88, 4C9L.A.1418, 4CAB.A.537, 2CCY.A.129, 1CG8.B.142, 1CGN.A.128, 1CH4.A.147, 2CJ1.A.1300, 2CMM.A.155, 1CPQ.A.130, 1CRC.A.105, 4CZC.A.1337, 1D0C.A.500, 2D0Q.A.300, 3D1K.A.200, 3D1K.B.400, 4D3O.A.901, 2D5X.B.147, 3DAM.A.600, 3DAX.A.601, 3DBG.A.500, 3DE8.D.150, 1DGF.A.3000, 3DHG.D.507, 2DKK.A.430, 1DLY.A.144, 4DVQ.A.601, 1DW1.A.113, 4DWU.A.201, 1E2R.A.602, 2E39.A.401, 3E4W.A.501, 3E5L.A.1408, 2E84.A.1315, 3EAH.A.861, 1ECD.A.137, 4EGO.A.501, 3EJ8.A.1901, 3EJD.B.405, 4ENP.A.801, 4ENU.A.801, 1EQD.A.185, 4ESA.A.202, 4ESA.B.202, 1EUP.A.410, 4FB2.B.501, 2FDU.A.500, 1FHF.A.350, 1FHJ.B.147, 4FVC.A.201, 3G1Q.A.480, 4G2C.A.501, 2G3H.A.154, 3G46.A.147, 4G45.A.401, 3GAS.A.1290, 2GB8.A.295, 1GCV.A.141, 1GCV.B.137, 3GE3.A.502, 1GEJ.A.501, 4GEP.A.580, 2GGN.X.251, 1GJQ.A.602, 2GKM.A.144, 3GPH.A.500, 4GQS.B.501, 1GW2.A.350, 1GWS.A.615, 1GWU.A.1306, 2GYQ.A.401, 4H0K.A.200, 1H1X.A.1154, 4H8Q.A.201, 1H97.A.148, 2HBT.A.900, 3HC1.A.305, 3HC1.A.306, 1HDS.A.142, 3HF2.A.482, 3HF4.F.147, 1HJ4.B.601, 1HJ5.B.601, 2HMQ.B.115, 4HRR.A.201, 4HRR.B.201, 1HV4.A.151, 3HX9.A.300, 2HYS.A.201, 1HZU.A.601, 1I3D.A.147, 1I4Y.E.605, 4I8V.A.601, 2I96.A.129, 4IAM.A.501, 2IBN.B.706, 3IBD.A.500, 2IG3.A.700, 4IGO.A.1000, 2IIZ.A.400, 2INN.A.513, 1IOP.A.154, 3I

QB.A.500, 1IRD.B.347, 2ISA.A.486, 1IT2.A.147, 1ITH.A.143, 1IWH.A.142, 1IX4.A.300
, 1IZO.C.501, 2J0P.A.1342, 1J1L.A.1001, 2J7A.D.1001, 1JAF.A.130, 1JEB.D.147, 4JE
T.A.201, 4JS9.A.501, 2JXM.B.250, 4KF0.A.501, 2KII.A.182, 3KX4.A.999, 1L0L.D.242,
4L1Y.A.300, 4L1Z.A.300, 4L2M.A.201, 4L3H.A.402, 2L4D.A.107, 4L54.A.501, 2L8M.A.
416, 1LC1.A.105, 1LGA.A.396, 1LH2.A.154, 1LH6.A.154, 1LH7.A.154, 1LHT.A.155, 2LH
2.A.154, 2LHB.A.151, 3LL8.A.506, 3LMX.M.600, 4M26.C.401, 1M54.F.1620, 1M56.A.100
2, 1M7S.D.600, 4M71.B.403, 1M85.A.1001, 1MBA.A.148, 1MGN.A.154, 2MHR.A.119, 3MM3
.A.501, 3MM6.B.570, 3MMB.A.580, 3MMO.A.1004, 4MM0.A.401, 1MN1.A.396, 3MOL.B.185,
1MYF.A.154, 3MYM.A.139, 3MYN.A.139, 3MZS.A.500, 3N3R.A.1500, 4N4M.A.616, 4N4N.C
.601, 3N8Y.B.601, 1N97.B.603, 3NA0.A.601, 4NK2.A.700, 4NKW.A.600, 3NN2.A.239, 3N
NF.A.600, 3NNL.A.600, 2NNJ.A.500, 2NOX.C.500, 2NP1.A.350, 1NR6.A.500, 3NTG.D.601
, 3NU1.A.302, 3O5C.B.402, 2O68.A.401, 1OAE.A.1125, 3OCD.C.401, 3OCD.D.401, 1OCZ.
A.515, 1ODO.A.1407, 3OFU.A.417, 1OG5.A.501, 1OIK.A.1302, 2OIF.A.163, 4OQR.A.501,
1OR4.A.180, 2OYY.A.201, 1OZW.B.300, 4OZ5.A.201, 1P3T.A.300, 3P3X.A.501, 2P85.A.
500, 3PCJ.R.600, 3PM0.A.900, 2PMS.A.347, 3PT8.A.500, 3PT8.B.500, 3Q14.A.501, 1Q5
E.A.440, 2Q8P.A.300, 3Q9K.A.605, 2Q9F.A.602, 1QGJ.A.1350, 3QGP.A.200, 1QJS.A.500
, 3QPI.A.1001, 3QQR.B.163, 2QRW.A.700, 2QSS.A.142, 1QWL.B.550, 3QY6.A.264, 3QZM.
B.201, 3QZX.A.200, 2R1H.D.148, 4R21.A.600, 2R79.A.500, 3R9B.A.501, 2RCL.B.600, 2
RDN.A.1, 2RF7.D.1, 2RFB.A.410, 2RI4.A.142, 3RIW.A.305, 4RKM.K.809, 4RKM.L.813, 3
RUR.A.200, 1S05.A.130, 1S13.A.300, 1S1F.A.430, 1S61.A.144, 1S69.A.125, 3S66.A.14
2, 3S79.A.600, 1SCH.A.300, 1SI8.A.501, 3SIK.A.154, 1SMJ.C.472, 1SOG.A.296, 1SPG.
A.144, 1SPG.B.148, 1SQ3.A.903, 1T47.B.430, 1T85.A.417, 3TBG.A.800, 1TMX.A.861, 3
TM8.A.902, 3TM8.A.903, 2TOH.A.501, 4TRI.A.501, 3TTV.A.760, 3TTW.A.760, 3TTX.B.76
0, 1TWN.A.300, 3TYW.A.501, 1U5U.A.999, 4U9D.D.201, 3U9J.A.200, 3U9M.A.201, 3UA1.
A.508, 4UBS.A.501, 1UC3.A.150, 3UHB.A.147, 3UHD.B.147, 1UMO.A.1172, 3UOI.I.200,
3UT2.A.1500, 1UX8.A.700, 1V4U.A.144, 1V4U.B.147, 1V54.A.516, 1V9Z.B.1140, 3VED.A
.401, 2VHD.A.401, 3VNO.A.501, 3VOL.A.401, 1VRE.A.148, 3VRF.B.201, 2VZW.A.1206, 3
W08.A.501, 3W4U.A.201, 3W8M.A.201, 1W92.A.1149, 3WCU.A.200, 3WCU.C.200, 3WCU.B.2
01, 3WCU.D.201, 3WFB.B.802, 4WG2.A.603, 2WIV.A.1553, 3WNU.A.801, 1WOW.A.300, 1WR
A.A.401, 3WXO.A.801, 4WXO.B.301, 2WY4.A.150, 2X66.A.1359, 1X8V.A.470, 1X9F.A.160
, 1X9F.B.160, 1X9F.C.160, 1X9F.D.160, 2XBK.A.1398, 2XF2.A.690, 2XMO.A.1128, 2XN8
.A.1434, 2XQ1.B.1503, 1Y01.B.142, 2Y4F.A.389, 2YGX.D.450, 2YL1.A.128, 1YMC.A.154
, 2Z3U.A.500, 2Z36.A.450, 2Z6F.A.3747, 2Z6N.A.150, 2Z6N.B.150, 2ZDO.A.200, 2ZFO.
D.200, 3ZK5.A.1407, 3ZKY.A.1332, 2ZPG.A.300, 2ZVU.A.300, 2ZZS.2.220, 2FMS.A.342,
4GXK.A.405, 3JPP.A.340, 2PFQ.A.1, 4TUR.A.404, 4ADB.A.1405, 3AGB.A.1, 4AYO.A.502
, 4BDR.A.902, 3C7E.A.489, 4DF9.A.501, 4G1K.B.301, 2GEZ.A.401, 1GEN.A.304, 1GUU.A
.1090, 1GV2.A.1192, 2HU3.A.9002, 1HXN.A.2, 4I2F.A.602, 3IFV.C.408, 2IY6.B.1540,
2J5W.A.3045, 2JHN.A.1298, 4KXW.A.1013, 4KZV.A.304, 3LP5.A.251, 4PFI.A.401, 4QFE.
B.305, 1QHU.A.437, 3STH.A.501, 1SU3.A.911, 3U21.A.500, 3VS8.A.501, 1W16.A.1002,
1W9W.A.900, 1ZDN.A.157, 4EJY.A.301, 1EWN.A.501, 3IOW.A.296, 4KLI.A.404, 4KLI.A.4
05, 4MO4.A.707, 1ORN.A.224, 3OSN.A.423, 4P4M.A.403, 1RZT.A.2001, 4TUP.A.402, 2A5
F.B.1326, 3AGC.A.1, 3AJN.A.136, 4AK1.A.1702, 2AU7.A.206, 4C1O.A.1731, 4C3X.A.561
, 3C7F.A.804, 4CCY.A.1298, 4CIT.A.1454, 3CYM.A.501, 2DDA.A.301, 3E9L.A.1, 3ELF.A
.351, 2FPR.B.505, 1G3K.A.500, 3GA5.B.701, 3GED.A.251, 2GJU.A.2001, 4GNJ.B.302, 2
GTW.B.3005, 1GV5.A.1142, 4H83.A.401, 4HUR.A.316, 4I29.A.601, 4I2A.A.602, 4IIB.A.
944, 3IJP.B.301, 3IMM.B.2, 4IQZ.A.316, 3IRS.A.290, 1JAY.A.215, 2JHJ.B.1296, 4JVL
.B.304, 1JZ7.A.3104, 4KA7.A.805, 3KRS.A.301, 4LG8.A.601, 4LIZ.A.202, 1M4Y.A.252,
3M9Y.B.254, 4M9B.A.201, 3MJ6.A.503, 4MMB.A.602, 4MMF.A.602, 3MQG.A.193, 4NAW.B.
303, 3NNB.A.401, 4NSJ.A.222, 3OLJ.A.1, 2OPL.A.187, 4P33.A.401, 4PCG.C.304, 3PGO.
A.147, 4PSR.A.619, 1PYF.A.315, 3PZR.A.373, 3PZS.A.287, 4Q5K.A.302, 1QNJ.A.280, 3
QXT.A.133, 3ROL.D.124, 4R3N.B.401, 2RGI.B.98, 3SJL.A.402, 4TKX.L.706, 4U99.A.203
, 1UD2.A.1002, 3UWP.A.424, 2V3U.A.1263, 2V79.A.1116, 1VMF.A.134, 3W5N.A.1210, 3W
C3.A.502, 3WNO.A.802, 2WW2.A.800, 4XCZ.A.405, 4XCZ.A.406, 1XKN.A.700, 1Y7W.B.282
, 3ZX3.C.522

[1] "Cluster 2"
4A7K.A.952, 3ASE.A.156, 3AYK.A.170, 2BCN.A.295, 2BIB.A.1550, 3BUD.A.1048, 3C10.A
.101, 2CEA.A.1603, 2CFU.A.1001, 2DDY.A.177, 4DHL.B.501, 4DV8.A.801, 1E4C.S.999,
4E5V.B.401, 3EII.A.301, 2FV9.B.4, 2GC2.A.401, 2GC3.A.402, 1GKP.B.1460, 2GMN.A.80
2, 3H8F.E.501, 2HD1.A.101, 4HGX.A.301, 3HR1.A.1, 1HTY.A.1102, 2ICS.A.400, 4ICQ.A
.501, 4ICQ.B.502, 4IGN.B.401, 1ITU.A.402, 2J0E.B.1265, 4JAA.A.501, 1JWQ.A.1001,
4JX5.D.1103, 3K5X.A.402, 1K9Z.A.402, 3KR5.E.1001, 4KYH.A.202, 1LCP.A.489, 2LQ6.A
.401, 3LS6.A.303, 4N27.C.201, 3N9R.A.308, 3O64.A.1, 2OUN.B.404, 2OW6.A.3001, 2OW
7.A.5001, 4PKT.A.802, 4PKW.A.801, 1Q3K.B.300, 1Q74.A.304, 3Q9B.A.345, 3QAY.A.180
, 2QFR.A.434, 1QIP.A.902, 1QIP.D.903, 4R6T.A.1003, 4R6T.D.1001, 4R76.A.1001, 4R7
M.C.1001, 4R7M.J.1003, 3S2J.A.402, 3S2L.A.402, 3S2M.A.402, 3S2M.A.403, 3S2N.A.40
2, 3S2N.A.403, 3SFW.A.501, 1T64.A.388, 1TQU.A.1400, 3V93.D.701, 3VH9.A.301, 2VQM
.A.1411, 3VUS.A.401, 2W3Z.A.1312, 2WEY.A.1772, 1XJS.A.150, 1XP3.A.301, 1Y13.A.17
4, 1Y93.A.264, 2ALW.A.5001, 4BBP.A.1316, 3BLB.A.1047, 1BLL.E.489, 2BNO.B.1201, 3
BXM.A.1751, 3D4Y.A.1047, 3D4Z.A.1046, 3D52.A.1046, 3D51.A.1046, 3DDF.A.3001, 3DD
G.A.3001, 2DDF.A.1, 1DE5.A.450, 3DFF.A.274, 4DO0.A.502, 3DX1.A.1048, 3DX2.A.1046
, 3DX3.A.1047, 3DX4.A.1047, 3E38.A.2, 3E49.B.500, 3E8R.B.2, 3EJP.A.1047, 3EJQ.A.
1047, 3EJR.A.1047, 3EJU.A.1047, 2F18.A.1805, 2F1A.A.1805, 2F1B.A.1804, 2F7O.A.50
01, 2F7P.A.5001, 2F7Q.A.5001, 2F7R.A.5001, 2F92.F.1003, 2FQP.B.100, 4H1S.A.603,
3HPS.A.701, 2I57.D.507, 3IBM.A.200, 4IE5.A.601, 1IM5.A.400, 3ISI.X.3001, 3ITU.A.
1, 3IVT.A.500, 4JDG.A.401, 1KAE.A.1101, 3KMC.B.2, 3KME.B.2, 1KQ3.A.401, 3KR5.A.1
001, 1KRM.A.501, 1LCP.A.488, 3LGP.A.1, 3LX3.A.201, 4M6R.B.301, 4NUR.A.701, 4NZ3.
A.501, 3OOJ.A.1, 3O64.B.485, 3O93.A.192, 2OB3.B.904, 4OJV.A.403, 4OJV.A.404, 4OJ
X.A.403, 4OJX.A.404, 4OP4.A.302, 2OQL.A.401, 1OS9.F.926, 2P18.A.301, 1PTM.A.330,
1PTM.B.331, 2PU1.A.500, 3QAY.D.180, 2QF7.A.1157, 4QGE.A.602, 1QH3.A.262, 1QIP.B
.901, 1R33.A.1163, 1R55.A.201, 4R6T.B.1003, 4R6T.F.1001, 4R76.A.1003, 4R76.E.100
1, 4R7M.C.1003, 3RCQ.A.1, 4RLO.B.302, 1RTQ.A.701, 3SJG.A.1751, 1SNN.A.402, 1SR9.
B.703, 4TO8.A.301, 2V9N.A.1275, 3W0T.A.201, 3W0T.B.201, 3W0U.A.201, 2WHG.B.1263,
4X2T.A.701, 4X2T.G.702, 2Y33.A.900, 1YGD.A.142, 2ZZW.A.362, 3A4K.A.301, 4AAB.B.
1156, 4AAB.B.1157, 2AGQ.A.4002, 2AQ4.A.302, 4AQX.D.1525, 4AQX.D.1526, 2BCV.A.576
, 4BDY.A.1381, 4BE1.A.1382, 4C2U.A.1666, 4CEI.A.2234, 1CWO.N.202, 1CWO.A.203, 4D
6N.A.1188, 4D6N.F.1196, 4D6O.D.1196, 4D6O.D.1197, 4DF4.A.901, 4DF8.A.903, 4DLG.A
.902, 4DOA.A.401, 4EEY.A.502, 2EZ6.A.501, 3F2B.A.5, 4F5P.A.401, 2FMS.A.340, 1G9Z
.C.902, 3GDX.A.347, 3GDX.A.348, 3GPL.A.800, 3GQC.B.203, 4IR9.F.402, 4IRD.F.903,
2ISO.A.339, 2ISP.A.339, 2IS4.A.1001, 3JPN.A.339, 3JPR.A.339, 3JPT.A.339, 3K57.A.
1001, 4K98.A.602, 4K99.A.602, 3LK9.A.339, 4LOX.A.401, 4M30.A.501, 4M30.A.502, 4M
47.A.402, 3M8R.A.2, 3MAQ.A.1001, 3MBY.A.339, 4MFF.A.401, 1MOW.D.374, 3MQY.A.500,
4NCB.B.702, 4NCB.B.703, 4O3N.A.503, 3OS0.A.394, 3OYA.A.396, 3OYA.A.397, 3OYC.A.
396, 3OYC.A.397, 3OYD.A.397, 3OYE.A.396, 3OYF.A.396, 3OYF.A.397, 3OYG.A.397, 3OY
H.A.396, 3OYH.A.397, 2OZM.A.904, 2PFN.A.950, 4PGQ.A.400, 4PQU.A.602, 4QCL.A.1302
, 1QSY.A.1001, 1QTM.A.1001, 4R8U.B.402, 3RJF.A.340, 3RJK.A.340, 3S3M.A.396, 3S3M
.A.397, 3SI6.A.905, 1SKR.A.4001, 3SPY.A.904, 3SV3.A.836, 1T7P.A.4001, 3TFS.A.340
, 1TKO.A.991, 1TK8.A.901, 4TUQ.A.402, 3TWH.A.401, 4UB3.A.401, 3UQ2.A.1, 2W35.A.1
224, 2XCP.A.1004, 2XCA.A.3000, 2XCA.A.3001, 1YVP.A.1001, 1ZBI.A.302, 1ZBL.A.202,
1ZBL.B.204, 3A06.A.500, 4A01.A.1770, 4A01.A.1771, 1A49.H.5334, 3A7D.A.300, 4ABZ
.A.1210, 4ACO.A.1205, 4ACF.A.1480, 2AE8.C.1009, 2AG1.B.611, 1AJB.A.452, 3AJO.A.1
83, 3AXK.A.478, 1AZS.C.403, 3B1X.A.301, 1B7T.A.836, 1B8C.A.308, 3B8I.A.289, 2BJI
.A.2277, 2BKU.A.221, 3BM4.B.304, 3BNY.D.701, 3BRB.A.10, 1BWV.C.490, 2BW7.A.2201,
3BWY.A.300, 4BYF.C.1000, 1BZY.A.901, 2C31.A.1553, 2C3P.A.2237, 4C5A.B.331, 3CB3
.A.501, 4CEO.A.1251, 1CG0.A.435, 1CG1.A.435, 1CG4.A.435, 1CH8.A.434, 1CIB.A.434,
2CJE.A.1268, 3CP6.A.501, 3CRR.A.324, 3CT2.A.401, 4CTA.B.401, 1CUL.C.396, 3CWH.A
.392, 4CW7.A.1002, 3CXO.A.500, 3CXO.B.500, 4CYM.A.1199, 4D2I.A.1478, 3D46.A.501,
3D47.A.501, 1DAK.A.901, 4DBH.A.401, 3DFY.A.401, 4DFD.B.301, 2DGN.A.1454, 4DH5.A
.402, 3DHD.A.502, 1DIE.A.399, 4DN1.B.401, 3DOE.A.193, 1DQN.A.451, 3DUF.A.1368, 2

DUA.A.292, 3DVA.A.1368, 2DW6.A.2001, 2DW6.D.2004, 4DWB.A.508, 4DXJ.A.401, 4DXJ.A
.402, 3DYG.A.3004, 3DYH.B.4002, 3DYS.A.902, 2E0A.A.500, 4E1E.A.403, 1E4E.A.365,
4E4F.A.504, 2E8W.A.1202, 1E9I.A.1431, 2E91.A.1302, 4EA0.A.301, 4EA0.A.302, 4EA0.
A.303, 1EBG.A.438, 1EBG.A.439, 1EBH.A.438, 1EC7.A.498, 1EC9.A.498, 1ECB.A.507, 1
ECQ.A.498, 1EFL.B.1604, 3EFQ.A.3003, 3EFQ.B.4002, 3EG5.A.180, 3EGT.A.3003, 3EKG.
A.601, 1ELZ.A.452, 3EQI.A.3, 3ES8.A.393, 1EXM.A.407, 3EYA.H.613, 3EZ3.B.1104, 4F
0Q.A.501, 1F2U.A.902, 3F78.C.1, 4F71.A.301, 1F8I.A.451, 3FA5.A.282, 3FD5.A.395,
3FD5.A.396, 3FD5.B.397, 3FD6.B.395, 3FDG.A.357, 3FE4.B.902, 4FFL.A.904, 4FFL.A.9
05, 4FFL.A.906, 4FFO.A.904, 4FFR.A.403, 2FG5.A.301, 4FI4.A.501, 3FLK.A.401, 3FLK
.A.405, 2FN0.A.701, 2FPR.A.503, 3FPA.C.901, 3FPB.A.1000, 3FQI.A.1000, 1FTN.A.300
, 3FTQ.A.371, 3FYY.A.402, 1G3B.A.501, 2G4J.A.392, 3G5A.B.307, 4G61.A.302, 2G9Y.A
.452, 2G9Z.A.704, 2G9Z.B.701, 1GAG.A.201, 4GA3.A.1004, 2GCQ.A.435, 2GGE.A.400, 4
GIS.A.405, 4GIU.A.402, 4GME.A.501, 2GQS.A.240, 2GQ3.A.1000, 2GT4.B.401, 4GT3.A.4
03, 3GY1.A.500, 4GYI.A.402, 1H1D.A.300, 4H1Z.A.401, 3H4L.A.701, 2HCJ.A.998, 3HDG
.B.201, 4HE1.A.403, 4HE1.A.404, 4HE2.A.405, 4HGR.A.201, 4HHL.A.402, 3HJN.A.501,
3HPF.A.402, 3HQD.A.501, 3HQP.B.502, 3HVH.A.265, 3HVI.A.1, 3HVK.A.1, 2HWG.A.901,
2HXU.A.601, 3HXX.A.445, 4I2B.A.602, 3I3O.A.306, 4I3Y.A.302, 4I3Y.A.304, 1I6I.A.5
01, 3I6E.A.386, 3IBA.A.401, 3IBA.A.402, 3ICZ.A.401, 2IDX.A.603, 4IEE.A.501, 4IFW
.A.502, 1IGW.A.441, 1II0.A.593, 1II9.A.593, 3IIE.A.501, 4IJQ.A.304, 2IK2.B.287,
2IK2.B.289, 2IK2.B.290, 2IK7.A.287, 2IOA.B.5004, 4IOK.A.604, 4IT1.A.501, 2IUC.B.
1008, 1IV4.A.1572, 4IX4.A.602, 1J34.C.501, 2J5X.A.200, 1JAH.A.168, 2JCS.A.1211,
2JI6.A.1567, 2JI8.A.1567, 1JP4.A.701, 1JP4.A.702, 3JUK.C.307, 4K33.A.802, 3K5H.A
.401, 3K5H.A.402, 1K9Y.A.401, 1K9Y.A.403, 1K9Y.A.402, 4K9N.A.601, 1KA2.A.501, 4K
CT.A.1001, 4KCU.A.1001, 4KCV.A.1001, 4KCW.A.1001, 3KDN.A.500, 1KEK.A.2237, 3KEU.
A.400, 1KH7.A.452, 1KHK.A.452, 1KHZ.B.310, 3KHQ.A.1, 1KKR.A.501, 4KMQ.A.1102, 1K
O5.A.1001, 1KP8.A.550, 4KQX.A.405, 4KQX.A.406, 3KR4.C.1004, 3KRO.D.3002, 4KS0.A.
1001, 4KUX.A.701, 3KWS.B.401, 4KWD.A.403, 4KX3.A.302, 3L0Y.A.257, 4L2X.F.403, 4L
2X.F.405, 1L8A.A.888, 4L9Y.B.403, 4LA7.B.601, 4LF1.A.801, 4LNI.E.505, 1LON.A.145
4, 3LV0.A.264, 3LVV.A.695, 3LVV.A.697, 4LZ3.A.406, 3M00.A.550, 1M1B.B.999, 3M1Y.
A.300, 4M69.A.403, 4M6U.A.401, 1MB9.B.601, 1MBZ.A.603, 1MC1.A.603, 1MEZ.A.1453,
4MFG.A.201, 4MIT.A.202, 1MMA.A.998, 1MNS.A.360, 4MPO.A.206, 4MPO.B.204, 4MPO.C.2
05, 4MPO.E.201, 3MQT.H.626, 1MRS.A.300, 4NOG.A.403, 1N1Z.A.702, 1N20.A.703, 1N24
.A.701, 1N24.A.703, 3N3T.A.802, 1N8I.A.900, 4NEH.B.703, 1NHT.A.435, 3NJL.A.501,
4NM3.A.405, 3NO1.A.397, 1NUW.A.2497, 1NUX.A.2342, 1NUY.A.2343, 3NZG.A.507, 4NZO.
A.404, 2O1O.A.501, 2O1O.A.503, 4O4D.A.401, 1OAD.A.392, 2ODB.A.205, 3OES.A.202, 1
OFH.B.453, 4OKM.A.902, 4OKZ.A.901, 4OKZ.A.902, 3OP2.A.500, 3OPS.A.500, 3OPS.A.50
1, 2OQY.A.401, 1ORK.A.223, 4ORK.A.502, 2OUN.A.403, 1OVM.A.601, 1OW2.A.401, 2OX4.
A.402, 3OYZ.A.500, 1OZF.A.699, 1OZH.A.1405, 3OZM.D.390, 3OZY.B.390, 3P0X.A.430,
3P3B.A.393, 2P3N.A.1758, 3P41.A.297, 3P5R.A.901, 1P7T.A.1000, 1P9B.A.1600, 3P93.
A.406, 4PAL.A.110, 3PDE.A.311, 1PFK.A.325, 1PFK.A.327, 4PFK.A.327, 2PGN.A.610, 2
PLS.G.603, 2PLS.J.604, 2PP3.A.901, 1PUN.A.130, 3PUV.A.1501, 4PU5.A.502, 1PYD.A.5
59, 1PYM.A.1003, 2PZA.A.6242, 2Q1A.X.294, 2Q1D.X.294, 4Q1V.A.803, 6Q21.D.173, 3Q
30.A.600, 3Q30.A.601, 3Q46.A.305, 3Q46.A.306, 2Q58.A.5, 1Q9S.A.201, 3Q9L.A.700,
1QC5.A.601, 1QC5.B.602, 4QE5.A.401, 4QEH.A.402, 1QF4.A.433, 1QF5.A.433, 2QGY.A.7
01, 1QMZ.A.383, 3QPE.A.393, 3QPE.B.393, 4QPM.A.1502, 2QQ0.B.452, 3QQV.A.382, 1QS
0.A.501, 2QTV.B.210, 3QU4.A.225, 2QX0.A.162, 3R1M.A.402, 3R25.A.402, 2R9V.A.504,
3RBM.A.1002, 3RBM.D.1003, 2RDX.A.378, 3RIM.A.1001, 4RJJ.A.602, 4RJK.H.602, 4RN3
.A.301, 4RNH.A.1501, 4ROP.A.504, 1RQI.A.605, 3RUV.A.544, 1S1C.A.300, 3S9I.A.743,
3SAD.A.801, 3SB0.A.801, 3SBF.A.402, 3SE1.A.182, 1SHQ.A.479, 3SH6.A.176, 3SOP.A.
401, 1SOJ.A.2123, 3SSN.A.501, 3ST8.A.496, 3T0Z.A.401, 3T1Q.A.198, 3T2E.A.409, 3T
6C.A.501, 3T8O.A.564, 1T9B.A.1699, 1TE6.A.640, 1TND.A.352, 4TQ4.A.401, 3TTE.A.36
1, 3TTE.B.361, 3TW6.C.2002, 3TZF.A.279, 4U0M.A.503, 3U2E.B.1, 3U2E.B.4, 3UJ2.A.4
31, 3UJR.A.501, 4USI.A.1151, 4USJ.C.302, 3UXL.A.360, 2V0N.A.601, 4V1T.A.1776, 4V
1T.A.1778, 2V3W.A.1528, 1V5F.A.1603, 1V5G.A.1603, 2V5K.A.301, 1V8K.A.501, 1VA6.A

.522, 1VA6.A.524, 2VBI.A.1000, 2VBV.A.1136, 3VD3.A.3001, 2VDM.B.2001, 3VKB.A.701
, 3VMM.A.501, 2VPR.A.1207, 2VP0.A.1209, 2VQD.A.1449, 3VR6.B.602, 2VWT.A.301, 3VY
T.C.602, 2VZB.A.1001, 2W00.B.1894, 3W2W.A.904, 1W5T.B.701, 3W7F.A.303, 1W88.C.13
68, 4WB8.A.402, 1WC6.C.2202, 1WDD.A.1476, 3WDL.B.902, 2WEF.A.402, 4WF7.A.601, 4W
K0.B.501, 4WK4.B.501, 1WL6.A.801, 3WO0.A.502, 3WO0.A.503, 4WRR.A.401, 2WX5.L.128
2, 2X3J.A.1590, 2X5Z.A.602, 2XAM.B.1030, 2XCL.A.480, 1XEF.A.801, 2XH4.A.1439, 5X
IM.A.395, 5XIN.A.395, 1XIN.A.395, 6XIM.A.395, 8XIM.A.395, 2XIM.A.395, 2XJC.A.149
9, 1XLC.A.399, 2XZW.A.202, 1XZ8.A.180, 1Y9I.A.601, 1YHM.B.1401, 1YHY.A.699, 1YIO
.A.212, 1YVE.I.602, 1YYQ.B.701, 1Z0K.A.1201, 2Z4W.A.1302, 2Z4Y.B.1301, 2Z7H.A.13
01, 3ZCB.A.301, 3ZDY.B.2001, 1ZVW.A.4001, 2ZVJ.A.300, 3ZXW.A.476, 3ZYC.A.1750, 3
A4K.C.301, 3AUO.A.577, 4BDZ.A.1380, 4D6O.A.1186, 4D6O.A.1187, 4DLE.A.901, 2HVI.D
.878, 3ICE.E.502, 4J9O.A.502, 3MDA.A.577, 4N41.E.101, 4NLK.A.402, 4NM1.A.401, 2P
YJ.B.9002, 3Q8P.B.423, 3TFR.A.340, 1TFW.B.1601, 2A5G.A.231, 4A7Y.A.952, 4ACF.D.1
480, 3ALO.A.1, 1AQF.A.534, 2AUU.A.201, 3AU9.B.602, 3B03.D.1001, 2B8W.A.595, 2B9J
.A.600, 2BBT.A.3, 4BCL.A.367, 4BCL.A.368, 4BCL.A.369, 2BHW.B.605, 1BHO.1.901, 4B
JR.A.1517, 4BJU.A.998, 2BKK.A.1266, 2BOZ.M.1304, 1BS1.A.901, 2BVN.A.1395, 2BW7.D
.2201, 4BYF.A.1000, 4BYG.A.996, 3C14.C.403, 2C43.A.1317, 3CBT.A.301, 3CK5.D.400,
 3CRL.A.2000, 1CUL.A.1006, 3CX7.A.378, 4CYI.D.1000, 2D33.A.525, 4DFX.E.403, 3DHF
.A.502, 3DKL.A.502, 4DL8.A.305, 4DLC.A.303, 3DUF.C.1368, 3DV0.A.1368, 1E14.M.130
3, 1E1R.A.601, 1E1R.F.601, 4E1E.A.402, 1E6D.M.1303, 2E8T.B.1303, 2E8W.A.1201, 2E
8X.A.1302, 1E9I.B.1431, 1E9I.D.1433, 2E91.B.1303, 2E92.B.1303, 3EA4.A.699, 3EF1.
A.1, 3EFQ.B.4003, 3EHB.A.562, 4EKD.A.407, 3ENI.A.375, 3ENI.A.378, 3EOJ.A.375, 2E
WG.B.4003, 3EYA.A.613, 3FDG.B.356, 4FFR.A.406, 2FKA.A.9001, 1FMW.A.800, 1FQJ.A.3
52, 2G07.A.601, 2G08.A.500, 3G8D.B.1002, 4GA3.A.1003, 3GFT.F.202, 1GIM.A.435, 2G
TP.A.401, 2GZD.A.950, 4HE0.A.402, 4HE0.A.403, 4HGQ.C.201, 4HGR.B.201, 3HIY.A.401
, 2HO4.B.301, 3HU2.D.801, 3HZH.A.202, 3IOO.A.502, 2I19.A.3002, 4I40.B.301, 2IK2.
A.289, 4IN7.M.411, 2IOA.A.5002, 4J5I.F.402, 1JB0.A.1011, 1JB0.A.1106, 1JB0.A.111
7, 1JB0.A.1121, 1JB0.A.1122, 1JB0.A.1128, 1JB0.A.1132, 1JB0.A.1133, 1JB0.A.1136,
 1JB0.B.1201, 1JB0.B.1202, 1JB0.B.1203, 1JB0.B.1204, 1JB0.B.1206, 1JB0.B.1207, 1
JB0.B.1211, 1JB0.B.1214, 1JB0.B.1215, 1JB0.B.1221, 1JB0.B.1223, 1JB0.B.1225, 1JB
0.B.1234, 1JB0.B.1239, 1JB0.L.1501, 1JB0.L.1502, 2JCM.A.1490, 2JCS.B.1211, 4JLZ.
B.502, 1JYX.A.3002, 4K81.B.203, 4KCV.B.1001, 1KJ8.B.394, 1KK8.A.997, 3KRF.D.901,
 3KRO.A.3003, 3L0C.A.257, 3L8F.A.401, 4LCZ.A.314, 3LMG.A.202, 4LNI.D.503, 4LNI.D
.505, 4LRJ.A.302, 4LRZ.A.302, 3LUZ.A.264, 3MCO.A.427, 3MCO.B.426, 4MFE.C.1104, 3
MGA.B.405, 4MGH.A.1302, 4MKU.A.209, 3MLE.C.222, 1MNZ.A.389, 4N0G.B.402, 1N22.B.7
06, 1N24.B.706, 4NCJ.A.903, 4NDN.A.401, 4NEH.A.1102, 4NM5.A.406, 3NNS.A.401, 4NN
N.Z.301, 2O1X.A.2001, 3O5T.A.299, 2O56.A.2001, 3O61.A.202, 4OEC.A.401, 2OI2.A.40
0, 2OQY.B.402, 2PAN.A.851, 1PPW.A.401, 2PSN.A.701, 4PTK.A.302, 2PUL.A.400, 3PUW.
A.1501, 3Q1O.D.400, 2Q58.A.3, 3Q7P.B.257, 3QQV.A.381, 1QSH.B.147, 4QTD.A.426, 4Q
VP.V.302, 4QXD.A.304, 1R03.A.301, 4R02.Y.302, 4R17.K.302, 3RBM.B.1001, 3RLG.A.28
6, 1RLT.A.805, 3RRA.A.406, 1RZH.L.854, 1RZH.M.851, 1RZH.M.853, 1SOJ.C.2127, 3SQS
.A.450, 3SS8.A.302, 3T34.A.1002, 3T5P.F.301, 3T9E.A.603, 3TAV.A.266, 4TQ4.C.402,
 4TVU.B.601, 4TXZ.B.502, 4U0N.A.401, 3U2E.A.2, 4UB6.B.603, 4UB6.B.607, 4UB6.B.61
0, 4UB6.B.616, 4UB6.C.504, 4UB6.C.506, 4UB6.C.507, 4UB6.C.509, 4UB6.C.510, 4UB6.
C.512, 4UB6.C.513, 4UB6.C.514, 4UB6.D.402, 4UM8.D.2001, 2UXR.A.1405, 2V54.B.1205
, 3VAD.A.402, 2VP0.B.1209, 3VTH.A.805, 2W00.A.1887, 1W5T.A.701, 3WBZ.F.403, 3WQM
.A.403, 3WU2.A.405, 3WU2.A.410, 3WU2.B.604, 3WU2.B.605, 3WU2.C.502, 3WU2.C.510,
3WU2.D.402, 3WU2.b.615, 3WU2.b.616, 3WU2.b.617, 3WU2.c.902, 1XBT.D.4194, 1XZ8.B.
180, 2YBE.A.1417, 1YF6.M.853, 1YF6.M.856, 1YQ7.A.908, 1Z5B.A.2001, 1ZCA.A.383, 1
ZM7.D.400, 2ZRW.A.702, 4BXO.B.1216, 2FKC.A.248, 3GII.A.415, 4K4I.A.603, 3KHL.A.4
15, 4KHN.A.1002, 4KHW.A.1003, 3QEP.A.906, 4QWE.A.703, 1R7M.A.304, 1TW8.C.803, 3U
IQ.A.905, 2WTF.B.1509, 1A25.A.292, 1A25.B.292, 1AF4.A.276, 4AIO.A.1890, 1ALA.A.4
02, 4B4F.B.607, 4B7M.B.1471, 4B7R.B.502, 2BAT.A.601, 2BD3.A.260, 3BOW.A.718, 4BT
X.A.1763, 2BW7.A.2202, 4CCE.A.4001, 2CDP.B.1140, 2CHI.A.218, 5CHY.A.401, 1CJY.A.

951, 3CK7.B.720, 3CKC.B.700, 4CPO.A.1466, 1CXV.B.6, 2D00.B.1002, 3D4G.H.484, 2DE
W.X.903, 2DEW.X.904, 1DM5.A.1131, 1DM5.C.1133, 1DQ1.A.238, 4DWW.A.301, 1E35.B.26
0, 3E3R.B.195, 1E5J.A.402, 1E8U.A.1002, 3E9T.A.6, 2EA7.B.452, 1EE6.A.300, 2ERQ.B
.702, 2EXK.D.2004, 1EZS.C.650, 3F19.A.266, 2F20.B.1001, 1F2N.A.1002, 1F2N.C.1001
, 4FGC.C.203, 3FHA.B.704, 3FRP.A.628, 4GDI.C.507, 4GGB.A.401, 1GQM.E.1089, 4GZS.
A.501, 4H1Q.A.304, 4H2E.B.304, 1HFY.A.124, 1HFZ.C.124, 3HLH.D.315, 4HSZ.B.101, 2
HTV.A.995, 2HTY.C.993, 4HZY.A.507, 4I5K.A.501, 1IME.B.278, 1J0Y.D.701, 1JAO.A.99
6, 1JI3.A.401, 1JIW.P.489, 1JRF.A.48, 4JUC.B.601, 3K1A.B.524, 3K37.A.467, 4K3Y.D
.604, 3K5T.A.802, 3K9X.D.249, 3KF9.A.303, 3KL6.A.3, 1KLJ.H.900, 4KNA.A.504, 4KS2
.A.501, 1KVO.A.192, 4KVK.A.712, 1KWH.A.800, 1L7L.A.201, 3L95.X.2001, 3LNF.B.305,
4LXF.A.701, 4MDV.A.402, 3MIN.D.524, 1MMP.B.3, 1MR8.A.102, 1MTV.A.480, 1N28.A.12
6, 1N28.A.128, 4N2F.A.703, 1NGO.A.1002, 1NGO.C.1001, 1NKQ.A.260, 4NRE.A.716, 4NR
E.A.717, 4NUP.A.301, 2NVO.A.533, 1NXO.A.902, 4O1Q.A.401, 3O9J.A.995, 2OO4.B.5004
, 2OO4.B.5006, 1OS2.C.473, 3OSH.A.121, 2OW1.B.447, 3OX6.E.502, 2P5V.C.1002, 4PHK
.A.304, 3PKO.D.280, 1PK8.F.817, 4PKH.J.1201, 4PLS.A.305, 3PRT.A.404, 1PYT.C.650,
3Q2L.A.703, 1Q3A.B.472, 3Q3K.A.262, 1QL9.A.480, 3QNI.B.400, 4QN3.B.501, 3QQZ.A.
324, 1QUO.B.702, 1R1Z.B.315, 2R8Y.C.203, 4R83.D.501, 2RHP.A.2, 2RHP.A.10, 2RHP.A
.18, 2RHP.A.20, 2RHP.A.28, 2RJP.A.2, 2RJP.A.3, 2RLD.C.121, 1ROS.A.404, 1ROS.B.50
3, 1SOE.A.1293, 1S1D.B.1002, 3SHI.G.305, 3SJS.A.222, 1SMP.A.478, 3SOB.B.1, 1SRR.
A.531, 1STB.A.150, 1TAD.A.352, 3TH3.L.145, 1TLD.A.480, 3U1R.A.700, 4UB6.O.301, 3
UBH.A.852, 3V03.A.584, 1V0Z.A.1477, 1V7V.A.1001, 1VFO.B.1002, 2VME.A.500, 3VRQ.A
.401, 1W3M.C.3014, 4W4Q.A.401, 3W9T.A.1006, 3W9T.C.1010, 2WG8.A.201, 3WIU.B.1002
, 2WVX.C.801, 1WVM.A.604, 1Y4A.E.1001, 2YFS.A.1711, 2YGM.A.1417, 2YN5.A.6365, 1Y
O8.A.1188, 1YO8.A.1197, 2Z8S.B.641, 2Z8S.B.647, 3ZHG.B.1323, 2ZID.A.882, 2ZJ6.A.
625, 2ZKT.A.413, 2ZRQ.A.7, 1ZTQ.B.565, 2ZUX.B.638, 2ZUY.A.627, 2ZWO.A.400, 2ZWO.
A.403, 2ZWO.B.402, 4ABT.B.1287, 2AOQ.A.232, 2AOR.A.401, 2AOR.A.402, 2ASD.A.415,
2ASD.A.416, 3AVX.A.3001, 3BQ1.A.4001, 3COW.A.304, 1DMU.A.300, 1DMU.A.302, 4DTP.A
.1002, 3DVO.A.340, 3EH8.A.302, 4ELV.A.908, 1FOO.B.761, 4F4W.A.402, 4F4W.B.403, 4
FJ9.A.1002, 4FJK.A.1002, 4FJL.A.1002, 4FJM.A.1002, 2GIJ.A.401, 3GV5.B.424, 3IAY.
A.1, 2IBK.A.401, 2IBK.A.402, 2IMW.P.406, 4J2A.A.1002, 4J2B.A.1002, 2JEJ.A.1344,
1JX4.A.4001, 1JXL.A.1402, 4K4H.A.607, 4K4H.E.602, 4K4I.A.606, 3KHR.A.416, 4KLD.A
.402, 4KYW.A.302, 4LQO.A.402, 3LZJ.A.905, 4M3Z.A.1002, 3M9N.B.4003, 3M9O.B.4001,
3M9O.B.4003, 3MXB.B.173, 3MXB.R.175, 1N3E.C.492, 1N3E.F.491, 1N3F.D.499, 1N3F.F
.497, 3O3G.A.1, 3ODH.A.195, 1OUP.A.300, 3QET.A.905, 4QWB.A.403, 1R7M.B.536, 3RAX
.A.415, 1RYS.A.801, 1SOO.A.401, 1S9F.A.4001, 3SLP.B.227, 3SPZ.A.905, 3SUN.A.897,
1TW8.A.801, 2WTF.B.1510, 3A09.A.601, 3A7Q.B.5001, 2A8K.B.404, 2AER.L.3008, 2AEP
.A.601, 1AFO.A.484, 1AFO.A.486, 1AG9.A.200, 1AG9.B.1000, 4AIE.A.1540, 3AMR.A.909
, 3AMR.A.910, 4AQ1.A.1925, 4AQ1.A.1926, 4AR9.A.1732, 4ARF.A.1723, 3ASI.A.2001, 1
AWB.A.1, 4AWD.A.1321, 3AYU.A.418, 3AZX.B.301, 3B00.B.301, 3B0I.A.124, 3B1U.A.901
, 1B4N.A.620, 3B4N.A.702, 4B4F.A.607, 1B8L.A.110, 1B9T.A.500, 3BC9.A.702, 3BC9.A
.705, 3BCD.A.708, 3BH4.B.1, 1BLI.A.600, 2BU3.A.1242, 2BV2.B.1085, 4BZ4.A.1233, 1
C3H.D.8003, 1C3H.F.8001, 4C9F.B.401, 4CAG.A.602, 2CDP.A.1140, 2CDP.B.1139, 1CGE.
A.305, 3CK7.D.730, 3CKC.A.600, 1CLC.A.650, 1CLC.A.652, 5CNA.C.240, 4CPY.A.1466,
1CRU.A.503, 4CUB.A.2645, 2CYY.A.2002, 2D00.D.1001, 3D4G.B.484, 3D6E.A.202, 3DAS.
A.351, 2DCJ.A.1003, 2DDU.A.1, 2DEW.X.901, 2DF7.C.5904, 2DIE.A.779, 4DK4.A.302, 4
DKB.A.302, 4DLK.A.403, 4DOU.A.1001, 4DOU.A.1002, 2DSN.B.2012, 2E9B.A.741, 3EAD.A
.1003, 3EF2.A.304, 3EF2.A.305, 3EHB.A.563, 4EJ7.B.402, 1ELT.A.300, 5ENL.A.438, 4
EPU.A.601, 3ESQ.A.213, 2EXH.D.2004, 1EX9.A.286, 2F3C.E.242, 4F8Z.A.409, 1FBL.A.9
96, 2FIB.A.412, 2FPS.A.503, 3FP8.E.601, 3FU1.A.301, 3G4E.A.1, 1G5N.A.402, 4G60.A
.302, 4G62.A.302, 1G87.A.615, 1G9K.A.700, 4GDJ.A.507, 3GG1.A.502, 2GGM.B.402, 4G
I6.A.601, 2GJP.A.1486, 2GKO.A.611, 3GK2.A.92, 3GN9.A.201, 4GQ7.A.301, 2GSM.A.300
7, 1GVK.B.1246, 4GW3.A.401, 4GZT.B.510, 2HOK.B.410, 1H3G.A.701, 4H3X.A.304, 1H5V
.A.306, 1H71.P.501, 1H71.P.502, 1H9H.E.1246, 3HB2.P.482, 1HDF.A.1101, 3HI7.A.802
, 1HL5.D.156, 1HM9.A.1901, 4HOW.A.704, 4HPN.A.401, 1HQV.A.999, 1HVX.A.517, 1HYO.

A.1006, 2HYU.A.502, 2HYV.A.608, 3I4I.A.1001, 2I4B.A.454, 3I57.A.186, 1I76.A.996, 4I8H.A.301, 3I98.A.627, 3IBZ.A.192, 4IHM.A.403, 1IME.A.278, 1IOD.G.503, 3IOX.A.903, 2IUF.E.1697, 2IXT.A.1311, 1J0H.A.601, 1J11.A.701, 1J1N.B.493, 1J35.C.501, 4J7M.A.403, 1J9K.A.301, 1J9L.B.1303, 4JCM.A.706, 1JE5.B.502, 1JI3.B.403, 2JKE.A.1728, 2JKH.A.1245, 3JXS.A.302, 4JZB.A.402, 4JZE.H.302, 4JZX.A.403, 3K37.B.467, 4K3K.B.401, 1K7I.A.483, 1K7Q.A.485, 4K70.B.1002, 4K89.A.408, 4K9P.A.601, 1KA1.A.401, 2KAY.B.188, 1KAP.P.617, 1KAP.P.618, 1KAP.P.620, 3KCG.H.500, 3KF9.C.304, 4KKF.A.703, 3KM5.A.2011, 3KMV.A.163, 4KPP.A.501, 3KQA.B.421, 4KS1.A.501, 4KTY.A.802, 1KU0.A.703, 4KXY.A.707, 3KZP.A.240, 2L51.A.207, 1L9M.A.702, 3L9I.C.1148, 3LHM.A.131, 4LJ3.B.403, 4LLS.A.303, 4LLT.A.303, 3LNH.A.303, 3LNI.A.303, 1LOC.E.688, 3LPD.A.342, 4LQR.A.202, 4LVN.A.704, 2M0P.A.1201, 3M1H.C.2001, 3MBR.X.300, 4MB1.A.602, 1MCT.A.246, 1MDU.A.403, 3MHF.A.328, 2MIN.B.525, 4MKM.A.403, 2ML1.A.201, 2ML1.A.202, 2ML1.A.204, 2ML2.A.201, 2ML3.A.203, 2ML3.A.204, 3MMZ.A.500, 3MVS.A.211, 3MVS.A.214, 3MVS.A.215, 4MWL.A.512, 3MW3.A.301, 4N20.A.705, 4N2B.A.705, 4N2G.A.705, 4N2I.A.704, 4N2I.A.705, 3N4E.A.500, 1N7V.A.601, 3N8G.A.1002, 4NAM.A.801, 4NAS.A.503, 1NBW.A.650, 4NEH.B.701, 3NIF.D.2002, 3NJH.B.502, 3NKQ.A.1003, 1NKG.A.800, 1NNL.B.2002, 4NOT.A.302, 1NRW.A.903, 1NSC.A.468, 1NUD.A.703, 4NUQ.A.301, 4NUZ.A.1001, 1NX1.A.3, 2NXP.A.600, 1O9I.C.269, 1OAH.B.1526, 1OB0.A.501, 3OJY.A.555, 3OJY.B.538, 1OM6.A.701, 1OM8.A.705, 3OM5.B.1, 3OM6.B.1, 3OTJ.E.1000, 4OUL.B.1201, 3P4G.A.402, 3P4G.A.403, 3P4G.A.404, 3P4G.A.405, 3P4G.A.406, 3P4G.A.407, 3P4G.A.408, 3P4G.A.409, 3P4G.A.410, 3P4G.A.412, 3P4G.B.411, 3P95.A.1, 3PGB.A.903, 4PHN.A.302, 3PK0.A.280, 4PLS.A.301, 2PNY.A.228, 3POJ.B.1, 2POO.A.805, 3PPE.A.401, 2PQY.A.500, 1PVY.B.603, 1PW9.A.404, 1PZ7.A.701, 2PZ0.A.501, 2Q16.A.200, 3Q2L.B.703, 3Q5I.A.528, 3Q8F.A.736, 1QCO.A.1002, 4QD2.E.302, 3QGV.A.504, 2QIM.A.158, 1QLB.A.1658, 4QN7.A.501, 2QUB.A.614, 2QUB.A.616, 2QUB.A.618, 4QU6.A.904, 3QXG.B.230, 4R12.A.809, 2R8Y.A.201, 3R8Y.A.242, 2RA3.A.1, 2RA3.B.1, 3RQ0.A.301, 1RXO.B.477, 1S0B.A.1292, 3S5U.E.221, 3S6J.A.4, 2SAS.A.187, 1SBH.A.290, 1SCB.A.276, 3SLE.B.401, 3SOO.D.97, 3SVL.A.201, 3T3P.B.2003, 1T5S.A.1004, 1TCM.B.687, 3TEW.A.800, 3TEW.A.801, 1TO2.E.450, 4TSH.B.1502, 1TU5.A.902, 1TYE.A.1406, 3U1R.A.704, 3U1R.A.705, 3U1R.A.706, 3U1R.A.707, 3UBF.A.754, 1UF3.D.913, 1UH3.A.1002, 1UKS.A.688, 1ULV.A.2001, 1ULV.A.2002, 1ULV.A.2003, 4UM9.B.2002, 4UP4.A.502, 1UPS.A.501, 1UTM.A.247, 2UWF.A.1369, 1UX6.B.2003, 1UX6.B.2007, 1UX6.B.2010, 1UYX.A.1133, 1UYX.A.1134, 1V0Z.B.1477, 3V96.B.304, 1VCL.A.1004, 1VCL.B.1001, 1VCL.B.1002, 2VCC.A.1917, 2VDR.B.2002, 2VJ3.A.1533, 1VL9.A.125, 2VL8.A.1545, 2VNG.B.1214, 3VOC.A.501, 3VTO.A.302, 3VV3.A.401, 2W1W.B.1135, 4WA3.A.503, 2WFK.A.1250, 3WH3.A.501, 3WN6.A.502, 2WO9.B.1272, 2WOB.E.1161, 1WPC.A.502, 2WQS.A.2415, 1WRZ.A.154, 2WW3.A.800, 3X17.A.602, 1XKD.A.1005, 2XR9.A.1869, 2XSG.B.1772, 2XVT.F.1137, 2Y09.A.1242, 1Y6W.A.149, 2Y6D.A.1266, 1Y7B.A.3001, 1Y9Z.A.604, 1Y9Z.B.605, 2YAY.A.1267, 2YEQ.A.1526, 2YGL.A.1413, 1YI7.A.3001, 1YO8.A.1183, 1YO8.A.1185, 1YO8.A.1193, 1YO8.A.1199, 1YO8.A.1201, 1YO8.A.1203, 1YO8.A.1205, 1YO8.A.1206, 1YO8.A.1208, 1YO8.A.1209, 1YS6.A.1001, 2Z2X.A.1007, 1Z6O.A.5302, 1Z70.X.3001, 2Z8X.A.620, 2Z8X.A.622, 2Z8X.A.623, 2Z8X.A.624, 2Z8S.B.643, 2ZE0.A.552, 3ZHG.D.1323, 2ZPR.A.2001, 2ZQ0.A.901, 2ZUX.A.630, 2ZUY.A.626, 3ZWH.A.501, 2ZWP.A.402, 4AWD.B.1321, 1B80.B.351, 4B9C.A.1151, 3BMV.A.685, 4BM1.A.401, 1DED.B.5004, 2FHF.A.2401, 3HOO.A.401, 1HFX.A.124, 3ILF.A.278, 4J3V.A.920, 4J3W.A.907, 4JGL.A.202, 1K12.A.160, 4L73.B.402, 1LLP.A.351, 4LPL.A.1101, 4LQR.A.201, 1LY8.A.9001, 1MVE.A.400, 3SRG.A.1357, 1URX.A.1300, 2A1X.A.450, 1A7E.A.119, 3ABM.A.516, 1B1X.A.690, 2B1X.E.502, 2B2O.B.1500, 4B2O.A.1267, 4B7G.A.3000, 3BFJ.M.1388, 4BGL.A.1001, 1BKA.A.693, 1BKA.A.694, 1BLF.A.700, 1BLF.A.701, 2BOY.G.1255, 2BQ8.X.1305, 2BQ8.X.1306, 2BUZ.B.1541, 2BUR.B.600, 2BV0.B.600, 1C7M.A.101, 2CAG.A.485, 1CE2.A.690, 1CE2.A.691, 4CHL.A.501, 1D9Y.A.310, 3DHG.A.502, 1DRY.A.332, 3DXU.A.360, 3E6S.B.200, 1EO9.B.600, 1FCD.C.902, 2FR7.A.501, 1FSL.A.144, 1FZ1.A.5001, 1FZ3.A.5002, 1FZ7.B.5003, 2G1M.A.600, 4G51.A.202, 4G51.B.202, 3GE3.A.501, 1GGF.B.760, 4GHF.B.401, 4GP5.A.602, 2H0V.A.501, 4H0W.A.701, 1H76.A.702, 1H76.A.703, 1HAC.B.200, 3HGI.A.281, 2HUO.A.301, 1I4Z.A.601, 4I4G.A.

601, 4I4H.A.601, 3IB0.A.999, 2INC.A.502, 3IXF.A.139, 1IZO.A.501, 1JNF.A.702, 1JN F.A.703, 4K0F.A.601, 4K9T.A.601, 4K9U.A.601, 4K9V.A.601, 4K9W.A.601, 4K9X.A.601, 3KT7.A.701, 1KW9.B.301, 1LCT.A.400, 1LFG.A.693, 1LFG.A.694, 1LKM.A.601, 3LXV.M.600, 4M1I.A.402, 3MDT.A.505, 4ME4.A.402, 1MMO.D.3, 1MMO.D.4, 1MTY.D.3, 3MZS.C.500, 3N2O.A.506, 2NOX.A.500, 1NX4.B.300, 3OOR.B.802, 2O7U.A.500, 1OCZ.A.516, 4OJ8.C.301, 1OQG.A.500, 1OQU.C.1009, 1OQU.C.1010, 1OVT.A.689, 3P3N.A.350, 3PCC.O.600, 3PCF.M.600, 3PCJ.O.600, 2PCC.A.296, 3PER.A.1001, 1PHG.A.417, 1PIU.A.402, 2Q0J.B.998, 3Q14.A.502, 3Q1G.A.1002, 3Q3M.A.509, 2QED.A.252, 3QFN.A.265, 3QFO.A.264, 2QPP.A.300, 3QY6.A.263, 3QY7.A.263, 3QY8.A.253, 3QYT.A.680, 1R1N.C.400, 3R1J.A.299, 4RC8.A.303, 2RDB.A.499, 3RNC.A.499, 3RNC.A.500, 3RNF.A.500, 3RNF.A.501, 1RYO.A.329, 1S9A.A.300, 1SYY.A.1319, 1SYY.A.1320, 1T0Q.A.499, 1T0Q.A.500, 1TFD.A.950, 3TMZ.A.501, 3TTX.A.760, 1U74.B.1101, 3UF9.A.315, 3VEZ.A.601, 1VFD.A.400, 3VMG.B.501, 2VUN.A.402, 3WEC.A.501, 3WFD.B.803, 1WRA.B.402, 2X9P.A.1398, 1XU5.A.1175, 1XVB.A.1170, 1XVF.B.1174, 1XVF.B.1175, 1XVG.A.529, 1Y8W.A.142, 1YGF.B.147, 1YUZ.A.301, 3ZK3.A.1311, 3ZK4.A.800, 4AC8.D.500, 1AHJ.A.208, 4ANP.A.1426, 4AUL.A.754, 2AWC.A.137, 4B2O.A.1266, 1B7Z.A.690, 1B7Z.A.691, 4BLY.A.500, 4BM1.A.500, 1BS3.A.202, 3BXD.A.302, 1CG5.A.142, 1CGO.A.128, 3CHH.A.501, 1D06.A.501, 1D2V.A.605, 1DRT.A.325, 3E65.A.901, 3EE4.A.317, 3EH5.A.801, 1EOB.B.600, 1EYS.C.609, 1EYS.C.612, 1F9B.A.690, 4FAG.A.401, 2FDG.A.300, 1FRV.B.537, 1FT5.A.214, 1FZ1.B.5003, 1GBU.B.148, 3GCF.D.501, 1GVH.A.1398, 1GY9.A.300, 1H2K.A.1350, 1H2L.A.1350, 1HDS.B.146, 1HRM.A.154, 1IBE.A.143, 3ICF.A.601, 3IVD.B.601, 4J1X.A.201, 2J2M.A.501, 3K9V.A.520, 4L7Y.B.201, 4M26.B.401, 3MPS.D.172, 1MQV.A.150, 1MXR.A.1003, 1N04.A.688, 3N1Y.A.503, 3N1Y.A.504, 4N71.A.201, 3NKT.A.369, 3O0F.A.304, 3O32.A.300, 3O6J.A.300, 1ODN.A.1326, 2OGI.A.301, 4OJ8.B.301, 1OM4.A.750, 1OUT.A.143, 1OUT.B.148, 3OWO.B.384, 3PCA.M.600, 3PCC.N.600, 3PCJ.N.600, 3PCK.M.600, 2PHD.B.370, 2PQ7.A.221, 1PRC.M.607, 2Q0J.A.998, 3Q3O.A.502, 1QFC.A.401, 1QHW.A.433, 3QY8.A.252, 2R2F.A.320, 4S1C.A.801, 3SCF.C.203, 1SP8.A.500, 3T4V.A.300, 3TKT.A.431, 1TKP.D.302, 3UFK.A.920, 3UF9.B.315, 3UHK.A.147, 1UOF.A.1311, 1UTE.A.501, 3VER.A.601, 2VE3.B.1444, 1VHB.A.150, 3VSI.B.401, 2VV6.C.1259, 1VZ4.A.1299, 1W2A.X.1302, 3W54.B.502, 3WAQ.A.201, 4WWZ.B.301, 1WZD.A.901, 2XMO.B.556, 1XZ5.A.142, 1XZW.A.429, 2YOI.A.1350, 1Y5F.B.147, 1Y5J.B.147, 2YDE.A.501, 1YFW.A.300, 1ZJ9.B.1569, 2ZYQ.A.301, 2ZZI.B.209, 3HW8.A.577, 3HW8.A.580, 1ORP.A.224, 4ORJ.A.305, 3PML.B.7, 1Q81.K.8346, 1VQ7.Q.9148, 2A7L.A.201, 2A9Y.A.2002, 3ASQ.B.701, 3AST.B.701, 4BR6.A.401, 4BVN.A.1360, 4C7A.B.1159, 4CSH.A.1169, 3CZJ.A.3102, 4CZN.A.1371, 4D1I.A.600, 4D1J.E.604, 4D9T.A.901, 3DR3.A.335, 2DV1.A.1000, 3EEB.A.211, 1EZ1.B.1002, 4F3Y.A.301, 4FEW.D.304, 4FMT.D.301, 3FZQ.B.274, 3G1N.B.5002, 1G5I.C.902, 4GDK.E.301, 4GRX.A.501, 2GTW.E.3006, 4GY9.A.207, 3H7K.A.387, 3HYS.A.267, 2HZY.A.1201, 4IOW.D.602, 3I2W.A.304, 4I2R.C.602, 3I44.A.477, 3IAQ.A.3103, 3IJP.A.301, 3IMX.A.467, 3IWK.D.504, 1IYN.A.298, 2J5W.A.3044, 2J5A.A.1109, 4JDO.G.301, 2JLN.A.1471, 3JS4.A.208, 1JZ7.C.3103, 1JZ8.D.3104, 3K6A.E.178, 1KAO.A.501, 4KA5.A.801, 4KA8.A.806, 3KED.A.951, 1L2T.A.1502, 4L3H.A.404, 3LJQ.A.597, 1LZS.A.131, 4M4V.A.505, 4M9U.A.402, 3MC1.A.301, 1MGV.A.501, 4MJD.A.203, 4MM7.A.601, 4MM9.A.602, 3MPQ.A.751, 4MPT.A.402, 3MUZ.1.3102, 4N3M.A.403, 3N3O.A.1000, 4NPJ.B.701, 4NRH.C.401, 4O1G.A.401, 2O34.A.503, 2O34.B.502, 4ODI.B.301, 3OEC.A.300, 2OKQ.A.119, 2ONP.G.707, 3OTK.A.586, 4OUC.A.801, 2OYC.A.305, 4PMO.A.310, 4PUV.A.405, 4PV3.A.201, 3Q94.A.310, 3Q9B.A.344, 1QOP.B.501, 4QVS.A.502, 2QZ7.A.193, 1RW9.A.900, 1S5C.A.241, 3S9J.A.401, 1SOO.A.547, 1TQY.H.1094, 3TXF.A.1138, 1UD8.A.1001, 2V4B.B.1562, 1VI6.A.208, 3VS8.C.501, 3VW7.A.2012, 3WNM.A.802, 1WPG.D.1300, 1WQR.A.131, 2WUW.E.1277, 2WU2.A.1590, 2WV7.B.401, 3WXO.A.805, 1X0G.A.1002, 1X0G.C.1001, 1X7D.A.1501, 1X7U.A.1000, 2X7J.A.1581, 1XAR.A.100, 1XDF.A.401, 4XEL.A.201, 2XZK.B.507, 2Y8K.A.1527, 1YAP.A.501, 1YQ2.A.7501, 2Z2F.A.2001, 1ZH8.A.329, 2ZJ9.A.1, 2ZN8.A.995, 2ZND.A.195, 3ZPQ.A.1360, 3ZQ5.A.1530, 2BCU.A.577, 2BCQ.A.1, 2BCR.A.604, 4ED3.A.502, 4KYW.A.303, 4P4P.A.401, 1Q81.A.8378, 1VQ8.J.9146, 1VQ8.Q.9148, 2A5D.B.326, 4A6U.B.1460, 4AFK.A.1507, 2AHR.C.1259, 2AMF.A.850, 4AMJ.A.1360, 3AR7.A.1000, 2AUT.D.605, 4B1M.A.1680, 3B

34.A.951, 1B57.A.364, 2BER.A.1649, 3BGA.A.6, 3BIA.X.117, 3BIB.X.117, 3BLJ.B.701, 3BOS.B.302, 2BS2.A.1658, 4BVO.A.1396, 3C17.A.323, 4CCY.A.1297, 2CD7.A.1132, 3CR
N.A.131, 4D1J.E.602, 4D1J.G.603, 2D4E.A.1901, 4D7C.A.1544, 3D9R.A.135, 2DDB.A.30
1, 2DDB.C.302, 4DEL.A.401, 4DF9.B.501, 4DOU.A.1004, 4DW8.A.304, 2E54.A.1004, 4E6
P.A.301, 4E6P.D.301, 2E7U.A.1003, 3E85.A.162, 3E85.A.163, 1EAS.A.5, 4EAE.A.302,
4EEK.A.302, 4EEL.A.302, 2EHQ.A.1540, 3EIF.A.1033, 3EIY.A.177, 3EUW.A.343, 3F3C.A
.752, 4FDZ.B.301, 4FET.A.301, 4FEX.A.303, 2FM1.D.344, 2FV7.A.403, 1G5H.A.901, 1G
5I.A.901, 4G8T.C.502, 4GAF.B.505, 3GIR.A.373, 4GIB.A.301, 4GKI.D.303, 3GOD.B.327
, 4GRX.B.501, 1GV2.A.1191, 3GZA.A.471, 3HON.A.203, 3HON.A.204, 1H16.A.9001, 3H12
.A.500, 1HBN.A.1561, 3HIJ.B.295, 3HSS.A.268, 3HVI.A.265, 1HX6.A.705, 2HZG.A.1101
, 3I01.M.730, 4IOW.B.603, 3I2W.B.304, 3IAN.A.1, 3IC3.C.100, 3ICF.A.515, 3IFV.A.4
02, 3IGZ.B.601, 2IJA.A.401, 3IPO.A.436, 3IPP.A.438, 3IWJ.A.505, 1IYN.A.297, 2IZV
.A.1430, 4J07.A.201, 2J80.A.1134, 2JBW.C.1367, 4JB3.A.301, 4JDO.A.301, 4JEX.B.51
1, 2JHN.B.1299, 1JMM.A.3001, 4JN7.A.401, 3JS4.D.208, 1JTP.A.501, 1JTP.L.503, 4JT
E.C.301, 4JTF.C.303, 4JTG.C.302, 4JTH.C.303, 4JTJ.C.302, 4JTK.C.302, 1JZ7.A.3102
, 1JZN.A.1139, 3K1U.A.412, 1K2X.A.801, 1KSU.A.810, 1L5B.A.302, 3L7X.A.142, 3LG1.
A.530, 4LGN.A.827, 1LLA.A.631, 4LL2.A.301, 4M48.A.701, 4M48.A.702, 4M4U.A.501, 4
M60.A.502, 3MQD.A.500, 3MYV.A.502, 3NOU.A.208, 3N83.G.707, 4N9S.A.402, 4N9V.A.40
4, 3NGJ.A.249, 3NMB.A.1, 4NPI.A.602, 4NRH.A.401, 4O0C.A.401, 2O34.A.501, 4O47.A.
401, 1O68.A.274, 3OEC.C.300, 4OF8.A.301, 3ON4.C.189, 3OND.A.509, 3OPK.A.301, 2OY
N.A.201, 1POZ.A.1633, 4PD6.A.502, 4PMO.A.311, 4PMO.A.312, 2POC.A.5001, 4PYJ.A.30
1, 1Q3X.A.800, 1Q6X.A.1002, 4Q69.A.501, 2QJY.R.2001, 2QKF.C.283, 2QV6.A.303, 4QX
K.A.402, 1R4P.A.4003, 4R6K.A.501, 2R85.A.600, 3RU5.A.133, 1RWH.A.900, 1SOA.A.150
1, 1S5D.A.241, 1S5E.A.241, 1S82.A.4, 3SIB.A.223, 3SSB.A.995, 1T3M.A.801, 1T64.A.
392, 1T8U.B.701, 3TDQ.A.117, 4TKX.L.705, 1TQY.B.1091, 3TSH.A.604, 1U7H.B.912, 1U
D2.A.1001, 3UES.B.503, 3UF4.A.601, 3USL.A.752, 1V0H.X.252, 2V4V.A.3052, 2V4B.A.1
566, 3VGL.A.323, 2VNZ.X.9252, 1W15.A.2002, 1W9S.A.1141, 3WAY.A.914, 2WCB.A.101,
2WDQ.A.1590, 4WFX.A.504, 3WG7.N.605, 2WGE.A.1426, 1WKY.A.504, 2WOF.A.1728, 2WOI.
D.1489, 2WQK.A.254, 2WWF.B.214, 2X7J.B.1581, 2X8J.A.1317, 4X9K.A.401, 2XNA.C.121
6, 2XZI.A.502, 1YOP.A.1810, 2Y5F.A.1245, 2YNQ.A.1392, 1YYA.A.1001, 2ZHJ.A.321, 2
ZJ9.B.2, 2ZND.A.196, 1ZOD.A.435, 1ZOR.A.1001, 1ZOR.B.1002, 2ZQ3.A.160, 3ZWF.B.13
65

[1] "Cluster 3"

1F30.B.201, 4FUK.B.402, 4KJG.A.1001, 4MCS.A.814, 2MLS.A.302, 3OOF.A.302, 1P6D.A.
247, 1ZEB.A.901, 3B3C.A.502, 3BON.A.500, 2C1G.A.1465, 3C88.A.450, 3C8A.A.450, 3C
8B.A.450, 2CB8.B.1090, 4CVR.A.1160, 4CVT.A.1160, 4CWM.A.432, 4CXV.A.432, 3DDA.A.
450, 2DEA.A.402, 3DFK.A.300, 4DJ4.B.403, 1EB6.A.178, 4ELC.A.501, 3EZT.B.401, 1FT
7.A.502, 4FW5.D.301, 4FW6.A.301, 4FW7.D.301, 3GBO.A.302, 2GSO.A.1001, 4GSZ.A.402
, 4GTW.A.1010, 4GTW.A.1011, 1GW6.A.1615, 2GYQ.A.407, 4ICQ.A.502, 2IMA.A.500, 2IW
0.A.1255, 3K5X.A.403, 1KH9.B.450, 3KR5.B.1004, 1L0Y.D.704, 4L3T.B.1101, 4LCF.A.3
11, 4LCG.A.301, 4LCH.A.301, 3LE9.A.1, 1LFW.A.1002, 1LOK.A.901, 1LOK.A.902, 4MCP.
A.801, 4MCQ.A.801, 4MCR.A.801, 3MK1.A.901, 4MTU.A.201, 1MZB.A.201, 4NGM.A.818, 4
NGQ.A.817, 4NGT.A.811, 4NY2.A.501, 4O5O.A.301, 4OC1.A.814, 4OME.A.815, 2OUN.A.40
2, 2OUQ.A.402, 3P3E.A.400, 3P3G.A.301, 1P5X.A.246, 2PLI.B.707, 4PPZ.A.602, 3PS1.
A.301, 3PS2.A.301, 3PS3.A.301, 2PTY.A.500, 4Q3J.A.401, 3QIY.A.431, 3QJ0.A.431, 3
QM3.A.355, 1QTW.A.302, 3QW7.A.501, 3QW8.A.501, 1R1I.A.1001, 1R1J.A.1001, 2RH6.A.
2, 1ROR.A.601, 3S2L.A.403, 1SDX.A.677, 1SHN.B.481, 3T3W.A.301, 1TXR.A.501, 3U1Y.
A.400, 3U79.H.110, 3V77.D.301, 3V94.A.702, 2V9L.A.1275, 3VPB.A.503, 4WB7.A.503,
3WT4.A.502, 1XVX.A.315, 1ZED.A.903, 2ZXC.A.647, 4X2T.L.701, 4BDZ.A.1381, 4BEO.A.
1381, 1G9Z.D.901, 4IEM.C.401, 4IRC.A.402, 1MOW.A.373, 1MUH.A.479, 4NCB.A.702, 4N
CB.C.101, 4O3S.A.503, 3OYB.A.397, 3OYE.A.397, 3OYJ.A.397, 3OYL.A.397, 3OYN.A.397
, 1RVC.A.401, 3S3O.A.397, 3S3N.A.397, 3SM4.E.15, 2VBN.E.1026, 1YTU.A.428, 1A49.A
.534, 3A7E.A.215, 4ACF.A.1481, 2AL1.B.438, 3AU9.A.601, 4AZW.A.1453, 3BZN.A.501,
4C5A.A.330, 4C5B.A.1313, 4C5C.A.1313, 3CFX.A.703, 3CVJ.C.243, 4CWB.A.1159, 1DAW.

A.342, 1DAY.A.341, 1DAY.A.342, 4DFX.E.404, 3DG6.A.2001, 4DHP.A.303, 4DWB.A.507, 1EC8.A.498, 1EFK.A.604, 2EGH.A.900, 1ELX.B.452, 4EOM.A.302, 3ETH.A.401, 3ETH.A.4 02, 3ETJ.A.402, 1EYZ.A.401, 1EYZ.A.402, 1F1Z.A.2002, 2FN1.A.504, 2FUV.A.901, 3GN 6.A.321, 4GOK.B.202, 3GQ8.A.692, 4H2H.A.401, 2HGS.A.501, 3HPF.A.401, 3HWO.A.1702 , 3I4K.A.385, 4IAC.A.401, 4IAD.A.401, 1IOW.A.330, 2IO8.A.7001, 4IZG.A.414, 2JOL. A.1688, 4J1O.A.401, 1J7L.A.302, 4J7L.A.402, 4K1O.D.404, 3KAL.A.502, 4KI8.E.602, 1KJ8.A.393, 1KJ8.A.394, 1KJ9.A.393, 1KJI.A.393, 1KJI.A.394, 1KJJ.A.394, 3KRO.D.3 001, 1LP4.A.341, 3LVV.A.696, 3MOO.A.551, 4M3A.A.402, 4MDB.A.402, 3NA5.A.547, 1NF S.A.401, 2NOM.A.402, 1NUW.A.2498, 1NUY.A.2341, 4NZN.A.403, 4O4D.A.402, 4OAV.B.80 3, 4OHF.B.503, 3OLP.A.547, 2ONS.A.702, 4ORK.A.501, 4OVN.A.202, 4OVN.A.204, 1P43. A.438, 3PFR.A.456, 4PFY.A.602, 1POW.B.610, 4PU5.A.501, 2PYW.A.500, 1PYX.A.1003, 4Q4C.A.403, 4QPM.A.1503, 2QVH.A.401, 4QXD.B.301, 3R75.A.700, 4RAD.D.302, 1RC5.A. 761, 3RLH.A.286, 4RUB.B.491, 3T12.A.198, 3T2D.A.411, 1T5T.A.1005, 3T7A.A.601, 3T DW.A.502, 3TMO.A.265, 4TYO.A.501, 3UJR.B.501, 3UJS.A.600, 3UJS.B.600, 1UMG.A.401 , 4UOP.A.1612, 3V4S.B.402, 1VA6.A.523, 3VA8.A.425, 3VAT.A.502, 3VC6.A.501, 2VPQ. A.1450, 2VPQ.A.1451, 1WOH.A.1000, 1W7V.A.1441, 4WB8.A.403, 4WH2.A.403, 4WH3.A.40 3, 4WK2.B.501, 3WNZ.A.503, 3WQQ.A.502, 3WQR.A.502, 2XHO.A.1439, 1Z2O.X.1296, 2Z4 X.B.1206, 3GV7.B.871, 4GZ2.A.402, 2HVI.A.878, 1MOW.A.371, 4OIN.D.2003, 2OTJ.3.80 78, 3V6J.A.403, 2A9F.B.800, 4CS3.A.1464, 4DCK.B.203, 1ECB.D.507, 3EW9.A.501, 4FV Q.A.902, 4FZL.B.302, 4GA3.A.1002, 4GIR.B.401, 4GX4.B.402, 1HJK.B.452, 3TLM.A.100 5, 2XGZ.B.1439, 1Z2P.X.1296, 2Z4W.B.1303, 4C5A.B.330, 4E1E.A.401, 1E4E.A.360, 3F PA.A.901, 4HYP.B.302, 3IG8.A.696, 2IO7.B.5003, 1L3R.E.391, 1MFO.A.1453, 4N57.B.4 02, 1OL5.A.1389, 4OVN.A.203, 4OVN.B.203, 3PUG.A.601, 2PUI.A.401, 3QHR.A.298, 4QX D.A.302, 4R9U.C.302, 3T9E.A.601, 4UAK.A.502, 1YYZ.A.340, 2I3P.B.1, 4K4H.E.604, 3 ALA.E.1764, 4AQ8.D.1238, 6CGT.A.685, 2CLT.B.1301, 2DPK.A.4001, 1DTH.A.903, 3E1I. C.502, 3GE4.B.167, 4GQR.A.502, 1JKU.B.2272, 1JKU.E.5272, 4JWU.A.502, 3K7L.A.701, 4LMF.B.302, 2LP2.A.202, 2PRK.A.281, 1RE3.C.408, 2RGB.A.601, 1RTM.2.3, 1S1E.A.22 5, 1ULV.A.2006, 3VOT.A.504, 1WD9.A.901, 2AGO.A.403, 1AZO.B.283, 2BQR.A.2002, 3CO W.A.301, 4DU3.A.1003, 3EH8.D.303, 2FLD.A.601, 3GIM.A.415, 2I3P.A.154, 2I3Q.B.81, 3LDY.A.145, 3MIS.A.2, 1N3E.D.493, 4NLG.A.401, 3PR4.A.343, 1Q9Y.A.939, 3QEX.A.90 7, 1R7M.A.306, 3A13.E.445, 1A25.A.290, 2A3W.R.336, 4A6S.C.1122, 3A7Q.A.4003, 3A7 Q.A.4004, 3A9Q.G.214, 1AFA.2.2, 3AKA.A.173, 1ANW.A.351, 4AQA.A.1210, 1ATN.D.265, 1AUI.B.502, 1AVA.A.501, 3B1T.A.903, 3B2Z.D.3, 1BAG.A.432, 2BB4.A.260, 3BBY.A.21 5, 1BCJ.2.3, 2BLO.C.1155, 3BOW.A.717, 4BQ3.B.1803, 2BW7.B.2202, 2C1O.A.1773, 4CA G.A.608, 4CAG.A.609, 2CDP.C.1139, 1CFF.A.152, 3CGA.B.102, 3CGT.A.685, 2CHN.A.171 7, 3CK9.A.710, 1CR8.A.45, 4CRR.A.1386, 3DOY.A.93, 2D3L.A.503, 3D94.A.2, 1DAN.L.1 55, 2DBX.B.701, 4DD8.B.1001, 1DE4.C.801, 1DGL.B.301, 2DIE.A.778, 1DJX.A.2, 1DSY. A.501, 2DW2.A.703, 3E1I.C.501, 2E6V.C.10, 1E8A.B.1090, 3E9T.B.3, 4ECG.A.502, 4EJ 7.A.403, 1ESV.S.395, 3ESR.A.213, 1F9O.A.2000, 3FAX.A.4, 4FDI.A.603, 1FHF.A.501, 2FH2.B.2001, 2FHF.A.2405, 3FLP.B.302, 1FZC.B.2, 1FZD.E.1, 4G0D.B.503, 1G5N.A.407 , 4G60.A.301, 4G62.A.301, 1G7Y.F.254, 4G9L.A.304, 1GCY.A.529, 2GDF.B.302, 3GIN.A .2, 3GK2.A.93, 3GN4.B.204, 1GU6.A.1479, 2HOK.A.410, 1H5V.A.305, 1HFZ.A.124, 1HFZ .D.124, 3HGP.A.250, 2HIH.A.603, 2HTV.B.993, 3HTL.X.1, 2HYW.B.514, 1I22.A.198, 2I I1.B.400, 3IJ8.A.497, 4ILW.D.305, 4IMM.B.402, 2IWV.B.1281, 1J1N.A.493, 2J1T.A.11 54, 2J7A.A.1007, 2J7A.B.1007, 2J7A.E.1006, 2JBK.A.803, 4JDZ.B.703, 2JHL.F.1298, 1JXN.B.246, 4JX1.A.504, 2K0F.A.153, 3K5S.A.218, 3K5T.A.803, 4K5W.A.201, 1K7Q.A.4 81, 3K7L.A.702, 3K7L.A.703, 3K7N.A.701, 3K7N.A.702, 3K7N.A.703, 1K9I.F.903, 2KAY .A.186, 2KAY.B.187, 4KDV.A.202, 1KTW.B.8, 4KTR.D.814, 4KTR.G.806, 1KWV.B.604, 3L 2Y.A.302, 1L8S.B.314, 1L9N.B.704, 3LCP.D.158, 1LGN.B.302, 1LN8.A.201, 3LPD.A.341 , 4LP7.A.301, 2LV6.A.203, 1M56.A.1007, 4M5I.A.203, 1M63.F.502, 4MHX.A.601, 4MIV. E.600, 4MVF.A.603, 4MVF.A.604, 4N20.A.706, 4N25.A.707, 1N2K.A.600, 4N2I.A.706, 1 N47.A.252, 4NDD.B.401, 4NHD.B.403, 1NIW.A.1003, 1NKQ.F.560, 2NQA.B.902, 1NTO.A.3 001, 1NUD.A.701, 3NWK.B.238, 1NX2.A.3, 2OA8.C.302, 1OBR.A.404, 4OKH.A.901, 3OM7. B.1, 4OMD.D.605, 2OO4.A.5003, 1OT5.A.601, 3OUU.B.455, 2OX9.A.803, 2OXE.A.600, 2O

ZN.B.401, 2P37.C.239, 1P8J.G.3014, 4P99.A.501, 4P99.B.509, 4P99.D.508, 4PIB.C.20
4, 4PKG.A.403, 2PMY.A.103, 4POS.E.401, 2PPL.A.482, 3PVN.B.5003, 3PVN.B.5004, 2Q1
6.B.200, 2Q17.E.315, 2Q1C.X.294, 3Q3L.A.436, 4Q6P.A.509, 3QQZ.A.326, 2QT6.A.3713
, 2QT7.B.201, 1R1Z.C.410, 1R1Z.C.415, 2R28.A.1003, 3RHT.A.257, 2RHP.A.8, 2RJP.B.
3, 3RRD.A.239, 3RRW.A.271, 3RTT.A.267, 1S2N.A.1291, 2SEC.E.276, 1SL6.A.3, 1SU3.B
.907, 1SVN.A.276, 1TT2.A.502, 4TVU.A.600, 4U2A.A.301, 1U94.A.701, 3UBR.B.476, 1U
H2.A.1002, 3UNX.A.280, 3USU.A.264, 3V03.A.586, 1VFP.A.995, 3VI3.A.2001, 3VI3.A.2
003, 3VI3.A.2004, 2VVE.B.1338, 2VXJ.G.200, 2VY0.A.1298, 1W0P.A.1780, 1WBL.D.303,
4WBQ.B.601, 1WC5.C.2100, 3WCT.B.203, 3WIU.A.1004, 2WL3.A.1290, 1WMY.B.203, 3WN6
.B.501, 1XJL.B.342, 2XVT.C.1137, 1Y3N.A.493, 1Y4D.E.1001, 2YN3.B.6361, 1YO8.A.11
92, 1YO8.A.1195, 1YO8.A.1202, 1Z3U.A.497, 2Z49.A.1002, 2Z57.A.1006, 2Z8Z.A.618,
2Z8S.A.642, 2Z8S.A.646, 2Z8S.A.648, 2Z8S.B.646, 2ZEY.B.152, 2ZJ7.A.619, 2ZN9.A.9
02, 2ZUX.A.638, 2ZUY.A.628, 2ZWP.A.401, 2M3S.A.204, 1N48.A.502, 4QWD.A.703, 1A25
.B.291, 1AMY.A.501, 4AOC.B.1129, 4AYU.A.205, 3B8Z.A.902, 3BVH.B.1, 1CGU.A.685, 1
CGW.A.692, 3DEM.A.3001, 3DR2.A.707, 1DX5.I.1001, 3HR4.B.201, 3HR6.A.1, 4I5L.B.60
2, 4I5L.E.701, 4IT5.A.301, 2J5W.A.3042, 2JBH.A.1228, 2KXV.A.201, 1KXQ.A.4001, 1L
GN.A.301, 3LJT.A.902, 3LND.A.208, 2LRO.A.202, 3ONR.A.72, 2P0R.A.1003, 3SG4.A.453
, 3T3P.A.2004, 3TH2.L.152, 4U6D.A.501, 4U6D.B.502, 1UOV.A.1420, 4WKE.A.502, 4X9Y
.A.502, 1Z32.X.497, 2AGQ.A.4001, 4AQU.A.1154, 4AQU.A.1156, 2ATL.B.1415, 3AVW.A.3
001, 4DTM.A.1002, 4DTU.A.1002, 4DU1.A.1003, 4DU3.A.1002, 4E3S.A.1002, 4ED0.A.503
, 3EH8.A.301, 3FD2.A.375, 4FK0.A.1002, 2FLD.B.602, 1G9Y.A.801, 1G9Y.B.802, 4J2D.
A.1002, 4J2E.A.1002, 4K4G.A.602, 4K4G.A.603, 4K4H.A.602, 4K4I.A.605, 3KHR.B.1416
, 4KHU.A.1002, 4KLD.A.403, 3MQ6.A.1, 3MX9.A.363, 3MXB.A.175, 3NCI.A.905, 3NDK.A.
905, 3NE6.A.904, 3NGI.A.905, 3ODH.B.195, 2ODI.B.702, 2Q10.B.702, 3QEP.A.905, 3QE
R.A.905, 3QEV.A.905, 3QNN.A.903, 3QNO.A.903, 4QWA.A.403, 3RAX.A.416, 3RB4.A.415,
1SOM.A.402, 4UAW.A.402, 4UAW.A.403, 4UB4.A.401, 4UB4.A.402, 2VBJ.A.1154, 2VBJ.A
.1155, 2VBO.A.1154, 2VBO.A.1155, 2VS7.A.1183, 2WTF.A.1511, 1A25.B.290, 3A24.A.12
68, 1A2Q.A.295, 2A3Y.A.601, 4A3X.A.1268, 4A3Z.A.2344, 3A4U.A.286, 4A42.A.1690, 4
A4A.A.1925, 4A5G.A.1309, 1A75.A.109, 1A75.A.110, 1A8A.A.407, 1A8B.A.407, 3A8R.A.
401, 2AAO.A.293, 2AAO.A.295, 2AAO.A.296, 3ACH.A.204, 2AEF.A.602, 4AE2.A.246, 5AE
R.A.200, 1AF0.A.476, 3AIE.A.4001, 1AJP.B.558, 1AK9.A.295, 3AKB.A.171, 3AKB.A.172
, 3AKB.A.175, 3ALU.A.201, 3AMR.A.905, 3AMR.A.907, 3AMR.A.908, 1ANW.B.353, 4AOC.A
.1129, 4AOC.E.1129, 4APX.B.1238, 4APX.B.1240, 4AQ1.A.1923, 4AQ1.A.1924, 4AQ1.C.1
923, 4AQA.A.1208, 4AQA.A.1209, 4AQE.A.1207, 4AQE.A.1209, 4AQJ.A.1097, 4AQO.A.188
1, 4AR1.A.1723, 4ASL.A.1268, 3ATG.A.301, 4ATE.A.1275, 1AVA.A.500, 1AVS.A.93, 1AV
S.A.94, 4AVS.A.207, 1AX0.A.290, 1AXN.A.353, 3AXD.A.3002, 1AYP.A.301, 2AYH.A.417,
4AYU.D.205, 2AZZ.A.125, 3AZY.A.301, 3B00.A.124, 3B0X.A.578, 3B0X.A.579, 3B2Z.A.
2, 3B4N.B.701, 1B8R.A.109, 3B8Z.A.903, 2B96.A.124, 3BC9.A.701, 3BEU.A.249, 1BF2.
A.751, 3BFM.A.235, 1BGP.A.501, 1BH6.A.501, 3BI1.A.1753, 1BIW.B.803, 2BIB.A.1551,
2BIB.A.1552, 1BJF.A.402, 4BJ0.A.1167, 1BLI.A.500, 3BNG.A.508, 2BOI.A.300, 2BOI.
B.600, 2BOJ.A.1116, 1BQB.A.351, 1BQB.A.352, 4BQ2.A.1797, 1BSW.A.900, 1BTU.A.260,
1BU3.A.109, 1BU3.A.110, 2BV4.A.300, 2BV4.B.300, 2BWR.A.500, 3BYK.A.474, 4BY5.A.
1183, 4BY5.A.1184, 4BY5.A.1185, 2C0T.A.1507, 2C10.B.1777, 3C1Q.A.2, 3C1Q.B.1, 2C
26.A.302, 3C3Y.A.238, 2C4X.A.1252, 2C4X.A.1253, 2C4F.L.1143, 1C8T.B.264, 1C9N.A.
277, 4CAG.A.603, 4CAG.A.604, 4CAG.A.610, 1CB8.A.3000, 2CBL.A.352, 4CBU.G.1150, 4
CBU.G.1151, 2CCL.B.1060, 2CCL.B.1061, 2CCM.A.1192, 2CCM.A.1194, 4CCD.A.3669, 2CD
O.A.1139, 2CDP.A.1139, 4CE8.A.998, 4CE8.B.998, 2CFT.A.1297, 4CFQ.A.501, 4CFQ.A.5
02, 4CFY.A.301, 3CHJ.A.338, 3CHK.A.503, 3CIO.K.402, 3CIP.G.201, 4CI7.A.1505, 1CJ
Y.A.950, 3CK7.A.710, 2CKI.A.997, 3CLN.A.152, 2CM5.A.1678, 2CM5.A.1679, 2CM6.A.16
79, 2CM6.A.1680, 2CN3.A.1778, 4COU.A.1270, 1CP9.B.554, 1CPN.A.209, 4CP0.A.1294,
4CP1.A.1294, 4CPV.A.109, 4CPV.A.110, 1CRU.A.501, 4CRQ.A.260, 2CT9.A.301, 2CT9.A.
302, 4CT3.A.1170, 4CTE.B.280, 4CUO.A.1326, 1CVR.A.504, 3CZT.X.93, 4CZN.A.1370, 1
D0L.A.400, 4D0E.A.1532, 1D2J.A.1, 1D2S.A.401, 1D3C.A.687, 4DA2.A.301, 1DB5.A.198
, 2DBX.D.702, 1DBI.A.701, 1DBI.A.703, 1DBI.A.704, 1DBN.A.301, 3DBK.A.303, 2DCK.A

.1001, 4DD8.A.1001, 2DEW.X.900, 3DEM.A.5001, 3DEM.A.8001, 2DIE.A.780, 4DIR.A.101
, 4DIR.A.102, 1DJX.B.2, 1DJY.B.2, 4DKB.A.301, 4DLK.B.401, 3DNZ.A.403, 3DO1.A.401
, 2DPK.A.3001, 1DTL.A.201, 1DTL.A.203, 2DUR.A.1, 4DUQ.A.101, 4DUQ.A.102, 1DVI.A.
271, 1DVI.A.273, 2DW0.A.701, 2DW0.A.702, 2DW0.A.703, 4DZ3.A.202, 4DZ3.B.202, 2E2
6.A.601, 2E26.A.602, 2E26.A.604, 2E26.A.605, 2E3X.A.802, 2E39.A.501, 2E4T.A.701,
1E43.A.502, 1E8A.A.1089, 1E8A.A.1090, 2E8Y.A.741, 2E85.A.1002, 3E9T.B.4, 3EAD.B
.1003, 3ECQ.A.2001, 3ECQ.B.2000, 1EDH.A.302, 1EDM.B.2, 3EDF.A.602, 2EGD.A.301, 2
EGD.A.302, 2EHB.A.1001, 2EHB.A.1004, 2EIG.A.1102, 4ENZ.A.1110, 2ERO.A.702, 3ETO.
A.2001, 3ETO.A.2003, 3EU3.A.1, 2EWE.A.701, 2EWE.A.703, 2EXH.A.2001, 2EXH.B.2002,
1EXR.A.1000, 1EXR.A.1002, 1EXR.A.1004, 3EXM.A.301, 4F0Z.B.201, 4F0Z.B.202, 4F0Z
.B.203, 4F0Z.B.204, 1F6S.A.201, 1FAT.A.255, 3FCS.A.2004, 3FED.A.1755, 2FH1.A.200
1, 2FH1.A.2002, 1FIB.A.500, 3FIA.A.201, 2FI1.A.191, 3FLP.A.301, 3FLP.A.302, 2FMJ
.A.338, 2FMD.A.301, 1FNY.A.500, 3FO3.A.527, 2FPW.A.503, 1FS7.A.651, 2FVY.A.311,
1FX5.A.251, 1FXH.B.1002, 3FZO.A.400, 1FZD.A.1, 4FZM.A.301, 4G01.A.300, 4G1M.A.20
08, 3G5C.A.801, 1G5N.A.401, 1G7Y.A.254, 1G8I.A.1599, 1G8I.B.1600, 1G8K.A.5008, 1
G8K.C.5108, 1G9G.A.630, 1G9K.A.701, 1G9K.A.702, 1G9K.A.704, 1G9K.A.705, 3GBO.A.3
01, 1GCA.A.351, 1GCY.A.528, 3GDC.A.401, 4GER.A.403, 1GGZ.A.149, 1GGZ.A.150, 1GGZ
.A.151, 2GGM.A.401, 4GGF.A.101, 4GGF.C.204, 4GGF.L.204, 2GJP.A.1487, 2GJP.A.1488
, 2GKO.A.610, 1GK9.B.1579, 1GKF.B.1571, 4GM5.A.404, 1GO7.P.1482, 1GO8.P.1482, 1G
PL.A.500, 3GPE.A.501, 3GPE.A.502, 2GSK.A.1, 2GSK.A.2, 1GSL.A.251, 4GUK.A.207, 1G
W2.A.502, 1GWT.A.502, 1GWU.A.1308, 1GX2.A.1310, 2GXS.A.601, 1GZC.A.290, 4H1Q.B.3
04, 1H2G.B.1558, 4H2A.A.805, 4H2B.A.604, 2H2N.B.1001, 1H3G.A.700, 1H3G.B.701, 1H
6X.A.1162, 1H6Y.A.1161, 2H61.D.817, 1H80.A.1493, 3HB2.P.481, 3HB2.P.484, 3HB3.A.
563, 1HDF.A.1102, 3HDB.A.621, 3HDB.A.622, 3HDL.A.306, 2HD9.A.2001, 4HHO.A.401, 4
HHQ.A.401, 3HI7.A.803, 3HJR.A.601, 3HJR.A.602, 1HPL.A.960, 4HPH.A.701, 1HQL.A.30
2, 1HQV.A.998, 2HQ8.A.201, 2HQ8.A.202, 2HQ8.A.203, 3HR4.D.202, 3HR9.A.401, 1HT6.
A.500, 1HT9.A.1001, 1HVX.A.516, 1HVX.A.518, 3HX4.A.601, 3HX4.A.602, 3HX4.A.603,
3HX4.A.604, 3HX6.A.1, 1HY7.A.304, 2HYK.A.477, 2HYV.A.605, 2HYV.A.607, 2HYW.A.503
, 3HZ3.A.1, 4I2Y.A.501, 4I2Y.A.503, 4I35.A.511, 4I35.A.514, 4I35.A.515, 3I57.B.1
86, 4I5L.B.601, 4I5L.E.702, 2I7A.A.2, 4I75.A.401, 1I82.A.192, 4IAI.A.401, 4IAI.A
.402, 4IAU.A.800, 3IBZ.A.193, 4ICB.A.76, 4ICB.A.77, 2ID4.A.901, 2ID4.A.906, 4IEF
.B.702, 3IGO.A.602, 3IGO.A.603, 4IHM.A.406, 2II1.C.400, 3IKW.A.1, 4IK8.A.502, 3I
K2.A.514, 4IMM.A.407, 3IPV.A.252, 2IPL.A.502, 4IRZ.A.2007, 4IT5.D.202, 4ITC.A.12
01, 4ITC.A.1202, 4IU3.B.301, 4IU3.B.302, 2J1V.A.1152, 1J1A.A.201, 2J1A.A.1769, 2
J1G.B.1290, 2J22.A.1150, 1J55.A.102, 2J5Z.A.1277, 2J7A.A.1006, 1J83.A.4001, 1J9L
.A.1301, 4JA8.A.503, 1JC9.A.301, 2JDY.A.1116, 2JD9.A.1146, 4JDZ.B.701, 2JDN.A.88
1, 2JDN.B.881, 2JDN.C.881, 2JE7.A.1241, 2JEC.A.240, 4JGU.A.901, 1JI1.A.2003, 1JI
A.A.134, 1JIW.P.485, 4JJJ.A.704, 1JK3.A.404, 1JKU.A.1272, 2JKA.A.1727, 3JQ1.B.1,
3JQW.A.1001, 2JT6.A.258, 4JWQ.A.202, 1JX9.B.601, 3JXS.A.301, 4JX1.E.505, 3K21.A
.192, 3K21.A.193, 3K21.A.194, 1K7I.A.480, 4K70.B.1003, 1K94.A.998, 1K94.A.999, 1
K96.A.91, 1K9I.I.1201, 1K9J.A.403, 1K9K.A.400, 1K9U.A.1001, 1K9U.A.1002, 4K90.A.
702, 1KAP.P.614, 1KAP.P.615, 1KAP.P.621, 3KCP.A.701, 4KDW.A.201, 3KF9.C.302, 3KH
E.A.192, 3KHE.A.193, 3KHE.A.194, 3KHE.A.195, 4KKK.A.701, 3KLK.A.1, 3KLL.A.1, 3KM
5.A.2012, 3KM5.B.2011, 1KQU.A.301, 3KQR.A.205, 3KQR.C.206, 4KTY.A.801, 4KTY.A.80
3, 1KVO.A.191, 1KVW.A.124, 3KWU.A.901, 3KWU.A.902, 4KWU.A.1109, 2KYF.A.109, 3L2Y
.A.301, 3L2Y.C.302, 4L73.A.403, 1L9N.A.700, 1L9N.A.702, 3LCP.C.159, 4LDC.A.502,
1LE6.A.461, 1LEN.A.184, 1LHW.A.401, 3LI6.A.149, 3LI6.D.150, 4LJ3.A.402, 4LM8.A.8
11, 4LMH.A.811, 3LND.B.208, 3LNF.A.302, 3LNI.A.302, 3LNP.A.472, 1LOC.A.228, 4LOR
.A.301, 4LOS.A.401, 1LPB.B.453, 3LPA.A.361, 3LPC.A.360, 3LPC.A.361, 3LPC.A.362,
4LP7.B.301, 1LQV.C.34, 2LRP.A.202, 2LTN.A.191, 1LVU.A.8001, 1LVU.D.8002, 4LVN.A.
702, 4LXO.A.1601, 4LXO.B.1601, 3M0W.I.202, 4M00.A.801, 4M00.A.803, 4M02.A.701, 3
M1H.A.2001, 3M1H.A.2002, 4M2P.A.301, 3M5Q.A.372, 4M5E.A.505, 4M5E.A.506, 1M63.B.
503, 4M7H.A.501, 1M8T.A.1001, 1MCX.A.349, 1MCX.A.351, 1MCX.A.352, 1MDW.B.4, 4MEW
.A.501, 4MGQ.A.601, 3MHZ.A.736, 1MKU.A.124, 1MKV.A.124, 2ML1.A.205, 2ML2.A.203,

2ML2.A.204, 2ML3.A.201, 2ML3.A.202, 3MMZ.A.501, 4MNO.A.302, 4MNO.A.303, 1MPX.A.6
38, 3MSE.B.180, 4MSP.A.201, 3MVS.A.212, 3MVS.A.213, 3MVS.A.216, 1MVQ.A.238, 1MXE
.A.506, 4N1G.A.202, 4N1G.A.203, 4N1G.A.204, 4N2B.A.707, 4N2B.A.709, 4N2G.A.704,
4N2L.A.704, 4N2L.A.707, 4N2N.A.704, 3N35.A.290, 1N47.B.252, 3N4E.B.500, 4N5X.A.2
01, 4N5X.A.203, 4N5X.A.204, 4N5X.A.205, 3N5A.A.101, 3N5A.A.102, 1N9E.A.803, 4NEH
.A.1101, 4NEH.A.1103, 1NIW.A.1004, 1NIW.C.1007, 1NIW.E.1009, 1NIW.G.1014, 1NLS.A
.240, 1NNL.A.2001, 3NNG.A.402, 3NOL.A.263, 1NPC.A.319, 1NPC.A.321, 3NQX.A.511, 3
NQZ.B.520, 4NUQ.A.303, 2NXP.C.602, 2NXP.D.603, 2NZM.A.405, 2O04.A.402, 3O0V.A.1,
3O0W.A.377, 3O4Y.A.198, 2O5G.A.401, 2O5G.A.402, 2O5G.A.403, 2O5G.A.404, 2O72.A.
401, 2O72.A.402, 2O8O.A.1009, 1O8F.A.1353, 1O8P.A.1149, 2O9Q.A.501, 1OAC.A.803,
1OAH.A.1525, 1OAH.A.1526, 1OBO.A.502, 2OBH.A.1001, 4ODG.A.202, 3OEA.A.200, 1OF3.
A.1174, 1OFL.A.528, 1OH4.A.1186, 1OHZ.B.1057, 1OHZ.B.1058, 2OKX.A.4001, 4OKH.A.9
02, 4OKH.A.904, 4OKH.B.901, 2OLG.A.2001, 3OM2.A.1, 3OM4.A.1, 3OM5.A.1, 3OM6.A.1,
3OM7.A.1, 3OMI.A.613, 4OMC.A.605, 4OMC.A.606, 4OMC.A.607, 2OO4.A.5001, 2OPO.A.3
01, 2OPO.A.302, 1OUX.A.402, 2OVU.A.238, 3OWF.A.151, 2OW9.B.610, 3OX6.B.501, 3OX6
.B.502, 2OX9.A.804, 1OYG.A.500, 4OY7.G.302, 2OZN.B.402, 2P0R.A.1002, 2P2K.A.239,
5P2P.A.125, 3P4G.A.411, 3P4G.A.413, 3P4G.B.405, 4P5X.A.1001, 3P6B.A.205, 1P8J.A
.3001, 4P99.A.514, 4P99.B.517, 4P99.C.505, 1PA2.A.308, 1PAM.A.688, 5PAL.A.110, 5
PAL.A.111, 3PDD.A.193, 3PDD.A.194, 4PEO.X.103, 4PEO.A.102, 2PEL.A.237, 4PET.A.40
3, 4PEU.A.401, 3PF2.A.1, 3PGB.A.902, 3PGV.A.267, 4PHJ.A.302, 4PHJ.A.303, 4PHJ.A.
304, 4PHN.B.303, 4PIB.C.201, 4PKG.G.1201, 4PKI.A.403, 4PLS.B.301, 2PMY.B.104, 3P
M8.A.1, 3PM8.A.514, 3PM8.B.1, 1PMH.X.300, 1PMJ.X.300, 1POA.A.201, 1POB.A.801, 1P
OC.A.501, 1POE.A.801, 3POJ.A.1, 2PPL.A.478, 3PPE.A.402, 3PPE.A.403, 2PQX.A.500,
1PT2.A.500, 1PVA.A.110, 1PVA.A.111, 3PVN.E.5009, 3PVN.M.5026, 2PVB.A.110, 2PVB.A
.111, 2Q17.A.315, 2Q1F.A.2001, 3Q2L.A.701, 3Q2N.A.303, 1Q3A.A.468, 3Q3U.A.340, 3
Q5I.A.1, 3Q5I.A.524, 3Q5I.A.525, 3Q5I.A.526, 1Q5P.A.271, 4Q6O.A.502, 3QC6.X.1, 4
QF4.A.202, 1QGJ.A.2002, 3QGM.A.503, 1QI3.A.451, 1QNW.A.302, 2QNG.A.201, 1QPK.A.4
51, 2QQM.A.1, 2QQO.A.1, 3QR0.A.1000, 3QRB.A.302, 3QRB.A.303, 3QRX.A.171, 3QRX.A.
172, 1QTX.A.152, 1QTX.A.153, 1QTX.A.154, 2QUB.A.617, 2QUB.A.619, 2QUB.A.620, 2QU
B.A.621, 2QUB.G.616, 2QUB.K.614, 2QV6.A.302, 1QX2.A.1001, 1QX2.A.1005, 1QX2.B.10
06, 1R0R.E.302, 1R1Z.A.285, 1R1Z.A.286, 2R2I.A.500, 2R2I.A.501, 2R2I.A.502, 1R55
.A.515, 3R5V.B.201, 1R64.A.701, 2R8Z.N.214, 4R83.B.501, 2R9F.A.366, 2RAN.A.324,
2RDZ.A.1501, 1RFJ.A.1001, 1RFJ.A.1003, 1RFJ.A.1004, 2RF7.A.1501, 2RHP.A.29, 2RJP
.C.3, 1RLW.A.400, 1RLW.A.401, 3RM1.A.102, 1RP8.A.500, 1RRO.A.109, 1RRO.A.110, 1R
U4.A.1, 3RUP.A.1006, 1RWY.A.421, 1RWY.A.422, 1S01.A.295, 1S02.A.276, 1S1E.A.226,
3S18.A.229, 1S3P.A.210, 3S5U.A.221, 1S6B.A.401, 1S6B.B.402, 1S6C.A.217, 1S6C.A.
218, 1SBF.A.601, 1SCH.A.301, 2SCP.A.190, 2SCP.A.191, 2SCP.A.192, 1SGT.A.246, 1SH
7.A.1291, 3SIB.A.221, 3SIB.A.222, 3SJG.A.1753, 1SL4.A.407, 1SL6.D.3, 1SL6.E.3, 1
SL7.A.301, 1SL8.A.669, 1SL8.A.670, 1SL8.A.671, 3SNY.A.97, 1SNC.A.150, 1SNN.B.503
, 3SO1.A.97, 1SRA.A.302, 3SRE.A.1356, 1ST3.A.270, 1SU4.A.995, 1SUD.A.295, 3SXQ.A
.526, 3T3P.B.2002, 1T44.G.702, 1TCF.A.160, 1TCF.A.163, 1TE2.A.702, 1TF4.A.3002,
1THM.A.301, 1THM.A.302, 3TI7.A.353, 3TI7.A.354, 3TI7.A.355, 3TI9.A.353, 3TI9.A.3
54, 3TI9.A.355, 1TKF.A.905, 1TM7.E.470, 1TN4.A.162, 4TNC.A.164, 5TNC.A.163, 3TOY
.A.361, 4TSH.B.1501, 3TTQ.A.2867, 1TU5.A.903, 4TVU.C.600, 3TZ1.A.1, 1U0A.A.5004,
3U1R.A.701, 3U1R.A.702, 4U36.A.302, 3U4X.A.237, 4U65.E.301, 3UBG.A.902, 3UBH.A.
857, 3UCP.A.912, 3UJO.B.304, 1UKG.A.1262, 3UL4.B.66, 3UL4.B.67, 4UM9.A.2001, 4UM
9.A.2003, 1UNE.A.124, 1UP8.A.599, 1UPS.B.501, 3USU.C.266, 2UWP.A.1246, 1UWW.A.11
92, 1UX6.B.2001, 1UX6.B.2005, 1UX6.B.2008, 1UX6.B.2009, 1UX6.B.2011, 1UX7.A.1135
, 3UXF.A.2, 1UXX.X.1130, 1UY4.A.1147, 1UYZ.A.1132, 4UZU.A.1483, 4UZU.A.1485, 1V0
A.A.1177, 2V0B.A.1241, 4V29.B.1178, 4V29.B.1179, 3V6N.A.229, 3V9M.A.205, 1VCL.A.
1001, 1VCL.A.1002, 1VCL.A.1005, 2VDQ.B.2002, 1VL9.A.124, 3VLV.A.503, 2VN5.B.102,
2VN6.B.1067, 2VN7.A.650, 2VNV.A.302, 2VNV.B.302, 2VPT.A.1235, 3VRR.A.401, 2VUC.
B.990, 2VUC.C.991, 2VUD.C.1118, 2VUD.D.1118, 2VVD.A.1328, 2VVE.A.1336, 3VV3.A.40
2, 3VYV.A.303, 2VZP.A.1128, 1W0N.A.1132, 2W08.A.206, 1W3M.E.3013, 2W46.A.1148, 2

W47.A.1137, 3W57.A.201, 3W57.A.203, 1W7C.A.803, 2W87.A.1149, 3WA5.A.504, 2WBX.A.
1103, 1WBF.A.303, 1WC0.A.2100, 1WC5.A.2100, 3WCS.A.1003, 3WCT.F.203, 3WDH.A.801,
2WD6.A.1765, 3WFD.B.806, 4WF7.A.600, 3WH2.A.302, 3WHI.A.401, 3WHT.B.501, 4WJK.B
.502, 1WKY.A.503, 4WK7.A.502, 4WK7.A.503, 3WLC.A.501, 3WLC.A.503, 3WN6.A.503, 2W
N3.A.400, 2WN3.C.400, 2WND.A.102, 2WNO.A.1251, 2WNP.F.1298, 2WOY.A.2415, 1WP6.A.
502, 1WPC.A.501, 2WPH.S.1246, 2WR9.A.1131, 2WR9.D.1132, 3WU2.c.901, 2WW8.A.1001,
2WW8.A.1002, 2WZ8.A.1149, 2WZA.A.2415, 2WZE.B.1552, 1X1J.A.2000, 3X17.A.603, 4X
DQ.A.301, 2XFG.B.1619, 2XFD.A.1112, 1XJO.A.905, 2XOM.A.1169, 1XVJ.A.422, 1Y1X.A.
201, 1Y1X.A.202, 2Y3N.B.1067, 2Y3N.B.1068, 1Y4J.A.1001, 2Y5I.A.101, 1Y6O.A.207,
2Y6L.A.1168, 1Y93.A.267, 1Y9Z.A.603, 2YAY.A.1266, 2YA2.A.1691, 2YFS.A.1712, 2YIH
.A.1520, 2YKK.A.1520, 2YLJ.A.1308, 2YN5.A.6364, 1YO8.A.1186, 1YO8.A.1187, 1YO8.A
.1189, 1YO8.A.1190, 1YO8.A.1198, 1YO8.A.1200, 1YO8.A.1207, 2YOA.A.1001, 2YOA.A.1
002, 1YU6.A.401, 1YUT.A.197, 1YXH.A.1001, 2Z2X.A.1002, 2Z2X.A.1003, 1Z3J.A.267,
2Z30.A.1002, 2Z30.A.1004, 2Z30.A.1005, 2Z48.A.1104, 2Z49.A.1003, 2Z49.A.1004, 2Z
49.A.1005, 1Z70.X.3002, 2Z8X.A.618, 2Z8X.A.619, 2Z8X.A.621, 2Z8X.A.625, 2Z8X.A.6
27, 2Z8Z.A.623, 1ZCM.A.1001, 3ZDV.A.200, 2ZEZ.A.200, 1ZED.A.906, 2ZEX.A.406, 2ZF
D.A.227, 2ZFD.A.228, 2ZFD.A.229, 2ZKM.X.800, 2ZN9.A.901, 2ZP4.A.124, 2ZUX.A.631,
2ZUX.A.632, 2ZUX.A.635, 2ZUX.A.636, 2ZUY.A.622, 2ZUY.A.625, 2ZVD.A.628, 2ZVD.C.
620, 2ZWO.A.402, 2ZWP.A.404, 2ZWP.B.402, 3ZYP.A.1220, 3ZYR.A.401, 2ZYH.A.700, 2Z
ZJ.A.239, 4QQZ.A.1002, 1A8E.A.339, 3AK9.A.168, 3AK9.C.168, 4AM5.A.1161, 4AQ2.E.8
00, 4AQ6.D.837, 1B0L.A.694, 1B3E.A.400, 4BMT.A.1324, 8CAT.A.507, 2CSG.A.504, 1D3
K.A.339, 1DLT.A.400, 1DSN.A.400, 3DU3.M.500, 3DUQ.M.500, 3E1M.A.301, 3E1M.C.300,
3E1N.B.300, 3EMR.A.400, 1EYS.M.607, 3FGS.A.402, 1FQE.A.500, 1FZ6.B.5004, 3GCF.A
.501, 3GJB.A.320, 3GVY.A.162, 2GYQ.B.404, 1H43.A.1315, 1H44.A.1326, 4H99.M.402,
4H9L.M.402, 4HBH.M.402, 3HF8.A.400, 4HR4.A.402, 1IEJ.A.333, 3IS8.B.162, 1J30.A.4
01, 1J30.B.404, 2J8C.M.1307, 4JO0.A.601, 1JQF.A.500, 4KF1.A.402, 4KVR.A.302, 1LK
O.A.600, 4M26.D.401, 3MPS.F.173, 3MPS.G.172, 1N7W.A.339, 4NB8.A.501, 1NF4.A.200,
2O0C.A.500, 2OHJ.D.2501, 3PCH.M.600, 3PWF.A.201, 3PWF.A.202, 3PWF.B.201, 3PWF.B
.202, 3PZA.A.173, 3PZA.B.173, 3Q1G.D.1002, 4QDF.A.402, 3QHB.B.182, 3QJV.A.801, 3
QVD.H.173, 3R0G.A.200, 1R2F.A.401, 4RC8.A.302, 3RI7.A.1, 3RMK.D.495, 1RVJ.M.857,
1RZH.M.857, 1SP8.B.500, 1SQI.A.1450, 1SQY.A.701, 1TH3.D.2003, 1TKP.A.304, 1TKP.
B.303, 1UMX.M.1306, 2UW1.A.1360, 2UW1.B.1359, 1UZR.A.1293, 2V27.A.1268, 3V83.F.7
03, 3VEO.A.602, 1VFE.A.400, 1VRN.M.500, 3VVA.A.501, 1VZ4.D.1301, 2VZB.A.6204, 1W
69.A.1350, 2YAV.A.402, 1YUX.A.302, 4QQZ.G.1002, 3AK9.J.168, 4AQ2.B.800, 4AQ6.F.8
37, 2BJJ.X.694, 1EH3.A.400, 1GVC.A.1253, 3MPS.D.1, 2O84.X.500, 1OQH.A.500, 3PCC.
M.600, 3TOD.A.694, 3V83.A.703, 3E6S.A.200, 2GIG.A.501, 1Q81.C.8345, 4TUP.A.401,
1WTE.A.1001, 1XSP.A.576, 4D1I.D.602, 4DKA.A.201, 4DMI.C.203, 4DNL.A.300, 4DXK.A.
502, 2FBB.A.131, 4G8T.D.502, 2HZL.A.500, 4IBO.A.402, 4IIL.A.402, 4JP4.A.305, 1JZ
3.B.3102, 3KQC.A.303, 4KQ7.B.502, 1LTM.A.400, 4LUG.A.301, 3MMD.A.410, 4MM7.A.602
, 3MNC.A.280, 3NUQ.A.283, 1O68.D.274, 4O7J.A.201, 3OP0.A.2, 3PDG.A.99, 3PFV.A.1,
3Q2G.A.701, 3U0F.A.410, 3UCY.A.100, 3UES.A.503, 3UTO.A.902, 3V7Z.A.404, 2VX5.A.
1421, 3W5D.A.1001, 1W9W.A.901, 3WGV.A.2005, 4X26.A.402, 2XRM.A.401, 2Y8K.A.1528,
2YEQ.A.582, 2ZHI.A.321, 2ZND.A.194, 2B2N.A.345, 4D1I.G.602, 1Y4A.E.1002, 3PNC.A
.2, 3CBT.A.420, 4CVU.A.1998, 3INJ.C.601, 4LLH.B.602, 1MMX.A.349, 1NZA.A.104, 3ZX
0.C.522, 1BPZ.A.341, 3C5G.A.803, 1CZ0.C.606, 2FMP.A.1340, 4FZX.B.101, 4FZX.A.101
, 4FZY.C.101, 4FZY.D.101, 2ISO.A.340, 1JJ2.0.8338, 3JPO.A.340, 3JPQ.A.340, 3JPR.
A.340, 4M9L.A.403, 1SA3.A.401, 1VQ8.0.9117, 3A6V.A.1005, 3A6V.B.1006, 3AR4.A.100
0, 3ATU.A.6267, 1AVT.A.301, 3B8X.B.668, 1BA0.A.490, 4DCC.A.302, 2DDA.B.302, 4DD8
.B.1005, 4DD8.D.1005, 3E3T.A.243, 2E5X.A.303, 3ED4.A.519, 4FMT.B.301, 2GA4.A.713
, 3GQ9.A.1, 2HIO.B.240, 3HLT.A.269, 3HSC.A.490, 3HSC.A.491, 4HUC.A.509, 1HVX.A.5
19, 3IC9.B.491, 3MZG.B.211, 4O52.A.301, 4O57.A.301, 4O5H.A.605, 1QHU.A.438, 2QR7
.A.1000, 2QSV.A.222, 1QUS.A.400, 2QZ7.A.194, 3R0L.D.125, 3SLZ.B.130, 4USW.A.1468
, 4UZU.A.1491, 1V7T.A.406, 2VPB.A.1398, 2WCP.A.501, 4WED.A.601, 3WFA.A.801, 3WNK
.A.813, 3WXO.A.806, 3WZ1.A.402, 1YQ2.C.7503

[1] "Cluster 4"
1A1G.A.201, 1A1I.A.201, 1A6Y.A.550, 1A6Y.A.551, 2A66.A.401, 1A73.A.202, 4AA6.A.253, 3AU0.B.579, 2B3J.A.2001, 2C7A.A.1641, 2C7A.A.1642, 3CBB.A.1001, 4CIS.A.300, 3COQ.A.1002, 1CYQ.A.601, 1CYQ.A.602, 2DRP.A.171, 2DRP.A.172, 1DSZ.A.1121, 1DSZ.A.1122, 1DSZ.B.1222, 3EPH.A.1, 3EQT.A.1, 2ER8.A.104, 2ER8.A.105, 4ESJ.A.301, 1F2I.G.1201, 1F2I.G.1202, 1F4S.P.64, 1F4S.P.65, 1FFY.A.1001, 2FF0.A.1001, 2FF0.A.1002, 1G2D.C.302, 1G2D.C.303, 1G2F.C.301, 1G2F.C.302, 1G2F.C.303, 3G9M.A.526, 2GAT.A.67, 4GAT.A.67, 6GAT.A.67, 4GLX.A.601, 3GOX.A.301, 3GOX.A.302, 4GZN.C.203, 3HAX.C.201, 2HAN.A.351, 2HAN.A.352, 2HAN.B.353, 2HAN.B.354, 4HC9.A.401, 4HC9.A.402, 4HCC.A.504, 2HGH.A.191, 2HGH.A.192, 2HGH.A.193, 4HN5.A.601, 4HN6.A.602, 4HP3.C.201, 4HP3.C.202, 1HWT.D.138, 2I13.A.502, 2I13.A.503, 2I13.A.506, 2I13.B.507, 2I13.B.510, 1I3J.A.100, 4IFD.J.1106, 2IHX.A.235, 2IHX.A.236, 4IQR.A.402, 4IQR.A.403, 2IVH.A.1577, 2JP9.A.131, 2JP9.A.132, 2JP9.A.133, 2JP9.A.134, 2JZW.A.56, 1K3X.A.501, 1K82.A.450, 2KAE.A.175, 1KB2.A.150, 1KB2.A.151, 3KDE.C.78, 2KKF.A.2001, 2KKF.A.2002, 2KMK.A.83, 2KMK.A.84, 2KMK.A.85, 3KMP.A.2, 1LAT.A.1514, 1LAT.A.1515, 4LJ0.A.501, 4LJ0.A.502, 4LJ0.A.503, 1LLM.C.301, 1LLM.C.302, 4LMG.A.201, 1LO1.A.195, 1LO1.A.196, 3LRR.A.1, 2LT7.A.701, 2LT7.A.702, 2LT7.A.703, 3M7K.A.143, 3M7K.A.144, 4M8O.A.1303, 3M9E.A.208, 3M9E.B.209, 4M9E.A.503, 4M9E.A.504, 4M9E.A.505, 4M9V.C.201, 4M9V.C.202, 1MEY.C.88, 1MEY.C.89, 1MEY.C.90, 3MLN.A.501, 4MTD.A.201, 3NCU.A.1, 4NDH.A.402, 2NLL.A.250, 2NLL.B.450, 2NLL.B.451, 4NM6.A.2002, 2NQ9.A.401, 2O6M.A.601, 4O6A.A.601, 3O9X.A.132, 1ODH.A.1171, 3OD8.A.200, 2OFI.A.302, 4OLN.A.101, 4OLN.A.102, 4OND.A.101, 4OND.A.102, 4OOR.A.102, 2OPF.A.501, 3OYM.A.393, 1OZJ.A.145, 1P47.A.203, 3PIH.A.917, 4PZI.A.1101, 4PZI.A.1102, 4QEN.A.802, 4QEN.A.803, 4QEN.A.804, 4QEN.A.805, 3QMD.A.300, 3QMD.A.301, 3QSV.A.1, 1QUM.A.301, 4R2A.A.503, 4R2A.A.504, 4R2A.A.505, 4R2Q.A.503, 4R2S.A.501, 1R4O.A.526, 3S14.A.1735, 1TDZ.A.1001, 1TF3.A.102, 1TF3.A.2, 1TF3.A.3, 4TNT.A.701, 4TNT.A.702, 3TS2.A.1, 3TS2.A.2, 3U6P.A.300, 1UBD.C.501, 1UBD.C.502, 1UBD.C.503, 1UBD.C.504, 3UK3.C.967, 3UK3.C.968, 3VD6.C.501, 2XQC.A.1138, 2XQC.D.1141, 2YKG.A.927, 1YUI.A.64, 1ZAA.C.203, 1ZGW.A.500, 1ZNS.A.1500, 258L.A.500, 2A1K.A.1, 3A1B.A.1, 3A1B.A.2, 3A1B.A.3, 1A1R.A.901, 4A24.A.601, 4A24.A.602, 4A2C.A.1349, 4A2V.A.1000, 3A32.A.708, 1A42.A.262, 3A43.A.701, 4A46.A.65, 2A5V.A.401, 1A5T.A.501, 2A51.A.54, 2A51.A.55, 2A5H.A.421, 2A5H.B.421, 3A6F.C.301, 3A6G.A.301, 3A6J.A.301, 3A6J.D.301, 2A6H.D.7458, 2A6H.D.7412, 1A71.A.401, 1A71.A.402, 1A72.A.376, 1A7I.A.83, 1A7T.A.251, 4A7K.A.950, 2A8D.A.1230, 3A9J.C.1, 3A9K.C.1, 1AAF.A.56, 1AAF.A.57, 2AA4.A.1001, 4AA1.A.1615, 2AB3.A.30, 2AC3.A.531, 2ADR.A.162, 2ADR.A.163, 1ADN.A.93, 4ADN.A.1223, 1AF2.A.296, 2AFU.A.391, 2AFW.A.996, 2AFX.A.996, 2AFZ.A.391, 4AF1.A.500, 2AFM.A.391, 3AII.A.1001, 4AI5.A.200, 4AIA.A.200, 4AIG.A.999, 1AJB.A.451, 4AJX.H.1030, 2AKL.A.117, 3ALR.A.601, 3ALR.A.602, 1AM6.A.262, 2AMT.B.2900, 1ANI.A.451, 1ANJ.B.451, 2AP1.A.304, 2APS.A.400, 2AQP.A.201, 1ARD.A.1, 1ARE.A.1, 1ARF.A.1, 4AR1.A.1722, 4AR9.B.1731, 4ARE.A.1790, 4ARF.A.1722, 3ASK.A.501, 3ASL.A.3, 2AS9.A.666, 2AS9.B.222, 2ASH.A.400, 3AUK.A.390, 2AU3.A.501, 4AU7.A.1248, 4AUQ.B.1299, 4AUQ.B.1300, 3AVR.A.1502, 2AW1.A.262, 4AWY.B.3228, 3AX1.A.601, 1AXG.A.401, 3AXS.A.401, 4AX0.B.3228, 4AX1.B.3228, 4AXD.A.700, 3AY2.A.1001, 2AYD.A.369, 2AYJ.A.57, 4AY8.A.600, 4AYC.A.1484, 4AYC.A.1485, 1AZM.A.261, 2AZH.A.150, 2B0O.E.698, 2B0P.A.400, 4B29.A.1205, 2B3Z.A.1360, 1B4E.A.405, 3B4F.A.262, 3B4N.B.712, 2B44.A.400, 2B5W.A.800, 3B5Q.A.500, 3B6P.A.800, 4B6D.A.1340, 4B6D.A.1341, 3B7R.L.701, 1B8T.A.193, 1B8T.A.195, 1B8Y.A.301, 2B83.C.3353, 2B8T.A.1218, 3B92.A.502, 2B9D.A.1002, 2BA1.A.201, 1BBO.A.60, 1BBO.A.61, 1BCD.A.262, 3BET.A.262, 2BE7.D.1108, 4BF1.A.270, 4BF6.A.1262, 3BHX.A.1752, 4BHW.A.1, 4BHW.A.4, 1BI0.A.291, 3BI0.A.1752, 3BI1.A.1752, 3BJI.A.1, 3BJI.B.2, 3BKN.A.201, 3BKN.A.202, 3BL0.A.262, 3BL1.A.262, 3BL5.A.300, 2BL6.A.1059, 2BL6.A.1060, 4BLB.B.910, 4BM9.A.1466, 4BM9.A.1469, 1BN1.A.262, 1BN3.A.262, 1BN4.A.262, 1BNL.A.179, 1BNM.A.262, 1BNN.A.262, 1BNQ.A.262, 1BNT.A.262, 1BNU.A.262, 1BNV.A.262, 1BNW.A.262, 2BNM.A.1199, 2BNN.A.1200, 3BO5.A.301, 3BO5.A.302, 3BO5.A.303, 3BO5.A.304, 3BOC.A.1001, 3BOF.A.701, 3BOL.A.701, 4BOL.A.1261, 1BP

3.A.500, 2BP0.A.1341, 3BQ5.A.800, 3BQ6.A.800, 1BS4.A.2001, 4BS9.A.1782, 1BTK.A.1
, 3BT0.C.375, 4BT7.A.301, 1BUD.A.800, 4BUE.A.2162, 1BV3.A.262, 2BY0.A.1209, 1BYF
.A.302, 3BYR.A.501, 1BZM.A.261, 2BZ1.A.1174, 4C09.A.351, 3C10.A.102, 2C1I.A.1465
, 4C1D.A.501, 4C1D.A.502, 4C1E.A.501, 4C1E.A.502, 4C1F.A.501, 4C1F.A.502, 4C1G.A
.300, 4C1G.A.301, 4C1G.B.301, 4C1Q.A.493, 1C2G.A.409, 2C20.A.601, 3C2S.A.448, 2C
2F.A.1211, 4C2P.A.701, 1C3I.B.260, 3C37.A.301, 2C36.A.1311, 4C3E.A.201, 4C3T.A.3
00, 4C40.B.600, 4C40.D.500, 3C52.B.401, 3C5K.A.201, 3C5K.A.202, 3C5K.A.203, 4C5W
.A.403, 3C63.A.107, 3C63.B.107, 3C63.C.107, 3C63.D.107, 3C6W.A.2, 2C6A.A.336, 2C
6C.A.1752, 1C7K.A.133, 3C7P.A.262, 2C7N.A.499, 1C8T.A.260, 3C8Z.A.413, 4C81.A.12
40, 4C8E.A.1162, 2C9S.A.1155, 1C9Q.A.999, 3CA2.A.264, 1CAI.A.262, 1CAK.A.262, 1C
AQ.A.301, 4CA1.A.283, 4CA1.A.284, 2CBD.A.262, 4CBY.A.2035, 1CCT.A.262, 4CCG.X.13
74, 4CCG.X.1375, 1CD0.B.376, 1CD0.B.377, 2CDC.A.1372, 4CDG.A.1643, 2CEX.C.1306,
3CE1.A.202, 2CFU.A.1002, 1CG2.A.502, 1CG2.C.502, 3CG7.A.299, 1CGL.A.302, 3CHQ.A.
701, 3CHS.A.701, 3CHV.A.301, 3CHV.A.302, 1CIL.A.262, 1CIM.A.262, 1CIN.A.262, 2CJ
L.A.1217, 2CJS.C.201, 2CJS.C.202, 3CJP.A.301, 2CKL.A.1104, 2CKL.A.1105, 2CKL.B.1
115, 2CKL.B.1116, 2CKI.A.999, 1CL4.A.81, 1CLC.A.653, 2CLT.A.1202, 3CMR.A.450, 1C
NG.A.1, 1CNH.A.1, 1CNI.A.1, 1CNJ.A.1, 3CNG.A.508, 1CNW.A.262, 1CNX.A.262, 1CNY.A
.262, 2C0T.A.201, 2C0T.A.401, 1C04.A.43, 3C0S.A.501, 3C0S.A.502, 2C08.A.201, 2C0
8.A.401, 2C0N.A.201, 2C0R.A.201, 2C0R.A.401, 4C0I.A.652, 4CPD.A.1200, 4CPD.A.130
0, 1CQR.B.2301, 3CQZ.B.3007, 3CQZ.I.3004, 3CQZ.L.3005, 4CQ0.A.1262, 2CQE.A.622,
2CQE.A.822, 2CQF.A.330, 2CRW.A.401, 1CRA.A.262, 2CR8.A.401, 2CRC.A.401, 2CRR.A.4
01, 2CSV.A.200, 2CSV.A.400, 2CSY.A.201, 2CSY.A.401, 2CSZ.A.201, 2CSZ.A.401, 3CSK
.A.712, 3CSQ.A.335, 2CS2.A.200, 2CS3.A.200, 2CS3.A.400, 2CS7.A.201, 2CS8.A.401,
2CS8.A.601, 2CSH.A.200, 2CSH.A.300, 2CSH.A.400, 1CTT.A.296, 1CTU.A.296, 2CT0.A.2
01, 2CT0.A.401, 2CT1.A.201, 2CT1.A.401, 2CT2.A.201, 2CT2.A.401, 2CT5.A.201, 2CT7
.A.201, 2CT7.A.401, 2CTD.A.201, 2CTD.A.401, 2CTT.A.201, 2CTT.A.401, 2CTU.A.201,
2CU8.A.201, 2CU8.A.401, 2CUP.A.201, 2CUP.A.401, 2CUP.A.601, 2CUQ.A.201, 2CUQ.A.4
01, 2CUR.A.201, 2CUR.A.401, 1CVE.A.262, 4CVR.A.1158, 3CX3.A.314, 3CXK.A.201, 3CX
L.A.500, 3CXL.A.501, 1CXV.A.3, 1CXX.A.1, 4CYK.A.42, 1CZM.A.261, 3CZV.A.262, 2D0W
.B.1207, 1D0C.A.900, 1D0Q.B.151, 4D0Y.A.1239, 1D1S.B.376, 1D1T.A.375, 1D1T.A.376
, 1D1T.A.401, 4D1N.A.900, 3D2N.A.101, 3D2N.A.102, 3D2Q.A.303, 3D2Q.A.304, 3D2Z.A
.262, 2D5B.A.501, 4D6S.A.1338, 3D7F.A.1752, 3D7V.A.2, 2D74.A.1001, 2D74.B.1002,
2D8X.A.201, 2D8X.A.401, 2D8Y.A.201, 2D8Y.A.401, 2D8Z.A.201, 2D8Z.A.401, 3D8W.A.2
62, 2D8Q.A.201, 2D8R.A.401, 2D8S.A.201, 2D8S.A.401, 2D8T.A.201, 2D8T.A.401, 2D8U
.A.201, 2D8U.A.401, 2D8V.A.201, 1D9D.A.1, 2D9G.A.201, 2D9H.A.201, 2D9H.A.401, 2D
9K.A.401, 2D9K.A.601, 2D9L.A.401, 2D9M.A.1085, 2D9N.A.256, 2D9N.A.456, 4D9W.A.40
8, 3DAZ.A.262, 2DAN.A.201, 2DAR.A.201, 2DAR.A.401, 2DAS.A.101, 3DBH.B.1, 3DBK.A.
302, 2DB6.A.201, 2DB6.A.401, 4DB3.A.401, 1DCA.A.262, 3DCC.A.262, 1DCQ.A.600, 1DD
6.A.502, 1DD6.A.503, 3DD0.A.262, 3DD8.A.262, 1DDZ.A.1, 1DDZ.A.2, 3DDT.A.46, 1DE6
.A.450, 1DEH.A.376, 1DFE.A.38, 3DFM.A.402, 4DF9.A.503, 2DFV.A.1001, 3DGD.C.128,
2DGE.A.1001, 2DH3.A.601, 3DH1.A.201, 3DHA.A.256, 3DI4.B.286, 2DID.A.201, 2DID.A.
401, 2DIP.A.201, 2DIP.A.401, 2DJ7.A.201, 2DJ7.A.401, 2DJ8.A.201, 2DJ8.A.401, 2DJ
A.A.201, 2DJA.A.401, 2DJB.A.201, 2DJB.A.401, 1DK4.A.290, 2DKT.A.191, 2DKT.A.241,
2DKT.A.291, 2DKT.A.341, 2DKT.A.391, 2DKT.A.441, 2DKD.B.922, 2DLK.A.201, 2DLK.A.
401, 1DL6.A.60, 2DL0.A.201, 2DL0.A.401, 2DLQ.A.200, 2DLQ.A.300, 2DLQ.A.400, 2DLQ
.A.500, 4DLA.A.401, 4DLA.A.402, 1DMT.A.755, 1DMX.A.280, 1DMY.A.280, 3DM0.A.131,
2DMD.A.191, 2DMD.A.241, 2DMD.A.291, 2DMI.A.200, 2DMI.A.300, 2DMJ.A.200, 1D05.A.2
8, 2D00.A.501, 2D00.A.502, 1DPM.A.801, 2DPH.A.1001, 2DQ4.A.502, 2DQ6.A.900, 4DR8
.A.201, 1DSV.A.171, 2DS7.A.100, 2DSN.B.2002, 1DTH.A.901, 1DVB.A.194, 1DVP.A.401,
1DVP.A.402, 3DWD.A.501, 2DW2.A.700, 1DY0.A.401, 4DYG.B.307, 4DZ7.A.301, 1E0E.A.
147, 3E1W.A.230, 3E1Z.A.111, 2E2Z.A.101, 3E24.A.230, 3E2C.A.200, 3E2I.A.200, 3E2
U.E.102, 4E2X.A.501, 1E3I.A.380, 1E3I.A.381, 1E3J.A.901, 1E3J.A.902, 1E3L.A.380,
3E3F.A.230, 3E3I.A.230, 1E46.S.999, 1E4B.S.999, 1E4U.A.79, 1E4U.A.80, 3E4Z.B.2,
2E47.A.172, 2E5R.A.201, 2E5R.A.401, 2E5S.A.201, 2E5S.A.401, 2E6R.A.201, 2E6R.A.

401, 2E6S.A.201, 2E6S.A.401, 2E6S.A.601, 1E67.A.129, 3E6U.A.501, 3E6U.C.502, 3E6U.B.503, 2E6I.A.201, 2E7Y.A.1301, 3E73.A.501, 1E7L.A.1165, 2E72.A.201, 2E73.A.201, 2E73.A.401, 2E9H.A.301, 1E9P.B.153, 1E9Q.B.153, 2EA5.A.201, 2EA5.A.401, 2EA6.A.201, 2EA6.A.401, 2EBL.A.191, 2EBL.A.241, 2EBT.A.100, 2EBT.A.200, 2EBT.A.300, 2EBV.A.201, 3EB5.A.1001, 3EBE.A.500, 3EBI.A.1, 2EBQ.A.201, 2EBR.A.201, 2ECJ.A.201, 2ECJ.A.401, 2ECI.A.201, 2ECI.A.401, 2ECT.A.201, 2ECT.A.401, 2ECV.A.201, 2ECV.A.401, 2ECW.A.201, 2ECW.A.401, 2ECY.A.201, 2ECY.A.401, 2ECG.A.201, 2ECG.A.401, 2ECL.A.201, 2ECL.A.401, 2ECL.A.601, 2ECM.A.201, 2ECM.A.401, 2ECN.A.201, 2ECN.A.401, 1ED9.A.451, 3EDI.A.210, 2EER.A.501, 1EE2.A.1300, 1EE2.A.1301, 1EE8.A.501, 3EED.A.194, 3EER.A.2004, 2EE8.A.301, 2EE8.A.501, 2EE8.A.701, 4EEX.A.402, 4EEX.B.402, 4EEZ.A.401, 4EEZ.A.402, 1EF4.A.56, 3EFO.A.766, 3EFO.B.1034, 3EFT.A.262, 4EFS.A.301, 2EGQ.A.200, 2EGQ.A.300, 2EG4.A.301, 2EG4.B.302, 2EGM.A.200, 2EGM.A.300, 2EGP.A.200, 2EGP.A.400, 4EGU.A.202, 3EH1.A.1269, 3EH2.A.800, 3EHX.A.264, 2EHE.A.200, 2EHE.A.300, 1EI6.A.409, 1EI6.B.408, 2EJ4.A.401, 1EKJ.A.4001, 1EKJ.C.4003, 1EKM.A.701, 2ELN.A.181, 2ELO.A.181, 2ELQ.A.181, 2ELR.A.181, 2ELS.A.181, 2ELT.A.181, 2ELU.A.181, 2ELV.A.181, 2ELW.A.181, 2ELX.A.181, 2ELY.A.200, 2ELZ.A.200, 1ELY.A.451, 2EL4.A.200, 2EL5.A.200, 2EL6.A.200, 2ELI.A.201, 2ELI.A.401, 2ELM.A.181, 2EMJ.A.201, 2EMI.A.201, 2EMX.A.201, 2EMY.A.201, 2EMZ.A.201, 2EM0.A.200, 2EM1.A.201, 2EM2.A.201, 2EM4.A.201, 2EM5.A.201, 2EM6.A.201, 2EM7.A.201, 2EM8.A.201, 2EM9.A.201, 2EMA.A.201, 2EMB.A.201, 2EMC.A.201, 2EME.A.201, 2EMF.A.201, 2EMG.A.201, 2EMH.A.201, 2EMK.A.201, 2EML.A.201, 2EMM.A.201, 2EMP.A.201, 2ENT.A.200, 2ENV.A.200, 2ENV.A.300, 1EN7.A.401, 2ENZ.A.300, 2ENZ.A.400, 2EN1.A.201, 2EN2.A.201, 2EN4.A.201, 2EN6.A.181, 2EN7.A.181, 2EN8.A.181, 2EN9.A.181, 2ENA.A.181, 2ENC.A.181, 2ENE.A.181, 2ENF.A.181, 2ENH.A.181, 2ENN.A.300, 2ENN.A.400, 2EOJ.A.201, 2EOI.A.201, 2EOQ.A.201, 2EOR.A.201, 2EOS.A.201, 2EOU.A.201, 2EOV.A.201, 2EOW.A.201, 2EOX.A.201, 2EOY.A.201, 2EOZ.A.201, 1EOU.A.300, 2EO4.A.201, 2EOD.A.300, 2EOD.A.400, 2EOE.A.201, 2EOF.A.201, 2EOG.A.201, 2EOH.A.201, 2EOK.A.201, 2EOL.A.201, 2EOM.A.201, 2EON.A.201, 2EOO.A.201, 2EOP.A.201, 2EPR.A.201, 2EPS.A.201, 2EPT.A.201, 2EPU.A.201, 2EPV.A.201, 2EPW.A.201, 2EPX.A.201, 2EPY.A.201, 2EPZ.A.201, 2EP0.A.201, 2EP1.A.201, 2EP2.A.201, 2EP3.A.201, 2EP4.A.200, 2EP4.A.300, 2EPA.A.300, 2EPA.A.400, 2EPC.A.201, 3EPZ.A.701, 2EPP.A.201, 2EPQ.A.201, 2EQW.A.201, 3EQN.B.757, 2EQ0.A.201, 2EQ1.A.201, 2EQ2.A.201, 2EQ3.A.201, 2EQ4.A.201, 2EQE.A.201, 2EQF.A.201, 2EQG.A.201, 1ESK.A.55, 1ESP.A.323, 2ESL.A.4, 4ETS.A.302, 1EU3.B.402, 1EU4.A.400, 2EU3.A.262, 4EVB.A.204, 3EWF.A.400, 2EWB.A.489, 2EXU.A.501, 1EXK.A.80, 1EXK.A.81, 4EXS.A.302, 1EYW.A.402, 3EYX.A.1, 4EYL.A.303, 4EYU.A.1702, 1EZM.A.302, 2F0Y.B.501, 3F0D.A.163, 3F0F.A.165, 1F18.A.155, 1F1G.A.4002, 2F14.A.1262, 1F2W.A.262, 1F35.A.306, 2F3B.A.341, 4F3W.A.201, 1F4T.A.369, 3F4X.A.262, 2F4M.A.501, 3F5L.A.1001, 1F62.A.52, 1F62.A.53, 3F7B.B.301, 3F7L.A.203, 3F7U.A.260, 4F7O.B.301, 1F81.A.88, 1F8F.A.372, 1F8F.A.373, 1F9X.A.999, 3F9O.A.309, 2F9I.B.601, 4F9C.B.401, 4F9V.A.401, 1FAQ.A.1, 1FAQ.A.2, 4FAI.A.401, 1FBX.A.3316, 4FBE.A.403, 3FCQ.A.322, 2FC6.A.201, 2FC7.A.201, 2FC7.A.401, 2FE3.A.201, 2FEA.A.1302, 2FEJ.A.1, 3FFP.X.262, 2FGY.A.620, 2FGY.A.621, 4FGL.A.301, 2FHX.A.317, 3FID.A.298, 3FID.A.299, 3FIE.A.428, 2FIF.B.901, 3FJU.A.502, 3FJU.A.507, 1FKW.A.400, 4FKB.A.401, 4FKD.A.102, 4FKE.A.1024, 4FKK.A.1025, 3FL2.A.1001, 3FL2.A.1002, 1FLJ.A.262, 3FLF.A.2004, 3FLO.B.1, 3FLO.B.2, 4FMN.B.901, 4FMN.B.902, 4FMP.A.400, 1FN9.A.1001, 2FNF.X.1, 2FNF.X.2, 2FOQ.A.262, 2FOS.A.262, 2FOU.A.262, 2FOV.A.262, 2FOY.A.301, 4FO9.A.501, 1FP0.A.90, 2FPR.A.502, 3FPC.A.353, 3FPL.A.352, 1FQL.A.262, 1FQM.A.262, 3FQM.A.901, 1FR2.B.301, 1FR7.A.262, 2FR5.A.147, 2FR6.A.147, 4FRC.A.302, 2FSA.A.501, 3FTN.A.354, 3FTN.D.357, 3FTW.A.701, 1FU9.A.37, 3FUN.A.701, 2FU8.A.401, 2FU9.A.401, 4FU5.A.302, 3FV4.A.321, 3FVP.A.321, 3FVZ.A.821, 4FVD.A.201, 4FVL.A.501, 4FVN.A.302, 4FVO.A.302, 4FVY.A.805, 3FW3.A.300, 1FWQ.A.124, 4FWE.A.901, 4FWE.A.902, 4FWU.A.401, 3FXP.A.3000, 2FYG.A.302, 4FYY.B.201, 2FZW.A.375, 2FZW.B.376, 2G0D.A.416, 3G1P.A.300, 1G25.A.66, 1G25.A.67, 3G27.A.97, 4G26.A.1001, 2G2N.A.1001, 4G3M.B.401, 1G47.A.999, 1G48.A.262, 3G42.D.500, 1G4K.A.301, 2G45.A.401, 1G52.A.262, 1G54.A.262, 1G

5C.A.1001, 2G54.A.1100, 2G6Q.A.400, 1G71.A.348, 4G7A.A.301, 2G84.A.506, 2G9T.A.9
99, 2G9Y.B.451, 1G9K.A.600, 3GA3.A.1, 2GA3.A.451, 3GAY.B.328, 2GAG.D.101, 2GAH.D
.101, 3GC9.B.603, 1GDC.A.73, 1GDC.A.74, 2GD8.A.262, 4GER.A.401, 2GFJ.B.401, 2GFE
.A.869, 2GFO.A.1200, 4GGJ.A.301, 1GI4.A.409, 3GI1.A.501, 3GIQ.A.482, 3GIQ.A.483,
4GIZ.C.201, 3GJ3.B.300, 3GJ4.D.300, 3GJ5.B.300, 3GJ7.D.300, 3GJ8.B.300, 1GKR.A.
1452, 2GMN.A.801, 2GMN.A.805, 2GMW.A.300, 4GNE.A.1501, 4GNE.A.1502, 4GNE.A.1503,
4GNE.A.1504, 1GO8.P.1486, 2GQJ.A.200, 2GQJ.A.300, 4GQT.B.502, 3GRB.A.129, 4GR0.
A.301, 4GR3.A.301, 4GR8.A.301, 4GRI.A.501, 2GSN.A.1000, 3GTT.A.155, 1GUP.A.350,
4GU1.A.905, 4GUA.A.1719, 4GUT.A.904, 3GV4.A.203, 1GVY.A.1425, 2GVI.A.301, 4GVE.A
.601, 1GYT.J.600, 1GZH.A.1293, 3GZE.B.2, 3GZK.A.539, 2GZL.A.900, 3H0L.E.902, 3H0
N.A.201, 4H0F.A.401, 1H19.A.701, 2H15.A.262, 4H12.A.1801, 4H12.A.1802, 4H12.A.18
03, 4H1Q.A.302, 1H2B.A.1362, 3H2P.B.154, 4H2K.B.1001, 2H39.A.352, 4H30.A.301, 4H
3S.A.901, 2H4N.A.262, 3H5A.B.360, 3H5N.A.500, 4H57.A.405, 2H6E.A.500, 1H7N.A.134
2, 3H7H.A.119, 1H7V.A.61, 3H8V.A.401, 4H84.A.301, 3H90.A.293, 3H99.A.601, 1H9Q.A
.262, 4H9D.A.201, 3HB2.P.486, 2HB9.A.401, 2HBA.A.101, 1HC7.A.490, 3HCI.A.1000, 1
HCP.A.98, 1HCP.A.99, 3HCJ.A.1000, 3HCS.A.303, 2HCS.A.1, 2HCS.A.2, 4HCG.A.202, 1H
DY.A.376, 2HD6.A.262, 2HDP.A.492, 2HDP.A.493, 4HDH.A.1002, 4HEW.A.301, 4HEY.A.30
1, 3HFF.A.154, 2HF1.A.102, 4HF3.A.301, 3HI2.A.121, 4HI8.B.101, 4HI8.B.102, 2HJN.
A.315, 3HJT.A.1, 2HJH.A.800, 1HK8.A.1589, 3HKN.A.261, 3HKO.A.701, 3HKQ.A.261, 3H
KT.A.261, 3HKU.A.261, 3HLJ.A.272, 2HL4.A.262, 4HMA.A.301, 3HNA.A.501, 3HNA.A.502
, 3HNA.A.503, 3HNA.A.504, 3HNI.A.107, 3HNI.G.107, 3HNI.H.107, 3HNJ.A.107, 3HNJ.B
.107, 3HNJ.C.107, 3HNJ.D.107, 2HNC.A.263, 4HNO.A.301, 2HOC.A.263, 3HPH.A.220, 2H
QH.E.1500, 2HRV.A.143, 3HS4.A.301, 1HSO.A.1376, 1HSZ.A.1376, 2HSI.A.283, 4HSU.A.
904, 1HT0.A.1376, 1HTD.A.401, 4HT0.A.301, 4HT2.A.301, 4HTB.A.401, 1HU8.A.501, 1H
UG.A.261, 3HUG.D.109, 4HU1.A.301, 2HU9.A.132, 4HVL.A.504, 1HXP.A.350, 1HXR.A.200
, 1HY7.A.302, 1HY7.B.801, 1HZ5.B.105, 2HZ8.A.117, 2I0O.A.579, 2I0O.A.581, 3I1U.A
.401, 4I1F.A.503, 4I1F.A.504, 4I1F.A.508, 4I1H.A.507, 3I2D.A.1, 4I28.A.602, 2I2X
.A.524, 2I3H.A.1001, 3I3T.A.700, 3I4C.A.400, 3I4C.A.500, 1I50.A.3006, 1I50.C.300
2, 2I5O.A.336, 2I50.A.336, 2I50.A.337, 2I50.A.338, 4I51.A.3005, 1I6N.A.401, 1I6P
.A.301, 1I73.A.998, 1I73.A.999, 1I76.A.999, 4I7C.A.601, 1I8J.B.400, 1I8Z.A.262,
3I9F.B.3, 2I9W.A.200, 2I9W.A.201, 1IA6.A.1264, 1IA9.A.2001, 1IAG.A.999, 1IAU.A.5
04, 1IB5.A.201, 1IBB.A.201, 1IBI.A.195, 1IBI.A.196, 3IBI.A.262, 3IBL.A.262, 3IBN
.A.262, 3IBU.A.262, 2IBI.A.1, 4IBY.A.301, 4ICR.A.501, 2IDA.A.103, 2IDA.A.104, 3I
EW.B.801, 1IF5.A.262, 1IF9.A.262, 3IFJ.A.201, 3IFJ.B.202, 3IFU.A.181, 3IGP.A.262
, 2IGI.A.1001, 2IGI.A.1004, 4II1.A.901, 3IJF.X.147, 4IJD.A.501, 4IJD.A.502, 4ILO
.A.301, 4ILX.A.301, 2IMZ.A.501, 2IMZ.B.502, 1IML.A.77, 1IML.A.78, 3IMI.A.201, 2I
MR.A.500, 1INN.B.167, 2INN.B.514, 3IO2.A.202, 2IOI.A.3001, 4IOU.D.1001, 1IQ8.A.6
00, 2IQJ.A.301, 4IR0.A.201, 1IRN.A.55, 3IR9.B.501, 3IRB.A.201, 1IS8.B.3109, 3ISI
.X.3000, 1ITU.A.401, 2IT4.A.561, 4ITO.A.301, 4ITP.A.302, 3IUF.A.1, 2IUC.A.1002,
2IUC.B.1007, 4IUM.A.501, 4IUQ.A.301, 4IUW.A.701, 2IWE.A.1129, 3IXE.B.301, 3IXE.B
.302, 4IXJ.A.301, 1IYM.A.182, 1IYM.A.183, 2IYB.E.1422, 2IYB.E.1423, 2J1Y.A.1290,
4J1V.A.301, 1J2O.A.115, 1J2O.A.116, 1J2T.A.302, 2J21.A.1289, 4J3D.B.302, 4J4M.A
.301, 2J6A.A.1138, 2J7U.A.1884, 2J7J.A.1088, 1J98.A.300, 1J9Y.A.1004, 2J9R.A.119
4, 2J9U.B.1162, 1JAO.A.999, 1JAZ.A.401, 2JA1.A.1192, 4JBG.A.401, 2JBG.B.1577, 1J
D0.A.901, 1JD5.A.501, 4JD1.B.202, 4JE6.A.200, 4JEA.A.202, 4JEA.B.202, 4JEA.C.202
, 4JEA.D.202, 4JEB.B.202, 2JHG.A.401, 4JH2.A.201, 4JH2.B.201, 4JIJ.A.302, 4JIU.A
.201, 4JIV.D.101, 2JIG.A.1253, 2JIG.B.1252, 1JJ9.A.999, 1JJD.A.102, 1JJD.A.103,
1JJD.A.104, 1JJE.B.251, 1JJT.B.251, 4JLW.A.401, 4JLX.A.501, 2JLP.A.226, 2JM0.A.2
01, 2JM0.A.401, 2JM1.A.2, 2JM3.A.92, 4JMY.A.201, 2JMD.A.65, 1JN7.A.37, 2JOX.A.10
8, 2JOX.A.109, 2JOX.A.110, 1JOC.A.300, 4JOM.A.1004, 4JPA.A.301, 1JQ5.A.371, 2JQ5
.A.129, 2JR7.A.85, 2JRJ.A.62, 2JRJ.A.63, 2JSD.A.275, 4JSA.A.301, 4JSR.A.401, 4JS
W.A.301, 4JSZ.A.301, 2JTG.A.88, 1JT1.A.400, 1JTK.A.137, 2JTN.A.183, 2JTN.A.184,
2JTN.A.185, 2JTN.A.186, 3JUE.A.999, 2JUN.A.220, 2JUN.A.221, 2JUN.A.222, 2JUN.A.2
23, 1JV0.A.261, 2JVX.A.29, 1JVB.A.400, 1JVB.A.500, 3JV7.A.501, 3JV7.A.502, 3JVH.

A.163, 2JVN.A.400, 2JWO.A.488, 2JWO.A.489, 1JW9.B.250, 3JWP.A.2001, 2JW6.A.601, 3JXP.A.320, 4JXE.A.501, 4JXE.A.502, 1JY8.A.300, 1JZS.A.1101, 2JZ8.A.150, 4JZO.A.802, 1K07.A.1, 2K0A.A.108, 2K0A.A.109, 2K0A.A.110, 2K1P.A.96, 2K16.A.940, 2K16.A.941, 4K1R.A.502, 1K24.A.401, 3K2F.A.262, 1K2Y.X.500, 2K2C.A.138, 2K2C.A.139, 2K2C.A.141, 2K2C.A.142, 2K2C.A.143, 2K2D.A.80, 4K2H.A.201, 4K2H.B.201, 3K34.A.1002, 3K35.C.317, 2K4X.A.56, 3K5K.A.1194, 3K5K.A.1197, 3K5K.B.1195, 3K5K.B.1196, 2K5C.A.96, 4K5N.A.1101, 3K6I.A.202, 3K6J.A.800, 1K7H.A.478, 1K7I.A.486, 3K7H.B.1001, 2K7R.A.129, 4K7D.A.501, 4K7D.A.502, 4K7D.A.503, 4K7D.A.504, 4K7D.A.505, 4K7D.A.506, 4K7D.A.507, 4K7D.A.508, 4K7W.B.101, 1K81.A.144, 1K83.I.3003, 2K8D.A.155, 2K9H.A.101, 2K9H.A.102, 2KAK.A.130, 2KAK.A.150, 2KAK.A.170, 1KBE.A.2, 3KB1.A.302, 3KBF.A.159, 2KDP.A.1, 2KDX.A.120, 3KE1.A.163, 3KED.A.875, 3KEE.A.2000, 1KEV.B.353, 2KEM.A.195, 1KFI.A.700, 2KFN.A.1, 2KGG.A.53, 2KGG.A.54, 2KGO.A.109, 1KH4.A.451, 1KH5.A.451, 1KH7.A.451, 1KHK.A.451, 3KHI.A.301, 2KIK.B.50, 2KIZ.A.70, 2KI7.B.124, 2KJE.A.501, 2KJE.A.502, 1KK1.A.411, 2KKT.A.500, 2KKH.A.201, 2KKR.A.500, 1KLR.A.31, 1KLS.A.31, 3KNE.A.500, 3KNV.A.201, 3KNV.A.202, 2KN9.A.82, 4KNI.A.301, 4KNJ.A.301, 4KNM.A.301, 1KOL.A.1001, 1KOL.A.1002, 1KOQ.A.301, 4KP5.A.301, 2KPI.A.150, 4KP8.A.301, 3KQI.A.71, 3KQI.A.72, 2KQ9.A.113, 2KQB.A.1001, 2KR1.A.65, 1KTO.A.405, 1KU0.A.701, 2KU3.A.63, 2KU3.A.64, 4KUJ.A.301, 4KUV.A.301, 4KUW.A.301, 4KUY.A.301, 2KVF.A.83, 3KV4.A.448, 3KV5.A.489, 3KV5.A.490, 3KVE.B.489, 4KVO.A.301, 1KWG.A.806, 1KWQ.A.262, 3KWO.B.161, 3KWO.C.161, 2KWJ.A.501, 2KWJ.A.601, 2KWJ.A.701, 2KWJ.A.801, 4KX8.A.1001, 4KXB.A.1001, 4KXC.A.1001, 4KXQ.A.601, 3KYC.B.641, 2KZY.A.63, 2KZM.A.1, 2L0Z.A.486, 3L00.A.180, 1L0Y.B.706, 3L0V.A.1, 2L0B.A.143, 2L0B.A.161, 4L05.A.203, 3L11.A.601, 3L11.A.602, 3L14.A.262, 1L1O.F.2, 3L22.A.1, 4L3J.A.402, 2L5U.A.62, 2L5U.A.63, 4L56.A.401, 4L58.A.102, 4L5O.A.303, 2L6Y.A.239, 2L6Z.B.37, 1L6J.A.500, 2L6L.A.201, 2L6M.A.201, 4L6H.A.801, 4L6O.A.801, 4L6T.A.301, 2L7X.A.106, 2L7X.A.107, 1L7O.B.301, 2L75.A.155, 2L75.A.156, 2L7P.A.201, 4L7X.A.101, 3L8H.A.901, 2L80.A.124, 2L8E.A.829, 2L9Z.A.403, 3L9Y.A.155, 3LAS.A.167, 3LAT.A.215, 2LAU.A.82, 4LAO.A.401, 1LBU.A.214, 2LBM.A.1, 2LBM.A.3, 4LBU.A.402, 3LCZ.A.54, 2LCE.A.200, 2LCE.A.300, 2LCQ.A.162, 1LD3.A.500, 1LDE.A.375, 1LDY.A.375, 3LE9.B.2, 3LEA.A.485, 4LEF.A.302, 4LEV.A.601, 2LFD.A.400, 2LGV.A.109, 2LGV.A.110, 2LGV.A.111, 1LG5.A.262, 2LGG.A.380, 2LGG.A.381, 2LGG.A.382, 4LGJ.A.301, 2LHN.A.501, 2LHN.A.502, 2LHN.A.503, 4LHI.A.301, 1LI5.B.964, 1LIQ.A.28, 2LI8.A.187, 2LI8.A.188, 2LI9.A.18, 4LIM.A.401, 2LJX.A.200, 2LJZ.A.201, 3LJU.X.401, 4LJO.A.1101, 4LJO.A.1102, 4LJO.A.1104, 4LJP.A.1101, 4LJP.A.1103, 4LJQ.B.1105, 4LJQ.A.1101, 3LKM.A.904, 2LKO.A.32, 2LK5.A.37, 4LK9.A.401, 1LLU.B.343, 3LMI.B.1002, 4LMY.A.202, 2LN0.A.401, 2LN0.A.501, 2LN0.A.601, 2LN0.A.701, 2LO2.A.101, 2LO3.A.101, 2LO4.A.300, 4LO9.A.401, 4LOE.C.401, 4LOF.A.401, 3LPE.B.60, 1LPV.A.53, 1LPV.A.54, 4LP6.A.310, 3LQB.A.201, 3LQH.A.1001, 3LQH.A.1002, 4LQG.A.802, 3LRQ.C.100, 4LR2.A.505, 2LRI.C.101, 2LRI.C.102, 3LS1.A.1, 3LS9.A.457, 3LSC.A.458, 3LT8.A.80, 2LUY.A.300, 2LUY.A.301, 3LUB.A.302, 2LUA.A.101, 2LUA.A.102, 2LUA.A.103, 4LU3.A.301, 2LUL.A.201, 2LVU.A.101, 1LV3.A.66, 2LV2.A.101, 2LV2.A.102, 2LV9.A.201, 2LV9.A.202, 2LVR.A.101, 2LVT.A.101, 2LWW.A.501, 2LWW.A.502, 2LWW.A.503, 4LW9.A.203, 4LW9.C.202, 4LW9.D.201, 4LW9.K.203, 3LXE.A.261, 2LXD.A.201, 2LXD.A.202, 2LXH.C.900, 4LXL.A.403, 3LYR.A.1, 4LY4.D.301, 2LZU.A.201, 2LZU.A.202, 3M04.A.501, 3M0A.D.401, 3M0N.A.201, 2M0D.A.101, 2M0E.A.101, 2M0F.A.101, 4M0W.A.401, 2M1S.A.100, 3M14.A.501, 3M15.A.107, 3M15.B.107, 3M1D.A.1000, 2M13.A.601, 2M13.A.602, 3M1W.A.500, 1M2K.A.999, 1M2O.A.800, 3M2N.A.263, 3M2X.A.500, 3M2Y.A.500, 4M2R.A.301, 4M2V.A.301, 2M3Z.A.101, 2M3Z.A.102, 1M3V.A.123, 1M3V.A.124, 3M3X.A.262, 2M3H.A.102, 2M3L.A.201, 4M3O.A.300, 4M3P.A.701, 3M40.A.262, 3M4C.B.108, 1M4M.A.502, 2M48.A.501, 2M48.A.502, 2M48.A.503, 2M48.A.504, 3M5E.A.262, 3M5S.A.500, 1M65.A.300, 1M6H.A.1376, 3M67.A.263, 3M6I.A.402, 1M6W.A.1376, 2M6M.A.201, 2M6M.A.202, 2M7Q.A.101, 2M7Q.A.102, 3M79.D.107, 2M7A.A.100, 3M8T.A.300, 3M8T.A.301, 2M85.A.801, 2M85.A.802, 2M9Y.A.401, 2M9Y.A.402, 3M96.A.262, 3M98.A.262, 1M9O.A.78, 2M9A.A.101, 2M9A.A.103, 2M9A.A.102, 3MA2.A.295, 2MA5.A.101, 2MA5.A.102, 2MA6.A.101, 2MA6.A.102, 3MBG.A.3,

3MBG.A.1, 3MBM.A.163, 1MBX.B.211, 4MB7.A.301, 1MC5.A.376, 3MDW.A.455, 3MDZ.A.263
, 2MD7.B.101, 2MD7.B.102, 2MDG.A.101, 2MDG.A.102, 4MDM.A.301, 1MEA.A.29, 3MEK.A.
500, 3MEK.A.501, 3MEK.A.502, 3MEN.D.400, 3MEQ.A.401, 3MEQ.A.501, 4ME3.A.301, 3MF
1.A.1000, 1MGO.A.375, 1MGO.A.376, 1MGO.B.375, 4MG3.A.201, 3MHC.A.262, 3MHH.E.97,
 3MHI.A.262, 3MHL.A.262, 3MHM.A.262, 3MHS.A.472, 3MHS.A.473, 3MHS.A.474, 3MHS.A.
475, 3MHS.A.476, 3MHS.A.477, 3MHS.E.97, 4MHN.A.400, 4MHQ.A.501, 4MHY.A.400, 3MI9
.C.87, 3MI9.C.88, 4MI5.A.802, 4MI5.A.803, 4MI5.A.804, 4MI5.A.805, 4MI5.A.806, 4M
I5.A.807, 2MIU.A.301, 2MIU.A.302, 2MIU.A.303, 3MJH.B.70, 4MJ7.A.201, 3MKG.A.155,
 3MKV.B.426, 2MKD.A.301, 3ML2.A.262, 1ML9.A.1, 1ML9.A.2, 1ML9.A.3, 3ML5.A.263, 4
MLT.A.301, 1MM3.A.62, 1MM3.A.63, 3MMF.A.262, 1MMR.A.1, 2MNY.A.401, 2MNY.A.402, 3
MNA.A.262, 3MND.A.153, 1MNC.A.282, 3MNU.A.262, 1MOO.A.262, 4MO8.A.301, 1MP0.A.37
6, 3MPZ.A.300, 3MP2.A.1, 1MQ0.B.147, 1MR1.C.601, 2MRE.B.301, 3MS3.A.401, 1MS7.B.
3001, 4MSG.A.1401, 4MSX.A.501, 3MTW.A.2, 4MT2.A.67, 4MT2.A.68, 4MTW.E.401, 4MTY.
A.301, 2MUM.A.301, 2MUM.A.302, 2MUQ.A.101, 1MVH.A.501, 3MWM.A.140, 3MWM.A.141, 3
MWM.A.142, 2MWX.A.201, 4MWP.E.401, 1MXG.A.437, 4MXJ.E.401, 3MYQ.A.262, 1MZ8.B.60
0, 1MZB.A.202, 4MZN.E.401, 3MZC.A.262, 4N0G.A.401, 4N0L.A.401, 4N0N.A.501, 4N0N.
A.503, 1N0Z.A.46, 4N0X.B.301, 3N2C.E.425, 3N2P.A.262, 3N3J.A.262, 3N3K.A.1, 4N4E
.E.401, 4N4F.A.1401, 4N4F.A.1402, 3N4B.A.262, 1N5N.A.401, 4N5P.E.405, 4N66.E.501
, 3N67.A.900, 1N8K.A.375, 1N8K.A.376, 1N92.A.375, 3NA7.A.300, 3NB5.A.261, 1NCS.A
.61, 1ND1.A.400, 1NEE.A.136, 3NGJ.A.250, 3NHE.A.1, 3NIS.A.1, 3NIS.A.2, 3NIS.A.3,
 3NIT.A.2, 3NI5.A.262, 1NJ1.A.513, 1NJG.A.401, 4NJ5.A.801, 4NJ5.A.803, 3NKQ.A.10
01, 1NKU.A.188, 3NKM.A.1001, 4NL4.H.802, 4NL4.H.803, 2NMX.A.301, 2NNV.A.262, 3NN
Q.A.201, 2NN1.A.301, 2NN7.A.301, 4NN2.A.401, 4NN2.A.402, 4NN2.A.403, 2NNG.A.262,
 2NNO.A.262, 1NO5.B.571, 4NOS.A.3000, 3NQ5.A.503, 3NQY.B.520, 3NQZ.B.1, 4NQ4.A.3
02, 4NQ5.A.301, 4NQ5.A.302, 4NQ6.A.301, 4NQ6.A.302, 4NQ7.A.301, 4NQ7.A.302, 4NQY
.A.501, 4NS5.A.401, 1NTO.A.500, 4NTM.A.201, 4NTN.A.201, 2NUP.B.1100, 1NUI.A.501,
 2NYT.A.2000, 3NY1.A.4, 3NY1.A.5, 3NY1.A.6, 3NY3.A.1, 3NY3.A.2, 3NY3.A.3, 1NZJ.A
.700, 4NZG.A.201, 300M.A.151, 2003.A.201, 2003.A.202, 2010.A.86, 2010.A.87, 2013
.A.190, 2013.A.191, 401K.A.301, 3036.A.1, 3036.A.2, 203K.A.401, 204Z.A.262, 304N
.A.2002, 204H.B.401, 204Q.A.2402, 2053.B.314, 4062.A.1001, 4064.A.2001, 4064.A.2
002, 4064.A.2003, 4064.A.2004, 406I.A.601, 3070.A.500, 3070.A.501, 307A.A.500, 3
07A.A.501, 307U.A.428, 10AL.A.152, 40AQ.A.403, 40BI.A.201, 30CA.A.300, 30CQ.A.18
4, 40CM.E.401, 20DX.A.156, 20D1.A.902, 40DR.A.202, 40DR.B.202, 20FK.A.201, 20GW.
A.500, 40GE.A.1201, 20HX.A.401, 10HL.A.400, 20H3.A.300, 30IL.A.262, 20I0.A.2, 20
IK.B.201, 40IF.A.701, 40IW.F.501, 10J7.B.1389, 30J3.J.902, 30J6.A.150, 30J7.A.11
4, 10KL.A.262, 10KM.A.262, 10KN.A.262, 20M1.B.801, 10NW.A.801, 300I.A.232, 300I.
A.233, 300I.A.234, 2004.A.6001, 10Q5.A.600, 30Q6.A.375, 10QJ.A.183, 20RW.A.401,
 30RU.A.250, 10S0.A.600, 20SM.A.262, 20SF.A.262, 40TE.A.304, 20U2.A.490, 20UI.A.3
61, 20VX.A.444, 20VZ.A.444, 10VX.A.61, 20W9.B.606, 20WA.A.201, 40WF.G.401, 10X7.
A.402, 20XZ.A.264, 10XN.A.1001, 30XF.A.440, 20X8.B.3, 30Y0.A.262, 10YW.A.801, 30
YQ.A.262, 30YS.A.262, 10ZB.I.50, 20ZU.A.800, 1P0F.A.2502, 2P09.A.200, 1P1R.A.375
, 1P1V.A.201, 3P1V.A.427, 3P2A.C.151, 3P3H.A.261, 3P3J.A.261, 1P42.A.501, 1P42.A
.502, 1P42.A.503, 3P44.A.261, 1P4Q.B.301, 1P5D.X.500, 3P55.A.261, 3P58.A.262, 3P
5A.A.262, 3P5L.A.262, 2P53.A.401, 2P57.A.201, 1P6B.A.406, 1P6O.A.401, 1P7A.A.38,
 3P8B.A.101, 1P9R.A.601, 4P9C.A.201, 4P9C.B.201, 1PAA.A.160, 1PB0.A.1301, 1PB0.A
.1303, 3PB4.X.400, 3PB6.X.400, 3PB7.X.400, 3PB8.X.400, 3PB9.X.400, 3PBB.A.391, 3
PBE.A.391, 3PBJ.D.31, 1PCX.A.950, 1PE8.A.317, 1PEG.A.4, 1PFT.A.51, 1PG5.B.500, 1
PGU.A.616, 2PG3.A.300, 4PHT.B.601, 3PII.A.340, 3PJN.A.187, 3PJN.A.188, 1PL8.A.40
2, 3PLW.A.187, 3PLW.A.188, 2PLI.B.709, 2PLI.D.702, 3PN3.A.1001, 3PN3.A.1002, 3PN
3.A.1009, 3PN3.A.1010, 3PN3.B.1011, 3PNU.A.336, 2POU.A.262, 2POW.A.262, 3PO6.A.1
, 2POI.A.100, 2PPT.A.300, 1PQ4.A.1002, 4PQ7.A.301, 2PQ8.A.501, 4PQT.A.501, 2PRS.
A.501, 1PS7.A.331, 3PSQ.B.321, 3PT9.A.1, 3PT9.A.2, 3PTM.A.1001, 1PTR.A.1, 4PTB.A
.901, 4PTB.A.902, 2PUY.A.355, 2PUY.A.356, 3PU7.A.155, 3PUQ.A.2, 3PUR.A.3, 1PV9.A
.402, 2PVX.A.901, 2PVE.A.301, 1PXE.A.64, 3PYK.A.262, 4PYX.A.301, 3PZC.A.1000, 3P

ZC.B.1000, 1PZW.A.100, 4PZH.A.302, 1Q08.A.401, 1Q08.A.402, 1Q08.B.403, 1Q08.B.404, 3Q01.A.1, 1Q0E.A.153, 4Q09.A.301, 4Q0L.A.301, 1Q1A.A.701, 3Q1D.A.201, 3Q1D.A.202, 2Q1B.A.400, 2Q1Q.A.262, 1Q2L.A.963, 2Q2L.A.1001, 1Q3A.A.465, 1Q3K.A.301, 3Q43.A.1, 3Q44.A.1, 1Q5W.A.32, 1Q68.A.201, 1Q69.A.207, 3Q6V.A.2, 2Q6E.A.501, 4Q6D.A.301, 4Q6E.A.301, 3Q7C.A.1, 4Q7R.A.301, 4Q7R.A.302, 4Q7R.A.303, 4Q7R.A.306, 4Q7R.B.302, 3Q87.A.126, 3Q94.A.301, 1QBH.A.364, 4QBG.B.301, 2QDT.A.401, 2QDT.A.402, 4QEF.A.301, 3QE3.A.356, 1QF2.A.320, 1QF8.A.216, 4QF2.A.1801, 4QF2.A.1802, 4QF3.A.2001, 4QF3.A.2002, 3QGV.A.500, 3QH5.A.321, 3QHD.A.163, 2QIC.A.300, 2QIC.A.400, 2QIN.A.2002, 2QJS.A.2002, 3QJ5.A.376, 1QJI.A.1201, 2QKD.A.501, 3QL9.A.1, 3QL9.A.3, 2QL0.A.53, 2QL1.A.1, 3QNA.A.122, 2QN0.A.431, 4QN1.A.1501, 2QO8.A.262, 2QOA.A.262, 2QP6.A.262, 4QP5.A.401, 2QQ4.A.139, 4QQ4.A.2001, 2QSW.A.201, 4QSI.A.301, 4QSJ.A.301, 1QTW.A.301, 1QUA.A.999, 3QU1.A.501, 3QU1.A.503, 3QU1.A.505, 3QU1.B.502, 1QV6.A.375, 1QV7.A.375, 3QVY.A.500, 3QVZ.D.500, 3QW0.C.500, 1QWY.A.400, 3QWP.A.500, 3QWP.A.501, 1QYB.A.401, 1QYP.A.58, 3QYK.A.262, 3R0D.A.428, 1R1H.A.1001, 3R16.A.262, 3R17.B.262, 4R1X.B.501, 1R22.A.502, 3R2N.A.135, 4R2Y.A.101, 4R2Y.A.102, 4R2Y.A.103, 4R2Y.B.102, 1R37.A.500, 1R3N.B.501, 3R3L.A.585, 2R3A.A.300, 2R3A.A.301, 2R3A.A.302, 2R3A.A.303, 1R4V.A.202, 1R5T.A.150, 2R59.A.701, 3R6F.A.132, 1R79.A.201, 1R79.A.401, 1R9P.A.135, 4R9G.A.505, 1RAY.A.262, 3RBU.A.1752, 3RC6.A.1, 3RCM.A.288, 3RF4.B.119, 4RF1.A.1901, 2RGV.A.146, 1RGO.A.221, 1RGO.A.222, 2RHQ.A.1, 3RHG.A.368, 2RHK.C.502, 2RI7.A.501, 2RI7.A.502, 3RIY.A.1001, 1RJ6.A.601, 3RJ7.A.300, 1RJQ.A.601, 1RJW.A.401, 1RJW.A.402, 2RJP.B.1, 4RL0.A.301, 1RM8.A.501, 1RMD.A.117, 1RMD.A.118, 1RMD.A.119, 1RMD.A.120, 3RMQ.A.114, 4RM5.D.300, 2RMN.A.1, 1RNI.A.256, 4RN4.A.301, 2RO1.A.201, 2RO1.A.301, 2ROW.A.601, 2ROW.A.602, 2RPR.A.201, 2RPC.A.201, 2RPC.A.401, 2RPC.A.601, 2RPC.A.801, 2RPP.A.201, 2RPP.A.401, 3RQD.A.390, 4RQT.A.401, 4RQT.A.402, 4RQU.B.402, 2RR4.A.501, 3RSM.A.500, 3RSN.A.200, 2RSD.A.901, 2RSH.A.101, 2RSI.A.101, 2RSI.A.102, 2RSI.A.103, 2RSJ.A.101, 2RSJ.A.102, 2RT9.A.701, 2RT9.A.702, 3RUI.A.1, 1RUR.L.601, 1RUT.X.603, 1RUT.X.604, 4RUW.A.501, 4RV9.A.501, 1RXR.A.213, 1RXR.A.214, 3RYM.A.106, 3RYV.B.262, 3RYX.B.262, 3RYY.A.1, 3RYZ.A.1, 3RYJ.B.262, 3RZV.A.1, 3RZV.A.2, 3RZ0.B.262, 3RZ1.B.262, 3RZ5.A.1, 3RZ7.A.1, 3RZ8.A.1, 1S1G.A.152, 3S2Q.B.501, 3S2E.A.500, 3S2E.A.501, 3S2E.G.500, 3S2F.E.500, 1S3G.A.219, 1S4B.P.1, 1S4I.A.802, 3S71.B.262, 3S72.B.262, 3S73.B.262, 3S75.B.262, 3S76.A.1, 3S77.B.262, 3S8P.A.400, 3S9T.A.262, 3SAX.A.262, 3SAP.A.262, 3SBH.A.262, 3SBI.A.262, 3SD9.A.2, 1SE0.A.201, 3SEY.C.373, 3SFH.A.403, 3SI2.A.601, 3SJG.A.1752, 1SLM.A.257, 1SML.A.271, 3SOU.A.7, 3SOU.B.8, 3SP1.A.481, 3SP4.A.601, 1SRD.B.156, 1SRK.A.36, 1SRP.A.920, 2SRT.A.257, 3SSB.A.999, 1SU3.A.913, 3SU6.A.5, 3SUB.A.161, 1SVM.A.700, 3SV6.A.4, 1SW1.A.401, 3SWR.A.3, 1SX1.A.23, 3SXK.B.3, 3SZY.A.501, 3T01.A.502, 3T02.A.501, 1T3A.A.422, 1T3K.A.201, 3T33.A.411, 1T4W.A.201, 3T5U.A.262, 3T5Z.A.262, 3T6P.A.1001, 3T6P.A.1002, 3T6P.A.1003, 3T6R.A.1, 3T6R.A.3, 3T73.A.410, 3T74.A.410, 3T7E.A.252, 3T7L.A.1, 3T7L.A.2, 3T8G.A.411, 3T8F.A.411, 1T8H.A.275, 3T80.D.301, 3T82.A.261, 3T84.A.261, 3T85.A.261, 3T87.A.326, 3T8C.A.326, 3T8D.A.325, 3T8H.A.326, 3T90.A.502, 1T9H.A.411, 1T9R.A.1, 3T92.A.122, 3T92.A.124, 1TAF.A.2003, 1TBN.A.1, 1TBN.A.2, 3TBG.A.601, 1TEQ.X.262, 3TEN.A.205, 1TFI.A.51, 3TGN.A.147, 3TGN.A.148, 3TG0.B.505, 3TG4.A.435, 3TG4.A.436, 3TG4.A.437, 1THJ.A.214, 1THJ.B.214, 1THL.A.324, 3TIO.A.1, 3TIO.B.185, 3TIO.D.185, 3TIO.E.185, 1TJL.A.200, 1TKF.A.901, 1TKF.A.902, 1TKH.A.901, 2TMN.E.321, 6TMN.E.322, 3TMJ.A.262, 1TOT.A.53, 1TOT.A.54, 1TT5.B.1014, 3TTY.A.676, 1TTM.A.262, 3TTC.A.1, 3TTC.A.2, 1TWF.J.3001, 4TWJ.A.301, 3TWO.A.349, 4TYT.A.302, 4TZC.A.501, 4TZU.A.503, 1U05.A.500, 1U0A.A.5005, 1U0L.A.298, 1U10.A.400, 1U10.A.601, 4U10.A.401, 1U1H.A.766, 3U1L.A.241, 1U2N.A.441, 1U3L.A.701, 1U3T.A.375, 1U3T.A.376, 1U3U.A.375, 1U3U.A.376, 1U3V.A.376, 1U3W.A.375, 3U31.A.276, 1U40.A.160, 1U4G.A.9800, 4U4L.A.301, 4U4L.A.302, 1U5K.A.300, 1U5S.B.138, 1U5S.B.139, 3U52.A.514, 3U5N.A.1, 3U5N.A.2, 3U7L.A.350, 1U85.A.34, 1U86.A.36, 3U9G.A.226, 3U9G.A.227, 3U9G.A.228, 3U9G.A.229, 4UA4.A.301, 4UA4.A.302, 4UA4.B.303, 1UAQ.A.200, 3UCJ.A.228, 3UCK.A.228, 3UCM.A.228, 3UCN.A.228, 3UCO.A.228, 1UD9.B.508, 1UD9.C.509, 3UDZ.B.800

, 3UEH.A.143, 3UEY.A.4, 3UEE.A.143, 3UEI.A.143, 3UEJ.A.301, 3UEJ.A.302, 4UF0.A.2
269, 3UFF.A.1, 3UGD.A.2, 3UIK.A.341, 3UJZ.A.1, 3UKO.A.400, 3UKO.A.401, 1UL4.A.13
9, 3UNG.C.903, 3UNT.A.400, 3UN6.A.325, 4UOV.A.298, 4UP0.A.1383, 4UP0.A.1384, 1US
N.A.257, 2USN.A.257, 4UTV.A.1299, 1UUF.A.401, 1UUF.A.402, 3UVC.A.301, 3UVC.B.303
, 3UVI.A.387, 2UVL.A.1336, 1UW0.A.1118, 3UW2.A.474, 3UW4.A.401, 3UWA.A.200, 1UX1
.A.1132, 3UX8.A.1001, 2UX1.K.1173, 2UYV.B.1276, 1UZF.A.701, 2UZG.A.131, 2UZG.A.1
32, 2UZG.A.133, 1V0D.A.401, 2V08.A.1090, 2V0C.A.1815, 2V0C.A.1816, 4V0R.A.1001,
2V1X.A.1595, 1V13.B.200, 3V1C.A.101, 3V1E.A.102, 3V1F.A.704, 3V1F.B.703, 4V1T.A.
776, 2V29.A.1276, 2V2A.A.1275, 4V2W.A.502, 4V2Y.A.150, 3V3G.B.301, 1V47.A.601, 3
V4K.A.2, 1V4P.A.1001, 1V51.A.602, 1V54.F.99, 1V5N.A.201, 1V5N.A.401, 1V5R.A.201,
 3V5G.A.262, 1V6G.A.201, 1V6G.A.401, 3V7M.A.509, 1V7Z.A.301, 3V7X.A.2001, 2V8G.C
.500, 1V87.A.201, 1V87.A.401, 2V9I.B.1275, 1V9E.B.260, 3V96.B.302, 1V9X.A.200, 2
V9E.A.1276, 2V9E.B.1276, 2V9K.A.1533, 1VA1.A.100, 1VA2.A.100, 1VA3.A.100, 3VBD.A
.2001, 1VDD.D.233, 3VDP.A.201, 2VES.A.1297, 2VES.C.1302, 1VFY.A.300, 1VFY.A.301,
 2VF7.A.1845, 2VF7.B.1844, 1VGN.A.301, 1VGN.A.302, 3VGL.A.322, 1VHH.A.400, 3VHS.
A.51, 3VHS.B.51, 3VHT.B.401, 1VJ0.A.400, 1VJE.A.167, 2VJE.B.1491, 2VJE.B.1492, 2
VKR.A.106, 1VK9.A.143, 2VL6.A.1266, 2VM5.A.1245, 2VNF.A.1247, 3VOV.A.401, 3VOW.A
.201, 2VO9.B.501, 3VPB.E.100, 3VPE.A.301, 2VP7.A.1399, 2VPD.A.1399, 2VPG.A.1400,
 2VPG.A.1401, 1VQ0.A.300, 2VQM.A.1412, 1VQ2.A.701, 1VQ2.A.702, 3VQZ.A.301, 2VQG.
C.1091, 2VRS.A.1328, 2VRW.B.1565, 2VRW.B.1566, 3VRK.A.301, 2VR6.A.1156, 2VRD.A.1
062, 1VSH.A.281, 1VSR.A.201, 3VTH.A.801, 3VTH.A.802, 3VUW.E.801, 2VUT.I.1713, 2V
VB.X.1268, 2VXI.B.201, 2VXX.A.201, 2VXX.B.202, 1VYK.A.1150, 1VYX.A.1061, 1VYX.A.
1062, 1VZY.A.1291, 2W0T.A.125, 3W0F.A.301, 2W0D.A.1269, 2W3N.B.1234, 2W3Q.A.1231
, 1W4R.A.400, 2W4L.A.1170, 2W5V.B.1377, 2W5X.A.1378, 2W5Z.A.4970, 1W5O.A.1339, 3
W5K.B.502, 3W5K.B.503, 3W5K.B.504, 2W57.B.201, 4W6Z.A.401, 4W6Z.A.402, 1W8P.B.10
30, 1WAA.A.1090, 1WAA.B.1090, 1WAA.D.1090, 1WAA.E.1089, 4WAI.A.101, 4WAJ.A.301,
2WBT.A.1130, 2WBT.A.1131, 3WBH.B.505, 2WB0.X.601, 2WB0.X.602, 2WCB.A.100, 2WCB.B
.100, 2WD2.A.1262, 2WD3.A.1263, 4WD6.A.302, 4WD8.B.303, 4WD8.C.303, 2WEJ.A.1262,
 1WE9.A.201, 1WE9.A.401, 1WEE.A.201, 1WEE.A.401, 1WEM.A.201, 1WEM.A.401, 1WEN.A.
201, 1WEN.A.401, 1WEO.A.201, 1WEO.A.401, 1WEP.A.201, 1WEP.A.401, 1WEQ.A.201, 1WE
Q.A.401, 1WES.A.201, 1WES.A.401, 1WEV.A.201, 1WEV.A.401, 1WEW.A.201, 1WEW.A.401,
 2WEH.A.1262, 2WEO.A.1262, 1WFE.A.201, 1WFE.A.401, 1WFF.A.201, 1WFF.A.401, 1WFH.
A.201, 1WFH.A.401, 1WFK.A.200, 1WFL.A.201, 1WFL.A.401, 3WF8.A.401, 1WFP.A.401, 1
WFZ.A.201, 1WG2.A.200, 1WG2.A.400, 2WGX.A.1300, 1WGE.A.201, 4WH6.A.1203, 2WHG.A.
1263, 1WIG.A.201, 1WIG.A.401, 1WII.A.201, 1WIL.A.201, 1WIL.A.401, 1WIM.A.201, 1W
IM.A.401, 3WID.A.1001, 1WIR.A.201, 1WJ2.A.470, 2WJV.A.1, 2WJV.A.3, 1WJA.A.56, 1W
JB.A.56, 1WJP.A.301, 1WJP.A.501, 1WJP.A.701, 1WJV.A.201, 1WJV.A.401, 1WKQ.B.202,
 4WK7.A.501, 4WKE.A.501, 3WL3.A.301, 3WLE.A.402, 3WLF.A.402, 1WN5.A.1001, 1WNU.A
.1001, 3WNQ.A.501, 1WO3.A.26, 1WO4.A.26, 2WO8.C.1268, 2WOJ.A.1353, 4WOK.A.401, 3
WRG.A.702, 3WS6.C.201, 1WUQ.A.1001, 1WUR.A.1001, 2WVJ.A.1193, 1WWD.A.57, 3WWL.A.
102, 1WWR.D.204, 2WWO.A.1162, 3WXC.A.302, 1WY2.B.407, 1WYH.A.201, 1WYH.A.401, 1W
YS.A.201, 1WYS.A.401, 1X0T.A.150, 3X17.A.601, 4X2Z.A.400, 1X31.D.1006, 1X3C.A.20
1, 1X3H.A.201, 1X3H.A.401, 1X3Z.A.999, 2X3B.A.1341, 2X4H.B.1141, 2X4H.B.1142, 1X
4I.A.201, 1X4I.A.401, 1X4J.A.201, 1X4J.A.401, 1X4K.A.201, 1X4K.A.401, 1X4L.A.201
, 1X4L.A.401, 1X4S.A.201, 1X4S.A.401, 1X4U.A.201, 1X4U.A.401, 1X4V.A.201, 1X4V.A
.401, 1X4W.A.201, 1X4W.A.401, 1X5W.A.201, 2X5R.A.1126, 1X61.A.201, 1X61.A.401, 1
X62.A.201, 1X62.A.401, 1X63.A.201, 1X63.A.401, 1X64.A.201, 1X64.A.401, 1X6E.A.40
1, 1X6F.A.201, 1X6H.A.401, 1X6M.A.200, 2X7S.A.1265, 2X7T.A.1263, 2X7U.A.1261, 2X
7M.A.1174, 2X7M.A.1175, 2X8Y.A.1616, 2X8Z.A.1616, 2X95.A.1615, 2X96.A.1617, 1XAF
.A.501, 2XAA.C.1346, 1XB0.A.403, 1XB8.A.1001, 2XB4.A.1224, 2XBL.A.1197, 2XBL.C.1
196, 1XC3.A.302, 1XCR.A.1001, 2XCM.E.1222, 2XCM.E.1223, 2XEU.A.1065, 1XEG.A.262,
 1XEM.A.401, 1XER.A.106, 1XF7.A.30, 4XIW.A.401, 4XIX.B.401, 2XIG.A.1151, 1XJH.A.
63, 1XKI.A.1003, 1XLL.A.399, 2XML.A.1349, 1XOX.A.999, 2XOC.A.993, 2XOC.A.994, 2X
OC.A.995, 2XOC.B.991, 2XOC.B.992, 1XPA.A.220, 1XPG.A.1887, 1XPZ.A.262, 1XQ0.A.26

2, 2XQV.A.401, 1XRT.A.1423, 1XRU.A.501, 1XSO.A.152, 2XS4.A.998, 1XTG.A.426, 1XTM.B.501, 1XUC.A.1261, 1XUF.A.246, 1XUJ.A.246, 1XWH.A.356, 1XWY.A.401, 2XXH.B.1303, 1XYD.A.94, 1XYD.B.94, 1Y02.A.161, 1Y02.A.162, 1Y0J.A.244, 2Y1N.A.1436, 2Y1N.A.1437, 1Y23.A.1001, 2Y20.C.1331, 2Y28.B.1181, 1Y3G.E.321, 2Y4Y.A.1172, 2Y4Y.C.1172, 2Y43.A.1097, 2Y43.A.1098, 2Y6C.A.1267, 2Y6D.A.1267, 1Y7W.B.285, 1Y8F.A.702, 1Y8J.A.800, 1Y8Q.B.642, 1Y93.A.265, 1YB0.B.160, 2YB5.A.1213, 1YC2.A.402, 1YC2.C.406, 1YC5.A.1001, 1YE3.A.375, 1YEJ.L.605, 2YHO.A.1001, 2YHO.A.1002, 1YHC.A.601, 2YHW.A.1719, 2YHY.A.2000, 2YIK.A.1533, 1YLK.A.401, 1YM3.A.301, 1YO7.A.201, 1YOP.A.84, 1YOU.A.301, 2YPU.A.1998, 1YQD.A.1000, 1YQD.A.2000, 2YQL.A.201, 2YQL.A.401, 2YQM.A.201, 2YQM.A.401, 2YQP.A.201, 2YQQ.A.201, 2YQQ.A.401, 2YRJ.A.200, 2YRT.A.201, 2YRT.A.401, 2YRC.A.200, 2YRD.A.200, 2YRE.A.401, 2YRE.A.501, 2YRE.A.601, 2YRE.A.701, 2YRG.A.201, 2YRG.A.401, 2YRH.A.200, 2YRK.A.201, 2YRM.A.201, 2YSJ.A.201, 2YSJ.A.401, 2YSV.A.201, 1YSB.A.501, 2YS2.A.201, 2YSA.A.181, 2YSL.A.401, 2YSM.A.301, 2YSM.A.501, 2YSM.A.701, 2YSM.A.901, 2YSO.A.181, 2YSP.A.181, 2YTH.A.201, 2YTG.A.201, 2YTP.A.181, 2YTQ.A.201, 2YTR.A.201, 2YTS.A.201, 2YTT.A.181, 2YT5.A.201, 2YT5.A.401, 2YT9.A.201, 2YT9.A.203, 2YTA.A.201, 2YTB.A.301, 2YTD.A.201, 2YTE.A.201, 2YTF.A.201, 2YTI.A.201, 2YTJ.A.201, 2YTK.A.201, 2YTM.A.181, 2YTN.A.201, 2YTO.A.201, 2YUU.A.201, 2YUU.A.401, 2YU4.A.201, 2YU8.A.201, 2YUC.A.201, 2YUC.A.401, 2YVR.A.1001, 2YVR.A.1002, 2YWW.A.504, 2YXO.A.501, 2YYR.A.401, 2YYR.A.402, 2YZ3.A.301, 2YZ5.B.1501, 1Z05.A.406, 2Z2S.B.204, 2Z2Y.A.2001, 2Z2Y.D.2004, 2Z26.A.400, 2Z3H.A.2001, 2Z3G.A.2001, 1Z3A.A.301, 2Z3I.A.2001, 2Z3J.A.2001, 2Z45.A.1001, 2Z45.B.1004, 1Z5H.A.2001, 1Z60.A.2, 1Z6R.A.501, 1Z6U.A.1, 1Z6U.A.2, 1Z83.B.642, 1Z84.A.603, 1Z84.A.604, 1Z8R.A.151, 2Z9J.A.902, 1Z9G.E.1005, 1Z9N.A.201, 1Z9Y.A.300, 2Z94.A.901, 2Z9K.A.901, 2Z9L.A.701, 2ZC0.B.408, 1ZDP.E.1005, 2ZEP.A.391, 1ZE8.A.263, 2ZED.A.391, 2ZEE.A.391, 2ZEF.A.391, 2ZEG.A.391, 2ZEH.A.391, 2ZEL.A.391, 2ZEM.A.391, 2ZEN.A.391, 2ZEO.A.391, 1ZFD.A.71, 1ZFK.A.1300, 3ZFJ.A.1159, 3ZFK.A.401, 1ZFO.A.31, 1ZFQ.A.600, 1ZGE.A.1000, 1ZGF.A.400, 3ZGO.A.400, 1ZH1.A.199, 1ZIN.A.219, 1ZLH.A.555, 3ZME.A.313, 1ZNB.A.1, 3ZNC.A.1, 1ZNF.A.27, 3ZNF.A.31, 3ZNI.A.1428, 3ZNI.A.1429, 1ZNM.A.29, 5ZNF.A.31, 7ZNF.A.31, 2ZNC.A.1, 2ZNE.B.993, 2ZNR.A.1, 2ZNR.A.2, 1ZP5.A.999, 3ZP9.A.1009, 3ZPC.B.401, 3ZPG.A.1358, 3ZQ6.A.1323, 1ZR9.A.117, 1ZRP.A.54, 1ZS0.A.999, 1ZSB.A.262, 1ZSC.A.262, 2ZTX.A.501, 3ZTG.A.1336, 3ZTG.A.1337, 1ZTQ.A.550, 1ZU1.A.129, 1ZU1.A.130, 1ZUD.1.501, 3ZUK.A.1664, 2ZU2.A.5517, 1ZVX.A.999, 3ZVS.A.1159, 3ZVS.A.1160, 1ZW8.A.66, 1ZW8.A.67, 3ZXH.A.301, 1ZXV.B.9002, 1ZXZ.A.198, 2ZXG.A.900, 1ZY7.A.801, 3ZYQ.A.1222, 3ZYQ.A.1223, 1ZZU.A.900, 2ZZE.A.753, 2ZZF.A.754, 2FLC.A.248, 4FLW.A.802, 1YJW.7.8044, 2A19.B.1642, 3ARA.A.166, 3C15.A.29, 1CLK.A.780, 2D32.B.2524, 2E8A.A.501, 1G8G.A.521, 2H5N.B.1001, 4HJH.A.552, 4HN2.A.404, 1IV4.D.1564, 2J4E.A.1002, 3KGX.A.503, 3LD0.J.54, 3M42.A.1, 4NNN.N.202, 2NOG.A.9220, 2O4G.B.800, 4O6I.B.602, 4OKE.B.203, 4OKK.A.204, 3OPK.C.401, 4QDG.A.402, 1T9Z.A.273, 3TAV.A.269, 1WOH.A.1001, 1XLB.A.399, 2XRI.A.1337, 2YFD.B.1145, 1YQ2.E.7005, 1YYZ.A.341, 1BRW.A.3001, 4R5O.A.509, 1SBH.A.291, 4AM5.A.1162, 1AOR.A.606, 1B2O.B.55, 2B5H.A.501, 1B71.A.192, 1BE7.A.55, 1BFY.A.55, 1BIQ.A.377, 2BKB.C.1193, 2BOY.E.1255, 1BOU.B.501, 4C4U.I.201, 3CF4.A.808, 1CJX.B.629, 2CKF.C.501, 3D19.F.302, 3D3L.A.801, 3DBY.A.306, 2DE6.B.501, 3E2T.A.1, 1E3D.A.269, 4EB5.D.201, 4F1E.P.201, 4FWI.B.401, 1FZO.A.5002, 2GBX.E.455, 3GC1.A.605, 3GKE.A.501, 3GL0.A.501, 3GL2.A.501, 2GPC.B.195, 1GUP.C.351, 2GYQ.A.402, 2HMK.A.451, 4HSL.A.202, 3I01.B.800, 1I4Z.E.605, 4ILT.A.301, 1JI5.A.152, 1JI5.B.151, 1JIG.A.402, 1JIG.B.401, 2JI3.A.1127, 1JQK.B.801, 1JRO.G.3001, 4K9F.A.101, 4KUO.D.101, 4KWL.A.301, 4KX6.N.301, 1LNB.E.900, 1N1Q.A.516, 1N1Q.B.515, 3N9Y.D.151, 3NA0.C.150, 3NA0.D.150, 4NBA.A.501, 4NBF.A.501, 4NBG.A.501, 1NF6.A.200, 1NO3.A.858, 2OHJ.A.502, 2OHJ.A.511, 2PCD.M.600, 2PUZ.A.500, 2Q0J.B.997, 3Q36.A.458, 4QDD.A.401, 1QGH.H.157, 4QLW.B.201, 3QVD.G.173, 1R2F.A.400, 1R9X.A.501, 4REU.B.202, 1SHR.B.801, 1T9O.A.255, 3U9M.A.202, 1ULI.A.600, 3V7P.A.429, 1VCX.A.54, 3VMH.C.501, 2W3S.E.1464, 2WLB.B.619, 1WQL.A.502, 2XSO.G.901, 2YFI.A.901, 2YFI.G.900, 1YK5.A.54, 1YUX.A.303, 2ZZI.A.208, 4FZY.A.201, 4FZY.B.201, 2AA0.A.2003, 2EHS.A.201

, 3FFZ.B.1302, 4FHA.A.402, 4FLK.A.504, 4FO2.E.101, 1G8I.B.1595, 1GW1.A.1423, 4IQ
Z.E.305, 1MAU.A.499, 4NT8.A.206, 2OCC.A.519, 1ODZ.A.1427, 4OVZ.B.903, 1W7Z.A.103
2, 2WHM.A.1425, 2X2E.A.1747
[1] "Cluster 5"
4LMG.B.202, 3P57.P.122, 1V15.A.1132, 3VD0.A.401, 3A1Z.C.226, 2A97.B.2437, 1AJD.A
.450, 1ALH.B.450, 1ANI.A.450, 2ANH.A.452, 2ANU.B.405, 2ANU.B.505, 2APO.B.501, 1B
ON.A.1002, 3BOZ.A.22, 1BTG.B.902, 4C98.A.1266, 3CE9.B.400, 2CEA.B.1606, 2CIH.A.2
13, 4DLF.A.404, 1DSQ.A.144, 2E1W.A.400, 2E84.A.558, 2EC7.A.50, 4EGE.A.412, 1ENQ.
C.238, 3EYV.L.220, 1F30.H.201, 1F30.I.201, 2F4L.A.1400, 1F5F.A.252, 3FGG.A.161,
3GJN.B.600, 3GZE.B.14, 3H66.B.500, 3H90.A.291, 3H90.D.5, 3HDB.A.620, 2HD1.B.101,
 4HDT.A.400, 4IOZ.A.504, 3ID7.A.402, 4IGM.A.401, 4IGM.F.401, 3ISO.B.220, 2IUC.B.
1006, 1JDI.A.301, 1JM7.B.143, 1JOE.A.205, 2K2G.A.2, 4KEQ.A.301, 1KH5.B.950, 1KHN
.A.452, 2KO8.C.54, 1KQ0.A.479, 3KVE.C.489, 2L1U.A.144, 3L8Y.A.301, 1L9Y.A.2, 3LZ
E.A.201, 3M7P.A.953, 2MQ1.A.101, 1MVH.A.503, 1N4P.H.378, 3NQ5.A.508, 4NRZ.B.301,
 1NYR.A.1002, 2O3Z.A.501, 2O36.A.690, 2OGJ.A.418, 1OLP.A.1373, 1OLP.D.1374, 1OS2
.B.369, 2OXW.A.264, 1P4Q.B.302, 1P91.B.2301, 2PJT.D.302, 3PJN.A.186, 3PJN.B.186,
 1Q74.D.304, 2QFP.A.434, 2QQ4.I.139, 1QX1.A.2004, 3R2J.A.301, 3RAM.D.998, 3SFW.A
.502, 3SOW.A.7, 3SPU.D.1004, 3T02.A.502, 1TF9.A.901, 3TVX.A.900, 3U24.A.594, 1UD
V.A.101, 4UNI.C.1697, 1UR6.B.79, 2V8V.B.1455, 3V94.E.702, 2VZ5.A.1131, 2W88.C.10
7, 2W9M.A.1565, 2WC0.B.3012, 4X2T.D.702, 2X3C.A.1342, 2X4H.A.1140, 2XR1.A.1638,
1YEW.G.662, 1Z3J.A.264, 1ZKN.C.601, 1ZKX.A.423, 3ZNR.B.101, 3ZNS.A.101, 3ZTV.A.1
599, 1ZZM.A.403, 2BPF.A.339, 3KK2.A.601, 1TTT.B.407, 2CN8.A.1504, 4NU1.A.404, 3R
BM.C.1002, 4DQP.A.903, 3E54.A.702, 3ICE.B.1001, 2NVQ.A.2001, 4P0P.A.601, 2PYJ.A.
9004, 3S14.A.2001, 1V14.C.1134, 2W9C.A.1344, 2W9C.B.1342, 3ZC0.B.197, 2AEK.A.804
, 2AKZ.A.440, 2AQX.A.600, 2AQX.A.601, 3AQC.D.327, 4AVQ.C.902, 2BM1.A.1690, 1BPM.
A.488, 1BR2.A.997, 3BU5.A.301, 3CNX.C.170, 3CR3.B.1213, 3CRL.B.2001, 2CW6.A.401,
 4CW7.C.1002, 4CYI.B.1000, 4CYU.A.171, 1D1C.A.998, 2D33.C.2525, 3D7M.A.356, 2DEJ
.A.402, 2DH4.A.341, 3DLS.C.11, 1DOA.A.199, 4DPG.F.604, 4DPM.D.401, 1E1Q.A.601, 1
E1Q.F.601, 2E6B.A.301, 2E92.A.1302, 2E92.B.1304, 2E95.A.1301, 3EPS.A.1606, 3EQL.
N.1528, 4F38.A.202, 1F4H.D.3001, 3FA4.A.401, 2FDR.A.1001, 3FR8.B.1, 3FYY.B.402,
3G2F.B.901, 2G5H.B.501, 2G74.A.185, 2G83.B.358, 3G9D.B.299, 1G9X.B.1301, 2GHT.B.
257, 2GHQ.B.257, 2GWC.A.1, 1H8H.F.601, 3HBO.A.701, 1HBN.A.1558, 3HQP.M.502, 2HT6
.B.444, 4HV6.B.201, 2I19.B.4004, 4I1O.A.201, 4I1O.C.201, 2I5R.B.302, 2ICJ.A.400,
 3IG8.A.697, 3IJQ.A.386, 2IK2.A.290, 4IL6.C.505, 4IL6.c.506, 2IO7.B.5004, 2IOA.A
.5001, 1IV2.F.1574, 4J99.D.803, 1JFG.B.703, 2JK1.A.1144, 4K6T.E.412, 3KA9.A.189,
 1KK8.B.998, 3KRF.A.904, 1LOO.A.602, 3LCB.B.579, 4LRT.A.403, 1LVH.A.801, 3M1Y.D.
300, 4M1W.A.201, 4MFE.D.1105, 4MKS.A.502, 3MQT.A.1243, 3MWC.A.400, 1N5K.B.413, 3
N9V.A.61, 1NEL.A.438, 3NIZ.A.312, 1NV3.A.2341, 2O1U.A.302, 2O1X.B.2002, 2O56.F.2
006, 3O7L.B.352, 3OAC.D.3002, 4OAU.C.803, 3OHM.A.402, 2OI5.B.5000, 4OVN.B.202, 4
P0V.A.404, 1P7L.A.388, 4P9D.C.202, 4PFQ.C.400, 1PKG.A.1481, 2PUN.B.401, 1PYX.A.1
002, 2Q58.A.4, 1Q8Y.A.809, 2Q80.A.401, 4QJB.A.301, 4QLQ.V.301, 3QU2.C.225, 3QU9.
A.227, 4QVY.K.302, 4QXD.B.302, 4QXD.B.304, 4R3A.A.400, 2R5T.A.600, 3RBM.B.1002,
1RC5.D.764, 2RIO.A.1102, 1RLT.C.807, 4S17.D.501, 1S4E.B.1600, 3SRD.C.603, 3SRF.D
.533, 1SVW.B.301, 3SY8.A.401, 3SZ5.A.220, 4TQ3.A.402, 3TW6.D.2002, 4UON.B.401, 3
U87.A.402, 3U87.B.403, 3UGJ.A.2006, 4UM8.B.2001, 2UXR.B.1398, 2V0N.B.502, 2VHQ.A
.1328, 3VI4.B.502, 2VKQ.A.1288, 3VTI.C.401, 3VYT.C.601, 1W9L.A.1748, 3W9T.B.511,
 1WBQ.C.1441, 3WEG.A.402, 3WEJ.A.402, 3WGU.C.2003, 3WKA.A.601, 1WQ1.R.168, 1WQA.
A.456, 3WU2.C.511, 1WUU.C.394, 1XMJ.A.2, 1YXI.A.342, 1YXO.A.5001, 1Z5B.B.2003, 1
ZCW.A.501, 2ZCQ.A.453, 2ZEV.A.1302, 2ZEV.B.1304, 1ZH4.A.201, 1ZOT.A.901, 2ZRW.D.
702, 3ZXT.A.1278, 1ZYK.A.702, 4EFJ.A.402, 1BCZ.A.410, 2C10.C.1773, 3DFO.A.720, 2
FH3.B.2001, 3IJE.A.4004, 3IKQ.A.403, 2K0E.A.153, 4KZW.B.400, 1LT9.B.1, 2MOK.A.20
2, 2MOK.A.203, 1MPR.A.428, 1NX1.A.4, 2OX9.B.803, 1QDO.B.243, 1QLK.B.93, 2RHP.A.2
7, 1SDD.A.2184, 3VI3.B.2001, 2VRO.D.1006, 2AUO.H.1415, 4BXO.A.2050, 2C2R.A.1344,
 2C28.A.1344, 4DTJ.A.1003, 4DTU.A.1003, 4EFJ.C.101, 4EFJ.A.401, 3GIJ.B.1415, 2GI

H.B.401, 4J2A.A.1006, 4K4H.M.605, 4K4I.E.606, 3KHL.B.1417, 4KHW.A.1005, 1M5X.C.801, 3M9M.B.354, 3NHG.A.909, 2NOF.A.328, 3OOR.A.237, 4Q0W.B.1001, 3QER.A.906, 3RBD.A.415, 4RI8.A.1101, 4RIC.B.1101, 3SQ2.A.906, 3V20.A.302, 2WTF.A.1512, 1A85.A.996, 1AEI.D.317, 1AEI.A.317, 1AFB.2.3, 4AG4.A.5002, 3AKB.A.173, 3ATS.A.380, 2AZ1.D.202, 1B1G.A.77, 3B2Z.C.3, 2B30.A.302, 4B7U.B.401, 1B90.A.930, 3B90.B.702, 3BCF.A.705, 4BCU.A.201, 3BOW.B.404, 3BS6.A.1, 4BTX.A.1764, 4BWE.A.503, 4BY6.A.3080, 2C11.C.1739, 4CAG.A.606, 2CDP.C.1140, 2CLT.A.1102, 2COL.B.801, 1CVM.A.802, 3D4G.A.484, 3D4G.A.485, 3D6E.B.202, 1D8F.A.305, 3DEM.B.6001, 3DF0.A.717, 3DF0.B.601, 4DLK.B.402, 2DW2.B.711, 2E0X.B.701, 2E3X.A.801, 2E6V.E.12, 3EAD.C.1001, 2EAA.C.904, 4EJ7.A.402, 4EM6.B.604, 4EMU.B.401, 1ESL.A.164, 2EXJ.D.2004, 2FH3.A.1001, 2FH3.A.1003, 1FZA.C.1, 1G5N.A.403, 3GE4.A.167, 4GEJ.A.201, 3GG1.B.503, 4GGB.A.402, 3GHG.L.601, 4GH8.B.203, 1GQM.A.1090, 1GQM.C.1088, 3GRI.B.700, 1GU6.A.1480, 2GXS.B.604, 2HOK.A.402, 2HOK.A.408, 2HOL.A.1015, 1H71.P.500, 3HDB.A.657, 2HF4.A.902, 2HTY.A.991, 4I9F.A.401, 2IAP.A.402, 3IAE.B.572, 2IEZ.A.219, 4IEF.H.704, 3IJE.B.4002, 3IRH.A.457, 1IT4.A.200, 2IWV.A.1284, 2J0T.C.1268, 2J1G.F.1291, 2J3G.A.1289, 4JBE.A.503, 1JED.B.535, 2JF9.P.1014, 4JP8.A.706, 4JWS.A.502, 1JX6.A.401, 2K0J.A.503, 4K1C.B.504, 3K39.F.1000, 3K8Y.A.167, 2KBM.B.101, 3KQA.C.420, 4KS3.A.502, 1KTW.A.5, 1KVO.D.192, 4L76.B.402, 4L76.D.402, 4L76.E.402, 1LGC.C.301, 4LIN.A.1301, 4LLF.O.401, 3LMW.A.9, 4LN6.G.403, 1LWU.K.4, 1LWU.C.5, 3MOW.G.202, 2M29.A.401, 4M7K.H.301, 3M83.B.408, 4M8D.B.305, 2M98.A.201, 4MBE.D.201, 3MDO.A.391, 4MDV.A.404, 2MG9.A.101, 1MTS.A.480, 1N28.B.127, 4N25.A.705, 4N2D.A.705, 3N9V.A.64, 4N96.B.401, 4NAS.C.501, 1NGS.B.681, 2NP0.A.1293, 4NUP.C.301, 4NUP.C.304, 4NVR.C.401, 2NWH.A.402, 1OLP.A.1372, 1OS2.A.872, 2OVX.B.447, 2OVX.B.450, 4OVG.A.404, 3P1O.A.234, 2P5V.B.1008, 2P5V.C.1001, 2P5W.B.701, 4POS.B.401, 1PZ8.C.703, 3Q2N.B.304, 1Q3A.A.467, 4Q4Y.1.5006, 1Q7B.B.9003, 1QDO.C.245, 1QDO.D.247, 1QLK.A.93, 1QMD.A.404, 4QN3.A.501, 2QVF.B.703, 3QWU.B.602, 2R1D.I.3000, 2R8Y.I.209, 3RBX.A.601, 1RFN.A.500, 2RHP.A.15, 2RHP.A.16, 1RJV.A.112, 3RRY.A.202, 4RSR.A.404, 1SOE.A.1292, 3S5U.F.220, 3S55.D.280, 1S6I.A.192, 1SAV.A.321, 3SH5.A.196, 3SHI.M.305, 3SNZ.A.97, 1SPJ.A.300, 1SRR.C.532, 1SUI.B.306, 1SUS.C.307, 1TCF.A.164, 2TEC.E.344, 3U24.A.602, 1UBN.A.277, 3UBH.A.855, 1UEA.A.4, 1UG9.A.2002, 2VB6.B.1148, 1VE5.B.2003, 2VVD.A.1329, 1WOO.A.1781, 1W1X.A.1479, 2W67.B.1590, 3WCV.B.203, 1WD9.A.902, 4WFE.A.306, 2WG8.C.201, 2WII.A.1643, 4WIW.C.701, 2WJS.A.5001, 2WJS.A.5002, 3WMW.A.401, 2WM4.A.1430, 3WNX.A.501, 3WNX.A.502, 2WW0.E.800, 1X36.A.269, 2XJO.B.1175, 1XJL.A.342, 2XSG.A.1772, 2XTJ.A.1423, 1Y1A.A.501, 1Y6P.A.217, 1Y6P.B.218, 2YGM.B.1418, 1YN8.B.1008, 2Z2D.A.267, 2ZUX.A.629, 2ZUX.A.637, 2ZUY.A.621, 2ZWP.B.401, 4AC8.B.500, 4C4U.B.201, 4CMY.A.1164, 4CMY.B.1165, 4D8F.A.401, 1DGG.A.2000, 4DHL.A.502, 1E2U.A.701, 3FE5.A.1, 3FG1.B.1500, 3FM3.A.452, 1FRF.L.565, 1GNL.A.1545, 1GUP.D.351, 3GZY.A.701, 1H2A.L.1004, 1HJF.A.1310, 1HJG.A.1307, 1I4Y.B.602, 1I4Z.D.604, 1I4Z.G.607, 2IBN.A.704, 2INP.A.3, 2ITB.B.501, 2J2F.E.371, 1KBP.A.438, 4KBP.A.438, 1LGT.A.500, 1LKD.A.500, 3LMX.O.600, 1NX8.A.300, 2OHH.B.1501, 2OHH.E.3501, 2OHJ.A.501, 1OQ9.A.365, 3PCB.O.600, 3PCJ.M.600, 2PHD.C.370, 1PIY.A.376, 1PIY.A.377, 3PM5.A.1002, 2PT2.A.400, 3Q1G.A.1001, 3Q1G.B.1001, 1QFC.A.402, 3QFM.A.264, 2R2F.B.320, 1RA5.A.500, 4RC5.A.1003, 1RSR.A.1003, 1SQ3.B.906, 1SQ3.C.908, 3T81.B.606, 4TOA.A.206, 4TOA.B.207, 3U52.B.512, 3U9M.E.205, 1ULJ.E.600, 2VHL.A.1398, 3VTI.B.803, 1W69.A.1349, 1W9M.A.1555, 3WHN.B.201, 2XRX.G.1461, 1XZW.B.929, 1Y4T.A.650, 1YKM.J.600, 1ZZ9.C.199, 3EPG.A.421, 8ICP.A.341, 2IHM.A.900, 2ISP.A.342, 4KHS.A.1009, 4KHU.A.1006, 3KNT.A.208, 4NLN.A.402, 1QVG.L.8347, 1U8R.D.4104, 3UXP.A.339, 1YIT.0.8538, 4A22.C.1346, 1A5S.B.2000, 3ASP.A.701, 3AST.A.701, 4B1L.A.1679, 3BFT.A.1007, 3BX1.B.284, 3C17.B.326, 2C9R.A.1103, 3C9F.B.603, 2C9A.A.1289, 4D1I.C.600, 4D1I.F.600, 4D1I.H.600, 3D32.A.120, 2D4E.C.1905, 2DDA.C.303, 3DKI.B.324, 3EPZ.A.1, 2EPF.C.307, 3EUW.B.343, 4FEW.B.303, 3FGH.A.180, 4FMT.A.301, 3G1N.A.5001, 4G1K.A.301, 1G8G.A.522, 1G8G.A.525, 1G8G.A.527, 1G8H.A.526, 3GCD.A.215, 3GVF.A.178, 4GY9.A.206, 4HKT.B.410, 4HMM.B.302, 3HVY.C.427, 4I2B.A.601, 3I4Q.A.177, 3IAP.D.3103, 3IAQ.B.3104, 3IJ6.B.313, 4IJK.B.301, 3IWK.B.504, 4J0Y.A.501, 4J1I.A

.502, 4JDO.D.301, 4JHG.A.205, 4JVL.A.703, 1JYX.B.3103, 1JZN.D.4139, 4K70.B.1011,
 1KNR.A.542, 4L3F.H.401, 4L73.A.404, 4L73.A.406, 4LDZ.A.205, 4LH7.A.404, 3N83.A.
701, 4NAW.N.304, 2NWH.A.404, 4NWH.A.201, 1O4Z.B.1005, 2O5W.C.171, 1OA8.A.1690, 3
OB8.B.3003, 4OBO.A.402, 3OO2.B.383, 2OSW.B.602, 2OSY.B.602, 3OTK.C.587, 3P8O.A.1
87, 3PYM.B.504, 1Q1Q.A.351, 1Q1Z.A.313, 4Q4X.1.5004, 3Q9E.L.343, 2QF2.A.700, 4QF
E.G.304, 2QZ7.B.196, 4R6C.A.213, 3RN0.A.402, 3S30.A.383, 3T2P.B.3102, 3T34.A.100
3, 1T64.B.1392, 3TXJ.A.1138, 3UA6.B.2, 2VDX.A.1385, 3VD5.C.3101, 3VD5.D.3102, 1V
IZ.B.239, 3W6P.A.804, 2WCF.B.1091, 2WD0.A.601, 2WG8.B.202, 2WO0.B.1728, 2WOI.B.1
491, 2WWH.B.212, 3WXO.A.807, 3WXO.A.808, 4WXG.A.502, 2X1Z.M.1163, 2X7J.D.1581, 2
YO0.A.1359, 1YAO.A.501, 2ZHJ.A.320, 3ZLY.A.1384, 4ZNB.A.3, 2ZXK.A.1, 3ZX2.B.521,
 3FKR.B.408, 4M5P.A.505
[1] "Cluster 6"
3AAK.A.992, 4BLB.D.910, 4CPA.I.308, 3CSQ.B.335, 1D1S.B.406, 4DWZ.A.302, 1F30.E.2
01, 2GA6.D.998, 3H90.C.2, 4IOD.A.502, 4JBS.B.1009, 4K0D.B.203, 1LOY.B.702, 4LTE.
A.1101, 3M3B.A.156, 3M4B.B.108, 1NL4.B.500, 1Q3A.B.469, 2QLA.A.500, 1RLY.A.61, 3
U94.B.259, 2V8V.A.1456, 3V94.D.702, 1VKG.A.400, 2DI2.A.30, 4FC5.E.305, 1GLC.F.16
9, 2MQ1.A.103, 2OX8.A.5, 1ANJ.A.450, 4BLL.A.1322, 3BWI.A.450, 4CBY.A.2034, 4COQ.
B.299, 1DK4.A.291, 1DK4.B.590, 4DYO.A.501, 3E4A.A.2000, 4EWL.A.403, 4FW3.A.301,
1G12.A.200, 2G9Y.B.450, 3GJ9.B.127, 2GO4.B.602, 3GSH.A.101, 1H48.A.900, 1H8L.A.9
99, 1JJE.B.261, 2JT2.A.336, 1K07.A.2, 4L3T.A.1101, 3LMS.A.309, 1LNF.E.800, 4LQY.
A.507, 3MN8.A.999, 4MN6.A.401, 4ONX.A.201, 2OOT.A.1751, 2OX8.A.4, 3PN6.B.202, 3Q
9B.E.345, 3R8B.B.122, 3R8B.D.122, 3R8B.F.122, 3R8B.P.122, 3RBU.A.1751, 1TXR.A.50
2, 1UXB.A.1367, 2VME.A.501, 2VQG.C.1092, 4WD8.C.302, 3WT4.A.501, 4X2T.E.701, 1XJ
O.A.901, 1YH8.A.501, 4D6N.F.1197, 4DFP.A.901, 4G7O.D.2003, 3LK9.A.340, 4M2Z.A.50
1, 3R7P.A.317, 3A58.B.401, 2A9F.A.801, 1AO0.A.469, 2ATX.A.201, 3C41.J.603, 4CZK.
A.1335, 3DYG.A.3002, 1E2A.C.106, 2EWG.A.3002, 3FFU.A.156, 3FFU.B.155, 3FHY.B.404
, 4FHX.A.402, 2FOL.A.202, 3G15.A.605, 3G9D.A.298, 4GMJ.B.302, 1GUS.A.1069, 4GWS.
A.402, 4GYZ.I.402, 1H65.A.282, 3HDG.E.204, 3HWX.A.602, 4HYV.A.1001, 4IFW.A.503,
3IJQ.B.386, 2IOA.B.5003, 2IUT.A.1724, 2J1L.A.1195, 1JCT.A.498, 1KFS.B.2, 4KMQ.A.
1103, 3KUD.A.171, 3LD0.5.54, 2LVJ.A.101, 1MBZ.A.604, 3MLE.A.222, 3N3T.B.803, 4NH
0.A.1402, 2NOM.A.401, 1NV7.A.3341, 4OGE.A.1204, 2OPM.A.907, 3POW.A.471, 4P9D.A.2
02, 3PCR.B.1231, 3PLS.A.1, 3PMG.A.562, 3PP1.A.410, 2PUI.B.401, 3Q8U.E.159, 3RUW.
D.544, 4S17.A.501, 1T5S.A.1005, 3TXA.A.801, 3U7F.B.1, 3UGV.A.500, 4UM9.B.2001, 2
W8D.A.1636, 3WBH.B.503, 3WQS.B.502, 2Y4M.A.400, 2YWF.A.701, 2W9C.B.1343, 4KI8.C.
602, 4M2Z.B.301, 4FFO.A.903, 4FFR.A.404, 3G9D.A.299, 4GX6.B.402, 1JR4.A.300, 4KI
8.A.602, 4LTZ.A.404, 3MJX.A.901, 3OTB.A.401, 4RUB.A.491, 2VBI.B.1000, 3A6P.H.117
8, 4DPV.N.12, 4IFD.J.1105, 4M30.B.302, 1N56.A.401, 2O5I.D.8001, 1QVG.O.8010, 4W9
M.E.902, 1A49.B.1134, 1A49.C.1734, 4ACF.B.1481, 4AN9.A.1383, 3AQC.D.328, 1AZT.A.
403, 2BBO.A.2, 3C5P.D.302, 3CNX.B.170, 1CQP.A.310, 3CWH.A.391, 4CYU.A.170, 2D32.
A.1523, 2D33.B.1524, 1DIE.A.398, 2DW7.A.2001, 2DW7.B.2002, 3DYF.A.3002, 3DYF.A.3
004, 3EA5.C.221, 1ELY.A.452, 4FOP.A.501, 2F1I.A.501, 4FHY.A.402, 4FMC.B.203, 3GT
8.C.13, 1GXB.C.1346, 1GY3.A.1298, 3HBO.B.702, 3HQP.A.502, 3HYL.A.675, 4I1O.E.201
, 3IAF.A.572, 3IAF.B.572, 2IO7.A.5002, 4IYN.A.804, 1JM6.A.4601, 4JVJ.F.405, 4K10
.A.405, 4LF2.D.601, 4LNI.D.504, 3LZ9.A.852, 3MQT.W.395, 1MXA.A.411, 4NOG.A.405,
1N8W.B.1900, 1NUZ.A.2342, 3OAB.A.904, 4OAU.C.802, 3OB8.D.3001, 4POV.A.403, 4P9D.
B.202, 4PYK.A.302, 1Q19.A.504, 3RBM.A.1003, 2RD5.C.1001, 4RJK.B.602, 3RUW.A.544,
 4S17.C.502, 1S6P.A.601, 1SBJ.A.163, 3SBE.A.501, 3TW6.B.2002, 4U03.A.504, 4U81.A
.501, 3UJ2.H.430, 4UOR.C.699, 2VON.A.502, 3WQP.J.501, 2X9H.A.3001, 1XF9.B.11, 1Y
8P.A.501, 1Y9I.B.602, 1YHM.A.401, 1ZCA.B.1383, 1EQR.B.902, 3JZM.A.701, 1SO2.C.47
5, 2Z2P.A.1003, 2ASJ.B.1415, 1BSS.A.433, 3GIK.A.415, 4K4G.I.602, 4K4I.I.604, 3OO
L.A.237, 3RBE.A.415, 4RIC.A.1101, 1SON.A.403, 3S9H.A.906, 3SQ1.A.907, 3A7Q.A.400
1, 2AAO.B.299, 1AFD.2.2, 1ALA.A.400, 1AR1.A.561, 1AVX.A.700, 1AXK.B.395, 1B09.C.
301, 1B09.C.302, 2BBM.A.182, 3BJU.C.606, 3BJU.C.608, 3BRX.A.328, 2BTW.B.400, 3BX
K.A.152, 2C11.A.1738, 4CAG.A.601, 2CHO.A.1717, 2CLT.A.1101, 1CTR.A.149, 1CTR.A.1

50, 2D3P.A.237, 2D7F.A.240, 2DDY.A.175, 3E3R.B.193, 2E6V.D.11, 1EAK.D.998, 1EGZ.B.300, 4EJ7.C.403, 4ELF.F.201, 1ESP.A.320, 1ETH.A.449, 1EXZ.C.805, 1F4M.E.105, 3FAX.A.1223, 3FCS.D.2002, 2FH2.B.2003, 2FH3.A.1002, 2FHC.A.2405, 1FIH.B.3, 3FLP.L.302, 4FU4.A.505, 3FW0.A.823, 1FZC.C.1, 1FZD.G.1, 4G0D.A.503, 1G1Q.C.803, 4G9L.B.306, 2GA9.D.480, 3GCW.E.1, 4GKX.B.302, 1GYK.A.1206, 2H0K.A.401, 3HR4.H.202, 2HTY.G.997, 1HVD.A.602, 1IH0.A.2, 3IJE.A.4007, 3IJE.A.4008, 1IVE.A.470, 2IWV.B.1284, 1IXX.D.124, 2J3U.C.1292, 2J64.C.1222, 1J84.A.366, 2JAL.B.1447, 4JDZ.B.704, 1JN2.P.238, 4JP8.A.704, 3JQL.A.121, 2K2F.A.94, 1K90.D.801, 1K90.E.802, 1K9I.A.407, 3KO0.H.302, 3KQA.A.420, 4L06.B.501, 4L06.C.501, 4L06.D.501, 4L76.A.402, 2LAN.A.301, 3LCP.D.159, 1LGC.E.301, 1LGN.A.302, 4LIN.E.1301, 3LND.A.210, 1LQD.B.1, 4LT6.A.602, 1LU1.A.301, 1LWJ.A.883, 1LWU.B.1, 1LWU.F.6, 3M0W.H.202, 1M1J.C.501, 3M83.E.407, 3M8D.A.710, 4MBE.A.202, 4MDV.B.404, 2ML3.A.206, 2MLR.A.305, 2MLS.A.304, 2MLS.A.305, 3MW3.A.302, 1N29.A.125, 3NP5.A.732, 1NX0.A.900, 4OJQ.B.1002, 2OKX.A.4004, 4OMD.F.604, 1OTN.A.250, 3OX5.B.502, 3OX5.C.501, 2OXE.B.600, 2OYH.B.2, 4P99.A.505, 4P99.D.512, 4PJ0.A.601, 3POG.B.1, 4POQ.C.401, 4POQ.E.401, 1Q3A.C.478, 4Q6O.A.501, 1QNI.D.903, 1QOO.A.302, 2QVF.B.704, 3QWU.A.601, 2RHP.A.11, 2RHP.A.24, 2RHP.A.25, 2RJP.B.4, 2RJQ.A.5, 3RMK.E.313, 3RYD.C.267, 1SOC.A.1879, 3S5U.A.220, 3S5U.D.220, 4SBV.A.261, 4SBV.C.261, 2SNI.E.276, 1SW8.A.81, 1SZB.B.1004, 1TD7.A.1001, 1TN3.A.183, 1TNQ.A.91, 3TRP.A.360, 4TVU.B.600, 1TYE.B.1402, 3UJO.D.304, 4UM8.A.2004, 4UM8.B.2003, 1UZJ.C.3648, 2V3T.A.1264, 1VAH.A.500, 1VFP.B.995, 3VU1.A.1001, 1W0P.A.1781, 2W2O.E.1333, 1W2M.A.1442, 3W9T.G.506, 3W9T.B.508, 1WDA.A.902, 4WFF.A.304, 3WHU.A.501, 4WK2.B.502, 1WMZ.B.203, 1WT9.A.1001, 2WW0.C.800, 2WW0.F.800, 3WYN.B.402, 2WZE.A.1552, 2X0G.B.1149, 1XFE.A.100, 1YAX.C.1003, 2YN3.C.6370, 1YO8.A.1184, 1Z3J.A.266, 2ZOC.A.501, 2ZOC.A.502, 2ZWO.A.401, 1ZZH.A.401, 3CFR.A.910, 4DTS.A.1002, 3FSP.A.501, 2G8K.A.401, 3GIL.B.1417, 4K4H.I.602, 3KHH.B.1416, 4LQ0.A.403, 1M5X.A.802, 1N56.B.403, 3NE6.A.905, 3ODH.A.196, 4PTF.A.1202, 2RDJ.B.353, 1A2X.A.160, 1A2X.A.161, 3A7Q.A.4002, 2A8K.C.403, 3AAJ.A.991, 1AEI.A.319, 5AER.A.201, 5AER.A.202, 1AFD.3.2, 1AJ4.A.163, 1AMY.A.502, 1ANN.A.319, 1ANW.B.354, 1ATN.D.264, 1AWB.A.278, 3B1T.A.900, 1B47.A.351, 4B7R.A.502, 1BAG.A.431, 3BJU.A.608, 3BOW.A.719, 3BOW.B.403, 4BTW.B.1764, 4BY5.C.1187, 4BY5.D.1185, 2C0O.B.1507, 1C9U.B.1002, 4CAG.A.605, 4CAJ.B.1325, 1CFF.A.149, 1CFF.A.150, 3CGA.A.102, 1CGU.A.686, 3CGT.A.686, 3CHK.A.502, 1CYG.A.682, 1D2L.A.46, 2D3P.C.241, 1D7F.A.5003, 1DCY.A.198, 2DDY.A.174, 3DF0.A.715, 3DF0.A.716, 3DF0.B.604, 1DJY.A.2, 2DPK.A.2001, 4DRZ.A.202, 2E3X.A.803, 3E3R.B.194, 4E52.A.403, 2E6W.A.300, 2E6W.A.301, 3E78.A.601, 1E8U.B.1003, 2E85.B.1004, 3E9T.A.1, 3EAD.B.1002, 3EAD.B.1004, 3ECQ.A.2000, 3EDD.A.700, 3EHJ.A.1, 3EHJ.B.1, 4ELF.C.201, 4ELG.A.202, 4ELG.F.202, 2EQD.A.701, 2ERO.B.702, 1ESP.A.322, 1EUB.A.278, 2EXI.D.3004, 1F6S.E.205, 3FCU.B.2002, 1FDK.A.124, 4FGC.B.204, 1FI5.A.162, 3FLP.B.301, 3FLT.A.302, 4FL4.C.401, 4FL4.C.402, 4FU4.B.505, 1GOH.A.290, 3G5C.B.802, 3GIN.A.1, 2GJR.A.1488, 4GKX.B.301, 1GQM.A.1089, 3GQF.D.154, 4GTW.A.1012, 2H0K.A.407, 1H4B.A.1085, 1H4B.A.1086, 1HDF.B.1101, 3HLI.D.315, 2HQ8.B.303, 2HYW.A.505, 2I08.A.200, 4I5N.B.601, 3IA7.A.402, 3IA7.B.402, 4IAV.A.420, 3IGO.A.601, 2IWV.D.1286, 1IXX.B.124, 1IZJ.A.1002, 2J1G.D.1289, 1JBA.A.500, 1JBA.A.501, 4JBE.A.502, 1JC2.A.3, 4JDZ.B.702, 4JE0.A.401, 1JL5.A.2004, 3JQ5.A.201, 1JSA.A.500, 1JSA.A.501, 3JTX.B.396, 4K1C.A.506, 4K1C.A.509, 2K7O.A.103, 3K8L.B.800, 1K90.F.804, 1K96.A.92, 2KAY.A.185, 1KB0.A.801, 3KF9.A.302, 1KIT.A.803, 1KMB.1.2, 1KTW.A.3, 1KV9.A.802, 4KVJ.A.714, 1KX1.A.222, 1KX1.E.501, 1KX1.E.502, 1KX1.F.601, 2KYF.A.110, 2KZ2.A.149, 1L6R.A.901, 1LBX.A.290, 3LCP.B.279, 1LGC.A.301, 1LHV.A.401, 1LMJ.A.101, 1LMJ.A.102, 4LMF.A.303, 4LMF.A.304, 2LMT.A.149, 2LMT.A.150, 4LMH.D.811, 1LPG.B.1, 2LP2.A.203, 2LP3.A.201, 1LTJ.B.2, 2LV6.A.204, 2LVK.A.102, 1LWJ.B.10, 2M0K.A.201, 1M34.B.2299, 2M5E.A.2001, 1M63.B.502, 4M7Z.B.409, 1M8T.C.1003, 1MAC.B.389, 4MBZ.H.401, 4MDV.B.403, 2ML1.A.206, 2ML2.A.202, 2ML2.A.205, 2ML3.A.205, 3MXW.A.402, 1N28.B.125, 4N25.A.706, 4N85.A.502, 1NAE.A.900, 4NDD.B.402, 4NEN.A.1115, 1NHE.A.805, 4NHF.B.301, 1NIW.A.1001, 1NIW.A.1002, 1NIW.C.1005, 3NN7.A.503, 3NOM.B.263, 1NUB.A.301, 1NUB.A.302, 1NUD.A.702, 1NX2.A

.4, 1NX3.A.4, 3O5S.A.243, 1ODB.F.1092, 2OKX.B.4002, 3OX6.A.501, 3OX6.A.502, 2POR .B.1004, 4P5W.A.1001, 4P99.A.512, 2PF2.A.174, 1PK8.A.817, 4PLM.A.504, 3PM8.B.514 , 2POJ.A.266, 4POQ.A.401, 2PPL.A.481, 1PTK.A.281, 4Q4W.1.905, 1Q7B.A.9002, 1Q8H. A.72, 1QNI.E.903, 3QRX.A.170, 3QRX.A.173, 4R9X.A.302, 3RBX.A.600, 2RDZ.B.1502, 2 RHP.A.1176, 2RHP.A.26, 3RV2.A.405, 3S5U.C.220, 2SAS.A.186, 2SAS.A.188, 1SCV.A.16 2, 3SO0.F.98, 3SO1.B.97, 1TCF.A.162, 1TEC.E.343, 1THL.A.3233, 3TRQ.A.358, 3TRQ.A .360, 3TSK.A.304, 1TTX.A.110, 1TTX.A.111, 3TUY.C.157, 1TYE.A.1405, 1TYE.A.1407, 1U7W.A.501, 1UG9.A.2006, 1UH3.A.1001, 3UJQ.D.305, 1ULV.A.2004, 4UM8.C.2004, 4UM9 .C.2001, 4UM9.C.2002, 1UMS.A.3, 3USU.H.272, 1UZJ.A.1648, 1UZJ.C.3649, 3V03.A.585 , 1V1G.A.1211, 1V2I.A.1001, 2V3T.B.1264, 1V3D.B.2001, 1V3J.A.687, 1V3J.B.690, 1V 3L.B.689, 2VB6.B.1151, 1VFO.A.1001, 3VI3.A.2002, 2VKH.A.1543, 2VZP.B.1129, 1W2M. C.1441, 2W2N.E.1334, 2W3J.A.1139, 3W9T.A.1005, 3W9T.A.1008, 3W9T.C.1009, 3W9T.D. 503, 3W9T.D.504, 4WBQ.B.602, 4WF7.D.600, 3WHU.B.502, 1WMD.A.1001, 2WO9.D.1270, 1 WRL.D.104, 3WU2.O.301, 2WVX.B.801, 2WVZ.B.800, 2WW0.A.800, 2WYS.A.1554, 1XJL.A.3 46, 1XJL.B.340, 2XQX.B.1949, 2XRM.A.405, 1YCM.A.266, 2YDP.A.502, 1YO8.A.1211, 1Y UU.A.197, 1YUU.B.199, 2Z2D.A.266, 1Z6C.A.247, 2Z8S.A.644, 2Z8S.A.647, 2Z8S.B.642 , 2ZBA.A.461, 1ZFS.A.104, 1ZIV.A.1, 2ZJ7.A.627, 2ZKT.B.413, 2ZN9.B.901, 2ZUY.A.6 24, 4BYA.A.77, 1J35.C.505, 2M3S.A.201, 3ZDY.C.2004, 1AZO.A.284, 1BSU.A.301, 4RNO .A.502, 1SOO.A.402, 1A25.A.291, 2A3Y.A.600, 2A40.B.1273, 1AFA.1.2, 1AFB.1.227, 1 AFB.1.2, 4AK8.A.1326, 1ANX.B.321, 4AOC.A.1130, 4AQ8.D.1239, 2ASP.A.600, 4AVS.A.2 06, 4AW7.A.1599, 4AWN.A.301, 4AYO.A.500, 4AYP.A.500, 4AYQ.A.500, 4AYR.A.500, 4AY U.A.206, 1B9A.A.110, 3B9G.A.318, 3B9X.A.400, 4B97.A.1152, 4B9P.A.1167, 1BCH.1.2, 1BCH.2.2, 3BJE.B.402, 3BMV.A.684, 4BM1.A.402, 2BOI.A.200, 2BOJ.A.1117, 2BOQ.A.1 352, 2BP6.A.801, 2BPE.B.1245, 2BV4.A.200, 1C0G.S.129, 2C40.A.1311, 4CE8.A.997, 1 CGT.A.685, 1CGV.A.692, 1CGX.A.692, 1CGY.A.692, 2CKI.A.998, 1CLC.A.651, 1CVR.A.50 3, 1CXL.A.689, 4CZN.A.1369, 3D1M.A.190, 1D3C.A.688, 3D34.A.228, 3DCQ.A.115, 3DE8 .A.109, 3DED.A.505, 3DED.C.508, 2DG1.A.3001, 3DHP.A.497, 4DLK.A.402, 4DOE.A.502, 1DSY.A.502, 2DSO.B.1002, 1DTU.A.688, 1DV8.A.1001, 1DV8.A.1003, 1DVI.A.274, 2E39 .A.502, 4E52.B.401, 3EF2.A.303, 3EF2.B.303, 1EO5.A.689, 3EPW.A.1001, 3EPX.A.1001 , 3ER9.B.901, 2EWE.A.702, 4EW9.A.203, 4EW9.A.204, 4EW9.B.203, 2F3Y.A.1174, 4FCS. A.403, 2FF2.A.601, 1FIF.A.1, 1FIF.A.2, 1FIH.A.2, 3FM1.A.352, 3FM4.A.352, 3FMU.A. 352, 1FO3.A.700, 1FWX.B.4903, 3FZO.B.400, 1G1T.A.160, 1G43.A.200, 3G5I.A.501, 1G 87.A.616, 3G81.A.401, 1G94.A.800, 2GGU.A.401, 2GGX.A.401, 4GNC.A.301, 2GUY.A.601 , 1GYK.A.1205, 3GZK.A.538, 1H3J.A.1346, 1H80.A.1492, 4HPK.A.1102, 1HUP.A.301, 1H UP.A.302, 1HXO.A.500, 2HYV.A.601, 4I5N.E.701, 1I75.A.1688, 4I70.A.401, 4I71.A.40 2, 4I72.A.401, 4I73.A.401, 1I82.A.193, 1IA6.A.1263, 2IE7.A.401, 4IEF.B.703, 4IEF .H.703, 3IKP.B.401, 3IKR.A.401, 1IOD.G.506, 4IT5.D.201, 2IWK.A.1607, 1J34.A.511, 1J34.B.512, 1J34.C.505, 1JAE.A.500, 4JA8.B.502, 2JDM.B.1116, 2JDN.B.882, 3JQW.A .1002, 1JZN.A.1138, 1K72.A.779, 1K9I.B.502, 1K9J.A.401, 1K9J.A.402, 1KCK.A.691, 1KCL.A.1692, 3KMB.1.2, 3KQR.A.206, 2KRD.C.90, 1KSC.A.500, 1KWT.A.502, 1KWU.A.503 , 1KWV.A.503, 1KWW.A.503, 1KWX.A.503, 1KWZ.A.503, 1KWZ.B.604, 1KXO.A.504, 1KXH.A .800, 1KXR.A.1, 1KZA.1.502, 1KZD.1.502, 2KZ2.A.150, 4KZO.A.501, 4KZO.B.501, 4KZV .A.302, 4KZV.A.303, 4KZW.A.302, 1LLP.A.352, 1LQV.C.45, 1LQV.D.47, 2LRO.A.201, 2M 3S.A.202, 2M3S.A.203, 3M5Q.A.371, 2MAS.A.316, 1MCX.A.347, 1MDW.A.1, 3MKM.A.501, 3MKN.A.501, 1MN2.A.371, 2MSB.A.1, 2MSB.B.2, 1MUQ.B.202, 1MXG.A.438, 3N1F.A.5, 3N 1G.A.192, 4N32.A.402, 4N33.A.404, 4N34.A.402, 4N35.A.404, 4N36.A.402, 4N37.A.402 , 4N38.A.403, 4N4E.E.402, 1NBC.B.156, 4NEH.A.1104, 4NPK.A.801, 2NQI.A.401, 4NVR. A.401, 2NXP.B.601, 2NZM.A.407, 2O8O.A.1010, 2ORJ.A.401, 2OS9.A.401, 1OT1.A.1693, 1OUR.A.401, 1OUX.A.401, 1OVP.A.116, 2OX9.A.801, 2OX9.A.802, 2OX9.B.804, 3P5G.A. 500, 3P5H.A.500, 3P5I.A.500, 3P7G.A.1, 3P7H.A.1, 3PAK.A.300, 3PDD.A.192, 1PEZ.A. 891, 4PEO.A.103, 3PF2.A.2, 4PHJ.A.301, 4PHN.A.304, 1PJ9.A.891, 3POE.A.2, 2POR.A. 303, 1PW9.A.401, 1PW9.B.402, 1PWB.B.401, 2PWA.A.1280, 3Q13.A.601, 3Q3U.A.341, 3Q 4W.A.225, 1Q8F.A.2001, 4QB1.A.202, 4QB6.A.203, 3QEE.A.21, 1QNI.A.901, 2QVM.A.100 1, 2R9F.A.365, 1RDI.1.2, 1RDJ.1.2, 1RDK.1.2, 1RDL.1.227, 1RDL.1.2, 1RDN.1.2, 1RD

0.1.2, 1RP8.A.501, 3RQI.A.181, 1RTM.1.2, 3S82.B.405, 3SBQ.A.703, 1SH7.A.1290, 1SL5.A.402, 1SL6.A.2, 1SMD.A.497, 1SNN.A.403, 3SWB.A.91, 1T44.G.701, 3T8I.C.400, 3TEC.E.344, 1TF4.B.3003, 3TH4.L.149, 1TLQ.A.190, 1TN3.A.182, 4TV9.A.503, 4U6B.C.501, 1UA7.A.601, 1UOV.A.1419, 1UQX.A.1115, 1UV4.A.1294, 1UZK.A.2512, 1V0A.A.1176, 4V0C.D.201, 3VM7.A.501, 2VMH.A.3050, 2VMI.A.3050, 2VNV.A.301, 2VUC.B.991, 2VUV.A.200, 2VUZ.A.1130, 3VV3.A.403, 3VYK.A.1007, 1W0Y.L.1156, 2W08.A.205, 1W3H.B.1348, 2W87.A.1148, 3WC3.A.501, 2WHK.A.1339, 3WH2.A.301, 4WK0.A.501, 1WMD.A.1002, 3WMS.A.801, 3WMT.A.606, 1WMY.A.202, 1WMZ.A.201, 1WMZ.A.202, 1WMZ.B.202, 2WNX.A.1163, 3WN6.A.501, 2WR9.A.1132, 2WZS.A.800, 2XFG.A.1447, 1XKD.A.1006, 2XR6.A.1390, 2XR6.A.1391, 1Y6W.A.150, 1Y9I.A.501, 1YCM.A.267, 1YO8.A.1182, 1YOE.A.1001, 1YUT.B.200, 1ZCM.A.1002, 2ZIC.A.944, 3ZQX.A.1146, 2ZWP.B.403, 3FD2.A.374, 4K4G.I.603, 4K4H.A.604, 4K4I.I.603, 1N48.A.501, 3NAE.A.906, 3NHG.A.908, 3QEW.A.905, 3QEX.A.905, 3RAX.B.1416, 3S9H.A.905, 3SCX.A.906, 3SQ1.A.903, 3SQ2.A.904, 1T9I.C.801, 1T9I.C.802, 3A4U.A.287, 4A41.A.2494, 4A5G.A.1308, 4A6O.A.2346, 4A6S.A.1122, 2AAO.A.294, 1AFD.1.2, 3AFG.A.541, 4AFA.A.1267, 4AFB.A.1267, 4AFC.A.1267, 1AJ4.A.164, 1ALC.A.200, 3ALT.A.201, 4AL9.A.1122, 3AMR.A.901, 3AMR.A.906, 1ANN.A.320, 4ANR.A.400, 1AP4.A.90, 2ARY.B.404, 3AR2.A.1001, 4ASM.B.1359, 3AUJ.A.1603, 3B0K.A.201, 1B1G.A.76, 3B2Z.B.2, 2B6N.A.300, 1B9O.A.124, 4B96.A.1155, 4B9F.A.153, 1BCJ.2.2, 2BF6.A.1693, 1BJ3.B.124, 1BJF.A.403, 1BJF.A.404, 3BJU.A.606, 1BLI.A.700, 4BLK.A.400, 2BO2.A.155, 2BO2.A.156, 2BOJ.C.1117, 3BPS.E.1, 2BP6.A.802, 2BP6.C.805, 2BPE.A.1245, 1BQB.A.353, 3BSG.A.501, 1BYF.A.201, 2C0T.B.1507, 4C0K.A.1615, 3C7F.A.803, 4CAJ.C.1323, 2CCM.A.1193, 1CGE.A.304, 1CGT.A.686, 4CGT.A.685, 2CHN.B.1716, 3CIO.K.401, 1CIU.A.684, 1CKK.A.151, 1CKK.A.153, 2CL8.B.1245, 4COV.A.1269, 4COY.A.1270, 1CPM.A.215, 4CPB.A.1122, 4CPB.D.1123, 3CQO.A.4001, 3CQO.A.4004, 1CXE.A.690, 4D0E.A.1531, 4D0E.A.1533, 3D1M.A.2, 1D2V.A.600, 1DB4.A.198, 3DBZ.A.401, 3DCQ.A.116, 3DCQ.B.116, 2DCK.A.1002, 2DEW.X.902, 3DED.A.504, 3DED.B.506, 3DEM.B.4001, 4DH2.B.101, 4DH2.B.102, 2DIJ.A.689, 1DJW.B.2, 4DLK.A.401, 3DSL.A.2, 2DSO.A.1001, 1DTL.A.202, 1DV8.A.1002, 4DZT.A.302, 1E43.A.504, 3EDF.A.603, 3EHU.A.500, 3EIF.A.1, 2EJN.A.1003, 3EKI.A.601, 4ELG.B.202, 4ELG.C.202, 1EMN.A.2225, 1EO7.A.689, 2ERO.A.701, 2ERV.A.195, 1ESL.A.163, 1ESP.A.319, 3ETO.A.2002, 2EV5.A.1150, 1EXR.A.1001, 3FAW.A.4, 3FCS.A.2008, 2FF3.A.701, 1FHF.A.502, 2FH1.A.2003, 4FHP.A.402, 1FI6.A.100, 1FIF.B.2, 1FIF.C.2, 3FLT.A.301, 3FMU.A.351, 1FPW.A.502, 4FVL.A.505, 1FWX.D.4903, 1G0H.A.291, 4G0N.A.202, 1G1Q.A.801, 1G5N.A.408, 1GEN.A.302, 4GER.A.402, 4GER.A.404, 2GGX.C.401, 1GGZ.A.152, 3GIS.Y.1002, 3GIS.Z.1003, 4GKY.A.302, 3GQ8.A.1, 2GVU.A.500, 2GVV.A.500, 1GWU.A.1307, 4GWI.A.204, 2H2T.B.322, 3H2W.A.538, 1HDH.A.1528, 3HDL.A.307, 4HEX.A.204, 1HFZ.B.124, 4HHR.A.703, 3HLH.B.315, 3HLI.B.315, 1HQV.A.997, 3HR4.F.202, 1HT6.A.501, 2HYW.A.501, 1I22.B.197, 4I2Y.B.504, 4I5K.A.502, 1I8A.A.192, 2IAW.A.401, 2IAX.A.401, 2IAO.A.401, 4IEF.D.704, 4IEF.F.703, 3IJ9.A.497, 3INM.A.521, 1IOD.A.501, 3IQT.A.1, 4IRZ.A.2005, 4IRZ.A.2006, 4IT5.B.201, 4IT5.C.201, 3IUC.A.2, 2IWV.D.1283, 2IWA.A.501, 1J1D.A.202, 1J34.C.504, 1J55.A.101, 4JCL.A.701, 2JDY.B.1117, 2JDA.A.1146, 2JDM.B.1115, 2JDM.C.1115, 1JI1.A.2001, 1JI1.A.2002, 2JKX.A.1641, 4JRF.A.601, 4JSD.A.603, 1JUG.A.126, 2JUO.A.500, 4K70.A.1003, 3K8K.A.710, 3K8L.A.700, 3K8L.A.710, 3KCP.A.702, 3KEZ.B.501, 3KF9.A.301, 3KF9.A.304, 4KHO.A.1001, 1KIC.B.328, 1KIE.B.328, 2KPN.A.762, 1KP4.A.200, 4KVL.A.703, 1KWW.B.602, 1KWZ.A.504, 1KX0.C.703, 1KX1.F.602, 1KXT.A.4001, 1KZM.A.501, 4L03.B.502, 1L6R.B.903, 4LHK.A.303, 4LHN.A.302, 3LI3.A.402, 3LI4.A.316, 4LJ3.A.403, 4LJH.A.201, 4LMF.D.303, 2LMV.A.150, 3LND.A.209, 1LPZ.B.1, 1LQV.C.42, 2LUX.A.202, 1LYC.B.9002, 2M28.A.301, 2M55.A.301, 4M5H.A.305, 1M63.B.501, 4M65.A.404, 1MAC.A.388, 2MLR.A.303, 1MN1.A.371, 4MNO.A.301, 4MSP.A.202, 2MTE.A.101, 2MTE.A.102, 1MXD.A.731, 4MZA.A.612, 3N1F.A.6, 3N1G.B.190, 4N2G.A.703, 4N4E.E.404, 4N6F.A.302, 4N7A.A.605, 4NHF.F.301, 1NIW.E.1010, 3NJH.C.503, 1NL0.G.905, 1NL1.A.204, 1NL2.A.205, 3NOK.A.257, 1NPC.A.322, 4NPK.A.802, 4NUQ.A.302, 1NYA.A.501, 1NZI.A.1001, 2NZM.A.406, 1O4Y.A.700, 4O6N.A.401, 4O6N.B.401, 1O9I.A.269, 1O9I.E.269, 2O04.A.5002, 1OUX.B.404, 1OVS.A.402, 1OVS.B.404, 4OV2.A.201, 2OW2.B.446, 4P5F.B.501, 2P69

.A.305, 3P7F.D.1, 4P99.A.515, 1PA2.A.307, 1PAM.A.687, 3PAQ.A.300, 3PAR.A.300, 4PHK.B.305, 1PJ9.A.890, 1PJX.A.491, 1PVY.A.503, 4Q1U.A.402, 3Q9K.A.606, 4QB2.A.202, 3QED.A.348, 1QGJ.A.2001, 1QNI.C.901, 1R17.A.599, 4R1D.A.601, 3R5Q.A.1001, 4RDQ.A.504, 4RDQ.B.502, 1RF1.E.462, 4RGP.B.302, 1S2N.A.1290, 3S5U.D.221, 3SBR.A.703, 1SCH.A.302, 1SH7.A.1292, 3SO0.A.97, 1SRA.A.301, 3SRE.A.1357, 1SU3.A.904, 1SU4.A.996, 1SZB.A.1001, 1SZB.A.1003, 3T3P.A.2005, 3T3P.A.2006, 3T3P.A.2007, 3TBD.A.401, 1TDQ.B.127, 1TDQ.B.128, 1TF4.A.3001, 3TH4.L.148, 1TLG.A.201, 1TMQ.A.1001, 3TO5.A.131, 3TRP.A.358, 4TV8.A.503, 4U65.E.302, 4U6B.A.501, 3U8D.A.202, 3UBG.A.901, 3UBH.A.856, 1UKT.B.690, 4UM9.A.2002, 4UM9.A.2004, 1USR.B.1573, 1UX6.B.2006, 1UX6.B.2014, 1UX7.A.1134, 1UZJ.B.2648, 4V0C.C.201, 4V29.A.1178, 1V3E.A.4001, 1V3J.A.688, 2V72.A.1139, 3VI3.C.2001, 2VN5.B.101, 2VN6.B.1066, 2VNG.A.1213, 2VZP.A.1129, 2VZQ.A.1130, 2VZR.B.1132, 1W0P.A.1779, 1W0Y.H.1259, 2W1Q.A.1947, 2W1S.A.1946, 2W2M.E.1334, 3W57.A.202, 3W5M.A.1201, 3W5N.A.1201, 2W86.A.1148, 2W86.A.1149, 1WDC.C.501, 2WG7.A.1126, 3WH3.A.500, 3WHD.A.501, 3WHT.B.502, 4WJK.A.503, 4WK0.A.502, 4WK0.A.504, 3WNO.A.801, 3WNP.A.801, 1WPC.A.503, 2WQ8.A.1641, 2WR9.B.1129, 3WU2.A.401, 2WVZ.A.800, 1WZL.A.1601, 2WZS.F.800, 1X2T.A.603, 2XFD.A.1111, 2XFE.A.1113, 1XO5.A.1, 1XO5.A.5, 1XYD.A.93, 2Y4Q.A.800, 2Y5I.A.102, 2Y8K.A.1526, 1YDY.A.904, 2YEQ.A.1525, 2YFU.A.1141, 2YHG.A.1936, 1YO8.A.1194, 1YO8.A.1196, 1YO8.A.1204, 1YO8.A.1210, 1YRO.A.124, 1YUT.A.198, 1YUT.B.199, 1YUU.B.200, 2Z48.A.1007, 2Z48.A.1102, 2Z48.A.1103, 2Z48.A.1205, 2Z49.A.1001, 1Z6C.A.246, 3ZDV.B.200, 2ZFD.A.230, 1ZH2.B.201, 3ZHG.A.1323, 1ZIV.A.2, 1ZJA.A.7001, 3ZO9.A.1589, 2ZUX.A.633, 2ZUX.A.634, 2ZUY.A.623, 3ZUC.A.1154, 3ZYH.A.1123, 2ZZV.A.501, 3E1I.B.502, 1EUB.A.277, 4K6T.G.408, 1KTW.A.496, 1KTW.A.9, 4L06.A.501, 1LRW.A.702, 4QNP.A.506, 2QPS.A.501, 3R6Q.H.469, 1T9H.A.414, 1W52.X.602, 3DHI.A.601, 1DMH.A.400, 4E2P.A.401, 1FZ6.B.5003, 4GAM.F.602, 2HMO.A.450, 4KVQ.A.302, 4N71.D.201, 3N9T.A.292, 1NNT.A.333, 3O0R.B.803, 4P1B.D.502, 2RDB.A.500, 1SQD.A.500, 3VE1.B.702, 3VVA.B.502, 3W54.A.502, 3WFC.B.803, 2ZI8.A.701, 4AC8.A.500, 1B1X.A.691, 3E1M.B.301, 1F9B.A.691, 2FKZ.C.1601, 3HH8.A.1, 3IS8.A.161, 3IS8.E.162, 1LKO.A.601, 1LKP.A.601, 1LTV.A.900, 4N71.A.202, 3PCH.R.600, 1PFR.B.503, 4PG0.A.302, 3Q1G.B.1002, 3QHB.A.182, 3QVD.C.175, 4RC6.A.301, 1SQ3.A.902, 2UW1.A.1359, 2VZB.B.6204, 3W54.A.501, 3W54.B.501, 1XVE.A.1170, 1YUZ.A.302, 2YU1.A.600, 1YV1.B.301, 4NLZ.A.402, 1Q81.A.8353, 1QVG.0.8317, 1VQ9.Q.9148, 2E5X.A.302, 1GOF.A.702, 3HIJ.A.294, 4IB0.A.401, 3N80.B.602, 2PKC.A.280, 3U27.A.306, 1W8O.A.1654, 1WX5.A.282, 4C0K.A.1614, 3DYQ.B.901, 1G8H.A.523, 3GCD.B.215, 3IO1.A.501, 4JC0.D.406, 4L73.B.405, 4ODN.A.207, 1QY1.A.204, 3ZDU.A.353, 1AG9.A.400, 1AG9.A.401, 4B6C.B.1257, 1GOH.A.702, 3RWK.X.521, 4DOA.A.402, 4DOB.A.402, 2FMQ.A.340, 3JPT.A.340, 4KHY.A.1013, 4NLK.A.403, 2PXI.A.340, 1U8R.A.1101, 1VQ9.C.9104, 2A5G.B.242, 4B6C.A.1257, 2BWU.A.1441, 4C1P.A.1728, 4CBY.B.2036, 1D7U.A.435, 4DD8.A.1005, 3G8Q.D.302, 4G8T.B.502, 4GKI.F.303, 1GVH.A.1399, 4H41.B.405, 3IC9.A.491, 2JBW.A.1368, 3KZW.D.498, 4LCZ.B.320, 4MVJ.A.401, 4NLQ.A.910, 4NT1.A.301, 4NTL.A.311, 4NYP.A.1001, 4O0C.B.402, 4O54.A.302, 2P6Z.A.402, 3R2H.A.156, 1SVY.A.2, 1TC8.A.121, 3VD3.D.3101, 2WD0.C.601, 3WGU.A.2005, 2WWG.B.213, 1YCE.B.201, 1YCE.a.201, 1ZEL.A.401, 3C0W.A.303, 3C5G.B.808, 2FLD.C.603, 4FZX.C.201, 4KLI.A.403, 3MBY.A.340, 2OTL.A.8545, 1Q81.N.8347, 1TW8.B.902, 4UAY.A.403, 3UXP.A.338, 1VQ5.A.9145, 1VQ9.M.9147, 1YIJ.0.8519, 1YJ9.0.8517, 1A5U.G.4732, 2AB8.A.2003, 4ATF.A.500, 3B1Q.C.332, 4B52.A.1305, 3BC9.A.704, 1BGP.A.502, 3BH4.A.489, 4BY5.B.1188, 4CBY.A.2036, 4D1I.B.600, 2DIE.A.781, 4DMI.A.202, 4F4R.A.501, 4FUS.A.828, 2GFH.A.249, 2GJP.A.1489, 3GQ9.A.692, 4H83.B.402, 1HBN.D.1561, 2HEU.A.4001, 1HNO.A.1800, 2HZG.B.1102, 2ID4.A.907, 2IM2.A.3001, 4IQL.A.407, 4J43.A.902, 2JBA.A.1127, 4JQR.A.301, 1JZ4.A.3101, 3KQB.A.303, 3KZW.A.497, 4KZW.A.304, 3ME4.B.2, 4MPY.C.503, 3MV1.3.3101, 4NLV.A.911, 1O04.A.6601, 1OB0.A.504, 4OMG.A.402, 3OQ8.A.460, 3OQ8.C.460, 2P3Z.A.501, 2P6Z.B.401, 2P6Z.B.403, 4PCG.A.302, 3Q2H.A.701, 3Q9C.A.344, 4Q92.C.502, 3R2H.A.157, 3SIS.B.3001, 3SIT.A.3000, 3TAY.A.1, 4TMW.B.902, 4TMX.A.902, 3USZ.A.902, 4US3.A.701, 1V8Z.A.389, 3VD5.B.3101, 3VDC.A.3103, 3VHS.B.52, 1VK1.A.302, 2W4M.A.1245, 3W5N.A.1211, 4WFZ.A.501, 3WGV.C.2005, 2WGM.u.

201, 2WOF.A.1727, 1WPC.A.504, 2WPC.A.1491, 1WX5.C.282, 1XAR.B.201, 2YDG.A.1130, 2YFO.A.1743, 2YXU.B.2406, 1Z45.A.702, 1ZDN.B.157, 3ZK1.D.90, 3ZK2.L.90, 1ZUD.1.7
01
[1] "Cluster 7"
2L1G.A.88, 4LJ0.B.502, 4LMG.D.202, 4MTD.A.202, 4OND.E.101, 4QCL.A.1303, 1QUM.A.3
02, 830C.A.272, 456C.A.272, 3A1Z.A.226, 4A37.A.376, 4A3W.A.1159, 3A52.A.1001, 4A
69.A.500, 2A7G.E.401, 1A7T.A.252, 2A7M.A.252, 4A7B.A.1276, 4A7Y.A.951, 4A7Z.A.95
0, 1A85.A.999, 1A86.A.999, 2A8H.A.486, 4A94.B.501, 2AB7.A.30, 1AFO.A.472, 1AH7.A
.246, 1AH7.A.248, 3AHT.A.1001, 3AIG.A.999, 2AIG.P.999, 2AIO.A.315, 1ALK.A.451, 1
ANJ.B.450, 2ANP.A.501, 2ANP.A.502, 4AR8.A.1731, 4AR9.A.1731, 1AST.A.999, 4ASQ.A.
1615, 4ASR.A.1615, 1ATL.A.401, 4AWY.B.3229, 4AWZ.A.3230, 4AX0.B.3229, 4AX1.B.322
9, 3AYU.A.415, 1B0N.A.1001, 2B13.B.401, 3B2Z.A.1, 1B3D.B.301, 3B35.A.292, 3B3C.A
.501, 3B4N.A.711, 3B4N.B.715, 1B57.A.360, 1B57.A.361, 4B52.A.401, 4B6Z.C.385, 3B
7S.A.701, 3B7U.X.701, 1B8J.A.451, 3B8Z.A.901, 1BA9.A.154, 2BH3.A.1001, 2BH3.A.10
02, 3BHX.A.1751, 3BI0.A.1751, 3BI1.A.1751, 1BIW.B.801, 2BIB.A.1549, 4BIN.A.500,
3BJC.A.876, 4BJH.A.423, 3BKK.A.701, 3BKL.A.701, 3BKN.B.201, 3BKN.B.202, 3BKQ.X.5
00, 1BLL.E.488, 4BLB.A.910, 2BMI.A.272, 2BNN.B.1200, 2BNO.A.1201, 1BOR.A.58, 3BO
O.A.500, 2BO9.A.999, 4BPO.A.1314, 2BP8.B.1340, 1BQO.B.301, 1BS8.A.2001, 4BT4.A.3
01, 4BT5.A.301, 4BT6.A.1257, 3BUB.A.1047, 3BUI.A.1046, 3BVT.A.1048, 3BVU.A.1048,
3BVV.A.1047, 3BVW.A.1046, 3BVX.A.1046, 4BXK.A.1620, 1BYF.B.302, 4BZ3.B.502, 4BZ
5.A.500, 4BZR.A.1630, 4BZS.A.701, 3C0Z.B.101, 4C1H.A.351, 2C20.B.601, 4C24.A.301
, 4C2N.A.701, 4C2O.A.1629, 1C3R.A.501, 1C3S.A.951, 3C52.A.401, 4C5W.A.402, 2C6P.
A.1751, 2C6P.A.1752, 2C6C.A.1751, 4C6L.A.2823, 4C6L.A.2824, 4C8I.B.1161, 1CAM.A.
262, 2CA2.A.264, 4CA5.A.1001, 4CA6.A.1001, 4CA7.A.1616, 4CA8.A.1620, 1CBX.A.309,
2CEA.E.1603, 1CG2.A.500, 1CG2.A.501, 1CGL.A.301, 3CHO.A.701, 2CHI.A.212, 3CKI.A
.501, 2CLB.A.1175, 4COG.A.401, 4COQ.A.299, 1CP7.A.901, 1CP7.A.902, 1CPX.A.308, 5
CPA.A.308, 6CPA.A.308, 7CPA.A.308, 8CPA.A.308, 3CV5.A.1047, 4CVR.A.1159, 4CVT.A.
1158, 4CVT.A.1159, 4CWM.A.433, 1CXV.A.1, 3CZN.A.1102, 3CZS.A.1102, 3D0N.A.262, 4
D0Y.B.1239, 3D10.A.95, 1D1T.C.407, 2D1N.A.270, 2D1O.A.257, 3D4U.A.309, 1D5J.A.30
1, 1D7X.B.801, 1D8D.A.1001, 1DCE.B.900, 4DD8.A.1002, 4DEF.A.401, 4DEL.A.402, 3DF
M.A.401, 3DHA.A.255, 3DID.A.130, 1DK4.B.591, 2DKD.A.921, 4DLM.A.401, 1DO5.B.29,
4DPE.A.301, 4DPR.A.701, 1DQS.A.402, 4DR9.A.201, 3DSX.B.332, 2DSN.A.2001, 2DVT.A.
1501, 2DVX.A.1501, 3DWB.A.771, 2DWO.A.700, 3DYC.A.451, 4DYK.A.501, 4DYO.A.502, 1
DZW.P.999, 4DZH.A.504, 3E0L.A.1452, 3E2D.A.602, 2E2D.A.500, 3E30.B.1001, 3E32.B.
1001, 3E33.B.1001, 3E34.B.1001, 3E37.B.1001, 3E38.A.1, 1E48.S.999, 1E49.S.999, 3
E4A.B.2000, 2E46.A.172, 3EBG.A.1, 3EBH.A.1, 1EC5.B.50, 1EC5.C.50, 1ED9.A.450, 3E
DZ.B.2, 3EER.A.2001, 2EG6.A.400, 2EG7.A.400, 2EG8.A.400, 3EHY.A.264, 1EI6.B.409,
4EJ5.A.501, 2EK8.A.1002, 2EK9.A.1002, 3ELF.A.352, 1ELX.B.451, 3ELM.A.300, 6ENL.
A.438, 3EQN.A.757, 2ERP.A.700, 2EV6.B.2151, 1EW9.A.450, 3EWC.A.372, 3EWD.A.371,
3EWJ.B.2, 4EYF.A.303, 1EZ2.A.402, 1F0J.A.1101, 3F15.A.264, 3F16.A.264, 3F17.A.26
4, 3F18.A.264, 3F19.A.264, 3F1A.A.264, 3F28.A.321, 3F2P.A.2005, 3F3Q.A.104, 1F57
.A.310, 4F7O.A.301, 1FA5.A.1200, 1FA5.A.1201, 3FB4.A.217, 3FDK.A.401, 3FFZ.A.130
0, 2FGN.A.248, 3FGD.A.321, 3FH4.A.301, 3FJU.A.999, 1FKX.A.400, 1FLS.A.166, 4FL7.
A.301, 4FMP.B.400, 1FT7.A.501, 3FU1.B.201, 1FUA.A.216, 3FUK.A.701, 4FU4.A.502, 4
FUA.A.216, 3FVL.A.1309, 2FV5.A.3, 2FVM.D.601, 4FW3.B.300, 4FW4.C.301, 4FW5.A.301
, 4FW7.A.301, 2FYV.A.2003, 4FYQ.A.1012, 4FYR.A.1013, 4FYT.A.1012, 1G05.B.801, 1G
49.B.801, 3G42.A.500, 4G9L.B.301, 4G9L.B.302, 2GA3.A.450, 4GBD.A.502, 1GE7.B.200
, 2GFK.A.401, 2GFJ.A.401, 3GIP.A.484, 1GKP.A.1461, 1GKR.A.1453, 4GK8.A.302, 4GM5
.A.401, 2GO4.A.601, 2GSN.A.1001, 2GSO.A.1000, 4GTM.B.501, 4GTO.B.501, 4GTP.B.501
, 4GTQ.B.501, 4GTR.B.501, 4GTV.B.401, 1GVF.A.288, 2GVI.A.302, 1GYT.G.600, 1GYT.G
.601, 3GZE.A.1, 4H01.A.601, 4H1Q.A.301, 4H2E.A.301, 4H2G.A.601, 4H2K.A.1001, 4H3
X.A.301, 1H48.C.900, 4H49.A.301, 4H76.A.301, 4H82.A.301, 3H90.B.294, 1H9N.A.262,
1HA5.C.4221, 2HBV.A.401, 2HBM.A.1001, 3HC4.L.401, 3HC8.A.864, 2HC9.A.701, 2HC9.
A.702, 1HDU.E.1308, 1HEE.E.1308, 1HFC.A.275, 1HFS.A.257, 2HH5.A.702, 1HI9.A.300,

2HIH.A.601, 1HJK.A.451, 3HK5.A.430, 3HK8.A.430, 3HK9.A.431, 3HKA.A.430, 1HLK.B.1004, 1HOV.A.166, 2HPT.A.950, 1HR6.B.501, 1HR7.B.501, 1HS6.A.701, 2HSE.B.954, 3HTR.B.118, 2HUC.A.248, 3HWP.A.295, 4HWO.A.701, 4HWP.A.701, 4HWR.A.701, 4HWS.A.701, 3HY7.A.901, 3HY9.A.901, 3HYG.A.901, 1HYT.A.805, 1HZ5.A.103, 1HZY.A.402, 1HZY.B.401, 2IOO.A.580, 4I11.A.502, 4I2F.A.603, 2I3C.A.314, 2I47.C.804, 3I9F.A.1, 4IAV.A.401, 4ICR.A.502, 3ID7.A.401, 4IE0.A.601, 4IE4.A.601, 4IE6.A.601, 2IEJ.B.939, 4IE7.A.601, 1IF6.A.262, 1IGB.A.502, 4IG2.A.401, 4IHM.A.402, 2ILP.A.500, 3IQ6.B.201, 3IQ6.C.201, 2IQ6.A.293, 4ISM.A.201, 3IT7.A.183, 3ITC.A.501, 3ITC.A.502, 2IXD.A.1234, 4IXN.A.401, 1IY7.A.308, 2J0T.A.1267, 2J13.A.1236, 1J2U.A.301, 4J4K.A.402, 4J5F.A.301, 4J5H.A.301, 4J5H.A.302, 1J79.B.400, 2J83.A.999, 1J9Y.A.1003, 2J9A.A.1493, 2JAZ.B.600, 1JAP.A.999, 1JAQ.A.999, 4JBS.A.1008, 2JBJ.A.1752, 1JCQ.B.1001, 1JCS.B.1001, 1JCZ.A.901, 4JD1.A.201, 4JE7.A.202, 4JH8.A.201, 4JH8.B.201, 1JI3.A.402, 1JIW.P.481, 2JIG.A.1252, 2JIH.B.1554, 1JJE.A.251, 1JJE.A.252, 1JJT.A.251, 1JJT.A.252, 4JJI.A.402, 4JJJ.A.718, 1JK3.A.400, 1JK9.C.302, 2JLP.B.226, 1JML.A.102, 2JNE.A.150, 2JNE.A.200, 4JP4.A.301, 1JPU.A.371, 2JSD.A.276, 2JT5.A.256, 2JT5.A.257, 2JT6.A.256, 4JYW.A.801, 4JZ0.A.801, 1K07.B.3, 3K2G.A.400, 2K2C.A.140, 2K4W.A.156, 1K4P.A.1004, 1K53.A.1003, 4K5L.A.1101, 4K5M.A.1101, 4K5O.A.1103, 4K5P.A.1101, 4K7W.A.102, 4K90.A.701, 1KAE.B.1102, 1KAP.P.613, 4KA7.A.801, 4KAP.A.301, 4KAY.A.601, 2KBX.B.299, 1KBC.A.999, 1KBE.A.1, 3KDS.E.996, 1KEQ.A.280, 4KEQ.A.302, 4KFT.C.303, 1KH4.A.450, 1KH5.A.450, 2KIK.A.50, 3KM8.A.400, 3KNS.A.229, 1KR6.A.405, 3KR5.G.1004, 1KRO.A.405, 3KRY.A.1999, 1KS7.A.405, 3KS3.A.262, 4KTX.A.501, 3KWO.C.162, 1KYS.A.301, 3L0T.B.2, 1L10.C.1, 4L2L.A.701, 3L3N.A.701, 4L9P.B.601, 1LAM.A.488, 1LAM.A.489, 4LCQ.A.601, 4LCQ.A.602, 1LD7.B.1001, 1LD8.B.1001, 4LE6.A.405, 4LE6.A.406, 4LEF.A.301, 1LFW.A.1001, 2LFF.A.502, 1LGD.A.262, 3LGG.B.512, 3LJT.A.901, 3LJZ.A.999, 3LK8.A.264, 1LND.E.800, 4LNB.B.602, 3LPE.D.60, 4LP6.A.302, 3LQ0.A.999, 4LQG.A.801, 4LQY.A.506, 3LTV.A.1001, 3LUB.B.302, 4LV4.A.401, 2LVH.A.101, 4LW9.A.201, 4LW9.I.201, 4LW9.R.201, 3M2Z.A.500, 2M30.A.201, 2M30.B.201, 3M4C.D.108, 3M4C.D.109, 1M4L.A.1308, 1M5E.A.1705, 3M52.B.116, 3M79.A.107, 3M79.B.107, 3M79.C.107, 3MA2.D.294, 3MA2.A.294, 3MAX.A.379, 4MBG.B.602, 4MCA.B.1000, 3MDJ.A.1000, 3MDU.A.454, 1MH2.A.201, 3MK1.A.902, 3MKV.B.425, 4MKH.A.301, 4MKT.A.701, 1MMB.A.999, 1MMP.A.1, 1MMP.A.2, 1MNC.A.281, 3MO2.D.6, 4MRQ.A.501, 4MSM.C.501, 3MVI.A.901, 3MWM.B.142, 1MXD.A.727, 1MZC.B.1001, 4N07.B.305, 3N2U.A.264, 3N2V.A.264, 4N27.A.201, 3N21.A.401, 1N4Q.B.378, 1N4Q.L.378, 3N9S.A.309, 4NAQ.A.1027, 4NGE.A.1101, 4NGE.E.101, 3NJ9.A.262, 3NKQ.A.1002, 3NKR.A.1002, 3NKN.A.1001, 3NKN.A.1002, 3NKO.A.1002, 1NPC.A.323, 2NQH.A.301, 2NQH.A.303, 4NTK.A.201, 1NW2.D.6004, 1NW2.F.6006, 3NX7.A.264, 3NXQ.A.650, 4NYY.B.501, 2NZE.A.401, 1O1S.B.1001, 3O2X.A.1999, 4O2I.A.401, 4O3A.A.303, 4O3A.B.302, 4O3A.B.303, 2O4Q.A.2401, 1O86.A.701, 1O8A.A.701, 3O90.B.192, 4O98.B.401, 2OB3.A.901, 2OC2.A.701, 3OD4.A.1350, 1OEZ.W.1154, 3OHL.A.4, 3OHL.A.5, 3OHO.A.5, 4OJA.A.202, 2OKL.A.601, 4OKO.A.401, 4ON1.A.400, 4ONG.H.302, 4ONX.B.201, 4OPN.A.201, 4OPN.B.201, 2OR4.A.1751, 1OS9.A.901, 4OTE.B.302, 4OUI.A.501, 2OW0.A.444, 2OW1.A.444, 4OXD.C.301, 2OY2.A.999, 3P24.A.999, 2P2L.A.901, 3P3C.A.401, 1P5X.A.248, 1P6D.A.246, 1P6D.A.248, 1P6E.A.248, 3P76.A.274, 4P9C.J.201, 2PAJ.A.493, 1PB0.A.1302, 1PE5.A.317, 1PE7.A.317, 2PIY.A.400, 2PIZ.A.400, 2PJT.A.302, 2PJ0.A.400, 2PJ3.A.400, 2PJ5.A.400, 2PJ7.A.400, 2PJ8.B.500, 2PJA.B.500, 2PJB.A.400, 2PJC.A.400, 1PL6.A.402, 2PLI.C.701, 2PLM.A.407, 1PMI.A.445, 3PN4.A.1001, 3PNU.A.337, 4PPZ.A.601, 4PQA.A.403, 4PQA.A.404, 1PS6.A.330, 1PSZ.A.1000, 3PSQ.A.326, 3PSQ.B.328, 1PTW.A.501, 4PUC.A.602, 2PVV.A.1752, 2PVW.A.1751, 1PV9.A.401, 1PVW.A.401, 4PVO.A.402, 3PW3.A.406, 4PXY.A.302, 3PZ1.B.332, 3PZ4.B.1, 3Q2G.A.401, 3Q2H.A.401, 1Q3K.B.301, 3Q4R.A.201, 3Q4R.A.202, 2Q5B.A.107, 2Q5B.C.107, 3Q6X.B.271, 3Q75.B.521, 3Q78.B.521, 3Q7A.B.521, 4Q7R.A.307, 3Q9F.B.344, 4QA0.A.401, 4QA1.A.403, 4QA2.A.403, 4QA4.A.502, 4QA5.A.401, 4QA6.A.401, 1QBQ.B.1000, 3QBU.A.294, 1QH5.A.262, 1QIN.A.401, 1QIN.B.301, 3QIZ.A.431, 1QJJ.A.250, 2QLA.B.502, 2QLA.C.501, 2QLA.D.503, 1QMU.A.999, 2QPJ.A.1, 3QW0.A.500, 3QW0.B.500, 3QW0.D.500, 3QW1.C.500, 1QXL.A.400, 2R2D.A.277, 2R2D.A.278, 1R3N.A.500,

1R3N.A.501, 1R42.A.804, 1R43.A.500, 1R43.A.501, 2RFH.A.1308, 3RF4.A.118, 2RH6.A.1, 1RJ5.A.601, 2RJP.A.1, 2RJQ.A.1, 1RK6.A.601, 4RL2.A.301, 4RL2.B.302, 1RM8.A.500, 1RMZ.A.264, 1ROS.A.400, 1RTQ.A.702, 3RTS.A.264, 3RTT.A.264, 3RYM.B.107, 1S0E.A.1291, 1S63.B.1001, 1S64.B.378, 3S9C.A.503, 1SA4.B.439, 1SA5.B.438, 3SEY.E.373, 3SFX.B.521, 3SFY.B.521, 1SHN.A.485, 1SHN.B.482, 3SI0.A.362, 3SJX.A.1751, 3SKS.A.568, 1SML.A.270, 3SNG.A.401, 3SNG.A.402, 3SPU.C.1010, 1SQM.A.1001, 3SZZ.A.502, 1T0A.A.661, 3T00.A.501, 3T00.A.502, 1T3A.B.422, 2TCL.A.170, 1TF9.A.902, 3TGN.B.147, 3TG0.A.501, 3TG0.A.502, 1TKH.A.902, 1TKJ.A.901, 1TKJ.A.902, 4TLN.A.321, 1TLP.E.322, 5TLN.A.321, 1TMN.E.322, 4TMN.E.322, 1TN6.B.1001, 1TN7.B.1001, 1TN8.B.1001, 1TNB.B.378, 1TNO.B.378, 1TNU.B.378, 1TNY.B.378, 1TNZ.B.378, 3TOL.D.107, 3TOL.B.107, 1TQS.A.1401, 1TQT.A.1301, 1TQV.A.1300, 1TQW.A.1400, 3TS4.A.301, 3TT4.A.302, 3TVC.A.501, 4TYT.A.301, 3U04.A.200, 3U24.A.595, 1U7J.A.50, 1U7J.B.150, 1U7M.A.54, 1U7M.B.154, 3U79.D.110, 3U79.E.110, 3U9W.A.2001, 3U93.B.259, 3UBF.A.1, 3UCT.B.102, 1UEA.C.1, 3UHM.A.300, 1URA.A.451, 2USH.A.601, 2USH.A.602, 1UUP.A.2222, 3UWB.A.200, 1UXA.B.1367, 2UX1.A.1174, 2UX1.C.1173, 1UZE.A.701, 3V0A.A.1297, 3V1E.A.101, 2V29.A.1274, 2V29.A.1275, 2V5W.A.1380, 2V5X.A.1377, 3V77.A.301, 3V77.A.302, 2V77.A.1042, 2V9I.A.1277, 3V96.B.301, 2V9E.A.1275, 2V9G.A.1276, 2V9M.A.1275, 3VAT.A.501, 2VES.A.1295, 3VH9.A.302, 2VJ8.A.1611, 3VPE.A.302, 2VQQ.A.1411, 3VQZ.A.302, 3VTG.A.301, 2VUN.A.401, 2VXI.A.201, 2VXI.A.202, 2VXX.C.202, 2W0D.A.1264, 2W15.A.1203, 1W22.A.1375, 2W5V.A.1377, 2W5X.B.1378, 3W5K.B.501, 2W57.B.202, 3W6H.A.302, 1WAA.A.1091, 1WAA.F.1091, 3WAW.A.913, 3WAX.A.912, 3WAY.A.911, 3WBH.A.505, 4WB7.A.502, 4WCM.A.538, 4WD6.A.301, 4WD6.B.302, 4WD8.A.301, 4WD8.A.302, 3WE7.A.301, 2WKN.B.412, 2WM1.A.1333, 4WNC.O.402, 3WOJ.A.805, 2WO8.A.1268, 2WO9.B.1268, 2WO9.C.1269, 2WOA.A.1270, 1WPP.A.602, 3WS9.B.801, 1WUO.A.301, 3WV3.A.301, 2WXU.A.1375, 3WXC.A.301, 2WXT.A.1371, 1WY2.A.405, 1WY2.A.406, 4X2T.B.1002, 2X4H.A.1142, 2X90.A.1618, 2X91.A.1619, 2X92.A.1615, 2X93.A.1615, 2X94.A.1616, 2X97.A.1616, 2X98.A.1475, 2X98.A.1476, 1XAF.B.507, 1XBU.A.901, 1XBU.A.902, 1XGE.A.400, 2XHM.A.1616, 4XIX.A.401, 2XIG.B.1150, 2XIG.C.1153, 1XJO.A.902, 2XL9.B.1269, 1XM8.A.700, 1XP3.A.302, 2XPY.A.1673, 2XR9.A.1867, 2XS4.A.999, 1XXW.A.201, 2XXO.B.1340, 2XX7.B.1264, 2XXG.A.1339, 2XYD.A.1620, 2Y28.A.1181, 2Y2B.B.1180, 2Y3D.B.149, 2Y6D.A.1268, 1Y7W.A.283, 2YD0.A.1946, 1YHC.B.602, 2YJP.A.1272, 2YJP.C.1270, 1YQY.A.781, 2Z2Y.C.2003, 2Z24.B.400, 2Z25.B.401, 2Z29.B.400, 2Z2D.A.265, 2Z3I.C.2003, 2Z72.A.401, 2Z72.A.402, 2ZBM.A.401, 2ZBM.A.402, 1ZED.A.904, 1ZG7.A.400, 1ZG8.A.400, 1ZG9.A.400, 2ZIR.B.901, 2ZIS.B.901, 2ZKW.A.401, 1ZNB.A.2, 2ZNE.B.991, 2ZO4.A.319, 2ZTG.A.902, 3ZU0.A.1595, 3ZU0.A.1596, 3ZU0.B.1588, 3ZU0.B.1589, 2ZWR.A.208, 2ZWR.A.209, 1ZXC.A.201, 3ZXH.A.300, 3ZXO.A.1579, 1ZZM.A.401, 4GNX.C.701, 4L8H.R.105, 2AQ2.B.1001, 2AYK.A.171, 1B8T.A.194, 4BLD.D.910, 1DGS.B.2701, 3DNG.B.998, 2FKM.X.500, 4FXO.A.301, 2FZ6.A.201, 2H6H.B.1001, 2H6F.B.1001, 4H82.B.300, 3H90.D.6, 2JMD.A.66, 2KBX.B.298, 2KIZ.A.71, 2KV1.A.125, 2KVH.A.84, 4LW9.E.204, 2LXH.C.901, 2MQ1.A.102, 1MVH.A.502, 4N07.A.309, 1NDV.A.400, 1OS2.F.769, 2OW2.A.444, 2OX8.B.5, 2POJ.A.265, 1Q9U.B.402, 1R87.A.905, 3U7K.A.350, 3UBF.A.6, 1UD9.B.510, 1V9P.B.2701, 2VW4.A.503, 4WD7.C.302, 3WIE.B.1001, 2WKN.A.412, 2X4H.A.1141, 2X5C.A.1128, 2XAM.B.700, 2Z45.A.1003, 2G8H.A.301, 2VBL.C.1026, 3SH1.A.222, 4DFM.A.903, 3EPH.B.2, 2G8F.A.301, 4IEM.A.502, 2OTJ.0.8044, 1QVG.0.8067, 3SNN.A.906, 1AJD.B.952, 3ATT.A.379, 3AUA.A.601, 4AZW.A.1452, 3B9T.A.485, 4BCL.A.370, 4BCL.A.371, 2BJI.A.2278, 2BVC.A.503, 3CTL.A.601, 3D19.A.301, 2D32.A.1524, 1DAM.A.901, 3DGB.A.2001, 1DXI.A.390, 2EB6.A.1001, 7ENL.A.438, 4EOO.A.301, 3F5U.A.297, 2F9R.B.602, 3FXG.B.501, 3FYY.A.401, 1G65.G.301, 4G61.A.301, 3GIE.A.1, 3GQ7.A.692, 2GQ3.A.1002, 2GQ3.B.1003, 4GWZ.A.402, 4GX6.A.402, 3I6E.B.386, 4IAC.A.402, 4IL6.j.102, 4IL8.A.501, 1IV2.B.1562, 1IV4.E.1565, 1J7U.A.301, 1JB0.A.1126, 1JB0.A.1131, 1JB0.B.1224, 1JB0.B.1236, 1KJI.B.394, 3KR4.A.1004, 1L2O.B.902, 3LD0.8.54, 4LNI.A.503, 1LP4.A.342, 4LYJ.A.201, 2M32.A.401, 4ML9.B.301, 1NOW.A.401, 1N8W.A.900, 4NHO.A.1403, 4NHO.B.1403, 4NZN.A.404, 4O1P.C.903, 4OTP.A.502, 2POC.B.5, 4PV4.A.501, 3Q2O.B.384, 1Q9L.C.218, 2QB8.B.401, 2QVU.A.340, 4QYI.E.203, 4R7O.E.402, 1SJA.B.701, 1SJB.A.1

001, 3SRF.G.533, 3T80.B.160, 4U0M.B.503, 3U2E.A.3, 4U0R.A.699, 3V3Z.L.302, 3V4S.A.402, 2VBI.G.1000, 2VPQ.B.1451, 1W88.A.1368, 2WB4.B.502, 3WNW.A.201, 3WQP.H.501, 3WU2.B.606, 3WU2.B.609, 3WU2.B.612, 3WU2.B.617, 2XTI.A.1551, 2ZDR.A.1001, 3ICE.D.1001, 4IR1.A.902, 1Q81.4.8078, 1RVB.B.302, 4UN4.B.2367, 3ALN.A.406, 1CUL.A.1007, 1D2E.D.504, 3DYF.B.4002, 2E8W.B.1203, 3EN9.A.600, 1GRV.A.490, 3LGH.A.150, 1MXB.A.411, 2O1V.A.755, 1RK2.C.320, 3VN9.A.402, 2VOS.A.1491, 2WCJ.A.1144, 1Z6K.A.275, 2EX5.X.802, 1G9Y.C.803, 4K4G.M.603, 3KHH.A.416, 3KHH.B.1417, 3KHL.B.1415, 3MQ6.A.340, 3NDK.A.906, 4Q10.A.1001, 1RYS.B.803, 2VA2.B.1343, 1AEI.A.318, 1AEI.A.320, 3AFG.B.541, 1AWB.B.279, 3AYU.A.419, 4B7U.D.401, 1B9V.A.500, 2BTW.A.400, 3C5I.A.371, 4CBX.G.1151, 1CIZ.A.305, 3D4G.B.485, 2DDF.A.475, 1DJZ.A.2, 2DSN.A.2011, 2E30.A.300, 1EA7.A.315, 1EGZ.C.300, 4EJ7.C.402, 3ELM.A.303, 7EST.E.11, 2EXH.C.2003, 1F5R.A.800, 3FLT.B.302, 2FYC.B.404, 1FZD.F.1, 3G20.A.201, 1G5N.A.404, 4G64.B.301, 1GMM.A.1132, 3HQ8.A.402, 2HT5.A.995, 2HYW.A.502, 1IJL.B.202, 4ILW.F.305, 2J7A.D.1007, 4JDZ.A.702, 4JQG.A.305, 2K0J.A.501, 4KPP.B.501, 4KTP.B.804, 1KX1.C.301, 3L9I.C.1151, 4LM8.A.812, 1M63.B.500, 4MIV.C.601, 2ML2.A.206, 1NGS.A.681, 3NHH.A.151, 4NIY.A.301, 4NPK.A.803, 1NW1.A.430, 1OLP.B.1372, 4OMC.E.607, 1OT5.A.602, 2OVX.B.449, 2OVZ.A.449, 4OVY.A.409, 3P2P.A.125, 3POW.A.900, 1PZ7.B.702, 2Q1F.B.2002, 3Q3L.B.437, 4Q4X.1.5006, 3RG0.A.1, 2RHP.A.22, 1RM8.A.504, 1SEL.A.277, 3SO0.A.98, 3SO0.B.97, 3SON.A.201, 1SPU.A.802, 2TBV.C.388, 2TCL.A.174, 1TFX.B.4007, 1TK2.A.1276, 1UG9.A.2003, 1V1G.A.1209, 1V3J.B.689, 1V6C.A.505, 3VI4.C.2004, 2W1W.A.1132, 1W2M.E.1442, 1WD9.A.900, 3WIV.B.1004, 1WY9.A.148, 2YC2.B.201, 1Z3U.B.497, 2ZOC.A.504, 2ZUY.A.629, 3FVI.D.125, 4MIV.D.601, 4DU4.A.1003, 4EBC.A.504, 2GIH.A.402, 3QEV.A.906, 4WUZ.B.301, 2AER.L.3005, 2B00.A.252, 4CP1.A.1297, 1D8M.A.304, 3EDD.A.701, 1EN7.A.403, 3FHA.A.705, 1HOV.A.168, 3HR4.H.203, 2HYW.A.506, 2II1.C.404, 1IJL.A.203, 2J60.C.1277, 1JHN.A.900, 1JYI.D.408, 4KS4.A.502, 1LPK.B.1, 1M1J.B.503, 3O82.B.544, 4OO2.C.601, 4P99.B.540, 2Q04.C.211, 1QD6.C.2, 1QHD.A.603, 1RK9.A.112, 4TQO.H.701, 1TRQ.A.106, 1UCN.A.1162, 4UM9.D.2002, 2UX1.K.174, 3WIU.B.1004, 4WK4.B.502, 1XZO.A.1014, 1XZO.B.1018, 2Z2D.A.268, 1ZH2.A.202, 4QQZ.C.1001, 4AIQ.A.1746, 4AM4.A.1161, 1B06.A.322, 2BIW.C.1492, 1BIQ.A.376, 1BIQ.B.376, 4BMT.A.1323, 2BUT.B.1541, 2BUU.B.1541, 2BUV.B.1541, 2BUW.B.600, 2BUM.B.1541, 2BUQ.B.1541, 3CEI.A.500, 3CI8.A.6, 1CJX.A.629, 2CKF.A.501, 2CW3.A.524, 4D8F.B.402, 2DE6.A.501, 1DLM.A.400, 4DVH.A.301, 3E13.X.326, 3E1N.C.300, 3E1Q.A.301, 4ELR.A.401, 1EO2.B.600, 3ESF.A.198, 1EYS.C.611, 3FG3.D.1500, 3FG4.A.1500, 2FKZ.C.1600, 2FLO.A.1602, 3FM3.B.552, 3FVB.A.164, 1FYZ.B.5004, 1FZ0.B.5004, 4G2D.A.402, 4GAM.F.601, 3GHQ.K.300, 1GN6.A.999, 1GUQ.A.351, 3HHX.A.281, 3HHY.A.281, 3HJ8.A.281, 3HJQ.A.281, 3HJS.A.281, 4HJL.A.502, 3HKP.A.281, 1HLM.A.159, 1HMD.A.115, 2HMN.A.450, 2HMQ.C.115, 1HSE.A.400, 2HTN.A.301, 4HVR.A.201, 3I4Y.A.281, 3I51.A.281, 4IEZ.A.501, 3IS8.A.163, 2ITB.A.502, 2IW4.B.1311, 2J2F.A.371, 4J6C.A.502, 1JI5.D.153, 4JPY.A.301, 4KEZ.A.401, 4KVQ.A.301, 1KW6.B.301, 4L7Y.A.201, 4NB8.B.501, 4NB9.A.501, 4NBC.A.501, 4NBG.C.501, 4NBH.A.501, 1NFT.A.333, 1NFV.A.200, 1NX4.A.300, 3O5U.A.300, 3O6R.A.300, 1OQU.B.1004, 2OWT.A.324, 4P1B.D.501, 4P9G.A.401, 3PCD.M.600, 3PCE.N.600, 3PCF.N.600, 3PCN.P.600, 2PCD.N.600, 1PIZ.A.376, 1Q0C.B.500, 2QFR.A.433, 3QFM.B.264, 1QNN.A.201, 3QVD.B.173, 2R1N.A.800, 3R2R.A.155, 3R2R.A.156, 4RC6.A.302, 4REU.A.201, 4REU.A.202, 1RNR.A.402, 1RSV.A.1004, 1SIO.A.321, 1SQ3.A.901, 1TDW.A.425, 4TOH.A.202, 4V06.A.1491, 2VHL.A.1397, 2VP1.A.1347, 2W7W.A.1195, 1XIK.B.377, 1XM8.A.701, 2XRX.M.1461, 2XSH.C.901, 2XSH.G.900, 1YCH.A.501, 1YFX.A.300, 2YFJ.C.901, 1Z6O.M.300, 2FKZ.A.1600, 2FLO.B.1605, 2JD7.0.203, 1MOJ.A.302, 1OQU.A.1001, 1OVB.A.300, 3T81.B.607, 3VV9.C.502, 2XRX.Q.1461, 2XSO.O.900, 1YKP.F.2600, 4DOC.A.402, 4KHN.A.1010, 4KHU.A.1008, 3AR8.A.1000, 4CBY.C.2037, 3DYQ.A.901, 3FKR.A.409, 1IP3.A.999, 1JED.A.525, 4KAF.A.404, 4MVJ.D.402, 2NQL.A.401, 1PX3.A.3101, 4Q4B.A.530, 1S81.A.2, 4TMV.A.903, 3UA6.A.147, 3VD3.C.3101, 3VDA.A.3101, 4XOO.B.401, 3ZPR.B.1, 2BCV.A.579, 1DIZ.A.825, 4FZZ.A.201, 2GIG.A.502, 2GIJ.F.403, 8ICK.A.341, 1JJ2.A.8345, 4KHY.A.1006, 1SUZ.A.403, 1TX3.A.801, 1VQ8.M.9147, 1YJW.A.8545, 2A65.A.751, 1A7T.A.255, 4A87.A.1162, 2ABS.A.1003, 4ATF.C.500, 2B2N.B.1001, 2BB

H.A.301, 4BEM.A.201, 4BEM.B.201, 4BEM.J.201, 1BUN.A.121, 3C7X.A.1001, 3CB8.A.820, 4CD5.A.1420, 4CD5.A.1421, 4CFY.A.302, 3CKI.A.502, 3CTP.A.402, 3CZJ.A.3101, 4D1J.A.601, 4D77.A.1543, 2DDB.B.303, 4DUW.A.3101, 4DUX.A.3101, 3DYM.A.3101, 3DYO.A.3101, 3DYP.A.3101, 1EBU.B.902, 1EJA.A.246, 3EPR.A.266, 4EXR.A.301, 4EZE.A.302, 1F6D.A.1378, 2FBL.A.304, 4FEV.B.303, 3FFZ.A.1302, 4FHA.B.402, 4FLL.A.504, 4FO2.Q.101, 4FXZ.A.603, 3GOT.B.437, 3GBV.B.1, 2GG8.A.503, 2GKO.A.614, 1GNY.A.1244, 1GVF.A.289, 3GVK.A.916, 4GVO.A.702, 4H7O.C.302, 1H80.A.1497, 4H83.A.402, 4H83.E.401, 2HIG.B.488, 1HXN.A.4, 3I3B.A.3101, 3I3D.A.3101, 3IMM.C.3, 4IQZ.A.317, 3IRD.A.301, 4J4B.A.902, 4J4B.B.303, 2JBW.D.1367, 4JEX.A.512, 1JJU.C.996, 4JNQ.A.402, 4JPA.A.305, 3JU4.A.7, 1JYN.A.3101, 1JYV.A.3101, 1JYW.A.3101, 1JZ2.A.3101, 1JZ3.A.3101, 1JZ6.A.3101, 1JZ7.A.3101, 1JZ8.A.3101, 3KEU.A.402, 3KWM.A.225, 4LDE.A.1402, 4LHL.A.301, 3LKB.A.394, 3MAX.A.381, 4MAT.A.501, 4MB4.A.604, 4MMB.A.601, 4MMF.A.601, 3MPN.A.751, 4MPY.A.503, 1MUQ.A.206, 3MUZ.1.3101, 3MV0.1.3101, 3MV1.1.3101, 3MX6.A.261, 3N3R.A.1000, 1NSX.A.1401, 1O4Z.A.1004, 4O4V.A.301, 1O68.E.274, 1OA8.C.1691, 1OAF.A.1252, 4OFI.A.801, 4OMC.A.608, 3ONF.A.507, 4OUA.A.403, 2OZ3.B.2001, 2P3Z.B.501, 2PFL.A.2001, 3PNX.C.169, 3PNX.E.168, 4PV3.A.202, 1PX3.B.3101, 1PX4.A.3101, 3PZJ.A.301, 1Q20.A.313, 1QHU.A.436, 4QKU.A.501, 3QS4.A.751, 3QS5.A.751, 3QS6.A.751, 3QST.A.253, 4QTO.A.501, 4QTO.B.501, 2QWL.A.589, 4R7U.A.504, 3RGA.A.312, 1SK4.A.342, 1SU4.A.997, 3T09.A.3101, 1T64.A.391, 3T8J.A.401, 3TAV.A.267, 4TMW.A.902, 1TO2.E.451, 3TYP.A.155, 1U4J.B.1001, 3UNX.A.281, 1V54.A.3519, 3V5U.A.701, 3V5U.A.703, 3VD3.A.3101, 3VD4.A.3101, 3VD5.A.3101, 3VD7.B.3101, 3VD9.A.3101, 3VDA.B.3101, 3VDC.A.3101, 3VDG.A.502, 1VI6.C.208, 3W0L.C.502, 1W9S.A.1142, 3WA2.X.702, 3WGU.C.2007, 2WGM.A.201, 2WGM.B.201, 2WOI.A.1491, 2WPC.C.1492, 2WWH.C.213, 2WX5.H.1252, 3WZ1.A.401, 1XFF.A.5301, 2Z5D.B.180, 3ZK1.A.90, 3ZK1.B.90, 1ZNB.A.3, 3ZQS.B.1295, 3ZYV.A.2337
[1] "Cluster 8"
2JZW.A.57, 3CQJ.A.285, 4CXO.A.453, 4CXV.A.433, 2DVU.A.1501, 2EG7.A.401, 4ENL.A.438, 2EWB.A.488, 1EYW.A.401, 4EYF.A.302, 1H4N.A.262, 4ILW.D.301, 1J79.A.400, 4JS6.A.301, 1KMG.A.154, 3KNS.A.228, 3L6N.A.301, 2LFF.A.500, 3MJM.B.401, 3MTW.A.1, 3N2C.A.425, 2NQH.A.302, 1ONW.A.800, 1P6B.A.401, 2Q02.C.300, 3Q6X.A.3, 3QDF.A.266, 1QF0.A.320, 1QF1.A.320, 1QTW.A.303, 2QVV.A.403, 3RHG.A.367, 3SXK.A.4, 3TOM.B.108, 1UIO.A.400, 1VFL.A.501, 3W52.A.311, 2XF4.A.1211, 2XS3.A.999, 2Z24.A.400, 2Z25.A.400, 2Z25.B.400, 2Z26.A.401, 2Z27.A.400, 2Z27.B.400, 2Z28.A.400, 2Z28.B.400, 2Z29.A.400, 2Z2A.A.400, 2Z2B.A.338, 1Z60.A.1, 1ZZM.A.402, 3F2D.A.5, 3IE1.D.442, 4MTD.B.201, 3A3O.B.65, 1A7I.A.82, 1ADB.A.375, 3AF5.A.665, 2AF2.B.154, 2ANH.A.451, 2AXR.A.501, 3B1B.A.378, 2B5L.C.3001, 1B8T.A.196, 1BAW.A.107, 1BH5.A.201, 1BQQ.M.289, 3BYW.D.1, 3COY.A.401, 3C0Z.A.101, 2CBN.A.402, 2CG3.Z.1, 3CHP.A.701, 2CIH.A.212, 3CPA.A.308, 3CQJ.B.285, 4CWM.B.433, 1D1T.B.406, 3D3X.A.428, 3D68.A.501, 1D8M.B.801, 3DLJ.A.2001, 3DLJ.B.2002, 1DXW.A.301, 2E26.A.603, 3E50.A.1, 2EC7.A.51, 4EGE.A.411, 3EII.D.301, 2EIM.C.262, 1ELX.A.451, 1ELZ.A.451, 1EPW.A.1291, 4FOR.A.501, 3FDK.A.402, 4FUK.A.401, 4G1P.A.501, 2GLQ.A.2002, 2GSU.A.1001, 4H00.A.601, 4H01.A.602, 2H42.A.501, 3H8F.B.501, 3H90.A.292, 4HGX.B.301, 1HOV.A.165, 1HP7.A.401, 1HYI.A.66, 4IGN.A.401, 3II1.A.571, 2IMC.A.600, 3ITM.A.1, 4J3D.A.301, 1JM7.A.123, 2JRP.A.150, 4JSS.A.301, 4K6T.B.403, 4K7S.B.101, 1KAR.A.501, 1KAR.B.502, 1KHL.A.451, 4KJG.B.1001, 2KVG.A.85, 2LOZ.A.487, 1LG6.A.262, 3LSF.H.2, 2M7Y.A.101, 3MKV.A.425, 4MLX.A.301, 3MO2.D.5, 1MWO.A.438, 4MZ7.A.701, 4N07.B.306, 3N2C.D.426, 2O1Q.B.145, 3O90.A.192, 4O98.A.401, 2OC7.A.901, 1OLP.A.1375, 1P5X.A.247, 3PJN.A.189, 2PTW.A.500, 4PVT.A.404, 3PW3.C.406, 3PW3.D.406, 1PYT.B.350, 3Q31.A.1, 4Q7R.A.305, 3QU6.A.114, 3QZC.A.2, 1R5X.A.122, 4R7M.D.1001, 1RO9.A.529, 3S6L.B.185, 1SMP.A.472, 1TOA.B.760, 1TM6.A.23, 4TQT.A.501, 4U9D.A.205, 3U94.A.259, 3UBF.A.7, 1UUP.D.5222, 1UXA.C.1367, 2VQH.B.1089, 3VUV.A.501, 2VXX.D.201, 3WC5.A.404, 3WI2.B.801, 2WWO.A.1165, 4X2T.D.701, 1XAF.A.503, 2XR1.A.1639, 2XY9.A.1628, 2Y2E.A.1180, 1YIX.A.601, 1YIX.B.603, 2Z2D.A.264, 1Z5R.C.600, 2ZNE.B.992, 1ZSW.A.315, 3ZTV.A.1598, 3ZUQ.A.1440, 3FSY.A.334, 1NUY.A.2342, 4DLG.A.903, 3HO1.X.22, 4IRK.A.402,

2OTL.A.8066, 1VQ7.0.8066, 1YJ9.0.8067, 521P.A.168, 4AG5.B.1588, 3AJP.A.183, 3AL N.B.406, 1AM4.D.679, 1AR1.A.560, 1AZT.B.406, 3BPD.G.126, 1CEE.A.180, 3CIK.A.690, 4D0L.B.2001, 3D19.D.301, 3D19.E.301, 4DVG.A.201, 2DW7.L.2012, 3E40.A.501, 2E8W. B.1204, 4EOP.D.501, 3EQB.A.9002, 3F74.C.1, 4FMA.F.402, 3FPA.B.901, 3GFT.E.202, 3 GOL.A.580, 1GQ9.B.1242, 3GT8.D.14, 4GZM.A.1001, 3H8A.C.1431, 2HAW.A.1001, 2HAW.A .1002, 4HNS.A.201, 3HYT.A.802, 4HYP.C.302, 4I40.A.301, 3IAP.A.3001, 4IL6.C.512, 4IL6.b.616, 1IOV.A.330, 2I07.A.5001, 4JVJ.F.403, 4JVJ.F.404, 4K6T.B.411, 3KRP.D. 903, 3KZ1.E.550, 3LAW.B.1401, 4LCZ.A.316, 1MAB.A.602, 3MG8.G.241, 4MPO.G.203, 4M PO.G.204, 3NCO.C.218, 4NNN.K.302, 2O1X.C.2003, 2O1X.D.2004, 2OPM.A.908, 3OPS.D.5 01, 2OQY.C.402, 2P8E.A.306, 4PRV.A.402, 1Q3H.A.674, 4QEH.A.401, 1ROZ.D.674, 4R9U .D.302, 4RAB.C.303, 3RYW.B.2003, 1SVT.E.601, 3T5P.H.301, 4U3W.A.503, 4UOR.K.699, 4UUX.A.401, 3VHX.G.185, 2VWI.B.1293, 1W1W.A.2001, 1W85.E.1368, 2WCJ.A.1146, 2WH E.A.1222, 3WIG.A.402, 3WNW.J.201, 3WU2.B.616, 3WU2.C.513, 1XD2.A.167, 1YMO.A.402 , 2Z2P.A.1001, 2ZRY.D.702, 3QHQ.A.230, 3TRQ.A.359, 2BOD.A.502, 4G3I.A.401, 4K4I. E.603, 3KHG.A.415, 4KHU.A.1003, 2NOL.A.328, 3OOR.A.236, 4Q0W.A.1001, 4QWD.A.702, 3QZ7.A.363, 3RBD.B.1415, 4AAH.A.702, 4AC8.B.1311, 1ALA.A.401, 1AWB.B.280, 1AXK. A.395, 2BD4.A.260, 1BK9.A.200, 2BZ6.H.1260, 3C14.A.29, 1C9P.A.501, 1D8M.B.804, 1 DM5.E.1135, 3E1I.B.503, 4EJ7.A.404, 1FBL.A.993, 1FBL.A.994, 4FVL.A.506, 4G1M.B.2 001, 3GG1.A.503, 4H82.C.305, 1HKB.A.923, 3HQ8.B.402, 1HVD.A.600, 2II1.D.401, 4IL W.F.304, 2IO4.B.701, 3IS5.F.1, 1ITC.A.1500, 1IVG.B.470, 1J24.A.1001, 1JDC.A.452, 1K6S.A.302, 1K6S.B.301, 1KVY.A.124, 4KW7.A.402, 4L41.B.201, 2LMV.A.151, 4MC7.A. 503, 4MIX.A.2501, 1MWN.A.100, 4N2I.A.707, 1N41.A.410, 1NMB.N.478, 3NSJ.A.702, 4N UY.A.1001, 3OHO.A.1, 4OKH.C.903, 2OVZ.B.449, 3OXQ.D.516, 2OZR.F.4030, 4P99.A.533 , 2PC6.B.303, 1PEX.A.502, 4PIB.B.203, 4POQ.G.401, 4POR.E.401, 2PR3.A.901, 4Q4X.1 .5007, 1QMD.B.405, 3R4I.D.342, 2RJP.D.2, 3RMK.B.308, 3RRV.C.255, 1SEL.B.277, 2TB V.A.388, 1TFX.A.1007, 4UM9.B.2003, 3UMJ.A.902, 2V5C.A.1625, 3V96.B.305, 3VEQ.B.3 01, 2VME.E.500, 3VOB.A.401, 2W0Q.B.803, 2W1W.B.1134, 3W9T.B.509, 3W9T.B.510, 1XJ L.A.344, 1Y7O.B.1004, 1YAX.A.1002, 2YA9.A.1303, 2YDP.B.502, 2YN3.D.6355, 1Z3J.A. 268, 2BKB.A.1193, 2CW2.A.402, 4F2N.B.300, 4FFK.A.301, 2GOJ.A.198, 2GPC.A.195, 3H 1S.A.1001, 1ISA.A.193, 4L2B.A.201, 4L2C.A.201, 3LIO.A.5000, 1MY6.B.200, 2R1K.A.8 00, 3TQJ.A.1001, 1UNF.X.1239, 1WB7.A.212, 1WB8.A.212, 1ZA5.B.393, 4QQW.G.1002, 1 AFR.B.454, 2BI4.A.1384, 4BMT.B.1323, 4BMT.B.1324, 4CVP.A.1155, 3DHG.A.501, 1DTO. A.1601, 3E1N.D.301, 3E1N.H.300, 2FKZ.A.1601, 3FMR.B.401, 1FZH.B.5004, 2GBX.A.456 , 1GUP.A.351, 3I4V.A.281, 3ICF.A.602, 1IDS.C.208, 3IS8.N.162, 2ITB.A.501, 4IWK.F .201, 1JYB.A.600, 3KCY.A.1350, 4KEV.D.401, 3LKT.M.600, 1MOJ.B.301, 1N7X.A.339, 1 NFV.A.201, 1NFV.M.200, 3NJZ.A.369, 1NNF.A.401, 1O7P.A.1453, 1OQ4.B.364, 1OS7.C.3 02, 2P6B.C.513, 3PCA.N.600, 3PCE.M.600, 3PCK.Q.600, 3PCL.R.600, 1PFR.A.502, 1PIU .A.401, 2QJE.A.692, 3R2M.A.155, 1RSR.A.1004, 1SQ3.C.907, 1T47.A.431, 4TOA.B.203, 4TOE.A.203, 3USS.B.212, 2VC7.D.1315, 1W2N.A.312, 2XSO.K.900, 2Z4G.A.503, 2ZQX.A .501, 4DOC.A.404, 3E45.A.260, 2ISP.A.340, 3JPS.A.340, 4M47.A.403, 2OTJ.R.8537, 1 Q81.M.8380, 1YIT.0.8517, 2A2A.C.3158, 3AXG.A.3005, 3C17.A.324, 4CH8.D.1580, 1CM5 .A.1056, 4D9U.A.901, 1DI4.A.501, 3DR3.A.336, 3DYQ.A.902, 2E7U.A.1002, 1EBU.A.901 , 4ENZ.A.1112, 1F7T.A.472, 1F7T.C.474, 4FOI.A.1005, 4FOI.A.1006, 2FQE.A.901, 3GA 5.A.700, 2GG2.A.703, 3H1V.X.600, 4HCH.A.407, 3HVU.D.182, 4HXV.A.403, 3I3D.C.3101 , 3IAQ.C.3101, 3IGQ.F.801, 4J2H.A.306, 2J5W.A.3043, 4JRX.D.301, 3K13.A.647, 4K7V .A.407, 3L27.B.3, 1MXO.D.901, 3NRB.C.287, 4O4W.A.301, 3OB8.A.3006, 4ODI.A.301, 3 POJ.A.711, 3PJ0.C.367, 2POC.D.5004, 1PX4.A.3104, 1QJS.A.512, 1QJS.A.513, 1QJS.A. 523, 2QZ7.A.195, 4R3W.B.402, 1R4P.F.4004, 3T2Q.D.3101, 3UA7.A.145, 1UD2.A.1003, 3V6N.A.232, 3VD7.A.3101, 3W0L.A.502, 1W90.A.1154, 2WOI.D.1488, 3WV2.A.305, 1XAR. B.200, 1XC6.A.8001, 1YCE.A.201, 1YCE.C.201, 3B1N.A.403, 2BL2.A.1157, 2DKB.A.436, 3MS8.A.401, 3MUI.A.401, 1MVO.A.202, 1N82.A.401, 2PPL.A.480, 2Q8X.A.401

Normal vs Compressed Enrichments: p-adjusted <= 0.05

Robert M Flight

2016-11-10 15:21:40

Contents

Purpose 1

Tables 1

all . . . 1
ca . . . 13
fe . . . 17
mg . . . 28
na . . . 28
zn . . . 29

Purpose

Generate PDF output of the gene ontology enrichment results.

Tables

all

Table S149: all 4 ligands

					normal					compress				
id	description	type	IPR.group	consistent	p	padjust	metal	perc	sig	p	padjust	metal	perc	sig
GO:0008270	zinc ion binding	MF	J	TRUE	$3.040e-14$	$6.627e-11$	zn	0.9827	TRUE	1	1	zn	1.0000	FALSE
GO:0046914	transition metal ion binding	MF	J	TRUE	$2.388e-06$	$1.041e-03$	zn	0.9234	TRUE	1	1	fe	0.3333	FALSE

Table S150: all 5 ligands

					normal					compress				
id	description	type	IPR.group	consistent	p	padjust	metal	perc	sig	p	padjust	metal	perc	sig
GO:0044699	single-organism process	BP	AS	TRUE	$2.530e-05$	$4.297e-03$	fe	0.3564	TRUE	$1.000e+00$	$1.000e+00$	zn	0.4938	FALSE
GO:0044710	single-organism metabolic process	BP	AS	TRUE	$7.700e-05$	$1.106e-02$	fe	0.3793	TRUE	$1.000e+00$	$1.000e+00$	zn	0.5000	FALSE
GO:0004784	superoxide dismutase activity	MF	AU	TRUE	$1.306e-04$	$1.459e-02$	fe	0.9310	TRUE	$1.000e+00$	$1.000e+00$	NA	NA	FALSE

GO:0016721	oxidoreductase activity, acting on superoxide radicals as acceptor	MF	AU	TRUE	$1.306e-04$	$1.459e-02$	fe	0.9310	TRUE	$1.000e+00$	$1.000e+00$	NA	NA	FALSE
GO:0015979	photosynthesis	BP	AV	TRUE	$2.280e-04$	$2.366e-02$	mg	0.8293	TRUE	$1.000e+00$	$1.000e+00$	ca	0.5000	FALSE
GO:0044711	single-organism biosynthetic process	BP	AY	TRUE	$5.032e-04$	$4.700e-02$	zn	0.4719	TRUE	$9.998e-01$	$1.000e+00$	zn	0.4615	FALSE
GO:0097159	organic cyclic compound binding	MF	AZ	TRUE	$1.868e-18$	$8.723e-16$	fe	0.5253	TRUE	$1.000e+00$	$1.000e+00$	zn	0.4524	FALSE
GO:1901363	heterocyclic compound binding	MF	AZ	TRUE	$1.868e-18$	$8.723e-16$	fe	0.5253	TRUE	$1.000e+00$	$1.000e+00$	zn	0.4524	FALSE
GO:0004601	peroxidase activity	MF	BD	TRUE	$6.908e-08$	$1.843e-05$	fe	0.7925	TRUE	$1.000e+00$	$1.000e+00$	NA	NA	FALSE
GO:0006979	response to oxidative stress	BP	BD	TRUE	$4.712e-06$	$8.802e-04$	fe	0.7500	TRUE	$1.000e+00$	$1.000e+00$	zn	1.0000	FALSE
GO:0016209	antioxidant activity	MF	BD	TRUE	$4.577e-12$	$1.710e-09$	fe	0.8313	TRUE	$1.000e+00$	$1.000e+00$	NA	NA	FALSE
GO:0016684	oxidoreductase activity, acting on peroxide as acceptor	MF	BD	TRUE	$6.908e-08$	$1.843e-05$	fe	0.7925	TRUE	$1.000e+00$	$1.000e+00$	NA	NA	FALSE
GO:0004096	catalase activity	MF	BO	TRUE	$4.536e-04$	$4.459e-02$	fe	0.8000	TRUE	$1.000e+00$	$1.000e+00$	NA	NA	FALSE
GO:0015669	gas transport	BP	BP	TRUE	$1.328e-04$	$1.459e-02$	fe	0.9535	TRUE	$1.000e+00$	$1.000e+00$	fe	0.5000	FALSE
GO:0015671	oxygen transport	BP	BP	TRUE	$1.328e-04$	$1.459e-02$	fe	0.9535	TRUE	$1.000e+00$	$1.000e+00$	fe	0.5000	FALSE
GO:0019825	oxygen binding	MF	BP	TRUE	$2.499e-07$	$5.835e-05$	fe	0.9722	TRUE	$1.000e+00$	$1.000e+00$	fe	0.3333	FALSE
GO:0016491	oxidoreductase activity	MF	F	TRUE	$3.237e-06$	$6.719e-04$	fe	0.7286	TRUE	$1.000e+00$	$1.000e+00$	fe	0.4595	FALSE
GO:0055114	oxidation-reduction process	BP	F	TRUE	$4.779e-05$	$7.440e-03$	fe	0.6626	TRUE	$1.000e+00$	$1.000e+00$	zn	0.4706	FALSE
GO:0020037	heme binding	MF	O	TRUE	$2.660e-19$	$2.485e-16$	fe	0.9020	TRUE	$1.000e+00$	$1.000e+00$	zn	0.6667	FALSE
GO:0046906	tetrapyrrole binding	MF	O	TRUE	$1.052e-19$	$1.965e-16$	fe	0.8889	TRUE	$1.000e+00$	$1.000e+00$	zn	0.6667	FALSE
GO:0006873	cellular ion homeostasis	BP	AW	TRUE	$9.999e-01$	$1.000e+00$	fe	0.4545	FALSE	$6.369e-04$	$2.704e-02$	zn	0.5333	TRUE
GO:0006875	cellular metal ion homeostasis	BP	AW	TRUE	$9.999e-01$	$1.000e+00$	fe	0.4545	FALSE	$6.369e-04$	$2.704e-02$	zn	0.5333	TRUE
GO:0006879	cellular iron ion homeostasis	BP	AW	TRUE	$9.999e-01$	$1.000e+00$	fe	0.4545	FALSE	$6.369e-04$	$2.704e-02$	zn	0.5333	TRUE
GO:0019725	cellular homeostasis	BP	AW	TRUE	$9.999e-01$	$1.000e+00$	fe	0.4615	FALSE	$3.675e-04$	$2.452e-02$	zn	0.5882	TRUE
GO:0030001	metal ion transport	BP	AW	TRUE	$9.999e-01$	$1.000e+00$	fe	0.3636	FALSE	$6.369e-04$	$2.704e-02$	fe	0.4000	TRUE
GO:0030003	cellular cation homeostasis	BP	AW	TRUE	$9.999e-01$	$1.000e+00$	fe	0.4545	FALSE	$6.369e-04$	$2.704e-02$	zn	0.5333	TRUE
GO:0042592	homeostatic process	BP	AW	TRUE	$9.999e-01$	$1.000e+00$	fe	0.4615	FALSE	$3.675e-04$	$2.452e-02$	zn	0.5882	TRUE
GO:0046916	cellular transition metal ion homeostasis	BP	AW	TRUE	$9.999e-01$	$1.000e+00$	fe	0.4545	FALSE	$6.369e-04$	$2.704e-02$	zn	0.5333	TRUE
GO:0050801	ion homeostasis	BP	AW	TRUE	$9.999e-01$	$1.000e+00$	fe	0.4545	FALSE	$6.369e-04$	$2.704e-02$	zn	0.5333	TRUE
GO:0055065	metal ion homeostasis	BP	AW	TRUE	$9.999e-01$	$1.000e+00$	fe	0.4545	FALSE	$6.369e-04$	$2.704e-02$	zn	0.5333	TRUE
GO:0055072	iron ion homeostasis	BP	AW	TRUE	$9.999e-01$	$1.000e+00$	fe	0.4545	FALSE	$6.369e-04$	$2.704e-02$	zn	0.5333	TRUE
GO:0055076	transition metal ion homeostasis	BP	AW	TRUE	$9.999e-01$	$1.000e+00$	fe	0.4545	FALSE	$6.369e-04$	$2.704e-02$	zn	0.5333	TRUE
GO:0055080	cation homeostasis	BP	AW	TRUE	$9.999e-01$	$1.000e+00$	fe	0.4545	FALSE	$6.369e-04$	$2.704e-02$	zn	0.5333	TRUE
GO:0065008	regulation of biological quality	BP	AW	TRUE	$9.998e-01$	$1.000e+00$	fe	0.4000	FALSE	$4.642e-04$	$2.704e-02$	zn	0.5833	TRUE
GO:0098771	inorganic ion homeostasis	BP	AW	TRUE	$9.999e-01$	$1.000e+00$	fe	0.4545	FALSE	$6.369e-04$	$2.704e-02$	zn	0.5333	TRUE
GO:0001071	nucleic acid binding transcription factor activity	MF	AX	FALSE	$1.000e+00$	$1.000e+00$	zn	1.0000	FALSE	$1.515e-04$	$1.436e-02$	zn	1.0000	TRUE
GO:0003700	transcription factor activity, sequence-specific DNA binding	MF	AX	FALSE	$1.000e+00$	$1.000e+00$	zn	1.0000	FALSE	$1.515e-04$	$1.436e-02$	zn	1.0000	TRUE

GO:0006355	regulation of transcription, DNA-templated	BP	AX	FALSE	$1.000e+00$	$1.000e+00$	fe	0.5000	FALSE	$1.768e-04$	$1.436e-02$	zn	1.0000	TRUE
GO:0010468	regulation of gene expression	BP	AX	FALSE	$1.000e+00$	$1.000e+00$	fe	0.5000	FALSE	$1.768e-04$	$1.436e-02$	zn	1.0000	TRUE
GO:0010556	regulation of macromolecule biosynthetic process	BP	AX	FALSE	$9.999e-01$	$1.000e+00$	fe	0.4000	FALSE	$4.329e-04$	$2.608e-02$	zn	1.0000	TRUE
GO:0019219	regulation of nucleobase-containing compound metabolic process	BP	AX	FALSE	$9.999e-01$	$1.000e+00$	fe	0.4000	FALSE	$4.329e-04$	$2.608e-02$	zn	1.0000	TRUE
GO:0051252	regulation of RNA metabolic process	BP	AX	FALSE	$1.000e+00$	$1.000e+00$	fe	0.5000	FALSE	$1.768e-04$	$1.436e-02$	zn	1.0000	TRUE
GO:0060255	regulation of macromolecule metabolic process	BP	AX	FALSE	$1.000e+00$	$1.000e+00$	zn	0.4286	FALSE	$2.924e-04$	$2.101e-02$	zn	1.0000	TRUE
GO:0080090	regulation of primary metabolic process	BP	AX	FALSE	$1.000e+00$	$1.000e+00$	zn	0.4286	FALSE	$2.924e-04$	$2.101e-02$	zn	1.0000	TRUE
GO:1903506	regulation of nucleic acid-templated transcription	BP	AX	FALSE	$1.000e+00$	$1.000e+00$	fe	0.5000	FALSE	$1.768e-04$	$1.436e-02$	zn	1.0000	TRUE
GO:2000112	regulation of cellular macromolecule biosynthetic process	BP	AX	FALSE	$9.999e-01$	$1.000e+00$	fe	0.4000	FALSE	$4.329e-04$	$2.608e-02$	zn	1.0000	TRUE
GO:2001141	regulation of RNA biosynthetic process	BP	AX	FALSE	$1.000e+00$	$1.000e+00$	fe	0.5000	FALSE	$1.768e-04$	$1.436e-02$	zn	1.0000	TRUE
GO:0004180	carboxypeptidase activity	MF	BC	TRUE	$1.000e+00$	$1.000e+00$	ca	1.0000	FALSE	$1.496e-09$	$1.397e-06$	zn	1.0000	TRUE
GO:0004181	metallocarboxypeptidase activity	MF	BC	TRUE	$1.000e+00$	$1.000e+00$	ca	1.0000	FALSE	$1.496e-09$	$1.397e-06$	zn	1.0000	TRUE
GO:0005509	calcium ion binding	MF	BI	TRUE	$1.000e+00$	$1.000e+00$	ca	0.8750	FALSE	$9.744e-06$	$2.022e-03$	ca	0.7447	TRUE
GO:0008235	metalloexopeptidase activity	MF	BQ	TRUE	$1.000e+00$	$1.000e+00$	zn	0.6875	FALSE	$3.094e-05$	$3.873e-03$	zn	0.9091	TRUE
GO:0008238	exopeptidase activity	MF	BQ	TRUE	$1.000e+00$	$1.000e+00$	zn	0.7826	FALSE	$1.585e-07$	$9.872e-05$	zn	0.9394	TRUE
GO:0008283	cell proliferation	BP	BR	TRUE	$1.000e+00$	$1.000e+00$	NA	NA	FALSE	$2.188e-05$	$3.144e-03$	zn	1.0000	TRUE
GO:0018342	protein prenylation	BP	BR	TRUE	$1.000e+00$	$1.000e+00$	NA	NA	FALSE	$3.802e-07$	$1.421e-04$	zn	1.0000	TRUE
GO:0018343	protein farnesylation	BP	BR	TRUE	$1.000e+00$	$1.000e+00$	NA	NA	FALSE	$2.188e-05$	$3.144e-03$	zn	1.0000	TRUE
GO:0042127	regulation of cell proliferation	BP	BR	TRUE	$1.000e+00$	$1.000e+00$	NA	NA	FALSE	$2.188e-05$	$3.144e-03$	zn	1.0000	TRUE
GO:0097354	prenylation	BP	BR	TRUE	$1.000e+00$	$1.000e+00$	NA	NA	FALSE	$3.802e-07$	$1.421e-04$	zn	1.0000	TRUE
GO:0043167	ion binding	MF	BW	TRUE	$9.993e-01$	$1.000e+00$	fe	0.3124	FALSE	$9.969e-04$	$3.880e-02$	zn	0.5625	TRUE
GO:0043169	cation binding	MF	BW	TRUE	$9.998e-01$	$1.000e+00$	fe	0.3654	FALSE	$2.501e-04$	$1.947e-02$	zn	0.6022	TRUE
GO:0046872	metal ion binding	MF	BW	TRUE	$9.993e-01$	$1.000e+00$	fe	0.3663	FALSE	$9.921e-04$	$3.880e-02$	zn	0.6133	TRUE
GO:0044238	primary metabolic process	BP	BX	TRUE	$9.994e-01$	$1.000e+00$	zn	0.3761	FALSE	$9.171e-04$	$3.724e-02$	zn	0.6732	TRUE
GO:0052689	carboxylic ester hydrolase activity	MF	N	TRUE	$9.999e-01$	$1.000e+00$	ca	0.5714	FALSE	$7.457e-04$	$3.096e-02$	ca	0.5000	TRUE
GO:0006508	proteolysis	BP	V	TRUE	$1.000e+00$	$1.000e+00$	zn	0.5119	FALSE	$3.493e-05$	$4.078e-03$	zn	0.8557	TRUE
GO:0008233	peptidase activity	MF	V	TRUE	$1.000e+00$	$1.000e+00$	zn	0.5291	FALSE	$1.308e-05$	$2.444e-03$	zn	0.8614	TRUE
GO:0008237	metallopeptidase activity	MF	V	TRUE	$1.000e+00$	$1.000e+00$	zn	0.6692	FALSE	$3.595e-06$	$8.395e-04$	zn	0.8941	TRUE
GO:0019538	protein metabolic process	BP	V	TRUE	$1.000e+00$	$1.000e+00$	zn	0.4828	FALSE	$1.039e-06$	$3.233e-04$	zn	0.8167	TRUE
GO:0043170	macromolecule metabolic process	BP	V	TRUE	$1.000e+00$	$1.000e+00$	zn	0.4313	FALSE	$1.468e-06$	$3.918e-04$	zn	0.7917	TRUE

GO:0070011	peptidase activity, acting on L-amino acid peptides	MF	V	TRUE	$1.000e+00$	$1.000e+00$	zn	0.5291	FALSE	$3.110e-05$	$3.873e-03$	zn	0.8586	TRUE

Table S151: all 6 ligands

					normal					compress				
id	description	type	IPR.group	consistent	p	padjust	metal	perc	sig	p	padjust	metal	perc	sig
GO:0004601	peroxidase activity	MF	AP	TRUE	$1.283e-04$	$1.274e-02$	fe	0.9123	TRUE	$1.000e+00$	$1.000e+00$	NA	NA	FALSE
GO:0006979	response to oxidative stress	BP	AP	TRUE	$2.424e-04$	$1.932e-02$	fe	0.9057	TRUE	$1.000e+00$	$1.000e+00$	NA	NA	FALSE
GO:0016209	antioxidant activity	MF	AP	TRUE	$1.283e-04$	$1.274e-02$	fe	0.9123	TRUE	$1.000e+00$	$1.000e+00$	NA	NA	FALSE
GO:0016684	oxidoreductase activity, acting on peroxide as acceptor	MF	AP	TRUE	$1.283e-04$	$1.274e-02$	fe	0.9123	TRUE	$1.000e+00$	$1.000e+00$	NA	NA	FALSE
GO:0020037	heme binding	MF	AR	TRUE	$2.053e-28$	$2.243e-25$	fe	0.9750	TRUE	$1.000e+00$	$1.000e+00$	fe	0.5833	FALSE
GO:0046906	tetrapyrrole binding	MF	AR	TRUE	$2.053e-28$	$2.243e-25$	fe	0.9750	TRUE	$1.000e+00$	$1.000e+00$	fe	0.5833	FALSE
GO:0016791	phosphatase activity	MF	AY	TRUE	$5.142e-05$	$7.379e-03$	mg	0.8043	TRUE	$1.000e+00$	$1.000e+00$	mg	0.5000	FALSE
GO:0042578	phosphoric ester hydrolase activity	MF	AY	TRUE	$1.702e-05$	$3.718e-03$	mg	0.6774	TRUE	$1.000e+00$	$1.000e+00$	ca	0.2500	FALSE
GO:0036094	small molecule binding	MF	F	TRUE	$1.519e-04$	$1.348e-02$	mg	0.8878	TRUE	$9.999e-01$	$1.000e+00$	mg	0.7073	FALSE
GO:0019825	oxygen binding	MF	I	TRUE	$4.858e-05$	$7.379e-03$	fe	1.0000	TRUE	$1.000e+00$	$1.000e+00$	fe	0.5000	FALSE
GO:0005525	GTP binding	MF	V	TRUE	$3.936e-08$	$1.229e-05$	mg	0.9871	TRUE	$1.000e+00$	$1.000e+00$	mg	1.0000	FALSE
GO:0007154	cell communication	BP	V	TRUE	$8.547e-05$	$1.099e-02$	mg	0.8246	TRUE	$1.000e+00$	$1.000e+00$	ca	0.4000	FALSE
GO:0007165	signal transduction	BP	V	TRUE	$3.354e-05$	$6.606e-03$	mg	0.8294	TRUE	$1.000e+00$	$1.000e+00$	mg	0.4444	FALSE
GO:0007264	small GTPase mediated signal transduction	BP	V	TRUE	$1.196e-04$	$1.274e-02$	mg	0.9908	TRUE	$1.000e+00$	$1.000e+00$	mg	0.7500	FALSE
GO:0019001	guanyl nucleotide binding	MF	V	TRUE	$8.999e-09$	$3.933e-06$	mg	0.9879	TRUE	$1.000e+00$	$1.000e+00$	mg	1.0000	FALSE
GO:0023052	signaling	BP	V	TRUE	$2.476e-04$	$1.932e-02$	mg	0.8294	TRUE	$9.999e-01$	$1.000e+00$	ca	0.4545	FALSE
GO:0032561	guanyl ribonucleotide binding	MF	V	TRUE	$3.936e-08$	$1.229e-05$	mg	0.9871	TRUE	$1.000e+00$	$1.000e+00$	mg	1.0000	FALSE
GO:0035556	intracellular signal transduction	BP	V	TRUE	$5.403e-05$	$7.379e-03$	mg	0.9603	TRUE	$1.000e+00$	$1.000e+00$	mg	0.6000	FALSE
GO:0044700	single organism signaling	BP	V	TRUE	$9.532e-05$	$1.157e-02$	mg	0.8294	TRUE	$1.000e+00$	$1.000e+00$	ca	0.4000	FALSE
GO:0050794	regulation of cellular process	BP	V	TRUE	$3.628e-05$	$6.606e-03$	mg	0.7926	TRUE	$1.000e+00$	$1.000e+00$	ca	0.3636	FALSE
GO:0050896	response to stimulus	BP	V	TRUE	$5.124e-05$	$7.379e-03$	mg	0.6043	TRUE	$1.000e+00$	$1.000e+00$	ca	0.2857	FALSE
GO:0016462	pyrophosphatase activity	MF	Y	TRUE	$2.252e-04$	$1.892e-02$	mg	0.9135	TRUE	$1.000e+00$	$1.000e+00$	ca	0.2500	FALSE
GO:0016817	hydrolase activity, acting on acid anhydrides	MF	Y	TRUE	$1.542e-04$	$1.348e-02$	mg	0.9159	TRUE	$1.000e+00$	$1.000e+00$	ca	0.2500	FALSE
GO:0016818	hydrolase activity, acting on acid anhydrides, in phosphorus-containing anhydrides	MF	Y	TRUE	$1.542e-04$	$1.348e-02$	mg	0.9159	TRUE	$1.000e+00$	$1.000e+00$	ca	0.2500	FALSE
GO:0017111	nucleoside-triphosphatase activity	MF	Y	TRUE	$1.261e-05$	$3.061e-03$	mg	0.9432	TRUE	$1.000e+00$	$1.000e+00$	mg	1.0000	FALSE
GO:0009055	electron carrier activity	MF	Z	TRUE	$1.320e-07$	$3.606e-05$	fe	0.9585	TRUE	$1.000e+00$	$1.000e+00$	fe	1.0000	FALSE
GO:0005509	calcium ion binding	MF	AB	TRUE	$1.000e+00$	$1.000e+00$	ca	0.7639	FALSE	$6.091e-23$	$1.331e-19$	ca	0.8507	TRUE
GO:0006873	cellular ion homeostasis	BP	AK	TRUE	$1.000e+00$	$1.000e+00$	fe	0.5625	FALSE	$6.995e-05$	$5.458e-03$	fe	0.8462	TRUE
GO:0006875	cellular metal ion homeostasis	BP	AK	TRUE	$1.000e+00$	$1.000e+00$	fe	0.5625	FALSE	$6.995e-05$	$5.458e-03$	fe	0.8462	TRUE
GO:0006879	cellular iron ion homeostasis	BP	AK	TRUE	$1.000e+00$	$1.000e+00$	fe	0.5625	FALSE	$6.995e-05$	$5.458e-03$	fe	0.8462	TRUE

GO:0019725	cellular homeostasis	BP	AK	TRUE	$1.000e+00$	$1.000e+00$	fe	0.5882	FALSE	$1.076e-04$	$7.346e-03$	fe	0.8462	TRUE
GO:0030003	cellular cation homeostasis	BP	AK	TRUE	$1.000e+00$	$1.000e+00$	fe	0.5625	FALSE	$6.995e-05$	$5.458e-03$	fe	0.8462	TRUE
GO:0042592	homeostatic process	BP	AK	TRUE	$1.000e+00$	$1.000e+00$	fe	0.5882	FALSE	$1.076e-04$	$7.346e-03$	fe	0.8462	TRUE
GO:0046916	cellular transition metal ion homeostasis	BP	AK	TRUE	$1.000e+00$	$1.000e+00$	fe	0.5625	FALSE	$6.995e-05$	$5.458e-03$	fe	0.8462	TRUE
GO:0048878	chemical homeostasis	BP	AK	TRUE	$1.000e+00$	$1.000e+00$	fe	0.5882	FALSE	$1.076e-04$	$7.346e-03$	fe	0.8462	TRUE
GO:0050801	ion homeostasis	BP	AK	TRUE	$1.000e+00$	$1.000e+00$	fe	0.5625	FALSE	$6.995e-05$	$5.458e-03$	fe	0.8462	TRUE
GO:0055065	metal ion homeostasis	BP	AK	TRUE	$1.000e+00$	$1.000e+00$	fe	0.5625	FALSE	$6.995e-05$	$5.458e-03$	fe	0.8462	TRUE
GO:0055072	iron ion homeostasis	BP	AK	TRUE	$1.000e+00$	$1.000e+00$	fe	0.5625	FALSE	$6.995e-05$	$5.458e-03$	fe	0.8462	TRUE
GO:0055076	transition metal ion homeostasis	BP	AK	TRUE	$1.000e+00$	$1.000e+00$	fe	0.5625	FALSE	$6.995e-05$	$5.458e-03$	fe	0.8462	TRUE
GO:0055080	cation homeostasis	BP	AK	TRUE	$1.000e+00$	$1.000e+00$	fe	0.5625	FALSE	$6.995e-05$	$5.458e-03$	fe	0.8462	TRUE
GO:0055082	cellular chemical homeostasis	BP	AK	TRUE	$1.000e+00$	$1.000e+00$	fe	0.5882	FALSE	$1.076e-04$	$7.346e-03$	fe	0.8462	TRUE
GO:0098771	inorganic ion homeostasis	BP	AK	TRUE	$1.000e+00$	$1.000e+00$	fe	0.5625	FALSE	$6.995e-05$	$5.458e-03$	fe	0.8462	TRUE
GO:0008199	ferric iron binding	MF	AN	TRUE	$1.000e+00$	$1.000e+00$	fe	0.5333	FALSE	$2.442e-07$	$1.067e-04$	fe	0.9412	TRUE
GO:0008484	sulfuric ester hydrolase activity	MF	AU	TRUE	$1.000e+00$	$1.000e+00$	NA	NA	FALSE	$6.020e-05$	$5.458e-03$	ca	0.4000	TRUE
GO:0009767	photosynthetic electron transport chain	BP	BC	TRUE	$1.000e+00$	$1.000e+00$	fe	0.5000	FALSE	$2.843e-08$	$1.553e-05$	fe	1.0000	TRUE
GO:0009772	photosynthetic electron transport in photosystem II	BP	BC	TRUE	$1.000e+00$	$1.000e+00$	mg	1.0000	FALSE	$5.018e-09$	$3.655e-06$	fe	1.0000	TRUE
GO:0015979	photosynthesis	BP	BC	TRUE	$9.999e-01$	$1.000e+00$	fe	0.9333	FALSE	$4.770e-04$	$2.895e-02$	fe	1.0000	TRUE
GO:0019684	photosynthesis, light reaction	BP	BC	TRUE	$1.000e+00$	$1.000e+00$	fe	0.8000	FALSE	$1.055e-06$	$3.293e-04$	fe	1.0000	TRUE
GO:0045156	electron transporter, transferring electrons within the cyclic electron transport pathway of photosynthesis activity	MF	BC	TRUE	$1.000e+00$	$1.000e+00$	mg	1.0000	FALSE	$5.018e-09$	$3.655e-06$	fe	1.0000	TRUE
GO:0043169	cation binding	MF	BO	TRUE	$1.000e+00$	$1.000e+00$	fe	0.5008	FALSE	$1.381e-06$	$3.773e-04$	ca	0.4452	TRUE
GO:0046872	metal ion binding	MF	BO	TRUE	$1.000e+00$	$1.000e+00$	fe	0.5052	FALSE	$3.578e-07$	$1.303e-04$	ca	0.4375	TRUE
GO:0004175	endopeptidase activity	MF	BS	TRUE	$9.999e-01$	$1.000e+00$	ca	0.7656	FALSE	$2.209e-04$	$1.419e-02$	ca	0.6538	TRUE
GO:0006508	proteolysis	BP	BS	TRUE	$1.000e+00$	$1.000e+00$	ca	0.7391	FALSE	$5.856e-05$	$5.458e-03$	ca	0.6207	TRUE
GO:0008233	peptidase activity	MF	BS	TRUE	$1.000e+00$	$1.000e+00$	ca	0.7162	FALSE	$6.621e-06$	$1.607e-03$	ca	0.6061	TRUE
GO:0070011	peptidase activity, acting on L-amino acid peptides	MF	BS	TRUE	$1.000e+00$	$1.000e+00$	ca	0.7260	FALSE	$1.217e-05$	$2.658e-03$	ca	0.5938	TRUE
GO:0015091	ferric iron transmembrane transporter activity	MF	J	TRUE	$1.000e+00$	$1.000e+00$	NA	NA	FALSE	$6.020e-05$	$5.458e-03$	fe	1.0000	TRUE
GO:0072510	trivalent inorganic cation transmembrane transporter activity	MF	J	TRUE	$1.000e+00$	$1.000e+00$	NA	NA	FALSE	$6.020e-05$	$5.458e-03$	fe	1.0000	TRUE
GO:0016701	oxidoreductase activity, acting on single donors with incorporation of molecular oxygen	MF	L	TRUE	$1.000e+00$	$1.000e+00$	fe	0.8750	FALSE	$5.028e-05$	$5.458e-03$	fe	0.9000	TRUE

GO:0016702	oxidoreductase activity, acting on single donors with incorporation of molecular oxygen, incorporation of two atoms of oxygen	MF	L	TRUE	$1.000e+00$	$1.000e+00$	fe	0.8333	FALSE	$5.397e-05$	$5.458e-03$	fe	1.0000	TRUE
GO:0051213	dioxygenase activity	MF	L	TRUE	$1.000e+00$	$1.000e+00$	fe	0.8333	FALSE	$3.484e-05$	$5.458e-03$	fe	1.0000	TRUE
GO:0016810	hydrolase activity, acting on carbon-nitrogen (but not peptide) bonds	MF	O	TRUE	$9.999e-01$	$1.000e+00$	ca	0.5714	FALSE	$4.345e-04$	$2.712e-02$	zn	0.7500	TRUE
GO:0019538	protein metabolic process	BP	R	TRUE	$9.999e-01$	$1.000e+00$	mg	0.4255	FALSE	$1.496e-04$	$9.907e-03$	ca	0.4444	TRUE
GO:0044238	primary metabolic process	BP	U	TRUE	$9.995e-01$	$1.000e+00$	mg	0.5463	FALSE	$8.014e-04$	$4.732e-02$	ca	0.3403	TRUE

Table S152: all 7 ligands

					normal					compress				
id	description	type	IPR.group	consistent	p	padjust	metal	perc	sig	p	padjust	metal	perc	sig
GO:0042743	hydrogen peroxide metabolic process	BP	B	TRUE	$2.118e-08$	$1.110e-06$	ca	1.0000	TRUE	1.0000	1	NA	NA	FALSE
GO:0042744	hydrogen peroxide catabolic process	BP	B	TRUE	$2.118e-08$	$1.110e-06$	ca	1.0000	TRUE	1.0000	1	NA	NA	FALSE
GO:0044248	cellular catabolic process	BP	B	TRUE	$2.218e-04$	$8.302e-03$	ca	1.0000	TRUE	1.0000	1	ca	0.8571	FALSE
GO:0072593	reactive oxygen species metabolic process	BP	B	TRUE	$4.605e-09$	$3.016e-07$	ca	1.0000	TRUE	1.0000	1	NA	NA	FALSE
GO:0097159	organic cyclic compound binding	MF	E	TRUE	$6.496e-10$	$5.106e-08$	ca	0.9394	TRUE	1.0000	1	ca	0.6364	FALSE
GO:1901363	heterocyclic compound binding	MF	E	TRUE	$6.496e-10$	$5.106e-08$	ca	0.9394	TRUE	1.0000	1	ca	0.6364	FALSE
GO:0003824	catalytic activity	MF	J	TRUE	$7.968e-07$	$3.684e-05$	ca	0.9383	TRUE	1.0000	1	ca	0.8421	FALSE
GO:0008150	biological process	BP	J	TRUE	$3.394e-05$	$1.334e-03$	ca	0.9241	TRUE	1.0000	1	ca	0.8380	FALSE
GO:0008152	metabolic process	BP	J	TRUE	$2.446e-07$	$1.201e-05$	ca	0.9167	TRUE	1.0000	1	ca	0.8222	FALSE
GO:0004601	peroxidase activity	MF	L	TRUE	$1.280e-14$	$2.516e-12$	ca	1.0000	TRUE	1.0000	1	ca	1.0000	FALSE
GO:0006950	response to stress	BP	L	TRUE	$2.053e-10$	$2.305e-08$	ca	0.9600	TRUE	1.0000	1	ca	0.5556	FALSE
GO:0006979	response to oxidative stress	BP	L	TRUE	$6.568e-16$	$5.163e-13$	ca	1.0000	TRUE	1.0000	1	NA	NA	FALSE
GO:0016209	antioxidant activity	MF	L	TRUE	$1.280e-14$	$2.516e-12$	ca	1.0000	TRUE	1.0000	1	ca	1.0000	FALSE
GO:0016491	oxidoreductase activity	MF	L	TRUE	$5.426e-09$	$3.281e-07$	ca	1.0000	TRUE	1.0000	1	ca	0.9091	FALSE
GO:0016684	oxidoreductase activity, acting on peroxide as acceptor	MF	L	TRUE	$1.280e-14$	$2.516e-12$	ca	1.0000	TRUE	1.0000	1	ca	1.0000	FALSE
GO:0020037	heme binding	MF	L	TRUE	$1.153e-12$	$1.673e-10$	ca	1.0000	TRUE	1.0000	1	ca	1.0000	FALSE
GO:0046906	tetrapyrrole binding	MF	L	TRUE	$1.277e-12$	$1.673e-10$	ca	1.0000	TRUE	1.0000	1	ca	1.0000	FALSE
GO:0050896	response to stimulus	BP	L	TRUE	$3.699e-10$	$3.634e-08$	ca	0.9655	TRUE	1.0000	1	ca	0.7333	FALSE
GO:0055114	oxidation-reduction process	BP	L	TRUE	$1.549e-09$	$1.107e-07$	ca	1.0000	TRUE	1.0000	1	ca	0.9091	FALSE
GO:0044699	single-organism process	BP	Q	TRUE	$6.479e-04$	$2.122e-02$	ca	1.0000	TRUE	0.9997	1	ca	0.8551	FALSE
GO:0044710	single-organism metabolic process	BP	Q	TRUE	$3.682e-04$	$1.258e-02$	ca	1.0000	TRUE	0.9999	1	ca	0.8163	FALSE
GO:0005975	carbohydrate metabolic process	BP	R	FALSE	$1.514e-06$	$6.609e-05$	ca	0.9706	TRUE	1.0000	1	ca	0.8684	FALSE

GO:0004553	hydrolase activity, hydrolyzing O-glycosyl compounds	MF	T	TRUE	1.229e-05	5.083e-04	ca	0.9048	TRUE	1.0000	1	ca	0.8235	FALSE
GO:0016798	hydrolase activity, acting on glycosyl bonds	MF	T	FALSE	3.450e-04	1.233e-02	ca	0.9048	TRUE	0.9999	1	ca	0.8750	FALSE

Table S153: all all ligands

					normal					compress				
id	description	type	IPR.group	consistent	p	padjust	metal	perc	sig	p	padjust	metal	perc	sig
GO:0044281	small molecule metabolic process	BP	AJ	FALSE	1.064e-03	3.229e-02	zn	0.3566	TRUE	9.993e-01	1.000e+00	mg	0.2703	FALSE
GO:0005506	iron ion binding	MF	AM	FALSE	1.095e-04	4.854e-03	fe	0.8641	TRUE	9.999e-01	1.000e+00	fe	0.6806	FALSE
GO:0004784	superoxide dismutase activity	MF	AO	TRUE	5.844e-04	2.038e-02	fe	0.8286	TRUE	1.000e+00	1.000e+00	NA	*NA*	FALSE
GO:0016721	oxidoreductase activity, acting on superoxide radicals as acceptor	MF	AO	TRUE	5.844e-04	2.038e-02	fe	0.8286	TRUE	1.000e+00	1.000e+00	NA	*NA*	FALSE
GO:0004096	catalase activity	MF	AQ	TRUE	1.059e-04	4.758e-03	fe	0.8372	TRUE	1.000e+00	1.000e+00	NA	*NA*	FALSE
GO:0003677	DNA binding	MF	AU	TRUE	4.870e-04	1.755e-02	zn	0.5185	TRUE	9.997e-01	1.000e+00	ca	0.2973	FALSE
GO:0000166	nucleotide binding	MF	AX	TRUE	1.116e-06	9.150e-05	mg	0.7027	TRUE	1.000e+00	1.000e+00	mg	0.5652	FALSE
GO:0001882	nucleoside binding	MF	AX	FALSE	8.041e-06	5.381e-04	mg	0.7744	TRUE	1.000e+00	1.000e+00	mg	0.6842	FALSE
GO:0001883	purine nucleoside binding	MF	AX	FALSE	1.043e-05	6.217e-04	mg	0.7769	TRUE	1.000e+00	1.000e+00	mg	0.6842	FALSE
GO:0017076	purine nucleotide binding	MF	AX	FALSE	1.932e-06	1.408e-04	mg	0.7811	TRUE	1.000e+00	1.000e+00	mg	0.6842	FALSE
GO:0032549	ribonucleoside binding	MF	AX	FALSE	9.565e-06	6.150e-04	mg	0.7754	TRUE	1.000e+00	1.000e+00	mg	0.6842	FALSE
GO:0032550	purine ribonucleoside binding	MF	AX	FALSE	1.043e-05	6.217e-04	mg	0.7769	TRUE	1.000e+00	1.000e+00	mg	0.6842	FALSE
GO:0032553	ribonucleotide binding	MF	AX	FALSE	2.536e-05	1.320e-03	mg	0.7714	TRUE	1.000e+00	1.000e+00	mg	0.6582	FALSE
GO:0032555	purine ribonucleotide binding	MF	AX	FALSE	9.565e-06	6.150e-04	mg	0.7754	TRUE	1.000e+00	1.000e+00	mg	0.6842	FALSE
GO:0035639	purine ribonucleoside triphosphate binding	MF	AX	FALSE	1.043e-05	6.217e-04	mg	0.7769	TRUE	1.000e+00	1.000e+00	mg	0.6842	FALSE
GO:0036094	small molecule binding	MF	AX	TRUE	1.041e-07	1.312e-05	mg	0.6864	TRUE	1.000e+00	1.000e+00	mg	0.5455	FALSE
GO:0043168	anion binding	MF	AX	FALSE	8.686e-05	4.069e-03	mg	0.7048	TRUE	9.999e-01	1.000e+00	mg	0.5437	FALSE
GO:0097367	carbohydrate derivative binding	MF	AX	FALSE	1.098e-05	6.431e-04	mg	0.7589	TRUE	1.000e+00	1.000e+00	mg	0.6582	FALSE
GO:1901265	nucleoside phosphate binding	MF	AX	TRUE	1.116e-06	9.150e-05	mg	0.7027	TRUE	1.000e+00	1.000e+00	mg	0.5652	FALSE
GO:0043565	sequence-specific DNA binding	MF	AY	TRUE	6.909e-05	3.381e-03	zn	0.9556	TRUE	1.000e+00	1.000e+00	NA	*NA*	FALSE
GO:0044260	cellular macromolecule metabolic process	BP	AZ	FALSE	4.301e-04	1.567e-02	zn	0.5269	TRUE	9.997e-01	1.000e+00	mg	0.3250	FALSE
GO:0009058	biosynthetic process	BP	BO	FALSE	4.425e-08	6.595e-06	zn	0.4138	TRUE	1.000e+00	1.000e+00	mg	0.3367	FALSE
GO:0018130	heterocycle biosynthetic process	BP	BO	FALSE	5.144e-07	4.558e-05	zn	0.5418	TRUE	1.000e+00	1.000e+00	mg	0.3077	FALSE
GO:0019438	aromatic compound biosynthetic process	BP	BO	FALSE	1.384e-05	7.826e-04	zn	0.5300	TRUE	1.000e+00	1.000e+00	mg	0.3056	FALSE
GO:0034654	nucleobase-containing compound biosynthetic process	BP	BO	FALSE	9.639e-04	3.090e-02	zn	0.5934	TRUE	9.995e-01	1.000e+00	zn	0.3333	FALSE

GO:0044249	cellular biosynthetic process	BP	BO	TRUE	$4.419e-08$	$6.595e-06$	zn	0.4264	TRUE	$1.000e+00$	$1.000e+00$	mg	0.3297	FALSE
GO:0044271	cellular nitrogen compound biosynthetic process	BP	BO	FALSE	$4.495e-07$	$4.211e-05$	zn	0.5201	TRUE	$1.000e+00$	$1.000e+00$	mg	0.3462	FALSE
GO:1901362	organic cyclic compound biosynthetic process	BP	BO	FALSE	$9.997e-08$	$1.311e-05$	zn	0.5235	TRUE	$1.000e+00$	$1.000e+00$	mg	0.3077	FALSE
GO:1901576	organic substance biosynthetic process	BP	BO	FALSE	$5.416e-09$	$1.110e-06$	zn	0.4275	TRUE	$1.000e+00$	$1.000e+00$	mg	0.3529	FALSE
GO:0072524	pyridine-containing compound metabolic process	BP	BU	TRUE	$1.518e-03$	$4.483e-02$	mg	0.4430	TRUE	$9.996e-01$	$1.000e+00$	mg	0.5000	FALSE
GO:0009055	electron carrier activity	MF	BV	TRUE	$8.795e-13$	$3.605e-10$	fe	0.7901	TRUE	$1.000e+00$	$1.000e+00$	fe	0.5385	FALSE
GO:0016829	lyase activity	MF	CD	FALSE	$2.776e-04$	$1.097e-02$	zn	0.5072	TRUE	$9.999e-01$	$1.000e+00$	zn	0.4054	FALSE
GO:0006139	nucleobase-containing compound metabolic process	BP	CJ	FALSE	$1.249e-07$	$1.517e-05$	zn	0.4707	TRUE	$1.000e+00$	$1.000e+00$	mg	0.3077	FALSE
GO:0006725	cellular aromatic compound metabolic process	BP	CJ	FALSE	$1.675e-06$	$1.248e-04$	zn	0.4220	TRUE	$1.000e+00$	$1.000e+00$	fe	0.3008	FALSE
GO:0034641	cellular nitrogen compound metabolic process	BP	CJ	TRUE	$8.436e-11$	$2.359e-08$	zn	0.4413	TRUE	$1.000e+00$	$1.000e+00$	mg	0.3211	FALSE
GO:0046483	heterocycle metabolic process	BP	CJ	FALSE	$8.634e-11$	$2.359e-08$	zn	0.4542	TRUE	$1.000e+00$	$1.000e+00$	mg	0.3093	FALSE
GO:0090304	nucleic acid metabolic process	BP	CJ	TRUE	$2.364e-07$	$2.500e-05$	zn	0.5664	TRUE	$1.000e+00$	$1.000e+00$	mg	0.3077	FALSE
GO:1901360	organic cyclic compound metabolic process	BP	CJ	FALSE	$1.596e-09$	$3.737e-07$	zn	0.4332	TRUE	$1.000e+00$	$1.000e+00$	mg	0.2909	FALSE
GO:0015077	monovalent inorganic cation transmembrane transporter activity	MF	DE	TRUE	$1.273e-03$	$3.796e-02$	fe	0.4242	TRUE	$9.997e-01$	$1.000e+00$	ca	0.7500	FALSE
GO:0015980	energy derivation by oxidation of organic compounds	BP	DE	TRUE	$8.924e-05$	$4.121e-03$	fe	0.6923	TRUE	$1.000e+00$	$1.000e+00$	ca	0.5000	FALSE
GO:0045333	cellular respiration	BP	DE	TRUE	$1.871e-04$	$7.865e-03$	fe	0.7377	TRUE	$1.000e+00$	$1.000e+00$	ca	0.5000	FALSE
GO:0003676	nucleic acid binding	MF	DJ	FALSE	$6.285e-13$	$2.944e-10$	zn	0.6762	TRUE	$1.000e+00$	$1.000e+00$	mg	0.3548	FALSE
GO:0008270	zinc ion binding	MF	DL	FALSE	$1.400e-13$	$7.653e-11$	zn	0.9031	TRUE	$1.000e+00$	$1.000e+00$	zn	0.7604	FALSE
GO:0006915	apoptotic process	BP	DN	TRUE	$3.081e-04$	$1.175e-02$	zn	0.9474	TRUE	$1.000e+00$	$1.000e+00$	NA	NA	FALSE
GO:0008219	cell death	BP	DN	TRUE	$2.489e-04$	$9.952e-03$	zn	0.9487	TRUE	$1.000e+00$	$1.000e+00$	NA	NA	FALSE
GO:0012501	programmed cell death	BP	DN	TRUE	$3.081e-04$	$1.175e-02$	zn	0.9474	TRUE	$1.000e+00$	$1.000e+00$	NA	NA	FALSE
GO:0016265	death	BP	DN	TRUE	$2.489e-04$	$9.952e-03$	zn	0.9487	TRUE	$1.000e+00$	$1.000e+00$	NA	NA	FALSE
GO:0004601	peroxidase activity	MF	DU	TRUE	$8.941e-04$	$2.932e-02$	fe	0.7015	TRUE	$9.996e-01$	$1.000e+00$	ca	1.0000	FALSE
GO:0016209	antioxidant activity	MF	DU	TRUE	$8.030e-06$	$5.381e-04$	fe	0.7235	TRUE	$1.000e+00$	$1.000e+00$	ca	1.0000	FALSE
GO:0016684	oxidoreductase activity, acting on peroxide as acceptor	MF	DU	TRUE	$8.941e-04$	$2.932e-02$	fe	0.7015	TRUE	$9.996e-01$	$1.000e+00$	ca	1.0000	FALSE
GO:0015669	gas transport	BP	DY	TRUE	$3.135e-07$	$3.115e-05$	fe	0.9837	TRUE	$1.000e+00$	$1.000e+00$	fe	0.4000	FALSE
GO:0015671	oxygen transport	BP	DY	TRUE	$3.135e-07$	$3.115e-05$	fe	0.9837	TRUE	$1.000e+00$	$1.000e+00$	fe	0.4000	FALSE
GO:0019825	oxygen binding	MF	DY	TRUE	$5.360e-11$	$1.757e-08$	fe	0.9894	TRUE	$1.000e+00$	$1.000e+00$	fe	0.4286	FALSE
GO:0006351	transcription, DNA-templated	BP	E	FALSE	$3.784e-04$	$1.394e-02$	zn	0.8298	TRUE	$9.999e-01$	$1.000e+00$	zn	0.7857	FALSE
GO:0009889	regulation of biosynthetic process	BP	E	FALSE	$1.008e-03$	$3.090e-02$	zn	0.8496	TRUE	$9.996e-01$	$1.000e+00$	zn	0.7857	FALSE
GO:0010467	gene expression	BP	E	FALSE	$2.878e-05$	$1.474e-03$	zn	0.7166	TRUE	$1.000e+00$	$1.000e+00$	zn	0.5000	FALSE
GO:0016070	RNA metabolic process	BP	E	FALSE	$9.083e-07$	$7.838e-05$	zn	0.7717	TRUE	$1.000e+00$	$1.000e+00$	zn	0.6316	FALSE

GO:0019219	regulation of nucleobase-containing compound metabolic process	BP	E	FALSE	$9.988e-04$	$3.090e-02$	zn	0.8504	TRUE	$9.996e-01$	$1.000e+00$	zn	0.8462	FALSE
GO:0019222	regulation of metabolic process	BP	E	FALSE	$7.086e-04$	$2.420e-02$	zn	0.8378	TRUE	$9.997e-01$	$1.000e+00$	zn	0.8125	FALSE
GO:0031323	regulation of cellular metabolic process	BP	E	FALSE	$6.277e-04$	$2.167e-02$	zn	0.8392	TRUE	$9.997e-01$	$1.000e+00$	zn	0.8000	FALSE
GO:0031326	regulation of cellular biosynthetic process	BP	E	FALSE	$1.008e-03$	$3.090e-02$	zn	0.8496	TRUE	$9.996e-01$	$1.000e+00$	zn	0.7857	FALSE
GO:0032774	RNA biosynthetic process	BP	E	FALSE	$1.366e-04$	$5.973e-03$	zn	0.8255	TRUE	$9.999e-01$	$1.000e+00$	zn	0.7857	FALSE
GO:0051171	regulation of nitrogen compound metabolic process	BP	E	FALSE	$8.941e-04$	$2.932e-02$	zn	0.8507	TRUE	$9.996e-01$	$1.000e+00$	zn	0.7857	FALSE
GO:0051252	regulation of RNA metabolic process	BP	E	FALSE	$1.622e-03$	$4.748e-02$	zn	0.8699	TRUE	$9.993e-01$	$1.000e+00$	zn	0.8462	FALSE
GO:0097659	nucleic acid-templated transcription	BP	E	FALSE	$3.784e-04$	$1.394e-02$	zn	0.8298	TRUE	$9.999e-01$	$1.000e+00$	zn	0.7857	FALSE
GO:0003924	GTPase activity	MF	EJ	TRUE	$1.556e-04$	$6.713e-03$	mg	0.8065	TRUE	$1.000e+00$	$1.000e+00$	ca	0.5000	FALSE
GO:0016705	oxidoreductase activity, acting on paired donors, with incorporation or reduction of molecular oxygen	MF	EN	FALSE	$1.073e-03$	$3.229e-02$	fe	0.9388	TRUE	$9.995e-01$	$1.000e+00$	fe	0.8400	FALSE
GO:0072593	reactive oxygen species metabolic process	BP	F	TRUE	$2.465e-04$	$9.952e-03$	fe	0.4821	TRUE	$9.999e-01$	$1.000e+00$	zn	1.0000	FALSE
GO:0016835	carbon-oxygen lyase activity	MF	FK	TRUE	$5.596e-04$	$1.995e-02$	zn	0.5867	TRUE	$9.998e-01$	$1.000e+00$	mg	0.4375	FALSE
GO:0016836	hydro-lyase activity	MF	FK	TRUE	$9.803e-04$	$3.090e-02$	zn	0.6529	TRUE	$9.996e-01$	$1.000e+00$	zn	0.5000	FALSE
GO:0009059	macromolecule biosynthetic process	BP	FQ	TRUE	$7.731e-05$	$3.728e-03$	zn	0.6743	TRUE	$1.000e+00$	$1.000e+00$	zn	0.4688	FALSE
GO:0034645	cellular macromolecule biosynthetic process	BP	FQ	TRUE	$1.057e-04$	$4.758e-03$	zn	0.6744	TRUE	$9.999e-01$	$1.000e+00$	zn	0.4688	FALSE
GO:0016491	oxidoreductase activity	MF	FU	TRUE	$4.185e-07$	$4.036e-05$	fe	0.5982	TRUE	$1.000e+00$	$1.000e+00$	fe	0.5000	FALSE
GO:1901564	organonitrogen compound metabolic process	BP	G	FALSE	$2.098e-04$	$8.707e-03$	mg	0.3582	TRUE	$9.999e-01$	$1.000e+00$	mg	0.3421	FALSE
GO:1901566	organonitrogen compound biosynthetic process	BP	G	TRUE	$2.530e-05$	$1.320e-03$	mg	0.3933	TRUE	$1.000e+00$	$1.000e+00$	mg	0.4865	FALSE
GO:0016053	organic acid biosynthetic process	BP	GL	TRUE	$9.988e-04$	$3.090e-02$	mg	0.3543	TRUE	$9.996e-01$	$1.000e+00$	mg	0.3077	FALSE
GO:0044283	small molecule biosynthetic process	BP	GL	TRUE	$1.993e-05$	$1.108e-03$	mg	0.4000	TRUE	$1.000e+00$	$1.000e+00$	mg	0.3333	FALSE
GO:0046394	carboxylic acid biosynthetic process	BP	GL	TRUE	$9.988e-04$	$3.090e-02$	mg	0.3543	TRUE	$9.996e-01$	$1.000e+00$	mg	0.3077	FALSE
GO:0005525	GTP binding	MF	GN	TRUE	$9.955e-09$	$1.813e-06$	mg	0.9250	TRUE	$1.000e+00$	$1.000e+00$	mg	0.5833	FALSE
GO:0007154	cell communication	BP	GN	TRUE	$2.308e-07$	$2.500e-05$	mg	0.6030	TRUE	$1.000e+00$	$1.000e+00$	ca	0.6000	FALSE
GO:0007165	signal transduction	BP	GN	TRUE	$3.297e-08$	$5.405e-06$	mg	0.6199	TRUE	$1.000e+00$	$1.000e+00$	ca	0.5484	FALSE
GO:0007264	small GTPase mediated signal transduction	BP	GN	FALSE	$8.089e-05$	$3.844e-03$	mg	0.9429	TRUE	$1.000e+00$	$1.000e+00$	mg	0.5000	FALSE
GO:0019001	guanyl nucleotide binding	MF	GN	TRUE	$4.442e-10$	$1.120e-07$	mg	0.9224	TRUE	$1.000e+00$	$1.000e+00$	mg	0.5833	FALSE
GO:0023052	signaling	BP	GN	TRUE	$1.180e-06$	$9.436e-05$	mg	0.6067	TRUE	$1.000e+00$	$1.000e+00$	ca	0.5946	FALSE

GO:0032561	guanyl ribonucleotide binding	MF	GN	TRUE	$8.474e-09$	$1.634e-06$	mg	0.9204	TRUE	$1.000e+00$	$1.000e+00$	mg	0.5833	FALSE
GO:0035556	intracellular signal transduction	BP	GN	TRUE	$8.837e-08$	$1.207e-05$	mg	0.7658	TRUE	$1.000e+00$	$1.000e+00$	ca	0.3529	FALSE
GO:0044700	single organism signaling	BP	GN	TRUE	$2.033e-07$	$2.381e-05$	mg	0.6123	TRUE	$1.000e+00$	$1.000e+00$	ca	0.5882	FALSE
GO:0050789	regulation of biological process	BP	GN	FALSE	$2.857e-04$	$1.115e-02$	zn	0.4425	TRUE	$9.998e-01$	$1.000e+00$	zn	0.3951	FALSE
GO:0050794	regulation of cellular process	BP	GN	TRUE	$2.047e-06$	$1.459e-04$	zn	0.4454	TRUE	$1.000e+00$	$1.000e+00$	zn	0.4545	FALSE
GO:0050896	response to stimulus	BP	GN	TRUE	$2.192e-06$	$1.530e-04$	mg	0.3825	TRUE	$1.000e+00$	$1.000e+00$	ca	0.4860	FALSE
GO:0051716	cellular response to stimulus	BP	GN	TRUE	$5.070e-09$	$1.108e-06$	mg	0.5203	TRUE	$1.000e+00$	$1.000e+00$	ca	0.5094	FALSE
GO:0020037	heme binding	MF	GR	FALSE	$1.519e-37$	$1.660e-34$	fe	0.9093	TRUE	$1.000e+00$	$1.000e+00$	ca	0.5366	FALSE
GO:0046906	tetrapyrrole binding	MF	GR	FALSE	$2.556e-37$	$2.095e-34$	fe	0.9037	TRUE	$1.000e+00$	$1.000e+00$	ca	0.5476	FALSE
GO:0044711	single-organism biosynthetic process	BP	K	FALSE	$3.551e-04$	$1.339e-02$	mg	0.4465	TRUE	$9.998e-01$	$1.000e+00$	mg	0.3571	FALSE
GO:0016462	pyrophosphatase activity	MF	U	TRUE	$3.311e-05$	$1.670e-03$	mg	0.7654	TRUE	$1.000e+00$	$1.000e+00$	mg	0.4706	FALSE
GO:0016817	hydrolase activity, acting on acid anhydrides	MF	U	TRUE	$1.020e-05$	$6.217e-04$	mg	0.7447	TRUE	$1.000e+00$	$1.000e+00$	mg	0.4706	FALSE
GO:0016818	hydrolase activity, acting on acid anhydrides, in phosphorus-containing anhydrides	MF	U	TRUE	$2.246e-05$	$1.207e-03$	mg	0.7692	TRUE	$1.000e+00$	$1.000e+00$	mg	0.4706	FALSE
GO:0017111	nucleoside-triphosphatase activity	MF	U	TRUE	$1.573e-06$	$1.212e-04$	mg	0.8039	TRUE	$1.000e+00$	$1.000e+00$	mg	0.7000	FALSE
GO:0016567	protein ubiquitination	BP	V	TRUE	$7.233e-04$	$2.445e-02$	zn	0.9706	TRUE	$1.000e+00$	$1.000e+00$	NA	NA	FALSE
GO:0032446	protein modification by small protein conjugation	BP	V	TRUE	$1.624e-04$	$6.915e-03$	zn	0.9512	TRUE	$1.000e+00$	$1.000e+00$	NA	NA	FALSE
GO:0070647	protein modification by small protein conjugation or removal	BP	V	TRUE	$5.580e-05$	$2.772e-03$	zn	0.9565	TRUE	$1.000e+00$	$1.000e+00$	NA	NA	FALSE
GO:0004252	serine-type endopeptidase activity	MF	AB	FALSE	$1.000e+00$	$1.000e+00$	ca	0.7222	FALSE	$2.949e-05$	$1.443e-03$	ca	0.7609	TRUE
GO:0008236	serine-type peptidase activity	MF	AB	FALSE	$1.000e+00$	$1.000e+00$	ca	0.6500	FALSE	$3.792e-05$	$1.751e-03$	ca	0.7143	TRUE
GO:0017171	serine hydrolase activity	MF	AB	FALSE	$1.000e+00$	$1.000e+00$	ca	0.6500	FALSE	$3.792e-05$	$1.751e-03$	ca	0.7143	TRUE
GO:0005543	phospholipid binding	MF	AT	TRUE	$1.000e+00$	$1.000e+00$	ca	1.0000	FALSE	$1.410e-06$	$9.439e-05$	ca	1.0000	TRUE
GO:0005544	calcium-dependent phospholipid binding	MF	AT	TRUE	$1.000e+00$	$1.000e+00$	ca	1.0000	FALSE	$1.410e-06$	$9.439e-05$	ca	1.0000	TRUE
GO:0008289	lipid binding	MF	AT	TRUE	$1.000e+00$	$1.000e+00$	ca	0.7500	FALSE	$4.523e-07$	$3.296e-05$	ca	1.0000	TRUE
GO:0005975	carbohydrate metabolic process	BP	AW	FALSE	$1.000e+00$	$1.000e+00$	ca	0.3741	FALSE	$1.303e-06$	$9.089e-05$	ca	0.6203	TRUE
GO:0008235	metalloexopeptidase activity	MF	BI	TRUE	$1.000e+00$	$1.000e+00$	zn	0.5417	FALSE	$2.102e-08$	$2.088e-06$	zn	0.8571	TRUE
GO:0008238	exopeptidase activity	MF	BI	TRUE	$1.000e+00$	$1.000e+00$	zn	0.7000	FALSE	$1.848e-11$	$6.061e-09$	zn	0.8605	TRUE
GO:0016758	transferase activity, transferring hexosyl groups	MF	BX	TRUE	$9.999e-01$	$1.000e+00$	ca	0.8000	FALSE	$3.507e-04$	$1.278e-02$	ca	1.0000	TRUE
GO:0017000	antibiotic biosynthetic process	BP	CH	TRUE	$1.000e+00$	$1.000e+00$	fe	1.0000	FALSE	$2.301e-04$	$9.202e-03$	ca	1.0000	TRUE
GO:0018342	protein prenylation	BP	CI	TRUE	$1.000e+00$	$1.000e+00$	NA	NA	FALSE	$2.280e-09$	$2.876e-07$	zn	1.0000	TRUE
GO:0018343	protein farnesylation	BP	CI	TRUE	$1.000e+00$	$1.000e+00$	NA	NA	FALSE	$3.328e-07$	$2.538e-05$	zn	1.0000	TRUE
GO:0097354	prenylation	BP	CI	TRUE	$1.000e+00$	$1.000e+00$	NA	NA	FALSE	$2.280e-09$	$2.876e-07$	zn	1.0000	TRUE

GO:0050263	ribosylpyrimidine nucleosidase activity	MF	CO	TRUE	$1.000e+00$	$1.000e+00$	NA	NA	FALSE	$1.330e-03$	$4.448e-02$	ca	1.0000	TRUE
GO:0065008	regulation of biological quality	BP	CQ	TRUE	$9.999e-01$	$1.000e+00$	fe	0.2778	FALSE	$1.661e-04$	$6.807e-03$	fe	0.3878	TRUE
GO:0004175	endopeptidase activity	MF	DZ	FALSE	$1.000e+00$	$1.000e+00$	zn	0.4300	FALSE	$2.016e-07$	$1.574e-05$	ca	0.5203	TRUE
GO:0004222	metalloendopeptidase activity	MF	DZ	FALSE	$9.994e-01$	$1.000e+00$	zn	0.6053	FALSE	$1.075e-03$	$3.633e-02$	zn	0.6143	TRUE
GO:0006508	proteolysis	BP	DZ	FALSE	$1.000e+00$	$1.000e+00$	zn	0.5094	FALSE	$3.218e-13$	$1.759e-10$	zn	0.5673	TRUE
GO:0008233	peptidase activity	MF	DZ	FALSE	$1.000e+00$	$1.000e+00$	zn	0.5000	FALSE	$2.065e-16$	$2.257e-13$	zn	0.5508	TRUE
GO:0008237	metallopeptidase activity	MF	DZ	FALSE	$1.000e+00$	$1.000e+00$	zn	0.6390	FALSE	$1.158e-11$	$4.220e-09$	zn	0.7295	TRUE
GO:0019538	protein metabolic process	BP	DZ	FALSE	$1.000e+00$	$1.000e+00$	zn	0.5016	FALSE	$4.564e-09$	$5.160e-07$	zn	0.5154	TRUE
GO:0070011	peptidase activity, acting on L-amino acid peptides	MF	DZ	FALSE	$1.000e+00$	$1.000e+00$	zn	0.4934	FALSE	$4.736e-16$	$3.882e-13$	zn	0.5489	TRUE
GO:0004180	carboxypeptidase activity	MF	EH	TRUE	$1.000e+00$	$1.000e+00$	ca	0.5000	FALSE	$9.027e-13$	$3.700e-10$	zn	0.9091	TRUE
GO:0004181	metallocarboxypeptidase activity	MF	EH	TRUE	$1.000e+00$	$1.000e+00$	ca	0.5000	FALSE	$9.027e-13$	$3.700e-10$	zn	0.9091	TRUE
GO:0004620	phospholipase activity	MF	EL	TRUE	$1.000e+00$	$1.000e+00$	ca	0.4762	FALSE	$3.211e-08$	$2.925e-06$	ca	0.7692	TRUE
GO:0004623	phospholipase A2 activity	MF	EL	TRUE	$1.000e+00$	$1.000e+00$	ca	0.7000	FALSE	$1.185e-09$	$1.618e-07$	ca	0.8182	TRUE
GO:0006820	anion transport	BP	EL	TRUE	$1.000e+00$	$1.000e+00$	ca	0.7000	FALSE	$8.500e-11$	$2.534e-08$	ca	0.7917	TRUE
GO:0006869	lipid transport	BP	EL	TRUE	$1.000e+00$	$1.000e+00$	ca	0.6364	FALSE	$2.915e-09$	$3.414e-07$	ca	0.8182	TRUE
GO:0010876	lipid localization	BP	EL	TRUE	$1.000e+00$	$1.000e+00$	ca	0.6364	FALSE	$2.915e-09$	$3.414e-07$	ca	0.8182	TRUE
GO:0015711	organic anion transport	BP	EL	TRUE	$1.000e+00$	$1.000e+00$	ca	0.7000	FALSE	$3.194e-10$	$7.480e-08$	ca	0.7826	TRUE
GO:0015718	monocarboxylic acid transport	BP	EL	TRUE	$1.000e+00$	$1.000e+00$	ca	0.7000	FALSE	$1.185e-09$	$1.618e-07$	ca	0.8182	TRUE
GO:0015849	organic acid transport	BP	EL	TRUE	$1.000e+00$	$1.000e+00$	ca	0.7000	FALSE	$3.194e-10$	$7.480e-08$	ca	0.7826	TRUE
GO:0015908	fatty acid transport	BP	EL	TRUE	$1.000e+00$	$1.000e+00$	ca	0.7000	FALSE	$1.185e-09$	$1.618e-07$	ca	0.8182	TRUE
GO:0015909	long-chain fatty acid transport	BP	EL	TRUE	$1.000e+00$	$1.000e+00$	ca	0.7000	FALSE	$1.185e-09$	$1.618e-07$	ca	0.8182	TRUE
GO:0016042	lipid catabolic process	BP	EL	TRUE	$1.000e+00$	$1.000e+00$	ca	0.5714	FALSE	$1.028e-07$	$8.646e-06$	ca	0.8095	TRUE
GO:0016298	lipase activity	MF	EL	TRUE	$1.000e+00$	$1.000e+00$	ca	0.4783	FALSE	$1.003e-07$	$8.646e-06$	ca	0.7692	TRUE
GO:0032309	icosanoid secretion	BP	EL	TRUE	$1.000e+00$	$1.000e+00$	ca	0.7000	FALSE	$1.185e-09$	$1.618e-07$	ca	0.8182	TRUE
GO:0046717	acid secretion	BP	EL	TRUE	$1.000e+00$	$1.000e+00$	ca	0.7000	FALSE	$1.185e-09$	$1.618e-07$	ca	0.8182	TRUE
GO:0046903	secretion	BP	EL	TRUE	$1.000e+00$	$1.000e+00$	ca	0.5556	FALSE	$4.783e-09$	$5.228e-07$	ca	0.8077	TRUE
GO:0046942	carboxylic acid transport	BP	EL	TRUE	$1.000e+00$	$1.000e+00$	ca	0.7000	FALSE	$3.194e-10$	$7.480e-08$	ca	0.7826	TRUE
GO:0050482	arachidonic acid secretion	BP	EL	TRUE	$1.000e+00$	$1.000e+00$	ca	0.7000	FALSE	$1.185e-09$	$1.618e-07$	ca	0.8182	TRUE
GO:0052689	carboxylic ester hydrolase activity	MF	EL	TRUE	$1.000e+00$	$1.000e+00$	ca	0.6471	FALSE	$1.261e-13$	$8.271e-11$	ca	0.8235	TRUE
GO:0071715	icosanoid transport	BP	EL	TRUE	$1.000e+00$	$1.000e+00$	ca	0.7000	FALSE	$1.185e-09$	$1.618e-07$	ca	0.8182	TRUE
GO:1901571	fatty acid derivative transport	BP	EL	TRUE	$1.000e+00$	$1.000e+00$	ca	0.7000	FALSE	$1.185e-09$	$1.618e-07$	ca	0.8182	TRUE
GO:1903963	arachidonate transport	BP	EL	TRUE	$1.000e+00$	$1.000e+00$	ca	0.7000	FALSE	$1.185e-09$	$1.618e-07$	ca	0.8182	TRUE
GO:0005509	calcium ion binding	MF	EU	FALSE	$1.000e+00$	$1.000e+00$	ca	0.7427	FALSE	$2.058e-66$	$6.747e-63$	ca	0.8973	TRUE
GO:0005976	polysaccharide metabolic process	BP	EW	TRUE	$1.000e+00$	$1.000e+00$	ca	0.4706	FALSE	$7.703e-09$	$8.148e-07$	ca	0.8800	TRUE
GO:0006073	cellular glucan metabolic process	BP	EY	TRUE	$1.000e+00$	$1.000e+00$	ca	1.0000	FALSE	$3.084e-08$	$2.889e-06$	ca	0.9231	TRUE
GO:0008810	cellulase activity	MF	EY	TRUE	$1.000e+00$	$1.000e+00$	ca	1.0000	FALSE	$6.132e-05$	$2.578e-03$	ca	0.8571	TRUE
GO:0009251	glucan catabolic process	BP	EY	TRUE	$1.000e+00$	$1.000e+00$	ca	1.0000	FALSE	$5.440e-05$	$2.317e-03$	ca	0.8750	TRUE

GO:0030243	cellulose metabolic process	BP	EY	TRUE	$1.000e+00$	$1.000e+00$	ca	1.0000	FALSE	$5.440e-05$	$2.317e-03$	ca	0.8750	TRUE
GO:0030245	cellulose catabolic process	BP	EY	TRUE	$1.000e+00$	$1.000e+00$	ca	1.0000	FALSE	$5.440e-05$	$2.317e-03$	ca	0.8750	TRUE
GO:0044042	glucan metabolic process	BP	EY	TRUE	$1.000e+00$	$1.000e+00$	ca	1.0000	FALSE	$3.084e-08$	$2.889e-06$	ca	0.9231	TRUE
GO:0044247	cellular polysaccharide catabolic process	BP	EY	TRUE	$1.000e+00$	$1.000e+00$	ca	1.0000	FALSE	$5.440e-05$	$2.317e-03$	ca	0.8750	TRUE
GO:0044264	cellular polysaccharide metabolic process	BP	EY	TRUE	$1.000e+00$	$1.000e+00$	ca	0.4286	FALSE	$8.685e-06$	$4.450e-04$	ca	0.9231	TRUE
GO:0051273	beta-glucan metabolic process	BP	EY	TRUE	$1.000e+00$	$1.000e+00$	ca	1.0000	FALSE	$5.440e-05$	$2.317e-03$	ca	0.8750	TRUE
GO:0051275	beta-glucan catabolic process	BP	EY	TRUE	$1.000e+00$	$1.000e+00$	ca	1.0000	FALSE	$5.440e-05$	$2.317e-03$	ca	0.8750	TRUE
GO:0006644	phospholipid metabolic process	BP	FI	TRUE	$1.000e+00$	$1.000e+00$	ca	0.3429	FALSE	$5.788e-07$	$4.126e-05$	ca	0.7000	TRUE
GO:0006873	cellular ion homeostasis	BP	FL	TRUE	$1.000e+00$	$1.000e+00$	fe	0.4762	FALSE	$3.443e-06$	$1.851e-04$	fe	0.6129	TRUE
GO:0006875	cellular metal ion homeostasis	BP	FL	TRUE	$1.000e+00$	$1.000e+00$	fe	0.4762	FALSE	$3.443e-06$	$1.851e-04$	fe	0.6129	TRUE
GO:0006879	cellular iron ion homeostasis	BP	FL	TRUE	$1.000e+00$	$1.000e+00$	fe	0.4878	FALSE	$2.417e-06$	$1.496e-04$	fe	0.6129	TRUE
GO:0008199	ferric iron binding	MF	FL	TRUE	$1.000e+00$	$1.000e+00$	fe	0.5714	FALSE	$1.071e-07$	$8.780e-06$	fe	0.6857	TRUE
GO:0019725	cellular homeostasis	BP	FL	TRUE	$1.000e+00$	$1.000e+00$	fe	0.4314	FALSE	$2.685e-06$	$1.630e-04$	fe	0.5429	TRUE
GO:0030003	cellular cation homeostasis	BP	FL	TRUE	$1.000e+00$	$1.000e+00$	fe	0.4762	FALSE	$3.443e-06$	$1.851e-04$	fe	0.6129	TRUE
GO:0042592	homeostatic process	BP	FL	TRUE	$1.000e+00$	$1.000e+00$	fe	0.3860	FALSE	$1.595e-05$	$7.925e-04$	fe	0.5429	TRUE
GO:0046916	cellular transition metal ion homeostasis	BP	FL	TRUE	$1.000e+00$	$1.000e+00$	fe	0.4878	FALSE	$2.417e-06$	$1.496e-04$	fe	0.6129	TRUE
GO:0048878	chemical homeostasis	BP	FL	TRUE	$1.000e+00$	$1.000e+00$	fe	0.4490	FALSE	$3.168e-05$	$1.505e-03$	fe	0.6129	TRUE
GO:0050801	ion homeostasis	BP	FL	TRUE	$1.000e+00$	$1.000e+00$	fe	0.4762	FALSE	$3.443e-06$	$1.851e-04$	fe	0.6129	TRUE
GO:0055065	metal ion homeostasis	BP	FL	TRUE	$1.000e+00$	$1.000e+00$	fe	0.4762	FALSE	$3.443e-06$	$1.851e-04$	fe	0.6129	TRUE
GO:0055072	iron ion homeostasis	BP	FL	TRUE	$1.000e+00$	$1.000e+00$	fe	0.4878	FALSE	$2.417e-06$	$1.496e-04$	fe	0.6129	TRUE
GO:0055076	transition metal ion homeostasis	BP	FL	TRUE	$1.000e+00$	$1.000e+00$	fe	0.4878	FALSE	$2.417e-06$	$1.496e-04$	fe	0.6129	TRUE
GO:0055080	cation homeostasis	BP	FL	TRUE	$1.000e+00$	$1.000e+00$	fe	0.4762	FALSE	$3.443e-06$	$1.851e-04$	fe	0.6129	TRUE
GO:0055082	cellular chemical homeostasis	BP	FL	TRUE	$1.000e+00$	$1.000e+00$	fe	0.4490	FALSE	$3.168e-05$	$1.505e-03$	fe	0.6129	TRUE
GO:0098771	inorganic ion homeostasis	BP	FL	TRUE	$1.000e+00$	$1.000e+00$	fe	0.4762	FALSE	$3.443e-06$	$1.851e-04$	fe	0.6129	TRUE
GO:0007155	cell adhesion	BP	FP	TRUE	$9.998e-01$	$1.000e+00$	ca	0.6042	FALSE	$4.176e-04$	$1.488e-02$	ca	0.7778	TRUE
GO:0022610	biological adhesion	BP	FP	TRUE	$9.999e-01$	$1.000e+00$	ca	0.6042	FALSE	$2.101e-04$	$8.505e-03$	ca	0.7500	TRUE
GO:0008484	sulfuric ester hydrolase activity	MF	FV	TRUE	$9.999e-01$	$1.000e+00$	ca	0.3333	FALSE	$6.402e-04$	$2.187e-02$	ca	0.4286	TRUE
GO:0009250	glucan biosynthetic process	BP	GH	TRUE	$1.000e+00$	$1.000e+00$	NA	NA	FALSE	$2.535e-04$	$9.445e-03$	ca	1.0000	TRUE
GO:0046527	glucosyltransferase activity	MF	GH	TRUE	$1.000e+00$	$1.000e+00$	NA	NA	FALSE	$2.535e-04$	$9.445e-03$	ca	1.0000	TRUE
GO:0015091	ferric iron transmembrane transporter activity	MF	GK	TRUE	$1.000e+00$	$1.000e+00$	NA	NA	FALSE	$2.535e-04$	$9.445e-03$	fe	1.0000	TRUE
GO:0072510	trivalent inorganic cation transmembrane transporter activity	MF	GK	TRUE	$1.000e+00$	$1.000e+00$	NA	NA	FALSE	$2.535e-04$	$9.445e-03$	fe	1.0000	TRUE

GO:0016708	oxidoreductase activity, acting on paired donors, with incorporation or reduction of molecular oxygen, NAD(P)H as one donor, and incorporation of two atoms of oxygen into one donor	MF	GQ	TRUE	$1.000e+00$	$1.000e+00$	fe	1.0000	FALSE	$3.288e-04$	$1.211e-02$	fe	0.9000	TRUE
GO:0016717	oxidoreductase activity, acting on paired donors, with oxidation of a pair of donors resulting in the reduction of molecular oxygen to two molecules of water	MF	GS	TRUE	$1.000e+00$	$1.000e+00$	NA	NA	FALSE	$2.535e-04$	$9.445e-03$	fe	1.0000	TRUE
GO:0045300	acyl-[acyl-carrier-protein] desaturase activity	MF	GS	TRUE	$1.000e+00$	$1.000e+00$	NA	NA	FALSE	$2.535e-04$	$9.445e-03$	fe	1.0000	TRUE
GO:0030246	carbohydrate binding	MF	GZ	TRUE	$1.000e+00$	$1.000e+00$	ca	0.5741	FALSE	$1.048e-18$	$1.719e-15$	ca	0.9394	TRUE
GO:0033036	macromolecule localization	BP	HJ	TRUE	$1.000e+00$	$1.000e+00$	mg	0.3077	FALSE	$6.407e-06$	$3.335e-04$	ca	0.8276	TRUE
GO:0071702	organic substance transport	BP	HJ	TRUE	$1.000e+00$	$1.000e+00$	mg	0.2941	FALSE	$5.875e-06$	$3.107e-04$	ca	0.7647	TRUE
GO:0051213	dioxygenase activity	MF	HQ	TRUE	$9.998e-01$	$1.000e+00$	fe	0.8947	FALSE	$5.164e-04$	$1.782e-02$	fe	0.9565	TRUE
GO:0000041	transition metal ion transport	BP	I	TRUE	$9.999e-01$	$1.000e+00$	fe	0.4054	FALSE	$3.913e-04$	$1.410e-02$	fe	0.6522	TRUE
GO:0006811	ion transport	BP	I	TRUE	$1.000e+00$	$1.000e+00$	mg	0.3889	FALSE	$7.213e-08$	$6.392e-06$	ca	0.4364	TRUE
GO:0006826	iron ion transport	BP	I	TRUE	$9.998e-01$	$1.000e+00$	fe	0.4286	FALSE	$4.656e-04$	$1.624e-02$	fe	0.6818	TRUE
GO:0030001	metal ion transport	BP	I	TRUE	$9.999e-01$	$1.000e+00$	mg	0.3273	FALSE	$1.563e-04$	$6.487e-03$	fe	0.5161	TRUE
GO:0000272	polysaccharide catabolic process	BP	L	TRUE	$1.000e+00$	$1.000e+00$	ca	0.8750	FALSE	$1.252e-07$	$1.001e-05$	ca	0.9412	TRUE

ca

Table S154: ca 5 ligands

					normal			compress		
	description	type	IPR.group	consistent	p	padjust	sig	p	padjust	sig
GO:0003824	catalytic activity	MF	A	TRUE	$2.015e-05$	0.01649	TRUE	1	1	FALSE

Table S155: ca 6 ligands

					normal			compress		
id	description	type	IPR.group	consistent	p	padjust	sig	p	padjust	sig
GO:0003824	catalytic activity	MF	M	TRUE	$2.017e-06$	0.001443	TRUE	$1.000e+00$	$1.000e+00$	FALSE
GO:0008150	biological process	BP	M	TRUE	$2.928e-06$	0.001443	TRUE	$1.000e+00$	$1.000e+00$	FALSE
GO:0008152	metabolic process	BP	M	TRUE	$5.931e-06$	0.001949	TRUE	$1.000e+00$	$1.000e+00$	FALSE
GO:0044238	primary metabolic process	BP	M	TRUE	$1.326e-04$	0.026139	TRUE	$9.999e-01$	$1.000e+00$	FALSE
GO:0071704	organic substance metabolic process	BP	M	TRUE	$2.669e-05$	0.006580	TRUE	$1.000e+00$	$1.000e+00$	FALSE

GO:0005509	calcium ion binding	MF	J	TRUE	$1.000e+00$	1.000000	FALSE	$6.185e-08$	$6.098e-05$	TRUE

Table S156: ca 7 ligands

					normal			compress		
id	description	type	IPR.group	consistent	p	padjust	sig	p	padjust	sig
GO:0003824	catalytic activity	MF	C	TRUE	$8.684e-08$	$3.740e-06$	TRUE	1.0000000	1.00000	FALSE
GO:0008150	biological process	BP	C	TRUE	$9.188e-06$	$3.332e-04$	TRUE	0.9999972	1.00000	FALSE
GO:0008152	metabolic process	BP	C	TRUE	$9.670e-08$	$3.919e-06$	TRUE	1.0000000	1.00000	FALSE
GO:0042743	hydrogen peroxide metabolic process	BP	D	TRUE	$2.935e-08$	$1.348e-06$	TRUE	1.0000000	1.00000	FALSE
GO:0042744	hydrogen peroxide catabolic process	BP	D	TRUE	$2.935e-08$	$1.348e-06$	TRUE	1.0000000	1.00000	FALSE
GO:0044248	cellular catabolic process	BP	D	TRUE	$1.367e-04$	$4.096e-03$	TRUE	0.9999822	1.00000	FALSE
GO:0072593	reactive oxygen species metabolic process	BP	D	TRUE	$6.532e-09$	$3.462e-07$	TRUE	1.0000000	1.00000	FALSE
GO:0044699	single-organism process	BP	H	TRUE	$7.993e-05$	$2.503e-03$	TRUE	0.9999724	1.00000	FALSE
GO:0044710	single-organism metabolic process	BP	H	TRUE	$3.329e-05$	$1.092e-03$	TRUE	0.9999902	1.00000	FALSE
GO:0044763	single-organism cellular process	BP	H	TRUE	$1.352e-03$	$3.726e-02$	TRUE	0.9994981	1.00000	FALSE
GO:0004601	peroxidase activity	MF	J	TRUE	$2.193e-14$	$3.777e-12$	TRUE	1.0000000	1.00000	FALSE
GO:0006950	response to stress	BP	J	TRUE	$9.316e-12$	$9.169e-10$	TRUE	1.0000000	1.00000	FALSE
GO:0006979	response to oxidative stress	BP	J	TRUE	$1.129e-15$	$7.778e-13$	TRUE	1.0000000	1.00000	FALSE
GO:0016209	antioxidant activity	MF	J	TRUE	$2.193e-14$	$3.777e-12$	TRUE	1.0000000	1.00000	FALSE
GO:0016491	oxidoreductase activity	MF	J	TRUE	$3.547e-09$	$2.036e-07$	TRUE	1.0000000	1.00000	FALSE
GO:0016684	oxidoreductase activity, acting on peroxide as acceptor	MF	J	TRUE	$2.193e-14$	$3.777e-12$	TRUE	1.0000000	1.00000	FALSE
GO:0020037	heme binding	MF	J	TRUE	$1.984e-12$	$2.553e-10$	TRUE	1.0000000	1.00000	FALSE
GO:0046906	tetrapyrrole binding	MF	J	TRUE	$2.224e-12$	$2.553e-10$	TRUE	1.0000000	1.00000	FALSE
GO:0050896	response to stimulus	BP	J	TRUE	$5.655e-11$	$3.896e-09$	TRUE	1.0000000	1.00000	FALSE
GO:0055114	oxidation-reduction process	BP	J	TRUE	$9.988e-10$	$6.256e-08$	TRUE	1.0000000	1.00000	FALSE
GO:0097159	organic cyclic compound binding	MF	J	TRUE	$2.141e-11$	$1.639e-09$	TRUE	1.0000000	1.00000	FALSE
GO:1901363	heterocyclic compound binding	MF	J	TRUE	$2.141e-11$	$1.639e-09$	TRUE	1.0000000	1.00000	FALSE
GO:0005975	carbohydrate metabolic process	BP	N	TRUE	$7.141e-07$	$2.733e-05$	TRUE	0.9999998	1.00000	FALSE
GO:0004553	hydrolase activity, hydrolyzing O-glycosyl compounds	MF	O	TRUE	$2.342e-05$	$8.068e-04$	TRUE	0.9999956	1.00000	FALSE
GO:0016798	hydrolase activity, acting on glycosyl bonds	MF	O	TRUE	$7.643e-04$	$2.194e-02$	TRUE	0.9997830	1.00000	FALSE
GO:0005509	calcium ion binding	MF	L	TRUE	$9.999e-01$	$1.000e+00$	FALSE	0.0002297	0.04299	TRUE
GO:0043167	ion binding	MF	L	TRUE	$9.999e-01$	$1.000e+00$	FALSE	0.0002496	0.04299	TRUE
GO:0043169	cation binding	MF	L	TRUE	$9.999e-01$	$1.000e+00$	FALSE	0.0002124	0.04299	TRUE
GO:0046872	metal ion binding	MF	L	TRUE	$9.999e-01$	$1.000e+00$	FALSE	0.0001779	0.04299	TRUE

Table S157: ca all ligands

id	description	type	IPR.group	consistent	normal p	padjust	sig	compress p	padjust	sig
GO:0097159	organic cyclic compound binding	MF	AC	TRUE	$5.401e-04$	$1.617e-02$	TRUE	$9.997e-01$	$1.000e+00$	FALSE
GO:1901363	heterocyclic compound binding	MF	AC	TRUE	$5.401e-04$	$1.617e-02$	TRUE	$9.997e-01$	$1.000e+00$	FALSE
GO:0044711	single-organism biosynthetic process	BP	AG	TRUE	$2.548e-03$	$4.709e-02$	TRUE	$9.990e-01$	$1.000e+00$	FALSE
GO:1901576	organic substance biosynthetic process	BP	AG	TRUE	$4.427e-04$	$1.509e-02$	TRUE	$9.998e-01$	$1.000e+00$	FALSE
GO:0004222	metalloendopeptidase activity	MF	AN	FALSE	$1.041e-03$	$2.705e-02$	TRUE	$9.995e-01$	$1.000e+00$	FALSE
GO:0008237	metallopeptidase activity	MF	AN	FALSE	$2.337e-03$	$4.374e-02$	TRUE	$9.989e-01$	$1.000e+00$	FALSE
GO:0009987	cellular process	BP	AP	TRUE	$1.280e-04$	$7.556e-03$	TRUE	$9.999e-01$	$1.000e+00$	FALSE
GO:0044237	cellular metabolic process	BP	AP	TRUE	$1.127e-03$	$2.860e-02$	TRUE	$9.993e-01$	$1.000e+00$	FALSE
GO:0044763	single-organism cellular process	BP	AP	TRUE	$1.914e-03$	$4.276e-02$	TRUE	$9.988e-01$	$1.000e+00$	FALSE
GO:0006139	nucleobase-containing compound metabolic process	BP	AT	TRUE	$1.048e-03$	$2.705e-02$	TRUE	$9.996e-01$	$1.000e+00$	FALSE
GO:0006259	DNA metabolic process	BP	AT	TRUE	$1.885e-03$	$4.275e-02$	TRUE	$9.994e-01$	$1.000e+00$	FALSE
GO:0006725	cellular aromatic compound metabolic process	BP	AT	TRUE	$7.655e-05$	$4.982e-03$	TRUE	$1.000e+00$	$1.000e+00$	FALSE
GO:0006807	nitrogen compound metabolic process	BP	AT	TRUE	$5.670e-05$	$3.944e-03$	TRUE	$1.000e+00$	$1.000e+00$	FALSE
GO:0034641	cellular nitrogen compound metabolic process	BP	AT	TRUE	$4.184e-04$	$1.509e-02$	TRUE	$9.998e-01$	$1.000e+00$	FALSE
GO:0046483	heterocycle metabolic process	BP	AT	TRUE	$3.707e-04$	$1.509e-02$	TRUE	$9.998e-01$	$1.000e+00$	FALSE
GO:0090304	nucleic acid metabolic process	BP	AT	TRUE	$8.627e-04$	$2.348e-02$	TRUE	$9.997e-01$	$1.000e+00$	FALSE
GO:1901360	organic cyclic compound metabolic process	BP	AT	TRUE	$9.939e-05$	$6.200e-03$	TRUE	$1.000e+00$	$1.000e+00$	FALSE
GO:0008270	zinc ion binding	MF	AW	TRUE	$3.648e-05$	$3.944e-03$	TRUE	$1.000e+00$	$1.000e+00$	FALSE
GO:0046914	transition metal ion binding	MF	AW	TRUE	$2.042e-04$	$1.054e-02$	TRUE	$9.999e-01$	$1.000e+00$	FALSE
GO:0016043	cellular component organization	BP	AX	TRUE	$1.308e-03$	$3.059e-02$	TRUE	$9.996e-01$	$1.000e+00$	FALSE
GO:0071840	cellular component organization or biogenesis	BP	AX	TRUE	$1.308e-03$	$3.059e-02$	TRUE	$9.996e-01$	$1.000e+00$	FALSE
GO:0044707	single-multicellular organism process	BP	B	TRUE	$2.125e-03$	$4.283e-02$	TRUE	$9.994e-01$	$1.000e+00$	FALSE
GO:0006928	movement of cell or subcellular component	BP	BC	TRUE	$2.146e-03$	$4.283e-02$	TRUE	$9.997e-01$	$1.000e+00$	FALSE
GO:0016477	cell migration	BP	BC	TRUE	$8.448e-04$	$2.348e-02$	TRUE	$9.999e-01$	$1.000e+00$	FALSE
GO:0030334	regulation of cell migration	BP	BC	TRUE	$2.291e-03$	$4.340e-02$	TRUE	$1.000e+00$	$1.000e+00$	FALSE
GO:0040011	locomotion	BP	BC	TRUE	$1.288e-03$	$3.059e-02$	TRUE	$9.998e-01$	$1.000e+00$	FALSE
GO:0040012	regulation of locomotion	BP	BC	TRUE	$2.291e-03$	$4.340e-02$	TRUE	$1.000e+00$	$1.000e+00$	FALSE
GO:0048870	cell motility	BP	BC	TRUE	$8.448e-04$	$2.348e-02$	TRUE	$9.999e-01$	$1.000e+00$	FALSE

GO:0051270	regulation of cellular component movement	BP	BC	TRUE	$2.291e-03$	$4.340e-02$	TRUE	$1.000e+00$	$1.000e+00$	FALSE
GO:0051674	localization of cell	BP	BC	TRUE	$8.448e-04$	$2.348e-02$	TRUE	$9.999e-01$	$1.000e+00$	FALSE
GO:2000145	regulation of cell motility	BP	BC	TRUE	$2.291e-03$	$4.340e-02$	TRUE	$1.000e+00$	$1.000e+00$	FALSE
GO:0042743	hydrogen peroxide metabolic process	BP	BK	TRUE	$3.654e-04$	$1.509e-02$	TRUE	$1.000e+00$	$1.000e+00$	FALSE
GO:0042744	hydrogen peroxide catabolic process	BP	BK	TRUE	$3.654e-04$	$1.509e-02$	TRUE	$1.000e+00$	$1.000e+00$	FALSE
GO:0072593	reactive oxygen species metabolic process	BP	BK	TRUE	$5.796e-05$	$3.944e-03$	TRUE	$1.000e+00$	$1.000e+00$	FALSE
GO:0004553	hydrolase activity, hydrolyzing O-glycosyl compounds	MF	BL	TRUE	$1.896e-07$	$3.154e-05$	TRUE	$1.000e+00$	$1.000e+00$	FALSE
GO:0016798	hydrolase activity, acting on glycosyl bonds	MF	BL	FALSE	$1.312e-04$	$7.556e-03$	TRUE	$9.999e-01$	$1.000e+00$	FALSE
GO:0016829	lyase activity	MF	BO	TRUE	$1.195e-03$	$2.982e-02$	TRUE	$9.998e-01$	$1.000e+00$	FALSE
GO:0048037	cofactor binding	MF	BP	TRUE	$8.478e-04$	$2.348e-02$	TRUE	$9.998e-01$	$1.000e+00$	FALSE
GO:0050662	coenzyme binding	MF	BP	TRUE	$2.146e-03$	$4.283e-02$	TRUE	$9.997e-01$	$1.000e+00$	FALSE
GO:0016787	hydrolase activity	MF	BQ	TRUE	$2.594e-08$	$4.855e-06$	TRUE	$1.000e+00$	$1.000e+00$	FALSE
GO:0006900	membrane budding	BP	BT	TRUE	$4.839e-04$	$1.509e-02$	TRUE	$1.000e+00$	$1.000e+00$	FALSE
GO:0016032	viral process	BP	BT	TRUE	$5.439e-05$	$3.944e-03$	TRUE	$1.000e+00$	$1.000e+00$	FALSE
GO:0016050	vesicle organization	BP	BT	TRUE	$2.146e-03$	$4.283e-02$	TRUE	$9.997e-01$	$1.000e+00$	FALSE
GO:0019058	viral life cycle	BP	BT	TRUE	$5.439e-05$	$3.944e-03$	TRUE	$1.000e+00$	$1.000e+00$	FALSE
GO:0019068	virion assembly	BP	BT	TRUE	$2.146e-03$	$4.283e-02$	TRUE	$9.997e-01$	$1.000e+00$	FALSE
GO:0019076	viral release from host cell	BP	BT	TRUE	$4.839e-04$	$1.509e-02$	TRUE	$1.000e+00$	$1.000e+00$	FALSE
GO:0044403	symbiosis, encompassing mutualism through parasitism	BP	BT	TRUE	$5.439e-05$	$3.944e-03$	TRUE	$1.000e+00$	$1.000e+00$	FALSE
GO:0044419	interspecies interaction between organisms	BP	BT	TRUE	$5.439e-05$	$3.944e-03$	TRUE	$1.000e+00$	$1.000e+00$	FALSE
GO:0044764	multi-organism cellular process	BP	BT	TRUE	$5.439e-05$	$3.944e-03$	TRUE	$1.000e+00$	$1.000e+00$	FALSE
GO:0044766	multi-organism transport	BP	BT	TRUE	$4.839e-04$	$1.509e-02$	TRUE	$1.000e+00$	$1.000e+00$	FALSE
GO:0044803	multi-organism membrane organization	BP	BT	TRUE	$1.571e-04$	$8.401e-03$	TRUE	$1.000e+00$	$1.000e+00$	FALSE
GO:0046753	non-lytic viral release	BP	BT	TRUE	$4.839e-04$	$1.509e-02$	TRUE	$1.000e+00$	$1.000e+00$	FALSE
GO:0046755	viral budding	BP	BT	TRUE	$4.839e-04$	$1.509e-02$	TRUE	$1.000e+00$	$1.000e+00$	FALSE
GO:0046761	viral budding from plasma membrane	BP	BT	TRUE	$4.839e-04$	$1.509e-02$	TRUE	$1.000e+00$	$1.000e+00$	FALSE
GO:0046794	transport of virus	BP	BT	TRUE	$4.839e-04$	$1.509e-02$	TRUE	$1.000e+00$	$1.000e+00$	FALSE
GO:0051704	multi-organism process	BP	BT	TRUE	$1.506e-05$	$2.254e-03$	TRUE	$1.000e+00$	$1.000e+00$	FALSE
GO:0061024	membrane organization	BP	BT	TRUE	$2.701e-04$	$1.264e-02$	TRUE	$1.000e+00$	$1.000e+00$	FALSE
GO:1902579	multi-organism localization	BP	BT	TRUE	$4.839e-04$	$1.509e-02$	TRUE	$1.000e+00$	$1.000e+00$	FALSE
GO:1902590	multi-organism organelle organization	BP	BT	TRUE	$4.839e-04$	$1.509e-02$	TRUE	$1.000e+00$	$1.000e+00$	FALSE
GO:1902592	multi-organism membrane budding	BP	BT	TRUE	$4.839e-04$	$1.509e-02$	TRUE	$1.000e+00$	$1.000e+00$	FALSE
GO:0006082	organic acid metabolic process	BP	C	TRUE	$2.125e-03$	$4.283e-02$	TRUE	$9.994e-01$	$1.000e+00$	FALSE
GO:0008652	cellular amino acid biosynthetic process	BP	C	TRUE	$2.146e-03$	$4.283e-02$	TRUE	$9.997e-01$	$1.000e+00$	FALSE

GO:0043436	oxoacid metabolic process	BP	C	TRUE	$2.125e-03$	$4.283e-02$	TRUE	$9.994e-01$	$1.000e+00$	FALSE
GO:0044283	small molecule biosynthetic process	BP	C	TRUE	$1.011e-03$	$2.703e-02$	TRUE	$9.998e-01$	$1.000e+00$	FALSE
GO:1901605	alpha-amino acid metabolic process	BP	C	TRUE	$1.288e-03$	$3.059e-02$	TRUE	$9.998e-01$	$1.000e+00$	FALSE
GO:0004175	endopeptidase activity	MF	F	FALSE	$2.228e-04$	$1.076e-02$	TRUE	$9.999e-01$	$1.000e+00$	FALSE
GO:0006508	proteolysis	BP	F	FALSE	$4.245e-05$	$3.944e-03$	TRUE	$1.000e+00$	$1.000e+00$	FALSE
GO:0008233	peptidase activity	MF	F	FALSE	$2.162e-04$	$1.076e-02$	TRUE	$9.999e-01$	$1.000e+00$	FALSE
GO:0019538	protein metabolic process	BP	F	FALSE	$2.876e-05$	$3.909e-03$	TRUE	$1.000e+00$	$1.000e+00$	FALSE
GO:0043170	macromolecule metabolic process	BP	F	FALSE	$3.849e-05$	$3.944e-03$	TRUE	$1.000e+00$	$1.000e+00$	FALSE
GO:0070011	peptidase activity, acting on L-amino acid peptides	MF	F	FALSE	$1.514e-04$	$8.392e-03$	TRUE	$9.999e-01$	$1.000e+00$	FALSE
GO:0004308	exo-alpha-sialidase activity	MF	G	TRUE	$2.139e-08$	$4.575e-06$	TRUE	$1.000e+00$	$1.000e+00$	FALSE
GO:0016997	alpha-sialidase activity	MF	G	TRUE	$2.139e-08$	$4.575e-06$	TRUE	$1.000e+00$	$1.000e+00$	FALSE
GO:0006950	response to stress	BP	L	TRUE	$4.333e-04$	$1.509e-02$	TRUE	$9.998e-01$	$1.000e+00$	FALSE
GO:0044281	small molecule metabolic process	BP	Q	TRUE	$1.540e-03$	$3.547e-02$	TRUE	$9.994e-01$	$1.000e+00$	FALSE
GO:0000287	magnesium ion binding	MF	R	TRUE	$3.134e-05$	$3.909e-03$	TRUE	$1.000e+00$	$1.000e+00$	FALSE
GO:0003824	catalytic activity	MF	T	TRUE	$4.609e-14$	$6.900e-11$	TRUE	$1.000e+00$	$1.000e+00$	FALSE
GO:0008150	biological process	BP	T	FALSE	$1.299e-11$	$6.480e-09$	TRUE	$1.000e+00$	$1.000e+00$	FALSE
GO:0008152	metabolic process	BP	T	FALSE	$7.850e-12$	$5.876e-09$	TRUE	$1.000e+00$	$1.000e+00$	FALSE
GO:0044238	primary metabolic process	BP	T	FALSE	$1.268e-09$	$3.798e-07$	TRUE	$1.000e+00$	$1.000e+00$	FALSE
GO:0071704	organic substance metabolic process	BP	T	FALSE	$5.256e-11$	$1.967e-08$	TRUE	$1.000e+00$	$1.000e+00$	FALSE
GO:0005488	binding	MF	AR	TRUE	$1.000e+00$	$1.000e+00$	FALSE	$1.888e-05$	$5.652e-03$	TRUE
GO:0005509	calcium ion binding	MF	AR	FALSE	$1.000e+00$	$1.000e+00$	FALSE	$2.871e-11$	$4.297e-08$	TRUE
GO:0043167	ion binding	MF	AR	TRUE	$1.000e+00$	$1.000e+00$	FALSE	$3.318e-06$	$1.655e-03$	TRUE
GO:0043169	cation binding	MF	AR	TRUE	$1.000e+00$	$1.000e+00$	FALSE	$3.117e-06$	$1.655e-03$	TRUE
GO:0030246	carbohydrate binding	MF	BF	TRUE	$1.000e+00$	$1.000e+00$	FALSE	$1.564e-05$	$5.652e-03$	TRUE

fe

Table S158: fe 5 ligands

					normal			compress		
id	description	type	IPR.group	consistent	p	padjust	sig	p	padjust	sig
GO:0016209	antioxidant activity	MF	G	TRUE	$3.869e-04$	$3.381e-02$	TRUE	$1.000e+00$	1.0000000	FALSE
GO:0020037	heme binding	MF	K	TRUE	$1.047e-11$	$1.144e-09$	TRUE	$1.000e+00$	1.0000000	FALSE
GO:0046906	tetrapyrrole binding	MF	K	TRUE	$1.047e-11$	$1.144e-09$	TRUE	$1.000e+00$	1.0000000	FALSE
GO:0097159	organic cyclic compound binding	MF	K	TRUE	$4.753e-12$	$1.039e-09$	TRUE	$1.000e+00$	1.0000000	FALSE
GO:1901363	heterocyclic compound binding	MF	K	TRUE	$4.753e-12$	$1.039e-09$	TRUE	$1.000e+00$	1.0000000	FALSE
GO:0009987	cellular process	BP	C	TRUE	$1.000e+00$	$1.000e+00$	FALSE	$7.160e-05$	0.0027835	TRUE
GO:0051213	dioxygenase activity	MF	D	TRUE	$1.000e+00$	$1.000e+00$	FALSE	$9.308e-05$	0.0027835	TRUE
GO:0000041	transition metal ion transport	BP	E	TRUE	$1.000e+00$	$1.000e+00$	FALSE	$5.334e-04$	0.0068552	TRUE

GO:0006811	ion transport	BP	E	TRUE	$1.000e+00$	$1.000e+00$	FALSE	$7.132e-05$	0.0027835	TRUE
GO:0006812	cation transport	BP	E	TRUE	$1.000e+00$	$1.000e+00$	FALSE	$7.132e-05$	0.0027835	TRUE
GO:0006826	iron ion transport	BP	E	TRUE	$1.000e+00$	$1.000e+00$	FALSE	$5.334e-04$	0.0068552	TRUE
GO:0006873	cellular ion homeostasis	BP	E	TRUE	$1.000e+00$	$1.000e+00$	FALSE	$1.465e-04$	0.0027835	TRUE
GO:0006875	cellular metal ion homeostasis	BP	E	TRUE	$1.000e+00$	$1.000e+00$	FALSE	$1.465e-04$	0.0027835	TRUE
GO:0006879	cellular iron ion homeostasis	BP	E	TRUE	$1.000e+00$	$1.000e+00$	FALSE	$1.465e-04$	0.0027835	TRUE
GO:0019725	cellular homeostasis	BP	E	TRUE	$1.000e+00$	$1.000e+00$	FALSE	$2.735e-04$	0.0041220	TRUE
GO:0030001	metal ion transport	BP	E	TRUE	$1.000e+00$	$1.000e+00$	FALSE	$7.132e-05$	0.0027835	TRUE
GO:0030003	cellular cation homeostasis	BP	E	TRUE	$1.000e+00$	$1.000e+00$	FALSE	$1.465e-04$	0.0027835	TRUE
GO:0042592	homeostatic process	BP	E	TRUE	$1.000e+00$	$1.000e+00$	FALSE	$2.735e-04$	0.0041220	TRUE
GO:0046916	cellular transition metal ion homeostasis	BP	E	TRUE	$1.000e+00$	$1.000e+00$	FALSE	$1.465e-04$	0.0027835	TRUE
GO:0048878	chemical homeostasis	BP	E	TRUE	$1.000e+00$	$1.000e+00$	FALSE	$2.735e-04$	0.0041220	TRUE
GO:0050801	ion homeostasis	BP	E	TRUE	$1.000e+00$	$1.000e+00$	FALSE	$1.465e-04$	0.0027835	TRUE
GO:0055065	metal ion homeostasis	BP	E	TRUE	$1.000e+00$	$1.000e+00$	FALSE	$1.465e-04$	0.0027835	TRUE
GO:0055072	iron ion homeostasis	BP	E	TRUE	$1.000e+00$	$1.000e+00$	FALSE	$1.465e-04$	0.0027835	TRUE
GO:0055076	transition metal ion homeostasis	BP	E	TRUE	$1.000e+00$	$1.000e+00$	FALSE	$1.465e-04$	0.0027835	TRUE
GO:0055080	cation homeostasis	BP	E	TRUE	$1.000e+00$	$1.000e+00$	FALSE	$1.465e-04$	0.0027835	TRUE
GO:0055082	cellular chemical homeostasis	BP	E	TRUE	$1.000e+00$	$1.000e+00$	FALSE	$2.735e-04$	0.0041220	TRUE
GO:0065007	biological regulation	BP	E	TRUE	$9.999e-01$	$1.000e+00$	FALSE	$7.700e-04$	0.0096143	TRUE
GO:0065008	regulation of biological quality	BP	E	TRUE	$9.998e-01$	$1.000e+00$	FALSE	$1.800e-03$	0.0218444	TRUE
GO:0098771	inorganic ion homeostasis	BP	E	TRUE	$1.000e+00$	$1.000e+00$	FALSE	$1.465e-04$	0.0027835	TRUE
GO:0009132	nucleoside diphosphate metabolic process	BP	F	TRUE	$9.999e-01$	$1.000e+00$	FALSE	$2.920e-03$	0.0319004	TRUE
GO:0009186	deoxyribonucleoside diphosphate metabolic process	BP	F	TRUE	$9.999e-01$	$1.000e+00$	FALSE	$2.920e-03$	0.0319004	TRUE
GO:0008199	ferric iron binding	MF	J	TRUE	$1.000e+00$	$1.000e+00$	FALSE	$4.742e-04$	0.0064764	TRUE
GO:0009056	catabolic process	BP	M	TRUE	$9.997e-01$	$1.000e+00$	FALSE	$2.123e-03$	0.0250737	TRUE
GO:0044248	cellular catabolic process	BP	M	TRUE	$1.000e+00$	$1.000e+00$	FALSE	$3.406e-04$	0.0049607	TRUE
GO:0044238	primary metabolic process	BP	N	TRUE	$1.000e+00$	$1.000e+00$	FALSE	$1.056e-04$	0.0027835	TRUE
GO:0044281	small molecule metabolic process	BP	N	TRUE	$1.000e+00$	$1.000e+00$	FALSE	$2.194e-04$	0.0039942	TRUE
GO:0071704	organic substance metabolic process	BP	N	TRUE	$9.999e-01$	$1.000e+00$	FALSE	$3.569e-04$	0.0050307	TRUE
GO:1901360	organic cyclic compound metabolic process	BP	N	TRUE	$9.995e-01$	$1.000e+00$	FALSE	$2.849e-03$	0.0319004	TRUE
GO:0006725	cellular aromatic compound metabolic process	BP	O	TRUE	$1.000e+00$	$1.000e+00$	FALSE	$3.472e-07$	0.0001517	TRUE

GO:0016708	oxidoreductase activity, acting on paired donors, with incorporation or reduction of molecular oxygen, NAD(P)H as one donor, and incorporation of two atoms of oxygen into one donor	MF	R	TRUE	1.000e+00	1.000e+00	FALSE	1.090e-05	0.0011913	TRUE
GO:0019439	aromatic compound catabolic process	BP	R	TRUE	1.000e+00	1.000e+00	FALSE	3.371e-06	0.0004911	TRUE
GO:0051536	iron-sulfur cluster binding	MF	R	TRUE	1.000e+00	1.000e+00	FALSE	7.132e-05	0.0027835	TRUE
GO:0051537	2 iron, 2 sulfur cluster binding	MF	R	TRUE	1.000e+00	1.000e+00	FALSE	2.919e-06	0.0004911	TRUE
GO:0051540	metal cluster binding	MF	R	TRUE	1.000e+00	1.000e+00	FALSE	7.132e-05	0.0027835	TRUE
GO:0044763	single-organism cellular process	BP	S	TRUE	1.000e+00	1.000e+00	FALSE	2.536e-04	0.0041220	TRUE

Table S159: fe 6 ligands

					normal			compress		
id	description	type	IPR.group	consistent	p	padjust	sig	p	padjust	sig
GO:0005488	binding	MF	U	TRUE	6.980e-14	8.543e-12	TRUE	1.000e+00	1.000e+00	FALSE
GO:0020037	heme binding	MF	U	TRUE	8.729e-37	1.336e-34	TRUE	1.000e+00	1.000e+00	FALSE
GO:0046906	tetrapyrrole binding	MF	U	TRUE	8.729e-37	1.336e-34	TRUE	1.000e+00	1.000e+00	FALSE
GO:0097159	organic cyclic compound binding	MF	U	TRUE	7.191e-38	2.200e-35	TRUE	1.000e+00	1.000e+00	FALSE
GO:1901363	heterocyclic compound binding	MF	U	TRUE	7.191e-38	2.200e-35	TRUE	1.000e+00	1.000e+00	FALSE
GO:0019825	oxygen binding	MF	V	TRUE	1.611e-04	1.643e-02	TRUE	1.000e+00	1.000e+00	FALSE
GO:0009055	electron carrier activity	MF	X	TRUE	4.916e-04	4.298e-02	TRUE	9.998e-01	1.000e+00	FALSE
GO:0016830	carbon-carbon lyase activity	MF	AD	TRUE	9.999e-01	1.000e+00	FALSE	3.649e-03	2.538e-02	TRUE
GO:0071771	aldehyde decarbonylase activity	MF	AD	TRUE	1.000e+00	1.000e+00	FALSE	9.838e-04	9.122e-03	TRUE
GO:0009132	nucleoside diphosphate metabolic process	BP	AE	TRUE	9.999e-01	1.000e+00	FALSE	8.304e-04	8.197e-03	TRUE
GO:0009165	nucleotide biosynthetic process	BP	AE	TRUE	1.000e+00	1.000e+00	FALSE	4.005e-04	4.625e-03	TRUE
GO:0009186	deoxyribonucleoside diphosphate metabolic process	BP	AE	TRUE	9.999e-01	1.000e+00	FALSE	8.304e-04	8.197e-03	TRUE
GO:0009262	deoxyribonucleotide metabolic process	BP	AE	TRUE	1.000e+00	1.000e+00	FALSE	4.005e-04	4.625e-03	TRUE
GO:0009263	deoxyribonucleotide biosynthetic process	BP	AE	TRUE	1.000e+00	1.000e+00	FALSE	4.005e-04	4.625e-03	TRUE
GO:0018130	heterocycle biosynthetic process	BP	AE	TRUE	9.995e-01	1.000e+00	FALSE	4.088e-03	2.780e-02	TRUE
GO:0019438	aromatic compound biosynthetic process	BP	AE	TRUE	9.995e-01	1.000e+00	FALSE	4.088e-03	2.780e-02	TRUE
GO:0034654	nucleobase-containing compound biosynthetic process	BP	AE	TRUE	9.997e-01	1.000e+00	FALSE	2.587e-03	2.328e-02	TRUE

GO:0090407	organophosphate biosynthetic process	BP	AE	TRUE	$1.000e+00$	$1.000e+00$	FALSE	$4.005e-04$	$4.625e-03$	TRUE
GO:1901137	carbohydrate derivative biosynthetic process	BP	AE	TRUE	$1.000e+00$	$1.000e+00$	FALSE	$4.005e-04$	$4.625e-03$	TRUE
GO:1901293	nucleoside phosphate biosynthetic process	BP	AE	TRUE	$1.000e+00$	$1.000e+00$	FALSE	$4.005e-04$	$4.625e-03$	TRUE
GO:0018575	chlorocatechol 1,2-dioxygenase activity	MF	AF	TRUE	$1.000e+00$	$1.000e+00$	FALSE	$9.838e-04$	$9.122e-03$	TRUE
GO:0006520	cellular amino acid metabolic process	BP	B	TRUE	$9.999e-01$	$1.000e+00$	FALSE	$8.291e-04$	$8.197e-03$	TRUE
GO:0009072	aromatic amino acid family metabolic process	BP	B	TRUE	$1.000e+00$	$1.000e+00$	FALSE	$1.302e-04$	$1.695e-03$	TRUE
GO:0006082	organic acid metabolic process	BP	D	TRUE	$9.999e-01$	$1.000e+00$	FALSE	$6.140e-04$	$6.479e-03$	TRUE
GO:0019752	carboxylic acid metabolic process	BP	D	TRUE	$9.999e-01$	$1.000e+00$	FALSE	$6.140e-04$	$6.479e-03$	TRUE
GO:0043436	oxoacid metabolic process	BP	D	TRUE	$9.999e-01$	$1.000e+00$	FALSE	$6.140e-04$	$6.479e-03$	TRUE
GO:0044238	primary metabolic process	BP	D	TRUE	$9.994e-01$	$1.000e+00$	FALSE	$2.118e-03$	$1.935e-02$	TRUE
GO:0044281	small molecule metabolic process	BP	D	TRUE	$1.000e+00$	$1.000e+00$	FALSE	$2.807e-05$	$4.521e-04$	TRUE
GO:0071704	organic substance metabolic process	BP	D	TRUE	$9.997e-01$	$1.000e+00$	FALSE	$8.512e-04$	$8.269e-03$	TRUE
GO:1901360	organic cyclic compound metabolic process	BP	D	TRUE	$9.999e-01$	$1.000e+00$	FALSE	$5.310e-04$	$5.908e-03$	TRUE
GO:0006725	cellular aromatic compound metabolic process	BP	E	TRUE	$1.000e+00$	$1.000e+00$	FALSE	$3.519e-08$	$2.692e-06$	TRUE
GO:0003868	4-hydroxyphenylpyruvate dioxygenase activity	MF	F	TRUE	$1.000e+00$	$1.000e+00$	FALSE	$9.838e-04$	$9.122e-03$	TRUE
GO:0003824	catalytic activity	MF	G	TRUE	$1.000e+00$	$1.000e+00$	FALSE	$1.230e-04$	$1.637e-03$	TRUE
GO:0008150	biological process	BP	G	TRUE	$1.000e+00$	$1.000e+00$	FALSE	$1.063e-06$	$2.324e-05$	TRUE
GO:0008152	metabolic process	BP	G	TRUE	$1.000e+00$	$1.000e+00$	FALSE	$9.975e-05$	$1.357e-03$	TRUE
GO:0044699	single-organism process	BP	G	TRUE	$1.000e+00$	$1.000e+00$	FALSE	$8.750e-06$	$1.513e-04$	TRUE
GO:0044710	single-organism metabolic process	BP	G	TRUE	$9.998e-01$	$1.000e+00$	FALSE	$4.093e-04$	$4.639e-03$	TRUE
GO:0055114	oxidation-reduction process	BP	G	TRUE	$9.997e-01$	$1.000e+00$	FALSE	$6.388e-04$	$6.626e-03$	TRUE
GO:0001816	cytokine production	BP	H	TRUE	$9.999e-01$	$1.000e+00$	FALSE	$3.649e-03$	$2.538e-02$	TRUE
GO:0001817	regulation of cytokine production	BP	H	TRUE	$9.999e-01$	$1.000e+00$	FALSE	$3.649e-03$	$2.538e-02$	TRUE
GO:0002376	immune system process	BP	H	TRUE	$9.999e-01$	$1.000e+00$	FALSE	$3.649e-03$	$2.538e-02$	TRUE
GO:0004175	endopeptidase activity	MF	H	TRUE	$9.999e-01$	$1.000e+00$	FALSE	$3.649e-03$	$2.538e-02$	TRUE
GO:0004252	serine-type endopeptidase activity	MF	H	TRUE	$9.999e-01$	$1.000e+00$	FALSE	$3.649e-03$	$2.538e-02$	TRUE
GO:0006955	immune response	BP	H	TRUE	$9.999e-01$	$1.000e+00$	FALSE	$3.649e-03$	$2.538e-02$	TRUE
GO:0006959	humoral immune response	BP	H	TRUE	$9.999e-01$	$1.000e+00$	FALSE	$3.649e-03$	$2.538e-02$	TRUE
GO:0008233	peptidase activity	MF	H	TRUE	$9.999e-01$	$1.000e+00$	FALSE	$3.649e-03$	$2.538e-02$	TRUE
GO:0008236	serine-type peptidase activity	MF	H	TRUE	$9.999e-01$	$1.000e+00$	FALSE	$3.649e-03$	$2.538e-02$	TRUE
GO:0009620	response to fungus	BP	H	TRUE	$9.999e-01$	$1.000e+00$	FALSE	$3.649e-03$	$2.538e-02$	TRUE
GO:0017171	serine hydrolase activity	MF	H	TRUE	$9.999e-01$	$1.000e+00$	FALSE	$3.649e-03$	$2.538e-02$	TRUE

GO:0019730	antimicrobial humoral response	BP	H	TRUE	$9.999e-01$	$1.000e+00$	FALSE	$3.649e-03$	$2.538e-02$	TRUE
GO:0019731	antibacterial humoral response	BP	H	TRUE	$9.999e-01$	$1.000e+00$	FALSE	$3.649e-03$	$2.538e-02$	TRUE
GO:0019732	antifungal humoral response	BP	H	TRUE	$9.999e-01$	$1.000e+00$	FALSE	$3.649e-03$	$2.538e-02$	TRUE
GO:0044707	single-multicellular organism process	BP	H	TRUE	$9.999e-01$	$1.000e+00$	FALSE	$3.649e-03$	$2.538e-02$	TRUE
GO:0050832	defense response to fungus	BP	H	TRUE	$9.999e-01$	$1.000e+00$	FALSE	$3.649e-03$	$2.538e-02$	TRUE
GO:0051239	regulation of multicellular organismal process	BP	H	TRUE	$9.999e-01$	$1.000e+00$	FALSE	$3.649e-03$	$2.538e-02$	TRUE
GO:0070011	peptidase activity, acting on L-amino acid peptides	MF	H	TRUE	$9.999e-01$	$1.000e+00$	FALSE	$3.649e-03$	$2.538e-02$	TRUE
GO:0009987	cellular process	BP	I	TRUE	$1.000e+00$	$1.000e+00$	FALSE	$2.871e-12$	$1.095e-09$	TRUE
GO:0044237	cellular metabolic process	BP	I	TRUE	$1.000e+00$	$1.000e+00$	FALSE	$1.051e-06$	$2.324e-05$	TRUE
GO:0044763	single-organism cellular process	BP	I	TRUE	$1.000e+00$	$1.000e+00$	FALSE	$1.236e-09$	$1.261e-07$	TRUE
GO:0000041	transition metal ion transport	BP	L	TRUE	$1.000e+00$	$1.000e+00$	FALSE	$4.881e-05$	$6.790e-04$	TRUE
GO:0006811	ion transport	BP	L	TRUE	$1.000e+00$	$1.000e+00$	FALSE	$4.881e-05$	$6.790e-04$	TRUE
GO:0006812	cation transport	BP	L	TRUE	$1.000e+00$	$1.000e+00$	FALSE	$4.881e-05$	$6.790e-04$	TRUE
GO:0006826	iron ion transport	BP	L	TRUE	$1.000e+00$	$1.000e+00$	FALSE	$4.881e-05$	$6.790e-04$	TRUE
GO:0006873	cellular ion homeostasis	BP	L	TRUE	$1.000e+00$	$1.000e+00$	FALSE	$4.251e-07$	$1.301e-05$	TRUE
GO:0006875	cellular metal ion homeostasis	BP	L	TRUE	$1.000e+00$	$1.000e+00$	FALSE	$4.251e-07$	$1.301e-05$	TRUE
GO:0006879	cellular iron ion homeostasis	BP	L	TRUE	$1.000e+00$	$1.000e+00$	FALSE	$4.251e-07$	$1.301e-05$	TRUE
GO:0019725	cellular homeostasis	BP	L	TRUE	$1.000e+00$	$1.000e+00$	FALSE	$8.209e-07$	$2.010e-05$	TRUE
GO:0030001	metal ion transport	BP	L	TRUE	$1.000e+00$	$1.000e+00$	FALSE	$4.881e-05$	$6.790e-04$	TRUE
GO:0030003	cellular cation homeostasis	BP	L	TRUE	$1.000e+00$	$1.000e+00$	FALSE	$4.251e-07$	$1.301e-05$	TRUE
GO:0042592	homeostatic process	BP	L	TRUE	$1.000e+00$	$1.000e+00$	FALSE	$8.209e-07$	$2.010e-05$	TRUE
GO:0046916	cellular transition metal ion homeostasis	BP	L	TRUE	$1.000e+00$	$1.000e+00$	FALSE	$4.251e-07$	$1.301e-05$	TRUE
GO:0048878	chemical homeostasis	BP	L	TRUE	$1.000e+00$	$1.000e+00$	FALSE	$8.209e-07$	$2.010e-05$	TRUE
GO:0050801	ion homeostasis	BP	L	TRUE	$1.000e+00$	$1.000e+00$	FALSE	$4.251e-07$	$1.301e-05$	TRUE
GO:0055065	metal ion homeostasis	BP	L	TRUE	$1.000e+00$	$1.000e+00$	FALSE	$4.251e-07$	$1.301e-05$	TRUE
GO:0055072	iron ion homeostasis	BP	L	TRUE	$1.000e+00$	$1.000e+00$	FALSE	$4.251e-07$	$1.301e-05$	TRUE
GO:0055076	transition metal ion homeostasis	BP	L	TRUE	$1.000e+00$	$1.000e+00$	FALSE	$4.251e-07$	$1.301e-05$	TRUE
GO:0055080	cation homeostasis	BP	L	TRUE	$1.000e+00$	$1.000e+00$	FALSE	$4.251e-07$	$1.301e-05$	TRUE
GO:0055082	cellular chemical homeostasis	BP	L	TRUE	$1.000e+00$	$1.000e+00$	FALSE	$8.209e-07$	$2.010e-05$	TRUE
GO:0065007	biological regulation	BP	L	TRUE	$1.000e+00$	$1.000e+00$	FALSE	$5.900e-07$	$1.720e-05$	TRUE
GO:0065008	regulation of biological quality	BP	L	TRUE	$1.000e+00$	$1.000e+00$	FALSE	$7.416e-06$	$1.464e-04$	TRUE
GO:0098771	inorganic ion homeostasis	BP	L	TRUE	$1.000e+00$	$1.000e+00$	FALSE	$4.251e-07$	$1.301e-05$	TRUE
GO:0009767	photosynthetic electron transport chain	BP	M	TRUE	$1.000e+00$	$1.000e+00$	FALSE	$5.925e-11$	$7.252e-09$	TRUE

GO:0009772	photosynthetic electron transport in photosystem II	BP	M	TRUE	$1.000e+00$	$1.000e+00$	FALSE	$5.366e-12$	$1.095e-09$	TRUE
GO:0015979	photosynthesis	BP	M	TRUE	$1.000e+00$	$1.000e+00$	FALSE	$7.416e-06$	$1.464e-04$	TRUE
GO:0019684	photosynthesis, light reaction	BP	M	TRUE	$1.000e+00$	$1.000e+00$	FALSE	$5.248e-09$	$4.588e-07$	TRUE
GO:0045156	electron transporter, transferring electrons within the cyclic electron transport pathway of photosynthesis activity	MF	M	TRUE	$1.000e+00$	$1.000e+00$	FALSE	$5.366e-12$	$1.095e-09$	TRUE
GO:0009056	catabolic process	BP	N	TRUE	$9.994e-01$	$1.000e+00$	FALSE	$2.727e-03$	$2.418e-02$	TRUE
GO:0016701	oxidoreductase activity, acting on single donors with incorporation of molecular oxygen	MF	P	TRUE	$1.000e+00$	$1.000e+00$	FALSE	$4.293e-06$	$9.060e-05$	TRUE
GO:0016702	oxidoreductase activity, acting on single donors with incorporation of molecular oxygen, incorporation of two atoms of oxygen	MF	P	TRUE	$1.000e+00$	$1.000e+00$	FALSE	$8.892e-07$	$2.093e-05$	TRUE
GO:0051213	dioxygenase activity	MF	P	TRUE	$1.000e+00$	$1.000e+00$	FALSE	$1.320e-07$	$8.977e-06$	TRUE
GO:0016787	hydrolase activity	MF	S	TRUE	$1.000e+00$	$1.000e+00$	FALSE	$4.204e-05$	$6.596e-04$	TRUE
GO:0005381	iron ion transmembrane transporter activity	MF	W	TRUE	$1.000e+00$	$1.000e+00$	FALSE	$9.147e-06$	$1.513e-04$	TRUE
GO:0015091	ferric iron transmembrane transporter activity	MF	W	TRUE	$1.000e+00$	$1.000e+00$	FALSE	$9.147e-06$	$1.513e-04$	TRUE
GO:0046873	metal ion transmembrane transporter activity	MF	W	TRUE	$1.000e+00$	$1.000e+00$	FALSE	$9.147e-06$	$1.513e-04$	TRUE
GO:0046915	transition metal ion transmembrane transporter activity	MF	W	TRUE	$1.000e+00$	$1.000e+00$	FALSE	$9.147e-06$	$1.513e-04$	TRUE
GO:0072510	trivalent inorganic cation transmembrane transporter activity	MF	W	TRUE	$1.000e+00$	$1.000e+00$	FALSE	$9.147e-06$	$1.513e-04$	TRUE
GO:0008199	ferric iron binding	MF	Y	TRUE	$1.000e+00$	$1.000e+00$	FALSE	$9.095e-12$	$1.392e-09$	TRUE

Table S160: fe all ligands

					normal			compress		
id	description	type	IPR.group	consistent	p	padjust	sig	p	padjust	sig
GO:0015669	gas transport	BP	AB	TRUE	$1.576e-04$	$8.997e-03$	TRUE	$1.000e+00$	$1.000e+00$	FALSE
GO:0015671	oxygen transport	BP	AB	TRUE	$1.576e-04$	$8.997e-03$	TRUE	$1.000e+00$	$1.000e+00$	FALSE
GO:0019825	oxygen binding	MF	AB	TRUE	$9.988e-07$	$1.235e-04$	TRUE	$1.000e+00$	$1.000e+00$	FALSE
GO:0005488	binding	MF	AE	TRUE	$2.192e-14$	$3.253e-12$	TRUE	$1.000e+00$	$1.000e+00$	FALSE
GO:0020037	heme binding	MF	AE	TRUE	$9.126e-43$	$1.693e-40$	TRUE	$1.000e+00$	$1.000e+00$	FALSE
GO:0046906	tetrapyrrole binding	MF	AE	TRUE	$9.126e-43$	$1.693e-40$	TRUE	$1.000e+00$	$1.000e+00$	FALSE
GO:0097159	organic cyclic compound binding	MF	AE	TRUE	$4.448e-44$	$1.650e-41$	TRUE	$1.000e+00$	$1.000e+00$	FALSE

GO:1901363	heterocyclic compound binding	MF	AE	TRUE	$4.448e-44$	$1.650e-41$	TRUE	$1.000e+00$	$1.000e+00$	FALSE
GO:0004601	peroxidase activity	MF	P	TRUE	$3.453e-05$	$2.847e-03$	TRUE	$1.000e+00$	$1.000e+00$	FALSE
GO:0006979	response to oxidative stress	BP	P	TRUE	$9.629e-05$	$7.145e-03$	TRUE	$1.000e+00$	$1.000e+00$	FALSE
GO:0016209	antioxidant activity	MF	P	TRUE	$1.188e-06$	$1.259e-04$	TRUE	$1.000e+00$	$1.000e+00$	FALSE
GO:0016684	oxidoreductase activity, acting on peroxide as acceptor	MF	P	TRUE	$3.453e-05$	$2.847e-03$	TRUE	$1.000e+00$	$1.000e+00$	FALSE
GO:0009055	electron carrier activity	MF	S	TRUE	$1.280e-04$	$8.635e-03$	TRUE	$1.000e+00$	$1.000e+00$	FALSE
GO:0009767	photosynthetic electron transport chain	BP	A	TRUE	$1.000e+00$	$1.000e+00$	FALSE	$6.373e-11$	$6.756e-09$	TRUE
GO:0009772	photosynthetic electron transport in photosystem II	BP	A	TRUE	$1.000e+00$	$1.000e+00$	FALSE	$5.791e-12$	$7.161e-10$	TRUE
GO:0019684	photosynthesis, light reaction	BP	A	TRUE	$1.000e+00$	$1.000e+00$	FALSE	$1.641e-08$	$3.929e-07$	TRUE
GO:0045156	electron transporter, transferring electrons within the cyclic electron transport pathway of photosynthesis activity	MF	A	TRUE	$1.000e+00$	$1.000e+00$	FALSE	$5.791e-12$	$7.161e-10$	TRUE
GO:0001816	cytokine production	BP	AD	TRUE	$1.000e+00$	$1.000e+00$	FALSE	$4.258e-04$	$3.472e-03$	TRUE
GO:0001817	regulation of cytokine production	BP	AD	TRUE	$1.000e+00$	$1.000e+00$	FALSE	$4.258e-04$	$3.472e-03$	TRUE
GO:0002376	immune system process	BP	AD	TRUE	$1.000e+00$	$1.000e+00$	FALSE	$4.258e-04$	$3.472e-03$	TRUE
GO:0004175	endopeptidase activity	MF	AD	TRUE	$1.000e+00$	$1.000e+00$	FALSE	$1.179e-03$	$8.580e-03$	TRUE
GO:0004252	serine-type endopeptidase activity	MF	AD	TRUE	$1.000e+00$	$1.000e+00$	FALSE	$4.258e-04$	$3.472e-03$	TRUE
GO:0006955	immune response	BP	AD	TRUE	$1.000e+00$	$1.000e+00$	FALSE	$4.258e-04$	$3.472e-03$	TRUE
GO:0006959	humoral immune response	BP	AD	TRUE	$1.000e+00$	$1.000e+00$	FALSE	$4.258e-04$	$3.472e-03$	TRUE
GO:0008233	peptidase activity	MF	AD	TRUE	$1.000e+00$	$1.000e+00$	FALSE	$1.576e-04$	$1.720e-03$	TRUE
GO:0008236	serine-type peptidase activity	MF	AD	TRUE	$1.000e+00$	$1.000e+00$	FALSE	$4.258e-04$	$3.472e-03$	TRUE
GO:0009605	response to external stimulus	BP	AD	TRUE	$9.998e-01$	$1.000e+00$	FALSE	$2.542e-03$	$1.626e-02$	TRUE
GO:0009607	response to biotic stimulus	BP	AD	TRUE	$9.998e-01$	$1.000e+00$	FALSE	$2.542e-03$	$1.626e-02$	TRUE
GO:0009617	response to bacterium	BP	AD	TRUE	$9.998e-01$	$1.000e+00$	FALSE	$2.542e-03$	$1.626e-02$	TRUE
GO:0009620	response to fungus	BP	AD	TRUE	$1.000e+00$	$1.000e+00$	FALSE	$4.258e-04$	$3.472e-03$	TRUE
GO:0017171	serine hydrolase activity	MF	AD	TRUE	$1.000e+00$	$1.000e+00$	FALSE	$4.258e-04$	$3.472e-03$	TRUE
GO:0019730	antimicrobial humoral response	BP	AD	TRUE	$1.000e+00$	$1.000e+00$	FALSE	$4.258e-04$	$3.472e-03$	TRUE
GO:0019731	antibacterial humoral response	BP	AD	TRUE	$1.000e+00$	$1.000e+00$	FALSE	$4.258e-04$	$3.472e-03$	TRUE
GO:0019732	antifungal humoral response	BP	AD	TRUE	$1.000e+00$	$1.000e+00$	FALSE	$4.258e-04$	$3.472e-03$	TRUE
GO:0042742	defense response to bacterium	BP	AD	TRUE	$9.998e-01$	$1.000e+00$	FALSE	$2.542e-03$	$1.626e-02$	TRUE
GO:0043207	response to external biotic stimulus	BP	AD	TRUE	$9.998e-01$	$1.000e+00$	FALSE	$2.542e-03$	$1.626e-02$	TRUE
GO:0044707	single-multicellular organism process	BP	AD	TRUE	$1.000e+00$	$1.000e+00$	FALSE	$4.258e-04$	$3.472e-03$	TRUE

GO:0050832	defense response to fungus	BP	AD	TRUE	$1.000e+00$	$1.000e+00$	FALSE	$4.258e-04$	$3.472e-03$	TRUE
GO:0051239	regulation of multicellular organismal process	BP	AD	TRUE	$1.000e+00$	$1.000e+00$	FALSE	$4.258e-04$	$3.472e-03$	TRUE
GO:0051704	multi-organism process	BP	AD	TRUE	$9.998e-01$	$1.000e+00$	FALSE	$2.542e-03$	$1.626e-02$	TRUE
GO:0051707	response to other organism	BP	AD	TRUE	$9.998e-01$	$1.000e+00$	FALSE	$2.542e-03$	$1.626e-02$	TRUE
GO:0070011	peptidase activity, acting on L-amino acid peptides	MF	AD	TRUE	$1.000e+00$	$1.000e+00$	FALSE	$1.576e-04$	$1.720e-03$	TRUE
GO:0098542	defense response to other organism	BP	AD	TRUE	$9.998e-01$	$1.000e+00$	FALSE	$2.542e-03$	$1.626e-02$	TRUE
GO:0016717	oxidoreductase activity, acting on paired donors, with oxidation of a pair of donors resulting in the reduction of molecular oxygen to two molecules of water	MF	AF	TRUE	$1.000e+00$	$1.000e+00$	FALSE	$8.855e-06$	$1.217e-04$	TRUE
GO:0045300	acyl-[acyl-carrier-protein] desaturase activity	MF	AF	TRUE	$1.000e+00$	$1.000e+00$	FALSE	$8.855e-06$	$1.217e-04$	TRUE
GO:0016787	hydrolase activity	MF	AG	TRUE	$9.991e-01$	$1.000e+00$	FALSE	$3.294e-03$	$2.071e-02$	TRUE
GO:0006807	nitrogen compound metabolic process	BP	AH	TRUE	$9.993e-01$	$1.000e+00$	FALSE	$1.843e-03$	$1.294e-02$	TRUE
GO:0016830	carbon-carbon lyase activity	MF	AJ	TRUE	$9.998e-01$	$1.000e+00$	FALSE	$2.542e-03$	$1.626e-02$	TRUE
GO:0071771	aldehyde decarbonylase activity	MF	AJ	TRUE	$1.000e+00$	$1.000e+00$	FALSE	$4.258e-04$	$3.472e-03$	TRUE
GO:0032501	multicellular organismal process	BP	AK	TRUE	$9.992e-01$	$1.000e+00$	FALSE	$7.812e-03$	$4.713e-02$	TRUE
GO:0018575	chlorocatechol 1,2-dioxygenase activity	MF	AL	TRUE	$9.996e-01$	$1.000e+00$	FALSE	$8.198e-03$	$4.793e-02$	TRUE
GO:0032787	monocarboxylic acid metabolic process	BP	AN	TRUE	$9.997e-01$	$1.000e+00$	FALSE	$2.042e-03$	$1.416e-02$	TRUE
GO:0006082	organic acid metabolic process	BP	AP	FALSE	$1.000e+00$	$1.000e+00$	FALSE	$2.605e-05$	$3.222e-04$	TRUE
GO:0019752	carboxylic acid metabolic process	BP	AP	FALSE	$1.000e+00$	$1.000e+00$	FALSE	$1.999e-05$	$2.557e-04$	TRUE
GO:0043436	oxoacid metabolic process	BP	AP	FALSE	$1.000e+00$	$1.000e+00$	FALSE	$2.605e-05$	$3.222e-04$	TRUE
GO:0044238	primary metabolic process	BP	AP	TRUE	$1.000e+00$	$1.000e+00$	FALSE	$4.378e-07$	$9.282e-06$	TRUE
GO:0044281	small molecule metabolic process	BP	AP	FALSE	$1.000e+00$	$1.000e+00$	FALSE	$6.556e-09$	$1.737e-07$	TRUE
GO:0071704	organic substance metabolic process	BP	AP	TRUE	$1.000e+00$	$1.000e+00$	FALSE	$7.689e-07$	$1.585e-05$	TRUE
GO:1901360	organic cyclic compound metabolic process	BP	AP	FALSE	$1.000e+00$	$1.000e+00$	FALSE	$1.268e-05$	$1.710e-04$	TRUE
GO:0051536	iron-sulfur cluster binding	MF	AQ	TRUE	$9.987e-01$	$1.000e+00$	FALSE	$5.197e-03$	$3.187e-02$	TRUE
GO:0051540	metal cluster binding	MF	AQ	TRUE	$9.987e-01$	$1.000e+00$	FALSE	$5.197e-03$	$3.187e-02$	TRUE
GO:0006558	L-phenylalanine metabolic process	BP	B	TRUE	$1.000e+00$	$1.000e+00$	FALSE	$9.528e-04$	$7.000e-03$	TRUE

GO:0006559	L-phenylalanine catabolic process	BP	B	TRUE	$1.000e+00$	$1.000e+00$	FALSE	$9.528e-04$	$7.000e-03$	TRUE
GO:0009074	aromatic amino acid family catabolic process	BP	B	TRUE	$9.996e-01$	$1.000e+00$	FALSE	$8.198e-03$	$4.793e-02$	TRUE
GO:1902221	erythrose 4-phosphate/phosphoenolpyruvate family amino acid metabolic process	BP	B	TRUE	$1.000e+00$	$1.000e+00$	FALSE	$9.528e-04$	$7.000e-03$	TRUE
GO:1902222	erythrose 4-phosphate/phosphoenolpyruvate family amino acid catabolic process	BP	B	TRUE	$1.000e+00$	$1.000e+00$	FALSE	$9.528e-04$	$7.000e-03$	TRUE
GO:0006520	cellular amino acid metabolic process	BP	C	FALSE	$1.000e+00$	$1.000e+00$	FALSE	$1.686e-04$	$1.814e-03$	TRUE
GO:0009072	aromatic amino acid family metabolic process	BP	C	TRUE	$1.000e+00$	$1.000e+00$	FALSE	$1.544e-05$	$2.046e-04$	TRUE
GO:0005381	iron ion transmembrane transporter activity	MF	D	TRUE	$1.000e+00$	$1.000e+00$	FALSE	$8.855e-06$	$1.217e-04$	TRUE
GO:0015091	ferric iron transmembrane transporter activity	MF	D	TRUE	$1.000e+00$	$1.000e+00$	FALSE	$8.855e-06$	$1.217e-04$	TRUE
GO:0046873	metal ion transmembrane transporter activity	MF	D	TRUE	$1.000e+00$	$1.000e+00$	FALSE	$8.855e-06$	$1.217e-04$	TRUE
GO:0046915	transition metal ion transmembrane transporter activity	MF	D	TRUE	$1.000e+00$	$1.000e+00$	FALSE	$8.855e-06$	$1.217e-04$	TRUE
GO:0072510	trivalent inorganic cation transmembrane transporter activity	MF	D	TRUE	$1.000e+00$	$1.000e+00$	FALSE	$8.855e-06$	$1.217e-04$	TRUE
GO:0016708	oxidoreductase activity, acting on paired donors, with incorporation or reduction of molecular oxygen, NAD(P)H as one donor, and incorporation of two atoms of oxygen into one donor	MF	E	TRUE	$1.000e+00$	$1.000e+00$	FALSE	$4.317e-06$	$8.009e-05$	TRUE
GO:0006139	nucleobase-containing compound metabolic process	BP	G	FALSE	$9.996e-01$	$1.000e+00$	FALSE	$1.706e-03$	$1.217e-02$	TRUE
GO:0006753	nucleoside phosphate metabolic process	BP	G	TRUE	$1.000e+00$	$1.000e+00$	FALSE	$1.119e-04$	$1.258e-03$	TRUE
GO:0006793	phosphorus metabolic process	BP	G	TRUE	$9.998e-01$	$1.000e+00$	FALSE	$9.270e-04$	$7.000e-03$	TRUE
GO:0006796	phosphate-containing compound metabolic process	BP	G	TRUE	$9.999e-01$	$1.000e+00$	FALSE	$6.855e-04$	$5.325e-03$	TRUE
GO:0009117	nucleotide metabolic process	BP	G	TRUE	$1.000e+00$	$1.000e+00$	FALSE	$3.347e-04$	$3.356e-03$	TRUE
GO:0009132	nucleoside diphosphate metabolic process	BP	G	TRUE	$1.000e+00$	$1.000e+00$	FALSE	$2.059e-06$	$3.918e-05$	TRUE
GO:0009165	nucleotide biosynthetic process	BP	G	TRUE	$1.000e+00$	$1.000e+00$	FALSE	$6.233e-06$	$1.005e-04$	TRUE

GO:0009186	deoxyribonucleoside diphosphate metabolic process	BP	G	TRUE	$1.000e+00$	$1.000e+00$	FALSE	$2.059e-06$	$3.918e-05$	TRUE
GO:0009262	deoxyribonucleotide metabolic process	BP	G	TRUE	$1.000e+00$	$1.000e+00$	FALSE	$6.233e-06$	$1.005e-04$	TRUE
GO:0009263	deoxyribonucleotide biosynthetic process	BP	G	TRUE	$1.000e+00$	$1.000e+00$	FALSE	$6.233e-06$	$1.005e-04$	TRUE
GO:0018130	heterocycle biosynthetic process	BP	G	FALSE	$9.999e-01$	$1.000e+00$	FALSE	$4.963e-04$	$3.918e-03$	TRUE
GO:0019438	aromatic compound biosynthetic process	BP	G	FALSE	$9.999e-01$	$1.000e+00$	FALSE	$4.963e-04$	$3.918e-03$	TRUE
GO:0019637	organophosphate metabolic process	BP	G	TRUE	$1.000e+00$	$1.000e+00$	FALSE	$2.476e-04$	$2.516e-03$	TRUE
GO:0034654	nucleobase-containing compound biosynthetic process	BP	G	FALSE	$1.000e+00$	$1.000e+00$	FALSE	$2.188e-04$	$2.286e-03$	TRUE
GO:0044271	cellular nitrogen compound biosynthetic process	BP	G	FALSE	$9.979e-01$	$1.000e+00$	FALSE	$8.203e-03$	$4.793e-02$	TRUE
GO:0044711	single-organism biosynthetic process	BP	G	FALSE	$9.979e-01$	$1.000e+00$	FALSE	$8.203e-03$	$4.793e-02$	TRUE
GO:0055086	nucleobase-containing small molecule metabolic process	BP	G	FALSE	$1.000e+00$	$1.000e+00$	FALSE	$2.476e-04$	$2.516e-03$	TRUE
GO:0090407	organophosphate biosynthetic process	BP	G	TRUE	$1.000e+00$	$1.000e+00$	FALSE	$6.233e-06$	$1.005e-04$	TRUE
GO:1901135	carbohydrate derivative metabolic process	BP	G	TRUE	$9.999e-01$	$1.000e+00$	FALSE	$4.963e-04$	$3.918e-03$	TRUE
GO:1901137	carbohydrate derivative biosynthetic process	BP	G	TRUE	$1.000e+00$	$1.000e+00$	FALSE	$6.233e-06$	$1.005e-04$	TRUE
GO:1901293	nucleoside phosphate biosynthetic process	BP	G	TRUE	$1.000e+00$	$1.000e+00$	FALSE	$6.233e-06$	$1.005e-04$	TRUE
GO:1901362	organic cyclic compound biosynthetic process	BP	G	FALSE	$9.991e-01$	$1.000e+00$	FALSE	$4.171e-03$	$2.601e-02$	TRUE
GO:0051537	2 iron, 2 sulfur cluster binding	MF	H	TRUE	$1.000e+00$	$1.000e+00$	FALSE	$4.489e-05$	$5.372e-04$	TRUE
GO:0000041	transition metal ion transport	BP	I	TRUE	$1.000e+00$	$1.000e+00$	FALSE	$1.572e-08$	$3.888e-07$	TRUE
GO:0006811	ion transport	BP	I	TRUE	$1.000e+00$	$1.000e+00$	FALSE	$2.671e-09$	$7.339e-08$	TRUE
GO:0006812	cation transport	BP	I	TRUE	$1.000e+00$	$1.000e+00$	FALSE	$2.671e-09$	$7.339e-08$	TRUE
GO:0006826	iron ion transport	BP	I	TRUE	$1.000e+00$	$1.000e+00$	FALSE	$1.572e-08$	$3.888e-07$	TRUE
GO:0006873	cellular ion homeostasis	BP	I	TRUE	$1.000e+00$	$1.000e+00$	FALSE	$2.574e-10$	$1.061e-08$	TRUE
GO:0006875	cellular metal ion homeostasis	BP	I	TRUE	$1.000e+00$	$1.000e+00$	FALSE	$2.574e-10$	$1.061e-08$	TRUE
GO:0006879	cellular iron ion homeostasis	BP	I	TRUE	$1.000e+00$	$1.000e+00$	FALSE	$2.574e-10$	$1.061e-08$	TRUE
GO:0019725	cellular homeostasis	BP	I	TRUE	$1.000e+00$	$1.000e+00$	FALSE	$7.724e-10$	$2.605e-08$	TRUE
GO:0030001	metal ion transport	BP	I	TRUE	$1.000e+00$	$1.000e+00$	FALSE	$2.671e-09$	$7.339e-08$	TRUE
GO:0030003	cellular cation homeostasis	BP	I	TRUE	$1.000e+00$	$1.000e+00$	FALSE	$2.574e-10$	$1.061e-08$	TRUE
GO:0042592	homeostatic process	BP	I	TRUE	$1.000e+00$	$1.000e+00$	FALSE	$7.724e-10$	$2.605e-08$	TRUE
GO:0046916	cellular transition metal ion homeostasis	BP	I	TRUE	$1.000e+00$	$1.000e+00$	FALSE	$2.574e-10$	$1.061e-08$	TRUE
GO:0048878	chemical homeostasis	BP	I	TRUE	$1.000e+00$	$1.000e+00$	FALSE	$7.724e-10$	$2.605e-08$	TRUE

GO:0050801	ion homeostasis	BP	I	TRUE	$1.000e+00$	$1.000e+00$	FALSE	$2.574e-10$	$1.061e-08$	TRUE
GO:0055065	metal ion homeostasis	BP	I	TRUE	$1.000e+00$	$1.000e+00$	FALSE	$2.574e-10$	$1.061e-08$	TRUE
GO:0055072	iron ion homeostasis	BP	I	TRUE	$1.000e+00$	$1.000e+00$	FALSE	$2.574e-10$	$1.061e-08$	TRUE
GO:0055076	transition metal ion homeostasis	BP	I	TRUE	$1.000e+00$	$1.000e+00$	FALSE	$2.574e-10$	$1.061e-08$	TRUE
GO:0055080	cation homeostasis	BP	I	TRUE	$1.000e+00$	$1.000e+00$	FALSE	$2.574e-10$	$1.061e-08$	TRUE
GO:0055082	cellular chemical homeostasis	BP	I	TRUE	$1.000e+00$	$1.000e+00$	FALSE	$7.724e-10$	$2.605e-08$	TRUE
GO:0065007	biological regulation	BP	I	FALSE	$1.000e+00$	$1.000e+00$	FALSE	$1.061e-09$	$3.424e-08$	TRUE
GO:0065008	regulation of biological quality	BP	I	TRUE	$1.000e+00$	$1.000e+00$	FALSE	$3.032e-08$	$7.030e-07$	TRUE
GO:0098771	inorganic ion homeostasis	BP	I	TRUE	$1.000e+00$	$1.000e+00$	FALSE	$2.574e-10$	$1.061e-08$	TRUE
GO:0009056	catabolic process	BP	M	TRUE	$1.000e+00$	$1.000e+00$	FALSE	$8.567e-05$	$9.780e-04$	TRUE
GO:0019439	aromatic compound catabolic process	BP	M	TRUE	$1.000e+00$	$1.000e+00$	FALSE	$7.296e-06$	$1.152e-04$	TRUE
GO:0044248	cellular catabolic process	BP	M	TRUE	$1.000e+00$	$1.000e+00$	FALSE	$1.751e-04$	$1.856e-03$	TRUE
GO:0006631	fatty acid metabolic process	BP	O	TRUE	$9.999e-01$	$1.000e+00$	FALSE	$1.478e-03$	$1.065e-02$	TRUE
GO:0044255	cellular lipid metabolic process	BP	O	TRUE	$9.992e-01$	$1.000e+00$	FALSE	$5.909e-03$	$3.594e-02$	TRUE
GO:0006725	cellular aromatic compound metabolic process	BP	R	FALSE	$1.000e+00$	$1.000e+00$	FALSE	$2.113e-12$	$3.920e-10$	TRUE
GO:0009987	cellular process	BP	T	TRUE	$1.000e+00$	$1.000e+00$	FALSE	$3.973e-16$	$2.948e-13$	TRUE
GO:0044237	cellular metabolic process	BP	T	TRUE	$1.000e+00$	$1.000e+00$	FALSE	$1.476e-07$	$3.220e-06$	TRUE
GO:0044763	single-organism cellular process	BP	T	TRUE	$1.000e+00$	$1.000e+00$	FALSE	$7.939e-14$	$2.946e-11$	TRUE
GO:0015979	photosynthesis	BP	U	TRUE	$1.000e+00$	$1.000e+00$	FALSE	$1.891e-05$	$2.461e-04$	TRUE
GO:0008199	ferric iron binding	MF	V	TRUE	$1.000e+00$	$1.000e+00$	FALSE	$3.480e-13$	$8.608e-11$	TRUE
GO:0016701	oxidoreductase activity, acting on single donors with incorporation of molecular oxygen	MF	X	TRUE	$1.000e+00$	$1.000e+00$	FALSE	$8.214e-05$	$9.523e-04$	TRUE
GO:0016702	oxidoreductase activity, acting on single donors with incorporation of molecular oxygen, incorporation of two atoms of oxygen	MF	X	TRUE	$1.000e+00$	$1.000e+00$	FALSE	$4.280e-05$	$5.206e-04$	TRUE
GO:0051213	dioxygenase activity	MF	X	TRUE	$1.000e+00$	$1.000e+00$	FALSE	$1.496e-09$	$4.625e-08$	TRUE
GO:0003868	4-hydroxyphenylpyruvate dioxygenase activity	MF	Y	TRUE	$1.000e+00$	$1.000e+00$	FALSE	$4.258e-04$	$3.472e-03$	TRUE
GO:0003824	catalytic activity	MF	Z	TRUE	$1.000e+00$	$1.000e+00$	FALSE	$6.928e-05$	$8.159e-04$	TRUE
GO:0008150	biological process	BP	Z	TRUE	$1.000e+00$	$1.000e+00$	FALSE	$1.248e-07$	$2.806e-06$	TRUE
GO:0008152	metabolic process	BP	Z	TRUE	$9.997e-01$	$1.000e+00$	FALSE	$6.889e-04$	$5.325e-03$	TRUE
GO:0044699	single-organism process	BP	Z	TRUE	$1.000e+00$	$1.000e+00$	FALSE	$1.049e-06$	$2.104e-05$	TRUE
GO:0044710	single-organism metabolic process	BP	Z	TRUE	$9.991e-01$	$1.000e+00$	FALSE	$1.849e-03$	$1.294e-02$	TRUE
GO:0055114	oxidation-reduction process	BP	Z	TRUE	$9.985e-01$	$1.000e+00$	FALSE	$2.783e-03$	$1.765e-02$	TRUE

mg

Table S161: mg 6 ligands

id	description	type	IPR.group	consistent	normal p	padjust	sig	compress p	padjust	sig
GO:0005525	GTP binding	MF	Q	TRUE	$9.602e-05$	0.04475	TRUE	1	1	FALSE
GO:0019001	guanyl nucleotide binding	MF	Q	TRUE	$3.752e-05$	0.04475	TRUE	1	1	FALSE
GO:0032561	guanyl ribonucleotide binding	MF	Q	TRUE	$9.602e-05$	0.04475	TRUE	1	1	FALSE

Table S162: mg all ligands

id	description	type	IPR.group	consistent	normal p	padjust	sig	compress p	padjust	sig
GO:0007154	cell communication	BP	C	TRUE	$2.189e-05$	0.005754	TRUE	1.0000	1	FALSE
GO:0007165	signal transduction	BP	C	TRUE	$2.189e-05$	0.005754	TRUE	1.0000	1	FALSE
GO:0023052	signaling	BP	C	TRUE	$2.189e-05$	0.005754	TRUE	1.0000	1	FALSE
GO:0035556	intracellular signal transduction	BP	C	TRUE	$1.054e-04$	0.015110	TRUE	1.0000	1	FALSE
GO:0044700	single organism signaling	BP	C	TRUE	$2.189e-05$	0.005754	TRUE	1.0000	1	FALSE
GO:0050789	regulation of biological process	BP	C	TRUE	$4.659e-05$	0.010497	TRUE	1.0000	1	FALSE
GO:0050794	regulation of cellular process	BP	C	TRUE	$2.121e-05$	0.005754	TRUE	1.0000	1	FALSE
GO:0051716	cellular response to stimulus	BP	C	TRUE	$2.233e-04$	0.029346	TRUE	0.9999	1	FALSE
GO:0065007	biological regulation	BP	C	TRUE	$9.192e-05$	0.014496	TRUE	1.0000	1	FALSE
GO:0005525	GTP binding	MF	X	TRUE	$8.492e-05$	0.014496	TRUE	1.0000	1	FALSE
GO:0019001	guanyl nucleotide binding	MF	X	TRUE	$1.626e-05$	0.005754	TRUE	1.0000	1	FALSE
GO:0032561	guanyl ribonucleotide binding	MF	X	TRUE	$8.492e-05$	0.014496	TRUE	1.0000	1	FALSE

na

Table S163: na 5 ligands

id	description	type	IPR.group	consistent	normal p	padjust	sig	compress p	padjust	sig
GO:0006811	ion transport	BP	A	TRUE	1	1	FALSE	0.0002186	0.03617	TRUE
GO:0004620	phospholipase activity	MF	H	TRUE	1	1	FALSE	0.0007490	0.03617	TRUE
GO:0004623	phospholipase A2 activity	MF	H	TRUE	1	1	FALSE	0.0007490	0.03617	TRUE
GO:0006820	anion transport	BP	H	TRUE	1	1	FALSE	0.0007490	0.03617	TRUE
GO:0015711	organic anion transport	BP	H	TRUE	1	1	FALSE	0.0007490	0.03617	TRUE
GO:0015718	monocarboxylic acid transport	BP	H	TRUE	1	1	FALSE	0.0007490	0.03617	TRUE
GO:0015849	organic acid transport	BP	H	TRUE	1	1	FALSE	0.0007490	0.03617	TRUE

GO:0015908	fatty acid transport	BP	H	TRUE	1	1	FALSE	0.0007490	0.03617	TRUE
GO:0015909	long-chain fatty acid transport	BP	H	TRUE	1	1	FALSE	0.0007490	0.03617	TRUE
GO:0016042	lipid catabolic process	BP	H	TRUE	1	1	FALSE	0.0007490	0.03617	TRUE
GO:0032309	icosanoid secretion	BP	H	TRUE	1	1	FALSE	0.0007490	0.03617	TRUE
GO:0046717	acid secretion	BP	H	TRUE	1	1	FALSE	0.0007490	0.03617	TRUE
GO:0046942	carboxylic acid transport	BP	H	TRUE	1	1	FALSE	0.0007490	0.03617	TRUE
GO:0050482	arachidonic acid secretion	BP	H	TRUE	1	1	FALSE	0.0007490	0.03617	TRUE
GO:0071715	icosanoid transport	BP	H	TRUE	1	1	FALSE	0.0007490	0.03617	TRUE
GO:1901571	fatty acid derivative transport	BP	H	TRUE	1	1	FALSE	0.0007490	0.03617	TRUE
GO:1903963	arachidonate transport	BP	H	TRUE	1	1	FALSE	0.0007490	0.03617	TRUE

Table S164: na 6 ligands

					normal			compress		
	description	type	IPR.group	consistent	p	padjust	sig	p	padjust	sig
GO:0005509	calcium ion binding	MF	A	TRUE	1	1	FALSE	$7.221e-06$	0.005871	TRUE

Table S165: na all ligands

					normal			compress		
	description	type	IPR.group	consistent	p	padjust	sig	p	padjust	sig
GO:0005509	calcium ion binding	MF	E	TRUE	1	1	FALSE	$2.505e-05$	0.03314	TRUE

zn

Table S166: zn 4 ligands

					normal			compress		
id	description	type	IPR.group	consistent	p	padjust	sig	p	padjust	sig
GO:0008270	zinc ion binding	MF	K	TRUE	$6.308e-06$	$2.021e-03$	TRUE	$1.000e+00$	1.000000	FALSE
GO:0043169	cation binding	MF	K	TRUE	$1.332e-07$	$7.111e-05$	TRUE	$1.000e+00$	1.000000	FALSE
GO:0046872	metal ion binding	MF	K	TRUE	$1.332e-07$	$7.111e-05$	TRUE	$1.000e+00$	1.000000	FALSE
GO:0008235	metalloexopeptidase activity	MF	A	TRUE	$1.000e+00$	$1.000e+00$	FALSE	$9.302e-05$	0.029803	TRUE
GO:0008233	peptidase activity	MF	J	TRUE	$1.000e+00$	$1.000e+00$	FALSE	$5.227e-05$	0.020934	TRUE
GO:0070011	peptidase activity, acting on L-amino acid peptides	MF	J	TRUE	$1.000e+00$	$1.000e+00$	FALSE	$3.727e-05$	0.019900	TRUE
GO:0008238	exopeptidase activity	MF	L	TRUE	$1.000e+00$	$1.000e+00$	FALSE	$9.556e-06$	0.008337	TRUE
GO:0016787	hydrolase activity	MF	O	TRUE	$1.000e+00$	$1.000e+00$	FALSE	$1.041e-05$	0.008337	TRUE

Table S167: zn 5 ligands

					normal			compress		
id	description	type	IPR.group	consistent	p	padjust	sig	p	padjust	sig

GO:0009056	catabolic process	BP	A	TRUE	$2.853e-04$	$1.613e-02$	TRUE	$9.999e-01$	1.0000000	FALSE
GO:0044281	small molecule metabolic process	BP	AL	TRUE	$3.150e-04$	$1.613e-02$	TRUE	$9.999e-01$	1.0000000	FALSE
GO:0055086	nucleobase-containing small molecule metabolic process	BP	AQ	TRUE	$5.853e-07$	$2.034e-04$	TRUE	$1.000e+00$	1.0000000	FALSE
GO:0016810	hydrolase activity, acting on carbon-nitrogen (but not peptide) bonds	MF	AR	TRUE	$7.027e-05$	$9.737e-03$	TRUE	$1.000e+00$	1.0000000	FALSE
GO:0006725	cellular aromatic compound metabolic process	BP	AS	TRUE	$2.389e-04$	$1.613e-02$	TRUE	$9.999e-01$	1.0000000	FALSE
GO:0006807	nitrogen compound metabolic process	BP	AS	TRUE	$7.460e-04$	$2.894e-02$	TRUE	$9.997e-01$	1.0000000	FALSE
GO:0009058	biosynthetic process	BP	AS	TRUE	$1.257e-03$	$4.688e-02$	TRUE	$9.995e-01$	1.0000000	FALSE
GO:0034641	cellular nitrogen compound metabolic process	BP	AS	TRUE	$1.357e-04$	$1.316e-02$	TRUE	$1.000e+00$	1.0000000	FALSE
GO:0044711	single-organism biosynthetic process	BP	AS	TRUE	$6.289e-07$	$2.034e-04$	TRUE	$1.000e+00$	1.0000000	FALSE
GO:0046483	heterocycle metabolic process	BP	AS	TRUE	$1.029e-04$	$1.221e-02$	TRUE	$1.000e+00$	1.0000000	FALSE
GO:1901360	organic cyclic compound metabolic process	BP	AS	TRUE	$7.027e-05$	$9.737e-03$	TRUE	$1.000e+00$	1.0000000	FALSE
GO:0003824	catalytic activity	MF	B	TRUE	$6.973e-08$	$6.764e-05$	TRUE	$1.000e+00$	1.0000000	FALSE
GO:0016787	hydrolase activity	MF	B	FALSE	$1.117e-05$	$2.168e-03$	TRUE	$1.000e+00$	1.0000000	FALSE
GO:0006753	nucleoside phosphate metabolic process	BP	J	TRUE	$1.619e-04$	$1.332e-02$	TRUE	$1.000e+00$	1.0000000	FALSE
GO:0006793	phosphorus metabolic process	BP	J	TRUE	$3.404e-04$	$1.613e-02$	TRUE	$9.999e-01$	1.0000000	FALSE
GO:0006796	phosphate-containing compound metabolic process	BP	J	TRUE	$3.404e-04$	$1.613e-02$	TRUE	$9.999e-01$	1.0000000	FALSE
GO:0009123	nucleoside monophosphate metabolic process	BP	J	TRUE	$3.825e-04$	$1.613e-02$	TRUE	$1.000e+00$	1.0000000	FALSE
GO:0009126	purine nucleoside monophosphate metabolic process	BP	J	TRUE	$3.825e-04$	$1.613e-02$	TRUE	$1.000e+00$	1.0000000	FALSE
GO:0009161	ribonucleoside monophosphate metabolic process	BP	J	TRUE	$3.825e-04$	$1.613e-02$	TRUE	$1.000e+00$	1.0000000	FALSE
GO:0009167	purine ribonucleoside monophosphate metabolic process	BP	J	TRUE	$3.825e-04$	$1.613e-02$	TRUE	$1.000e+00$	1.0000000	FALSE
GO:0019637	organophosphate metabolic process	BP	J	TRUE	$1.785e-04$	$1.332e-02$	TRUE	$1.000e+00$	1.0000000	FALSE
GO:0044710	single-organism metabolic process	BP	M	TRUE	$4.764e-04$	$1.925e-02$	TRUE	$9.998e-01$	1.0000000	FALSE
GO:0044763	single-organism cellular process	BP	M	TRUE	$1.084e-05$	$2.168e-03$	TRUE	$1.000e+00$	1.0000000	FALSE
GO:0072524	pyridine-containing compound metabolic process	BP	O	TRUE	$1.305e-03$	$4.688e-02$	TRUE	$1.000e+00$	1.0000000	FALSE
GO:1901564	organonitrogen compound metabolic process	BP	R	TRUE	$1.132e-04$	$1.221e-02$	TRUE	$1.000e+00$	1.0000000	FALSE

GO:1901566	organonitrogen compound biosynthetic process	BP	R	TRUE	$1.785e-04$	$1.332e-02$	TRUE	$1.000e+00$	1.0000000	FALSE
GO:0004151	dihydroorotase activity	MF	Z	TRUE	$3.825e-04$	$1.613e-02$	TRUE	$1.000e+00$	1.0000000	FALSE
GO:0018342	protein prenylation	BP	AB	TRUE	$1.000e+00$	$1.000e+00$	FALSE	$1.329e-04$	0.0322286	TRUE
GO:0097354	prenylation	BP	AB	TRUE	$1.000e+00$	$1.000e+00$	FALSE	$1.329e-04$	0.0322286	TRUE
GO:0004180	carboxypeptidase activity	MF	U	TRUE	$1.000e+00$	$1.000e+00$	FALSE	$9.080e-07$	0.0004404	TRUE
GO:0004181	metallocarboxypeptidase activity	MF	U	TRUE	$1.000e+00$	$1.000e+00$	FALSE	$9.080e-07$	0.0004404	TRUE

Table S168: zn all ligands

					normal			compress		
id	description	type	IPR.group	consistent	p	padjust	sig	p	padjust	sig
GO:0009058	biosynthetic process	BP	AD	TRUE	$4.052e-04$	$3.241e-02$	TRUE	$9.998e-01$	$1.000e+00$	FALSE
GO:0018130	heterocycle biosynthetic process	BP	AD	TRUE	$1.821e-04$	$1.675e-02$	TRUE	$9.999e-01$	$1.000e+00$	FALSE
GO:0044249	cellular biosynthetic process	BP	AD	TRUE	$3.753e-04$	$3.139e-02$	TRUE	$9.998e-01$	$1.000e+00$	FALSE
GO:0044271	cellular nitrogen compound biosynthetic process	BP	AD	TRUE	$4.471e-05$	$4.839e-03$	TRUE	$1.000e+00$	$1.000e+00$	FALSE
GO:1901362	organic cyclic compound biosynthetic process	BP	AD	TRUE	$1.647e-04$	$1.595e-02$	TRUE	$9.999e-01$	$1.000e+00$	FALSE
GO:0006139	nucleobase-containing compound metabolic process	BP	AP	TRUE	$1.762e-06$	$2.494e-04$	TRUE	$1.000e+00$	$1.000e+00$	FALSE
GO:0006725	cellular aromatic compound metabolic process	BP	AP	TRUE	$3.206e-07$	$5.363e-05$	TRUE	$1.000e+00$	$1.000e+00$	FALSE
GO:0006807	nitrogen compound metabolic process	BP	AP	TRUE	$1.396e-05$	$1.712e-03$	TRUE	$1.000e+00$	$1.000e+00$	FALSE
GO:0034641	cellular nitrogen compound metabolic process	BP	AP	TRUE	$1.939e-07$	$3.965e-05$	TRUE	$1.000e+00$	$1.000e+00$	FALSE
GO:0046483	heterocycle metabolic process	BP	AP	TRUE	$2.284e-07$	$4.202e-05$	TRUE	$1.000e+00$	$1.000e+00$	FALSE
GO:0090304	nucleic acid metabolic process	BP	AP	TRUE	$1.064e-05$	$1.398e-03$	TRUE	$1.000e+00$	$1.000e+00$	FALSE
GO:1901360	organic cyclic compound metabolic process	BP	AP	TRUE	$1.843e-07$	$3.965e-05$	TRUE	$1.000e+00$	$1.000e+00$	FALSE
GO:0003677	DNA binding	MF	AT	TRUE	$5.647e-04$	$4.329e-02$	TRUE	$9.998e-01$	$1.000e+00$	FALSE
GO:0051716	cellular response to stimulus	BP	B	TRUE	$2.385e-05$	$2.743e-03$	TRUE	$1.000e+00$	$1.000e+00$	FALSE
GO:0003676	nucleic acid binding	MF	G	TRUE	$1.031e-15$	$1.897e-12$	TRUE	$1.000e+00$	$1.000e+00$	FALSE
GO:0097159	organic cyclic compound binding	MF	G	TRUE	$9.102e-13$	$5.583e-10$	TRUE	$1.000e+00$	$1.000e+00$	FALSE
GO:1901363	heterocyclic compound binding	MF	G	TRUE	$9.102e-13$	$5.583e-10$	TRUE	$1.000e+00$	$1.000e+00$	FALSE
GO:0005515	protein binding	MF	J	TRUE	$3.548e-04$	$3.108e-02$	TRUE	$9.999e-01$	$1.000e+00$	FALSE
GO:0016740	transferase activity	MF	M	TRUE	$6.089e-05$	$6.224e-03$	TRUE	$1.000e+00$	$1.000e+00$	FALSE
GO:0033554	cellular response to stress	BP	N	TRUE	$5.985e-04$	$4.405e-02$	TRUE	$1.000e+00$	$1.000e+00$	FALSE
GO:0008270	zinc ion binding	MF	Q	TRUE	$3.921e-07$	$6.012e-05$	TRUE	$1.000e+00$	$1.000e+00$	FALSE

GO:0004463	leukotriene-A4 hydrolase activity	MF	AC	TRUE	$1.000e+00$	$1.000e+00$	FALSE	$4.006e-04$	$1.675e-02$	TRUE
GO:0016801	hydrolase activity, acting on ether bonds	MF	AC	TRUE	$1.000e+00$	$1.000e+00$	FALSE	$4.006e-04$	$1.675e-02$	TRUE
GO:0016803	ether hydrolase activity	MF	AC	TRUE	$1.000e+00$	$1.000e+00$	FALSE	$4.006e-04$	$1.675e-02$	TRUE
GO:0005506	iron ion binding	MF	AF	FALSE	$9.996e-01$	$1.000e+00$	FALSE	$1.194e-03$	$4.226e-02$	TRUE
GO:0006873	cellular ion homeostasis	BP	AR	TRUE	$1.000e+00$	$1.000e+00$	FALSE	$1.795e-04$	$8.054e-03$	TRUE
GO:0006875	cellular metal ion homeostasis	BP	AR	TRUE	$1.000e+00$	$1.000e+00$	FALSE	$1.795e-04$	$8.054e-03$	TRUE
GO:0006879	cellular iron ion homeostasis	BP	AR	TRUE	$1.000e+00$	$1.000e+00$	FALSE	$8.740e-05$	$5.026e-03$	TRUE
GO:0008199	ferric iron binding	MF	AR	TRUE	$1.000e+00$	$1.000e+00$	FALSE	$8.740e-05$	$5.026e-03$	TRUE
GO:0019725	cellular homeostasis	BP	AR	TRUE	$1.000e+00$	$1.000e+00$	FALSE	$3.657e-05$	$2.804e-03$	TRUE
GO:0030003	cellular cation homeostasis	BP	AR	TRUE	$1.000e+00$	$1.000e+00$	FALSE	$1.795e-04$	$8.054e-03$	TRUE
GO:0042592	homeostatic process	BP	AR	TRUE	$9.999e-01$	$1.000e+00$	FALSE	$7.523e-04$	$3.004e-02$	TRUE
GO:0046916	cellular transition metal ion homeostasis	BP	AR	TRUE	$1.000e+00$	$1.000e+00$	FALSE	$8.740e-05$	$5.026e-03$	TRUE
GO:0048878	chemical homeostasis	BP	AR	TRUE	$1.000e+00$	$1.000e+00$	FALSE	$1.795e-04$	$8.054e-03$	TRUE
GO:0050801	ion homeostasis	BP	AR	TRUE	$1.000e+00$	$1.000e+00$	FALSE	$1.795e-04$	$8.054e-03$	TRUE
GO:0055065	metal ion homeostasis	BP	AR	TRUE	$1.000e+00$	$1.000e+00$	FALSE	$1.795e-04$	$8.054e-03$	TRUE
GO:0055072	iron ion homeostasis	BP	AR	TRUE	$1.000e+00$	$1.000e+00$	FALSE	$8.740e-05$	$5.026e-03$	TRUE
GO:0055076	transition metal ion homeostasis	BP	AR	TRUE	$1.000e+00$	$1.000e+00$	FALSE	$8.740e-05$	$5.026e-03$	TRUE
GO:0055080	cation homeostasis	BP	AR	TRUE	$1.000e+00$	$1.000e+00$	FALSE	$1.795e-04$	$8.054e-03$	TRUE
GO:0055082	cellular chemical homeostasis	BP	AR	TRUE	$1.000e+00$	$1.000e+00$	FALSE	$1.795e-04$	$8.054e-03$	TRUE
GO:0098771	inorganic ion homeostasis	BP	AR	TRUE	$1.000e+00$	$1.000e+00$	FALSE	$1.795e-04$	$8.054e-03$	TRUE
GO:0008235	metalloexopeptidase activity	MF	AS	TRUE	$1.000e+00$	$1.000e+00$	FALSE	$7.810e-13$	$1.198e-10$	TRUE
GO:0008238	exopeptidase activity	MF	AU	TRUE	$1.000e+00$	$1.000e+00$	FALSE	$1.477e-16$	$2.718e-14$	TRUE
GO:0008241	peptidyl-dipeptidase activity	MF	AV	TRUE	$9.999e-01$	$1.000e+00$	FALSE	$7.610e-04$	$3.004e-02$	TRUE
GO:0008283	cell proliferation	BP	AW	TRUE	$1.000e+00$	$1.000e+00$	FALSE	$8.098e-05$	$5.026e-03$	TRUE
GO:0042127	regulation of cell proliferation	BP	AW	TRUE	$1.000e+00$	$1.000e+00$	FALSE	$8.098e-05$	$5.026e-03$	TRUE
GO:0009405	pathogenesis	BP	AY	TRUE	$1.000e+00$	$1.000e+00$	FALSE	$7.043e-06$	$5.891e-04$	TRUE
GO:0004175	endopeptidase activity	MF	AZ	TRUE	$1.000e+00$	$1.000e+00$	FALSE	$9.603e-07$	$8.835e-05$	TRUE
GO:0004222	metalloendopeptidase activity	MF	AZ	FALSE	$1.000e+00$	$1.000e+00$	FALSE	$3.466e-06$	$3.037e-04$	TRUE
GO:0006508	proteolysis	BP	AZ	TRUE	$1.000e+00$	$1.000e+00$	FALSE	$6.097e-21$	$2.805e-18$	TRUE
GO:0008233	peptidase activity	MF	AZ	TRUE	$1.000e+00$	$1.000e+00$	FALSE	$1.055e-23$	$1.942e-20$	TRUE
GO:0008237	metallopeptidase activity	MF	AZ	TRUE	$1.000e+00$	$1.000e+00$	FALSE	$1.002e-21$	$6.148e-19$	TRUE
GO:0070011	peptidase activity, acting on L-amino acid peptides	MF	AZ	TRUE	$1.000e+00$	$1.000e+00$	FALSE	$2.306e-23$	$2.121e-20$	TRUE
GO:0016787	hydrolase activity	MF	BD	FALSE	$1.000e+00$	$1.000e+00$	FALSE	$8.267e-19$	$3.042e-16$	TRUE
GO:0016791	phosphatase activity	MF	BE	TRUE	$1.000e+00$	$1.000e+00$	FALSE	$2.416e-05$	$1.932e-03$	TRUE
GO:0018342	protein prenylation	BP	BH	TRUE	$1.000e+00$	$1.000e+00$	FALSE	$4.332e-11$	$5.313e-09$	TRUE
GO:0018343	protein farnesylation	BP	BH	TRUE	$1.000e+00$	$1.000e+00$	FALSE	$1.754e-08$	$1.899e-06$	TRUE
GO:0097354	prenylation	BP	BH	TRUE	$1.000e+00$	$1.000e+00$	FALSE	$4.332e-11$	$5.313e-09$	TRUE
GO:0019538	protein metabolic process	BP	BL	TRUE	$1.000e+00$	$1.000e+00$	FALSE	$1.182e-16$	$2.417e-14$	TRUE

GO:0043170	macromolecule metabolic process	BP	BL	TRUE	$1.000e+00$	$1.000e+00$	FALSE	$1.239e-07$	$1.200e-05$	TRUE
GO:0044238	primary metabolic process	BP	BL	TRUE	$1.000e+00$	$1.000e+00$	FALSE	$8.486e-09$	$9.758e-07$	TRUE
GO:0071704	organic substance metabolic process	BP	BL	TRUE	$1.000e+00$	$1.000e+00$	FALSE	$3.553e-08$	$3.632e-06$	TRUE
GO:0065008	regulation of biological quality	BP	BT	TRUE	$1.000e+00$	$1.000e+00$	FALSE	$7.226e-05$	$5.026e-03$	TRUE
GO:0004180	carboxypeptidase activity	MF	D	TRUE	$1.000e+00$	$1.000e+00$	FALSE	$7.799e-17$	$1.794e-14$	TRUE
GO:0004181	metallocarboxypeptidase activity	MF	D	TRUE	$1.000e+00$	$1.000e+00$	FALSE	$7.799e-17$	$1.794e-14$	TRUE
GO:0004970	ionotropic glutamate receptor activity	MF	O	TRUE	$1.000e+00$	$1.000e+00$	FALSE	$8.326e-04$	$3.004e-02$	TRUE
GO:0005230	extracellular ligand-gated ion channel activity	MF	O	TRUE	$1.000e+00$	$1.000e+00$	FALSE	$8.326e-04$	$3.004e-02$	TRUE
GO:0008066	glutamate receptor activity	MF	O	TRUE	$1.000e+00$	$1.000e+00$	FALSE	$8.326e-04$	$3.004e-02$	TRUE
GO:0015276	ligand-gated ion channel activity	MF	O	TRUE	$1.000e+00$	$1.000e+00$	FALSE	$8.326e-04$	$3.004e-02$	TRUE
GO:0022834	ligand-gated channel activity	MF	O	TRUE	$1.000e+00$	$1.000e+00$	FALSE	$8.326e-04$	$3.004e-02$	TRUE
GO:0042060	wound healing	BP	R	TRUE	$9.998e-01$	$1.000e+00$	FALSE	$1.342e-03$	$4.661e-02$	TRUE
